# Ligand Binding Site Structure Shapes Folding, Assembly and Degradation of Homomeric Protein Complexes

**DOI:** 10.1016/j.jmb.2019.07.014

**Published:** 2019-09-06

**Authors:** György Abrusán, Joseph A. Marsh

**Affiliations:** MRC Human Genetics Unit, Institute of Genetics and Molecular Medicine, University of Edinburgh, Crewe Road, Edinburgh, EH4 2XU, UK

**Keywords:** quaternary structure, ligand binding, protein folding, complex assembly, chaperones, SBS, singlechain binding site, MBS, multichain binding site, PDB, Protein Data Bank, GO, Gene Ontology

## Abstract

Ligand binding site structure has profound consequences for the evolution of function of protein complexes, particularly in homomers—complexes comprising multiple copies of the same protein. Previously, we have shown that homomers with multichain binding sites (MBSs) are characterized by more conserved binding sites and quaternary structure, and qualitatively different allosteric pathways than homomers with single-chain binding sites (SBSs) or monomers. Here, using computational methods, we show that the folds of single-domain MBS and SBS homomers are different, and SBS homomers are likely to be folded cotranslationally, while MBS homomers are more likely to form post-translationally and rely on more advanced folding-assistance and quality control mechanisms, which include chaperonins. In addition, our findings demonstrate that MBS homomers are qualitatively different from monomers, while SBS homomers are much less distinct, supporting the hypothesis that the evolution of quaternary structure in SBS homomers is significantly influenced by stochastic processes.

## Graphical abstract

**Figure:**
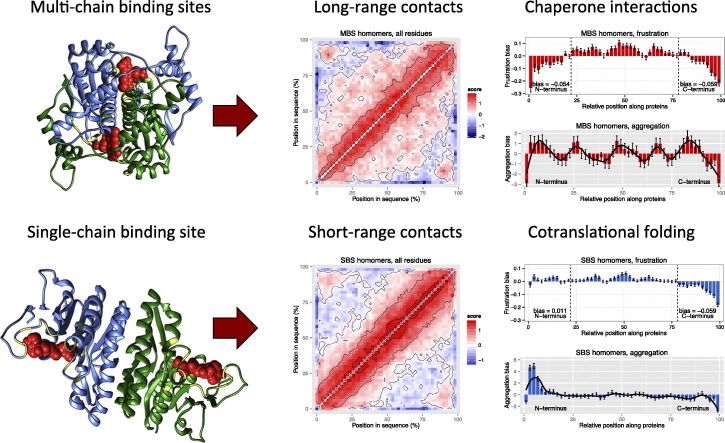

## Introduction

Most proteins do not function in isolation but form complexes, either with their own copies (homomers) or with different proteins (heteromers). It is generally assumed that complex formation is necessary to perform the biological functions of the proteins that form them [1], [2] and that these proteins would not be able to perform their functions as monomers. There are several, highly intuitive arguments that support this assumption: protein complexes are ubiquitous across living organisms, the interfaces of protein complexes are conserved, and protein quaternary structure and assembly pathways are also often conserved [3], [4]. However, there are also strong arguments against the assumption that complex formation is always functionally important, particularly for homomers. First, structure determination methods like x-ray crystallography are capable of producing biologically irrelevant crystal packing artifacts. Second, as Lynch [5], [6] has pointed out, unlike most genomic traits (e.g.*,* genome size, the fraction of coding sequence in a genome, recombination rate and mutation rate), the quaternary structure of homomers does not scale with organismal complexity or effective population size, indicating that its evolution does not depend on the strength of selection and is likely to be strongly influenced by neutral processes [7], [8]. Finally, recent experimental work has demonstrated than in many homomers, quaternary structure is volatile, and a few point mutations (or just one) are often sufficient to change it radically, and even to trigger the formation of large supramolecular assemblies [9], [10].

**Fig. 1:**
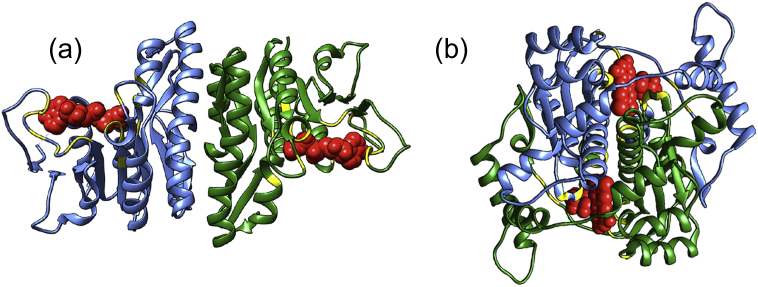
Examples of homomers with different binding site structure. (A) Dimer with single-chain binding sites (SBSs): isopentenyl phosphate kinase from *Thermoplasma acidophilum* (PDB ID: 3LKK). The ligands (ATP) bind only residues of a single protein chain. (B) Dimer with an multichain binding site (MBS): oxygen-insensitive-NADPH-nitroreductase from *Escherichia coli* (PDB ID: 1F5V). The ligands (FMN) interact with both chains of the dimer. In both examples, the ligands are shown in red, and the ligand-binding residues are highlighted in yellow.

Recently, we have examined whether the ligand binding sites of protein complexes—and thus their biological functions—evolve differently than in monomers [11]. We have found that ligand binding sites of homomers have a profound effect on their evolution. The binding sites of homomers with single-chain sites (SBSs; Fig. 1A), that is, ligand binding sites with residues restricted to a single protein chain, evolve at similar rates as in monomers. In contrast, homomers with multichain binding sites (MBSs; Fig. 1B), where the binding site residues are distributed across multiple chains, evolve much slower [11] and bind more similar ligands [12]. In addition, we have shown that in the case of homomers binding cofactors and metals, the quaternary structure of MBS complexes evolves much slower than for SBS complexes. This suggest that complex formation has little effect on biochemical activity/ligand binding of SBS homomers *per se*, and their quaternary structure may be related to other processes like degradation [13], [14], [15] where the exact topology may not matter and is likely to evolve neutrally, as suggested by Lynch [7].

Based on our previous observations, we hypothesized that the structure of ligand binding sites is likely to have major consequences for complex assembly. In the case of single-chain binding sites, their topology is encoded in the sequence; thus, the accuracy of the assembly may have little effect on the function of SBS homomers as they may perform their biological roles with different topologies or even as monomers. In contrast, for complexes with MBSs, inaccurate assembly will be likely to result in dysfunctional complexes, and thus more elaborate mechanisms of quality control should be necessary to guarantee their correct assembly. In addition, our recent findings indicate that the allosteric pathways of MBS and SBS homomers are qualitatively different, and the frequency of allostery is much higher in MBS compared to SBS homomers [16]. This indicates that that these protein classes have different dynamical properties, and that this is likely to be the result of differences in their folds and protein folding.

Here, through computational analysis of thousands of protein structures, we investigate whether several fundamental aspects of protein folding, chaperone interactions, complex assembly, and protein degradation are different in MBS homomers, SBS homomers and monomers. Our results demonstrate that most aspects of folding, interactions with the proteostasis network and sequence/structure signatures of interactions with chaperones are different in MBS homomers compared to SBS homomers or monomers when proteins with a single domain are considered, and support the hypothesis that quaternary structure is much more influenced by neutral processes in SBS homomers than in MBS homomers.

## Results

### MBS homomers have more long-range residue interactions than SBS homomers

The folded state of a protein is the result of a network of non-covalent residue interactions, and is close to the energy minimum of the possible topologies for a given sequence [17]. We investigated the patterns of non-covalent residue interactions by analyzing all non-redundant MBS and SBS homomers of the Protein Data Bank (PDB), with sequence similarity less than 50% (see Methods). We divided our data set into two categories: proteins with a single PFAM domain and proteins with more than one PFAM domain, and analyzed them separately. This distinction is crucial because in multidomain proteins, different functions like ligand binding or protein–protein interfaces can be located in separate domains, and thus, different domains can be under different selective pressures. Altogether we identified 700 MBS and 2036 SBS homomers with a single domain (Supplementary Table 1), and 319 MBS and 920 SBS homomers with multiple domains (Supplementary Table 2), which satisfy the criteria of sequence coverage higher than 50% by a PDB entry, and resolution better than 3 Å (see Methods and also Supplementary Data).

**Fig. 2:**
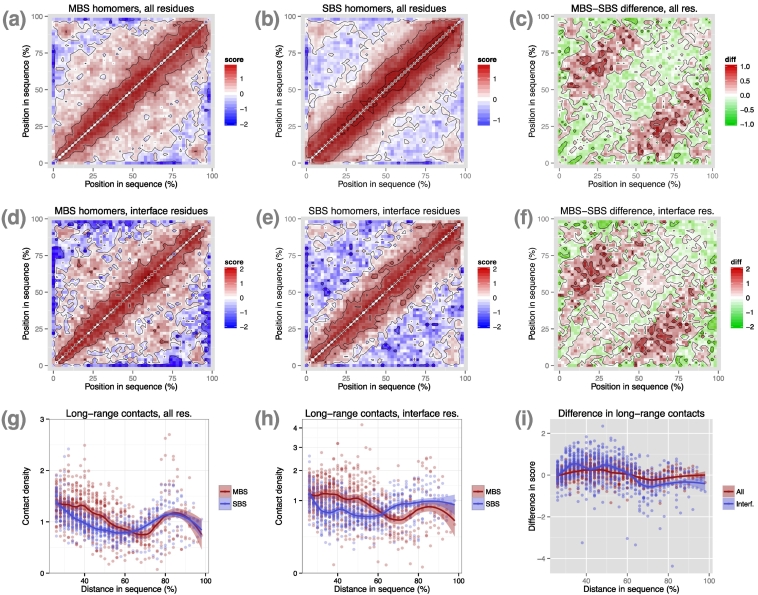
Single-domain MBS homomers have higher frequencies of long-range residue contacts than single-domain SBS homomers. (A and B) Matrices of contact scores in MBS and SBS homomers, using all residues. (C) Differences in contact scores between MBS and SBS homomers, all residues. MBS homomers have consistently more long-range contacts than SBS homomers. (D and E) Contact score matrices of interface residues. (F) The pattern of the differences in long-range interface contact scores is qualitatively similar and even more pronounced than for all residues. (G) Relationships between residue distance and contact density (i.e., exp(contact score)) for residues more than 25% distant in the sequence using all residues. The difference between MBS and SBS homomers is highly significant (distances 25%–60%; *p* = 6.9e − 16, ANCOVA). (H) The same as in panel G, for interface residues. Note that the *y*-axis is square-root transformed, due to outliers. The difference is highly significant (distances 25%–60%; *p* = 1.4e − 23, ANCOVA). (I) Interface residues show a higher enrichment in long-range contact scores than all residues (distances 25%–60%; *p* = 1.3e − 04, ANCOVA). On panels A‐F, minimum values, and on panels C,E, maximum values were set, to prevent the biasing of color scales by a small number of outliers.

For proteins with a single domain, we found significantly more long-range residue interactions in MBS homomers than in SBS homomers (Fig. 2). Residue–residue interactions (nrint) were calculated with the RINerator tool [18] (see Methods); we divided each protein into 50 bins and calculated an interaction score for each cell of the 50 × 50 matrix, which corresponds to the logarithm of the sum of non-covalent contacts between the corresponding sequence regions (see Methods). MBS homomers have a broader distribution of long-range contacts (Fig. 2A, B), which is most pronounced between residues more distant than 25% of the sequence length (Fig. 2C), and the difference from SBS homomers is highly significant (Fig. 2G, *p* = 6.9e − 16, ANCOVA for distances 25%–60%). In addition, the structures of MBS homomers are characterized by an excess of interactions between their N- and C- termini (Fig. 2A, C, G). Since we have recently found that, in allosteric MBS homomers, signal transduction pathways cross the protein–protein interface [16], we repeated the analysis using the non-covalent interactions of interface residues (with all residues). The results show that interface residues show a similar, even more pronounced pattern (Fig. 2D–F), which, despite the smaller data set is even more significant (Fig. 2H, *p* = 1.4e − 23, ANCOVA for distances 25%–60%; note that the *y*-axis is square-root transformed due to outliers). The comparison of the enrichment of interface and all residues shows that interface contacts are significantly more enriched in long-range contacts than the full set of residues (Fig. 2I, *p* = 1.3e − 04, ANCOVA for distances 25%–60%).

In the case of proteins with multiple domains, we performed a similar analysis of residue interaction enrichment on their protein domains. We used two different domain annotation schemes, PFAM domains and CATH domains [19]; for the latter, we used only domains that are continuous in their sequence. For a fair comparison with single-domain proteins, we only used domains that contain residues that bind BioLiP ligands and also interface residues. Similar to single-domain proteins, the sequence regions corresponding to their PFAM/CATH domains were divided into 50 bins (Fig. S1). The results show that in the case of PFAM domains (439 in MBS and 879 in SBS homomers), the enrichment of long-range residue interactions of interface residues is weaker than for single-domain proteins, although it is still significant (Fig. S1A–C, G; *p* = 6.68e − 05, ANCOVA on distances 25%–60%), and the pattern is somewhat less pronounced on all residues (not shown). For CATH domains, however (374 in MBS and 649 in SBS homomers), the difference between MBS and SBS homomers is not significant (Fig. S1D–F, H; *p* = 0.67, ANCOVA on distances 25%–60%). The difference between PFAM and CATH domains is highly significant (Fig. S1I, *p* = 1.7e − 13, ANCOVA on distances 25%–60%; see also Fig. S2 for a comparison of the location of the PFAM and CATH domains used).

### Residue interactions of SBS homomers and monomers are similar

**Fig. 3:**
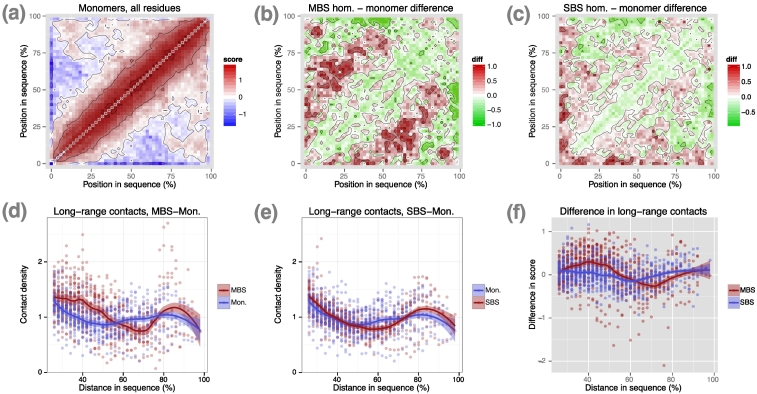
In single-domain proteins, the residue interaction pattern of monomers is qualitatively similar to SBS homomers. (A) Matrix of contact scores in monomers, using all residues. (B and C) Differences in contact scores between MBS homomers, SBS homomers, and monomers. MBS homomers have more long-range contacts than monomers, while in the case of SBS homomers, the difference is much smaller. (D) Relationships between residue distance and contact density (i.e., exp(contact score)) for residues more than 25% distant in the sequence using all residues. The difference between MBS homomers and monomomers is highly significant (distances 25%–60%; *p* = 2.3e − 18, ANCOVA). (E) The difference is not significant between SBS homomers and monomers (distances 25%–60%; *p* = 0.135, ANCOVA)). (F) Residues of MBS homomers show a significantly higher enrichment in long-range contact scores than residues of SBS homomers, when compared to monomers (distances 25%–60%; *p* = 3.0e − 22, ANCOVA). Similarly to Fig. 2, on panels B and C, minimum and maximum values (− 1 and 1, respectively) were set to prevent the biasing of color scales by a few outliers. The original score differences are presented in panel F.

Next, we examined whether the non-covalent contact patterns of MBS or SBS homomers are different from monomers. Using similar criteria as for homomers (see Methods), we identified 2060 non-redundant monomers (max. 50% sequence similarity) with a single PFAM domain and 963 multidomain monomers. We calculated their residue interaction matrices and compared them to the matrices of homomers. For single-domain proteins, the residue interaction matrices show that MBS homomers have more long-range contacts than monomers (Fig. 3A, B), while there is little difference between SBS homomers and monomers, although monomers do appear to have their residues with long-range contacts located closer to their C-termini (Fig. 3A,C). The difference between MBS homomers and monomers is highly significant (Fig. 3D; *p* = 2.3e − 18, ANCOVA for distances 25%–60%), while it is not significant for SBS homomers *versus* monomers (Fig. 3E; *p* = 0.135, ANCOVA for distances 25%–60%). The difference from monomers is significantly larger for MBS homomers than for SBS homomers (Fig. 3F; *p* = 3.0e − 22, ANCOVA on distances 25%–60%).

In multidomain monomers, similar to multidomain homomers, we used ligand-binding PFAM and CATH domains (all residues of the domains). The differences from homomers are considerably smaller than in single-domain proteins (Fig. S3). In the case of PFAM domains (470 in MBS homomers and 1269 in monomers), a modest enrichment in long-range contacts can be seen both for MBS homomers (Fig. S3A and B, *p* = 5.49e − 09, ANCOVA for distances 50%–80%) and a negligible enrichment in SBS homomers (not shown). In the case of CATH domains (398 in MBS homomers and 969 in monomers), we found no significant differences from monomers (Fig. 2C and D; *p* = 0.34; ANCOVA for distances 50%–80%), although a weak trend appears to be present (Fig. 2C). The difference between PFAM and CATH domains is highly significant (Fig. 2E, distances 50%–80%; *p* = 2.8e − 14; ANCOVA).

### The sequence and domain diversity is not lower in MBS homomers than in SBS homomers

**Fig. 4:**
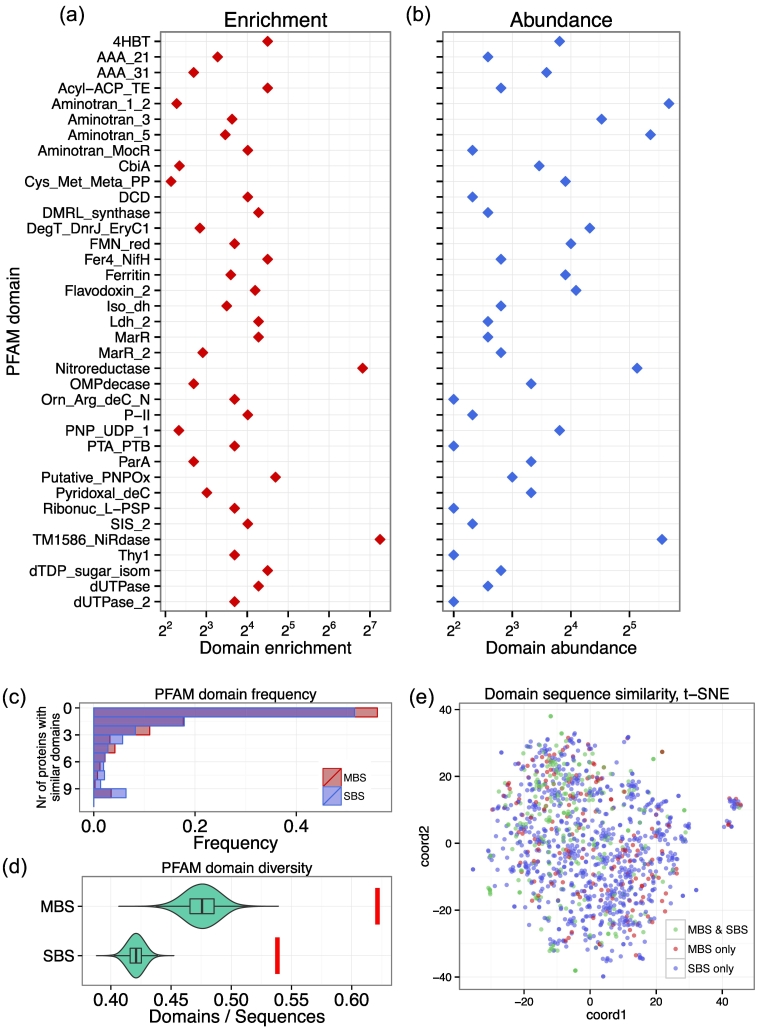
Characteristics of conserved domains in MBS and SBS homomers with a single PFAM domain. (A) PFAM domains that are significantly enriched in MBS homomers (FDR-corrected *p* < 0.05) compared to SBS homomers. For plotting, in the case of domains that are absent in SBS homomers, we used 1 as their abundance in SBS homomers (but not for significance testing). (B) The abundance of significantly enriched PFAM domains in MBS homomers. (C) The frequency distribution of PFAM domains does not differ qualitatively in MBS and SBS homomers: in both cases, more than 50% of domains are present in only a single protein, and ~ 80% of domains are present in three or less proteins. Note that domains present in more than 10 proteins are included in bin with 10, and domain overlaps (i.e., more than one different domains mapping to the same sequence region) were permitted. (D) MBS homomers have a somewhat higher observed diversity (0.62, red vertical bar) of PFAM domains than SBS homomers (0.54). Similarly, their expected diversity obtained through Monte-Carlo simulations (violin plots) is higher than in SBS homomers. Diversity was measured as the number of domains per the number of sequences. (E) Two-dimensional (t-SNE) representation of the sequence space of PFAM domains of homomers. Neither domains specific for MBS or SBS homomers nor domains present in both of them form separate clusters, but are distributed relatively evenly across the sequence space. (See also Fig. S4 for their full sequences.) Taken together, these results indicate that the differences between MBs and SBS homomers are not caused by the more limited diversity of domains in MBS homomers.

If the diversity of sequences is dramatically lower in single-domain MBS homomers than SBS homomers and is dominated by a few conserved domains with many long-range contacts, this could result in patterns that simply reflect the characteristics of a few domains, and not MBS homomers in general. To exclude this possibility, we compared the PFAM domain composition and sequence similarity of single-domain homomers with several methods (in addition to using non-redundant proteins). First, using all PFAM domains that map to a particular sequence (thus if two PFAM domains map to the same region, both were used), we tested whether there are PFAM domains that are highly enriched in MBS homomers compared to SBS homomers. We found that 37 domains are significantly (*p* < 0.05, tests of proportions) enriched in MBS homomers (Fig. 4A). The Nitroreductase and TM1586_NiRdase domains, which are related and map to the same sequence regions, are particularly highly enriched (Fig. 4A). However, their high enrichment does not result in a high frequency: only 5% (35) of MBS sequences contain a Nitroreductase domain, and 6.7% (47) contain a TM1586_NiRdase domain (and these are the same sequences). The most abundant domain among the significantly enriched domains is the Aminotran_1_2 domain (Fig. 4B), which is present in 7.3% (51) of MBS sequences, but it shows only a modest enrichment compared to SBS sequences (Fig. 4A). Next we tested whether there is a consistent difference between the number of sequences that share the same domain, and found that there is no qualitative difference between the two types of homomers: in both cases, approximately 50% of domains are present in only a single sequence, and ~ 80% of domains are present in three or less sequences (Fig. 4C). The comparison of domain diversity (number of domains divided by number of sequences) shows that MBS homomers have a somewhat higher diversity than SBS homomers (Fig. 4D, red bars). To account for differences in domain frequency distributions of the two homomer types, we also performed Monte-Carlo simulations to test the expected domain diversity when the sequences are sampled randomly. The results show that both in MBS homomers and SBS homomers, the expected diversity is somewhat lower than observed; thus, a small number of sequences contributes disproportionally more to diversity than the average, but MBS homomers remain more diverse than SBS homomers (Fig. 4D).

The above tests did not take into account the sequence similarity of PFAM domains. To exclude the possibility that the sequences and PFAM domains of MBS homomers are more similar to each other than the sequences and domains of SBS homomers (despite the 50% clustering cutoff), we used an alignment-free method to compare the sequence similarity of PFAM domains, and also the full sequences of MBS and SBS homomers. Using their seed alignments, we transformed every PFAM domain alignment into a feature vector with 400 elements, where the values of the vectors are all possible dipeptide frequencies. Next we clustered the vectors with t-SNE [20], with 2 dimensions and perplexity 5, for visualization (Fig. 4E). The results show that neither the domains specific to MBS or SBS homomers, nor the ones present in both homomer types form distinct clusters but are instead distributed relatively evenly across the sequence space (Fig. 4E). We performed the same analysis for the individual sequences of MBS and SBS homomers (Fig. S4, dimensionality 2, perplexity 5), and our results show that the sequences of the two types of homomers do not form distinct clusters. In fact, SBS homomers show more pronounced clustering than MBS homomers. (Note that in the case of short sequences, the dipeptide frequencies of the feature vectors can be dominated by zeroes, and thus, clusters might be formed by the lack of certain dipeptides rather than their frequencies in the actual sequences.) Taken together, these findings indicate that the differences between MBS and SBS homomers are not caused by the high frequency of a few domains that bias the data set in MBS homomers, but are general characteristics of MBS homomers.

### Gene Ontology analysis of protein–protein interactions

**Fig. 5:**
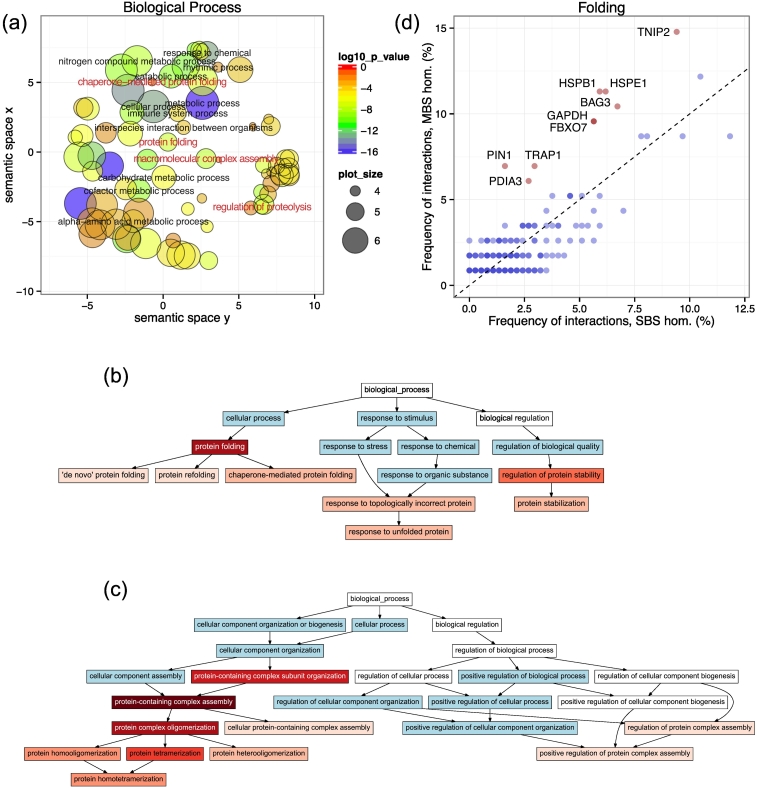
Folding, assembly and degradation related GO terms are enriched in the interactome of human MBS homomers. (A) Summary (made with REVIGO) of the main categories of the Biological Process GO terms that are significantly enriched in the interactors of MBS homomers compared to the interactors of all homomers (MBS + SBS). Altogether, more than 600 terms are significantly enriched, many of them related to protein folding, assembly and proteolysis (highlighted with red). These include “chaperone mediated protein folding,” “protein folding,” “macromolecular complex assembly” and “regulation of proteolysis.” (see supplementary data for the full GO enrichment results). (B) Graph of the significantly enriched terms related to protein folding and protein stabilization. The intensity of red corresponds to significance, while terms in blue are also (highly) significant but are too high level to be considered as folding related. (C) Graph of the significantly enriched terms related to assembly of protein complexes. Color coding is similar to panel B. (D) Frequencies of interactions of folding related genes in MBS and SBs homomers. A gene with *x* = 4% and *y* = 5% interacts with 4% of SBS homomers and 5% of MBS homomers. Most genes fall close to the *x* = *y* line, that is, have a similar frequency of interactions in MBS homomers and SBS homomers; however, nine genes form a separate cluster (highlighted with red) and are more distant from the parity line than 3 standard deviations of the remaining genes. These genes include an HSP70/HSC70 chaperone regulator BAG3, a small chaperone protein HSPB1, a co-chaperonin HSPE1 and a 75 kDa heat shock protein TRAP1 (see also Supplementary Table 3).

The folding rate of proteins is correlated with the complexity of their fold (contact order); proteins with more long-range contacts fold slower [21], [22], and the more complex a protein fold is, the more prone it is to misfolding and dependent on chaperones for correct folding [23]. Thus, the higher frequency of long-range residue interactions in MBS homomers (Fig. 2, Fig. 3) indicates that the topologies of the folds of MBS homomers are more difficult to fold (and also fold slower) than SBS homomers or monomers, at least in single-domain proteins. This suggests that their interactions with chaperones and other proteins that assist folding are likely to be different or more frequent. To investigate this, we tested whether Gene Ontology (GO) terms related to folding, chaperone and complex assembly have different frequencies in the proteins that interact with MBS and SBS homomers. We used the protein–protein interactions from the BioGRID database [24] (v3.5.169) to identify the interactomes of human MBS and SBS homomers. Altogether, 196 human MBS homomers and 614 SBS homomers have protein–protein interactions in BioGRID, which interact with 3368 and 8056 genes, respectively (no distinction was made between single- and multidomain proteins). GO enrichment analysis of the interacting genes (see Methods) indicates that more than 600 Biological Process-related terms are significantly enriched in the interactome of MBS homomers (*p* < 0.05, with FDR correction), which cover a broad range of biological processes (see Fig. 5A for a summary of the significantly enriched terms using REVIGO [25], and Supplementary Data for the full results). Several of the enriched terms are related to high-level terms like protein folding and complex assembly, but also regulation of proteolysis (Fig. 5A, highlighted with red). Examination of the hierarchies of significantly enriched of GO terms (Fig. 5B and C, Fig. S5) indicates that genes related to most aspects of protein stabilization, refolding, chaperone-mediated folding, response to unfolding, oligomerization, and ubiquitin-dependent proteolysis are overrepresented among the interactors of MBS homomers (Fig. 5B and C, Fig. S5).

To find out which genes associated with the enriched terms might preferentially interact with MBS homomers, for each gene that has a GO term in the significantly enriched categories of folding, assembly or regulation of degradation, we examined the percentage of their interacting genes among MBS and SBS homomers (Figs. 5D and S6). The majority of genes interact with a similar and typically small fraction (less than 5%) of genes in both MBS and SBS homomers (i.e. are located close to the diagonal). However, for folding-related genes, a cluster of nine genes has a substantially higher frequency of interactions with MBS homomers than with SBS homomers (Fig. 5D, labeled genes highlighted with red; see also Supplementary Table3) as their distance from the diagonal is larger than three standard deviations of the remaining genes. These genes include BAG3, a regulator/co-chaperone of HSP70 [26]; HSPB1 (HSP27), a small chaperone protein that can form large oligomers [27]; HSPE1 (HSP10), a mitochondrial co-chaperonin of HSP60 that is structurally highly similar to the prokaryotic GroEL/ES complex [28]; a mitochondrial chaperone TRAP1 (HSP75) that belongs to the HSP90 family of chaperones [29]; and PDIA3 (ERp57), a multifunctional protein that is responsible for reorganization of disulfide bonds but is also involved in the unfolded protein response of the endoplasmatic reticulum [30] and the maintenance of the steady-state concentration of prion protein [31]. These nine genes, while representing only 4% of the 213 genes with folding related (enriched) GO terms, interact with 49% of the 115 MBS homomers that do interact with such genes. For assembly or proteolysis-related genes, no clear clusters could be identified that interact with a substantially higher fraction of MBS homomers than SBS homomers (Fig. S6). However, we note that HSPE1 and GAPDH are also involved in the regulation of proteolysis (Fig. S6B, highlighted with red).

The vast majority of mitochondrial proteins are encoded in the nucleus and are synthesized in the cytoplasm. In order to reach the matrix or inner membrane of the mitochondrion, these proteins must be transported there in an unfolded state through membrane channels, with the aid of chaperones [32], [33]. Since two of the chaperones with high frequency of MBS interactions are mitochondrial (HSPE1, TRAP1), we also performed a cellular component GO analysis of the human homomers, to examine whether MBS homomers are enriched in mitochondria. The analysis shows that this is the case, and four cellular component terms are significantly enriched (see Table S4 and Supplementary Data): mitochondrial matrix (*p* = 0.015), transporter complex (*p* = 0.015), ion channel complex (*p* = 0.015) and transmembrane transporter complex (*p* = 0.015), indicating that a higher fraction of MBS homomers than SBS homomers is transported to mitochondria. However, despite their significant enrichment, only a minority of MBS homomers are targeted to the mitochondrial matrix (17.4%), or organelle lumens (29.6%, see Table S4).

### Structural and sequence signatures of chaperone interactions and folding

Despite their rapid growth, protein–protein interaction databases are still highly incomplete, even for the most studied species like humans, and also contain considerable experimental noise [34], [35]. Moreover, the majority of proteins in our data set were non-human, originating from a diverse set of organisms, with a large fraction being prokaryotic. Therefore, we examined whether the structures and sequences of MBS homomers also indicate differences in their chaperone interactions and complex assembly, as the GO analysis of their interactome indicates for human proteins. Most chaperones interact with a broad range of target proteins, recognizing general patterns in the sequences or structures, like aggregation-prone regions or frustration, rather than well-defined interaction motifs. Trigger factor and the HSP70/DnaK group of chaperones, which act as a central hub of the chaperone network in most organisms [23] (except Archaea), recognize aggregation-prone and hydrophobic regions of proteins [36], [37]. The HSP90 chaperone group recognizes conformational instability in its clients [38], and several microbial chaperones like Spy, Skp and SurA have been recently shown to interact with frustrated regions of proteins and folding intermediates [39], [40] (see Ref. [41] for a recent review of frustration and folding). The factors determining the target specificity of chaperonins like GroEL/GroES, TRIC or HSP60 are less clear, but aggregation-prone sequences are known to be involved, and the sequences of these proteins were shown to display signs of periodicity [42], due to interactions with the chaperonin cage.

#### Frustration

**Fig. 6:**
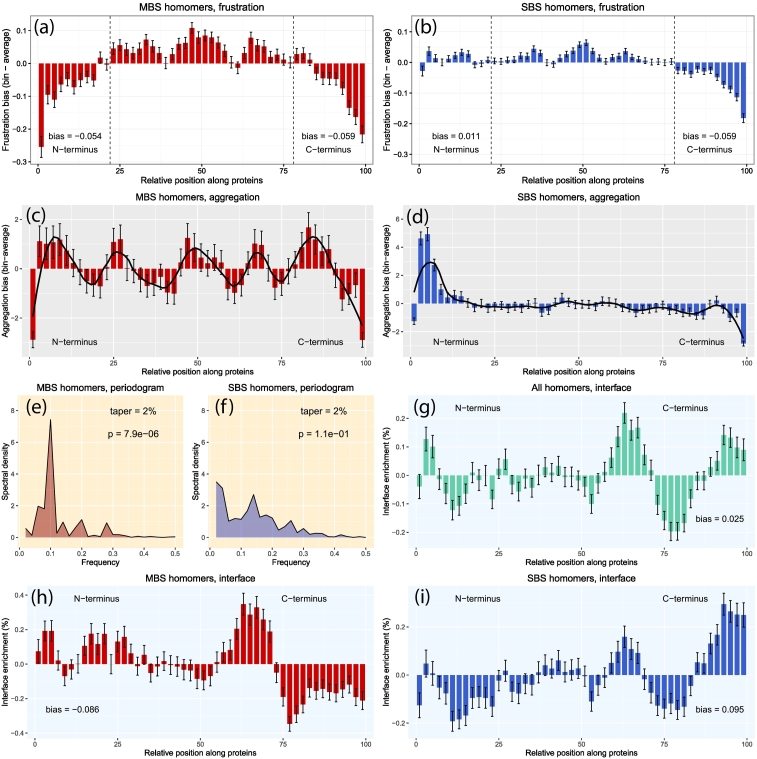
The sequence and structural signatures of chaperone interactions and complex assembly are different in single-domain MBS and SBS homomers. (A and B) Distribution of frustration bias along the protein sequences (lower is more frustrated). MBS homomers have significantly elevated levels of frustration at both termini of the sequence, but there is no difference between the two termini (*p* = 0.19, randomization test), while in SBS homomers, only the C terminus is frustrated (*p* < 0.0001, rand. test). (C and D) The distribution of aggregation propensity along the protein sequences. Bias was measured as the difference from the average of the proteins. In MBS homomers, the pattern is characterized by clear periodicity, although of modest magnitude, while in SBS homomers, it is characterized with N-terminal enrichment (*p* < 0.0001, rand. test). (E and F) The periodograms of aggregation bias indicate that the periodicity is highly significant in MBS homomers but not in SBS homomers (Fisher's *G* tests). (G, H and I) Distributions of interface enrichment in all BioLiP homomers (G), MBS homomers (H) and SBS homomers (I). MBS homomers show a depletion of interface residues at the C terminus (*p* = 0.0001, rand. test), while SBS homomers show an enrichment of interface residues (*p* = 0.0001, rand. test), indicating that cotranslational assembly influences the location of the interface only in SBS homomers. In the pooled data set of homomers (G), there is no significant C-terminal bias (*p* = 0.1, rand. test). On every plot, whiskers represent standard deviations.

First, we examined whether the distribution of frustrated residues is different in single-domain homomers and monomers. We used Fustratometer2 [43] to determine the degree of configurational frustration for every intramolecular interacting residue pair in the longest chain of PDB entries. We divided each protein into 50 bins, and for each bin, we calculated the difference of the average frustration of the residues that fall into the bin from the entire protein average and determined the significance of differences through randomization (see Methods). In MBS homomers, highly frustrated residues (i.e., with negative frustration bias) are located both at the N- and C-termini (Fig. 6A), and there is no significant difference between the two termini (*p* = 0.19, randomization test, see Methods). In SBS homomers, a comparable enrichment of frustrated residues is present only toward the C-termini (Fig. 6B, *p* < 0.0001, rand. test). For monomers, none of the termini are strongly enriched in frustrated residues, although the C-terminal region is somewhat more frustrated than the N-terminal region (Fig. S7A, *p* = 0.0002, rand. test). Since frustration is a property of interactions between residue pairs, we also calculated matrices of frustration bias in order to obtain more detailed information on the distribution of frustration (Fig. S8, see also Methods). The matrices indicate that the residues of the most frustrated interactions are typically located close within the protein sequences, for example, within helices (i.e., cells with negative bias fall close to the diagonal), while long-range interactions are generally less frustrated (have more positive bias) than the average, particularly in MBS homomers (Fig. S8A–C). The difference in frustration bias between the long-range residue interactions of MBS and SBS homomers is highly significant (Fig. S8D, *p* = 3.18e-34, ANCOVA for distances 20%–60%).

#### Aggregation

We predicted the aggregation propensity of each single-domain protein sequence with TANGO [44] and calculated the difference from the protein average, similar as for frustration (Fig. 6C, D and Fig. S7B). MBS and SBS homomers show qualitative differences: MBS homomers show no enrichment at any of the termini (*p* = 0.348, rand. test), but are characterized by clear periodicity with a frequency 0.1 (~ 10 bins; Fig. 6C and E, *p* = 7.8e − 06, Fisher's *G* test). In contrast, SBS homomers have a significant N-terminal enrichment (Fig. 6D, *p* < 0.0001, rand. test) but no periodicity (Fig. 6D and F, *p* = 0.11, Fisher's *G* test). Monomers are characterized by strong N-terminal enrichment (Fig. S7B *p* < 0.0001, rand. test) and no significant periodicity (Fig. S7C, *p* = 0.127, Fisher's *G* test). Note that aggregation-prone regions are typically located in a few short regions in the sequences, and the periodicity we observe is not periodicity within individual sequences, but an average pattern over many, indicating that aggregation-prone regions are typically enriched (or depleted) within certain locations of the sequences (i.e., different peaks are present in different sequences). These findings are consistent with our observation that MBS homomers interact more frequently with chaperonins than SBS homomers (or monomers), as indicated by the GO analysis (Fig. 5).

#### Interface bias

Recently, it has been reported that cotranslational assembly is frequent in protein complexes [45], [46]. Ribosome profiling experiments indicate that, in addition to their role in folding, HSP70 chaperones play a key role in cotranslational assembly, while computational work shows that in the case of homomers, cotranslational assembly results in evolutionary constraints on the location of interface residues: it selects for their biased distribution, overrepresented in the C-termini of the protein sequences to allow for folding before assembly, as the interface residues of these proteins will be translated last [46]. We hypothesized that the MBS and SBS homomers assemble differently, for several reasons. First, their GO enrichment and aggregation pattern suggest frequent interactions of MBS homomers with chaperonins, but folding in the chaperonin cage effectively makes cotranslational assembly impossible. Second, previous analyses suggest that frustrated regions are often located near interfaces [47]; thus, the differences in the enrichment of frustrated residues between MBS and SBS homomers suggest fundamental differences in the location of interface residues as well. Third, our GO analysis indicates a significant enrichment of assembly-related proteins among the interactors of MBS homomers (Fig. 5C).

We calculated interface enrichment for both homomer types, using a similar method to that used by Natan *et al*. [46] (see also Methods) and found that MBS homomers and SBS homomers have qualitatively different patterns of interface enrichment (Fig. 6G-I). SBS homomers show a similar C-terminal enrichment as reported (Fig. 6I, *p* = 0.0001, rand. test), suggesting that they fold (and assemble) cotranslationally. In contrast, MBS homomers show the opposite pattern and are characterized by C-terminal depletion of interface residues (Fig. 6H, *p* = 0.0001, rand. test), and their interface residues form several peaks along the sequence (Fig. 6H), which is more consistent with post-translational folding and assembly. (The combined data set of MBS and SBS homomers shows no significant enrichment at the C-terminus).

#### Disulfide bridge frequency

**Fig. 7:**
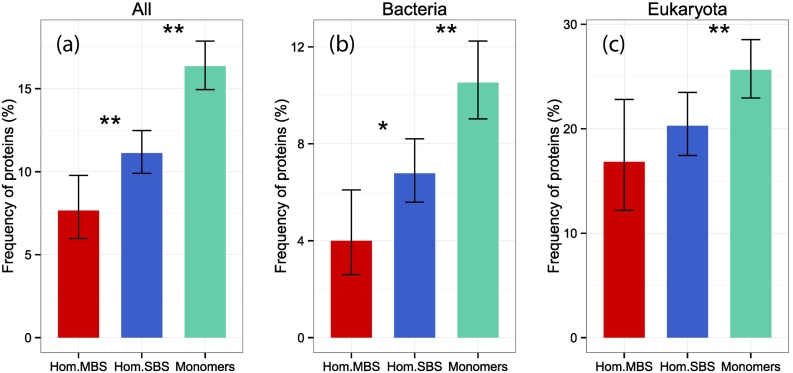
Frequencies of complexes with disulfide bridges. All proteins. (B) Bacterial proteins. (C) Eukaryotic proteins. SBS homomers have significantly fewer disulfide bridges than monomers, while the difference between MBS and SBS homomers is significant in the pooled data set and bacteria. (Note that the number of eukaryotic proteins is much smaller than the number of Bacterial proteins.) *p < 0.05, **p < 0.005; tests of proportions.

Finally, we examined whether the frequency of disulfide bridges, another determinant of protein stability, is also different between MBS and SBS homomers and monomers. The endoplasmatic reticulum foldase PDIA3, which is one of the nine folding related genes with higher frequency of interactions in MBS homomers (see Fig. 5D), is also involved in the remodeling of disulfide bridges in proteins. We calculated the frequency of disulfide bridges with DSSP for all single-domain structures and found that their frequency is significantly lower in MBS homomers than in SBS homomers, and in SBS homomers than in monomers (Fig. 7). We also note that the frequency of disulfide bridges is lower in prokaryotes than in eukaryotes, as reported previously [48]. While the overall frequency of proteins with disulfide bridges is low in all quaternary structure categories, this finding is consistent with our previous observations that MBS complexes are more flexible than SBS complexes [11], and that allostery is enriched in MBS homomers [16], as allostery requires flexibility.

### MBS homomers are degraded more slowly than either SBS homomers or monomers

**Fig. 8:**
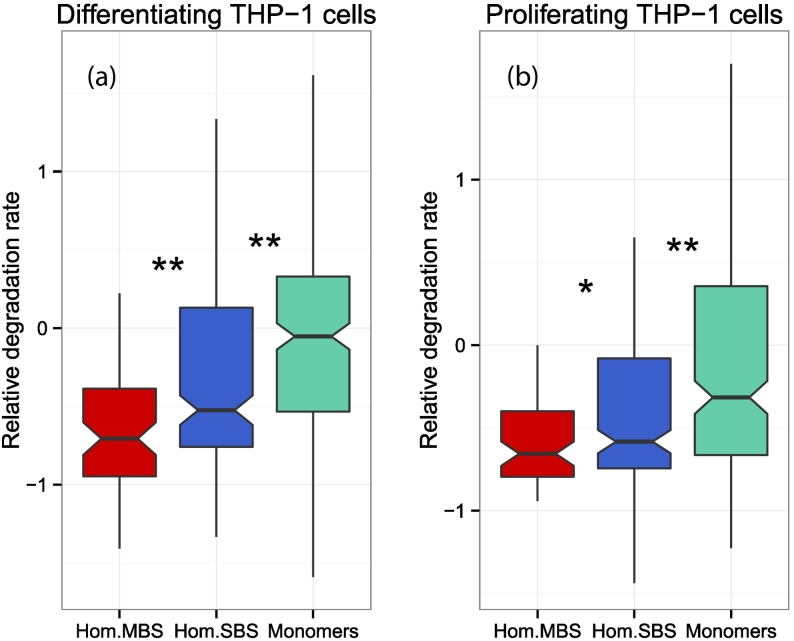
Relative degradation rates of homomers and monomers in human THP-1 leukemia cells. (A) Differentiating cells. (B) Proliferating cells. In both cases, MBS homomers are degraded significantly slower than SBS homomers, and SBS homomers are degraded significantly slower than monomers. *p < 0.05, **p < 0.005; Wilcoxon tests.

The high number of enriched GO terms related to proteolysis and its regulation (Figs. 5A and S4) suggests that the final stage of protein metabolism—degradation—is also likely to be different for MBS and SBS homomers. Mallik and Kundu [15] have recently demonstrated that contact order, that is, the average distance of interacting residues in the protein sequence and oligomerization status are key regulators of degradation rate. Proteins with higher contact order (more long-range residue interactions) that form complexes are degraded at a lower rate than monomers, possibly due to the higher mechanical stability of their folds, and burying disordered and ubiquitylation sites in interfaces. Using a human data set of differentiating and proliferating THP-1 leukemia cells [49], we examined whether there are differences in the degradation rate of MBS and SBS homomers. We found that the degradation is slowest in MBS homomers and fastest in monomers (Fig. 8), which is consistent with the higher fraction of long-range interactions in MBS homomers. However, as MBS homomers are more flexible than SBS homomers or monomers [11], mechanical resistance to proteolysis is unlikely to be the main cause of the difference.

## Discussion

Our results indicate that MBS homomers are a distinct class of complexes, which function, are metabolized, and evolve differently from SBS homomers or monomers. Protein complexes have so far been classified based on their geometric properties, like symmetry and topology [50], [51]. Functional aspects, like the structure of their binding site, folding, degradation or interactions with other components of the proteostasis network, have largely been neglected. Our results, together with our previous findings on binding-site evolution [11] and allostery [16], indicate that these properties are of fundamental importance. Thus, MBS and SBS dimers are likely to be more different from each other than SBS dimers and tetramers (or other SBS topologies), which suggests that the binding site structure of their natural ligands, e.g. “polydesmic” vs. “monodesmic” (δεσμός: binding, πολοί: many, μόνος: single) could be added as an additional layer of complexity to the “periodic table of protein complexes” [51]

The key steps of proteostasis, that is, folding, chaperone interactions, assembly and degradation, are likely to follow qualitatively different paths in MBS homomers compared to SBS homomers or monomers. Our results are consistent with the hypothesis that the factor driving the difference between the two complex types is the structure of their binding sites, and that MBS homomers require more assistance from the proteostasis network than SBS homomers for correct folding and assembly. Approximately 70% of proteins are folded on the ribosome [23] and interact mainly with the ribosome-associated proteins like trigger factor [52], [53] (TF) in prokaryotes or nascent-chain/ribosome-associated complexes [23], [54] (NAC/RAC) in eukaryotes during folding. In the case of SBS homomers, the C-terminal enrichment of their interface residues indicates that they are more frequently folded and assembled cotranslationally (Fig. 6I) on the ribosome. Cotranslational folding also means that regions that are buried in the folded protein, like the hydrophobic core and aggregation-prone motifs, are located closer to the N-terminus of the sequence [55], and the clear enrichment of aggregation-prone regions close to the N-terminus both in SBS homomers and monomers is in agreement with this. Besides cotranslational assembly, the pattern of residue–residue interactions (Fig. 2) also suggests that SBS homomers fall largely to this category: they are characterized by a lower frequency of long-range contacts, and therefore, their likelihood of misfolding is probably lower than for MBS homomers, and makes interactions with other chaperones and chaperonins less crucial. In addition, trigger factor prevents misfolding by binding to partially folded regions and preventing the formation of incorrect long-range contacts [56], [57]; thus, proteins with fewer long-range contacts are more likely to be folded on the ribosome. The lower frequency of interactions with chaperones that are more downstream of the chaperone pathways, like (mitochondrial) chaperonins, HSP90, or small chaperones (Fig. 5), also supports this hypothesis.

In contrast, MBS homomers are more likely to fall into the remaining 30% of proteins that need the assistance of chaperones and chaperonins that are more downstream in the chaperone pathways and are folded/assembled post-translationally. This is supported by several findings: the higher frequency of long-range residue interactions indicates that their folds have more complex topologies (Fig. 2) and thus may have a higher likelihood of misfolding; in humans, GO terms related to folding and assembly are significantly enriched among their interacting proteins (Fig. 5B,C); and a higher frequency of MBS homomers interacts with chaperonins and other HSP90/small chaperones (Fig. 5D). This is in agreement with the distribution of aggregation-prone sequences: in MBS homomers, aggregation propensity is not biased toward the N-terminus but shows a clear (albeit low amplitude) periodicity (Fig. 6C, E). Periodicity has been reported as one characteristic of substrates of GroEL/ES chaperonins [42] due to interactions with the subunits of the chaperonin cage. However, as periodicity is likely to be the consequence of interactions with a cyclic, multimeric molecular machine, other explanations are also possible, such as interactions with the hexameric prefoldin (Pfd) that in eukaryotes shuttles proteins to the TRiC chaperonin complex [58]. Both frustrated and interface residues are enriched at N-termini, indicating that MBS homomers are not selected to bury their N-terminal residues in the hydrophobic core and are less likely to be folded and assembled cotranslationally (Fig. 6A, H). Finally, in eukaryotes, proteins that are transported to organelles like mitochondria cannot fold and assemble cotranslationally, as these proteins must pass membranes in an unfolded state [33], [59], and in human, we found a significant enrichment of MBS homomers among the proteins of mitochondrial matrix (Table S4). However, the majority of proteins in our data set are bacterial, and even in humans, only a minority of MBS homomers (~ 30%) are transported to organelles (Table S4). Thus, it is unlikely that this is the main evolutionary force that drives the structural differences between the two types of homomers.

Cotranslational folding and assembly have received considerable attention in recent years [45], [60], [61], [62], [63], [64]. However, it is still unclear what fraction of the proteome is actually folded or assembled cotranslationally, due to the lack of large-scale analyses. Mathematical modeling predicts that in *Escherichia coli* one-third of the proteome is likely to fold cotranslationaly [65], while, based on a small set of 31 proteins, Duncan and Mata [66] estimated that ~ 38% of heteromers may assemble cotranslationaly. These studies indicate that contranslational folding and assembly are frequent, but further, high-throughput studies and novel methodologies are needed to estimate their frequency. Our results indicate that—in addition to cellular location in eukaryotes—the type of the ligand-binding site is likely to influence (and predict) whether a protein folds and assembles cotranslationally.

These findings also support the hypothesis that the evolution of quaternary structure of SBS homomers is largely stochastic [7], [11] and is governed by factors like reducing degradation rates, rather than selection for biochemical function per se. The comparison of degradation rates indicates that homomers are generally degraded at lower rates than monomers (Fig. 7, see also Mallik and Kundu [15]); however, MBS homomers are degraded at even lower rates than SBS homomers. As MBS complexes are generally more flexible than SBS complexes or monomers [11] and their interactomes are enriched for proteins regulating degradation (Fig. S4), their low degradation rate is probably not caused by their more rigid folds but is likely to be actively regulated. The evolutionary force that drives their lower degradation rates might be the high cost of folding and assembly, as most chaperones/chaperonins require ATP to assist folding. This suggests that proteins and complexes that are energetically more costly to build have a longer lifespan in the cell.

Taken together, our findings indicate that, at least in the case of proteins with a single domain, ligand binding site structure has major consequences for the topology of protein folds, assembly and the interactions of proteins with the proteostasis network. Surprisingly, in the case of multidomain proteins, the differences between fold topologies are much weaker, and the choice of domain annotation has a qualitative effect on the results. This is most likely due to the separation of functions between different domains, that is, ligand binding, protein–protein interactions and other, for example, related to protein motions or allostery can be performed by different domains and can be subjected to different selective pressures.

## Methods

### Data sources and data preparation

We used the February 2017 freeze of the cross-ref uniprot–pdb mappings and discarded all protein sequences that lack a PDB entry in the BioLiP database of protein ligands. We excluded PDB entries of viruses, helical and fibril-forming entries, and, since BioLiP is based on the asymmetric unit, entries that have different quaternary structure in the biounit and the asymmetric unit. Next we determined the PFAM conserved domains in every Uniprot sequence with HMMER [67], with a minimum bitscore cutoff of 22, and a minimum *e*-value of 0.001. The CATH domain assignments were downloaded from the CATH database [19]; we only used domains that are continuous in their sequence. We divided full protein sequences into two categories: those with only one dominant PFAM domain (i.e., sequences with conserved domains that map only to one region of the sequence) and the ones with more than one dominant domain (with domains mapping to different regions of the sequence). We kept only those sequences that have an entry with resolution lower than 3 Å and with sequence coverage higher than 50%. Next, to remove redundancies, we clustered the two sets with uclust [68] at 50% sequence similarity. Finally, we discarded sequences that have a heteromeric entry in the PDB and determined the quaternary structure and binding site type of the remaining proteins using the biological units in the PDB. If a sequence has at least one homomeric entry, it was classified as a homomer, while if it only has monomeric entries, it was classified as a monomer. Binding site type was determined based on BioLiP; if any of the PDB entries has a small-molecule ligand (i.e., not peptide or nucleotide) that binds two or more protein chains, it was classified as a MBS homomer; otherwise, it was classified as a SBS homomer (or monomer). From the PDB entries mapping to each sequence, we selected the biounit with highest coverage and highest resolution.

### Calculation of residue interaction scores

First, we preprocessed each structure with the dock-prep tool of Chimera [69] in order to complete side chains and remove residues with alternative positions having low occupancies. Next we processed the structures with the RepairPDB tool of FoldX [70], to correct torsion angles, remove clashes and minimize the structure. Finally, the numbers of non-covalent residue interactions in each structure were calculated with the RINerator tool [18], which determines the contacts based on all atoms (including hydrogens), and the ones closer than 0.25 Å are considered to be in contact. For each residue pair, we used the total interaction scores (all_all) in the *_nrint.ea. files. To obtain the interaction matrices, we first aligned the sequence of the PDB entry to the Uniprot sequence with the Needle tool of the EMBOSS software package [71]. Second, we divided the length of every protein into 50 bins, and the interactions of every interacting residue pair, divided by the length of the sequence of their PDB entry (normalized_interactions) were added to the corresponding the position in a 50 × 50 matrix. Interactions between residues less than five amino acids apart were not used to avoid biasing the data set by a large number of contacts within helices. The contact density for each cell in the matrix was calculated as [(500/nr_of_structures) * ∑ normalized_interactions], where 500 is a scaling factor. The contact score was calculated as the natural logarithm of contact density; in the case of multi-domain proteins, the number of domains was used instead of structures.

### Analyses of protein domain similarity and diversity

We used a method that allows the visualization of the sequence space of thousands of protein alignments/sequences. For every PFAM domain, its seed alignment was downloaded from the PFAM database. We removed the gaps from the alignments and transformed the alignments into a feature vector by calculating the frequencies of all possible dipeptides in the sequences of the alignments. Finally, the feature vectors were clustered using the Barnes-Hut tSNE algorithm [20], using dimensionality 2 and perplexity 5 for visualization.

The observed diversity of protein domains was calculated as the ratio of the number of domains and protein sequences, allowing for overlapping domains. The expected diversity was determined with Monte-Carlo simulations, by randomly sampling the proteins of MBS and SBS homomers 10,000 times, and calculating the ratio of domains/sequences in each sample.

### GO analysis of protein–protein interactions

Human protein–protein interactions were downloaded from the BioGRID database [24] (v3.5.169). We determined the quaternary structure and binding site type for each human protein in the PDB, and if present in the BioGRID database, we identified the proteins that interact with MBS and SBS homomers. We downloaded the GO annotations of human genes and the full hierarchy of GO terms (go.obo) from the GO Resource [72], [73]. For every gene that interacts with either an MBS or SBS homomer, we determined their full list of Biological Process GO terms using their GO annotations, and through recursively processing the hierarchy of go terms to the top of the ontology tree using the “is_a” relationships between the terms. Finally, we determined the terms enriched in MBS homomers with Gene Merge [74] using the combined data set of MBS and SBS homomers as the reference set. Unlike Gene Merge, which discards terms that have only a single gene in the study group (MBS homomers), we used all terms in FDR correction; thus, our significances are more stringent than the ones reported by GeneMerge, and the lists of significant terms are somewhat shorter (see Supplementary Data, *p*-values files). Note that in the case of interactome analysis, for a fraction of GO terms, it is not possible to estimate their correct enrichment, because all human genes (present in BioGRID) with the given GO term might be interacting with homomers. This can result in situations where the number of interacting genes is the same in the full population (MBS + SBS) and the study group (MBS), leading to “significance” that is caused by differences in the sample sizes rather than a real biological effect. The full list of these problematic GO terms is available in the Supplementary Data; however, only one of the significantly enriched terms was problematic (GO:0071550—death-inducing signaling complex assembly), and none of the folding/assembly/proteolysis associated terms. Visualizations of GO enrichments were made with in house Perl scripts using GraphViz, and REVIGO [25]. In the analysis of the Cellular Component GO terms, we used the homomers themselves not their interactome.

### Frustration and aggregation analysis

From each protein complex structure, we selected the longest chain and processed it with Frustratometer2 [43] to identify configurational frustration between the interacting residues. We calculated the average frustration level of the protein as the average frustration of all residue pairs. Next, the length of each protein was divided into 50 bins, and the frustration of each bin was calculated as the average frustration of all interactions of all residues that fall into the bin. Finally, frustration bias of each bin was calculated as the difference from the protein average.

The matrices of frustration were calculated using a comparable method to the residue interactions. For each residue pair of single-domain proteins that has a non-covalent interaction, we determined their coordinate in the 50 × 50 matrix and calculated their frustration bias, as

[(500/nr_of_structures) * ∑ normalized_frustration], where normalized frustration is the frustration bias of every residue pair falling into a cell, divided by the number of residues in the protein chain. The frustration score was calculated as the natural logarithm of frustration bias.

Aggregation propensity of proteins was determined with Tango [44]. Similar to frustration, the average aggregation propensity was calculated for each protein, and the differences from the average were determined for 50 bins along each protein sequence. The periodicity of aggregation bias (periodograms) was calculated with the multitaper R package using 2% taper, the significance of periodicity was determined using Fisher's *G* test function of the GeneCycle R package.

The enrichment of frustrated and aggregation-prone residues in C-termini was calculated as follows. Each protein was split into N- and C terminal halves (bins 1–25 and 26–50); for each half, the sum of the difference from the protein average of every bin was calculated, both for frustration and aggregation. Next using 10,000 replications, we randomly resampled the proteins and determined the C- or N-terminal enrichment of frustration/aggregation in each of the 10,000 samples. Significance was calculated as [*x*/(*y* − 1)], where *y* is 10,000 and *x* is the number of samples with N-terminal enrichment. A similar, reverse procedure was used to calculate N-terminal enrichment. Standard deviations (whiskers) for each bin were calculated using a similar randomization procedure: we randomly resampled the proteins 10,000 times, and SD was calculated for each bin as sqrt[∑(*d*_s_ − *d*_p_)/(*N* − 1)], where *d*_s_ is the difference from average of the bin in every sample, d_p_ is the difference from average in the original list of proteins, and *N* is the number of samples (10,000).

### Estimation of interface bias

The distribution of interface residues along the protein sequence was calculated similarly as in Natan *et al*. [46]. Briefly, for every residue of the complexes, we calculated its solvent-accessible surface in complex and in monomer, using AREAIMOL from the CCP4 package; interface area was calculated as the difference between the two. Similar to frustration and aggregation, the protein sequences were divided into 50 bins and the amount of surface and interface area falling into every bin was calculated. Interface enrichment was calculated as [(interface_bin_/interface_total_)/(surface_bin_/surface_total_)] − 1, that is, as the ratio of relative interface and surface area for every bin. Confidence intervals and N/C-terminal enrichment of interface residues was calculated as above, except that instead of a difference from the protein average, the ratio of interface and surface areas was used for the N- and C-termini.

## Appendix A Supplementary data

Supplementary figures Image 1

Supplementary tables Image 2

Supplementary material Image 3
