# Supplementary material for: Ligand Binding Site Structure Shapes Folding, Assembly and Degradation of Homomeric Protein Complexes
Source: J Mol Biol. 2019 Sep 6;431(19):3871–88. doi: 10.1016/j.jmb.2019.07.014 (PMC6739599; doi:10.1016/j.jmb.2019.07.014)
Supplement: Supplementary file 1 — Supplementary figures [file mmc1.pdf]

Supplementary Figures

to

Ligand binding site structure shapes folding, assembly and degradation of homomeric protein complexes.

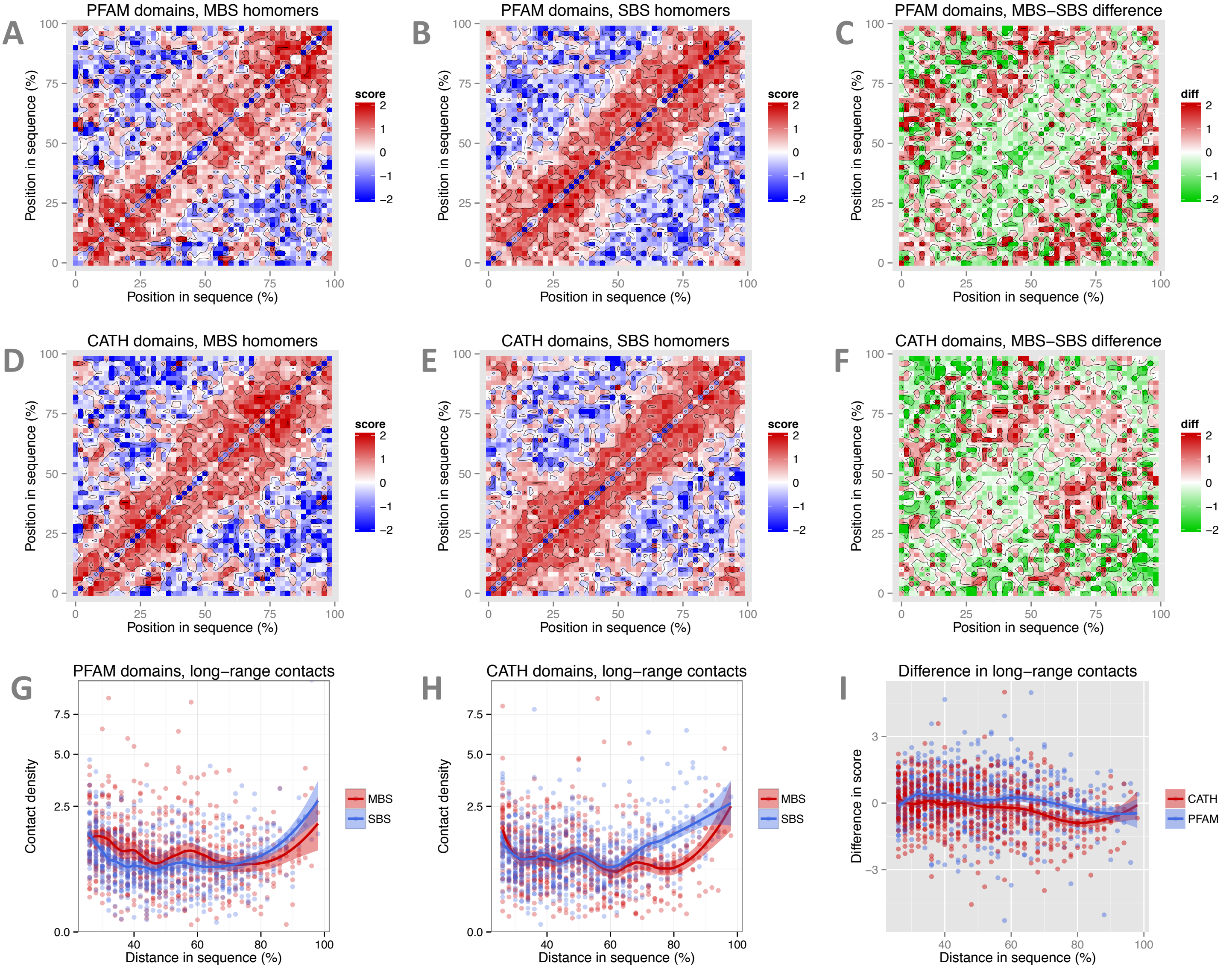

Figure S1. Frequencies of residue contacts of interface residues in ligand binding domains of multi-domain MBS and SBS homomers. **A and B)** Matrices of contact scores in the PFAM domains of MBS and SBS homomers, **C)** Differences in in contact scores between MBS and SBS homomers, using PFAM domains. Interface residues of MBS homomers have much more long-range contacts than SBS homomers. **D and E)** Contact score matrices in CATH domains. **F)** The differences in long-range contact scores between MBS and SBS homomers using CATH domains. The pattern is much less clear than in PFAM domains. **G)** Relationships between residue distance and contact density (exp(contact score)), for residues more than 25% distant in the sequence, using PFAM domains. The difference between MBS and SBS homomers is moderately significant, (distances 25-60%; p= 6.68e-05, ANCOVA). Note that the y-axis is square-root transformed, due to outliers. **H)** In the case of CATH domains the difference is not significant (distances 25-60%; p= 0.67, ANCOVA). **I)** Comparison of enrichment of long range contacts in PFAM and CATH domains. PFAM domains show a highly significant difference in long-range contact scores compared to CATH domains (distances 25-60%; p = 1.7e-13, ANCOVA). On panels A-F minimum and maximum values (-2 and 2) were set, to prevent the biasing of color scales by a small number of outliers. The original contact densities are plotted on panels G and H, score-differences on panel I.

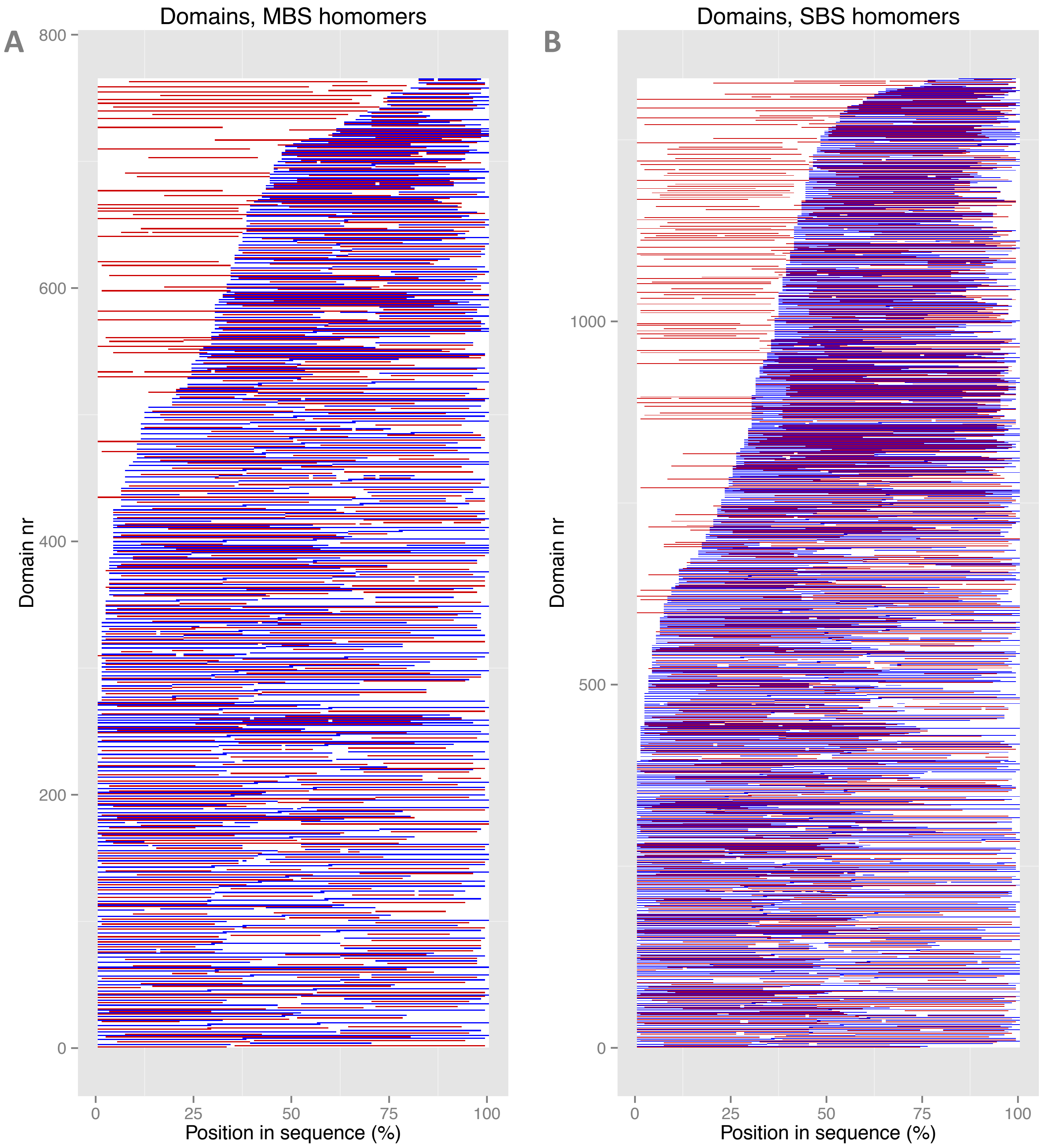

Figure S2. Comparison of the locations of all ligand binding PFAM and CATH domains (having interface residues) in multidomain homomers. **A)** MBS homomers **B)** SBS homomers. On both panels red lines represent PFAM domains and blue lines represent CATH domains. Note that discontinuous CATH domains were not used. For every PDB entry, their PFAM domains are followed immediately by their CATH domains, thus if domains 1,2,3 of 1abc entry are its PFAM domains, than its CATH domains will start at domain number 4. The PDB entries were ordered by the location of their first CATH domain, to enable a better visual comparison of the two.

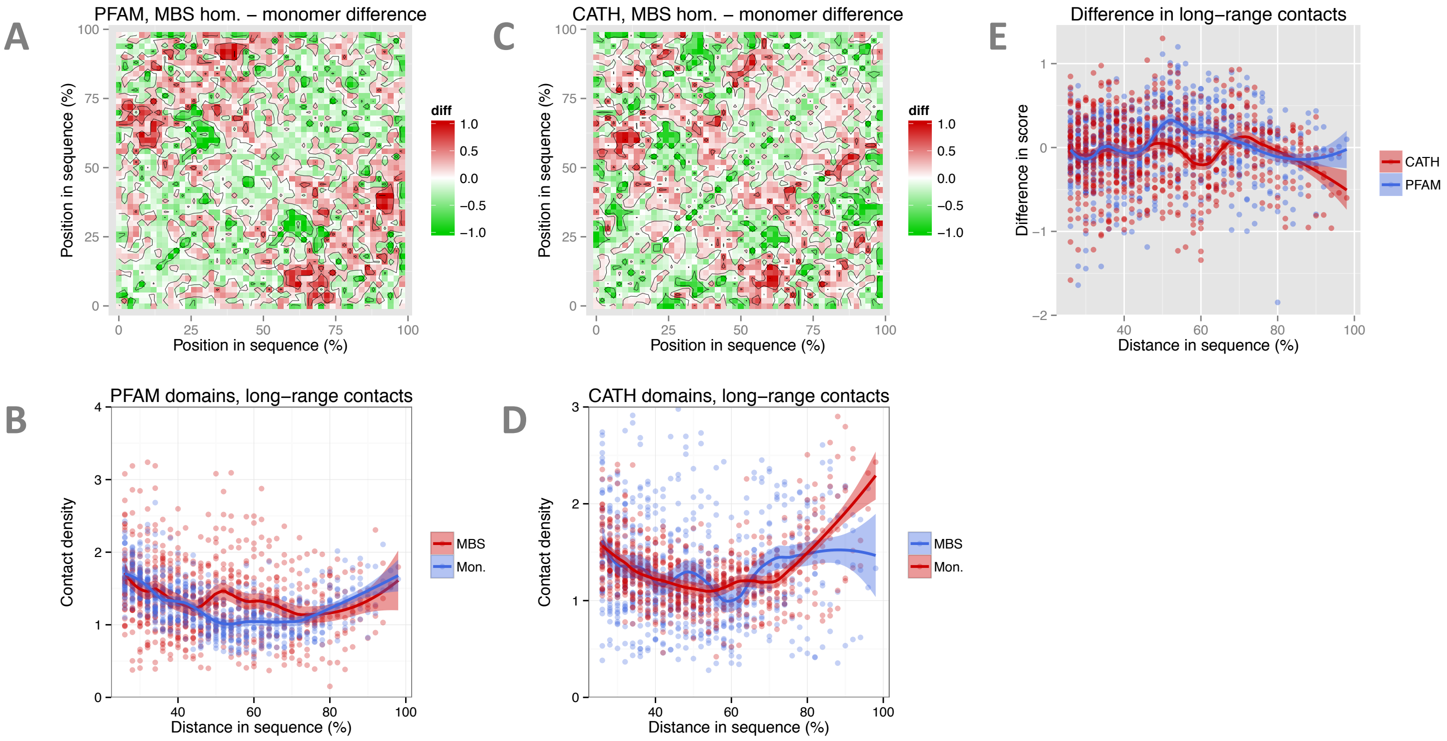

Figure S3. Comparison of residue contact patterns of multi-domain monomers and MBS homomers, using all residues of their ligand binding domains. **A and B)** Differences in in contact scores between multi-domain MBS homomers, and monomers, using their PFAM domains. MBS monomers have significantly more long-range contacts (50-80% distance in sequence) than monomers (p = 5.59e-09, ANCOVA, panel B). **C and D)** The differences in contact scores are not significantly different when CATH domains are used (p = 0.34, ANCOVA, panel D) although a trend is present (panel C). **F)** The comparison of the two domain assignment methods shows that PFAM domains of MBS homomers are more enriched in long-range contacts than CATH domains (distances 50-80%; p= 2.8e-14, ANCOVA). Note that only continuous CATH domains were used, which do not necessarily contain a highly conserved region. On panels A and B minimum and maximum values (-1 and 1, respectively), were set, to prevent biasing of color scales by a few outliers.

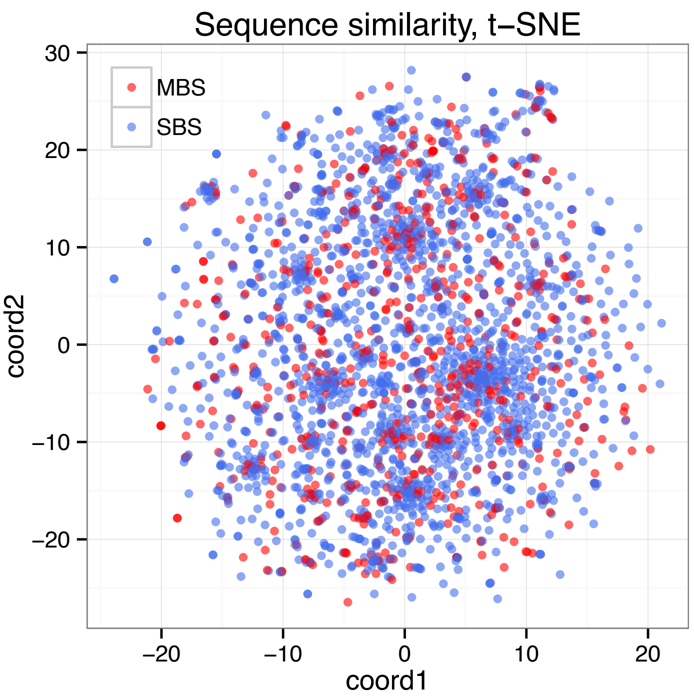

Figure S4. Two-dimensional (t-SNE) representation of the sequence space of homomers. The sequences of both MBS homomers and SBS homomers are distributed across the entire sequence space, and neither of them form separate clusters.

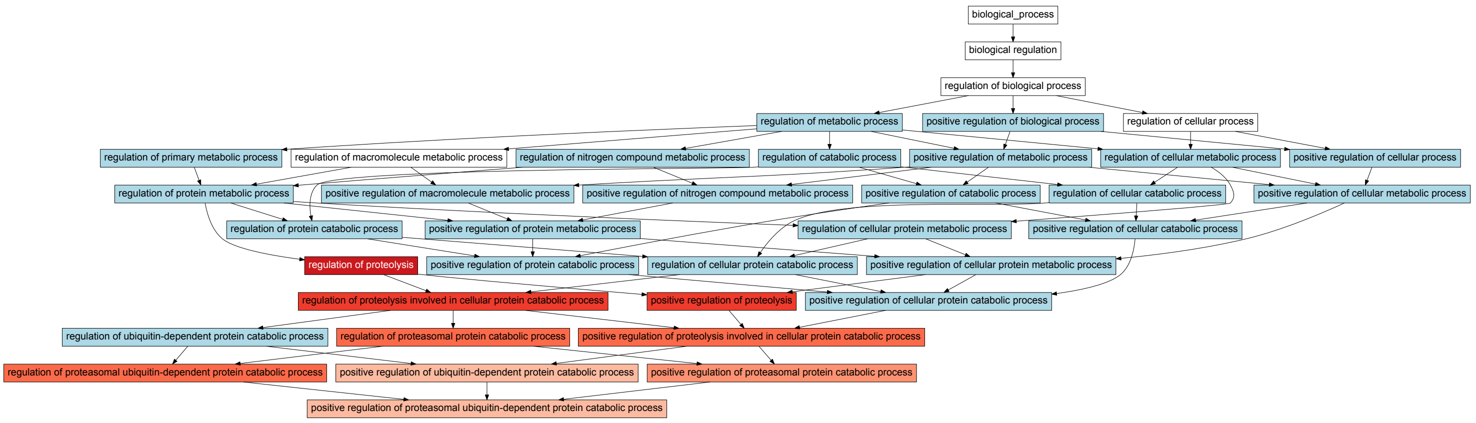

Figure S5. Tree of the terms related to regulation of proteolysis, that are significantly enriched in the interactors of MBS homomers. The intensity of red corresponds to significance, blue corresponds to terms that are also significantly enriched, but are too general to be considered proteolysis related.

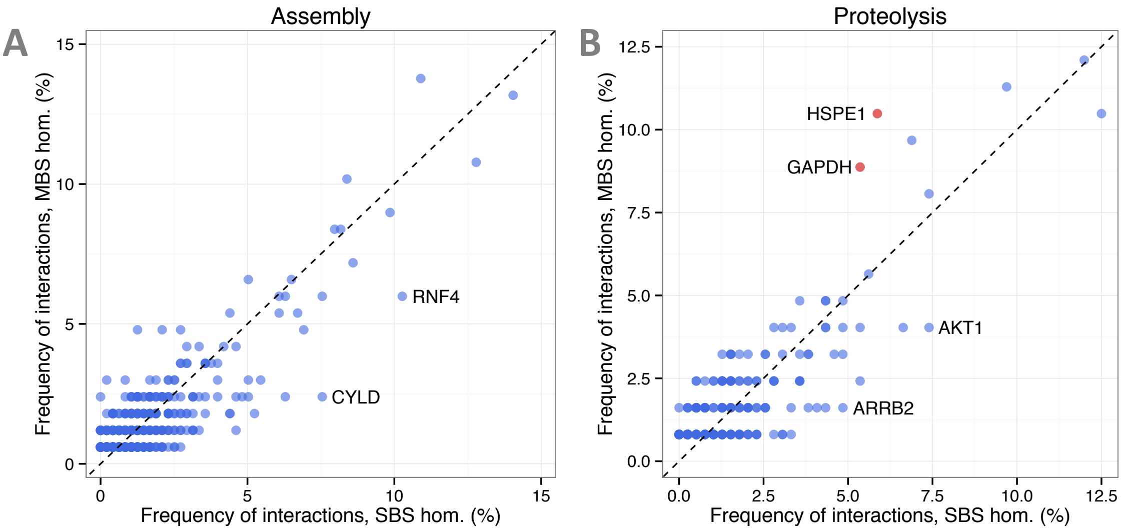

Figure S6. Frequencies of interactions of enriched assembly and proteolysis ("regulation of proteolysis") related genes in MBS and SBS homomers. Unlike in the case of folding related interactors (see Figure 5), no clusters of genes could be identified that interact with MBS homomers with much higher frequencies than with SBS homomers. Note that the HSPE1 and GAPDH genes are also present among the proteolysis related genes.

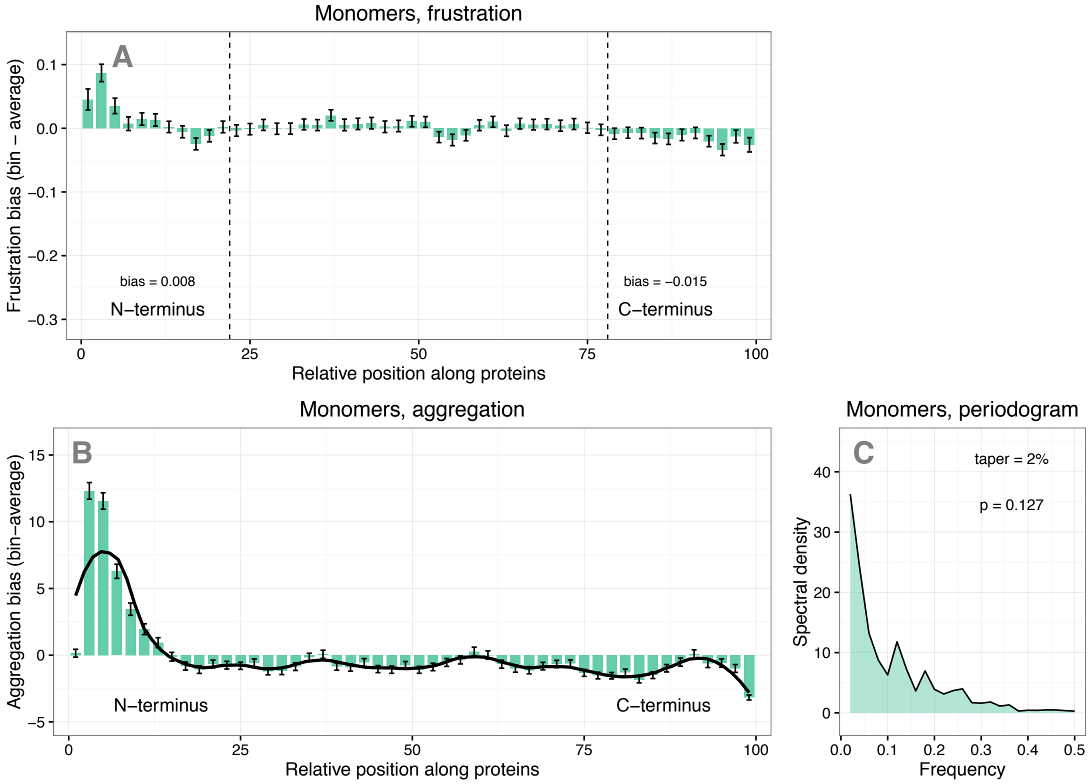

Figure S7. Frustration and aggregation patterns in singeldomain monomers. **A)** Frustration bias, **B)** Aggregation bias, **C)** Periodogram of aggregation bias. Residues at the N-terminus are somewhat less frustrated than the rest of the sequence (A, $p = 0.0002$), while aggregation propensity is higher at the N-termnius (B, $p < 0.0001$) but no clear periodicity is present (C, $p = 0.127$, Fisher's G test).

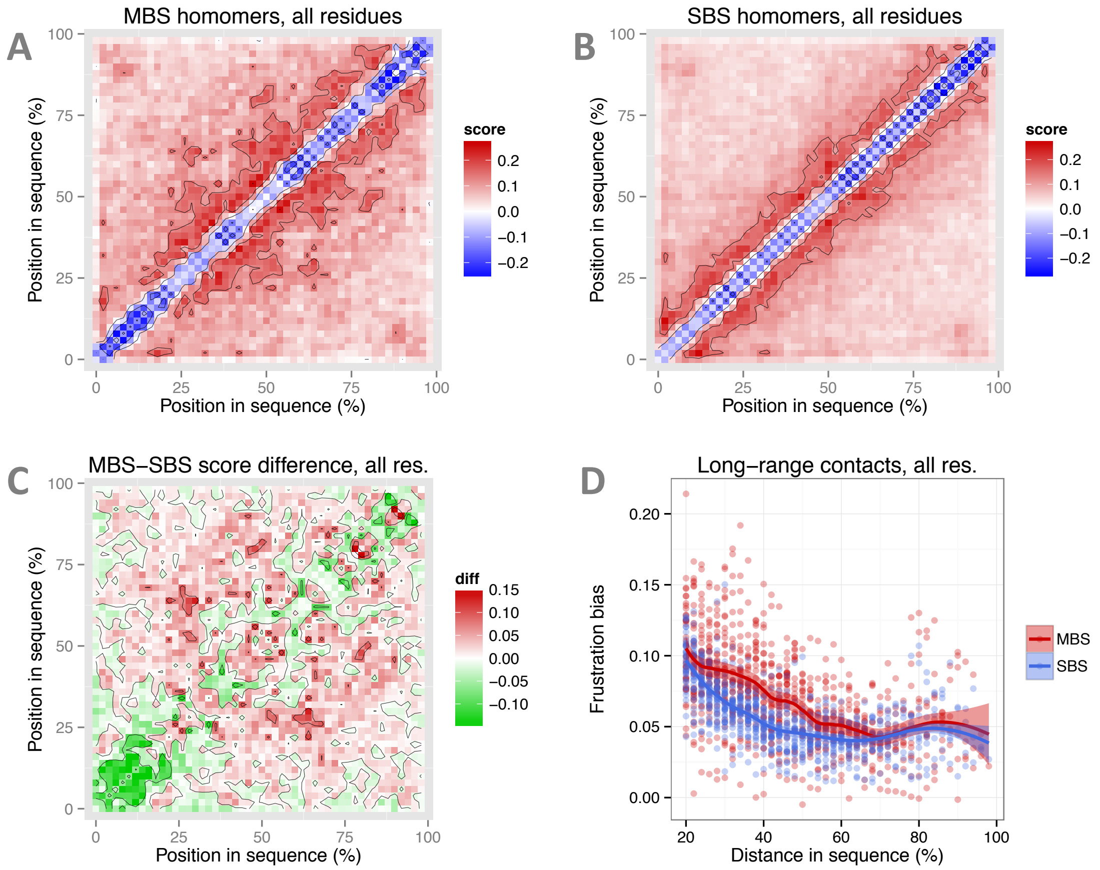

Figure S8. The matrices of frustration bias (singledomain proteins). **A)** MBS homomers, **B)** SBS homomers **C)** Difference between MBS and SBS homomers. **D)** Frustration bias of log-range contacts. On panel C -0.15 was the lowest permitted value, to keep the scale balanced (symmetrical). The most frustrated (more negative) contacts are short-range contacts both in MBS and SBS homomers (A and B). MBS homomers are more frustrated at their N-terminus (C, see also Figure 6), and have less frustrated long-range contacts (panel D, p=3.18e-34, ANCOVA on distances between 20-60%).
