# Supplementary material for: Ligand Binding Site Structure Shapes Folding, Assembly and Degradation of Homomeric Protein Complexes
Source: J Mol Biol. 2019 Sep 6;431(19):3871–88. doi: 10.1016/j.jmb.2019.07.014 (PMC6739599; doi:10.1016/j.jmb.2019.07.014)
Supplement: Supplementary file 3 — Supplementary material [file mmc3.zip › GeneOntology.tar.bz2!/GO/GO_output.C.txt.html]

 GeneMerge Output - output.C.txt

### GeneMerge v1.4

### Castillo-Davis, C.I. 2015. GeneMerge v1.4 - post-genomic data analysis

Output File Name: output.C.txt   
Gene Association File: terms.txt   
Description File: descriptions.txt   
Population File: genes\_all.txt   
Study File: genes\_multichain.txt  
Custom FDR: 0.5%  

|  |  |  |  |  |  |  |  |  |  |  |  |  |  |  |  |  |  |  |  |  |  |  |  |  |  |  |  |  |  |  |  |  |  |  |  |  |  |  |  |  |
| --- | --- | --- | --- | --- | --- | --- | --- | --- | --- | --- | --- | --- | --- | --- | --- | --- | --- | --- | --- | --- | --- | --- | --- | --- | --- | --- | --- | --- | --- | --- | --- | --- | --- | --- | --- | --- | --- | --- | --- | --- |
| **GMRG Term** | **Pop Frequency** | **Pop Fraction** | **Study Fraction** | ***P*-value** | **Bon. Corr. *P*-value** | **10% FDR** | **5% FDR** | **1% FDR** | **0.5% FDR** | **Description** | **Contributing genes** |  |  |  |  |  |  |  |  |  |  |  |  |  |  |  |  |  |  |  |  |  |  |  |  |  |  |  |  |  |
| GO:0005759 | 0.0975954738330976 | 69/707 | 30/172 | 0.00017942057513031 | 0.0547232754147445 | T | T | T | F | mitochondrial matrix | AADAT | ABAT | ACAD8 | ACADM | ACADS | ACADSB | BCAT2 | CS | DECR1 | FECH | GLRX2 | GLRX5 | GLS | GLUD1 | GOT2 | GSR | GSTK1 | HADH | IDH2 | IVD | LRRK2 | ME2 | MMAB | OAT | RIDA | RNASEL | SIRT5 | SOD1 | THEM4 | TYMS |
 GO:1990351 | 0.0226308345120226 | 16/707 | 11/172 | 0.000183202388390608 | 0.0558767284591354 | T | T | T | F | transporter complex | CHRNA1 | CHRNA2 | CHRNA3 | CHRNA7 | GLRA3 | GRIA2 | HCN4 | KCNK1 | KCNK2 | NOS1 | PDE4B | GO:0034702 | 0.0226308345120226 | 16/707 | 11/172 | 0.000183202388390608 | 0.0558767284591354 | T | T | T | F | ion channel complex | CHRNA1 | CHRNA2 | CHRNA3 | CHRNA7 | GLRA3 | GRIA2 | HCN4 | KCNK1 | KCNK2 | NOS1 | PDE4B | GO:1902495 | 0.0226308345120226 | 16/707 | 11/172 | 0.000183202388390608 | 0.0558767284591354 | T | T | T | F | transmembrane transporter complex | CHRNA1 | CHRNA2 | CHRNA3 | CHRNA7 | GLRA3 | GRIA2 | HCN4 | KCNK1 | KCNK2 | NOS1 | PDE4B | GO:0043233 | 0.209335219236209 | 148/707 | 51/172 | 0.00115183765092106 | 0.351310483530923 | T | T | F | F | organelle lumen | AADAT | ABAT | ACAD8 | ACADM | ACADS | ACADSB | APRT | BCAT2 | CAT | CES1 | CS | CTSK | CYR61 | DECR1 | DEFA5 | FABP5 | FECH | GLRX2 | GLRX5 | GLS | GLUD1 | GOT2 | GPI | GSR | GSTK1 | GSTP1 | GYG1 | HADH | IDH2 | INS | IVD | LRRK2 | ME2 | MIF | MMAB | MMP9 | OAT | PGAM1 | PGLYRP1 | PKM | PPT1 | PTGES | PYGL | RIDA | RNASEL | S100A12 | S100A7 | SIRT5 | SOD1 | THEM4 | TYMS | GO:0031974 | 0.209335219236209 | 148/707 | 51/172 | 0.00115183765092106 | 0.351310483530923 | T | T | F | F | membrane-enclosed lumen | AADAT | ABAT | ACAD8 | ACADM | ACADS | ACADSB | APRT | BCAT2 | CAT | CES1 | CS | CTSK | CYR61 | DECR1 | DEFA5 | FABP5 | FECH | GLRX2 | GLRX5 | GLS | GLUD1 | GOT2 | GPI | GSR | GSTK1 | GSTP1 | GYG1 | HADH | IDH2 | INS | IVD | LRRK2 | ME2 | MIF | MMAB | MMP9 | OAT | PGAM1 | PGLYRP1 | PKM | PPT1 | PTGES | PYGL | RIDA | RNASEL | S100A12 | S100A7 | SIRT5 | SOD1 | THEM4 | TYMS | GO:0070013 | 0.209335219236209 | 148/707 | 51/172 | 0.00115183765092106 | 0.351310483530923 | T | T | F | F | intracellular organelle lumen | AADAT | ABAT | ACAD8 | ACADM | ACADS | ACADSB | APRT | BCAT2 | CAT | CES1 | CS | CTSK | CYR61 | DECR1 | DEFA5 | FABP5 | FECH | GLRX2 | GLRX5 | GLS | GLUD1 | GOT2 | GPI | GSR | GSTK1 | GSTP1 | GYG1 | HADH | IDH2 | INS | IVD | LRRK2 | ME2 | MIF | MMAB | MMP9 | OAT | PGAM1 | PGLYRP1 | PKM | PPT1 | PTGES | PYGL | RIDA | RNASEL | S100A12 | S100A7 | SIRT5 | SOD1 | THEM4 | TYMS | GO:0098797 | 0.0311173974540311 | 22/707 | 12/172 | 0.00190212787146243 | 0.58014900079604 | T | F | F | F | plasma membrane protein complex | BIRC2 | CHRNA1 | CHRNA2 | CHRNA3 | CHRNA7 | GLRA3 | GRIA2 | HCN4 | KCNK1 | KCNK2 | NOS1 | PDE4B | GO:0044444 | 0.817538896746818 | 578/707 | 153/172 | 0.00258554064121045 | 0.788589895569187 | T | F | F | F | cytoplasmic part | AADAT | ABAT | ACACB | ACAD8 | ACADM | ACADS | ACADSB | ACOT13 | ADH1C | ADH5 | ADSL | AHCY | AK1 | ALOX5AP | AOC3 | APLP1 | APRT | AQP5 | ASL | ATIC | BCAT1 | BCAT2 | BCL6 | BIRC2 | BRD4 | CAD | CAT | CBS | CES1 | CNDP2 | COQ9 | CS | CSNK1D | CTH | CTSK | CYGB | CYP51A1 | CYR61 | DCPS | DDC | DECR1 | DEFA5 | DUT | FABP5 | FBP1 | FECH | FIGNL1 | GAD1 | GALNT2 | GALT | GBP1 | GGPS1 | GLO1 | GLRX2 | GLRX5 | GLS | GLTP | GLUD1 | GMDS | GMPR2 | GNPDA1 | GNPNAT1 | GOT1 | GOT2 | GPI | GRIA2 | GSR | GSTA1 | GSTA2 | GSTA3 | GSTA4 | GSTK1 | GSTP1 | GYG1 | HADH | HCN4 | HDC | HINT1 | HMGCR | HS2ST1 | HSD11B1 | IDH2 | INS | ITK | IVD | IYD | KCNK1 | KCNK2 | KYAT1 | LRRK2 | LTC4S | MB | ME2 | MELK | MIF | MMAB | MMP9 | NAGA | NAMPT | NAPEPLD | NGB | NMRAL1 | NOS1 | NOS3 | NQO1 | NQO2 | NUDT5 | OAT | OLR1 | PAH | PANK1 | PANK3 | PAPSS1 | PDE4B | PFKP | PGAM1 | PGD | PGLYRP1 | PKM | PLA2G1B | PLA2G2A | PPT1 | PRKG1 | PSAT1 | PTGES | PYGL | PYGM | RHO | RIDA | RNASEL | RRM1 | S100A12 | S100A7 | S100B | SAT1 | SCLY | SEPT3 | SET | SGPL1 | SHMT1 | SIRT5 | SOD1 | TDO2 | THEM4 | TKT | TLR7 | TLR8 | TULP1 | TYMS | UMPS | UPP1 | UPP2 | UXS1 | GO:0005892 | 0.00565770862800566 | 4/707 | 4/172 | 0.00341094166719713 | 1 | T | F | F | F | acetylcholine-gated channel complex | CHRNA1 | CHRNA2 | CHRNA3 | CHRNA7 | GO:0044429 | 0.127298444130127 | 90/707 | 33/172 | 0.00346691161630489 | 1 | T | F | F | F | mitochondrial part | AADAT | ABAT | ACACB | ACAD8 | ACADM | ACADS | ACADSB | BCAT2 | CAT | COQ9 | CS | DECR1 | FECH | GLRX2 | GLRX5 | GLS | GLUD1 | GOT2 | GSR | GSTK1 | HADH | IDH2 | IVD | LRRK2 | ME2 | MMAB | OAT | RIDA | RNASEL | SIRT5 | SOD1 | THEM4 | TYMS | GO:1904813 | 0.0226308345120226 | 16/707 | 9/172 | 0.00562168839298825 | 1 | T | F | F | F | ficolin-1-rich granule lumen | CAT | GPI | GSTP1 | GYG1 | MIF | MMP9 | PGAM1 | PKM | PYGL | GO:0045211 | 0.0155586987270156 | 11/707 | 7/172 | 0.00604368138427495 | 1 | T | F | F | F | postsynaptic membrane | CHRNA1 | CHRNA2 | CHRNA3 | CHRNA7 | FABP5 | GLRA3 | GRIA2 | GO:0043005 | 0.0792079207920792 | 56/707 | 22/172 | 0.00696297128653189 | 1 | T | F | F | F | neuron projection | ABAT | ACADM | AHCY | CHRNA1 | CHRNA2 | CHRNA3 | CHRNA7 | CSNK1D | CYGB | DDC | GLRA3 | GLRX2 | GLRX5 | GPI | GRIA2 | KCNK1 | LRRK2 | NOS1 | NQO1 | PDE4B | PPT1 | SEPT3 | GO:0042995 | 0.117397454031117 | 83/707 | 30/172 | 0.006981652651954 | 1 | T | F | F | F | cell projection | ABAT | ACADM | AHCY | AOC3 | AQP5 | CAD | CHRNA1 | CHRNA2 | CHRNA3 | CHRNA7 | CSNK1D | CYGB | DDC | GLRA3 | GLRX2 | GLRX5 | GPI | GRIA2 | KCNK1 | KCNK2 | LRRK2 | NOS1 | NQO1 | PDE4B | PKM | PPT1 | RRM1 | S100B | SEPT3 | TULP1 | GO:0043025 | 0.0396039603960396 | 28/707 | 13/172 | 0.00767011361713821 | 1 | F | F | F | F | neuronal cell body | CAD | CHRNA3 | CYGB | DDC | GLRX2 | GLRX5 | KCNK2 | LRRK2 | NQO1 | PPT1 | RRM1 | S100B | SOD1 | GO:0097458 | 0.145685997171146 | 103/707 | 35/172 | 0.0109960004697419 | 1 | F | F | F | F | neuron part | ABAT | ACADM | AHCY | CAD | CHRNA1 | CHRNA2 | CHRNA3 | CHRNA7 | CSNK1D | CYGB | DDC | FABP5 | GAD1 | GLRA3 | GLRX2 | GLRX5 | GOT1 | GOT2 | GPI | GRIA2 | KCNK1 | KCNK2 | LRRK2 | NAPEPLD | NGB | NOS1 | NQO1 | PDE4B | PPT1 | RHO | RRM1 | S100B | SEPT3 | SOD1 | TULP1 | GO:0005739 | 0.161244695898161 | 114/707 | 38/172 | 0.0113838988390555 | 1 | F | F | F | F | mitochondrion | ABAT | ACACB | ACADM | ACADS | ACADSB | ACOT13 | ADH5 | BCAT1 | BCAT2 | COQ9 | CS | DCPS | DECR1 | DUT | FECH | GLRX2 | GLRX5 | GLS | GLUD1 | GOT2 | GSR | GSTK1 | GSTP1 | HADH | IDH2 | IVD | KYAT1 | LRRK2 | ME2 | NGB | NOS1 | OAT | PKM | PTGES | SIRT5 | SOD1 | THEM4 | TYMS | GO:0120025 | 0.111739745403112 | 79/707 | 28/172 | 0.0125356630823095 | 1 | F | F | F | F | plasma membrane bounded cell projection | ABAT | ACADM | AHCY | AOC3 | AQP5 | CHRNA1 | CHRNA2 | CHRNA3 | CHRNA7 | CSNK1D | CYGB | DDC | GLRA3 | GLRX2 | GLRX5 | GPI | GRIA2 | KCNK1 | KCNK2 | LRRK2 | NOS1 | NQO1 | PDE4B | PKM | PPT1 | S100B | SEPT3 | TULP1 | GO:0043235 | 0.0212164073550212 | 15/707 | 8/172 | 0.0137232320936619 | 1 | F | F | F | F | receptor complex | BIRC2 | CHRNA1 | CHRNA2 | CHRNA3 | CHRNA7 | GRIA2 | OLR1 | TLR7 | GO:0008076 | 0.00424328147100424 | 3/707 | 3/172 | 0.0142088930988558 | 1 | F | F | F | F | voltage-gated potassium channel complex | HCN4 | KCNK1 | KCNK2 | GO:0034703 | 0.0141442715700141 | 10/707 | 6/172 | 0.0165408910044221 | 1 | F | F | F | F | cation channel complex | GRIA2 | HCN4 | KCNK1 | KCNK2 | NOS1 | PDE4B | GO:0005635 | 0.0113154172560113 | 8/707 | 5/172 | 0.0236310718710597 | 1 | F | F | F | F | nuclear envelope | ALOX5AP | LTC4S | NAPEPLD | RRM1 | TPR | GO:0044456 | 0.0678925035360679 | 48/707 | 18/172 | 0.0245995168417442 | 1 | F | F | F | F | synapse part | CAD | CHRNA1 | CHRNA2 | CHRNA3 | CHRNA7 | DDC | FABP5 | GAD1 | GLRA3 | GOT1 | GRIA2 | KCNK2 | LRRK2 | NOS1 | PDE4B | PPT1 | SEPT3 | TULP1 | GO:0045202 | 0.0452616690240453 | 32/707 | 13/172 | 0.0276128863178619 | 1 | F | F | F | F | synapse | CHRNA1 | CHRNA2 | CHRNA3 | CHRNA7 | FABP5 | GAD1 | GLRA3 | GRIA2 | KCNK1 | LRRK2 | NOS1 | PDE4B | TULP1 | GO:0044297 | 0.0466760961810467 | 33/707 | 13/172 | 0.0359919840817142 | 1 | F | F | F | F | cell body | CAD | CHRNA3 | CYGB | DDC | GLRX2 | GLRX5 | KCNK2 | LRRK2 | NQO1 | PPT1 | RRM1 | S100B | SOD1 | GO:0034774 | 0.0565770862800566 | 40/707 | 15/172 | 0.0392860860098012 | 1 | F | F | F | F | secretory granule lumen | APRT | CAT | DEFA5 | FABP5 | GPI | GSTP1 | GYG1 | INS | MIF | PGAM1 | PGLYRP1 | PKM | PYGL | S100A12 | S100A7 | GO:0031967 | 0.0127298444130127 | 9/707 | 5/172 | 0.042827915334639 | 1 | F | F | F | F | organelle envelope | ALOX5AP | LTC4S | NAPEPLD | RRM1 | TPR | GO:0031975 | 0.0127298444130127 | 9/707 | 5/172 | 0.042827915334639 | 1 | F | F | F | F | envelope | ALOX5AP | LTC4S | NAPEPLD | RRM1 | TPR | GO:0034705 | 0.00565770862800566 | 4/707 | 3/172 | 0.0466027473938346 | 1 | F | F | F | F | potassium channel complex | HCN4 | KCNK1 | KCNK2 | GO:0098802 | 0.016973125884017 | 12/707 | 6/172 | 0.0467233851019001 | 1 | F | F | F | F | plasma membrane receptor complex | BIRC2 | CHRNA1 | CHRNA2 | CHRNA3 | CHRNA7 | GRIA2 | GO:0031983 | 0.057991513437058 | 41/707 | 15/172 | 0.0488831363217037 | 1 | F | F | F | F | vesicle lumen | APRT | CAT | DEFA5 | FABP5 | GPI | GSTP1 | GYG1 | INS | MIF | PGAM1 | PGLYRP1 | PKM | PYGL | S100A12 | S100A7 | GO:0060205 | 0.057991513437058 | 41/707 | 15/172 | 0.0488831363217037 | 1 | F | F | F | F | cytoplasmic vesicle lumen | APRT | CAT | DEFA5 | FABP5 | GPI | GSTP1 | GYG1 | INS | MIF | PGAM1 | PGLYRP1 | PKM | PYGL | S100A12 | S100A7 | GO:0036020 | 0.00282885431400283 | 2/707 | 2/172 | 0.0589251154981874 | 1 | F | F | F | F | endolysosome membrane | TLR7 | TLR8 | GO:0044305 | 0.00282885431400283 | 2/707 | 2/172 | 0.0589251154981874 | 1 | F | F | F | F | calyx of Held | KCNK2 | NOS1 | GO:0060076 | 0.00282885431400283 | 2/707 | 2/172 | 0.0589251154981874 | 1 | F | F | F | F | excitatory synapse | GRIA2 | PDE4B | GO:0033116 | 0.00282885431400283 | 2/707 | 2/172 | 0.0589251154981874 | 1 | F | F | F | F | endoplasmic reticulum-Golgi intermediate compartment membrane | CSNK1D | INS | GO:0000323 | 0.0268741159830269 | 19/707 | 8/172 | 0.0647937918435002 | 1 | F | F | F | F | lytic vacuole | CAT | CTSK | GOT1 | LRRK2 | NAGA | PPT1 | SOD1 | TLR7 | GO:0005764 | 0.0268741159830269 | 19/707 | 8/172 | 0.0647937918435002 | 1 | F | F | F | F | lysosome | CAT | CTSK | GOT1 | LRRK2 | NAGA | PPT1 | SOD1 | TLR7 | GO:0005743 | 0.0226308345120226 | 16/707 | 7/172 | 0.0678262683762245 | 1 | F | F | F | F | mitochondrial inner membrane | COQ9 | FECH | GOT2 | LRRK2 | SIRT5 | THEM4 | TYMS | GO:0098796 | 0.0466760961810467 | 33/707 | 12/172 | 0.0783737598247462 | 1 | F | F | F | F | membrane protein complex | BIRC2 | CHRNA1 | CHRNA2 | CHRNA3 | CHRNA7 | GLRA3 | GRIA2 | HCN4 | KCNK1 | KCNK2 | NOS1 | PDE4B | GO:0097060 | 0.024045261669024 | 17/707 | 7/172 | 0.0923382069705048 | 1 | F | F | F | F | synaptic membrane | CHRNA1 | CHRNA2 | CHRNA3 | CHRNA7 | FABP5 | GLRA3 | GRIA2 | GO:0005902 | 0.00707213578500707 | 5/707 | 3/172 | 0.0958156014294358 | 1 | F | F | F | F | microvillus | AOC3 | AQP5 | LRRK2 | GO:0000228 | 0.00707213578500707 | 5/707 | 3/172 | 0.0958156014294358 | 1 | F | F | F | F | nuclear chromosome | BRD4 | FIGNL1 | HDAC8 | GO:0031970 | 0.0155586987270156 | 11/707 | 5/172 | 0.102429437261733 | 1 | F | F | F | F | organelle envelope lumen | CAT | PTGES | SIRT5 | SOD1 | THEM4 | GO:0005758 | 0.0113154172560113 | 8/707 | 4/172 | 0.103503652710604 | 1 | F | F | F | F | mitochondrial intermembrane space | CAT | SIRT5 | SOD1 | THEM4 | GO:0098793 | 0.0297029702970297 | 21/707 | 8/172 | 0.111266568010772 | 1 | F | F | F | F | presynapse | CAD | GAD1 | GOT1 | KCNK2 | LRRK2 | NOS1 | SEPT3 | TULP1 | GO:0000139 | 0.0495049504950495 | 35/707 | 12/172 | 0.115868561030713 | 1 | F | F | F | F | Golgi membrane | CSNK1D | GALNT2 | GBP1 | GNPNAT1 | HS2ST1 | INS | NAPEPLD | NOS3 | RHO | TLR7 | TLR8 | UXS1 | GO:0044433 | 0.132956152758133 | 94/707 | 28/172 | 0.117044427103769 | 1 | F | F | F | F | cytoplasmic vesicle part | APRT | CAT | CTSK | DEFA5 | FABP5 | GAD1 | GNPNAT1 | GPI | GRIA2 | GSTP1 | GYG1 | INS | IYD | LRRK2 | MIF | MMP9 | NAPEPLD | NOS3 | OLR1 | PGAM1 | PGLYRP1 | PKM | PYGL | RHO | S100A12 | S100A7 | TLR7 | TLR8 | GO:0005576 | 0.138613861386139 | 98/707 | 29/172 | 0.119694134415951 | 1 | F | F | F | F | extracellular region | APRT | CAT | CCL5 | CTSK | DEFA5 | FABP5 | FCN2 | GALNT2 | GBP1 | GPI | GSTP1 | GYG1 | INS | MIF | MMP9 | OLR1 | PGAM1 | PGLYRP1 | PKM | PLA2G1B | PLA2G2A | PPT1 | PYGL | S100A12 | S100A7 | S100B | SOD1 | TPSAB1 | TULP1 | GO:0031226 | 0.0650636492220651 | 46/707 | 15/172 | 0.121316640638588 | 1 | F | F | F | F | intrinsic component of plasma membrane | AQP5 | CHRNA1 | CHRNA2 | CHRNA3 | CHRNA7 | GLRA3 | GRIA2 | HCN4 | IYD | KCNK1 | KCNK10 | KCNK2 | OLR1 | RHO | SLC14A1 | GO:0044428 | 0.243281471004243 | 172/707 | 48/172 | 0.124504299124357 | 1 | F | F | F | F | nuclear part | ACADM | ACADS | ADH1C | ALOX5AP | APRT | ATAD2 | BCL6 | BRD4 | CAD | CNDP2 | CSNK1D | CTSK | CYGB | DCPS | DECR1 | DUT | FABP5 | FIGNL1 | GLRX2 | GOT1 | GPI | HADH | HDAC8 | HINT1 | IYD | KYAT1 | L3MBTL3 | LTC4S | MIF | NAMPT | NAPEPLD | NOS1 | NQO2 | NR0B2 | OAT | OLR1 | PPARD | PTGES | RNASEL | RRM1 | SEPHS1 | SET | SLC14A1 | SOD1 | TKT | TPR | TYMS | UPP1 | GO:0030425 | 0.0353606789250354 | 25/707 | 9/172 | 0.12716193058022 | 1 | F | F | F | F | dendrite | CHRNA3 | GLRA3 | GLRX2 | GLRX5 | GRIA2 | KCNK1 | LRRK2 | NQO1 | PPT1 | GO:0005887 | 0.0608203677510608 | 43/707 | 14/172 | 0.133506856957501 | 1 | F | F | F | F | integral component of plasma membrane | AQP5 | CHRNA1 | CHRNA2 | CHRNA3 | CHRNA7 | GLRA3 | GRIA2 | IYD | KCNK1 | KCNK10 | KCNK2 | OLR1 | RHO | SLC14A1 | GO:0014069 | 0.016973125884017 | 12/707 | 5/172 | 0.142219474518773 | 1 | F | F | F | F | postsynaptic density | CHRNA3 | FABP5 | GRIA2 | NOS1 | PDE4B | GO:0099572 | 0.016973125884017 | 12/707 | 5/172 | 0.142219474518773 | 1 | F | F | F | F | postsynaptic specialization | CHRNA3 | FABP5 | GRIA2 | NOS1 | PDE4B | GO:0098794 | 0.016973125884017 | 12/707 | 5/172 | 0.142219474518773 | 1 | F | F | F | F | postsynapse | CHRNA7 | GRIA2 | LRRK2 | NOS1 | PDE4B | GO:0032839 | 0.00424328147100424 | 3/707 | 2/172 | 0.14835756029688 | 1 | F | F | F | F | dendrite cytoplasm | LRRK2 | SOD1 | GO:0031904 | 0.00424328147100424 | 3/707 | 2/172 | 0.14835756029688 | 1 | F | F | F | F | endosome lumen | CTSK | INS | GO:0034704 | 0.00424328147100424 | 3/707 | 2/172 | 0.14835756029688 | 1 | F | F | F | F | calcium channel complex | NOS1 | PDE4B | GO:0043195 | 0.00424328147100424 | 3/707 | 2/172 | 0.14835756029688 | 1 | F | F | F | F | terminal bouton | CAD | LRRK2 | GO:0005891 | 0.00424328147100424 | 3/707 | 2/172 | 0.14835756029688 | 1 | F | F | F | F | voltage-gated calcium channel complex | NOS1 | PDE4B | GO:0016234 | 0.00424328147100424 | 3/707 | 2/172 | 0.14835756029688 | 1 | F | F | F | F | inclusion body | LRRK2 | TPR | GO:0042622 | 0.00424328147100424 | 3/707 | 2/172 | 0.14835756029688 | 1 | F | F | F | F | photoreceptor outer segment membrane | NAPEPLD | RHO | GO:0044446 | 0.585572842998586 | 414/707 | 107/172 | 0.151767984543698 | 1 | F | F | F | F | intracellular organelle part | AADAT | ABAT | ACACB | ACAD8 | ACADM | ACADS | ACADSB | ACOT13 | ADH1C | ALOX5AP | APRT | ATAD2 | BCAT2 | BCL6 | BRD4 | CAD | CAT | CES1 | CNDP2 | COQ9 | CS | CSNK1D | CTSK | CYGB | CYP51A1 | CYR61 | DCPS | DECR1 | DEFA5 | DUT | FABP5 | FECH | FIGNL1 | GAD1 | GALNT2 | GBP1 | GLRX2 | GLRX5 | GLS | GLUD1 | GNPNAT1 | GOT1 | GOT2 | GPI | GRIA2 | GSR | GSTK1 | GSTP1 | GYG1 | HADH | HDAC8 | HINT1 | HMGCR | HS2ST1 | HSD11B1 | IDH2 | INS | IVD | IYD | KCNK2 | KYAT1 | L3MBTL3 | LRRK2 | LTC4S | ME2 | MIF | MMAB | MMP9 | NAMPT | NAPEPLD | NOS1 | NOS3 | NQO2 | NR0B2 | OAT | OLR1 | PDE4B | PGAM1 | PGLYRP1 | PKM | PLA2G2A | PPARD | PPT1 | PTGES | PYGL | RHO | RIDA | RNASEL | RRM1 | S100A12 | S100A7 | SEPHS1 | SEPT3 | SET | SGPL1 | SIRT5 | SLC14A1 | SOD1 | THEM4 | TKT | TLR7 | TLR8 | TPR | TYMS | UPP1 | UPP2 | UXS1 | GO:0019866 | 0.0268741159830269 | 19/707 | 7/172 | 0.154042716882935 | 1 | F | F | F | F | organelle inner membrane | COQ9 | FECH | GOT2 | LRRK2 | SIRT5 | THEM4 | TYMS | GO:1903561 | 0.298444130127298 | 211/707 | 57/172 | 0.161060348016399 | 1 | F | F | F | F | extracellular vesicle | ADH5 | AHCY | AK1 | APRT | AQP5 | ASL | ATAD2 | ATIC | CAD | CAT | CNDP2 | CS | CTH | DDC | DUT | FABP5 | FBP1 | FCN2 | FIGNL1 | GLO1 | GMDS | GNPDA1 | GOT1 | GOT2 | GPI | GSR | GSTA1 | GSTA2 | GSTA3 | GSTK1 | GSTP1 | HINT1 | IDH2 | LRRK2 | MB | MIF | MMP9 | NAGA | NAMPT | NAPEPLD | NQO2 | NUDT5 | PFKP | PGAM1 | PGD | PGLYRP1 | PKM | PLA2G2A | PPT1 | PSAT1 | PYGL | PYGM | RIDA | SHMT1 | SOD1 | TKT | UXS1 | GO:0070062 | 0.298444130127298 | 211/707 | 57/172 | 0.161060348016399 | 1 | F | F | F | F | extracellular exosome | ADH5 | AHCY | AK1 | APRT | AQP5 | ASL | ATAD2 | ATIC | CAD | CAT | CNDP2 | CS | CTH | DDC | DUT | FABP5 | FBP1 | FCN2 | FIGNL1 | GLO1 | GMDS | GNPDA1 | GOT1 | GOT2 | GPI | GSR | GSTA1 | GSTA2 | GSTA3 | GSTK1 | GSTP1 | HINT1 | IDH2 | LRRK2 | MB | MIF | MMP9 | NAGA | NAMPT | NAPEPLD | NQO2 | NUDT5 | PFKP | PGAM1 | PGD | PGLYRP1 | PKM | PLA2G2A | PPT1 | PSAT1 | PYGL | PYGM | RIDA | SHMT1 | SOD1 | TKT | UXS1 | GO:0043230 | 0.298444130127298 | 211/707 | 57/172 | 0.161060348016399 | 1 | F | F | F | F | extracellular organelle | ADH5 | AHCY | AK1 | APRT | AQP5 | ASL | ATAD2 | ATIC | CAD | CAT | CNDP2 | CS | CTH | DDC | DUT | FABP5 | FBP1 | FCN2 | FIGNL1 | GLO1 | GMDS | GNPDA1 | GOT1 | GOT2 | GPI | GSR | GSTA1 | GSTA2 | GSTA3 | GSTK1 | GSTP1 | HINT1 | IDH2 | LRRK2 | MB | MIF | MMP9 | NAGA | NAMPT | NAPEPLD | NQO2 | NUDT5 | PFKP | PGAM1 | PGD | PGLYRP1 | PKM | PLA2G2A | PPT1 | PSAT1 | PYGL | PYGM | RIDA | SHMT1 | SOD1 | TKT | UXS1 | GO:0033267 | 0.0325318246110325 | 23/707 | 8/172 | 0.171798636706329 | 1 | F | F | F | F | axon part | CAD | GAD1 | GOT1 | KCNK2 | LRRK2 | NOS1 | SOD1 | TULP1 | GO:0005773 | 0.0325318246110325 | 23/707 | 8/172 | 0.171798636706329 | 1 | F | F | F | F | vacuole | CAT | CTSK | GOT1 | LRRK2 | NAGA | PPT1 | SOD1 | TLR7 | GO:0044463 | 0.1004243281471 | 71/707 | 21/172 | 0.172643229769932 | 1 | F | F | F | F | cell projection part | CAD | CHRNA3 | GAD1 | GLRA3 | GLRX2 | GLRX5 | GOT1 | GPI | GRIA2 | KCNK1 | KCNK2 | LRRK2 | NAPEPLD | NOS1 | NQO1 | PDE4B | PPT1 | RHO | SOD1 | THEM4 | TULP1 | GO:0120038 | 0.1004243281471 | 71/707 | 21/172 | 0.172643229769932 | 1 | F | F | F | F | plasma membrane bounded cell projection part | CAD | CHRNA3 | GAD1 | GLRA3 | GLRX2 | GLRX5 | GOT1 | GPI | GRIA2 | KCNK1 | KCNK2 | LRRK2 | NAPEPLD | NOS1 | NQO1 | PDE4B | PPT1 | RHO | SOD1 | THEM4 | TULP1 | GO:0044432 | 0.0848656294200849 | 60/707 | 18/172 | 0.179514706814819 | 1 | F | F | F | F | endoplasmic reticulum part | ALOX5AP | CES1 | CYP51A1 | CYR61 | GALNT2 | GRIA2 | HMGCR | HSD11B1 | INS | KCNK2 | LRRK2 | LTC4S | NOS1 | PLA2G2A | PTGES | SGPL1 | TLR7 | TLR8 | GO:0044421 | 0.369165487977369 | 261/707 | 69/172 | 0.181543026234946 | 1 | F | F | F | F | extracellular region part | ADH5 | AHCY | AK1 | APLP1 | APRT | AQP5 | ASL | ATAD2 | ATIC | CAD | CAT | CCL5 | CES1 | CNDP2 | CS | CTH | CTSK | CYR61 | DDC | DEFA5 | DUT | FABP5 | FBP1 | FCN2 | FIGNL1 | GLO1 | GMDS | GNPDA1 | GOT1 | GOT2 | GPI | GSR | GSTA1 | GSTA2 | GSTA3 | GSTK1 | GSTP1 | HINT1 | IDH2 | INS | LRRK2 | MB | MIF | MMP9 | NAGA | NAMPT | NAPEPLD | NQO2 | NUDT5 | PFKP | PGAM1 | PGD | PGLYRP1 | PKM | PLA2G1B | PLA2G2A | PPT1 | PSAT1 | PYGL | PYGM | RIDA | S100A7 | S100B | SHMT1 | SOD1 | TKT | TPSAB1 | TPSB2 | UXS1 | GO:0005886 | 0.233380480905233 | 165/707 | 45/172 | 0.182736832193344 | 1 | F | F | F | F | plasma membrane | ADH1C | AOC3 | APLP1 | AQP5 | ATIC | CAT | CHRNA1 | CHRNA2 | CHRNA3 | CHRNA7 | CSNK1D | CYP51A1 | FABP5 | GAD1 | GBP1 | GLO1 | GLRA3 | GOT2 | GPI | GRIA2 | GSTP1 | HCN4 | HDAC8 | HINT1 | IYD | KCNK1 | KCNK10 | KCNK2 | LRRK2 | MELK | NAMPT | NOS1 | NOS3 | OLR1 | PLA2G2A | PRKG1 | RHO | S100A12 | SEPHS1 | SLC14A1 | SOD1 | THEM4 | TLR7 | TLR8 | TULP1 | GO:0043227 | 0.708628005657709 | 501/707 | 127/172 | 0.187016796208379 | 1 | F | F | F | F | membrane-bounded organelle | ABAT | ACACB | ACADM | ACADS | ACADSB | ACOT13 | ADH5 | AHCY | AK1 | ALOX5AP | AOC3 | APRT | AQP5 | ASL | ATAD2 | ATIC | BCAT1 | BCAT2 | BCL6 | BIRC2 | BRD4 | CAD | CAT | CBS | CES1 | CNDP2 | COQ9 | CS | CSNK1D | CTH | CTSK | CYP51A1 | DCPS | DDC | DECR1 | DEFA5 | DUT | FABP5 | FBP1 | FCN2 | FECH | FIGNL1 | GALNT2 | GALT | GBP1 | GLO1 | GLRX2 | GLRX5 | GLS | GLUD1 | GMDS | GNPDA1 | GNPNAT1 | GOT1 | GOT2 | GPI | GSR | GSTA1 | GSTA2 | GSTA3 | GSTK1 | GSTP1 | HADH | HDAC8 | HINT1 | HMGCR | IDH2 | INS | ITK | IVD | KCNK1 | KCNK2 | KYAT1 | L3MBTL3 | LRRK2 | LTC4S | MB | ME2 | MELK | MIF | MMP9 | NAGA | NAMPT | NAPEPLD | NGB | NMRAL1 | NOS1 | NOS3 | NQO2 | NR0B2 | NUDT5 | OAT | OLR1 | PDE4B | PFKP | PGAM1 | PGD | PGLYRP1 | PKM | PLA2G1B | PLA2G2A | PPARD | PPT1 | PRKG1 | PSAT1 | PTGES | PYGL | PYGM | RHO | RIDA | S100A12 | S100A7 | S100B | SET | SGPL1 | SHMT1 | SIRT5 | SLC14A1 | SOD1 | THEM4 | TKT | TLR7 | TPR | TYMS | UMPS | UPP1 | UXS1 | GO:0030133 | 0.0183875530410184 | 13/707 | 5/172 | 0.18756294143613 | 1 | F | F | F | F | transport vesicle | DDC | DEFA5 | INS | PDE4B | PPT1 | GO:0043679 | 0.0183875530410184 | 13/707 | 5/172 | 0.18756294143613 | 1 | F | F | F | F | axon terminus | GAD1 | GOT1 | KCNK2 | NOS1 | TULP1 | GO:0044440 | 0.0282885431400283 | 20/707 | 7/172 | 0.190574505184527 | 1 | F | F | F | F | endosomal part | CTSK | GNPNAT1 | INS | LRRK2 | NAPEPLD | TLR7 | TLR8 | GO:0030054 | 0.074964639321075 | 53/707 | 16/172 | 0.191135498265628 | 1 | F | F | F | F | cell junction | CAT | CHRNA1 | CHRNA2 | CHRNA3 | CHRNA7 | FABP5 | GLRA3 | GRIA2 | ITK | KCNK1 | LRRK2 | NAMPT | RHO | S100A7 | SEPT3 | TULP1 | GO:0005829 | 0.533239038189533 | 377/707 | 97/172 | 0.200465322992814 | 1 | F | F | F | F | cytosol | ACACB | ACOT13 | ADH1C | ADH5 | ADSL | AHCY | AK1 | APRT | ASL | ATIC | BCAT1 | BIRC2 | BRD4 | CAD | CAT | CBS | CES1 | CNDP2 | CSNK1D | CTH | CYGB | DCPS | DDC | DECR1 | FABP5 | FBP1 | GALT | GBP1 | GGPS1 | GLO1 | GLS | GLTP | GMDS | GMPR2 | GNPDA1 | GNPNAT1 | GOT1 | GPI | GSR | GSTA1 | GSTA2 | GSTA3 | GSTA4 | GSTK1 | GSTP1 | GYG1 | HDC | HINT1 | IDH2 | ITK | KYAT1 | LRRK2 | MB | MIF | NAMPT | NAPEPLD | NGB | NMRAL1 | NOS1 | NOS3 | NQO1 | NQO2 | NUDT5 | PAH | PANK1 | PANK3 | PAPSS1 | PDE4B | PFKP | PGAM1 | PGD | PKM | PLA2G1B | PPT1 | PRKG1 | PSAT1 | PYGL | PYGM | RIDA | RNASEL | RRM1 | S100A12 | S100A7 | SAT1 | SCLY | SET | SHMT1 | SIRT5 | SOD1 | TDO2 | THEM4 | TKT | TULP1 | TYMS | UMPS | UPP1 | UPP2 | GO:0043226 | 0.734087694483734 | 519/707 | 131/172 | 0.200976258863421 | 1 | F | F | F | F | organelle | ABAT | ACACB | ACADM | ACADS | ACADSB | ACOT13 | ADH5 | AHCY | AK1 | ALOX5AP | AOC3 | APRT | AQP5 | ASL | ATAD2 | ATIC | BCAT1 | BCAT2 | BCL6 | BIRC2 | BRD4 | CAD | CAT | CBS | CES1 | CHRNA3 | CNDP2 | COQ9 | CS | CSNK1D | CTH | CTSK | CYP51A1 | DCPS | DDC | DECR1 | DEFA5 | DUT | FABP5 | FBP1 | FCN2 | FECH | FIGNL1 | GALNT2 | GALT | GBP1 | GLO1 | GLRX2 | GLRX5 | GLS | GLUD1 | GMDS | GNPDA1 | GNPNAT1 | GOT1 | GOT2 | GPI | GRIA2 | GSR | GSTA1 | GSTA2 | GSTA3 | GSTK1 | GSTP1 | HADH | HDAC8 | HINT1 | HMGCR | IDH2 | INS | ITK | IVD | KCNK1 | KCNK2 | KYAT1 | L3MBTL3 | LRRK2 | LTC4S | MB | ME2 | MELK | MIF | MMP9 | NAGA | NAMPT | NAPEPLD | NGB | NMRAL1 | NOS1 | NOS3 | NQO2 | NR0B2 | NUDT5 | OAT | OLR1 | PDE4B | PFKP | PGAM1 | PGD | PGLYRP1 | PKM | PLA2G1B | PLA2G2A | PPARD | PPT1 | PRKG1 | PSAT1 | PTGES | PYGL | PYGM | RHO | RIDA | S100A12 | S100A7 | S100B | SEPT3 | SET | SGPL1 | SHMT1 | SIRT5 | SLC14A1 | SOD1 | THEM4 | TKT | TLR7 | TPR | TULP1 | TYMS | UMPS | UPP1 | UXS1 | GO:0005654 | 0.173974540311174 | 123/707 | 34/172 | 0.20298677209971 | 1 | F | F | F | F | nucleoplasm | ACADS | ADH1C | APRT | ATAD2 | BCL6 | BRD4 | CAD | CNDP2 | CSNK1D | CTSK | DCPS | DECR1 | DUT | FABP5 | GLRX2 | GOT1 | GPI | HADH | HDAC8 | IYD | KYAT1 | MIF | NAPEPLD | NOS1 | NQO2 | NR0B2 | OAT | OLR1 | PPARD | SET | SOD1 | TKT | TPR | UPP1 | GO:0044431 | 0.0650636492220651 | 46/707 | 14/172 | 0.203442325199023 | 1 | F | F | F | F | Golgi apparatus part | CSNK1D | DEFA5 | GALNT2 | GBP1 | GNPNAT1 | HS2ST1 | INS | LRRK2 | NAPEPLD | NOS3 | RHO | TLR7 | TLR8 | UXS1 | GO:0044422 | 0.603960396039604 | 427/707 | 109/172 | 0.204201391994724 | 1 | F | F | F | F | organelle part | AADAT | ABAT | ACACB | ACAD8 | ACADM | ACADS | ACADSB | ACOT13 | ADH1C | ALOX5AP | APRT | ATAD2 | BCAT2 | BCL6 | BRD4 | CAD | CAT | CES1 | CHRNA1 | CNDP2 | COQ9 | CS | CSNK1D | CTSK | CYGB | CYP51A1 | CYR61 | DCPS | DECR1 | DEFA5 | DUT | FABP5 | FECH | FIGNL1 | GAD1 | GALNT2 | GBP1 | GLRX2 | GLRX5 | GLS | GLUD1 | GNPNAT1 | GOT1 | GOT2 | GPI | GRIA2 | GSR | GSTK1 | GSTP1 | GYG1 | HADH | HDAC8 | HINT1 | HMGCR | HS2ST1 | HSD11B1 | IDH2 | INS | IVD | IYD | KCNK2 | KYAT1 | L3MBTL3 | LRRK2 | LTC4S | ME2 | MIF | MMAB | MMP9 | NAMPT | NAPEPLD | NOS1 | NOS3 | NQO2 | NR0B2 | OAT | OLR1 | PDE4B | PGAM1 | PGLYRP1 | PKM | PLA2G2A | PPARD | PPT1 | PTGES | PYGL | RHO | RIDA | RNASEL | RRM1 | S100A12 | S100A7 | SEPHS1 | SEPT3 | SET | SGPL1 | SIRT5 | SLC14A1 | SOD1 | THEM4 | TKT | TLR7 | TLR8 | TPR | TULP1 | TYMS | UPP1 | UPP2 | UXS1 | GO:0043231 | 0.541725601131542 | 383/707 | 98/172 | 0.223654446970863 | 1 | F | F | F | F | intracellular membrane-bounded organelle | ABAT | ACACB | ACADM | ACADS | ACADSB | ACOT13 | ADH5 | AHCY | ALOX5AP | AOC3 | AQP5 | ATAD2 | BCAT1 | BCAT2 | BCL6 | BIRC2 | BRD4 | CAD | CAT | CBS | CES1 | COQ9 | CS | CSNK1D | CTSK | CYP51A1 | DCPS | DECR1 | DUT | FABP5 | FBP1 | FECH | FIGNL1 | GALNT2 | GALT | GBP1 | GLO1 | GLRX2 | GLRX5 | GLS | GLUD1 | GNPNAT1 | GOT1 | GOT2 | GSR | GSTK1 | GSTP1 | HADH | HDAC8 | HINT1 | HMGCR | IDH2 | ITK | IVD | KCNK1 | KCNK2 | KYAT1 | L3MBTL3 | LRRK2 | LTC4S | ME2 | MELK | NAGA | NAPEPLD | NGB | NMRAL1 | NOS1 | NOS3 | NR0B2 | NUDT5 | OAT | OLR1 | PFKP | PGD | PKM | PLA2G2A | PPARD | PPT1 | PRKG1 | PTGES | RHO | RIDA | S100A12 | S100A7 | S100B | SET | SGPL1 | SHMT1 | SIRT5 | SLC14A1 | SOD1 | THEM4 | TKT | TLR7 | TPR | TYMS | UMPS | UPP1 | GO:0005789 | 0.0608203677510608 | 43/707 | 13/172 | 0.223688106150607 | 1 | F | F | F | F | endoplasmic reticulum membrane | ALOX5AP | CYP51A1 | GALNT2 | GRIA2 | HMGCR | HSD11B1 | KCNK2 | LTC4S | PLA2G2A | PTGES | SGPL1 | TLR7 | TLR8 | GO:0098858 | 0.0099009900990099 | 7/707 | 3/172 | 0.228983949828319 | 1 | F | F | F | F | actin-based cell projection | AOC3 | AQP5 | LRRK2 | GO:0008021 | 0.0099009900990099 | 7/707 | 3/172 | 0.228983949828319 | 1 | F | F | F | F | synaptic vesicle | DDC | PDE4B | PPT1 | GO:0001917 | 0.0099009900990099 | 7/707 | 3/172 | 0.228983949828319 | 1 | F | F | F | F | photoreceptor inner segment | NOS1 | RHO | TULP1 | GO:0031982 | 0.352192362093352 | 249/707 | 65/172 | 0.235138986594135 | 1 | F | F | F | F | vesicle | ADH5 | AHCY | AK1 | AOC3 | APRT | AQP5 | ASL | ATAD2 | ATIC | CAD | CAT | CNDP2 | CS | CTH | DDC | DEFA5 | DUT | FABP5 | FBP1 | FCN2 | FIGNL1 | GLO1 | GMDS | GNPDA1 | GNPNAT1 | GOT1 | GOT2 | GPI | GSR | GSTA1 | GSTA2 | GSTA3 | GSTK1 | GSTP1 | HINT1 | IDH2 | INS | KCNK1 | LRRK2 | MB | MIF | MMP9 | NAGA | NAMPT | NAPEPLD | NQO2 | NUDT5 | PDE4B | PFKP | PGAM1 | PGD | PGLYRP1 | PKM | PLA2G1B | PLA2G2A | PPT1 | PSAT1 | PYGL | PYGM | RIDA | SHMT1 | SOD1 | TKT | TLR7 | UXS1 | GO:0044306 | 0.0198019801980198 | 14/707 | 5/172 | 0.237298466868555 | 1 | F | F | F | F | neuron projection terminus | GAD1 | GOT1 | KCNK2 | NOS1 | TULP1 | GO:0045121 | 0.0353606789250354 | 25/707 | 8/172 | 0.244101208138331 | 1 | F | F | F | F | membrane raft | BIRC2 | CHRNA3 | CHRNA7 | LRRK2 | NOS1 | NOS3 | OLR1 | PPT1 | GO:0098857 | 0.0353606789250354 | 25/707 | 8/172 | 0.244101208138331 | 1 | F | F | F | F | membrane microdomain | BIRC2 | CHRNA3 | CHRNA7 | LRRK2 | NOS1 | NOS3 | OLR1 | PPT1 | GO:0098589 | 0.0353606789250354 | 25/707 | 8/172 | 0.244101208138331 | 1 | F | F | F | F | membrane region | BIRC2 | CHRNA3 | CHRNA7 | LRRK2 | NOS1 | NOS3 | OLR1 | PPT1 | GO:0098590 | 0.0891089108910891 | 63/707 | 18/172 | 0.248142918854418 | 1 | F | F | F | F | plasma membrane region | AQP5 | CHRNA1 | CHRNA2 | CHRNA3 | CHRNA7 | FABP5 | GLRA3 | GPI | GRIA2 | KCNK1 | KCNK2 | LRRK2 | NAPEPLD | NOS1 | NOS3 | RHO | SLC14A1 | THEM4 | GO:0030315 | 0.00565770862800566 | 4/707 | 2/172 | 0.250112373199934 | 1 | F | F | F | F | T-tubule | GOT2 | NOS1 | GO:0001750 | 0.00565770862800566 | 4/707 | 2/172 | 0.250112373199934 | 1 | F | F | F | F | photoreceptor outer segment | RHO | TULP1 | GO:0005783 | 0.0792079207920792 | 56/707 | 16/172 | 0.266535860792332 | 1 | F | F | F | F | endoplasmic reticulum | ALOX5AP | AOC3 | AQP5 | CAT | CES1 | CYP51A1 | GNPNAT1 | HMGCR | LRRK2 | LTC4S | NOS1 | PLA2G2A | S100A7 | SET | SGPL1 | TLR7 | GO:0042579 | 0.0311173974540311 | 22/707 | 7/172 | 0.272391162819898 | 1 | F | F | F | F | microbody | CAT | GSTK1 | HMGCR | IDH2 | RIDA | SOD1 | TKT | GO:0005794 | 0.0693069306930693 | 49/707 | 14/172 | 0.286896444169865 | 1 | F | F | F | F | Golgi apparatus | AOC3 | BCL6 | CAT | CSNK1D | GALNT2 | GALT | GBP1 | GNPNAT1 | LRRK2 | NAPEPLD | NOS3 | PPT1 | PRKG1 | RHO | GO:0043204 | 0.0212164073550212 | 15/707 | 5/172 | 0.290148742378516 | 1 | F | F | F | F | perikaryon | GLRA3 | GOT2 | KCNK1 | LRRK2 | NGB | GO:0031966 | 0.0424328147100424 | 30/707 | 9/172 | 0.292326111513656 | 1 | F | F | F | F | mitochondrial membrane | ACACB | ACADM | COQ9 | FECH | GOT2 | LRRK2 | SIRT5 | THEM4 | TYMS | GO:0070382 | 0.0113154172560113 | 8/707 | 3/172 | 0.304272128099004 | 1 | F | F | F | F | exocytic vesicle | DDC | PDE4B | PPT1 | GO:0044309 | 0.0113154172560113 | 8/707 | 3/172 | 0.304272128099004 | 1 | F | F | F | F | neuron spine | GRIA2 | NOS1 | PDE4B | GO:0043197 | 0.0113154172560113 | 8/707 | 3/172 | 0.304272128099004 | 1 | F | F | F | F | dendritic spine | GRIA2 | NOS1 | PDE4B | GO:0099503 | 0.0268741159830269 | 19/707 | 6/172 | 0.305451574422033 | 1 | F | F | F | F | secretory vesicle | DDC | PDE4B | PLA2G1B | PLA2G2A | PPT1 | SOD1 | GO:0005777 | 0.0268741159830269 | 19/707 | 6/172 | 0.305451574422033 | 1 | F | F | F | F | peroxisome | CAT | GSTK1 | IDH2 | RIDA | SOD1 | TKT | GO:0016607 | 0.016973125884017 | 12/707 | 4/172 | 0.329801078730482 | 1 | F | F | F | F | nuclear speck | ACADM | CYGB | NAMPT | TKT | GO:0016021 | 0.14992927864215 | 106/707 | 28/172 | 0.332770271195143 | 1 | F | F | F | F | integral component of membrane | ALOX5AP | AOC3 | APLP1 | AQP5 | CHRNA1 | CHRNA2 | CHRNA3 | CHRNA7 | CYP51A1 | GALNT2 | GLRA3 | GRIA2 | HMGCR | HS2ST1 | HSD11B1 | IYD | KCNK1 | KCNK10 | KCNK2 | LTC4S | OLR1 | PTGES | RHO | SGPL1 | SLC14A1 | TLR7 | TLR8 | UXS1 | GO:0044464 | 0.968882602545969 | 685/707 | 168/172 | 0.346809007670149 | 1 | F | F | F | F | cell part | AADAT | ABAT | ACACB | ACAD8 | ACADM | ACADS | ACADSB | ACOT13 | ADH1C | ADH5 | ADSL | AHCY | AK1 | ALOX5AP | AOC3 | APLP1 | APRT | AQP5 | ASL | ATAD2 | ATIC | BCAT1 | BCAT2 | BCL6 | BIRC2 | BRD4 | CAD | CAT | CBS | CES1 | CHRNA1 | CHRNA2 | CHRNA3 | CHRNA7 | CNDP2 | COQ9 | CS | CSAD | CSNK1D | CTH | CTSK | CYGB | CYP51A1 | CYR61 | DCPS | DDC | DECR1 | DEFA5 | DUT | FABP5 | FBP1 | FECH | FIGNL1 | GAD1 | GALNT2 | GALT | GBP1 | GGPS1 | GLO1 | GLRA3 | GLRX2 | GLRX5 | GLS | GLTP | GLUD1 | GMDS | GMPR2 | GNPDA1 | GNPNAT1 | GOT1 | GOT2 | GPI | GRIA2 | GSR | GSTA1 | GSTA2 | GSTA3 | GSTA4 | GSTK1 | GSTP1 | GYG1 | HADH | HCN4 | HDAC8 | HDC | HINT1 | HMGCR | HS2ST1 | HSD11B1 | IDH2 | INS | ITK | IVD | IYD | KCNK1 | KCNK10 | KCNK2 | KYAT1 | L3MBTL3 | LRRK2 | LTC4S | MB | ME2 | MELK | MIF | MMAB | MMP9 | NAGA | NAMPT | NAPEPLD | NGB | NMRAL1 | NOS1 | NOS3 | NQO1 | NQO2 | NR0B2 | NUDT5 | OAT | OLR1 | PAH | PANK1 | PANK3 | PAPSS1 | PDE4B | PFKP | PGAM1 | PGD | PGLYRP1 | PKM | PLA2G1B | PLA2G2A | PPARD | PPT1 | PRKG1 | PSAT1 | PTGES | PYGL | PYGM | RHO | RIDA | RNASEL | RRM1 | S100A12 | S100A7 | S100B | SAT1 | SCLY | SEPHS1 | SEPT3 | SET | SGPL1 | SHMT1 | SIRT5 | SLC14A1 | SOD1 | TDO2 | THEM4 | TKT | TLR7 | TLR8 | TPR | TULP1 | TYMS | UMPS | UPP1 | UPP2 | UXS1 | GO:0031301 | 0.00707213578500707 | 5/707 | 2/172 | 0.35297688771354 | 1 | F | F | F | F | integral component of organelle membrane | GALNT2 | SGPL1 | GO:0000118 | 0.00707213578500707 | 5/707 | 2/172 | 0.35297688771354 | 1 | F | F | F | F | histone deacetylase complex | HDAC8 | HINT1 | GO:0062023 | 0.0339462517680339 | 24/707 | 7/172 | 0.361579115096014 | 1 | F | F | F | F | collagen-containing extracellular matrix | APLP1 | CYR61 | MMP9 | PKM | S100A7 | TPSAB1 | TPSB2 | GO:0031090 | 0.220650636492221 | 156/707 | 40/172 | 0.368388484018171 | 1 | F | F | F | F | organelle membrane | ACACB | ACADM | ALOX5AP | CAT | COQ9 | CSNK1D | CYP51A1 | FABP5 | FECH | GAD1 | GALNT2 | GBP1 | GNPNAT1 | GOT2 | GPI | GRIA2 | HMGCR | HS2ST1 | HSD11B1 | INS | IYD | KCNK2 | LRRK2 | LTC4S | NAPEPLD | NOS1 | NOS3 | OLR1 | PLA2G2A | PTGES | RHO | SEPHS1 | SGPL1 | SIRT5 | THEM4 | TLR7 | TLR8 | TPR | TYMS | UXS1 | GO:0060170 | 0.0127298444130127 | 9/707 | 3/172 | 0.380386070842799 | 1 | F | F | F | F | ciliary membrane | GPI | NAPEPLD | RHO | GO:0044853 | 0.0183875530410184 | 13/707 | 4/172 | 0.393018028639066 | 1 | F | F | F | F | plasma membrane raft | CHRNA3 | CHRNA7 | NOS1 | NOS3 | GO:0044424 | 0.934936350777935 | 661/707 | 162/172 | 0.413041397022712 | 1 | F | F | F | F | intracellular part | AADAT | ABAT | ACACB | ACAD8 | ACADM | ACADS | ACADSB | ACOT13 | ADH1C | ADH5 | ADSL | AHCY | AK1 | ALOX5AP | AOC3 | APLP1 | APRT | AQP5 | ASL | ATAD2 | ATIC | BCAT1 | BCAT2 | BCL6 | BIRC2 | BRD4 | CAD | CAT | CBS | CES1 | CNDP2 | COQ9 | CS | CSAD | CSNK1D | CTH | CTSK | CYGB | CYP51A1 | CYR61 | DCPS | DDC | DECR1 | DEFA5 | DUT | FABP5 | FBP1 | FECH | FIGNL1 | GAD1 | GALNT2 | GALT | GBP1 | GGPS1 | GLO1 | GLRX2 | GLRX5 | GLS | GLTP | GLUD1 | GMDS | GMPR2 | GNPDA1 | GNPNAT1 | GOT1 | GOT2 | GPI | GRIA2 | GSR | GSTA1 | GSTA2 | GSTA3 | GSTA4 | GSTK1 | GSTP1 | GYG1 | HADH | HCN4 | HDAC8 | HDC | HINT1 | HMGCR | HS2ST1 | HSD11B1 | IDH2 | INS | ITK | IVD | IYD | KCNK1 | KCNK2 | KYAT1 | L3MBTL3 | LRRK2 | LTC4S | MB | ME2 | MELK | MIF | MMAB | MMP9 | NAGA | NAMPT | NAPEPLD | NGB | NMRAL1 | NOS1 | NOS3 | NQO1 | NQO2 | NR0B2 | NUDT5 | OAT | OLR1 | PAH | PANK1 | PANK3 | PAPSS1 | PDE4B | PFKP | PGAM1 | PGD | PGLYRP1 | PKM | PLA2G1B | PLA2G2A | PPARD | PPT1 | PRKG1 | PSAT1 | PTGES | PYGL | PYGM | RHO | RIDA | RNASEL | RRM1 | S100A12 | S100A7 | S100B | SAT1 | SCLY | SEPHS1 | SEPT3 | SET | SGPL1 | SHMT1 | SIRT5 | SLC14A1 | SOD1 | TDO2 | THEM4 | TKT | TLR7 | TLR8 | TPR | TULP1 | TYMS | UMPS | UPP1 | UPP2 | UXS1 | GO:0005634 | 0.301272984441301 | 213/707 | 53/172 | 0.445725153791551 | 1 | F | F | F | F | nucleus | ACACB | ACADM | ACADS | ACOT13 | AHCY | ATAD2 | BCL6 | BIRC2 | BRD4 | CAD | CBS | CS | CSNK1D | DCPS | DECR1 | DUT | FABP5 | FBP1 | FIGNL1 | GLO1 | GLRX2 | GLRX5 | GOT1 | GSTP1 | HDAC8 | HINT1 | ITK | KCNK2 | L3MBTL3 | LRRK2 | MELK | NMRAL1 | NOS1 | NOS3 | NR0B2 | NUDT5 | PFKP | PGD | PKM | PPARD | PPT1 | RIDA | S100A12 | S100A7 | S100B | SET | SHMT1 | SIRT5 | SOD1 | TPR | TYMS | UMPS | UPP1 | GO:0016363 | 0.00848656294200849 | 6/707 | 2/172 | 0.450419768271332 | 1 | F | F | F | F | nuclear matrix | CAD | RNASEL | GO:0030666 | 0.00848656294200849 | 6/707 | 2/172 | 0.450419768271332 | 1 | F | F | F | F | endocytic vesicle membrane | GRIA2 | NOS3 | GO:0120111 | 0.00848656294200849 | 6/707 | 2/172 | 0.450419768271332 | 1 | F | F | F | F | neuron projection cytoplasm | LRRK2 | SOD1 | GO:0032580 | 0.00848656294200849 | 6/707 | 2/172 | 0.450419768271332 | 1 | F | F | F | F | Golgi cisterna membrane | GALNT2 | UXS1 | GO:0005796 | 0.00848656294200849 | 6/707 | 2/172 | 0.450419768271332 | 1 | F | F | F | F | Golgi lumen | DEFA5 | INS | GO:0031012 | 0.0367751060820368 | 26/707 | 7/172 | 0.45283447283523 | 1 | F | F | F | F | extracellular matrix | APLP1 | CYR61 | MMP9 | PKM | S100A7 | TPSAB1 | TPSB2 | GO:0031224 | 0.164073550212164 | 116/707 | 29/172 | 0.468242023026836 | 1 | F | F | F | F | intrinsic component of membrane | ALOX5AP | AOC3 | APLP1 | AQP5 | CHRNA1 | CHRNA2 | CHRNA3 | CHRNA7 | CYP51A1 | GALNT2 | GLRA3 | GRIA2 | HCN4 | HMGCR | HS2ST1 | HSD11B1 | IYD | KCNK1 | KCNK10 | KCNK2 | LTC4S | OLR1 | PTGES | RHO | SGPL1 | SLC14A1 | TLR7 | TLR8 | UXS1 | GO:0016020 | 0.415841584158416 | 294/707 | 72/172 | 0.500730335734593 | 1 | F | F | F | F | membrane | ACACB | ACADM | ADH1C | ALOX5AP | AOC3 | APLP1 | AQP5 | ATIC | BIRC2 | CAD | CAT | CHRNA1 | CHRNA2 | CHRNA3 | CHRNA7 | COQ9 | CSNK1D | CYP51A1 | FABP5 | FECH | GAD1 | GALNT2 | GBP1 | GLO1 | GLRA3 | GLTP | GNPNAT1 | GOT2 | GPI | GRIA2 | GSTK1 | GSTP1 | GYG1 | HCN4 | HDAC8 | HINT1 | HMGCR | HS2ST1 | HSD11B1 | INS | IYD | KCNK1 | KCNK10 | KCNK2 | LRRK2 | LTC4S | MELK | NAMPT | NAPEPLD | NOS1 | NOS3 | OLR1 | PFKP | PGAM1 | PLA2G2A | PPT1 | PRKG1 | PTGES | RHO | S100A12 | SEPHS1 | SGPL1 | SIRT5 | SLC14A1 | SOD1 | THEM4 | TLR7 | TLR8 | TPR | TULP1 | TYMS | UXS1 | GO:0043229 | 0.602545968882603 | 426/707 | 104/172 | 0.511107243179026 | 1 | F | F | F | F | intracellular organelle | ABAT | ACACB | ACADM | ACADS | ACADSB | ACOT13 | ADH5 | AHCY | ALOX5AP | AOC3 | AQP5 | ATAD2 | BCAT1 | BCAT2 | BCL6 | BIRC2 | BRD4 | CAD | CAT | CBS | CES1 | COQ9 | CS | CSNK1D | CTSK | CYP51A1 | DCPS | DDC | DECR1 | DEFA5 | DUT | FABP5 | FBP1 | FECH | FIGNL1 | GALNT2 | GALT | GBP1 | GLO1 | GLRX2 | GLRX5 | GLS | GLUD1 | GNPNAT1 | GOT1 | GOT2 | GSR | GSTK1 | GSTP1 | HADH | HDAC8 | HINT1 | HMGCR | IDH2 | INS | ITK | IVD | KCNK1 | KCNK2 | KYAT1 | L3MBTL3 | LRRK2 | LTC4S | ME2 | MELK | NAGA | NAPEPLD | NGB | NMRAL1 | NOS1 | NOS3 | NR0B2 | NUDT5 | OAT | OLR1 | PDE4B | PFKP | PGD | PKM | PLA2G1B | PLA2G2A | PPARD | PPT1 | PRKG1 | PTGES | RHO | RIDA | S100A12 | S100A7 | S100B | SEPT3 | SET | SGPL1 | SHMT1 | SIRT5 | SLC14A1 | SOD1 | THEM4 | TKT | TLR7 | TPR | TYMS | UMPS | UPP1 | GO:0044445 | 0.0155586987270156 | 11/707 | 3/172 | 0.524782351747333 | 1 | F | F | F | F | cytosolic part | LRRK2 | PFKP | RRM1 | GO:0005819 | 0.0155586987270156 | 11/707 | 3/172 | 0.524782351747333 | 1 | F | F | F | F | spindle | ACOT13 | CSNK1D | TPR | GO:0061695 | 0.0099009900990099 | 7/707 | 2/172 | 0.538994095648595 | 1 | F | F | F | F | transferase complex, transferring phosphorus-containing groups | PFKP | PKM | GO:0030018 | 0.0099009900990099 | 7/707 | 2/172 | 0.538994095648595 | 1 | F | F | F | F | Z disc | NOS1 | PDE4B | GO:0032838 | 0.0099009900990099 | 7/707 | 2/172 | 0.538994095648595 | 1 | F | F | F | F | plasma membrane bounded cell projection cytoplasm | LRRK2 | SOD1 | GO:0005929 | 0.0099009900990099 | 7/707 | 2/172 | 0.538994095648595 | 1 | F | F | F | F | cilium | PKM | TULP1 | GO:0044459 | 0.157001414427157 | 111/707 | 27/172 | 0.542746320762698 | 1 | F | F | F | F | plasma membrane part | AQP5 | BIRC2 | CHRNA1 | CHRNA2 | CHRNA3 | CHRNA7 | FABP5 | GLRA3 | GOT2 | GPI | GRIA2 | GSR | HCN4 | IYD | KCNK1 | KCNK10 | KCNK2 | LRRK2 | NAPEPLD | NOS1 | NOS3 | OLR1 | PDE4B | RHO | SLC14A1 | THEM4 | TLR8 | GO:0048471 | 0.0636492220650637 | 45/707 | 11/172 | 0.553224277254944 | 1 | F | F | F | F | perinuclear region of cytoplasm | APLP1 | CSNK1D | FIGNL1 | GALNT2 | HCN4 | NMRAL1 | NOS1 | PLA2G2A | PTGES | S100B | SET | GO:0098552 | 0.0282885431400283 | 20/707 | 5/172 | 0.559478284412609 | 1 | F | F | F | F | side of membrane | BIRC2 | GRIA2 | GSR | LRRK2 | TLR8 | GO:0031410 | 0.0876944837340877 | 62/707 | 15/172 | 0.563204885584923 | 1 | F | F | F | F | cytoplasmic vesicle | AHCY | AOC3 | DDC | DEFA5 | GNPNAT1 | INS | KCNK1 | LRRK2 | NAPEPLD | PDE4B | PLA2G1B | PLA2G2A | PPT1 | SOD1 | TLR7 | GO:0097708 | 0.0876944837340877 | 62/707 | 15/172 | 0.563204885584923 | 1 | F | F | F | F | intracellular vesicle | AHCY | AOC3 | DDC | DEFA5 | GNPNAT1 | INS | KCNK1 | LRRK2 | NAPEPLD | PDE4B | PLA2G1B | PLA2G2A | PPT1 | SOD1 | TLR7 | GO:0099568 | 0.0226308345120226 | 16/707 | 4/172 | 0.572208968678283 | 1 | F | F | F | F | cytoplasmic region | GAD1 | LRRK2 | MELK | SOD1 | GO:0010008 | 0.0226308345120226 | 16/707 | 4/172 | 0.572208968678283 | 1 | F | F | F | F | endosome membrane | GNPNAT1 | NAPEPLD | TLR7 | TLR8 | GO:0044441 | 0.0226308345120226 | 16/707 | 4/172 | 0.572208968678283 | 1 | F | F | F | F | ciliary part | GPI | NAPEPLD | RHO | TULP1 | GO:0005694 | 0.0226308345120226 | 16/707 | 4/172 | 0.572208968678283 | 1 | F | F | F | F | chromosome | BIRC2 | BRD4 | FIGNL1 | HDAC8 | GO:0031965 | 0.0226308345120226 | 16/707 | 4/172 | 0.572208968678283 | 1 | F | F | F | F | nuclear membrane | ALOX5AP | LTC4S | SEPHS1 | TPR | GO:0030424 | 0.0226308345120226 | 16/707 | 4/172 | 0.572208968678283 | 1 | F | F | F | F | axon | ACADM | DDC | LRRK2 | PPT1 | GO:0009897 | 0.016973125884017 | 12/707 | 3/172 | 0.589776109419685 | 1 | F | F | F | F | external side of plasma membrane | GRIA2 | GSR | TLR8 | GO:0030141 | 0.016973125884017 | 12/707 | 3/172 | 0.589776109419685 | 1 | F | F | F | F | secretory granule | PLA2G1B | PLA2G2A | SOD1 | GO:0005768 | 0.0353606789250354 | 25/707 | 6/172 | 0.59448425956553 | 1 | F | F | F | F | endosome | AOC3 | GNPNAT1 | KCNK1 | LRRK2 | NAPEPLD | TLR7 | GO:0031253 | 0.0297029702970297 | 21/707 | 5/172 | 0.607919293221242 | 1 | F | F | F | F | cell projection membrane | GPI | KCNK1 | NAPEPLD | RHO | THEM4 | GO:0005901 | 0.0113154172560113 | 8/707 | 2/172 | 0.617234751498561 | 1 | F | F | F | F | caveola | NOS1 | NOS3 | GO:1904724 | 0.0113154172560113 | 8/707 | 2/172 | 0.617234751498561 | 1 | F | F | F | F | tertiary granule lumen | MMP9 | PGLYRP1 | GO:0031300 | 0.0113154172560113 | 8/707 | 2/172 | 0.617234751498561 | 1 | F | F | F | F | intrinsic component of organelle membrane | GALNT2 | SGPL1 | GO:0098562 | 0.0113154172560113 | 8/707 | 2/172 | 0.617234751498561 | 1 | F | F | F | F | cytoplasmic side of membrane | BIRC2 | LRRK2 | GO:0005856 | 0.0367751060820368 | 26/707 | 6/172 | 0.636979195584531 | 1 | F | F | F | F | cytoskeleton | GBP1 | HINT1 | NOS1 | NOS3 | S100A12 | SEPT3 | GO:0012506 | 0.0551626591230552 | 39/707 | 9/172 | 0.637890539201002 | 1 | F | F | F | F | vesicle membrane | FABP5 | GAD1 | GBP1 | GRIA2 | IYD | LRRK2 | NOS1 | NOS3 | OLR1 | GO:0043202 | 0.0183875530410184 | 13/707 | 3/172 | 0.648803533653854 | 1 | F | F | F | F | lysosomal lumen | CTSK | GYG1 | PPT1 | GO:1902494 | 0.0495049504950495 | 35/707 | 8/172 | 0.648926092491411 | 1 | F | F | F | F | catalytic complex | ABAT | GMPR2 | HDAC8 | HINT1 | PFKP | PKM | RRM1 | TPR | GO:0031903 | 0.0127298444130127 | 9/707 | 2/172 | 0.684905803114472 | 1 | F | F | F | F | microbody membrane | CAT | HMGCR | GO:0005778 | 0.0127298444130127 | 9/707 | 2/172 | 0.684905803114472 | 1 | F | F | F | F | peroxisomal membrane | CAT | HMGCR | GO:0031984 | 0.0127298444130127 | 9/707 | 2/172 | 0.684905803114472 | 1 | F | F | F | F | organelle subcompartment | GALNT2 | LRRK2 | GO:0098791 | 0.0127298444130127 | 9/707 | 2/172 | 0.684905803114472 | 1 | F | F | F | F | Golgi subcompartment | GALNT2 | LRRK2 | GO:0005938 | 0.0127298444130127 | 9/707 | 2/172 | 0.684905803114472 | 1 | F | F | F | F | cell cortex | GAD1 | MELK | GO:0005775 | 0.0325318246110325 | 23/707 | 5/172 | 0.695033351052256 | 1 | F | F | F | F | vacuolar lumen | CTSK | FABP5 | GYG1 | PPT1 | S100A7 | GO:0005788 | 0.0198019801980198 | 14/707 | 3/172 | 0.70157562828297 | 1 | F | F | F | F | endoplasmic reticulum lumen | CES1 | CYR61 | INS | GO:0005615 | 0.107496463932108 | 76/707 | 17/172 | 0.708610347725389 | 1 | F | F | F | F | extracellular space | CCL5 | CES1 | CTSK | DEFA5 | GSTP1 | INS | LRRK2 | MIF | MMP9 | PGLYRP1 | PLA2G1B | PLA2G2A | S100A7 | S100B | SOD1 | TPSAB1 | TPSB2 | GO:0009986 | 0.0396039603960396 | 28/707 | 6/172 | 0.71367734653217 | 1 | F | F | F | F | cell surface | AOC3 | CHRNA1 | GOT2 | KCNK2 | MIF | PLA2G1B | GO:0005765 | 0.0141442715700141 | 10/707 | 2/172 | 0.742494061939378 | 1 | F | F | F | F | lysosomal membrane | TLR7 | TLR8 | GO:0098852 | 0.0141442715700141 | 10/707 | 2/172 | 0.742494061939378 | 1 | F | F | F | F | lytic vacuole membrane | TLR7 | TLR8 | GO:1990234 | 0.0212164073550212 | 15/707 | 3/172 | 0.748128789201436 | 1 | F | F | F | F | transferase complex | ABAT | PFKP | PKM | GO:0032991 | 0.207920792079208 | 147/707 | 33/172 | 0.757715163395311 | 1 | F | F | F | F | protein-containing complex | ABAT | BIRC2 | CAD | CHRNA1 | CHRNA2 | CHRNA3 | CHRNA7 | DCPS | FCN2 | GLRA3 | GMPR2 | GOT2 | GRIA2 | GSTP1 | HCN4 | HDAC8 | HINT1 | KCNK1 | KCNK2 | LRRK2 | NOS1 | NR0B2 | OLR1 | PDE4B | PFKP | PKM | PPARD | RRM1 | SEPT3 | SET | SOD1 | TLR7 | TPR | GO:0044437 | 0.0480905233380481 | 34/707 | 7/172 | 0.760951719259595 | 1 | F | F | F | F | vacuolar part | CTSK | FABP5 | GYG1 | PPT1 | S100A7 | TLR7 | TLR8 | GO:0019867 | 0.0226308345120226 | 16/707 | 3/172 | 0.788725670860686 | 1 | F | F | F | F | outer membrane | ACACB | LRRK2 | LTC4S | GO:0031968 | 0.0226308345120226 | 16/707 | 3/172 | 0.788725670860686 | 1 | F | F | F | F | organelle outer membrane | ACACB | LRRK2 | LTC4S | GO:0044449 | 0.0155586987270156 | 11/707 | 2/172 | 0.790874441982147 | 1 | F | F | F | F | contractile fiber part | NOS1 | PDE4B | GO:0098588 | 0.144271570014144 | 102/707 | 22/172 | 0.794438432291343 | 1 | F | F | F | F | bounding membrane of organelle | ACACB | CAT | CSNK1D | FABP5 | GAD1 | GALNT2 | GBP1 | GNPNAT1 | GPI | GRIA2 | HMGCR | HS2ST1 | INS | LRRK2 | LTC4S | NAPEPLD | NOS3 | OLR1 | RHO | TLR7 | TLR8 | UXS1 | GO:0098805 | 0.101838755304102 | 72/707 | 15/172 | 0.80769019337096 | 1 | F | F | F | F | whole membrane | ACACB | CAT | FABP5 | GAD1 | GNPNAT1 | GRIA2 | HMGCR | LRRK2 | LTC4S | NAPEPLD | NOS3 | OLR1 | RHO | TLR7 | TLR8 | GO:0044439 | 0.024045261669024 | 17/707 | 3/172 | 0.823774787629154 | 1 | F | F | F | F | peroxisomal part | CAT | GSTK1 | HMGCR | GO:0016324 | 0.024045261669024 | 17/707 | 3/172 | 0.823774787629154 | 1 | F | F | F | F | apical plasma membrane | AQP5 | KCNK1 | KCNK2 | GO:0044438 | 0.024045261669024 | 17/707 | 3/172 | 0.823774787629154 | 1 | F | F | F | F | microbody part | CAT | GSTK1 | HMGCR | GO:0005769 | 0.016973125884017 | 12/707 | 2/172 | 0.831094108494725 | 1 | F | F | F | F | early endosome | AOC3 | NAPEPLD | GO:0005774 | 0.016973125884017 | 12/707 | 2/172 | 0.831094108494725 | 1 | F | F | F | F | vacuolar membrane | TLR7 | TLR8 | GO:0044425 | 0.275813295615276 | 195/707 | 43/172 | 0.833577717253653 | 1 | F | F | F | F | membrane part | ALOX5AP | AOC3 | APLP1 | AQP5 | BIRC2 | CHRNA1 | CHRNA2 | CHRNA3 | CHRNA7 | CYP51A1 | FABP5 | GALNT2 | GLRA3 | GOT2 | GPI | GRIA2 | GSR | HCN4 | HMGCR | HS2ST1 | HSD11B1 | IYD | KCNK1 | KCNK10 | KCNK2 | LRRK2 | LTC4S | NAPEPLD | NOS1 | NOS3 | OLR1 | PDE4B | PLA2G2A | PPT1 | PTGES | RHO | SGPL1 | SLC14A1 | THEM4 | TLR7 | TLR8 | TPR | UXS1 | GO:0030659 | 0.0523338048090523 | 37/707 | 7/172 | 0.837536437615793 | 1 | F | F | F | F | cytoplasmic vesicle membrane | FABP5 | GAD1 | GRIA2 | IYD | LRRK2 | NOS3 | OLR1 | GO:0031907 | 0.0183875530410184 | 13/707 | 2/172 | 0.864237849374863 | 1 | F | F | F | F | microbody lumen | CAT | GSTK1 | GO:0005741 | 0.0183875530410184 | 13/707 | 2/172 | 0.864237849374863 | 1 | F | F | F | F | mitochondrial outer membrane | ACACB | LRRK2 | GO:0035578 | 0.0183875530410184 | 13/707 | 2/172 | 0.864237849374863 | 1 | F | F | F | F | azurophil granule lumen | FABP5 | S100A7 | GO:0005782 | 0.0183875530410184 | 13/707 | 2/172 | 0.864237849374863 | 1 | F | F | F | F | peroxisomal matrix | CAT | GSTK1 | GO:0043228 | 0.125884016973126 | 89/707 | 18/172 | 0.864632663564337 | 1 | F | F | F | F | non-membrane-bounded organelle | ACOT13 | BIRC2 | BRD4 | CSNK1D | DCPS | FIGNL1 | GBP1 | HDAC8 | HINT1 | L3MBTL3 | NOS1 | NOS3 | S100A12 | SEPT3 | SET | SLC14A1 | TPR | TYMS | GO:0043232 | 0.125884016973126 | 89/707 | 18/172 | 0.864632663564337 | 1 | F | F | F | F | intracellular non-membrane-bounded organelle | ACOT13 | BIRC2 | BRD4 | CSNK1D | DCPS | FIGNL1 | GBP1 | HDAC8 | HINT1 | L3MBTL3 | NOS1 | NOS3 | S100A12 | SEPT3 | SET | SLC14A1 | TPR | TYMS | GO:0005813 | 0.0212164073550212 | 15/707 | 2/172 | 0.913381978072996 | 1 | F | F | F | F | centrosome | CSNK1D | PDE4B | GO:1990904 | 0.0226308345120226 | 16/707 | 2/172 | 0.931190021960537 | 1 | F | F | F | F | ribonucleoprotein complex | DCPS | LRRK2 | GO:0099513 | 0.0226308345120226 | 16/707 | 2/172 | 0.931190021960537 | 1 | F | F | F | F | polymeric cytoskeletal fiber | CSNK1D | UPP2 | GO:0005737 | 0.340876944837341 | 241/707 | 51/172 | 0.934576663024876 | 1 | F | F | F | F | cytoplasm | AOC3 | APRT | ASL | BIRC2 | CAD | CBS | CSAD | CSNK1D | CTH | DCPS | FABP5 | FBP1 | FIGNL1 | GAD1 | GALT | GBP1 | GLO1 | GLUD1 | GMDS | GNPDA1 | GOT1 | GSTP1 | HADH | HDAC8 | KYAT1 | LRRK2 | MELK | NAGA | NOS1 | NOS3 | NQO1 | NR0B2 | OAT | PFKP | PGAM1 | PKM | PRKG1 | PYGL | PYGM | RIDA | S100A12 | S100A7 | S100B | SEPHS1 | SET | SOD1 | TLR7 | TPR | TYMS | UMPS | UXS1 | GO:0016604 | 0.0396039603960396 | 28/707 | 4/172 | 0.939347645485638 | 1 | F | F | F | F | nuclear body | ACADM | CYGB | NAMPT | TKT | GO:0005911 | 0.024045261669024 | 17/707 | 2/172 | 0.945512053204796 | 1 | F | F | F | F | cell-cell junction | ITK | RHO | GO:0005924 | 0.0254596888260255 | 18/707 | 2/172 | 0.956980056483791 | 1 | F | F | F | F | cell-substrate adherens junction | CAT | S100A7 | GO:0030055 | 0.0254596888260255 | 18/707 | 2/172 | 0.956980056483791 | 1 | F | F | F | F | cell-substrate junction | CAT | S100A7 | GO:0005925 | 0.0254596888260255 | 18/707 | 2/172 | 0.956980056483791 | 1 | F | F | F | F | focal adhesion | CAT | S100A7 | GO:0099081 | 0.0268741159830269 | 19/707 | 2/172 | 0.966126653139635 | 1 | F | F | F | F | supramolecular polymer | CSNK1D | UPP2 | GO:0030667 | 0.0268741159830269 | 19/707 | 2/172 | 0.966126653139635 | 1 | F | F | F | F | secretory granule membrane | FABP5 | OLR1 | GO:0099512 | 0.0268741159830269 | 19/707 | 2/172 | 0.966126653139635 | 1 | F | F | F | F | supramolecular fiber | CSNK1D | UPP2 | GO:0099080 | 0.0268741159830269 | 19/707 | 2/172 | 0.966126653139635 | 1 | F | F | F | F | supramolecular complex | CSNK1D | UPP2 | GO:0044451 | 0.0594059405940594 | 42/707 | 6/172 | 0.966243765959103 | 1 | F | F | F | F | nucleoplasm part | ACADM | CYGB | HDAC8 | HINT1 | NAMPT | TKT | GO:0005815 | 0.0282885431400283 | 20/707 | 2/172 | 0.973395774932895 | 1 | F | F | F | F | microtubule organizing center | CSNK1D | PDE4B | GO:0005912 | 0.0297029702970297 | 21/707 | 2/172 | 0.979154046544124 | 1 | F | F | F | F | adherens junction | CAT | S100A7 | GO:0005730 | 0.0396039603960396 | 28/707 | 3/172 | 0.981746229141491 | 1 | F | F | F | F | nucleolus | L3MBTL3 | SLC14A1 | TYMS | GO:0070161 | 0.0325318246110325 | 23/707 | 2/172 | 0.987283918276743 | 1 | F | F | F | F | anchoring junction | CAT | S100A7 | GO:0044427 | 0.0424328147100424 | 30/707 | 3/172 | 0.98838367505159 | 1 | F | F | F | F | chromosomal part | BCL6 | PPARD | TPR | GO:0044430 | 0.0678925035360679 | 48/707 | 6/172 | 0.98888262371703 | 1 | F | F | F | F | cytoskeletal part | ACOT13 | CSNK1D | PDE4B | SEPT3 | TPR | UPP2 | GO:0005575 | 1 | 707/707 | 172/172 | 1 | 1 | F | F | F | F | cellular\_component | AADAT | ABAT | ACACB | ACAD8 | ACADM | ACADS | ACADSB | ACOT13 | ADH1C | ADH5 | ADSL | AHCY | AK1 | ALOX5AP | AOC3 | APLP1 | APRT | AQP5 | ASL | ATAD2 | ATIC | BCAT1 | BCAT2 | BCL6 | BIRC2 | BRD4 | CAD | CAT | CBS | CCL5 | CES1 | CHRNA1 | CHRNA2 | CHRNA3 | CHRNA7 | CNDP2 | COQ9 | CS | CSAD | CSNK1D | CTH | CTSK | CYGB | CYP51A1 | CYR61 | DCPS | DDC | DECR1 | DEFA5 | DUT | FABP5 | FBP1 | FCN2 | FECH | FIGNL1 | GAD1 | GALNT2 | GALT | GBP1 | GGPS1 | GLO1 | GLRA3 | GLRX2 | GLRX5 | GLS | GLTP | GLUD1 | GMDS | GMPR2 | GNPDA1 | GNPNAT1 | GOT1 | GOT2 | GPI | GRIA2 | GSR | GSTA1 | GSTA2 | GSTA3 | GSTA4 | GSTK1 | GSTP1 | GYG1 | HADH | HCN4 | HDAC8 | HDC | HINT1 | HMGCR | HS2ST1 | HSD11B1 | IDH2 | INS | ITK | IVD | IYD | KCNK1 | KCNK10 | KCNK2 | KYAT1 | L3MBTL3 | LRRK2 | LTC4S | MB | ME2 | MELK | MIF | MMAB | MMP9 | NAGA | NAMPT | NAPEPLD | NGB | NMRAL1 | NOS1 | NOS3 | NQO1 | NQO2 | NR0B2 | NUDT5 | OAT | OLR1 | PAH | PANK1 | PANK3 | PAPSS1 | PDE4B | PFKP | PGAM1 | PGD | PGLYRP1 | PKM | PLA2G1B | PLA2G2A | PPARD | PPT1 | PRKG1 | PSAT1 | PTGES | PYGL | PYGM | RHO | RIDA | RNASEL | RRM1 | S100A12 | S100A7 | S100B | SAT1 | SCLY | SEPHS1 | SEPT3 | SET | SGPL1 | SHMT1 | SIRT5 | SLC14A1 | SOD1 | TDO2 | THEM4 | TKT | TLR7 | TLR8 | TPR | TPSAB1 | TPSB2 | TULP1 | TYMS | UMPS | UPP1 | UPP2 | UXS1 | GO:0097057 | 0.00141442715700141 | 1/707 | 1/172 | NA | NA | NA | NA | NA | NA | TRAF2-GSTP1 complex | GSTP1 | GO:0099501 | 0.00424328147100424 | 3/707 | 1/172 | NA | NA | NA | NA | NA | NA | exocytic vesicle membrane | LRRK2 | GO:0055037 | 0.00565770862800566 | 4/707 | 1/172 | NA | NA | NA | NA | NA | NA | recycling endosome | KCNK1 | GO:0097386 | 0.00707213578500707 | 5/707 | 1/172 | NA | NA | NA | NA | NA | NA | glial cell projection | KCNK2 | GO:0099240 | 0.00282885431400283 | 2/707 | 1/172 | NA | NA | NA | NA | NA | NA | intrinsic component of synaptic membrane | CHRNA1 | GO:0042383 | 0.00424328147100424 | 3/707 | 1/172 | NA | NA | NA | NA | NA | NA | sarcolemma | NOS1 | GO:1902937 | 0.00141442715700141 | 1/707 | 1/172 | NA | NA | NA | NA | NA | NA | inward rectifier potassium channel complex | KCNK1 | GO:0030426 | 0.00707213578500707 | 5/707 | 1/172 | NA | NA | NA | NA | NA | NA | growth cone | LRRK2 | GO:0000932 | 0.00424328147100424 | 3/707 | 1/172 | NA | NA | NA | NA | NA | NA | P-body | DCPS | GO:0071944 | 0.00282885431400283 | 2/707 | 1/172 | NA | NA | NA | NA | NA | NA | cell periphery | PANK1 | GO:0030176 | 0.00565770862800566 | 4/707 | 1/172 | NA | NA | NA | NA | NA | NA | integral component of endoplasmic reticulum membrane | SGPL1 | GO:1902560 | 0.00282885431400283 | 2/707 | 1/172 | NA | NA | NA | NA | NA | NA | GMP reductase complex | GMPR2 | GO:0070971 | 0.00424328147100424 | 3/707 | 1/172 | NA | NA | NA | NA | NA | NA | endoplasmic reticulum exit site | LRRK2 | GO:0005875 | 0.00424328147100424 | 3/707 | 1/172 | NA | NA | NA | NA | NA | NA | microtubule associated complex | TPR | GO:0005882 | 0.00565770862800566 | 4/707 | 1/172 | NA | NA | NA | NA | NA | NA | intermediate filament | UPP2 | GO:0060342 | 0.00141442715700141 | 1/707 | 1/172 | NA | NA | NA | NA | NA | NA | photoreceptor inner segment membrane | RHO | GO:0035631 | 0.00141442715700141 | 1/707 | 1/172 | NA | NA | NA | NA | NA | NA | CD40 receptor complex | BIRC2 | GO:0005767 | 0.00282885431400283 | 2/707 | 1/172 | NA | NA | NA | NA | NA | NA | secondary lysosome | LRRK2 | GO:1990909 | 0.00141442715700141 | 1/707 | 1/172 | NA | NA | NA | NA | NA | NA | Wnt signalosome | LRRK2 | GO:0044448 | 0.0099009900990099 | 7/707 | 1/172 | NA | NA | NA | NA | NA | NA | cell cortex part | SEPT3 | GO:0032144 | 0.00141442715700141 | 1/707 | 1/172 | NA | NA | NA | NA | NA | NA | 4-aminobutyrate transaminase complex | ABAT | GO:0071682 | 0.00424328147100424 | 3/707 | 1/172 | NA | NA | NA | NA | NA | NA | endocytic vesicle lumen | PGLYRP1 | GO:0000794 | 0.00424328147100424 | 3/707 | 1/172 | NA | NA | NA | NA | NA | NA | condensed nuclear chromosome | BRD4 | GO:0005640 | 0.00424328147100424 | 3/707 | 1/172 | NA | NA | NA | NA | NA | NA | nuclear outer membrane | LTC4S | GO:0044216 | 0.00424328147100424 | 3/707 | 1/172 | NA | NA | NA | NA | NA | NA | other organism cell | TPR | GO:0044450 | 0.0113154172560113 | 8/707 | 1/172 | NA | NA | NA | NA | NA | NA | microtubule organizing center part | PDE4B | GO:0043657 | 0.00424328147100424 | 3/707 | 1/172 | NA | NA | NA | NA | NA | NA | host cell | TPR | GO:0005604 | 0.00424328147100424 | 3/707 | 1/172 | NA | NA | NA | NA | NA | NA | basement membrane | APLP1 | GO:0032156 | 0.00141442715700141 | 1/707 | 1/172 | NA | NA | NA | NA | NA | NA | septin cytoskeleton | SEPT3 | GO:0031105 | 0.00141442715700141 | 1/707 | 1/172 | NA | NA | NA | NA | NA | NA | septin complex | SEPT3 | GO:0048786 | 0.00141442715700141 | 1/707 | 1/172 | NA | NA | NA | NA | NA | NA | presynaptic active zone | GAD1 | GO:0060077 | 0.00141442715700141 | 1/707 | 1/172 | NA | NA | NA | NA | NA | NA | inhibitory synapse | GAD1 | GO:0044217 | 0.00707213578500707 | 5/707 | 1/172 | NA | NA | NA | NA | NA | NA | other organism part | TPR | GO:0097449 | 0.00141442715700141 | 1/707 | 1/172 | NA | NA | NA | NA | NA | NA | astrocyte projection | KCNK2 | GO:0032009 | 0.00282885431400283 | 2/707 | 1/172 | NA | NA | NA | NA | NA | NA | early phagosome | TLR7 | GO:0030665 | 0.00848656294200849 | 6/707 | 1/172 | NA | NA | NA | NA | NA | NA | clathrin-coated vesicle membrane | GAD1 | GO:0098936 | 0.00282885431400283 | 2/707 | 1/172 | NA | NA | NA | NA | NA | NA | intrinsic component of postsynaptic membrane | CHRNA1 | GO:0042405 | 0.00141442715700141 | 1/707 | 1/172 | NA | NA | NA | NA | NA | NA | nuclear inclusion body | TPR | GO:0005945 | 0.00141442715700141 | 1/707 | 1/172 | NA | NA | NA | NA | NA | NA | 6-phosphofructokinase complex | PFKP | GO:0097013 | 0.00282885431400283 | 2/707 | 1/172 | NA | NA | NA | NA | NA | NA | phagocytic vesicle lumen | PGLYRP1 | GO:0030173 | 0.00141442715700141 | 1/707 | 1/172 | NA | NA | NA | NA | NA | NA | integral component of Golgi membrane | GALNT2 | GO:0099523 | 0.00424328147100424 | 3/707 | 1/172 | NA | NA | NA | NA | NA | NA | presynaptic cytosol | LRRK2 | GO:0070821 | 0.00565770862800566 | 4/707 | 1/172 | NA | NA | NA | NA | NA | NA | tertiary granule membrane | OLR1 | GO:0048770 | 0.0099009900990099 | 7/707 | 1/172 | NA | NA | NA | NA | NA | NA | pigment granule | AHCY | GO:0030662 | 0.0099009900990099 | 7/707 | 1/172 | NA | NA | NA | NA | NA | NA | coated vesicle membrane | GAD1 | GO:0099699 | 0.00282885431400283 | 2/707 | 1/172 | NA | NA | NA | NA | NA | NA | integral component of synaptic membrane | CHRNA1 | GO:0098843 | 0.00141442715700141 | 1/707 | 1/172 | NA | NA | NA | NA | NA | NA | postsynaptic endocytic zone | GRIA2 | GO:0031227 | 0.00707213578500707 | 5/707 | 1/172 | NA | NA | NA | NA | NA | NA | intrinsic component of endoplasmic reticulum membrane | SGPL1 | GO:0099569 | 0.00141442715700141 | 1/707 | 1/172 | NA | NA | NA | NA | NA | NA | presynaptic cytoskeleton | SEPT3 | GO:0097381 | 0.00424328147100424 | 3/707 | 1/172 | NA | NA | NA | NA | NA | NA | photoreceptor disc membrane | RHO | GO:0005667 | 0.0113154172560113 | 8/707 | 1/172 | NA | NA | NA | NA | NA | NA | transcription factor complex | PPARD | GO:0030286 | 0.00141442715700141 | 1/707 | 1/172 | NA | NA | NA | NA | NA | NA | dynein complex | TPR | GO:0008328 | 0.00282885431400283 | 2/707 | 1/172 | NA | NA | NA | NA | NA | NA | ionotropic glutamate receptor complex | GRIA2 | GO:0044798 | 0.0099009900990099 | 7/707 | 1/172 | NA | NA | NA | NA | NA | NA | nuclear transcription factor complex | PPARD | GO:0044754 | 0.00282885431400283 | 2/707 | 1/172 | NA | NA | NA | NA | NA | NA | autolysosome | LRRK2 | GO:0045098 | 0.00141442715700141 | 1/707 | 1/172 | NA | NA | NA | NA | NA | NA | type III intermediate filament | UPP2 | GO:0098978 | 0.0127298444130127 | 9/707 | 1/172 | NA | NA | NA | NA | NA | NA | glutamatergic synapse | LRRK2 | GO:0099522 | 0.00424328147100424 | 3/707 | 1/172 | NA | NA | NA | NA | NA | NA | region of cytosol | LRRK2 | GO:0005793 | 0.00282885431400283 | 2/707 | 1/172 | NA | NA | NA | NA | NA | NA | endoplasmic reticulum-Golgi intermediate compartment | GNPNAT1 | GO:0035580 | 0.0113154172560113 | 8/707 | 1/172 | NA | NA | NA | NA | NA | NA | specific granule lumen | PGLYRP1 | GO:0001726 | 0.00565770862800566 | 4/707 | 1/172 | NA | NA | NA | NA | NA | NA | ruffle | S100B | GO:0005802 | 0.00848656294200849 | 6/707 | 1/172 | NA | NA | NA | NA | NA | NA | trans-Golgi network | LRRK2 | GO:0072686 | 0.00848656294200849 | 6/707 | 1/172 | NA | NA | NA | NA | NA | NA | mitotic spindle | TPR | GO:0005776 | 0.00707213578500707 | 5/707 | 1/172 | NA | NA | NA | NA | NA | NA | autophagosome | LRRK2 | GO:0000803 | 0.00141442715700141 | 1/707 | 1/172 | NA | NA | NA | NA | NA | NA | sex chromosome | BIRC2 | GO:0005874 | 0.0155586987270156 | 11/707 | 1/172 | NA | NA | NA | NA | NA | NA | microtubule | CSNK1D | GO:0036464 | 0.00848656294200849 | 6/707 | 1/172 | NA | NA | NA | NA | NA | NA | cytoplasmic ribonucleoprotein granule | DCPS | GO:0016323 | 0.0127298444130127 | 9/707 | 1/172 | NA | NA | NA | NA | NA | NA | basolateral plasma membrane | SLC14A1 | GO:0005940 | 0.00141442715700141 | 1/707 | 1/172 | NA | NA | NA | NA | NA | NA | septin ring | SEPT3 | GO:0034399 | 0.00141442715700141 | 1/707 | 1/172 | NA | NA | NA | NA | NA | NA | nuclear periphery | TPR | GO:0000785 | 0.0226308345120226 | 16/707 | 1/172 | NA | NA | NA | NA | NA | NA | chromatin | PPARD | GO:0000930 | 0.00282885431400283 | 2/707 | 1/172 | NA | NA | NA | NA | NA | NA | gamma-tubulin complex | PDE4B | GO:0000790 | 0.0127298444130127 | 9/707 | 1/172 | NA | NA | NA | NA | NA | NA | nuclear chromatin | PPARD | GO:0031256 | 0.0127298444130127 | 9/707 | 1/172 | NA | NA | NA | NA | NA | NA | leading edge membrane | THEM4 | GO:0098855 | 0.00141442715700141 | 1/707 | 1/172 | NA | NA | NA | NA | NA | NA | HCN channel complex | HCN4 | GO:0005798 | 0.00424328147100424 | 3/707 | 1/172 | NA | NA | NA | NA | NA | NA | Golgi-associated vesicle | LRRK2 | GO:0005868 | 0.00141442715700141 | 1/707 | 1/172 | NA | NA | NA | NA | NA | NA | cytoplasmic dynein complex | TPR | GO:0031526 | 0.00282885431400283 | 2/707 | 1/172 | NA | NA | NA | NA | NA | NA | brush border membrane | KCNK1 | GO:0005770 | 0.0113154172560113 | 8/707 | 1/172 | NA | NA | NA | NA | NA | NA | late endosome | GNPNAT1 | GO:0005643 | 0.00424328147100424 | 3/707 | 1/172 | NA | NA | NA | NA | NA | NA | nuclear pore | TPR | GO:0015630 | 0.00707213578500707 | 5/707 | 1/172 | NA | NA | NA | NA | NA | NA | microtubule cytoskeleton | SEPT3 | GO:0098948 | 0.00141442715700141 | 1/707 | 1/172 | NA | NA | NA | NA | NA | NA | intrinsic component of postsynaptic specialization membrane | CHRNA1 | GO:0005971 | 0.00282885431400283 | 2/707 | 1/172 | NA | NA | NA | NA | NA | NA | ribonucleoside-diphosphate reductase complex | RRM1 | GO:0035579 | 0.00848656294200849 | 6/707 | 1/172 | NA | NA | NA | NA | NA | NA | specific granule membrane | OLR1 | GO:0005811 | 0.00848656294200849 | 6/707 | 1/172 | NA | NA | NA | NA | NA | NA | lipid droplet | SET | GO:0044615 | 0.00141442715700141 | 1/707 | 1/172 | NA | NA | NA | NA | NA | NA | nuclear pore nuclear basket | TPR | GO:0012505 | 0.00141442715700141 | 1/707 | 1/172 | NA | NA | NA | NA | NA | NA | endomembrane system | ACACB | GO:0045335 | 0.00282885431400283 | 2/707 | 1/172 | NA | NA | NA | NA | NA | NA | phagocytic vesicle | TLR7 | GO:0005657 | 0.00141442715700141 | 1/707 | 1/172 | NA | NA | NA | NA | NA | NA | replication fork | BCL6 | GO:1902912 | 0.00141442715700141 | 1/707 | 1/172 | NA | NA | NA | NA | NA | NA | pyruvate kinase complex | PKM | GO:0031594 | 0.00282885431400283 | 2/707 | 1/172 | NA | NA | NA | NA | NA | NA | neuromuscular junction | CHRNA1 | GO:0090575 | 0.0099009900990099 | 7/707 | 1/172 | NA | NA | NA | NA | NA | NA | RNA polymerase II transcription factor complex | PPARD | GO:0005876 | 0.00424328147100424 | 3/707 | 1/172 | NA | NA | NA | NA | NA | NA | spindle microtubule | CSNK1D | GO:0015629 | 0.00707213578500707 | 5/707 | 1/172 | NA | NA | NA | NA | NA | NA | actin cytoskeleton | GBP1 | GO:0031228 | 0.00141442715700141 | 1/707 | 1/172 | NA | NA | NA | NA | NA | NA | intrinsic component of Golgi membrane | GALNT2 | GO:0016935 | 0.00141442715700141 | 1/707 | 1/172 | NA | NA | NA | NA | NA | NA | glycine-gated chloride channel complex | GLRA3 | GO:0032473 | 0.00282885431400283 | 2/707 | 1/172 | NA | NA | NA | NA | NA | NA | cytoplasmic side of mitochondrial outer membrane | LRRK2 | GO:0035770 | 0.00848656294200849 | 6/707 | 1/172 | NA | NA | NA | NA | NA | NA | ribonucleoprotein granule | DCPS | GO:0097487 | 0.00141442715700141 | 1/707 | 1/172 | NA | NA | NA | NA | NA | NA | multivesicular body, internal vesicle | LRRK2 | GO:0034707 | 0.00282885431400283 | 2/707 | 1/172 | NA | NA | NA | NA | NA | NA | chloride channel complex | GLRA3 | GO:0044753 | 0.00141442715700141 | 1/707 | 1/172 | NA | NA | NA | NA | NA | NA | amphisome | LRRK2 | GO:0030660 | 0.00282885431400283 | 2/707 | 1/172 | NA | NA | NA | NA | NA | NA | Golgi-associated vesicle membrane | RHO | GO:0005641 | 0.00424328147100424 | 3/707 | 1/172 | NA | NA | NA | NA | NA | NA | nuclear envelope lumen | PTGES | GO:0098878 | 0.00282885431400283 | 2/707 | 1/172 | NA | NA | NA | NA | NA | NA | neurotransmitter receptor complex | GRIA2 | GO:0030658 | 0.0099009900990099 | 7/707 | 1/172 | NA | NA | NA | NA | NA | NA | transport vesicle membrane | LRRK2 | GO:1904115 | 0.00565770862800566 | 4/707 | 1/172 | NA | NA | NA | NA | NA | NA | axon cytoplasm | SOD1 | GO:1990204 | 0.00707213578500707 | 5/707 | 1/172 | NA | NA | NA | NA | NA | NA | oxidoreductase complex | RRM1 | GO:0099055 | 0.00282885431400283 | 2/707 | 1/172 | NA | NA | NA | NA | NA | NA | integral component of postsynaptic membrane | CHRNA1 | GO:0030427 | 0.00707213578500707 | 5/707 | 1/172 | NA | NA | NA | NA | NA | NA | site of polarized growth | LRRK2 | GO:0030672 | 0.00424328147100424 | 3/707 | 1/172 | NA | NA | NA | NA | NA | NA | synaptic vesicle membrane | LRRK2 | GO:0044454 | 0.0212164073550212 | 15/707 | 1/172 | NA | NA | NA | NA | NA | NA | nuclear chromosome part | PPARD | GO:1990425 | 0.00141442715700141 | 1/707 | 1/172 | NA | NA | NA | NA | NA | NA | ryanodine receptor complex | NOS1 | GO:0000776 | 0.00424328147100424 | 3/707 | 1/172 | NA | NA | NA | NA | NA | NA | kinetochore | TPR | GO:0061202 | 0.00141442715700141 | 1/707 | 1/172 | NA | NA | NA | NA | NA | NA | clathrin-sculpted gamma-aminobutyric acid transport vesicle membrane | GAD1 | GO:0000793 | 0.00707213578500707 | 5/707 | 1/172 | NA | NA | NA | NA | NA | NA | condensed chromosome | BRD4 | GO:0005581 | 0.00848656294200849 | 6/707 | 1/172 | NA | NA | NA | NA | NA | NA | collagen trimer | FCN2 | GO:0099060 | 0.00141442715700141 | 1/707 | 1/172 | NA | NA | NA | NA | NA | NA | integral component of postsynaptic specialization membrane | CHRNA1 | GO:0005623 | 0.00424328147100424 | 3/707 | 1/172 | NA | NA | NA | NA | NA | NA | cell | CCL5 | GO:0009898 | 0.00707213578500707 | 5/707 | 1/172 | NA | NA | NA | NA | NA | NA | cytoplasmic side of plasma membrane | BIRC2 | GO:0044455 | 0.00707213578500707 | 5/707 | 1/172 | NA | NA | NA | NA | NA | NA | mitochondrial membrane part | LRRK2 | GO:0036021 | 0.00141442715700141 | 1/707 | 1/172 | NA | NA | NA | NA | NA | NA | endolysosome lumen | CTSK | GO:0032281 | 0.00141442715700141 | 1/707 | 1/172 | NA | NA | NA | NA | NA | NA | AMPA glutamate receptor complex | GRIA2 | GO:0032587 | 0.00848656294200849 | 6/707 | 1/172 | NA | NA | NA | NA | NA | NA | ruffle membrane | THEM4 | GO:0019898 | 0.0183875530410184 | 13/707 | 1/172 | NA | NA | NA | NA | NA | NA | extrinsic component of membrane | TPR | GO:0030139 | 0.0099009900990099 | 7/707 | 1/172 | NA | NA | NA | NA | NA | NA | endocytic vesicle | TLR7 | GO:0031901 | 0.00707213578500707 | 5/707 | 1/172 | NA | NA | NA | NA | NA | NA | early endosome membrane | NAPEPLD | GO:0005795 | 0.00282885431400283 | 2/707 | 1/172 | NA | NA | NA | NA | NA | NA | Golgi stack | GALNT2 | GO:0042470 | 0.0099009900990099 | 7/707 | 1/172 | NA | NA | NA | NA | NA | NA | melanosome | AHCY | GO:0016529 | 0.00848656294200849 | 6/707 | 1/172 | NA | NA | NA | NA | NA | NA | sarcoplasmic reticulum | NOS1 | GO:0001741 | 0.00141442715700141 | 1/707 | 1/172 | NA | NA | NA | NA | NA | NA | XY body | BIRC2 | GO:0009925 | 0.00282885431400283 | 2/707 | 1/172 | NA | NA | NA | NA | NA | NA | basal plasma membrane | AQP5 | GO:0072562 | 0.0127298444130127 | 9/707 | 1/172 | NA | NA | NA | NA | NA | NA | blood microparticle | FCN2 | GO:0031045 | 0.00141442715700141 | 1/707 | 1/172 | NA | NA | NA | NA | NA | NA | dense core granule | SOD1 | GO:0099400 | 0.00141442715700141 | 1/707 | 1/172 | NA | NA | NA | NA | NA | NA | caveola neck | LRRK2 |

Total number of genes: 707   
Total number of Study genes: 172   
Total number of Study gene GMRG terms (pop non-singletons): 345 (305)   
FDR Threshold *P*-values: [10% = 0.00711], [5% = 0.00166], [1% = 0.00019], [0.5% = NA]   
Genes with GMRG information: 172   
Genes with no GMRG information:   

These are:
