# Supplementary material for: Ligand Binding Site Structure Shapes Folding, Assembly and Degradation of Homomeric Protein Complexes
Source: J Mol Biol. 2019 Sep 6;431(19):3871–88. doi: 10.1016/j.jmb.2019.07.014 (PMC6739599; doi:10.1016/j.jmb.2019.07.014)
Supplement: Supplementary file 3 — Supplementary material [file mmc3.zip › GeneOntology.tar.bz2!/GO/GO_output.P.interactome.txt.html]

 GeneMerge Output - output.P.txt

### GeneMerge v1.4

### Castillo-Davis, C.I. 2015. GeneMerge v1.4 - post-genomic data analysis

Output File Name: output.P.txt   
Gene Association File: terms.txt   
Description File: descriptions.txt   
Population File: genes\_all.txt   
Study File: genes\_multichain.txt  
Custom FDR: 0.5%  

|  |  |  |  |  |  |  |  |  |  |  |  |  |  |  |  |  |  |  |  |  |  |  |  |  |  |  |  |  |  |  |  |  |  |  |  |  |  |  |  |  |  |  |  |  |  |  |  |  |  |  |  |  |  |  |  |  |  |  |  |  |  |  |  |  |  |  |  |  |  |  |  |  |  |  |  |  |  |  |  |  |  |  |  |  |  |  |  |  |  |  |  |  |  |  |  |  |  |  |  |  |  |  |  |  |  |  |  |  |  |  |  |  |  |  |  |  |  |  |  |  |  |  |  |  |  |  |  |  |  |  |  |  |  |  |  |  |  |  |  |  |  |  |  |  |  |  |  |  |  |  |  |  |  |  |  |  |  |  |  |  |  |  |  |  |  |  |  |  |  |  |  |  |  |  |  |  |  |  |  |  |  |  |  |  |  |  |  |  |  |  |  |  |  |  |  |  |  |  |  |  |  |  |  |  |  |  |  |  |  |  |  |  |  |  |  |  |  |  |  |  |  |  |  |  |  |  |  |  |  |  |  |  |  |  |  |  |  |  |  |  |  |  |  |  |  |  |  |  |  |  |  |  |  |  |  |  |  |  |  |  |  |  |  |  |  |  |  |  |  |  |  |  |  |  |  |  |  |  |  |  |  |  |  |  |  |  |  |  |  |  |  |  |  |  |  |  |  |  |  |  |  |  |  |  |  |  |  |  |  |  |  |  |  |  |  |  |  |  |  |  |  |  |  |  |  |  |  |  |  |  |  |  |  |  |  |  |  |  |  |  |  |  |  |  |  |  |  |  |  |  |  |  |  |  |  |  |  |  |  |  |  |  |  |  |  |  |  |  |  |  |  |  |  |  |  |  |  |  |  |  |  |  |  |  |  |  |  |  |  |  |  |  |  |  |  |  |  |  |  |  |  |  |  |  |  |  |  |  |  |  |  |  |  |  |  |  |  |  |  |  |  |  |  |  |  |  |  |  |  |  |  |  |  |  |  |  |  |  |  |  |  |  |  |  |  |  |  |  |  |  |  |  |  |  |  |  |  |  |  |  |  |  |  |  |  |  |  |  |  |  |  |  |  |  |  |  |  |  |  |  |  |  |  |  |  |
| --- | --- | --- | --- | --- | --- | --- | --- | --- | --- | --- | --- | --- | --- | --- | --- | --- | --- | --- | --- | --- | --- | --- | --- | --- | --- | --- | --- | --- | --- | --- | --- | --- | --- | --- | --- | --- | --- | --- | --- | --- | --- | --- | --- | --- | --- | --- | --- | --- | --- | --- | --- | --- | --- | --- | --- | --- | --- | --- | --- | --- | --- | --- | --- | --- | --- | --- | --- | --- | --- | --- | --- | --- | --- | --- | --- | --- | --- | --- | --- | --- | --- | --- | --- | --- | --- | --- | --- | --- | --- | --- | --- | --- | --- | --- | --- | --- | --- | --- | --- | --- | --- | --- | --- | --- | --- | --- | --- | --- | --- | --- | --- | --- | --- | --- | --- | --- | --- | --- | --- | --- | --- | --- | --- | --- | --- | --- | --- | --- | --- | --- | --- | --- | --- | --- | --- | --- | --- | --- | --- | --- | --- | --- | --- | --- | --- | --- | --- | --- | --- | --- | --- | --- | --- | --- | --- | --- | --- | --- | --- | --- | --- | --- | --- | --- | --- | --- | --- | --- | --- | --- | --- | --- | --- | --- | --- | --- | --- | --- | --- | --- | --- | --- | --- | --- | --- | --- | --- | --- | --- | --- | --- | --- | --- | --- | --- | --- | --- | --- | --- | --- | --- | --- | --- | --- | --- | --- | --- | --- | --- | --- | --- | --- | --- | --- | --- | --- | --- | --- | --- | --- | --- | --- | --- | --- | --- | --- | --- | --- | --- | --- | --- | --- | --- | --- | --- | --- | --- | --- | --- | --- | --- | --- | --- | --- | --- | --- | --- | --- | --- | --- | --- | --- | --- | --- | --- | --- | --- | --- | --- | --- | --- | --- | --- | --- | --- | --- | --- | --- | --- | --- | --- | --- | --- | --- | --- | --- | --- | --- | --- | --- | --- | --- | --- | --- | --- | --- | --- | --- | --- | --- | --- | --- | --- | --- | --- | --- | --- | --- | --- | --- | --- | --- | --- | --- | --- | --- | --- | --- | --- | --- | --- | --- | --- | --- | --- | --- | --- | --- | --- | --- | --- | --- | --- | --- | --- | --- | --- | --- | --- | --- | --- | --- | --- | --- | --- | --- | --- | --- | --- | --- | --- | --- | --- | --- | --- | --- | --- | --- | --- | --- | --- | --- | --- | --- | --- | --- | --- | --- | --- | --- | --- | --- | --- | --- | --- | --- | --- | --- | --- | --- | --- | --- | --- | --- | --- | --- | --- | --- | --- | --- | --- | --- | --- | --- | --- | --- | --- | --- | --- | --- | --- | --- | --- | --- | --- | --- | --- | --- | --- | --- | --- | --- | --- | --- | --- | --- | --- | --- | --- | --- | --- | --- | --- | --- | --- | --- | --- | --- | --- | --- | --- | --- | --- | --- | --- | --- | --- | --- | --- | --- | --- | --- | --- | --- | --- | --- | --- | --- | --- | --- | --- | --- | --- | --- | --- | --- | --- | --- | --- | --- | --- | --- | --- | --- | --- | --- | --- | --- | --- | --- | --- | --- | --- | --- | --- | --- | --- | --- | --- | --- | --- | --- | --- | --- | --- | --- | --- | --- | --- | --- | --- | --- | --- | --- | --- |
| **GMRG Term** | **Pop Frequency** | **Pop Fraction** | **Study Fraction** | ***P*-value** | **Bon. Corr. *P*-value** | **10% FDR** | **5% FDR** | **1% FDR** | **0.5% FDR** | **Description** | **Contributing genes** |  |  |  |  |  |  |  |  |  |  |  |  |  |  |  |  |  |  |  |  |  |  |  |  |  |  |  |  |  |  |  |  |  |  |  |  |  |  |  |  |  |  |  |  |  |  |  |  |  |  |  |  |  |  |  |  |  |  |  |  |  |  |  |  |  |  |  |  |  |  |  |  |  |  |  |  |  |  |  |  |  |  |  |  |  |  |  |  |  |  |  |  |  |  |  |  |  |  |  |  |  |  |  |  |  |  |  |  |  |  |  |  |  |  |  |  |  |  |  |  |  |  |  |  |  |  |  |  |  |  |  |  |  |  |  |  |  |  |  |  |  |  |  |  |  |  |  |  |  |  |  |  |  |  |  |  |  |  |  |  |  |  |  |  |  |  |  |  |  |  |  |  |  |  |  |  |  |  |  |  |  |  |  |  |  |  |  |  |  |  |  |  |  |  |  |  |  |  |  |  |  |  |  |  |  |  |  |  |  |  |  |  |  |  |  |  |  |  |  |  |  |  |  |  |  |  |  |  |  |  |  |  |  |  |  |  |  |  |  |  |  |  |  |  |  |  |  |  |  |  |  |  |  |  |  |  |  |  |  |  |  |  |  |  |  |  |  |  |  |  |  |  |  |  |  |  |  |  |  |  |  |  |  |  |  |  |  |  |  |  |  |  |  |  |  |  |  |  |  |  |  |  |  |  |  |  |  |  |  |  |  |  |  |  |  |  |  |  |  |  |  |  |  |  |  |  |  |  |  |  |  |  |  |  |  |  |  |  |  |  |  |  |  |  |  |  |  |  |  |  |  |  |  |  |  |  |  |  |  |  |  |  |  |  |  |  |  |  |  |  |  |  |  |  |  |  |  |  |  |  |  |  |  |  |  |  |  |  |  |  |  |  |  |  |  |  |  |  |  |  |  |  |  |  |  |  |  |  |  |  |  |  |  |  |  |  |  |  |  |  |  |  |  |  |  |  |  |  |  |  |  |  |  |  |  |  |  |  |  |  |  |  |  |  |  |  |  |  |  |  |  |  |  |  |  |  |  |  |  |  |  |  |  |  |  |  |  |  |  |  |  |  |  |  |
| GO:0044281 | 0.106029358243358 | 874/8243 | 475/3250 | 2.8237471937139e-21 | 2.68114796043135e-17 | T | T | T | T | small molecule metabolic process | AARS | AARSD1 | AASDH | AASDHPPT | AASS | ABAT | ABCD3 | ABCG1 | ABHD14B | ACAA1 | ACAA2 | ACACA | ACAD8 | ACAD9 | ACADS | ACADSB | ACADVL | ACAT1 | ACAT2 | ACCS | ACLY | ACO1 | ACO2 | ACOT13 | ACOT7 | ACOT9 | ACOX1 | ACSL1 | ACSL4 | ACSL5 | ACSS2 | ACY1 | ADA | ADCYAP1 | ADH1A | ADH1B | ADH5 | ADK | ADSS | ADTRP | AHCY | AHCYL1 | AICDA | AIMP2 | AK1 | AK2 | AK4 | AKR1A1 | AKR1B1 | AKR1B10 | AKR1C2 | AKT1 | AKT2 | ALDH1B1 | ALDH4A1 | ALDH5A1 | ALDH6A1 | ALDH7A1 | ALDH8A1 | ALDOA | ALDOC | ALOX12 | ALOX5 | ALOX5AP | AMD1 | AMDHD2 | APOA1 | APOD | APOE | APOL2 | APP | APRT | ASL | ASNS | ASPDH | ASS1 | ATIC | ATP6V1A | ATP6V1B1 | BCAT2 | BCKDHB | BDH1 | BRCA1 | BSG | CALM1 | CARS | CAT | CBR1 | CBR4 | CBS | CD36 | CD74 | CDA | CEBPA | CEL | CES1 | CFTR | CLPX | CMPK1 | COQ2 | COQ3 | COQ4 | COQ5 | COQ6 | COQ7 | COQ9 | CPT1A | CPT2 | CROT | CRY1 | CTH | CTPS1 | CYB5R3 | CYP1A1 | CYP1A2 | CYP2C18 | DARS | DBH | DBI | DBT | DCK | DCTPP1 | DCXR | DECR1 | DEGS1 | DERA | DGUOK | DHCR24 | DHRS4 | DLAT | DLD | DLG2 | DLST | DPEP1 | DPM1 | DUT | ECH1 | ECHDC2 | ECHS1 | ECI1 | ECI2 | EGLN1 | EGLN3 | EHHADH | ENO1 | ENO2 | ENO3 | ENOPH1 | ENTPD7 | EPRS | ERLIN2 | ESD | ETFA | ETFB | ETFDH | EXTL2 | FABP3 | FABP5 | FAH | FAR2 | FARSB | FASN | FBP1 | FBP2 | FDPS | FDXR | FGF2 | FGFR1 | FH | FIGNL1 | FOLH1 | FOLR1 | FPGS | G6PD | GAA | GAD2 | GALE | GAPDH | GAPDHS | GARS | GART | GCDH | GDA | GDPGP1 | GFPT1 | GFPT2 | GGPS1 | GGT1 | GLO1 | GLRX | GLRX5 | GLS | GLS2 | GLUD2 | GLUL | GMDS | GMPR | GMPS | GNAI3 | GNPDA1 | GNPDA2 | GOT1 | GOT1L1 | GOT2 | GPD1 | GPD1L | GPI | GPT2 | GPX1 | GPX4 | GRHPR | GSR | GSS | GSTA1 | GSTM4 | GSTO1 | GSTO2 | GSTP1 | GSTZ1 | HAAO | HADH | HAGH | HARS | HIBADH | HIBCH | HIF1A | HINT1 | HLCS | HMGCL | HNF4A | HPRT1 | HSD17B10 | HSD17B4 | HSD17B8 | HSD3B7 | HSPA1B | HSPA8 | IARS | IDH1 | IDH2 | IDH3B | IDH3G | IDI2 | IMPA1 | IMPA2 | IMPDH2 | INSIG1 | INSIG2 | IP6K1 | IREB2 | ISYNA1 | ITPA | IVD | KCNAB2 | KDM1A | KDSR | KYNU | L2HGDH | LARS | LDHA | LDHAL6A | LDHAL6B | LDHB | LDHC | LDHD | LGMN | LIMA1 | LPIN3 | LRRK2 | LTC4S | LYPLA1 | LYPLA2 | MAN2B1 | MAPK1 | MAPK14 | MAPK3 | MAT2A | MAT2B | MCAT | MCCC1 | MDH1 | MDH2 | ME1 | MECP2 | MGST1 | MIF | MMAB | MME | MPI | MPST | MTAP | MTHFD1 | MTMR7 | MTRR | MVD | MYH6 | MYO5A | NAGK | NANP | NDUFA9 | NDUFS1 | NDUFS6 | NFS1 | NIT2 | NME1 | NME2 | NME4 | NMNAT1 | NMNAT3 | NMRK2 | NNT | NOS2 | NOS3 | NR0B2 | NR5A2 | NT5C2 | NUDT13 | NUDT18 | NUDT19 | NUDT3 | NUDT9 | OAT | OGDH | OLA1 | ONECUT1 | OPA1 | OSBPL1A | OTC | OXCT1 | P4HA1 | P4HA2 | P4HB | PADI4 | PAH | PARK7 | PARP1 | PC | PCBD1 | PCSK9 | PDE4D | PDHA1 | PDHA2 | PDHB | PDHX | PDK2 | PDSS1 | PDSS2 | PDXK | PEPD | PER2 | PFAS | PFKL | PFKM | PFKP | PGAM1 | PGAM2 | PGD | PGK1 | PGK2 | PGM1 | PGM2 | PGM5 | PGP | PHGDH | PIK3CA | PLA2G10 | PLA2G1B | PLCG1 | PLOD2 | PNKP | PNP | PPA1 | PPA2 | PPARD | PPARG | PPARGC1A | PPAT | PPM1K | PRDX4 | PRKAA1 | PRKAA2 | PRKAB2 | PRKAG1 | PRKAR2B | PRTFDC1 | PSAT1 | PTEN | PTGDS | PTGES | PTGES2 | PTGES3 | PTGR1 | PYCR1 | PYCR2 | QARS | QDPR | RAN | RARS2 | RBP1 | RDH11 | RDH12 | RDH13 | RDH14 | RDH5 | REXO2 | RFK | RGN | RHOA | RNASEH2B | RPE | RPIA | RRM1 | RRM2 | RRM2B | RXRA | SARS | SCLY | SCO2 | SDHA | SDHB | SEPHS1 | SEPHS2 | SFXN1 | SGPL1 | SHMT1 | SHMT2 | SLC16A1 | SLC25A1 | SLC25A10 | SLC25A11 | SLC25A13 | SLC25A32 | SLC2A9 | SNCA | SNX17 | SOAT1 | SORD | SPR | SPTLC2 | ST3GAL1 | STAT5A | SUCLA2 | SUCLG2 | SULT1A3 | TALDO1 | TARS | TARS2 | TDO2 | TECR | THEM4 | TK1 | TK2 | TKT | TNF | TNFRSF1A | TPI1 | TPST2 | TXN | TXN2 | TXNRD1 | TYMS | UBIAD1 | UCK2 | UGGT1 | UGP2 | UMPS | UPP2 | VARS | VCP | VDAC1 | VDR | VKORC1 | WARS | WDTC1 | XBP1 |
 GO:0044237 | 0.538274899915079 | 4437/8243 | 1956/3250 | 4.89092986819296e-21 | 4.64393790984921e-17 | T | T | T | T | cellular metabolic process | AARS | AARSD1 | AASDH | AASDHPPT | AASS | ABAT | ABCD3 | ABCE1 | ABHD14B | ABHD17A | ABL1 | ABL2 | ACAA1 | ACAA2 | ACACA | ACAD8 | ACAD9 | ACADS | ACADSB | ACADVL | ACAT1 | ACAT2 | ACCS | ACD | ACE2 | ACLY | ACO1 | ACO2 | ACOT13 | ACOT7 | ACOT9 | ACOX1 | ACP1 | ACSL1 | ACSL4 | ACSL5 | ACSS2 | ACTB | ACTL6A | ACTN2 | ACY1 | ADA | ADCK2 | ADCY6 | ADCYAP1 | ADH1A | ADH1B | ADH5 | ADK | ADORA2A | ADPRH | ADRB2 | ADRM1 | ADSL | ADSS | ADTRP | AFF4 | AFG3L2 | AGK | AGL | AGO1 | AGO2 | AHCY | AHCYL1 | AHSG | AICDA | AIMP2 | AK1 | AK2 | AK4 | AKR1A1 | AKR1B1 | AKR1B10 | AKR1B15 | AKR1C2 | AKT1 | AKT2 | AKT3 | ALAS1 | ALB | ALDH1B1 | ALDH4A1 | ALDH5A1 | ALDH6A1 | ALDH7A1 | ALDH8A1 | ALDH9A1 | ALDOA | ALDOC | ALOX12 | ALOX5 | ALOX5AP | AMD1 | AMDHD2 | AMFR | ANAPC4 | ANXA1 | ANXA7 | AOC2 | APBB1 | APC | APCS | APEH | APEX1 | APEX2 | APOA1 | APOE | APP | APRT | AR | ARAF | ARF1 | ARF4 | ARHGAP35 | ARID4B | ARMC8 | ARNT | ARNT2 | ARRB1 | ARRB2 | ASB12 | ASB15 | ASB16 | ASB6 | ASB9 | ASCC1 | ASCC2 | ASGR1 | ASGR2 | ASL | ASNS | ASPDH | ASS1 | ATAD5 | ATCAY | ATF2 | ATF6B | ATG2B | ATG4C | ATG5 | ATIC | ATM | ATOH1 | ATP13A2 | ATP1A1 | ATP6V1A | ATP6V1B1 | ATRX | ATXN1 | ATXN2 | AUP1 | AURKB | B3GAT3 | B3GNT3 | B4GALT3 | BABAM1 | BACE1 | BAG3 | BAG6 | BANF1 | BAX | BCAT2 | BCKDHB | BCL10 | BCL11A | BCL2 | BCOR | BDH1 | BIRC2 | BIRC3 | BIRC5 | BIRC7 | BIRC8 | BLVRB | BMI1 | BMPR1A | BNIPL | BRAF | BRCA1 | BRD4 | BSG | BTBD9 | BTK | BTRC | BUD13 | BUD31 | BYSL | C1QBP | C4A | C9orf64 | CAB39 | CACNG2 | CACNG4 | CACNG5 | CALCOCO2 | CALM1 | CALU | CAMK1D | CAMK2A | CAMK2B | CAMKV | CAND1 | CAPN1 | CARM1 | CARS | CASP3 | CAT | CAV1 | CBFB | CBL | CBLB | CBLC | CBR1 | CBR4 | CBS | CBX4 | CBX8 | CCAR2 | CCDC155 | CCDC22 | CCDC36 | CCDC8 | CCDC88A | CCNB1 | CCNT1 | CCNT2 | CCP110 | CCR5 | CCS | CD2BP2 | CD36 | CD40LG | CD74 | CD81 | CDA | CDC14B | CDC16 | CDC20 | CDC25A | CDC25B | CDC25C | CDC27 | CDC34 | CDC42 | CDC5L | CDC73 | CDK15 | CDK16 | CDK19 | CDK2 | CDK4 | CDK5 | CDK5R1 | CDK8 | CDK9 | CDKL3 | CDKN1A | CEBPA | CEL | CENPF | CES1 | CFL1 | CFTR | CHAF1A | CHCHD10 | CHD1L | CHDH | CHEK2 | CHGB | CHMP2A | CHRAC1 | CHRM3 | CHST4 | CILP2 | CISD2 | CKAP4 | CKB | CKM | CKMT1A | CLK1 | CLPX | CLTC | CMBL | CMPK1 | CNDP2 | COG3 | COL2A1 | COL4A2 | COL4A3BP | COMMD1 | COMT | COPS2 | COPS5 | COPS6 | COQ2 | COQ3 | COQ4 | COQ5 | COQ6 | COQ7 | COQ9 | COX11 | COX15 | COX17 | COX20 | COX5A | COX6A1 | COX6C | COX7A2 | CPNE3 | CPSF7 | CPT1A | CPT2 | CRBN | CREB1 | CREBBP | CRKL | CRMP1 | CROT | CRY1 | CRY2 | CRYAB | CRYZ | CSNK1A1 | CSNK1E | CSNK2A2 | CSNK2B | CSTF2T | CT45A5 | CTBP1 | CTCF | CTDP1 | CTH | CTNNB1 | CTPS1 | CTSA | CUL1 | CUL2 | CUL3 | CUL4B | CUL5 | CUL7 | CWC15 | CXCL8 | CYB5B | CYB5R3 | CYC1 | CYCS | CYLD | CYP1A1 | CYP1A2 | CYP2C18 | DAD1 | DAP3 | DAPK1 | DAPP1 | DARS | DBH | DBI | DBT | DCAF12 | DCAF7 | DCAF8 | DCAKD | DCK | DCLK1 | DCN | DCP2 | DCTPP1 | DCUN1D1 | DCXR | DDA1 | DDB1 | DDB2 | DDIT4 | DDOST | DDRGK1 | DDX1 | DDX39A | DDX39B | DDX3X | DDX6 | DECR1 | DEGS1 | DERA | DERL1 | DGKE | DGKZ | DGUOK | DHRS4 | DHRS7B | DHX15 | DHX36 | DHX9 | DIDO1 | DIS3 | DLAT | DLD | DLG2 | DLG3 | DLG4 | DLST | DMAP1 | DMD | DNA2 | DNAJA3 | DNAJB2 | DNAJC10 | DNAJC30 | DNASE2 | DNASE2B | DNPEP | DPEP1 | DPF2 | DPM1 | DRG2 | DROSHA | DSE | DTX1 | DTX2 | DUS3L | DUSP1 | DUSP12 | DUSP13 | DUSP14 | DUSP16 | DUSP19 | DUSP22 | DUSP7 | DUT | DVL1 | DVL2 | DYNLL1 | DYRK2 | DZIP3 | E2F1 | E2F2 | E2F3 | EAF2 | ECD | ECH1 | ECHDC2 | ECHS1 | ECI1 | ECI2 | EDC4 | EDEM1 | EED | EEF1A1 | EEF1A1P5 | EEF1A2 | EEF1B2 | EEF1D | EEF1G | EEF2 | EEF2KMT | EFEMP1 | EFTUD2 | EGFR | EGLN1 | EGLN3 | EHHADH | EHMT1 | EHMT2 | EIF1AX | EIF2AK3 | EIF2AK4 | EIF2B1 | EIF2B3 | EIF2B5 | EIF2S1 | EIF2S2 | EIF3A | EIF3D | EIF3E | EIF3H | EIF3I | EIF4A3 | EIF4E | EIF4E2 | EIF4EBP1 | EIF4G3 | EIF4H | EIF5 | EIF5A | EIF5B | EIF6 | ELAC2 | ELAVL1 | ELAVL4 | ELF3 | ENDOG | ENO1 | ENO2 | ENO3 | ENOPH1 | ENTPD7 | EP300 | EPHA4 | EPHA6 | EPHB6 | EPRS | ERAL1 | ERBB2 | ERBB3 | ERCC5 | ERG | ERLIN2 | ESD | ESR1 | ESR2 | ESRRA | ESRRB | ESRRG | ETFA | ETFB | ETFDH | ETS1 | EVA1A | EXOSC1 | EXOSC5 | EXOSC6 | EXOSC9 | EXTL2 | EYA2 | EYA4 | EZH2 | EZR | F5 | FABP1 | FABP3 | FABP5 | FABP7 | FAF1 | FAF2 | FAH | FAM20B | FAM98A | FANCA | FANCC | FANCG | FANCI | FANCM | FAR2 | FARSB | FASN | FBP1 | FBP2 | FBXL12 | FBXO11 | FBXO25 | FBXO32 | FBXO6 | FBXO7 | FBXW11 | FBXW5 | FDPS | FDXR | FECH | FEN1 | FGF2 | FGFR1 | FGFR3 | FH | FIGNL1 | FIS1 | FKBP14 | FKBP1A | FKBP1B | FKBP2 | FKBP5 | FKBP7 | FKBP8 | FN1 | FOLH1 | FOLR1 | FOS | FOXK1 | FOXK2 | FOXM1 | FOXO1 | FPGS | FTO | FTSJ3 | FUBP1 | FUBP3 | FUNDC2 | FUS | FYN | FZR1 | G3BP1 | G6PD | GAA | GAD2 | GAK | GALNS | GALNT7 | GANAB | GAPDH | GAPDHS | GAR1 | GARS | GART | GATA1 | GBE1 | GCDH | GDA | GEMIN4 | GEMIN5 | GEMIN7 | GFPT1 | GFPT2 | GGA1 | GGCT | GGPS1 | GGT1 | GGTLC1 | GINS2 | GINS3 | GINS4 | GLA | GLCE | GLO1 | GLRX | GLRX3 | GLRX5 | GLS | GLS2 | GLUD2 | GLUL | GMDS | GMPPB | GMPR | GMPS | GNAI3 | GNAS | GNPDA1 | GNPDA2 | GNPNAT1 | GNS | GOLGA2 | GOLPH3 | GOT1 | GOT1L1 | GOT2 | GPC5 | GPD1 | GPD1L | GPI | GPS2 | GPT2 | GPX1 | GPX4 | GRB2 | GRHPR | GRIA1 | GRK1 | GRK5 | GSK3A | GSK3B | GSPT1 | GSPT2 | GSR | GSS | GSTA1 | GSTA2 | GSTA3 | GSTA4 | GSTA5 | GSTK1 | GSTM4 | GSTO1 | GSTO2 | GSTP1 | GSTT1 | GSTT2 | GSTT2B | GSTZ1 | GTF2A1 | GTF2E2 | GTF2F1 | GTF2F2 | GTF2I | GTF3C2 | GUSB | GYG1 | GYG2 | GYS1 | H1F0 | H2AFX | H3F3A | HAAO | HADH | HAGH | HAP1 | HARS | HBEGF | HBZ | HCCS | HDAC1 | HDAC11 | HDAC2 | HDAC3 | HDAC4 | HDAC5 | HDAC6 | HDAC7 | HDAC9 | HECW1 | HECW2 | HELZ2 | HERC1 | HERC2 | HEXA | HEXB | HGS | HIBADH | HIBCH | HIF1A | HIF1AN | HINT1 | HIPK4 | HIST1H2BA | HIST1H3A | HIST1H4A | HIST2H3A | HIST3H3 | HK1 | HK2 | HLCS | HMBS | HMGB1 | HMGB2 | HMGB3 | HMGCL | HMOX1 | HNF4A | HNF4G | HNRNPA1 | HNRNPA1L2 | HNRNPA2B1 | HNRNPD | HNRNPF | HNRNPH1 | HNRNPH2 | HNRNPH3 | HNRNPK | HNRNPL | HNRNPM | HNRNPU | HOXB9 | HPRT1 | HPX | HRAS | HS2ST1 | HS6ST1 | HSD17B10 | HSD17B14 | HSD17B4 | HSD17B6 | HSD17B8 | HSD3B7 | HSF1 | HSF4 | HSP90AA1 | HSP90AB1 | HSP90B1 | HSPA14 | HSPA1B | HSPA5 | HSPA8 | HSPA9 | HSPD1 | HSPG2 | HTR1B | HTRA2 | HUWE1 | IARS | ICK | IDE | IDH1 | IDH2 | IDH3B | IDH3G | IDI2 | IFI16 | IGF1R | IGFBP7 | IK | IKBKB | IKBKE | IKBKG | IL3RA | ILF2 | ILF3 | ILK | IMP4 | IMPA1 | IMPA2 | IMPDH2 | ING5 | INO80B | INO80C | INSIG1 | INSIG2 | INSR | IP6K1 | IREB2 | IRF1 | IRF4 | IRF7 | ISG15 | ISYNA1 | ITCH | ITGAV | ITK | ITPA | IVD | JAK1 | JAK3 | JMJD6 | JOSD2 | JUN | KAT5 | KAT7 | KAT8 | KBTBD7 | KCMF1 | KCNAB2 | KCTD17 | KCTD2 | KDM1A | KDM2A | KDM3B | KDM4D | KDM5B | KDM5C | KDM8 | KDR | KDSR | KEAP1 | KHDRBS1 | KIAA0391 | KLF5 | KLHL20 | KLHL22 | KMT2A | KMT2B | KMT2C | KPNA1 | KPNA2 | KPNB1 | KRAS | KSR1 | KYNU | L2HGDH | LACTB2 | LAMB1 | LARP4 | LARP7 | LARS | LAT | LATS1 | LCMT2 | LDHA | LDHAL6A | LDHAL6B | LDHB | LDHC | LDHD | LEF1 | LGALS1 | LGALS3 | LGALS8 | LGMN | LIG4 | LMO2 | LMO7 | LNX1 | LOXL2 | LPIN3 | LRRC7 | LRRK1 | LRRK2 | LRSAM1 | LSM1 | LSM12 | LSM3 | LSM4 | LSM5 | LSM8 | LTBP1 | LTC4S | LUM | LYG2 | LYPLA1 | LYPLA2 | LYRM7 | LZTR1 | MAD2L1 | MAN2B1 | MAOA | MAP2K1 | MAP2K3 | MAP2K4 | MAP2K6 | MAP2K7 | MAP3K1 | MAP3K13 | MAP3K14 | MAP3K2 | MAP3K3 | MAP3K4 | MAP4K1 | MAPK1 | MAPK14 | MAPK15 | MAPK3 | MAPK4 | MAPK6 | MAPK7 | MAPK8 | MAPK9 | MAPT | MARCH5 | MAST1 | MAST3 | MAT2A | MAT2B | MATK | MBD3 | MBP | MC1R | MCAT | MCCC1 | MCFD2 | MCM2 | MCM3 | MCM4 | MCM5 | MCM6 | MCM7 | MCM9 | MCMBP | MCTS1 | MDC1 | MDH1 | MDH2 | MDM2 | MDM4 | MDN1 | ME1 | MECP2 | MED1 | MED12 | MED14 | MED17 | MED20 | MED21 | MED23 | MED24 | MED31 | MED4 | MED6 | MELK | MEPCE | METAP2 | MGMT | MGST1 | MIB1 | MIF | MITF | MKNK2 | MLH1 | MLLT1 | MLLT3 | MMAB | MME | MOCOS | MORF4L1 | MORF4L2 | MOV10 | MPC2 | MPG | MPHOSPH6 | MPI | MPPE1 | MPST | MRGBP | MRM1 | MRPL12 | MRPL14 | MRPL19 | MRPL28 | MRPL38 | MRPL4 | MRPL43 | MRPL45 | MRPL46 | MRPL9 | MRPS5 | MRRF | MSH6 | MTA1 | MTA3 | MTAP | MTERF1 | MTHFD1 | MTMR14 | MTMR7 | MTPN | MTRR | MVD | MYBBP1A | MYC | MYH6 | MYO5A | NAE1 | NAGK | NAMPT | NANP | NANS | NAP1L1 | NAPRT | NBAS | NCBP1 | NCK1 | NCOA1 | NCOA3 | NCOR1 | NDUFA10 | NDUFA13 | NDUFA5 | NDUFA8 | NDUFA9 | NDUFAF1 | NDUFAF2 | NDUFAF5 | NDUFAF7 | NDUFB1 | NDUFS1 | NDUFS2 | NDUFS3 | NDUFS6 | NDUFS7 | NDUFS8 | NDUFV1 | NDUFV3 | NEDD4 | NEDD8 | NEK1 | NEK7 | NELFB | NELFCD | NELFE | NEURL4 | NFATC1 | NFE2L2 | NFKBIA | NFS1 | NHLRC3 | NIPBL | NIT2 | NKX2-1 | NLK | NME1 | NME2 | NME4 | NMNAT1 | NMNAT3 | NMRAL1 | NMRK2 | NNT | NOB1 | NOD2 | NOP10 | NOP56 | NOS2 | NOS3 | NOSIP | NPEPPS | NPLOC4 | NPM1 | NPR1 | NQO1 | NQO2 | NR0B2 | NR1H2 | NR1H3 | NR1I2 | NR3C1 | NR4A1 | NR5A2 | NRBF2 | NSFL1C | NSMCE4A | NSUN2 | NSUN6 | NT5C2 | NT5DC2 | NTMT1 | NTRK1 | NUBP1 | NUBP2 | NUCB1 | NUDT13 | NUDT18 | NUDT19 | NUDT3 | NUDT9 | NUFIP1 | NUP107 | NUP153 | NUP160 | NUP205 | NUP214 | NUP85 | NUP88 | NUP93 | NUP98 | NUPR1 | OAS2 | OAT | OAZ3 | OBSL1 | OGDH | OGFOD1 | OGT | OLA1 | OPA1 | OPTN | OSBPL1A | OTC | OTUB1 | OTUB2 | OTUD4 | OTUD5 | OVGP1 | OXA1L | OXCT1 | OXSR1 | P2RX1 | P4HA1 | P4HA2 | P4HB | PA2G4 | PABPC1 | PABPC3 | PABPC4 | PADI4 | PAFAH1B1 | PAH | PAICS | PAIP1 | PAK2 | PAK6 | PALB2 | PAN2 | PAPSS1 | PAPSS2 | PARK7 | PARP1 | PARP12 | PARP14 | PARP6 | PAX8 | PAXIP1 | PC | PCBD1 | PCBP1 | PCBP2 | PCF11 | PCGF1 | PCGF5 | PCGF6 | PCMT1 | PCMTD1 | PCMTD2 | PCNA | PCSK9 | PCTP | PCYT2 | PDE12 | PDE4D | PDF | PDHA1 | PDHA2 | PDHB | PDHX | PDIA6 | PDK2 | PDSS1 | PDSS2 | PDXK | PEBP1 | PELI1 | PEPD | PER1 | PER2 | PEX5 | PFAS | PFKL | PFKM | PFKP | PGAM1 | PGAM2 | PGAM5 | PGD | PGK1 | PGK2 | PGM1 | PGM2 | PGM3 | PGP | PHB | PHF10 | PHGDH | PHKA1 | PHOSPHO1 | PI4K2A | PIAS1 | PIAS3 | PICK1 | PIGU | PIH1D1 | PIK3C2A | PIK3CA | PIK3CG | PIK3R1 | PIK3R3 | PIKFYVE | PIM1 | PIN1 | PINX1 | PIP4K2A | PIPSL | PIR | PJA1 | PKM | PKN2 | PLA2G10 | PLA2G12A | PLA2G1B | PLA2G2A | PLA2G6 | PLCG1 | PLK1 | PLOD2 | PLSCR1 | PML | PMPCA | PMPCB | PNKP | PNP | POLA2 | POLD1 | POLDIP3 | POLE2 | POLE3 | POLM | POLR2A | POLR2E | POLR2L | POLR2M | POM121 | POT1 | POU5F1 | PPA1 | PPA2 | PPARD | PPARG | PPARGC1A | PPAT | PPEF1 | PPIA | PPIB | PPID | PPIE | PPIF | PPIL3 | PPM1A | PPM1B | PPM1D | PPM1H | PPM1K | PPME1 | PPOX | PPP1CA | PPP1CB | PPP1CC | PPP1R11 | PPP1R12A | PPP1R2 | PPP1R3B | PPP1R3C | PPP1R8 | PPP2CA | PPP2CB | PPP2R1A | PPP2R1B | PPP2R2A | PPP2R2D | PPP2R5D | PPP3CC | PPP4R2 | PPP5C | PPP6C | PPT1 | PPTC7 | PRCC | PRDX1 | PRDX2 | PRDX3 | PRDX4 | PRDX5 | PRDX6 | PRKAA1 | PRKAA2 | PRKAB2 | PRKACA | PRKAG1 | PRKAR2B | PRKCD | PRKCE | PRKCSH | PRKCZ | PRKD1 | PRKD2 | PRKD3 | PRKDC | PRMT2 | PRMT3 | PRMT6 | PRPF19 | PRPF40A | PRPF8 | PRSS23 | PRTFDC1 | PSAP | PSAT1 | PSEN1 | PSEN2 | PSMA1 | PSMA2 | PSMA3 | PSMA5 | PSMA6 | PSMB5 | PSMB6 | PSMB7 | PSMB8 | PSMC1 | PSMC2 | PSMC3 | PSMC4 | PSMC5 | PSMC6 | PSMD1 | PSMD10 | PSMD11 | PSMD12 | PSMD14 | PSMD2 | PSMD4 | PSMD9 | PSME2 | PSME3 | PTEN | PTGDS | PTGES | PTGES2 | PTGES3 | PTGR1 | PTK2 | PTMA | PTMS | PTPMT1 | PTPN1 | PTPN14 | PTPN21 | PTPN22 | PTPN23 | PTPN4 | PTPN6 | PTPRA | PTPRD | PTPRF | PTPRK | PTPRO | PTPRR | PTTG1 | PUM1 | PUS1 | PUS7 | PXDN | PYCR1 | PYCR2 | PYGB | PYGL | QARS | QDPR | QSOX1 | RAB11A | RAB11B | RAB1A | RAB1B | RAB24 | RAB2A | RAB31 | RAB5A | RAB7A | RAD21 | RAD23A | RAD23B | RAD51 | RAD51AP1 | RAE1 | RAF1 | RAN | RANBP9 | RANGAP1 | RARA | RARG | RARS2 | RB1CC1 | RBBP4 | RBBP6 | RBM17 | RBM25 | RBM3 | RBM4 | RBM8A | RBMX | RBP1 | RBPJ | RBX1 | RC3H1 | RDH11 | RDH12 | RDH13 | RDH14 | RDH5 | RELA | REPIN1 | REST | REXO2 | RFC1 | RFC2 | RFC3 | RFC4 | RFC5 | RFK | RGN | RHBDD1 | RHO | RHOA | RHOT1 | RIF1 | RING1 | RIOK1 | RIPK1 | RIPK2 | RIPK3 | RIPK4 | RMND1 | RNASE3 | RNASEH2A | RNASEH2B | RNF123 | RNF126 | RNF139 | RNF181 | RNF183 | RNF185 | RNF19A | RNF2 | RNF25 | RNF31 | RNF4 | RNF8 | RPA2 | RPA3 | RPAIN | RPE | RPL10 | RPL10A | RPL12 | RPL18A | RPL22 | RPL23 | RPL23A | RPL24 | RPL30 | RPL36AL | RPL37A | RPL38 | RPL6 | RPL7 | RPL7A | RPL9 | RPLP0 | RPLP0P6 | RPLP1 | RPLP2 | RPN1 | RPN2 | RPP14 | RPS12 | RPS13 | RPS14 | RPS15A | RPS19 | RPS2 | RPS20 | RPS24 | RPS29 | RPS3 | RPS4X | RPS6KA1 | RPS6KA2 | RPS6KB2 | RPS8 | RRM1 | RRM2 | RRM2B | RRP7A | RRS1 | RTCB | RTF1 | RTFDC1 | RUNX1T1 | RUNX2 | RUVBL1 | RUVBL2 | RXRA | RXRB | RXRG | S100A12 | S100A9 | SAE1 | SAP18 | SARS | SART1 | SART3 | SAT1 | SAT2 | SBDS | SCAF1 | SCCPDH | SCG2 | SCG3 | SCLY | SCO2 | SCYL2 | SDC1 | SDC4 | SDHA | SDHB | SDHD | SEL1L | SENP3 | SENP6 | SENP7 | SENP8 | SEPHS1 | SEPHS2 | SET | SETDB1 | SF1 | SF3B6 | SFPQ | SFXN1 | SGPL1 | SGSH | SGTA | SGTB | SH3PXD2A | SHC1 | SHMT1 | SHMT2 | SIAH1 | SIN3A | SIRT1 | SIRT3 | SIRT5 | SIRT6 | SIRT7 | SKI | SKIV2L | SLC16A1 | SLC25A1 | SLC25A10 | SLC25A13 | SLC25A32 | SLC25A4 | SLC2A9 | SMAD2 | SMAD3 | SMAD4 | SMAD5 | SMARCB1 | SMC1A | SMC3 | SMG5 | SMG6 | SMG8 | SMN1 | SMURF1 | SMURF2 | SNCA | SND1 | SNF8 | SNRNP27 | SNRPA | SNRPA1 | SNRPB | SNRPB2 | SNRPD1 | SNRPD3 | SNRPE | SNW1 | SNX3 | SOAT1 | SOCS1 | SOCS2 | SOCS6 | SOD1 | SOD2 | SORD | SP1 | SP100 | SPATA2 | SPI1 | SPIN1 | SPOP | SPR | SPRR2A | SPRTN | SPSB3 | SPTAN1 | SPTBN1 | SPTBN2 | SPTLC2 | SQSTM1 | SRC | SRPK1 | SRPK2 | SRSF11 | SRSF12 | SRSF5 | SSB | ST3GAL1 | ST3GAL6 | ST6GALNAC5 | ST8SIA4 | STARD7 | STAT3 | STAT5A | STBD1 | STK11 | STK24 | STK25 | STK26 | STK3 | STK38 | STK40 | STOML2 | STUB1 | STYX | SUCLA2 | SUCLG2 | SULF2 | SULT1A3 | SUMO1 | SUMO1P1 | SUMO2 | SUMO3 | SUPT4H1 | SUPT5H | SUZ12 | SWAP70 | SYVN1 | TAB1 | TAB2 | TADA2B | TAF12 | TAF15 | TAF1A | TALDO1 | TAMM41 | TAOK3 | TARDBP | TARS | TARS2 | TATDN1 | TAZ | TBL1X | TBRG4 | TCEA1 | TCEA2 | TCERG1 | TCF4 | TCIRG1 | TDO2 | TDP1 | TECR | TERF1 | TERF2 | TERF2IP | TESK2 | TFCP2 | TFIP11 | TGFB1 | TGFBR3 | TGM2 | TGOLN2 | THADA | THBS1 | THEM4 | THOC2 | THOC6 | THOC7 | THOP1 | THRA | TIMM50 | TIMP1 | TINF2 | TK1 | TK2 | TKT | TLR3 | TMBIM6 | TMEM132A | TMEM189-UBE2V1 | TMEM258 | TMTC3 | TNF | TNFAIP3 | TNFRSF1A | TNIP2 | TNK2 | TNKS | TNKS2 | TNP1 | TOE1 | TOLLIP | TOMM20 | TOMM22 | TOMM40 | TOP1 | TOP2A | TOP3A | TOR1A | TP53 | TP53RK | TP73 | TPI1 | TPR | TPST2 | TPTE | TRA2A | TRAF2 | TRAF3 | TRAF6 | TRAPPC8 | TREX1 | TRIB2 | TRIB3 | TRIM21 | TRIM23 | TRIM25 | TRIM28 | TRIM29 | TRIM32 | TRIM33 | TRIM41 | TRIM47 | TRIM63 | TRIM69 | TRIM7 | TRIM9 | TRIP12 | TRIP13 | TRMT44 | TRMT6 | TRMT61B | TRNT1 | TRUB2 | TSC22D1 | TSFM | TSG101 | TSN | TSPAN17 | TTC5 | TTLL1 | TUFM | TWF1 | TXN2 | TXNL4A | TXNRD1 | TYMS | U2AF2 | UAP1 | UAP1L1 | UBA1 | UBA2 | UBA3 | UBA5 | UBA6 | UBAC1 | UBASH3A | UBB | UBC | UBE2A | UBE2B | UBE2C | UBE2D1 | UBE2D2 | UBE2D3 | UBE2H | UBE2I | UBE2J1 | UBE2K | UBE2L3 | UBE2L6 | UBE2M | UBE2N | UBE2O | UBE2Q2 | UBE2R2 | UBE2S | UBE2V1 | UBE2W | UBE3A | UBE3B | UBIAD1 | UBL4A | UBQLN1 | UBQLN2 | UBQLN4 | UBR4 | UBR5 | UBXN1 | UBXN7 | UCHL1 | UCHL3 | UCHL5 | UCK2 | UFC1 | UFM1 | UGDH | UGGT1 | UGP2 | UGT8 | ULK1 | UMPS | UNKL | UPF2 | UPF3B | UPP2 | UQCRB | UQCRC2 | UQCRFS1 | UQCRQ | USP11 | USP14 | USP15 | USP16 | USP17L2 | USP19 | USP2 | USP20 | USP21 | USP26 | USP28 | USP39 | USP4 | USP42 | USP46 | USP47 | USP5 | USP50 | USP53 | USP7 | USP8 | USP9X | USP9Y | USPL1 | UTP14A | UTP6 | UXS1 | VAC14 | VAPA | VAPB | VARS | VAV1 | VCAN | VCP | VCPIP1 | VDAC1 | VDR | VGF | VHL | VKORC1 | VPS18 | VPS37D | VPS4B | VTA1 | WARS | WDR12 | WDR4 | WDR41 | WDR45B | WDR48 | WDR82 | WDTC1 | WDYHV1 | WIPI1 | WIPI2 | WNK1 | WNK4 | WSB1 | WT1 | WTAP | WWP2 | XBP1 | XIAP | XPNPEP1 | XPO5 | XRCC1 | XRCC3 | XRCC5 | XRCC6 | XYLT1 | YAP1 | YBEY | YEATS2 | YES1 | YME1L1 | YWHAB | YWHAE | YY1 | ZAP70 | ZBTB16 | ZBTB7A | ZBTB7B | ZC3HC1 | ZDHHC17 | ZDHHC23 | ZFYVE26 | ZMPSTE24 | ZNF217 | ZNF598 | ZNF638 | ZNRF4 | ZRANB2 | GO:0008152 | 0.577095717578551 | 4757/8243 | 2078/3250 | 1.18544419954203e-20 | 1.12557926746516e-16 | T | T | T | T | metabolic process | A2M | AARS | AARSD1 | AASDH | AASDHPPT | AASS | ABAT | ABCD3 | ABCE1 | ABCG1 | ABHD14B | ABHD17A | ABL1 | ABL2 | ACAA1 | ACAA2 | ACACA | ACAD8 | ACAD9 | ACADS | ACADSB | ACADVL | ACAT1 | ACAT2 | ACBD3 | ACCS | ACD | ACE2 | ACLY | ACO1 | ACO2 | ACOT13 | ACOT7 | ACOT9 | ACOX1 | ACP1 | ACSL1 | ACSL4 | ACSL5 | ACSS2 | ACTB | ACTL6A | ACTN2 | ACY1 | ADA | ADAMTS4 | ADCK2 | ADCY6 | ADCYAP1 | ADH1A | ADH1B | ADH5 | ADK | ADORA2A | ADPRH | ADRB2 | ADRM1 | ADSL | ADSS | ADTRP | AFF4 | AFG3L2 | AGK | AGL | AGO1 | AGO2 | AHCY | AHCYL1 | AHSG | AICDA | AIFM1 | AIMP2 | AK1 | AK2 | AK4 | AKR1A1 | AKR1B1 | AKR1B10 | AKR1B15 | AKR1C2 | AKT1 | AKT2 | AKT3 | ALAS1 | ALB | ALDH16A1 | ALDH1B1 | ALDH4A1 | ALDH5A1 | ALDH6A1 | ALDH7A1 | ALDH8A1 | ALDH9A1 | ALDOA | ALDOC | ALOX12 | ALOX5 | ALOX5AP | AMD1 | AMDHD2 | AMFR | ANAPC4 | ANXA1 | ANXA2 | ANXA7 | AOC2 | AP3D1 | APBB1 | APC | APCS | APEH | APEX1 | APEX2 | APOA1 | APOD | APOE | APOL2 | APP | APRT | AR | ARAF | ARF1 | ARF4 | ARHGAP35 | ARID4B | ARMC8 | ARNT | ARNT2 | ARRB1 | ARRB2 | ASB12 | ASB15 | ASB16 | ASB6 | ASB9 | ASCC1 | ASCC2 | ASGR1 | ASGR2 | ASL | ASMTL | ASNS | ASPDH | ASS1 | ATAD5 | ATCAY | ATF2 | ATF6B | ATG2B | ATG4C | ATG5 | ATIC | ATM | ATOH1 | ATP13A2 | ATP1A1 | ATP6V1A | ATP6V1B1 | ATRX | ATXN1 | ATXN2 | AUP1 | AURKB | AZU1 | B3GAT3 | B3GNT3 | B4GALT3 | BABAM1 | BACE1 | BAG2 | BAG3 | BAG6 | BANF1 | BAX | BCAT2 | BCKDHB | BCL10 | BCL11A | BCL2 | BCOR | BDH1 | BIRC2 | BIRC3 | BIRC5 | BIRC7 | BIRC8 | BLVRB | BMI1 | BMPR1A | BNIPL | BRAF | BRCA1 | BRD4 | BSG | BTBD9 | BTK | BTN2A1 | BTRC | BUD13 | BUD31 | BYSL | C1QBP | C1R | C4A | C9orf64 | CAB39 | CACNG2 | CACNG4 | CACNG5 | CALCOCO2 | CALM1 | CALR | CALU | CAMK1D | CAMK2A | CAMK2B | CAMKV | CAND1 | CAPN1 | CAPN2 | CAPNS1 | CARM1 | CARS | CASP10 | CASP2 | CASP3 | CASP7 | CASP8 | CASP9 | CAT | CAV1 | CBFB | CBL | CBLB | CBLC | CBR1 | CBR4 | CBS | CBX4 | CBX8 | CCAR2 | CCDC155 | CCDC22 | CCDC36 | CCDC8 | CCDC88A | CCNB1 | CCNT1 | CCNT2 | CCP110 | CCR5 | CCS | CD2BP2 | CD36 | CD40LG | CD74 | CD81 | CDA | CDC14B | CDC16 | CDC20 | CDC25A | CDC25B | CDC25C | CDC27 | CDC34 | CDC42 | CDC5L | CDC73 | CDK15 | CDK16 | CDK19 | CDK2 | CDK4 | CDK5 | CDK5R1 | CDK8 | CDK9 | CDKL3 | CDKN1A | CEBPA | CEBPE | CEL | CENPF | CES1 | CFL1 | CFTR | CHAF1A | CHCHD10 | CHD1L | CHDH | CHEK2 | CHGB | CHMP2A | CHP1 | CHRAC1 | CHRM3 | CHST4 | CILP2 | CISD2 | CISD3 | CKAP4 | CKB | CKM | CKMT1A | CLK1 | CLPX | CLTC | CLU | CMBL | CMPK1 | CNDP1 | CNDP2 | COG3 | COL2A1 | COL4A2 | COL4A3BP | COL5A1 | COMMD1 | COMT | COMTD1 | COPS2 | COPS5 | COPS6 | COQ2 | COQ3 | COQ4 | COQ5 | COQ6 | COQ7 | COQ9 | COX11 | COX15 | COX17 | COX20 | COX5A | COX6A1 | COX6C | COX7A2 | CPNE3 | CPSF7 | CPT1A | CPT2 | CPVL | CRBN | CREB1 | CREBBP | CRK | CRKL | CRMP1 | CROT | CRY1 | CRY2 | CRYAB | CRYZ | CS | CSNK1A1 | CSNK1E | CSNK2A2 | CSNK2B | CSTF2T | CT45A5 | CTBP1 | CTCF | CTDP1 | CTH | CTNNB1 | CTPS1 | CTSA | CTSC | CTSD | CTSL | CTSV | CUL1 | CUL2 | CUL3 | CUL4B | CUL5 | CUL7 | CWC15 | CXCL8 | CYB5B | CYB5R1 | CYB5R3 | CYC1 | CYCS | CYLD | CYP1A1 | CYP1A2 | CYP2C18 | DAD1 | DAP3 | DAPK1 | DAPP1 | DARS | DBH | DBI | DBT | DCAF12 | DCAF7 | DCAF8 | DCAKD | DCK | DCLK1 | DCN | DCP2 | DCTPP1 | DCUN1D1 | DCXR | DDA1 | DDB1 | DDB2 | DDI1 | DDIT4 | DDOST | DDRGK1 | DDX1 | DDX39A | DDX39B | DDX3X | DDX6 | DECR1 | DEGS1 | DERA | DERL1 | DGKE | DGKZ | DGUOK | DHCR24 | DHRS4 | DHRS7B | DHX15 | DHX36 | DHX9 | DIDO1 | DIS3 | DLAT | DLD | DLG2 | DLG3 | DLG4 | DLST | DMAP1 | DMD | DNA2 | DNAJA3 | DNAJB11 | DNAJB2 | DNAJC10 | DNAJC30 | DNASE2 | DNASE2B | DNPEP | DPEP1 | DPF2 | DPM1 | DPP9 | DRG2 | DROSHA | DSE | DTX1 | DTX2 | DUS3L | DUSP1 | DUSP12 | DUSP13 | DUSP14 | DUSP16 | DUSP19 | DUSP22 | DUSP7 | DUT | DVL1 | DVL2 | DYNLL1 | DYRK2 | DZIP3 | E2F1 | E2F2 | E2F3 | EAF2 | ECD | ECH1 | ECHDC2 | ECHS1 | ECI1 | ECI2 | ECSIT | EDA | EDC4 | EDEM1 | EED | EEF1A1 | EEF1A1P5 | EEF1A2 | EEF1B2 | EEF1D | EEF1G | EEF2 | EEF2KMT | EFEMP1 | EFTUD2 | EGFR | EGLN1 | EGLN3 | EHHADH | EHMT1 | EHMT2 | EIF1AX | EIF2AK3 | EIF2AK4 | EIF2B1 | EIF2B3 | EIF2B5 | EIF2S1 | EIF2S2 | EIF3A | EIF3D | EIF3E | EIF3H | EIF3I | EIF4A3 | EIF4E | EIF4E2 | EIF4EBP1 | EIF4G3 | EIF4H | EIF5 | EIF5A | EIF5B | EIF6 | ELAC2 | ELAVL1 | ELAVL4 | ELF3 | ENDOG | ENO1 | ENO2 | ENO3 | ENOPH1 | ENTPD7 | EP300 | EPHA4 | EPHA6 | EPHB6 | EPRS | ERAL1 | ERBB2 | ERBB3 | ERCC5 | ERG | ERLIN2 | ESD | ESR1 | ESR2 | ESRRA | ESRRB | ESRRG | ETFA | ETFB | ETFDH | ETS1 | EVA1A | EXOSC1 | EXOSC5 | EXOSC6 | EXOSC9 | EXTL2 | EYA2 | EYA4 | EZH2 | EZR | F5 | F9 | FABP1 | FABP3 | FABP5 | FABP7 | FADD | FAF1 | FAF2 | FAH | FAHD1 | FAM20B | FAM86C1 | FAM98A | FANCA | FANCC | FANCG | FANCI | FANCM | FAR2 | FARSB | FASN | FBP1 | FBP2 | FBXL12 | FBXO11 | FBXO25 | FBXO32 | FBXO6 | FBXO7 | FBXW11 | FBXW5 | FDPS | FDXR | FECH | FEN1 | FGB | FGF2 | FGFR1 | FGFR3 | FGL2 | FH | FIGNL1 | FIS1 | FKBP14 | FKBP1A | FKBP1B | FKBP2 | FKBP5 | FKBP7 | FKBP8 | FN1 | FOLH1 | FOLR1 | FOS | FOXA1 | FOXK1 | FOXK2 | FOXM1 | FOXO1 | FPGS | FTH1 | FTL | FTO | FTSJ3 | FUBP1 | FUBP3 | FUNDC2 | FUS | FYN | FZR1 | G3BP1 | G6PD | GAA | GAD2 | GAK | GALE | GALNS | GALNT7 | GANAB | GAPDH | GAPDHS | GAR1 | GARS | GART | GATA1 | GBE1 | GCDH | GDA | GDPGP1 | GEMIN4 | GEMIN5 | GEMIN7 | GFOD1 | GFPT1 | GFPT2 | GGA1 | GGCT | GGPS1 | GGT1 | GGTLC1 | GINS2 | GINS3 | GINS4 | GLA | GLB1L2 | GLCE | GLO1 | GLRX | GLRX3 | GLRX5 | GLS | GLS2 | GLUD2 | GLUL | GMDS | GMPPB | GMPR | GMPS | GNAI3 | GNAS | GNPDA1 | GNPDA2 | GNPNAT1 | GNS | GOLGA2 | GOLPH3 | GOT1 | GOT1L1 | GOT2 | GPC5 | GPD1 | GPD1L | GPI | GPS2 | GPT2 | GPX1 | GPX4 | GPX7 | GRB2 | GRHPR | GRIA1 | GRK1 | GRK5 | GSK3A | GSK3B | GSPT1 | GSPT2 | GSR | GSS | GSTA1 | GSTA2 | GSTA3 | GSTA4 | GSTA5 | GSTK1 | GSTM4 | GSTO1 | GSTO2 | GSTP1 | GSTT1 | GSTT2 | GSTT2B | GSTZ1 | GTF2A1 | GTF2E2 | GTF2F1 | GTF2F2 | GTF2I | GTF3C2 | GUSB | GYG1 | GYG2 | GYS1 | GZMA | GZMK | H1F0 | H2AFX | H3F3A | HAAO | HADH | HAGH | HAP1 | HARS | HBEGF | HBZ | HCCS | HDAC1 | HDAC11 | HDAC2 | HDAC3 | HDAC4 | HDAC5 | HDAC6 | HDAC7 | HDAC9 | HECW1 | HECW2 | HELZ2 | HERC1 | HERC2 | HEXA | HEXB | HGS | HIBADH | HIBCH | HIF1A | HIF1AN | HINT1 | HIPK4 | HIST1H2BA | HIST1H3A | HIST1H4A | HIST2H3A | HIST3H3 | HK1 | HK2 | HLCS | HMBS | HMGB1 | HMGB2 | HMGB3 | HMGCL | HMMR | HMOX1 | HNF4A | HNF4G | HNRNPA1 | HNRNPA1L2 | HNRNPA2B1 | HNRNPD | HNRNPF | HNRNPH1 | HNRNPH2 | HNRNPH3 | HNRNPK | HNRNPL | HNRNPM | HNRNPU | HOXB9 | HPRT1 | HPX | HRAS | HS2ST1 | HS6ST1 | HSD17B10 | HSD17B14 | HSD17B4 | HSD17B6 | HSD17B8 | HSD3B7 | HSDL2 | HSF1 | HSF4 | HSP90AA1 | HSP90AB1 | HSP90B1 | HSPA14 | HSPA1B | HSPA5 | HSPA8 | HSPA9 | HSPD1 | HSPG2 | HTATIP2 | HTR1B | HTRA1 | HTRA2 | HUWE1 | IARS | ICK | IDE | IDH1 | IDH2 | IDH3A | IDH3B | IDH3G | IDI2 | IFI16 | IGF1R | IGFBP7 | IK | IKBKB | IKBKE | IKBKG | IL3RA | ILF2 | ILF3 | ILK | IMP4 | IMPA1 | IMPA2 | IMPDH2 | ING5 | INO80B | INO80C | INSIG1 | INSIG2 | INSR | IP6K1 | IREB2 | IRF1 | IRF4 | IRF7 | ISG15 | ISYNA1 | ITCH | ITGAV | ITK | ITPA | IVD | JAK1 | JAK3 | JMJD6 | JOSD2 | JUN | KAT5 | KAT7 | KAT8 | KBTBD7 | KCMF1 | KCNAB1 | KCNAB2 | KCTD17 | KCTD2 | KDM1A | KDM2A | KDM3B | KDM4D | KDM5B | KDM5C | KDM8 | KDR | KDSR | KEAP1 | KHDRBS1 | KIAA0391 | KLF5 | KLHL20 | KLHL22 | KMT2A | KMT2B | KMT2C | KPNA1 | KPNA2 | KPNB1 | KRAS | KSR1 | KYNU | L2HGDH | LACTB2 | LAMB1 | LAP3 | LARP4 | LARP7 | LARS | LAT | LATS1 | LCMT2 | LDHA | LDHAL6A | LDHAL6B | LDHB | LDHC | LDHD | LEF1 | LGALS1 | LGALS3 | LGALS8 | LGMN | LIG4 | LIMA1 | LMO2 | LMO7 | LNX1 | LOXL2 | LPIN3 | LRRC7 | LRRK1 | LRRK2 | LRSAM1 | LSM1 | LSM12 | LSM3 | LSM4 | LSM5 | LSM8 | LTBP1 | LTC4S | LUM | LYG2 | LYPLA1 | LYPLA2 | LYRM7 | LYZL1 | LYZL2 | LZTR1 | MAD2L1 | MAN2B1 | MAOA | MAP2K1 | MAP2K3 | MAP2K4 | MAP2K6 | MAP2K7 | MAP3K1 | MAP3K13 | MAP3K14 | MAP3K2 | MAP3K3 | MAP3K4 | MAP4K1 | MAPK1 | MAPK14 | MAPK15 | MAPK3 | MAPK4 | MAPK6 | MAPK7 | MAPK8 | MAPK9 | MAPT | MARCH5 | MASP1 | MASP2 | MAST1 | MAST3 | MAT2A | MAT2B | MATK | MBD3 | MBP | MC1R | MCAT | MCCC1 | MCFD2 | MCM2 | MCM3 | MCM4 | MCM5 | MCM6 | MCM7 | MCM9 | MCMBP | MCTS1 | MDC1 | MDH1 | MDH2 | MDM2 | MDM4 | MDN1 | ME1 | MECP2 | MED1 | MED12 | MED14 | MED17 | MED20 | MED21 | MED23 | MED24 | MED31 | MED4 | MED6 | MELK | MEPCE | METAP2 | METTL13 | METTL23 | MGMT | MGST1 | MIB1 | MIF | MITF | MKNK2 | MLH1 | MLLT1 | MLLT3 | MMAB | MME | MMP26 | MOCOS | MORF4L1 | MORF4L2 | MOV10 | MPC2 | MPG | MPHOSPH6 | MPI | MPPE1 | MPST | MRGBP | MRM1 | MRPL12 | MRPL14 | MRPL19 | MRPL28 | MRPL38 | MRPL4 | MRPL43 | MRPL45 | MRPL46 | MRPL9 | MRPS5 | MRRF | MSH6 | MTA1 | MTA3 | MTAP | MTERF1 | MTHFD1 | MTMR14 | MTMR7 | MTPN | MTRR | MVD | MYBBP1A | MYC | MYH6 | MYO1E | MYO5A | NAE1 | NAGK | NAMPT | NANP | NANS | NAP1L1 | NAPRT | NBAS | NCBP1 | NCEH1 | NCK1 | NCOA1 | NCOA3 | NCOR1 | NDUFA10 | NDUFA13 | NDUFA5 | NDUFA8 | NDUFA9 | NDUFAF1 | NDUFAF2 | NDUFAF5 | NDUFAF7 | NDUFB1 | NDUFS1 | NDUFS2 | NDUFS3 | NDUFS6 | NDUFS7 | NDUFS8 | NDUFV1 | NDUFV3 | NEDD4 | NEDD8 | NEK1 | NEK7 | NELFB | NELFCD | NELFE | NEURL4 | NFATC1 | NFE2L2 | NFKBIA | NFS1 | NHLRC3 | NIPBL | NIT1 | NIT2 | NKX2-1 | NLK | NME1 | NME2 | NME4 | NMNAT1 | NMNAT3 | NMRAL1 | NMRK2 | NNT | NOB1 | NOD2 | NOP10 | NOP56 | NOS2 | NOS3 | NOSIP | NPEPL1 | NPEPPS | NPLOC4 | NPM1 | NPR1 | NQO1 | NQO2 | NR0B2 | NR1H2 | NR1H3 | NR1I2 | NR3C1 | NR4A1 | NR5A2 | NRBF2 | NSFL1C | NSMCE4A | NSUN2 | NSUN6 | NT5C2 | NT5DC2 | NTMT1 | NTRK1 | NUBP1 | NUBP2 | NUCB1 | NUDT13 | NUDT18 | NUDT19 | NUDT3 | NUDT9 | NUFIP1 | NUP107 | NUP153 | NUP160 | NUP205 | NUP214 | NUP85 | NUP88 | NUP93 | NUP98 | NUPR1 | OAS2 | OAT | OAZ3 | OBSL1 | OGDH | OGFOD1 | OGT | OLA1 | ONECUT1 | OPA1 | OPTN | OSBPL1A | OTC | OTUB1 | OTUB2 | OTUD4 | OTUD5 | OVGP1 | OXA1L | OXCT1 | OXNAD1 | OXSR1 | P2RX1 | P4HA1 | P4HA2 | P4HB | PA2G4 | PABPC1 | PABPC3 | PABPC4 | PADI4 | PAFAH1B1 | PAH | PAICS | PAIP1 | PAK2 | PAK6 | PALB2 | PAN2 | PAPLN | PAPSS1 | PAPSS2 | PARK7 | PARP1 | PARP12 | PARP14 | PARP6 | PAWR | PAX8 | PAXIP1 | PC | PCBD1 | PCBP1 | PCBP2 | PCF11 | PCGF1 | PCGF5 | PCGF6 | PCMT1 | PCMTD1 | PCMTD2 | PCNA | PCSK9 | PCTP | PCYT2 | PDCD6IP | PDE12 | PDE4D | PDF | PDHA1 | PDHA2 | PDHB | PDHX | PDIA3 | PDIA4 | PDIA6 | PDK2 | PDSS1 | PDSS2 | PDXK | PEBP1 | PELI1 | PEPD | PER1 | PER2 | PEX5 | PFAS | PFKL | PFKM | PFKP | PGAM1 | PGAM2 | PGAM5 | PGD | PGK1 | PGK2 | PGM1 | PGM2 | PGM3 | PGM5 | PGP | PHB | PHF10 | PHGDH | PHKA1 | PHOSPHO1 | PI4K2A | PIAS1 | PIAS3 | PICK1 | PIGU | PIH1D1 | PIK3C2A | PIK3CA | PIK3CG | PIK3R1 | PIK3R3 | PIKFYVE | PIM1 | PIN1 | PINX1 | PIP4K2A | PIPSL | PIR | PITPNA | PITPNB | PJA1 | PKM | PKN2 | PLA2G10 | PLA2G12A | PLA2G1B | PLA2G2A | PLA2G6 | PLBD2 | PLCG1 | PLK1 | PLOD2 | PLSCR1 | PML | PMPCA | PMPCB | PNKP | PNP | POLA2 | POLD1 | POLDIP3 | POLE2 | POLE3 | POLM | POLR2A | POLR2E | POLR2L | POLR2M | POM121 | POT1 | POU5F1 | PPA1 | PPA2 | PPARD | PPARG | PPARGC1A | PPAT | PPEF1 | PPIA | PPIB | PPID | PPIE | PPIF | PPIL3 | PPM1A | PPM1B | PPM1D | PPM1H | PPM1K | PPME1 | PPOX | PPP1CA | PPP1CB | PPP1CC | PPP1R11 | PPP1R12A | PPP1R2 | PPP1R3B | PPP1R3C | PPP1R8 | PPP2CA | PPP2CB | PPP2R1A | PPP2R1B | PPP2R2A | PPP2R2D | PPP2R5D | PPP3CC | PPP4R2 | PPP5C | PPP6C | PPT1 | PPTC7 | PRCC | PRCP | PRDX1 | PRDX2 | PRDX3 | PRDX4 | PRDX5 | PRDX6 | PREP | PREPL | PRKAA1 | PRKAA2 | PRKAB2 | PRKACA | PRKAG1 | PRKAR2B | PRKCD | PRKCE | PRKCSH | PRKCZ | PRKD1 | PRKD2 | PRKD3 | PRKDC | PRMT2 | PRMT3 | PRMT6 | PROZ | PRPF19 | PRPF40A | PRPF8 | PRSS16 | PRSS23 | PRTFDC1 | PSAP | PSAT1 | PSEN1 | PSEN2 | PSMA1 | PSMA2 | PSMA3 | PSMA5 | PSMA6 | PSMB5 | PSMB6 | PSMB7 | PSMB8 | PSMC1 | PSMC2 | PSMC3 | PSMC4 | PSMC5 | PSMC6 | PSMD1 | PSMD10 | PSMD11 | PSMD12 | PSMD14 | PSMD2 | PSMD4 | PSMD9 | PSME2 | PSME3 | PTEN | PTGDS | PTGES | PTGES2 | PTGES3 | PTGR1 | PTK2 | PTMA | PTMS | PTPMT1 | PTPN1 | PTPN14 | PTPN21 | PTPN22 | PTPN23 | PTPN4 | PTPN6 | PTPRA | PTPRD | PTPRF | PTPRK | PTPRO | PTPRR | PTTG1 | PUM1 | PUS1 | PUS7 | PXDN | PYCR1 | PYCR2 | PYGB | PYGL | QARS | QDPR | QSOX1 | RAB11A | RAB11B | RAB1A | RAB1B | RAB24 | RAB2A | RAB31 | RAB5A | RAB7A | RAD21 | RAD23A | RAD23B | RAD51 | RAD51AP1 | RAE1 | RAF1 | RAN | RANBP9 | RANGAP1 | RARA | RARG | RARS2 | RB1CC1 | RBBP4 | RBBP6 | RBM17 | RBM25 | RBM3 | RBM4 | RBM8A | RBMX | RBP1 | RBPJ | RBX1 | RC3H1 | RDH11 | RDH12 | RDH13 | RDH14 | RDH5 | RELA | REPIN1 | REST | REXO2 | RFC1 | RFC2 | RFC3 | RFC4 | RFC5 | RFK | RGN | RHBDD1 | RHO | RHOA | RHOT1 | RIF1 | RING1 | RIOK1 | RIPK1 | RIPK2 | RIPK3 | RIPK4 | RMND1 | RNASE3 | RNASEH2A | RNASEH2B | RNF123 | RNF126 | RNF139 | RNF181 | RNF183 | RNF185 | RNF19A | RNF2 | RNF25 | RNF31 | RNF4 | RNF8 | RNPEP | RPA2 | RPA3 | RPAIN | RPE | RPIA | RPL10 | RPL10A | RPL12 | RPL18A | RPL22 | RPL23 | RPL23A | RPL24 | RPL30 | RPL36AL | RPL37A | RPL38 | RPL6 | RPL7 | RPL7A | RPL9 | RPLP0 | RPLP0P6 | RPLP1 | RPLP2 | RPN1 | RPN2 | RPP14 | RPS12 | RPS13 | RPS14 | RPS15A | RPS19 | RPS2 | RPS20 | RPS24 | RPS29 | RPS3 | RPS4X | RPS6KA1 | RPS6KA2 | RPS6KB2 | RPS8 | RRM1 | RRM2 | RRM2B | RRP7A | RRS1 | RTCB | RTF1 | RTFDC1 | RTN4IP1 | RUNX1T1 | RUNX2 | RUVBL1 | RUVBL2 | RXRA | RXRB | RXRG | S100A12 | S100A9 | SAE1 | SAP18 | SARS | SART1 | SART3 | SAT1 | SAT2 | SBDS | SCAF1 | SCCPDH | SCG2 | SCG3 | SCLY | SCO2 | SCYL2 | SDC1 | SDC4 | SDHA | SDHB | SDHD | SDR39U1 | SEC63 | SEL1L | SELENBP1 | SENP3 | SENP6 | SENP7 | SENP8 | SEPHS1 | SEPHS2 | SERPINH1 | SET | SETDB1 | SF1 | SF3B6 | SFPQ | SFXN1 | SGPL1 | SGSH | SGTA | SGTB | SH3PXD2A | SHC1 | SHMT1 | SHMT2 | SIAH1 | SIN3A | SIRT1 | SIRT3 | SIRT5 | SIRT6 | SIRT7 | SKI | SKIV2L | SLC16A1 | SLC25A1 | SLC25A10 | SLC25A11 | SLC25A13 | SLC25A32 | SLC25A4 | SLC2A5 | SLC2A9 | SLC3A2 | SLC9A1 | SMAD2 | SMAD3 | SMAD4 | SMAD5 | SMARCB1 | SMC1A | SMC3 | SMG5 | SMG6 | SMG8 | SMN1 | SMURF1 | SMURF2 | SNCA | SND1 | SNF8 | SNRNP27 | SNRPA | SNRPA1 | SNRPB | SNRPB2 | SNRPD1 | SNRPD3 | SNRPE | SNW1 | SNX17 | SNX3 | SOAT1 | SOCS1 | SOCS2 | SOCS6 | SOD1 | SOD2 | SORD | SP1 | SP100 | SPATA2 | SPI1 | SPIN1 | SPOP | SPR | SPRR2A | SPRTN | SPSB3 | SPTAN1 | SPTBN1 | SPTBN2 | SPTLC2 | SQSTM1 | SRC | SRPK1 | SRPK2 | SRSF11 | SRSF12 | SRSF5 | SRXN1 | SSB | ST3GAL1 | ST3GAL6 | ST6GALNAC5 | ST8SIA4 | STARD7 | STAT3 | STAT5A | STBD1 | STK11 | STK24 | STK25 | STK26 | STK3 | STK38 | STK40 | STOML2 | STUB1 | STYX | SUCLA2 | SUCLG2 | SULF2 | SULT1A3 | SUMO1 | SUMO1P1 | SUMO2 | SUMO3 | SUPT4H1 | SUPT5H | SUZ12 | SWAP70 | SYVN1 | TAB1 | TAB2 | TADA2B | TAF12 | TAF15 | TAF1A | TALDO1 | TAMM41 | TANK | TAOK3 | TARDBP | TARS | TARS2 | TATDN1 | TAZ | TBL1X | TBRG4 | TCEA1 | TCEA2 | TCERG1 | TCF4 | TCIRG1 | TDO2 | TDP1 | TECR | TERF1 | TERF2 | TERF2IP | TESK2 | TFCP2 | TFIP11 | TGFB1 | TGFBR3 | TGM2 | TGOLN2 | THADA | THBS1 | THEM4 | THOC2 | THOC6 | THOC7 | THOP1 | THRA | TIMM50 | TIMP1 | TINF2 | TK1 | TK2 | TKT | TLR3 | TMBIM6 | TMEM132A | TMEM189-UBE2V1 | TMEM258 | TMTC3 | TMX4 | TNF | TNFAIP3 | TNFRSF1A | TNIP2 | TNK2 | TNKS | TNKS2 | TNP1 | TNXB | TOE1 | TOLLIP | TOMM20 | TOMM22 | TOMM40 | TOP1 | TOP2A | TOP3A | TOR1A | TP53 | TP53RK | TP73 | TPI1 | TPR | TPSAB1 | TPSB2 | TPST2 | TPTE | TRA2A | TRAF2 | TRAF3 | TRAF6 | TRAPPC8 | TREX1 | TRIB2 | TRIB3 | TRIM21 | TRIM23 | TRIM25 | TRIM28 | TRIM29 | TRIM32 | TRIM33 | TRIM41 | TRIM47 | TRIM63 | TRIM69 | TRIM7 | TRIM9 | TRIP12 | TRIP13 | TRMT44 | TRMT6 | TRMT61B | TRNT1 | TRUB2 | TSC22D1 | TSFM | TSG101 | TSN | TSPAN17 | TTC19 | TTC5 | TTLL1 | TUFM | TWF1 | TXN | TXN2 | TXNDC17 | TXNL4A | TXNRD1 | TYMS | U2AF2 | UAP1 | UAP1L1 | UBA1 | UBA2 | UBA3 | UBA5 | UBA6 | UBAC1 | UBASH3A | UBB | UBC | UBE2A | UBE2B | UBE2C | UBE2D1 | UBE2D2 | UBE2D3 | UBE2H | UBE2I | UBE2J1 | UBE2K | UBE2L3 | UBE2L6 | UBE2M | UBE2N | UBE2O | UBE2Q2 | UBE2R2 | UBE2S | UBE2V1 | UBE2W | UBE3A | UBE3B | UBIAD1 | UBL4A | UBQLN1 | UBQLN2 | UBQLN4 | UBR4 | UBR5 | UBXN1 | UBXN7 | UCHL1 | UCHL3 | UCHL5 | UCK2 | UFC1 | UFM1 | UGDH | UGGT1 | UGP2 | UGT8 | ULK1 | UMPS | UNKL | UPF2 | UPF3B | UPP2 | UQCRB | UQCRC2 | UQCRFS1 | UQCRQ | USP11 | USP14 | USP15 | USP16 | USP17L2 | USP19 | USP2 | USP20 | USP21 | USP26 | USP28 | USP39 | USP4 | USP42 | USP46 | USP47 | USP5 | USP50 | USP53 | USP7 | USP8 | USP9X | USP9Y | USPL1 | UTP14A | UTP6 | UXS1 | VAC14 | VAPA | VAPB | VARS | VASH2 | VAT1 | VAV1 | VCAM1 | VCAN | VCP | VCPIP1 | VDAC1 | VDR | VGF | VHL | VKORC1 | VPS18 | VPS37D | VPS4B | VTA1 | WARS | WDR12 | WDR4 | WDR41 | WDR45B | WDR48 | WDR82 | WDTC1 | WDYHV1 | WIPI1 | WIPI2 | WNK1 | WNK4 | WSB1 | WT1 | WTAP | WWOX | WWP2 | XBP1 | XIAP | XPNPEP1 | XPO5 | XRCC1 | XRCC3 | XRCC5 | XRCC6 | XYLT1 | YAP1 | YBEY | YEATS2 | YES1 | YME1L1 | YWHAB | YWHAE | YWHAH | YY1 | ZAP70 | ZBTB16 | ZBTB25 | ZBTB7A | ZBTB7B | ZC3HC1 | ZDHHC17 | ZDHHC23 | ZFYVE26 | ZMPSTE24 | ZNF217 | ZNF598 | ZNF638 | ZNRF4 | ZRANB2 | GO:0071704 | 0.541307776295038 | 4462/8243 | 1947/3250 | 1.06616543245232e-17 | 1.01232407811347e-13 | T | T | T | T | organic substance metabolic process | A2M | AARS | AARSD1 | AASDH | AASDHPPT | AASS | ABAT | ABCD3 | ABCG1 | ABHD14B | ABHD17A | ABL1 | ABL2 | ACAA1 | ACAA2 | ACACA | ACAD8 | ACAD9 | ACADS | ACADSB | ACADVL | ACAT1 | ACAT2 | ACBD3 | ACCS | ACD | ACE2 | ACLY | ACO1 | ACO2 | ACOT13 | ACOT7 | ACOT9 | ACOX1 | ACP1 | ACSL1 | ACSL4 | ACSL5 | ACSS2 | ACTB | ACTL6A | ACTN2 | ACY1 | ADA | ADAMTS4 | ADCK2 | ADCY6 | ADCYAP1 | ADH1A | ADH1B | ADH5 | ADK | ADORA2A | ADPRH | ADRB2 | ADRM1 | ADSL | ADSS | ADTRP | AFF4 | AFG3L2 | AGK | AGL | AGO1 | AGO2 | AHCY | AHCYL1 | AHSG | AICDA | AIMP2 | AK1 | AK2 | AK4 | AKR1A1 | AKR1B1 | AKR1B10 | AKR1B15 | AKR1C2 | AKT1 | AKT2 | AKT3 | ALAS1 | ALB | ALDH1B1 | ALDH4A1 | ALDH5A1 | ALDH6A1 | ALDH7A1 | ALDH8A1 | ALDH9A1 | ALDOA | ALDOC | ALOX12 | ALOX5 | ALOX5AP | AMD1 | AMDHD2 | AMFR | ANAPC4 | ANXA1 | ANXA2 | AOC2 | APBB1 | APC | APCS | APEH | APEX1 | APEX2 | APOA1 | APOD | APOE | APOL2 | APP | APRT | AR | ARAF | ARF1 | ARF4 | ARHGAP35 | ARID4B | ARMC8 | ARNT | ARRB1 | ARRB2 | ASB12 | ASB15 | ASB16 | ASB6 | ASB9 | ASCC1 | ASCC2 | ASGR1 | ASGR2 | ASL | ASNS | ASPDH | ASS1 | ATAD5 | ATF2 | ATF6B | ATG4C | ATG5 | ATIC | ATM | ATOH1 | ATP13A2 | ATP6V1A | ATP6V1B1 | ATRX | ATXN1 | ATXN2 | AUP1 | AURKB | AZU1 | B3GAT3 | B3GNT3 | B4GALT3 | BABAM1 | BACE1 | BAG2 | BAG6 | BANF1 | BAX | BCAT2 | BCKDHB | BCL10 | BCL11A | BCL2 | BCOR | BDH1 | BIRC2 | BIRC3 | BIRC5 | BIRC7 | BIRC8 | BLVRB | BMI1 | BMPR1A | BRAF | BRCA1 | BRD4 | BSG | BTBD9 | BTK | BTN2A1 | BTRC | BUD13 | BUD31 | BYSL | C1QBP | C1R | C4A | C9orf64 | CAB39 | CACNG2 | CACNG4 | CACNG5 | CALM1 | CALR | CALU | CAMK1D | CAMK2A | CAMK2B | CAMKV | CAND1 | CAPN1 | CAPN2 | CAPNS1 | CARM1 | CARS | CASP10 | CASP2 | CASP3 | CASP7 | CASP8 | CASP9 | CAT | CAV1 | CBFB | CBL | CBLB | CBLC | CBR1 | CBR4 | CBS | CBX4 | CBX8 | CCAR2 | CCDC155 | CCDC22 | CCDC36 | CCDC8 | CCDC88A | CCNB1 | CCNT1 | CCNT2 | CCP110 | CCR5 | CCS | CD2BP2 | CD36 | CD40LG | CD74 | CD81 | CDA | CDC14B | CDC16 | CDC20 | CDC25A | CDC25B | CDC25C | CDC27 | CDC34 | CDC42 | CDC5L | CDC73 | CDK15 | CDK16 | CDK19 | CDK2 | CDK4 | CDK5 | CDK5R1 | CDK8 | CDK9 | CDKL3 | CDKN1A | CEBPA | CEBPE | CEL | CENPF | CES1 | CFL1 | CFTR | CHAF1A | CHD1L | CHDH | CHEK2 | CHGB | CHP1 | CHRAC1 | CHRM3 | CHST4 | CISD3 | CKAP4 | CKB | CKM | CKMT1A | CLK1 | CLPX | CLTC | CLU | CMPK1 | CNDP1 | CNDP2 | COG3 | COL2A1 | COL4A2 | COL4A3BP | COL5A1 | COMMD1 | COMT | COPS2 | COPS5 | COPS6 | COQ2 | COQ3 | COQ4 | COQ5 | COQ6 | COQ7 | COQ9 | COX15 | CPNE3 | CPSF7 | CPT1A | CPT2 | CPVL | CRBN | CREB1 | CREBBP | CRK | CRKL | CRMP1 | CROT | CRY1 | CRY2 | CRYAB | CS | CSNK1A1 | CSNK1E | CSNK2A2 | CSNK2B | CSTF2T | CT45A5 | CTBP1 | CTCF | CTDP1 | CTH | CTNNB1 | CTPS1 | CTSA | CTSC | CTSD | CTSL | CTSV | CUL1 | CUL2 | CUL3 | CUL4B | CUL5 | CUL7 | CWC15 | CXCL8 | CYB5R1 | CYB5R3 | CYC1 | CYCS | CYLD | CYP1A1 | CYP1A2 | CYP2C18 | DAD1 | DAP3 | DAPK1 | DAPP1 | DARS | DBH | DBI | DBT | DCAF12 | DCAF7 | DCAF8 | DCAKD | DCK | DCLK1 | DCN | DCP2 | DCTPP1 | DCUN1D1 | DCXR | DDA1 | DDB1 | DDB2 | DDI1 | DDOST | DDRGK1 | DDX1 | DDX39A | DDX39B | DDX3X | DDX6 | DECR1 | DEGS1 | DERA | DERL1 | DGKE | DGKZ | DGUOK | DHCR24 | DHRS4 | DHRS7B | DHX15 | DHX36 | DHX9 | DIDO1 | DIS3 | DLAT | DLD | DLG2 | DLG3 | DLG4 | DLST | DMAP1 | DMD | DNA2 | DNAJA3 | DNAJB11 | DNAJB2 | DNAJC10 | DNAJC30 | DNASE2 | DNASE2B | DNPEP | DPEP1 | DPF2 | DPM1 | DPP9 | DRG2 | DROSHA | DSE | DTX1 | DTX2 | DUS3L | DUSP1 | DUSP12 | DUSP13 | DUSP14 | DUSP16 | DUSP19 | DUSP22 | DUSP7 | DUT | DVL1 | DVL2 | DYRK2 | DZIP3 | E2F1 | E2F2 | E2F3 | EAF2 | ECD | ECH1 | ECHDC2 | ECHS1 | ECI1 | ECI2 | EDA | EDC4 | EDEM1 | EED | EEF1A1 | EEF1A1P5 | EEF1A2 | EEF1B2 | EEF1D | EEF1G | EEF2 | EEF2KMT | EFEMP1 | EFTUD2 | EGFR | EGLN1 | EGLN3 | EHHADH | EHMT1 | EHMT2 | EIF2AK3 | EIF2AK4 | EIF2S1 | EIF3E | EIF3H | EIF4A3 | EIF4H | EIF5A | ELAC2 | ELAVL1 | ELAVL4 | ELF3 | ENDOG | ENO1 | ENO2 | ENO3 | ENOPH1 | ENTPD7 | EP300 | EPHA4 | EPHA6 | EPHB6 | EPRS | ERAL1 | ERBB2 | ERBB3 | ERCC5 | ERG | ERLIN2 | ESD | ESR1 | ESR2 | ESRRA | ESRRB | ESRRG | ETFA | ETFB | ETFDH | ETS1 | EVA1A | EXOSC1 | EXOSC5 | EXOSC6 | EXOSC9 | EXTL2 | EYA2 | EYA4 | EZH2 | EZR | F5 | F9 | FABP1 | FABP3 | FABP5 | FABP7 | FADD | FAF1 | FAF2 | FAH | FAM20B | FAM98A | FANCA | FANCC | FANCG | FANCI | FANCM | FAR2 | FARSB | FASN | FBP1 | FBP2 | FBXL12 | FBXO11 | FBXO25 | FBXO32 | FBXO6 | FBXO7 | FBXW11 | FBXW5 | FDPS | FDXR | FECH | FEN1 | FGB | FGF2 | FGFR1 | FGFR3 | FGL2 | FH | FIGNL1 | FKBP14 | FKBP1A | FKBP1B | FKBP2 | FKBP5 | FKBP7 | FKBP8 | FN1 | FOLH1 | FOLR1 | FOS | FOXK1 | FOXK2 | FOXM1 | FOXO1 | FPGS | FTO | FTSJ3 | FUBP1 | FUBP3 | FUS | FYN | FZR1 | G3BP1 | G6PD | GAA | GAD2 | GAK | GALE | GALNS | GALNT7 | GANAB | GAPDH | GAPDHS | GAR1 | GARS | GART | GATA1 | GBE1 | GCDH | GDA | GDPGP1 | GEMIN4 | GEMIN5 | GEMIN7 | GFPT1 | GFPT2 | GGA1 | GGCT | GGPS1 | GGT1 | GGTLC1 | GINS2 | GINS3 | GINS4 | GLA | GLB1L2 | GLCE | GLO1 | GLRX | GLRX3 | GLRX5 | GLS | GLS2 | GLUD2 | GLUL | GMDS | GMPPB | GMPR | GMPS | GNAI3 | GNAS | GNPDA1 | GNPDA2 | GNPNAT1 | GNS | GOLGA2 | GOLPH3 | GOT1 | GOT1L1 | GOT2 | GPC5 | GPD1 | GPD1L | GPI | GPS2 | GPT2 | GPX1 | GPX4 | GRB2 | GRHPR | GRIA1 | GRK1 | GRK5 | GSK3A | GSK3B | GSPT1 | GSPT2 | GSR | GSS | GSTA1 | GSTA2 | GSTA3 | GSTA4 | GSTA5 | GSTK1 | GSTM4 | GSTO1 | GSTO2 | GSTP1 | GSTT1 | GSTT2 | GSTT2B | GSTZ1 | GTF2A1 | GTF2E2 | GTF2F1 | GTF2F2 | GTF2I | GTF3C2 | GUSB | GYG1 | GYG2 | GYS1 | GZMA | GZMK | H1F0 | H2AFX | H3F3A | HAAO | HADH | HAGH | HARS | HBEGF | HCCS | HDAC1 | HDAC11 | HDAC2 | HDAC3 | HDAC4 | HDAC5 | HDAC6 | HDAC7 | HDAC9 | HECW1 | HECW2 | HELZ2 | HERC1 | HERC2 | HEXA | HEXB | HGS | HIBADH | HIBCH | HIF1A | HIF1AN | HINT1 | HIPK4 | HIST1H2BA | HIST1H3A | HIST1H4A | HIST2H3A | HIST3H3 | HK1 | HK2 | HLCS | HMBS | HMGB1 | HMGB2 | HMGB3 | HMGCL | HMMR | HMOX1 | HNF4A | HNF4G | HNRNPA1 | HNRNPA1L2 | HNRNPA2B1 | HNRNPD | HNRNPF | HNRNPH1 | HNRNPH2 | HNRNPH3 | HNRNPK | HNRNPL | HNRNPM | HNRNPU | HOXB9 | HPRT1 | HPX | HRAS | HS2ST1 | HS6ST1 | HSD17B10 | HSD17B14 | HSD17B4 | HSD17B6 | HSD17B8 | HSD3B7 | HSF1 | HSF4 | HSP90AA1 | HSP90AB1 | HSP90B1 | HSPA14 | HSPA1B | HSPA5 | HSPA8 | HSPD1 | HSPG2 | HTR1B | HTRA1 | HTRA2 | HUWE1 | IARS | ICK | IDE | IDH1 | IDH2 | IDH3A | IDH3B | IDH3G | IDI2 | IFI16 | IGF1R | IGFBP7 | IK | IKBKB | IKBKE | IKBKG | IL3RA | ILF2 | ILF3 | ILK | IMP4 | IMPA1 | IMPA2 | IMPDH2 | ING5 | INO80B | INO80C | INSIG1 | INSIG2 | INSR | IP6K1 | IREB2 | IRF1 | IRF4 | IRF7 | ISG15 | ISYNA1 | ITCH | ITGAV | ITK | ITPA | IVD | JAK1 | JAK3 | JMJD6 | JOSD2 | JUN | KAT5 | KAT7 | KAT8 | KBTBD7 | KCMF1 | KCNAB2 | KCTD17 | KCTD2 | KDM1A | KDM2A | KDM3B | KDM4D | KDM5B | KDM5C | KDM8 | KDR | KDSR | KEAP1 | KHDRBS1 | KIAA0391 | KLF5 | KLHL20 | KLHL22 | KMT2A | KMT2B | KMT2C | KPNA1 | KPNA2 | KPNB1 | KRAS | KSR1 | KYNU | L2HGDH | LACTB2 | LAMB1 | LAP3 | LARP4 | LARP7 | LARS | LAT | LATS1 | LCMT2 | LDHA | LDHAL6A | LDHAL6B | LDHB | LDHC | LDHD | LEF1 | LGALS1 | LGALS3 | LGMN | LIG4 | LIMA1 | LMO2 | LMO7 | LNX1 | LOXL2 | LPIN3 | LRRC7 | LRRK1 | LRRK2 | LRSAM1 | LSM1 | LSM12 | LSM3 | LSM4 | LSM5 | LSM8 | LTBP1 | LTC4S | LUM | LYG2 | LYPLA1 | LYPLA2 | LZTR1 | MAD2L1 | MAN2B1 | MAOA | MAP2K1 | MAP2K3 | MAP2K4 | MAP2K6 | MAP2K7 | MAP3K1 | MAP3K13 | MAP3K14 | MAP3K2 | MAP3K3 | MAP3K4 | MAP4K1 | MAPK1 | MAPK14 | MAPK15 | MAPK3 | MAPK4 | MAPK6 | MAPK7 | MAPK8 | MAPK9 | MAPT | MARCH5 | MASP1 | MASP2 | MAST1 | MAST3 | MATK | MBD3 | MBP | MC1R | MCAT | MCCC1 | MCFD2 | MCM2 | MCM3 | MCM4 | MCM5 | MCM6 | MCM7 | MCM9 | MCMBP | MDC1 | MDH1 | MDH2 | MDM2 | MDM4 | MDN1 | ME1 | MECP2 | MED1 | MED12 | MED14 | MED17 | MED20 | MED21 | MED23 | MED24 | MED31 | MED4 | MED6 | MELK | MEPCE | METAP2 | MGMT | MGST1 | MIB1 | MIF | MITF | MKNK2 | MLH1 | MLLT1 | MLLT3 | MMAB | MME | MMP26 | MOCOS | MORF4L1 | MORF4L2 | MOV10 | MPC2 | MPG | MPHOSPH6 | MPI | MPPE1 | MPST | MRGBP | MRM1 | MRPL12 | MRPL14 | MRPL19 | MRPL28 | MRPL38 | MRPL4 | MRPL43 | MRPL45 | MRPL46 | MRPL9 | MRPS5 | MRRF | MSH6 | MTA1 | MTA3 | MTAP | MTERF1 | MTHFD1 | MTMR14 | MTMR7 | MTPN | MTRR | MVD | MYC | MYH6 | MYO5A | NAE1 | NAGK | NAMPT | NANP | NANS | NAP1L1 | NAPRT | NBAS | NCBP1 | NCEH1 | NCK1 | NCOA1 | NCOA3 | NCOR1 | NDUFA9 | NDUFAF5 | NDUFAF7 | NDUFS1 | NDUFS6 | NEDD4 | NEDD8 | NEK1 | NEK7 | NELFB | NELFCD | NELFE | NEURL4 | NFATC1 | NFE2L2 | NFKBIA | NFS1 | NHLRC3 | NIPBL | NIT2 | NKX2-1 | NLK | NME1 | NME2 | NME4 | NMNAT1 | NMNAT3 | NMRAL1 | NMRK2 | NNT | NOB1 | NOD2 | NOP10 | NOP56 | NOS2 | NOS3 | NOSIP | NPEPL1 | NPEPPS | NPLOC4 | NPM1 | NPR1 | NR0B2 | NR1H2 | NR1H3 | NR1I2 | NR3C1 | NR4A1 | NR5A2 | NRBF2 | NSFL1C | NSMCE4A | NSUN2 | NSUN6 | NT5C2 | NTMT1 | NTRK1 | NUCB1 | NUDT13 | NUDT18 | NUDT19 | NUDT3 | NUDT9 | NUFIP1 | NUP107 | NUP153 | NUP160 | NUP205 | NUP214 | NUP85 | NUP88 | NUP93 | NUP98 | NUPR1 | OAS2 | OAT | OAZ3 | OBSL1 | OGDH | OGFOD1 | OGT | OLA1 | ONECUT1 | OPA1 | OSBPL1A | OTC | OTUB1 | OTUB2 | OTUD4 | OTUD5 | OVGP1 | OXA1L | OXCT1 | OXSR1 | P2RX1 | P4HA1 | P4HA2 | P4HB | PA2G4 | PABPC1 | PABPC3 | PABPC4 | PADI4 | PAFAH1B1 | PAH | PAICS | PAK2 | PAK6 | PALB2 | PAN2 | PAPLN | PAPSS1 | PAPSS2 | PARK7 | PARP1 | PARP12 | PARP14 | PARP6 | PAWR | PAX8 | PAXIP1 | PC | PCBD1 | PCBP1 | PCBP2 | PCF11 | PCGF1 | PCGF5 | PCGF6 | PCMT1 | PCMTD1 | PCMTD2 | PCNA | PCSK9 | PCTP | PCYT2 | PDCD6IP | PDE12 | PDE4D | PDF | PDHA1 | PDHA2 | PDHB | PDHX | PDIA3 | PDIA6 | PDK2 | PDSS1 | PDSS2 | PDXK | PEBP1 | PELI1 | PEPD | PER1 | PER2 | PEX5 | PFAS | PFKL | PFKM | PFKP | PGAM1 | PGAM2 | PGAM5 | PGD | PGK1 | PGK2 | PGM1 | PGM2 | PGM3 | PGM5 | PGP | PHB | PHF10 | PHGDH | PHKA1 | PHOSPHO1 | PI4K2A | PIAS1 | PIAS3 | PICK1 | PIGU | PIH1D1 | PIK3C2A | PIK3CA | PIK3CG | PIK3R1 | PIK3R3 | PIKFYVE | PIM1 | PIN1 | PINX1 | PIP4K2A | PIPSL | PIR | PITPNA | PITPNB | PJA1 | PKM | PKN2 | PLA2G10 | PLA2G12A | PLA2G1B | PLA2G2A | PLA2G6 | PLBD2 | PLCG1 | PLK1 | PLOD2 | PLSCR1 | PML | PMPCA | PMPCB | PNKP | PNP | POLA2 | POLD1 | POLDIP3 | POLE2 | POLE3 | POLM | POLR2A | POLR2E | POLR2L | POLR2M | POM121 | POT1 | POU5F1 | PPA1 | PPA2 | PPARD | PPARG | PPARGC1A | PPAT | PPEF1 | PPIA | PPIB | PPID | PPIE | PPIF | PPIL3 | PPM1A | PPM1B | PPM1D | PPM1H | PPM1K | PPME1 | PPOX | PPP1CA | PPP1CB | PPP1CC | PPP1R11 | PPP1R12A | PPP1R2 | PPP1R3B | PPP1R3C | PPP1R8 | PPP2CA | PPP2CB | PPP2R1A | PPP2R1B | PPP2R2A | PPP2R2D | PPP2R5D | PPP3CC | PPP4R2 | PPP5C | PPP6C | PPT1 | PPTC7 | PRCC | PRCP | PRDX3 | PRDX4 | PRDX6 | PREP | PREPL | PRKAA1 | PRKAA2 | PRKAB2 | PRKACA | PRKAG1 | PRKAR2B | PRKCD | PRKCE | PRKCSH | PRKCZ | PRKD1 | PRKD2 | PRKD3 | PRKDC | PRMT2 | PRMT3 | PRMT6 | PROZ | PRPF19 | PRPF40A | PRPF8 | PRSS16 | PRSS23 | PRTFDC1 | PSAP | PSAT1 | PSEN1 | PSEN2 | PSMA1 | PSMA2 | PSMA3 | PSMA5 | PSMA6 | PSMB5 | PSMB6 | PSMB7 | PSMB8 | PSMC1 | PSMC2 | PSMC3 | PSMC4 | PSMC5 | PSMC6 | PSMD1 | PSMD10 | PSMD11 | PSMD12 | PSMD14 | PSMD2 | PSMD4 | PSMD9 | PSME2 | PSME3 | PTEN | PTGDS | PTGES | PTGES2 | PTGES3 | PTGR1 | PTK2 | PTMA | PTMS | PTPMT1 | PTPN1 | PTPN14 | PTPN21 | PTPN22 | PTPN23 | PTPN4 | PTPN6 | PTPRA | PTPRD | PTPRF | PTPRK | PTPRO | PTPRR | PTTG1 | PUM1 | PUS1 | PUS7 | PYCR1 | PYCR2 | PYGB | PYGL | QARS | QDPR | QSOX1 | RAB11A | RAB11B | RAB1A | RAB1B | RAB2A | RAB31 | RAB5A | RAB7A | RAD21 | RAD23A | RAD23B | RAD51 | RAD51AP1 | RAE1 | RAF1 | RAN | RANBP9 | RANGAP1 | RARA | RARG | RARS2 | RBBP4 | RBBP6 | RBM17 | RBM25 | RBM3 | RBM4 | RBM8A | RBMX | RBP1 | RBPJ | RBX1 | RC3H1 | RDH11 | RDH12 | RDH13 | RDH14 | RDH5 | RELA | REPIN1 | REST | REXO2 | RFC1 | RFC2 | RFC3 | RFC4 | RFC5 | RFK | RGN | RHBDD1 | RHO | RHOA | RHOT1 | RIF1 | RING1 | RIOK1 | RIPK1 | RIPK2 | RIPK3 | RIPK4 | RMND1 | RNASE3 | RNASEH2A | RNASEH2B | RNF123 | RNF126 | RNF139 | RNF181 | RNF183 | RNF185 | RNF19A | RNF2 | RNF25 | RNF31 | RNF4 | RNF8 | RNPEP | RPA2 | RPA3 | RPAIN | RPE | RPIA | RPL10 | RPL10A | RPL12 | RPL18A | RPL22 | RPL23 | RPL23A | RPL24 | RPL30 | RPL36AL | RPL37A | RPL38 | RPL6 | RPL7 | RPL7A | RPL9 | RPLP0 | RPLP0P6 | RPLP1 | RPLP2 | RPN1 | RPN2 | RPP14 | RPS12 | RPS13 | RPS14 | RPS15A | RPS19 | RPS2 | RPS20 | RPS24 | RPS29 | RPS3 | RPS4X | RPS6KA1 | RPS6KA2 | RPS6KB2 | RPS8 | RRM1 | RRM2 | RRM2B | RRP7A | RRS1 | RTCB | RTF1 | RTFDC1 | RUNX2 | RUVBL1 | RUVBL2 | RXRA | RXRB | RXRG | S100A9 | SAE1 | SAP18 | SARS | SART1 | SART3 | SAT1 | SAT2 | SBDS | SCAF1 | SCCPDH | SCG2 | SCG3 | SCLY | SCO2 | SCYL2 | SDC1 | SDC4 | SDHA | SDHB | SEL1L | SENP3 | SENP6 | SENP7 | SENP8 | SEPHS1 | SEPHS2 | SERPINH1 | SET | SETDB1 | SF1 | SF3B6 | SFPQ | SGPL1 | SGSH | SGTA | SGTB | SHC1 | SHMT1 | SHMT2 | SIAH1 | SIN3A | SIRT1 | SIRT3 | SIRT5 | SIRT6 | SIRT7 | SKI | SKIV2L | SLC16A1 | SLC25A1 | SLC25A10 | SLC25A11 | SLC25A13 | SLC25A32 | SLC2A5 | SLC2A9 | SLC3A2 | SLC9A1 | SMAD2 | SMAD3 | SMAD4 | SMAD5 | SMARCB1 | SMC1A | SMC3 | SMG5 | SMG6 | SMG8 | SMN1 | SMURF1 | SMURF2 | SNCA | SND1 | SNRNP27 | SNRPA | SNRPA1 | SNRPB | SNRPB2 | SNRPD1 | SNRPD3 | SNRPE | SNW1 | SNX17 | SNX3 | SOAT1 | SOCS1 | SOCS2 | SOCS6 | SOD1 | SORD | SP1 | SP100 | SPATA2 | SPI1 | SPIN1 | SPOP | SPR | SPRR2A | SPRTN | SPSB3 | SPTAN1 | SPTBN1 | SPTBN2 | SPTLC2 | SQSTM1 | SRC | SRPK1 | SRPK2 | SRSF11 | SRSF12 | SRSF5 | SSB | ST3GAL1 | ST3GAL6 | ST6GALNAC5 | ST8SIA4 | STARD7 | STAT3 | STAT5A | STBD1 | STK11 | STK24 | STK25 | STK26 | STK3 | STK38 | STK40 | STOML2 | STUB1 | STYX | SUCLA2 | SUCLG2 | SULF2 | SULT1A3 | SUMO1 | SUMO1P1 | SUMO2 | SUMO3 | SUPT4H1 | SUPT5H | SUZ12 | SWAP70 | SYVN1 | TAB1 | TAB2 | TADA2B | TAF12 | TAF15 | TAF1A | TALDO1 | TAMM41 | TANK | TAOK3 | TARDBP | TARS | TARS2 | TATDN1 | TAZ | TBL1X | TBRG4 | TCEA1 | TCEA2 | TCERG1 | TCF4 | TCIRG1 | TDO2 | TDP1 | TECR | TERF1 | TERF2 | TERF2IP | TESK2 | TFCP2 | TFIP11 | TGFB1 | TGFBR3 | TGM2 | TGOLN2 | THADA | THBS1 | THEM4 | THOC2 | THOC6 | THOC7 | THOP1 | THRA | TIMM50 | TIMP1 | TINF2 | TK1 | TK2 | TKT | TLR3 | TMEM132A | TMEM189-UBE2V1 | TMEM258 | TMTC3 | TNF | TNFAIP3 | TNFRSF1A | TNIP2 | TNK2 | TNKS | TNKS2 | TNP1 | TOE1 | TOLLIP | TOMM20 | TOP1 | TOP2A | TOP3A | TOR1A | TP53 | TP53RK | TP73 | TPI1 | TPR | TPSAB1 | TPSB2 | TPST2 | TPTE | TRA2A | TRAF2 | TRAF3 | TRAF6 | TREX1 | TRIB2 | TRIB3 | TRIM21 | TRIM23 | TRIM25 | TRIM28 | TRIM29 | TRIM32 | TRIM33 | TRIM41 | TRIM47 | TRIM63 | TRIM69 | TRIM7 | TRIM9 | TRIP12 | TRIP13 | TRMT44 | TRMT6 | TRMT61B | TRNT1 | TRUB2 | TSC22D1 | TSFM | TSG101 | TSN | TSPAN17 | TTC5 | TTLL1 | TUFM | TWF1 | TXN | TXN2 | TXNL4A | TXNRD1 | TYMS | U2AF2 | UAP1 | UAP1L1 | UBA1 | UBA2 | UBA3 | UBA5 | UBA6 | UBAC1 | UBB | UBC | UBE2A | UBE2B | UBE2C | UBE2D1 | UBE2D2 | UBE2D3 | UBE2H | UBE2I | UBE2J1 | UBE2K | UBE2L3 | UBE2L6 | UBE2M | UBE2N | UBE2O | UBE2Q2 | UBE2R2 | UBE2S | UBE2V1 | UBE2W | UBE3A | UBE3B | UBIAD1 | UBL4A | UBQLN1 | UBQLN2 | UBQLN4 | UBR4 | UBR5 | UBXN1 | UBXN7 | UCHL1 | UCHL3 | UCHL5 | UCK2 | UFC1 | UFM1 | UGDH | UGGT1 | UGP2 | UGT8 | ULK1 | UMPS | UNKL | UPF2 | UPF3B | UPP2 | UQCRC2 | USP11 | USP14 | USP15 | USP16 | USP17L2 | USP19 | USP2 | USP20 | USP21 | USP26 | USP28 | USP39 | USP4 | USP42 | USP46 | USP47 | USP5 | USP50 | USP53 | USP7 | USP8 | USP9X | USP9Y | USPL1 | UTP14A | UTP6 | UXS1 | VAC14 | VAPA | VAPB | VARS | VASH2 | VCAM1 | VCAN | VCP | VCPIP1 | VDAC1 | VDR | VGF | VHL | VKORC1 | VPS37D | VPS4B | WARS | WDR12 | WDR4 | WDR45B | WDR48 | WDR82 | WDTC1 | WDYHV1 | WIPI1 | WIPI2 | WNK1 | WNK4 | WSB1 | WT1 | WTAP | WWOX | WWP2 | XBP1 | XIAP | XPNPEP1 | XPO5 | XRCC1 | XRCC3 | XRCC5 | XRCC6 | XYLT1 | YAP1 | YBEY | YEATS2 | YES1 | YME1L1 | YWHAB | YWHAE | YWHAH | YY1 | ZAP70 | ZBTB16 | ZBTB25 | ZBTB7A | ZBTB7B | ZC3HC1 | ZDHHC17 | ZDHHC23 | ZFYVE26 | ZMPSTE24 | ZNF217 | ZNF598 | ZNF638 | ZNRF4 | ZRANB2 | GO:0042221 | 0.191313842047798 | 1577/8243 | 769/3250 | 3.61706303393724e-17 | 3.43440135072341e-13 | T | T | T | T | response to chemical | ABAT | ABCD3 | ABCG1 | ABL1 | ABL2 | ACAA2 | ACACA | ACAT1 | ACO1 | ACSL1 | ACSL4 | ACTA1 | ACTB | ACTG1 | ACTR2 | ACTR3 | ADA | ADCY6 | ADCYAP1 | ADH5 | ADORA2A | ADRB2 | ADSL | ADSS | ADTRP | AES | AGER | AGL | AHCY | AHCYL1 | AICDA | AIFM1 | AK4 | AKAP8 | AKAP9 | AKR1B1 | AKR1C2 | AKT1 | AKT2 | ALOX12 | ALOX5AP | ANK3 | ANXA1 | ANXA11 | ANXA2 | ANXA5 | ANXA7 | APBB1 | APEX1 | APLP1 | APOA1 | APOD | APOE | APP | APRT | AR | ARNT2 | ARPC1B | ARRB1 | ASCL1 | ASNS | ASS1 | ATF2 | ATG5 | ATIC | ATM | ATP13A2 | ATP1A1 | ATP2A2 | ATP6V1A | ATRX | BACE1 | BAG3 | BAG4 | BAX | BCAR1 | BCAR3 | BCAT2 | BCKDHB | BCL10 | BCL11A | BCL2 | BCL2L1 | BIRC2 | BMPR1A | BRAF | BRCA1 | BSG | BSPRY | BTK | BTRC | C1QA | CACNA1A | CACNG2 | CACNG4 | CALCOCO2 | CALM1 | CALM2 | CALM3 | CALR | CAPN2 | CARD9 | CARM1 | CASP3 | CASP7 | CASP8 | CASP9 | CAT | CAV1 | CBL | CBX8 | CCL21 | CCL22 | CCNB1 | CCR4 | CCR5 | CCS | CD27 | CD36 | CD40 | CD6 | CD81 | CDC34 | CDC42EP4 | CDC5L | CDC73 | CDH1 | CDK19 | CDK2 | CDK4 | CDK5 | CDK9 | CDKN1A | CEBPA | CEBPE | CENPF | CES1 | CFL1 | CFTR | CHEK2 | CHRND | CHRNE | CHRNG | CLDN18 | CLIC4 | CLU | COL18A1 | COL2A1 | COL4A1 | COL4A2 | COL6A1 | COL6A2 | COMT | CPNE3 | CPT1A | CREB1 | CRIP1 | CRK | CRKL | CRY1 | CRYAB | CSN1S1 | CSNK2B | CTH | CTNNB1 | CTPS1 | CTSC | CTSL | CTSV | CUTA | CXCL11 | CXCL5 | CXCL6 | CXCL8 | CYC1 | CYCS | CYP1A1 | CYP1A2 | DAB1 | DAD1 | DAPK1 | DAXX | DBH | DCN | DDIT4 | DDOST | DDX1 | DDX3X | DENND4C | DERL1 | DHX15 | DHX36 | DHX9 | DLG2 | DLG4 | DMAP1 | DNAJA1 | DNAJB1 | DNAJB2 | DNM1L | DPEP1 | DTX1 | DUSP1 | DUSP22 | E2F1 | EED | EEF1A1 | EEF1A2 | EEF2 | EFTUD2 | EGFR | EGLN1 | EHD4 | EHMT1 | EIF2AK3 | EIF2B1 | EIF2B3 | EIF2B5 | EIF2S1 | EIF4A3 | EIF4E | EIF4EBP1 | EIF6 | ENDOG | ENO3 | EPHA4 | EPRS | ERBB2 | ERRFI1 | ESR1 | ESRRA | ETS1 | EZH2 | EZR | FABP1 | FABP3 | FADD | FAF2 | FANCC | FASLG | FASN | FBP1 | FBXO32 | FBXO6 | FECH | FGB | FHL2 | FIS1 | FKBP1B | FLNB | FLOT1 | FOLR1 | FOS | FOXA1 | FOXO1 | FUS | FYN | G6PD | GAD2 | GAPDH | GART | GATA1 | GBP2 | GBP3 | GBP5 | GBP6 | GDI1 | GFPT2 | GGT1 | GLP1R | GLRB | GLUL | GNA12 | GNAI1 | GNAI2 | GNAO1 | GNAS | GNB1 | GNG12 | GNPNAT1 | GOT1 | GOT2 | GPD1 | GPI | GPX1 | GPX4 | GPX7 | GRAMD1A | GSK3A | GSK3B | GSR | GSS | GSTO1 | GSTO2 | GSTP1 | H2AFZ | HAAO | HADH | HAVCR2 | HAX1 | HCN1 | HCN2 | HCN3 | HDAC2 | HDAC4 | HDAC5 | HDAC6 | HDAC9 | HDGF | HID1 | HIF1A | HK2 | HLCS | HMGB1 | HMGB2 | HMGCL | HMOX1 | HNF4A | HNRNPA1 | HNRNPD | HNRNPL | HNRNPU | HPRT1 | HSF1 | HSP90AA1 | HSP90AB1 | HSP90B1 | HSPA14 | HSPA1B | HSPA1L | HSPA2 | HSPA4 | HSPA4L | HSPA5 | HSPA8 | HSPA9 | HSPB1 | HSPB2 | HSPD1 | HSPE1 | HSPH1 | HTR1B | HTR3A | HTRA2 | ICAM1 | ID2 | IDH1 | IFI16 | IFIT1 | IFITM3 | IFNA2 | IFNAR1 | IGBP1 | IGF1R | IGFBP7 | IKBKB | IKBKE | IL3RA | IMPDH2 | INSIG1 | INSIG2 | INSR | IPO5 | IQGAP1 | IREB2 | IRF1 | ISG15 | ITGA4 | ITGB1BP1 | ITPR2 | ITPR3 | JAK1 | JAK3 | JUN | JUNB | JUND | JUP | KAT5 | KAT7 | KCNIP2 | KCNK1 | KDM1A | KDM3B | KDM5B | KDR | KEAP1 | KIF5B | KLF5 | KLHL20 | KLHL22 | KRAS | KYNU | LAMP3 | LARS | LDHA | LEF1 | LGALS1 | LGMN | LIG4 | LIMS1 | LMNA | LMO2 | LOXL2 | LPAR1 | LPIN3 | LRP6 | LRRK2 | LUM | MAP2K3 | MAP2K4 | MAP2K6 | MAP2K7 | MAP4K1 | MAPK1 | MAPK14 | MAPK3 | MAPK4 | MAPK6 | MAPK7 | MAPK8 | MAPK9 | MAPT | MAS1 | MAT2A | MAVS | MBD3 | MBP | MCM2 | MCM7 | MDM2 | MDM4 | ME1 | MED1 | MGMT | MGST1 | MKNK2 | MME | MPST | MSN | MTAP | MYC | MYD88 | MYO1C | MYO5A | MYO6 | NAMPT | NCOA1 | NCOA2 | NCOA3 | NDRG1 | NDUFA13 | NDUFA9 | NEDD4 | NEDD8 | NEUROD1 | NFATC2 | NFE2L2 | NFKB2 | NFKBIA | NKX2-1 | NME1 | NME2 | NMNAT3 | NNT | NOD2 | NOS2 | NOS3 | NPEPPS | NQO1 | NR0B2 | NR1H2 | NR1H3 | NR3C1 | NR4A1 | NR5A2 | NRP2 | NTRK1 | NUCB1 | NUPR1 | OGT | OPA1 | OPTN | OTC | OTUB1 | OTUD5 | OXCT1 | OXSR1 | P2RX1 | P2RX2 | P2RX4 | P4HB | PAK2 | PARK7 | PARP1 | PAX8 | PAX9 | PCNA | PCSK9 | PDCD10 | PDCD6 | PDE12 | PDE4D | PDIA3 | PDK2 | PEBP1 | PEF1 | PELI1 | PER1 | PFAS | PFKL | PFKP | PGAM2 | PGK1 | PHB | PHB2 | PIAS3 | PICK1 | PIK3CA | PIK3CG | PIK3R1 | PKM | PLA2G10 | PLA2G1B | PLCG1 | PLSCR1 | PML | PNP | PPARD | PPARG | PPARGC1A | PPAT | PPIF | PPM1A | PPOX | PPP1CA | PPP1R12A | PPP1R14A | PPP2CA | PPP2CB | PPP2R1A | PPP2R2A | PPP5C | PRDX1 | PRDX2 | PRDX3 | PRDX5 | PRDX6 | PRKAA1 | PRKAA2 | PRKACA | PRKAR1A | PRKAR2B | PRKCD | PRKCE | PRKCZ | PRKD1 | PRKD2 | PRKDC | PRPF8 | PSAP | PSEN1 | PSMD14 | PTEN | PTGES | PTGIR | PTGR1 | PTN | PTPN1 | PTPN14 | PTPN22 | PTPN23 | PTPN4 | PTPN6 | PTPRK | PXN | PYCR1 | PYCR2 | QDPR | RAB10 | RAB31 | RAB35 | RAD23B | RAD51 | RAE1 | RANGAP1 | RAP1A | RAP1B | RARA | RARG | RB1 | RBBP4 | RBM4 | RBMX | RC3H1 | RDX | REL | RELA | REST | RFC3 | RGS2 | RHBDD1 | RHOA | RIF1 | RIPK1 | RIPK2 | RIPK3 | RNF4 | ROMO1 | RPL10A | RPL18A | RPL23 | RPN2 | RPS3 | RPTOR | RRM2B | RUNX2 | RUVBL2 | RXRA | S100A13 | S100A7 | S100B | S100P | SARM1 | SCFD1 | SDC1 | SEC31A | SELL | SERPINH1 | SETDB1 | SH3GL2 | SHC1 | SHMT1 | SHMT2 | SIN3A | SIPA1 | SIRT1 | SLAMF8 | SLC12A2 | SLC16A1 | SLC25A13 | SLC25A24 | SLC25A5 | SLC2A5 | SLC3A2 | SLC9A1 | SMAD2 | SMAD3 | SMAD4 | SMAD5 | SMAD9 | SMO | SNAI2 | SNCA | SNW1 | SNX6 | SOCS1 | SOCS2 | SOD1 | SOD2 | SORD | SORT1 | SOX2 | SP1 | SP100 | SPI1 | SPINT2 | SPRY2 | SRC | SRP14 | SRP19 | SRSF5 | SRXN1 | ST3GAL6 | STAT1 | STAT3 | STAT6 | STAU1 | STIM1 | STIP1 | STK24 | STK25 | STK26 | STUB1 | STXBP1 | STXBP2 | SULT1A3 | SUMO1 | SYP | TANK | TBC1D4 | TBXA2R | TDO2 | TERF1 | TGFB1 | TGFBR3 | THBS1 | THRA | TIMP1 | TIMP2 | TLR3 | TMBIM6 | TMEM173 | TNF | TNFAIP3 | TNIP2 | TNKS | TOMM20 | TOP1 | TP53 | TP73 | TPCN2 | TPM1 | TPR | TRADD | TRAF2 | TRAF6 | TREX1 | TRIB3 | TRIM21 | TRIM25 | TRIM32 | TRIM41 | TRIM63 | TUFM | TWF2 | TWIST1 | TXN2 | TXNIP | TXNRD1 | TYMS | UBE2B | UBE2D3 | UBE2K | UBE2L3 | UBE2W | UBE3A | UBQLN1 | UMPS | UQCRC2 | USP47 | USP8 | VCAM1 | VCP | VGF | VIM | VN1R1 | VPS4B | VSTM2A | WAS | WBP2 | WDR35 | WDTC1 | WNK1 | WNT2 | WT1 | WWOX | XAF1 | XBP1 | XPO1 | XRCC1 | XRCC3 | XRCC5 | YAP1 | YES1 | YWHAG | YY1 | ZBTB7B | ZFAND6 | ZNF277 | GO:0009987 | 0.870678151158559 | 7177/8243 | 2952/3250 | 3.76050309743061e-17 | 3.57059769101037e-13 | T | T | T | T | cellular process | A2M | AAR2 | AARS | AARSD1 | AASDH | AASDHPPT | AASS | ABAT | ABCB7 | ABCD3 | ABCE1 | ABCG1 | ABHD14B | ABHD17A | ABI3BP | ABL1 | ABL2 | ABLIM1 | ACAA1 | ACAA2 | ACACA | ACAD8 | ACAD9 | ACADS | ACADSB | ACADVL | ACAT1 | ACAT2 | ACCS | ACD | ACE2 | ACKR2 | ACLY | ACO1 | ACO2 | ACOT13 | ACOT7 | ACOT9 | ACOX1 | ACP1 | ACSL1 | ACSL4 | ACSL5 | ACSS2 | ACTA1 | ACTA2 | ACTB | ACTG1 | ACTL6A | ACTN1 | ACTN2 | ACTN4 | ACTR1A | ACTR2 | ACTR3 | ACY1 | ADA | ADAMTS4 | ADCK2 | ADCY6 | ADCYAP1 | ADD3 | ADH1A | ADH1B | ADH5 | ADK | ADORA2A | ADPRH | ADRB2 | ADRM1 | ADSL | ADSS | ADTRP | AEBP2 | AES | AFF4 | AFG3L2 | AGER | AGK | AGL | AGO1 | AGO2 | AGR2 | AGTRAP | AHCY | AHCYL1 | AHI1 | AHNAK | AHSG | AICDA | AIFM1 | AIMP2 | AK1 | AK2 | AK4 | AKAP8 | AKAP9 | AKR1A1 | AKR1B1 | AKR1B10 | AKR1B15 | AKR1C2 | AKT1 | AKT2 | AKT3 | AKTIP | ALAS1 | ALB | ALDH1B1 | ALDH4A1 | ALDH5A1 | ALDH6A1 | ALDH7A1 | ALDH8A1 | ALDH9A1 | ALDOA | ALDOC | ALOX12 | ALOX5 | ALOX5AP | AMD1 | AMDHD2 | AMFR | ANAPC4 | ANK1 | ANK3 | ANKRD28 | ANKRD49 | ANKS4B | ANP32A | ANXA1 | ANXA11 | ANXA2 | ANXA5 | ANXA6 | ANXA7 | AOC2 | AP1G1 | AP1M1 | AP3D1 | AP3M1 | APBB1 | APBB2 | APC | APCS | APEH | APEX1 | APEX2 | API5 | APLP1 | APOA1 | APOD | APOE | APP | APRT | AR | ARAF | ARF1 | ARF4 | ARFGAP1 | ARHGAP1 | ARHGAP19 | ARHGAP26 | ARHGAP35 | ARHGDIA | ARHGEF7 | ARHGEF9 | ARID4B | ARL4D | ARMC8 | ARNT | ARNT2 | ARPC1A | ARPC1B | ARPC2 | ARPC3 | ARPC4 | ARPC5 | ARR3 | ARRB1 | ARRB2 | ASB12 | ASB15 | ASB16 | ASB6 | ASB9 | ASCC1 | ASCC2 | ASCL1 | ASGR1 | ASGR2 | ASL | ASNS | ASPDH | ASS1 | ATAD3A | ATAD5 | ATCAY | ATF2 | ATF6 | ATF6B | ATG2B | ATG4C | ATG5 | ATIC | ATL3 | ATM | ATOH1 | ATOX1 | ATP12A | ATP13A2 | ATP1A1 | ATP1B3 | ATP2A2 | ATP2B2 | ATP4A | ATP6V1A | ATP6V1B1 | ATP6V1C1 | ATP6V1D | ATP6V1E1 | ATP9A | ATPAF2 | ATRX | ATXN1 | ATXN10 | ATXN2 | AUP1 | AURKB | AVIL | AXIN1 | AZU1 | B3GAT3 | B3GNT3 | B4GALT3 | BABAM1 | BACE1 | BAG1 | BAG2 | BAG3 | BAG4 | BAG5 | BAG6 | BAIAP2 | BAIAP2L1 | BANF1 | BANP | BASP1 | BAX | BBC3 | BCAR1 | BCAR3 | BCAT2 | BCKDHB | BCL10 | BCL11A | BCL2 | BCL2L1 | BCL2L13 | BCL2L2 | BCL6 | BCOR | BCS1L | BDH1 | BDKRB2 | BIN1 | BIRC2 | BIRC3 | BIRC5 | BIRC7 | BIRC8 | BLVRB | BLZF1 | BMI1 | BMPR1A | BNIPL | BRAF | BRCA1 | BRD2 | BRD3 | BRD4 | BRD9 | BSG | BSPRY | BTBD9 | BTK | BTN2A1 | BTRC | BUD13 | BUD31 | BYSL | C1QA | C1QBP | C1QL1 | C1QTNF1 | C4A | C9orf64 | CA8 | CAB39 | CABP4 | CACNA1A | CACNG2 | CACNG4 | CACNG5 | CALCOCO2 | CALM1 | CALM2 | CALM3 | CALR | CALU | CAMK1D | CAMK2A | CAMK2B | CAMKV | CAND1 | CANX | CAP2 | CAPG | CAPN1 | CAPN2 | CAPNS1 | CAPZA1 | CAPZA2 | CAPZB | CARD9 | CARM1 | CARS | CASP10 | CASP2 | CASP3 | CASP7 | CASP8 | CASP9 | CAT | CAV1 | CBFA2T3 | CBFB | CBL | CBLB | CBLC | CBR1 | CBR4 | CBS | CBX4 | CBX7 | CBX8 | CBY1 | CCAR2 | CCDC120 | CCDC155 | CCDC22 | CCDC36 | CCDC68 | CCDC69 | CCDC8 | CCDC88A | CCHCR1 | CCL21 | CCL22 | CCNB1 | CCNDBP1 | CCNT1 | CCNT2 | CCNY | CCP110 | CCR1 | CCR3 | CCR4 | CCR5 | CCS | CCT2 | CCT3 | CCT4 | CCT5 | CCT6A | CCT6B | CCT7 | CCT8 | CD109 | CD2 | CD209 | CD22 | CD27 | CD2BP2 | CD36 | CD40 | CD40LG | CD6 | CD74 | CD79B | CD81 | CDA | CDC14B | CDC16 | CDC20 | CDC25A | CDC25B | CDC25C | CDC27 | CDC34 | CDC37 | CDC42 | CDC42EP3 | CDC42EP4 | CDC5L | CDC73 | CDH1 | CDH5 | CDK15 | CDK16 | CDK19 | CDK2 | CDK4 | CDK5 | CDK5R1 | CDK8 | CDK9 | CDKL3 | CDKN1A | CDKN2C | CEBPA | CEBPE | CEL | CENPF | CENPJ | CENPV | CEP135 | CEP152 | CEP290 | CEP350 | CEP55 | CEP57 | CEP70 | CEP89 | CES1 | CETN3 | CFL1 | CFL2 | CFTR | CGN | CGRRF1 | CHAF1A | CHCHD10 | CHCHD2 | CHCHD3 | CHD1L | CHD3 | CHD4 | CHD8 | CHDH | CHEK2 | CHGB | CHMP2A | CHORDC1 | CHP1 | CHRAC1 | CHRD | CHRM2 | CHRM3 | CHRNA9 | CHRND | CHRNE | CHRNG | CHST4 | CILP2 | CIRBP | CISD2 | CKAP4 | CKAP5 | CKB | CKM | CKMT1A | CKS1B | CKS2 | CLDN18 | CLGN | CLIC1 | CLIC3 | CLIC4 | CLK1 | CLNK | CLNS1A | CLPB | CLPX | CLSTN1 | CLTC | CLU | CLUH | CMBL | CMPK1 | CNDP2 | CNTRL | CNTROB | COA3 | COG3 | COL12A1 | COL14A1 | COL18A1 | COL2A1 | COL4A1 | COL4A2 | COL4A3BP | COL5A1 | COL6A1 | COL6A2 | COMMD1 | COMT | COPB1 | COPG1 | COPS2 | COPS5 | COPS6 | COQ2 | COQ3 | COQ4 | COQ5 | COQ6 | COQ7 | COQ9 | CORO2B | CORT | COTL1 | COX11 | COX15 | COX17 | COX19 | COX20 | COX5A | COX6A1 | COX6C | COX7A2 | CPLX1 | CPLX3 | CPNE3 | CPSF7 | CPT1A | CPT2 | CRBN | CREB1 | CREB3 | CREBBP | CRIP1 | CRK | CRKL | CRMP1 | CROCC | CROT | CRY1 | CRY2 | CRYAB | CRYZ | CSE1L | CSNK1A1 | CSNK1E | CSNK2A2 | CSNK2B | CSTF2T | CT45A5 | CTBP1 | CTCF | CTDP1 | CTH | CTNNB1 | CTNNBIP1 | CTNND1 | CTPS1 | CTSA | CTSC | CTSD | CTSL | CTSV | CTTN | CUL1 | CUL2 | CUL3 | CUL4B | CUL5 | CUL7 | CUTC | CWC15 | CXCL11 | CXCL5 | CXCL6 | CXCL8 | CYB5B | CYB5R1 | CYB5R3 | CYC1 | CYCS | CYLD | CYP1A1 | CYP1A2 | CYP2C18 | DAB1 | DAB2 | DAD1 | DAP3 | DAPK1 | DAPP1 | DARS | DAXX | DAZAP1 | DBF4B | DBH | DBI | DBN1 | DBNL | DBT | DCAF12 | DCAF7 | DCAF8 | DCAKD | DCK | DCLK1 | DCN | DCP2 | DCTN1 | DCTPP1 | DCUN1D1 | DCXR | DDA1 | DDB1 | DDB2 | DDIT4 | DDOST | DDRGK1 | DDX1 | DDX39A | DDX39B | DDX3X | DDX6 | DECR1 | DEDD | DEGS1 | DENND4C | DEPDC7 | DERA | DERL1 | DES | DGKE | DGKZ | DGUOK | DHCR24 | DHRS4 | DHRS7B | DHX15 | DHX36 | DHX9 | DIABLO | DIAPH3 | DIDO1 | DIS3 | DISC1 | DLAT | DLD | DLG2 | DLG3 | DLG4 | DLST | DMAP1 | DMD | DMRT3 | DNA2 | DNAAF2 | DNAAF5 | DNAH9 | DNAJA1 | DNAJA2 | DNAJA3 | DNAJA4 | DNAJB1 | DNAJB11 | DNAJB2 | DNAJC10 | DNAJC19 | DNAJC30 | DNAJC7 | DNASE2 | DNASE2B | DNM1L | DNPEP | DOCK5 | DOCK7 | DOK1 | DPEP1 | DPF2 | DPM1 | DPYSL5 | DRG2 | DROSHA | DSE | DSTN | DTX1 | DTX2 | DUS3L | DUSP1 | DUSP12 | DUSP13 | DUSP14 | DUSP16 | DUSP19 | DUSP22 | DUSP7 | DUT | DVL1 | DVL2 | DVL3 | DYM | DYNC1H1 | DYNC1I1 | DYNC1I2 | DYNC1LI1 | DYNLL1 | DYNLT1 | DYRK2 | DZIP3 | E2F1 | E2F2 | E2F3 | E2F4 | E2F6 | EAF2 | ECD | ECH1 | ECHDC2 | ECHS1 | ECI1 | ECI2 | ECSIT | EDA | EDC4 | EDEM1 | EED | EEF1A1 | EEF1A1P5 | EEF1A2 | EEF1B2 | EEF1D | EEF1G | EEF2 | EEF2KMT | EFEMP1 | EFEMP2 | EFTUD2 | EGFR | EGLN1 | EGLN3 | EHD4 | EHHADH | EHMT1 | EHMT2 | EID1 | EIF1AX | EIF2AK3 | EIF2AK4 | EIF2B1 | EIF2B3 | EIF2B5 | EIF2S1 | EIF2S2 | EIF3A | EIF3D | EIF3E | EIF3H | EIF3I | EIF4A3 | EIF4E | EIF4E2 | EIF4EBP1 | EIF4G3 | EIF4H | EIF5 | EIF5A | EIF5B | EIF6 | ELAC2 | ELAVL1 | ELAVL3 | ELAVL4 | ELF3 | ELF4 | EMC2 | EML4 | ENDOG | ENO1 | ENO2 | ENO3 | ENOPH1 | ENTPD7 | EP300 | EPHA4 | EPHA6 | EPHB6 | EPPIN | EPPK1 | EPRS | ERAL1 | ERBB2 | ERBB3 | ERCC5 | ERF | ERG | ERLIN2 | ERRFI1 | ESD | ESR1 | ESR2 | ESRRA | ESRRB | ESRRG | ETFA | ETFB | ETFDH | ETS1 | EVA1A | EVC2 | EXOC6 | EXOSC1 | EXOSC5 | EXOSC6 | EXOSC9 | EXTL2 | EYA2 | EYA4 | EZH2 | EZR | F5 | FABP1 | FABP3 | FABP5 | FABP7 | FADD | FAF1 | FAF2 | FAH | FAM118B | FAM129B | FAM161B | FAM20B | FAM3C | FAM49B | FAM83D | FAM83H | FAM98A | FAM9B | FANCA | FANCC | FANCG | FANCI | FANCM | FAR2 | FARSB | FASLG | FASN | FBF1 | FBP1 | FBP2 | FBXL12 | FBXO11 | FBXO25 | FBXO32 | FBXO6 | FBXO7 | FBXW11 | FBXW5 | FCGR1A | FCHSD1 | FCN1 | FDPS | FDXR | FECH | FEN1 | FERMT2 | FGB | FGF2 | FGFR1 | FGFR1OP | FGFR3 | FGL2 | FH | FHL1 | FHL2 | FIBP | FIGNL1 | FIS1 | FKBP14 | FKBP1A | FKBP1B | FKBP2 | FKBP5 | FKBP7 | FKBP8 | FLNB | FLOT1 | FN1 | FOLH1 | FOLR1 | FOPNL | FOS | FOXA1 | FOXD4 | FOXD4L6 | FOXI2 | FOXJ1 | FOXK1 | FOXK2 | FOXM1 | FOXO1 | FPGS | FRAS1 | FREM2 | FSCN1 | FSD1 | FTH1 | FTL | FTO | FTSJ3 | FUBP1 | FUBP3 | FUNDC2 | FUS | FYN | FZR1 | G3BP1 | G3BP2 | G6PD | GAA | GABPB1 | GABRA6 | GABRR1 | GAD2 | GADD45G | GAK | GALNS | GALNT7 | GANAB | GAP43 | GAPDH | GAPDHS | GAPVD1 | GAR1 | GARS | GART | GATA1 | GBE1 | GBP2 | GBP3 | GBP5 | GBP6 | GCDH | GDA | GDF9 | GDI1 | GDI2 | GEMIN4 | GEMIN5 | GEMIN7 | GFAP | GFPT1 | GFPT2 | GGA1 | GGCT | GGPS1 | GGT1 | GGTLC1 | GHDC | GHITM | GIGYF1 | GINS2 | GINS3 | GINS4 | GIT1 | GJA1 | GKAP1 | GLA | GLCE | GLMN | GLO1 | GLP1R | GLRB | GLRX | GLRX3 | GLRX5 | GLS | GLS2 | GLUD2 | GLUL | GMDS | GMPPB | GMPR | GMPS | GNA12 | GNA13 | GNA15 | GNAI1 | GNAI2 | GNAI3 | GNAO1 | GNAQ | GNAS | GNAZ | GNB1 | GNB2 | GNG12 | GNG5 | GNL1 | GNPDA1 | GNPDA2 | GNPNAT1 | GNS | GOLGA2 | GOLPH3 | GOPC | GORASP2 | GOT1 | GOT1L1 | GOT2 | GPC5 | GPD1 | GPD1L | GPI | GPS2 | GPSM1 | GPT2 | GPX1 | GPX4 | GPX7 | GRAMD1A | GRB2 | GRB7 | GRHPR | GRIA1 | GRID2 | GRIK2 | GRIP1 | GRK1 | GRK5 | GRPEL1 | GSK3A | GSK3B | GSPT1 | GSPT2 | GSR | GSS | GSTA1 | GSTA2 | GSTA3 | GSTA4 | GSTA5 | GSTK1 | GSTM4 | GSTO1 | GSTO2 | GSTP1 | GSTT1 | GSTT2 | GSTT2B | GSTZ1 | GTF2A1 | GTF2E2 | GTF2F1 | GTF2F2 | GTF2I | GTF3C2 | GTSF1 | GUSB | GYG1 | GYG2 | GYS1 | GZMA | H1F0 | H1FX | H2AFV | H2AFX | H2AFZ | H3F3A | HAAO | HADH | HAGH | HAP1 | HARS | HAVCR2 | HAX1 | HBEGF | HBZ | HCCS | HCN1 | HCN2 | HCN3 | HDAC1 | HDAC11 | HDAC2 | HDAC3 | HDAC4 | HDAC5 | HDAC6 | HDAC7 | HDAC9 | HDGF | HEBP1 | HECW1 | HECW2 | HELZ | HELZ2 | HEPACAM2 | HERC1 | HERC2 | HEXA | HEXB | HGS | HIBADH | HIBCH | HIF1A | HIF1AN | HINT1 | HIPK4 | HIRIP3 | HIST1H1A | HIST1H2AB | HIST1H2BA | HIST1H3A | HIST1H4A | HIST2H3A | HIST3H3 | HK1 | HK2 | HLA-A | HLA-B | HLA-DQA1 | HLA-DRA | HLA-DRB1 | HLA-DRB3 | HLA-DRB4 | HLA-DRB5 | HLCS | HLX | HMBS | HMGB1 | HMGB2 | HMGB3 | HMGCL | HMOX1 | HNF1A | HNF4A | HNF4G | HNRNPA1 | HNRNPA1L2 | HNRNPA2B1 | HNRNPAB | HNRNPD | HNRNPDL | HNRNPF | HNRNPH1 | HNRNPH2 | HNRNPH3 | HNRNPK | HNRNPL | HNRNPM | HNRNPU | HOXA5 | HOXB5 | HOXB9 | HOXD10 | HPRT1 | HPX | HRAS | HRNR | HS2ST1 | HS6ST1 | HSD17B10 | HSD17B14 | HSD17B4 | HSD17B6 | HSD17B8 | HSD3B7 | HSF1 | HSF4 | HSP90AA1 | HSP90AA5P | HSP90AB1 | HSP90AB3P | HSP90AB4P | HSP90B1 | HSPA14 | HSPA1B | HSPA1L | HSPA2 | HSPA4 | HSPA4L | HSPA5 | HSPA8 | HSPA9 | HSPB1 | HSPBP1 | HSPD1 | HSPE1 | HSPG2 | HSPH1 | HTATIP2 | HTR1B | HTR3A | HTR3E | HTRA1 | HTRA2 | HTT | HUWE1 | IARS | IBTK | ICAM1 | ICK | ID2 | IDE | IDH1 | IDH2 | IDH3B | IDH3G | IDI2 | IFI16 | IFIT1 | IFITM3 | IFNA2 | IFNAR1 | IFT122 | IGBP1 | IGF1R | IGFBP7 | IGHV3-13 | IGHV3-23 | IGHV3-30 | IGKV3-20 | IGLC7 | IGSF21 | IGSF8 | IK | IKBKB | IKBKE | IKBKG | IKZF5 | IL13RA2 | IL32 | IL3RA | ILF2 | ILF3 | ILK | IMMT | IMP4 | IMPA1 | IMPA2 | IMPDH2 | ING1 | ING5 | INO80B | INO80C | INSIG1 | INSIG2 | INSR | IP6K1 | IPO4 | IPO5 | IPO7 | IPO8 | IQCB1 | IQGAP1 | IREB2 | IRF1 | IRF4 | IRF7 | ISG15 | ISLR | ISYNA1 | ITCH | ITGA4 | ITGA7 | ITGAV | ITGB1BP1 | ITGB3 | ITGB3BP | ITGB5 | ITK | ITPA | ITPR1 | ITPR2 | ITPR3 | IVD | JAK1 | JAK3 | JMJD6 | JOSD2 | JUN | JUNB | JUND | JUP | KANK2 | KAT5 | KAT7 | KAT8 | KBTBD7 | KCMF1 | KCNA4 | KCNAB1 | KCNAB2 | KCNIP2 | KCNS3 | KCTD12 | KCTD17 | KCTD18 | KCTD2 | KCTD3 | KDM1A | KDM2A | KDM3B | KDM4D | KDM5B | KDM5C | KDM8 | KDR | KDSR | KEAP1 | KHDRBS1 | KIAA0391 | KIF11 | KIF13B | KIF2C | KIF5B | KIF5C | KIF6 | KIFC3 | KIR3DS1 | KLC1 | KLC2 | KLF5 | KLF6 | KLHL20 | KLHL22 | KLRC2 | KMT2A | KMT2B | KMT2C | KPNA1 | KPNA2 | KPNB1 | KRAS | KRT17 | KRT31 | KRT40 | KRT71 | KSR1 | KYNU | L2HGDH | L3MBTL1 | L3MBTL2 | L3MBTL3 | L3MBTL4 | LACTB2 | LAMB1 | LANCL1 | LARP4 | LARP7 | LARS | LAT | LATS1 | LCMT2 | LCP1 | LCP2 | LDHA | LDHAL6A | LDHAL6B | LDHB | LDHC | LDHD | LEF1 | LEMD2 | LEMD3 | LETM1 | LGALS1 | LGALS3 | LGALS3BP | LGALS7 | LGALS8 | LGMN | LGR4 | LIG4 | LIMA1 | LIMS1 | LMAN2L | LMNA | LMNB1 | LMO2 | LMO7 | LNX1 | LOXL2 | LPAR1 | LPAR2 | LPAR4 | LPIN3 | LRP6 | LRPPRC | LRRC7 | LRRK1 | LRRK2 | LRRN2 | LRSAM1 | LSM1 | LSM12 | LSM3 | LSM4 | LSM5 | LSM8 | LTBP1 | LTBR | LTC4S | LUC7L2 | LUM | LURAP1 | LYG2 | LYPLA1 | LYPLA2 | LYRM7 | LZTR1 | MAD1L1 | MAD2L1 | MAGI1 | MAL2 | MAN2B1 | MAOA | MAP1B | MAP2K1 | MAP2K3 | MAP2K4 | MAP2K6 | MAP2K7 | MAP3K1 | MAP3K13 | MAP3K14 | MAP3K2 | MAP3K3 | MAP3K4 | MAP4K1 | MAPK1 | MAPK14 | MAPK15 | MAPK3 | MAPK4 | MAPK6 | MAPK7 | MAPK8 | MAPK8IP3 | MAPK9 | MAPKAP1 | MAPT | MARCH5 | MAS1 | MAST1 | MAST3 | MAT2A | MAT2B | MATK | MAVS | MBD3 | MBP | MC1R | MCAT | MCC | MCCC1 | MCFD2 | MCM2 | MCM3 | MCM4 | MCM5 | MCM6 | MCM7 | MCM9 | MCMBP | MCOLN3 | MCTS1 | MDC1 | MDFI | MDH1 | MDH2 | MDM2 | MDM4 | MDN1 | ME1 | MECP2 | MED1 | MED12 | MED14 | MED17 | MED20 | MED21 | MED23 | MED24 | MED31 | MED4 | MED6 | MELK | MEPCE | METAP2 | METRN | METRNL | MEX3C | MGMT | MGST1 | MIB1 | MICA | MICU1 | MICU2 | MIF | MITF | MKNK2 | MKS1 | MLH1 | MLLT1 | MLLT11 | MLLT3 | MLST8 | MMAB | MME | MMP26 | MMRN1 | MOB1A | MOB1B | MOCOS | MORF4L1 | MORF4L2 | MOV10 | MPC2 | MPG | MPHOSPH6 | MPI | MPP2 | MPPE1 | MPST | MRGBP | MRM1 | MRPL12 | MRPL14 | MRPL19 | MRPL28 | MRPL38 | MRPL4 | MRPL43 | MRPL45 | MRPL46 | MRPL9 | MRPS5 | MRRF | MSH6 | MSN | MTA1 | MTA3 | MTAP | MTERF1 | MTHFD1 | MTMR14 | MTMR7 | MTNR1A | MTPN | MTRR | MTSS1 | MVD | MYBBP1A | MYC | MYD88 | MYH11 | MYH6 | MYL2 | MYL6 | MYO1B | MYO1C | MYO1E | MYO5A | MYO6 | MYOC | NACC1 | NAE1 | NAGK | NAMPT | NANOG | NANP | NANS | NAP1L1 | NAP1L4 | NAPRT | NBAS | NCAPH2 | NCBP1 | NCDN | NCK1 | NCOA1 | NCOA2 | NCOA3 | NCOA4 | NCOR1 | NDRG1 | NDUFA10 | NDUFA13 | NDUFA5 | NDUFA8 | NDUFA9 | NDUFAF1 | NDUFAF2 | NDUFAF3 | NDUFAF4 | NDUFAF5 | NDUFAF7 | NDUFB1 | NDUFS1 | NDUFS2 | NDUFS3 | NDUFS6 | NDUFS7 | NDUFS8 | NDUFV1 | NDUFV3 | NEBL | NEDD4 | NEDD8 | NEK1 | NEK7 | NELFB | NELFCD | NELFE | NELL1 | NEURL4 | NEUROD1 | NF2 | NFATC1 | NFATC2 | NFE2L2 | NFKB2 | NFKBIA | NFS1 | NGB | NHLRC3 | NID1 | NIF3L1 | NIN | NIPBL | NIT2 | NKX2-1 | NLK | NMB | NME1 | NME2 | NME4 | NMNAT1 | NMNAT3 | NMRAL1 | NMRK2 | NNT | NOB1 | NOD2 | NOP10 | NOP56 | NOS1AP | NOS2 | NOS3 | NOSIP | NPEPPS | NPHP3 | NPLOC4 | NPM1 | NPR1 | NPTX1 | NQO1 | NQO2 | NR0B2 | NR1H2 | NR1H3 | NR1I2 | NR3C1 | NR4A1 | NR5A2 | NRBF2 | NRP2 | NRXN1 | NSFL1C | NSL1 | NSMCE4A | NSUN2 | NSUN6 | NT5C2 | NT5DC2 | NTF3 | NTMT1 | NTRK1 | NUBP1 | NUBP2 | NUCB1 | NUDC | NUDT13 | NUDT18 | NUDT19 | NUDT3 | NUDT9 | NUFIP1 | NUMA1 | NUP107 | NUP153 | NUP160 | NUP205 | NUP214 | NUP85 | NUP88 | NUP93 | NUP98 | NUPR1 | NUSAP1 | OAS2 | OAT | OAZ3 | OBSL1 | OFD1 | OGDH | OGFOD1 | OGT | OLA1 | ONECUT1 | OPA1 | OPTN | OR5F1 | ORM1 | OSBPL1A | OTC | OTUB1 | OTUB2 | OTUD4 | OTUD5 | OVGP1 | OXA1L | OXCT1 | OXSR1 | P2RX1 | P2RX2 | P2RX4 | P4HA1 | P4HA2 | P4HB | PA2G4 | PABPC1 | PABPC3 | PABPC4 | PACS2 | PACSIN3 | PADI4 | PAFAH1B1 | PAG1 | PAH | PAICS | PAIP1 | PAK2 | PAK6 | PALB2 | PAN2 | PANX3 | PAPSS1 | PAPSS2 | PARD3 | PARD6A | PARK7 | PARP1 | PARP12 | PARP14 | PARP6 | PATZ1 | PAWR | PAX8 | PAX9 | PAXIP1 | PC | PCBD1 | PCBP1 | PCBP2 | PCDHB16 | PCF11 | PCGF1 | PCGF5 | PCGF6 | PCM1 | PCMT1 | PCMTD1 | PCMTD2 | PCNA | PCSK9 | PCTP | PCYT2 | PDCD10 | PDCD6 | PDCD6IP | PDCL | PDE12 | PDE4D | PDE4DIP | PDE6G | PDF | PDHA1 | PDHA2 | PDHB | PDHX | PDIA3 | PDIA4 | PDIA6 | PDK2 | PDLIM5 | PDSS1 | PDSS2 | PDXK | PEBP1 | PEF1 | PELI1 | PEPD | PER1 | PER2 | PEX11B | PEX19 | PEX5 | PFAS | PFDN2 | PFKL | PFKM | PFKP | PFN1 | PGAM1 | PGAM2 | PGAM5 | PGD | PGK1 | PGK2 | PGM1 | PGM2 | PGM3 | PGM5 | PGP | PGRMC1 | PHB | PHB2 | PHF10 | PHF21A | PHGDH | PHKA1 | PHOSPHO1 | PI4K2A | PIAS1 | PIAS3 | PICK1 | PIGU | PIH1D1 | PIK3C2A | PIK3CA | PIK3CG | PIK3R1 | PIK3R3 | PIKFYVE | PIM1 | PIN1 | PINX1 | PIP4K2A | PIPSL | PIR | PITPNA | PITPNB | PJA1 | PKIG | PKM | PKN2 | PLA2G10 | PLA2G12A | PLA2G1B | PLA2G2A | PLA2G6 | PLAUR | PLCG1 | PLEC | PLEKHF1 | PLK1 | PLOD2 | PLS3 | PLSCR1 | PLXNA1 | PLXNA2 | PLXNB2 | PML | PMP2 | PMPCA | PMPCB | PNKP | PNP | POC1B | POC5 | POLA2 | POLD1 | POLDIP2 | POLDIP3 | POLE2 | POLE3 | POLM | POLR2A | POLR2E | POLR2L | POLR2M | POM121 | POT1 | POU5F1 | PPA1 | PPA2 | PPARD | PPARG | PPARGC1A | PPAT | PPEF1 | PPIA | PPIB | PPID | PPIE | PPIF | PPIL3 | PPM1A | PPM1B | PPM1D | PPM1H | PPM1K | PPME1 | PPOX | PPP1CA | PPP1CB | PPP1CC | PPP1R11 | PPP1R12A | PPP1R12B | PPP1R14A | PPP1R2 | PPP1R3B | PPP1R3C | PPP1R8 | PPP2CA | PPP2CB | PPP2R1A | PPP2R1B | PPP2R2A | PPP2R2D | PPP2R5D | PPP3CC | PPP4R2 | PPP5C | PPP6C | PPP6R1 | PPT1 | PPTC7 | PQLC1 | PRCC | PRCP | PRDX1 | PRDX2 | PRDX3 | PRDX4 | PRDX5 | PRDX6 | PRELID1 | PRG2 | PRKAA1 | PRKAA2 | PRKAB2 | PRKACA | PRKAG1 | PRKAR1A | PRKAR2B | PRKCD | PRKCE | PRKCSH | PRKCZ | PRKD1 | PRKD2 | PRKD3 | PRKDC | PRMT2 | PRMT3 | PRMT6 | PROX1 | PRPF19 | PRPF40A | PRPF8 | PRRC2C | PRSS23 | PRTFDC1 | PSAP | PSAT1 | PSEN1 | PSEN2 | PSMA1 | PSMA2 | PSMA3 | PSMA5 | PSMA6 | PSMB5 | PSMB6 | PSMB7 | PSMB8 | PSMC1 | PSMC2 | PSMC3 | PSMC4 | PSMC5 | PSMC6 | PSMD1 | PSMD10 | PSMD11 | PSMD12 | PSMD14 | PSMD2 | PSMD4 | PSMD9 | PSME2 | PSME3 | PSMG1 | PSMG4 | PSTPIP1 | PTEN | PTGDS | PTGER3 | PTGES | PTGES2 | PTGES3 | PTGIR | PTGR1 | PTK2 | PTMA | PTMS | PTN | PTPMT1 | PTPN1 | PTPN14 | PTPN21 | PTPN22 | PTPN23 | PTPN4 | PTPN6 | PTPRA | PTPRD | PTPRF | PTPRK | PTPRO | PTPRR | PTRH2 | PTTG1 | PUM1 | PUS1 | PUS7 | PXDN | PXN | PYCR1 | PYCR2 | PYGB | PYGL | QARS | QDPR | QSOX1 | RAB10 | RAB11A | RAB11B | RAB11FIP2 | RAB18 | RAB1A | RAB1B | RAB21 | RAB24 | RAB29 | RAB2A | RAB31 | RAB32 | RAB35 | RAB3GAP1 | RAB3IP | RAB5A | RAB5B | RAB7A | RAB9A | RABL2A | RABL3 | RAC1 | RAD21 | RAD23A | RAD23B | RAD51 | RAD51AP1 | RADIL | RAE1 | RAF1 | RAI14 | RAN | RANBP9 | RANGAP1 | RAP1A | RAP1B | RAP1GDS1 | RAPGEF4 | RARA | RARG | RARS2 | RASD1 | RASL11B | RASSF10 | RASSF7 | RASSF9 | RB1 | RB1CC1 | RBBP4 | RBBP6 | RBM17 | RBM25 | RBM3 | RBM4 | RBM8A | RBMX | RBP1 | RBPJ | RBX1 | RC3H1 | RCC1 | RDH11 | RDH12 | RDH13 | RDH14 | RDH5 | RDX | RECK | REL | RELA | REPIN1 | REST | REXO2 | RFC1 | RFC2 | RFC3 | RFC4 | RFC5 | RFK | RFXANK | RGN | RGS1 | RGS2 | RGS20 | RHBDD1 | RHO | RHOA | RHOJ | RHOT1 | RHOT2 | RHOU | RHPN1 | RIC3 | RIC8B | RIF1 | RING1 | RIOK1 | RIPK1 | RIPK2 | RIPK3 | RIPK4 | RIT1 | RMND1 | RNASE3 | RNASEH2A | RNASEH2B | RNF123 | RNF126 | RNF139 | RNF181 | RNF183 | RNF185 | RNF19A | RNF2 | RNF25 | RNF31 | RNF4 | RNF8 | ROMO1 | RP2 | RPA2 | RPA3 | RPAIN | RPE | RPGRIP1 | RPGRIP1L | RPL10 | RPL10A | RPL12 | RPL18A | RPL22 | RPL23 | RPL23A | RPL24 | RPL30 | RPL36AL | RPL37A | RPL38 | RPL6 | RPL7 | RPL7A | RPL9 | RPLP0 | RPLP0P6 | RPLP1 | RPLP2 | RPN1 | RPN2 | RPP14 | RPS10P5 | RPS12 | RPS13 | RPS14 | RPS15A | RPS19 | RPS2 | RPS20 | RPS24 | RPS29 | RPS3 | RPS4X | RPS6KA1 | RPS6KA2 | RPS6KB2 | RPS8 | RPTOR | RRM1 | RRM2 | RRM2B | RRP7A | RRS1 | RSU1 | RTCB | RTF1 | RTFDC1 | RTKN | RTN4 | RUNX1 | RUNX1T1 | RUNX2 | RUVBL1 | RUVBL2 | RXRA | RXRB | RXRG | S100A1 | S100A11 | S100A12 | S100A13 | S100A2 | S100A4 | S100A6 | S100A7 | S100A9 | S100B | S100P | SAE1 | SAG | SAMD9 | SAP18 | SARM1 | SARS | SART1 | SART3 | SAT1 | SAT2 | SBDS | SCAF1 | SCARA3 | SCCPDH | SCEL | SCFD1 | SCG2 | SCG3 | SCLT1 | SCLY | SCO2 | SCYL2 | SDC1 | SDC4 | SDCBP | SDCBP2 | SDHA | SDHB | SDHD | SEC13 | SEC16A | SEC24B | SEC24C | SEC31A | SEL1L | SELL | SEMA7A | SENP3 | SENP6 | SENP7 | SENP8 | SEPHS1 | SEPHS2 | SEPT1 | SEPT10 | SEPT11 | SEPT12 | SEPT14 | SEPT2 | SEPT3 | SEPT4 | SEPT5 | SEPT6 | SEPT7 | SEPT8 | SEPT9 | SERPINB2 | SERPINB3 | SERPINB5 | SERPINB6 | SERPINE2 | SERPINH1 | SET | SETDB1 | SF1 | SF3B6 | SFMBT1 | SFN | SFPQ | SFXN1 | SGPL1 | SGSH | SGTA | SGTB | SH2B1 | SH3GL1 | SH3GL2 | SH3GL3 | SH3KBP1 | SH3PXD2A | SHC1 | SHKBP1 | SHMT1 | SHMT2 | SIAH1 | SIN3A | SIPA1 | SIPA1L1 | SIRT1 | SIRT3 | SIRT5 | SIRT6 | SIRT7 | SKA1 | SKA3 | SKI | SKIV2L | SLAMF1 | SLAMF8 | SLC12A2 | SLC16A1 | SLC25A1 | SLC25A10 | SLC25A13 | SLC25A24 | SLC25A32 | SLC25A4 | SLC25A5 | SLC25A6 | SLC2A5 | SLC2A9 | SLC35F6 | SLC39A4 | SLC3A2 | SLC9A1 | SLC9A3R1 | SLC9A3R2 | SLTM | SMAD2 | SMAD3 | SMAD4 | SMAD5 | SMAD9 | SMARCA2 | SMARCA4 | SMARCB1 | SMARCC1 | SMARCC2 | SMARCE1 | SMC1A | SMC3 | SMG5 | SMG6 | SMG8 | SMN1 | SMO | SMTNL2 | SMURF1 | SMURF2 | SNAI2 | SNAP29 | SNCA | SND1 | SNF8 | SNRNP27 | SNRPA | SNRPA1 | SNRPB | SNRPB2 | SNRPD1 | SNRPD3 | SNRPE | SNW1 | SNX17 | SNX2 | SNX3 | SNX6 | SOAT1 | SOCS1 | SOCS2 | SOCS6 | SOD1 | SOD2 | SORD | SORT1 | SOX2 | SP1 | SP100 | SPACA1 | SPACA3 | SPATA2 | SPATA20 | SPATA24 | SPC25 | SPDL1 | SPEN | SPERT | SPG21 | SPI1 | SPICE1 | SPIN1 | SPINT2 | SPOP | SPR | SPRR2A | SPRTN | SPRY2 | SPSB3 | SPTAN1 | SPTBN1 | SPTBN2 | SPTLC2 | SQSTM1 | SRC | SRGAP2 | SRGAP3 | SRI | SRP14 | SRP19 | SRPK1 | SRPK2 | SRPRB | SRSF11 | SRSF12 | SRSF5 | SRXN1 | SSB | SSSCA1 | SSX2IP | ST13 | ST3GAL1 | ST3GAL6 | ST6GALNAC5 | ST8SIA4 | STAC | STAG2 | STARD13 | STARD7 | STAT1 | STAT3 | STAT4 | STAT5A | STAT6 | STAU1 | STBD1 | STIL | STIM1 | STIP1 | STK11 | STK24 | STK25 | STK26 | STK3 | STK38 | STK40 | STMN1 | STOM | STOML2 | STRAP | STRBP | STUB1 | STX5 | STXBP1 | STXBP2 | STYX | SUB1 | SUCLA2 | SUCLG2 | SUGT1 | SULF2 | SULT1A3 | SUMO1 | SUMO1P1 | SUMO2 | SUMO3 | SUN2 | SUPT4H1 | SUPT5H | SUSD5 | SUZ12 | SVIL | SWAP70 | SYDE1 | SYN1 | SYNE2 | SYNJ2BP | SYP | SYVN1 | TAB1 | TAB2 | TADA2B | TAF12 | TAF15 | TAF1A | TAGLN | TAGLN2 | TALDO1 | TAMM41 | TANGO2 | TANK | TAOK3 | TARDBP | TARS | TARS2 | TAS2R41 | TAS2R60 | TAS2R7 | TATDN1 | TAZ | TBC1D23 | TBC1D4 | TBCA | TBCE | TBL1X | TBRG4 | TBXA2R | TCEA1 | TCEA2 | TCERG1 | TCF21 | TCF4 | TCIRG1 | TCOF1 | TCP1 | TCTN2 | TCTN3 | TDO2 | TDP1 | TDRD3 | TECR | TEKT2 | TEKT4 | TERF1 | TERF2 | TERF2IP | TESK2 | TEX264 | TFCP2 | TFDP1 | TFG | TFIP11 | TGFB1 | TGFBR3 | TGM2 | TGOLN2 | THADA | THBS1 | THEM4 | THOC2 | THOC6 | THOC7 | THOP1 | THRA | TIA1 | TIAL1 | TIMM13 | TIMM21 | TIMM50 | TIMMDC1 | TIMP1 | TIMP2 | TINF2 | TK1 | TK2 | TKT | TLE1 | TLE3 | TLR3 | TM9SF4 | TMBIM6 | TMED9 | TMEM11 | TMEM132A | TMEM17 | TMEM173 | TMEM189-UBE2V1 | TMEM216 | TMEM219 | TMEM231 | TMEM258 | TMEM30A | TMEM70 | TMOD3 | TMOD4 | TMTC3 | TMX2 | TMX4 | TNF | TNFAIP3 | TNFRSF12A | TNFRSF1A | TNFRSF8 | TNFSF12 | TNFSF14 | TNIP2 | TNK2 | TNKS | TNKS2 | TNN | TNNT1 | TNP1 | TNPO1 | TNXB | TOE1 | TOLLIP | TOMM20 | TOMM22 | TOMM40 | TOP1 | TOP2A | TOP3A | TOR1A | TP53 | TP53RK | TP63 | TP73 | TPCN2 | TPD52 | TPI1 | TPM1 | TPM2 | TPM3 | TPM4 | TPR | TPSAB1 | TPST2 | TPTE | TRA2A | TRADD | TRAF1 | TRAF2 | TRAF3 | TRAF3IP1 | TRAF6 | TRAP1 | TRAPPC8 | TREML2 | TREX1 | TRIAP1 | TRIB2 | TRIB3 | TRIM21 | TRIM23 | TRIM25 | TRIM28 | TRIM29 | TRIM32 | TRIM33 | TRIM34 | TRIM35 | TRIM41 | TRIM47 | TRIM63 | TRIM69 | TRIM7 | TRIM9 | TRIP12 | TRIP13 | TRIP4 | TRIP6 | TRMT44 | TRMT6 | TRMT61B | TRNT1 | TRUB2 | TSC22D1 | TSFM | TSG101 | TSGA10 | TSKU | TSN | TSPAN17 | TSPYL2 | TTC1 | TTC19 | TTC5 | TTLL1 | TUBA1A | TUBA1C | TUBA4A | TUBB | TUBB2A | TUBB2B | TUBB3 | TUBB4A | TUBB4B | TUBB6 | TUBB8 | TUBG1 | TUBGCP3 | TUFM | TUFT1 | TULP3 | TWF1 | TWF2 | TWIST1 | TXLNA | TXN | TXN2 | TXNDC11 | TXNDC15 | TXNDC17 | TXNDC5 | TXNIP | TXNL4A | TXNRD1 | TYMS | U2AF2 | UAP1 | UAP1L1 | UBA1 | UBA2 | UBA3 | UBA5 | UBA6 | UBAC1 | UBAP2L | UBASH3A | UBB | UBC | UBE2A | UBE2B | UBE2C | UBE2D1 | UBE2D2 | UBE2D3 | UBE2H | UBE2I | UBE2J1 | UBE2K | UBE2L3 | UBE2L6 | UBE2M | UBE2N | UBE2O | UBE2Q2 | UBE2R2 | UBE2S | UBE2V1 | UBE2W | UBE3A | UBE3B | UBIAD1 | UBL4A | UBQLN1 | UBQLN2 | UBQLN4 | UBR4 | UBR5 | UBXN1 | UBXN10 | UBXN7 | UCHL1 | UCHL3 | UCHL5 | UCK2 | UFC1 | UFM1 | UGDH | UGGT1 | UGP2 | UGT8 | ULK1 | UMPS | UNC119 | UNK | UNKL | UPF2 | UPF3B | UPP2 | UQCC1 | UQCRB | UQCRC2 | UQCRFS1 | UQCRQ | USP11 | USP14 | USP15 | USP16 | USP17L2 | USP19 | USP2 | USP20 | USP21 | USP26 | USP28 | USP39 | USP4 | USP42 | USP46 | USP47 | USP5 | USP50 | USP53 | USP7 | USP8 | USP9X | USP9Y | USPL1 | UTP14A | UTP6 | UXS1 | VAC14 | VAPA | VAPB | VARS | VASH2 | VASP | VAT1 | VAV1 | VBP1 | VCAM1 | VCAN | VCL | VCP | VCPIP1 | VDAC1 | VDAC2 | VDAC3 | VDR | VGF | VHL | VIM | VKORC1 | VN1R1 | VPS18 | VPS26A | VPS29 | VPS37D | VPS4B | VPS72 | VPS8 | VSTM2A | VTA1 | VTN | WARS | WAS | WBP2 | WDR1 | WDR12 | WDR19 | WDR35 | WDR4 | WDR41 | WDR45B | WDR48 | WDR77 | WDR82 | WDTC1 | WDYHV1 | WIPI1 | WIPI2 | WNK1 | WNK4 | WNT16 | WNT2 | WSB1 | WT1 | WTAP | WWOX | WWP2 | XAF1 | XBP1 | XIAP | XIRP2 | XPNPEP1 | XPO5 | XRCC1 | XRCC3 | XRCC5 | XRCC6 | XYLT1 | YAP1 | YBEY | YEATS2 | YES1 | YKT6 | YME1L1 | YWHAB | YWHAE | YWHAG | YWHAH | YWHAQ | YWHAZ | YY1 | ZAP70 | ZBTB16 | ZBTB17 | ZBTB33 | ZBTB42 | ZBTB7A | ZBTB7B | ZC3HC1 | ZDHHC17 | ZDHHC23 | ZFAND5 | ZFAND6 | ZFP41 | ZFYVE19 | ZFYVE26 | ZHX1 | ZIC1 | ZMPSTE24 | ZMYND10 | ZNF207 | ZNF217 | ZNF24 | ZNF277 | ZNF443 | ZNF488 | ZNF598 | ZNF622 | ZNF638 | ZNRF4 | ZRANB2 | ZSCAN2 | ZW10 | ZWINT | GO:0006082 | 0.0560475555016378 | 462/8243 | 266/3250 | 3.97503557087434e-16 | 3.77429627454519e-12 | T | T | T | T | organic acid metabolic process | AARS | AARSD1 | AASDH | AASDHPPT | AASS | ABAT | ABCD3 | ACAA1 | ACAA2 | ACACA | ACAD8 | ACAD9 | ACADS | ACADSB | ACADVL | ACAT1 | ACAT2 | ACCS | ACLY | ACO1 | ACO2 | ACOT7 | ACOX1 | ACSL1 | ACSL4 | ACSL5 | ACSS2 | ACY1 | ADH1A | ADH1B | ADSS | ADTRP | AHCY | AIMP2 | AKR1A1 | AKR1C2 | ALDH4A1 | ALDH5A1 | ALDH6A1 | ALDH7A1 | ALDH8A1 | ALOX12 | ALOX5 | ALOX5AP | AMDHD2 | ASL | ASNS | ASS1 | ATP6V1B1 | BCAT2 | BCKDHB | BRCA1 | BSG | CARS | CBR1 | CBR4 | CBS | CD36 | CD74 | CEL | CES1 | CPT1A | CPT2 | CROT | CTH | CTPS1 | CYB5R3 | CYP1A1 | CYP1A2 | CYP2C18 | DARS | DBT | DCXR | DECR1 | DEGS1 | DLAT | DLD | DLST | DPEP1 | ECH1 | ECHDC2 | ECHS1 | ECI1 | ECI2 | EGLN1 | EGLN3 | EHHADH | ENOPH1 | EPRS | ETFA | ETFB | ETFDH | FABP3 | FAH | FARSB | FASN | FH | FOLH1 | FOLR1 | FPGS | GAD2 | GARS | GART | GCDH | GFPT1 | GFPT2 | GGT1 | GLO1 | GLS | GLS2 | GLUD2 | GLUL | GMPS | GNPDA1 | GNPDA2 | GOT1 | GOT1L1 | GOT2 | GPT2 | GPX1 | GPX4 | GRHPR | GSS | GSTA1 | GSTM4 | GSTO1 | GSTO2 | GSTP1 | GSTZ1 | HAAO | HADH | HAGH | HARS | HIBADH | HIBCH | HIF1A | HLCS | HMGCL | HNF4A | HSD17B10 | HSD17B4 | HSD17B8 | HSD3B7 | IARS | IDH1 | IDH2 | IDH3B | IDH3G | IREB2 | IVD | KYNU | L2HGDH | LARS | LDHA | LDHAL6A | LDHAL6B | LDHB | LDHC | LDHD | LPIN3 | LTC4S | LYPLA1 | LYPLA2 | MAPK14 | MAPK3 | MCAT | MCCC1 | MDH1 | MDH2 | ME1 | MECP2 | MGST1 | MIF | MPST | MTAP | MTHFD1 | MTRR | MYO5A | NAGK | NANP | NDUFS6 | NFS1 | NIT2 | NOS2 | NOS3 | NR5A2 | NUDT19 | OAT | OGDH | OSBPL1A | OTC | P4HA1 | P4HA2 | P4HB | PADI4 | PAH | PARK7 | PC | PCBD1 | PDHA1 | PDHA2 | PDHB | PDHX | PEPD | PER2 | PFAS | PGD | PHGDH | PLA2G10 | PLA2G1B | PLOD2 | PPA1 | PPA2 | PPARD | PPARG | PPARGC1A | PPAT | PPM1K | PRDX4 | PRKAA1 | PRKAA2 | PRKAB2 | PRKAG1 | PRKAR2B | PSAT1 | PTGDS | PTGES | PTGES2 | PTGES3 | PTGR1 | PYCR1 | PYCR2 | QARS | QDPR | RARS2 | RBP1 | RGN | SARS | SCLY | SDHA | SDHB | SEPHS1 | SEPHS2 | SGPL1 | SHMT1 | SHMT2 | SLC16A1 | SLC25A32 | SLC2A9 | SNCA | SORD | ST3GAL1 | STAT5A | SUCLA2 | SUCLG2 | TARS | TARS2 | TDO2 | TECR | THEM4 | TNFRSF1A | TXN2 | VARS | VDAC1 | WARS | XBP1 | GO:0055114 | 0.0635690889239355 | 524/8243 | 295/3250 | 5.63307495460377e-16 | 5.34860466939628e-12 | T | T | T | T | oxidation-reduction process | AASS | ABCD3 | ACAA1 | ACAA2 | ACAD8 | ACAD9 | ACADS | ACADSB | ACADVL | ACAT1 | ACAT2 | ACOX1 | ACSS2 | ADH1A | ADH1B | ADH5 | ADSL | AGL | AIFM1 | AKR1A1 | AKR1B1 | AKR1B10 | AKR1B15 | AKR1C2 | AKT1 | AKT2 | ALDH16A1 | ALDH1B1 | ALDH4A1 | ALDH5A1 | ALDH6A1 | ALDH7A1 | ALDH8A1 | ALDH9A1 | ALOX12 | ALOX5 | AOC2 | APEX1 | APOA1 | ASPDH | BCKDHB | BDH1 | BLVRB | CALM1 | CAT | CBR1 | CBR4 | CBS | CCS | CHDH | COQ6 | COQ7 | COQ9 | COX11 | COX15 | COX20 | COX5A | COX6A1 | COX6C | COX7A2 | CPT1A | CPT2 | CROT | CRYZ | CTBP1 | CYB5B | CYB5R1 | CYB5R3 | CYC1 | CYCS | CYP1A1 | CYP1A2 | CYP2C18 | DBH | DCXR | DECR1 | DEGS1 | DHCR24 | DHRS4 | DHRS7B | DLD | DNAJC10 | DUS3L | ECH1 | ECHDC2 | ECHS1 | ECI1 | ECI2 | ECSIT | EGLN1 | EGLN3 | EHHADH | ETFA | ETFB | ETFDH | FAR2 | FASN | FDXR | FTH1 | FTL | FTO | G6PD | GAA | GBE1 | GCDH | GFOD1 | GFPT1 | GFPT2 | GLRX | GLRX3 | GLRX5 | GLUD2 | GMPR | GNAS | GPD1L | GPX1 | GPX4 | GPX7 | GRHPR | GSK3A | GSK3B | GSR | GSTK1 | GSTO1 | GSTO2 | GYG1 | GYG2 | GYS1 | HAAO | HADH | HCCS | HIBADH | HIBCH | HIF1AN | HMOX1 | HSD17B10 | HSD17B14 | HSD17B4 | HSD17B6 | HSD17B8 | HSD3B7 | HSDL2 | HTATIP2 | IDH1 | IDH3B | IMPDH2 | IVD | JMJD6 | KCNAB1 | KCNAB2 | KDM1A | KDM2A | KDM3B | KDM4D | KDM5B | KDM5C | KDM8 | KDSR | L2HGDH | LDHA | LDHAL6A | LDHAL6B | LDHB | LDHC | LDHD | LOXL2 | LYRM7 | MAOA | MAPK14 | MCAT | MDH2 | ME1 | MECP2 | MGST1 | MTHFD1 | MTRR | MYBBP1A | MYC | NDUFA10 | NDUFA13 | NDUFA5 | NDUFA8 | NDUFA9 | NDUFAF1 | NDUFAF2 | NDUFAF5 | NDUFB1 | NDUFS1 | NDUFS2 | NDUFS3 | NDUFS6 | NDUFS7 | NDUFS8 | NDUFV1 | NDUFV3 | NNT | NOS2 | NOS3 | NQO1 | NQO2 | OGFOD1 | OXA1L | OXNAD1 | P4HA1 | P4HA2 | P4HB | PAH | PARK7 | PCBD1 | PDIA3 | PDIA4 | PDIA6 | PER2 | PFKM | PGD | PGM1 | PGM2 | PHGDH | PHKA1 | PIR | PLOD2 | PPARD | PPARG | PPARGC1A | PPOX | PPP1CA | PPP1CB | PPP1CC | PPP1R2 | PPP1R3B | PPP1R3C | PRDX1 | PRDX2 | PRDX3 | PRDX4 | PRDX5 | PRDX6 | PRKAA1 | PTGES | PTGES2 | PTGR1 | PXDN | PYCR1 | PYCR2 | PYGB | PYGL | QDPR | QSOX1 | RDH11 | RDH12 | RDH13 | RDH14 | RDH5 | RRM1 | RRM2 | RRM2B | RTN4IP1 | SCCPDH | SCO2 | SDHA | SDHB | SDHD | SDR39U1 | SELENBP1 | SIRT3 | SLC25A10 | SLC25A13 | SNCA | SOD1 | SOD2 | SORD | SPR | SRXN1 | STBD1 | STK40 | TAZ | TBRG4 | TDO2 | TECR | TMX4 | TTC19 | TXN | TXN2 | TXNDC17 | TXNRD1 | UGDH | UGP2 | UQCRB | UQCRC2 | UQCRFS1 | UQCRQ | VAT1 | VCAM1 | VKORC1 | WWOX | GO:1901564 | 0.352784180516802 | 2908/8243 | 1312/3250 | 4.2959760978096e-15 | 4.07902930487021e-11 | T | T | T | T | organonitrogen compound metabolic process | A2M | AARS | AARSD1 | AASDH | AASDHPPT | AASS | ABAT | ABCG1 | ABHD14B | ABHD17A | ABL1 | ABL2 | ACACA | ACAT1 | ACCS | ACE2 | ACLY | ACOT13 | ACOT7 | ACOT9 | ACP1 | ACSL1 | ACSL4 | ACSL5 | ACSS2 | ACTB | ACTL6A | ACTN2 | ACY1 | ADA | ADAMTS4 | ADCK2 | ADCY6 | ADCYAP1 | ADH5 | ADK | ADORA2A | ADPRH | ADRB2 | ADRM1 | ADSL | ADSS | AFG3L2 | AGK | AGO2 | AHCY | AHCYL1 | AHSG | AIMP2 | AK1 | AK2 | AK4 | AKR1A1 | AKR1B1 | AKT1 | AKT2 | AKT3 | ALAS1 | ALB | ALDH4A1 | ALDH5A1 | ALDH6A1 | ALDH7A1 | ALDH9A1 | ALDOA | AMD1 | AMFR | ANAPC4 | ANXA1 | ANXA2 | AOC2 | APBB1 | APC | APCS | APEH | APOA1 | APOE | APOL2 | APP | APRT | AR | ARAF | ARF4 | ARID4B | ARMC8 | ARRB1 | ARRB2 | ASB12 | ASB15 | ASB16 | ASB6 | ASB9 | ASGR1 | ASGR2 | ASL | ASNS | ASPDH | ASS1 | ATF2 | ATG4C | ATG5 | ATIC | ATM | ATP13A2 | ATP6V1A | ATP6V1B1 | AUP1 | AURKB | AZU1 | B3GAT3 | B3GNT3 | B4GALT3 | BABAM1 | BACE1 | BAG2 | BAG6 | BAX | BCAT2 | BCL10 | BCL11A | BCL2 | BCOR | BIRC2 | BIRC3 | BIRC5 | BIRC7 | BIRC8 | BMI1 | BMPR1A | BRAF | BRCA1 | BRD4 | BTBD9 | BTK | BTRC | C1QBP | C1R | C4A | CAB39 | CALM1 | CALR | CALU | CAMK1D | CAMK2A | CAMK2B | CAMKV | CAND1 | CAPN1 | CAPN2 | CAPNS1 | CARM1 | CARS | CASP10 | CASP2 | CASP3 | CASP7 | CASP8 | CASP9 | CAT | CBFB | CBL | CBLB | CBLC | CBR4 | CBS | CBX4 | CBX8 | CCDC22 | CCDC8 | CCNB1 | CCNT1 | CCP110 | CCR5 | CCS | CD36 | CDA | CDC14B | CDC16 | CDC20 | CDC25A | CDC25B | CDC25C | CDC27 | CDC34 | CDC42 | CDC73 | CDK15 | CDK16 | CDK19 | CDK2 | CDK4 | CDK5 | CDK5R1 | CDK8 | CDK9 | CDKL3 | CEBPA | CEBPE | CEL | CES1 | CFL1 | CFTR | CHDH | CHEK2 | CHGB | CHRM3 | CHST4 | CISD3 | CKAP4 | CKB | CKM | CKMT1A | CLK1 | CLPX | CLTC | CMPK1 | CNDP1 | CNDP2 | COG3 | COL2A1 | COL4A3BP | COMMD1 | COMT | COPS2 | COPS5 | COPS6 | COX15 | CPNE3 | CPT1A | CPVL | CRBN | CREB1 | CREBBP | CRKL | CROT | CRY1 | CRY2 | CRYAB | CSNK1A1 | CSNK1E | CSNK2A2 | CSNK2B | CTBP1 | CTDP1 | CTH | CTNNB1 | CTPS1 | CTSA | CTSC | CTSD | CTSL | CTSV | CUL1 | CUL2 | CUL3 | CUL4B | CUL5 | CUL7 | CYC1 | CYCS | CYLD | CYP1A1 | CYP1A2 | DAD1 | DAPK1 | DAPP1 | DARS | DBH | DBI | DCAF12 | DCAF7 | DCAF8 | DCAKD | DCK | DCLK1 | DCN | DCUN1D1 | DCXR | DDA1 | DDB1 | DDB2 | DDI1 | DDOST | DDRGK1 | DEGS1 | DERL1 | DGUOK | DLAT | DLD | DLG2 | DLG3 | DLG4 | DLST | DMAP1 | DMD | DNAJB11 | DNAJB2 | DNAJC10 | DNAJC30 | DNPEP | DPEP1 | DPF2 | DPM1 | DPP9 | DRG2 | DSE | DTX1 | DTX2 | DUSP1 | DUSP12 | DUSP13 | DUSP14 | DUSP16 | DUSP19 | DUSP22 | DUSP7 | DUT | DYRK2 | DZIP3 | EDEM1 | EED | EEF1A1 | EEF1A1P5 | EEF1A2 | EEF2KMT | EFEMP1 | EGFR | EGLN1 | EGLN3 | EHHADH | EHMT1 | EHMT2 | EIF2AK3 | EIF2AK4 | EIF2S1 | EIF3H | EIF4H | ENOPH1 | EP300 | EPHA4 | EPHA6 | EPHB6 | EPRS | ERBB2 | ERBB3 | ERG | ERLIN2 | ESD | ESR1 | EVA1A | EXTL2 | EYA2 | EYA4 | EZH2 | F5 | F9 | FABP5 | FADD | FAF1 | FAF2 | FAH | FAM20B | FAM98A | FAR2 | FARSB | FASN | FBXL12 | FBXO11 | FBXO25 | FBXO32 | FBXO6 | FBXO7 | FBXW11 | FBXW5 | FECH | FGB | FGF2 | FGFR1 | FGFR3 | FGL2 | FIGNL1 | FKBP14 | FKBP1A | FKBP1B | FKBP2 | FKBP5 | FKBP7 | FKBP8 | FN1 | FOLH1 | FOLR1 | FOXK1 | FOXK2 | FOXO1 | FPGS | FYN | FZR1 | G6PD | GAD2 | GAK | GALNT7 | GANAB | GAPDH | GARS | GART | GCDH | GDA | GEMIN5 | GFPT1 | GFPT2 | GGA1 | GGCT | GGT1 | GGTLC1 | GLA | GLCE | GLO1 | GLRX | GLRX3 | GLRX5 | GLS | GLS2 | GLUD2 | GLUL | GMPR | GMPS | GNAI3 | GOLGA2 | GOLPH3 | GOT1 | GOT1L1 | GOT2 | GPC5 | GPD1L | GPS2 | GPT2 | GPX1 | GPX4 | GRB2 | GRK1 | GRK5 | GSK3A | GSK3B | GSPT1 | GSPT2 | GSR | GSS | GSTA1 | GSTA2 | GSTA3 | GSTA4 | GSTA5 | GSTK1 | GSTM4 | GSTO1 | GSTO2 | GSTP1 | GSTT1 | GSTT2 | GSTT2B | GSTZ1 | GZMA | GZMK | H3F3A | HAAO | HAGH | HARS | HBEGF | HCCS | HDAC1 | HDAC11 | HDAC2 | HDAC3 | HDAC4 | HDAC5 | HDAC6 | HDAC7 | HDAC9 | HECW1 | HECW2 | HERC1 | HERC2 | HEXA | HEXB | HGS | HIF1A | HIF1AN | HIPK4 | HIST1H2BA | HIST1H3A | HIST1H4A | HIST2H3A | HLCS | HMBS | HMGCL | HMOX1 | HNF4A | HPRT1 | HPX | HRAS | HS2ST1 | HS6ST1 | HSD17B4 | HSD17B8 | HSF1 | HSF4 | HSP90B1 | HSPA14 | HSPA1B | HSPA5 | HSPA8 | HSPD1 | HSPG2 | HTRA1 | HTRA2 | HUWE1 | IARS | ICK | IDE | IDH1 | IDH2 | IFI16 | IGF1R | IGFBP7 | IKBKB | IKBKE | IKBKG | IL3RA | ILF3 | ILK | IMPDH2 | ING5 | INO80B | INO80C | INSR | IREB2 | IRF4 | IRF7 | ISG15 | ITCH | ITGAV | ITK | JAK1 | JAK3 | JMJD6 | JOSD2 | KAT5 | KAT7 | KAT8 | KBTBD7 | KCMF1 | KCNAB2 | KCTD17 | KCTD2 | KDM1A | KDM2A | KDM3B | KDM4D | KDM5B | KDM5C | KDM8 | KDR | KDSR | KEAP1 | KLHL20 | KLHL22 | KMT2A | KMT2B | KMT2C | KRAS | KSR1 | KYNU | L2HGDH | LAMB1 | LAP3 | LARP4 | LARS | LAT | LATS1 | LCMT2 | LDHA | LDHC | LEF1 | LGALS1 | LGMN | LMO7 | LNX1 | LOXL2 | LPIN3 | LRRC7 | LRRK1 | LRRK2 | LRSAM1 | LTBP1 | LUM | LYPLA1 | LYPLA2 | LZTR1 | MAD2L1 | MAN2B1 | MAOA | MAP2K1 | MAP2K3 | MAP2K4 | MAP2K6 | MAP2K7 | MAP3K1 | MAP3K13 | MAP3K14 | MAP3K2 | MAP3K3 | MAP3K4 | MAP4K1 | MAPK1 | MAPK14 | MAPK15 | MAPK3 | MAPK4 | MAPK6 | MAPK7 | MAPK8 | MAPK9 | MAPT | MARCH5 | MASP1 | MASP2 | MAST1 | MAST3 | MATK | MBD3 | MBP | MCFD2 | MDH1 | MDH2 | MDM2 | MDM4 | ME1 | MECP2 | MED1 | MED12 | MED20 | MED21 | MED31 | MELK | METAP2 | MGST1 | MIB1 | MKNK2 | MMAB | MME | MMP26 | MOCOS | MORF4L1 | MORF4L2 | MPC2 | MPI | MPPE1 | MPST | MRGBP | MRPL28 | MRPL43 | MRPL9 | MRRF | MTA1 | MTA3 | MTAP | MTHFD1 | MTMR14 | MTMR7 | MTPN | MTRR | MVD | MYC | MYH6 | NAE1 | NAMPT | NAPRT | NCK1 | NCOA1 | NCOA3 | NDUFAF5 | NDUFAF7 | NDUFS1 | NEDD4 | NEDD8 | NEK1 | NEK7 | NEURL4 | NFE2L2 | NFKBIA | NFS1 | NHLRC3 | NIT2 | NLK | NME1 | NME2 | NME4 | NMNAT1 | NMNAT3 | NMRAL1 | NMRK2 | NNT | NOD2 | NOS2 | NOS3 | NOSIP | NPEPL1 | NPEPPS | NPLOC4 | NPR1 | NSFL1C | NT5C2 | NTMT1 | NTRK1 | NUCB1 | NUP107 | NUP153 | NUP160 | NUP205 | NUP214 | NUP85 | NUP88 | NUP93 | NUP98 | NUPR1 | OAT | OAZ3 | OBSL1 | OGDH | OGFOD1 | OGT | OLA1 | OPA1 | OTC | OTUB1 | OTUB2 | OTUD4 | OTUD5 | OXSR1 | P2RX1 | P4HA1 | P4HA2 | P4HB | PABPC4 | PADI4 | PAFAH1B1 | PAH | PAICS | PAK2 | PAK6 | PAPLN | PAPSS1 | PAPSS2 | PARK7 | PARP1 | PARP12 | PARP14 | PARP6 | PAWR | PAXIP1 | PCBD1 | PCBP2 | PCGF1 | PCGF5 | PCGF6 | PCMT1 | PCMTD1 | PCMTD2 | PCNA | PCSK9 | PCTP | PDCD6IP | PDF | PDHA1 | PDHA2 | PDHB | PDHX | PDIA3 | PDIA6 | PDK2 | PDXK | PEBP1 | PELI1 | PEPD | PER1 | PER2 | PEX5 | PFAS | PGAM5 | PGK1 | PGM3 | PGP | PHB | PHF10 | PHGDH | PHKA1 | PHOSPHO1 | PIAS1 | PIAS3 | PICK1 | PIGU | PIK3CA | PIK3CG | PIK3R1 | PIM1 | PIN1 | PJA1 | PKN2 | PLA2G10 | PLA2G12A | PLA2G1B | PLA2G2A | PLA2G6 | PLK1 | PLOD2 | PLSCR1 | PML | PMPCA | PMPCB | PNP | POLE3 | POM121 | PPA1 | PPA2 | PPARD | PPARG | PPAT | PPEF1 | PPIA | PPIB | PPID | PPIE | PPIF | PPIL3 | PPM1A | PPM1B | PPM1D | PPM1H | PPM1K | PPME1 | PPOX | PPP1CA | PPP1CB | PPP1CC | PPP1R11 | PPP1R12A | PPP1R3B | PPP1R3C | PPP2CA | PPP2CB | PPP2R1A | PPP2R1B | PPP2R2A | PPP2R2D | PPP2R5D | PPP3CC | PPP4R2 | PPP5C | PPP6C | PPT1 | PPTC7 | PRCP | PRDX3 | PRDX4 | PREP | PREPL | PRKAA1 | PRKAA2 | PRKAB2 | PRKACA | PRKAG1 | PRKCD | PRKCE | PRKCSH | PRKCZ | PRKD1 | PRKD2 | PRKD3 | PRKDC | PRMT2 | PRMT3 | PRMT6 | PROZ | PRPF19 | PRSS16 | PRSS23 | PRTFDC1 | PSAP | PSAT1 | PSEN1 | PSEN2 | PSMA1 | PSMA2 | PSMA3 | PSMA5 | PSMA6 | PSMB5 | PSMB6 | PSMB7 | PSMB8 | PSMC1 | PSMC2 | PSMC3 | PSMC4 | PSMC5 | PSMC6 | PSMD1 | PSMD10 | PSMD11 | PSMD12 | PSMD14 | PSMD2 | PSMD4 | PSMD9 | PSME2 | PSME3 | PTEN | PTGES | PTK2 | PTPMT1 | PTPN1 | PTPN14 | PTPN21 | PTPN22 | PTPN23 | PTPN4 | PTPN6 | PTPRA | PTPRD | PTPRF | PTPRK | PTPRO | PTPRR | PTTG1 | PYCR1 | PYCR2 | QARS | QDPR | QSOX1 | RAB11A | RAB11B | RAB1A | RAB1B | RAB2A | RAB5A | RAB7A | RAD23A | RAD23B | RAE1 | RAF1 | RAN | RANBP9 | RANGAP1 | RARA | RARS2 | RBBP6 | RBMX | RBX1 | RC3H1 | RELA | REST | RFK | RHBDD1 | RHO | RHOA | RHOT1 | RING1 | RIOK1 | RIPK1 | RIPK2 | RIPK3 | RIPK4 | RMND1 | RNF123 | RNF126 | RNF139 | RNF181 | RNF183 | RNF185 | RNF19A | RNF2 | RNF25 | RNF31 | RNF4 | RNF8 | RNPEP | RPL10 | RPL10A | RPL12 | RPL18A | RPL22 | RPL23 | RPL23A | RPL24 | RPL30 | RPL36AL | RPL37A | RPL38 | RPL6 | RPL7 | RPL7A | RPL9 | RPLP0 | RPLP0P6 | RPLP1 | RPLP2 | RPN1 | RPN2 | RPS12 | RPS13 | RPS14 | RPS15A | RPS19 | RPS2 | RPS20 | RPS24 | RPS29 | RPS3 | RPS4X | RPS6KA1 | RPS6KA2 | RPS6KB2 | RPS8 | RRM1 | RTF1 | RUVBL1 | RUVBL2 | S100A9 | SAE1 | SARS | SAT1 | SAT2 | SCG2 | SCG3 | SCLY | SCO2 | SCYL2 | SDC1 | SDC4 | SEL1L | SENP3 | SENP6 | SENP7 | SENP8 | SEPHS1 | SEPHS2 | SERPINH1 | SETDB1 | SFPQ | SGPL1 | SGSH | SGTA | SGTB | SHC1 | SHMT1 | SHMT2 | SIAH1 | SIN3A | SIRT1 | SIRT3 | SIRT5 | SIRT6 | SIRT7 | SLC25A1 | SLC25A13 | SLC25A32 | SMAD2 | SMAD3 | SMAD4 | SMAD5 | SMURF1 | SMURF2 | SNCA | SNRPB | SNRPD3 | SNX3 | SOCS1 | SOCS2 | SOCS6 | SOD1 | SPATA2 | SPI1 | SPOP | SPR | SPRR2A | SPSB3 | SPTAN1 | SPTBN1 | SPTBN2 | SPTLC2 | SQSTM1 | SRC | SRPK1 | SRPK2 | ST3GAL1 | ST3GAL6 | ST6GALNAC5 | ST8SIA4 | STARD7 | STAT5A | STK11 | STK24 | STK25 | STK26 | STK3 | STK38 | STK40 | STOML2 | STUB1 | STYX | SUCLA2 | SUCLG2 | SULF2 | SULT1A3 | SUMO1 | SUMO1P1 | SUMO2 | SUMO3 | SUZ12 | SYVN1 | TAB1 | TAB2 | TADA2B | TAF12 | TAMM41 | TANK | TAOK3 | TARS | TARS2 | TBL1X | TBRG4 | TCIRG1 | TDO2 | TECR | TESK2 | TGFB1 | TGFBR3 | TGM2 | TGOLN2 | THBS1 | THEM4 | THOP1 | TIMM50 | TIMP1 | TK1 | TK2 | TLR3 | TMEM132A | TMEM189-UBE2V1 | TMEM258 | TMTC3 | TNF | TNFAIP3 | TNIP2 | TNK2 | TNKS | TNKS2 | TOLLIP | TOMM20 | TOP1 | TOR1A | TP53 | TP53RK | TPR | TPSAB1 | TPSB2 | TPST2 | TPTE | TRAF2 | TRAF3 | TRAF6 | TREX1 | TRIB2 | TRIB3 | TRIM21 | TRIM23 | TRIM25 | TRIM28 | TRIM32 | TRIM33 | TRIM41 | TRIM47 | TRIM63 | TRIM69 | TRIM7 | TRIM9 | TRIP12 | TSG101 | TSPAN17 | TTLL1 | TWF1 | TYMS | UBA1 | UBA2 | UBA3 | UBA5 | UBA6 | UBAC1 | UBB | UBC | UBE2A | UBE2B | UBE2C | UBE2D1 | UBE2D2 | UBE2D3 | UBE2H | UBE2I | UBE2J1 | UBE2K | UBE2L3 | UBE2L6 | UBE2M | UBE2N | UBE2O | UBE2Q2 | UBE2R2 | UBE2S | UBE2V1 | UBE2W | UBE3A | UBE3B | UBL4A | UBQLN1 | UBQLN2 | UBQLN4 | UBR4 | UBR5 | UBXN1 | UBXN7 | UCHL1 | UCHL3 | UCHL5 | UCK2 | UFC1 | UFM1 | UGDH | UGGT1 | UGT8 | ULK1 | UMPS | UNKL | UPP2 | UQCRC2 | USP11 | USP14 | USP15 | USP16 | USP17L2 | USP19 | USP2 | USP20 | USP21 | USP26 | USP28 | USP39 | USP4 | USP42 | USP46 | USP47 | USP5 | USP50 | USP53 | USP7 | USP8 | USP9X | USP9Y | USPL1 | VAPA | VAPB | VARS | VASH2 | VCAM1 | VCAN | VCP | VCPIP1 | VGF | VHL | VKORC1 | VPS37D | VPS4B | WARS | WDR45B | WDR48 | WDR82 | WDTC1 | WDYHV1 | WIPI1 | WIPI2 | WNK1 | WNK4 | WSB1 | WWP2 | XBP1 | XIAP | XPNPEP1 | XYLT1 | YBEY | YEATS2 | YES1 | YME1L1 | YWHAB | YWHAE | YY1 | ZAP70 | ZBTB16 | ZC3HC1 | ZDHHC17 | ZDHHC23 | ZMPSTE24 | ZNF598 | ZNRF4 | GO:0050896 | 0.346233167536091 | 2854/8243 | 1290/3250 | 4.33080443515007e-15 | 4.112098811175e-11 | T | T | T | T | response to stimulus | A2M | ABAT | ABCD3 | ABCE1 | ABCG1 | ABL1 | ABL2 | ACAA2 | ACACA | ACADVL | ACAT1 | ACD | ACKR2 | ACO1 | ACO2 | ACSL1 | ACSL4 | ACTA1 | ACTA2 | ACTB | ACTG1 | ACTL6A | ACTN4 | ACTR2 | ACTR3 | ADA | ADCY6 | ADCYAP1 | ADH5 | ADORA2A | ADRB2 | ADSL | ADSS | ADTRP | AES | AFG3L2 | AGER | AGL | AGR2 | AGTRAP | AHCY | AHCYL1 | AHSG | AICDA | AIFM1 | AIRE | AK4 | AKAP8 | AKAP9 | AKR1B1 | AKR1C2 | AKT1 | AKT2 | ALB | ALOX12 | ALOX5AP | AMFR | ANK3 | ANKS4B | ANXA1 | ANXA11 | ANXA2 | ANXA5 | ANXA7 | APBB1 | APC | APCS | APEX1 | APEX2 | APLP1 | APOA1 | APOD | APOE | APOL2 | APP | APRT | AR | ARF1 | ARF4 | ARFGAP1 | ARHGAP35 | ARNT | ARNT2 | ARPC1B | ARRB1 | ARRB2 | ASCL1 | ASGR1 | ASNS | ASS1 | ATAD5 | ATF2 | ATF6 | ATF6B | ATG5 | ATIC | ATM | ATOX1 | ATP13A2 | ATP1A1 | ATP2A2 | ATP6V1A | ATRX | AUP1 | AURKB | AZU1 | BABAM1 | BACE1 | BAG3 | BAG4 | BAG6 | BAIAP2 | BAIAP2L1 | BANF1 | BAX | BBC3 | BCAR1 | BCAR3 | BCAT2 | BCKDHB | BCL10 | BCL11A | BCL2 | BCL2L1 | BCL2L2 | BCL6 | BDKRB2 | BIRC2 | BMPR1A | BRAF | BRCA1 | BRD4 | BSG | BSPRY | BTK | BTRC | C1QA | C1QBP | C1R | C4A | CAB39 | CABP4 | CACNA1A | CACNG2 | CACNG4 | CALCOCO2 | CALM1 | CALM2 | CALM3 | CALR | CAMK1D | CAMK2A | CAPN2 | CAPZA1 | CAPZA2 | CARD9 | CARM1 | CASP2 | CASP3 | CASP7 | CASP8 | CASP9 | CAT | CAV1 | CBFA2T3 | CBL | CBS | CBX8 | CCAR2 | CCDC130 | CCDC155 | CCL21 | CCL22 | CCNB1 | CCR1 | CCR3 | CCR4 | CCR5 | CCS | CCT5 | CD209 | CD27 | CD36 | CD40 | CD40LG | CD6 | CD74 | CD79B | CD81 | CDC14B | CDC25A | CDC25C | CDC34 | CDC37 | CDC42EP4 | CDC5L | CDC73 | CDH1 | CDK19 | CDK2 | CDK4 | CDK5 | CDK9 | CDKN1A | CEBPA | CEBPE | CENPF | CES1 | CFL1 | CFTR | CHAF1A | CHD1L | CHEK2 | CHP1 | CHRM2 | CHRNA9 | CHRND | CHRNE | CHRNG | CHST4 | CIRBP | CLDN18 | CLIC4 | CLNK | CLPB | CLU | COL18A1 | COL2A1 | COL4A1 | COL4A2 | COL4A3BP | COL6A1 | COL6A2 | COLEC10 | COMT | COPS2 | COPS5 | COPS6 | COTL1 | CPNE3 | CPT1A | CREB1 | CREB3 | CREBBP | CRIP1 | CRK | CRKL | CRNN | CRP | CRY1 | CRY2 | CRYAB | CSMD1 | CSN1S1 | CSNK1E | CSNK2B | CTH | CTNNB1 | CTPS1 | CTSC | CTSL | CTSV | CUL1 | CUL2 | CUL4B | CUL7 | CUTA | CXCL11 | CXCL5 | CXCL6 | CXCL8 | CYC1 | CYCS | CYLD | CYP1A1 | CYP1A2 | DAB1 | DAD1 | DAPK1 | DAXX | DBH | DBNL | DCLK1 | DCN | DCTN1 | DCTPP1 | DDB1 | DDB2 | DDIT4 | DDOST | DDRGK1 | DDX1 | DDX3X | DENND4C | DERL1 | DHCR24 | DHX15 | DHX36 | DHX9 | DLG2 | DLG4 | DMAP1 | DMD | DNA2 | DNAJA1 | DNAJA2 | DNAJA3 | DNAJA4 | DNAJB1 | DNAJB11 | DNAJB2 | DNAJC10 | DNM1L | DPEP1 | DROSHA | DTX1 | DUSP1 | DUSP22 | DYRK2 | E2F1 | E2F4 | ECSIT | EDA | EDEM1 | EED | EEF1A1 | EEF1A2 | EEF1D | EEF1G | EEF2 | EFTUD2 | EGFR | EGLN1 | EGLN3 | EHD4 | EHMT1 | EHMT2 | EIF2AK3 | EIF2AK4 | EIF2B1 | EIF2B3 | EIF2B5 | EIF2S1 | EIF4A3 | EIF4E | EIF4EBP1 | EIF6 | ELF3 | ELF4 | ENDOG | ENO1 | ENO3 | EP300 | EPHA4 | EPPIN | EPPK1 | EPRS | ERBB2 | ERBB3 | ERCC5 | ERLIN2 | ERRFI1 | ESR1 | ESRRA | ETFDH | ETS1 | EXOSC5 | EXOSC9 | EXTL2 | EYA2 | EYA4 | EZH2 | EZR | F9 | FABP1 | FABP3 | FADD | FAF2 | FANCA | FANCC | FANCG | FANCI | FANCM | FASLG | FASN | FBP1 | FBXO32 | FBXO6 | FBXW11 | FCGR1A | FCN1 | FECH | FEN1 | FGB | FGF2 | FHL2 | FIGNL1 | FIS1 | FKBP14 | FKBP1B | FKBP5 | FKBPL | FLNB | FLOT1 | FN1 | FOLR1 | FOS | FOXA1 | FOXJ1 | FOXM1 | FOXO1 | FTH1 | FUS | FYN | FZR1 | G6PD | GAD2 | GAP43 | GAPDH | GART | GATA1 | GBP2 | GBP3 | GBP5 | GBP6 | GDI1 | GFAP | GFPT1 | GFPT2 | GGT1 | GINS2 | GINS4 | GLP1R | GLRB | GLUL | GMPR | GNA12 | GNAI1 | GNAI2 | GNAO1 | GNAQ | GNAS | GNB1 | GNG12 | GNL1 | GNPNAT1 | GORASP2 | GOT1 | GOT2 | GPD1 | GPI | GPS2 | GPX1 | GPX4 | GPX7 | GRAMD1A | GRB2 | GRIK2 | GRK1 | GSK3A | GSK3B | GSR | GSS | GSTO1 | GSTO2 | GSTP1 | GTF2F1 | GZMA | H2AFX | H2AFZ | HAAO | HADH | HAVCR2 | HAX1 | HCN1 | HCN2 | HCN3 | HDAC2 | HDAC3 | HDAC4 | HDAC5 | HDAC6 | HDAC9 | HDGF | HERC2 | HEXIM1 | HID1 | HIF1A | HIF1AN | HIST1H2BA | HIST1H4A | HIST3H3 | HK2 | HLA-A | HLA-B | HLA-DQA1 | HLA-DRA | HLA-DRB3 | HLA-DRB4 | HLCS | HMGB1 | HMGB2 | HMGB3 | HMGCL | HMOX1 | HNF4A | HNRNPA1 | HNRNPD | HNRNPL | HNRNPU | HPRT1 | HRAS | HSF1 | HSF4 | HSP90AA1 | HSP90AB1 | HSP90B1 | HSPA14 | HSPA1B | HSPA1L | HSPA2 | HSPA4 | HSPA4L | HSPA5 | HSPA8 | HSPA9 | HSPB1 | HSPB2 | HSPD1 | HSPE1 | HSPG2 | HSPH1 | HTR1B | HTR3A | HTRA2 | HUWE1 | ICAM1 | ID2 | IDH1 | IFI16 | IFIT1 | IFITM3 | IFNA2 | IFNAR1 | IGBP1 | IGF1R | IGFBP7 | IGHV3-13 | IGHV3-23 | IGHV3-30 | IGKV3-20 | IGLC7 | IKBKB | IKBKE | IKBKG | IL13RA2 | IL32 | IL3RA | ILF3 | IMPDH2 | INO80B | INO80C | INSIG1 | INSIG2 | INSR | IPO5 | IPO7 | IQGAP1 | IREB2 | IRF1 | IRF4 | IRF7 | ISG15 | ITCH | ITGA4 | ITGB1BP1 | ITGB3 | ITK | ITPR1 | ITPR2 | ITPR3 | JAK1 | JAK3 | JUN | JUNB | JUND | JUP | KAT5 | KAT7 | KCNIP2 | KCNK1 | KDM1A | KDM2A | KDM3B | KDM4D | KDM5B | KDR | KEAP1 | KIF5B | KIR3DL2 | KIR3DS1 | KLF5 | KLHL20 | KLHL22 | KLRC2 | KRAS | KYNU | LAMP3 | LARS | LAT | LCP2 | LDHA | LEF1 | LGALS1 | LGALS3 | LGALS3BP | LGALS8 | LGMN | LGR4 | LIG4 | LIMS1 | LMNA | LMO2 | LOXL2 | LPAR1 | LPIN3 | LRP6 | LRRK2 | LSM5 | LTBR | LUM | LYG2 | LYZL1 | LYZL2 | MAP2K1 | MAP2K3 | MAP2K4 | MAP2K6 | MAP2K7 | MAP3K1 | MAP3K13 | MAP3K14 | MAP3K2 | MAP3K3 | MAP3K4 | MAP4K1 | MAPK1 | MAPK14 | MAPK15 | MAPK3 | MAPK4 | MAPK6 | MAPK7 | MAPK8 | MAPK8IP3 | MAPK9 | MAPT | MAS1 | MASP1 | MASP2 | MAT2A | MATR3 | MAVS | MBD3 | MBP | MC1R | MCM2 | MCM7 | MCM9 | MCTS1 | MDC1 | MDM2 | MDM4 | ME1 | MECP2 | MED1 | METRNL | MGMT | MGST1 | MICA | MICU1 | MIF | MKNK2 | MLH1 | MME | MORF4L1 | MORF4L2 | MPG | MPST | MSH6 | MSN | MTA1 | MTAP | MTPN | MTSS1 | MYBBP1A | MYC | MYD88 | MYH6 | MYO1C | MYO5A | MYO6 | MYOC | NAMPT | NAPRT | NCK1 | NCOA1 | NCOA2 | NCOA3 | NDRG1 | NDUFA13 | NDUFA9 | NDUFS2 | NDUFS8 | NEDD4 | NEDD8 | NEK1 | NEUROD1 | NFATC2 | NFE2L2 | NFKB2 | NFKBIA | NIPBL | NKX2-1 | NME1 | NME2 | NMNAT1 | NMNAT3 | NNT | NOD2 | NOS2 | NOS3 | NPEPPS | NPLOC4 | NPM1 | NQO1 | NR0B2 | NR1H2 | NR1H3 | NR3C1 | NR4A1 | NR5A2 | NRBF2 | NRP2 | NSMCE4A | NTRK1 | NUCB1 | NUPR1 | OAS2 | OGT | OPA1 | OPTN | OR5F1 | ORM1 | OTC | OTUB1 | OTUD4 | OTUD5 | OXCT1 | OXSR1 | P2RX1 | P2RX2 | P2RX4 | P4HB | PADI4 | PAG1 | PAK2 | PAK6 | PALB2 | PARK7 | PARP1 | PARP14 | PAX8 | PAX9 | PAXIP1 | PCBP2 | PCNA | PCSK9 | PDCD10 | PDCD6 | PDE12 | PDE4D | PDE6G | PDIA3 | PDIA4 | PDIA6 | PDK2 | PEBP1 | PEF1 | PELI1 | PER1 | PER2 | PFAS | PFKL | PFKP | PGAM2 | PGK1 | PHB | PHB2 | PIAS1 | PIAS3 | PICK1 | PIK3CA | PIK3CG | PIK3R1 | PKM | PLA2G10 | PLA2G1B | PLA2G2A | PLA2G6 | PLCG1 | PLEC | PLGRKT | PLK1 | PLOD2 | PLSCR1 | PML | PNKP | PNMA1 | PNP | POLD1 | POLE2 | POLE3 | POLM | POLR2A | POLR2E | POLR2L | POU5F1 | PPARD | PPARG | PPARGC1A | PPAT | PPEF1 | PPID | PPIE | PPIF | PPM1A | PPM1D | PPOX | PPP1CA | PPP1CB | PPP1CC | PPP1R11 | PPP1R12A | PPP1R14A | PPP2CA | PPP2CB | PPP2R1A | PPP2R2A | PPP5C | PPP6C | PPT1 | PRCP | PRDX1 | PRDX2 | PRDX3 | PRDX4 | PRDX5 | PRDX6 | PRELID1 | PRG2 | PRKAA1 | PRKAA2 | PRKACA | PRKAR1A | PRKAR2B | PRKCD | PRKCE | PRKCZ | PRKD1 | PRKD2 | PRKDC | PRMT6 | PROX1 | PRPF19 | PRPF8 | PSAP | PSEN1 | PSEN2 | PSMA1 | PSMA2 | PSMA3 | PSMA5 | PSMA6 | PSMB5 | PSMB6 | PSMB7 | PSMB8 | PSMC1 | PSMC2 | PSMC3 | PSMC4 | PSMC5 | PSMC6 | PSMD1 | PSMD10 | PSMD11 | PSMD12 | PSMD14 | PSMD2 | PSMD4 | PSMD9 | PSME2 | PSME3 | PSTPIP1 | PTEN | PTGER3 | PTGES | PTGIR | PTGR1 | PTN | PTPN1 | PTPN14 | PTPN22 | PTPN23 | PTPN4 | PTPN6 | PTPRF | PTPRK | PTTG1 | PXDN | PXN | PYCR1 | PYCR2 | PYGL | QDPR | RAB10 | RAB11B | RAB1A | RAB29 | RAB31 | RAB35 | RAB3GAP1 | RAC1 | RAD21 | RAD23A | RAD23B | RAD51 | RAD51AP1 | RAE1 | RAF1 | RANGAP1 | RAP1A | RAP1B | RARA | RARG | RB1 | RBBP4 | RBBP6 | RBM17 | RBM3 | RBM4 | RBMX | RBPJ | RBX1 | RC3H1 | RDH13 | RDH5 | RDX | REL | RELA | REST | RFC1 | RFC2 | RFC3 | RFC4 | RFC5 | RGS1 | RGS2 | RHBDD1 | RHO | RHOA | RIF1 | RIPK1 | RIPK2 | RIPK3 | RNASE3 | RNASEH2A | RNF139 | RNF183 | RNF185 | RNF4 | RNF8 | ROMO1 | RPA2 | RPA3 | RPAIN | RPGRIP1 | RPL10A | RPL18A | RPL23 | RPL30 | RPN2 | RPS15A | RPS19 | RPS3 | RPS6KA1 | RPTOR | RRM1 | RRM2B | RUNX2 | RUVBL1 | RUVBL2 | RXRA | S100A12 | S100A13 | S100A7 | S100A9 | S100B | S100P | SAG | SARM1 | SCARA3 | SCEL | SCFD1 | SCG2 | SDC1 | SDC4 | SEC16A | SEC31A | SEL1L | SELL | SEMA7A | SERPINB2 | SERPINB6 | SERPINE2 | SERPINH1 | SETDB1 | SFN | SFPQ | SGTA | SGTB | SH3GL2 | SHC1 | SHMT1 | SHMT2 | SIN3A | SIPA1 | SIRT1 | SIRT5 | SIRT6 | SLAMF1 | SLAMF8 | SLC12A2 | SLC16A1 | SLC25A13 | SLC25A24 | SLC25A5 | SLC25A6 | SLC2A5 | SLC39A4 | SLC3A2 | SLC9A1 | SMAD2 | SMAD3 | SMAD4 | SMAD5 | SMAD9 | SMC1A | SMC3 | SMO | SMURF1 | SNAI2 | SNCA | SNW1 | SNX3 | SNX6 | SOCS1 | SOCS2 | SOCS6 | SOD1 | SOD2 | SORD | SORT1 | SOX2 | SP1 | SP100 | SP2 | SPACA3 | SPI1 | SPINT2 | SPRTN | SPRY2 | SQSTM1 | SRC | SRP14 | SRP19 | SRPK1 | SRPK2 | SRPRB | SRSF5 | SRXN1 | ST13 | ST3GAL6 | STAC | STAT1 | STAT3 | STAT6 | STAU1 | STIM1 | STIP1 | STK11 | STK24 | STK25 | STK26 | STK3 | STMN1 | STOML2 | STUB1 | STXBP1 | STXBP2 | SULF2 | SULT1A3 | SUMO1 | SYP | SYVN1 | TAB1 | TAB2 | TANK | TAOK3 | TARDBP | TAS2R41 | TAS2R60 | TAS2R7 | TBC1D4 | TBXA2R | TCEA1 | TCF12 | TCIRG1 | TDO2 | TDP1 | TERF1 | TERF2 | TERF2IP | TFDP1 | TFIP11 | TGFB1 | TGFBR3 | THBS1 | THRA | TIAL1 | TIMP1 | TIMP2 | TLR3 | TM9SF4 | TMBIM6 | TMEM173 | TMEM189-UBE2V1 | TMTC3 | TNF | TNFAIP3 | TNFRSF1A | TNFRSF8 | TNFSF12 | TNFSF14 | TNIP2 | TNKS | TNP1 | TOLLIP | TOMM20 | TOP1 | TOP2A | TOR1A | TP53 | TP63 | TP73 | TPCN2 | TPM1 | TPR | TPSAB1 | TRADD | TRAF2 | TRAF3 | TRAF6 | TREX1 | TRIAP1 | TRIB3 | TRIM14 | TRIM21 | TRIM23 | TRIM25 | TRIM28 | TRIM29 | TRIM32 | TRIM34 | TRIM35 | TRIM41 | TRIM63 | TRIP12 | TRIP13 | TSC22D3 | TSC22D4 | TSPYL2 | TTC5 | TUBB | TUBB4B | TUFM | TWF2 | TWIST1 | TXN | TXN2 | TXNIP | TXNRD1 | TYMS | UBA1 | UBA5 | UBB | UBC | UBE2A | UBE2B | UBE2D3 | UBE2J1 | UBE2K | UBE2L3 | UBE2L6 | UBE2N | UBE2V1 | UBE2W | UBE3A | UBQLN1 | UBQLN2 | UBR5 | UCHL1 | UCHL5 | UFC1 | UFM1 | UGGT1 | ULK1 | UMPS | UNC119 | UQCRC2 | USP14 | USP16 | USP19 | USP2 | USP28 | USP46 | USP47 | USP53 | USP7 | USP8 | VAPB | VAV1 | VCAM1 | VCP | VDAC1 | VDAC3 | VGF | VHL | VIM | VN1R1 | VPS4B | VSIG4 | VSTM2A | VTN | WAS | WBP2 | WDR35 | WDR45B | WDR48 | WDTC1 | WIPI1 | WIPI2 | WNK1 | WNT16 | WNT2 | WT1 | WWOX | XAF1 | XBP1 | XIAP | XPO1 | XRCC1 | XRCC3 | XRCC5 | XRCC6 | YAP1 | YES1 | YWHAE | YWHAG | YY1 | ZAP70 | ZBTB17 | ZBTB42 | ZBTB7A | ZBTB7B | ZFAND6 | ZFYVE26 | ZMPSTE24 | ZNF277 | GO:0019752 | 0.0501031177969186 | 413/8243 | 239/3250 | 6.34026040034854e-15 | 6.02007725013094e-11 | T | T | T | T | carboxylic acid metabolic process | AARS | AARSD1 | AASDH | AASDHPPT | AASS | ABAT | ABCD3 | ACAA1 | ACAA2 | ACACA | ACAD9 | ACADS | ACADSB | ACADVL | ACAT1 | ACAT2 | ACCS | ACLY | ACO1 | ACO2 | ACOT7 | ACOX1 | ACSL1 | ACSL4 | ACSL5 | ACSS2 | ACY1 | ADH1A | ADH1B | ADSS | AIMP2 | AKR1A1 | AKR1C2 | ALDH4A1 | ALDH5A1 | ALDH6A1 | ALDH7A1 | ALDH8A1 | ALOX12 | ALOX5 | ALOX5AP | ASL | ASNS | ASS1 | ATP6V1B1 | BCAT2 | BRCA1 | BSG | CARS | CBR1 | CBR4 | CBS | CD36 | CD74 | CES1 | CPT1A | CPT2 | CROT | CTH | CTPS1 | CYB5R3 | CYP1A1 | CYP1A2 | CYP2C18 | DARS | DECR1 | DEGS1 | DLAT | DLD | DLST | DPEP1 | ECH1 | ECHDC2 | ECHS1 | ECI1 | ECI2 | EGLN1 | EGLN3 | EHHADH | ENOPH1 | EPRS | ETFA | ETFB | ETFDH | FABP3 | FAH | FARSB | FASN | FH | FOLH1 | FPGS | GAD2 | GARS | GART | GCDH | GFPT1 | GFPT2 | GGT1 | GLO1 | GLS | GLS2 | GLUD2 | GLUL | GMPS | GOT1 | GOT1L1 | GOT2 | GPT2 | GPX1 | GPX4 | GRHPR | GSS | GSTA1 | GSTM4 | GSTO1 | GSTO2 | GSTP1 | HAAO | HADH | HAGH | HARS | HIBCH | HIF1A | HLCS | HNF4A | HSD17B4 | HSD17B8 | HSD3B7 | IARS | IDH1 | IDH2 | IDH3B | IDH3G | IREB2 | IVD | KYNU | L2HGDH | LARS | LDHA | LDHAL6A | LDHAL6B | LDHB | LDHC | LTC4S | LYPLA1 | LYPLA2 | MAPK14 | MAPK3 | MCAT | MCCC1 | MDH1 | MDH2 | ME1 | MECP2 | MGST1 | MIF | MPST | MTAP | MTHFD1 | MTRR | MYO5A | NANP | NDUFS6 | NIT2 | NOS2 | NOS3 | NR5A2 | OAT | OGDH | OSBPL1A | OTC | P4HA1 | P4HA2 | P4HB | PADI4 | PAH | PARK7 | PC | PDHA1 | PDHA2 | PDHB | PDHX | PEPD | PER2 | PFAS | PGD | PHGDH | PLA2G10 | PLA2G1B | PLOD2 | PPA1 | PPA2 | PPARD | PPARG | PPARGC1A | PPAT | PRDX4 | PRKAA1 | PRKAA2 | PRKAB2 | PRKAG1 | PRKAR2B | PSAT1 | PTGDS | PTGES | PTGES2 | PTGES3 | PTGR1 | PYCR1 | PYCR2 | QARS | QDPR | RARS2 | RBP1 | RGN | SARS | SCLY | SDHA | SDHB | SEPHS1 | SEPHS2 | SGPL1 | SHMT1 | SHMT2 | SLC16A1 | SLC25A32 | SNCA | ST3GAL1 | SUCLA2 | SUCLG2 | TARS | TARS2 | TECR | THEM4 | TNFRSF1A | VARS | VDAC1 | WARS | XBP1 | GO:0043436 | 0.0501031177969186 | 413/8243 | 239/3250 | 6.34026040034854e-15 | 6.02007725013094e-11 | T | T | T | T | oxoacid metabolic process | AARS | AARSD1 | AASDH | AASDHPPT | AASS | ABAT | ABCD3 | ACAA1 | ACAA2 | ACACA | ACAD9 | ACADS | ACADSB | ACADVL | ACAT1 | ACAT2 | ACCS | ACLY | ACO1 | ACO2 | ACOT7 | ACOX1 | ACSL1 | ACSL4 | ACSL5 | ACSS2 | ACY1 | ADH1A | ADH1B | ADSS | AIMP2 | AKR1A1 | AKR1C2 | ALDH4A1 | ALDH5A1 | ALDH6A1 | ALDH7A1 | ALDH8A1 | ALOX12 | ALOX5 | ALOX5AP | ASL | ASNS | ASS1 | ATP6V1B1 | BCAT2 | BRCA1 | BSG | CARS | CBR1 | CBR4 | CBS | CD36 | CD74 | CES1 | CPT1A | CPT2 | CROT | CTH | CTPS1 | CYB5R3 | CYP1A1 | CYP1A2 | CYP2C18 | DARS | DECR1 | DEGS1 | DLAT | DLD | DLST | DPEP1 | ECH1 | ECHDC2 | ECHS1 | ECI1 | ECI2 | EGLN1 | EGLN3 | EHHADH | ENOPH1 | EPRS | ETFA | ETFB | ETFDH | FABP3 | FAH | FARSB | FASN | FH | FOLH1 | FPGS | GAD2 | GARS | GART | GCDH | GFPT1 | GFPT2 | GGT1 | GLO1 | GLS | GLS2 | GLUD2 | GLUL | GMPS | GOT1 | GOT1L1 | GOT2 | GPT2 | GPX1 | GPX4 | GRHPR | GSS | GSTA1 | GSTM4 | GSTO1 | GSTO2 | GSTP1 | HAAO | HADH | HAGH | HARS | HIBCH | HIF1A | HLCS | HNF4A | HSD17B4 | HSD17B8 | HSD3B7 | IARS | IDH1 | IDH2 | IDH3B | IDH3G | IREB2 | IVD | KYNU | L2HGDH | LARS | LDHA | LDHAL6A | LDHAL6B | LDHB | LDHC | LTC4S | LYPLA1 | LYPLA2 | MAPK14 | MAPK3 | MCAT | MCCC1 | MDH1 | MDH2 | ME1 | MECP2 | MGST1 | MIF | MPST | MTAP | MTHFD1 | MTRR | MYO5A | NANP | NDUFS6 | NIT2 | NOS2 | NOS3 | NR5A2 | OAT | OGDH | OSBPL1A | OTC | P4HA1 | P4HA2 | P4HB | PADI4 | PAH | PARK7 | PC | PDHA1 | PDHA2 | PDHB | PDHX | PEPD | PER2 | PFAS | PGD | PHGDH | PLA2G10 | PLA2G1B | PLOD2 | PPA1 | PPA2 | PPARD | PPARG | PPARGC1A | PPAT | PRDX4 | PRKAA1 | PRKAA2 | PRKAB2 | PRKAG1 | PRKAR2B | PSAT1 | PTGDS | PTGES | PTGES2 | PTGES3 | PTGR1 | PYCR1 | PYCR2 | QARS | QDPR | RARS2 | RBP1 | RGN | SARS | SCLY | SDHA | SDHB | SEPHS1 | SEPHS2 | SGPL1 | SHMT1 | SHMT2 | SLC16A1 | SLC25A32 | SNCA | ST3GAL1 | SUCLA2 | SUCLG2 | TARS | TARS2 | TECR | THEM4 | TNFRSF1A | VARS | VDAC1 | WARS | XBP1 | GO:0042493 | 0.0584738566056048 | 482/8243 | 271/3250 | 1.21281394175234e-14 | 1.15156683769385e-10 | T | T | T | T | response to drug | ABAT | ABCD3 | ABL1 | ACSL1 | ACTB | ACTR2 | ADA | ADCY6 | ADCYAP1 | ADORA2A | AIFM1 | AK4 | ANXA1 | APEX1 | APOA1 | APOD | ARRB1 | ASCL1 | ASNS | ASS1 | ATG5 | ATP1A1 | ATRX | BCAR3 | BCL11A | BCL2 | BCL2L1 | BIRC2 | BRAF | BRCA1 | CACNG4 | CALM1 | CALM3 | CALR | CAPN2 | CARD9 | CASP3 | CASP8 | CASP9 | CAT | CAV1 | CBL | CBX8 | CCNB1 | CD27 | CD40 | CDH1 | CDK2 | CDK4 | CDK9 | CDKN1A | CENPF | CFTR | CHEK2 | CHRND | CHRNE | CHRNG | CLDN18 | COL18A1 | COMT | CPT1A | CREB1 | CRK | CRYAB | CSN1S1 | CTNNB1 | CTPS1 | CYP1A1 | DAB1 | DAD1 | DAXX | DBH | DDIT4 | DMAP1 | DPEP1 | DUSP1 | EEF2 | EFTUD2 | EGFR | EGLN1 | EIF4E | EIF4EBP1 | ENO3 | ERRFI1 | ETS1 | EZH2 | FABP1 | FABP3 | FADD | FBP1 | FBXO32 | FECH | FKBP1B | FOLR1 | FOS | FOXO1 | FYN | G6PD | GAD2 | GNA12 | GNAI1 | GNAO1 | GNAS | GOT2 | GPI | GPX1 | GSTP1 | HADH | HCN3 | HDAC2 | HDAC4 | HDAC5 | HDAC6 | HDAC9 | HLCS | HMGB2 | HMOX1 | HNRNPA1 | HNRNPD | HPRT1 | HSF1 | HSP90B1 | HSPA5 | HTR1B | HTR3A | ICAM1 | IGFBP7 | IREB2 | ITPR2 | JUN | JUNB | JUND | JUP | KAT7 | KCNK1 | KDM3B | KDR | KYNU | LDHA | LGALS1 | LRRK2 | MAP2K6 | MAP4K1 | MAPK1 | MAPK14 | MAPK3 | MAPK7 | MAS1 | MCM7 | MDM2 | MGMT | MGST1 | MYC | MYO6 | NCOA1 | NCOA2 | NEUROD1 | NFATC2 | NFE2L2 | NFKBIA | NME1 | NOD2 | NOS2 | NQO1 | NR1H3 | NR3C1 | NTRK1 | NUCB1 | OPA1 | OTC | OXCT1 | P2RX1 | P2RX2 | P2RX4 | PARK7 | PCNA | PDCD10 | PEBP1 | PFAS | PNP | PPARG | PPARGC1A | PPAT | PPIF | PPOX | PPP1R12A | PPP1R14A | PPP2CB | PPP2R2A | PRDX3 | PRKAA1 | PRKAA2 | PRKAR2B | PRKCD | PRKCE | PSMD14 | PTEN | PTGR1 | PTN | PTPN22 | QDPR | RAD51 | RAP1A | RAP1B | RARA | RELA | REST | RGS2 | RHOA | RIPK1 | RIPK2 | RIPK3 | RPL10A | RPL18A | RPN2 | RPS3 | SDC1 | SELL | SETDB1 | SIN3A | SIRT1 | SLAMF8 | SNCA | SOD1 | SORD | SPI1 | SRC | SRP14 | SRP19 | STAT1 | STAT3 | STAT6 | STK24 | STK25 | STK26 | SULT1A3 | TBXA2R | TDO2 | TERF1 | TGFB1 | THBS1 | TIMP2 | TLR3 | TMBIM6 | TNF | TNFAIP3 | TOP1 | TP53 | TP73 | TRAF2 | TREX1 | TRIM41 | TUFM | TXN2 | TXNIP | TYMS | UBE2B | UBE3A | UMPS | UQCRC2 | USP47 | USP8 | VCAM1 | VIM | WBP2 | WDR35 | XPO1 | XRCC1 | XRCC5 | YAP1 | ZNF277 | GO:0006091 | 0.0280237777508189 | 231/8243 | 147/3250 | 4.28089964978789e-14 | 4.0647142174736e-10 | T | T | T | T | generation of precursor metabolites and energy | ACADVL | ACAT1 | ACO2 | ACOX1 | ACSS2 | ADH1A | ADH1B | ADH5 | ADSL | AGL | AKR1A1 | AKR1B1 | AKT1 | AKT2 | ALDH1B1 | ALDH4A1 | ALDH5A1 | ALDOA | ALDOC | AOC2 | CALM1 | CAT | CEBPA | CHCHD10 | COQ9 | COX11 | COX15 | COX17 | COX20 | COX5A | COX6A1 | COX6C | COX7A2 | CROT | CYC1 | CYCS | CYP1A2 | DEGS1 | DLD | DLST | ENO1 | ENO2 | ENO3 | ETFA | ETFB | ETFDH | FDXR | FECH | GAA | GAPDH | GAPDHS | GBE1 | GFPT1 | GFPT2 | GLRX | GLRX3 | GLRX5 | GNAS | GNPDA1 | GPI | GSK3A | GSK3B | GSR | GYG1 | GYG2 | GYS1 | HAAO | HK1 | HK2 | HSD17B6 | IDH3B | LDHA | LOXL2 | LYRM7 | MDH2 | ME1 | MECP2 | MYBBP1A | MYC | NDUFA10 | NDUFA13 | NDUFA5 | NDUFA8 | NDUFA9 | NDUFAF1 | NDUFAF2 | NDUFB1 | NDUFS1 | NDUFS2 | NDUFS3 | NDUFS6 | NDUFS7 | NDUFS8 | NDUFV1 | NDUFV3 | NQO1 | NQO2 | OGDH | OXA1L | P4HA2 | PER2 | PFKL | PFKM | PFKP | PGAM1 | PGAM2 | PGK1 | PGK2 | PGM1 | PGM2 | PHGDH | PHKA1 | PKM | PPARD | PPARGC1A | PPP1CA | PPP1CB | PPP1CC | PPP1R2 | PPP1R3B | PPP1R3C | PTGES2 | PYGB | PYGL | QDPR | RUNX1T1 | SDHA | SDHB | SDHD | SIRT3 | SLC25A13 | SLC25A4 | SNCA | STBD1 | STK40 | STOML2 | TAZ | TBRG4 | TPI1 | TREX1 | TXNRD1 | UGP2 | UQCRB | UQCRC2 | UQCRFS1 | UQCRQ | VGF | GO:0009056 | 0.148732257673177 | 1226/8243 | 601/3250 | 9.52455515148996e-14 | 9.04356511633971e-10 | T | T | T | T | catabolic process | AASS | ABAT | ABCD3 | ABHD17A | ABL1 | ACAA1 | ACAA2 | ACAD8 | ACADS | ACADSB | ACADVL | ACAT1 | ACAT2 | ACOT7 | ACOX1 | ACSL1 | ADA | ADH5 | ADRM1 | ADTRP | AGL | AGO1 | AHCY | AICDA | AKR1A1 | AKR1B10 | AKT1 | ALDH1B1 | ALDH4A1 | ALDH5A1 | ALDH6A1 | ALDH7A1 | ALDH8A1 | ALDOA | ALDOC | AMDHD2 | AMFR | ANAPC4 | ANXA2 | ANXA7 | APOA1 | APOE | ARMC8 | ARRB1 | ARRB2 | ASPDH | ATCAY | ATG2B | ATG4C | ATG5 | ATM | AUP1 | AURKB | BACE1 | BAG3 | BAG6 | BCAT2 | BCKDHB | BDH1 | BIRC2 | BLVRB | BNIPL | BTRC | CALCOCO2 | CALM1 | CAPN1 | CASP3 | CAT | CBL | CBLC | CBS | CCNB1 | CD36 | CDA | CDC16 | CDC20 | CDC27 | CDC34 | CDK2 | CDK5 | CEL | CES1 | CHDH | CHEK2 | CHMP2A | CHP1 | CISD2 | CLPX | CLTC | COMT | CPT1A | CPT2 | CRBN | CROT | CRYZ | CSNK2A2 | CSNK2B | CTH | CTNNB1 | CTSD | CTSL | CUL1 | CUL2 | CUL3 | CUL4B | CUL5 | CUL7 | CYLD | CYP1A1 | CYP1A2 | CYP2C18 | DBH | DBT | DCN | DCP2 | DCTPP1 | DCXR | DDB1 | DDX6 | DECR1 | DERA | DERL1 | DIS3 | DLD | DLST | DNA2 | DNAJB2 | DNAJC10 | DNASE2 | DNASE2B | DPEP1 | DROSHA | DUT | DYNLL1 | ECH1 | ECHDC2 | ECHS1 | ECI1 | ECI2 | EDC4 | EDEM1 | EHHADH | EIF3E | EIF4A3 | ENDOG | ENO1 | ENO2 | ENO3 | ENTPD7 | ERLIN2 | ESD | ETFA | ETFB | ETFDH | EVA1A | EXOSC1 | EXOSC5 | EXOSC6 | EXOSC9 | FABP1 | FABP3 | FABP5 | FABP7 | FAF1 | FAF2 | FAH | FBXL12 | FBXO11 | FBXO6 | FBXO7 | FBXW11 | FBXW5 | FEN1 | FGF2 | FIS1 | FOXO1 | FUNDC2 | FZR1 | GAA | GAD2 | GALE | GALNS | GAPDH | GAPDHS | GCDH | GDA | GGT1 | GGTLC1 | GLA | GLS | GLS2 | GLUD2 | GLUL | GNPDA1 | GNPDA2 | GNS | GOT1 | GOT2 | GPC5 | GPD1 | GPD1L | GPI | GPT2 | GPX1 | GSK3A | GSPT1 | GSPT2 | GSTM4 | GSTO1 | GSTZ1 | GUSB | H1F0 | HAAO | HADH | HAGH | HAP1 | HBZ | HDAC6 | HECW1 | HECW2 | HELZ2 | HERC2 | HEXA | HEXB | HGS | HIBADH | HIBCH | HINT1 | HK1 | HK2 | HMGB1 | HMGB2 | HMGCL | HMMR | HMOX1 | HNRNPD | HPRT1 | HSD17B10 | HSD17B14 | HSD17B4 | HSD17B6 | HSD3B7 | HSP90AA1 | HSP90B1 | HSPA1B | HSPA5 | HSPA8 | HSPG2 | HTRA2 | HUWE1 | IDE | IFI16 | IMPA1 | IMPA2 | ISG15 | ITCH | ITPA | IVD | KAT5 | KCTD17 | KCTD2 | KEAP1 | KLHL20 | KLHL22 | KPNA1 | KPNB1 | KYNU | LDHA | LDHD | LGALS8 | LGMN | LNX1 | LPIN3 | LRRK2 | LRSAM1 | LSM1 | LSM3 | LSM4 | LSM5 | LUM | LYG2 | LYPLA1 | LYPLA2 | MAD2L1 | MAN2B1 | MAOA | MCAT | MCCC1 | MDM2 | MLH1 | MME | MMP26 | MPST | MTA1 | MTAP | MTMR14 | MTMR7 | MTRR | NAGK | NBAS | NCBP1 | NCEH1 | NEDD4 | NEDD8 | NFE2L2 | NHLRC3 | NOS2 | NOS3 | NPEPPS | NPLOC4 | NR1I2 | NRBF2 | NSFL1C | NT5C2 | NUDT18 | NUDT19 | NUDT3 | NUDT9 | OAS2 | OAT | OGDH | OPTN | OTC | OVGP1 | OXCT1 | PABPC1 | PABPC4 | PADI4 | PAFAH1B1 | PAH | PAN2 | PARK7 | PCBD1 | PCBP2 | PCSK9 | PDE12 | PDE4D | PELI1 | PEPD | PFKL | PFKM | PFKP | PGAM1 | PGAM2 | PGAM5 | PGK1 | PGK2 | PGM1 | PGM2 | PHKA1 | PJA1 | PKM | PLA2G10 | PLA2G12A | PLA2G1B | PLA2G2A | PLA2G6 | PLBD2 | PLCG1 | PLK1 | PML | PNP | PPARD | PPARGC1A | PPAT | PPM1K | PPP1R11 | PPP1R8 | PPP2CA | PPP2CB | PPP2R1A | PPP2R2A | PPT1 | PRDX1 | PRDX2 | PRDX3 | PRDX4 | PRDX5 | PRDX6 | PRKAA1 | PRKAA2 | PRKAB2 | PRKAG1 | PRPF19 | PRSS16 | PRTFDC1 | PSAP | PSMA1 | PSMA2 | PSMA3 | PSMA5 | PSMA6 | PSMB5 | PSMB6 | PSMB7 | PSMB8 | PSMC1 | PSMC2 | PSMC3 | PSMC4 | PSMC5 | PSMC6 | PSMD1 | PSMD10 | PSMD11 | PSMD12 | PSMD14 | PSMD2 | PSMD4 | PSMD9 | PSME2 | PSME3 | PTEN | PTPN22 | PTPN23 | PTTG1 | PXDN | PYGB | PYGL | QDPR | RAB1A | RAB1B | RAB24 | RAB7A | RAD23A | RAD23B | RB1CC1 | RBBP6 | RBM8A | RBX1 | RC3H1 | RHBDD1 | RIPK1 | RNASE3 | RNASEH2A | RNASEH2B | RNF126 | RNF139 | RNF185 | RNF19A | RNF4 | RNF8 | RPE | RPL10 | RPL10A | RPL12 | RPL18A | RPL22 | RPL23 | RPL23A | RPL24 | RPL30 | RPL37A | RPL38 | RPL6 | RPL7 | RPL7A | RPL9 | RPLP0 | RPLP1 | RPLP2 | RPS12 | RPS13 | RPS14 | RPS15A | RPS19 | RPS2 | RPS20 | RPS24 | RPS29 | RPS3 | RPS4X | RPS8 | S100A9 | SAT1 | SAT2 | SDC1 | SDC4 | SEL1L | SGPL1 | SGSH | SGTA | SGTB | SHMT1 | SHMT2 | SIAH1 | SIRT1 | SKIV2L | SLC9A1 | SMG5 | SMG6 | SMG8 | SMURF1 | SMURF2 | SND1 | SNF8 | SNX17 | SOCS6 | SORD | SPOP | SPSB3 | SQSTM1 | SRC | SSB | STBD1 | STK11 | STUB1 | SULT1A3 | SYVN1 | TBL1X | TCIRG1 | TDO2 | TGFB1 | TMBIM6 | TOLLIP | TOMM20 | TOMM22 | TOMM40 | TOR1A | TP53 | TPI1 | TRAF2 | TRAPPC8 | TREX1 | TRIM25 | TRIM32 | TRIM9 | TRIP12 | TSG101 | TXN2 | UBA6 | UBB | UBC | UBE2A | UBE2B | UBE2C | UBE2D1 | UBE2D2 | UBE2D3 | UBE2H | UBE2I | UBE2J1 | UBE2K | UBE2L3 | UBE2L6 | UBE2R2 | UBE2S | UBE2W | UBE3A | UBE3B | UBQLN1 | UBQLN2 | UBQLN4 | UBR4 | UBXN1 | UCHL1 | UCHL3 | UCHL5 | UGGT1 | ULK1 | UPF2 | UPF3B | UPP2 | USP11 | USP14 | USP15 | USP16 | USP17L2 | USP19 | USP2 | USP20 | USP21 | USP26 | USP28 | USP4 | USP42 | USP46 | USP47 | USP5 | USP50 | USP7 | USP8 | USP9Y | VCAN | VCP | VDAC1 | VPS18 | VPS37D | VPS4B | VTA1 | WDR41 | WDR45B | WIPI1 | WIPI2 | WWP2 | XBP1 | XPNPEP1 | YME1L1 | YWHAH | ZMPSTE24 | ZNRF4 | GO:0051716 | 0.222491811233774 | 1834/8243 | 859/3250 | 1.512113185253e-13 | 1.43575146939773e-09 | T | T | T | T | cellular response to stimulus | ABCG1 | ABL1 | ABL2 | ACAA2 | ACACA | ACADVL | ACD | ACTA1 | ACTB | ACTG1 | ACTL6A | ACTR2 | ACTR3 | ADCY6 | ADCYAP1 | ADRB2 | ADSS | ADTRP | AGER | AGR2 | AICDA | AIFM1 | AKAP8 | AKAP9 | AKR1B1 | AKR1C2 | AKT1 | AKT2 | ALB | ALOX12 | ALOX5AP | AMFR | ANK3 | ANKS4B | ANXA1 | ANXA5 | APBB1 | APC | APEX1 | APEX2 | APLP1 | APOA1 | APOD | APP | APRT | AR | ARFGAP1 | ARHGAP35 | ARNT | ASCL1 | ASGR1 | ASNS | ASS1 | ATAD5 | ATF2 | ATF6 | ATF6B | ATG5 | ATIC | ATM | ATP13A2 | ATP1A1 | ATP2A2 | ATP6V1A | ATRX | AUP1 | AURKB | BABAM1 | BACE1 | BAG3 | BAG4 | BAG6 | BAX | BBC3 | BCAR1 | BCAT2 | BCL10 | BCL11A | BCL2 | BCL2L1 | BCL2L2 | BCL6 | BMPR1A | BRAF | BRCA1 | BRD4 | BSPRY | BTK | BTRC | CAB39 | CACNA1A | CALR | CAPN2 | CARM1 | CASP2 | CASP3 | CASP7 | CASP8 | CASP9 | CAT | CAV1 | CBL | CBS | CBX8 | CCAR2 | CCDC155 | CCL21 | CCL22 | CCNB1 | CCR5 | CCS | CD36 | CD40 | CD81 | CDC14B | CDC25A | CDC25C | CDC34 | CDC37 | CDC42EP4 | CDC5L | CDC73 | CDH1 | CDK19 | CDK2 | CDK4 | CDK5 | CDK9 | CDKN1A | CEBPA | CEBPE | CFTR | CHAF1A | CHD1L | CHEK2 | CHP1 | CLIC4 | CLPB | COL2A1 | COL4A1 | COL4A2 | COL4A3BP | COL6A1 | COMT | COPS2 | COPS5 | COPS6 | CPNE3 | CPT1A | CREB1 | CREB3 | CREBBP | CRIP1 | CRK | CRKL | CRY1 | CRY2 | CRYAB | CSNK1E | CTH | CTNNB1 | CTSL | CTSV | CUL1 | CUL2 | CUL4B | CUL7 | CXCL11 | CXCL5 | CXCL6 | CXCL8 | CYCS | CYP1A1 | CYP1A2 | DAPK1 | DAXX | DCTN1 | DCTPP1 | DDB1 | DDB2 | DDIT4 | DDRGK1 | DDX1 | DDX3X | DENND4C | DERL1 | DHX36 | DHX9 | DLG2 | DLG4 | DMAP1 | DNA2 | DNAJA1 | DNAJB1 | DNAJB11 | DNAJB2 | DNAJC10 | DNM1L | DPEP1 | DTX1 | DUSP1 | DUSP22 | DYRK2 | E2F1 | E2F4 | EDEM1 | EED | EEF1A1 | EEF1D | EEF2 | EFTUD2 | EGFR | EGLN1 | EGLN3 | EHD4 | EHMT2 | EIF2AK3 | EIF2AK4 | EIF2B5 | EIF2S1 | EIF4A3 | EIF4E | EIF4EBP1 | ENDOG | EP300 | EPHA4 | EPRS | ERBB2 | ERCC5 | ERLIN2 | ERRFI1 | ESR1 | ETS1 | EXTL2 | EYA2 | EYA4 | EZH2 | EZR | FABP1 | FADD | FAF2 | FANCA | FANCC | FANCG | FANCI | FANCM | FASN | FBP1 | FBXO32 | FBXO6 | FBXW11 | FECH | FEN1 | FGB | FIGNL1 | FIS1 | FKBP14 | FLNB | FLOT1 | FOLR1 | FOS | FOXM1 | FOXO1 | FUS | FYN | FZR1 | G6PD | GAP43 | GAPDH | GATA1 | GBP2 | GBP3 | GBP5 | GBP6 | GFAP | GFPT1 | GFPT2 | GINS2 | GINS4 | GLP1R | GLUL | GNAI1 | GNB1 | GNL1 | GNPNAT1 | GORASP2 | GOT1 | GPD1 | GPS2 | GPX1 | GPX7 | GRAMD1A | GRB2 | GRK1 | GSK3A | GSK3B | GSR | GSTO1 | GSTO2 | GSTP1 | H2AFX | H2AFZ | HAVCR2 | HAX1 | HCN1 | HCN2 | HCN3 | HDAC2 | HDAC3 | HDAC4 | HDAC5 | HDAC6 | HDAC9 | HDGF | HERC2 | HIF1A | HIF1AN | HIST1H4A | HIST3H3 | HK2 | HMGB1 | HMGB2 | HMOX1 | HNRNPA1 | HNRNPD | HNRNPL | HNRNPU | HRAS | HSF1 | HSF4 | HSP90AA1 | HSP90AB1 | HSP90B1 | HSPA14 | HSPA1B | HSPA1L | HSPA2 | HSPA5 | HSPA8 | HSPA9 | HSPB1 | HTR1B | HTR3A | HTRA2 | HUWE1 | ICAM1 | ID2 | IFI16 | IFIT1 | IFNAR1 | IGF1R | IGFBP7 | IKBKB | IKBKE | IKBKG | IL3RA | IMPDH2 | INO80B | INO80C | INSIG1 | INSIG2 | INSR | IPO5 | IQGAP1 | IREB2 | IRF1 | IRF7 | ISG15 | ITGA4 | ITGB1BP1 | ITPR1 | ITPR2 | JUN | JUNB | JUND | JUP | KAT5 | KAT7 | KDM1A | KDM2A | KDM4D | KDM5B | KDR | KEAP1 | KIF5B | KLF5 | KLHL22 | LARS | LEF1 | LGALS1 | LGMN | LIG4 | LIMS1 | LMNA | LMO2 | LPAR1 | LPIN3 | LRP6 | LRRK2 | LTBR | MAP2K1 | MAP2K3 | MAP2K4 | MAP2K6 | MAP2K7 | MAP3K1 | MAP3K13 | MAP3K14 | MAP3K2 | MAP3K3 | MAP3K4 | MAP4K1 | MAPK1 | MAPK14 | MAPK15 | MAPK3 | MAPK4 | MAPK6 | MAPK7 | MAPK8 | MAPK8IP3 | MAPK9 | MAPT | MAT2A | MAVS | MC1R | MCM2 | MCM7 | MCM9 | MCTS1 | MDC1 | MDM2 | MDM4 | MED1 | MGMT | MGST1 | MICA | MIF | MKNK2 | MLH1 | MME | MORF4L1 | MORF4L2 | MPG | MSH6 | MSN | MTA1 | MTPN | MTSS1 | MYBBP1A | MYC | MYD88 | MYO1C | MYO5A | MYO6 | NAMPT | NCK1 | NCOA1 | NCOA2 | NCOA3 | NDRG1 | NDUFA13 | NEDD4 | NEK1 | NEUROD1 | NFATC2 | NFE2L2 | NFKBIA | NIPBL | NME1 | NME2 | NOD2 | NOS2 | NPEPPS | NPLOC4 | NPM1 | NQO1 | NR1H2 | NR1H3 | NR3C1 | NR4A1 | NR5A2 | NRBF2 | NRP2 | NSMCE4A | NTRK1 | NUPR1 | OPA1 | OPTN | OTUB1 | OXSR1 | P2RX4 | P4HB | PAK2 | PAK6 | PALB2 | PARK7 | PARP1 | PAX8 | PAX9 | PAXIP1 | PCNA | PCSK9 | PDCD10 | PDCD6 | PDE12 | PDE4D | PDE6G | PDIA3 | PDIA4 | PDIA6 | PDK2 | PFKP | PGK1 | PHB | PHB2 | PICK1 | PIK3CA | PIK3CG | PIK3R1 | PKM | PLA2G10 | PLA2G1B | PLA2G6 | PLCG1 | PLK1 | PML | PNKP | POLD1 | POLE2 | POLE3 | POLM | POLR2A | POLR2E | POLR2L | PPARD | PPARG | PPARGC1A | PPAT | PPID | PPIE | PPIF | PPM1A | PPM1D | PPP1CA | PPP1R12A | PPP1R14A | PPP2CB | PPP5C | PRDX1 | PRDX2 | PRDX3 | PRDX5 | PRDX6 | PRKAA1 | PRKAA2 | PRKACA | PRKAR1A | PRKAR2B | PRKCD | PRKCE | PRKCZ | PRKD1 | PRKD2 | PRKDC | PRMT6 | PRPF19 | PRPF8 | PSAP | PSEN1 | PSMA1 | PSMA2 | PSMA3 | PSMA5 | PSMA6 | PSMB5 | PSMB6 | PSMB7 | PSMB8 | PSMC1 | PSMC2 | PSMC3 | PSMC4 | PSMC5 | PSMC6 | PSMD1 | PSMD10 | PSMD11 | PSMD12 | PSMD14 | PSMD2 | PSMD4 | PSMD9 | PSME2 | PSME3 | PTEN | PTN | PTPN1 | PTPN14 | PTPN22 | PTPN23 | PTPN4 | PTPN6 | PTPRF | PTPRK | PTTG1 | PXN | PYCR1 | PYCR2 | QDPR | RAB10 | RAB11B | RAB31 | RAB35 | RAB3GAP1 | RAC1 | RAD21 | RAD23A | RAD23B | RAD51 | RAD51AP1 | RAE1 | RANGAP1 | RAP1A | RAP1B | RARA | RARG | RB1 | RBBP6 | RBM17 | RBM4 | RBMX | RBPJ | RBX1 | RC3H1 | RDX | RELA | REST | RFC1 | RFC2 | RFC3 | RFC4 | RFC5 | RHBDD1 | RHO | RHOA | RIF1 | RIPK1 | RIPK2 | RIPK3 | RNASEH2A | RNF139 | RNF183 | RNF185 | RNF8 | ROMO1 | RPA2 | RPA3 | RPAIN | RPL23 | RPS3 | RPS6KA1 | RPTOR | RRM2B | RUNX2 | RUVBL1 | RUVBL2 | S100B | SAG | SEC16A | SEC31A | SEL1L | SERPINB6 | SFN | SFPQ | SGTA | SGTB | SH3GL2 | SHC1 | SHMT1 | SHMT2 | SIN3A | SIPA1 | SIRT1 | SIRT6 | SLAMF8 | SLC12A2 | SLC16A1 | SLC25A24 | SLC25A5 | SLC2A5 | SLC39A4 | SLC9A1 | SMAD3 | SMAD4 | SMAD5 | SMAD9 | SMC1A | SMC3 | SMO | SMURF1 | SNAI2 | SNCA | SNW1 | SNX6 | SOCS1 | SOCS2 | SOD1 | SOD2 | SP1 | SP100 | SPI1 | SPINT2 | SPRTN | SPRY2 | SQSTM1 | SRC | SRPRB | SRSF5 | SRXN1 | ST3GAL6 | STAC | STAT1 | STAT3 | STAT6 | STAU1 | STIP1 | STK11 | STK24 | STK25 | STK26 | STK3 | STOML2 | STUB1 | STXBP1 | STXBP2 | SULT1A3 | SUMO1 | SYP | SYVN1 | TAB1 | TAB2 | TANK | TAOK3 | TARDBP | TBC1D4 | TBXA2R | TCEA1 | TDP1 | TERF2 | TERF2IP | TFDP1 | TFIP11 | TGFB1 | THBS1 | TLR3 | TMBIM6 | TMEM173 | TMEM189-UBE2V1 | TMTC3 | TNF | TNFAIP3 | TNFRSF1A | TNFRSF8 | TNFSF14 | TNIP2 | TNKS | TNP1 | TOP2A | TOR1A | TP53 | TP63 | TP73 | TPM1 | TPR | TRADD | TRAF2 | TRAF6 | TREX1 | TRIAP1 | TRIB3 | TRIM25 | TRIM28 | TRIM41 | TRIP12 | TRIP13 | TSPYL2 | TTC5 | TWF2 | TWIST1 | TXN2 | TXNIP | TXNRD1 | UBA1 | UBA5 | UBB | UBC | UBE2A | UBE2B | UBE2D3 | UBE2J1 | UBE2K | UBE2L3 | UBE2L6 | UBE2N | UBE2V1 | UBE2W | UBQLN1 | UBQLN2 | UBR5 | UCHL5 | UFC1 | UFM1 | UGGT1 | ULK1 | UMPS | USP16 | USP19 | USP28 | USP47 | USP7 | USP8 | VAPB | VCAM1 | VCP | VHL | VIM | VSTM2A | WAS | WBP2 | WDR35 | WDR45B | WDR48 | WDTC1 | WIPI1 | WIPI2 | WNK1 | WNT16 | WNT2 | WT1 | WWOX | XBP1 | XIAP | XRCC1 | XRCC3 | XRCC5 | XRCC6 | YAP1 | YES1 | YWHAE | YWHAG | YY1 | ZBTB17 | ZBTB42 | ZBTB7A | ZFAND6 | ZFYVE26 | ZMPSTE24 | ZNF277 | GO:0051186 | 0.0312992842411743 | 258/8243 | 159/3250 | 2.18335874306335e-13 | 2.07309912653865e-09 | T | T | T | T | cofactor metabolic process | AASDHPPT | ACACA | ACAT1 | ACLY | ACOT13 | ACOT7 | ACOT9 | ACSL1 | ACSL4 | ACSL5 | ACSS2 | AHCY | AHCYL1 | AKR1A1 | AKR1B1 | AKR1B10 | AKR1C2 | AKT1 | ALAS1 | ALDH5A1 | AMD1 | ASPDH | ATIC | BLVRB | CALM1 | CAT | CBR4 | CNDP2 | COQ2 | COQ3 | COQ4 | COQ5 | COQ6 | COQ7 | COQ9 | COX15 | CROT | CYP1A1 | CYP1A2 | DBI | DCAKD | DCXR | DLAT | DLD | DLST | DPEP1 | EGFR | FAR2 | FASN | FDXR | FECH | FOLH1 | FOLR1 | FPGS | G6PD | GART | GCDH | GGCT | GGT1 | GGTLC1 | GLO1 | GOT1 | GOT2 | GPD1L | GPT2 | GPX1 | GPX4 | GSR | GSS | GSTA1 | GSTA2 | GSTA3 | GSTA4 | GSTA5 | GSTK1 | GSTM4 | GSTP1 | GSTT1 | GSTT2 | GSTT2B | GSTZ1 | HAAO | HAGH | HBZ | HLCS | HMBS | HMGCL | HMOX1 | HPX | HSD17B4 | HSD17B8 | HSP90AA1 | IDH1 | IDH2 | IREB2 | KCNAB2 | KYNU | L2HGDH | LDHA | MAT2A | MAT2B | MCCC1 | MDH1 | MDH2 | ME1 | MGST1 | MMAB | MOCOS | MPC2 | MTHFD1 | MTRR | MVD | NAMPT | NAPRT | NDUFA9 | NFS1 | NMNAT1 | NMNAT3 | NMRK2 | NNT | NOS3 | OGDH | PARK7 | PC | PCBD1 | PDHA1 | PDHA2 | PDHB | PDHX | PDSS1 | PDSS2 | PDXK | PNP | PPOX | PPT1 | PRDX1 | PRDX2 | PRDX3 | PRDX4 | PRDX5 | PRDX6 | PSAT1 | PTGES | PXDN | QDPR | RFK | SHMT1 | SHMT2 | SLC25A1 | SLC25A32 | SOD1 | SPR | SUCLA2 | SUCLG2 | TECR | THEM4 | TYMS | UBIAD1 | VCP | GO:0044238 | 0.475555016377532 | 3920/8243 | 1705/3250 | 3.64824796524587e-13 | 3.46401144300095e-09 | T | T | T | T | primary metabolic process | A2M | AARS | AARSD1 | AASDH | AASDHPPT | AASS | ABCD3 | ABCG1 | ABHD14B | ABHD17A | ABL1 | ABL2 | ACAA1 | ACAA2 | ACACA | ACAD8 | ACAD9 | ACADS | ACADSB | ACADVL | ACAT1 | ACAT2 | ACBD3 | ACD | ACE2 | ACLY | ACO1 | ACO2 | ACOT13 | ACOT7 | ACOT9 | ACOX1 | ACP1 | ACSL1 | ACSL4 | ACSL5 | ACTB | ACTL6A | ACTN2 | ACY1 | ADA | ADAMTS4 | ADCK2 | ADCYAP1 | ADH1A | ADH1B | ADH5 | ADK | ADORA2A | ADPRH | ADRB2 | ADRM1 | ADSS | ADTRP | AFG3L2 | AGK | AGL | AGO1 | AGO2 | AHCY | AHCYL1 | AHSG | AICDA | AIMP2 | AK1 | AK2 | AK4 | AKR1A1 | AKR1B1 | AKR1B10 | AKR1B15 | AKR1C2 | AKT1 | AKT2 | AKT3 | ALB | ALDH1B1 | ALDH4A1 | ALDH5A1 | ALDH6A1 | ALDH7A1 | ALDH8A1 | ALDOA | ALDOC | ALOX12 | ALOX5 | ALOX5AP | AMD1 | AMDHD2 | AMFR | ANAPC4 | ANXA1 | ANXA2 | APBB1 | APC | APCS | APEH | APEX1 | APEX2 | APOA1 | APOD | APOE | APOL2 | APP | APRT | AR | ARAF | ARF4 | ARID4B | ARMC8 | ARNT | ARRB1 | ARRB2 | ASB12 | ASB15 | ASB16 | ASB6 | ASB9 | ASCC1 | ASCC2 | ASGR1 | ASGR2 | ASL | ASNS | ASPDH | ASS1 | ATAD5 | ATF2 | ATG4C | ATG5 | ATIC | ATM | ATP13A2 | ATP6V1A | ATP6V1B1 | ATRX | ATXN1 | ATXN2 | AUP1 | AURKB | AZU1 | B3GAT3 | B3GNT3 | B4GALT3 | BABAM1 | BACE1 | BAG2 | BAG6 | BANF1 | BAX | BCL10 | BCL11A | BCL2 | BCOR | BIRC2 | BIRC3 | BIRC5 | BIRC7 | BIRC8 | BMI1 | BMPR1A | BRAF | BRCA1 | BRD4 | BTK | BTN2A1 | BTRC | BUD13 | BUD31 | BYSL | C1QBP | C1R | C4A | C9orf64 | CAB39 | CALM1 | CALR | CALU | CAMK1D | CAMK2A | CAMK2B | CAMKV | CAND1 | CAPN1 | CAPN2 | CAPNS1 | CARM1 | CARS | CASP10 | CASP2 | CASP3 | CASP7 | CASP8 | CASP9 | CAT | CAV1 | CBFB | CBL | CBLB | CBLC | CBR1 | CBS | CBX4 | CBX8 | CCAR2 | CCDC155 | CCDC22 | CCDC36 | CCDC8 | CCNB1 | CCNT1 | CCP110 | CCR5 | CCS | CD2BP2 | CD36 | CD40LG | CDA | CDC14B | CDC16 | CDC20 | CDC25A | CDC25B | CDC25C | CDC27 | CDC34 | CDC42 | CDC5L | CDC73 | CDK15 | CDK16 | CDK19 | CDK2 | CDK4 | CDK5 | CDK5R1 | CDK8 | CDK9 | CDKL3 | CEBPA | CEBPE | CEL | CES1 | CFL1 | CFTR | CHAF1A | CHD1L | CHEK2 | CHGB | CHRM3 | CHST4 | CISD3 | CKAP4 | CLK1 | CLPX | CLTC | CLU | CMPK1 | CNDP1 | CNDP2 | COG3 | COL2A1 | COL4A3BP | COMMD1 | COMT | COPS2 | COPS5 | COPS6 | COQ2 | COQ3 | CPNE3 | CPSF7 | CPT1A | CPT2 | CPVL | CRBN | CREB1 | CREBBP | CRK | CRKL | CRMP1 | CROT | CRY1 | CRY2 | CRYAB | CS | CSNK1A1 | CSNK1E | CSNK2A2 | CSNK2B | CSTF2T | CT45A5 | CTBP1 | CTCF | CTDP1 | CTH | CTNNB1 | CTPS1 | CTSA | CTSC | CTSD | CTSL | CTSV | CUL1 | CUL2 | CUL3 | CUL4B | CUL5 | CUL7 | CWC15 | CYB5R1 | CYB5R3 | CYCS | CYLD | CYP1A1 | CYP1A2 | CYP2C18 | DAD1 | DAPK1 | DAPP1 | DARS | DBI | DCAF12 | DCAF7 | DCAF8 | DCK | DCLK1 | DCN | DCP2 | DCTPP1 | DCUN1D1 | DCXR | DDA1 | DDB1 | DDB2 | DDI1 | DDOST | DDRGK1 | DDX1 | DDX39A | DDX39B | DDX3X | DECR1 | DEGS1 | DERA | DERL1 | DGKE | DGKZ | DGUOK | DHCR24 | DHRS4 | DHRS7B | DHX15 | DHX36 | DHX9 | DIS3 | DLAT | DLD | DLG2 | DLG3 | DLG4 | DLST | DMAP1 | DNA2 | DNAJB11 | DNAJB2 | DNAJC10 | DNASE2 | DNASE2B | DNPEP | DPEP1 | DPF2 | DPM1 | DPP9 | DRG2 | DROSHA | DSE | DTX1 | DTX2 | DUS3L | DUSP1 | DUSP12 | DUSP13 | DUSP14 | DUSP16 | DUSP19 | DUSP22 | DUSP7 | DUT | DYRK2 | DZIP3 | ECD | ECH1 | ECHDC2 | ECHS1 | ECI1 | ECI2 | EDC4 | EDEM1 | EED | EEF1A1 | EEF1A1P5 | EEF1A2 | EEF2KMT | EFEMP1 | EFTUD2 | EGFR | EGLN1 | EGLN3 | EHHADH | EHMT1 | EHMT2 | EIF2AK3 | EIF2AK4 | EIF2S1 | EIF3H | EIF4A3 | EIF4H | ELAC2 | ELAVL1 | ELAVL4 | ENDOG | ENO1 | ENO2 | ENO3 | ENOPH1 | ENTPD7 | EP300 | EPHA4 | EPHA6 | EPHB6 | EPRS | ERBB2 | ERBB3 | ERCC5 | ERG | ERLIN2 | ESR1 | ETFA | ETFB | ETFDH | EVA1A | EXOSC1 | EXOSC5 | EXOSC6 | EXOSC9 | EXTL2 | EYA2 | EYA4 | EZH2 | F5 | F9 | FABP1 | FABP3 | FABP5 | FABP7 | FADD | FAF1 | FAF2 | FAH | FAHD1 | FAM20B | FAM98A | FANCA | FANCC | FANCG | FANCI | FANCM | FAR2 | FARSB | FASN | FBP1 | FBP2 | FBXL12 | FBXO11 | FBXO25 | FBXO32 | FBXO6 | FBXO7 | FBXW11 | FBXW5 | FDPS | FDXR | FEN1 | FGB | FGF2 | FGFR1 | FGFR3 | FGL2 | FH | FIGNL1 | FKBP14 | FKBP1A | FKBP1B | FKBP2 | FKBP5 | FKBP7 | FKBP8 | FN1 | FOLH1 | FOS | FOXK1 | FOXK2 | FOXM1 | FOXO1 | FPGS | FTO | FTSJ3 | FUS | FYN | FZR1 | G3BP1 | G6PD | GAA | GAD2 | GAK | GALNT7 | GANAB | GAPDH | GAPDHS | GAR1 | GARS | GART | GBE1 | GCDH | GDA | GDPGP1 | GEMIN4 | GEMIN5 | GEMIN7 | GFPT1 | GFPT2 | GGA1 | GGPS1 | GGT1 | GGTLC1 | GINS2 | GINS3 | GINS4 | GLA | GLB1L2 | GLCE | GLO1 | GLRX | GLRX3 | GLRX5 | GLS | GLS2 | GLUD2 | GLUL | GMDS | GMPR | GMPS | GNAI3 | GNAS | GNPDA1 | GNPDA2 | GOLGA2 | GOT1 | GOT1L1 | GOT2 | GPC5 | GPD1 | GPD1L | GPI | GPS2 | GPT2 | GPX1 | GPX4 | GRB2 | GRK1 | GRK5 | GSK3A | GSK3B | GSPT1 | GSPT2 | GSR | GSS | GSTA1 | GSTP1 | GTF2F1 | GTF2F2 | GUSB | GYG1 | GYG2 | GYS1 | GZMA | GZMK | H1F0 | H2AFX | H3F3A | HAAO | HADH | HARS | HBEGF | HCCS | HDAC1 | HDAC11 | HDAC2 | HDAC3 | HDAC4 | HDAC5 | HDAC6 | HDAC7 | HDAC9 | HECW1 | HECW2 | HELZ2 | HERC1 | HERC2 | HEXA | HEXB | HGS | HIBCH | HIF1A | HIF1AN | HINT1 | HIPK4 | HIST1H2BA | HIST1H3A | HIST1H4A | HIST2H3A | HIST3H3 | HK1 | HK2 | HLCS | HMBS | HMGB1 | HMGB2 | HMGB3 | HMGCL | HNF4A | HNRNPA1 | HNRNPA1L2 | HNRNPA2B1 | HNRNPD | HNRNPF | HNRNPH1 | HNRNPH2 | HNRNPH3 | HNRNPK | HNRNPL | HNRNPM | HNRNPU | HPRT1 | HPX | HRAS | HS2ST1 | HS6ST1 | HSD17B10 | HSD17B14 | HSD17B4 | HSD17B6 | HSD17B8 | HSD3B7 | HSF1 | HSF4 | HSP90AA1 | HSP90AB1 | HSP90B1 | HSPA14 | HSPA1B | HSPA5 | HSPA8 | HSPD1 | HSPG2 | HTRA1 | HTRA2 | HUWE1 | IARS | ICK | IDE | IDH1 | IDH2 | IDH3A | IDH3B | IDH3G | IDI2 | IFI16 | IGF1R | IGFBP7 | IK | IKBKB | IKBKE | IKBKG | IL3RA | ILF3 | ILK | IMP4 | IMPA1 | IMPA2 | IMPDH2 | ING5 | INO80B | INO80C | INSIG1 | INSIG2 | INSR | IP6K1 | IREB2 | IRF4 | IRF7 | ISG15 | ISYNA1 | ITCH | ITGAV | ITK | ITPA | IVD | JAK1 | JAK3 | JMJD6 | JOSD2 | KAT5 | KAT7 | KAT8 | KBTBD7 | KCMF1 | KCNAB2 | KCTD17 | KCTD2 | KDM1A | KDM2A | KDM3B | KDM4D | KDM5B | KDM5C | KDM8 | KDR | KDSR | KEAP1 | KHDRBS1 | KIAA0391 | KLHL20 | KLHL22 | KMT2A | KMT2B | KMT2C | KPNA1 | KPNA2 | KPNB1 | KRAS | KSR1 | KYNU | L2HGDH | LACTB2 | LAMB1 | LAP3 | LARP4 | LARP7 | LARS | LAT | LATS1 | LCMT2 | LDHA | LDHAL6A | LDHAL6B | LDHB | LDHC | LEF1 | LGALS1 | LGALS3 | LGMN | LIG4 | LIMA1 | LMO2 | LMO7 | LNX1 | LOXL2 | LPIN3 | LRRC7 | LRRK1 | LRRK2 | LRSAM1 | LSM1 | LSM12 | LSM3 | LSM4 | LSM5 | LSM8 | LTBP1 | LTC4S | LYPLA1 | LYPLA2 | LZTR1 | MAD2L1 | MAN2B1 | MAP2K1 | MAP2K3 | MAP2K4 | MAP2K6 | MAP2K7 | MAP3K1 | MAP3K13 | MAP3K14 | MAP3K2 | MAP3K3 | MAP3K4 | MAP4K1 | MAPK1 | MAPK14 | MAPK15 | MAPK3 | MAPK4 | MAPK6 | MAPK7 | MAPK8 | MAPK9 | MAPT | MARCH5 | MASP1 | MASP2 | MAST1 | MAST3 | MATK | MBD3 | MBP | MC1R | MCAT | MCFD2 | MCM2 | MCM3 | MCM4 | MCM5 | MCM6 | MCM7 | MCM9 | MDC1 | MDH1 | MDH2 | MDM2 | MDM4 | MDN1 | ME1 | MECP2 | MED1 | MED12 | MED20 | MED21 | MED31 | MELK | MEPCE | METAP2 | MGMT | MIB1 | MKNK2 | MLH1 | MME | MMP26 | MORF4L1 | MORF4L2 | MOV10 | MPG | MPHOSPH6 | MPI | MPPE1 | MPST | MRGBP | MRM1 | MRPL28 | MRPL43 | MRPL9 | MRRF | MSH6 | MTA1 | MTA3 | MTAP | MTHFD1 | MTMR14 | MTMR7 | MTRR | MVD | MYC | MYH6 | NAE1 | NAGK | NANP | NANS | NCBP1 | NCK1 | NCOA1 | NCOA3 | NDUFAF5 | NDUFAF7 | NDUFS1 | NDUFS6 | NEDD4 | NEDD8 | NEK1 | NEK7 | NEURL4 | NFE2L2 | NFKBIA | NFS1 | NHLRC3 | NIPBL | NIT2 | NKX2-1 | NLK | NME1 | NME2 | NME4 | NMNAT1 | NMNAT3 | NMRK2 | NNT | NOB1 | NOD2 | NOP10 | NOP56 | NOS2 | NOS3 | NOSIP | NPEPL1 | NPEPPS | NPLOC4 | NPM1 | NPR1 | NR0B2 | NR1H2 | NR1H3 | NR1I2 | NR5A2 | NSFL1C | NSMCE4A | NSUN2 | NSUN6 | NT5C2 | NTMT1 | NTRK1 | NUCB1 | NUDT13 | NUDT18 | NUDT19 | NUDT3 | NUDT9 | NUFIP1 | NUP107 | NUP153 | NUP160 | NUP205 | NUP214 | NUP85 | NUP88 | NUP93 | NUP98 | NUPR1 | OAS2 | OAT | OBSL1 | OGDH | OGFOD1 | OGT | OLA1 | ONECUT1 | OPA1 | OSBPL1A | OTC | OTUB1 | OTUB2 | OTUD4 | OTUD5 | OVGP1 | OXSR1 | P2RX1 | P4HA1 | P4HA2 | P4HB | PA2G4 | PABPC1 | PABPC3 | PABPC4 | PADI4 | PAFAH1B1 | PAK2 | PAK6 | PALB2 | PAN2 | PAPLN | PARK7 | PARP1 | PARP12 | PARP14 | PARP6 | PAWR | PAXIP1 | PC | PCBP1 | PCBP2 | PCF11 | PCGF1 | PCGF5 | PCGF6 | PCMT1 | PCMTD1 | PCMTD2 | PCNA | PCSK9 | PDCD6IP | PDE12 | PDE4D | PDF | PDHA1 | PDHA2 | PDHB | PDIA3 | PDIA6 | PDK2 | PEBP1 | PELI1 | PEPD | PER1 | PER2 | PEX5 | PFAS | PFKM | PGAM1 | PGAM2 | PGAM5 | PGD | PGK1 | PGK2 | PGM1 | PGM2 | PGM3 | PGM5 | PGP | PHB | PHF10 | PHGDH | PHKA1 | PI4K2A | PIAS1 | PIAS3 | PICK1 | PIGU | PIH1D1 | PIK3C2A | PIK3CA | PIK3CG | PIK3R1 | PIK3R3 | PIKFYVE | PIM1 | PIN1 | PINX1 | PIP4K2A | PIPSL | PITPNA | PITPNB | PJA1 | PKN2 | PLA2G10 | PLA2G12A | PLA2G1B | PLA2G2A | PLA2G6 | PLCG1 | PLK1 | PLOD2 | PML | PMPCA | PMPCB | PNKP | PNP | POLA2 | POLD1 | POLDIP3 | POLE2 | POLE3 | POLM | POLR2A | POLR2E | POLR2L | POM121 | POT1 | POU5F1 | PPA1 | PPA2 | PPARD | PPARG | PPARGC1A | PPAT | PPEF1 | PPIA | PPIB | PPID | PPIE | PPIF | PPIL3 | PPM1A | PPM1B | PPM1D | PPM1H | PPM1K | PPME1 | PPP1CA | PPP1CB | PPP1CC | PPP1R11 | PPP1R12A | PPP1R2 | PPP1R3B | PPP1R3C | PPP1R8 | PPP2CA | PPP2CB | PPP2R1A | PPP2R1B | PPP2R2A | PPP2R2D | PPP2R5D | PPP3CC | PPP4R2 | PPP5C | PPP6C | PPT1 | PPTC7 | PRCC | PRCP | PRDX3 | PRDX4 | PRDX6 | PREP | PREPL | PRKAA1 | PRKAA2 | PRKAB2 | PRKACA | PRKAG1 | PRKAR2B | PRKCD | PRKCE | PRKCSH | PRKCZ | PRKD1 | PRKD2 | PRKD3 | PRKDC | PRMT2 | PRMT3 | PRMT6 | PROZ | PRPF19 | PRPF40A | PRPF8 | PRSS16 | PRSS23 | PRTFDC1 | PSAP | PSAT1 | PSEN1 | PSEN2 | PSMA1 | PSMA2 | PSMA3 | PSMA5 | PSMA6 | PSMB5 | PSMB6 | PSMB7 | PSMB8 | PSMC1 | PSMC2 | PSMC3 | PSMC4 | PSMC5 | PSMC6 | PSMD1 | PSMD10 | PSMD11 | PSMD12 | PSMD14 | PSMD2 | PSMD4 | PSMD9 | PSME2 | PSME3 | PTEN | PTGDS | PTGES | PTGES2 | PTGES3 | PTGR1 | PTK2 | PTPMT1 | PTPN1 | PTPN14 | PTPN21 | PTPN22 | PTPN23 | PTPN4 | PTPN6 | PTPRA | PTPRD | PTPRF | PTPRK | PTPRO | PTPRR | PTTG1 | PUM1 | PUS1 | PUS7 | PYCR1 | PYCR2 | PYGB | PYGL | QARS | QDPR | QSOX1 | RAB11A | RAB11B | RAB1A | RAB1B | RAB2A | RAB5A | RAB7A | RAD21 | RAD23A | RAD23B | RAD51 | RAD51AP1 | RAE1 | RAF1 | RAN | RANBP9 | RANGAP1 | RARA | RARS2 | RBBP6 | RBM17 | RBM25 | RBM3 | RBM4 | RBM8A | RBMX | RBP1 | RBX1 | RC3H1 | RDH11 | RDH12 | RDH13 | RDH14 | RDH5 | RELA | REST | REXO2 | RFC1 | RFC2 | RFC3 | RFC4 | RFC5 | RHBDD1 | RHO | RHOA | RHOT1 | RIF1 | RING1 | RIOK1 | RIPK1 | RIPK2 | RIPK3 | RIPK4 | RMND1 | RNASE3 | RNASEH2A | RNASEH2B | RNF123 | RNF126 | RNF139 | RNF181 | RNF183 | RNF185 | RNF19A | RNF2 | RNF25 | RNF31 | RNF4 | RNF8 | RNPEP | RPA2 | RPA3 | RPAIN | RPE | RPIA | RPL10 | RPL10A | RPL12 | RPL18A | RPL22 | RPL23 | RPL23A | RPL24 | RPL30 | RPL36AL | RPL37A | RPL38 | RPL6 | RPL7 | RPL7A | RPL9 | RPLP0 | RPLP0P6 | RPLP1 | RPLP2 | RPN1 | RPN2 | RPP14 | RPS12 | RPS13 | RPS14 | RPS15A | RPS19 | RPS2 | RPS20 | RPS24 | RPS29 | RPS3 | RPS4X | RPS6KA1 | RPS6KA2 | RPS6KB2 | RPS8 | RRM1 | RRM2 | RRM2B | RRP7A | RRS1 | RTCB | RTF1 | RTFDC1 | RUVBL1 | RUVBL2 | RXRA | S100A9 | SAE1 | SAP18 | SARS | SART1 | SART3 | SBDS | SCAF1 | SCCPDH | SCG2 | SCG3 | SCLY | SCO2 | SCYL2 | SDC1 | SDC4 | SDHA | SDHB | SDHD | SEL1L | SENP3 | SENP6 | SENP7 | SENP8 | SEPHS1 | SEPHS2 | SERPINH1 | SETDB1 | SF1 | SF3B6 | SFPQ | SGPL1 | SGSH | SGTA | SGTB | SHC1 | SHMT1 | SHMT2 | SIAH1 | SIN3A | SIRT1 | SIRT3 | SIRT5 | SIRT6 | SIRT7 | SLC16A1 | SLC25A1 | SLC25A10 | SLC25A11 | SLC25A13 | SLC25A32 | SLC2A5 | SLC3A2 | SMAD2 | SMAD3 | SMAD4 | SMAD5 | SMARCB1 | SMC1A | SMC3 | SMG5 | SMG6 | SMURF1 | SMURF2 | SNCA | SND1 | SNRNP27 | SNRPA | SNRPA1 | SNRPB | SNRPB2 | SNRPD1 | SNRPD3 | SNRPE | SNW1 | SNX3 | SOAT1 | SOCS1 | SOCS2 | SOCS6 | SORD | SP100 | SPATA2 | SPI1 | SPOP | SPRR2A | SPRTN | SPSB3 | SPTAN1 | SPTBN1 | SPTBN2 | SPTLC2 | SQSTM1 | SRC | SRPK1 | SRPK2 | SRSF11 | SRSF12 | SRSF5 | SSB | ST3GAL1 | ST3GAL6 | ST6GALNAC5 | ST8SIA4 | STAT3 | STBD1 | STK11 | STK24 | STK25 | STK26 | STK3 | STK38 | STK40 | STOML2 | STUB1 | STYX | SUCLA2 | SUCLG2 | SULF2 | SULT1A3 | SUMO1 | SUMO1P1 | SUMO2 | SUMO3 | SUPT5H | SUZ12 | SWAP70 | SYVN1 | TAB1 | TAB2 | TADA2B | TAF12 | TALDO1 | TANK | TAOK3 | TARDBP | TARS | TARS2 | TATDN1 | TAZ | TBL1X | TBRG4 | TCEA1 | TCIRG1 | TDP1 | TERF1 | TERF2 | TERF2IP | TESK2 | TFCP2 | TFIP11 | TGFB1 | TGFBR3 | TGM2 | TGOLN2 | THADA | THBS1 | THEM4 | THOC2 | THOC6 | THOC7 | THOP1 | TIMM50 | TIMP1 | TINF2 | TK1 | TK2 | TKT | TLR3 | TMEM132A | TMEM189-UBE2V1 | TMEM258 | TMTC3 | TNF | TNFAIP3 | TNFRSF1A | TNIP2 | TNK2 | TNKS | TNKS2 | TNP1 | TOE1 | TOLLIP | TOMM20 | TOP1 | TOP2A | TOP3A | TOR1A | TP53 | TP53RK | TP73 | TPI1 | TPR | TPSAB1 | TPSB2 | TPST2 | TPTE | TRA2A | TRAF2 | TRAF3 | TRAF6 | TREX1 | TRIB2 | TRIB3 | TRIM21 | TRIM23 | TRIM25 | TRIM28 | TRIM32 | TRIM33 | TRIM41 | TRIM47 | TRIM63 | TRIM69 | TRIM7 | TRIM9 | TRIP12 | TRIP13 | TRMT44 | TRMT6 | TRMT61B | TRNT1 | TRUB2 | TSG101 | TSN | TSPAN17 | TTC5 | TTLL1 | TWF1 | TXNL4A | TXNRD1 | TYMS | U2AF2 | UBA1 | UBA2 | UBA3 | UBA5 | UBA6 | UBAC1 | UBB | UBC | UBE2A | UBE2B | UBE2C | UBE2D1 | UBE2D2 | UBE2D3 | UBE2H | UBE2I | UBE2J1 | UBE2K | UBE2L3 | UBE2L6 | UBE2M | UBE2N | UBE2O | UBE2Q2 | UBE2R2 | UBE2S | UBE2V1 | UBE2W | UBE3A | UBE3B | UBL4A | UBQLN1 | UBQLN2 | UBQLN4 | UBR4 | UBR5 | UBXN1 | UBXN7 | UCHL1 | UCHL3 | UCHL5 | UCK2 | UFC1 | UFM1 | UGDH | UGGT1 | UGP2 | UGT8 | ULK1 | UMPS | UNKL | UPF3B | UPP2 | UQCRC2 | USP11 | USP14 | USP15 | USP16 | USP17L2 | USP19 | USP2 | USP20 | USP21 | USP26 | USP28 | USP39 | USP4 | USP42 | USP46 | USP47 | USP5 | USP50 | USP53 | USP7 | USP8 | USP9X | USP9Y | USPL1 | UTP14A | UTP6 | VAPA | VAPB | VARS | VASH2 | VCAN | VCP | VCPIP1 | VDR | VGF | VHL | VKORC1 | VPS37D | VPS4B | WARS | WDR12 | WDR4 | WDR45B | WDR48 | WDR82 | WDTC1 | WDYHV1 | WIPI1 | WIPI2 | WNK1 | WNK4 | WSB1 | WT1 | WTAP | WWOX | WWP2 | XBP1 | XIAP | XPNPEP1 | XPO5 | XRCC1 | XRCC3 | XRCC5 | XRCC6 | XYLT1 | YBEY | YEATS2 | YES1 | YME1L1 | YWHAB | YWHAE | YY1 | ZAP70 | ZBTB16 | ZBTB7A | ZC3HC1 | ZDHHC17 | ZDHHC23 | ZFYVE26 | ZMPSTE24 | ZNF598 | ZNF638 | ZNRF4 | ZRANB2 | GO:0010033 | 0.155647215819483 | 1283/8243 | 620/3250 | 1.19796342062188e-12 | 1.13746626788048e-08 | T | T | T | T | response to organic substance | ABAT | ABCD3 | ABCG1 | ABL1 | ABL2 | ACACA | ACAT1 | ACSL1 | ACSL4 | ACTA1 | ACTB | ACTG1 | ACTR2 | ACTR3 | ADA | ADCY6 | ADCYAP1 | ADH5 | ADORA2A | ADRB2 | ADSS | ADTRP | AES | AGER | AGL | AICDA | AIFM1 | AKAP8 | AKAP9 | AKR1B1 | AKR1C2 | AKT1 | AKT2 | ALOX12 | ANXA1 | ANXA2 | ANXA5 | ANXA7 | APEX1 | APLP1 | APP | APRT | AR | ARNT2 | ARPC1B | ASCL1 | ASNS | ASS1 | ATIC | ATM | ATP1A1 | ATP2A2 | ATRX | BACE1 | BAG3 | BAG4 | BAX | BCAR1 | BCAT2 | BCL10 | BCL11A | BCL2 | BCL2L1 | BIRC2 | BMPR1A | BRAF | BRCA1 | BSG | BSPRY | BTK | BTRC | CACNA1A | CACNG4 | CALCOCO2 | CALM1 | CALM3 | CALR | CAPN2 | CARD9 | CARM1 | CASP3 | CASP7 | CASP8 | CASP9 | CAT | CAV1 | CBL | CCL21 | CCL22 | CCNB1 | CCR5 | CD27 | CD36 | CD40 | CD6 | CD81 | CDC34 | CDC42EP4 | CDC5L | CDC73 | CDH1 | CDK19 | CDK4 | CDK5 | CDK9 | CDKN1A | CEBPA | CEBPE | CFL1 | CFTR | CHEK2 | CLDN18 | CLU | COL2A1 | COL4A1 | COL4A2 | COL6A1 | COL6A2 | COMT | CPNE3 | CPT1A | CREB1 | CRIP1 | CRK | CRKL | CRY1 | CRYAB | CSN1S1 | CSNK2B | CTH | CTNNB1 | CTSC | CTSL | CTSV | CXCL11 | CXCL5 | CXCL6 | CXCL8 | CYC1 | CYP1A1 | CYP1A2 | DAPK1 | DAXX | DBH | DCN | DDIT4 | DDOST | DDX1 | DENND4C | DERL1 | DHX15 | DHX36 | DHX9 | DMAP1 | DNAJA1 | DNAJB1 | DNAJB2 | DNM1L | DTX1 | DUSP1 | DUSP22 | E2F1 | EED | EEF1A1 | EEF2 | EFTUD2 | EGFR | EHD4 | EIF2B1 | EIF2B3 | EIF2B5 | EIF4A3 | EIF4E | EIF4EBP1 | EIF6 | ENDOG | EPHA4 | EPRS | ERBB2 | ERRFI1 | ESR1 | ESRRA | ETS1 | EZH2 | EZR | FABP3 | FADD | FAF2 | FASLG | FASN | FBXO32 | FBXO6 | FECH | FGB | FHL2 | FIS1 | FKBP1B | FLNB | FLOT1 | FOLR1 | FOS | FOXA1 | FOXO1 | FYN | G6PD | GAPDH | GART | GATA1 | GBP2 | GBP3 | GBP5 | GBP6 | GFPT2 | GGT1 | GLP1R | GLRB | GLUL | GNAI1 | GNAO1 | GNB1 | GNG12 | GNPNAT1 | GOT1 | GOT2 | GPD1 | GPI | GPX4 | GRAMD1A | GSK3A | GSK3B | GSS | GSTP1 | H2AFZ | HADH | HAVCR2 | HAX1 | HCN1 | HCN2 | HCN3 | HDAC2 | HDAC4 | HDAC5 | HDAC6 | HDAC9 | HDGF | HID1 | HIF1A | HK2 | HLCS | HMGB1 | HMGB2 | HMGCL | HNF4A | HNRNPD | HNRNPL | HNRNPU | HPRT1 | HSF1 | HSP90AA1 | HSP90AB1 | HSP90B1 | HSPA14 | HSPA1B | HSPA1L | HSPA2 | HSPA4 | HSPA4L | HSPA5 | HSPA8 | HSPA9 | HSPB1 | HSPB2 | HSPD1 | HSPE1 | HSPH1 | HTR1B | HTR3A | HTRA2 | ICAM1 | IDH1 | IFI16 | IFIT1 | IFITM3 | IFNA2 | IFNAR1 | IGBP1 | IGF1R | IGFBP7 | IKBKB | IKBKE | IL3RA | IMPDH2 | INSIG1 | INSIG2 | INSR | IPO5 | IQGAP1 | IREB2 | IRF1 | ISG15 | ITGA4 | ITGB1BP1 | ITPR2 | JAK3 | JUN | JUNB | JUND | JUP | KAT5 | KAT7 | KDM1A | KDM5B | KDR | KEAP1 | KIF5B | KLF5 | KLHL20 | KLHL22 | KRAS | KYNU | LAMP3 | LARS | LDHA | LEF1 | LGALS1 | LGMN | LIMS1 | LMO2 | LPAR1 | LPIN3 | LRP6 | LRRK2 | LUM | MAP2K3 | MAP2K4 | MAP2K6 | MAP2K7 | MAP4K1 | MAPK1 | MAPK14 | MAPK3 | MAPK4 | MAPK6 | MAPK7 | MAPK8 | MAPK9 | MAPT | MAS1 | MAT2A | MAVS | MBD3 | MCM2 | MCM7 | MDM2 | ME1 | MED1 | MGMT | MGST1 | MME | MSN | MTAP | MYC | MYD88 | MYO1C | MYO5A | NAMPT | NCOA1 | NCOA2 | NCOA3 | NDUFA13 | NDUFA9 | NEDD8 | NEUROD1 | NFE2L2 | NFKB2 | NFKBIA | NKX2-1 | NME1 | NME2 | NMNAT3 | NOD2 | NOS2 | NOS3 | NQO1 | NR0B2 | NR1H2 | NR1H3 | NR3C1 | NR4A1 | NR5A2 | NRP2 | NTRK1 | OGT | OPA1 | OPTN | OTC | OTUB1 | OTUD5 | OXCT1 | OXSR1 | P2RX1 | P2RX2 | P2RX4 | P4HB | PAK2 | PARK7 | PARP1 | PAX8 | PAX9 | PCNA | PCSK9 | PDCD10 | PDE12 | PDE4D | PDIA3 | PEBP1 | PELI1 | PER1 | PFKL | PFKP | PHB | PHB2 | PIAS3 | PIK3CA | PIK3CG | PIK3R1 | PKM | PLA2G10 | PLA2G1B | PLCG1 | PLSCR1 | PML | PPARD | PPARG | PPARGC1A | PPAT | PPM1A | PPP2CA | PPP2R1A | PPP2R2A | PPP5C | PRDX2 | PRDX3 | PRKAA1 | PRKAA2 | PRKACA | PRKAR1A | PRKAR2B | PRKCD | PRKCE | PRKCZ | PRKD1 | PRKD2 | PRKDC | PRPF8 | PSAP | PSEN1 | PSMD14 | PTEN | PTGES | PTGIR | PTN | PTPN1 | PTPN14 | PTPN22 | PTPN23 | PTPN4 | PTPN6 | QDPR | RAB10 | RAB31 | RAB35 | RAD23B | RAD51 | RAE1 | RANGAP1 | RAP1A | RAP1B | RARA | RARG | RBBP4 | RBMX | RC3H1 | RDX | REL | RELA | REST | RFC3 | RGS2 | RHBDD1 | RHOA | RIF1 | RIPK1 | RIPK2 | RPL10A | RPL23 | RPS3 | RPTOR | RRM2B | RUNX2 | RUVBL2 | RXRA | S100A7 | S100B | S100P | SARM1 | SDC1 | SELL | SERPINH1 | SETDB1 | SH3GL2 | SHC1 | SHMT1 | SHMT2 | SIN3A | SIRT1 | SLC12A2 | SLC16A1 | SLC25A5 | SLC2A5 | SLC3A2 | SLC9A1 | SMAD2 | SMAD3 | SMAD4 | SMAD5 | SMAD9 | SMO | SNAI2 | SNCA | SNW1 | SNX6 | SOCS1 | SOCS2 | SOD1 | SORD | SORT1 | SOX2 | SP1 | SP100 | SPI1 | SPINT2 | SPRY2 | SRC | SRSF5 | ST3GAL6 | STAT1 | STAT3 | STIP1 | STUB1 | STXBP1 | STXBP2 | SULT1A3 | SYP | TANK | TBC1D4 | TBXA2R | TDO2 | TGFB1 | TGFBR3 | THBS1 | THRA | TIMP1 | TIMP2 | TLR3 | TMBIM6 | TMEM173 | TNF | TNFAIP3 | TNIP2 | TOMM20 | TP53 | TP73 | TPCN2 | TPR | TRADD | TRAF6 | TREX1 | TRIB3 | TRIM21 | TRIM25 | TRIM32 | TRIM41 | TRIM63 | TUFM | TWF2 | TWIST1 | TXN2 | TXNIP | TYMS | UBE2K | UBE2L3 | UBE2W | UBE3A | USP8 | VCAM1 | VGF | VIM | VN1R1 | VPS4B | VSTM2A | WAS | WBP2 | WDR35 | WDTC1 | WNK1 | WNT2 | WT1 | WWOX | XAF1 | XBP1 | XRCC1 | XRCC3 | XRCC5 | YAP1 | YES1 | YWHAG | YY1 | ZBTB7B | ZFAND6 | GO:1901575 | 0.128472643455053 | 1059/8243 | 523/3250 | 1.22622685683463e-12 | 1.16430240056448e-08 | T | T | T | T | organic substance catabolic process | AASS | ABAT | ABCD3 | ABHD17A | ACAA1 | ACAA2 | ACAD8 | ACADS | ACADSB | ACADVL | ACAT1 | ACAT2 | ACOT7 | ACOX1 | ADA | ADH5 | ADRM1 | ADTRP | AGL | AGO1 | AHCY | AICDA | AKR1A1 | AKR1B10 | AKT1 | ALDH1B1 | ALDH4A1 | ALDH5A1 | ALDH6A1 | ALDH7A1 | ALDH8A1 | ALDOA | ALDOC | AMDHD2 | AMFR | ANAPC4 | ANXA2 | APOA1 | APOE | ARMC8 | ARRB1 | ARRB2 | ASPDH | ATM | AUP1 | AURKB | BACE1 | BAG6 | BCAT2 | BCKDHB | BDH1 | BIRC2 | BLVRB | BTRC | CALM1 | CAPN1 | CASP3 | CBL | CBLC | CBS | CCNB1 | CDA | CDC16 | CDC20 | CDC27 | CDC34 | CDK2 | CDK5 | CEL | CES1 | CHDH | CHEK2 | CHP1 | CLPX | CLTC | COMT | CPT1A | CPT2 | CRBN | CROT | CTH | CTNNB1 | CTSD | CUL1 | CUL2 | CUL3 | CUL4B | CUL5 | CUL7 | CYLD | CYP1A1 | CYP1A2 | DBH | DBT | DCN | DCP2 | DCTPP1 | DCXR | DDB1 | DDX6 | DECR1 | DERA | DERL1 | DIS3 | DLD | DLST | DNA2 | DNAJB2 | DNAJC10 | DNASE2 | DNASE2B | DROSHA | DUT | ECH1 | ECHDC2 | ECHS1 | ECI1 | ECI2 | EDC4 | EDEM1 | EHHADH | EIF3E | EIF4A3 | ENDOG | ENO1 | ENO2 | ENO3 | ENTPD7 | ERLIN2 | ESD | ETFA | ETFB | ETFDH | EXOSC1 | EXOSC5 | EXOSC6 | EXOSC9 | FABP1 | FABP3 | FABP5 | FABP7 | FAF1 | FAF2 | FAH | FBXL12 | FBXO11 | FBXO6 | FBXO7 | FBXW11 | FBXW5 | FEN1 | FGF2 | FZR1 | GAA | GAD2 | GALE | GALNS | GAPDH | GAPDHS | GCDH | GDA | GGT1 | GGTLC1 | GLA | GLS | GLS2 | GLUD2 | GLUL | GNPDA1 | GNPDA2 | GNS | GOT1 | GOT2 | GPC5 | GPD1 | GPD1L | GPI | GPT2 | GPX1 | GSK3A | GSPT1 | GSPT2 | GSTZ1 | GUSB | H1F0 | HAAO | HADH | HAGH | HDAC6 | HECW1 | HECW2 | HELZ2 | HERC2 | HEXA | HEXB | HIBADH | HIBCH | HINT1 | HK1 | HK2 | HMGB1 | HMGB2 | HMGCL | HMMR | HMOX1 | HNRNPD | HPRT1 | HSD17B10 | HSD17B14 | HSD17B4 | HSD17B6 | HSD3B7 | HSP90B1 | HSPA1B | HSPA5 | HSPG2 | HTRA2 | HUWE1 | IDE | IMPA1 | IMPA2 | ISG15 | ITCH | ITPA | IVD | KAT5 | KCTD17 | KCTD2 | KEAP1 | KLHL20 | KLHL22 | KPNA1 | KPNB1 | KYNU | LDHA | LDHD | LGMN | LNX1 | LPIN3 | LRSAM1 | LSM1 | LSM3 | LSM4 | LSM5 | LUM | LYG2 | LYPLA1 | LYPLA2 | MAD2L1 | MAN2B1 | MAOA | MCAT | MCCC1 | MDM2 | MLH1 | MPST | MTA1 | MTAP | MTMR7 | MTRR | NAGK | NBAS | NCBP1 | NCEH1 | NEDD4 | NEDD8 | NFE2L2 | NHLRC3 | NOS2 | NOS3 | NPEPPS | NPLOC4 | NSFL1C | NT5C2 | NUDT18 | NUDT19 | NUDT3 | NUDT9 | OAS2 | OAT | OGDH | OTC | OVGP1 | OXCT1 | PABPC1 | PABPC4 | PADI4 | PAFAH1B1 | PAH | PAN2 | PCBD1 | PCBP2 | PCSK9 | PDE12 | PDE4D | PELI1 | PFKL | PFKM | PFKP | PGAM1 | PGAM2 | PGK1 | PGK2 | PGM1 | PGM2 | PHKA1 | PJA1 | PKM | PLA2G10 | PLA2G12A | PLA2G1B | PLA2G2A | PLA2G6 | PLBD2 | PLCG1 | PLK1 | PML | PNP | PPARD | PPAT | PPM1K | PPP1R11 | PPP1R8 | PPP2CA | PPP2CB | PPP2R1A | PPP2R2A | PPT1 | PRDX6 | PRPF19 | PRSS16 | PRTFDC1 | PSAP | PSMA1 | PSMA2 | PSMA3 | PSMA5 | PSMA6 | PSMB5 | PSMB6 | PSMB7 | PSMB8 | PSMC1 | PSMC2 | PSMC3 | PSMC4 | PSMC5 | PSMC6 | PSMD1 | PSMD10 | PSMD11 | PSMD12 | PSMD14 | PSMD2 | PSMD4 | PSMD9 | PSME2 | PSME3 | PTEN | PTPN23 | PTTG1 | PYGB | PYGL | QDPR | RAB7A | RAD23A | RAD23B | RBBP6 | RBM8A | RBX1 | RC3H1 | RHBDD1 | RIPK1 | RNASE3 | RNASEH2A | RNASEH2B | RNF126 | RNF139 | RNF185 | RNF19A | RNF4 | RNF8 | RPE | RPL10 | RPL10A | RPL12 | RPL18A | RPL22 | RPL23 | RPL23A | RPL24 | RPL30 | RPL37A | RPL38 | RPL6 | RPL7 | RPL7A | RPL9 | RPLP0 | RPLP1 | RPLP2 | RPS12 | RPS13 | RPS14 | RPS15A | RPS19 | RPS2 | RPS20 | RPS24 | RPS29 | RPS3 | RPS4X | RPS8 | SAT1 | SAT2 | SDC1 | SDC4 | SEL1L | SGPL1 | SGSH | SGTA | SGTB | SHMT1 | SHMT2 | SIAH1 | SIRT1 | SKIV2L | SLC9A1 | SMG5 | SMG6 | SMG8 | SMURF1 | SMURF2 | SND1 | SNX17 | SOCS6 | SORD | SPOP | SPSB3 | SQSTM1 | SSB | STBD1 | STUB1 | SULT1A3 | SYVN1 | TBL1X | TCIRG1 | TDO2 | TGFB1 | TOLLIP | TOR1A | TPI1 | TRAF2 | TREX1 | TRIM25 | TRIM32 | TRIM9 | TRIP12 | TSG101 | TXN2 | UBA6 | UBB | UBC | UBE2A | UBE2B | UBE2C | UBE2D1 | UBE2D2 | UBE2D3 | UBE2H | UBE2I | UBE2J1 | UBE2K | UBE2L3 | UBE2L6 | UBE2R2 | UBE2S | UBE2W | UBE3A | UBE3B | UBQLN1 | UBQLN2 | UBQLN4 | UBR4 | UBXN1 | UCHL1 | UCHL3 | UCHL5 | UGGT1 | UPF2 | UPF3B | UPP2 | USP11 | USP14 | USP15 | USP16 | USP17L2 | USP19 | USP2 | USP20 | USP21 | USP26 | USP28 | USP4 | USP42 | USP46 | USP47 | USP5 | USP50 | USP7 | USP8 | USP9Y | VCAN | VCP | VPS37D | VPS4B | WWP2 | XBP1 | XPNPEP1 | YME1L1 | YWHAH | ZMPSTE24 | ZNRF4 | GO:0043067 | 0.119252699259978 | 983/8243 | 489/3250 | 1.78761338847086e-12 | 1.69733891235309e-08 | T | T | T | T | regulation of programmed cell death | AARS | ABL1 | ACAA2 | ACTN1 | ACTN2 | ACTN4 | ADA | ADCYAP1 | ADORA2A | AES | AGAP2 | AHI1 | AIFM1 | AKR1B1 | AKT1 | AKT2 | ALB | ALOX12 | ANXA1 | ANXA5 | APBB1 | APBB2 | APC | APEX1 | API5 | APOE | AR | ARAF | ARF4 | ARHGDIA | ARHGEF7 | ARHGEF9 | ARNT2 | ARRB1 | ARRB2 | ASCL1 | ASNS | ATAD3A | ATAD5 | ATF2 | ATF6 | ATG5 | ATM | ATOH1 | ATOX1 | AURKB | AZU1 | BACE1 | BAG1 | BAG3 | BAG4 | BAG5 | BAG6 | BAX | BBC3 | BCAR1 | BCL10 | BCL2 | BCL2L1 | BCL2L13 | BCL2L2 | BCL6 | BDKRB2 | BIN1 | BIRC2 | BIRC3 | BIRC5 | BIRC7 | BIRC8 | BMPR1A | BRAF | BRCA1 | BTK | C1QBP | CALR | CAMK1D | CAMK2A | CAPN2 | CARD9 | CASP10 | CASP2 | CASP3 | CASP8 | CASP9 | CAT | CAV1 | CBL | CBX4 | CCAR2 | CCL21 | CCR5 | CD27 | CD40 | CD40LG | CD74 | CDC34 | CDK19 | CDK4 | CDK5 | CDK5R1 | CDKN1A | CFL1 | CHCHD10 | CHD8 | CHEK2 | CLU | COL18A1 | COL2A1 | COPS5 | CREB1 | CREB3 | CREBBP | CRYAB | CSNK2A2 | CTH | CTNNB1 | CTSC | CTTN | CYCS | CYLD | DAB2 | DAD1 | DAPK1 | DBH | DDB1 | DDRGK1 | DDX3X | DEDD | DHCR24 | DIABLO | DNAJA1 | DNAJA3 | DNM1L | DPEP1 | DUSP1 | E2F1 | E2F3 | EEF1A2 | EGFR | EGLN3 | EIF2AK3 | EIF2B5 | EIF2S1 | ENDOG | ENO1 | ERBB3 | ERCC5 | ESR1 | EYA2 | EYA4 | FABP1 | FADD | FAF1 | FAM129B | FASLG | FGB | FGFR1 | FHL2 | FIGNL1 | FIS1 | FKBP8 | FOXA1 | FOXO1 | FYN | G6PD | GADD45G | GAPDH | GATA1 | GDF9 | GLO1 | GLS2 | GNA13 | GNAI2 | GNAI3 | GOLPH3 | GPI | GPX1 | GPX4 | GRID2 | GRIK2 | GRK5 | GSK3A | GSK3B | GSTP1 | GZMA | HAX1 | HDAC1 | HDAC2 | HDAC3 | HDAC4 | HDAC6 | HIF1A | HMGB1 | HMGB2 | HMOX1 | HNRNPK | HOXA5 | HRAS | HSF1 | HSP90AB1 | HSP90B1 | HSPA1B | HSPA5 | HSPA9 | HSPB1 | HSPD1 | HSPE1 | HTATIP2 | HTRA2 | HTT | HYPK | ICAM1 | IGBP1 | IGF1R | IKBKB | ILK | ING5 | IRF7 | ITCH | ITGA4 | ITGAV | ITGB3BP | JAK3 | JUN | KANK2 | KDM1A | KDR | KLHL20 | KRAS | LAMP3 | LANCL1 | LATS1 | LDHA | LEF1 | LGALS1 | LGALS3 | LGMN | LIG4 | LMNA | LPAR1 | LRP6 | LRRK2 | LTBR | MAD2L1 | MAP2K4 | MAP2K6 | MAPK7 | MAPK8 | MAPK9 | MAPT | MDM2 | MDM4 | MECP2 | MED1 | MELK | MGMT | MIF | MLLT11 | MTCH2 | MYBBP1A | MYC | MYD88 | NAE1 | NCK1 | NCOA1 | NDRG1 | NDUFA13 | NDUFS3 | NEUROD1 | NF2 | NFE2L2 | NFKBIA | NME1 | NME2 | NMNAT1 | NNT | NOD2 | NOS3 | NPM1 | NQO1 | NQO2 | NTF3 | NTRK1 | NUPR1 | OPA1 | P2RX1 | P4HB | PA2G4 | PAK2 | PALB2 | PAM16 | PARK7 | PARP1 | PAWR | PAX8 | PCSK9 | PDCD10 | PDCD6 | PDIA3 | PELI1 | PHB | PHB2 | PIAS1 | PIH1D1 | PIK3CA | PIK3CG | PIK3R1 | PIM1 | PIN1 | PLA2G6 | PLAUR | PLCG1 | PLEKHF1 | PLK1 | PML | PNMA1 | PPARD | PPARG | PPARGC1A | PPID | PPIF | PPP1CA | PPP2R1A | PPP2R1B | PPP3CC | PPT1 | PRDX2 | PRDX3 | PRDX5 | PRELID1 | PRKAA1 | PRKAA2 | PRKCD | PRKCZ | PRKDC | PRMT2 | PSEN1 | PSEN2 | PSMC5 | PSMD10 | PSME3 | PTCRA | PTEN | PTK2 | PTN | PTPMT1 | PTPN1 | PTRH2 | QARS | RAF1 | RARA | RARG | RB1CC1 | RBM25 | RELA | REST | RGN | RHBDD1 | RHOA | RIPK1 | RIPK2 | RIPK3 | RNF183 | RPL10 | RPS3 | RPS6KA1 | RPS6KA2 | RRM2B | RTKN | RTN4 | S100A9 | S100B | SAP18 | SARM1 | SCG2 | SEPT4 | SEPT7 | SERPINB2 | SET | SFN | SFPQ | SHC1 | SIAH1 | SIN3A | SIRT1 | SIRT5 | SLC25A4 | SLC25A5 | SLC35F6 | SLC9A1 | SLC9A3R1 | SMAD3 | SMO | SNAI2 | SNCA | SNX6 | SOCS2 | SOD1 | SOD2 | SORT1 | SOX2 | SP100 | SPATA2 | SPRY2 | SQSTM1 | SRC | SRPK2 | STAT1 | STAT3 | STIL | STK26 | STK3 | STK40 | STXBP1 | SYVN1 | TARDBP | TCP1 | TCTN3 | TFDP1 | TGFB1 | TGM2 | THBS1 | THOC6 | THRA | TIA1 | TIMP1 | TLE1 | TLR3 | TMBIM6 | TMEM219 | TNF | TNFAIP3 | TNFRSF12A | TNFRSF1A | TNFRSF8 | TNFSF12 | TNFSF14 | TNIP2 | TOMM22 | TOP2A | TP53 | TP63 | TP73 | TRADD | TRAF1 | TRAF2 | TRAF3 | TRAF6 | TRAP1 | TRIAP1 | TRIM32 | TRIM35 | TSC22D3 | TWIST1 | TXNDC5 | TXNIP | UBB | UBC | UBE2B | UBE2M | UBQLN1 | UFM1 | USP17L2 | USP42 | USP47 | VAV1 | VCP | VDAC2 | VDR | VHL | WDR35 | WT1 | WWOX | XBP1 | XIAP | YAP1 | YME1L1 | YWHAB | YWHAE | YWHAG | YWHAH | YWHAQ | YWHAZ | ZBTB16 | ZBTB7A | ZC3HC1 | ZFAND6 | ZNF420 | ZNF622 | GO:0044283 | 0.0395487079946621 | 326/8243 | 190/3250 | 1.80705429777747e-12 | 1.71579805573971e-08 | T | T | T | T | small molecule biosynthetic process | AASDHPPT | AASS | ABAT | ABCD3 | ACAA2 | ACACA | ACAT1 | ACAT2 | ACCS | ACLY | ACOT7 | ACSS2 | ADA | ADK | AKR1A1 | AKR1B1 | ALDH8A1 | ALDOA | ALDOC | ALOX12 | ALOX5 | ALOX5AP | AMD1 | APOA1 | APOE | APRT | ASL | ASNS | ASPDH | ASS1 | BCAT2 | BDH1 | BRCA1 | CBR4 | CBS | CD74 | CDA | CES1 | CFTR | COQ2 | COQ3 | COQ4 | COQ5 | COQ6 | COQ7 | COQ9 | CRY1 | CTH | CYB5R3 | CYP1A1 | CYP1A2 | DBH | DCK | DCTPP1 | DEGS1 | DERA | DHCR24 | DUT | ENO1 | ENO2 | ENO3 | ENOPH1 | FASN | FBP1 | FBP2 | FDPS | FDXR | FGF2 | FOLH1 | G6PD | GAPDH | GAPDHS | GDA | GGPS1 | GGT1 | GLS | GLS2 | GLUD2 | GLUL | GMPS | GOT1 | GOT1L1 | GOT2 | GPD1 | GPI | GPT2 | GPX1 | GPX4 | GSTM4 | HAAO | HINT1 | HMGCL | HPRT1 | HSD17B4 | HSD17B8 | HSD3B7 | IDI2 | IMPA1 | IMPA2 | IMPDH2 | INSIG1 | INSIG2 | IP6K1 | ISYNA1 | ITPA | KYNU | LDHC | LTC4S | MCAT | MDH1 | MDH2 | MGST1 | MIF | MMAB | MTAP | MTHFD1 | MTRR | MVD | MYO5A | NANP | NDUFA9 | NME1 | NME2 | NME4 | NT5C2 | NUDT18 | NUDT3 | NUDT9 | OAT | OSBPL1A | OTC | PAH | PARK7 | PC | PCBD1 | PDE4D | PDSS1 | PDSS2 | PER2 | PGAM1 | PGAM2 | PGD | PGK1 | PGK2 | PGM1 | PGP | PHGDH | PLA2G1B | PLCG1 | PLOD2 | PNP | PPARGC1A | PRKAA1 | PRKAA2 | PRKAB2 | PRKAG1 | PRTFDC1 | PSAT1 | PTGDS | PTGES | PTGES3 | PYCR1 | PYCR2 | QDPR | RBP1 | RFK | RGN | SEPHS1 | SEPHS2 | SHMT1 | SHMT2 | SLC25A1 | SLC25A10 | SLC25A11 | SLC25A13 | SORD | SPR | SPTLC2 | TALDO1 | TECR | TK1 | TK2 | TKT | TPI1 | UBIAD1 | UCK2 | UMPS | UPP2 | VCP | XBP1 | GO:0033554 | 0.135387601601359 | 1116/8243 | 546/3250 | 2.80750915187274e-12 | 2.66572993970317e-08 | T | T | T | T | cellular response to stress | ABL1 | ACAA2 | ACADVL | ACD | ACTL6A | AGR2 | AIFM1 | AKR1B1 | AKT1 | ALB | AMFR | ANKS4B | ANXA1 | APBB1 | APC | APEX1 | APEX2 | APOA1 | APOD | ARFGAP1 | ARNT | ASNS | ASS1 | ATAD5 | ATF2 | ATF6 | ATF6B | ATG5 | ATM | ATP13A2 | ATP2A2 | ATRX | AUP1 | BABAM1 | BAG3 | BAG6 | BAX | BBC3 | BCL2 | BCL2L1 | BCL2L2 | BCL6 | BRCA1 | BRD4 | BTK | BTRC | CAB39 | CALR | CARM1 | CASP2 | CASP3 | CASP9 | CAT | CAV1 | CBL | CBS | CBX8 | CCAR2 | CCDC155 | CCNB1 | CCS | CD36 | CDC14B | CDC25C | CDC37 | CDC5L | CDK2 | CDK9 | CDKN1A | CFTR | CHAF1A | CHD1L | CHEK2 | CLPB | COL4A3BP | COMT | COPS2 | COPS5 | COPS6 | CREB3 | CREBBP | CRIP1 | CRK | CRKL | CRY1 | CRY2 | CRYAB | CSNK1E | CTH | CTSV | CUL1 | CUL2 | CUL4B | CUL7 | CXCL8 | CYCS | DAPK1 | DAXX | DCTN1 | DCTPP1 | DDB1 | DDB2 | DDIT4 | DDRGK1 | DDX1 | DDX3X | DERL1 | DHX36 | DMAP1 | DNA2 | DNAJA1 | DNAJB1 | DNAJB11 | DNAJB2 | DNAJC10 | DPEP1 | DYRK2 | E2F1 | E2F4 | EDEM1 | EEF2 | EGFR | EGLN1 | EGLN3 | EHMT2 | EIF2AK3 | EIF2AK4 | EIF2B5 | EIF2S1 | EIF4EBP1 | ENDOG | EP300 | ERCC5 | ERLIN2 | ERRFI1 | ETS1 | EXTL2 | EYA2 | EYA4 | EZH2 | FABP1 | FAF2 | FANCA | FANCC | FANCG | FANCI | FANCM | FBXO6 | FBXW11 | FEN1 | FKBP14 | FLOT1 | FOLR1 | FOS | FOXM1 | FOXO1 | FZR1 | G6PD | GAP43 | GFAP | GFPT1 | GINS2 | GINS4 | GLUL | GNB1 | GNL1 | GORASP2 | GPS2 | GPX1 | GPX7 | GRB2 | GSK3A | GSK3B | GSR | GSTP1 | H2AFX | HDAC2 | HDAC3 | HDAC6 | HDGF | HERC2 | HIF1A | HIF1AN | HIST1H4A | HIST3H3 | HK2 | HMOX1 | HNRNPA1 | HNRNPD | HNRNPL | HRAS | HSF1 | HSF4 | HSP90AA1 | HSP90B1 | HSPA14 | HSPA1B | HSPA1L | HSPA2 | HSPA5 | HSPA8 | HSPA9 | HTRA2 | HUWE1 | ICAM1 | ID2 | IFI16 | IKBKB | IKBKE | IKBKG | INO80B | INO80C | INSIG1 | INSIG2 | IREB2 | IRF7 | ISG15 | ITPR1 | JUN | KAT5 | KAT7 | KDM2A | KDM4D | LARS | LIG4 | LMNA | LRRK2 | MAP2K1 | MAP2K3 | MAP2K4 | MAP2K6 | MAP2K7 | MAP3K1 | MAP3K13 | MAP3K14 | MAP3K2 | MAP3K3 | MAP3K4 | MAP4K1 | MAPK1 | MAPK14 | MAPK15 | MAPK3 | MAPK7 | MAPK8 | MAPK8IP3 | MAPK9 | MAPT | MC1R | MCM7 | MCM9 | MCTS1 | MDC1 | MDM2 | MDM4 | MGMT | MGST1 | MICA | MIF | MLH1 | MORF4L1 | MORF4L2 | MPG | MSH6 | MTA1 | MTSS1 | MYBBP1A | MYC | MYO6 | NCK1 | NDRG1 | NEDD4 | NEK1 | NFATC2 | NFE2L2 | NFKBIA | NIPBL | NME2 | NOD2 | NPEPPS | NPLOC4 | NPM1 | NQO1 | NRBF2 | NSMCE4A | NUPR1 | OPA1 | OPTN | OTUB1 | OXSR1 | P4HB | PAK2 | PAK6 | PALB2 | PARK7 | PARP1 | PAXIP1 | PCNA | PCSK9 | PDCD6 | PDIA3 | PDIA4 | PDIA6 | PDK2 | PGK1 | PHB2 | PICK1 | PIK3R1 | PLA2G6 | PLK1 | PML | PNKP | POLD1 | POLE2 | POLE3 | POLM | POLR2A | POLR2E | POLR2L | PPARD | PPARG | PPARGC1A | PPIE | PPIF | PPM1D | PPP1CA | PPP2CB | PPP5C | PRDX1 | PRDX2 | PRDX3 | PRDX5 | PRDX6 | PRKAA1 | PRKAA2 | PRKACA | PRKCD | PRKCE | PRKD1 | PRKDC | PRMT6 | PRPF19 | PSEN1 | PSMA1 | PSMA2 | PSMA3 | PSMA5 | PSMA6 | PSMB5 | PSMB6 | PSMB7 | PSMB8 | PSMC1 | PSMC2 | PSMC3 | PSMC4 | PSMC5 | PSMC6 | PSMD1 | PSMD10 | PSMD11 | PSMD12 | PSMD14 | PSMD2 | PSMD4 | PSMD9 | PSME2 | PSME3 | PTEN | PTN | PTPN1 | PTPRF | PTPRK | PTTG1 | PXN | PYCR1 | PYCR2 | RAD21 | RAD23A | RAD23B | RAD51 | RAD51AP1 | RBBP6 | RBM17 | RBM4 | RBPJ | RBX1 | RELA | RFC1 | RFC2 | RFC3 | RFC4 | RFC5 | RHBDD1 | RHOA | RIF1 | RIPK1 | RIPK2 | RIPK3 | RNASEH2A | RNF139 | RNF183 | RNF185 | RNF8 | ROMO1 | RPA2 | RPA3 | RPAIN | RPS3 | RPS6KA1 | RPTOR | RRM2B | RUVBL1 | RUVBL2 | S100B | SEC16A | SEC31A | SEL1L | SERPINB6 | SFN | SFPQ | SGTA | SGTB | SHC1 | SIN3A | SIPA1 | SIRT1 | SIRT6 | SLC25A24 | SLC39A4 | SLC9A1 | SMC1A | SMC3 | SMURF1 | SNCA | SNW1 | SOD1 | SOD2 | SP100 | SPRTN | SQSTM1 | SRC | SRPRB | SRXN1 | STAC | STAT6 | STAU1 | STK11 | STK24 | STK25 | STK26 | STK3 | STOML2 | STUB1 | SULT1A3 | SUMO1 | SYVN1 | TAB1 | TAB2 | TANK | TAOK3 | TARDBP | TCEA1 | TDP1 | TERF2 | TERF2IP | TFDP1 | TFIP11 | THBS1 | TMBIM6 | TMEM189-UBE2V1 | TMTC3 | TNF | TNFAIP3 | TNFRSF1A | TNIP2 | TNP1 | TOP2A | TOR1A | TP53 | TP63 | TP73 | TPM1 | TPR | TRAF2 | TRAF6 | TREX1 | TRIAP1 | TRIB3 | TRIM25 | TRIM28 | TRIP12 | TRIP13 | TSPYL2 | TTC5 | TWIST1 | TXNRD1 | UBA1 | UBA5 | UBB | UBC | UBE2A | UBE2B | UBE2D3 | UBE2J1 | UBE2K | UBE2L6 | UBE2N | UBE2V1 | UBE2W | UBQLN1 | UBQLN2 | UBR5 | UCHL5 | UFC1 | UFM1 | UGGT1 | ULK1 | USP16 | USP19 | USP28 | USP47 | USP7 | VAPB | VCP | VHL | WDR45B | WDR48 | WIPI1 | WIPI2 | WNT16 | XBP1 | XIAP | XRCC1 | XRCC3 | XRCC5 | XRCC6 | YAP1 | YWHAE | YY1 | ZBTB17 | ZBTB42 | ZBTB7A | ZFYVE26 | ZMPSTE24 | ZNF277 | GO:0042981 | 0.117432973432003 | 968/8243 | 481/3250 | 3.56839716635995e-12 | 3.38819310945877e-08 | T | T | T | T | regulation of apoptotic process | AARS | ABL1 | ACAA2 | ACTN1 | ACTN2 | ACTN4 | ADA | ADCYAP1 | ADORA2A | AES | AGAP2 | AHI1 | AIFM1 | AKR1B1 | AKT1 | AKT2 | ALB | ALOX12 | ANXA1 | ANXA5 | APBB1 | APBB2 | APC | APEX1 | API5 | APOE | AR | ARAF | ARF4 | ARHGDIA | ARHGEF7 | ARHGEF9 | ARNT2 | ARRB1 | ARRB2 | ASCL1 | ASNS | ATAD3A | ATAD5 | ATF2 | ATF6 | ATG5 | ATM | ATOH1 | ATOX1 | AURKB | AZU1 | BACE1 | BAG1 | BAG3 | BAG4 | BAG5 | BAG6 | BAX | BBC3 | BCAR1 | BCL10 | BCL2 | BCL2L1 | BCL2L13 | BCL2L2 | BCL6 | BDKRB2 | BIN1 | BIRC2 | BIRC3 | BIRC5 | BIRC7 | BIRC8 | BMPR1A | BRAF | BRCA1 | BTK | C1QBP | CALR | CAMK1D | CAMK2A | CAPN2 | CARD9 | CASP10 | CASP2 | CASP3 | CASP8 | CASP9 | CAT | CAV1 | CBL | CBX4 | CCAR2 | CCL21 | CCR5 | CD27 | CD40 | CD40LG | CD74 | CDC34 | CDK19 | CDK4 | CDK5 | CDK5R1 | CDKN1A | CFL1 | CHCHD10 | CHD8 | CHEK2 | CLU | COL18A1 | COL2A1 | COPS5 | CREB1 | CREB3 | CREBBP | CRYAB | CSNK2A2 | CTH | CTNNB1 | CTSC | CTTN | CYCS | CYLD | DAB2 | DAD1 | DAPK1 | DBH | DDB1 | DDRGK1 | DDX3X | DEDD | DHCR24 | DIABLO | DNAJA1 | DNM1L | DPEP1 | DUSP1 | E2F1 | E2F3 | EEF1A2 | EGFR | EGLN3 | EIF2AK3 | EIF2B5 | EIF2S1 | ENDOG | ENO1 | ERBB3 | ERCC5 | ESR1 | EYA2 | EYA4 | FABP1 | FADD | FAF1 | FAM129B | FASLG | FGB | FGFR1 | FHL2 | FIGNL1 | FIS1 | FKBP8 | FOXA1 | FOXO1 | FYN | G6PD | GADD45G | GAPDH | GATA1 | GDF9 | GLO1 | GLS2 | GNA13 | GNAI2 | GNAI3 | GOLPH3 | GPI | GPX1 | GRID2 | GRIK2 | GRK5 | GSK3A | GSK3B | GSTP1 | GZMA | HAX1 | HDAC1 | HDAC2 | HDAC3 | HDAC4 | HIF1A | HMGB1 | HMGB2 | HMOX1 | HNRNPK | HOXA5 | HRAS | HSF1 | HSP90AB1 | HSP90B1 | HSPA1B | HSPA5 | HSPA9 | HSPB1 | HSPD1 | HSPE1 | HTATIP2 | HTRA2 | HTT | HYPK | ICAM1 | IGBP1 | IGF1R | IKBKB | ILK | ING5 | IRF7 | ITCH | ITGA4 | ITGAV | ITGB3BP | JAK3 | JUN | KDM1A | KDR | KLHL20 | KRAS | LAMP3 | LANCL1 | LATS1 | LDHA | LEF1 | LGALS1 | LGALS3 | LGMN | LIG4 | LMNA | LPAR1 | LRP6 | LRRK2 | LTBR | MAD2L1 | MAP2K4 | MAP2K6 | MAPK7 | MAPK8 | MAPK9 | MAPT | MDM2 | MDM4 | MECP2 | MED1 | MELK | MGMT | MIF | MLLT11 | MTCH2 | MYBBP1A | MYC | MYD88 | NAE1 | NCK1 | NCOA1 | NDRG1 | NDUFA13 | NDUFS3 | NEUROD1 | NF2 | NFE2L2 | NFKBIA | NME1 | NME2 | NMNAT1 | NNT | NOD2 | NOS3 | NPM1 | NQO1 | NQO2 | NTF3 | NTRK1 | NUPR1 | OPA1 | P2RX1 | P4HB | PA2G4 | PAK2 | PALB2 | PAM16 | PARK7 | PARP1 | PAWR | PAX8 | PCSK9 | PDCD10 | PDCD6 | PDIA3 | PHB | PHB2 | PIAS1 | PIH1D1 | PIK3CA | PIK3CG | PIK3R1 | PIM1 | PIN1 | PLA2G6 | PLAUR | PLCG1 | PLEKHF1 | PLK1 | PML | PNMA1 | PPARD | PPARG | PPARGC1A | PPID | PPIF | PPP1CA | PPP2R1A | PPP2R1B | PPP3CC | PPT1 | PRDX2 | PRDX3 | PRDX5 | PRELID1 | PRKAA1 | PRKAA2 | PRKCD | PRKCZ | PRKDC | PRMT2 | PSEN1 | PSEN2 | PSMD10 | PSME3 | PTCRA | PTEN | PTK2 | PTN | PTPMT1 | PTPN1 | PTRH2 | QARS | RAF1 | RARA | RARG | RB1CC1 | RBM25 | RELA | REST | RGN | RHBDD1 | RHOA | RIPK1 | RIPK2 | RIPK3 | RNF183 | RPL10 | RPS3 | RPS6KA1 | RPS6KA2 | RRM2B | RTKN | RTN4 | S100A9 | S100B | SAP18 | SARM1 | SCG2 | SEPT4 | SEPT7 | SERPINB2 | SET | SFN | SFPQ | SHC1 | SIAH1 | SIN3A | SIRT1 | SIRT5 | SLC25A5 | SLC35F6 | SLC9A1 | SLC9A3R1 | SMAD3 | SMO | SNAI2 | SNCA | SNX6 | SOCS2 | SOD1 | SOD2 | SORT1 | SOX2 | SP100 | SPRY2 | SQSTM1 | SRC | SRPK2 | STAT1 | STAT3 | STIL | STK26 | STK3 | STK40 | STXBP1 | SYVN1 | TARDBP | TCP1 | TCTN3 | TFDP1 | TGFB1 | TGM2 | THBS1 | THOC6 | THRA | TIA1 | TIMP1 | TLE1 | TLR3 | TMBIM6 | TMEM219 | TNF | TNFAIP3 | TNFRSF12A | TNFRSF1A | TNFRSF8 | TNFSF12 | TNFSF14 | TNIP2 | TOMM22 | TOP2A | TP53 | TP63 | TP73 | TRADD | TRAF1 | TRAF2 | TRAF3 | TRAF6 | TRAP1 | TRIAP1 | TRIM32 | TRIM35 | TSC22D3 | TWIST1 | TXNDC5 | TXNIP | UBB | UBC | UBE2B | UBE2M | UBQLN1 | UFM1 | USP17L2 | USP42 | USP47 | VAV1 | VCP | VDAC2 | VDR | VHL | WDR35 | WT1 | WWOX | XBP1 | XIAP | YAP1 | YME1L1 | YWHAB | YWHAE | YWHAG | YWHAH | YWHAQ | YWHAZ | ZBTB16 | ZBTB7A | ZC3HC1 | ZFAND6 | ZNF420 | ZNF622 | GO:0010941 | 0.128715273565449 | 1061/8243 | 521/3250 | 4.69107704612647e-12 | 4.45417765529708e-08 | T | T | T | T | regulation of cell death | AARS | ABL1 | ACAA2 | ACTN1 | ACTN2 | ACTN4 | ADA | ADCYAP1 | ADORA2A | AES | AGAP2 | AGR2 | AHI1 | AIFM1 | AIMP2 | AKR1B1 | AKT1 | AKT2 | ALB | ALOX12 | ANXA1 | ANXA5 | APBB1 | APBB2 | APC | APEX1 | API5 | APOE | AR | ARAF | ARF4 | ARHGDIA | ARHGEF7 | ARHGEF9 | ARNT2 | ARRB1 | ARRB2 | ASCL1 | ASNS | ATAD3A | ATAD5 | ATF2 | ATF6 | ATG5 | ATM | ATOH1 | ATOX1 | ATP13A2 | AURKB | AZU1 | BACE1 | BAG1 | BAG3 | BAG4 | BAG5 | BAG6 | BAX | BBC3 | BCAR1 | BCL10 | BCL2 | BCL2L1 | BCL2L13 | BCL2L2 | BCL6 | BDKRB2 | BIN1 | BIRC2 | BIRC3 | BIRC5 | BIRC7 | BIRC8 | BMPR1A | BRAF | BRCA1 | BTK | C1QA | C1QBP | CALR | CAMK1D | CAMK2A | CAPN2 | CARD9 | CASP10 | CASP2 | CASP3 | CASP8 | CASP9 | CAT | CAV1 | CBL | CBX4 | CCAR2 | CCL21 | CCR5 | CD27 | CD36 | CD40 | CD40LG | CD74 | CDC34 | CDK19 | CDK4 | CDK5 | CDK5R1 | CDKN1A | CFL1 | CHCHD10 | CHD8 | CHEK2 | CHMP2A | CHP1 | CLU | COL18A1 | COL2A1 | COPS5 | CREB1 | CREB3 | CREBBP | CRYAB | CSNK2A2 | CTH | CTNNB1 | CTSC | CTTN | CYCS | CYLD | DAB2 | DAD1 | DAPK1 | DAXX | DBH | DDB1 | DDIT4 | DDRGK1 | DDX3X | DEDD | DHCR24 | DIABLO | DNAJA1 | DNAJA3 | DNM1L | DPEP1 | DUSP1 | E2F1 | E2F3 | EEF1A2 | EGFR | EGLN1 | EGLN3 | EIF2AK3 | EIF2B5 | EIF2S1 | ENDOG | ENO1 | ERBB3 | ERCC5 | ESR1 | EYA2 | EYA4 | FABP1 | FADD | FAF1 | FAM129B | FASLG | FBXO7 | FGB | FGF2 | FGFR1 | FHL2 | FIGNL1 | FIS1 | FKBP8 | FOS | FOXA1 | FOXO1 | FTH1 | FYN | G6PD | GADD45G | GAPDH | GATA1 | GDF9 | GLO1 | GLS2 | GNA13 | GNAI2 | GNAI3 | GOLPH3 | GPI | GPX1 | GPX4 | GRID2 | GRIK2 | GRK5 | GSK3A | GSK3B | GSTP1 | GZMA | HAX1 | HDAC1 | HDAC2 | HDAC3 | HDAC4 | HDAC6 | HIF1A | HMGB1 | HMGB2 | HMOX1 | HNRNPK | HOXA5 | HRAS | HSF1 | HSP90AB1 | HSP90B1 | HSPA1B | HSPA5 | HSPA9 | HSPB1 | HSPD1 | HSPE1 | HTATIP2 | HTRA2 | HTT | HYPK | ICAM1 | IGBP1 | IGF1R | IKBKB | IKBKG | ILK | ING1 | ING5 | IRF7 | ITCH | ITGA4 | ITGAV | ITGB3BP | JAK3 | JUN | KANK2 | KDM1A | KDR | KIR3DL2 | KLHL20 | KRAS | LAMP3 | LANCL1 | LATS1 | LDHA | LEF1 | LGALS1 | LGALS3 | LGMN | LIG4 | LMNA | LPAR1 | LRP6 | LRRK2 | LTBR | MAD2L1 | MAP2K4 | MAP2K6 | MAPK7 | MAPK8 | MAPK9 | MAPT | MDM2 | MDM4 | MECP2 | MED1 | MELK | MGMT | MIF | MLLT11 | MTCH2 | MYBBP1A | MYC | MYD88 | NAE1 | NCK1 | NCOA1 | NDRG1 | NDUFA13 | NDUFS3 | NEUROD1 | NF2 | NFE2L2 | NFKBIA | NME1 | NME2 | NMNAT1 | NNT | NOD2 | NOS3 | NPM1 | NQO1 | NQO2 | NTF3 | NTRK1 | NUPR1 | OPA1 | P2RX1 | P4HB | PA2G4 | PAK2 | PALB2 | PAM16 | PARK7 | PARP1 | PAWR | PAX8 | PCSK9 | PDCD10 | PDCD6 | PDE12 | PDIA3 | PELI1 | PHB | PHB2 | PIAS1 | PIH1D1 | PIK3CA | PIK3CG | PIK3R1 | PIM1 | PIN1 | PLA2G6 | PLAUR | PLCG1 | PLEKHF1 | PLK1 | PML | PNMA1 | PPARD | PPARG | PPARGC1A | PPID | PPIF | PPP1CA | PPP2R1A | PPP2R1B | PPP3CC | PPT1 | PRDX2 | PRDX3 | PRDX5 | PRELID1 | PRKAA1 | PRKAA2 | PRKCD | PRKCZ | PRKD1 | PRKDC | PRMT2 | PSAP | PSEN1 | PSEN2 | PSMC1 | PSMC5 | PSMD10 | PSME3 | PTCRA | PTEN | PTK2 | PTN | PTPMT1 | PTPN1 | PTRH2 | PYCR1 | QARS | RAB29 | RAF1 | RARA | RARG | RB1CC1 | RBM25 | REL | RELA | REST | RGN | RHBDD1 | RHOA | RIPK1 | RIPK2 | RIPK3 | RNF183 | RPL10 | RPS3 | RPS6KA1 | RPS6KA2 | RRM2B | RTKN | RTN4 | S100A9 | S100B | SAP18 | SARM1 | SCG2 | SEPT4 | SEPT7 | SERPINB2 | SET | SFN | SFPQ | SHC1 | SIAH1 | SIN3A | SIRT1 | SIRT5 | SLC25A24 | SLC25A4 | SLC25A5 | SLC35F6 | SLC9A1 | SLC9A3R1 | SMAD3 | SMAD4 | SMO | SNAI2 | SNCA | SNX6 | SOCS2 | SOD1 | SOD2 | SORT1 | SOX2 | SP100 | SPATA2 | SPRY2 | SQSTM1 | SRC | SRGAP2 | SRPK2 | STAT1 | STAT3 | STIL | STK26 | STK3 | STK40 | STXBP1 | SULT1A3 | SYVN1 | TARDBP | TCP1 | TCTN3 | TFDP1 | TGFB1 | TGM2 | THBS1 | THOC6 | THRA | TIA1 | TIMP1 | TLE1 | TLR3 | TMBIM6 | TMEM219 | TNF | TNFAIP3 | TNFRSF12A | TNFRSF1A | TNFRSF8 | TNFSF12 | TNFSF14 | TNIP2 | TOMM22 | TOP2A | TP53 | TP63 | TP73 | TRADD | TRAF1 | TRAF2 | TRAF3 | TRAF6 | TRAP1 | TRIAP1 | TRIM32 | TRIM35 | TSC22D3 | TWIST1 | TXN | TXNDC5 | TXNIP | UBB | UBC | UBE2B | UBE2M | UBQLN1 | UFM1 | USP17L2 | USP42 | USP47 | VAV1 | VCP | VDAC2 | VDR | VHL | VPS4B | WDR35 | WNT16 | WT1 | WWOX | XBP1 | XIAP | YAP1 | YME1L1 | YWHAB | YWHAE | YWHAG | YWHAH | YWHAQ | YWHAZ | ZBTB16 | ZBTB7A | ZC3HC1 | ZFAND6 | ZNF420 | ZNF622 | ZNF746 | GO:0044248 | 0.131262889724615 | 1082/8243 | 530/3250 | 4.9642897013138e-12 | 4.71359307139746e-08 | T | T | T | T | cellular catabolic process | AASS | ABAT | ABCD3 | ABL1 | ACAA1 | ACAA2 | ACAD8 | ACADS | ACADSB | ACADVL | ACAT1 | ACAT2 | ACOT7 | ACOX1 | ACSL1 | ADA | ADH5 | ADRM1 | ADTRP | AGL | AGO1 | AHCY | AICDA | AKR1A1 | AKR1B10 | ALDH1B1 | ALDH4A1 | ALDH5A1 | ALDH6A1 | ALDH7A1 | ALDH8A1 | AMDHD2 | AMFR | ANAPC4 | ANXA7 | APOA1 | APOE | ARMC8 | ARRB1 | ARRB2 | ASPDH | ATCAY | ATG2B | ATG4C | ATG5 | ATM | AUP1 | AURKB | BAG3 | BAG6 | BCAT2 | BCKDHB | BDH1 | BIRC2 | BLVRB | BNIPL | BTRC | CALCOCO2 | CALM1 | CAPN1 | CASP3 | CAT | CBL | CBLC | CBS | CCNB1 | CD36 | CDA | CDC16 | CDC20 | CDC27 | CDC34 | CDK2 | CDK5 | CEL | CHDH | CHEK2 | CHMP2A | CISD2 | CLTC | COMT | CPT1A | CPT2 | CRBN | CROT | CRYZ | CSNK2A2 | CSNK2B | CTH | CTNNB1 | CUL1 | CUL2 | CUL3 | CUL4B | CUL5 | CUL7 | CYLD | CYP1A1 | CYP1A2 | CYP2C18 | DBH | DBT | DCN | DCP2 | DCTPP1 | DCXR | DDB1 | DDX6 | DECR1 | DERA | DERL1 | DIS3 | DLD | DLST | DNA2 | DNAJB2 | DNAJC10 | DNASE2 | DNASE2B | DPEP1 | DROSHA | DUT | DYNLL1 | ECH1 | ECHDC2 | ECHS1 | ECI1 | ECI2 | EDC4 | EDEM1 | EHHADH | EIF3E | EIF4A3 | ENDOG | ENTPD7 | ERLIN2 | ESD | ETFA | ETFB | ETFDH | EVA1A | EXOSC1 | EXOSC5 | EXOSC6 | EXOSC9 | FABP1 | FABP3 | FABP5 | FABP7 | FAF1 | FAF2 | FAH | FBXL12 | FBXO11 | FBXO6 | FBXO7 | FBXW11 | FBXW5 | FEN1 | FIS1 | FOXO1 | FUNDC2 | FZR1 | GAA | GAD2 | GALNS | GCDH | GDA | GGT1 | GGTLC1 | GLA | GLS | GLS2 | GLUD2 | GLUL | GNPDA1 | GNPDA2 | GNS | GOT1 | GOT2 | GPI | GPT2 | GPX1 | GSK3A | GSPT1 | GSPT2 | GSTM4 | GSTO1 | GSTZ1 | GUSB | H1F0 | HAAO | HADH | HAGH | HAP1 | HBZ | HDAC6 | HECW1 | HECW2 | HELZ2 | HERC2 | HEXA | HEXB | HGS | HIBADH | HIBCH | HINT1 | HMGB1 | HMGB2 | HMGCL | HMOX1 | HNRNPD | HPRT1 | HSD17B10 | HSD17B4 | HSP90AA1 | HSP90B1 | HSPA1B | HSPA5 | HSPA8 | HTRA2 | HUWE1 | IDE | IFI16 | ISG15 | ITCH | ITPA | IVD | KAT5 | KCTD17 | KCTD2 | KLHL20 | KLHL22 | KPNA1 | KPNB1 | KYNU | LDHD | LGALS8 | LGMN | LNX1 | LPIN3 | LRRK2 | LRSAM1 | LSM1 | LSM3 | LSM4 | LSM5 | LUM | LYG2 | MAD2L1 | MAOA | MCAT | MCCC1 | MDM2 | MLH1 | MME | MPST | MTA1 | MTAP | MTMR14 | MTRR | NAGK | NBAS | NCBP1 | NEDD4 | NEDD8 | NFE2L2 | NHLRC3 | NOS2 | NOS3 | NPEPPS | NPLOC4 | NR1I2 | NRBF2 | NSFL1C | NT5C2 | NUDT18 | NUDT19 | NUDT3 | NUDT9 | OAS2 | OAT | OGDH | OPTN | OTC | OVGP1 | OXCT1 | PABPC1 | PABPC4 | PADI4 | PAH | PAN2 | PARK7 | PCBD1 | PCBP2 | PCSK9 | PDE12 | PDE4D | PELI1 | PFKM | PGAM5 | PGM1 | PGM2 | PHKA1 | PLCG1 | PLK1 | PML | PNP | PPARD | PPARGC1A | PPAT | PPM1K | PPP1R11 | PPP1R8 | PPP2CA | PPP2CB | PPP2R1A | PPP2R2A | PPT1 | PRDX1 | PRDX2 | PRDX3 | PRDX4 | PRDX5 | PRDX6 | PRKAA1 | PRKAA2 | PRKAB2 | PRKAG1 | PRTFDC1 | PSAP | PSMA1 | PSMA2 | PSMA3 | PSMA5 | PSMA6 | PSMB5 | PSMB6 | PSMB7 | PSMB8 | PSMC1 | PSMC2 | PSMC3 | PSMC4 | PSMC5 | PSMC6 | PSMD1 | PSMD10 | PSMD11 | PSMD12 | PSMD14 | PSMD2 | PSMD4 | PSMD9 | PSME2 | PSME3 | PTPN22 | PTPN23 | PTTG1 | PXDN | PYGB | PYGL | QDPR | RAB1A | RAB1B | RAB24 | RAB7A | RAD23A | RAD23B | RB1CC1 | RBBP6 | RBM8A | RBX1 | RC3H1 | RIPK1 | RNASE3 | RNASEH2A | RNASEH2B | RNF126 | RNF185 | RNF19A | RNF4 | RNF8 | RPL10 | RPL10A | RPL12 | RPL18A | RPL22 | RPL23 | RPL23A | RPL24 | RPL30 | RPL37A | RPL38 | RPL6 | RPL7 | RPL7A | RPL9 | RPLP0 | RPLP1 | RPLP2 | RPS12 | RPS13 | RPS14 | RPS15A | RPS19 | RPS2 | RPS20 | RPS24 | RPS29 | RPS3 | RPS4X | RPS8 | S100A9 | SEL1L | SGPL1 | SGSH | SGTA | SGTB | SHMT1 | SHMT2 | SIAH1 | SIRT1 | SKIV2L | SMG5 | SMG6 | SMG8 | SMURF1 | SMURF2 | SND1 | SNF8 | SORD | SPOP | SPSB3 | SQSTM1 | SRC | SSB | STBD1 | STK11 | STUB1 | SULT1A3 | SYVN1 | TBL1X | TCIRG1 | TDO2 | TGFB1 | TMBIM6 | TOLLIP | TOMM20 | TOMM22 | TOMM40 | TP53 | TPI1 | TRAPPC8 | TREX1 | TRIM25 | TRIM32 | TRIM9 | TRIP12 | TSG101 | TXN2 | UBA6 | UBB | UBC | UBE2A | UBE2B | UBE2C | UBE2D1 | UBE2D2 | UBE2D3 | UBE2H | UBE2I | UBE2J1 | UBE2K | UBE2L3 | UBE2L6 | UBE2R2 | UBE2S | UBE2W | UBE3A | UBE3B | UBQLN1 | UBQLN2 | UBQLN4 | UBR4 | UBXN1 | UCHL1 | UCHL3 | UCHL5 | ULK1 | UPF2 | UPF3B | UPP2 | USP11 | USP14 | USP15 | USP16 | USP17L2 | USP19 | USP2 | USP20 | USP21 | USP26 | USP28 | USP4 | USP42 | USP46 | USP47 | USP5 | USP50 | USP7 | USP8 | USP9Y | VCAN | VCP | VDAC1 | VPS18 | VPS37D | VPS4B | VTA1 | WDR41 | WDR45B | WIPI1 | WIPI2 | WWP2 | XBP1 | XPNPEP1 | YME1L1 | ZMPSTE24 | ZNRF4 | GO:0006807 | 0.481742084192648 | 3971/8243 | 1714/3250 | 1.28387437815476e-11 | 1.21903872205795e-07 | T | T | T | T | nitrogen compound metabolic process | A2M | AARS | AARSD1 | AASDH | AASDHPPT | AASS | ABAT | ABCG1 | ABHD14B | ABHD17A | ABL1 | ABL2 | ACACA | ACAT1 | ACCS | ACD | ACE2 | ACLY | ACOT13 | ACOT7 | ACOT9 | ACP1 | ACSL1 | ACSL4 | ACSL5 | ACSS2 | ACTB | ACTL6A | ACTN2 | ACY1 | ADA | ADAMTS4 | ADCK2 | ADCY6 | ADCYAP1 | ADH5 | ADK | ADORA2A | ADPRH | ADRB2 | ADRM1 | ADSL | ADSS | AFF4 | AFG3L2 | AGK | AGO1 | AGO2 | AHCY | AHCYL1 | AHSG | AICDA | AIMP2 | AK1 | AK2 | AK4 | AKR1A1 | AKR1B1 | AKT1 | AKT2 | AKT3 | ALAS1 | ALB | ALDH4A1 | ALDH5A1 | ALDH6A1 | ALDH7A1 | ALDH9A1 | ALDOA | AMD1 | AMDHD2 | AMFR | ANAPC4 | ANXA1 | ANXA2 | AOC2 | APBB1 | APC | APCS | APEH | APEX1 | APEX2 | APOA1 | APOE | APOL2 | APP | APRT | AR | ARAF | ARF4 | ARHGAP35 | ARID4B | ARMC8 | ARNT | ARRB1 | ARRB2 | ASB12 | ASB15 | ASB16 | ASB6 | ASB9 | ASCC1 | ASCC2 | ASGR1 | ASGR2 | ASL | ASNS | ASPDH | ASS1 | ATAD5 | ATF2 | ATF6B | ATG4C | ATG5 | ATIC | ATM | ATOH1 | ATP13A2 | ATP6V1A | ATP6V1B1 | ATRX | ATXN1 | ATXN2 | AUP1 | AURKB | AZU1 | B3GAT3 | B3GNT3 | B4GALT3 | BABAM1 | BACE1 | BAG2 | BAG6 | BANF1 | BAX | BCAT2 | BCL10 | BCL11A | BCL2 | BCOR | BIRC2 | BIRC3 | BIRC5 | BIRC7 | BIRC8 | BLVRB | BMI1 | BMPR1A | BRAF | BRCA1 | BRD4 | BTBD9 | BTK | BTRC | BUD13 | BUD31 | BYSL | C1QBP | C1R | C4A | C9orf64 | CAB39 | CALM1 | CALR | CALU | CAMK1D | CAMK2A | CAMK2B | CAMKV | CAND1 | CAPN1 | CAPN2 | CAPNS1 | CARM1 | CARS | CASP10 | CASP2 | CASP3 | CASP7 | CASP8 | CASP9 | CAT | CBFB | CBL | CBLB | CBLC | CBR4 | CBS | CBX4 | CBX8 | CCAR2 | CCDC155 | CCDC22 | CCDC36 | CCDC8 | CCNB1 | CCNT1 | CCNT2 | CCP110 | CCR5 | CCS | CD2BP2 | CD36 | CD40LG | CDA | CDC14B | CDC16 | CDC20 | CDC25A | CDC25B | CDC25C | CDC27 | CDC34 | CDC42 | CDC5L | CDC73 | CDK15 | CDK16 | CDK19 | CDK2 | CDK4 | CDK5 | CDK5R1 | CDK8 | CDK9 | CDKL3 | CDKN1A | CEBPA | CEBPE | CEL | CENPF | CES1 | CFL1 | CFTR | CHAF1A | CHD1L | CHDH | CHEK2 | CHGB | CHRAC1 | CHRM3 | CHST4 | CISD3 | CKAP4 | CKB | CKM | CKMT1A | CLK1 | CLPX | CLTC | CMPK1 | CNDP1 | CNDP2 | COG3 | COL2A1 | COL4A2 | COL4A3BP | COMMD1 | COMT | COPS2 | COPS5 | COPS6 | COX15 | CPNE3 | CPSF7 | CPT1A | CPVL | CRBN | CREB1 | CREBBP | CRKL | CRMP1 | CROT | CRY1 | CRY2 | CRYAB | CSNK1A1 | CSNK1E | CSNK2A2 | CSNK2B | CSTF2T | CT45A5 | CTBP1 | CTCF | CTDP1 | CTH | CTNNB1 | CTPS1 | CTSA | CTSC | CTSD | CTSL | CTSV | CUL1 | CUL2 | CUL3 | CUL4B | CUL5 | CUL7 | CWC15 | CYC1 | CYCS | CYLD | CYP1A1 | CYP1A2 | DAD1 | DAPK1 | DAPP1 | DARS | DBH | DBI | DCAF12 | DCAF7 | DCAF8 | DCAKD | DCK | DCLK1 | DCN | DCP2 | DCTPP1 | DCUN1D1 | DCXR | DDA1 | DDB1 | DDB2 | DDI1 | DDOST | DDRGK1 | DDX1 | DDX39A | DDX39B | DDX3X | DDX6 | DEGS1 | DERA | DERL1 | DGUOK | DHX15 | DHX36 | DHX9 | DIDO1 | DIS3 | DLAT | DLD | DLG2 | DLG3 | DLG4 | DLST | DMAP1 | DMD | DNA2 | DNAJB11 | DNAJB2 | DNAJC10 | DNAJC30 | DNASE2 | DNASE2B | DNPEP | DPEP1 | DPF2 | DPM1 | DPP9 | DRG2 | DROSHA | DSE | DTX1 | DTX2 | DUS3L | DUSP1 | DUSP12 | DUSP13 | DUSP14 | DUSP16 | DUSP19 | DUSP22 | DUSP7 | DUT | DVL1 | DVL2 | DYRK2 | DZIP3 | E2F1 | E2F2 | E2F3 | EAF2 | ECD | EDC4 | EDEM1 | EED | EEF1A1 | EEF1A1P5 | EEF1A2 | EEF2KMT | EFEMP1 | EFTUD2 | EGFR | EGLN1 | EGLN3 | EHHADH | EHMT1 | EHMT2 | EIF2AK3 | EIF2AK4 | EIF2S1 | EIF3E | EIF3H | EIF4A3 | EIF4H | ELAC2 | ELAVL1 | ELAVL4 | ELF3 | ENDOG | ENOPH1 | ENTPD7 | EP300 | EPHA4 | EPHA6 | EPHB6 | EPRS | ERBB2 | ERBB3 | ERCC5 | ERG | ERLIN2 | ESD | ESR1 | ESR2 | ESRRA | ESRRB | ESRRG | ETS1 | EVA1A | EXOSC1 | EXOSC5 | EXOSC6 | EXOSC9 | EXTL2 | EYA2 | EYA4 | EZH2 | F5 | F9 | FABP5 | FADD | FAF1 | FAF2 | FAH | FAM20B | FAM98A | FANCA | FANCC | FANCG | FANCI | FANCM | FAR2 | FARSB | FASN | FBXL12 | FBXO11 | FBXO25 | FBXO32 | FBXO6 | FBXO7 | FBXW11 | FBXW5 | FECH | FEN1 | FGB | FGF2 | FGFR1 | FGFR3 | FGL2 | FIGNL1 | FKBP14 | FKBP1A | FKBP1B | FKBP2 | FKBP5 | FKBP7 | FKBP8 | FN1 | FOLH1 | FOLR1 | FOS | FOXK1 | FOXK2 | FOXM1 | FOXO1 | FPGS | FTO | FTSJ3 | FUBP1 | FUBP3 | FUS | FYN | FZR1 | G3BP1 | G6PD | GAD2 | GAK | GALNT7 | GANAB | GAPDH | GAR1 | GARS | GART | GATA1 | GCDH | GDA | GEMIN4 | GEMIN5 | GEMIN7 | GFPT1 | GFPT2 | GGA1 | GGCT | GGT1 | GGTLC1 | GINS2 | GINS3 | GINS4 | GLA | GLCE | GLO1 | GLRX | GLRX3 | GLRX5 | GLS | GLS2 | GLUD2 | GLUL | GMDS | GMPPB | GMPR | GMPS | GNAI3 | GNAS | GNPDA1 | GNPDA2 | GNPNAT1 | GOLGA2 | GOLPH3 | GOT1 | GOT1L1 | GOT2 | GPC5 | GPD1L | GPS2 | GPT2 | GPX1 | GPX4 | GRB2 | GRHPR | GRK1 | GRK5 | GSK3A | GSK3B | GSPT1 | GSPT2 | GSR | GSS | GSTA1 | GSTA2 | GSTA3 | GSTA4 | GSTA5 | GSTK1 | GSTM4 | GSTO1 | GSTO2 | GSTP1 | GSTT1 | GSTT2 | GSTT2B | GSTZ1 | GTF2A1 | GTF2E2 | GTF2F1 | GTF2F2 | GTF2I | GTF3C2 | GZMA | GZMK | H1F0 | H2AFX | H3F3A | HAAO | HAGH | HARS | HBEGF | HCCS | HDAC1 | HDAC11 | HDAC2 | HDAC3 | HDAC4 | HDAC5 | HDAC6 | HDAC7 | HDAC9 | HECW1 | HECW2 | HELZ2 | HERC1 | HERC2 | HEXA | HEXB | HGS | HIF1A | HIF1AN | HINT1 | HIPK4 | HIST1H2BA | HIST1H3A | HIST1H4A | HIST2H3A | HIST3H3 | HLCS | HMBS | HMGB1 | HMGB2 | HMGB3 | HMGCL | HMOX1 | HNF4A | HNF4G | HNRNPA1 | HNRNPA1L2 | HNRNPA2B1 | HNRNPD | HNRNPF | HNRNPH1 | HNRNPH2 | HNRNPH3 | HNRNPK | HNRNPL | HNRNPM | HNRNPU | HOXB9 | HPRT1 | HPX | HRAS | HS2ST1 | HS6ST1 | HSD17B10 | HSD17B4 | HSD17B8 | HSF1 | HSF4 | HSP90AA1 | HSP90AB1 | HSP90B1 | HSPA14 | HSPA1B | HSPA5 | HSPA8 | HSPD1 | HSPG2 | HTRA1 | HTRA2 | HUWE1 | IARS | ICK | IDE | IDH1 | IDH2 | IFI16 | IGF1R | IGFBP7 | IK | IKBKB | IKBKE | IKBKG | IL3RA | ILF2 | ILF3 | ILK | IMP4 | IMPDH2 | ING5 | INO80B | INO80C | INSR | IREB2 | IRF1 | IRF4 | IRF7 | ISG15 | ITCH | ITGAV | ITK | ITPA | JAK1 | JAK3 | JMJD6 | JOSD2 | JUN | KAT5 | KAT7 | KAT8 | KBTBD7 | KCMF1 | KCNAB2 | KCTD17 | KCTD2 | KDM1A | KDM2A | KDM3B | KDM4D | KDM5B | KDM5C | KDM8 | KDR | KDSR | KEAP1 | KHDRBS1 | KIAA0391 | KLF5 | KLHL20 | KLHL22 | KMT2A | KMT2B | KMT2C | KPNA1 | KPNA2 | KPNB1 | KRAS | KSR1 | KYNU | L2HGDH | LACTB2 | LAMB1 | LAP3 | LARP4 | LARP7 | LARS | LAT | LATS1 | LCMT2 | LDHA | LDHC | LEF1 | LGALS1 | LGALS3 | LGMN | LIG4 | LMO2 | LMO7 | LNX1 | LOXL2 | LPIN3 | LRRC7 | LRRK1 | LRRK2 | LRSAM1 | LSM1 | LSM12 | LSM3 | LSM4 | LSM5 | LSM8 | LTBP1 | LUM | LYPLA1 | LYPLA2 | LZTR1 | MAD2L1 | MAN2B1 | MAOA | MAP2K1 | MAP2K3 | MAP2K4 | MAP2K6 | MAP2K7 | MAP3K1 | MAP3K13 | MAP3K14 | MAP3K2 | MAP3K3 | MAP3K4 | MAP4K1 | MAPK1 | MAPK14 | MAPK15 | MAPK3 | MAPK4 | MAPK6 | MAPK7 | MAPK8 | MAPK9 | MAPT | MARCH5 | MASP1 | MASP2 | MAST1 | MAST3 | MATK | MBD3 | MBP | MC1R | MCFD2 | MCM2 | MCM3 | MCM4 | MCM5 | MCM6 | MCM7 | MCM9 | MDC1 | MDH1 | MDH2 | MDM2 | MDM4 | MDN1 | ME1 | MECP2 | MED1 | MED12 | MED14 | MED17 | MED20 | MED21 | MED23 | MED24 | MED31 | MED4 | MED6 | MELK | MEPCE | METAP2 | MGMT | MGST1 | MIB1 | MITF | MKNK2 | MLH1 | MLLT1 | MLLT3 | MMAB | MME | MMP26 | MOCOS | MORF4L1 | MORF4L2 | MOV10 | MPC2 | MPG | MPHOSPH6 | MPI | MPPE1 | MPST | MRGBP | MRM1 | MRPL12 | MRPL28 | MRPL43 | MRPL9 | MRRF | MSH6 | MTA1 | MTA3 | MTAP | MTERF1 | MTHFD1 | MTMR14 | MTMR7 | MTPN | MTRR | MVD | MYC | MYH6 | MYO1E | NAE1 | NAGK | NAMPT | NANS | NAPRT | NBAS | NCBP1 | NCK1 | NCOA1 | NCOA3 | NCOR1 | NDUFAF5 | NDUFAF7 | NDUFS1 | NEDD4 | NEDD8 | NEK1 | NEK7 | NELFB | NELFCD | NELFE | NEURL4 | NFATC1 | NFE2L2 | NFKBIA | NFS1 | NHLRC3 | NIPBL | NIT1 | NIT2 | NLK | NME1 | NME2 | NME4 | NMNAT1 | NMNAT3 | NMRAL1 | NMRK2 | NNT | NOB1 | NOD2 | NOP10 | NOP56 | NOS2 | NOS3 | NOSIP | NPEPL1 | NPEPPS | NPLOC4 | NPM1 | NPR1 | NQO1 | NR0B2 | NR1H2 | NR1H3 | NR1I2 | NR3C1 | NR4A1 | NR5A2 | NRBF2 | NSFL1C | NSMCE4A | NSUN2 | NSUN6 | NT5C2 | NTMT1 | NTRK1 | NUCB1 | NUDT13 | NUDT18 | NUDT3 | NUDT9 | NUFIP1 | NUP107 | NUP153 | NUP160 | NUP205 | NUP214 | NUP85 | NUP88 | NUP93 | NUP98 | NUPR1 | OAS2 | OAT | OAZ3 | OBSL1 | OGDH | OGFOD1 | OGT | OLA1 | OPA1 | OTC | OTUB1 | OTUB2 | OTUD4 | OTUD5 | OXSR1 | P2RX1 | P4HA1 | P4HA2 | P4HB | PA2G4 | PABPC1 | PABPC3 | PABPC4 | PADI4 | PAFAH1B1 | PAH | PAICS | PAK2 | PAK6 | PALB2 | PAN2 | PAPLN | PAPSS1 | PAPSS2 | PARK7 | PARP1 | PARP12 | PARP14 | PARP6 | PAWR | PAX8 | PAXIP1 | PCBD1 | PCBP1 | PCBP2 | PCF11 | PCGF1 | PCGF5 | PCGF6 | PCMT1 | PCMTD1 | PCMTD2 | PCNA | PCSK9 | PCTP | PDCD6IP | PDE12 | PDE4D | PDF | PDHA1 | PDHA2 | PDHB | PDHX | PDIA3 | PDIA6 | PDK2 | PDXK | PEBP1 | PELI1 | PEPD | PER1 | PER2 | PEX5 | PFAS | PGAM5 | PGK1 | PGM3 | PGP | PHB | PHF10 | PHGDH | PHKA1 | PHOSPHO1 | PIAS1 | PIAS3 | PICK1 | PIGU | PIH1D1 | PIK3CA | PIK3CG | PIK3R1 | PIM1 | PIN1 | PINX1 | PIR | PJA1 | PKN2 | PLA2G10 | PLA2G12A | PLA2G1B | PLA2G2A | PLA2G6 | PLK1 | PLOD2 | PLSCR1 | PML | PMPCA | PMPCB | PNKP | PNP | POLA2 | POLD1 | POLDIP3 | POLE2 | POLE3 | POLM | POLR2A | POLR2E | POLR2L | POLR2M | POM121 | POT1 | POU5F1 | PPA1 | PPA2 | PPARD | PPARG | PPARGC1A | PPAT | PPEF1 | PPIA | PPIB | PPID | PPIE | PPIF | PPIL3 | PPM1A | PPM1B | PPM1D | PPM1H | PPM1K | PPME1 | PPOX | PPP1CA | PPP1CB | PPP1CC | PPP1R11 | PPP1R12A | PPP1R3B | PPP1R3C | PPP1R8 | PPP2CA | PPP2CB | PPP2R1A | PPP2R1B | PPP2R2A | PPP2R2D | PPP2R5D | PPP3CC | PPP4R2 | PPP5C | PPP6C | PPT1 | PPTC7 | PRCC | PRCP | PRDX3 | PRDX4 | PREP | PREPL | PRKAA1 | PRKAA2 | PRKAB2 | PRKACA | PRKAG1 | PRKCD | PRKCE | PRKCSH | PRKCZ | PRKD1 | PRKD2 | PRKD3 | PRKDC | PRMT2 | PRMT3 | PRMT6 | PROZ | PRPF19 | PRPF40A | PRPF8 | PRSS16 | PRSS23 | PRTFDC1 | PSAP | PSAT1 | PSEN1 | PSEN2 | PSMA1 | PSMA2 | PSMA3 | PSMA5 | PSMA6 | PSMB5 | PSMB6 | PSMB7 | PSMB8 | PSMC1 | PSMC2 | PSMC3 | PSMC4 | PSMC5 | PSMC6 | PSMD1 | PSMD10 | PSMD11 | PSMD12 | PSMD14 | PSMD2 | PSMD4 | PSMD9 | PSME2 | PSME3 | PTEN | PTGES | PTGES3 | PTK2 | PTMA | PTPMT1 | PTPN1 | PTPN14 | PTPN21 | PTPN22 | PTPN23 | PTPN4 | PTPN6 | PTPRA | PTPRD | PTPRF | PTPRK | PTPRO | PTPRR | PTTG1 | PUM1 | PUS1 | PUS7 | PYCR1 | PYCR2 | QARS | QDPR | QSOX1 | RAB11A | RAB11B | RAB1A | RAB1B | RAB2A | RAB5A | RAB7A | RAD21 | RAD23A | RAD23B | RAD51 | RAD51AP1 | RAE1 | RAF1 | RAN | RANBP9 | RANGAP1 | RARA | RARG | RARS2 | RBBP6 | RBM17 | RBM25 | RBM3 | RBM4 | RBM8A | RBMX | RBPJ | RBX1 | RC3H1 | RELA | REST | REXO2 | RFC1 | RFC2 | RFC3 | RFC4 | RFC5 | RFK | RHBDD1 | RHO | RHOA | RHOT1 | RIF1 | RING1 | RIOK1 | RIPK1 | RIPK2 | RIPK3 | RIPK4 | RMND1 | RNASE3 | RNASEH2A | RNASEH2B | RNF123 | RNF126 | RNF139 | RNF181 | RNF183 | RNF185 | RNF19A | RNF2 | RNF25 | RNF31 | RNF4 | RNF8 | RNPEP | RPA2 | RPA3 | RPAIN | RPL10 | RPL10A | RPL12 | RPL18A | RPL22 | RPL23 | RPL23A | RPL24 | RPL30 | RPL36AL | RPL37A | RPL38 | RPL6 | RPL7 | RPL7A | RPL9 | RPLP0 | RPLP0P6 | RPLP1 | RPLP2 | RPN1 | RPN2 | RPP14 | RPS12 | RPS13 | RPS14 | RPS15A | RPS19 | RPS2 | RPS20 | RPS24 | RPS29 | RPS3 | RPS4X | RPS6KA1 | RPS6KA2 | RPS6KB2 | RPS8 | RRM1 | RRM2 | RRM2B | RRP7A | RRS1 | RTCB | RTF1 | RTFDC1 | RUNX2 | RUVBL1 | RUVBL2 | RXRA | RXRB | RXRG | S100A9 | SAE1 | SAP18 | SARS | SART1 | SART3 | SAT1 | SAT2 | SBDS | SCAF1 | SCG2 | SCG3 | SCLY | SCO2 | SCYL2 | SDC1 | SDC4 | SEC63 | SEL1L | SENP3 | SENP6 | SENP7 | SENP8 | SEPHS1 | SEPHS2 | SERPINH1 | SETDB1 | SF1 | SF3B6 | SFPQ | SGPL1 | SGSH | SGTA | SGTB | SHC1 | SHMT1 | SHMT2 | SIAH1 | SIN3A | SIRT1 | SIRT3 | SIRT5 | SIRT6 | SIRT7 | SKI | SKIV2L | SLC25A1 | SLC25A13 | SLC25A32 | SMAD2 | SMAD3 | SMAD4 | SMAD5 | SMARCB1 | SMC1A | SMC3 | SMG5 | SMG6 | SMG8 | SMN1 | SMURF1 | SMURF2 | SNCA | SND1 | SNRNP27 | SNRPA | SNRPA1 | SNRPB | SNRPB2 | SNRPD1 | SNRPD3 | SNRPE | SNW1 | SNX3 | SOCS1 | SOCS2 | SOCS6 | SOD1 | SP1 | SP100 | SPATA2 | SPI1 | SPIN1 | SPOP | SPR | SPRR2A | SPRTN | SPSB3 | SPTAN1 | SPTBN1 | SPTBN2 | SPTLC2 | SQSTM1 | SRC | SRPK1 | SRPK2 | SRSF11 | SRSF12 | SRSF5 | SSB | ST3GAL1 | ST3GAL6 | ST6GALNAC5 | ST8SIA4 | STARD7 | STAT3 | STAT5A | STK11 | STK24 | STK25 | STK26 | STK3 | STK38 | STK40 | STOML2 | STUB1 | STYX | SUCLA2 | SUCLG2 | SULF2 | SULT1A3 | SUMO1 | SUMO1P1 | SUMO2 | SUMO3 | SUPT4H1 | SUPT5H | SUZ12 | SWAP70 | SYVN1 | TAB1 | TAB2 | TADA2B | TAF12 | TAF15 | TAF1A | TAMM41 | TANK | TAOK3 | TARDBP | TARS | TARS2 | TATDN1 | TBL1X | TBRG4 | TCEA1 | TCEA2 | TCERG1 | TCF4 | TCIRG1 | TDO2 | TDP1 | TECR | TERF1 | TERF2 | TERF2IP | TESK2 | TFCP2 | TFIP11 | TGFB1 | TGFBR3 | TGM2 | TGOLN2 | THADA | THBS1 | THEM4 | THOC2 | THOC6 | THOC7 | THOP1 | THRA | TIMM50 | TIMP1 | TINF2 | TK1 | TK2 | TLR3 | TMEM132A | TMEM189-UBE2V1 | TMEM258 | TMTC3 | TNF | TNFAIP3 | TNIP2 | TNK2 | TNKS | TNKS2 | TNP1 | TOE1 | TOLLIP | TOMM20 | TOP1 | TOP2A | TOP3A | TOR1A | TP53 | TP53RK | TP73 | TPR | TPSAB1 | TPSB2 | TPST2 | TPTE | TRA2A | TRAF2 | TRAF3 | TRAF6 | TREX1 | TRIB2 | TRIB3 | TRIM21 | TRIM23 | TRIM25 | TRIM28 | TRIM29 | TRIM32 | TRIM33 | TRIM41 | TRIM47 | TRIM63 | TRIM69 | TRIM7 | TRIM9 | TRIP12 | TRIP13 | TRMT44 | TRMT6 | TRMT61B | TRNT1 | TRUB2 | TSC22D1 | TSG101 | TSN | TSPAN17 | TTC5 | TTLL1 | TWF1 | TXNL4A | TXNRD1 | TYMS | U2AF2 | UAP1 | UAP1L1 | UBA1 | UBA2 | UBA3 | UBA5 | UBA6 | UBAC1 | UBB | UBC | UBE2A | UBE2B | UBE2C | UBE2D1 | UBE2D2 | UBE2D3 | UBE2H | UBE2I | UBE2J1 | UBE2K | UBE2L3 | UBE2L6 | UBE2M | UBE2N | UBE2O | UBE2Q2 | UBE2R2 | UBE2S | UBE2V1 | UBE2W | UBE3A | UBE3B | UBL4A | UBQLN1 | UBQLN2 | UBQLN4 | UBR4 | UBR5 | UBXN1 | UBXN7 | UCHL1 | UCHL3 | UCHL5 | UCK2 | UFC1 | UFM1 | UGDH | UGGT1 | UGP2 | UGT8 | ULK1 | UMPS | UNKL | UPF2 | UPF3B | UPP2 | UQCRC2 | USP11 | USP14 | USP15 | USP16 | USP17L2 | USP19 | USP2 | USP20 | USP21 | USP26 | USP28 | USP39 | USP4 | USP42 | USP46 | USP47 | USP5 | USP50 | USP53 | USP7 | USP8 | USP9X | USP9Y | USPL1 | UTP14A | UTP6 | UXS1 | VAPA | VAPB | VARS | VASH2 | VCAM1 | VCAN | VCP | VCPIP1 | VDR | VGF | VHL | VKORC1 | VPS37D | VPS4B | WARS | WDR12 | WDR4 | WDR45B | WDR48 | WDR82 | WDTC1 | WDYHV1 | WIPI1 | WIPI2 | WNK1 | WNK4 | WSB1 | WT1 | WTAP | WWP2 | XBP1 | XIAP | XPNPEP1 | XPO5 | XRCC1 | XRCC3 | XRCC5 | XRCC6 | XYLT1 | YAP1 | YBEY | YEATS2 | YES1 | YME1L1 | YWHAB | YWHAE | YY1 | ZAP70 | ZBTB16 | ZBTB7A | ZBTB7B | ZC3HC1 | ZDHHC17 | ZDHHC23 | ZFYVE26 | ZMPSTE24 | ZNF217 | ZNF598 | ZNF638 | ZNRF4 | ZRANB2 | GO:0065003 | 0.124954506854301 | 1030/8243 | 505/3250 | 1.49141215884557e-11 | 1.41609584482387e-07 | T | T | T | T | protein-containing complex assembly | AAR2 | AASS | ACACA | ACAD9 | ACAT1 | ACD | ACOT13 | ACTN2 | ADRB2 | ADRM1 | ADSL | AGO1 | AGO2 | AHNAK | AIMP2 | ALDH5A1 | ALDOA | ALOX5AP | AMFR | ANKRD28 | ANKS4B | ANXA2 | ANXA5 | ANXA6 | APC | APCS | APOA1 | APOE | AR | ARF1 | ARPC2 | ARPC4 | ATL3 | ATPAF2 | ATRX | ATXN10 | ATXN2 | AXIN1 | BAX | BCL10 | BCS1L | BIN1 | BIRC2 | BIRC3 | BRAF | BRD2 | C1QBP | C1QTNF1 | CAB39 | CALR | CAND1 | CAPG | CAPZA1 | CAPZA2 | CASP8 | CAT | CAV1 | CBR4 | CBY1 | CCNB1 | CCT2 | CCT3 | CCT6B | CCT8 | CD2BP2 | CD40 | CD74 | CD79B | CDA | CDC73 | CENPF | CENPJ | CENPV | CEP57 | CHAF1A | CHD1L | CHMP2A | CHP1 | CIRBP | CKAP5 | CLGN | CLNS1A | CLTC | CLU | COA3 | COL6A1 | COL6A2 | COX11 | COX15 | COX17 | COX19 | COX20 | COX7A2 | CPSF7 | CPT1A | CREBBP | CRYAB | CRYZ | CSNK1A1 | CSNK2B | CTH | CTNNB1 | CTSC | CTTN | CUL1 | CUL3 | CUL4B | CUTC | CYLD | DARS | DAXX | DBNL | DCTPP1 | DCXR | DDB1 | DDB2 | DDX1 | DDX39B | DDX3X | DDX6 | DECR1 | DERL1 | DHRS4 | DHX9 | DIAPH3 | DLG4 | DMD | DNAAF2 | DNAAF5 | DNM1L | DVL2 | DYNC1H1 | ECSIT | EHD4 | EIF2AK3 | EIF2S1 | EIF3A | EIF3D | EIF3E | EIF3H | EIF3I | EIF4H | EIF5 | EIF6 | ELAVL1 | EP300 | EPPIN | EPRS | ERAL1 | ERCC5 | F5 | FADD | FANCA | FANCC | FARSB | FBP1 | FERMT2 | FGB | FIS1 | FKBP1A | FLOT1 | FN1 | FOLR1 | FSD1 | FUS | G3BP1 | G3BP2 | GBP5 | GEMIN4 | GEMIN5 | GEMIN7 | GLRB | GLS | GLUL | GNB1 | GOLGA2 | GOPC | GPX4 | GRB2 | GRB7 | GRHPR | GRIA1 | GSK3B | H1F0 | H1FX | H2AFX | H3F3A | HBZ | HCCS | HCN1 | HDAC1 | HIST1H1A | HIST1H2BA | HIST1H3A | HIST1H4A | HIST2H3A | HIST3H3 | HLA-DRA | HLA-DRB1 | HMGB2 | HMGCL | HMOX1 | HPRT1 | HRAS | HSD17B10 | HSD17B8 | HSF1 | HSF4 | HSP90AA1 | HSP90AB1 | HSPA4 | HSPD1 | HTRA2 | IDE | IGF1R | IKBKE | IKZF5 | ILK | IMPDH2 | INSR | IPO4 | ITGB1BP1 | ITGB3BP | ITPR3 | JMJD6 | JUP | KAT5 | KCNA4 | KCNS3 | KCTD12 | KCTD17 | KCTD18 | KCTD2 | KCTD3 | KHDRBS1 | KMT2A | LEF1 | LETM1 | LIMS1 | LNX1 | LRRK2 | LSM3 | LSM4 | LUC7L2 | LYRM7 | MAGI1 | MAPK3 | MAPT | MAT2A | MCCC1 | MCFD2 | MCM2 | MCTS1 | MDM2 | MDM4 | MDN1 | ME1 | MED6 | MGST1 | MICU1 | MIF | MITF | MPP2 | MYC | MYH11 | NACC1 | NAP1L1 | NAP1L4 | NCBP1 | NCK1 | NDUFA10 | NDUFA13 | NDUFA5 | NDUFA8 | NDUFA9 | NDUFAF1 | NDUFAF2 | NDUFAF3 | NDUFAF4 | NDUFAF5 | NDUFAF7 | NDUFB1 | NDUFS1 | NDUFS2 | NDUFS3 | NDUFS6 | NDUFS7 | NDUFS8 | NDUFV1 | NDUFV3 | NFS1 | NME2 | NOD2 | NPM1 | NUFIP1 | NUP107 | NUP153 | NUP205 | NUP93 | NUP98 | NUPR1 | OAT | OBSL1 | OGFOD1 | OPA1 | OTC | OXA1L | P2RX1 | P2RX2 | P2RX4 | P4HB | PADI4 | PANX3 | PARD3 | PARP1 | PCBD1 | PDCD6 | PDCD6IP | PDCL | PDE4DIP | PDSS1 | PDSS2 | PEF1 | PEX11B | PFKL | PFKM | PIAS1 | PIH1D1 | PIK3C2A | PIN1 | PKM | PML | POLE3 | PPARGC1A | PPAT | PPID | PPP2R1A | PPP6C | PPP6R1 | PRKAA1 | PRKACA | PRKCZ | PRPF19 | PRPF8 | PSMD10 | PSMD11 | PSMD4 | PSMD9 | PSMG1 | PSMG4 | PSTPIP1 | PTGES3 | PTK2 | PXN | RAB1A | RAB1B | RAC1 | RAD23B | RAD51 | RAF1 | RANBP9 | RAP1GDS1 | RBBP4 | RBMX | RBX1 | RC3H1 | RCC1 | RIC3 | RIPK1 | RIPK3 | RPA2 | RPA3 | RPL10 | RPL12 | RPL23A | RPL24 | RPL38 | RPL6 | RPLP0 | RPLP0P6 | RPS10P5 | RPS14 | RPS19 | RRM1 | RRM2 | RRP7A | RRS1 | RTN4 | RUVBL1 | RUVBL2 | RXRA | SART1 | SART3 | SBDS | SCFD1 | SCO2 | SEC13 | SEC16A | SEC24B | SEC24C | SEC31A | SEPT11 | SEPT4 | SEPT7 | SEPT9 | SET | SF1 | SH3GL2 | SHKBP1 | SHMT1 | SHMT2 | SKI | SLC9A1 | SLC9A3R1 | SLC9A3R2 | SMAD2 | SMAD3 | SMAD4 | SMARCA4 | SMARCB1 | SMN1 | SNAP29 | SNCA | SNRPB | SNRPD1 | SNRPD3 | SNRPE | SNX2 | SOAT1 | SOD2 | SQSTM1 | SRC | SRP19 | SRPK1 | SRPK2 | SRSF12 | SRSF5 | ST13 | STOM | STOML2 | STRAP | STX5 | STXBP1 | TAF12 | TAZ | TBCA | TCF4 | TCP1 | TDO2 | TEKT2 | TFG | TGFB1 | TGFBR3 | TGM2 | TIMM21 | TIMMDC1 | TK1 | TLE1 | TLE3 | TM9SF4 | TMEM70 | TNF | TNFAIP3 | TNFRSF1A | TOMM20 | TOR1A | TP53 | TP63 | TP73 | TPR | TRADD | TRAF1 | TRAF2 | TRIM21 | TRIM28 | TRIM34 | TRMT61B | TSPYL2 | TTC19 | TXNL4A | UBB | UBC | UBE2C | UBE2K | UBE2S | UGDH | UQCC1 | UQCRB | USP16 | USP39 | USP4 | UXS1 | VASP | VBP1 | VCP | VTN | WAS | WDR77 | XRCC6 | YAP1 | YME1L1 | YWHAB | ZMYND10 | ZNF207 | ZW10 | GO:1901565 | 0.0612641028751668 | 505/8243 | 271/3250 | 1.71281644808197e-11 | 1.62631921745383e-07 | T | T | T | T | organonitrogen compound catabolic process | AASS | ABAT | ABHD17A | ACAD8 | ACADSB | ACAT1 | ACOT7 | ADA | AHCY | AICDA | AKT1 | ALDH4A1 | ALDH5A1 | ALDH6A1 | ALDH7A1 | ALDH8A1 | AMFR | ANAPC4 | ANXA2 | APOE | ARMC8 | ARRB1 | ARRB2 | ASPDH | AUP1 | AURKB | BACE1 | BAG6 | BCAT2 | BCKDHB | BIRC2 | BLVRB | BTRC | CBS | CCNB1 | CDA | CDC16 | CDC20 | CDC27 | CDC34 | CDK2 | CHDH | CHEK2 | CHP1 | CLPX | CLTC | COMT | CRBN | CTH | CTNNB1 | CTSD | CUL1 | CUL2 | CUL3 | CUL4B | CUL5 | DBH | DBT | DCN | DCTPP1 | DDB1 | DERA | DERL1 | DLD | DLST | DNAJB2 | DNAJC10 | DUT | EDEM1 | ERLIN2 | FAF1 | FAF2 | FAH | FBXL12 | FBXO6 | FBXW11 | FBXW5 | FGF2 | FZR1 | GAD2 | GALNS | GCDH | GDA | GGT1 | GGTLC1 | GLA | GLS | GLS2 | GLUD2 | GLUL | GNS | GOT1 | GOT2 | GPC5 | GPT2 | GPX1 | GSK3A | GSTZ1 | GUSB | HAAO | HECW1 | HECW2 | HERC2 | HEXA | HEXB | HIBADH | HIBCH | HINT1 | HMGCL | HMMR | HMOX1 | HPRT1 | HSD17B10 | HSP90B1 | HSPA5 | HSPG2 | HTRA2 | IDE | ITCH | ITPA | IVD | KAT5 | KCTD17 | KCTD2 | KEAP1 | KLHL20 | KLHL22 | KYNU | LRSAM1 | LUM | LYG2 | LYPLA1 | LYPLA2 | MAD2L1 | MAOA | MCCC1 | MDM2 | MPST | MTA1 | MTAP | MTRR | NEDD4 | NFE2L2 | NHLRC3 | NOS2 | NOS3 | NPEPPS | NPLOC4 | NSFL1C | NT5C2 | NUDT18 | NUDT3 | NUDT9 | OAT | OGDH | OTC | OVGP1 | PADI4 | PAH | PCBD1 | PCBP2 | PCSK9 | PDE4D | PELI1 | PJA1 | PLK1 | PML | PNP | PPAT | PPM1K | PPP2CB | PPT1 | PRPF19 | PRSS16 | PRTFDC1 | PSAP | PSMA1 | PSMA2 | PSMA3 | PSMA5 | PSMA6 | PSMB5 | PSMB6 | PSMB7 | PSMB8 | PSMC1 | PSMC2 | PSMC3 | PSMC4 | PSMC5 | PSMC6 | PSMD1 | PSMD10 | PSMD11 | PSMD12 | PSMD14 | PSMD2 | PSMD4 | PSMD9 | PSME2 | PSME3 | PTTG1 | QDPR | RAB7A | RAD23A | RAD23B | RBX1 | RHBDD1 | RIPK1 | RNF126 | RNF139 | RNF185 | RNF4 | SAT1 | SAT2 | SDC1 | SDC4 | SEL1L | SGPL1 | SGSH | SGTA | SGTB | SHMT1 | SHMT2 | SIAH1 | SIRT1 | SLC9A1 | SMURF1 | SMURF2 | SOCS6 | SPOP | SPSB3 | STUB1 | SULT1A3 | SYVN1 | TBL1X | TCIRG1 | TDO2 | TGFB1 | TOR1A | TRAF2 | TRIM25 | TRIM9 | TXN2 | UBB | UBC | UBE2A | UBE2B | UBE2C | UBE2D1 | UBE2D3 | UBE2H | UBE2I | UBE2J1 | UBE2R2 | UBE2S | UBE2W | UBE3A | UBQLN1 | UBQLN2 | UBXN1 | UCHL1 | UCHL3 | UGGT1 | UPP2 | USP19 | VCAN | VCP | WWP2 | XPNPEP1 | YME1L1 | GO:0009894 | 0.0858910590804319 | 708/8243 | 362/3250 | 2.81130827915241e-11 | 2.66933721105521e-07 | T | T | T | T | regulation of catabolic process | ABL1 | ABL2 | ADRB2 | AGAP2 | AGO2 | AIFM1 | AKT1 | AKT2 | ANP32A | ANXA2 | APC | APEX1 | APOE | ARAF | ARNT | ASB9 | ATM | ATP13A2 | ATP6V1A | ATP6V1B1 | ATP6V1C1 | ATP6V1D | ATP6V1E1 | AXIN1 | BAG2 | BAG3 | BAG5 | BAG6 | BANP | BAX | BCL2 | BTRC | CALCOCO2 | CAPN1 | CAPNS1 | CASP3 | CAV1 | CBFA2T3 | CCAR2 | CCDC22 | CD81 | CDC37 | CDH1 | CDK4 | CDK5 | CDK5R1 | CEBPA | CHEK2 | CIRBP | CISD2 | CLU | COMMD1 | CPT1A | CSNK1A1 | CSNK1E | CSNK2A2 | CTSA | CTSC | CTTN | CUL4B | DAB2 | DAPK1 | DCN | DCP2 | DDA1 | DDB1 | DDIT4 | DDRGK1 | DHX36 | DHX9 | DIS3 | DNAJB2 | DNM1L | DVL1 | E2F1 | ECD | EDEM1 | EEF1A1 | EEF1A2 | EGFR | EGLN1 | EIF2AK4 | EIF4E | EIF4G3 | EIF6 | ELAVL1 | ENDOG | EP300 | EPHA4 | EPM2A | ESRRB | EXOSC1 | EXOSC5 | EXOSC6 | EXOSC9 | EZR | FABP1 | FAF1 | FAM192A | FAM83D | FBP1 | FBXO7 | FOXO1 | FTO | FXR2 | FYN | FZR1 | GAPDH | GAPDHS | GFAP | GGA1 | GLMN | GNA12 | GNAI3 | GOLGA2 | GPD1 | GPSM1 | GPX1 | GSK3A | GSK3B | HAX1 | HDAC4 | HDAC6 | HECW1 | HECW2 | HERC1 | HGS | HIF1A | HK2 | HMGB1 | HMOX1 | HNRNPD | HNRNPM | HNRNPU | HSF1 | HSP90AA1 | HSP90AB1 | HSP90AB3P | HSP90AB4P | HSPA1B | HSPA8 | HSPB1 | HSPBP1 | HTRA2 | HTT | IDE | IDH1 | IFI16 | IGF2BP1 | IKBKG | INSR | ITCH | ITPR1 | KAT8 | KDR | KEAP1 | KLHL22 | LAMP3 | LARS | LATS1 | LRPPRC | LRRK2 | LRSAM1 | MAD2L1 | MAPK14 | MAPK15 | MAPK3 | MAPK8 | MAPK9 | MAPT | MDM2 | MDM4 | MLST8 | MOV10 | MSN | MYD88 | NAMPT | NBAS | NCOR1 | NDUFA13 | NEDD4 | NELL1 | NFE2L2 | NMNAT1 | NNT | NOS2 | NPM1 | NUP107 | NUP153 | NUP160 | NUP205 | NUP214 | NUP85 | NUP88 | NUP93 | NUP98 | NUPR1 | OAZ3 | OGT | OPTN | PABPC1 | PABPC4 | PACSIN3 | PAIP1 | PAM16 | PARK7 | PCSK9 | PDE12 | PGAM1 | PHB | PIAS1 | PIK3CA | PIK3CG | PIKFYVE | PIN1 | PIP4K2A | PLEKHF1 | PLK1 | PML | POLDIP2 | POM121 | PPARGC1A | PPP1CA | PPP1CB | PPP1R3B | PRKAA1 | PRKAA2 | PRKAB2 | PRKACA | PRKAG1 | PRKCD | PRKCE | PRKD1 | PSAP | PSEN1 | PSMA1 | PSMA2 | PSMA3 | PSMA5 | PSMA6 | PSMB5 | PSMB6 | PSMB7 | PSMB8 | PSMC1 | PSMC2 | PSMC3 | PSMC4 | PSMC5 | PSMC6 | PSMD1 | PSMD10 | PSMD11 | PSMD12 | PSMD14 | PSMD2 | PSMD4 | PSMD9 | PSME2 | PSME3 | PTEN | PTK2 | PTPN1 | PTPN22 | PUM1 | QSOX1 | RAB1B | RAB3GAP1 | RAB5A | RAB7A | RAD23A | RAD23B | RAE1 | RB1CC1 | RBX1 | RC3H1 | RDX | RELA | RGN | RIPK2 | RNF139 | RNF185 | RNF19A | RPL23 | RPTOR | SCFD1 | SDCBP | SEC13 | SERPINE2 | SET | SGTA | SIRT1 | SIRT6 | SMAD3 | SMARCC1 | SMG6 | SMURF1 | SMURF2 | SNCA | SNF8 | SNX12 | SNX3 | SNX6 | SOGA3 | SPTLC2 | STAT3 | STK11 | STUB1 | STX5 | STYX | SUMO1 | SUMO2 | SUPT5H | TAB2 | TARDBP | TBRG4 | THRA | TIMP1 | TIMP2 | TMTC3 | TNF | TNFAIP3 | TNFSF12 | TNPO1 | TPCN2 | TPR | TREX1 | TRIB2 | TRIB3 | TRIM21 | TRIM32 | TWIST1 | UBB | UBC | UBE2J1 | UBE2K | UBE3A | UBQLN1 | UBQLN2 | UBQLN4 | UBXN1 | UCHL1 | UCHL5 | ULK1 | USP14 | USP19 | USP5 | USP7 | USP8 | VCP | VDAC1 | VIM | VPS26A | VPS29 | WDR41 | WWP2 | XBP1 | XPO1 | YWHAB | YWHAZ | ZBTB7A | ZMPSTE24 | GO:0006950 | 0.220914715516196 | 1821/8243 | 838/3250 | 5.30499631150387e-11 | 5.03709399777292e-07 | T | T | T | T | response to stress | ABAT | ABCE1 | ABL1 | ACAA2 | ACADVL | ACAT1 | ACD | ACKR2 | ACO2 | ACTL6A | ACTN4 | ACTR2 | ADA | ADCYAP1 | ADH5 | ADORA2A | ADRB2 | ADSL | AGER | AGR2 | AGTRAP | AHCY | AHSG | AICDA | AIFM1 | AKAP8 | AKR1B1 | AKT1 | ALB | AMFR | ANKS4B | ANXA1 | ANXA7 | APBB1 | APC | APCS | APEX1 | APEX2 | APOA1 | APOD | APOE | APOL2 | APP | ARF4 | ARFGAP1 | ARNT | ARNT2 | ASNS | ASS1 | ATAD5 | ATF2 | ATF6 | ATF6B | ATG5 | ATM | ATOX1 | ATP13A2 | ATP2A2 | ATRX | AUP1 | AZU1 | BABAM1 | BAG3 | BAG6 | BAX | BBC3 | BCL10 | BCL2 | BCL2L1 | BCL2L2 | BCL6 | BDKRB2 | BIRC2 | BRCA1 | BRD4 | BTK | BTRC | C1QA | C1QBP | C1R | C4A | CAB39 | CALR | CAMK1D | CAMK2A | CAPN2 | CAPZA1 | CAPZA2 | CARD9 | CARM1 | CASP2 | CASP3 | CASP8 | CASP9 | CAT | CAV1 | CBFA2T3 | CBL | CBS | CBX8 | CCAR2 | CCDC155 | CCL21 | CCL22 | CCNB1 | CCR1 | CCR3 | CCR4 | CCR5 | CCS | CD209 | CD36 | CD40 | CD40LG | CD6 | CD74 | CDC14B | CDC25C | CDC37 | CDC5L | CDK2 | CDK4 | CDK5 | CDK9 | CDKN1A | CEBPE | CFTR | CHAF1A | CHD1L | CHEK2 | CHST4 | CIRBP | CLPB | CLU | COL18A1 | COL4A3BP | COLEC10 | COMT | COPS2 | COPS5 | COPS6 | COTL1 | CREB1 | CREB3 | CREBBP | CRIP1 | CRK | CRKL | CRNN | CRP | CRY1 | CRY2 | CRYAB | CSNK1E | CTH | CTSV | CUL1 | CUL2 | CUL4B | CUL7 | CXCL11 | CXCL5 | CXCL6 | CXCL8 | CYCS | CYLD | CYP1A1 | CYP1A2 | DAPK1 | DAXX | DBH | DCN | DCTN1 | DCTPP1 | DDB1 | DDB2 | DDIT4 | DDRGK1 | DDX1 | DDX3X | DERL1 | DHCR24 | DHX36 | DHX9 | DMAP1 | DNA2 | DNAJA1 | DNAJA2 | DNAJA3 | DNAJA4 | DNAJB1 | DNAJB11 | DNAJB2 | DNAJC10 | DNM1L | DPEP1 | DROSHA | DUSP1 | DYRK2 | E2F1 | E2F4 | ECSIT | EDEM1 | EEF2 | EGFR | EGLN1 | EGLN3 | EHMT2 | EIF2AK3 | EIF2AK4 | EIF2B1 | EIF2B3 | EIF2B5 | EIF2S1 | EIF4E | EIF4EBP1 | ELF3 | ELF4 | ENDOG | EP300 | EPPIN | EPPK1 | ERBB2 | ERBB3 | ERCC5 | ERLIN2 | ERRFI1 | ETFDH | ETS1 | EXOSC5 | EXTL2 | EYA2 | EYA4 | EZH2 | FABP1 | FADD | FAF2 | FANCA | FANCC | FANCG | FANCI | FANCM | FBXO6 | FBXW11 | FCN1 | FEN1 | FGB | FGF2 | FIS1 | FKBP14 | FKBP1B | FLOT1 | FN1 | FOLR1 | FOS | FOXM1 | FOXO1 | FYN | FZR1 | G6PD | GAP43 | GAPDH | GBP3 | GBP5 | GBP6 | GFAP | GFPT1 | GINS2 | GINS4 | GLP1R | GLUL | GMPR | GNAO1 | GNB1 | GNL1 | GORASP2 | GOT1 | GPI | GPS2 | GPX1 | GPX4 | GPX7 | GRB2 | GRIK2 | GSK3A | GSK3B | GSR | GSS | GSTP1 | H2AFX | HAVCR2 | HDAC2 | HDAC3 | HDAC4 | HDAC5 | HDAC6 | HDAC9 | HDGF | HERC2 | HEXIM1 | HIF1A | HIF1AN | HIST1H2BA | HIST1H4A | HIST3H3 | HK2 | HMGB1 | HMGB2 | HMGB3 | HMGCL | HMOX1 | HNRNPA1 | HNRNPD | HNRNPL | HRAS | HSF1 | HSF4 | HSP90AA1 | HSP90AB1 | HSP90B1 | HSPA14 | HSPA1B | HSPA1L | HSPA2 | HSPA4 | HSPA4L | HSPA5 | HSPA8 | HSPA9 | HSPB1 | HSPB2 | HSPD1 | HSPE1 | HSPG2 | HSPH1 | HTRA2 | HUWE1 | ICAM1 | ID2 | IDH1 | IFI16 | IFIT1 | IFITM3 | IFNA2 | IGFBP7 | IGHV3-13 | IGHV3-23 | IGHV3-30 | IGKV3-20 | IGLC7 | IKBKB | IKBKE | IKBKG | IL32 | ILF3 | INO80B | INO80C | INSIG1 | INSIG2 | IPO7 | IREB2 | IRF1 | IRF4 | IRF7 | ISG15 | ITCH | ITGB3 | ITK | ITPR1 | ITPR2 | JAK3 | JUN | KAT5 | KAT7 | KDM2A | KDM4D | KIR3DL2 | KIR3DS1 | KLRC2 | KRAS | LARS | LAT | LDHA | LGALS1 | LGALS3 | LGALS3BP | LGR4 | LIG4 | LMNA | LOXL2 | LRRK2 | LYG2 | LYZL1 | LYZL2 | MAP2K1 | MAP2K3 | MAP2K4 | MAP2K6 | MAP2K7 | MAP3K1 | MAP3K13 | MAP3K14 | MAP3K2 | MAP3K3 | MAP3K4 | MAP4K1 | MAPK1 | MAPK14 | MAPK15 | MAPK3 | MAPK7 | MAPK8 | MAPK8IP3 | MAPK9 | MAPT | MASP1 | MASP2 | MATR3 | MAVS | MC1R | MCM7 | MCM9 | MCTS1 | MDC1 | MDM2 | MDM4 | MECP2 | METRNL | MGMT | MGST1 | MICA | MICU1 | MIF | MLH1 | MORF4L1 | MORF4L2 | MPG | MSH6 | MTA1 | MTSS1 | MYBBP1A | MYC | MYD88 | MYH6 | MYO6 | NAPRT | NCK1 | NDRG1 | NDUFS2 | NDUFS8 | NEDD4 | NEK1 | NFATC2 | NFE2L2 | NFKBIA | NIPBL | NME2 | NMNAT1 | NMNAT3 | NOD2 | NOS2 | NOS3 | NPEPPS | NPLOC4 | NPM1 | NQO1 | NRBF2 | NSMCE4A | NTRK1 | NUPR1 | OAS2 | OPA1 | OPTN | ORM1 | OTUB1 | OTUD4 | OXCT1 | OXSR1 | P2RX2 | P2RX4 | P4HB | PADI4 | PAK2 | PAK6 | PALB2 | PARK7 | PARP1 | PARP14 | PAXIP1 | PCBP2 | PCNA | PCSK9 | PDCD10 | PDCD6 | PDE12 | PDIA3 | PDIA4 | PDIA6 | PDK2 | PEBP1 | PER2 | PGK1 | PHB2 | PICK1 | PIK3CG | PIK3R1 | PKM | PLA2G1B | PLA2G2A | PLA2G6 | PLEC | PLGRKT | PLK1 | PLOD2 | PLSCR1 | PML | PNKP | PNMA1 | POLD1 | POLE2 | POLE3 | POLM | POLR2A | POLR2E | POLR2L | POU5F1 | PPARD | PPARG | PPARGC1A | PPIE | PPIF | PPM1D | PPP1CA | PPP1R11 | PPP2CB | PPP5C | PPP6C | PRCP | PRDX1 | PRDX2 | PRDX3 | PRDX4 | PRDX5 | PRDX6 | PRG2 | PRKAA1 | PRKAA2 | PRKACA | PRKCD | PRKCE | PRKCZ | PRKD1 | PRKDC | PRMT6 | PRPF19 | PSEN1 | PSEN2 | PSMA1 | PSMA2 | PSMA3 | PSMA5 | PSMA6 | PSMB5 | PSMB6 | PSMB7 | PSMB8 | PSMC1 | PSMC2 | PSMC3 | PSMC4 | PSMC5 | PSMC6 | PSMD1 | PSMD10 | PSMD11 | PSMD12 | PSMD14 | PSMD2 | PSMD4 | PSMD9 | PSME2 | PSME3 | PSTPIP1 | PTEN | PTGER3 | PTGES | PTGIR | PTN | PTPN1 | PTPN6 | PTPRF | PTPRK | PTTG1 | PXDN | PXN | PYCR1 | PYCR2 | RAB1A | RAC1 | RAD21 | RAD23A | RAD23B | RAD51 | RAD51AP1 | RAF1 | RANGAP1 | RBBP6 | RBM17 | RBM3 | RBM4 | RBPJ | RBX1 | REL | RELA | REST | RFC1 | RFC2 | RFC3 | RFC4 | RFC5 | RHBDD1 | RHOA | RIF1 | RIPK1 | RIPK2 | RIPK3 | RNASE3 | RNASEH2A | RNF139 | RNF183 | RNF185 | RNF8 | ROMO1 | RPA2 | RPA3 | RPAIN | RPL30 | RPS19 | RPS3 | RPS6KA1 | RPTOR | RRM2B | RUVBL1 | RUVBL2 | S100A12 | S100A7 | S100A9 | S100B | SARM1 | SCARA3 | SCFD1 | SCG2 | SDC1 | SDC4 | SEC16A | SEC31A | SEL1L | SEMA7A | SERPINB2 | SERPINB6 | SERPINH1 | SFN | SFPQ | SGTA | SGTB | SHC1 | SIN3A | SIPA1 | SIRT1 | SIRT6 | SLAMF1 | SLAMF8 | SLC12A2 | SLC25A24 | SLC39A4 | SLC9A1 | SMAD2 | SMAD3 | SMAD4 | SMC1A | SMC3 | SMURF1 | SNCA | SNW1 | SOCS6 | SOD1 | SOD2 | SORD | SOX2 | SP1 | SP100 | SPACA3 | SPRTN | SQSTM1 | SRC | SRPK1 | SRPK2 | SRPRB | SRXN1 | STAC | STAT1 | STAT3 | STAT6 | STAU1 | STK11 | STK24 | STK25 | STK26 | STK3 | STOML2 | STUB1 | SULF2 | SULT1A3 | SUMO1 | SYVN1 | TAB1 | TAB2 | TANK | TAOK3 | TARDBP | TBXA2R | TCEA1 | TCIRG1 | TDP1 | TERF2 | TERF2IP | TFDP1 | TFIP11 | TGFB1 | TGFBR3 | THBS1 | THRA | TIAL1 | TLR3 | TM9SF4 | TMBIM6 | TMEM173 | TMEM189-UBE2V1 | TMTC3 | TNF | TNFAIP3 | TNFRSF1A | TNIP2 | TNP1 | TOLLIP | TOP2A | TOR1A | TP53 | TP63 | TP73 | TPM1 | TPR | TPSAB1 | TRAF2 | TRAF3 | TRAF6 | TREX1 | TRIAP1 | TRIB3 | TRIM14 | TRIM21 | TRIM23 | TRIM25 | TRIM28 | TRIM29 | TRIM32 | TRIM34 | TRIM35 | TRIP12 | TRIP13 | TSC22D3 | TSC22D4 | TSPYL2 | TTC5 | TUBB | TUBB4B | TWIST1 | TXN2 | TXNIP | TXNRD1 | UBA1 | UBA5 | UBB | UBC | UBE2A | UBE2B | UBE2D3 | UBE2J1 | UBE2K | UBE2L6 | UBE2N | UBE2V1 | UBE2W | UBQLN1 | UBQLN2 | UBR5 | UCHL1 | UCHL5 | UFC1 | UFM1 | UGGT1 | ULK1 | USP14 | USP16 | USP19 | USP28 | USP46 | USP47 | USP7 | VAPB | VCAM1 | VCP | VDAC1 | VDAC3 | VGF | VHL | VSIG4 | WAS | WDR45B | WDR48 | WIPI1 | WIPI2 | WNT16 | XBP1 | XIAP | XRCC1 | XRCC3 | XRCC5 | XRCC6 | YAP1 | YWHAE | YY1 | ZBTB17 | ZBTB42 | ZBTB7A | ZFYVE26 | ZMPSTE24 | ZNF277 | GO:0051259 | 0.0391847628290671 | 323/8243 | 184/3250 | 6.27058416590662e-11 | 5.95391966552833e-07 | T | T | T | T | protein complex oligomerization | AASS | ACACA | ACAT1 | ACOT13 | ACTN2 | ADSL | AHNAK | ALDH5A1 | ALDOA | ALOX5AP | AMFR | ANXA2 | ANXA5 | ANXA6 | APC | AR | ATL3 | ATXN10 | BAX | BCL10 | BIRC2 | BIRC3 | BRAF | C1QTNF1 | CAB39 | CAT | CAV1 | CBR4 | CBY1 | CD74 | CD79B | CDA | CEP57 | CHMP2A | CHP1 | COL6A1 | COL6A2 | CPSF7 | CPT1A | CRYAB | CRYZ | CTH | CUTC | DCTPP1 | DCXR | DECR1 | DERL1 | DHRS4 | DNM1L | DVL2 | EHD4 | EIF2AK3 | ELAVL1 | EPPIN | FARSB | FBP1 | FIS1 | FLOT1 | FSD1 | FUS | GBP5 | GLRB | GLS | GLUL | GNB1 | GOLGA2 | GOPC | GRB2 | GRHPR | HBZ | HCN1 | HIST1H3A | HIST1H4A | HIST3H3 | HMGCL | HMOX1 | HPRT1 | HRAS | HSD17B10 | HSD17B8 | HSF1 | HSF4 | HTRA2 | IDE | IGF1R | IKBKE | IKZF5 | ILK | IMPDH2 | INSR | ITPR3 | JMJD6 | JUP | KCNA4 | KCNS3 | KCTD12 | KCTD17 | KCTD18 | KCTD2 | KCTD3 | KHDRBS1 | LETM1 | LIMS1 | LNX1 | MAPT | MAT2A | MCCC1 | ME1 | MGST1 | MICU1 | MIF | MPP2 | NACC1 | NME2 | NOD2 | NPM1 | NUFIP1 | OAT | OPA1 | OTC | OXA1L | P2RX1 | P2RX2 | P2RX4 | PANX3 | PCBD1 | PDCD6IP | PDSS1 | PDSS2 | PEX11B | PFKL | PFKM | PKM | PPAT | PRKAA1 | PRKCZ | RAD51 | RBMX | RCC1 | RIPK1 | RIPK3 | RPS19 | RRM1 | RRM2 | RXRA | SEPT11 | SEPT4 | SEPT7 | SEPT9 | SHKBP1 | SHMT1 | SHMT2 | SKI | SLC9A1 | SMAD4 | SNCA | SNX2 | SOD2 | SQSTM1 | ST13 | STOM | STOML2 | TDO2 | TGM2 | TK1 | TNFAIP3 | TOR1A | TP53 | TP63 | TP73 | TRADD | TRAF2 | TRIM21 | TRIM28 | TRIM34 | TRMT61B | UGDH | USP16 | UXS1 | VASP | VCP | XRCC6 | YME1L1 | YWHAB | GO:0051246 | 0.199320635690889 | 1643/8243 | 763/3250 | 6.28298905526489e-11 | 5.96569810797401e-07 | T | T | T | T | regulation of protein metabolic process | A2M | AARS | ABL1 | ACO1 | ACSL1 | ACTB | ADCY6 | ADCYAP1 | ADORA2A | ADRB2 | ADRM1 | ADTRP | AGER | AGO1 | AGO2 | AHSG | AIFM1 | AIMP2 | AIRE | AKAP8 | AKAP9 | AKT1 | AKT2 | AKTIP | ALOX12 | ANXA2 | APC | APCS | APOA1 | APOD | APOE | APP | AR | ARAF | ARNT | ARR3 | ARRB1 | ARRB2 | ASPSCR1 | ATG5 | ATM | ATP13A2 | ATRX | ATXN2 | AXIN1 | AZU1 | B3GAT3 | BAG2 | BAG4 | BAG5 | BAG6 | BAX | BBC3 | BCAR3 | BCL10 | BCL2 | BCL2L13 | BCL6 | BCOR | BDKRB2 | BIN1 | BIRC2 | BIRC3 | BIRC5 | BIRC7 | BIRC8 | BMI1 | BMPR1A | BRAF | BRCA1 | BRD4 | BTRC | C1QBP | C1QL4 | C1QTNF1 | C4A | CAB39 | CALM1 | CALM2 | CALM3 | CALR | CARD9 | CASP10 | CASP3 | CASP8 | CASP9 | CAV1 | CBFA2T3 | CBL | CBLB | CBLC | CCAR2 | CCDC22 | CCDC88A | CCL21 | CCL22 | CCNB1 | CCNT1 | CCNT2 | CCNY | CCNYL1 | CCR1 | CD109 | CD27 | CD36 | CD40 | CD40LG | CD74 | CD81 | CDC14B | CDC20 | CDC25A | CDC25B | CDC25C | CDC37 | CDK4 | CDK5 | CDK5R1 | CDK9 | CDKN1A | CDKN2C | CEBPA | CHEK2 | CHORDC1 | CHP1 | CHRNA9 | CIRBP | CKS1B | CKS2 | CLPX | CLU | CNDP1 | COA3 | COMMD1 | COPS5 | CRK | CRKL | CRY1 | CRY2 | CRYAB | CSNK1A1 | CSNK1E | CSNK2A2 | CSNK2B | CTBP1 | CTCF | CTNNB1 | CTSC | CUL3 | CYCS | CYLD | DAB1 | DAB2 | DAPK1 | DAXX | DBF4B | DBNDD2 | DBNL | DCUN1D1 | DDA1 | DDIT4 | DDRGK1 | DDX1 | DDX39B | DDX3X | DERL1 | DHCR24 | DHX36 | DHX9 | DIABLO | DLG3 | DLG4 | DMD | DNAJA1 | DNAJB2 | DNAJC10 | DOCK7 | DPEP1 | DUSP1 | DUSP14 | DUSP16 | DUSP19 | DUSP22 | DUSP7 | DVL1 | DVL2 | DVL3 | EDEM1 | EED | EEF2 | EGFR | EGLN3 | EHD4 | EID1 | EIF2AK3 | EIF2AK4 | EIF2B5 | EIF2S1 | EIF3A | EIF3D | EIF3E | EIF3H | EIF4A3 | EIF4E | EIF4E2 | EIF4EBP1 | EIF4ENIF1 | EIF4G3 | EIF4H | EIF5 | EIF5A | EIF5B | EIF6 | ELAVL1 | ENO1 | EP300 | EPHA4 | EPPIN | EPRS | ERBB2 | ERBB3 | ERRFI1 | EZH2 | EZR | FABP1 | FADD | FAF1 | FAM192A | FAM83D | FANCI | FANCM | FASLG | FBXO7 | FBXW11 | FGB | FGF2 | FGFR1 | FGFR1OP | FGFR3 | FIS1 | FKBP1A | FKBP1B | FKBP8 | FLOT1 | FN1 | FOXJ1 | FOXM1 | FOXO1 | FTO | FXR2 | FYN | FZR1 | G6PD | GADD45G | GAPDH | GATA1 | GDF9 | GEMIN5 | GLMN | GNA12 | GNAI2 | GNAQ | GOLGA2 | GPD1L | GPI | GPS2 | GPX1 | GRB2 | GRB7 | GRIK2 | GSK3A | GSK3B | GSTP1 | HAP1 | HAVCR2 | HAX1 | HBEGF | HDAC1 | HDAC2 | HDAC3 | HDAC4 | HDAC5 | HDAC6 | HEXIM1 | HGS | HMGB1 | HMOX1 | HNRNPD | HNRNPL | HPX | HRAS | HSF1 | HSP90AA1 | HSP90AB1 | HSP90AB3P | HSP90AB4P | HSP90B1 | HSPA1B | HSPA2 | HSPA5 | HSPB1 | HSPBP1 | HSPD1 | HSPE1 | HTRA2 | HTT | IBTK | ICAM1 | IDE | IFI16 | IFNA2 | IGBP1 | IGF1R | IGF2BP1 | IKBKB | IKBKG | ILF3 | ILK | INSR | IPO5 | IPO7 | IQGAP1 | IREB2 | IRF1 | IRF4 | ISG15 | ITCH | ITGAV | ITGB1BP1 | ITGB3 | JUN | KAT5 | KAT7 | KDM1A | KDM4D | KDR | KEAP1 | KHDRBS1 | KLHDC10 | KMT2A | KMT2B | KRAS | KSR1 | LAMP3 | LARP4 | LAT | LATS1 | LCP2 | LDLRAD4 | LEF1 | LEMD2 | LGALS3 | LGMN | LMNA | LPAR1 | LRP6 | LRPPRC | LRRK1 | LRRK2 | LTBR | MAD2L1 | MAP2K1 | MAP2K3 | MAP2K4 | MAP2K6 | MAP2K7 | MAP3K1 | MAP3K13 | MAP3K14 | MAP3K2 | MAP3K3 | MAP3K4 | MAP4K1 | MAPK1 | MAPK14 | MAPK3 | MAPK7 | MAPK8 | MAPK8IP3 | MAPK9 | MAPKAP1 | MAPT | MAS1 | MAVS | MBP | MDM2 | MECP2 | MGMT | MIF | MKNK2 | MLLT1 | MLST8 | MOB1B | MOV10 | MSN | MTA1 | MTPN | MYC | MYOC | NCBP1 | NCK1 | NCOR1 | NDUFA13 | NEDD8 | NEK1 | NELFE | NELL1 | NF2 | NFE2L2 | NFKBIA | NIPBL | NMNAT1 | NNT | NOD2 | NOS1AP | NPM1 | NQO2 | NR1H2 | NR1H3 | NTF3 | NTRK1 | NUPR1 | OGFOD1 | OGT | OTUB1 | OTUD4 | OXSR1 | P2RX1 | PA2G4 | PABPC1 | PACSIN3 | PAFAH1B1 | PAIP1 | PAK2 | PAK6 | PAPLN | PARD3 | PARD6A | PARK7 | PARP14 | PAWR | PAXIP1 | PCSK9 | PDCD10 | PDCD6 | PDE4D | PDE6G | PEBP1 | PEF1 | PELI1 | PER1 | PER2 | PHB | PHB2 | PIAS1 | PIAS3 | PIH1D1 | PIK3CA | PIK3CG | PIK3R3 | PIN1 | PINX1 | PKIG | PLA2G10 | PLA2G1B | PLA2G2A | PLA2G6 | PLAUR | PLGRKT | PLK1 | PLXNB2 | PML | PNKP | POLDIP3 | PPARG | PPARGC1A | PPIB | PPME1 | PPP1CA | PPP1R11 | PPP1R12A | PPP1R14A | PPP1R16A | PPP1R2 | PPP1R3B | PPP1R3C | PPP1R8 | PPP2CA | PPP2R1A | PPP2R1B | PPP2R2A | PPP2R2D | PPP2R5D | PPP4R2 | PPP6R1 | PRDX1 | PRDX2 | PRDX3 | PRDX5 | PRELID1 | PRKAA1 | PRKAA2 | PRKACA | PRKAG1 | PRKAR1A | PRKAR2B | PRKCD | PRKCE | PRKCZ | PRKD1 | PRKD2 | PRKDC | PRMT3 | PRMT6 | PROX1 | PRRC1 | PSAP | PSEN1 | PSMA3 | PSMB8 | PSMC1 | PSMC2 | PSMC3 | PSMC4 | PSMC5 | PSMC6 | PSMD10 | PSMD14 | PSME2 | PSME3 | PTEN | PTK2 | PTN | PTPN1 | PTPN22 | PTPN6 | PTPRR | PTTG1 | PUM1 | PUS7 | QARS | RAB1A | RAB1B | RAB3GAP1 | RAC1 | RAD23A | RAD23B | RAF1 | RANBP9 | RAP1A | RAP1B | RARA | RB1 | RB1CC1 | RBM3 | RBM4 | RBM8A | RBX1 | RC3H1 | RDX | RECK | RELA | REST | RGN | RGS2 | RHBDD1 | RHOA | RIF1 | RIPK1 | RIPK2 | RIPK3 | RMND1 | RNF139 | RNF185 | RNF19A | RPL10 | RPL23 | RPL38 | RPLP1 | RPS14 | RPS2 | RPS3 | RPS4X | RPS6KA1 | RPS6KB2 | RPTOR | RTF1 | RUVBL2 | RXRA | S100A12 | S100A7 | S100A9 | SAG | SART3 | SDC4 | SDCBP | SEMA7A | SEPT4 | SERPINB2 | SERPINB3 | SERPINB5 | SERPINB6 | SERPINB8 | SERPINE2 | SERPINH1 | SET | SFN | SGTA | SHC1 | SHMT1 | SHMT2 | SIN3A | SIRT1 | SIRT3 | SKI | SLAMF1 | SLC9A3R1 | SMAD3 | SMAD4 | SMARCB1 | SMARCC1 | SMG8 | SMURF1 | SNAI2 | SNCA | SNF8 | SNTA1 | SNW1 | SNX12 | SNX6 | SOAT1 | SOCS1 | SOD1 | SOX2 | SPI1 | SPINK7 | SPINT2 | SPOP | SPRTN | SPRY2 | SQSTM1 | SRC | STAT3 | STK11 | STK24 | STK25 | STK26 | STK3 | STK38 | STK40 | STRAP | STUB1 | STYX | SUMO1 | SUMO2 | SWAP70 | SYNJ2BP | TAB1 | TAB2 | TACO1 | TADA2B | TANK | TAOK3 | TARDBP | TCOF1 | TERF2IP | TGFB1 | TGFBR3 | THBS1 | TIA1 | TIMP1 | TIMP2 | TINF2 | TLR3 | TMTC3 | TNF | TNFAIP3 | TNFRSF1A | TNFRSF8 | TNFSF14 | TNK2 | TNP1 | TOLLIP | TP53 | TP63 | TP73 | TPR | TRADD | TRAF2 | TRAF3 | TRAF3IP1 | TRAF6 | TRAP1 | TRIAP1 | TRIB2 | TRIB3 | TRIM21 | TRIM32 | TRIP12 | TRUB2 | TSFM | TSG101 | TSPYL2 | TWF1 | TWIST1 | TXN | TYMS | U2AF2 | UBB | UBC | UBE2B | UBE2C | UBE2D1 | UBE2I | UBE2J1 | UBE2K | UBE2L3 | UBE2N | UBE2S | UBE2V1 | UBE3A | UBQLN1 | UBQLN2 | UBQLN4 | UBR5 | UBXN1 | UCHL1 | UCHL5 | UNC119 | UNK | UPF3B | UQCC1 | USP14 | USP16 | USP17L2 | USP19 | USP4 | USP47 | USP5 | USP50 | USP7 | VCP | VIM | VTN | WARS | WBP2 | WDR35 | WDR48 | WNK1 | WNT16 | XIAP | XRCC1 | XRCC5 | XRCC6 | YES1 | YTHDF1 | YWHAB | YWHAE | YWHAG | ZBTB7B | ZMPSTE24 | ZNF598 | ZNF622 | GO:0006457 | 0.0202596142181245 | 167/8243 | 107/3250 | 7.18086433352514e-11 | 6.81823068468212e-07 | T | T | T | T | protein folding | AMFR | APCS | ATF6 | BAG1 | BAG2 | BAG3 | BAG4 | BAG5 | CALR | CANX | CCT2 | CCT3 | CCT4 | CCT5 | CCT6A | CCT6B | CCT7 | CCT8 | CD74 | CDC37 | CHORDC1 | CLGN | CLPX | CLU | CRYAB | CSNK2A2 | CSNK2B | DERL1 | DNAJA1 | DNAJA2 | DNAJA3 | DNAJA4 | DNAJB1 | DNAJB11 | DNAJB2 | DNAJC10 | DNAJC19 | DNAJC7 | EMC2 | FKBP1A | FKBP1B | FKBP5 | GAK | GANAB | GNAI1 | GNAI2 | GNAI3 | GNAO1 | GNAZ | GNB1 | GNB2 | GRPEL1 | HSP90AA1 | HSP90AA5P | HSP90AB1 | HSP90AB3P | HSP90AB4P | HSP90B1 | HSPA14 | HSPA1B | HSPA1L | HSPA2 | HSPA4L | HSPA5 | HSPA8 | HSPA9 | HSPB1 | HSPBP1 | HSPD1 | HSPE1 | HSPH1 | LMAN2L | NUDC | P4HB | PDCL | PDIA3 | PDIA4 | PDIA6 | PEX19 | PFDN2 | PPIA | PPIB | PPID | PPIE | PPIF | PPIL3 | PRDX4 | PRKCSH | PSMC1 | PTGES3 | RAD23B | RIC3 | RP2 | RUVBL2 | SGTA | ST13 | TBCA | TBCE | TCP1 | TOR1A | TRAP1 | TTC1 | UBXN1 | UGGT1 | VAPA | VBP1 | VCP | GO:0009628 | 0.0780055804925391 | 643/8243 | 331/3250 | 7.83705673415515e-11 | 7.44128536908032e-07 | T | T | T | T | response to abiotic stimulus | ABAT | ACAA2 | ACADVL | ACTA1 | ACTG1 | ACTN4 | ADA | ADRB2 | ADSL | ADSS | AGER | AGTRAP | AHCY | AIFM1 | AKAP9 | AKR1B1 | AKT1 | AKT2 | ANXA1 | ANXA7 | APP | ARNT | ARNT2 | ARRB1 | ARRB2 | ASCL1 | ASNS | ATF2 | ATM | ATP1A1 | ATP2A2 | ATP6V1A | AURKB | BABAM1 | BACE1 | BAG3 | BAX | BCL10 | BCL2 | BCL2L1 | BDKRB2 | BIRC2 | BRAF | BRCA1 | CAB39 | CABP4 | CAPN2 | CASP2 | CASP3 | CASP8 | CASP9 | CAT | CAV1 | CBFA2T3 | CBL | CCAR2 | CCNB1 | CCR4 | CD40 | CDC25A | CDK4 | CDK5 | CDKN1A | CHEK2 | CHP1 | CHRNA9 | CIRBP | CLPB | COL18A1 | CREB1 | CREBBP | CRIP1 | CRNN | CRY1 | CRY2 | CRYAB | CUL4B | CYP1A1 | DAXX | DBH | DCN | DDB1 | DDB2 | DDIT4 | DDX3X | DHX36 | DMD | DNAJA1 | DNAJA2 | DNAJA3 | DNAJA4 | DNM1L | DUSP1 | E2F1 | EEF1A2 | EEF1D | EGFR | EGLN1 | EGLN3 | EIF2AK3 | EIF2AK4 | EIF2B1 | EIF2B3 | EIF2B5 | EIF2S1 | EIF4EBP1 | ENDOG | EP300 | ERCC5 | ERRFI1 | ETS1 | FABP1 | FADD | FANCG | FECH | FEN1 | FIGNL1 | FIS1 | FKBPL | FOS | FOXO1 | FYN | GMPR | GNAQ | GNB1 | GOT1 | GPI | GPX1 | GRB2 | GRK1 | H2AFX | HDAC2 | HDAC4 | HIF1A | HK2 | HMOX1 | HNRNPD | HRAS | HSF1 | HSF4 | HSP90AA1 | HSP90B1 | HSPA14 | HSPA1B | HSPA1L | HSPA2 | HSPA5 | HSPA8 | HSPA9 | HSPD1 | HTR1B | HTRA2 | ICAM1 | ID2 | IFI16 | IGFBP7 | IREB2 | IRF1 | ITPR1 | ITPR2 | JUN | JUNB | JUND | JUP | KAT5 | KDM1A | KDM4D | KRAS | LDHA | LGMN | LIG4 | LMNA | LOXL2 | LPAR1 | LTBR | MAP2K4 | MAP2K7 | MAP3K1 | MAP3K14 | MAP3K2 | MAP3K4 | MAPK14 | MAPK3 | MAPK8 | MAPK9 | MAPT | MC1R | MDM2 | MDM4 | MECP2 | METRNL | MGMT | MICA | MME | MSH6 | MTA1 | MTPN | MYC | MYD88 | NAMPT | NDRG1 | NEDD4 | NFKBIA | NIPBL | NOS2 | NOS3 | NPEPPS | NPM1 | NQO1 | NTRK1 | OPA1 | OXSR1 | P2RX2 | P4HB | PARP1 | PAXIP1 | PCNA | PDCD6 | PDE6G | PEBP1 | PER1 | PER2 | PGK1 | PHB2 | PIAS1 | PICK1 | PIK3R1 | PKM | PLOD2 | PML | PNKP | POLD1 | POLE3 | PPARD | PPARG | PPARGC1A | PPID | PPM1D | PPP1CA | PPP1CB | PPP1CC | PRKAA1 | PRKACA | PRKCE | PRKDC | PSEN2 | PTEN | PTN | PTPRK | RAB11B | RAC1 | RAD51 | RAD51AP1 | RAF1 | RBM3 | RBM4 | RDH13 | RELA | REST | RHBDD1 | RHO | RHOA | RNF8 | RRM1 | RUVBL2 | S100A13 | S100B | SAG | SCARA3 | SCEL | SCFD1 | SERPINB6 | SERPINE2 | SIPA1 | SIRT1 | SLC12A2 | SLC9A1 | SMAD3 | SMAD4 | SMC1A | SNAI2 | SOD1 | SORD | SPRTN | SRC | STAC | STAT1 | STK11 | STUB1 | SUMO1 | TANK | TGFB1 | TGFBR3 | THBS1 | THRA | TLR3 | TM9SF4 | TNF | TNFRSF1A | TNFRSF8 | TNFSF14 | TP53 | TPR | TREX1 | TRIAP1 | TRIM32 | TRIM63 | TSC22D3 | TSC22D4 | TWIST1 | TXN | TXN2 | TXNIP | UBE2A | UBE2B | UBQLN1 | UNC119 | USP2 | USP28 | USP47 | USP53 | VCAM1 | VCP | VGF | XRCC1 | XRCC5 | XRCC6 | YAP1 | YWHAE | YY1 | ZMPSTE24 | GO:1901700 | 0.101540701201019 | 837/8243 | 417/3250 | 8.20771940406547e-11 | 7.79322957416016e-07 | T | T | T | T | response to oxygen-containing compound | ABAT | ABL1 | ABL2 | ACACA | ACSL1 | ACTB | ACTR2 | ADA | ADCY6 | ADCYAP1 | ADH5 | ADRB2 | AGER | AICDA | AIFM1 | AKAP8 | AKAP9 | AKR1B1 | AKR1C2 | AKT1 | AKT2 | ANXA1 | ANXA5 | APEX1 | APLP1 | APOD | APOE | APP | APRT | AR | ARNT2 | ARPC1B | ASCL1 | ASNS | ASS1 | ATF2 | ATM | ATP1A1 | ATP2A2 | BACE1 | BCL11A | BCL2 | BCL2L1 | BIRC2 | BRAF | BRCA1 | BSG | BTK | CACNA1A | CACNG4 | CALM1 | CALM3 | CALR | CAPN2 | CARD9 | CARM1 | CASP3 | CASP8 | CASP9 | CAT | CAV1 | CBL | CBX8 | CCL21 | CCNB1 | CCR5 | CD27 | CD36 | CD40 | CD6 | CDC5L | CDC73 | CDH1 | CDK19 | CDK2 | CDK4 | CDK5 | CDKN1A | CEBPE | CFL1 | CFTR | CHEK2 | CLDN18 | COL18A1 | COL4A1 | COL6A1 | COL6A2 | COMT | CPT1A | CREB1 | CRK | CRY1 | CRYAB | CSN1S1 | CSNK2B | CTNNB1 | CTSV | CXCL11 | CXCL5 | CXCL6 | CXCL8 | CYC1 | CYP1A1 | CYP1A2 | DAPK1 | DCN | DDIT4 | DENND4C | DHX36 | DMAP1 | DNM1L | DPEP1 | DUSP1 | E2F1 | EEF2 | EFTUD2 | EGFR | EGLN1 | EIF2B1 | EIF2B3 | EIF2B5 | EIF4E | EIF4EBP1 | EIF6 | ENDOG | EPHA4 | EPRS | ERRFI1 | ESR1 | ESRRA | ETS1 | EZH2 | EZR | FABP1 | FABP3 | FASLG | FBXO32 | FECH | FIS1 | FKBP1B | FOLR1 | FOS | FOXA1 | FOXO1 | FYN | G6PD | GGT1 | GLP1R | GLRB | GLUL | GNAI1 | GNAO1 | GNB1 | GNG12 | GOT1 | GOT2 | GPD1 | GPI | GPX1 | GPX4 | GRAMD1A | GSK3A | GSK3B | GSS | GSTP1 | H2AFZ | HADH | HAVCR2 | HCN1 | HCN2 | HCN3 | HDAC2 | HDAC5 | HDAC6 | HDAC9 | HID1 | HLCS | HMGB1 | HMGB2 | HMGCL | HMOX1 | HNF4A | HNRNPD | HNRNPL | HSF1 | HSP90B1 | HSPA5 | HTR1B | HTR3A | HTRA2 | ICAM1 | IFNAR1 | IGF1R | IGFBP7 | INSIG1 | INSIG2 | INSR | IPO5 | IQGAP1 | IREB2 | ITGA4 | ITPR2 | JUN | JUNB | JUND | JUP | KAT5 | KAT7 | KDM1A | KLF5 | KLHL22 | LARS | LDHA | LGALS1 | LGMN | LPAR1 | LPIN3 | LRP6 | LRRK2 | MAP2K4 | MAP2K6 | MAP4K1 | MAPK1 | MAPK14 | MAPK3 | MAPK7 | MAPK8 | MAPK9 | MAPT | MBD3 | MDM2 | ME1 | MGMT | MGST1 | MSN | MTAP | MYO5A | NAMPT | NCOA1 | NCOA2 | NCOA3 | NDUFA13 | NDUFA9 | NEUROD1 | NFE2L2 | NFKB2 | NFKBIA | NME1 | NME2 | NOD2 | NOS2 | NOS3 | NQO1 | NR0B2 | NR1H2 | NR1H3 | NR3C1 | NR4A1 | NTRK1 | OGT | OPA1 | OTC | OTUD5 | OXCT1 | P2RX1 | P2RX2 | P2RX4 | PARK7 | PARP1 | PCNA | PCSK9 | PDCD10 | PDE4D | PDK2 | PEBP1 | PELI1 | PER1 | PFKL | PHB2 | PIK3CA | PIK3CG | PIK3R1 | PKM | PLA2G1B | PPARD | PPARG | PPARGC1A | PPAT | PPIF | PPP2CB | PPP5C | PRDX2 | PRDX3 | PRDX5 | PRKAA1 | PRKAA2 | PRKACA | PRKAR1A | PRKAR2B | PRKCD | PRKCE | PRKCZ | PRKD1 | PRKDC | PRPF8 | PSEN1 | PSMD14 | PTEN | PTGES | PTGIR | PTN | PTPN22 | PTPRK | PXN | QDPR | RAB10 | RAB31 | RAD51 | RANGAP1 | RAP1A | RAP1B | RARA | RARG | RBBP4 | RELA | RGS2 | RHOA | RIPK1 | RIPK2 | RIPK3 | ROMO1 | RPL10A | RPL23 | RPS3 | RPTOR | RUVBL2 | RXRA | S100A7 | SARM1 | SDC1 | SELL | SETDB1 | SHMT1 | SHMT2 | SIN3A | SIPA1 | SIRT1 | SLC2A5 | SLC9A1 | SMAD2 | SMO | SNCA | SNW1 | SNX6 | SOCS1 | SOCS2 | SOD1 | SOD2 | SORT1 | SP1 | SPI1 | SRC | SRSF5 | STAT1 | STAT3 | STAT6 | STK24 | STK25 | STK26 | STXBP1 | SULT1A3 | TBC1D4 | TBXA2R | TDO2 | TGFB1 | TGFBR3 | THBS1 | TIMP1 | TMBIM6 | TNF | TNFAIP3 | TNIP2 | TOMM20 | TP53 | TPCN2 | TPM1 | TRAF2 | TRAF6 | TREX1 | TRIB3 | TRIM25 | TRIM41 | TUFM | TWF2 | TXN2 | TXNIP | TYMS | UBE3A | USP8 | VCAM1 | VCP | VGF | VIM | WBP2 | WDR35 | WDTC1 | WNT2 | WT1 | XBP1 | XRCC1 | XRCC5 | YAP1 | YES1 | YWHAG | YY1 | ZBTB7B | ZNF277 | GO:0032268 | 0.193618828096567 | 1596/8243 | 742/3250 | 9.92209802913034e-11 | 9.42103207865926e-07 | T | T | T | T | regulation of cellular protein metabolic process | A2M | AARS | ABL1 | ACO1 | ACSL1 | ACTB | ADCY6 | ADCYAP1 | ADORA2A | ADRB2 | ADRM1 | ADTRP | AGER | AGO1 | AGO2 | AHSG | AIFM1 | AIMP2 | AIRE | AKAP8 | AKAP9 | AKT1 | AKT2 | AKTIP | ALOX12 | ANXA2 | APC | APOA1 | APOE | APP | AR | ARAF | ARNT | ARR3 | ARRB1 | ARRB2 | ASPSCR1 | ATG5 | ATM | ATP13A2 | ATRX | ATXN2 | AXIN1 | AZU1 | B3GAT3 | BAG2 | BAG4 | BAG5 | BAG6 | BAX | BBC3 | BCAR3 | BCL10 | BCL2 | BCL2L13 | BCL6 | BCOR | BDKRB2 | BIN1 | BIRC2 | BIRC3 | BIRC5 | BIRC7 | BIRC8 | BMI1 | BMPR1A | BRAF | BRCA1 | BRD4 | BTRC | C1QBP | C1QL4 | C1QTNF1 | C4A | CAB39 | CALM1 | CALM2 | CALM3 | CALR | CARD9 | CASP10 | CASP3 | CASP8 | CASP9 | CAV1 | CBFA2T3 | CBL | CBLB | CBLC | CCAR2 | CCDC22 | CCDC88A | CCL21 | CCL22 | CCNB1 | CCNT1 | CCNT2 | CCNY | CCNYL1 | CCR1 | CD109 | CD27 | CD36 | CD40 | CD40LG | CD74 | CD81 | CDC14B | CDC20 | CDC25A | CDC25B | CDC25C | CDC37 | CDK4 | CDK5 | CDK5R1 | CDK9 | CDKN1A | CDKN2C | CEBPA | CHEK2 | CHORDC1 | CHP1 | CHRNA9 | CIRBP | CKS1B | CKS2 | CLPX | CLU | CNDP1 | COA3 | COMMD1 | COPS5 | CRK | CRKL | CRY1 | CRY2 | CRYAB | CSNK1A1 | CSNK1E | CSNK2A2 | CSNK2B | CTBP1 | CTCF | CTNNB1 | CTSC | CUL3 | CYCS | CYLD | DAB1 | DAB2 | DAPK1 | DAXX | DBF4B | DBNDD2 | DBNL | DCUN1D1 | DDA1 | DDIT4 | DDRGK1 | DDX1 | DDX39B | DDX3X | DERL1 | DHCR24 | DHX36 | DHX9 | DIABLO | DLG3 | DLG4 | DMD | DNAJA1 | DNAJB2 | DNAJC10 | DOCK7 | DPEP1 | DUSP1 | DUSP14 | DUSP16 | DUSP19 | DUSP22 | DUSP7 | DVL1 | DVL2 | DVL3 | EDEM1 | EED | EEF2 | EGFR | EGLN3 | EHD4 | EID1 | EIF2AK3 | EIF2AK4 | EIF2B5 | EIF2S1 | EIF3A | EIF3D | EIF3E | EIF3H | EIF4A3 | EIF4E | EIF4E2 | EIF4EBP1 | EIF4ENIF1 | EIF4G3 | EIF4H | EIF5 | EIF5A | EIF5B | EIF6 | ELAVL1 | ENO1 | EP300 | EPHA4 | EPPIN | EPRS | ERBB2 | ERBB3 | ERRFI1 | EZH2 | EZR | FABP1 | FADD | FAF1 | FAM192A | FAM83D | FANCI | FANCM | FASLG | FBXO7 | FBXW11 | FGB | FGF2 | FGFR1 | FGFR1OP | FGFR3 | FIS1 | FKBP1A | FKBP1B | FKBP8 | FLOT1 | FN1 | FOXM1 | FOXO1 | FTO | FXR2 | FYN | FZR1 | G6PD | GADD45G | GAPDH | GATA1 | GDF9 | GEMIN5 | GLMN | GNA12 | GNAI2 | GNAQ | GOLGA2 | GPD1L | GPI | GPS2 | GPX1 | GRB2 | GRB7 | GRIK2 | GSK3A | GSK3B | GSTP1 | HAVCR2 | HAX1 | HBEGF | HDAC2 | HDAC3 | HDAC4 | HDAC5 | HDAC6 | HEXIM1 | HGS | HMGB1 | HNRNPD | HNRNPL | HPX | HRAS | HSF1 | HSP90AA1 | HSP90AB1 | HSP90AB3P | HSP90AB4P | HSP90B1 | HSPA1B | HSPA2 | HSPA5 | HSPB1 | HSPBP1 | HSPD1 | HSPE1 | HTRA2 | HTT | IBTK | ICAM1 | IDE | IFI16 | IFNA2 | IGBP1 | IGF1R | IGF2BP1 | IKBKB | IKBKG | ILF3 | ILK | INSR | IPO5 | IPO7 | IQGAP1 | IREB2 | ISG15 | ITCH | ITGB1BP1 | ITGB3 | JUN | KAT5 | KAT7 | KDM1A | KDM4D | KDR | KEAP1 | KHDRBS1 | KLHDC10 | KMT2A | KMT2B | KRAS | KSR1 | LAMP3 | LARP4 | LAT | LATS1 | LCP2 | LDLRAD4 | LEF1 | LEMD2 | LGALS3 | LGMN | LMNA | LPAR1 | LRP6 | LRPPRC | LRRK1 | LRRK2 | LTBR | MAD2L1 | MAP2K1 | MAP2K3 | MAP2K4 | MAP2K6 | MAP2K7 | MAP3K1 | MAP3K13 | MAP3K14 | MAP3K2 | MAP3K3 | MAP3K4 | MAP4K1 | MAPK1 | MAPK14 | MAPK3 | MAPK8 | MAPK8IP3 | MAPK9 | MAPKAP1 | MAPT | MAS1 | MAVS | MBP | MDM2 | MECP2 | MIF | MKNK2 | MLLT1 | MLST8 | MOB1B | MOV10 | MSN | MTA1 | MTPN | MYC | MYOC | NCBP1 | NCK1 | NCOR1 | NDUFA13 | NEK1 | NELFE | NELL1 | NF2 | NFE2L2 | NFKBIA | NIPBL | NMNAT1 | NNT | NOD2 | NOS1AP | NPM1 | NQO2 | NR1H2 | NR1H3 | NTF3 | NTRK1 | NUPR1 | OGFOD1 | OGT | OTUB1 | OTUD4 | OXSR1 | P2RX1 | PA2G4 | PABPC1 | PACSIN3 | PAFAH1B1 | PAIP1 | PAK2 | PAK6 | PAPLN | PARD3 | PARD6A | PARK7 | PARP14 | PAXIP1 | PCSK9 | PDCD10 | PDCD6 | PDE4D | PDE6G | PEBP1 | PEF1 | PELI1 | PER1 | PER2 | PHB | PHB2 | PIAS1 | PIAS3 | PIH1D1 | PIK3CA | PIK3CG | PIK3R3 | PIN1 | PINX1 | PKIG | PLA2G10 | PLA2G1B | PLA2G2A | PLA2G6 | PLAUR | PLGRKT | PLK1 | PLXNB2 | PML | PNKP | POLDIP3 | PPARG | PPARGC1A | PPIB | PPME1 | PPP1CA | PPP1R11 | PPP1R12A | PPP1R14A | PPP1R16A | PPP1R2 | PPP1R3B | PPP1R3C | PPP1R8 | PPP2CA | PPP2R1A | PPP2R1B | PPP2R2A | PPP2R2D | PPP2R5D | PPP4R2 | PPP6R1 | PRDX1 | PRDX2 | PRDX3 | PRDX5 | PRELID1 | PRKAA1 | PRKAA2 | PRKACA | PRKAG1 | PRKAR1A | PRKAR2B | PRKCD | PRKCE | PRKCZ | PRKD1 | PRKD2 | PRKDC | PRMT3 | PRMT6 | PROX1 | PRRC1 | PSAP | PSEN1 | PSMC1 | PSMC2 | PSMC3 | PSMC4 | PSMC5 | PSMC6 | PSMD10 | PSMD14 | PSME2 | PSME3 | PTEN | PTK2 | PTN | PTPN1 | PTPN22 | PTPN6 | PTPRR | PTTG1 | PUM1 | PUS7 | QARS | RAB1A | RAB3GAP1 | RAC1 | RAD23A | RAD23B | RAF1 | RANBP9 | RAP1A | RAP1B | RARA | RB1 | RB1CC1 | RBM3 | RBM4 | RBM8A | RBX1 | RC3H1 | RDX | RECK | RELA | REST | RGN | RGS2 | RHBDD1 | RHOA | RIF1 | RIPK1 | RIPK2 | RIPK3 | RMND1 | RNF139 | RNF185 | RNF19A | RPL10 | RPL23 | RPL38 | RPLP1 | RPS14 | RPS2 | RPS3 | RPS4X | RPS6KA1 | RPS6KB2 | RPTOR | RTF1 | RUVBL2 | RXRA | S100A12 | S100A7 | S100A9 | SAG | SART3 | SDC4 | SDCBP | SEMA7A | SEPT4 | SERPINB2 | SERPINB3 | SERPINB5 | SERPINB6 | SERPINB8 | SERPINE2 | SERPINH1 | SET | SFN | SGTA | SHC1 | SHMT1 | SHMT2 | SIN3A | SIRT1 | SIRT3 | SKI | SLAMF1 | SLC9A3R1 | SMAD3 | SMAD4 | SMARCB1 | SMARCC1 | SMG8 | SMURF1 | SNAI2 | SNCA | SNF8 | SNTA1 | SNW1 | SNX12 | SNX6 | SOCS1 | SOD1 | SOX2 | SPI1 | SPINK7 | SPINT2 | SPRTN | SPRY2 | SQSTM1 | SRC | STAT3 | STK11 | STK24 | STK25 | STK26 | STK3 | STK38 | STK40 | STRAP | STUB1 | STYX | SUMO1 | SUMO2 | SWAP70 | SYNJ2BP | TAB1 | TAB2 | TACO1 | TADA2B | TANK | TAOK3 | TARDBP | TCOF1 | TERF2IP | TGFB1 | TGFBR3 | THBS1 | TIA1 | TIMP1 | TIMP2 | TINF2 | TLR3 | TMTC3 | TNF | TNFAIP3 | TNFRSF1A | TNFSF14 | TNK2 | TNP1 | TOLLIP | TP53 | TP73 | TPR | TRADD | TRAF2 | TRAF3IP1 | TRAF6 | TRAP1 | TRIAP1 | TRIB2 | TRIB3 | TRIM21 | TRIM32 | TRIP12 | TRUB2 | TSFM | TSG101 | TSPYL2 | TWF1 | TWIST1 | TXN | TYMS | U2AF2 | UBB | UBC | UBE2B | UBE2C | UBE2D1 | UBE2I | UBE2J1 | UBE2K | UBE2L3 | UBE2N | UBE2S | UBE2V1 | UBE3A | UBQLN1 | UBQLN2 | UBQLN4 | UBR5 | UBXN1 | UCHL1 | UCHL5 | UNC119 | UNK | UPF3B | UQCC1 | USP14 | USP16 | USP17L2 | USP19 | USP4 | USP47 | USP5 | USP50 | USP7 | VCP | VIM | VTN | WARS | WBP2 | WDR35 | WDR48 | WNK1 | WNT16 | XIAP | XRCC1 | XRCC5 | XRCC6 | YES1 | YTHDF1 | YWHAB | YWHAE | YWHAG | ZBTB7B | ZMPSTE24 | ZNF598 | ZNF622 | GO:1901576 | 0.182821788183914 | 1507/8243 | 703/3250 | 1.73517362197048e-10 | 1.64754735406097e-06 | T | T | T | T | organic substance biosynthetic process | AASDHPPT | AASS | ABAT | ABCD3 | ACAA2 | ACACA | ACAT1 | ACAT2 | ACBD3 | ACCS | ACE2 | ACLY | ACOT7 | ACSL1 | ACSL4 | ACSL5 | ACSS2 | ADA | ADCY6 | ADK | ADRM1 | ADSL | ADSS | AFF4 | AGK | AGL | AGO2 | AK1 | AK2 | AK4 | AKR1A1 | AKR1B1 | AKR1B15 | AKT1 | AKT2 | ALAS1 | ALDH7A1 | ALDH8A1 | ALDH9A1 | ALDOA | ALDOC | ALOX12 | ALOX5 | ALOX5AP | AMD1 | AMDHD2 | APOA1 | APOE | APRT | AR | ARF1 | ARHGAP35 | ARNT | ARRB1 | ARRB2 | ASL | ASNS | ASPDH | ASS1 | ATAD5 | ATF6B | ATIC | ATM | ATOH1 | B3GAT3 | B3GNT3 | B4GALT3 | BAX | BCAT2 | BCL10 | BDH1 | BRCA1 | CBFB | CBR4 | CBS | CCDC88A | CCNB1 | CCNT1 | CCNT2 | CD74 | CDA | CDC73 | CDK2 | CDK4 | CDK8 | CDK9 | CDKN1A | CEBPA | CEBPE | CENPF | CES1 | CFTR | CHAF1A | CHDH | CHRAC1 | CKM | CMPK1 | CNDP2 | COL4A2 | COL4A3BP | COL5A1 | COPS2 | COPS5 | COQ2 | COQ3 | COQ4 | COQ5 | COQ6 | COQ7 | COQ9 | COX15 | CPNE3 | CPSF7 | CREB1 | CREBBP | CRY1 | CSNK2A2 | CSNK2B | CSTF2T | CTDP1 | CTH | CTPS1 | CYB5R1 | CYB5R3 | CYC1 | CYP1A1 | CYP1A2 | DAP3 | DARS | DBH | DCAKD | DCK | DCN | DCTPP1 | DCXR | DEGS1 | DERA | DGKE | DGKZ | DGUOK | DHCR24 | DHRS7B | DHX9 | DIDO1 | DLAT | DLD | DMD | DNA2 | DNAJA3 | DNAJC30 | DPM1 | DRG2 | DSE | DTX1 | DUT | DVL1 | DVL2 | E2F1 | E2F2 | E2F3 | EAF2 | ECD | EEF1A1 | EEF1A1P5 | EEF1A2 | EEF1B2 | EEF1D | EEF1G | EEF2 | EGFR | EIF4H | EIF5A | ELF3 | ENO1 | ENO2 | ENO3 | ENOPH1 | ERAL1 | ESD | ESR1 | ESR2 | ESRRA | ESRRB | ESRRG | ETS1 | EXTL2 | FABP5 | FAM20B | FAR2 | FARSB | FASN | FBP1 | FBP2 | FDPS | FDXR | FECH | FEN1 | FGF2 | FOLH1 | FOS | FPGS | FUBP1 | FUBP3 | G6PD | GAPDH | GAPDHS | GAR1 | GARS | GART | GATA1 | GBE1 | GCDH | GDA | GEMIN5 | GFPT1 | GFPT2 | GGCT | GGPS1 | GGT1 | GGTLC1 | GLCE | GLS | GLS2 | GLUD2 | GLUL | GMDS | GMPPB | GMPR | GMPS | GNPDA1 | GNPDA2 | GNPNAT1 | GOLPH3 | GOT1 | GOT1L1 | GOT2 | GPC5 | GPD1 | GPD1L | GPI | GPT2 | GPX1 | GPX4 | GSPT1 | GSPT2 | GSS | GSTA1 | GSTA2 | GSTA3 | GSTA4 | GSTK1 | GSTM4 | GSTO1 | GSTO2 | GSTP1 | GSTT1 | GSTT2B | GSTZ1 | GTF2A1 | GTF2E2 | GTF2F1 | GTF2F2 | GTF2I | GTF3C2 | GYG1 | GYG2 | GYS1 | HAAO | HAGH | HARS | HEXA | HEXB | HIF1A | HINT1 | HIST1H4A | HMBS | HMGCL | HNF4A | HNF4G | HOXB9 | HPRT1 | HS2ST1 | HS6ST1 | HSD17B14 | HSD17B4 | HSD17B6 | HSD17B8 | HSD3B7 | HSF1 | HSP90AA1 | HSP90AB1 | HSPA14 | HSPG2 | IDH2 | IDI2 | ILF2 | IMPA1 | IMPA2 | IMPDH2 | ING5 | INSIG1 | INSIG2 | IP6K1 | IREB2 | IRF1 | IRF7 | ISG15 | ISYNA1 | ITPA | JUN | KAT5 | KAT7 | KDSR | KLF5 | KMT2A | KYNU | LARP4 | LCMT2 | LDHC | LEF1 | LIG4 | LMO2 | LPIN3 | LTC4S | LUM | MAPK1 | MAPK3 | MC1R | MCAT | MCM2 | MCM3 | MCM4 | MCM5 | MCM6 | MCM7 | MCM9 | MCMBP | MDH1 | MDH2 | ME1 | MECP2 | MED1 | MED12 | MED14 | MED17 | MED20 | MED21 | MED23 | MED24 | MED31 | MED4 | MED6 | MGST1 | MIF | MITF | MLLT1 | MLLT3 | MMAB | MOCOS | MPC2 | MPI | MPPE1 | MRPL12 | MRPL14 | MRPL19 | MRPL28 | MRPL38 | MRPL4 | MRPL43 | MRPL45 | MRPL46 | MRPL9 | MRPS5 | MRRF | MTAP | MTERF1 | MTHFD1 | MTMR14 | MTMR7 | MTRR | MVD | MYO5A | NAGK | NAMPT | NANP | NANS | NAP1L1 | NAPRT | NCBP1 | NCOA1 | NCOR1 | NDUFA9 | NELFB | NELFCD | NELFE | NFATC1 | NFE2L2 | NFS1 | NME1 | NME2 | NME4 | NMNAT1 | NMNAT3 | NMRAL1 | NMRK2 | NOP10 | NPLOC4 | NPR1 | NR0B2 | NR1H2 | NR1H3 | NR1I2 | NR3C1 | NR4A1 | NR5A2 | NRBF2 | NT5C2 | NUDT18 | NUDT3 | NUDT9 | NUP98 | OAT | OAZ3 | OSBPL1A | OTC | OXA1L | P2RX1 | PABPC4 | PAH | PAICS | PAPSS1 | PAPSS2 | PARK7 | PARP1 | PAWR | PAX8 | PC | PCBD1 | PCF11 | PCNA | PCTP | PCYT2 | PDE4D | PDF | PDHA1 | PDHA2 | PDHB | PDHX | PDSS1 | PDSS2 | PDXK | PER2 | PFAS | PGAM1 | PGAM2 | PGD | PGK1 | PGK2 | PGM1 | PGM2 | PGM3 | PGP | PHB | PHGDH | PHOSPHO1 | PI4K2A | PIGU | PIK3C2A | PIK3CA | PIK3CG | PIK3R1 | PIK3R3 | PIKFYVE | PINX1 | PIP4K2A | PIR | PLA2G10 | PLA2G12A | PLA2G1B | PLA2G2A | PLA2G6 | PLCG1 | PLOD2 | PLSCR1 | PNKP | PNP | POLA2 | POLD1 | POLE2 | POLE3 | POLM | POLR2A | POLR2E | POLR2L | POLR2M | POT1 | POU5F1 | PPARD | PPARG | PPARGC1A | PPAT | PPIA | PPM1D | PPOX | PPP1R3C | PPP5C | PPT1 | PRKAA1 | PRKAA2 | PRKAB2 | PRKAG1 | PRKD1 | PRKD2 | PRKD3 | PRPF19 | PRTFDC1 | PSAT1 | PTEN | PTGDS | PTGES | PTGES3 | PTMA | PTMS | PTPMT1 | PTTG1 | PYCR1 | PYCR2 | PYGL | QDPR | RAB5A | RARA | RARG | RARS2 | RBBP4 | RBBP6 | RBMX | RBP1 | RBPJ | RELA | REPIN1 | RFC1 | RFC2 | RFC3 | RFC4 | RFC5 | RFK | RGN | RMND1 | RNASEH2A | RPA2 | RPA3 | RPAIN | RPL10 | RPL10A | RPL12 | RPL18A | RPL22 | RPL23 | RPL23A | RPL24 | RPL30 | RPL36AL | RPL37A | RPL38 | RPL6 | RPL7 | RPL7A | RPL9 | RPLP0 | RPLP0P6 | RPLP1 | RPLP2 | RPS12 | RPS13 | RPS14 | RPS15A | RPS19 | RPS2 | RPS20 | RPS24 | RPS29 | RPS3 | RPS4X | RPS6KB2 | RPS8 | RRM1 | RRM2 | RRM2B | RTF1 | RUNX2 | RXRA | RXRB | RXRG | SARS | SAT1 | SCAF1 | SCCPDH | SDC1 | SDC4 | SEPHS1 | SEPHS2 | SET | SGPL1 | SHMT1 | SHMT2 | SIN3A | SIRT1 | SIRT7 | SKI | SLC25A1 | SLC25A10 | SLC25A11 | SLC25A13 | SMAD4 | SMG5 | SMG6 | SMN1 | SNCA | SNRPB | SNRPD3 | SNRPE | SNW1 | SORD | SP1 | SPI1 | SPIN1 | SPR | SPRTN | SPTLC2 | ST3GAL1 | ST3GAL6 | ST6GALNAC5 | ST8SIA4 | STARD7 | STAT3 | STOML2 | SUCLA2 | SUPT4H1 | SUPT5H | TAF12 | TAF15 | TAF1A | TALDO1 | TAMM41 | TARDBP | TARS | TAZ | TCEA1 | TCEA2 | TCERG1 | TCF4 | TECR | TERF1 | TERF2 | TERF2IP | TFCP2 | TGFB1 | THRA | TK1 | TK2 | TKT | TNF | TOP1 | TOP3A | TP53 | TPI1 | TREX1 | TRIM25 | TRIM28 | TRIM29 | TRIP13 | TSC22D1 | TSFM | TUFM | TYMS | UAP1 | UAP1L1 | UBB | UBC | UBE2L6 | UBIAD1 | UCK2 | UGDH | UGP2 | UGT8 | UMPS | UPP2 | USPL1 | UXS1 | VAC14 | VAPA | VAPB | VCAN | VCP | VDR | WARS | XBP1 | XYLT1 | YAP1 | ZBTB7A | ZBTB7B | ZNF217 | ZNF598 | GO:0051247 | 0.120708479922358 | 995/8243 | 484/3250 | 1.99257798730313e-10 | 1.89195279894432e-06 | T | T | T | T | positive regulation of protein metabolic process | ABL1 | ACSL1 | ADCY6 | ADCYAP1 | ADRB2 | ADRM1 | ADTRP | AGER | AIFM1 | AIMP2 | AKAP8 | AKAP9 | AKT1 | AKT2 | AKTIP | ALOX12 | ANXA2 | APOE | APP | AR | ARAF | ARNT | ARRB1 | ARRB2 | ASPSCR1 | ATM | AXIN1 | AZU1 | BAG2 | BAG4 | BAG6 | BAX | BBC3 | BCAR3 | BCL10 | BCL2 | BCL2L13 | BCL6 | BIRC2 | BIRC3 | BIRC7 | BMI1 | BMPR1A | BRAF | BRCA1 | BRD4 | BTRC | C1QBP | C1QTNF1 | CAB39 | CALM1 | CALM2 | CALM3 | CARD9 | CASP10 | CASP3 | CASP8 | CASP9 | CAV1 | CBFA2T3 | CCDC22 | CCDC88A | CCL21 | CCL22 | CCNB1 | CCNT1 | CCNT2 | CCNY | CCNYL1 | CCR1 | CD27 | CD36 | CD40 | CD40LG | CD74 | CD81 | CDC14B | CDC20 | CDC25B | CDK4 | CDK5 | CDK5R1 | CDK9 | CDKN1A | CEBPA | CHEK2 | CHP1 | CIRBP | CKS1B | CKS2 | CLPX | CLU | COA3 | COMMD1 | CRK | CRKL | CSNK1A1 | CSNK1E | CSNK2B | CTBP1 | CTNNB1 | CTSC | CUL3 | CYCS | DAB1 | DAB2 | DAPK1 | DAXX | DBF4B | DBNL | DCUN1D1 | DDRGK1 | DDX39B | DDX3X | DERL1 | DHX36 | DHX9 | DIABLO | DLG3 | DLG4 | DNAJB2 | DOCK7 | DUSP19 | DUSP22 | DUSP7 | DVL1 | DVL2 | DVL3 | EDEM1 | EED | EEF2 | EGFR | EGLN3 | EHD4 | EIF2AK3 | EIF2AK4 | EIF2B5 | EIF3D | EIF3E | EIF4A3 | EIF5A | EIF6 | ELAVL1 | ENO1 | EPHA4 | ERBB2 | ERBB3 | EZH2 | EZR | FADD | FAF1 | FANCI | FANCM | FASLG | FBXW11 | FGB | FGF2 | FGFR1 | FGFR3 | FIS1 | FKBP1A | FLOT1 | FN1 | FXR2 | FYN | FZR1 | GADD45G | GATA1 | GDF9 | GLMN | GNAI2 | GOLGA2 | GSK3A | GSK3B | HAVCR2 | HAX1 | HBEGF | HDAC2 | HDAC3 | HDAC4 | HDAC6 | HMGB1 | HMOX1 | HNRNPD | HNRNPL | HPX | HRAS | HSF1 | HSP90AA1 | HSP90AB1 | HSPA1B | HSPA2 | HSPA5 | HSPB1 | HSPBP1 | HSPD1 | HSPE1 | HTRA2 | ICAM1 | IFI16 | IFNA2 | IGF1R | IKBKG | ILK | INSR | IQGAP1 | IRF1 | IRF4 | ITGB1BP1 | ITGB3 | JUN | KAT5 | KAT7 | KDM1A | KDR | KEAP1 | KHDRBS1 | KLHDC10 | KMT2A | KRAS | KSR1 | LARP4 | LAT | LATS1 | LCP2 | LGMN | LMNA | LPAR1 | LRRK1 | LRRK2 | LTBR | MAP2K1 | MAP2K3 | MAP2K4 | MAP2K6 | MAP2K7 | MAP3K1 | MAP3K13 | MAP3K14 | MAP3K2 | MAP3K3 | MAP3K4 | MAP4K1 | MAPK1 | MAPK14 | MAPK3 | MAPK7 | MAPK8 | MAPK8IP3 | MAPK9 | MAPKAP1 | MAPT | MAS1 | MAVS | MBP | MDM2 | MECP2 | MIF | MLST8 | MSN | MTA1 | MTPN | MYC | NCK1 | NDUFA13 | NEK1 | NELFE | NFE2L2 | NFKBIA | NIPBL | NMNAT1 | NOD2 | NOS1AP | NPM1 | NQO2 | NR1H2 | NR1H3 | NTF3 | NTRK1 | NUPR1 | OGT | OXSR1 | P2RX1 | PABPC1 | PACSIN3 | PAIP1 | PAK2 | PAK6 | PARK7 | PARP14 | PAWR | PAXIP1 | PCSK9 | PDCD10 | PDCD6 | PDE6G | PEF1 | PELI1 | PHB | PHB2 | PIAS1 | PIAS3 | PIH1D1 | PIK3CA | PIK3CG | PIK3R3 | PIN1 | PLA2G10 | PLA2G1B | PLA2G2A | PLA2G6 | PLAUR | PLGRKT | PLK1 | PML | POLDIP3 | PPARG | PPARGC1A | PPP1R12A | PPP2CA | PPP2R5D | PRDX2 | PRELID1 | PRKAA1 | PRKAA2 | PRKACA | PRKAG1 | PRKAR1A | PRKAR2B | PRKCD | PRKCE | PRKCZ | PRKD1 | PRKD2 | PROX1 | PRRC1 | PSAP | PSEN1 | PSMC1 | PSMC2 | PSMC3 | PSMC4 | PSMC5 | PSMC6 | PSMD10 | PSMD14 | PSME2 | PSME3 | PTEN | PTK2 | PTPN1 | PTPN22 | RAB1A | RAB1B | RAB3GAP1 | RAC1 | RAD23A | RAF1 | RAP1A | RAP1B | RB1CC1 | RBM3 | RBX1 | RDX | RELA | REST | RGN | RHBDD1 | RHOA | RIF1 | RIPK1 | RIPK2 | RIPK3 | RMND1 | RNF139 | RNF185 | RNF19A | RPLP1 | RPS2 | RPS3 | RPS4X | RPS6KB2 | RPTOR | RTF1 | RUVBL2 | RXRA | S100A12 | S100A7 | S100A9 | SART3 | SDC4 | SDCBP | SEMA7A | SEPT4 | SERPINB3 | SGTA | SHC1 | SIRT1 | SLAMF1 | SMAD3 | SMAD4 | SMARCB1 | SMURF1 | SNAI2 | SNCA | SNW1 | SOAT1 | SOD1 | SOX2 | SPRTN | SPRY2 | SQSTM1 | SRC | STAT3 | STK11 | STK24 | STK25 | STK26 | STK3 | STUB1 | SUMO1 | SUMO2 | TAB1 | TAB2 | TADA2B | TANK | TAOK3 | TERF2IP | TGFB1 | THBS1 | TIMP2 | TLR3 | TMTC3 | TNF | TNFAIP3 | TNFRSF1A | TNFRSF8 | TNK2 | TNP1 | TOLLIP | TP53 | TP73 | TRADD | TRAF2 | TRAF6 | TRIB2 | TRIB3 | TRIM32 | TRUB2 | TXN | UBB | UBC | UBE2C | UBE2D1 | UBE2I | UBE2K | UBE2L3 | UBE2N | UBE2S | UBE2V1 | UBE3A | UBQLN1 | UBQLN2 | UNC119 | UPF3B | UQCC1 | USP16 | USP5 | USP50 | VCP | VIM | VTN | WBP2 | WDR35 | WDR48 | WNK1 | WNT16 | XIAP | XRCC5 | XRCC6 | YES1 | YTHDF1 | ZBTB7B | ZNF622 | GO:0042176 | 0.0325124347931578 | 268/8243 | 156/3250 | 2.13324350498492e-10 | 2.02551470798318e-06 | T | T | T | T | regulation of protein catabolic process | AGAP2 | AKT1 | ANXA2 | APC | APOE | ARAF | ASB9 | ATP13A2 | AXIN1 | BAG2 | BAG5 | BAG6 | BANP | BTRC | CAV1 | CBFA2T3 | CCAR2 | CCDC22 | CD81 | CDH1 | CEBPA | CHEK2 | CLU | COMMD1 | CSNK1A1 | CSNK1E | CSNK2A2 | CTSC | CUL4B | DAB2 | DDA1 | DDB1 | DDRGK1 | DNAJB2 | DVL1 | EDEM1 | EGFR | EGLN1 | EPHA4 | EZR | FAF1 | FAM192A | FAM83D | FOXO1 | FYN | FZR1 | GGA1 | GLMN | GNA12 | GPX1 | GSK3A | GSK3B | HECW1 | HECW2 | HGS | HSP90AA1 | HSP90AB1 | HSP90AB3P | HSP90AB4P | HSPA1B | HSPBP1 | IDE | ITCH | KEAP1 | LAMP3 | LATS1 | LRRK2 | MAD2L1 | MAPK9 | MDM2 | MDM4 | MSN | NDUFA13 | NEDD4 | NELL1 | NFE2L2 | NOS2 | NUPR1 | OAZ3 | OGT | PACSIN3 | PARK7 | PCSK9 | PHB | PIAS1 | PIN1 | PLK1 | PML | PRKACA | PSEN1 | PSMC1 | PSMC2 | PSMC3 | PSMC4 | PSMC5 | PSMC6 | PSMD1 | PSMD10 | PSMD14 | PSMD2 | PSME2 | PSME3 | PTEN | PTK2 | RAB7A | RAD23A | RAD23B | RBX1 | RDX | RELA | RGN | RNF139 | RNF185 | RNF19A | RPL23 | SDCBP | SERPINE2 | SGTA | SMAD3 | SMARCC1 | SMURF1 | SMURF2 | SNF8 | SNX12 | SNX3 | STUB1 | STX5 | STYX | SUMO1 | SUMO2 | TIMP1 | TIMP2 | TMTC3 | TNF | TNFAIP3 | TNFSF12 | TRIB2 | TRIB3 | TRIM32 | UBB | UBE2J1 | UBE2K | UBE3A | UBQLN1 | UBQLN2 | UBQLN4 | UBXN1 | UCHL5 | USP14 | USP19 | USP5 | USP7 | USP8 | VCP | WWP2 | XPO1 | GO:0043069 | 0.0715758825670266 | 590/8243 | 305/3250 | 2.57953480975462e-10 | 2.44926830186201e-06 | T | T | T | T | negative regulation of programmed cell death | AARS | ABL1 | ACAA2 | ADA | ADCYAP1 | ADORA2A | AGAP2 | AHI1 | AKR1B1 | AKT1 | AKT2 | ALB | ALOX12 | ANXA1 | ANXA5 | APBB2 | API5 | APOE | AR | ARAF | ARF4 | ARHGDIA | ARNT2 | ARRB1 | ARRB2 | ASCL1 | ASNS | ATAD3A | ATAD5 | ATG5 | ATOH1 | ATOX1 | AURKB | AZU1 | BAG1 | BAG3 | BAG4 | BAG5 | BAG6 | BAX | BCL10 | BCL2 | BCL2L1 | BCL2L2 | BCL6 | BDKRB2 | BIRC2 | BIRC3 | BIRC5 | BIRC7 | BIRC8 | BRAF | BRCA1 | CAMK1D | CASP2 | CASP3 | CASP8 | CAT | CAV1 | CBL | CBX4 | CCAR2 | CCL21 | CCR5 | CD27 | CD40LG | CD74 | CDKN1A | CFL1 | CHD8 | CLU | COL2A1 | COPS5 | CREB3 | CRYAB | CSNK2A2 | CTH | CTNNB1 | CTTN | DAB2 | DAD1 | DAPK1 | DDB1 | DDRGK1 | DDX3X | DHCR24 | DNAJA1 | DNAJA3 | DPEP1 | DUSP1 | EGFR | ENO1 | ERBB3 | ERCC5 | EYA2 | EYA4 | FABP1 | FADD | FAM129B | FASLG | FGB | FHL2 | FIGNL1 | FKBP8 | FOXO1 | FYN | GATA1 | GLO1 | GNAI2 | GNAI3 | GOLPH3 | GPI | GPX1 | GPX4 | GRIK2 | GRK5 | GSK3B | GSTP1 | HAX1 | HDAC1 | HDAC2 | HDAC3 | HIF1A | HMGB2 | HMOX1 | HNRNPK | HRAS | HSF1 | HSP90AB1 | HSP90B1 | HSPA1B | HSPA5 | HSPA9 | HSPB1 | HSPD1 | HTATIP2 | HTRA2 | HTT | HYPK | ICAM1 | IGBP1 | IGF1R | IKBKB | ILK | IRF7 | ITCH | ITGAV | JAK3 | JUN | KANK2 | KDM1A | KDR | KLHL20 | KRAS | LAMP3 | LEF1 | LGALS3 | LGMN | LIG4 | LMNA | LRP6 | LRRK2 | MAD2L1 | MAP2K4 | MAPK7 | MAPK8 | MDM2 | MDM4 | MECP2 | MED1 | MGMT | MIF | MYC | MYD88 | NDUFA13 | NDUFS3 | NEUROD1 | NFE2L2 | NFKBIA | NME2 | NMNAT1 | NNT | NOD2 | NOS3 | NPM1 | NQO1 | NTF3 | NTRK1 | OPA1 | PA2G4 | PAK2 | PALB2 | PAM16 | PARK7 | PAX8 | PDCD10 | PELI1 | PHB2 | PIAS1 | PIH1D1 | PIK3CA | PIK3CG | PIK3R1 | PIM1 | PIN1 | PLAUR | PLK1 | PPARD | PPARGC1A | PPIF | PPT1 | PRDX2 | PRDX3 | PRDX5 | PRELID1 | PRKAA1 | PRKAA2 | PRKCD | PRKCZ | PRKDC | PSEN1 | PSMC5 | PSMD10 | PSME3 | PTCRA | PTEN | PTK2 | PTPN1 | PTRH2 | QARS | RAF1 | RARA | RB1CC1 | RELA | RGN | RHBDD1 | RHOA | RIPK1 | RIPK2 | RPL10 | RPS6KA1 | RRM2B | SCG2 | SERPINB2 | SET | SFN | SHC1 | SIN3A | SIRT1 | SIRT5 | SLC25A4 | SLC25A5 | SLC35F6 | SLC9A1 | SMAD3 | SMO | SNAI2 | SNCA | SNX6 | SOCS2 | SOD1 | SOD2 | SPRY2 | SQSTM1 | SRC | STAT3 | STIL | STK40 | STXBP1 | SYVN1 | THBS1 | THOC6 | TIMP1 | TLE1 | TMBIM6 | TNF | TNFAIP3 | TNFSF14 | TNIP2 | TP53 | TRADD | TRAF2 | TRAF6 | TRAP1 | TRIAP1 | TRIM32 | TSC22D3 | TWIST1 | TXNDC5 | UBB | UBC | UBE2B | UFM1 | USP47 | VDAC2 | VHL | WT1 | XBP1 | XIAP | YAP1 | YME1L1 | YWHAE | YWHAZ | ZC3HC1 | ZFAND6 | GO:0070887 | 0.127380807958268 | 1050/8243 | 507/3250 | 2.78672396587344e-10 | 2.64599440559683e-06 | T | T | T | T | cellular response to chemical stimulus | ABL1 | ABL2 | ACAA2 | ACACA | ACTA1 | ACTB | ACTG1 | ACTR2 | ACTR3 | ADCY6 | ADCYAP1 | ADRB2 | ADTRP | AGER | AICDA | AIFM1 | AKAP8 | AKAP9 | AKR1B1 | AKR1C2 | AKT1 | AKT2 | ALOX12 | ALOX5AP | ANK3 | ANXA1 | ANXA5 | APEX1 | APLP1 | APP | APRT | AR | ASNS | ASS1 | ATIC | ATM | ATP13A2 | ATP1A1 | ATP2A2 | ATP6V1A | ATRX | BACE1 | BAG3 | BAG4 | BAX | BCAR1 | BCAT2 | BCL11A | BCL2 | BCL2L1 | BMPR1A | BRAF | BRCA1 | BSPRY | BTK | BTRC | CACNA1A | CALR | CAPN2 | CASP3 | CASP7 | CASP8 | CASP9 | CAT | CAV1 | CBL | CBX8 | CCL21 | CCL22 | CCNB1 | CCR5 | CCS | CD36 | CD40 | CDC34 | CDC42EP4 | CDC5L | CDC73 | CDH1 | CDK19 | CDK2 | CDK4 | CDK5 | CDK9 | CEBPA | CEBPE | CFTR | CHEK2 | CLIC4 | COL2A1 | COL4A1 | COL4A2 | COL6A1 | CPNE3 | CPT1A | CREB1 | CRIP1 | CRK | CRKL | CTH | CTNNB1 | CTSL | CXCL11 | CXCL5 | CXCL6 | CXCL8 | CYCS | CYP1A1 | CYP1A2 | DAPK1 | DAXX | DDIT4 | DDX3X | DENND4C | DHX36 | DHX9 | DLG2 | DLG4 | DNM1L | DPEP1 | DTX1 | DUSP1 | DUSP22 | E2F1 | EED | EEF1A1 | EEF2 | EFTUD2 | EGFR | EHD4 | EIF2AK3 | EIF2S1 | EIF4A3 | EIF4E | EIF4EBP1 | ENDOG | EPHA4 | EPRS | ERBB2 | ERRFI1 | ESR1 | ETS1 | EZH2 | EZR | FABP1 | FANCC | FASN | FBP1 | FBXO32 | FECH | FGB | FIS1 | FLNB | FLOT1 | FOLR1 | FOS | FOXO1 | FUS | FYN | G6PD | GAPDH | GATA1 | GBP2 | GBP3 | GBP5 | GBP6 | GFPT2 | GLP1R | GNAI1 | GNB1 | GNPNAT1 | GOT1 | GPD1 | GPX1 | GPX7 | GRAMD1A | GSK3A | GSK3B | GSR | GSTO1 | GSTO2 | GSTP1 | H2AFZ | HAVCR2 | HAX1 | HCN1 | HCN2 | HCN3 | HDAC2 | HDAC4 | HDAC5 | HDAC6 | HDAC9 | HDGF | HIF1A | HK2 | HMGB1 | HMGB2 | HMOX1 | HNRNPA1 | HNRNPD | HNRNPU | HSF1 | HSP90AB1 | HSP90B1 | HSPA14 | HSPA1B | HSPA1L | HSPA2 | HSPA5 | HSPA8 | HSPA9 | HSPB1 | HTR1B | HTR3A | HTRA2 | ICAM1 | ID2 | IFI16 | IFIT1 | IFNAR1 | IGF1R | IGFBP7 | IKBKB | IL3RA | IMPDH2 | INSIG1 | INSR | IPO5 | IQGAP1 | IREB2 | IRF1 | ITGA4 | ITGB1BP1 | ITPR2 | JUN | JUNB | JUND | JUP | KAT5 | KDM1A | KDM5B | KDR | KEAP1 | KIF5B | KLF5 | KLHL22 | LARS | LEF1 | LGALS1 | LGMN | LIG4 | LIMS1 | LMNA | LMO2 | LPAR1 | LPIN3 | LRP6 | LRRK2 | MAP2K3 | MAP2K4 | MAP2K6 | MAP4K1 | MAPK1 | MAPK14 | MAPK3 | MAPK4 | MAPK6 | MAPK7 | MAPK8 | MAPK9 | MAPT | MAT2A | MAVS | MCM2 | MCM7 | MDM2 | MDM4 | MED1 | MGMT | MGST1 | MKNK2 | MME | MSN | MYC | MYO1C | MYO5A | NAMPT | NCOA1 | NCOA2 | NCOA3 | NDRG1 | NDUFA13 | NEUROD1 | NFE2L2 | NME1 | NME2 | NOD2 | NOS2 | NPEPPS | NQO1 | NR1H2 | NR1H3 | NR3C1 | NR4A1 | NR5A2 | NRP2 | NTRK1 | OPA1 | OPTN | OTUB1 | OXSR1 | P2RX4 | P4HB | PAK2 | PARK7 | PARP1 | PAX8 | PAX9 | PCNA | PCSK9 | PDCD10 | PDE12 | PDE4D | PDIA3 | PDK2 | PFKP | PGK1 | PHB | PHB2 | PICK1 | PIK3CA | PIK3CG | PIK3R1 | PKM | PLA2G10 | PLA2G1B | PLCG1 | PML | PPARD | PPARG | PPARGC1A | PPAT | PPIF | PPM1A | PPP1R12A | PPP1R14A | PRDX1 | PRDX2 | PRDX3 | PRDX5 | PRDX6 | PRKAA1 | PRKAA2 | PRKACA | PRKAR1A | PRKAR2B | PRKCD | PRKCE | PRKCZ | PRKD1 | PRKD2 | PRKDC | PRPF8 | PSAP | PSEN1 | PTEN | PTN | PTPN1 | PTPN14 | PTPN22 | PTPN23 | PTPN4 | PTPN6 | PTPRK | PXN | PYCR1 | PYCR2 | QDPR | RAB10 | RAB31 | RAB35 | RAD23B | RAD51 | RAE1 | RANGAP1 | RAP1A | RAP1B | RARA | RARG | RB1 | RBMX | RC3H1 | RDX | RELA | REST | RHBDD1 | RHOA | RIF1 | RIPK1 | RIPK2 | RIPK3 | ROMO1 | RPL23 | RPS3 | RPTOR | RUNX2 | RUVBL2 | S100B | SH3GL2 | SHC1 | SHMT1 | SHMT2 | SIN3A | SIPA1 | SIRT1 | SLAMF8 | SLC12A2 | SLC16A1 | SLC25A24 | SLC25A5 | SLC2A5 | SLC9A1 | SMAD3 | SMAD4 | SMAD5 | SMAD9 | SMO | SNAI2 | SNCA | SNW1 | SNX6 | SOCS1 | SOCS2 | SOD1 | SOD2 | SP1 | SPI1 | SPINT2 | SPRY2 | SRC | SRSF5 | SRXN1 | ST3GAL6 | STAT1 | STAT3 | STAT6 | STAU1 | STIP1 | STUB1 | STXBP1 | STXBP2 | SULT1A3 | SUMO1 | SYP | TANK | TBC1D4 | TBXA2R | TGFB1 | THBS1 | TLR3 | TMBIM6 | TMEM173 | TNF | TNFAIP3 | TNIP2 | TNKS | TP53 | TPM1 | TPR | TRADD | TRAF2 | TRAF6 | TREX1 | TRIB3 | TRIM25 | TRIM41 | TWF2 | TWIST1 | TXNRD1 | UBE2D3 | UBE2K | UBE2L3 | UBE2W | UBQLN1 | UMPS | USP8 | VCAM1 | VCP | VIM | VSTM2A | WAS | WBP2 | WDR35 | WDTC1 | WNK1 | WNT2 | WT1 | WWOX | XBP1 | XRCC5 | YAP1 | YES1 | YWHAG | YY1 | ZFAND6 | ZNF277 | GO:0043066 | 0.069998786849448 | 577/8243 | 299/3250 | 2.79566045795297e-10 | 2.65447960482635e-06 | T | T | T | T | negative regulation of apoptotic process | AARS | ABL1 | ACAA2 | ADA | ADCYAP1 | ADORA2A | AGAP2 | AHI1 | AKR1B1 | AKT1 | AKT2 | ALB | ALOX12 | ANXA1 | ANXA5 | APBB2 | API5 | APOE | AR | ARAF | ARF4 | ARHGDIA | ARNT2 | ARRB1 | ARRB2 | ASCL1 | ASNS | ATAD3A | ATAD5 | ATG5 | ATOH1 | ATOX1 | AURKB | AZU1 | BAG1 | BAG3 | BAG4 | BAG5 | BAG6 | BAX | BCL10 | BCL2 | BCL2L1 | BCL2L2 | BCL6 | BDKRB2 | BIRC2 | BIRC3 | BIRC5 | BIRC7 | BIRC8 | BRAF | BRCA1 | CAMK1D | CASP2 | CASP3 | CASP8 | CAT | CAV1 | CBL | CBX4 | CCAR2 | CCL21 | CCR5 | CD27 | CD40LG | CD74 | CDKN1A | CFL1 | CHD8 | CLU | COL2A1 | COPS5 | CREB3 | CRYAB | CSNK2A2 | CTH | CTNNB1 | CTTN | DAB2 | DAD1 | DAPK1 | DDB1 | DDRGK1 | DDX3X | DHCR24 | DNAJA1 | DPEP1 | DUSP1 | EGFR | ENO1 | ERBB3 | ERCC5 | EYA2 | EYA4 | FABP1 | FADD | FAM129B | FASLG | FGB | FHL2 | FIGNL1 | FKBP8 | FOXO1 | FYN | GATA1 | GLO1 | GNAI2 | GNAI3 | GOLPH3 | GPI | GPX1 | GRIK2 | GRK5 | GSK3B | GSTP1 | HAX1 | HDAC1 | HDAC2 | HDAC3 | HIF1A | HMGB2 | HMOX1 | HNRNPK | HRAS | HSF1 | HSP90AB1 | HSP90B1 | HSPA1B | HSPA5 | HSPA9 | HSPB1 | HSPD1 | HTATIP2 | HTRA2 | HTT | HYPK | ICAM1 | IGBP1 | IGF1R | IKBKB | ILK | IRF7 | ITCH | ITGAV | JAK3 | JUN | KDM1A | KDR | KLHL20 | KRAS | LAMP3 | LEF1 | LGALS3 | LGMN | LIG4 | LMNA | LRP6 | LRRK2 | MAD2L1 | MAP2K4 | MAPK7 | MAPK8 | MDM2 | MDM4 | MECP2 | MED1 | MGMT | MIF | MYC | MYD88 | NDUFA13 | NDUFS3 | NEUROD1 | NFE2L2 | NFKBIA | NME2 | NMNAT1 | NNT | NOD2 | NOS3 | NPM1 | NQO1 | NTF3 | NTRK1 | OPA1 | PA2G4 | PAK2 | PALB2 | PAM16 | PARK7 | PAX8 | PDCD10 | PHB2 | PIAS1 | PIH1D1 | PIK3CA | PIK3CG | PIK3R1 | PIM1 | PIN1 | PLAUR | PLK1 | PPARD | PPARGC1A | PPIF | PPT1 | PRDX2 | PRDX3 | PRDX5 | PRELID1 | PRKAA1 | PRKAA2 | PRKCD | PRKCZ | PRKDC | PSEN1 | PSMD10 | PSME3 | PTCRA | PTEN | PTK2 | PTPN1 | PTRH2 | QARS | RAF1 | RARA | RB1CC1 | RELA | RGN | RHBDD1 | RHOA | RIPK1 | RIPK2 | RPL10 | RPS6KA1 | RRM2B | SCG2 | SERPINB2 | SET | SFN | SHC1 | SIN3A | SIRT1 | SIRT5 | SLC25A5 | SLC35F6 | SLC9A1 | SMAD3 | SMO | SNAI2 | SNCA | SNX6 | SOCS2 | SOD1 | SOD2 | SPRY2 | SQSTM1 | SRC | STAT3 | STIL | STK40 | STXBP1 | SYVN1 | THBS1 | THOC6 | TIMP1 | TLE1 | TMBIM6 | TNF | TNFAIP3 | TNFSF14 | TNIP2 | TP53 | TRADD | TRAF2 | TRAF6 | TRAP1 | TRIAP1 | TRIM32 | TSC22D3 | TWIST1 | TXNDC5 | UBB | UBC | UBE2B | UFM1 | USP47 | VDAC2 | VHL | WT1 | XBP1 | XIAP | YAP1 | YME1L1 | YWHAE | YWHAZ | ZC3HC1 | ZFAND6 | GO:0010638 | 0.0534999393424724 | 441/8243 | 237/3250 | 2.92289313559998e-10 | 2.77528703225218e-06 | T | T | T | T | positive regulation of organelle organization | ABL1 | ACD | ACTN2 | ACTR2 | ACTR3 | ADRB2 | AKAP8 | AKAP9 | AKT2 | ANAPC4 | ANK1 | ANXA1 | ANXA2 | APOA1 | ARHGAP35 | ARPC1A | ARPC1B | ARPC2 | ARPC3 | ARPC4 | ARPC5 | ARRB1 | ATL3 | ATM | ATRX | AURKB | BAG4 | BAIAP2 | BAIAP2L1 | BAX | BBC3 | BCL2 | BCL6 | BIN1 | BRAF | BRCA1 | BRD4 | CALCOCO2 | CAPG | CASP8 | CCDC88A | CCL21 | CCNB1 | CCP110 | CCT2 | CCT3 | CCT4 | CCT5 | CCT6A | CCT7 | CCT8 | CDC37 | CDC42 | CDC42EP3 | CDC42EP4 | CDK5 | CDK5R1 | CDK9 | CENPJ | CEP135 | CFL1 | CFL2 | CHCHD10 | CROCC | CSNK2A2 | CTBP1 | CTNNB1 | CTTN | CUL3 | DCN | DCTN1 | DHX36 | DNM1L | DSTN | DYNC1H1 | DYNLL1 | E2F1 | EED | FBXO7 | FCHSD1 | FEN1 | FIS1 | FSCN1 | GRB2 | GSK3A | GSK3B | HAP1 | HAX1 | HDAC6 | HIF1A | HK2 | HNRNPA1 | HNRNPA2B1 | HNRNPD | HNRNPU | HRAS | HSPA1B | HSPA1L | HTRA2 | HTT | HUWE1 | ICAM1 | INSR | ITGB1BP1 | KAT7 | KCTD17 | KDM1A | KDR | KIF5B | KMT2A | LCP1 | LIG4 | LMNA | LPAR1 | LRRK2 | LRSAM1 | MAP2K7 | MAP3K4 | MAPK1 | MAPK15 | MAPK3 | MAPK8 | MAPK9 | MAPT | MARCH5 | MECP2 | MLLT11 | MSN | MYO1C | MYOC | NCK1 | NEK7 | NELFE | NF2 | NIN | NIPBL | NPEPPS | NSFL1C | NTF3 | NUMA1 | NUSAP1 | OGT | OPA1 | OPTN | PAN2 | PARK7 | PAXIP1 | PDCD6IP | PEBP1 | PFDN2 | PFN1 | PHB2 | PIH1D1 | PIP4K2A | PLA2G6 | PLAUR | PML | PNKP | POT1 | PPARGC1A | PPIF | PPP3CC | PRKAA1 | PRKCE | PRKD1 | PRKD2 | PROX1 | PXN | RAB3GAP1 | RAC1 | RAD21 | RB1 | RHOA | RHOU | RIF1 | RNF31 | RPS3 | RTF1 | RUVBL2 | SAE1 | SART3 | SDC1 | SDC4 | SDCBP | SEPT7 | SEPT9 | SETDB1 | SFN | SFPQ | SH2B1 | SIN3A | SIRT1 | SIRT6 | SMAD3 | SMAD4 | SMARCB1 | SMURF1 | SNAI2 | SNW1 | SRC | SWAP70 | TADA2B | TCP1 | TERF1 | TFDP1 | TGFB1 | TMED9 | TNF | TNKS | TNKS2 | TP53 | TP63 | TP73 | TPM1 | TPR | TRIM28 | UBE2B | UBE2C | UBE2D3 | UBE2L3 | UBE2N | ULK1 | VASP | VPS4B | WAS | WBP2 | WDR1 | WDR35 | XRCC5 | YWHAB | YWHAE | YWHAG | YWHAH | YWHAQ | YWHAZ | ZBTB7B | ZMYND10 | GO:0009058 | 0.185854664563872 | 1532/8243 | 712/3250 | 3.04609756812957e-10 | 2.89226964093903e-06 | T | T | T | T | biosynthetic process | AASDHPPT | AASS | ABAT | ABCD3 | ACAA2 | ACACA | ACAT1 | ACAT2 | ACBD3 | ACCS | ACE2 | ACLY | ACOT7 | ACSL1 | ACSL4 | ACSL5 | ACSS2 | ADA | ADCY6 | ADK | ADRM1 | ADSL | ADSS | AFF4 | AGK | AGL | AGO2 | AK1 | AK2 | AK4 | AKR1A1 | AKR1B1 | AKR1B15 | AKT1 | AKT2 | ALAS1 | ALDH7A1 | ALDH8A1 | ALDH9A1 | ALDOA | ALDOC | ALOX12 | ALOX5 | ALOX5AP | AMD1 | AMDHD2 | APOA1 | APOE | APRT | AR | ARF1 | ARHGAP35 | ARNT | ARRB1 | ARRB2 | ASL | ASNS | ASPDH | ASS1 | ATAD5 | ATF6B | ATIC | ATM | ATOH1 | B3GAT3 | B3GNT3 | B4GALT3 | BAX | BCAT2 | BCL10 | BDH1 | BRCA1 | CBFB | CBR4 | CBS | CCDC88A | CCNB1 | CCNT1 | CCNT2 | CD74 | CDA | CDC73 | CDK2 | CDK4 | CDK8 | CDK9 | CDKN1A | CEBPA | CEBPE | CENPF | CES1 | CFTR | CHAF1A | CHDH | CHRAC1 | CKM | CMPK1 | CNDP2 | COL4A2 | COL4A3BP | COL5A1 | COPS2 | COPS5 | COQ2 | COQ3 | COQ4 | COQ5 | COQ6 | COQ7 | COQ9 | COX15 | CPNE3 | CPSF7 | CREB1 | CREBBP | CRY1 | CSNK2A2 | CSNK2B | CSTF2T | CTDP1 | CTH | CTPS1 | CYB5R1 | CYB5R3 | CYC1 | CYP1A1 | CYP1A2 | DAP3 | DARS | DBH | DCAKD | DCK | DCN | DCTPP1 | DCXR | DEGS1 | DERA | DGKE | DGKZ | DGUOK | DHCR24 | DHRS7B | DHX9 | DIDO1 | DLAT | DLD | DMD | DNA2 | DNAJA3 | DNAJC30 | DPM1 | DRG2 | DSE | DTX1 | DUT | DVL1 | DVL2 | E2F1 | E2F2 | E2F3 | EAF2 | ECD | EEF1A1 | EEF1A1P5 | EEF1A2 | EEF1B2 | EEF1D | EEF1G | EEF2 | EGFR | EIF4H | EIF5A | ELF3 | ENO1 | ENO2 | ENO3 | ENOPH1 | ERAL1 | ESD | ESR1 | ESR2 | ESRRA | ESRRB | ESRRG | ETS1 | EXTL2 | FABP5 | FAM20B | FAR2 | FARSB | FASN | FBP1 | FBP2 | FDPS | FDXR | FECH | FEN1 | FGF2 | FOLH1 | FOS | FPGS | FUBP1 | FUBP3 | G6PD | GAD2 | GAPDH | GAPDHS | GAR1 | GARS | GART | GATA1 | GBE1 | GCDH | GDA | GEMIN5 | GFPT1 | GFPT2 | GGCT | GGPS1 | GGT1 | GGTLC1 | GLCE | GLS | GLS2 | GLUD2 | GLUL | GMDS | GMPPB | GMPR | GMPS | GNPDA1 | GNPDA2 | GNPNAT1 | GOLPH3 | GOT1 | GOT1L1 | GOT2 | GPC5 | GPD1 | GPD1L | GPI | GPT2 | GPX1 | GPX4 | GSPT1 | GSPT2 | GSS | GSTA1 | GSTA2 | GSTA3 | GSTA4 | GSTK1 | GSTM4 | GSTO1 | GSTO2 | GSTP1 | GSTT1 | GSTT2B | GSTZ1 | GTF2A1 | GTF2E2 | GTF2F1 | GTF2F2 | GTF2I | GTF3C2 | GYG1 | GYG2 | GYS1 | HAAO | HAGH | HARS | HEXA | HEXB | HIF1A | HINT1 | HIST1H4A | HMBS | HMGCL | HNF4A | HNF4G | HOXB9 | HPRT1 | HS2ST1 | HS6ST1 | HSD17B14 | HSD17B4 | HSD17B6 | HSD17B8 | HSD3B7 | HSF1 | HSP90AA1 | HSP90AB1 | HSPA14 | HSPG2 | IDH2 | IDI2 | ILF2 | IMPA1 | IMPA2 | IMPDH2 | ING5 | INSIG1 | INSIG2 | IP6K1 | IREB2 | IRF1 | IRF7 | ISG15 | ISYNA1 | ITPA | JUN | KAT5 | KAT7 | KDSR | KLF5 | KMT2A | KYNU | LARP4 | LCMT2 | LDHC | LEF1 | LIG4 | LMO2 | LPIN3 | LTC4S | LUM | MAPK1 | MAPK3 | MAT2A | MAT2B | MC1R | MCAT | MCM2 | MCM3 | MCM4 | MCM5 | MCM6 | MCM7 | MCM9 | MCMBP | MDH1 | MDH2 | ME1 | MECP2 | MED1 | MED12 | MED14 | MED17 | MED20 | MED21 | MED23 | MED24 | MED31 | MED4 | MED6 | MGST1 | MIF | MITF | MLLT1 | MLLT3 | MMAB | MOCOS | MPC2 | MPI | MPPE1 | MPST | MRPL12 | MRPL14 | MRPL19 | MRPL28 | MRPL38 | MRPL4 | MRPL43 | MRPL45 | MRPL46 | MRPL9 | MRPS5 | MRRF | MTAP | MTERF1 | MTHFD1 | MTMR14 | MTMR7 | MTRR | MVD | MYO5A | NAGK | NAMPT | NANP | NANS | NAP1L1 | NAPRT | NCBP1 | NCOA1 | NCOR1 | NDUFA9 | NELFB | NELFCD | NELFE | NFATC1 | NFE2L2 | NFS1 | NME1 | NME2 | NME4 | NMNAT1 | NMNAT3 | NMRAL1 | NMRK2 | NOP10 | NOS2 | NOS3 | NPLOC4 | NPR1 | NQO1 | NR0B2 | NR1H2 | NR1H3 | NR1I2 | NR3C1 | NR4A1 | NR5A2 | NRBF2 | NT5C2 | NUDT18 | NUDT3 | NUDT9 | NUP98 | OAT | OAZ3 | OSBPL1A | OTC | OXA1L | P2RX1 | PABPC4 | PAH | PAICS | PAPSS1 | PAPSS2 | PARK7 | PARP1 | PAWR | PAX8 | PC | PCBD1 | PCF11 | PCNA | PCTP | PCYT2 | PDE4D | PDF | PDHA1 | PDHA2 | PDHB | PDHX | PDSS1 | PDSS2 | PDXK | PER2 | PFAS | PGAM1 | PGAM2 | PGD | PGK1 | PGK2 | PGM1 | PGM2 | PGM3 | PGP | PHB | PHGDH | PHOSPHO1 | PI4K2A | PIGU | PIK3C2A | PIK3CA | PIK3CG | PIK3R1 | PIK3R3 | PIKFYVE | PINX1 | PIP4K2A | PIR | PLA2G10 | PLA2G12A | PLA2G1B | PLA2G2A | PLA2G6 | PLCG1 | PLOD2 | PLSCR1 | PNKP | PNP | POLA2 | POLD1 | POLE2 | POLE3 | POLM | POLR2A | POLR2E | POLR2L | POLR2M | POT1 | POU5F1 | PPARD | PPARG | PPARGC1A | PPAT | PPIA | PPM1D | PPOX | PPP1R3C | PPP5C | PPT1 | PRKAA1 | PRKAA2 | PRKAB2 | PRKAG1 | PRKD1 | PRKD2 | PRKD3 | PRPF19 | PRTFDC1 | PSAT1 | PTEN | PTGDS | PTGES | PTGES3 | PTMA | PTMS | PTPMT1 | PTTG1 | PYCR1 | PYCR2 | PYGL | QDPR | RAB5A | RARA | RARG | RARS2 | RBBP4 | RBBP6 | RBMX | RBP1 | RBPJ | RELA | REPIN1 | RFC1 | RFC2 | RFC3 | RFC4 | RFC5 | RFK | RGN | RMND1 | RNASEH2A | RPA2 | RPA3 | RPAIN | RPL10 | RPL10A | RPL12 | RPL18A | RPL22 | RPL23 | RPL23A | RPL24 | RPL30 | RPL36AL | RPL37A | RPL38 | RPL6 | RPL7 | RPL7A | RPL9 | RPLP0 | RPLP0P6 | RPLP1 | RPLP2 | RPS12 | RPS13 | RPS14 | RPS15A | RPS19 | RPS2 | RPS20 | RPS24 | RPS29 | RPS3 | RPS4X | RPS6KB2 | RPS8 | RRM1 | RRM2 | RRM2B | RTF1 | RUNX2 | RXRA | RXRB | RXRG | SARS | SAT1 | SCAF1 | SCCPDH | SDC1 | SDC4 | SEPHS1 | SEPHS2 | SERPINH1 | SET | SGPL1 | SHMT1 | SHMT2 | SIN3A | SIRT1 | SIRT7 | SKI | SLC25A1 | SLC25A10 | SLC25A11 | SLC25A13 | SMAD4 | SMG5 | SMG6 | SMN1 | SNCA | SNRPB | SNRPD3 | SNRPE | SNW1 | SOD1 | SORD | SP1 | SPI1 | SPIN1 | SPR | SPRTN | SPTLC2 | ST3GAL1 | ST3GAL6 | ST6GALNAC5 | ST8SIA4 | STARD7 | STAT3 | STOML2 | SUCLA2 | SUPT4H1 | SUPT5H | TAF12 | TAF15 | TAF1A | TALDO1 | TAMM41 | TARDBP | TARS | TAZ | TCEA1 | TCEA2 | TCERG1 | TCF4 | TECR | TERF1 | TERF2 | TERF2IP | TFCP2 | TGFB1 | THRA | TK1 | TK2 | TKT | TNF | TOP1 | TOP3A | TP53 | TPI1 | TREX1 | TRIM25 | TRIM28 | TRIM29 | TRIP13 | TSC22D1 | TSFM | TUFM | TYMS | UAP1 | UAP1L1 | UBB | UBC | UBE2L6 | UBIAD1 | UCK2 | UGDH | UGP2 | UGT8 | UMPS | UPP2 | USPL1 | UXS1 | VAC14 | VAPA | VAPB | VCAN | VCP | VDR | WARS | XBP1 | XYLT1 | YAP1 | ZBTB7A | ZBTB7B | ZNF217 | ZNF598 | GO:0051130 | 0.0934125925027296 | 770/8243 | 384/3250 | 4.44372567799389e-10 | 4.2193175312552e-06 | T | T | T | T | positive regulation of cellular component organization | ABL1 | ABL2 | ACD | ACE2 | ACTN2 | ACTN4 | ACTR2 | ACTR3 | ADCYAP1 | ADRB2 | AHI1 | AKAP8 | AKAP9 | AKT2 | ANAPC4 | ANK1 | ANXA1 | ANXA2 | APBB1 | APC | APOA1 | APOE | APP | AR | ARF1 | ARHGAP35 | ARHGDIA | ARHGEF7 | ARPC1A | ARPC1B | ARPC2 | ARPC3 | ARPC4 | ARPC5 | ARRB1 | ARRB2 | ATAD1 | ATL3 | ATM | ATRX | AURKB | AVIL | BAG4 | BAIAP2 | BAIAP2L1 | BAX | BBC3 | BCL2 | BCL6 | BIN1 | BRAF | BRCA1 | BRD4 | C1QBP | CALCOCO2 | CALR | CAMK1D | CAMK2B | CAND1 | CAPG | CAPN2 | CASP8 | CAV1 | CBL | CCDC88A | CCL21 | CCNB1 | CCP110 | CCT2 | CCT3 | CCT4 | CCT5 | CCT6A | CCT7 | CCT8 | CD36 | CDC20 | CDC34 | CDC37 | CDC42 | CDC42EP3 | CDC42EP4 | CDK5 | CDK5R1 | CDK9 | CDKL3 | CENPJ | CEP135 | CFL1 | CFL2 | CHCHD10 | CHD1L | CLSTN1 | CLU | COLGALT1 | CREB1 | CRK | CRKL | CROCC | CSNK2A2 | CTBP1 | CTNNB1 | CTTN | CUL3 | CUL4B | CUL7 | DAB2 | DBN1 | DCN | DCTN1 | DDB1 | DDB2 | DHX36 | DLG4 | DNM1L | DOCK5 | DSTN | DVL1 | DVL2 | DVL3 | DYNC1H1 | DYNLL1 | E2F1 | EED | EIF5A | ENDOG | EP300 | EPHA4 | ERCC5 | ESR1 | EZH2 | FAF1 | FAM98A | FBXO7 | FCHSD1 | FEN1 | FGB | FGFR1 | FIS1 | FKBP1B | FLOT1 | FN1 | FSCN1 | FYN | GBP5 | GDI1 | GOLGA4 | GPX1 | GRB2 | GRID2 | GRIP1 | GSK3A | GSK3B | HAP1 | HAX1 | HDAC4 | HDAC6 | HIF1A | HIST1H1A | HK2 | HNRNPA1 | HNRNPA2B1 | HNRNPD | HNRNPK | HNRNPU | HRAS | HSF1 | HSP90AA1 | HSPA1B | HSPA1L | HSPA5 | HTRA2 | HTT | HUWE1 | ICAM1 | IDE | IGF1R | ILK | INSR | IQGAP1 | ITGB1BP1 | KAT7 | KCTD17 | KDM1A | KDR | KIF5B | KMT2A | LCP1 | LIG4 | LIMS1 | LMNA | LPAR1 | LRRC7 | LRRK2 | LRSAM1 | MAP1B | MAP2K1 | MAP2K7 | MAP3K13 | MAP3K4 | MAPK1 | MAPK14 | MAPK15 | MAPK3 | MAPK6 | MAPK8 | MAPK9 | MAPT | MARCH5 | MECP2 | METRN | MIB1 | MLLT11 | MSN | MYO1C | MYOC | NCK1 | NEK7 | NELFE | NF2 | NFATC2 | NFE2L2 | NIN | NIPBL | NME1 | NME2 | NPEPPS | NR1H2 | NRXN1 | NSFL1C | NTF3 | NTRK1 | NUMA1 | NUSAP1 | OBSL1 | OGT | OPA1 | OPTN | PAFAH1B1 | PAN2 | PARK7 | PARP1 | PARP6 | PAXIP1 | PCSK9 | PDCD6IP | PEBP1 | PFDN2 | PFN1 | PHB2 | PICK1 | PIH1D1 | PIP4K2A | PLA2G6 | PLAUR | PLXNA1 | PLXNA2 | PLXNB2 | PML | PNKP | POT1 | PPARG | PPARGC1A | PPIF | PPP3CC | PPT1 | PRKAA1 | PRKCD | PRKCE | PRKD1 | PRKD2 | PROX1 | PSEN1 | PSMC2 | PSMC3 | PSMC4 | PSMC5 | PSMC6 | PTN | PTPN23 | PTPRD | PXN | RAB11A | RAB21 | RAB31 | RAB3GAP1 | RAC1 | RAD21 | RAP1A | RB1 | RBX1 | RGS2 | RHOA | RHOU | RIF1 | RNF31 | RPA2 | RPA3 | RPS3 | RTF1 | RUVBL2 | S100A9 | SAE1 | SART3 | SCYL2 | SDC1 | SDC4 | SDCBP | SEMA7A | SEPT7 | SEPT9 | SETDB1 | SFN | SFPQ | SH2B1 | SH3GL1 | SH3GL2 | SIN3A | SIRT1 | SIRT6 | SMAD3 | SMAD4 | SMARCB1 | SMURF1 | SNAI2 | SNCA | SNW1 | SNX3 | SRC | STK11 | STK25 | STUB1 | SUMO1 | SWAP70 | TADA2B | TCP1 | TERF1 | TFDP1 | TGFB1 | THBS2 | TMED9 | TMEM30A | TNF | TNFRSF12A | TNFSF14 | TNKS | TNKS2 | TOR1A | TP53 | TP63 | TP73 | TPM1 | TPR | TRIM28 | TSG101 | TUBB2B | TWF1 | TWF2 | UBE2B | UBE2C | UBE2D3 | UBE2L3 | UBE2N | ULK1 | USP50 | VASP | VCP | VPS4B | VTN | WARS | WAS | WBP2 | WDR1 | WDR35 | XRCC5 | YWHAB | YWHAE | YWHAG | YWHAH | YWHAQ | YWHAZ | ZBTB7B | ZMYND10 | ZMYND8 | GO:0032270 | 0.117311658376805 | 967/8243 | 470/3250 | 4.48675941640062e-10 | 4.26017806587239e-06 | T | T | T | T | positive regulation of cellular protein metabolic process | ABL1 | ACSL1 | ADCY6 | ADCYAP1 | ADRB2 | ADRM1 | ADTRP | AGER | AIFM1 | AIMP2 | AKAP8 | AKAP9 | AKT1 | AKT2 | AKTIP | ALOX12 | ANXA2 | APOE | APP | AR | ARAF | ARNT | ARRB1 | ARRB2 | ASPSCR1 | ATM | AXIN1 | AZU1 | BAG2 | BAG4 | BAG6 | BAX | BBC3 | BCAR3 | BCL10 | BCL2 | BCL2L13 | BCL6 | BIRC2 | BIRC3 | BIRC7 | BMI1 | BMPR1A | BRAF | BRCA1 | BRD4 | BTRC | C1QBP | C1QTNF1 | CAB39 | CALM1 | CALM2 | CALM3 | CARD9 | CASP10 | CASP8 | CASP9 | CAV1 | CBFA2T3 | CCDC22 | CCDC88A | CCL21 | CCL22 | CCNB1 | CCNT1 | CCNT2 | CCNY | CCNYL1 | CCR1 | CD27 | CD36 | CD40 | CD40LG | CD74 | CD81 | CDC14B | CDC20 | CDC25B | CDK4 | CDK5 | CDK5R1 | CDK9 | CDKN1A | CEBPA | CHEK2 | CHP1 | CIRBP | CKS1B | CKS2 | CLPX | CLU | COA3 | COMMD1 | CRK | CRKL | CSNK1A1 | CSNK1E | CSNK2B | CTBP1 | CTNNB1 | CTSC | CUL3 | CYCS | DAB1 | DAB2 | DAPK1 | DAXX | DBF4B | DBNL | DCUN1D1 | DDRGK1 | DDX39B | DDX3X | DERL1 | DHX36 | DHX9 | DIABLO | DLG3 | DLG4 | DNAJB2 | DOCK7 | DUSP19 | DUSP22 | DUSP7 | DVL1 | DVL2 | DVL3 | EDEM1 | EED | EEF2 | EGFR | EGLN3 | EHD4 | EIF2AK3 | EIF2AK4 | EIF2B5 | EIF3D | EIF3E | EIF4A3 | EIF5A | EIF6 | ELAVL1 | ENO1 | EPHA4 | ERBB2 | ERBB3 | EZH2 | EZR | FADD | FAF1 | FANCI | FANCM | FASLG | FBXW11 | FGB | FGF2 | FGFR1 | FGFR3 | FIS1 | FKBP1A | FLOT1 | FN1 | FXR2 | FYN | FZR1 | GADD45G | GATA1 | GDF9 | GNAI2 | GOLGA2 | GSK3A | GSK3B | HAVCR2 | HAX1 | HBEGF | HDAC2 | HDAC3 | HDAC4 | HDAC6 | HMGB1 | HNRNPD | HNRNPL | HPX | HRAS | HSF1 | HSP90AA1 | HSP90AB1 | HSPA1B | HSPA2 | HSPA5 | HSPBP1 | HSPD1 | HSPE1 | HTRA2 | ICAM1 | IFI16 | IFNA2 | IGF1R | IKBKG | ILK | INSR | IQGAP1 | ITGB1BP1 | ITGB3 | JUN | KAT5 | KAT7 | KDM1A | KDR | KEAP1 | KHDRBS1 | KLHDC10 | KMT2A | KRAS | KSR1 | LARP4 | LAT | LATS1 | LCP2 | LGMN | LMNA | LPAR1 | LRRK1 | LRRK2 | LTBR | MAP2K1 | MAP2K3 | MAP2K4 | MAP2K6 | MAP2K7 | MAP3K1 | MAP3K13 | MAP3K14 | MAP3K2 | MAP3K3 | MAP3K4 | MAP4K1 | MAPK1 | MAPK14 | MAPK3 | MAPK8IP3 | MAPK9 | MAPKAP1 | MAPT | MAS1 | MAVS | MBP | MDM2 | MECP2 | MIF | MLST8 | MSN | MTA1 | MYC | NCK1 | NDUFA13 | NEK1 | NELFE | NFE2L2 | NFKBIA | NIPBL | NMNAT1 | NOD2 | NOS1AP | NPM1 | NQO2 | NR1H2 | NR1H3 | NTF3 | NTRK1 | NUPR1 | OGT | OXSR1 | P2RX1 | PABPC1 | PACSIN3 | PAIP1 | PAK2 | PAK6 | PARK7 | PARP14 | PAXIP1 | PCSK9 | PDCD10 | PDCD6 | PDE6G | PEF1 | PELI1 | PHB | PHB2 | PIAS1 | PIAS3 | PIH1D1 | PIK3CA | PIK3CG | PIK3R3 | PIN1 | PLA2G10 | PLA2G1B | PLA2G2A | PLA2G6 | PLAUR | PLGRKT | PLK1 | PML | POLDIP3 | PPARG | PPARGC1A | PPP1R12A | PPP2CA | PPP2R5D | PRDX2 | PRELID1 | PRKAA1 | PRKAA2 | PRKACA | PRKAG1 | PRKAR1A | PRKAR2B | PRKCD | PRKCE | PRKCZ | PRKD1 | PRKD2 | PROX1 | PRRC1 | PSAP | PSEN1 | PSMC1 | PSMC2 | PSMC3 | PSMC4 | PSMC5 | PSMC6 | PSMD10 | PSMD14 | PSME2 | PSME3 | PTEN | PTK2 | PTPN1 | PTPN22 | RAB1A | RAB3GAP1 | RAC1 | RAD23A | RAF1 | RAP1A | RAP1B | RB1CC1 | RBM3 | RBX1 | RDX | REST | RGN | RHBDD1 | RHOA | RIF1 | RIPK1 | RIPK2 | RIPK3 | RMND1 | RNF139 | RNF185 | RNF19A | RPLP1 | RPS2 | RPS3 | RPS4X | RPS6KB2 | RPTOR | RTF1 | RUVBL2 | RXRA | S100A12 | S100A7 | S100A9 | SART3 | SDC4 | SDCBP | SEMA7A | SEPT4 | SERPINB3 | SGTA | SHC1 | SIRT1 | SLAMF1 | SMAD3 | SMAD4 | SMARCB1 | SMURF1 | SNAI2 | SNCA | SNW1 | SOD1 | SOX2 | SPRTN | SPRY2 | SQSTM1 | SRC | STAT3 | STK11 | STK24 | STK25 | STK26 | STK3 | STUB1 | SUMO1 | SUMO2 | TAB1 | TAB2 | TADA2B | TANK | TAOK3 | TERF2IP | TGFB1 | THBS1 | TIMP2 | TLR3 | TMTC3 | TNF | TNFAIP3 | TNFRSF1A | TNK2 | TNP1 | TOLLIP | TP53 | TP73 | TRADD | TRAF2 | TRAF6 | TRIB2 | TRIB3 | TRIM32 | TRUB2 | TXN | UBB | UBC | UBE2C | UBE2D1 | UBE2I | UBE2K | UBE2L3 | UBE2N | UBE2S | UBE2V1 | UBE3A | UBQLN1 | UBQLN2 | UNC119 | UPF3B | UQCC1 | USP16 | USP5 | USP50 | VCP | VIM | VTN | WBP2 | WDR35 | WDR48 | WNK1 | WNT16 | XIAP | XRCC5 | XRCC6 | YES1 | YTHDF1 | ZBTB7B | ZNF622 | GO:0060548 | 0.0781268955477375 | 644/8243 | 328/3250 | 4.85115206969923e-10 | 4.60616889017942e-06 | T | T | T | T | negative regulation of cell death | AARS | ABL1 | ACAA2 | ADA | ADCYAP1 | ADORA2A | AGAP2 | AGR2 | AHI1 | AKR1B1 | AKT1 | AKT2 | ALB | ALOX12 | ANXA1 | ANXA5 | APBB2 | API5 | APOE | AR | ARAF | ARF4 | ARHGDIA | ARNT2 | ARRB1 | ARRB2 | ASCL1 | ASNS | ATAD3A | ATAD5 | ATG5 | ATOH1 | ATOX1 | ATP13A2 | AURKB | AZU1 | BAG1 | BAG3 | BAG4 | BAG5 | BAG6 | BAX | BCL10 | BCL2 | BCL2L1 | BCL2L2 | BCL6 | BDKRB2 | BIRC2 | BIRC3 | BIRC5 | BIRC7 | BIRC8 | BRAF | BRCA1 | CAMK1D | CASP2 | CASP3 | CASP8 | CAT | CAV1 | CBL | CBX4 | CCAR2 | CCL21 | CCR5 | CD27 | CD40LG | CD74 | CDK5 | CDKN1A | CFL1 | CHD8 | CHMP2A | CLU | COL2A1 | COPS5 | CREB1 | CREB3 | CRYAB | CSNK2A2 | CTH | CTNNB1 | CTTN | DAB2 | DAD1 | DAPK1 | DDB1 | DDRGK1 | DDX3X | DHCR24 | DNAJA1 | DNAJA3 | DPEP1 | DUSP1 | EGFR | ENO1 | ERBB3 | ERCC5 | EYA2 | EYA4 | FABP1 | FADD | FAM129B | FASLG | FBXO7 | FGB | FGF2 | FHL2 | FIGNL1 | FKBP8 | FOXO1 | FTH1 | FYN | GATA1 | GLO1 | GNAI2 | GNAI3 | GOLPH3 | GPI | GPX1 | GPX4 | GRIK2 | GRK5 | GSK3B | GSTP1 | HAX1 | HDAC1 | HDAC2 | HDAC3 | HIF1A | HMGB2 | HMOX1 | HNRNPK | HRAS | HSF1 | HSP90AB1 | HSP90B1 | HSPA1B | HSPA5 | HSPA9 | HSPB1 | HSPD1 | HTATIP2 | HTRA2 | HTT | HYPK | ICAM1 | IGBP1 | IGF1R | IKBKB | IKBKG | ILK | IRF7 | ITCH | ITGAV | JAK3 | JUN | KANK2 | KDM1A | KDR | KIR3DL2 | KLHL20 | KRAS | LAMP3 | LEF1 | LGALS3 | LGMN | LIG4 | LMNA | LRP6 | LRRK2 | MAD2L1 | MAP2K4 | MAPK7 | MAPK8 | MDM2 | MDM4 | MECP2 | MED1 | MGMT | MIF | MYC | MYD88 | NCK1 | NDUFA13 | NDUFS3 | NEUROD1 | NFE2L2 | NFKBIA | NME2 | NMNAT1 | NNT | NOD2 | NOS3 | NPM1 | NQO1 | NTF3 | NTRK1 | OPA1 | PA2G4 | PAK2 | PALB2 | PAM16 | PARK7 | PAX8 | PDCD10 | PDE12 | PELI1 | PHB2 | PIAS1 | PIH1D1 | PIK3CA | PIK3CG | PIK3R1 | PIM1 | PIN1 | PLAUR | PLK1 | PPARD | PPARGC1A | PPIF | PPT1 | PRDX2 | PRDX3 | PRDX5 | PRELID1 | PRKAA1 | PRKAA2 | PRKCD | PRKCZ | PRKD1 | PRKDC | PSAP | PSEN1 | PSMC1 | PSMC5 | PSMD10 | PSME3 | PTCRA | PTEN | PTK2 | PTPN1 | PTRH2 | PYCR1 | QARS | RAF1 | RARA | RB1CC1 | REL | RELA | RGN | RHBDD1 | RHOA | RIPK1 | RIPK2 | RPL10 | RPS6KA1 | RRM2B | SCG2 | SERPINB2 | SET | SFN | SHC1 | SIN3A | SIRT1 | SIRT5 | SLC25A4 | SLC25A5 | SLC35F6 | SLC9A1 | SMAD3 | SMAD4 | SMO | SNAI2 | SNCA | SNX6 | SOCS2 | SOD1 | SOD2 | SPRY2 | SQSTM1 | SRC | SRGAP2 | STAT3 | STIL | STK40 | STXBP1 | SULT1A3 | SYVN1 | THBS1 | THOC6 | TIMP1 | TLE1 | TMBIM6 | TNF | TNFAIP3 | TNFSF14 | TNIP2 | TP53 | TRADD | TRAF2 | TRAF6 | TRAP1 | TRIAP1 | TRIM32 | TSC22D3 | TWIST1 | TXN | TXNDC5 | UBB | UBC | UBE2B | UFM1 | USP47 | VDAC2 | VHL | VPS4B | WNT16 | WT1 | XBP1 | XIAP | YAP1 | YME1L1 | YWHAE | YWHAZ | ZC3HC1 | ZFAND6 | GO:0070647 | 0.0800679364309111 | 660/8243 | 335/3250 | 5.18357907659444e-10 | 4.92180833322642e-06 | T | T | T | T | protein modification by small protein conjugation or removal | ACTB | ACTL6A | ADRB2 | ADRM1 | AKT1 | AMFR | ANAPC4 | APC | AR | ARRB1 | ARRB2 | ASB12 | ASB15 | ASB16 | ASB6 | ASB9 | BABAM1 | BCL10 | BCL11A | BCL2 | BCOR | BIRC2 | BIRC3 | BIRC7 | BIRC8 | BMI1 | BRCA1 | BTRC | CAND1 | CBFB | CBL | CBLB | CBLC | CBX4 | CBX8 | CCDC22 | CCP110 | CDC16 | CDC20 | CDC25A | CDC27 | CDC34 | CDC42 | CDC73 | CFTR | COMMD1 | COPS2 | COPS5 | COPS6 | CRBN | CTNNB1 | CUL1 | CUL2 | CUL3 | CUL4B | CUL5 | CUL7 | CYLD | DCAF12 | DCAF7 | DCAF8 | DCUN1D1 | DDB1 | DDB2 | DDRGK1 | DERL1 | DTX1 | DTX2 | DZIP3 | EP300 | ESR1 | FBXL12 | FBXO11 | FBXO25 | FBXO32 | FBXO6 | FBXO7 | FBXW11 | FBXW5 | FOXK1 | FOXK2 | FZR1 | HDAC4 | HDAC6 | HECW1 | HECW2 | HERC1 | HERC2 | HGS | HIF1A | HIST1H2BA | HUWE1 | IKBKG | INO80B | INO80C | ITCH | JOSD2 | KCMF1 | KEAP1 | KLHL20 | KLHL22 | LMO7 | LNX1 | LRSAM1 | LZTR1 | MARCH5 | MDM2 | MDM4 | MED1 | MED12 | MED20 | MED21 | MED31 | MIB1 | MYC | NAE1 | NEDD4 | NEDD8 | NEURL4 | NFE2L2 | NFKBIA | NHLRC3 | NOSIP | NPEPPS | NUP107 | NUP153 | NUP160 | NUP205 | NUP214 | NUP85 | NUP88 | NUP93 | NUP98 | OGT | OTUB1 | OTUB2 | OTUD4 | OTUD5 | PCGF1 | PCGF5 | PCGF6 | PCNA | PELI1 | PEX5 | PIAS1 | PIAS3 | PJA1 | PLK1 | POM121 | PPP1R11 | PRKDC | PRPF19 | PSMA1 | PSMA2 | PSMA3 | PSMA5 | PSMA6 | PSMB5 | PSMB6 | PSMB7 | PSMB8 | PSMC1 | PSMC2 | PSMC3 | PSMC4 | PSMC5 | PSMC6 | PSMD1 | PSMD10 | PSMD11 | PSMD12 | PSMD14 | PSMD2 | PSMD4 | PSMD9 | PSME2 | PSME3 | PTEN | RAD23A | RAD23B | RAE1 | RANGAP1 | RBBP6 | RBX1 | RC3H1 | RHOA | RHOT1 | RING1 | RIPK1 | RNF123 | RNF126 | RNF139 | RNF181 | RNF183 | RNF185 | RNF19A | RNF2 | RNF25 | RNF31 | RNF4 | RNF8 | RTF1 | RUVBL1 | SAE1 | SENP3 | SENP6 | SENP7 | SENP8 | SHMT2 | SIAH1 | SIRT1 | SMAD2 | SMAD3 | SMAD4 | SMURF1 | SMURF2 | SNX3 | SOCS1 | SOCS2 | SOCS6 | SPATA2 | SPOP | SPSB3 | STUB1 | SUMO1 | SUMO1P1 | SUMO2 | SUMO3 | SUZ12 | SYVN1 | TAB1 | TADA2B | THOP1 | TMEM189-UBE2V1 | TNFAIP3 | TNIP2 | TNKS | TNKS2 | TOMM20 | TOR1A | TP53 | TPR | TRAF2 | TRAF3 | TRAF6 | TRIM21 | TRIM23 | TRIM25 | TRIM28 | TRIM32 | TRIM33 | TRIM41 | TRIM47 | TRIM63 | TRIM69 | TRIM7 | TRIM9 | TRIP12 | TSG101 | TSPAN17 | UBA1 | UBA2 | UBA3 | UBA5 | UBA6 | UBAC1 | UBB | UBC | UBE2A | UBE2B | UBE2C | UBE2D1 | UBE2D2 | UBE2D3 | UBE2H | UBE2I | UBE2J1 | UBE2K | UBE2L3 | UBE2L6 | UBE2M | UBE2N | UBE2O | UBE2Q2 | UBE2R2 | UBE2S | UBE2V1 | UBE2W | UBE3A | UBE3B | UBR4 | UBR5 | UCHL1 | UCHL3 | UCHL5 | UFC1 | UFM1 | UNKL | USP11 | USP14 | USP15 | USP16 | USP17L2 | USP19 | USP2 | USP20 | USP21 | USP26 | USP28 | USP39 | USP4 | USP42 | USP46 | USP47 | USP5 | USP50 | USP53 | USP7 | USP8 | USP9X | USP9Y | USPL1 | VCP | VCPIP1 | VHL | WDR48 | WDTC1 | WSB1 | WWP2 | XIAP | YY1 | ZBTB16 | ZC3HC1 | ZNF598 | ZNRF4 | GO:0019221 | 0.0493752274657285 | 407/8243 | 220/3250 | 6.65198239976978e-10 | 6.31605728858141e-06 | T | T | T | T | cytokine-mediated signaling pathway | ACKR2 | ACSL1 | ACTN4 | AKT1 | ALOX5 | ANXA1 | ANXA2 | ARF1 | BAG4 | BCL2 | BCL2L1 | BCL6 | BIRC2 | BIRC3 | BIRC5 | BTRC | CAMK2A | CAMK2B | CANX | CAPZA1 | CASP3 | CBL | CCL21 | CCL22 | CCR1 | CCR3 | CCR4 | CCR5 | CD27 | CD36 | CD40 | CD40LG | CD74 | CDC42 | CDKN1A | CEBPA | CFL1 | CLDN18 | CRK | CRKL | CSNK2B | CUL1 | CXCL11 | CXCL5 | CXCL6 | CXCL8 | DOK1 | EDA | FASLG | FBXW11 | FCGR1A | FGF2 | FN1 | FOS | FOXO1 | FSCN1 | FYN | GBP2 | GRB2 | GSTA2 | GSTO1 | HIF1A | HIST1H3A | HIST2H3A | HLA-A | HLA-B | HLA-DQA1 | HLA-DRA | HLA-DRB1 | HLA-DRB3 | HLA-DRB4 | HLA-DRB5 | HMOX1 | HNRNPA2B1 | HNRNPDL | HNRNPF | HSP90AA1 | HSP90B1 | HSPA8 | HSPA9 | ICAM1 | IFIT1 | IFITM3 | IFNA2 | IFNAR1 | IKBKB | IKBKG | IL13RA2 | IL32 | IL3RA | ILK | IRF1 | IRF4 | IRF7 | ISG15 | JAK1 | JAK3 | JUNB | KLF6 | KRAS | LCP1 | LIMS1 | LMNB1 | LTBR | MAOA | MAP3K14 | MAP3K3 | MAPK3 | MIF | MSN | MTAP | MYC | MYD88 | NANOG | NFKBIA | NOD2 | NOS2 | OAS2 | OXSR1 | P4HB | PAK2 | PELI1 | PIAS3 | PIK3CA | PIK3R1 | PIM1 | PITPNA | PML | PPIA | PRKACA | PRKCD | PSMA1 | PSMA2 | PSMA3 | PSMA5 | PSMA6 | PSMB5 | PSMB6 | PSMB7 | PSMB8 | PSMC1 | PSMC2 | PSMC3 | PSMC4 | PSMC5 | PSMC6 | PSMD1 | PSMD10 | PSMD11 | PSMD12 | PSMD14 | PSMD2 | PSMD4 | PSMD9 | PSME2 | PSME3 | PTPN6 | RAP1B | RBX1 | RELA | RHOU | RIPK1 | RIPK2 | RPLP0 | SDC1 | SERPINB2 | SHC1 | SIRT1 | SMAD4 | SMARCA4 | SNRPA1 | SOCS1 | SOCS2 | SOD1 | SOD2 | SOX2 | SP100 | SPI1 | SQSTM1 | STAT1 | STAT3 | STAT4 | STAT5A | STAT6 | TAB1 | TAB2 | TALDO1 | TCP1 | TGFB1 | TIMP1 | TNF | TNFRSF12A | TNFRSF1A | TNFRSF8 | TNFSF12 | TNFSF14 | TNIP2 | TOLLIP | TP53 | TRADD | TRAF2 | TRAF3 | TRAF6 | TREX1 | TRIM21 | TRIM25 | TRIM34 | TWIST1 | TXLNA | TXNDC17 | UBB | UBC | UBE2N | UBE2V1 | VAV1 | VCAM1 | VIM | WNK1 | XAF1 | YWHAZ | GO:0043933 | 0.145820696348417 | 1202/8243 | 570/3250 | 6.86795348993832e-10 | 6.52112183869644e-06 | T | T | T | T | protein-containing complex subunit organization | AAR2 | AASS | ABCE1 | ABCG1 | ACACA | ACAD9 | ACAT1 | ACD | ACOT13 | ACTN2 | ADRB2 | ADRM1 | ADSL | AGO1 | AGO2 | AHNAK | AIMP2 | ALB | ALDH5A1 | ALDOA | ALOX5AP | AMFR | ANKRD28 | ANKS4B | ANXA2 | ANXA5 | ANXA6 | APC | APCS | APEH | APOA1 | APOE | AR | ARF1 | ARPC2 | ARPC4 | ATL3 | ATPAF2 | ATRX | ATXN10 | ATXN2 | AXIN1 | BAX | BCL10 | BCS1L | BIN1 | BIRC2 | BIRC3 | BRAF | BRD2 | C1QBP | C1QTNF1 | CAB39 | CALR | CAND1 | CAPG | CAPZA1 | CAPZA2 | CASP8 | CAT | CAV1 | CBR4 | CBY1 | CCNB1 | CCT2 | CCT3 | CCT6B | CCT8 | CD2BP2 | CD40 | CD74 | CD79B | CDA | CDC73 | CENPF | CENPJ | CENPV | CEP57 | CFL1 | CFL2 | CHAF1A | CHCHD10 | CHD1L | CHMP2A | CHP1 | CIRBP | CKAP5 | CLGN | CLNS1A | CLTC | CLU | COA3 | COL12A1 | COL14A1 | COL2A1 | COL5A1 | COL6A1 | COL6A2 | COX11 | COX15 | COX17 | COX19 | COX20 | COX7A2 | CPSF7 | CPT1A | CREBBP | CRYAB | CRYZ | CSNK1A1 | CSNK2B | CTCF | CTH | CTNNB1 | CTSC | CTTN | CUL1 | CUL3 | CUL4B | CUTC | CYLD | DAP3 | DARS | DAXX | DBNL | DCTPP1 | DCXR | DDB1 | DDB2 | DDIT4 | DDX1 | DDX39B | DDX3X | DDX6 | DECR1 | DERL1 | DHRS4 | DHX9 | DIAPH3 | DLG4 | DMD | DNAAF2 | DNAAF5 | DNM1L | DSTN | DVL1 | DVL2 | DVL3 | DYNC1H1 | ECSIT | EHD4 | EIF2AK3 | EIF2S1 | EIF3A | EIF3D | EIF3E | EIF3H | EIF3I | EIF4H | EIF5 | EIF6 | ELAVL1 | EP300 | EPPIN | EPRS | ERAL1 | ERCC5 | F5 | FADD | FANCA | FANCC | FARSB | FBP1 | FERMT2 | FGB | FIS1 | FKBP1A | FLOT1 | FN1 | FOLR1 | FSD1 | FUS | G3BP1 | G3BP2 | GAK | GBP5 | GEMIN4 | GEMIN5 | GEMIN7 | GLRB | GLS | GLUL | GNB1 | GOLGA2 | GOPC | GPX4 | GRB2 | GRB7 | GRHPR | GRIA1 | GSK3B | GSPT1 | GSPT2 | H1F0 | H1FX | H2AFX | H3F3A | HBZ | HCCS | HCN1 | HDAC1 | HDAC6 | HIST1H1A | HIST1H2BA | HIST1H3A | HIST1H4A | HIST2H3A | HIST3H3 | HLA-DRA | HLA-DRB1 | HMGB2 | HMGCL | HMOX1 | HPRT1 | HRAS | HSD17B10 | HSD17B8 | HSF1 | HSF4 | HSP90AA1 | HSP90AB1 | HSPA4 | HSPA8 | HSPD1 | HTRA2 | IDE | IGF1R | IKBKE | IKZF5 | ILK | IMPDH2 | INSR | IPO4 | ITGB1BP1 | ITGB3BP | ITPR3 | JMJD6 | JUP | KAT5 | KCNA4 | KCNS3 | KCTD12 | KCTD17 | KCTD18 | KCTD2 | KCTD3 | KHDRBS1 | KIF2C | KIF5B | KLC1 | KMT2A | LEF1 | LETM1 | LIMS1 | LNX1 | LOXL2 | LRP6 | LRRK2 | LSM3 | LSM4 | LUC7L2 | LUM | LYRM7 | MAGI1 | MAPK3 | MAPT | MAT2A | MCCC1 | MCFD2 | MCM2 | MCTS1 | MDM2 | MDM4 | MDN1 | ME1 | MED6 | MGST1 | MICU1 | MIF | MITF | MPP2 | MRPL12 | MRPL14 | MRPL19 | MRPL28 | MRPL38 | MRPL4 | MRPL43 | MRPL45 | MRPL46 | MRPL9 | MRPS5 | MRRF | MYC | MYH11 | NACC1 | NAP1L1 | NAP1L4 | NCBP1 | NCK1 | NDUFA10 | NDUFA13 | NDUFA5 | NDUFA8 | NDUFA9 | NDUFAF1 | NDUFAF2 | NDUFAF3 | NDUFAF4 | NDUFAF5 | NDUFAF7 | NDUFB1 | NDUFS1 | NDUFS2 | NDUFS3 | NDUFS6 | NDUFS7 | NDUFS8 | NDUFV1 | NDUFV3 | NFS1 | NME2 | NOD2 | NPM1 | NUFIP1 | NUP107 | NUP153 | NUP205 | NUP93 | NUP98 | NUPR1 | OAT | OBSL1 | OGFOD1 | OPA1 | OTC | OXA1L | P2RX1 | P2RX2 | P2RX4 | P4HA1 | P4HB | PADI4 | PANX3 | PARD3 | PARP1 | PCBD1 | PDCD6 | PDCD6IP | PDCL | PDE4DIP | PDSS1 | PDSS2 | PEF1 | PEX11B | PFKL | PFKM | PIAS1 | PIH1D1 | PIK3C2A | PIN1 | PKM | PLA2G2A | PLOD2 | PML | POLE3 | PPARGC1A | PPAT | PPID | PPP1CA | PPP2R1A | PPP6C | PPP6R1 | PRKAA1 | PRKACA | PRKCZ | PRPF19 | PRPF8 | PSMD10 | PSMD11 | PSMD4 | PSMD9 | PSMG1 | PSMG4 | PSTPIP1 | PTGES3 | PTK2 | PTMA | PXN | RAB1A | RAB1B | RAC1 | RAD23B | RAD51 | RAF1 | RANBP9 | RAP1GDS1 | RBBP4 | RBMX | RBX1 | RC3H1 | RCC1 | RIC3 | RIPK1 | RIPK3 | RNF8 | RPA2 | RPA3 | RPL10 | RPL12 | RPL23 | RPL23A | RPL24 | RPL38 | RPL6 | RPLP0 | RPLP0P6 | RPS10P5 | RPS14 | RPS19 | RRM1 | RRM2 | RRP7A | RRS1 | RTN4 | RUVBL1 | RUVBL2 | RXRA | SART1 | SART3 | SBDS | SCFD1 | SCO2 | SEC13 | SEC16A | SEC24B | SEC24C | SEC31A | SEPT11 | SEPT4 | SEPT7 | SEPT9 | SERPINH1 | SET | SF1 | SH3GL1 | SH3GL2 | SHKBP1 | SHMT1 | SHMT2 | SKI | SLC9A1 | SLC9A3R1 | SLC9A3R2 | SMAD2 | SMAD3 | SMAD4 | SMARCA4 | SMARCB1 | SMARCC1 | SMARCC2 | SMARCE1 | SMN1 | SNAP29 | SNCA | SNRPB | SNRPD1 | SNRPD3 | SNRPE | SNX2 | SOAT1 | SOD2 | SQSTM1 | SRC | SRP19 | SRPK1 | SRPK2 | SRSF12 | SRSF5 | ST13 | STMN1 | STOM | STOML2 | STRAP | STX5 | STXBP1 | TAF12 | TAZ | TBCA | TCF4 | TCP1 | TDO2 | TEKT2 | TFG | TFIP11 | TGFB1 | TGFBR3 | TGM2 | TIMM21 | TIMMDC1 | TK1 | TLE1 | TLE3 | TM9SF4 | TMEM70 | TNF | TNFAIP3 | TNFRSF1A | TNP1 | TNXB | TOMM20 | TOR1A | TP53 | TP63 | TP73 | TPR | TRADD | TRAF1 | TRAF2 | TRIM21 | TRIM28 | TRIM34 | TRMT61B | TSG101 | TSPYL2 | TTC19 | TWF1 | TWF2 | TXNL4A | UBB | UBC | UBE2C | UBE2K | UBE2S | UBQLN1 | UGDH | UQCC1 | UQCRB | USP16 | USP39 | USP4 | UXS1 | VASP | VBP1 | VCP | VPS4B | VPS72 | VTA1 | VTN | WAS | WDR1 | WDR77 | WIPI2 | XRCC6 | YAP1 | YME1L1 | YWHAB | ZMYND10 | ZNF207 | ZW10 | GO:0080090 | 0.260099478345263 | 2144/8243 | 963/3250 | 1.0187029538154e-09 | 9.67258454647719e-06 | T | T | T | T | regulation of primary metabolic process | A2M | AARS | ABAT | ABCE1 | ABCG1 | ABL1 | ACACA | ACADVL | ACD | ACO1 | ACOX1 | ACSL1 | ACTB | ACTR2 | ADCY6 | ADCYAP1 | ADORA2A | ADRB2 | ADRM1 | ADTRP | AGAP2 | AGER | AGO1 | AGO2 | AHCYL1 | AHNAK | AHSG | AIFM1 | AIMP2 | AIRE | AKAP8 | AKAP9 | AKT1 | AKT2 | AKTIP | ALAS1 | ALOX12 | ANXA1 | ANXA2 | APBB1 | APC | APCS | APOA1 | APOD | APOE | APP | AR | ARAF | ARF1 | ARNT | ARR3 | ARRB1 | ARRB2 | ASPSCR1 | ATAD5 | ATCAY | ATG5 | ATM | ATP13A2 | ATP1A1 | ATRX | ATXN2 | AURKB | AXIN1 | AZU1 | B3GAT3 | BABAM1 | BAG2 | BAG4 | BAG5 | BAG6 | BAX | BBC3 | BCAR3 | BCL10 | BCL2 | BCL2L13 | BCL6 | BCOR | BDKRB2 | BIN1 | BIRC2 | BIRC3 | BIRC5 | BIRC7 | BIRC8 | BMI1 | BMPR1A | BRAF | BRCA1 | BRD4 | BTRC | C1QBP | C1QL4 | C1QTNF1 | C4A | CAB39 | CALM1 | CALM2 | CALM3 | CALR | CAPN2 | CARD9 | CARM1 | CASP10 | CASP3 | CASP8 | CASP9 | CAV1 | CBFA2T3 | CBL | CBLB | CBLC | CBX8 | CCAR2 | CCDC22 | CCDC88A | CCL21 | CCL22 | CCNB1 | CCNT1 | CCNT2 | CCNY | CCNYL1 | CCR1 | CCT2 | CCT3 | CCT4 | CCT5 | CCT6A | CCT7 | CCT8 | CD109 | CD27 | CD36 | CD40 | CD40LG | CD74 | CD81 | CDA | CDC14B | CDC20 | CDC25A | CDC25B | CDC25C | CDC37 | CDC73 | CDK2 | CDK4 | CDK5 | CDK5R1 | CDK9 | CDKN1A | CDKN2C | CEBPA | CHEK2 | CHORDC1 | CHP1 | CHRNA9 | CIRBP | CKS1B | CKS2 | CLK1 | CLPX | CLTC | CLU | CNDP1 | COA3 | COMMD1 | COMT | COPS5 | COX11 | CPSF7 | CPT1A | CPT2 | CREB1 | CREBBP | CRK | CRKL | CRY1 | CRY2 | CRYAB | CSNK1A1 | CSNK1E | CSNK2A2 | CSNK2B | CTBP1 | CTCF | CTNNB1 | CTSC | CUL3 | CYCS | CYLD | CYP1A1 | DAB1 | DAB2 | DAPK1 | DAXX | DAZAP1 | DBF4B | DBNDD2 | DBNL | DCP2 | DCUN1D1 | DDA1 | DDIT4 | DDRGK1 | DDX1 | DDX39B | DDX3X | DERL1 | DGKZ | DHCR24 | DHX36 | DHX9 | DIABLO | DLG3 | DLG4 | DMD | DNAJA1 | DNAJB2 | DNAJC10 | DNM1L | DOCK7 | DPEP1 | DROSHA | DUSP1 | DUSP12 | DUSP14 | DUSP16 | DUSP19 | DUSP22 | DUSP7 | DVL1 | DVL2 | DVL3 | DYRK2 | ECD | EDEM1 | EED | EEF1A2 | EEF2 | EGFR | EGLN3 | EHD4 | EID1 | EIF2AK3 | EIF2AK4 | EIF2B5 | EIF2S1 | EIF3A | EIF3D | EIF3E | EIF3H | EIF4A3 | EIF4E | EIF4E2 | EIF4EBP1 | EIF4ENIF1 | EIF4G3 | EIF4H | EIF5 | EIF5A | EIF5B | EIF6 | ELAVL1 | ENDOG | ENO1 | EP300 | EPHA4 | EPPIN | EPRS | ERBB2 | ERBB3 | ERLIN2 | ERRFI1 | ESR1 | ESRRB | EXOSC6 | EYA2 | EYA4 | EZH2 | EZR | FABP1 | FABP3 | FABP5 | FADD | FAF1 | FAM129B | FAM192A | FAM3C | FAM83D | FANCI | FANCM | FASLG | FASN | FBP1 | FBXO7 | FBXW11 | FDPS | FGB | FGF2 | FGFR1 | FGFR1OP | FGFR3 | FHL2 | FIGNL1 | FIS1 | FKBP1A | FKBP1B | FKBP8 | FLOT1 | FN1 | FOXJ1 | FOXM1 | FOXO1 | FTO | FUS | FXR2 | FYN | FZR1 | G6PD | GADD45G | GAPDH | GAPDHS | GATA1 | GDF9 | GEMIN5 | GGPS1 | GLMN | GNA12 | GNAI2 | GNAQ | GOLGA2 | GPD1 | GPD1L | GPI | GPS2 | GPX1 | GRB2 | GRB7 | GRIK2 | GSK3A | GSK3B | GSTP1 | GSTZ1 | GZMA | H1F0 | H1FX | H2AFX | HAP1 | HAVCR2 | HAX1 | HBEGF | HDAC1 | HDAC2 | HDAC3 | HDAC4 | HDAC5 | HDAC6 | HELZ2 | HEXIM1 | HGS | HIF1A | HIST1H1A | HMGB1 | HMGB2 | HMOX1 | HNF4A | HNRNPA1 | HNRNPA2B1 | HNRNPD | HNRNPF | HNRNPH1 | HNRNPK | HNRNPL | HNRNPU | HPRT1 | HPX | HRAS | HSF1 | HSP90AA1 | HSP90AB1 | HSP90AB3P | HSP90AB4P | HSP90B1 | HSPA1B | HSPA2 | HSPA5 | HSPB1 | HSPBP1 | HSPD1 | HSPE1 | HTRA2 | HTT | IBTK | ICAM1 | ID2 | IDE | IDH1 | IFI16 | IFNA2 | IGBP1 | IGF1R | IGF2BP1 | IGFBP7 | IKBKB | IKBKG | ILF3 | ILK | INSIG1 | INSIG2 | INSR | IPO5 | IPO7 | IQGAP1 | IREB2 | IRF1 | IRF4 | ISG15 | ITCH | ITGAV | ITGB1BP1 | ITGB3 | JMJD6 | JUN | KAT5 | KAT7 | KDM1A | KDM4D | KDR | KEAP1 | KHDRBS1 | KLHDC10 | KMT2A | KMT2B | KPNA1 | KPNA2 | KPNB1 | KRAS | KSR1 | LAMP3 | LARP4 | LAT | LATS1 | LCP2 | LDLRAD4 | LEF1 | LEMD2 | LGALS3 | LGMN | LMNA | LPAR1 | LRP6 | LRPPRC | LRRK1 | LRRK2 | LTBR | MAD2L1 | MAP2K1 | MAP2K3 | MAP2K4 | MAP2K6 | MAP2K7 | MAP3K1 | MAP3K13 | MAP3K14 | MAP3K2 | MAP3K3 | MAP3K4 | MAP4K1 | MAPK1 | MAPK14 | MAPK15 | MAPK3 | MAPK7 | MAPK8 | MAPK8IP3 | MAPK9 | MAPKAP1 | MAPT | MAS1 | MAVS | MBD3 | MBP | MDM2 | ME1 | MECP2 | MED1 | MGMT | MIF | MKNK2 | MLH1 | MLLT1 | MLST8 | MOB1B | MOV10 | MSH6 | MSN | MTA1 | MTPN | MVD | MYC | MYOC | NCBP1 | NCK1 | NCOA1 | NCOA2 | NCOR1 | NCOR2 | NDUFA13 | NEDD8 | NEK1 | NEK7 | NELFE | NELL1 | NF2 | NFE2L2 | NFKBIA | NIPBL | NMNAT1 | NNT | NOD2 | NOS1AP | NPM1 | NQO1 | NQO2 | NR1H2 | NR1H3 | NRBF2 | NTF3 | NTRK1 | NUP107 | NUP153 | NUP160 | NUP205 | NUP214 | NUP85 | NUP88 | NUP93 | NUP98 | NUPR1 | OAS2 | OAZ3 | OGFOD1 | OGT | OTUB1 | OTUD4 | OXSR1 | P2RX1 | PA2G4 | PABPC1 | PACSIN3 | PAFAH1B1 | PAIP1 | PAK2 | PAK6 | PAM16 | PAPLN | PARD3 | PARD6A | PARK7 | PARP1 | PARP14 | PAWR | PAXIP1 | PCNA | PCSK9 | PDCD10 | PDCD6 | PDE4D | PDE6G | PDK2 | PEBP1 | PEF1 | PELI1 | PER1 | PER2 | PGAM1 | PGP | PHB | PHB2 | PIAS1 | PIAS3 | PIH1D1 | PIK3CA | PIK3CG | PIK3R1 | PIK3R3 | PIN1 | PINX1 | PKIG | PLA2G10 | PLA2G1B | PLA2G2A | PLA2G6 | PLAUR | PLGRKT | PLK1 | PLXNB2 | PML | PNKP | POLDIP3 | POLR2A | POM121 | POT1 | PPARD | PPARG | PPARGC1A | PPIB | PPME1 | PPP1CA | PPP1CB | PPP1R11 | PPP1R12A | PPP1R14A | PPP1R16A | PPP1R2 | PPP1R3B | PPP1R3C | PPP1R8 | PPP2CA | PPP2R1A | PPP2R1B | PPP2R2A | PPP2R2D | PPP2R5D | PPP4R2 | PPP6R1 | PRDX1 | PRDX2 | PRDX3 | PRDX5 | PRDX6 | PRELID1 | PRKAA1 | PRKAA2 | PRKAB2 | PRKACA | PRKAG1 | PRKAR1A | PRKAR2B | PRKCD | PRKCE | PRKCZ | PRKD1 | PRKD2 | PRKDC | PRMT3 | PRMT6 | PROX1 | PRPF19 | PRRC1 | PSAP | PSEN1 | PSMA1 | PSMA2 | PSMA3 | PSMA5 | PSMA6 | PSMB5 | PSMB6 | PSMB7 | PSMB8 | PSMC1 | PSMC2 | PSMC3 | PSMC4 | PSMC5 | PSMC6 | PSMD1 | PSMD10 | PSMD11 | PSMD12 | PSMD14 | PSMD2 | PSMD4 | PSMD9 | PSME2 | PSME3 | PTEN | PTK2 | PTN | PTPN1 | PTPN22 | PTPN6 | PTPRR | PTTG1 | PUM1 | PUS7 | QARS | RAB1A | RAB1B | RAB3GAP1 | RAC1 | RAD23A | RAD23B | RAD51 | RAD51AP1 | RAE1 | RAF1 | RAN | RANBP9 | RAP1A | RAP1B | RARA | RB1 | RB1CC1 | RBM17 | RBM25 | RBM3 | RBM4 | RBM8A | RBMX | RBX1 | RC3H1 | RDX | RECK | RELA | REST | RGN | RGS2 | RHBDD1 | RHOA | RIF1 | RIOK1 | RIPK1 | RIPK2 | RIPK3 | RMND1 | RNF139 | RNF185 | RNF19A | RNF8 | RPA2 | RPL10 | RPL23 | RPL38 | RPLP1 | RPS13 | RPS14 | RPS2 | RPS3 | RPS4X | RPS6KA1 | RPS6KB2 | RPTOR | RTF1 | RUVBL2 | RXRA | S100A12 | S100A7 | S100A9 | SAG | SAP18 | SART3 | SDC4 | SDCBP | SEC13 | SEMA7A | SEPT4 | SERPINB2 | SERPINB3 | SERPINB5 | SERPINB6 | SERPINB8 | SERPINE2 | SERPINH1 | SET | SF1 | SFN | SGTA | SHC1 | SHMT1 | SHMT2 | SIN3A | SIRT1 | SIRT3 | SIRT6 | SKI | SLAMF1 | SLC9A3R1 | SLTM | SMAD3 | SMAD4 | SMARCB1 | SMARCC1 | SMG5 | SMG6 | SMG8 | SMURF1 | SNAI2 | SNCA | SNF8 | SNRPA | SNTA1 | SNW1 | SNX12 | SNX6 | SOAT1 | SOCS1 | SOCS2 | SOCS6 | SOD1 | SOX2 | SP1 | SPI1 | SPINK7 | SPINT2 | SPOP | SPRTN | SPRY2 | SQSTM1 | SRC | SRPK1 | SRPK2 | SRSF12 | SRSF5 | STAT3 | STAT6 | STK11 | STK24 | STK25 | STK26 | STK3 | STK38 | STK40 | STOML2 | STRAP | STUB1 | STYX | SUMO1 | SUMO2 | SUPT5H | SWAP70 | SYNJ2BP | TAB1 | TAB2 | TACO1 | TADA2B | TANK | TAOK3 | TARDBP | TAZ | TBL1X | TCEA1 | TCOF1 | TCP1 | TERF1 | TERF2 | TERF2IP | TFIP11 | TGFB1 | TGFBR3 | THBS1 | TIA1 | TIMP1 | TIMP2 | TINF2 | TLR3 | TMBIM6 | TMTC3 | TNF | TNFAIP3 | TNFRSF1A | TNFRSF8 | TNFSF14 | TNK2 | TNKS | TNKS2 | TNP1 | TOLLIP | TP53 | TP63 | TP73 | TPR | TRA2A | TRADD | TRAF2 | TRAF3 | TRAF3IP1 | TRAF6 | TRAP1 | TREX1 | TRIAP1 | TRIB2 | TRIB3 | TRIM21 | TRIM28 | TRIM32 | TRIP12 | TRUB2 | TSFM | TSG101 | TSPYL2 | TWF1 | TWIST1 | TXN | TXNRD1 | TYMS | U2AF2 | UBB | UBC | UBE2B | UBE2C | UBE2D1 | UBE2I | UBE2J1 | UBE2K | UBE2L3 | UBE2N | UBE2S | UBE2V1 | UBE3A | UBQLN1 | UBQLN2 | UBQLN4 | UBR5 | UBXN1 | UCHL1 | UCHL5 | UNC119 | UNK | UPF3B | UQCC1 | USP14 | USP16 | USP17L2 | USP19 | USP4 | USP47 | USP5 | USP50 | USP7 | USP9X | VCP | VDR | VIM | VTN | WARS | WAS | WBP2 | WDR35 | WDR48 | WDTC1 | WNK1 | WNT16 | WT1 | WTAP | XIAP | XRCC1 | XRCC5 | XRCC6 | YES1 | YTHDF1 | YWHAB | YWHAE | YWHAG | ZBTB7A | ZBTB7B | ZMPSTE24 | ZNF598 | ZNF622 | GO:0044249 | 0.170811597719277 | 1408/8243 | 655/3250 | 1.68978632163546e-09 | 1.60445211239287e-05 | T | T | T | T | cellular biosynthetic process | AASDHPPT | AASS | ABAT | ABCD3 | ACACA | ACAT1 | ACCS | ACLY | ACOT7 | ACSL1 | ACSL4 | ACSL5 | ACSS2 | ADA | ADCY6 | ADK | ADRM1 | ADSL | ADSS | AFF4 | AGK | AGL | AGO2 | AK1 | AK2 | AK4 | AKR1A1 | AKR1B1 | AKR1B15 | AKT1 | AKT2 | ALAS1 | ALDH7A1 | ALDH8A1 | ALDH9A1 | ALDOA | ALOX12 | ALOX5 | ALOX5AP | AMD1 | AMDHD2 | APOA1 | APOE | APRT | AR | ARF1 | ARHGAP35 | ARNT | ARRB1 | ARRB2 | ASL | ASNS | ASPDH | ASS1 | ATAD5 | ATF6B | ATIC | ATM | ATOH1 | B3GAT3 | B3GNT3 | B4GALT3 | BAX | BCAT2 | BDH1 | BRCA1 | CBFB | CBR4 | CBS | CCDC88A | CCNB1 | CCNT1 | CCNT2 | CD74 | CDA | CDC73 | CDK2 | CDK4 | CDK8 | CDK9 | CDKN1A | CEBPA | CENPF | CHAF1A | CHDH | CHRAC1 | CMPK1 | CNDP2 | COL4A2 | COL4A3BP | COPS2 | COPS5 | COQ2 | COQ3 | COQ4 | COQ5 | COQ6 | COQ7 | COQ9 | COX15 | CPNE3 | CPSF7 | CREB1 | CREBBP | CSNK2A2 | CSNK2B | CSTF2T | CTDP1 | CTH | CTPS1 | CYC1 | CYP1A1 | CYP1A2 | DAP3 | DARS | DBH | DCAKD | DCK | DCN | DCTPP1 | DEGS1 | DERA | DGKE | DGUOK | DHRS7B | DHX9 | DIDO1 | DLAT | DLD | DMD | DNA2 | DNAJA3 | DNAJC30 | DPM1 | DRG2 | DSE | DTX1 | DUT | DVL1 | DVL2 | E2F1 | E2F2 | E2F3 | EAF2 | ECD | EEF1A1 | EEF1A1P5 | EEF1A2 | EEF1B2 | EEF1D | EEF1G | EEF2 | EGFR | EIF4H | EIF5A | ELF3 | ENOPH1 | ERAL1 | ESD | ESR1 | ESR2 | ESRRA | ESRRB | ESRRG | ETS1 | EXTL2 | FABP5 | FAM20B | FARSB | FASN | FBP1 | FBP2 | FDPS | FDXR | FECH | FEN1 | FGF2 | FOLH1 | FOS | FPGS | FUBP1 | FUBP3 | GAD2 | GAR1 | GARS | GART | GATA1 | GBE1 | GCDH | GDA | GEMIN5 | GFPT1 | GFPT2 | GGCT | GGPS1 | GGT1 | GGTLC1 | GLCE | GLS | GLS2 | GLUD2 | GLUL | GMDS | GMPPB | GMPR | GMPS | GNPDA1 | GNPDA2 | GNPNAT1 | GOLPH3 | GOT1 | GOT1L1 | GOT2 | GPI | GPT2 | GPX1 | GPX4 | GSPT1 | GSPT2 | GSS | GSTA1 | GSTA2 | GSTA3 | GSTA4 | GSTK1 | GSTM4 | GSTO1 | GSTO2 | GSTP1 | GSTT1 | GSTT2B | GSTZ1 | GTF2A1 | GTF2E2 | GTF2F1 | GTF2F2 | GTF2I | GTF3C2 | GYG1 | GYG2 | GYS1 | HAAO | HAGH | HARS | HEXB | HIF1A | HINT1 | HIST1H4A | HMBS | HMGCL | HNF4A | HNF4G | HOXB9 | HPRT1 | HS2ST1 | HSD17B14 | HSD17B4 | HSD17B6 | HSD17B8 | HSD3B7 | HSF1 | HSP90AA1 | HSP90AB1 | HSPA14 | IDH2 | IDI2 | ILF2 | IMPA1 | IMPA2 | IMPDH2 | ING5 | IREB2 | IRF1 | IRF7 | ISG15 | ISYNA1 | ITPA | JUN | KAT5 | KAT7 | KDSR | KLF5 | KMT2A | KYNU | LARP4 | LDHC | LEF1 | LIG4 | LMO2 | LPIN3 | LTC4S | LUM | MAPK1 | MAPK3 | MAT2A | MAT2B | MC1R | MCAT | MCM2 | MCM3 | MCM4 | MCM5 | MCM6 | MCM7 | MCM9 | MCMBP | ME1 | MECP2 | MED1 | MED12 | MED14 | MED17 | MED20 | MED21 | MED23 | MED24 | MED31 | MED4 | MED6 | MGST1 | MIF | MITF | MLLT1 | MLLT3 | MMAB | MOCOS | MPC2 | MPI | MPPE1 | MPST | MRPL12 | MRPL14 | MRPL19 | MRPL28 | MRPL38 | MRPL4 | MRPL43 | MRPL45 | MRPL46 | MRPL9 | MRPS5 | MRRF | MTAP | MTERF1 | MTHFD1 | MTMR14 | MTMR7 | MTRR | MVD | MYO5A | NAGK | NAMPT | NANP | NANS | NAP1L1 | NAPRT | NCBP1 | NCOA1 | NCOR1 | NDUFA9 | NELFB | NELFCD | NELFE | NFATC1 | NFE2L2 | NFS1 | NME1 | NME2 | NME4 | NMNAT1 | NMNAT3 | NMRAL1 | NMRK2 | NOP10 | NOS2 | NOS3 | NPLOC4 | NPR1 | NQO1 | NR0B2 | NR1H2 | NR1H3 | NR1I2 | NR3C1 | NR4A1 | NR5A2 | NRBF2 | NT5C2 | NUDT18 | NUDT3 | NUDT9 | NUP98 | OAT | OAZ3 | OSBPL1A | OTC | OXA1L | P2RX1 | PABPC4 | PAH | PAICS | PAPSS1 | PAPSS2 | PARK7 | PARP1 | PAX8 | PCBD1 | PCF11 | PCNA | PCTP | PCYT2 | PDE4D | PDF | PDHA1 | PDHA2 | PDHB | PDHX | PDSS1 | PDSS2 | PDXK | PER2 | PFAS | PGM1 | PGM2 | PGM3 | PGP | PHB | PHGDH | PHOSPHO1 | PI4K2A | PIGU | PIK3C2A | PIK3CA | PIK3CG | PIK3R1 | PIK3R3 | PIKFYVE | PINX1 | PIP4K2A | PIR | PLA2G1B | PLA2G6 | PLOD2 | PLSCR1 | PNKP | PNP | POLA2 | POLD1 | POLE2 | POLE3 | POLM | POLR2A | POLR2E | POLR2L | POLR2M | POT1 | POU5F1 | PPARD | PPARG | PPARGC1A | PPAT | PPIA | PPM1D | PPOX | PPP1R3C | PPP5C | PPT1 | PRKAA1 | PRKAA2 | PRKAB2 | PRKAG1 | PRKD1 | PRKD2 | PRKD3 | PRTFDC1 | PSAT1 | PTEN | PTGDS | PTGES | PTGES3 | PTMA | PTMS | PTPMT1 | PTTG1 | PYCR1 | PYCR2 | QDPR | RAB5A | RARA | RARG | RARS2 | RBBP4 | RBBP6 | RBMX | RBP1 | RBPJ | RELA | REPIN1 | RFC1 | RFC2 | RFC3 | RFC4 | RFC5 | RFK | RGN | RMND1 | RNASEH2A | RPA2 | RPA3 | RPAIN | RPL10 | RPL10A | RPL12 | RPL18A | RPL22 | RPL23 | RPL23A | RPL24 | RPL30 | RPL36AL | RPL37A | RPL38 | RPL6 | RPL7 | RPL7A | RPL9 | RPLP0 | RPLP0P6 | RPLP1 | RPLP2 | RPS12 | RPS13 | RPS14 | RPS15A | RPS19 | RPS2 | RPS20 | RPS24 | RPS29 | RPS3 | RPS4X | RPS6KB2 | RPS8 | RRM1 | RRM2 | RRM2B | RTF1 | RUNX2 | RXRA | RXRB | RXRG | SARS | SAT1 | SCAF1 | SCCPDH | SEPHS1 | SEPHS2 | SET | SGPL1 | SHMT1 | SHMT2 | SIN3A | SIRT1 | SIRT7 | SKI | SLC25A1 | SLC25A13 | SMAD4 | SMG5 | SMG6 | SMN1 | SNCA | SNRPB | SNRPD3 | SNRPE | SNW1 | SOD1 | SP1 | SPI1 | SPIN1 | SPR | SPRTN | SPTLC2 | ST3GAL1 | ST3GAL6 | ST6GALNAC5 | ST8SIA4 | STARD7 | STAT3 | STOML2 | SUCLA2 | SUPT4H1 | SUPT5H | TAF12 | TAF15 | TAF1A | TAMM41 | TARDBP | TARS | TAZ | TCEA1 | TCEA2 | TCERG1 | TCF4 | TECR | TERF1 | TERF2 | TERF2IP | TFCP2 | TGFB1 | THRA | TK1 | TK2 | TKT | TOP1 | TOP3A | TP53 | TPI1 | TREX1 | TRIM25 | TRIM28 | TRIM29 | TRIP13 | TSC22D1 | TSFM | TUFM | TYMS | UAP1 | UAP1L1 | UBB | UBC | UBE2L6 | UBIAD1 | UCK2 | UGDH | UGP2 | UGT8 | UMPS | UPP2 | USPL1 | UXS1 | VAC14 | VAPA | VAPB | VCAN | VCP | VDR | WARS | XBP1 | XYLT1 | YAP1 | ZBTB7A | ZBTB7B | ZNF217 | ZNF598 | GO:0044260 | 0.354239961179182 | 2920/8243 | 1277/3250 | 1.95934521735386e-09 | 1.86039828387749e-05 | T | T | T | T | cellular macromolecule metabolic process | ABHD17A | ABL1 | ABL2 | ACD | ACE2 | ACP1 | ACTB | ACTL6A | ACTN2 | ADCK2 | ADH5 | ADORA2A | ADPRH | ADRB2 | ADRM1 | AFF4 | AFG3L2 | AGL | AGO1 | AGO2 | AHSG | AICDA | AKT1 | AKT2 | AKT3 | ALB | AMFR | ANAPC4 | ANXA1 | APBB1 | APC | APCS | APEX1 | APEX2 | APOA1 | APOE | APP | AR | ARAF | ARF4 | ARHGAP35 | ARID4B | ARMC8 | ARNT | ARRB1 | ARRB2 | ASB12 | ASB15 | ASB16 | ASB6 | ASB9 | ASCC1 | ASCC2 | ASGR1 | ASGR2 | ATAD5 | ATF2 | ATF6B | ATG5 | ATM | ATOH1 | ATP13A2 | ATRX | AUP1 | AURKB | B3GAT3 | B3GNT3 | B4GALT3 | BABAM1 | BACE1 | BAG6 | BANF1 | BCL10 | BCL11A | BCL2 | BCOR | BIRC2 | BIRC3 | BIRC5 | BIRC7 | BIRC8 | BMI1 | BMPR1A | BRAF | BRCA1 | BRD4 | BTK | BTRC | C4A | CAB39 | CACNG2 | CACNG4 | CACNG5 | CALM1 | CALU | CAMK1D | CAMK2A | CAMK2B | CAMKV | CAND1 | CAPN1 | CARM1 | CASP3 | CAT | CAV1 | CBFB | CBL | CBLB | CBLC | CBS | CBX4 | CBX8 | CCDC155 | CCDC22 | CCDC36 | CCDC8 | CCDC88A | CCNB1 | CCNT1 | CCNT2 | CCP110 | CCR5 | CD36 | CD40LG | CD81 | CDC14B | CDC16 | CDC20 | CDC25A | CDC25B | CDC25C | CDC27 | CDC34 | CDC42 | CDC5L | CDC73 | CDK15 | CDK16 | CDK19 | CDK2 | CDK4 | CDK5 | CDK5R1 | CDK8 | CDK9 | CDKL3 | CEBPA | CEL | CENPF | CFL1 | CFTR | CHAF1A | CHD1L | CHEK2 | CHGB | CHRAC1 | CHRM3 | CHST4 | CKAP4 | CLK1 | CLTC | COG3 | COL2A1 | COL4A2 | COMMD1 | COPS2 | COPS5 | COPS6 | CPNE3 | CRBN | CREB1 | CREBBP | CRKL | CRY1 | CRY2 | CRYAB | CSNK1A1 | CSNK1E | CSNK2A2 | CSNK2B | CTBP1 | CTCF | CTDP1 | CTH | CTNNB1 | CUL1 | CUL2 | CUL3 | CUL4B | CUL5 | CUL7 | CXCL8 | CYCS | CYLD | DAD1 | DAP3 | DAPK1 | DAPP1 | DARS | DBI | DCAF12 | DCAF7 | DCAF8 | DCLK1 | DCN | DCP2 | DCTPP1 | DCUN1D1 | DDA1 | DDB1 | DDB2 | DDOST | DDRGK1 | DDX1 | DDX6 | DERL1 | DGUOK | DHX9 | DIDO1 | DIS3 | DLD | DLG2 | DLG3 | DLG4 | DLST | DMAP1 | DNA2 | DNAJA3 | DNAJB2 | DNAJC10 | DNASE2 | DNASE2B | DPF2 | DPM1 | DRG2 | DROSHA | DSE | DTX1 | DTX2 | DUSP1 | DUSP12 | DUSP13 | DUSP14 | DUSP16 | DUSP19 | DUSP22 | DUSP7 | DUT | DVL1 | DVL2 | DYRK2 | DZIP3 | E2F1 | EAF2 | ECD | EDC4 | EDEM1 | EED | EEF1A1 | EEF1A1P5 | EEF1A2 | EEF1B2 | EEF1D | EEF1G | EEF2 | EEF2KMT | EFEMP1 | EGFR | EGLN1 | EGLN3 | EHHADH | EHMT1 | EHMT2 | EIF2AK3 | EIF2AK4 | EIF2S1 | EIF3E | EIF4A3 | EIF4H | EIF5A | ELF3 | ENDOG | EP300 | EPHA4 | EPHA6 | EPHB6 | ERAL1 | ERBB2 | ERBB3 | ERCC5 | ERG | ERLIN2 | ESR1 | ETS1 | EVA1A | EXOSC1 | EXOSC5 | EXOSC6 | EXOSC9 | EXTL2 | EYA2 | EYA4 | EZH2 | EZR | F5 | FAF1 | FAF2 | FAM20B | FAM98A | FANCA | FANCC | FANCG | FANCI | FANCM | FARSB | FBXL12 | FBXO11 | FBXO25 | FBXO32 | FBXO6 | FBXO7 | FBXW11 | FBXW5 | FEN1 | FGF2 | FGFR1 | FGFR3 | FKBP14 | FKBP1A | FKBP1B | FKBP2 | FKBP5 | FKBP7 | FKBP8 | FN1 | FOLH1 | FOS | FOXK1 | FOXK2 | FOXM1 | FOXO1 | FTO | FTSJ3 | FUBP1 | FUBP3 | FYN | FZR1 | GAA | GAK | GALNT7 | GANAB | GAPDH | GAR1 | GATA1 | GBE1 | GEMIN5 | GFPT1 | GFPT2 | GGA1 | GGT1 | GINS2 | GINS3 | GINS4 | GLCE | GLRX | GLRX5 | GLUL | GNAS | GOLGA2 | GOLPH3 | GPS2 | GPX1 | GRB2 | GRIA1 | GRK1 | GRK5 | GSK3A | GSK3B | GSPT1 | GSPT2 | GTF2A1 | GTF2E2 | GTF2F1 | GTF2F2 | GTF2I | GTF3C2 | GYG1 | GYG2 | GYS1 | H1F0 | H2AFX | H3F3A | HARS | HBEGF | HCCS | HDAC1 | HDAC11 | HDAC2 | HDAC3 | HDAC4 | HDAC5 | HDAC6 | HDAC7 | HDAC9 | HECW1 | HECW2 | HELZ2 | HERC1 | HERC2 | HEXB | HGS | HIF1A | HIF1AN | HIPK4 | HIST1H2BA | HIST1H3A | HIST1H4A | HIST2H3A | HIST3H3 | HLCS | HMBS | HMGB1 | HMGB2 | HMGB3 | HNRNPD | HOXB9 | HPX | HRAS | HS2ST1 | HS6ST1 | HSD17B10 | HSF1 | HSF4 | HSP90AA1 | HSP90AB1 | HSP90B1 | HSPA14 | HSPA1B | HSPA5 | HSPD1 | HSPG2 | HTR1B | HTRA2 | HUWE1 | ICK | IGF1R | IGFBP7 | IKBKB | IKBKE | IKBKG | IL3RA | ILF2 | ILF3 | ILK | ING5 | INO80B | INO80C | INSR | IRF1 | IRF4 | IRF7 | ISG15 | ITCH | ITGAV | ITK | JAK1 | JAK3 | JMJD6 | JOSD2 | JUN | KAT5 | KAT7 | KAT8 | KBTBD7 | KCMF1 | KCTD17 | KCTD2 | KDM1A | KDM2A | KDM3B | KDM4D | KDM5B | KDM5C | KDM8 | KDR | KEAP1 | KIAA0391 | KLF5 | KLHL20 | KLHL22 | KMT2A | KMT2B | KMT2C | KPNA1 | KPNA2 | KPNB1 | KRAS | KSR1 | L2HGDH | LAMB1 | LARP4 | LAT | LATS1 | LCMT2 | LEF1 | LGALS1 | LGMN | LIG4 | LMO2 | LMO7 | LNX1 | LOXL2 | LRRC7 | LRRK1 | LRRK2 | LRSAM1 | LSM1 | LSM3 | LSM4 | LSM5 | LTBP1 | LYG2 | LYPLA1 | LYPLA2 | LZTR1 | MAD2L1 | MAN2B1 | MAP2K1 | MAP2K3 | MAP2K4 | MAP2K6 | MAP2K7 | MAP3K1 | MAP3K13 | MAP3K14 | MAP3K2 | MAP3K3 | MAP3K4 | MAP4K1 | MAPK1 | MAPK14 | MAPK15 | MAPK3 | MAPK4 | MAPK6 | MAPK7 | MAPK8 | MAPK9 | MAPT | MARCH5 | MAST1 | MAST3 | MATK | MBD3 | MBP | MC1R | MCFD2 | MCM2 | MCM3 | MCM4 | MCM5 | MCM6 | MCM7 | MCM9 | MCMBP | MDC1 | MDH2 | MDM2 | MDM4 | MECP2 | MED1 | MED12 | MED20 | MED21 | MED31 | MED4 | MELK | MEPCE | METAP2 | MGMT | MIB1 | MITF | MKNK2 | MLH1 | MLLT1 | MLLT3 | MORF4L1 | MORF4L2 | MPG | MPI | MPPE1 | MRGBP | MRM1 | MRPL12 | MRPL14 | MRPL19 | MRPL28 | MRPL38 | MRPL4 | MRPL43 | MRPL45 | MRPL46 | MRPL9 | MRPS5 | MRRF | MSH6 | MTA1 | MTA3 | MTMR14 | MTMR7 | MTRR | MYC | MYH6 | NAE1 | NAP1L1 | NBAS | NCBP1 | NCK1 | NCOA1 | NCOA3 | NCOR1 | NDUFAF5 | NDUFAF7 | NEDD4 | NEDD8 | NEK1 | NEK7 | NELFB | NELFCD | NELFE | NEURL4 | NFATC1 | NFE2L2 | NFKBIA | NFS1 | NHLRC3 | NIPBL | NLK | NME1 | NME2 | NOD2 | NOP10 | NOS2 | NOSIP | NPEPPS | NPLOC4 | NPM1 | NPR1 | NR3C1 | NSFL1C | NSMCE4A | NSUN2 | NSUN6 | NTMT1 | NTRK1 | NUCB1 | NUP107 | NUP153 | NUP160 | NUP205 | NUP214 | NUP85 | NUP88 | NUP93 | NUP98 | NUPR1 | OAS2 | OBSL1 | OGDH | OGFOD1 | OGT | OTUB1 | OTUB2 | OTUD4 | OTUD5 | OXA1L | OXSR1 | P4HA1 | P4HA2 | P4HB | PABPC1 | PABPC4 | PADI4 | PAK2 | PAK6 | PALB2 | PAN2 | PARK7 | PARP1 | PARP12 | PARP14 | PARP6 | PAX8 | PAXIP1 | PCBP2 | PCGF1 | PCGF5 | PCGF6 | PCMT1 | PCMTD1 | PCMTD2 | PCNA | PCSK9 | PDE12 | PDF | PDIA6 | PDK2 | PEBP1 | PELI1 | PER1 | PER2 | PEX5 | PFKM | PGAM5 | PGM1 | PGM2 | PGM3 | PGP | PHB | PHF10 | PHKA1 | PIAS1 | PIAS3 | PICK1 | PIGU | PIK3CA | PIK3CG | PIK3R1 | PIM1 | PIN1 | PINX1 | PIR | PJA1 | PKN2 | PLK1 | PLOD2 | PML | PMPCA | PMPCB | PNKP | POLA2 | POLD1 | POLE2 | POLE3 | POLM | POLR2A | POLR2E | POLR2L | POM121 | POT1 | POU5F1 | PPA2 | PPARD | PPARG | PPEF1 | PPIA | PPIB | PPID | PPIE | PPIF | PPIL3 | PPM1A | PPM1B | PPM1D | PPM1H | PPM1K | PPME1 | PPP1CA | PPP1CB | PPP1CC | PPP1R11 | PPP1R12A | PPP1R2 | PPP1R3B | PPP1R3C | PPP1R8 | PPP2CA | PPP2CB | PPP2R1A | PPP2R1B | PPP2R2A | PPP2R2D | PPP2R5D | PPP3CC | PPP4R2 | PPP5C | PPP6C | PPT1 | PPTC7 | PRDX3 | PRDX4 | PRKAA1 | PRKAA2 | PRKAB2 | PRKACA | PRKAG1 | PRKCD | PRKCE | PRKCSH | PRKCZ | PRKD1 | PRKD2 | PRKD3 | PRKDC | PRMT2 | PRMT3 | PRMT6 | PRPF19 | PRSS23 | PSEN1 | PSEN2 | PSMA1 | PSMA2 | PSMA3 | PSMA5 | PSMA6 | PSMB5 | PSMB6 | PSMB7 | PSMB8 | PSMC1 | PSMC2 | PSMC3 | PSMC4 | PSMC5 | PSMC6 | PSMD1 | PSMD10 | PSMD11 | PSMD12 | PSMD14 | PSMD2 | PSMD4 | PSMD9 | PSME2 | PSME3 | PTEN | PTGES3 | PTK2 | PTMA | PTMS | PTPMT1 | PTPN1 | PTPN14 | PTPN21 | PTPN22 | PTPN23 | PTPN4 | PTPN6 | PTPRA | PTPRD | PTPRF | PTPRK | PTPRO | PTPRR | PTTG1 | PYGB | PYGL | QSOX1 | RAB11A | RAB11B | RAB1A | RAB1B | RAB2A | RAB31 | RAB5A | RAB7A | RAD21 | RAD23A | RAD23B | RAD51 | RAD51AP1 | RAE1 | RAF1 | RAN | RANBP9 | RANGAP1 | RARA | RARS2 | RBBP4 | RBBP6 | RBM17 | RBM8A | RBMX | RBX1 | RC3H1 | RELA | REPIN1 | REST | RFC1 | RFC2 | RFC3 | RFC4 | RFC5 | RHBDD1 | RHO | RHOA | RHOT1 | RIF1 | RING1 | RIOK1 | RIPK1 | RIPK2 | RIPK3 | RIPK4 | RMND1 | RNASE3 | RNASEH2A | RNASEH2B | RNF123 | RNF126 | RNF139 | RNF181 | RNF183 | RNF185 | RNF19A | RNF2 | RNF25 | RNF31 | RNF4 | RNF8 | RPA2 | RPA3 | RPAIN | RPL10 | RPL10A | RPL12 | RPL18A | RPL22 | RPL23 | RPL23A | RPL24 | RPL30 | RPL36AL | RPL37A | RPL38 | RPL6 | RPL7 | RPL7A | RPL9 | RPLP0 | RPLP0P6 | RPLP1 | RPLP2 | RPN1 | RPN2 | RPS12 | RPS13 | RPS14 | RPS15A | RPS19 | RPS2 | RPS20 | RPS24 | RPS29 | RPS3 | RPS4X | RPS6KA1 | RPS6KA2 | RPS6KB2 | RPS8 | RRM1 | RRM2 | RRM2B | RTF1 | RTFDC1 | RUVBL1 | RUVBL2 | S100A9 | SAE1 | SARS | SCAF1 | SCG2 | SCG3 | SCYL2 | SEL1L | SENP3 | SENP6 | SENP7 | SENP8 | SEPHS1 | SET | SETDB1 | SFPQ | SGSH | SGTA | SGTB | SHC1 | SHMT2 | SIAH1 | SIN3A | SIRT1 | SIRT3 | SIRT5 | SIRT6 | SIRT7 | SKI | SKIV2L | SMAD2 | SMAD3 | SMAD4 | SMAD5 | SMARCB1 | SMC1A | SMC3 | SMG5 | SMG6 | SMG8 | SMURF1 | SMURF2 | SNCA | SND1 | SNRPB | SNRPD3 | SNX3 | SOCS1 | SOCS2 | SOCS6 | SP1 | SP100 | SPATA2 | SPI1 | SPIN1 | SPOP | SPRR2A | SPRTN | SPSB3 | SPTAN1 | SPTBN1 | SPTBN2 | SQSTM1 | SRC | SRPK1 | SRPK2 | SSB | ST3GAL1 | ST3GAL6 | ST6GALNAC5 | ST8SIA4 | STAT3 | STBD1 | STK11 | STK24 | STK25 | STK26 | STK3 | STK38 | STK40 | STOML2 | STUB1 | STYX | SULF2 | SULT1A3 | SUMO1 | SUMO1P1 | SUMO2 | SUMO3 | SUPT4H1 | SUPT5H | SUZ12 | SWAP70 | SYVN1 | TAB1 | TAB2 | TADA2B | TAF12 | TAF15 | TAF1A | TAOK3 | TARDBP | TARS | TATDN1 | TBL1X | TBRG4 | TCEA1 | TCERG1 | TCIRG1 | TDP1 | TERF1 | TERF2 | TERF2IP | TESK2 | TFCP2 | TFIP11 | TGFB1 | TGFBR3 | TGM2 | TGOLN2 | THADA | THBS1 | THOP1 | THRA | TIMM50 | TIMP1 | TINF2 | TK1 | TK2 | TLR3 | TMEM132A | TMEM189-UBE2V1 | TMEM258 | TMTC3 | TNF | TNFAIP3 | TNIP2 | TNK2 | TNKS | TNKS2 | TNP1 | TOLLIP | TOMM20 | TOP1 | TOP2A | TOP3A | TOR1A | TP53 | TP53RK | TP73 | TPR | TPST2 | TPTE | TRAF2 | TRAF3 | TRAF6 | TREX1 | TRIB2 | TRIB3 | TRIM21 | TRIM23 | TRIM25 | TRIM28 | TRIM29 | TRIM32 | TRIM33 | TRIM41 | TRIM47 | TRIM63 | TRIM69 | TRIM7 | TRIM9 | TRIP12 | TRIP13 | TRMT44 | TRMT6 | TRMT61B | TSC22D1 | TSFM | TSG101 | TSN | TSPAN17 | TTC5 | TTLL1 | TUFM | TWF1 | TYMS | UBA1 | UBA2 | UBA3 | UBA5 | UBA6 | UBAC1 | UBB | UBC | UBE2A | UBE2B | UBE2C | UBE2D1 | UBE2D2 | UBE2D3 | UBE2H | UBE2I | UBE2J1 | UBE2K | UBE2L3 | UBE2L6 | UBE2M | UBE2N | UBE2O | UBE2Q2 | UBE2R2 | UBE2S | UBE2V1 | UBE2W | UBE3A | UBE3B | UBL4A | UBQLN1 | UBQLN2 | UBQLN4 | UBR4 | UBR5 | UBXN1 | UBXN7 | UCHL1 | UCHL3 | UCHL5 | UFC1 | UFM1 | UGDH | UGGT1 | UGP2 | ULK1 | UNKL | UPF2 | UPF3B | UQCRC2 | USP11 | USP14 | USP15 | USP16 | USP17L2 | USP19 | USP2 | USP20 | USP21 | USP26 | USP28 | USP39 | USP4 | USP42 | USP46 | USP47 | USP5 | USP50 | USP53 | USP7 | USP8 | USP9X | USP9Y | USPL1 | VCAN | VCP | VCPIP1 | VGF | VHL | VKORC1 | VPS37D | VPS4B | WARS | WDR4 | WDR45B | WDR48 | WDR82 | WDTC1 | WDYHV1 | WIPI1 | WIPI2 | WNK1 | WNK4 | WSB1 | WTAP | WWP2 | XBP1 | XIAP | XRCC1 | XRCC3 | XRCC5 | XRCC6 | XYLT1 | YEATS2 | YES1 | YME1L1 | YWHAB | YWHAE | YY1 | ZAP70 | ZBTB16 | ZBTB7A | ZBTB7B | ZC3HC1 | ZDHHC17 | ZDHHC23 | ZFYVE26 | ZMPSTE24 | ZNF217 | ZNF598 | ZNRF4 | GO:0065008 | 0.251728739536576 | 2075/8243 | 932/3250 | 2.27855396246811e-09 | 2.16348698736347e-05 | T | T | T | T | regulation of biological quality | AARS | AARSD1 | AASDH | ABAT | ABCB7 | ABCG1 | ABL1 | ABL2 | ACAA2 | ACACA | ACADVL | ACD | ACE2 | ACKR2 | ACO1 | ACOT13 | ACOX1 | ACSL1 | ACTA2 | ACTB | ACTG1 | ACTN2 | ACTR2 | ACTR3 | ADA | ADCY6 | ADCYAP1 | ADD3 | ADH1A | ADH1B | ADH5 | ADORA2A | ADRB2 | ADTRP | AFG3L2 | AFM | AGER | AGR2 | AGTRAP | AKAP9 | AKR1B1 | AKR1B10 | AKR1B15 | AKR1C2 | AKT1 | AKT2 | AKT3 | ALB | ALDH5A1 | ALDH7A1 | ALDH8A1 | ALDH9A1 | ALDOA | ALOX12 | ANK3 | ANP32A | ANXA1 | ANXA2 | ANXA5 | ANXA6 | ANXA7 | AP3D1 | APC | APEX1 | APOA1 | APOE | APP | APRT | AR | ARF1 | ARHGAP35 | ARHGEF7 | ARNT | ARPC1A | ARPC1B | ARPC2 | ARPC3 | ARPC4 | ARPC5 | ARRB1 | ARRB2 | ASGR2 | ASPSCR1 | ASS1 | ATAD1 | ATF2 | ATG5 | ATM | ATOX1 | ATP12A | ATP13A2 | ATP1A1 | ATP1B3 | ATP2A2 | ATP2B2 | ATP4A | ATP6V1A | ATP6V1B1 | ATP6V1C1 | ATP6V1D | ATP6V1E1 | ATP9A | ATRX | AURKB | AVIL | AZU1 | BACE1 | BAG2 | BAG3 | BAG4 | BAG5 | BAG6 | BAIAP2 | BAIAP2L1 | BAX | BBC3 | BCAT2 | BCL10 | BCL2 | BCL2L1 | BDKRB2 | BIN1 | BRAF | BTBD9 | BTK | BTRC | C1QTNF1 | CACNA1A | CACNG2 | CACNG4 | CACNG5 | CALM1 | CALM2 | CALM3 | CALR | CAMK2A | CAMK2B | CAND1 | CAPG | CAPZA1 | CAPZA2 | CAPZB | CASP3 | CASP8 | CAV1 | CCAR2 | CCDC22 | CCL21 | CCR1 | CCR3 | CCR4 | CCR5 | CCT2 | CCT3 | CCT4 | CCT5 | CCT6A | CCT7 | CCT8 | CD36 | CD40 | CD74 | CD81 | CDC20 | CDC37 | CDC42 | CDC42EP3 | CDC42EP4 | CDC42SE2 | CDC73 | CDK16 | CDK4 | CDK5 | CDKN1A | CEBPA | CEL | CES1 | CFL1 | CFL2 | CFTR | CHCHD10 | CHDH | CHEK2 | CHP1 | CHRM2 | CHRM3 | CHRNA9 | CHRND | CHRNE | CHRNG | CIRBP | CKB | CLDN18 | CLIC1 | CLIC4 | CLNS1A | CLSTN1 | CLU | COG3 | COL2A1 | COL4A3BP | COMMD1 | COMT | COPA | COX11 | COX19 | CPLX1 | CPLX3 | CPT1A | CPT2 | CREB1 | CREBBP | CRK | CROCC | CRP | CRY1 | CRY2 | CRYAB | CSMD1 | CTNNB1 | CTNNBIP1 | CTNND1 | CTSA | CTTN | CUL1 | CUL3 | CUTC | CXCL11 | CXCL6 | CYLD | CYP1A1 | DBH | DBN1 | DCN | DCP2 | DCTN1 | DDB1 | DECR1 | DERL1 | DHX36 | DHX9 | DIS3 | DLD | DLG2 | DLG3 | DLG4 | DMD | DNA2 | DNAJB2 | DNAJC10 | DNM1L | DOCK5 | DOCK7 | DSTN | DVL1 | DVL3 | DYNC1H1 | DYNLL1 | E2F1 | E2F4 | EGFR | EGLN1 | EHMT1 | EIF2AK4 | EIF4A3 | ELAVL1 | ENDOG | EP300 | EPHA4 | ESR1 | ESRRG | ETS1 | EXOSC1 | EXOSC5 | EXOSC6 | EXOSC9 | EYA2 | EZH2 | EZR | F5 | F9 | FABP3 | FABP5 | FADD | FAF1 | FAM129B | FANCC | FASLG | FBXO7 | FBXW11 | FCHSD1 | FDXR | FEN1 | FERMT2 | FGB | FGF2 | FGFR1 | FH | FHL1 | FIS1 | FKBP1A | FKBP1B | FKBPL | FLNB | FLOT1 | FN1 | FOXA1 | FOXO1 | FTH1 | FTL | FTO | FXR2 | FYN | G3BP2 | GAA | GABRA6 | GABRR1 | GAD2 | GADD45G | GAPDH | GAR1 | GATA1 | GDF9 | GFAP | GIT1 | GJA1 | GLA | GLP1R | GLRB | GLRX | GLRX3 | GLRX5 | GLUL | GNA12 | GNA13 | GNA15 | GNAI2 | GNAQ | GNAS | GNB1 | GOLPH3 | GOPC | GOT1 | GOT2 | GPD1L | GPI | GPX1 | GRB2 | GRIA1 | GRID2 | GRIK2 | GRIP1 | GSK3A | GSK3B | GSR | GSTO1 | GTF2I | H3F3A | HAAO | HADH | HAP1 | HAX1 | HCN1 | HCN2 | HCN3 | HDAC1 | HDAC2 | HDAC3 | HDAC6 | HDAC9 | HEXB | HIF1A | HIST1H3A | HIST1H4A | HIST2H3A | HIST3H3 | HK1 | HK2 | HMGB1 | HMGB2 | HMOX1 | HNF1A | HNF4A | HNRNPA1 | HNRNPA2B1 | HNRNPD | HNRNPM | HNRNPU | HOXA5 | HOXB6 | HPX | HRAS | HRNR | HSD17B14 | HSD17B4 | HSD17B6 | HSD17B8 | HSF1 | HSP90AA1 | HSP90AB1 | HSP90B1 | HSPA14 | HSPA1B | HSPA5 | HSPA8 | HSPA9 | HSPB1 | HSPD1 | HTR1B | HTR3A | HTR3E | HTT | HYPK | IARS | IBTK | ICAM1 | ID2 | IDE | IFNA2 | IGF1R | IGF2BP1 | IKBKE | IMMT | INSIG1 | INSR | IP6K1 | IREB2 | IRF1 | IRF4 | ISG15 | ITGA7 | ITGAV | ITGB1BP1 | ITGB3 | ITPR1 | ITPR2 | ITPR3 | JAK3 | JUN | JUP | KCNIP2 | KCNK1 | KCNK16 | KCNS3 | KCTD17 | KDM1A | KDM5B | KDR | KEAP1 | KHDRBS1 | KIF5B | KRAS | LARS | LAT | LATS1 | LETM1 | LGALS1 | LGMN | LGR4 | LIMA1 | LMNA | LPAR1 | LPAR2 | LPAR4 | LRP6 | LRRC7 | LRRK1 | LRRK2 | LTBP1 | MAOA | MAP2K6 | MAP2K7 | MAP3K4 | MAPK1 | MAPK14 | MAPK15 | MAPK3 | MAPK8 | MAPT | MBP | MCOLN3 | MDFI | MDM2 | MDM4 | MECP2 | MED1 | METRNL | MEX3C | MICU1 | MICU2 | MIF | MKS1 | MLLT11 | MME | MMRN1 | MOV10 | MPC2 | MPP2 | MSN | MTHFD1 | MTPN | MTX1 | MYC | MYD88 | MYH6 | MYL2 | MYO1C | MYO5A | MYOC | NAMPT | NCDN | NCK1 | NCOA1 | NCOA4 | NDUFAF2 | NDUFS1 | NEDD4 | NEDD8 | NEK7 | NEUROD1 | NF2 | NFE2L2 | NFKBIA | NLK | NMB | NME1 | NME2 | NNT | NOD2 | NOP10 | NOS1AP | NOS2 | NOS3 | NPM1 | NPR1 | NQO1 | NR0B2 | NR1H2 | NR1H3 | NR5A2 | NRXN1 | NUBP1 | NUP214 | OAS2 | OGT | OPA1 | OTC | OXCT1 | P2RX1 | P2RX2 | P2RX4 | P4HB | PABPC1 | PABPC4 | PAFAH1B1 | PAH | PAIP1 | PAPSS2 | PARK7 | PARP1 | PAX8 | PCNA | PCSK9 | PCTP | PDCD10 | PDCD6IP | PDE12 | PDE4D | PDIA3 | PDIA4 | PDIA6 | PDK2 | PDSS2 | PEBP1 | PER2 | PEX11B | PEX19 | PFKL | PFKM | PFN1 | PGAM5 | PHB | PHB2 | PHF21A | PIAS3 | PICK1 | PIH1D1 | PIK3C2A | PIK3CA | PIK3CG | PIK3R1 | PIN1 | PINX1 | PLA2G10 | PLA2G1B | PLA2G6 | PLAUR | PLCG1 | PLK1 | PLSCR1 | PLXNA1 | PLXNA2 | PLXNB2 | PML | PNKP | POC1B | POLA2 | POLD1 | POLE2 | POLE3 | POLR2M | POT1 | POTEF | POTEI | PPA2 | PPARD | PPARG | PPARGC1A | PPAT | PPIB | PPIF | PPP1CA | PPP3CC | PPT1 | PQLC1 | PRCP | PRDX1 | PRDX2 | PRDX3 | PRDX4 | PRDX5 | PRDX6 | PRELID1 | PREPL | PRKAA1 | PRKAA2 | PRKAB2 | PRKACA | PRKAR1A | PRKAR2B | PRKCD | PRKCE | PRKCZ | PRKD1 | PRKDC | PROZ | PRPF40A | PSEN1 | PSMA1 | PSMA2 | PSMA3 | PSMA5 | PSMA6 | PSMB5 | PSMB6 | PSMB7 | PSMB8 | PSMC1 | PSMC2 | PSMC3 | PSMC4 | PSMC5 | PSMC6 | PSMD1 | PSMD10 | PSMD11 | PSMD12 | PSMD14 | PSMD2 | PSMD4 | PSMD9 | PSME2 | PSME3 | PTEN | PTGER3 | PTGES2 | PTGES3 | PTGIR | PTK2 | PTN | PTPN6 | PUM1 | PYCR1 | PYGL | QSOX1 | RAB11A | RAB11B | RAB11FIP2 | RAB1A | RAB3GAP1 | RAB5A | RAB7A | RAC1 | RAD23A | RAD51 | RAF1 | RANGAP1 | RAP1A | RAP1B | RAP1GDS1 | RAPGEF4 | RARA | RARG | RB1 | RB1CC1 | RBP1 | RBPJ | RC3H1 | RDH11 | RDH12 | RDH13 | RDH14 | RDH5 | RDX | REST | RFC1 | RFC2 | RFC3 | RFC4 | RFC5 | RGN | RGS2 | RHOA | RHOJ | RHOT1 | RHOT2 | RHOU | RIC3 | RIF1 | RIPK3 | RNF139 | RNPEP | RPA2 | RPA3 | RPL23 | RPS24 | RPTOR | RTN4 | S100A13 | S100A7 | S100A9 | S100B | SART3 | SCO2 | SEC16A | SEL1L | SEPT5 | SEPT7 | SERPINB2 | SERPINE2 | SET | SFN | SFXN1 | SFXN4 | SGPL1 | SH2B1 | SH3GL1 | SH3KBP1 | SIAH1 | SIN3A | SIPA1L1 | SIRT1 | SIRT3 | SIRT6 | SLAMF8 | SLC12A2 | SLC16A1 | SLC25A4 | SLC25A5 | SLC25A6 | SLC2A5 | SLC35F6 | SLC39A4 | SLC9A1 | SLC9A3R1 | SMAD2 | SMAD3 | SMAD4 | SMARCA4 | SMARCB1 | SMG5 | SMG6 | SMO | SNCA | SNF8 | SNTA1 | SNX6 | SOAT1 | SOCS2 | SOD1 | SOD2 | SP100 | SPI1 | SPR | SPTAN1 | SPTBN1 | SPTBN2 | SQSTM1 | SRC | SRI | STAT1 | STAT3 | STAT5A | STAT6 | STAU1 | STIM1 | STK11 | STK3 | STMN1 | STOML2 | STRAP | STUB1 | STXBP1 | SUGT1 | SUMO1 | SUN2 | SWAP70 | SYN1 | SYNE2 | SYP | SYVN1 | TARDBP | TARS2 | TBL1X | TBRG4 | TBXA2R | TCIRG1 | TCP1 | TERF1 | TERF2 | TERF2IP | TFDP1 | TFIP11 | TGFB1 | TGM2 | THADA | THBS1 | THEM4 | THRA | TINF2 | TLE3 | TM9SF4 | TMBIM6 | TMEM30A | TMOD3 | TMOD4 | TMX2 | TMX4 | TNF | TNFAIP3 | TNFSF14 | TNIP2 | TNKS | TNKS2 | TNPO1 | TOR1A | TP53 | TP63 | TP73 | TPCN2 | TPM1 | TPR | TRAF6 | TREX1 | TRIAP1 | TRIM21 | TRIM32 | TSC22D3 | TWF1 | TWF2 | TXN | TXN2 | TXNDC11 | TXNDC15 | TXNDC5 | TXNRD1 | UBAP2L | UBB | UBC | UBE2B | UMPS | USP19 | USP2 | USP28 | USP4 | USP46 | USP47 | USP53 | USP7 | USP8 | USP9X | VAPB | VARS | VASP | VAV1 | VCP | VDR | VGF | VHL | VIM | VKORC1 | VPS18 | VSTM2A | VTN | WAS | WDR1 | WDR35 | WDR48 | WDTC1 | WNK1 | WNK4 | WWP2 | XBP1 | XIAP | XPO1 | XRCC1 | XRCC3 | XRCC5 | XRCC6 | YAP1 | YES1 | YRDC | YWHAB | YWHAE | YWHAG | YWHAH | YWHAQ | YWHAZ | ZBTB7B | ZMPSTE24 | ZMYND8 | ZNF207 | GO:1901605 | 0.0115249302438433 | 95/8243 | 66/3250 | 2.45923817274419e-09 | 2.33504664502061e-05 | T | T | T | T | alpha-amino acid metabolic process | AASDHPPT | AASS | ADSS | ALDH4A1 | ALDH5A1 | ALDH7A1 | ASL | ASNS | ASS1 | CBS | CTH | CTPS1 | DLD | DLST | DPEP1 | EGLN1 | EGLN3 | ENOPH1 | FAH | FPGS | GAD2 | GART | GCDH | GFPT1 | GFPT2 | GGT1 | GLS | GLS2 | GLUD2 | GLUL | GMPS | GOT1 | GOT1L1 | GOT2 | GPT2 | HAAO | KYNU | MECP2 | MPST | MTAP | MTHFD1 | MTRR | NIT2 | NOS2 | NOS3 | OAT | OGDH | OTC | P4HA1 | P4HA2 | P4HB | PADI4 | PFAS | PHGDH | PLOD2 | PPAT | PRDX4 | PSAT1 | PYCR1 | PYCR2 | SARS | SEPHS1 | SEPHS2 | SHMT1 | SHMT2 | SLC25A32 | GO:0014070 | 0.0558049253912411 | 460/8243 | 242/3250 | 2.69640061410034e-09 | 2.56023238308828e-05 | T | T | T | T | response to organic cyclic compound | ABCD3 | ABL1 | ACAT1 | ACSL1 | ACTA1 | ACTB | ACTR2 | ADA | ADCY6 | ADCYAP1 | ADORA2A | ADSS | ADTRP | AGL | AIFM1 | AKAP9 | AKT1 | ANXA1 | ANXA7 | APEX1 | APLP1 | APP | AR | ARNT2 | ARPC1B | ASCL1 | ASNS | ASS1 | ATP1A1 | BCL2 | BCL2L1 | BIRC2 | BRAF | BRCA1 | BSG | BTRC | CACNG4 | CALM1 | CALM3 | CALR | CARD9 | CARM1 | CASP3 | CASP7 | CASP8 | CASP9 | CAT | CAV1 | CBL | CCNB1 | CCR5 | CD36 | CD40 | CDC5L | CDH1 | CDK4 | CDKN1A | CEBPA | CFTR | CHEK2 | COMT | CPT1A | CRYAB | CSN1S1 | CSNK2B | CTNNB1 | CTSV | CYP1A1 | CYP1A2 | DDIT4 | DDX1 | DHX36 | DHX9 | DNM1L | DUSP1 | EEF2 | EFTUD2 | EGFR | EIF4A3 | EIF4E | EIF4EBP1 | ENDOG | ERRFI1 | ESR1 | ESRRA | ETS1 | EZH2 | EZR | FADD | FBXO32 | FECH | FIS1 | FKBP1B | FLOT1 | FOLR1 | FOS | FOXA1 | FOXO1 | G6PD | GGT1 | GNAI1 | GNAO1 | GNB1 | GOT1 | GOT2 | GPD1 | GPI | GPX4 | GRAMD1A | GSTP1 | H2AFZ | HCN1 | HCN2 | HCN3 | HDAC2 | HDAC5 | HID1 | HLCS | HMGB2 | HNRNPD | HNRNPU | HSF1 | HSP90B1 | HSPA5 | HTR1B | HTR3A | ICAM1 | IDH1 | IFIT1 | IFNA2 | IGFBP7 | INSIG1 | IREB2 | ITPR2 | JUN | JUNB | JUND | JUP | KAT5 | KDM1A | KLF5 | KRAS | KYNU | LDHA | LGALS1 | LRP6 | LRRK2 | LUM | MAPK1 | MAPK3 | MAVS | MBD3 | MDM2 | MED1 | MGMT | MSN | MTAP | NAMPT | NCOA1 | NCOA2 | NCOA3 | NEDD8 | NFKBIA | NME1 | NOD2 | NQO1 | NR1H3 | NR3C1 | OPA1 | OTC | P2RX1 | P2RX2 | P2RX4 | PAK2 | PARP1 | PCNA | PDE12 | PDE4D | PEBP1 | PELI1 | PER1 | PIK3CG | PPARG | PPARGC1A | PPP2R2A | PRKAA1 | PRKACA | PRKAR2B | PRKCE | PTEN | PTGES | PTN | RAD51 | RAE1 | RAP1A | RAP1B | RARA | RELA | REST | RHOA | RIPK2 | RUVBL2 | S100B | SDC1 | SELL | SHMT1 | SHMT2 | SIN3A | SLC16A1 | SLC3A2 | SLC9A1 | SMAD2 | SMAD5 | SMO | SNCA | SOCS2 | SOD1 | SRC | STAT1 | STAT3 | STXBP1 | SULT1A3 | TBXA2R | TGFB1 | THBS1 | TLR3 | TMEM173 | TNF | TOMM20 | TPCN2 | TRIM25 | TRIM63 | TXN2 | TXNIP | TYMS | UBE2L3 | UBE3A | USP8 | VGF | WBP2 | WT1 | YAP1 | GO:1901137 | 0.0260827368676453 | 215/8243 | 127/3250 | 2.98872431930155e-09 | 2.83779374117682e-05 | T | T | T | T | carbohydrate derivative biosynthetic process | ACACA | ACAT1 | ACLY | ACOT7 | ACSL1 | ACSL4 | ACSL5 | ACSS2 | ADA | ADCY6 | ADK | ADSL | ADSS | AK1 | AK2 | AK4 | AKR1A1 | ALDOA | AMD1 | AMDHD2 | APRT | ATIC | B3GAT3 | B3GNT3 | B4GALT3 | CBR4 | CDA | CMPK1 | CTPS1 | CYC1 | DCAKD | DCK | DCN | DCXR | DLAT | DLD | DNAJC30 | DPM1 | DSE | DUT | EXTL2 | FAM20B | FASN | G6PD | GART | GCDH | GFPT1 | GFPT2 | GLCE | GMDS | GMPPB | GMPS | GNPDA1 | GNPDA2 | GNPNAT1 | GOLPH3 | GPC5 | HEXA | HPRT1 | HS2ST1 | HS6ST1 | HSD17B8 | HSPG2 | IMPDH2 | LCMT2 | LDHC | LUM | MPC2 | MPI | MPPE1 | MTAP | NAGK | NANP | NANS | NME1 | NME2 | NME4 | NPR1 | PAICS | PAPSS1 | PAPSS2 | PDHA1 | PDHA2 | PDHB | PDHX | PFAS | PGM3 | PIGU | PPAT | PPT1 | PRTFDC1 | PYGL | RFK | RRM1 | RRM2 | RRM2B | SCCPDH | SDC1 | SDC4 | SHMT1 | SLC25A1 | SLC25A13 | SORD | ST3GAL1 | ST3GAL6 | ST6GALNAC5 | ST8SIA4 | STOML2 | TECR | TGFB1 | TK1 | TK2 | TKT | TPI1 | TREX1 | TYMS | UAP1 | UAP1L1 | UCK2 | UGDH | UGP2 | UGT8 | UMPS | UPP2 | UXS1 | VCAN | XYLT1 | GO:0030162 | 0.0548344049496543 | 452/8243 | 238/3250 | 3.31524716772808e-09 | 3.14782718575781e-05 | T | T | T | T | regulation of proteolysis | A2M | ADORA2A | ADRM1 | AGER | AHSG | AIFM1 | AKT1 | ALOX12 | ANXA2 | APOE | APP | ARAF | ARRB1 | ARRB2 | ATP13A2 | AXIN1 | BAG2 | BAG5 | BAG6 | BAX | BBC3 | BCL10 | BCL2L13 | BIN1 | BIRC2 | BIRC3 | BIRC5 | BIRC7 | BIRC8 | BTRC | C4A | CASP10 | CASP8 | CASP9 | CAV1 | CBFA2T3 | CCAR2 | CCDC22 | CD109 | CD27 | CDK5 | CEBPA | CLPX | CLU | COMMD1 | CRYAB | CSNK1A1 | CSNK1E | CSNK2A2 | CTSC | CYCS | DAB2 | DAPK1 | DDA1 | DDRGK1 | DDX3X | DHCR24 | DIABLO | DNAJB2 | DPEP1 | DVL1 | EDEM1 | EGLN3 | EIF2AK3 | ENO1 | EPHA4 | EPPIN | FABP1 | FADD | FAM192A | FASLG | FBXW11 | FIS1 | FN1 | FYN | GAPDH | GLMN | GNA12 | GPI | GPX1 | GSK3A | GSK3B | HDAC1 | HDAC2 | HDAC6 | HMGB1 | HSF1 | HSP90AB1 | HSP90AB3P | HSP90AB4P | HSPA1B | HSPBP1 | HSPD1 | HSPE1 | HTRA2 | IDE | IFI16 | IGBP1 | ITCH | KEAP1 | LAMP3 | LATS1 | LEF1 | LGMN | LRRK2 | MAPK14 | MAPK3 | MAPK9 | MAPT | MBP | MDM2 | MGMT | MYC | NDUFA13 | NEDD8 | NFE2L2 | NR1H2 | NUPR1 | OGT | OTUD4 | P2RX1 | PACSIN3 | PAK2 | PAPLN | PARK7 | PDCD6 | PEBP1 | PIAS1 | PIH1D1 | PLAUR | PLGRKT | PLK1 | PML | PPARG | PRDX3 | PRDX5 | PRELID1 | PRKACA | PSEN1 | PSMA3 | PSMB8 | PSMC1 | PSMC2 | PSMC3 | PSMC4 | PSMC5 | PSMC6 | PSMD10 | PSMD14 | PSME2 | PSME3 | PTEN | PTK2 | PTTG1 | RAD23A | RAD23B | RAF1 | RBX1 | RECK | REST | RGN | RHBDD1 | RHOA | RIPK1 | RIPK2 | RNF139 | RNF185 | RNF19A | RPL23 | RPS3 | RPS6KA1 | S100A9 | SART3 | SDCBP | SERPINB2 | SERPINB3 | SERPINB5 | SERPINB6 | SERPINB8 | SERPINE2 | SERPINH1 | SFN | SGTA | SIRT1 | SMAD3 | SMARCC1 | SMURF1 | SNCA | SNX12 | SOX2 | SPINK7 | SPINT2 | SPOP | SRC | STAT3 | STUB1 | STYX | SUMO1 | SUMO2 | TANK | THBS1 | TIMP1 | TIMP2 | TMTC3 | TNF | TNFSF14 | TNP1 | TP63 | TRADD | TRAF2 | TRAF3 | TRIAP1 | TRIB2 | TRIB3 | TRIM21 | TRIM32 | UBB | UBE2J1 | UBE2K | UBE3A | UBQLN1 | UBQLN2 | UBQLN4 | UBXN1 | UCHL5 | USP14 | USP17L2 | USP19 | USP47 | USP5 | USP50 | USP7 | VCP | VTN | WDR35 | WDR48 | XIAP | YWHAE | GO:0006520 | 0.0178333131141575 | 147/8243 | 93/3250 | 3.34397808312257e-09 | 3.17510718992488e-05 | T | T | T | T | cellular amino acid metabolic process | AARS | AARSD1 | AASDH | AASDHPPT | AASS | ACY1 | ADSS | AIMP2 | ALDH4A1 | ALDH5A1 | ALDH6A1 | ALDH7A1 | ASL | ASNS | ASS1 | CARS | CBS | CTH | CTPS1 | DARS | DLD | DLST | DPEP1 | EGLN1 | EGLN3 | ENOPH1 | EPRS | FAH | FARSB | FPGS | GAD2 | GARS | GART | GCDH | GFPT1 | GFPT2 | GGT1 | GLS | GLS2 | GLUD2 | GLUL | GMPS | GOT1 | GOT1L1 | GOT2 | GPT2 | GSS | HAAO | HARS | HNF4A | IARS | KYNU | LARS | MECP2 | MPST | MTAP | MTHFD1 | MTRR | NIT2 | NOS2 | NOS3 | OAT | OGDH | OTC | P4HA1 | P4HA2 | P4HB | PADI4 | PEPD | PFAS | PHGDH | PLOD2 | PPA1 | PPA2 | PPAT | PRDX4 | PSAT1 | PYCR1 | PYCR2 | QARS | QDPR | RARS2 | SARS | SCLY | SEPHS1 | SEPHS2 | SHMT1 | SHMT2 | SLC25A32 | TARS | TARS2 | VARS | WARS | GO:0007165 | 0.283149338832949 | 2334/8243 | 1037/3250 | 3.40816207748048e-09 | 3.23604989256771e-05 | T | T | T | T | signal transduction | ABL1 | ABL2 | ACADVL | ACKR2 | ACSL1 | ACTB | ACTG1 | ACTL6A | ACTN2 | ACTN4 | ACTR2 | ACTR3 | ADCY6 | ADCYAP1 | ADORA2A | ADRB2 | ADTRP | AES | AGER | AGO1 | AGO2 | AGTRAP | AHCYL1 | AHI1 | AIFM1 | AKAP8 | AKAP9 | AKR1B1 | AKR1C2 | AKT1 | AKT2 | AKT3 | ALOX5 | AMFR | ANK1 | ANK3 | ANP32A | ANXA1 | ANXA2 | ANXA5 | ANXA6 | APBB1 | APBB2 | APC | APOA1 | APOE | APP | AR | ARAF | ARF1 | ARF4 | ARFGAP1 | ARHGAP1 | ARHGAP19 | ARHGAP26 | ARHGAP35 | ARHGDIA | ARHGEF7 | ARHGEF9 | ARNT | ARPC1A | ARPC1B | ARPC2 | ARPC3 | ARPC4 | ARPC5 | ARR3 | ARRB1 | ARRB2 | ASB15 | ASB16 | ASB6 | ASB9 | ASCL1 | ASGR2 | ASNS | ATAD5 | ATF6 | ATF6B | ATM | ATOH1 | ATP2A2 | ATP6V1A | ATP6V1B1 | ATP6V1C1 | ATP6V1D | ATP6V1E1 | ATRX | AXIN1 | AZU1 | BABAM1 | BAG1 | BAG3 | BAG4 | BAG6 | BAIAP2 | BAX | BBC3 | BCAR1 | BCAR3 | BCL10 | BCL2 | BCL2L1 | BCL2L2 | BCL6 | BDKRB2 | BIRC2 | BIRC3 | BIRC5 | BMPR1A | BRAF | BRCA1 | BSG | BTK | BTN2A1 | BTRC | C1QBP | CA8 | CAB39 | CABP4 | CALM1 | CALM2 | CALM3 | CALR | CAMK2A | CAMK2B | CANX | CAP2 | CAPZA1 | CARD9 | CARM1 | CASP10 | CASP2 | CASP3 | CASP8 | CASP9 | CAV1 | CBL | CBLB | CBLC | CCAR2 | CCDC68 | CCDC88A | CCL21 | CCL22 | CCNB1 | CCNY | CCR1 | CCR3 | CCR4 | CCR5 | CD2 | CD209 | CD27 | CD36 | CD40 | CD40LG | CD6 | CD74 | CD79B | CD81 | CDA | CDC14B | CDC25C | CDC37 | CDC42 | CDC42EP3 | CDC42EP4 | CDC5L | CDC73 | CDH5 | CDK2 | CDK4 | CDK5R1 | CDKN1A | CEBPA | CENPJ | CEP57 | CFL1 | CGN | CHD8 | CHEK2 | CHP1 | CHRD | CHRM2 | CHRM3 | CHRNA9 | CHRND | CHRNE | CHRNG | CLDN18 | CLIC1 | CLIC3 | CLNK | CLTC | COL4A1 | COL4A2 | COL4A3BP | COPS2 | CORT | CPNE3 | CREB1 | CREB3 | CREBBP | CRIP1 | CRK | CRKL | CRY1 | CRY2 | CRYAB | CSNK1A1 | CSNK1E | CSNK2A2 | CSNK2B | CTH | CTNNB1 | CTNNBIP1 | CTNND1 | CTSL | CUL1 | CUL2 | CUL3 | CUL5 | CUL7 | CXCL11 | CXCL5 | CXCL6 | CXCL8 | CYLD | DAB1 | DAB2 | DAP3 | DAPK1 | DAPP1 | DAXX | DBNL | DCLK1 | DCTN1 | DDB1 | DDIT4 | DDX3X | DEDD | DEPDC7 | DERL1 | DGKE | DGKZ | DIABLO | DIDO1 | DISC1 | DLG2 | DLG3 | DLG4 | DMD | DNAJA1 | DNAJA3 | DNAJB11 | DNAJC10 | DOCK5 | DOCK7 | DOK1 | DPF2 | DPYSL5 | DRG2 | DTX1 | DTX2 | DUSP1 | DUSP22 | DUSP7 | DVL1 | DVL2 | DVL3 | DYRK2 | E2F1 | E2F2 | E2F4 | EDA | EDEM1 | EFEMP1 | EGFR | EIF2AK3 | EIF2B1 | EIF2B3 | EIF2B5 | EIF2S1 | EIF4EBP1 | EP300 | EPHA4 | EPHA6 | EPHB6 | ERBB2 | ERBB3 | ERG | ERLIN2 | ESR1 | ESR2 | ESRRA | ESRRB | ESRRG | EVC2 | EXTL2 | EYA2 | EZR | FADD | FASLG | FBXW11 | FCGR1A | FCN1 | FERMT2 | FGB | FGF2 | FGFR1 | FGFR3 | FHL2 | FIBP | FIS1 | FKBP14 | FKBP1B | FKBP8 | FLNB | FLOT1 | FN1 | FOS | FOXA1 | FOXM1 | FOXO1 | FSCN1 | FYN | FZR1 | G3BP1 | G3BP2 | GABRA6 | GABRR1 | GAP43 | GAPVD1 | GBP2 | GDF9 | GDI1 | GDI2 | GFPT1 | GIGYF1 | GIT1 | GJA1 | GKAP1 | GLP1R | GLRB | GMDS | GNA12 | GNA13 | GNA15 | GNAI1 | GNAI2 | GNAI3 | GNAO1 | GNAQ | GNAS | GNAZ | GNB1 | GNB2 | GNG12 | GNG5 | GNL1 | GOT1 | GPS2 | GPX1 | GRB2 | GRB7 | GRIA1 | GRID2 | GRIK2 | GRIP1 | GRK1 | GRK5 | GSK3A | GSK3B | GSTA2 | GSTO1 | GTF2F1 | GTF2F2 | GTF2I | HAP1 | HAVCR2 | HBEGF | HDAC4 | HDGF | HEBP1 | HGS | HIF1A | HINT1 | HIST1H3A | HIST2H3A | HLA-A | HLA-B | HLA-DQA1 | HLA-DRA | HLA-DRB1 | HLA-DRB3 | HLA-DRB4 | HLA-DRB5 | HMGB1 | HMOX1 | HNF4A | HNF4G | HNRNPA1 | HNRNPA2B1 | HNRNPDL | HNRNPF | HNRNPH1 | HNRNPK | HNRNPM | HRAS | HSF1 | HSP90AA1 | HSP90AB1 | HSP90B1 | HSPA5 | HSPA8 | HSPA9 | HSPB1 | HSPD1 | HTR1B | HTR3A | HTR3E | HTRA2 | ICAM1 | ICK | IDE | IFI16 | IFIT1 | IFITM3 | IFNA2 | IFNAR1 | IFT122 | IGBP1 | IGF1R | IGHV3-13 | IGHV3-23 | IGHV3-30 | IGKV3-20 | IGLC7 | IKBKB | IKBKE | IKBKG | IL13RA2 | IL32 | IL3RA | ILK | IMPA1 | IMPA2 | INSIG1 | INSIG2 | INSR | IPO7 | IPO8 | IQGAP1 | IRF1 | IRF4 | IRF7 | ISG15 | ITCH | ITGA4 | ITGA7 | ITGAV | ITGB1BP1 | ITGB3 | ITGB3BP | ITGB5 | ITK | ITPR1 | ITPR2 | ITPR3 | JAK1 | JAK3 | JMJD6 | JUN | JUNB | KAT5 | KAT7 | KBTBD7 | KCNIP2 | KDR | KHDRBS1 | KIF13B | KLF6 | KLRC2 | KPNA1 | KPNA2 | KPNB1 | KRAS | KRT17 | KSR1 | LANCL1 | LAT | LATS1 | LCP1 | LCP2 | LEF1 | LGALS3BP | LGMN | LGR4 | LIMS1 | LMNA | LMNB1 | LPAR1 | LPAR2 | LPAR4 | LRP6 | LRRC7 | LRRK2 | LRRN2 | LTBP1 | LTBR | MAGI1 | MAOA | MAP2K1 | MAP2K3 | MAP2K4 | MAP2K6 | MAP2K7 | MAP3K1 | MAP3K13 | MAP3K14 | MAP3K2 | MAP3K3 | MAP3K4 | MAP4K1 | MAPK1 | MAPK14 | MAPK15 | MAPK3 | MAPK4 | MAPK6 | MAPK7 | MAPK8 | MAPK9 | MAPKAP1 | MAS1 | MAST1 | MAST3 | MATK | MAVS | MBP | MC1R | MCC | MDM2 | MDM4 | MED1 | MED12 | MELK | MIB1 | MIF | MITF | MKNK2 | MKS1 | MLH1 | MLLT11 | MLST8 | MOB1A | MOB1B | MOV10 | MSH6 | MSN | MTA1 | MTAP | MTNR1A | MTSS1 | MYBBP1A | MYC | MYD88 | MYH6 | MYO1C | MYO1E | MYO6 | MYOC | NAE1 | NAMPT | NANOG | NCBP1 | NCK1 | NCOA1 | NCOA3 | NCOA4 | NDRG1 | NDUFA13 | NEDD4 | NEK1 | NEUROD1 | NFATC1 | NFATC2 | NFE2L2 | NFKB2 | NFKBIA | NLK | NMB | NME2 | NOD2 | NOS2 | NOS3 | NPHP3 | NPM1 | NPR1 | NR0B2 | NR1H2 | NR1H3 | NR1I2 | NR3C1 | NR4A1 | NR5A2 | NRP2 | NTF3 | NTRK1 | NUP93 | NUPR1 | OAS2 | OGT | ONECUT1 | OPTN | OR5F1 | OXSR1 | P2RX1 | P2RX2 | P2RX4 | P4HB | PAG1 | PAK2 | PAK6 | PARD3 | PARD6A | PARK7 | PARP1 | PAWR | PAX8 | PAXIP1 | PCNA | PDCD10 | PDCD6 | PDCL | PDE4D | PDE6G | PDIA6 | PDK2 | PEBP1 | PELI1 | PEX11B | PFN1 | PGAM2 | PHB | PIAS1 | PIAS3 | PICK1 | PIK3C2A | PIK3CA | PIK3CG | PIK3R1 | PIK3R3 | PIKFYVE | PIM1 | PITPNA | PKIG | PKN2 | PLA2G1B | PLA2G6 | PLAUR | PLCG1 | PLK1 | PLXNA1 | PLXNA2 | PLXNB2 | PML | POLR2A | POLR2E | POLR2L | POLR2M | PPARD | PPARG | PPARGC1A | PPIA | PPM1D | PPP1R12A | PPP1R12B | PPP2CA | PPP2R1A | PPP2R5D | PPP3CC | PPP5C | PRKAA1 | PRKAA2 | PRKAB2 | PRKACA | PRKAG1 | PRKAR1A | PRKAR2B | PRKCD | PRKCE | PRKCSH | PRKCZ | PRKD1 | PRKD2 | PRKD3 | PRKDC | PRMT2 | PRPF19 | PSAP | PSEN1 | PSEN2 | PSMA1 | PSMA2 | PSMA3 | PSMA5 | PSMA6 | PSMB5 | PSMB6 | PSMB7 | PSMB8 | PSMC1 | PSMC2 | PSMC3 | PSMC4 | PSMC5 | PSMC6 | PSMD1 | PSMD10 | PSMD11 | PSMD12 | PSMD14 | PSMD2 | PSMD4 | PSMD9 | PSME2 | PSME3 | PSTPIP1 | PTEN | PTGER3 | PTGES | PTGES3 | PTGIR | PTK2 | PTN | PTPN1 | PTPN22 | PTPN6 | PTPRA | PTPRD | PTPRF | PTPRK | PTPRR | PXN | RAB10 | RAB11A | RAB11B | RAB18 | RAB1A | RAB1B | RAB21 | RAB24 | RAB29 | RAB2A | RAB31 | RAB32 | RAB35 | RAB5A | RAB5B | RAB7A | RAB9A | RABL2A | RABL3 | RAC1 | RADIL | RAF1 | RAN | RANBP9 | RANGAP1 | RAP1A | RAP1B | RAPGEF4 | RARA | RARG | RASD1 | RASL11B | RASSF10 | RASSF7 | RASSF9 | RB1 | RBPJ | RBX1 | RC3H1 | RDX | RECK | REL | RELA | RFXANK | RGS1 | RGS2 | RGS20 | RHO | RHOA | RHOJ | RHOT1 | RHOT2 | RHOU | RHPN1 | RIC8B | RIPK1 | RIPK2 | RIPK3 | RIT1 | RNF31 | RNF4 | RPLP0 | RPS19 | RPS6KA1 | RPS6KA2 | RPS6KB2 | RPTOR | RSU1 | RTF1 | RTKN | RUNX2 | RXRA | RXRB | RXRG | S100A1 | S100A11 | S100A6 | S100A9 | SAG | SARM1 | SCARA3 | SCG2 | SDC1 | SDCBP | SDCBP2 | SEC31A | SEL1L | SEMA7A | SEPT2 | SERPINB2 | SETDB1 | SFN | SGPL1 | SH2B1 | SH3GL1 | SH3GL2 | SH3GL3 | SHC1 | SIPA1 | SIPA1L1 | SIRT1 | SKI | SLC12A2 | SLC39A4 | SLC9A1 | SLC9A3R1 | SMAD2 | SMAD3 | SMAD4 | SMAD5 | SMAD9 | SMARCA4 | SMARCC1 | SMO | SMURF1 | SMURF2 | SNAI2 | SNRPA1 | SNW1 | SNX17 | SNX3 | SOCS1 | SOCS2 | SOCS6 | SOD1 | SOD2 | SORT1 | SOX2 | SP100 | SPEN | SPG21 | SPI1 | SPIN1 | SPTAN1 | SPTBN1 | SPTBN2 | SQSTM1 | SRC | SRGAP2 | SRGAP3 | SRI | SRPK1 | SRPK2 | SRPRB | STAC | STARD13 | STAT1 | STAT3 | STAT4 | STAT5A | STAT6 | STIL | STK11 | STK24 | STK25 | STK26 | STK3 | STK38 | STMN1 | STOML2 | STUB1 | SUB1 | SULF2 | SULT1A3 | SUSD5 | SYDE1 | SYNJ2BP | SYVN1 | TAB1 | TAB2 | TALDO1 | TANK | TAOK3 | TAS2R41 | TAS2R60 | TAS2R7 | TBXA2R | TCIRG1 | TCP1 | TCTN2 | TCTN3 | TFDP1 | TGFB1 | TGFBR3 | THEM4 | THRA | TIA1 | TIAL1 | TIMP1 | TLE1 | TLE3 | TLR3 | TMBIM6 | TMEM17 | TMEM173 | TMEM231 | TNF | TNFAIP3 | TNFRSF12A | TNFRSF1A | TNFRSF8 | TNFSF12 | TNFSF14 | TNIP2 | TNK2 | TNKS | TNKS2 | TOLLIP | TP53 | TP63 | TP73 | TPCN2 | TPTE | TRADD | TRAF1 | TRAF2 | TRAF3 | TRAF6 | TREX1 | TRIAP1 | TRIB3 | TRIM21 | TRIM25 | TRIM28 | TRIM34 | TRIM63 | TRIP4 | TSPAN17 | TSPYL2 | TUFT1 | TULP3 | TWIST1 | TXLNA | TXN | TXNDC17 | TXNIP | TXNRD1 | UBB | UBC | UBE2D1 | UBE2D2 | UBE2D3 | UBE2K | UBE2N | UBE2V1 | UBE3A | UBR5 | ULK1 | UNC119 | USP15 | USP28 | USP50 | USP8 | USP9X | USP9Y | VAC14 | VAPB | VAV1 | VCAM1 | VCP | VDR | VIM | VN1R1 | VPS26A | VPS29 | WAS | WBP2 | WDR12 | WDR19 | WIPI1 | WNK1 | WNK4 | WNT16 | WNT2 | WSB1 | WWOX | XAF1 | XBP1 | XIAP | YAP1 | YES1 | YWHAB | YWHAE | YWHAH | YWHAZ | ZAP70 | ZBTB17 | ZBTB33 | ZFAND5 | ZMPSTE24 | ZNF622 | GO:1901566 | 0.0655101298071091 | 540/8243 | 277/3250 | 5.04883035915679e-09 | 4.79386442601937e-05 | T | T | T | T | organonitrogen compound biosynthetic process | AASDHPPT | AASS | ABAT | ACACA | ACAT1 | ACCS | ACLY | ACOT7 | ACSL1 | ACSL4 | ACSL5 | ACSS2 | ADA | ADCY6 | ADK | ADSL | ADSS | AGK | AGO2 | AK1 | AK2 | AK4 | AKR1A1 | AKT1 | ALAS1 | ALDH7A1 | ALDH9A1 | ALDOA | AMD1 | APOA1 | APOE | APRT | ASL | ASNS | ASPDH | ASS1 | ATIC | B3GAT3 | B3GNT3 | B4GALT3 | BCAT2 | BCL10 | CBR4 | CBS | CDA | CEBPA | CHDH | CKM | CMPK1 | CNDP2 | COL4A3BP | COPS5 | COX15 | CSNK2A2 | CSNK2B | CTH | CTPS1 | CYC1 | DARS | DBH | DCAKD | DCK | DCN | DEGS1 | DGUOK | DLAT | DLD | DMD | DNAJC30 | DRG2 | DSE | DUT | EEF1A1 | EEF1A1P5 | EEF1A2 | EGFR | EIF4H | ENOPH1 | ESD | EXTL2 | FABP5 | FAM20B | FARSB | FASN | FECH | FOLH1 | FPGS | GART | GCDH | GEMIN5 | GGCT | GGT1 | GGTLC1 | GLCE | GLS | GLS2 | GLUD2 | GLUL | GMPR | GMPS | GOLPH3 | GOT1 | GOT1L1 | GOT2 | GPC5 | GPT2 | GSPT1 | GSPT2 | GSS | GSTA1 | GSTA2 | GSTA3 | GSTA4 | GSTK1 | GSTM4 | GSTO1 | GSTO2 | GSTP1 | GSTT1 | GSTT2B | GSTZ1 | HAAO | HAGH | HARS | HEXA | HIF1A | HMBS | HPRT1 | HS2ST1 | HS6ST1 | HSD17B8 | HSPA14 | HSPG2 | IDH2 | IMPDH2 | IREB2 | KDSR | KYNU | LARP4 | LCMT2 | LDHC | LPIN3 | LUM | ME1 | MGST1 | MMAB | MOCOS | MPC2 | MPI | MRPL28 | MRPL43 | MRPL9 | MRRF | MTAP | MTHFD1 | MTRR | NAMPT | NAPRT | NFS1 | NME1 | NME2 | NME4 | NMNAT1 | NMNAT3 | NMRAL1 | NMRK2 | NPR1 | OAT | OAZ3 | OTC | P2RX1 | PABPC4 | PAH | PAICS | PAPSS1 | PAPSS2 | PAWR | PCBD1 | PCTP | PDF | PDHA1 | PDHA2 | PDHB | PDHX | PFAS | PHGDH | PHOSPHO1 | PLOD2 | PLSCR1 | PNP | PPARG | PPAT | PPOX | PPT1 | PRKD1 | PRKD2 | PRKD3 | PRTFDC1 | PSAT1 | PYCR1 | PYCR2 | QDPR | RARS2 | RFK | RMND1 | RPL10 | RPL10A | RPL12 | RPL18A | RPL22 | RPL23 | RPL23A | RPL24 | RPL30 | RPL36AL | RPL37A | RPL38 | RPL6 | RPL7 | RPL7A | RPL9 | RPLP0 | RPLP0P6 | RPLP1 | RPLP2 | RPS12 | RPS13 | RPS14 | RPS15A | RPS19 | RPS2 | RPS20 | RPS24 | RPS29 | RPS3 | RPS4X | RPS6KB2 | RPS8 | SARS | SAT1 | SDC1 | SDC4 | SEPHS1 | SEPHS2 | SGPL1 | SHMT1 | SHMT2 | SLC25A1 | SLC25A13 | SNCA | SPR | SPTLC2 | ST3GAL1 | ST3GAL6 | ST6GALNAC5 | ST8SIA4 | STARD7 | STOML2 | SUCLA2 | TAMM41 | TARS | TECR | TGFB1 | TK1 | TK2 | TREX1 | TYMS | UCK2 | UGDH | UGT8 | UMPS | UPP2 | VAPA | VAPB | VCAN | WARS | XYLT1 | GO:1990748 | 0.00752153342229771 | 62/8243 | 47/3250 | 5.57461153896513e-09 | 5.29309365624739e-05 | T | T | T | T | cellular detoxification | ADH5 | AKR1A1 | AKR1B10 | ALB | ALOX5AP | APOE | CAT | CCS | ESD | FABP1 | FANCC | GPX1 | GPX4 | GPX7 | GSR | GSTA1 | GSTK1 | GSTO1 | GSTO2 | GSTP1 | GSTT1 | GSTZ1 | HBZ | KDM3B | LTC4S | MGST1 | NNT | NOS3 | NQO1 | PARK7 | PRDX1 | PRDX2 | PRDX3 | PRDX4 | PRDX5 | PRDX6 | PXDN | RDH11 | RDH12 | S100A9 | SOD1 | SOD2 | SRXN1 | TXN | TXNDC17 | TXNRD1 | UBIAD1 | GO:1903320 | 0.0213514497149096 | 176/8243 | 107/3250 | 5.89856136551018e-09 | 5.60068401655192e-05 | T | T | T | T | regulation of protein modification by small protein conjugation or removal | ABL1 | ADRB2 | AIMP2 | AKT1 | ARNT | ARRB1 | ARRB2 | ATG5 | AXIN1 | BAG2 | BAG5 | BCL10 | BIRC2 | BIRC3 | BMI1 | BRCA1 | CAV1 | CDC14B | CDC20 | CDK5 | CDK9 | CHP1 | COMMD1 | CRY1 | CTNNB1 | CUL3 | DAXX | DCUN1D1 | DERL1 | DNAJA1 | DNAJB2 | FANCI | FANCM | FKBP1A | FYN | FZR1 | GLMN | GOLGA2 | GPS2 | GSK3A | HDAC4 | HSP90AA1 | HSP90AB1 | HSPA1B | HSPA5 | HSPBP1 | ISG15 | ITCH | KDM1A | LRRK2 | MAD2L1 | MAPK9 | MTA1 | NOD2 | OGT | OTUB1 | OTUD4 | PARK7 | PAXIP1 | PDCD6 | PEF1 | PELI1 | PER2 | PIAS1 | PIAS3 | PIN1 | PINX1 | PLK1 | PRKCE | PRMT3 | PSEN1 | PSMD10 | PTEN | PTPN22 | RAB1A | RELA | RIPK2 | RPL23 | RPS2 | RPS3 | SART3 | SEPT4 | SPRTN | STUB1 | TANK | TNFAIP3 | TOLLIP | TRAF6 | TRIB3 | TRIM21 | TRIP12 | U2AF2 | UBB | UBE2C | UBE2D1 | UBE2I | UBE2L3 | UBE2N | UBE2S | UBE3A | UBQLN1 | UBR5 | UBXN1 | USP4 | VCP | WDR48 | XIAP | GO:0046364 | 0.0050952323183307 | 42/8243 | 35/3250 | 6.03209269006283e-09 | 5.72747200921466e-05 | T | T | T | T | monosaccharide biosynthetic process | AKR1B1 | ALDOA | ALDOC | CRY1 | ENO1 | ENO2 | ENO3 | FBP1 | FBP2 | G6PD | GAPDH | GAPDHS | GOT1 | GOT2 | GPD1 | GPI | MDH1 | MDH2 | PC | PER2 | PGAM1 | PGAM2 | PGD | PGK1 | PGK2 | PGM1 | PPARGC1A | SLC25A1 | SLC25A10 | SLC25A11 | SLC25A13 | SORD | TALDO1 | TKT | TPI1 | GO:0071310 | 0.105301467912168 | 868/8243 | 421/3250 | 6.08796902995229e-09 | 5.7805265939397e-05 | T | T | T | T | cellular response to organic substance | ABL1 | ABL2 | ACACA | ACTA1 | ACTB | ACTG1 | ACTR2 | ACTR3 | ADCY6 | ADCYAP1 | ADRB2 | ADTRP | AGER | AICDA | AIFM1 | AKAP8 | AKAP9 | AKR1B1 | AKR1C2 | AKT1 | AKT2 | ALOX12 | ANXA1 | ANXA5 | APEX1 | APLP1 | APP | APRT | AR | ASNS | ASS1 | ATIC | ATM | ATP1A1 | BACE1 | BAG3 | BAG4 | BAX | BCAR1 | BCAT2 | BCL11A | BCL2L1 | BMPR1A | BRAF | BRCA1 | BSPRY | BTK | BTRC | CACNA1A | CAPN2 | CASP3 | CASP7 | CASP8 | CASP9 | CAT | CAV1 | CBL | CCL21 | CCL22 | CCNB1 | CCR5 | CD36 | CD40 | CDC34 | CDC42EP4 | CDC5L | CDC73 | CDH1 | CDK19 | CDK4 | CDK5 | CDK9 | CEBPA | CEBPE | CFTR | CHEK2 | COL2A1 | COL4A1 | COL4A2 | COL6A1 | CPNE3 | CPT1A | CREB1 | CRK | CRKL | CTH | CTNNB1 | CTSL | CXCL11 | CXCL5 | CXCL6 | CXCL8 | CYP1A1 | DAPK1 | DAXX | DDIT4 | DENND4C | DHX9 | DNM1L | DTX1 | DUSP1 | DUSP22 | E2F1 | EED | EEF1A1 | EEF2 | EGFR | EHD4 | EIF4A3 | EIF4E | EIF4EBP1 | ENDOG | EPHA4 | EPRS | ERBB2 | ERRFI1 | ESR1 | EZH2 | EZR | FASN | FBXO32 | FECH | FGB | FIS1 | FLNB | FLOT1 | FOLR1 | FOS | FOXO1 | FYN | GAPDH | GATA1 | GBP2 | GBP3 | GBP5 | GBP6 | GFPT2 | GLP1R | GNAI1 | GNB1 | GNPNAT1 | GOT1 | GPD1 | GRAMD1A | GSK3A | GSK3B | GSTP1 | H2AFZ | HAVCR2 | HAX1 | HCN1 | HCN2 | HCN3 | HDAC2 | HDAC4 | HDAC5 | HDAC6 | HDAC9 | HDGF | HIF1A | HK2 | HMGB1 | HMGB2 | HNRNPD | HNRNPU | HSF1 | HSP90AB1 | HSP90B1 | HSPA14 | HSPA1B | HSPA1L | HSPA2 | HSPA5 | HSPA8 | HSPA9 | HSPB1 | HTR1B | HTR3A | HTRA2 | ICAM1 | IFI16 | IFIT1 | IFNAR1 | IGF1R | IGFBP7 | IKBKB | IL3RA | IMPDH2 | INSIG1 | INSR | IPO5 | IQGAP1 | IREB2 | IRF1 | ITGA4 | ITGB1BP1 | ITPR2 | JUN | JUNB | JUND | JUP | KAT5 | KDM1A | KDM5B | KDR | KEAP1 | KIF5B | KLF5 | KLHL22 | LARS | LEF1 | LGALS1 | LGMN | LIMS1 | LMO2 | LPAR1 | LPIN3 | LRP6 | LRRK2 | MAP2K3 | MAP2K4 | MAP2K6 | MAP4K1 | MAPK1 | MAPK14 | MAPK3 | MAPK4 | MAPK6 | MAPK7 | MAPK8 | MAPK9 | MAPT | MAT2A | MAVS | MCM2 | MCM7 | MDM2 | MED1 | MGMT | MME | MSN | MYO1C | MYO5A | NAMPT | NCOA1 | NCOA2 | NCOA3 | NDUFA13 | NEUROD1 | NFE2L2 | NME1 | NME2 | NOD2 | NOS2 | NR1H2 | NR1H3 | NR3C1 | NR4A1 | NR5A2 | NRP2 | NTRK1 | OPA1 | OPTN | OTUB1 | OXSR1 | P2RX4 | P4HB | PAK2 | PARK7 | PARP1 | PAX8 | PAX9 | PCSK9 | PDCD10 | PDE12 | PDE4D | PDIA3 | PFKP | PHB | PHB2 | PIK3CA | PIK3CG | PIK3R1 | PKM | PLA2G10 | PLA2G1B | PLCG1 | PML | PPARD | PPARG | PPARGC1A | PPAT | PPM1A | PRKAA1 | PRKAA2 | PRKACA | PRKAR1A | PRKAR2B | PRKCD | PRKCE | PRKCZ | PRKD1 | PRKD2 | PRKDC | PRPF8 | PSAP | PSEN1 | PTEN | PTN | PTPN1 | PTPN14 | PTPN22 | PTPN23 | PTPN4 | PTPN6 | RAB10 | RAB31 | RAB35 | RAD23B | RAD51 | RAE1 | RANGAP1 | RAP1A | RAP1B | RARA | RARG | RBMX | RC3H1 | RDX | RELA | REST | RHBDD1 | RHOA | RIF1 | RIPK1 | RIPK2 | RPL23 | RPS3 | RPTOR | RUNX2 | RUVBL2 | SH3GL2 | SHC1 | SHMT1 | SHMT2 | SIN3A | SIRT1 | SLC12A2 | SLC16A1 | SLC25A5 | SLC2A5 | SLC9A1 | SMAD3 | SMAD4 | SMAD5 | SMAD9 | SMO | SNAI2 | SNCA | SNW1 | SNX6 | SOCS1 | SOCS2 | SOD1 | SP1 | SPI1 | SPINT2 | SPRY2 | SRC | SRSF5 | ST3GAL6 | STAT1 | STAT3 | STIP1 | STUB1 | STXBP1 | STXBP2 | SULT1A3 | SYP | TANK | TBC1D4 | TBXA2R | TGFB1 | THBS1 | TLR3 | TMBIM6 | TMEM173 | TNF | TNFAIP3 | TNIP2 | TP53 | TPR | TRADD | TRAF6 | TREX1 | TRIB3 | TRIM25 | TRIM41 | TWF2 | TWIST1 | UBE2K | UBE2L3 | UBE2W | USP8 | VCAM1 | VIM | VSTM2A | WAS | WDR35 | WDTC1 | WNK1 | WNT2 | WT1 | WWOX | XBP1 | XRCC5 | YAP1 | YES1 | YWHAG | YY1 | ZFAND6 | GO:0051128 | 0.184884144122286 | 1524/8243 | 700/3250 | 6.26060596196646e-09 | 5.94444536088715e-05 | T | T | T | T | regulation of cellular component organization | ABHD17A | ABL1 | ABL2 | ACAA2 | ACD | ACE2 | ACTN2 | ACTN4 | ACTR2 | ACTR3 | ADCY6 | ADCYAP1 | ADD3 | ADRB2 | ADTRP | AHI1 | AICDA | AIFM1 | AKAP8 | AKAP9 | AKT1 | AKT2 | ALB | ALDOA | ANAPC4 | ANK1 | ANXA1 | ANXA2 | ANXA7 | APBB1 | APBB2 | APC | APOA1 | APOD | APOE | APP | AR | ARF1 | ARF4 | ARFGAP1 | ARHGAP35 | ARHGDIA | ARHGEF7 | ARHGEF9 | ARPC1A | ARPC1B | ARPC2 | ARPC3 | ARPC4 | ARPC5 | ARRB1 | ARRB2 | ATAD1 | ATAD3A | ATG5 | ATL3 | ATM | ATP13A2 | ATRX | ATXN2 | AURKB | AVIL | BAG3 | BAG4 | BAG5 | BAIAP2 | BAIAP2L1 | BAX | BBC3 | BCAR1 | BCL11A | BCL2 | BCL2L1 | BCL6 | BCOR | BIN1 | BIRC2 | BLZF1 | BRAF | BRCA1 | BRD4 | BTBD9 | C1QBP | CALCOCO2 | CALM1 | CALM3 | CALR | CAMK1D | CAMK2B | CAMKV | CAND1 | CAPG | CAPN2 | CAPZA1 | CAPZA2 | CAPZB | CARM1 | CASP8 | CAV1 | CBFB | CBL | CCAR2 | CCDC36 | CCDC8 | CCDC88A | CCL21 | CCNB1 | CCP110 | CCT2 | CCT3 | CCT4 | CCT5 | CCT6A | CCT7 | CCT8 | CD22 | CD36 | CDA | CDC14B | CDC16 | CDC20 | CDC25C | CDC27 | CDC34 | CDC37 | CDC42 | CDC42EP3 | CDC42EP4 | CDC42SE2 | CDK5 | CDK5R1 | CDK9 | CDKL3 | CDKN1A | CDKN2C | CENPF | CENPJ | CENPV | CEP135 | CEP70 | CFL1 | CFL2 | CHCHD10 | CHD1L | CHMP2A | CHORDC1 | CLIC4 | CLSTN1 | CLTC | CLU | CNTRL | CNTROB | COL5A1 | COLGALT1 | CPLX1 | CPLX3 | CRBN | CREB1 | CRK | CRKL | CRMP1 | CROCC | CRYAB | CSNK2A2 | CTBP1 | CTCF | CTDP1 | CTNNB1 | CTNNBIP1 | CTTN | CUL3 | CUL4B | CUL7 | CYLD | DAB1 | DAB2 | DBN1 | DCN | DCP2 | DCTN1 | DDB1 | DDB2 | DDX39B | DDX3X | DGUOK | DHX36 | DLG4 | DNAJA4 | DNAJB1 | DNAJB2 | DNM1L | DOCK5 | DSTN | DUSP1 | DUSP22 | DVL1 | DVL2 | DVL3 | DYNC1H1 | DYNC1LI1 | DYNLL1 | E2F1 | EAF2 | EED | EGFR | EGLN1 | EHD4 | EID1 | EIF4EBP1 | EIF5A | ENDOG | ENO1 | EP300 | EPHA4 | ERBB2 | ERCC5 | ESR1 | ESR2 | ETS1 | EXOSC9 | EZH2 | EZR | FAF1 | FAM98A | FBP1 | FBXO7 | FBXW5 | FCHSD1 | FEN1 | FERMT2 | FGB | FGFR1 | FGFR1OP | FHL1 | FIS1 | FKBP1B | FLOT1 | FN1 | FSCN1 | FSD1 | FYN | FZR1 | G6PD | GAK | GAP43 | GBP5 | GDF9 | GDI1 | GFAP | GJA1 | GNA12 | GNA13 | GNAI1 | GOLGA4 | GOLPH3 | GPX1 | GRB2 | GRID2 | GRIP1 | GSK3A | GSK3B | H1F0 | H1FX | H3F3A | HAP1 | HAX1 | HBEGF | HDAC2 | HDAC4 | HDAC5 | HDAC6 | HECW1 | HECW2 | HEXB | HIF1A | HIST1H1A | HIST3H3 | HK2 | HMGB1 | HNF4A | HNRNPA1 | HNRNPA2B1 | HNRNPD | HNRNPK | HNRNPU | HRAS | HSF1 | HSP90AA1 | HSPA1B | HSPA1L | HSPA2 | HSPA5 | HSPA8 | HTRA2 | HTT | HUWE1 | ICAM1 | IDE | IGF1R | IGFBP7 | IK | IKBKB | ILK | ING1 | INSIG1 | INSR | IQGAP1 | ITCH | ITGA7 | ITGB1BP1 | ITGB3 | KAT7 | KCTD17 | KDM1A | KDM4D | KDR | KIF11 | KIF13B | KIF5B | KLF5 | KLHL22 | KMT2A | KMT2B | L3MBTL1 | LARP4 | LATS1 | LCP1 | LDLRAD4 | LEF1 | LGALS1 | LGALS3 | LIG4 | LIMA1 | LIMS1 | LMNA | LPAR1 | LRFN1 | LRRC7 | LRRK2 | LRSAM1 | MAD1L1 | MAD2L1 | MAP1B | MAP2K1 | MAP2K7 | MAP3K13 | MAP3K4 | MAPK1 | MAPK14 | MAPK15 | MAPK3 | MAPK6 | MAPK8 | MAPK9 | MAPT | MARCH5 | MAVS | MCM2 | MDM1 | MDM2 | MECP2 | METRN | MIB1 | MLLT11 | MLST8 | MOV10 | MSN | MTPN | MYC | MYL2 | MYO1C | MYO5A | MYOC | NCBP1 | NCK1 | NDUFA13 | NDUFS3 | NEDD4 | NEK7 | NELFE | NF2 | NFATC2 | NFE2L2 | NIN | NIPBL | NME1 | NME2 | NMNAT1 | NPEPPS | NPM1 | NPR1 | NR1H2 | NR1H3 | NRP2 | NRXN1 | NSFL1C | NTF3 | NTRK1 | NUBP1 | NUMA1 | NUSAP1 | OBSL1 | OGFOD1 | OGT | OPA1 | OPTN | OTUB1 | P2RX4 | PACSIN3 | PAFAH1B1 | PAM16 | PAN2 | PARK7 | PARP1 | PARP6 | PAXIP1 | PCSK9 | PDCD6IP | PDE4DIP | PDLIM5 | PEBP1 | PEX5 | PFDN2 | PFN1 | PHB | PHB2 | PICK1 | PIH1D1 | PIK3R1 | PIKFYVE | PIN1 | PINX1 | PIP4K2A | PLA2G6 | PLAUR | PLK1 | PLXNA1 | PLXNA2 | PLXNB2 | PML | PNKP | POT1 | PPARD | PPARG | PPARGC1A | PPIF | PPM1A | PPP2CA | PPP2R1A | PPP3CC | PPT1 | PRELID1 | PRKAA1 | PRKAA2 | PRKACA | PRKAR1A | PRKCD | PRKCE | PRKCZ | PRKD1 | PRKD2 | PRMT6 | PROX1 | PRPF40A | PSEN1 | PSMC2 | PSMC3 | PSMC4 | PSMC5 | PSMC6 | PSMD10 | PTEN | PTK2 | PTN | PTPN1 | PTPN23 | PTPRD | PTPRF | PTPRO | PTTG1 | PXN | QRICH1 | RAB11A | RAB1B | RAB21 | RAB29 | RAB31 | RAB3GAP1 | RAB5A | RAB5B | RAB7A | RAC1 | RAD21 | RAE1 | RAF1 | RAP1A | RAP1B | RASSF7 | RB1 | RBX1 | RCC1 | RDX | RGS2 | RHOA | RHOJ | RHOT1 | RHOT2 | RHOU | RIF1 | RNF31 | RNF4 | RPA2 | RPA3 | RPS3 | RPS6KA1 | RPTOR | RTF1 | RTN4 | RTN4IP1 | RUNX1 | RUVBL2 | S100A13 | S100A9 | S100B | SAE1 | SARM1 | SART3 | SCFD1 | SCYL2 | SDC1 | SDC4 | SDCBP | SEC24B | SEMA7A | SENP6 | SEPT11 | SEPT7 | SEPT8 | SEPT9 | SERPINE2 | SET | SETDB1 | SFN | SFPQ | SH2B1 | SH3GL1 | SH3GL2 | SH3GL3 | SH3KBP1 | SIN3A | SIPA1 | SIPA1L1 | SIRT1 | SIRT6 | SKA1 | SKA3 | SKI | SLC25A5 | SLC35F6 | SLC9A1 | SLC9A3R1 | SMAD3 | SMAD4 | SMARCA2 | SMARCA4 | SMARCB1 | SMC1A | SMC3 | SMG5 | SMG6 | SMURF1 | SNAI2 | SNCA | SNW1 | SNX12 | SNX17 | SNX3 | SOCS2 | SPDL1 | SPI1 | SPICE1 | SPRY2 | SPTAN1 | SPTBN1 | SPTBN2 | SQSTM1 | SRC | STAG2 | STIL | STK11 | STK24 | STK25 | STMN1 | STUB1 | STX5 | STXBP1 | SUMO1 | SWAP70 | SYDE1 | SYNE2 | SYNJ2BP | TADA2B | TBC1D15 | TBC1D22B | TBC1D4 | TCP1 | TERF1 | TERF2 | TERF2IP | TFDP1 | TFIP11 | TGFB1 | THBS1 | THBS2 | THRA | TINF2 | TMED9 | TMEM30A | TMOD3 | TMOD4 | TNF | TNFRSF12A | TNFRSF1A | TNFSF14 | TNK2 | TNKS | TNKS2 | TOP2A | TOR1A | TP53 | TP63 | TP73 | TPM1 | TPR | TRAF3IP1 | TREX1 | TRIAP1 | TRIM28 | TRIM32 | TRIM9 | TRIP12 | TRIP13 | TSG101 | TSPYL2 | TUBA1A | TUBB | TUBB2B | TUBB4A | TWF1 | TWF2 | TWIST1 | UBE2B | UBE2C | UBE2D3 | UBE2L3 | UBE2N | UBQLN2 | UBQLN4 | UBR5 | ULK1 | UNC119 | USP17L2 | USP46 | USP47 | USP50 | USP7 | VASP | VAT1 | VCP | VDAC1 | VDAC2 | VDAC3 | VIM | VPS18 | VPS4B | VTN | WARS | WAS | WBP2 | WDR1 | WDR35 | WT1 | XPO1 | XRCC1 | XRCC3 | XRCC5 | YWHAB | YWHAE | YWHAG | YWHAH | YWHAQ | YWHAZ | YY1 | ZBTB7B | ZMPSTE24 | ZMYM5 | ZMYND10 | ZMYND8 | ZNF207 | ZW10 | GO:0072522 | 0.0107970399126532 | 89/8243 | 62/3250 | 6.34678366202942e-09 | 6.02627108709694e-05 | T | T | T | T | purine-containing compound biosynthetic process | ACACA | ACAT1 | ACLY | ACOT7 | ACSL1 | ACSL4 | ACSL5 | ACSS2 | ADA | ADCY6 | ADK | ADSL | ADSS | AK1 | AK2 | AK4 | ALDOA | AMD1 | APRT | ATIC | CBR4 | CYC1 | DCAKD | DCK | DGUOK | DLAT | DLD | DNAJC30 | FASN | GART | GCDH | GMPR | GMPS | HPRT1 | HSD17B8 | IMPDH2 | LDHC | MPC2 | MTAP | MTHFD1 | NME1 | NME2 | NME4 | NPR1 | PAICS | PAPSS1 | PAPSS2 | PDHA1 | PDHA2 | PDHB | PDHX | PFAS | PNP | PPAT | PPT1 | PRTFDC1 | SHMT1 | SLC25A1 | SLC25A13 | STOML2 | TECR | TGFB1 | GO:1901135 | 0.0471915564721582 | 389/8243 | 208/3250 | 6.64792783200959e-09 | 6.3122074764931e-05 | T | T | T | T | carbohydrate derivative metabolic process | ABHD14B | ACACA | ACAT1 | ACLY | ACOT13 | ACOT7 | ACOT9 | ACSL1 | ACSL4 | ACSL5 | ACSS2 | ADA | ADCY6 | ADCYAP1 | ADK | ADSL | ADSS | AK1 | AK2 | AK4 | AKR1A1 | AKR1B1 | AKR1B10 | AKR1C2 | AKT1 | ALDH5A1 | ALDOA | ALDOC | AMD1 | AMDHD2 | AMFR | APRT | ATIC | ATP6V1A | ATP6V1B1 | B3GAT3 | B3GNT3 | B4GALT3 | BAX | CBR4 | CDA | CHST4 | CLPX | CMPK1 | COL2A1 | CROT | CTPS1 | CTSA | CYC1 | DBI | DCAKD | DCK | DCN | DCXR | DGUOK | DLAT | DLD | DLG2 | DLST | DNAJC30 | DPM1 | DSE | DUT | EDEM1 | EXTL2 | FAM20B | FAR2 | FASN | FBP1 | FBP2 | FIGNL1 | G6PD | GANAB | GART | GCDH | GFPT1 | GFPT2 | GLA | GLCE | GMDS | GMPPB | GMPR | GMPS | GNAI3 | GNPDA1 | GNPDA2 | GNPNAT1 | GOLPH3 | GPC5 | GPD1 | GPI | HEXA | HEXB | HIF1A | HK1 | HK2 | HMGCL | HPRT1 | HS2ST1 | HS6ST1 | HSD17B4 | HSD17B8 | HSPA1B | HSPA8 | HSPG2 | IMPDH2 | LCMT2 | LDHC | LRRK2 | LUM | MAN2B1 | MPC2 | MPI | MPPE1 | MTAP | MVD | MYH6 | NAGK | NANP | NANS | NDUFS1 | NME1 | NME2 | NME4 | NMNAT1 | NPR1 | NT5C2 | OGDH | OLA1 | OPA1 | PAICS | PAPSS1 | PAPSS2 | PARK7 | PARP1 | PDHA1 | PDHA2 | PDHB | PDHX | PFAS | PFKL | PFKM | PFKP | PGD | PGM3 | PIGU | PIM1 | PPARD | PPAT | PPT1 | PRKCSH | PRTFDC1 | PSAP | PYGL | RAN | RFK | RHOA | RNASEH2B | RNF139 | RNF185 | RPE | RPIA | RRM1 | RRM2 | RRM2B | SCCPDH | SDC1 | SDC4 | SEL1L | SGSH | SHMT1 | SLC25A1 | SLC25A13 | SORD | ST3GAL1 | ST3GAL6 | ST6GALNAC5 | ST8SIA4 | STOML2 | SUCLA2 | SUCLG2 | SULF2 | SULT1A3 | SYVN1 | TALDO1 | TECR | TGFB1 | THEM4 | TK1 | TK2 | TKT | TPI1 | TPST2 | TREX1 | TYMS | UAP1 | UAP1L1 | UCK2 | UGDH | UGGT1 | UGP2 | UGT8 | UMPS | UPP2 | UXS1 | VCAN | VCP | XYLT1 | GO:0022607 | 0.185248089287881 | 1527/8243 | 700/3250 | 9.52857383289087e-09 | 9.04738085432988e-05 | T | T | T | T | cellular component assembly | AAR2 | AASS | ABL1 | ABLIM1 | ACACA | ACAD9 | ACAT1 | ACD | ACOT13 | ACTB | ACTG1 | ACTN1 | ACTN2 | ACTN4 | ACTR2 | ACTR3 | ADD3 | ADRB2 | ADRM1 | ADSL | AGO1 | AGO2 | AHI1 | AHNAK | AIMP2 | ALDH5A1 | ALDOA | ALOX5AP | AMFR | ANK3 | ANKRD28 | ANKS4B | ANXA2 | ANXA5 | ANXA6 | APC | APCS | APOA1 | APOE | APP | AR | ARF1 | ARHGEF7 | ARPC2 | ARPC4 | ARRB1 | ATG2B | ATG4C | ATG5 | ATL3 | ATP6V1D | ATPAF2 | ATRX | ATXN10 | ATXN2 | AURKB | AVIL | AXIN1 | BAG3 | BAG4 | BAG6 | BAIAP2 | BAIAP2L1 | BAX | BCL10 | BCL2 | BCS1L | BIN1 | BIRC2 | BIRC3 | BIRC5 | BRAF | BRD2 | C1QBP | C1QTNF1 | CAB39 | CALR | CAND1 | CAPG | CAPZA1 | CAPZA2 | CASP8 | CAT | CAV1 | CBR4 | CBY1 | CCDC155 | CCDC69 | CCDC88A | CCL21 | CCNB1 | CCP110 | CCT2 | CCT3 | CCT6B | CCT8 | CD2BP2 | CD40 | CD74 | CD79B | CDA | CDC20 | CDC42 | CDC73 | CDH1 | CDH5 | CDK2 | CDK5 | CEL | CENPF | CENPJ | CENPV | CEP135 | CEP152 | CEP290 | CEP57 | CEP89 | CHAF1A | CHD1L | CHEK2 | CHMP2A | CHP1 | CIRBP | CKAP5 | CLGN | CLNS1A | CLTC | CLU | CNTROB | COA3 | COL6A1 | COL6A2 | COX11 | COX15 | COX17 | COX19 | COX20 | COX7A2 | CPSF7 | CPT1A | CREBBP | CRKL | CRYAB | CRYZ | CSNK1A1 | CSNK2B | CTCF | CTH | CTNNB1 | CTNND1 | CTSC | CTTN | CUL1 | CUL3 | CUL4B | CUTC | CYLD | DARS | DAXX | DBNL | DCTN1 | DCTPP1 | DCXR | DDB1 | DDB2 | DDX1 | DDX39B | DDX3X | DDX6 | DECR1 | DERL1 | DHRS4 | DHX9 | DIAPH3 | DISC1 | DLG4 | DMD | DNA2 | DNAAF2 | DNAAF5 | DNM1L | DVL1 | DVL2 | DYNC1H1 | DYNLL1 | E2F4 | ECSIT | EFEMP2 | EHD4 | EIF2AK3 | EIF2S1 | EIF3A | EIF3D | EIF3E | EIF3H | EIF3I | EIF4H | EIF5 | EIF6 | ELAVL1 | EP300 | EPPIN | EPPK1 | EPRS | ERAL1 | ERCC5 | EZR | F5 | FADD | FANCA | FANCC | FARSB | FBF1 | FBP1 | FERMT2 | FGB | FIS1 | FKBP1A | FLOT1 | FN1 | FOLR1 | FOPNL | FOXJ1 | FSCN1 | FSD1 | FUS | G3BP1 | G3BP2 | GAK | GBP5 | GEMIN4 | GEMIN5 | GEMIN7 | GJA1 | GLRB | GLRX3 | GLS | GLUL | GNB1 | GOLGA2 | GOLPH3 | GOPC | GORASP2 | GPX4 | GRB2 | GRB7 | GRHPR | GRIA1 | GRID2 | GSK3B | H1F0 | H1FX | H2AFX | H3F3A | HBZ | HCCS | HCN1 | HDAC1 | HDAC3 | HDAC6 | HDAC7 | HGS | HIST1H1A | HIST1H2BA | HIST1H3A | HIST1H4A | HIST2H3A | HIST3H3 | HLA-DRA | HLA-DRB1 | HMGB2 | HMGCL | HMOX1 | HPRT1 | HRAS | HSD17B10 | HSD17B8 | HSF1 | HSF4 | HSP90AA1 | HSP90AB1 | HSP90B1 | HSPA4 | HSPA9 | HSPD1 | HTRA2 | ICK | IDE | IFT122 | IGF1R | IKBKE | IKZF5 | ILK | IMPDH2 | INSR | IPO4 | IQCB1 | ITGB1BP1 | ITGB3BP | ITGB5 | ITPR3 | JMJD6 | JUP | KAT5 | KCNA4 | KCNS3 | KCTD12 | KCTD17 | KCTD18 | KCTD2 | KCTD3 | KHDRBS1 | KIF11 | KLF5 | KMT2A | KPNB1 | LCP1 | LEF1 | LETM1 | LIMA1 | LIMS1 | LNX1 | LPAR1 | LRRK2 | LSM3 | LSM4 | LUC7L2 | LYRM7 | MAGI1 | MAPK3 | MAPT | MAT2A | MCCC1 | MCFD2 | MCM2 | MCTS1 | MDM2 | MDM4 | MDN1 | ME1 | MECP2 | MED6 | MGST1 | MICU1 | MIF | MITF | MKS1 | MLH1 | MPP2 | MTSS1 | MYC | MYH11 | MYH6 | MYL2 | MYO1B | NACC1 | NAP1L1 | NAP1L4 | NCBP1 | NCK1 | NCOR1 | NDUFA10 | NDUFA13 | NDUFA5 | NDUFA8 | NDUFA9 | NDUFAF1 | NDUFAF2 | NDUFAF3 | NDUFAF4 | NDUFAF5 | NDUFAF7 | NDUFB1 | NDUFS1 | NDUFS2 | NDUFS3 | NDUFS6 | NDUFS7 | NDUFS8 | NDUFV1 | NDUFV3 | NEK1 | NEK7 | NFS1 | NME2 | NOD2 | NPHP3 | NPM1 | NRXN1 | NSFL1C | NUBP1 | NUBP2 | NUFIP1 | NUMA1 | NUP107 | NUP153 | NUP205 | NUP85 | NUP93 | NUP98 | NUPR1 | OAT | OBSL1 | OFD1 | OGFOD1 | ONECUT1 | OPA1 | OTC | OXA1L | P2RX1 | P2RX2 | P2RX4 | P4HB | PACS2 | PADI4 | PAFAH1B1 | PANX3 | PARD3 | PARD6A | PARP1 | PAWR | PCBD1 | PCDHB16 | PCM1 | PDCD10 | PDCD6 | PDCD6IP | PDCL | PDE4DIP | PDSS1 | PDSS2 | PEF1 | PEX11B | PFKL | PFKM | PGM5 | PIAS1 | PIH1D1 | PIK3C2A | PIKFYVE | PIN1 | PKM | PKN2 | PLEC | PLS3 | PLXNB2 | PML | POC1B | POLE3 | POT1 | PPARGC1A | PPAT | PPID | PPP2R1A | PPP6C | PPP6R1 | PRKAA1 | PRKACA | PRKCZ | PROX1 | PRPF19 | PRPF8 | PSAP | PSEN1 | PSMD10 | PSMD11 | PSMD4 | PSMD9 | PSMG1 | PSMG4 | PSTPIP1 | PTEN | PTGES3 | PTK2 | PTPN23 | PTPRD | PTPRK | PTPRO | PXN | RAB11A | RAB1A | RAB1B | RAB29 | RAB32 | RAB7A | RAC1 | RAD23B | RAD51 | RAF1 | RANBP9 | RAP1A | RAP1B | RAP1GDS1 | RB1CC1 | RBBP4 | RBMX | RBX1 | RC3H1 | RCC1 | RDX | RHOA | RHOJ | RHOU | RIC3 | RIPK1 | RIPK3 | RPA2 | RPA3 | RPGRIP1L | RPL10 | RPL12 | RPL23A | RPL24 | RPL38 | RPL6 | RPLP0 | RPLP0P6 | RPS10P5 | RPS14 | RPS19 | RPS3 | RRM1 | RRM2 | RRP7A | RRS1 | RTKN | RTN4 | RUVBL1 | RUVBL2 | RXRA | SART1 | SART3 | SBDS | SCFD1 | SCLT1 | SCO2 | SDCBP | SEC13 | SEC16A | SEC24B | SEC24C | SEC31A | SEPT11 | SEPT2 | SEPT4 | SEPT6 | SEPT7 | SEPT9 | SET | SF1 | SH2B1 | SH3GL2 | SHKBP1 | SHMT1 | SHMT2 | SKI | SLC9A1 | SLC9A3R1 | SLC9A3R2 | SMAD2 | SMAD3 | SMAD4 | SMARCA4 | SMARCB1 | SMN1 | SNAP29 | SNCA | SNF8 | SNRPB | SNRPD1 | SNRPD3 | SNRPE | SNX2 | SOAT1 | SOD2 | SPACA1 | SPERT | SPICE1 | SPTBN1 | SPTBN2 | SQSTM1 | SRC | SRGAP2 | SRP19 | SRPK1 | SRPK2 | SRSF12 | SRSF5 | SSX2IP | ST13 | STK25 | STK26 | STOM | STOML2 | STRAP | STX5 | STXBP1 | SWAP70 | TAF12 | TAZ | TBCA | TCF4 | TCP1 | TCTN2 | TCTN3 | TDO2 | TEKT2 | TEKT4 | TERF1 | TESK2 | TFG | TGFB1 | TGFBR3 | TGM2 | TIMM21 | TIMMDC1 | TINF2 | TK1 | TLE1 | TLE3 | TM9SF4 | TMEM17 | TMEM216 | TMEM231 | TMEM70 | TMOD3 | TMOD4 | TNF | TNFAIP3 | TNFRSF1A | TNKS | TNXB | TOMM20 | TOR1A | TP53 | TP63 | TP73 | TPR | TRADD | TRAF1 | TRAF2 | TRAPPC8 | TRIM21 | TRIM28 | TRIM34 | TRIP13 | TRIP6 | TRMT61B | TSG101 | TSGA10 | TSPYL2 | TTC19 | TTLL1 | TUBB | TUBB8 | TUBGCP3 | TXNL4A | UBB | UBC | UBE2B | UBE2C | UBE2K | UBE2S | UBQLN1 | UBQLN2 | UBXN10 | UGDH | UGT8 | ULK1 | UQCC1 | UQCRB | USP16 | USP39 | USP4 | UXS1 | VASP | VBP1 | VCL | VCP | VCPIP1 | VPS37D | VPS4B | VTA1 | VTN | WAS | WDR1 | WDR35 | WDR45B | WDR77 | WIPI1 | WIPI2 | XRCC3 | XRCC6 | YAP1 | YME1L1 | YWHAB | YWHAZ | ZMYND10 | ZNF207 | ZW10 | GO:0070646 | 0.0270532573092321 | 223/8243 | 129/3250 | 1.36281194996634e-08 | 0.000129398994649304 | T | T | T | T | protein modification by small protein removal | ACTB | ACTL6A | ADRB2 | ADRM1 | APC | AR | ARRB2 | BABAM1 | BIRC2 | BIRC3 | BRCA1 | CCP110 | CDC20 | CDC25A | CFTR | COPS2 | COPS5 | COPS6 | CYLD | DDB2 | EP300 | ESR1 | FOXK1 | FOXK2 | HGS | HIF1A | IKBKG | INO80B | INO80C | JOSD2 | KEAP1 | MDM2 | MDM4 | MYC | NEDD8 | NFKBIA | OGT | OTUB1 | OTUB2 | OTUD4 | OTUD5 | PSMA1 | PSMA2 | PSMA3 | PSMA5 | PSMA6 | PSMB5 | PSMB6 | PSMB7 | PSMB8 | PSMC1 | PSMC2 | PSMC3 | PSMC4 | PSMC5 | PSMC6 | PSMD1 | PSMD10 | PSMD11 | PSMD12 | PSMD14 | PSMD2 | PSMD4 | PSMD9 | PSME2 | PSME3 | PTEN | RAD23A | RAD23B | RHOA | RHOT1 | RIPK1 | RNF123 | RUVBL1 | SENP3 | SENP6 | SENP7 | SENP8 | SHMT2 | SMAD2 | SMAD3 | SMAD4 | SMURF2 | SNX3 | SPATA2 | TAB1 | TADA2B | TNFAIP3 | TNIP2 | TOMM20 | TOR1A | TP53 | TRAF2 | TRAF3 | TRAF6 | UBB | UBC | UBE2D1 | UCHL1 | UCHL3 | UCHL5 | USP11 | USP14 | USP15 | USP16 | USP17L2 | USP19 | USP2 | USP20 | USP21 | USP26 | USP28 | USP39 | USP4 | USP42 | USP46 | USP47 | USP5 | USP50 | USP53 | USP7 | USP8 | USP9X | USP9Y | USPL1 | VCP | VCPIP1 | WDR48 | YY1 | GO:0016054 | 0.0174693679485624 | 144/8243 | 90/3250 | 1.43608850487367e-08 | 0.000136356603537755 | T | T | T | T | organic acid catabolic process | AASS | ABAT | ABCD3 | ACAA1 | ACAA2 | ACAD8 | ACADS | ACADSB | ACADVL | ACAT1 | ACAT2 | ACOX1 | ADTRP | AHCY | AKR1A1 | ALDH4A1 | ALDH5A1 | ALDH6A1 | ALDH7A1 | ALDH8A1 | AMDHD2 | BCAT2 | BCKDHB | CBS | CEL | CPT1A | CPT2 | CROT | CTH | DBT | DCXR | DECR1 | DLD | DLST | ECH1 | ECHDC2 | ECHS1 | ECI1 | ECI2 | EHHADH | ETFA | ETFB | ETFDH | FAH | GAD2 | GCDH | GLS | GLS2 | GLUD2 | GLUL | GNPDA1 | GNPDA2 | GOT1 | GOT2 | GPT2 | GSTZ1 | HAAO | HADH | HIBADH | HIBCH | HMGCL | HSD17B10 | HSD17B4 | IVD | KYNU | LDHD | LPIN3 | MCAT | MCCC1 | MPST | MTRR | NAGK | NOS2 | NOS3 | NUDT19 | OAT | OGDH | OTC | PADI4 | PAH | PCBD1 | PPARD | PPAT | PPM1K | QDPR | SHMT1 | SHMT2 | SORD | TDO2 | TXN2 | GO:0051603 | 0.0473128715273565 | 390/8243 | 207/3250 | 1.59904848633635e-08 | 0.000151829653777636 | T | T | T | T | proteolysis involved in cellular protein catabolic process | ADRM1 | AMFR | ANAPC4 | ARMC8 | ARRB1 | ARRB2 | AUP1 | AURKB | BAG6 | BIRC2 | BTRC | CAPN2 | CASP8 | CBL | CBLC | CCNB1 | CDC16 | CDC20 | CDC27 | CDC34 | CDK2 | CLPX | CPVL | CRBN | CTNNB1 | CTSA | CTSC | CTSL | CTSV | CUL1 | CUL2 | CUL3 | CUL4B | CUL5 | CUL7 | CYLD | DDB1 | DERL1 | DNAJB2 | DNAJC10 | EDEM1 | ERLIN2 | FAF1 | FAF2 | FBXL12 | FBXO11 | FBXO6 | FBXO7 | FBXW11 | FBXW5 | FZR1 | GSK3A | GZMA | HDAC6 | HECW1 | HECW2 | HERC2 | HSP90B1 | HSPA5 | HUWE1 | IDE | ISG15 | ITCH | KAT5 | KCTD17 | KCTD2 | KEAP1 | KLHL20 | KLHL22 | LGMN | LNX1 | MAD2L1 | MDM2 | MTA1 | NEDD4 | NEDD8 | NFE2L2 | NHLRC3 | NPLOC4 | NSFL1C | PCBP2 | PDCD6IP | PELI1 | PLK1 | PML | PPP1R11 | PPP2CB | PRPF19 | PSMA1 | PSMA2 | PSMA3 | PSMA5 | PSMA6 | PSMB5 | PSMB6 | PSMB7 | PSMB8 | PSMC1 | PSMC2 | PSMC3 | PSMC4 | PSMC5 | PSMC6 | PSMD1 | PSMD10 | PSMD11 | PSMD12 | PSMD14 | PSMD2 | PSMD4 | PSMD9 | PSME2 | PSME3 | PTPN23 | PTTG1 | RAD23A | RAD23B | RBBP6 | RBX1 | RHBDD1 | RNF123 | RNF126 | RNF139 | RNF185 | RNF19A | RNF4 | RNF8 | SEL1L | SGTA | SGTB | SIAH1 | SIRT1 | SMURF1 | SMURF2 | SOCS6 | SPOP | SPSB3 | SQSTM1 | STUB1 | SYVN1 | TBL1X | TNFAIP3 | TOLLIP | TOR1A | TRIM25 | TRIM32 | TRIM9 | TRIP12 | TSG101 | UBA6 | UBB | UBC | UBE2A | UBE2B | UBE2C | UBE2D1 | UBE2D2 | UBE2D3 | UBE2H | UBE2I | UBE2J1 | UBE2K | UBE2L3 | UBE2L6 | UBE2R2 | UBE2S | UBE2W | UBE3A | UBE3B | UBQLN1 | UBQLN2 | UBQLN4 | UBR4 | UBXN1 | UCHL1 | UCHL3 | UCHL5 | UGGT1 | USP11 | USP14 | USP15 | USP16 | USP17L2 | USP19 | USP2 | USP20 | USP21 | USP26 | USP28 | USP4 | USP42 | USP46 | USP47 | USP5 | USP50 | USP7 | USP8 | USP9X | USP9Y | VCP | VPS37D | VPS4B | WWP2 | XBP1 | YME1L1 | ZMPSTE24 | ZNRF4 | GO:0045862 | 0.0286303530268106 | 236/8243 | 135/3250 | 1.64963244489941e-08 | 0.000156632600643199 | T | T | T | T | positive regulation of proteolysis | ADRM1 | AGER | AIFM1 | AKT1 | ALOX12 | APOE | APP | ARRB1 | AXIN1 | BAG2 | BAG6 | BAX | BBC3 | BCL10 | BCL2L13 | BTRC | CASP10 | CASP8 | CASP9 | CAV1 | CBFA2T3 | CCDC22 | CEBPA | CLPX | CLU | CSNK1A1 | CSNK1E | CTSC | CYCS | DAB2 | DAPK1 | DDRGK1 | DDX3X | DIABLO | DNAJB2 | DVL1 | EDEM1 | EGLN3 | EIF2AK3 | ENO1 | EPHA4 | FADD | FASLG | FBXW11 | FIS1 | FN1 | FYN | GSK3A | GSK3B | HDAC2 | HMGB1 | HSF1 | HSPA1B | HSPBP1 | HSPD1 | HSPE1 | HTRA2 | IFI16 | KEAP1 | LGMN | LRRK2 | MAPK14 | MAPK3 | MAPK9 | MAPT | MBP | MDM2 | MYC | NDUFA13 | NFE2L2 | NUPR1 | OGT | P2RX1 | PACSIN3 | PDCD6 | PIAS1 | PLGRKT | PLK1 | PML | PPARG | PRELID1 | PSEN1 | PSMC1 | PSMC2 | PSMC3 | PSMC4 | PSMC5 | PSMC6 | PSMD10 | PSMD14 | PSME2 | PSME3 | PTEN | PTK2 | RAD23A | RBX1 | REST | RGN | RHBDD1 | RHOA | RIPK1 | RIPK2 | RNF139 | RNF185 | RNF19A | RPS3 | S100A9 | SART3 | SERPINB3 | SGTA | SIRT1 | SMAD3 | SMURF1 | SNCA | SRC | STAT3 | STUB1 | SUMO1 | SUMO2 | TANK | TMTC3 | TNF | TNP1 | TRADD | TRAF2 | TRIB2 | TRIB3 | TRIM32 | UBQLN1 | UBQLN2 | USP5 | USP50 | VCP | WDR35 | WDR48 | GO:0051188 | 0.014193861458207 | 117/8243 | 76/3250 | 1.71390959680375e-08 | 0.000162735716216516 | T | T | T | T | cofactor biosynthetic process | ACACA | ACAT1 | ACLY | ACOT7 | ACSL1 | ACSL4 | ACSL5 | ACSS2 | ALAS1 | ASPDH | ATIC | CBR4 | CNDP2 | COQ2 | COQ3 | COQ4 | COQ5 | COQ6 | COQ7 | COQ9 | COX15 | CYP1A1 | CYP1A2 | DCAKD | DLAT | DLD | FASN | FDXR | FECH | FPGS | GART | GCDH | GGCT | GGT1 | GSS | HAAO | HAGH | HMBS | HSD17B8 | IDH2 | IREB2 | KYNU | MAT2A | MAT2B | ME1 | MMAB | MOCOS | MPC2 | MTHFD1 | NAMPT | NAPRT | NDUFA9 | NFS1 | NMNAT1 | NMNAT3 | NMRK2 | PCBD1 | PDHA1 | PDHA2 | PDHB | PDHX | PDSS1 | PDSS2 | PDXK | PNP | PPOX | PPT1 | PSAT1 | QDPR | RFK | SLC25A1 | SOD1 | SPR | SUCLA2 | TECR | UBIAD1 | GO:0051262 | 0.0118888754094383 | 98/8243 | 66/3250 | 1.77309206171632e-08 | 0.000168355091259965 | T | T | T | T | protein tetramerization | AASS | ACACA | ACOT13 | ACTN2 | ADSL | ALDH5A1 | ALDOA | ANXA2 | CAT | CBR4 | CBY1 | CD74 | CDA | CPSF7 | CRYZ | CTH | CUTC | DCTPP1 | DCXR | DECR1 | DHRS4 | DNM1L | FARSB | FBP1 | GBP5 | GLS | GOLGA2 | HCN1 | HIST1H3A | HIST1H4A | HIST3H3 | HMGCL | HPRT1 | HSD17B10 | HSD17B8 | IDE | IGF1R | IMPDH2 | INSR | ME1 | OXA1L | PCBD1 | PDSS1 | PDSS2 | PFKL | PKM | PPAT | RCC1 | RPS19 | RRM1 | RRM2 | RXRA | SHMT1 | SHMT2 | SNCA | SOD2 | ST13 | TDO2 | TK1 | TP53 | TP63 | TP73 | USP16 | UXS1 | VASP | XRCC6 | GO:0036211 | 0.230013344656072 | 1896/8243 | 851/3250 | 2.02376149132968e-08 | 0.000192156153601753 | T | T | T | T | protein modification process | ABHD17A | ABL1 | ABL2 | ACP1 | ACTB | ACTL6A | ACTN2 | ADCK2 | ADH5 | ADPRH | ADRB2 | ADRM1 | AHSG | AKT1 | AKT2 | AKT3 | ALB | AMFR | ANAPC4 | ANXA1 | APBB1 | APC | APOA1 | APOE | APP | AR | ARAF | ARF4 | ARID4B | ARRB1 | ARRB2 | ASB12 | ASB15 | ASB16 | ASB6 | ASB9 | ASGR1 | ASGR2 | ATF2 | ATG5 | ATM | ATP13A2 | AURKB | B3GAT3 | B3GNT3 | B4GALT3 | BABAM1 | BAG6 | BCL10 | BCL11A | BCL2 | BCOR | BIRC2 | BIRC3 | BIRC5 | BIRC7 | BIRC8 | BMI1 | BMPR1A | BRAF | BRCA1 | BRD4 | BTK | BTRC | C4A | CAB39 | CALM1 | CALU | CAMK1D | CAMK2A | CAMK2B | CAMKV | CAND1 | CARM1 | CBFB | CBL | CBLB | CBLC | CBX4 | CBX8 | CCDC22 | CCDC8 | CCNB1 | CCNT1 | CCP110 | CCR5 | CDC14B | CDC16 | CDC20 | CDC25A | CDC25B | CDC25C | CDC27 | CDC34 | CDC42 | CDC73 | CDK15 | CDK16 | CDK19 | CDK2 | CDK4 | CDK5 | CDK5R1 | CDK8 | CDK9 | CDKL3 | CEL | CFL1 | CFTR | CHEK2 | CHGB | CHRM3 | CHST4 | CKAP4 | CLK1 | COG3 | COMMD1 | COPS2 | COPS5 | COPS6 | CPNE3 | CRBN | CREB1 | CREBBP | CRKL | CRY1 | CRY2 | CRYAB | CSNK1A1 | CSNK1E | CSNK2A2 | CSNK2B | CTBP1 | CTDP1 | CTH | CTNNB1 | CUL1 | CUL2 | CUL3 | CUL4B | CUL5 | CUL7 | CYCS | CYLD | DAD1 | DAPK1 | DAPP1 | DBI | DCAF12 | DCAF7 | DCAF8 | DCLK1 | DCN | DCUN1D1 | DDA1 | DDB1 | DDB2 | DDOST | DDRGK1 | DERL1 | DGUOK | DLD | DLG2 | DLG3 | DLG4 | DLST | DMAP1 | DPF2 | DPM1 | DTX1 | DTX2 | DUSP1 | DUSP12 | DUSP13 | DUSP14 | DUSP16 | DUSP19 | DUSP22 | DUSP7 | DYRK2 | DZIP3 | EDEM1 | EED | EEF2KMT | EFEMP1 | EGFR | EGLN1 | EGLN3 | EHHADH | EHMT1 | EHMT2 | EIF2AK3 | EIF2AK4 | EIF2S1 | EP300 | EPHA4 | EPHA6 | EPHB6 | ERBB2 | ERBB3 | ERG | ESR1 | EVA1A | EXTL2 | EYA2 | EYA4 | EZH2 | F5 | FAM20B | FAM98A | FBXL12 | FBXO11 | FBXO25 | FBXO32 | FBXO6 | FBXO7 | FBXW11 | FBXW5 | FGF2 | FGFR1 | FGFR3 | FKBP14 | FKBP1A | FKBP1B | FKBP2 | FKBP5 | FKBP7 | FKBP8 | FN1 | FOLH1 | FOXK1 | FOXK2 | FOXO1 | FYN | FZR1 | GAK | GALNT7 | GAPDH | GFPT1 | GFPT2 | GLRX | GLRX5 | GLUL | GOLGA2 | GPS2 | GPX1 | GRB2 | GRK1 | GRK5 | GSK3A | GSK3B | GSPT1 | HBEGF | HCCS | HDAC1 | HDAC11 | HDAC2 | HDAC3 | HDAC4 | HDAC5 | HDAC6 | HDAC7 | HDAC9 | HECW1 | HECW2 | HERC1 | HERC2 | HGS | HIF1A | HIF1AN | HIPK4 | HIST1H2BA | HLCS | HMBS | HRAS | HSF1 | HSF4 | HSP90B1 | HUWE1 | ICK | IGF1R | IGFBP7 | IKBKB | IKBKE | IKBKG | IL3RA | ILF3 | ILK | ING5 | INO80B | INO80C | INSR | IRF4 | ISG15 | ITCH | ITGAV | ITK | JAK1 | JAK3 | JMJD6 | JOSD2 | KAT5 | KAT7 | KAT8 | KBTBD7 | KCMF1 | KDM1A | KDM2A | KDM3B | KDM4D | KDM5B | KDM5C | KDM8 | KDR | KEAP1 | KLHL20 | KLHL22 | KMT2A | KMT2B | KMT2C | KRAS | KSR1 | LAMB1 | LAT | LATS1 | LCMT2 | LEF1 | LGALS1 | LMO7 | LNX1 | LOXL2 | LRRC7 | LRRK1 | LRRK2 | LRSAM1 | LTBP1 | LYPLA1 | LYPLA2 | LZTR1 | MAN2B1 | MAP2K1 | MAP2K3 | MAP2K4 | MAP2K6 | MAP2K7 | MAP3K1 | MAP3K13 | MAP3K14 | MAP3K2 | MAP3K3 | MAP3K4 | MAP4K1 | MAPK1 | MAPK14 | MAPK15 | MAPK3 | MAPK4 | MAPK6 | MAPK7 | MAPK8 | MAPK9 | MAPT | MARCH5 | MAST1 | MAST3 | MATK | MBD3 | MBP | MCFD2 | MDH2 | MDM2 | MDM4 | MECP2 | MED1 | MED12 | MED20 | MED21 | MED31 | MELK | METAP2 | MIB1 | MKNK2 | MORF4L1 | MORF4L2 | MPI | MPPE1 | MRGBP | MTA1 | MTA3 | MTMR14 | MTMR7 | MYC | MYH6 | NAE1 | NCK1 | NCOA1 | NCOA3 | NDUFAF5 | NDUFAF7 | NEDD4 | NEDD8 | NEK1 | NEK7 | NEURL4 | NFE2L2 | NFKBIA | NFS1 | NHLRC3 | NLK | NME2 | NOD2 | NOS2 | NOSIP | NPEPPS | NPR1 | NTMT1 | NTRK1 | NUCB1 | NUP107 | NUP153 | NUP160 | NUP205 | NUP214 | NUP85 | NUP88 | NUP93 | NUP98 | NUPR1 | OBSL1 | OGDH | OGFOD1 | OGT | OTUB1 | OTUB2 | OTUD4 | OTUD5 | OXSR1 | P4HA1 | P4HA2 | P4HB | PADI4 | PAK2 | PAK6 | PARK7 | PARP1 | PARP12 | PARP14 | PARP6 | PAXIP1 | PCGF1 | PCGF5 | PCGF6 | PCMT1 | PCMTD1 | PCMTD2 | PCNA | PCSK9 | PDF | PDIA6 | PDK2 | PEBP1 | PELI1 | PER1 | PER2 | PEX5 | PGAM5 | PGM3 | PGP | PHB | PHF10 | PHKA1 | PIAS1 | PIAS3 | PICK1 | PIGU | PIK3CA | PIK3CG | PIK3R1 | PIM1 | PIN1 | PJA1 | PKN2 | PLK1 | PLOD2 | PML | POLE3 | POM121 | PPA2 | PPEF1 | PPIA | PPIB | PPID | PPIE | PPIF | PPIL3 | PPM1A | PPM1B | PPM1D | PPM1H | PPM1K | PPME1 | PPP1CA | PPP1CB | PPP1CC | PPP1R11 | PPP1R12A | PPP1R3B | PPP1R3C | PPP2CA | PPP2CB | PPP2R1A | PPP2R1B | PPP2R2A | PPP2R2D | PPP2R5D | PPP3CC | PPP4R2 | PPP5C | PPP6C | PPT1 | PPTC7 | PRDX3 | PRDX4 | PRKAA1 | PRKAA2 | PRKAB2 | PRKACA | PRKAG1 | PRKCD | PRKCE | PRKCSH | PRKCZ | PRKD1 | PRKD2 | PRKD3 | PRKDC | PRMT2 | PRMT3 | PRMT6 | PRPF19 | PRSS23 | PSEN1 | PSMA1 | PSMA2 | PSMA3 | PSMA5 | PSMA6 | PSMB5 | PSMB6 | PSMB7 | PSMB8 | PSMC1 | PSMC2 | PSMC3 | PSMC4 | PSMC5 | PSMC6 | PSMD1 | PSMD10 | PSMD11 | PSMD12 | PSMD14 | PSMD2 | PSMD4 | PSMD9 | PSME2 | PSME3 | PTEN | PTK2 | PTPMT1 | PTPN1 | PTPN14 | PTPN21 | PTPN22 | PTPN23 | PTPN4 | PTPN6 | PTPRA | PTPRD | PTPRF | PTPRK | PTPRO | PTPRR | QSOX1 | RAB11A | RAB11B | RAB1A | RAB1B | RAB2A | RAB5A | RAD23A | RAD23B | RAE1 | RAF1 | RANBP9 | RANGAP1 | RARA | RBBP6 | RBX1 | RC3H1 | REST | RHBDD1 | RHO | RHOA | RHOT1 | RING1 | RIOK1 | RIPK1 | RIPK2 | RIPK3 | RIPK4 | RNF123 | RNF126 | RNF139 | RNF181 | RNF183 | RNF185 | RNF19A | RNF2 | RNF25 | RNF31 | RNF4 | RNF8 | RPN1 | RPN2 | RPS6KA1 | RPS6KA2 | RPS6KB2 | RTF1 | RUVBL1 | RUVBL2 | S100A9 | SAE1 | SCG2 | SCG3 | SCYL2 | SEL1L | SENP3 | SENP6 | SENP7 | SENP8 | SEPHS1 | SETDB1 | SFPQ | SHC1 | SHMT2 | SIAH1 | SIN3A | SIRT1 | SIRT3 | SIRT5 | SIRT6 | SIRT7 | SMAD2 | SMAD3 | SMAD4 | SMAD5 | SMURF1 | SMURF2 | SNRPB | SNRPD3 | SNX3 | SOCS1 | SOCS2 | SOCS6 | SPATA2 | SPI1 | SPOP | SPRR2A | SPSB3 | SPTAN1 | SPTBN1 | SPTBN2 | SQSTM1 | SRC | SRPK1 | SRPK2 | ST3GAL1 | ST3GAL6 | ST6GALNAC5 | ST8SIA4 | STK11 | STK24 | STK25 | STK26 | STK3 | STK38 | STK40 | STUB1 | STYX | SULT1A3 | SUMO1 | SUMO1P1 | SUMO2 | SUMO3 | SUZ12 | SYVN1 | TAB1 | TAB2 | TADA2B | TAF12 | TAOK3 | TBL1X | TBRG4 | TESK2 | TGFB1 | TGFBR3 | TGM2 | TGOLN2 | THBS1 | THOP1 | TIMM50 | TIMP1 | TLR3 | TMEM132A | TMEM189-UBE2V1 | TMEM258 | TMTC3 | TNF | TNFAIP3 | TNIP2 | TNK2 | TNKS | TNKS2 | TOMM20 | TOP1 | TOR1A | TP53 | TP53RK | TPR | TPST2 | TPTE | TRAF2 | TRAF3 | TRAF6 | TRIB2 | TRIB3 | TRIM21 | TRIM23 | TRIM25 | TRIM28 | TRIM32 | TRIM33 | TRIM41 | TRIM47 | TRIM63 | TRIM69 | TRIM7 | TRIM9 | TRIP12 | TSG101 | TSPAN17 | TTLL1 | TWF1 | UBA1 | UBA2 | UBA3 | UBA5 | UBA6 | UBAC1 | UBB | UBC | UBE2A | UBE2B | UBE2C | UBE2D1 | UBE2D2 | UBE2D3 | UBE2H | UBE2I | UBE2J1 | UBE2K | UBE2L3 | UBE2L6 | UBE2M | UBE2N | UBE2O | UBE2Q2 | UBE2R2 | UBE2S | UBE2V1 | UBE2W | UBE3A | UBE3B | UBL4A | UBR4 | UBR5 | UBXN7 | UCHL1 | UCHL3 | UCHL5 | UFC1 | UFM1 | UGGT1 | ULK1 | UNKL | USP11 | USP14 | USP15 | USP16 | USP17L2 | USP19 | USP2 | USP20 | USP21 | USP26 | USP28 | USP39 | USP4 | USP42 | USP46 | USP47 | USP5 | USP50 | USP53 | USP7 | USP8 | USP9X | USP9Y | USPL1 | VCAN | VCP | VCPIP1 | VGF | VHL | VKORC1 | WDR45B | WDR48 | WDR82 | WDTC1 | WDYHV1 | WIPI1 | WIPI2 | WNK1 | WNK4 | WSB1 | WWP2 | XIAP | YEATS2 | YES1 | YWHAB | YWHAE | YY1 | ZAP70 | ZBTB16 | ZC3HC1 | ZDHHC17 | ZDHHC23 | ZMPSTE24 | ZNF598 | ZNRF4 | GO:0006464 | 0.230013344656072 | 1896/8243 | 851/3250 | 2.02376149132968e-08 | 0.000192156153601753 | T | T | T | T | cellular protein modification process | ABHD17A | ABL1 | ABL2 | ACP1 | ACTB | ACTL6A | ACTN2 | ADCK2 | ADH5 | ADPRH | ADRB2 | ADRM1 | AHSG | AKT1 | AKT2 | AKT3 | ALB | AMFR | ANAPC4 | ANXA1 | APBB1 | APC | APOA1 | APOE | APP | AR | ARAF | ARF4 | ARID4B | ARRB1 | ARRB2 | ASB12 | ASB15 | ASB16 | ASB6 | ASB9 | ASGR1 | ASGR2 | ATF2 | ATG5 | ATM | ATP13A2 | AURKB | B3GAT3 | B3GNT3 | B4GALT3 | BABAM1 | BAG6 | BCL10 | BCL11A | BCL2 | BCOR | BIRC2 | BIRC3 | BIRC5 | BIRC7 | BIRC8 | BMI1 | BMPR1A | BRAF | BRCA1 | BRD4 | BTK | BTRC | C4A | CAB39 | CALM1 | CALU | CAMK1D | CAMK2A | CAMK2B | CAMKV | CAND1 | CARM1 | CBFB | CBL | CBLB | CBLC | CBX4 | CBX8 | CCDC22 | CCDC8 | CCNB1 | CCNT1 | CCP110 | CCR5 | CDC14B | CDC16 | CDC20 | CDC25A | CDC25B | CDC25C | CDC27 | CDC34 | CDC42 | CDC73 | CDK15 | CDK16 | CDK19 | CDK2 | CDK4 | CDK5 | CDK5R1 | CDK8 | CDK9 | CDKL3 | CEL | CFL1 | CFTR | CHEK2 | CHGB | CHRM3 | CHST4 | CKAP4 | CLK1 | COG3 | COMMD1 | COPS2 | COPS5 | COPS6 | CPNE3 | CRBN | CREB1 | CREBBP | CRKL | CRY1 | CRY2 | CRYAB | CSNK1A1 | CSNK1E | CSNK2A2 | CSNK2B | CTBP1 | CTDP1 | CTH | CTNNB1 | CUL1 | CUL2 | CUL3 | CUL4B | CUL5 | CUL7 | CYCS | CYLD | DAD1 | DAPK1 | DAPP1 | DBI | DCAF12 | DCAF7 | DCAF8 | DCLK1 | DCN | DCUN1D1 | DDA1 | DDB1 | DDB2 | DDOST | DDRGK1 | DERL1 | DGUOK | DLD | DLG2 | DLG3 | DLG4 | DLST | DMAP1 | DPF2 | DPM1 | DTX1 | DTX2 | DUSP1 | DUSP12 | DUSP13 | DUSP14 | DUSP16 | DUSP19 | DUSP22 | DUSP7 | DYRK2 | DZIP3 | EDEM1 | EED | EEF2KMT | EFEMP1 | EGFR | EGLN1 | EGLN3 | EHHADH | EHMT1 | EHMT2 | EIF2AK3 | EIF2AK4 | EIF2S1 | EP300 | EPHA4 | EPHA6 | EPHB6 | ERBB2 | ERBB3 | ERG | ESR1 | EVA1A | EXTL2 | EYA2 | EYA4 | EZH2 | F5 | FAM20B | FAM98A | FBXL12 | FBXO11 | FBXO25 | FBXO32 | FBXO6 | FBXO7 | FBXW11 | FBXW5 | FGF2 | FGFR1 | FGFR3 | FKBP14 | FKBP1A | FKBP1B | FKBP2 | FKBP5 | FKBP7 | FKBP8 | FN1 | FOLH1 | FOXK1 | FOXK2 | FOXO1 | FYN | FZR1 | GAK | GALNT7 | GAPDH | GFPT1 | GFPT2 | GLRX | GLRX5 | GLUL | GOLGA2 | GPS2 | GPX1 | GRB2 | GRK1 | GRK5 | GSK3A | GSK3B | GSPT1 | HBEGF | HCCS | HDAC1 | HDAC11 | HDAC2 | HDAC3 | HDAC4 | HDAC5 | HDAC6 | HDAC7 | HDAC9 | HECW1 | HECW2 | HERC1 | HERC2 | HGS | HIF1A | HIF1AN | HIPK4 | HIST1H2BA | HLCS | HMBS | HRAS | HSF1 | HSF4 | HSP90B1 | HUWE1 | ICK | IGF1R | IGFBP7 | IKBKB | IKBKE | IKBKG | IL3RA | ILF3 | ILK | ING5 | INO80B | INO80C | INSR | IRF4 | ISG15 | ITCH | ITGAV | ITK | JAK1 | JAK3 | JMJD6 | JOSD2 | KAT5 | KAT7 | KAT8 | KBTBD7 | KCMF1 | KDM1A | KDM2A | KDM3B | KDM4D | KDM5B | KDM5C | KDM8 | KDR | KEAP1 | KLHL20 | KLHL22 | KMT2A | KMT2B | KMT2C | KRAS | KSR1 | LAMB1 | LAT | LATS1 | LCMT2 | LEF1 | LGALS1 | LMO7 | LNX1 | LOXL2 | LRRC7 | LRRK1 | LRRK2 | LRSAM1 | LTBP1 | LYPLA1 | LYPLA2 | LZTR1 | MAN2B1 | MAP2K1 | MAP2K3 | MAP2K4 | MAP2K6 | MAP2K7 | MAP3K1 | MAP3K13 | MAP3K14 | MAP3K2 | MAP3K3 | MAP3K4 | MAP4K1 | MAPK1 | MAPK14 | MAPK15 | MAPK3 | MAPK4 | MAPK6 | MAPK7 | MAPK8 | MAPK9 | MAPT | MARCH5 | MAST1 | MAST3 | MATK | MBD3 | MBP | MCFD2 | MDH2 | MDM2 | MDM4 | MECP2 | MED1 | MED12 | MED20 | MED21 | MED31 | MELK | METAP2 | MIB1 | MKNK2 | MORF4L1 | MORF4L2 | MPI | MPPE1 | MRGBP | MTA1 | MTA3 | MTMR14 | MTMR7 | MYC | MYH6 | NAE1 | NCK1 | NCOA1 | NCOA3 | NDUFAF5 | NDUFAF7 | NEDD4 | NEDD8 | NEK1 | NEK7 | NEURL4 | NFE2L2 | NFKBIA | NFS1 | NHLRC3 | NLK | NME2 | NOD2 | NOS2 | NOSIP | NPEPPS | NPR1 | NTMT1 | NTRK1 | NUCB1 | NUP107 | NUP153 | NUP160 | NUP205 | NUP214 | NUP85 | NUP88 | NUP93 | NUP98 | NUPR1 | OBSL1 | OGDH | OGFOD1 | OGT | OTUB1 | OTUB2 | OTUD4 | OTUD5 | OXSR1 | P4HA1 | P4HA2 | P4HB | PADI4 | PAK2 | PAK6 | PARK7 | PARP1 | PARP12 | PARP14 | PARP6 | PAXIP1 | PCGF1 | PCGF5 | PCGF6 | PCMT1 | PCMTD1 | PCMTD2 | PCNA | PCSK9 | PDF | PDIA6 | PDK2 | PEBP1 | PELI1 | PER1 | PER2 | PEX5 | PGAM5 | PGM3 | PGP | PHB | PHF10 | PHKA1 | PIAS1 | PIAS3 | PICK1 | PIGU | PIK3CA | PIK3CG | PIK3R1 | PIM1 | PIN1 | PJA1 | PKN2 | PLK1 | PLOD2 | PML | POLE3 | POM121 | PPA2 | PPEF1 | PPIA | PPIB | PPID | PPIE | PPIF | PPIL3 | PPM1A | PPM1B | PPM1D | PPM1H | PPM1K | PPME1 | PPP1CA | PPP1CB | PPP1CC | PPP1R11 | PPP1R12A | PPP1R3B | PPP1R3C | PPP2CA | PPP2CB | PPP2R1A | PPP2R1B | PPP2R2A | PPP2R2D | PPP2R5D | PPP3CC | PPP4R2 | PPP5C | PPP6C | PPT1 | PPTC7 | PRDX3 | PRDX4 | PRKAA1 | PRKAA2 | PRKAB2 | PRKACA | PRKAG1 | PRKCD | PRKCE | PRKCSH | PRKCZ | PRKD1 | PRKD2 | PRKD3 | PRKDC | PRMT2 | PRMT3 | PRMT6 | PRPF19 | PRSS23 | PSEN1 | PSMA1 | PSMA2 | PSMA3 | PSMA5 | PSMA6 | PSMB5 | PSMB6 | PSMB7 | PSMB8 | PSMC1 | PSMC2 | PSMC3 | PSMC4 | PSMC5 | PSMC6 | PSMD1 | PSMD10 | PSMD11 | PSMD12 | PSMD14 | PSMD2 | PSMD4 | PSMD9 | PSME2 | PSME3 | PTEN | PTK2 | PTPMT1 | PTPN1 | PTPN14 | PTPN21 | PTPN22 | PTPN23 | PTPN4 | PTPN6 | PTPRA | PTPRD | PTPRF | PTPRK | PTPRO | PTPRR | QSOX1 | RAB11A | RAB11B | RAB1A | RAB1B | RAB2A | RAB5A | RAD23A | RAD23B | RAE1 | RAF1 | RANBP9 | RANGAP1 | RARA | RBBP6 | RBX1 | RC3H1 | REST | RHBDD1 | RHO | RHOA | RHOT1 | RING1 | RIOK1 | RIPK1 | RIPK2 | RIPK3 | RIPK4 | RNF123 | RNF126 | RNF139 | RNF181 | RNF183 | RNF185 | RNF19A | RNF2 | RNF25 | RNF31 | RNF4 | RNF8 | RPN1 | RPN2 | RPS6KA1 | RPS6KA2 | RPS6KB2 | RTF1 | RUVBL1 | RUVBL2 | S100A9 | SAE1 | SCG2 | SCG3 | SCYL2 | SEL1L | SENP3 | SENP6 | SENP7 | SENP8 | SEPHS1 | SETDB1 | SFPQ | SHC1 | SHMT2 | SIAH1 | SIN3A | SIRT1 | SIRT3 | SIRT5 | SIRT6 | SIRT7 | SMAD2 | SMAD3 | SMAD4 | SMAD5 | SMURF1 | SMURF2 | SNRPB | SNRPD3 | SNX3 | SOCS1 | SOCS2 | SOCS6 | SPATA2 | SPI1 | SPOP | SPRR2A | SPSB3 | SPTAN1 | SPTBN1 | SPTBN2 | SQSTM1 | SRC | SRPK1 | SRPK2 | ST3GAL1 | ST3GAL6 | ST6GALNAC5 | ST8SIA4 | STK11 | STK24 | STK25 | STK26 | STK3 | STK38 | STK40 | STUB1 | STYX | SULT1A3 | SUMO1 | SUMO1P1 | SUMO2 | SUMO3 | SUZ12 | SYVN1 | TAB1 | TAB2 | TADA2B | TAF12 | TAOK3 | TBL1X | TBRG4 | TESK2 | TGFB1 | TGFBR3 | TGM2 | TGOLN2 | THBS1 | THOP1 | TIMM50 | TIMP1 | TLR3 | TMEM132A | TMEM189-UBE2V1 | TMEM258 | TMTC3 | TNF | TNFAIP3 | TNIP2 | TNK2 | TNKS | TNKS2 | TOMM20 | TOP1 | TOR1A | TP53 | TP53RK | TPR | TPST2 | TPTE | TRAF2 | TRAF3 | TRAF6 | TRIB2 | TRIB3 | TRIM21 | TRIM23 | TRIM25 | TRIM28 | TRIM32 | TRIM33 | TRIM41 | TRIM47 | TRIM63 | TRIM69 | TRIM7 | TRIM9 | TRIP12 | TSG101 | TSPAN17 | TTLL1 | TWF1 | UBA1 | UBA2 | UBA3 | UBA5 | UBA6 | UBAC1 | UBB | UBC | UBE2A | UBE2B | UBE2C | UBE2D1 | UBE2D2 | UBE2D3 | UBE2H | UBE2I | UBE2J1 | UBE2K | UBE2L3 | UBE2L6 | UBE2M | UBE2N | UBE2O | UBE2Q2 | UBE2R2 | UBE2S | UBE2V1 | UBE2W | UBE3A | UBE3B | UBL4A | UBR4 | UBR5 | UBXN7 | UCHL1 | UCHL3 | UCHL5 | UFC1 | UFM1 | UGGT1 | ULK1 | UNKL | USP11 | USP14 | USP15 | USP16 | USP17L2 | USP19 | USP2 | USP20 | USP21 | USP26 | USP28 | USP39 | USP4 | USP42 | USP46 | USP47 | USP5 | USP50 | USP53 | USP7 | USP8 | USP9X | USP9Y | USPL1 | VCAN | VCP | VCPIP1 | VGF | VHL | VKORC1 | WDR45B | WDR48 | WDR82 | WDTC1 | WDYHV1 | WIPI1 | WIPI2 | WNK1 | WNK4 | WSB1 | WWP2 | XIAP | YEATS2 | YES1 | YWHAB | YWHAE | YY1 | ZAP70 | ZBTB16 | ZC3HC1 | ZDHHC17 | ZDHHC23 | ZMPSTE24 | ZNF598 | ZNRF4 | GO:1903362 | 0.0219580249909014 | 181/8243 | 108/3250 | 2.14689710826925e-08 | 0.000203847880430165 | T | T | T | T | regulation of cellular protein catabolic process | AKT1 | ANXA2 | APOE | ARAF | ATP13A2 | AXIN1 | BAG2 | BAG5 | BAG6 | BTRC | CAV1 | CBFA2T3 | CCAR2 | CCDC22 | CD81 | CEBPA | CLU | COMMD1 | CSNK1A1 | CSNK1E | CSNK2A2 | CTSC | DAB2 | DDA1 | DDRGK1 | DNAJB2 | DVL1 | EDEM1 | EPHA4 | EZR | FAF1 | FAM192A | GLMN | GNA12 | GPX1 | GSK3A | GSK3B | HSP90AA1 | HSP90AB1 | HSP90AB3P | HSP90AB4P | HSPA1B | HSPBP1 | KEAP1 | LAMP3 | LATS1 | LRRK2 | MAPK9 | MDM2 | MSN | NELL1 | NFE2L2 | NUPR1 | OGT | PARK7 | PCSK9 | PIAS1 | PLK1 | PML | PRKACA | PSEN1 | PSMC1 | PSMC2 | PSMC3 | PSMC4 | PSMC5 | PSMC6 | PSMD10 | PSMD14 | PSME2 | PSME3 | PTEN | PTK2 | RAD23A | RAD23B | RBX1 | RDX | RGN | RNF139 | RNF185 | RNF19A | RPL23 | SDCBP | SGTA | SMARCC1 | SMURF1 | STUB1 | STYX | SUMO1 | SUMO2 | TMTC3 | TNFAIP3 | TRIB2 | TRIB3 | UBB | UBE2J1 | UBE2K | UBE3A | UBQLN1 | UBQLN2 | UBQLN4 | UBXN1 | UCHL5 | USP14 | USP19 | USP5 | USP7 | VCP | GO:0010035 | 0.0411258037122407 | 339/8243 | 183/3250 | 2.20644091999371e-08 | 0.000209501565353403 | T | T | T | T | response to inorganic substance | ABAT | ABL1 | ACO1 | ACTA1 | ACTG1 | ADA | AHCYL1 | AIFM1 | AKR1B1 | AKT1 | ALOX5AP | ANK3 | ANXA1 | ANXA11 | ANXA5 | ANXA7 | APBB1 | APEX1 | APP | ASCL1 | ASS1 | ATF2 | ATIC | ATP13A2 | ATRX | BACE1 | BCL2 | BRAF | BSG | C1QA | CACNG2 | CALM1 | CALM2 | CALM3 | CALR | CAPN2 | CASP3 | CASP8 | CASP9 | CAT | CAV1 | CBX8 | CCNB1 | CDH1 | CDK2 | CDK4 | CEBPA | CLIC4 | COL18A1 | CPNE3 | CREB1 | CRIP1 | CRK | CRYAB | CUTA | CYP1A1 | CYP1A2 | DAXX | DHX36 | DLG2 | DLG4 | DPEP1 | DUSP1 | EEF1A2 | EEF2 | EGFR | EGLN1 | EIF2AK3 | EIF2B5 | EIF2S1 | EIF4A3 | ENDOG | ETS1 | EZH2 | FABP1 | FBP1 | FECH | FGB | FIS1 | FKBP1B | FOS | FOXO1 | FUS | FYN | G6PD | GART | GDI1 | GNAO1 | GOT1 | GPI | GPX1 | GSS | HAAO | HDAC2 | HDAC6 | HIF1A | HMOX1 | HNRNPA1 | HNRNPD | HSF1 | HSPA5 | ICAM1 | ID2 | IQGAP1 | IREB2 | ITPR3 | JUN | JUNB | KCNIP2 | KDR | LDHA | LGMN | LIG4 | LOXL2 | LRRK2 | MAPK1 | MAPK3 | MAPK7 | MAPK8 | MAPK9 | MAPT | MDM2 | NDRG1 | NEDD4 | NFE2L2 | NQO1 | NTRK1 | OTC | P2RX4 | PARK7 | PARP1 | PCNA | PDCD10 | PDCD6 | PEBP1 | PEF1 | PGAM2 | PPARGC1A | PPIF | PPP1CA | PPP2CA | PPP2CB | PPP5C | PRDX3 | PRKAA1 | PRKAA2 | PRKCD | PTEN | PTGES | QDPR | RELA | RIPK1 | RIPK3 | RPS3 | S100A13 | SDC1 | SEC31A | SIPA1 | SIRT1 | SLC25A13 | SLC25A24 | SNCA | SOD1 | SOD2 | SORD | SRC | STAT1 | STAT6 | STIM1 | STK24 | STK25 | STK26 | SUMO1 | THBS1 | TNFAIP3 | TRAF2 | TREX1 | TXNIP | UBE2D3 | VCAM1 | VCP | WNK1 | ZNF277 | GO:0031329 | 0.0723037728982167 | 596/8243 | 299/3250 | 2.24068521933101e-08 | 0.000212753061575479 | T | T | T | T | regulation of cellular catabolic process | ABL1 | ABL2 | ADRB2 | AGO2 | AIFM1 | AKT1 | AKT2 | ANP32A | ANXA2 | APEX1 | APOE | ARAF | ARNT | ATM | ATP13A2 | ATP6V1A | ATP6V1B1 | ATP6V1C1 | ATP6V1D | ATP6V1E1 | AXIN1 | BAG2 | BAG3 | BAG5 | BAG6 | BAX | BCL2 | BTRC | CALCOCO2 | CAPN1 | CAPNS1 | CASP3 | CAV1 | CBFA2T3 | CCAR2 | CCDC22 | CD81 | CDC37 | CDK5 | CDK5R1 | CEBPA | CIRBP | CISD2 | CLU | COMMD1 | CPT1A | CSNK1A1 | CSNK1E | CSNK2A2 | CTSA | CTSC | CTTN | DAB2 | DAPK1 | DCN | DCP2 | DDA1 | DDRGK1 | DHX36 | DHX9 | DIS3 | DNAJB2 | DNM1L | DVL1 | E2F1 | EDEM1 | EEF1A1 | EEF1A2 | EIF2AK4 | EIF4E | EIF4G3 | ELAVL1 | ENDOG | EP300 | EPHA4 | EPM2A | ESRRB | EXOSC1 | EXOSC5 | EXOSC6 | EXOSC9 | EZR | FABP1 | FAF1 | FAM192A | FBXO7 | FOXO1 | FTO | FXR2 | GAPDH | GAPDHS | GFAP | GLMN | GNA12 | GNAI3 | GOLGA2 | GPD1 | GPSM1 | GPX1 | GSK3A | GSK3B | HAX1 | HDAC6 | HERC1 | HIF1A | HK2 | HMGB1 | HMOX1 | HNRNPD | HNRNPM | HNRNPU | HSF1 | HSP90AA1 | HSP90AB1 | HSP90AB3P | HSP90AB4P | HSPA1B | HSPA8 | HSPB1 | HSPBP1 | HTRA2 | HTT | IDH1 | IFI16 | IGF2BP1 | IKBKG | INSR | ITCH | ITPR1 | KAT8 | KDR | KEAP1 | KLHL22 | LAMP3 | LARS | LATS1 | LRPPRC | LRRK2 | LRSAM1 | MAPK14 | MAPK15 | MAPK3 | MAPK8 | MAPK9 | MAPT | MDM2 | MLST8 | MOV10 | MSN | MYD88 | NAMPT | NBAS | NEDD4 | NELL1 | NFE2L2 | NMNAT1 | NNT | NPM1 | NUP214 | NUPR1 | OGT | OPTN | PABPC1 | PABPC4 | PACSIN3 | PAIP1 | PAM16 | PARK7 | PCSK9 | PDE12 | PGAM1 | PIAS1 | PIK3CA | PIK3CG | PIKFYVE | PIP4K2A | PLEKHF1 | PLK1 | PML | POLDIP2 | PPP1CA | PPP1CB | PPP1R3B | PRKAA1 | PRKAA2 | PRKAB2 | PRKACA | PRKAG1 | PRKCD | PRKD1 | PSAP | PSEN1 | PSMA1 | PSMA2 | PSMA3 | PSMA5 | PSMA6 | PSMB5 | PSMB6 | PSMB7 | PSMB8 | PSMC1 | PSMC2 | PSMC3 | PSMC4 | PSMC5 | PSMC6 | PSMD1 | PSMD10 | PSMD11 | PSMD12 | PSMD14 | PSMD2 | PSMD4 | PSMD9 | PSME2 | PSME3 | PTEN | PTK2 | PTPN1 | PTPN22 | PUM1 | QSOX1 | RAB1B | RAB3GAP1 | RAB5A | RAD23A | RAD23B | RB1CC1 | RBX1 | RC3H1 | RDX | RGN | RIPK2 | RNF139 | RNF185 | RNF19A | RPL23 | RPTOR | SCFD1 | SDCBP | SET | SGTA | SIRT1 | SMARCC1 | SMG6 | SMURF1 | SNCA | SNX6 | SOGA3 | SPTLC2 | STAT3 | STK11 | STUB1 | STYX | SUMO1 | SUMO2 | SUPT5H | TAB2 | TARDBP | TBRG4 | TIMP1 | TIMP2 | TMTC3 | TNF | TNFAIP3 | TNPO1 | TPCN2 | TRIB2 | TRIB3 | TRIM21 | TWIST1 | UBB | UBC | UBE2J1 | UBE2K | UBE3A | UBQLN1 | UBQLN2 | UBQLN4 | UBXN1 | UCHL1 | UCHL5 | ULK1 | USP14 | USP19 | USP5 | USP7 | VCP | VDAC1 | VIM | VPS26A | VPS29 | WDR41 | XBP1 | XPO1 | YWHAB | YWHAZ | ZMPSTE24 | GO:0006732 | 0.0183185733349509 | 151/8243 | 93/3250 | 2.36971969395524e-08 | 0.00022500488494105 | T | T | T | T | coenzyme metabolic process | AASDHPPT | ACACA | ACAT1 | ACLY | ACOT13 | ACOT7 | ACOT9 | ACSL1 | ACSL4 | ACSL5 | ACSS2 | AHCY | AHCYL1 | AMD1 | ASPDH | ATIC | CBR4 | COQ2 | COQ3 | COQ4 | COQ5 | COQ6 | COQ7 | COQ9 | CROT | DBI | DCAKD | DCXR | DLAT | DLD | DLST | FAR2 | FASN | FDXR | FOLH1 | FOLR1 | FPGS | G6PD | GART | GCDH | GPD1L | HAAO | HMGCL | HSD17B4 | HSD17B8 | IDH1 | IDH2 | KCNAB2 | KYNU | LDHA | MAT2A | MAT2B | MDH1 | MDH2 | ME1 | MOCOS | MPC2 | MTHFD1 | MTRR | MVD | NAMPT | NAPRT | NDUFA9 | NFS1 | NMNAT1 | NMNAT3 | NMRK2 | NNT | OGDH | PCBD1 | PDHA1 | PDHA2 | PDHB | PDHX | PDSS1 | PDSS2 | PDXK | PNP | PPT1 | QDPR | RFK | SHMT1 | SHMT2 | SLC25A1 | SLC25A32 | SPR | SUCLA2 | SUCLG2 | TECR | THEM4 | TYMS | UBIAD1 | VCP | GO:0016579 | 0.0254761615916535 | 210/8243 | 122/3250 | 2.40255259290817e-08 | 0.00022812236869663 | T | T | T | T | protein deubiquitination | ACTB | ACTL6A | ADRB2 | ADRM1 | APC | AR | ARRB2 | BABAM1 | BIRC2 | BIRC3 | BRCA1 | CCP110 | CDC20 | CDC25A | CFTR | COPS5 | CYLD | DDB2 | EP300 | ESR1 | FOXK1 | FOXK2 | HGS | HIF1A | IKBKG | INO80B | INO80C | JOSD2 | KEAP1 | MDM2 | MDM4 | MYC | NEDD8 | NFKBIA | OGT | OTUB1 | OTUB2 | OTUD4 | OTUD5 | PSMA1 | PSMA2 | PSMA3 | PSMA5 | PSMA6 | PSMB5 | PSMB6 | PSMB7 | PSMB8 | PSMC1 | PSMC2 | PSMC3 | PSMC4 | PSMC5 | PSMC6 | PSMD1 | PSMD10 | PSMD11 | PSMD12 | PSMD14 | PSMD2 | PSMD4 | PSMD9 | PSME2 | PSME3 | PTEN | RAD23A | RAD23B | RHOA | RHOT1 | RIPK1 | RNF123 | RUVBL1 | SENP8 | SHMT2 | SMAD2 | SMAD3 | SMAD4 | SMURF2 | SNX3 | SPATA2 | TAB1 | TADA2B | TNFAIP3 | TNIP2 | TOMM20 | TP53 | TRAF2 | TRAF3 | TRAF6 | UBB | UBC | UBE2D1 | UCHL1 | UCHL3 | UCHL5 | USP11 | USP14 | USP15 | USP16 | USP17L2 | USP19 | USP2 | USP20 | USP21 | USP26 | USP28 | USP39 | USP4 | USP42 | USP46 | USP47 | USP5 | USP50 | USP53 | USP7 | USP8 | USP9X | USP9Y | VCP | VCPIP1 | WDR48 | YY1 | GO:0006099 | 0.00351813660075215 | 29/8243 | 26/3250 | 2.56702353401771e-08 | 0.000243738884554982 | T | T | T | T | tricarboxylic acid cycle | ACO1 | ACO2 | CS | DLAT | DLD | DLST | FAHD1 | FH | IDH1 | IDH2 | IDH3A | IDH3B | IDH3G | IREB2 | MDH1 | MDH2 | NNT | OGDH | PDHA1 | PDHA2 | PDHB | SDHA | SDHB | SDHD | SUCLA2 | SUCLG2 | GO:0071478 | 0.0138299162926119 | 114/8243 | 74/3250 | 2.76125624211781e-08 | 0.000262181280189087 | T | T | T | T | cellular response to radiation | AKT2 | ATM | AURKB | BAX | BCL2L1 | CASP9 | CDC25A | CDKN1A | CHEK2 | CREBBP | CRIP1 | CRY1 | CRY2 | CRYAB | CUL4B | DDB1 | DDB2 | DHX36 | EEF1D | EIF2AK4 | EIF2S1 | EP300 | FIGNL1 | GNB1 | GRB2 | GRK1 | H2AFX | HRAS | HSF1 | HSPA5 | IFI16 | KDM1A | KDM4D | LIG4 | MAPK14 | MC1R | MDM2 | MGMT | MME | MYC | NAMPT | NEDD4 | NIPBL | NPM1 | PARP1 | PCNA | PDE6G | PIK3R1 | POLD1 | POLE3 | PPID | PTN | PTPRK | RAD51 | RAD51AP1 | RHBDD1 | RHO | RUVBL2 | SAG | SIRT1 | SNAI2 | STK11 | TANK | TGFB1 | TP53 | TREX1 | TRIAP1 | USP28 | USP47 | XRCC5 | XRCC6 | YAP1 | YY1 | ZMPSTE24 | GO:2001233 | 0.0368797767802984 | 304/8243 | 166/3250 | 3.56768838574417e-08 | 0.000338752012226409 | T | T | T | T | regulation of apoptotic signaling pathway | ACAA2 | ADORA2A | AKT1 | AR | ARRB2 | ATAD5 | BAG5 | BAX | BBC3 | BCL10 | BCL2 | BCL2L1 | BCL2L2 | BDKRB2 | BRCA1 | CASP2 | CASP8 | CAV1 | CCAR2 | CD74 | CHCHD10 | CLU | COL2A1 | CREB3 | CSNK2A2 | CTH | CTNNB1 | CTSC | CTTN | CYLD | DBH | DDX3X | DNAJA1 | DNM1L | E2F1 | EIF2AK3 | ENO1 | EYA2 | EYA4 | FADD | FAF1 | FASLG | FGB | FGFR1 | FIGNL1 | FIS1 | FYN | GATA1 | GNAI2 | GNAI3 | GPX1 | GSK3A | GSK3B | GSTP1 | HDAC1 | HIF1A | HMGB2 | HMOX1 | HNRNPK | HSPA1B | HSPB1 | HTRA2 | HTT | ICAM1 | ING5 | ITGAV | KDM1A | LGALS3 | LMNA | LRRK2 | LTBR | MAPK7 | MAPK8 | MAPK9 | MDM2 | MIF | MLLT11 | NCK1 | NDUFA13 | NDUFS3 | NFE2L2 | NOS3 | OPA1 | P4HB | PAK2 | PAM16 | PARK7 | PARP1 | PDIA3 | PIH1D1 | PLA2G6 | PLAUR | PLEKHF1 | PML | PPIF | PPP1CA | PPP2R1A | PPP2R1B | PPP3CC | PRDX2 | PRELID1 | PRKCD | PSEN1 | PSMD10 | PSME3 | PTEN | PTPMT1 | PTPN1 | QARS | RAF1 | RB1CC1 | RELA | RIPK1 | RIPK3 | RNF183 | RPS3 | RRM2B | S100A9 | SCG2 | SEPT4 | SFN | SFPQ | SIAH1 | SIRT1 | SLC25A5 | SLC35F6 | SLC9A3R1 | SMAD3 | SNAI2 | SOD1 | SOD2 | SP100 | SRC | STK3 | SYVN1 | TFDP1 | THBS1 | TMBIM6 | TNF | TNFAIP3 | TNFRSF12A | TNFSF12 | TP53 | TP63 | TP73 | TRADD | TRAF1 | TRAF2 | TRAP1 | TRIAP1 | TRIM32 | UBB | UBQLN1 | USP47 | VDAC2 | WDR35 | WWOX | XBP1 | YAP1 | YWHAB | YWHAE | YWHAG | YWHAH | YWHAQ | YWHAZ | ZC3HC1 | GO:0009108 | 0.0104330947470581 | 86/8243 | 59/3250 | 3.59828384197848e-08 | 0.000341657050795856 | T | T | T | T | coenzyme biosynthetic process | ACACA | ACAT1 | ACLY | ACOT7 | ACSL1 | ACSL4 | ACSL5 | ACSS2 | ASPDH | ATIC | CBR4 | COQ2 | COQ3 | COQ4 | COQ5 | COQ6 | COQ7 | COQ9 | DCAKD | DLAT | DLD | FASN | FDXR | FPGS | GART | GCDH | HAAO | HSD17B8 | IDH2 | KYNU | MAT2A | MAT2B | ME1 | MOCOS | MPC2 | MTHFD1 | NAMPT | NAPRT | NDUFA9 | NFS1 | NMNAT1 | NMNAT3 | NMRK2 | PCBD1 | PDHA1 | PDHA2 | PDHB | PDHX | PDSS1 | PDSS2 | PDXK | PNP | PPT1 | QDPR | RFK | SLC25A1 | SPR | TECR | UBIAD1 | GO:0005975 | 0.0283877229164139 | 234/8243 | 133/3250 | 3.60046869390974e-08 | 0.00034186450248673 | T | T | T | T | carbohydrate metabolic process | AGL | AKR1A1 | AKR1B1 | AKT1 | AKT2 | ALDH1B1 | ALDH5A1 | ALDOA | ALDOC | AMDHD2 | APOD | B3GNT3 | B4GALT3 | BRAF | CALM1 | CHST4 | COQ2 | COQ3 | CPT1A | CRY1 | CS | DCXR | DLAT | ENO1 | ENO2 | ENO3 | FABP5 | FBP1 | FBP2 | G6PD | GAA | GALNT7 | GANAB | GAPDH | GAPDHS | GBE1 | GDPGP1 | GLA | GLB1L2 | GLO1 | GNPDA1 | GNPDA2 | GOT1 | GOT2 | GPD1 | GPD1L | GPI | GSK3A | GSK3B | GUSB | GYG1 | GYG2 | GYS1 | HEXA | HK1 | HK2 | HS2ST1 | IDH1 | IDH2 | IDH3A | IDH3G | IMPA1 | IMPA2 | INSR | ISYNA1 | LDHAL6A | LDHAL6B | LDHB | LDHC | MAN2B1 | MAPK14 | MDH1 | MDH2 | ME1 | MECP2 | MPI | MTMR7 | NAGK | NANP | NANS | ONECUT1 | OVGP1 | PC | PDHA1 | PDHA2 | PDHB | PDK2 | PER2 | PFKM | PGAM1 | PGAM2 | PGD | PGK1 | PGK2 | PGM1 | PGM2 | PGM3 | PGM5 | PGP | PHKA1 | PIK3CA | PPARD | PPARGC1A | PPP1CA | PPP1CB | PPP1CC | PPP1R2 | PPP1R3B | PPP1R3C | PRKAA1 | PTEN | PYGB | PYGL | RPE | RPIA | SLC25A1 | SLC25A10 | SLC25A11 | SLC25A13 | SLC2A5 | SLC3A2 | SORD | ST3GAL6 | ST6GALNAC5 | ST8SIA4 | STBD1 | STK40 | TALDO1 | TKT | TNF | TPI1 | UGP2 | WDTC1 | GO:0098754 | 0.00800679364309111 | 66/8243 | 48/3250 | 3.78126011579484e-08 | 0.00035903064799472 | T | T | T | T | detoxification | ADH5 | AKR1A1 | AKR1B10 | ALB | ALOX5AP | APOE | CAT | CCS | ESD | FABP1 | FANCC | GPX1 | GPX4 | GPX7 | GSR | GSTA1 | GSTK1 | GSTO1 | GSTO2 | GSTP1 | GSTT1 | GSTZ1 | HBZ | KDM3B | LTC4S | MGST1 | NFE2L2 | NNT | NOS3 | NQO1 | PARK7 | PRDX1 | PRDX2 | PRDX3 | PRDX4 | PRDX5 | PRDX6 | PXDN | RDH11 | RDH12 | S100A9 | SOD1 | SOD2 | SRXN1 | TXN | TXNDC17 | TXNRD1 | UBIAD1 | GO:0001775 | 0.0757005944437705 | 624/8243 | 310/3250 | 4.27650003296404e-08 | 0.000406053678129936 | T | T | T | T | cell activation | ABL1 | ACAA1 | ACLY | ACTN1 | ACTR2 | ADA | ADORA2A | AGL | AHSG | AICDA | ALDOA | ALDOC | ALOX5 | ANXA1 | ANXA2 | AP1M1 | APEH | APP | APRT | ARMC8 | ARPC5 | ARRB1 | ARRB2 | ATAD5 | ATP6V1D | AZU1 | BAG6 | BAX | BCL2 | BCL6 | BRAF | BTK | CAB39 | CAND1 | CAPN1 | CASP8 | CAT | CBFB | CBL | CCT2 | CCT8 | CD2 | CD22 | CD36 | CD40 | CD40LG | CD79B | CDA | CEP290 | CKAP4 | CLU | COPB1 | COTL1 | CPNE3 | CSNK2B | CTNNB1 | CTPS1 | CTSA | CTSC | CTSD | CXCL6 | CXCL8 | CYB5R3 | DBNL | DDOST | DDX3X | DEGS1 | DERA | DGKE | DGKZ | DNAJA3 | DYNC1H1 | DYNC1LI1 | DYNLL1 | DYNLT1 | EEF1A1 | EEF2 | EGFR | EIF2AK4 | ELF4 | EP300 | EXOSC6 | FABP5 | FADD | FAF2 | FCN1 | FGL2 | FKBP1A | FKBP1B | FTH1 | FTL | FYN | GAA | GALNS | GDI2 | GHDC | GLA | GNA12 | GNA13 | GNA15 | GNAQ | GNB1 | GNS | GPI | GPS2 | GSTP1 | GUSB | GYG1 | HAVCR2 | HDAC4 | HDAC5 | HDAC9 | HEXB | HMGB1 | HNF1A | HPRT1 | HRNR | HSP90AA1 | HSP90AB1 | HSPA1B | HSPA8 | HSPD1 | HUWE1 | ICAM1 | ID2 | IDH1 | IFNA2 | IGBP1 | ILF2 | IMPDH2 | IQGAP1 | IRF1 | IRF4 | ITGA4 | ITGAV | ITGB3 | ITK | ITPR1 | ITPR2 | ITPR3 | JAK3 | JMJD6 | JUN | JUP | KCMF1 | KCNAB2 | KIF13B | KIR3DS1 | KLF6 | KPNB1 | LAT | LCP1 | LCP2 | LEF1 | LGALS1 | LGALS3 | LIG4 | LRRC7 | LTBR | MAN2B1 | MAPK1 | MAPK14 | MAPK3 | MAPT | MGST1 | MICA | MIF | MLH1 | MME | MSH6 | MSN | NAMPT | NAPRT | NCAPH2 | NCK1 | NDRG1 | NEDD4 | NFKB2 | NHLRC3 | NIT2 | NME2 | NTRK1 | ONECUT1 | ORM1 | P2RX1 | P2RX4 | PA2G4 | PATZ1 | PDXK | PFKL | PGAM1 | PGM1 | PGM2 | PGRMC1 | PI4K2A | PIK3CA | PIK3CG | PIK3R1 | PKM | PLAUR | PLSCR1 | PNP | POLM | PPIA | PPIE | PRCP | PRDX1 | PRDX2 | PRDX4 | PRDX6 | PRG2 | PRKCD | PRKCE | PRKDC | PSAP | PSEN1 | PSMA2 | PSMA5 | PSMB7 | PSMC2 | PSMC3 | PSMD1 | PSMD11 | PSMD12 | PSMD14 | PSMD2 | PTGES2 | PTPN22 | PTPN6 | PYGB | PYGL | QSOX1 | RAB10 | RAB18 | RAB24 | RAB29 | RAB31 | RAB5B | RAB7A | RAC1 | RAF1 | RAP1A | RAP1B | RBPJ | RC3H1 | RHOA | RIPK2 | RIPK3 | RNASE3 | RNF8 | RUNX2 | S100A11 | S100A12 | S100A13 | S100A7 | S100A9 | S100P | SDCBP | SELL | SERPINB3 | SERPINB6 | SLAMF1 | SLC2A5 | SMAD3 | SMAD4 | SMO | SNAP29 | SNCA | SPACA3 | SPI1 | SPTAN1 | SRC | SRP14 | STBD1 | STOM | STOML2 | STXBP2 | SWAP70 | TCIRG1 | TGFB1 | TIMP1 | TIMP2 | TLR3 | TMEM173 | TMEM30A | TNF | TNFSF14 | TOLLIP | TPD52 | TRAF6 | TREML2 | TREX1 | TUBB | TUBB4B | TXLNA | TXNDC5 | UBR4 | VAPA | VAT1 | VAV1 | VCAM1 | VCL | VCP | WAS | XRCC5 | XRCC6 | YWHAZ | ZAP70 | ZBTB7A | ZBTB7B | GO:0045321 | 0.0680577459662744 | 561/8243 | 282/3250 | 4.59796741214041e-08 | 0.000436577005782732 | T | T | T | T | leukocyte activation | ABL1 | ACAA1 | ACLY | ACTR2 | ADA | AGL | AHSG | AICDA | ALDOA | ALDOC | ALOX5 | ANXA1 | ANXA2 | AP1M1 | APEH | APRT | ARMC8 | ARPC5 | ATAD5 | ATP6V1D | AZU1 | BAG6 | BAX | BCL2 | BCL6 | BRAF | BTK | CAB39 | CAND1 | CAPN1 | CASP8 | CAT | CBFB | CBL | CCT2 | CCT8 | CD2 | CD22 | CD36 | CD40 | CD40LG | CD79B | CDA | CEP290 | CKAP4 | CLU | COPB1 | COTL1 | CPNE3 | CSNK2B | CTNNB1 | CTPS1 | CTSA | CTSC | CTSD | CXCL6 | CXCL8 | CYB5R3 | DBNL | DDOST | DDX3X | DEGS1 | DERA | DNAJA3 | DYNC1H1 | DYNC1LI1 | DYNLL1 | DYNLT1 | EEF1A1 | EEF2 | EIF2AK4 | ELF4 | EP300 | EXOSC6 | FABP5 | FADD | FAF2 | FCN1 | FGL2 | FKBP1A | FKBP1B | FTH1 | FTL | FYN | GAA | GALNS | GDI2 | GHDC | GLA | GNS | GPI | GPS2 | GSTP1 | GUSB | GYG1 | HAVCR2 | HDAC4 | HDAC5 | HDAC9 | HEXB | HMGB1 | HNF1A | HPRT1 | HRNR | HSP90AA1 | HSP90AB1 | HSPA1B | HSPA8 | HSPD1 | HUWE1 | ICAM1 | ID2 | IDH1 | IFNA2 | IGBP1 | ILF2 | IMPDH2 | IQGAP1 | IRF1 | IRF4 | ITGA4 | ITGAV | ITK | JAK3 | JMJD6 | JUN | JUP | KCMF1 | KCNAB2 | KIF13B | KIR3DS1 | KLF6 | KPNB1 | LAT | LCP1 | LCP2 | LEF1 | LGALS1 | LGALS3 | LIG4 | LRRC7 | LTBR | MAN2B1 | MAPK1 | MAPK14 | MAPT | MGST1 | MICA | MIF | MLH1 | MME | MSH6 | MSN | NAMPT | NAPRT | NCAPH2 | NCK1 | NDRG1 | NEDD4 | NHLRC3 | NIT2 | NME2 | NTRK1 | ONECUT1 | ORM1 | P2RX1 | PA2G4 | PATZ1 | PDXK | PFKL | PGAM1 | PGM1 | PGM2 | PGRMC1 | PI4K2A | PIK3CG | PIK3R1 | PKM | PLAUR | PNP | POLM | PPIA | PPIE | PRCP | PRDX1 | PRDX2 | PRDX4 | PRDX6 | PRG2 | PRKCD | PRKCE | PRKDC | PSAP | PSEN1 | PSMA2 | PSMA5 | PSMB7 | PSMC2 | PSMC3 | PSMD1 | PSMD11 | PSMD12 | PSMD14 | PSMD2 | PTGES2 | PTPN22 | PTPN6 | PYGB | PYGL | QSOX1 | RAB10 | RAB18 | RAB24 | RAB29 | RAB31 | RAB5B | RAB7A | RAC1 | RAP1A | RAP1B | RBPJ | RC3H1 | RHOA | RIPK2 | RIPK3 | RNASE3 | RNF8 | RUNX2 | S100A11 | S100A12 | S100A13 | S100A7 | S100A9 | S100P | SDCBP | SELL | SERPINB3 | SERPINB6 | SLAMF1 | SLC2A5 | SMAD3 | SNAP29 | SNCA | SPACA3 | SPI1 | SPTAN1 | SRP14 | STBD1 | STOM | STOML2 | STXBP2 | SWAP70 | TCIRG1 | TGFB1 | TIMP2 | TLR3 | TMEM173 | TMEM30A | TNF | TNFSF14 | TOLLIP | TPD52 | TRAF6 | TREML2 | TREX1 | TUBB | TUBB4B | TXLNA | TXNDC5 | UBR4 | VAPA | VAT1 | VAV1 | VCAM1 | VCL | VCP | WAS | XRCC5 | XRCC6 | ZAP70 | ZBTB7A | ZBTB7B | GO:0098869 | 0.00655101298071091 | 54/8243 | 41/3250 | 4.95174953601952e-08 | 0.000470168618445054 | T | T | T | T | cellular oxidant detoxification | ALB | ALOX5AP | APOE | CAT | CCS | FABP1 | FANCC | GPX1 | GPX4 | GPX7 | GSR | GSTA1 | GSTK1 | GSTO1 | GSTO2 | GSTP1 | GSTT1 | GSTZ1 | HBZ | KDM3B | LTC4S | MGST1 | NNT | NOS3 | NQO1 | PARK7 | PRDX1 | PRDX2 | PRDX3 | PRDX4 | PRDX5 | PRDX6 | PXDN | S100A9 | SOD1 | SOD2 | SRXN1 | TXN | TXNDC17 | TXNRD1 | UBIAD1 | GO:0031399 | 0.136358122042946 | 1124/8243 | 525/3250 | 5.64765589881448e-08 | 0.000536244927592435 | T | T | T | T | regulation of protein modification process | ABL1 | ACSL1 | ACTB | ADCY6 | ADCYAP1 | ADORA2A | ADRB2 | ADTRP | AGER | AIMP2 | AKAP8 | AKAP9 | AKT1 | AKT2 | AKTIP | ANXA2 | APC | APOA1 | APOE | APP | AR | ARAF | ARNT | ARR3 | ARRB1 | ARRB2 | ASPSCR1 | ATG5 | ATM | ATRX | AXIN1 | AZU1 | B3GAT3 | BAG2 | BAG4 | BAG5 | BAX | BCAR3 | BCL10 | BCL2 | BCL6 | BCOR | BDKRB2 | BIRC2 | BIRC3 | BIRC7 | BMI1 | BMPR1A | BRAF | BRCA1 | BRD4 | C1QL4 | C1QTNF1 | CAB39 | CALM1 | CALM2 | CALM3 | CARD9 | CASP3 | CAV1 | CBL | CBLB | CBLC | CCAR2 | CCDC88A | CCL21 | CCL22 | CCNB1 | CCNT1 | CCNT2 | CCNY | CCNYL1 | CCR1 | CD109 | CD27 | CD36 | CD40 | CD40LG | CD74 | CD81 | CDC14B | CDC20 | CDC25A | CDC25B | CDC25C | CDC37 | CDK4 | CDK5 | CDK5R1 | CDK9 | CDKN1A | CDKN2C | CEBPA | CHEK2 | CHORDC1 | CHP1 | CHRNA9 | CKS1B | CKS2 | CLU | COMMD1 | COPS5 | CRK | CRKL | CRY1 | CRY2 | CSNK2B | CTBP1 | CTCF | CTNNB1 | CUL3 | CYLD | DAB1 | DAB2 | DAXX | DBF4B | DBNDD2 | DBNL | DCUN1D1 | DDIT4 | DDRGK1 | DDX3X | DERL1 | DLG3 | DLG4 | DMD | DNAJA1 | DNAJB2 | DNAJC10 | DOCK7 | DUSP1 | DUSP14 | DUSP16 | DUSP19 | DUSP22 | DUSP7 | DVL1 | DVL2 | DVL3 | EED | EGFR | EHD4 | EID1 | EIF2AK3 | EIF2AK4 | EIF3A | EP300 | EPHA4 | ERBB2 | ERBB3 | ERRFI1 | EZH2 | EZR | FAF1 | FAM83D | FANCI | FANCM | FBXO7 | FGB | FGF2 | FGFR1 | FGFR1OP | FGFR3 | FKBP1A | FKBP1B | FKBP8 | FLOT1 | FN1 | FOXM1 | FOXO1 | FYN | FZR1 | G6PD | GADD45G | GATA1 | GDF9 | GLMN | GNAI2 | GNAQ | GOLGA2 | GPD1L | GPS2 | GRB2 | GRIK2 | GSK3A | GSK3B | GSTP1 | HAVCR2 | HAX1 | HBEGF | HDAC2 | HDAC3 | HDAC4 | HDAC5 | HDAC6 | HEXIM1 | HGS | HMGB1 | HPX | HRAS | HSF1 | HSP90AA1 | HSP90AB1 | HSP90B1 | HSPA1B | HSPA2 | HSPA5 | HSPB1 | HSPBP1 | HTT | IBTK | ICAM1 | IFNA2 | IGBP1 | IGF1R | IKBKB | IKBKG | ILK | INSR | IPO5 | IPO7 | IQGAP1 | ISG15 | ITCH | ITGB1BP1 | ITGB3 | JUN | KAT5 | KAT7 | KDM1A | KDM4D | KDR | KLHDC10 | KMT2A | KMT2B | KRAS | KSR1 | LAT | LATS1 | LCP2 | LDLRAD4 | LEMD2 | LGALS3 | LMNA | LPAR1 | LRP6 | LRRK1 | LRRK2 | LTBR | MAD2L1 | MAP2K1 | MAP2K3 | MAP2K4 | MAP2K6 | MAP2K7 | MAP3K1 | MAP3K13 | MAP3K14 | MAP3K2 | MAP3K3 | MAP3K4 | MAP4K1 | MAPK1 | MAPK14 | MAPK3 | MAPK8 | MAPK8IP3 | MAPK9 | MAPKAP1 | MAPT | MAS1 | MAVS | MECP2 | MIF | MLLT1 | MLST8 | MOB1B | MTA1 | MYC | MYOC | NCK1 | NCOR1 | NEK1 | NELFE | NF2 | NIPBL | NMNAT1 | NNT | NOD2 | NOS1AP | NPM1 | NQO2 | NTF3 | NTRK1 | NUPR1 | OGT | OTUB1 | OTUD4 | OXSR1 | PAFAH1B1 | PAK2 | PAK6 | PARD3 | PARD6A | PARK7 | PARP14 | PAXIP1 | PDCD10 | PDCD6 | PDE4D | PDE6G | PEBP1 | PEF1 | PELI1 | PER1 | PER2 | PHB | PHB2 | PIAS1 | PIAS3 | PIH1D1 | PIK3CA | PIK3CG | PIK3R3 | PIN1 | PINX1 | PKIG | PLA2G1B | PLA2G2A | PLA2G6 | PLAUR | PLK1 | PLXNB2 | PML | PNKP | PPARGC1A | PPIB | PPME1 | PPP1R11 | PPP1R12A | PPP1R14A | PPP1R16A | PPP1R2 | PPP1R3B | PPP1R3C | PPP1R8 | PPP2CA | PPP2R1A | PPP2R1B | PPP2R2A | PPP2R2D | PPP2R5D | PPP4R2 | PPP6R1 | PRDX1 | PRDX2 | PRKAA1 | PRKAA2 | PRKACA | PRKAG1 | PRKAR1A | PRKAR2B | PRKCD | PRKCE | PRKCZ | PRKD1 | PRKD2 | PRKDC | PRMT3 | PRMT6 | PROX1 | PRRC1 | PSAP | PSEN1 | PSMD10 | PTEN | PTK2 | PTN | PTPN1 | PTPN22 | PTPN6 | PTPRR | QARS | RAB1A | RAB3GAP1 | RAC1 | RAF1 | RANBP9 | RAP1A | RAP1B | RB1 | RB1CC1 | RELA | RGN | RGS2 | RHOA | RIF1 | RIPK1 | RIPK2 | RIPK3 | RPL23 | RPLP1 | RPS2 | RPS3 | RPTOR | RTF1 | RUVBL2 | S100A12 | S100A7 | SAG | SART3 | SDC4 | SDCBP | SEMA7A | SEPT4 | SERPINB3 | SET | SFN | SHC1 | SIN3A | SIRT1 | SIRT3 | SKI | SLAMF1 | SLC9A3R1 | SMAD4 | SMARCB1 | SMG8 | SNAI2 | SNCA | SNF8 | SNTA1 | SNW1 | SNX6 | SOCS1 | SOD1 | SOX2 | SPI1 | SPRTN | SPRY2 | SQSTM1 | SRC | STAT3 | STK11 | STK24 | STK25 | STK26 | STK3 | STK38 | STK40 | STRAP | STUB1 | STYX | SWAP70 | SYNJ2BP | TAB1 | TAB2 | TADA2B | TANK | TAOK3 | TARDBP | TERF2IP | TGFB1 | TGFBR3 | THBS1 | TIMP2 | TINF2 | TLR3 | TNF | TNFAIP3 | TNFRSF1A | TNK2 | TOLLIP | TP53 | TP73 | TRAF2 | TRAF3IP1 | TRAF6 | TRIB2 | TRIB3 | TRIM21 | TRIP12 | TSG101 | TSPYL2 | TWF1 | TWIST1 | TXN | U2AF2 | UBB | UBC | UBE2B | UBE2C | UBE2D1 | UBE2I | UBE2K | UBE2L3 | UBE2N | UBE2S | UBE2V1 | UBE3A | UBQLN1 | UBR5 | UBXN1 | UCHL1 | UNC119 | USP17L2 | USP4 | VCP | VTN | WARS | WBP2 | WDR48 | WNK1 | WNT16 | XIAP | XRCC1 | XRCC5 | XRCC6 | YES1 | YWHAB | YWHAE | YWHAG | ZBTB7B | ZMPSTE24 | ZNF622 | GO:1903050 | 0.0195317238869344 | 161/8243 | 97/3250 | 5.75139460373695e-08 | 0.000546094917624823 | T | T | T | T | regulation of proteolysis involved in cellular protein catabolic process | AKT1 | APOE | ARAF | AXIN1 | BAG2 | BAG5 | BAG6 | BTRC | CAV1 | CBFA2T3 | CCAR2 | CCDC22 | CEBPA | CLU | COMMD1 | CSNK1A1 | CSNK1E | CSNK2A2 | CTSC | DAB2 | DDA1 | DDRGK1 | DNAJB2 | DVL1 | EDEM1 | EPHA4 | FAM192A | GLMN | GNA12 | GPX1 | GSK3A | GSK3B | HSP90AB1 | HSP90AB3P | HSP90AB4P | HSPA1B | HSPBP1 | KEAP1 | LAMP3 | LATS1 | LRRK2 | MAPK9 | MDM2 | NFE2L2 | NUPR1 | OGT | PARK7 | PIAS1 | PLK1 | PML | PRKACA | PSEN1 | PSMC1 | PSMC2 | PSMC3 | PSMC4 | PSMC5 | PSMC6 | PSMD10 | PSMD14 | PSME2 | PSME3 | PTEN | PTK2 | RAD23A | RAD23B | RBX1 | RGN | RNF139 | RNF185 | RNF19A | RPL23 | SDCBP | SGTA | SMARCC1 | SMURF1 | STUB1 | STYX | SUMO1 | SUMO2 | TMTC3 | TRIB2 | TRIB3 | UBB | UBE2J1 | UBE2K | UBE3A | UBQLN1 | UBQLN2 | UBQLN4 | UBXN1 | UCHL5 | USP14 | USP19 | USP5 | USP7 | VCP | GO:0080134 | 0.112944316389664 | 931/8243 | 442/3250 | 7.26741037043385e-08 | 0.000690040614672694 | T | T | T | T | regulation of response to stress | A2M | ABAT | ABCE1 | ABL1 | ACE2 | ACTR2 | ADA | ADCYAP1 | ADORA2A | ADTRP | AGER | AGR2 | AHSG | AKT1 | AKT3 | ALOX12 | ANXA1 | ANXA2 | ANXA5 | AP1G1 | APBB1 | APCS | APOA1 | APOD | APOE | APP | ARRB2 | ATAD5 | ATF6 | ATF6B | ATM | AXIN1 | BABAM1 | BAG1 | BAG2 | BAG3 | BAG4 | BAG5 | BAG6 | BAX | BCL10 | BCL2 | BCL2L1 | BCL6 | BDKRB2 | BIRC2 | BIRC3 | BIRC7 | BMPR1A | BRAF | BRCA1 | BRD4 | BTK | BTRC | C1QA | C1QBP | C1QTNF1 | CAMK2A | CAMK2B | CARD9 | CASP8 | CASP9 | CAV1 | CBX8 | CCAR2 | CCL21 | CD109 | CD209 | CD27 | CD36 | CD40LG | CD6 | CD74 | CDC37 | CDH5 | CDK19 | CDK9 | CDKN1A | CEBPA | CHCHD2 | CHEK2 | CHORDC1 | CLU | COPS5 | CREB3 | CREBBP | CRK | CRY1 | CRYAB | CTNNB1 | CTSL | CUL1 | CXCL6 | CYLD | DAXX | DBNL | DDX39B | DHX9 | DNAJA1 | DNAJB1 | DNAJC7 | DROSHA | DUSP1 | DUSP19 | DUSP22 | DVL2 | DVL3 | EDEM1 | EGFR | EIF2AK3 | EIF2AK4 | ENDOG | ENO1 | EP300 | EPHA4 | EPPK1 | ERRFI1 | ESR1 | ETS1 | EYA2 | EYA4 | EZR | FADD | FAM19A3 | FANCA | FBXO7 | FBXW11 | FCN1 | FGB | FGF2 | FIGNL1 | FKBP1B | FLOT1 | FOXM1 | FOXO1 | FUS | FYN | GADD45G | GBP5 | GGT1 | GPS2 | GPX1 | GPX4 | GRIK2 | GSK3B | GSTP1 | H2AFX | HAVCR2 | HBEGF | HDAC3 | HDAC6 | HEXIM1 | HIF1A | HMGB1 | HMGB2 | HMGB3 | HNRNPK | HPX | HRAS | HSF1 | HSP90AA1 | HSP90AB1 | HSP90B1 | HSPA1B | HSPA1L | HSPA5 | HSPA8 | HSPB1 | HSPD1 | HSPH1 | HTRA1 | HTRA2 | IFI16 | IFIT1 | IGBP1 | IGF1R | IKBKB | IKBKE | IKBKG | INSIG1 | IRF1 | IRF4 | IRF7 | ITCH | JAK1 | KDM1A | KDM4D | KLHDC10 | KRAS | LANCL1 | LGMN | LGR4 | LMNA | LRRK2 | LRSAM1 | LTBR | MAP2K1 | MAP2K4 | MAP2K6 | MAP2K7 | MAP3K1 | MAP3K13 | MAP3K2 | MAP3K3 | MAP3K4 | MAP4K1 | MAPK1 | MAPK14 | MAPK3 | MAPK7 | MAPK8IP3 | MAPKAP1 | MAPT | MAS1 | MATR3 | MAVS | MDM2 | MED1 | METRNL | MGMT | MICA | MIF | MLST8 | MMP26 | MYC | MYD88 | NAMPT | NCK1 | NCOR1 | NFE2L2 | NFKBIA | NOD2 | NOS2 | NOS3 | NPLOC4 | NPM1 | NR1H2 | NR1H3 | NSMCE4A | NUP107 | NUP153 | NUP160 | NUP205 | NUP214 | NUP85 | NUP88 | NUP93 | NUP98 | OPA1 | OPTN | OTUB1 | OTUD4 | P4HB | PAFAH1B1 | PAK2 | PARK7 | PARP1 | PARP14 | PAWR | PAXIP1 | PCBP2 | PCNA | PDCD10 | PELI1 | PER1 | PIAS1 | PIK3CG | PIK3R1 | PLA2G2A | PLAUR | PLSCR1 | PML | POM121 | PPARD | PPARG | PPM1B | PPP4R2 | PRDX1 | PRDX2 | PRKACA | PRKCD | PRKCE | PRKDC | PSAP | PSMA1 | PSMA2 | PSMA3 | PSMA5 | PSMA6 | PSMB5 | PSMB6 | PSMB7 | PSMB8 | PSMC1 | PSMC2 | PSMC3 | PSMC4 | PSMC5 | PSMC6 | PSMD1 | PSMD10 | PSMD11 | PSMD12 | PSMD14 | PSMD2 | PSMD4 | PSMD9 | PSME2 | PSME3 | PTEN | PTGER3 | PTGES3 | PTPN1 | PTPN22 | PTPN6 | PTPRF | PUM1 | PYCR1 | QARS | RAD51 | RAD51AP1 | RAE1 | RAF1 | RB1 | RB1CC1 | RELA | RGN | RIF1 | RIPK1 | RIPK2 | RNASEH2B | RNF183 | RNF185 | RNF8 | RPA2 | RPA3 | RPS19 | RPS3 | RPTOR | RTN4 | S100A1 | S100A12 | S100A9 | SARM1 | SCARA3 | SDCBP | SEC13 | SEMA7A | SERPINB2 | SERPINB3 | SERPINE2 | SFPQ | SGTA | SIN3A | SIRT1 | SIRT6 | SLAMF1 | SLAMF8 | SLC25A6 | SMAD3 | SNAI2 | SNCA | SNX6 | SOCS1 | SOD1 | SOD2 | SPATA2 | SRC | STAT1 | STK24 | STK25 | STK26 | STK3 | SUMO1 | SYVN1 | TAB1 | TAB2 | TANK | TAOK3 | TBC1D23 | TBXA2R | TERF2 | TERF2IP | TFIP11 | TGFBR3 | TGM2 | THBS1 | TLR3 | TMBIM6 | TMEM173 | TNF | TNFAIP3 | TNFRSF12A | TNFRSF1A | TNIP2 | TPR | TRADD | TRAF2 | TRAF3 | TRAF3IP1 | TRAF6 | TRAP1 | TREX1 | TRIAP1 | TRIM28 | TRIM32 | TRIP12 | TWIST1 | TXN | UBB | UBC | UBE2D1 | UBE2D2 | UBE2D3 | UBE2J1 | UBE2K | UBE2N | UBE2V1 | UBQLN1 | UBQLN2 | UBR5 | UBXN1 | USP14 | USP17L2 | USP19 | USP47 | VAV1 | VSIG4 | VTN | WAS | WDFY1 | WNT16 | XBP1 | XIAP | XRCC1 | XRCC5 | XRCC6 | YWHAE | ZBTB7B | ZMPSTE24 | ZNF277 | ZNF622 | GO:0043412 | 0.242751425451899 | 2001/8243 | 889/3250 | 9.32949546727394e-08 | 0.000885835594617661 | T | T | T | T | macromolecule modification | AARS | ABHD17A | ABL1 | ABL2 | ACP1 | ACTB | ACTL6A | ACTN2 | ADCK2 | ADH5 | ADPRH | ADRB2 | ADRM1 | AHSG | AICDA | AKT1 | AKT2 | AKT3 | ALB | AMFR | ANAPC4 | ANXA1 | APBB1 | APC | APEX1 | APOA1 | APOE | APP | AR | ARAF | ARF4 | ARID4B | ARRB1 | ARRB2 | ASB12 | ASB15 | ASB16 | ASB6 | ASB9 | ASCC1 | ASCC2 | ASGR1 | ASGR2 | ATF2 | ATG5 | ATM | ATP13A2 | ATRX | AURKB | B3GAT3 | B3GNT3 | B4GALT3 | BABAM1 | BAG6 | BCL10 | BCL11A | BCL2 | BCOR | BIRC2 | BIRC3 | BIRC5 | BIRC7 | BIRC8 | BMI1 | BMPR1A | BRAF | BRCA1 | BRD4 | BTK | BTRC | C4A | C9orf64 | CAB39 | CALM1 | CALU | CAMK1D | CAMK2A | CAMK2B | CAMKV | CAND1 | CARM1 | CBFB | CBL | CBLB | CBLC | CBX4 | CBX8 | CCDC22 | CCDC8 | CCNB1 | CCNT1 | CCP110 | CCR5 | CDC14B | CDC16 | CDC20 | CDC25A | CDC25B | CDC25C | CDC27 | CDC34 | CDC42 | CDC73 | CDK15 | CDK16 | CDK19 | CDK2 | CDK4 | CDK5 | CDK5R1 | CDK8 | CDK9 | CDKL3 | CEL | CFL1 | CFTR | CHEK2 | CHGB | CHRM3 | CHST4 | CKAP4 | CLK1 | COG3 | COMMD1 | COPS2 | COPS5 | COPS6 | CPNE3 | CRBN | CREB1 | CREBBP | CRKL | CRY1 | CRY2 | CRYAB | CSNK1A1 | CSNK1E | CSNK2A2 | CSNK2B | CTBP1 | CTCF | CTDP1 | CTH | CTNNB1 | CUL1 | CUL2 | CUL3 | CUL4B | CUL5 | CUL7 | CYCS | CYLD | DAD1 | DAPK1 | DAPP1 | DBI | DCAF12 | DCAF7 | DCAF8 | DCLK1 | DCN | DCUN1D1 | DDA1 | DDB1 | DDB2 | DDOST | DDRGK1 | DERL1 | DGUOK | DLD | DLG2 | DLG3 | DLG4 | DLST | DMAP1 | DPF2 | DPM1 | DTX1 | DTX2 | DUS3L | DUSP1 | DUSP12 | DUSP13 | DUSP14 | DUSP16 | DUSP19 | DUSP22 | DUSP7 | DYRK2 | DZIP3 | EDEM1 | EED | EEF2KMT | EFEMP1 | EGFR | EGLN1 | EGLN3 | EHHADH | EHMT1 | EHMT2 | EIF2AK3 | EIF2AK4 | EIF2S1 | EP300 | EPHA4 | EPHA6 | EPHB6 | ERBB2 | ERBB3 | ERG | ESR1 | EVA1A | EXOSC5 | EXOSC6 | EXTL2 | EYA2 | EYA4 | EZH2 | F5 | FAM20B | FAM98A | FBXL12 | FBXO11 | FBXO25 | FBXO32 | FBXO6 | FBXO7 | FBXW11 | FBXW5 | FGF2 | FGFR1 | FGFR3 | FKBP14 | FKBP1A | FKBP1B | FKBP2 | FKBP5 | FKBP7 | FKBP8 | FN1 | FOLH1 | FOS | FOXK1 | FOXK2 | FOXO1 | FTO | FTSJ3 | FYN | FZR1 | GAK | GALNT7 | GAPDH | GAR1 | GFPT1 | GFPT2 | GLRX | GLRX5 | GLUL | GNAS | GOLGA2 | GPS2 | GPX1 | GRB2 | GRK1 | GRK5 | GSK3A | GSK3B | GSPT1 | HBEGF | HCCS | HDAC1 | HDAC11 | HDAC2 | HDAC3 | HDAC4 | HDAC5 | HDAC6 | HDAC7 | HDAC9 | HECW1 | HECW2 | HERC1 | HERC2 | HGS | HIF1A | HIF1AN | HIPK4 | HIST1H2BA | HLCS | HMBS | HRAS | HSD17B10 | HSF1 | HSF4 | HSP90B1 | HUWE1 | ICK | IGF1R | IGFBP7 | IKBKB | IKBKE | IKBKG | IL3RA | ILF3 | ILK | ING5 | INO80B | INO80C | INSR | IRF4 | ISG15 | ITCH | ITGAV | ITK | JAK1 | JAK3 | JMJD6 | JOSD2 | KAT5 | KAT7 | KAT8 | KBTBD7 | KCMF1 | KDM1A | KDM2A | KDM3B | KDM4D | KDM5B | KDM5C | KDM8 | KDR | KEAP1 | KIAA0391 | KLHL20 | KLHL22 | KMT2A | KMT2B | KMT2C | KRAS | KSR1 | LAMB1 | LAT | LATS1 | LCMT2 | LEF1 | LGALS1 | LMO7 | LNX1 | LOXL2 | LRRC7 | LRRK1 | LRRK2 | LRSAM1 | LTBP1 | LYPLA1 | LYPLA2 | LZTR1 | MAN2B1 | MAP2K1 | MAP2K3 | MAP2K4 | MAP2K6 | MAP2K7 | MAP3K1 | MAP3K13 | MAP3K14 | MAP3K2 | MAP3K3 | MAP3K4 | MAP4K1 | MAPK1 | MAPK14 | MAPK15 | MAPK3 | MAPK4 | MAPK6 | MAPK7 | MAPK8 | MAPK9 | MAPT | MARCH5 | MAST1 | MAST3 | MATK | MBD3 | MBP | MCFD2 | MDH2 | MDM2 | MDM4 | MECP2 | MED1 | MED12 | MED20 | MED21 | MED31 | MELK | MEPCE | METAP2 | MGMT | MIB1 | MKNK2 | MORF4L1 | MORF4L2 | MPG | MPI | MPPE1 | MRGBP | MRM1 | MTA1 | MTA3 | MTMR14 | MTMR7 | MTRR | MYC | MYH6 | NAE1 | NCK1 | NCOA1 | NCOA3 | NDUFAF5 | NDUFAF7 | NEDD4 | NEDD8 | NEK1 | NEK7 | NEURL4 | NFE2L2 | NFKBIA | NFS1 | NHLRC3 | NLK | NME2 | NOD2 | NOP10 | NOS2 | NOSIP | NPEPPS | NPR1 | NSUN2 | NSUN6 | NTMT1 | NTRK1 | NUCB1 | NUP107 | NUP153 | NUP160 | NUP205 | NUP214 | NUP85 | NUP88 | NUP93 | NUP98 | NUPR1 | OBSL1 | OGDH | OGFOD1 | OGT | OTUB1 | OTUB2 | OTUD4 | OTUD5 | OXSR1 | P4HA1 | P4HA2 | P4HB | PADI4 | PAK2 | PAK6 | PARK7 | PARP1 | PARP12 | PARP14 | PARP6 | PAXIP1 | PCGF1 | PCGF5 | PCGF6 | PCMT1 | PCMTD1 | PCMTD2 | PCNA | PCSK9 | PDF | PDIA6 | PDK2 | PEBP1 | PELI1 | PER1 | PER2 | PEX5 | PGAM5 | PGM3 | PGP | PHB | PHF10 | PHKA1 | PIAS1 | PIAS3 | PICK1 | PIGU | PIK3CA | PIK3CG | PIK3R1 | PIM1 | PIN1 | PJA1 | PKN2 | PLK1 | PLOD2 | PML | POLE3 | POM121 | PPA2 | PPEF1 | PPIA | PPIB | PPID | PPIE | PPIF | PPIL3 | PPM1A | PPM1B | PPM1D | PPM1H | PPM1K | PPME1 | PPP1CA | PPP1CB | PPP1CC | PPP1R11 | PPP1R12A | PPP1R3B | PPP1R3C | PPP2CA | PPP2CB | PPP2R1A | PPP2R1B | PPP2R2A | PPP2R2D | PPP2R5D | PPP3CC | PPP4R2 | PPP5C | PPP6C | PPT1 | PPTC7 | PRDX3 | PRDX4 | PRKAA1 | PRKAA2 | PRKAB2 | PRKACA | PRKAG1 | PRKCD | PRKCE | PRKCSH | PRKCZ | PRKD1 | PRKD2 | PRKD3 | PRKDC | PRMT2 | PRMT3 | PRMT6 | PRPF19 | PRSS23 | PSEN1 | PSMA1 | PSMA2 | PSMA3 | PSMA5 | PSMA6 | PSMB5 | PSMB6 | PSMB7 | PSMB8 | PSMC1 | PSMC2 | PSMC3 | PSMC4 | PSMC5 | PSMC6 | PSMD1 | PSMD10 | PSMD11 | PSMD12 | PSMD14 | PSMD2 | PSMD4 | PSMD9 | PSME2 | PSME3 | PTEN | PTK2 | PTPMT1 | PTPN1 | PTPN14 | PTPN21 | PTPN22 | PTPN23 | PTPN4 | PTPN6 | PTPRA | PTPRD | PTPRF | PTPRK | PTPRO | PTPRR | PUS1 | PUS7 | QSOX1 | RAB11A | RAB11B | RAB1A | RAB1B | RAB2A | RAB5A | RAD23A | RAD23B | RAE1 | RAF1 | RANBP9 | RANGAP1 | RARA | RBBP6 | RBX1 | RC3H1 | REST | RHBDD1 | RHO | RHOA | RHOT1 | RING1 | RIOK1 | RIPK1 | RIPK2 | RIPK3 | RIPK4 | RNF123 | RNF126 | RNF139 | RNF181 | RNF183 | RNF185 | RNF19A | RNF2 | RNF25 | RNF31 | RNF4 | RNF8 | RPN1 | RPN2 | RPS6KA1 | RPS6KA2 | RPS6KB2 | RTF1 | RUVBL1 | RUVBL2 | S100A9 | SAE1 | SCG2 | SCG3 | SCYL2 | SEL1L | SENP3 | SENP6 | SENP7 | SENP8 | SEPHS1 | SEPHS2 | SETDB1 | SFPQ | SHC1 | SHMT2 | SIAH1 | SIN3A | SIRT1 | SIRT3 | SIRT5 | SIRT6 | SIRT7 | SMAD2 | SMAD3 | SMAD4 | SMAD5 | SMURF1 | SMURF2 | SNRPB | SNRPD3 | SNX3 | SOCS1 | SOCS2 | SOCS6 | SPATA2 | SPI1 | SPOP | SPRR2A | SPSB3 | SPTAN1 | SPTBN1 | SPTBN2 | SQSTM1 | SRC | SRPK1 | SRPK2 | SSB | ST3GAL1 | ST3GAL6 | ST6GALNAC5 | ST8SIA4 | STK11 | STK24 | STK25 | STK26 | STK3 | STK38 | STK40 | STUB1 | STYX | SULT1A3 | SUMO1 | SUMO1P1 | SUMO2 | SUMO3 | SUZ12 | SYVN1 | TAB1 | TAB2 | TADA2B | TAF12 | TAOK3 | TBL1X | TBRG4 | TESK2 | TGFB1 | TGFBR3 | TGM2 | TGOLN2 | THADA | THBS1 | THOP1 | TIMM50 | TIMP1 | TLR3 | TMEM132A | TMEM189-UBE2V1 | TMEM258 | TMTC3 | TNF | TNFAIP3 | TNIP2 | TNK2 | TNKS | TNKS2 | TOMM20 | TOP1 | TOR1A | TP53 | TP53RK | TPR | TPST2 | TPTE | TRAF2 | TRAF3 | TRAF6 | TREX1 | TRIB2 | TRIB3 | TRIM21 | TRIM23 | TRIM25 | TRIM28 | TRIM32 | TRIM33 | TRIM41 | TRIM47 | TRIM63 | TRIM69 | TRIM7 | TRIM9 | TRIP12 | TRMT44 | TRMT6 | TRMT61B | TRUB2 | TSG101 | TSPAN17 | TTLL1 | TWF1 | UBA1 | UBA2 | UBA3 | UBA5 | UBA6 | UBAC1 | UBB | UBC | UBE2A | UBE2B | UBE2C | UBE2D1 | UBE2D2 | UBE2D3 | UBE2H | UBE2I | UBE2J1 | UBE2K | UBE2L3 | UBE2L6 | UBE2M | UBE2N | UBE2O | UBE2Q2 | UBE2R2 | UBE2S | UBE2V1 | UBE2W | UBE3A | UBE3B | UBL4A | UBR4 | UBR5 | UBXN7 | UCHL1 | UCHL3 | UCHL5 | UFC1 | UFM1 | UGGT1 | ULK1 | UNKL | USP11 | USP14 | USP15 | USP16 | USP17L2 | USP19 | USP2 | USP20 | USP21 | USP26 | USP28 | USP39 | USP4 | USP42 | USP46 | USP47 | USP5 | USP50 | USP53 | USP7 | USP8 | USP9X | USP9Y | USPL1 | VCAN | VCP | VCPIP1 | VGF | VHL | VKORC1 | WDR4 | WDR45B | WDR48 | WDR82 | WDTC1 | WDYHV1 | WIPI1 | WIPI2 | WNK1 | WNK4 | WSB1 | WTAP | WWP2 | XIAP | YEATS2 | YES1 | YWHAB | YWHAE | YY1 | ZAP70 | ZBTB16 | ZC3HC1 | ZDHHC17 | ZDHHC23 | ZMPSTE24 | ZNF598 | ZNRF4 | GO:0048519 | 0.367705932306199 | 3031/8243 | 1307/3250 | 9.94440507174124e-08 | 0.000944221261561831 | T | T | T | T | negative regulation of biological process | A2M | AARS | ABAT | ABCE1 | ABCG1 | ABHD17A | ABL1 | ABL2 | ACAA2 | ACADVL | ACD | ACOT13 | ACTN1 | ACTN2 | ACTN4 | ADA | ADCY6 | ADCYAP1 | ADD3 | ADORA2A | ADRB2 | ADTRP | AEBP2 | AES | AGAP2 | AGER | AGO1 | AGO2 | AGR2 | AHI1 | AHSG | AICDA | AIMP2 | AKAP8 | AKR1B1 | AKT1 | AKT2 | AKT3 | ALB | ALOX12 | ANK3 | ANXA1 | ANXA2 | ANXA5 | ANXA6 | ANXA7 | APBB1 | APBB2 | APC | APCS | APEX1 | API5 | APLP1 | APOA1 | APOD | APOE | APP | AR | ARAF | ARF1 | ARF4 | ARHGAP1 | ARHGAP35 | ARHGDIA | ARHGEF7 | ARNT2 | ARRB1 | ARRB2 | ASCL1 | ASNS | ASS1 | ATAD1 | ATAD3A | ATAD5 | ATCAY | ATF2 | ATF6B | ATG5 | ATM | ATOH1 | ATOX1 | ATP13A2 | ATP1A1 | ATP2A2 | ATRX | ATXN1 | ATXN2 | AURKB | AVIL | AXIN1 | AZU1 | BABAM1 | BACE1 | BACH2 | BAG1 | BAG2 | BAG3 | BAG4 | BAG5 | BAG6 | BANF1 | BANP | BASP1 | BAX | BCL10 | BCL11A | BCL2 | BCL2L1 | BCL2L2 | BCL6 | BCL6B | BCOR | BDKRB2 | BIN1 | BIRC2 | BIRC3 | BIRC5 | BIRC7 | BIRC8 | BMI1 | BMPR1A | BNIPL | BRAF | BRCA1 | BTK | BTRC | C1QBP | C1QL4 | C1QTNF1 | C4A | CAB39 | CALM1 | CALM2 | CALM3 | CALR | CAMK1D | CAPG | CAPZA1 | CAPZA2 | CAPZB | CARM1 | CASP2 | CASP3 | CASP8 | CAT | CAV1 | CBFA2T3 | CBFB | CBL | CBLB | CBLC | CBX4 | CBX7 | CBX8 | CBY1 | CCAR2 | CCDC22 | CCDC8 | CCL21 | CCNB1 | CCNT1 | CCP110 | CCR1 | CCR5 | CD109 | CD22 | CD27 | CD2BP2 | CD36 | CD40LG | CD74 | CDA | CDC14B | CDC20 | CDC25C | CDC34 | CDC42 | CDC73 | CDH1 | CDH5 | CDK2 | CDK4 | CDK5 | CDK5R1 | CDK9 | CDKL3 | CDKN1A | CDKN2C | CEBPA | CENPF | CENPJ | CFL1 | CGRRF1 | CHCHD3 | CHD8 | CHEK2 | CHMP2A | CHORDC1 | CHP1 | CHRD | CHRNA9 | CIRBP | CLDN18 | CLIC4 | CLTC | CLU | CMTM5 | COL18A1 | COL2A1 | COL4A2 | COL5A1 | COMMD1 | COMT | COPS2 | COPS5 | COX11 | CRBN | CREB1 | CREB3 | CREBBP | CRK | CRKL | CRMP1 | CRP | CRY1 | CRY2 | CRYAB | CSNK1A1 | CSNK2A2 | CSNK2B | CTBP1 | CTCF | CTDP1 | CTH | CTNNB1 | CTNNBIP1 | CTNND1 | CTSA | CTTN | CUL1 | CUL2 | CUL3 | CUL5 | CXCL8 | CYLD | DAB1 | DAB2 | DAD1 | DAPK1 | DAXX | DBNDD2 | DCLK1 | DCN | DCP2 | DDB1 | DDIT4 | DDRGK1 | DDX39B | DDX3X | DDX6 | DGUOK | DHCR24 | DHX36 | DHX9 | DIS3 | DLG2 | DLG3 | DLG4 | DMAP1 | DMD | DNA2 | DNAJA1 | DNAJA3 | DNAJA4 | DNAJB1 | DNAJB2 | DNAJC10 | DNM1L | DOCK5 | DOCK7 | DPEP1 | DPF2 | DTX1 | DUSP1 | DUSP14 | DUSP16 | DUSP19 | DUSP22 | DUSP7 | DVL1 | DVL2 | DVL3 | DYNLL1 | DYNLT1 | DYRK2 | E2F1 | E2F2 | E2F3 | E2F4 | E2F6 | EAF2 | EDC4 | EED | EFEMP1 | EGFR | EGLN1 | EHMT1 | EHMT2 | EID1 | EIF2AK3 | EIF2AK4 | EIF2S1 | EIF3A | EIF3E | EIF4A3 | EIF4E | EIF4E2 | EIF4EBP1 | EIF4ENIF1 | EIF4G3 | EIF6 | ELAVL1 | ELF3 | ENO1 | EP300 | EPHA4 | EPM2A | EPPIN | EPPK1 | EPRS | ERBB2 | ERBB3 | ERCC5 | ERF | ERLIN2 | ERRFI1 | ESR1 | ESR2 | ESRRA | ESRRB | ETS1 | EXOSC1 | EXOSC5 | EXOSC6 | EXOSC9 | EYA2 | EYA4 | EZH2 | EZR | FABP1 | FABP3 | FABP5 | FABP7 | FADD | FAF1 | FAM129B | FAM192A | FAM19A3 | FAM3C | FASLG | FBP1 | FBXO6 | FBXO7 | FBXW11 | FCN1 | FGB | FGF2 | FGFR1 | FGFR1OP | FGFR3 | FHL1 | FHL2 | FIGNL1 | FKBP1A | FKBP1B | FKBP8 | FN1 | FOXA1 | FOXJ1 | FOXK1 | FOXM1 | FOXO1 | FTH1 | FTO | FXR2 | FYN | FZR1 | G3BP1 | G3BP2 | G6PD | GAK | GAPDH | GATA1 | GDF9 | GDI1 | GFAP | GJA1 | GLA | GLMN | GLO1 | GLRX3 | GNAI2 | GNAI3 | GNAO1 | GNAQ | GOLGA2 | GOLPH3 | GOPC | GOT1 | GPD1L | GPI | GPS2 | GPSM1 | GPX1 | GPX4 | GRB2 | GRB7 | GRIA1 | GRID2 | GRIK2 | GRK5 | GSK3A | GSK3B | GSPT1 | GSPT2 | GSTO1 | GSTP1 | GTF2I | GZMA | H1F0 | H1FX | H2AFX | H3F3A | HADH | HAP1 | HAVCR2 | HAX1 | HBEGF | HBZ | HDAC1 | HDAC2 | HDAC3 | HDAC4 | HDAC5 | HDAC6 | HDAC7 | HDAC9 | HDGF | HECW1 | HECW2 | HELZ | HELZ2 | HERC1 | HEXIM1 | HGS | HIF1A | HIF1AN | HIST1H1A | HIST1H2AB | HIST1H3A | HIST1H4A | HIST2H3A | HIST3H3 | HIVEP1 | HK2 | HLX | HMGB1 | HMGB2 | HMOX1 | HNF4A | HNRNPA1 | HNRNPA2B1 | HNRNPD | HNRNPK | HNRNPL | HNRNPU | HOXA5 | HRAS | HSF1 | HSF4 | HSP90AB1 | HSP90AB3P | HSP90AB4P | HSP90B1 | HSPA1B | HSPA2 | HSPA5 | HSPA8 | HSPA9 | HSPB1 | HSPD1 | HSPG2 | HTATIP2 | HTR1B | HTRA1 | HTRA2 | HTT | HYPK | IBTK | ICAM1 | ID2 | IDE | IDH2 | IFI16 | IFIT1 | IFITM3 | IFNA2 | IFT122 | IGBP1 | IGF1R | IGF2BP1 | IGFBP7 | IK | IKBKB | IKBKE | IKBKG | IKZF5 | IL13RA2 | ILF3 | ILK | ING1 | ING5 | INSIG1 | INSIG2 | IP6K1 | IPO5 | IPO7 | IQCB1 | IQGAP1 | IREB2 | IRF1 | IRF4 | IRF7 | ISG15 | ITCH | ITGAV | ITGB1BP1 | ITGB3 | ITPR1 | ITPR2 | ITPR3 | JAK3 | JUN | JUNB | JUND | JUP | KANK2 | KAT5 | KAT7 | KAT8 | KCNAB1 | KDM1A | KDM4D | KDM5B | KDM5C | KDR | KEAP1 | KHDRBS1 | KIR3DL2 | KLF5 | KLHL20 | KLHL22 | KMT2A | KMT2B | KRAS | L3MBTL1 | L3MBTL2 | LAMP3 | LARS | LATS1 | LDLRAD4 | LEF1 | LEMD2 | LEMD3 | LETM1 | LGALS1 | LGALS3 | LGMN | LGR4 | LIG4 | LIMA1 | LIMS1 | LMNA | LOXL2 | LPAR1 | LRP6 | LRPPRC | LRRK1 | LRRK2 | LRSAM1 | LSM1 | LSM3 | LSM4 | LSM5 | LTBP1 | LYPLA1 | LZTR1 | MAD1L1 | MAD2L1 | MAF1 | MAP1B | MAP2K1 | MAP2K4 | MAP2K6 | MAP3K3 | MAPK1 | MAPK14 | MAPK15 | MAPK7 | MAPK8 | MAPKAP1 | MAPT | MARCH5 | MAS1 | MAVS | MBD3 | MBP | MC1R | MCC | MCM2 | MCOLN3 | MDC1 | MDFI | MDM1 | MDM2 | MDM4 | MECP2 | MED1 | MED31 | MEOX2 | MEPCE | METRNL | MGA | MGMT | MIB1 | MICA | MIF | MITF | MLH1 | MLLT1 | MLLT3 | MLST8 | MMP26 | MORF4L1 | MORF4L2 | MOV10 | MSH6 | MTA1 | MTA3 | MTSS1 | MXD1 | MYBBP1A | MYC | MYD88 | MYL2 | MYO1D | MYOC | NACC1 | NAE1 | NAMPT | NANOG | NBAS | NCBP1 | NCK1 | NCOA2 | NCOR1 | NCOR2 | NDRG1 | NDUFA13 | NDUFAF2 | NDUFS3 | NEDD4 | NELFB | NELFCD | NELFE | NELL1 | NEUROD1 | NF2 | NFATC1 | NFATC2 | NFE2L2 | NFKBIA | NIF3L1 | NIPBL | NKRF | NKX2-1 | NLK | NMB | NME1 | NME2 | NMNAT1 | NMRK2 | NNT | NOD2 | NOS2 | NOS3 | NOSIP | NOSTRIN | NPHP3 | NPLOC4 | NPM1 | NPR1 | NQO1 | NR0B2 | NR1H2 | NR1H3 | NR1I2 | NR3C1 | NR4A1 | NRXN1 | NSFL1C | NSUN2 | NTF3 | NTRK1 | NUBP1 | NUDT6 | NUP153 | NUPR1 | OAZ3 | OGT | ONECUT1 | OPA1 | OPTN | ORM1 | OTUB1 | OTUD4 | OTUD5 | OVGP1 | OXA1L | OXSR1 | P2RX4 | PA2G4 | PABPC1 | PACSIN3 | PAFAH1B1 | PAG1 | PAIP1 | PAK2 | PALB2 | PAM16 | PAN2 | PAPLN | PARD3 | PARD6A | PARK7 | PARP1 | PARP14 | PATZ1 | PAWR | PAX8 | PAX9 | PAXIP1 | PC | PCBP2 | PCGF1 | PCGF6 | PCM1 | PCNA | PCSK9 | PCTP | PDCD10 | PDCD6 | PDCL | PDE12 | PDE4D | PEBP1 | PELI1 | PER1 | PER2 | PEX5 | PFKL | PFN1 | PGAM5 | PGK1 | PGP | PHB | PHB2 | PHC3 | PHF10 | PHF21A | PIAS1 | PIAS3 | PICK1 | PIH1D1 | PIK3CA | PIK3CG | PIK3R1 | PIM1 | PIN1 | PINX1 | PKIG | PLA2G10 | PLA2G6 | PLAUR | PLCG1 | PLK1 | PLSCR1 | PLXNA1 | PLXNA2 | PLXNB2 | PML | PNKP | POLDIP2 | POT1 | POU2F3 | POU5F1 | PPARD | PPARG | PPARGC1A | PPID | PPIF | PPM1A | PPM1B | PPM1D | PPME1 | PPP1R11 | PPP1R14A | PPP1R2 | PPP1R8 | PPP2CA | PPP2CB | PPP2R1A | PPP2R2A | PPP2R5D | PPT1 | PRCC | PRDX2 | PRDX3 | PRDX4 | PRDX5 | PRELID1 | PRKAA1 | PRKAA2 | PRKAB2 | PRKACA | PRKAG1 | PRKAR1A | PRKAR2B | PRKCD | PRKCE | PRKCZ | PRKD1 | PRKDC | PRMT2 | PRMT3 | PRMT6 | PROX1 | PSAP | PSEN1 | PSMA1 | PSMA2 | PSMA3 | PSMA5 | PSMA6 | PSMB5 | PSMB6 | PSMB7 | PSMB8 | PSMC1 | PSMC2 | PSMC3 | PSMC4 | PSMC5 | PSMC6 | PSMD1 | PSMD10 | PSMD11 | PSMD12 | PSMD14 | PSMD2 | PSMD4 | PSMD9 | PSME2 | PSME3 | PTCRA | PTEN | PTGER3 | PTGES | PTGIR | PTK2 | PTN | PTPN1 | PTPN14 | PTPN22 | PTPN23 | PTPN6 | PTPRK | PTPRO | PTPRR | PTRH2 | PTTG1 | PUM1 | PUS7 | PXDN | PYCR1 | QARS | QSOX1 | RAB29 | RAB7A | RAC1 | RAD21 | RAD51 | RAF1 | RANBP9 | RANGAP1 | RAP1A | RAP1B | RARA | RARG | RASD1 | RASL11B | RB1 | RB1CC1 | RBBP4 | RBM3 | RBM4 | RBM8A | RBMX | RBPJ | RBX1 | RC3H1 | RDX | RECK | REL | RELA | REST | RFC1 | RGN | RGS1 | RGS2 | RGS20 | RHBDD1 | RHOA | RIF1 | RING1 | RIPK1 | RIPK2 | RNASEH2B | RNF126 | RNF139 | RNF2 | RNF8 | RPA2 | RPGRIP1L | RPL10 | RPL10A | RPL12 | RPL18A | RPL22 | RPL23 | RPL23A | RPL24 | RPL30 | RPL37A | RPL38 | RPL6 | RPL7 | RPL7A | RPL9 | RPLP0 | RPLP1 | RPLP2 | RPS12 | RPS13 | RPS14 | RPS15A | RPS19 | RPS2 | RPS20 | RPS24 | RPS29 | RPS3 | RPS4X | RPS6KA1 | RPS6KA2 | RPS8 | RPTOR | RRM2 | RRM2B | RTF1 | RTKN | RTN4 | RUNX1 | RUNX1T1 | RUNX2 | RUVBL2 | RXRA | S100A1 | S100A11 | S100B | SAG | SAMD7 | SAP18 | SARM1 | SARS | SCFD1 | SCG2 | SCYL2 | SDC4 | SDCBP | SEMA7A | SEPT7 | SERPINB2 | SERPINB3 | SERPINB5 | SERPINB6 | SERPINB8 | SERPINE2 | SERPINH1 | SET | SETDB1 | SF1 | SFMBT1 | SFN | SFPQ | SGTA | SH3GL2 | SH3GL3 | SH3KBP1 | SHC1 | SHMT1 | SIAH1 | SIN3A | SIPA1 | SIRT1 | SIRT3 | SIRT5 | SIRT6 | SIRT7 | SKI | SKIV2L | SLAMF1 | SLAMF8 | SLC25A4 | SLC25A5 | SLC35F6 | SLC9A1 | SLC9A3R1 | SMAD2 | SMAD3 | SMAD4 | SMAD5 | SMARCA2 | SMARCA4 | SMARCB1 | SMARCC1 | SMARCC2 | SMARCE1 | SMC1A | SMC3 | SMG5 | SMG6 | SMG8 | SMO | SMURF1 | SMURF2 | SNAI2 | SNCA | SND1 | SNTA1 | SNW1 | SNX12 | SNX3 | SNX6 | SOCS1 | SOCS2 | SOCS6 | SOD1 | SOD2 | SORT1 | SOX2 | SP100 | SP2 | SPDL1 | SPEN | SPERT | SPI1 | SPINK7 | SPINT2 | SPRY2 | SPTAN1 | SPTBN1 | SPTBN2 | SQSTM1 | SRC | SRGAP2 | SRGAP3 | SRI | SRPK1 | SRPK2 | SRSF12 | SSB | ST13 | STAG2 | STARD13 | STAT1 | STAT3 | STAT6 | STIL | STK11 | STK24 | STK26 | STK3 | STK38 | STK40 | STMN1 | STRAP | STUB1 | STXBP1 | STYX | SULF2 | SULT1A3 | SUMO1 | SUPT4H1 | SUPT5H | SUZ12 | SWAP70 | SYNJ2BP | SYVN1 | TAB2 | TAGLN3 | TANK | TAOK3 | TARDBP | TBC1D4 | TBL1X | TBRG4 | TBXA2R | TCERG1 | TCF21 | TCF25 | TCP10L | TERF1 | TERF2 | TERF2IP | TES | TFDP1 | TFIP11 | TGFB1 | TGFBR3 | THADA | THAP11 | THAP7 | THBS1 | THBS2 | THEM4 | THOC6 | THRA | TIA1 | TIMP1 | TIMP2 | TINF2 | TLE1 | TLE3 | TLR3 | TMBIM6 | TMOD3 | TMOD4 | TNF | TNFAIP3 | TNFRSF1A | TNFRSF8 | TNFSF14 | TNIP2 | TNK2 | TNKS | TNKS2 | TNN | TNNT1 | TNP1 | TOP2A | TP53 | TP63 | TP73 | TPCN2 | TPM1 | TPR | TRADD | TRAF2 | TRAF3 | TRAF3IP1 | TRAF6 | TRAP1 | TREX1 | TRIAP1 | TRIB2 | TRIB3 | TRIM14 | TRIM21 | TRIM25 | TRIM28 | TRIM29 | TRIM32 | TRIM33 | TRIM35 | TRIM63 | TRIM9 | TRIP12 | TRIP13 | TSC22D3 | TSC22D4 | TSG101 | TSKU | TSPYL2 | TUBB4A | TULP3 | TWF1 | TWF2 | TWIST1 | TXN | TXNDC5 | TXNIP | TYMS | U2AF2 | UBA3 | UBASH3A | UBB | UBC | UBE2B | UBE2D1 | UBE2D3 | UBE2I | UBE2J1 | UBE2L6 | UBQLN1 | UBQLN2 | UBQLN4 | UBR5 | UBXN1 | UCHL1 | UCHL5 | UFM1 | ULK1 | UNC119 | UNK | UPF2 | UPF3B | USP14 | USP17L2 | USP19 | USP2 | USP28 | USP4 | USP47 | USP7 | USP9X | VAPA | VAT1 | VCL | VDAC1 | VDAC2 | VDR | VHL | VIM | VPS4B | VPS72 | VSIG4 | VTN | WARS | WAS | WDR35 | WDR77 | WDTC1 | WNK1 | WNK4 | WNT16 | WT1 | WWOX | WWP2 | XBP1 | XIAP | XPO1 | XRCC1 | XRCC5 | XRCC6 | YAF2 | YAP1 | YEATS2 | YME1L1 | YRDC | YWHAB | YWHAE | YWHAG | YWHAH | YWHAQ | YWHAZ | YY1 | ZBTB16 | ZBTB17 | ZBTB21 | ZBTB33 | ZBTB42 | ZBTB7A | ZBTB7B | ZC3H4 | ZC3HC1 | ZFAND6 | ZFYVE19 | ZHX1 | ZMPSTE24 | ZMYND8 | ZNF207 | ZNF217 | ZNF24 | ZNF746 | ZW10 | ZWINT | GO:0033043 | 0.10809171418173 | 891/8243 | 424/3250 | 1.00060963390698e-07 | 0.000950078847394678 | T | T | T | T | regulation of organelle organization | ABL1 | ABL2 | ACAA2 | ACD | ACTN2 | ACTR2 | ACTR3 | ADD3 | ADRB2 | AICDA | AKAP8 | AKAP9 | AKT1 | AKT2 | ANAPC4 | ANK1 | ANXA1 | ANXA2 | APC | APOA1 | ARF1 | ARHGAP35 | ARHGDIA | ARHGEF9 | ARPC1A | ARPC1B | ARPC2 | ARPC3 | ARPC4 | ARPC5 | ARRB1 | ARRB2 | ATG5 | ATL3 | ATM | ATP13A2 | ATRX | ATXN2 | AURKB | AVIL | BAG3 | BAG4 | BAIAP2 | BAIAP2L1 | BAX | BBC3 | BCL2 | BCL2L1 | BCL6 | BCOR | BIN1 | BRAF | BRCA1 | BRD4 | CALCOCO2 | CALR | CAPG | CAPN2 | CAPZA1 | CAPZA2 | CAPZB | CASP8 | CCDC36 | CCDC8 | CCDC88A | CCL21 | CCNB1 | CCP110 | CCT2 | CCT3 | CCT4 | CCT5 | CCT6A | CCT7 | CCT8 | CDC14B | CDC16 | CDC20 | CDC25C | CDC27 | CDC37 | CDC42 | CDC42EP3 | CDC42EP4 | CDK5 | CDK5R1 | CDK9 | CENPF | CENPJ | CENPV | CEP135 | CEP70 | CFL1 | CFL2 | CHCHD10 | CHMP2A | CHORDC1 | CLIC4 | CLTC | CLU | CNTRL | CNTROB | CPLX1 | CPLX3 | CRK | CROCC | CSNK2A2 | CTBP1 | CTCF | CTNNB1 | CTTN | CUL3 | CUL7 | CYLD | DCN | DCP2 | DCTN1 | DHX36 | DNM1L | DSTN | DUSP1 | DYNC1H1 | DYNC1LI1 | DYNLL1 | E2F1 | EED | EID1 | EZR | FBXO7 | FBXW5 | FCHSD1 | FEN1 | FIS1 | FSCN1 | FSD1 | FZR1 | GAP43 | GNAI1 | GOLPH3 | GPX1 | GRB2 | GRID2 | GSK3A | GSK3B | H1F0 | H1FX | HAP1 | HAX1 | HDAC5 | HDAC6 | HECW2 | HIF1A | HIST1H1A | HK2 | HNRNPA1 | HNRNPA2B1 | HNRNPD | HNRNPU | HRAS | HSPA1B | HSPA1L | HTRA2 | HTT | HUWE1 | ICAM1 | IK | ILK | INSIG1 | INSR | ITGB1BP1 | KAT7 | KCTD17 | KDM1A | KDM4D | KDR | KIF11 | KIF5B | KLHL22 | KMT2A | KMT2B | L3MBTL1 | LATS1 | LCP1 | LIG4 | LIMA1 | LMNA | LPAR1 | LRFN1 | LRRK2 | LRSAM1 | MAD1L1 | MAD2L1 | MAP1B | MAP2K1 | MAP2K7 | MAP3K4 | MAPK1 | MAPK15 | MAPK3 | MAPK8 | MAPK9 | MAPT | MARCH5 | MAVS | MCM2 | MDM1 | MECP2 | MLLT11 | MLST8 | MSN | MTPN | MYC | MYO1C | MYO5A | MYOC | NCK1 | NEK7 | NELFE | NF2 | NIN | NIPBL | NPEPPS | NPM1 | NRXN1 | NSFL1C | NTF3 | NUBP1 | NUMA1 | NUSAP1 | OBSL1 | OGT | OPA1 | OPTN | OTUB1 | PAFAH1B1 | PAM16 | PAN2 | PARK7 | PARP1 | PAXIP1 | PDCD6IP | PDE4DIP | PEBP1 | PFDN2 | PFN1 | PHB2 | PICK1 | PIH1D1 | PIK3R1 | PIKFYVE | PIN1 | PINX1 | PIP4K2A | PLA2G6 | PLAUR | PLK1 | PML | PNKP | POT1 | PPARGC1A | PPIF | PPP3CC | PRELID1 | PRKAA1 | PRKAA2 | PRKAR1A | PRKCD | PRKCE | PRKD1 | PRKD2 | PRMT6 | PROX1 | PSMD10 | PTK2 | PTPRD | PTTG1 | PXN | RAB1B | RAB3GAP1 | RAB5A | RAB7A | RAC1 | RAD21 | RAE1 | RASSF7 | RB1 | RCC1 | RDX | RHOA | RHOJ | RHOT1 | RHOT2 | RHOU | RIF1 | RNF31 | RNF4 | RPS3 | RTF1 | RUVBL2 | S100A9 | SAE1 | SART3 | SCFD1 | SDC1 | SDC4 | SDCBP | SEC24B | SENP6 | SEPT7 | SEPT8 | SEPT9 | SET | SETDB1 | SFN | SFPQ | SH2B1 | SIN3A | SIRT1 | SIRT6 | SKA1 | SKA3 | SKI | SLC25A5 | SLC35F6 | SLC9A1 | SMAD3 | SMAD4 | SMARCB1 | SMC1A | SMC3 | SMG5 | SMG6 | SMURF1 | SNAI2 | SNCA | SNW1 | SPDL1 | SPI1 | SPICE1 | SPTAN1 | SPTBN1 | SPTBN2 | SQSTM1 | SRC | STAG2 | STIL | STMN1 | STX5 | STXBP1 | SWAP70 | SYDE1 | SYNE2 | TADA2B | TBC1D15 | TBC1D22B | TBC1D4 | TCP1 | TERF1 | TERF2 | TERF2IP | TFDP1 | TGFB1 | TINF2 | TMED9 | TMOD3 | TMOD4 | TNF | TNKS | TNKS2 | TOP2A | TP53 | TP63 | TP73 | TPM1 | TPR | TRAF3IP1 | TREX1 | TRIAP1 | TRIM28 | TRIM9 | TRIP12 | TRIP13 | TSG101 | TUBB4A | TWF1 | TWF2 | TWIST1 | UBE2B | UBE2C | UBE2D3 | UBE2L3 | UBE2N | UBQLN2 | UBQLN4 | UBR5 | ULK1 | USP17L2 | USP7 | VASP | VAT1 | VDAC1 | VDAC3 | VPS18 | VPS4B | WAS | WBP2 | WDR1 | WDR35 | XPO1 | XRCC1 | XRCC3 | XRCC5 | YWHAB | YWHAE | YWHAG | YWHAH | YWHAQ | YWHAZ | ZBTB7B | ZMPSTE24 | ZMYND10 | ZMYND8 | ZNF207 | ZW10 | GO:1901698 | 0.0622346233167536 | 513/8243 | 259/3250 | 1.04210754201121e-07 | 0.000989481111139644 | T | T | T | T | response to nitrogen compound | ABL1 | ACTA1 | ACTB | ACTR2 | ADA | ADCY6 | ADORA2A | ADRB2 | ADSS | ADTRP | AGER | AIFM1 | AKAP9 | AKR1B1 | AKT1 | AKT2 | ANXA1 | ANXA5 | APEX1 | APLP1 | APP | APRT | ASCL1 | ASNS | ASS1 | ATRX | BACE1 | BCL11A | BCL2L1 | BIRC2 | BRAF | BRCA1 | BSG | CACNA1A | CACNG4 | CALM1 | CALM3 | CAPN2 | CARD9 | CARM1 | CASP3 | CASP7 | CAT | CAV1 | CD36 | CD40 | CD81 | CDC5L | CDH1 | CDK2 | CDK4 | CDK5 | CDKN1A | CFL1 | CFTR | COL4A1 | COL6A1 | CREB1 | CRK | CRY1 | CTNNB1 | CYC1 | DAXX | DBH | DDX1 | DENND4C | DHX15 | DHX36 | DHX9 | DPEP1 | DUSP1 | EEF2 | EFTUD2 | EGFR | EGLN1 | EIF2B1 | EIF2B3 | EIF2B5 | EIF6 | EPHA4 | EPRS | ERRFI1 | EZH2 | EZR | FABP3 | FADD | FIS1 | FLOT1 | FOLR1 | FOS | FOXO1 | FYN | GLP1R | GLRB | GNAI1 | GNAO1 | GNB1 | GOT1 | GOT2 | GPD1 | GPI | GSK3A | GSK3B | GSS | GSTP1 | HADH | HCN1 | HCN2 | HCN3 | HDAC2 | HDAC5 | HDAC9 | HLCS | HNRNPD | HNRNPL | HPRT1 | HSF1 | HSP90B1 | HSPA5 | HTR1B | HTR3A | ICAM1 | IFIT1 | IFNA2 | IGF1R | INSIG2 | INSR | IPO5 | IQGAP1 | IREB2 | ITGA4 | ITPR2 | JUN | JUNB | JUND | JUP | KAT7 | KDM1A | KLF5 | KLHL22 | KYNU | LARS | LDHA | LGMN | LPIN3 | LRP6 | LRRK2 | MAPK1 | MAPK14 | MAPK3 | MAVS | MDM2 | MGMT | MGST1 | MYD88 | MYO5A | NAMPT | NFE2L2 | NFKBIA | NME1 | NME2 | NOD2 | NQO1 | NR4A1 | NTRK1 | OGT | OPA1 | OTC | P2RX1 | P2RX2 | P2RX4 | PARP1 | PCNA | PCSK9 | PDE12 | PDE4D | PEBP1 | PELI1 | PER1 | PIK3CG | PIK3R1 | PKM | PLA2G1B | PPARG | PPARGC1A | PPAT | PPP2R2A | PRKAA1 | PRKACA | PRKAR1A | PRKAR2B | PRKCD | PRKCE | PRKCZ | PRKDC | PSEN1 | PTEN | PTN | PTPN22 | QDPR | RAB10 | RAB31 | RAD51 | RANGAP1 | RAP1A | RAP1B | RBBP4 | RELA | RGS2 | RHOA | RIPK2 | RPL23 | RPTOR | RRM2B | SDC1 | SELL | SHMT1 | SHMT2 | SIN3A | SIRT1 | SLC3A2 | SLC9A1 | SNCA | SNX6 | SOCS1 | SOD1 | SORT1 | SP1 | SRC | SRSF5 | STAT1 | STAT6 | SULT1A3 | TBC1D4 | TDO2 | TGFB1 | TGFBR3 | TIMP1 | TLR3 | TMBIM6 | TMEM173 | TNF | TNFAIP3 | TOMM20 | TP53 | TP73 | TRAF2 | TREX1 | TRIB3 | TRIM41 | TYMS | VCAM1 | VGF | VIM | WDTC1 | WT1 | XBP1 | YWHAG | ZBTB7B | GO:0009968 | 0.0915928666747543 | 755/8243 | 365/3250 | 1.13460813482153e-07 | 0.00107731042401304 | T | T | T | T | negative regulation of signal transduction | ABL1 | ABL2 | ACAA2 | ADA | ADRB2 | AES | AHSG | AKT1 | APC | APLP1 | APOA1 | APOD | APOE | AR | ARHGAP35 | ARHGEF7 | ARRB1 | ARRB2 | ATAD5 | ATF6B | ATM | AXIN1 | BAG5 | BAX | BCL2 | BCL2L1 | BCL2L2 | BCL6 | BDKRB2 | BRCA1 | BTRC | C1QBP | C1QL4 | CALM1 | CALM2 | CALM3 | CALR | CASP8 | CAV1 | CBL | CBLB | CBLC | CBY1 | CCAR2 | CCDC22 | CD109 | CD22 | CD74 | CDC34 | CDC42 | CHD8 | CHP1 | CHRD | CHRNA9 | CLU | COL2A1 | CREB3 | CRY1 | CRY2 | CSNK1A1 | CSNK2A2 | CTH | CTNNB1 | CTNNBIP1 | CTNND1 | CTTN | CUL3 | CXCL8 | CYLD | DAB1 | DAB2 | DCN | DDIT4 | DDX3X | DNAJA1 | DUSP1 | DUSP14 | DUSP16 | DUSP19 | DUSP22 | DUSP7 | DVL1 | DVL2 | DVL3 | DYRK2 | EGFR | EIF3A | EIF4A3 | ENO1 | EPM2A | ERBB2 | ERBB3 | ERRFI1 | ESR1 | EYA2 | EYA4 | EZH2 | EZR | FADD | FAM129B | FASLG | FBP1 | FBXW11 | FGB | FHL2 | FIGNL1 | FKBP1A | FKBP1B | FOXM1 | FOXO1 | FYN | G3BP1 | GATA1 | GNAI2 | GNAI3 | GPD1L | GPS2 | GPX1 | GRB2 | GSK3A | GSK3B | GSTO1 | GSTP1 | HBEGF | HDAC1 | HDAC3 | HDAC7 | HECW1 | HGS | HIF1A | HIF1AN | HMGB2 | HMOX1 | HSPA1B | HSPA5 | HSPB1 | HTRA1 | HTRA2 | HTT | ICAM1 | IFT122 | IGBP1 | IGF1R | INSIG1 | IRF4 | ITCH | ITGAV | ITGB1BP1 | ITPR1 | KANK2 | KDM1A | LATS1 | LDLRAD4 | LEMD2 | LEMD3 | LGALS3 | LGMN | LGR4 | LMNA | LPAR1 | LRP6 | LRRK2 | LTBP1 | LZTR1 | MAPK14 | MAPK7 | MAPKAP1 | MCC | MDFI | MDM2 | MIF | MLLT3 | MYC | MYOC | NCK1 | NCOR1 | NCOR2 | NDUFA13 | NDUFS3 | NEDD4 | NEUROD1 | NF2 | NFATC1 | NFE2L2 | NFKBIA | NKX2-1 | NLK | NOS3 | NPHP3 | NPLOC4 | NR1H2 | NR1H3 | ONECUT1 | OPA1 | OPTN | OTUD4 | PAFAH1B1 | PAM16 | PARK7 | PARP14 | PAWR | PDCD6 | PDE4D | PEBP1 | PER1 | PHB | PHB2 | PIH1D1 | PIN1 | PLAUR | PPARG | PPIF | PPM1A | PPM1B | PPP2CA | PPP2CB | PPP2R1A | PRDX2 | PRELID1 | PRKAA1 | PRKAA2 | PRKACA | PRKCD | PRKCZ | PSEN1 | PSMA1 | PSMA2 | PSMA3 | PSMA5 | PSMA6 | PSMB5 | PSMB6 | PSMB7 | PSMB8 | PSMC1 | PSMC2 | PSMC3 | PSMC4 | PSMC5 | PSMC6 | PSMD1 | PSMD10 | PSMD11 | PSMD12 | PSMD14 | PSMD2 | PSMD4 | PSMD9 | PSME2 | PSME3 | PTEN | PTGIR | PTPN1 | PTPN22 | PTPN6 | PTPRO | PTPRR | PXDN | QARS | RAB7A | RAF1 | RANBP9 | RASL11B | RB1 | RB1CC1 | RBX1 | RELA | RGS1 | RGS2 | RGS20 | RHOA | RIPK1 | RNF126 | RPGRIP1L | RRM2B | RUNX2 | RUVBL2 | SARM1 | SCG2 | SCYL2 | SERPINB3 | SERPINE2 | SH3GL2 | SH3KBP1 | SIAH1 | SIRT1 | SIRT3 | SKI | SLAMF1 | SLC25A5 | SLC35F6 | SLC9A3R1 | SMAD2 | SMAD3 | SMAD4 | SMARCA4 | SMURF1 | SMURF2 | SNAI2 | SNCA | SNX6 | SOCS1 | SOCS2 | SOCS6 | SOD2 | SOX2 | SPERT | SPRY2 | SRC | SRI | STAT1 | STK11 | STK3 | STK38 | STMN1 | STRAP | STUB1 | SULF2 | SYNJ2BP | SYVN1 | TANK | TAOK3 | TCF21 | TGFB1 | TGFBR3 | THBS1 | THEM4 | TIMP2 | TLE1 | TLE3 | TLR3 | TMBIM6 | TNF | TNFAIP3 | TNN | TP63 | TRADD | TRAF2 | TRAF3IP1 | TRAP1 | TREX1 | TRIAP1 | TRIB3 | TRIM32 | TRIM33 | TSG101 | TSKU | TULP3 | TWIST1 | UBASH3A | UBB | UBC | UBE2B | UBQLN1 | UCHL1 | USP47 | VDAC2 | VHL | WWOX | WWP2 | XBP1 | YAP1 | YWHAB | ZBTB7A | ZC3HC1 | GO:0019538 | 0.291641392696834 | 2404/8243 | 1053/3250 | 1.14554956796172e-07 | 0.00108769931477966 | T | T | T | T | protein metabolic process | A2M | ABCG1 | ABHD17A | ABL1 | ABL2 | ACACA | ACE2 | ACP1 | ACTB | ACTL6A | ACTN2 | ACY1 | ADAMTS4 | ADCK2 | ADH5 | ADORA2A | ADPRH | ADRB2 | ADRM1 | AFG3L2 | AGO2 | AHSG | AKT1 | AKT2 | AKT3 | ALB | AMFR | ANAPC4 | ANXA1 | ANXA2 | APBB1 | APC | APCS | APEH | APOA1 | APOE | APOL2 | APP | AR | ARAF | ARF4 | ARID4B | ARMC8 | ARRB1 | ARRB2 | ASB12 | ASB15 | ASB16 | ASB6 | ASB9 | ASGR1 | ASGR2 | ATF2 | ATG4C | ATG5 | ATM | ATP13A2 | AUP1 | AURKB | AZU1 | B3GAT3 | B3GNT3 | B4GALT3 | BABAM1 | BACE1 | BAG2 | BAG6 | BCL10 | BCL11A | BCL2 | BCOR | BIRC2 | BIRC3 | BIRC5 | BIRC7 | BIRC8 | BMI1 | BMPR1A | BRAF | BRCA1 | BRD4 | BTK | BTRC | C1QBP | C1R | C4A | CAB39 | CALM1 | CALR | CALU | CAMK1D | CAMK2A | CAMK2B | CAMKV | CAND1 | CAPN1 | CAPN2 | CAPNS1 | CARM1 | CASP10 | CASP2 | CASP3 | CASP7 | CASP8 | CASP9 | CAT | CBFB | CBL | CBLB | CBLC | CBX4 | CBX8 | CCDC22 | CCDC8 | CCNB1 | CCNT1 | CCP110 | CCR5 | CCS | CD36 | CDC14B | CDC16 | CDC20 | CDC25A | CDC25B | CDC25C | CDC27 | CDC34 | CDC42 | CDC73 | CDK15 | CDK16 | CDK19 | CDK2 | CDK4 | CDK5 | CDK5R1 | CDK8 | CDK9 | CDKL3 | CEBPE | CEL | CES1 | CFL1 | CFTR | CHEK2 | CHGB | CHRM3 | CHST4 | CISD3 | CKAP4 | CLK1 | CLPX | CLTC | CNDP1 | CNDP2 | COG3 | COL2A1 | COMMD1 | COPS2 | COPS5 | COPS6 | CPNE3 | CPVL | CRBN | CREB1 | CREBBP | CRKL | CRY1 | CRY2 | CRYAB | CSNK1A1 | CSNK1E | CSNK2A2 | CSNK2B | CTBP1 | CTDP1 | CTH | CTNNB1 | CTSA | CTSC | CTSD | CTSL | CTSV | CUL1 | CUL2 | CUL3 | CUL4B | CUL5 | CUL7 | CYCS | CYLD | DAD1 | DAPK1 | DAPP1 | DARS | DBI | DCAF12 | DCAF7 | DCAF8 | DCLK1 | DCN | DCUN1D1 | DDA1 | DDB1 | DDB2 | DDI1 | DDOST | DDRGK1 | DERL1 | DGUOK | DLD | DLG2 | DLG3 | DLG4 | DLST | DMAP1 | DNAJB11 | DNAJB2 | DNAJC10 | DNPEP | DPEP1 | DPF2 | DPM1 | DPP9 | DRG2 | DSE | DTX1 | DTX2 | DUSP1 | DUSP12 | DUSP13 | DUSP14 | DUSP16 | DUSP19 | DUSP22 | DUSP7 | DYRK2 | DZIP3 | EDEM1 | EED | EEF1A1 | EEF1A1P5 | EEF1A2 | EEF2KMT | EFEMP1 | EGFR | EGLN1 | EGLN3 | EHHADH | EHMT1 | EHMT2 | EIF2AK3 | EIF2AK4 | EIF2S1 | EIF3H | EIF4H | EP300 | EPHA4 | EPHA6 | EPHB6 | ERBB2 | ERBB3 | ERG | ERLIN2 | ESR1 | EVA1A | EXTL2 | EYA2 | EYA4 | EZH2 | F5 | F9 | FADD | FAF1 | FAF2 | FAM20B | FAM98A | FARSB | FBXL12 | FBXO11 | FBXO25 | FBXO32 | FBXO6 | FBXO7 | FBXW11 | FBXW5 | FGB | FGF2 | FGFR1 | FGFR3 | FGL2 | FKBP14 | FKBP1A | FKBP1B | FKBP2 | FKBP5 | FKBP7 | FKBP8 | FN1 | FOLH1 | FOXK1 | FOXK2 | FOXO1 | FYN | FZR1 | GAK | GALNT7 | GANAB | GAPDH | GEMIN5 | GFPT1 | GFPT2 | GGA1 | GGT1 | GGTLC1 | GLCE | GLRX | GLRX3 | GLRX5 | GLUL | GOLGA2 | GPS2 | GPX1 | GRB2 | GRK1 | GRK5 | GSK3A | GSK3B | GSPT1 | GSPT2 | GZMA | GZMK | H3F3A | HARS | HBEGF | HCCS | HDAC1 | HDAC11 | HDAC2 | HDAC3 | HDAC4 | HDAC5 | HDAC6 | HDAC7 | HDAC9 | HECW1 | HECW2 | HERC1 | HERC2 | HEXB | HGS | HIF1A | HIF1AN | HIPK4 | HIST1H2BA | HIST1H3A | HIST1H4A | HIST2H3A | HLCS | HMBS | HPX | HRAS | HS2ST1 | HS6ST1 | HSF1 | HSF4 | HSP90B1 | HSPA14 | HSPA5 | HSPD1 | HSPG2 | HTRA1 | HTRA2 | HUWE1 | ICK | IDE | IFI16 | IGF1R | IGFBP7 | IKBKB | IKBKE | IKBKG | IL3RA | ILF3 | ILK | ING5 | INO80B | INO80C | INSR | IRF4 | IRF7 | ISG15 | ITCH | ITGAV | ITK | JAK1 | JAK3 | JMJD6 | JOSD2 | KAT5 | KAT7 | KAT8 | KBTBD7 | KCMF1 | KCTD17 | KCTD2 | KDM1A | KDM2A | KDM3B | KDM4D | KDM5B | KDM5C | KDM8 | KDR | KEAP1 | KLHL20 | KLHL22 | KMT2A | KMT2B | KMT2C | KRAS | KSR1 | L2HGDH | LAMB1 | LAP3 | LARP4 | LAT | LATS1 | LCMT2 | LEF1 | LGALS1 | LGMN | LMO7 | LNX1 | LOXL2 | LRRC7 | LRRK1 | LRRK2 | LRSAM1 | LTBP1 | LYPLA1 | LYPLA2 | LZTR1 | MAD2L1 | MAN2B1 | MAP2K1 | MAP2K3 | MAP2K4 | MAP2K6 | MAP2K7 | MAP3K1 | MAP3K13 | MAP3K14 | MAP3K2 | MAP3K3 | MAP3K4 | MAP4K1 | MAPK1 | MAPK14 | MAPK15 | MAPK3 | MAPK4 | MAPK6 | MAPK7 | MAPK8 | MAPK9 | MAPT | MARCH5 | MASP1 | MASP2 | MAST1 | MAST3 | MATK | MBD3 | MBP | MCFD2 | MDH2 | MDM2 | MDM4 | MECP2 | MED1 | MED12 | MED20 | MED21 | MED31 | MELK | METAP2 | MIB1 | MKNK2 | MME | MMP26 | MORF4L1 | MORF4L2 | MPI | MPPE1 | MRGBP | MRPL28 | MRPL43 | MRPL9 | MRRF | MTA1 | MTA3 | MTMR14 | MTMR7 | MYC | MYH6 | NAE1 | NCK1 | NCOA1 | NCOA3 | NDUFAF5 | NDUFAF7 | NEDD4 | NEDD8 | NEK1 | NEK7 | NEURL4 | NFE2L2 | NFKBIA | NFS1 | NHLRC3 | NLK | NME2 | NOD2 | NOS2 | NOSIP | NPEPL1 | NPEPPS | NPLOC4 | NPR1 | NSFL1C | NTMT1 | NTRK1 | NUCB1 | NUP107 | NUP153 | NUP160 | NUP205 | NUP214 | NUP85 | NUP88 | NUP93 | NUP98 | NUPR1 | OBSL1 | OGDH | OGFOD1 | OGT | OTUB1 | OTUB2 | OTUD4 | OTUD5 | OXSR1 | P4HA1 | P4HA2 | P4HB | PABPC4 | PADI4 | PAK2 | PAK6 | PAPLN | PARK7 | PARP1 | PARP12 | PARP14 | PARP6 | PAWR | PAXIP1 | PCBP2 | PCGF1 | PCGF5 | PCGF6 | PCMT1 | PCMTD1 | PCMTD2 | PCNA | PCSK9 | PDCD6IP | PDF | PDIA3 | PDIA6 | PDK2 | PEBP1 | PELI1 | PEPD | PER1 | PER2 | PEX5 | PGAM5 | PGK1 | PGM3 | PGP | PHB | PHF10 | PHKA1 | PIAS1 | PIAS3 | PICK1 | PIGU | PIK3CA | PIK3CG | PIK3R1 | PIM1 | PIN1 | PJA1 | PKN2 | PLK1 | PLOD2 | PML | PMPCA | PMPCB | POLE3 | POM121 | PPA2 | PPARD | PPEF1 | PPIA | PPIB | PPID | PPIE | PPIF | PPIL3 | PPM1A | PPM1B | PPM1D | PPM1H | PPM1K | PPME1 | PPP1CA | PPP1CB | PPP1CC | PPP1R11 | PPP1R12A | PPP1R3B | PPP1R3C | PPP2CA | PPP2CB | PPP2R1A | PPP2R1B | PPP2R2A | PPP2R2D | PPP2R5D | PPP3CC | PPP4R2 | PPP5C | PPP6C | PPT1 | PPTC7 | PRCP | PRDX3 | PRDX4 | PREP | PREPL | PRKAA1 | PRKAA2 | PRKAB2 | PRKACA | PRKAG1 | PRKCD | PRKCE | PRKCSH | PRKCZ | PRKD1 | PRKD2 | PRKD3 | PRKDC | PRMT2 | PRMT3 | PRMT6 | PROZ | PRPF19 | PRSS16 | PRSS23 | PSEN1 | PSEN2 | PSMA1 | PSMA2 | PSMA3 | PSMA5 | PSMA6 | PSMB5 | PSMB6 | PSMB7 | PSMB8 | PSMC1 | PSMC2 | PSMC3 | PSMC4 | PSMC5 | PSMC6 | PSMD1 | PSMD10 | PSMD11 | PSMD12 | PSMD14 | PSMD2 | PSMD4 | PSMD9 | PSME2 | PSME3 | PTEN | PTK2 | PTPMT1 | PTPN1 | PTPN14 | PTPN21 | PTPN22 | PTPN23 | PTPN4 | PTPN6 | PTPRA | PTPRD | PTPRF | PTPRK | PTPRO | PTPRR | PTTG1 | QSOX1 | RAB11A | RAB11B | RAB1A | RAB1B | RAB2A | RAB5A | RAB7A | RAD23A | RAD23B | RAE1 | RAF1 | RANBP9 | RANGAP1 | RARA | RARS2 | RBBP6 | RBMX | RBX1 | RC3H1 | RELA | REST | RHBDD1 | RHO | RHOA | RHOT1 | RING1 | RIOK1 | RIPK1 | RIPK2 | RIPK3 | RIPK4 | RMND1 | RNF123 | RNF126 | RNF139 | RNF181 | RNF183 | RNF185 | RNF19A | RNF2 | RNF25 | RNF31 | RNF4 | RNF8 | RNPEP | RPL10 | RPL10A | RPL12 | RPL18A | RPL22 | RPL23 | RPL23A | RPL24 | RPL30 | RPL36AL | RPL37A | RPL38 | RPL6 | RPL7 | RPL7A | RPL9 | RPLP0 | RPLP0P6 | RPLP1 | RPLP2 | RPN1 | RPN2 | RPS12 | RPS13 | RPS14 | RPS15A | RPS19 | RPS2 | RPS20 | RPS24 | RPS29 | RPS3 | RPS4X | RPS6KA1 | RPS6KA2 | RPS6KB2 | RPS8 | RTF1 | RUVBL1 | RUVBL2 | S100A9 | SAE1 | SARS | SCG2 | SCG3 | SCYL2 | SEL1L | SENP3 | SENP6 | SENP7 | SENP8 | SEPHS1 | SERPINH1 | SETDB1 | SFPQ | SGSH | SGTA | SGTB | SHC1 | SHMT2 | SIAH1 | SIN3A | SIRT1 | SIRT3 | SIRT5 | SIRT6 | SIRT7 | SMAD2 | SMAD3 | SMAD4 | SMAD5 | SMURF1 | SMURF2 | SNCA | SNRPB | SNRPD3 | SNX3 | SOCS1 | SOCS2 | SOCS6 | SPATA2 | SPI1 | SPOP | SPRR2A | SPSB3 | SPTAN1 | SPTBN1 | SPTBN2 | SQSTM1 | SRC | SRPK1 | SRPK2 | ST3GAL1 | ST3GAL6 | ST6GALNAC5 | ST8SIA4 | STK11 | STK24 | STK25 | STK26 | STK3 | STK38 | STK40 | STOML2 | STUB1 | STYX | SULF2 | SULT1A3 | SUMO1 | SUMO1P1 | SUMO2 | SUMO3 | SUZ12 | SYVN1 | TAB1 | TAB2 | TADA2B | TAF12 | TANK | TAOK3 | TARS | TBL1X | TBRG4 | TCIRG1 | TESK2 | TGFB1 | TGFBR3 | TGM2 | TGOLN2 | THBS1 | THOP1 | TIMM50 | TIMP1 | TLR3 | TMEM132A | TMEM189-UBE2V1 | TMEM258 | TMTC3 | TNF | TNFAIP3 | TNIP2 | TNK2 | TNKS | TNKS2 | TOLLIP | TOMM20 | TOP1 | TOR1A | TP53 | TP53RK | TPR | TPSAB1 | TPSB2 | TPST2 | TPTE | TRAF2 | TRAF3 | TRAF6 | TRIB2 | TRIB3 | TRIM21 | TRIM23 | TRIM25 | TRIM28 | TRIM32 | TRIM33 | TRIM41 | TRIM47 | TRIM63 | TRIM69 | TRIM7 | TRIM9 | TRIP12 | TSG101 | TSPAN17 | TTLL1 | TWF1 | UBA1 | UBA2 | UBA3 | UBA5 | UBA6 | UBAC1 | UBB | UBC | UBE2A | UBE2B | UBE2C | UBE2D1 | UBE2D2 | UBE2D3 | UBE2H | UBE2I | UBE2J1 | UBE2K | UBE2L3 | UBE2L6 | UBE2M | UBE2N | UBE2O | UBE2Q2 | UBE2R2 | UBE2S | UBE2V1 | UBE2W | UBE3A | UBE3B | UBL4A | UBQLN1 | UBQLN2 | UBQLN4 | UBR4 | UBR5 | UBXN1 | UBXN7 | UCHL1 | UCHL3 | UCHL5 | UFC1 | UFM1 | UGDH | UGGT1 | ULK1 | UNKL | UQCRC2 | USP11 | USP14 | USP15 | USP16 | USP17L2 | USP19 | USP2 | USP20 | USP21 | USP26 | USP28 | USP39 | USP4 | USP42 | USP46 | USP47 | USP5 | USP50 | USP53 | USP7 | USP8 | USP9X | USP9Y | USPL1 | VASH2 | VCAN | VCP | VCPIP1 | VGF | VHL | VKORC1 | VPS37D | VPS4B | WARS | WDR45B | WDR48 | WDR82 | WDTC1 | WDYHV1 | WIPI1 | WIPI2 | WNK1 | WNK4 | WSB1 | WWP2 | XBP1 | XIAP | XPNPEP1 | XYLT1 | YBEY | YEATS2 | YES1 | YME1L1 | YWHAB | YWHAE | YY1 | ZAP70 | ZBTB16 | ZC3HC1 | ZDHHC17 | ZDHHC23 | ZMPSTE24 | ZNF598 | ZNRF4 | GO:0010646 | 0.244449836224675 | 2015/8243 | 894/3250 | 1.15122530213154e-07 | 0.0010930884243739 | T | T | T | T | regulation of cell communication | A2M | ABAT | ABCE1 | ABL1 | ABL2 | ACAA2 | ACTN2 | ACTN4 | ADA | ADCYAP1 | ADORA2A | ADRB2 | AES | AGER | AGO1 | AGR2 | AHSG | AKAP9 | AKR1B1 | AKR1C2 | AKT1 | AKT3 | ANK3 | ANXA1 | ANXA5 | APC | APLP1 | APOA1 | APOD | APOE | APP | AR | ARAF | ARF1 | ARFGAP1 | ARHGAP1 | ARHGAP19 | ARHGAP26 | ARHGAP35 | ARHGDIA | ARHGEF7 | ARHGEF9 | ARNT | ARRB1 | ARRB2 | ASCL1 | ATAD1 | ATAD5 | ATF6 | ATF6B | ATM | ATP2A2 | AURKB | AXIN1 | BACE1 | BAG4 | BAG5 | BAIAP2L1 | BANP | BAX | BBC3 | BCL10 | BCL2 | BCL2L1 | BCL2L2 | BCL6 | BDKRB2 | BIRC2 | BIRC3 | BIRC7 | BIRC8 | BMPR1A | BRAF | BRCA1 | BRD4 | BTRC | BUD31 | C1QBP | C1QL4 | C1QTNF1 | CACNA1A | CACNG2 | CACNG4 | CACNG5 | CALM1 | CALM2 | CALM3 | CALR | CAMK2A | CAMK2B | CAPN1 | CARD9 | CARM1 | CASP10 | CASP2 | CASP8 | CAT | CAV1 | CBFB | CBL | CBLB | CBLC | CBY1 | CCAR2 | CCDC22 | CCL21 | CCL22 | CCNY | CCR1 | CD109 | CD22 | CD27 | CD36 | CD40 | CD40LG | CD74 | CD81 | CDC20 | CDC34 | CDC37 | CDC42 | CDC42SE2 | CDC73 | CDH1 | CDK16 | CDK2 | CDK4 | CDK5 | CDK5R1 | CEL | CEP55 | CFTR | CHCHD10 | CHD3 | CHD4 | CHD8 | CHEK2 | CHGB | CHP1 | CHRD | CHRM2 | CHRNA9 | CLEC11A | CLNK | CLSTN1 | CLU | CMTM5 | COL2A1 | COPA | COPS5 | CORT | CPLX1 | CPLX3 | CPT1A | CREB1 | CREB3 | CREBBP | CRK | CRKL | CRY1 | CRY2 | CSNK1A1 | CSNK1E | CSNK2A2 | CSNK2B | CTH | CTNNB1 | CTNNBIP1 | CTNND1 | CTSC | CTTN | CUL3 | CXCL11 | CXCL5 | CXCL6 | CXCL8 | CYLD | DAB1 | DAB2 | DAPK1 | DAXX | DBH | DBN1 | DBNL | DCN | DDIT4 | DDRGK1 | DDX1 | DDX3X | DENND1A | DEPDC7 | DGKE | DHX36 | DISC1 | DLG2 | DLG3 | DLG4 | DMD | DNA2 | DNAJA1 | DNM1L | DOK1 | DTX1 | DUSP1 | DUSP14 | DUSP16 | DUSP19 | DUSP22 | DUSP7 | DVL1 | DVL2 | DVL3 | DYNLL1 | DYNLT1 | DYRK2 | E2F1 | EDA | EEF1D | EFEMP1 | EGFR | EGLN1 | EHMT1 | EHMT2 | EIF2AK3 | EIF2AK4 | EIF3A | EIF4A3 | EIF4E | ELF3 | ENO1 | EP300 | EPHA4 | EPM2A | ERBB2 | ERBB3 | ERRFI1 | ESR1 | ESR2 | EYA2 | EYA4 | EZH2 | EZR | FABP5 | FADD | FAF1 | FAM129B | FAM3C | FAM83D | FANCA | FASLG | FBP1 | FBRS | FBXW11 | FGB | FGF2 | FGFR1 | FGFR3 | FHL2 | FIGNL1 | FIS1 | FKBP1A | FKBP1B | FKBP8 | FLOT1 | FN1 | FOLR1 | FOXA1 | FOXM1 | FOXO1 | FYN | G3BP1 | GADD45G | GATA1 | GDF9 | GDI1 | GDI2 | GFAP | GIT1 | GJA1 | GKAP1 | GLP1R | GLUL | GNA12 | GNA13 | GNAI1 | GNAI2 | GNAI3 | GNAQ | GNAS | GOLPH3 | GOLT1B | GOT1 | GPC5 | GPD1L | GPI | GPS2 | GPX1 | GRB2 | GRB7 | GRIA1 | GRID2 | GRIK2 | GRK1 | GRK5 | GSK3A | GSK3B | GSTO1 | GSTP1 | HADH | HAP1 | HAVCR2 | HAX1 | HBEGF | HDAC1 | HDAC2 | HDAC3 | HDAC6 | HDAC7 | HDGF | HECW1 | HEXIM1 | HGS | HIF1A | HIF1AN | HINT1 | HIPK4 | HMGB1 | HMGB2 | HMOX1 | HNF4A | HNRNPK | HPX | HRAS | HSF1 | HSP90AA1 | HSP90AB1 | HSPA1B | HSPA5 | HSPB1 | HTR1B | HTRA1 | HTRA2 | HTT | ICAM1 | IFNA2 | IFT122 | IGBP1 | IGF1R | IKBKB | IKBKE | IKBKG | IL32 | ILK | ING5 | INSIG1 | INSR | IQGAP1 | IRF1 | IRF4 | IRF7 | ITCH | ITGAV | ITGB1BP1 | ITGB3 | ITPR1 | ITPR2 | ITPR3 | JAK1 | JAK3 | JUN | JUP | KANK2 | KAT5 | KCNS3 | KDM1A | KDM5B | KDR | KHDRBS1 | KIF5B | KLHDC10 | KLHL22 | KRAS | KSR1 | L3MBTL1 | LARS | LASP1 | LAT | LATS1 | LDLRAD4 | LEF1 | LEMD2 | LEMD3 | LGALS1 | LGALS3 | LGMN | LGR4 | LIMS1 | LMNA | LPAR1 | LPAR4 | LRP6 | LRRC7 | LRRK1 | LRRK2 | LTBP1 | LTBR | LURAP1 | LZTR1 | MAOA | MAP2K1 | MAP2K3 | MAP2K4 | MAP2K6 | MAP2K7 | MAP3K1 | MAP3K13 | MAP3K14 | MAP3K2 | MAP3K3 | MAP3K4 | MAP4K1 | MAPK1 | MAPK14 | MAPK3 | MAPK7 | MAPK8 | MAPK8IP3 | MAPK9 | MAPKAP1 | MAPT | MAS1 | MAVS | MBD3 | MC1R | MCC | MDFI | MDM2 | MDM4 | MECP2 | MED1 | METAP2 | METRNL | MIF | MKS1 | MLLT11 | MLLT3 | MLST8 | MME | MPC2 | MPP2 | MYC | MYD88 | MYO1C | MYO5A | MYOC | NAMPT | NCDN | NCK1 | NCOA1 | NCOA3 | NCOR1 | NCOR2 | NDUFA13 | NDUFAF2 | NDUFS3 | NEDD4 | NELFE | NEUROD1 | NF2 | NFATC1 | NFE2L2 | NFKBIA | NKX2-1 | NLK | NMB | NMNAT1 | NOD2 | NOS1AP | NOS2 | NOS3 | NPHP3 | NPLOC4 | NPR1 | NPTX1 | NQO2 | NR0B2 | NR1H2 | NR1H3 | NRXN1 | NTF3 | NTRK1 | NUMA1 | NUP93 | OGT | ONECUT1 | OPA1 | OPTN | OTUD4 | OXCT1 | P2RX1 | P2RX2 | P2RX4 | P4HB | PAFAH1B1 | PAG1 | PAK2 | PAK6 | PAM16 | PARK7 | PARP1 | PARP14 | PAWR | PAX8 | PCSK9 | PDCD10 | PDCD6 | PDCL | PDE4D | PDE6G | PDIA3 | PDK2 | PEBP1 | PELI1 | PER1 | PER2 | PFKL | PFKM | PHB | PHB2 | PIAS1 | PICK1 | PIGU | PIH1D1 | PIK3CA | PIK3CG | PIK3R1 | PIN1 | PIP4K2A | PLA2G1B | PLA2G2A | PLA2G6 | PLAUR | PLCG1 | PLEKHF1 | PLSCR1 | PML | PPARD | PPARG | PPARGC1A | PPEF1 | PPIF | PPM1A | PPM1B | PPP1CA | PPP1R2 | PPP2CA | PPP2CB | PPP2R1A | PPP2R1B | PPP3CC | PPP5C | PRCP | PRDX1 | PRDX2 | PRELID1 | PREPL | PRKAA1 | PRKAA2 | PRKAB2 | PRKACA | PRKAG1 | PRKAR2B | PRKCD | PRKCE | PRKCZ | PRKD1 | PRKD2 | PRMT2 | PRMT6 | PSAP | PSEN1 | PSMA1 | PSMA2 | PSMA3 | PSMA5 | PSMA6 | PSMB5 | PSMB6 | PSMB7 | PSMB8 | PSMC1 | PSMC2 | PSMC3 | PSMC4 | PSMC5 | PSMC6 | PSMD1 | PSMD10 | PSMD11 | PSMD12 | PSMD14 | PSMD2 | PSMD4 | PSMD9 | PSME2 | PSME3 | PTEN | PTGIR | PTK2 | PTN | PTPMT1 | PTPN1 | PTPN22 | PTPN23 | PTPN6 | PTPRA | PTPRO | PTPRR | PUM1 | PXDN | PXN | QARS | RAB11A | RAB29 | RAB3GAP1 | RAB5A | RAB7A | RAC1 | RAF1 | RANBP9 | RAP1A | RAP1B | RAPGEF4 | RARA | RASL11B | RB1 | RB1CC1 | RBBP4 | RBPJ | RBX1 | RC3H1 | RDH11 | RDX | RECK | REL | RELA | REST | RFC2 | RFC3 | RFC4 | RFC5 | RGN | RGS1 | RGS2 | RGS20 | RHO | RHOA | RHOJ | RHOT1 | RHOT2 | RHOU | RIC8B | RIPK1 | RIPK2 | RIPK3 | RNF126 | RNF183 | RNF19A | RNF31 | RPA2 | RPA3 | RPGRIP1L | RPL23 | RPS3 | RPTOR | RRM2B | RRS1 | RTN4 | RUNX1 | RUNX2 | RUVBL1 | RUVBL2 | S100A12 | S100A13 | S100A4 | S100A7 | S100A9 | S100B | SAG | SARM1 | SCEL | SCG2 | SCYL2 | SDCBP | SEC13 | SEMA7A | SEPT4 | SEPT5 | SERPINB3 | SERPINE2 | SFN | SFPQ | SH2B1 | SH3GL1 | SH3GL2 | SH3KBP1 | SHC1 | SHKBP1 | SIAH1 | SIPA1 | SIPA1L1 | SIRT1 | SIRT3 | SKI | SLAMF1 | SLC16A1 | SLC25A4 | SLC25A5 | SLC25A6 | SLC35F6 | SLC9A1 | SLC9A3R1 | SMAD2 | SMAD3 | SMAD4 | SMARCA4 | SMARCB1 | SMO | SMURF1 | SMURF2 | SNAI2 | SNCA | SNF8 | SNW1 | SNX3 | SNX6 | SOCS1 | SOCS2 | SOCS6 | SOD1 | SOD2 | SOX2 | SP100 | SPATA2 | SPERT | SPIN1 | SPRY2 | SPTBN1 | SQSTM1 | SRC | SRGAP2 | SRGAP3 | SRI | SSX2IP | STARD13 | STAT1 | STAT3 | STAU1 | STK11 | STK25 | STK3 | STK38 | STK40 | STMN1 | STRAP | STUB1 | STXBP1 | STYX | SULF2 | SUMO1 | SYDE1 | SYN1 | SYNJ2BP | SYP | SYVN1 | TAB1 | TAB2 | TAF12 | TAF15 | TANK | TAOK3 | TARDBP | TBL1X | TCF21 | TERF2IP | TFDP1 | TFG | TGFB1 | TGFBR3 | TGM2 | THBS1 | THEM4 | THRA | TIMP1 | TIMP2 | TLE1 | TLE3 | TLR3 | TMBIM6 | TMEM9B | TNF | TNFAIP3 | TNFRSF12A | TNFRSF1A | TNFSF12 | TNFSF14 | TNIP2 | TNKS | TNKS2 | TNN | TOP3A | TOR1A | TP53 | TP53RK | TP63 | TP73 | TRADD | TRAF1 | TRAF2 | TRAF3IP1 | TRAF6 | TRAP1 | TREX1 | TRIAP1 | TRIB2 | TRIB3 | TRIM25 | TRIM32 | TRIM33 | TRIP6 | TSG101 | TSKU | TSPYL2 | TTC5 | TUBB2B | TULP3 | TWIST1 | TXLNA | TXN | UBA5 | UBASH3A | UBB | UBC | UBE2B | UBE2I | UBE2K | UBE2N | UBE2O | UBE2V1 | UBQLN1 | UBQLN2 | UBR5 | UCHL1 | UFM1 | USP17L2 | USP20 | USP46 | USP47 | USP8 | VAPA | VAV1 | VCP | VDAC2 | VGF | VHL | VPS18 | VTN | WBP2 | WDFY1 | WDR35 | WNK1 | WNT16 | WNT2 | WWOX | WWP2 | XBP1 | XIAP | YAP1 | YWHAB | YWHAE | YWHAG | YWHAH | YWHAQ | YWHAZ | ZAP70 | ZBTB7A | ZBTB7B | ZC3HC1 | ZDHHC17 | ZFAND6 | ZIC1 | ZMPSTE24 | ZMYND8 | ZNF622 | GO:0019319 | 0.0046099720975373 | 38/8243 | 31/3250 | 1.21319317301696e-07 | 0.0011519269177796 | T | T | T | T | hexose biosynthetic process | AKR1B1 | ALDOA | ALDOC | CRY1 | ENO1 | ENO2 | ENO3 | FBP1 | FBP2 | GAPDH | GAPDHS | GOT1 | GOT2 | GPD1 | GPI | MDH1 | MDH2 | PC | PER2 | PGAM1 | PGAM2 | PGK1 | PGK2 | PGM1 | PPARGC1A | SLC25A1 | SLC25A10 | SLC25A11 | SLC25A13 | SORD | TPI1 | GO:0006979 | 0.0296008734683974 | 244/8243 | 136/3250 | 1.26048773513052e-07 | 0.00119683310450643 | T | T | T | T | response to oxidative stress | ABL1 | ADA | AIFM1 | AKT1 | ANXA1 | APEX1 | APOD | APOE | APP | ATOX1 | ATP13A2 | ATP2A2 | BCL2 | BTK | CAPN2 | CASP3 | CAT | CBX8 | CCS | CD36 | CDK2 | CRK | CRYAB | CYCS | DAPK1 | DHCR24 | DPEP1 | DUSP1 | EEF2 | EGFR | EGLN1 | EIF2S1 | ETFDH | ETS1 | EZH2 | FABP1 | FANCC | FKBP1B | FOS | FOXO1 | FYN | G6PD | GNAO1 | GPX1 | GPX4 | GPX7 | GSR | GSS | GSTP1 | HDAC2 | HDAC6 | HMOX1 | HNRNPD | HSF1 | HSPA1B | HTRA2 | IDH1 | JUN | LDHA | LRRK2 | MAPK1 | MAPK3 | MAPK7 | MAPK8 | MAPK9 | MAPT | MDM2 | MGMT | MGST1 | NAPRT | NDUFS2 | NDUFS8 | NFE2L2 | NME2 | NQO1 | OXSR1 | PARK7 | PARP1 | PCNA | PDCD10 | PDK2 | PEBP1 | PNKP | PPARGC1A | PPIF | PPP2CB | PRDX1 | PRDX2 | PRDX3 | PRDX4 | PRDX5 | PRDX6 | PRKAA1 | PRKAA2 | PRKCD | PRKD1 | PSEN1 | PSMB5 | PTPRK | PXDN | PXN | PYCR1 | PYCR2 | RELA | RIPK1 | RIPK3 | ROMO1 | RPS3 | RRM2B | S100A7 | SCARA3 | SDC1 | SIRT1 | SLC25A24 | SNCA | SOD1 | SOD2 | SP1 | SRC | SRXN1 | STAT1 | STAT6 | STAU1 | STK24 | STK25 | STK26 | TNFAIP3 | TOR1A | TPM1 | TRAF2 | TREX1 | TXN2 | TXNIP | TXNRD1 | XRCC1 | ZNF277 | GO:0046677 | 0.02426301103967 | 200/8243 | 115/3250 | 1.30969971916014e-07 | 0.00124355988334256 | T | T | T | T | response to antibiotic | ABAT | ABL1 | ACTR2 | ADA | ADCY6 | ADCYAP1 | AIFM1 | ANXA1 | APEX1 | BCL2 | BCL2L1 | BIRC2 | CAPN2 | CASP3 | CASP8 | CASP9 | CAT | CBL | CBX8 | CCR4 | CD27 | CFTR | CLDN18 | CRIP1 | CRK | CRYAB | CYP1A1 | DAXX | DMAP1 | DUSP1 | EEF2 | EHMT1 | EIF4EBP1 | ENDOG | ETS1 | EZH2 | FABP1 | FECH | FKBP1B | FOXO1 | FYN | G6PD | GNAI1 | GNAO1 | GOT2 | GPX1 | GSTP1 | HDAC2 | HDAC6 | HID1 | HMOX1 | HNRNPA1 | HNRNPD | HSF1 | HSP90AA1 | HSPA5 | HTR1B | HTR3A | ICAM1 | ITPR2 | JAK1 | JUN | KAT7 | KDM1A | KDM5B | LDHA | MAPK7 | MDM2 | MGMT | NFE2L2 | NQO1 | OPA1 | OXCT1 | PARK7 | PCNA | PDCD10 | PEBP1 | PPIF | PPP2CB | PRDX3 | PRKAA1 | PRKCD | PRKCE | PSMD14 | PTEN | RARA | RELA | RGS2 | RHOA | RIPK1 | RIPK3 | RPL10A | RPL23 | RPS3 | SDC1 | SETDB1 | SIRT1 | SLC9A1 | SOD1 | SPI1 | SRC | STAT1 | STAT3 | STAT6 | STK24 | STK25 | STK26 | TBXA2R | TNFAIP3 | TP53 | TUFM | TXNIP | TYMS | VCAM1 | ZNF277 | GO:0009152 | 0.00909862913987626 | 75/8243 | 52/3250 | 1.36222119935737e-07 | 0.00129342902878983 | T | T | T | T | purine ribonucleotide biosynthetic process | ACACA | ACAT1 | ACLY | ACOT7 | ACSL1 | ACSL4 | ACSL5 | ACSS2 | ADCY6 | ADK | ADSL | ADSS | AK1 | AK2 | AK4 | ALDOA | APRT | ATIC | CBR4 | CYC1 | DCAKD | DLAT | DLD | DNAJC30 | FASN | GART | GCDH | GMPS | HPRT1 | HSD17B8 | IMPDH2 | LDHC | MPC2 | NME1 | NME2 | NME4 | NPR1 | PAICS | PAPSS1 | PAPSS2 | PDHA1 | PDHA2 | PDHB | PDHX | PFAS | PPAT | PPT1 | SLC25A1 | SLC25A13 | STOML2 | TECR | TGFB1 | GO:0009719 | 0.0803105665413078 | 662/8243 | 324/3250 | 1.41508249055868e-07 | 0.00134362082478547 | T | T | T | T | response to endogenous stimulus | ABCG1 | ABL1 | ACACA | ACAT1 | ACTA1 | ACTB | ACTR2 | ADCY6 | ADCYAP1 | ADRB2 | ADTRP | AGER | AGL | AIFM1 | AKAP8 | AKAP9 | AKR1B1 | AKR1C2 | AKT1 | AKT2 | ANXA1 | ANXA2 | ANXA5 | APEX1 | APLP1 | APP | APRT | AR | ARNT2 | ARPC1B | ASCL1 | ASNS | ASS1 | ATP1A1 | BACE1 | BAG4 | BCL11A | BCL2 | BCL2L1 | BMPR1A | BRAF | BRCA1 | BSG | CACNA1A | CALM1 | CALM3 | CALR | CAPN2 | CASP3 | CASP7 | CASP8 | CASP9 | CAT | CAV1 | CBL | CCL21 | CD36 | CD81 | CDC5L | CDH1 | CDK4 | CDK5 | CDKN1A | CFTR | CHEK2 | COL2A1 | COL4A1 | COL4A2 | COL6A1 | CREB1 | CRK | CRKL | CRY1 | CRYAB | CSN1S1 | CSNK2B | CTNNB1 | CTSL | CTSV | CXCL8 | CYC1 | CYP1A2 | DDIT4 | DENND4C | DUSP1 | DUSP22 | E2F1 | EEF1A1 | EEF2 | EGFR | EIF2B1 | EIF2B3 | EIF2B5 | EIF4A3 | EIF4E | EIF4EBP1 | EIF6 | ENDOG | EPHA4 | EPRS | ERBB2 | ERRFI1 | ESR1 | ESRRA | ETS1 | EZH2 | EZR | FABP3 | FBXO32 | FECH | FGB | FHL2 | FIS1 | FOLR1 | FOS | FOXA1 | FOXO1 | FYN | GATA1 | GGT1 | GLP1R | GNAI1 | GNB1 | GOT1 | GOT2 | GPD1 | GPI | GPX4 | GSK3A | GSK3B | GSTP1 | H2AFZ | HADH | HCN1 | HCN2 | HCN3 | HDAC2 | HDAC4 | HDAC5 | HDAC9 | HMGB2 | HNRNPD | HNRNPU | HSF1 | HSP90B1 | HSPA5 | HTR1B | ICAM1 | IDH1 | IGF1R | IGFBP7 | INSIG2 | INSR | IPO5 | IQGAP1 | IREB2 | ITGA4 | ITGB1BP1 | ITPR2 | JUN | JUNB | JUND | JUP | KAT5 | KDM1A | KDM5B | KLF5 | KLHL22 | KRAS | LARS | LGMN | LIMS1 | LMO2 | LPIN3 | LRP6 | LRRK2 | MAPK1 | MAPK3 | MAPK7 | MAPT | MAS1 | MBD3 | MCM7 | MDM2 | ME1 | MED1 | MSN | MTAP | MYD88 | MYO5A | NAMPT | NCOA1 | NCOA2 | NCOA3 | NFE2L2 | NKX2-1 | NME1 | NME2 | NOD2 | NOS2 | NOS3 | NQO1 | NR1H3 | NR3C1 | NR4A1 | NTRK1 | OGT | OPA1 | OTC | OXCT1 | P2RX4 | PARP1 | PAX8 | PCNA | PCSK9 | PDE4D | PEBP1 | PIAS3 | PIK3CG | PIK3R1 | PKM | PLA2G1B | PLCG1 | PPARG | PPARGC1A | PPAT | PPM1A | PRKAA1 | PRKAA2 | PRKACA | PRKAR1A | PRKAR2B | PRKCD | PRKCE | PRKCZ | PRKDC | PSEN1 | PTEN | PTN | PTPN22 | QDPR | RAB10 | RAB31 | RAB35 | RAD51 | RANGAP1 | RAP1A | RAP1B | RARA | RBBP4 | RDX | RELA | REST | RHOA | RIPK2 | RPL23 | RPTOR | RUNX2 | RUVBL2 | S100B | SDC1 | SH3GL2 | SHMT1 | SHMT2 | SIN3A | SIRT1 | SLC9A1 | SMAD3 | SMAD4 | SMAD9 | SMC1A | SNAI2 | SNCA | SNX6 | SOCS1 | SOCS2 | SOD1 | SORD | SORT1 | SP1 | SPINT2 | SRC | SRSF5 | STAT1 | STAT3 | STXBP1 | SULT1A3 | TBC1D4 | TBXA2R | TGFB1 | TGFBR3 | THBS1 | TIMP1 | TIMP2 | TNF | TOMM20 | TP53 | TPR | TREX1 | TRIB3 | TRIM41 | TRIM63 | TXN2 | TXNIP | TYMS | UBE2L3 | UBE3A | USP8 | VCAM1 | VGF | VIM | VSTM2A | WBP2 | WDTC1 | WNT2 | WT1 | WWOX | XBP1 | YAP1 | YES1 | YWHAG | YY1 | ZBTB7B | ZMPSTE24 | GO:0031396 | 0.0188038335557443 | 155/8243 | 93/3250 | 1.42025471543721e-07 | 0.00134853185230764 | T | T | T | T | regulation of protein ubiquitination | ABL1 | ADRB2 | AIMP2 | AKT1 | ARRB1 | ARRB2 | ATG5 | AXIN1 | BAG2 | BAG5 | BCL10 | BIRC2 | BIRC3 | BMI1 | BRCA1 | CAV1 | CDC14B | CDC20 | CDK5 | CDK9 | CHP1 | COMMD1 | CRY1 | CUL3 | DAXX | DCUN1D1 | DERL1 | DNAJA1 | DNAJB2 | FANCI | FANCM | FKBP1A | FYN | FZR1 | GLMN | GOLGA2 | GPS2 | GSK3A | HSP90AA1 | HSP90AB1 | HSPA1B | HSPA5 | HSPBP1 | ISG15 | KDM1A | LRRK2 | MAD2L1 | MAPK9 | MTA1 | NOD2 | OGT | OTUB1 | PARK7 | PAXIP1 | PDCD6 | PEF1 | PELI1 | PER2 | PIN1 | PINX1 | PLK1 | PRKCE | PRMT3 | PSEN1 | PSMD10 | PTEN | PTPN22 | RAB1A | RIPK2 | RPL23 | RPS2 | RPS3 | SEPT4 | SPRTN | STUB1 | TNFAIP3 | TRAF6 | TRIB3 | TRIP12 | U2AF2 | UBB | UBE2C | UBE2D1 | UBE2L3 | UBE2N | UBE2S | UBE3A | UBQLN1 | UBR5 | UBXN1 | USP4 | WDR48 | XIAP | GO:0061136 | 0.0163775324517773 | 135/8243 | 83/3250 | 1.49805654401949e-07 | 0.00142240468854651 | T | T | T | T | regulation of proteasomal protein catabolic process | AKT1 | APOE | ARAF | BAG2 | BAG5 | BAG6 | BTRC | CAV1 | CBFA2T3 | CCAR2 | CEBPA | CLU | COMMD1 | CSNK1A1 | CSNK1E | DAB2 | DDA1 | DDRGK1 | DNAJB2 | DVL1 | EDEM1 | FAM192A | GLMN | GNA12 | GPX1 | GSK3A | GSK3B | HSP90AB1 | HSP90AB3P | HSP90AB4P | HSPA1B | HSPBP1 | KEAP1 | LAMP3 | LRRK2 | MAPK9 | MDM2 | NFE2L2 | NUPR1 | OGT | PARK7 | PIAS1 | PLK1 | PRKACA | PSEN1 | PSMC1 | PSMC2 | PSMC3 | PSMC4 | PSMC5 | PSMC6 | PSMD10 | PSMD14 | PSME2 | PSME3 | RAD23A | RAD23B | RBX1 | RNF185 | RNF19A | SDCBP | SGTA | SMARCC1 | STUB1 | STYX | SUMO1 | SUMO2 | TMTC3 | TRIB2 | TRIB3 | UBB | UBE2J1 | UBE2K | UBQLN1 | UBQLN2 | UBQLN4 | UBXN1 | UCHL5 | USP14 | USP19 | USP5 | USP7 | VCP | GO:1901701 | 0.0638117190343322 | 526/8243 | 264/3250 | 1.51900177580366e-07 | 0.00144229218612557 | T | T | T | T | cellular response to oxygen-containing compound | ABL1 | ABL2 | ACACA | ACTB | ACTR2 | ADCY6 | ADRB2 | AGER | AICDA | AIFM1 | AKAP8 | AKAP9 | AKR1B1 | AKR1C2 | AKT1 | AKT2 | ANXA1 | ANXA5 | APEX1 | APLP1 | APP | APRT | AR | ASS1 | ATM | BACE1 | BCL11A | BCL2L1 | BRCA1 | BTK | CACNA1A | CAPN2 | CASP9 | CAV1 | CBX8 | CCNB1 | CCR5 | CD36 | CD40 | CDC5L | CDC73 | CDH1 | CDK19 | CDK2 | CDK4 | CDK5 | CEBPE | CFTR | CHEK2 | COL4A1 | COL6A1 | CPT1A | CREB1 | CRK | CTNNB1 | CXCL11 | CXCL5 | CXCL6 | CXCL8 | DAPK1 | DDIT4 | DENND4C | DHX36 | DNM1L | DPEP1 | E2F1 | EGFR | EIF4E | EIF4EBP1 | ENDOG | EPHA4 | EPRS | ERRFI1 | ESR1 | ETS1 | EZH2 | EZR | FABP1 | FBXO32 | FECH | FIS1 | FOLR1 | FOS | FOXO1 | FYN | GLP1R | GNAI1 | GNB1 | GOT1 | GPD1 | GRAMD1A | GSK3A | GSK3B | GSTP1 | H2AFZ | HAVCR2 | HCN1 | HCN2 | HCN3 | HDAC2 | HDAC5 | HDAC6 | HDAC9 | HMGB1 | HMGB2 | HNRNPD | HSF1 | HSP90B1 | HSPA5 | HTRA2 | ICAM1 | IGF1R | INSIG1 | INSR | IPO5 | IREB2 | ITGA4 | ITPR2 | JUN | JUP | KAT5 | KDM1A | KLF5 | KLHL22 | LARS | LGALS1 | LGMN | LPAR1 | LPIN3 | LRP6 | LRRK2 | MAP2K4 | MAP2K6 | MAP4K1 | MAPK1 | MAPK14 | MAPK3 | MAPK7 | MAPK8 | MAPK9 | MAPT | MDM2 | MGST1 | MSN | MYO5A | NAMPT | NCOA3 | NDUFA13 | NEUROD1 | NFE2L2 | NME1 | NME2 | NOD2 | NOS2 | NQO1 | NR1H2 | NR1H3 | NR3C1 | NR4A1 | NTRK1 | OPA1 | P2RX4 | PARK7 | PARP1 | PCNA | PCSK9 | PDE4D | PDK2 | PHB2 | PIK3CA | PIK3CG | PIK3R1 | PKM | PLA2G1B | PPARD | PPARG | PPARGC1A | PPAT | PPIF | PRDX3 | PRDX5 | PRKAA1 | PRKAA2 | PRKACA | PRKAR1A | PRKAR2B | PRKCD | PRKCE | PRKCZ | PRKD1 | PRKDC | PRPF8 | PSEN1 | PTEN | PTN | PTPN22 | PTPRK | PXN | RAB10 | RAB31 | RAD51 | RANGAP1 | RAP1A | RAP1B | RARA | RARG | RELA | RHOA | RIPK1 | RIPK2 | RIPK3 | ROMO1 | RPL23 | RPS3 | RPTOR | RUVBL2 | SHMT1 | SHMT2 | SIN3A | SIPA1 | SIRT1 | SLC2A5 | SLC9A1 | SMO | SNCA | SNW1 | SNX6 | SOCS1 | SOD1 | SP1 | SPI1 | SRC | SRSF5 | STAT1 | STAT6 | SULT1A3 | TBC1D4 | TBXA2R | TGFB1 | TNF | TNFAIP3 | TNIP2 | TP53 | TPM1 | TRAF2 | TRAF6 | TREX1 | TRIB3 | TRIM41 | TWF2 | USP8 | VCAM1 | VCP | VIM | WDR35 | WDTC1 | WNT2 | WT1 | XBP1 | XRCC5 | YAP1 | YES1 | YWHAG | ZNF277 | GO:0044267 | 0.255732136358122 | 2108/8243 | 931/3250 | 1.57384146091455e-07 | 0.00149436246713836 | T | T | T | T | cellular protein metabolic process | ABHD17A | ABL1 | ABL2 | ACP1 | ACTB | ACTL6A | ACTN2 | ADCK2 | ADH5 | ADORA2A | ADPRH | ADRB2 | ADRM1 | AFG3L2 | AGO2 | AHSG | AKT1 | AKT2 | AKT3 | ALB | AMFR | ANAPC4 | ANXA1 | APBB1 | APC | APCS | APOA1 | APOE | APP | AR | ARAF | ARF4 | ARID4B | ARRB1 | ARRB2 | ASB12 | ASB15 | ASB16 | ASB6 | ASB9 | ASGR1 | ASGR2 | ATF2 | ATG5 | ATM | ATP13A2 | AURKB | B3GAT3 | B3GNT3 | B4GALT3 | BABAM1 | BACE1 | BAG6 | BCL10 | BCL11A | BCL2 | BCOR | BIRC2 | BIRC3 | BIRC5 | BIRC7 | BIRC8 | BMI1 | BMPR1A | BRAF | BRCA1 | BRD4 | BTK | BTRC | C4A | CAB39 | CALM1 | CALU | CAMK1D | CAMK2A | CAMK2B | CAMKV | CAND1 | CARM1 | CBFB | CBL | CBLB | CBLC | CBX4 | CBX8 | CCDC22 | CCDC8 | CCNB1 | CCNT1 | CCP110 | CCR5 | CDC14B | CDC16 | CDC20 | CDC25A | CDC25B | CDC25C | CDC27 | CDC34 | CDC42 | CDC73 | CDK15 | CDK16 | CDK19 | CDK2 | CDK4 | CDK5 | CDK5R1 | CDK8 | CDK9 | CDKL3 | CEL | CFL1 | CFTR | CHEK2 | CHGB | CHRM3 | CHST4 | CKAP4 | CLK1 | CLTC | COG3 | COMMD1 | COPS2 | COPS5 | COPS6 | CPNE3 | CRBN | CREB1 | CREBBP | CRKL | CRY1 | CRY2 | CRYAB | CSNK1A1 | CSNK1E | CSNK2A2 | CSNK2B | CTBP1 | CTDP1 | CTH | CTNNB1 | CUL1 | CUL2 | CUL3 | CUL4B | CUL5 | CUL7 | CYCS | CYLD | DAD1 | DAPK1 | DAPP1 | DARS | DBI | DCAF12 | DCAF7 | DCAF8 | DCLK1 | DCN | DCUN1D1 | DDA1 | DDB1 | DDB2 | DDOST | DDRGK1 | DERL1 | DGUOK | DLD | DLG2 | DLG3 | DLG4 | DLST | DMAP1 | DPF2 | DPM1 | DRG2 | DTX1 | DTX2 | DUSP1 | DUSP12 | DUSP13 | DUSP14 | DUSP16 | DUSP19 | DUSP22 | DUSP7 | DYRK2 | DZIP3 | EDEM1 | EED | EEF1A1 | EEF1A1P5 | EEF1A2 | EEF2KMT | EFEMP1 | EGFR | EGLN1 | EGLN3 | EHHADH | EHMT1 | EHMT2 | EIF2AK3 | EIF2AK4 | EIF2S1 | EIF4H | EP300 | EPHA4 | EPHA6 | EPHB6 | ERBB2 | ERBB3 | ERG | ESR1 | EVA1A | EXTL2 | EYA2 | EYA4 | EZH2 | F5 | FAM20B | FAM98A | FARSB | FBXL12 | FBXO11 | FBXO25 | FBXO32 | FBXO6 | FBXO7 | FBXW11 | FBXW5 | FGF2 | FGFR1 | FGFR3 | FKBP14 | FKBP1A | FKBP1B | FKBP2 | FKBP5 | FKBP7 | FKBP8 | FN1 | FOLH1 | FOXK1 | FOXK2 | FOXO1 | FYN | FZR1 | GAK | GALNT7 | GAPDH | GEMIN5 | GFPT1 | GFPT2 | GGA1 | GGT1 | GLRX | GLRX5 | GLUL | GOLGA2 | GPS2 | GPX1 | GRB2 | GRK1 | GRK5 | GSK3A | GSK3B | GSPT1 | GSPT2 | H3F3A | HARS | HBEGF | HCCS | HDAC1 | HDAC11 | HDAC2 | HDAC3 | HDAC4 | HDAC5 | HDAC6 | HDAC7 | HDAC9 | HECW1 | HECW2 | HERC1 | HERC2 | HEXB | HGS | HIF1A | HIF1AN | HIPK4 | HIST1H2BA | HIST1H3A | HIST1H4A | HIST2H3A | HLCS | HMBS | HRAS | HSF1 | HSF4 | HSP90B1 | HSPA14 | HSPG2 | HTRA2 | HUWE1 | ICK | IGF1R | IGFBP7 | IKBKB | IKBKE | IKBKG | IL3RA | ILF3 | ILK | ING5 | INO80B | INO80C | INSR | IRF4 | ISG15 | ITCH | ITGAV | ITK | JAK1 | JAK3 | JMJD6 | JOSD2 | KAT5 | KAT7 | KAT8 | KBTBD7 | KCMF1 | KDM1A | KDM2A | KDM3B | KDM4D | KDM5B | KDM5C | KDM8 | KDR | KEAP1 | KLHL20 | KLHL22 | KMT2A | KMT2B | KMT2C | KRAS | KSR1 | L2HGDH | LAMB1 | LARP4 | LAT | LATS1 | LCMT2 | LEF1 | LGALS1 | LGMN | LMO7 | LNX1 | LOXL2 | LRRC7 | LRRK1 | LRRK2 | LRSAM1 | LTBP1 | LYPLA1 | LYPLA2 | LZTR1 | MAN2B1 | MAP2K1 | MAP2K3 | MAP2K4 | MAP2K6 | MAP2K7 | MAP3K1 | MAP3K13 | MAP3K14 | MAP3K2 | MAP3K3 | MAP3K4 | MAP4K1 | MAPK1 | MAPK14 | MAPK15 | MAPK3 | MAPK4 | MAPK6 | MAPK7 | MAPK8 | MAPK9 | MAPT | MARCH5 | MAST1 | MAST3 | MATK | MBD3 | MBP | MCFD2 | MDH2 | MDM2 | MDM4 | MECP2 | MED1 | MED12 | MED20 | MED21 | MED31 | MELK | METAP2 | MIB1 | MKNK2 | MORF4L1 | MORF4L2 | MPI | MPPE1 | MRGBP | MRPL28 | MRPL43 | MRPL9 | MRRF | MTA1 | MTA3 | MTMR14 | MTMR7 | MYC | MYH6 | NAE1 | NCK1 | NCOA1 | NCOA3 | NDUFAF5 | NDUFAF7 | NEDD4 | NEDD8 | NEK1 | NEK7 | NEURL4 | NFE2L2 | NFKBIA | NFS1 | NHLRC3 | NLK | NME2 | NOD2 | NOS2 | NOSIP | NPEPPS | NPR1 | NTMT1 | NTRK1 | NUCB1 | NUP107 | NUP153 | NUP160 | NUP205 | NUP214 | NUP85 | NUP88 | NUP93 | NUP98 | NUPR1 | OBSL1 | OGDH | OGFOD1 | OGT | OTUB1 | OTUB2 | OTUD4 | OTUD5 | OXSR1 | P4HA1 | P4HA2 | P4HB | PABPC4 | PADI4 | PAK2 | PAK6 | PARK7 | PARP1 | PARP12 | PARP14 | PARP6 | PAXIP1 | PCGF1 | PCGF5 | PCGF6 | PCMT1 | PCMTD1 | PCMTD2 | PCNA | PCSK9 | PDF | PDIA6 | PDK2 | PEBP1 | PELI1 | PER1 | PER2 | PEX5 | PGAM5 | PGM3 | PGP | PHB | PHF10 | PHKA1 | PIAS1 | PIAS3 | PICK1 | PIGU | PIK3CA | PIK3CG | PIK3R1 | PIM1 | PIN1 | PJA1 | PKN2 | PLK1 | PLOD2 | PML | PMPCA | PMPCB | POLE3 | POM121 | PPA2 | PPEF1 | PPIA | PPIB | PPID | PPIE | PPIF | PPIL3 | PPM1A | PPM1B | PPM1D | PPM1H | PPM1K | PPME1 | PPP1CA | PPP1CB | PPP1CC | PPP1R11 | PPP1R12A | PPP1R3B | PPP1R3C | PPP2CA | PPP2CB | PPP2R1A | PPP2R1B | PPP2R2A | PPP2R2D | PPP2R5D | PPP3CC | PPP4R2 | PPP5C | PPP6C | PPT1 | PPTC7 | PRDX3 | PRDX4 | PRKAA1 | PRKAA2 | PRKAB2 | PRKACA | PRKAG1 | PRKCD | PRKCE | PRKCSH | PRKCZ | PRKD1 | PRKD2 | PRKD3 | PRKDC | PRMT2 | PRMT3 | PRMT6 | PRPF19 | PRSS23 | PSEN1 | PSEN2 | PSMA1 | PSMA2 | PSMA3 | PSMA5 | PSMA6 | PSMB5 | PSMB6 | PSMB7 | PSMB8 | PSMC1 | PSMC2 | PSMC3 | PSMC4 | PSMC5 | PSMC6 | PSMD1 | PSMD10 | PSMD11 | PSMD12 | PSMD14 | PSMD2 | PSMD4 | PSMD9 | PSME2 | PSME3 | PTEN | PTK2 | PTPMT1 | PTPN1 | PTPN14 | PTPN21 | PTPN22 | PTPN23 | PTPN4 | PTPN6 | PTPRA | PTPRD | PTPRF | PTPRK | PTPRO | PTPRR | QSOX1 | RAB11A | RAB11B | RAB1A | RAB1B | RAB2A | RAB5A | RAB7A | RAD23A | RAD23B | RAE1 | RAF1 | RANBP9 | RANGAP1 | RARA | RARS2 | RBBP6 | RBX1 | RC3H1 | REST | RHBDD1 | RHO | RHOA | RHOT1 | RING1 | RIOK1 | RIPK1 | RIPK2 | RIPK3 | RIPK4 | RMND1 | RNF123 | RNF126 | RNF139 | RNF181 | RNF183 | RNF185 | RNF19A | RNF2 | RNF25 | RNF31 | RNF4 | RNF8 | RPL10 | RPL10A | RPL12 | RPL18A | RPL22 | RPL23 | RPL23A | RPL24 | RPL30 | RPL36AL | RPL37A | RPL38 | RPL6 | RPL7 | RPL7A | RPL9 | RPLP0 | RPLP0P6 | RPLP1 | RPLP2 | RPN1 | RPN2 | RPS12 | RPS13 | RPS14 | RPS15A | RPS19 | RPS2 | RPS20 | RPS24 | RPS29 | RPS3 | RPS4X | RPS6KA1 | RPS6KA2 | RPS6KB2 | RPS8 | RTF1 | RUVBL1 | RUVBL2 | S100A9 | SAE1 | SARS | SCG2 | SCG3 | SCYL2 | SEL1L | SENP3 | SENP6 | SENP7 | SENP8 | SEPHS1 | SETDB1 | SFPQ | SHC1 | SHMT2 | SIAH1 | SIN3A | SIRT1 | SIRT3 | SIRT5 | SIRT6 | SIRT7 | SMAD2 | SMAD3 | SMAD4 | SMAD5 | SMURF1 | SMURF2 | SNCA | SNRPB | SNRPD3 | SNX3 | SOCS1 | SOCS2 | SOCS6 | SPATA2 | SPI1 | SPOP | SPRR2A | SPSB3 | SPTAN1 | SPTBN1 | SPTBN2 | SQSTM1 | SRC | SRPK1 | SRPK2 | ST3GAL1 | ST3GAL6 | ST6GALNAC5 | ST8SIA4 | STK11 | STK24 | STK25 | STK26 | STK3 | STK38 | STK40 | STOML2 | STUB1 | STYX | SULT1A3 | SUMO1 | SUMO1P1 | SUMO2 | SUMO3 | SUZ12 | SYVN1 | TAB1 | TAB2 | TADA2B | TAF12 | TAOK3 | TARS | TBL1X | TBRG4 | TCIRG1 | TESK2 | TGFB1 | TGFBR3 | TGM2 | TGOLN2 | THBS1 | THOP1 | TIMM50 | TIMP1 | TLR3 | TMEM132A | TMEM189-UBE2V1 | TMEM258 | TMTC3 | TNF | TNFAIP3 | TNIP2 | TNK2 | TNKS | TNKS2 | TOMM20 | TOP1 | TOR1A | TP53 | TP53RK | TPR | TPST2 | TPTE | TRAF2 | TRAF3 | TRAF6 | TRIB2 | TRIB3 | TRIM21 | TRIM23 | TRIM25 | TRIM28 | TRIM32 | TRIM33 | TRIM41 | TRIM47 | TRIM63 | TRIM69 | TRIM7 | TRIM9 | TRIP12 | TSG101 | TSPAN17 | TTLL1 | TWF1 | UBA1 | UBA2 | UBA3 | UBA5 | UBA6 | UBAC1 | UBB | UBC | UBE2A | UBE2B | UBE2C | UBE2D1 | UBE2D2 | UBE2D3 | UBE2H | UBE2I | UBE2J1 | UBE2K | UBE2L3 | UBE2L6 | UBE2M | UBE2N | UBE2O | UBE2Q2 | UBE2R2 | UBE2S | UBE2V1 | UBE2W | UBE3A | UBE3B | UBL4A | UBR4 | UBR5 | UBXN7 | UCHL1 | UCHL3 | UCHL5 | UFC1 | UFM1 | UGGT1 | ULK1 | UNKL | UQCRC2 | USP11 | USP14 | USP15 | USP16 | USP17L2 | USP19 | USP2 | USP20 | USP21 | USP26 | USP28 | USP39 | USP4 | USP42 | USP46 | USP47 | USP5 | USP50 | USP53 | USP7 | USP8 | USP9X | USP9Y | USPL1 | VCAN | VCP | VCPIP1 | VGF | VHL | VKORC1 | WARS | WDR45B | WDR48 | WDR82 | WDTC1 | WDYHV1 | WIPI1 | WIPI2 | WNK1 | WNK4 | WSB1 | WWP2 | XIAP | YEATS2 | YES1 | YME1L1 | YWHAB | YWHAE | YY1 | ZAP70 | ZBTB16 | ZC3HC1 | ZDHHC17 | ZDHHC23 | ZMPSTE24 | ZNF598 | ZNRF4 | GO:0006511 | 0.0417323789882324 | 344/8243 | 182/3250 | 1.63264075801222e-07 | 0.0015501923997326 | T | T | T | T | ubiquitin-dependent protein catabolic process | ADRM1 | AMFR | ANAPC4 | ARMC8 | ARRB1 | ARRB2 | AUP1 | AURKB | BAG6 | BIRC2 | BTRC | CBL | CBLC | CCNB1 | CDC16 | CDC20 | CDC27 | CDC34 | CDK2 | CRBN | CTNNB1 | CUL1 | CUL2 | CUL3 | CUL4B | CUL5 | CUL7 | CYLD | DDB1 | DERL1 | DNAJB2 | DNAJC10 | EDEM1 | ERLIN2 | FAF1 | FAF2 | FBXL12 | FBXO11 | FBXO6 | FBXO7 | FBXW11 | FBXW5 | FZR1 | GSK3A | HDAC6 | HECW1 | HECW2 | HERC2 | HSP90B1 | HSPA5 | HUWE1 | ITCH | KAT5 | KCTD17 | KCTD2 | KLHL20 | KLHL22 | LNX1 | MAD2L1 | MDM2 | MTA1 | NEDD4 | NEDD8 | NFE2L2 | NHLRC3 | NPLOC4 | NSFL1C | PCBP2 | PELI1 | PLK1 | PML | PPP1R11 | PPP2CB | PSMA1 | PSMA2 | PSMA3 | PSMA5 | PSMA6 | PSMB5 | PSMB6 | PSMB7 | PSMB8 | PSMC1 | PSMC2 | PSMC3 | PSMC4 | PSMC5 | PSMC6 | PSMD1 | PSMD10 | PSMD11 | PSMD12 | PSMD14 | PSMD2 | PSMD4 | PSMD9 | PSME2 | PSME3 | PTPN23 | PTTG1 | RAD23A | RAD23B | RBBP6 | RBX1 | RNF126 | RNF185 | RNF19A | RNF4 | RNF8 | SEL1L | SGTA | SGTB | SIAH1 | SIRT1 | SMURF1 | SMURF2 | SPOP | SPSB3 | SQSTM1 | STUB1 | SYVN1 | TBL1X | TOLLIP | TRIM25 | TRIM32 | TRIM9 | TRIP12 | TSG101 | UBA6 | UBB | UBC | UBE2A | UBE2B | UBE2C | UBE2D1 | UBE2D2 | UBE2D3 | UBE2H | UBE2I | UBE2J1 | UBE2K | UBE2L3 | UBE2L6 | UBE2R2 | UBE2S | UBE2W | UBE3A | UBE3B | UBQLN1 | UBQLN2 | UBQLN4 | UBR4 | UBXN1 | UCHL1 | UCHL3 | UCHL5 | USP11 | USP14 | USP15 | USP16 | USP17L2 | USP19 | USP2 | USP20 | USP21 | USP26 | USP28 | USP4 | USP42 | USP46 | USP47 | USP5 | USP50 | USP7 | USP8 | USP9Y | VCP | VPS37D | VPS4B | WWP2 | XBP1 | ZNRF4 | GO:0043648 | 0.00424602693194225 | 35/8243 | 29/3250 | 1.64500091418681e-07 | 0.00156192836802038 | T | T | T | T | dicarboxylic acid metabolic process | ACLY | ADSS | ALDH5A1 | ASL | ASS1 | DLD | DLST | FH | FPGS | GGT1 | GLUD2 | GLUL | GOT1 | GOT2 | GPT2 | GRHPR | HAAO | IDH1 | IDH2 | L2HGDH | MDH1 | MDH2 | ME1 | NIT2 | OGDH | SDHA | SDHB | SUCLA2 | SUCLG2 | GO:0006164 | 0.00958388936066966 | 79/8243 | 54/3250 | 1.65324918554561e-07 | 0.00156976010167555 | T | T | T | T | purine nucleotide biosynthetic process | ACACA | ACAT1 | ACLY | ACOT7 | ACSL1 | ACSL4 | ACSL5 | ACSS2 | ADA | ADCY6 | ADK | ADSL | ADSS | AK1 | AK2 | AK4 | ALDOA | APRT | ATIC | CBR4 | CYC1 | DCAKD | DLAT | DLD | DNAJC30 | FASN | GART | GCDH | GMPS | HPRT1 | HSD17B8 | IMPDH2 | LDHC | MPC2 | MTHFD1 | NME1 | NME2 | NME4 | NPR1 | PAICS | PAPSS1 | PAPSS2 | PDHA1 | PDHA2 | PDHB | PDHX | PFAS | PPAT | PPT1 | SLC25A1 | SLC25A13 | STOML2 | TECR | TGFB1 | GO:0006508 | 0.0879534150188038 | 725/8243 | 351/3250 | 1.70256638065383e-07 | 0.00161658677843081 | T | T | T | T | proteolysis | ACE2 | ACTB | ACTL6A | ACY1 | ADAMTS4 | ADRB2 | ADRM1 | AFG3L2 | AMFR | ANAPC4 | APC | APEH | AR | ARMC8 | ARRB1 | ARRB2 | ATG4C | AUP1 | AURKB | AZU1 | BABAM1 | BACE1 | BAG6 | BIRC2 | BIRC3 | BRCA1 | BTRC | C1R | CAPN1 | CAPN2 | CAPNS1 | CASP10 | CASP2 | CASP3 | CASP7 | CASP8 | CASP9 | CBL | CBLC | CCNB1 | CCP110 | CDC16 | CDC20 | CDC25A | CDC27 | CDC34 | CDK2 | CES1 | CFTR | CLPX | CNDP1 | CNDP2 | COPS2 | COPS5 | COPS6 | CPVL | CRBN | CTNNB1 | CTSA | CTSC | CTSD | CTSL | CTSV | CUL1 | CUL2 | CUL3 | CUL4B | CUL5 | CUL7 | CYLD | DDB1 | DDB2 | DDI1 | DERL1 | DLD | DNAJB2 | DNAJC10 | DNPEP | DPEP1 | DPP9 | EDEM1 | EIF3H | EP300 | ERLIN2 | ESR1 | F9 | FADD | FAF1 | FAF2 | FBXL12 | FBXO11 | FBXO6 | FBXO7 | FBXW11 | FBXW5 | FGB | FGL2 | FOLH1 | FOXK1 | FOXK2 | FZR1 | GGT1 | GGTLC1 | GSK3A | GZMA | GZMK | HDAC6 | HECW1 | HECW2 | HERC2 | HGS | HIF1A | HIST1H2BA | HSP90B1 | HSPA5 | HTRA1 | HTRA2 | HUWE1 | IDE | IFI16 | IKBKG | INO80B | INO80C | ISG15 | ITCH | JOSD2 | KAT5 | KCTD17 | KCTD2 | KDM8 | KEAP1 | KLHL20 | KLHL22 | LAP3 | LGMN | LNX1 | MAD2L1 | MASP1 | MASP2 | MDM2 | MDM4 | METAP2 | MME | MMP26 | MTA1 | MYC | NEDD4 | NEDD8 | NFE2L2 | NFKBIA | NHLRC3 | NPEPL1 | NPEPPS | NPLOC4 | NSFL1C | NUP98 | OGT | OTUB1 | OTUB2 | OTUD4 | OTUD5 | PAPLN | PARK7 | PARP1 | PCBP2 | PCSK9 | PDCD6IP | PDIA3 | PELI1 | PEPD | PGK1 | PLK1 | PML | PMPCA | PMPCB | PPP1R11 | PPP2CB | PRCP | PREP | PREPL | PROZ | PRPF19 | PRSS16 | PRSS23 | PSEN1 | PSEN2 | PSMA1 | PSMA2 | PSMA3 | PSMA5 | PSMA6 | PSMB5 | PSMB6 | PSMB7 | PSMB8 | PSMC1 | PSMC2 | PSMC3 | PSMC4 | PSMC5 | PSMC6 | PSMD1 | PSMD10 | PSMD11 | PSMD12 | PSMD14 | PSMD2 | PSMD4 | PSMD9 | PSME2 | PSME3 | PTEN | PTPN23 | PTTG1 | RAD23A | RAD23B | RBBP6 | RBMX | RBX1 | RELA | RHBDD1 | RHOA | RHOT1 | RIPK1 | RIPK2 | RNF123 | RNF126 | RNF139 | RNF185 | RNF19A | RNF4 | RNF8 | RNPEP | RUVBL1 | SEL1L | SENP3 | SENP6 | SENP7 | SENP8 | SGTA | SGTB | SHMT2 | SIAH1 | SIRT1 | SMAD2 | SMAD3 | SMAD4 | SMURF1 | SMURF2 | SNX3 | SOCS6 | SPATA2 | SPOP | SPSB3 | SQSTM1 | STOML2 | STUB1 | SYVN1 | TAB1 | TADA2B | TANK | TBL1X | TGFB1 | THOP1 | TNFAIP3 | TNIP2 | TOLLIP | TOMM20 | TOR1A | TP53 | TPSAB1 | TPSB2 | TRAF2 | TRAF3 | TRAF6 | TRIM25 | TRIM32 | TRIM9 | TRIP12 | TSG101 | UBA3 | UBA6 | UBB | UBC | UBE2A | UBE2B | UBE2C | UBE2D1 | UBE2D2 | UBE2D3 | UBE2H | UBE2I | UBE2J1 | UBE2K | UBE2L3 | UBE2L6 | UBE2N | UBE2R2 | UBE2S | UBE2W | UBE3A | UBE3B | UBQLN1 | UBQLN2 | UBQLN4 | UBR4 | UBXN1 | UCHL1 | UCHL3 | UCHL5 | UGGT1 | UQCRC2 | USP11 | USP14 | USP15 | USP16 | USP17L2 | USP19 | USP2 | USP20 | USP21 | USP26 | USP28 | USP39 | USP4 | USP42 | USP46 | USP47 | USP5 | USP50 | USP53 | USP7 | USP8 | USP9X | USP9Y | USPL1 | VASH2 | VCP | VCPIP1 | VHL | VPS37D | VPS4B | WDR48 | WWP2 | XBP1 | XPNPEP1 | YBEY | YME1L1 | YY1 | ZMPSTE24 | ZNRF4 | GO:0023051 | 0.247240082494238 | 2038/8243 | 902/3250 | 1.71338115508207e-07 | 0.00162685540675042 | T | T | T | T | regulation of signaling | A2M | ABAT | ABCE1 | ABL1 | ABL2 | ACAA2 | ACE2 | ACTN2 | ACTN4 | ADA | ADCYAP1 | ADORA2A | ADRB2 | AES | AGER | AGO1 | AGR2 | AHCYL1 | AHSG | AKAP9 | AKR1B1 | AKR1C2 | AKT1 | AKT3 | ANXA1 | ANXA2 | ANXA5 | APC | APLP1 | APOA1 | APOD | APOE | APP | AR | ARAF | ARF1 | ARFGAP1 | ARHGAP1 | ARHGAP19 | ARHGAP26 | ARHGAP35 | ARHGDIA | ARHGEF7 | ARHGEF9 | ARNT | ARRB1 | ARRB2 | ASCL1 | ATAD1 | ATAD5 | ATF6 | ATF6B | ATM | ATP1A1 | ATP1B3 | ATP2A2 | ATP2B2 | AURKB | AXIN1 | BACE1 | BAG4 | BAG5 | BAIAP2L1 | BANP | BAX | BBC3 | BCL10 | BCL2 | BCL2L1 | BCL2L2 | BCL6 | BDKRB2 | BIRC2 | BIRC3 | BIRC7 | BIRC8 | BMPR1A | BRAF | BRCA1 | BRD4 | BTRC | BUD31 | C1QBP | C1QL4 | C1QTNF1 | CACNA1A | CACNG2 | CACNG4 | CACNG5 | CALM1 | CALM2 | CALM3 | CALR | CAMK2A | CAMK2B | CAPN1 | CARD9 | CARM1 | CASP10 | CASP2 | CASP8 | CAT | CAV1 | CBFB | CBL | CBLB | CBLC | CBY1 | CCAR2 | CCDC22 | CCL21 | CCL22 | CCNY | CCR1 | CD109 | CD22 | CD27 | CD36 | CD40 | CD40LG | CD74 | CD81 | CDC20 | CDC34 | CDC37 | CDC42 | CDC42SE2 | CDC73 | CDH1 | CDK16 | CDK2 | CDK4 | CDK5 | CDK5R1 | CEL | CEP55 | CFTR | CHCHD10 | CHD3 | CHD4 | CHD8 | CHEK2 | CHGB | CHP1 | CHRD | CHRM2 | CHRNA9 | CLEC11A | CLNK | CLSTN1 | CLU | CMTM5 | COL2A1 | COPA | COPS5 | CORT | CPLX1 | CPLX3 | CPT1A | CREB1 | CREB3 | CREBBP | CRK | CRKL | CRY1 | CRY2 | CSNK1A1 | CSNK1E | CSNK2A2 | CSNK2B | CTH | CTNNB1 | CTNNBIP1 | CTNND1 | CTSC | CTTN | CUL3 | CXCL11 | CXCL5 | CXCL6 | CXCL8 | CYLD | DAB1 | DAB2 | DAPK1 | DAXX | DBH | DBN1 | DBNL | DCN | DDIT4 | DDRGK1 | DDX1 | DDX3X | DENND1A | DEPDC7 | DGKE | DHX36 | DISC1 | DLG2 | DLG3 | DLG4 | DMD | DNA2 | DNAJA1 | DNM1L | DOK1 | DTX1 | DUSP1 | DUSP14 | DUSP16 | DUSP19 | DUSP22 | DUSP7 | DVL1 | DVL2 | DVL3 | DYNLL1 | DYNLT1 | DYRK2 | E2F1 | EDA | EEF1D | EFEMP1 | EGFR | EGLN1 | EHMT1 | EHMT2 | EIF2AK3 | EIF2AK4 | EIF3A | EIF4A3 | EIF4E | ELF3 | ENO1 | EP300 | EPHA4 | EPM2A | ERBB2 | ERBB3 | ERRFI1 | ESR1 | ESR2 | EYA2 | EYA4 | EZH2 | EZR | FABP5 | FADD | FAF1 | FAM129B | FAM3C | FAM83D | FANCA | FASLG | FBP1 | FBRS | FBXW11 | FGB | FGF2 | FGFR1 | FGFR3 | FHL2 | FIGNL1 | FIS1 | FKBP1A | FKBP1B | FKBP8 | FLOT1 | FN1 | FOLR1 | FOXA1 | FOXM1 | FOXO1 | FYN | G3BP1 | GADD45G | GATA1 | GDF9 | GDI1 | GDI2 | GFAP | GIT1 | GJA1 | GKAP1 | GLP1R | GLUL | GNA12 | GNA13 | GNAI1 | GNAI2 | GNAI3 | GNAQ | GNAS | GOLPH3 | GOLT1B | GOT1 | GPC5 | GPD1L | GPI | GPS2 | GPX1 | GRB2 | GRB7 | GRIA1 | GRID2 | GRIK2 | GRK1 | GRK5 | GSK3A | GSK3B | GSTO1 | GSTP1 | HADH | HAP1 | HAVCR2 | HAX1 | HBEGF | HDAC1 | HDAC2 | HDAC3 | HDAC6 | HDAC7 | HDGF | HECW1 | HEXIM1 | HGS | HIF1A | HIF1AN | HINT1 | HIPK4 | HMGB1 | HMGB2 | HMOX1 | HNF4A | HNRNPK | HPX | HRAS | HSF1 | HSP90AA1 | HSP90AB1 | HSPA1B | HSPA5 | HSPB1 | HTR1B | HTRA1 | HTRA2 | HTT | ICAM1 | IFNA2 | IFT122 | IGBP1 | IGF1R | IKBKB | IKBKE | IKBKG | IL32 | ILK | ING5 | INSIG1 | INSR | IQGAP1 | IRF1 | IRF4 | IRF7 | ITCH | ITGAV | ITGB1BP1 | ITGB3 | ITPR1 | ITPR2 | ITPR3 | JAK1 | JAK3 | JUN | JUP | KANK2 | KAT5 | KCNS3 | KDM1A | KDM5B | KDR | KHDRBS1 | KIF5B | KLHDC10 | KLHL22 | KRAS | KSR1 | L3MBTL1 | LARS | LASP1 | LAT | LATS1 | LDLRAD4 | LEF1 | LEMD2 | LEMD3 | LGALS1 | LGALS3 | LGMN | LGR4 | LIMS1 | LMNA | LMO7 | LPAR1 | LPAR4 | LRP6 | LRRC7 | LRRK1 | LRRK2 | LTBP1 | LTBR | LURAP1 | LZTR1 | MAOA | MAP2K1 | MAP2K3 | MAP2K4 | MAP2K6 | MAP2K7 | MAP3K1 | MAP3K13 | MAP3K14 | MAP3K2 | MAP3K3 | MAP3K4 | MAP4K1 | MAPK1 | MAPK14 | MAPK3 | MAPK7 | MAPK8 | MAPK8IP3 | MAPK9 | MAPKAP1 | MAPT | MAS1 | MAVS | MBD3 | MC1R | MCC | MDFI | MDM2 | MDM4 | MECP2 | MED1 | METAP2 | METRNL | MIF | MKS1 | MLLT11 | MLLT3 | MLST8 | MME | MPC2 | MPP2 | MYC | MYD88 | MYO1C | MYO5A | MYOC | NAMPT | NCDN | NCK1 | NCOA1 | NCOA3 | NCOR1 | NCOR2 | NDUFA13 | NDUFAF2 | NDUFS3 | NEDD4 | NELFE | NEUROD1 | NF2 | NFATC1 | NFE2L2 | NFKBIA | NKX2-1 | NLK | NMB | NMNAT1 | NOD2 | NOS1AP | NOS2 | NOS3 | NPHP3 | NPLOC4 | NPR1 | NPTX1 | NQO2 | NR0B2 | NR1H2 | NR1H3 | NRXN1 | NTF3 | NTRK1 | NUMA1 | NUP93 | OGT | ONECUT1 | OPA1 | OPTN | OTUD4 | OXCT1 | P2RX1 | P2RX2 | P2RX4 | P4HB | PAFAH1B1 | PAG1 | PAK2 | PAK6 | PAM16 | PARK7 | PARP1 | PARP14 | PAWR | PAX8 | PCSK9 | PDCD10 | PDCD6 | PDCL | PDE4D | PDE6G | PDIA3 | PDK2 | PEBP1 | PELI1 | PER1 | PER2 | PFKL | PFKM | PHB | PHB2 | PIAS1 | PICK1 | PIGU | PIH1D1 | PIK3CA | PIK3CG | PIK3R1 | PIN1 | PIP4K2A | PLA2G1B | PLA2G2A | PLA2G6 | PLAUR | PLCG1 | PLEKHF1 | PLSCR1 | PML | PPARD | PPARG | PPARGC1A | PPEF1 | PPIF | PPM1A | PPM1B | PPP1CA | PPP1R2 | PPP2CA | PPP2CB | PPP2R1A | PPP2R1B | PPP3CC | PPP5C | PRCP | PRDX1 | PRDX2 | PRELID1 | PREPL | PRKAA1 | PRKAA2 | PRKAB2 | PRKACA | PRKAG1 | PRKAR2B | PRKCD | PRKCE | PRKCZ | PRKD1 | PRKD2 | PRMT2 | PRMT6 | PSAP | PSEN1 | PSMA1 | PSMA2 | PSMA3 | PSMA5 | PSMA6 | PSMB5 | PSMB6 | PSMB7 | PSMB8 | PSMC1 | PSMC2 | PSMC3 | PSMC4 | PSMC5 | PSMC6 | PSMD1 | PSMD10 | PSMD11 | PSMD12 | PSMD14 | PSMD2 | PSMD4 | PSMD9 | PSME2 | PSME3 | PTEN | PTGIR | PTK2 | PTN | PTPMT1 | PTPN1 | PTPN22 | PTPN23 | PTPN6 | PTPRA | PTPRO | PTPRR | PUM1 | PXDN | PXN | QARS | RAB11A | RAB29 | RAB3GAP1 | RAB5A | RAB7A | RAC1 | RAF1 | RANBP9 | RAP1A | RAP1B | RAPGEF4 | RARA | RASL11B | RB1 | RB1CC1 | RBBP4 | RBPJ | RBX1 | RC3H1 | RDH11 | RDX | RECK | REL | RELA | REST | RFC2 | RFC3 | RFC4 | RFC5 | RGN | RGS1 | RGS2 | RGS20 | RHO | RHOA | RHOJ | RHOT1 | RHOT2 | RHOU | RIC8B | RIPK1 | RIPK2 | RIPK3 | RNF126 | RNF183 | RNF19A | RNF31 | RPA2 | RPA3 | RPGRIP1L | RPL23 | RPS3 | RPTOR | RRM2B | RRS1 | RTN4 | RUNX1 | RUNX2 | RUVBL1 | RUVBL2 | S100A12 | S100A13 | S100A4 | S100A7 | S100A9 | S100B | SAG | SARM1 | SCEL | SCG2 | SCYL2 | SDCBP | SEC13 | SEMA7A | SEPT4 | SEPT5 | SERPINB3 | SERPINE2 | SFN | SFPQ | SH2B1 | SH3GL1 | SH3GL2 | SH3KBP1 | SHC1 | SHKBP1 | SIAH1 | SIPA1 | SIPA1L1 | SIRT1 | SIRT3 | SKI | SLAMF1 | SLC16A1 | SLC25A4 | SLC25A5 | SLC25A6 | SLC35F6 | SLC9A1 | SLC9A3R1 | SMAD2 | SMAD3 | SMAD4 | SMARCA4 | SMARCB1 | SMO | SMURF1 | SMURF2 | SNAI2 | SNCA | SNF8 | SNW1 | SNX3 | SNX6 | SOCS1 | SOCS2 | SOCS6 | SOD1 | SOD2 | SOX2 | SP100 | SPATA2 | SPERT | SPIN1 | SPRY2 | SPTBN1 | SQSTM1 | SRC | SRGAP2 | SRGAP3 | SRI | SSX2IP | STARD13 | STAT1 | STAT3 | STAU1 | STIM1 | STK11 | STK25 | STK3 | STK38 | STK40 | STMN1 | STRAP | STUB1 | STXBP1 | STYX | SULF2 | SUMO1 | SYDE1 | SYN1 | SYNJ2BP | SYP | SYVN1 | TAB1 | TAB2 | TAF12 | TAF15 | TANK | TAOK3 | TARDBP | TBL1X | TCF21 | TERF2IP | TFDP1 | TFG | TGFB1 | TGFBR3 | TGM2 | THBS1 | THEM4 | THRA | TIMP1 | TIMP2 | TLE1 | TLE3 | TLR3 | TMBIM6 | TMEM65 | TMEM9B | TNF | TNFAIP3 | TNFRSF12A | TNFRSF1A | TNFSF12 | TNFSF14 | TNIP2 | TNKS | TNKS2 | TNN | TOP3A | TOR1A | TP53 | TP53RK | TP63 | TP73 | TRADD | TRAF1 | TRAF2 | TRAF3IP1 | TRAF6 | TRAP1 | TREX1 | TRIAP1 | TRIB2 | TRIB3 | TRIM25 | TRIM32 | TRIM33 | TRIP6 | TSG101 | TSKU | TSPYL2 | TTC5 | TUBB2B | TULP3 | TWIST1 | TXLNA | TXN | UBA5 | UBASH3A | UBB | UBC | UBE2B | UBE2I | UBE2K | UBE2N | UBE2O | UBE2V1 | UBQLN1 | UBQLN2 | UBR5 | UCHL1 | UFM1 | USP17L2 | USP20 | USP46 | USP47 | USP8 | VAPA | VAV1 | VCP | VDAC2 | VGF | VHL | VPS18 | VTN | WBP2 | WDFY1 | WDR35 | WNK1 | WNT16 | WNT2 | WWOX | WWP2 | XBP1 | XIAP | YAP1 | YWHAB | YWHAE | YWHAG | YWHAH | YWHAQ | YWHAZ | ZAP70 | ZBTB7A | ZBTB7B | ZC3HC1 | ZDHHC17 | ZFAND6 | ZIC1 | ZMPSTE24 | ZMYND8 | ZNF622 | GO:0060828 | 0.0213514497149096 | 176/8243 | 103/3250 | 1.7958969816872e-07 | 0.001705204184112 | T | T | T | T | regulation of canonical Wnt signaling pathway | AES | APC | APOE | AXIN1 | BIRC8 | BTRC | CAV1 | CCAR2 | CCNY | CHD8 | CSNK1A1 | CSNK1E | CTNNB1 | CTNND1 | CUL3 | CYLD | DAB2 | DDX3X | DVL1 | DVL2 | DVL3 | EDA | EGFR | FOLR1 | FOXO1 | G3BP1 | GNAQ | GSK3A | GSK3B | HDAC1 | HECW1 | ILK | JUP | LATS1 | LGR4 | LRP6 | LRRK1 | LRRK2 | MAPK14 | MCC | MKS1 | MLLT3 | NPHP3 | PIN1 | PPM1A | PPP1CA | PSEN1 | PSMA1 | PSMA2 | PSMA3 | PSMA5 | PSMA6 | PSMB5 | PSMB6 | PSMB7 | PSMB8 | PSMC1 | PSMC2 | PSMC3 | PSMC4 | PSMC5 | PSMC6 | PSMD1 | PSMD10 | PSMD11 | PSMD12 | PSMD14 | PSMD2 | PSMD4 | PSMD9 | PSME2 | PSME3 | PTPRO | RBPJ | RBX1 | RECK | RUVBL1 | RUVBL2 | SCEL | SCYL2 | SLC9A3R1 | SMAD3 | SMURF2 | SNAI2 | SOX2 | SRC | STK11 | STK3 | SULF2 | TBL1X | TLE1 | TLE3 | TNKS | TNKS2 | TNN | UBE2B | UBR5 | USP47 | USP8 | VCP | WNK1 | XIAP | YAP1 | GO:0009057 | 0.0838287031420599 | 691/8243 | 336/3250 | 1.86592678000106e-07 | 0.001771697477611 | T | T | T | T | macromolecule catabolic process | ABHD17A | ADRM1 | AGL | AGO1 | AKT1 | AMFR | ANAPC4 | ANXA2 | APOE | ARMC8 | ARRB1 | ARRB2 | ATM | AUP1 | AURKB | BACE1 | BAG6 | BIRC2 | BTRC | CALM1 | CAPN1 | CASP3 | CBL | CBLC | CCNB1 | CDC16 | CDC20 | CDC27 | CDC34 | CDK2 | CDK5 | CHEK2 | CHP1 | CLPX | CLTC | CRBN | CTNNB1 | CTSD | CUL1 | CUL2 | CUL3 | CUL4B | CUL5 | CUL7 | CYLD | DCN | DCP2 | DDB1 | DDX6 | DERL1 | DIS3 | DNA2 | DNAJB2 | DNAJC10 | DNASE2 | DNASE2B | DROSHA | EDC4 | EDEM1 | EIF3E | EIF4A3 | ENDOG | ERLIN2 | EXOSC1 | EXOSC5 | EXOSC6 | EXOSC9 | FAF1 | FAF2 | FBXL12 | FBXO11 | FBXO6 | FBXO7 | FBXW11 | FBXW5 | FEN1 | FGF2 | FZR1 | GAA | GALNS | GNS | GPC5 | GSK3A | GSPT1 | GSPT2 | GUSB | H1F0 | HDAC6 | HECW1 | HECW2 | HELZ2 | HERC2 | HEXA | HEXB | HMGB1 | HMGB2 | HMMR | HNRNPD | HSP90B1 | HSPA1B | HSPA5 | HSPG2 | HTRA2 | HUWE1 | IDE | ISG15 | ITCH | KAT5 | KCTD17 | KCTD2 | KEAP1 | KLHL20 | KLHL22 | KPNA1 | KPNB1 | LGMN | LNX1 | LRSAM1 | LSM1 | LSM3 | LSM4 | LSM5 | LUM | LYG2 | LYPLA1 | LYPLA2 | MAD2L1 | MDM2 | MLH1 | MTA1 | NBAS | NCBP1 | NEDD4 | NEDD8 | NFE2L2 | NHLRC3 | NPLOC4 | NSFL1C | OAS2 | OVGP1 | PABPC1 | PABPC4 | PAN2 | PCBP2 | PCSK9 | PDE12 | PELI1 | PFKM | PGM1 | PGM2 | PHKA1 | PJA1 | PLK1 | PML | PPP1R11 | PPP1R8 | PPP2CA | PPP2CB | PPP2R1A | PPP2R2A | PPT1 | PRPF19 | PRSS16 | PSMA1 | PSMA2 | PSMA3 | PSMA5 | PSMA6 | PSMB5 | PSMB6 | PSMB7 | PSMB8 | PSMC1 | PSMC2 | PSMC3 | PSMC4 | PSMC5 | PSMC6 | PSMD1 | PSMD10 | PSMD11 | PSMD12 | PSMD14 | PSMD2 | PSMD4 | PSMD9 | PSME2 | PSME3 | PTPN23 | PTTG1 | PYGB | PYGL | RAB7A | RAD23A | RAD23B | RBBP6 | RBM8A | RBX1 | RC3H1 | RHBDD1 | RIPK1 | RNASE3 | RNASEH2A | RNASEH2B | RNF126 | RNF139 | RNF185 | RNF19A | RNF4 | RNF8 | RPL10 | RPL10A | RPL12 | RPL18A | RPL22 | RPL23 | RPL23A | RPL24 | RPL30 | RPL37A | RPL38 | RPL6 | RPL7 | RPL7A | RPL9 | RPLP0 | RPLP1 | RPLP2 | RPS12 | RPS13 | RPS14 | RPS15A | RPS19 | RPS2 | RPS20 | RPS24 | RPS29 | RPS3 | RPS4X | RPS8 | SDC1 | SDC4 | SEL1L | SGSH | SGTA | SGTB | SIAH1 | SIRT1 | SKIV2L | SLC9A1 | SMG5 | SMG6 | SMG8 | SMURF1 | SMURF2 | SND1 | SOCS6 | SPOP | SPSB3 | SQSTM1 | SSB | STBD1 | STUB1 | SYVN1 | TBL1X | TCIRG1 | TGFB1 | TOLLIP | TOR1A | TRAF2 | TREX1 | TRIM25 | TRIM32 | TRIM9 | TRIP12 | TSG101 | UBA6 | UBB | UBC | UBE2A | UBE2B | UBE2C | UBE2D1 | UBE2D2 | UBE2D3 | UBE2H | UBE2I | UBE2J1 | UBE2K | UBE2L3 | UBE2L6 | UBE2R2 | UBE2S | UBE2W | UBE3A | UBE3B | UBQLN1 | UBQLN2 | UBQLN4 | UBR4 | UBXN1 | UCHL1 | UCHL3 | UCHL5 | UGGT1 | UPF2 | UPF3B | USP11 | USP14 | USP15 | USP16 | USP17L2 | USP19 | USP2 | USP20 | USP21 | USP26 | USP28 | USP4 | USP42 | USP46 | USP47 | USP5 | USP50 | USP7 | USP8 | USP9Y | VCAN | VCP | VPS37D | VPS4B | WWP2 | XBP1 | YME1L1 | ZMPSTE24 | ZNRF4 | GO:0019941 | 0.0423389542642242 | 349/8243 | 184/3250 | 1.92694561998556e-07 | 0.00182963486617629 | T | T | T | T | modification-dependent protein catabolic process | ADRM1 | AMFR | ANAPC4 | ARMC8 | ARRB1 | ARRB2 | AUP1 | AURKB | BAG6 | BIRC2 | BTRC | CBL | CBLC | CCNB1 | CDC16 | CDC20 | CDC27 | CDC34 | CDK2 | CRBN | CTNNB1 | CUL1 | CUL2 | CUL3 | CUL4B | CUL5 | CUL7 | CYLD | DDB1 | DERL1 | DNAJB2 | DNAJC10 | EDEM1 | ERLIN2 | FAF1 | FAF2 | FBXL12 | FBXO11 | FBXO6 | FBXO7 | FBXW11 | FBXW5 | FZR1 | GSK3A | HDAC6 | HECW1 | HECW2 | HERC2 | HSP90B1 | HSPA5 | HUWE1 | ISG15 | ITCH | KAT5 | KCTD17 | KCTD2 | KLHL20 | KLHL22 | LNX1 | MAD2L1 | MDM2 | MTA1 | NEDD4 | NEDD8 | NFE2L2 | NHLRC3 | NPLOC4 | NSFL1C | PCBP2 | PELI1 | PLK1 | PML | PPP1R11 | PPP2CB | PSMA1 | PSMA2 | PSMA3 | PSMA5 | PSMA6 | PSMB5 | PSMB6 | PSMB7 | PSMB8 | PSMC1 | PSMC2 | PSMC3 | PSMC4 | PSMC5 | PSMC6 | PSMD1 | PSMD10 | PSMD11 | PSMD12 | PSMD14 | PSMD2 | PSMD4 | PSMD9 | PSME2 | PSME3 | PTPN23 | PTTG1 | RAD23A | RAD23B | RBBP6 | RBX1 | RNF126 | RNF185 | RNF19A | RNF4 | RNF8 | SEL1L | SGTA | SGTB | SIAH1 | SIRT1 | SMURF1 | SMURF2 | SPOP | SPSB3 | SQSTM1 | STUB1 | SYVN1 | TBL1X | TOLLIP | TRIM25 | TRIM32 | TRIM9 | TRIP12 | TSG101 | UBA6 | UBB | UBC | UBE2A | UBE2B | UBE2C | UBE2D1 | UBE2D2 | UBE2D3 | UBE2H | UBE2I | UBE2J1 | UBE2K | UBE2L3 | UBE2L6 | UBE2R2 | UBE2S | UBE2W | UBE3A | UBE3B | UBQLN1 | UBQLN2 | UBQLN4 | UBR4 | UBXN1 | UCHL1 | UCHL3 | UCHL5 | USP11 | USP14 | USP15 | USP16 | USP17L2 | USP19 | USP2 | USP20 | USP21 | USP26 | USP28 | USP4 | USP42 | USP46 | USP47 | USP5 | USP50 | USP7 | USP8 | USP9Y | VCP | VPS37D | VPS4B | WWP2 | XBP1 | ZMPSTE24 | ZNRF4 | GO:0048522 | 0.373771685066117 | 3081/8243 | 1324/3250 | 2.12464611147302e-07 | 0.00201735148284363 | T | T | T | T | positive regulation of cellular process | ABAT | ABHD14B | ABL1 | ABL2 | ACACA | ACD | ACE2 | ACLY | ACSL1 | ACTA2 | ACTL6A | ACTN1 | ACTN2 | ACTN4 | ACTR2 | ACTR3 | ADA | ADCY6 | ADCYAP1 | ADORA2A | ADRB2 | ADRM1 | ADTRP | AES | AGAP2 | AGER | AGO1 | AGO2 | AGR2 | AHI1 | AICDA | AIFM1 | AIMP2 | AIRE | AKAP8 | AKAP9 | AKR1B1 | AKR1C2 | AKT1 | AKT2 | AKT3 | AKTIP | ALOX12 | ANAPC4 | ANK1 | ANK3 | ANKRD49 | ANXA1 | ANXA2 | ANXA5 | AP1G1 | AP3D1 | APBB1 | APBB2 | APC | APEX1 | APOA1 | APOE | APP | AR | ARAF | ARF1 | ARF4 | ARHGAP1 | ARHGAP35 | ARHGDIA | ARHGEF7 | ARHGEF9 | ARID3B | ARID4B | ARNT | ARNT2 | ARPC1A | ARPC1B | ARPC2 | ARPC3 | ARPC4 | ARPC5 | ARRB1 | ARRB2 | ASCL1 | ASNS | ASPSCR1 | ASS1 | ATAD1 | ATAD5 | ATF2 | ATF6 | ATF6B | ATL3 | ATM | ATOH1 | ATP13A2 | ATRX | AURKB | AVIL | AXIN1 | AZU1 | BABAM1 | BACE1 | BAG2 | BAG3 | BAG4 | BAG6 | BAIAP2 | BAIAP2L1 | BANP | BAX | BBC3 | BCAR1 | BCAR3 | BCL10 | BCL2 | BCL2L1 | BCL2L13 | BCL6 | BIN1 | BIRC2 | BIRC3 | BIRC5 | BIRC7 | BIRC8 | BMI1 | BMPR1A | BRAF | BRCA1 | BRD4 | BTK | BTRC | BUD31 | C1QA | C1QBP | C1QTNF1 | CAB39 | CACNG2 | CACNG4 | CACNG5 | CALCOCO2 | CALM1 | CALM2 | CALM3 | CALR | CAMK1D | CAMK2A | CAMK2B | CAND1 | CAPG | CAPN1 | CAPN2 | CAPNS1 | CARD9 | CARM1 | CASP10 | CASP2 | CASP3 | CASP8 | CASP9 | CAT | CAV1 | CBFA2T3 | CBFB | CBL | CBX8 | CCAR2 | CCDC22 | CCDC88A | CCL21 | CCL22 | CCNB1 | CCNT1 | CCNT2 | CCNY | CCNYL1 | CCP110 | CCR1 | CCR3 | CCT2 | CCT3 | CCT4 | CCT5 | CCT6A | CCT7 | CCT8 | CD2 | CD209 | CD27 | CD36 | CD40 | CD40LG | CD6 | CD74 | CD81 | CDC14B | CDC20 | CDC25A | CDC25B | CDC25C | CDC34 | CDC37 | CDC42 | CDC42EP3 | CDC42EP4 | CDC5L | CDC73 | CDH1 | CDH5 | CDK19 | CDK2 | CDK4 | CDK5 | CDK5R1 | CDK8 | CDK9 | CDKL3 | CDKN1A | CEBPA | CEBPE | CENPJ | CENPV | CEP135 | CEP290 | CFL1 | CFL2 | CFTR | CHCHD10 | CHCHD2 | CHD1L | CHD8 | CHEK2 | CHMP2A | CHP1 | CHRD | CIRBP | CKS1B | CKS2 | CLEC11A | CLIC1 | CLNK | CLPX | CLSTN1 | CLU | COA3 | COL18A1 | COLGALT1 | COMMD1 | COMT | COPS5 | COX17 | CPNE3 | CPT1A | CREB1 | CREB3 | CREBBP | CRIP2 | CRK | CRKL | CROCC | CRP | CSNK1A1 | CSNK1E | CSNK2A2 | CSNK2B | CTBP1 | CTCF | CTH | CTNNB1 | CTNNBIP1 | CTSC | CTTN | CUL3 | CUL4B | CUL7 | CXCL11 | CXCL5 | CXCL8 | CYCS | CYLD | CYP1A1 | DAB1 | DAB2 | DAPK1 | DAXX | DAZAP1 | DBF4B | DBN1 | DBNL | DCN | DCTN1 | DCUN1D1 | DDB1 | DDB2 | DDIT4 | DDRGK1 | DDX1 | DDX39B | DDX3X | DERL1 | DGKZ | DHX36 | DHX9 | DIABLO | DISC1 | DLG3 | DLG4 | DNA2 | DNAJA1 | DNAJA2 | DNAJA3 | DNAJB2 | DNM1L | DOCK5 | DOCK7 | DOK1 | DPF2 | DSTN | DTX1 | DUSP1 | DUSP12 | DUSP19 | DUSP22 | DUSP7 | DVL1 | DVL2 | DVL3 | DYNC1H1 | DYNC1LI1 | DYNLL1 | DYRK2 | E2F1 | E2F2 | E2F3 | E2F4 | E2F6 | EAF2 | EBF1 | ECD | EDA | EDEM1 | EED | EEF1A2 | EEF1D | EEF2 | EGFR | EGLN1 | EGLN3 | EHD4 | EIF2AK3 | EIF2AK4 | EIF2B5 | EIF2S1 | EIF3D | EIF3E | EIF4A3 | EIF4E | EIF4EBP1 | EIF5A | EIF6 | ELAVL1 | ELF3 | ELF4 | ENDOG | ENO1 | EP300 | EPHA4 | EPM2A | ERBB2 | ERBB3 | ERCC5 | ERG | ERLIN2 | ESR1 | ESR2 | ESRRA | ESRRB | ESRRG | ETS1 | EXOSC6 | EXOSC9 | EYA2 | EYA4 | EZH2 | EZR | FABP1 | FABP3 | FABP5 | FADD | FAF1 | FAM129B | FAM19A3 | FAM49B | FAM83D | FAM83H | FAM98A | FANCI | FANCM | FASLG | FASN | FBRS | FBXO7 | FBXW11 | FCHSD1 | FCN1 | FEN1 | FGB | FGF2 | FGFR1 | FGFR1OP | FGFR3 | FHL2 | FIS1 | FKBP1A | FKBP1B | FKBP8 | FLOT1 | FN1 | FOS | FOXA1 | FOXJ1 | FOXK1 | FOXK2 | FOXM1 | FOXO1 | FSCN1 | FTO | FUBP3 | FUS | FXR2 | FYN | FZR1 | GABPB1 | GADD45G | GAPDH | GAPDHS | GATA1 | GBP5 | GDF9 | GDI1 | GFAP | GJA1 | GKAP1 | GLMN | GLUL | GNA13 | GNAI2 | GNAI3 | GNAS | GNG5 | GOLGA2 | GOLGA4 | GOLPH3 | GOLT1B | GOT1 | GPD1 | GPI | GPS2 | GPSM1 | GPX1 | GRB2 | GRB7 | GRID2 | GRIK2 | GRIP1 | GRK5 | GSK3A | GSK3B | GSTO1 | GSTP1 | GTF2A1 | GTF2F1 | GTF2F2 | GZMA | H1F0 | H1FX | H2AFX | H2AFZ | H3F3A | HAP1 | HAVCR2 | HAX1 | HBEGF | HDAC1 | HDAC2 | HDAC3 | HDAC4 | HDAC5 | HDAC6 | HDAC7 | HDAC9 | HELZ2 | HEXB | HEXIM1 | HGS | HIF1A | HIF1AN | HINT1 | HIST1H1A | HK1 | HK2 | HLX | HMGB1 | HMGB2 | HMGB3 | HMOX1 | HNF1A | HNF4A | HNF4G | HNRNPA1 | HNRNPA2B1 | HNRNPAB | HNRNPD | HNRNPK | HNRNPL | HNRNPU | HOXA5 | HOXB5 | HOXB9 | HOXD10 | HPRT1 | HPX | HRAS | HSF1 | HSF4 | HSP90AA1 | HSP90AB1 | HSPA1B | HSPA1L | HSPA2 | HSPA5 | HSPB1 | HSPBP1 | HSPD1 | HSPE1 | HSPH1 | HTATIP2 | HTR1B | HTRA1 | HTRA2 | HTT | HUWE1 | ICAM1 | ID2 | IDE | IFI16 | IFNA2 | IGF1R | IGHV3-13 | IGHV3-23 | IGHV3-30 | IGLC7 | IKBKB | IKBKE | IKBKG | IKZF5 | ILF2 | ILF3 | ILK | ING1 | ING5 | INSIG1 | INSIG2 | INSR | IQGAP1 | IRF1 | IRF4 | IRF7 | ISG15 | ITCH | ITGA4 | ITGAV | ITGB1BP1 | ITGB3 | ITGB3BP | ITPR3 | JAK3 | JMJD6 | JUN | JUNB | JUND | JUP | KAT5 | KAT7 | KAT8 | KCTD17 | KDM1A | KDM4D | KDM5B | KDM8 | KDR | KEAP1 | KHDRBS1 | KIF5B | KLF5 | KLF6 | KLHDC10 | KLHL22 | KMT2A | KMT2B | KMT2C | KRAS | KSR1 | LAMB1 | LARP4 | LARS | LASP1 | LAT | LATS1 | LCP1 | LCP2 | LDHA | LEF1 | LGALS1 | LGALS3 | LGMN | LGR4 | LIG4 | LIMS1 | LMNA | LMO2 | LMO7 | LOXL2 | LPAR1 | LPAR4 | LPIN3 | LRP6 | LRRC7 | LRRK1 | LRRK2 | LRSAM1 | LTBR | LUM | LURAP1 | MAD2L1 | MAOA | MAP1B | MAP2K1 | MAP2K3 | MAP2K4 | MAP2K6 | MAP2K7 | MAP3K1 | MAP3K13 | MAP3K14 | MAP3K2 | MAP3K3 | MAP3K4 | MAP4K1 | MAPK1 | MAPK14 | MAPK15 | MAPK3 | MAPK6 | MAPK7 | MAPK8 | MAPK8IP3 | MAPK9 | MAPKAP1 | MAPT | MARCH5 | MARCKSL1 | MAS1 | MATK | MAVS | MBP | MC1R | MCTS1 | MDM2 | MDM4 | MECP2 | MED1 | MED12 | MED14 | MED17 | MED20 | MED21 | MED23 | MED24 | MED31 | MED4 | MED6 | MELK | MEOX2 | MEPCE | METRN | METRNL | METTL23 | MGMT | MIB1 | MIF | MITF | MLH1 | MLLT11 | MLLT3 | MLST8 | MME | MOB1B | MORF4L2 | MOV10 | MPC2 | MPP2 | MRPL12 | MSH6 | MSN | MTA1 | MTA3 | MTCH2 | MTPN | MVD | MYBBP1A | MYC | MYD88 | MYO1C | MYO6 | MYOC | NACC1 | NAMPT | NANOG | NAP1L1 | NCBP1 | NCK1 | NCOA1 | NCOA2 | NCOA3 | NCOA4 | NDUFA13 | NEDD4 | NEK1 | NEK7 | NELFE | NELL1 | NEUROD1 | NF2 | NFATC1 | NFATC2 | NFE2L2 | NFKB2 | NFKBIA | NIF3L1 | NIN | NIPBL | NKRF | NKX2-1 | NMB | NMD3 | NME1 | NME2 | NMNAT1 | NNT | NOD2 | NOS1AP | NOS2 | NOS3 | NPEPPS | NPM1 | NPR1 | NQO1 | NQO2 | NR0B2 | NR1H2 | NR1H3 | NR1I2 | NR3C1 | NR4A1 | NR5A2 | NRP2 | NRXN1 | NSFL1C | NSMCE4A | NTF3 | NTRK1 | NUFIP1 | NUMA1 | NUP85 | NUP93 | NUP98 | NUPR1 | NUSAP1 | OBSL1 | OGT | ONECUT1 | OPA1 | OPTN | ORM1 | OTUD4 | OXCT1 | OXSR1 | P2RX1 | P2RX2 | P2RX4 | PA2G4 | PABPC1 | PACSIN3 | PAFAH1B1 | PAG1 | PAIP1 | PAK2 | PAK6 | PAN2 | PANX3 | PARD3 | PARK7 | PARP1 | PARP14 | PARP6 | PATZ1 | PAWR | PAX8 | PAX9 | PAXIP1 | PCBD1 | PCBP1 | PCGF5 | PCNA | PCSK9 | PDCD10 | PDCD6 | PDCD6IP | PDCL | PDE6G | PDF | PDIA3 | PEBP1 | PEF1 | PELI1 | PER1 | PER2 | PFDN2 | PFKM | PFN1 | PGK1 | PGK2 | PHB | PHB2 | PHF10 | PIAS1 | PIAS3 | PICK1 | PIH1D1 | PIK3C2A | PIK3CA | PIK3CG | PIK3R1 | PIK3R3 | PIM1 | PIN1 | PIP4K2A | PKN2 | PLA2G10 | PLA2G1B | PLA2G2A | PLA2G6 | PLAUR | PLCG1 | PLEKHF1 | PLGRKT | PLK1 | PLSCR1 | PLXNA1 | PLXNA2 | PLXNB2 | PML | PNKP | PNMA1 | PNP | POLDIP2 | POLDIP3 | POLR2A | POT1 | POU2F3 | POU5F1 | PPARD | PPARG | PPARGC1A | PPIA | PPID | PPIF | PPM1A | PPP1CA | PPP1CC | PPP1R12A | PPP2CA | PPP2R1A | PPP2R1B | PPP2R5D | PPP3CC | PPP5C | PPT1 | PRDX2 | PRDX3 | PRDX6 | PRELID1 | PRKAA1 | PRKAA2 | PRKACA | PRKAG1 | PRKAR1A | PRKAR2B | PRKCD | PRKCE | PRKCZ | PRKD1 | PRKD2 | PRKDC | PRMT2 | PROX1 | PRPF19 | PRRC1 | PSAP | PSEN1 | PSEN2 | PSMA1 | PSMA2 | PSMA3 | PSMA5 | PSMA6 | PSMB5 | PSMB6 | PSMB7 | PSMB8 | PSMC1 | PSMC2 | PSMC3 | PSMC4 | PSMC5 | PSMC6 | PSMD1 | PSMD10 | PSMD11 | PSMD12 | PSMD14 | PSMD2 | PSMD4 | PSMD9 | PSME2 | PSME3 | PTEN | PTGES2 | PTGES3 | PTK2 | PTN | PTPN1 | PTPN22 | PTPN23 | PTPN6 | PTPRD | PTRH2 | PUM1 | PXN | RAB11A | RAB1A | RAB1B | RAB21 | RAB29 | RAB31 | RAB3GAP1 | RAB5A | RAB7A | RAB9A | RAC1 | RAD21 | RAD23A | RAD51 | RAF1 | RAN | RAP1A | RAP1B | RARA | RARG | RASSF10 | RB1 | RB1CC1 | RBM3 | RBM4 | RBMX | RBPJ | RBX1 | RC3H1 | RDX | RECK | REL | RELA | REST | RFC1 | RFC2 | RFC3 | RFC4 | RFC5 | RFXANK | RGN | RGS2 | RHBDD1 | RHOA | RHOJ | RHOU | RIF1 | RIOK1 | RIPK1 | RIPK2 | RIPK3 | RMND1 | RNASEH2B | RNF139 | RNF183 | RNF185 | RNF19A | RNF31 | RNF4 | RNF8 | ROMO1 | RPA2 | RPA3 | RPL23 | RPLP1 | RPS15A | RPS19 | RPS2 | RPS3 | RPS4X | RPS6KA1 | RPS6KA2 | RPS6KB2 | RPTOR | RTF1 | RTN4 | RUNX1 | RUNX2 | RUVBL1 | RUVBL2 | RXRA | RXRB | RXRG | S100A12 | S100A13 | S100A4 | S100A6 | S100A7 | S100A9 | S100B | SAE1 | SAP18 | SARM1 | SART1 | SART3 | SCEL | SCG2 | SCYL2 | SDC1 | SDC4 | SDCBP | SEC13 | SEMA7A | SEPT4 | SEPT7 | SEPT9 | SERPINB3 | SERPINE2 | SETDB1 | SFN | SFPQ | SGTA | SGTB | SH2B1 | SH3GL1 | SH3GL2 | SH3GL3 | SHC1 | SHKBP1 | SHMT2 | SIAH1 | SIN3A | SIRT1 | SIRT3 | SIRT6 | SIRT7 | SKI | SLAMF1 | SLC25A5 | SLC35F6 | SLC9A1 | SLC9A3R1 | SMAD2 | SMAD3 | SMAD4 | SMAD5 | SMAD9 | SMARCA2 | SMARCA4 | SMARCB1 | SMARCC1 | SMARCC2 | SMARCE1 | SMO | SMURF1 | SMURF2 | SNAI2 | SNCA | SNF8 | SNW1 | SNX3 | SOAT1 | SOCS1 | SOCS2 | SOD1 | SOD2 | SORT1 | SOX2 | SP1 | SP100 | SPACA3 | SPEN | SPI1 | SPIN1 | SPRTN | SPRY2 | SPTBN1 | SPTLC2 | SQSTM1 | SRC | SRI | SRPK2 | SRSF5 | STAT1 | STAT3 | STAT4 | STAT5A | STAT6 | STAU1 | STK11 | STK24 | STK25 | STK26 | STK3 | STMN1 | STOML2 | STUB1 | STXBP1 | SUB1 | SULF2 | SUMO1 | SUMO2 | SUN2 | SUPT4H1 | SUPT5H | SUZ12 | SVIL | SWAP70 | SYDE1 | SYNE2 | TAB1 | TAB2 | TADA2B | TAF12 | TAF15 | TANK | TAOK3 | TARDBP | TAZ | TBL1X | TBRG4 | TCEA1 | TCEA2 | TCERG1 | TCF12 | TCF21 | TCF4 | TCIRG1 | TCP1 | TCTN3 | TDRD3 | TERF1 | TERF2IP | TFCP2 | TFDP1 | TFG | TGFB1 | TGFBR3 | TGM2 | THBS1 | THBS2 | THRA | TIA1 | TIAL1 | TIMP1 | TIMP2 | TLR3 | TMED9 | TMEM173 | TMEM30A | TMEM9B | TMOD3 | TMTC3 | TNF | TNFAIP3 | TNFRSF12A | TNFRSF1A | TNFRSF8 | TNFSF12 | TNFSF14 | TNIP2 | TNK2 | TNKS | TNKS2 | TNP1 | TOLLIP | TOMM22 | TOP2A | TOR1A | TP53 | TP63 | TP73 | TPM1 | TPR | TRA2A | TRADD | TRAF2 | TRAF6 | TRIAP1 | TRIB2 | TRIB3 | TRIM21 | TRIM25 | TRIM28 | TRIM32 | TRIM35 | TRIP4 | TRIP6 | TRUB2 | TSG101 | TTC5 | TUBB2B | TWF1 | TWF2 | TWIST1 | TXN | TXNIP | U2AF2 | UBB | UBC | UBE2A | UBE2B | UBE2C | UBE2D1 | UBE2D3 | UBE2I | UBE2K | UBE2L3 | UBE2M | UBE2N | UBE2O | UBE2S | UBE2V1 | UBE3A | UBQLN1 | UBQLN2 | UBR5 | ULK1 | UNC119 | UPF3B | UQCC1 | USP16 | USP17L2 | USP19 | USP2 | USP21 | USP47 | USP5 | USP50 | USP7 | USP8 | USP9X | VAPA | VASH2 | VASP | VAV1 | VCAM1 | VCP | VDR | VHL | VIM | VPS4B | VSTM2A | VTN | WARS | WAS | WBP2 | WDFY1 | WDR1 | WDR35 | WDR48 | WDR77 | WNK1 | WNT16 | WNT2 | WT1 | WWOX | WWP2 | XBP1 | XIAP | XPO5 | XRCC1 | XRCC3 | XRCC5 | XRCC6 | YAF2 | YAP1 | YES1 | YTHDF1 | YWHAB | YWHAE | YWHAG | YWHAH | YWHAQ | YWHAZ | YY1 | ZAP70 | ZBTB16 | ZBTB17 | ZBTB7B | ZDHHC17 | ZIC1 | ZMYND10 | ZMYND8 | ZNF24 | ZNF622 | ZNF746 | GO:0032446 | 0.0621133082615553 | 512/8243 | 257/3250 | 2.22003369155317e-07 | 0.00210792199012974 | T | T | T | T | protein modification by small protein conjugation | AKT1 | AMFR | ANAPC4 | ARRB1 | ARRB2 | ASB12 | ASB15 | ASB16 | ASB6 | ASB9 | BCL10 | BCL11A | BCL2 | BCOR | BIRC2 | BIRC3 | BIRC7 | BIRC8 | BMI1 | BRCA1 | BTRC | CAND1 | CBFB | CBL | CBLB | CBLC | CBX4 | CBX8 | CCDC22 | CDC16 | CDC20 | CDC27 | CDC34 | CDC42 | CDC73 | COMMD1 | CRBN | CTNNB1 | CUL1 | CUL2 | CUL3 | CUL4B | CUL5 | CUL7 | DCAF12 | DCAF7 | DCAF8 | DCUN1D1 | DDB1 | DDB2 | DDRGK1 | DERL1 | DTX1 | DTX2 | DZIP3 | FBXL12 | FBXO11 | FBXO25 | FBXO32 | FBXO6 | FBXO7 | FBXW11 | FBXW5 | FZR1 | HDAC4 | HDAC6 | HECW1 | HECW2 | HERC1 | HERC2 | HIF1A | HIST1H2BA | HUWE1 | ITCH | KCMF1 | KEAP1 | KLHL20 | KLHL22 | LMO7 | LNX1 | LRSAM1 | LZTR1 | MARCH5 | MDM2 | MED1 | MED12 | MED20 | MED21 | MED31 | MIB1 | NAE1 | NEDD4 | NEDD8 | NEURL4 | NFE2L2 | NHLRC3 | NOSIP | NPEPPS | NUP107 | NUP153 | NUP160 | NUP205 | NUP214 | NUP85 | NUP88 | NUP93 | NUP98 | PCGF1 | PCGF5 | PCGF6 | PCNA | PELI1 | PEX5 | PIAS1 | PIAS3 | PJA1 | PLK1 | POM121 | PPP1R11 | PRKDC | PRPF19 | PSMA1 | PSMA2 | PSMA3 | PSMA5 | PSMA6 | PSMB5 | PSMB6 | PSMB7 | PSMB8 | PSMC1 | PSMC2 | PSMC3 | PSMC4 | PSMC5 | PSMC6 | PSMD1 | PSMD10 | PSMD11 | PSMD12 | PSMD14 | PSMD2 | PSMD4 | PSMD9 | PSME2 | PSME3 | RAE1 | RANGAP1 | RBBP6 | RBX1 | RC3H1 | RING1 | RNF123 | RNF126 | RNF139 | RNF181 | RNF183 | RNF185 | RNF19A | RNF2 | RNF25 | RNF31 | RNF4 | RNF8 | RTF1 | SAE1 | SENP6 | SIAH1 | SIRT1 | SMURF1 | SMURF2 | SOCS1 | SOCS2 | SOCS6 | SPOP | SPSB3 | STUB1 | SUMO1 | SUMO1P1 | SUMO2 | SUMO3 | SUZ12 | SYVN1 | THOP1 | TMEM189-UBE2V1 | TNFAIP3 | TNKS | TNKS2 | TPR | TRAF2 | TRAF3 | TRAF6 | TRIM21 | TRIM23 | TRIM25 | TRIM28 | TRIM32 | TRIM33 | TRIM41 | TRIM47 | TRIM63 | TRIM69 | TRIM7 | TRIM9 | TRIP12 | TSG101 | TSPAN17 | UBA1 | UBA2 | UBA3 | UBA5 | UBA6 | UBAC1 | UBB | UBC | UBE2A | UBE2B | UBE2C | UBE2D1 | UBE2D2 | UBE2D3 | UBE2H | UBE2I | UBE2J1 | UBE2K | UBE2L3 | UBE2L6 | UBE2M | UBE2N | UBE2O | UBE2Q2 | UBE2R2 | UBE2S | UBE2V1 | UBE2W | UBE3A | UBE3B | UBR4 | UBR5 | UCHL3 | UFC1 | UFM1 | UNKL | USP5 | USP7 | USP9X | VCP | VCPIP1 | VHL | WDTC1 | WSB1 | WWP2 | XIAP | ZBTB16 | ZC3HC1 | ZNF598 | ZNRF4 | GO:0009260 | 0.0105544098022565 | 87/8243 | 58/3250 | 2.23458070510607e-07 | 0.00212173437949821 | T | T | T | T | ribonucleotide biosynthetic process | ACACA | ACAT1 | ACLY | ACOT7 | ACSL1 | ACSL4 | ACSL5 | ACSS2 | ADCY6 | ADK | ADSL | ADSS | AK1 | AK2 | AK4 | ALDOA | APRT | ATIC | CBR4 | CMPK1 | CTPS1 | CYC1 | DCAKD | DLAT | DLD | DNAJC30 | FASN | GART | GCDH | GMPS | HPRT1 | HSD17B8 | IMPDH2 | LDHC | MPC2 | NME1 | NME2 | NME4 | NPR1 | PAICS | PAPSS1 | PAPSS2 | PDHA1 | PDHA2 | PDHB | PDHX | PFAS | PPAT | PPT1 | RFK | SLC25A1 | SLC25A13 | STOML2 | TECR | TGFB1 | UCK2 | UMPS | UPP2 | GO:1901293 | 0.016984107727769 | 140/8243 | 85/3250 | 2.35275765760035e-07 | 0.00223394339589153 | T | T | T | T | nucleoside phosphate biosynthetic process | ACACA | ACAT1 | ACLY | ACOT7 | ACSL1 | ACSL4 | ACSL5 | ACSS2 | ADA | ADCY6 | ADK | ADSL | ADSS | AK1 | AK2 | AK4 | ALDOA | APRT | ASPDH | ATIC | CBR4 | CMPK1 | CTPS1 | CYC1 | DCAKD | DCK | DGUOK | DLAT | DLD | DNAJC30 | DUT | FASN | GARS | GART | GCDH | GMPS | HAAO | HPRT1 | HSD17B8 | IDH2 | IMPDH2 | KYNU | LDHC | MAPK1 | ME1 | MPC2 | MTHFD1 | NAMPT | NAPRT | NME1 | NME2 | NME4 | NMNAT1 | NMNAT3 | NMRK2 | NPR1 | PAICS | PAPSS1 | PAPSS2 | PDHA1 | PDHA2 | PDHB | PDHX | PFAS | PNKP | PNP | PPAT | PPT1 | RFK | RRM1 | RRM2 | RRM2B | SHMT1 | SLC25A1 | SLC25A13 | STOML2 | TAMM41 | TECR | TGFB1 | TK1 | TK2 | TYMS | UCK2 | UMPS | UPP2 | GO:0009165 | 0.0167414776173723 | 138/8243 | 84/3250 | 2.36371825958844e-07 | 0.00224435048747923 | T | T | T | T | nucleotide biosynthetic process | ACACA | ACAT1 | ACLY | ACOT7 | ACSL1 | ACSL4 | ACSL5 | ACSS2 | ADA | ADCY6 | ADK | ADSL | ADSS | AK1 | AK2 | AK4 | ALDOA | APRT | ASPDH | ATIC | CBR4 | CMPK1 | CTPS1 | CYC1 | DCAKD | DCK | DGUOK | DLAT | DLD | DNAJC30 | DUT | FASN | GARS | GART | GCDH | GMPS | HAAO | HPRT1 | HSD17B8 | IDH2 | IMPDH2 | KYNU | LDHC | MAPK1 | ME1 | MPC2 | MTHFD1 | NAMPT | NAPRT | NME1 | NME2 | NME4 | NMNAT1 | NMNAT3 | NMRK2 | NPR1 | PAICS | PAPSS1 | PAPSS2 | PDHA1 | PDHA2 | PDHB | PDHX | PFAS | PNKP | PNP | PPAT | PPT1 | RFK | RRM1 | RRM2 | RRM2B | SHMT1 | SLC25A1 | SLC25A13 | STOML2 | TAMM41 | TECR | TGFB1 | TK2 | TYMS | UCK2 | UMPS | UPP2 | GO:0046390 | 0.0110396700230499 | 91/8243 | 60/3250 | 2.50880320330131e-07 | 0.0023821086415346 | T | T | T | T | ribose phosphate biosynthetic process | ACACA | ACAT1 | ACLY | ACOT7 | ACSL1 | ACSL4 | ACSL5 | ACSS2 | ADCY6 | ADK | ADSL | ADSS | AK1 | AK2 | AK4 | ALDOA | APRT | ATIC | CBR4 | CMPK1 | CTPS1 | CYC1 | DCAKD | DLAT | DLD | DNAJC30 | FASN | G6PD | GART | GCDH | GMPS | HPRT1 | HSD17B8 | IMPDH2 | LDHC | MPC2 | NME1 | NME2 | NME4 | NPR1 | PAICS | PAPSS1 | PAPSS2 | PDHA1 | PDHA2 | PDHB | PDHX | PFAS | PPAT | PPT1 | PYGL | RFK | SLC25A1 | SLC25A13 | STOML2 | TECR | TGFB1 | UCK2 | UMPS | UPP2 | GO:0104004 | 0.0245056411500667 | 202/8243 | 115/3250 | 2.70023005957494e-07 | 0.00256386844156641 | T | T | T | T | cellular response to environmental stimulus | ADSS | AKR1B1 | AKT1 | AKT2 | ASCL1 | ATM | ATP1A1 | AURKB | BAG3 | BAX | BCL10 | BCL2L1 | CAB39 | CASP2 | CASP3 | CASP8 | CASP9 | CD40 | CDC25A | CDKN1A | CHEK2 | CHP1 | CREBBP | CRIP1 | CRY1 | CRY2 | CRYAB | CUL4B | DDB1 | DDB2 | DDX3X | DHX36 | EEF1D | EGFR | EIF2AK4 | EIF2S1 | EP300 | ERRFI1 | FADD | FIGNL1 | GNB1 | GOT1 | GRB2 | GRK1 | H2AFX | HDAC4 | HRAS | HSF1 | HSPA5 | IFI16 | IRF1 | KDM1A | KDM4D | LIG4 | LTBR | MAP2K4 | MAP3K1 | MAP3K14 | MAP3K2 | MAPK14 | MAPK3 | MAPK8 | MC1R | MDM2 | MGMT | MME | MTPN | MYC | MYD88 | NAMPT | NEDD4 | NIPBL | NPM1 | OXSR1 | PARP1 | PCNA | PDE6G | PIK3R1 | POLD1 | POLE3 | PPID | PTEN | PTN | PTPRK | RAB11B | RAC1 | RAD51 | RAD51AP1 | REST | RHBDD1 | RHO | RUVBL2 | SAG | SERPINB6 | SIPA1 | SIRT1 | SLC9A1 | SNAI2 | STK11 | TANK | TGFB1 | TLR3 | TNFRSF1A | TNFRSF8 | TNFSF14 | TP53 | TREX1 | TRIAP1 | USP28 | USP47 | XRCC5 | XRCC6 | YAP1 | YY1 | ZMPSTE24 | GO:0071214 | 0.0245056411500667 | 202/8243 | 115/3250 | 2.70023005957494e-07 | 0.00256386844156641 | T | T | T | T | cellular response to abiotic stimulus | ADSS | AKR1B1 | AKT1 | AKT2 | ASCL1 | ATM | ATP1A1 | AURKB | BAG3 | BAX | BCL10 | BCL2L1 | CAB39 | CASP2 | CASP3 | CASP8 | CASP9 | CD40 | CDC25A | CDKN1A | CHEK2 | CHP1 | CREBBP | CRIP1 | CRY1 | CRY2 | CRYAB | CUL4B | DDB1 | DDB2 | DDX3X | DHX36 | EEF1D | EGFR | EIF2AK4 | EIF2S1 | EP300 | ERRFI1 | FADD | FIGNL1 | GNB1 | GOT1 | GRB2 | GRK1 | H2AFX | HDAC4 | HRAS | HSF1 | HSPA5 | IFI16 | IRF1 | KDM1A | KDM4D | LIG4 | LTBR | MAP2K4 | MAP3K1 | MAP3K14 | MAP3K2 | MAPK14 | MAPK3 | MAPK8 | MC1R | MDM2 | MGMT | MME | MTPN | MYC | MYD88 | NAMPT | NEDD4 | NIPBL | NPM1 | OXSR1 | PARP1 | PCNA | PDE6G | PIK3R1 | POLD1 | POLE3 | PPID | PTEN | PTN | PTPRK | RAB11B | RAC1 | RAD51 | RAD51AP1 | REST | RHBDD1 | RHO | RUVBL2 | SAG | SERPINB6 | SIPA1 | SIRT1 | SLC9A1 | SNAI2 | STK11 | TANK | TGFB1 | TLR3 | TNFRSF1A | TNFRSF8 | TNFSF14 | TP53 | TREX1 | TRIAP1 | USP28 | USP47 | XRCC5 | XRCC6 | YAP1 | YY1 | ZMPSTE24 | GO:1903322 | 0.0127380807958268 | 105/8243 | 67/3250 | 3.28504822493467e-07 | 0.00311915328957547 | T | T | T | T | positive regulation of protein modification by small protein conjugation or removal | ADRB2 | AIMP2 | ARNT | ARRB1 | ARRB2 | AXIN1 | BCL10 | BIRC2 | BIRC3 | BMI1 | BRCA1 | CAV1 | CDC14B | CDC20 | CDK9 | COMMD1 | CUL3 | DCUN1D1 | DERL1 | FANCI | FANCM | FKBP1A | FZR1 | GOLGA2 | GSK3A | HDAC4 | HSPA5 | HSPBP1 | KDM1A | LRRK2 | MAPK9 | MTA1 | NOD2 | PAXIP1 | PDCD6 | PEF1 | PELI1 | PIAS1 | PIAS3 | PIN1 | PLK1 | PSMD10 | PTEN | PTPN22 | RAB1A | RIPK2 | RPS2 | SART3 | SEPT4 | SPRTN | STUB1 | TANK | TOLLIP | TRAF6 | TRIB3 | UBB | UBE2C | UBE2D1 | UBE2I | UBE2L3 | UBE2N | UBE2S | UBE3A | UBQLN1 | VCP | WDR48 | XIAP | GO:0009636 | 0.0299648186339925 | 247/8243 | 136/3250 | 3.30017449550941e-07 | 0.00313351568348619 | T | T | T | T | response to toxic substance | ABAT | ABL1 | ACTB | ACTR2 | ADA | ADCYAP1 | ADORA2A | AIFM1 | ANXA1 | APEX1 | ASNS | ASS1 | BAX | BCL2 | BCL2L1 | BIRC2 | CALM1 | CALM3 | CAPN2 | CASP3 | CASP8 | CAT | CBL | CBX8 | CCNB1 | CD27 | CDH1 | CDK4 | CDKN1A | CES1 | CHRND | CHRNE | CHRNG | CLDN18 | CREB1 | CRK | CRYAB | CYP1A1 | DAXX | DBH | DHX15 | DMAP1 | DUSP1 | EEF2 | EHMT1 | EIF4EBP1 | ETS1 | EZH2 | FABP1 | FECH | FIS1 | FKBP1B | FOS | FOXO1 | FYN | G6PD | GNAO1 | GOT2 | GPX1 | GSTP1 | HCN3 | HDAC2 | HDAC6 | HDAC9 | HMOX1 | HNRNPA1 | HPRT1 | HSF1 | HTR1B | HTR3A | HTRA2 | ICAM1 | ITPR2 | JUN | KCNK1 | KDM1A | KDM3B | KDM5B | KDR | LDHA | MAPK1 | MAPK3 | MAPK7 | MBP | MDM2 | MGMT | MPST | NFE2L2 | NQO1 | NTRK1 | NUCB1 | NUPR1 | OXCT1 | PARK7 | PCNA | PDCD10 | PEBP1 | PPARGC1A | PPIF | PPP2CB | PRDX3 | PRKAA1 | PRKCD | PRKCE | PSMD14 | PTEN | RARA | RELA | RGS2 | RHOA | RIPK1 | RIPK3 | RPL10A | RPS3 | SCFD1 | SDC1 | SETDB1 | SIRT1 | SOD1 | SPI1 | SRC | STAT1 | STAT3 | STAT6 | STK24 | STK25 | STK26 | TBXA2R | TNF | TNFAIP3 | TUFM | TXNIP | TYMS | VCAM1 | WDR35 | ZNF277 | GO:0034599 | 0.0180759432245542 | 149/8243 | 89/3250 | 3.49166468613199e-07 | 0.00331533561948233 | T | T | T | T | cellular response to oxidative stress | ABL1 | AIFM1 | AKT1 | ANXA1 | APEX1 | ATP13A2 | ATP2A2 | BTK | CAT | CBX8 | CCS | CD36 | CDK2 | CRK | CYCS | DAPK1 | DPEP1 | EGFR | EIF2S1 | ETS1 | EZH2 | FABP1 | FANCC | FOS | FOXO1 | G6PD | GPX1 | GPX7 | GSR | GSTP1 | HDAC2 | HDAC6 | HNRNPD | HSF1 | HSPA1B | HTRA2 | JUN | LRRK2 | MAPK1 | MAPK3 | MAPK7 | MAPK8 | MAPK9 | MAPT | MDM2 | MGMT | MGST1 | NFE2L2 | NME2 | NQO1 | PARK7 | PARP1 | PCNA | PDK2 | PPARGC1A | PPIF | PRDX1 | PRDX2 | PRDX3 | PRDX5 | PRDX6 | PRKAA1 | PRKAA2 | PRKCD | PRKD1 | PTPRK | PXN | PYCR1 | PYCR2 | RELA | RIPK1 | RIPK3 | ROMO1 | RPS3 | SIRT1 | SLC25A24 | SNCA | SOD1 | SOD2 | SRC | SRXN1 | STAT6 | STAU1 | TNFAIP3 | TPM1 | TRAF2 | TREX1 | TXNRD1 | ZNF277 | GO:0048523 | 0.329249059808322 | 2714/8243 | 1174/3250 | 3.7214916828256e-07 | 0.00353355635284291 | T | T | T | T | negative regulation of cellular process | A2M | AARS | ABAT | ABCE1 | ABCG1 | ABL1 | ABL2 | ACAA2 | ACADVL | ACD | ACTN1 | ACTN4 | ADA | ADCY6 | ADCYAP1 | ADD3 | ADORA2A | ADRB2 | ADTRP | AEBP2 | AES | AGAP2 | AGO1 | AGO2 | AGR2 | AHI1 | AHSG | AICDA | AIMP2 | AKR1B1 | AKT1 | AKT2 | AKT3 | ALB | ALOX12 | ANXA1 | ANXA2 | ANXA5 | ANXA6 | APBB1 | APBB2 | APC | APCS | APEX1 | API5 | APLP1 | APOA1 | APOD | APOE | APP | AR | ARAF | ARF1 | ARF4 | ARHGAP1 | ARHGAP35 | ARHGDIA | ARHGEF7 | ARNT2 | ARRB1 | ARRB2 | ASCL1 | ASNS | ASS1 | ATAD1 | ATAD3A | ATAD5 | ATCAY | ATF2 | ATF6B | ATG5 | ATM | ATOH1 | ATOX1 | ATP13A2 | ATP1A1 | ATRX | ATXN1 | ATXN2 | AURKB | AVIL | AXIN1 | AZU1 | BABAM1 | BACH2 | BAG1 | BAG2 | BAG3 | BAG4 | BAG5 | BAG6 | BASP1 | BAX | BCL10 | BCL11A | BCL2 | BCL2L1 | BCL2L2 | BCL6 | BCL6B | BCOR | BDKRB2 | BIN1 | BIRC2 | BIRC3 | BIRC5 | BIRC7 | BIRC8 | BMI1 | BMPR1A | BNIPL | BRAF | BRCA1 | BTK | BTRC | C1QBP | C1QL4 | C1QTNF1 | C4A | CAB39 | CALM1 | CALM2 | CALM3 | CALR | CAMK1D | CAPG | CAPZA1 | CAPZA2 | CAPZB | CARM1 | CASP2 | CASP3 | CASP8 | CAT | CAV1 | CBFA2T3 | CBFB | CBL | CBLB | CBLC | CBX4 | CBX7 | CBX8 | CBY1 | CCAR2 | CCDC22 | CCDC8 | CCL21 | CCNB1 | CCNT1 | CCP110 | CCR5 | CD109 | CD22 | CD27 | CD2BP2 | CD36 | CD40LG | CD74 | CDA | CDC14B | CDC20 | CDC25C | CDC34 | CDC42 | CDC73 | CDH1 | CDK2 | CDK4 | CDK5 | CDK5R1 | CDK9 | CDKL3 | CDKN1A | CDKN2C | CEBPA | CENPF | CENPJ | CFL1 | CGRRF1 | CHCHD3 | CHD8 | CHEK2 | CHMP2A | CHORDC1 | CHP1 | CHRD | CHRNA9 | CIRBP | CLDN18 | CLIC4 | CLU | CMTM5 | COL18A1 | COL2A1 | COL5A1 | COMT | COPS2 | COPS5 | COX11 | CRBN | CREB1 | CREB3 | CREBBP | CRK | CRKL | CRMP1 | CRP | CRY1 | CRY2 | CRYAB | CSNK1A1 | CSNK2A2 | CSNK2B | CTBP1 | CTCF | CTDP1 | CTH | CTNNB1 | CTNNBIP1 | CTNND1 | CTSA | CTTN | CUL1 | CUL2 | CUL3 | CUL5 | CXCL8 | CYLD | DAB1 | DAB2 | DAD1 | DAPK1 | DAXX | DBNDD2 | DCN | DCP2 | DDB1 | DDIT4 | DDRGK1 | DDX39B | DDX3X | DDX6 | DGUOK | DHCR24 | DHX36 | DHX9 | DLG2 | DLG3 | DLG4 | DMAP1 | DMD | DNA2 | DNAJA1 | DNAJA3 | DNAJA4 | DNAJB1 | DNAJB2 | DNAJC10 | DNM1L | DPEP1 | DPF2 | DTX1 | DUSP1 | DUSP14 | DUSP16 | DUSP19 | DUSP22 | DUSP7 | DVL1 | DVL2 | DVL3 | DYNLL1 | DYNLT1 | DYRK2 | E2F1 | E2F2 | E2F3 | E2F4 | E2F6 | EAF2 | EED | EFEMP1 | EGFR | EHMT1 | EHMT2 | EID1 | EIF2AK3 | EIF2AK4 | EIF2S1 | EIF3A | EIF3E | EIF4A3 | EIF4E | EIF4E2 | EIF4EBP1 | EIF4ENIF1 | EIF4G3 | EIF6 | ELAVL1 | ELF3 | ENO1 | EP300 | EPHA4 | EPM2A | EPPIN | EPPK1 | EPRS | ERBB2 | ERBB3 | ERCC5 | ERF | ERLIN2 | ERRFI1 | ESR1 | ESR2 | ESRRA | ESRRB | ETS1 | EYA2 | EYA4 | EZH2 | EZR | FABP1 | FABP3 | FABP7 | FADD | FAM129B | FAM192A | FAM19A3 | FAM3C | FASLG | FBP1 | FBXO6 | FBXO7 | FBXW11 | FGB | FGF2 | FGFR1 | FGFR1OP | FHL1 | FHL2 | FIGNL1 | FKBP1A | FKBP1B | FKBP8 | FN1 | FOXA1 | FOXJ1 | FOXK1 | FOXM1 | FOXO1 | FTH1 | FTO | FXR2 | FYN | FZR1 | G3BP1 | G6PD | GAK | GAPDH | GATA1 | GDF9 | GDI1 | GFAP | GJA1 | GLA | GLMN | GLO1 | GNAI2 | GNAI3 | GNAQ | GOLGA2 | GOLPH3 | GOPC | GPD1L | GPI | GPS2 | GPX1 | GPX4 | GRB2 | GRB7 | GRIA1 | GRIK2 | GRK5 | GSK3A | GSK3B | GSTO1 | GSTP1 | GZMA | H1F0 | H1FX | H2AFX | H3F3A | HADH | HAP1 | HAVCR2 | HAX1 | HBEGF | HBZ | HDAC1 | HDAC2 | HDAC3 | HDAC4 | HDAC5 | HDAC6 | HDAC7 | HDAC9 | HDGF | HECW1 | HERC1 | HEXIM1 | HGS | HIF1A | HIF1AN | HIST1H1A | HIST1H2AB | HIST1H3A | HIST1H4A | HIST2H3A | HIST3H3 | HIVEP1 | HK2 | HLX | HMGB1 | HMGB2 | HMOX1 | HNF4A | HNRNPA1 | HNRNPA2B1 | HNRNPD | HNRNPK | HNRNPL | HNRNPU | HOXA5 | HRAS | HSF1 | HSF4 | HSP90AB1 | HSP90AB3P | HSP90AB4P | HSP90B1 | HSPA1B | HSPA2 | HSPA5 | HSPA8 | HSPA9 | HSPB1 | HSPD1 | HTATIP2 | HTR1B | HTRA1 | HTRA2 | HTT | HYPK | IBTK | ICAM1 | ID2 | IDE | IDH2 | IFI16 | IFNA2 | IFT122 | IGBP1 | IGF1R | IGF2BP1 | IGFBP7 | IK | IKBKB | IKBKG | IKZF5 | IL13RA2 | ILF3 | ILK | ING1 | ING5 | INSIG1 | INSIG2 | IPO5 | IPO7 | IQCB1 | IQGAP1 | IREB2 | IRF1 | IRF4 | IRF7 | ISG15 | ITCH | ITGAV | ITGB1BP1 | ITGB3 | ITPR1 | ITPR2 | ITPR3 | JAK3 | JUN | JUNB | JUND | JUP | KANK2 | KAT5 | KAT7 | KAT8 | KDM1A | KDM4D | KDM5B | KDM5C | KDR | KHDRBS1 | KIR3DL2 | KLF5 | KLHL20 | KLHL22 | KMT2A | KRAS | L3MBTL1 | L3MBTL2 | LAMP3 | LARS | LATS1 | LDLRAD4 | LEF1 | LEMD2 | LEMD3 | LETM1 | LGALS1 | LGALS3 | LGMN | LGR4 | LIG4 | LIMA1 | LIMS1 | LMNA | LOXL2 | LPAR1 | LRP6 | LRPPRC | LRRK1 | LRRK2 | LRSAM1 | LSM1 | LTBP1 | LYPLA1 | LZTR1 | MAD1L1 | MAD2L1 | MAF1 | MAP2K1 | MAP2K4 | MAP2K6 | MAP3K3 | MAPK1 | MAPK14 | MAPK15 | MAPK7 | MAPK8 | MAPKAP1 | MAPT | MARCH5 | MAS1 | MBD3 | MBP | MCC | MCM2 | MCOLN3 | MDC1 | MDFI | MDM1 | MDM2 | MDM4 | MECP2 | MED1 | MED31 | MEOX2 | MEPCE | MGA | MGMT | MIB1 | MICA | MIF | MITF | MLH1 | MLLT1 | MLLT3 | MLST8 | MORF4L1 | MORF4L2 | MOV10 | MSH6 | MTA1 | MTA3 | MTSS1 | MXD1 | MYBBP1A | MYC | MYD88 | MYL2 | MYO1D | MYOC | NACC1 | NAE1 | NAMPT | NANOG | NBAS | NCK1 | NCOA2 | NCOR1 | NCOR2 | NDRG1 | NDUFA13 | NDUFAF2 | NDUFS3 | NEDD4 | NELFB | NELFCD | NELFE | NELL1 | NEUROD1 | NF2 | NFATC1 | NFATC2 | NFE2L2 | NFKBIA | NIF3L1 | NIPBL | NKRF | NKX2-1 | NLK | NMB | NME1 | NME2 | NMNAT1 | NMRK2 | NNT | NOD2 | NOS3 | NOSTRIN | NPHP3 | NPLOC4 | NPM1 | NPR1 | NQO1 | NR0B2 | NR1H2 | NR1H3 | NR1I2 | NR3C1 | NR4A1 | NSUN2 | NTF3 | NTRK1 | NUBP1 | NUDT6 | NUPR1 | OGT | ONECUT1 | OPA1 | OPTN | OTUB1 | OTUD4 | OVGP1 | PA2G4 | PABPC1 | PACSIN3 | PAFAH1B1 | PAG1 | PAIP1 | PAK2 | PALB2 | PAM16 | PAPLN | PARD3 | PARD6A | PARK7 | PARP1 | PARP14 | PATZ1 | PAWR | PAX8 | PAX9 | PCGF1 | PCGF6 | PCM1 | PCNA | PCSK9 | PDCD10 | PDCD6 | PDCL | PDE12 | PDE4D | PEBP1 | PELI1 | PER1 | PER2 | PEX5 | PFKL | PFN1 | PGP | PHB | PHB2 | PHC3 | PHF10 | PHF21A | PIAS1 | PIAS3 | PICK1 | PIH1D1 | PIK3CA | PIK3CG | PIK3R1 | PIM1 | PIN1 | PINX1 | PKIG | PLA2G6 | PLAUR | PLCG1 | PLK1 | PLXNA1 | PLXNA2 | PLXNB2 | PML | PNKP | POLDIP2 | POT1 | POU5F1 | PPARD | PPARG | PPARGC1A | PPID | PPIF | PPM1A | PPM1B | PPM1D | PPME1 | PPP1R11 | PPP1R14A | PPP1R2 | PPP1R8 | PPP2CA | PPP2CB | PPP2R1A | PPP2R5D | PPT1 | PRCC | PRDX2 | PRDX3 | PRDX4 | PRDX5 | PRELID1 | PRKAA1 | PRKAA2 | PRKAB2 | PRKACA | PRKAG1 | PRKAR1A | PRKAR2B | PRKCD | PRKCE | PRKCZ | PRKD1 | PRKDC | PRMT2 | PRMT3 | PRMT6 | PROX1 | PSAP | PSEN1 | PSMA1 | PSMA2 | PSMA3 | PSMA5 | PSMA6 | PSMB5 | PSMB6 | PSMB7 | PSMB8 | PSMC1 | PSMC2 | PSMC3 | PSMC4 | PSMC5 | PSMC6 | PSMD1 | PSMD10 | PSMD11 | PSMD12 | PSMD14 | PSMD2 | PSMD4 | PSMD9 | PSME2 | PSME3 | PTCRA | PTEN | PTGES | PTGIR | PTK2 | PTN | PTPN1 | PTPN14 | PTPN22 | PTPN23 | PTPN6 | PTPRK | PTPRO | PTPRR | PTRH2 | PTTG1 | PUM1 | PUS7 | PXDN | PYCR1 | QARS | QSOX1 | RAB29 | RAB7A | RAC1 | RAD21 | RAD51 | RAF1 | RANBP9 | RAP1A | RAP1B | RARA | RARG | RASD1 | RASL11B | RB1 | RB1CC1 | RBBP4 | RBM3 | RBM4 | RBMX | RBPJ | RBX1 | RC3H1 | RDX | RECK | REL | RELA | REST | RFC1 | RGN | RGS1 | RGS2 | RGS20 | RHBDD1 | RHOA | RIF1 | RING1 | RIPK1 | RIPK2 | RNF126 | RNF139 | RNF2 | RNF8 | RPA2 | RPGRIP1L | RPL10 | RPL23 | RPL24 | RPS13 | RPS14 | RPS3 | RPS6KA1 | RPS6KA2 | RPTOR | RRM2 | RRM2B | RTF1 | RTN4 | RUNX1 | RUNX1T1 | RUNX2 | RUVBL2 | RXRA | S100A1 | S100A11 | S100B | SAG | SAP18 | SARM1 | SARS | SCFD1 | SCG2 | SCYL2 | SDC4 | SDCBP | SEMA7A | SEPT7 | SERPINB2 | SERPINB3 | SERPINB5 | SERPINB6 | SERPINB8 | SERPINE2 | SERPINH1 | SET | SF1 | SFMBT1 | SFN | SFPQ | SGTA | SH3GL2 | SH3GL3 | SH3KBP1 | SHC1 | SHMT1 | SIAH1 | SIN3A | SIPA1 | SIRT1 | SIRT3 | SIRT5 | SIRT6 | SIRT7 | SKI | SLAMF1 | SLAMF8 | SLC25A4 | SLC25A5 | SLC35F6 | SLC9A1 | SLC9A3R1 | SMAD2 | SMAD3 | SMAD4 | SMAD5 | SMARCA2 | SMARCA4 | SMARCB1 | SMARCC1 | SMARCC2 | SMARCE1 | SMC1A | SMC3 | SMG6 | SMO | SMURF1 | SMURF2 | SNAI2 | SNCA | SNTA1 | SNW1 | SNX12 | SNX3 | SNX6 | SOCS1 | SOCS2 | SOCS6 | SOD1 | SOD2 | SORT1 | SOX2 | SP100 | SP2 | SPDL1 | SPEN | SPERT | SPI1 | SPINK7 | SPINT2 | SPRY2 | SPTAN1 | SPTBN1 | SPTBN2 | SQSTM1 | SRC | SRGAP2 | SRGAP3 | SRI | SRSF12 | ST13 | STAG2 | STARD13 | STAT1 | STAT3 | STAT6 | STIL | STK11 | STK24 | STK26 | STK3 | STK38 | STK40 | STMN1 | STRAP | STUB1 | STXBP1 | STYX | SULF2 | SULT1A3 | SUMO1 | SUPT4H1 | SUPT5H | SUZ12 | SWAP70 | SYNJ2BP | SYVN1 | TAB2 | TAGLN3 | TANK | TAOK3 | TARDBP | TBC1D4 | TBL1X | TBRG4 | TBXA2R | TCERG1 | TCF21 | TCF25 | TCP10L | TERF1 | TERF2 | TERF2IP | TES | TFDP1 | TFIP11 | TGFB1 | TGFBR3 | THAP11 | THAP7 | THBS1 | THEM4 | THOC6 | THRA | TIA1 | TIMP1 | TIMP2 | TINF2 | TLE1 | TLE3 | TLR3 | TMBIM6 | TMOD3 | TMOD4 | TNF | TNFAIP3 | TNFRSF1A | TNFRSF8 | TNFSF14 | TNIP2 | TNKS | TNKS2 | TNN | TNP1 | TOP2A | TP53 | TP63 | TP73 | TPCN2 | TPM1 | TPR | TRADD | TRAF2 | TRAF3IP1 | TRAF6 | TRAP1 | TREX1 | TRIAP1 | TRIB2 | TRIB3 | TRIM21 | TRIM28 | TRIM29 | TRIM32 | TRIM33 | TRIM35 | TRIM9 | TRIP12 | TRIP13 | TSC22D3 | TSC22D4 | TSG101 | TSKU | TSPYL2 | TUBB4A | TULP3 | TWF1 | TWF2 | TWIST1 | TXN | TXNDC5 | TXNIP | TYMS | U2AF2 | UBA3 | UBASH3A | UBB | UBC | UBE2B | UBE2D1 | UBE2D3 | UBE2I | UBE2J1 | UBQLN1 | UBQLN2 | UBQLN4 | UBR5 | UBXN1 | UCHL1 | UCHL5 | UFM1 | ULK1 | UNC119 | UNK | USP14 | USP17L2 | USP19 | USP2 | USP28 | USP4 | USP47 | USP7 | USP9X | VAT1 | VCL | VDAC1 | VDAC2 | VDR | VHL | VIM | VPS4B | VPS72 | VSIG4 | VTN | WARS | WAS | WDR35 | WDR77 | WDTC1 | WNK1 | WNT16 | WT1 | WWOX | WWP2 | XBP1 | XIAP | XPO1 | XRCC1 | XRCC5 | XRCC6 | YAF2 | YAP1 | YEATS2 | YME1L1 | YWHAB | YWHAE | YWHAG | YWHAH | YWHAQ | YWHAZ | YY1 | ZBTB16 | ZBTB17 | ZBTB21 | ZBTB33 | ZBTB42 | ZBTB7A | ZBTB7B | ZC3H4 | ZC3HC1 | ZFAND6 | ZFYVE19 | ZHX1 | ZMPSTE24 | ZMYND8 | ZNF207 | ZNF217 | ZNF24 | ZNF746 | ZW10 | ZWINT | GO:0034976 | 0.0213514497149096 | 176/8243 | 102/3250 | 3.98021344762251e-07 | 0.00377921266851757 | T | T | T | T | response to endoplasmic reticulum stress | ACADVL | AGR2 | AIFM1 | AMFR | ANKS4B | ARFGAP1 | ASNS | ATF6 | ATF6B | ATP2A2 | AUP1 | BAG6 | BAX | BBC3 | BCL2 | CALR | CFTR | COL4A3BP | CREB3 | CTH | CUL7 | CXCL8 | DCTN1 | DDRGK1 | DERL1 | DNAJB11 | DNAJB2 | DNAJC10 | EDEM1 | EEF2 | EIF2AK3 | EIF2AK4 | EIF2B5 | EIF2S1 | ERLIN2 | EXTL2 | FAF2 | FBXO6 | FKBP14 | FLOT1 | GFPT1 | GORASP2 | GSK3A | GSK3B | HDGF | HSP90B1 | HSPA5 | ITPR1 | JUN | LMNA | NCK1 | NFE2L2 | NPLOC4 | NRBF2 | P4HB | PDIA3 | PDIA4 | PDIA6 | PIK3R1 | PLA2G6 | PML | PPP2CB | PSMC6 | PTPN1 | RHBDD1 | RNF139 | RNF183 | RNF185 | SEC16A | SEC31A | SEL1L | SGTA | SGTB | SHC1 | SRPRB | STUB1 | SULT1A3 | SYVN1 | TARDBP | THBS1 | TMBIM6 | TMTC3 | TOR1A | TP53 | TRAF2 | TRIB3 | TRIM25 | TSPYL2 | UBA5 | UBE2J1 | UBE2K | UBQLN1 | UBQLN2 | UFC1 | UFM1 | UGGT1 | USP19 | VAPB | VCP | WIPI1 | XBP1 | ZBTB17 | GO:0002376 | 0.153584859881111 | 1266/8243 | 579/3250 | 4.10493267235855e-07 | 0.00389763357240444 | T | T | T | T | immune system process | ABCE1 | ABL1 | ACAA1 | ACKR2 | ACLY | ACTB | ACTG1 | ACTR1A | ACTR2 | ACTR3 | ADA | ADSS | AGER | AGL | AHCY | AHSG | AICDA | AIRE | AKAP8 | AKT1 | ALDOA | ALDOC | ALOX5 | ANXA1 | ANXA2 | AP1B1 | AP1G1 | AP1M1 | AP3D1 | APCS | APEH | APP | APRT | ARF1 | ARMC8 | ARPC1A | ARPC1B | ARPC2 | ARPC3 | ARPC4 | ARPC5 | ATAD5 | ATG5 | ATP1B3 | ATP6V1D | AZU1 | BAG6 | BAIAP2 | BAX | BCAR1 | BCL10 | BCL2 | BCL6 | BIRC2 | BIRC3 | BMPR1A | BRAF | BSG | BTK | BTN2A1 | BTRC | C1QA | C1QBP | C1R | C4A | CAB39 | CALR | CAND1 | CANX | CAPN1 | CAPZA1 | CAPZA2 | CAPZB | CARD9 | CASP3 | CASP8 | CAT | CAV1 | CBFB | CBL | CCL21 | CCL22 | CCR1 | CCR3 | CCR4 | CCR5 | CCT2 | CCT8 | CD2 | CD209 | CD22 | CD27 | CD36 | CD40 | CD40LG | CD6 | CD74 | CD79B | CD81 | CDA | CDC42 | CEP290 | CHST4 | CKAP4 | CLNK | CLTC | CLU | COL4A3BP | COLEC10 | COPB1 | COTL1 | CPNE3 | CREBBP | CRIP1 | CRK | CRKL | CRP | CSNK2B | CTNNB1 | CTPS1 | CTSA | CTSC | CTSD | CTSL | CTSV | CUL1 | CXCL11 | CXCL5 | CXCL6 | CXCL8 | CYB5R3 | CYLD | DBH | DBNL | DCTN1 | DDIT4 | DDOST | DDX1 | DDX3X | DEGS1 | DERA | DHX36 | DHX9 | DNAJA3 | DYNC1H1 | DYNC1I1 | DYNC1I2 | DYNC1LI1 | DYNLL1 | DYNLT1 | ECSIT | EDA | EEF1A1 | EEF2 | EIF2AK4 | EIF2B1 | EIF2B3 | EIF2B5 | ELF4 | EP300 | EPPIN | ETS1 | EXOSC5 | EXOSC6 | EXOSC9 | FABP5 | FADD | FAF2 | FANCC | FASLG | FBXW11 | FCGR1A | FCN1 | FGB | FGL2 | FKBP1A | FKBP1B | FN1 | FOXJ1 | FTH1 | FTL | FYN | GAA | GALNS | GAPDH | GBP2 | GBP3 | GBP6 | GDI2 | GHDC | GLA | GNL1 | GNS | GPI | GPS2 | GRB2 | GRB7 | GSTP1 | GUSB | GYG1 | GZMA | HAVCR2 | HDAC4 | HDAC5 | HDAC9 | HEXB | HEXIM1 | HIF1A | HIST1H2BA | HLA-A | HLA-B | HLA-DQA1 | HLA-DRA | HLA-DRB1 | HLA-DRB3 | HLA-DRB4 | HLA-DRB5 | HMGB1 | HMGB2 | HMGB3 | HMOX1 | HNF1A | HOXB6 | HPRT1 | HRAS | HRNR | HSD3B7 | HSP90AA1 | HSP90AB1 | HSP90B1 | HSPA1B | HSPA8 | HSPD1 | HUWE1 | ICAM1 | ID2 | IDH1 | IFI16 | IFIT1 | IFITM3 | IFNA2 | IGBP1 | IGF1R | IGHV3-13 | IGHV3-23 | IGHV3-30 | IGKV3-20 | IGLC7 | IKBKB | IKBKE | IKBKG | IL13RA2 | IL32 | ILF2 | ILF3 | IMPDH2 | IPO7 | IQGAP1 | IREB2 | IRF1 | IRF4 | IRF7 | ISG15 | ITCH | ITGA4 | ITGAV | ITGB3 | ITGB5 | ITK | JAK3 | JMJD6 | JUN | JUP | KCMF1 | KCNAB2 | KIF11 | KIF13B | KIF2C | KIR3DS1 | KLC1 | KLC2 | KLF6 | KLRC2 | KPNB1 | KRAS | LAMP3 | LAT | LCP1 | LCP2 | LEF1 | LGALS1 | LGALS3 | LGMN | LGR4 | LIG4 | LRRC7 | LTBR | MAN2B1 | MAP2K6 | MAP3K1 | MAP3K14 | MAPK1 | MAPK14 | MAPK3 | MAPT | MASP1 | MASP2 | MATR3 | MAVS | MBP | MCM3AP | MGST1 | MICA | MIF | MLH1 | MME | MSH6 | MSN | MTHFD1 | MYD88 | MYO1C | NAMPT | NAPRT | NCAPH2 | NCK1 | NDRG1 | NEDD4 | NFATC2 | NFKB2 | NFKBIA | NHLRC3 | NIT2 | NME2 | NOD2 | NOS2 | NTRK1 | NUP85 | OAS2 | ONECUT1 | OPTN | ORM1 | OSBPL1A | OTUB1 | OTUD4 | P2RX1 | PA2G4 | PADI4 | PAG1 | PAK2 | PARP14 | PATZ1 | PCBP2 | PDE12 | PDE4D | PDIA3 | PDXK | PFKL | PGAM1 | PGM1 | PGM2 | PGRMC1 | PI4K2A | PIK3CA | PIK3CG | PIK3R1 | PKM | PLA2G1B | PLA2G2A | PLA2G6 | PLAUR | PLCG1 | PLSCR1 | PML | PNMA1 | PNP | POLM | PPARG | PPIA | PPIE | PPP6C | PRCP | PRDX1 | PRDX2 | PRDX4 | PRDX6 | PRELID1 | PRG2 | PRKACA | PRKCD | PRKCE | PRKD1 | PRKD2 | PRKDC | PSAP | PSEN1 | PSMA1 | PSMA2 | PSMA3 | PSMA5 | PSMA6 | PSMB5 | PSMB6 | PSMB7 | PSMB8 | PSMC1 | PSMC2 | PSMC3 | PSMC4 | PSMC5 | PSMC6 | PSMD1 | PSMD10 | PSMD11 | PSMD12 | PSMD14 | PSMD2 | PSMD4 | PSMD9 | PSME2 | PSME3 | PSTPIP1 | PTGES2 | PTK2 | PTMS | PTPN22 | PTPN6 | PTPRO | PXDN | PYGB | PYGL | QSOX1 | RAB10 | RAB18 | RAB24 | RAB29 | RAB31 | RAB32 | RAB35 | RAB5B | RAB7A | RAC1 | RAF1 | RAP1A | RAP1B | RBPJ | RC3H1 | RELA | RGS1 | RHOA | RIPK1 | RIPK2 | RIPK3 | RNASE3 | RNF31 | RNF8 | ROMO1 | RPL30 | RPS19 | RPS24 | RUNX2 | S100A1 | S100A11 | S100A12 | S100A13 | S100A7 | S100A9 | S100B | S100P | SARM1 | SBDS | SCARA3 | SCG2 | SDC1 | SDC4 | SDCBP | SEC13 | SEC24B | SEC24C | SEC31A | SELL | SEMA7A | SERPINB3 | SERPINB6 | SFPQ | SH3GL2 | SHC1 | SIN3A | SIRT1 | SLAMF1 | SLAMF8 | SLC12A2 | SLC16A1 | SLC2A5 | SLC3A2 | SMAD3 | SNAP29 | SNCA | SOD1 | SP2 | SPACA3 | SPG21 | SPI1 | SPTAN1 | SPTBN2 | SQSTM1 | SRC | SRP14 | SRPK1 | SRPK2 | STAT1 | STBD1 | STK11 | STOM | STOML2 | STXBP2 | SWAP70 | TAB1 | TAB2 | TANK | TCF12 | TCIRG1 | TGFB1 | TGFBR3 | THBS1 | TIMP2 | TLR3 | TMEM173 | TMEM30A | TNF | TNFAIP3 | TNFSF12 | TNFSF14 | TNIP2 | TOLLIP | TPD52 | TRAF3 | TRAF6 | TREML2 | TREX1 | TRIM14 | TRIM21 | TRIM23 | TRIM25 | TRIM28 | TRIM29 | TRIM32 | TRIM34 | TRIM35 | TUBB | TUBB4B | TXLNA | TXNDC5 | UBB | UBC | UBE2D1 | UBE2D2 | UBE2D3 | UBE2N | UBE2V1 | UBR4 | USP14 | VAPA | VAT1 | VAV1 | VCAM1 | VCL | VCP | VSIG4 | VTN | WAS | WDR1 | WNK1 | XBP1 | XRCC5 | XRCC6 | YES1 | ZAP70 | ZBTB7A | ZBTB7B | GO:0051049 | 0.123741356302317 | 1020/8243 | 475/3250 | 4.39864000656994e-07 | 0.00417650868623815 | T | T | T | T | regulation of transport | ABAT | ABCG1 | ABL1 | ABL2 | ACAA2 | ACE2 | ACTB | ACTN2 | ACTN4 | ADA | ADCYAP1 | ADORA2A | ADRB2 | ADTRP | AGER | AHCYL1 | AHI1 | AHNAK | AHSG | AKAP9 | AKT1 | AKT2 | ANK3 | ANXA1 | ANXA2 | ANXA5 | AP1G1 | APBB1 | APOA1 | APOD | APOE | APP | ARF1 | ARFGAP1 | ARFIP1 | ARHGAP1 | ARHGEF7 | ARRB1 | ARRB2 | ASPSCR1 | ATAD1 | ATG5 | ATP13A2 | ATP1A1 | ATP1B3 | ATP2A2 | ATXN2 | AZU1 | B3GAT3 | BACE1 | BAG3 | BAG4 | BAX | BBC3 | BCL2 | BIN1 | BRAF | BTBD9 | C1QTNF1 | C4A | CAB39 | CACNA1A | CACNG2 | CACNG4 | CACNG5 | CALM1 | CALM2 | CALM3 | CALR | CAMK1D | CAMK2A | CAMK2B | CAPN1 | CASP8 | CAV1 | CBL | CCL21 | CCR1 | CD2 | CD22 | CD36 | CD40 | CD40LG | CD74 | CDH1 | CDK16 | CDK5 | CDK5R1 | CEP290 | CFTR | CHCHD10 | CHMP2A | CHP1 | CHRM2 | CLIC1 | CLIC3 | CLIC4 | CLU | COMMD1 | COMT | COX17 | CPLX1 | CPLX3 | CPT1A | CRBN | CREB1 | CRP | CRY1 | CRY2 | CRYAB | CSNK2A2 | CTNNB1 | CXCL11 | DAB2 | DAPK1 | DHX36 | DHX9 | DLG2 | DLG3 | DLG4 | DMAP1 | DMD | DNAJA1 | DNAJC30 | DNM1L | DVL1 | DYNC1H1 | DYNLL1 | E2F1 | EDEM1 | EGFR | EHD4 | EPPIN | ERBB2 | ERBB3 | EZR | FABP3 | FABP5 | FCN1 | FGB | FGFR1 | FHL1 | FIS1 | FKBP1A | FKBP1B | FLOT1 | FN1 | FYN | G6PD | GAPDH | GAPVD1 | GDF9 | GDI1 | GFAP | GIT1 | GLMN | GLP1R | GLRX | GLUL | GNAI2 | GNAO1 | GNAS | GOLPH3 | GOPC | GPD1L | GPI | GPS2 | GRIA1 | GSK3A | GSK3B | GSTO1 | HADH | HAP1 | HAVCR2 | HAX1 | HCN1 | HCN2 | HCN3 | HDAC3 | HDAC9 | HECW1 | HECW2 | HGS | HIF1A | HIST1H1A | HK1 | HK2 | HMGB1 | HMOX1 | HNF4A | HNRNPK | HRAS | HSPA1L | HSPA2 | HSPA8 | HTR1B | HTR3A | HTRA2 | HTT | HUWE1 | ICAM1 | IDH2 | IFNA2 | IL13RA2 | INSIG1 | INSR | IPO5 | ITGAV | ITGB1BP1 | ITGB3 | ITPR1 | ITPR2 | ITPR3 | JUP | KCNA4 | KCNAB1 | KCNAB2 | KCNIP2 | KCNK16 | KCNS3 | KDM5B | KHDRBS1 | KIF5B | KMT2A | LCP1 | LGALS3 | LGR4 | LRRC7 | LRRK2 | LRSAM1 | LYPLA1 | MAP1B | MAP2K1 | MAP2K6 | MAPK1 | MAPK14 | MAPK15 | MAPK3 | MAPK8 | MAVS | MBP | MDM2 | MIB1 | MIF | MPC2 | MSN | MYO1C | MYO5A | MYO6 | NCOR1 | NDUFAF2 | NEDD4 | NEUROD1 | NFE2L2 | NFKBIA | NMB | NOD2 | NOS1AP | NOS2 | NOS3 | NPEPPS | NPR1 | NR0B2 | NR1H2 | NR1H3 | NRXN1 | NTF3 | NUCB1 | NUMA1 | NUP153 | NUP214 | OAS2 | OAZ3 | ORM1 | OXCT1 | OXSR1 | P2RX1 | P2RX2 | P2RX4 | PACSIN3 | PANX3 | PARK7 | PAX8 | PCM1 | PCSK9 | PDCD10 | PDCD6IP | PDE4D | PER1 | PER2 | PFKL | PFKM | PICK1 | PIK3CG | PIK3R1 | PKIG | PLA2G10 | PLA2G1B | PLA2G6 | PLCG1 | PML | PPARD | PPARG | PPARGC1A | PPIA | PPID | PPIF | PPM1A | PPP1CC | PPP1R11 | PPP1R12A | PPP3CC | PPT1 | PRELID1 | PREPL | PRKAA1 | PRKACA | PRKCD | PRKCE | PRKCZ | PRKD1 | PSEN1 | PSEN2 | PSMD9 | PTEN | PTGER3 | PTPN1 | PTPN14 | PTPN22 | PTPN23 | PTPN6 | RAB10 | RAB11A | RAB11B | RAB21 | RAB29 | RAB31 | RAB3GAP1 | RAB5A | RAB5B | RAB7A | RAB9A | RAC1 | RAN | RANGAP1 | RAP1A | RAP1B | RAPGEF4 | RBM4 | RDX | REST | RGN | RGS2 | RHBDD1 | RHOA | RHOT1 | RHOU | RIPK1 | RIPK2 | RNF139 | RNF31 | S100A1 | SAE1 | SARAF | SCFD1 | SCYL2 | SDC1 | SDC4 | SDCBP | SEC16A | SEC24B | SEPT1 | SEPT4 | SEPT5 | SEPT8 | SERPINE2 | SFN | SH3GL1 | SH3GL2 | SH3GL3 | SIRT1 | SIRT3 | SIRT6 | SLC16A1 | SLC25A4 | SLC25A5 | SLC25A6 | SLC35F6 | SLC9A1 | SLC9A3R1 | SMAD3 | SMAD4 | SMO | SNCA | SNF8 | SNTA1 | SNX12 | SNX17 | SNX3 | SNX6 | SOCS1 | SOD1 | SP100 | SPACA3 | SPTBN1 | SRC | SRI | STAC | STIM1 | STOM | STXBP1 | STXBP2 | SUMO1 | SYN1 | SYNJ2BP | SYP | TARDBP | TBC1D4 | TFDP1 | TGFB1 | THADA | THBS1 | TM9SF4 | TMBIM6 | TMEM30A | TNF | TNFAIP3 | TNFRSF1A | TNK2 | TOR1A | TP53 | TP63 | TP73 | TPCN2 | TPR | TRAF2 | TRAF6 | TRIAP1 | TRIB3 | TRIM28 | TRIM9 | TSG101 | TWIST1 | TXN | UBE2D3 | UBE2J1 | UBE2L3 | UBQLN1 | UBQLN2 | UBR5 | UNC119 | USP17L2 | USP46 | USP50 | VPS18 | VPS4B | VTN | WDR1 | WNK1 | WNK4 | WWP2 | XBP1 | XPO1 | XPO4 | XPO5 | YES1 | YRDC | YWHAB | YWHAE | YWHAG | YWHAH | YWHAQ | YWHAZ | ZIC1 | ZMPSTE24 | GO:0016043 | 0.381050588378018 | 3141/8243 | 1345/3250 | 4.41796729006757e-07 | 0.00419485994191916 | T | T | T | T | cellular component organization | A2M | AAR2 | AASS | ABCD3 | ABCE1 | ABCG1 | ABI3BP | ABL1 | ABLIM1 | ACAA1 | ACAA2 | ACACA | ACAD9 | ACAT1 | ACD | ACOT13 | ACOX1 | ACTA1 | ACTB | ACTG1 | ACTL6A | ACTN1 | ACTN2 | ACTN4 | ACTR2 | ACTR3 | ADAMTS4 | ADCYAP1 | ADD3 | ADORA2A | ADRB2 | ADRM1 | ADSL | AEBP2 | AFG3L2 | AGER | AGK | AGO1 | AGO2 | AHI1 | AHNAK | AIFM1 | AIMP2 | AKAP8 | AKAP9 | AKT1 | AKT2 | AKT3 | AKTIP | ALAS1 | ALB | ALDH5A1 | ALDOA | ALOX5AP | AMFR | ANK1 | ANK3 | ANKRD28 | ANKS4B | ANXA1 | ANXA2 | ANXA5 | ANXA6 | ANXA7 | AP1G1 | AP1M1 | AP3D1 | APBB1 | APBB2 | APC | APCS | APEH | APEX1 | APOA1 | APOD | APOE | APP | AR | ARF1 | ARHGAP26 | ARHGAP35 | ARHGEF7 | ARID4B | ARPC1A | ARPC1B | ARPC2 | ARPC3 | ARPC4 | ARPC5 | ARRB1 | ARRB2 | ATAD3A | ATCAY | ATF2 | ATG2B | ATG4C | ATG5 | ATL3 | ATM | ATP13A2 | ATP2A2 | ATP6V1D | ATP9A | ATPAF2 | ATRX | ATXN10 | ATXN2 | AURKB | AVIL | AXIN1 | BABAM1 | BAG3 | BAG4 | BAG5 | BAG6 | BAIAP2 | BAIAP2L1 | BANF1 | BANP | BAX | BBC3 | BCAR1 | BCL10 | BCL2 | BCL2L1 | BCL6 | BCOR | BCS1L | BIN1 | BIRC2 | BIRC3 | BIRC5 | BLZF1 | BMI1 | BRAF | BRCA1 | BRD2 | BRD3 | BRD4 | BRD9 | BSG | C1QA | C1QBP | C1QL1 | C1QTNF1 | CAB39 | CABP4 | CACNG2 | CACNG4 | CACNG5 | CALM1 | CALM3 | CALR | CAMK2A | CAND1 | CAP2 | CAPG | CAPN1 | CAPN2 | CAPNS1 | CAPZA1 | CAPZA2 | CAPZB | CARM1 | CASP3 | CASP8 | CAT | CAV1 | CBL | CBR4 | CBX4 | CBX7 | CBX8 | CBY1 | CCAR2 | CCDC120 | CCDC155 | CCDC36 | CCDC68 | CCDC69 | CCDC8 | CCDC88A | CCL21 | CCNB1 | CCP110 | CCR5 | CCT2 | CCT3 | CCT6B | CCT8 | CD2 | CD2BP2 | CD36 | CD40 | CD74 | CD79B | CDA | CDC14B | CDC20 | CDC25B | CDC42 | CDC42EP3 | CDC42EP4 | CDC73 | CDH1 | CDH5 | CDK16 | CDK2 | CDK4 | CDK5 | CDK5R1 | CEBPA | CEL | CENPF | CENPJ | CENPV | CEP135 | CEP152 | CEP290 | CEP350 | CEP55 | CEP57 | CEP89 | CETN3 | CFL1 | CFL2 | CFTR | CHAF1A | CHCHD10 | CHCHD2 | CHCHD3 | CHD1L | CHD3 | CHD4 | CHD8 | CHEK2 | CHMP2A | CHP1 | CHRAC1 | CHRM2 | CIRBP | CISD2 | CKAP5 | CKS2 | CLGN | CLIC4 | CLNS1A | CLSTN1 | CLTC | CLU | CLUH | CNTROB | COA3 | COG3 | COL12A1 | COL14A1 | COL18A1 | COL2A1 | COL4A1 | COL4A2 | COL4A3BP | COL5A1 | COL6A1 | COL6A2 | COPS2 | COPS5 | COPS6 | CORO2B | COX11 | COX15 | COX17 | COX19 | COX20 | COX7A2 | CPSF7 | CPT1A | CREB1 | CREBBP | CRK | CRKL | CRMP1 | CROCC | CROT | CRYAB | CRYZ | CSNK1A1 | CSNK2B | CTBP1 | CTCF | CTH | CTNNB1 | CTNND1 | CTSC | CTSL | CTSV | CTTN | CUL1 | CUL3 | CUL4B | CUL7 | CUTC | CYCS | CYLD | DAB1 | DAB2 | DAP3 | DARS | DAXX | DBN1 | DBNL | DCLK1 | DCN | DCTN1 | DCTPP1 | DCXR | DDB1 | DDB2 | DDIT4 | DDX1 | DDX39B | DDX3X | DDX6 | DECR1 | DERL1 | DES | DHRS4 | DHX36 | DHX9 | DIAPH3 | DISC1 | DLG2 | DLG3 | DLG4 | DMAP1 | DMD | DNA2 | DNAAF2 | DNAAF5 | DNAH9 | DNAJA3 | DNM1L | DOCK7 | DPF2 | DSTN | DVL1 | DVL2 | DVL3 | DYM | DYNC1H1 | DYNC1LI1 | DYNLL1 | DYNLT1 | E2F1 | E2F4 | ECH1 | ECI2 | ECSIT | EED | EFEMP2 | EGFR | EHD4 | EHHADH | EHMT1 | EHMT2 | EIF2AK3 | EIF2S1 | EIF3A | EIF3D | EIF3E | EIF3H | EIF3I | EIF4H | EIF5 | EIF6 | ELAVL1 | ELF3 | EML4 | EP300 | EPHA4 | EPPIN | EPPK1 | EPRS | ERAL1 | ERBB2 | ERCC5 | ESR1 | ESRRA | ETS1 | EYA2 | EYA4 | EZH2 | EZR | F5 | FADD | FAF2 | FAM118B | FAM161B | FAM83H | FANCA | FANCC | FANCG | FANCM | FARSB | FBF1 | FBP1 | FBXO7 | FCGR1A | FCHSD1 | FEN1 | FERMT2 | FGB | FGF2 | FGFR1OP | FIGNL1 | FIS1 | FKBP1A | FLNB | FLOT1 | FN1 | FOLR1 | FOPNL | FOXA1 | FOXJ1 | FSCN1 | FSD1 | FUNDC2 | FUS | FYN | G3BP1 | G3BP2 | GAA | GABPB1 | GAK | GAP43 | GAPDH | GAPVD1 | GAR1 | GATA1 | GBP5 | GEMIN4 | GEMIN5 | GEMIN7 | GFAP | GGCT | GINS2 | GINS4 | GJA1 | GLRB | GLRX3 | GLS | GLUL | GNAI3 | GNAO1 | GNB1 | GOLGA2 | GOLPH3 | GOPC | GORASP2 | GPX4 | GRAMD1A | GRB2 | GRB7 | GRHPR | GRIA1 | GRID2 | GRIP1 | GSK3A | GSK3B | GSPT1 | GSPT2 | GSTK1 | H1F0 | H1FX | H2AFV | H2AFX | H2AFZ | H3F3A | HAP1 | HAX1 | HBEGF | HBZ | HCCS | HCN1 | HDAC1 | HDAC11 | HDAC2 | HDAC3 | HDAC4 | HDAC5 | HDAC6 | HDAC7 | HDAC9 | HEPACAM2 | HERC1 | HEXB | HGS | HIPK4 | HIRIP3 | HIST1H1A | HIST1H2AB | HIST1H2BA | HIST1H3A | HIST1H4A | HIST2H3A | HIST3H3 | HK2 | HLA-DRA | HLA-DRB1 | HLCS | HMGB1 | HMGB2 | HMGB3 | HMGCL | HMOX1 | HNRNPA2B1 | HNRNPU | HPRT1 | HRAS | HRNR | HSD17B10 | HSD17B4 | HSD17B8 | HSF1 | HSF4 | HSP90AA1 | HSP90AB1 | HSP90B1 | HSPA2 | HSPA4 | HSPA8 | HSPA9 | HSPD1 | HSPG2 | HTRA1 | HTRA2 | HTT | HUWE1 | ICAM1 | ICK | ID2 | IDE | IDH1 | IFT122 | IGF1R | IGHV3-13 | IGHV3-23 | IGHV3-30 | IGLC7 | IGSF21 | IKBKB | IKBKE | IKZF5 | ILK | IMMT | IMPDH2 | ING5 | INO80B | INO80C | INSR | IPO4 | IQCB1 | IRF4 | ITGA4 | ITGA7 | ITGAV | ITGB1BP1 | ITGB3 | ITGB3BP | ITGB5 | ITPA | ITPR3 | JMJD6 | JUN | JUP | KAT5 | KAT7 | KAT8 | KCNA4 | KCNIP2 | KCNS3 | KCTD12 | KCTD17 | KCTD18 | KCTD2 | KCTD3 | KDM1A | KDM2A | KDM3B | KDM4D | KDM5B | KDM5C | KDM8 | KDR | KHDRBS1 | KIF11 | KIF2C | KIF5B | KIF5C | KIFC3 | KLC1 | KLF5 | KLHL20 | KLHL22 | KMT2A | KMT2B | KMT2C | KPNB1 | KRAS | KRT17 | KRT31 | KRT71 | L3MBTL1 | L3MBTL2 | L3MBTL3 | L3MBTL4 | LAMB1 | LARP4 | LARS | LATS1 | LCP1 | LEF1 | LEMD2 | LEMD3 | LETM1 | LGMN | LIG4 | LIMA1 | LIMS1 | LMAN2L | LMNA | LNX1 | LOXL2 | LPAR1 | LRP6 | LRRC7 | LRRK2 | LSM3 | LSM4 | LUC7L2 | LUM | LURAP1 | LYRM7 | MAD2L1 | MAGI1 | MAP1B | MAPK14 | MAPK15 | MAPK3 | MAPK8 | MAPK8IP3 | MAPKAP1 | MAPT | MAST1 | MAST3 | MAT2A | MBD3 | MBP | MCCC1 | MCFD2 | MCM2 | MCM4 | MCM6 | MCM7 | MCM9 | MCMBP | MCTS1 | MDM2 | MDM4 | MDN1 | ME1 | MECP2 | MED1 | MED6 | MGST1 | MICU1 | MIF | MITF | MKS1 | MLH1 | MMP26 | MORF4L1 | MORF4L2 | MPP2 | MRGBP | MRPL12 | MRPL14 | MRPL19 | MRPL28 | MRPL38 | MRPL4 | MRPL43 | MRPL45 | MRPL46 | MRPL9 | MRPS5 | MRRF | MSH6 | MSN | MTA1 | MTA3 | MTERF1 | MTPN | MTSS1 | MYC | MYH11 | MYH6 | MYL2 | MYO1B | MYO1C | MYO1E | MYO5A | MYOC | NACC1 | NAP1L1 | NAP1L4 | NCAPH2 | NCBP1 | NCDN | NCK1 | NCOA1 | NCOA3 | NCOR1 | NDRG1 | NDUFA10 | NDUFA13 | NDUFA5 | NDUFA8 | NDUFA9 | NDUFAF1 | NDUFAF2 | NDUFAF3 | NDUFAF4 | NDUFAF5 | NDUFAF7 | NDUFB1 | NDUFS1 | NDUFS2 | NDUFS3 | NDUFS6 | NDUFS7 | NDUFS8 | NDUFV1 | NDUFV3 | NEBL | NEDD4 | NEK1 | NEK7 | NF2 | NFKB2 | NFS1 | NID1 | NIN | NIPBL | NME2 | NOD2 | NOP10 | NOS1AP | NOS2 | NOS3 | NPHP3 | NPLOC4 | NPM1 | NPTX1 | NR3C1 | NRXN1 | NSFL1C | NSL1 | NTF3 | NTMT1 | NTRK1 | NUBP1 | NUBP2 | NUDT19 | NUFIP1 | NUMA1 | NUP107 | NUP153 | NUP205 | NUP85 | NUP93 | NUP98 | NUPR1 | NUSAP1 | OAT | OBSL1 | OFD1 | OGFOD1 | OGT | ONECUT1 | OPA1 | OPTN | OTC | OXA1L | P2RX1 | P2RX2 | P2RX4 | P4HA1 | P4HB | PACS2 | PACSIN3 | PADI4 | PAFAH1B1 | PAK6 | PANX3 | PARD3 | PARD6A | PARK7 | PARP1 | PAWR | PAXIP1 | PCBD1 | PCDHB16 | PCGF1 | PCGF5 | PCGF6 | PCM1 | PCNA | PDCD10 | PDCD6 | PDCD6IP | PDCL | PDE4DIP | PDSS1 | PDSS2 | PEF1 | PER1 | PER2 | PEX11B | PEX19 | PEX5 | PFKL | PFKM | PFN1 | PGM5 | PHB | PHB2 | PHF10 | PHF21A | PHGDH | PI4K2A | PIAS1 | PICK1 | PIH1D1 | PIK3C2A | PIKFYVE | PIN1 | PINX1 | PITPNA | PITPNB | PKM | PKN2 | PLA2G1B | PLA2G2A | PLEC | PLEKHF1 | PLK1 | PLOD2 | PLS3 | PLSCR1 | PLXNB2 | PML | PMP2 | POC1B | POLA2 | POLD1 | POLDIP2 | POLE2 | POLE3 | POT1 | PPARGC1A | PPAT | PPIA | PPID | PPIF | PPM1D | PPP1CA | PPP1R12A | PPP2CA | PPP2CB | PPP2R1A | PPP2R2A | PPP3CC | PPP6C | PPP6R1 | PPT1 | PQLC1 | PRDX3 | PRDX4 | PRELID1 | PRKAA1 | PRKAA2 | PRKACA | PRKAR1A | PRKCD | PRKCE | PRKCZ | PRKD1 | PRKDC | PRMT2 | PRMT3 | PRMT6 | PROX1 | PRPF19 | PRPF40A | PRPF8 | PSAP | PSEN1 | PSEN2 | PSMD10 | PSMD11 | PSMD4 | PSMD9 | PSMG1 | PSMG4 | PSTPIP1 | PTEN | PTGES3 | PTK2 | PTMA | PTPN1 | PTPN23 | PTPRD | PTPRF | PTPRK | PTPRO | PTTG1 | PXDN | PXN | RAB10 | RAB11A | RAB18 | RAB1A | RAB1B | RAB29 | RAB2A | RAB31 | RAB32 | RAB35 | RAB3GAP1 | RAB5A | RAB5B | RAB7A | RAC1 | RAD21 | RAD23B | RAD51 | RADIL | RAE1 | RAF1 | RAN | RANBP9 | RAP1A | RAP1B | RAP1GDS1 | RARG | RB1 | RB1CC1 | RBBP4 | RBMX | RBX1 | RC3H1 | RCC1 | RDX | RECK | RELA | REST | RFC1 | RFC2 | RFC3 | RFC4 | RFC5 | RHOA | RHOJ | RHOT1 | RHOT2 | RHOU | RIC3 | RIF1 | RING1 | RIPK1 | RIPK3 | RNF19A | RNF2 | RNF8 | ROMO1 | RP2 | RPA2 | RPA3 | RPGRIP1L | RPL10 | RPL12 | RPL23 | RPL23A | RPL24 | RPL38 | RPL6 | RPLP0 | RPLP0P6 | RPS10P5 | RPS14 | RPS19 | RPS3 | RPTOR | RRM1 | RRM2 | RRP7A | RRS1 | RTF1 | RTKN | RTN4 | RUVBL1 | RUVBL2 | RXRA | S100A6 | S100B | SAMD9 | SART1 | SART3 | SBDS | SCARA3 | SCFD1 | SCLT1 | SCO2 | SDC4 | SDCBP | SEC13 | SEC16A | SEC24B | SEC24C | SEC31A | SEPT11 | SEPT2 | SEPT4 | SEPT6 | SEPT7 | SEPT9 | SERPINB5 | SERPINE2 | SERPINH1 | SET | SETDB1 | SF1 | SFMBT1 | SFN | SFPQ | SH2B1 | SH3GL1 | SH3GL2 | SH3KBP1 | SH3PXD2A | SHC1 | SHKBP1 | SHMT1 | SHMT2 | SIN3A | SIPA1 | SIPA1L1 | SIRT1 | SIRT3 | SIRT5 | SIRT6 | SIRT7 | SKI | SLC12A2 | SLC16A1 | SLC25A4 | SLC25A5 | SLC35F6 | SLC9A1 | SLC9A3R1 | SLC9A3R2 | SMAD2 | SMAD3 | SMAD4 | SMARCA2 | SMARCA4 | SMARCB1 | SMARCC1 | SMARCC2 | SMARCE1 | SMC1A | SMC3 | SMG5 | SMG6 | SMN1 | SMTNL2 | SMURF1 | SNAI2 | SNAP29 | SNCA | SNF8 | SNRPB | SNRPD1 | SNRPD3 | SNRPE | SNX2 | SNX3 | SOAT1 | SOD1 | SOD2 | SORT1 | SOX2 | SP100 | SPACA1 | SPACA3 | SPC25 | SPDL1 | SPERT | SPI1 | SPICE1 | SPIN1 | SPINT2 | SPRY2 | SPTAN1 | SPTBN1 | SPTBN2 | SQSTM1 | SRC | SRGAP2 | SRP19 | SRPK1 | SRPK2 | SRSF12 | SRSF5 | SSX2IP | ST13 | STAG2 | STAT3 | STAU1 | STIL | STK11 | STK24 | STK25 | STK26 | STMN1 | STOM | STOML2 | STRAP | STX5 | STXBP1 | SULF2 | SUMO1 | SUMO1P1 | SUN2 | SUPT4H1 | SUZ12 | SVIL | SWAP70 | SYN1 | SYNE2 | SYNJ2BP | SYP | TADA2B | TAF12 | TANGO2 | TARDBP | TAZ | TBC1D23 | TBCA | TBCE | TBL1X | TCF4 | TCP1 | TCTN2 | TCTN3 | TDO2 | TDRD3 | TEKT2 | TEKT4 | TERF1 | TERF2 | TERF2IP | TESK2 | TFDP1 | TFG | TFIP11 | TGFB1 | TGFBR3 | TGM2 | TGOLN2 | THBS1 | THEM4 | TIMM13 | TIMM21 | TIMM50 | TIMMDC1 | TIMP1 | TIMP2 | TINF2 | TK1 | TLE1 | TLE3 | TM9SF4 | TMED9 | TMEM11 | TMEM17 | TMEM216 | TMEM231 | TMEM30A | TMEM70 | TMOD3 | TMOD4 | TNF | TNFAIP3 | TNFRSF1A | TNKS | TNN | TNNT1 | TNP1 | TNXB | TOMM20 | TOMM22 | TOP1 | TOP2A | TOP3A | TOR1A | TP53 | TP63 | TP73 | TPCN2 | TPM1 | TPM2 | TPM3 | TPM4 | TPR | TPSAB1 | TRADD | TRAF1 | TRAF2 | TRAPPC8 | TREX1 | TRIAP1 | TRIM21 | TRIM28 | TRIM32 | TRIM34 | TRIP13 | TRIP6 | TRMT61B | TSG101 | TSGA10 | TSKU | TSPYL2 | TTC19 | TTLL1 | TUBA1A | TUBA1C | TUBA4A | TUBB | TUBB2A | TUBB2B | TUBB3 | TUBB4A | TUBB4B | TUBB6 | TUBB8 | TUBG1 | TUBGCP3 | TWF1 | TWF2 | TXNL4A | UBB | UBC | UBE2A | UBE2B | UBE2C | UBE2D1 | UBE2D2 | UBE2D3 | UBE2K | UBE2N | UBE2S | UBQLN1 | UBQLN2 | UBXN10 | UGDH | UGT8 | ULK1 | UNK | UQCC1 | UQCRB | USP15 | USP16 | USP21 | USP39 | USP4 | USP50 | USP7 | USP8 | USP9X | USPL1 | UXS1 | VAPA | VAPB | VASP | VAV1 | VBP1 | VCAM1 | VCAN | VCL | VCP | VCPIP1 | VDR | VHL | VIM | VPS18 | VPS37D | VPS4B | VPS72 | VPS8 | VTA1 | VTN | WAS | WDR1 | WDR19 | WDR35 | WDR45B | WDR77 | WDR82 | WDTC1 | WIPI1 | WIPI2 | WT1 | XIRP2 | XRCC1 | XRCC3 | XRCC5 | XRCC6 | YAP1 | YEATS2 | YKT6 | YME1L1 | YWHAB | YWHAE | YWHAG | YWHAH | YWHAQ | YWHAZ | ZBTB7A | ZFAND6 | ZMPSTE24 | ZMYND10 | ZNF207 | ZW10 | ZWINT | GO:0031400 | 0.0497391726313235 | 410/8243 | 210/3250 | 4.65542480413674e-07 | 0.00442032585152784 | T | T | T | T | negative regulation of protein modification process | ABL1 | ADORA2A | AKT1 | APC | APOE | ARRB1 | ARRB2 | ATG5 | BAG2 | BAG5 | BAX | BCOR | BDKRB2 | BRCA1 | C1QL4 | CALM1 | CALM2 | CALM3 | CASP3 | CAV1 | CBL | CBLB | CBLC | CCNB1 | CD109 | CDK5 | CDKN1A | CDKN2C | CEBPA | CHORDC1 | CHP1 | CHRNA9 | CRKL | CRY1 | CRY2 | CTBP1 | CTNNB1 | CYLD | DBNDD2 | DDIT4 | DMD | DNAJA1 | DNAJC10 | DUSP1 | DUSP14 | DUSP16 | DUSP19 | DUSP22 | DUSP7 | DVL1 | EIF3A | ERRFI1 | EZR | FBXO7 | FGFR1OP | FKBP1A | FKBP1B | FKBP8 | FOXM1 | FOXO1 | FYN | G6PD | GLMN | GNAI2 | GNAQ | GPD1L | GPS2 | GSK3B | GSTP1 | HDAC2 | HDAC3 | HEXIM1 | HSPA1B | HSPB1 | IBTK | IGBP1 | IGF1R | IKBKB | ILK | IPO5 | IPO7 | ISG15 | ITCH | ITGB1BP1 | JUN | KDM1A | KDM4D | LATS1 | LDLRAD4 | LEMD2 | LGALS3 | LRP6 | LRRK1 | LRRK2 | MAD2L1 | MAPT | MAS1 | MECP2 | MLLT1 | MYC | NCK1 | NCOR1 | NF2 | NNT | NPM1 | NTF3 | OGT | OTUB1 | PAFAH1B1 | PAK2 | PARD3 | PARD6A | PARK7 | PARP14 | PDE4D | PEBP1 | PER1 | PER2 | PHB | PIAS3 | PIH1D1 | PIN1 | PINX1 | PKIG | PLK1 | PNKP | PPARGC1A | PPME1 | PPP1R11 | PPP1R14A | PPP1R2 | PPP1R8 | PPP2CA | PPP2R1A | PPP2R5D | PRKAA1 | PRKAA2 | PRKAR1A | PRKAR2B | PRKCD | PRKCE | PRKCZ | PRKDC | PRMT3 | PRMT6 | PSEN1 | PSMD10 | PTEN | PTN | PTPN1 | PTPN22 | PTPN6 | PTPRR | QARS | RANBP9 | RB1 | RELA | RGN | RGS2 | RPL23 | RPS3 | RPTOR | SAG | SERPINB3 | SET | SFN | SIN3A | SIRT1 | SIRT3 | SKI | SLC9A3R1 | SMAD4 | SMARCB1 | SNCA | SNTA1 | SNX6 | SOCS1 | SPI1 | SPRY2 | STK38 | STRAP | SWAP70 | SYNJ2BP | TAOK3 | TARDBP | TERF2IP | TGFB1 | TINF2 | TNF | TNFAIP3 | TRAF3IP1 | TRIB2 | TRIB3 | TRIM21 | TRIP12 | TSG101 | TWIST1 | U2AF2 | UBE2B | UBR5 | UBXN1 | UCHL1 | USP17L2 | USP4 | WARS | WNK1 | XRCC1 | YWHAB | YWHAE | YWHAG | GO:0006094 | 0.0043673419871406 | 36/8243 | 29/3250 | 5.26525516398827e-07 | 0.00499935977820686 | T | T | T | T | gluconeogenesis | ALDOA | ALDOC | CRY1 | ENO1 | ENO2 | ENO3 | FBP1 | FBP2 | GAPDH | GAPDHS | GOT1 | GOT2 | GPD1 | GPI | MDH1 | MDH2 | PC | PER2 | PGAM1 | PGAM2 | PGK1 | PGK2 | PGM1 | PPARGC1A | SLC25A1 | SLC25A10 | SLC25A11 | SLC25A13 | TPI1 | GO:0048583 | 0.288729831372073 | 2380/8243 | 1037/3250 | 5.69863093953407e-07 | 0.0054108500770876 | T | T | T | T | regulation of response to stimulus | A2M | ABAT | ABCE1 | ABL1 | ABL2 | ACAA2 | ACE2 | ACTB | ACTG1 | ACTN2 | ACTN4 | ACTR2 | ACTR3 | ADA | ADCYAP1 | ADORA2A | ADRB2 | ADTRP | AES | AGER | AGO1 | AGR2 | AHSG | AKAP9 | AKR1B1 | AKR1C2 | AKT1 | AKT3 | ALOX12 | ANXA1 | ANXA2 | ANXA5 | AP1G1 | APBB1 | APC | APCS | APLP1 | APOA1 | APOD | APOE | APP | AR | ARAF | ARFGAP1 | ARHGAP1 | ARHGAP19 | ARHGAP26 | ARHGAP35 | ARHGDIA | ARHGEF7 | ARHGEF9 | ARNT | ARPC1A | ARPC1B | ARPC2 | ARPC3 | ARPC4 | ARPC5 | ARRB1 | ARRB2 | ASCL1 | ATAD5 | ATF6 | ATF6B | ATG5 | ATM | ATP2A2 | AURKB | AXIN1 | AZU1 | BABAM1 | BACE1 | BAG1 | BAG2 | BAG3 | BAG4 | BAG5 | BAG6 | BAIAP2 | BAIAP2L1 | BANP | BAX | BBC3 | BCAR1 | BCL10 | BCL2 | BCL2L1 | BCL2L2 | BCL6 | BDKRB2 | BIRC2 | BIRC3 | BIRC7 | BIRC8 | BMPR1A | BRAF | BRCA1 | BRD4 | BTK | BTN2A1 | BTRC | BUD31 | C1QA | C1QBP | C1QL4 | C1QTNF1 | C1R | C4A | CACNG2 | CACNG4 | CACNG5 | CALM1 | CALM2 | CALM3 | CALR | CAMK1D | CAMK2A | CAMK2B | CAPN1 | CARD9 | CARM1 | CASP10 | CASP2 | CASP8 | CASP9 | CAT | CAV1 | CBFB | CBL | CBLB | CBLC | CBX8 | CBY1 | CCAR2 | CCDC22 | CCL21 | CCL22 | CCNY | CCR1 | CCR4 | CD109 | CD209 | CD22 | CD27 | CD36 | CD40 | CD40LG | CD6 | CD74 | CD79B | CD81 | CDC34 | CDC37 | CDC42 | CDC42SE2 | CDC73 | CDH5 | CDK16 | CDK19 | CDK2 | CDK4 | CDK5 | CDK5R1 | CDK9 | CDKN1A | CEBPA | CEP55 | CFTR | CHCHD10 | CHCHD2 | CHD3 | CHD4 | CHD8 | CHEK2 | CHGB | CHORDC1 | CHP1 | CHRD | CHRNA9 | CLEC11A | CLNK | CLU | CMTM5 | COL2A1 | COLEC10 | COPA | COPS5 | CORT | CREB3 | CREBBP | CRK | CRKL | CRP | CRY1 | CRY2 | CRYAB | CSNK1A1 | CSNK1E | CSNK2A2 | CSNK2B | CTDP1 | CTH | CTNNB1 | CTNNBIP1 | CTNND1 | CTSC | CTSL | CTTN | CUL1 | CUL3 | CXCL11 | CXCL5 | CXCL6 | CXCL8 | CYLD | DAB1 | DAB2 | DAPK1 | DAXX | DBH | DBNL | DCN | DDIT4 | DDRGK1 | DDX1 | DDX39B | DDX3X | DENND1A | DEPDC7 | DHX36 | DHX9 | DISC1 | DLG2 | DLG3 | DLG4 | DMD | DNA2 | DNAJA1 | DNAJB1 | DNAJC7 | DNASE2 | DNM1L | DOK1 | DROSHA | DTX1 | DUSP1 | DUSP14 | DUSP16 | DUSP19 | DUSP22 | DUSP7 | DVL1 | DVL2 | DVL3 | DYNLL1 | DYNLT1 | DYRK2 | E2F1 | EDA | EDEM1 | EEF1D | EFEMP1 | EGFR | EHMT1 | EHMT2 | EIF2AK3 | EIF2AK4 | EIF2B1 | EIF2B3 | EIF2B5 | EIF3A | EIF4A3 | ELF3 | ENDOG | ENO1 | EP300 | EPHA4 | EPM2A | EPPK1 | ERBB2 | ERBB3 | ERRFI1 | ESR1 | ESR2 | ESRRA | ETS1 | EXOSC6 | EYA2 | EYA4 | EZH2 | EZR | FABP5 | FABP7 | FADD | FAF1 | FAM129B | FAM19A3 | FAM3C | FAM49B | FAM83D | FANCA | FASLG | FBP1 | FBRS | FBXO7 | FBXW11 | FCGR1A | FCN1 | FGB | FGF2 | FGFR1 | FGFR3 | FHL2 | FIGNL1 | FIS1 | FKBP1A | FKBP1B | FKBP8 | FLOT1 | FN1 | FOLR1 | FOS | FOXA1 | FOXJ1 | FOXM1 | FOXO1 | FUS | FYN | G3BP1 | G6PD | GADD45G | GATA1 | GBP5 | GDF9 | GDI1 | GDI2 | GGT1 | GIT1 | GJA1 | GKAP1 | GLRX3 | GNA12 | GNA13 | GNAI1 | GNAI2 | GNAI3 | GNAQ | GOLPH3 | GOLT1B | GOT1 | GPC5 | GPD1L | GPI | GPS2 | GPX1 | GPX4 | GRB2 | GRB7 | GRIA1 | GRID2 | GRIK2 | GRK1 | GRK5 | GSK3A | GSK3B | GSTO1 | GSTP1 | H2AFX | HAP1 | HAVCR2 | HAX1 | HBEGF | HDAC1 | HDAC2 | HDAC3 | HDAC6 | HDAC7 | HDGF | HECW1 | HEXIM1 | HGS | HIF1A | HIF1AN | HINT1 | HIPK4 | HK1 | HLA-A | HLA-B | HLA-DQA1 | HLA-DRA | HLA-DRB1 | HLA-DRB3 | HLA-DRB4 | HLA-DRB5 | HLX | HMGB1 | HMGB2 | HMGB3 | HMOX1 | HNRNPK | HPX | HRAS | HSF1 | HSP90AA1 | HSP90AB1 | HSP90B1 | HSPA1B | HSPA1L | HSPA5 | HSPA8 | HSPB1 | HSPD1 | HSPH1 | HTR1B | HTRA1 | HTRA2 | HTT | ICAM1 | IFI16 | IFIT1 | IFNA2 | IFT122 | IGBP1 | IGF1R | IGHV3-13 | IGHV3-23 | IGHV3-30 | IGKV3-20 | IGLC7 | IKBKB | IKBKE | IKBKG | IL13RA2 | IL32 | ILK | ING5 | INSIG1 | INSR | IQGAP1 | IRF1 | IRF4 | IRF7 | ITCH | ITGA4 | ITGAV | ITGB1BP1 | ITGB3 | ITK | ITPR1 | JAK1 | JAK3 | JUN | JUP | KANK2 | KAT5 | KDM1A | KDM4D | KDR | KHDRBS1 | KIF5B | KIR3DL2 | KLHDC10 | KLHL22 | KMT2A | KRAS | KSR1 | L3MBTL1 | LANCL1 | LARS | LASP1 | LAT | LATS1 | LCP2 | LDLRAD4 | LEF1 | LEMD2 | LEMD3 | LGALS1 | LGALS3 | LGMN | LGR4 | LIMS1 | LMNA | LPAR1 | LPAR4 | LRP6 | LRRC7 | LRRK1 | LRRK2 | LRSAM1 | LTBP1 | LTBR | LURAP1 | LZTR1 | MAOA | MAP2K1 | MAP2K3 | MAP2K4 | MAP2K6 | MAP2K7 | MAP3K1 | MAP3K13 | MAP3K14 | MAP3K2 | MAP3K3 | MAP3K4 | MAP4K1 | MAPK1 | MAPK14 | MAPK3 | MAPK7 | MAPK8 | MAPK8IP3 | MAPK9 | MAPKAP1 | MAPT | MAS1 | MASP1 | MASP2 | MATR3 | MAVS | MBD3 | MC1R | MCC | MDFI | MDM2 | MDM4 | MED1 | METAP2 | METRNL | MGMT | MICA | MIF | MKS1 | MLH1 | MLLT11 | MLLT3 | MLST8 | MMP26 | MPC2 | MSH6 | MTPN | MYC | MYD88 | MYO1C | MYO5A | MYOC | NAMPT | NCK1 | NCOA1 | NCOA3 | NCOR1 | NCOR2 | NDUFA13 | NDUFAF2 | NDUFS3 | NEDD4 | NELFE | NEUROD1 | NF2 | NFATC1 | NFATC2 | NFE2L2 | NFKBIA | NKX2-1 | NLK | NMB | NMNAT1 | NOD2 | NOS1AP | NOS2 | NOS3 | NPHP3 | NPLOC4 | NPM1 | NPR1 | NPTX1 | NQO2 | NR1H2 | NR1H3 | NRXN1 | NSMCE4A | NTF3 | NTRK1 | NUMA1 | NUP107 | NUP153 | NUP160 | NUP205 | NUP214 | NUP85 | NUP88 | NUP93 | NUP98 | OGT | ONECUT1 | OPA1 | OPTN | OTUB1 | OTUD4 | OXCT1 | OXSR1 | P2RX2 | P2RX4 | P4HB | PAFAH1B1 | PAG1 | PAK2 | PAK6 | PAM16 | PARK7 | PARP1 | PARP14 | PAWR | PAXIP1 | PCBP2 | PCNA | PCSK9 | PDCD10 | PDCD6 | PDCL | PDE4D | PDE6G | PDIA3 | PDK2 | PEBP1 | PELI1 | PER1 | PER2 | PHB | PHB2 | PIANP | PIAS1 | PIGU | PIH1D1 | PIK3CA | PIK3CG | PIK3R1 | PIN1 | PIP4K2A | PLA2G1B | PLA2G2A | PLA2G6 | PLAUR | PLCG1 | PLEKHF1 | PLSCR1 | PML | POM121 | PPARD | PPARG | PPARGC1A | PPEF1 | PPIF | PPM1A | PPM1B | PPP1CA | PPP1R2 | PPP2CA | PPP2CB | PPP2R1A | PPP2R1B | PPP3CC | PPP4R2 | PPP5C | PRCP | PRDX1 | PRDX2 | PRELID1 | PRKAA1 | PRKAA2 | PRKAB2 | PRKACA | PRKAG1 | PRKCD | PRKCE | PRKCZ | PRKD1 | PRKD2 | PRKDC | PRMT2 | PRMT6 | PSAP | PSEN1 | PSMA1 | PSMA2 | PSMA3 | PSMA5 | PSMA6 | PSMB5 | PSMB6 | PSMB7 | PSMB8 | PSMC1 | PSMC2 | PSMC3 | PSMC4 | PSMC5 | PSMC6 | PSMD1 | PSMD10 | PSMD11 | PSMD12 | PSMD14 | PSMD2 | PSMD4 | PSMD9 | PSME2 | PSME3 | PTEN | PTGER3 | PTGES3 | PTGIR | PTK2 | PTN | PTPMT1 | PTPN1 | PTPN22 | PTPN6 | PTPRF | PTPRO | PTPRR | PUM1 | PXDN | PXN | PYCR1 | QARS | RAB29 | RAB7A | RAC1 | RAD51 | RAD51AP1 | RAE1 | RAF1 | RANBP9 | RAP1A | RAP1B | RARA | RASL11B | RB1 | RB1CC1 | RBBP4 | RBPJ | RBX1 | RC3H1 | RDH11 | RDX | RECK | REL | RELA | RFC2 | RFC3 | RFC4 | RFC5 | RGN | RGS1 | RGS2 | RGS20 | RHO | RHOA | RHOJ | RHOT1 | RHOT2 | RHOU | RIC8B | RIF1 | RIPK1 | RIPK2 | RIPK3 | RNASEH2B | RNF126 | RNF183 | RNF185 | RNF31 | RNF8 | RPA2 | RPA3 | RPGRIP1L | RPL23 | RPS19 | RPS3 | RPTOR | RRM2B | RRS1 | RTN4 | RUNX1 | RUNX2 | RUVBL1 | RUVBL2 | S100A1 | S100A12 | S100A13 | S100A4 | S100A7 | S100A9 | S100B | SAG | SARM1 | SCARA3 | SCEL | SCG2 | SCYL2 | SDCBP | SEC13 | SELL | SEMA7A | SEPT4 | SERPINB2 | SERPINB3 | SERPINE2 | SFN | SFPQ | SGTA | SH2B1 | SH3GL1 | SH3GL2 | SH3KBP1 | SHC1 | SHKBP1 | SIAH1 | SIN3A | SIPA1 | SIPA1L1 | SIRT1 | SIRT3 | SIRT6 | SKI | SLAMF1 | SLAMF8 | SLC25A5 | SLC25A6 | SLC35F6 | SLC9A1 | SLC9A3R1 | SMAD2 | SMAD3 | SMAD4 | SMARCA4 | SMARCB1 | SMO | SMURF1 | SMURF2 | SNAI2 | SNCA | SNF8 | SNW1 | SNX3 | SNX6 | SOCS1 | SOCS2 | SOCS6 | SOD1 | SOD2 | SOX2 | SP100 | SPATA2 | SPERT | SPG21 | SPIN1 | SPRY2 | SPTBN1 | SQSTM1 | SRC | SRGAP2 | SRGAP3 | SRI | SSX2IP | STARD13 | STAT1 | STAT3 | STAT6 | STK11 | STK24 | STK25 | STK26 | STK3 | STK38 | STK40 | STMN1 | STOML2 | STRAP | STUB1 | STXBP1 | STXBP2 | STYX | SULF2 | SUMO1 | SWAP70 | SYDE1 | SYNJ2BP | SYP | SYVN1 | TAB1 | TAB2 | TAF12 | TAF15 | TANK | TAOK3 | TBC1D23 | TBL1X | TBXA2R | TCF21 | TERF2 | TERF2IP | TFDP1 | TFG | TFIP11 | TGFB1 | TGFBR3 | TGM2 | THBS1 | THEM4 | THRA | TIMP1 | TIMP2 | TLE1 | TLE3 | TLR3 | TMBIM6 | TMEM173 | TMEM9B | TNF | TNFAIP3 | TNFRSF12A | TNFRSF1A | TNFSF12 | TNFSF14 | TNIP2 | TNKS | TNKS2 | TNN | TNNT1 | TOP3A | TOR1A | TP53 | TP53RK | TP63 | TP73 | TPR | TRADD | TRAF1 | TRAF2 | TRAF3 | TRAF3IP1 | TRAF6 | TRAP1 | TREML2 | TREX1 | TRIAP1 | TRIB2 | TRIB3 | TRIM25 | TRIM28 | TRIM32 | TRIM33 | TRIM63 | TRIP12 | TRIP6 | TSG101 | TSKU | TSPYL2 | TTC5 | TUBB2B | TULP3 | TWF1 | TWIST1 | TXLNA | TXN | UBA5 | UBASH3A | UBB | UBC | UBE2B | UBE2D1 | UBE2D2 | UBE2D3 | UBE2I | UBE2J1 | UBE2K | UBE2N | UBE2O | UBE2V1 | UBQLN1 | UBQLN2 | UBR5 | UBXN1 | UCHL1 | UFM1 | USP14 | USP17L2 | USP19 | USP20 | USP47 | USP8 | VAPA | VAV1 | VCAM1 | VCP | VDAC2 | VGF | VHL | VSIG4 | VTN | WAS | WBP2 | WDFY1 | WDR35 | WNK1 | WNT16 | WNT2 | WWOX | WWP2 | XBP1 | XIAP | XRCC1 | XRCC5 | XRCC6 | YAP1 | YES1 | YWHAB | YWHAE | YWHAG | YWHAH | YWHAQ | YWHAZ | YY1 | ZAP70 | ZBTB7A | ZBTB7B | ZC3HC1 | ZDHHC17 | ZFAND6 | ZIC1 | ZMPSTE24 | ZMYND8 | ZNF277 | ZNF622 | GO:0009966 | 0.222855756399369 | 1837/8243 | 815/3250 | 5.71302763423302e-07 | 0.00542451973870425 | T | T | T | T | regulation of signal transduction | A2M | ABAT | ABCE1 | ABL1 | ABL2 | ACAA2 | ACTN2 | ACTN4 | ADA | ADCYAP1 | ADORA2A | ADRB2 | AES | AGER | AGO1 | AGR2 | AHSG | AKAP9 | AKR1B1 | AKR1C2 | AKT1 | AKT3 | APC | APLP1 | APOA1 | APOD | APOE | APP | AR | ARAF | ARFGAP1 | ARHGAP1 | ARHGAP19 | ARHGAP26 | ARHGAP35 | ARHGDIA | ARHGEF7 | ARHGEF9 | ARNT | ARRB1 | ARRB2 | ASCL1 | ATAD5 | ATF6 | ATF6B | ATM | AURKB | AXIN1 | BAG4 | BAG5 | BAIAP2L1 | BANP | BAX | BBC3 | BCL10 | BCL2 | BCL2L1 | BCL2L2 | BCL6 | BDKRB2 | BIRC2 | BIRC3 | BIRC7 | BIRC8 | BMPR1A | BRAF | BRCA1 | BRD4 | BTRC | BUD31 | C1QBP | C1QL4 | C1QTNF1 | CACNG2 | CACNG4 | CACNG5 | CALM1 | CALM2 | CALM3 | CALR | CAPN1 | CARD9 | CARM1 | CASP10 | CASP2 | CASP8 | CAT | CAV1 | CBFB | CBL | CBLB | CBLC | CBY1 | CCAR2 | CCDC22 | CCL21 | CCL22 | CCNY | CCR1 | CD109 | CD22 | CD27 | CD36 | CD40 | CD40LG | CD74 | CD81 | CDC34 | CDC37 | CDC42 | CDC42SE2 | CDC73 | CDK2 | CDK4 | CDK5 | CDK5R1 | CEP55 | CHCHD10 | CHD3 | CHD4 | CHD8 | CHEK2 | CHGB | CHP1 | CHRD | CHRNA9 | CLEC11A | CLNK | CLU | CMTM5 | COL2A1 | COPA | COPS5 | CORT | CREB3 | CREBBP | CRK | CRKL | CRY1 | CRY2 | CSNK1A1 | CSNK1E | CSNK2A2 | CSNK2B | CTH | CTNNB1 | CTNNBIP1 | CTNND1 | CTSC | CTTN | CUL3 | CXCL11 | CXCL5 | CXCL6 | CXCL8 | CYLD | DAB1 | DAB2 | DAPK1 | DAXX | DBH | DBNL | DCN | DDIT4 | DDRGK1 | DDX1 | DDX3X | DENND1A | DEPDC7 | DHX36 | DISC1 | DLG2 | DLG3 | DLG4 | DMD | DNA2 | DNAJA1 | DNM1L | DOK1 | DTX1 | DUSP1 | DUSP14 | DUSP16 | DUSP19 | DUSP22 | DUSP7 | DVL1 | DVL2 | DVL3 | DYNLT1 | DYRK2 | E2F1 | EDA | EEF1D | EFEMP1 | EGFR | EHMT1 | EHMT2 | EIF2AK3 | EIF3A | EIF4A3 | ELF3 | ENO1 | EP300 | EPHA4 | EPM2A | ERBB2 | ERBB3 | ERRFI1 | ESR1 | ESR2 | EYA2 | EYA4 | EZH2 | EZR | FABP5 | FADD | FAF1 | FAM129B | FAM3C | FAM83D | FANCA | FASLG | FBP1 | FBRS | FBXW11 | FGB | FGF2 | FGFR1 | FGFR3 | FHL2 | FIGNL1 | FIS1 | FKBP1A | FKBP1B | FKBP8 | FLOT1 | FN1 | FOLR1 | FOXA1 | FOXM1 | FOXO1 | FYN | G3BP1 | GADD45G | GATA1 | GDF9 | GDI1 | GDI2 | GIT1 | GJA1 | GKAP1 | GNA12 | GNA13 | GNAI1 | GNAI2 | GNAI3 | GNAQ | GOLPH3 | GOLT1B | GOT1 | GPC5 | GPD1L | GPI | GPS2 | GPX1 | GRB2 | GRB7 | GRIA1 | GRIK2 | GRK1 | GRK5 | GSK3A | GSK3B | GSTO1 | GSTP1 | HAP1 | HAVCR2 | HAX1 | HBEGF | HDAC1 | HDAC2 | HDAC3 | HDAC6 | HDAC7 | HDGF | HECW1 | HEXIM1 | HGS | HIF1A | HIF1AN | HINT1 | HIPK4 | HMGB1 | HMGB2 | HMOX1 | HNRNPK | HPX | HRAS | HSF1 | HSP90AA1 | HSP90AB1 | HSPA1B | HSPA5 | HSPB1 | HTRA1 | HTRA2 | HTT | ICAM1 | IFNA2 | IFT122 | IGBP1 | IGF1R | IKBKB | IKBKE | IKBKG | IL32 | ILK | ING5 | INSIG1 | INSR | IQGAP1 | IRF1 | IRF4 | IRF7 | ITCH | ITGAV | ITGB1BP1 | ITGB3 | ITPR1 | JAK1 | JAK3 | JUN | JUP | KANK2 | KAT5 | KDM1A | KDR | KHDRBS1 | KLHDC10 | KLHL22 | KRAS | KSR1 | L3MBTL1 | LARS | LASP1 | LAT | LATS1 | LDLRAD4 | LEF1 | LEMD2 | LEMD3 | LGALS1 | LGALS3 | LGMN | LGR4 | LIMS1 | LMNA | LPAR1 | LPAR4 | LRP6 | LRRC7 | LRRK1 | LRRK2 | LTBP1 | LTBR | LURAP1 | LZTR1 | MAOA | MAP2K1 | MAP2K3 | MAP2K4 | MAP2K6 | MAP2K7 | MAP3K1 | MAP3K13 | MAP3K14 | MAP3K2 | MAP3K3 | MAP3K4 | MAP4K1 | MAPK1 | MAPK14 | MAPK3 | MAPK7 | MAPK8 | MAPK8IP3 | MAPK9 | MAPKAP1 | MAPT | MAS1 | MAVS | MBD3 | MC1R | MCC | MDFI | MDM2 | MDM4 | MED1 | METAP2 | METRNL | MIF | MKS1 | MLLT11 | MLLT3 | MLST8 | MYC | MYD88 | MYO1C | MYO5A | MYOC | NAMPT | NCK1 | NCOA1 | NCOA3 | NCOR1 | NCOR2 | NDUFA13 | NDUFS3 | NEDD4 | NELFE | NEUROD1 | NF2 | NFATC1 | NFE2L2 | NFKBIA | NKX2-1 | NLK | NMB | NMNAT1 | NOD2 | NOS1AP | NOS3 | NPHP3 | NPLOC4 | NPR1 | NPTX1 | NQO2 | NR1H2 | NR1H3 | NRXN1 | NTF3 | NTRK1 | NUMA1 | NUP93 | OGT | ONECUT1 | OPA1 | OPTN | OTUD4 | P2RX2 | P2RX4 | P4HB | PAFAH1B1 | PAG1 | PAK2 | PAK6 | PAM16 | PARK7 | PARP1 | PARP14 | PAWR | PCSK9 | PDCD10 | PDCD6 | PDCL | PDE4D | PDE6G | PDIA3 | PDK2 | PEBP1 | PELI1 | PER1 | PHB | PHB2 | PIAS1 | PIGU | PIH1D1 | PIK3CA | PIK3CG | PIK3R1 | PIN1 | PIP4K2A | PLA2G1B | PLA2G2A | PLA2G6 | PLAUR | PLEKHF1 | PLSCR1 | PML | PPARD | PPARG | PPARGC1A | PPEF1 | PPIF | PPM1A | PPM1B | PPP1CA | PPP1R2 | PPP2CA | PPP2CB | PPP2R1A | PPP2R1B | PPP3CC | PPP5C | PRCP | PRDX1 | PRDX2 | PRELID1 | PRKAA1 | PRKAA2 | PRKAB2 | PRKACA | PRKAG1 | PRKCD | PRKCE | PRKCZ | PRKD1 | PRKD2 | PRMT2 | PRMT6 | PSAP | PSEN1 | PSMA1 | PSMA2 | PSMA3 | PSMA5 | PSMA6 | PSMB5 | PSMB6 | PSMB7 | PSMB8 | PSMC1 | PSMC2 | PSMC3 | PSMC4 | PSMC5 | PSMC6 | PSMD1 | PSMD10 | PSMD11 | PSMD12 | PSMD14 | PSMD2 | PSMD4 | PSMD9 | PSME2 | PSME3 | PTEN | PTGIR | PTK2 | PTN | PTPMT1 | PTPN1 | PTPN22 | PTPN6 | PTPRO | PTPRR | PUM1 | PXDN | PXN | QARS | RAB29 | RAB7A | RAC1 | RAF1 | RANBP9 | RAP1A | RAP1B | RASL11B | RB1 | RB1CC1 | RBBP4 | RBPJ | RBX1 | RC3H1 | RDH11 | RDX | RECK | REL | RELA | RFC2 | RFC3 | RFC4 | RFC5 | RGN | RGS1 | RGS2 | RGS20 | RHO | RHOA | RHOJ | RHOT1 | RHOT2 | RHOU | RIC8B | RIPK1 | RIPK2 | RIPK3 | RNF126 | RNF183 | RNF31 | RPA2 | RPA3 | RPGRIP1L | RPL23 | RPS3 | RPTOR | RRM2B | RRS1 | RTN4 | RUNX1 | RUNX2 | RUVBL1 | RUVBL2 | S100A12 | S100A13 | S100A4 | S100A7 | S100A9 | S100B | SAG | SARM1 | SCEL | SCG2 | SCYL2 | SDCBP | SEC13 | SEMA7A | SEPT4 | SERPINB3 | SERPINE2 | SFN | SFPQ | SH2B1 | SH3GL1 | SH3GL2 | SH3KBP1 | SHC1 | SHKBP1 | SIAH1 | SIPA1 | SIPA1L1 | SIRT1 | SIRT3 | SKI | SLAMF1 | SLC25A5 | SLC35F6 | SLC9A1 | SLC9A3R1 | SMAD2 | SMAD3 | SMAD4 | SMARCA4 | SMARCB1 | SMO | SMURF1 | SMURF2 | SNAI2 | SNCA | SNF8 | SNW1 | SNX3 | SNX6 | SOCS1 | SOCS2 | SOCS6 | SOD1 | SOD2 | SOX2 | SP100 | SPATA2 | SPERT | SPIN1 | SPRY2 | SPTBN1 | SQSTM1 | SRC | SRGAP2 | SRGAP3 | SRI | SSX2IP | STARD13 | STAT1 | STAT3 | STK11 | STK25 | STK3 | STK38 | STK40 | STMN1 | STRAP | STUB1 | STYX | SULF2 | SUMO1 | SYDE1 | SYNJ2BP | SYP | SYVN1 | TAB1 | TAB2 | TAF12 | TAF15 | TANK | TAOK3 | TBL1X | TCF21 | TERF2IP | TFDP1 | TFG | TGFB1 | TGFBR3 | TGM2 | THBS1 | THEM4 | THRA | TIMP1 | TIMP2 | TLE1 | TLE3 | TLR3 | TMBIM6 | TMEM9B | TNF | TNFAIP3 | TNFRSF12A | TNFRSF1A | TNFSF12 | TNFSF14 | TNIP2 | TNKS | TNKS2 | TNN | TOP3A | TP53 | TP53RK | TP63 | TP73 | TRADD | TRAF1 | TRAF2 | TRAF3IP1 | TRAF6 | TRAP1 | TREX1 | TRIAP1 | TRIB2 | TRIB3 | TRIM25 | TRIM32 | TRIM33 | TRIP6 | TSG101 | TSKU | TSPYL2 | TTC5 | TULP3 | TWIST1 | TXLNA | TXN | UBA5 | UBASH3A | UBB | UBC | UBE2B | UBE2I | UBE2K | UBE2N | UBE2O | UBE2V1 | UBQLN1 | UBQLN2 | UBR5 | UCHL1 | UFM1 | USP17L2 | USP20 | USP47 | USP8 | VAPA | VAV1 | VCP | VDAC2 | VGF | VHL | VTN | WBP2 | WDFY1 | WDR35 | WNK1 | WNT16 | WNT2 | WWOX | WWP2 | XBP1 | XIAP | YAP1 | YWHAB | YWHAE | YWHAG | YWHAH | YWHAQ | YWHAZ | ZAP70 | ZBTB7A | ZBTB7B | ZC3HC1 | ZDHHC17 | ZFAND6 | ZIC1 | ZMPSTE24 | ZMYND8 | ZNF622 | GO:0023057 | 0.0979012495450685 | 807/8243 | 383/3250 | 6.51301434644857e-07 | 0.00618410712195292 | T | T | T | T | negative regulation of signaling | ABL1 | ABL2 | ACAA2 | ADA | ADRB2 | AES | AHSG | AKT1 | ANXA5 | APC | APLP1 | APOA1 | APOD | APOE | AR | ARF1 | ARHGAP35 | ARHGEF7 | ARRB1 | ARRB2 | ATAD1 | ATAD5 | ATF6B | ATM | AXIN1 | BAG5 | BAX | BCL2 | BCL2L1 | BCL2L2 | BCL6 | BDKRB2 | BRAF | BRCA1 | BTRC | C1QBP | C1QL4 | CALM1 | CALM2 | CALM3 | CALR | CASP8 | CAV1 | CBL | CBLB | CBLC | CBY1 | CCAR2 | CCDC22 | CD109 | CD22 | CD74 | CDC34 | CDC42 | CHD8 | CHP1 | CHRD | CHRNA9 | CLU | COL2A1 | CREB3 | CRY1 | CRY2 | CSNK1A1 | CSNK2A2 | CTH | CTNNB1 | CTNNBIP1 | CTNND1 | CTTN | CUL3 | CXCL8 | CYLD | DAB1 | DAB2 | DCN | DDIT4 | DDX3X | DNAJA1 | DUSP1 | DUSP14 | DUSP16 | DUSP19 | DUSP22 | DUSP7 | DVL1 | DVL2 | DVL3 | DYRK2 | EGFR | EIF3A | EIF4A3 | ENO1 | EPM2A | ERBB2 | ERBB3 | ERRFI1 | ESR1 | EYA2 | EYA4 | EZH2 | EZR | FADD | FAM129B | FASLG | FBP1 | FBXW11 | FGB | FHL2 | FIGNL1 | FKBP1A | FKBP1B | FOXM1 | FOXO1 | FYN | G3BP1 | GATA1 | GNAI2 | GNAI3 | GPD1L | GPS2 | GPX1 | GRB2 | GRIA1 | GRIK2 | GSK3A | GSK3B | GSTO1 | GSTP1 | HADH | HBEGF | HDAC1 | HDAC3 | HDAC7 | HECW1 | HGS | HIF1A | HIF1AN | HMGB2 | HMOX1 | HSPA1B | HSPA5 | HSPB1 | HTR1B | HTRA1 | HTRA2 | HTT | ICAM1 | IFT122 | IGBP1 | IGF1R | INSIG1 | IRF4 | ITCH | ITGAV | ITGB1BP1 | ITPR1 | KANK2 | KDM1A | LATS1 | LDLRAD4 | LEMD2 | LEMD3 | LGALS3 | LGMN | LGR4 | LMNA | LPAR1 | LRP6 | LRRK2 | LTBP1 | LZTR1 | MAPK14 | MAPK7 | MAPKAP1 | MCC | MDFI | MDM2 | MIF | MLLT3 | MYC | MYOC | NCK1 | NCOR1 | NCOR2 | NDUFA13 | NDUFAF2 | NDUFS3 | NEDD4 | NEUROD1 | NF2 | NFATC1 | NFE2L2 | NFKBIA | NKX2-1 | NLK | NMB | NOS3 | NPHP3 | NPLOC4 | NR1H2 | NR1H3 | ONECUT1 | OPA1 | OPTN | OTUD4 | PAFAH1B1 | PAM16 | PARK7 | PARP14 | PAWR | PCSK9 | PDCD6 | PDE4D | PEBP1 | PER1 | PFKL | PHB | PHB2 | PICK1 | PIH1D1 | PIN1 | PLA2G6 | PLAUR | PPARG | PPIF | PPM1A | PPM1B | PPP2CA | PPP2CB | PPP2R1A | PRDX2 | PRELID1 | PRKAA1 | PRKAA2 | PRKACA | PRKCD | PRKCZ | PSEN1 | PSMA1 | PSMA2 | PSMA3 | PSMA5 | PSMA6 | PSMB5 | PSMB6 | PSMB7 | PSMB8 | PSMC1 | PSMC2 | PSMC3 | PSMC4 | PSMC5 | PSMC6 | PSMD1 | PSMD10 | PSMD11 | PSMD12 | PSMD14 | PSMD2 | PSMD4 | PSMD9 | PSME2 | PSME3 | PTEN | PTGIR | PTPN1 | PTPN22 | PTPN6 | PTPRO | PTPRR | PXDN | QARS | RAB7A | RAF1 | RANBP9 | RAP1A | RAP1B | RASL11B | RB1 | RB1CC1 | RBX1 | RELA | REST | RGS1 | RGS2 | RGS20 | RHOA | RIPK1 | RNF126 | RPGRIP1L | RRM2B | RUNX2 | RUVBL2 | SARM1 | SCG2 | SCYL2 | SERPINB3 | SERPINE2 | SH3GL2 | SH3KBP1 | SIAH1 | SIRT1 | SIRT3 | SKI | SLAMF1 | SLC25A5 | SLC35F6 | SLC9A3R1 | SMAD2 | SMAD3 | SMAD4 | SMARCA4 | SMURF1 | SMURF2 | SNAI2 | SNCA | SNX6 | SOCS1 | SOCS2 | SOCS6 | SOD2 | SOX2 | SPERT | SPRY2 | SRC | SRI | STAT1 | STK11 | STK3 | STK38 | STMN1 | STRAP | STUB1 | STXBP1 | SULF2 | SYNJ2BP | SYVN1 | TANK | TAOK3 | TCF21 | TGFB1 | TGFBR3 | THBS1 | THEM4 | TIMP2 | TLE1 | TLE3 | TLR3 | TMBIM6 | TNF | TNFAIP3 | TNN | TP63 | TRADD | TRAF2 | TRAF3IP1 | TRAP1 | TREX1 | TRIAP1 | TRIB3 | TRIM32 | TRIM33 | TSG101 | TSKU | TULP3 | TWIST1 | UBASH3A | UBB | UBC | UBE2B | UBQLN1 | UCHL1 | USP47 | VDAC2 | VHL | WWOX | WWP2 | XBP1 | YAP1 | YWHAB | ZBTB7A | ZC3HC1 | GO:0015980 | 0.0103117796918598 | 85/8243 | 56/3250 | 6.54324225175816e-07 | 0.00621280851804437 | T | T | T | T | energy derivation by oxidation of organic compounds | ACADVL | ADSL | AGL | AKT1 | AKT2 | CALM1 | CAT | COX15 | COX20 | COX5A | COX6A1 | COX6C | CYCS | CYP1A2 | GAA | GBE1 | GFPT1 | GFPT2 | GNAS | GSK3A | GSK3B | GYG1 | GYG2 | GYS1 | LYRM7 | MDH2 | MYC | NDUFAF2 | NDUFS1 | NDUFS7 | NDUFS8 | NDUFV1 | OXA1L | PER2 | PFKM | PGM1 | PGM2 | PHKA1 | PPARGC1A | PPP1CA | PPP1CB | PPP1CC | PPP1R2 | PPP1R3B | PPP1R3C | PYGB | PYGL | SDHB | SIRT3 | SLC25A13 | STBD1 | STK40 | TBRG4 | UGP2 | UQCRB | UQCRC2 | GO:0043632 | 0.0431881596506126 | 356/8243 | 185/3250 | 6.57448099655861e-07 | 0.0062424697062324 | T | T | T | T | modification-dependent macromolecule catabolic process | ADRM1 | AMFR | ANAPC4 | ARMC8 | ARRB1 | ARRB2 | AUP1 | AURKB | BAG6 | BIRC2 | BTRC | CBL | CBLC | CCNB1 | CDC16 | CDC20 | CDC27 | CDC34 | CDK2 | CRBN | CTNNB1 | CUL1 | CUL2 | CUL3 | CUL4B | CUL5 | CUL7 | CYLD | DDB1 | DERL1 | DNAJB2 | DNAJC10 | EDEM1 | ERLIN2 | EXOSC9 | FAF1 | FAF2 | FBXL12 | FBXO11 | FBXO6 | FBXO7 | FBXW11 | FBXW5 | FZR1 | GSK3A | HDAC6 | HECW1 | HECW2 | HERC2 | HSP90B1 | HSPA5 | HUWE1 | ISG15 | ITCH | KAT5 | KCTD17 | KCTD2 | KLHL20 | KLHL22 | LNX1 | MAD2L1 | MDM2 | MTA1 | NEDD4 | NEDD8 | NFE2L2 | NHLRC3 | NPLOC4 | NSFL1C | PCBP2 | PELI1 | PLK1 | PML | PPP1R11 | PPP2CB | PSMA1 | PSMA2 | PSMA3 | PSMA5 | PSMA6 | PSMB5 | PSMB6 | PSMB7 | PSMB8 | PSMC1 | PSMC2 | PSMC3 | PSMC4 | PSMC5 | PSMC6 | PSMD1 | PSMD10 | PSMD11 | PSMD12 | PSMD14 | PSMD2 | PSMD4 | PSMD9 | PSME2 | PSME3 | PTPN23 | PTTG1 | RAD23A | RAD23B | RBBP6 | RBX1 | RNF126 | RNF185 | RNF19A | RNF4 | RNF8 | SEL1L | SGTA | SGTB | SIAH1 | SIRT1 | SMURF1 | SMURF2 | SPOP | SPSB3 | SQSTM1 | STUB1 | SYVN1 | TBL1X | TOLLIP | TRIM25 | TRIM32 | TRIM9 | TRIP12 | TSG101 | UBA6 | UBB | UBC | UBE2A | UBE2B | UBE2C | UBE2D1 | UBE2D2 | UBE2D3 | UBE2H | UBE2I | UBE2J1 | UBE2K | UBE2L3 | UBE2L6 | UBE2R2 | UBE2S | UBE2W | UBE3A | UBE3B | UBQLN1 | UBQLN2 | UBQLN4 | UBR4 | UBXN1 | UCHL1 | UCHL3 | UCHL5 | USP11 | USP14 | USP15 | USP16 | USP17L2 | USP19 | USP2 | USP20 | USP21 | USP26 | USP28 | USP4 | USP42 | USP46 | USP47 | USP5 | USP50 | USP7 | USP8 | USP9Y | VCP | VPS37D | VPS4B | WWP2 | XBP1 | ZMPSTE24 | ZNRF4 | GO:0044282 | 0.0279024626956205 | 230/8243 | 127/3250 | 6.58160675306953e-07 | 0.00624923561203952 | T | T | T | T | small molecule catabolic process | AASS | ABAT | ABCD3 | ACAA1 | ACAA2 | ACAD8 | ACADS | ACADSB | ACADVL | ACAT1 | ACAT2 | ACOX1 | ADA | ADH5 | ADTRP | AHCY | AICDA | AKR1A1 | AKR1B10 | ALDH1B1 | ALDH4A1 | ALDH5A1 | ALDH6A1 | ALDH7A1 | ALDH8A1 | AMDHD2 | APOE | BCAT2 | BCKDHB | BDH1 | CBS | CDA | CEL | CPT1A | CPT2 | CROT | CTH | CYP1A1 | DBT | DCXR | DECR1 | DERA | DLD | DLST | ECH1 | ECHDC2 | ECHS1 | ECI1 | ECI2 | EHHADH | ENTPD7 | ESD | ETFA | ETFB | ETFDH | FAH | GAD2 | GALE | GCDH | GLS | GLS2 | GLUD2 | GLUL | GNPDA1 | GNPDA2 | GOT1 | GOT2 | GPT2 | GSTZ1 | HAAO | HADH | HAGH | HIBADH | HIBCH | HMGCL | HPRT1 | HSD17B10 | HSD17B4 | HSD3B7 | IMPA1 | IMPA2 | IVD | KYNU | LDHD | LPIN3 | MCAT | MCCC1 | MPST | MTAP | MTMR7 | MTRR | NAGK | NOS2 | NOS3 | NUDT18 | NUDT19 | NUDT3 | NUDT9 | OAT | OGDH | OTC | OXCT1 | PADI4 | PAH | PCBD1 | PFKL | PFKM | PFKP | PGM1 | PGM2 | PNP | PPARD | PPAT | PPM1K | PRTFDC1 | PTEN | QDPR | RPE | SHMT1 | SHMT2 | SNX17 | SORD | SULT1A3 | TDO2 | TPI1 | TXN2 | UPP2 | GO:1901606 | 0.00521654737352906 | 43/8243 | 33/3250 | 6.73594807380181e-07 | 0.00639578269607482 | T | T | T | T | alpha-amino acid catabolic process | AASS | ACAT1 | AHCY | ALDH4A1 | ALDH7A1 | ALDH8A1 | BCAT2 | CBS | DLD | DLST | FAH | GAD2 | GCDH | GLS | GLS2 | GLUD2 | GLUL | GOT1 | GOT2 | GPT2 | GSTZ1 | HAAO | KYNU | MTRR | NOS2 | NOS3 | OAT | OGDH | PADI4 | PPAT | SHMT1 | SHMT2 | TDO2 | GO:0010648 | 0.0976586194346718 | 805/8243 | 382/3250 | 6.86780058792683e-07 | 0.00652097665823653 | T | T | T | T | negative regulation of cell communication | ABL1 | ABL2 | ACAA2 | ADA | ADRB2 | AES | AHSG | AKT1 | ANXA5 | APC | APLP1 | APOA1 | APOD | APOE | AR | ARF1 | ARHGAP35 | ARHGEF7 | ARRB1 | ARRB2 | ATAD1 | ATAD5 | ATF6B | ATM | AXIN1 | BAG5 | BAX | BCL2 | BCL2L1 | BCL2L2 | BCL6 | BDKRB2 | BRAF | BRCA1 | BTRC | C1QBP | C1QL4 | CALM1 | CALM2 | CALM3 | CALR | CASP8 | CAV1 | CBL | CBLB | CBLC | CBY1 | CCAR2 | CCDC22 | CD109 | CD22 | CD74 | CDC34 | CDC42 | CHD8 | CHP1 | CHRD | CHRNA9 | CLU | COL2A1 | CREB3 | CRY1 | CRY2 | CSNK1A1 | CSNK2A2 | CTH | CTNNB1 | CTNNBIP1 | CTNND1 | CTTN | CUL3 | CXCL8 | CYLD | DAB1 | DAB2 | DCN | DDIT4 | DDX3X | DNAJA1 | DUSP1 | DUSP14 | DUSP16 | DUSP19 | DUSP22 | DUSP7 | DVL1 | DVL2 | DVL3 | DYRK2 | EGFR | EIF3A | EIF4A3 | ENO1 | EPM2A | ERBB2 | ERBB3 | ERRFI1 | ESR1 | EYA2 | EYA4 | EZH2 | EZR | FADD | FAM129B | FASLG | FBP1 | FBXW11 | FGB | FHL2 | FIGNL1 | FKBP1A | FKBP1B | FOXM1 | FOXO1 | FYN | G3BP1 | GATA1 | GNAI2 | GNAI3 | GPD1L | GPS2 | GPX1 | GRB2 | GRIA1 | GRIK2 | GSK3A | GSK3B | GSTO1 | GSTP1 | HADH | HBEGF | HDAC1 | HDAC3 | HDAC7 | HECW1 | HGS | HIF1A | HIF1AN | HMGB2 | HMOX1 | HSPA1B | HSPA5 | HSPB1 | HTR1B | HTRA1 | HTRA2 | HTT | ICAM1 | IFT122 | IGBP1 | IGF1R | INSIG1 | IRF4 | ITCH | ITGAV | ITGB1BP1 | ITPR1 | KANK2 | KDM1A | LATS1 | LDLRAD4 | LEMD2 | LEMD3 | LGALS3 | LGMN | LGR4 | LMNA | LPAR1 | LRP6 | LRRK2 | LTBP1 | LZTR1 | MAPK14 | MAPK7 | MAPKAP1 | MCC | MDFI | MDM2 | MIF | MLLT3 | MYC | MYOC | NCK1 | NCOR1 | NCOR2 | NDUFA13 | NDUFAF2 | NDUFS3 | NEDD4 | NEUROD1 | NF2 | NFATC1 | NFE2L2 | NFKBIA | NKX2-1 | NLK | NMB | NOS3 | NPHP3 | NPLOC4 | NR1H2 | NR1H3 | ONECUT1 | OPA1 | OPTN | OTUD4 | PAFAH1B1 | PAM16 | PARK7 | PARP14 | PAWR | PDCD6 | PDE4D | PEBP1 | PER1 | PFKL | PHB | PHB2 | PICK1 | PIH1D1 | PIN1 | PLA2G6 | PLAUR | PPARG | PPIF | PPM1A | PPM1B | PPP2CA | PPP2CB | PPP2R1A | PRDX2 | PRELID1 | PRKAA1 | PRKAA2 | PRKACA | PRKCD | PRKCZ | PSEN1 | PSMA1 | PSMA2 | PSMA3 | PSMA5 | PSMA6 | PSMB5 | PSMB6 | PSMB7 | PSMB8 | PSMC1 | PSMC2 | PSMC3 | PSMC4 | PSMC5 | PSMC6 | PSMD1 | PSMD10 | PSMD11 | PSMD12 | PSMD14 | PSMD2 | PSMD4 | PSMD9 | PSME2 | PSME3 | PTEN | PTGIR | PTPN1 | PTPN22 | PTPN6 | PTPRO | PTPRR | PXDN | QARS | RAB7A | RAF1 | RANBP9 | RAP1A | RAP1B | RASL11B | RB1 | RB1CC1 | RBX1 | RELA | REST | RGS1 | RGS2 | RGS20 | RHOA | RIPK1 | RNF126 | RPGRIP1L | RRM2B | RUNX2 | RUVBL2 | SARM1 | SCG2 | SCYL2 | SERPINB3 | SERPINE2 | SH3GL2 | SH3KBP1 | SIAH1 | SIRT1 | SIRT3 | SKI | SLAMF1 | SLC25A5 | SLC35F6 | SLC9A3R1 | SMAD2 | SMAD3 | SMAD4 | SMARCA4 | SMURF1 | SMURF2 | SNAI2 | SNCA | SNX6 | SOCS1 | SOCS2 | SOCS6 | SOD2 | SOX2 | SPERT | SPRY2 | SRC | SRI | STAT1 | STK11 | STK3 | STK38 | STMN1 | STRAP | STUB1 | STXBP1 | SULF2 | SYNJ2BP | SYVN1 | TANK | TAOK3 | TCF21 | TGFB1 | TGFBR3 | THBS1 | THEM4 | TIMP2 | TLE1 | TLE3 | TLR3 | TMBIM6 | TNF | TNFAIP3 | TNN | TP63 | TRADD | TRAF2 | TRAF3IP1 | TRAP1 | TREX1 | TRIAP1 | TRIB3 | TRIM32 | TRIM33 | TSG101 | TSKU | TULP3 | TWIST1 | UBASH3A | UBB | UBC | UBE2B | UBQLN1 | UCHL1 | USP47 | VDAC2 | VHL | WWOX | WWP2 | XBP1 | YAP1 | YWHAB | ZBTB7A | ZC3HC1 | GO:0050778 | 0.0570180759432246 | 470/8243 | 236/3250 | 6.88762703685178e-07 | 0.00653980187149076 | T | T | T | T | positive regulation of immune response | ABL1 | ACTB | ACTG1 | ACTR2 | ACTR3 | ADA | ANXA1 | AP1G1 | APCS | ARPC1A | ARPC1B | ARPC2 | ARPC3 | ARPC4 | ARPC5 | ATAD5 | BAG6 | BAIAP2 | BAX | BCAR1 | BCL10 | BCL2 | BIRC2 | BIRC3 | BRAF | BTK | BTN2A1 | BTRC | C1QA | C1QBP | C1R | C4A | CARD9 | CASP8 | CAV1 | CD209 | CD36 | CD40 | CD74 | CD79B | CDC42 | CLU | COLEC10 | CREBBP | CRK | CRKL | CRP | CTSL | CUL1 | CYLD | DDX1 | DHX36 | DHX9 | EIF2AK4 | EIF2B1 | EIF2B3 | EIF2B5 | EP300 | EXOSC6 | FADD | FAM49B | FBXW11 | FCGR1A | FCN1 | FGB | FLOT1 | FYN | GBP5 | GRB2 | HAVCR2 | HEXIM1 | HK1 | HLA-A | HLA-B | HLA-DQA1 | HLA-DRA | HLA-DRB1 | HLA-DRB3 | HLA-DRB4 | HLA-DRB5 | HLX | HMGB1 | HMGB2 | HMGB3 | HPX | HRAS | HSP90AA1 | HSP90AB1 | HSP90B1 | HSPA1B | HSPD1 | IFI16 | IGHV3-13 | IGHV3-23 | IGHV3-30 | IGKV3-20 | IGLC7 | IKBKB | IKBKE | IKBKG | IRF7 | ITCH | ITK | KRAS | LAT | LCP2 | LGMN | MAP2K6 | MAP3K1 | MAPK1 | MAPK14 | MAPK3 | MASP1 | MASP2 | MATR3 | MAVS | MED1 | MICA | MIF | MLH1 | MSH6 | MYD88 | MYO1C | NCK1 | NFATC2 | NFKBIA | NOD2 | NR1H3 | PAG1 | PAK2 | PARK7 | PAXIP1 | PDE4D | PELI1 | PHB | PIK3CA | PIK3R1 | PLA2G1B | PLA2G6 | PLCG1 | PLSCR1 | PRKACA | PRKCD | PRKCE | PRKCZ | PRKD2 | PRKDC | PSEN1 | PSMA1 | PSMA2 | PSMA3 | PSMA5 | PSMA6 | PSMB5 | PSMB6 | PSMB7 | PSMB8 | PSMC1 | PSMC2 | PSMC3 | PSMC4 | PSMC5 | PSMC6 | PSMD1 | PSMD10 | PSMD11 | PSMD12 | PSMD14 | PSMD2 | PSMD4 | PSMD9 | PSME2 | PSME3 | PTK2 | PTPN22 | PTPN6 | PUM1 | RAB29 | RAC1 | RAF1 | RAP1A | RARA | RC3H1 | RELA | RIF1 | RIPK1 | RIPK2 | RNF31 | RPS19 | RPS3 | RTN4 | S100A1 | S100A9 | SCARA3 | SEMA7A | SFPQ | SIN3A | SIRT1 | SPG21 | SRC | STAT6 | STK11 | STOML2 | STXBP1 | TAB1 | TAB2 | TANK | TGFB1 | TLR3 | TMEM173 | TNF | TNFAIP3 | TNIP2 | TRAF2 | TRAF3 | TRAF6 | TREX1 | UBB | UBC | UBE2D1 | UBE2D2 | UBE2D3 | UBE2K | UBE2N | UBE2V1 | USP17L2 | VAV1 | VSIG4 | WAS | WDFY1 | WNK1 | XBP1 | XRCC5 | XRCC6 | YES1 | ZAP70 | GO:0035556 | 0.121557685308747 | 1002/8243 | 466/3250 | 7.02054494023241e-07 | 0.00666600742075067 | T | T | T | T | intracellular signal transduction | ABL1 | ACKR2 | ACTN2 | ADCY6 | ADCYAP1 | ADORA2A | ADRB2 | ADTRP | AIFM1 | AKR1B1 | AKT1 | AKT2 | AKT3 | ANP32A | APBB2 | APOA1 | APOE | ARAF | ARHGAP1 | ARHGDIA | ARHGEF7 | ASB15 | ASB16 | ASB6 | ASB9 | ATAD5 | ATM | ATP2A2 | ATRX | AZU1 | BABAM1 | BAG6 | BAX | BBC3 | BCAR3 | BCL10 | BCL2 | BCL2L1 | BCL2L2 | BCL6 | BIRC2 | BIRC3 | BRAF | BRCA1 | BTK | BTRC | C1QBP | CA8 | CAB39 | CALM1 | CALM2 | CALM3 | CARD9 | CARM1 | CASP2 | CASP3 | CASP8 | CASP9 | CCDC68 | CCDC88A | CCNB1 | CCR1 | CCR3 | CCR4 | CCR5 | CD209 | CD36 | CD40 | CDC14B | CDC25C | CDC42 | CDC42EP3 | CDC42EP4 | CDC5L | CDK2 | CDKN1A | CFL1 | CHEK2 | CHP1 | CLNK | CRIP1 | CRKL | CRYAB | CUL1 | CUL2 | CUL3 | CUL5 | CXCL11 | CXCL8 | CYLD | DAB1 | DAPK1 | DBNL | DCLK1 | DDIT4 | DDX3X | DEPDC7 | DGKE | DGKZ | DIABLO | DLG2 | DLG3 | DLG4 | DMD | DNAJA3 | DNAJC10 | DOCK5 | DOCK7 | DOK1 | DUSP1 | DUSP7 | DVL1 | DVL2 | DVL3 | DYRK2 | E2F1 | E2F2 | E2F4 | EGFR | EIF2AK3 | EIF4EBP1 | EP300 | ERBB2 | ERBB3 | EZR | FBXW11 | FGF2 | FGFR1 | FGFR3 | FIS1 | FKBP1B | FKBP8 | FLOT1 | FOXM1 | FYN | FZR1 | G3BP1 | G3BP2 | GDI1 | GDI2 | GLP1R | GNA12 | GNA13 | GNAI2 | GNAQ | GNAS | GNB1 | GPS2 | GPX1 | GRB2 | GRIP1 | GSK3B | GSTO1 | HBEGF | HDAC4 | HINT1 | HMOX1 | HRAS | HSF1 | HSPB1 | HTRA2 | ICK | IFI16 | IFT122 | IGF1R | IKBKB | IKBKE | IKBKG | IL3RA | ILK | IRF7 | ITCH | ITGAV | ITGB1BP1 | ITK | ITPR1 | ITPR2 | ITPR3 | JAK1 | JAK3 | JUN | KAT5 | KAT7 | KBTBD7 | KDR | KPNB1 | KRAS | KSR1 | LAT | LATS1 | LCP1 | LCP2 | LGR4 | LRRC7 | LRRK2 | MAP2K1 | MAP2K3 | MAP2K4 | MAP2K6 | MAP2K7 | MAP3K1 | MAP3K13 | MAP3K14 | MAP3K2 | MAP3K3 | MAP3K4 | MAP4K1 | MAPK1 | MAPK14 | MAPK15 | MAPK3 | MAPK4 | MAPK6 | MAPK7 | MAPK8 | MAPK9 | MAPKAP1 | MAS1 | MAST1 | MAST3 | MBP | MC1R | MDM2 | MDM4 | MED1 | MELK | MIF | MKNK2 | MLH1 | MLLT11 | MLST8 | MOB1A | MOB1B | MSH6 | MYBBP1A | MYC | MYO6 | NDRG1 | NEK1 | NEUROD1 | NFATC1 | NFATC2 | NFKB2 | NFKBIA | NLK | NME2 | NOD2 | NOS2 | NOS3 | NPR1 | NR5A2 | NTRK1 | NUPR1 | OGT | OXSR1 | P2RX4 | PAG1 | PAK2 | PAK6 | PARK7 | PAXIP1 | PCNA | PDCD10 | PDE4D | PDK2 | PEBP1 | PIK3C2A | PIK3CA | PIK3CG | PIK3R1 | PIK3R3 | PIKFYVE | PKN2 | PLA2G1B | PLCG1 | PLK1 | PML | POLR2M | PPM1D | PPP2CA | PPP2R1A | PPP3CC | PPP5C | PRKAA1 | PRKAA2 | PRKACA | PRKAR1A | PRKAR2B | PRKCD | PRKCE | PRKCSH | PRKCZ | PRKD1 | PRKD2 | PRKD3 | PRKDC | PRPF19 | PSEN1 | PSEN2 | PSMA1 | PSMA2 | PSMA3 | PSMA5 | PSMA6 | PSMB5 | PSMB6 | PSMB7 | PSMB8 | PSMC1 | PSMC2 | PSMC3 | PSMC4 | PSMC5 | PSMC6 | PSMD1 | PSMD10 | PSMD11 | PSMD12 | PSMD14 | PSMD2 | PSMD4 | PSMD9 | PSME2 | PSME3 | PTEN | PTGER3 | PTGIR | PTK2 | PTPN6 | PTPRA | RAB10 | RAB11A | RAB11B | RAB18 | RAB1A | RAB1B | RAB21 | RAB24 | RAB29 | RAB2A | RAB31 | RAB32 | RAB35 | RAB5A | RAB5B | RAB7A | RAB9A | RABL2A | RABL3 | RAC1 | RAF1 | RANBP9 | RAP1A | RAP1B | RAPGEF4 | RASD1 | RB1 | RBX1 | RDX | REL | RELA | RFXANK | RHOA | RHOJ | RHOT1 | RHOT2 | RHOU | RIPK1 | RIPK2 | RIPK3 | RIT1 | RNF31 | RPS6KA1 | RPS6KA2 | RPS6KB2 | RPTOR | RTKN | S100A1 | SCG2 | SDCBP | SDCBP2 | SETDB1 | SFN | SH2B1 | SHC1 | SIPA1 | SIRT1 | SLC9A1 | SLC9A3R1 | SMAD2 | SMAD4 | SNW1 | SOCS1 | SOCS2 | SOCS6 | SP100 | SPTAN1 | SPTBN1 | SPTBN2 | SQSTM1 | SRC | SRPK1 | SRPK2 | STAC | STK11 | STK24 | STK25 | STK26 | STK3 | STK38 | STMN1 | SULT1A3 | SYNJ2BP | TAB1 | TAB2 | TANK | TAOK3 | TBXA2R | TFDP1 | TGFB1 | TGFBR3 | THEM4 | TLR3 | TMBIM6 | TMEM173 | TNF | TNFAIP3 | TNFRSF1A | TNIP2 | TNK2 | TOLLIP | TP53 | TP63 | TP73 | TPCN2 | TRADD | TRAF2 | TRAF6 | TREX1 | TRIAP1 | TRIB3 | TRIM28 | TUFT1 | UBB | UBC | UBE2K | UBE2N | UBE2V1 | USP28 | USP50 | USP8 | VAV1 | VCAM1 | WAS | WNK1 | WNK4 | WSB1 | WWOX | XBP1 | YAP1 | YWHAB | YWHAE | ZAP70 | ZBTB33 | ZMPSTE24 | ZNF622 | GO:0032434 | 0.0110396700230499 | 91/8243 | 59/3250 | 7.48612671607291e-07 | 0.00710807731691123 | T | T | T | T | regulation of proteasomal ubiquitin-dependent protein catabolic process | AKT1 | ARAF | BAG2 | BAG5 | BAG6 | CAV1 | CBFA2T3 | CCAR2 | CEBPA | CLU | COMMD1 | CSNK1A1 | CSNK1E | DAB2 | DDA1 | DDRGK1 | DNAJB2 | DVL1 | GLMN | GNA12 | GSK3A | GSK3B | HSP90AB1 | HSPA1B | HSPBP1 | KEAP1 | LRRK2 | MAPK9 | MDM2 | NFE2L2 | OGT | PARK7 | PIAS1 | PLK1 | PSEN1 | PSMD10 | RAD23A | RAD23B | RBX1 | RNF19A | SDCBP | SGTA | SMARCC1 | STUB1 | STYX | SUMO1 | SUMO2 | TRIB2 | TRIB3 | UBE2K | UBQLN1 | UBQLN2 | UBQLN4 | UBXN1 | UCHL5 | USP14 | USP5 | USP7 | VCP | GO:0032787 | 0.0289942981924057 | 239/8243 | 131/3250 | 7.59759879626326e-07 | 0.00721392005705197 | T | T | T | T | monocarboxylic acid metabolic process | AASDH | AASDHPPT | ABCD3 | ACAA1 | ACAA2 | ACACA | ACAD9 | ACADS | ACADSB | ACADVL | ACAT1 | ACAT2 | ACLY | ACOT7 | ACOX1 | ACSL1 | ACSL4 | ACSL5 | ACSS2 | ADH1A | ADH1B | AKR1C2 | ALDH4A1 | ALDH5A1 | ALDH8A1 | ALOX12 | ALOX5 | ALOX5AP | ATP6V1B1 | BRCA1 | BSG | CBR1 | CBR4 | CD36 | CD74 | CES1 | CPT1A | CPT2 | CROT | CYP1A1 | CYP1A2 | CYP2C18 | DECR1 | DEGS1 | DLAT | DLD | ECH1 | ECHDC2 | ECHS1 | ECI1 | ECI2 | EHHADH | ETFA | ETFB | ETFDH | FABP3 | FASN | GCDH | GGT1 | GLO1 | GOT2 | GPX1 | GPX4 | GRHPR | GSTA1 | GSTM4 | GSTP1 | HAAO | HADH | HAGH | HIBCH | HIF1A | HLCS | HSD17B4 | HSD17B8 | HSD3B7 | IDH1 | IDH2 | IVD | KYNU | LDHA | LDHAL6B | LDHB | LDHC | LTC4S | LYPLA1 | LYPLA2 | MAPK14 | MAPK3 | MCAT | MCCC1 | ME1 | MGST1 | MIF | MYO5A | NDUFS6 | NR5A2 | OSBPL1A | PARK7 | PC | PDHA1 | PDHA2 | PDHB | PDHX | PER2 | PGD | PHGDH | PLA2G10 | PLA2G1B | PPARD | PPARG | PPARGC1A | PRKAA1 | PRKAA2 | PRKAB2 | PRKAG1 | PRKAR2B | PTGDS | PTGES | PTGES2 | PTGES3 | PTGR1 | RBP1 | SGPL1 | SLC16A1 | SNCA | TECR | THEM4 | TNFRSF1A | VDAC1 | XBP1 | GO:0050776 | 0.0770350600509523 | 635/8243 | 308/3250 | 8.49554174908347e-07 | 0.00806651689075475 | T | T | T | T | regulation of immune response | A2M | ABCE1 | ABL1 | ACTB | ACTG1 | ACTR2 | ACTR3 | ADA | ADCYAP1 | AGER | ANXA1 | AP1G1 | APCS | APOA1 | APOE | ARPC1A | ARPC1B | ARPC2 | ARPC3 | ARPC4 | ARPC5 | ARRB2 | ATAD5 | ATG5 | BAG6 | BAIAP2 | BAX | BCAR1 | BCL10 | BCL2 | BCL6 | BIRC2 | BIRC3 | BRAF | BTK | BTN2A1 | BTRC | C1QA | C1QBP | C1R | C4A | CALM1 | CARD9 | CASP8 | CAV1 | CBFB | CD209 | CD22 | CD36 | CD40 | CD40LG | CD74 | CD79B | CD81 | CDC37 | CDC42 | CLU | COL2A1 | COLEC10 | CREBBP | CRK | CRKL | CRP | CTSL | CUL1 | CYLD | DDX1 | DHX36 | DHX9 | DNASE2 | DUSP22 | EIF2AK4 | EIF2B1 | EIF2B3 | EIF2B5 | EP300 | ESR1 | EXOSC6 | EZR | FADD | FAM49B | FBXW11 | FCGR1A | FCN1 | FGB | FKBP1A | FLOT1 | FOS | FOXJ1 | FYN | GBP5 | GPS2 | GPX1 | GRB2 | HAVCR2 | HEXIM1 | HK1 | HLA-A | HLA-B | HLA-DQA1 | HLA-DRA | HLA-DRB1 | HLA-DRB3 | HLA-DRB4 | HLA-DRB5 | HLX | HMGB1 | HMGB2 | HMGB3 | HMOX1 | HPX | HRAS | HSP90AA1 | HSP90AB1 | HSP90B1 | HSPA1B | HSPD1 | ICAM1 | IFI16 | IFNA2 | IGHV3-13 | IGHV3-23 | IGHV3-30 | IGKV3-20 | IGLC7 | IKBKB | IKBKE | IKBKG | IL13RA2 | IRF1 | IRF4 | IRF7 | ITCH | ITGA4 | ITK | JAK1 | JAK3 | JUN | KIR3DL2 | KRAS | LAT | LCP2 | LGALS3 | LGMN | LGR4 | MAP2K4 | MAP2K6 | MAP2K7 | MAP3K1 | MAPK1 | MAPK14 | MAPK3 | MAPK8 | MAPK9 | MASP1 | MASP2 | MATR3 | MAVS | MED1 | MICA | MIF | MLH1 | MSH6 | MYD88 | MYO1C | NCK1 | NFATC1 | NFATC2 | NFKBIA | NOD2 | NPLOC4 | NR1H2 | NR1H3 | OTUD4 | PAG1 | PAK2 | PARK7 | PARP14 | PAWR | PAXIP1 | PDE4D | PELI1 | PHB | PHB2 | PIANP | PIAS1 | PIK3CA | PIK3R1 | PLA2G1B | PLA2G6 | PLCG1 | PLSCR1 | PPARG | PRKACA | PRKCD | PRKCE | PRKCZ | PRKD2 | PRKDC | PSEN1 | PSMA1 | PSMA2 | PSMA3 | PSMA5 | PSMA6 | PSMB5 | PSMB6 | PSMB7 | PSMB8 | PSMC1 | PSMC2 | PSMC3 | PSMC4 | PSMC5 | PSMC6 | PSMD1 | PSMD10 | PSMD11 | PSMD12 | PSMD14 | PSMD2 | PSMD4 | PSMD9 | PSME2 | PSME3 | PTK2 | PTPN1 | PTPN22 | PTPN6 | PUM1 | RAB29 | RAC1 | RAF1 | RAP1A | RARA | RC3H1 | RELA | RIF1 | RIPK1 | RIPK2 | RIPK3 | RNF31 | RPS19 | RPS3 | RTN4 | RUNX1 | S100A1 | S100A9 | SARM1 | SCARA3 | SELL | SEMA7A | SFPQ | SHC1 | SIN3A | SIRT1 | SLAMF8 | SMAD3 | SNX6 | SOCS1 | SPG21 | SRC | STAT1 | STAT6 | STK11 | STOML2 | STXBP1 | STXBP2 | SUMO1 | TAB1 | TAB2 | TANK | TGFB1 | TLR3 | TMEM173 | TNF | TNFAIP3 | TNIP2 | TRAF2 | TRAF3 | TRAF6 | TREML2 | TREX1 | UBASH3A | UBB | UBC | UBE2D1 | UBE2D2 | UBE2D3 | UBE2K | UBE2N | UBE2V1 | UBQLN1 | USP17L2 | VAV1 | VCAM1 | VSIG4 | VTN | WAS | WDFY1 | WNK1 | XBP1 | XIAP | XRCC5 | XRCC6 | YES1 | ZAP70 | ZBTB7B | GO:1903364 | 0.0127380807958268 | 105/8243 | 66/3250 | 8.98229594455241e-07 | 0.00852868999935251 | T | T | T | T | positive regulation of cellular protein catabolic process | AKT1 | APOE | AXIN1 | BAG2 | BAG6 | CAV1 | CBFA2T3 | CCDC22 | CD81 | CEBPA | CLU | CSNK1A1 | CSNK1E | CTSC | DAB2 | DDRGK1 | DNAJB2 | DVL1 | EDEM1 | EZR | FAF1 | GSK3A | GSK3B | HSP90AA1 | HSPA1B | HSPBP1 | KEAP1 | LRRK2 | MAPK9 | MDM2 | MSN | NFE2L2 | NUPR1 | PCSK9 | PIAS1 | PLK1 | PSEN1 | PSMC1 | PSMC2 | PSMC3 | PSMC4 | PSMC5 | PSMC6 | PSMD10 | PTEN | PTK2 | RAD23A | RBX1 | RDX | RGN | RNF139 | RNF185 | RNF19A | SGTA | SMURF1 | STUB1 | SUMO1 | SUMO2 | TMTC3 | TNFAIP3 | TRIB2 | TRIB3 | UBQLN1 | UBQLN2 | USP5 | VCP | GO:0009314 | 0.0329976950139512 | 272/8243 | 146/3250 | 9.34786564118623e-07 | 0.00887579842630632 | T | T | T | T | response to radiation | AKT1 | AKT2 | ANXA1 | APP | ARRB1 | ASNS | ATM | AURKB | BABAM1 | BACE1 | BAX | BCL2 | BCL2L1 | BRAF | BRCA1 | CABP4 | CASP3 | CASP9 | CAT | CBL | CCAR2 | CCR4 | CDC25A | CDK5 | CDKN1A | CHEK2 | CIRBP | CREB1 | CREBBP | CRIP1 | CRY1 | CRY2 | CRYAB | CUL4B | DBH | DDB1 | DDB2 | DHX36 | DUSP1 | EEF1D | EGFR | EIF2AK4 | EIF2S1 | EP300 | ERCC5 | FANCG | FECH | FEN1 | FIGNL1 | FKBPL | FOS | GNAQ | GNB1 | GPX1 | GRB2 | GRK1 | H2AFX | HIF1A | HRAS | HSF1 | HSPA5 | ICAM1 | ID2 | IFI16 | JUN | JUNB | JUND | KAT5 | KDM1A | KDM4D | KRAS | LIG4 | MAP2K7 | MAP3K4 | MAPK14 | MAPK8 | MC1R | MDM2 | MECP2 | MGMT | MME | MSH6 | MTA1 | MYC | NAMPT | NEDD4 | NIPBL | NPM1 | NTRK1 | PARP1 | PAXIP1 | PCNA | PDE6G | PER1 | PER2 | PIAS1 | PIK3R1 | PML | PNKP | POLD1 | POLE3 | PPID | PPM1D | PPP1CA | PPP1CB | PPP1CC | PRKAA1 | PRKDC | PTN | PTPRK | RAD51 | RAD51AP1 | RBM4 | RDH13 | RELA | RHBDD1 | RHO | RNF8 | RRM1 | RUVBL2 | SAG | SCARA3 | SIRT1 | SMC1A | SNAI2 | SPRTN | STK11 | TANK | TGFB1 | TP53 | TREX1 | TRIAP1 | TRIM32 | TXN | UBE2A | UBE2B | UNC119 | USP2 | USP28 | USP47 | VCAM1 | XRCC5 | XRCC6 | YAP1 | YY1 | ZMPSTE24 | GO:0030111 | 0.0277811476404222 | 229/8243 | 126/3250 | 9.39821928074374e-07 | 0.00892360920706618 | T | T | T | T | regulation of Wnt signaling pathway | ABL1 | AES | APC | APOE | APP | AXIN1 | BIRC8 | BTRC | CAV1 | CBFB | CBY1 | CCAR2 | CCNY | CDC73 | CHD8 | CSNK1A1 | CSNK1E | CTNNB1 | CTNNBIP1 | CTNND1 | CUL3 | CYLD | DAB2 | DDX3X | DISC1 | DVL1 | DVL2 | DVL3 | EDA | EGFR | ESR1 | FOLR1 | FOXO1 | G3BP1 | GNAQ | GSK3A | GSK3B | HDAC1 | HECW1 | ILK | JUP | LATS1 | LEF1 | LGR4 | LRP6 | LRRK1 | LRRK2 | MAPK14 | MCC | MDFI | MKS1 | MLLT3 | NFATC1 | NLK | NPHP3 | PIN1 | PPM1A | PPP1CA | PPP2CA | PPP2R1A | PSEN1 | PSMA1 | PSMA2 | PSMA3 | PSMA5 | PSMA6 | PSMB5 | PSMB6 | PSMB7 | PSMB8 | PSMC1 | PSMC2 | PSMC3 | PSMC4 | PSMC5 | PSMC6 | PSMD1 | PSMD10 | PSMD11 | PSMD12 | PSMD14 | PSMD2 | PSMD4 | PSMD9 | PSME2 | PSME3 | PTPRO | RBPJ | RBX1 | RECK | RUNX1 | RUVBL1 | RUVBL2 | SCEL | SCYL2 | SKI | SLC9A3R1 | SMAD3 | SMARCA4 | SMURF2 | SNAI2 | SNX3 | SOX2 | SPERT | SPIN1 | SRC | STK11 | STK3 | SULF2 | TBL1X | TLE1 | TLE3 | TNFAIP3 | TNKS | TNKS2 | TNN | TSKU | UBE2B | UBR5 | USP47 | USP8 | VCP | WNK1 | WWOX | XIAP | YAP1 | GO:0046395 | 0.0149217517893971 | 123/8243 | 75/3250 | 9.43475723233478e-07 | 0.00895830199210187 | T | T | T | T | carboxylic acid catabolic process | AASS | ABAT | ABCD3 | ACAA1 | ACAA2 | ACAD8 | ACADS | ACADVL | ACAT1 | ACAT2 | ACOX1 | ADTRP | AKR1A1 | ALDH4A1 | ALDH5A1 | ALDH6A1 | ALDH7A1 | AMDHD2 | CBS | CEL | CPT1A | CPT2 | CROT | DCXR | DECR1 | DLD | DLST | ECH1 | ECHDC2 | ECHS1 | ECI1 | ECI2 | EHHADH | ETFA | ETFB | ETFDH | FAH | GAD2 | GCDH | GLS | GLS2 | GLUD2 | GLUL | GNPDA1 | GNPDA2 | GOT1 | GOT2 | GPT2 | GSTZ1 | HADH | HIBADH | HIBCH | HMGCL | HSD17B4 | IVD | LDHD | LPIN3 | MCAT | MCCC1 | NAGK | NOS2 | NOS3 | NUDT19 | OAT | OGDH | OTC | PADI4 | PAH | PCBD1 | PPARD | PPAT | QDPR | SHMT1 | SHMT2 | SORD | GO:0035722 | 0.004852602207934 | 40/8243 | 31/3250 | 1.01646800969572e-06 | 0.00965136375206084 | T | T | T | T | interleukin-12-mediated signaling pathway | ANXA2 | ARF1 | CAPZA1 | CDC42 | CFL1 | GSTA2 | GSTO1 | HNRNPA2B1 | HNRNPDL | HNRNPF | HSPA9 | JAK1 | LCP1 | LMNB1 | MIF | MSN | MTAP | P4HB | PAK2 | PITPNA | PPIA | PSME2 | RAP1B | RPLP0 | SERPINB2 | SNRPA1 | SOD1 | SOD2 | STAT4 | TALDO1 | TCP1 | GO:0032269 | 0.0816450321484896 | 673/8243 | 324/3250 | 1.04065350974986e-06 | 0.00988100507507493 | T | T | T | T | negative regulation of cellular protein metabolic process | A2M | ABL1 | ADORA2A | AGO1 | AGO2 | AHSG | AKT1 | ANXA2 | APC | APOE | APP | ARRB1 | ARRB2 | ATG5 | ATP13A2 | BAG2 | BAG5 | BAG6 | BAX | BCOR | BDKRB2 | BIN1 | BIRC2 | BIRC3 | BIRC5 | BIRC7 | BIRC8 | BRCA1 | C1QL4 | C4A | CALM1 | CALM2 | CALM3 | CALR | CASP3 | CAV1 | CBL | CBLB | CBLC | CCAR2 | CCNB1 | CD109 | CD27 | CDK5 | CDKN1A | CDKN2C | CEBPA | CHORDC1 | CHP1 | CHRNA9 | CIRBP | CRKL | CRY1 | CRY2 | CRYAB | CSNK2A2 | CTBP1 | CTNNB1 | CYLD | DAPK1 | DBNDD2 | DDIT4 | DDRGK1 | DDX3X | DHCR24 | DHX36 | DMD | DNAJA1 | DNAJC10 | DPEP1 | DUSP1 | DUSP14 | DUSP16 | DUSP19 | DUSP22 | DUSP7 | DVL1 | EIF2AK3 | EIF2AK4 | EIF2S1 | EIF3A | EIF3E | EIF4A3 | EIF4E | EIF4E2 | EIF4EBP1 | EIF4ENIF1 | EIF6 | EPHA4 | EPPIN | EPRS | ERRFI1 | EZR | FABP1 | FAM192A | FBXO7 | FGFR1OP | FKBP1A | FKBP1B | FKBP8 | FOXM1 | FOXO1 | FTO | FXR2 | FYN | G6PD | GAPDH | GLMN | GNAI2 | GNAQ | GPD1L | GPI | GPS2 | GPX1 | GRB7 | GSK3B | GSTP1 | HDAC2 | HDAC3 | HDAC6 | HEXIM1 | HNRNPD | HSP90AB1 | HSP90AB3P | HSP90AB4P | HSPA1B | HSPB1 | IBTK | IDE | IFI16 | IGBP1 | IGF1R | IGF2BP1 | IKBKB | ILF3 | ILK | IPO5 | IPO7 | IREB2 | ISG15 | ITCH | ITGB1BP1 | JUN | KDM1A | KDM4D | LAMP3 | LATS1 | LDLRAD4 | LEF1 | LEMD2 | LGALS3 | LRP6 | LRRK1 | LRRK2 | MAD2L1 | MAPT | MAS1 | MDM2 | MECP2 | MIF | MLLT1 | MOV10 | MYC | NCK1 | NCOR1 | NELL1 | NF2 | NNT | NPM1 | NR1H2 | NTF3 | OGT | OTUB1 | PAFAH1B1 | PAK2 | PAPLN | PARD3 | PARD6A | PARK7 | PARP14 | PDE4D | PEBP1 | PER1 | PER2 | PHB | PIAS3 | PIH1D1 | PIN1 | PINX1 | PKIG | PLAUR | PLK1 | PML | PNKP | PPARGC1A | PPME1 | PPP1R11 | PPP1R14A | PPP1R2 | PPP1R8 | PPP2CA | PPP2R1A | PPP2R5D | PRDX3 | PRDX5 | PRKAA1 | PRKAA2 | PRKAR1A | PRKAR2B | PRKCD | PRKCE | PRKCZ | PRKDC | PRMT3 | PRMT6 | PSEN1 | PSMD10 | PTEN | PTN | PTPN1 | PTPN22 | PTPN6 | PTPRR | PTTG1 | PUM1 | PUS7 | QARS | RAF1 | RANBP9 | RARA | RB1 | RBM3 | RBM4 | RC3H1 | RECK | RELA | RGN | RGS2 | RNF139 | RPL23 | RPS3 | RPS6KA1 | RPTOR | SAG | SDCBP | SERPINB2 | SERPINB3 | SERPINB5 | SERPINB6 | SERPINB8 | SERPINE2 | SERPINH1 | SET | SFN | SGTA | SHMT1 | SIN3A | SIRT1 | SIRT3 | SKI | SLC9A3R1 | SMAD4 | SMARCB1 | SMARCC1 | SNCA | SNTA1 | SNX12 | SNX6 | SOCS1 | SPI1 | SPINK7 | SPINT2 | SPRY2 | SRC | STAT3 | STK38 | STRAP | STYX | SWAP70 | SYNJ2BP | TAOK3 | TARDBP | TERF2IP | TGFB1 | THBS1 | TIA1 | TIMP1 | TIMP2 | TINF2 | TNF | TNFAIP3 | TNFSF14 | TPR | TRAF3IP1 | TRIAP1 | TRIB2 | TRIB3 | TRIM21 | TRIP12 | TSG101 | TWIST1 | TYMS | U2AF2 | UBE2B | UBE2J1 | UBR5 | UBXN1 | UCHL1 | UCHL5 | UNK | USP14 | USP17L2 | USP19 | USP4 | USP47 | USP7 | VTN | WARS | WNK1 | XIAP | XRCC1 | YWHAB | YWHAE | YWHAG | GO:1903052 | 0.0106757248574548 | 88/8243 | 57/3250 | 1.20670383873394e-06 | 0.0114576529487788 | T | T | T | T | positive regulation of proteolysis involved in cellular protein catabolic process | AKT1 | AXIN1 | BAG2 | BAG6 | CAV1 | CBFA2T3 | CCDC22 | CEBPA | CLU | CSNK1A1 | CSNK1E | CTSC | DAB2 | DDRGK1 | DNAJB2 | DVL1 | EDEM1 | GSK3A | GSK3B | HSPA1B | HSPBP1 | KEAP1 | LRRK2 | MAPK9 | MDM2 | NFE2L2 | NUPR1 | PIAS1 | PLK1 | PSEN1 | PSMC1 | PSMC2 | PSMC3 | PSMC4 | PSMC5 | PSMC6 | PSMD10 | PTEN | PTK2 | RAD23A | RBX1 | RGN | RNF139 | RNF185 | RNF19A | SGTA | SMURF1 | STUB1 | SUMO1 | SUMO2 | TMTC3 | TRIB2 | TRIB3 | UBQLN1 | UBQLN2 | USP5 | VCP | GO:0002684 | 0.0753366492781754 | 621/8243 | 301/3250 | 1.23682431193315e-06 | 0.0117436468418053 | T | T | T | T | positive regulation of immune system process | ABL1 | ACTB | ACTG1 | ACTR2 | ACTR3 | ADA | AGER | AKT1 | ANXA1 | AP1G1 | AP3D1 | APCS | APP | ARNT | ARPC1A | ARPC1B | ARPC2 | ARPC3 | ARPC4 | ARPC5 | ATAD5 | BAG6 | BAIAP2 | BAX | BCAR1 | BCL10 | BCL2 | BCL6 | BIRC2 | BIRC3 | BRAF | BTK | BTN2A1 | BTRC | C1QA | C1QBP | C1R | C4A | CALR | CAMK1D | CARD9 | CASP8 | CAV1 | CBFB | CCL21 | CCR1 | CD2 | CD209 | CD27 | CD36 | CD40 | CD40LG | CD6 | CD74 | CD79B | CD81 | CDC42 | CEBPA | CLU | COLEC10 | CREB1 | CREB3 | CREBBP | CRK | CRKL | CRP | CTNNBIP1 | CTSL | CUL1 | CXCL8 | CYLD | DDX1 | DHX36 | DHX9 | DNAJA3 | EIF2AK4 | EIF2B1 | EIF2B3 | EIF2B5 | EP300 | ETS1 | EXOSC6 | FADD | FAM19A3 | FAM49B | FBXW11 | FCGR1A | FCN1 | FGB | FLOT1 | FOS | FOXJ1 | FYN | GATA1 | GBP5 | GNAS | GPI | GRB2 | HAVCR2 | HAX1 | HEXIM1 | HIF1A | HK1 | HLA-A | HLA-B | HLA-DQA1 | HLA-DRA | HLA-DRB1 | HLA-DRB3 | HLA-DRB4 | HLA-DRB5 | HLX | HMGB1 | HMGB2 | HMGB3 | HMOX1 | HOXA5 | HPX | HRAS | HSP90AA1 | HSP90AB1 | HSP90B1 | HSPA1B | HSPD1 | HSPH1 | ICAM1 | ID2 | IFI16 | IGHV3-13 | IGHV3-23 | IGHV3-30 | IGKV3-20 | IGLC7 | IKBKB | IKBKE | IKBKG | IL13RA2 | IRF7 | ISG15 | ITCH | ITGA4 | ITK | JAK3 | JUN | KRAS | LAT | LCP2 | LEF1 | LGALS1 | LGALS3 | LGMN | LRRK2 | MAP2K6 | MAP3K1 | MAPK1 | MAPK14 | MAPK3 | MASP1 | MASP2 | MATR3 | MAVS | MED1 | MICA | MIF | MLH1 | MSH6 | MYD88 | MYO1C | NCK1 | NFATC2 | NFKBIA | NOD2 | NR1H3 | OXSR1 | P2RX4 | PAG1 | PAK2 | PARK7 | PAXIP1 | PDE4D | PELI1 | PHB | PIK3CA | PIK3R1 | PLA2G1B | PLA2G6 | PLCG1 | PLSCR1 | PNP | PRKACA | PRKCD | PRKCE | PRKCZ | PRKD2 | PRKDC | PSEN1 | PSMA1 | PSMA2 | PSMA3 | PSMA5 | PSMA6 | PSMB5 | PSMB6 | PSMB7 | PSMB8 | PSMC1 | PSMC2 | PSMC3 | PSMC4 | PSMC5 | PSMC6 | PSMD1 | PSMD10 | PSMD11 | PSMD12 | PSMD14 | PSMD2 | PSMD4 | PSMD9 | PSME2 | PSME3 | PTK2 | PTPN22 | PTPN6 | PUM1 | RAB29 | RAC1 | RAF1 | RAP1A | RARA | RB1 | RC3H1 | RELA | RHOA | RIF1 | RIPK1 | RIPK2 | RNF31 | RPS19 | RPS3 | RTN4 | RUNX1 | S100A1 | S100A7 | S100A9 | SART1 | SCARA3 | SEMA7A | SFPQ | SIN3A | SIRT1 | SNX6 | SOCS1 | SPACA3 | SPG21 | SRC | STAT1 | STAT3 | STAT6 | STK11 | STOML2 | STXBP1 | STXBP2 | SWAP70 | TAB1 | TAB2 | TANK | TGFB1 | THBS1 | TLR3 | TMEM173 | TNF | TNFAIP3 | TNFSF14 | TNIP2 | TRAF2 | TRAF3 | TRAF6 | TREX1 | UBB | UBC | UBE2D1 | UBE2D2 | UBE2D3 | UBE2K | UBE2N | UBE2V1 | USP17L2 | VAV1 | VCAM1 | VSIG4 | WAS | WDFY1 | WNK1 | XBP1 | XRCC5 | XRCC6 | YES1 | ZAP70 | ZBTB16 | ZBTB7B | GO:0016032 | 0.0604148974887784 | 498/8243 | 247/3250 | 1.31702454086877e-06 | 0.012505148015549 | T | T | T | T | viral process | ABCE1 | AP1B1 | AP1G1 | AP1M1 | AP3M1 | APOE | ARF1 | ASGR1 | ASGR2 | ATF7 | ATG5 | BANF1 | BAX | BCL2L1 | BIN1 | BRD2 | BRD4 | BTRC | C1QBP | CALCOCO2 | CARM1 | CASP8 | CAV1 | CCNT1 | CCNT2 | CCR3 | CD209 | CDC25C | CDC42 | CEBPA | CHMP2A | COPB1 | COPS6 | CREB1 | CREB3 | CREBBP | CTBP1 | CTNNB1 | CUL1 | CUL2 | CUL5 | CUL7 | DAXX | DDB1 | DDX3X | DDX6 | DERL1 | DHX9 | DYNC1I2 | DYNC1LI1 | DYNLL1 | DYNLT1 | EIF2AK4 | EIF3A | EIF3D | EIF4E | EIF4H | EP300 | FADD | FDPS | FKBP8 | FYN | GPS2 | GRB2 | H2AFX | HLA-DRA | HNRNPA1 | HNRNPK | HPX | HSPA8 | HSPD1 | HTATIP2 | IFIT1 | IK | IKBKB | IKBKE | IKBKG | INSR | IPO5 | IPO7 | IQGAP1 | IRF7 | ISG15 | KAT5 | KDR | KEAP1 | KLC1 | KPNA1 | KPNA2 | KPNB1 | LIG4 | LRSAM1 | LTBR | MAPK1 | MAPK3 | MAVS | MCTS1 | MDM2 | MICA | MSH6 | NEDD4 | NFE2L2 | NFKBIA | NPM1 | NRP2 | NUP107 | NUP153 | NUP160 | NUP205 | NUP214 | NUP85 | NUP88 | NUP93 | NUP98 | NXF1 | OGT | PACS1 | PACS2 | PAK2 | PARD6A | PC | PCBP1 | PCBP2 | PCNA | PDCD6IP | PIK3R1 | PLCG1 | PML | POLR2E | POM121 | PPIA | PPID | PRMT6 | PSMA3 | PSMB5 | PSMB6 | PSMB7 | PSMB8 | PSMC3 | RAB1A | RAB1B | RAB29 | RAB5A | RAB7A | RAC1 | RAD23A | RAE1 | RAN | RB1 | RBX1 | RCC1 | RELA | RHOA | RIPK1 | RPL10 | RPL10A | RPL12 | RPL18A | RPL22 | RPL23 | RPL23A | RPL24 | RPL30 | RPL37A | RPL38 | RPL6 | RPL7 | RPL7A | RPL9 | RPLP0 | RPLP1 | RPLP2 | RPS12 | RPS13 | RPS14 | RPS15A | RPS19 | RPS2 | RPS20 | RPS24 | RPS29 | RPS3 | RPS4X | RPS8 | RPTOR | RXRA | SEC13 | SEPT6 | SET | SGTA | SHC1 | SIRT1 | SLC25A4 | SLC25A5 | SLC25A6 | SMAD3 | SMARCB1 | SND1 | SNW1 | SP1 | SP100 | SPEN | SRC | SRPK1 | SSB | STAT1 | STAT3 | STAU1 | SUMO1 | TANK | TGFB1 | TMEM173 | TNFRSF1A | TNPO1 | TOP1 | TP53 | TP73 | TPR | TRIM23 | TRIM25 | TRIM28 | TSG101 | TYMS | UBB | UBC | UBE2I | UBE3A | UBR4 | UBR5 | UBXN1 | USP7 | VAC14 | VAPA | VAPB | VCP | VDAC1 | VIM | VPS29 | VPS37D | VPS4B | VTA1 | WDR48 | XPO1 | XRCC5 | XRCC6 | YWHAB | YWHAE | GO:0044403 | 0.0604148974887784 | 498/8243 | 247/3250 | 1.31702454086877e-06 | 0.012505148015549 | T | T | T | T | symbiont process | ABCE1 | AP1B1 | AP1G1 | AP1M1 | AP3M1 | APOE | ARF1 | ASGR1 | ASGR2 | ATF7 | ATG5 | BANF1 | BAX | BCL2L1 | BIN1 | BRD2 | BRD4 | BTRC | C1QBP | CALCOCO2 | CARM1 | CASP8 | CAV1 | CCNT1 | CCNT2 | CCR3 | CD209 | CDC25C | CDC42 | CEBPA | CHMP2A | COPB1 | COPS6 | CREB1 | CREB3 | CREBBP | CTBP1 | CTNNB1 | CUL1 | CUL2 | CUL5 | CUL7 | DAXX | DDB1 | DDX3X | DDX6 | DERL1 | DHX9 | DYNC1I2 | DYNC1LI1 | DYNLL1 | DYNLT1 | EIF2AK4 | EIF3A | EIF3D | EIF4E | EIF4H | EP300 | FADD | FDPS | FKBP8 | FYN | GPS2 | GRB2 | H2AFX | HLA-DRA | HNRNPA1 | HNRNPK | HPX | HSPA8 | HSPD1 | HTATIP2 | IFIT1 | IK | IKBKB | IKBKE | IKBKG | INSR | IPO5 | IPO7 | IQGAP1 | IRF7 | ISG15 | KAT5 | KDR | KEAP1 | KLC1 | KPNA1 | KPNA2 | KPNB1 | LIG4 | LRSAM1 | LTBR | MAPK1 | MAPK3 | MAVS | MCTS1 | MDM2 | MICA | MSH6 | NEDD4 | NFE2L2 | NFKBIA | NPM1 | NRP2 | NUP107 | NUP153 | NUP160 | NUP205 | NUP214 | NUP85 | NUP88 | NUP93 | NUP98 | NXF1 | OGT | PACS1 | PACS2 | PAK2 | PARD6A | PC | PCBP1 | PCBP2 | PCNA | PDCD6IP | PIK3R1 | PLCG1 | PML | POLR2E | POM121 | PPIA | PPID | PRMT6 | PSMA3 | PSMB5 | PSMB6 | PSMB7 | PSMB8 | PSMC3 | RAB1A | RAB1B | RAB29 | RAB5A | RAB7A | RAC1 | RAD23A | RAE1 | RAN | RB1 | RBX1 | RCC1 | RELA | RHOA | RIPK1 | RPL10 | RPL10A | RPL12 | RPL18A | RPL22 | RPL23 | RPL23A | RPL24 | RPL30 | RPL37A | RPL38 | RPL6 | RPL7 | RPL7A | RPL9 | RPLP0 | RPLP1 | RPLP2 | RPS12 | RPS13 | RPS14 | RPS15A | RPS19 | RPS2 | RPS20 | RPS24 | RPS29 | RPS3 | RPS4X | RPS8 | RPTOR | RXRA | SEC13 | SEPT6 | SET | SGTA | SHC1 | SIRT1 | SLC25A4 | SLC25A5 | SLC25A6 | SMAD3 | SMARCB1 | SND1 | SNW1 | SP1 | SP100 | SPEN | SRC | SRPK1 | SSB | STAT1 | STAT3 | STAU1 | SUMO1 | TANK | TGFB1 | TMEM173 | TNFRSF1A | TNPO1 | TOP1 | TP53 | TP73 | TPR | TRIM23 | TRIM25 | TRIM28 | TSG101 | TYMS | UBB | UBC | UBE2I | UBE3A | UBR4 | UBR5 | UBXN1 | USP7 | VAC14 | VAPA | VAPB | VCP | VDAC1 | VIM | VPS29 | VPS37D | VPS4B | VTA1 | WDR48 | XPO1 | XRCC5 | XRCC6 | YWHAB | YWHAE | GO:2001234 | 0.0207448744389179 | 171/8243 | 98/3250 | 1.3593315089388e-06 | 0.0129068526773739 | T | T | T | T | negative regulation of apoptotic signaling pathway | ACAA2 | AKT1 | AR | ARRB2 | ATAD5 | BAG5 | BAX | BCL2 | BCL2L1 | BCL2L2 | BDKRB2 | BRCA1 | CASP8 | CCAR2 | CD74 | CLU | COL2A1 | CREB3 | CSNK2A2 | CTH | CTNNB1 | CTTN | DDX3X | DNAJA1 | ENO1 | EYA2 | EYA4 | FADD | FASLG | FGB | FIGNL1 | FYN | GATA1 | GNAI2 | GNAI3 | GPX1 | GSTP1 | HDAC1 | HIF1A | HMGB2 | HMOX1 | HSPA1B | HSPB1 | HTRA2 | HTT | ICAM1 | ITGAV | KDM1A | LGALS3 | LMNA | LRRK2 | MAPK7 | MDM2 | MIF | NDUFA13 | NDUFS3 | NFE2L2 | NOS3 | OPA1 | PAM16 | PARK7 | PIH1D1 | PLAUR | PPIF | PRDX2 | PRELID1 | PSEN1 | PSMD10 | PSME3 | PTPN1 | QARS | RAF1 | RB1CC1 | RELA | RIPK1 | RRM2B | SCG2 | SIRT1 | SLC25A5 | SLC35F6 | SNAI2 | SOD2 | SRC | SYVN1 | THBS1 | TMBIM6 | TNF | TNFAIP3 | TRADD | TRAF2 | TRAP1 | TRIAP1 | TRIM32 | USP47 | VDAC2 | XBP1 | YAP1 | ZC3HC1 | GO:0044272 | 0.0116462452990416 | 96/8243 | 61/3250 | 1.35950364684739e-06 | 0.012908487126816 | T | T | T | T | sulfur compound biosynthetic process | ACACA | ACAT1 | ACLY | ACSL1 | ACSL4 | ACSL5 | ACSS2 | AKR1A1 | AMD1 | B3GNT3 | B4GALT3 | CBR4 | CBS | CNDP2 | CTH | DCN | DLAT | DLD | DSE | ESD | FASN | GCDH | GGCT | GGT1 | GGTLC1 | GLCE | GSS | GSTA1 | GSTA2 | GSTA3 | GSTA4 | GSTK1 | GSTM4 | GSTO1 | GSTO2 | GSTP1 | GSTT1 | GSTT2B | GSTZ1 | HAGH | HSD17B8 | LUM | MAT2A | MAT2B | MGST1 | MPC2 | MPST | PAPSS1 | PAPSS2 | PDHA1 | PDHA2 | PDHB | PDHX | PPT1 | SLC25A1 | ST3GAL1 | ST3GAL6 | TECR | UGDH | VCAN | XYLT1 | GO:0031647 | 0.0253548465364552 | 209/8243 | 116/3250 | 1.41677074203776e-06 | 0.0134522381956485 | T | T | T | T | regulation of protein stability | AFM | APOA1 | ASGR2 | ATP1B3 | BAG2 | BAG3 | BAG5 | BAG6 | BCL2 | BTRC | CALR | CASP3 | CCAR2 | CCT2 | CCT3 | CCT4 | CCT5 | CCT6A | CCT7 | CCT8 | CD81 | CDC37 | CDC73 | CDKN1A | CHEK2 | CHP1 | CLU | COG3 | CREB1 | CREBBP | CRYAB | CTSA | CUL3 | DERL1 | DVL1 | DVL3 | EP300 | EPHA4 | FBXO7 | FBXW11 | FKBPL | FLOT1 | GAPDH | GNAQ | HDAC3 | HDAC6 | HSP90AA1 | HSP90AB1 | HSPA1B | HSPA8 | HSPD1 | HTT | HYPK | KHDRBS1 | KRAS | LMNA | LRRK2 | MAPK1 | MDM2 | MDM4 | NF2 | NLK | PARK7 | PDCD10 | PEX19 | PFN1 | PHB | PHB2 | PIK3R1 | PIN1 | PINX1 | PLK1 | PPARGC1A | PPIB | PRKCD | PRKD1 | PRKDC | PTEN | PTGES3 | RAD23A | RNF139 | RPL23 | RTN4 | SEC16A | SEL1L | SIAH1 | SIRT1 | SIRT6 | SMAD3 | SMO | SNCA | SRC | STK3 | STUB1 | STXBP1 | SUGT1 | SUMO1 | SYVN1 | TBL1X | TCP1 | TNIP2 | TPR | TREX1 | TRIM21 | UBE2B | USP19 | USP2 | USP28 | USP4 | USP47 | USP7 | USP8 | USP9X | VHL | XBP1 | ZNF207 | GO:0009266 | 0.0131020259614218 | 108/8243 | 67/3250 | 1.48240917659187e-06 | 0.0140754751317398 | T | T | T | T | response to temperature stimulus | ACADVL | ADRB2 | AKT1 | ARRB2 | ATP2A2 | BAG3 | CASP8 | CDKN1A | CIRBP | CLPB | CRNN | DAXX | DHX36 | DNAJA1 | DNAJA2 | DNAJA3 | DNAJA4 | EIF2AK3 | EIF2AK4 | EIF2B1 | EIF2B3 | EIF2B5 | EIF2S1 | FOS | FOXO1 | GMPR | HDAC2 | HMOX1 | HSF1 | HSF4 | HSP90AA1 | HSPA14 | HSPA1B | HSPA1L | HSPA2 | HSPA5 | HSPA8 | HSPA9 | HSPD1 | HTR1B | HTRA2 | IGFBP7 | MAP2K7 | MAPT | METRNL | MICA | NFKBIA | NOS3 | NTRK1 | PDCD6 | PEBP1 | PPARG | PPARGC1A | PRKAA1 | PRKACA | RBM3 | SLC9A1 | SOD1 | STAC | STUB1 | SUMO1 | THBS1 | THRA | TPR | VCP | VGF | YWHAE | GO:2000058 | 0.0138299162926119 | 114/8243 | 70/3250 | 1.49950317874576e-06 | 0.014237782682191 | T | T | T | T | regulation of ubiquitin-dependent protein catabolic process | AKT1 | ARAF | AXIN1 | BAG2 | BAG5 | BAG6 | CAV1 | CBFA2T3 | CCAR2 | CCDC22 | CEBPA | CLU | COMMD1 | CSNK1A1 | CSNK1E | CSNK2A2 | DAB2 | DDA1 | DDRGK1 | DNAJB2 | DVL1 | GLMN | GNA12 | GSK3A | GSK3B | HSP90AB1 | HSPA1B | HSPBP1 | KEAP1 | LATS1 | LRRK2 | MAPK9 | MDM2 | NFE2L2 | OGT | PARK7 | PIAS1 | PLK1 | PML | PSEN1 | PSMD10 | PTEN | PTK2 | RAD23A | RAD23B | RBX1 | RNF139 | RNF19A | RPL23 | SDCBP | SGTA | SMARCC1 | SMURF1 | STUB1 | STYX | SUMO1 | SUMO2 | TRIB2 | TRIB3 | UBE2K | UBE3A | UBQLN1 | UBQLN2 | UBQLN4 | UBXN1 | UCHL5 | USP14 | USP5 | USP7 | VCP | GO:0071840 | 0.385539245420357 | 3178/8243 | 1354/3250 | 1.68143358383271e-06 | 0.0159652118784916 | T | T | T | T | cellular component organization or biogenesis | A2M | AAR2 | AASS | ABCD3 | ABCE1 | ABCG1 | ABI3BP | ABL1 | ABLIM1 | ACAA1 | ACAA2 | ACACA | ACAD9 | ACAT1 | ACD | ACOT13 | ACOX1 | ACTA1 | ACTB | ACTG1 | ACTL6A | ACTN1 | ACTN2 | ACTN4 | ACTR2 | ACTR3 | ADAMTS4 | ADCYAP1 | ADD3 | ADORA2A | ADRB2 | ADRM1 | ADSL | AEBP2 | AFG3L2 | AGER | AGK | AGO1 | AGO2 | AHI1 | AHNAK | AIFM1 | AIMP2 | AKAP8 | AKAP9 | AKT1 | AKT2 | AKT3 | AKTIP | ALAS1 | ALB | ALDH5A1 | ALDOA | ALOX5AP | AMFR | ANK1 | ANK3 | ANKRD28 | ANKS4B | ANXA1 | ANXA2 | ANXA5 | ANXA6 | ANXA7 | AP1G1 | AP1M1 | AP3D1 | APBB1 | APBB2 | APC | APCS | APEH | APEX1 | APOA1 | APOD | APOE | APP | AR | ARF1 | ARHGAP26 | ARHGAP35 | ARHGEF7 | ARID4B | ARPC1A | ARPC1B | ARPC2 | ARPC3 | ARPC4 | ARPC5 | ARRB1 | ARRB2 | ATAD3A | ATCAY | ATF2 | ATG2B | ATG4C | ATG5 | ATL3 | ATM | ATP13A2 | ATP2A2 | ATP6V1D | ATP9A | ATPAF2 | ATRX | ATXN10 | ATXN2 | AURKB | AVIL | AXIN1 | BABAM1 | BAG3 | BAG4 | BAG5 | BAG6 | BAIAP2 | BAIAP2L1 | BANF1 | BANP | BAX | BBC3 | BCAR1 | BCL10 | BCL2 | BCL2L1 | BCL6 | BCOR | BCS1L | BIN1 | BIRC2 | BIRC3 | BIRC5 | BLZF1 | BMI1 | BRAF | BRCA1 | BRD2 | BRD3 | BRD4 | BRD9 | BSG | C1QA | C1QBP | C1QL1 | C1QTNF1 | CAB39 | CABP4 | CACNG2 | CACNG4 | CACNG5 | CALM1 | CALM3 | CALR | CAMK2A | CAND1 | CAP2 | CAPG | CAPN1 | CAPN2 | CAPNS1 | CAPZA1 | CAPZA2 | CAPZB | CARM1 | CASP3 | CASP8 | CAT | CAV1 | CBL | CBR4 | CBX4 | CBX7 | CBX8 | CBY1 | CCAR2 | CCDC120 | CCDC155 | CCDC36 | CCDC68 | CCDC69 | CCDC8 | CCDC88A | CCL21 | CCNB1 | CCP110 | CCR5 | CCT2 | CCT3 | CCT6B | CCT8 | CD2 | CD2BP2 | CD36 | CD40 | CD74 | CD79B | CDA | CDC14B | CDC20 | CDC25B | CDC42 | CDC42EP3 | CDC42EP4 | CDC73 | CDH1 | CDH5 | CDK16 | CDK2 | CDK4 | CDK5 | CDK5R1 | CEBPA | CEL | CENPF | CENPJ | CENPV | CEP135 | CEP152 | CEP290 | CEP350 | CEP55 | CEP57 | CEP89 | CETN3 | CFL1 | CFL2 | CFTR | CHAF1A | CHCHD10 | CHCHD2 | CHCHD3 | CHD1L | CHD3 | CHD4 | CHD8 | CHEK2 | CHMP2A | CHP1 | CHRAC1 | CHRM2 | CIRBP | CISD2 | CKAP5 | CKS2 | CLGN | CLIC4 | CLNS1A | CLSTN1 | CLTC | CLU | CLUH | CNTROB | COA3 | COG3 | COL12A1 | COL14A1 | COL18A1 | COL2A1 | COL4A1 | COL4A2 | COL4A3BP | COL5A1 | COL6A1 | COL6A2 | COPS2 | COPS5 | COPS6 | CORO2B | COX11 | COX15 | COX17 | COX19 | COX20 | COX7A2 | CPSF7 | CPT1A | CREB1 | CREBBP | CRK | CRKL | CRMP1 | CROCC | CROT | CRYAB | CRYZ | CSNK1A1 | CSNK2B | CTBP1 | CTCF | CTH | CTNNB1 | CTNND1 | CTSC | CTSL | CTSV | CTTN | CUL1 | CUL3 | CUL4B | CUL7 | CUTC | CYCS | CYLD | DAB1 | DAB2 | DAP3 | DARS | DAXX | DBN1 | DBNL | DCLK1 | DCN | DCTN1 | DCTPP1 | DCXR | DDB1 | DDB2 | DDIT4 | DDX1 | DDX39B | DDX3X | DDX6 | DECR1 | DERL1 | DES | DHRS4 | DHX36 | DHX9 | DIAPH3 | DISC1 | DLG2 | DLG3 | DLG4 | DMAP1 | DMD | DNA2 | DNAAF2 | DNAAF5 | DNAH9 | DNAJA3 | DNM1L | DOCK7 | DPF2 | DROSHA | DSTN | DVL1 | DVL2 | DVL3 | DYM | DYNC1H1 | DYNC1LI1 | DYNLL1 | DYNLT1 | E2F1 | E2F4 | ECH1 | ECI2 | ECSIT | EED | EFEMP2 | EGFR | EHD4 | EHHADH | EHMT1 | EHMT2 | EIF2AK3 | EIF2S1 | EIF3A | EIF3D | EIF3E | EIF3H | EIF3I | EIF4H | EIF5 | EIF6 | ELAVL1 | ELF3 | EML4 | EP300 | EPHA4 | EPPIN | EPPK1 | EPRS | ERAL1 | ERBB2 | ERCC5 | ESR1 | ESRRA | ETS1 | EYA2 | EYA4 | EZH2 | EZR | F5 | FADD | FAF2 | FAM118B | FAM161B | FAM83H | FANCA | FANCC | FANCG | FANCM | FARSB | FBF1 | FBP1 | FBXO7 | FCGR1A | FCHSD1 | FEN1 | FERMT2 | FGB | FGF2 | FGFR1OP | FIGNL1 | FIS1 | FKBP1A | FLNB | FLOT1 | FN1 | FOLR1 | FOPNL | FOXA1 | FOXJ1 | FSCN1 | FSD1 | FUNDC2 | FUS | FYN | G3BP1 | G3BP2 | GAA | GABPB1 | GAK | GAP43 | GAPDH | GAPVD1 | GAR1 | GATA1 | GBP5 | GEMIN4 | GEMIN5 | GEMIN7 | GFAP | GGCT | GINS2 | GINS4 | GJA1 | GLRB | GLRX3 | GLS | GLUL | GNAI3 | GNAO1 | GNB1 | GOLGA2 | GOLPH3 | GOPC | GORASP2 | GPX4 | GRAMD1A | GRB2 | GRB7 | GRHPR | GRIA1 | GRID2 | GRIP1 | GSK3A | GSK3B | GSPT1 | GSPT2 | GSTK1 | H1F0 | H1FX | H2AFV | H2AFX | H2AFZ | H3F3A | HAP1 | HAX1 | HBEGF | HBZ | HCCS | HCN1 | HDAC1 | HDAC11 | HDAC2 | HDAC3 | HDAC4 | HDAC5 | HDAC6 | HDAC7 | HDAC9 | HEATR3 | HEPACAM2 | HERC1 | HEXB | HGS | HIPK4 | HIRIP3 | HIST1H1A | HIST1H2AB | HIST1H2BA | HIST1H3A | HIST1H4A | HIST2H3A | HIST3H3 | HK2 | HLA-DRA | HLA-DRB1 | HLCS | HMGB1 | HMGB2 | HMGB3 | HMGCL | HMOX1 | HNRNPA2B1 | HNRNPU | HPRT1 | HRAS | HRNR | HSD17B10 | HSD17B4 | HSD17B8 | HSF1 | HSF4 | HSP90AA1 | HSP90AB1 | HSP90B1 | HSPA2 | HSPA4 | HSPA8 | HSPA9 | HSPD1 | HSPG2 | HTRA1 | HTRA2 | HTT | HUWE1 | ICAM1 | ICK | ID2 | IDE | IDH1 | IFT122 | IGF1R | IGHV3-13 | IGHV3-23 | IGHV3-30 | IGLC7 | IGSF21 | IKBKB | IKBKE | IKZF5 | ILK | IMMT | IMPDH2 | ING5 | INO80B | INO80C | INSR | IPO4 | IQCB1 | IRF4 | ITGA4 | ITGA7 | ITGAV | ITGB1BP1 | ITGB3 | ITGB3BP | ITGB5 | ITPA | ITPR3 | JMJD6 | JUN | JUP | KAT5 | KAT7 | KAT8 | KCNA4 | KCNIP2 | KCNS3 | KCTD12 | KCTD17 | KCTD18 | KCTD2 | KCTD3 | KDM1A | KDM2A | KDM3B | KDM4D | KDM5B | KDM5C | KDM8 | KDR | KHDRBS1 | KIF11 | KIF2C | KIF5B | KIF5C | KIFC3 | KLC1 | KLF5 | KLHL20 | KLHL22 | KMT2A | KMT2B | KMT2C | KPNB1 | KRAS | KRT17 | KRT31 | KRT71 | L3MBTL1 | L3MBTL2 | L3MBTL3 | L3MBTL4 | LAMB1 | LARP4 | LARS | LATS1 | LCP1 | LEF1 | LEMD2 | LEMD3 | LETM1 | LGMN | LIG4 | LIMA1 | LIMS1 | LMAN2L | LMNA | LNX1 | LOXL2 | LPAR1 | LRP6 | LRRC7 | LRRK2 | LSM3 | LSM4 | LUC7L2 | LUM | LURAP1 | LYRM7 | MAD2L1 | MAGI1 | MAP1B | MAPK14 | MAPK15 | MAPK3 | MAPK8 | MAPK8IP3 | MAPKAP1 | MAPT | MAST1 | MAST3 | MAT2A | MBD3 | MBP | MCCC1 | MCFD2 | MCM2 | MCM4 | MCM6 | MCM7 | MCM9 | MCMBP | MCTS1 | MDM2 | MDM4 | MDN1 | ME1 | MECP2 | MED1 | MED6 | MGST1 | MICU1 | MIF | MITF | MKS1 | MLH1 | MMP26 | MORF4L1 | MORF4L2 | MPP2 | MRGBP | MRPL12 | MRPL14 | MRPL19 | MRPL28 | MRPL38 | MRPL4 | MRPL43 | MRPL45 | MRPL46 | MRPL9 | MRPS5 | MRRF | MSH6 | MSN | MTA1 | MTA3 | MTERF1 | MTPN | MTSS1 | MYBBP1A | MYC | MYH11 | MYH6 | MYL2 | MYO1B | MYO1C | MYO1E | MYO5A | MYOC | NACC1 | NAP1L1 | NAP1L4 | NCAPH2 | NCBP1 | NCDN | NCK1 | NCOA1 | NCOA3 | NCOR1 | NDRG1 | NDUFA10 | NDUFA13 | NDUFA5 | NDUFA8 | NDUFA9 | NDUFAF1 | NDUFAF2 | NDUFAF3 | NDUFAF4 | NDUFAF5 | NDUFAF7 | NDUFB1 | NDUFS1 | NDUFS2 | NDUFS3 | NDUFS6 | NDUFS7 | NDUFS8 | NDUFV1 | NDUFV3 | NEBL | NEDD4 | NEK1 | NEK7 | NF2 | NFKB2 | NFS1 | NID1 | NIN | NIPBL | NME2 | NOD2 | NOP10 | NOS1AP | NOS2 | NOS3 | NPHP3 | NPLOC4 | NPM1 | NPTX1 | NR3C1 | NRXN1 | NSFL1C | NSL1 | NTF3 | NTMT1 | NTRK1 | NUBP1 | NUBP2 | NUDT19 | NUFIP1 | NUMA1 | NUP107 | NUP153 | NUP205 | NUP85 | NUP93 | NUP98 | NUPR1 | NUSAP1 | OAT | OBSL1 | OFD1 | OGFOD1 | OGT | ONECUT1 | OPA1 | OPTN | OTC | OXA1L | P2RX1 | P2RX2 | P2RX4 | P4HA1 | P4HB | PACS2 | PACSIN3 | PADI4 | PAFAH1B1 | PAK6 | PANX3 | PARD3 | PARD6A | PARK7 | PARP1 | PAWR | PAXIP1 | PCBD1 | PCDHB16 | PCGF1 | PCGF5 | PCGF6 | PCM1 | PCNA | PDCD10 | PDCD6 | PDCD6IP | PDCL | PDE4DIP | PDSS1 | PDSS2 | PEF1 | PER1 | PER2 | PEX11B | PEX19 | PEX5 | PFKL | PFKM | PFN1 | PGM5 | PHB | PHB2 | PHF10 | PHF21A | PHGDH | PI4K2A | PIAS1 | PICK1 | PIH1D1 | PIK3C2A | PIKFYVE | PIN1 | PINX1 | PITPNA | PITPNB | PKM | PKN2 | PLA2G1B | PLA2G2A | PLEC | PLEKHF1 | PLK1 | PLOD2 | PLS3 | PLSCR1 | PLXNB2 | PML | PMP2 | POC1B | POLA2 | POLD1 | POLDIP2 | POLE2 | POLE3 | POT1 | PPARGC1A | PPAT | PPIA | PPID | PPIF | PPM1D | PPP1CA | PPP1R12A | PPP2CA | PPP2CB | PPP2R1A | PPP2R2A | PPP3CC | PPP6C | PPP6R1 | PPT1 | PQLC1 | PRDX3 | PRDX4 | PRELID1 | PRKAA1 | PRKAA2 | PRKACA | PRKAR1A | PRKCD | PRKCE | PRKCZ | PRKD1 | PRKDC | PRMT2 | PRMT3 | PRMT6 | PROX1 | PRPF19 | PRPF40A | PRPF8 | PSAP | PSEN1 | PSEN2 | PSMD10 | PSMD11 | PSMD4 | PSMD9 | PSMG1 | PSMG4 | PSTPIP1 | PTEN | PTGES3 | PTK2 | PTMA | PTPN1 | PTPN23 | PTPRD | PTPRF | PTPRK | PTPRO | PTTG1 | PXDN | PXN | RAB10 | RAB11A | RAB18 | RAB1A | RAB1B | RAB29 | RAB2A | RAB31 | RAB32 | RAB35 | RAB3GAP1 | RAB5A | RAB5B | RAB7A | RAC1 | RAD21 | RAD23B | RAD51 | RADIL | RAE1 | RAF1 | RAN | RANBP9 | RAP1A | RAP1B | RAP1GDS1 | RARG | RB1 | RB1CC1 | RBBP4 | RBMX | RBX1 | RC3H1 | RCC1 | RDX | RECK | RELA | REST | RFC1 | RFC2 | RFC3 | RFC4 | RFC5 | RHOA | RHOJ | RHOT1 | RHOT2 | RHOU | RIC3 | RIF1 | RING1 | RIOK1 | RIPK1 | RIPK3 | RNF19A | RNF2 | RNF8 | ROMO1 | RP2 | RPA2 | RPA3 | RPGRIP1L | RPL10 | RPL12 | RPL23 | RPL23A | RPL24 | RPL38 | RPL6 | RPL7 | RPLP0 | RPLP0P6 | RPS10P5 | RPS14 | RPS19 | RPS24 | RPS3 | RPTOR | RRM1 | RRM2 | RRP7A | RRS1 | RTF1 | RTKN | RTN4 | RUVBL1 | RUVBL2 | RXRA | S100A6 | S100B | SAMD9 | SART1 | SART3 | SBDS | SCARA3 | SCFD1 | SCLT1 | SCO2 | SDC4 | SDCBP | SEC13 | SEC16A | SEC24B | SEC24C | SEC31A | SEPT11 | SEPT2 | SEPT4 | SEPT6 | SEPT7 | SEPT9 | SERPINB5 | SERPINE2 | SERPINH1 | SET | SETDB1 | SF1 | SFMBT1 | SFN | SFPQ | SH2B1 | SH3GL1 | SH3GL2 | SH3KBP1 | SH3PXD2A | SHC1 | SHKBP1 | SHMT1 | SHMT2 | SIN3A | SIPA1 | SIPA1L1 | SIRT1 | SIRT3 | SIRT5 | SIRT6 | SIRT7 | SKI | SLC12A2 | SLC16A1 | SLC25A4 | SLC25A5 | SLC35F6 | SLC9A1 | SLC9A3R1 | SLC9A3R2 | SMAD2 | SMAD3 | SMAD4 | SMARCA2 | SMARCA4 | SMARCB1 | SMARCC1 | SMARCC2 | SMARCE1 | SMC1A | SMC3 | SMG5 | SMG6 | SMN1 | SMTNL2 | SMURF1 | SNAI2 | SNAP29 | SNCA | SNF8 | SNRPB | SNRPD1 | SNRPD3 | SNRPE | SNX2 | SNX3 | SOAT1 | SOD1 | SOD2 | SORT1 | SOX2 | SP100 | SPACA1 | SPACA3 | SPC25 | SPDL1 | SPERT | SPI1 | SPICE1 | SPIN1 | SPINT2 | SPRY2 | SPTAN1 | SPTBN1 | SPTBN2 | SQSTM1 | SRC | SRGAP2 | SRP19 | SRPK1 | SRPK2 | SRSF12 | SRSF5 | SSX2IP | ST13 | STAG2 | STAT3 | STAU1 | STIL | STK11 | STK24 | STK25 | STK26 | STMN1 | STOM | STOML2 | STRAP | STX5 | STXBP1 | SULF2 | SUMO1 | SUMO1P1 | SUN2 | SUPT4H1 | SUZ12 | SVIL | SWAP70 | SYN1 | SYNE2 | SYNJ2BP | SYP | TADA2B | TAF12 | TANGO2 | TARDBP | TAZ | TBC1D23 | TBCA | TBCE | TBL1X | TCF4 | TCP1 | TCTN2 | TCTN3 | TDO2 | TDRD3 | TEKT2 | TEKT4 | TERF1 | TERF2 | TERF2IP | TESK2 | TFDP1 | TFG | TFIP11 | TGFB1 | TGFBR3 | TGM2 | TGOLN2 | THBS1 | THEM4 | TIMM13 | TIMM21 | TIMM50 | TIMMDC1 | TIMP1 | TIMP2 | TINF2 | TK1 | TLE1 | TLE3 | TM9SF4 | TMED9 | TMEM11 | TMEM17 | TMEM216 | TMEM231 | TMEM30A | TMEM70 | TMOD3 | TMOD4 | TNF | TNFAIP3 | TNFRSF1A | TNKS | TNN | TNNT1 | TNP1 | TNXB | TOMM20 | TOMM22 | TOP1 | TOP2A | TOP3A | TOR1A | TP53 | TP63 | TP73 | TPCN2 | TPM1 | TPM2 | TPM3 | TPM4 | TPR | TPSAB1 | TRADD | TRAF1 | TRAF2 | TRAPPC8 | TREX1 | TRIAP1 | TRIM21 | TRIM28 | TRIM32 | TRIM34 | TRIP13 | TRIP6 | TRMT61B | TSG101 | TSGA10 | TSKU | TSPYL2 | TTC19 | TTLL1 | TUBA1A | TUBA1C | TUBA4A | TUBB | TUBB2A | TUBB2B | TUBB3 | TUBB4A | TUBB4B | TUBB6 | TUBB8 | TUBG1 | TUBGCP3 | TWF1 | TWF2 | TXNL4A | UBB | UBC | UBE2A | UBE2B | UBE2C | UBE2D1 | UBE2D2 | UBE2D3 | UBE2K | UBE2N | UBE2S | UBQLN1 | UBQLN2 | UBXN10 | UGDH | UGT8 | ULK1 | UNK | UQCC1 | UQCRB | USP15 | USP16 | USP21 | USP39 | USP4 | USP50 | USP7 | USP8 | USP9X | USPL1 | UXS1 | VAPA | VAPB | VASP | VAV1 | VBP1 | VCAM1 | VCAN | VCL | VCP | VCPIP1 | VDR | VHL | VIM | VPS18 | VPS37D | VPS4B | VPS72 | VPS8 | VTA1 | VTN | WAS | WDR1 | WDR12 | WDR19 | WDR35 | WDR45B | WDR77 | WDR82 | WDTC1 | WIPI1 | WIPI2 | WT1 | XIRP2 | XPO1 | XRCC1 | XRCC3 | XRCC5 | XRCC6 | YAP1 | YEATS2 | YKT6 | YME1L1 | YWHAB | YWHAE | YWHAG | YWHAH | YWHAQ | YWHAZ | ZBTB7A | ZFAND6 | ZMPSTE24 | ZMYND10 | ZNF207 | ZNF622 | ZW10 | ZWINT | GO:0048518 | 0.417202474827126 | 3439/8243 | 1458/3250 | 1.74804230670198e-06 | 0.0165976617021353 | T | T | T | T | positive regulation of biological process | ABAT | ABCG1 | ABHD14B | ABHD17A | ABI3BP | ABL1 | ABL2 | ACACA | ACD | ACE2 | ACLY | ACSL1 | ACTA1 | ACTA2 | ACTB | ACTG1 | ACTG2 | ACTL6A | ACTN1 | ACTN2 | ACTN4 | ACTR2 | ACTR3 | ADA | ADCY6 | ADCYAP1 | ADORA2A | ADRB2 | ADRM1 | ADTRP | AES | AGAP2 | AGER | AGO1 | AGO2 | AGR2 | AHCYL1 | AHI1 | AHSG | AICDA | AIFM1 | AIMP2 | AIRE | AKAP8 | AKAP9 | AKR1B1 | AKR1C2 | AKT1 | AKT2 | AKT3 | AKTIP | ALOX12 | ANAPC4 | ANK1 | ANK3 | ANKRD49 | ANXA1 | ANXA2 | ANXA5 | AP1G1 | AP3D1 | APBB1 | APBB2 | APC | APCS | APEX1 | APOA1 | APOE | APP | AR | ARAF | ARF1 | ARF4 | ARHGAP1 | ARHGAP35 | ARHGDIA | ARHGEF7 | ARHGEF9 | ARID3B | ARID4B | ARNT | ARNT2 | ARPC1A | ARPC1B | ARPC2 | ARPC3 | ARPC4 | ARPC5 | ARRB1 | ARRB2 | ASB9 | ASCL1 | ASNS | ASPSCR1 | ASS1 | ATAD1 | ATAD5 | ATF2 | ATF6 | ATF6B | ATG5 | ATL3 | ATM | ATOH1 | ATP13A2 | ATP1A1 | ATP1B3 | ATP2A2 | ATRX | AURKB | AVIL | AXIN1 | AZU1 | B3GAT3 | BABAM1 | BACE1 | BAG2 | BAG3 | BAG4 | BAG6 | BAIAP2 | BAIAP2L1 | BANP | BASP1 | BAX | BBC3 | BCAR1 | BCAR3 | BCL10 | BCL11A | BCL2 | BCL2L1 | BCL2L13 | BCL6 | BIN1 | BIRC2 | BIRC3 | BIRC5 | BIRC7 | BIRC8 | BMI1 | BMPR1A | BRAF | BRCA1 | BRD4 | BTK | BTN2A1 | BTRC | BUD31 | C1QA | C1QBP | C1QTNF1 | C1R | C4A | CAB39 | CACNG2 | CACNG4 | CACNG5 | CALCOCO2 | CALM1 | CALM2 | CALM3 | CALR | CAMK1D | CAMK2A | CAMK2B | CAND1 | CAPG | CAPN1 | CAPN2 | CAPNS1 | CARD9 | CARM1 | CASP10 | CASP2 | CASP3 | CASP8 | CASP9 | CAT | CAV1 | CBFA2T3 | CBFB | CBL | CBX8 | CCAR2 | CCDC22 | CCDC88A | CCL21 | CCL22 | CCNB1 | CCNT1 | CCNT2 | CCNY | CCNYL1 | CCP110 | CCR1 | CCR3 | CCR4 | CCS | CCT2 | CCT3 | CCT4 | CCT5 | CCT6A | CCT7 | CCT8 | CD2 | CD209 | CD27 | CD36 | CD40 | CD40LG | CD6 | CD74 | CD79B | CD81 | CDC14B | CDC20 | CDC25A | CDC25B | CDC25C | CDC34 | CDC37 | CDC42 | CDC42EP3 | CDC42EP4 | CDC5L | CDC73 | CDH1 | CDH5 | CDK19 | CDK2 | CDK4 | CDK5 | CDK5R1 | CDK8 | CDK9 | CDKL3 | CDKN1A | CEBPA | CEBPE | CENPJ | CENPV | CEP135 | CEP290 | CFL1 | CFL2 | CFTR | CHCHD10 | CHCHD2 | CHD1L | CHD8 | CHEK2 | CHMP2A | CHP1 | CHRD | CHRM3 | CIRBP | CKS1B | CKS2 | CLEC11A | CLIC1 | CLNK | CLPX | CLSTN1 | CLU | COA3 | COL18A1 | COLEC10 | COLGALT1 | COMMD1 | COMT | COPS5 | COX17 | CPNE3 | CPT1A | CPT2 | CREB1 | CREB3 | CREBBP | CRIP2 | CRK | CRKL | CROCC | CRP | CSNK1A1 | CSNK1E | CSNK2A2 | CSNK2B | CTBP1 | CTCF | CTDP1 | CTH | CTNNB1 | CTNNBIP1 | CTSC | CTSL | CTTN | CUL1 | CUL3 | CUL4B | CUL7 | CXCL11 | CXCL5 | CXCL8 | CYCS | CYLD | CYP1A1 | DAB1 | DAB2 | DAPK1 | DAXX | DAZAP1 | DBF4B | DBH | DBN1 | DBNL | DCN | DCTN1 | DCUN1D1 | DDB1 | DDB2 | DDIT4 | DDRGK1 | DDX1 | DDX39B | DDX3X | DECR1 | DERL1 | DGKZ | DHX36 | DHX9 | DIABLO | DISC1 | DLG3 | DLG4 | DMAP1 | DMD | DNA2 | DNAJA1 | DNAJA2 | DNAJA3 | DNAJB2 | DNM1L | DOCK5 | DOCK7 | DOK1 | DPF2 | DROSHA | DSTN | DTX1 | DUSP1 | DUSP12 | DUSP19 | DUSP22 | DUSP7 | DVL1 | DVL2 | DVL3 | DYNC1H1 | DYNC1LI1 | DYNLL1 | DYRK2 | E2F1 | E2F2 | E2F3 | E2F4 | E2F6 | EAF2 | EBF1 | ECD | EDA | EDEM1 | EED | EEF1A2 | EEF1D | EEF2 | EGFR | EGLN1 | EGLN3 | EHD4 | EHMT1 | EIF2AK3 | EIF2AK4 | EIF2B1 | EIF2B3 | EIF2B5 | EIF2S1 | EIF3D | EIF3E | EIF4A3 | EIF4E | EIF4EBP1 | EIF5A | EIF6 | ELAVL1 | ELF3 | ELF4 | EMILIN2 | ENDOG | ENO1 | EP300 | EPHA4 | EPM2A | ERBB2 | ERBB3 | ERCC5 | ERG | ERLIN2 | ESR1 | ESR2 | ESRRA | ESRRB | ESRRG | ETS1 | EXOSC6 | EXOSC9 | EYA2 | EYA4 | EZH2 | EZR | FABP1 | FABP3 | FABP5 | FADD | FAF1 | FAM129B | FAM19A3 | FAM49B | FAM83D | FAM83H | FAM98A | FANCI | FANCM | FASLG | FASN | FBRS | FBXO7 | FBXW11 | FCGR1A | FCHSD1 | FCN1 | FEN1 | FGB | FGF2 | FGFR1 | FGFR1OP | FGFR3 | FH | FHL1 | FHL2 | FIS1 | FKBP1A | FKBP1B | FKBP8 | FLOT1 | FN1 | FOS | FOXA1 | FOXJ1 | FOXK1 | FOXK2 | FOXM1 | FOXO1 | FSCN1 | FTO | FUBP1 | FUBP3 | FUS | FXR2 | FYN | FZR1 | G6PD | GABPB1 | GADD45G | GAPDH | GAPDHS | GATA1 | GBP5 | GDF9 | GDI1 | GFAP | GGA1 | GJA1 | GKAP1 | GLMN | GLRX | GLUL | GNA13 | GNAI1 | GNAI2 | GNAI3 | GNAS | GNG5 | GOLGA2 | GOLGA4 | GOLPH3 | GOLT1B | GOT1 | GPD1 | GPD1L | GPI | GPS2 | GPSM1 | GPX1 | GRB2 | GRB7 | GRID2 | GRIK2 | GRIP1 | GRK5 | GSK3A | GSK3B | GSTO1 | GSTP1 | GTF2A1 | GTF2F1 | GTF2F2 | GZMA | H1F0 | H1FX | H2AFX | H2AFZ | H3F3A | HADH | HAP1 | HAVCR2 | HAX1 | HBEGF | HDAC1 | HDAC2 | HDAC3 | HDAC4 | HDAC5 | HDAC6 | HDAC7 | HDAC9 | HECW1 | HECW2 | HELZ2 | HEXB | HEXIM1 | HGS | HIF1A | HIF1AN | HINT1 | HIST1H1A | HK1 | HK2 | HLA-A | HLA-B | HLA-DQA1 | HLA-DRA | HLA-DRB1 | HLA-DRB3 | HLA-DRB4 | HLA-DRB5 | HLX | HMGB1 | HMGB2 | HMGB3 | HMOX1 | HNF1A | HNF4A | HNF4G | HNRNPA1 | HNRNPA2B1 | HNRNPAB | HNRNPD | HNRNPK | HNRNPL | HNRNPU | HOXA5 | HOXB5 | HOXB9 | HOXD10 | HPRT1 | HPX | HRAS | HSF1 | HSF4 | HSP90AA1 | HSP90AB1 | HSP90B1 | HSPA1B | HSPA1L | HSPA2 | HSPA5 | HSPB1 | HSPBP1 | HSPD1 | HSPE1 | HSPH1 | HTATIP2 | HTR1B | HTR3A | HTRA1 | HTRA2 | HTT | HUWE1 | ICAM1 | ID2 | IDE | IFI16 | IFIT1 | IFNA2 | IGF1R | IGF2BP1 | IGHV3-13 | IGHV3-23 | IGHV3-30 | IGKV3-20 | IGLC7 | IKBKB | IKBKE | IKBKG | IKZF5 | IL13RA2 | ILF2 | ILF3 | ILK | ING1 | ING5 | INSIG1 | INSIG2 | INSR | IPO5 | IQGAP1 | IRF1 | IRF4 | IRF7 | ISG15 | ITCH | ITGA4 | ITGAV | ITGB1BP1 | ITGB3 | ITGB3BP | ITK | ITPR3 | JAK1 | JAK3 | JMJD6 | JUN | JUNB | JUND | JUP | KAT5 | KAT7 | KAT8 | KCNIP2 | KCTD17 | KDM1A | KDM4D | KDM5B | KDM8 | KDR | KEAP1 | KHDRBS1 | KIF5B | KLF5 | KLF6 | KLHDC10 | KLHL22 | KMT2A | KMT2B | KMT2C | KRAS | KSR1 | LAMB1 | LAMP3 | LARP4 | LARS | LASP1 | LAT | LATS1 | LCP1 | LCP2 | LDHA | LEF1 | LGALS1 | LGALS3 | LGMN | LGR4 | LIG4 | LIMS1 | LMNA | LMO2 | LMO7 | LOXL2 | LPAR1 | LPAR4 | LPIN3 | LRP6 | LRRC7 | LRRK1 | LRRK2 | LRSAM1 | LTBR | LUM | LURAP1 | MAD2L1 | MAOA | MAP1B | MAP2K1 | MAP2K3 | MAP2K4 | MAP2K6 | MAP2K7 | MAP3K1 | MAP3K13 | MAP3K14 | MAP3K2 | MAP3K3 | MAP3K4 | MAP4K1 | MAPK1 | MAPK14 | MAPK15 | MAPK3 | MAPK6 | MAPK7 | MAPK8 | MAPK8IP3 | MAPK9 | MAPKAP1 | MAPT | MARCH5 | MARCKSL1 | MAS1 | MASP1 | MASP2 | MATK | MATR3 | MAVS | MBP | MC1R | MCTS1 | MDM2 | MDM4 | MECP2 | MED1 | MED12 | MED14 | MED17 | MED20 | MED21 | MED23 | MED24 | MED31 | MED4 | MED6 | MELK | MEOX2 | MEPCE | METRN | METRNL | METTL23 | MGMT | MIB1 | MICA | MIF | MITF | MLH1 | MLLT11 | MLLT3 | MLST8 | MME | MMRN1 | MOB1B | MORF4L2 | MOV10 | MPC2 | MPP2 | MRPL12 | MSH6 | MSN | MTA1 | MTA3 | MTCH2 | MTPN | MVD | MYBBP1A | MYC | MYD88 | MYO1C | MYO6 | MYOC | NACC1 | NAMPT | NANOG | NAP1L1 | NBAS | NCBP1 | NCK1 | NCOA1 | NCOA2 | NCOA3 | NCOA4 | NCOR1 | NCOR2 | NDUFA13 | NEDD4 | NEK1 | NEK7 | NELFB | NELFCD | NELFE | NELL1 | NEUROD1 | NF2 | NFATC1 | NFATC2 | NFE2L2 | NFKB2 | NFKBIA | NID1 | NIF3L1 | NIN | NIPBL | NKRF | NKX2-1 | NMB | NMD3 | NME1 | NME2 | NMNAT1 | NNT | NOD2 | NOP10 | NOS1AP | NOS2 | NOS3 | NPEPPS | NPM1 | NPR1 | NQO1 | NQO2 | NR0B2 | NR1H2 | NR1H3 | NR1I2 | NR3C1 | NR4A1 | NR5A2 | NRP2 | NRXN1 | NSFL1C | NSMCE4A | NTF3 | NTRK1 | NUFIP1 | NUMA1 | NUP85 | NUP93 | NUP98 | NUPR1 | NUSAP1 | OAZ3 | OBSL1 | OGT | ONECUT1 | OPA1 | OPTN | ORM1 | OTUD4 | OXCT1 | OXSR1 | P2RX1 | P2RX2 | P2RX4 | P4HB | PA2G4 | PABPC1 | PACSIN3 | PAFAH1B1 | PAG1 | PAIP1 | PAK2 | PAK6 | PAN2 | PANX3 | PARD3 | PARD6A | PARK7 | PARP1 | PARP14 | PARP6 | PATZ1 | PAWR | PAX8 | PAX9 | PAXIP1 | PC | PCBD1 | PCBP1 | PCGF5 | PCM1 | PCNA | PCSK9 | PDCD10 | PDCD6 | PDCD6IP | PDCL | PDE12 | PDE4D | PDE6G | PDF | PDIA3 | PEBP1 | PEF1 | PELI1 | PER1 | PER2 | PFDN2 | PFKM | PFN1 | PGK1 | PGK2 | PHB | PHB2 | PHF10 | PIAS1 | PIAS3 | PICK1 | PIH1D1 | PIK3C2A | PIK3CA | PIK3CG | PIK3R1 | PIK3R3 | PIM1 | PIN1 | PINX1 | PIP4K2A | PKM | PKN2 | PLA2G10 | PLA2G1B | PLA2G2A | PLA2G6 | PLAUR | PLCG1 | PLEKHF1 | PLGRKT | PLK1 | PLSCR1 | PLXNA1 | PLXNA2 | PLXNB2 | PML | PNKP | PNMA1 | PNP | POLDIP2 | POLDIP3 | POLR2A | POLR2E | POLR2L | POT1 | POU2F3 | POU5F1 | PPARD | PPARG | PPARGC1A | PPIA | PPIB | PPID | PPIE | PPIF | PPM1A | PPM1K | PPP1CA | PPP1CC | PPP1R12A | PPP2CA | PPP2R1A | PPP2R1B | PPP2R5D | PPP3CC | PPP5C | PPT1 | PRDX2 | PRDX3 | PRDX6 | PRELID1 | PRKAA1 | PRKAA2 | PRKAB2 | PRKACA | PRKAG1 | PRKAR1A | PRKAR2B | PRKCD | PRKCE | PRKCZ | PRKD1 | PRKD2 | PRKDC | PRMT2 | PROX1 | PRPF19 | PRRC1 | PSAP | PSEN1 | PSEN2 | PSMA1 | PSMA2 | PSMA3 | PSMA5 | PSMA6 | PSMB5 | PSMB6 | PSMB7 | PSMB8 | PSMC1 | PSMC2 | PSMC3 | PSMC4 | PSMC5 | PSMC6 | PSMD1 | PSMD10 | PSMD11 | PSMD12 | PSMD14 | PSMD2 | PSMD4 | PSMD9 | PSME2 | PSME3 | PTEN | PTGER3 | PTGES2 | PTGES3 | PTK2 | PTN | PTPN1 | PTPN22 | PTPN23 | PTPN6 | PTPRD | PTRH2 | PUM1 | PXN | RAB11A | RAB11FIP2 | RAB1A | RAB1B | RAB21 | RAB29 | RAB31 | RAB3GAP1 | RAB5A | RAB7A | RAB9A | RAC1 | RAD21 | RAD23A | RAD51 | RAF1 | RAN | RAP1A | RAP1B | RARA | RARG | RASSF10 | RB1 | RB1CC1 | RBM3 | RBM4 | RBMX | RBPJ | RBX1 | RC3H1 | RDX | RECK | REL | RELA | REST | RFC1 | RFC2 | RFC3 | RFC4 | RFC5 | RFK | RFXANK | RGN | RGS2 | RHBDD1 | RHOA | RHOJ | RHOU | RIF1 | RIOK1 | RIPK1 | RIPK2 | RIPK3 | RIPK4 | RMND1 | RNASEH2B | RNF139 | RNF183 | RNF185 | RNF19A | RNF25 | RNF31 | RNF4 | RNF8 | ROMO1 | RPA2 | RPA3 | RPL23 | RPLP1 | RPS15A | RPS19 | RPS2 | RPS3 | RPS4X | RPS6KA1 | RPS6KA2 | RPS6KB2 | RPTOR | RSU1 | RTF1 | RTN4 | RUNX1 | RUNX2 | RUVBL1 | RUVBL2 | RXRA | RXRB | RXRG | S100A1 | S100A12 | S100A13 | S100A4 | S100A6 | S100A7 | S100A9 | S100B | SAE1 | SAP18 | SARM1 | SART1 | SART3 | SCARA3 | SCEL | SCG2 | SCYL2 | SDC1 | SDC4 | SDCBP | SEC13 | SEC16A | SEMA7A | SEPT4 | SEPT7 | SEPT9 | SERPINB3 | SERPINE2 | SETDB1 | SFN | SFPQ | SGTA | SGTB | SH2B1 | SH3GL1 | SH3GL2 | SH3GL3 | SHC1 | SHKBP1 | SHMT2 | SIAH1 | SIN3A | SIRT1 | SIRT3 | SIRT6 | SIRT7 | SKI | SLAMF1 | SLC25A5 | SLC25A6 | SLC35F6 | SLC9A1 | SLC9A3R1 | SMAD2 | SMAD3 | SMAD4 | SMAD5 | SMAD9 | SMARCA2 | SMARCA4 | SMARCB1 | SMARCC1 | SMARCC2 | SMARCE1 | SMC3 | SMO | SMURF1 | SMURF2 | SNAI2 | SNCA | SNF8 | SNW1 | SNX3 | SNX6 | SOAT1 | SOCS1 | SOCS2 | SOD1 | SOD2 | SORT1 | SOX2 | SP1 | SP100 | SPACA3 | SPEN | SPG21 | SPI1 | SPIN1 | SPRTN | SPRY2 | SPTBN1 | SPTLC2 | SQSTM1 | SRC | SRI | SRPK1 | SRPK2 | SRSF5 | STAC | STAT1 | STAT3 | STAT4 | STAT5A | STAT6 | STAU1 | STIM1 | STK11 | STK24 | STK25 | STK26 | STK3 | STMN1 | STOM | STOML2 | STUB1 | STX5 | STXBP1 | STXBP2 | SUB1 | SULF2 | SUMO1 | SUMO2 | SUN2 | SUPT4H1 | SUPT5H | SUZ12 | SVIL | SWAP70 | SYDE1 | SYNE2 | TAB1 | TAB2 | TADA2B | TAF12 | TAF15 | TAF1A | TANK | TAOK3 | TARDBP | TAZ | TBC1D23 | TBL1X | TBRG4 | TBXA2R | TCEA1 | TCEA2 | TCERG1 | TCF12 | TCF21 | TCF4 | TCIRG1 | TCP1 | TCTN3 | TDRD3 | TERF1 | TERF2 | TERF2IP | TFCP2 | TFDP1 | TFG | TGFB1 | TGFBR3 | TGM2 | THBS1 | THBS2 | THRA | TIA1 | TIAL1 | TIMP1 | TIMP2 | TLE1 | TLR3 | TM9SF4 | TMED9 | TMEM173 | TMEM30A | TMEM9B | TMOD3 | TMTC3 | TNF | TNFAIP3 | TNFRSF12A | TNFRSF1A | TNFRSF8 | TNFSF12 | TNFSF14 | TNIP2 | TNK2 | TNKS | TNKS2 | TNP1 | TOLLIP | TOMM22 | TOP2A | TOR1A | TP53 | TP63 | TP73 | TPM1 | TPR | TRA2A | TRADD | TRAF1 | TRAF2 | TRAF3 | TRAF6 | TREX1 | TRIAP1 | TRIB2 | TRIB3 | TRIM14 | TRIM21 | TRIM25 | TRIM28 | TRIM32 | TRIM34 | TRIM35 | TRIP4 | TRIP6 | TRUB2 | TSG101 | TTC5 | TUBB2B | TWF1 | TWF2 | TWIST1 | TXN | TXNIP | U2AF2 | UBB | UBC | UBE2A | UBE2B | UBE2C | UBE2D1 | UBE2D2 | UBE2D3 | UBE2I | UBE2K | UBE2L3 | UBE2M | UBE2N | UBE2O | UBE2S | UBE2V1 | UBE3A | UBQLN1 | UBQLN2 | UBR5 | ULK1 | UNC119 | UPF3B | UQCC1 | USP16 | USP17L2 | USP19 | USP2 | USP21 | USP47 | USP5 | USP50 | USP7 | USP8 | USP9X | VAPA | VAPB | VASH2 | VASP | VAV1 | VCAM1 | VCP | VDR | VHL | VIM | VPS4B | VSIG4 | VSTM2A | VTN | WARS | WAS | WBP2 | WDFY1 | WDR1 | WDR35 | WDR48 | WDR77 | WNK1 | WNK4 | WNT16 | WNT2 | WT1 | WWOX | WWP2 | XBP1 | XIAP | XPO4 | XPO5 | XRCC1 | XRCC3 | XRCC5 | XRCC6 | YAF2 | YAP1 | YES1 | YTHDF1 | YWHAB | YWHAE | YWHAG | YWHAH | YWHAQ | YWHAZ | YY1 | ZAP70 | ZBTB16 | ZBTB17 | ZBTB7A | ZBTB7B | ZDHHC17 | ZIC1 | ZMPSTE24 | ZMYND10 | ZMYND8 | ZNF24 | ZNF622 | ZNF746 | GO:0016051 | 0.00982651947106636 | 81/8243 | 53/3250 | 1.79237282024432e-06 | 0.0170185799282198 | T | T | T | T | carbohydrate biosynthetic process | AGL | AKR1B1 | AKT1 | AKT2 | ALDOA | ALDOC | B3GNT3 | CRY1 | ENO1 | ENO2 | ENO3 | FBP1 | FBP2 | G6PD | GAPDH | GAPDHS | GBE1 | GOT1 | GOT2 | GPD1 | GPI | GYG1 | GYG2 | GYS1 | HS2ST1 | IMPA1 | IMPA2 | ISYNA1 | MDH1 | MDH2 | NANS | PC | PER2 | PGAM1 | PGAM2 | PGD | PGK1 | PGK2 | PGM1 | PGM2 | PGP | PPARGC1A | PPP1R3C | SLC25A1 | SLC25A10 | SLC25A11 | SLC25A13 | SORD | ST6GALNAC5 | TALDO1 | TKT | TPI1 | UGP2 | GO:0080135 | 0.0609001577095718 | 502/8243 | 248/3250 | 1.85703353977822e-06 | 0.0176325334601942 | T | T | T | T | regulation of cellular response to stress | ABL1 | ACTR2 | AGER | AGR2 | AKT1 | AKT3 | APBB1 | APP | ATAD5 | ATF6 | ATF6B | ATM | AXIN1 | BABAM1 | BAG1 | BAG2 | BAG3 | BAG4 | BAG5 | BAG6 | BAX | BCL2 | BCL2L1 | BCL6 | BDKRB2 | BIRC7 | BMPR1A | BRAF | BRCA1 | CAMK2A | CAMK2B | CARD9 | CASP9 | CAV1 | CBX8 | CCAR2 | CCL21 | CD27 | CD36 | CD40LG | CD74 | CDK9 | CHCHD2 | CHEK2 | CHORDC1 | CLU | COPS5 | CREB3 | CREBBP | CRY1 | CRYAB | CTNNB1 | CYLD | DAXX | DBNL | DDX39B | DHX9 | DNAJA1 | DNAJB1 | DNAJC7 | DUSP1 | DUSP19 | DUSP22 | DVL2 | DVL3 | EDEM1 | EGFR | EIF2AK3 | EIF2AK4 | ENDOG | ENO1 | EP300 | EPHA4 | EYA2 | EYA4 | EZR | FBXO7 | FIGNL1 | FKBP1B | FOXM1 | FOXO1 | FUS | FYN | GADD45G | GPS2 | GPX1 | GRIK2 | GSK3B | GSTP1 | H2AFX | HDAC3 | HDAC6 | HIF1A | HMGB1 | HNRNPK | HRAS | HSF1 | HSP90AA1 | HSP90AB1 | HSPA1B | HSPA1L | HSPA5 | HSPA8 | HSPB1 | HSPH1 | HTRA2 | IGBP1 | IGF1R | INSIG1 | ITCH | KDM1A | KDM4D | KLHDC10 | KRAS | LANCL1 | LRRK2 | LTBR | MAP2K1 | MAP2K4 | MAP2K7 | MAP3K13 | MAP3K2 | MAP3K3 | MAP3K4 | MAP4K1 | MAPK1 | MAPK3 | MAPK7 | MAPK8IP3 | MAPKAP1 | MAPT | MDM2 | MGMT | MIF | MLST8 | MYC | NAMPT | NCK1 | NCOR1 | NFE2L2 | NOD2 | NPM1 | NSMCE4A | NUP107 | NUP153 | NUP160 | NUP205 | NUP214 | NUP85 | NUP88 | NUP93 | NUP98 | OPA1 | OTUB1 | P4HB | PAFAH1B1 | PARK7 | PARP1 | PAWR | PAXIP1 | PCNA | PDCD10 | PER1 | PIK3R1 | PML | POM121 | PPP4R2 | PRDX1 | PRKCD | PRKDC | PSAP | PSMD10 | PTEN | PTGES3 | PTPN1 | PTPN22 | PTPRF | PYCR1 | QARS | RAD51 | RAD51AP1 | RAE1 | RB1CC1 | RGN | RIF1 | RIPK1 | RIPK2 | RNASEH2B | RNF183 | RNF185 | RNF8 | RPA2 | RPA3 | RPS3 | RPTOR | SDCBP | SEC13 | SERPINB3 | SFPQ | SGTA | SIRT1 | SIRT6 | SLAMF1 | SNAI2 | SOD1 | SOD2 | STK24 | STK25 | STK26 | STK3 | SYVN1 | TAOK3 | TERF2 | TERF2IP | TFIP11 | TGFBR3 | TLR3 | TMBIM6 | TNF | TPR | TRAF2 | TRAF6 | TRAP1 | TRIAP1 | TRIM28 | TRIM32 | TRIP12 | TWIST1 | TXN | UBE2J1 | UBE2N | UBE2V1 | UBQLN1 | UBQLN2 | UBR5 | UBXN1 | USP14 | USP19 | USP47 | WAS | WNT16 | XBP1 | XRCC1 | YWHAE | ZBTB7B | ZMPSTE24 | ZNF277 | ZNF622 | GO:0009725 | 0.0513162683489021 | 423/8243 | 213/3250 | 1.90255960187679e-06 | 0.0180648034198202 | T | T | T | T | response to hormone | ABCG1 | ACACA | ACAT1 | ACTA1 | ADCY6 | ADCYAP1 | ADTRP | AGL | AIFM1 | AKAP8 | AKR1B1 | AKR1C2 | AKT1 | AKT2 | ANXA1 | ANXA2 | ANXA5 | APEX1 | APRT | AR | ARNT2 | ARPC1B | ASNS | ASS1 | ATP1A1 | BCL2 | BRAF | BSG | CALM1 | CALM3 | CALR | CASP3 | CASP8 | CASP9 | CAT | CAV1 | CBL | CCL21 | CDC5L | CDK4 | CDKN1A | CHEK2 | CREB1 | CRK | CRY1 | CRYAB | CSN1S1 | CSNK2B | CTNNB1 | CTSL | CTSV | CYC1 | CYP1A2 | DDIT4 | DENND4C | DUSP1 | EEF2 | EGFR | EIF2B1 | EIF2B3 | EIF2B5 | EIF4E | EIF4EBP1 | EIF6 | ENDOG | EPRS | ERRFI1 | ESR1 | ESRRA | ETS1 | EZH2 | FABP3 | FBXO32 | FECH | FGB | FHL2 | FOS | FOXA1 | FOXO1 | FYN | GATA1 | GGT1 | GLP1R | GNAI1 | GNB1 | GOT1 | GOT2 | GPI | GPX4 | GSK3A | GSTP1 | H2AFZ | HADH | HDAC4 | HDAC5 | HDAC9 | HMGB2 | HNRNPD | HNRNPU | HSF1 | HTR1B | ICAM1 | IDH1 | IGFBP7 | INSIG2 | INSR | IQGAP1 | JUN | JUNB | JUND | KAT5 | KRAS | LMO2 | LPIN3 | LRP6 | MAS1 | MBD3 | MDM2 | ME1 | MED1 | MSN | MTAP | MYO5A | NCOA1 | NCOA2 | NCOA3 | NFE2L2 | NKX2-1 | NME1 | NME2 | NOS2 | NOS3 | NQO1 | NR1H3 | NR3C1 | NR4A1 | OGT | OTC | OXCT1 | PARP1 | PAX8 | PCNA | PCSK9 | PEBP1 | PIAS3 | PIK3R1 | PKM | PLA2G1B | PPARG | PPARGC1A | PPAT | PRKAA1 | PRKAA2 | PRKACA | PRKAR1A | PRKAR2B | PRKCD | PRKCE | PRKCZ | PRKDC | PTEN | PTN | QDPR | RAB10 | RAB31 | RANGAP1 | RAP1B | RARA | RBBP4 | RDX | RELA | REST | RHOA | RUVBL2 | S100B | SDC1 | SIRT1 | SLC9A1 | SOCS2 | SORD | SORT1 | SP1 | SRC | SRSF5 | STAT1 | STAT3 | STXBP1 | TBC1D4 | TBXA2R | TGFB1 | TGFBR3 | THBS1 | TIMP1 | TIMP2 | TNF | TOMM20 | TRIB3 | TRIM63 | TXN2 | TXNIP | TYMS | UBE2L3 | UBE3A | USP8 | VGF | WBP2 | WDTC1 | WT1 | XBP1 | YAP1 | YWHAG | YY1 | ZBTB7B | GO:0000302 | 0.0171054227829674 | 141/8243 | 83/3250 | 2.00078360265206e-06 | 0.0189974403071813 | T | T | T | T | response to reactive oxygen species | ABL1 | ADA | AIFM1 | AKT1 | ANXA1 | APEX1 | APOD | APOE | BCL2 | BTK | CAPN2 | CASP3 | CAT | CBX8 | CDK2 | CRK | CRYAB | DPEP1 | DUSP1 | EEF2 | EGFR | EGLN1 | ETS1 | EZH2 | FABP1 | FKBP1B | FOS | FOXO1 | FYN | GNAO1 | GPX1 | GSTP1 | HDAC2 | HDAC6 | HMOX1 | HNRNPD | HSF1 | JUN | LDHA | MAPK1 | MAPK3 | MAPK7 | MAPK8 | MAPK9 | MAPT | MDM2 | NFE2L2 | NQO1 | PARK7 | PCNA | PDCD10 | PDK2 | PPARGC1A | PPIF | PPP2CB | PRDX3 | PRDX5 | PRKAA1 | PRKCD | PTPRK | PXN | RELA | RIPK1 | RIPK3 | ROMO1 | RPS3 | S100A7 | SDC1 | SIRT1 | SOD1 | SOD2 | SRC | STAT1 | STAT6 | STK24 | STK25 | STK26 | TNFAIP3 | TPM1 | TRAF2 | TREX1 | TXNIP | ZNF277 | GO:0044419 | 0.074851389057382 | 617/8243 | 298/3250 | 2.07077804377478e-06 | 0.0196620375256415 | T | T | T | T | interspecies interaction between organisms | ABCE1 | ACE2 | ANXA2 | AP1B1 | AP1G1 | AP1M1 | AP3M1 | APCS | APOE | ARF1 | ASGR1 | ASGR2 | ATF7 | ATG5 | AZU1 | BANF1 | BAX | BCL2L1 | BIN1 | BRD2 | BRD4 | BTRC | C1QBP | CALCOCO2 | CALM1 | CALM2 | CALM3 | CARM1 | CASP8 | CAV1 | CBL | CCNT1 | CCNT2 | CCR3 | CCR5 | CD209 | CD81 | CDC25C | CDC42 | CDH1 | CEBPA | CFL1 | CHMP2A | COPB1 | COPS6 | CREB1 | CREB3 | CREBBP | CTBP1 | CTDP1 | CTNNB1 | CTNND1 | CUL1 | CUL2 | CUL5 | CUL7 | DAXX | DDB1 | DDX39B | DDX3X | DDX6 | DERL1 | DHX9 | DYNC1I2 | DYNC1LI1 | DYNLL1 | DYNLT1 | EGFR | EIF2AK4 | EIF3A | EIF3D | EIF4E | EIF4H | EP300 | FADD | FDPS | FKBP8 | FN1 | FYN | GAPDH | GPS2 | GPX1 | GRB2 | H2AFX | HDAC1 | HLA-DRA | HNRNPA1 | HNRNPK | HPX | HSPA1B | HSPA8 | HSPD1 | HTATIP2 | ICAM1 | IDE | IFIT1 | IK | IKBKB | IKBKE | IKBKG | INSR | IPO5 | IPO7 | IQGAP1 | IRF7 | ISG15 | ITCH | ITGAV | ITGB3 | ITGB5 | JUN | KAT5 | KDR | KEAP1 | KLC1 | KPNA1 | KPNA2 | KPNB1 | LAMP3 | LEF1 | LIG4 | LRSAM1 | LTBR | MAPK1 | MAPK3 | MAVS | MCTS1 | MDM2 | MECP2 | MICA | MSH6 | NEDD4 | NFE2L2 | NFKBIA | NPM1 | NRP2 | NUP107 | NUP153 | NUP160 | NUP205 | NUP214 | NUP85 | NUP88 | NUP93 | NUP98 | NXF1 | OGT | PACS1 | PACS2 | PAK2 | PARD6A | PC | PCBP1 | PCBP2 | PCNA | PDCD6IP | PIK3R1 | PLCG1 | PML | POLR2E | POM121 | POU2F3 | PPIA | PPIB | PPID | PRMT6 | PSMA3 | PSMB5 | PSMB6 | PSMB7 | PSMB8 | PSMC3 | RAB1A | RAB1B | RAB29 | RAB5A | RAB7A | RAB9A | RAC1 | RAD23A | RAE1 | RAN | RB1 | RBX1 | RCC1 | RELA | REST | RHOA | RIPK1 | ROMO1 | RPL10 | RPL10A | RPL12 | RPL18A | RPL22 | RPL23 | RPL23A | RPL24 | RPL30 | RPL37A | RPL38 | RPL6 | RPL7 | RPL7A | RPL9 | RPLP0 | RPLP1 | RPLP2 | RPS12 | RPS13 | RPS14 | RPS15A | RPS19 | RPS2 | RPS20 | RPS24 | RPS29 | RPS3 | RPS4X | RPS8 | RPTOR | RXRA | S100A12 | S100A9 | SEC13 | SEPT6 | SERPINB3 | SET | SGTA | SHC1 | SIRT1 | SLAMF1 | SLC25A4 | SLC25A5 | SLC25A6 | SMAD3 | SMARCA4 | SMARCB1 | SMC3 | SND1 | SNW1 | SP1 | SP100 | SPEN | SRC | SRPK1 | SSB | STAT1 | STAT3 | STAU1 | STOM | SUGT1 | SUMO1 | TANK | TARDBP | TCP1 | TGFB1 | THOC2 | THOC6 | THOC7 | TMEM173 | TNFRSF1A | TNPO1 | TOP1 | TP53 | TP73 | TPR | TRIM23 | TRIM25 | TRIM28 | TSG101 | TYMS | UBB | UBC | UBE2I | UBE3A | UBR4 | UBR5 | UBXN1 | USP7 | VAC14 | VAPA | VAPB | VCP | VDAC1 | VIM | VPS18 | VPS29 | VPS37D | VPS4B | VTA1 | WDR48 | WWP2 | XPO1 | XRCC5 | XRCC6 | YWHAB | YWHAE | GO:0032879 | 0.182579158073517 | 1505/8243 | 673/3250 | 2.18242547716544e-06 | 0.0207221299056859 | T | T | T | T | regulation of localization | ABAT | ABCG1 | ABHD17A | ABL1 | ABL2 | ACAA2 | ACE2 | ACTB | ACTN1 | ACTN2 | ACTN4 | ADA | ADCYAP1 | ADORA2A | ADRB2 | ADTRP | AGER | AGO2 | AGR2 | AHCYL1 | AHI1 | AHNAK | AHSG | AIRE | AKAP9 | AKT1 | AKT2 | AKT3 | ALOX12 | ANK3 | ANXA1 | ANXA2 | ANXA5 | ANXA6 | AP1G1 | AP3D1 | APBB1 | APC | APEX1 | APOA1 | APOD | APOE | APP | AR | ARF1 | ARFGAP1 | ARFIP1 | ARHGAP1 | ARHGEF7 | ARRB1 | ARRB2 | ASPSCR1 | ATAD1 | ATCAY | ATG5 | ATM | ATP13A2 | ATP1A1 | ATP1B3 | ATP2A2 | ATXN2 | AZU1 | B3GAT3 | BACE1 | BAG3 | BAG4 | BAX | BBC3 | BCAR1 | BCL2 | BCL2L1 | BCL6 | BIN1 | BMPR1A | BRAF | BTBD9 | C1QBP | C1QTNF1 | C4A | CAB39 | CACNA1A | CACNG2 | CACNG4 | CACNG5 | CALM1 | CALM2 | CALM3 | CALR | CAMK1D | CAMK2A | CAMK2B | CAPN1 | CASP8 | CAV1 | CBL | CCDC88A | CCL21 | CCR1 | CCR5 | CCT2 | CCT3 | CCT4 | CCT5 | CCT6A | CCT7 | CCT8 | CD2 | CD22 | CD36 | CD40 | CD40LG | CD74 | CD81 | CDH1 | CDK16 | CDK5 | CDK5R1 | CENPJ | CEP135 | CEP290 | CFTR | CHCHD10 | CHMP2A | CHP1 | CHRD | CHRM2 | CLDN18 | CLIC1 | CLIC3 | CLIC4 | CLTC | CLU | COL18A1 | COMMD1 | COMT | COX17 | CPLX1 | CPLX3 | CPNE3 | CPT1A | CRBN | CREB1 | CREB3 | CRK | CRKL | CROCC | CRP | CRY1 | CRY2 | CRYAB | CSNK1E | CSNK2A2 | CSNK2B | CTNNB1 | CXCL11 | CXCL8 | CYLD | DAB2 | DAPK1 | DBN1 | DCLK1 | DCN | DCP2 | DDRGK1 | DHX36 | DHX9 | DLG2 | DLG3 | DLG4 | DMAP1 | DMD | DNAJA1 | DNAJC30 | DNM1L | DOCK5 | DOCK7 | DPEP1 | DUSP1 | DUSP22 | DVL1 | DVL3 | DYNC1H1 | DYNLL1 | E2F1 | EDEM1 | EGFR | EHD4 | EIF2AK3 | EPPIN | EPPK1 | ERBB2 | ERBB3 | ETS1 | EZR | FABP3 | FABP5 | FADD | FAM83H | FASLG | FCN1 | FGB | FGF2 | FGFR1 | FGFR1OP | FHL1 | FIS1 | FKBP1A | FKBP1B | FLOT1 | FN1 | FTO | FYN | G6PD | GAPDH | GAPVD1 | GDF9 | GDI1 | GFAP | GIT1 | GLMN | GLP1R | GLRX | GLUL | GNA12 | GNA13 | GNAI1 | GNAI2 | GNAO1 | GNAS | GOLPH3 | GOPC | GPD1L | GPI | GPS2 | GRB7 | GRIA1 | GSK3A | GSK3B | GSTO1 | GSTP1 | HADH | HAP1 | HAVCR2 | HAX1 | HBEGF | HCN1 | HCN2 | HCN3 | HDAC3 | HDAC4 | HDAC5 | HDAC6 | HDAC7 | HDAC9 | HECW1 | HECW2 | HGS | HIF1A | HIST1H1A | HK1 | HK2 | HMGB1 | HMOX1 | HNF4A | HNRNPK | HNRNPU | HRAS | HSP90AA1 | HSP90AB1 | HSP90AB3P | HSP90AB4P | HSPA1L | HSPA2 | HSPA5 | HSPA8 | HSPB1 | HTR1B | HTR3A | HTRA2 | HTT | HUWE1 | IBTK | ICAM1 | IDH2 | IFNA2 | IGBP1 | IGF1R | IKBKE | IL13RA2 | ILK | INSIG1 | INSR | IPO5 | IQGAP1 | ITGA4 | ITGAV | ITGB1BP1 | ITGB3 | ITPR1 | ITPR2 | ITPR3 | JUN | JUP | KAT7 | KCNA4 | KCNAB1 | KCNAB2 | KCNIP2 | KCNK16 | KCNS3 | KDM1A | KDM5B | KDR | KHDRBS1 | KIF5B | KMT2A | LAMB1 | LCP1 | LDLRAD4 | LEF1 | LETM1 | LGALS3 | LGMN | LGR4 | LMNA | LPAR1 | LRRC7 | LRRK2 | LRSAM1 | LYPLA1 | MAD1L1 | MAP1B | MAP2K1 | MAP2K3 | MAP2K6 | MAP3K3 | MAPK1 | MAPK14 | MAPK15 | MAPK3 | MAPK8 | MAPT | MAVS | MBP | MCC | MCOLN3 | MDM2 | MECP2 | MEOX2 | MIB1 | MIF | MITF | MPC2 | MSN | MYO1C | MYO5A | MYO6 | MYOC | NCK1 | NCOR1 | NDUFAF2 | NEDD4 | NEUROD1 | NF2 | NFE2L2 | NFKBIA | NIPBL | NKX2-1 | NMB | NMD3 | NOD2 | NOP10 | NOS1AP | NOS2 | NOS3 | NPEPPS | NPR1 | NR0B2 | NR1H2 | NR1H3 | NRP2 | NRXN1 | NSFL1C | NTF3 | NUCB1 | NUMA1 | NUP153 | NUP214 | OAS2 | OAZ3 | ONECUT1 | ORM1 | OXCT1 | OXSR1 | P2RX1 | P2RX2 | P2RX4 | PACSIN3 | PANX3 | PARD3 | PARD6A | PARK7 | PARP1 | PAX8 | PCM1 | PCSK9 | PDCD10 | PDCD6 | PDCD6IP | PDE4D | PER1 | PER2 | PFKL | PFKM | PFN1 | PICK1 | PIK3C2A | PIK3CG | PIK3R1 | PIK3R3 | PIN1 | PINX1 | PKIG | PKN2 | PLA2G10 | PLA2G1B | PLA2G6 | PLCG1 | PLK1 | PLXNA1 | PLXNA2 | PLXNB2 | PML | PPARD | PPARG | PPARGC1A | PPIA | PPID | PPIF | PPM1A | PPP1CC | PPP1R11 | PPP1R12A | PPP3CC | PPT1 | PRCP | PRELID1 | PREPL | PRKAA1 | PRKAA2 | PRKACA | PRKCD | PRKCE | PRKCZ | PRKD1 | PRKD2 | PROX1 | PSEN1 | PSEN2 | PSMD9 | PTEN | PTGER3 | PTK2 | PTN | PTPN1 | PTPN14 | PTPN22 | PTPN23 | PTPN6 | PTPRK | PTPRO | PTPRR | RAB10 | RAB11A | RAB11B | RAB11FIP2 | RAB21 | RAB29 | RAB31 | RAB3GAP1 | RAB5A | RAB5B | RAB7A | RAB9A | RAC1 | RAF1 | RAN | RANGAP1 | RAP1A | RAP1B | RAPGEF4 | RB1 | RBM4 | RDX | RECK | REST | RGN | RGS2 | RHBDD1 | RHOA | RHOJ | RHOT1 | RHOU | RIPK1 | RIPK2 | RIPK3 | RNF139 | RNF31 | RPS19 | RTN4 | RUVBL1 | RUVBL2 | S100A1 | S100A7 | SAE1 | SARAF | SCFD1 | SCYL2 | SDC1 | SDC4 | SDCBP | SEC16A | SEC24B | SEMA7A | SEPT1 | SEPT4 | SEPT5 | SEPT7 | SEPT8 | SERPINB3 | SERPINE2 | SFN | SH3GL1 | SH3GL2 | SH3GL3 | SIN3A | SIRT1 | SIRT3 | SIRT6 | SLAMF8 | SLC16A1 | SLC25A4 | SLC25A5 | SLC25A6 | SLC35F6 | SLC9A1 | SLC9A3R1 | SMAD3 | SMAD4 | SMO | SMURF2 | SNAI2 | SNCA | SNF8 | SNTA1 | SNX12 | SNX17 | SNX3 | SNX6 | SOCS1 | SOD1 | SOD2 | SP1 | SP100 | SPACA3 | SPINT2 | SPRY2 | SPTBN1 | SQSTM1 | SRC | SRGAP2 | SRGAP3 | SRI | SSX2IP | STAC | STARD13 | STAT3 | STAT5A | STIM1 | STK11 | STK24 | STK26 | STMN1 | STOM | STRAP | STXBP1 | STXBP2 | SUMO1 | SUN2 | SWAP70 | SYDE1 | SYN1 | SYNE2 | SYNJ2BP | SYP | TARDBP | TBC1D4 | TBXA2R | TCP1 | TERF1 | TFDP1 | TGFB1 | TGFBR3 | THADA | THBS1 | TIMP1 | TM9SF4 | TMBIM6 | TMEM231 | TMEM30A | TNF | TNFAIP3 | TNFRSF1A | TNFSF14 | TNK2 | TNN | TOR1A | TP53 | TP63 | TP73 | TPCN2 | TPM1 | TPR | TRADD | TRAF2 | TRAF6 | TRIAP1 | TRIB3 | TRIM28 | TRIM29 | TRIM32 | TRIM9 | TRIP6 | TSG101 | TUBB2B | TWIST1 | TXN | UBE2D3 | UBE2J1 | UBE2L3 | UBQLN1 | UBQLN2 | UBR5 | UNC119 | USP17L2 | USP46 | USP50 | USP8 | VCL | VPS18 | VPS4B | VSTM2A | VTN | WAS | WDR1 | WNK1 | WNK4 | WWP2 | XBP1 | XPO1 | XPO4 | XPO5 | YES1 | YRDC | YWHAB | YWHAE | YWHAG | YWHAH | YWHAQ | YWHAZ | ZIC1 | ZMPSTE24 | ZMYND8 | GO:0051173 | 0.23086255004246 | 1903/8243 | 837/3250 | 2.18893817231986e-06 | 0.0207839679461771 | T | T | T | T | positive regulation of nitrogen compound metabolic process | ABAT | ABHD14B | ABL1 | ACD | ACSL1 | ACTA2 | ACTL6A | ACTN1 | ACTN2 | ACTN4 | ACTR2 | ACTR3 | ADCY6 | ADCYAP1 | ADRB2 | ADRM1 | ADTRP | AGER | AGO1 | AGO2 | AHI1 | AICDA | AIFM1 | AIMP2 | AIRE | AKAP8 | AKAP9 | AKT1 | AKT2 | AKTIP | ALOX12 | ANKRD49 | ANXA2 | APBB1 | APEX1 | APOE | APP | AR | ARAF | ARF4 | ARID3B | ARID4B | ARNT | ARNT2 | ARRB1 | ARRB2 | ASCL1 | ASPSCR1 | ASS1 | ATAD5 | ATF2 | ATF6 | ATF6B | ATM | ATOH1 | ATRX | AURKB | AXIN1 | AZU1 | BABAM1 | BAG2 | BAG4 | BAG6 | BANP | BAX | BBC3 | BCAR3 | BCL10 | BCL2 | BCL2L13 | BCL6 | BIRC2 | BIRC3 | BIRC7 | BMI1 | BMPR1A | BRAF | BRCA1 | BRD4 | BTRC | BUD31 | C1QBP | C1QTNF1 | CAB39 | CALM1 | CALM2 | CALM3 | CAND1 | CAPN2 | CARD9 | CARM1 | CASP10 | CASP3 | CASP8 | CASP9 | CAV1 | CBFA2T3 | CBFB | CBX8 | CCAR2 | CCDC22 | CCDC88A | CCL21 | CCL22 | CCNB1 | CCNT1 | CCNT2 | CCNY | CCNYL1 | CCR1 | CCT2 | CCT3 | CCT4 | CCT5 | CCT6A | CCT7 | CCT8 | CD27 | CD36 | CD40 | CD40LG | CD74 | CD81 | CDC14B | CDC20 | CDC25B | CDC5L | CDC73 | CDH1 | CDK2 | CDK4 | CDK5 | CDK5R1 | CDK8 | CDK9 | CDKN1A | CEBPA | CEBPE | CEP290 | CHCHD10 | CHCHD2 | CHD8 | CHEK2 | CHP1 | CIRBP | CKS1B | CKS2 | CLPX | CLU | COA3 | COMMD1 | COMT | COPS5 | CREB1 | CREB3 | CREBBP | CRK | CRKL | CSNK1A1 | CSNK1E | CSNK2B | CTBP1 | CTCF | CTNNB1 | CTSC | CUL3 | CYCS | DAB1 | DAB2 | DAPK1 | DAXX | DAZAP1 | DBF4B | DBNL | DCN | DCUN1D1 | DDRGK1 | DDX39B | DDX3X | DERL1 | DHX36 | DHX9 | DIABLO | DLG3 | DLG4 | DNAJB2 | DOCK7 | DPF2 | DTX1 | DUSP19 | DUSP22 | DUSP7 | DVL1 | DVL2 | DVL3 | E2F1 | E2F2 | E2F3 | E2F4 | E2F6 | EAF2 | EBF1 | ECD | EDEM1 | EED | EEF2 | EGFR | EGLN1 | EGLN3 | EHD4 | EIF2AK3 | EIF2AK4 | EIF2B5 | EIF3D | EIF3E | EIF4A3 | EIF5A | EIF6 | ELAVL1 | ELF3 | ELF4 | ENDOG | ENO1 | EP300 | EPHA4 | ERBB2 | ERBB3 | ERG | ERLIN2 | ESR1 | ESR2 | ESRRA | ESRRB | ESRRG | ETS1 | EXOSC6 | EXOSC9 | EYA2 | EYA4 | EZH2 | EZR | FADD | FAF1 | FAM129B | FANCI | FANCM | FASLG | FBXW11 | FGB | FGF2 | FGFR1 | FGFR3 | FHL2 | FIS1 | FKBP1A | FLOT1 | FN1 | FOS | FOXA1 | FOXJ1 | FOXK1 | FOXK2 | FOXM1 | FOXO1 | FTO | FUBP3 | FUS | FXR2 | FYN | FZR1 | GABPB1 | GADD45G | GATA1 | GDF9 | GLMN | GNAI2 | GOLGA2 | GPS2 | GRIP1 | GSK3A | GSK3B | GTF2A1 | GTF2F1 | GTF2F2 | H1F0 | H1FX | H2AFX | H2AFZ | HAVCR2 | HAX1 | HBEGF | HDAC1 | HDAC2 | HDAC3 | HDAC4 | HDAC5 | HDAC6 | HELZ2 | HEXB | HIF1A | HIST1H1A | HMGB1 | HMGB2 | HMGB3 | HMOX1 | HNF1A | HNF4A | HNF4G | HNRNPA1 | HNRNPA2B1 | HNRNPAB | HNRNPD | HNRNPK | HNRNPL | HNRNPU | HOXA5 | HOXB5 | HOXB9 | HOXD10 | HPRT1 | HPX | HRAS | HSF1 | HSF4 | HSP90AA1 | HSP90AB1 | HSPA1B | HSPA2 | HSPA5 | HSPB1 | HSPBP1 | HSPD1 | HSPE1 | HTATIP2 | HTRA2 | ICAM1 | ID2 | IFI16 | IFNA2 | IGF1R | IKBKB | IKBKG | IKZF5 | ILF2 | ILF3 | ILK | ING1 | ING5 | INSIG1 | INSIG2 | INSR | IQGAP1 | IRF1 | IRF4 | IRF7 | ITGB1BP1 | ITGB3 | JMJD6 | JUN | JUNB | JUND | JUP | KAT5 | KAT7 | KAT8 | KDM1A | KDM4D | KDM8 | KDR | KEAP1 | KHDRBS1 | KLF5 | KLF6 | KLHDC10 | KMT2A | KMT2B | KMT2C | KRAS | KSR1 | LARP4 | LAT | LATS1 | LCP2 | LEF1 | LGMN | LGR4 | LMNA | LMO2 | LMO7 | LPAR1 | LPIN3 | LRP6 | LRRK1 | LRRK2 | LTBR | LUM | MAP2K1 | MAP2K3 | MAP2K4 | MAP2K6 | MAP2K7 | MAP3K1 | MAP3K13 | MAP3K14 | MAP3K2 | MAP3K3 | MAP3K4 | MAP4K1 | MAPK1 | MAPK14 | MAPK15 | MAPK3 | MAPK7 | MAPK8 | MAPK8IP3 | MAPK9 | MAPKAP1 | MAPT | MAS1 | MAVS | MBP | MC1R | MDM2 | MECP2 | MED1 | MED12 | MED14 | MED17 | MED20 | MED21 | MED23 | MED24 | MED31 | MED4 | MED6 | MEOX2 | METTL23 | MGMT | MIF | MITF | MLH1 | MLLT11 | MLLT3 | MLST8 | MORF4L2 | MOV10 | MRPL12 | MSH6 | MSN | MTA1 | MTA3 | MTPN | MYC | MYO6 | NAMPT | NANOG | NCBP1 | NCK1 | NCOA1 | NCOA2 | NCOA3 | NCOA4 | NDUFA13 | NEK1 | NEK7 | NELFE | NEUROD1 | NFATC1 | NFATC2 | NFE2L2 | NFKB2 | NFKBIA | NIF3L1 | NIPBL | NKRF | NKX2-1 | NMD3 | NME2 | NMNAT1 | NOD2 | NOS1AP | NOS2 | NOS3 | NPM1 | NQO2 | NR1H2 | NR1H3 | NR1I2 | NR3C1 | NR4A1 | NR5A2 | NTF3 | NTRK1 | NUFIP1 | NUP85 | NUP98 | NUPR1 | OGT | ONECUT1 | OTUD4 | OXSR1 | P2RX1 | P2RX4 | PABPC1 | PACSIN3 | PAIP1 | PAK2 | PAK6 | PARK7 | PARP1 | PARP14 | PATZ1 | PAWR | PAX8 | PAX9 | PAXIP1 | PCBD1 | PCBP1 | PCGF5 | PCNA | PCSK9 | PDCD10 | PDCD6 | PDE6G | PEBP1 | PEF1 | PELI1 | PER1 | PER2 | PFKM | PHB | PHB2 | PHF10 | PIAS1 | PIAS3 | PIH1D1 | PIK3CA | PIK3CG | PIK3R1 | PIK3R3 | PIN1 | PLA2G10 | PLA2G1B | PLA2G2A | PLA2G6 | PLAUR | PLGRKT | PLK1 | PLSCR1 | PML | PNKP | POLDIP3 | POLR2A | POT1 | POU2F3 | POU5F1 | PPARD | PPARG | PPARGC1A | PPM1A | PPP1R12A | PPP2CA | PPP2R5D | PRDX2 | PRDX6 | PRELID1 | PRKAA1 | PRKAA2 | PRKACA | PRKAG1 | PRKAR1A | PRKAR2B | PRKCD | PRKCE | PRKCZ | PRKD1 | PRKD2 | PRKDC | PRMT2 | PROX1 | PRPF19 | PRRC1 | PSAP | PSEN1 | PSMC1 | PSMC2 | PSMC3 | PSMC4 | PSMC5 | PSMC6 | PSMD10 | PSMD14 | PSMD9 | PSME2 | PSME3 | PTEN | PTGES2 | PTGES3 | PTK2 | PTPN1 | PTPN22 | PUM1 | RAB1A | RAB1B | RAB3GAP1 | RAC1 | RAD23A | RAD51 | RAF1 | RAN | RAP1A | RAP1B | RARA | RARG | RB1 | RB1CC1 | RBM3 | RBMX | RBPJ | RBX1 | RC3H1 | RDX | REL | RELA | REST | RFC1 | RFC2 | RFC3 | RFC4 | RFC5 | RFXANK | RGN | RHBDD1 | RHOA | RIF1 | RIOK1 | RIPK1 | RIPK2 | RIPK3 | RMND1 | RNF139 | RNF185 | RNF19A | RNF4 | RNF8 | RPLP1 | RPS2 | RPS3 | RPS4X | RPS6KA1 | RPS6KB2 | RPTOR | RTF1 | RUNX1 | RUNX2 | RUVBL1 | RUVBL2 | RXRA | RXRB | RXRG | S100A12 | S100A7 | S100A9 | SART3 | SDC4 | SDCBP | SEMA7A | SEPT4 | SERPINB3 | SFPQ | SGTA | SHC1 | SIN3A | SIRT1 | SIRT6 | SIRT7 | SKI | SLAMF1 | SLC9A1 | SMAD2 | SMAD3 | SMAD4 | SMAD5 | SMAD9 | SMARCA2 | SMARCA4 | SMARCB1 | SMARCC1 | SMARCC2 | SMARCE1 | SMO | SMURF1 | SNAI2 | SNCA | SNW1 | SOAT1 | SOD1 | SOX2 | SP1 | SP100 | SPI1 | SPIN1 | SPRTN | SPRY2 | SQSTM1 | SRC | SRSF5 | STAT1 | STAT3 | STAT4 | STAT6 | STK11 | STK24 | STK25 | STK26 | STK3 | STUB1 | SUB1 | SUMO1 | SUMO2 | SUPT4H1 | SUPT5H | TAB1 | TAB2 | TADA2B | TAF12 | TAF15 | TANK | TAOK3 | TARDBP | TAZ | TBL1X | TCEA1 | TCEA2 | TCERG1 | TCF12 | TCF21 | TCF4 | TCP1 | TDRD3 | TERF2IP | TFCP2 | TFDP1 | TGFB1 | THBS1 | THRA | TIMP2 | TLR3 | TMEM173 | TMTC3 | TNF | TNFAIP3 | TNFRSF1A | TNFRSF8 | TNIP2 | TNK2 | TNKS | TNKS2 | TNP1 | TOLLIP | TOP2A | TP53 | TP63 | TP73 | TRA2A | TRADD | TRAF2 | TRAF6 | TRIAP1 | TRIB2 | TRIB3 | TRIM28 | TRIM32 | TRIP4 | TRUB2 | TSG101 | TTC5 | TWIST1 | TXN | U2AF2 | UBB | UBC | UBE2B | UBE2C | UBE2D1 | UBE2I | UBE2K | UBE2L3 | UBE2N | UBE2S | UBE2V1 | UBE3A | UBQLN1 | UBQLN2 | UNC119 | UPF3B | UQCC1 | USP16 | USP21 | USP5 | USP50 | USP7 | USP9X | VCP | VDR | VHL | VIM | VTN | WAS | WBP2 | WDR35 | WDR48 | WDR77 | WNK1 | WNT16 | WNT2 | WT1 | WWOX | WWP2 | XBP1 | XIAP | XRCC1 | XRCC5 | XRCC6 | YAF2 | YAP1 | YES1 | YTHDF1 | YWHAH | YY1 | ZBTB16 | ZBTB17 | ZBTB7B | ZIC1 | ZNF24 | ZNF622 | ZNF746 | GO:0051248 | 0.0844352784180517 | 696/8243 | 332/3250 | 2.22345815449921e-06 | 0.02111173517697 | T | T | T | T | negative regulation of protein metabolic process | A2M | ABL1 | ADORA2A | AGO1 | AGO2 | AHSG | AKT1 | ANXA2 | APC | APCS | APOD | APOE | APP | ARRB1 | ARRB2 | ATG5 | ATP13A2 | BAG2 | BAG5 | BAG6 | BAX | BCOR | BDKRB2 | BIN1 | BIRC2 | BIRC3 | BIRC5 | BIRC7 | BIRC8 | BRCA1 | C1QL4 | C4A | CALM1 | CALM2 | CALM3 | CALR | CASP3 | CAV1 | CBL | CBLB | CBLC | CCAR2 | CCNB1 | CD109 | CD27 | CDK5 | CDKN1A | CDKN2C | CEBPA | CHORDC1 | CHP1 | CHRNA9 | CIRBP | CLU | CRKL | CRY1 | CRY2 | CRYAB | CSNK2A2 | CTBP1 | CTNNB1 | CYLD | DAPK1 | DBNDD2 | DDIT4 | DDRGK1 | DDX3X | DHCR24 | DHX36 | DMD | DNAJA1 | DNAJC10 | DPEP1 | DUSP1 | DUSP14 | DUSP16 | DUSP19 | DUSP22 | DUSP7 | DVL1 | EIF2AK3 | EIF2AK4 | EIF2S1 | EIF3A | EIF3E | EIF4A3 | EIF4E | EIF4E2 | EIF4EBP1 | EIF4ENIF1 | EIF6 | EPHA4 | EPPIN | EPRS | ERRFI1 | EZR | FABP1 | FAM192A | FBXO7 | FGFR1OP | FKBP1A | FKBP1B | FKBP8 | FOXJ1 | FOXM1 | FOXO1 | FTO | FXR2 | FYN | G6PD | GAPDH | GLMN | GNAI2 | GNAQ | GPD1L | GPI | GPS2 | GPX1 | GRB7 | GSK3B | GSTP1 | HAP1 | HBEGF | HDAC2 | HDAC3 | HDAC6 | HEXIM1 | HNRNPD | HSP90AB1 | HSP90AB3P | HSP90AB4P | HSPA1B | HSPB1 | IBTK | IDE | IFI16 | IGBP1 | IGF1R | IGF2BP1 | IKBKB | ILF3 | ILK | IPO5 | IPO7 | IREB2 | ISG15 | ITCH | ITGAV | ITGB1BP1 | ITGB3 | JUN | KDM1A | KDM4D | LAMP3 | LATS1 | LDLRAD4 | LEF1 | LEMD2 | LGALS3 | LRP6 | LRRK1 | LRRK2 | MAD2L1 | MAPT | MAS1 | MDM2 | MECP2 | MIF | MLLT1 | MOV10 | MYC | NCK1 | NCOR1 | NELL1 | NF2 | NNT | NPM1 | NR1H2 | NTF3 | OGT | OTUB1 | PAFAH1B1 | PAK2 | PAPLN | PARD3 | PARD6A | PARK7 | PARP14 | PDE4D | PEBP1 | PER1 | PER2 | PHB | PIAS3 | PIH1D1 | PIN1 | PINX1 | PKIG | PLAUR | PLK1 | PML | PNKP | PPARGC1A | PPME1 | PPP1R11 | PPP1R14A | PPP1R2 | PPP1R8 | PPP2CA | PPP2R1A | PPP2R5D | PRDX3 | PRDX5 | PRKAA1 | PRKAA2 | PRKAR1A | PRKAR2B | PRKCD | PRKCE | PRKCZ | PRKDC | PRMT3 | PRMT6 | PSEN1 | PSMD10 | PTEN | PTN | PTPN1 | PTPN22 | PTPN6 | PTPRR | PTTG1 | PUM1 | PUS7 | QARS | RAF1 | RANBP9 | RARA | RB1 | RBM3 | RBM4 | RC3H1 | RECK | RELA | RGN | RGS2 | RNF139 | RPL23 | RPS3 | RPS6KA1 | RPTOR | SAG | SDCBP | SERPINB2 | SERPINB3 | SERPINB5 | SERPINB6 | SERPINB8 | SERPINE2 | SERPINH1 | SET | SFN | SGTA | SHMT1 | SIN3A | SIRT1 | SIRT3 | SKI | SLC9A3R1 | SMAD4 | SMARCB1 | SMARCC1 | SNCA | SNTA1 | SNX12 | SNX6 | SOCS1 | SPI1 | SPINK7 | SPINT2 | SPRY2 | SRC | STAT3 | STK38 | STRAP | STYX | SWAP70 | SYNJ2BP | TAOK3 | TARDBP | TERF2IP | TGFB1 | THBS1 | TIA1 | TIMP1 | TIMP2 | TINF2 | TNF | TNFAIP3 | TNFSF14 | TPR | TRAF3IP1 | TRIAP1 | TRIB2 | TRIB3 | TRIM21 | TRIP12 | TSG101 | TWIST1 | TYMS | U2AF2 | UBE2B | UBE2J1 | UBR5 | UBXN1 | UCHL1 | UCHL5 | UNK | USP14 | USP17L2 | USP19 | USP4 | USP47 | USP7 | VTN | WARS | WNK1 | XIAP | XRCC1 | YWHAB | YWHAE | YWHAG | GO:0051054 | 0.0151643818997938 | 125/8243 | 75/3250 | 2.2751076654343e-06 | 0.0216021472832986 | T | T | T | T | positive regulation of DNA metabolic process | ACD | ACTR2 | AKT1 | APBB1 | ATAD5 | ATM | ATRX | AURKB | BABAM1 | BRCA1 | CBX8 | CCT2 | CCT3 | CCT4 | CCT5 | CCT6A | CCT7 | CCT8 | CD40 | CDK2 | CTNNB1 | DHX36 | DHX9 | EGFR | EXOSC6 | EYA2 | EYA4 | FOXM1 | FUS | H2AFX | HMGB1 | HNRNPA1 | HNRNPA2B1 | HNRNPD | KDM4D | MAP2K7 | MAP3K4 | MAPK1 | MAPK15 | MAPK3 | MECP2 | MGMT | MLH1 | MSH6 | MYC | NEK7 | OTUD4 | PARP1 | PAXIP1 | PCNA | PML | PNKP | POT1 | PPARGC1A | PRKCD | RAD51 | RIF1 | RNF8 | RPS3 | SIRT1 | SIRT6 | STAT6 | TCP1 | TGFB1 | TNKS | TNKS2 | TRIM28 | UBE2B | UBE2N | USP7 | USP9X | WAS | WT1 | XRCC1 | XRCC5 | GO:0035690 | 0.0245056411500667 | 202/8243 | 112/3250 | 2.29520112844456e-06 | 0.0217929347145811 | T | T | T | T | cellular response to drug | ABL1 | ACTB | ADCY6 | AIFM1 | ANXA1 | APEX1 | ASS1 | ATRX | BCL11A | BRAF | BRCA1 | CASP9 | CBX8 | CDH1 | CDK2 | CDK4 | CFTR | CHEK2 | CRK | CTNNB1 | DAXX | DDIT4 | DPEP1 | EFTUD2 | EGFR | EIF4E | EIF4EBP1 | ERRFI1 | ETS1 | EZH2 | FABP1 | FBP1 | FBXO32 | FECH | FOLR1 | FOXO1 | FYN | GNAI1 | HCN3 | HDAC2 | HDAC6 | HMOX1 | HNRNPA1 | HNRNPD | HSF1 | HSP90B1 | HSPA5 | HTR1B | ICAM1 | ITPR2 | JUP | KDR | LRRK2 | MAP4K1 | MAPK1 | MAPK3 | MAPK7 | MDM2 | MYC | NFE2L2 | NME1 | NOD2 | NOS2 | NQO1 | NR3C1 | NTRK1 | OPA1 | P2RX4 | PARK7 | PCNA | PPARGC1A | PPAT | PPIF | PPP1R12A | PPP1R14A | PRKAA1 | PRKAA2 | PRKCD | PRKCE | PTEN | PTPN22 | QDPR | RAD51 | RAP1A | RAP1B | RELA | REST | RIPK1 | RIPK2 | RIPK3 | RPS3 | SIN3A | SIRT1 | SLAMF8 | SOD1 | SPI1 | SRC | STAT6 | SULT1A3 | TGFB1 | TLR3 | TNF | TNFAIP3 | TP53 | TRAF2 | TREX1 | TRIM41 | UMPS | USP8 | VIM | WDR35 | ZNF277 | GO:0043068 | 0.0525294189008856 | 433/8243 | 217/3250 | 2.35258151242601e-06 | 0.0223377614604849 | T | T | T | T | positive regulation of programmed cell death | ABL1 | ADORA2A | AES | AIFM1 | AKT1 | ALOX12 | ANXA1 | ANXA5 | APBB1 | APBB2 | APC | ARHGEF7 | ARHGEF9 | ARRB1 | ARRB2 | ASCL1 | ATF2 | ATF6 | ATM | BACE1 | BAX | BBC3 | BCL10 | BCL2 | BCL2L1 | BCL2L13 | BCL6 | BIN1 | C1QBP | CAMK1D | CAMK2A | CAPN2 | CASP10 | CASP2 | CASP3 | CASP8 | CASP9 | CAV1 | CCAR2 | CD40 | CD40LG | CDC34 | CDK19 | CDK4 | CDK5 | CDK5R1 | CHCHD10 | CHEK2 | CLU | COL18A1 | CREB1 | CTNNB1 | CTSC | CYCS | CYLD | DAPK1 | DDX3X | DIABLO | DNAJA1 | DNM1L | DUSP1 | E2F1 | E2F3 | EEF1A2 | EGLN3 | EIF2AK3 | EIF2B5 | EIF2S1 | ENDOG | FADD | FAF1 | FASLG | FIS1 | FOXA1 | FOXO1 | GADD45G | GAPDH | GNA13 | GRIK2 | GSK3A | GSK3B | GZMA | HDAC4 | HDAC6 | HMGB1 | HMOX1 | HOXA5 | HSF1 | HSPD1 | HSPE1 | HTRA2 | HTT | ING5 | ITGA4 | ITGB3BP | JUN | LATS1 | LDHA | LPAR1 | LRRK2 | LTBR | MAP2K4 | MAP2K6 | MAPK8 | MAPK9 | MAPT | MELK | MLLT11 | MTCH2 | MYBBP1A | MYC | NCK1 | NCOA1 | NDUFA13 | NEUROD1 | NQO1 | NQO2 | NTRK1 | NUPR1 | P2RX1 | PAK2 | PARK7 | PAWR | PCSK9 | PDCD6 | PDIA3 | PIN1 | PLA2G6 | PLAUR | PLCG1 | PLEKHF1 | PML | PNMA1 | PPARG | PPARGC1A | PPID | PPIF | PPP1CA | PPP2R1A | PPP2R1B | PPP3CC | PRELID1 | PRKCD | PRKDC | PRMT2 | PSEN1 | PSEN2 | PTEN | PTN | PTRH2 | RARG | REST | RHOA | RIPK1 | RIPK2 | RIPK3 | RNF183 | RPS3 | RPS6KA2 | S100A9 | S100B | SAP18 | SEPT4 | SEPT7 | SFN | SFPQ | SIAH1 | SIRT1 | SLC9A1 | SLC9A3R1 | SMAD3 | SNCA | SOD1 | SOD2 | SORT1 | SQSTM1 | SRC | SRPK2 | STK3 | TCTN3 | TFDP1 | TGFB1 | TGM2 | THBS1 | TIA1 | TLR3 | TNF | TNFRSF12A | TNFRSF1A | TNFRSF8 | TNFSF12 | TOMM22 | TOP2A | TP53 | TP63 | TP73 | TRADD | TRAF2 | TRAF6 | TRIM35 | TXNIP | UBB | UBC | UBE2M | VAV1 | VCP | VDR | WDR35 | WWOX | YWHAB | YWHAE | YWHAG | YWHAH | YWHAQ | YWHAZ | ZBTB16 | ZNF622 | GO:0045732 | 0.0186825185005459 | 154/8243 | 89/3250 | 2.55280232313477e-06 | 0.0242388580581647 | T | T | T | T | positive regulation of protein catabolic process | AKT1 | APC | APOE | ASB9 | AXIN1 | BAG2 | BAG6 | CAV1 | CBFA2T3 | CCDC22 | CD81 | CEBPA | CLU | CSNK1A1 | CSNK1E | CTSC | CUL4B | DAB2 | DDB1 | DDRGK1 | DNAJB2 | DVL1 | EDEM1 | EZR | FAF1 | FOXO1 | FZR1 | GGA1 | GSK3A | GSK3B | HECW1 | HECW2 | HSP90AA1 | HSPA1B | HSPBP1 | IDE | ITCH | KEAP1 | LRRK2 | MAPK9 | MDM2 | MSN | NDUFA13 | NEDD4 | NFE2L2 | NUPR1 | OAZ3 | PACSIN3 | PCSK9 | PIAS1 | PLK1 | PSEN1 | PSMC1 | PSMC2 | PSMC3 | PSMC4 | PSMC5 | PSMC6 | PSMD10 | PTEN | PTK2 | RAB7A | RAD23A | RBX1 | RDX | RGN | RNF139 | RNF185 | RNF19A | SGTA | SMURF1 | SMURF2 | SNF8 | STUB1 | STX5 | SUMO1 | SUMO2 | TMTC3 | TNF | TNFAIP3 | TNFSF12 | TRIB2 | TRIB3 | TRIM32 | UBQLN1 | UBQLN2 | USP5 | VCP | WWP2 | GO:0019438 | 0.0879534150188038 | 725/8243 | 344/3250 | 2.66944007105341e-06 | 0.0253463334746521 | T | T | T | T | aromatic compound biosynthetic process | ACACA | ACAT1 | ACLY | ACOT7 | ACSL1 | ACSL4 | ACSL5 | ACSS2 | ADA | ADCY6 | ADK | ADRM1 | ADSL | ADSS | AFF4 | AK1 | AK2 | AK4 | ALAS1 | ALDOA | AMD1 | AMDHD2 | APRT | AR | ARHGAP35 | ARNT | ARRB1 | ARRB2 | ASPDH | ATF6B | ATIC | ATOH1 | BAX | CBFB | CBR4 | CCNB1 | CCNT1 | CCNT2 | CDA | CDC73 | CDK4 | CDK8 | CDK9 | CDKN1A | CEBPA | CENPF | CHRAC1 | CMPK1 | COL4A2 | COPS2 | COPS5 | COX15 | CPSF7 | CREB1 | CREBBP | CSTF2T | CTDP1 | CTPS1 | CYC1 | DBH | DCAKD | DCK | DCTPP1 | DERA | DGUOK | DHX9 | DIDO1 | DLAT | DLD | DNAJC30 | DTX1 | DUT | DVL1 | DVL2 | E2F1 | E2F2 | E2F3 | EAF2 | ECD | ELF3 | ESR1 | ESR2 | ESRRA | ESRRB | ESRRG | ETS1 | FASN | FECH | FOS | FPGS | FUBP1 | FUBP3 | GAR1 | GARS | GART | GATA1 | GCDH | GDA | GFPT1 | GFPT2 | GMDS | GMPPB | GMPR | GMPS | GNPDA1 | GNPDA2 | GNPNAT1 | GPX1 | GTF2A1 | GTF2E2 | GTF2F1 | GTF2F2 | GTF2I | GTF3C2 | HAAO | HIF1A | HINT1 | HIST1H4A | HMBS | HNF4A | HNF4G | HOXB9 | HPRT1 | HSD17B8 | HSF1 | HSP90AA1 | HSP90AB1 | IDH2 | ILF2 | IMPDH2 | IREB2 | IRF1 | IRF7 | ISG15 | ITPA | JUN | KLF5 | KMT2A | KYNU | LDHC | LEF1 | LIG4 | LMO2 | MAPK1 | MAPK3 | MC1R | ME1 | MECP2 | MED1 | MED12 | MED14 | MED17 | MED20 | MED21 | MED23 | MED24 | MED31 | MED4 | MED6 | MITF | MLLT1 | MLLT3 | MMAB | MPC2 | MPI | MRPL12 | MTAP | MTERF1 | MTHFD1 | MYO5A | NAGK | NAMPT | NANS | NAPRT | NCBP1 | NCOA1 | NCOR1 | NELFB | NELFCD | NELFE | NFATC1 | NFE2L2 | NME1 | NME2 | NME4 | NMNAT1 | NMNAT3 | NMRK2 | NOP10 | NPLOC4 | NPR1 | NR0B2 | NR1H2 | NR1H3 | NR1I2 | NR3C1 | NR4A1 | NR5A2 | NRBF2 | NT5C2 | NUDT18 | NUDT3 | NUDT9 | PAH | PAICS | PAPSS1 | PAPSS2 | PARP1 | PAX8 | PCBD1 | PCF11 | PCNA | PDE4D | PDHA1 | PDHA2 | PDHB | PDHX | PFAS | PGM3 | PHB | PINX1 | PIR | PNKP | PNP | POLA2 | POLD1 | POLE2 | POLE3 | POLM | POLR2A | POLR2E | POLR2L | POLR2M | POT1 | POU5F1 | PPARD | PPARG | PPARGC1A | PPAT | PPIA | PPM1D | PPOX | PPP5C | PPT1 | PRTFDC1 | PSAT1 | PTEN | PTGES3 | PTMA | PTTG1 | QDPR | RARA | RARG | RBMX | RBPJ | RELA | RFC1 | RFC2 | RFC3 | RFC4 | RFC5 | RFK | RPA2 | RPA3 | RRM1 | RRM2 | RRM2B | RTF1 | RUNX2 | RXRA | RXRB | RXRG | SARS | SCAF1 | SHMT1 | SIRT1 | SIRT7 | SKI | SLC25A1 | SLC25A13 | SMAD4 | SMG5 | SMG6 | SMN1 | SNCA | SNRPB | SNRPD3 | SNRPE | SNW1 | SP1 | SPI1 | SPIN1 | SPR | SPRTN | STAT3 | STOML2 | SUCLA2 | SUPT4H1 | SUPT5H | TAF12 | TAF15 | TAF1A | TAMM41 | TARDBP | TCEA1 | TCEA2 | TCERG1 | TCF4 | TECR | TERF1 | TERF2 | TERF2IP | TFCP2 | TGFB1 | THRA | TK1 | TK2 | TP53 | TREX1 | TRIM25 | TRIM28 | TRIM29 | TRIP13 | TSC22D1 | TYMS | UAP1 | UAP1L1 | UBB | UBC | UBE2L6 | UCK2 | UGDH | UGP2 | UMPS | UPP2 | USPL1 | UXS1 | VCP | VDR | XBP1 | YAP1 | ZBTB7A | ZBTB7B | ZNF217 | GO:0010498 | 0.0305713939099842 | 252/8243 | 135/3250 | 2.75914675856839e-06 | 0.0261980984726069 | T | T | T | T | proteasomal protein catabolic process | AMFR | ANAPC4 | ARMC8 | ARRB1 | ARRB2 | AUP1 | AURKB | BAG6 | BIRC2 | BTRC | CCNB1 | CDC16 | CDC20 | CDC27 | CDC34 | CDK2 | CRBN | CTNNB1 | CUL1 | CUL2 | CUL3 | CUL4B | CUL5 | DDB1 | DERL1 | DNAJB2 | DNAJC10 | EDEM1 | ERLIN2 | FAF1 | FAF2 | FBXL12 | FBXO6 | FBXW11 | FBXW5 | FZR1 | GSK3A | HECW1 | HECW2 | HERC2 | HSP90B1 | HSPA5 | ITCH | KAT5 | KCTD17 | KCTD2 | KEAP1 | KLHL20 | KLHL22 | MAD2L1 | MDM2 | MTA1 | NEDD4 | NFE2L2 | NHLRC3 | NPLOC4 | NSFL1C | PCBP2 | PELI1 | PLK1 | PML | PPP2CB | PRPF19 | PSMA1 | PSMA2 | PSMA3 | PSMA5 | PSMA6 | PSMB5 | PSMB6 | PSMB7 | PSMB8 | PSMC1 | PSMC2 | PSMC3 | PSMC4 | PSMC5 | PSMC6 | PSMD1 | PSMD10 | PSMD11 | PSMD12 | PSMD14 | PSMD2 | PSMD4 | PSMD9 | PSME2 | PSME3 | PTTG1 | RAD23A | RAD23B | RBX1 | RHBDD1 | RNF126 | RNF139 | RNF185 | RNF4 | SEL1L | SGTA | SGTB | SIAH1 | SIRT1 | SMURF1 | SMURF2 | SOCS6 | SPOP | SPSB3 | STUB1 | SYVN1 | TBL1X | TOR1A | TRIM25 | TRIM9 | UBB | UBC | UBE2A | UBE2B | UBE2C | UBE2D1 | UBE2D3 | UBE2H | UBE2I | UBE2J1 | UBE2R2 | UBE2S | UBE2W | UBE3A | UBQLN1 | UBQLN2 | UBXN1 | UCHL1 | UGGT1 | USP19 | VCP | WWP2 | GO:0071495 | 0.0570180759432246 | 470/8243 | 233/3250 | 2.78889728740722e-06 | 0.0264805797439316 | T | T | T | T | cellular response to endogenous stimulus | ABL1 | ACACA | ACTA1 | ACTB | ACTR2 | ADCY6 | ADCYAP1 | ADRB2 | ADTRP | AGER | AIFM1 | AKAP8 | AKAP9 | AKR1B1 | AKR1C2 | AKT1 | AKT2 | ANXA1 | ANXA5 | APEX1 | APLP1 | APP | APRT | AR | ASNS | ASS1 | ATP1A1 | BACE1 | BAG4 | BCL11A | BCL2L1 | BMPR1A | BRAF | BRCA1 | CACNA1A | CAPN2 | CASP3 | CASP7 | CASP9 | CAV1 | CBL | CD36 | CDC5L | CDH1 | CDK4 | CDK5 | CFTR | CHEK2 | COL2A1 | COL4A1 | COL4A2 | COL6A1 | CREB1 | CRK | CRKL | CTNNB1 | CTSL | CXCL8 | DDIT4 | DENND4C | DUSP1 | DUSP22 | E2F1 | EEF1A1 | EEF2 | EGFR | EIF4A3 | EIF4E | EIF4EBP1 | EPHA4 | EPRS | ERBB2 | ERRFI1 | ESR1 | EZH2 | EZR | FBXO32 | FECH | FGB | FIS1 | FOLR1 | FOS | FOXO1 | FYN | GATA1 | GLP1R | GNB1 | GOT1 | GPD1 | GSK3A | GSK3B | GSTP1 | H2AFZ | HCN1 | HCN2 | HCN3 | HDAC2 | HDAC4 | HDAC5 | HDAC9 | HNRNPD | HNRNPU | HSF1 | HSP90B1 | HSPA5 | HTR1B | ICAM1 | IGF1R | IGFBP7 | INSR | IPO5 | IQGAP1 | IREB2 | ITGA4 | ITGB1BP1 | ITPR2 | JUN | JUNB | JUND | JUP | KAT5 | KDM1A | KDM5B | KLF5 | KLHL22 | LARS | LGMN | LIMS1 | LMO2 | LPIN3 | LRRK2 | MAPK1 | MAPK3 | MAPK7 | MAPT | MCM7 | MDM2 | MED1 | MSN | MYO5A | NAMPT | NCOA1 | NCOA2 | NCOA3 | NFE2L2 | NOD2 | NR3C1 | NR4A1 | NTRK1 | OPA1 | P2RX4 | PARP1 | PAX8 | PCSK9 | PDE4D | PIK3CG | PIK3R1 | PKM | PLA2G1B | PLCG1 | PPARG | PPARGC1A | PPAT | PPM1A | PRKAA1 | PRKAA2 | PRKACA | PRKAR1A | PRKAR2B | PRKCD | PRKCE | PRKCZ | PRKDC | PSEN1 | PTEN | PTN | PTPN22 | RAB10 | RAB31 | RAB35 | RAD51 | RANGAP1 | RAP1A | RAP1B | RDX | RELA | REST | RIPK2 | RPL23 | RPTOR | RUNX2 | RUVBL2 | SH3GL2 | SHMT1 | SHMT2 | SIN3A | SLC9A1 | SMAD3 | SMAD4 | SMAD9 | SMC1A | SNAI2 | SNCA | SNX6 | SOCS1 | SOCS2 | SOD1 | SP1 | SPINT2 | SRC | SRSF5 | STAT1 | STAT3 | SULT1A3 | TBC1D4 | TGFB1 | TNF | TP53 | TRIB3 | TRIM41 | UBE2L3 | USP8 | VCAM1 | VIM | VSTM2A | WDTC1 | WNT2 | WT1 | WWOX | XBP1 | YES1 | YWHAG | ZMPSTE24 | GO:0044271 | 0.11100327550649 | 915/8243 | 425/3250 | 2.79722544925819e-06 | 0.0265596556407065 | T | T | T | T | cellular nitrogen compound biosynthetic process | ACACA | ACAT1 | ACLY | ACOT7 | ACSL1 | ACSL4 | ACSL5 | ACSS2 | ADA | ADCY6 | ADK | ADRM1 | ADSL | ADSS | AFF4 | AGK | AGO2 | AK1 | AK2 | AK4 | AKT1 | ALAS1 | ALDH7A1 | ALDH9A1 | ALDOA | AMD1 | AMDHD2 | APRT | AR | ARHGAP35 | ARNT | ARRB1 | ARRB2 | ASL | ASPDH | ASS1 | ATF6B | ATIC | ATOH1 | B4GALT3 | BAX | CBFB | CBR4 | CCNB1 | CCNT1 | CCNT2 | CDA | CDC73 | CDK4 | CDK8 | CDK9 | CDKN1A | CEBPA | CENPF | CHDH | CHRAC1 | CMPK1 | CNDP2 | COL4A2 | COPS2 | COPS5 | COX15 | CPSF7 | CREB1 | CREBBP | CSTF2T | CTDP1 | CTPS1 | CYC1 | DARS | DBH | DCAKD | DCK | DCTPP1 | DEGS1 | DERA | DGUOK | DHX9 | DIDO1 | DLAT | DLD | DMD | DNAJC30 | DRG2 | DTX1 | DUT | DVL1 | DVL2 | E2F1 | E2F2 | E2F3 | EAF2 | ECD | EEF1A1 | EEF1A1P5 | EEF1A2 | EGFR | EIF4H | ELF3 | ESR1 | ESR2 | ESRRA | ESRRB | ESRRG | ETS1 | FARSB | FASN | FECH | FOS | FPGS | FUBP1 | FUBP3 | GAR1 | GARS | GART | GATA1 | GCDH | GDA | GEMIN5 | GFPT1 | GFPT2 | GGCT | GGT1 | GGTLC1 | GMDS | GMPPB | GMPR | GMPS | GNPDA1 | GNPDA2 | GNPNAT1 | GPX1 | GSPT1 | GSPT2 | GSS | GTF2A1 | GTF2E2 | GTF2F1 | GTF2F2 | GTF2I | GTF3C2 | HAAO | HAGH | HARS | HIF1A | HINT1 | HIST1H4A | HMBS | HNF4A | HNF4G | HOXB9 | HPRT1 | HSD17B8 | HSF1 | HSP90AA1 | HSP90AB1 | HSPA14 | IDH2 | ILF2 | IMPDH2 | IREB2 | IRF1 | IRF7 | ISG15 | ITPA | JUN | KLF5 | KMT2A | KYNU | LARP4 | LDHC | LEF1 | LIG4 | LMO2 | MAPK1 | MAPK3 | ME1 | MECP2 | MED1 | MED12 | MED14 | MED17 | MED20 | MED21 | MED23 | MED24 | MED31 | MED4 | MED6 | MITF | MLLT1 | MLLT3 | MMAB | MPC2 | MPI | MRPL12 | MRPL28 | MRPL43 | MRPL9 | MRRF | MTAP | MTERF1 | MTHFD1 | NAGK | NAMPT | NANS | NAPRT | NCBP1 | NCOA1 | NCOR1 | NELFB | NELFCD | NELFE | NFATC1 | NFE2L2 | NME1 | NME2 | NME4 | NMNAT1 | NMNAT3 | NMRAL1 | NMRK2 | NOP10 | NOS2 | NOS3 | NPLOC4 | NPR1 | NQO1 | NR0B2 | NR1H2 | NR1H3 | NR1I2 | NR3C1 | NR4A1 | NR5A2 | NRBF2 | NT5C2 | NUDT18 | NUDT3 | NUDT9 | OAZ3 | OTC | P2RX1 | PABPC4 | PAICS | PAPSS1 | PAPSS2 | PARP1 | PAX8 | PCBD1 | PCF11 | PCNA | PDE4D | PDF | PDHA1 | PDHA2 | PDHB | PDHX | PFAS | PGM3 | PHB | PINX1 | PIR | PNKP | PNP | POLA2 | POLD1 | POLE2 | POLE3 | POLM | POLR2A | POLR2E | POLR2L | POLR2M | POT1 | POU5F1 | PPARD | PPARG | PPARGC1A | PPAT | PPIA | PPM1D | PPOX | PPP5C | PPT1 | PRTFDC1 | PSAT1 | PTEN | PTGES3 | PTMA | PTTG1 | QDPR | RARA | RARG | RARS2 | RBMX | RBPJ | RELA | RFC1 | RFC2 | RFC3 | RFC4 | RFC5 | RFK | RMND1 | RPA2 | RPA3 | RPL10 | RPL10A | RPL12 | RPL18A | RPL22 | RPL23 | RPL23A | RPL24 | RPL30 | RPL36AL | RPL37A | RPL38 | RPL6 | RPL7 | RPL7A | RPL9 | RPLP0 | RPLP0P6 | RPLP1 | RPLP2 | RPS12 | RPS13 | RPS14 | RPS15A | RPS19 | RPS2 | RPS20 | RPS24 | RPS29 | RPS3 | RPS4X | RPS6KB2 | RPS8 | RRM1 | RRM2 | RRM2B | RTF1 | RUNX2 | RXRA | RXRB | RXRG | SARS | SAT1 | SCAF1 | SHMT1 | SIRT1 | SIRT7 | SKI | SLC25A1 | SLC25A13 | SMAD4 | SMG5 | SMG6 | SMN1 | SNRPB | SNRPD3 | SNRPE | SNW1 | SP1 | SPI1 | SPIN1 | SPR | SPRTN | SPTLC2 | ST8SIA4 | STAT3 | STOML2 | SUCLA2 | SUPT4H1 | SUPT5H | TAF12 | TAF15 | TAF1A | TAMM41 | TARDBP | TARS | TCEA1 | TCEA2 | TCERG1 | TCF4 | TECR | TERF1 | TERF2 | TERF2IP | TFCP2 | TGFB1 | THRA | TK1 | TK2 | TP53 | TREX1 | TRIM25 | TRIM28 | TRIM29 | TRIP13 | TSC22D1 | TYMS | UAP1 | UAP1L1 | UBB | UBC | UBE2L6 | UCK2 | UGDH | UGP2 | UGT8 | UMPS | UPP2 | USPL1 | UXS1 | VCP | VDR | WARS | XBP1 | YAP1 | ZBTB7A | ZBTB7B | ZNF217 | GO:1901699 | 0.0374863520562902 | 309/8243 | 161/3250 | 2.87764365729135e-06 | 0.0273232265259814 | T | T | T | T | cellular response to nitrogen compound | ABL1 | ACTA1 | ACTB | ACTR2 | ADCY6 | ADRB2 | AGER | AIFM1 | AKAP9 | AKR1B1 | AKT1 | AKT2 | ANXA5 | APEX1 | APLP1 | APP | APRT | ASS1 | ATRX | BACE1 | BCL11A | BCL2L1 | BRCA1 | CACNA1A | CAPN2 | CASP3 | CASP7 | CAV1 | CD36 | CDC5L | CDH1 | CDK2 | CDK4 | CDK5 | CFTR | COL4A1 | COL6A1 | CREB1 | CRK | CTNNB1 | DAXX | DENND4C | DHX9 | DPEP1 | EGFR | EPHA4 | EPRS | ERRFI1 | EZH2 | EZR | FIS1 | FLOT1 | FOLR1 | FOXO1 | FYN | GLP1R | GNB1 | GOT1 | GPD1 | GSK3A | GSK3B | GSTP1 | HCN1 | HCN2 | HCN3 | HDAC2 | HDAC5 | HDAC9 | HNRNPD | HSF1 | HSP90B1 | HSPA5 | HTR1B | ICAM1 | IFIT1 | IGF1R | INSR | IPO5 | ITGA4 | ITPR2 | JUP | KDM1A | KLF5 | KLHL22 | LARS | LGMN | LPIN3 | LRRK2 | MAPK1 | MAPK3 | MAVS | MDM2 | MYO5A | NAMPT | NFE2L2 | NOD2 | NR4A1 | NTRK1 | OPA1 | P2RX4 | PARP1 | PCSK9 | PDE12 | PDE4D | PIK3CG | PIK3R1 | PKM | PLA2G1B | PPARG | PPARGC1A | PPAT | PRKAA1 | PRKACA | PRKAR1A | PRKAR2B | PRKCD | PRKCZ | PRKDC | PSEN1 | PTEN | PTPN22 | RAB10 | RAB31 | RAD51 | RANGAP1 | RAP1A | RAP1B | RELA | RIPK2 | RPL23 | RPTOR | SHMT1 | SHMT2 | SIN3A | SLC9A1 | SNCA | SNX6 | SOCS1 | SOD1 | SP1 | SRC | SRSF5 | STAT1 | STAT6 | SULT1A3 | TBC1D4 | TGFB1 | TLR3 | TMEM173 | TNF | TP53 | TRAF2 | TREX1 | TRIB3 | TRIM41 | VCAM1 | VIM | WDTC1 | WT1 | XBP1 | YWHAG | GO:0051260 | 0.0230498604876865 | 190/8243 | 106/3250 | 2.90620288314563e-06 | 0.0275943963754677 | T | T | T | T | protein homooligomerization | ACACA | ACAT1 | ACOT13 | ACTN2 | ALDH5A1 | ALDOA | ALOX5AP | ANXA5 | ANXA6 | APC | ATL3 | ATXN10 | BAX | BCL10 | C1QTNF1 | CAT | CAV1 | CBR4 | CBY1 | CD79B | CDA | CEP57 | CHMP2A | CPT1A | CRYAB | CRYZ | CTH | DCTPP1 | DCXR | DECR1 | DERL1 | DNM1L | EHD4 | EIF2AK3 | ELAVL1 | FBP1 | FIS1 | FLOT1 | FSD1 | FUS | GBP5 | GLS | GLUL | GOLGA2 | GOPC | HCN1 | HMOX1 | HPRT1 | HSD17B10 | HSF1 | HSF4 | HTRA2 | IDE | IKBKE | IMPDH2 | ITPR3 | JMJD6 | KCNA4 | KCNS3 | KCTD12 | KCTD17 | KCTD18 | KCTD2 | KCTD3 | LETM1 | LNX1 | MGST1 | MICU1 | MIF | MPP2 | NACC1 | OTC | P2RX1 | P2RX2 | P2RX4 | PCBD1 | PDCD6IP | PEX11B | PFKL | PKM | PPAT | RAD51 | RBMX | RIPK1 | RIPK3 | RXRA | SEPT4 | SHKBP1 | SHMT1 | SHMT2 | SKI | SMAD4 | SOD2 | ST13 | STOM | TDO2 | TGM2 | TK1 | TOR1A | TP53 | TP63 | TRAF2 | TRMT61B | USP16 | VASP | VCP | GO:0002274 | 0.0469489263617615 | 387/8243 | 196/3250 | 3.03349886707749e-06 | 0.0288030717429008 | T | T | T | T | myeloid leukocyte activation | ACAA1 | ACLY | ACTR2 | AGL | AHSG | ALDOA | ALDOC | ALOX5 | ANXA2 | AP1M1 | APEH | APRT | ARMC8 | ARPC5 | ATP6V1D | AZU1 | CAB39 | CAND1 | CAPN1 | CAT | CBL | CCT2 | CCT8 | CD36 | CDA | CEP290 | CKAP4 | CLU | COPB1 | COTL1 | CPNE3 | CSNK2B | CTSA | CTSC | CTSD | CXCL6 | CXCL8 | CYB5R3 | DBNL | DDOST | DDX3X | DEGS1 | DERA | DYNC1H1 | DYNC1LI1 | DYNLL1 | DYNLT1 | EEF1A1 | EEF2 | FABP5 | FAF2 | FCN1 | FGL2 | FTH1 | FTL | GAA | GALNS | GDI2 | GHDC | GLA | GNS | GPI | GSTP1 | GUSB | GYG1 | HAVCR2 | HEXB | HMGB1 | HRNR | HSP90AA1 | HSP90AB1 | HSPA1B | HSPA8 | HUWE1 | IDH1 | ILF2 | IMPDH2 | IQGAP1 | IRF4 | ITGAV | JMJD6 | JUN | JUP | KCMF1 | KCNAB2 | KPNB1 | LAT | LCP2 | LGALS3 | LRRC7 | LTBR | MAN2B1 | MAPK1 | MAPK14 | MAPT | MGST1 | MIF | MME | NAMPT | NAPRT | NDRG1 | NHLRC3 | NIT2 | NME2 | ORM1 | P2RX1 | PA2G4 | PDXK | PFKL | PGAM1 | PGM1 | PGM2 | PGRMC1 | PI4K2A | PIK3CG | PKM | PLAUR | PNP | PPIA | PPIE | PRCP | PRDX4 | PRDX6 | PRG2 | PRKCD | PRKCE | PSAP | PSEN1 | PSMA2 | PSMA5 | PSMB7 | PSMC2 | PSMC3 | PSMD1 | PSMD11 | PSMD12 | PSMD14 | PSMD2 | PTGES2 | PTPN6 | PYGB | PYGL | QSOX1 | RAB10 | RAB18 | RAB24 | RAB31 | RAB5B | RAB7A | RAC1 | RAP1A | RAP1B | RBPJ | RHOA | RNASE3 | S100A11 | S100A12 | S100A13 | S100A7 | S100A9 | S100P | SDCBP | SELL | SERPINB3 | SERPINB6 | SLAMF1 | SLC2A5 | SNAP29 | SNCA | SPACA3 | SPI1 | SPTAN1 | SRP14 | STBD1 | STOM | STXBP2 | TCIRG1 | TGFB1 | TIMP2 | TLR3 | TMEM173 | TMEM30A | TNF | TOLLIP | TRAF6 | TREX1 | TUBB | TUBB4B | TXNDC5 | UBR4 | VAPA | VAT1 | VCL | VCP | XRCC5 | XRCC6 | GO:0006749 | 0.00376076671114885 | 31/8243 | 25/3250 | 3.20948642150572e-06 | 0.0304740735721968 | T | T | T | T | glutathione metabolic process | ALDH5A1 | DPEP1 | G6PD | GGT1 | GLO1 | GPX1 | GPX4 | GSR | GSTA1 | GSTA2 | GSTA3 | GSTA4 | GSTA5 | GSTK1 | GSTM4 | GSTP1 | GSTT1 | GSTT2 | GSTT2B | GSTZ1 | IDH1 | MGST1 | PARK7 | PTGES | SOD1 | GO:0010821 | 0.0162562173965789 | 134/8243 | 79/3250 | 3.23675186501121e-06 | 0.0307329589582814 | T | T | T | T | regulation of mitochondrion organization | ACAA2 | AKT1 | ARRB2 | ATP13A2 | BAG3 | BAG4 | BAX | BBC3 | BCL2 | BCL2L1 | CASP8 | CDC37 | CHCHD10 | CLU | CSNK2A2 | CTTN | DCN | DNM1L | E2F1 | FBXO7 | FIS1 | GOLPH3 | GPX1 | GSK3A | GSK3B | HAX1 | HDAC6 | HIF1A | HK2 | HSPA1L | HTRA2 | HTT | HUWE1 | KDR | LMNA | LRRK2 | MAPK8 | MAPT | MARCH5 | MLLT11 | NPEPPS | OPA1 | OPTN | PAM16 | PARK7 | PLA2G6 | PLAUR | PPARGC1A | PPIF | PPP3CC | PRELID1 | PRKAA1 | PSMD10 | RHOT1 | RHOT2 | RHOU | RNF31 | SAE1 | SFN | SLC25A5 | SLC35F6 | SMURF1 | SQSTM1 | TFDP1 | TP53 | TP63 | TP73 | TRIAP1 | UBE2D3 | UBE2L3 | VAT1 | VDAC1 | WDR35 | YWHAB | YWHAE | YWHAG | YWHAH | YWHAQ | YWHAZ | GO:0007166 | 0.15297828460512 | 1261/8243 | 570/3250 | 3.35060042525142e-06 | 0.0318139510377622 | T | T | T | T | cell surface receptor signaling pathway | ABL1 | ACKR2 | ACSL1 | ACTB | ACTG1 | ACTN4 | ACTR2 | ACTR3 | ADORA2A | ADRB2 | AES | AGER | AGO1 | AGO2 | AHI1 | AKT1 | AKT2 | ALOX5 | ANXA1 | ANXA2 | APC | APOA1 | APP | ARF1 | ARF4 | ARHGDIA | ARHGEF7 | ARPC1A | ARPC1B | ARPC2 | ARPC3 | ARPC4 | ARPC5 | ARRB2 | ASCL1 | ASGR2 | ATOH1 | ATP6V1A | ATP6V1B1 | ATP6V1C1 | ATP6V1D | ATP6V1E1 | AXIN1 | BAG1 | BAG3 | BAG4 | BAG6 | BAIAP2 | BAX | BCAR1 | BCL10 | BCL2 | BCL2L1 | BCL2L2 | BCL6 | BDKRB2 | BIRC2 | BIRC3 | BIRC5 | BMPR1A | BRAF | BSG | BTK | BTN2A1 | BTRC | CALM1 | CAMK2A | CAMK2B | CANX | CAPZA1 | CARD9 | CASP10 | CASP2 | CASP3 | CASP8 | CASP9 | CBL | CBLB | CBLC | CCAR2 | CCL21 | CCL22 | CCNY | CCR1 | CCR3 | CCR4 | CCR5 | CD2 | CD209 | CD27 | CD36 | CD40 | CD40LG | CD6 | CD74 | CD79B | CD81 | CDA | CDC37 | CDC42 | CDC73 | CDH5 | CDK5R1 | CDKN1A | CEBPA | CENPJ | CEP57 | CFL1 | CGN | CHD8 | CHRD | CLDN18 | CLNK | CLTC | COL4A1 | COL4A2 | CPNE3 | CREB1 | CREBBP | CRK | CRKL | CSNK1A1 | CSNK1E | CSNK2A2 | CSNK2B | CTNNB1 | CTNNBIP1 | CTNND1 | CUL1 | CUL3 | CUL5 | CXCL11 | CXCL5 | CXCL6 | CXCL8 | CYLD | DAB2 | DAPK1 | DAXX | DDB1 | DDIT4 | DDX3X | DEDD | DIABLO | DISC1 | DOK1 | DTX1 | DTX2 | DUSP22 | DVL1 | DVL2 | DVL3 | DYRK2 | EDA | EFEMP1 | EGFR | EIF2AK3 | EIF2B1 | EIF2B3 | EIF2B5 | EIF4EBP1 | EP300 | EPHA4 | EPHA6 | EPHB6 | ERBB2 | ERBB3 | EVC2 | EYA2 | FADD | FASLG | FBXW11 | FCGR1A | FCN1 | FERMT2 | FGF2 | FGFR1 | FGFR3 | FIBP | FKBP8 | FN1 | FOS | FOXA1 | FOXO1 | FSCN1 | FYN | GBP2 | GDF9 | GIGYF1 | GIT1 | GLP1R | GMDS | GNAI2 | GNAO1 | GNAQ | GNB1 | GOT1 | GRB2 | GRB7 | GRIA1 | GRID2 | GRIK2 | GRK5 | GSK3A | GSK3B | GSTA2 | GSTO1 | GTF2F1 | GTF2F2 | HAP1 | HBEGF | HIF1A | HIST1H3A | HIST2H3A | HLA-A | HLA-B | HLA-DQA1 | HLA-DRA | HLA-DRB1 | HLA-DRB3 | HLA-DRB4 | HLA-DRB5 | HMOX1 | HNRNPA1 | HNRNPA2B1 | HNRNPDL | HNRNPF | HNRNPH1 | HNRNPM | HRAS | HSP90AA1 | HSP90AB1 | HSP90B1 | HSPA8 | HSPA9 | HSPB1 | ICAM1 | IDE | IFIT1 | IFITM3 | IFNA2 | IFNAR1 | IFT122 | IGF1R | IGHV3-13 | IGHV3-23 | IGHV3-30 | IGKV3-20 | IGLC7 | IKBKB | IKBKG | IL13RA2 | IL32 | IL3RA | ILK | INSR | IQGAP1 | IRF1 | IRF4 | IRF7 | ISG15 | ITCH | ITGA4 | ITGA7 | ITGAV | ITGB1BP1 | ITGB3 | ITGB5 | ITK | JAK1 | JAK3 | JMJD6 | JUN | JUNB | KDR | KHDRBS1 | KLF6 | KRAS | LAT | LCP1 | LCP2 | LEF1 | LGR4 | LIMS1 | LMNB1 | LRP6 | LRRK2 | LTBP1 | LTBR | MAGI1 | MAOA | MAP2K4 | MAP2K7 | MAP3K1 | MAP3K14 | MAP3K3 | MAPK1 | MAPK14 | MAPK3 | MAPK8 | MAPK9 | MATK | MCC | MED12 | MIB1 | MIF | MITF | MKNK2 | MKS1 | MLLT11 | MOV10 | MSN | MTAP | MTSS1 | MYC | MYD88 | MYH6 | MYO1C | MYO1E | MYOC | NAMPT | NANOG | NCBP1 | NCK1 | NDUFA13 | NFATC1 | NFATC2 | NFKBIA | NLK | NME2 | NOD2 | NOS2 | NOS3 | NPHP3 | NPR1 | NR0B2 | NRP2 | NTF3 | NTRK1 | OAS2 | ONECUT1 | OXSR1 | P2RX1 | P2RX2 | P2RX4 | P4HB | PAG1 | PAK2 | PARD3 | PARD6A | PARP1 | PDCD6 | PDE4D | PDE6G | PDK2 | PELI1 | PFN1 | PGAM2 | PIAS1 | PIAS3 | PIK3C2A | PIK3CA | PIK3R1 | PIK3R3 | PIM1 | PITPNA | PLA2G6 | PLAUR | PLCG1 | PLXNA1 | PLXNA2 | PLXNB2 | PML | POLR2A | POLR2E | POLR2L | PPIA | PRKAA1 | PRKAA2 | PRKACA | PRKCD | PRKCE | PRKCZ | PRKD1 | PRKD2 | PSEN1 | PSEN2 | PSMA1 | PSMA2 | PSMA3 | PSMA5 | PSMA6 | PSMB5 | PSMB6 | PSMB7 | PSMB8 | PSMC1 | PSMC2 | PSMC3 | PSMC4 | PSMC5 | PSMC6 | PSMD1 | PSMD10 | PSMD11 | PSMD12 | PSMD14 | PSMD2 | PSMD4 | PSMD9 | PSME2 | PSME3 | PTEN | PTK2 | PTN | PTPN1 | PTPN22 | PTPN6 | PTPRA | PTPRD | PTPRF | PTPRK | PTPRR | PXN | RAC1 | RAF1 | RAP1A | RAP1B | RARG | RBPJ | RBX1 | RC3H1 | RECK | RELA | RHOA | RHOU | RIPK1 | RIPK2 | RNF31 | RPLP0 | RPS19 | RTF1 | RUNX2 | SAG | SDC1 | SDCBP | SEL1L | SEMA7A | SEPT2 | SERPINB2 | SGPL1 | SH2B1 | SHC1 | SIPA1L1 | SIRT1 | SKI | SLC9A3R1 | SMAD2 | SMAD3 | SMAD4 | SMAD5 | SMAD9 | SMARCA4 | SMARCC1 | SMO | SMURF1 | SMURF2 | SNAI2 | SNRPA1 | SNW1 | SNX3 | SOCS1 | SOCS2 | SOCS6 | SOD1 | SOD2 | SORT1 | SOX2 | SP100 | SPEN | SPG21 | SPI1 | SPIN1 | SQSTM1 | SRC | STAT1 | STAT3 | STAT4 | STAT5A | STAT6 | STIL | STK11 | STMN1 | STOML2 | STUB1 | SULF2 | SULT1A3 | SUSD5 | TAB1 | TAB2 | TALDO1 | TCIRG1 | TCP1 | TCTN2 | TCTN3 | TGFB1 | TGFBR3 | TIA1 | TIAL1 | TIMP1 | TLE1 | TLE3 | TLR3 | TMEM17 | TMEM231 | TNF | TNFRSF12A | TNFRSF1A | TNFRSF8 | TNFSF12 | TNFSF14 | TNIP2 | TNK2 | TNKS | TNKS2 | TOLLIP | TP53 | TP63 | TRADD | TRAF2 | TRAF3 | TRAF6 | TREX1 | TRIM21 | TRIM25 | TRIM34 | TSPAN17 | TULP3 | TWIST1 | TXLNA | TXNDC17 | TXNIP | UBB | UBC | UBE2D1 | UBE2D3 | UBE2N | UBE2V1 | USP15 | USP9X | USP9Y | VAV1 | VCAM1 | VIM | VPS26A | VPS29 | WAS | WDR12 | WDR19 | WNK1 | WNT16 | WNT2 | WWOX | XAF1 | XIAP | YES1 | YWHAZ | ZAP70 | ZBTB33 | ZFAND5 | GO:0030522 | 0.0157709571757855 | 130/8243 | 77/3250 | 3.35883281496654e-06 | 0.0318921175781073 | T | T | T | T | intracellular receptor signaling pathway | ACTN4 | AR | ARNT | BRCA1 | CALR | CARM1 | CASP8 | CRKL | CRY1 | CRY2 | CTNNB1 | CYLD | DAXX | DNAJA1 | ESR1 | ESR2 | ESRRA | ESRRB | ESRRG | FHL2 | GRIP1 | IKBKG | IRF7 | ITCH | KAT5 | MAP2K6 | MED1 | NCOA1 | NCOA3 | NCOA4 | NEDD4 | NFKBIA | NOD2 | NR1H2 | NR1H3 | NR1I2 | NR3C1 | NR4A1 | NR5A2 | PHB | PIAS1 | PIM1 | PML | PPARD | PPARG | PPARGC1A | RAN | RARA | RARG | RB1 | RELA | RHOA | RIPK2 | RNF4 | RXRA | RXRB | RXRG | SNW1 | SRC | STAT3 | TAB1 | TAB2 | THRA | TMEM173 | TNFAIP3 | TRAF6 | TRIP4 | UBB | UBC | UBE2N | UBE2V1 | UBE3A | UBR5 | VDR | WBP2 | YAP1 | YWHAH | GO:0097193 | 0.0135872861822152 | 112/8243 | 68/3250 | 3.73938607915539e-06 | 0.0355054708215804 | T | T | T | T | intrinsic apoptotic signaling pathway | ABL1 | AIFM1 | ATAD5 | ATM | BAG6 | BAX | BBC3 | BCL2 | BCL2L1 | BCL2L2 | BRCA1 | CASP2 | CASP3 | CASP9 | CDKN1A | CHEK2 | CRIP1 | CUL1 | CUL2 | CUL3 | CUL5 | DDIT4 | DDX3X | DIABLO | DNAJC10 | DYRK2 | E2F1 | E2F2 | EP300 | GPX1 | HINT1 | HMOX1 | HRAS | HTRA2 | IFI16 | IKBKE | ITPR1 | MELK | MLH1 | MLLT11 | MSH6 | MYBBP1A | NUPR1 | PDCD10 | PDK2 | PIK3R1 | PML | PRKCD | PRKDC | SFN | SIRT1 | SNW1 | STK11 | STK24 | STK25 | TMBIM6 | TNF | TNFRSF1A | TP53 | TP63 | TP73 | TRAF2 | TRIB3 | UBE2K | USP28 | WWOX | XBP1 | ZNF622 | GO:0071482 | 0.00812810869828946 | 67/8243 | 45/3250 | 3.74140341898154e-06 | 0.0355246254632297 | T | T | T | T | cellular response to light stimulus | AKT2 | AURKB | BAX | CASP9 | CDC25A | CDKN1A | CREBBP | CRIP1 | CRY1 | CRY2 | CUL4B | DDB1 | DDB2 | DHX36 | EIF2AK4 | EIF2S1 | EP300 | GNB1 | GRK1 | KDM1A | MC1R | MDM2 | MME | MYC | NEDD4 | NPM1 | PARP1 | PCNA | PDE6G | PIK3R1 | POLD1 | PPID | PTN | PTPRK | RHBDD1 | RHO | RUVBL2 | SAG | SIRT1 | STK11 | TP53 | TRIAP1 | USP28 | USP47 | YY1 | GO:0065009 | 0.223219701564964 | 1840/8243 | 809/3250 | 3.78770564018205e-06 | 0.0359642650535285 | T | T | T | T | regulation of molecular function | A2M | ABCE1 | ABL1 | ABL2 | ACD | ACSL1 | ACTB | ACTN2 | ACTN4 | ADCY6 | ADCYAP1 | ADORA2A | ADPRH | ADRB2 | ADRM1 | AES | AGAP1 | AGAP2 | AGER | AHCYL1 | AHNAK | AHSA1 | AHSG | AIFM1 | AIMP2 | AKAP9 | AKT1 | AKT2 | AKTIP | ALOX12 | ALOX5AP | AMFR | ANK3 | ANXA1 | ANXA2 | ANXA5 | APC | APCS | APOA1 | APOE | APP | AR | ARF1 | ARF4 | ARFGAP1 | ARHGAP1 | ARHGAP19 | ARHGAP26 | ARHGAP35 | ARHGDIA | ARHGEF7 | ARRB1 | ARRB2 | ATF2 | ATM | ATP13A2 | ATP1B3 | AUP1 | AURKB | AXIN1 | AZU1 | B3GAT3 | BAG1 | BAG2 | BAG3 | BAG4 | BAG5 | BAX | BBC3 | BCL10 | BCL2 | BCL2L13 | BCL6 | BIN1 | BIRC2 | BIRC3 | BIRC5 | BIRC7 | BIRC8 | BMI1 | BRAF | BTK | BTRC | BUD31 | C4A | CAB39 | CACNG2 | CACNG4 | CACNG5 | CALM1 | CALM2 | CALM3 | CAMK1D | CAMK2A | CAND1 | CAP2 | CAPN1 | CARM1 | CASP10 | CASP3 | CASP8 | CASP9 | CAT | CAV1 | CBL | CBLB | CBLC | CBX8 | CCAR2 | CCDC22 | CCDC8 | CCDC88A | CCL21 | CCL22 | CCNB1 | CCNT1 | CCNT2 | CCNY | CCNYL1 | CCS | CCT2 | CCT4 | CD109 | CD27 | CD2BP2 | CD36 | CD40 | CD40LG | CD74 | CD81 | CDC14B | CDC20 | CDC25A | CDC25B | CDC25C | CDC37 | CDK4 | CDK5 | CDK5R1 | CDK9 | CDKN1A | CDKN2C | CEBPA | CFTR | CHCHD10 | CHGB | CHORDC1 | CHP1 | CKS1B | CKS2 | CLEC11A | CLPX | CLU | CMTM5 | COMMD1 | COPA | COPS5 | CORT | COX11 | COX17 | COX6A1 | CRBN | CRK | CRKL | CRMP1 | CRY2 | CRYAB | CSNK1A1 | CSNK1E | CSNK2B | CTCF | CTH | CTNNB1 | CTNNBIP1 | CTSA | CXCL11 | CXCL5 | CXCL6 | CXCL8 | CYCS | CYLD | DAB1 | DAB2 | DAPK1 | DAXX | DBF4B | DBNDD2 | DBNL | DCUN1D1 | DDRGK1 | DDX3X | DENND1A | DERL1 | DGKZ | DHCR24 | DHX9 | DIABLO | DISC1 | DLG2 | DLG3 | DLG4 | DMD | DNAJA1 | DNAJA2 | DNAJA3 | DNAJB1 | DNAJB11 | DNAJB2 | DNAJC10 | DNAJC19 | DNAJC9 | DNM1L | DOCK7 | DPEP1 | DUSP1 | DUSP12 | DUSP14 | DUSP16 | DUSP19 | DUSP22 | DUSP7 | DVL1 | DVL2 | DVL3 | DYNLL1 | E2F1 | ECSIT | EDA | EEF1A2 | EFEMP1 | EGFR | EGLN1 | EGLN3 | EIF2AK3 | EIF2AK4 | EIF2S1 | EIF3D | EIF3E | EIF4A3 | EIF5 | ELMOD3 | EP300 | EPHA4 | EPPIN | ERBB2 | ERBB3 | ERRFI1 | ESR1 | ESR2 | EZH2 | FABP1 | FADD | FAF1 | FAF2 | FAM129B | FAM192A | FAM3C | FANCA | FASLG | FBRS | FBXO7 | FGF2 | FGFR1 | FGFR1OP | FGFR3 | FHL1 | FIS1 | FKBP1A | FKBP1B | FLOT1 | FN1 | FOS | FOXA1 | FOXJ1 | FYN | FZR1 | G3BP2 | GADD45G | GAPDH | GAPVD1 | GATA1 | GDF9 | GDI1 | GDI2 | GIT1 | GLA | GLP1R | GLRX | GNA13 | GNA15 | GNAI2 | GNAI3 | GNAO1 | GNAQ | GNAS | GOLGA2 | GOPC | GPD1L | GPI | GPS2 | GPSM1 | GPX1 | GRIA1 | GRPEL1 | GSK3A | GSK3B | GSTO1 | GSTP1 | GTF2F1 | GZMA | H1F0 | HAP1 | HAVCR2 | HBEGF | HDAC1 | HDAC2 | HDAC4 | HDAC5 | HDAC6 | HDAC9 | HDGF | HECW1 | HECW2 | HEXIM1 | HGS | HIF1A | HIST1H2BA | HMGB1 | HMGB2 | HMOX1 | HNRNPA2B1 | HNRNPD | HNRNPL | HNRNPU | HRAS | HSF1 | HSP90AA1 | HSP90AB1 | HSP90B1 | HSPA1B | HSPA2 | HSPA5 | HSPB1 | HSPB2 | HSPBP1 | HSPD1 | HSPE1 | HSPH1 | HTR3A | HTRA2 | HTT | IBTK | ICAM1 | ID2 | IFI16 | IFIT1 | IFNA2 | IGBP1 | IGF1R | IKBKB | IKBKG | IL32 | ILK | INSR | IPO5 | IPO7 | IQGAP1 | IRF4 | ITCH | ITGA4 | ITGB1BP1 | ITGB3 | ITK | JUN | JUP | KCNAB1 | KCNIP2 | KDM1A | KDM4D | KEAP1 | KIF5B | KMT2A | KRAS | KSR1 | LAMP3 | LARS | LAT | LATS1 | LCP2 | LEF1 | LGALS3 | LGMN | LGR4 | LIMS1 | LPAR1 | LPAR2 | LRP6 | LRRC7 | LRRK2 | LTC4S | LYPLA1 | MAD2L1 | MAP2K1 | MAP2K3 | MAP2K4 | MAP2K6 | MAP2K7 | MAP3K1 | MAP3K13 | MAP3K14 | MAP3K2 | MAP3K3 | MAP3K4 | MAP4K1 | MAPK1 | MAPK14 | MAPK15 | MAPK3 | MAPK7 | MAPK8 | MAPK8IP3 | MAPK9 | MAPKAP1 | MAPT | MAS1 | MAT2B | MAVS | MBP | MCM2 | MDFI | MDM2 | MED1 | MEPCE | METRNL | MGMT | MIF | MLLT1 | MLST8 | MOB1B | MSH6 | MTPN | MTRR | MYC | MYD88 | MYH6 | MYO1D | MYO5A | NAMPT | NCBP1 | NCK1 | NCOA3 | NDUFA13 | NEDD4 | NEK1 | NEK7 | NEUROD1 | NF2 | NFKB2 | NFKBIA | NIPBL | NMB | NMD3 | NME1 | NOD2 | NOS1AP | NOS2 | NOS3 | NOSIP | NOSTRIN | NPM1 | NPTX1 | NQO1 | NR0B2 | NR1H2 | NR1H3 | NRBF2 | NTF3 | NTRK1 | NUPR1 | OAS2 | OAZ3 | OXA1L | OXSR1 | P2RX1 | PAFAH1B1 | PAK2 | PAK6 | PAM16 | PAPLN | PARK7 | PARP1 | PAXIP1 | PCBD1 | PCNA | PCSK9 | PDCD10 | PDCD6 | PDE4D | PDE6G | PEBP1 | PELI1 | PER2 | PEX19 | PFN1 | PGAM5 | PHB2 | PIH1D1 | PIK3CA | PIK3CG | PIK3R1 | PIK3R3 | PIM1 | PIN1 | PINX1 | PKIG | PLA2G10 | PLA2G1B | PLA2G6 | PLAUR | PLCG1 | PLK1 | PLSCR1 | PLXNA1 | PLXNA2 | PLXNB2 | PML | PNKP | POT1 | PPARG | PPARGC1A | PPIF | PPM1K | PPME1 | PPP1CA | PPP1R11 | PPP1R12A | PPP1R12B | PPP1R14A | PPP1R16A | PPP1R2 | PPP1R3B | PPP1R3C | PPP1R8 | PPP2CA | PPP2CB | PPP2R1A | PPP2R1B | PPP2R2A | PPP2R2D | PPP2R5D | PPP4R2 | PPP6R1 | PPT1 | PRDX2 | PRDX3 | PRDX5 | PRELID1 | PRKAA1 | PRKAB2 | PRKACA | PRKAG1 | PRKAR1A | PRKAR2B | PRKCD | PRKCE | PRKCZ | PRKD1 | PRKD2 | PRMT2 | PROX1 | PRRC1 | PSAP | PSEN1 | PSEN2 | PSMA3 | PSMA6 | PSMB8 | PSMD1 | PSMD10 | PSMD14 | PSMD2 | PSME2 | PSME3 | PTEN | PTGES3 | PTK2 | PTN | PTPN1 | PTPN22 | PTPN6 | PTPRF | PTTG1 | QARS | RAB1A | RAB3GAP1 | RAB9A | RAF1 | RAN | RANBP3 | RANGAP1 | RAP1A | RAP1GDS1 | RARA | RB1 | RDX | RECK | RELA | REST | RFC1 | RFC2 | RFC3 | RFC4 | RFC5 | RFK | RGN | RGS1 | RGS2 | RGS20 | RHOA | RIC8B | RING1 | RIPK1 | RIPK2 | RIPK3 | RIPK4 | RNF2 | RNF25 | RNF31 | RP2 | RPA2 | RPL23 | RPLP1 | RPS2 | RPS3 | RPS6KA1 | RPTOR | RSU1 | RTKN | RUVBL2 | S100A1 | S100A12 | S100A9 | SAE1 | SAG | SCG2 | SDC4 | SERPINB2 | SERPINB3 | SERPINB5 | SERPINB6 | SERPINB8 | SERPINE2 | SERPINH1 | SET | SFN | SH3PXD2A | SHC1 | SIN3A | SIPA1 | SIPA1L1 | SIRT1 | SKI | SLAMF8 | SLC9A1 | SLC9A3R1 | SMAD2 | SMAD3 | SMAD4 | SMARCA4 | SMARCB1 | SMG6 | SMG8 | SMO | SNCA | SNF8 | SNX6 | SOCS1 | SOCS2 | SOCS6 | SOD1 | SORT1 | SOX2 | SP100 | SPINK7 | SPINT2 | SPR | SPRY2 | SRC | SRGAP2 | SRGAP3 | SRI | STAC | STARD13 | STAT3 | STIM1 | STK11 | STK24 | STK25 | STK26 | STK3 | STK38 | STMN1 | STOM | STUB1 | STYX | SUGT1 | SUMO1 | SUMO3 | SYDE1 | TAB1 | TAB2 | TAF12 | TANK | TAOK3 | TBC1D15 | TBC1D22B | TBC1D4 | TCEA1 | TCP1 | TERF1 | TERF2 | TERF2IP | TFIP11 | TGFB1 | TGFBR3 | THADA | THBS1 | TIMP1 | TIMP2 | TLR3 | TMBIM6 | TMEM173 | TNF | TNFAIP3 | TNFRSF8 | TNFSF12 | TNFSF14 | TNK2 | TNKS | TP53 | TP63 | TP73 | TPM1 | TPM2 | TRADD | TRAF1 | TRAF2 | TRAF3 | TRAF6 | TREX1 | TRIAP1 | TRIB2 | TRIB3 | TRIM14 | TRIM21 | TRIM23 | TRIM25 | TRIM28 | TRIM32 | TRIM34 | TSG101 | TSPYL2 | TWIST1 | TXLNA | TXN | TXNIP | UBA2 | UBB | UBC | UBE2C | UBE2I | UBE2L3 | UBE2N | UBE2S | UBE2V1 | UBQLN1 | UBXN1 | UCHL1 | UCHL5 | UNC119 | USP14 | USP17L2 | USP47 | USP50 | USP7 | VAV1 | VCP | VDR | VGF | VTN | WARS | WAS | WDR35 | WDTC1 | WNK1 | WNK4 | WNT2 | WWP2 | XIAP | XRCC1 | XRCC5 | XRCC6 | YWHAB | YWHAE | YWHAG | YWHAH | ZBTB7A | ZMPSTE24 | ZNF622 | GO:0002682 | 0.10954749484411 | 903/8243 | 419/3250 | 3.78972161953185e-06 | 0.0359834067774549 | T | T | T | T | regulation of immune system process | A2M | ABCE1 | ABL1 | ACTB | ACTG1 | ACTR2 | ACTR3 | ADA | ADCYAP1 | ADORA2A | ADTRP | AGER | AGO1 | AIRE | AKT1 | ANXA1 | AP1G1 | AP3D1 | APCS | APOA1 | APOD | APOE | APP | ARNT | ARPC1A | ARPC1B | ARPC2 | ARPC3 | ARPC4 | ARPC5 | ARRB2 | ATAD5 | ATG5 | ATM | BAG6 | BAIAP2 | BAX | BCAR1 | BCL10 | BCL2 | BCL6 | BIRC2 | BIRC3 | BRAF | BTK | BTN2A1 | BTRC | C1QA | C1QBP | C1R | C4A | CALM1 | CALR | CAMK1D | CARD9 | CASP3 | CASP8 | CAV1 | CBFB | CCL21 | CCR1 | CD2 | CD209 | CD22 | CD27 | CD36 | CD40 | CD40LG | CD6 | CD74 | CD79B | CD81 | CDC37 | CDC42 | CDC73 | CEBPA | CLDN18 | CLU | COL2A1 | COLEC10 | CREB1 | CREB3 | CREBBP | CRK | CRKL | CRP | CTNNB1 | CTNNBIP1 | CTSL | CUL1 | CXCL6 | CXCL8 | CYLD | DDX1 | DHX36 | DHX9 | DNAJA3 | DNASE2 | DPF2 | DROSHA | DTX1 | DUSP1 | DUSP22 | EIF2AK4 | EIF2B1 | EIF2B3 | EIF2B5 | EIF6 | EP300 | ERBB2 | ESR1 | ESRRA | ETS1 | EXOSC6 | EZR | FADD | FAM19A3 | FAM49B | FANCA | FBXO7 | FBXW11 | FCGR1A | FCN1 | FGB | FKBP1A | FLOT1 | FOS | FOXJ1 | FYN | GATA1 | GBP5 | GGT1 | GLMN | GNAS | GPI | GPS2 | GPX1 | GRB2 | H3F3A | HAVCR2 | HAX1 | HDAC1 | HEXIM1 | HIF1A | HIST1H3A | HIST1H4A | HIST2H3A | HK1 | HLA-A | HLA-B | HLA-DQA1 | HLA-DRA | HLA-DRB1 | HLA-DRB3 | HLA-DRB4 | HLA-DRB5 | HLX | HMGB1 | HMGB2 | HMGB3 | HMOX1 | HOXA5 | HPX | HRAS | HSP90AA1 | HSP90AB1 | HSP90B1 | HSPA1B | HSPA9 | HSPD1 | HSPH1 | HTRA1 | ICAM1 | ID2 | IFI16 | IFIT1 | IFNA2 | IGHV3-13 | IGHV3-23 | IGHV3-30 | IGKV3-20 | IGLC7 | IKBKB | IKBKE | IKBKG | IL13RA2 | IRF1 | IRF4 | IRF7 | ISG15 | ITCH | ITGA4 | ITK | JAK1 | JAK3 | JUN | KIR3DL2 | KMT2A | KMT2B | KMT2C | KRAS | L3MBTL1 | LAT | LCP2 | LEF1 | LGALS1 | LGALS3 | LGMN | LGR4 | LMO2 | LRRK2 | MAD1L1 | MAP2K4 | MAP2K6 | MAP2K7 | MAP3K1 | MAPK1 | MAPK14 | MAPK3 | MAPK8 | MAPK9 | MASP1 | MASP2 | MATR3 | MAVS | MED1 | MICA | MIF | MITF | MLH1 | MOV10 | MSH6 | MSN | MYC | MYD88 | MYL9 | MYO1C | NCK1 | NFATC1 | NFATC2 | NFE2L2 | NFKBIA | NME1 | NME2 | NOD2 | NPLOC4 | NR1H2 | NR1H3 | ORM1 | OTUD4 | OXSR1 | P2RX4 | PAG1 | PAK2 | PARK7 | PARP14 | PAWR | PAXIP1 | PCBP2 | PDE4D | PELI1 | PHB | PHB2 | PIANP | PIAS1 | PIAS3 | PIK3CA | PIK3R1 | PLA2G10 | PLA2G1B | PLA2G6 | PLCG1 | PLSCR1 | PNP | PPARG | PPM1B | PRDX2 | PRELID1 | PRKACA | PRKAR1A | PRKCD | PRKCE | PRKCZ | PRKD2 | PRKDC | PRMT6 | PSEN1 | PSMA1 | PSMA2 | PSMA3 | PSMA5 | PSMA6 | PSMB5 | PSMB6 | PSMB7 | PSMB8 | PSMC1 | PSMC2 | PSMC3 | PSMC4 | PSMC5 | PSMC6 | PSMD1 | PSMD10 | PSMD11 | PSMD12 | PSMD14 | PSMD2 | PSMD4 | PSMD9 | PSME2 | PSME3 | PTK2 | PTPN1 | PTPN22 | PTPN6 | PUM1 | PUS7 | RAB29 | RAC1 | RAF1 | RAP1A | RARA | RARG | RB1 | RC3H1 | RELA | RHOA | RIF1 | RIPK1 | RIPK2 | RIPK3 | RNF31 | RPS19 | RPS3 | RTN4 | RUNX1 | S100A1 | S100A7 | S100A9 | SARM1 | SART1 | SCARA3 | SDC4 | SELL | SEMA7A | SFPQ | SHC1 | SIN3A | SIRT1 | SLAMF8 | SMAD3 | SNCA | SNX6 | SOCS1 | SOCS6 | SOD1 | SPACA3 | SPG21 | SPI1 | SRC | STAT1 | STAT3 | STAT6 | STK11 | STOML2 | STXBP1 | STXBP2 | SUMO1 | SWAP70 | TAB1 | TAB2 | TANK | TCF12 | TGFB1 | THBS1 | TLR3 | TMBIM6 | TMEM173 | TNF | TNFAIP3 | TNFSF14 | TNIP2 | TP73 | TRAF2 | TRAF3 | TRAF3IP1 | TRAF6 | TREML2 | TREX1 | TSC22D3 | UBASH3A | UBB | UBC | UBE2D1 | UBE2D2 | UBE2D3 | UBE2K | UBE2N | UBE2V1 | UBQLN1 | USP17L2 | VAV1 | VCAM1 | VSIG4 | VTN | WAS | WDFY1 | WNK1 | XBP1 | XIAP | XRCC5 | XRCC6 | YAP1 | YES1 | ZAP70 | ZBTB16 | ZBTB7B | ZMPSTE24 | GO:0043161 | 0.0288729831372073 | 238/8243 | 128/3250 | 3.82908326709995e-06 | 0.036357145621114 | T | T | T | T | proteasome-mediated ubiquitin-dependent protein catabolic process | AMFR | ANAPC4 | ARMC8 | ARRB1 | ARRB2 | AUP1 | AURKB | BAG6 | BIRC2 | BTRC | CCNB1 | CDC16 | CDC20 | CDC27 | CDC34 | CDK2 | CRBN | CTNNB1 | CUL1 | CUL2 | CUL3 | CUL4B | CUL5 | DDB1 | DERL1 | DNAJB2 | DNAJC10 | EDEM1 | ERLIN2 | FAF1 | FAF2 | FBXL12 | FBXO6 | FBXW11 | FBXW5 | FZR1 | GSK3A | HECW1 | HECW2 | HERC2 | HSP90B1 | HSPA5 | ITCH | KAT5 | KCTD17 | KCTD2 | KLHL20 | KLHL22 | MAD2L1 | MDM2 | MTA1 | NEDD4 | NFE2L2 | NHLRC3 | NPLOC4 | NSFL1C | PCBP2 | PELI1 | PLK1 | PML | PPP2CB | PSMA1 | PSMA2 | PSMA3 | PSMA5 | PSMA6 | PSMB5 | PSMB6 | PSMB7 | PSMB8 | PSMC1 | PSMC2 | PSMC3 | PSMC4 | PSMC5 | PSMC6 | PSMD1 | PSMD10 | PSMD11 | PSMD12 | PSMD14 | PSMD2 | PSMD4 | PSMD9 | PSME2 | PSME3 | PTTG1 | RAD23A | RAD23B | RBX1 | RNF126 | RNF185 | RNF4 | SEL1L | SGTA | SGTB | SIAH1 | SIRT1 | SMURF1 | SMURF2 | SPOP | SPSB3 | STUB1 | SYVN1 | TBL1X | TRIM25 | TRIM9 | UBB | UBC | UBE2A | UBE2B | UBE2C | UBE2D1 | UBE2D3 | UBE2H | UBE2I | UBE2J1 | UBE2R2 | UBE2S | UBE2W | UBE3A | UBQLN1 | UBQLN2 | UBXN1 | UCHL1 | USP19 | VCP | WWP2 | GO:0070201 | 0.0555622952808444 | 458/8243 | 227/3250 | 3.84222553393363e-06 | 0.0364819314446998 | T | T | T | T | regulation of establishment of protein localization | ABAT | ABL1 | ADORA2A | ADTRP | AGER | AKT2 | ANK3 | ANXA1 | ANXA5 | APBB1 | APOA1 | APOD | ARF1 | ARFIP1 | ARRB1 | ATAD1 | ATG5 | ATP13A2 | B3GAT3 | BAG3 | BAG4 | BBC3 | BCL2 | CACNA1A | CASP8 | CCT2 | CCT3 | CCT4 | CCT5 | CCT6A | CCT7 | CCT8 | CD2 | CD22 | CD36 | CD40 | CD40LG | CDH1 | CDK16 | CDK5 | CDK5R1 | CENPJ | CEP135 | CEP290 | CFTR | CHP1 | CPT1A | CRP | CSNK2A2 | DHX36 | DHX9 | DMAP1 | DNAJA1 | DNM1L | DYNLL1 | E2F1 | EDEM1 | EGFR | ERBB2 | EZR | FCN1 | FGB | FIS1 | FKBP1B | FN1 | FYN | GAPDH | GAPVD1 | GDI1 | GLMN | GLP1R | GLUL | GOLPH3 | GOPC | GPI | GSK3A | GSK3B | HADH | HAVCR2 | HAX1 | HDAC3 | HDAC6 | HDAC9 | HIF1A | HK1 | HMGB1 | HNF4A | HRAS | HSPA1L | HSPA8 | HTRA2 | HUWE1 | IDH2 | IFNA2 | INSIG1 | IPO5 | ITGB1BP1 | ITGB3 | ITPR1 | ITPR2 | ITPR3 | JUP | KCNS3 | KIF5B | LCP1 | LGR4 | LRRK2 | LYPLA1 | MAPK1 | MAPK14 | MAPK3 | MAPK8 | MAPT | MAVS | MBP | MDM2 | MIF | MPC2 | MYO1C | NDUFAF2 | NEUROD1 | NOD2 | NOS2 | NPEPPS | NR0B2 | NR1H2 | NR1H3 | NUCB1 | ORM1 | OXCT1 | PANX3 | PARK7 | PCM1 | PDCD10 | PER2 | PFKL | PFKM | PIK3R1 | PKIG | PLA2G1B | PLA2G6 | PML | PPARD | PPIA | PPID | PPM1A | PPP1R11 | PPP3CC | PRKAA1 | PRKACA | PRKCD | PRKCE | PRKCZ | PSEN1 | PSMD9 | PTPN1 | PTPN14 | PTPN22 | PTPN23 | RAB11A | RAB29 | RAN | RANGAP1 | RAP1A | RAPGEF4 | REST | RHOU | RIPK2 | RNF31 | SAE1 | SCFD1 | SEC16A | SFN | SIRT3 | SLC16A1 | SLC25A4 | SLC25A5 | SLC25A6 | SMAD3 | SMO | SNX12 | SNX3 | SOCS1 | SP100 | SPTBN1 | SRC | SRI | STOM | TARDBP | TCP1 | TERF1 | TFDP1 | TGFB1 | TM9SF4 | TMBIM6 | TMEM30A | TNF | TNFAIP3 | TP53 | TP63 | TP73 | TPR | TRAF2 | TRAF6 | TRIM28 | TSG101 | TWIST1 | TXN | UBE2D3 | UBE2J1 | UBE2L3 | UBR5 | USP17L2 | USP46 | USP50 | WWP2 | XBP1 | XPO1 | XPO4 | XPO5 | YWHAB | YWHAE | YWHAG | YWHAH | YWHAQ | YWHAZ | ZIC1 | GO:1900034 | 0.00837073880868616 | 69/8243 | 46/3250 | 3.9947526225137e-06 | 0.0379301761507676 | T | T | T | T | regulation of cellular response to heat | ATM | BAG1 | BAG2 | BAG3 | BAG4 | BAG5 | CAMK2A | CAMK2B | CCAR2 | CHORDC1 | CREBBP | CRYAB | DNAJB1 | DNAJC7 | EP300 | GSK3B | HSF1 | HSP90AA1 | HSP90AB1 | HSPA1B | HSPA1L | HSPA8 | HSPH1 | MAPK1 | MAPK3 | MAPT | MLST8 | NUP107 | NUP153 | NUP160 | NUP205 | NUP214 | NUP85 | NUP88 | NUP93 | NUP98 | POM121 | PTGES3 | RAE1 | RPA2 | RPA3 | RPTOR | SEC13 | SIRT1 | TPR | YWHAE | GO:0009893 | 0.266650491325974 | 2198/8243 | 955/3250 | 3.99558798758668e-06 | 0.0379381079421355 | T | T | T | T | positive regulation of metabolic process | ABAT | ABCG1 | ABHD14B | ABL1 | ABL2 | ACACA | ACD | ACE2 | ACLY | ACSL1 | ACTA1 | ACTA2 | ACTB | ACTG2 | ACTL6A | ACTN1 | ACTN2 | ACTN4 | ACTR2 | ACTR3 | ADCY6 | ADCYAP1 | ADRB2 | ADRM1 | ADTRP | AGAP2 | AGER | AGO1 | AGO2 | AGR2 | AHI1 | AICDA | AIFM1 | AIMP2 | AIRE | AKAP8 | AKAP9 | AKT1 | AKT2 | AKTIP | ALOX12 | ANK3 | ANKRD49 | ANXA1 | ANXA2 | APBB1 | APC | APEX1 | APOA1 | APOE | APP | AR | ARAF | ARF4 | ARID3B | ARID4B | ARNT | ARNT2 | ARRB1 | ARRB2 | ASB9 | ASCL1 | ASPSCR1 | ASS1 | ATAD1 | ATAD5 | ATF2 | ATF6 | ATF6B | ATM | ATOH1 | ATRX | AURKB | AXIN1 | AZU1 | BABAM1 | BAG2 | BAG3 | BAG4 | BAG6 | BANP | BAX | BBC3 | BCAR3 | BCL10 | BCL11A | BCL2 | BCL2L13 | BCL6 | BIRC2 | BIRC3 | BIRC7 | BMI1 | BMPR1A | BRAF | BRCA1 | BRD4 | BTRC | BUD31 | C1QBP | C1QTNF1 | CAB39 | CALCOCO2 | CALM1 | CALM2 | CALM3 | CALR | CAMK1D | CAND1 | CAPN2 | CARD9 | CARM1 | CASP10 | CASP3 | CASP8 | CASP9 | CAV1 | CBFA2T3 | CBFB | CBX8 | CCAR2 | CCDC22 | CCDC88A | CCL21 | CCL22 | CCNB1 | CCNT1 | CCNT2 | CCNY | CCNYL1 | CCR1 | CCS | CCT2 | CCT3 | CCT4 | CCT5 | CCT6A | CCT7 | CCT8 | CD27 | CD36 | CD40 | CD40LG | CD74 | CD81 | CDC14B | CDC20 | CDC25B | CDC37 | CDC5L | CDC73 | CDH1 | CDK2 | CDK4 | CDK5 | CDK5R1 | CDK8 | CDK9 | CDKN1A | CEBPA | CEBPE | CEP290 | CHCHD10 | CHCHD2 | CHD8 | CHEK2 | CHP1 | CIRBP | CKS1B | CKS2 | CLPX | CLU | COA3 | COMMD1 | COMT | COPS5 | COX17 | CPT1A | CPT2 | CREB1 | CREB3 | CREBBP | CRK | CRKL | CRP | CSNK1A1 | CSNK1E | CSNK2B | CTBP1 | CTCF | CTNNB1 | CTSC | CUL3 | CUL4B | CYCS | DAB1 | DAB2 | DAPK1 | DAXX | DAZAP1 | DBF4B | DBH | DBNL | DCN | DCUN1D1 | DDB1 | DDRGK1 | DDX39B | DDX3X | DECR1 | DERL1 | DGKZ | DHX36 | DHX9 | DIABLO | DLG3 | DLG4 | DNA2 | DNAJB2 | DOCK7 | DPF2 | DROSHA | DTX1 | DUSP12 | DUSP19 | DUSP22 | DUSP7 | DVL1 | DVL2 | DVL3 | DYNC1H1 | DYRK2 | E2F1 | E2F2 | E2F3 | E2F4 | E2F6 | EAF2 | EBF1 | ECD | EDA | EDEM1 | EED | EEF1A2 | EEF2 | EGFR | EGLN1 | EGLN3 | EHD4 | EHMT1 | EIF2AK3 | EIF2AK4 | EIF2B5 | EIF3D | EIF3E | EIF4A3 | EIF5A | EIF6 | ELAVL1 | ELF3 | ELF4 | ENDOG | ENO1 | EP300 | EPHA4 | EPM2A | ERBB2 | ERBB3 | ERG | ERLIN2 | ESR1 | ESR2 | ESRRA | ESRRB | ESRRG | ETS1 | EXOSC6 | EXOSC9 | EYA2 | EYA4 | EZH2 | EZR | FABP1 | FABP3 | FABP5 | FADD | FAF1 | FAM129B | FAM98A | FANCI | FANCM | FASLG | FASN | FBXO7 | FBXW11 | FGB | FGF2 | FGFR1 | FGFR3 | FH | FHL2 | FIS1 | FKBP1A | FLOT1 | FN1 | FOS | FOXA1 | FOXJ1 | FOXK1 | FOXK2 | FOXM1 | FOXO1 | FTO | FUBP1 | FUBP3 | FUS | FXR2 | FYN | FZR1 | GABPB1 | GADD45G | GAPDHS | GATA1 | GDF9 | GGA1 | GJA1 | GLMN | GNAI2 | GNAI3 | GNAS | GOLGA2 | GPD1 | GPS2 | GPSM1 | GRB2 | GRIP1 | GSK3A | GSK3B | GSTP1 | GTF2A1 | GTF2F1 | GTF2F2 | H1F0 | H1FX | H2AFX | H2AFZ | HADH | HAVCR2 | HAX1 | HBEGF | HDAC1 | HDAC2 | HDAC3 | HDAC4 | HDAC5 | HDAC6 | HECW1 | HECW2 | HELZ2 | HEXB | HGS | HIF1A | HIST1H1A | HK2 | HMGB1 | HMGB2 | HMGB3 | HMOX1 | HNF1A | HNF4A | HNF4G | HNRNPA1 | HNRNPA2B1 | HNRNPAB | HNRNPD | HNRNPK | HNRNPL | HNRNPU | HOXA5 | HOXB5 | HOXB9 | HOXD10 | HPRT1 | HPX | HRAS | HSF1 | HSF4 | HSP90AA1 | HSP90AB1 | HSPA1B | HSPA2 | HSPA5 | HSPB1 | HSPBP1 | HSPD1 | HSPE1 | HSPH1 | HTATIP2 | HTRA2 | HTT | ICAM1 | ID2 | IDE | IFI16 | IFNA2 | IGF1R | IGF2BP1 | IKBKB | IKBKG | IKZF5 | ILF2 | ILF3 | ILK | ING1 | ING5 | INSIG1 | INSIG2 | INSR | IQGAP1 | IRF1 | IRF4 | IRF7 | ITCH | ITGB1BP1 | ITGB3 | JMJD6 | JUN | JUNB | JUND | JUP | KAT5 | KAT7 | KAT8 | KDM1A | KDM4D | KDM5B | KDM8 | KDR | KEAP1 | KHDRBS1 | KLF5 | KLF6 | KLHDC10 | KMT2A | KMT2B | KMT2C | KRAS | KSR1 | LAMP3 | LARP4 | LAT | LATS1 | LCP2 | LEF1 | LGMN | LGR4 | LIMS1 | LMNA | LMO2 | LMO7 | LPAR1 | LPIN3 | LRP6 | LRRK1 | LRRK2 | LRSAM1 | LTBR | LUM | MAP2K1 | MAP2K3 | MAP2K4 | MAP2K6 | MAP2K7 | MAP3K1 | MAP3K13 | MAP3K14 | MAP3K2 | MAP3K3 | MAP3K4 | MAP4K1 | MAPK1 | MAPK14 | MAPK15 | MAPK3 | MAPK7 | MAPK8 | MAPK8IP3 | MAPK9 | MAPKAP1 | MAPT | MAS1 | MAVS | MBP | MC1R | MDM2 | MECP2 | MED1 | MED12 | MED14 | MED17 | MED20 | MED21 | MED23 | MED24 | MED31 | MED4 | MED6 | MEOX2 | METTL23 | MGMT | MIF | MITF | MLH1 | MLLT11 | MLLT3 | MLST8 | MOB1B | MORF4L2 | MOV10 | MRPL12 | MSH6 | MSN | MTA1 | MTA3 | MTPN | MYBBP1A | MYC | MYD88 | MYO1C | MYO6 | NAMPT | NANOG | NBAS | NCBP1 | NCK1 | NCOA1 | NCOA2 | NCOA3 | NCOA4 | NCOR1 | NCOR2 | NDUFA13 | NEDD4 | NEK1 | NEK7 | NELFE | NEUROD1 | NFATC1 | NFATC2 | NFE2L2 | NFKB2 | NFKBIA | NIF3L1 | NIPBL | NKRF | NKX2-1 | NMD3 | NME2 | NMNAT1 | NNT | NOD2 | NOS1AP | NOS2 | NOS3 | NPM1 | NQO2 | NR0B2 | NR1H2 | NR1H3 | NR1I2 | NR3C1 | NR4A1 | NR5A2 | NTF3 | NTRK1 | NUFIP1 | NUP85 | NUP98 | NUPR1 | OAZ3 | OGT | ONECUT1 | OPTN | OTUD4 | OXSR1 | P2RX1 | P2RX4 | PABPC1 | PACSIN3 | PAIP1 | PAK2 | PAK6 | PARK7 | PARP1 | PARP14 | PATZ1 | PAWR | PAX8 | PAX9 | PAXIP1 | PCBD1 | PCBP1 | PCGF5 | PCNA | PCSK9 | PDCD10 | PDCD6 | PDE6G | PEBP1 | PEF1 | PELI1 | PER1 | PER2 | PFKM | PGK1 | PGK2 | PHB | PHB2 | PHF10 | PIAS1 | PIAS3 | PICK1 | PIH1D1 | PIK3CA | PIK3CG | PIK3R1 | PIK3R3 | PIN1 | PIP4K2A | PLA2G10 | PLA2G1B | PLA2G2A | PLA2G6 | PLAUR | PLEKHF1 | PLGRKT | PLK1 | PLSCR1 | PML | PNKP | POLDIP3 | POLR2A | POLR2E | POLR2L | POT1 | POU2F3 | POU5F1 | PPARD | PPARG | PPARGC1A | PPM1A | PPM1K | PPP1R12A | PPP2CA | PPP2R5D | PRDX2 | PRDX6 | PRELID1 | PRKAA1 | PRKAA2 | PRKAB2 | PRKACA | PRKAG1 | PRKAR1A | PRKAR2B | PRKCD | PRKCE | PRKCZ | PRKD1 | PRKD2 | PRKDC | PRMT2 | PROX1 | PRPF19 | PRRC1 | PSAP | PSEN1 | PSMC1 | PSMC2 | PSMC3 | PSMC4 | PSMC5 | PSMC6 | PSMD10 | PSMD14 | PSMD9 | PSME2 | PSME3 | PTEN | PTGES2 | PTGES3 | PTK2 | PTPN1 | PTPN22 | PUM1 | RAB1A | RAB1B | RAB29 | RAB3GAP1 | RAB7A | RAC1 | RAD23A | RAD51 | RAF1 | RAN | RAP1A | RAP1B | RARA | RARG | RB1 | RB1CC1 | RBM3 | RBMX | RBPJ | RBX1 | RC3H1 | RDX | REL | RELA | REST | RFC1 | RFC2 | RFC3 | RFC4 | RFC5 | RFK | RFXANK | RGN | RHBDD1 | RHOA | RIF1 | RIOK1 | RIPK1 | RIPK2 | RIPK3 | RMND1 | RNF139 | RNF185 | RNF19A | RNF4 | RNF8 | ROMO1 | RPL23 | RPLP1 | RPS19 | RPS2 | RPS3 | RPS4X | RPS6KA1 | RPS6KB2 | RPTOR | RTF1 | RUNX1 | RUNX2 | RUVBL1 | RUVBL2 | RXRA | RXRB | RXRG | S100A1 | S100A12 | S100A7 | S100A9 | SART3 | SCYL2 | SDC4 | SDCBP | SEMA7A | SEPT4 | SERPINB3 | SETDB1 | SFPQ | SGTA | SHC1 | SIN3A | SIRT1 | SIRT6 | SIRT7 | SKI | SLAMF1 | SLC9A1 | SMAD2 | SMAD3 | SMAD4 | SMAD5 | SMAD9 | SMARCA2 | SMARCA4 | SMARCB1 | SMARCC1 | SMARCC2 | SMARCE1 | SMO | SMURF1 | SMURF2 | SNAI2 | SNCA | SNF8 | SNW1 | SOAT1 | SOD1 | SOX2 | SP1 | SP100 | SPI1 | SPIN1 | SPRTN | SPRY2 | SPTLC2 | SQSTM1 | SRC | SRPK2 | SRSF5 | STAT1 | STAT3 | STAT4 | STAT6 | STK11 | STK24 | STK25 | STK26 | STK3 | STOML2 | STUB1 | STX5 | SUB1 | SUMO1 | SUMO2 | SUPT4H1 | SUPT5H | TAB1 | TAB2 | TADA2B | TAF12 | TAF15 | TAF1A | TANK | TAOK3 | TARDBP | TAZ | TBL1X | TCEA1 | TCEA2 | TCERG1 | TCF12 | TCF21 | TCF4 | TCP1 | TDRD3 | TERF2 | TERF2IP | TFCP2 | TFDP1 | TGFB1 | THBS1 | THRA | TIMP2 | TLE1 | TLR3 | TMEM173 | TMTC3 | TNF | TNFAIP3 | TNFRSF1A | TNFRSF8 | TNFSF12 | TNIP2 | TNK2 | TNKS | TNKS2 | TNP1 | TOLLIP | TOP2A | TP53 | TP63 | TP73 | TRA2A | TRADD | TRAF2 | TRAF6 | TRIAP1 | TRIB2 | TRIB3 | TRIM21 | TRIM28 | TRIM32 | TRIP4 | TRUB2 | TSG101 | TTC5 | TWIST1 | TXN | U2AF2 | UBB | UBC | UBE2B | UBE2C | UBE2D1 | UBE2I | UBE2K | UBE2L3 | UBE2N | UBE2S | UBE2V1 | UBE3A | UBQLN1 | UBQLN2 | UBR5 | ULK1 | UNC119 | UPF3B | UQCC1 | USP16 | USP21 | USP5 | USP50 | USP7 | USP9X | VCP | VDR | VHL | VIM | VSTM2A | VTN | WARS | WAS | WBP2 | WDR35 | WDR48 | WDR77 | WNK1 | WNT16 | WNT2 | WT1 | WWOX | WWP2 | XBP1 | XIAP | XRCC1 | XRCC5 | XRCC6 | YAF2 | YAP1 | YES1 | YTHDF1 | YWHAH | YY1 | ZBTB16 | ZBTB17 | ZBTB7B | ZIC1 | ZMPSTE24 | ZNF24 | ZNF622 | ZNF746 | GO:0016567 | 0.0553196651704477 | 456/8243 | 226/3250 | 4.05272067694692e-06 | 0.038480582827611 | T | T | T | T | protein ubiquitination | AKT1 | AMFR | ANAPC4 | ARRB1 | ARRB2 | ASB12 | ASB15 | ASB16 | ASB6 | ASB9 | BCL10 | BCL2 | BCOR | BIRC2 | BIRC3 | BIRC7 | BIRC8 | BMI1 | BRCA1 | BTRC | CAND1 | CBFB | CBL | CBLB | CBLC | CBX8 | CCDC22 | CDC16 | CDC20 | CDC27 | CDC34 | CDC42 | CDC73 | COMMD1 | CRBN | CTNNB1 | CUL1 | CUL2 | CUL3 | CUL4B | CUL5 | CUL7 | DCAF12 | DCAF7 | DCAF8 | DDB1 | DDB2 | DERL1 | DTX1 | DTX2 | DZIP3 | FBXL12 | FBXO11 | FBXO25 | FBXO32 | FBXO6 | FBXO7 | FBXW11 | FBXW5 | FZR1 | HDAC6 | HECW1 | HECW2 | HERC1 | HERC2 | HIF1A | HIST1H2BA | HUWE1 | ITCH | KCMF1 | KEAP1 | KLHL20 | KLHL22 | LMO7 | LNX1 | LRSAM1 | LZTR1 | MARCH5 | MDM2 | MED1 | MED12 | MED20 | MED21 | MED31 | MIB1 | NEDD4 | NEDD8 | NEURL4 | NFE2L2 | NHLRC3 | NOSIP | NPEPPS | PCGF1 | PCGF5 | PCGF6 | PCNA | PELI1 | PEX5 | PJA1 | PLK1 | PPP1R11 | PRKDC | PRPF19 | PSMA1 | PSMA2 | PSMA3 | PSMA5 | PSMA6 | PSMB5 | PSMB6 | PSMB7 | PSMB8 | PSMC1 | PSMC2 | PSMC3 | PSMC4 | PSMC5 | PSMC6 | PSMD1 | PSMD10 | PSMD11 | PSMD12 | PSMD14 | PSMD2 | PSMD4 | PSMD9 | PSME2 | PSME3 | RBBP6 | RBX1 | RC3H1 | RING1 | RNF123 | RNF126 | RNF139 | RNF181 | RNF183 | RNF185 | RNF19A | RNF2 | RNF25 | RNF31 | RNF4 | RNF8 | RTF1 | SAE1 | SIAH1 | SIRT1 | SMURF1 | SMURF2 | SOCS1 | SOCS2 | SOCS6 | SPOP | SPSB3 | STUB1 | SUZ12 | SYVN1 | THOP1 | TMEM189-UBE2V1 | TNFAIP3 | TNKS | TNKS2 | TRAF2 | TRAF3 | TRAF6 | TRIM21 | TRIM23 | TRIM25 | TRIM28 | TRIM32 | TRIM33 | TRIM41 | TRIM47 | TRIM63 | TRIM69 | TRIM7 | TRIM9 | TRIP12 | TSG101 | TSPAN17 | UBA1 | UBA6 | UBAC1 | UBB | UBC | UBE2A | UBE2B | UBE2C | UBE2D1 | UBE2D2 | UBE2D3 | UBE2H | UBE2I | UBE2J1 | UBE2K | UBE2L3 | UBE2L6 | UBE2M | UBE2N | UBE2O | UBE2Q2 | UBE2R2 | UBE2S | UBE2V1 | UBE2W | UBE3A | UBE3B | UBR4 | UBR5 | UCHL3 | UNKL | USP5 | USP7 | USP9X | VCP | VCPIP1 | VHL | WDTC1 | WSB1 | WWP2 | XIAP | ZBTB16 | ZC3HC1 | ZNF598 | ZNRF4 | GO:0010243 | 0.0583525415504064 | 481/8243 | 237/3250 | 4.06537986017909e-06 | 0.0386007817724004 | T | T | T | T | response to organonitrogen compound | ABL1 | ACTA1 | ACTB | ACTR2 | ADA | ADCY6 | ADORA2A | ADRB2 | ADSS | ADTRP | AGER | AIFM1 | AKAP9 | AKR1B1 | AKT1 | AKT2 | ANXA1 | ANXA5 | APEX1 | APLP1 | APP | APRT | ASCL1 | ASNS | ASS1 | BACE1 | BCL11A | BCL2L1 | BIRC2 | BRAF | BRCA1 | BSG | CACNA1A | CACNG4 | CALM1 | CALM3 | CAPN2 | CARD9 | CARM1 | CASP3 | CASP7 | CAT | CAV1 | CD36 | CD40 | CD81 | CDC5L | CDH1 | CDK4 | CDK5 | CDKN1A | CFL1 | CFTR | COL4A1 | COL6A1 | CREB1 | CRK | CRY1 | CTNNB1 | CYC1 | DBH | DENND4C | DHX15 | DUSP1 | EEF2 | EFTUD2 | EGFR | EIF2B1 | EIF2B3 | EIF2B5 | EIF6 | EPHA4 | EPRS | ERRFI1 | EZH2 | EZR | FABP3 | FADD | FIS1 | FOLR1 | FOS | FOXO1 | FYN | GLP1R | GLRB | GNAI1 | GNAO1 | GNB1 | GOT1 | GOT2 | GPD1 | GPI | GSK3A | GSK3B | GSS | GSTP1 | HADH | HCN1 | HCN2 | HCN3 | HDAC2 | HDAC5 | HDAC9 | HLCS | HNRNPD | HNRNPL | HPRT1 | HSF1 | HSP90B1 | HSPA5 | HTR1B | HTR3A | ICAM1 | IGF1R | INSIG2 | INSR | IPO5 | IQGAP1 | IREB2 | ITGA4 | ITPR2 | JUN | JUNB | JUND | JUP | KAT7 | KDM1A | KLF5 | KLHL22 | KYNU | LARS | LDHA | LGMN | LPIN3 | LRP6 | LRRK2 | MAPK1 | MAPK14 | MAPK3 | MDM2 | MGMT | MGST1 | MYD88 | MYO5A | NAMPT | NFE2L2 | NFKBIA | NME1 | NME2 | NOD2 | NR4A1 | NTRK1 | OGT | OPA1 | OTC | P2RX1 | P2RX2 | P2RX4 | PARP1 | PCNA | PCSK9 | PDE4D | PEBP1 | PER1 | PIK3CG | PIK3R1 | PKM | PLA2G1B | PPARG | PPARGC1A | PPAT | PPP2R2A | PRKAA1 | PRKACA | PRKAR1A | PRKAR2B | PRKCD | PRKCE | PRKCZ | PRKDC | PSEN1 | PTEN | PTN | PTPN22 | QDPR | RAB10 | RAB31 | RAD51 | RANGAP1 | RAP1A | RAP1B | RBBP4 | RELA | RGS2 | RHOA | RIPK2 | RPL23 | RPTOR | RRM2B | SDC1 | SELL | SHMT1 | SHMT2 | SIN3A | SIRT1 | SLC9A1 | SNCA | SNX6 | SOCS1 | SOD1 | SORT1 | SP1 | SRC | SRSF5 | STAT1 | SULT1A3 | TBC1D4 | TGFB1 | TGFBR3 | TIMP1 | TMBIM6 | TNF | TNFAIP3 | TOMM20 | TP53 | TP73 | TRIB3 | TRIM41 | TYMS | VCAM1 | VGF | VIM | WDTC1 | WT1 | XBP1 | YWHAG | ZBTB7B | GO:0043065 | 0.0520441586800922 | 429/8243 | 214/3250 | 4.18482553960827e-06 | 0.0397349184985806 | T | T | T | T | positive regulation of apoptotic process | ABL1 | ADORA2A | AES | AIFM1 | AKT1 | ALOX12 | ANXA1 | ANXA5 | APBB1 | APBB2 | APC | ARHGEF7 | ARHGEF9 | ARRB1 | ARRB2 | ASCL1 | ATF2 | ATF6 | ATM | BACE1 | BAX | BBC3 | BCL10 | BCL2 | BCL2L1 | BCL2L13 | BCL6 | BIN1 | C1QBP | CAMK1D | CAMK2A | CAPN2 | CASP10 | CASP2 | CASP3 | CASP8 | CASP9 | CAV1 | CCAR2 | CD40 | CD40LG | CDC34 | CDK19 | CDK4 | CDK5 | CDK5R1 | CHCHD10 | CHEK2 | CLU | COL18A1 | CREB1 | CTNNB1 | CTSC | CYCS | CYLD | DAPK1 | DDX3X | DIABLO | DNAJA1 | DNM1L | DUSP1 | E2F1 | E2F3 | EEF1A2 | EGLN3 | EIF2AK3 | EIF2B5 | EIF2S1 | ENDOG | FADD | FAF1 | FASLG | FIS1 | FOXA1 | FOXO1 | GADD45G | GAPDH | GNA13 | GRIK2 | GSK3A | GSK3B | GZMA | HDAC4 | HMGB1 | HMOX1 | HOXA5 | HSF1 | HSPD1 | HSPE1 | HTRA2 | HTT | ING5 | ITGA4 | ITGB3BP | JUN | LATS1 | LDHA | LPAR1 | LTBR | MAP2K4 | MAP2K6 | MAPK8 | MAPK9 | MAPT | MELK | MLLT11 | MTCH2 | MYBBP1A | MYC | NCK1 | NCOA1 | NDUFA13 | NEUROD1 | NQO1 | NQO2 | NUPR1 | P2RX1 | PAK2 | PARK7 | PAWR | PCSK9 | PDCD6 | PDIA3 | PIN1 | PLA2G6 | PLAUR | PLCG1 | PLEKHF1 | PML | PNMA1 | PPARG | PPARGC1A | PPID | PPIF | PPP1CA | PPP2R1A | PPP2R1B | PPP3CC | PRELID1 | PRKCD | PRKDC | PRMT2 | PSEN1 | PSEN2 | PTEN | PTN | PTRH2 | RARG | REST | RHOA | RIPK1 | RIPK2 | RIPK3 | RNF183 | RPS3 | RPS6KA2 | S100A9 | S100B | SAP18 | SEPT4 | SEPT7 | SFN | SFPQ | SIAH1 | SIRT1 | SLC9A1 | SLC9A3R1 | SMAD3 | SNCA | SOD1 | SOD2 | SORT1 | SQSTM1 | SRC | SRPK2 | STK3 | TCTN3 | TFDP1 | TGFB1 | TGM2 | THBS1 | TIA1 | TLR3 | TNF | TNFRSF12A | TNFRSF1A | TNFRSF8 | TNFSF12 | TOMM22 | TOP2A | TP53 | TP63 | TP73 | TRADD | TRAF2 | TRAF6 | TRIM35 | TXNIP | UBB | UBC | UBE2M | VAV1 | VCP | VDR | WDR35 | WWOX | YWHAB | YWHAE | YWHAG | YWHAH | YWHAQ | YWHAZ | ZBTB16 | ZNF622 | GO:0051289 | 0.00679364309110761 | 56/8243 | 39/3250 | 4.27219889965296e-06 | 0.0405645285522048 | T | T | T | T | protein homotetramerization | ACACA | ACOT13 | ACTN2 | ALDH5A1 | ALDOA | CAT | CBR4 | CBY1 | CDA | CRYZ | CTH | DCTPP1 | DCXR | DECR1 | DNM1L | FBP1 | GBP5 | GLS | GOLGA2 | HCN1 | HPRT1 | HSD17B10 | IDE | IMPDH2 | PCBD1 | PFKL | PKM | PPAT | RXRA | SHMT1 | SHMT2 | SOD2 | ST13 | TDO2 | TK1 | TP53 | TP63 | USP16 | VASP | GO:0018130 | 0.0874681547980104 | 721/8243 | 341/3250 | 4.28594727267638e-06 | 0.0406950693540623 | T | T | T | T | heterocycle biosynthetic process | ACACA | ACAT1 | ACLY | ACOT7 | ACSL1 | ACSL4 | ACSL5 | ACSS2 | ADA | ADCY6 | ADK | ADRM1 | ADSL | ADSS | AFF4 | AK1 | AK2 | AK4 | ALAS1 | ALDOA | AMD1 | AMDHD2 | APRT | AR | ARHGAP35 | ARNT | ARRB1 | ARRB2 | ASPDH | ATF6B | ATIC | ATOH1 | BAX | CBFB | CBR4 | CCNB1 | CCNT1 | CCNT2 | CDA | CDC73 | CDK4 | CDK8 | CDK9 | CDKN1A | CEBPA | CENPF | CHRAC1 | CMPK1 | COL4A2 | COPS2 | COPS5 | COX15 | CPSF7 | CREB1 | CREBBP | CSTF2T | CTDP1 | CTPS1 | CYC1 | DCAKD | DCK | DCTPP1 | DERA | DGUOK | DHX9 | DIDO1 | DLAT | DLD | DNAJC30 | DTX1 | DUT | DVL1 | DVL2 | E2F1 | E2F2 | E2F3 | EAF2 | ECD | ELF3 | ESR1 | ESR2 | ESRRA | ESRRB | ESRRG | ETS1 | FASN | FECH | FOS | FPGS | FUBP1 | FUBP3 | GAR1 | GARS | GART | GATA1 | GCDH | GDA | GFPT1 | GFPT2 | GMDS | GMPPB | GMPR | GMPS | GNPDA1 | GNPDA2 | GNPNAT1 | GPX1 | GTF2A1 | GTF2E2 | GTF2F1 | GTF2F2 | GTF2I | GTF3C2 | HAAO | HIF1A | HINT1 | HIST1H4A | HMBS | HNF4A | HNF4G | HOXB9 | HPRT1 | HSD17B8 | HSF1 | HSP90AA1 | HSP90AB1 | IDH2 | ILF2 | IMPDH2 | IREB2 | IRF1 | IRF7 | ISG15 | ITPA | JUN | KLF5 | KMT2A | KYNU | LDHC | LEF1 | LIG4 | LMO2 | MAPK1 | MAPK3 | ME1 | MECP2 | MED1 | MED12 | MED14 | MED17 | MED20 | MED21 | MED23 | MED24 | MED31 | MED4 | MED6 | MITF | MLLT1 | MLLT3 | MMAB | MOCOS | MPC2 | MPI | MRPL12 | MTAP | MTERF1 | MTHFD1 | NAGK | NAMPT | NANS | NAPRT | NCBP1 | NCOA1 | NCOR1 | NELFB | NELFCD | NELFE | NFATC1 | NFE2L2 | NFS1 | NME1 | NME2 | NME4 | NMNAT1 | NMNAT3 | NMRK2 | NOP10 | NPLOC4 | NPR1 | NR0B2 | NR1H2 | NR1H3 | NR1I2 | NR3C1 | NR4A1 | NR5A2 | NRBF2 | NT5C2 | NUDT18 | NUDT3 | NUDT9 | PAICS | PAPSS1 | PAPSS2 | PARP1 | PAX8 | PCBD1 | PCF11 | PCNA | PDE4D | PDHA1 | PDHA2 | PDHB | PDHX | PFAS | PGM3 | PHB | PINX1 | PIR | PNKP | PNP | POLA2 | POLD1 | POLE2 | POLE3 | POLM | POLR2A | POLR2E | POLR2L | POLR2M | POT1 | POU5F1 | PPARD | PPARG | PPARGC1A | PPAT | PPIA | PPM1D | PPOX | PPP5C | PPT1 | PRTFDC1 | PSAT1 | PTEN | PTGES3 | PTMA | PTTG1 | QDPR | RARA | RARG | RBMX | RBPJ | RELA | RFC1 | RFC2 | RFC3 | RFC4 | RFC5 | RFK | RPA2 | RPA3 | RRM1 | RRM2 | RRM2B | RTF1 | RUNX2 | RXRA | RXRB | RXRG | SARS | SCAF1 | SHMT1 | SIRT1 | SIRT7 | SKI | SLC25A1 | SLC25A13 | SMAD4 | SMG5 | SMG6 | SMN1 | SNRPB | SNRPD3 | SNRPE | SNW1 | SP1 | SPI1 | SPIN1 | SPR | SPRTN | STAT3 | STOML2 | SUCLA2 | SUPT4H1 | SUPT5H | TAF12 | TAF15 | TAF1A | TAMM41 | TARDBP | TCEA1 | TCEA2 | TCERG1 | TCF4 | TECR | TERF1 | TERF2 | TERF2IP | TFCP2 | TGFB1 | THRA | TK1 | TK2 | TP53 | TREX1 | TRIM25 | TRIM28 | TRIM29 | TRIP13 | TSC22D1 | TYMS | UAP1 | UAP1L1 | UBB | UBC | UBE2L6 | UCK2 | UGDH | UGP2 | UMPS | UPP2 | USPL1 | UXS1 | VCP | VDR | XBP1 | YAP1 | ZBTB7A | ZBTB7B | ZNF217 | GO:0051098 | 0.0334829552347446 | 276/8243 | 145/3250 | 4.99209027713117e-06 | 0.0473998971813605 | T | T | T | T | regulation of binding | ABL1 | ACD | ACTB | ADRB2 | AES | AGER | AKT1 | AKTIP | AMFR | ANXA2 | APOE | APP | ARHGEF7 | ARRB1 | ARRB2 | AURKB | BAG2 | BAX | BCL2 | CALM2 | CALM3 | CARM1 | CAV1 | CD36 | CDK5 | CDK9 | CRBN | CRK | CRMP1 | CSNK1A1 | CSNK1E | CSNK2B | CTNNB1 | CTNNBIP1 | CYLD | DAB2 | DDRGK1 | DERL1 | DHX9 | DISC1 | DNAJB2 | DVL1 | E2F1 | EIF2S1 | EIF3D | EIF3E | EIF4A3 | EP300 | EPHA4 | FAM129B | FAM192A | FKBP1A | FLOT1 | GATA1 | GOLGA2 | GPSM1 | GSK3B | GTF2F1 | GZMA | H1F0 | HDAC2 | HDAC4 | HDAC5 | HIST1H2BA | HMGB1 | HMGB2 | HMOX1 | HNRNPL | HSF1 | HSPA5 | ID2 | IFI16 | IFIT1 | IRF4 | ITGA4 | ITGB1BP1 | JUN | KDM1A | KDM4D | LEF1 | LGALS3 | LRRK2 | MAPK3 | MAPK8 | MDFI | MEPCE | NCBP1 | NEUROD1 | NFKBIA | NIPBL | NMD3 | NME1 | PARK7 | PARP1 | PCBD1 | PCSK9 | PER2 | PEX19 | PIN1 | PINX1 | PLAUR | PLK1 | PPARG | PPP1CA | PPP2CA | PPP2CB | PRKACA | PRKCD | PSEN1 | PTPRF | RAN | RARA | RB1 | RIPK2 | RUVBL2 | SIN3A | SKI | SMAD2 | SMAD3 | SMAD4 | SMO | SP100 | SRC | SRI | STK3 | STMN1 | STUB1 | STYX | SUMO1 | SUMO3 | TFIP11 | TGFB1 | TGFBR3 | TMBIM6 | TMEM173 | TNKS | TRAF2 | TRAF6 | TRIB3 | TRIM28 | TWIST1 | TXN | VTN | ZBTB7A | ZMPSTE24 | GO:1901362 | 0.096688098993085 | 797/8243 | 373/3250 | 5.15504656475049e-06 | 0.0489471671323059 | T | T | T | T | organic cyclic compound biosynthetic process | ACAA2 | ACACA | ACAT1 | ACAT2 | ACBD3 | ACLY | ACOT7 | ACSL1 | ACSL4 | ACSL5 | ACSS2 | ADA | ADCY6 | ADK | ADRM1 | ADSL | ADSS | AFF4 | AK1 | AK2 | AK4 | AKR1B1 | AKR1B15 | ALAS1 | ALDOA | AMD1 | AMDHD2 | APOA1 | APOE | APRT | AR | ARHGAP35 | ARNT | ARRB1 | ARRB2 | ASPDH | ATF6B | ATIC | ATOH1 | BAX | CBFB | CBR4 | CCNB1 | CCNT1 | CCNT2 | CDA | CDC73 | CDK4 | CDK8 | CDK9 | CDKN1A | CEBPA | CENPF | CES1 | CFTR | CHRAC1 | CMPK1 | COL4A2 | COPS2 | COPS5 | COX15 | CPSF7 | CREB1 | CREBBP | CSTF2T | CTDP1 | CTPS1 | CYB5R1 | CYB5R3 | CYC1 | DBH | DCAKD | DCK | DCTPP1 | DERA | DGUOK | DHCR24 | DHX9 | DIDO1 | DLAT | DLD | DNAJC30 | DTX1 | DUT | DVL1 | DVL2 | E2F1 | E2F2 | E2F3 | EAF2 | ECD | ELF3 | ESR1 | ESR2 | ESRRA | ESRRB | ESRRG | ETS1 | FASN | FDPS | FDXR | FECH | FOS | FPGS | FUBP1 | FUBP3 | G6PD | GAR1 | GARS | GART | GATA1 | GCDH | GDA | GFPT1 | GFPT2 | GGPS1 | GMDS | GMPPB | GMPR | GMPS | GNPDA1 | GNPDA2 | GNPNAT1 | GPX1 | GTF2A1 | GTF2E2 | GTF2F1 | GTF2F2 | GTF2I | GTF3C2 | HAAO | HIF1A | HINT1 | HIST1H4A | HMBS | HNF4A | HNF4G | HOXB9 | HPRT1 | HSD17B14 | HSD17B4 | HSD17B6 | HSD17B8 | HSD3B7 | HSF1 | HSP90AA1 | HSP90AB1 | IDH2 | IDI2 | ILF2 | IMPDH2 | INSIG1 | INSIG2 | IREB2 | IRF1 | IRF7 | ISG15 | ITPA | JUN | KLF5 | KMT2A | KYNU | LDHC | LEF1 | LIG4 | LMO2 | MAPK1 | MAPK3 | MC1R | ME1 | MECP2 | MED1 | MED12 | MED14 | MED17 | MED20 | MED21 | MED23 | MED24 | MED31 | MED4 | MED6 | MITF | MLLT1 | MLLT3 | MMAB | MOCOS | MPC2 | MPI | MRPL12 | MTAP | MTERF1 | MTHFD1 | MVD | MYO5A | NAGK | NAMPT | NANS | NAPRT | NCBP1 | NCOA1 | NCOR1 | NELFB | NELFCD | NELFE | NFATC1 | NFE2L2 | NFS1 | NME1 | NME2 | NME4 | NMNAT1 | NMNAT3 | NMRK2 | NOP10 | NPLOC4 | NPR1 | NR0B2 | NR1H2 | NR1H3 | NR1I2 | NR3C1 | NR4A1 | NR5A2 | NRBF2 | NT5C2 | NUDT18 | NUDT3 | NUDT9 | OSBPL1A | PAH | PAICS | PAPSS1 | PAPSS2 | PARP1 | PAX8 | PCBD1 | PCF11 | PCNA | PDE4D | PDHA1 | PDHA2 | PDHB | PDHX | PFAS | PGM3 | PHB | PINX1 | PIR | PNKP | PNP | POLA2 | POLD1 | POLE2 | POLE3 | POLM | POLR2A | POLR2E | POLR2L | POLR2M | POT1 | POU5F1 | PPARD | PPARG | PPARGC1A | PPAT | PPIA | PPM1D | PPOX | PPP5C | PPT1 | PRKAA1 | PRKAA2 | PRTFDC1 | PSAT1 | PTEN | PTGES3 | PTMA | PTTG1 | QDPR | RARA | RARG | RBMX | RBPJ | RELA | RFC1 | RFC2 | RFC3 | RFC4 | RFC5 | RFK | RPA2 | RPA3 | RRM1 | RRM2 | RRM2B | RTF1 | RUNX2 | RXRA | RXRB | RXRG | SARS | SCAF1 | SHMT1 | SIRT1 | SIRT7 | SKI | SLC25A1 | SLC25A13 | SMAD4 | SMG5 | SMG6 | SMN1 | SNCA | SNRPB | SNRPD3 | SNRPE | SNW1 | SP1 | SPI1 | SPIN1 | SPR | SPRTN | STAT3 | STOML2 | SUCLA2 | SUPT4H1 | SUPT5H | TAF12 | TAF15 | TAF1A | TAMM41 | TARDBP | TCEA1 | TCEA2 | TCERG1 | TCF4 | TECR | TERF1 | TERF2 | TERF2IP | TFCP2 | TGFB1 | THRA | TK1 | TK2 | TP53 | TREX1 | TRIM25 | TRIM28 | TRIM29 | TRIP13 | TSC22D1 | TYMS | UAP1 | UAP1L1 | UBB | UBC | UBE2L6 | UCK2 | UGDH | UGP2 | UMPS | UPP2 | USPL1 | UXS1 | VCP | VDR | XBP1 | YAP1 | ZBTB7A | ZBTB7B | ZNF217 | GO:0009063 | 0.00594443770471916 | 49/8243 | 35/3250 | 5.36889752411419e-06 | 0.0509776819914642 | T | T | T | T | cellular amino acid catabolic process | AASS | ABAT | ACAD8 | ALDH4A1 | ALDH5A1 | ALDH6A1 | ALDH7A1 | CBS | DLD | DLST | FAH | GCDH | GLS | GLS2 | GOT1 | GOT2 | GPT2 | GSTZ1 | HIBADH | HIBCH | HMGCL | IVD | MCCC1 | NOS2 | NOS3 | OAT | OGDH | OTC | PADI4 | PAH | PCBD1 | PPAT | QDPR | SHMT1 | SHMT2 | GO:0051726 | 0.101298071090622 | 835/8243 | 389/3250 | 5.500839880241e-06 | 0.0522304746628883 | T | T | T | T | regulation of cell cycle | ABL1 | ACTB | ACTR1A | ADCYAP1 | AICDA | AKAP9 | AKT1 | AKT2 | ANAPC4 | ANXA1 | APBB1 | APBB2 | APC | APEX1 | APP | ASCL1 | ASNS | ATAD5 | ATF2 | ATM | ATRX | AURKB | BABAM1 | BAX | BCL2 | BCL2L1 | BCL6 | BIN1 | BIRC2 | BMI1 | BRCA1 | BRD4 | BTRC | CAB39 | CALM1 | CALM2 | CALM3 | CALR | CARM1 | CASP2 | CASP3 | CCAR2 | CCDC36 | CCDC8 | CCNB1 | CCNDBP1 | CCNT1 | CCNT2 | CCNY | CCNYL1 | CCP110 | CDC14B | CDC16 | CDC20 | CDC25A | CDC25B | CDC25C | CDC27 | CDC37 | CDC42 | CDC73 | CDK15 | CDK16 | CDK19 | CDK2 | CDK4 | CDK5 | CDK5R1 | CDK8 | CDK9 | CDKL3 | CDKN1A | CDKN2C | CEBPA | CENPF | CENPJ | CENPV | CEP135 | CEP152 | CEP290 | CEP57 | CEP70 | CGRRF1 | CHEK2 | CHMP2A | CHORDC1 | CKAP5 | CKS1B | CKS2 | CLIC1 | CLTC | CNTRL | COPS5 | CRY1 | CSNK1E | CSNK2A2 | CTBP1 | CTCF | CTNNB1 | CUL1 | CUL2 | CUL3 | CUL4B | CUL5 | CUL7 | CXCL8 | CYLD | CYP1A1 | DBF4B | DCTN1 | DDB1 | DDRGK1 | DDX39B | DDX3X | DHCR24 | DNA2 | DUSP1 | DYNC1H1 | DYNC1I2 | DYNC1LI1 | DYNLL1 | E2F1 | E2F2 | E2F3 | E2F4 | E2F6 | ECD | EED | EGFR | EHMT1 | EHMT2 | EIF2AK4 | EIF4E | EIF4EBP1 | EP300 | EZH2 | FAM83D | FBXL12 | FBXO6 | FBXO7 | FBXW5 | FEN1 | FGFR1 | FGFR1OP | FHL1 | FIGNL1 | FOXA1 | FOXM1 | FSD1 | FZR1 | GADD45G | GIT1 | GNAI1 | GRK5 | H2AFX | HECW2 | HEXIM1 | HMMR | HNRNPU | HRAS | HSF1 | HSP90AA1 | HSP90AB1 | HSPA1B | HSPA2 | HTRA2 | ID2 | IK | ILK | INSR | IPO5 | IPO7 | IQGAP1 | IRF1 | JUN | JUNB | JUND | KANK2 | KHDRBS1 | KIF11 | KLHL22 | L3MBTL1 | L3MBTL2 | LATS1 | LGMN | LRP6 | MAD1L1 | MAD2L1 | MAP2K1 | MAP2K6 | MAPK14 | MAPK15 | MDC1 | MDM1 | MDM2 | MDM4 | MECP2 | MED1 | MEPCE | MGA | MIF | MLST8 | MSH6 | MTA3 | MYBBP1A | MYC | NAE1 | NEK7 | NEUROD1 | NF2 | NIPBL | NPM1 | NR4A1 | NSFL1C | NSUN2 | NUBP1 | NUDT6 | NUMA1 | NUP214 | NUPR1 | NUSAP1 | OBSL1 | OFD1 | PA2G4 | PAFAH1B1 | PAXIP1 | PCGF6 | PCM1 | PCNA | PDCD6IP | PEBP1 | PER2 | PHB2 | PHC3 | PIN1 | PINX1 | PKN2 | PLK1 | PML | POLDIP2 | PPM1A | PPP2R1A | PRCC | PRKAA1 | PRKAA2 | PRKAB2 | PRKACA | PRKAG1 | PRKAR1A | PRKAR2B | PRKCE | PRKDC | PRMT2 | PROX1 | PRPF40A | PSMA1 | PSMA2 | PSMA3 | PSMA5 | PSMA6 | PSMB5 | PSMB6 | PSMB7 | PSMB8 | PSMC1 | PSMC2 | PSMC3 | PSMC4 | PSMC5 | PSMC6 | PSMD1 | PSMD10 | PSMD11 | PSMD12 | PSMD14 | PSMD2 | PSMD4 | PSMD9 | PSME2 | PSME3 | PTEN | PTPN6 | PTPRK | PTTG1 | PUM1 | RAB11A | RAD21 | RAD23A | RAD51 | RAE1 | RARA | RB1 | RBBP4 | RBX1 | RCC1 | RDX | RHOA | RING1 | RIPK1 | RNASEH2B | RNF2 | RNF4 | RPA2 | RPA3 | RPL23 | RPL24 | RPS15A | RPS6KA2 | RPTOR | RRM2 | SDCBP | SENP6 | SEPT7 | SFN | SFPQ | SH2B1 | SIN3A | SIPA1 | SIRT1 | SLC9A3R1 | SMAD3 | SMC1A | SMC3 | SND1 | SOX2 | SPDL1 | SPICE1 | SRC | SRPK2 | SRSF5 | STAG2 | STAT3 | STIL | STK11 | SUZ12 | SVIL | TAOK3 | TARDBP | TBRG4 | TERF1 | TERF2 | TFDP1 | TGFB1 | THBS1 | TIMP2 | TMOD3 | TNF | TNFAIP3 | TNKS | TOP2A | TP53 | TP73 | TPR | TRIAP1 | TRIM21 | TRIM32 | TRIM35 | TRIP13 | TSG101 | TSPYL2 | TUBA1A | TUBA4A | TUBB | TUBB4A | TUBB4B | TUBG1 | UBA3 | UBE2B | UBE2C | UBE2D1 | USP16 | USP17L2 | USP19 | USP2 | USP28 | USP47 | VPS4B | XPO1 | XRCC3 | YAF2 | YWHAE | YWHAG | ZBTB17 | ZFYVE19 | ZFYVE26 | ZMPSTE24 | ZNF207 | ZW10 | ZWINT | GO:2000278 | 0.0103117796918598 | 85/8243 | 54/3250 | 5.50199780107133e-06 | 0.0522414691211723 | T | T | T | T | regulation of DNA biosynthetic process | ACD | ARRB2 | ATM | AURKB | CCT2 | CCT3 | CCT4 | CCT5 | CCT6A | CCT7 | CCT8 | CTNNB1 | DCP2 | DDX39B | DUSP1 | FAM129B | FGF2 | HNRNPA1 | HNRNPA2B1 | HNRNPD | HNRNPU | HSP90AA1 | HSP90AB1 | MAP2K7 | MAP3K4 | MAPK1 | MAPK15 | MAPK3 | MYC | NEK7 | PINX1 | PNKP | POT1 | PPARG | PRKD2 | PTGES3 | RFC2 | RFC3 | RFC4 | RFC5 | RGN | SH2B1 | SMG5 | SMG6 | SRC | TCP1 | TERF1 | TERF2 | TFDP1 | TINF2 | TNKS | TNKS2 | TP53 | XRCC5 | GO:0007006 | 0.0103117796918598 | 85/8243 | 54/3250 | 5.50199780107133e-06 | 0.0522414691211723 | T | T | T | T | mitochondrial membrane organization | ACAA2 | AFG3L2 | AGK | ATF2 | BAX | BBC3 | BCL2 | BCL2L1 | CALM1 | CALM3 | CAMK2A | CASP8 | CHCHD10 | CHCHD3 | E2F1 | EYA2 | GSK3A | GSK3B | HK2 | HSP90AA1 | HSPA4 | IMMT | LETM1 | MAPK8 | NDUFA13 | OPA1 | OXA1L | PPIF | PPP3CC | RHOT1 | RHOT2 | ROMO1 | SFN | SLC25A5 | SLC35F6 | SLC9A1 | SNCA | STAT3 | TAZ | TFDP1 | THEM4 | TIMM13 | TIMM50 | TMEM11 | TOMM22 | TP53 | TP63 | TP73 | YWHAB | YWHAE | YWHAG | YWHAH | YWHAQ | YWHAZ | GO:0031398 | 0.0110396700230499 | 91/8243 | 57/3250 | 5.79665464402233e-06 | 0.055039235844992 | T | T | T | T | positive regulation of protein ubiquitination | ADRB2 | AIMP2 | ARRB1 | ARRB2 | AXIN1 | BCL10 | BIRC2 | BIRC3 | BMI1 | BRCA1 | CAV1 | CDC14B | CDC20 | CDK9 | COMMD1 | CUL3 | DCUN1D1 | DERL1 | FANCI | FANCM | FKBP1A | FZR1 | GOLGA2 | GSK3A | HSPA5 | HSPBP1 | KDM1A | LRRK2 | MAPK9 | MTA1 | NOD2 | PAXIP1 | PDCD6 | PEF1 | PELI1 | PIN1 | PLK1 | PSMD10 | PTEN | PTPN22 | RAB1A | RIPK2 | RPS2 | SEPT4 | SPRTN | STUB1 | TRAF6 | TRIB3 | UBB | UBE2C | UBE2D1 | UBE2L3 | UBE2N | UBE2S | UBE3A | UBQLN1 | XIAP | GO:0022900 | 0.0129807109062235 | 107/8243 | 65/3250 | 5.9381742743515e-06 | 0.0563829647349675 | T | T | T | T | electron transport chain | ADH5 | AKR1A1 | AKR1B1 | ALDH4A1 | ALDH5A1 | AOC2 | COQ9 | COX11 | COX15 | COX5A | COX6A1 | COX6C | COX7A2 | CYC1 | CYCS | CYP1A2 | DEGS1 | DLD | ETFA | ETFB | ETFDH | GLRX | GLRX3 | GLRX5 | GSR | HAAO | HSD17B6 | IDH3B | LOXL2 | ME1 | MECP2 | MYBBP1A | NDUFA10 | NDUFA13 | NDUFA5 | NDUFA8 | NDUFA9 | NDUFAF1 | NDUFAF2 | NDUFB1 | NDUFS1 | NDUFS2 | NDUFS3 | NDUFS6 | NDUFS7 | NDUFS8 | NDUFV1 | NDUFV3 | NQO1 | NQO2 | P4HA2 | PHGDH | PPARGC1A | PTGES2 | QDPR | SDHA | SDHB | SDHD | SNCA | TAZ | TXNRD1 | UQCRB | UQCRC2 | UQCRFS1 | UQCRQ | GO:0007005 | 0.0264466820332403 | 218/8243 | 118/3250 | 6.05760060642387e-06 | 0.0575169177579946 | T | T | T | T | mitochondrion organization | ACAA2 | AFG3L2 | AGK | AKT1 | AKT3 | ALAS1 | ATAD3A | ATCAY | ATF2 | ATG2B | ATG5 | BAX | BBC3 | BCL2 | BCL2L1 | BCS1L | CALM1 | CALM3 | CAMK2A | CASP8 | CCAR2 | CDK5 | CEBPA | CEP89 | CHCHD10 | CHCHD2 | CHCHD3 | CISD2 | CLU | CLUH | COL4A3BP | COX7A2 | CYCS | DNAJA3 | DNM1L | E2F1 | ESRRA | EYA2 | FANCG | FBXO7 | FIS1 | FUNDC2 | GABPB1 | GGCT | GSK3A | GSK3B | HAP1 | HAX1 | HK2 | HMGCL | HSD17B10 | HSP90AA1 | HSPA4 | HSPD1 | HTRA2 | IMMT | JUN | LETM1 | LRRK2 | MAPK8 | MAPT | MTERF1 | NDUFA13 | NDUFS1 | NDUFS6 | NOS3 | OPA1 | OXA1L | PARK7 | PARP1 | PHB | PHB2 | POLDIP2 | PPARGC1A | PPIF | PPP2CB | PPP3CC | PRDX3 | RAB29 | RAB32 | RB1CC1 | RHOT1 | RHOT2 | ROMO1 | SEPT4 | SFN | SIRT3 | SIRT5 | SLC25A4 | SLC25A5 | SLC35F6 | SLC9A1 | SNCA | SOD2 | SQSTM1 | STAT3 | STOML2 | SYNJ2BP | TAZ | TFDP1 | THEM4 | TIMM13 | TIMM50 | TMEM11 | TOMM22 | TP53 | TP63 | TP73 | WDR45B | WIPI1 | WIPI2 | YME1L1 | YWHAB | YWHAE | YWHAG | YWHAH | YWHAQ | YWHAZ | GO:0044092 | 0.0823729224796797 | 679/8243 | 322/3250 | 6.09624114701667e-06 | 0.0578838096909232 | T | T | T | T | negative regulation of molecular function | A2M | ABCE1 | ABL1 | ACTB | ACTN2 | ADCYAP1 | ADORA2A | ADRB2 | AES | AHSG | AKAP9 | AKT1 | ANK3 | ANXA1 | ANXA2 | ANXA5 | APC | APCS | APOA1 | APOE | APP | ARRB1 | ARRB2 | AURKB | BAG2 | BAG4 | BAG5 | BAX | BIN1 | BIRC2 | BIRC3 | BIRC5 | BIRC7 | BIRC8 | BTRC | C4A | CAB39 | CALM1 | CALM2 | CALM3 | CAND1 | CARM1 | CASP3 | CASP8 | CAT | CAV1 | CBL | CBLB | CBLC | CCAR2 | CCDC22 | CCDC8 | CD109 | CD27 | CD2BP2 | CDKN1A | CDKN2C | CEBPA | CHORDC1 | CHP1 | COMMD1 | COX11 | CRMP1 | CRY2 | CRYAB | CSNK1E | CTNNBIP1 | CYLD | DAB2 | DBNDD2 | DDX3X | DHCR24 | DISC1 | DLG2 | DLG3 | DNAJA1 | DNAJB2 | DPEP1 | DUSP1 | DUSP14 | DUSP16 | DUSP19 | DUSP22 | DUSP7 | DVL1 | E2F1 | EGLN1 | EIF2AK4 | EIF2S1 | EIF4A3 | EPPIN | ERRFI1 | ESR1 | ESR2 | EZH2 | FABP1 | FAF1 | FAF2 | FAM192A | FBXO7 | FGFR1OP | FKBP1A | FKBP1B | FOXJ1 | G3BP2 | GAPDH | GATA1 | GLA | GNAI2 | GNAI3 | GNAQ | GOLGA2 | GOPC | GPI | GPS2 | GPSM1 | GPX1 | GSK3A | GSK3B | GSTO1 | GSTP1 | GTF2F1 | GZMA | HAVCR2 | HDAC2 | HDAC4 | HDAC6 | HDAC9 | HECW1 | HECW2 | HEXIM1 | HMOX1 | HNRNPU | HRAS | HSPA5 | HSPB1 | HSPBP1 | IBTK | ID2 | IFI16 | IFIT1 | IGBP1 | IGF1R | IKBKB | ILK | IPO5 | IPO7 | IQGAP1 | ITCH | ITGA4 | ITGB1BP1 | JUN | KCNAB1 | KDM1A | KEAP1 | LAMP3 | LATS1 | LEF1 | LGALS3 | LRP6 | LRRK2 | MAD2L1 | MAPK3 | MAPK7 | MAPK8 | MAPT | MCM2 | MDFI | MDM2 | MEPCE | MLLT1 | MTRR | MYO1D | NCK1 | NEDD4 | NF2 | NFKBIA | NOS3 | NOSIP | NPM1 | NQO1 | NR0B2 | OAZ3 | OXA1L | OXSR1 | PAK2 | PAM16 | PAPLN | PARK7 | PAXIP1 | PCSK9 | PDE6G | PEBP1 | PELI1 | PER2 | PEX19 | PHB2 | PIH1D1 | PIM1 | PIN1 | PINX1 | PKIG | PLA2G10 | PLAUR | PLK1 | POT1 | PPARG | PPARGC1A | PPIF | PPME1 | PPP1CA | PPP1R11 | PPP1R12A | PPP1R12B | PPP1R14A | PPP1R2 | PPP1R8 | PPP2CA | PPP2R1A | PRDX2 | PRDX3 | PRDX5 | PRKAR1A | PRKAR2B | PRKCD | PRKCE | PRKCZ | PRMT2 | PROX1 | PSEN1 | PSMD10 | PTEN | PTN | PTPN1 | PTPN22 | PTPN6 | PTPRF | PTTG1 | QARS | RAB9A | RAF1 | RB1 | RDX | RECK | RGN | RGS2 | RNF2 | RPL23 | RPS6KA1 | RPTOR | RTKN | RUVBL2 | SAG | SERPINB2 | SERPINB3 | SERPINB5 | SERPINB6 | SERPINB8 | SERPINE2 | SERPINH1 | SET | SFN | SIN3A | SIRT1 | SLC9A3R1 | SMO | SNCA | SNX6 | SOCS1 | SORT1 | SP100 | SPINK7 | SPINT2 | SPRY2 | SRC | SRI | STK38 | STMN1 | STUB1 | STYX | SUGT1 | SUMO1 | SUMO3 | TAOK3 | TERF1 | TERF2 | TFIP11 | THADA | THBS1 | TIMP1 | TIMP2 | TMBIM6 | TNF | TNFAIP3 | TNFSF14 | TNK2 | TNKS | TP53 | TRAF3 | TRIAP1 | TRIB2 | TRIB3 | TRIM21 | TSG101 | TWIST1 | TXNIP | UBQLN1 | UBXN1 | UCHL1 | UCHL5 | USP14 | USP17L2 | USP47 | USP7 | VTN | WARS | WDTC1 | WNK1 | WWP2 | XIAP | YWHAE | YWHAG | GO:0031325 | 0.245784301831857 | 2026/8243 | 883/3250 | 6.30825111312312e-06 | 0.059896844319104 | T | T | T | T | positive regulation of cellular metabolic process | ABAT | ABHD14B | ABL1 | ACACA | ACD | ACE2 | ACLY | ACSL1 | ACTA2 | ACTL6A | ACTN1 | ACTN2 | ACTN4 | ACTR2 | ACTR3 | ADCY6 | ADCYAP1 | ADRB2 | ADRM1 | ADTRP | AGAP2 | AGER | AGO1 | AGO2 | AHI1 | AICDA | AIFM1 | AIMP2 | AIRE | AKAP8 | AKAP9 | AKT1 | AKT2 | AKTIP | ALOX12 | ANKRD49 | ANXA1 | ANXA2 | APBB1 | APEX1 | APOA1 | APOE | APP | AR | ARAF | ARF4 | ARID3B | ARID4B | ARNT | ARNT2 | ARRB1 | ARRB2 | ASCL1 | ASPSCR1 | ASS1 | ATAD1 | ATAD5 | ATF2 | ATF6 | ATF6B | ATM | ATOH1 | ATRX | AURKB | AXIN1 | AZU1 | BABAM1 | BAG2 | BAG3 | BAG4 | BAG6 | BANP | BAX | BBC3 | BCAR3 | BCL10 | BCL2 | BCL2L13 | BCL6 | BIRC2 | BIRC3 | BIRC7 | BMI1 | BMPR1A | BRAF | BRCA1 | BRD4 | BTRC | BUD31 | C1QBP | C1QTNF1 | CAB39 | CALCOCO2 | CALM1 | CALM2 | CALM3 | CAND1 | CAPN2 | CARD9 | CARM1 | CASP10 | CASP3 | CASP8 | CASP9 | CAV1 | CBFA2T3 | CBFB | CBX8 | CCAR2 | CCDC22 | CCDC88A | CCL21 | CCL22 | CCNB1 | CCNT1 | CCNT2 | CCNY | CCNYL1 | CCR1 | CCT2 | CCT3 | CCT4 | CCT5 | CCT6A | CCT7 | CCT8 | CD27 | CD36 | CD40 | CD40LG | CD74 | CD81 | CDC14B | CDC20 | CDC25B | CDC37 | CDC5L | CDC73 | CDH1 | CDK2 | CDK4 | CDK5 | CDK5R1 | CDK8 | CDK9 | CDKN1A | CEBPA | CEBPE | CEP290 | CHCHD10 | CHCHD2 | CHD8 | CHEK2 | CHP1 | CIRBP | CKS1B | CKS2 | CLPX | CLU | COA3 | COMMD1 | COMT | COPS5 | COX17 | CPT1A | CREB1 | CREB3 | CREBBP | CRK | CRKL | CRP | CSNK1A1 | CSNK1E | CSNK2B | CTBP1 | CTCF | CTNNB1 | CTSC | CUL3 | CYCS | DAB1 | DAB2 | DAPK1 | DAXX | DAZAP1 | DBF4B | DBNL | DCN | DCUN1D1 | DDRGK1 | DDX39B | DDX3X | DERL1 | DGKZ | DHX36 | DHX9 | DIABLO | DLG3 | DLG4 | DNA2 | DNAJB2 | DOCK7 | DPF2 | DTX1 | DUSP12 | DUSP19 | DUSP22 | DUSP7 | DVL1 | DVL2 | DVL3 | DYRK2 | E2F1 | E2F2 | E2F3 | E2F4 | E2F6 | EAF2 | EBF1 | ECD | EDEM1 | EED | EEF1A2 | EEF2 | EGFR | EGLN1 | EGLN3 | EHD4 | EIF2AK3 | EIF2AK4 | EIF2B5 | EIF3D | EIF3E | EIF4A3 | EIF5A | EIF6 | ELAVL1 | ELF3 | ELF4 | ENDOG | ENO1 | EP300 | EPHA4 | EPM2A | ERBB2 | ERBB3 | ERG | ERLIN2 | ESR1 | ESR2 | ESRRA | ESRRB | ESRRG | ETS1 | EXOSC6 | EXOSC9 | EYA2 | EYA4 | EZH2 | EZR | FABP1 | FABP3 | FADD | FAF1 | FAM129B | FANCI | FANCM | FASLG | FASN | FBXO7 | FBXW11 | FGB | FGF2 | FGFR1 | FGFR3 | FHL2 | FIS1 | FKBP1A | FLOT1 | FN1 | FOS | FOXA1 | FOXJ1 | FOXK1 | FOXK2 | FOXM1 | FOXO1 | FTO | FUBP3 | FUS | FXR2 | FYN | FZR1 | GABPB1 | GADD45G | GAPDHS | GATA1 | GDF9 | GLMN | GNAI2 | GNAI3 | GOLGA2 | GPD1 | GPS2 | GPSM1 | GRB2 | GRIP1 | GSK3A | GSK3B | GSTP1 | GTF2A1 | GTF2F1 | GTF2F2 | H1F0 | H1FX | H2AFX | H2AFZ | HAVCR2 | HAX1 | HBEGF | HDAC1 | HDAC2 | HDAC3 | HDAC4 | HDAC5 | HDAC6 | HELZ2 | HEXB | HIF1A | HIST1H1A | HK2 | HMGB1 | HMGB2 | HMGB3 | HMOX1 | HNF1A | HNF4A | HNF4G | HNRNPA1 | HNRNPA2B1 | HNRNPAB | HNRNPD | HNRNPK | HNRNPL | HNRNPU | HOXA5 | HOXB5 | HOXB9 | HOXD10 | HPRT1 | HPX | HRAS | HSF1 | HSF4 | HSP90AA1 | HSP90AB1 | HSPA1B | HSPA2 | HSPA5 | HSPB1 | HSPBP1 | HSPD1 | HSPE1 | HTATIP2 | HTRA2 | HTT | ICAM1 | ID2 | IFI16 | IFNA2 | IGF1R | IKBKB | IKBKG | IKZF5 | ILF2 | ILF3 | ILK | ING1 | ING5 | INSIG1 | INSIG2 | INSR | IQGAP1 | IRF1 | IRF4 | IRF7 | ITCH | ITGB1BP1 | ITGB3 | JMJD6 | JUN | JUNB | JUND | JUP | KAT5 | KAT7 | KAT8 | KDM1A | KDM4D | KDM8 | KDR | KEAP1 | KHDRBS1 | KLF5 | KLF6 | KLHDC10 | KMT2A | KMT2B | KMT2C | KRAS | KSR1 | LARP4 | LAT | LATS1 | LCP2 | LEF1 | LGMN | LGR4 | LMNA | LMO2 | LMO7 | LPAR1 | LPIN3 | LRP6 | LRRK1 | LRRK2 | LRSAM1 | LTBR | LUM | MAP2K1 | MAP2K3 | MAP2K4 | MAP2K6 | MAP2K7 | MAP3K1 | MAP3K13 | MAP3K14 | MAP3K2 | MAP3K3 | MAP3K4 | MAP4K1 | MAPK1 | MAPK14 | MAPK15 | MAPK3 | MAPK7 | MAPK8IP3 | MAPK9 | MAPKAP1 | MAPT | MAS1 | MAVS | MBP | MC1R | MDM2 | MECP2 | MED1 | MED12 | MED14 | MED17 | MED20 | MED21 | MED23 | MED24 | MED31 | MED4 | MED6 | MEOX2 | METTL23 | MGMT | MIF | MITF | MLH1 | MLLT11 | MLLT3 | MLST8 | MOB1B | MORF4L2 | MOV10 | MRPL12 | MSH6 | MSN | MTA1 | MTA3 | MYC | MYO6 | NAMPT | NANOG | NCBP1 | NCK1 | NCOA1 | NCOA2 | NCOA3 | NCOA4 | NDUFA13 | NEK1 | NEK7 | NELFE | NEUROD1 | NFATC1 | NFATC2 | NFE2L2 | NFKB2 | NFKBIA | NIF3L1 | NIPBL | NKRF | NKX2-1 | NMD3 | NME2 | NMNAT1 | NNT | NOD2 | NOS1AP | NOS2 | NOS3 | NPM1 | NQO2 | NR1H2 | NR1H3 | NR1I2 | NR3C1 | NR4A1 | NR5A2 | NTF3 | NTRK1 | NUFIP1 | NUP85 | NUP98 | NUPR1 | OGT | ONECUT1 | OPTN | OTUD4 | OXSR1 | P2RX1 | P2RX4 | PABPC1 | PACSIN3 | PAIP1 | PAK2 | PAK6 | PARK7 | PARP1 | PARP14 | PATZ1 | PAWR | PAX8 | PAX9 | PAXIP1 | PCBD1 | PCBP1 | PCGF5 | PCNA | PCSK9 | PDCD10 | PDCD6 | PDE6G | PEBP1 | PEF1 | PELI1 | PER1 | PER2 | PFKM | PGK1 | PGK2 | PHB | PHB2 | PHF10 | PIAS1 | PIAS3 | PICK1 | PIH1D1 | PIK3CA | PIK3CG | PIK3R1 | PIK3R3 | PIN1 | PIP4K2A | PLA2G10 | PLA2G1B | PLA2G2A | PLA2G6 | PLAUR | PLEKHF1 | PLGRKT | PLK1 | PLSCR1 | PML | PNKP | POLDIP3 | POLR2A | POT1 | POU2F3 | POU5F1 | PPARD | PPARG | PPARGC1A | PPM1A | PPP1R12A | PPP2CA | PPP2R5D | PRDX2 | PRDX6 | PRELID1 | PRKAA1 | PRKAA2 | PRKACA | PRKAG1 | PRKAR1A | PRKAR2B | PRKCD | PRKCE | PRKCZ | PRKD1 | PRKD2 | PRKDC | PRMT2 | PROX1 | PRPF19 | PRRC1 | PSAP | PSEN1 | PSMC1 | PSMC2 | PSMC3 | PSMC4 | PSMC5 | PSMC6 | PSMD10 | PSMD14 | PSMD9 | PSME2 | PSME3 | PTEN | PTGES2 | PTGES3 | PTK2 | PTPN1 | PTPN22 | PUM1 | RAB1A | RAB1B | RAB29 | RAB3GAP1 | RAC1 | RAD23A | RAD51 | RAF1 | RAN | RAP1A | RAP1B | RARA | RARG | RB1 | RB1CC1 | RBM3 | RBMX | RBPJ | RBX1 | RC3H1 | RDX | REL | RELA | REST | RFC1 | RFC2 | RFC3 | RFC4 | RFC5 | RFXANK | RGN | RHBDD1 | RHOA | RIF1 | RIOK1 | RIPK1 | RIPK2 | RIPK3 | RMND1 | RNF139 | RNF185 | RNF19A | RNF4 | RNF8 | ROMO1 | RPLP1 | RPS2 | RPS3 | RPS4X | RPS6KA1 | RPS6KB2 | RPTOR | RTF1 | RUNX1 | RUNX2 | RUVBL1 | RUVBL2 | RXRA | RXRB | RXRG | S100A12 | S100A7 | S100A9 | SART3 | SCYL2 | SDC4 | SDCBP | SEMA7A | SEPT4 | SERPINB3 | SFPQ | SGTA | SHC1 | SIN3A | SIRT1 | SIRT6 | SIRT7 | SKI | SLAMF1 | SLC9A1 | SMAD2 | SMAD3 | SMAD4 | SMAD5 | SMAD9 | SMARCA2 | SMARCA4 | SMARCB1 | SMARCC1 | SMARCC2 | SMARCE1 | SMO | SMURF1 | SNAI2 | SNCA | SNW1 | SOAT1 | SOD1 | SOX2 | SP1 | SP100 | SPI1 | SPIN1 | SPRTN | SPRY2 | SPTLC2 | SQSTM1 | SRC | SRSF5 | STAT1 | STAT3 | STAT4 | STAT6 | STK11 | STK24 | STK25 | STK26 | STK3 | STOML2 | STUB1 | SUB1 | SUMO1 | SUMO2 | SUPT4H1 | SUPT5H | TAB1 | TAB2 | TADA2B | TAF12 | TAF15 | TANK | TAOK3 | TARDBP | TAZ | TBL1X | TCEA1 | TCEA2 | TCERG1 | TCF12 | TCF21 | TCF4 | TCP1 | TDRD3 | TERF2IP | TFCP2 | TFDP1 | TGFB1 | THBS1 | THRA | TIMP2 | TLR3 | TMEM173 | TMTC3 | TNF | TNFAIP3 | TNFRSF1A | TNFRSF8 | TNIP2 | TNK2 | TNKS | TNKS2 | TNP1 | TOLLIP | TOP2A | TP53 | TP63 | TP73 | TRA2A | TRADD | TRAF2 | TRAF6 | TRIAP1 | TRIB2 | TRIB3 | TRIM21 | TRIM28 | TRIM32 | TRIP4 | TRUB2 | TSG101 | TTC5 | TWIST1 | TXN | U2AF2 | UBB | UBC | UBE2B | UBE2C | UBE2D1 | UBE2I | UBE2K | UBE2L3 | UBE2N | UBE2S | UBE2V1 | UBE3A | UBQLN1 | UBQLN2 | ULK1 | UNC119 | UPF3B | UQCC1 | USP16 | USP21 | USP5 | USP50 | USP7 | USP9X | VCP | VDR | VHL | VIM | VTN | WAS | WBP2 | WDR35 | WDR48 | WDR77 | WNK1 | WNT16 | WNT2 | WT1 | WWOX | WWP2 | XBP1 | XIAP | XRCC1 | XRCC5 | XRCC6 | YAF2 | YAP1 | YES1 | YTHDF1 | YWHAH | YY1 | ZBTB16 | ZBTB17 | ZBTB7B | ZIC1 | ZNF24 | ZNF622 | ZNF746 | GO:2001252 | 0.016984107727769 | 140/8243 | 81/3250 | 6.73304128030686e-06 | 0.0639302269565137 | T | T | T | T | positive regulation of chromosome organization | ACD | AKAP8 | ANAPC4 | ARRB1 | ATM | ATRX | AURKB | BCL6 | BRCA1 | BRD4 | CCNB1 | CCT2 | CCT3 | CCT4 | CCT5 | CCT6A | CCT7 | CCT8 | CDK9 | CTBP1 | CTNNB1 | CUL3 | DHX36 | EED | FEN1 | HNRNPA1 | HNRNPA2B1 | HNRNPD | HNRNPU | KAT7 | KDM1A | KMT2A | LIG4 | LMNA | LRRK2 | MAP2K7 | MAP3K4 | MAPK1 | MAPK15 | MAPK3 | MECP2 | NEK7 | NELFE | NIPBL | NUMA1 | OGT | PAXIP1 | PIH1D1 | PML | PNKP | POT1 | PPARGC1A | PRKD1 | PRKD2 | RAD21 | RB1 | RIF1 | RTF1 | RUVBL2 | SART3 | SETDB1 | SFPQ | SIN3A | SIRT1 | SIRT6 | SMAD4 | SMARCB1 | SNAI2 | SNW1 | TADA2B | TCP1 | TERF1 | TGFB1 | TNKS | TNKS2 | TPR | TRIM28 | UBE2N | WBP2 | XRCC5 | ZBTB7B | GO:0034654 | 0.0835860730316632 | 689/8243 | 326/3250 | 6.80085767409264e-06 | 0.0645741436155096 | T | T | T | T | nucleobase-containing compound biosynthetic process | ACACA | ACAT1 | ACLY | ACOT7 | ACSL1 | ACSL4 | ACSL5 | ACSS2 | ADA | ADCY6 | ADK | ADRM1 | ADSL | ADSS | AFF4 | AK1 | AK2 | AK4 | ALDOA | AMD1 | AMDHD2 | APRT | AR | ARHGAP35 | ARNT | ARRB1 | ARRB2 | ASPDH | ATF6B | ATIC | ATOH1 | BAX | CBFB | CBR4 | CCNB1 | CCNT1 | CCNT2 | CDA | CDC73 | CDK4 | CDK8 | CDK9 | CDKN1A | CEBPA | CENPF | CHRAC1 | CMPK1 | COL4A2 | COPS2 | COPS5 | CPSF7 | CREB1 | CREBBP | CSTF2T | CTDP1 | CTPS1 | CYC1 | DCAKD | DCK | DCTPP1 | DERA | DGUOK | DHX9 | DIDO1 | DLAT | DLD | DNAJC30 | DTX1 | DUT | DVL1 | DVL2 | E2F1 | E2F2 | E2F3 | EAF2 | ECD | ELF3 | ESR1 | ESR2 | ESRRA | ESRRB | ESRRG | ETS1 | FASN | FOS | FUBP1 | FUBP3 | GAR1 | GARS | GART | GATA1 | GCDH | GDA | GFPT1 | GFPT2 | GMDS | GMPPB | GMPR | GMPS | GNPDA1 | GNPDA2 | GNPNAT1 | GPX1 | GTF2A1 | GTF2E2 | GTF2F1 | GTF2F2 | GTF2I | GTF3C2 | HAAO | HIF1A | HINT1 | HIST1H4A | HNF4A | HNF4G | HOXB9 | HPRT1 | HSD17B8 | HSF1 | HSP90AA1 | HSP90AB1 | IDH2 | ILF2 | IMPDH2 | IRF1 | IRF7 | ISG15 | ITPA | JUN | KLF5 | KMT2A | KYNU | LDHC | LEF1 | LIG4 | LMO2 | MAPK1 | MAPK3 | ME1 | MECP2 | MED1 | MED12 | MED14 | MED17 | MED20 | MED21 | MED23 | MED24 | MED31 | MED4 | MED6 | MITF | MLLT1 | MLLT3 | MPC2 | MPI | MRPL12 | MTAP | MTERF1 | MTHFD1 | NAGK | NAMPT | NANS | NAPRT | NCBP1 | NCOA1 | NCOR1 | NELFB | NELFCD | NELFE | NFATC1 | NFE2L2 | NME1 | NME2 | NME4 | NMNAT1 | NMNAT3 | NMRK2 | NOP10 | NPLOC4 | NPR1 | NR0B2 | NR1H2 | NR1H3 | NR1I2 | NR3C1 | NR4A1 | NR5A2 | NRBF2 | NT5C2 | NUDT18 | NUDT3 | NUDT9 | PAICS | PAPSS1 | PAPSS2 | PARP1 | PAX8 | PCF11 | PCNA | PDE4D | PDHA1 | PDHA2 | PDHB | PDHX | PFAS | PGM3 | PHB | PINX1 | PIR | PNKP | PNP | POLA2 | POLD1 | POLE2 | POLE3 | POLM | POLR2A | POLR2E | POLR2L | POLR2M | POT1 | POU5F1 | PPARD | PPARG | PPARGC1A | PPAT | PPIA | PPM1D | PPP5C | PPT1 | PRTFDC1 | PTEN | PTGES3 | PTMA | PTTG1 | RARA | RARG | RBMX | RBPJ | RELA | RFC1 | RFC2 | RFC3 | RFC4 | RFC5 | RFK | RPA2 | RPA3 | RRM1 | RRM2 | RRM2B | RTF1 | RUNX2 | RXRA | RXRB | RXRG | SARS | SCAF1 | SHMT1 | SIRT1 | SIRT7 | SKI | SLC25A1 | SLC25A13 | SMAD4 | SMG5 | SMG6 | SMN1 | SNRPB | SNRPD3 | SNRPE | SNW1 | SP1 | SPI1 | SPIN1 | SPRTN | STAT3 | STOML2 | SUPT4H1 | SUPT5H | TAF12 | TAF15 | TAF1A | TAMM41 | TARDBP | TCEA1 | TCEA2 | TCERG1 | TCF4 | TECR | TERF1 | TERF2 | TERF2IP | TFCP2 | TGFB1 | THRA | TK1 | TK2 | TP53 | TREX1 | TRIM25 | TRIM28 | TRIM29 | TRIP13 | TSC22D1 | TYMS | UAP1 | UAP1L1 | UBB | UBC | UBE2L6 | UCK2 | UGDH | UGP2 | UMPS | UPP2 | USPL1 | UXS1 | VCP | VDR | XBP1 | YAP1 | ZBTB7A | ZBTB7B | ZNF217 | GO:0043535 | 0.00824942375348781 | 68/8243 | 45/3250 | 6.85692533923504e-06 | 0.0651065060960367 | T | T | T | T | regulation of blood vessel endothelial cell migration | ABL1 | AKT1 | AKT3 | ALOX12 | ANXA1 | APOE | CD40 | CSNK2B | ETS1 | FGF2 | FGFR1 | HDAC5 | HDAC7 | HDAC9 | HIF1A | HMGB1 | HMOX1 | HSPB1 | ITGB1BP1 | JUP | KDR | MAP2K3 | MAP3K3 | MAPK14 | MECP2 | MEOX2 | NFE2L2 | NOS3 | P2RX4 | PDCD10 | PIK3C2A | PLCG1 | PPARG | PRCP | PRKD1 | PRKD2 | RHOA | RHOJ | SIRT1 | SP1 | STARD13 | STAT5A | TBXA2R | TGFB1 | THBS1 | GO:0019725 | 0.057867281329613 | 477/8243 | 234/3250 | 6.99743733886404e-06 | 0.066440667532514 | T | T | T | T | cellular homeostasis | ABCB7 | ABL1 | ABL2 | ACD | ACKR2 | ACO1 | ADCYAP1 | AFG3L2 | ALDOA | ANXA2 | ANXA6 | ANXA7 | APEX1 | APOE | APP | ARF1 | ATG5 | ATOX1 | ATP12A | ATP13A2 | ATP1A1 | ATP1B3 | ATP2A2 | ATP2B2 | ATP4A | ATP6V1A | ATP6V1B1 | ATP6V1C1 | ATP6V1D | ATP6V1E1 | BAG3 | BAX | BCL2 | BDKRB2 | C1QTNF1 | CACNA1A | CALM1 | CALM2 | CALM3 | CALR | CAND1 | CAV1 | CCDC22 | CCL21 | CCR1 | CCR3 | CCR4 | CCR5 | CD36 | CD40 | CDK5 | CFL2 | CFTR | CHP1 | CHRNA9 | CKB | CLIC4 | CLNS1A | COX19 | CUL1 | CXCL11 | DCTN1 | DLD | DLG4 | DMD | DNA2 | DNAJB2 | DNAJC10 | E2F4 | EGLN1 | ENDOG | ESR1 | FASLG | FEN1 | FGF2 | FIS1 | FKBP1B | FOXO1 | FTH1 | FTL | GAA | GLP1R | GLRX | GLRX3 | GLRX5 | GNA13 | GNA15 | GNB1 | GOT1 | GPX1 | GRIA1 | GRIK2 | GSR | GSTO1 | GTF2I | HAAO | HAP1 | HEXB | HIF1A | HK1 | HK2 | HMGB1 | HMOX1 | HPX | HTR1B | HTT | IBTK | ICAM1 | IDE | IGF1R | IMMT | IREB2 | ITGAV | ITPR1 | ITPR2 | ITPR3 | KCTD17 | LETM1 | LGALS1 | LPAR1 | LPAR2 | LPAR4 | LRP6 | LRRK2 | MAPK1 | MAPK3 | MCOLN3 | MICU1 | MICU2 | MYC | MYO5A | NCOA4 | NEDD8 | NEUROD1 | NFE2L2 | NMB | NME1 | NME2 | NNT | NOS2 | NOS3 | NUBP1 | OPA1 | P2RX1 | P2RX2 | P2RX4 | P4HB | PCNA | PDIA3 | PDIA4 | PDIA6 | PFKM | PIK3CA | PIK3CG | PIK3R1 | PLA2G1B | PLA2G6 | PLCG1 | PML | POLA2 | POLD1 | POLE2 | POLE3 | PPARGC1A | PPT1 | PRDX1 | PRDX2 | PRDX3 | PRDX4 | PRDX5 | PRDX6 | PRKAA1 | PRKAA2 | PRKACA | PRKCE | PSEN1 | PTGER3 | PTGES2 | PTGIR | QSOX1 | RAB7A | RAP1A | RAP1GDS1 | RFC1 | RFC2 | RFC3 | RFC4 | RFC5 | RGN | RHOT1 | RHOT2 | RIC3 | RPA2 | RPA3 | RTN4 | SCO2 | SIN3A | SIRT1 | SLAMF8 | SLC12A2 | SLC39A4 | SLC9A1 | SLC9A3R1 | SMAD3 | SMAD4 | SNCA | SOD1 | SRI | STIM1 | STOML2 | SWAP70 | TBXA2R | TCIRG1 | TGFB1 | TGM2 | THADA | TM9SF4 | TMBIM6 | TMX2 | TMX4 | TPCN2 | TRIM32 | TXN | TXN2 | TXNDC11 | TXNDC15 | TXNDC5 | TXNRD1 | VAPB | VDR | WDR35 | WDTC1 | XBP1 | YWHAE | GO:0031324 | 0.191799102268592 | 1581/8243 | 700/3250 | 7.12218642428435e-06 | 0.0676251600985799 | T | T | T | T | negative regulation of cellular metabolic process | A2M | ABCE1 | ABL1 | ACADVL | ACD | ADORA2A | AEBP2 | AES | AGO1 | AGO2 | AHSG | AKT1 | ANXA2 | APBB1 | APC | APCS | APEX1 | APOD | APOE | APP | AR | ARHGAP35 | ARRB1 | ARRB2 | ASCL1 | ATCAY | ATF2 | ATG5 | ATM | ATP13A2 | ATP1A1 | ATRX | ATXN1 | ATXN2 | AURKB | BACH2 | BAG2 | BAG3 | BAG4 | BAG5 | BAG6 | BASP1 | BAX | BCL11A | BCL2 | BCL6 | BCL6B | BCOR | BDKRB2 | BIN1 | BIRC2 | BIRC3 | BIRC5 | BIRC7 | BIRC8 | BMI1 | BRCA1 | BTRC | C1QBP | C1QL4 | C4A | CALM1 | CALM2 | CALM3 | CALR | CASP3 | CAV1 | CBFA2T3 | CBFB | CBL | CBLB | CBLC | CBX4 | CBX7 | CBX8 | CBY1 | CCAR2 | CCDC8 | CCNB1 | CCNT1 | CD109 | CD27 | CD2BP2 | CD36 | CDA | CDC73 | CDK2 | CDK5 | CDK5R1 | CDK9 | CDKN1A | CDKN2C | CEBPA | CENPF | CENPJ | CHCHD3 | CHD8 | CHEK2 | CHORDC1 | CHP1 | CHRNA9 | CIRBP | CLU | COMT | COPS2 | COX11 | CREB1 | CREBBP | CRKL | CRY1 | CRY2 | CRYAB | CSNK2A2 | CTBP1 | CTCF | CTNNB1 | CTNNBIP1 | CTSA | CUL3 | CYLD | DAB2 | DAPK1 | DAXX | DBNDD2 | DCP2 | DDIT4 | DDRGK1 | DDX3X | DHCR24 | DHX36 | DHX9 | DLG2 | DLG3 | DLG4 | DMAP1 | DMD | DNAJA1 | DNAJA3 | DNAJB1 | DNAJC10 | DPEP1 | DPF2 | DUSP1 | DUSP14 | DUSP16 | DUSP19 | DUSP22 | DUSP7 | DVL1 | DYNLL1 | E2F1 | E2F2 | E2F3 | E2F4 | E2F6 | EED | EHMT1 | EHMT2 | EID1 | EIF2AK3 | EIF2AK4 | EIF2S1 | EIF3A | EIF3E | EIF4A3 | EIF4E | EIF4E2 | EIF4EBP1 | EIF4ENIF1 | EIF4G3 | EIF6 | ELAVL1 | ELF3 | ENO1 | EP300 | EPHA4 | EPPIN | EPRS | ERF | ERLIN2 | ERRFI1 | ESR1 | ESR2 | ESRRA | EZH2 | EZR | FABP1 | FAM129B | FAM192A | FAM3C | FASLG | FBP1 | FBXO7 | FBXW11 | FGFR1 | FGFR1OP | FHL2 | FKBP1A | FKBP1B | FKBP8 | FOXA1 | FOXJ1 | FOXK1 | FOXM1 | FOXO1 | FTO | FXR2 | FYN | G6PD | GAPDH | GATA1 | GLA | GLMN | GNAI2 | GNAQ | GOLGA2 | GPD1L | GPI | GPS2 | GPX1 | GRB7 | GSK3A | GSK3B | GSTP1 | GZMA | H1F0 | H1FX | H3F3A | HAP1 | HBEGF | HBZ | HDAC1 | HDAC2 | HDAC3 | HDAC4 | HDAC5 | HDAC6 | HDAC7 | HDAC9 | HDGF | HERC1 | HEXIM1 | HIF1A | HIF1AN | HIST1H1A | HIST1H3A | HIST1H4A | HIST2H3A | HIVEP1 | HK2 | HMGB1 | HMGB2 | HMOX1 | HNRNPA1 | HNRNPA2B1 | HNRNPD | HNRNPK | HNRNPL | HNRNPU | HSF1 | HSF4 | HSP90AB1 | HSP90AB3P | HSP90AB4P | HSPA1B | HSPA8 | HSPB1 | HTRA2 | IBTK | ID2 | IDE | IFI16 | IFNA2 | IGBP1 | IGF1R | IGF2BP1 | IKBKB | IKZF5 | ILF3 | ILK | INSIG1 | INSIG2 | IPO5 | IPO7 | IQGAP1 | IREB2 | IRF1 | IRF7 | ISG15 | ITCH | ITGAV | ITGB1BP1 | ITGB3 | JAK3 | JUN | JUNB | JUND | KANK2 | KAT5 | KAT7 | KAT8 | KDM1A | KDM4D | KDM5B | KDM5C | KHDRBS1 | KLF5 | KLHL22 | KMT2A | L3MBTL1 | LAMP3 | LARS | LATS1 | LDLRAD4 | LEF1 | LEMD2 | LGALS3 | LGR4 | LIMS1 | LOXL2 | LRP6 | LRPPRC | LRRK1 | LRRK2 | MAD2L1 | MAF1 | MAPK14 | MAPT | MAS1 | MBD3 | MDFI | MDM2 | MDM4 | MECP2 | MED1 | MEPCE | MIF | MITF | MLH1 | MLLT1 | MORF4L1 | MORF4L2 | MOV10 | MSH6 | MTA1 | MTA3 | MXD1 | MYBBP1A | MYC | MYD88 | MYO1D | NACC1 | NAMPT | NANOG | NBAS | NCK1 | NCOA2 | NCOR1 | NCOR2 | NDUFA13 | NEDD4 | NELFB | NELFCD | NELFE | NELL1 | NF2 | NFATC2 | NIF3L1 | NIPBL | NKRF | NKX2-1 | NMNAT1 | NNT | NOSTRIN | NPM1 | NR0B2 | NR1H2 | NR1H3 | NR1I2 | NR3C1 | NTF3 | OGT | OPTN | OTUB1 | PA2G4 | PABPC1 | PAFAH1B1 | PAIP1 | PAK2 | PAM16 | PAPLN | PARD3 | PARD6A | PARK7 | PARP1 | PARP14 | PATZ1 | PAWR | PAX9 | PCGF1 | PCGF6 | PCSK9 | PDE12 | PDE4D | PEBP1 | PER1 | PER2 | PGP | PHB | PHB2 | PHF10 | PHF21A | PIAS1 | PIAS3 | PIH1D1 | PIK3CA | PIK3CG | PIN1 | PINX1 | PKIG | PLAUR | PLK1 | PML | PNKP | POLDIP2 | POT1 | POU5F1 | PPARD | PPARG | PPARGC1A | PPID | PPIF | PPM1A | PPM1D | PPME1 | PPP1R11 | PPP1R14A | PPP1R2 | PPP1R8 | PPP2CA | PPP2R1A | PPP2R5D | PRDX2 | PRDX3 | PRDX5 | PRKAA1 | PRKAA2 | PRKAR1A | PRKAR2B | PRKCD | PRKCE | PRKCZ | PRKDC | PRMT2 | PRMT3 | PRMT6 | PROX1 | PSEN1 | PSMC5 | PSMD10 | PTEN | PTK2 | PTN | PTPN1 | PTPN22 | PTPN6 | PTPRK | PTPRR | PTTG1 | PUM1 | PUS7 | QARS | QSOX1 | RAF1 | RANBP9 | RARA | RARG | RASD1 | RB1 | RBM3 | RBM4 | RBMX | RBPJ | RC3H1 | RECK | REL | RELA | REST | RFC1 | RGN | RGS2 | RHOA | RIF1 | RING1 | RNF139 | RNF2 | RNF8 | RPL10 | RPL23 | RPS13 | RPS14 | RPS3 | RPS6KA1 | RPTOR | RTF1 | RUNX1 | RUNX1T1 | RUNX2 | RUVBL2 | RXRA | S100A1 | S100A11 | SAG | SAP18 | SARS | SCFD1 | SDCBP | SERPINB2 | SERPINB3 | SERPINB5 | SERPINB6 | SERPINB8 | SERPINE2 | SERPINH1 | SET | SF1 | SFMBT1 | SFN | SFPQ | SGTA | SHC1 | SHMT1 | SIN3A | SIRT1 | SIRT3 | SIRT5 | SIRT6 | SIRT7 | SKI | SLC9A3R1 | SMAD2 | SMAD3 | SMAD4 | SMAD5 | SMARCA2 | SMARCA4 | SMARCB1 | SMARCC1 | SMARCC2 | SMARCE1 | SMC1A | SMC3 | SMG6 | SMO | SMURF2 | SNAI2 | SNCA | SNTA1 | SNW1 | SNX12 | SNX6 | SOCS1 | SOX2 | SP100 | SP2 | SPEN | SPI1 | SPINK7 | SPINT2 | SPRY2 | SQSTM1 | SRC | SRSF12 | STAG2 | STAT1 | STAT3 | STAT6 | STK38 | STRAP | STYX | SUMO1 | SUPT4H1 | SUPT5H | SUZ12 | SWAP70 | SYNJ2BP | TAB2 | TAGLN3 | TAOK3 | TARDBP | TBL1X | TCERG1 | TCF21 | TCF25 | TCP10L | TERF1 | TERF2 | TERF2IP | TFIP11 | TGFB1 | THAP11 | THAP7 | THBS1 | THRA | TIA1 | TIMP1 | TIMP2 | TINF2 | TLE1 | TLE3 | TMBIM6 | TNF | TNFAIP3 | TNFSF14 | TNKS | TNKS2 | TNP1 | TP53 | TP63 | TPR | TRAF3IP1 | TRAF6 | TRAP1 | TRIAP1 | TRIB2 | TRIB3 | TRIM21 | TRIM28 | TRIM29 | TRIM33 | TRIP12 | TSC22D4 | TSG101 | TSPYL2 | TWIST1 | TXN | TXNIP | TYMS | U2AF2 | UBA3 | UBB | UBC | UBE2B | UBE2D1 | UBE2D3 | UBE2I | UBE2J1 | UBQLN2 | UBQLN4 | UBR5 | UBXN1 | UCHL1 | UCHL5 | UNK | USP14 | USP17L2 | USP19 | USP2 | USP4 | USP47 | USP7 | USP9X | VDAC1 | VDR | VHL | VPS72 | VTN | WARS | WDR35 | WDTC1 | WNK1 | WT1 | WWOX | WWP2 | XIAP | XPO1 | XRCC1 | XRCC5 | XRCC6 | YAF2 | YAP1 | YEATS2 | YWHAB | YWHAE | YWHAG | YWHAQ | YY1 | ZBTB16 | ZBTB21 | ZBTB33 | ZBTB42 | ZBTB7A | ZBTB7B | ZC3H4 | ZHX1 | ZMPSTE24 | ZMYND8 | ZNF217 | ZNF24 | ZNF746 | GO:0043536 | 0.0053378624287274 | 44/8243 | 32/3250 | 7.3585683226232e-06 | 0.0698696062233073 | T | T | T | T | positive regulation of blood vessel endothelial cell migration | ABL1 | AKT1 | AKT3 | ALOX12 | ANXA1 | CD40 | ETS1 | FGF2 | FGFR1 | HDAC7 | HDAC9 | HIF1A | HMGB1 | HMOX1 | HSPB1 | KDR | MAP2K3 | MAP3K3 | MAPK14 | NFE2L2 | NOS3 | P2RX4 | PIK3C2A | PLCG1 | PRKD1 | PRKD2 | RHOJ | SIRT1 | SP1 | STAT5A | TGFB1 | THBS1 | GO:0055086 | 0.0270532573092321 | 223/8243 | 120/3250 | 7.38396986316284e-06 | 0.0701107938507312 | T | T | T | T | nucleobase-containing small molecule metabolic process | ABHD14B | ACACA | ACAT1 | ACLY | ACOT13 | ACOT7 | ACOT9 | ADA | ADCYAP1 | ADK | ADSS | AHCY | AICDA | AK1 | AK2 | AK4 | ALDH6A1 | AMD1 | APRT | ASPDH | ATIC | ATP6V1A | ATP6V1B1 | CDA | CLPX | CMPK1 | CROT | CTPS1 | DBI | DCK | DCTPP1 | DCXR | DERA | DGUOK | DLG2 | DLST | DUT | ENTPD7 | EXTL2 | FAR2 | FASN | FIGNL1 | G6PD | GDA | GFPT1 | GFPT2 | GLRX | GMDS | GMPR | GMPS | GNAI3 | GPD1L | GPX1 | GSR | HINT1 | HMGCL | HPRT1 | HSD17B4 | HSPA1B | HSPA8 | IDH1 | IDH2 | IMPDH2 | ITPA | KCNAB2 | KDM1A | LDHA | LRRK2 | MAPK1 | MDH1 | MDH2 | MTAP | MVD | MYH6 | NDUFS1 | NME1 | NME2 | NME4 | NMNAT1 | NMNAT3 | NMRK2 | NNT | NT5C2 | NUDT13 | NUDT18 | NUDT3 | NUDT9 | OGDH | OLA1 | OPA1 | PARK7 | PARP1 | PDE4D | PNKP | PNP | PPAT | PRTFDC1 | RAN | REXO2 | RHOA | RNASEH2B | RRM1 | RRM2 | RRM2B | SCO2 | SUCLA2 | SUCLG2 | SULT1A3 | THEM4 | TK1 | TK2 | TPST2 | TXNRD1 | TYMS | UCK2 | UGGT1 | UGP2 | UMPS | UPP2 | VCP | GO:0043170 | 0.437583404100449 | 3607/8243 | 1518/3250 | 7.4714167674718e-06 | 0.0709411022071447 | T | T | T | T | macromolecule metabolic process | A2M | AARS | AARSD1 | ABCG1 | ABHD17A | ABL1 | ABL2 | ACACA | ACD | ACE2 | ACP1 | ACTB | ACTL6A | ACTN2 | ACY1 | ADAMTS4 | ADCK2 | ADH5 | ADORA2A | ADPRH | ADRB2 | ADRM1 | AFF4 | AFG3L2 | AGL | AGO1 | AGO2 | AHCYL1 | AHSG | AICDA | AIMP2 | AKT1 | AKT2 | AKT3 | ALB | AMFR | ANAPC4 | ANXA1 | ANXA2 | APBB1 | APC | APCS | APEH | APEX1 | APEX2 | APOA1 | APOE | APOL2 | APP | AR | ARAF | ARF4 | ARHGAP35 | ARID4B | ARMC8 | ARNT | ARRB1 | ARRB2 | ASB12 | ASB15 | ASB16 | ASB6 | ASB9 | ASCC1 | ASCC2 | ASGR1 | ASGR2 | ATAD5 | ATF2 | ATF6B | ATG4C | ATG5 | ATM | ATOH1 | ATP13A2 | ATRX | ATXN1 | ATXN2 | AUP1 | AURKB | AZU1 | B3GAT3 | B3GNT3 | B4GALT3 | BABAM1 | BACE1 | BAG2 | BAG6 | BANF1 | BAX | BCL10 | BCL11A | BCL2 | BCOR | BIRC2 | BIRC3 | BIRC5 | BIRC7 | BIRC8 | BMI1 | BMPR1A | BRAF | BRCA1 | BRD4 | BTK | BTRC | BUD13 | BUD31 | BYSL | C1QBP | C1R | C4A | C9orf64 | CAB39 | CACNG2 | CACNG4 | CACNG5 | CALM1 | CALR | CALU | CAMK1D | CAMK2A | CAMK2B | CAMKV | CAND1 | CAPN1 | CAPN2 | CAPNS1 | CARM1 | CARS | CASP10 | CASP2 | CASP3 | CASP7 | CASP8 | CASP9 | CAT | CAV1 | CBFB | CBL | CBLB | CBLC | CBS | CBX4 | CBX8 | CCAR2 | CCDC155 | CCDC22 | CCDC36 | CCDC8 | CCDC88A | CCNB1 | CCNT1 | CCNT2 | CCP110 | CCR5 | CCS | CD2BP2 | CD36 | CD40LG | CD81 | CDC14B | CDC16 | CDC20 | CDC25A | CDC25B | CDC25C | CDC27 | CDC34 | CDC42 | CDC5L | CDC73 | CDK15 | CDK16 | CDK19 | CDK2 | CDK4 | CDK5 | CDK5R1 | CDK8 | CDK9 | CDKL3 | CDKN1A | CEBPA | CEBPE | CEL | CENPF | CES1 | CFL1 | CFTR | CHAF1A | CHD1L | CHEK2 | CHGB | CHRAC1 | CHRM3 | CHST4 | CISD3 | CKAP4 | CLK1 | CLPX | CLTC | CNDP1 | CNDP2 | COG3 | COL2A1 | COL4A2 | COL5A1 | COMMD1 | COPS2 | COPS5 | COPS6 | CPNE3 | CPSF7 | CPVL | CRBN | CREB1 | CREBBP | CRKL | CRY1 | CRY2 | CRYAB | CSNK1A1 | CSNK1E | CSNK2A2 | CSNK2B | CSTF2T | CT45A5 | CTBP1 | CTCF | CTDP1 | CTH | CTNNB1 | CTSA | CTSC | CTSD | CTSL | CTSV | CUL1 | CUL2 | CUL3 | CUL4B | CUL5 | CUL7 | CWC15 | CXCL8 | CYCS | CYLD | DAD1 | DAP3 | DAPK1 | DAPP1 | DARS | DBI | DCAF12 | DCAF7 | DCAF8 | DCLK1 | DCN | DCP2 | DCTPP1 | DCUN1D1 | DDA1 | DDB1 | DDB2 | DDI1 | DDOST | DDRGK1 | DDX1 | DDX39A | DDX39B | DDX3X | DDX6 | DERL1 | DGUOK | DHRS7B | DHX15 | DHX36 | DHX9 | DIDO1 | DIS3 | DLD | DLG2 | DLG3 | DLG4 | DLST | DMAP1 | DNA2 | DNAJA3 | DNAJB11 | DNAJB2 | DNAJC10 | DNASE2 | DNASE2B | DNPEP | DPEP1 | DPF2 | DPM1 | DPP9 | DRG2 | DROSHA | DSE | DTX1 | DTX2 | DUS3L | DUSP1 | DUSP12 | DUSP13 | DUSP14 | DUSP16 | DUSP19 | DUSP22 | DUSP7 | DUT | DVL1 | DVL2 | DYRK2 | DZIP3 | E2F1 | E2F2 | E2F3 | EAF2 | ECD | EDA | EDC4 | EDEM1 | EED | EEF1A1 | EEF1A1P5 | EEF1A2 | EEF1B2 | EEF1D | EEF1G | EEF2 | EEF2KMT | EFEMP1 | EFTUD2 | EGFR | EGLN1 | EGLN3 | EHHADH | EHMT1 | EHMT2 | EIF2AK3 | EIF2AK4 | EIF2S1 | EIF3E | EIF3H | EIF4A3 | EIF4H | EIF5A | ELAC2 | ELAVL1 | ELAVL4 | ELF3 | ENDOG | EP300 | EPHA4 | EPHA6 | EPHB6 | EPRS | ERAL1 | ERBB2 | ERBB3 | ERCC5 | ERG | ERLIN2 | ESR1 | ESR2 | ESRRA | ESRRB | ESRRG | ETS1 | EVA1A | EXOSC1 | EXOSC5 | EXOSC6 | EXOSC9 | EXTL2 | EYA2 | EYA4 | EZH2 | EZR | F5 | F9 | FADD | FAF1 | FAF2 | FAM20B | FAM98A | FANCA | FANCC | FANCG | FANCI | FANCM | FARSB | FASN | FBXL12 | FBXO11 | FBXO25 | FBXO32 | FBXO6 | FBXO7 | FBXW11 | FBXW5 | FEN1 | FGB | FGF2 | FGFR1 | FGFR3 | FGL2 | FKBP14 | FKBP1A | FKBP1B | FKBP2 | FKBP5 | FKBP7 | FKBP8 | FN1 | FOLH1 | FOS | FOXK1 | FOXK2 | FOXM1 | FOXO1 | FTO | FTSJ3 | FUBP1 | FUBP3 | FUS | FYN | FZR1 | G3BP1 | GAA | GAK | GALNT7 | GANAB | GAPDH | GAR1 | GARS | GATA1 | GBE1 | GEMIN4 | GEMIN5 | GEMIN7 | GFPT1 | GFPT2 | GGA1 | GGT1 | GGTLC1 | GINS2 | GINS3 | GINS4 | GLCE | GLRX | GLRX3 | GLRX5 | GLUL | GNAS | GOLGA2 | GOLPH3 | GPC5 | GPS2 | GPX1 | GRB2 | GRIA1 | GRK1 | GRK5 | GSK3A | GSK3B | GSPT1 | GSPT2 | GTF2A1 | GTF2E2 | GTF2F1 | GTF2F2 | GTF2I | GTF3C2 | GYG1 | GYG2 | GYS1 | GZMA | GZMK | H1F0 | H2AFX | H3F3A | HARS | HBEGF | HCCS | HDAC1 | HDAC11 | HDAC2 | HDAC3 | HDAC4 | HDAC5 | HDAC6 | HDAC7 | HDAC9 | HECW1 | HECW2 | HELZ2 | HERC1 | HERC2 | HEXA | HEXB | HGS | HIF1A | HIF1AN | HIPK4 | HIST1H2BA | HIST1H3A | HIST1H4A | HIST2H3A | HIST3H3 | HLCS | HMBS | HMGB1 | HMGB2 | HMGB3 | HNF4A | HNF4G | HNRNPA1 | HNRNPA1L2 | HNRNPA2B1 | HNRNPD | HNRNPF | HNRNPH1 | HNRNPH2 | HNRNPH3 | HNRNPK | HNRNPL | HNRNPM | HNRNPU | HOXB9 | HPX | HRAS | HS2ST1 | HS6ST1 | HSD17B10 | HSF1 | HSF4 | HSP90AA1 | HSP90AB1 | HSP90B1 | HSPA14 | HSPA1B | HSPA5 | HSPA8 | HSPD1 | HSPG2 | HTR1B | HTRA1 | HTRA2 | HUWE1 | IARS | ICK | IDE | IFI16 | IGF1R | IGFBP7 | IK | IKBKB | IKBKE | IKBKG | IL3RA | ILF2 | ILF3 | ILK | IMP4 | ING5 | INO80B | INO80C | INSR | IRF1 | IRF4 | IRF7 | ISG15 | ITCH | ITGAV | ITK | JAK1 | JAK3 | JMJD6 | JOSD2 | JUN | KAT5 | KAT7 | KAT8 | KBTBD7 | KCMF1 | KCTD17 | KCTD2 | KDM1A | KDM2A | KDM3B | KDM4D | KDM5B | KDM5C | KDM8 | KDR | KEAP1 | KHDRBS1 | KIAA0391 | KLF5 | KLHL20 | KLHL22 | KMT2A | KMT2B | KMT2C | KPNA1 | KPNA2 | KPNB1 | KRAS | KSR1 | L2HGDH | LACTB2 | LAMB1 | LAP3 | LARP4 | LARP7 | LARS | LAT | LATS1 | LCMT2 | LEF1 | LGALS1 | LGALS3 | LGMN | LIG4 | LMO2 | LMO7 | LNX1 | LOXL2 | LRRC7 | LRRK1 | LRRK2 | LRSAM1 | LSM1 | LSM12 | LSM3 | LSM4 | LSM5 | LSM8 | LTBP1 | LUM | LYG2 | LYPLA1 | LYPLA2 | LZTR1 | MAD2L1 | MAN2B1 | MAP2K1 | MAP2K3 | MAP2K4 | MAP2K6 | MAP2K7 | MAP3K1 | MAP3K13 | MAP3K14 | MAP3K2 | MAP3K3 | MAP3K4 | MAP4K1 | MAPK1 | MAPK14 | MAPK15 | MAPK3 | MAPK4 | MAPK6 | MAPK7 | MAPK8 | MAPK9 | MAPT | MARCH5 | MASP1 | MASP2 | MAST1 | MAST3 | MATK | MBD3 | MBP | MC1R | MCFD2 | MCM2 | MCM3 | MCM4 | MCM5 | MCM6 | MCM7 | MCM9 | MCMBP | MDC1 | MDH2 | MDM2 | MDM4 | MDN1 | MECP2 | MED1 | MED12 | MED14 | MED17 | MED20 | MED21 | MED23 | MED24 | MED31 | MED4 | MED6 | MELK | MEPCE | METAP2 | MGMT | MIB1 | MITF | MKNK2 | MLH1 | MLLT1 | MLLT3 | MME | MMP26 | MORF4L1 | MORF4L2 | MOV10 | MPG | MPHOSPH6 | MPI | MPPE1 | MRGBP | MRM1 | MRPL12 | MRPL14 | MRPL19 | MRPL28 | MRPL38 | MRPL4 | MRPL43 | MRPL45 | MRPL46 | MRPL9 | MRPS5 | MRRF | MSH6 | MTA1 | MTA3 | MTERF1 | MTMR14 | MTMR7 | MTRR | MYC | MYH6 | NAE1 | NAP1L1 | NBAS | NCBP1 | NCK1 | NCOA1 | NCOA3 | NCOR1 | NDUFAF5 | NDUFAF7 | NEDD4 | NEDD8 | NEK1 | NEK7 | NELFB | NELFCD | NELFE | NEURL4 | NFATC1 | NFE2L2 | NFKBIA | NFS1 | NHLRC3 | NIPBL | NLK | NME1 | NME2 | NMNAT1 | NOB1 | NOD2 | NOP10 | NOP56 | NOS2 | NOSIP | NPEPL1 | NPEPPS | NPLOC4 | NPM1 | NPR1 | NR0B2 | NR1H2 | NR1H3 | NR1I2 | NR3C1 | NR4A1 | NR5A2 | NRBF2 | NSFL1C | NSMCE4A | NSUN2 | NSUN6 | NTMT1 | NTRK1 | NUCB1 | NUFIP1 | NUP107 | NUP153 | NUP160 | NUP205 | NUP214 | NUP85 | NUP88 | NUP93 | NUP98 | NUPR1 | OAS2 | OBSL1 | OGDH | OGFOD1 | OGT | OTUB1 | OTUB2 | OTUD4 | OTUD5 | OXA1L | OXSR1 | P4HA1 | P4HA2 | P4HB | PA2G4 | PABPC1 | PABPC3 | PABPC4 | PADI4 | PAK2 | PAK6 | PALB2 | PAN2 | PAPLN | PARK7 | PARP1 | PARP12 | PARP14 | PARP6 | PAWR | PAX8 | PAXIP1 | PCBP1 | PCBP2 | PCF11 | PCGF1 | PCGF5 | PCGF6 | PCMT1 | PCMTD1 | PCMTD2 | PCNA | PCSK9 | PDCD6IP | PDE12 | PDF | PDIA3 | PDIA6 | PDK2 | PEBP1 | PELI1 | PEPD | PER1 | PER2 | PEX5 | PFKM | PGAM5 | PGK1 | PGM1 | PGM2 | PGM3 | PGP | PHB | PHF10 | PHKA1 | PIAS1 | PIAS3 | PICK1 | PIGU | PIH1D1 | PIK3CA | PIK3CG | PIK3R1 | PIM1 | PIN1 | PINX1 | PIR | PJA1 | PKN2 | PLK1 | PLOD2 | PML | PMPCA | PMPCB | PNKP | POLA2 | POLD1 | POLDIP3 | POLE2 | POLE3 | POLM | POLR2A | POLR2E | POLR2L | POLR2M | POM121 | POT1 | POU5F1 | PPA1 | PPA2 | PPARD | PPARG | PPARGC1A | PPEF1 | PPIA | PPIB | PPID | PPIE | PPIF | PPIL3 | PPM1A | PPM1B | PPM1D | PPM1H | PPM1K | PPME1 | PPP1CA | PPP1CB | PPP1CC | PPP1R11 | PPP1R12A | PPP1R2 | PPP1R3B | PPP1R3C | PPP1R8 | PPP2CA | PPP2CB | PPP2R1A | PPP2R1B | PPP2R2A | PPP2R2D | PPP2R5D | PPP3CC | PPP4R2 | PPP5C | PPP6C | PPT1 | PPTC7 | PRCC | PRCP | PRDX3 | PRDX4 | PREP | PREPL | PRKAA1 | PRKAA2 | PRKAB2 | PRKACA | PRKAG1 | PRKCD | PRKCE | PRKCSH | PRKCZ | PRKD1 | PRKD2 | PRKD3 | PRKDC | PRMT2 | PRMT3 | PRMT6 | PROZ | PRPF19 | PRPF40A | PRPF8 | PRSS16 | PRSS23 | PSEN1 | PSEN2 | PSMA1 | PSMA2 | PSMA3 | PSMA5 | PSMA6 | PSMB5 | PSMB6 | PSMB7 | PSMB8 | PSMC1 | PSMC2 | PSMC3 | PSMC4 | PSMC5 | PSMC6 | PSMD1 | PSMD10 | PSMD11 | PSMD12 | PSMD14 | PSMD2 | PSMD4 | PSMD9 | PSME2 | PSME3 | PTEN | PTGES3 | PTK2 | PTMA | PTMS | PTPMT1 | PTPN1 | PTPN14 | PTPN21 | PTPN22 | PTPN23 | PTPN4 | PTPN6 | PTPRA | PTPRD | PTPRF | PTPRK | PTPRO | PTPRR | PTTG1 | PUM1 | PUS1 | PUS7 | PYGB | PYGL | QARS | QSOX1 | RAB11A | RAB11B | RAB1A | RAB1B | RAB2A | RAB31 | RAB5A | RAB7A | RAD21 | RAD23A | RAD23B | RAD51 | RAD51AP1 | RAE1 | RAF1 | RAN | RANBP9 | RANGAP1 | RARA | RARG | RARS2 | RBBP4 | RBBP6 | RBM17 | RBM25 | RBM3 | RBM4 | RBM8A | RBMX | RBPJ | RBX1 | RC3H1 | RELA | REPIN1 | REST | REXO2 | RFC1 | RFC2 | RFC3 | RFC4 | RFC5 | RHBDD1 | RHO | RHOA | RHOT1 | RIF1 | RING1 | RIOK1 | RIPK1 | RIPK2 | RIPK3 | RIPK4 | RMND1 | RNASE3 | RNASEH2A | RNASEH2B | RNF123 | RNF126 | RNF139 | RNF181 | RNF183 | RNF185 | RNF19A | RNF2 | RNF25 | RNF31 | RNF4 | RNF8 | RNPEP | RPA2 | RPA3 | RPAIN | RPL10 | RPL10A | RPL12 | RPL18A | RPL22 | RPL23 | RPL23A | RPL24 | RPL30 | RPL36AL | RPL37A | RPL38 | RPL6 | RPL7 | RPL7A | RPL9 | RPLP0 | RPLP0P6 | RPLP1 | RPLP2 | RPN1 | RPN2 | RPP14 | RPS12 | RPS13 | RPS14 | RPS15A | RPS19 | RPS2 | RPS20 | RPS24 | RPS29 | RPS3 | RPS4X | RPS6KA1 | RPS6KA2 | RPS6KB2 | RPS8 | RRM1 | RRM2 | RRM2B | RRP7A | RRS1 | RTCB | RTF1 | RTFDC1 | RUNX2 | RUVBL1 | RUVBL2 | RXRA | RXRB | RXRG | S100A9 | SAE1 | SAP18 | SARS | SART1 | SART3 | SBDS | SCAF1 | SCG2 | SCG3 | SCYL2 | SDC1 | SDC4 | SEL1L | SENP3 | SENP6 | SENP7 | SENP8 | SEPHS1 | SEPHS2 | SERPINH1 | SET | SETDB1 | SF1 | SF3B6 | SFPQ | SGSH | SGTA | SGTB | SHC1 | SHMT2 | SIAH1 | SIN3A | SIRT1 | SIRT3 | SIRT5 | SIRT6 | SIRT7 | SKI | SKIV2L | SMAD2 | SMAD3 | SMAD4 | SMAD5 | SMARCB1 | SMC1A | SMC3 | SMG5 | SMG6 | SMG8 | SMN1 | SMURF1 | SMURF2 | SNCA | SND1 | SNRNP27 | SNRPA | SNRPA1 | SNRPB | SNRPB2 | SNRPD1 | SNRPD3 | SNRPE | SNW1 | SNX3 | SOCS1 | SOCS2 | SOCS6 | SP1 | SP100 | SPATA2 | SPI1 | SPIN1 | SPOP | SPRR2A | SPRTN | SPSB3 | SPTAN1 | SPTBN1 | SPTBN2 | SQSTM1 | SRC | SRPK1 | SRPK2 | SRSF11 | SRSF12 | SRSF5 | SSB | ST3GAL1 | ST3GAL6 | ST6GALNAC5 | ST8SIA4 | STAT3 | STBD1 | STK11 | STK24 | STK25 | STK26 | STK3 | STK38 | STK40 | STOML2 | STUB1 | STYX | SULF2 | SULT1A3 | SUMO1 | SUMO1P1 | SUMO2 | SUMO3 | SUPT4H1 | SUPT5H | SUZ12 | SWAP70 | SYVN1 | TAB1 | TAB2 | TADA2B | TAF12 | TAF15 | TAF1A | TANK | TAOK3 | TARDBP | TARS | TARS2 | TATDN1 | TBL1X | TBRG4 | TCEA1 | TCEA2 | TCERG1 | TCF4 | TCIRG1 | TDP1 | TERF1 | TERF2 | TERF2IP | TESK2 | TFCP2 | TFIP11 | TGFB1 | TGFBR3 | TGM2 | TGOLN2 | THADA | THBS1 | THOC2 | THOC6 | THOC7 | THOP1 | THRA | TIMM50 | TIMP1 | TINF2 | TK1 | TK2 | TLR3 | TMEM132A | TMEM189-UBE2V1 | TMEM258 | TMTC3 | TNF | TNFAIP3 | TNIP2 | TNK2 | TNKS | TNKS2 | TNP1 | TOE1 | TOLLIP | TOMM20 | TOP1 | TOP2A | TOP3A | TOR1A | TP53 | TP53RK | TP73 | TPR | TPSAB1 | TPSB2 | TPST2 | TPTE | TRA2A | TRAF2 | TRAF3 | TRAF6 | TREX1 | TRIB2 | TRIB3 | TRIM21 | TRIM23 | TRIM25 | TRIM28 | TRIM29 | TRIM32 | TRIM33 | TRIM41 | TRIM47 | TRIM63 | TRIM69 | TRIM7 | TRIM9 | TRIP12 | TRIP13 | TRMT44 | TRMT6 | TRMT61B | TRNT1 | TRUB2 | TSC22D1 | TSFM | TSG101 | TSN | TSPAN17 | TTC5 | TTLL1 | TUFM | TWF1 | TXNL4A | TYMS | U2AF2 | UBA1 | UBA2 | UBA3 | UBA5 | UBA6 | UBAC1 | UBB | UBC | UBE2A | UBE2B | UBE2C | UBE2D1 | UBE2D2 | UBE2D3 | UBE2H | UBE2I | UBE2J1 | UBE2K | UBE2L3 | UBE2L6 | UBE2M | UBE2N | UBE2O | UBE2Q2 | UBE2R2 | UBE2S | UBE2V1 | UBE2W | UBE3A | UBE3B | UBL4A | UBQLN1 | UBQLN2 | UBQLN4 | UBR4 | UBR5 | UBXN1 | UBXN7 | UCHL1 | UCHL3 | UCHL5 | UFC1 | UFM1 | UGDH | UGGT1 | UGP2 | ULK1 | UNKL | UPF2 | UPF3B | UQCRC2 | USP11 | USP14 | USP15 | USP16 | USP17L2 | USP19 | USP2 | USP20 | USP21 | USP26 | USP28 | USP39 | USP4 | USP42 | USP46 | USP47 | USP5 | USP50 | USP53 | USP7 | USP8 | USP9X | USP9Y | USPL1 | UTP14A | UTP6 | VARS | VASH2 | VCAN | VCP | VCPIP1 | VDR | VGF | VHL | VKORC1 | VPS37D | VPS4B | WARS | WDR12 | WDR4 | WDR45B | WDR48 | WDR82 | WDTC1 | WDYHV1 | WIPI1 | WIPI2 | WNK1 | WNK4 | WSB1 | WT1 | WTAP | WWP2 | XBP1 | XIAP | XPNPEP1 | XPO5 | XRCC1 | XRCC3 | XRCC5 | XRCC6 | XYLT1 | YAP1 | YBEY | YEATS2 | YES1 | YME1L1 | YWHAB | YWHAE | YY1 | ZAP70 | ZBTB16 | ZBTB25 | ZBTB7A | ZBTB7B | ZC3HC1 | ZDHHC17 | ZDHHC23 | ZFYVE26 | ZMPSTE24 | ZNF217 | ZNF598 | ZNF638 | ZNRF4 | ZRANB2 | GO:0034641 | 0.24335800072789 | 2006/8243 | 874/3250 | 7.69805408835923e-06 | 0.0730930235689708 | T | T | T | T | cellular nitrogen compound metabolic process | AARS | AARSD1 | ABHD14B | ABL1 | ACACA | ACAT1 | ACD | ACLY | ACOT13 | ACOT7 | ACOT9 | ACSL1 | ACSL4 | ACSL5 | ACSS2 | ACTL6A | ADA | ADCY6 | ADCYAP1 | ADK | ADRM1 | ADSL | ADSS | AFF4 | AGK | AGO1 | AGO2 | AHCY | AHCYL1 | AICDA | AIMP2 | AK1 | AK2 | AK4 | AKT1 | ALAS1 | ALDH5A1 | ALDH6A1 | ALDH7A1 | ALDH9A1 | ALDOA | AMD1 | AMDHD2 | APBB1 | APEH | APEX1 | APEX2 | APP | APRT | AR | ARHGAP35 | ARID4B | ARNT | ARRB1 | ARRB2 | ASCC1 | ASCC2 | ASL | ASPDH | ASS1 | ATAD5 | ATF6B | ATIC | ATM | ATOH1 | ATP6V1A | ATP6V1B1 | ATRX | ATXN1 | ATXN2 | B4GALT3 | BABAM1 | BACE1 | BANF1 | BAX | BLVRB | BRCA1 | BTBD9 | BTRC | BUD13 | BUD31 | BYSL | C1QBP | C9orf64 | CARS | CASP3 | CBFB | CBR4 | CBS | CCAR2 | CCDC155 | CCDC36 | CCNB1 | CCNT1 | CCNT2 | CD2BP2 | CD40LG | CDA | CDC14B | CDC34 | CDC5L | CDC73 | CDK2 | CDK4 | CDK8 | CDK9 | CDKN1A | CEBPA | CENPF | CHAF1A | CHD1L | CHDH | CHEK2 | CHRAC1 | CLPX | CMPK1 | CNDP2 | COL4A2 | COL4A3BP | COPS2 | COPS5 | COPS6 | COX15 | CPSF7 | CPT1A | CREB1 | CREBBP | CRMP1 | CROT | CRY2 | CSNK1E | CSTF2T | CT45A5 | CTCF | CTDP1 | CTPS1 | CUL4B | CWC15 | CYC1 | DARS | DBH | DBI | DCAKD | DCK | DCP2 | DCTPP1 | DCXR | DDB1 | DDB2 | DDX1 | DDX39A | DDX39B | DDX3X | DDX6 | DEGS1 | DERA | DGUOK | DHX15 | DHX36 | DHX9 | DIDO1 | DIS3 | DLAT | DLD | DLG2 | DLST | DMAP1 | DMD | DNA2 | DNAJC30 | DNASE2 | DNASE2B | DNPEP | DPEP1 | DRG2 | DROSHA | DTX1 | DUS3L | DUT | DVL1 | DVL2 | E2F1 | E2F2 | E2F3 | EAF2 | ECD | EDC4 | EED | EEF1A1 | EEF1A1P5 | EEF1A2 | EFTUD2 | EGFR | EHMT1 | EHMT2 | EIF3E | EIF4A3 | EIF4H | ELAC2 | ELAVL1 | ELAVL4 | ELF3 | ENDOG | ENTPD7 | EP300 | EPRS | ERCC5 | ESR1 | ESR2 | ESRRA | ESRRB | ESRRG | ETS1 | EXOSC1 | EXOSC5 | EXOSC6 | EXOSC9 | EXTL2 | EYA2 | EYA4 | EZH2 | FANCA | FANCC | FANCG | FANCI | FANCM | FAR2 | FARSB | FASN | FBXO6 | FECH | FEN1 | FIGNL1 | FOLR1 | FOS | FOXM1 | FPGS | FTO | FTSJ3 | FUBP1 | FUBP3 | FUS | FZR1 | G3BP1 | G6PD | GAR1 | GARS | GART | GATA1 | GCDH | GDA | GEMIN4 | GEMIN5 | GEMIN7 | GFPT1 | GFPT2 | GGCT | GGT1 | GGTLC1 | GINS2 | GINS3 | GINS4 | GLO1 | GLRX | GMDS | GMPPB | GMPR | GMPS | GNAI3 | GNAS | GNPDA1 | GNPDA2 | GNPNAT1 | GPD1L | GPX1 | GPX4 | GRHPR | GSPT1 | GSPT2 | GSR | GSS | GSTA1 | GSTA2 | GSTA3 | GSTA4 | GSTA5 | GSTK1 | GSTM4 | GSTP1 | GSTT1 | GSTT2 | GSTT2B | GSTZ1 | GTF2A1 | GTF2E2 | GTF2F1 | GTF2F2 | GTF2I | GTF3C2 | H1F0 | H2AFX | HAAO | HAGH | HARS | HDAC6 | HELZ2 | HERC2 | HIF1A | HINT1 | HIST1H4A | HIST3H3 | HMBS | HMGB1 | HMGB2 | HMGB3 | HMGCL | HMOX1 | HNF4A | HNF4G | HNRNPA1 | HNRNPA1L2 | HNRNPA2B1 | HNRNPD | HNRNPF | HNRNPH1 | HNRNPH2 | HNRNPH3 | HNRNPK | HNRNPL | HNRNPM | HNRNPU | HOXB9 | HPRT1 | HSD17B10 | HSD17B4 | HSD17B8 | HSF1 | HSP90AA1 | HSP90AB1 | HSPA14 | HSPA1B | HSPA8 | HSPD1 | HTRA2 | HUWE1 | IARS | IDE | IDH1 | IDH2 | IK | ILF2 | IMP4 | IMPDH2 | INO80B | INO80C | IREB2 | IRF1 | IRF7 | ISG15 | ITPA | JMJD6 | JUN | KAT5 | KCNAB2 | KDM1A | KDM2A | KDM4D | KHDRBS1 | KIAA0391 | KLF5 | KMT2A | KPNA1 | KPNA2 | KPNB1 | KYNU | LACTB2 | LARP4 | LARP7 | LARS | LCMT2 | LDHA | LDHC | LEF1 | LGALS3 | LIG4 | LMO2 | LRRK2 | LSM1 | LSM12 | LSM3 | LSM4 | LSM5 | LSM8 | MAOA | MAPK1 | MAPK3 | MAPT | MC1R | MCM2 | MCM3 | MCM4 | MCM5 | MCM6 | MCM7 | MCM9 | MDC1 | MDH1 | MDH2 | MDN1 | ME1 | MECP2 | MED1 | MED12 | MED14 | MED17 | MED20 | MED21 | MED23 | MED24 | MED31 | MED4 | MED6 | MEPCE | MGMT | MGST1 | MITF | MLH1 | MLLT1 | MLLT3 | MMAB | MME | MORF4L1 | MORF4L2 | MOV10 | MPC2 | MPG | MPHOSPH6 | MPI | MPST | MRM1 | MRPL12 | MRPL28 | MRPL43 | MRPL9 | MRRF | MSH6 | MTA1 | MTAP | MTERF1 | MTHFD1 | MTRR | MVD | MYH6 | NAGK | NAMPT | NANS | NAPRT | NBAS | NCBP1 | NCOA1 | NCOR1 | NDUFS1 | NELFB | NELFCD | NELFE | NFATC1 | NFE2L2 | NIPBL | NME1 | NME2 | NME4 | NMNAT1 | NMNAT3 | NMRAL1 | NMRK2 | NNT | NOB1 | NOP10 | NOP56 | NOS2 | NOS3 | NPLOC4 | NPM1 | NPR1 | NQO1 | NR0B2 | NR1H2 | NR1H3 | NR1I2 | NR3C1 | NR4A1 | NR5A2 | NRBF2 | NSMCE4A | NSUN2 | NSUN6 | NT5C2 | NUDT13 | NUDT18 | NUDT3 | NUDT9 | NUFIP1 | OAS2 | OAZ3 | OGDH | OLA1 | OPA1 | OTC | OTUB1 | P2RX1 | PA2G4 | PABPC1 | PABPC3 | PABPC4 | PAICS | PALB2 | PAN2 | PAPSS1 | PAPSS2 | PARK7 | PARP1 | PAX8 | PAXIP1 | PCBD1 | PCBP1 | PCBP2 | PCF11 | PCGF5 | PCGF6 | PCNA | PDE12 | PDE4D | PDF | PDHA1 | PDHA2 | PDHB | PDHX | PDXK | PFAS | PGM3 | PHB | PICK1 | PIH1D1 | PINX1 | PIR | PNKP | PNP | POLA2 | POLD1 | POLDIP3 | POLE2 | POLE3 | POLM | POLR2A | POLR2E | POLR2L | POLR2M | POT1 | POU5F1 | PPA1 | PPA2 | PPARD | PPARG | PPARGC1A | PPAT | PPIA | PPIE | PPIL3 | PPM1D | PPOX | PPP1R8 | PPP2CA | PPP2R1A | PPP2R2A | PPP4R2 | PPP5C | PPT1 | PRCC | PRKACA | PRKDC | PRMT6 | PRPF19 | PRPF40A | PRPF8 | PRTFDC1 | PSAT1 | PSEN1 | PSEN2 | PSMD14 | PTEN | PTGES | PTGES3 | PTMA | PTTG1 | PUM1 | PUS1 | PUS7 | QARS | QDPR | RAD21 | RAD23A | RAD23B | RAD51 | RAD51AP1 | RAN | RARA | RARG | RARS2 | RBBP6 | RBM17 | RBM25 | RBM3 | RBM4 | RBM8A | RBMX | RBPJ | RBX1 | RC3H1 | RELA | REXO2 | RFC1 | RFC2 | RFC3 | RFC4 | RFC5 | RFK | RHOA | RIF1 | RIOK1 | RMND1 | RNASE3 | RNASEH2A | RNASEH2B | RNF8 | RPA2 | RPA3 | RPAIN | RPL10 | RPL10A | RPL12 | RPL18A | RPL22 | RPL23 | RPL23A | RPL24 | RPL30 | RPL36AL | RPL37A | RPL38 | RPL6 | RPL7 | RPL7A | RPL9 | RPLP0 | RPLP0P6 | RPLP1 | RPLP2 | RPP14 | RPS12 | RPS13 | RPS14 | RPS15A | RPS19 | RPS2 | RPS20 | RPS24 | RPS29 | RPS3 | RPS4X | RPS6KB2 | RPS8 | RRM1 | RRM2 | RRM2B | RRP7A | RRS1 | RTCB | RTF1 | RTFDC1 | RUNX2 | RUVBL1 | RUVBL2 | RXRA | RXRB | RXRG | SAP18 | SARS | SART1 | SART3 | SAT1 | SAT2 | SBDS | SCAF1 | SCO2 | SENP3 | SEPHS1 | SEPHS2 | SF1 | SF3B6 | SFPQ | SGPL1 | SHMT1 | SHMT2 | SIRT1 | SIRT6 | SIRT7 | SKI | SKIV2L | SLC25A1 | SLC25A13 | SLC25A32 | SMAD2 | SMAD3 | SMAD4 | SMARCB1 | SMC1A | SMC3 | SMG5 | SMG6 | SMG8 | SMN1 | SND1 | SNRNP27 | SNRPA | SNRPA1 | SNRPB | SNRPB2 | SNRPD1 | SNRPD3 | SNRPE | SNW1 | SOD1 | SP1 | SP100 | SPI1 | SPIN1 | SPR | SPRTN | SPTLC2 | SRPK1 | SRPK2 | SRSF11 | SRSF12 | SRSF5 | SSB | ST8SIA4 | STAT3 | STOML2 | STUB1 | SUCLA2 | SUCLG2 | SULT1A3 | SUMO1 | SUPT4H1 | SUPT5H | SWAP70 | TAF12 | TAF15 | TAF1A | TAMM41 | TAOK3 | TARDBP | TARS | TARS2 | TATDN1 | TBRG4 | TCEA1 | TCEA2 | TCERG1 | TCF4 | TDO2 | TDP1 | TECR | TERF1 | TERF2 | TERF2IP | TFCP2 | TFIP11 | TGFB1 | THADA | THEM4 | THOC2 | THOC6 | THOC7 | THOP1 | THRA | TINF2 | TK1 | TK2 | TMEM189-UBE2V1 | TNP1 | TOE1 | TOP1 | TOP2A | TOP3A | TP53 | TP53RK | TP73 | TPST2 | TRA2A | TREX1 | TRIM25 | TRIM28 | TRIM29 | TRIP12 | TRIP13 | TRMT44 | TRMT6 | TRMT61B | TRNT1 | TRUB2 | TSC22D1 | TSN | TTC5 | TXNL4A | TXNRD1 | TYMS | U2AF2 | UAP1 | UAP1L1 | UBB | UBC | UBE2A | UBE2B | UBE2D3 | UBE2L6 | UBE2N | UBE2V1 | UBE2W | UBR5 | UCHL5 | UCK2 | UGDH | UGGT1 | UGP2 | UGT8 | UMPS | UPF2 | UPF3B | UPP2 | USP14 | USP28 | USP39 | USP47 | USP7 | USPL1 | UTP14A | UTP6 | UXS1 | VARS | VCP | VDR | WARS | WDR12 | WDR4 | WDR48 | WT1 | WTAP | XBP1 | XPO5 | XRCC1 | XRCC3 | XRCC5 | XRCC6 | YAP1 | YBEY | YY1 | ZBTB7A | ZBTB7B | ZFYVE26 | ZMPSTE24 | ZNF217 | ZNF638 | ZRANB2 | GO:0030163 | 0.0377289821666869 | 311/8243 | 160/3250 | 8.02100197448442e-06 | 0.0761594137477296 | T | T | T | T | protein catabolic process | ABHD17A | AKT1 | AMFR | ANAPC4 | ANXA2 | APOE | ARMC8 | ARRB1 | ARRB2 | AUP1 | AURKB | BACE1 | BAG6 | BIRC2 | BTRC | CCNB1 | CDC16 | CDC20 | CDC27 | CDC34 | CDK2 | CHEK2 | CLPX | CLTC | CRBN | CTNNB1 | CTSD | CUL1 | CUL2 | CUL3 | CUL4B | CUL5 | DDB1 | DERL1 | DNAJB2 | DNAJC10 | EDEM1 | ERLIN2 | FAF1 | FAF2 | FBXL12 | FBXO6 | FBXW11 | FBXW5 | FZR1 | GSK3A | HECW1 | HECW2 | HERC2 | HSP90B1 | HSPA5 | HTRA2 | IDE | ITCH | KAT5 | KCTD17 | KCTD2 | KEAP1 | KLHL20 | KLHL22 | LRSAM1 | LYPLA1 | LYPLA2 | MAD2L1 | MDM2 | MTA1 | NEDD4 | NFE2L2 | NHLRC3 | NPLOC4 | NSFL1C | PCBP2 | PCSK9 | PELI1 | PJA1 | PLK1 | PML | PPP2CB | PPT1 | PRPF19 | PRSS16 | PSMA1 | PSMA2 | PSMA3 | PSMA5 | PSMA6 | PSMB5 | PSMB6 | PSMB7 | PSMB8 | PSMC1 | PSMC2 | PSMC3 | PSMC4 | PSMC5 | PSMC6 | PSMD1 | PSMD10 | PSMD11 | PSMD12 | PSMD14 | PSMD2 | PSMD4 | PSMD9 | PSME2 | PSME3 | PTTG1 | RAB7A | RAD23A | RAD23B | RBX1 | RHBDD1 | RIPK1 | RNF126 | RNF139 | RNF185 | RNF4 | SEL1L | SGSH | SGTA | SGTB | SIAH1 | SIRT1 | SMURF1 | SMURF2 | SOCS6 | SPOP | SPSB3 | STUB1 | SYVN1 | TBL1X | TCIRG1 | TOR1A | TRAF2 | TRIM25 | TRIM9 | UBB | UBC | UBE2A | UBE2B | UBE2C | UBE2D1 | UBE2D3 | UBE2H | UBE2I | UBE2J1 | UBE2R2 | UBE2S | UBE2W | UBE3A | UBQLN1 | UBQLN2 | UBXN1 | UCHL1 | UCHL3 | UGGT1 | USP19 | VCP | WWP2 | YME1L1 | GO:1901800 | 0.00921994419507461 | 76/8243 | 49/3250 | 8.15593305031449e-06 | 0.0774405843127361 | T | T | T | T | positive regulation of proteasomal protein catabolic process | AKT1 | BAG2 | BAG6 | CAV1 | CBFA2T3 | CEBPA | CLU | CSNK1A1 | CSNK1E | DAB2 | DDRGK1 | DNAJB2 | DVL1 | EDEM1 | GSK3A | GSK3B | HSPA1B | HSPBP1 | KEAP1 | LRRK2 | MAPK9 | MDM2 | NFE2L2 | NUPR1 | PIAS1 | PLK1 | PSEN1 | PSMC1 | PSMC2 | PSMC3 | PSMC4 | PSMC5 | PSMC6 | PSMD10 | RAD23A | RBX1 | RNF185 | RNF19A | SGTA | STUB1 | SUMO1 | SUMO2 | TMTC3 | TRIB2 | TRIB3 | UBQLN1 | UBQLN2 | USP5 | VCP | GO:1902749 | 0.0208661894941162 | 172/8243 | 96/3250 | 8.25240894486865e-06 | 0.0783566229315278 | T | T | T | T | regulation of cell cycle G2/M phase transition | ACTR1A | AKAP9 | APP | ATAD5 | ATM | AURKB | BABAM1 | BRCA1 | BRD4 | CCNB1 | CCP110 | CDC14B | CDC25A | CDC25B | CDC25C | CDK2 | CDK4 | CENPF | CENPJ | CEP135 | CEP152 | CEP290 | CEP57 | CEP70 | CKAP5 | CNTRL | CSNK1E | CUL1 | DBF4B | DCTN1 | DYNC1H1 | DYNC1I2 | DYNLL1 | FGFR1OP | FHL1 | FZR1 | HMMR | HSP90AA1 | HSPA2 | MECP2 | MSH6 | MTA3 | NAE1 | NPM1 | OFD1 | PAFAH1B1 | PAXIP1 | PCM1 | PINX1 | PLK1 | PPP2R1A | PRKACA | PRKAR2B | PSMA1 | PSMA2 | PSMA3 | PSMA5 | PSMA6 | PSMB5 | PSMB6 | PSMB7 | PSMB8 | PSMC1 | PSMC2 | PSMC3 | PSMC4 | PSMC5 | PSMC6 | PSMD1 | PSMD10 | PSMD11 | PSMD12 | PSMD14 | PSMD2 | PSMD4 | PSMD9 | PSME2 | PSME3 | RAB11A | RAD21 | RBX1 | RNASEH2B | SIN3A | TAOK3 | TP53 | TUBA1A | TUBA4A | TUBB | TUBB4A | TUBB4B | TUBG1 | USP47 | VPS4B | YWHAE | YWHAG | ZFYVE19 | GO:2000573 | 0.00691495814630596 | 57/8243 | 39/3250 | 8.39933417736808e-06 | 0.07975167801411 | T | T | T | T | positive regulation of DNA biosynthetic process | ACD | ARRB2 | ATM | AURKB | CCT2 | CCT3 | CCT4 | CCT5 | CCT6A | CCT7 | CCT8 | CTNNB1 | DDX39B | FGF2 | HNRNPA1 | HNRNPA2B1 | HNRNPD | HSP90AA1 | HSP90AB1 | MAP2K7 | MAP3K4 | MAPK1 | MAPK15 | MAPK3 | MYC | NEK7 | PNKP | POT1 | PRKD2 | PTGES3 | RFC2 | RFC3 | RFC4 | RFC5 | SRC | TCP1 | TNKS | TNKS2 | XRCC5 | GO:0009892 | 0.217396578915443 | 1792/8243 | 786/3250 | 8.62189212710609e-06 | 0.0818648657468723 | T | T | T | T | negative regulation of metabolic process | A2M | ABCE1 | ABL1 | ACADVL | ACD | ACOT13 | ADORA2A | AEBP2 | AES | AGAP2 | AGO1 | AGO2 | AHSG | AICDA | AKT1 | ANXA2 | ANXA7 | APBB1 | APC | APCS | APEX1 | APOD | APOE | APP | AR | ARHGAP35 | ARRB1 | ARRB2 | ASCL1 | ATCAY | ATF2 | ATG5 | ATM | ATP13A2 | ATP1A1 | ATRX | ATXN1 | ATXN2 | AURKB | BACH2 | BAG2 | BAG3 | BAG4 | BAG5 | BAG6 | BANP | BASP1 | BAX | BCL11A | BCL2 | BCL6 | BCL6B | BCOR | BDKRB2 | BIN1 | BIRC2 | BIRC3 | BIRC5 | BIRC7 | BIRC8 | BMI1 | BRCA1 | BTRC | C1QBP | C1QL4 | C4A | CALM1 | CALM2 | CALM3 | CALR | CASP3 | CAV1 | CBFA2T3 | CBFB | CBL | CBLB | CBLC | CBX4 | CBX7 | CBX8 | CBY1 | CCAR2 | CCDC8 | CCNB1 | CCNT1 | CCR1 | CD109 | CD27 | CD2BP2 | CD36 | CDA | CDC73 | CDK2 | CDK5 | CDK5R1 | CDK9 | CDKN1A | CDKN2C | CEBPA | CENPF | CENPJ | CHCHD3 | CHD8 | CHEK2 | CHORDC1 | CHP1 | CHRNA9 | CIRBP | CLTC | CLU | COMT | COPS2 | COX11 | CREB1 | CREBBP | CRKL | CRY1 | CRY2 | CRYAB | CSNK2A2 | CTBP1 | CTCF | CTNNB1 | CTNNBIP1 | CTSA | CUL3 | CYLD | DAB2 | DAPK1 | DAXX | DBNDD2 | DCP2 | DDIT4 | DDRGK1 | DDX3X | DDX6 | DHCR24 | DHX36 | DHX9 | DIS3 | DLG2 | DLG3 | DLG4 | DMAP1 | DMD | DNAJA1 | DNAJA3 | DNAJB1 | DNAJC10 | DOCK7 | DPEP1 | DPF2 | DUSP1 | DUSP14 | DUSP16 | DUSP19 | DUSP22 | DUSP7 | DVL1 | DYNLL1 | E2F1 | E2F2 | E2F3 | E2F4 | E2F6 | EDC4 | EED | EGFR | EHMT1 | EHMT2 | EID1 | EIF2AK3 | EIF2AK4 | EIF2S1 | EIF3A | EIF3E | EIF4A3 | EIF4E | EIF4E2 | EIF4EBP1 | EIF4ENIF1 | EIF4G3 | EIF6 | ELAVL1 | ELF3 | ENO1 | EP300 | EPHA4 | EPPIN | EPRS | ERF | ERLIN2 | ERRFI1 | ESR1 | ESR2 | ESRRA | EXOSC1 | EXOSC5 | EXOSC6 | EXOSC9 | EZH2 | EZR | FABP1 | FAM129B | FAM192A | FAM3C | FASLG | FBP1 | FBXO7 | FBXW11 | FGFR1 | FGFR1OP | FHL2 | FKBP1A | FKBP1B | FKBP8 | FOXA1 | FOXJ1 | FOXK1 | FOXM1 | FOXO1 | FTO | FXR2 | FYN | G6PD | GAPDH | GATA1 | GLA | GLMN | GNAI2 | GNAQ | GOLGA2 | GOT1 | GPD1L | GPI | GPS2 | GPX1 | GRB7 | GSK3A | GSK3B | GSPT1 | GSPT2 | GSTP1 | GZMA | H1F0 | H1FX | H3F3A | HAP1 | HAVCR2 | HBEGF | HBZ | HDAC1 | HDAC2 | HDAC3 | HDAC4 | HDAC5 | HDAC6 | HDAC7 | HDAC9 | HDGF | HELZ | HELZ2 | HERC1 | HEXIM1 | HIF1A | HIF1AN | HIST1H1A | HIST1H3A | HIST1H4A | HIST2H3A | HIVEP1 | HK2 | HMGB1 | HMGB2 | HMOX1 | HNRNPA1 | HNRNPA2B1 | HNRNPD | HNRNPK | HNRNPL | HNRNPU | HRAS | HSF1 | HSF4 | HSP90AB1 | HSP90AB3P | HSP90AB4P | HSPA1B | HSPA8 | HSPB1 | HTRA2 | IBTK | ID2 | IDE | IFI16 | IFNA2 | IGBP1 | IGF1R | IGF2BP1 | IKBKB | IKZF5 | ILF3 | ILK | INSIG1 | INSIG2 | IP6K1 | IPO5 | IPO7 | IQGAP1 | IREB2 | IRF1 | IRF7 | ISG15 | ITCH | ITGAV | ITGB1BP1 | ITGB3 | JAK3 | JUN | JUNB | JUND | KANK2 | KAT5 | KAT7 | KAT8 | KDM1A | KDM4D | KDM5B | KDM5C | KDR | KHDRBS1 | KLF5 | KLHL22 | KMT2A | KMT2B | L3MBTL1 | LAMP3 | LARS | LATS1 | LDLRAD4 | LEF1 | LEMD2 | LGALS3 | LGMN | LGR4 | LIMS1 | LOXL2 | LRP6 | LRPPRC | LRRK1 | LRRK2 | LSM1 | LSM3 | LSM4 | LSM5 | MAD2L1 | MAF1 | MAP2K1 | MAP2K6 | MAPK14 | MAPT | MAS1 | MBD3 | MDFI | MDM2 | MDM4 | MECP2 | MED1 | MEPCE | MIF | MITF | MLH1 | MLLT1 | MORF4L1 | MORF4L2 | MOV10 | MSH6 | MTA1 | MTA3 | MXD1 | MYBBP1A | MYC | MYD88 | MYO1D | NACC1 | NAMPT | NANOG | NBAS | NCBP1 | NCK1 | NCOA2 | NCOR1 | NCOR2 | NDUFA13 | NEDD4 | NELFB | NELFCD | NELFE | NELL1 | NF2 | NFATC2 | NIF3L1 | NIPBL | NKRF | NKX2-1 | NME1 | NMNAT1 | NNT | NOS2 | NOSIP | NOSTRIN | NPM1 | NR0B2 | NR1H2 | NR1H3 | NR1I2 | NR3C1 | NTF3 | OGT | OPTN | OTUB1 | OXA1L | PA2G4 | PABPC1 | PAFAH1B1 | PAIP1 | PAK2 | PAM16 | PAN2 | PAPLN | PARD3 | PARD6A | PARK7 | PARP1 | PARP14 | PATZ1 | PAWR | PAX9 | PC | PCGF1 | PCGF6 | PCSK9 | PCTP | PDCD10 | PDE12 | PDE4D | PEBP1 | PER1 | PER2 | PGAM5 | PGP | PHB | PHB2 | PHF10 | PHF21A | PIAS1 | PIAS3 | PIH1D1 | PIK3CA | PIK3CG | PIN1 | PINX1 | PKIG | PLAUR | PLK1 | PML | PNKP | POLDIP2 | POT1 | POU5F1 | PPARD | PPARG | PPARGC1A | PPID | PPIF | PPM1A | PPM1D | PPME1 | PPP1R11 | PPP1R14A | PPP1R2 | PPP1R8 | PPP2CA | PPP2R1A | PPP2R2A | PPP2R5D | PRDX2 | PRDX3 | PRDX5 | PRKAA1 | PRKAA2 | PRKAR1A | PRKAR2B | PRKCD | PRKCE | PRKCZ | PRKDC | PRMT2 | PRMT3 | PRMT6 | PROX1 | PSEN1 | PSMC5 | PSMD10 | PTEN | PTK2 | PTN | PTPN1 | PTPN22 | PTPN6 | PTPRK | PTPRR | PTRH2 | PTTG1 | PUM1 | PUS7 | QARS | QSOX1 | RAF1 | RANBP9 | RAP1A | RARA | RARG | RASD1 | RB1 | RBBP4 | RBM3 | RBM4 | RBM8A | RBMX | RBPJ | RC3H1 | RECK | REL | RELA | REST | RFC1 | RGN | RGS2 | RHOA | RIF1 | RING1 | RNASEH2B | RNF139 | RNF2 | RNF8 | RPL10 | RPL10A | RPL12 | RPL18A | RPL22 | RPL23 | RPL23A | RPL24 | RPL30 | RPL37A | RPL38 | RPL6 | RPL7 | RPL7A | RPL9 | RPLP0 | RPLP1 | RPLP2 | RPS12 | RPS13 | RPS14 | RPS15A | RPS19 | RPS2 | RPS20 | RPS24 | RPS29 | RPS3 | RPS4X | RPS6KA1 | RPS8 | RPTOR | RTF1 | RUNX1 | RUNX1T1 | RUNX2 | RUVBL2 | RXRA | S100A1 | S100A11 | SAG | SAMD7 | SAP18 | SARS | SCFD1 | SDCBP | SERPINB2 | SERPINB3 | SERPINB5 | SERPINB6 | SERPINB8 | SERPINE2 | SERPINH1 | SET | SF1 | SFMBT1 | SFN | SFPQ | SGTA | SHC1 | SHMT1 | SIN3A | SIRT1 | SIRT3 | SIRT5 | SIRT6 | SIRT7 | SKI | SKIV2L | SLAMF8 | SLC9A3R1 | SMAD2 | SMAD3 | SMAD4 | SMAD5 | SMARCA2 | SMARCA4 | SMARCB1 | SMARCC1 | SMARCC2 | SMARCE1 | SMC1A | SMC3 | SMG5 | SMG6 | SMG8 | SMO | SMURF2 | SNAI2 | SNCA | SND1 | SNTA1 | SNW1 | SNX12 | SNX3 | SNX6 | SOCS1 | SOD1 | SOX2 | SP100 | SP2 | SPEN | SPI1 | SPINK7 | SPINT2 | SPRY2 | SQSTM1 | SRC | SRSF12 | SSB | STAG2 | STAT1 | STAT3 | STAT6 | STK11 | STK38 | STRAP | STYX | SUMO1 | SUPT4H1 | SUPT5H | SUZ12 | SWAP70 | SYNJ2BP | TAB2 | TAGLN3 | TAOK3 | TARDBP | TBL1X | TCERG1 | TCF21 | TCF25 | TCP10L | TERF1 | TERF2 | TERF2IP | TFIP11 | TGFB1 | THAP11 | THAP7 | THBS1 | THRA | TIA1 | TIMP1 | TIMP2 | TINF2 | TLE1 | TLE3 | TMBIM6 | TNF | TNFAIP3 | TNFSF14 | TNKS | TNKS2 | TNP1 | TP53 | TP63 | TPR | TRAF3IP1 | TRAF6 | TRAP1 | TRIAP1 | TRIB2 | TRIB3 | TRIM21 | TRIM28 | TRIM29 | TRIM33 | TRIP12 | TSC22D4 | TSG101 | TSPYL2 | TWIST1 | TXN | TXNIP | TYMS | U2AF2 | UBA3 | UBB | UBC | UBE2B | UBE2D1 | UBE2D3 | UBE2I | UBE2J1 | UBQLN2 | UBQLN4 | UBR5 | UBXN1 | UCHL1 | UCHL5 | UNK | UPF2 | UPF3B | USP14 | USP17L2 | USP19 | USP2 | USP4 | USP47 | USP7 | USP9X | VDAC1 | VDR | VHL | VPS72 | VTN | WARS | WDR35 | WDTC1 | WNK1 | WT1 | WWOX | WWP2 | XIAP | XPO1 | XRCC1 | XRCC5 | XRCC6 | YAF2 | YAP1 | YEATS2 | YWHAB | YWHAE | YWHAG | YWHAQ | YY1 | ZBTB16 | ZBTB21 | ZBTB33 | ZBTB42 | ZBTB7A | ZBTB7B | ZC3H4 | ZHX1 | ZMPSTE24 | ZMYND8 | ZNF217 | ZNF24 | ZNF746 | GO:0042391 | 0.0245056411500667 | 202/8243 | 110/3250 | 8.65896150616064e-06 | 0.0822168395009953 | T | T | T | T | regulation of membrane potential | ABAT | ABL1 | ACTN2 | ADCYAP1 | ADORA2A | ADRB2 | AKAP9 | AKT1 | AKT2 | ALOX12 | ANK3 | APP | ARRB2 | ATP1A1 | ATP1B3 | ATP2A2 | BAX | BCL2 | BCL2L1 | BIN1 | CACNA1A | CACNG2 | CACNG4 | CAV1 | CD36 | CDK5 | CFTR | CHCHD10 | CHRNA9 | CHRND | CHRNE | CHRNG | CLIC1 | DCN | DLD | DLG4 | DMD | DVL1 | EIF4A3 | FHL1 | FKBP1B | GABRA6 | GABRR1 | GJA1 | GLRB | GLRX | GNAQ | GOT1 | GPD1L | GRIA1 | GRID2 | GRIK2 | GSK3A | GSK3B | HCN1 | HCN2 | HCN3 | HTR3A | HTR3E | JUN | JUP | KCNIP2 | KCNK1 | KCNK16 | KDR | KIF5B | LRRK2 | MAPT | MECP2 | MLLT11 | MPP2 | MYOC | NDUFS1 | NEDD4 | NNT | NOS1AP | NRXN1 | P2RX1 | P2RX2 | P2RX4 | PARK7 | PARP1 | PIAS3 | PPA2 | PRDX3 | PRELID1 | PRKCZ | PSEN1 | PTEN | PTN | PYCR1 | RAB3GAP1 | SH3GL1 | SLC9A1 | SNCA | SNTA1 | SOD1 | SRC | SRI | STOML2 | SUMO1 | UBB | USP53 | VCP | WDR1 | WWP2 | YWHAE | YWHAH | ZMPSTE24 | ZMYND8 | GO:0009226 | 0.00230498604876865 | 19/8243 | 17/3250 | 8.87178370994205e-06 | 0.0842375863258998 | T | T | T | T | nucleotide-sugar biosynthetic process | AMDHD2 | GFPT1 | GFPT2 | GMDS | GMPPB | GNPDA1 | GNPDA2 | GNPNAT1 | MPI | NAGK | NANS | PGM3 | UAP1 | UAP1L1 | UGDH | UGP2 | UXS1 | GO:0010564 | 0.0661167050831008 | 545/8243 | 263/3250 | 9.28533665989817e-06 | 0.0881642715857331 | T | T | T | T | regulation of cell cycle process | ACTR1A | AICDA | AKAP9 | AKT1 | AKT2 | ANAPC4 | ANXA1 | APC | APEX1 | APP | ATAD5 | ATM | ATRX | AURKB | BABAM1 | BAX | BCL2 | BCL2L1 | BCL6 | BIN1 | BMI1 | BRCA1 | BRD4 | BTRC | CALM1 | CALM2 | CALM3 | CALR | CARM1 | CASP2 | CCDC36 | CCDC8 | CCNB1 | CCP110 | CDC14B | CDC16 | CDC20 | CDC25A | CDC25B | CDC25C | CDC27 | CDC42 | CDC73 | CDK2 | CDK4 | CDK5 | CDK5R1 | CDK9 | CDKN1A | CDKN2C | CENPF | CENPJ | CENPV | CEP135 | CEP152 | CEP290 | CEP57 | CEP70 | CHEK2 | CHMP2A | CHORDC1 | CKAP5 | CLTC | CNTRL | CSNK1E | CSNK2A2 | CTCF | CTNNB1 | CUL1 | CUL3 | CUL4B | CUL7 | CYP1A1 | DBF4B | DCTN1 | DDB1 | DDRGK1 | DDX3X | DUSP1 | DYNC1H1 | DYNC1I2 | DYNC1LI1 | DYNLL1 | E2F1 | E2F4 | E2F6 | ECD | EED | EGFR | EHMT1 | EHMT2 | EP300 | EZH2 | FAM83D | FBXO7 | FBXW5 | FEN1 | FGFR1 | FGFR1OP | FHL1 | FOXM1 | FSD1 | FZR1 | GIT1 | GNAI1 | HECW2 | HMMR | HNRNPU | HSP90AA1 | HSP90AB1 | HSPA1B | HSPA2 | ID2 | IK | INSR | KANK2 | KIF11 | KLHL22 | L3MBTL1 | L3MBTL2 | MAD1L1 | MAD2L1 | MAPK15 | MDM1 | MDM2 | MDM4 | MECP2 | MED1 | MEPCE | MGA | MIF | MSH6 | MTA3 | MYBBP1A | NAE1 | NEUROD1 | NIPBL | NPM1 | NSFL1C | NUBP1 | NUMA1 | NUSAP1 | OBSL1 | OFD1 | PAFAH1B1 | PAXIP1 | PCGF6 | PCM1 | PCNA | PDCD6IP | PEBP1 | PHB2 | PHC3 | PIN1 | PINX1 | PKN2 | PLK1 | PML | PPP2R1A | PRKACA | PRKAR1A | PRKAR2B | PRKCE | PRKDC | PRMT2 | PRPF40A | PSMA1 | PSMA2 | PSMA3 | PSMA5 | PSMA6 | PSMB5 | PSMB6 | PSMB7 | PSMB8 | PSMC1 | PSMC2 | PSMC3 | PSMC4 | PSMC5 | PSMC6 | PSMD1 | PSMD10 | PSMD11 | PSMD12 | PSMD14 | PSMD2 | PSMD4 | PSMD9 | PSME2 | PSME3 | PTEN | PTPN6 | PTTG1 | RAB11A | RAD21 | RAD51 | RAE1 | RB1 | RBBP4 | RBX1 | RCC1 | RDX | RHOA | RING1 | RIPK1 | RNASEH2B | RNF2 | RNF4 | RPA2 | RPL23 | RPTOR | RRM2 | SENP6 | SEPT7 | SFN | SFPQ | SH2B1 | SIN3A | SMC1A | SMC3 | SND1 | SPDL1 | SPICE1 | STAG2 | STIL | SUZ12 | SVIL | TAOK3 | TERF1 | TERF2 | TFDP1 | TGFB1 | TMOD3 | TNKS | TP53 | TP73 | TPR | TRIAP1 | TRIP13 | TUBA1A | TUBA4A | TUBB | TUBB4A | TUBB4B | TUBG1 | UBE2B | UBE2C | UBE2D1 | USP19 | USP47 | VPS4B | XPO1 | XRCC3 | YAF2 | YWHAE | YWHAG | ZBTB17 | ZFYVE19 | ZFYVE26 | ZMPSTE24 | ZNF207 | ZW10 | GO:0044265 | 0.07206114278782 | 594/8243 | 284/3250 | 1.01745005847365e-05 | 0.0966068830520726 | T | T | T | T | cellular macromolecule catabolic process | ADRM1 | AGO1 | AMFR | ANAPC4 | ARMC8 | ARRB1 | ARRB2 | ATM | AUP1 | AURKB | BAG6 | BIRC2 | BTRC | CASP3 | CBL | CBLC | CCNB1 | CDC16 | CDC20 | CDC27 | CDC34 | CDK2 | CHEK2 | CLTC | CRBN | CTNNB1 | CUL1 | CUL2 | CUL3 | CUL4B | CUL5 | CUL7 | CYLD | DCP2 | DDB1 | DDX6 | DERL1 | DIS3 | DNA2 | DNAJB2 | DNAJC10 | DNASE2 | DNASE2B | DROSHA | EDC4 | EDEM1 | EIF3E | EIF4A3 | ENDOG | ERLIN2 | EXOSC1 | EXOSC5 | EXOSC6 | EXOSC9 | FAF1 | FAF2 | FBXL12 | FBXO11 | FBXO6 | FBXO7 | FBXW11 | FBXW5 | FEN1 | FZR1 | GSK3A | GSPT1 | GSPT2 | H1F0 | HDAC6 | HECW1 | HECW2 | HELZ2 | HERC2 | HMGB1 | HMGB2 | HNRNPD | HSP90B1 | HSPA1B | HSPA5 | HTRA2 | HUWE1 | ISG15 | ITCH | KAT5 | KCTD17 | KCTD2 | KLHL20 | KLHL22 | KPNA1 | KPNB1 | LNX1 | LSM1 | LSM3 | LSM4 | LSM5 | LYG2 | MAD2L1 | MDM2 | MLH1 | MTA1 | NBAS | NCBP1 | NEDD4 | NEDD8 | NFE2L2 | NHLRC3 | NPLOC4 | NSFL1C | OAS2 | PABPC1 | PABPC4 | PAN2 | PCBP2 | PCSK9 | PDE12 | PELI1 | PLK1 | PML | PPP1R11 | PPP1R8 | PPP2CA | PPP2CB | PPP2R1A | PPP2R2A | PPT1 | PSMA1 | PSMA2 | PSMA3 | PSMA5 | PSMA6 | PSMB5 | PSMB6 | PSMB7 | PSMB8 | PSMC1 | PSMC2 | PSMC3 | PSMC4 | PSMC5 | PSMC6 | PSMD1 | PSMD10 | PSMD11 | PSMD12 | PSMD14 | PSMD2 | PSMD4 | PSMD9 | PSME2 | PSME3 | PTPN23 | PTTG1 | RAB7A | RAD23A | RAD23B | RBBP6 | RBM8A | RBX1 | RC3H1 | RIPK1 | RNASE3 | RNASEH2A | RNASEH2B | RNF126 | RNF185 | RNF19A | RNF4 | RNF8 | RPL10 | RPL10A | RPL12 | RPL18A | RPL22 | RPL23 | RPL23A | RPL24 | RPL30 | RPL37A | RPL38 | RPL6 | RPL7 | RPL7A | RPL9 | RPLP0 | RPLP1 | RPLP2 | RPS12 | RPS13 | RPS14 | RPS15A | RPS19 | RPS2 | RPS20 | RPS24 | RPS29 | RPS3 | RPS4X | RPS8 | SEL1L | SGSH | SGTA | SGTB | SIAH1 | SIRT1 | SKIV2L | SMG5 | SMG6 | SMG8 | SMURF1 | SMURF2 | SND1 | SPOP | SPSB3 | SQSTM1 | SSB | STUB1 | SYVN1 | TBL1X | TCIRG1 | TOLLIP | TREX1 | TRIM25 | TRIM32 | TRIM9 | TRIP12 | TSG101 | UBA6 | UBB | UBC | UBE2A | UBE2B | UBE2C | UBE2D1 | UBE2D2 | UBE2D3 | UBE2H | UBE2I | UBE2J1 | UBE2K | UBE2L3 | UBE2L6 | UBE2R2 | UBE2S | UBE2W | UBE3A | UBE3B | UBQLN1 | UBQLN2 | UBQLN4 | UBR4 | UBXN1 | UCHL1 | UCHL3 | UCHL5 | UPF2 | UPF3B | USP11 | USP14 | USP15 | USP16 | USP17L2 | USP19 | USP2 | USP20 | USP21 | USP26 | USP28 | USP4 | USP42 | USP46 | USP47 | USP5 | USP50 | USP7 | USP8 | USP9Y | VCP | VPS37D | VPS4B | WWP2 | XBP1 | YME1L1 | ZMPSTE24 | ZNRF4 | GO:0097190 | 0.0232924905980832 | 192/8243 | 105/3250 | 1.07684474145981e-05 | 0.102246408201609 | T | T | T | T | apoptotic signaling pathway | ABL1 | AIFM1 | ANXA6 | ATAD5 | ATM | BAG3 | BAG6 | BAX | BBC3 | BCL2 | BCL2L1 | BCL2L2 | BRCA1 | BTK | CASP10 | CASP2 | CASP3 | CASP8 | CASP9 | CAV1 | CD27 | CDKN1A | CHEK2 | CRIP1 | CUL1 | CUL2 | CUL3 | CUL5 | DAP3 | DAPK1 | DAXX | DDIT4 | DDX3X | DEDD | DIABLO | DIDO1 | DNAJC10 | DPF2 | DYRK2 | E2F1 | E2F2 | EP300 | ERBB3 | EYA2 | FADD | FASLG | FGFR3 | GPX1 | GSK3A | GSK3B | HINT1 | HMOX1 | HRAS | HTRA2 | IFI16 | IKBKE | ITGAV | ITPR1 | MELK | MKNK2 | MLH1 | MLLT11 | MSH6 | MYBBP1A | NDUFA13 | NUPR1 | P2RX4 | PAWR | PDCD10 | PDCD6 | PDK2 | PIK3R1 | PML | PPARD | PRKCD | PRKDC | RIPK1 | RIPK3 | SFN | SGPL1 | SIRT1 | SMAD3 | SNW1 | SORT1 | STK11 | STK24 | STK25 | TGFB1 | TLR3 | TMBIM6 | TNF | TNFRSF12A | TNFRSF1A | TNFSF12 | TP53 | TP63 | TP73 | TRADD | TRAF2 | TRIB3 | UBE2K | USP28 | WWOX | XBP1 | ZNF622 | GO:0002253 | 0.043309474705811 | 357/8243 | 180/3250 | 1.09269018153588e-05 | 0.103750932736832 | T | T | T | T | activation of immune response | ABL1 | ACTB | ACTG1 | ACTR2 | ACTR3 | APCS | ARPC1A | ARPC1B | ARPC2 | ARPC3 | ARPC4 | ARPC5 | BAG6 | BAIAP2 | BAX | BCAR1 | BCL10 | BCL2 | BIRC2 | BIRC3 | BRAF | BTK | BTN2A1 | BTRC | C1QA | C1QBP | C1R | C4A | CARD9 | CASP8 | CD209 | CD36 | CD79B | CDC42 | CLU | COLEC10 | CREBBP | CRK | CRKL | CRP | CTSL | CUL1 | CYLD | EIF2B1 | EIF2B3 | EIF2B5 | EP300 | FADD | FBXW11 | FCGR1A | FCN1 | FGB | FYN | GRB2 | HAVCR2 | HEXIM1 | HLA-DQA1 | HLA-DRA | HLA-DRB1 | HLA-DRB3 | HLA-DRB4 | HLA-DRB5 | HMGB1 | HRAS | HSP90AA1 | HSP90AB1 | HSP90B1 | HSPD1 | IFI16 | IGHV3-13 | IGHV3-23 | IGHV3-30 | IGKV3-20 | IGLC7 | IKBKB | IKBKE | IKBKG | IRF7 | ITCH | ITK | KRAS | LAT | LCP2 | LGMN | MAP2K6 | MAP3K1 | MAPK1 | MAPK3 | MASP1 | MASP2 | MATR3 | MAVS | MYD88 | MYO1C | NCK1 | NFATC2 | NFKBIA | NOD2 | PAG1 | PAK2 | PDE4D | PIK3CA | PIK3R1 | PLA2G6 | PLCG1 | PRKACA | PRKCD | PRKCE | PRKD2 | PRKDC | PSEN1 | PSMA1 | PSMA2 | PSMA3 | PSMA5 | PSMA6 | PSMB5 | PSMB6 | PSMB7 | PSMB8 | PSMC1 | PSMC2 | PSMC3 | PSMC4 | PSMC5 | PSMC6 | PSMD1 | PSMD10 | PSMD11 | PSMD12 | PSMD14 | PSMD2 | PSMD4 | PSMD9 | PSME2 | PSME3 | PTK2 | PTPN22 | PTPN6 | RAC1 | RAF1 | RC3H1 | RELA | RIPK1 | RIPK2 | RNF31 | S100A1 | S100A9 | SCARA3 | SFPQ | SIN3A | SPG21 | SRC | STK11 | STOML2 | TAB1 | TAB2 | TANK | TLR3 | TMEM173 | TNFAIP3 | TNIP2 | TRAF3 | TRAF6 | TREX1 | UBB | UBC | UBE2D1 | UBE2D2 | UBE2D3 | UBE2N | UBE2V1 | VAV1 | VSIG4 | WAS | WNK1 | XRCC5 | XRCC6 | YES1 | ZAP70 | GO:0010038 | 0.0280237777508189 | 231/8243 | 123/3250 | 1.12244311165348e-05 | 0.106575973451498 | T | T | T | T | response to metal ion | ABAT | ACO1 | ACTA1 | ACTG1 | AHCYL1 | AKT1 | ALOX5AP | ANK3 | ANXA11 | ANXA5 | ANXA7 | APBB1 | APP | ASCL1 | ASS1 | ATP13A2 | BACE1 | BCL2 | BRAF | BSG | C1QA | CACNG2 | CALM1 | CALM2 | CALM3 | CALR | CASP3 | CASP8 | CASP9 | CAT | CAV1 | CCNB1 | CDH1 | CDK4 | CEBPA | CLIC4 | CPNE3 | CREB1 | CRIP1 | CUTA | CYP1A1 | CYP1A2 | DAXX | DLG2 | DLG4 | DPEP1 | DUSP1 | EGFR | EIF2AK3 | EIF2B5 | EIF2S1 | ENDOG | FBP1 | FECH | FGB | FOS | FUS | G6PD | GDI1 | GOT1 | GPI | GSS | HAAO | HIF1A | HMOX1 | HNRNPD | HSF1 | HSPA5 | ICAM1 | ID2 | IQGAP1 | IREB2 | ITPR3 | JUN | JUNB | KCNIP2 | LGMN | LIG4 | LOXL2 | LRRK2 | MAPK1 | MAPK3 | MAPK8 | MAPK9 | MAPT | MDM2 | NDRG1 | NEDD4 | NQO1 | OTC | P2RX4 | PARP1 | PCNA | PDCD6 | PEBP1 | PEF1 | PGAM2 | PPARGC1A | PPIF | PPP1CA | PPP2CA | PPP2CB | PPP5C | PRKAA1 | PRKAA2 | PTEN | PTGES | QDPR | S100A13 | SDC1 | SEC31A | SLC25A13 | SLC25A24 | SNCA | SOD1 | SORD | STIM1 | SUMO1 | THBS1 | TXNIP | UBE2D3 | VCAM1 | WNK1 | GO:0048584 | 0.162804804076186 | 1342/8243 | 599/3250 | 1.2503456579667e-05 | 0.118720320223938 | T | T | T | T | positive regulation of response to stimulus | ABAT | ABL1 | ACTB | ACTG1 | ACTN4 | ACTR2 | ACTR3 | ADA | ADCYAP1 | ADORA2A | ADRB2 | AGER | AGO1 | AGR2 | AKR1B1 | AKR1C2 | AKT1 | AKT3 | ANXA1 | AP1G1 | APBB1 | APCS | APOA1 | APOE | APP | AR | ARHGAP1 | ARNT | ARPC1A | ARPC1B | ARPC2 | ARPC3 | ARPC4 | ARPC5 | ARRB1 | ARRB2 | ASCL1 | ATAD5 | ATF6 | ATM | AXIN1 | AZU1 | BABAM1 | BAG4 | BAG6 | BAIAP2 | BAX | BBC3 | BCAR1 | BCL10 | BCL2 | BCL2L1 | BIRC2 | BIRC3 | BIRC7 | BIRC8 | BMPR1A | BRAF | BRCA1 | BRD4 | BTK | BTN2A1 | BTRC | BUD31 | C1QA | C1QBP | C1QTNF1 | C1R | C4A | CACNG2 | CACNG4 | CALM1 | CALM2 | CALM3 | CALR | CAMK1D | CARD9 | CASP10 | CASP2 | CASP8 | CAT | CAV1 | CBL | CBX8 | CCAR2 | CCDC22 | CCL21 | CCL22 | CCR1 | CCR4 | CD209 | CD27 | CD36 | CD40 | CD40LG | CD6 | CD74 | CD79B | CD81 | CDC42 | CDC73 | CDK19 | CEBPA | CFTR | CHCHD10 | CLNK | CLU | COLEC10 | CREB3 | CREBBP | CRK | CRKL | CRP | CSNK1E | CSNK2B | CTH | CTNNB1 | CTSC | CTSL | CUL1 | CXCL8 | CYLD | DAB2 | DAXX | DBNL | DCN | DDRGK1 | DDX1 | DDX3X | DHX36 | DHX9 | DISC1 | DLG4 | DNM1L | DOK1 | DUSP19 | DUSP22 | DUSP7 | DVL1 | DVL2 | DVL3 | DYNLL1 | E2F1 | EDA | EDEM1 | EEF1D | EGFR | EIF2AK4 | EIF2B1 | EIF2B3 | EIF2B5 | ELF3 | ENDOG | EP300 | EPHA4 | ERBB2 | ERBB3 | ESR1 | ESRRA | ETS1 | EXOSC6 | EYA2 | EYA4 | EZH2 | FABP5 | FADD | FAF1 | FAM19A3 | FAM49B | FASLG | FBXW11 | FCGR1A | FCN1 | FGB | FGF2 | FGFR1 | FGFR3 | FIS1 | FKBP1A | FKBP1B | FKBP8 | FLOT1 | FOXA1 | FOXM1 | FUS | FYN | GADD45G | GBP5 | GDF9 | GJA1 | GKAP1 | GNAI2 | GOLPH3 | GOLT1B | GOT1 | GPS2 | GPX1 | GRB2 | GRB7 | GSK3A | GSK3B | GSTO1 | H2AFX | HAP1 | HAVCR2 | HAX1 | HBEGF | HDAC2 | HDAC3 | HDAC6 | HEXIM1 | HIF1A | HINT1 | HK1 | HLA-A | HLA-B | HLA-DQA1 | HLA-DRA | HLA-DRB1 | HLA-DRB3 | HLA-DRB4 | HLA-DRB5 | HLX | HMGB1 | HMGB2 | HMGB3 | HMOX1 | HPX | HRAS | HSF1 | HSP90AA1 | HSP90AB1 | HSP90B1 | HSPA1B | HSPB1 | HSPD1 | HTRA2 | HTT | ICAM1 | IFI16 | IFNA2 | IGF1R | IGHV3-13 | IGHV3-23 | IGHV3-30 | IGKV3-20 | IGLC7 | IKBKB | IKBKE | IKBKG | ILK | ING5 | INSR | IQGAP1 | IRF7 | ITCH | ITGB1BP1 | ITGB3 | ITK | JUN | JUP | KDM4D | KDR | KHDRBS1 | KIF5B | KLHDC10 | KLHL22 | KMT2A | KRAS | KSR1 | LARS | LASP1 | LAT | LCP2 | LGALS1 | LGMN | LGR4 | LIMS1 | LPAR1 | LPAR4 | LRRK1 | LRRK2 | LRSAM1 | LTBR | LURAP1 | MAOA | MAP2K1 | MAP2K3 | MAP2K4 | MAP2K6 | MAP2K7 | MAP3K1 | MAP3K13 | MAP3K14 | MAP3K2 | MAP3K3 | MAP3K4 | MAP4K1 | MAPK1 | MAPK14 | MAPK3 | MAPK8 | MAPK8IP3 | MAPK9 | MAS1 | MASP1 | MASP2 | MATR3 | MAVS | MC1R | MED1 | MGMT | MICA | MIF | MLH1 | MLLT11 | MLLT3 | MLST8 | MPC2 | MSH6 | MYC | MYD88 | MYO1C | MYOC | NCK1 | NEDD4 | NELFE | NFATC2 | NFE2L2 | NFKBIA | NMNAT1 | NOD2 | NOS1AP | NPR1 | NQO2 | NR1H3 | NRXN1 | NSMCE4A | NTF3 | NTRK1 | NUMA1 | NUP93 | OPA1 | OPTN | OXCT1 | OXSR1 | P2RX2 | P2RX4 | PAFAH1B1 | PAG1 | PAK2 | PARK7 | PARP1 | PARP14 | PAWR | PAXIP1 | PCNA | PDCD10 | PDCL | PDE4D | PDE6G | PDIA3 | PEBP1 | PELI1 | PHB | PHB2 | PIH1D1 | PIK3CA | PIK3CG | PIK3R1 | PIN1 | PLA2G1B | PLA2G2A | PLA2G6 | PLAUR | PLCG1 | PLEKHF1 | PLSCR1 | PML | PPARD | PPIF | PPM1A | PPP1CA | PPP2R1A | PPP2R1B | PPP3CC | PPP5C | PRDX2 | PRKAA1 | PRKACA | PRKCD | PRKCE | PRKCZ | PRKD1 | PRKD2 | PRKDC | PSAP | PSEN1 | PSMA1 | PSMA2 | PSMA3 | PSMA5 | PSMA6 | PSMB5 | PSMB6 | PSMB7 | PSMB8 | PSMC1 | PSMC2 | PSMC3 | PSMC4 | PSMC5 | PSMC6 | PSMD1 | PSMD10 | PSMD11 | PSMD12 | PSMD14 | PSMD2 | PSMD4 | PSMD9 | PSME2 | PSME3 | PTEN | PTGER3 | PTK2 | PTPN1 | PTPN22 | PTPN6 | PUM1 | RAB29 | RAC1 | RAF1 | RAP1A | RAP1B | RARA | RB1CC1 | RBPJ | RC3H1 | RECK | REL | RELA | RGN | RHOA | RIF1 | RIPK1 | RIPK2 | RIPK3 | RNF183 | RNF185 | RNF31 | RNF8 | RPL23 | RPS19 | RPS3 | RPTOR | RTN4 | RUVBL1 | S100A1 | S100A12 | S100A13 | S100A4 | S100A7 | S100A9 | S100B | SARM1 | SCARA3 | SCEL | SCG2 | SDCBP | SEC13 | SEMA7A | SEPT4 | SFN | SFPQ | SGTA | SH2B1 | SHC1 | SHKBP1 | SIAH1 | SIN3A | SIRT1 | SKI | SLAMF1 | SLC25A6 | SLC9A1 | SLC9A3R1 | SMAD2 | SMAD3 | SMAD4 | SMARCA4 | SMARCB1 | SMO | SMURF2 | SNCA | SNW1 | SOCS2 | SOD1 | SOX2 | SPG21 | SPIN1 | SPRY2 | SRC | SRI | STAT3 | STAT6 | STK11 | STK25 | STK3 | STOML2 | STXBP1 | SULF2 | SWAP70 | TAB1 | TAB2 | TANK | TAOK3 | TBL1X | TBXA2R | TERF2IP | TFDP1 | TFG | TGFB1 | TGFBR3 | TGM2 | THBS1 | TIMP2 | TLR3 | TMEM173 | TMEM9B | TNF | TNFAIP3 | TNFRSF12A | TNFRSF1A | TNFSF12 | TNFSF14 | TNIP2 | TNKS | TNKS2 | TP53 | TP63 | TP73 | TRADD | TRAF2 | TRAF3 | TRAF6 | TREX1 | TRIM25 | TRIM28 | TRIM32 | TRIP6 | TUBB2B | TXN | UBB | UBC | UBE2B | UBE2D1 | UBE2D2 | UBE2D3 | UBE2I | UBE2K | UBE2N | UBE2O | UBE2V1 | UBQLN1 | UBQLN2 | UBR5 | USP17L2 | USP47 | USP8 | VAPA | VAV1 | VCP | VSIG4 | VTN | WAS | WBP2 | WDFY1 | WDR35 | WNK1 | WNT16 | WWOX | XBP1 | XIAP | XRCC1 | XRCC5 | XRCC6 | YAP1 | YES1 | YWHAB | YWHAE | YWHAG | YWHAH | YWHAQ | YWHAZ | ZAP70 | ZBTB7B | ZDHHC17 | ZNF622 | GO:0002275 | 0.0425815843746209 | 351/8243 | 177/3250 | 1.27978375574104e-05 | 0.121515467607612 | T | T | T | T | myeloid cell activation involved in immune response | ACAA1 | ACLY | ACTR2 | AGL | AHSG | ALDOA | ALDOC | ALOX5 | ANXA2 | AP1M1 | APEH | APRT | ARMC8 | ARPC5 | ATP6V1D | AZU1 | CAB39 | CAND1 | CAPN1 | CAT | CBL | CCT2 | CCT8 | CD36 | CDA | CEP290 | CKAP4 | COPB1 | COTL1 | CPNE3 | CSNK2B | CTSA | CTSC | CTSD | CYB5R3 | DBNL | DDOST | DDX3X | DEGS1 | DERA | DYNC1H1 | DYNC1LI1 | DYNLL1 | DYNLT1 | EEF1A1 | EEF2 | FABP5 | FAF2 | FCN1 | FGL2 | FTH1 | FTL | GAA | GALNS | GDI2 | GHDC | GLA | GNS | GPI | GSTP1 | GUSB | GYG1 | HAVCR2 | HEXB | HMGB1 | HRNR | HSP90AA1 | HSP90AB1 | HSPA1B | HSPA8 | HUWE1 | IDH1 | ILF2 | IMPDH2 | IQGAP1 | ITGAV | JUP | KCMF1 | KCNAB2 | KPNB1 | LAT | LGALS3 | LRRC7 | MAN2B1 | MAPK1 | MAPK14 | MGST1 | MIF | MME | NAPRT | NHLRC3 | NIT2 | NME2 | ORM1 | P2RX1 | PA2G4 | PDXK | PFKL | PGAM1 | PGM1 | PGM2 | PGRMC1 | PI4K2A | PIK3CG | PKM | PLAUR | PNP | PPIA | PPIE | PRCP | PRDX4 | PRDX6 | PRG2 | PRKCD | PRKCE | PSAP | PSEN1 | PSMA2 | PSMA5 | PSMB7 | PSMC2 | PSMC3 | PSMD1 | PSMD11 | PSMD12 | PSMD14 | PSMD2 | PTGES2 | PTPN6 | PYGB | PYGL | QSOX1 | RAB10 | RAB18 | RAB24 | RAB31 | RAB5B | RAB7A | RAC1 | RAP1A | RAP1B | RHOA | RNASE3 | S100A11 | S100A12 | S100A13 | S100A7 | S100A9 | S100P | SDCBP | SELL | SERPINB3 | SERPINB6 | SLAMF1 | SLC2A5 | SNAP29 | SPTAN1 | SRP14 | STBD1 | STOM | STXBP2 | TCIRG1 | TIMP2 | TMEM173 | TMEM30A | TOLLIP | TREX1 | TUBB | TUBB4B | TXNDC5 | UBR4 | VAPA | VAT1 | VCL | VCP | XRCC5 | XRCC6 | GO:1904951 | 0.0342108455659347 | 282/8243 | 146/3250 | 1.29628266911509e-05 | 0.123082039432477 | T | T | T | T | positive regulation of establishment of protein localization | ABAT | ABL1 | ADORA2A | AGER | AKT2 | ANK3 | APBB1 | ARF1 | ARRB1 | ATP13A2 | B3GAT3 | BAG3 | BBC3 | BCL2 | CASP8 | CCT2 | CCT3 | CCT4 | CCT5 | CCT6A | CCT7 | CCT8 | CD2 | CDH1 | CDK5 | CDK5R1 | CENPJ | CEP135 | CEP290 | CFTR | CHP1 | CSNK2A2 | DHX36 | DHX9 | DMAP1 | DNM1L | DYNLL1 | E2F1 | EDEM1 | EGFR | ERBB2 | EZR | FCN1 | FGB | FIS1 | FYN | GAPDH | GLMN | GLUL | GOLPH3 | GPI | GSK3A | GSK3B | HAVCR2 | HDAC3 | HIF1A | HK1 | HMGB1 | HRAS | HSPA1L | HTRA2 | HUWE1 | IPO5 | ITGB1BP1 | JUP | KIF5B | LRRK2 | MAPK1 | MAPK14 | MAPK3 | MAPK8 | MAVS | MBP | MDM2 | MIF | MPC2 | MYO1C | NOD2 | NPEPPS | NR0B2 | NR1H2 | ORM1 | OXCT1 | PANX3 | PCM1 | PDCD10 | PFKM | PIK3R1 | PLA2G1B | PLA2G6 | PPARD | PPIA | PPID | PPM1A | PPP3CC | PRKAA1 | PRKACA | PRKCD | PRKCE | PRKCZ | PSEN1 | PSMD9 | PTPN22 | PTPN23 | RAB29 | RAN | RHOU | RIPK2 | RNF31 | SAE1 | SEC16A | SFN | SIRT3 | SMAD3 | SMO | SPTBN1 | SRC | SRI | STOM | TARDBP | TCP1 | TFDP1 | TGFB1 | TM9SF4 | TMEM30A | TNF | TP53 | TP63 | TP73 | TPR | TRIM28 | TSG101 | TWIST1 | UBE2D3 | UBE2L3 | UBR5 | USP50 | XBP1 | XPO4 | YWHAB | YWHAE | YWHAG | YWHAH | YWHAQ | YWHAZ | ZIC1 | GO:1902532 | 0.038820817663472 | 320/8243 | 163/3250 | 1.37428129044723e-05 | 0.130488008527964 | T | T | T | T | negative regulation of intracellular signal transduction | ABL1 | ABL2 | AKT1 | APLP1 | APOE | ARHGAP35 | ARRB1 | ARRB2 | ATAD5 | ATM | BAG5 | BCL2 | BCL2L1 | BCL2L2 | BCL6 | BDKRB2 | C1QBP | C1QL4 | CALM1 | CALM2 | CALM3 | CASP8 | CAV1 | CBLC | CCAR2 | CCDC22 | CD22 | CD74 | CDC34 | CHP1 | CHRNA9 | CLU | CREB3 | CUL3 | CYLD | DDIT4 | DDX3X | DNAJA1 | DUSP1 | DUSP14 | DUSP16 | DUSP19 | DUSP22 | DUSP7 | DYRK2 | EIF3A | ENO1 | EPM2A | ERRFI1 | ESR1 | EZR | FBP1 | FBXW11 | FHL2 | FIGNL1 | FKBP1A | FKBP1B | FOXM1 | FOXO1 | GNAI2 | GPD1L | GPS2 | GPX1 | GSK3A | GSK3B | GSTO1 | GSTP1 | HDAC1 | HDAC3 | HDAC7 | HIF1A | HSPB1 | HTRA2 | IGBP1 | IGF1R | ITCH | ITGB1BP1 | ITPR1 | KDM1A | LEMD2 | LPAR1 | LRRK2 | LZTR1 | MAPK7 | MAPKAP1 | MDM2 | MIF | MYC | MYOC | NCOR1 | NDUFA13 | NDUFS3 | NF2 | NFE2L2 | NPLOC4 | OPA1 | OPTN | PAFAH1B1 | PARK7 | PDCD6 | PDE4D | PEBP1 | PER1 | PHB | PIN1 | PLAUR | PPIF | PPM1A | PPM1B | PPP2CA | PPP2CB | PPP2R1A | PRKAA1 | PRKAA2 | PRKCD | PSMD10 | PTEN | PTPN1 | PTPN22 | PTPN6 | PTPRR | QARS | RANBP9 | RELA | RGS2 | RHOA | RIPK1 | RRM2B | SERPINB3 | SERPINE2 | SIRT1 | SIRT3 | SLC9A3R1 | SMAD4 | SNAI2 | SOD2 | SPRY2 | SRC | SRI | STAT1 | STK11 | STK38 | STMN1 | SYNJ2BP | SYVN1 | TANK | TAOK3 | THBS1 | THEM4 | TIMP2 | TLE1 | TMBIM6 | TNFAIP3 | TRAP1 | TRIAP1 | TRIB3 | TRIM32 | TWIST1 | UBE2B | UCHL1 | USP47 | VDAC2 | XBP1 | GO:0009064 | 0.00521654737352906 | 43/8243 | 31/3250 | 1.37548210564281e-05 | 0.130602025930785 | T | T | T | T | glutamine family amino acid metabolic process | ALDH4A1 | ALDH5A1 | ASL | ASNS | ASS1 | CTPS1 | FAH | FPGS | GAD2 | GFPT1 | GFPT2 | GGT1 | GLS | GLS2 | GLUD2 | GLUL | GMPS | GOT1 | GOT2 | MECP2 | NIT2 | NOS2 | NOS3 | OAT | OTC | PADI4 | PFAS | PHGDH | PPAT | PYCR1 | PYCR2 | GO:0038093 | 0.0197743539973311 | 163/8243 | 91/3250 | 1.39056808903193e-05 | 0.132034440053582 | T | T | T | T | Fc receptor signaling pathway | ABL1 | ACTB | ACTG1 | ACTR2 | ACTR3 | ARPC1A | ARPC1B | ARPC2 | ARPC3 | ARPC4 | ARPC5 | BAIAP2 | BCL10 | BTK | BTRC | CALM1 | CDC42 | CRK | CUL1 | FBXW11 | FCGR1A | FOS | FYN | GRB2 | HSP90AA1 | HSP90AB1 | IGHV3-13 | IGHV3-23 | IGHV3-30 | IGKV3-20 | IGLC7 | IKBKB | IKBKG | ITK | JUN | LAT | LCP2 | MAP2K4 | MAP2K7 | MAP3K1 | MAPK1 | MAPK3 | MAPK8 | MAPK9 | MYO1C | NCK1 | NFATC1 | NFATC2 | PAK2 | PIK3CA | PIK3R1 | PLA2G6 | PLCG1 | PRKCD | PRKCE | PSMA1 | PSMA2 | PSMA3 | PSMA5 | PSMA6 | PSMB5 | PSMB6 | PSMB7 | PSMB8 | PSMC1 | PSMC2 | PSMC3 | PSMC4 | PSMC5 | PSMC6 | PSMD1 | PSMD10 | PSMD11 | PSMD12 | PSMD14 | PSMD2 | PSMD4 | PSMD9 | PSME2 | PSME3 | PTK2 | RAC1 | RELA | SHC1 | SRC | TAB1 | TAB2 | TRAF6 | VAV1 | WAS | YES1 | GO:1902531 | 0.1323547252214 | 1091/8243 | 494/3250 | 1.4021033224622e-05 | 0.133129710467786 | T | T | T | T | regulation of intracellular signal transduction | A2M | ABL1 | ABL2 | ACTN4 | ADA | ADCYAP1 | ADRB2 | AGER | AGO1 | AKR1C2 | AKT1 | AKT3 | APLP1 | APOA1 | APOE | APP | AR | ARAF | ARFGAP1 | ARHGAP1 | ARHGAP19 | ARHGAP26 | ARHGAP35 | ARHGDIA | ARHGEF7 | ARHGEF9 | ARRB1 | ARRB2 | ATAD5 | ATM | AURKB | AXIN1 | BAG4 | BAG5 | BANP | BAX | BCL10 | BCL2 | BCL2L1 | BCL2L2 | BCL6 | BDKRB2 | BIRC2 | BIRC3 | BIRC7 | BRAF | BRCA1 | BRD4 | BTRC | C1QBP | C1QL4 | C1QTNF1 | CALM1 | CALM2 | CALM3 | CALR | CARD9 | CASP10 | CASP8 | CAT | CAV1 | CBL | CBLC | CCAR2 | CCDC22 | CCL21 | CCL22 | CCR1 | CD22 | CD27 | CD36 | CD40 | CD40LG | CD74 | CD81 | CDC34 | CDC42 | CDC42SE2 | CDK2 | CDK5 | CDK5R1 | CEP55 | CHD3 | CHD4 | CHEK2 | CHP1 | CHRNA9 | CLU | COPS5 | CREB3 | CRK | CRKL | CSNK2A2 | CSNK2B | CTH | CTNNB1 | CUL3 | CYLD | DAXX | DBNL | DCN | DDIT4 | DDRGK1 | DDX1 | DDX3X | DENND1A | DEPDC7 | DHX36 | DMD | DNA2 | DNAJA1 | DNM1L | DUSP1 | DUSP14 | DUSP16 | DUSP19 | DUSP22 | DUSP7 | DVL2 | DVL3 | DYRK2 | EDA | EEF1D | EGFR | EHMT1 | EHMT2 | EIF2AK3 | EIF3A | ENO1 | EP300 | EPHA4 | EPM2A | ERBB2 | ERBB3 | ERRFI1 | ESR1 | EZH2 | EZR | FADD | FAM83D | FASLG | FBP1 | FBXW11 | FGB | FGF2 | FGFR1 | FGFR3 | FHL2 | FIGNL1 | FIS1 | FKBP1A | FKBP1B | FLOT1 | FN1 | FOXM1 | FOXO1 | FYN | GADD45G | GDF9 | GDI1 | GDI2 | GJA1 | GNA12 | GNA13 | GNAI1 | GNAI2 | GOLPH3 | GOLT1B | GPD1L | GPS2 | GPX1 | GRB2 | GRIK2 | GSK3A | GSK3B | GSTO1 | GSTP1 | HAP1 | HAVCR2 | HAX1 | HBEGF | HDAC1 | HDAC2 | HDAC3 | HDAC7 | HEXIM1 | HGS | HIF1A | HINT1 | HIPK4 | HMGB1 | HMOX1 | HNRNPK | HRAS | HSP90AA1 | HSP90AB1 | HSPA1B | HSPB1 | HTRA2 | HTT | ICAM1 | IGBP1 | IGF1R | IKBKB | IKBKE | IKBKG | ILK | ING5 | INSR | IQGAP1 | ITCH | ITGB1BP1 | ITPR1 | JUN | KANK2 | KAT5 | KDM1A | KDR | KLHDC10 | KLHL22 | KRAS | KSR1 | L3MBTL1 | LARS | LEMD2 | LEMD3 | LGALS1 | LIMS1 | LPAR1 | LPAR4 | LRRK1 | LRRK2 | LTBR | LURAP1 | LZTR1 | MAP2K1 | MAP2K3 | MAP2K4 | MAP2K6 | MAP2K7 | MAP3K1 | MAP3K13 | MAP3K14 | MAP3K2 | MAP3K3 | MAP3K4 | MAP4K1 | MAPK1 | MAPK14 | MAPK3 | MAPK7 | MAPK8IP3 | MAPKAP1 | MAPT | MAS1 | MAVS | MBD3 | MC1R | MDM2 | MDM4 | MIF | MLST8 | MYC | MYD88 | MYO5A | MYOC | NCK1 | NCOR1 | NDUFA13 | NDUFS3 | NEDD4 | NELFE | NF2 | NFE2L2 | NFKBIA | NMNAT1 | NOD2 | NOS1AP | NPLOC4 | NPR1 | NQO2 | NTF3 | NTRK1 | OGT | OPA1 | OPTN | P2RX2 | P2RX4 | P4HB | PAFAH1B1 | PAK2 | PAK6 | PARK7 | PARP1 | PDCD10 | PDCD6 | PDE4D | PDE6G | PDK2 | PEBP1 | PELI1 | PER1 | PHB | PHB2 | PIH1D1 | PIK3CA | PIK3CG | PIK3R1 | PIN1 | PIP4K2A | PLA2G1B | PLA2G2A | PLA2G6 | PLAUR | PLEKHF1 | PML | PPARD | PPIF | PPM1A | PPM1B | PPP2CA | PPP2CB | PPP2R1A | PPP5C | PRDX1 | PRDX2 | PRKAA1 | PRKAA2 | PRKAB2 | PRKACA | PRKAG1 | PRKCD | PRKCE | PRKCZ | PRKD1 | PRKD2 | PRMT6 | PSAP | PSEN1 | PSMD10 | PTEN | PTK2 | PTPMT1 | PTPN1 | PTPN22 | PTPN6 | PTPRR | PUM1 | QARS | RAC1 | RAF1 | RANBP9 | RAP1A | RAP1B | RB1CC1 | RBBP4 | RC3H1 | RDX | REL | RELA | RFC2 | RFC3 | RFC4 | RFC5 | RGN | RGS2 | RHOA | RHOJ | RHOT1 | RHOT2 | RHOU | RIPK1 | RIPK2 | RIPK3 | RNF183 | RNF31 | RPA2 | RPA3 | RPL23 | RPS3 | RPTOR | RRM2B | RRS1 | RTN4 | S100A12 | S100A13 | S100A4 | S100A7 | S100A9 | S100B | SDCBP | SEC13 | SEMA7A | SEPT4 | SERPINB3 | SERPINE2 | SFPQ | SHC1 | SIAH1 | SIPA1 | SIPA1L1 | SIRT1 | SIRT3 | SLAMF1 | SLC9A1 | SLC9A3R1 | SMAD4 | SNAI2 | SNF8 | SOD1 | SOD2 | SOX2 | SPRY2 | SQSTM1 | SRC | SRGAP2 | SRGAP3 | SRI | SSX2IP | STARD13 | STAT1 | STK11 | STK25 | STK3 | STK38 | STK40 | STMN1 | STYX | SYDE1 | SYNJ2BP | SYVN1 | TAB1 | TAB2 | TAF12 | TAF15 | TANK | TAOK3 | TERF2IP | TFG | TGFB1 | TGFBR3 | TGM2 | THBS1 | THEM4 | TIMP2 | TLE1 | TLR3 | TMBIM6 | TMEM9B | TNF | TNFAIP3 | TNFRSF1A | TNFSF14 | TNIP2 | TOP3A | TP53 | TP53RK | TP63 | TP73 | TRADD | TRAF2 | TRAF6 | TRAP1 | TRIAP1 | TRIB2 | TRIB3 | TRIM25 | TRIM32 | TRIP6 | TSG101 | TTC5 | TWIST1 | TXN | UBB | UBC | UBE2B | UBE2I | UBE2N | UBE2V1 | UBQLN1 | UCHL1 | USP17L2 | USP47 | VAPA | VAV1 | VDAC2 | WNT16 | XBP1 | XIAP | ZAP70 | ZDHHC17 | ZFAND6 | ZMPSTE24 | ZNF622 | GO:0048585 | 0.115613247604028 | 953/8243 | 436/3250 | 1.42365741987672e-05 | 0.135176272017294 | T | T | T | T | negative regulation of response to stimulus | A2M | ABAT | ABL1 | ABL2 | ACAA2 | ADA | ADCYAP1 | ADORA2A | ADRB2 | ADTRP | AES | AGER | AHSG | AKT1 | AKT3 | ALOX12 | ANXA1 | ANXA2 | ANXA5 | APC | APCS | APLP1 | APOA1 | APOD | APOE | AR | ARHGAP35 | ARHGEF7 | ARRB1 | ARRB2 | ATAD5 | ATF6B | ATM | AXIN1 | BACE1 | BAG5 | BAX | BCL2 | BCL2L1 | BCL2L2 | BCL6 | BDKRB2 | BRCA1 | BTRC | C1QBP | C1QL4 | C1QTNF1 | CALM1 | CALM2 | CALM3 | CALR | CASP8 | CAV1 | CBL | CBLB | CBLC | CBY1 | CCAR2 | CCDC22 | CD109 | CD22 | CD74 | CDC34 | CDC42 | CDH5 | CDKN1A | CHD8 | CHEK2 | CHP1 | CHRD | CHRNA9 | CLU | COL2A1 | CREB3 | CRK | CRY1 | CRY2 | CSNK1A1 | CSNK2A2 | CTH | CTNNB1 | CTNNBIP1 | CTNND1 | CTTN | CUL3 | CXCL8 | CYLD | DAB1 | DAB2 | DCN | DDIT4 | DDX39B | DDX3X | DNAJA1 | DUSP1 | DUSP14 | DUSP16 | DUSP19 | DUSP22 | DUSP7 | DVL1 | DVL2 | DVL3 | DYRK2 | EGFR | EIF3A | EIF4A3 | ENO1 | EPHA4 | EPM2A | EPPK1 | ERBB2 | ERBB3 | ERRFI1 | ESR1 | ETS1 | EYA2 | EYA4 | EZH2 | EZR | FABP7 | FADD | FAM129B | FAM19A3 | FASLG | FBP1 | FBXO7 | FBXW11 | FGB | FGF2 | FHL2 | FIGNL1 | FKBP1A | FKBP1B | FOXJ1 | FOXM1 | FOXO1 | FYN | G3BP1 | GATA1 | GNAI2 | GNAI3 | GPD1L | GPS2 | GPX1 | GRB2 | GRID2 | GSK3A | GSK3B | GSTO1 | GSTP1 | HAVCR2 | HBEGF | HDAC1 | HDAC3 | HDAC7 | HECW1 | HGS | HIF1A | HIF1AN | HLX | HMGB2 | HMOX1 | HSF1 | HSPA1B | HSPA5 | HSPB1 | HTRA1 | HTRA2 | HTT | ICAM1 | IFI16 | IFNA2 | IFT122 | IGBP1 | IGF1R | IL13RA2 | INSIG1 | IRF4 | ITCH | ITGAV | ITGB1BP1 | ITPR1 | JAK3 | KANK2 | KDM1A | LATS1 | LDLRAD4 | LEMD2 | LEMD3 | LGALS3 | LGMN | LGR4 | LMNA | LPAR1 | LRP6 | LRRK2 | LTBP1 | LZTR1 | MAP3K3 | MAPK14 | MAPK7 | MAPKAP1 | MCC | MDFI | MDM2 | METRNL | MICA | MIF | MLLT3 | MMP26 | MYC | MYOC | NAMPT | NCK1 | NCOR1 | NCOR2 | NDUFA13 | NDUFAF2 | NDUFS3 | NEDD4 | NEUROD1 | NF2 | NFATC1 | NFE2L2 | NFKBIA | NKX2-1 | NLK | NOS3 | NPHP3 | NPLOC4 | NR1H2 | NR1H3 | NRXN1 | ONECUT1 | OPA1 | OPTN | OTUB1 | OTUD4 | PAFAH1B1 | PAM16 | PARK7 | PARP14 | PAWR | PCBP2 | PDCD6 | PDE4D | PEBP1 | PER1 | PHB | PHB2 | PIH1D1 | PIN1 | PLAUR | PPARD | PPARG | PPIF | PPM1A | PPM1B | PPP2CA | PPP2CB | PPP2R1A | PRDX2 | PRELID1 | PRKAA1 | PRKAA2 | PRKACA | PRKCD | PRKCZ | PRKDC | PSAP | PSEN1 | PSMA1 | PSMA2 | PSMA3 | PSMA5 | PSMA6 | PSMB5 | PSMB6 | PSMB7 | PSMB8 | PSMC1 | PSMC2 | PSMC3 | PSMC4 | PSMC5 | PSMC6 | PSMD1 | PSMD10 | PSMD11 | PSMD12 | PSMD14 | PSMD2 | PSMD4 | PSMD9 | PSME2 | PSME3 | PTEN | PTGIR | PTPN1 | PTPN22 | PTPN6 | PTPRO | PTPRR | PXDN | PYCR1 | QARS | RAB7A | RAF1 | RANBP9 | RASL11B | RB1 | RB1CC1 | RBX1 | RC3H1 | RELA | RGS1 | RGS2 | RGS20 | RHOA | RIF1 | RIPK1 | RNF126 | RPGRIP1L | RPS19 | RPS3 | RRM2B | RUNX2 | RUVBL2 | SARM1 | SCG2 | SCYL2 | SEMA7A | SERPINB2 | SERPINB3 | SERPINE2 | SGTA | SH3GL2 | SH3KBP1 | SIAH1 | SIRT1 | SIRT3 | SKI | SLAMF1 | SLAMF8 | SLC25A5 | SLC35F6 | SLC9A3R1 | SMAD2 | SMAD3 | SMAD4 | SMARCA4 | SMURF1 | SMURF2 | SNAI2 | SNCA | SNX6 | SOCS1 | SOCS2 | SOCS6 | SOD1 | SOD2 | SOX2 | SPERT | SPRY2 | SRC | SRI | STAT1 | STAT6 | STK11 | STK3 | STK38 | STMN1 | STRAP | STUB1 | SULF2 | SYNJ2BP | SYVN1 | TANK | TAOK3 | TCF21 | TERF2 | TFIP11 | TGFB1 | TGFBR3 | THBS1 | THEM4 | TIMP2 | TLE1 | TLE3 | TLR3 | TMBIM6 | TNF | TNFAIP3 | TNFRSF1A | TNN | TP63 | TRADD | TRAF2 | TRAF3IP1 | TRAP1 | TREX1 | TRIAP1 | TRIB3 | TRIM32 | TRIM33 | TRIP12 | TSG101 | TSKU | TULP3 | TWIST1 | TXN | UBASH3A | UBB | UBC | UBE2B | UBE2J1 | UBQLN1 | UBR5 | UBXN1 | UCHL1 | USP14 | USP47 | VDAC2 | VHL | VSIG4 | VTN | WWOX | WWP2 | XBP1 | XRCC1 | YAP1 | YWHAB | ZBTB7A | ZBTB7B | ZC3HC1 | GO:0050790 | 0.168263981560112 | 1387/8243 | 617/3250 | 1.48617110750825e-05 | 0.141111946657908 | T | T | T | T | regulation of catalytic activity | A2M | ABCE1 | ABL1 | ABL2 | ACD | ACSL1 | ACTB | ADCY6 | ADCYAP1 | ADORA2A | ADPRH | ADRB2 | ADRM1 | AGAP1 | AGAP2 | AGER | AHSA1 | AHSG | AIFM1 | AIMP2 | AKAP9 | AKT1 | AKT2 | ALOX12 | ALOX5AP | ANXA1 | ANXA2 | ANXA5 | APC | APCS | APOA1 | APOE | APP | AR | ARF1 | ARF4 | ARFGAP1 | ARHGAP1 | ARHGAP19 | ARHGAP26 | ARHGAP35 | ARHGDIA | ARHGEF7 | ARRB1 | ARRB2 | ATM | ATP13A2 | ATP1B3 | AUP1 | AURKB | AXIN1 | AZU1 | B3GAT3 | BAG1 | BAG2 | BAG3 | BAG4 | BAG5 | BAX | BBC3 | BCL10 | BCL2 | BCL2L13 | BCL6 | BIN1 | BIRC2 | BIRC3 | BIRC5 | BIRC7 | BIRC8 | BMI1 | BRAF | C4A | CAB39 | CALM1 | CALM2 | CALM3 | CAND1 | CAP2 | CAPN1 | CASP10 | CASP3 | CASP8 | CASP9 | CAV1 | CBL | CBLB | CBLC | CBX8 | CCAR2 | CCDC8 | CCDC88A | CCL21 | CCL22 | CCNB1 | CCNT1 | CCNT2 | CCNY | CCNYL1 | CCS | CCT2 | CCT4 | CD109 | CD27 | CD2BP2 | CD40 | CD40LG | CD74 | CD81 | CDC14B | CDC20 | CDC25A | CDC25B | CDC25C | CDC37 | CDK4 | CDK5 | CDK5R1 | CDKN1A | CDKN2C | CEBPA | CHCHD10 | CHORDC1 | CHP1 | CKS1B | CKS2 | CLPX | CLU | COX11 | COX17 | COX6A1 | CRK | CRKL | CRY2 | CRYAB | CSNK2B | CTNNB1 | CTSA | CYCS | DAB1 | DAPK1 | DAXX | DBF4B | DBNDD2 | DBNL | DCUN1D1 | DDRGK1 | DDX3X | DENND1A | DGKZ | DHCR24 | DHX9 | DIABLO | DLG2 | DLG3 | DLG4 | DNAJA1 | DNAJA2 | DNAJA3 | DNAJB1 | DNAJB11 | DNAJB2 | DNAJC10 | DNAJC19 | DNAJC9 | DNM1L | DOCK7 | DPEP1 | DUSP1 | DUSP12 | DUSP14 | DUSP16 | DUSP19 | DUSP22 | DUSP7 | DVL1 | DVL2 | DVL3 | DYNLL1 | ECSIT | EEF1A2 | EGFR | EGLN1 | EGLN3 | EIF2AK3 | EIF5 | ELMOD3 | EPHA4 | EPPIN | ERBB2 | ERBB3 | ERRFI1 | ESR1 | EZH2 | FABP1 | FADD | FAF1 | FAF2 | FASLG | FBXO7 | FGF2 | FGFR1 | FGFR1OP | FGFR3 | FIS1 | FKBP1A | FKBP1B | FN1 | FOXJ1 | FYN | FZR1 | GADD45G | GAPDH | GAPVD1 | GDI1 | GDI2 | GIT1 | GLA | GLP1R | GNA13 | GNA15 | GNAI2 | GNAI3 | GNAO1 | GNAQ | GNAS | GOLGA2 | GPI | GPS2 | GPSM1 | GPX1 | GRPEL1 | GSK3A | GSK3B | GSTP1 | GTF2F1 | GZMA | HDAC1 | HDAC6 | HDAC9 | HEXIM1 | HGS | HIF1A | HMGB1 | HMGB2 | HNRNPA2B1 | HNRNPD | HNRNPU | HRAS | HSF1 | HSP90AA1 | HSP90AB1 | HSP90B1 | HSPA2 | HSPB1 | HSPB2 | HSPBP1 | HSPD1 | HSPE1 | HSPH1 | HTRA2 | HTT | IBTK | ICAM1 | IFI16 | IFIT1 | IGBP1 | IGF1R | IKBKB | IKBKG | ILK | INSR | IPO5 | IPO7 | IQGAP1 | ITGB1BP1 | ITGB3 | ITK | JUN | KRAS | KSR1 | LAMP3 | LARS | LAT | LATS1 | LCP2 | LEF1 | LGALS3 | LGMN | LGR4 | LIMS1 | LPAR1 | LPAR2 | LRP6 | LRRK2 | LTC4S | LYPLA1 | MAD2L1 | MAP2K1 | MAP2K3 | MAP2K4 | MAP2K6 | MAP2K7 | MAP3K1 | MAP3K13 | MAP3K14 | MAP3K2 | MAP3K3 | MAP3K4 | MAP4K1 | MAPK1 | MAPK14 | MAPK15 | MAPK3 | MAPK7 | MAPK8 | MAPK8IP3 | MAPKAP1 | MAPT | MAS1 | MAT2B | MBP | MCM2 | MDM2 | MGMT | MIF | MLLT1 | MLST8 | MOB1B | MSH6 | MTRR | MYC | MYH6 | MYO1D | NCK1 | NDUFA13 | NEK1 | NEK7 | NF2 | NOD2 | NOS1AP | NOS2 | NOS3 | NOSIP | NOSTRIN | NPM1 | NQO1 | NR1H2 | NR1H3 | NRBF2 | NTF3 | NTRK1 | NUPR1 | OAS2 | OAZ3 | OXA1L | OXSR1 | P2RX1 | PAFAH1B1 | PAK2 | PAK6 | PAM16 | PAPLN | PARK7 | PARP1 | PCNA | PDCD10 | PDCD6 | PDE6G | PEBP1 | PFN1 | PGAM5 | PIH1D1 | PIK3CA | PIK3CG | PIK3R1 | PIK3R3 | PIN1 | PINX1 | PKIG | PLA2G1B | PLAUR | PLCG1 | PLK1 | PLSCR1 | PLXNA1 | PLXNA2 | PLXNB2 | PML | PNKP | POT1 | PPARG | PPIF | PPM1K | PPME1 | PPP1R11 | PPP1R12A | PPP1R12B | PPP1R14A | PPP1R16A | PPP1R2 | PPP1R3B | PPP1R3C | PPP1R8 | PPP2CA | PPP2R1A | PPP2R1B | PPP2R2A | PPP2R2D | PPP2R5D | PPP4R2 | PPP6R1 | PPT1 | PRDX2 | PRDX3 | PRDX5 | PRELID1 | PRKAA1 | PRKAB2 | PRKACA | PRKAG1 | PRKAR1A | PRKAR2B | PRKCD | PRKCE | PRKCZ | PRKD1 | PRKD2 | PROX1 | PRRC1 | PSAP | PSEN1 | PSEN2 | PSMA3 | PSMB8 | PSMD1 | PSMD10 | PSMD14 | PSMD2 | PSME2 | PSME3 | PTEN | PTGES3 | PTK2 | PTN | PTPN1 | PTPN22 | PTPN6 | PTTG1 | QARS | RAB1A | RAB3GAP1 | RAF1 | RANBP3 | RANGAP1 | RAP1A | RAP1GDS1 | RB1 | RDX | RECK | REST | RFC1 | RFC2 | RFC3 | RFC4 | RFC5 | RFK | RGN | RGS1 | RGS2 | RGS20 | RHOA | RIC8B | RING1 | RIPK1 | RIPK2 | RIPK3 | RP2 | RPA2 | RPL23 | RPLP1 | RPS2 | RPS3 | RPS6KA1 | RPTOR | RSU1 | RTKN | S100A1 | S100A12 | S100A9 | SAE1 | SAG | SDC4 | SERPINB2 | SERPINB3 | SERPINB5 | SERPINB6 | SERPINB8 | SERPINE2 | SERPINH1 | SET | SFN | SH3PXD2A | SHC1 | SIPA1 | SIPA1L1 | SIRT1 | SLAMF8 | SLC9A3R1 | SMAD3 | SMG6 | SMG8 | SNCA | SNF8 | SNX6 | SOCS1 | SOCS2 | SOCS6 | SOD1 | SORT1 | SOX2 | SPINK7 | SPINT2 | SPR | SPRY2 | SRC | SRGAP2 | SRGAP3 | STARD13 | STAT3 | STIM1 | STK11 | STK24 | STK25 | STK26 | STK3 | STK38 | STUB1 | STYX | SUGT1 | SUMO1 | SYDE1 | TAB1 | TAB2 | TANK | TAOK3 | TBC1D15 | TBC1D22B | TBC1D4 | TCEA1 | TCP1 | TERF1 | TERF2 | TFIP11 | TGFB1 | THADA | THBS1 | TIMP1 | TIMP2 | TLR3 | TMBIM6 | TNF | TNFAIP3 | TNFSF14 | TNK2 | TNKS | TP53 | TP63 | TP73 | TPM1 | TPM2 | TRADD | TRAF2 | TRAF6 | TREX1 | TRIAP1 | TRIB2 | TRIB3 | TRIM23 | TSG101 | TSPYL2 | TXN | TXNIP | UBA2 | UBB | UBC | UBE2C | UBE2I | UBE2L3 | UBE2N | UBE2S | UBE2V1 | UBXN1 | UCHL1 | UCHL5 | UNC119 | USP14 | USP17L2 | USP47 | USP50 | VAV1 | VCP | VDR | VTN | WARS | WAS | WDR35 | WDTC1 | WNK1 | XIAP | XRCC1 | XRCC5 | XRCC6 | YWHAB | YWHAE | YWHAG | ZNF622 | GO:0051172 | 0.177362610699988 | 1462/8243 | 648/3250 | 1.49434687239228e-05 | 0.141888235533647 | T | T | T | T | negative regulation of nitrogen compound metabolic process | A2M | ABCE1 | ABL1 | ACD | ADORA2A | AEBP2 | AES | AGO1 | AGO2 | AHSG | AKT1 | ANXA2 | APBB1 | APC | APCS | APEX1 | APOD | APOE | APP | AR | ARHGAP35 | ARRB1 | ARRB2 | ASCL1 | ATCAY | ATF2 | ATG5 | ATM | ATP13A2 | ATRX | ATXN1 | AURKB | BACH2 | BAG2 | BAG3 | BAG5 | BAG6 | BASP1 | BAX | BCL11A | BCL6 | BCL6B | BCOR | BDKRB2 | BIN1 | BIRC2 | BIRC3 | BIRC5 | BIRC7 | BIRC8 | BMI1 | BRCA1 | BTRC | C1QBP | C1QL4 | C4A | CALM1 | CALM2 | CALM3 | CALR | CASP3 | CAV1 | CBFA2T3 | CBFB | CBL | CBLB | CBLC | CBX4 | CBX7 | CBX8 | CBY1 | CCAR2 | CCNB1 | CCNT1 | CD109 | CD27 | CD36 | CDA | CDC73 | CDK2 | CDK5 | CDK5R1 | CDK9 | CDKN1A | CDKN2C | CEBPA | CENPF | CENPJ | CHCHD3 | CHD8 | CHORDC1 | CHP1 | CHRNA9 | CIRBP | CLTC | CLU | COMT | COPS2 | CREB1 | CREBBP | CRKL | CRY1 | CRY2 | CRYAB | CSNK2A2 | CTBP1 | CTCF | CTNNB1 | CTNNBIP1 | CUL3 | CYLD | DAB2 | DAPK1 | DAXX | DBNDD2 | DCP2 | DDIT4 | DDRGK1 | DDX3X | DHCR24 | DHX36 | DHX9 | DMAP1 | DMD | DNAJA1 | DNAJA3 | DNAJB1 | DNAJC10 | DPEP1 | DPF2 | DUSP1 | DUSP14 | DUSP16 | DUSP19 | DUSP22 | DUSP7 | DVL1 | E2F1 | E2F2 | E2F3 | E2F4 | E2F6 | EED | EHMT1 | EHMT2 | EID1 | EIF2AK3 | EIF2AK4 | EIF2S1 | EIF3A | EIF3E | EIF4A3 | EIF4E | EIF4E2 | EIF4EBP1 | EIF4ENIF1 | EIF6 | ELAVL1 | ELF3 | ENO1 | EP300 | EPHA4 | EPPIN | EPRS | ERF | ERRFI1 | ESR1 | ESR2 | ESRRA | EZH2 | EZR | FABP1 | FAM129B | FAM192A | FASLG | FBP1 | FBXO7 | FBXW11 | FGFR1 | FGFR1OP | FHL2 | FKBP1A | FKBP1B | FKBP8 | FOXA1 | FOXJ1 | FOXK1 | FOXM1 | FOXO1 | FTO | FXR2 | FYN | G6PD | GAPDH | GATA1 | GLA | GLMN | GNAI2 | GNAQ | GPD1L | GPI | GPS2 | GPX1 | GRB7 | GSK3A | GSK3B | GSTP1 | GZMA | H1F0 | H1FX | H3F3A | HAP1 | HBEGF | HBZ | HDAC1 | HDAC2 | HDAC3 | HDAC4 | HDAC5 | HDAC6 | HDAC7 | HDAC9 | HDGF | HEXIM1 | HIF1AN | HIST1H1A | HIST1H3A | HIST1H4A | HIST2H3A | HIVEP1 | HMGB1 | HMGB2 | HNRNPA1 | HNRNPA2B1 | HNRNPD | HNRNPK | HNRNPL | HNRNPU | HSF1 | HSF4 | HSP90AB1 | HSP90AB3P | HSP90AB4P | HSPA1B | HSPA8 | HSPB1 | IBTK | ID2 | IDE | IFI16 | IFNA2 | IGBP1 | IGF1R | IGF2BP1 | IKBKB | IKZF5 | ILF3 | ILK | IPO5 | IPO7 | IREB2 | IRF1 | IRF7 | ISG15 | ITCH | ITGAV | ITGB1BP1 | ITGB3 | JAK3 | JUN | JUNB | JUND | KANK2 | KAT5 | KAT7 | KAT8 | KDM1A | KDM4D | KDM5B | KDM5C | KHDRBS1 | KLF5 | KMT2A | L3MBTL1 | LAMP3 | LATS1 | LDLRAD4 | LEF1 | LEMD2 | LGALS3 | LGR4 | LIMS1 | LOXL2 | LRP6 | LRPPRC | LRRK1 | LRRK2 | MAD2L1 | MAF1 | MAPK14 | MAPT | MAS1 | MBD3 | MDFI | MDM2 | MDM4 | MECP2 | MED1 | MEPCE | MIF | MITF | MLH1 | MLLT1 | MORF4L1 | MORF4L2 | MOV10 | MSH6 | MTA1 | MTA3 | MXD1 | MYBBP1A | MYC | MYD88 | NACC1 | NANOG | NBAS | NCK1 | NCOA2 | NCOR1 | NCOR2 | NDUFA13 | NEDD4 | NELFB | NELFCD | NELFE | NELL1 | NF2 | NFATC2 | NIF3L1 | NIPBL | NKRF | NKX2-1 | NMNAT1 | NNT | NOSTRIN | NPM1 | NR0B2 | NR1H2 | NR1H3 | NR1I2 | NR3C1 | NTF3 | OGT | OTUB1 | PA2G4 | PABPC1 | PAFAH1B1 | PAIP1 | PAK2 | PAM16 | PAPLN | PARD3 | PARD6A | PARK7 | PARP1 | PARP14 | PATZ1 | PAWR | PAX9 | PCGF1 | PCGF6 | PDE4D | PEBP1 | PER1 | PER2 | PHB | PHB2 | PHF10 | PHF21A | PIAS1 | PIAS3 | PIH1D1 | PIN1 | PINX1 | PKIG | PLAUR | PLK1 | PML | PNKP | POT1 | POU5F1 | PPARD | PPARG | PPARGC1A | PPID | PPM1A | PPM1D | PPME1 | PPP1R11 | PPP1R14A | PPP1R2 | PPP1R8 | PPP2CA | PPP2R1A | PPP2R5D | PRDX3 | PRDX5 | PRKAA1 | PRKAA2 | PRKAR1A | PRKAR2B | PRKCD | PRKCE | PRKCZ | PRKDC | PRMT2 | PRMT3 | PRMT6 | PROX1 | PSEN1 | PSMC5 | PSMD10 | PTEN | PTN | PTPN1 | PTPN22 | PTPN6 | PTPRK | PTPRR | PTTG1 | PUM1 | PUS7 | QARS | RAF1 | RANBP9 | RARA | RARG | RASD1 | RB1 | RBM3 | RBM4 | RBMX | RBPJ | RC3H1 | RECK | REL | RELA | REST | RFC1 | RGN | RGS2 | RIF1 | RING1 | RNF139 | RNF2 | RNF8 | RPL10 | RPL23 | RPS13 | RPS14 | RPS3 | RPS6KA1 | RPTOR | RTF1 | RUNX1 | RUNX1T1 | RUNX2 | RUVBL2 | RXRA | S100A1 | SAG | SAP18 | SARS | SDCBP | SERPINB2 | SERPINB3 | SERPINB5 | SERPINB6 | SERPINB8 | SERPINE2 | SERPINH1 | SET | SF1 | SFMBT1 | SFN | SFPQ | SGTA | SHC1 | SHMT1 | SIN3A | SIRT1 | SIRT3 | SIRT5 | SIRT6 | SIRT7 | SKI | SLC9A3R1 | SMAD2 | SMAD3 | SMAD4 | SMAD5 | SMARCA2 | SMARCA4 | SMARCB1 | SMARCC1 | SMARCC2 | SMARCE1 | SMG6 | SMO | SMURF2 | SNAI2 | SNCA | SNTA1 | SNW1 | SNX12 | SNX6 | SOCS1 | SOX2 | SP100 | SP2 | SPEN | SPI1 | SPINK7 | SPINT2 | SPRY2 | SQSTM1 | SRC | SRSF12 | STAT1 | STAT3 | STAT6 | STK38 | STRAP | STYX | SUMO1 | SUPT4H1 | SUPT5H | SUZ12 | SWAP70 | SYNJ2BP | TAGLN3 | TAOK3 | TARDBP | TBL1X | TCERG1 | TCF21 | TCF25 | TCP10L | TERF1 | TERF2 | TERF2IP | TFIP11 | TGFB1 | THAP11 | THAP7 | THBS1 | THRA | TIA1 | TIMP1 | TIMP2 | TINF2 | TLE1 | TLE3 | TMBIM6 | TNF | TNFAIP3 | TNFSF14 | TNKS | TNKS2 | TNP1 | TP53 | TP63 | TPR | TRAF3IP1 | TRAF6 | TRIAP1 | TRIB2 | TRIB3 | TRIM21 | TRIM28 | TRIM29 | TRIM33 | TRIP12 | TSC22D4 | TSG101 | TWIST1 | TXN | TXNIP | TYMS | U2AF2 | UBA3 | UBB | UBC | UBE2B | UBE2D1 | UBE2D3 | UBE2I | UBE2J1 | UBR5 | UBXN1 | UCHL1 | UCHL5 | UNK | USP14 | USP17L2 | USP19 | USP2 | USP4 | USP47 | USP7 | USP9X | VDR | VHL | VPS72 | VTN | WARS | WDR35 | WDTC1 | WNK1 | WT1 | WWOX | WWP2 | XIAP | XPO1 | XRCC1 | XRCC5 | XRCC6 | YAF2 | YAP1 | YEATS2 | YWHAB | YWHAE | YWHAG | YWHAQ | YY1 | ZBTB16 | ZBTB21 | ZBTB33 | ZBTB42 | ZBTB7A | ZBTB7B | ZC3H4 | ZHX1 | ZMPSTE24 | ZMYND8 | ZNF217 | ZNF24 | ZNF746 | GO:0043122 | 0.0192890937765377 | 159/8243 | 89/3250 | 1.500093058774e-05 | 0.142433835930591 | T | T | T | T | regulation of I-kappaB kinase/NF-kappaB signaling | ABL1 | AKT1 | BCL10 | BIRC2 | BIRC3 | BRD4 | BTRC | CARD9 | CASP10 | CASP8 | CCDC22 | CCL21 | CD36 | CD40 | CD74 | CTH | CTNNB1 | DDRGK1 | DDX1 | DHX36 | EDA | EEF1D | ESR1 | FADD | FASLG | FKBP1A | FYN | GJA1 | GOLT1B | GSTP1 | HDAC1 | HMOX1 | HSPB1 | IKBKB | IKBKE | IKBKG | LGALS1 | LPAR1 | LTBR | LURAP1 | MAP3K14 | MAP3K3 | MAVS | MYD88 | NOD2 | OPTN | PELI1 | PER1 | PPM1A | PPM1B | PPP5C | PRKCE | PRKD1 | REL | RELA | RHOA | RIPK1 | RIPK2 | RNF31 | S100A12 | S100A13 | S100A4 | S100B | SIRT1 | SQSTM1 | STAT1 | TAB2 | TANK | TERF2IP | TFG | TGM2 | TLE1 | TLR3 | TMEM9B | TNF | TNFAIP3 | TNFRSF1A | TNIP2 | TRADD | TRAF2 | TRAF6 | TRIM25 | TRIM32 | UBE2I | UBE2N | UBE2V1 | VAPA | ZDHHC17 | ZFAND6 | GO:0010563 | 0.0429455295402159 | 354/8243 | 178/3250 | 1.50482875777335e-05 | 0.14288349055058 | T | T | T | T | negative regulation of phosphorus metabolic process | ABL1 | ADORA2A | AHSG | AKT1 | APC | APOE | ARRB1 | ARRB2 | BAG4 | BAX | BDKRB2 | C1QL4 | CALM1 | CALM2 | CALM3 | CASP3 | CAV1 | CBL | CBLB | CBLC | CCDC8 | CCNB1 | CD109 | CD2BP2 | CDA | CDKN1A | CDKN2C | CEBPA | CHORDC1 | CHP1 | CHRNA9 | COX11 | CRKL | CRY2 | CYLD | DBNDD2 | DDIT4 | DLG2 | DLG3 | DMD | DNAJA1 | DNAJC10 | DUSP1 | DUSP14 | DUSP16 | DUSP19 | DUSP22 | DUSP7 | DVL1 | DYNLL1 | EIF3A | ERRFI1 | EZR | FBXO7 | FGFR1OP | FKBP1A | FKBP1B | FKBP8 | FOXM1 | FOXO1 | GNAI2 | GNAQ | GPD1L | GPS2 | GSK3B | GSTP1 | HDAC3 | HEXIM1 | HNRNPU | HSPB1 | IBTK | IGBP1 | IGF1R | IKBKB | ILK | IPO5 | IPO7 | IQGAP1 | ITCH | ITGB1BP1 | JUN | LATS1 | LDLRAD4 | LEMD2 | LGALS3 | LRP6 | LRRK1 | LRRK2 | MAPT | MAS1 | MLLT1 | MYC | MYO1D | NCK1 | NCOR1 | NF2 | NNT | NPM1 | NTF3 | PAFAH1B1 | PAK2 | PARD3 | PARD6A | PARK7 | PARP14 | PDE12 | PDE4D | PEBP1 | PER1 | PHB | PIN1 | PKIG | PLK1 | PPARGC1A | PPIF | PPME1 | PPP1R11 | PPP1R14A | PPP1R2 | PPP1R8 | PPP2CA | PPP2R1A | PPP2R5D | PRDX3 | PRKAR1A | PRKAR2B | PRKCD | PRKCZ | PRKDC | PSEN1 | PSMD10 | PTEN | PTN | PTPN1 | PTPN22 | PTPN6 | PTPRR | QARS | RANBP9 | RB1 | RGN | RGS2 | RHOA | RPTOR | SAG | SERPINB3 | SET | SFN | SIRT1 | SIRT3 | SLC9A3R1 | SMAD4 | SNCA | SNX6 | SOCS1 | SPRY2 | STK38 | STRAP | SWAP70 | SYNJ2BP | TAOK3 | TARDBP | TERF2IP | TGFB1 | TNF | TNFAIP3 | TRAF3IP1 | TRIB2 | TRIB3 | TSG101 | TWIST1 | UBE2B | UCHL1 | WARS | WNK1 | YWHAB | YWHAE | YWHAG | GO:0045936 | 0.0429455295402159 | 354/8243 | 178/3250 | 1.50482875777335e-05 | 0.14288349055058 | T | T | T | T | negative regulation of phosphate metabolic process | ABL1 | ADORA2A | AHSG | AKT1 | APC | APOE | ARRB1 | ARRB2 | BAG4 | BAX | BDKRB2 | C1QL4 | CALM1 | CALM2 | CALM3 | CASP3 | CAV1 | CBL | CBLB | CBLC | CCDC8 | CCNB1 | CD109 | CD2BP2 | CDA | CDKN1A | CDKN2C | CEBPA | CHORDC1 | CHP1 | CHRNA9 | COX11 | CRKL | CRY2 | CYLD | DBNDD2 | DDIT4 | DLG2 | DLG3 | DMD | DNAJA1 | DNAJC10 | DUSP1 | DUSP14 | DUSP16 | DUSP19 | DUSP22 | DUSP7 | DVL1 | DYNLL1 | EIF3A | ERRFI1 | EZR | FBXO7 | FGFR1OP | FKBP1A | FKBP1B | FKBP8 | FOXM1 | FOXO1 | GNAI2 | GNAQ | GPD1L | GPS2 | GSK3B | GSTP1 | HDAC3 | HEXIM1 | HNRNPU | HSPB1 | IBTK | IGBP1 | IGF1R | IKBKB | ILK | IPO5 | IPO7 | IQGAP1 | ITCH | ITGB1BP1 | JUN | LATS1 | LDLRAD4 | LEMD2 | LGALS3 | LRP6 | LRRK1 | LRRK2 | MAPT | MAS1 | MLLT1 | MYC | MYO1D | NCK1 | NCOR1 | NF2 | NNT | NPM1 | NTF3 | PAFAH1B1 | PAK2 | PARD3 | PARD6A | PARK7 | PARP14 | PDE12 | PDE4D | PEBP1 | PER1 | PHB | PIN1 | PKIG | PLK1 | PPARGC1A | PPIF | PPME1 | PPP1R11 | PPP1R14A | PPP1R2 | PPP1R8 | PPP2CA | PPP2R1A | PPP2R5D | PRDX3 | PRKAR1A | PRKAR2B | PRKCD | PRKCZ | PRKDC | PSEN1 | PSMD10 | PTEN | PTN | PTPN1 | PTPN22 | PTPN6 | PTPRR | QARS | RANBP9 | RB1 | RGN | RGS2 | RHOA | RPTOR | SAG | SERPINB3 | SET | SFN | SIRT1 | SIRT3 | SLC9A3R1 | SMAD4 | SNCA | SNX6 | SOCS1 | SPRY2 | STK38 | STRAP | SWAP70 | SYNJ2BP | TAOK3 | TARDBP | TERF2IP | TGFB1 | TNF | TNFAIP3 | TRAF3IP1 | TRIB2 | TRIB3 | TSG101 | TWIST1 | UBE2B | UCHL1 | WARS | WNK1 | YWHAB | YWHAE | YWHAG | GO:0042542 | 0.0110396700230499 | 91/8243 | 56/3250 | 1.50636325597815e-05 | 0.143029191155125 | T | T | T | T | response to hydrogen peroxide | ABL1 | ADA | AIFM1 | ANXA1 | APEX1 | BCL2 | CAPN2 | CASP3 | CAT | CBX8 | CRK | CRYAB | DUSP1 | EEF2 | ETS1 | EZH2 | FABP1 | FKBP1B | FOXO1 | FYN | GNAO1 | GPX1 | HDAC2 | HDAC6 | HMOX1 | HSF1 | JUN | LDHA | MAPK7 | MDM2 | NFE2L2 | NQO1 | PARK7 | PCNA | PDCD10 | PPIF | PPP2CB | PRDX3 | PRKAA1 | PRKCD | RELA | RIPK1 | RIPK3 | RPS3 | SDC1 | SIRT1 | SOD1 | SRC | STAT1 | STAT6 | STK24 | STK25 | STK26 | TNFAIP3 | TXNIP | ZNF277 | GO:0010605 | 0.200048526022079 | 1649/8243 | 725/3250 | 1.51405158326942e-05 | 0.143759197831432 | T | T | T | T | negative regulation of macromolecule metabolic process | A2M | ABCE1 | ABL1 | ACD | ADORA2A | AEBP2 | AES | AGO1 | AGO2 | AHSG | AICDA | AKT1 | ANXA2 | ANXA7 | APBB1 | APC | APCS | APEX1 | APOD | APOE | APP | AR | ARHGAP35 | ARRB1 | ARRB2 | ASCL1 | ATF2 | ATG5 | ATM | ATP13A2 | ATRX | ATXN1 | ATXN2 | AURKB | BACH2 | BAG2 | BAG3 | BAG5 | BAG6 | BASP1 | BAX | BCL11A | BCL6 | BCL6B | BCOR | BDKRB2 | BIN1 | BIRC2 | BIRC3 | BIRC5 | BIRC7 | BIRC8 | BMI1 | BRCA1 | BTRC | C1QBP | C1QL4 | C4A | CALM1 | CALM2 | CALM3 | CALR | CASP3 | CAV1 | CBFA2T3 | CBFB | CBL | CBLB | CBLC | CBX4 | CBX7 | CBX8 | CBY1 | CCAR2 | CCNB1 | CCNT1 | CCR1 | CD109 | CD27 | CD36 | CDC73 | CDK2 | CDK5 | CDK5R1 | CDK9 | CDKN1A | CDKN2C | CEBPA | CENPF | CENPJ | CHCHD3 | CHD8 | CHEK2 | CHORDC1 | CHP1 | CHRNA9 | CIRBP | CLTC | CLU | COPS2 | CREB1 | CREBBP | CRKL | CRY1 | CRY2 | CRYAB | CSNK2A2 | CTBP1 | CTCF | CTNNB1 | CTNNBIP1 | CUL3 | CYLD | DAB2 | DAPK1 | DAXX | DBNDD2 | DCP2 | DDIT4 | DDRGK1 | DDX3X | DDX6 | DHCR24 | DHX36 | DHX9 | DIS3 | DLG4 | DMAP1 | DMD | DNAJA1 | DNAJA3 | DNAJB1 | DNAJC10 | DPEP1 | DPF2 | DUSP1 | DUSP14 | DUSP16 | DUSP19 | DUSP22 | DUSP7 | DVL1 | E2F1 | E2F2 | E2F3 | E2F4 | E2F6 | EDC4 | EED | EHMT1 | EHMT2 | EID1 | EIF2AK3 | EIF2AK4 | EIF2S1 | EIF3A | EIF3E | EIF4A3 | EIF4E | EIF4E2 | EIF4EBP1 | EIF4ENIF1 | EIF6 | ELAVL1 | ELF3 | ENO1 | EP300 | EPHA4 | EPPIN | EPRS | ERF | ERRFI1 | ESR1 | ESR2 | ESRRA | EXOSC1 | EXOSC5 | EXOSC6 | EXOSC9 | EZH2 | EZR | FABP1 | FAM129B | FAM192A | FASLG | FBP1 | FBXO7 | FBXW11 | FGFR1 | FGFR1OP | FHL2 | FKBP1A | FKBP1B | FKBP8 | FOXA1 | FOXJ1 | FOXK1 | FOXM1 | FOXO1 | FTO | FXR2 | FYN | G6PD | GAPDH | GATA1 | GLMN | GNAI2 | GNAQ | GPD1L | GPI | GPS2 | GPX1 | GRB7 | GSK3A | GSK3B | GSPT1 | GSPT2 | GSTP1 | GZMA | H1F0 | H1FX | H3F3A | HAP1 | HAVCR2 | HBEGF | HBZ | HDAC1 | HDAC2 | HDAC3 | HDAC4 | HDAC5 | HDAC6 | HDAC7 | HDAC9 | HDGF | HELZ | HELZ2 | HEXIM1 | HIF1A | HIF1AN | HIST1H1A | HIST1H3A | HIST1H4A | HIST2H3A | HIVEP1 | HMGB1 | HMGB2 | HNRNPA1 | HNRNPA2B1 | HNRNPD | HNRNPK | HNRNPL | HNRNPU | HRAS | HSF1 | HSF4 | HSP90AB1 | HSP90AB3P | HSP90AB4P | HSPA1B | HSPA8 | HSPB1 | IBTK | ID2 | IDE | IFI16 | IFNA2 | IGBP1 | IGF1R | IGF2BP1 | IKBKB | IKZF5 | ILF3 | ILK | IPO5 | IPO7 | IREB2 | IRF1 | IRF7 | ISG15 | ITCH | ITGAV | ITGB1BP1 | ITGB3 | JAK3 | JUN | JUNB | JUND | KANK2 | KAT5 | KAT7 | KAT8 | KDM1A | KDM4D | KDM5B | KDM5C | KDR | KHDRBS1 | KLF5 | KMT2A | KMT2B | L3MBTL1 | LAMP3 | LATS1 | LDLRAD4 | LEF1 | LEMD2 | LGALS3 | LGMN | LGR4 | LIMS1 | LOXL2 | LRP6 | LRPPRC | LRRK1 | LRRK2 | LSM1 | LSM3 | LSM4 | LSM5 | MAD2L1 | MAF1 | MAP2K1 | MAPK14 | MAPT | MAS1 | MBD3 | MDFI | MDM2 | MDM4 | MECP2 | MED1 | MEPCE | MIF | MITF | MLH1 | MLLT1 | MORF4L1 | MORF4L2 | MOV10 | MSH6 | MTA1 | MTA3 | MXD1 | MYBBP1A | MYC | MYD88 | NACC1 | NANOG | NBAS | NCBP1 | NCK1 | NCOA2 | NCOR1 | NCOR2 | NDUFA13 | NEDD4 | NELFB | NELFCD | NELFE | NELL1 | NF2 | NFATC2 | NIF3L1 | NIPBL | NKRF | NKX2-1 | NME1 | NMNAT1 | NNT | NOS2 | NOSTRIN | NPM1 | NR0B2 | NR1H2 | NR1H3 | NR1I2 | NR3C1 | NTF3 | OGT | OPTN | OTUB1 | PA2G4 | PABPC1 | PAFAH1B1 | PAIP1 | PAK2 | PAM16 | PAN2 | PAPLN | PARD3 | PARD6A | PARK7 | PARP1 | PARP14 | PATZ1 | PAWR | PAX9 | PC | PCGF1 | PCGF6 | PCSK9 | PDCD10 | PDE12 | PDE4D | PEBP1 | PER1 | PER2 | PHB | PHB2 | PHF10 | PHF21A | PIAS1 | PIAS3 | PIH1D1 | PIN1 | PINX1 | PKIG | PLAUR | PLK1 | PML | PNKP | POT1 | POU5F1 | PPARD | PPARG | PPARGC1A | PPID | PPM1A | PPM1D | PPME1 | PPP1R11 | PPP1R14A | PPP1R2 | PPP1R8 | PPP2CA | PPP2R1A | PPP2R2A | PPP2R5D | PRDX3 | PRDX5 | PRKAA1 | PRKAA2 | PRKAR1A | PRKAR2B | PRKCD | PRKCE | PRKCZ | PRKDC | PRMT2 | PRMT3 | PRMT6 | PROX1 | PSEN1 | PSMC5 | PSMD10 | PTEN | PTN | PTPN1 | PTPN22 | PTPN6 | PTPRK | PTPRR | PTRH2 | PTTG1 | PUM1 | PUS7 | QARS | RAF1 | RANBP9 | RARA | RARG | RASD1 | RB1 | RBBP4 | RBM3 | RBM4 | RBM8A | RBMX | RBPJ | RC3H1 | RECK | REL | RELA | REST | RFC1 | RGN | RGS2 | RIF1 | RING1 | RNASEH2B | RNF139 | RNF2 | RNF8 | RPL10 | RPL10A | RPL12 | RPL18A | RPL22 | RPL23 | RPL23A | RPL24 | RPL30 | RPL37A | RPL38 | RPL6 | RPL7 | RPL7A | RPL9 | RPLP0 | RPLP1 | RPLP2 | RPS12 | RPS13 | RPS14 | RPS15A | RPS19 | RPS2 | RPS20 | RPS24 | RPS29 | RPS3 | RPS4X | RPS6KA1 | RPS8 | RPTOR | RTF1 | RUNX1 | RUNX1T1 | RUNX2 | RUVBL2 | RXRA | S100A1 | S100A11 | SAG | SAMD7 | SAP18 | SARS | SDCBP | SERPINB2 | SERPINB3 | SERPINB5 | SERPINB6 | SERPINB8 | SERPINE2 | SERPINH1 | SET | SF1 | SFMBT1 | SFN | SFPQ | SGTA | SHC1 | SHMT1 | SIN3A | SIRT1 | SIRT3 | SIRT5 | SIRT6 | SIRT7 | SKI | SKIV2L | SLC9A3R1 | SMAD2 | SMAD3 | SMAD4 | SMAD5 | SMARCA2 | SMARCA4 | SMARCB1 | SMARCC1 | SMARCC2 | SMARCE1 | SMC1A | SMC3 | SMG5 | SMG6 | SMG8 | SMO | SMURF2 | SNAI2 | SNCA | SND1 | SNTA1 | SNW1 | SNX12 | SNX6 | SOCS1 | SOX2 | SP100 | SP2 | SPEN | SPI1 | SPINK7 | SPINT2 | SPRY2 | SQSTM1 | SRC | SRSF12 | SSB | STAG2 | STAT1 | STAT3 | STAT6 | STK38 | STRAP | STYX | SUMO1 | SUPT4H1 | SUPT5H | SUZ12 | SWAP70 | SYNJ2BP | TAGLN3 | TAOK3 | TARDBP | TBL1X | TCERG1 | TCF21 | TCF25 | TCP10L | TERF1 | TERF2 | TERF2IP | TFIP11 | TGFB1 | THAP11 | THAP7 | THBS1 | THRA | TIA1 | TIMP1 | TIMP2 | TINF2 | TLE1 | TLE3 | TMBIM6 | TNF | TNFAIP3 | TNFSF14 | TNKS | TNKS2 | TNP1 | TP53 | TP63 | TPR | TRAF3IP1 | TRAF6 | TRIAP1 | TRIB2 | TRIB3 | TRIM21 | TRIM28 | TRIM29 | TRIM33 | TRIP12 | TSC22D4 | TSG101 | TSPYL2 | TWIST1 | TXN | TXNIP | TYMS | U2AF2 | UBA3 | UBB | UBC | UBE2B | UBE2D1 | UBE2D3 | UBE2I | UBE2J1 | UBQLN2 | UBR5 | UBXN1 | UCHL1 | UCHL5 | UNK | UPF2 | UPF3B | USP14 | USP17L2 | USP19 | USP2 | USP4 | USP47 | USP7 | USP9X | VDR | VHL | VPS72 | VTN | WARS | WDR35 | WDTC1 | WNK1 | WT1 | WWOX | WWP2 | XIAP | XPO1 | XRCC1 | XRCC5 | XRCC6 | YAF2 | YAP1 | YEATS2 | YWHAB | YWHAE | YWHAG | YWHAQ | YY1 | ZBTB16 | ZBTB21 | ZBTB33 | ZBTB42 | ZBTB7A | ZBTB7B | ZC3H4 | ZHX1 | ZMPSTE24 | ZMYND8 | ZNF217 | ZNF24 | ZNF746 | GO:0044093 | 0.132476040276598 | 1092/8243 | 494/3250 | 1.58227731772625e-05 | 0.150237231318108 | T | T | T | T | positive regulation of molecular function | ABL1 | ABL2 | ACD | ACSL1 | ACTN2 | ACTN4 | ADCY6 | ADCYAP1 | ADPRH | ADRB2 | ADRM1 | AGAP1 | AGAP2 | AGER | AHSA1 | AIFM1 | AIMP2 | AKAP9 | AKT1 | AKT2 | AKTIP | ALOX12 | ALOX5AP | AMFR | ANK3 | ANXA2 | APOA1 | APOE | APP | AR | ARF1 | ARF4 | ARFGAP1 | ARHGAP1 | ARHGAP19 | ARHGAP26 | ARHGAP35 | ARHGDIA | ARHGEF7 | ARRB1 | ATF2 | ATP1B3 | AURKB | AXIN1 | AZU1 | B3GAT3 | BAX | BBC3 | BCL10 | BCL2 | BCL2L13 | BIRC7 | BMI1 | BRAF | BTK | BUD31 | CAB39 | CACNG2 | CACNG4 | CALM1 | CALM2 | CALM3 | CAMK1D | CAMK2A | CARM1 | CASP10 | CASP3 | CASP8 | CASP9 | CAT | CAV1 | CCDC88A | CCL21 | CCL22 | CCNB1 | CCNT1 | CCNT2 | CCNY | CCNYL1 | CCS | CCT2 | CCT4 | CD36 | CD40 | CD40LG | CD74 | CD81 | CDC14B | CDC20 | CDC25B | CDK5 | CDK5R1 | CDK9 | CDKN1A | CFTR | CHCHD10 | CHP1 | CKS1B | CKS2 | CLPX | CLU | COPS5 | COX17 | CRBN | CRK | CRKL | CTH | CTNNB1 | CTSA | CYCS | DAB1 | DAPK1 | DAXX | DBF4B | DBNL | DCUN1D1 | DDRGK1 | DDX3X | DENND1A | DERL1 | DGKZ | DHX9 | DIABLO | DLG3 | DLG4 | DMD | DNAJA1 | DNAJA2 | DNAJB1 | DNAJB11 | DNAJB2 | DNAJC10 | DNAJC19 | DNAJC9 | DNM1L | DOCK7 | DUSP12 | DUSP19 | DUSP7 | DVL2 | DVL3 | DYNLL1 | EDA | EEF1A2 | EGFR | EGLN3 | EIF2AK3 | EIF2S1 | EIF3D | EIF3E | EIF5 | ELMOD3 | EP300 | EPHA4 | ERBB2 | ERBB3 | ERRFI1 | ESR1 | ESR2 | EZH2 | FABP1 | FADD | FAM129B | FASLG | FGF2 | FGFR1 | FGFR3 | FIS1 | FKBP1A | FLOT1 | FN1 | FOXA1 | FOXJ1 | FYN | FZR1 | GADD45G | GAPVD1 | GDI1 | GDI2 | GIT1 | GLRX | GNA13 | GNA15 | GNAI2 | GNAI3 | GNAO1 | GNAQ | GNAS | GOLGA2 | GPSM1 | GSK3A | GSK3B | GSTO1 | GTF2F1 | H1F0 | HAP1 | HDAC4 | HDAC5 | HIF1A | HIST1H2BA | HMGB1 | HMGB2 | HNRNPA2B1 | HNRNPD | HNRNPL | HNRNPU | HRAS | HSF1 | HSP90AA1 | HSP90AB1 | HSPA1B | HSPA2 | HSPB2 | HSPD1 | HSPE1 | HTR3A | HTRA2 | HTT | ICAM1 | IFI16 | IKBKB | IKBKG | ILK | INSR | IQGAP1 | IRF4 | ITGB1BP1 | ITGB3 | ITK | JUN | JUP | KCNIP2 | KDM1A | KDM4D | KIF5B | KMT2A | KRAS | LARS | LAT | LCP2 | LGALS3 | LGMN | LIMS1 | LPAR1 | LPAR2 | LRP6 | LRRK2 | LTC4S | MAP2K1 | MAP2K3 | MAP2K4 | MAP2K6 | MAP2K7 | MAP3K1 | MAP3K13 | MAP3K14 | MAP3K2 | MAP3K3 | MAP3K4 | MAP4K1 | MAPK1 | MAPK14 | MAPK15 | MAPK3 | MAPK8 | MAPK8IP3 | MAPKAP1 | MAPT | MAS1 | MAVS | MBP | MED1 | MIF | MLST8 | MOB1B | MSH6 | MTPN | MYC | MYD88 | NCBP1 | NCOA3 | NDUFA13 | NEK1 | NEK7 | NEUROD1 | NFKB2 | NIPBL | NMD3 | NME1 | NOD2 | NOS1AP | NOS2 | NOS3 | NPM1 | NR1H2 | NR1H3 | NTF3 | NTRK1 | NUPR1 | OXSR1 | P2RX1 | PAK2 | PAK6 | PARK7 | PARP1 | PCNA | PDCD10 | PDCD6 | PDE6G | PFN1 | PGAM5 | PHB2 | PIH1D1 | PIK3CA | PIK3CG | PIN1 | PINX1 | PLA2G1B | PLAUR | PLCG1 | PLK1 | PLSCR1 | PML | PNKP | POT1 | PPARG | PPARGC1A | PPM1K | PPP1R12A | PPP1R12B | PPP2CA | PPP2CB | PRDX2 | PRDX3 | PRELID1 | PRKAA1 | PRKACA | PRKAG1 | PRKAR1A | PRKAR2B | PRKCD | PRKCE | PRKCZ | PRKD1 | PRKD2 | PROX1 | PRRC1 | PSAP | PSEN1 | PSEN2 | PSMA6 | PSMD10 | PSMD14 | PSME2 | PSME3 | PTEN | PTGES3 | PTK2 | PTPN1 | RAB1A | RAB3GAP1 | RAF1 | RAN | RANBP3 | RANGAP1 | RAP1A | RAP1GDS1 | RARA | RB1 | RELA | REST | RFC1 | RFC2 | RFC3 | RFC4 | RFC5 | RFK | RGN | RGS1 | RGS2 | RGS20 | RHOA | RIC8B | RIPK1 | RIPK2 | RIPK3 | RIPK4 | RNF25 | RNF31 | RP2 | RPA2 | RPLP1 | RPS2 | RPS3 | RPTOR | RSU1 | S100A1 | S100A12 | S100A9 | SAE1 | SDC4 | SERPINB3 | SH3PXD2A | SHC1 | SIPA1 | SIPA1L1 | SIRT1 | SKI | SLC9A1 | SMAD3 | SMARCA4 | SMARCB1 | SMO | SNCA | SOD1 | SPRY2 | SRC | SRGAP2 | SRGAP3 | STAC | STARD13 | STAT3 | STIM1 | STK11 | STK24 | STK25 | STK26 | STK3 | STMN1 | STUB1 | SUMO1 | SYDE1 | TAB1 | TAB2 | TAF12 | TANK | TAOK3 | TBC1D15 | TBC1D22B | TBC1D4 | TCEA1 | TCP1 | TERF2 | TERF2IP | TGFB1 | THBS1 | TIMP2 | TLR3 | TMEM173 | TNF | TNFRSF8 | TNKS | TP73 | TPM1 | TRADD | TRAF1 | TRAF2 | TRAF6 | TRIB3 | TRIM14 | TRIM21 | TRIM23 | TRIM25 | TRIM28 | TRIM32 | TRIM34 | TWIST1 | TXN | UBA2 | UBB | UBC | UBE2C | UBE2I | UBE2L3 | UBE2N | UBE2S | UBE2V1 | UNC119 | USP17L2 | USP50 | VAV1 | VCP | VDR | VTN | WDR35 | WNK1 | WNK4 | WNT2 | XRCC1 | XRCC5 | XRCC6 | YWHAB | ZBTB7A | ZNF622 | GO:0010604 | 0.244328521169477 | 2014/8243 | 874/3250 | 1.6417670244484e-05 | 0.155885778971376 | T | T | T | T | positive regulation of macromolecule metabolic process | ABHD14B | ABL1 | ACD | ACSL1 | ACTA1 | ACTA2 | ACTB | ACTG2 | ACTL6A | ACTN1 | ACTN2 | ACTN4 | ACTR2 | ACTR3 | ADCY6 | ADCYAP1 | ADRB2 | ADRM1 | ADTRP | AGER | AGO1 | AGO2 | AGR2 | AHI1 | AICDA | AIFM1 | AIMP2 | AIRE | AKAP8 | AKAP9 | AKT1 | AKT2 | AKTIP | ALOX12 | ANK3 | ANKRD49 | ANXA2 | APBB1 | APEX1 | APOE | APP | AR | ARAF | ARF4 | ARID3B | ARID4B | ARNT | ARNT2 | ARRB1 | ARRB2 | ASCL1 | ASPSCR1 | ATAD1 | ATAD5 | ATF2 | ATF6 | ATF6B | ATM | ATOH1 | ATRX | AURKB | AXIN1 | AZU1 | BABAM1 | BAG2 | BAG4 | BAG6 | BANP | BAX | BBC3 | BCAR3 | BCL10 | BCL11A | BCL2 | BCL2L13 | BCL6 | BIRC2 | BIRC3 | BIRC7 | BMI1 | BMPR1A | BRAF | BRCA1 | BRD4 | BTRC | BUD31 | C1QBP | C1QTNF1 | CAB39 | CALM1 | CALM2 | CALM3 | CALR | CAND1 | CARD9 | CARM1 | CASP10 | CASP3 | CASP8 | CASP9 | CAV1 | CBFA2T3 | CBFB | CBX8 | CCAR2 | CCDC22 | CCDC88A | CCL21 | CCL22 | CCNB1 | CCNT1 | CCNT2 | CCNY | CCNYL1 | CCR1 | CCT2 | CCT3 | CCT4 | CCT5 | CCT6A | CCT7 | CCT8 | CD27 | CD36 | CD40 | CD40LG | CD74 | CD81 | CDC14B | CDC20 | CDC25B | CDC5L | CDC73 | CDH1 | CDK2 | CDK4 | CDK5 | CDK5R1 | CDK8 | CDK9 | CDKN1A | CEBPA | CEBPE | CEP290 | CHCHD10 | CHCHD2 | CHD8 | CHEK2 | CHP1 | CIRBP | CKS1B | CKS2 | CLPX | CLU | COA3 | COMMD1 | COPS5 | CREB1 | CREB3 | CREBBP | CRK | CRKL | CRP | CSNK1A1 | CSNK1E | CSNK2B | CTBP1 | CTCF | CTNNB1 | CTSC | CUL3 | CYCS | DAB1 | DAB2 | DAPK1 | DAXX | DAZAP1 | DBF4B | DBNL | DCN | DCUN1D1 | DDRGK1 | DDX39B | DDX3X | DERL1 | DHX36 | DHX9 | DIABLO | DLG3 | DLG4 | DNA2 | DNAJB2 | DOCK7 | DPF2 | DROSHA | DTX1 | DUSP19 | DUSP22 | DUSP7 | DVL1 | DVL2 | DVL3 | DYRK2 | E2F1 | E2F2 | E2F3 | E2F4 | E2F6 | EAF2 | EBF1 | ECD | EDA | EDEM1 | EED | EEF2 | EGFR | EGLN1 | EGLN3 | EHD4 | EIF2AK3 | EIF2AK4 | EIF2B5 | EIF3D | EIF3E | EIF4A3 | EIF5A | EIF6 | ELAVL1 | ELF3 | ELF4 | ENDOG | ENO1 | EP300 | EPHA4 | ERBB2 | ERBB3 | ERG | ERLIN2 | ESR1 | ESR2 | ESRRA | ESRRB | ESRRG | ETS1 | EXOSC6 | EXOSC9 | EYA2 | EYA4 | EZH2 | EZR | FADD | FAF1 | FAM129B | FAM98A | FANCI | FANCM | FASLG | FBXW11 | FGB | FGF2 | FGFR1 | FGFR3 | FHL2 | FIS1 | FKBP1A | FLOT1 | FN1 | FOS | FOXA1 | FOXJ1 | FOXK1 | FOXK2 | FOXM1 | FOXO1 | FTO | FUBP1 | FUBP3 | FUS | FXR2 | FYN | FZR1 | GABPB1 | GADD45G | GATA1 | GDF9 | GLMN | GNAI2 | GOLGA2 | GPS2 | GRIP1 | GSK3A | GSK3B | GTF2A1 | GTF2F1 | GTF2F2 | H1F0 | H1FX | H2AFX | H2AFZ | HAVCR2 | HAX1 | HBEGF | HDAC1 | HDAC2 | HDAC3 | HDAC4 | HDAC5 | HDAC6 | HELZ2 | HEXB | HGS | HIF1A | HIST1H1A | HMGB1 | HMGB2 | HMGB3 | HMOX1 | HNF1A | HNF4A | HNF4G | HNRNPA1 | HNRNPA2B1 | HNRNPAB | HNRNPD | HNRNPK | HNRNPL | HNRNPU | HOXA5 | HOXB5 | HOXB9 | HOXD10 | HPX | HRAS | HSF1 | HSF4 | HSP90AA1 | HSP90AB1 | HSPA1B | HSPA2 | HSPA5 | HSPB1 | HSPBP1 | HSPD1 | HSPE1 | HSPH1 | HTATIP2 | HTRA2 | ICAM1 | ID2 | IFI16 | IFNA2 | IGF1R | IGF2BP1 | IKBKB | IKBKG | IKZF5 | ILF2 | ILF3 | ILK | ING1 | ING5 | INSIG1 | INSIG2 | INSR | IQGAP1 | IRF1 | IRF4 | IRF7 | ITCH | ITGB1BP1 | ITGB3 | JMJD6 | JUN | JUNB | JUND | JUP | KAT5 | KAT7 | KAT8 | KDM1A | KDM4D | KDM5B | KDM8 | KDR | KEAP1 | KHDRBS1 | KLF5 | KLF6 | KLHDC10 | KMT2A | KMT2B | KMT2C | KRAS | KSR1 | LAMP3 | LARP4 | LAT | LATS1 | LCP2 | LEF1 | LGMN | LGR4 | LIMS1 | LMNA | LMO2 | LMO7 | LPAR1 | LPIN3 | LRP6 | LRRK1 | LRRK2 | LTBR | LUM | MAP2K1 | MAP2K3 | MAP2K4 | MAP2K6 | MAP2K7 | MAP3K1 | MAP3K13 | MAP3K14 | MAP3K2 | MAP3K3 | MAP3K4 | MAP4K1 | MAPK1 | MAPK14 | MAPK15 | MAPK3 | MAPK7 | MAPK8 | MAPK8IP3 | MAPK9 | MAPKAP1 | MAPT | MAS1 | MAVS | MBP | MC1R | MDM2 | MECP2 | MED1 | MED12 | MED14 | MED17 | MED20 | MED21 | MED23 | MED24 | MED31 | MED4 | MED6 | MEOX2 | METTL23 | MGMT | MIF | MITF | MLH1 | MLLT11 | MLLT3 | MLST8 | MORF4L2 | MOV10 | MRPL12 | MSH6 | MSN | MTA1 | MTA3 | MTPN | MYBBP1A | MYC | MYD88 | MYO1C | MYO6 | NAMPT | NANOG | NBAS | NCBP1 | NCK1 | NCOA1 | NCOA2 | NCOA3 | NCOA4 | NCOR1 | NCOR2 | NDUFA13 | NEK1 | NEK7 | NELFE | NEUROD1 | NFATC1 | NFATC2 | NFE2L2 | NFKB2 | NFKBIA | NIF3L1 | NIPBL | NKRF | NKX2-1 | NMD3 | NME2 | NMNAT1 | NOD2 | NOS1AP | NOS3 | NPM1 | NQO2 | NR0B2 | NR1H2 | NR1H3 | NR1I2 | NR3C1 | NR4A1 | NR5A2 | NTF3 | NTRK1 | NUFIP1 | NUP85 | NUP98 | NUPR1 | OGT | ONECUT1 | OTUD4 | OXSR1 | P2RX1 | PABPC1 | PACSIN3 | PAIP1 | PAK2 | PAK6 | PARK7 | PARP1 | PARP14 | PATZ1 | PAWR | PAX8 | PAX9 | PAXIP1 | PCBD1 | PCBP1 | PCGF5 | PCNA | PCSK9 | PDCD10 | PDCD6 | PDE6G | PEF1 | PELI1 | PER1 | PER2 | PFKM | PHB | PHB2 | PHF10 | PIAS1 | PIAS3 | PICK1 | PIH1D1 | PIK3CA | PIK3CG | PIK3R1 | PIK3R3 | PIN1 | PLA2G10 | PLA2G1B | PLA2G2A | PLA2G6 | PLAUR | PLGRKT | PLK1 | PLSCR1 | PML | PNKP | POLDIP3 | POLR2A | POLR2E | POLR2L | POT1 | POU2F3 | POU5F1 | PPARD | PPARG | PPARGC1A | PPM1A | PPP1R12A | PPP2CA | PPP2R5D | PRDX2 | PRDX6 | PRELID1 | PRKAA1 | PRKAA2 | PRKACA | PRKAG1 | PRKAR1A | PRKAR2B | PRKCD | PRKCE | PRKCZ | PRKD1 | PRKD2 | PRKDC | PRMT2 | PROX1 | PRPF19 | PRRC1 | PSAP | PSEN1 | PSMC1 | PSMC2 | PSMC3 | PSMC4 | PSMC5 | PSMC6 | PSMD10 | PSMD14 | PSMD9 | PSME2 | PSME3 | PTEN | PTGES2 | PTGES3 | PTK2 | PTPN1 | PTPN22 | PUM1 | RAB1A | RAB1B | RAB29 | RAB3GAP1 | RAC1 | RAD23A | RAD51 | RAF1 | RAN | RAP1A | RAP1B | RARA | RARG | RB1 | RB1CC1 | RBM3 | RBMX | RBPJ | RBX1 | RC3H1 | RDX | REL | RELA | REST | RFC1 | RFC2 | RFC3 | RFC4 | RFC5 | RFXANK | RGN | RHBDD1 | RHOA | RIF1 | RIOK1 | RIPK1 | RIPK2 | RIPK3 | RMND1 | RNF139 | RNF185 | RNF19A | RNF4 | RNF8 | RPL23 | RPLP1 | RPS2 | RPS3 | RPS4X | RPS6KA1 | RPS6KB2 | RPTOR | RTF1 | RUNX1 | RUNX2 | RUVBL1 | RUVBL2 | RXRA | RXRB | RXRG | S100A12 | S100A7 | S100A9 | SART3 | SCYL2 | SDC4 | SDCBP | SEMA7A | SEPT4 | SERPINB3 | SETDB1 | SFPQ | SGTA | SHC1 | SIN3A | SIRT1 | SIRT6 | SIRT7 | SKI | SLAMF1 | SLC9A1 | SMAD2 | SMAD3 | SMAD4 | SMAD5 | SMAD9 | SMARCA2 | SMARCA4 | SMARCB1 | SMARCC1 | SMARCC2 | SMARCE1 | SMO | SMURF1 | SNAI2 | SNCA | SNF8 | SNW1 | SOAT1 | SOD1 | SOX2 | SP1 | SP100 | SPI1 | SPIN1 | SPRTN | SPRY2 | SQSTM1 | SRC | SRPK2 | SRSF5 | STAT1 | STAT3 | STAT4 | STAT6 | STK11 | STK24 | STK25 | STK26 | STK3 | STOML2 | STUB1 | SUB1 | SUMO1 | SUMO2 | SUPT4H1 | SUPT5H | TAB1 | TAB2 | TADA2B | TAF12 | TAF15 | TAF1A | TANK | TAOK3 | TARDBP | TBL1X | TCEA1 | TCEA2 | TCERG1 | TCF12 | TCF21 | TCF4 | TCP1 | TDRD3 | TERF2 | TERF2IP | TFCP2 | TFDP1 | TGFB1 | THBS1 | THRA | TIMP2 | TLE1 | TLR3 | TMEM173 | TMTC3 | TNF | TNFAIP3 | TNFRSF1A | TNFRSF8 | TNIP2 | TNK2 | TNKS | TNKS2 | TNP1 | TOLLIP | TOP2A | TP53 | TP63 | TP73 | TRA2A | TRADD | TRAF2 | TRAF6 | TRIAP1 | TRIB2 | TRIB3 | TRIM28 | TRIM32 | TRIP4 | TRUB2 | TSG101 | TTC5 | TWIST1 | TXN | U2AF2 | UBB | UBC | UBE2B | UBE2C | UBE2D1 | UBE2I | UBE2K | UBE2L3 | UBE2N | UBE2S | UBE2V1 | UBE3A | UBQLN1 | UBQLN2 | UBR5 | UNC119 | UPF3B | UQCC1 | USP16 | USP21 | USP5 | USP50 | USP7 | USP9X | VCP | VDR | VHL | VIM | VSTM2A | VTN | WARS | WAS | WBP2 | WDR35 | WDR48 | WDR77 | WNK1 | WNT16 | WNT2 | WT1 | WWOX | WWP2 | XBP1 | XIAP | XRCC1 | XRCC5 | XRCC6 | YAF2 | YAP1 | YES1 | YTHDF1 | YWHAH | YY1 | ZBTB16 | ZBTB17 | ZBTB7B | ZIC1 | ZMPSTE24 | ZNF24 | ZNF622 | ZNF746 | GO:0045935 | 0.0226859153220915 | 187/8243 | 102/3250 | 1.66190626690068e-05 | 0.15779800004222 | T | T | T | T | positive regulation of nucleobase-containing compound metabolic process | ACD | ACTR2 | AKT1 | APBB1 | ATAD5 | ATM | ATRX | AURKB | BABAM1 | BAX | BRCA1 | CBX8 | CCNB1 | CCT2 | CCT3 | CCT4 | CCT5 | CCT6A | CCT7 | CCT8 | CD40 | CDC73 | CDK2 | CIRBP | CTNNB1 | DAZAP1 | DHX36 | DHX9 | EGFR | ENDOG | EXOSC6 | EYA2 | EYA4 | FOXM1 | FUS | H2AFX | HMGB1 | HMGB2 | HNRNPA1 | HNRNPA2B1 | HNRNPD | HRAS | HSF1 | KDM4D | MAP2K1 | MAP2K7 | MAP3K4 | MAPK1 | MAPK15 | MAPK3 | MECP2 | MGMT | MLH1 | MSH6 | MYC | NCBP1 | NEK7 | NUP98 | OTUD4 | PARP1 | PAXIP1 | PCNA | PIK3R1 | PML | PNKP | POLR2A | POT1 | PPARGC1A | PRDX6 | PRKCD | PRPF19 | RAD51 | RBM3 | RBMX | RELA | RIF1 | RIOK1 | RIPK1 | RNF8 | RPS3 | SIRT1 | SIRT6 | SNW1 | SRSF5 | STAT6 | TCEA1 | TCP1 | TGFB1 | TNKS | TNKS2 | TP53 | TRA2A | TRIM28 | U2AF2 | UBE2B | UBE2N | USP7 | USP9X | WAS | WT1 | XRCC1 | XRCC5 | GO:0010942 | 0.0567754458328279 | 468/8243 | 228/3250 | 1.67979265262737e-05 | 0.159496312366969 | T | T | T | T | positive regulation of cell death | ABL1 | ADORA2A | AES | AIFM1 | AIMP2 | AKT1 | ALOX12 | ANXA1 | ANXA5 | APBB1 | APBB2 | APC | ARHGEF7 | ARHGEF9 | ARRB1 | ARRB2 | ASCL1 | ATF2 | ATF6 | ATM | BACE1 | BAX | BBC3 | BCL10 | BCL2 | BCL2L1 | BCL2L13 | BCL6 | BIN1 | C1QA | C1QBP | CAMK1D | CAMK2A | CAPN2 | CASP10 | CASP2 | CASP3 | CASP8 | CASP9 | CAV1 | CCAR2 | CD36 | CD40 | CD40LG | CDC34 | CDK19 | CDK4 | CDK5 | CDK5R1 | CDKN1A | CHCHD10 | CHEK2 | CLU | COL18A1 | CREB1 | CTNNB1 | CTSC | CYCS | CYLD | DAPK1 | DAXX | DDIT4 | DDX3X | DIABLO | DNAJA1 | DNM1L | DUSP1 | E2F1 | E2F3 | EEF1A2 | EGLN3 | EIF2AK3 | EIF2B5 | EIF2S1 | ENDOG | FADD | FAF1 | FASLG | FIS1 | FOS | FOXA1 | FOXO1 | FYN | GADD45G | GAPDH | GNA13 | GRIK2 | GSK3A | GSK3B | GZMA | HDAC4 | HDAC6 | HMGB1 | HMOX1 | HOXA5 | HSF1 | HSPD1 | HSPE1 | HTRA2 | HTT | ING5 | ITGA4 | ITGB3BP | JUN | LATS1 | LDHA | LPAR1 | LRRK2 | LTBR | MAP2K4 | MAP2K6 | MAPK8 | MAPK9 | MAPT | MELK | MLLT11 | MTCH2 | MYBBP1A | MYC | NCK1 | NCOA1 | NDUFA13 | NEUROD1 | NQO1 | NQO2 | NTRK1 | NUPR1 | P2RX1 | PAK2 | PARK7 | PARP1 | PAWR | PCSK9 | PDCD6 | PDIA3 | PHB | PIN1 | PLA2G6 | PLAUR | PLCG1 | PLEKHF1 | PML | PNMA1 | PPARG | PPARGC1A | PPID | PPIF | PPP1CA | PPP2R1A | PPP2R1B | PPP3CC | PRELID1 | PRKCD | PRKDC | PRMT2 | PSEN1 | PSEN2 | PTEN | PTN | PTRH2 | RARG | REST | RHOA | RIPK1 | RIPK2 | RIPK3 | RNF183 | RPS3 | RPS6KA2 | S100A9 | S100B | SAP18 | SEPT4 | SEPT7 | SFN | SFPQ | SIAH1 | SIRT1 | SLC9A1 | SLC9A3R1 | SMAD3 | SNCA | SOD1 | SOD2 | SORT1 | SQSTM1 | SRC | SRPK2 | STK3 | TCTN3 | TFDP1 | TGFB1 | TGM2 | THBS1 | TIA1 | TLR3 | TNF | TNFRSF12A | TNFRSF1A | TNFRSF8 | TNFSF12 | TOMM22 | TOP2A | TP53 | TP63 | TP73 | TRADD | TRAF2 | TRAF6 | TRIM35 | TXNIP | UBB | UBC | UBE2M | VAV1 | VCP | VDR | WDR35 | WWOX | YWHAB | YWHAE | YWHAG | YWHAH | YWHAQ | YWHAZ | ZBTB16 | ZNF622 | ZNF746 | GO:0045088 | 0.0357879412835133 | 295/8243 | 151/3250 | 2.04153861979161e-05 | 0.193844091949214 | T | T | T | T | regulation of innate immune response | A2M | ABCE1 | AP1G1 | APOE | ARRB2 | BCL10 | BIRC2 | BIRC3 | BTK | BTRC | C1QBP | CARD9 | CASP8 | CAV1 | CD209 | CD36 | CDC37 | CREBBP | CRK | CTSL | CUL1 | CYLD | DHX9 | EP300 | ESR1 | FADD | FBXW11 | FCN1 | FGB | FLOT1 | FYN | GBP5 | GPS2 | HAVCR2 | HEXIM1 | HMGB1 | HMGB2 | HMGB3 | HPX | HRAS | HSP90AB1 | HSP90B1 | HSPA1B | HSPD1 | IFI16 | IKBKB | IKBKE | IKBKG | IRF1 | IRF4 | IRF7 | ITCH | JAK1 | KRAS | LGMN | LGR4 | MAP2K6 | MAP3K1 | MATR3 | MAVS | MED1 | MICA | MYD88 | NFKBIA | NOD2 | NPLOC4 | NR1H2 | NR1H3 | OTUD4 | PAK2 | PARP14 | PELI1 | PIAS1 | PLSCR1 | PPARG | PRKACA | PRKCD | PRKCE | PRKDC | PSMA1 | PSMA2 | PSMA3 | PSMA5 | PSMA6 | PSMB5 | PSMB6 | PSMB7 | PSMB8 | PSMC1 | PSMC2 | PSMC3 | PSMC4 | PSMC5 | PSMC6 | PSMD1 | PSMD10 | PSMD11 | PSMD12 | PSMD14 | PSMD2 | PSMD4 | PSMD9 | PSME2 | PSME3 | PTPN1 | PTPN22 | PTPN6 | PUM1 | RAF1 | RELA | RIPK1 | RIPK2 | RPS19 | RTN4 | S100A1 | S100A9 | SARM1 | SCARA3 | SFPQ | SIN3A | SLAMF8 | SOCS1 | SRC | STAT1 | SUMO1 | TAB1 | TAB2 | TANK | TLR3 | TMEM173 | TNFAIP3 | TNIP2 | TRAF3 | TRAF6 | TREX1 | UBB | UBC | UBE2D1 | UBE2D2 | UBE2D3 | UBE2K | UBE2N | UBE2V1 | UBQLN1 | USP17L2 | VAV1 | VSIG4 | WDFY1 | XIAP | XRCC5 | XRCC6 | GO:0010389 | 0.019410408831736 | 160/8243 | 89/3250 | 2.08659570809296e-05 | 0.198122262483427 | T | T | T | T | regulation of G2/M transition of mitotic cell cycle | ACTR1A | AKAP9 | APP | ATM | AURKB | BRCA1 | BRD4 | CCNB1 | CCP110 | CDC25A | CDC25B | CDC25C | CDK2 | CDK4 | CENPF | CENPJ | CEP135 | CEP152 | CEP290 | CEP57 | CEP70 | CKAP5 | CNTRL | CSNK1E | CUL1 | DBF4B | DCTN1 | DYNC1H1 | DYNC1I2 | DYNLL1 | FGFR1OP | FHL1 | HMMR | HSP90AA1 | HSPA2 | MECP2 | MSH6 | MTA3 | NAE1 | OFD1 | PAFAH1B1 | PCM1 | PINX1 | PLK1 | PPP2R1A | PRKACA | PRKAR2B | PSMA1 | PSMA2 | PSMA3 | PSMA5 | PSMA6 | PSMB5 | PSMB6 | PSMB7 | PSMB8 | PSMC1 | PSMC2 | PSMC3 | PSMC4 | PSMC5 | PSMC6 | PSMD1 | PSMD10 | PSMD11 | PSMD12 | PSMD14 | PSMD2 | PSMD4 | PSMD9 | PSME2 | PSME3 | RAB11A | RAD21 | RBX1 | RNASEH2B | SIN3A | TAOK3 | TUBA1A | TUBA4A | TUBB | TUBB4A | TUBB4B | TUBG1 | USP47 | VPS4B | YWHAE | YWHAG | ZFYVE19 | GO:0043467 | 0.0131020259614218 | 108/8243 | 64/3250 | 2.17329072163513e-05 | 0.206353954019255 | T | T | T | T | regulation of generation of precursor metabolites and energy | AKT1 | AKT2 | ARNT | CBFA2T3 | CCNB1 | CHCHD10 | CISD1 | COX17 | COX7A2 | DDIT4 | DNAJC30 | DYRK2 | ECD | EIF6 | ESRRB | FBP1 | GAPDHS | GPD1 | GSK3A | GSK3B | HDAC4 | HIF1A | IDE | INSR | NCOR1 | NOS2 | NUP107 | NUP153 | NUP160 | NUP205 | NUP214 | NUP85 | NUP88 | NUP93 | NUP98 | OGT | PARK7 | PDE12 | PGAM1 | PGK1 | PGK2 | PIK3CA | POM121 | PPARGC1A | PPIF | PPP1CA | PPP1CB | PPP1R3B | PRELID1 | PRKAA1 | PRKAA2 | PRKAG1 | RAE1 | RHOA | SEC13 | SHMT2 | SIRT6 | SNCA | STAT3 | TPR | TRAP1 | TREX1 | VCP | ZBTB7A | GO:0022408 | 0.0111609850782482 | 92/8243 | 56/3250 | 2.38309353497116e-05 | 0.226274731145512 | T | T | T | T | negative regulation of cell-cell adhesion | ABAT | ABL1 | ADORA2A | ADTRP | AKT1 | ALOX12 | ANXA1 | APOA1 | ASS1 | BCL6 | C1QTNF1 | CASP3 | CBFB | CCL21 | CD74 | CDH1 | DTX1 | DUSP22 | ERBB2 | FOXJ1 | GLMN | HAVCR2 | HLX | HMGB1 | IFNA2 | IRF1 | ITCH | JAK3 | LGALS3 | MAD1L1 | MAPK7 | MBP | NF2 | PAG1 | PAWR | PELI1 | PRDX2 | PRKAR1A | PRKCD | PTK2 | PTPN22 | PTPN6 | RC3H1 | RDX | RUNX1 | SDC4 | SERPINE2 | SNAI2 | SOCS1 | SOCS6 | SPINT2 | SWAP70 | TGFB1 | VSIG4 | WNK1 | ZBTB7B | GO:0033209 | 0.0106757248574548 | 88/8243 | 54/3250 | 2.39427434168214e-05 | 0.22733634874272 | T | T | T | T | tumor necrosis factor-mediated signaling pathway | ACTN4 | BAG4 | BIRC2 | BIRC3 | CD27 | CD40 | CD40LG | CLDN18 | EDA | IKBKB | ILK | LIMS1 | LTBR | MAP3K14 | NFKBIA | PIAS3 | PSMA1 | PSMA2 | PSMA3 | PSMA5 | PSMA6 | PSMB5 | PSMB6 | PSMB7 | PSMB8 | PSMC1 | PSMC2 | PSMC3 | PSMC4 | PSMC5 | PSMC6 | PSMD1 | PSMD10 | PSMD11 | PSMD12 | PSMD14 | PSMD2 | PSMD4 | PSMD9 | PSME2 | PSME3 | RELA | RIPK1 | STAT1 | TNF | TNFRSF12A | TNFRSF1A | TNFRSF8 | TNFSF12 | TNFSF14 | TRADD | TRAF2 | TRAF3 | TXNDC17 | GO:0043299 | 0.0418536940434308 | 345/8243 | 173/3250 | 2.42045914113175e-05 | 0.22982259545046 | T | T | T | T | leukocyte degranulation | ACAA1 | ACLY | ACTR2 | AGL | AHSG | ALDOA | ALDOC | ALOX5 | ANXA2 | AP1M1 | APEH | APRT | ARMC8 | ARPC5 | ATP6V1D | AZU1 | CAB39 | CAND1 | CAPN1 | CAT | CBL | CCT2 | CCT8 | CD36 | CDA | CEP290 | CKAP4 | COPB1 | COTL1 | CPNE3 | CSNK2B | CTSA | CTSC | CTSD | CYB5R3 | DBNL | DDOST | DDX3X | DEGS1 | DERA | DYNC1H1 | DYNC1LI1 | DYNLL1 | DYNLT1 | EEF1A1 | EEF2 | FABP5 | FAF2 | FCN1 | FGL2 | FTH1 | FTL | GAA | GALNS | GDI2 | GHDC | GLA | GNS | GPI | GSTP1 | GUSB | GYG1 | HEXB | HMGB1 | HRNR | HSP90AA1 | HSP90AB1 | HSPA1B | HSPA8 | HUWE1 | IDH1 | ILF2 | IMPDH2 | IQGAP1 | ITGAV | JUP | KCMF1 | KCNAB2 | KPNB1 | LAT | LGALS3 | LRRC7 | MAN2B1 | MAPK1 | MAPK14 | MGST1 | MIF | MME | NAPRT | NHLRC3 | NIT2 | NME2 | ORM1 | P2RX1 | PA2G4 | PDXK | PFKL | PGAM1 | PGM1 | PGM2 | PGRMC1 | PI4K2A | PIK3CG | PKM | PLAUR | PNP | PPIA | PPIE | PRCP | PRDX4 | PRDX6 | PRG2 | PRKCD | PSAP | PSEN1 | PSMA2 | PSMA5 | PSMB7 | PSMC2 | PSMC3 | PSMD1 | PSMD11 | PSMD12 | PSMD14 | PSMD2 | PTGES2 | PTPN6 | PYGB | PYGL | QSOX1 | RAB10 | RAB18 | RAB24 | RAB31 | RAB5B | RAB7A | RAC1 | RAP1A | RAP1B | RHOA | RNASE3 | S100A11 | S100A12 | S100A13 | S100A7 | S100A9 | S100P | SDCBP | SELL | SERPINB3 | SERPINB6 | SLC2A5 | SNAP29 | SPTAN1 | SRP14 | STBD1 | STOM | STXBP2 | TCIRG1 | TIMP2 | TMEM173 | TMEM30A | TOLLIP | TUBB | TUBB4B | TXNDC5 | UBR4 | VAPA | VAT1 | VCL | VCP | XRCC5 | XRCC6 | GO:0002366 | 0.048162076913745 | 397/8243 | 196/3250 | 2.42396988307276e-05 | 0.230155940397759 | T | T | T | T | leukocyte activation involved in immune response | ABL1 | ACAA1 | ACLY | ACTR2 | ADA | AGL | AHSG | AICDA | ALDOA | ALDOC | ALOX5 | ANXA2 | AP1M1 | APEH | APRT | ARMC8 | ARPC5 | ATAD5 | ATP6V1D | AZU1 | CAB39 | CAND1 | CAPN1 | CAT | CBL | CCT2 | CCT8 | CD36 | CD40LG | CDA | CEP290 | CKAP4 | COPB1 | COTL1 | CPNE3 | CSNK2B | CTSA | CTSC | CTSD | CYB5R3 | DBNL | DDOST | DDX3X | DEGS1 | DERA | DYNC1H1 | DYNC1LI1 | DYNLL1 | DYNLT1 | EEF1A1 | EEF2 | EIF2AK4 | EXOSC6 | FABP5 | FAF2 | FCN1 | FGL2 | FTH1 | FTL | GAA | GALNS | GDI2 | GHDC | GLA | GNS | GPI | GSTP1 | GUSB | GYG1 | HAVCR2 | HEXB | HMGB1 | HRNR | HSP90AA1 | HSP90AB1 | HSPA1B | HSPA8 | HSPD1 | HUWE1 | ICAM1 | IDH1 | IFNA2 | ILF2 | IMPDH2 | IQGAP1 | ITGAV | JUP | KCMF1 | KCNAB2 | KPNB1 | LAT | LCP1 | LEF1 | LGALS1 | LGALS3 | LIG4 | LRRC7 | MAN2B1 | MAPK1 | MAPK14 | MGST1 | MIF | MLH1 | MME | MSH6 | NAPRT | NHLRC3 | NIT2 | NME2 | ORM1 | P2RX1 | PA2G4 | PDXK | PFKL | PGAM1 | PGM1 | PGM2 | PGRMC1 | PI4K2A | PIK3CG | PKM | PLAUR | PNP | PPIA | PPIE | PRCP | PRDX4 | PRDX6 | PRG2 | PRKCD | PRKCE | PSAP | PSEN1 | PSMA2 | PSMA5 | PSMB7 | PSMC2 | PSMC3 | PSMD1 | PSMD11 | PSMD12 | PSMD14 | PSMD2 | PTGES2 | PTPN6 | PYGB | PYGL | QSOX1 | RAB10 | RAB18 | RAB24 | RAB31 | RAB5B | RAB7A | RAC1 | RAP1A | RAP1B | RC3H1 | RHOA | RNASE3 | RNF8 | S100A11 | S100A12 | S100A13 | S100A7 | S100A9 | S100P | SDCBP | SELL | SERPINB3 | SERPINB6 | SLAMF1 | SLC2A5 | SNAP29 | SPTAN1 | SRP14 | STBD1 | STOM | STXBP2 | SWAP70 | TCIRG1 | TIMP2 | TMEM173 | TMEM30A | TOLLIP | TREX1 | TUBB | TUBB4B | TXNDC5 | UBR4 | VAPA | VAT1 | VCL | VCP | XRCC5 | XRCC6 | GO:0051051 | 0.0334829552347446 | 276/8243 | 142/3250 | 2.5724786140012e-05 | 0.244256844399414 | T | T | T | T | negative regulation of transport | ABAT | ACAA2 | ACTN2 | ADA | ADCYAP1 | ADTRP | AKT1 | AKT2 | ANK3 | ANXA1 | ANXA5 | APOA1 | APOD | APP | ARHGAP1 | ATG5 | ATXN2 | BAG3 | BAG4 | BCL2 | BIN1 | BRAF | CAB39 | CALM1 | CALM2 | CALM3 | CAV1 | CD22 | CD36 | CD74 | CDK5 | CHP1 | COMMD1 | COMT | CRBN | CRY1 | CRYAB | DLG4 | EPPIN | ERBB3 | EZR | FABP5 | FKBP1A | FKBP1B | FN1 | GDI1 | GNAI2 | GNAO1 | GOPC | GSK3A | GSTO1 | HADH | HDAC9 | HECW1 | HECW2 | HMGB1 | HMOX1 | HTR1B | ICAM1 | IDH2 | IFNA2 | IL13RA2 | INSIG1 | ITGAV | ITGB1BP1 | ITGB3 | KCNAB1 | LGALS3 | LGR4 | LRRK2 | LRSAM1 | LYPLA1 | MAP1B | NDUFAF2 | NEDD4 | NMB | NOS3 | NR1H2 | NR1H3 | NUP153 | OAZ3 | OXSR1 | PACSIN3 | PARK7 | PCSK9 | PFKL | PKIG | PLA2G10 | PML | PPIF | PPP1R11 | PRKCE | PRKD1 | PSEN1 | PSMD9 | PTEN | PTGER3 | PTPN22 | RAB7A | RAC1 | RANGAP1 | RAP1A | RAP1B | REST | RGS2 | SDCBP | SERPINE2 | SH3GL3 | SIRT6 | SLC25A5 | SLC35F6 | SLC9A3R1 | SNCA | SNX12 | SNX3 | SP100 | SRI | SUMO1 | TBC1D4 | TGFB1 | THADA | THBS1 | TMBIM6 | TNF | TNFAIP3 | TNFRSF1A | TPR | TRIM9 | TWIST1 | TXN | UBE2J1 | UBQLN1 | UBQLN2 | UNC119 | USP17L2 | VPS4B | WNK1 | WNK4 | WWP2 | YRDC | YWHAE | YWHAQ | GO:0002263 | 0.04852602207934 | 400/8243 | 197/3250 | 2.79185796229737e-05 | 0.265086913520135 | T | T | T | T | cell activation involved in immune response | ABL1 | ACAA1 | ACLY | ACTR2 | ADA | AGL | AHSG | AICDA | ALDOA | ALDOC | ALOX5 | ANXA2 | AP1M1 | APEH | APP | APRT | ARMC8 | ARPC5 | ATAD5 | ATP6V1D | AZU1 | CAB39 | CAND1 | CAPN1 | CAT | CBL | CCT2 | CCT8 | CD36 | CD40LG | CDA | CEP290 | CKAP4 | COPB1 | COTL1 | CPNE3 | CSNK2B | CTSA | CTSC | CTSD | CYB5R3 | DBNL | DDOST | DDX3X | DEGS1 | DERA | DYNC1H1 | DYNC1LI1 | DYNLL1 | DYNLT1 | EEF1A1 | EEF2 | EIF2AK4 | EXOSC6 | FABP5 | FAF2 | FCN1 | FGL2 | FTH1 | FTL | GAA | GALNS | GDI2 | GHDC | GLA | GNS | GPI | GSTP1 | GUSB | GYG1 | HAVCR2 | HEXB | HMGB1 | HRNR | HSP90AA1 | HSP90AB1 | HSPA1B | HSPA8 | HSPD1 | HUWE1 | ICAM1 | IDH1 | IFNA2 | ILF2 | IMPDH2 | IQGAP1 | ITGAV | JUP | KCMF1 | KCNAB2 | KPNB1 | LAT | LCP1 | LEF1 | LGALS1 | LGALS3 | LIG4 | LRRC7 | MAN2B1 | MAPK1 | MAPK14 | MGST1 | MIF | MLH1 | MME | MSH6 | NAPRT | NHLRC3 | NIT2 | NME2 | ORM1 | P2RX1 | PA2G4 | PDXK | PFKL | PGAM1 | PGM1 | PGM2 | PGRMC1 | PI4K2A | PIK3CG | PKM | PLAUR | PNP | PPIA | PPIE | PRCP | PRDX4 | PRDX6 | PRG2 | PRKCD | PRKCE | PSAP | PSEN1 | PSMA2 | PSMA5 | PSMB7 | PSMC2 | PSMC3 | PSMD1 | PSMD11 | PSMD12 | PSMD14 | PSMD2 | PTGES2 | PTPN6 | PYGB | PYGL | QSOX1 | RAB10 | RAB18 | RAB24 | RAB31 | RAB5B | RAB7A | RAC1 | RAP1A | RAP1B | RC3H1 | RHOA | RNASE3 | RNF8 | S100A11 | S100A12 | S100A13 | S100A7 | S100A9 | S100P | SDCBP | SELL | SERPINB3 | SERPINB6 | SLAMF1 | SLC2A5 | SNAP29 | SPTAN1 | SRP14 | STBD1 | STOM | STXBP2 | SWAP70 | TCIRG1 | TIMP2 | TMEM173 | TMEM30A | TOLLIP | TREX1 | TUBB | TUBB4B | TXNDC5 | UBR4 | VAPA | VAT1 | VCL | VCP | XRCC5 | XRCC6 | GO:0010647 | 0.127866068179061 | 1054/8243 | 476/3250 | 2.88382688202706e-05 | 0.273819362448469 | T | T | T | T | positive regulation of cell communication | ABAT | ABL1 | ACTN4 | ADA | ADCYAP1 | ADORA2A | ADRB2 | AGER | AGO1 | AGR2 | AKR1B1 | AKR1C2 | AKT1 | AKT3 | ANK3 | APOA1 | APOE | APP | AR | ARHGAP1 | ARHGEF7 | ARNT | ARRB1 | ARRB2 | ASCL1 | ATF6 | ATM | AXIN1 | BAG4 | BAX | BBC3 | BCL10 | BCL2 | BCL2L1 | BIRC2 | BIRC3 | BIRC7 | BIRC8 | BMPR1A | BRAF | BRD4 | BUD31 | C1QBP | C1QTNF1 | CACNG2 | CACNG4 | CACNG5 | CALM1 | CALM2 | CALM3 | CALR | CARD9 | CASP10 | CASP2 | CASP8 | CAT | CAV1 | CBL | CCAR2 | CCDC22 | CCL21 | CCL22 | CCR1 | CD27 | CD36 | CD40 | CD40LG | CD74 | CD81 | CDC73 | CDK5 | CFTR | CHCHD10 | CLNK | CLSTN1 | CREB1 | CREBBP | CRK | CRKL | CSNK1E | CSNK2B | CTH | CTNNB1 | CTSC | CYLD | DAB2 | DAXX | DBNL | DCN | DDRGK1 | DDX1 | DDX3X | DHX36 | DISC1 | DLG4 | DNM1L | DOK1 | DUSP19 | DUSP22 | DUSP7 | DVL1 | DVL2 | DVL3 | DYNLL1 | E2F1 | EDA | EEF1D | EGFR | EIF2AK4 | ELF3 | EP300 | EPHA4 | ERBB2 | ERBB3 | ESR1 | EZH2 | FABP5 | FADD | FAF1 | FASLG | FGB | FGF2 | FGFR1 | FGFR3 | FIS1 | FKBP1A | FKBP8 | FLOT1 | FOXA1 | FYN | GADD45G | GDF9 | GFAP | GJA1 | GKAP1 | GLUL | GNAI2 | GOLPH3 | GOLT1B | GOT1 | GPS2 | GPX1 | GRB2 | GRB7 | GRIK2 | GSK3A | GSK3B | GSTO1 | HAP1 | HAVCR2 | HAX1 | HBEGF | HDAC2 | HDAC3 | HDAC6 | HEXIM1 | HIF1A | HINT1 | HMGB1 | HMOX1 | HPX | HRAS | HSF1 | HSP90AA1 | HSP90AB1 | HSPA1B | HTRA2 | HTT | ICAM1 | IFNA2 | IGF1R | IKBKB | IKBKE | IKBKG | ILK | ING5 | INSR | IQGAP1 | IRF7 | ITGB1BP1 | ITGB3 | ITPR3 | JUN | JUP | KDR | KHDRBS1 | KIF5B | KLHDC10 | KLHL22 | KRAS | KSR1 | LARS | LASP1 | LAT | LGALS1 | LGMN | LGR4 | LIMS1 | LPAR1 | LPAR4 | LRRK1 | LRRK2 | LTBR | LURAP1 | MAOA | MAP2K1 | MAP2K3 | MAP2K4 | MAP2K6 | MAP2K7 | MAP3K1 | MAP3K13 | MAP3K14 | MAP3K2 | MAP3K3 | MAP3K4 | MAP4K1 | MAPK1 | MAPK14 | MAPK3 | MAPK8 | MAPK8IP3 | MAPK9 | MAS1 | MAVS | MC1R | MECP2 | MED1 | MIF | MLLT11 | MLLT3 | MLST8 | MME | MPC2 | MPP2 | MYD88 | MYO1C | MYOC | NCK1 | NEDD4 | NELFE | NMB | NMNAT1 | NOD2 | NOS1AP | NPR1 | NQO2 | NR0B2 | NR1H3 | NRXN1 | NTF3 | NTRK1 | NUMA1 | NUP93 | OPA1 | OXCT1 | P2RX2 | P2RX4 | PAFAH1B1 | PAG1 | PAK2 | PARK7 | PARP1 | PARP14 | PDCD10 | PDCL | PDE6G | PDIA3 | PEBP1 | PELI1 | PFKM | PHB | PHB2 | PIH1D1 | PIK3CA | PIK3CG | PIK3R1 | PIN1 | PLA2G1B | PLA2G2A | PLA2G6 | PLAUR | PLEKHF1 | PML | PPARD | PPIF | PPM1A | PPP1CA | PPP2R1A | PPP2R1B | PPP3CC | PPP5C | PRDX2 | PRKAA1 | PRKCD | PRKCE | PRKCZ | PRKD1 | PRKD2 | PSAP | PSEN1 | PSMA1 | PSMA2 | PSMA3 | PSMA5 | PSMA6 | PSMB5 | PSMB6 | PSMB7 | PSMB8 | PSMC1 | PSMC2 | PSMC3 | PSMC4 | PSMC5 | PSMC6 | PSMD1 | PSMD10 | PSMD11 | PSMD12 | PSMD14 | PSMD2 | PSMD4 | PSMD9 | PSME2 | PSME3 | PTEN | PTK2 | PTN | PTPN1 | PTPN22 | PTPN23 | PTPN6 | PUM1 | RAB29 | RAB3GAP1 | RAC1 | RAF1 | RAP1A | RAP1B | RB1CC1 | RBPJ | RC3H1 | RECK | REL | RELA | RHOA | RIPK1 | RIPK2 | RIPK3 | RNF183 | RNF31 | RPL23 | RPS3 | RPTOR | RTN4 | RUVBL1 | S100A12 | S100A13 | S100A4 | S100A7 | S100A9 | S100B | SARM1 | SCEL | SDCBP | SEC13 | SEMA7A | SEPT4 | SERPINE2 | SFN | SFPQ | SH2B1 | SHC1 | SHKBP1 | SIAH1 | SIRT1 | SIRT3 | SKI | SLAMF1 | SLC9A1 | SLC9A3R1 | SMAD2 | SMAD3 | SMAD4 | SMARCA4 | SMARCB1 | SMO | SMURF2 | SNCA | SNW1 | SOCS2 | SOD1 | SOX2 | SPIN1 | SPRY2 | SQSTM1 | SRC | SRI | STAT3 | STAU1 | STK11 | STK25 | STK3 | STXBP1 | SULF2 | TAB1 | TAB2 | TAOK3 | TARDBP | TBL1X | TERF2IP | TFDP1 | TFG | TGFB1 | TGFBR3 | TGM2 | THBS1 | TIMP2 | TLR3 | TMEM9B | TNF | TNFAIP3 | TNFRSF12A | TNFRSF1A | TNFSF12 | TNFSF14 | TNIP2 | TNKS | TNKS2 | TP53 | TP63 | TP73 | TRADD | TRAF2 | TRAF6 | TRIM25 | TRIM32 | TRIP6 | TXN | UBB | UBC | UBE2B | UBE2I | UBE2K | UBE2N | UBE2O | UBE2V1 | UBQLN2 | UBR5 | USP17L2 | USP47 | USP8 | VAPA | VAV1 | VCP | VTN | WBP2 | WDFY1 | WDR35 | WNK1 | WNT16 | WWOX | XBP1 | XIAP | YAP1 | YWHAB | YWHAE | YWHAG | YWHAH | YWHAQ | YWHAZ | ZAP70 | ZBTB7B | ZDHHC17 | ZNF622 | GO:0060341 | 0.0723037728982167 | 596/8243 | 282/3250 | 2.96514239334435e-05 | 0.281540270248046 | T | T | T | T | regulation of cellular localization | ABHD17A | ABL1 | ACTB | ACTN2 | ADORA2A | AGR2 | AKT1 | AKT2 | ANK3 | ANXA2 | APC | APOD | AR | ARF1 | ARHGAP1 | ARRB1 | ATAD1 | ATG5 | ATP13A2 | ATP2A2 | B3GAT3 | BACE1 | BAG3 | BAG4 | BAX | BBC3 | BCL2 | BCL2L1 | BRAF | BTBD9 | CACNG2 | CALM1 | CALM2 | CALM3 | CAMK2A | CASP8 | CAV1 | CCDC88A | CCT2 | CCT3 | CCT4 | CCT5 | CCT6A | CCT7 | CCT8 | CD36 | CDH1 | CDK16 | CDK5 | CDK5R1 | CEP290 | CFTR | CHP1 | CHRM2 | CLDN18 | CLTC | COMMD1 | CPLX1 | CPLX3 | CRKL | CROCC | CRYAB | CSNK1E | CSNK2A2 | CTNNB1 | CXCL11 | CYLD | DAB2 | DBN1 | DCLK1 | DDRGK1 | DHX36 | DHX9 | DMAP1 | DMD | DNAJA1 | DNAJC30 | DNM1L | DVL1 | DVL3 | DYNC1H1 | DYNLL1 | E2F1 | EDEM1 | EGFR | EIF2AK3 | ERBB2 | EZR | FIS1 | FKBP1A | FKBP1B | FYN | GDI1 | GIT1 | GNAI1 | GOPC | GPD1L | GSK3A | GSK3B | GSTO1 | HAP1 | HAX1 | HDAC3 | HIF1A | HMOX1 | HNRNPU | HRAS | HSP90AA1 | HSP90AB1 | HSP90AB3P | HSP90AB4P | HSPA1L | HTR1B | HTRA2 | HTT | HUWE1 | IL13RA2 | INSIG1 | IPO5 | IQGAP1 | ITGB1BP1 | ITGB3 | JUP | KAT7 | KCNAB2 | KDM1A | KHDRBS1 | KIF5B | LCP1 | LGALS3 | LMNA | LRRK2 | LYPLA1 | MAD1L1 | MAP1B | MAP2K1 | MAPK1 | MAPK14 | MAPK15 | MAPK3 | MAPK8 | MAPT | MAVS | MDM2 | MPC2 | MSN | MYO1C | MYO5A | NDUFAF2 | NEDD4 | NF2 | NIPBL | NMD3 | NPEPPS | NRXN1 | NSFL1C | NUCB1 | NUMA1 | NUP153 | NUP214 | OXCT1 | P2RX1 | P2RX2 | P2RX4 | PARD3 | PARD6A | PARK7 | PARP1 | PCM1 | PCSK9 | PDCD10 | PDE4D | PER2 | PIK3R1 | PIN1 | PINX1 | PKIG | PLA2G1B | PLA2G6 | PLCG1 | PLK1 | PML | PPM1A | PPP1CC | PPP1R12A | PPP3CC | PREPL | PRKAA1 | PRKAA2 | PRKACA | PRKCD | PRKCE | PRKCZ | PSEN1 | PTEN | PTN | PTPN1 | PTPN14 | PTPN23 | PTPN6 | RAB11A | RAB11B | RAB11FIP2 | RAB21 | RAB29 | RAB3GAP1 | RAB5A | RAN | RANGAP1 | RAP1A | RAP1B | RAPGEF4 | RB1 | RBM4 | RDX | RHOT1 | RHOU | RNF139 | RNF31 | RTN4 | SAE1 | SCFD1 | SEC16A | SEC24B | SEPT5 | SFN | SH3GL1 | SIN3A | SMAD3 | SMO | SNCA | SNX12 | SNX3 | SNX6 | SP100 | SPTBN1 | SQSTM1 | SRC | SRI | STAC | STK11 | STOM | STXBP1 | STXBP2 | SYN1 | SYP | TARDBP | TCP1 | TERF1 | TFDP1 | TGFB1 | TM9SF4 | TMBIM6 | TMEM30A | TNF | TOR1A | TP53 | TP63 | TP73 | TPR | TRIM28 | TRIM29 | TSG101 | TXN | UBE2D3 | UBE2J1 | UBE2L3 | UBR5 | USP17L2 | USP46 | VPS18 | XPO1 | XPO4 | XPO5 | YWHAB | YWHAE | YWHAG | YWHAH | YWHAQ | YWHAZ | ZIC1 | ZMYND8 | GO:0006725 | 0.216304743418658 | 1783/8243 | 777/3250 | 3.04372869994261e-05 | 0.289002040059551 | T | T | T | T | cellular aromatic compound metabolic process | AARS | AARSD1 | ABHD14B | ABL1 | ACACA | ACAT1 | ACD | ACLY | ACOT13 | ACOT7 | ACOT9 | ACSL1 | ACSL4 | ACSL5 | ACSS2 | ACTL6A | ADA | ADCY6 | ADCYAP1 | ADK | ADRM1 | ADSL | ADSS | AFF4 | AGO1 | AGO2 | AHCY | AHCYL1 | AICDA | AIMP2 | AK1 | AK2 | AK4 | AKR1A1 | AKR1B1 | AKR1B10 | AKR1C2 | ALAS1 | ALDH6A1 | ALDOA | AMD1 | AMDHD2 | AOC2 | APBB1 | APEX1 | APEX2 | APP | APRT | AR | ARHGAP35 | ARID4B | ARNT | ARRB1 | ARRB2 | ASCC1 | ASCC2 | ASPDH | ATAD5 | ATF6B | ATIC | ATM | ATOH1 | ATP6V1A | ATP6V1B1 | ATRX | ATXN1 | ATXN2 | BABAM1 | BANF1 | BAX | BCL2 | BRCA1 | BTBD9 | BTRC | BUD13 | BUD31 | BYSL | C1QBP | C9orf64 | CARS | CASP3 | CBFB | CBR4 | CBS | CCAR2 | CCDC155 | CCDC36 | CCNB1 | CCNT1 | CCNT2 | CD2BP2 | CD40LG | CDA | CDC14B | CDC34 | CDC5L | CDC73 | CDK2 | CDK4 | CDK8 | CDK9 | CDKN1A | CEBPA | CENPF | CHAF1A | CHD1L | CHEK2 | CHRAC1 | CLPX | CMPK1 | COL4A2 | COMT | COPS2 | COPS5 | COPS6 | COX15 | CPSF7 | CREB1 | CREBBP | CRMP1 | CROT | CRY2 | CSNK1E | CSTF2T | CT45A5 | CTCF | CTDP1 | CTPS1 | CUL4B | CWC15 | CYC1 | CYP1A1 | CYP1A2 | DARS | DBH | DBI | DCAKD | DCK | DCP2 | DCTPP1 | DCXR | DDB1 | DDB2 | DDX1 | DDX39A | DDX39B | DDX3X | DERA | DGUOK | DHX15 | DHX36 | DHX9 | DIDO1 | DIS3 | DLAT | DLD | DLG2 | DLST | DMAP1 | DNA2 | DNAJC30 | DNASE2 | DNASE2B | DROSHA | DTX1 | DUS3L | DUT | DVL1 | DVL2 | E2F1 | E2F2 | E2F3 | EAF2 | ECD | EDC4 | EED | EFTUD2 | EHMT1 | EHMT2 | EIF4A3 | ELAC2 | ELAVL1 | ELAVL4 | ELF3 | ENDOG | ENTPD7 | EP300 | EPRS | ERCC5 | ESR1 | ESR2 | ESRRA | ESRRB | ESRRG | ETS1 | EXOSC1 | EXOSC5 | EXOSC6 | EXOSC9 | EXTL2 | EYA2 | EYA4 | EZH2 | FANCA | FANCC | FANCG | FANCI | FANCM | FAR2 | FARSB | FASN | FBXO6 | FECH | FEN1 | FIGNL1 | FOLH1 | FOLR1 | FOS | FOXM1 | FPGS | FTO | FTSJ3 | FUBP1 | FUBP3 | FUS | FZR1 | G3BP1 | G6PD | GAR1 | GARS | GART | GATA1 | GCDH | GDA | GEMIN4 | GEMIN5 | GEMIN7 | GFPT1 | GFPT2 | GINS2 | GINS3 | GINS4 | GLRX | GMDS | GMPPB | GMPR | GMPS | GNAI3 | GNAS | GNPDA1 | GNPDA2 | GNPNAT1 | GOT2 | GPD1L | GPX1 | GSR | GSTM4 | GTF2A1 | GTF2E2 | GTF2F1 | GTF2F2 | GTF2I | GTF3C2 | H1F0 | H2AFX | HAAO | HARS | HELZ2 | HERC2 | HIF1A | HINT1 | HIST1H4A | HIST3H3 | HMBS | HMGB1 | HMGB2 | HMGB3 | HMGCL | HMOX1 | HNF4A | HNF4G | HNRNPA1 | HNRNPA1L2 | HNRNPA2B1 | HNRNPD | HNRNPF | HNRNPH1 | HNRNPH2 | HNRNPH3 | HNRNPK | HNRNPL | HNRNPM | HNRNPU | HOXB9 | HPRT1 | HPX | HSD17B10 | HSD17B4 | HSD17B8 | HSF1 | HSP90AA1 | HSP90AB1 | HSPA1B | HSPA8 | HSPD1 | HUWE1 | IARS | IDH1 | IDH2 | IK | ILF2 | IMP4 | IMPDH2 | INO80B | INO80C | IREB2 | IRF1 | IRF7 | ISG15 | ITPA | JMJD6 | JUN | KAT5 | KCNAB2 | KDM1A | KDM2A | KDM4D | KHDRBS1 | KIAA0391 | KLF5 | KMT2A | KPNA1 | KPNA2 | KPNB1 | KYNU | LACTB2 | LARP7 | LARS | LCMT2 | LDHA | LDHC | LEF1 | LGALS3 | LIG4 | LMO2 | LRRK2 | LSM1 | LSM12 | LSM3 | LSM4 | LSM5 | LSM8 | MAPK1 | MAPK3 | MAPT | MC1R | MCM2 | MCM3 | MCM4 | MCM5 | MCM6 | MCM7 | MCM9 | MDC1 | MDH1 | MDH2 | MDN1 | ME1 | MECP2 | MED1 | MED12 | MED14 | MED17 | MED20 | MED21 | MED23 | MED24 | MED31 | MED4 | MED6 | MEPCE | MGMT | MITF | MLH1 | MLLT1 | MLLT3 | MMAB | MORF4L1 | MORF4L2 | MOV10 | MPC2 | MPG | MPHOSPH6 | MPI | MRM1 | MRPL12 | MSH6 | MTA1 | MTAP | MTERF1 | MTHFD1 | MTPN | MTRR | MVD | MYH6 | MYO5A | NAGK | NAMPT | NANS | NAPRT | NCBP1 | NCOA1 | NCOR1 | NDUFS1 | NELFB | NELFCD | NELFE | NFATC1 | NFE2L2 | NIPBL | NME1 | NME2 | NME4 | NMNAT1 | NMNAT3 | NMRK2 | NNT | NOB1 | NOP10 | NOP56 | NPLOC4 | NPM1 | NPR1 | NR0B2 | NR1H2 | NR1H3 | NR1I2 | NR3C1 | NR4A1 | NR5A2 | NRBF2 | NSMCE4A | NSUN2 | NSUN6 | NT5C2 | NUDT13 | NUDT18 | NUDT3 | NUDT9 | NUFIP1 | OAS2 | OGDH | OLA1 | OPA1 | OTUB1 | PA2G4 | PABPC1 | PABPC3 | PABPC4 | PAH | PAICS | PALB2 | PAN2 | PAPSS1 | PAPSS2 | PARK7 | PARP1 | PAX8 | PAXIP1 | PCBD1 | PCBP1 | PCBP2 | PCF11 | PCGF5 | PCGF6 | PCNA | PDE12 | PDE4D | PDHA1 | PDHA2 | PDHB | PDHX | PFAS | PGM3 | PHB | PICK1 | PIH1D1 | PINX1 | PIR | PNKP | PNP | POLA2 | POLD1 | POLDIP3 | POLE2 | POLE3 | POLM | POLR2A | POLR2E | POLR2L | POLR2M | POT1 | POU5F1 | PPA1 | PPA2 | PPARD | PPARG | PPARGC1A | PPAT | PPIA | PPIE | PPIL3 | PPM1D | PPOX | PPP1R8 | PPP2CA | PPP2R1A | PPP4R2 | PPP5C | PPT1 | PRCC | PRKACA | PRKDC | PRMT6 | PRPF19 | PRPF40A | PRPF8 | PRTFDC1 | PSAT1 | PSMD14 | PTEN | PTGES3 | PTMA | PTTG1 | PUM1 | PUS1 | PUS7 | QARS | QDPR | RAD21 | RAD23A | RAD23B | RAD51 | RAD51AP1 | RAN | RARA | RARG | RARS2 | RBBP6 | RBM17 | RBM25 | RBM3 | RBM4 | RBM8A | RBMX | RBPJ | RBX1 | RELA | REXO2 | RFC1 | RFC2 | RFC3 | RFC4 | RFC5 | RFK | RHOA | RIF1 | RIOK1 | RNASE3 | RNASEH2A | RNASEH2B | RNF8 | RPA2 | RPA3 | RPAIN | RPL10A | RPL7 | RPL7A | RPP14 | RPS14 | RPS19 | RPS2 | RPS24 | RPS3 | RPS8 | RRM1 | RRM2 | RRM2B | RRP7A | RRS1 | RTCB | RTF1 | RTFDC1 | RUNX2 | RUVBL1 | RUVBL2 | RXRA | RXRB | RXRG | SAP18 | SARS | SART1 | SART3 | SBDS | SCAF1 | SCO2 | SENP3 | SEPHS1 | SEPHS2 | SF1 | SF3B6 | SFPQ | SHMT1 | SHMT2 | SIRT1 | SIRT6 | SIRT7 | SKI | SLC25A1 | SLC25A13 | SLC25A32 | SMAD2 | SMAD3 | SMAD4 | SMARCB1 | SMC1A | SMC3 | SMG5 | SMG6 | SMN1 | SNCA | SND1 | SNRNP27 | SNRPA | SNRPA1 | SNRPB | SNRPB2 | SNRPD1 | SNRPD3 | SNRPE | SNW1 | SP1 | SP100 | SPI1 | SPIN1 | SPR | SPRTN | SRPK1 | SRPK2 | SRSF11 | SRSF12 | SRSF5 | SSB | STAT3 | STOML2 | STUB1 | SUCLA2 | SUCLG2 | SULT1A3 | SUMO1 | SUPT4H1 | SUPT5H | SWAP70 | TAF12 | TAF15 | TAF1A | TAMM41 | TAOK3 | TARDBP | TARS | TARS2 | TATDN1 | TBRG4 | TCEA1 | TCEA2 | TCERG1 | TCF4 | TDP1 | TECR | TERF1 | TERF2 | TERF2IP | TFCP2 | TFIP11 | TGFB1 | THADA | THEM4 | THOC2 | THOC6 | THOC7 | THRA | TINF2 | TK1 | TK2 | TMEM189-UBE2V1 | TNP1 | TOE1 | TOP1 | TOP2A | TOP3A | TP53 | TP53RK | TP73 | TPST2 | TRA2A | TREX1 | TRIM25 | TRIM28 | TRIM29 | TRIP12 | TRIP13 | TRMT44 | TRMT6 | TRMT61B | TRNT1 | TRUB2 | TSC22D1 | TSN | TTC5 | TXNL4A | TXNRD1 | TYMS | U2AF2 | UAP1 | UAP1L1 | UBB | UBC | UBE2A | UBE2B | UBE2D3 | UBE2L6 | UBE2N | UBE2V1 | UBE2W | UBR5 | UCHL5 | UCK2 | UGDH | UGGT1 | UGP2 | UMPS | UPF3B | UPP2 | USP14 | USP28 | USP39 | USP47 | USP7 | USPL1 | UTP14A | UTP6 | UXS1 | VARS | VCP | VDR | WARS | WDR12 | WDR4 | WDR48 | WT1 | WTAP | XBP1 | XPO5 | XRCC1 | XRCC3 | XRCC5 | XRCC6 | YAP1 | YBEY | YY1 | ZBTB7A | ZBTB7B | ZFYVE26 | ZMPSTE24 | ZNF217 | ZNF638 | ZRANB2 | GO:0033143 | 0.00740021836709936 | 61/8243 | 40/3250 | 3.0661810841136e-05 | 0.291133893936586 | T | T | T | T | regulation of intracellular steroid hormone receptor signaling pathway | AR | ARRB2 | AXIN1 | BRCA1 | CALR | CARM1 | CBFB | CRY1 | CRY2 | DAB2 | DDRGK1 | EP300 | ESR1 | FOXA1 | HDAC1 | HDAC6 | KANK2 | LATS1 | MED1 | NCOR1 | NCOR2 | PARK7 | PARP1 | PER1 | PHB | PHB2 | PRMT2 | RHOA | RUNX1 | SIRT1 | SMARCA4 | SRC | TCF21 | TP63 | UBA5 | UBE2I | UFM1 | WBP2 | YAP1 | ZBTB7A | GO:0043312 | 0.0406405434914473 | 335/8243 | 168/3250 | 3.14911572265449e-05 | 0.299008537866044 | T | T | T | T | neutrophil degranulation | ACAA1 | ACLY | ACTR2 | AGL | AHSG | ALDOA | ALDOC | ALOX5 | ANXA2 | AP1M1 | APEH | APRT | ARMC8 | ARPC5 | ATP6V1D | AZU1 | CAB39 | CAND1 | CAPN1 | CAT | CCT2 | CCT8 | CD36 | CDA | CEP290 | CKAP4 | COPB1 | COTL1 | CPNE3 | CSNK2B | CTSA | CTSC | CTSD | CYB5R3 | DBNL | DDOST | DDX3X | DEGS1 | DERA | DYNC1H1 | DYNC1LI1 | DYNLL1 | DYNLT1 | EEF1A1 | EEF2 | FABP5 | FAF2 | FCN1 | FGL2 | FTH1 | FTL | GAA | GALNS | GDI2 | GHDC | GLA | GNS | GPI | GSTP1 | GUSB | GYG1 | HEXB | HMGB1 | HRNR | HSP90AA1 | HSP90AB1 | HSPA1B | HSPA8 | HUWE1 | IDH1 | ILF2 | IMPDH2 | IQGAP1 | ITGAV | JUP | KCMF1 | KCNAB2 | KPNB1 | LGALS3 | LRRC7 | MAN2B1 | MAPK1 | MAPK14 | MGST1 | MIF | MME | NAPRT | NHLRC3 | NIT2 | NME2 | ORM1 | P2RX1 | PA2G4 | PDXK | PFKL | PGAM1 | PGM1 | PGM2 | PGRMC1 | PKM | PLAUR | PNP | PPIA | PPIE | PRCP | PRDX4 | PRDX6 | PRG2 | PRKCD | PSAP | PSEN1 | PSMA2 | PSMA5 | PSMB7 | PSMC2 | PSMC3 | PSMD1 | PSMD11 | PSMD12 | PSMD14 | PSMD2 | PTGES2 | PTPN6 | PYGB | PYGL | QSOX1 | RAB10 | RAB18 | RAB24 | RAB31 | RAB5B | RAB7A | RAC1 | RAP1A | RAP1B | RHOA | RNASE3 | S100A11 | S100A12 | S100A7 | S100A9 | S100P | SDCBP | SELL | SERPINB3 | SERPINB6 | SLC2A5 | SNAP29 | SPTAN1 | SRP14 | STBD1 | STOM | STXBP2 | TCIRG1 | TIMP2 | TMEM173 | TMEM30A | TOLLIP | TUBB | TUBB4B | TXNDC5 | UBR4 | VAPA | VAT1 | VCL | VCP | XRCC5 | XRCC6 | GO:0042326 | 0.03603057139391 | 297/8243 | 151/3250 | 3.22025702139649e-05 | 0.305763404181597 | T | T | T | T | negative regulation of phosphorylation | ABL1 | ADORA2A | AHSG | AKT1 | APC | APOE | ARRB1 | ARRB2 | BAX | BDKRB2 | C1QL4 | CALM1 | CALM2 | CALM3 | CASP3 | CAV1 | CBL | CBLB | CBLC | CCNB1 | CD109 | CDKN1A | CDKN2C | CEBPA | CHORDC1 | CHP1 | CHRNA9 | COX11 | CRKL | CYLD | DBNDD2 | DDIT4 | DMD | DNAJA1 | DNAJC10 | DUSP1 | DUSP14 | DUSP16 | DUSP19 | DUSP22 | DUSP7 | DVL1 | DYNLL1 | EIF3A | ERRFI1 | EZR | FBXO7 | FGFR1OP | FKBP8 | FOXM1 | FOXO1 | GNAQ | GPD1L | GPS2 | GSTP1 | HDAC3 | HEXIM1 | HNRNPU | HSPB1 | IBTK | IGBP1 | IGF1R | ILK | IPO5 | IPO7 | ITCH | ITGB1BP1 | JUN | LATS1 | LDLRAD4 | LEMD2 | LRP6 | LRRK1 | LRRK2 | MAPT | MAS1 | MLLT1 | MYC | NCK1 | NCOR1 | NF2 | NNT | NPM1 | NTF3 | PAFAH1B1 | PAK2 | PARD3 | PARD6A | PARK7 | PARP14 | PDE12 | PDE4D | PEBP1 | PER1 | PHB | PIN1 | PKIG | PLK1 | PPARGC1A | PPIF | PPP2CA | PPP2R1A | PPP2R5D | PRDX3 | PRKAR1A | PRKAR2B | PRKCD | PRKCZ | PRKDC | PSEN1 | PSMD10 | PTEN | PTPN1 | PTPN22 | PTPN6 | PTPRR | QARS | RANBP9 | RB1 | RGN | RGS2 | RHOA | RPTOR | SERPINB3 | SFN | SIRT1 | SIRT3 | SLC9A3R1 | SMAD4 | SNCA | SNX6 | SOCS1 | SPRY2 | STK38 | STRAP | SYNJ2BP | TAOK3 | TARDBP | TERF2IP | TGFB1 | TNFAIP3 | TRAF3IP1 | TRIB2 | TRIB3 | TSG101 | TWIST1 | UBE2B | UCHL1 | WARS | WNK1 | YWHAG | GO:0002757 | 0.0382142423874803 | 315/8243 | 159/3250 | 3.29495255048998e-05 | 0.312855744669023 | T | T | T | T | immune response-activating signal transduction | ABL1 | ACTB | ACTG1 | ACTR2 | ACTR3 | ARPC1A | ARPC1B | ARPC2 | ARPC3 | ARPC4 | ARPC5 | BAG6 | BAIAP2 | BAX | BCAR1 | BCL10 | BCL2 | BIRC2 | BIRC3 | BRAF | BTK | BTN2A1 | BTRC | CARD9 | CASP8 | CD209 | CD36 | CD79B | CDC42 | CREBBP | CRK | CRKL | CTSL | CUL1 | CYLD | EIF2B1 | EIF2B3 | EIF2B5 | EP300 | FADD | FBXW11 | FCGR1A | FCN1 | FGB | FYN | GRB2 | HAVCR2 | HLA-DQA1 | HLA-DRA | HLA-DRB1 | HLA-DRB3 | HLA-DRB4 | HLA-DRB5 | HMGB1 | HRAS | HSP90AA1 | HSP90AB1 | HSP90B1 | HSPD1 | IGHV3-13 | IGHV3-23 | IGHV3-30 | IGKV3-20 | IGLC7 | IKBKB | IKBKE | IKBKG | IRF7 | ITCH | ITK | KRAS | LAT | LCP2 | LGMN | MAP2K6 | MAP3K1 | MAPK1 | MAPK3 | MYD88 | MYO1C | NCK1 | NFATC2 | NFKBIA | NOD2 | PAG1 | PAK2 | PDE4D | PIK3CA | PIK3R1 | PLA2G6 | PLCG1 | PRKACA | PRKCD | PRKCE | PRKD2 | PSEN1 | PSMA1 | PSMA2 | PSMA3 | PSMA5 | PSMA6 | PSMB5 | PSMB6 | PSMB7 | PSMB8 | PSMC1 | PSMC2 | PSMC3 | PSMC4 | PSMC5 | PSMC6 | PSMD1 | PSMD10 | PSMD11 | PSMD12 | PSMD14 | PSMD2 | PSMD4 | PSMD9 | PSME2 | PSME3 | PTK2 | PTPN22 | PTPN6 | RAC1 | RAF1 | RC3H1 | RELA | RIPK1 | RIPK2 | RNF31 | S100A1 | S100A9 | SCARA3 | SPG21 | SRC | STK11 | STOML2 | TAB1 | TAB2 | TANK | TLR3 | TMEM173 | TNFAIP3 | TNIP2 | TRAF3 | TRAF6 | UBB | UBC | UBE2D1 | UBE2D2 | UBE2D3 | UBE2N | UBE2V1 | VAV1 | WAS | WNK1 | YES1 | ZAP70 | GO:0071417 | 0.0346961057867281 | 286/8243 | 146/3250 | 3.33079362595673e-05 | 0.316258854784592 | T | T | T | T | cellular response to organonitrogen compound | ABL1 | ACTA1 | ACTB | ACTR2 | ADCY6 | ADRB2 | AGER | AKAP9 | AKR1B1 | AKT1 | AKT2 | ANXA5 | APEX1 | APLP1 | APP | APRT | ASS1 | BACE1 | BCL11A | BCL2L1 | BRCA1 | CACNA1A | CAPN2 | CASP3 | CASP7 | CAV1 | CD36 | CDC5L | CDH1 | CDK4 | CDK5 | CFTR | COL4A1 | COL6A1 | CREB1 | CRK | CTNNB1 | DENND4C | EGFR | EPHA4 | EPRS | ERRFI1 | EZH2 | EZR | FIS1 | FOLR1 | FOXO1 | FYN | GLP1R | GNB1 | GOT1 | GPD1 | GSK3A | GSK3B | GSTP1 | HCN1 | HCN2 | HCN3 | HDAC2 | HDAC5 | HDAC9 | HNRNPD | HSF1 | HSP90B1 | HSPA5 | HTR1B | ICAM1 | IGF1R | INSR | IPO5 | ITGA4 | ITPR2 | JUP | KDM1A | KLF5 | KLHL22 | LARS | LGMN | LPIN3 | LRRK2 | MAPK1 | MAPK3 | MDM2 | MYO5A | NAMPT | NFE2L2 | NOD2 | NR4A1 | NTRK1 | OPA1 | P2RX4 | PARP1 | PCSK9 | PDE4D | PIK3CG | PIK3R1 | PKM | PLA2G1B | PPARG | PPARGC1A | PPAT | PRKAA1 | PRKACA | PRKAR1A | PRKAR2B | PRKCD | PRKCZ | PRKDC | PSEN1 | PTEN | PTPN22 | RAB10 | RAB31 | RAD51 | RANGAP1 | RAP1A | RAP1B | RELA | RIPK2 | RPL23 | RPTOR | SHMT1 | SHMT2 | SIN3A | SLC9A1 | SNCA | SNX6 | SOCS1 | SOD1 | SP1 | SRC | SRSF5 | STAT1 | SULT1A3 | TBC1D4 | TGFB1 | TNF | TP53 | TRIB3 | TRIM41 | VCAM1 | VIM | WDTC1 | WT1 | XBP1 | YWHAG | GO:1903829 | 0.0296008734683974 | 244/8243 | 127/3250 | 3.40439457814372e-05 | 0.323247265194747 | T | T | T | T | positive regulation of cellular protein localization | ABHD17A | AGR2 | AKT1 | AKT2 | ANK3 | APC | B3GAT3 | BAG3 | BBC3 | BCL2 | CACNG2 | CASP8 | CCDC88A | CCT2 | CCT3 | CCT4 | CCT5 | CCT6A | CCT7 | CCT8 | CDH1 | CDK5 | CDK5R1 | CEP290 | CHP1 | CROCC | CSNK2A2 | CYLD | DDRGK1 | DMAP1 | E2F1 | EDEM1 | EGFR | EIF2AK3 | ERBB2 | EZR | FIS1 | FYN | GNAI1 | GPD1L | GSK3A | GSK3B | HDAC3 | HRAS | HSP90AB1 | HSPA1L | HTRA2 | HUWE1 | IPO5 | IQGAP1 | ITGB1BP1 | JUP | KAT7 | KIF5B | LGALS3 | MAPK1 | MAPK14 | MAPK8 | MAPT | MAVS | MDM2 | MSN | MYO1C | NIPBL | NMD3 | NPEPPS | NUMA1 | PARD6A | PARK7 | PARP1 | PCM1 | PDCD10 | PIK3R1 | PINX1 | PLK1 | PPM1A | PPP3CC | PRKAA1 | PRKAA2 | PRKACA | PRKCD | PRKCE | PSEN1 | PTN | RAB11A | RAB11FIP2 | RAB29 | RAN | RDX | RHOU | RNF31 | RTN4 | SAE1 | SEC16A | SFN | SMAD3 | SMO | SPTBN1 | SQSTM1 | SRC | STAC | STK11 | STOM | TARDBP | TCP1 | TFDP1 | TGFB1 | TM9SF4 | TMEM30A | TNF | TP53 | TP63 | TP73 | TPR | TRIM28 | TSG101 | UBE2D3 | UBE2L3 | UBR5 | XPO4 | YWHAB | YWHAE | YWHAG | YWHAH | YWHAQ | YWHAZ | ZIC1 | GO:0033044 | 0.0333616401795463 | 275/8243 | 141/3250 | 3.42607652370495e-05 | 0.325305965925785 | T | T | T | T | regulation of chromosome organization | ACD | AICDA | AKAP8 | ANAPC4 | APC | ARRB1 | ATG5 | ATM | ATRX | AURKB | BCL6 | BCOR | BRCA1 | BRD4 | CCNB1 | CCT2 | CCT3 | CCT4 | CCT5 | CCT6A | CCT7 | CCT8 | CDC20 | CDC27 | CDK9 | CENPF | CENPV | CTBP1 | CTCF | CTNNB1 | CUL3 | DCP2 | DHX36 | DUSP1 | DYNC1LI1 | EED | EID1 | FEN1 | H1F0 | H1FX | HDAC5 | HECW2 | HIST1H1A | HNRNPA1 | HNRNPA2B1 | HNRNPD | HNRNPU | IK | KAT7 | KDM1A | KDM4D | KLHL22 | KMT2A | KMT2B | LIG4 | LMNA | LRRK2 | MAD1L1 | MAD2L1 | MAP2K7 | MAP3K4 | MAPK1 | MAPK15 | MAPK3 | MAPK8 | MAPT | MCM2 | MECP2 | MYC | NEK7 | NELFE | NIPBL | NUMA1 | OGT | OTUB1 | PARP1 | PAXIP1 | PIH1D1 | PINX1 | PLK1 | PML | PNKP | POT1 | PPARGC1A | PRKD1 | PRKD2 | PRMT6 | PTTG1 | RAD21 | RB1 | RIF1 | RNF4 | RTF1 | RUVBL2 | SART3 | SENP6 | SET | SETDB1 | SFPQ | SIN3A | SIRT1 | SIRT6 | SKI | SMAD4 | SMARCB1 | SMG5 | SMG6 | SNAI2 | SNCA | SNW1 | SPDL1 | SPI1 | SRC | TADA2B | TCP1 | TERF1 | TERF2 | TERF2IP | TGFB1 | TINF2 | TNKS | TNKS2 | TOP2A | TPR | TRIM28 | TRIP12 | TRIP13 | TWIST1 | UBE2B | UBE2N | UBR5 | USP17L2 | USP7 | WBP2 | XRCC1 | XRCC3 | XRCC5 | ZBTB7B | ZMPSTE24 | ZNF207 | ZW10 | GO:0031401 | 0.0883173601843989 | 728/8243 | 338/3250 | 3.44212032603161e-05 | 0.326829324956701 | T | T | T | T | positive regulation of protein modification process | ABL1 | ACSL1 | ADCY6 | ADCYAP1 | ADRB2 | ADTRP | AGER | AIMP2 | AKAP8 | AKAP9 | AKT1 | AKT2 | AKTIP | ANXA2 | APOE | APP | AR | ARAF | ARNT | ARRB1 | ARRB2 | ASPSCR1 | ATM | AXIN1 | AZU1 | BAG4 | BCAR3 | BCL10 | BCL2 | BCL6 | BIRC2 | BIRC3 | BIRC7 | BMI1 | BMPR1A | BRAF | BRCA1 | BRD4 | C1QTNF1 | CAB39 | CALM1 | CALM2 | CALM3 | CARD9 | CAV1 | CCDC88A | CCL21 | CCL22 | CCNB1 | CCNT1 | CCNT2 | CCNY | CCNYL1 | CCR1 | CD27 | CD36 | CD40 | CD40LG | CD74 | CD81 | CDC14B | CDC20 | CDC25B | CDK5 | CDK5R1 | CDK9 | CDKN1A | CHEK2 | CHP1 | CKS1B | CKS2 | CLU | COMMD1 | CRK | CRKL | CSNK2B | CTBP1 | CTNNB1 | CUL3 | DAB1 | DAB2 | DAXX | DBF4B | DBNL | DCUN1D1 | DDRGK1 | DDX3X | DERL1 | DLG3 | DLG4 | DOCK7 | DUSP19 | DUSP22 | DUSP7 | DVL1 | DVL2 | DVL3 | EED | EGFR | EHD4 | EPHA4 | ERBB2 | ERBB3 | EZH2 | FANCI | FANCM | FGB | FGF2 | FGFR1 | FGFR3 | FKBP1A | FLOT1 | FYN | FZR1 | GADD45G | GATA1 | GDF9 | GNAI2 | GOLGA2 | GSK3A | HAVCR2 | HAX1 | HBEGF | HDAC2 | HDAC3 | HDAC4 | HDAC6 | HMGB1 | HPX | HRAS | HSF1 | HSP90AA1 | HSP90AB1 | HSPA2 | HSPA5 | HSPBP1 | ICAM1 | IFNA2 | IGF1R | IKBKG | ILK | INSR | IQGAP1 | ITGB1BP1 | ITGB3 | JUN | KAT5 | KAT7 | KDM1A | KDR | KLHDC10 | KMT2A | KRAS | KSR1 | LAT | LATS1 | LCP2 | LMNA | LPAR1 | LRRK1 | LRRK2 | LTBR | MAP2K1 | MAP2K3 | MAP2K4 | MAP2K6 | MAP2K7 | MAP3K1 | MAP3K13 | MAP3K14 | MAP3K2 | MAP3K3 | MAP3K4 | MAP4K1 | MAPK1 | MAPK14 | MAPK3 | MAPK8IP3 | MAPK9 | MAPKAP1 | MAS1 | MAVS | MECP2 | MIF | MLST8 | MTA1 | NEK1 | NELFE | NIPBL | NMNAT1 | NOD2 | NOS1AP | NQO2 | NTF3 | NTRK1 | NUPR1 | OGT | OXSR1 | PAK2 | PAK6 | PARK7 | PARP14 | PAXIP1 | PDCD10 | PDCD6 | PDE6G | PEF1 | PELI1 | PHB | PHB2 | PIAS1 | PIAS3 | PIH1D1 | PIK3CA | PIK3CG | PIK3R3 | PIN1 | PLA2G1B | PLA2G2A | PLA2G6 | PLAUR | PLK1 | PPARGC1A | PPP1R12A | PPP2CA | PPP2R5D | PRDX2 | PRKAA1 | PRKAA2 | PRKACA | PRKAG1 | PRKAR1A | PRKAR2B | PRKCD | PRKCE | PRKCZ | PRKD1 | PRKD2 | PROX1 | PRRC1 | PSAP | PSEN1 | PSMD10 | PTEN | PTK2 | PTPN1 | PTPN22 | RAB1A | RAB3GAP1 | RAC1 | RAF1 | RAP1A | RAP1B | RB1CC1 | RHOA | RIF1 | RIPK1 | RIPK2 | RIPK3 | RPLP1 | RPS2 | RPS3 | RPTOR | RTF1 | RUVBL2 | S100A12 | S100A7 | SART3 | SDC4 | SDCBP | SEMA7A | SEPT4 | SHC1 | SIRT1 | SLAMF1 | SMAD4 | SMARCB1 | SNAI2 | SNCA | SNW1 | SOD1 | SOX2 | SPRTN | SPRY2 | SQSTM1 | SRC | STAT3 | STK11 | STK24 | STK25 | STK26 | STK3 | STUB1 | TAB1 | TAB2 | TADA2B | TANK | TAOK3 | TERF2IP | TGFB1 | THBS1 | TIMP2 | TLR3 | TNF | TNFRSF1A | TNK2 | TOLLIP | TP53 | TP73 | TRAF2 | TRAF6 | TRIB3 | TXN | UBB | UBC | UBE2C | UBE2D1 | UBE2I | UBE2K | UBE2L3 | UBE2N | UBE2S | UBE2V1 | UBE3A | UBQLN1 | UNC119 | VCP | VTN | WBP2 | WDR48 | WNK1 | WNT16 | XIAP | XRCC5 | XRCC6 | YES1 | ZBTB7B | ZNF622 | GO:0043603 | 0.0379716122770836 | 313/8243 | 158/3250 | 3.47227820180374e-05 | 0.329692815261265 | T | T | T | T | cellular amide metabolic process | ACACA | ACAT1 | ACLY | ACOT13 | ACOT7 | ACOT9 | ACSL1 | ACSL4 | ACSL5 | ACSS2 | AGK | AGO2 | AKT1 | ALDH5A1 | APEH | ASL | ASS1 | B4GALT3 | BACE1 | CBR4 | CEBPA | CNDP2 | COL4A3BP | COPS5 | DARS | DBI | DEGS1 | DLAT | DLD | DLST | DMD | DNPEP | DPEP1 | DRG2 | EEF1A1 | EEF1A1P5 | EEF1A2 | EGFR | EIF4H | FAR2 | FARSB | FASN | FOLR1 | FPGS | G6PD | GCDH | GEMIN5 | GGCT | GGT1 | GGTLC1 | GLO1 | GPX1 | GPX4 | GSPT1 | GSPT2 | GSR | GSS | GSTA1 | GSTA2 | GSTA3 | GSTA4 | GSTA5 | GSTK1 | GSTM4 | GSTP1 | GSTT1 | GSTT2 | GSTT2B | GSTZ1 | HAGH | HARS | HMGCL | HSD17B4 | HSD17B8 | HSPA14 | HTRA2 | IDE | IDH1 | LARP4 | MGST1 | MME | MPC2 | MRPL28 | MRPL43 | MRPL9 | MRRF | MTHFD1 | MTRR | MVD | NMRAL1 | OGDH | OTC | P2RX1 | PABPC4 | PARK7 | PDF | PDHA1 | PDHA2 | PDHB | PDHX | PPP2CA | PPP2R1A | PPT1 | PSEN1 | PSEN2 | PTGES | RARS2 | RMND1 | RPL10 | RPL10A | RPL12 | RPL18A | RPL22 | RPL23 | RPL23A | RPL24 | RPL30 | RPL36AL | RPL37A | RPL38 | RPL6 | RPL7 | RPL7A | RPL9 | RPLP0 | RPLP0P6 | RPLP1 | RPLP2 | RPS12 | RPS13 | RPS14 | RPS15A | RPS19 | RPS2 | RPS20 | RPS24 | RPS29 | RPS3 | RPS4X | RPS6KB2 | RPS8 | SARS | SGPL1 | SHMT1 | SHMT2 | SLC25A1 | SLC25A32 | SOD1 | SPTLC2 | ST8SIA4 | SUCLA2 | SUCLG2 | TARS | TECR | THEM4 | THOP1 | UGT8 | WARS | GO:1904886 | 0.00133446560718185 | 11/8243 | 11/3250 | 3.54258379960099e-05 | 0.336368331772114 | T | T | T | T | beta-catenin destruction complex disassembly | APC | AXIN1 | CAV1 | CSNK1A1 | CTNNB1 | DVL1 | DVL2 | DVL3 | GSK3B | LRP6 | PPP1CA | GO:0090263 | 0.0120101904646367 | 99/8243 | 59/3250 | 3.57023999038875e-05 | 0.338994287087411 | T | T | T | T | positive regulation of canonical Wnt signaling pathway | AXIN1 | BIRC8 | CAV1 | CCAR2 | CSNK1E | DDX3X | EDA | EGFR | ILK | JUP | LGR4 | LRRK1 | LRRK2 | PIN1 | PPM1A | PSMA1 | PSMA2 | PSMA3 | PSMA5 | PSMA6 | PSMB5 | PSMB6 | PSMB7 | PSMB8 | PSMC1 | PSMC2 | PSMC3 | PSMC4 | PSMC5 | PSMC6 | PSMD1 | PSMD10 | PSMD11 | PSMD12 | PSMD14 | PSMD2 | PSMD4 | PSMD9 | PSME2 | PSME3 | RBPJ | RECK | RUVBL1 | SCEL | SMAD3 | SMURF2 | SRC | SULF2 | TBL1X | TNKS | TNKS2 | UBE2B | UBR5 | USP47 | USP8 | VCP | WNK1 | XIAP | YAP1 | GO:0032210 | 0.00582312264952081 | 48/8243 | 33/3250 | 3.57904298237479e-05 | 0.339830131176486 | T | T | T | T | regulation of telomere maintenance via telomerase | ACD | ATM | AURKB | CCT2 | CCT3 | CCT4 | CCT5 | CCT6A | CCT7 | CCT8 | CTNNB1 | DCP2 | HNRNPA1 | HNRNPU | MAP2K7 | MAP3K4 | MAPK1 | MAPK15 | MAPK3 | NEK7 | PINX1 | PNKP | POT1 | SMG5 | SMG6 | SRC | TCP1 | TERF1 | TERF2 | TINF2 | TNKS | TNKS2 | XRCC5 | GO:0031347 | 0.0542278296736625 | 447/8243 | 217/3250 | 3.58525633457038e-05 | 0.340420088967458 | T | T | T | T | regulation of defense response | A2M | ABCE1 | ACE2 | ADA | ADCYAP1 | ADORA2A | AGER | AHSG | ANXA1 | AP1G1 | APCS | APOA1 | APOD | APOE | APP | ARRB2 | ATM | BCL10 | BCL6 | BIRC2 | BIRC3 | BRD4 | BTK | BTRC | C1QA | C1QBP | CARD9 | CASP8 | CAV1 | CD209 | CD36 | CD6 | CDC37 | CDH5 | CDK19 | CEBPA | CREBBP | CRK | CTSL | CUL1 | CXCL6 | CYLD | DHX9 | DROSHA | EGFR | EIF2AK4 | EP300 | ESR1 | ETS1 | FADD | FAM19A3 | FANCA | FBXW11 | FCN1 | FGB | FLOT1 | FYN | GBP5 | GGT1 | GPS2 | GPX1 | GPX4 | GSTP1 | HAVCR2 | HEXIM1 | HMGB1 | HMGB2 | HMGB3 | HPX | HRAS | HSP90AB1 | HSP90B1 | HSPA1B | HSPD1 | HTRA1 | IFI16 | IFIT1 | IKBKB | IKBKE | IKBKG | IRF1 | IRF4 | IRF7 | ITCH | JAK1 | KRAS | LGMN | LGR4 | LRRK2 | LRSAM1 | MAP2K6 | MAP3K1 | MAPK14 | MAPK3 | MAPK7 | MAS1 | MATR3 | MAVS | MED1 | METRNL | MICA | MMP26 | MYD88 | NFKBIA | NOD2 | NOS2 | NPLOC4 | NR1H2 | NR1H3 | OPTN | OTUD4 | PAK2 | PARK7 | PARP14 | PCBP2 | PELI1 | PER1 | PIAS1 | PIK3CG | PLA2G2A | PLSCR1 | PPARD | PPARG | PPM1B | PRKACA | PRKCD | PRKCE | PRKDC | PSMA1 | PSMA2 | PSMA3 | PSMA5 | PSMA6 | PSMB5 | PSMB6 | PSMB7 | PSMB8 | PSMC1 | PSMC2 | PSMC3 | PSMC4 | PSMC5 | PSMC6 | PSMD1 | PSMD10 | PSMD11 | PSMD12 | PSMD14 | PSMD2 | PSMD4 | PSMD9 | PSME2 | PSME3 | PTGER3 | PTPN1 | PTPN22 | PTPN6 | PUM1 | RAF1 | RB1 | RELA | RIPK1 | RIPK2 | RPS19 | RTN4 | S100A1 | S100A12 | S100A9 | SARM1 | SCARA3 | SEMA7A | SFPQ | SIN3A | SLAMF8 | SLC25A6 | SMAD3 | SNCA | SNX6 | SOCS1 | SOD1 | SPATA2 | SRC | STAT1 | SUMO1 | TAB1 | TAB2 | TANK | TBC1D23 | TGM2 | TLR3 | TMEM173 | TNF | TNFAIP3 | TNFRSF1A | TNIP2 | TRADD | TRAF3 | TRAF3IP1 | TRAF6 | TREX1 | UBB | UBC | UBE2D1 | UBE2D2 | UBE2D3 | UBE2K | UBE2N | UBE2V1 | UBQLN1 | USP17L2 | VAV1 | VSIG4 | WDFY1 | XIAP | XRCC5 | XRCC6 | ZMPSTE24 | GO:0043123 | 0.0157709571757855 | 130/8243 | 74/3250 | 3.59542395138885e-05 | 0.341385504184371 | T | T | T | T | positive regulation of I-kappaB kinase/NF-kappaB signaling | ABL1 | AKT1 | BCL10 | BIRC2 | BIRC3 | BRD4 | CARD9 | CASP10 | CASP8 | CCDC22 | CCL21 | CD36 | CD40 | CD74 | CTH | CTNNB1 | DDRGK1 | DDX1 | DHX36 | EDA | EEF1D | FADD | FASLG | FKBP1A | FYN | GJA1 | GOLT1B | HMOX1 | IKBKB | IKBKE | IKBKG | LGALS1 | LPAR1 | LTBR | LURAP1 | MAP3K14 | MAP3K3 | MAVS | MYD88 | NOD2 | PELI1 | PPM1A | PPP5C | PRKCE | PRKD1 | REL | RELA | RHOA | RIPK1 | RIPK2 | RNF31 | S100A12 | S100A13 | S100A4 | S100B | TAB2 | TERF2IP | TFG | TGM2 | TLR3 | TMEM9B | TNF | TNFRSF1A | TNIP2 | TRADD | TRAF2 | TRAF6 | TRIM25 | TRIM32 | UBE2I | UBE2N | UBE2V1 | VAPA | ZDHHC17 | GO:0002764 | 0.0410044886570423 | 338/8243 | 169/3250 | 3.68231403510703e-05 | 0.349635717633412 | T | T | T | T | immune response-regulating signaling pathway | ABL1 | ACTB | ACTG1 | ACTR2 | ACTR3 | ARPC1A | ARPC1B | ARPC2 | ARPC3 | ARPC4 | ARPC5 | BAG6 | BAIAP2 | BAX | BCAR1 | BCL10 | BCL2 | BIRC2 | BIRC3 | BRAF | BTK | BTN2A1 | BTRC | CALM1 | CARD9 | CASP8 | CD209 | CD36 | CD40 | CD79B | CDC42 | CREBBP | CRK | CRKL | CTSL | CUL1 | CYLD | EIF2B1 | EIF2B3 | EIF2B5 | EP300 | FADD | FBXW11 | FCGR1A | FCN1 | FGB | FOS | FYN | GRB2 | HAVCR2 | HLA-DQA1 | HLA-DRA | HLA-DRB1 | HLA-DRB3 | HLA-DRB4 | HLA-DRB5 | HMGB1 | HRAS | HSP90AA1 | HSP90AB1 | HSP90B1 | HSPD1 | IGHV3-13 | IGHV3-23 | IGHV3-30 | IGKV3-20 | IGLC7 | IKBKB | IKBKE | IKBKG | IRF7 | ITCH | ITK | JUN | KRAS | LAT | LCP2 | LGMN | MAP2K4 | MAP2K6 | MAP2K7 | MAP3K1 | MAPK1 | MAPK3 | MAPK8 | MAPK9 | MYD88 | MYO1C | NCK1 | NFATC1 | NFATC2 | NFKBIA | NOD2 | PAG1 | PAK2 | PDE4D | PIK3CA | PIK3R1 | PLA2G6 | PLCG1 | PRKACA | PRKCD | PRKCE | PRKD2 | PSEN1 | PSMA1 | PSMA2 | PSMA3 | PSMA5 | PSMA6 | PSMB5 | PSMB6 | PSMB7 | PSMB8 | PSMC1 | PSMC2 | PSMC3 | PSMC4 | PSMC5 | PSMC6 | PSMD1 | PSMD10 | PSMD11 | PSMD12 | PSMD14 | PSMD2 | PSMD4 | PSMD9 | PSME2 | PSME3 | PTK2 | PTPN22 | PTPN6 | RAC1 | RAF1 | RC3H1 | RELA | RIPK1 | RIPK2 | RNF31 | S100A1 | S100A9 | SCARA3 | SHC1 | SPG21 | SRC | STK11 | STOML2 | TAB1 | TAB2 | TANK | TLR3 | TMEM173 | TNFAIP3 | TNIP2 | TRAF3 | TRAF6 | UBB | UBC | UBE2D1 | UBE2D2 | UBE2D3 | UBE2N | UBE2V1 | VAV1 | WAS | WNK1 | YES1 | ZAP70 | GO:0009967 | 0.118282178818391 | 975/8243 | 442/3250 | 3.74265286237567e-05 | 0.35536488928257 | T | T | T | T | positive regulation of signal transduction | ABAT | ABL1 | ACTN4 | ADA | ADCYAP1 | ADORA2A | ADRB2 | AGER | AGO1 | AGR2 | AKR1B1 | AKR1C2 | AKT1 | AKT3 | APOA1 | APOE | APP | AR | ARHGAP1 | ARNT | ARRB1 | ARRB2 | ASCL1 | ATF6 | ATM | AXIN1 | BAG4 | BAX | BBC3 | BCL10 | BCL2 | BCL2L1 | BIRC2 | BIRC3 | BIRC7 | BIRC8 | BMPR1A | BRAF | BRD4 | BUD31 | C1QBP | C1QTNF1 | CACNG2 | CACNG4 | CALM1 | CALM2 | CALM3 | CALR | CARD9 | CASP10 | CASP2 | CASP8 | CAT | CAV1 | CBL | CCAR2 | CCDC22 | CCL21 | CCL22 | CCR1 | CD27 | CD36 | CD40 | CD40LG | CD74 | CD81 | CDC73 | CHCHD10 | CLNK | CREBBP | CRK | CRKL | CSNK1E | CSNK2B | CTH | CTNNB1 | CTSC | CYLD | DAB2 | DAXX | DBNL | DCN | DDRGK1 | DDX1 | DDX3X | DHX36 | DISC1 | DLG4 | DNM1L | DOK1 | DUSP19 | DUSP22 | DUSP7 | DVL1 | DVL2 | DVL3 | E2F1 | EDA | EEF1D | EGFR | ELF3 | EP300 | EPHA4 | ERBB2 | ERBB3 | ESR1 | EZH2 | FABP5 | FADD | FAF1 | FASLG | FGB | FGF2 | FGFR1 | FGFR3 | FIS1 | FKBP1A | FKBP8 | FLOT1 | FOXA1 | FYN | GADD45G | GDF9 | GJA1 | GKAP1 | GNAI2 | GOLPH3 | GOLT1B | GOT1 | GPS2 | GPX1 | GRB2 | GRB7 | GSK3A | GSK3B | GSTO1 | HAP1 | HAVCR2 | HAX1 | HBEGF | HDAC2 | HDAC3 | HDAC6 | HEXIM1 | HIF1A | HINT1 | HMGB1 | HMOX1 | HPX | HRAS | HSF1 | HSP90AA1 | HSP90AB1 | HSPA1B | HTRA2 | HTT | ICAM1 | IFNA2 | IGF1R | IKBKB | IKBKE | IKBKG | ILK | ING5 | INSR | IQGAP1 | IRF7 | ITGB1BP1 | ITGB3 | JUN | JUP | KDR | KHDRBS1 | KLHDC10 | KLHL22 | KRAS | KSR1 | LARS | LASP1 | LAT | LGALS1 | LGR4 | LIMS1 | LPAR1 | LPAR4 | LRRK1 | LRRK2 | LTBR | LURAP1 | MAOA | MAP2K1 | MAP2K3 | MAP2K4 | MAP2K6 | MAP2K7 | MAP3K1 | MAP3K13 | MAP3K14 | MAP3K2 | MAP3K3 | MAP3K4 | MAP4K1 | MAPK1 | MAPK14 | MAPK3 | MAPK8 | MAPK8IP3 | MAPK9 | MAS1 | MAVS | MC1R | MED1 | MIF | MLLT11 | MLLT3 | MLST8 | MYD88 | MYO1C | MYOC | NCK1 | NEDD4 | NELFE | NMNAT1 | NOD2 | NOS1AP | NPR1 | NQO2 | NR1H3 | NRXN1 | NTF3 | NTRK1 | NUMA1 | NUP93 | OPA1 | P2RX2 | P2RX4 | PAFAH1B1 | PAG1 | PAK2 | PARK7 | PARP1 | PARP14 | PDCD10 | PDCL | PDE6G | PDIA3 | PEBP1 | PELI1 | PHB | PHB2 | PIH1D1 | PIK3CA | PIK3CG | PIK3R1 | PIN1 | PLA2G1B | PLA2G2A | PLA2G6 | PLAUR | PLEKHF1 | PML | PPARD | PPIF | PPM1A | PPP1CA | PPP2R1A | PPP2R1B | PPP3CC | PPP5C | PRDX2 | PRKAA1 | PRKCD | PRKCE | PRKCZ | PRKD1 | PRKD2 | PSAP | PSEN1 | PSMA1 | PSMA2 | PSMA3 | PSMA5 | PSMA6 | PSMB5 | PSMB6 | PSMB7 | PSMB8 | PSMC1 | PSMC2 | PSMC3 | PSMC4 | PSMC5 | PSMC6 | PSMD1 | PSMD10 | PSMD11 | PSMD12 | PSMD14 | PSMD2 | PSMD4 | PSMD9 | PSME2 | PSME3 | PTEN | PTK2 | PTPN1 | PTPN22 | PTPN6 | PUM1 | RAB29 | RAC1 | RAF1 | RAP1A | RAP1B | RB1CC1 | RBPJ | RC3H1 | RECK | REL | RELA | RHOA | RIPK1 | RIPK2 | RIPK3 | RNF183 | RNF31 | RPL23 | RPS3 | RPTOR | RTN4 | RUVBL1 | S100A12 | S100A13 | S100A4 | S100A7 | S100A9 | S100B | SARM1 | SCEL | SDCBP | SEC13 | SEMA7A | SEPT4 | SFN | SFPQ | SH2B1 | SHC1 | SHKBP1 | SIAH1 | SIRT1 | SKI | SLAMF1 | SLC9A1 | SLC9A3R1 | SMAD2 | SMAD3 | SMAD4 | SMARCA4 | SMARCB1 | SMO | SMURF2 | SNW1 | SOCS2 | SOD1 | SOX2 | SPIN1 | SPRY2 | SRC | STAT3 | STK11 | STK25 | STK3 | SULF2 | TAB1 | TAB2 | TAOK3 | TBL1X | TERF2IP | TFDP1 | TFG | TGFB1 | TGFBR3 | TGM2 | THBS1 | TIMP2 | TLR3 | TMEM9B | TNF | TNFAIP3 | TNFRSF12A | TNFRSF1A | TNFSF12 | TNFSF14 | TNIP2 | TNKS | TNKS2 | TP53 | TP63 | TP73 | TRADD | TRAF2 | TRAF6 | TRIM25 | TRIM32 | TRIP6 | TXN | UBB | UBC | UBE2B | UBE2I | UBE2K | UBE2N | UBE2O | UBE2V1 | UBQLN2 | UBR5 | USP17L2 | USP47 | USP8 | VAPA | VAV1 | VCP | VTN | WBP2 | WDFY1 | WDR35 | WNK1 | WNT16 | WWOX | XBP1 | XIAP | YAP1 | YWHAB | YWHAE | YWHAG | YWHAH | YWHAQ | YWHAZ | ZAP70 | ZBTB7B | ZDHHC17 | ZNF622 | GO:0048511 | 0.0198956690525294 | 164/8243 | 90/3250 | 3.78269568934963e-05 | 0.359166955703747 | T | T | T | T | rhythmic process | AHCY | ANXA1 | ARRB1 | ARRB2 | ASS1 | BTBD9 | BTRC | CASP2 | CASP3 | CCAR2 | CDK4 | CDK5 | CDK5R1 | CPT1A | CREB1 | CREBBP | CRY1 | CRY2 | CSNK1E | DHX9 | EGFR | EP300 | ESR1 | ETS1 | EZH2 | FBXW11 | GFPT1 | GSK3B | HDAC1 | HDAC2 | HDAC3 | HEBP1 | HNRNPD | HNRNPL | HNRNPU | HSPA5 | ID2 | IMPDH2 | JUN | KDM5B | KDM5C | KMT2A | LGR4 | MAP2K6 | MAPK8 | MAPK9 | MTA1 | MTNR1A | MYBBP1A | NAMPT | NCOA1 | NCOA2 | NCOR1 | NDUFA9 | NFKB2 | NKX2-1 | NOS2 | NOS3 | NTRK1 | OGT | PCNA | PER1 | PER2 | PML | PPARG | PPARGC1A | PPP1CA | PPP1CB | PPP1CC | PRKAA1 | PRKAA2 | PRKDC | PROX1 | PTEN | PTN | RBM4 | SFPQ | SGPL1 | SIN3A | SIRT1 | SP1 | SRC | TOP1 | TOP2A | TP53 | TWIST1 | TYMS | UBE3A | USP2 | USP9X | GO:0048524 | 0.0105544098022565 | 87/8243 | 53/3250 | 3.7909189642511e-05 | 0.359947755655642 | T | T | T | T | positive regulation of viral process | ANXA2 | APOE | CCNT1 | CD74 | CDK9 | CFL1 | CHMP2A | CTDP1 | DDB1 | DDX3X | DHX9 | EP300 | GTF2F1 | GTF2F2 | IFIT1 | JUN | LEF1 | LGALS1 | NELFB | NELFCD | NELFE | NR5A2 | P4HB | PABPC1 | PC | PDE12 | PKN2 | POLR2A | POLR2E | POLR2L | PPIA | PPIB | PPID | PPIE | RAB7A | RAD23A | SMARCA4 | SMARCB1 | SMC3 | SNW1 | SP1 | SRPK1 | SRPK2 | STAU1 | STOM | SUPT4H1 | SUPT5H | TOP2A | TRIM21 | TSG101 | VAPA | VAPB | VPS4B | GO:0043271 | 0.00982651947106636 | 81/8243 | 50/3250 | 3.79863944031747e-05 | 0.360680814858144 | T | T | T | T | negative regulation of ion transport | ABAT | ACTN2 | ADCYAP1 | AKT1 | AKT2 | ANK3 | BCL2 | BIN1 | CAB39 | CALM1 | CALM2 | CALM3 | CAV1 | COMMD1 | CRBN | EPPIN | FKBP1A | FKBP1B | GNAO1 | GOPC | GSTO1 | HECW1 | HECW2 | HTR1B | ICAM1 | KCNAB1 | NEDD4 | NOS3 | OXSR1 | PACSIN3 | PCSK9 | PPIF | PRKCE | PTEN | RGS2 | SERPINE2 | SLC9A3R1 | SNCA | SRI | SUMO1 | TGFB1 | THADA | THBS1 | TMBIM6 | TWIST1 | UBQLN1 | WNK1 | WNK4 | YWHAE | YWHAQ | GO:0046483 | 0.214242387480286 | 1766/8243 | 769/3250 | 3.86844559852017e-05 | 0.36730890957949 | T | T | T | T | heterocycle metabolic process | AARS | AARSD1 | ABHD14B | ABL1 | ACACA | ACAT1 | ACD | ACLY | ACOT13 | ACOT7 | ACOT9 | ACSL1 | ACSL4 | ACSL5 | ACSS2 | ACTL6A | ADA | ADCY6 | ADCYAP1 | ADK | ADRM1 | ADSL | ADSS | AFF4 | AGO1 | AGO2 | AHCY | AHCYL1 | AICDA | AIMP2 | AK1 | AK2 | AK4 | ALAS1 | ALDH6A1 | ALDOA | ALOX12 | AMD1 | AMDHD2 | APBB1 | APEX1 | APEX2 | APP | APRT | AR | ARHGAP35 | ARID4B | ARNT | ARRB1 | ARRB2 | ASCC1 | ASCC2 | ASPDH | ATAD5 | ATF6B | ATIC | ATM | ATOH1 | ATP6V1A | ATP6V1B1 | ATRX | ATXN1 | ATXN2 | BABAM1 | BANF1 | BAX | BRCA1 | BTBD9 | BTRC | BUD13 | BUD31 | BYSL | C1QBP | C9orf64 | CARS | CASP3 | CBFB | CBR4 | CBS | CCAR2 | CCDC155 | CCDC36 | CCNB1 | CCNT1 | CCNT2 | CD2BP2 | CD40LG | CDA | CDC14B | CDC34 | CDC5L | CDC73 | CDK2 | CDK4 | CDK8 | CDK9 | CDKN1A | CEBPA | CENPF | CHAF1A | CHD1L | CHEK2 | CHRAC1 | CLPX | CMPK1 | COL4A2 | COPS2 | COPS5 | COPS6 | COX15 | CPSF7 | CREB1 | CREBBP | CRMP1 | CROT | CRY2 | CSNK1E | CSTF2T | CT45A5 | CTCF | CTDP1 | CTPS1 | CUL4B | CWC15 | CYC1 | CYP1A1 | CYP1A2 | DARS | DBI | DCAKD | DCK | DCP2 | DCTPP1 | DCXR | DDB1 | DDB2 | DDX1 | DDX39A | DDX39B | DDX3X | DERA | DGUOK | DHX15 | DHX36 | DHX9 | DIDO1 | DIS3 | DLAT | DLD | DLG2 | DLST | DMAP1 | DNA2 | DNAJC30 | DNASE2 | DNASE2B | DPEP1 | DROSHA | DTX1 | DUS3L | DUT | DVL1 | DVL2 | E2F1 | E2F2 | E2F3 | EAF2 | ECD | EDC4 | EED | EFTUD2 | EHMT1 | EHMT2 | EIF4A3 | ELAC2 | ELAVL1 | ELAVL4 | ELF3 | ENDOG | ENTPD7 | EP300 | EPRS | ERCC5 | ESR1 | ESR2 | ESRRA | ESRRB | ESRRG | ETS1 | EXOSC1 | EXOSC5 | EXOSC6 | EXOSC9 | EXTL2 | EYA2 | EYA4 | EZH2 | FANCA | FANCC | FANCG | FANCI | FANCM | FAR2 | FARSB | FASN | FBXO6 | FECH | FEN1 | FIGNL1 | FOLH1 | FOLR1 | FOS | FOXM1 | FPGS | FTO | FTSJ3 | FUBP1 | FUBP3 | FUS | FZR1 | G3BP1 | G6PD | GAR1 | GARS | GART | GATA1 | GCDH | GDA | GEMIN4 | GEMIN5 | GEMIN7 | GFPT1 | GFPT2 | GINS2 | GINS3 | GINS4 | GLRX | GMDS | GMPPB | GMPR | GMPS | GNAI3 | GNAS | GNPDA1 | GNPDA2 | GNPNAT1 | GPD1L | GPX1 | GSR | GTF2A1 | GTF2E2 | GTF2F1 | GTF2F2 | GTF2I | GTF3C2 | H1F0 | H2AFX | HAAO | HARS | HELZ2 | HERC2 | HIF1A | HINT1 | HIST1H4A | HIST3H3 | HMBS | HMGB1 | HMGB2 | HMGB3 | HMGCL | HMOX1 | HNF4A | HNF4G | HNRNPA1 | HNRNPA1L2 | HNRNPA2B1 | HNRNPD | HNRNPF | HNRNPH1 | HNRNPH2 | HNRNPH3 | HNRNPK | HNRNPL | HNRNPM | HNRNPU | HOXB9 | HPRT1 | HPX | HSD17B10 | HSD17B4 | HSD17B8 | HSF1 | HSP90AA1 | HSP90AB1 | HSPA1B | HSPA8 | HSPD1 | HUWE1 | IARS | IDH1 | IDH2 | IK | ILF2 | IMP4 | IMPDH2 | INO80B | INO80C | IREB2 | IRF1 | IRF7 | ISG15 | ITPA | JMJD6 | JUN | KAT5 | KCNAB2 | KDM1A | KDM2A | KDM4D | KHDRBS1 | KIAA0391 | KLF5 | KMT2A | KPNA1 | KPNA2 | KPNB1 | KYNU | LACTB2 | LARP7 | LARS | LCMT2 | LDHA | LDHC | LEF1 | LGALS3 | LIG4 | LMO2 | LRRK2 | LSM1 | LSM12 | LSM3 | LSM4 | LSM5 | LSM8 | MAPK1 | MAPK3 | MAPT | MC1R | MCM2 | MCM3 | MCM4 | MCM5 | MCM6 | MCM7 | MCM9 | MDC1 | MDH1 | MDH2 | MDN1 | ME1 | MECP2 | MED1 | MED12 | MED14 | MED17 | MED20 | MED21 | MED23 | MED24 | MED31 | MED4 | MED6 | MEPCE | MGMT | MITF | MLH1 | MLLT1 | MLLT3 | MMAB | MME | MOCOS | MORF4L1 | MORF4L2 | MOV10 | MPC2 | MPG | MPHOSPH6 | MPI | MRM1 | MRPL12 | MSH6 | MTA1 | MTAP | MTERF1 | MTHFD1 | MTRR | MVD | MYH6 | NAGK | NAMPT | NANS | NAPRT | NCBP1 | NCOA1 | NCOR1 | NDUFS1 | NELFB | NELFCD | NELFE | NFATC1 | NFE2L2 | NFS1 | NIPBL | NME1 | NME2 | NME4 | NMNAT1 | NMNAT3 | NMRK2 | NNT | NOB1 | NOP10 | NOP56 | NPLOC4 | NPM1 | NPR1 | NR0B2 | NR1H2 | NR1H3 | NR1I2 | NR3C1 | NR4A1 | NR5A2 | NRBF2 | NSMCE4A | NSUN2 | NSUN6 | NT5C2 | NUDT13 | NUDT18 | NUDT3 | NUDT9 | NUFIP1 | OAS2 | OGDH | OLA1 | OPA1 | OTUB1 | PA2G4 | PABPC1 | PABPC3 | PABPC4 | PAICS | PALB2 | PAN2 | PAPSS1 | PAPSS2 | PARK7 | PARP1 | PAX8 | PAXIP1 | PCBD1 | PCBP1 | PCBP2 | PCF11 | PCGF5 | PCGF6 | PCNA | PDE12 | PDE4D | PDHA1 | PDHA2 | PDHB | PDHX | PDXK | PFAS | PGM3 | PHB | PICK1 | PIH1D1 | PINX1 | PIR | PNKP | PNP | POLA2 | POLD1 | POLDIP3 | POLE2 | POLE3 | POLM | POLR2A | POLR2E | POLR2L | POLR2M | POT1 | POU5F1 | PPA1 | PPA2 | PPARD | PPARG | PPARGC1A | PPAT | PPIA | PPIE | PPIL3 | PPM1D | PPOX | PPP1R8 | PPP2CA | PPP2R1A | PPP4R2 | PPP5C | PPT1 | PRCC | PRKACA | PRKDC | PRMT6 | PRPF19 | PRPF40A | PRPF8 | PRTFDC1 | PSAT1 | PSMD14 | PTEN | PTGES3 | PTMA | PTTG1 | PUM1 | PUS1 | PUS7 | QARS | QDPR | RAD21 | RAD23A | RAD23B | RAD51 | RAD51AP1 | RAN | RARA | RARG | RARS2 | RBBP6 | RBM17 | RBM25 | RBM3 | RBM4 | RBM8A | RBMX | RBPJ | RBX1 | RELA | REXO2 | RFC1 | RFC2 | RFC3 | RFC4 | RFC5 | RFK | RHOA | RIF1 | RIOK1 | RNASE3 | RNASEH2A | RNASEH2B | RNF8 | RPA2 | RPA3 | RPAIN | RPL10A | RPL7 | RPL7A | RPP14 | RPS14 | RPS19 | RPS2 | RPS24 | RPS3 | RPS8 | RRM1 | RRM2 | RRM2B | RRP7A | RRS1 | RTCB | RTF1 | RTFDC1 | RUNX2 | RUVBL1 | RUVBL2 | RXRA | RXRB | RXRG | SAP18 | SARS | SART1 | SART3 | SBDS | SCAF1 | SCO2 | SENP3 | SEPHS1 | SEPHS2 | SF1 | SF3B6 | SFPQ | SHMT1 | SHMT2 | SIRT1 | SIRT6 | SIRT7 | SKI | SLC25A1 | SLC25A13 | SLC25A32 | SMAD2 | SMAD3 | SMAD4 | SMARCB1 | SMC1A | SMC3 | SMG5 | SMG6 | SMN1 | SND1 | SNRNP27 | SNRPA | SNRPA1 | SNRPB | SNRPB2 | SNRPD1 | SNRPD3 | SNRPE | SNW1 | SP1 | SP100 | SPI1 | SPIN1 | SPR | SPRTN | SRPK1 | SRPK2 | SRSF11 | SRSF12 | SRSF5 | SSB | STAT3 | STOML2 | STUB1 | SUCLA2 | SUCLG2 | SULT1A3 | SUMO1 | SUPT4H1 | SUPT5H | SWAP70 | TAF12 | TAF15 | TAF1A | TAMM41 | TAOK3 | TARDBP | TARS | TARS2 | TATDN1 | TBRG4 | TCEA1 | TCEA2 | TCERG1 | TCF4 | TDP1 | TECR | TERF1 | TERF2 | TERF2IP | TFCP2 | TFIP11 | TGFB1 | THADA | THEM4 | THOC2 | THOC6 | THOC7 | THRA | TINF2 | TK1 | TK2 | TMEM189-UBE2V1 | TNP1 | TOE1 | TOP1 | TOP2A | TOP3A | TP53 | TP53RK | TP73 | TPST2 | TRA2A | TREX1 | TRIM25 | TRIM28 | TRIM29 | TRIP12 | TRIP13 | TRMT44 | TRMT6 | TRMT61B | TRNT1 | TRUB2 | TSC22D1 | TSN | TTC5 | TXNL4A | TXNRD1 | TYMS | U2AF2 | UAP1 | UAP1L1 | UBB | UBC | UBE2A | UBE2B | UBE2D3 | UBE2L6 | UBE2N | UBE2V1 | UBE2W | UBR5 | UCHL5 | UCK2 | UGDH | UGGT1 | UGP2 | UMPS | UPF3B | UPP2 | USP14 | USP28 | USP39 | USP47 | USP7 | USPL1 | UTP14A | UTP6 | UXS1 | VARS | VCP | VDR | WARS | WDR12 | WDR4 | WDR48 | WT1 | WTAP | XBP1 | XPO5 | XRCC1 | XRCC3 | XRCC5 | XRCC6 | YAP1 | YBEY | YY1 | ZBTB7A | ZBTB7B | ZFYVE26 | ZMPSTE24 | ZNF217 | ZNF638 | ZRANB2 | GO:0002283 | 0.0407618585466456 | 336/8243 | 168/3250 | 3.88125457160743e-05 | 0.368525121574126 | T | T | T | T | neutrophil activation involved in immune response | ACAA1 | ACLY | ACTR2 | AGL | AHSG | ALDOA | ALDOC | ALOX5 | ANXA2 | AP1M1 | APEH | APRT | ARMC8 | ARPC5 | ATP6V1D | AZU1 | CAB39 | CAND1 | CAPN1 | CAT | CCT2 | CCT8 | CD36 | CDA | CEP290 | CKAP4 | COPB1 | COTL1 | CPNE3 | CSNK2B | CTSA | CTSC | CTSD | CYB5R3 | DBNL | DDOST | DDX3X | DEGS1 | DERA | DYNC1H1 | DYNC1LI1 | DYNLL1 | DYNLT1 | EEF1A1 | EEF2 | FABP5 | FAF2 | FCN1 | FGL2 | FTH1 | FTL | GAA | GALNS | GDI2 | GHDC | GLA | GNS | GPI | GSTP1 | GUSB | GYG1 | HEXB | HMGB1 | HRNR | HSP90AA1 | HSP90AB1 | HSPA1B | HSPA8 | HUWE1 | IDH1 | ILF2 | IMPDH2 | IQGAP1 | ITGAV | JUP | KCMF1 | KCNAB2 | KPNB1 | LGALS3 | LRRC7 | MAN2B1 | MAPK1 | MAPK14 | MGST1 | MIF | MME | NAPRT | NHLRC3 | NIT2 | NME2 | ORM1 | P2RX1 | PA2G4 | PDXK | PFKL | PGAM1 | PGM1 | PGM2 | PGRMC1 | PKM | PLAUR | PNP | PPIA | PPIE | PRCP | PRDX4 | PRDX6 | PRG2 | PRKCD | PSAP | PSEN1 | PSMA2 | PSMA5 | PSMB7 | PSMC2 | PSMC3 | PSMD1 | PSMD11 | PSMD12 | PSMD14 | PSMD2 | PTGES2 | PTPN6 | PYGB | PYGL | QSOX1 | RAB10 | RAB18 | RAB24 | RAB31 | RAB5B | RAB7A | RAC1 | RAP1A | RAP1B | RHOA | RNASE3 | S100A11 | S100A12 | S100A7 | S100A9 | S100P | SDCBP | SELL | SERPINB3 | SERPINB6 | SLC2A5 | SNAP29 | SPTAN1 | SRP14 | STBD1 | STOM | STXBP2 | TCIRG1 | TIMP2 | TMEM173 | TMEM30A | TOLLIP | TUBB | TUBB4B | TXNDC5 | UBR4 | VAPA | VAT1 | VCL | VCP | XRCC5 | XRCC6 | GO:0043269 | 0.0391847628290671 | 323/8243 | 162/3250 | 4.31694269649973e-05 | 0.409893709032649 | T | T | T | T | regulation of ion transport | ABAT | ABL1 | ACE2 | ACTN2 | ACTN4 | ADCYAP1 | ADORA2A | ADRB2 | AHCYL1 | AHNAK | AKAP9 | AKT1 | AKT2 | ANK3 | ANXA2 | APOA1 | APOE | APP | ARF1 | ARRB2 | ATG5 | ATP1A1 | ATP1B3 | BAX | BCL2 | BIN1 | CAB39 | CACNA1A | CACNG2 | CACNG4 | CACNG5 | CALM1 | CALM2 | CALM3 | CAMK2A | CAMK2B | CAPN1 | CAV1 | CCR1 | CFTR | CHCHD10 | CHP1 | CLIC1 | CLIC3 | CLIC4 | COMMD1 | COX17 | CRBN | CRY2 | CTNNB1 | CXCL11 | DAPK1 | DLG2 | DLG3 | DLG4 | DMD | DNAJC30 | EPPIN | FABP3 | FGFR1 | FHL1 | FKBP1A | FKBP1B | FYN | G6PD | GLRX | GNAI2 | GNAO1 | GOPC | GPD1L | GRIA1 | GSTO1 | HAP1 | HCN1 | HCN2 | HCN3 | HECW1 | HECW2 | HSPA2 | HTR1B | HTR3A | HTT | ICAM1 | KCNA4 | KCNAB1 | KCNAB2 | KCNIP2 | KCNK16 | KCNS3 | KIF5B | LGALS3 | LRRC7 | MAP2K6 | MIF | MYO5A | NCOR1 | NEDD4 | NOS1AP | NOS3 | OXSR1 | P2RX1 | P2RX2 | P2RX4 | PACSIN3 | PARK7 | PCSK9 | PDE4D | PER1 | PER2 | PIK3CG | PLA2G10 | PLA2G1B | PLA2G6 | PLCG1 | PML | PPARGC1A | PPIF | PRELID1 | PRKACA | PRKCD | PRKCE | PSEN1 | PSEN2 | PTEN | PTPN6 | RAB11B | RAB3GAP1 | RGN | RGS2 | RHOA | RIPK1 | S100A1 | SARAF | SERPINE2 | SLC9A1 | SLC9A3R1 | SNCA | SNTA1 | SNX6 | SRI | STAC | STIM1 | STOM | STXBP1 | SUMO1 | TGFB1 | THADA | THBS1 | TMBIM6 | TOR1A | TPCN2 | TRIAP1 | TWIST1 | UBQLN1 | WDR1 | WNK1 | WNK4 | WWP2 | YWHAE | YWHAH | YWHAQ | ZMPSTE24 | GO:0019220 | 0.120465849811962 | 993/8243 | 449/3250 | 4.37382094385187e-05 | 0.415294298618735 | T | T | T | T | regulation of phosphate metabolic process | ABL1 | ACSL1 | ACTB | ADCY6 | ADCYAP1 | ADORA2A | ADRB2 | ADTRP | AGAP2 | AGER | AHSG | AKAP8 | AKAP9 | AKT1 | AKT2 | AKTIP | ANXA2 | APC | APOA1 | APOE | APP | AR | ARAF | ARF1 | ARR3 | ARRB1 | ARRB2 | ATM | AXIN1 | AZU1 | B3GAT3 | BAG4 | BAX | BCAR3 | BCL10 | BCL2 | BDKRB2 | BIRC7 | BMPR1A | BRAF | BRD4 | C1QL4 | C1QTNF1 | CAB39 | CALM1 | CALM2 | CALM3 | CARD9 | CASP3 | CAV1 | CBL | CBLB | CBLC | CCDC8 | CCDC88A | CCL21 | CCL22 | CCNB1 | CCNT1 | CCNT2 | CCNY | CCNYL1 | CCR1 | CD109 | CD27 | CD2BP2 | CD36 | CD40 | CD40LG | CD74 | CD81 | CDA | CDC25A | CDC25B | CDC25C | CDC37 | CDK4 | CDK5 | CDK5R1 | CDK9 | CDKN1A | CDKN2C | CEBPA | CHEK2 | CHORDC1 | CHP1 | CHRNA9 | CKS1B | CKS2 | CLU | COPS5 | COX11 | COX7A2 | CRK | CRKL | CRY2 | CSNK2B | CTNNB1 | CYLD | DAB1 | DAB2 | DAXX | DBF4B | DBNDD2 | DBNL | DDIT4 | DDRGK1 | DDX3X | DGKZ | DLG2 | DLG3 | DLG4 | DMD | DNAJA1 | DNAJC10 | DNAJC30 | DNM1L | DOCK7 | DUSP1 | DUSP12 | DUSP14 | DUSP16 | DUSP19 | DUSP22 | DUSP7 | DVL1 | DVL2 | DVL3 | DYNLL1 | EEF1A2 | EGFR | EHD4 | EIF2AK3 | EIF2AK4 | EIF3A | EPHA4 | ERBB2 | ERBB3 | ERRFI1 | EZH2 | EZR | FAF1 | FAM83D | FBXO7 | FGB | FGF2 | FGFR1 | FGFR1OP | FGFR3 | FKBP1A | FKBP1B | FKBP8 | FLOT1 | FN1 | FOXM1 | FOXO1 | FYN | GADD45G | GATA1 | GDF9 | GLMN | GNAI2 | GNAQ | GPD1L | GPS2 | GRB2 | GRIK2 | GSK3B | GSTP1 | HAVCR2 | HAX1 | HBEGF | HDAC2 | HDAC3 | HDAC6 | HEXIM1 | HGS | HMGB1 | HNRNPU | HPX | HRAS | HSF1 | HSP90AA1 | HSP90AB1 | HSP90B1 | HSPA2 | HSPB1 | HTT | IBTK | ICAM1 | IFNA2 | IGBP1 | IGF1R | IKBKB | IKBKG | ILK | INSR | IPO5 | IPO7 | IQGAP1 | ITCH | ITGB1BP1 | ITGB3 | JUN | KDM4D | KDR | KLHDC10 | KRAS | KSR1 | LAT | LATS1 | LCP2 | LDLRAD4 | LEMD2 | LGALS3 | LPAR1 | LRP6 | LRRK1 | LRRK2 | LTBR | MAP2K1 | MAP2K3 | MAP2K4 | MAP2K6 | MAP2K7 | MAP3K1 | MAP3K13 | MAP3K14 | MAP3K2 | MAP3K3 | MAP3K4 | MAP4K1 | MAPK1 | MAPK14 | MAPK3 | MAPK8IP3 | MAPKAP1 | MAPT | MAS1 | MAVS | MCM7 | ME1 | MIF | MLLT1 | MLST8 | MOB1B | MYC | MYO1D | MYOC | NCK1 | NCOR1 | NEK1 | NELFE | NF2 | NMNAT1 | NNT | NOD2 | NPM1 | NQO2 | NRBF2 | NTF3 | NTRK1 | OXSR1 | PAFAH1B1 | PAK2 | PAK6 | PARD3 | PARD6A | PARK7 | PARP14 | PDCD10 | PDE12 | PDE4D | PDE6G | PEBP1 | PER1 | PGK1 | PGK2 | PHB | PHB2 | PIH1D1 | PIK3CA | PIK3CG | PIK3R1 | PIK3R3 | PIN1 | PKIG | PLA2G1B | PLA2G2A | PLA2G6 | PLAUR | PLK1 | PLXNB2 | PML | PPARGC1A | PPIF | PPME1 | PPP1R11 | PPP1R12A | PPP1R14A | PPP1R16A | PPP1R2 | PPP1R3B | PPP1R3C | PPP1R8 | PPP2CA | PPP2R1A | PPP2R1B | PPP2R2A | PPP2R2D | PPP2R5D | PPP4R2 | PPP6R1 | PRDX1 | PRDX2 | PRDX3 | PRKAA1 | PRKACA | PRKAG1 | PRKAR1A | PRKAR2B | PRKCD | PRKCE | PRKCZ | PRKD1 | PRKD2 | PRKDC | PROX1 | PRRC1 | PSAP | PSEN1 | PSMD10 | PTEN | PTGES3 | PTK2 | PTN | PTPN1 | PTPN22 | PTPN6 | PTPRR | QARS | RAC1 | RAF1 | RANBP9 | RAP1A | RAP1B | RB1 | RB1CC1 | RGN | RGS2 | RHOA | RIPK1 | RIPK2 | RIPK3 | RPLP1 | RPS3 | RPTOR | S100A12 | S100A7 | SAG | SDC4 | SDCBP | SEMA7A | SERPINB3 | SET | SFN | SHC1 | SHMT2 | SIRT1 | SIRT3 | SLAMF1 | SLAMF8 | SLC9A3R1 | SMAD3 | SMAD4 | SMG5 | SMG6 | SMG8 | SNCA | SNF8 | SNX6 | SOCS1 | SOCS2 | SOCS6 | SOD1 | SOX2 | SPRY2 | SQSTM1 | SRC | STAT3 | STK11 | STK24 | STK25 | STK26 | STK3 | STK38 | STK40 | STOML2 | STRAP | STYX | SWAP70 | SYNJ2BP | TAB1 | TAB2 | TAOK3 | TARDBP | TAZ | TERF2IP | TGFB1 | TGFBR3 | THBS1 | TIMP2 | TLR3 | TNF | TNFAIP3 | TNFRSF1A | TNK2 | TP53 | TP73 | TRAF2 | TRAF3IP1 | TRAF6 | TRIB2 | TRIB3 | TSG101 | TSPYL2 | TWF1 | TWIST1 | TXN | UBB | UBC | UBE2B | UBE2K | UBE2N | UBE2V1 | UCHL1 | UNC119 | VCP | VTN | WARS | WNK1 | WNT16 | XRCC5 | XRCC6 | YES1 | YWHAB | YWHAE | YWHAG | ZNF622 | GO:1901360 | 0.229285454324882 | 1890/8243 | 819/3250 | 4.46228760968691e-05 | 0.423694208539772 | T | T | T | T | organic cyclic compound metabolic process | AARS | AARSD1 | ABCG1 | ABHD14B | ABL1 | ACAA1 | ACAA2 | ACACA | ACAT1 | ACAT2 | ACBD3 | ACD | ACLY | ACOT13 | ACOT7 | ACOT9 | ACSL1 | ACSL4 | ACSL5 | ACSS2 | ACTL6A | ADA | ADCY6 | ADCYAP1 | ADK | ADRM1 | ADSL | ADSS | AFF4 | AGO1 | AGO2 | AHCY | AHCYL1 | AICDA | AIMP2 | AK1 | AK2 | AK4 | AKR1A1 | AKR1B1 | AKR1B10 | AKR1B15 | AKR1C2 | ALAS1 | ALDH6A1 | ALDOA | ALOX12 | AMD1 | AMDHD2 | AOC2 | APBB1 | APEX1 | APEX2 | APOA1 | APOE | APOL2 | APP | APRT | AR | ARHGAP35 | ARID4B | ARNT | ARRB1 | ARRB2 | ASCC1 | ASCC2 | ASPDH | ATAD5 | ATF6B | ATIC | ATM | ATOH1 | ATP6V1A | ATP6V1B1 | ATRX | ATXN1 | ATXN2 | BABAM1 | BANF1 | BAX | BCL2 | BRCA1 | BTBD9 | BTRC | BUD13 | BUD31 | BYSL | C1QBP | C9orf64 | CARS | CASP3 | CAT | CBFB | CBR4 | CBS | CCAR2 | CCDC155 | CCDC36 | CCNB1 | CCNT1 | CCNT2 | CD2BP2 | CD40LG | CDA | CDC14B | CDC34 | CDC5L | CDC73 | CDK2 | CDK4 | CDK8 | CDK9 | CDKN1A | CEBPA | CENPF | CES1 | CFTR | CHAF1A | CHD1L | CHEK2 | CHRAC1 | CLPX | CMPK1 | COL4A2 | COMT | COPS2 | COPS5 | COPS6 | COX15 | CPSF7 | CREB1 | CREBBP | CRMP1 | CROT | CRY2 | CSNK1E | CSTF2T | CT45A5 | CTCF | CTDP1 | CTPS1 | CUL4B | CWC15 | CYB5R1 | CYB5R3 | CYC1 | CYP1A1 | CYP1A2 | DARS | DBH | DBI | DCAKD | DCK | DCP2 | DCTPP1 | DCXR | DDB1 | DDB2 | DDX1 | DDX39A | DDX39B | DDX3X | DERA | DGUOK | DHCR24 | DHRS4 | DHX15 | DHX36 | DHX9 | DIDO1 | DIS3 | DLAT | DLD | DLG2 | DLST | DMAP1 | DNA2 | DNAJC30 | DNASE2 | DNASE2B | DROSHA | DTX1 | DUS3L | DUT | DVL1 | DVL2 | E2F1 | E2F2 | E2F3 | EAF2 | ECD | EDC4 | EED | EFTUD2 | EHMT1 | EHMT2 | EIF4A3 | ELAC2 | ELAVL1 | ELAVL4 | ELF3 | ENDOG | ENTPD7 | EP300 | EPRS | ERCC5 | ERLIN2 | ESR1 | ESR2 | ESRRA | ESRRB | ESRRG | ETS1 | EXOSC1 | EXOSC5 | EXOSC6 | EXOSC9 | EXTL2 | EYA2 | EYA4 | EZH2 | FANCA | FANCC | FANCG | FANCI | FANCM | FAR2 | FARSB | FASN | FBXO6 | FDPS | FDXR | FECH | FEN1 | FGFR1 | FIGNL1 | FOLH1 | FOLR1 | FOS | FOXM1 | FPGS | FTO | FTSJ3 | FUBP1 | FUBP3 | FUS | FZR1 | G3BP1 | G6PD | GAR1 | GARS | GART | GATA1 | GCDH | GDA | GEMIN4 | GEMIN5 | GEMIN7 | GFPT1 | GFPT2 | GGPS1 | GINS2 | GINS3 | GINS4 | GLRX | GMDS | GMPPB | GMPR | GMPS | GNAI3 | GNAS | GNPDA1 | GNPDA2 | GNPNAT1 | GOT2 | GPD1L | GPX1 | GSR | GSTM4 | GTF2A1 | GTF2E2 | GTF2F1 | GTF2F2 | GTF2I | GTF3C2 | H1F0 | H2AFX | HAAO | HARS | HELZ2 | HERC2 | HIF1A | HINT1 | HIST1H4A | HIST3H3 | HMBS | HMGB1 | HMGB2 | HMGB3 | HMGCL | HMOX1 | HNF4A | HNF4G | HNRNPA1 | HNRNPA1L2 | HNRNPA2B1 | HNRNPD | HNRNPF | HNRNPH1 | HNRNPH2 | HNRNPH3 | HNRNPK | HNRNPL | HNRNPM | HNRNPU | HOXB9 | HPRT1 | HPX | HSD17B10 | HSD17B14 | HSD17B4 | HSD17B6 | HSD17B8 | HSD3B7 | HSF1 | HSP90AA1 | HSP90AB1 | HSPA1B | HSPA8 | HSPD1 | HTRA2 | HUWE1 | IARS | IDH1 | IDH2 | IDI2 | IK | ILF2 | IMP4 | IMPDH2 | INO80B | INO80C | INSIG1 | INSIG2 | IREB2 | IRF1 | IRF7 | ISG15 | ITPA | JMJD6 | JUN | KAT5 | KCNAB2 | KDM1A | KDM2A | KDM4D | KHDRBS1 | KIAA0391 | KLF5 | KMT2A | KPNA1 | KPNA2 | KPNB1 | KYNU | LACTB2 | LARP7 | LARS | LCMT2 | LDHA | LDHC | LEF1 | LGALS3 | LGMN | LIG4 | LIMA1 | LMO2 | LRRK2 | LSM1 | LSM12 | LSM3 | LSM4 | LSM5 | LSM8 | MAPK1 | MAPK3 | MAPT | MC1R | MCM2 | MCM3 | MCM4 | MCM5 | MCM6 | MCM7 | MCM9 | MDC1 | MDH1 | MDH2 | MDN1 | ME1 | MECP2 | MED1 | MED12 | MED14 | MED17 | MED20 | MED21 | MED23 | MED24 | MED31 | MED4 | MED6 | MEPCE | MGMT | MITF | MLH1 | MLLT1 | MLLT3 | MMAB | MME | MOCOS | MORF4L1 | MORF4L2 | MOV10 | MPC2 | MPG | MPHOSPH6 | MPI | MRM1 | MRPL12 | MSH6 | MTA1 | MTAP | MTERF1 | MTHFD1 | MTPN | MTRR | MVD | MYH6 | MYO5A | NAGK | NAMPT | NANS | NAPRT | NCBP1 | NCOA1 | NCOR1 | NDUFS1 | NELFB | NELFCD | NELFE | NFATC1 | NFE2L2 | NFS1 | NIPBL | NME1 | NME2 | NME4 | NMNAT1 | NMNAT3 | NMRK2 | NNT | NOB1 | NOP10 | NOP56 | NPLOC4 | NPM1 | NPR1 | NR0B2 | NR1H2 | NR1H3 | NR1I2 | NR3C1 | NR4A1 | NR5A2 | NRBF2 | NSMCE4A | NSUN2 | NSUN6 | NT5C2 | NUDT13 | NUDT18 | NUDT3 | NUDT9 | NUFIP1 | OAS2 | OGDH | OLA1 | OPA1 | OSBPL1A | OTUB1 | PA2G4 | PABPC1 | PABPC3 | PABPC4 | PAH | PAICS | PALB2 | PAN2 | PAPSS1 | PAPSS2 | PARK7 | PARP1 | PAX8 | PAXIP1 | PCBD1 | PCBP1 | PCBP2 | PCF11 | PCGF5 | PCGF6 | PCNA | PCSK9 | PDE12 | PDE4D | PDHA1 | PDHA2 | PDHB | PDHX | PDXK | PFAS | PGM3 | PHB | PICK1 | PIH1D1 | PINX1 | PIR | PNKP | PNP | POLA2 | POLD1 | POLDIP3 | POLE2 | POLE3 | POLM | POLR2A | POLR2E | POLR2L | POLR2M | POT1 | POU5F1 | PPA1 | PPA2 | PPARD | PPARG | PPARGC1A | PPAT | PPIA | PPIE | PPIL3 | PPM1D | PPOX | PPP1R8 | PPP2CA | PPP2R1A | PPP4R2 | PPP5C | PPT1 | PRCC | PRKAA1 | PRKAA2 | PRKACA | PRKDC | PRMT6 | PRPF19 | PRPF40A | PRPF8 | PRTFDC1 | PSAT1 | PSMD14 | PTEN | PTGES3 | PTMA | PTTG1 | PUM1 | PUS1 | PUS7 | QARS | QDPR | RAD21 | RAD23A | RAD23B | RAD51 | RAD51AP1 | RAN | RARA | RARG | RARS2 | RBBP6 | RBM17 | RBM25 | RBM3 | RBM4 | RBM8A | RBMX | RBPJ | RBX1 | RELA | REXO2 | RFC1 | RFC2 | RFC3 | RFC4 | RFC5 | RFK | RHOA | RIF1 | RIOK1 | RNASE3 | RNASEH2A | RNASEH2B | RNF8 | RPA2 | RPA3 | RPAIN | RPL10A | RPL7 | RPL7A | RPP14 | RPS14 | RPS19 | RPS2 | RPS24 | RPS3 | RPS8 | RRM1 | RRM2 | RRM2B | RRP7A | RRS1 | RTCB | RTF1 | RTFDC1 | RUNX2 | RUVBL1 | RUVBL2 | RXRA | RXRB | RXRG | SAP18 | SARS | SART1 | SART3 | SBDS | SCAF1 | SCO2 | SENP3 | SEPHS1 | SEPHS2 | SF1 | SF3B6 | SFPQ | SGPL1 | SHMT1 | SHMT2 | SIRT1 | SIRT6 | SIRT7 | SKI | SLC25A1 | SLC25A13 | SLC25A32 | SMAD2 | SMAD3 | SMAD4 | SMARCB1 | SMC1A | SMC3 | SMG5 | SMG6 | SMN1 | SNCA | SND1 | SNRNP27 | SNRPA | SNRPA1 | SNRPB | SNRPB2 | SNRPD1 | SNRPD3 | SNRPE | SNW1 | SOAT1 | SP1 | SP100 | SPI1 | SPIN1 | SPR | SPRTN | SRPK1 | SRPK2 | SRSF11 | SRSF12 | SRSF5 | SSB | STAT3 | STOML2 | STUB1 | SUCLA2 | SUCLG2 | SULT1A3 | SUMO1 | SUPT4H1 | SUPT5H | SWAP70 | TAF12 | TAF15 | TAF1A | TAMM41 | TAOK3 | TARDBP | TARS | TARS2 | TATDN1 | TBRG4 | TCEA1 | TCEA2 | TCERG1 | TCF4 | TDP1 | TECR | TERF1 | TERF2 | TERF2IP | TFCP2 | TFIP11 | TGFB1 | THADA | THEM4 | THOC2 | THOC6 | THOC7 | THRA | TINF2 | TK1 | TK2 | TMEM189-UBE2V1 | TNP1 | TOE1 | TOP1 | TOP2A | TOP3A | TP53 | TP53RK | TP73 | TPST2 | TRA2A | TREX1 | TRIM25 | TRIM28 | TRIM29 | TRIP12 | TRIP13 | TRMT44 | TRMT6 | TRMT61B | TRNT1 | TRUB2 | TSC22D1 | TSN | TTC5 | TXNL4A | TXNRD1 | TYMS | U2AF2 | UAP1 | UAP1L1 | UBB | UBC | UBE2A | UBE2B | UBE2D3 | UBE2L6 | UBE2N | UBE2V1 | UBE2W | UBR5 | UCHL5 | UCK2 | UGDH | UGGT1 | UGP2 | UMPS | UPF3B | UPP2 | USP14 | USP28 | USP39 | USP47 | USP7 | USPL1 | UTP14A | UTP6 | UXS1 | VARS | VCP | VDR | WARS | WDR12 | WDR4 | WDR48 | WT1 | WTAP | WWOX | XBP1 | XPO5 | XRCC1 | XRCC3 | XRCC5 | XRCC6 | YAP1 | YBEY | YY1 | ZBTB7A | ZBTB7B | ZFYVE26 | ZMPSTE24 | ZNF217 | ZNF638 | ZRANB2 | GO:1904356 | 0.00655101298071091 | 54/8243 | 36/3250 | 4.47875773523306e-05 | 0.425258046960379 | T | T | T | T | regulation of telomere maintenance via telomere lengthening | ACD | ATM | AURKB | CCT2 | CCT3 | CCT4 | CCT5 | CCT6A | CCT7 | CCT8 | CTNNB1 | DCP2 | DHX36 | HNRNPA1 | HNRNPA2B1 | HNRNPU | MAP2K7 | MAP3K4 | MAPK1 | MAPK15 | MAPK3 | NEK7 | PARP1 | PINX1 | PNKP | POT1 | SMG5 | SMG6 | SRC | TCP1 | TERF1 | TERF2 | TINF2 | TNKS | TNKS2 | XRCC5 | GO:0042592 | 0.102632536697804 | 846/8243 | 387/3250 | 4.67414804555341e-05 | 0.443810356925296 | T | T | T | T | homeostatic process | ABCB7 | ABCG1 | ABL1 | ABL2 | ACACA | ACADVL | ACD | ACKR2 | ACO1 | ACOX1 | ACTB | ACTG1 | ADCY6 | ADCYAP1 | ADRB2 | AFG3L2 | AKR1B1 | AKT1 | AKT3 | ALB | ALDOA | ALOX12 | ANK3 | ANXA1 | ANXA2 | ANXA6 | ANXA7 | AP3D1 | APEX1 | APOA1 | APOE | APP | ARF1 | ASGR2 | ASPSCR1 | ATG5 | ATM | ATOX1 | ATP12A | ATP13A2 | ATP1A1 | ATP1B3 | ATP2A2 | ATP2B2 | ATP4A | ATP6V1A | ATP6V1B1 | ATP6V1C1 | ATP6V1D | ATP6V1E1 | BAG3 | BAX | BCL2 | BDKRB2 | BTBD9 | C1QTNF1 | CACNA1A | CALM1 | CALM2 | CALM3 | CALR | CAND1 | CASP3 | CAV1 | CCDC22 | CCL21 | CCR1 | CCR3 | CCR4 | CCR5 | CD36 | CD40 | CDK5 | CEBPA | CFL2 | CFTR | CHP1 | CHRNA9 | CKB | CLIC4 | CLNS1A | COL2A1 | COL4A3BP | COMMD1 | COX11 | COX19 | CREBBP | CROCC | CRY1 | CRY2 | CSMD1 | CTNNB1 | CUL1 | CUTC | CXCL11 | CXCL6 | CYLD | DBH | DCN | DCTN1 | DDB1 | DLD | DLG4 | DMD | DNA2 | DNAJB2 | DNAJC10 | E2F4 | EGFR | EGLN1 | ENDOG | ESR1 | EZH2 | FABP3 | FABP5 | FADD | FANCC | FASLG | FEN1 | FGF2 | FH | FHL1 | FIS1 | FKBP1B | FOXA1 | FOXO1 | FTH1 | FTL | FTO | GAA | GAR1 | GATA1 | GLP1R | GLRX | GLRX3 | GLRX5 | GNA13 | GNA15 | GNAS | GNB1 | GOT1 | GPD1L | GPI | GPX1 | GRIA1 | GRIK2 | GSR | GSTO1 | GTF2I | HAAO | HAP1 | HDAC9 | HEXB | HIF1A | HIST1H4A | HIST3H3 | HK1 | HK2 | HMGB1 | HMOX1 | HNF1A | HNF4A | HOXB6 | HPX | HRNR | HSP90AA1 | HSP90AB1 | HSPB1 | HTR1B | HTT | IBTK | ICAM1 | IDE | IGF1R | IMMT | INSIG1 | INSR | IREB2 | ITGAV | ITPR1 | ITPR2 | ITPR3 | JAK3 | KCTD17 | KDR | KRAS | LAT | LETM1 | LGALS1 | LGR4 | LIMA1 | LPAR1 | LPAR2 | LPAR4 | LRP6 | LRRK1 | LRRK2 | MAPK1 | MAPK3 | MCOLN3 | METRNL | MEX3C | MICU1 | MICU2 | MKS1 | MLLT11 | MTHFD1 | MYC | MYO5A | MYOC | NCDN | NCOA4 | NEDD8 | NEUROD1 | NFE2L2 | NMB | NME1 | NME2 | NNT | NOD2 | NOP10 | NOS2 | NOS3 | NR1H2 | NR1H3 | NR5A2 | NUBP1 | OPA1 | OTC | P2RX1 | P2RX2 | P2RX4 | P4HB | PARK7 | PARP1 | PCNA | PCSK9 | PDIA3 | PDIA4 | PDIA6 | PDK2 | PFKM | PIK3CA | PIK3CG | PIK3R1 | PINX1 | PLA2G10 | PLA2G1B | PLA2G6 | PLCG1 | PML | POC1B | POLA2 | POLD1 | POLE2 | POLE3 | POT1 | POTEF | POTEI | PPARG | PPARGC1A | PPT1 | PRCP | PRDX1 | PRDX2 | PRDX3 | PRDX4 | PRDX5 | PRDX6 | PRKAA1 | PRKAA2 | PRKACA | PRKAR1A | PRKAR2B | PRKCE | PRKDC | PSEN1 | PTGER3 | PTGES2 | PTGES3 | PTGIR | PYGL | QSOX1 | RAB11A | RAB11FIP2 | RAB7A | RAC1 | RAD51 | RAP1A | RAP1GDS1 | RB1 | RBP1 | RC3H1 | RFC1 | RFC2 | RFC3 | RFC4 | RFC5 | RGN | RHOT1 | RHOT2 | RIC3 | RIF1 | RIPK3 | RPA2 | RPA3 | RPS24 | RTN4 | SART3 | SCO2 | SFN | SFXN1 | SFXN4 | SIN3A | SIRT1 | SIRT6 | SLAMF8 | SLC12A2 | SLC16A1 | SLC39A4 | SLC9A1 | SLC9A3R1 | SMAD3 | SMAD4 | SMG5 | SMG6 | SMO | SNCA | SOAT1 | SOD1 | SOD2 | SP100 | SRC | SRI | STAT1 | STAT3 | STAU1 | STIM1 | STK11 | STMN1 | STOML2 | STRAP | SWAP70 | TBXA2R | TCIRG1 | TERF1 | TERF2 | TERF2IP | TFIP11 | TGFB1 | TGM2 | THADA | TINF2 | TM9SF4 | TMBIM6 | TMX2 | TMX4 | TNFAIP3 | TNFSF14 | TP63 | TPCN2 | TRAF6 | TRIM32 | TXN | TXN2 | TXNDC11 | TXNDC15 | TXNDC5 | TXNRD1 | UBAP2L | UBB | VAPB | VDR | VGF | WDR35 | WDR48 | WDTC1 | WNK1 | WNK4 | XBP1 | XIAP | XRCC1 | XRCC3 | XRCC5 | XRCC6 | YAP1 | YWHAE | GO:0006790 | 0.0135872861822152 | 112/8243 | 65/3250 | 4.69469437208228e-05 | 0.445761230629212 | T | T | T | T | sulfur compound metabolic process | ABHD14B | ACACA | ACOT13 | ACOT7 | ACOT9 | AHCY | AHCYL1 | ALDH5A1 | AMD1 | CBS | CHST4 | CTH | DBI | DCN | DLD | DLST | DPEP1 | FAR2 | FASN | G6PD | GGT1 | GLO1 | GLRX3 | GPX1 | GPX4 | GSR | GSTA1 | GSTA2 | GSTA3 | GSTA4 | GSTA5 | GSTK1 | GSTM4 | GSTP1 | GSTT1 | GSTT2 | GSTT2B | GSTZ1 | HMGCL | HSD17B4 | HSPA9 | IDH1 | MGST1 | MPST | MTHFD1 | MTRR | MVD | NFS1 | NUBP1 | NUBP2 | OGDH | PAPSS1 | PAPSS2 | PARK7 | PAX8 | PHGDH | PTGES | SLC25A10 | SOD1 | STAT5A | SUCLA2 | SUCLG2 | SULT1A3 | THEM4 | TPST2 | GO:0034765 | 0.0265679970884387 | 219/8243 | 115/3250 | 4.81623174960854e-05 | 0.457301204625331 | T | T | T | T | regulation of ion transmembrane transport | ABL1 | ACTN2 | ACTN4 | ADRB2 | AHCYL1 | AHNAK | AKAP9 | AKT1 | AKT2 | ANK3 | APP | ARF1 | ATG5 | ATP1B3 | BAX | BIN1 | CAB39 | CACNA1A | CACNG2 | CACNG4 | CACNG5 | CALM1 | CALM2 | CALM3 | CAPN1 | CAV1 | CFTR | CHCHD10 | CHP1 | CLIC1 | CLIC3 | CLIC4 | COMMD1 | COX17 | CRBN | CXCL11 | DAPK1 | DLG2 | DLG3 | DLG4 | DMD | DNAJC30 | FHL1 | FKBP1A | FKBP1B | FYN | G6PD | GLRX | GOPC | GPD1L | GRIA1 | GSTO1 | HAP1 | HCN1 | HCN2 | HCN3 | HECW1 | HECW2 | HSPA2 | HTR3A | HTT | KCNA4 | KCNAB1 | KCNAB2 | KCNIP2 | KCNK16 | KCNS3 | KIF5B | LRRC7 | MYO5A | NEDD4 | NOS1AP | OXSR1 | PARK7 | PCSK9 | PDE4D | PER2 | PIK3CG | PLA2G6 | PLCG1 | PPARGC1A | PPIF | PRKACA | PRKCE | PSEN1 | PSEN2 | PTEN | PTPN6 | RGN | RGS2 | RIPK1 | S100A1 | SLC9A1 | SLC9A3R1 | SNCA | SNTA1 | SRI | STAC | STIM1 | STOM | SUMO1 | TGFB1 | THADA | THBS1 | TPCN2 | TWIST1 | UBQLN1 | WDR1 | WNK1 | WNK4 | WWP2 | YWHAE | YWHAH | YWHAQ | ZMPSTE24 | GO:0051276 | 0.0316632294067694 | 261/8243 | 134/3250 | 4.91704256049e-05 | 0.466873191118525 | T | T | T | T | chromosome organization | ABL1 | ACD | AIFM1 | AKAP8 | ANXA1 | APEX1 | ATM | ATRX | BAG6 | CCDC155 | CCDC36 | CDC20 | CENPF | CENPV | CHD1L | CHD3 | CHD4 | CHD8 | COPS2 | COPS5 | COPS6 | CUL4B | DDB1 | DDB2 | DDX1 | DDX3X | DHX36 | DHX9 | DNA2 | FANCM | FEN1 | G3BP1 | GAR1 | GATA1 | GINS2 | GINS4 | H1F0 | H1FX | H3F3A | HDAC2 | HIST1H1A | HIST1H3A | HIST1H4A | HIST3H3 | HMGB1 | HMGB2 | HMGB3 | HNRNPA2B1 | HSP90AA1 | HSP90AB1 | HSPA2 | ITPA | KLHL22 | LATS1 | LIG4 | MAD2L1 | MCM2 | MCM4 | MCM6 | MCM7 | MCM9 | MCMBP | MLH1 | MSH6 | MTERF1 | MYC | NCAPH2 | NIPBL | NOP10 | NSL1 | NUP107 | NUP98 | NUSAP1 | PARP1 | PCNA | PHB2 | PINX1 | PLK1 | POLA2 | POLD1 | POLE2 | POLE3 | POT1 | PPP2R1A | PRKDC | PTGES3 | PTTG1 | RAD21 | RAD23B | RAD51 | RAE1 | RAN | RBX1 | RFC1 | RFC2 | RFC3 | RFC4 | RFC5 | RIF1 | RPA2 | RPA3 | RUVBL1 | RUVBL2 | SIRT1 | SMC1A | SMC3 | SMG5 | SMG6 | SP100 | SRPK1 | STAG2 | TERF1 | TERF2 | TERF2IP | TFIP11 | TINF2 | TOP1 | TOP2A | TOP3A | TP53 | TREX1 | TRIP13 | TUBG1 | UBB | UBC | UBE2B | XRCC1 | XRCC3 | XRCC5 | XRCC6 | ZMPSTE24 | ZNF207 | ZW10 | ZWINT | GO:0051174 | 0.12058716486716 | 994/8243 | 449/3250 | 4.92458089912901e-05 | 0.4675889563723 | T | T | T | T | regulation of phosphorus metabolic process | ABL1 | ACSL1 | ACTB | ADCY6 | ADCYAP1 | ADORA2A | ADRB2 | ADTRP | AGAP2 | AGER | AHSG | AKAP8 | AKAP9 | AKT1 | AKT2 | AKTIP | ANXA2 | APC | APOA1 | APOE | APP | AR | ARAF | ARF1 | ARR3 | ARRB1 | ARRB2 | ATM | AXIN1 | AZU1 | B3GAT3 | BAG4 | BAX | BCAR3 | BCL10 | BCL2 | BDKRB2 | BIRC7 | BMPR1A | BRAF | BRD4 | C1QL4 | C1QTNF1 | CAB39 | CALM1 | CALM2 | CALM3 | CARD9 | CASP3 | CAV1 | CBL | CBLB | CBLC | CCDC8 | CCDC88A | CCL21 | CCL22 | CCNB1 | CCNT1 | CCNT2 | CCNY | CCNYL1 | CCR1 | CD109 | CD27 | CD2BP2 | CD36 | CD40 | CD40LG | CD74 | CD81 | CDA | CDC25A | CDC25B | CDC25C | CDC37 | CDK4 | CDK5 | CDK5R1 | CDK9 | CDKN1A | CDKN2C | CEBPA | CHEK2 | CHORDC1 | CHP1 | CHRNA9 | CKS1B | CKS2 | CLU | COPS5 | COX11 | COX7A2 | CRK | CRKL | CRY2 | CSNK2B | CTNNB1 | CYLD | DAB1 | DAB2 | DAXX | DBF4B | DBNDD2 | DBNL | DDIT4 | DDRGK1 | DDX3X | DGKZ | DLG2 | DLG3 | DLG4 | DMD | DNAJA1 | DNAJC10 | DNAJC30 | DNM1L | DOCK7 | DUSP1 | DUSP12 | DUSP14 | DUSP16 | DUSP19 | DUSP22 | DUSP7 | DVL1 | DVL2 | DVL3 | DYNLL1 | EEF1A2 | EGFR | EHD4 | EIF2AK3 | EIF2AK4 | EIF3A | EPHA4 | ERBB2 | ERBB3 | ERRFI1 | EZH2 | EZR | FAF1 | FAM83D | FBXO7 | FGB | FGF2 | FGFR1 | FGFR1OP | FGFR3 | FKBP1A | FKBP1B | FKBP8 | FLOT1 | FN1 | FOXM1 | FOXO1 | FYN | GADD45G | GATA1 | GDF9 | GLMN | GNAI2 | GNAQ | GPD1L | GPS2 | GRB2 | GRIK2 | GSK3B | GSTP1 | HAVCR2 | HAX1 | HBEGF | HDAC2 | HDAC3 | HDAC6 | HEXIM1 | HGS | HMGB1 | HNRNPU | HPX | HRAS | HSF1 | HSP90AA1 | HSP90AB1 | HSP90B1 | HSPA2 | HSPB1 | HTT | IBTK | ICAM1 | IFNA2 | IGBP1 | IGF1R | IKBKB | IKBKG | ILK | INSR | IPO5 | IPO7 | IQGAP1 | ITCH | ITGB1BP1 | ITGB3 | JUN | KDM4D | KDR | KLHDC10 | KRAS | KSR1 | LAT | LATS1 | LCP2 | LDLRAD4 | LEMD2 | LGALS3 | LPAR1 | LRP6 | LRRK1 | LRRK2 | LTBR | MAP2K1 | MAP2K3 | MAP2K4 | MAP2K6 | MAP2K7 | MAP3K1 | MAP3K13 | MAP3K14 | MAP3K2 | MAP3K3 | MAP3K4 | MAP4K1 | MAPK1 | MAPK14 | MAPK3 | MAPK8IP3 | MAPKAP1 | MAPT | MAS1 | MAVS | MCM7 | ME1 | MIF | MLLT1 | MLST8 | MOB1B | MYC | MYO1D | MYOC | NCK1 | NCOR1 | NEK1 | NELFE | NF2 | NMNAT1 | NNT | NOD2 | NPM1 | NQO2 | NRBF2 | NTF3 | NTRK1 | OXSR1 | PAFAH1B1 | PAK2 | PAK6 | PARD3 | PARD6A | PARK7 | PARP14 | PDCD10 | PDE12 | PDE4D | PDE6G | PEBP1 | PER1 | PGK1 | PGK2 | PHB | PHB2 | PIH1D1 | PIK3CA | PIK3CG | PIK3R1 | PIK3R3 | PIN1 | PKIG | PLA2G1B | PLA2G2A | PLA2G6 | PLAUR | PLK1 | PLXNB2 | PML | PPARGC1A | PPIF | PPME1 | PPP1R11 | PPP1R12A | PPP1R14A | PPP1R16A | PPP1R2 | PPP1R3B | PPP1R3C | PPP1R8 | PPP2CA | PPP2R1A | PPP2R1B | PPP2R2A | PPP2R2D | PPP2R5D | PPP4R2 | PPP6R1 | PRDX1 | PRDX2 | PRDX3 | PRKAA1 | PRKACA | PRKAG1 | PRKAR1A | PRKAR2B | PRKCD | PRKCE | PRKCZ | PRKD1 | PRKD2 | PRKDC | PROX1 | PRRC1 | PSAP | PSEN1 | PSMD10 | PTEN | PTGES3 | PTK2 | PTN | PTPN1 | PTPN22 | PTPN6 | PTPRR | QARS | RAC1 | RAF1 | RANBP9 | RAP1A | RAP1B | RB1 | RB1CC1 | RGN | RGS2 | RHOA | RIPK1 | RIPK2 | RIPK3 | RPLP1 | RPS3 | RPTOR | S100A12 | S100A7 | SAG | SDC4 | SDCBP | SEMA7A | SERPINB3 | SET | SFN | SHC1 | SHMT2 | SIRT1 | SIRT3 | SLAMF1 | SLAMF8 | SLC9A3R1 | SMAD3 | SMAD4 | SMG5 | SMG6 | SMG8 | SNCA | SNF8 | SNX6 | SOCS1 | SOCS2 | SOCS6 | SOD1 | SOX2 | SPRY2 | SQSTM1 | SRC | STAT3 | STK11 | STK24 | STK25 | STK26 | STK3 | STK38 | STK40 | STOML2 | STRAP | STYX | SWAP70 | SYNJ2BP | TAB1 | TAB2 | TAOK3 | TARDBP | TAZ | TERF2IP | TGFB1 | TGFBR3 | THBS1 | TIMP2 | TLR3 | TNF | TNFAIP3 | TNFRSF1A | TNK2 | TP53 | TP73 | TRAF2 | TRAF3IP1 | TRAF6 | TRIB2 | TRIB3 | TSG101 | TSPYL2 | TWF1 | TWIST1 | TXN | UBB | UBC | UBE2B | UBE2K | UBE2N | UBE2V1 | UCHL1 | UNC119 | VCP | VTN | WARS | WNK1 | WNT16 | XRCC5 | XRCC6 | YES1 | YWHAB | YWHAE | YWHAG | ZNF622 | GO:0036230 | 0.0417323789882324 | 344/8243 | 171/3250 | 4.9909031793861e-05 | 0.47388625688271 | T | T | T | T | granulocyte activation | ACAA1 | ACLY | ACTR2 | AGL | AHSG | ALDOA | ALDOC | ALOX5 | ANXA2 | AP1M1 | APEH | APRT | ARMC8 | ARPC5 | ATP6V1D | AZU1 | CAB39 | CAND1 | CAPN1 | CAT | CCT2 | CCT8 | CD36 | CDA | CEP290 | CKAP4 | COPB1 | COTL1 | CPNE3 | CSNK2B | CTSA | CTSC | CTSD | CXCL6 | CXCL8 | CYB5R3 | DBNL | DDOST | DDX3X | DEGS1 | DERA | DYNC1H1 | DYNC1LI1 | DYNLL1 | DYNLT1 | EEF1A1 | EEF2 | FABP5 | FAF2 | FCN1 | FGL2 | FTH1 | FTL | GAA | GALNS | GDI2 | GHDC | GLA | GNS | GPI | GSTP1 | GUSB | GYG1 | HEXB | HMGB1 | HRNR | HSP90AA1 | HSP90AB1 | HSPA1B | HSPA8 | HUWE1 | IDH1 | ILF2 | IMPDH2 | IQGAP1 | ITGAV | JUP | KCMF1 | KCNAB2 | KPNB1 | LGALS3 | LRRC7 | MAN2B1 | MAPK1 | MAPK14 | MGST1 | MIF | MME | NAPRT | NHLRC3 | NIT2 | NME2 | ORM1 | P2RX1 | PA2G4 | PDXK | PFKL | PGAM1 | PGM1 | PGM2 | PGRMC1 | PI4K2A | PKM | PLAUR | PNP | PPIA | PPIE | PRCP | PRDX4 | PRDX6 | PRG2 | PRKCD | PSAP | PSEN1 | PSMA2 | PSMA5 | PSMB7 | PSMC2 | PSMC3 | PSMD1 | PSMD11 | PSMD12 | PSMD14 | PSMD2 | PTGES2 | PTPN6 | PYGB | PYGL | QSOX1 | RAB10 | RAB18 | RAB24 | RAB31 | RAB5B | RAB7A | RAC1 | RAP1A | RAP1B | RHOA | RNASE3 | S100A11 | S100A12 | S100A7 | S100A9 | S100P | SDCBP | SELL | SERPINB3 | SERPINB6 | SLC2A5 | SNAP29 | SPTAN1 | SRP14 | STBD1 | STOM | STXBP2 | TCIRG1 | TIMP2 | TMEM173 | TMEM30A | TOLLIP | TUBB | TUBB4B | TXNDC5 | UBR4 | VAPA | VAT1 | VCL | VCP | XRCC5 | XRCC6 | GO:0033993 | 0.0536212543976708 | 442/8243 | 214/3250 | 5.00960964300663e-05 | 0.47566243560348 | T | T | T | T | response to lipid | ABCG1 | ABL1 | ABL2 | ACACA | ACSL1 | ACTA1 | ADCY6 | ADCYAP1 | ADH5 | ADTRP | AGL | AICDA | AIFM1 | AKAP8 | AKR1C2 | AKT1 | ALOX12 | ANXA1 | AR | ARNT2 | ARPC1B | ASCL1 | ASS1 | ATM | ATP1A1 | ATP2A2 | BCL2 | CALM1 | CALM3 | CALR | CAPN2 | CASP3 | CASP8 | CASP9 | CAT | CAV1 | CBL | CCL21 | CCNB1 | CCR5 | CD36 | CD40 | CD6 | CDC73 | CDK19 | CDK4 | CDKN1A | CEBPE | CFTR | COMT | CPT1A | CREB1 | CRYAB | CSN1S1 | CSNK2B | CTNNB1 | CTSV | CXCL11 | CXCL5 | CXCL6 | CXCL8 | CYP1A1 | CYP1A2 | DCN | DDIT4 | DNM1L | DUSP1 | E2F1 | EEF2 | EGFR | EIF4E | EIF4EBP1 | ENDOG | ERRFI1 | ESR1 | ESRRA | ETS1 | EZH2 | FABP3 | FASLG | FBXO32 | FECH | FIS1 | FOS | FOXA1 | FOXO1 | GGT1 | GNAI1 | GNB1 | GNG12 | GOT1 | GPI | GPX4 | GRAMD1A | GSTP1 | H2AFZ | HAVCR2 | HDAC2 | HDAC5 | HMGB1 | HMGB2 | HMGCL | HNRNPD | HNRNPU | HSF1 | HTR1B | HTRA2 | ICAM1 | IDH1 | IFNAR1 | IGFBP7 | INSIG1 | INSIG2 | IREB2 | JUN | JUNB | JUND | KAT5 | KRAS | LPAR1 | LRP6 | MAP4K1 | MAPK14 | MAPK8 | MBD3 | MDM2 | MED1 | MGST1 | MSN | MTAP | NCOA1 | NCOA2 | NCOA3 | NDUFA13 | NFKB2 | NME1 | NME2 | NOD2 | NOS2 | NOS3 | NQO1 | NR1H2 | NR1H3 | NR3C1 | OTUD5 | PARP1 | PCNA | PDE4D | PEBP1 | PELI1 | PHB2 | PPARD | PPARG | PPARGC1A | PPP5C | PRDX2 | PRDX3 | PRKAA1 | PRKAA2 | PRKCE | PRPF8 | PTEN | PTGES | PTGIR | PTN | PTPN22 | RARA | RARG | RELA | REST | RHOA | RUVBL2 | RXRA | S100A7 | S100B | SDC1 | SMAD2 | SMO | SNCA | SNW1 | SOCS2 | SRC | STAT3 | STXBP1 | TBXA2R | TGFB1 | TGFBR3 | THBS1 | THRA | TNF | TNFAIP3 | TNIP2 | TPCN2 | TRAF6 | TRIM25 | TRIM41 | TRIM63 | TWF2 | TXNIP | TYMS | UBE2L3 | UBE3A | USP8 | VCAM1 | VIM | VPS4B | WBP2 | WDR35 | WNT2 | XBP1 | XRCC5 | YAP1 | YES1 | YY1 | GO:0042119 | 0.0414897488778357 | 342/8243 | 170/3250 | 5.26047100705547e-05 | 0.499481722119916 | T | T | T | T | neutrophil activation | ACAA1 | ACLY | ACTR2 | AGL | AHSG | ALDOA | ALDOC | ALOX5 | ANXA2 | AP1M1 | APEH | APRT | ARMC8 | ARPC5 | ATP6V1D | AZU1 | CAB39 | CAND1 | CAPN1 | CAT | CCT2 | CCT8 | CD36 | CDA | CEP290 | CKAP4 | COPB1 | COTL1 | CPNE3 | CSNK2B | CTSA | CTSC | CTSD | CXCL6 | CXCL8 | CYB5R3 | DBNL | DDOST | DDX3X | DEGS1 | DERA | DYNC1H1 | DYNC1LI1 | DYNLL1 | DYNLT1 | EEF1A1 | EEF2 | FABP5 | FAF2 | FCN1 | FGL2 | FTH1 | FTL | GAA | GALNS | GDI2 | GHDC | GLA | GNS | GPI | GSTP1 | GUSB | GYG1 | HEXB | HMGB1 | HRNR | HSP90AA1 | HSP90AB1 | HSPA1B | HSPA8 | HUWE1 | IDH1 | ILF2 | IMPDH2 | IQGAP1 | ITGAV | JUP | KCMF1 | KCNAB2 | KPNB1 | LGALS3 | LRRC7 | MAN2B1 | MAPK1 | MAPK14 | MGST1 | MIF | MME | NAPRT | NHLRC3 | NIT2 | NME2 | ORM1 | P2RX1 | PA2G4 | PDXK | PFKL | PGAM1 | PGM1 | PGM2 | PGRMC1 | PKM | PLAUR | PNP | PPIA | PPIE | PRCP | PRDX4 | PRDX6 | PRG2 | PRKCD | PSAP | PSEN1 | PSMA2 | PSMA5 | PSMB7 | PSMC2 | PSMC3 | PSMD1 | PSMD11 | PSMD12 | PSMD14 | PSMD2 | PTGES2 | PTPN6 | PYGB | PYGL | QSOX1 | RAB10 | RAB18 | RAB24 | RAB31 | RAB5B | RAB7A | RAC1 | RAP1A | RAP1B | RHOA | RNASE3 | S100A11 | S100A12 | S100A7 | S100A9 | S100P | SDCBP | SELL | SERPINB3 | SERPINB6 | SLC2A5 | SNAP29 | SPTAN1 | SRP14 | STBD1 | STOM | STXBP2 | TCIRG1 | TIMP2 | TMEM173 | TMEM30A | TOLLIP | TUBB | TUBB4B | TXNDC5 | UBR4 | VAPA | VAT1 | VCL | VCP | XRCC5 | XRCC6 | GO:0051090 | 0.03603057139391 | 297/8243 | 150/3250 | 5.27914292286787e-05 | 0.501254620526305 | T | T | T | T | regulation of DNA-binding transcription factor activity | AGER | AKT1 | APP | AR | ARRB1 | ARRB2 | ATF2 | BCL10 | BTK | BTRC | BUD31 | CAMK1D | CAMK2A | CARM1 | CAT | CAV1 | CCDC22 | CD36 | CD40 | CD40LG | CHP1 | CLU | COMMD1 | COPS5 | CTH | CTNNB1 | CTNNBIP1 | CYLD | DDRGK1 | DHX9 | DVL2 | EDA | EGLN1 | EIF2AK4 | EP300 | ESR1 | ESR2 | EZH2 | FAF1 | FANCA | FLOT1 | FOS | FOXA1 | FOXJ1 | G3BP2 | HAVCR2 | HDAC2 | HDAC4 | HDAC5 | HMOX1 | HSPA1B | ICAM1 | ID2 | IKBKB | IKBKG | ITCH | JUN | JUP | KDM1A | KEAP1 | KRAS | LRP6 | MAP3K13 | MAPK1 | MAPK14 | MAPK3 | MAPK8 | MAPK9 | MAVS | MDFI | MTPN | MYD88 | NEUROD1 | NFKB2 | NFKBIA | NOD2 | NPM1 | NR0B2 | NTRK1 | PARK7 | PAXIP1 | PELI1 | PHB2 | PIM1 | PLA2G10 | PLA2G1B | PPARG | PPARGC1A | PRDX2 | PRDX3 | PRKCZ | PRKD1 | PRKD2 | PRMT2 | PROX1 | PSMA6 | PSMD10 | PTEN | RB1 | RELA | RIPK1 | RIPK2 | RIPK3 | RIPK4 | RNF2 | RNF25 | RNF31 | RPS3 | S100A12 | S100A9 | SIRT1 | SMAD3 | SMARCA4 | SMARCB1 | SMO | SP100 | SRI | STAT3 | STK3 | SUMO1 | TAB1 | TAB2 | TAF12 | TERF2IP | TGFB1 | TLR3 | TMEM173 | TNF | TNFAIP3 | TNFRSF8 | TRADD | TRAF1 | TRAF2 | TRAF3 | TRAF6 | TRIM14 | TRIM21 | TRIM25 | TRIM32 | TRIM34 | TWIST1 | UBB | UBC | UBE2I | UBE2N | UBE2V1 | USP7 | WNT2 | WWP2 | ZBTB7A | GO:2000026 | 0.138420477981317 | 1141/8243 | 510/3250 | 5.40330964012855e-05 | 0.513044250330205 | T | T | T | T | regulation of multicellular organismal development | ABL1 | ABL2 | ACTR2 | ADA | ADCY6 | ADCYAP1 | ADRB2 | AGER | AGO1 | AGO2 | AHI1 | AHSG | AKT1 | AKT3 | ALOX12 | ANXA1 | ANXA2 | AP3D1 | APBB1 | APCS | APOA1 | APOE | APP | AR | ARF1 | ARHGAP35 | ARHGDIA | ARHGEF9 | ARNT | ARRB2 | ASCL1 | ATAD5 | ATF2 | ATOH1 | AVIL | BAG5 | BAG6 | BASP1 | BAX | BCL11A | BCL2 | BCL6 | BCOR | BIN1 | BMPR1A | BRAF | BRCA1 | BTK | C1QA | C1QBP | CALR | CAMK1D | CAMK2A | CAMK2B | CARM1 | CASP8 | CAV1 | CBFB | CCDC88A | CCNB1 | CCR1 | CCR3 | CD109 | CD2 | CD27 | CD40 | CD74 | CDC20 | CDC42 | CDC73 | CDH5 | CDK5 | CDK5R1 | CDKL3 | CENPF | CFL1 | CLDN18 | CLSTN1 | CLTC | COL4A2 | COL5A1 | CREB1 | CREBBP | CRK | CRKL | CRMP1 | CSNK1E | CTDP1 | CTH | CTNNB1 | CTNNBIP1 | CTSL | CTSV | CTTN | CUL7 | CXCL8 | CYLD | DAB1 | DAB2 | DBN1 | DCN | DDX39B | DDX6 | DGUOK | DHX36 | DISC1 | DLG4 | DMRT3 | DNM1L | DOCK7 | DPF2 | DROSHA | DTX1 | DVL1 | DVL2 | DVL3 | DYNLT1 | E2F1 | E2F2 | EAF2 | EED | EFEMP1 | EGFR | EGLN1 | EHMT1 | EIF2AK3 | EIF2AK4 | EIF4E | EIF4ENIF1 | EIF6 | EP300 | EPHA4 | ERBB2 | ERBB3 | ERRFI1 | ESR1 | ESRRA | ETS1 | EXOSC6 | EZH2 | FADD | FAM129B | FANCA | FASLG | FBXO7 | FGF2 | FGFR1 | FKBP1B | FKBPL | FLOT1 | FN1 | FOS | FOXA1 | FOXJ1 | FYN | G6PD | GAK | GAP43 | GATA1 | GDF9 | GDI1 | GFAP | GLUL | GNAS | GNG5 | GOLGA4 | GPX1 | GRID2 | GRIP1 | GSK3A | GSK3B | GTF2I | H3F3A | HAP1 | HAVCR2 | HAX1 | HDAC1 | HDAC2 | HDAC4 | HDAC5 | HDAC7 | HDAC9 | HECW1 | HECW2 | HGS | HIF1A | HIF1AN | HIST1H3A | HIST1H4A | HIST2H3A | HK2 | HLX | HMGB1 | HMGB2 | HMOX1 | HNF4A | HOXA5 | HSPA1B | HSPA5 | HSPA9 | HSPB1 | HSPG2 | HTATIP2 | ID2 | IDH2 | IFNA2 | IFT122 | IKBKB | ILK | INSR | IQGAP1 | IRF1 | IRF4 | IRF7 | ISG15 | ITCH | ITGB1BP1 | JAK1 | JAK3 | JUN | JUP | KDM1A | KDM5B | KDR | KEAP1 | KIF13B | KMT2A | KMT2B | KMT2C | L3MBTL1 | LDLRAD4 | LEF1 | LGALS1 | LGALS3 | LGR4 | LIG4 | LMO2 | LOXL2 | LPAR1 | LRFN1 | LRRC7 | LRRK2 | LSM1 | MAP1B | MAP2K1 | MAP3K13 | MAP3K3 | MAPK1 | MAPK14 | MAPK6 | MAPK7 | MAPT | MBP | MDM2 | MECP2 | MED1 | MED12 | MEOX2 | METRN | MIB1 | MITF | MKS1 | MLH1 | MLLT3 | MME | MOV10 | MSH6 | MTPN | MYC | MYH6 | MYL9 | NAP1L1 | NCK1 | NCOA1 | NCOA3 | NEDD4 | NELL1 | NEUROD1 | NF2 | NFATC1 | NFATC2 | NFE2L2 | NFKBIA | NIN | NIPBL | NKX2-1 | NME1 | NME2 | NOS3 | NPHP3 | NPR1 | NRXN1 | NTF3 | NTRK1 | NUMA1 | NUPR1 | OAS2 | OBSL1 | OPA1 | P2RX4 | PAFAH1B1 | PARD3 | PARD6A | PARP6 | PAX8 | PAX9 | PAXIP1 | PCM1 | PDCD10 | PDCD6 | PDLIM5 | PER2 | PFN1 | PGK1 | PHB2 | PHOSPHO1 | PIAS3 | PIK3C2A | PIK3R1 | PIM1 | PIN1 | PKM | PLCG1 | PLXNA1 | PLXNA2 | PLXNB2 | PML | PNP | PPARD | PPARG | PPP1CC | PPP1R12A | PPP2CA | PRDX2 | PRELID1 | PRKAA1 | PRKACA | PRKCZ | PRKD1 | PRKD2 | PRMT6 | PROX1 | PRPF19 | PSEN1 | PSMA1 | PSMA2 | PSMA3 | PSMA5 | PSMA6 | PSMB5 | PSMB6 | PSMB7 | PSMB8 | PSMC1 | PSMC2 | PSMC3 | PSMC4 | PSMC5 | PSMC6 | PSMD1 | PSMD10 | PSMD11 | PSMD12 | PSMD14 | PSMD2 | PSMD4 | PSMD9 | PSME2 | PSME3 | PTEN | PTK2 | PTN | PTPN6 | PTPRD | PTPRF | PTPRO | PUS7 | RAB11A | RAB21 | RAB29 | RAC1 | RAP1A | RARA | RARG | RASSF10 | RB1 | RBPJ | RC3H1 | RECK | RELA | REST | RGN | RGS2 | RHOA | RHOJ | RIF1 | RIPK1 | RIPK2 | RTN4 | RTN4IP1 | RUNX1 | RUNX2 | RXRA | S100A1 | S100A9 | S100B | SARM1 | SARS | SART1 | SDCBP | SEC24B | SEMA7A | SEPT7 | SERPINB3 | SERPINE2 | SFMBT1 | SFN | SH3GL3 | SIN3A | SIPA1L1 | SIRT1 | SIRT6 | SKI | SLAMF8 | SMAD2 | SMAD3 | SMAD4 | SMO | SMURF1 | SMURF2 | SNAI2 | SNCA | SNW1 | SNX3 | SOCS1 | SOCS2 | SOD1 | SOD2 | SOX2 | SP1 | SP100 | SPEN | SPI1 | SPRY2 | SRC | SRGAP2 | STARD13 | STAT1 | STAT3 | STAT6 | STIM1 | STK11 | STK24 | STK25 | STK3 | STRAP | SULF2 | SYDE1 | SYNJ2BP | TBXA2R | TCF12 | TCF4 | TGFB1 | TGFBR3 | THBS1 | THBS2 | TIMP1 | TIMP2 | TLR3 | TMEM30A | TNF | TNFAIP3 | TNFRSF12A | TNFRSF1A | TNFSF12 | TNN | TP63 | TP73 | TRADD | TRAF3IP1 | TRAF6 | TRIM32 | TRIP12 | TUBB2B | TULP3 | TWF1 | TWF2 | TWIST1 | USP19 | VASH2 | VDR | VIM | WARS | WDR1 | WDR77 | WNK1 | WNT2 | WT1 | XBP1 | XRCC5 | YAP1 | YWHAG | YWHAH | YWHAZ | YY1 | ZAP70 | ZBTB16 | ZBTB7B | ZMPSTE24 | ZMYND8 | GO:0035384 | 0.00327550649035545 | 27/8243 | 21/3250 | 5.57874694038844e-05 | 0.529702021989883 | T | T | T | T | thioester biosynthetic process | ACACA | ACAT1 | ACLY | ACSL1 | ACSL4 | ACSL5 | ACSS2 | CBR4 | DLAT | DLD | FASN | GCDH | HSD17B8 | MPC2 | PDHA1 | PDHA2 | PDHB | PDHX | PPT1 | SLC25A1 | TECR | GO:0071616 | 0.00327550649035545 | 27/8243 | 21/3250 | 5.57874694038844e-05 | 0.529702021989883 | T | T | T | T | acyl-CoA biosynthetic process | ACACA | ACAT1 | ACLY | ACSL1 | ACSL4 | ACSL5 | ACSS2 | CBR4 | DLAT | DLD | FASN | GCDH | HSD17B8 | MPC2 | PDHA1 | PDHA2 | PDHB | PDHX | PPT1 | SLC25A1 | TECR | GO:0009124 | 0.0041247118767439 | 34/8243 | 25/3250 | 5.58360204543947e-05 | 0.530163014214478 | T | T | T | T | nucleoside monophosphate biosynthetic process | ADA | ADK | ADSL | ADSS | APRT | ATIC | CMPK1 | DCK | DGUOK | DUT | GART | GMPS | HPRT1 | IMPDH2 | PAICS | PFAS | PPAT | RFK | SHMT1 | TK1 | TK2 | TYMS | UCK2 | UMPS | UPP2 | GO:0006611 | 0.0041247118767439 | 34/8243 | 25/3250 | 5.58360204543947e-05 | 0.530163014214478 | T | T | T | T | protein export from nucleus | ABCE1 | AHCYL1 | CALR | CCHCR1 | CHP1 | CSE1L | DUSP16 | EIF5A | EIF6 | NMD3 | NPM1 | NUP214 | NUP88 | NUTF2 | RAN | RANBP3 | RRS1 | SMURF1 | STYX | TGFB1 | TPR | XPO1 | XPO4 | XPO5 | XPO7 | GO:0023056 | 0.128593958510251 | 1060/8243 | 476/3250 | 5.78644553860406e-05 | 0.549423003890455 | T | T | T | T | positive regulation of signaling | ABAT | ABL1 | ACTN4 | ADA | ADCYAP1 | ADORA2A | ADRB2 | AGER | AGO1 | AGR2 | AKR1B1 | AKR1C2 | AKT1 | AKT3 | ANXA2 | APOA1 | APOE | APP | AR | ARHGAP1 | ARHGEF7 | ARNT | ARRB1 | ARRB2 | ASCL1 | ATF6 | ATM | AXIN1 | BAG4 | BAX | BBC3 | BCL10 | BCL2 | BCL2L1 | BIRC2 | BIRC3 | BIRC7 | BIRC8 | BMPR1A | BRAF | BRD4 | BUD31 | C1QBP | C1QTNF1 | CACNG2 | CACNG4 | CACNG5 | CALM1 | CALM2 | CALM3 | CALR | CARD9 | CASP10 | CASP2 | CASP8 | CAT | CAV1 | CBL | CCAR2 | CCDC22 | CCL21 | CCL22 | CCR1 | CD27 | CD36 | CD40 | CD40LG | CD74 | CD81 | CDC73 | CDK5 | CFTR | CHCHD10 | CLNK | CLSTN1 | CREB1 | CREBBP | CRK | CRKL | CSNK1E | CSNK2B | CTH | CTNNB1 | CTSC | CYLD | DAB2 | DAXX | DBNL | DCN | DDRGK1 | DDX1 | DDX3X | DHX36 | DISC1 | DLG4 | DNM1L | DOK1 | DUSP19 | DUSP22 | DUSP7 | DVL1 | DVL2 | DVL3 | DYNLL1 | E2F1 | EDA | EEF1D | EGFR | EIF2AK4 | ELF3 | EP300 | EPHA4 | ERBB2 | ERBB3 | ESR1 | EZH2 | FABP5 | FADD | FAF1 | FASLG | FGB | FGF2 | FGFR1 | FGFR3 | FIS1 | FKBP1A | FKBP8 | FLOT1 | FOXA1 | FYN | GADD45G | GDF9 | GFAP | GJA1 | GKAP1 | GLUL | GNAI2 | GOLPH3 | GOLT1B | GOT1 | GPS2 | GPX1 | GRB2 | GRB7 | GRIK2 | GSK3A | GSK3B | GSTO1 | HAP1 | HAVCR2 | HAX1 | HBEGF | HDAC2 | HDAC3 | HDAC6 | HEXIM1 | HIF1A | HINT1 | HMGB1 | HMOX1 | HPX | HRAS | HSF1 | HSP90AA1 | HSP90AB1 | HSPA1B | HTRA2 | HTT | ICAM1 | IFNA2 | IGF1R | IKBKB | IKBKE | IKBKG | ILK | ING5 | INSR | IQGAP1 | IRF7 | ITGB1BP1 | ITGB3 | ITPR3 | JUN | JUP | KDR | KHDRBS1 | KIF5B | KLHDC10 | KLHL22 | KRAS | KSR1 | LARS | LASP1 | LAT | LGALS1 | LGMN | LGR4 | LIMS1 | LPAR1 | LPAR4 | LRRK1 | LRRK2 | LTBR | LURAP1 | MAOA | MAP2K1 | MAP2K3 | MAP2K4 | MAP2K6 | MAP2K7 | MAP3K1 | MAP3K13 | MAP3K14 | MAP3K2 | MAP3K3 | MAP3K4 | MAP4K1 | MAPK1 | MAPK14 | MAPK3 | MAPK8 | MAPK8IP3 | MAPK9 | MAS1 | MAVS | MC1R | MECP2 | MED1 | MIF | MLLT11 | MLLT3 | MLST8 | MME | MPC2 | MPP2 | MYD88 | MYO1C | MYOC | NCK1 | NEDD4 | NELFE | NMB | NMNAT1 | NOD2 | NOS1AP | NPR1 | NQO2 | NR0B2 | NR1H3 | NRXN1 | NTF3 | NTRK1 | NUMA1 | NUP93 | OPA1 | OXCT1 | P2RX2 | P2RX4 | PAFAH1B1 | PAG1 | PAK2 | PARK7 | PARP1 | PARP14 | PDCD10 | PDCL | PDE6G | PDIA3 | PEBP1 | PELI1 | PFKM | PHB | PHB2 | PIH1D1 | PIK3CA | PIK3CG | PIK3R1 | PIN1 | PLA2G1B | PLA2G2A | PLA2G6 | PLAUR | PLEKHF1 | PML | PPARD | PPIF | PPM1A | PPP1CA | PPP2R1A | PPP2R1B | PPP3CC | PPP5C | PRDX2 | PRKAA1 | PRKCD | PRKCE | PRKCZ | PRKD1 | PRKD2 | PSAP | PSEN1 | PSMA1 | PSMA2 | PSMA3 | PSMA5 | PSMA6 | PSMB5 | PSMB6 | PSMB7 | PSMB8 | PSMC1 | PSMC2 | PSMC3 | PSMC4 | PSMC5 | PSMC6 | PSMD1 | PSMD10 | PSMD11 | PSMD12 | PSMD14 | PSMD2 | PSMD4 | PSMD9 | PSME2 | PSME3 | PTEN | PTK2 | PTN | PTPN1 | PTPN22 | PTPN23 | PTPN6 | PUM1 | RAB29 | RAB3GAP1 | RAC1 | RAF1 | RAP1A | RAP1B | RB1CC1 | RBPJ | RC3H1 | RECK | REL | RELA | RHOA | RIPK1 | RIPK2 | RIPK3 | RNF183 | RNF31 | RPL23 | RPS3 | RPTOR | RTN4 | RUVBL1 | S100A12 | S100A13 | S100A4 | S100A7 | S100A9 | S100B | SARM1 | SCEL | SDCBP | SEC13 | SEMA7A | SEPT4 | SERPINE2 | SFN | SFPQ | SH2B1 | SHC1 | SHKBP1 | SIAH1 | SIRT1 | SIRT3 | SKI | SLAMF1 | SLC9A1 | SLC9A3R1 | SMAD2 | SMAD3 | SMAD4 | SMARCA4 | SMARCB1 | SMO | SMURF2 | SNCA | SNW1 | SOCS2 | SOD1 | SOX2 | SPIN1 | SPRY2 | SQSTM1 | SRC | SRI | STAT3 | STAU1 | STK11 | STK25 | STK3 | STXBP1 | SULF2 | TAB1 | TAB2 | TAOK3 | TARDBP | TBL1X | TERF2IP | TFDP1 | TFG | TGFB1 | TGFBR3 | TGM2 | THBS1 | TIMP2 | TLR3 | TMEM9B | TNF | TNFAIP3 | TNFRSF12A | TNFRSF1A | TNFSF12 | TNFSF14 | TNIP2 | TNKS | TNKS2 | TP53 | TP63 | TP73 | TRADD | TRAF2 | TRAF6 | TRIM25 | TRIM32 | TRIP6 | TXN | UBB | UBC | UBE2B | UBE2I | UBE2K | UBE2N | UBE2O | UBE2V1 | UBQLN2 | UBR5 | USP17L2 | USP47 | USP8 | VAPA | VAV1 | VCP | VTN | WBP2 | WDFY1 | WDR35 | WNK1 | WNT16 | WWOX | XBP1 | XIAP | YAP1 | YWHAB | YWHAE | YWHAG | YWHAH | YWHAQ | YWHAZ | ZAP70 | ZBTB7B | ZDHHC17 | ZNF622 | GO:0006112 | 0.00545917748392576 | 45/8243 | 31/3250 | 5.81237819760232e-05 | 0.551885309862341 | T | T | T | T | energy reserve metabolic process | AGL | AKT1 | AKT2 | CALM1 | GAA | GBE1 | GFPT1 | GFPT2 | GNAS | GSK3A | GSK3B | GYG1 | GYG2 | GYS1 | MYC | PER2 | PFKM | PGM1 | PGM2 | PHKA1 | PPP1CA | PPP1CB | PPP1CC | PPP1R2 | PPP1R3B | PPP1R3C | PYGB | PYGL | STBD1 | STK40 | UGP2 | GO:0061077 | 0.00545917748392576 | 45/8243 | 31/3250 | 5.81237819760232e-05 | 0.551885309862341 | T | T | T | T | chaperone-mediated protein folding | BAG1 | CCT2 | CD74 | CHORDC1 | CLU | DNAJB1 | DNAJB2 | DNAJC7 | FKBP1A | FKBP1B | FKBP5 | GAK | HSPA14 | HSPA1B | HSPA1L | HSPA2 | HSPA5 | HSPA8 | HSPA9 | HSPB1 | HSPE1 | HSPH1 | PDIA4 | PEX19 | PPIB | PPID | PTGES3 | SGTA | ST13 | TOR1A | TRAP1 | GO:1901607 | 0.00545917748392576 | 45/8243 | 31/3250 | 5.81237819760232e-05 | 0.551885309862341 | T | T | T | T | alpha-amino acid biosynthetic process | AASDHPPT | AASS | ASL | ASNS | ASS1 | CBS | CTH | ENOPH1 | GGT1 | GLS | GLS2 | GLUD2 | GLUL | GOT1 | GOT1L1 | GOT2 | MTAP | MTHFD1 | MTRR | OAT | OTC | PAH | PHGDH | PLOD2 | PSAT1 | PYCR1 | PYCR2 | SEPHS1 | SEPHS2 | SHMT1 | SHMT2 | GO:0043618 | 0.0109183549678515 | 90/8243 | 54/3250 | 5.84030151913901e-05 | 0.554536629242249 | T | T | T | T | regulation of transcription from RNA polymerase II promoter in response to stress | ARNT | ATF6 | ATF6B | BAG3 | CREBBP | CUL2 | DNAJB1 | EGLN1 | EGLN3 | EP300 | HIF1A | HIF1AN | HMOX1 | HSF1 | HSF4 | HSPA5 | JUN | MAPK7 | NCK1 | NEDD4 | NFE2L2 | PSMA1 | PSMA2 | PSMA3 | PSMA5 | PSMA6 | PSMB5 | PSMB6 | PSMB7 | PSMB8 | PSMC1 | PSMC2 | PSMC3 | PSMC4 | PSMC5 | PSMC6 | PSMD1 | PSMD10 | PSMD11 | PSMD12 | PSMD14 | PSMD2 | PSMD4 | PSMD9 | PSME2 | PSME3 | RBPJ | RBX1 | SIN3A | TMBIM6 | TP53 | UBB | UBC | VHL | GO:1901654 | 0.0149217517893971 | 123/8243 | 70/3250 | 5.85063526676891e-05 | 0.555517818579708 | T | T | T | T | response to ketone | ACACA | ADCY6 | AIFM1 | AKAP8 | AKR1B1 | AKR1C2 | AKT1 | AR | ASS1 | BCL2L1 | CALM1 | CALM3 | CALR | CASP9 | CAV1 | CBL | CCL21 | CDC5L | CDK4 | CDKN1A | CFTR | CSN1S1 | CSNK2B | DDIT4 | DUSP1 | EGFR | EIF4E | EIF4EBP1 | ERRFI1 | FBXO32 | FECH | FOS | FOXO1 | GNAI1 | GNB1 | GPI | HNRNPD | HSF1 | ICAM1 | IGFBP7 | MAP4K1 | MSN | MTAP | NCOA1 | NCOA2 | NME1 | NR1H3 | NR3C1 | OPA1 | PARP1 | PCNA | PEBP1 | PPARG | PRKAA1 | PRKAA2 | PRKCE | PTN | RELA | SIN3A | SRC | TBXA2R | TGFB1 | TGFBR3 | THBS1 | TXNIP | TYMS | UBE3A | USP8 | WBP2 | YAP1 | GO:2001242 | 0.0149217517893971 | 123/8243 | 70/3250 | 5.85063526676891e-05 | 0.555517818579708 | T | T | T | T | regulation of intrinsic apoptotic signaling pathway | AKT1 | ATAD5 | BAG5 | BAX | BCL2 | BCL2L1 | BCL2L2 | BDKRB2 | CAV1 | CCAR2 | CD74 | CLU | CREB3 | CYLD | DDX3X | DNAJA1 | DNM1L | EIF2AK3 | ENO1 | FIGNL1 | FIS1 | GPX1 | HDAC1 | HIF1A | HNRNPK | HSPB1 | HTRA2 | KDM1A | LRRK2 | MAPK7 | MDM2 | MIF | NCK1 | NDUFA13 | NDUFS3 | NFE2L2 | OPA1 | P4HB | PARK7 | PARP1 | PLAUR | PLEKHF1 | PPIF | PTPMT1 | PTPN1 | RIPK3 | RNF183 | RPS3 | RRM2B | S100A9 | SEPT4 | SFPQ | SIAH1 | SIRT1 | SLC9A3R1 | SNAI2 | SOD1 | SOD2 | SRC | SYVN1 | TMBIM6 | TP53 | TRAP1 | TRIAP1 | TRIM32 | UBB | UBQLN1 | USP47 | VDAC2 | XBP1 | GO:0032204 | 0.00849205386388451 | 70/8243 | 44/3250 | 5.87925976162521e-05 | 0.558235714366314 | T | T | T | T | regulation of telomere maintenance | ACD | ATM | ATRX | AURKB | CCT2 | CCT3 | CCT4 | CCT5 | CCT6A | CCT7 | CCT8 | CTNNB1 | DCP2 | DHX36 | HNRNPA1 | HNRNPA2B1 | HNRNPD | HNRNPU | MAP2K7 | MAP3K4 | MAPK1 | MAPK15 | MAPK3 | MYC | NEK7 | PARP1 | PINX1 | PML | PNKP | POT1 | SIRT6 | SMG5 | SMG6 | SRC | TCP1 | TERF1 | TERF2 | TERF2IP | TINF2 | TNKS | TNKS2 | USP7 | XRCC1 | XRCC5 | GO:0032386 | 0.0366371466699017 | 302/8243 | 152/3250 | 5.89989833613138e-05 | 0.560195347015675 | T | T | T | T | regulation of intracellular transport | ACTN2 | ADORA2A | AKT2 | ANK3 | ANXA2 | APOD | ARF1 | ARHGAP1 | ATAD1 | ATP13A2 | ATP2A2 | B3GAT3 | BACE1 | BAG3 | BAG4 | BRAF | CALM1 | CALM3 | CAMK2A | CD36 | CDH1 | CDK5 | CDK5R1 | CEP290 | CHP1 | CHRM2 | CPLX1 | CPLX3 | CRYAB | CSNK2A2 | DAB2 | DHX9 | DMAP1 | DNAJC30 | DNM1L | DVL1 | DYNC1H1 | EDEM1 | ERBB2 | EZR | FIS1 | FYN | GDI1 | GIT1 | GSK3A | GSK3B | HAP1 | HAX1 | HDAC3 | HMOX1 | HRAS | HSPA1L | HTR1B | HTRA2 | HUWE1 | IL13RA2 | INSIG1 | IPO5 | ITGB1BP1 | ITGB3 | JUP | KHDRBS1 | KIF5B | LCP1 | LRRK2 | MAP1B | MAP2K1 | MAPK1 | MAPK14 | MAPK15 | MAPK3 | MAVS | MDM2 | MSN | MYO1C | NEDD4 | NPEPPS | NUCB1 | NUMA1 | NUP153 | NUP214 | P2RX1 | PARK7 | PCM1 | PCSK9 | PDCD10 | PIK3R1 | PKIG | PPM1A | PPP1CC | PPP1R12A | PREPL | PRKAA1 | PRKACA | PRKCD | PSEN1 | PTPN1 | PTPN14 | PTPN23 | RAB11B | RAB21 | RAB29 | RAB3GAP1 | RAB5A | RAN | RANGAP1 | RAP1A | RAP1B | RBM4 | RDX | RHOT1 | RHOU | RNF139 | RNF31 | SAE1 | SCFD1 | SEC16A | SEC24B | SEPT5 | SFN | SMAD3 | SMO | SNX12 | SNX3 | SNX6 | SP100 | SRC | STOM | STXBP1 | STXBP2 | SYN1 | SYP | TARDBP | TGFB1 | TM9SF4 | TMEM30A | TP53 | TPR | TRIM28 | TXN | UBE2D3 | UBE2J1 | UBE2L3 | UBR5 | USP17L2 | USP46 | VPS18 | XPO1 | XPO4 | XPO5 | YWHAE | ZIC1 | GO:0009408 | 0.00873468397428121 | 72/8243 | 45/3250 | 5.95150075336394e-05 | 0.565094996531906 | T | T | T | T | response to heat | AKT1 | ATP2A2 | BAG3 | CDKN1A | CLPB | CRNN | DAXX | DHX36 | DNAJA1 | DNAJA2 | DNAJA3 | DNAJA4 | EIF2B1 | EIF2B3 | EIF2B5 | EIF2S1 | HDAC2 | HMOX1 | HSF1 | HSF4 | HSP90AA1 | HSPA14 | HSPA1B | HSPA1L | HSPA2 | HSPA5 | HSPA8 | HSPA9 | HTRA2 | IGFBP7 | MAP2K7 | MAPT | MICA | NOS3 | PDCD6 | PEBP1 | PRKACA | SOD1 | STAC | STUB1 | SUMO1 | THBS1 | TPR | VCP | YWHAE | GO:0051291 | 0.0101904646366614 | 84/8243 | 51/3250 | 6.00413831052703e-05 | 0.570092932584542 | T | T | T | T | protein heterooligomerization | ANXA2 | BCL10 | BIRC2 | BIRC3 | BRAF | C1QTNF1 | CAB39 | CBR4 | CD74 | CHMP2A | COL6A1 | COL6A2 | CPSF7 | FARSB | GLRB | GNB1 | GRB2 | HBZ | HIST1H3A | HIST1H4A | HIST3H3 | HRAS | HSD17B8 | IDE | IKZF5 | ILK | INSR | ITPR3 | JUP | LIMS1 | MAT2A | MCCC1 | P2RX1 | PCBD1 | PDSS1 | PDSS2 | PRKAA1 | PRKCZ | RCC1 | RIPK1 | RIPK3 | RRM1 | RRM2 | SEPT11 | SEPT7 | SEPT9 | SQSTM1 | TRADD | TRAF2 | XRCC6 | YWHAB | GO:0045089 | 0.0280237777508189 | 231/8243 | 120/3250 | 6.21663014409126e-05 | 0.590269032181465 | T | T | T | T | positive regulation of innate immune response | AP1G1 | BCL10 | BIRC2 | BIRC3 | BTK | BTRC | CARD9 | CASP8 | CAV1 | CD209 | CD36 | CREBBP | CTSL | CUL1 | CYLD | DHX9 | EP300 | FADD | FBXW11 | FCN1 | FGB | FLOT1 | FYN | GBP5 | HAVCR2 | HEXIM1 | HMGB1 | HMGB2 | HMGB3 | HPX | HRAS | HSP90B1 | HSPA1B | HSPD1 | IFI16 | IKBKB | IKBKE | IKBKG | IRF7 | ITCH | KRAS | LGMN | MAP2K6 | MAP3K1 | MATR3 | MAVS | MED1 | MICA | MYD88 | NFKBIA | NOD2 | NR1H3 | PAK2 | PELI1 | PLSCR1 | PRKACA | PRKCD | PRKCE | PRKDC | PSMA1 | PSMA2 | PSMA3 | PSMA5 | PSMA6 | PSMB5 | PSMB6 | PSMB7 | PSMB8 | PSMC1 | PSMC2 | PSMC3 | PSMC4 | PSMC5 | PSMC6 | PSMD1 | PSMD10 | PSMD11 | PSMD12 | PSMD14 | PSMD2 | PSMD4 | PSMD9 | PSME2 | PSME3 | PTPN22 | PUM1 | RAF1 | RELA | RIPK1 | RIPK2 | RPS19 | RTN4 | S100A1 | S100A9 | SCARA3 | SFPQ | SIN3A | SRC | TAB1 | TAB2 | TANK | TLR3 | TMEM173 | TNFAIP3 | TNIP2 | TRAF3 | TRAF6 | UBB | UBC | UBE2D1 | UBE2D2 | UBE2D3 | UBE2K | UBE2N | UBE2V1 | USP17L2 | VAV1 | WDFY1 | XRCC5 | XRCC6 | GO:0097305 | 0.0167414776173723 | 138/8243 | 77/3250 | 6.41238519831759e-05 | 0.608855974580255 | T | T | T | T | response to alcohol | ABAT | ACACA | ACTR2 | ADCY6 | ADCYAP1 | AIFM1 | AKAP8 | AKR1C2 | AKT1 | BCL2L1 | BIRC2 | BRCA1 | CALM1 | CALM3 | CASP8 | CAT | CBL | CCL21 | CCR5 | CD27 | CDH1 | CDK4 | CDKN1A | CFTR | CLDN18 | CSN1S1 | CTNNB1 | DMAP1 | EEF2 | EIF4EBP1 | FECH | FOS | FYN | G6PD | GNAI1 | GNB1 | GOT2 | GRAMD1A | GSTP1 | HNRNPD | HTR1B | HTR3A | ICAM1 | IGFBP7 | IREB2 | ITPR2 | JUP | LRP6 | MAP4K1 | MGMT | NQO1 | OXCT1 | PARP1 | PEBP1 | PPARG | PRKAA1 | PRKAA2 | PRKCE | PSMD14 | PTEN | RAD51 | RARA | RGS2 | RHOA | RPL10A | SETDB1 | SMAD2 | SMO | SOD1 | SPI1 | STAT3 | TBXA2R | TGFB1 | TGFBR3 | TUFM | TYMS | VCAM1 | GO:0001933 | 0.033968215455538 | 280/8243 | 142/3250 | 6.4363735667036e-05 | 0.611133670158507 | T | T | T | T | negative regulation of protein phosphorylation | ABL1 | ADORA2A | AKT1 | APC | APOE | ARRB1 | ARRB2 | BAX | BDKRB2 | C1QL4 | CALM1 | CALM2 | CALM3 | CASP3 | CAV1 | CBL | CBLB | CBLC | CCNB1 | CD109 | CDKN1A | CDKN2C | CEBPA | CHORDC1 | CHP1 | CHRNA9 | CRKL | CYLD | DBNDD2 | DDIT4 | DMD | DNAJA1 | DNAJC10 | DUSP1 | DUSP14 | DUSP16 | DUSP19 | DUSP22 | DUSP7 | DVL1 | EIF3A | ERRFI1 | EZR | FBXO7 | FGFR1OP | FKBP8 | FOXM1 | FOXO1 | GNAQ | GPD1L | GPS2 | GSTP1 | HDAC3 | HEXIM1 | HSPB1 | IBTK | IGBP1 | IGF1R | ILK | IPO5 | IPO7 | ITCH | ITGB1BP1 | JUN | LATS1 | LDLRAD4 | LEMD2 | LRP6 | LRRK1 | LRRK2 | MAS1 | MLLT1 | MYC | NCK1 | NCOR1 | NF2 | NNT | NPM1 | NTF3 | PAFAH1B1 | PAK2 | PARD3 | PARD6A | PARK7 | PARP14 | PDE4D | PEBP1 | PER1 | PHB | PIN1 | PKIG | PLK1 | PPARGC1A | PPP2CA | PPP2R1A | PPP2R5D | PRKAR1A | PRKAR2B | PRKCD | PRKCZ | PRKDC | PSEN1 | PSMD10 | PTEN | PTPN1 | PTPN22 | PTPN6 | PTPRR | QARS | RANBP9 | RB1 | RGN | RGS2 | RPTOR | SERPINB3 | SFN | SIRT1 | SIRT3 | SLC9A3R1 | SMAD4 | SNCA | SNX6 | SOCS1 | SPRY2 | STK38 | STRAP | SYNJ2BP | TAOK3 | TARDBP | TERF2IP | TGFB1 | TNFAIP3 | TRAF3IP1 | TRIB2 | TRIB3 | TSG101 | TWIST1 | UBE2B | UCHL1 | WARS | WNK1 | YWHAG | GO:0002768 | 0.031541914351571 | 260/8243 | 133/3250 | 6.53447784106644e-05 | 0.620448671009258 | T | T | T | T | immune response-regulating cell surface receptor signaling pathway | ABL1 | ACTB | ACTG1 | ACTR2 | ACTR3 | ARPC1A | ARPC1B | ARPC2 | ARPC3 | ARPC4 | ARPC5 | BAG6 | BAIAP2 | BAX | BCAR1 | BCL10 | BCL2 | BRAF | BTK | BTN2A1 | BTRC | CALM1 | CARD9 | CD209 | CD40 | CD79B | CDC42 | CREBBP | CRK | CRKL | CUL1 | EIF2B1 | EIF2B3 | EIF2B5 | EP300 | FBXW11 | FCGR1A | FCN1 | FOS | FYN | GRB2 | HLA-DQA1 | HLA-DRA | HLA-DRB1 | HLA-DRB3 | HLA-DRB4 | HLA-DRB5 | HRAS | HSP90AA1 | HSP90AB1 | IGHV3-13 | IGHV3-23 | IGHV3-30 | IGKV3-20 | IGLC7 | IKBKB | IKBKG | ITK | JUN | KRAS | LAT | LCP2 | MAP2K4 | MAP2K7 | MAP3K1 | MAPK1 | MAPK3 | MAPK8 | MAPK9 | MYO1C | NCK1 | NFATC1 | NFATC2 | PAG1 | PAK2 | PDE4D | PIK3CA | PIK3R1 | PLA2G6 | PLCG1 | PRKACA | PRKCD | PRKCE | PRKD2 | PSEN1 | PSMA1 | PSMA2 | PSMA3 | PSMA5 | PSMA6 | PSMB5 | PSMB6 | PSMB7 | PSMB8 | PSMC1 | PSMC2 | PSMC3 | PSMC4 | PSMC5 | PSMC6 | PSMD1 | PSMD10 | PSMD11 | PSMD12 | PSMD14 | PSMD2 | PSMD4 | PSMD9 | PSME2 | PSME3 | PTK2 | PTPN22 | PTPN6 | RAC1 | RAF1 | RC3H1 | RELA | RIPK2 | RNF31 | SHC1 | SPG21 | SRC | STK11 | STOML2 | TAB1 | TAB2 | TRAF6 | UBE2N | VAV1 | WAS | WNK1 | YES1 | ZAP70 | GO:1904358 | 0.0043673419871406 | 36/8243 | 26/3250 | 6.581299260394e-05 | 0.624894364774411 | T | T | T | T | positive regulation of telomere maintenance via telomere lengthening | ACD | ATM | AURKB | CCT2 | CCT3 | CCT4 | CCT5 | CCT6A | CCT7 | CCT8 | CTNNB1 | DHX36 | HNRNPA1 | HNRNPA2B1 | MAP2K7 | MAP3K4 | MAPK1 | MAPK15 | MAPK3 | NEK7 | PNKP | POT1 | TCP1 | TNKS | TNKS2 | XRCC5 | GO:0007346 | 0.0577459662744147 | 476/8243 | 228/3250 | 6.79631115478206e-05 | 0.645309744146557 | T | T | T | T | regulation of mitotic cell cycle | ABL1 | ACTR1A | AKAP9 | AKT1 | ANAPC4 | ANXA1 | APC | APEX1 | APP | ASCL1 | ASNS | ATAD5 | ATF2 | ATM | ATRX | AURKB | BAX | BCL2 | BCL2L1 | BCL6 | BRCA1 | BRD4 | BTRC | CARM1 | CASP2 | CCDC8 | CCNB1 | CCP110 | CDC14B | CDC16 | CDC20 | CDC25A | CDC25B | CDC25C | CDC27 | CDC73 | CDK2 | CDK4 | CDK9 | CDKN1A | CDKN2C | CENPF | CENPJ | CEP135 | CEP152 | CEP290 | CEP57 | CEP70 | CHEK2 | CHMP2A | CKAP5 | CKS1B | CKS2 | CLTC | CNTRL | CSNK1E | CTNNB1 | CUL1 | CUL3 | CUL4B | CUL7 | CYLD | CYP1A1 | DBF4B | DCTN1 | DDB1 | DDX3X | DUSP1 | DYNC1H1 | DYNC1I2 | DYNC1LI1 | DYNLL1 | E2F1 | E2F4 | ECD | EGFR | EIF4E | EIF4EBP1 | EP300 | EZH2 | FBXO7 | FBXW5 | FGFR1 | FGFR1OP | FHL1 | FOXA1 | FSD1 | FZR1 | GNAI1 | HECW2 | HMMR | HNRNPU | HRAS | HSF1 | HSP90AA1 | HSPA1B | HSPA2 | ID2 | IK | INSR | IQGAP1 | KANK2 | KIF11 | KLHL22 | L3MBTL1 | LGMN | MAD1L1 | MAD2L1 | MDC1 | MDM2 | MDM4 | MECP2 | MEPCE | MSH6 | MTA3 | NAE1 | NEK7 | NIPBL | NSFL1C | NUMA1 | NUSAP1 | OBSL1 | OFD1 | PAFAH1B1 | PCM1 | PCNA | PDCD6IP | PEBP1 | PHB2 | PIN1 | PINX1 | PKN2 | PLK1 | PML | POLDIP2 | PPP2R1A | PRCC | PRKACA | PRKAR2B | PRKDC | PRMT2 | PSMA1 | PSMA2 | PSMA3 | PSMA5 | PSMA6 | PSMB5 | PSMB6 | PSMB7 | PSMB8 | PSMC1 | PSMC2 | PSMC3 | PSMC4 | PSMC5 | PSMC6 | PSMD1 | PSMD10 | PSMD11 | PSMD12 | PSMD14 | PSMD2 | PSMD4 | PSMD9 | PSME2 | PSME3 | PTEN | PTPN6 | PTTG1 | RAB11A | RAD21 | RAE1 | RB1 | RBX1 | RCC1 | RDX | RIPK1 | RNASEH2B | RPA2 | RPA3 | RPL24 | RPTOR | SDCBP | SEPT7 | SFN | SH2B1 | SIN3A | SIRT1 | SLC9A3R1 | SMAD3 | SMC1A | SMC3 | SPDL1 | STAG2 | TAOK3 | TFDP1 | TGFB1 | TIMP2 | TMOD3 | TNF | TNKS | TOP2A | TP53 | TP73 | TPR | TRIAP1 | TRIM35 | TRIP13 | TUBA1A | TUBA4A | TUBB | TUBB4A | TUBB4B | TUBG1 | UBE2C | UBE2D1 | USP17L2 | USP2 | USP47 | VPS4B | XRCC3 | YWHAE | YWHAG | ZFYVE19 | ZMPSTE24 | ZNF207 | ZW10 | ZWINT | GO:0002429 | 0.0286303530268106 | 236/8243 | 122/3250 | 7.17172006663322e-05 | 0.680954820326825 | T | T | T | T | immune response-activating cell surface receptor signaling pathway | ABL1 | ACTB | ACTG1 | ACTR2 | ACTR3 | ARPC1A | ARPC1B | ARPC2 | ARPC3 | ARPC4 | ARPC5 | BAG6 | BAIAP2 | BAX | BCAR1 | BCL10 | BCL2 | BRAF | BTK | BTN2A1 | BTRC | CARD9 | CD209 | CD79B | CDC42 | CREBBP | CRK | CRKL | CUL1 | EIF2B1 | EIF2B3 | EIF2B5 | EP300 | FBXW11 | FCGR1A | FCN1 | FYN | GRB2 | HLA-DQA1 | HLA-DRA | HLA-DRB1 | HLA-DRB3 | HLA-DRB4 | HLA-DRB5 | HRAS | HSP90AA1 | HSP90AB1 | IGHV3-13 | IGHV3-23 | IGHV3-30 | IGKV3-20 | IGLC7 | IKBKB | IKBKG | ITK | KRAS | LAT | LCP2 | MAPK1 | MAPK3 | MYO1C | NCK1 | NFATC2 | PAG1 | PAK2 | PDE4D | PIK3CA | PIK3R1 | PLA2G6 | PLCG1 | PRKACA | PRKCD | PRKCE | PRKD2 | PSEN1 | PSMA1 | PSMA2 | PSMA3 | PSMA5 | PSMA6 | PSMB5 | PSMB6 | PSMB7 | PSMB8 | PSMC1 | PSMC2 | PSMC3 | PSMC4 | PSMC5 | PSMC6 | PSMD1 | PSMD10 | PSMD11 | PSMD12 | PSMD14 | PSMD2 | PSMD4 | PSMD9 | PSME2 | PSME3 | PTK2 | PTPN22 | PTPN6 | RAC1 | RAF1 | RC3H1 | RELA | RIPK2 | RNF31 | SPG21 | SRC | STK11 | STOML2 | TAB1 | TAB2 | TRAF6 | UBE2N | VAV1 | WAS | WNK1 | YES1 | ZAP70 | GO:0016053 | 0.0180759432245542 | 149/8243 | 82/3250 | 7.23465534875813e-05 | 0.686930525364584 | T | T | T | T | organic acid biosynthetic process | AASDHPPT | AASS | ABAT | ABCD3 | ACACA | ACCS | ACLY | ACOT7 | ACSS2 | AKR1A1 | ALDH8A1 | ALOX12 | ALOX5 | ALOX5AP | ASL | ASNS | ASS1 | BCAT2 | BRCA1 | CBR4 | CBS | CD74 | CTH | CYP1A1 | CYP1A2 | DEGS1 | ENOPH1 | FASN | FOLH1 | GGT1 | GLS | GLS2 | GLUD2 | GLUL | GOT1 | GOT1L1 | GOT2 | GPT2 | GPX4 | GSTM4 | HAAO | HSD17B4 | HSD17B8 | HSD3B7 | KYNU | LDHC | LTC4S | MCAT | MGST1 | MIF | MTAP | MTHFD1 | MTRR | MYO5A | NANP | OAT | OSBPL1A | OTC | PAH | PARK7 | PER2 | PHGDH | PLA2G1B | PLOD2 | PRKAA1 | PRKAA2 | PRKAB2 | PRKAG1 | PSAT1 | PTGDS | PTGES | PTGES3 | PYCR1 | PYCR2 | RBP1 | RGN | SEPHS1 | SEPHS2 | SHMT1 | SHMT2 | TECR | XBP1 | GO:0006793 | 0.13089894455902 | 1079/8243 | 483/3250 | 7.4148264429887e-05 | 0.704037770761777 | T | T | T | T | phosphorus metabolic process | ABHD14B | ABL1 | ABL2 | ACACA | ACAT1 | ACLY | ACOT13 | ACOT7 | ACOT9 | ACP1 | ACTN2 | ADCK2 | ADCYAP1 | ADK | ADSS | AGK | AK1 | AK2 | AK4 | AKT1 | AKT2 | AKT3 | ALDH5A1 | ALDOA | ALDOC | APEX1 | APOA1 | APP | ARAF | ATM | ATP13A2 | ATP1A1 | ATP6V1A | ATP6V1B1 | AURKB | BCL2 | BIRC5 | BMPR1A | BRAF | BRD4 | BTK | BTRC | CAB39 | CALM1 | CAMK1D | CAMK2A | CAMK2B | CAMKV | CCNB1 | CCNT1 | CCR5 | CDC14B | CDC25A | CDC25B | CDC25C | CDK15 | CDK16 | CDK19 | CDK2 | CDK4 | CDK5 | CDK5R1 | CDK8 | CDK9 | CDKL3 | CFL1 | CHCHD10 | CHEK2 | CILP2 | CKB | CKM | CKMT1A | CLK1 | CLPX | CMPK1 | COL4A3BP | COPS2 | CPNE3 | CREB1 | CRKL | CROT | CRY1 | CRYAB | CSNK1A1 | CSNK1E | CSNK2A2 | CSNK2B | CTBP1 | CTDP1 | CUL1 | CUL3 | CYC1 | CYCS | DAPK1 | DAPP1 | DBI | DCAKD | DCK | DCLK1 | DCXR | DGKE | DGKZ | DGUOK | DLG2 | DLG3 | DLG4 | DLST | DUSP1 | DUSP12 | DUSP13 | DUSP14 | DUSP16 | DUSP19 | DUSP22 | DUSP7 | DYRK2 | EFEMP1 | EGFR | EIF2AK3 | EIF2AK4 | EIF2S1 | ENOPH1 | EPHA4 | EPHA6 | EPHB6 | ERBB2 | ERBB3 | ERG | EYA2 | EYA4 | FAM20B | FAR2 | FASN | FBP1 | FBP2 | FBXW11 | FGF2 | FGFR1 | FGFR3 | FIGNL1 | FYN | G6PD | GAK | GFPT1 | GFPT2 | GMPR | GNAI3 | GPD1 | GPD1L | GPI | GPS2 | GPX4 | GRB2 | GRK1 | GRK5 | GSK3A | GSK3B | HBEGF | HIPK4 | HK1 | HK2 | HMGCL | HPRT1 | HRAS | HSD17B4 | HSF1 | HSPA1B | HSPA8 | ICK | IDH1 | IDH2 | IDI2 | IGF1R | IKBKB | IKBKE | IKBKG | IL3RA | ILF3 | ILK | IMPA1 | IMPA2 | INSR | IP6K1 | ISYNA1 | ITGAV | ITK | JAK1 | JAK3 | KBTBD7 | KCNAB2 | KDR | KRAS | KSR1 | LAT | LATS1 | LDHA | LPIN3 | LRRC7 | LRRK1 | LRRK2 | MAP2K1 | MAP2K3 | MAP2K4 | MAP2K6 | MAP2K7 | MAP3K1 | MAP3K13 | MAP3K14 | MAP3K2 | MAP3K3 | MAP3K4 | MAP4K1 | MAPK1 | MAPK14 | MAPK15 | MAPK3 | MAPK4 | MAPK6 | MAPK7 | MAPK8 | MAPK9 | MAST1 | MAST3 | MATK | MBP | MDH1 | MDH2 | MECP2 | MED1 | MELK | MKNK2 | MOCOS | MTMR14 | MTMR7 | MVD | MYC | MYH6 | NAGK | NANP | NCK1 | NDUFS1 | NEK1 | NEK7 | NKX2-1 | NLK | NME1 | NME2 | NME4 | NMNAT1 | NMNAT3 | NMRK2 | NNT | NOD2 | NPR1 | NT5C2 | NT5DC2 | NTRK1 | NUDT3 | OGDH | OLA1 | OPA1 | OXSR1 | PAFAH1B1 | PAK2 | PAK6 | PAPSS1 | PAPSS2 | PARK7 | PARP1 | PCSK9 | PDK2 | PDXK | PEBP1 | PFKL | PFKM | PFKP | PGAM5 | PGK1 | PGK2 | PGM3 | PGP | PHKA1 | PHOSPHO1 | PI4K2A | PICK1 | PIK3C2A | PIK3CA | PIK3CG | PIK3R1 | PIK3R3 | PIKFYVE | PIM1 | PIP4K2A | PIPSL | PKN2 | PLA2G10 | PLA2G12A | PLA2G1B | PLA2G2A | PLA2G6 | PLCG1 | PLK1 | PML | PNKP | PPA1 | PPA2 | PPEF1 | PPM1A | PPM1B | PPM1D | PPM1H | PPM1K | PPP1CA | PPP1CB | PPP1CC | PPP1R12A | PPP1R3B | PPP1R3C | PPP2CA | PPP2CB | PPP2R1A | PPP2R1B | PPP2R2A | PPP2R2D | PPP2R5D | PPP3CC | PPP4R2 | PPP5C | PPP6C | PPTC7 | PRDX4 | PRKAA1 | PRKAA2 | PRKAB2 | PRKACA | PRKAG1 | PRKCD | PRKCE | PRKCZ | PRKD1 | PRKD2 | PRKD3 | PRKDC | PSMA1 | PSMA2 | PSMA3 | PSMA5 | PSMA6 | PSMB5 | PSMB6 | PSMB7 | PSMB8 | PSMC1 | PSMC2 | PSMC3 | PSMC4 | PSMC5 | PSMC6 | PSMD1 | PSMD10 | PSMD11 | PSMD12 | PSMD14 | PSMD2 | PSMD4 | PSMD9 | PSME2 | PSME3 | PTEN | PTK2 | PTPMT1 | PTPN1 | PTPN14 | PTPN21 | PTPN22 | PTPN23 | PTPN4 | PTPN6 | PTPRA | PTPRD | PTPRF | PTPRK | PTPRO | PTPRR | RAF1 | RAN | RANBP9 | RARA | RBX1 | REXO2 | RFK | RHO | RHOA | RIOK1 | RIPK1 | RIPK2 | RIPK3 | RIPK4 | RNASEH2B | RPS6KA1 | RPS6KA2 | RPS6KB2 | RRM2B | SCG2 | SCYL2 | SEPHS1 | SEPHS2 | SHC1 | SMAD2 | SMAD5 | SNCA | SOCS1 | SOCS2 | SOCS6 | SPTAN1 | SPTBN1 | SPTBN2 | SQSTM1 | SRC | SRPK1 | SRPK2 | ST3GAL1 | STAT3 | STK11 | STK24 | STK25 | STK26 | STK3 | STK38 | STK40 | STOML2 | STYX | SUCLA2 | SUCLG2 | SULT1A3 | TAB1 | TAB2 | TALDO1 | TAOK3 | TAZ | TBRG4 | TESK2 | TGFB1 | TGFBR3 | THEM4 | TIMM50 | TK1 | TK2 | TLR3 | TNF | TNIP2 | TNK2 | TNKS | TOLLIP | TOP1 | TP53RK | TPST2 | TPTE | TRAF6 | TRIB2 | TRIB3 | TRIM28 | TWF1 | TYMS | UBASH3A | UBB | UBC | UBE2N | UBE2V1 | UCK2 | UGP2 | ULK1 | UQCRB | UQCRC2 | USP15 | VCP | WNK1 | WNK4 | YES1 | YWHAB | YWHAE | ZAP70 | ZMPSTE24 | GO:0006796 | 0.13089894455902 | 1079/8243 | 483/3250 | 7.4148264429887e-05 | 0.704037770761777 | T | T | T | T | phosphate-containing compound metabolic process | ABHD14B | ABL1 | ABL2 | ACACA | ACAT1 | ACLY | ACOT13 | ACOT7 | ACOT9 | ACP1 | ACTN2 | ADCK2 | ADCYAP1 | ADK | ADSS | AGK | AK1 | AK2 | AK4 | AKT1 | AKT2 | AKT3 | ALDH5A1 | ALDOA | ALDOC | APEX1 | APOA1 | APP | ARAF | ATM | ATP13A2 | ATP1A1 | ATP6V1A | ATP6V1B1 | AURKB | BCL2 | BIRC5 | BMPR1A | BRAF | BRD4 | BTK | BTRC | CAB39 | CALM1 | CAMK1D | CAMK2A | CAMK2B | CAMKV | CCNB1 | CCNT1 | CCR5 | CDC14B | CDC25A | CDC25B | CDC25C | CDK15 | CDK16 | CDK19 | CDK2 | CDK4 | CDK5 | CDK5R1 | CDK8 | CDK9 | CDKL3 | CFL1 | CHCHD10 | CHEK2 | CILP2 | CKB | CKM | CKMT1A | CLK1 | CLPX | CMPK1 | COL4A3BP | COPS2 | CPNE3 | CREB1 | CRKL | CROT | CRY1 | CRYAB | CSNK1A1 | CSNK1E | CSNK2A2 | CSNK2B | CTBP1 | CTDP1 | CUL1 | CUL3 | CYC1 | CYCS | DAPK1 | DAPP1 | DBI | DCAKD | DCK | DCLK1 | DCXR | DGKE | DGKZ | DGUOK | DLG2 | DLG3 | DLG4 | DLST | DUSP1 | DUSP12 | DUSP13 | DUSP14 | DUSP16 | DUSP19 | DUSP22 | DUSP7 | DYRK2 | EFEMP1 | EGFR | EIF2AK3 | EIF2AK4 | EIF2S1 | ENOPH1 | EPHA4 | EPHA6 | EPHB6 | ERBB2 | ERBB3 | ERG | EYA2 | EYA4 | FAM20B | FAR2 | FASN | FBP1 | FBP2 | FBXW11 | FGF2 | FGFR1 | FGFR3 | FIGNL1 | FYN | G6PD | GAK | GFPT1 | GFPT2 | GMPR | GNAI3 | GPD1 | GPD1L | GPI | GPS2 | GPX4 | GRB2 | GRK1 | GRK5 | GSK3A | GSK3B | HBEGF | HIPK4 | HK1 | HK2 | HMGCL | HPRT1 | HRAS | HSD17B4 | HSF1 | HSPA1B | HSPA8 | ICK | IDH1 | IDH2 | IDI2 | IGF1R | IKBKB | IKBKE | IKBKG | IL3RA | ILF3 | ILK | IMPA1 | IMPA2 | INSR | IP6K1 | ISYNA1 | ITGAV | ITK | JAK1 | JAK3 | KBTBD7 | KCNAB2 | KDR | KRAS | KSR1 | LAT | LATS1 | LDHA | LPIN3 | LRRC7 | LRRK1 | LRRK2 | MAP2K1 | MAP2K3 | MAP2K4 | MAP2K6 | MAP2K7 | MAP3K1 | MAP3K13 | MAP3K14 | MAP3K2 | MAP3K3 | MAP3K4 | MAP4K1 | MAPK1 | MAPK14 | MAPK15 | MAPK3 | MAPK4 | MAPK6 | MAPK7 | MAPK8 | MAPK9 | MAST1 | MAST3 | MATK | MBP | MDH1 | MDH2 | MECP2 | MED1 | MELK | MKNK2 | MOCOS | MTMR14 | MTMR7 | MVD | MYC | MYH6 | NAGK | NANP | NCK1 | NDUFS1 | NEK1 | NEK7 | NKX2-1 | NLK | NME1 | NME2 | NME4 | NMNAT1 | NMNAT3 | NMRK2 | NNT | NOD2 | NPR1 | NT5C2 | NT5DC2 | NTRK1 | NUDT3 | OGDH | OLA1 | OPA1 | OXSR1 | PAFAH1B1 | PAK2 | PAK6 | PAPSS1 | PAPSS2 | PARK7 | PARP1 | PCSK9 | PDK2 | PDXK | PEBP1 | PFKL | PFKM | PFKP | PGAM5 | PGK1 | PGK2 | PGM3 | PGP | PHKA1 | PHOSPHO1 | PI4K2A | PICK1 | PIK3C2A | PIK3CA | PIK3CG | PIK3R1 | PIK3R3 | PIKFYVE | PIM1 | PIP4K2A | PIPSL | PKN2 | PLA2G10 | PLA2G12A | PLA2G1B | PLA2G2A | PLA2G6 | PLCG1 | PLK1 | PML | PNKP | PPA1 | PPA2 | PPEF1 | PPM1A | PPM1B | PPM1D | PPM1H | PPM1K | PPP1CA | PPP1CB | PPP1CC | PPP1R12A | PPP1R3B | PPP1R3C | PPP2CA | PPP2CB | PPP2R1A | PPP2R1B | PPP2R2A | PPP2R2D | PPP2R5D | PPP3CC | PPP4R2 | PPP5C | PPP6C | PPTC7 | PRDX4 | PRKAA1 | PRKAA2 | PRKAB2 | PRKACA | PRKAG1 | PRKCD | PRKCE | PRKCZ | PRKD1 | PRKD2 | PRKD3 | PRKDC | PSMA1 | PSMA2 | PSMA3 | PSMA5 | PSMA6 | PSMB5 | PSMB6 | PSMB7 | PSMB8 | PSMC1 | PSMC2 | PSMC3 | PSMC4 | PSMC5 | PSMC6 | PSMD1 | PSMD10 | PSMD11 | PSMD12 | PSMD14 | PSMD2 | PSMD4 | PSMD9 | PSME2 | PSME3 | PTEN | PTK2 | PTPMT1 | PTPN1 | PTPN14 | PTPN21 | PTPN22 | PTPN23 | PTPN4 | PTPN6 | PTPRA | PTPRD | PTPRF | PTPRK | PTPRO | PTPRR | RAF1 | RAN | RANBP9 | RARA | RBX1 | REXO2 | RFK | RHO | RHOA | RIOK1 | RIPK1 | RIPK2 | RIPK3 | RIPK4 | RNASEH2B | RPS6KA1 | RPS6KA2 | RPS6KB2 | RRM2B | SCG2 | SCYL2 | SEPHS1 | SEPHS2 | SHC1 | SMAD2 | SMAD5 | SNCA | SOCS1 | SOCS2 | SOCS6 | SPTAN1 | SPTBN1 | SPTBN2 | SQSTM1 | SRC | SRPK1 | SRPK2 | ST3GAL1 | STAT3 | STK11 | STK24 | STK25 | STK26 | STK3 | STK38 | STK40 | STOML2 | STYX | SUCLA2 | SUCLG2 | SULT1A3 | TAB1 | TAB2 | TALDO1 | TAOK3 | TAZ | TBRG4 | TESK2 | TGFB1 | TGFBR3 | THEM4 | TIMM50 | TK1 | TK2 | TLR3 | TNF | TNIP2 | TNK2 | TNKS | TOLLIP | TOP1 | TP53RK | TPST2 | TPTE | TRAF6 | TRIB2 | TRIB3 | TRIM28 | TWF1 | TYMS | UBASH3A | UBB | UBC | UBE2N | UBE2V1 | UCK2 | UGP2 | ULK1 | UQCRB | UQCRC2 | USP15 | VCP | WNK1 | WNK4 | YES1 | YWHAB | YWHAE | ZAP70 | ZMPSTE24 | GO:0006073 | 0.0046099720975373 | 38/8243 | 27/3250 | 7.5697500697343e-05 | 0.718747769121271 | T | T | T | T | cellular glucan metabolic process | AGL | AKT1 | AKT2 | CALM1 | GAA | GBE1 | GSK3A | GSK3B | GYG1 | GYG2 | GYS1 | PER2 | PFKM | PGM1 | PGM2 | PHKA1 | PPP1CA | PPP1CB | PPP1CC | PPP1R2 | PPP1R3B | PPP1R3C | PYGB | PYGL | STBD1 | STK40 | UGP2 | GO:0005977 | 0.0046099720975373 | 38/8243 | 27/3250 | 7.5697500697343e-05 | 0.718747769121271 | T | T | T | T | glycogen metabolic process | AGL | AKT1 | AKT2 | CALM1 | GAA | GBE1 | GSK3A | GSK3B | GYG1 | GYG2 | GYS1 | PER2 | PFKM | PGM1 | PGM2 | PHKA1 | PPP1CA | PPP1CB | PPP1CC | PPP1R2 | PPP1R3B | PPP1R3C | PYGB | PYGL | STBD1 | STK40 | UGP2 | GO:0044042 | 0.0046099720975373 | 38/8243 | 27/3250 | 7.5697500697343e-05 | 0.718747769121271 | T | T | T | T | glucan metabolic process | AGL | AKT1 | AKT2 | CALM1 | GAA | GBE1 | GSK3A | GSK3B | GYG1 | GYG2 | GYS1 | PER2 | PFKM | PGM1 | PGM2 | PHKA1 | PPP1CA | PPP1CB | PPP1CC | PPP1R2 | PPP1R3B | PPP1R3C | PYGB | PYGL | STBD1 | STK40 | UGP2 | GO:0045055 | 0.0542278296736625 | 447/8243 | 215/3250 | 8.00718578897917e-05 | 0.760282290663572 | T | T | T | T | regulated exocytosis | A2M | ACAA1 | ACLY | ACTN1 | ACTN2 | ACTN4 | ACTR2 | AGL | AHSG | ALB | ALDOA | ALDOC | ALOX5 | ANXA2 | ANXA5 | AP1M1 | APEH | APOA1 | APP | APRT | ARMC8 | ARPC5 | ATP6V1D | AZU1 | CAB39 | CALM1 | CAND1 | CAPN1 | CAT | CBL | CCT2 | CCT8 | CD109 | CD36 | CDA | CDK5 | CEP290 | CHP1 | CKAP4 | CLU | COPB1 | COTL1 | CPLX1 | CPLX3 | CPNE3 | CSNK2B | CTSA | CTSC | CTSD | CYB5R1 | CYB5R3 | DBNL | DDOST | DDX3X | DEGS1 | DERA | DYNC1H1 | DYNC1LI1 | DYNLL1 | DYNLT1 | EEF1A1 | EEF2 | F5 | FABP5 | FAF2 | FAM3C | FAM49B | FCN1 | FGB | FGL2 | FN1 | FTH1 | FTL | GAA | GALNS | GDI2 | GHDC | GLA | GNS | GPI | GSTP1 | GUSB | GYG1 | HEXB | HMGB1 | HRNR | HSP90AA1 | HSP90AB1 | HSPA1B | HSPA8 | HUWE1 | IDH1 | ILF2 | IMPDH2 | IQGAP1 | ISLR | ITGAV | ITGB3 | JUP | KCMF1 | KCNAB2 | KPNB1 | LAT | LGALS3 | LGALS3BP | LRRC7 | MAN2B1 | MAPK1 | MAPK14 | MGST1 | MIF | MME | MMRN1 | NAPRT | NHLRC3 | NIT2 | NME2 | OLA1 | ORM1 | P2RX1 | PA2G4 | PDXK | PFKL | PGAM1 | PGM1 | PGM2 | PGRMC1 | PI4K2A | PIK3CG | PKM | PLAUR | PNP | PPIA | PPIE | PRCP | PRDX4 | PRDX6 | PRG2 | PRKCD | PSAP | PSEN1 | PSMA2 | PSMA5 | PSMB7 | PSMC2 | PSMC3 | PSMD1 | PSMD11 | PSMD12 | PSMD14 | PSMD2 | PTGES2 | PTPN6 | PYGB | PYGL | QSOX1 | RAB10 | RAB11B | RAB11FIP2 | RAB18 | RAB24 | RAB31 | RAB5B | RAB7A | RAC1 | RAP1A | RAP1B | RAPGEF4 | RHOA | RNASE3 | S100A11 | S100A12 | S100A13 | S100A7 | S100A9 | S100P | SCCPDH | SCG3 | SDCBP | SELL | SERPINB3 | SERPINB6 | SLC2A5 | SNAP29 | SNCA | SOD1 | SPTAN1 | SRP14 | STBD1 | STOM | STXBP1 | STXBP2 | TAGLN2 | TCIRG1 | TEX264 | TGFB1 | THBS1 | TIMP1 | TIMP2 | TMEM173 | TMEM30A | TOLLIP | TRIM9 | TUBA4A | TUBB | TUBB4B | TXNDC5 | UBR4 | VAPA | VAT1 | VCL | VCP | WDR1 | XRCC5 | XRCC6 | GO:0034766 | 0.00667232803590926 | 55/8243 | 36/3250 | 8.06794223157235e-05 | 0.766051114887794 | T | T | T | T | negative regulation of ion transmembrane transport | ACTN2 | AKT1 | AKT2 | ANK3 | BIN1 | CAB39 | CALM1 | CALM2 | CALM3 | CAV1 | COMMD1 | CRBN | FKBP1A | FKBP1B | GOPC | GSTO1 | HECW1 | HECW2 | KCNAB1 | NEDD4 | OXSR1 | PCSK9 | PPIF | PRKCE | PTEN | RGS2 | SLC9A3R1 | SRI | SUMO1 | TGFB1 | THADA | THBS1 | TWIST1 | UBQLN1 | YWHAE | YWHAQ | GO:0035966 | 0.00667232803590926 | 55/8243 | 36/3250 | 8.06794223157235e-05 | 0.766051114887794 | T | T | T | T | response to topologically incorrect protein | BAG3 | BAX | CLU | DAXX | DERL1 | DNAJA1 | DNAJB1 | DNAJB2 | FAF2 | FBXO6 | HDAC6 | HSF1 | HSP90AA1 | HSP90AB1 | HSPA14 | HSPA1B | HSPA1L | HSPA2 | HSPA4 | HSPA4L | HSPA5 | HSPA8 | HSPA9 | HSPB1 | HSPB2 | HSPD1 | HSPE1 | HSPH1 | OPTN | PTPN1 | RHBDD1 | SERPINH1 | STUB1 | THBS1 | TMBIM6 | UBE2W | GO:0022898 | 0.0173480528933641 | 143/8243 | 79/3250 | 8.19117653486452e-05 | 0.777752211985386 | T | T | T | T | regulation of transmembrane transporter activity | ACTB | ACTN2 | ACTN4 | ADRB2 | AHCYL1 | AHNAK | AKAP9 | ANK3 | APP | ATP1B3 | BCL2 | CAB39 | CACNG2 | CACNG4 | CACNG5 | CALM1 | CALM2 | CALM3 | CAPN1 | CAV1 | CFTR | CHCHD10 | CHP1 | COX17 | DAPK1 | DLG2 | DLG3 | DLG4 | DMD | FHL1 | FKBP1A | FKBP1B | GLRX | GOPC | GPD1L | GRIA1 | GSTO1 | HAP1 | HECW1 | HECW2 | HSPA2 | HTR3A | HTT | KCNAB1 | KCNIP2 | KIF5B | LRRC7 | MYO5A | NEDD4 | NOS1AP | OXSR1 | PARK7 | PCSK9 | PDE4D | PLA2G6 | PPARGC1A | PPIF | PRKACA | PRKCE | PTEN | RGN | RIPK1 | S100A1 | SLC9A1 | SLC9A3R1 | SNCA | SRI | STAC | STIM1 | STOM | SUMO1 | THADA | TWIST1 | UBQLN1 | WNK1 | WNK4 | WWP2 | YWHAE | YWHAH | GO:0043620 | 0.0115249302438433 | 95/8243 | 56/3250 | 8.57345029076758e-05 | 0.814049105108382 | T | T | T | T | regulation of DNA-templated transcription in response to stress | ARNT | ATF6 | ATF6B | BAG3 | CREBBP | CUL2 | DNAJB1 | EGLN1 | EGLN3 | EP300 | HIF1A | HIF1AN | HMOX1 | HSF1 | HSF4 | HSPA5 | JUN | MAPK7 | NCK1 | NEDD4 | NFE2L2 | PSMA1 | PSMA2 | PSMA3 | PSMA5 | PSMA6 | PSMB5 | PSMB6 | PSMB7 | PSMB8 | PSMC1 | PSMC2 | PSMC3 | PSMC4 | PSMC5 | PSMC6 | PSMD1 | PSMD10 | PSMD11 | PSMD12 | PSMD14 | PSMD2 | PSMD4 | PSMD9 | PSME2 | PSME3 | RBPJ | RBX1 | RELA | RPS6KA1 | SIN3A | TMBIM6 | TP53 | UBB | UBC | VHL | GO:0050792 | 0.0189251486109426 | 156/8243 | 85/3250 | 8.65089747548329e-05 | 0.821402715297138 | T | T | T | T | regulation of viral process | AICDA | ANXA2 | APCS | APOE | BANF1 | BCL2 | CCNT1 | CD74 | CDK9 | CFL1 | CHMP2A | CTDP1 | CXCL8 | DDB1 | DDX3X | DHX9 | EIF2AK4 | EP300 | FCN1 | GTF2F1 | GTF2F2 | HDAC1 | IFI16 | IFIT1 | IFITM3 | IFNA2 | ILF3 | ISG15 | JUN | LAMP3 | LEF1 | LGALS1 | MAVS | NELFB | NELFCD | NELFE | NR5A2 | P4HB | PABPC1 | PC | PDE12 | PKN2 | PLSCR1 | PML | POLR2A | POLR2E | POLR2L | POU2F3 | PPIA | PPIB | PPID | PPIE | PROX1 | PSMC3 | RAB7A | RAD23A | REST | SETDB1 | SMARCA4 | SMARCB1 | SMC3 | SNF8 | SNW1 | SNX3 | SP1 | SRPK1 | SRPK2 | STAT1 | STAU1 | STOM | SUPT4H1 | SUPT5H | TARDBP | TNF | TOP2A | TRIM14 | TRIM21 | TRIM25 | TRIM28 | TRIM32 | TRIM35 | TSG101 | VAPA | VAPB | VPS4B | GO:0051338 | 0.0780055804925391 | 643/8243 | 299/3250 | 8.72866176741544e-05 | 0.828786434816096 | T | T | T | T | regulation of transferase activity | ABL1 | ACD | ACSL1 | ACTB | ADCY6 | ADCYAP1 | ADORA2A | ADRB2 | AGAP2 | AGER | AHSG | AKT1 | APC | APOE | APP | ARRB1 | ATM | AURKB | AXIN1 | AZU1 | BAG2 | BAG5 | BCL10 | BIRC7 | BMI1 | BRAF | CAB39 | CALM1 | CALM2 | CALM3 | CASP3 | CAV1 | CBL | CBLB | CBLC | CCDC88A | CCL21 | CCNB1 | CCNT1 | CCNT2 | CCNY | CCNYL1 | CCT2 | CCT4 | CD40 | CD40LG | CD74 | CD81 | CDC14B | CDC20 | CDC25A | CDC25B | CDC25C | CDC37 | CDK4 | CDK5 | CDK5R1 | CDKN1A | CDKN2C | CEBPA | CHORDC1 | CHP1 | CKS1B | CKS2 | CLU | COX11 | CRK | CRKL | CSNK2B | CTNNB1 | DAB1 | DAXX | DBF4B | DBNDD2 | DBNL | DCUN1D1 | DDX3X | DGKZ | DLG3 | DLG4 | DNAJA1 | DUSP1 | DUSP12 | DUSP14 | DUSP16 | DUSP19 | DUSP22 | DUSP7 | DVL1 | DVL2 | DVL3 | EEF1A2 | EGFR | EPHA4 | ERBB2 | ERBB3 | ERRFI1 | EZH2 | FAF1 | FBXO7 | FGF2 | FGFR1 | FGFR1OP | FGFR3 | FYN | FZR1 | GADD45G | GNAQ | GOLGA2 | GPS2 | GSK3A | GSK3B | GSTP1 | HEXIM1 | HGS | HNRNPA2B1 | HNRNPD | HNRNPU | HRAS | HSP90AA1 | HSP90AB1 | HSPB1 | HTT | IBTK | IGF1R | IKBKG | ILK | INSR | IPO5 | IPO7 | IQGAP1 | ITGB1BP1 | ITGB3 | KRAS | KSR1 | LAT | LATS1 | LCP2 | LPAR1 | LRP6 | LRRK2 | MAD2L1 | MAP2K1 | MAP2K3 | MAP2K4 | MAP2K6 | MAP2K7 | MAP3K1 | MAP3K13 | MAP3K14 | MAP3K2 | MAP3K3 | MAP3K4 | MAP4K1 | MAPK1 | MAPK14 | MAPK15 | MAPK3 | MAPK8IP3 | MAPKAP1 | MAPT | MAS1 | MIF | MLLT1 | MLST8 | MOB1B | MYC | NCK1 | NEK1 | NEK7 | NF2 | NOD2 | NPM1 | NRBF2 | NTF3 | NTRK1 | OXSR1 | PAK2 | PAK6 | PARK7 | PDCD10 | PDE6G | PIH1D1 | PIK3CA | PIK3CG | PIK3R1 | PIK3R3 | PIN1 | PINX1 | PKIG | PLA2G1B | PLK1 | PNKP | POT1 | PPARG | PPP2CA | PPP2R1A | PRDX2 | PRDX3 | PRKAA1 | PRKACA | PRKAG1 | PRKAR1A | PRKAR2B | PRKCD | PRKCZ | PRKD1 | PROX1 | PRRC1 | PSEN1 | PSMD10 | PTEN | PTGES3 | PTK2 | PTPN1 | PTPN22 | PTPN6 | QARS | RAB1A | RAF1 | RAP1A | RB1 | RFC2 | RFC3 | RFC4 | RFC5 | RGN | RGS2 | RHOA | RIPK1 | RIPK2 | RIPK3 | RPL23 | RPLP1 | RPS2 | RPS3 | RPTOR | S100A12 | SDC4 | SERPINB3 | SFN | SHC1 | SIRT1 | SLAMF8 | SLC9A3R1 | SMG6 | SMG8 | SNCA | SNF8 | SNX6 | SOCS1 | SOCS2 | SOCS6 | SOD1 | SPRY2 | SRC | STK11 | STK24 | STK25 | STK26 | STK3 | STK38 | STUB1 | TAB1 | TAB2 | TAOK3 | TCP1 | TERF1 | TGFB1 | THBS1 | TLR3 | TNF | TNFAIP3 | TNKS | TP53 | TP73 | TRAF2 | TRAF6 | TRIB2 | TRIB3 | TSG101 | TSPYL2 | TXN | UBB | UBC | UBE2C | UBE2I | UBE2L3 | UBE2N | UBE2S | UBE2V1 | UCHL1 | UNC119 | WARS | WNK1 | XRCC5 | XRCC6 | YWHAG | ZNF622 | GO:0006048 | 0.0012131505519835 | 10/8243 | 10/3250 | 9.00188037712991e-05 | 0.854728541808485 | T | T | T | T | UDP-N-acetylglucosamine biosynthetic process | AMDHD2 | GFPT1 | GFPT2 | GNPDA1 | GNPDA2 | GNPNAT1 | NAGK | PGM3 | UAP1 | UAP1L1 | GO:0006085 | 0.0012131505519835 | 10/8243 | 10/3250 | 9.00188037712991e-05 | 0.854728541808485 | T | T | T | T | acetyl-CoA biosynthetic process | ACAT1 | ACLY | ACSS2 | DLAT | DLD | MPC2 | PDHA1 | PDHA2 | PDHB | PDHX | GO:0051050 | 0.0686643212422662 | 566/8243 | 266/3250 | 9.0269638902771e-05 | 0.857110221381811 | T | T | T | T | positive regulation of transport | ABAT | ABCG1 | ABL1 | ACE2 | ACTB | ACTN2 | ACTN4 | ADCYAP1 | ADORA2A | ADRB2 | AGER | AHCYL1 | AHI1 | AHSG | AKAP9 | AKT1 | AKT2 | ANK3 | ANXA1 | ANXA2 | AP1G1 | APBB1 | APOA1 | APOE | ARF1 | ARHGEF7 | ARRB1 | ARRB2 | ATAD1 | ATG5 | ATP13A2 | ATP1B3 | AZU1 | B3GAT3 | BAG3 | BAX | BBC3 | BCL2 | BRAF | C1QTNF1 | C4A | CACNG2 | CACNG4 | CALM1 | CALM2 | CALM3 | CALR | CAMK1D | CAMK2A | CASP8 | CAV1 | CBL | CCL21 | CCR1 | CD2 | CD36 | CDH1 | CDK5 | CDK5R1 | CEP290 | CFTR | CHCHD10 | CHMP2A | CHP1 | CLU | COX17 | CREB1 | CSNK2A2 | CXCL11 | DAB2 | DHX36 | DHX9 | DMAP1 | DMD | DNM1L | DYNC1H1 | DYNLL1 | E2F1 | EDEM1 | EGFR | ERBB2 | EZR | FABP3 | FCN1 | FGB | FGFR1 | FHL1 | FIS1 | FLOT1 | FYN | G6PD | GAPDH | GLMN | GLRX | GLUL | GNAI2 | GOLPH3 | GPD1L | GPI | GPS2 | GSK3A | GSK3B | GSTO1 | HAP1 | HAVCR2 | HDAC3 | HGS | HIF1A | HIST1H1A | HK1 | HMGB1 | HNRNPK | HRAS | HSPA1L | HSPA2 | HTR3A | HTRA2 | HTT | HUWE1 | INSR | IPO5 | ITGAV | ITGB1BP1 | JUP | KCNIP2 | KHDRBS1 | KIF5B | KMT2A | LGALS3 | LRRK2 | MAP2K6 | MAPK1 | MAPK14 | MAPK3 | MAPK8 | MAVS | MBP | MDM2 | MIB1 | MIF | MPC2 | MSN | MYO1C | NEDD4 | NFE2L2 | NFKBIA | NMB | NOD2 | NOS1AP | NPEPPS | NPR1 | NR0B2 | NR1H2 | NR1H3 | NTF3 | NUMA1 | ORM1 | OXCT1 | P2RX1 | P2RX2 | P2RX4 | PANX3 | PARK7 | PCM1 | PCSK9 | PDCD10 | PDCD6IP | PFKM | PICK1 | PIK3R1 | PLA2G10 | PLA2G1B | PLA2G6 | PLCG1 | PPARD | PPARG | PPIA | PPID | PPM1A | PPP3CC | PPT1 | PRELID1 | PRKAA1 | PRKACA | PRKCD | PRKCE | PRKCZ | PSEN1 | PSMD9 | PTPN22 | PTPN23 | RAB21 | RAB29 | RAB31 | RAB3GAP1 | RAB5A | RAB7A | RAB9A | RAN | RAP1A | RDX | RGN | RHBDD1 | RHOU | RIPK2 | RNF31 | S100A1 | SAE1 | SCYL2 | SDC1 | SDC4 | SDCBP | SEC16A | SFN | SH3GL1 | SIRT1 | SIRT3 | SLC9A1 | SLC9A3R1 | SMAD3 | SMAD4 | SMO | SNCA | SNF8 | SOD1 | SPACA3 | SPTBN1 | SRC | SRI | STAC | STIM1 | STOM | STXBP1 | SUMO1 | TARDBP | TFDP1 | TGFB1 | TM9SF4 | TMEM30A | TNF | TOR1A | TP53 | TP63 | TP73 | TPR | TRIAP1 | TRIM28 | TSG101 | TWIST1 | UBE2D3 | UBE2L3 | UBR5 | USP50 | VPS4B | VTN | WNK1 | WNK4 | XBP1 | XPO4 | YWHAB | YWHAE | YWHAG | YWHAH | YWHAQ | YWHAZ | ZIC1 | GO:0023014 | 0.031177969185976 | 257/8243 | 131/3250 | 9.11375620304307e-05 | 0.86535115147894 | T | T | T | T | signal transduction by protein phosphorylation | ACTN2 | APOA1 | ARAF | BRAF | BTRC | CAB39 | CALM1 | CCR5 | CRKL | CRYAB | CUL1 | CUL3 | DLG2 | DLG3 | DLG4 | DUSP7 | EGFR | ERBB2 | ERBB3 | FBXW11 | FGF2 | FGFR1 | FGFR3 | FYN | GPS2 | GRB2 | HBEGF | HRAS | HSF1 | IKBKB | IKBKG | IL3RA | ILK | ITGAV | JAK1 | JAK3 | KBTBD7 | KDR | KRAS | KSR1 | LAT | LRRC7 | LRRK2 | MAP2K1 | MAP2K3 | MAP2K4 | MAP2K6 | MAP2K7 | MAP3K1 | MAP3K13 | MAP3K14 | MAP3K2 | MAP3K3 | MAP3K4 | MAP4K1 | MAPK1 | MAPK14 | MAPK15 | MAPK3 | MAPK4 | MAPK6 | MAPK7 | MAPK8 | MAPK9 | MBP | MED1 | MYC | NEK1 | NLK | NOD2 | OXSR1 | PAK2 | PAK6 | PEBP1 | PPP5C | PSMA1 | PSMA2 | PSMA3 | PSMA5 | PSMA6 | PSMB5 | PSMB6 | PSMB7 | PSMB8 | PSMC1 | PSMC2 | PSMC3 | PSMC4 | PSMC5 | PSMC6 | PSMD1 | PSMD10 | PSMD11 | PSMD12 | PSMD14 | PSMD2 | PSMD4 | PSMD9 | PSME2 | PSME3 | PTK2 | PTPRA | RAF1 | RANBP9 | RBX1 | RIPK1 | RIPK2 | SCG2 | SHC1 | SPTAN1 | SPTBN1 | SPTBN2 | STK24 | STK25 | STK26 | STK3 | SULT1A3 | TAB1 | TAB2 | TAOK3 | TGFB1 | TNF | TNIP2 | TRAF6 | UBB | UBC | UBE2N | UBE2V1 | WNK1 | YWHAB | YWHAE | GO:0031331 | 0.031177969185976 | 257/8243 | 131/3250 | 9.11375620304307e-05 | 0.86535115147894 | T | T | T | T | positive regulation of cellular catabolic process | ADRB2 | AGO2 | AKT1 | AKT2 | APOE | ARNT | ATM | AXIN1 | BAG2 | BAG3 | BAG6 | BAX | CALCOCO2 | CAV1 | CBFA2T3 | CCDC22 | CD81 | CDC37 | CEBPA | CLU | CPT1A | CSNK1A1 | CSNK1E | CTSC | DAB2 | DAPK1 | DCN | DDRGK1 | DHX36 | DNAJB2 | DVL1 | EDEM1 | EIF2AK4 | ENDOG | EPM2A | ESRRB | EZR | FABP1 | FAF1 | FBXO7 | FOXO1 | FTO | GAPDHS | GNAI3 | GPD1 | GPSM1 | GSK3A | GSK3B | HDAC6 | HIF1A | HK2 | HMGB1 | HMOX1 | HNRNPD | HSF1 | HSP90AA1 | HSPA1B | HSPBP1 | HTT | IKBKG | INSR | ITCH | KDR | KEAP1 | LRRK2 | LRSAM1 | MAPK3 | MAPK9 | MDM2 | MOV10 | MSN | NFE2L2 | NNT | NUPR1 | OPTN | PABPC1 | PACSIN3 | PARK7 | PCSK9 | PIAS1 | PIP4K2A | PLEKHF1 | PLK1 | PRKAA1 | PRKAA2 | PRKCD | PRKD1 | PSEN1 | PSMC1 | PSMC2 | PSMC3 | PSMC4 | PSMC5 | PSMC6 | PSMD10 | PTEN | PTK2 | PTPN1 | PUM1 | RAB3GAP1 | RAD23A | RBX1 | RC3H1 | RDX | RGN | RIPK2 | RNF139 | RNF185 | RNF19A | SGTA | SIRT1 | SMURF1 | SNCA | SPTLC2 | STK11 | STUB1 | SUMO1 | SUMO2 | SUPT5H | TMTC3 | TNF | TNFAIP3 | TRIB2 | TRIB3 | TRIM21 | TWIST1 | UBQLN1 | UBQLN2 | ULK1 | USP5 | VCP | GO:0051341 | 0.00764284847749606 | 63/8243 | 40/3250 | 9.14084244926174e-05 | 0.867922990557402 | T | T | T | T | regulation of oxidoreductase activity | ABL1 | ABL2 | AKT1 | APOE | CALM1 | CALM3 | CAV1 | CCS | CHCHD10 | COX17 | ECSIT | EGFR | GLA | GNAI2 | GNAI3 | GZMA | HDAC6 | HIF1A | HSP90AA1 | KRAS | LRRK2 | LYPLA1 | NOD2 | NOS1AP | NOS3 | NOSIP | NOSTRIN | OXA1L | PARK7 | PPM1K | RFK | RGN | RIPK3 | S100A1 | SLAMF8 | SNCA | SPR | TERF2 | TNF | VDR | GO:0046394 | 0.016620162562174 | 137/8243 | 76/3250 | 9.24893412123483e-05 | 0.878186294811248 | T | T | T | T | carboxylic acid biosynthetic process | AASDHPPT | AASS | ABAT | ABCD3 | ACACA | ACCS | ACLY | ACOT7 | ACSS2 | AKR1A1 | ALOX12 | ALOX5 | ALOX5AP | ASL | ASNS | ASS1 | BCAT2 | BRCA1 | CBR4 | CD74 | CYP1A1 | CYP1A2 | DEGS1 | ENOPH1 | FASN | FOLH1 | GLS | GLS2 | GLUD2 | GLUL | GOT1 | GOT1L1 | GOT2 | GPT2 | GPX4 | GSTM4 | HAAO | HSD17B4 | HSD17B8 | HSD3B7 | KYNU | LDHC | LTC4S | MCAT | MGST1 | MIF | MTAP | MTHFD1 | MTRR | MYO5A | NANP | OAT | OSBPL1A | OTC | PAH | PARK7 | PER2 | PHGDH | PLA2G1B | PLOD2 | PRKAA1 | PRKAA2 | PRKAB2 | PRKAG1 | PSAT1 | PTGDS | PTGES | PTGES3 | PYCR1 | PYCR2 | RBP1 | RGN | SHMT1 | SHMT2 | TECR | XBP1 | GO:0051052 | 0.0260827368676453 | 215/8243 | 112/3250 | 9.31137336706093e-05 | 0.884114901202436 | T | T | T | T | regulation of DNA metabolic process | ACD | ACTR2 | AKT1 | APBB1 | ATAD5 | ATM | ATRX | AURKB | BABAM1 | BCL6 | BRCA1 | CBX8 | CCT2 | CCT3 | CCT4 | CCT5 | CCT6A | CCT7 | CCT8 | CD40 | CDK2 | CDK9 | CTNNB1 | DCP2 | DHX36 | DHX9 | EGFR | EXOSC6 | EYA2 | EYA4 | FAM129B | FIGNL1 | FOXM1 | FUS | GZMA | H1F0 | H1FX | H2AFX | HIST1H1A | HMGB1 | HNRNPA1 | HNRNPA2B1 | HNRNPD | HNRNPU | HSF1 | KDM1A | KDM4D | KMT2A | KPNA1 | KPNA2 | MAP2K7 | MAP3K4 | MAPK1 | MAPK15 | MAPK3 | MBD3 | MECP2 | MGMT | MLH1 | MSH6 | MYC | NEK7 | NPM1 | OTUB1 | OTUD4 | PARP1 | PAXIP1 | PCNA | PIK3CA | PINX1 | PML | PNKP | POT1 | PPARGC1A | PPP4R2 | PRKCD | RAD51 | RAD51AP1 | RIF1 | RNF8 | RPA2 | RPS3 | SIRT1 | SIRT6 | SMG5 | SMG6 | SPI1 | SRC | STAT6 | TCP1 | TERF1 | TERF2 | TERF2IP | TFIP11 | TGFB1 | TINF2 | TNKS | TNKS2 | TRIM28 | TRIP12 | TWIST1 | UBE2B | UBE2N | UBE2V1 | UBR5 | USP7 | USP9X | WAS | WT1 | XRCC1 | XRCC5 | ZMPSTE24 | GO:0051972 | 0.0050952323183307 | 42/8243 | 29/3250 | 9.44948587352034e-05 | 0.897228683690756 | T | T | T | T | regulation of telomerase activity | ACD | ATM | AURKB | CCT2 | CCT4 | CTNNB1 | HNRNPA2B1 | HNRNPD | HSP90AA1 | HSP90AB1 | MAP2K7 | MAP3K4 | MAPK1 | MAPK15 | MAPK3 | MYC | NEK7 | PINX1 | PNKP | POT1 | PPARG | PTGES3 | SMG6 | SRC | TCP1 | TERF1 | TNKS | TP53 | XRCC5 | GO:0033365 | 0.0579885963848114 | 478/8243 | 228/3250 | 9.45136607017101e-05 | 0.897407208362737 | T | T | T | T | protein localization to organelle | ACAA1 | ACD | ACOX1 | AGK | AKT1 | AP3D1 | AP3M1 | AP4M1 | ARF4 | ARL5B | ATM | ATP6V1D | ATRX | AURKB | BANP | BAX | CACNG2 | CACNG4 | CACNG5 | CALM1 | CALM3 | CALR | CARM1 | CAT | CBLB | CD81 | CHCHD10 | CLU | COG3 | CROCC | CROT | CSE1L | CTCF | DDX1 | DDX3X | DHRS4 | DHX9 | DLG2 | DLG4 | DNAJA1 | DNAJC19 | DNM1L | DVL1 | ECH1 | ECI2 | EHHADH | ESR1 | EZH2 | FAM83D | FAM83H | FBXO7 | FIS1 | GAK | GBP2 | GBP5 | GGA1 | GRPEL1 | GSTK1 | HEATR3 | HGS | HK1 | HK2 | HMGCL | HNRNPU | HSD17B4 | HSP90AA1 | HSPA4 | HSPA8 | HSPD1 | IDE | IDH1 | IFT122 | IK | IPO11 | IPO4 | IPO5 | IPO7 | IPO8 | IPO9 | KPNA1 | KPNA2 | KPNB1 | LARS | LEMD2 | LMNA | LRRC7 | LRRK2 | LRSAM1 | MAPK15 | MARCH5 | MCM9 | MDM2 | MTCH2 | NCOA4 | NDUFA13 | NEDD4 | NIPBL | NOS2 | NUDT19 | NUP107 | NUP153 | NUP214 | NUP85 | NUP88 | NUP93 | NUP98 | NUTF2 | NXT2 | OBSL1 | OPTN | OXA1L | PACS1 | PAM16 | PCM1 | PEX19 | PEX5 | PHB2 | PIH1D1 | PIK3R1 | PIKFYVE | PINX1 | PLK1 | PML | POM121 | POT1 | RAB10 | RAB11A | RAB29 | RAB35 | RAB3GAP1 | RAB7A | RAD21 | RAN | RB1 | ROMO1 | RPA2 | RPAIN | RPL10 | RPL10A | RPL12 | RPL18A | RPL22 | RPL23 | RPL23A | RPL24 | RPL30 | RPL37A | RPL38 | RPL6 | RPL7 | RPL7A | RPL9 | RPLP0 | RPLP1 | RPLP2 | RPS12 | RPS13 | RPS14 | RPS15A | RPS19 | RPS2 | RPS20 | RPS24 | RPS29 | RPS3 | RPS4X | RPS8 | RRS1 | RTKN | RTN4 | RUVBL2 | SCG3 | SEC16A | SEC62 | SEC63 | SGTA | SGTB | SLC25A6 | SMO | SMURF1 | SNF8 | SPDL1 | SRP14 | SRP19 | SSB | STAT3 | STIL | SUMO1 | TERF2 | TERF2IP | TIA1 | TIMM13 | TIMM21 | TIMM23 | TIMM44 | TIMM50 | TINF2 | TMEM30A | TNFAIP3 | TNKS | TNKS2 | TNPO1 | TOLLIP | TOMM20 | TOMM22 | TOMM40 | TOR1A | TPR | TRAPPC8 | TSPAN17 | TULP3 | TXNIP | UBB | UBC | UBE2D1 | UBE2D2 | UBE2D3 | USP9X | VAPA | VPS37D | WDR35 | XPO1 | XRCC5 | YWHAE | ZBTB16 | ZBTB7A | ZFAND6 | ZW10 | GO:0009896 | 0.0366371466699017 | 302/8243 | 151/3250 | 9.47989509496489e-05 | 0.900116039266916 | T | T | T | T | positive regulation of catabolic process | ADRB2 | AGO2 | AKT1 | AKT2 | APC | APOE | ARNT | ASB9 | ATM | AXIN1 | BAG2 | BAG3 | BAG6 | BAX | CALCOCO2 | CAV1 | CBFA2T3 | CCDC22 | CD81 | CDC37 | CEBPA | CLU | CPT1A | CSNK1A1 | CSNK1E | CTSC | CUL4B | DAB2 | DAPK1 | DCN | DDB1 | DDRGK1 | DHX36 | DNAJB2 | DVL1 | EDEM1 | EIF2AK4 | ENDOG | EPM2A | ESRRB | EZR | FABP1 | FAF1 | FBXO7 | FOXO1 | FTO | FZR1 | GAPDHS | GGA1 | GNAI3 | GPD1 | GPSM1 | GSK3A | GSK3B | HDAC6 | HECW1 | HECW2 | HIF1A | HK2 | HMGB1 | HMOX1 | HNRNPD | HSF1 | HSP90AA1 | HSPA1B | HSPBP1 | HTT | IDE | IKBKG | INSR | ITCH | KDR | KEAP1 | LRRK2 | LRSAM1 | MAPK3 | MAPK9 | MDM2 | MOV10 | MSN | NDUFA13 | NEDD4 | NFE2L2 | NNT | NUPR1 | OAZ3 | OPTN | PABPC1 | PACSIN3 | PARK7 | PCSK9 | PIAS1 | PIP4K2A | PLEKHF1 | PLK1 | PRKAA1 | PRKAA2 | PRKCD | PRKCE | PRKD1 | PSEN1 | PSMC1 | PSMC2 | PSMC3 | PSMC4 | PSMC5 | PSMC6 | PSMD10 | PTEN | PTK2 | PTPN1 | PUM1 | RAB3GAP1 | RAB7A | RAD23A | RBX1 | RC3H1 | RDX | RGN | RIPK2 | RNF139 | RNF185 | RNF19A | SGTA | SIRT1 | SMURF1 | SMURF2 | SNCA | SNF8 | SPTLC2 | STK11 | STUB1 | STX5 | SUMO1 | SUMO2 | SUPT5H | TMTC3 | TNF | TNFAIP3 | TNFSF12 | TRIB2 | TRIB3 | TRIM21 | TRIM32 | TWIST1 | UBQLN1 | UBQLN2 | ULK1 | USP5 | VCP | WWP2 | GO:0070498 | 0.00982651947106636 | 81/8243 | 49/3250 | 9.49995895559917e-05 | 0.902021102834141 | T | T | T | T | interleukin-1-mediated signaling pathway | BTRC | CUL1 | FBXW11 | IKBKB | IKBKG | MAP3K3 | MAPK3 | MYD88 | NFKBIA | NOD2 | PELI1 | PSMA1 | PSMA2 | PSMA3 | PSMA5 | PSMA6 | PSMB5 | PSMB6 | PSMB7 | PSMB8 | PSMC1 | PSMC2 | PSMC3 | PSMC4 | PSMC5 | PSMC6 | PSMD1 | PSMD10 | PSMD11 | PSMD12 | PSMD14 | PSMD2 | PSMD4 | PSMD9 | PSME2 | PSME3 | RBX1 | RELA | RIPK2 | SQSTM1 | TAB1 | TAB2 | TNIP2 | TOLLIP | TRAF6 | UBB | UBC | UBE2N | UBE2V1 | GO:0002252 | 0.0741234987261919 | 611/8243 | 285/3250 | 9.82414510218837e-05 | 0.932802577452786 | T | T | T | T | immune effector process | ABCE1 | ABL1 | ACAA1 | ACLY | ACTB | ACTG1 | ACTR2 | ACTR3 | ADA | AGL | AHSG | AICDA | AIRE | ALDOA | ALDOC | ALOX5 | ANXA2 | AP1M1 | APCS | APEH | APP | APRT | ARMC8 | ARPC1A | ARPC1B | ARPC2 | ARPC3 | ARPC4 | ARPC5 | ATAD5 | ATP6V1D | AZU1 | BAIAP2 | BCL10 | BCL2 | C1QA | C1QBP | C1R | C4A | CAB39 | CAND1 | CAPN1 | CARD9 | CAT | CBL | CCT2 | CCT8 | CD27 | CD36 | CD40 | CD40LG | CD74 | CD81 | CDA | CDC42 | CEP290 | CKAP4 | CLU | COLEC10 | COPB1 | COTL1 | CPNE3 | CRK | CRP | CSNK2B | CTSA | CTSC | CTSD | CYB5R3 | DBH | DBNL | DDIT4 | DDOST | DDX1 | DDX3X | DEGS1 | DERA | DHX36 | DYNC1H1 | DYNC1LI1 | DYNLL1 | DYNLT1 | EEF1A1 | EEF2 | EIF2AK4 | EXOSC5 | EXOSC6 | FABP5 | FADD | FAF2 | FCGR1A | FCN1 | FGL2 | FTH1 | FTL | FYN | GAA | GALNS | GBP3 | GDI2 | GHDC | GLA | GNL1 | GNS | GPI | GRB2 | GSTP1 | GUSB | GYG1 | HAVCR2 | HEXB | HMGB1 | HPRT1 | HRNR | HSP90AA1 | HSP90AB1 | HSPA1B | HSPA8 | HSPD1 | HUWE1 | ICAM1 | IDH1 | IFI16 | IFIT1 | IFITM3 | IFNA2 | IGHV3-13 | IGHV3-23 | IGHV3-30 | IGKV3-20 | IGLC7 | IL13RA2 | ILF2 | ILF3 | IMPDH2 | IQGAP1 | IRF1 | IRF7 | ISG15 | ITCH | ITGAV | JUP | KCMF1 | KCNAB2 | KLRC2 | KPNB1 | LAT | LCP1 | LEF1 | LGALS1 | LGALS3 | LIG4 | LRRC7 | MAN2B1 | MAP3K14 | MAPK1 | MAPK14 | MAPK3 | MASP1 | MASP2 | MAVS | MGST1 | MICA | MIF | MLH1 | MME | MSH6 | MYO1C | NAPRT | NCK1 | NHLRC3 | NIT2 | NME2 | OAS2 | ORM1 | P2RX1 | PA2G4 | PCBP2 | PDE12 | PDXK | PFKL | PGAM1 | PGM1 | PGM2 | PGRMC1 | PI4K2A | PIK3CA | PIK3CG | PIK3R1 | PKM | PLA2G1B | PLA2G6 | PLAUR | PLCG1 | PLSCR1 | PML | PNP | PPIA | PPIE | PRCP | PRDX1 | PRDX2 | PRDX4 | PRDX6 | PRG2 | PRKCD | PRKCE | PSAP | PSEN1 | PSMA2 | PSMA5 | PSMB7 | PSMC2 | PSMC3 | PSMD1 | PSMD11 | PSMD12 | PSMD14 | PSMD2 | PTGES2 | PTK2 | PTPN6 | PYGB | PYGL | QSOX1 | RAB10 | RAB18 | RAB24 | RAB31 | RAB5B | RAB7A | RAC1 | RAP1A | RAP1B | RC3H1 | RELA | RHOA | RNASE3 | RNF8 | S100A11 | S100A12 | S100A13 | S100A7 | S100A9 | S100P | SCARA3 | SDCBP | SELL | SERPINB3 | SERPINB6 | SLAMF1 | SLC2A5 | SNAP29 | SPTAN1 | SRC | SRP14 | STAT1 | STBD1 | STOM | STXBP2 | SWAP70 | TCIRG1 | TIMP2 | TLR3 | TMEM173 | TMEM30A | TOLLIP | TREX1 | TRIM25 | TRIM34 | TUBB | TUBB4B | TXNDC5 | UBR4 | VAPA | VAT1 | VAV1 | VCL | VCP | VSIG4 | WAS | WDR1 | XRCC5 | XRCC6 | YES1 | GO:0050852 | 0.0161349023413806 | 133/8243 | 74/3250 | 0.000100120104570589 | 0.950640392897742 | T | T | T | T | T cell receptor signaling pathway | ABL1 | BCAR1 | BCL10 | BRAF | BTK | BTN2A1 | BTRC | CRKL | CUL1 | EIF2B1 | EIF2B3 | EIF2B5 | FBXW11 | FYN | HLA-DQA1 | HLA-DRA | HLA-DRB1 | HLA-DRB3 | HLA-DRB4 | HLA-DRB5 | HRAS | IKBKB | IKBKG | ITK | LAT | LCP2 | MAPK1 | NCK1 | PAG1 | PAK2 | PDE4D | PIK3CA | PIK3R1 | PLCG1 | PRKD2 | PSEN1 | PSMA1 | PSMA2 | PSMA3 | PSMA5 | PSMA6 | PSMB5 | PSMB6 | PSMB7 | PSMB8 | PSMC1 | PSMC2 | PSMC3 | PSMC4 | PSMC5 | PSMC6 | PSMD1 | PSMD10 | PSMD11 | PSMD12 | PSMD14 | PSMD2 | PSMD4 | PSMD9 | PSME2 | PSME3 | PTPN22 | RC3H1 | RELA | RIPK2 | RNF31 | STK11 | STOML2 | TAB2 | TRAF6 | UBE2N | WAS | WNK1 | ZAP70 | GO:0038095 | 0.0135872861822152 | 112/8243 | 64/3250 | 0.000101630973185124 | 0.964986090392753 | T | T | T | T | Fc-epsilon receptor signaling pathway | BCL10 | BTK | BTRC | CALM1 | CUL1 | FBXW11 | FOS | GRB2 | IGHV3-13 | IGHV3-23 | IGHV3-30 | IGKV3-20 | IGLC7 | IKBKB | IKBKG | ITK | JUN | LAT | LCP2 | MAP2K4 | MAP2K7 | MAP3K1 | MAPK1 | MAPK3 | MAPK8 | MAPK9 | NFATC1 | NFATC2 | PAK2 | PIK3CA | PIK3R1 | PLCG1 | PSMA1 | PSMA2 | PSMA3 | PSMA5 | PSMA6 | PSMB5 | PSMB6 | PSMB7 | PSMB8 | PSMC1 | PSMC2 | PSMC3 | PSMC4 | PSMC5 | PSMC6 | PSMD1 | PSMD10 | PSMD11 | PSMD12 | PSMD14 | PSMD2 | PSMD4 | PSMD9 | PSME2 | PSME3 | RAC1 | RELA | SHC1 | TAB1 | TAB2 | TRAF6 | VAV1 | GO:1901653 | 0.0197743539973311 | 163/8243 | 88/3250 | 0.000101692672475844 | 0.965571925158137 | T | T | T | T | cellular response to peptide | ADCY6 | ADRB2 | AGER | AKR1B1 | AKT1 | AKT2 | ANXA5 | APEX1 | APP | APRT | ASS1 | BACE1 | CACNA1A | CAV1 | CD36 | CDC5L | CDK4 | CDK5 | CREB1 | CRK | DENND4C | EPHA4 | EPRS | ERRFI1 | FIS1 | FOXO1 | FYN | GLP1R | GOT1 | GSK3A | GSK3B | GSTP1 | HDAC5 | HDAC9 | HSF1 | ICAM1 | IGF1R | INSR | ITGA4 | KLF5 | LGMN | LPIN3 | MDM2 | MYO5A | NAMPT | NFE2L2 | NOD2 | NR4A1 | NTRK1 | PARP1 | PCSK9 | PIK3R1 | PKM | PLA2G1B | PPARG | PPAT | PRKACA | PRKAR1A | PRKAR2B | PRKCD | PRKCZ | PRKDC | PSEN1 | PTEN | PTPN22 | RAB10 | RAB31 | RANGAP1 | RAP1B | RELA | RIPK2 | RPL23 | SLC9A1 | SNX6 | SP1 | SRC | SRSF5 | STAT1 | TBC1D4 | TGFB1 | TP53 | TRIB3 | TRIM41 | VCAM1 | VIM | WDTC1 | XBP1 | YWHAG | GO:0043281 | 0.0179546281693558 | 148/8243 | 81/3250 | 0.000102945438765454 | 0.977466941077984 | T | T | T | T | regulation of cysteine-type endopeptidase activity involved in apoptotic process | ADORA2A | AIFM1 | AKT1 | ALOX12 | ARRB1 | ARRB2 | BAX | BBC3 | BCL10 | BCL2L13 | BIRC2 | BIRC3 | BIRC7 | BIRC8 | CASP10 | CASP8 | CASP9 | CD27 | CRYAB | CYCS | DAPK1 | DDX3X | DHCR24 | DIABLO | DPEP1 | EGLN3 | EIF2AK3 | FABP1 | FADD | FASLG | FIS1 | GPI | GPX1 | HMGB1 | HSF1 | HSPD1 | HSPE1 | HTRA2 | IGBP1 | LAMP3 | LEF1 | MAPT | MDM2 | MGMT | MYC | NDUFA13 | P2RX1 | PAK2 | PARK7 | PDCD6 | PIH1D1 | PLAUR | PML | PPARG | PRDX3 | PRDX5 | RAF1 | REST | RHOA | RIPK1 | RPS3 | RPS6KA1 | S100A9 | SFN | SIRT1 | SMAD3 | SNCA | SOX2 | SRC | THBS1 | TNF | TNFSF14 | TP63 | TRADD | TRAF2 | TRIAP1 | USP47 | VCP | WDR35 | XIAP | YWHAE | GO:0008652 | 0.0053378624287274 | 44/8243 | 30/3250 | 0.000103145629090965 | 0.979367748218716 | T | T | T | T | cellular amino acid biosynthetic process | AASDHPPT | AASS | ABAT | ACCS | ASL | ASNS | ASS1 | BCAT2 | ENOPH1 | FOLH1 | GLS | GLS2 | GLUL | GOT1 | GOT1L1 | GOT2 | GPT2 | MTAP | MTHFD1 | MTRR | OAT | OTC | PAH | PHGDH | PLOD2 | PSAT1 | PYCR1 | PYCR2 | SHMT1 | SHMT2 | GO:0006259 | 0.0566541307776295 | 467/8243 | 223/3250 | 0.000103620855365916 | 0.983880021699369 | T | T | T | T | DNA metabolic process | ABL1 | ACD | ACTL6A | AICDA | APBB1 | APEX1 | APEX2 | ASCC1 | ASCC2 | ATAD5 | ATM | ATRX | BABAM1 | BANF1 | BRCA1 | CBS | CCDC155 | CCDC36 | CD40LG | CDC14B | CDC34 | CDC5L | CDK2 | CDK9 | CHAF1A | CHD1L | CHEK2 | COPS2 | COPS5 | COPS6 | CRY2 | CSNK1E | CTCF | CUL4B | DCTPP1 | DDB1 | DDB2 | DDX1 | DMAP1 | DNA2 | DNASE2 | EHMT1 | EHMT2 | ENDOG | EP300 | ERCC5 | EXOSC5 | EXOSC6 | EYA2 | EYA4 | EZH2 | FANCA | FANCC | FANCG | FANCI | FANCM | FBXO6 | FEN1 | FOS | FOXM1 | FTO | FZR1 | GAR1 | GINS2 | GINS3 | GINS4 | GNAS | H2AFX | HERC2 | HIST1H4A | HIST3H3 | HMGB1 | HMGB2 | HMGB3 | HSF1 | HSP90AA1 | HSP90AB1 | HSPD1 | HUWE1 | INO80B | INO80C | ISG15 | KAT5 | KDM2A | KDM4D | KPNA2 | LEF1 | LIG4 | MC1R | MCM2 | MCM3 | MCM4 | MCM5 | MCM6 | MCM7 | MCM9 | MDC1 | MGMT | MLH1 | MORF4L1 | MORF4L2 | MPG | MSH6 | MTA1 | MTRR | NIPBL | NME1 | NOP10 | NPLOC4 | NPM1 | NSMCE4A | OTUB1 | PALB2 | PARK7 | PARP1 | PAXIP1 | PCNA | PICK1 | PINX1 | PNKP | POLA2 | POLD1 | POLE2 | POLE3 | POLM | POLR2A | POLR2E | POLR2L | POT1 | PPIE | PPM1D | PPP5C | PRKDC | PRMT6 | PRPF19 | PSMD14 | PTGES3 | PTTG1 | RAD21 | RAD23A | RAD23B | RAD51 | RAD51AP1 | RAN | RBM17 | RBX1 | RFC1 | RFC2 | RFC3 | RFC4 | RFC5 | RIF1 | RNASEH2A | RNF8 | RPA2 | RPA3 | RPAIN | RPS3 | RRM2B | RTFDC1 | RUVBL1 | RUVBL2 | SFPQ | SIRT1 | SIRT6 | SMARCB1 | SMC1A | SMC3 | SMG5 | SMG6 | SP100 | SPRTN | STUB1 | SUMO1 | SWAP70 | TAOK3 | TATDN1 | TCEA1 | TDP1 | TERF1 | TERF2 | TERF2IP | TFIP11 | TINF2 | TK1 | TMEM189-UBE2V1 | TNP1 | TOP1 | TOP2A | TOP3A | TP53 | TP73 | TREX1 | TRIM25 | TRIM28 | TRIP12 | TRIP13 | TSN | TTC5 | UBB | UBC | UBE2A | UBE2B | UBE2D3 | UBE2L6 | UBE2N | UBE2V1 | UBE2W | UBR5 | UCHL5 | USP28 | USP47 | USP7 | VCP | WDR48 | XRCC1 | XRCC3 | XRCC5 | XRCC6 | YY1 | ZBTB7A | ZFYVE26 | ZMPSTE24 | GO:2001251 | 0.0133446560718185 | 110/8243 | 63/3250 | 0.0001048620646062 | 0.995665303435873 | T | T | T | T | negative regulation of chromosome organization | ACD | AICDA | APC | ATG5 | ATM | ATRX | AURKB | BCOR | BRCA1 | CDC20 | CENPF | CTBP1 | DCP2 | H1F0 | H1FX | HIST1H1A | HNRNPA1 | HNRNPU | IK | KDM1A | KDM4D | KLHL22 | MAD1L1 | MAD2L1 | MCM2 | MECP2 | OTUB1 | PARP1 | PIH1D1 | PINX1 | PLK1 | POT1 | PRMT6 | PTTG1 | RAD21 | SET | SIN3A | SIRT1 | SKI | SMARCB1 | SMG6 | SNCA | SPDL1 | SPI1 | SRC | TERF1 | TERF2 | TERF2IP | TINF2 | TNKS | TNKS2 | TOP2A | TPR | TRIP12 | TRIP13 | TWIST1 | UBE2B | UBR5 | USP17L2 | XRCC1 | XRCC5 | ZNF207 | ZW10 | GO:0032212 | 0.00400339682154555 | 33/8243 | 24/3250 | 0.000105591725369282 | 1 | T | T | T | T | positive regulation of telomere maintenance via telomerase | ACD | ATM | AURKB | CCT2 | CCT3 | CCT4 | CCT5 | CCT6A | CCT7 | CCT8 | CTNNB1 | HNRNPA1 | MAP2K7 | MAP3K4 | MAPK1 | MAPK15 | MAPK3 | NEK7 | PNKP | POT1 | TCP1 | TNKS | TNKS2 | XRCC5 | GO:0032880 | 0.0776416353269441 | 640/8243 | 297/3250 | 0.000110690733446662 | 1 | T | T | T | T | regulation of protein localization | ABAT | ABHD17A | ABL1 | ACTB | ACTN2 | ADCYAP1 | ADORA2A | ADTRP | AGER | AGR2 | AKT1 | AKT2 | ANK3 | ANXA1 | ANXA5 | APBB1 | APC | APOA1 | APOD | AR | ARF1 | ARFIP1 | ARRB1 | ATAD1 | ATCAY | ATG5 | ATP13A2 | B3GAT3 | BAG3 | BAG4 | BBC3 | BCL2 | BCL2L1 | CACNA1A | CACNG2 | CASP8 | CCDC88A | CCT2 | CCT3 | CCT4 | CCT5 | CCT6A | CCT7 | CCT8 | CD2 | CD22 | CD36 | CD40 | CD40LG | CDH1 | CDK16 | CDK5 | CDK5R1 | CENPJ | CEP135 | CEP290 | CFTR | CHP1 | CLDN18 | CLTC | COMMD1 | CPT1A | CRKL | CROCC | CRP | CSNK1E | CSNK2A2 | CTNNB1 | CYLD | DAB2 | DBN1 | DCLK1 | DDRGK1 | DHX36 | DHX9 | DMAP1 | DNAJA1 | DNM1L | DVL1 | DVL3 | DYNLL1 | E2F1 | EDEM1 | EGFR | EIF2AK3 | ERBB2 | EZR | FCN1 | FGB | FIS1 | FKBP1A | FKBP1B | FN1 | FYN | GAPDH | GAPVD1 | GDI1 | GLMN | GLP1R | GLUL | GNAI1 | GOLPH3 | GOPC | GPD1L | GPI | GSK3A | GSK3B | HADH | HAVCR2 | HAX1 | HDAC3 | HDAC6 | HDAC9 | HIF1A | HK1 | HMGB1 | HNF4A | HRAS | HSP90AA1 | HSP90AB1 | HSP90AB3P | HSP90AB4P | HSPA1L | HSPA8 | HTRA2 | HUWE1 | IDH2 | IFNA2 | INSIG1 | IPO5 | IQGAP1 | ITGB1BP1 | ITGB3 | ITPR1 | ITPR2 | ITPR3 | JUP | KAT7 | KCNAB2 | KCNS3 | KDM1A | KIF5B | LCP1 | LGALS3 | LGR4 | LMNA | LRRK2 | LYPLA1 | MAPK1 | MAPK14 | MAPK3 | MAPK8 | MAPT | MAVS | MBP | MDM2 | MIF | MPC2 | MSN | MYO1C | NDUFAF2 | NEUROD1 | NF2 | NIPBL | NMD3 | NOD2 | NOS2 | NPEPPS | NR0B2 | NR1H2 | NR1H3 | NSFL1C | NUCB1 | NUMA1 | ORM1 | OXCT1 | PANX3 | PARD6A | PARK7 | PARP1 | PCM1 | PDCD10 | PER2 | PFKL | PFKM | PIK3R1 | PIN1 | PINX1 | PKIG | PLA2G1B | PLA2G6 | PLK1 | PML | PPARD | PPIA | PPID | PPM1A | PPP1R11 | PPP3CC | PRKAA1 | PRKAA2 | PRKACA | PRKCD | PRKCE | PRKCZ | PSEN1 | PSMD9 | PTN | PTPN1 | PTPN14 | PTPN22 | PTPN23 | RAB11A | RAB11B | RAB11FIP2 | RAB29 | RAB9A | RAN | RANGAP1 | RAP1A | RAPGEF4 | RB1 | RDX | REST | RHOU | RIPK2 | RNF31 | RTN4 | SAE1 | SCFD1 | SEC16A | SFN | SIN3A | SIRT3 | SLC16A1 | SLC25A4 | SLC25A5 | SLC25A6 | SMAD3 | SMO | SNX12 | SNX3 | SOCS1 | SP100 | SPTBN1 | SQSTM1 | SRC | SRI | STAC | STK11 | STOM | SUMO1 | SWAP70 | TARDBP | TCP1 | TERF1 | TFDP1 | TGFB1 | TM9SF4 | TMBIM6 | TMEM231 | TMEM30A | TNF | TNFAIP3 | TOR1A | TP53 | TP63 | TP73 | TPR | TRAF2 | TRAF6 | TRIM28 | TRIM29 | TSG101 | TWIST1 | TXN | UBE2D3 | UBE2J1 | UBE2L3 | UBR5 | USP17L2 | USP46 | USP50 | USP8 | WWP2 | XBP1 | XPO1 | XPO4 | XPO5 | YWHAB | YWHAE | YWHAG | YWHAH | YWHAQ | YWHAZ | ZIC1 | ZMYND8 | GO:0006744 | 0.00157709571757855 | 13/8243 | 12/3250 | 0.000115403733367204 | 1 | T | T | T | T | ubiquinone biosynthetic process | COQ2 | COQ3 | COQ4 | COQ5 | COQ6 | COQ7 | COQ9 | FDXR | NDUFA9 | PDSS1 | PDSS2 | UBIAD1 | GO:1901663 | 0.00157709571757855 | 13/8243 | 12/3250 | 0.000115403733367204 | 1 | T | T | T | T | quinone biosynthetic process | COQ2 | COQ3 | COQ4 | COQ5 | COQ6 | COQ7 | COQ9 | FDXR | NDUFA9 | PDSS1 | PDSS2 | UBIAD1 | GO:0006996 | 0.178090501031178 | 1468/8243 | 642/3250 | 0.00011701381524531 | 1 | T | T | T | T | organelle organization | ABCD3 | ABL1 | ABLIM1 | ACAA1 | ACAA2 | ACD | ACOX1 | ACTB | ACTG1 | ACTN2 | ACTR2 | ACTR3 | AFG3L2 | AGK | AHI1 | AIFM1 | AKAP8 | AKAP9 | AKT1 | AKT3 | AKTIP | ALAS1 | ANK1 | ANK3 | ANKRD28 | ANXA1 | ANXA2 | AP1G1 | AP1M1 | AP3D1 | APEX1 | APOE | ARF1 | ARHGAP26 | ARHGAP35 | ARHGEF7 | ARPC1A | ARPC5 | ARRB1 | ATAD3A | ATCAY | ATF2 | ATG2B | ATG4C | ATG5 | ATL3 | ATM | ATP13A2 | ATP2A2 | ATP6V1D | ATRX | ATXN10 | ATXN2 | AURKB | AVIL | BAG3 | BAG5 | BAG6 | BAX | BBC3 | BCL2 | BCL2L1 | BCL6 | BCS1L | BIN1 | BIRC5 | BLZF1 | C1QBP | CALM1 | CALM3 | CALR | CAMK2A | CAPZA1 | CAPZA2 | CAPZB | CASP8 | CAT | CAV1 | CBY1 | CCAR2 | CCDC120 | CCDC155 | CCDC36 | CCDC68 | CCDC8 | CCDC88A | CCNB1 | CCP110 | CDC14B | CDC20 | CDC25B | CDC42 | CDK2 | CDK5 | CDK5R1 | CEBPA | CENPF | CENPJ | CENPV | CEP135 | CEP152 | CEP290 | CEP350 | CEP55 | CEP57 | CEP89 | CFL1 | CFL2 | CHCHD10 | CHCHD2 | CHCHD3 | CHD1L | CHD3 | CHD4 | CHD8 | CHEK2 | CHMP2A | CHP1 | CIRBP | CISD2 | CKAP5 | CKS2 | CLU | CLUH | CNTROB | COG3 | COL4A3BP | COPS2 | COPS5 | COPS6 | CORO2B | COX7A2 | CREB1 | CRMP1 | CROCC | CROT | CRYAB | CSNK1A1 | CTSC | CTTN | CUL3 | CUL4B | CUL7 | CYCS | DBNL | DCTN1 | DDB1 | DDB2 | DDX1 | DDX3X | DDX6 | DES | DHRS4 | DHX36 | DHX9 | DIAPH3 | DISC1 | DLG2 | DLG3 | DLG4 | DMD | DNA2 | DNAJA3 | DNM1L | DOCK7 | DVL1 | DYM | DYNC1H1 | DYNC1LI1 | DYNLL1 | DYNLT1 | E2F1 | E2F4 | ECH1 | ECI2 | EHHADH | EIF2AK3 | EIF2S1 | EIF6 | EML4 | EPPK1 | ESRRA | ETS1 | EYA2 | EZR | F5 | FAF2 | FAM118B | FAM161B | FAM83H | FANCA | FANCG | FANCM | FBF1 | FBXO7 | FEN1 | FGFR1OP | FIGNL1 | FIS1 | FLNB | FOLR1 | FOPNL | FOXJ1 | FSCN1 | FSD1 | FUNDC2 | G3BP1 | G3BP2 | GAA | GABPB1 | GAK | GAPDH | GAR1 | GATA1 | GFAP | GGCT | GINS2 | GINS4 | GNAI3 | GOLGA2 | GOLPH3 | GORASP2 | GRB7 | GRIA1 | GSK3A | GSK3B | GSTK1 | H1F0 | H1FX | H3F3A | HAP1 | HAX1 | HDAC2 | HDAC3 | HDAC6 | HEXB | HGS | HIST1H1A | HIST1H3A | HIST1H4A | HIST3H3 | HK2 | HMGB1 | HMGB2 | HMGB3 | HMGCL | HNRNPA2B1 | HSD17B10 | HSD17B4 | HSF1 | HSP90AA1 | HSP90AB1 | HSPA2 | HSPA4 | HSPD1 | HTRA2 | HTT | ICK | IDE | IDH1 | IFT122 | IKBKB | IMMT | IQCB1 | ITGB5 | ITPA | JUN | JUP | KIF11 | KIF2C | KIF5B | KIF5C | KIFC3 | KLC1 | KLHL20 | KLHL22 | KPNB1 | KRAS | KRT17 | KRT31 | KRT71 | LARP4 | LATS1 | LETM1 | LIG4 | LMAN2L | LMNA | LRRK2 | LSM3 | LSM4 | LURAP1 | MAD2L1 | MAP1B | MAPK15 | MAPK8 | MAPKAP1 | MAPT | MAST1 | MAST3 | MCFD2 | MCM2 | MCM4 | MCM6 | MCM7 | MCM9 | MCMBP | MCTS1 | MECP2 | MKS1 | MLH1 | MRRF | MSH6 | MSN | MTERF1 | MTSS1 | MYC | MYH11 | MYH6 | MYL2 | NCAPH2 | NCOR1 | NDUFA13 | NDUFS1 | NDUFS6 | NEK1 | NEK7 | NF2 | NIN | NIPBL | NOP10 | NOS1AP | NOS2 | NOS3 | NPHP3 | NPLOC4 | NPM1 | NSFL1C | NSL1 | NTMT1 | NUDT19 | NUMA1 | NUP107 | NUP153 | NUP205 | NUP93 | NUP98 | NUSAP1 | OBSL1 | OFD1 | OGFOD1 | ONECUT1 | OPA1 | OPTN | OXA1L | PACS2 | PACSIN3 | PAFAH1B1 | PAK6 | PARD3 | PARK7 | PARP1 | PCM1 | PCNA | PDCD10 | PDCD6 | PDCD6IP | PEF1 | PEX11B | PEX19 | PEX5 | PFN1 | PGM5 | PHB | PHB2 | PI4K2A | PIN1 | PINX1 | PITPNB | PLEC | PLEKHF1 | PLK1 | PML | POC1B | POLA2 | POLD1 | POLDIP2 | POLE2 | POLE3 | POT1 | PPARGC1A | PPIA | PPID | PPIF | PPP2CB | PPP2R1A | PPP3CC | PPP6C | PPP6R1 | PRDX3 | PRKAR1A | PRKCZ | PRKD1 | PRKDC | PROX1 | PRPF40A | PSEN1 | PTEN | PTGES3 | PTK2 | PTPN1 | PTPN23 | PTTG1 | RAB10 | RAB11A | RAB18 | RAB1A | RAB1B | RAB29 | RAB2A | RAB31 | RAB32 | RAB3GAP1 | RAB5B | RAB7A | RAC1 | RAD21 | RAD23B | RAD51 | RAE1 | RAF1 | RAN | RANBP9 | RAP1GDS1 | RB1CC1 | RBX1 | RC3H1 | RCC1 | RFC1 | RFC2 | RFC3 | RFC4 | RFC5 | RHOA | RHOJ | RHOT1 | RHOT2 | RHOU | RIF1 | RNF19A | ROMO1 | RPA2 | RPA3 | RPGRIP1L | RPS19 | RPS3 | RTKN | RTN4 | RUVBL1 | RUVBL2 | SAMD9 | SBDS | SCFD1 | SCLT1 | SDCBP | SEC13 | SEC16A | SEC24B | SEC24C | SEC31A | SEPT2 | SEPT4 | SEPT6 | SEPT7 | SERPINE2 | SF1 | SFN | SH3KBP1 | SHC1 | SIPA1 | SIPA1L1 | SIRT1 | SIRT3 | SIRT5 | SLC25A4 | SLC25A5 | SLC35F6 | SLC9A1 | SLC9A3R1 | SMC1A | SMC3 | SMG5 | SMG6 | SMTNL2 | SNAP29 | SNCA | SNF8 | SNX3 | SOD1 | SOD2 | SORT1 | SP100 | SPACA1 | SPC25 | SPDL1 | SPERT | SPICE1 | SPRY2 | SPTAN1 | SPTBN1 | SPTBN2 | SQSTM1 | SRC | SRGAP2 | SRPK1 | SRPK2 | SSX2IP | STAG2 | STAT3 | STIL | STK25 | STMN1 | STOML2 | STX5 | STXBP1 | SUMO1 | SUMO1P1 | SUN2 | SVIL | SYNE2 | SYNJ2BP | SYP | TANGO2 | TAZ | TBCE | TCTN2 | TCTN3 | TEKT2 | TEKT4 | TERF1 | TERF2 | TERF2IP | TESK2 | TFDP1 | TFG | TFIP11 | THEM4 | TIMM13 | TIMM50 | TINF2 | TMED9 | TMEM11 | TMEM17 | TMEM216 | TMEM231 | TMOD3 | TMOD4 | TNF | TNFAIP3 | TNKS | TNNT1 | TNP1 | TNXB | TOMM22 | TOP1 | TOP2A | TOP3A | TOR1A | TP53 | TP63 | TP73 | TPCN2 | TPM1 | TPR | TRAPPC8 | TREX1 | TRIM32 | TRIP13 | TSG101 | TTLL1 | TUBA1A | TUBA1C | TUBA4A | TUBB | TUBB2A | TUBB2B | TUBB3 | TUBB4A | TUBB4B | TUBB6 | TUBB8 | TUBG1 | TUBGCP3 | UBB | UBC | UBE2B | UBE2D1 | UBE2D2 | UBE2D3 | UBQLN1 | UBQLN2 | UBXN10 | UGT8 | ULK1 | USP16 | USP50 | USP8 | USP9X | USPL1 | VAPA | VAPB | VCP | VCPIP1 | VIM | VPS18 | VPS37D | VPS4B | VPS8 | VTA1 | WAS | WDR1 | WDR35 | WDR45B | WIPI1 | WIPI2 | XIRP2 | XRCC1 | XRCC3 | XRCC5 | XRCC6 | YME1L1 | YWHAB | YWHAE | YWHAG | YWHAH | YWHAQ | YWHAZ | ZFAND6 | ZMPSTE24 | ZMYND10 | ZNF207 | ZW10 | ZWINT | GO:0006986 | 0.00582312264952081 | 48/8243 | 32/3250 | 0.000118523049770615 | 1 | T | T | T | T | response to unfolded protein | BAG3 | BAX | DAXX | DERL1 | DNAJA1 | DNAJB1 | DNAJB2 | FAF2 | FBXO6 | HSF1 | HSP90AA1 | HSP90AB1 | HSPA14 | HSPA1B | HSPA1L | HSPA2 | HSPA4 | HSPA4L | HSPA5 | HSPA8 | HSPA9 | HSPB1 | HSPB2 | HSPD1 | HSPE1 | HSPH1 | OPTN | PTPN1 | RHBDD1 | SERPINH1 | THBS1 | TMBIM6 | GO:0052547 | 0.0321484896275628 | 265/8243 | 134/3250 | 0.000121805699708202 | 1 | T | T | T | T | regulation of peptidase activity | A2M | ADORA2A | ADRM1 | AGER | AHSG | AIFM1 | AKT1 | ALOX12 | APP | ARRB1 | ARRB2 | ATP13A2 | BAX | BBC3 | BCL10 | BCL2L13 | BIN1 | BIRC2 | BIRC3 | BIRC5 | BIRC7 | BIRC8 | C4A | CASP10 | CASP8 | CASP9 | CAV1 | CD109 | CD27 | CLPX | CRYAB | CYCS | DAPK1 | DDRGK1 | DDX3X | DHCR24 | DIABLO | DPEP1 | EGLN3 | EIF2AK3 | EPHA4 | EPPIN | FABP1 | FADD | FASLG | FIS1 | FN1 | FYN | GAPDH | GPI | GPX1 | HDAC1 | HMGB1 | HSF1 | HSPD1 | HSPE1 | HTRA2 | IFI16 | IGBP1 | LAMP3 | LEF1 | LGMN | MAPK14 | MAPK3 | MAPT | MBP | MDM2 | MGMT | MYC | NDUFA13 | P2RX1 | PAK2 | PAPLN | PARK7 | PDCD6 | PEBP1 | PIH1D1 | PLAUR | PML | PPARG | PRDX3 | PRDX5 | PRELID1 | PSMA3 | PSMB8 | PSMD14 | PSME2 | PSME3 | PTTG1 | RAF1 | RECK | REST | RHOA | RIPK1 | RIPK2 | RPS3 | RPS6KA1 | S100A9 | SERPINB2 | SERPINB3 | SERPINB5 | SERPINB6 | SERPINB8 | SERPINE2 | SERPINH1 | SFN | SIRT1 | SMAD3 | SNCA | SOX2 | SPINK7 | SPINT2 | SRC | STAT3 | TANK | THBS1 | TIMP1 | TIMP2 | TNF | TNFSF14 | TP63 | TRADD | TRAF2 | TRIAP1 | UBXN1 | UCHL5 | USP14 | USP47 | USP50 | VCP | VTN | WDR35 | XIAP | YWHAE | GO:0006090 | 0.00254761615916535 | 21/8243 | 17/3250 | 0.000123430207868222 | 1 | T | T | T | T | pyruvate metabolic process | BSG | DLAT | DLD | GLO1 | HAGH | LDHA | LDHAL6B | LDHB | LDHC | ME1 | PC | PDHA1 | PDHA2 | PDHB | PDHX | SLC16A1 | VDAC1 | GO:0030177 | 0.0146791216790004 | 121/8243 | 68/3250 | 0.000125794301815337 | 1 | T | T | T | T | positive regulation of Wnt signaling pathway | ABL1 | AXIN1 | BIRC8 | CAV1 | CCAR2 | CDC73 | CSNK1E | DAB2 | DDX3X | DISC1 | EDA | EGFR | ILK | JUP | LGR4 | LRRK1 | LRRK2 | MLLT3 | PIN1 | PPM1A | PSMA1 | PSMA2 | PSMA3 | PSMA5 | PSMA6 | PSMB5 | PSMB6 | PSMB7 | PSMB8 | PSMC1 | PSMC2 | PSMC3 | PSMC4 | PSMC5 | PSMC6 | PSMD1 | PSMD10 | PSMD11 | PSMD12 | PSMD14 | PSMD2 | PSMD4 | PSMD9 | PSME2 | PSME3 | RBPJ | RECK | RUVBL1 | SCEL | SKI | SMAD3 | SMARCA4 | SMURF2 | SPIN1 | SRC | SULF2 | TBL1X | TNFAIP3 | TNKS | TNKS2 | UBE2B | UBR5 | USP47 | USP8 | VCP | WNK1 | XIAP | YAP1 | GO:0032870 | 0.0270532573092321 | 223/8243 | 115/3250 | 0.000130491339218891 | 1 | T | T | T | T | cellular response to hormone stimulus | ACACA | ADCY6 | ADCYAP1 | ADTRP | AIFM1 | AKAP8 | AKR1C2 | AKT1 | AKT2 | ANXA1 | ANXA5 | APEX1 | APRT | AR | ASNS | ASS1 | ATP1A1 | CASP9 | CAV1 | CDC5L | CDK4 | CHEK2 | CREB1 | CRK | CTSL | DDIT4 | DENND4C | DUSP1 | EGFR | EIF4E | EIF4EBP1 | EPRS | ERRFI1 | ESR1 | FBXO32 | FECH | FGB | FOS | FOXO1 | FYN | GATA1 | GLP1R | GNB1 | GOT1 | GSK3A | GSTP1 | H2AFZ | HDAC4 | HDAC5 | HDAC9 | HNRNPD | HNRNPU | HSF1 | ICAM1 | IGFBP7 | INSR | JUN | JUNB | JUND | KAT5 | LMO2 | LPIN3 | MDM2 | MED1 | MSN | MYO5A | NCOA1 | NCOA2 | NCOA3 | NFE2L2 | NR3C1 | NR4A1 | PARP1 | PAX8 | PCSK9 | PIK3R1 | PKM | PLA2G1B | PPARG | PPARGC1A | PPAT | PRKAA1 | PRKAA2 | PRKACA | PRKAR1A | PRKAR2B | PRKCD | PRKCE | PRKCZ | PRKDC | PTEN | RAB10 | RAB31 | RANGAP1 | RAP1B | RDX | RELA | REST | RUVBL2 | SLC9A1 | SOCS2 | SP1 | SRC | SRSF5 | STAT1 | STAT3 | TBC1D4 | TGFB1 | TRIB3 | UBE2L3 | USP8 | WDTC1 | WT1 | XBP1 | YWHAG | GO:0051091 | 0.0230498604876865 | 190/8243 | 100/3250 | 0.000131158066307548 | 1 | T | T | T | T | positive regulation of DNA-binding transcription factor activity | AGER | AKT1 | APP | AR | ATF2 | BCL10 | BTK | BUD31 | CAMK1D | CAMK2A | CARM1 | CAT | CAV1 | CD36 | CD40 | CD40LG | CLU | COPS5 | CTH | CTNNB1 | DDRGK1 | DHX9 | DVL2 | EDA | EP300 | ESR1 | ESR2 | FLOT1 | FOXA1 | HDAC4 | HDAC5 | HSPA1B | ICAM1 | IKBKB | IKBKG | JUP | KDM1A | KRAS | LRP6 | MAP3K13 | MAVS | MTPN | MYD88 | NEUROD1 | NFKB2 | NOD2 | NPM1 | NTRK1 | PARK7 | PHB2 | PLA2G1B | PPARG | PPARGC1A | PRDX3 | PRKCZ | PRKD1 | PRKD2 | PSMA6 | PTEN | RELA | RIPK1 | RIPK2 | RIPK3 | RIPK4 | RNF25 | RNF31 | RPS3 | S100A12 | S100A9 | SMAD3 | SMARCA4 | SMARCB1 | SMO | STAT3 | STK3 | TAB1 | TAB2 | TAF12 | TERF2IP | TGFB1 | TLR3 | TMEM173 | TNF | TNFRSF8 | TRADD | TRAF1 | TRAF2 | TRAF6 | TRIM14 | TRIM21 | TRIM25 | TRIM32 | TRIM34 | UBB | UBC | UBE2I | UBE2N | UBE2V1 | WNT2 | ZBTB7A | GO:0032409 | 0.0183185733349509 | 151/8243 | 82/3250 | 0.000133325407835803 | 1 | T | T | T | T | regulation of transporter activity | ACTB | ACTN2 | ACTN4 | ADRB2 | AHCYL1 | AHNAK | AKAP9 | ANK3 | APP | ATP1B3 | BCL2 | CAB39 | CACNG2 | CACNG4 | CACNG5 | CALM1 | CALM2 | CALM3 | CAPN1 | CAV1 | CFTR | CHCHD10 | CHP1 | COX17 | DAPK1 | DLG2 | DLG3 | DLG4 | DMD | FHL1 | FKBP1A | FKBP1B | GLRX | GOPC | GPD1L | GRIA1 | GSTO1 | HAP1 | HECW1 | HECW2 | HSPA2 | HTR3A | HTT | KCNAB1 | KCNIP2 | KIF5B | KMT2A | LRRC7 | MYO5A | NEDD4 | NOS1AP | OXSR1 | PARK7 | PCSK9 | PDE4D | PLA2G6 | PPARG | PPARGC1A | PPIF | PRKACA | PRKCD | PRKCE | PTEN | RGN | RIPK1 | S100A1 | SLC9A1 | SLC9A3R1 | SNCA | SRI | STAC | STIM1 | STOM | SUMO1 | THADA | TWIST1 | UBQLN1 | WNK1 | WNK4 | WWP2 | YWHAE | YWHAH | GO:0046902 | 0.00655101298071091 | 54/8243 | 35/3250 | 0.0001362085162037 | 1 | T | T | T | T | regulation of mitochondrial membrane permeability | ACAA2 | ATF2 | BAX | BBC3 | BCL2 | BCL2L1 | CAMK2A | CASP8 | CHCHD10 | E2F1 | EYA2 | GSK3A | GSK3B | HK2 | MAPK8 | PPIF | PPP3CC | RHOT1 | RHOT2 | SFN | SLC25A5 | SLC35F6 | SLC9A1 | STAT3 | TFDP1 | THEM4 | TP53 | TP63 | TP73 | YWHAB | YWHAE | YWHAG | YWHAH | YWHAQ | YWHAZ | GO:0010822 | 0.0109183549678515 | 90/8243 | 53/3250 | 0.000136450623930157 | 1 | T | T | T | T | positive regulation of mitochondrion organization | BAX | BBC3 | BCL2 | CASP8 | CDC37 | CHCHD10 | CSNK2A2 | DCN | DNM1L | E2F1 | FBXO7 | FIS1 | GSK3A | GSK3B | HDAC6 | HIF1A | HK2 | HSPA1L | HTRA2 | HTT | HUWE1 | KDR | MAPK8 | MARCH5 | MLLT11 | NPEPPS | OPA1 | OPTN | PARK7 | PLA2G6 | PLAUR | PPARGC1A | PPIF | PPP3CC | PRKAA1 | RHOU | RNF31 | SAE1 | SFN | SMURF1 | TFDP1 | TP53 | TP63 | TP73 | UBE2D3 | UBE2L3 | WDR35 | YWHAB | YWHAE | YWHAG | YWHAH | YWHAQ | YWHAZ | GO:0034614 | 0.0109183549678515 | 90/8243 | 53/3250 | 0.000136450623930157 | 1 | T | T | T | T | cellular response to reactive oxygen species | ABL1 | AIFM1 | AKT1 | ANXA1 | APEX1 | BTK | CBX8 | CDK2 | CRK | DPEP1 | EGFR | ETS1 | EZH2 | FABP1 | FOS | FOXO1 | HDAC2 | HDAC6 | HNRNPD | HSF1 | JUN | MAPK1 | MAPK3 | MAPK7 | MAPK8 | MAPK9 | MAPT | MDM2 | NFE2L2 | NQO1 | PARK7 | PCNA | PDK2 | PPIF | PRDX3 | PRDX5 | PRKAA1 | PRKCD | PTPRK | PXN | RELA | RIPK1 | RIPK3 | ROMO1 | RPS3 | SIRT1 | SRC | STAT6 | TNFAIP3 | TPM1 | TRAF2 | TREX1 | ZNF277 | GO:0034097 | 0.0451292005337862 | 372/8243 | 181/3250 | 0.000136462566622806 | 1 | T | T | T | T | response to cytokine | ACSL4 | ACTG1 | ACTR2 | ACTR3 | AES | AKT1 | ANXA1 | ASS1 | ATIC | BAG4 | BCAT2 | BCL2 | BCL2L1 | BRCA1 | BSPRY | BTK | CALCOCO2 | CAPN2 | CASP3 | CASP8 | CCL21 | CCL22 | CD40 | CDC34 | CDC42EP4 | CDC5L | CDK4 | CDK9 | CEBPA | CREB1 | CRKL | CTH | CXCL8 | DAPK1 | DDOST | DHX9 | DTX1 | DUSP1 | EED | ENDOG | EPRS | ETS1 | FASN | FGB | FLNB | GAPDH | GBP2 | GBP3 | GBP5 | GBP6 | GFPT2 | GGT1 | GNAO1 | GNPNAT1 | GPD1 | GSK3A | GSK3B | GSS | HAX1 | HDAC4 | HDGF | HIF1A | HK2 | HNRNPU | HSP90AB1 | HSPA5 | HTRA2 | ICAM1 | IFI16 | IFIT1 | IFITM3 | IFNAR1 | IGBP1 | IKBKB | IKBKE | IL3RA | IMPDH2 | IRF1 | ISG15 | ITGA4 | JAK3 | JUN | JUNB | JUND | KDM5B | KEAP1 | KIF5B | KLF5 | KLHL20 | KYNU | LAMP3 | LEF1 | MAP2K7 | MAPK1 | MAPK14 | MAPK3 | MAPK8 | MAT2A | MCM2 | MME | MYD88 | MYO1C | NDUFA13 | NFE2L2 | NFKB2 | NMNAT3 | NOS2 | NR5A2 | NRP2 | OTUB1 | OXSR1 | P4HB | PDCD10 | PDE12 | PDIA3 | PFKP | PHB | PLA2G10 | PLSCR1 | PML | PPARGC1A | PRPF8 | PTGES | PTPN14 | PTPN23 | PTPN4 | PTPN6 | RAD23B | RARA | RARG | RBMX | RC3H1 | REL | RELA | RHOA | RIF1 | RIPK1 | RIPK2 | RPS3 | SHMT1 | SHMT2 | SIRT1 | SLC12A2 | SLC25A5 | SMAD3 | SNCA | SP100 | SPRY2 | SRC | ST3GAL6 | STAT1 | STAT3 | STIP1 | STXBP1 | STXBP2 | TANK | THBS1 | TIMP1 | TIMP2 | TLR3 | TMEM173 | TPR | TRADD | TRAF6 | TREX1 | TRIM21 | TRIM25 | TRIM32 | TRIM63 | TYMS | UBE2K | VCAM1 | VIM | WAS | WDR35 | WNK1 | XAF1 | XBP1 | XRCC5 | YY1 | ZFAND6 | GO:0050821 | 0.0160135872861822 | 132/8243 | 73/3250 | 0.000144035955408562 | 1 | T | T | T | T | protein stabilization | AFM | APOA1 | ATP1B3 | BAG2 | BAG3 | BAG5 | BAG6 | CALR | CCT2 | CCT3 | CCT4 | CCT5 | CCT6A | CCT7 | CCT8 | CDC37 | CDKN1A | CHEK2 | CHP1 | CLU | COG3 | CREB1 | CRYAB | DVL1 | DVL3 | EP300 | EPHA4 | FKBPL | FLOT1 | GAPDH | GNAQ | HSP90AA1 | HSP90AB1 | HSPA1B | HSPD1 | HYPK | MDM4 | NLK | PARK7 | PDCD10 | PEX19 | PFN1 | PHB | PHB2 | PIK3R1 | PIN1 | PPARGC1A | PPIB | PRKCD | PTEN | PTGES3 | RPL23 | RTN4 | SEC16A | SEL1L | SMAD3 | SMO | STK3 | STXBP1 | SUGT1 | SUMO1 | SYVN1 | TBL1X | TCP1 | TNIP2 | TREX1 | UBE2B | USP19 | USP2 | USP7 | USP9X | VHL | ZNF207 | GO:0038061 | 0.00703627320150431 | 58/8243 | 37/3250 | 0.000144360182333407 | 1 | T | T | T | T | NIK/NF-kappaB signaling | AKT1 | BIRC2 | BIRC3 | BTRC | CUL1 | FBXW11 | IKBKE | MAP3K14 | NFKB2 | PSMA1 | PSMA2 | PSMA3 | PSMA5 | PSMA6 | PSMB5 | PSMB6 | PSMB7 | PSMB8 | PSMC1 | PSMC2 | PSMC3 | PSMC4 | PSMC5 | PSMC6 | PSMD1 | PSMD10 | PSMD11 | PSMD12 | PSMD14 | PSMD2 | PSMD4 | PSMD9 | PSME2 | PSME3 | REL | RELA | RIPK3 | GO:0061418 | 0.00752153342229771 | 62/8243 | 39/3250 | 0.000149789549301549 | 1 | T | T | T | T | regulation of transcription from RNA polymerase II promoter in response to hypoxia | ARNT | CREBBP | CUL2 | EGLN1 | EGLN3 | EP300 | HIF1A | HIF1AN | NFE2L2 | PSMA1 | PSMA2 | PSMA3 | PSMA5 | PSMA6 | PSMB5 | PSMB6 | PSMB7 | PSMB8 | PSMC1 | PSMC2 | PSMC3 | PSMC4 | PSMC5 | PSMC6 | PSMD1 | PSMD10 | PSMD11 | PSMD12 | PSMD14 | PSMD2 | PSMD4 | PSMD9 | PSME2 | PSME3 | RBPJ | RBX1 | UBB | UBC | VHL | GO:2001243 | 0.00921994419507461 | 76/8243 | 46/3250 | 0.000151556844691533 | 1 | T | T | T | T | negative regulation of intrinsic apoptotic signaling pathway | AKT1 | ATAD5 | BAG5 | BCL2 | BCL2L1 | BCL2L2 | BDKRB2 | CCAR2 | CD74 | CLU | CREB3 | DDX3X | DNAJA1 | ENO1 | FIGNL1 | GPX1 | HDAC1 | HIF1A | HSPB1 | HTRA2 | KDM1A | LRRK2 | MAPK7 | MDM2 | MIF | NDUFA13 | NDUFS3 | NFE2L2 | OPA1 | PARK7 | PLAUR | PPIF | PTPN1 | RRM2B | SIRT1 | SNAI2 | SOD2 | SRC | SYVN1 | TMBIM6 | TRAP1 | TRIAP1 | TRIM32 | USP47 | VDAC2 | XBP1 | GO:0022904 | 0.00776416353269441 | 64/8243 | 40/3250 | 0.0001515657843193 | 1 | T | T | T | T | respiratory electron transport chain | ALDH5A1 | COQ9 | COX15 | COX5A | COX6A1 | COX6C | CYC1 | CYCS | DLD | ETFA | ETFB | ETFDH | MECP2 | MYBBP1A | NDUFA10 | NDUFA13 | NDUFA5 | NDUFA8 | NDUFA9 | NDUFAF1 | NDUFAF2 | NDUFB1 | NDUFS1 | NDUFS2 | NDUFS3 | NDUFS6 | NDUFS7 | NDUFS8 | NDUFV1 | NDUFV3 | PPARGC1A | SDHA | SDHB | SDHD | SNCA | TAZ | UQCRB | UQCRC2 | UQCRFS1 | UQCRQ | GO:2000060 | 0.00873468397428121 | 72/8243 | 44/3250 | 0.000153323765134337 | 1 | T | T | T | T | positive regulation of ubiquitin-dependent protein catabolic process | AKT1 | AXIN1 | BAG2 | CAV1 | CBFA2T3 | CCDC22 | CEBPA | CLU | CSNK1A1 | CSNK1E | DAB2 | DDRGK1 | DNAJB2 | DVL1 | GSK3A | GSK3B | HSPA1B | HSPBP1 | KEAP1 | LRRK2 | MAPK9 | MDM2 | NFE2L2 | PIAS1 | PLK1 | PSEN1 | PSMD10 | PTEN | PTK2 | RAD23A | RBX1 | RNF139 | RNF19A | SGTA | SMURF1 | STUB1 | SUMO1 | SUMO2 | TRIB2 | TRIB3 | UBQLN1 | UBQLN2 | USP5 | VCP | GO:1903827 | 0.0463423510857697 | 382/8243 | 185/3250 | 0.000158605085083362 | 1 | T | T | T | T | regulation of cellular protein localization | ABHD17A | ACTB | ACTN2 | AGR2 | AKT1 | AKT2 | ANK3 | APC | APOD | AR | ATAD1 | ATP13A2 | B3GAT3 | BAG3 | BAG4 | BBC3 | BCL2 | BCL2L1 | CACNG2 | CASP8 | CCDC88A | CCT2 | CCT3 | CCT4 | CCT5 | CCT6A | CCT7 | CCT8 | CD36 | CDH1 | CDK5 | CDK5R1 | CEP290 | CHP1 | CLDN18 | CLTC | COMMD1 | CRKL | CROCC | CSNK1E | CSNK2A2 | CTNNB1 | CYLD | DAB2 | DCLK1 | DDRGK1 | DMAP1 | DNAJA1 | DVL1 | DVL3 | E2F1 | EDEM1 | EGFR | EIF2AK3 | ERBB2 | EZR | FIS1 | FYN | GDI1 | GNAI1 | GOPC | GPD1L | GSK3A | GSK3B | HAX1 | HDAC3 | HRAS | HSP90AA1 | HSP90AB1 | HSP90AB3P | HSP90AB4P | HSPA1L | HTRA2 | HUWE1 | INSIG1 | IPO5 | IQGAP1 | ITGB1BP1 | ITGB3 | JUP | KAT7 | KCNAB2 | KDM1A | KIF5B | LCP1 | LGALS3 | LMNA | LRRK2 | LYPLA1 | MAPK1 | MAPK14 | MAPK8 | MAPT | MAVS | MDM2 | MSN | MYO1C | NF2 | NIPBL | NMD3 | NPEPPS | NSFL1C | NUCB1 | NUMA1 | PARD6A | PARK7 | PARP1 | PCM1 | PDCD10 | PIK3R1 | PIN1 | PINX1 | PKIG | PLK1 | PPM1A | PPP3CC | PRKAA1 | PRKAA2 | PRKACA | PRKCD | PRKCE | PRKCZ | PSEN1 | PTN | PTPN1 | PTPN14 | RAB11A | RAB11B | RAB11FIP2 | RAB29 | RAN | RANGAP1 | RAP1A | RB1 | RDX | RHOU | RNF31 | RTN4 | SAE1 | SEC16A | SFN | SIN3A | SMAD3 | SMO | SP100 | SPTBN1 | SQSTM1 | SRC | STAC | STK11 | STOM | TARDBP | TCP1 | TERF1 | TFDP1 | TGFB1 | TM9SF4 | TMEM30A | TNF | TOR1A | TP53 | TP63 | TP73 | TPR | TRIM28 | TRIM29 | TSG101 | TXN | UBE2D3 | UBE2J1 | UBE2L3 | UBR5 | USP17L2 | USP46 | XPO1 | XPO4 | XPO5 | YWHAB | YWHAE | YWHAG | YWHAH | YWHAQ | YWHAZ | ZIC1 | ZMYND8 | GO:0070932 | 0.0021836709935703 | 18/8243 | 15/3250 | 0.000174886008579412 | 1 | T | T | T | T | histone H3 deacetylation | HDAC1 | HDAC11 | HDAC2 | HDAC3 | HDAC4 | HDAC5 | HDAC6 | HDAC7 | HDAC9 | PER1 | PER2 | SFPQ | SIRT1 | SIRT6 | SIRT7 | GO:0009416 | 0.0215940798253063 | 178/8243 | 94/3250 | 0.000176114331224923 | 1 | T | T | T | T | response to light stimulus | AKT1 | AKT2 | APP | ARRB1 | ASNS | AURKB | BAX | BCL2 | BRAF | CABP4 | CASP3 | CASP9 | CAT | CCAR2 | CDC25A | CDK5 | CDKN1A | CIRBP | CREB1 | CREBBP | CRIP1 | CRY1 | CRY2 | CUL4B | DBH | DDB1 | DDB2 | DHX36 | DUSP1 | EGFR | EIF2AK4 | EIF2S1 | EP300 | ERCC5 | FECH | FEN1 | FOS | GNAQ | GNB1 | GPX1 | GRK1 | HIF1A | ID2 | KDM1A | KRAS | MAP2K7 | MAP3K4 | MAPK8 | MC1R | MDM2 | MECP2 | MME | MSH6 | MTA1 | MYC | NEDD4 | NPM1 | PARP1 | PCNA | PDE6G | PER1 | PER2 | PIAS1 | PIK3R1 | PML | POLD1 | PPID | PPP1CA | PPP1CB | PPP1CC | PRKAA1 | PTN | PTPRK | RBM4 | RDH13 | RELA | RHBDD1 | RHO | RUVBL2 | SAG | SCARA3 | SIRT1 | SPRTN | STK11 | TP53 | TRIAP1 | TRIM32 | UBE2A | UBE2B | UNC119 | USP2 | USP28 | USP47 | YY1 | GO:0042325 | 0.110639330340895 | 912/8243 | 410/3250 | 0.000180580060142048 | 1 | T | T | T | T | regulation of phosphorylation | ABL1 | ACSL1 | ACTB | ADCY6 | ADCYAP1 | ADORA2A | ADRB2 | ADTRP | AGAP2 | AGER | AHSG | AKAP8 | AKAP9 | AKT1 | AKT2 | AKTIP | ANXA2 | APC | APOA1 | APOE | APP | AR | ARAF | ARR3 | ARRB1 | ARRB2 | ATM | AXIN1 | AZU1 | BAG4 | BAX | BCAR3 | BCL10 | BCL2 | BDKRB2 | BIRC7 | BMPR1A | BRAF | BRD4 | C1QL4 | C1QTNF1 | CAB39 | CALM1 | CALM2 | CALM3 | CARD9 | CASP3 | CAV1 | CBL | CBLB | CBLC | CCDC88A | CCL21 | CCL22 | CCNB1 | CCNT1 | CCNT2 | CCNY | CCNYL1 | CCR1 | CD109 | CD27 | CD36 | CD40 | CD40LG | CD74 | CD81 | CDC25A | CDC25B | CDC25C | CDC37 | CDK4 | CDK5 | CDK5R1 | CDK9 | CDKN1A | CDKN2C | CEBPA | CHEK2 | CHORDC1 | CHP1 | CHRNA9 | CKS1B | CKS2 | CLU | COPS5 | COX11 | COX7A2 | CRK | CRKL | CSNK2B | CTNNB1 | CYLD | DAB1 | DAB2 | DAXX | DBF4B | DBNDD2 | DBNL | DDIT4 | DDRGK1 | DDX3X | DGKZ | DLG3 | DLG4 | DMD | DNAJA1 | DNAJC10 | DNAJC30 | DOCK7 | DUSP1 | DUSP12 | DUSP14 | DUSP16 | DUSP19 | DUSP22 | DUSP7 | DVL1 | DVL2 | DVL3 | DYNLL1 | EEF1A2 | EGFR | EHD4 | EIF2AK3 | EIF2AK4 | EIF3A | EPHA4 | ERBB2 | ERBB3 | ERRFI1 | EZH2 | EZR | FAF1 | FAM83D | FBXO7 | FGB | FGF2 | FGFR1 | FGFR1OP | FGFR3 | FKBP8 | FLOT1 | FN1 | FOXM1 | FOXO1 | FYN | GADD45G | GATA1 | GDF9 | GLMN | GNAI2 | GNAQ | GPD1L | GPS2 | GRB2 | GRIK2 | GSTP1 | HAVCR2 | HAX1 | HBEGF | HDAC2 | HDAC3 | HDAC6 | HEXIM1 | HGS | HMGB1 | HNRNPU | HPX | HRAS | HSF1 | HSP90AA1 | HSP90AB1 | HSPA2 | HSPB1 | HTT | IBTK | ICAM1 | IFNA2 | IGBP1 | IGF1R | IKBKB | IKBKG | ILK | INSR | IPO5 | IPO7 | IQGAP1 | ITCH | ITGB1BP1 | ITGB3 | JUN | KDM4D | KDR | KLHDC10 | KRAS | KSR1 | LAT | LATS1 | LCP2 | LDLRAD4 | LEMD2 | LPAR1 | LRP6 | LRRK1 | LRRK2 | LTBR | MAP2K1 | MAP2K3 | MAP2K4 | MAP2K6 | MAP2K7 | MAP3K1 | MAP3K13 | MAP3K14 | MAP3K2 | MAP3K3 | MAP3K4 | MAP4K1 | MAPK1 | MAPK14 | MAPK3 | MAPK8IP3 | MAPKAP1 | MAPT | MAS1 | MAVS | MCM7 | MIF | MLLT1 | MLST8 | MOB1B | MYC | MYOC | NCK1 | NCOR1 | NEK1 | NELFE | NF2 | NMNAT1 | NNT | NOD2 | NPM1 | NQO2 | NRBF2 | NTF3 | NTRK1 | OXSR1 | PAFAH1B1 | PAK2 | PAK6 | PARD3 | PARD6A | PARK7 | PARP14 | PDCD10 | PDE12 | PDE4D | PDE6G | PEBP1 | PER1 | PGK1 | PGK2 | PHB | PHB2 | PIH1D1 | PIK3CA | PIK3CG | PIK3R1 | PIK3R3 | PIN1 | PKIG | PLA2G1B | PLA2G2A | PLA2G6 | PLAUR | PLK1 | PLXNB2 | PML | PPARGC1A | PPIF | PPP1R14A | PPP2CA | PPP2R1A | PPP2R5D | PRDX1 | PRDX2 | PRDX3 | PRKAA1 | PRKACA | PRKAG1 | PRKAR1A | PRKAR2B | PRKCD | PRKCE | PRKCZ | PRKD1 | PRKD2 | PRKDC | PROX1 | PRRC1 | PSAP | PSEN1 | PSMD10 | PTEN | PTGES3 | PTK2 | PTPN1 | PTPN22 | PTPN6 | PTPRR | QARS | RAC1 | RAF1 | RANBP9 | RAP1A | RAP1B | RB1 | RB1CC1 | RGN | RGS2 | RHOA | RIPK1 | RIPK2 | RIPK3 | RPLP1 | RPS3 | RPTOR | S100A12 | S100A7 | SDC4 | SDCBP | SEMA7A | SERPINB3 | SFN | SHC1 | SHMT2 | SIRT1 | SIRT3 | SLAMF1 | SLAMF8 | SLC9A3R1 | SMAD4 | SMG8 | SNCA | SNF8 | SNX6 | SOCS1 | SOCS2 | SOCS6 | SOD1 | SOX2 | SPRY2 | SQSTM1 | SRC | STAT3 | STK11 | STK24 | STK25 | STK26 | STK3 | STK38 | STK40 | STRAP | STYX | SYNJ2BP | TAB1 | TAB2 | TAOK3 | TARDBP | TERF2IP | TGFB1 | TGFBR3 | THBS1 | TIMP2 | TLR3 | TNF | TNFAIP3 | TNFRSF1A | TNK2 | TP53 | TP73 | TRAF2 | TRAF3IP1 | TRAF6 | TRIB2 | TRIB3 | TSG101 | TSPYL2 | TWF1 | TWIST1 | TXN | UBB | UBC | UBE2B | UBE2K | UBE2N | UBE2V1 | UCHL1 | UNC119 | VCP | VTN | WARS | WNK1 | WNT16 | XRCC5 | XRCC6 | YES1 | YWHAG | ZNF622 | GO:0052548 | 0.0302074487443892 | 249/8243 | 126/3250 | 0.000184892910472562 | 1 | T | T | T | T | regulation of endopeptidase activity | A2M | ADORA2A | ADRM1 | AGER | AHSG | AIFM1 | AKT1 | ALOX12 | APP | ARRB1 | ARRB2 | ATP13A2 | BAX | BBC3 | BCL10 | BCL2L13 | BIN1 | BIRC2 | BIRC3 | BIRC5 | BIRC7 | BIRC8 | C4A | CASP10 | CASP8 | CASP9 | CD109 | CD27 | CRYAB | CYCS | DAPK1 | DDX3X | DHCR24 | DIABLO | DPEP1 | EGLN3 | EIF2AK3 | EPHA4 | EPPIN | FABP1 | FADD | FASLG | FIS1 | FYN | GAPDH | GPI | GPX1 | HDAC1 | HMGB1 | HSF1 | HSPD1 | HSPE1 | HTRA2 | IFI16 | IGBP1 | LAMP3 | LEF1 | LGMN | MAPT | MBP | MDM2 | MGMT | MYC | NDUFA13 | P2RX1 | PAK2 | PAPLN | PARK7 | PDCD6 | PEBP1 | PIH1D1 | PLAUR | PML | PPARG | PRDX3 | PRDX5 | PRELID1 | PSMA3 | PSMB8 | PSMD14 | PSME2 | PSME3 | PTTG1 | RAF1 | RECK | REST | RHOA | RIPK1 | RIPK2 | RPS3 | RPS6KA1 | S100A9 | SERPINB2 | SERPINB3 | SERPINB5 | SERPINB6 | SERPINB8 | SERPINE2 | SERPINH1 | SFN | SIRT1 | SMAD3 | SNCA | SOX2 | SPINK7 | SPINT2 | SRC | STAT3 | THBS1 | TIMP1 | TIMP2 | TNF | TNFSF14 | TP63 | TRADD | TRAF2 | TRIAP1 | UCHL5 | USP14 | USP47 | USP50 | VCP | VTN | WDR35 | XIAP | YWHAE | GO:0048732 | 0.0213514497149096 | 176/8243 | 93/3250 | 0.000184939421276798 | 1 | T | T | T | T | gland development | ABL1 | ACAT1 | ACO2 | ADA | ADCYAP1 | AK4 | ANXA1 | APOA1 | AR | ARHGAP35 | ASNS | ASS1 | BCL2 | BMPR1A | BRAF | CAV1 | CDH1 | CEBPA | CREB1 | CRKL | CSNK2A2 | CSNK2B | CTNNB1 | DDX39B | EDA | EGFR | ETS1 | EZH2 | FADD | FASN | FPGS | GNPNAT1 | HLX | HMGCL | HMOX1 | HNF1A | HNRNPD | HOXA5 | HOXB9 | INSR | JUN | KRAS | LEF1 | MAD1L1 | MAP2K1 | MAPK1 | MAPK3 | MED1 | MPST | NKX2-1 | ONECUT1 | OTC | PAX8 | PCNA | PCSK9 | PIK3CA | PKM | PRDX2 | PRKDC | PROX1 | PSEN1 | PTN | QDPR | RAF1 | RAP1A | RARA | RARG | RB1CC1 | RBPJ | RELA | RGN | RIPK3 | RPGRIP1L | SEC63 | SMAD2 | SMAD3 | SMAD4 | SMARCC1 | SOD1 | SOX2 | SRSF5 | SULF2 | TCF21 | TGFB1 | TGFBR3 | THRA | TYMS | UPF2 | VTN | WDR35 | WT1 | XBP1 | ZMPSTE24 | GO:0043687 | 0.0288729831372073 | 238/8243 | 121/3250 | 0.000192048598097505 | 1 | T | T | T | T | post-translational protein modification | AHSG | ALB | APOA1 | APOE | APP | ASB12 | ASB15 | ASB16 | ASB6 | ASB9 | ATG5 | BTRC | C4A | CALU | CAND1 | CCDC22 | CCDC8 | CHGB | CKAP4 | COMMD1 | COPS2 | COPS5 | COPS6 | CUL1 | CUL2 | CUL3 | CUL4B | CUL5 | CUL7 | DCAF7 | DCAF8 | DCUN1D1 | DDA1 | DDB1 | DDB2 | EVA1A | F5 | FBXL12 | FBXO11 | FBXO32 | FBXO6 | FBXO7 | FBXW11 | FBXW5 | FN1 | FOLH1 | HIF1A | HSP90B1 | IGFBP7 | KBTBD7 | KEAP1 | KLHL20 | KLHL22 | LAMB1 | LCMT2 | LGALS1 | LMO7 | LTBP1 | NAE1 | NEDD8 | NUCB1 | OBSL1 | P4HB | PCSK9 | PDIA6 | PRKCSH | PRSS23 | PSMA1 | PSMA2 | PSMA3 | PSMA5 | PSMA6 | PSMB5 | PSMB6 | PSMB7 | PSMB8 | PSMC1 | PSMC2 | PSMC3 | PSMC4 | PSMC5 | PSMC6 | PSMD1 | PSMD10 | PSMD11 | PSMD12 | PSMD14 | PSMD2 | PSMD4 | PSMD9 | PSME2 | PSME3 | QSOX1 | RAB11A | RAB11B | RAB1A | RAB1B | RAB2A | RAB5A | RBX1 | RHBDD1 | SCG2 | SCG3 | SENP8 | SOCS2 | SOCS6 | SPSB3 | TGOLN2 | TIMP1 | TMEM132A | UBA3 | UBE2M | UBXN7 | UCHL3 | VCAN | VGF | VHL | WDTC1 | WIPI2 | WSB1 | ZBTB16 | GO:1901990 | 0.0395487079946621 | 326/8243 | 160/3250 | 0.0001944377529065 | 1 | T | T | T | T | regulation of mitotic cell cycle phase transition | ACTR1A | AKAP9 | AKT1 | ANAPC4 | ANXA1 | APC | APEX1 | APP | ATAD5 | ATM | AURKB | BAX | BCL2 | BRCA1 | BRD4 | BTRC | CARM1 | CASP2 | CCNB1 | CCP110 | CDC14B | CDC16 | CDC20 | CDC25A | CDC25B | CDC25C | CDC27 | CDC73 | CDK2 | CDK4 | CDKN1A | CDKN2C | CENPF | CENPJ | CEP135 | CEP152 | CEP290 | CEP57 | CEP70 | CHEK2 | CKAP5 | CNTRL | CSNK1E | CUL1 | CUL3 | CUL4B | CYP1A1 | DBF4B | DCTN1 | DDB1 | DDX3X | DUSP1 | DYNC1H1 | DYNC1I2 | DYNC1LI1 | DYNLL1 | E2F1 | E2F4 | ECD | EGFR | EP300 | EZH2 | FBXO7 | FGFR1OP | FHL1 | FZR1 | HECW2 | HMMR | HSP90AA1 | HSPA2 | ID2 | IK | KANK2 | KLHL22 | MAD1L1 | MAD2L1 | MDM2 | MDM4 | MECP2 | MEPCE | MSH6 | MTA3 | NAE1 | OFD1 | PAFAH1B1 | PCM1 | PCNA | PHB2 | PINX1 | PLK1 | PML | PPP2R1A | PRKACA | PRKAR2B | PRKDC | PRMT2 | PSMA1 | PSMA2 | PSMA3 | PSMA5 | PSMA6 | PSMB5 | PSMB6 | PSMB7 | PSMB8 | PSMC1 | PSMC2 | PSMC3 | PSMC4 | PSMC5 | PSMC6 | PSMD1 | PSMD10 | PSMD11 | PSMD12 | PSMD14 | PSMD2 | PSMD4 | PSMD9 | PSME2 | PSME3 | PTEN | PTPN6 | RAB11A | RAD21 | RB1 | RBX1 | RDX | RIPK1 | RNASEH2B | RPA2 | RPTOR | SEPT7 | SFN | SIN3A | SPDL1 | TAOK3 | TFDP1 | TGFB1 | TMOD3 | TP53 | TPR | TRIAP1 | TRIP13 | TUBA1A | TUBA4A | TUBB | TUBB4A | TUBB4B | TUBG1 | UBE2C | UBE2D1 | USP47 | VPS4B | XRCC3 | YWHAE | YWHAG | ZFYVE19 | ZNF207 | ZW10 | GO:0090407 | 0.0343321606211331 | 283/8243 | 141/3250 | 0.000196775074043814 | 1 | T | T | T | T | organophosphate biosynthetic process | ACACA | ACAT1 | ACLY | ACOT7 | ACSL1 | ACSL4 | ACSL5 | ACSS2 | ADA | ADCY6 | ADK | ADSL | ADSS | AGK | AK1 | AK2 | AK4 | AKR1A1 | ALDOA | APOA1 | APRT | ARF1 | ASPDH | ATIC | ATM | CBR4 | CKM | CMPK1 | CPNE3 | CSNK2A2 | CSNK2B | CTPS1 | CYC1 | DCAKD | DCK | DCXR | DGKE | DGUOK | DLAT | DLD | DNAJC30 | DPM1 | DUT | FABP5 | FASN | FDPS | FGF2 | G6PD | GARS | GART | GCDH | GGPS1 | GMPS | HAAO | HEXB | HPRT1 | HSD17B8 | IDH2 | IDI2 | IMPA1 | IMPDH2 | IP6K1 | ISYNA1 | KYNU | LDHC | LPIN3 | MAPK1 | ME1 | MOCOS | MPC2 | MPPE1 | MTHFD1 | MTMR14 | MTMR7 | MVD | NAMPT | NAPRT | NFS1 | NME1 | NME2 | NME4 | NMNAT1 | NMNAT3 | NMRK2 | NPR1 | PAICS | PAPSS1 | PAPSS2 | PCTP | PCYT2 | PDHA1 | PDHA2 | PDHB | PDHX | PFAS | PHOSPHO1 | PI4K2A | PIGU | PIK3C2A | PIK3CA | PIK3CG | PIK3R1 | PIK3R3 | PIKFYVE | PIP4K2A | PLA2G6 | PLCG1 | PLSCR1 | PNKP | PNP | PPARD | PPAT | PPT1 | PTEN | PTPMT1 | PYGL | RAB5A | RFK | RRM1 | RRM2 | RRM2B | SHMT1 | SLC25A1 | SLC25A13 | SORD | SPTLC2 | STARD7 | STOML2 | TAMM41 | TAZ | TECR | TGFB1 | TK1 | TK2 | TKT | TPI1 | TYMS | UCK2 | UMPS | UPP2 | VAC14 | GO:0051347 | 0.052650733956084 | 434/8243 | 207/3250 | 0.00019894882902551 | 1 | T | T | T | T | positive regulation of transferase activity | ABL1 | ACD | ACSL1 | ADCY6 | ADCYAP1 | ADRB2 | AGAP2 | AGER | AKT1 | ARRB1 | AURKB | AXIN1 | AZU1 | BCL10 | BIRC7 | BMI1 | BRAF | CAB39 | CALM1 | CALM2 | CALM3 | CCDC88A | CCL21 | CCNB1 | CCNT1 | CCNT2 | CCNY | CCNYL1 | CCT2 | CCT4 | CD40 | CD40LG | CD74 | CD81 | CDC14B | CDC20 | CDC25B | CDK5 | CDK5R1 | CDKN1A | CKS1B | CKS2 | CLU | CRK | CRKL | CTNNB1 | DAB1 | DAXX | DBF4B | DBNL | DCUN1D1 | DDX3X | DGKZ | DLG3 | DLG4 | DUSP12 | DUSP19 | DUSP7 | DVL2 | DVL3 | EEF1A2 | EGFR | EPHA4 | ERBB2 | ERBB3 | EZH2 | FGF2 | FGFR1 | FGFR3 | FYN | FZR1 | GADD45G | GOLGA2 | GSK3A | HNRNPA2B1 | HNRNPD | HRAS | HSP90AA1 | HSP90AB1 | IKBKG | ILK | INSR | IQGAP1 | ITGB1BP1 | ITGB3 | KRAS | LAT | LCP2 | LPAR1 | LRRK2 | MAP2K1 | MAP2K3 | MAP2K4 | MAP2K6 | MAP2K7 | MAP3K1 | MAP3K13 | MAP3K14 | MAP3K2 | MAP3K3 | MAP3K4 | MAP4K1 | MAPK1 | MAPK14 | MAPK15 | MAPK3 | MAPK8IP3 | MAPKAP1 | MAPT | MAS1 | MIF | MLST8 | MOB1B | MYC | NEK1 | NEK7 | NOD2 | NTF3 | NTRK1 | OXSR1 | PAK2 | PAK6 | PARK7 | PDCD10 | PDE6G | PIH1D1 | PIK3CA | PIK3CG | PIN1 | PLA2G1B | PLK1 | PNKP | POT1 | PPP2CA | PRDX2 | PRKAA1 | PRKACA | PRKAG1 | PRKAR1A | PRKAR2B | PRKCD | PRKCZ | PRKD1 | PROX1 | PRRC1 | PSEN1 | PSMD10 | PTEN | PTGES3 | PTK2 | PTPN1 | RAB1A | RAF1 | RAP1A | RFC2 | RFC3 | RFC4 | RFC5 | RHOA | RIPK1 | RIPK2 | RIPK3 | RPLP1 | RPS2 | RPS3 | RPTOR | S100A12 | SDC4 | SHC1 | SIRT1 | SNCA | SOD1 | SPRY2 | SRC | STK11 | STK24 | STK25 | STK26 | STK3 | STUB1 | TAB1 | TAB2 | TAOK3 | TCP1 | TGFB1 | THBS1 | TLR3 | TNF | TNKS | TP73 | TRAF2 | TRAF6 | TRIB3 | TXN | UBB | UBC | UBE2C | UBE2I | UBE2L3 | UBE2N | UBE2S | UBE2V1 | UNC119 | WNK1 | XRCC5 | XRCC6 | ZNF622 | GO:0051239 | 0.205507703506005 | 1694/8243 | 732/3250 | 0.000203481007799525 | 1 | T | T | T | T | regulation of multicellular organismal process | ABAT | ABL1 | ABL2 | ACE2 | ACOT13 | ACSL1 | ACTR2 | ADA | ADCY6 | ADCYAP1 | ADORA2A | ADRB2 | ADTRP | AFG3L2 | AGER | AGO1 | AGO2 | AHCYL1 | AHI1 | AHSG | AKAP8 | AKAP9 | AKT1 | AKT3 | ALOX12 | ANXA1 | ANXA2 | ANXA5 | ANXA6 | AP3D1 | APBB1 | APC | APCS | APOA1 | APOD | APOE | APP | AR | ARF1 | ARHGAP35 | ARHGDIA | ARHGEF9 | ARNT | ARRB1 | ARRB2 | ASCL1 | ATAD5 | ATF2 | ATG5 | ATOH1 | ATP1A1 | ATP1B3 | ATP2A2 | ATP2B2 | AVIL | AZU1 | BAG5 | BAG6 | BASP1 | BAX | BCAR1 | BCL10 | BCL11A | BCL2 | BCL6 | BCOR | BDKRB2 | BIN1 | BIRC2 | BIRC3 | BMPR1A | BRAF | BRCA1 | BTK | C1QA | C1QBP | C1QTNF1 | CACNG2 | CACNG4 | CACNG5 | CALM1 | CALM2 | CALM3 | CALR | CAMK1D | CAMK2A | CAMK2B | CAPN2 | CARD9 | CARM1 | CASP8 | CAV1 | CBFB | CCDC88A | CCNB1 | CCR1 | CCR3 | CD109 | CD2 | CD27 | CD36 | CD40 | CD40LG | CD6 | CD74 | CDC20 | CDC42 | CDC73 | CDH5 | CDK4 | CDK5 | CDK5R1 | CDK9 | CDKL3 | CEBPA | CENPF | CFL1 | CHRD | CHRM2 | CHRM3 | CLDN18 | CLIC1 | CLSTN1 | CLTC | CLU | COL4A2 | COL5A1 | COMT | CPT2 | CREB1 | CREBBP | CRK | CRKL | CRMP1 | CRP | CRY1 | CSNK1E | CSNK2B | CTDP1 | CTH | CTNNB1 | CTNNBIP1 | CTSL | CTSV | CTTN | CUL7 | CXCL6 | CXCL8 | CYLD | DAB1 | DAB2 | DBH | DBN1 | DCN | DDX1 | DDX39B | DDX3X | DDX6 | DECR1 | DES | DGUOK | DHX36 | DHX9 | DISC1 | DLG4 | DMD | DMRT3 | DNM1L | DOCK5 | DOCK7 | DPF2 | DROSHA | DTX1 | DVL1 | DVL2 | DVL3 | DYNC1H1 | DYNLT1 | E2F1 | E2F2 | EAF2 | EED | EFEMP1 | EGFR | EGLN1 | EHMT1 | EIF2AK3 | EIF2AK4 | EIF4A3 | EIF4E | EIF4ENIF1 | EIF6 | ELAVL1 | ENO1 | EP300 | EPHA4 | EPPK1 | ERBB2 | ERBB3 | ERRFI1 | ESR1 | ESRRA | ESRRB | ESRRG | ETS1 | EXOSC6 | EZH2 | EZR | FABP5 | FADD | FAM129B | FAM49B | FANCA | FASLG | FBXO7 | FCN1 | FGB | FGF2 | FGFR1 | FH | FKBP1B | FKBPL | FLOT1 | FN1 | FOS | FOXA1 | FOXJ1 | FOXO1 | FTO | FYN | G6PD | GAA | GADD45G | GAK | GAP43 | GAPDH | GATA1 | GBP5 | GDF9 | GDI1 | GFAP | GJA1 | GLMN | GLP1R | GLRX3 | GLS | GLUL | GNAI2 | GNAO1 | GNAS | GNG5 | GOLGA4 | GPD1L | GPI | GPX1 | GRID2 | GRIP1 | GSK3A | GSK3B | GSTO1 | GSTP1 | GTF2I | H3F3A | HADH | HAP1 | HAVCR2 | HAX1 | HBEGF | HDAC1 | HDAC2 | HDAC3 | HDAC4 | HDAC5 | HDAC6 | HDAC7 | HDAC9 | HECW1 | HECW2 | HGS | HIF1A | HIF1AN | HIST1H3A | HIST1H4A | HIST2H3A | HK1 | HK2 | HLX | HMGB1 | HMGB2 | HMOX1 | HNF4A | HNRNPK | HOXA5 | HRAS | HSF1 | HSP90AB1 | HSPA1B | HSPA5 | HSPA9 | HSPB1 | HSPD1 | HSPG2 | HTATIP2 | HTRA2 | ICAM1 | ID2 | IDH2 | IFI16 | IFNA2 | IFT122 | IGF1R | IGF2BP1 | IKBKB | IKBKE | ILK | INSR | IP6K1 | IQGAP1 | IRF1 | IRF4 | IRF7 | ISG15 | ITCH | ITGAV | ITGB1BP1 | ITGB3 | ITPR1 | ITPR2 | ITPR3 | JAK1 | JAK3 | JUN | JUP | KAT5 | KCNIP2 | KCNK1 | KDM1A | KDM5B | KDR | KEAP1 | KIF13B | KMT2A | KMT2B | KMT2C | L3MBTL1 | LATS1 | LDLRAD4 | LEF1 | LGALS1 | LGALS3 | LGMN | LGR4 | LIG4 | LMNA | LMO2 | LOXL2 | LPAR1 | LRFN1 | LRRC7 | LRRK2 | LSM1 | LTBP1 | LUM | LURAP1 | MAP1B | MAP2K1 | MAP2K3 | MAP2K6 | MAP3K13 | MAP3K3 | MAPK1 | MAPK14 | MAPK3 | MAPK6 | MAPK7 | MAPT | MAVS | MBP | MC1R | MCC | MDM2 | MECP2 | MED1 | MED12 | MEOX2 | METRN | MIB1 | MIF | MITF | MKS1 | MLH1 | MLLT3 | MME | MOV10 | MSH6 | MTPN | MYC | MYD88 | MYH6 | MYL2 | MYL9 | NAP1L1 | NCK1 | NCOA1 | NCOA3 | NEDD4 | NELL1 | NEUROD1 | NF2 | NFATC1 | NFATC2 | NFE2L2 | NFKB2 | NFKBIA | NIN | NIPBL | NKX2-1 | NME1 | NME2 | NOD2 | NOS1AP | NOS2 | NOS3 | NPHP3 | NPLOC4 | NPR1 | NPTX1 | NR1H2 | NR1H3 | NRP2 | NRXN1 | NTF3 | NTRK1 | NUMA1 | NUPR1 | OAS2 | OBSL1 | OGT | OPA1 | ORM1 | OTUD5 | P2RX1 | P2RX4 | PAFAH1B1 | PANX3 | PARD3 | PARD6A | PARK7 | PARP1 | PARP6 | PAX8 | PAX9 | PAXIP1 | PCBP2 | PCM1 | PCSK9 | PCTP | PDCD10 | PDCD6 | PDE4D | PDLIM5 | PEBP1 | PELI1 | PER1 | PER2 | PFN1 | PGAM5 | PGK1 | PHB2 | PHOSPHO1 | PIAS3 | PIK3C2A | PIK3CA | PIK3CG | PIK3R1 | PIM1 | PIN1 | PKM | PLAUR | PLCG1 | PLXNA1 | PLXNA2 | PLXNB2 | PML | PNP | POLR2E | POLR2L | PPARD | PPARG | PPARGC1A | PPIB | PPM1B | PPP1CC | PPP1R11 | PPP1R12A | PPP1R12B | PPP2CA | PPP2R1A | PRCP | PRDX2 | PRDX4 | PRELID1 | PRKAA1 | PRKAB2 | PRKACA | PRKCD | PRKCE | PRKCZ | PRKD1 | PRKD2 | PRKDC | PRMT6 | PROX1 | PRPF19 | PSEN1 | PSMA1 | PSMA2 | PSMA3 | PSMA5 | PSMA6 | PSMB5 | PSMB6 | PSMB7 | PSMB8 | PSMC1 | PSMC2 | PSMC3 | PSMC4 | PSMC5 | PSMC6 | PSMD1 | PSMD10 | PSMD11 | PSMD12 | PSMD14 | PSMD2 | PSMD4 | PSMD9 | PSME2 | PSME3 | PTEN | PTGDS | PTGER3 | PTK2 | PTN | PTPN22 | PTPN23 | PTPN6 | PTPRD | PTPRF | PTPRO | PTPRR | PUM1 | PUS7 | RAB11A | RAB21 | RAB29 | RAC1 | RAP1A | RARA | RARG | RASSF10 | RB1 | RBPJ | RC3H1 | RECK | REL | RELA | REST | RGN | RGS2 | RHBDD1 | RHOA | RHOJ | RIF1 | RIPK1 | RIPK2 | RIPK3 | RPS19 | RPS3 | RTN4 | RTN4IP1 | RUNX1 | RUNX2 | RXRA | S100A1 | S100A9 | S100B | SARM1 | SARS | SART1 | SDCBP | SEC24B | SEMA7A | SEPT7 | SERPINB2 | SERPINB3 | SERPINE2 | SFMBT1 | SFN | SGPL1 | SH3GL1 | SH3GL3 | SIN3A | SIPA1L1 | SIRT1 | SIRT6 | SKI | SLAMF1 | SLAMF8 | SLC9A1 | SLC9A3R1 | SMAD2 | SMAD3 | SMAD4 | SMAD5 | SMO | SMURF1 | SMURF2 | SNAI2 | SNCA | SNTA1 | SNW1 | SNX3 | SNX6 | SOCS1 | SOCS2 | SOD1 | SOD2 | SOX2 | SP1 | SP100 | SPEN | SPI1 | SPRY2 | SPTBN1 | SRC | SRGAP2 | SRI | STARD13 | STAT1 | STAT3 | STAT5A | STAT6 | STIM1 | STK11 | STK24 | STK25 | STK3 | STRAP | SULF2 | SUMO1 | SYDE1 | SYNJ2BP | TBC1D23 | TBXA2R | TCF12 | TCF4 | TGFB1 | TGFBR3 | THBS1 | THBS2 | THRA | TIA1 | TIMP1 | TIMP2 | TLE3 | TLR3 | TMEM173 | TMEM30A | TMEM65 | TNF | TNFAIP3 | TNFRSF12A | TNFRSF1A | TNFRSF8 | TNFSF12 | TNKS2 | TNN | TNNT1 | TP63 | TP73 | TPM1 | TRADD | TRAF2 | TRAF3 | TRAF3IP1 | TRAF6 | TREX1 | TRIB2 | TRIM21 | TRIM25 | TRIM32 | TRIM63 | TRIP12 | TUBB2B | TULP3 | TWF1 | TWF2 | TWIST1 | UBASH3A | UBE2J1 | UBE2L6 | USP17L2 | USP19 | USP50 | VASH2 | VDR | VIM | VSIG4 | VTN | WARS | WDR1 | WDR77 | WNK1 | WNK4 | WNT2 | WT1 | XBP1 | XRCC5 | XRCC6 | YAP1 | YWHAE | YWHAG | YWHAH | YWHAZ | YY1 | ZAP70 | ZBTB16 | ZBTB7B | ZMPSTE24 | ZMYND8 | GO:0019882 | 0.019046463666141 | 157/8243 | 84/3250 | 0.000216882675201955 | 1 | T | T | T | T | antigen processing and presentation | ACTR1A | AP1B1 | AP1G1 | AP1M1 | AP3D1 | ARF1 | ATG5 | CALR | CANX | CAPZA1 | CAPZA2 | CAPZB | CD209 | CD36 | CD74 | CLTC | CTSD | CTSL | CTSV | DCTN1 | DYNC1H1 | DYNC1I1 | DYNC1I2 | DYNC1LI1 | DYNLL1 | FCGR1A | HLA-A | HLA-B | HLA-DQA1 | HLA-DRA | HLA-DRB1 | HLA-DRB3 | HLA-DRB4 | HLA-DRB5 | ICAM1 | IKBKB | IKBKG | ITGAV | ITGB5 | KIF11 | KIF2C | KLC1 | KLC2 | LGMN | OSBPL1A | PDIA3 | PSMA1 | PSMA2 | PSMA3 | PSMA5 | PSMA6 | PSMB5 | PSMB6 | PSMB7 | PSMB8 | PSMC1 | PSMC2 | PSMC3 | PSMC4 | PSMC5 | PSMC6 | PSMD1 | PSMD10 | PSMD11 | PSMD12 | PSMD14 | PSMD2 | PSMD4 | PSMD9 | PSME2 | PSME3 | RAB10 | RAB32 | RAB35 | RAB5B | RAB7A | SEC13 | SEC24B | SEC24C | SEC31A | SH3GL2 | SPTBN2 | TRAF6 | TREX1 | GO:0033048 | 0.00303287637995875 | 25/8243 | 19/3250 | 0.00022024312632481 | 1 | T | T | T | T | negative regulation of mitotic sister chromatid segregation | APC | ATM | ATRX | AURKB | CDC20 | CENPF | IK | KLHL22 | MAD1L1 | MAD2L1 | PLK1 | PTTG1 | RAD21 | SPDL1 | TNKS | TPR | TRIP13 | ZNF207 | ZW10 | GO:0033046 | 0.00303287637995875 | 25/8243 | 19/3250 | 0.00022024312632481 | 1 | T | T | T | T | negative regulation of sister chromatid segregation | APC | ATM | ATRX | AURKB | CDC20 | CENPF | IK | KLHL22 | MAD1L1 | MAD2L1 | PLK1 | PTTG1 | RAD21 | SPDL1 | TNKS | TPR | TRIP13 | ZNF207 | ZW10 | GO:0051985 | 0.00303287637995875 | 25/8243 | 19/3250 | 0.00022024312632481 | 1 | T | T | T | T | negative regulation of chromosome segregation | APC | ATM | ATRX | AURKB | CDC20 | CENPF | IK | KLHL22 | MAD1L1 | MAD2L1 | PLK1 | PTTG1 | RAD21 | SPDL1 | TNKS | TPR | TRIP13 | ZNF207 | ZW10 | GO:0045899 | 0.00109183549678515 | 9/8243 | 9/3250 | 0.000228699423096944 | 1 | T | T | T | T | positive regulation of RNA polymerase II transcriptional preinitiation complex assembly | CAND1 | CREB1 | ESR1 | PSMC2 | PSMC3 | PSMC4 | PSMC5 | PSMC6 | TP53 | GO:0009127 | 0.00181972582797525 | 15/8243 | 13/3250 | 0.000232375936773603 | 1 | T | T | T | T | purine nucleoside monophosphate biosynthetic process | ADA | ADK | ADSL | ADSS | APRT | ATIC | GART | GMPS | HPRT1 | IMPDH2 | PAICS | PFAS | PPAT | GO:0009168 | 0.00181972582797525 | 15/8243 | 13/3250 | 0.000232375936773603 | 1 | T | T | T | T | purine ribonucleoside monophosphate biosynthetic process | ADA | ADK | ADSL | ADSS | APRT | ATIC | GART | GMPS | HPRT1 | IMPDH2 | PAICS | PFAS | PPAT | GO:1902117 | 0.00667232803590926 | 55/8243 | 35/3250 | 0.000233384430506504 | 1 | T | T | T | T | positive regulation of organelle assembly | ARHGAP35 | CAPG | CCDC88A | CCP110 | CENPJ | CEP135 | CROCC | DYNC1H1 | DYNLL1 | FSCN1 | HAP1 | HTT | KCTD17 | LCP1 | LRSAM1 | MAPK15 | MAPK9 | MSN | NUMA1 | PAN2 | PDCD6IP | PIP4K2A | PROX1 | RAB3GAP1 | RHOA | SDC1 | SDC4 | SDCBP | SEPT7 | SEPT9 | SRC | TNF | ULK1 | VPS4B | ZMYND10 | GO:0051702 | 0.00667232803590926 | 55/8243 | 35/3250 | 0.000233384430506504 | 1 | T | T | T | T | interaction with symbiont | ANXA2 | APCS | APOE | AZU1 | CALM1 | CALM2 | CALM3 | CCNT1 | CFL1 | CTDP1 | DDB1 | EIF2AK4 | EP300 | FN1 | GAPDH | GPX1 | HDAC1 | HSPD1 | JUN | LEF1 | PC | POU2F3 | PPIB | PSMC3 | RAB9A | REST | SMARCA4 | SMARCB1 | SMC3 | SNW1 | SP1 | STOM | SUGT1 | TARDBP | VAPA | GO:0019884 | 0.016984107727769 | 140/8243 | 76/3250 | 0.000234820509350766 | 1 | T | T | T | T | antigen processing and presentation of exogenous antigen | ACTR1A | AP1B1 | AP1G1 | AP1M1 | AP3D1 | ARF1 | CALR | CANX | CAPZA1 | CAPZA2 | CAPZB | CD36 | CD74 | CLTC | CTSD | CTSL | CTSV | DCTN1 | DYNC1H1 | DYNC1I1 | DYNC1I2 | DYNC1LI1 | DYNLL1 | FCGR1A | HLA-A | HLA-B | HLA-DQA1 | HLA-DRA | HLA-DRB1 | HLA-DRB3 | HLA-DRB4 | HLA-DRB5 | IKBKB | IKBKG | ITGAV | ITGB5 | KIF11 | KIF2C | KLC1 | KLC2 | LGMN | OSBPL1A | PDIA3 | PSMA1 | PSMA2 | PSMA3 | PSMA5 | PSMA6 | PSMB5 | PSMB6 | PSMB7 | PSMB8 | PSMC1 | PSMC2 | PSMC3 | PSMC4 | PSMC5 | PSMC6 | PSMD1 | PSMD10 | PSMD11 | PSMD12 | PSMD14 | PSMD2 | PSMD4 | PSMD9 | PSME2 | PSME3 | RAB7A | SEC13 | SEC24B | SEC24C | SEC31A | SH3GL2 | SPTBN2 | TRAF6 | GO:0006110 | 0.00691495814630596 | 57/8243 | 36/3250 | 0.000237760974483191 | 1 | T | T | T | T | regulation of glycolytic process | ARNT | CBFA2T3 | DDIT4 | ECD | EIF6 | ESRRB | FBP1 | GAPDHS | GPD1 | HDAC4 | HIF1A | INSR | NCOR1 | NUP107 | NUP153 | NUP160 | NUP205 | NUP214 | NUP85 | NUP88 | NUP93 | NUP98 | OGT | PGAM1 | POM121 | PPARGC1A | PRKAA1 | PRKAA2 | PRKAG1 | RAE1 | SEC13 | SIRT6 | STAT3 | TPR | TREX1 | ZBTB7A | GO:2000377 | 0.00885599902947956 | 73/8243 | 44/3250 | 0.000239809027081772 | 1 | T | T | T | T | regulation of reactive oxygen species metabolic process | ACE2 | ARF4 | ATG5 | BCL2 | BIRC2 | BRCA1 | CD36 | CDKN1A | COQ7 | CRP | CRYAB | EGFR | EIF6 | FOXM1 | FOXO1 | G6PD | GNAI2 | GNAI3 | GRB2 | GSTP1 | HDAC6 | HIF1A | HK2 | MAPK14 | MAPT | NFE2L2 | NQO2 | PARK7 | PRCP | PRDX2 | PRKCD | RAC1 | RGN | RIPK1 | RIPK3 | ROMO1 | SIRT3 | SIRT5 | SOD1 | TGFB1 | THBS1 | TNF | TP53 | VDAC1 | GO:0032200 | 0.00885599902947956 | 73/8243 | 44/3250 | 0.000239809027081772 | 1 | T | T | T | T | telomere organization | ACD | APEX1 | ATM | DNA2 | FEN1 | GAR1 | H3F3A | HIST1H3A | HIST1H4A | HIST3H3 | HSP90AA1 | HSP90AB1 | NOP10 | PARP1 | PCNA | PINX1 | POLA2 | POLD1 | POLE2 | POLE3 | POT1 | PRKDC | PTGES3 | RAD51 | RFC1 | RFC2 | RFC3 | RFC4 | RFC5 | RIF1 | RPA2 | RPA3 | SMG5 | SMG6 | SP100 | TERF1 | TERF2 | TERF2IP | TFIP11 | TINF2 | XRCC1 | XRCC3 | XRCC5 | XRCC6 | GO:0034644 | 0.00715758825670266 | 59/8243 | 37/3250 | 0.000241050516461004 | 1 | T | T | T | T | cellular response to UV | AURKB | BAX | CASP9 | CDC25A | CDKN1A | CREBBP | CRIP1 | CUL4B | DDB1 | DDB2 | DHX36 | EIF2AK4 | EIF2S1 | EP300 | KDM1A | MC1R | MDM2 | MME | MYC | NEDD4 | NPM1 | PARP1 | PCNA | PIK3R1 | POLD1 | PPID | PTN | PTPRK | RHBDD1 | RUVBL2 | SIRT1 | STK11 | TP53 | TRIAP1 | USP28 | USP47 | YY1 | GO:0002218 | 0.0228072303772898 | 188/8243 | 98/3250 | 0.000242164502388121 | 1 | T | T | T | T | activation of innate immune response | BCL10 | BIRC2 | BIRC3 | BTK | BTRC | CARD9 | CASP8 | CD209 | CD36 | CREBBP | CTSL | CUL1 | CYLD | EP300 | FADD | FBXW11 | FCN1 | FGB | FYN | HAVCR2 | HEXIM1 | HMGB1 | HRAS | HSP90B1 | HSPD1 | IFI16 | IKBKB | IKBKE | IKBKG | IRF7 | ITCH | KRAS | LGMN | MAP2K6 | MAP3K1 | MATR3 | MAVS | MYD88 | NFKBIA | NOD2 | PAK2 | PRKACA | PRKCD | PRKCE | PRKDC | PSMA1 | PSMA2 | PSMA3 | PSMA5 | PSMA6 | PSMB5 | PSMB6 | PSMB7 | PSMB8 | PSMC1 | PSMC2 | PSMC3 | PSMC4 | PSMC5 | PSMC6 | PSMD1 | PSMD10 | PSMD11 | PSMD12 | PSMD14 | PSMD2 | PSMD4 | PSMD9 | PSME2 | PSME3 | RAF1 | RELA | RIPK1 | RIPK2 | S100A1 | S100A9 | SCARA3 | SFPQ | SIN3A | SRC | TAB1 | TAB2 | TANK | TLR3 | TMEM173 | TNFAIP3 | TNIP2 | TRAF3 | TRAF6 | UBB | UBC | UBE2D1 | UBE2D2 | UBE2D3 | UBE2N | UBE2V1 | XRCC5 | XRCC6 | GO:0032436 | 0.00740021836709936 | 61/8243 | 38/3250 | 0.000243324997522834 | 1 | T | T | T | T | positive regulation of proteasomal ubiquitin-dependent protein catabolic process | AKT1 | BAG2 | CAV1 | CBFA2T3 | CEBPA | CLU | CSNK1A1 | CSNK1E | DAB2 | DDRGK1 | DNAJB2 | DVL1 | GSK3A | GSK3B | HSPA1B | HSPBP1 | KEAP1 | LRRK2 | MAPK9 | MDM2 | NFE2L2 | PIAS1 | PLK1 | PSEN1 | PSMD10 | RAD23A | RBX1 | RNF19A | SGTA | STUB1 | SUMO1 | SUMO2 | TRIB2 | TRIB3 | UBQLN1 | UBQLN2 | USP5 | VCP | GO:0009411 | 0.0126167657406284 | 104/8243 | 59/3250 | 0.000244802020388139 | 1 | T | T | T | T | response to UV | AKT1 | AURKB | BAX | BCL2 | CASP3 | CASP9 | CAT | CCAR2 | CDC25A | CDKN1A | CIRBP | CREBBP | CRIP1 | CUL4B | DDB1 | DDB2 | DHX36 | EGFR | EIF2AK4 | EIF2S1 | EP300 | ERCC5 | FEN1 | GPX1 | KDM1A | MAP2K7 | MAP3K4 | MAPK8 | MC1R | MDM2 | MME | MSH6 | MYC | NEDD4 | NPM1 | PARP1 | PCNA | PIK3R1 | PML | POLD1 | PPID | PRKAA1 | PTN | PTPRK | RELA | RHBDD1 | RUVBL2 | SCARA3 | SIRT1 | SPRTN | STK11 | TP53 | TRIAP1 | TRIM32 | UBE2A | UBE2B | USP28 | USP47 | YY1 | GO:1903038 | 0.00788547858789276 | 65/8243 | 40/3250 | 0.000245127358800145 | 1 | T | T | T | T | negative regulation of leukocyte cell-cell adhesion | ADORA2A | ADTRP | AKT1 | ANXA1 | ASS1 | BCL6 | CASP3 | CBFB | CCL21 | CD74 | DTX1 | DUSP22 | ERBB2 | FOXJ1 | GLMN | HAVCR2 | HLX | HMGB1 | IFNA2 | IRF1 | ITCH | JAK3 | LGALS3 | MAD1L1 | PAG1 | PAWR | PELI1 | PRDX2 | PRKAR1A | PTPN22 | PTPN6 | RC3H1 | RUNX1 | SDC4 | SOCS1 | SOCS6 | TGFB1 | VSIG4 | WNK1 | ZBTB7B | GO:1900182 | 0.00788547858789276 | 65/8243 | 40/3250 | 0.000245127358800145 | 1 | T | T | T | T | positive regulation of protein localization to nucleus | AKT1 | BAG3 | CCT2 | CCT3 | CCT4 | CCT5 | CCT6A | CCT7 | CCT8 | CDH1 | DMAP1 | EIF2AK3 | FYN | HDAC3 | IPO5 | JUP | KAT7 | MAPK1 | MAPK14 | MAVS | NMD3 | PARK7 | PARP1 | PIK3R1 | PINX1 | PLK1 | PRKCD | PSEN1 | RAN | SMAD3 | SMO | SRC | STK11 | TARDBP | TCP1 | TGFB1 | TPR | TRIM28 | UBR5 | ZIC1 | GO:0043470 | 0.00788547858789276 | 65/8243 | 40/3250 | 0.000245127358800145 | 1 | T | T | T | T | regulation of carbohydrate catabolic process | ARNT | CBFA2T3 | DDIT4 | ECD | EIF6 | ESRRB | FBP1 | GAPDHS | GPD1 | GSK3A | HDAC4 | HIF1A | INSR | NCOR1 | NUP107 | NUP153 | NUP160 | NUP205 | NUP214 | NUP85 | NUP88 | NUP93 | NUP98 | OGT | PGAM1 | POM121 | PPARGC1A | PPP1CA | PPP1CB | PPP1R3B | PRKAA1 | PRKAA2 | PRKAG1 | RAE1 | SEC13 | SIRT6 | STAT3 | TPR | TREX1 | ZBTB7A | GO:0007179 | 0.00788547858789276 | 65/8243 | 40/3250 | 0.000245127358800145 | 1 | T | T | T | T | transforming growth factor beta receptor signaling pathway | APOA1 | ARRB2 | BMPR1A | CBL | CDH5 | CGN | CREB1 | DUSP22 | FERMT2 | FOS | GDF9 | ITGB5 | JUN | NLK | PARD3 | PARD6A | PARP1 | PML | PRKCZ | PTK2 | PTPRK | PXN | RHOA | SIRT1 | SKI | SMAD2 | SMAD3 | SMAD4 | SMAD5 | SMAD9 | SMURF1 | SRC | TAB1 | TGFB1 | TGFBR3 | UBB | UBC | USP15 | USP9X | USP9Y | GO:0002478 | 0.0167414776173723 | 138/8243 | 75/3250 | 0.00024554829558385 | 1 | T | T | T | T | antigen processing and presentation of exogenous peptide antigen | ACTR1A | AP1B1 | AP1G1 | AP1M1 | ARF1 | CALR | CANX | CAPZA1 | CAPZA2 | CAPZB | CD36 | CD74 | CLTC | CTSD | CTSL | CTSV | DCTN1 | DYNC1H1 | DYNC1I1 | DYNC1I2 | DYNC1LI1 | DYNLL1 | FCGR1A | HLA-A | HLA-B | HLA-DQA1 | HLA-DRA | HLA-DRB1 | HLA-DRB3 | HLA-DRB4 | HLA-DRB5 | IKBKB | IKBKG | ITGAV | ITGB5 | KIF11 | KIF2C | KLC1 | KLC2 | LGMN | OSBPL1A | PDIA3 | PSMA1 | PSMA2 | PSMA3 | PSMA5 | PSMA6 | PSMB5 | PSMB6 | PSMB7 | PSMB8 | PSMC1 | PSMC2 | PSMC3 | PSMC4 | PSMC5 | PSMC6 | PSMD1 | PSMD10 | PSMD11 | PSMD12 | PSMD14 | PSMD2 | PSMD4 | PSMD9 | PSME2 | PSME3 | RAB7A | SEC13 | SEC24B | SEC24C | SEC31A | SH3GL2 | SPTBN2 | TRAF6 | GO:0034033 | 0.0043673419871406 | 36/8243 | 25/3250 | 0.000250734446205997 | 1 | T | T | T | T | purine nucleoside bisphosphate biosynthetic process | ACACA | ACAT1 | ACLY | ACOT7 | ACSL1 | ACSL4 | ACSL5 | ACSS2 | CBR4 | DCAKD | DLAT | DLD | FASN | GCDH | HSD17B8 | MPC2 | PAPSS1 | PAPSS2 | PDHA1 | PDHA2 | PDHB | PDHX | PPT1 | SLC25A1 | TECR | GO:0034030 | 0.0043673419871406 | 36/8243 | 25/3250 | 0.000250734446205997 | 1 | T | T | T | T | ribonucleoside bisphosphate biosynthetic process | ACACA | ACAT1 | ACLY | ACOT7 | ACSL1 | ACSL4 | ACSL5 | ACSS2 | CBR4 | DCAKD | DLAT | DLD | FASN | GCDH | HSD17B8 | MPC2 | PAPSS1 | PAPSS2 | PDHA1 | PDHA2 | PDHB | PDHX | PPT1 | SLC25A1 | TECR | GO:0033866 | 0.0043673419871406 | 36/8243 | 25/3250 | 0.000250734446205997 | 1 | T | T | T | T | nucleoside bisphosphate biosynthetic process | ACACA | ACAT1 | ACLY | ACOT7 | ACSL1 | ACSL4 | ACSL5 | ACSS2 | CBR4 | DCAKD | DLAT | DLD | FASN | GCDH | HSD17B8 | MPC2 | PAPSS1 | PAPSS2 | PDHA1 | PDHA2 | PDHB | PDHX | PPT1 | SLC25A1 | TECR | GO:0051100 | 0.0144364915686037 | 119/8243 | 66/3250 | 0.000263989000924777 | 1 | T | T | T | T | negative regulation of binding | ACTB | ADRB2 | AES | AKT1 | ARRB2 | AURKB | BAG2 | BAX | CARM1 | CAV1 | CRMP1 | CSNK1E | CTNNBIP1 | DAB2 | DISC1 | DNAJB2 | DVL1 | E2F1 | EIF2S1 | EIF4A3 | FAM192A | GATA1 | GOLGA2 | GPSM1 | GSK3B | GTF2F1 | GZMA | HDAC2 | HMOX1 | HSPA5 | ID2 | IFI16 | IFIT1 | ITGA4 | ITGB1BP1 | JUN | KDM1A | LEF1 | LRRK2 | MAPK3 | MAPK8 | MDFI | MEPCE | NFKBIA | PARK7 | PCSK9 | PER2 | PEX19 | PIN1 | PPP1CA | PRKCD | PSEN1 | PTPRF | RUVBL2 | SIN3A | SMO | SP100 | SRI | STMN1 | STUB1 | STYX | SUMO1 | SUMO3 | TFIP11 | TMBIM6 | TNKS | GO:0071407 | 0.0285090379716123 | 235/8243 | 119/3250 | 0.000266325667378416 | 1 | T | T | T | T | cellular response to organic cyclic compound | ABL1 | ACTB | ACTR2 | ADCY6 | ADCYAP1 | ADTRP | AIFM1 | AKAP9 | AKT1 | ANXA1 | APEX1 | APLP1 | APP | AR | ASS1 | ATP1A1 | BRCA1 | BTRC | CASP3 | CASP7 | CASP8 | CASP9 | CAV1 | CCNB1 | CDC5L | CDH1 | CDK4 | CEBPA | CFTR | CHEK2 | CTNNB1 | CYP1A1 | DDIT4 | DHX9 | DNM1L | EGFR | EIF4E | EIF4EBP1 | ERRFI1 | ESR1 | EZH2 | EZR | FBXO32 | FECH | FIS1 | FLOT1 | FOLR1 | FOXO1 | GNAI1 | GNB1 | GPD1 | GRAMD1A | GSTP1 | H2AFZ | HCN1 | HCN2 | HCN3 | HDAC2 | HNRNPD | HNRNPU | HSF1 | HSP90B1 | HSPA5 | ICAM1 | IFIT1 | INSIG1 | ITPR2 | JUP | KAT5 | KDM1A | KLF5 | LGALS1 | LRP6 | LRRK2 | MAPK1 | MAPK3 | MAVS | MDM2 | MED1 | MGMT | MSN | NCOA3 | NOD2 | NR3C1 | P2RX4 | PAK2 | PDE12 | PDE4D | PIK3CG | PPARG | PPARGC1A | PRKACA | PTN | RAD51 | RAE1 | RAP1A | RAP1B | REST | RUVBL2 | SHMT1 | SHMT2 | SIN3A | SLC16A1 | SLC9A1 | SMAD5 | SMO | SNCA | SOD1 | SRC | STAT1 | STAT3 | SULT1A3 | TGFB1 | TLR3 | TMEM173 | TNF | UBE2L3 | USP8 | WT1 | GO:1903543 | 0.0014557806623802 | 12/8243 | 11/3250 | 0.000271783138805664 | 1 | T | T | T | T | positive regulation of exosomal secretion | ATP13A2 | CHMP2A | HGS | PDCD6IP | RAB7A | SDC1 | SDC4 | SDCBP | SNF8 | TSG101 | VPS4B | GO:0034695 | 0.0014557806623802 | 12/8243 | 11/3250 | 0.000271783138805664 | 1 | T | T | T | T | response to prostaglandin E | ACACA | ADCY6 | AKAP8 | AKT1 | CCL21 | GNB1 | PPARG | PRKAA1 | PRKAA2 | PRKCE | TGFBR3 | GO:1903541 | 0.0014557806623802 | 12/8243 | 11/3250 | 0.000271783138805664 | 1 | T | T | T | T | regulation of exosomal secretion | ATP13A2 | CHMP2A | HGS | PDCD6IP | RAB7A | SDC1 | SDC4 | SDCBP | SNF8 | TSG101 | VPS4B | GO:0046349 | 0.0014557806623802 | 12/8243 | 11/3250 | 0.000271783138805664 | 1 | T | T | T | T | amino sugar biosynthetic process | AMDHD2 | GFPT1 | GFPT2 | GNPDA1 | GNPDA2 | GNPNAT1 | NAGK | NANP | PGM3 | UAP1 | UAP1L1 | GO:0051223 | 0.0509523231833071 | 420/8243 | 200/3250 | 0.000283813558354557 | 1 | T | T | T | F | regulation of protein transport | ABAT | ABL1 | ADORA2A | ADTRP | AGER | AKT2 | ANK3 | ANXA1 | ANXA5 | APBB1 | APOA1 | APOD | ARF1 | ARFIP1 | ARRB1 | ATAD1 | ATG5 | ATP13A2 | B3GAT3 | BAG3 | BAG4 | CACNA1A | CD2 | CD22 | CD36 | CD40 | CD40LG | CDH1 | CDK16 | CDK5 | CDK5R1 | CEP290 | CFTR | CHP1 | CPT1A | CRP | CSNK2A2 | DHX36 | DHX9 | DMAP1 | DNAJA1 | DNM1L | DYNLL1 | EDEM1 | EGFR | ERBB2 | EZR | FCN1 | FGB | FIS1 | FKBP1B | FN1 | FYN | GAPDH | GAPVD1 | GDI1 | GLMN | GLP1R | GLUL | GOLPH3 | GOPC | GPI | GSK3A | GSK3B | HADH | HAVCR2 | HAX1 | HDAC3 | HDAC9 | HIF1A | HK1 | HMGB1 | HNF4A | HRAS | HSPA1L | HSPA8 | HTRA2 | HUWE1 | IDH2 | IFNA2 | INSIG1 | IPO5 | ITGB1BP1 | ITGB3 | ITPR1 | ITPR2 | ITPR3 | JUP | KCNS3 | KIF5B | LCP1 | LGR4 | LRRK2 | LYPLA1 | MAPK1 | MAPK14 | MAPK3 | MAVS | MBP | MDM2 | MIF | MPC2 | MYO1C | NDUFAF2 | NEUROD1 | NOD2 | NOS2 | NPEPPS | NR0B2 | NR1H2 | NR1H3 | NUCB1 | ORM1 | OXCT1 | PANX3 | PARK7 | PCM1 | PDCD10 | PER2 | PFKL | PFKM | PIK3R1 | PKIG | PLA2G1B | PLA2G6 | PML | PPARD | PPIA | PPID | PPM1A | PPP1R11 | PRKAA1 | PRKACA | PRKCD | PRKCE | PRKCZ | PSEN1 | PSMD9 | PTPN1 | PTPN14 | PTPN22 | PTPN23 | RAB11A | RAB29 | RAN | RANGAP1 | RAP1A | RAPGEF4 | REST | RHOU | RIPK2 | RNF31 | SAE1 | SCFD1 | SEC16A | SFN | SIRT3 | SLC16A1 | SLC25A4 | SLC25A5 | SLC25A6 | SMAD3 | SMO | SNX12 | SNX3 | SOCS1 | SP100 | SPTBN1 | SRC | SRI | STOM | TARDBP | TGFB1 | TM9SF4 | TMBIM6 | TMEM30A | TNF | TNFAIP3 | TP53 | TPR | TRAF2 | TRAF6 | TRIM28 | TSG101 | TWIST1 | TXN | UBE2D3 | UBE2J1 | UBE2L3 | UBR5 | USP17L2 | USP46 | USP50 | WWP2 | XBP1 | XPO1 | XPO4 | XPO5 | YWHAE | ZIC1 | GO:0070997 | 0.004852602207934 | 40/8243 | 27/3250 | 0.000296102290899122 | 1 | T | T | T | F | neuron death | AIFM1 | AKT2 | APP | BAG5 | BAX | BCL2 | BCL2L1 | CASP3 | CBL | CDK5 | DIABLO | ENDOG | ERBB3 | FADD | GAPDH | GRIK2 | HSPA5 | LIG4 | LRRK2 | NAE1 | NAMPT | PSEN1 | RB1 | SIAH1 | SLC9A1 | TP63 | USP53 | GO:0090090 | 0.0137086012374136 | 113/8243 | 63/3250 | 0.000297358730882525 | 1 | T | T | T | F | negative regulation of canonical Wnt signaling pathway | AES | APC | APOE | AXIN1 | CAV1 | CHD8 | CSNK1A1 | CTNND1 | CUL3 | CYLD | DAB2 | DVL1 | DVL2 | DVL3 | FOXO1 | G3BP1 | GSK3A | GSK3B | HDAC1 | HECW1 | LATS1 | LRP6 | MAPK14 | MCC | MLLT3 | NPHP3 | PSMA1 | PSMA2 | PSMA3 | PSMA5 | PSMA6 | PSMB5 | PSMB6 | PSMB7 | PSMB8 | PSMC1 | PSMC2 | PSMC3 | PSMC4 | PSMC5 | PSMC6 | PSMD1 | PSMD10 | PSMD11 | PSMD12 | PSMD14 | PSMD2 | PSMD4 | PSMD9 | PSME2 | PSME3 | PTPRO | RBX1 | RUVBL2 | SCYL2 | SLC9A3R1 | SNAI2 | SOX2 | STK11 | STK3 | TLE1 | TLE3 | TNN | GO:0031349 | 0.0354239961179182 | 292/8243 | 144/3250 | 0.000305730312534986 | 1 | T | T | T | F | positive regulation of defense response | AGER | AP1G1 | APP | BCL10 | BIRC2 | BIRC3 | BTK | BTRC | C1QA | CARD9 | CASP8 | CAV1 | CD209 | CD36 | CD6 | CDK19 | CEBPA | CREBBP | CTSL | CUL1 | CYLD | DHX9 | EGFR | EP300 | ETS1 | FADD | FAM19A3 | FBXW11 | FCN1 | FGB | FLOT1 | FYN | GBP5 | HAVCR2 | HEXIM1 | HMGB1 | HMGB2 | HMGB3 | HPX | HRAS | HSP90B1 | HSPA1B | HSPD1 | IFI16 | IKBKB | IKBKE | IKBKG | IRF7 | ITCH | KRAS | LGMN | LRRK2 | LRSAM1 | MAP2K6 | MAP3K1 | MAPK3 | MATR3 | MAVS | MED1 | MICA | MYD88 | NFKBIA | NOD2 | NR1H3 | OPTN | PAK2 | PARK7 | PELI1 | PIK3CG | PLA2G2A | PLSCR1 | PRKACA | PRKCD | PRKCE | PRKDC | PSMA1 | PSMA2 | PSMA3 | PSMA5 | PSMA6 | PSMB5 | PSMB6 | PSMB7 | PSMB8 | PSMC1 | PSMC2 | PSMC3 | PSMC4 | PSMC5 | PSMC6 | PSMD1 | PSMD10 | PSMD11 | PSMD12 | PSMD14 | PSMD2 | PSMD4 | PSMD9 | PSME2 | PSME3 | PTGER3 | PTPN22 | PUM1 | RAF1 | RELA | RIPK1 | RIPK2 | RPS19 | RTN4 | S100A1 | S100A12 | S100A9 | SCARA3 | SFPQ | SIN3A | SLC25A6 | SNCA | SRC | TAB1 | TAB2 | TANK | TGM2 | TLR3 | TMEM173 | TNF | TNFAIP3 | TNFRSF1A | TNIP2 | TRADD | TRAF3 | TRAF6 | UBB | UBC | UBE2D1 | UBE2D2 | UBE2D3 | UBE2K | UBE2N | UBE2V1 | USP17L2 | VAV1 | WDFY1 | XRCC5 | XRCC6 | GO:1902175 | 0.0026689312143637 | 22/8243 | 17/3250 | 0.000343012924653301 | 1 | T | T | T | F | regulation of oxidative stress-induced intrinsic apoptotic signaling pathway | AKT1 | BAG5 | GPX1 | HIF1A | HSPB1 | HTRA2 | MAPK7 | NFE2L2 | P4HB | PARK7 | PARP1 | SFPQ | SIRT1 | SOD1 | SOD2 | TRAP1 | UBQLN1 | GO:0051129 | 0.0561688705568361 | 463/8243 | 218/3250 | 0.000343210586309096 | 1 | T | T | T | F | negative regulation of cellular component organization | ACAA2 | ACD | ACTN4 | ADCY6 | ADD3 | ADTRP | AICDA | AKT1 | ALB | APC | APOA1 | APOD | APOE | APP | AR | ARHGDIA | ARRB2 | ATG5 | ATM | ATRX | ATXN2 | AURKB | AVIL | BAG3 | BAG4 | BAG5 | BCL11A | BCL2L1 | BCOR | BIRC2 | BRCA1 | CAPG | CAPZA1 | CAPZA2 | CAPZB | CARM1 | CAV1 | CCL21 | CCP110 | CDC20 | CDC42 | CDK5 | CDK5R1 | CDKL3 | CENPF | CHMP2A | CLU | CRBN | CRMP1 | CRYAB | CTBP1 | CTNNBIP1 | DAB1 | DCP2 | DDX3X | DGUOK | DLG4 | DNAJA4 | DNAJB1 | DNAJB2 | DNM1L | DUSP22 | EIF4EBP1 | EP300 | EPHA4 | FYN | GAK | GDI1 | GFAP | GPX1 | GSK3B | H1F0 | H1FX | HDAC2 | HDAC6 | HIST1H1A | HIST3H3 | HMGB1 | HNRNPA1 | HNRNPU | HSF1 | HSPA1B | HSPA2 | HSPA8 | HTRA2 | IK | IKBKB | INSIG1 | ITGB1BP1 | KDM1A | KDM4D | KLHL22 | LDLRAD4 | LGALS1 | LGALS3 | LIMA1 | LMNA | LPAR1 | LRRK2 | LRSAM1 | MAD1L1 | MAD2L1 | MAPT | MCM2 | MDM1 | MDM2 | MECP2 | MYOC | NMNAT1 | NPM1 | NR1H2 | NR1H3 | NUBP1 | OPA1 | OTUB1 | PACSIN3 | PAFAH1B1 | PAM16 | PARK7 | PARP1 | PCSK9 | PEX5 | PFN1 | PICK1 | PIH1D1 | PINX1 | PLK1 | POT1 | PPARGC1A | PPIF | PPM1A | PRELID1 | PRKAR1A | PRKCD | PRKCZ | PRKD1 | PRMT6 | PSEN1 | PSMD10 | PTEN | PTK2 | PTN | PTPRO | PTTG1 | RAB29 | RAB7A | RAC1 | RAD21 | RAF1 | RDX | RHOA | RTN4 | SCFD1 | SDCBP | SEMA7A | SET | SH3GL3 | SIN3A | SIRT1 | SKI | SLC25A5 | SLC35F6 | SMAD4 | SMARCB1 | SMG6 | SNCA | SPDL1 | SPI1 | SPRY2 | SPTAN1 | SPTBN1 | SPTBN2 | SRC | STMN1 | STXBP1 | SWAP70 | TBC1D4 | TERF1 | TERF2 | TERF2IP | TFIP11 | TGFB1 | THBS1 | THRA | TINF2 | TMOD3 | TMOD4 | TNF | TNFRSF1A | TNKS | TNKS2 | TOP2A | TPR | TRAF3IP1 | TRIAP1 | TRIM9 | TRIP12 | TRIP13 | TUBB4A | TWF1 | TWF2 | TWIST1 | UBE2B | UBQLN2 | UBQLN4 | UBR5 | ULK1 | UNC119 | USP17L2 | VAT1 | VDAC2 | VIM | WAS | XRCC1 | XRCC5 | YWHAH | ZNF207 | ZW10 | GO:0072594 | 0.0368797767802984 | 304/8243 | 149/3250 | 0.000345232579591957 | 1 | T | T | T | F | establishment of protein localization to organelle | ACAA1 | ACD | ACOX1 | AGK | AKT1 | AP3D1 | AP3M1 | AP4M1 | ATM | BAX | CACNG2 | CACNG4 | CACNG5 | CALM1 | CALM3 | CAT | CBLB | CLU | CROT | CSE1L | DHRS4 | DNAJC19 | ECH1 | ECI2 | EHHADH | FBXO7 | FIS1 | GRPEL1 | GSTK1 | HEATR3 | HGS | HK1 | HK2 | HMGCL | HSD17B4 | HSP90AA1 | HSPA4 | HSPA8 | HSPD1 | IDE | IDH1 | IFT122 | IPO11 | IPO4 | IPO5 | IPO7 | IPO8 | IPO9 | KPNA1 | KPNA2 | KPNB1 | LARS | LMNA | LRRC7 | LRSAM1 | NCOA4 | NDUFA13 | NEDD4 | NIPBL | NOS2 | NUDT19 | NUP107 | NUP153 | NUP214 | NUP85 | NUP88 | NUP93 | NUP98 | NUTF2 | NXT2 | OXA1L | PAM16 | PEX19 | PEX5 | PHB2 | PIH1D1 | PIK3R1 | PML | POM121 | POT1 | RAB10 | RAB11A | RAB3GAP1 | RAB7A | RAN | ROMO1 | RPAIN | RPL10 | RPL10A | RPL12 | RPL18A | RPL22 | RPL23 | RPL23A | RPL24 | RPL30 | RPL37A | RPL38 | RPL6 | RPL7 | RPL7A | RPL9 | RPLP0 | RPLP1 | RPLP2 | RPS12 | RPS13 | RPS14 | RPS15A | RPS19 | RPS2 | RPS20 | RPS24 | RPS29 | RPS3 | RPS4X | RPS8 | RUVBL2 | SEC62 | SEC63 | SGTA | SGTB | SLC25A6 | SMURF1 | SNF8 | SRP14 | SRP19 | STAT3 | TIMM13 | TIMM21 | TIMM23 | TIMM44 | TIMM50 | TNFAIP3 | TNPO1 | TOMM20 | TOMM22 | TOMM40 | TPR | TSPAN17 | TXNIP | UBB | UBC | UBE2D1 | UBE2D2 | UBE2D3 | USP9X | VPS37D | ZFAND6 | GO:1904063 | 0.0055804925391241 | 46/8243 | 30/3250 | 0.000346966829605671 | 1 | T | T | T | F | negative regulation of cation transmembrane transport | ACTN2 | ANK3 | BIN1 | CAB39 | CALM1 | CALM2 | CALM3 | CAV1 | COMMD1 | FKBP1A | FKBP1B | GSTO1 | HECW1 | HECW2 | KCNAB1 | NEDD4 | OXSR1 | PCSK9 | PPIF | PRKCE | PTEN | RGS2 | SLC9A3R1 | SRI | SUMO1 | TGFB1 | THADA | TWIST1 | UBQLN1 | YWHAE | GO:0034605 | 0.0055804925391241 | 46/8243 | 30/3250 | 0.000346966829605671 | 1 | T | T | T | F | cellular response to heat | ATP2A2 | BAG3 | CDKN1A | CLPB | DAXX | DHX36 | EIF2S1 | HDAC2 | HMOX1 | HSF1 | HSF4 | HSP90AA1 | HSPA14 | HSPA1B | HSPA1L | HSPA2 | HSPA5 | HSPA8 | HSPA9 | HTRA2 | MAPT | PDCD6 | PRKACA | STAC | STUB1 | SUMO1 | THBS1 | TPR | VCP | YWHAE | GO:0032206 | 0.0055804925391241 | 46/8243 | 30/3250 | 0.000346966829605671 | 1 | T | T | T | F | positive regulation of telomere maintenance | ACD | ATM | ATRX | AURKB | CCT2 | CCT3 | CCT4 | CCT5 | CCT6A | CCT7 | CCT8 | CTNNB1 | DHX36 | HNRNPA1 | HNRNPA2B1 | HNRNPD | MAP2K7 | MAP3K4 | MAPK1 | MAPK15 | MAPK3 | NEK7 | PML | PNKP | POT1 | SIRT6 | TCP1 | TNKS | TNKS2 | XRCC5 | GO:0036293 | 0.0222006551012981 | 183/8243 | 95/3250 | 0.000361701106016449 | 1 | T | T | T | F | response to decreased oxygen levels | ABAT | ACAA2 | ACTN4 | ADA | ADSL | AGER | AGTRAP | AHCY | AIFM1 | AKT1 | ARNT | ARNT2 | ATM | BCL2 | BIRC2 | CAPN2 | CASP3 | CAT | CAV1 | CBFA2T3 | CBL | CCNB1 | CREB1 | CREBBP | CRYAB | CYP1A1 | DDIT4 | DNM1L | E2F1 | EGLN1 | EGLN3 | EIF4EBP1 | ENDOG | EP300 | ETS1 | FABP1 | FIS1 | GNB1 | HIF1A | HK2 | HMOX1 | HSF1 | HSP90B1 | ICAM1 | IREB2 | ITPR1 | ITPR2 | LDHA | LMNA | LOXL2 | MDM2 | MDM4 | MECP2 | MYC | NAMPT | NDRG1 | NOS2 | NPEPPS | OPA1 | P2RX2 | P4HB | PGK1 | PHB2 | PICK1 | PKM | PLOD2 | PML | PPARD | PPARGC1A | PRKAA1 | PRKCE | PSEN2 | PTEN | PTN | RAF1 | REST | RHOA | S100B | SCFD1 | SIRT1 | SLC9A1 | SMAD3 | SMAD4 | SRC | STUB1 | TGFB1 | TGFBR3 | THBS1 | TM9SF4 | TP53 | TWIST1 | TXN2 | UBQLN1 | VCAM1 | XRCC1 | GO:0034762 | 0.0325124347931578 | 268/8243 | 133/3250 | 0.000366378733421997 | 1 | T | T | T | F | regulation of transmembrane transport | ABL1 | ACTB | ACTN2 | ACTN4 | ADRB2 | AHCYL1 | AHNAK | AKAP9 | AKT1 | AKT2 | ANK3 | APP | ARF1 | ASPSCR1 | ATG5 | ATP1B3 | BAX | BCL2 | BIN1 | BRAF | CAB39 | CACNA1A | CACNG2 | CACNG4 | CACNG5 | CALM1 | CALM2 | CALM3 | CAPN1 | CAV1 | CFTR | CHCHD10 | CHP1 | CLIC1 | CLIC3 | CLIC4 | COMMD1 | COX17 | CRBN | CXCL11 | DAPK1 | DLG2 | DLG3 | DLG4 | DMD | DNAJC30 | FABP5 | FHL1 | FKBP1A | FKBP1B | FYN | G6PD | GLRX | GOPC | GPD1L | GRIA1 | GSK3A | GSTO1 | HAP1 | HCN1 | HCN2 | HCN3 | HECW1 | HECW2 | HK2 | HSPA2 | HTR3A | HTT | INSR | KCNA4 | KCNAB1 | KCNAB2 | KCNIP2 | KCNK16 | KCNS3 | KIF5B | LRRC7 | MAPK14 | MYO5A | NEDD4 | NFE2L2 | NOS1AP | OAZ3 | OXSR1 | PARK7 | PCSK9 | PDE4D | PER2 | PIK3CG | PIK3R1 | PLA2G1B | PLA2G6 | PLCG1 | PPARGC1A | PPIF | PRKACA | PRKCE | PSEN1 | PSEN2 | PTEN | PTPN6 | RAP1A | RGN | RGS2 | RIPK1 | S100A1 | SIRT6 | SLC9A1 | SLC9A3R1 | SNCA | SNTA1 | SRI | STAC | STIM1 | STOM | SUMO1 | TGFB1 | THADA | THBS1 | TNF | TPCN2 | TRIB3 | TWIST1 | UBQLN1 | WDR1 | WNK1 | WNK4 | WWP2 | YES1 | YWHAE | YWHAH | YWHAQ | ZMPSTE24 | GO:0045454 | 0.00630838287031421 | 52/8243 | 33/3250 | 0.00037752744483975 | 1 | T | T | T | F | cell redox homeostasis | APEX1 | DLD | DNAJC10 | GLRX | GLRX3 | GLRX5 | GPX1 | GSR | NFE2L2 | NNT | NOS2 | NOS3 | P4HB | PDIA3 | PDIA4 | PDIA6 | PRDX1 | PRDX2 | PRDX3 | PRDX4 | PRDX5 | PRDX6 | PTGES2 | QSOX1 | SCO2 | TMX2 | TMX4 | TXN | TXN2 | TXNDC11 | TXNDC15 | TXNDC5 | TXNRD1 | GO:0000723 | 0.00849205386388451 | 70/8243 | 42/3250 | 0.000379003575263374 | 1 | T | T | T | F | telomere maintenance | ACD | APEX1 | ATM | DNA2 | FEN1 | GAR1 | HIST1H4A | HIST3H3 | HSP90AA1 | HSP90AB1 | NOP10 | PARP1 | PCNA | PINX1 | POLA2 | POLD1 | POLE2 | POLE3 | POT1 | PRKDC | PTGES3 | RAD51 | RFC1 | RFC2 | RFC3 | RFC4 | RFC5 | RIF1 | RPA2 | RPA3 | SMG5 | SMG6 | SP100 | TERF1 | TERF2 | TERF2IP | TFIP11 | TINF2 | XRCC1 | XRCC3 | XRCC5 | XRCC6 | GO:0034763 | 0.00824942375348781 | 68/8243 | 41/3250 | 0.000383554474444938 | 1 | T | T | T | F | negative regulation of transmembrane transport | ACTN2 | AKT1 | AKT2 | ANK3 | BIN1 | CAB39 | CALM1 | CALM2 | CALM3 | CAV1 | COMMD1 | CRBN | FABP5 | FKBP1A | FKBP1B | GOPC | GSK3A | GSTO1 | HECW1 | HECW2 | KCNAB1 | NEDD4 | OAZ3 | OXSR1 | PCSK9 | PPIF | PRKCE | PTEN | RGS2 | SIRT6 | SLC9A3R1 | SRI | SUMO1 | TGFB1 | THADA | THBS1 | TNF | TWIST1 | UBQLN1 | YWHAE | YWHAQ | GO:1900407 | 0.00655101298071091 | 54/8243 | 34/3250 | 0.000383650544260792 | 1 | T | T | T | F | regulation of cellular response to oxidative stress | AKT1 | BAG5 | CD36 | CTNNB1 | ENDOG | FBXO7 | FYN | GPX1 | HDAC6 | HIF1A | HSPB1 | HTRA2 | LANCL1 | LRRK2 | MAPK7 | MAPKAP1 | NFE2L2 | P4HB | PARK7 | PARP1 | PAWR | PSAP | PYCR1 | RGN | RIPK1 | SFPQ | SIRT1 | SOD1 | SOD2 | STK26 | TNF | TRAP1 | TXN | UBQLN1 | GO:1903747 | 0.00679364309110761 | 56/8243 | 35/3250 | 0.000387960640758905 | 1 | T | T | T | F | regulation of establishment of protein localization to mitochondrion | BAG3 | BAG4 | BBC3 | BCL2 | CASP8 | CSNK2A2 | DNAJA1 | E2F1 | GSK3A | HAX1 | HSPA1L | HTRA2 | HUWE1 | LRRK2 | MAPK8 | MAPT | NPEPPS | PPP3CC | PRKAA1 | RHOU | RNF31 | SAE1 | SFN | TFDP1 | TP53 | TP63 | TP73 | UBE2D3 | UBE2L3 | YWHAB | YWHAE | YWHAG | YWHAH | YWHAQ | YWHAZ | GO:0034404 | 0.00703627320150431 | 58/8243 | 36/3250 | 0.000390598634367205 | 1 | T | T | T | F | nucleobase-containing small molecule biosynthetic process | ACAT1 | ACOT7 | ADA | ADK | AMD1 | APRT | ASPDH | CDA | DCK | DCTPP1 | DERA | DUT | GDA | GMPS | GPX1 | HINT1 | HPRT1 | IMPDH2 | ITPA | MTAP | NME1 | NME2 | NME4 | NT5C2 | NUDT18 | NUDT3 | NUDT9 | PDE4D | PNP | PRTFDC1 | TK1 | TK2 | UCK2 | UMPS | UPP2 | VCP | GO:0008630 | 0.00703627320150431 | 58/8243 | 36/3250 | 0.000390598634367205 | 1 | T | T | T | F | intrinsic apoptotic signaling pathway in response to DNA damage | ABL1 | ATAD5 | ATM | BAG6 | BAX | BCL2 | BCL2L1 | BCL2L2 | BRCA1 | CASP2 | CASP9 | CHEK2 | CRIP1 | DDIT4 | DYRK2 | E2F1 | EP300 | HMOX1 | HTRA2 | IFI16 | IKBKE | MLH1 | MSH6 | NUPR1 | PIK3R1 | PML | PRKDC | SFN | SIRT1 | SNW1 | TNF | TNFRSF1A | TP53 | TP63 | TP73 | USP28 | GO:0051704 | 0.115613247604028 | 953/8243 | 424/3250 | 0.000405398723452344 | 1 | T | T | T | F | multi-organism process | ABAT | ABCE1 | ACE2 | ACTA2 | ADCYAP1 | AICDA | ANXA2 | ANXA7 | AP1B1 | AP1G1 | AP1M1 | AP3M1 | APCS | APOE | APP | ARF1 | ASGR1 | ASGR2 | ATF7 | ATG5 | ATXN1 | AZU1 | BAIAP2 | BAIAP2L1 | BANF1 | BAX | BCL10 | BCL2 | BCL2L1 | BIN1 | BRD2 | BRD4 | BTRC | BYSL | C1QBP | CALCOCO2 | CALM1 | CALM2 | CALM3 | CAPN2 | CARD9 | CARM1 | CASP8 | CAV1 | CBL | CCDC130 | CCL22 | CCNT1 | CCNT2 | CCR3 | CCR4 | CCR5 | CCT5 | CD209 | CD36 | CD40 | CD79B | CD81 | CDC25C | CDC42 | CDH1 | CEBPA | CEBPE | CFL1 | CHD8 | CHMP2A | CHRM2 | CLU | COMT | COPB1 | COPS6 | COTL1 | CREB1 | CREB3 | CREBBP | CRK | CRP | CTBP1 | CTDP1 | CTNNB1 | CTNND1 | CUL1 | CUL2 | CUL5 | CUL7 | CXCL11 | CXCL5 | CXCL6 | CXCL8 | CYP1A1 | DAXX | DCLK1 | DDB1 | DDIT4 | DDX1 | DDX39B | DDX3X | DDX6 | DERL1 | DHX36 | DHX9 | DNAJC9 | DROSHA | DVL1 | DYNC1I2 | DYNC1LI1 | DYNLL1 | DYNLT1 | EEF1G | EGFR | EIF2AK4 | EIF3A | EIF3D | EIF4E | EIF4H | ENO1 | EP300 | EPPIN | ETS1 | EXOSC5 | FADD | FCHSD1 | FDPS | FGB | FKBP5 | FKBP8 | FN1 | FOS | FYN | GAPDH | GBP3 | GBP5 | GBP6 | GLS | GOT2 | GPS2 | GPX1 | GRB2 | GTF2F1 | GZMA | H2AFX | HAVCR2 | HDAC1 | HEXB | HLA-DRA | HMGB2 | HNRNPA1 | HNRNPK | HPX | HRAS | HSP90AB1 | HSPA1B | HSPA8 | HSPB1 | HSPD1 | HTATIP2 | ICAM1 | IDE | IFI16 | IFIT1 | IFITM3 | IFNA2 | IFNAR1 | IGHV3-13 | IGHV3-23 | IGHV3-30 | IGKV3-20 | IGLC7 | IK | IKBKB | IKBKE | IKBKG | ILF3 | INSR | IPO5 | IPO7 | IQGAP1 | IRF1 | IRF4 | IRF7 | ISG15 | ITCH | ITGAV | ITGB3 | ITGB5 | JUN | KAT5 | KDR | KEAP1 | KLC1 | KPNA1 | KPNA2 | KPNB1 | KRAS | LAMP3 | LEF1 | LGALS8 | LIG4 | LRSAM1 | LSM5 | LTBR | LYG2 | LYZL1 | LYZL2 | MAP3K14 | MAPK1 | MAPK14 | MAPK3 | MAVS | MCTS1 | MDM2 | MECP2 | MICA | MLH1 | MSH6 | MYD88 | NAMPT | NCK1 | NEDD4 | NFE2L2 | NFKBIA | NOD2 | NOS2 | NPM1 | NRP2 | NRXN1 | NUP107 | NUP153 | NUP160 | NUP205 | NUP214 | NUP85 | NUP88 | NUP93 | NUP98 | NXF1 | OAS2 | OGT | OPTN | OVGP1 | P2RX1 | PACS1 | PACS2 | PAK2 | PARD6A | PC | PCBP1 | PCBP2 | PCM1 | PCNA | PDCD6IP | PDE12 | PIK3R1 | PLA2G1B | PLA2G2A | PLA2G6 | PLCG1 | PLSCR1 | PML | POLR2E | POM121 | POU2F3 | PPIA | PPIB | PPID | PPM1D | PPP1R11 | PRG2 | PRKCD | PRMT6 | PSMA2 | PSMA3 | PSMB5 | PSMB6 | PSMB7 | PSMB8 | PSMC3 | PTEN | PYGL | RAB1A | RAB1B | RAB29 | RAB5A | RAB7A | RAB9A | RAC1 | RAD23A | RAE1 | RAN | RARA | RB1 | RBPJ | RBX1 | RCC1 | RELA | REST | RGS1 | RHOA | RIPK1 | RIPK2 | RNASE3 | ROMO1 | RPL10 | RPL10A | RPL12 | RPL18A | RPL22 | RPL23 | RPL23A | RPL24 | RPL30 | RPL37A | RPL38 | RPL6 | RPL7 | RPL7A | RPL9 | RPLP0 | RPLP1 | RPLP2 | RPS12 | RPS13 | RPS14 | RPS15A | RPS19 | RPS2 | RPS20 | RPS24 | RPS29 | RPS3 | RPS4X | RPS8 | RPTOR | RXRA | S100A12 | S100A7 | S100A9 | SEC13 | SEPT5 | SEPT6 | SERPINB3 | SET | SGTA | SHC1 | SIRT1 | SLAMF1 | SLAMF8 | SLC25A4 | SLC25A5 | SLC25A6 | SMAD3 | SMARCA4 | SMARCB1 | SMC3 | SND1 | SNW1 | SNX3 | SP1 | SP100 | SPACA3 | SPEN | SRC | SRPK1 | SSB | ST13 | STAT1 | STAT3 | STAU1 | STMN1 | STOM | SUGT1 | SUMO1 | TANK | TARDBP | TCP1 | TGFB1 | THOC2 | THOC6 | THOC7 | TLR3 | TMEM173 | TNF | TNFRSF1A | TNP1 | TNPO1 | TOP1 | TP53 | TP73 | TPR | TREX1 | TRIM23 | TRIM25 | TRIM28 | TRIM34 | TSG101 | TYMS | UBB | UBC | UBE2I | UBE3A | UBR4 | UBR5 | UBXN1 | UMPS | USP7 | VAC14 | VAPA | VAPB | VCP | VDAC1 | VGF | VIM | VPS18 | VPS29 | VPS37D | VPS4B | VTA1 | WDR48 | WWP2 | XPO1 | XRCC5 | XRCC6 | YWHAB | YWHAE | GO:0001932 | 0.103117796918598 | 850/8243 | 381/3250 | 0.000413008635557323 | 1 | T | T | T | F | regulation of protein phosphorylation | ABL1 | ACSL1 | ACTB | ADCY6 | ADCYAP1 | ADORA2A | ADRB2 | ADTRP | AGER | AKAP8 | AKAP9 | AKT1 | AKT2 | AKTIP | ANXA2 | APC | APOA1 | APOE | APP | AR | ARAF | ARR3 | ARRB1 | ARRB2 | ATM | AXIN1 | AZU1 | BAG4 | BAX | BCAR3 | BCL2 | BDKRB2 | BIRC7 | BMPR1A | BRAF | BRD4 | C1QL4 | C1QTNF1 | CAB39 | CALM1 | CALM2 | CALM3 | CARD9 | CASP3 | CAV1 | CBL | CBLB | CBLC | CCDC88A | CCL21 | CCL22 | CCNB1 | CCNT1 | CCNT2 | CCNY | CCNYL1 | CCR1 | CD109 | CD27 | CD36 | CD40 | CD40LG | CD74 | CD81 | CDC25A | CDC25B | CDC25C | CDC37 | CDK4 | CDK5 | CDK5R1 | CDK9 | CDKN1A | CDKN2C | CEBPA | CHEK2 | CHORDC1 | CHP1 | CHRNA9 | CKS1B | CKS2 | CLU | COPS5 | CRK | CRKL | CSNK2B | CTNNB1 | CYLD | DAB1 | DAB2 | DAXX | DBF4B | DBNDD2 | DBNL | DDIT4 | DDRGK1 | DDX3X | DLG3 | DLG4 | DMD | DNAJA1 | DNAJC10 | DOCK7 | DUSP1 | DUSP14 | DUSP16 | DUSP19 | DUSP22 | DUSP7 | DVL1 | DVL2 | DVL3 | EGFR | EHD4 | EIF2AK3 | EIF2AK4 | EIF3A | EPHA4 | ERBB2 | ERBB3 | ERRFI1 | EZH2 | EZR | FAF1 | FAM83D | FBXO7 | FGB | FGF2 | FGFR1 | FGFR1OP | FGFR3 | FKBP8 | FLOT1 | FN1 | FOXM1 | FOXO1 | FYN | GADD45G | GATA1 | GDF9 | GNAI2 | GNAQ | GPD1L | GPS2 | GRB2 | GRIK2 | GSTP1 | HAVCR2 | HAX1 | HBEGF | HDAC2 | HDAC3 | HDAC6 | HEXIM1 | HGS | HMGB1 | HPX | HRAS | HSF1 | HSP90AA1 | HSP90AB1 | HSPA2 | HSPB1 | HTT | IBTK | ICAM1 | IFNA2 | IGBP1 | IGF1R | IKBKG | ILK | INSR | IPO5 | IPO7 | IQGAP1 | ITCH | ITGB1BP1 | ITGB3 | JUN | KDM4D | KDR | KLHDC10 | KRAS | KSR1 | LAT | LATS1 | LCP2 | LDLRAD4 | LEMD2 | LPAR1 | LRP6 | LRRK1 | LRRK2 | LTBR | MAP2K1 | MAP2K3 | MAP2K4 | MAP2K6 | MAP2K7 | MAP3K1 | MAP3K13 | MAP3K14 | MAP3K2 | MAP3K3 | MAP3K4 | MAP4K1 | MAPK1 | MAPK14 | MAPK3 | MAPK8IP3 | MAPKAP1 | MAS1 | MAVS | MIF | MLLT1 | MLST8 | MOB1B | MYC | MYOC | NCK1 | NCOR1 | NEK1 | NELFE | NF2 | NMNAT1 | NNT | NOD2 | NPM1 | NQO2 | NTF3 | NTRK1 | OXSR1 | PAFAH1B1 | PAK2 | PAK6 | PARD3 | PARD6A | PARK7 | PARP14 | PDCD10 | PDE4D | PDE6G | PEBP1 | PER1 | PHB | PHB2 | PIH1D1 | PIK3CA | PIK3CG | PIK3R3 | PIN1 | PKIG | PLA2G1B | PLA2G2A | PLA2G6 | PLAUR | PLK1 | PLXNB2 | PML | PPARGC1A | PPP2CA | PPP2R1A | PPP2R5D | PRDX1 | PRDX2 | PRKAA1 | PRKACA | PRKAG1 | PRKAR1A | PRKAR2B | PRKCD | PRKCE | PRKCZ | PRKD1 | PRKD2 | PRKDC | PROX1 | PRRC1 | PSAP | PSEN1 | PSMD10 | PTEN | PTK2 | PTPN1 | PTPN22 | PTPN6 | PTPRR | QARS | RAC1 | RAF1 | RANBP9 | RAP1A | RAP1B | RB1 | RB1CC1 | RGN | RGS2 | RHOA | RIPK1 | RIPK2 | RIPK3 | RPLP1 | RPS3 | RPTOR | S100A12 | S100A7 | SDC4 | SDCBP | SEMA7A | SERPINB3 | SFN | SHC1 | SIRT1 | SIRT3 | SLAMF1 | SLC9A3R1 | SMAD4 | SMG8 | SNCA | SNF8 | SNX6 | SOCS1 | SOD1 | SOX2 | SPRY2 | SQSTM1 | SRC | STAT3 | STK11 | STK24 | STK25 | STK26 | STK3 | STK38 | STK40 | STRAP | STYX | SYNJ2BP | TAB1 | TAB2 | TAOK3 | TARDBP | TERF2IP | TGFB1 | TGFBR3 | THBS1 | TIMP2 | TLR3 | TNF | TNFAIP3 | TNFRSF1A | TNK2 | TP53 | TP73 | TRAF2 | TRAF3IP1 | TRAF6 | TRIB2 | TRIB3 | TSG101 | TSPYL2 | TWF1 | TWIST1 | TXN | UBB | UBC | UBE2B | UBE2K | UBE2N | UBE2V1 | UCHL1 | UNC119 | VTN | WARS | WNK1 | WNT16 | XRCC5 | XRCC6 | YES1 | YWHAG | ZNF622 | GO:0061621 | 0.0029115613247604 | 24/8243 | 18/3250 | 0.000430545079988249 | 1 | T | T | T | F | canonical glycolysis | ALDOA | ALDOC | ENO1 | ENO2 | ENO3 | GAPDH | GAPDHS | GPI | HK1 | HK2 | PFKL | PFKM | PFKP | PGAM1 | PGAM2 | PGK1 | PKM | TPI1 | GO:2000116 | 0.0198956690525294 | 164/8243 | 86/3250 | 0.000441828530136864 | 1 | T | T | T | F | regulation of cysteine-type endopeptidase activity | ADORA2A | AIFM1 | AKT1 | ALOX12 | ARRB1 | ARRB2 | BAX | BBC3 | BCL10 | BCL2L13 | BIRC2 | BIRC3 | BIRC7 | BIRC8 | CASP10 | CASP8 | CASP9 | CD27 | CRYAB | CYCS | DAPK1 | DDX3X | DHCR24 | DIABLO | DPEP1 | EGLN3 | EIF2AK3 | FABP1 | FADD | FASLG | FIS1 | FYN | GPI | GPX1 | HMGB1 | HSF1 | HSPD1 | HSPE1 | HTRA2 | IFI16 | IGBP1 | LAMP3 | LEF1 | LGMN | MAPT | MDM2 | MGMT | MYC | NDUFA13 | P2RX1 | PAK2 | PARK7 | PDCD6 | PIH1D1 | PLAUR | PML | PPARG | PRDX3 | PRDX5 | RAF1 | REST | RHOA | RIPK1 | RIPK2 | RPS3 | RPS6KA1 | S100A9 | SFN | SIRT1 | SMAD3 | SNCA | SOX2 | SRC | THBS1 | TNF | TNFSF14 | TP63 | TRADD | TRAF2 | TRIAP1 | USP47 | USP50 | VCP | WDR35 | XIAP | YWHAE | GO:0000165 | 0.0293582433580007 | 242/8243 | 121/3250 | 0.000456534379462362 | 1 | T | T | T | F | MAPK cascade | ACTN2 | APOA1 | ARAF | BRAF | BTRC | CALM1 | CCR5 | CRKL | CRYAB | CUL1 | CUL3 | DLG2 | DLG3 | DLG4 | DUSP7 | EGFR | ERBB2 | ERBB3 | FBXW11 | FGF2 | FGFR1 | FGFR3 | FYN | GPS2 | GRB2 | HBEGF | HRAS | HSF1 | IKBKB | IKBKG | IL3RA | ILK | ITGAV | JAK1 | JAK3 | KBTBD7 | KDR | KRAS | KSR1 | LAT | LRRC7 | LRRK2 | MAP2K1 | MAP2K3 | MAP2K4 | MAP2K6 | MAP2K7 | MAP3K1 | MAP3K13 | MAP3K14 | MAP3K3 | MAP3K4 | MAPK1 | MAPK14 | MAPK15 | MAPK3 | MAPK4 | MAPK6 | MAPK7 | MAPK8 | MAPK9 | MBP | MED1 | MYC | NLK | NOD2 | PEBP1 | PPP5C | PSMA1 | PSMA2 | PSMA3 | PSMA5 | PSMA6 | PSMB5 | PSMB6 | PSMB7 | PSMB8 | PSMC1 | PSMC2 | PSMC3 | PSMC4 | PSMC5 | PSMC6 | PSMD1 | PSMD10 | PSMD11 | PSMD12 | PSMD14 | PSMD2 | PSMD4 | PSMD9 | PSME2 | PSME3 | PTK2 | PTPRA | RAF1 | RANBP9 | RBX1 | RIPK1 | RIPK2 | SCG2 | SHC1 | SPTAN1 | SPTBN1 | SPTBN2 | STK24 | STK26 | SULT1A3 | TAB1 | TAB2 | TAOK3 | TGFB1 | TNF | TNIP2 | TRAF6 | UBB | UBC | UBE2N | UBE2V1 | YWHAB | YWHAE | GO:0032412 | 0.0167414776173723 | 138/8243 | 74/3250 | 0.000460722252307755 | 1 | T | T | T | F | regulation of ion transmembrane transporter activity | ACTN2 | ACTN4 | ADRB2 | AHCYL1 | AHNAK | AKAP9 | ANK3 | APP | ATP1B3 | CAB39 | CACNG2 | CACNG4 | CACNG5 | CALM1 | CALM2 | CALM3 | CAPN1 | CAV1 | CFTR | CHCHD10 | CHP1 | COX17 | DAPK1 | DLG2 | DLG3 | DLG4 | DMD | FHL1 | FKBP1A | FKBP1B | GLRX | GOPC | GPD1L | GRIA1 | GSTO1 | HAP1 | HECW1 | HECW2 | HSPA2 | HTR3A | HTT | KCNAB1 | KCNIP2 | KIF5B | LRRC7 | MYO5A | NEDD4 | NOS1AP | OXSR1 | PCSK9 | PDE4D | PLA2G6 | PPARGC1A | PPIF | PRKACA | PRKCE | PTEN | RGN | RIPK1 | S100A1 | SLC9A1 | SLC9A3R1 | SRI | STAC | STIM1 | STOM | SUMO1 | THADA | UBQLN1 | WNK1 | WNK4 | WWP2 | YWHAE | YWHAH | GO:0010629 | 0.133325245662987 | 1099/8243 | 484/3250 | 0.000461740151858918 | 1 | T | T | T | F | negative regulation of gene expression | AEBP2 | AES | AGO1 | AGO2 | AKT1 | ANXA7 | APBB1 | APOE | APP | AR | ARHGAP35 | ASCL1 | ATF2 | ATM | ATRX | ATXN1 | AURKB | BACH2 | BAG3 | BASP1 | BCL11A | BCL6 | BCL6B | BCOR | BIRC2 | BIRC3 | BIRC5 | BIRC7 | BIRC8 | BMI1 | BRCA1 | BTRC | C1QBP | CALR | CAV1 | CBFA2T3 | CBFB | CBX4 | CBX7 | CBX8 | CBY1 | CCAR2 | CCNB1 | CCNT1 | CCR1 | CD36 | CDC73 | CDK2 | CDK5 | CDK5R1 | CDK9 | CEBPA | CENPF | CHCHD3 | CHD8 | CIRBP | COPS2 | CREB1 | CREBBP | CRKL | CRY1 | CRY2 | CRYAB | CTBP1 | CTCF | CTNNB1 | CTNNBIP1 | CUL3 | DAB2 | DAPK1 | DAXX | DCP2 | DDRGK1 | DDX3X | DDX6 | DHX36 | DIS3 | DMAP1 | DNAJA3 | DNAJB1 | DPF2 | DUSP22 | E2F1 | E2F2 | E2F3 | E2F4 | E2F6 | EDC4 | EED | EHMT1 | EHMT2 | EID1 | EIF2AK3 | EIF2AK4 | EIF2S1 | EIF3E | EIF4A3 | EIF4E | EIF4E2 | EIF4EBP1 | EIF4ENIF1 | EIF6 | ELF3 | ENO1 | EP300 | EPRS | ERF | ESR1 | ESR2 | ESRRA | EXOSC1 | EXOSC5 | EXOSC6 | EXOSC9 | EZH2 | EZR | FAM129B | FASLG | FBP1 | FBXW11 | FGFR1 | FHL2 | FOXA1 | FOXJ1 | FOXK1 | FOXM1 | FOXO1 | FTO | FXR2 | FYN | GAPDH | GATA1 | GPS2 | GRB7 | GSPT1 | GSPT2 | H1F0 | H3F3A | HAVCR2 | HBZ | HDAC1 | HDAC2 | HDAC3 | HDAC4 | HDAC5 | HDAC6 | HDAC7 | HDAC9 | HDGF | HELZ | HELZ2 | HEXIM1 | HIF1A | HIF1AN | HIST1H3A | HIST1H4A | HIST2H3A | HIVEP1 | HMGB1 | HMGB2 | HNRNPA2B1 | HNRNPD | HNRNPK | HNRNPL | HNRNPU | HRAS | HSF1 | HSF4 | HSPA1B | HSPA8 | ID2 | IFI16 | IFNA2 | IGBP1 | IGF2BP1 | IKZF5 | ILF3 | IREB2 | IRF1 | IRF7 | JUN | JUNB | JUND | KANK2 | KAT5 | KAT7 | KAT8 | KDM1A | KDM5B | KDM5C | KDR | KHDRBS1 | KLF5 | KMT2B | L3MBTL1 | LEF1 | LGMN | LGR4 | LIMS1 | LOXL2 | LRRK2 | LSM1 | LSM3 | LSM4 | LSM5 | MAF1 | MAP2K1 | MAPT | MBD3 | MDFI | MDM2 | MDM4 | MECP2 | MED1 | MEPCE | MIF | MITF | MLH1 | MORF4L1 | MORF4L2 | MOV10 | MTA1 | MTA3 | MXD1 | MYBBP1A | MYC | NACC1 | NBAS | NCBP1 | NCK1 | NCOA2 | NCOR1 | NCOR2 | NDUFA13 | NEDD4 | NELFB | NELFCD | NELFE | NFATC2 | NIPBL | NKRF | NKX2-1 | NME1 | NOS2 | NOSTRIN | NR0B2 | NR1H2 | NR1H3 | NR1I2 | NR3C1 | PA2G4 | PABPC1 | PAN2 | PARK7 | PARP1 | PARP14 | PATZ1 | PAWR | PAX9 | PC | PCGF1 | PCGF6 | PDCD10 | PDE12 | PER1 | PER2 | PHB | PHB2 | PHF10 | PHF21A | PIAS1 | PIAS3 | PKIG | PLK1 | PML | POU5F1 | PPARD | PPARG | PPID | PPM1A | PPM1D | PPP2CA | PPP2R1A | PPP2R2A | PRDX5 | PRKAA1 | PRKAA2 | PRMT2 | PRMT6 | PROX1 | PSEN1 | PSMC5 | PSMD10 | PTPN22 | PTPRK | PTRH2 | PUM1 | PUS7 | QARS | RARA | RARG | RASD1 | RB1 | RBBP4 | RBM3 | RBM4 | RBM8A | RBMX | RBPJ | RC3H1 | REL | RELA | REST | RFC1 | RGS2 | RIF1 | RING1 | RNASEH2B | RNF139 | RNF2 | RNF8 | RPL10 | RPL10A | RPL12 | RPL18A | RPL22 | RPL23 | RPL23A | RPL24 | RPL30 | RPL37A | RPL38 | RPL6 | RPL7 | RPL7A | RPL9 | RPLP0 | RPLP1 | RPLP2 | RPS12 | RPS13 | RPS14 | RPS15A | RPS19 | RPS2 | RPS20 | RPS24 | RPS29 | RPS3 | RPS4X | RPS8 | RTF1 | RUNX1 | RUNX1T1 | RUNX2 | RXRA | S100A1 | SAMD7 | SAP18 | SARS | SERPINE2 | SET | SFMBT1 | SFPQ | SHC1 | SHMT1 | SIN3A | SIRT1 | SIRT5 | SIRT6 | SIRT7 | SKI | SKIV2L | SMAD2 | SMAD3 | SMAD4 | SMAD5 | SMARCA2 | SMARCA4 | SMARCC2 | SMARCE1 | SMG5 | SMG6 | SMG8 | SMO | SMURF2 | SNAI2 | SNCA | SND1 | SNW1 | SNX12 | SNX6 | SOX2 | SP100 | SP2 | SPEN | SPI1 | SQSTM1 | SRC | SRSF12 | SSB | STAT1 | STAT3 | STAT6 | STRAP | SUMO1 | SUPT4H1 | SUPT5H | SUZ12 | TAGLN3 | TARDBP | TBL1X | TCERG1 | TCF21 | TCF25 | TCP10L | TERF2 | TGFB1 | THAP11 | THAP7 | THBS1 | THRA | TIA1 | TLE1 | TMBIM6 | TNF | TNP1 | TP53 | TP63 | TPR | TRAF6 | TRIB3 | TRIM28 | TRIM29 | TRIM33 | TSC22D4 | TSG101 | TWIST1 | TXN | TXNIP | TYMS | U2AF2 | UBA3 | UBB | UBC | UBE2D1 | UBE2D3 | UBE2I | UNK | UPF2 | UPF3B | USP17L2 | USP2 | USP47 | USP9X | VDR | VHL | VPS72 | WDR35 | WDTC1 | WT1 | WWOX | WWP2 | XIAP | XPO1 | XRCC5 | XRCC6 | YAF2 | YEATS2 | YWHAB | YWHAQ | YY1 | ZBTB16 | ZBTB21 | ZBTB33 | ZBTB42 | ZBTB7A | ZBTB7B | ZC3H4 | ZHX1 | ZMPSTE24 | ZNF217 | ZNF24 | ZNF746 | GO:0043086 | 0.0581099114400097 | 479/8243 | 224/3250 | 0.00046194085483588 | 1 | T | T | T | F | negative regulation of catalytic activity | A2M | ABCE1 | ABL1 | ADCYAP1 | ADORA2A | AHSG | AKAP9 | AKT1 | ANXA1 | ANXA2 | ANXA5 | APC | APCS | APOA1 | APOE | APP | ARRB1 | ARRB2 | BAG2 | BAG4 | BAG5 | BIN1 | BIRC2 | BIRC3 | BIRC5 | BIRC7 | BIRC8 | C4A | CAND1 | CASP3 | CASP8 | CAV1 | CBL | CBLB | CBLC | CCAR2 | CCDC8 | CD109 | CD27 | CD2BP2 | CDKN1A | CDKN2C | CEBPA | CHORDC1 | CHP1 | COX11 | CRY2 | CRYAB | DBNDD2 | DDX3X | DHCR24 | DLG2 | DLG3 | DNAJA1 | DPEP1 | DUSP1 | DUSP14 | DUSP16 | DUSP19 | DUSP22 | DUSP7 | DVL1 | EGLN1 | EPPIN | ERRFI1 | FABP1 | FAF2 | FBXO7 | FGFR1OP | FKBP1A | FKBP1B | GAPDH | GLA | GNAI2 | GNAI3 | GNAQ | GPI | GPS2 | GPSM1 | GPX1 | GSK3A | GSK3B | GSTP1 | GZMA | HDAC6 | HDAC9 | HEXIM1 | HNRNPU | HRAS | HSPB1 | HSPBP1 | IBTK | IFI16 | IFIT1 | IGBP1 | IGF1R | IKBKB | ILK | IPO5 | IPO7 | IQGAP1 | ITGB1BP1 | LAMP3 | LATS1 | LEF1 | LGALS3 | LRP6 | LRRK2 | MAD2L1 | MAPK7 | MAPT | MCM2 | MDM2 | MLLT1 | MTRR | MYO1D | NCK1 | NF2 | NOS3 | NOSIP | NPM1 | NQO1 | OAZ3 | OXA1L | PAK2 | PAM16 | PAPLN | PARK7 | PDE6G | PEBP1 | PIH1D1 | PINX1 | PKIG | PLAUR | PLK1 | POT1 | PPARG | PPIF | PPME1 | PPP1R11 | PPP1R12A | PPP1R12B | PPP1R14A | PPP1R2 | PPP1R8 | PPP2CA | PPP2R1A | PRDX3 | PRDX5 | PRKAR1A | PRKAR2B | PRKCD | PRKCZ | PSEN1 | PTEN | PTN | PTPN1 | PTPN22 | PTPN6 | PTTG1 | QARS | RAF1 | RB1 | RDX | RECK | RGN | RGS2 | RPL23 | RPS6KA1 | RPTOR | RTKN | SAG | SERPINB2 | SERPINB3 | SERPINB5 | SERPINB6 | SERPINB8 | SERPINE2 | SERPINH1 | SET | SFN | SIRT1 | SNCA | SNX6 | SOCS1 | SORT1 | SPINK7 | SPINT2 | SPRY2 | SRC | STK38 | STYX | TAOK3 | TERF1 | TERF2 | TFIP11 | THADA | THBS1 | TIMP1 | TIMP2 | TMBIM6 | TNF | TNFAIP3 | TNFSF14 | TNK2 | TP53 | TRIAP1 | TRIB2 | TRIB3 | TSG101 | TXNIP | UBXN1 | UCHL1 | UCHL5 | USP14 | USP17L2 | USP47 | VTN | WARS | WDTC1 | WNK1 | XIAP | YWHAE | YWHAG | GO:0007178 | 0.0105544098022565 | 87/8243 | 50/3250 | 0.000470420282258618 | 1 | T | T | T | F | transmembrane receptor protein serine/threonine kinase signaling pathway | APOA1 | ARRB2 | BMPR1A | CBL | CDH5 | CGN | CHRD | CREB1 | DUSP22 | FERMT2 | FOS | GDF9 | ITGB5 | JUN | LEF1 | LTBP1 | MAPK14 | MAPK3 | MYH6 | NLK | PARD3 | PARD6A | PARP1 | PML | PRKCZ | PTK2 | PTPRK | PXN | RHOA | RUNX2 | SIRT1 | SKI | SMAD2 | SMAD3 | SMAD4 | SMAD5 | SMAD9 | SMURF1 | SMURF2 | SRC | TAB1 | TGFB1 | TGFBR3 | UBB | UBC | UBE2D1 | UBE2D3 | USP15 | USP9X | USP9Y | GO:0043900 | 0.0312992842411743 | 258/8243 | 128/3250 | 0.000476055218267356 | 1 | T | T | T | F | regulation of multi-organism process | ADA | AGO2 | AICDA | ANXA2 | APCS | APOE | BANF1 | BCL2 | BIRC2 | BIRC3 | C1QBP | CALR | CAV1 | CCNT1 | CD36 | CD74 | CDK9 | CFL1 | CHMP2A | CTDP1 | CXCL6 | CXCL8 | DDB1 | DDX3X | DHX9 | EIF2AK4 | EP300 | FCN1 | GAPDH | GTF2F1 | GTF2F2 | HAVCR2 | HDAC1 | HDAC2 | HDAC4 | HTRA1 | IFI16 | IFIT1 | IFITM3 | IFNA2 | ILF3 | ISG15 | ITCH | ITGAV | JUN | LAMP3 | LEF1 | LGALS1 | LRSAM1 | MAPK3 | MAVS | MIF | NELFB | NELFCD | NELFE | NOS2 | NPLOC4 | NR5A2 | OPTN | OVGP1 | P4HB | PABPC1 | PC | PCBP2 | PDE12 | PKN2 | PLSCR1 | PML | POLR2A | POLR2E | POLR2L | POU2F3 | PPIA | PPIB | PPID | PPIE | PPM1B | PPP2R1A | PRDX2 | PRDX4 | PROX1 | PSMC3 | PUM1 | RAB7A | RAD23A | REST | RHBDD1 | RIPK2 | SETDB1 | SIN3A | SMARCA4 | SMARCB1 | SMC3 | SMURF2 | SNF8 | SNW1 | SNX3 | SP1 | SQSTM1 | SRPK1 | SRPK2 | STAT1 | STAU1 | STOM | SUPT4H1 | SUPT5H | SYDE1 | TARDBP | TIMP1 | TMEM173 | TNF | TNFAIP3 | TOP2A | TRAF3 | TRAF3IP1 | TRAF6 | TRIM14 | TRIM21 | TRIM25 | TRIM28 | TRIM32 | TRIM35 | TSG101 | USP17L2 | VAPA | VAPB | VPS4B | ZMPSTE24 | GO:0000209 | 0.0269319422540337 | 222/8243 | 112/3250 | 0.00047782476512433 | 1 | T | T | T | F | protein polyubiquitination | AMFR | ANAPC4 | BCL2 | BIRC2 | BRCA1 | BTRC | CBFB | CBL | CDC16 | CDC27 | CDC34 | CTNNB1 | CUL1 | CUL3 | DDB2 | DZIP3 | FBXL12 | FBXO11 | FBXO32 | FBXO6 | FBXO7 | FBXW11 | FBXW5 | FZR1 | HDAC6 | HECW1 | HECW2 | HUWE1 | ITCH | KLHL20 | LMO7 | LRSAM1 | MARCH5 | NEDD4 | NHLRC3 | NPEPPS | PELI1 | PRPF19 | PSMA1 | PSMA2 | PSMA3 | PSMA5 | PSMA6 | PSMB5 | PSMB6 | PSMB7 | PSMB8 | PSMC1 | PSMC2 | PSMC3 | PSMC4 | PSMC5 | PSMC6 | PSMD1 | PSMD10 | PSMD11 | PSMD12 | PSMD14 | PSMD2 | PSMD4 | PSMD9 | PSME2 | PSME3 | RBBP6 | RBX1 | RC3H1 | RNF126 | RNF183 | RNF19A | RNF31 | RNF4 | RNF8 | SIAH1 | SMURF1 | SMURF2 | STUB1 | SYVN1 | THOP1 | TMEM189-UBE2V1 | TNFAIP3 | TNKS | TNKS2 | TRAF2 | TRAF6 | TRIM21 | TRIM32 | TRIM69 | TRIP12 | UBB | UBC | UBE2A | UBE2B | UBE2C | UBE2D1 | UBE2D2 | UBE2D3 | UBE2H | UBE2K | UBE2L3 | UBE2N | UBE2O | UBE2Q2 | UBE2R2 | UBE2S | UBE2V1 | UBE2W | UBE3A | UBE3B | UBR5 | UNKL | WSB1 | WWP2 | GO:0043270 | 0.0164988475069756 | 136/8243 | 73/3250 | 0.00048286711716721 | 1 | T | T | T | F | positive regulation of ion transport | ABAT | ABL1 | ACE2 | ACTN2 | ACTN4 | ADORA2A | ADRB2 | AHCYL1 | AKAP9 | AKT1 | ANK3 | ANXA2 | APOA1 | APOE | ARF1 | ARRB2 | ATP1B3 | BAX | CACNG2 | CACNG4 | CALM1 | CALM2 | CALM3 | CAMK2A | CAV1 | CCR1 | CFTR | CHCHD10 | CHP1 | COX17 | CXCL11 | DMD | FABP3 | FHL1 | G6PD | GLRX | GPD1L | GSTO1 | HAP1 | HSPA2 | HTR3A | HTT | KCNIP2 | KIF5B | LGALS3 | MAP2K6 | MIF | NOS1AP | P2RX1 | P2RX2 | P2RX4 | PARK7 | PLA2G10 | PLA2G1B | PLA2G6 | PLCG1 | PRELID1 | PRKCD | PSEN1 | RAB3GAP1 | RGN | S100A1 | SLC9A1 | SLC9A3R1 | SNCA | SRI | STAC | STIM1 | STXBP1 | SUMO1 | TRIAP1 | WNK1 | WNK4 | GO:0051353 | 0.00448865704233895 | 37/8243 | 25/3250 | 0.000484599511050675 | 1 | T | T | T | F | positive regulation of oxidoreductase activity | ABL1 | ABL2 | AKT1 | APOE | CALM1 | CALM3 | CCS | CHCHD10 | COX17 | GNAI2 | GNAI3 | HIF1A | KRAS | NOD2 | NOS1AP | PARK7 | PPM1K | RFK | RGN | RIPK3 | S100A1 | SNCA | TERF2 | TNF | VDR | GO:0007568 | 0.0220793400460997 | 182/8243 | 94/3250 | 0.00048619853129349 | 1 | T | T | T | F | aging | ABAT | ADA | AKT1 | ALOX12 | AMFR | APEX1 | APOD | ASS1 | ATM | AURKB | BCL2 | C1QA | CALR | CANX | CARM1 | CASP2 | CASP9 | CAT | CDKN1A | CHEK2 | CISD2 | COL4A2 | CREB1 | CRYAB | CTSC | CTSV | CYP1A1 | DCN | DLD | DNAJA3 | EEF2 | EIF2B5 | EIF2S1 | ENDOG | ENO3 | FOS | GNAO1 | GPX4 | GRB2 | GSS | H2AFX | HRAS | HTRA2 | ICAM1 | ID2 | ILK | IREB2 | JUN | KYNU | LIMS1 | LOXL2 | MAP2K1 | MAPK1 | MAPK14 | MAPK3 | MBD3 | MIF | MME | NAMPT | NDUFS6 | NFE2L2 | NFKB2 | NPM1 | NQO1 | NTRK1 | OPA1 | PDE4D | PEBP1 | PML | PPARGC1A | PRKCD | PRMT6 | PTEN | RELA | RGN | ROMO1 | RPN2 | SEC63 | SIN3A | SIRT1 | SIRT3 | SLC12A2 | SNCA | SOD1 | STAT3 | TERF2 | TGFB1 | TIMP1 | TIMP2 | TP53 | TP63 | TYMS | VCAM1 | WNT16 | GO:0002758 | 0.0207448744389179 | 171/8243 | 89/3250 | 0.000490201800092402 | 1 | T | T | T | F | innate immune response-activating signal transduction | BCL10 | BIRC2 | BIRC3 | BTK | BTRC | CARD9 | CASP8 | CD209 | CD36 | CREBBP | CTSL | CUL1 | CYLD | EP300 | FADD | FBXW11 | FCN1 | FGB | FYN | HAVCR2 | HMGB1 | HRAS | HSP90B1 | HSPD1 | IKBKB | IKBKE | IKBKG | IRF7 | ITCH | KRAS | LGMN | MAP2K6 | MAP3K1 | MYD88 | NFKBIA | NOD2 | PAK2 | PRKACA | PRKCD | PRKCE | PSMA1 | PSMA2 | PSMA3 | PSMA5 | PSMA6 | PSMB5 | PSMB6 | PSMB7 | PSMB8 | PSMC1 | PSMC2 | PSMC3 | PSMC4 | PSMC5 | PSMC6 | PSMD1 | PSMD10 | PSMD11 | PSMD12 | PSMD14 | PSMD2 | PSMD4 | PSMD9 | PSME2 | PSME3 | RAF1 | RELA | RIPK1 | RIPK2 | S100A1 | S100A9 | SCARA3 | SRC | TAB1 | TAB2 | TANK | TLR3 | TMEM173 | TNFAIP3 | TNIP2 | TRAF3 | TRAF6 | UBB | UBC | UBE2D1 | UBE2D2 | UBE2D3 | UBE2N | UBE2V1 | GO:0022603 | 0.0755792793885721 | 623/8243 | 285/3250 | 0.000495865497258675 | 1 | T | T | T | F | regulation of anatomical structure morphogenesis | ABL1 | ACTN4 | ACTR2 | ADA | AGO1 | AGO2 | AHI1 | AKT3 | ALDOA | ALOX12 | ANXA1 | ANXA7 | APOA1 | APOE | AR | ARHGAP35 | ARHGDIA | ARHGEF7 | ARPC2 | ARRB2 | ATF2 | BAIAP2 | BASP1 | BAX | BCL11A | BCL2 | BCL6 | BCOR | BMPR1A | BRAF | BRCA1 | C1QBP | CALR | CAMK2B | CAPN2 | CAPZB | CARM1 | CCR3 | CD36 | CD40 | CDC42 | CDC42EP3 | CDC42EP4 | CDC42SE2 | CDH5 | CDK5 | CDK5R1 | CDKL3 | CFL1 | CLTC | COL4A2 | COL5A1 | CRK | CRKL | CTNNB1 | CTTN | CUL7 | CXCL8 | DAB1 | DAB2 | DBN1 | DCN | DHX36 | DLG4 | DMRT3 | DNM1L | DOCK5 | DVL1 | DVL2 | DVL3 | E2F2 | EGLN1 | EPHA4 | ERBB2 | ESR1 | ETS1 | EZR | FAM129B | FASLG | FERMT2 | FGB | FGF2 | FGFR1 | FIS1 | FKBPL | FLOT1 | FN1 | FOXJ1 | FYN | GDI1 | GLUL | GNA12 | GNA13 | GNG5 | GOLGA4 | GRIP1 | GSK3B | GTF2I | HDAC5 | HDAC7 | HDAC9 | HECW1 | HECW2 | HEXB | HGS | HIF1A | HIF1AN | HK2 | HMOX1 | HNF4A | HOXA5 | HSPB1 | HSPG2 | HTATIP2 | ICAM1 | ILK | ITGA7 | ITGB1BP1 | JAK1 | JUP | KDR | KIF13B | LARP4 | LGR4 | LIMS1 | LPAR1 | LRRK2 | MAP1B | MAP2K1 | MAP3K13 | MAP3K3 | MAPK14 | MAPK7 | MAPT | MARCH5 | MBP | MECP2 | MED1 | MED12 | MEOX2 | METRN | MKS1 | MLLT3 | MOV10 | MSN | MYC | MYOC | NEDD4 | NFATC2 | NFE2L2 | NIN | NOS3 | NPHP3 | NPR1 | OBSL1 | OPA1 | PAFAH1B1 | PARD6A | PARP6 | PAX8 | PAX9 | PDCD10 | PDCD6 | PDLIM5 | PFN1 | PGK1 | PHB2 | PIK3C2A | PIM1 | PKM | PLCG1 | PLXNA1 | PLXNA2 | PLXNB2 | PML | PPARG | PPARGC1A | PRKD1 | PRKD2 | PROX1 | PRPF40A | PSEN1 | PSMA1 | PSMA2 | PSMA3 | PSMA5 | PSMA6 | PSMB5 | PSMB6 | PSMB7 | PSMB8 | PSMC1 | PSMC2 | PSMC3 | PSMC4 | PSMC5 | PSMC6 | PSMD1 | PSMD10 | PSMD11 | PSMD12 | PSMD14 | PSMD2 | PSMD4 | PSMD9 | PSME2 | PSME3 | PTEN | PTK2 | PTN | PTPRD | PTPRO | QRICH1 | RAB11A | RAB21 | RAC1 | RAP1A | RBPJ | RC3H1 | RDX | RECK | RHOA | RHOJ | RHOU | RTN4 | RUNX1 | RUNX2 | RXRA | S100A1 | S100A13 | S100B | SARM1 | SARS | SEC24B | SEMA7A | SEPT7 | SH3KBP1 | SIPA1L1 | SIRT1 | SIRT6 | SLC9A3R1 | SMAD4 | SMO | SMURF1 | SMURF2 | SNAI2 | SP1 | SP100 | SPRY2 | SRC | STARD13 | STAT1 | STAT3 | STIM1 | STK11 | STK25 | SULF2 | SYNJ2BP | TBXA2R | TGFB1 | THBS1 | THBS2 | TLR3 | TNF | TNFAIP3 | TNFRSF12A | TNFRSF1A | TNFSF12 | TNFSF14 | TPM1 | TUBB2B | TWF2 | TWIST1 | VASH2 | VAT1 | VDR | WARS | WDR1 | WNK1 | WNT2 | WT1 | XBP1 | YWHAH | ZMPSTE24 | ZMYM5 | GO:1901652 | 0.0299648186339925 | 247/8243 | 123/3250 | 0.000505286646878519 | 1 | T | T | T | F | response to peptide | ADCY6 | ADRB2 | AGER | AKR1B1 | AKT1 | AKT2 | ANXA1 | ANXA5 | APEX1 | APP | APRT | ASS1 | BACE1 | BRAF | BSG | CACNA1A | CARD9 | CAT | CAV1 | CD36 | CD40 | CDC5L | CDK4 | CDK5 | CREB1 | CRK | CRY1 | CYC1 | DENND4C | EIF2B1 | EIF2B3 | EIF2B5 | EIF6 | EPHA4 | EPRS | ERRFI1 | FABP3 | FIS1 | FOXO1 | FYN | GLP1R | GNAI1 | GOT1 | GOT2 | GSK3A | GSK3B | GSTP1 | HADH | HDAC5 | HDAC9 | HNRNPL | HSF1 | ICAM1 | IGF1R | INSIG2 | INSR | IQGAP1 | ITGA4 | KAT7 | KLF5 | LGMN | LPIN3 | LRP6 | MAPK14 | MDM2 | MYO5A | NAMPT | NFE2L2 | NFKBIA | NME2 | NOD2 | NR4A1 | NTRK1 | OGT | OTC | PARP1 | PCSK9 | PIK3R1 | PKM | PLA2G1B | PPARG | PPAT | PRKACA | PRKAR1A | PRKAR2B | PRKCD | PRKCZ | PRKDC | PSEN1 | PTEN | PTPN22 | QDPR | RAB10 | RAB31 | RANGAP1 | RAP1B | RBBP4 | RELA | RIPK2 | RPL23 | SIRT1 | SLC9A1 | SNX6 | SORT1 | SP1 | SRC | SRSF5 | STAT1 | TBC1D4 | TGFB1 | TGFBR3 | TIMP1 | TNFAIP3 | TP53 | TRIB3 | TRIM41 | VCAM1 | VGF | VIM | WDTC1 | XBP1 | YWHAG | ZBTB7B | GO:2001236 | 0.0144364915686037 | 119/8243 | 65/3250 | 0.000517112103213825 | 1 | T | T | T | F | regulation of extrinsic apoptotic signaling pathway | AKT1 | AR | BCL10 | BCL2 | BCL2L1 | BRCA1 | CASP8 | CAV1 | COL2A1 | CTTN | CYLD | DBH | EYA2 | EYA4 | FADD | FAF1 | FASLG | FGB | FGFR1 | FYN | GATA1 | GPX1 | GSTP1 | HMGB2 | HMOX1 | HSPA1B | HTRA2 | HTT | ICAM1 | ITGAV | LGALS3 | LMNA | LTBR | MAPK7 | NOS3 | PAK2 | PARK7 | PDIA3 | PML | PPP1CA | PPP2R1A | PPP2R1B | PRDX2 | PSME3 | PTEN | RAF1 | RB1CC1 | RELA | RIPK1 | SCG2 | SNAI2 | SP100 | SRC | STK3 | THBS1 | TNF | TNFAIP3 | TNFRSF12A | TNFSF12 | TRADD | TRAF1 | TRAF2 | WWOX | YAP1 | ZC3HC1 | GO:0032201 | 0.00230498604876865 | 19/8243 | 15/3250 | 0.000526640432879913 | 1 | T | T | T | F | telomere maintenance via semi-conservative replication | ACD | DNA2 | FEN1 | PCNA | POLA2 | POLD1 | POLE2 | POLE3 | RFC1 | RFC2 | RFC3 | RFC4 | RFC5 | RPA2 | RPA3 | GO:0017004 | 0.00230498604876865 | 19/8243 | 15/3250 | 0.000526640432879913 | 1 | T | T | T | F | cytochrome complex assembly | BCS1L | COA3 | COX11 | COX15 | COX17 | COX19 | COX20 | HCCS | LYRM7 | OXA1L | SCO2 | TIMM21 | TTC19 | UQCC1 | UQCRB | GO:0043902 | 0.0157709571757855 | 130/8243 | 70/3250 | 0.000555408040195302 | 1 | T | T | T | F | positive regulation of multi-organism process | AGO2 | ANXA2 | APOE | C1QBP | CCNT1 | CD74 | CDK9 | CFL1 | CHMP2A | CTDP1 | DDB1 | DDX3X | DHX9 | EP300 | GAPDH | GTF2F1 | GTF2F2 | HAVCR2 | HDAC2 | HDAC4 | IFIT1 | JUN | LEF1 | LGALS1 | LRSAM1 | MAPK3 | MIF | NELFB | NELFCD | NELFE | NOS2 | NR5A2 | OPTN | P4HB | PABPC1 | PC | PDE12 | PKN2 | POLR2A | POLR2E | POLR2L | PPIA | PPIB | PPID | PPIE | PUM1 | RAB7A | RAD23A | RIPK2 | SMARCA4 | SMARCB1 | SMC3 | SMURF2 | SNW1 | SP1 | SRPK1 | SRPK2 | STAU1 | STOM | SUPT4H1 | SUPT5H | SYDE1 | TOP2A | TRAF6 | TRIM21 | TSG101 | USP17L2 | VAPA | VAPB | VPS4B | GO:0071897 | 0.00885599902947956 | 73/8243 | 43/3250 | 0.000565553100900242 | 1 | T | T | T | F | DNA biosynthetic process | CENPF | CHRAC1 | GAR1 | HSP90AA1 | HSP90AB1 | ISG15 | LIG4 | NOP10 | NPLOC4 | PCNA | PHB | PINX1 | POLA2 | POLD1 | POLE2 | POLE3 | POLM | POT1 | PPIA | PTGES3 | RFC1 | RFC2 | RFC3 | RFC4 | RFC5 | RPA2 | RPA3 | SIRT1 | SMG5 | SMG6 | SPRTN | TERF1 | TERF2 | TERF2IP | TK1 | TK2 | TREX1 | TRIM25 | TYMS | UBB | UBC | UBE2L6 | VCP | GO:0001666 | 0.0213514497149096 | 176/8243 | 91/3250 | 0.000566847223643321 | 1 | T | T | T | F | response to hypoxia | ABAT | ACAA2 | ACTN4 | ADA | ADSL | AGER | AGTRAP | AHCY | AKT1 | ARNT | ARNT2 | ATM | BCL2 | BIRC2 | CAPN2 | CASP3 | CAT | CAV1 | CBFA2T3 | CCNB1 | CREB1 | CREBBP | CRYAB | CYP1A1 | DDIT4 | DNM1L | E2F1 | EGLN1 | EGLN3 | EIF4EBP1 | ENDOG | EP300 | ETS1 | FABP1 | FIS1 | GNB1 | HIF1A | HK2 | HMOX1 | HSF1 | HSP90B1 | ICAM1 | IREB2 | ITPR1 | ITPR2 | LDHA | LMNA | LOXL2 | MDM2 | MDM4 | MECP2 | MYC | NDRG1 | NOS2 | NPEPPS | OPA1 | P2RX2 | P4HB | PGK1 | PHB2 | PKM | PLOD2 | PML | PPARD | PPARGC1A | PRKAA1 | PRKCE | PSEN2 | PTEN | PTN | RAF1 | REST | RHOA | S100B | SCFD1 | SIRT1 | SLC9A1 | SMAD3 | SMAD4 | SRC | STUB1 | TGFB1 | TGFBR3 | THBS1 | TM9SF4 | TP53 | TWIST1 | TXN2 | UBQLN1 | VCAM1 | XRCC1 | GO:1903706 | 0.0355453111731166 | 293/8243 | 143/3250 | 0.000568578212273911 | 1 | T | T | T | F | regulation of hemopoiesis | ABL1 | ADA | AGER | AGO1 | ANXA1 | AP3D1 | APCS | ARNT | BCL6 | BRAF | BTK | CASP8 | CBFB | CCR1 | CD2 | CD27 | CD74 | CDC73 | CLDN18 | CREB1 | CREBBP | CTNNB1 | CTNNBIP1 | CYLD | DHX36 | DPF2 | DROSHA | DTX1 | EIF6 | EP300 | ERBB2 | ESRRA | ETS1 | FADD | FANCA | FBXO7 | FOS | FOXJ1 | GATA1 | GNAS | H3F3A | HAX1 | HDAC1 | HIF1A | HIST1H3A | HIST1H4A | HIST2H3A | HLX | HMGB1 | HMGB2 | HOXA5 | HSPA1B | HSPA9 | ID2 | IFNA2 | IRF1 | IRF4 | IRF7 | ISG15 | ITCH | JAK3 | JUN | KMT2A | KMT2B | KMT2C | L3MBTL1 | LEF1 | LGALS1 | LGALS3 | LMO2 | MAPK14 | MED1 | MITF | MOV10 | MYC | MYL9 | NFATC2 | NFE2L2 | NFKBIA | NME1 | NME2 | PIAS3 | PIK3R1 | PNP | PRDX2 | PRELID1 | PRKCZ | PRMT6 | PSMA1 | PSMA2 | PSMA3 | PSMA5 | PSMA6 | PSMB5 | PSMB6 | PSMB7 | PSMB8 | PSMC1 | PSMC2 | PSMC3 | PSMC4 | PSMC5 | PSMC6 | PSMD1 | PSMD10 | PSMD11 | PSMD12 | PSMD14 | PSMD2 | PSMD4 | PSMD9 | PSME2 | PSME3 | PTPN6 | PUS7 | RARA | RARG | RB1 | RC3H1 | RHOA | RIPK1 | RIPK2 | RUNX1 | SART1 | SIN3A | SLAMF8 | SOCS1 | SOD1 | SPI1 | STAT1 | STAT3 | TCF12 | TGFB1 | THBS1 | TLR3 | TNF | TP73 | TRAF6 | XBP1 | YAP1 | ZAP70 | ZBTB16 | ZBTB7B | GO:0006002 | 0.000970520441586801 | 8/8243 | 8/3250 | 0.000580919108326817 | 1 | T | T | T | F | fructose 6-phosphate metabolic process | FBP1 | FBP2 | GFPT1 | GFPT2 | PFKL | PFKM | PFKP | TALDO1 | GO:0071550 | 0.000970520441586801 | 8/8243 | 8/3250 | 0.000580919108326817 | 1 | T | T | T | F | death-inducing signaling complex assembly | CASP8 | FADD | RAF1 | RIPK1 | TNF | TNFRSF1A | TRADD | TRAF2 | GO:0048002 | 0.0171054227829674 | 141/8243 | 75/3250 | 0.000581010574949572 | 1 | T | T | T | F | antigen processing and presentation of peptide antigen | ACTR1A | AP1B1 | AP1G1 | AP1M1 | ARF1 | CALR | CANX | CAPZA1 | CAPZA2 | CAPZB | CD36 | CD74 | CLTC | CTSD | CTSL | CTSV | DCTN1 | DYNC1H1 | DYNC1I1 | DYNC1I2 | DYNC1LI1 | DYNLL1 | FCGR1A | HLA-A | HLA-B | HLA-DQA1 | HLA-DRA | HLA-DRB1 | HLA-DRB3 | HLA-DRB4 | HLA-DRB5 | IKBKB | IKBKG | ITGAV | ITGB5 | KIF11 | KIF2C | KLC1 | KLC2 | LGMN | OSBPL1A | PDIA3 | PSMA1 | PSMA2 | PSMA3 | PSMA5 | PSMA6 | PSMB5 | PSMB6 | PSMB7 | PSMB8 | PSMC1 | PSMC2 | PSMC3 | PSMC4 | PSMC5 | PSMC6 | PSMD1 | PSMD10 | PSMD11 | PSMD12 | PSMD14 | PSMD2 | PSMD4 | PSMD9 | PSME2 | PSME3 | RAB7A | SEC13 | SEC24B | SEC24C | SEC31A | SH3GL2 | SPTBN2 | TRAF6 | GO:0032410 | 0.00545917748392576 | 45/8243 | 29/3250 | 0.000584579573717259 | 1 | T | T | T | F | negative regulation of transporter activity | ACTN2 | ANK3 | CAB39 | CALM1 | CALM2 | CALM3 | CAV1 | FKBP1A | FKBP1B | GOPC | GSTO1 | HECW1 | HECW2 | KCNAB1 | NEDD4 | OXSR1 | PCSK9 | PPIF | PRKCE | PTEN | SLC9A3R1 | SNCA | SRI | SUMO1 | THADA | TWIST1 | UBQLN1 | WWP2 | YWHAE | GO:1903201 | 0.00570180759432246 | 47/8243 | 30/3250 | 0.000599613994953867 | 1 | T | T | T | F | regulation of oxidative stress-induced cell death | AKT1 | BAG5 | CTNNB1 | ENDOG | FBXO7 | FYN | GPX1 | HDAC6 | HIF1A | HSPB1 | HTRA2 | LANCL1 | LRRK2 | MAPK7 | NFE2L2 | P4HB | PARK7 | PARP1 | PAWR | PSAP | PYCR1 | RIPK1 | SFPQ | SIRT1 | SOD1 | SOD2 | STK26 | TRAP1 | TXN | UBQLN1 | GO:0051346 | 0.031177969185976 | 257/8243 | 127/3250 | 0.000611864198754471 | 1 | T | T | F | F | negative regulation of hydrolase activity | A2M | ABCE1 | ABL1 | ADCYAP1 | ADORA2A | AHSG | AKT1 | ANXA1 | APCS | APOA1 | APP | ARRB1 | ARRB2 | BAG4 | BIN1 | BIRC2 | BIRC3 | BIRC5 | BIRC7 | BIRC8 | C4A | CCDC8 | CD109 | CD27 | CD2BP2 | CHP1 | CRY2 | CRYAB | DDX3X | DHCR24 | DLG2 | DLG3 | DPEP1 | EGLN1 | EPPIN | FABP1 | FKBP1A | FKBP1B | GAPDH | GNAI2 | GPI | GPS2 | GPSM1 | GPX1 | GSK3B | GZMA | HDAC9 | HRAS | IFI16 | IFIT1 | IGBP1 | IKBKB | IPO5 | IQGAP1 | LAMP3 | LEF1 | LGALS3 | LRRK2 | MAPK7 | MCM2 | MDM2 | MYO1D | NOS3 | OXA1L | PAK2 | PAM16 | PAPLN | PARK7 | PEBP1 | PIH1D1 | PLAUR | PPIF | PPME1 | PPP1R11 | PPP1R14A | PPP1R2 | PPP1R8 | PRDX3 | PRDX5 | PRKCZ | PTN | PTTG1 | RAF1 | RDX | RECK | RGN | RGS2 | RPS6KA1 | RTKN | SAG | SERPINB2 | SERPINB3 | SERPINB5 | SERPINB6 | SERPINB8 | SERPINE2 | SERPINH1 | SET | SFN | SIRT1 | SNCA | SORT1 | SPINK7 | SPINT2 | SPRY2 | SRC | TERF1 | TERF2 | THADA | THBS1 | TIMP1 | TIMP2 | TMBIM6 | TNF | TNFSF14 | TNK2 | TP53 | TRIAP1 | UBXN1 | UCHL5 | USP14 | USP17L2 | USP47 | VTN | WNK1 | XIAP | YWHAE | GO:0033108 | 0.00715758825670266 | 59/8243 | 36/3250 | 0.000624673949349288 | 1 | T | T | F | F | mitochondrial respiratory chain complex assembly | ACAD9 | BCS1L | COA3 | COX17 | COX19 | COX20 | ECSIT | LYRM7 | NDUFA10 | NDUFA13 | NDUFA5 | NDUFA8 | NDUFA9 | NDUFAF1 | NDUFAF2 | NDUFAF3 | NDUFAF4 | NDUFAF5 | NDUFAF7 | NDUFB1 | NDUFS1 | NDUFS2 | NDUFS3 | NDUFS6 | NDUFS7 | NDUFS8 | NDUFV1 | NDUFV3 | OXA1L | SCO2 | TAZ | TIMM21 | TIMMDC1 | TTC19 | UQCC1 | UQCRB | GO:0032411 | 0.00715758825670266 | 59/8243 | 36/3250 | 0.000624673949349288 | 1 | T | T | F | F | positive regulation of transporter activity | ACTN2 | ACTN4 | ADRB2 | AKAP9 | ANK3 | ATP1B3 | CACNG2 | CACNG4 | CALM1 | CALM2 | CALM3 | CFTR | CHCHD10 | CHP1 | COX17 | DMD | GLRX | GSTO1 | HAP1 | HSPA2 | HTR3A | HTT | KCNIP2 | KIF5B | KMT2A | NOS1AP | PARK7 | PRKCD | RGN | S100A1 | SLC9A1 | STAC | STIM1 | SUMO1 | WNK1 | WNK4 | GO:0071479 | 0.00667232803590926 | 55/8243 | 34/3250 | 0.000626938537955354 | 1 | T | T | F | F | cellular response to ionizing radiation | ATM | BCL2L1 | CDKN1A | CHEK2 | CRYAB | EEF1D | FIGNL1 | GRB2 | H2AFX | HRAS | HSF1 | HSPA5 | IFI16 | KDM1A | KDM4D | LIG4 | MAPK14 | MDM2 | MGMT | NAMPT | NIPBL | POLE3 | RAD51 | RAD51AP1 | SIRT1 | SNAI2 | TANK | TGFB1 | TP53 | TREX1 | XRCC5 | XRCC6 | YAP1 | ZMPSTE24 | GO:1902882 | 0.00667232803590926 | 55/8243 | 34/3250 | 0.000626938537955354 | 1 | T | T | F | F | regulation of response to oxidative stress | AKT1 | BAG5 | CD36 | CTNNB1 | ENDOG | FBXO7 | FYN | GPX1 | HDAC6 | HIF1A | HSPB1 | HTRA2 | LANCL1 | LRRK2 | MAPK7 | MAPKAP1 | NFE2L2 | P4HB | PARK7 | PARP1 | PAWR | PSAP | PYCR1 | RGN | RIPK1 | SFPQ | SIRT1 | SOD1 | SOD2 | STK26 | TNF | TRAP1 | TXN | UBQLN1 | GO:0009113 | 0.00133446560718185 | 11/8243 | 10/3250 | 0.000635948461531676 | 1 | T | T | F | F | purine nucleobase biosynthetic process | ADA | APRT | GART | GMPS | HPRT1 | MTHFD1 | PAICS | PPAT | PRTFDC1 | SHMT1 | GO:0009891 | 0.151401188887541 | 1248/8243 | 544/3250 | 0.000636664155003585 | 1 | T | T | F | F | positive regulation of biosynthetic process | ABCG1 | ABHD14B | ABL1 | ACD | ACTA2 | ACTL6A | ACTN1 | ACTN2 | ACTN4 | ACTR2 | ACTR3 | ADCYAP1 | ADRB2 | AGAP2 | AGER | AGO1 | AGO2 | AHI1 | AICDA | AIRE | AKT1 | AKT2 | ANKRD49 | ANXA1 | APBB1 | APEX1 | APOA1 | APOE | APP | AR | ARF4 | ARID3B | ARID4B | ARNT | ARNT2 | ARRB1 | ARRB2 | ASCL1 | ASS1 | ATAD5 | ATF2 | ATF6 | ATF6B | ATM | ATOH1 | ATRX | AURKB | AXIN1 | AZU1 | BANP | BCL10 | BIRC2 | BMPR1A | BRCA1 | BRD4 | BTRC | BUD31 | C1QBP | CAND1 | CAPN2 | CARM1 | CBFB | CBX8 | CCAR2 | CCL21 | CCNT1 | CCNT2 | CCT2 | CCT3 | CCT4 | CCT5 | CCT6A | CCT7 | CCT8 | CD36 | CD40 | CD74 | CD81 | CDC5L | CDC73 | CDH1 | CDK2 | CDK4 | CDK8 | CDK9 | CEBPA | CEBPE | CEP290 | CHCHD10 | CHCHD2 | CHD8 | CHEK2 | CHP1 | CIRBP | CLU | COA3 | COPS5 | CREB1 | CREB3 | CREBBP | CTCF | CTNNB1 | DAB2 | DAXX | DBF4B | DCN | DDRGK1 | DDX39B | DDX3X | DHX36 | DHX9 | DNA2 | DPF2 | DTX1 | DVL1 | DVL2 | DVL3 | DYRK2 | E2F1 | E2F2 | E2F3 | E2F4 | E2F6 | EAF2 | EBF1 | ECD | EEF2 | EGFR | EGLN1 | EIF2AK3 | EIF2AK4 | EIF2B5 | EIF3D | EIF3E | EIF4A3 | EIF5A | EIF6 | ELAVL1 | ELF3 | ELF4 | ENO1 | EP300 | ERBB2 | ERG | ERLIN2 | ESR1 | ESR2 | ESRRA | ESRRB | ESRRG | ETS1 | EXOSC9 | FABP3 | FADD | FAF1 | FAM129B | FBXW11 | FGF2 | FGFR1 | FHL2 | FOS | FOXA1 | FOXJ1 | FOXK1 | FOXK2 | FOXM1 | FOXO1 | FUBP3 | FUS | FXR2 | GABPB1 | GATA1 | GLMN | GOLGA2 | GPS2 | GRIP1 | GTF2A1 | GTF2F1 | GTF2F2 | H1F0 | H1FX | H2AFZ | HAX1 | HDAC1 | HDAC2 | HDAC3 | HDAC4 | HDAC5 | HDAC6 | HELZ2 | HEXB | HIF1A | HIST1H1A | HMGB1 | HMGB2 | HMGB3 | HMOX1 | HNF1A | HNF4A | HNF4G | HNRNPA1 | HNRNPA2B1 | HNRNPAB | HNRNPD | HNRNPK | HNRNPL | HNRNPU | HOXA5 | HOXB5 | HOXB9 | HOXD10 | HRAS | HSF1 | HSF4 | HSP90AA1 | HSP90AB1 | HSPA5 | HSPB1 | HSPH1 | HTATIP2 | ICAM1 | ID2 | IFI16 | IFNA2 | IKBKB | IKBKG | IKZF5 | ILF2 | ILF3 | ILK | ING1 | ING5 | INSIG1 | INSIG2 | INSR | IRF1 | IRF4 | IRF7 | ITGB1BP1 | JMJD6 | JUN | JUNB | JUND | JUP | KAT5 | KAT7 | KAT8 | KDM1A | KDM8 | KDR | KHDRBS1 | KLF5 | KLF6 | KMT2A | KMT2B | KMT2C | LARP4 | LEF1 | LGR4 | LMO2 | LMO7 | LPIN3 | LRP6 | LRRK2 | LUM | MAP2K1 | MAP2K3 | MAP2K4 | MAP2K6 | MAP2K7 | MAP3K2 | MAP3K4 | MAPK1 | MAPK14 | MAPK15 | MAPK3 | MAPK7 | MAS1 | MAVS | MC1R | MECP2 | MED1 | MED12 | MED14 | MED17 | MED20 | MED21 | MED23 | MED24 | MED31 | MED4 | MED6 | MEOX2 | METTL23 | MITF | MLLT11 | MLLT3 | MORF4L2 | MRPL12 | MTA1 | MTA3 | MTPN | MYC | MYO6 | NAMPT | NANOG | NCK1 | NCOA1 | NCOA2 | NCOA3 | NCOA4 | NEK7 | NELFE | NEUROD1 | NFATC1 | NFATC2 | NFE2L2 | NFKB2 | NFKBIA | NIF3L1 | NIPBL | NKRF | NKX2-1 | NMD3 | NME2 | NOD2 | NOS1AP | NOS2 | NOS3 | NPM1 | NR1H2 | NR1H3 | NR1I2 | NR3C1 | NR4A1 | NR5A2 | NUFIP1 | NUP85 | NUP98 | NUPR1 | OGT | ONECUT1 | P2RX4 | PABPC1 | PAIP1 | PARK7 | PARP1 | PATZ1 | PAWR | PAX8 | PAX9 | PAXIP1 | PCBD1 | PCBP1 | PCGF5 | PCNA | PER1 | PER2 | PFKM | PHB | PHF10 | PIAS1 | PIH1D1 | PIK3R1 | PIN1 | PLA2G1B | PLA2G6 | PLSCR1 | PML | PNKP | POLDIP3 | POT1 | POU2F3 | POU5F1 | PPARD | PPARG | PPARGC1A | PPM1A | PPP1R12A | PRKAA1 | PRKCD | PRKD1 | PRKD2 | PRKDC | PRMT2 | PROX1 | PSEN1 | PSMC2 | PSMC3 | PSMC4 | PSMC5 | PSMC6 | PSMD9 | PTGES2 | PTGES3 | RAB3GAP1 | RAC1 | RAF1 | RAN | RARA | RARG | RB1 | RBM3 | RBMX | RBPJ | REL | RELA | REST | RFC1 | RFC2 | RFC3 | RFC4 | RFC5 | RFXANK | RGN | RHOA | RIPK1 | RIPK2 | RIPK3 | RMND1 | RNF4 | RPS4X | RPS6KA1 | RPS6KB2 | RPTOR | RTF1 | RUNX1 | RUNX2 | RUVBL1 | RUVBL2 | RXRA | RXRB | RXRG | SFPQ | SHC1 | SIN3A | SIRT1 | SIRT7 | SKI | SLC9A1 | SMAD2 | SMAD3 | SMAD4 | SMAD5 | SMAD9 | SMARCA2 | SMARCA4 | SMARCB1 | SMARCC1 | SMARCC2 | SMARCE1 | SMO | SNCA | SNW1 | SOAT1 | SOX2 | SP1 | SP100 | SPI1 | SPIN1 | SQSTM1 | SRC | STAT1 | STAT3 | STAT4 | STAT6 | STOML2 | SUB1 | SUMO2 | SUPT4H1 | SUPT5H | TADA2B | TAF12 | TAF15 | TARDBP | TAZ | TBL1X | TCEA2 | TCERG1 | TCF12 | TCF21 | TCF4 | TCP1 | TDRD3 | TFCP2 | TFDP1 | TGFB1 | THBS1 | THRA | TLR3 | TMEM173 | TNF | TNFRSF1A | TNFRSF8 | TNIP2 | TNKS | TNKS2 | TOP2A | TP53 | TP63 | TP73 | TRAF6 | TRIAP1 | TRIM28 | TRIM32 | TRIP4 | TRUB2 | TSG101 | TTC5 | TWIST1 | UBB | UBC | UBE2L3 | UBE2V1 | UPF3B | UQCC1 | USP16 | USP21 | VCP | VDR | VHL | VIM | WAS | WBP2 | WDR77 | WNT2 | WT1 | WWOX | WWP2 | XBP1 | XRCC5 | XRCC6 | YAF2 | YAP1 | YES1 | YTHDF1 | YWHAH | YY1 | ZBTB16 | ZBTB17 | ZBTB7B | ZIC1 | ZNF24 | ZNF746 | GO:0001889 | 0.0106757248574548 | 88/8243 | 50/3250 | 0.000676560866549498 | 1 | T | T | F | F | liver development | ACAT1 | ACO2 | ADA | AK4 | ASNS | ASS1 | CEBPA | CSNK2A2 | CSNK2B | DDX39B | EGFR | EZH2 | FPGS | GNPNAT1 | HLX | HMGCL | HMOX1 | HNF1A | HNRNPD | JUN | KRAS | MED1 | MPST | ONECUT1 | OTC | PCNA | PCSK9 | PIK3CA | PKM | PROX1 | PTN | QDPR | RAP1A | RARA | RB1CC1 | RELA | RGN | RPGRIP1L | SEC63 | SMAD3 | SRSF5 | SULF2 | TGFB1 | TGFBR3 | TYMS | UPF2 | VTN | WDR35 | XBP1 | ZMPSTE24 | GO:1901136 | 0.0106757248574548 | 88/8243 | 50/3250 | 0.000676560866549498 | 1 | T | T | F | F | carbohydrate derivative catabolic process | ACAT1 | ACOT7 | ADA | AHCY | AICDA | AMDHD2 | CDA | CHP1 | DCN | DCTPP1 | DERA | DUT | EDEM1 | FBXO6 | FGF2 | GALNS | GLA | GNPDA1 | GNPDA2 | GNS | GPC5 | GPD1 | GPD1L | GUSB | HEXA | HEXB | HINT1 | HMMR | HPRT1 | HSPG2 | ITPA | LUM | LYG2 | MTAP | NUDT18 | NUDT3 | NUDT9 | OVGP1 | PDE4D | PGM2 | PNP | PRTFDC1 | PSAP | SDC1 | SDC4 | SGSH | SLC9A1 | TGFB1 | UPP2 | VCAN | GO:1901987 | 0.0420963241538275 | 347/8243 | 166/3250 | 0.000703784316885356 | 1 | T | T | F | F | regulation of cell cycle phase transition | ACTR1A | AKAP9 | AKT1 | ANAPC4 | ANXA1 | APC | APEX1 | APP | ATAD5 | ATM | AURKB | BABAM1 | BAX | BCL2 | BRCA1 | BRD4 | BTRC | CARM1 | CASP2 | CCNB1 | CCP110 | CDC14B | CDC16 | CDC20 | CDC25A | CDC25B | CDC25C | CDC27 | CDC73 | CDK2 | CDK4 | CDKN1A | CDKN2C | CENPF | CENPJ | CEP135 | CEP152 | CEP290 | CEP57 | CEP70 | CHEK2 | CKAP5 | CNTRL | CSNK1E | CUL1 | CUL3 | CUL4B | CYP1A1 | DBF4B | DCTN1 | DDB1 | DDRGK1 | DDX3X | DUSP1 | DYNC1H1 | DYNC1I2 | DYNC1LI1 | DYNLL1 | E2F1 | E2F4 | ECD | EGFR | EP300 | EZH2 | FAM83D | FBXO7 | FGFR1OP | FHL1 | FZR1 | HECW2 | HMMR | HSP90AA1 | HSPA2 | ID2 | IK | KANK2 | KLHL22 | MAD1L1 | MAD2L1 | MAPK15 | MDM2 | MDM4 | MECP2 | MEPCE | MSH6 | MTA3 | NAE1 | NPM1 | OFD1 | PAFAH1B1 | PAXIP1 | PCM1 | PCNA | PHB2 | PINX1 | PLK1 | PML | PPP2R1A | PRKACA | PRKAR2B | PRKDC | PRMT2 | PSMA1 | PSMA2 | PSMA3 | PSMA5 | PSMA6 | PSMB5 | PSMB6 | PSMB7 | PSMB8 | PSMC1 | PSMC2 | PSMC3 | PSMC4 | PSMC5 | PSMC6 | PSMD1 | PSMD10 | PSMD11 | PSMD12 | PSMD14 | PSMD2 | PSMD4 | PSMD9 | PSME2 | PSME3 | PTEN | PTPN6 | RAB11A | RAD21 | RB1 | RBX1 | RDX | RIPK1 | RNASEH2B | RPA2 | RPTOR | SEPT7 | SFN | SIN3A | SPDL1 | TAOK3 | TFDP1 | TGFB1 | TMOD3 | TP53 | TPR | TRIAP1 | TRIP13 | TUBA1A | TUBA4A | TUBB | TUBB4A | TUBB4B | TUBG1 | UBE2C | UBE2D1 | USP47 | VPS4B | XRCC3 | YWHAE | YWHAG | ZFYVE19 | ZNF207 | ZW10 | GO:0030178 | 0.0161349023413806 | 133/8243 | 71/3250 | 0.00070409316794848 | 1 | T | T | F | F | negative regulation of Wnt signaling pathway | AES | APC | APOE | AXIN1 | CAV1 | CBY1 | CHD8 | CSNK1A1 | CTNNBIP1 | CTNND1 | CUL3 | CYLD | DAB2 | DVL1 | DVL2 | DVL3 | FOXO1 | G3BP1 | GSK3A | GSK3B | HDAC1 | HECW1 | LATS1 | LRP6 | MAPK14 | MCC | MDFI | MLLT3 | NFATC1 | NLK | NPHP3 | PSMA1 | PSMA2 | PSMA3 | PSMA5 | PSMA6 | PSMB5 | PSMB6 | PSMB7 | PSMB8 | PSMC1 | PSMC2 | PSMC3 | PSMC4 | PSMC5 | PSMC6 | PSMD1 | PSMD10 | PSMD11 | PSMD12 | PSMD14 | PSMD2 | PSMD4 | PSMD9 | PSME2 | PSME3 | PTPRO | RBX1 | RUVBL2 | SCYL2 | SLC9A3R1 | SNAI2 | SOX2 | SPERT | STK11 | STK3 | TLE1 | TLE3 | TNN | TSKU | WWOX | GO:0043903 | 0.021472764770108 | 177/8243 | 91/3250 | 0.000721037179870008 | 1 | T | T | F | F | regulation of symbiosis, encompassing mutualism through parasitism | AICDA | ANXA2 | APCS | APOE | BANF1 | BCL2 | CAV1 | CCNT1 | CD36 | CD74 | CDK9 | CFL1 | CHMP2A | CTDP1 | CXCL6 | CXCL8 | DDB1 | DDX3X | DHX9 | EIF2AK4 | EP300 | FCN1 | GAPDH | GTF2F1 | GTF2F2 | HDAC1 | IFI16 | IFIT1 | IFITM3 | IFNA2 | ILF3 | ISG15 | ITGAV | JUN | LAMP3 | LEF1 | LGALS1 | MAVS | NELFB | NELFCD | NELFE | NR5A2 | P4HB | PABPC1 | PC | PDE12 | PKN2 | PLSCR1 | PML | POLR2A | POLR2E | POLR2L | POU2F3 | PPIA | PPIB | PPID | PPIE | PROX1 | PSMC3 | RAB7A | RAD23A | REST | SETDB1 | SMARCA4 | SMARCB1 | SMC3 | SNF8 | SNW1 | SNX3 | SP1 | SQSTM1 | SRPK1 | SRPK2 | STAT1 | STAU1 | STOM | SUPT4H1 | SUPT5H | TARDBP | TNF | TOP2A | TRIM14 | TRIM21 | TRIM25 | TRIM28 | TRIM32 | TRIM35 | TSG101 | VAPA | VAPB | VPS4B | GO:2000027 | 0.0188038335557443 | 155/8243 | 81/3250 | 0.000733978358956468 | 1 | T | T | F | F | regulation of animal organ morphogenesis | ABL1 | AHI1 | AR | ARRB2 | BASP1 | BAX | BCL2 | BCOR | BMPR1A | CARM1 | CDC42 | CLTC | CTNNB1 | DAB2 | DMRT3 | DVL1 | DVL2 | DVL3 | ESR1 | FGFR1 | GNG5 | LGR4 | MED1 | MED12 | MKS1 | MLLT3 | MYC | NPHP3 | PARD6A | PAX8 | PAX9 | PFN1 | PHB2 | PIM1 | PSMA1 | PSMA2 | PSMA3 | PSMA5 | PSMA6 | PSMB5 | PSMB6 | PSMB7 | PSMB8 | PSMC1 | PSMC2 | PSMC3 | PSMC4 | PSMC5 | PSMC6 | PSMD1 | PSMD10 | PSMD11 | PSMD12 | PSMD14 | PSMD2 | PSMD4 | PSMD9 | PSME2 | PSME3 | PTN | RAC1 | RBPJ | RHOA | RUNX2 | RXRA | SEC24B | SMAD4 | SMO | SMURF1 | SMURF2 | SNAI2 | STAT1 | SULF2 | TGFB1 | TNF | TNFAIP3 | TWIST1 | VDR | WNT2 | WT1 | XBP1 | GO:0006096 | 0.0038820817663472 | 32/8243 | 22/3250 | 0.000736264972499066 | 1 | T | T | F | F | glycolytic process | ALDOA | ALDOC | ENO1 | ENO2 | ENO3 | GAPDH | GAPDHS | GPI | HK1 | HK2 | LDHA | OGDH | PFKL | PFKM | PFKP | PGAM1 | PGAM2 | PGK1 | PGK2 | PGM1 | PKM | TPI1 | GO:0051973 | 0.0038820817663472 | 32/8243 | 22/3250 | 0.000736264972499066 | 1 | T | T | F | F | positive regulation of telomerase activity | ACD | AURKB | CCT2 | CCT4 | CTNNB1 | HNRNPA2B1 | HNRNPD | HSP90AA1 | HSP90AB1 | MAP2K7 | MAP3K4 | MAPK1 | MAPK15 | MAPK3 | MYC | NEK7 | PNKP | POT1 | PTGES3 | TCP1 | TNKS | XRCC5 | GO:0006631 | 0.0172267378381657 | 142/8243 | 75/3250 | 0.000762070194602798 | 1 | T | T | F | F | fatty acid metabolic process | AASDH | ABCD3 | ACAA1 | ACAA2 | ACAD9 | ACADS | ACADSB | ACADVL | ACAT1 | ACAT2 | ACOX1 | ACSL1 | ACSL4 | ACSL5 | AKR1C2 | ALDH5A1 | ALOX12 | ALOX5 | ALOX5AP | ATP6V1B1 | CBR1 | CD36 | CES1 | CPT1A | CPT2 | CROT | CYP1A1 | CYP1A2 | CYP2C18 | DECR1 | DLD | ECH1 | ECHDC2 | ECHS1 | ECI1 | ECI2 | EHHADH | ETFA | ETFB | ETFDH | FABP3 | FASN | GCDH | GGT1 | GPX1 | GPX4 | GSTA1 | GSTP1 | HADH | HIBCH | HSD17B4 | IVD | LTC4S | LYPLA1 | LYPLA2 | MAPK14 | MAPK3 | MCAT | NDUFS6 | PER2 | PLA2G10 | PPARD | PPARG | PPARGC1A | PRKAA1 | PRKAR2B | PTGDS | PTGES | PTGES2 | PTGES3 | PTGR1 | SGPL1 | SNCA | THEM4 | TNFRSF1A | GO:0090316 | 0.0140725464030086 | 116/8243 | 63/3250 | 0.000765257123386231 | 1 | T | T | F | F | positive regulation of intracellular protein transport | AKT2 | ANK3 | B3GAT3 | BAG3 | CDH1 | CDK5 | CDK5R1 | CEP290 | CHP1 | CSNK2A2 | DMAP1 | EDEM1 | ERBB2 | FIS1 | FYN | GSK3A | GSK3B | HDAC3 | HRAS | HSPA1L | HTRA2 | HUWE1 | IPO5 | ITGB1BP1 | JUP | KIF5B | MAPK1 | MAPK14 | MAVS | MDM2 | MYO1C | NPEPPS | PCM1 | PDCD10 | PIK3R1 | PPM1A | PRKAA1 | PRKACA | PRKCD | PSEN1 | RAB29 | RAN | RHOU | RNF31 | SAE1 | SEC16A | SFN | SMAD3 | SMO | STOM | TARDBP | TGFB1 | TM9SF4 | TMEM30A | TP53 | TPR | TRIM28 | UBE2D3 | UBE2L3 | UBR5 | XPO4 | YWHAE | ZIC1 | GO:0050793 | 0.177119980589591 | 1460/8243 | 630/3250 | 0.00076551653664851 | 1 | T | T | F | F | regulation of developmental process | ABCG1 | ABL1 | ABL2 | ACTN4 | ACTR2 | ADA | ADCY6 | ADCYAP1 | ADRB2 | AFG3L2 | AGER | AGO1 | AGO2 | AGR2 | AHI1 | AHSG | AKT1 | AKT3 | ALDOA | ALOX12 | ANXA1 | ANXA2 | ANXA7 | AP3D1 | APBB1 | APC | APCS | APOA1 | APOE | APP | AR | ARF1 | ARHGAP35 | ARHGDIA | ARHGEF7 | ARHGEF9 | ARNT | ARPC2 | ARRB2 | ASCL1 | ATAD5 | ATF2 | ATOH1 | AVIL | BAG5 | BAG6 | BAIAP2 | BASP1 | BAX | BCL11A | BCL2 | BCL6 | BCOR | BIN1 | BIRC2 | BMPR1A | BRAF | BRCA1 | BTK | C1QA | C1QBP | C1QL4 | CALR | CAMK1D | CAMK2A | CAMK2B | CAPN2 | CAPZB | CARM1 | CASP8 | CAV1 | CBFB | CCDC88A | CCNB1 | CCR1 | CCR3 | CD109 | CD2 | CD27 | CD36 | CD40 | CD74 | CDC20 | CDC42 | CDC42EP3 | CDC42EP4 | CDC42SE2 | CDC73 | CDH5 | CDK4 | CDK5 | CDK5R1 | CDK9 | CDKL3 | CDKN1A | CEBPA | CENPF | CFL1 | CHRD | CLDN18 | CLIC1 | CLSTN1 | CLTC | CMTM5 | COL4A2 | COL5A1 | CREB1 | CREBBP | CRK | CRKL | CRMP1 | CRP | CSNK1E | CTDP1 | CTH | CTNNB1 | CTNNBIP1 | CTSL | CTSV | CTTN | CUL7 | CXCL8 | CYLD | DAB1 | DAB2 | DBN1 | DCN | DDX39B | DDX6 | DGUOK | DHX36 | DISC1 | DLG4 | DMRT3 | DNM1L | DOCK5 | DOCK7 | DPF2 | DROSHA | DTX1 | DVL1 | DVL2 | DVL3 | DYNLT1 | E2F1 | E2F2 | EAF2 | EED | EFEMP1 | EGFR | EGLN1 | EHMT1 | EID1 | EIF2AK3 | EIF2AK4 | EIF4E | EIF4ENIF1 | EIF6 | ELAVL1 | EP300 | EPHA4 | EPPK1 | ERBB2 | ERBB3 | ERRFI1 | ESR1 | ESRRA | ESRRB | ETS1 | EXOSC6 | EZH2 | EZR | FADD | FAM129B | FANCA | FASLG | FBXO7 | FERMT2 | FGB | FGF2 | FGFR1 | FGFR3 | FIS1 | FKBP1B | FKBPL | FLOT1 | FN1 | FOS | FOXA1 | FOXJ1 | FOXM1 | FOXO1 | FTO | FYN | FZR1 | G6PD | GAK | GAP43 | GATA1 | GDF9 | GDI1 | GFAP | GLUL | GNA12 | GNA13 | GNAS | GNG5 | GOLGA4 | GPS2 | GPX1 | GRID2 | GRIP1 | GSK3A | GSK3B | GTF2I | H3F3A | HAP1 | HAVCR2 | HAX1 | HDAC1 | HDAC2 | HDAC3 | HDAC4 | HDAC5 | HDAC6 | HDAC7 | HDAC9 | HECW1 | HECW2 | HEXB | HGS | HIF1A | HIF1AN | HIST1H3A | HIST1H4A | HIST2H3A | HK2 | HLX | HMGB1 | HMGB2 | HMOX1 | HNF4A | HNRNPU | HOXA5 | HSF1 | HSF4 | HSP90AB1 | HSPA1B | HSPA5 | HSPA9 | HSPB1 | HSPG2 | HTATIP2 | HTRA2 | ICAM1 | ID2 | IDH2 | IFNA2 | IFT122 | IKBKB | ILK | INSIG1 | INSR | IQCB1 | IQGAP1 | IRF1 | IRF4 | IRF7 | ISG15 | ITCH | ITGA7 | ITGAV | ITGB1BP1 | ITGB3 | JAK1 | JAK3 | JUN | JUNB | JUND | JUP | KDM1A | KDM5B | KDR | KEAP1 | KIF13B | KLF5 | KMT2A | KMT2B | KMT2C | KRAS | L3MBTL1 | LARP4 | LATS1 | LDLRAD4 | LEF1 | LGALS1 | LGALS3 | LGMN | LGR4 | LIG4 | LIMS1 | LMNA | LMO2 | LOXL2 | LPAR1 | LRFN1 | LRRC7 | LRRK2 | LSM1 | MAP1B | MAP2K1 | MAP3K13 | MAP3K3 | MAPK1 | MAPK14 | MAPK6 | MAPK7 | MAPK9 | MAPT | MARCH5 | MBP | MDM2 | MECP2 | MED1 | MED12 | MEOX2 | METRN | METRNL | MEX3C | MIB1 | MIF | MITF | MKS1 | MLH1 | MLLT3 | MME | MORF4L2 | MOV10 | MSH6 | MSN | MTPN | MYC | MYH6 | MYL9 | MYOC | NAMPT | NANOG | NAP1L1 | NCK1 | NCOA1 | NCOA3 | NEDD4 | NELFB | NELL1 | NEUROD1 | NF2 | NFATC1 | NFATC2 | NFE2L2 | NFKBIA | NIN | NIPBL | NKX2-1 | NME1 | NME2 | NMRK2 | NOS3 | NPHP3 | NPR1 | NR1H2 | NR1H3 | NRXN1 | NTF3 | NTRK1 | NUMA1 | NUPR1 | OAS2 | OBSL1 | OPA1 | P2RX4 | PA2G4 | PAFAH1B1 | PARD3 | PARD6A | PARP1 | PARP6 | PAWR | PAX8 | PAX9 | PAXIP1 | PCM1 | PDCD10 | PDCD6 | PDLIM5 | PER2 | PFN1 | PGK1 | PHB2 | PHOSPHO1 | PIAS1 | PIAS3 | PIK3C2A | PIK3CA | PIK3R1 | PIM1 | PIN1 | PKM | PLA2G10 | PLA2G2A | PLCG1 | PLXNA1 | PLXNA2 | PLXNB2 | PML | PNP | PPARD | PPARG | PPARGC1A | PPIB | PPP1CC | PPP1R12A | PPP2CA | PPP2R1A | PRDX2 | PRELID1 | PRKAA1 | PRKACA | PRKCZ | PRKD1 | PRKD2 | PRKDC | PRMT6 | PROX1 | PRPF19 | PRPF40A | PSEN1 | PSMA1 | PSMA2 | PSMA3 | PSMA5 | PSMA6 | PSMB5 | PSMB6 | PSMB7 | PSMB8 | PSMC1 | PSMC2 | PSMC3 | PSMC4 | PSMC5 | PSMC6 | PSMD1 | PSMD10 | PSMD11 | PSMD12 | PSMD14 | PSMD2 | PSMD4 | PSMD9 | PSME2 | PSME3 | PTEN | PTK2 | PTN | PTPN6 | PTPRD | PTPRF | PTPRO | PUS7 | QRICH1 | RAB11A | RAB21 | RAB29 | RAC1 | RAF1 | RAP1A | RARA | RARG | RASSF10 | RB1 | RBM4 | RBPJ | RC3H1 | RDX | RECK | RELA | REST | RGN | RGS2 | RHOA | RHOJ | RHOU | RIF1 | RIPK1 | RIPK2 | RPS19 | RPS6KA1 | RTN4 | RTN4IP1 | RUNX1 | RUNX1T1 | RUNX2 | RXRA | S100A1 | S100A13 | S100A9 | S100B | SARM1 | SARS | SART1 | SDCBP | SEC24B | SEMA7A | SEPT7 | SERPINB3 | SERPINE2 | SFMBT1 | SFN | SGPL1 | SH3GL3 | SH3KBP1 | SIN3A | SIPA1L1 | SIRT1 | SIRT6 | SKI | SLAMF8 | SLC9A3R1 | SMAD2 | SMAD3 | SMAD4 | SMAD5 | SMO | SMURF1 | SMURF2 | SNAI2 | SNCA | SNW1 | SNX3 | SOCS1 | SOCS2 | SOD1 | SOD2 | SORT1 | SOX2 | SP1 | SP100 | SPEN | SPI1 | SPRY2 | SRC | SRGAP2 | STARD13 | STAT1 | STAT3 | STAT5A | STAT6 | STIM1 | STK11 | STK24 | STK25 | STK3 | STRAP | SULF2 | SUZ12 | SYDE1 | SYNJ2BP | TBXA2R | TCF12 | TCF4 | TERF2 | TGFB1 | TGFBR3 | THBS1 | THBS2 | TIMP1 | TIMP2 | TLR3 | TMEM30A | TNF | TNFAIP3 | TNFRSF12A | TNFRSF1A | TNFSF12 | TNFSF14 | TNKS2 | TNN | TP63 | TP73 | TPM1 | TRADD | TRAF3IP1 | TRAF6 | TRIB2 | TRIB3 | TRIM32 | TRIP12 | TRIP4 | TUBB2B | TULP3 | TWF1 | TWF2 | TWIST1 | USP19 | VASH2 | VAT1 | VDR | VHL | VIM | VSTM2A | WARS | WDR1 | WDR77 | WNK1 | WNT2 | WT1 | XBP1 | XRCC5 | YAP1 | YWHAG | YWHAH | YWHAZ | YY1 | ZAP70 | ZBTB16 | ZBTB7B | ZMPSTE24 | ZMYM5 | ZMYND8 | ZNF277 | GO:0051170 | 0.00970520441586801 | 80/8243 | 46/3250 | 0.000770421173836842 | 1 | T | T | F | F | import into nucleus | AKT1 | BACH2 | CBLB | CSE1L | GEMIN4 | GEMIN5 | GEMIN7 | HEATR3 | HNRNPA1 | HTATIP2 | IPO11 | IPO4 | IPO5 | IPO7 | IPO8 | IPO9 | KPNA1 | KPNA2 | KPNB1 | LMNA | NUP107 | NUP153 | NUP214 | NUP85 | NUP88 | NUP93 | NUP98 | NUTF2 | NXT2 | PHB2 | PIK3R1 | PML | POM121 | PRKAG1 | RAN | RPAIN | RPL23 | SMN1 | SNRPB | SNRPD1 | SNRPD3 | SNRPE | STAT3 | TNPO1 | TPR | TXNIP | GO:0051234 | 0.28824457115128 | 2376/8243 | 1001/3250 | 0.000778539788015726 | 1 | T | T | F | F | establishment of localization | A2M | ABCB7 | ABCB8 | ABCD3 | ABCE1 | ABCG1 | ABL1 | ACAA1 | ACACA | ACD | ACE2 | ACKR2 | ACLY | ACO1 | ACOX1 | ACSL1 | ACSL4 | ACTB | ACTG1 | ACTN1 | ACTN2 | ACTN4 | ACTR1A | ACTR2 | ACTR3 | ADA | ADCYAP1 | ADD3 | ADORA2A | ADPRH | ADRB2 | AFG3L2 | AFM | AGAP1 | AGAP2 | AGER | AGK | AGL | AGR2 | AHCYL1 | AHI1 | AHSG | AKAP8 | AKT1 | AKT2 | AKTIP | ALB | ALDOA | ALDOC | ALOX5 | ANK1 | ANK3 | ANP32A | ANXA1 | ANXA11 | ANXA2 | ANXA5 | ANXA6 | AP1B1 | AP1G1 | AP1M1 | AP3D1 | AP3M1 | AP4M1 | AP4S1 | APEH | APLP1 | APOA1 | APOD | APOE | APOL2 | APP | APPBP2 | APRT | ARF1 | ARF4 | ARF5 | ARFGAP1 | ARFIP1 | ARHGAP1 | ARL4D | ARL5B | ARMC8 | ARPC5 | ARR3 | ARRB1 | ARRB2 | ASGR1 | ASGR2 | ASPSCR1 | ATG4C | ATL3 | ATM | ATOX1 | ATP12A | ATP13A2 | ATP1A1 | ATP1B3 | ATP2A2 | ATP2B2 | ATP4A | ATP6V1A | ATP6V1B1 | ATP6V1C1 | ATP6V1D | ATP6V1E1 | ATP9A | ATXN1 | ATXN2 | AUP1 | AZU1 | BACH2 | BAG3 | BAG6 | BANF1 | BAX | BCL2 | BCL2L1 | BDKRB2 | BIN1 | BLZF1 | BRAF | BSPRY | BTK | C16orf70 | CA14 | CAB39 | CACNA1A | CACNG2 | CACNG4 | CACNG5 | CALM1 | CALM3 | CALR | CAMK2A | CAND1 | CANX | CAPN1 | CAPZA1 | CAPZA2 | CAPZB | CAT | CAV1 | CBL | CBLB | CCDC155 | CCDC22 | CCDC88A | CCHCR1 | CCL21 | CCNB1 | CCR1 | CCR5 | CCS | CCT2 | CCT3 | CCT4 | CCT5 | CCT6B | CCT7 | CCT8 | CD109 | CD209 | CD36 | CD40LG | CD6 | CD74 | CD81 | CDA | CDC37 | CDC42 | CDC42SE2 | CDK16 | CDK2 | CDK5 | CEBPE | CEL | CENPF | CEP290 | CEP55 | CFTR | CHMP2A | CHP1 | CHRM3 | CHRNA9 | CHRND | CHRNE | CHRNG | CKAP4 | CKAP5 | CLDN17 | CLIC1 | CLIC3 | CLIC4 | CLNS1A | CLSTN1 | CLTC | CLU | CNNM1 | COG3 | COL4A3BP | COMMD1 | COPA | COPB1 | COPE | COPG1 | COPS5 | COTL1 | COX11 | COX15 | COX17 | COX5A | COX6A1 | COX6C | COX7A2 | CPLX1 | CPLX3 | CPNE3 | CPT1A | CPT2 | CREB1 | CROCC | CROT | CSE1L | CSN1S1 | CSNK1E | CSNK2B | CTNNB1 | CTSA | CTSC | CTSD | CTTN | CUL3 | CUL5 | CUTC | CXCL8 | CYB5R1 | CYB5R3 | CYC1 | DAB2 | DBNL | DCLK1 | DCTN1 | DDOST | DDX19A | DDX19B | DDX39A | DDX39B | DDX3X | DEGS1 | DENND1A | DENND4C | DERA | DERL1 | DHRS4 | DHX9 | DLG2 | DLG4 | DNAJA1 | DNAJC19 | DNM1L | DOCK7 | DUSP16 | DVL1 | DYNC1H1 | DYNC1I1 | DYNC1I2 | DYNC1LI1 | DYNLL1 | DYNLT1 | ECH1 | ECI2 | EEF1A1 | EEF2 | EGFR | EHD4 | EHHADH | EIF2S1 | EIF2S2 | EIF4A3 | EIF4E | EIF4ENIF1 | EIF5A | EIF6 | EPRS | ERLIN2 | EXOC6 | EZR | F5 | F9 | FABP1 | FABP3 | FABP5 | FAF2 | FAM129B | FAM3C | FAM49B | FAM83D | FASLG | FBXO7 | FCGR1A | FCN1 | FGB | FGF2 | FGL2 | FIS1 | FKBP1A | FKBP1B | FLOT1 | FN1 | FOLR1 | FRAS1 | FTH1 | FTL | FYN | FYTTD1 | G3BP2 | GAA | GABRA6 | GABRR1 | GAD2 | GAK | GALNS | GAPVD1 | GBP5 | GDI1 | GDI2 | GEMIN4 | GEMIN5 | GEMIN7 | GGA1 | GHDC | GJA1 | GLA | GLRB | GLS | GLS2 | GLUL | GNS | GOLGA2 | GOLGA4 | GOLPH3 | GOLT1B | GOPC | GORASP2 | GOT2 | GPI | GPR107 | GRAMD1A | GRB2 | GRIA1 | GRID2 | GRIK2 | GRIP1 | GRPEL1 | GSTK1 | GSTP1 | GUSB | GYG1 | HAP1 | HBZ | HCN1 | HCN2 | HCN3 | HDAC6 | HEATR3 | HERC2 | HEXB | HGS | HID1 | HIF1A | HK1 | HK2 | HMGB1 | HMGCL | HNF1A | HNRNPA1 | HNRNPA1L2 | HNRNPA2B1 | HNRNPU | HPX | HRAS | HRNR | HSD17B4 | HSF1 | HSP90AA1 | HSP90AB1 | HSP90B1 | HSPA1B | HSPA2 | HSPA4 | HSPA5 | HSPA8 | HSPB1 | HSPD1 | HSPG2 | HSPH1 | HTATIP2 | HTR1B | HTR3A | HTR3E | HTT | HUWE1 | IBTK | ICK | IDE | IDH1 | IFIT1 | IFT122 | IGF2BP1 | IGHV3-13 | IGHV3-23 | IGHV3-30 | IGKV3-20 | IGLC7 | IKBKG | ILF2 | IMPDH2 | INSR | IPO11 | IPO4 | IPO5 | IPO7 | IPO8 | IPO9 | IQGAP1 | IREB2 | ISLR | ITGA4 | ITGAV | ITGB1BP1 | ITGB3 | ITPR1 | ITPR2 | ITPR3 | JUP | KCMF1 | KCNA4 | KCNAB1 | KCNAB2 | KCNIP2 | KCNK1 | KCNK16 | KCNS3 | KIF11 | KIF13B | KIF2C | KIF5B | KIF5C | KLC1 | KLC2 | KLHL20 | KPNA1 | KPNA2 | KPNB1 | LARS | LASP1 | LAT | LCP2 | LETM1 | LGALS3 | LGALS3BP | LIMA1 | LIMS1 | LMAN2L | LMNA | LOXL2 | LRP6 | LRPPRC | LRRC7 | LRRK2 | LRSAM1 | MAL2 | MAN2B1 | MAP1B | MAPK1 | MAPK14 | MAPK15 | MAPK3 | MAPK8IP3 | MAPT | MASP1 | MCC | MCFD2 | MCM3AP | MCOLN3 | MDM2 | MECP2 | MED1 | MGST1 | MIB1 | MICU1 | MICU2 | MIF | MLH1 | MME | MMGT1 | MMRN1 | MPC2 | MPPE1 | MTX1 | MYC | MYO1B | MYO1C | MYO1D | MYO1E | MYO5A | MYO6 | NAPRT | NBAS | NCBP1 | NCOA1 | NCOA2 | NCOA4 | NDUFA13 | NDUFA9 | NDUFS7 | NEDD4 | NEUROD1 | NGB | NHLRC3 | NIPBL | NIT2 | NMB | NMD3 | NME1 | NME2 | NME4 | NNT | NOD2 | NOS2 | NOSTRIN | NPLOC4 | NPM1 | NPR1 | NR1H3 | NR1I2 | NRXN1 | NSFL1C | NUDT19 | NUMA1 | NUP107 | NUP153 | NUP160 | NUP205 | NUP214 | NUP85 | NUP88 | NUP93 | NUP98 | NUSAP1 | NUTF2 | NXF1 | NXF2 | NXT2 | OAZ3 | OLA1 | OPA1 | OPTN | ORM1 | OSBPL1A | OXA1L | P2RX1 | P2RX2 | P2RX4 | PA2G4 | PACSIN3 | PAFAH1B1 | PAM16 | PANX3 | PARD3 | PARK7 | PCM1 | PCSK9 | PCTP | PDCD10 | PDCD6 | PDCD6IP | PDIA4 | PDXK | PEF1 | PEX19 | PEX5 | PFKL | PGAM1 | PGM1 | PGM2 | PGRMC1 | PHB | PHB2 | PI4K2A | PICK1 | PIH1D1 | PIK3C2A | PIK3CG | PIK3R1 | PIKFYVE | PINX1 | PITPNA | PITPNB | PKM | PLA2G10 | PLA2G12A | PLA2G1B | PLA2G2A | PLAUR | PLCG1 | PLEKHF2 | PLIN3 | PLK1 | PLSCR1 | PML | PMPCA | PMPCB | PNP | POLDIP3 | POM121 | POT1 | PPARD | PPARG | PPAT | PPIA | PPID | PPIE | PPT1 | PQLC1 | PRCP | PRDX4 | PRDX6 | PRELID1 | PRG2 | PRKAA2 | PRKAB2 | PRKAG1 | PRKCD | PRKCE | PRKCZ | PRKD1 | PROZ | PSAP | PSEN1 | PSEN2 | PSMA1 | PSMA2 | PSMA3 | PSMA5 | PSMA6 | PSMB5 | PSMB6 | PSMB7 | PSMB8 | PSMC1 | PSMC2 | PSMC3 | PSMC4 | PSMC5 | PSMC6 | PSMD1 | PSMD10 | PSMD11 | PSMD12 | PSMD14 | PSMD2 | PSMD4 | PSMD9 | PSME2 | PSME3 | PSTPIP1 | PTGES2 | PTK2 | PTPN23 | PTPN6 | PYGB | PYGL | QSOX1 | RAB10 | RAB11A | RAB11B | RAB11FIP2 | RAB18 | RAB1A | RAB1B | RAB21 | RAB24 | RAB29 | RAB2A | RAB31 | RAB32 | RAB35 | RAB3GAP1 | RAB3IP | RAB5A | RAB5B | RAB7A | RAB9A | RABGEF1 | RABL2A | RABL3 | RAC1 | RAE1 | RAF1 | RAN | RANBP3 | RAP1A | RAP1B | RAPGEF4 | RARA | RASSF9 | RBM8A | RBPJ | RDX | RHOA | RHOJ | RHOT1 | RHOT2 | RHOU | RNASE3 | RNF126 | RNF185 | ROMO1 | RP2 | RPAIN | RPL10 | RPL10A | RPL12 | RPL18A | RPL22 | RPL23 | RPL23A | RPL24 | RPL30 | RPL37A | RPL38 | RPL6 | RPL7 | RPL7A | RPL9 | RPLP0 | RPLP1 | RPLP2 | RPS12 | RPS13 | RPS14 | RPS15A | RPS19 | RPS2 | RPS20 | RPS24 | RPS29 | RPS3 | RPS4X | RPS8 | RRS1 | RUVBL2 | RXRA | S100A11 | S100A12 | S100A13 | S100A6 | S100A7 | S100A9 | S100P | SARAF | SCARA3 | SCCPDH | SCFD1 | SCG2 | SCG3 | SCO2 | SCYL2 | SDCBP | SDCBP2 | SEC13 | SEC16A | SEC22C | SEC24B | SEC24C | SEC31A | SEC62 | SEC63 | SEL1L | SELENBP1 | SELL | SERPINB3 | SERPINB6 | SERPINE2 | SFPQ | SFXN1 | SFXN4 | SGTA | SGTB | SH3GL3 | SH3KBP1 | SLAMF1 | SLC12A2 | SLC15A1 | SLC16A1 | SLC25A1 | SLC25A10 | SLC25A11 | SLC25A13 | SLC25A22 | SLC25A24 | SLC25A3 | SLC25A32 | SLC25A4 | SLC25A41 | SLC25A5 | SLC25A6 | SLC2A5 | SLC2A9 | SLC35F6 | SLC37A3 | SLC39A4 | SLC39A9 | SLC3A2 | SLC7A1 | SLC9A1 | SLC9A3R1 | SMAD2 | SMG5 | SMG6 | SMN1 | SMURF1 | SNAP29 | SNCA | SNF8 | SNRPB | SNRPD1 | SNRPD3 | SNRPE | SNX12 | SNX17 | SNX2 | SNX21 | SNX24 | SNX3 | SNX6 | SOAT1 | SOCS2 | SOD1 | SORT1 | SPDL1 | SPICE1 | SPRY2 | SPTAN1 | SPTBN1 | SPTBN2 | SQSTM1 | SRC | SRI | SRP14 | SRP19 | SRSF11 | SRSF5 | SSB | SSX2IP | STARD7 | STAT3 | STAT5A | STBD1 | STIM1 | STK25 | STOM | STOML2 | STX5 | STXBP1 | STXBP2 | STYX | SUN2 | SYN1 | SYNE2 | SYNJ2BP | SYP | SYVN1 | TAGLN2 | TANGO2 | TBC1D15 | TBC1D22B | TBC1D23 | TBC1D4 | TCIRG1 | TCP1 | TERF1 | TERF2 | TEX264 | TFG | TGFB1 | TGM2 | THBS1 | THOC2 | THOC6 | THOC7 | TIMM13 | TIMM21 | TIMM23 | TIMM44 | TIMM50 | TIMP1 | TIMP2 | TM9SF4 | TMED9 | TMEM173 | TMEM30A | TNFAIP3 | TNK2 | TNKS | TNPO1 | TOLLIP | TOMM20 | TOMM22 | TOMM40 | TOR1A | TPCN2 | TPD52 | TPR | TRAF3IP1 | TRAPPC8 | TREX1 | TRIAP1 | TRIM23 | TRIM9 | TRIP4 | TSC22D3 | TSG101 | TSPAN17 | TTYH3 | TUBA1A | TUBA1C | TUBA4A | TUBB | TUBB4B | TXLNA | TXNDC5 | TXNIP | U2AF2 | UBB | UBC | UBE2B | UBE2D1 | UBE2D2 | UBE2D3 | UBE2O | UBL4A | UBR4 | UCHL1 | UMPS | UNC119 | UPF2 | UPF3B | USP20 | USP9X | VAPA | VAPB | VAT1 | VCL | VCP | VDAC1 | VDAC2 | VDAC3 | VDR | VGF | VPS13B | VPS18 | VPS26A | VPS29 | VPS37D | VPS4B | VPS8 | VTA1 | VTN | WAS | WBP2 | WDR1 | WDR19 | WDR35 | WNK1 | WNK4 | WWP2 | XBP1 | XPO1 | XPO4 | XPO5 | XPO7 | XPOT | XRCC5 | XRCC6 | YKT6 | YME1L1 | YWHAB | YWHAE | YWHAG | YWHAH | YWHAZ | ZBTB7B | ZDHHC17 | ZDHHC23 | ZFAND6 | ZMPSTE24 | ZW10 | ZWINT | GO:0034694 | 0.0019410408831736 | 16/8243 | 13/3250 | 0.00078936143753291 | 1 | T | T | F | F | response to prostaglandin | ACACA | ADCY6 | AKAP8 | AKR1C2 | AKT1 | CCL21 | GNB1 | PPARG | PRKAA1 | PRKAA2 | PRKCE | TGFBR3 | YY1 | GO:0090087 | 0.0528933640664807 | 436/8243 | 204/3250 | 0.000793609496993085 | 1 | T | T | F | F | regulation of peptide transport | ABAT | ABL1 | ADCYAP1 | ADORA2A | ADTRP | AGER | AKT2 | ANK3 | ANXA1 | ANXA5 | APBB1 | APOA1 | APOD | ARF1 | ARFIP1 | ARHGEF7 | ARRB1 | ATAD1 | ATG5 | ATP13A2 | B3GAT3 | BAG3 | BAG4 | CACNA1A | CD2 | CD22 | CD36 | CD40 | CD40LG | CD74 | CDH1 | CDK16 | CDK5 | CDK5R1 | CEP290 | CFTR | CHP1 | CPT1A | CRP | CSNK2A2 | DHX36 | DHX9 | DMAP1 | DNAJA1 | DNM1L | DYNLL1 | EDEM1 | EGFR | ERBB2 | EZR | FCN1 | FGB | FIS1 | FKBP1B | FN1 | FYN | GAPDH | GAPVD1 | GDI1 | GLMN | GLP1R | GLUL | GOLPH3 | GOPC | GPI | GSK3A | GSK3B | HADH | HAVCR2 | HAX1 | HDAC3 | HDAC9 | HIF1A | HK1 | HMGB1 | HNF4A | HRAS | HSPA1L | HSPA8 | HTRA2 | HUWE1 | IDH2 | IFNA2 | INSIG1 | IPO5 | ITGB1BP1 | ITGB3 | ITPR1 | ITPR2 | ITPR3 | JUP | KCNS3 | KIF5B | LCP1 | LGR4 | LRRK2 | LYPLA1 | MAPK1 | MAPK14 | MAPK3 | MAVS | MBP | MDM2 | MIF | MPC2 | MYO1C | NDUFAF2 | NEUROD1 | NOD2 | NOS2 | NPEPPS | NR0B2 | NR1H2 | NR1H3 | NUCB1 | ORM1 | OXCT1 | PANX3 | PARK7 | PAX8 | PCM1 | PDCD10 | PER2 | PFKL | PFKM | PIK3R1 | PKIG | PLA2G1B | PLA2G6 | PML | PPARD | PPIA | PPID | PPM1A | PPP1R11 | PRKAA1 | PRKACA | PRKCD | PRKCE | PRKCZ | PSEN1 | PSMD9 | PTPN1 | PTPN14 | PTPN22 | PTPN23 | RAB11A | RAB29 | RAN | RANGAP1 | RAP1A | RAPGEF4 | REST | RHOU | RIPK2 | RNF31 | SAE1 | SCFD1 | SEC16A | SFN | SIRT3 | SLC16A1 | SLC25A4 | SLC25A5 | SLC25A6 | SMAD3 | SMO | SNX12 | SNX3 | SOCS1 | SP100 | SPTBN1 | SRC | SRI | STOM | TARDBP | TGFB1 | TM9SF4 | TMBIM6 | TMEM30A | TNF | TNFAIP3 | TP53 | TPR | TRAF2 | TRAF6 | TRIM28 | TSG101 | TWIST1 | TXN | UBE2D3 | UBE2J1 | UBE2L3 | UBR5 | USP17L2 | USP46 | USP50 | WWP2 | XBP1 | XPO1 | XPO4 | XPO5 | YWHAE | ZIC1 | GO:0070482 | 0.0245056411500667 | 202/8243 | 102/3250 | 0.000805996750354005 | 1 | T | T | F | F | response to oxygen levels | ABAT | ACAA2 | ACTN4 | ADA | ADSL | AGER | AGTRAP | AHCY | AIFM1 | AKT1 | ARNT | ARNT2 | ATM | ATP6V1A | BCL2 | BIRC2 | CAPN2 | CASP3 | CAT | CAV1 | CBFA2T3 | CBL | CCNB1 | CDK4 | CDKN1A | CREB1 | CREBBP | CRYAB | CYP1A1 | DDIT4 | DNM1L | E2F1 | EGLN1 | EGLN3 | EIF4EBP1 | ENDOG | EP300 | ETS1 | FABP1 | FIS1 | FOXO1 | GNB1 | HDAC2 | HIF1A | HK2 | HMOX1 | HSF1 | HSP90B1 | ICAM1 | IREB2 | ITPR1 | ITPR2 | LDHA | LMNA | LOXL2 | LPAR1 | MDM2 | MDM4 | MECP2 | MYC | NAMPT | NDRG1 | NOS2 | NPEPPS | OPA1 | P2RX2 | P4HB | PGK1 | PHB2 | PICK1 | PKM | PLOD2 | PML | PPARD | PPARG | PPARGC1A | PRKAA1 | PRKCE | PSEN2 | PTEN | PTN | RAF1 | REST | RHOA | S100B | SCFD1 | SIRT1 | SLC9A1 | SMAD3 | SMAD4 | SRC | STUB1 | TGFB1 | TGFBR3 | THBS1 | TM9SF4 | TP53 | TWIST1 | TXN2 | UBQLN1 | VCAM1 | XRCC1 | GO:0034767 | 0.00921994419507461 | 76/8243 | 44/3250 | 0.000817141288366137 | 1 | T | T | F | F | positive regulation of ion transmembrane transport | ABL1 | ACTN2 | ACTN4 | ADRB2 | AKAP9 | ANK3 | ARF1 | ATP1B3 | BAX | CACNG2 | CACNG4 | CALM1 | CALM2 | CALM3 | CFTR | CHCHD10 | CHP1 | COX17 | CXCL11 | DMD | G6PD | GLRX | GSTO1 | HAP1 | HSPA2 | HTR3A | HTT | KCNIP2 | KIF5B | NOS1AP | PARK7 | PLCG1 | PSEN1 | RGN | S100A1 | SLC9A1 | SLC9A3R1 | SNCA | SRI | STAC | STIM1 | SUMO1 | WNK1 | WNK4 | GO:1905819 | 0.00279024626956205 | 23/8243 | 17/3250 | 0.000831520588139276 | 1 | T | T | F | F | negative regulation of chromosome separation | APC | ATM | AURKB | CDC20 | CENPF | IK | KLHL22 | MAD1L1 | MAD2L1 | PLK1 | PTTG1 | RAD21 | SPDL1 | TPR | TRIP13 | ZNF207 | ZW10 | GO:2000816 | 0.00279024626956205 | 23/8243 | 17/3250 | 0.000831520588139276 | 1 | T | T | F | F | negative regulation of mitotic sister chromatid separation | APC | ATM | AURKB | CDC20 | CENPF | IK | KLHL22 | MAD1L1 | MAD2L1 | PLK1 | PTTG1 | RAD21 | SPDL1 | TPR | TRIP13 | ZNF207 | ZW10 | GO:0044772 | 0.0231711755428849 | 191/8243 | 97/3250 | 0.000838421958713585 | 1 | T | T | F | F | mitotic cell cycle phase transition | ACTR1A | AKAP9 | BTRC | CALM2 | CALM3 | CAMK2A | CCNB1 | CCNY | CCP110 | CDC25A | CDC25B | CDC25C | CDC27 | CDC34 | CDK2 | CDK4 | CDKN1A | CDKN2C | CENPJ | CEP135 | CEP152 | CEP290 | CEP57 | CEP70 | CHEK2 | CHMP2A | CKAP5 | CKS2 | CNTRL | CSNK1E | CTDP1 | CUL1 | CUL2 | CUL3 | CUL4B | CUL5 | DCTN1 | DYNC1H1 | DYNC1I2 | DYNLL1 | EIF4E | EIF4EBP1 | FBXL12 | FBXW11 | FGFR1OP | FOXM1 | GSPT1 | HSP90AA1 | KDM8 | KHDRBS1 | LATS1 | MCM2 | MCM3 | MCM4 | MCM5 | MCM6 | MCM7 | MELK | MYC | OFD1 | OPTN | PAFAH1B1 | PCM1 | PIAS1 | PLK1 | POLA2 | POLE2 | POLE3 | PPAT | PPM1D | PPME1 | PPP1CB | PPP1R12A | PPP1R12B | PPP2R1A | PPP2R2A | PPP2R2D | PPP6C | PRKACA | PRKAR2B | RB1 | RCC1 | RHOU | RPA2 | RPA3 | RPL24 | TUBA1A | TUBA4A | TUBB | TUBB4A | TUBB4B | TUBG1 | UBE2C | UBE2S | USP26 | YWHAE | YWHAG | GO:0043393 | 0.019410408831736 | 160/8243 | 83/3250 | 0.000852885161327293 | 1 | T | T | F | F | regulation of protein binding | ABL1 | ACTB | ADRB2 | AES | AKT1 | AKTIP | AMFR | ANXA2 | APOE | APP | ARHGEF7 | ARRB1 | ARRB2 | AURKB | BAG2 | BAX | BCL2 | CARM1 | CAV1 | CD36 | CDK5 | CRBN | CRK | CRMP1 | CSNK1E | CTNNBIP1 | CYLD | DAB2 | DERL1 | DISC1 | DNAJB2 | DVL1 | EIF2S1 | EP300 | EPHA4 | FAM192A | FKBP1A | FLOT1 | GOLGA2 | GPSM1 | GSK3B | GTF2F1 | HDAC4 | HDAC5 | HSF1 | HSPA5 | IFIT1 | ITGA4 | ITGB1BP1 | KDM1A | LEF1 | LGALS3 | LRRK2 | MAPK3 | MAPK8 | NMD3 | PARK7 | PCBD1 | PCSK9 | PIN1 | PLK1 | PPP1CA | PPP2CA | PPP2CB | PRKACA | PRKCD | PSEN1 | PTPRF | RAN | RIPK2 | RUVBL2 | SRC | STK3 | STMN1 | STUB1 | STYX | TFIP11 | TGFBR3 | TMBIM6 | TMEM173 | TRAF2 | TRIB3 | VTN | GO:0051101 | 0.0115249302438433 | 95/8243 | 53/3250 | 0.000854458453937153 | 1 | T | T | F | F | regulation of DNA binding | ACD | AGER | CALM2 | CALM3 | CSNK2B | CTNNB1 | CTNNBIP1 | DDRGK1 | E2F1 | EP300 | FAM129B | GATA1 | GZMA | H1F0 | HDAC2 | HMGB1 | HMGB2 | HMOX1 | ID2 | IFI16 | IRF4 | JUN | KDM1A | LEF1 | MAPK8 | MDFI | NEUROD1 | NFKBIA | NIPBL | NME1 | PARK7 | PARP1 | PER2 | PINX1 | PLAUR | PPARG | PSEN1 | RB1 | SIN3A | SKI | SMO | SP100 | SRI | SUMO1 | SUMO3 | TGFB1 | TNKS | TRAF6 | TRIM28 | TWIST1 | TXN | ZBTB7A | ZMPSTE24 | GO:0010557 | 0.145335436127623 | 1198/8243 | 522/3250 | 0.000870601085564536 | 1 | T | T | F | F | positive regulation of macromolecule biosynthetic process | ABHD14B | ABL1 | ACD | ACTA2 | ACTL6A | ACTN1 | ACTN2 | ACTN4 | ACTR2 | ACTR3 | ADCYAP1 | ADRB2 | AGER | AGO1 | AGO2 | AHI1 | AICDA | AIRE | AKT1 | AKT2 | ANKRD49 | APBB1 | APEX1 | APOE | APP | AR | ARF4 | ARID3B | ARID4B | ARNT | ARNT2 | ARRB1 | ARRB2 | ASCL1 | ATAD5 | ATF2 | ATF6 | ATF6B | ATM | ATOH1 | ATRX | AURKB | AXIN1 | AZU1 | BANP | BCL10 | BIRC2 | BMPR1A | BRCA1 | BRD4 | BTRC | BUD31 | C1QBP | CAND1 | CARM1 | CBFB | CCAR2 | CCL21 | CCNT1 | CCNT2 | CCT2 | CCT3 | CCT4 | CCT5 | CCT6A | CCT7 | CCT8 | CD40 | CD74 | CD81 | CDC5L | CDC73 | CDH1 | CDK2 | CDK4 | CDK8 | CDK9 | CEBPA | CEBPE | CEP290 | CHCHD10 | CHCHD2 | CHD8 | CHEK2 | CHP1 | CIRBP | COA3 | COPS5 | CREB1 | CREB3 | CREBBP | CTCF | CTNNB1 | DAB2 | DAXX | DBF4B | DCN | DDRGK1 | DDX39B | DDX3X | DHX36 | DHX9 | DNA2 | DPF2 | DTX1 | DVL1 | DVL2 | DVL3 | DYRK2 | E2F1 | E2F2 | E2F3 | E2F4 | E2F6 | EAF2 | EBF1 | ECD | EEF2 | EGFR | EGLN1 | EIF2AK3 | EIF2AK4 | EIF2B5 | EIF3D | EIF3E | EIF4A3 | EIF5A | EIF6 | ELAVL1 | ELF3 | ELF4 | EP300 | ERBB2 | ERG | ERLIN2 | ESR1 | ESR2 | ESRRA | ESRRB | ESRRG | ETS1 | EXOSC9 | FADD | FAF1 | FAM129B | FBXW11 | FGF2 | FGFR1 | FHL2 | FOS | FOXA1 | FOXJ1 | FOXK1 | FOXK2 | FOXM1 | FOXO1 | FUBP3 | FUS | FXR2 | GABPB1 | GATA1 | GLMN | GOLGA2 | GPS2 | GRIP1 | GTF2A1 | GTF2F1 | GTF2F2 | H1F0 | H1FX | H2AFZ | HAX1 | HDAC1 | HDAC2 | HDAC3 | HDAC4 | HDAC5 | HDAC6 | HELZ2 | HEXB | HIF1A | HIST1H1A | HMGB1 | HMGB2 | HMGB3 | HMOX1 | HNF1A | HNF4A | HNF4G | HNRNPA1 | HNRNPA2B1 | HNRNPAB | HNRNPD | HNRNPK | HNRNPL | HNRNPU | HOXA5 | HOXB5 | HOXB9 | HOXD10 | HRAS | HSF1 | HSF4 | HSP90AA1 | HSP90AB1 | HSPA5 | HSPB1 | HSPH1 | HTATIP2 | ID2 | IFI16 | IFNA2 | IKBKB | IKBKG | IKZF5 | ILF2 | ILF3 | ILK | ING1 | ING5 | INSIG1 | INSIG2 | INSR | IRF1 | IRF4 | IRF7 | ITGB1BP1 | JMJD6 | JUN | JUNB | JUND | JUP | KAT5 | KAT7 | KAT8 | KDM1A | KDM8 | KDR | KHDRBS1 | KLF5 | KLF6 | KMT2A | KMT2B | KMT2C | LARP4 | LEF1 | LGR4 | LMO2 | LMO7 | LPIN3 | LRP6 | LRRK2 | LUM | MAP2K1 | MAP2K3 | MAP2K4 | MAP2K6 | MAP2K7 | MAP3K2 | MAP3K4 | MAPK1 | MAPK14 | MAPK15 | MAPK3 | MAPK7 | MAS1 | MAVS | MC1R | MECP2 | MED1 | MED12 | MED14 | MED17 | MED20 | MED21 | MED23 | MED24 | MED31 | MED4 | MED6 | MEOX2 | METTL23 | MITF | MLLT11 | MLLT3 | MORF4L2 | MRPL12 | MTA1 | MTA3 | MTPN | MYC | MYO6 | NAMPT | NANOG | NCK1 | NCOA1 | NCOA2 | NCOA3 | NCOA4 | NEK7 | NELFE | NEUROD1 | NFATC1 | NFATC2 | NFE2L2 | NFKB2 | NFKBIA | NIF3L1 | NIPBL | NKRF | NKX2-1 | NMD3 | NME2 | NOD2 | NPM1 | NR1H2 | NR1H3 | NR1I2 | NR3C1 | NR4A1 | NR5A2 | NUFIP1 | NUP85 | NUP98 | NUPR1 | OGT | ONECUT1 | PABPC1 | PAIP1 | PARK7 | PARP1 | PATZ1 | PAWR | PAX8 | PAX9 | PAXIP1 | PCBD1 | PCBP1 | PCGF5 | PCNA | PER1 | PER2 | PFKM | PHB | PHF10 | PIAS1 | PIH1D1 | PIK3R1 | PIN1 | PLA2G1B | PLSCR1 | PML | PNKP | POLDIP3 | POT1 | POU2F3 | POU5F1 | PPARD | PPARG | PPARGC1A | PPM1A | PPP1R12A | PRKAA1 | PRKD1 | PRKD2 | PRKDC | PRMT2 | PROX1 | PSEN1 | PSMC2 | PSMC3 | PSMC4 | PSMC5 | PSMC6 | PSMD9 | PTGES2 | PTGES3 | RAB3GAP1 | RAC1 | RAF1 | RAN | RARA | RARG | RB1 | RBM3 | RBMX | RBPJ | REL | RELA | REST | RFC1 | RFC2 | RFC3 | RFC4 | RFC5 | RFXANK | RHOA | RIPK1 | RIPK2 | RIPK3 | RMND1 | RNF4 | RPS4X | RPS6KA1 | RPS6KB2 | RPTOR | RTF1 | RUNX1 | RUNX2 | RUVBL1 | RUVBL2 | RXRA | RXRB | RXRG | SFPQ | SHC1 | SIN3A | SIRT1 | SIRT7 | SKI | SLC9A1 | SMAD2 | SMAD3 | SMAD4 | SMAD5 | SMAD9 | SMARCA2 | SMARCA4 | SMARCB1 | SMARCC1 | SMARCC2 | SMARCE1 | SMO | SNW1 | SOAT1 | SOX2 | SP1 | SP100 | SPI1 | SPIN1 | SQSTM1 | SRC | STAT1 | STAT3 | STAT4 | STAT6 | STOML2 | SUB1 | SUMO2 | SUPT4H1 | SUPT5H | TADA2B | TAF12 | TAF15 | TARDBP | TBL1X | TCEA2 | TCERG1 | TCF12 | TCF21 | TCF4 | TCP1 | TDRD3 | TFCP2 | TFDP1 | TGFB1 | THBS1 | THRA | TLR3 | TMEM173 | TNF | TNFRSF1A | TNFRSF8 | TNIP2 | TNKS | TNKS2 | TOP2A | TP53 | TP63 | TP73 | TRAF6 | TRIAP1 | TRIM28 | TRIM32 | TRIP4 | TRUB2 | TSG101 | TTC5 | TWIST1 | UBB | UBC | UBE2L3 | UBE2V1 | UPF3B | UQCC1 | USP16 | USP21 | VDR | VHL | VIM | WAS | WBP2 | WDR77 | WNT2 | WT1 | WWOX | WWP2 | XBP1 | XRCC5 | XRCC6 | YAF2 | YAP1 | YES1 | YTHDF1 | YWHAH | YY1 | ZBTB16 | ZBTB17 | ZBTB7B | ZIC1 | ZNF24 | ZNF746 | GO:0043085 | 0.105422782967366 | 869/8243 | 386/3250 | 0.000871160819418171 | 1 | T | T | F | F | positive regulation of catalytic activity | ABL1 | ABL2 | ACD | ACSL1 | ADCY6 | ADCYAP1 | ADPRH | ADRB2 | ADRM1 | AGAP1 | AGAP2 | AGER | AHSA1 | AIFM1 | AIMP2 | AKT1 | AKT2 | ALOX12 | ALOX5AP | APOA1 | APOE | APP | ARF1 | ARF4 | ARFGAP1 | ARHGAP1 | ARHGAP19 | ARHGAP26 | ARHGAP35 | ARHGDIA | ARHGEF7 | ARRB1 | ATP1B3 | AURKB | AXIN1 | AZU1 | B3GAT3 | BAX | BBC3 | BCL10 | BCL2 | BCL2L13 | BIRC7 | BMI1 | BRAF | CAB39 | CALM1 | CALM2 | CALM3 | CASP10 | CASP3 | CASP8 | CASP9 | CAV1 | CCDC88A | CCL21 | CCL22 | CCNB1 | CCNT1 | CCNT2 | CCNY | CCNYL1 | CCS | CCT2 | CCT4 | CD40 | CD40LG | CD74 | CD81 | CDC14B | CDC20 | CDC25B | CDK5 | CDK5R1 | CDKN1A | CHCHD10 | CKS1B | CKS2 | CLPX | CLU | COX17 | CRK | CRKL | CTNNB1 | CTSA | CYCS | DAB1 | DAPK1 | DAXX | DBF4B | DBNL | DCUN1D1 | DDRGK1 | DDX3X | DENND1A | DGKZ | DHX9 | DIABLO | DLG3 | DLG4 | DNAJA1 | DNAJA2 | DNAJB1 | DNAJB11 | DNAJB2 | DNAJC10 | DNAJC19 | DNAJC9 | DNM1L | DOCK7 | DUSP12 | DUSP19 | DUSP7 | DVL2 | DVL3 | DYNLL1 | EEF1A2 | EGFR | EGLN3 | EIF2AK3 | EIF5 | ELMOD3 | EPHA4 | ERBB2 | ERBB3 | ERRFI1 | ESR1 | EZH2 | FABP1 | FADD | FASLG | FGF2 | FGFR1 | FGFR3 | FIS1 | FN1 | FOXJ1 | FYN | FZR1 | GADD45G | GAPVD1 | GDI1 | GDI2 | GIT1 | GNA13 | GNA15 | GNAI2 | GNAI3 | GNAO1 | GNAQ | GNAS | GOLGA2 | GSK3A | GSK3B | GTF2F1 | HIF1A | HMGB1 | HMGB2 | HNRNPA2B1 | HNRNPD | HNRNPU | HRAS | HSF1 | HSP90AA1 | HSP90AB1 | HSPA2 | HSPB2 | HSPD1 | HSPE1 | HTRA2 | ICAM1 | IFI16 | IKBKG | ILK | INSR | IQGAP1 | ITGB1BP1 | ITGB3 | ITK | JUN | KRAS | LARS | LAT | LCP2 | LGMN | LIMS1 | LPAR1 | LPAR2 | LRRK2 | LTC4S | MAP2K1 | MAP2K3 | MAP2K4 | MAP2K6 | MAP2K7 | MAP3K1 | MAP3K13 | MAP3K14 | MAP3K2 | MAP3K3 | MAP3K4 | MAP4K1 | MAPK1 | MAPK14 | MAPK15 | MAPK3 | MAPK8 | MAPK8IP3 | MAPKAP1 | MAPT | MAS1 | MBP | MIF | MLST8 | MOB1B | MSH6 | MYC | NDUFA13 | NEK1 | NEK7 | NOD2 | NOS1AP | NOS2 | NOS3 | NR1H2 | NR1H3 | NTF3 | NTRK1 | NUPR1 | OXSR1 | P2RX1 | PAK2 | PAK6 | PARK7 | PCNA | PDCD10 | PDCD6 | PDE6G | PFN1 | PGAM5 | PIH1D1 | PIK3CA | PIK3CG | PIN1 | PLA2G1B | PLCG1 | PLK1 | PLSCR1 | PML | PNKP | POT1 | PPARG | PPM1K | PPP1R12A | PPP1R12B | PPP2CA | PRDX2 | PRELID1 | PRKAA1 | PRKACA | PRKAG1 | PRKAR1A | PRKAR2B | PRKCD | PRKCE | PRKCZ | PRKD1 | PRKD2 | PROX1 | PRRC1 | PSAP | PSEN1 | PSEN2 | PSMD10 | PSMD14 | PSME2 | PSME3 | PTEN | PTGES3 | PTK2 | PTPN1 | RAB1A | RAB3GAP1 | RAF1 | RANBP3 | RANGAP1 | RAP1A | RAP1GDS1 | REST | RFC1 | RFC2 | RFC3 | RFC4 | RFC5 | RFK | RGN | RGS1 | RGS2 | RGS20 | RHOA | RIC8B | RIPK1 | RIPK2 | RIPK3 | RP2 | RPA2 | RPLP1 | RPS2 | RPS3 | RPTOR | RSU1 | S100A1 | S100A12 | S100A9 | SAE1 | SDC4 | SERPINB3 | SH3PXD2A | SHC1 | SIPA1 | SIPA1L1 | SIRT1 | SMAD3 | SNCA | SOD1 | SPRY2 | SRC | SRGAP2 | SRGAP3 | STARD13 | STAT3 | STIM1 | STK11 | STK24 | STK25 | STK26 | STK3 | STUB1 | SUMO1 | SYDE1 | TAB1 | TAB2 | TANK | TAOK3 | TBC1D15 | TBC1D22B | TBC1D4 | TCEA1 | TCP1 | TERF2 | TGFB1 | THBS1 | TIMP2 | TLR3 | TNF | TNKS | TP73 | TPM1 | TRADD | TRAF2 | TRAF6 | TRIB3 | TRIM23 | TXN | UBA2 | UBB | UBC | UBE2C | UBE2I | UBE2L3 | UBE2N | UBE2S | UBE2V1 | UNC119 | USP17L2 | USP50 | VAV1 | VCP | VDR | WDR35 | WNK1 | XRCC1 | XRCC5 | XRCC6 | YWHAB | ZNF622 | GO:0017038 | 0.0112823001334466 | 93/8243 | 52/3250 | 0.000888532728793696 | 1 | T | T | F | F | protein import | AKT1 | APOE | CBLB | CLU | CSE1L | DNAJC19 | GRPEL1 | HEATR3 | HSP90AA1 | HSPA4 | HSPD1 | IPO11 | IPO4 | IPO5 | IPO7 | IPO8 | IPO9 | KPNA1 | KPNA2 | KPNB1 | LMNA | NUP107 | NUP153 | NUP214 | NUP85 | NUP88 | NUP93 | NUP98 | NUTF2 | NXT2 | PAM16 | PEX19 | PEX5 | PHB2 | PIK3R1 | PML | POM121 | RAN | ROMO1 | RPAIN | RPL23 | STAT3 | TIMM21 | TIMM23 | TIMM44 | TIMM50 | TNPO1 | TOMM20 | TOMM22 | TOMM40 | TPR | TXNIP | GO:0032413 | 0.0046099720975373 | 38/8243 | 25/3250 | 0.000888624988243625 | 1 | T | T | F | F | negative regulation of ion transmembrane transporter activity | ACTN2 | ANK3 | CAB39 | CALM1 | CALM2 | CALM3 | CAV1 | FKBP1A | FKBP1B | GOPC | GSTO1 | HECW1 | HECW2 | KCNAB1 | NEDD4 | OXSR1 | PCSK9 | PRKCE | PTEN | SLC9A3R1 | SRI | SUMO1 | THADA | UBQLN1 | YWHAE | GO:0042180 | 0.0046099720975373 | 38/8243 | 25/3250 | 0.000888624988243625 | 1 | T | T | F | F | cellular ketone metabolic process | AKR1A1 | AKR1B1 | AKR1B10 | AKR1C2 | CBR4 | COQ2 | COQ3 | COQ4 | COQ5 | COQ6 | COQ7 | COQ9 | DHRS4 | FDXR | GLO1 | GOT2 | GPD1 | GPI | KDSR | NDUFA9 | PARK7 | PDSS1 | PDSS2 | TPI1 | UBIAD1 | GO:0042127 | 0.113065631444862 | 932/8243 | 412/3250 | 0.000906572041977634 | 1 | T | T | F | F | regulation of cell population proliferation | ABL1 | ABL2 | ACE2 | ADA | ADCYAP1 | ADORA2A | AGER | AIMP2 | AKR1B1 | AKR1C2 | AKT1 | AKT2 | AKT3 | ALOX12 | ANXA1 | ANXA2 | APC | APOD | APOE | APP | AR | ARNT | ARNT2 | ARRB1 | ASCL1 | ATAD5 | ATF2 | ATM | BAG6 | BAX | BCL2 | BCL2L1 | BCL6 | BIRC2 | BIRC5 | BIRC7 | BMI1 | BMPR1A | BNIPL | BRAF | BRCA1 | BTK | C1QL4 | CALR | CAPN1 | CAPNS1 | CARM1 | CASP3 | CAV1 | CBFA2T3 | CBX8 | CCDC88A | CCNB1 | CCR3 | CD109 | CD209 | CD22 | CD40 | CD40LG | CD6 | CD74 | CD81 | CDC20 | CDC25B | CDC73 | CDK2 | CDK4 | CDKN1A | CDKN2C | CEBPA | CGRRF1 | CHRD | CLEC11A | CLU | COL18A1 | COMT | COX17 | CREB1 | CRIP2 | CRKL | CSNK2B | CTBP1 | CTCF | CTNNB1 | CTNNBIP1 | CUL1 | CUL2 | CUL3 | CUL5 | CXCL11 | CXCL5 | CXCL8 | DBF4B | DBH | DDRGK1 | DDX39B | DHX9 | DISC1 | DLG3 | DNAJA2 | DNAJA3 | DNAJB2 | DUSP1 | DUSP22 | E2F1 | E2F3 | E2F4 | EAF2 | EGFR | EGLN3 | EIF5A | EPPK1 | ERBB2 | ERBB3 | ERRFI1 | ESR1 | ESRRA | ETS1 | EZH2 | FABP3 | FABP7 | FADD | FAM129B | FAM98A | FANCA | FASLG | FBRS | FGF2 | FGFR1 | FGFR1OP | FGFR3 | FN1 | FOXJ1 | FOXM1 | FOXO1 | FTH1 | FTO | FZR1 | GATA1 | GDF9 | GFAP | GLMN | GLUL | GNAI2 | GNAI3 | GNG5 | GPX1 | GRK5 | GSTP1 | HAVCR2 | HBEGF | HDAC1 | HDAC2 | HDAC4 | HGS | HIF1A | HIST1H2AB | HLX | HMGB1 | HMGB2 | HMOX1 | HNF4A | HNRNPU | HOXA5 | HRAS | HSF1 | HSF4 | HSPA1B | HTR1B | HTRA1 | ID2 | IDH2 | IFT122 | IGF1R | IGFBP7 | ILK | ING1 | ING5 | INSR | IRF1 | ITCH | ITGA4 | ITGAV | ITGB1BP1 | ITGB3 | JAK3 | JUN | JUP | KANK2 | KDM1A | KDM5B | KDR | KLF5 | KRAS | KSR1 | LAMB1 | LEF1 | LGALS3 | LGMN | LIG4 | LIMS1 | LMNA | LRRK2 | MAD1L1 | MAP2K1 | MAP3K3 | MAPK1 | MAPK14 | MAPK15 | MARCKSL1 | MAS1 | MATK | MCC | MCTS1 | MDM2 | MDM4 | MECP2 | MED1 | MED31 | MIF | MITF | MTA3 | MTSS1 | MVD | MYC | NACC1 | NAMPT | NANOG | NAP1L1 | NCK1 | NDRG1 | NELL1 | NF2 | NFATC2 | NFKBIA | NMB | NME1 | NME2 | NOD2 | NOS2 | NOS3 | NPM1 | NPR1 | NQO2 | NR4A1 | NR5A2 | NRP2 | NTF3 | NTRK1 | NUDT6 | NUPR1 | PAWR | PDCD10 | PDCD6 | PDF | PELI1 | PER2 | PHB | PHB2 | PIAS1 | PIM1 | PINX1 | PLA2G1B | PLCG1 | PML | PNP | PPARD | PPARG | PPARGC1A | PPM1D | PPP1CC | PRDX3 | PRDX4 | PRKAA1 | PRKAR1A | PRKCZ | PRKD1 | PRKD2 | PRKDC | PROX1 | PTEN | PTGES | PTGIR | PTK2 | PTN | PTPN14 | PTPN22 | PTPN6 | PTPRK | RAF1 | RARA | RARG | RASSF10 | RB1 | RBBP4 | RBPJ | RC3H1 | RELA | REST | RGN | RHBDD1 | RHOA | RIPK1 | RIPK2 | RIPK3 | RNASEH2B | RNF126 | RNF139 | ROMO1 | RPA3 | RPL23 | RPS15A | RPS3 | RPS4X | RPS6KA2 | RPTOR | RTN4 | RUNX2 | S100A11 | S100A13 | S100A6 | S100B | SAT1 | SCG2 | SDC4 | SDCBP | SERPINB3 | SERPINB5 | SERPINE2 | SF1 | SFN | SHC1 | SHMT2 | SIRT1 | SIRT6 | SKI | SLAMF1 | SLC25A5 | SLC35F6 | SLC9A3R1 | SMAD2 | SMAD3 | SMAD4 | SMARCA2 | SMO | SNAI2 | SOD2 | SOX2 | SP1 | SPINT2 | SPRY2 | SRPK2 | STAT1 | STAT3 | STAT5A | STAT6 | STK11 | STK3 | STRAP | SULF2 | SUZ12 | SYNJ2BP | TBRG4 | TCIRG1 | TES | TFDP1 | TGFB1 | TGFBR3 | TGM2 | THBS1 | TIAL1 | TIMP1 | TIMP2 | TINF2 | TNF | TNFAIP3 | TNFRSF8 | TNFSF12 | TNK2 | TNN | TP53 | TP63 | TP73 | TPM1 | TRAF6 | TRIM32 | TSG101 | TWIST1 | TXNIP | UBE2A | USP17L2 | VASH2 | VCAM1 | VDR | VHL | VSIG4 | VSTM2A | WARS | WDR48 | WDR77 | WNT2 | WT1 | XBP1 | XIAP | XRCC5 | XRCC6 | YAP1 | ZAP70 | ZBTB16 | ZBTB17 | ZBTB7B | ZMPSTE24 | GO:0048145 | 0.00800679364309111 | 66/8243 | 39/3250 | 0.00092450677313553 | 1 | T | T | F | F | regulation of fibroblast proliferation | ANXA2 | BAX | BMI1 | C1QL4 | CCNB1 | CD74 | CDC73 | CDK4 | CDKN1A | CREB1 | CTNNB1 | DHX9 | E2F1 | EGFR | ESR1 | FBRS | FN1 | FTH1 | GSTP1 | JUN | LIG4 | MED31 | MIF | MYC | NUPR1 | PAWR | PLA2G1B | PML | PPARG | PRKDC | RNASEH2B | S100A6 | SIRT6 | SKI | TGFB1 | TP53 | TRIM32 | WNT2 | ZMPSTE24 | GO:0022402 | 0.0799466213757127 | 659/8243 | 298/3250 | 0.000927880409127466 | 1 | T | T | F | F | cell cycle process | ABL1 | ACD | ACTR1A | ACTR2 | ACTR3 | AKAP8 | AKAP9 | ANK3 | ANXA11 | APBB1 | APBB2 | APC | ARF1 | ATAD5 | ATF2 | ATM | ATRX | AURKB | BAG6 | BANF1 | BAX | BCL2L1 | BIRC5 | BRCA1 | BRD4 | BTRC | CAB39 | CALM2 | CALM3 | CAMK2A | CARM1 | CASP2 | CCDC155 | CCDC36 | CCDC69 | CCNB1 | CCNY | CCP110 | CDC14B | CDC20 | CDC25A | CDC25B | CDC25C | CDC27 | CDC34 | CDK2 | CDK4 | CDKN1A | CDKN2C | CENPF | CENPJ | CEP135 | CEP152 | CEP290 | CEP55 | CEP57 | CEP70 | CETN3 | CFL1 | CGRRF1 | CHD3 | CHEK2 | CHMP2A | CKAP5 | CKS2 | CNTRL | CNTROB | CROCC | CSNK1E | CTDP1 | CUL1 | CUL2 | CUL3 | CUL4B | CUL5 | CUL7 | CXCL8 | DCTN1 | DHCR24 | DNA2 | DUSP1 | DYNC1H1 | DYNC1I2 | DYNLL1 | DYNLT1 | E2F1 | E2F4 | E2F6 | EIF2AK4 | EIF4E | EIF4EBP1 | EP300 | ESRRB | EZH2 | EZR | FANCA | FANCM | FBXL12 | FBXW11 | FEN1 | FGFR1OP | FIGNL1 | FOXM1 | GOLGA2 | GSPT1 | HDAC3 | HEPACAM2 | HRAS | HSF1 | HSP90AA1 | HSPA2 | HTT | ID2 | IK | ILK | IRF1 | KDM8 | KHDRBS1 | KIF11 | KIF2C | KLHL22 | KPNB1 | LATS1 | LMNA | MAD1L1 | MAD2L1 | MAP2K1 | MAP2K6 | MCM2 | MCM3 | MCM4 | MCM5 | MCM6 | MCM7 | MCMBP | MDC1 | MDM2 | MDM4 | MECP2 | MELK | MLH1 | MLST8 | MSH6 | MYC | NAE1 | NCAPH2 | NCOR1 | NEK7 | NIN | NIPBL | NPM1 | NSFL1C | NSL1 | NSUN2 | NTMT1 | NUMA1 | NUSAP1 | OFD1 | OPTN | PA2G4 | PAFAH1B1 | PARD6A | PCM1 | PCNA | PDCD6IP | PHB2 | PHGDH | PIAS1 | PINX1 | PLK1 | PML | POLA2 | POLD1 | POLE2 | POLE3 | PPAT | PPM1A | PPM1D | PPME1 | PPP1CB | PPP1R12A | PPP1R12B | PPP2CA | PPP2R1A | PPP2R2A | PPP2R2D | PPP6C | PRCC | PRKAA1 | PRKAA2 | PRKAB2 | PRKACA | PRKAG1 | PRKAR1A | PRKAR2B | PRKDC | PTTG1 | RAB11A | RAB35 | RAD21 | RAD51 | RAN | RB1 | RCC1 | RFC1 | RFC2 | RFC3 | RFC4 | RFC5 | RHOA | RHOU | RPA2 | RPA3 | RPL24 | RPS3 | RPTOR | RRM2 | RRS1 | RTFDC1 | RTKN | SBDS | SEPT1 | SEPT10 | SEPT11 | SEPT12 | SEPT14 | SEPT2 | SEPT3 | SEPT4 | SEPT5 | SEPT6 | SEPT7 | SEPT8 | SEPT9 | SFN | SIRT7 | SLC16A1 | SMAD3 | SMC1A | SMC3 | SOX2 | SPC25 | SPDL1 | SPICE1 | SPRY2 | SPTBN1 | SSX2IP | STAG2 | STIL | STK11 | STMN1 | SUN2 | SYNE2 | TAOK3 | TBCE | TBRG4 | TERF1 | TFDP1 | TGFB1 | THBS1 | TNKS | TOP2A | TOP3A | TP53 | TP73 | TPR | TRIAP1 | TRIP13 | TSG101 | TTC19 | TUBA1A | TUBA4A | TUBB | TUBB4A | TUBB4B | TUBB8 | TUBG1 | TUBGCP3 | TYMS | UBB | UBE2B | UBE2C | UBE2L3 | UBE2S | UNC119 | USP16 | USP17L2 | USP26 | USP8 | VCP | VPS4B | YWHAE | YWHAG | ZFYVE19 | ZFYVE26 | ZNF207 | ZW10 | ZWINT | GO:0060249 | 0.0226859153220915 | 187/8243 | 95/3250 | 0.000930629890946138 | 1 | T | T | F | F | anatomical structure homeostasis | ACACA | ACD | ACTB | ACTG1 | ADRB2 | AKR1B1 | AKT3 | ALB | ALDOA | APEX1 | ATM | ATP2B2 | BAG3 | BAX | BCL2 | CDK5 | CFL2 | COL2A1 | CROCC | CTNNB1 | DCTN1 | DMD | DNA2 | DNAJB2 | FEN1 | FH | GAA | GAR1 | GATA1 | GNAS | HAAO | HIF1A | HIST1H4A | HIST3H3 | HSP90AA1 | HSP90AB1 | HSPB1 | HTR1B | KRAS | LRRK1 | MKS1 | NCDN | NOD2 | NOP10 | NOS3 | P2RX1 | P2RX4 | PARP1 | PCNA | PFKM | PINX1 | POC1B | POLA2 | POLD1 | POLE2 | POLE3 | POT1 | POTEF | POTEI | PRDX1 | PRKAA1 | PRKDC | PTGES3 | RAC1 | RAD51 | RB1 | RFC1 | RFC2 | RFC3 | RFC4 | RFC5 | RIF1 | RPA2 | RPA3 | SMG5 | SMG6 | SMO | SOD1 | SP100 | SRC | STK11 | STRAP | TERF1 | TERF2 | TERF2IP | TFIP11 | TGFB1 | TINF2 | TRAF6 | TRIM32 | XRCC1 | XRCC3 | XRCC5 | XRCC6 | YAP1 | GO:0007623 | 0.0107970399126532 | 89/8243 | 50/3250 | 0.000959179805283344 | 1 | T | T | F | F | circadian rhythm | AHCY | ASS1 | BTBD9 | CDK4 | CPT1A | CREB1 | CRY1 | CRY2 | CSNK1E | EGFR | EP300 | GFPT1 | GSK3B | HDAC1 | HDAC2 | HDAC3 | HEBP1 | HNRNPD | HNRNPL | HNRNPU | ID2 | IMPDH2 | JUN | KMT2A | LGR4 | MTA1 | MTNR1A | MYBBP1A | NAMPT | NCOA2 | NCOR1 | NDUFA9 | NOS2 | NTRK1 | OGT | PER1 | PER2 | PML | PPARGC1A | PPP1CA | PPP1CB | PPP1CC | PROX1 | PTEN | RBM4 | SIRT1 | TOP1 | TP53 | TYMS | USP2 | GO:0051222 | 0.0300861336891908 | 248/8243 | 122/3250 | 0.000965629174045268 | 1 | T | T | F | F | positive regulation of protein transport | ABAT | ABL1 | ADORA2A | AGER | AKT2 | ANK3 | APBB1 | ARF1 | ARRB1 | ATP13A2 | B3GAT3 | BAG3 | CD2 | CDH1 | CDK5 | CDK5R1 | CEP290 | CFTR | CHP1 | CSNK2A2 | DHX36 | DHX9 | DMAP1 | DNM1L | DYNLL1 | EDEM1 | EGFR | ERBB2 | EZR | FCN1 | FGB | FIS1 | FYN | GAPDH | GLMN | GLUL | GOLPH3 | GPI | GSK3A | GSK3B | HAVCR2 | HDAC3 | HIF1A | HK1 | HMGB1 | HRAS | HSPA1L | HTRA2 | HUWE1 | IPO5 | ITGB1BP1 | JUP | KIF5B | LRRK2 | MAPK1 | MAPK14 | MAPK3 | MAVS | MBP | MDM2 | MIF | MPC2 | MYO1C | NOD2 | NPEPPS | NR0B2 | NR1H2 | ORM1 | OXCT1 | PANX3 | PCM1 | PDCD10 | PFKM | PIK3R1 | PLA2G1B | PLA2G6 | PPARD | PPIA | PPID | PPM1A | PRKAA1 | PRKACA | PRKCD | PRKCE | PRKCZ | PSEN1 | PSMD9 | PTPN22 | PTPN23 | RAB29 | RAN | RHOU | RIPK2 | RNF31 | SAE1 | SEC16A | SFN | SIRT3 | SMAD3 | SMO | SPTBN1 | SRC | SRI | STOM | TARDBP | TGFB1 | TM9SF4 | TMEM30A | TNF | TP53 | TPR | TRIM28 | TSG101 | TWIST1 | UBE2D3 | UBE2L3 | UBR5 | USP50 | XBP1 | XPO4 | YWHAE | ZIC1 | GO:0061620 | 0.00303287637995875 | 25/8243 | 18/3250 | 0.00097132153226729 | 1 | T | T | F | F | glycolytic process through glucose-6-phosphate | ALDOA | ALDOC | ENO1 | ENO2 | ENO3 | GAPDH | GAPDHS | GPI | HK1 | HK2 | PFKL | PFKM | PFKP | PGAM1 | PGAM2 | PGK1 | PKM | TPI1 | GO:0061615 | 0.00303287637995875 | 25/8243 | 18/3250 | 0.00097132153226729 | 1 | T | T | F | F | glycolytic process through fructose-6-phosphate | ALDOA | ALDOC | ENO1 | ENO2 | ENO3 | GAPDH | GAPDHS | GPI | HK1 | HK2 | PFKL | PFKM | PFKP | PGAM1 | PGAM2 | PGK1 | PKM | TPI1 | GO:0033045 | 0.00727890331190101 | 60/8243 | 36/3250 | 0.000974125646828998 | 1 | T | T | F | F | regulation of sister chromatid segregation | ANAPC4 | APC | ATM | ATRX | AURKB | CCNB1 | CDC20 | CDC27 | CENPF | CTCF | CTNNB1 | CUL3 | DUSP1 | DYNC1LI1 | FEN1 | HECW2 | HNRNPU | IK | KLHL22 | MAD1L1 | MAD2L1 | MAPK15 | NIPBL | NUMA1 | PLK1 | PTTG1 | RAD21 | RB1 | SFPQ | SPDL1 | TNKS | TPR | TRIP13 | XRCC3 | ZNF207 | ZW10 | GO:0050868 | 0.00703627320150431 | 58/8243 | 35/3250 | 0.000986525991486574 | 1 | T | T | F | F | negative regulation of T cell activation | ADORA2A | ANXA1 | BCL6 | CASP3 | CBFB | CD74 | DTX1 | DUSP22 | ERBB2 | FOXJ1 | GLMN | HAVCR2 | HLX | HMGB1 | IFNA2 | IRF1 | ITCH | JAK3 | LGALS3 | MAD1L1 | PAG1 | PAWR | PELI1 | PRDX2 | PRKAR1A | PTPN22 | PTPN6 | RC3H1 | RUNX1 | SDC4 | SOCS1 | SOCS6 | TGFB1 | VSIG4 | ZBTB7B | GO:0032387 | 0.00582312264952081 | 48/8243 | 30/3250 | 0.00100012845901891 | 1 | T | T | F | F | negative regulation of intracellular transport | APOD | ARHGAP1 | BAG3 | BAG4 | BRAF | CD36 | CDK5 | CHP1 | CRYAB | GDI1 | HMOX1 | IL13RA2 | INSIG1 | ITGB1BP1 | LRRK2 | MAP1B | NUP153 | PARK7 | PCSK9 | PKIG | RANGAP1 | RAP1A | RAP1B | SNX12 | SNX3 | SP100 | TPR | TXN | UBE2J1 | USP17L2 | GO:0032414 | 0.00655101298071091 | 54/8243 | 33/3250 | 0.00100294969887038 | 1 | T | T | F | F | positive regulation of ion transmembrane transporter activity | ACTN2 | ACTN4 | ADRB2 | AKAP9 | ANK3 | ATP1B3 | CACNG2 | CACNG4 | CALM1 | CALM2 | CALM3 | CFTR | CHCHD10 | CHP1 | COX17 | DMD | GLRX | GSTO1 | HAP1 | HSPA2 | HTR3A | HTT | KCNIP2 | KIF5B | NOS1AP | RGN | S100A1 | SLC9A1 | STAC | STIM1 | SUMO1 | WNK1 | WNK4 | GO:0006518 | 0.00655101298071091 | 54/8243 | 33/3250 | 0.00100294969887038 | 1 | T | T | F | F | peptide metabolic process | ALDH5A1 | APEH | BACE1 | DNPEP | DPEP1 | G6PD | GGT1 | GLO1 | GPX1 | GPX4 | GSR | GSTA1 | GSTA2 | GSTA3 | GSTA4 | GSTA5 | GSTK1 | GSTM4 | GSTP1 | GSTT1 | GSTT2 | GSTT2B | GSTZ1 | IDE | IDH1 | MGST1 | MME | PARK7 | PSEN1 | PSEN2 | PTGES | SOD1 | THOP1 | GO:0044070 | 0.00606575275991751 | 50/8243 | 31/3250 | 0.00100536096136291 | 1 | T | T | F | F | regulation of anion transport | ABAT | ACE2 | ADORA2A | AHCYL1 | AKT1 | AKT2 | APOA1 | APOE | CFTR | CRY2 | FABP3 | FGFR1 | GOPC | MAP2K6 | MIF | NCOR1 | P2RX4 | PER2 | PLA2G10 | PLA2G6 | PRELID1 | PRKCD | PSEN1 | RAB11B | RAB3GAP1 | RGS2 | RIPK1 | SNCA | STXBP1 | THBS1 | TRIAP1 | GO:1905330 | 0.014193861458207 | 117/8243 | 63/3250 | 0.00102781387279152 | 1 | T | T | F | F | regulation of morphogenesis of an epithelium | ABL1 | AHI1 | ALOX12 | AR | ARRB2 | CDC42 | CLTC | CTNNB1 | DAB2 | DVL1 | DVL2 | DVL3 | ESR1 | FGFR1 | FKBPL | LGR4 | MED12 | MKS1 | MLLT3 | NPHP3 | PARD6A | PAX8 | PFN1 | PHB2 | PSMA1 | PSMA2 | PSMA3 | PSMA5 | PSMA6 | PSMB5 | PSMB6 | PSMB7 | PSMB8 | PSMC1 | PSMC2 | PSMC3 | PSMC4 | PSMC5 | PSMC6 | PSMD1 | PSMD10 | PSMD11 | PSMD12 | PSMD14 | PSMD2 | PSMD4 | PSMD9 | PSME2 | PSME3 | PTEN | RAC1 | RHOA | RXRA | SEC24B | SIRT6 | SMO | SMURF1 | SMURF2 | SNAI2 | STAT1 | TGFB1 | TNF | WNT2 | GO:0009065 | 0.0021836709935703 | 18/8243 | 14/3250 | 0.00106894662040819 | 1 | T | T | F | F | glutamine family amino acid catabolic process | ALDH4A1 | FAH | GAD2 | GLS | GLS2 | GLUD2 | GLUL | GOT1 | GOT2 | NOS2 | NOS3 | OAT | PADI4 | PPAT | GO:0009083 | 0.0021836709935703 | 18/8243 | 14/3250 | 0.00106894662040819 | 1 | T | T | F | F | branched-chain amino acid catabolic process | ACAD8 | ACADSB | ACAT1 | ALDH6A1 | BCAT2 | BCKDHB | DBT | DLD | HIBADH | HIBCH | HSD17B10 | IVD | MCCC1 | PPM1K | GO:0099177 | 0.0285090379716123 | 235/8243 | 116/3250 | 0.00108834549895529 | 1 | T | T | F | F | regulation of trans-synaptic signaling | ABAT | ABL1 | ADCYAP1 | ADORA2A | ADRB2 | AGER | AKAP9 | APOE | APP | ARF1 | ARRB2 | ATAD1 | ATP2A2 | BACE1 | BRAF | CACNA1A | CACNG2 | CACNG4 | CACNG5 | CALM1 | CALM3 | CAMK2A | CAMK2B | CDC20 | CDH1 | CDK5 | CEL | CHRM2 | CLSTN1 | CPLX1 | CPLX3 | CREB1 | DBN1 | DGKE | DLG4 | DNM1L | DVL1 | EGFR | EGLN1 | EIF2AK4 | EIF4A3 | EIF4E | EPHA4 | FABP5 | FLOT1 | FYN | GFAP | GIT1 | GLUL | GNAI2 | GRIA1 | GRID2 | GRIK2 | GSK3B | HAP1 | HRAS | HTR1B | ITPR3 | KIF5B | KRAS | LGMN | LRRK2 | MAPK1 | MAPT | MECP2 | MME | MPP2 | NCDN | NPTX1 | NRXN1 | NTF3 | NTRK1 | P2RX1 | PICK1 | PLA2G6 | PLCG1 | PREPL | PRKACA | PRKAR2B | PRKCE | PRKCZ | PSEN1 | PSMC5 | PTEN | PTN | PTPRA | RAB11A | RAB3GAP1 | RAB5A | RAP1A | RAP1B | RARA | RHOT1 | RNF19A | S100B | SEPT5 | SERPINE2 | SH3GL1 | SIPA1L1 | SNCA | SQSTM1 | SRC | STAT3 | STAU1 | STXBP1 | SYN1 | SYP | TNF | TOR1A | TUBB2B | USP46 | USP8 | VPS18 | YWHAG | YWHAH | ZMYND8 | GO:0042026 | 0.00327550649035545 | 27/8243 | 19/3250 | 0.00109815220344985 | 1 | T | T | F | F | protein refolding | DNAJA2 | DNAJA4 | DNAJB2 | FKBP1A | FKBP1B | HSP90AA1 | HSPA14 | HSPA1B | HSPA1L | HSPA2 | HSPA5 | HSPA8 | HSPA9 | HSPD1 | PPIA | PPIB | PPID | PPIE | PPIF | GO:0045898 | 0.00157709571757855 | 13/8243 | 11/3250 | 0.00113186986869878 | 1 | T | T | F | F | regulation of RNA polymerase II transcriptional preinitiation complex assembly | CAND1 | CREB1 | ESR1 | HMGB1 | PSMC2 | PSMC3 | PSMC4 | PSMC5 | PSMC6 | THRA | TP53 | GO:0010869 | 0.00157709571757855 | 13/8243 | 11/3250 | 0.00113186986869878 | 1 | T | T | F | F | regulation of receptor biosynthetic process | HDAC1 | HDAC2 | HDAC6 | HIF1A | HNRNPK | HOXA5 | ITGAV | ITGB3 | NR1H3 | PARK7 | PPARG | GO:0071243 | 0.00157709571757855 | 13/8243 | 11/3250 | 0.00113186986869878 | 1 | T | T | F | F | cellular response to arsenic-containing substance | DAXX | DDX3X | DHX36 | GSTO1 | GSTO2 | HMOX1 | HNRNPA1 | HSF1 | MKNK2 | PPIF | VCP | GO:0042558 | 0.00157709571757855 | 13/8243 | 11/3250 | 0.00113186986869878 | 1 | T | T | F | F | pteridine-containing compound metabolic process | ATIC | FOLH1 | FOLR1 | FPGS | MTHFD1 | MTRR | QDPR | SHMT1 | SHMT2 | SLC25A32 | TYMS | GO:1902307 | 0.00157709571757855 | 13/8243 | 11/3250 | 0.00113186986869878 | 1 | T | T | F | F | positive regulation of sodium ion transmembrane transport | ACTN4 | ANK3 | ARF1 | ATP1B3 | CHP1 | DMD | GLRX | KIF5B | SLC9A1 | WNK1 | WNK4 | GO:0010948 | 0.0302074487443892 | 249/8243 | 122/3250 | 0.00116857712984426 | 1 | T | T | F | F | negative regulation of cell cycle process | APC | ATM | ATRX | AURKB | BABAM1 | BAX | BCL2 | BCL6 | BMI1 | BRCA1 | CALR | CARM1 | CASP2 | CCNB1 | CDC14B | CDC20 | CDC25C | CDC73 | CDK2 | CDK4 | CDK9 | CDKN1A | CDKN2C | CENPF | CHEK2 | CHMP2A | CUL1 | E2F1 | E2F4 | E2F6 | EED | EHMT1 | EHMT2 | EP300 | EZH2 | FBXO7 | FHL1 | FZR1 | HSP90AB1 | IK | KANK2 | KLHL22 | L3MBTL2 | MAD1L1 | MAD2L1 | MDM1 | MDM2 | MDM4 | MGA | MIF | MSH6 | NAE1 | NPM1 | NUBP1 | PCGF6 | PCNA | PHC3 | PINX1 | PLK1 | PML | PRKAR1A | PRKDC | PRMT2 | PSMA1 | PSMA2 | PSMA3 | PSMA5 | PSMA6 | PSMB5 | PSMB6 | PSMB7 | PSMB8 | PSMC1 | PSMC2 | PSMC3 | PSMC4 | PSMC5 | PSMC6 | PSMD1 | PSMD10 | PSMD11 | PSMD12 | PSMD14 | PSMD2 | PSMD4 | PSMD9 | PSME2 | PSME3 | PTEN | PTTG1 | RAD21 | RAD51 | RB1 | RBBP4 | RBX1 | RING1 | RIPK1 | RNF2 | RPA2 | RPL23 | RRM2 | SEPT7 | SFN | SMC1A | SMC3 | SPDL1 | STAG2 | SUZ12 | TAOK3 | TERF1 | TERF2 | TFDP1 | TNKS | TP53 | TPR | TRIAP1 | TRIP13 | USP47 | YAF2 | ZFYVE19 | ZNF207 | ZW10 | GO:0044770 | 0.023899065874075 | 197/8243 | 99/3250 | 0.00117132103278781 | 1 | T | T | F | F | cell cycle phase transition | ACTR1A | AKAP8 | AKAP9 | BTRC | CALM2 | CALM3 | CAMK2A | CCNB1 | CCNY | CCP110 | CDC25A | CDC25B | CDC25C | CDC27 | CDC34 | CDK2 | CDK4 | CDKN1A | CDKN2C | CENPJ | CEP135 | CEP152 | CEP290 | CEP57 | CEP70 | CHEK2 | CHMP2A | CKAP5 | CKS2 | CNTRL | CSNK1E | CTDP1 | CUL1 | CUL2 | CUL3 | CUL4B | CUL5 | DCTN1 | DYNC1H1 | DYNC1I2 | DYNLL1 | EIF4E | EIF4EBP1 | FBXL12 | FBXW11 | FGFR1OP | FOXM1 | GSPT1 | HSP90AA1 | KDM8 | KHDRBS1 | LATS1 | MCM2 | MCM3 | MCM4 | MCM5 | MCM6 | MCM7 | MELK | MYC | OFD1 | OPTN | PAFAH1B1 | PCM1 | PIAS1 | PLK1 | POLA2 | POLE2 | POLE3 | PPAT | PPM1D | PPME1 | PPP1CB | PPP1R12A | PPP1R12B | PPP2R1A | PPP2R2A | PPP2R2D | PPP6C | PRKACA | PRKAR2B | RB1 | RCC1 | RHOU | RPA2 | RPA3 | RPL24 | TUBA1A | TUBA4A | TUBB | TUBB4A | TUBB4B | TUBG1 | UBE2C | UBE2L3 | UBE2S | USP26 | YWHAE | YWHAG | GO:0043401 | 0.0116462452990416 | 96/8243 | 53/3250 | 0.00118464296469598 | 1 | T | T | F | F | steroid hormone mediated signaling pathway | AR | BRCA1 | CALR | CARM1 | CTNNB1 | DAXX | DNAJA1 | ESR1 | ESR2 | ESRRA | ESRRB | ESRRG | FHL2 | GRIP1 | HNF4A | HNF4G | KAT5 | LEF1 | MED1 | NCOA1 | NCOA3 | NCOA4 | NEDD4 | NR0B2 | NR1H2 | NR1H3 | NR1I2 | NR3C1 | NR4A1 | NR5A2 | PHB | PIAS1 | PPARD | PPARG | PPARGC1A | RAN | RARA | RARG | RB1 | RHOA | RNF4 | RXRA | RXRB | RXRG | SRC | THRA | TRIP4 | UBE3A | UBR5 | VDR | WBP2 | YAP1 | YWHAH | GO:0032091 | 0.00934125925027296 | 77/8243 | 44/3250 | 0.00118650776068854 | 1 | T | T | F | F | negative regulation of protein binding | ACTB | ADRB2 | AES | AKT1 | ARRB2 | AURKB | BAG2 | BAX | CARM1 | CAV1 | CRMP1 | CSNK1E | CTNNBIP1 | DAB2 | DISC1 | DNAJB2 | DVL1 | EIF2S1 | FAM192A | GOLGA2 | GPSM1 | GSK3B | GTF2F1 | HSPA5 | IFIT1 | ITGA4 | ITGB1BP1 | KDM1A | LEF1 | LRRK2 | MAPK3 | MAPK8 | PARK7 | PCSK9 | PIN1 | PPP1CA | PRKCD | PTPRF | RUVBL2 | STMN1 | STUB1 | STYX | TFIP11 | TMBIM6 | GO:0019222 | 0.462574305471309 | 3813/8243 | 1571/3250 | 0.001206743507938 | 1 | T | T | F | F | regulation of metabolic process | A2M | AARS | ABAT | ABCE1 | ABCG1 | ABHD14B | ABL1 | ABL2 | ACACA | ACADVL | ACD | ACE2 | ACLY | ACO1 | ACOT13 | ACOX1 | ACSL1 | ACTA1 | ACTA2 | ACTB | ACTG2 | ACTL6A | ACTN1 | ACTN2 | ACTN4 | ACTR2 | ACTR3 | ADCY6 | ADCYAP1 | ADORA2A | ADRB2 | ADRM1 | ADTRP | AEBP2 | AES | AFF1 | AFF4 | AGAP2 | AGER | AGO1 | AGO2 | AGR2 | AHCYL1 | AHI1 | AHNAK | AHSG | AICDA | AIFM1 | AIMP2 | AIRE | AKAP8 | AKAP9 | AKT1 | AKT2 | AKTIP | ALAS1 | ALOX12 | ANK3 | ANKRD49 | ANP32A | ANXA1 | ANXA2 | ANXA7 | APBB1 | APBB2 | APBB3 | APC | APCS | APEX1 | APOA1 | APOD | APOE | APP | AR | ARAF | ARF1 | ARF4 | ARHGAP35 | ARID3B | ARID4B | ARNT | ARNT2 | ARR3 | ARRB1 | ARRB2 | ASB9 | ASCC1 | ASCC2 | ASCL1 | ASPSCR1 | ASS1 | ATAD1 | ATAD5 | ATCAY | ATF2 | ATF6 | ATF6B | ATF7 | ATG5 | ATM | ATOH1 | ATP13A2 | ATP1A1 | ATP6V1A | ATP6V1B1 | ATP6V1C1 | ATP6V1D | ATP6V1E1 | ATRX | ATXN1 | ATXN2 | AURKB | AXIN1 | AZU1 | B3GAT3 | BABAM1 | BACH2 | BAG2 | BAG3 | BAG4 | BAG5 | BAG6 | BANP | BASP1 | BAX | BBC3 | BCAR3 | BCL10 | BCL11A | BCL2 | BCL2L13 | BCL6 | BCL6B | BCOR | BDKRB2 | BIN1 | BIRC2 | BIRC3 | BIRC5 | BIRC7 | BIRC8 | BLZF1 | BMI1 | BMPR1A | BOLA1 | BOLA3 | BRAF | BRCA1 | BRD2 | BRD3 | BRD4 | BTK | BTRC | BUD31 | C1QBP | C1QL4 | C1QTNF1 | C4A | CAB39 | CALCOCO2 | CALM1 | CALM2 | CALM3 | CALR | CAMK1D | CAMK2A | CAND1 | CAPN1 | CAPN2 | CAPNS1 | CARD9 | CARM1 | CASP10 | CASP3 | CASP8 | CASP9 | CAT | CAV1 | CBFA2T3 | CBFB | CBL | CBLB | CBLC | CBX4 | CBX7 | CBX8 | CBY1 | CCAR2 | CCDC22 | CCDC8 | CCDC88A | CCL21 | CCL22 | CCNB1 | CCNT1 | CCNT2 | CCNY | CCNYL1 | CCR1 | CCS | CCT2 | CCT3 | CCT4 | CCT5 | CCT6A | CCT7 | CCT8 | CD109 | CD27 | CD2BP2 | CD36 | CD40 | CD40LG | CD74 | CD81 | CDA | CDC14B | CDC20 | CDC25A | CDC25B | CDC25C | CDC37 | CDC5L | CDC73 | CDH1 | CDK2 | CDK4 | CDK5 | CDK5R1 | CDK8 | CDK9 | CDKN1A | CDKN2C | CEBPA | CEBPE | CENPF | CENPJ | CEP290 | CHCHD10 | CHCHD2 | CHCHD3 | CHD3 | CHD4 | CHD8 | CHEK2 | CHORDC1 | CHP1 | CHRD | CHRNA9 | CIRBP | CISD1 | CISD2 | CKS1B | CKS2 | CLK1 | CLPX | CLTC | CLU | CNDP1 | COA3 | COL2A1 | COMMD1 | COMT | COPS2 | COPS5 | COQ3 | COQ7 | COX11 | COX17 | COX7A2 | CPSF7 | CPT1A | CPT2 | CREB1 | CREB3 | CREBBP | CRIP1 | CRK | CRKL | CRP | CRY1 | CRY2 | CRYAB | CSNK1A1 | CSNK1E | CSNK2A2 | CSNK2B | CTBP1 | CTCF | CTDP1 | CTH | CTNNB1 | CTNNBIP1 | CTSA | CTSC | CTTN | CUL2 | CUL3 | CUL4B | CYCS | CYLD | CYP1A1 | CYP1A2 | DAB1 | DAB2 | DAPK1 | DAXX | DAZAP1 | DBF4B | DBH | DBNDD2 | DBNL | DCN | DCP2 | DCUN1D1 | DDA1 | DDB1 | DDIT4 | DDRGK1 | DDX1 | DDX39B | DDX3X | DDX6 | DECR1 | DERL1 | DGKZ | DHCR24 | DHX36 | DHX9 | DIABLO | DIS3 | DLG2 | DLG3 | DLG4 | DMAP1 | DMD | DMRT3 | DNA2 | DNAJA1 | DNAJA3 | DNAJB1 | DNAJB2 | DNAJC10 | DNAJC30 | DNM1L | DOCK7 | DPEP1 | DPF2 | DROSHA | DTX1 | DUSP1 | DUSP12 | DUSP14 | DUSP16 | DUSP19 | DUSP22 | DUSP7 | DVL1 | DVL2 | DVL3 | DYNC1H1 | DYNLL1 | DYRK2 | E2F1 | E2F2 | E2F3 | E2F4 | E2F6 | EAF2 | EBF1 | ECD | ECSIT | EDA | EDC4 | EDEM1 | EED | EEF1A1 | EEF1A2 | EEF2 | EFEMP1 | EGFR | EGLN1 | EGLN3 | EHD4 | EHMT1 | EHMT2 | EID1 | EIF2AK3 | EIF2AK4 | EIF2B5 | EIF2S1 | EIF3A | EIF3D | EIF3E | EIF3H | EIF4A3 | EIF4E | EIF4E2 | EIF4EBP1 | EIF4ENIF1 | EIF4G3 | EIF4H | EIF5 | EIF5A | EIF5B | EIF6 | ELAVL1 | ELF3 | ELF4 | ENDOG | ENO1 | EP300 | EPHA4 | EPM2A | EPPIN | EPRS | ERBB2 | ERBB3 | ERF | ERG | ERLIN2 | ERRFI1 | ESR1 | ESR2 | ESRRA | ESRRB | ESRRG | ETS1 | EWSR1 | EXOSC1 | EXOSC5 | EXOSC6 | EXOSC9 | EYA2 | EYA4 | EZH2 | EZR | FABP1 | FABP3 | FABP5 | FADD | FAF1 | FAM129B | FAM192A | FAM3C | FAM83D | FAM98A | FANCA | FANCI | FANCM | FASLG | FASN | FBP1 | FBXO7 | FBXW11 | FDPS | FGB | FGF2 | FGFR1 | FGFR1OP | FGFR3 | FH | FHL2 | FIGNL1 | FIS1 | FKBP1A | FKBP1B | FKBP8 | FLOT1 | FN1 | FOS | FOXA1 | FOXD4 | FOXD4L6 | FOXI2 | FOXJ1 | FOXK1 | FOXK2 | FOXM1 | FOXO1 | FTO | FUBP1 | FUBP3 | FUS | FXR2 | FYN | FZR1 | G3BP2 | G6PD | GABPB1 | GADD45G | GAPDH | GAPDHS | GATA1 | GDF9 | GEMIN5 | GFAP | GFPT1 | GGA1 | GGPS1 | GJA1 | GLA | GLMN | GLO1 | GNA12 | GNAI2 | GNAI3 | GNAQ | GNAS | GOLGA2 | GOT1 | GPD1 | GPD1L | GPI | GPS2 | GPSM1 | GPX1 | GRB2 | GRB7 | GRIK2 | GRIP1 | GSK3A | GSK3B | GSPT1 | GSPT2 | GSTP1 | GSTZ1 | GTF2A1 | GTF2F1 | GTF2F2 | GTF2I | GZMA | H1F0 | H1FX | H2AFX | H2AFZ | H3F3A | HADH | HAP1 | HAVCR2 | HAX1 | HBEGF | HBZ | HDAC1 | HDAC2 | HDAC3 | HDAC4 | HDAC5 | HDAC6 | HDAC7 | HDAC9 | HDGF | HECW1 | HECW2 | HELZ | HELZ2 | HERC1 | HEXB | HEXIM1 | HGS | HIF1A | HIF1AN | HINT1 | HIST1H1A | HIST1H3A | HIST1H4A | HIST2H3A | HIVEP1 | HK2 | HLX | HMGB1 | HMGB2 | HMGB3 | HMOX1 | HNF1A | HNF4A | HNF4G | HNRNPA1 | HNRNPA2B1 | HNRNPAB | HNRNPD | HNRNPF | HNRNPH1 | HNRNPK | HNRNPL | HNRNPM | HNRNPU | HOXA5 | HOXB5 | HOXB6 | HOXB9 | HOXD10 | HPRT1 | HPX | HRAS | HSF1 | HSF4 | HSP90AA1 | HSP90AB1 | HSP90AB3P | HSP90AB4P | HSP90B1 | HSPA1B | HSPA2 | HSPA5 | HSPA8 | HSPB1 | HSPBP1 | HSPD1 | HSPE1 | HSPH1 | HTATIP2 | HTRA1 | HTRA2 | HTT | IBTK | ICAM1 | ICK | ID2 | IDE | IDH1 | IFI16 | IFNA2 | IGBP1 | IGF1R | IGF2BP1 | IGFBP7 | IKBKB | IKBKG | IKZF5 | ILF2 | ILF3 | ILK | ING1 | ING5 | INSIG1 | INSIG2 | INSR | IP6K1 | IPO5 | IPO7 | IPO8 | IQGAP1 | IREB2 | IRF1 | IRF4 | IRF7 | ISG15 | ITCH | ITGAV | ITGB1BP1 | ITGB3 | ITGB3BP | ITPR1 | JAK3 | JMJD6 | JUN | JUNB | JUND | JUP | KANK2 | KAT5 | KAT7 | KAT8 | KDM1A | KDM2A | KDM4D | KDM5B | KDM5C | KDM8 | KDR | KEAP1 | KHDRBS1 | KLF5 | KLF6 | KLHDC10 | KLHL22 | KMT2A | KMT2B | KMT2C | KPNA1 | KPNA2 | KPNB1 | KRAS | KSR1 | L3MBTL1 | L3MBTL2 | L3MBTL3 | L3MBTL4 | LAMP3 | LARP4 | LARS | LAT | LATS1 | LCP2 | LDLRAD4 | LEF1 | LEMD2 | LEMD3 | LGALS3 | LGMN | LGR4 | LIMS1 | LMNA | LMO2 | LMO7 | LOXL2 | LPAR1 | LPIN3 | LRIF1 | LRP6 | LRPPRC | LRRK1 | LRRK2 | LRSAM1 | LSM1 | LSM3 | LSM4 | LSM5 | LTBR | LUM | LYPLA1 | LZTR1 | MAD2L1 | MAF1 | MAP2K1 | MAP2K3 | MAP2K4 | MAP2K6 | MAP2K7 | MAP3K1 | MAP3K13 | MAP3K14 | MAP3K2 | MAP3K3 | MAP3K4 | MAP4K1 | MAPK1 | MAPK14 | MAPK15 | MAPK3 | MAPK4 | MAPK6 | MAPK7 | MAPK8 | MAPK8IP3 | MAPK9 | MAPKAP1 | MAPT | MAS1 | MATR3 | MAVS | MBD3 | MBP | MC1R | MCM7 | MDFI | MDM2 | MDM4 | ME1 | MECP2 | MED1 | MED12 | MED14 | MED17 | MED20 | MED21 | MED23 | MED24 | MED31 | MED4 | MED6 | MEOX2 | MEPCE | METTL23 | MGA | MGMT | MIF | MITF | MKNK2 | MLH1 | MLLT1 | MLLT11 | MLLT3 | MLST8 | MOB1B | MORF4L1 | MORF4L2 | MOV10 | MRGBP | MRPL12 | MSH6 | MSN | MTA1 | MTA3 | MTERF1 | MTPN | MVD | MXD1 | MYBBP1A | MYC | MYD88 | MYH6 | MYO1C | MYO1D | MYO6 | MYOC | NACC1 | NAMPT | NANOG | NBAS | NCBP1 | NCK1 | NCOA1 | NCOA2 | NCOA3 | NCOA4 | NCOR1 | NCOR2 | NDUFA13 | NEDD4 | NEDD8 | NEK1 | NEK7 | NELFB | NELFCD | NELFE | NELL1 | NEUROD1 | NF2 | NFATC1 | NFATC2 | NFE2L2 | NFKB2 | NFKBIA | NIF3L1 | NIPBL | NKRF | NKX2-1 | NLK | NMD3 | NME1 | NME2 | NMNAT1 | NNT | NOD2 | NOS1AP | NOS2 | NOS3 | NOSIP | NOSTRIN | NPM1 | NQO1 | NQO2 | NR0B2 | NR1H2 | NR1H3 | NR1I2 | NR3C1 | NR4A1 | NR5A2 | NRBF2 | NTF3 | NTRK1 | NUFIP1 | NUMA1 | NUP107 | NUP153 | NUP160 | NUP205 | NUP214 | NUP85 | NUP88 | NUP93 | NUP98 | NUPR1 | OAS2 | OAZ3 | OGFOD1 | OGT | ONECUT1 | OPTN | OTUB1 | OTUD4 | OXA1L | OXSR1 | P2RX1 | P2RX4 | PA2G4 | PABPC1 | PABPC4 | PACSIN3 | PAFAH1B1 | PAIP1 | PAK2 | PAK6 | PAM16 | PAN2 | PAPLN | PARD3 | PARD6A | PARK7 | PARP1 | PARP14 | PATZ1 | PAWR | PAX8 | PAX9 | PAXIP1 | PC | PCBD1 | PCBP1 | PCGF1 | PCGF5 | PCGF6 | PCNA | PCSK9 | PCTP | PDCD10 | PDCD6 | PDE12 | PDE4D | PDE6G | PDK2 | PEBP1 | PEF1 | PELI1 | PER1 | PER2 | PFKM | PFN1 | PGAM1 | PGAM5 | PGK1 | PGK2 | PGP | PHB | PHB2 | PHF10 | PHF20L1 | PHF21A | PHGDH | PIAS1 | PIAS3 | PICK1 | PIH1D1 | PIK3CA | PIK3CG | PIK3R1 | PIK3R3 | PIKFYVE | PIM1 | PIN1 | PINX1 | PIP4K2A | PIR | PKIG | PLA2G10 | PLA2G1B | PLA2G2A | PLA2G6 | PLAUR | PLEKHF1 | PLGRKT | PLK1 | PLSCR1 | PLXNB2 | PML | PNKP | POLDIP2 | POLDIP3 | POLR2A | POLR2E | POLR2L | POM121 | POT1 | POU2F3 | POU5F1 | PPARD | PPARG | PPARGC1A | PPIB | PPID | PPIE | PPIF | PPM1A | PPM1D | PPM1K | PPME1 | PPP1CA | PPP1CB | PPP1CC | PPP1R11 | PPP1R12A | PPP1R14A | PPP1R16A | PPP1R2 | PPP1R3B | PPP1R3C | PPP1R8 | PPP2CA | PPP2CB | PPP2R1A | PPP2R1B | PPP2R2A | PPP2R2D | PPP2R5D | PPP4R2 | PPP6R1 | PRCP | PRDX1 | PRDX2 | PRDX3 | PRDX5 | PRDX6 | PRELID1 | PRKAA1 | PRKAA2 | PRKAB2 | PRKACA | PRKAG1 | PRKAR1A | PRKAR2B | PRKCD | PRKCE | PRKCZ | PRKD1 | PRKD2 | PRKDC | PRMT2 | PRMT3 | PRMT6 | PROX1 | PRPF19 | PRRC1 | PSAP | PSEN1 | PSMA1 | PSMA2 | PSMA3 | PSMA5 | PSMA6 | PSMB5 | PSMB6 | PSMB7 | PSMB8 | PSMC1 | PSMC2 | PSMC3 | PSMC4 | PSMC5 | PSMC6 | PSMD1 | PSMD10 | PSMD11 | PSMD12 | PSMD14 | PSMD2 | PSMD4 | PSMD9 | PSME2 | PSME3 | PTEN | PTGES2 | PTGES3 | PTK2 | PTN | PTPN1 | PTPN14 | PTPN22 | PTPN6 | PTPRK | PTPRR | PTRH2 | PTTG1 | PUM1 | PUS7 | QARS | QRICH1 | QSOX1 | RAB1A | RAB1B | RAB29 | RAB3GAP1 | RAB5A | RAB7A | RAC1 | RAD21 | RAD23A | RAD23B | RAD51 | RAD51AP1 | RAE1 | RAF1 | RAN | RANBP9 | RAP1A | RAP1B | RARA | RARG | RASD1 | RB1 | RB1CC1 | RBBP4 | RBBP6 | RBM17 | RBM25 | RBM3 | RBM4 | RBM8A | RBMX | RBPJ | RBX1 | RC3H1 | RDX | RECK | REL | RELA | REST | RFC1 | RFC2 | RFC3 | RFC4 | RFC5 | RFK | RFXANK | RGN | RGS2 | RHBDD1 | RHOA | RIF1 | RING1 | RIOK1 | RIPK1 | RIPK2 | RIPK3 | RIPK4 | RMND1 | RNASEH2B | RNF139 | RNF185 | RNF19A | RNF2 | RNF25 | RNF31 | RNF4 | RNF8 | ROMO1 | RPA2 | RPL10 | RPL10A | RPL12 | RPL18A | RPL22 | RPL23 | RPL23A | RPL24 | RPL30 | RPL37A | RPL38 | RPL6 | RPL7 | RPL7A | RPL9 | RPLP0 | RPLP1 | RPLP2 | RPS12 | RPS13 | RPS14 | RPS15A | RPS19 | RPS2 | RPS20 | RPS24 | RPS29 | RPS3 | RPS4X | RPS6KA1 | RPS6KB2 | RPS8 | RPTOR | RRM2 | RTF1 | RUNX1 | RUNX1T1 | RUNX2 | RUVBL1 | RUVBL2 | RXRA | RXRB | RXRG | S100A1 | S100A11 | S100A12 | S100A7 | S100A9 | SAG | SAMD7 | SAP18 | SARS | SART3 | SCFD1 | SCML2 | SCYL2 | SDC4 | SDCBP | SEC13 | SEMA7A | SEPT4 | SERPINB2 | SERPINB3 | SERPINB5 | SERPINB6 | SERPINB8 | SERPINE2 | SERPINH1 | SET | SETBP1 | SETDB1 | SF1 | SFMBT1 | SFN | SFPQ | SGTA | SH2B1 | SH3GL2 | SHC1 | SHMT1 | SHMT2 | SIN3A | SIRT1 | SIRT3 | SIRT5 | SIRT6 | SIRT7 | SKI | SKIV2L | SLAMF1 | SLAMF8 | SLC9A1 | SLC9A3R1 | SLTM | SMAD2 | SMAD3 | SMAD4 | SMAD5 | SMAD9 | SMARCA2 | SMARCA4 | SMARCB1 | SMARCC1 | SMARCC2 | SMARCE1 | SMC1A | SMC3 | SMG5 | SMG6 | SMG8 | SMO | SMURF1 | SMURF2 | SNAI2 | SNCA | SND1 | SNF8 | SNRPA | SNTA1 | SNW1 | SNX12 | SNX3 | SNX6 | SOAT1 | SOCS1 | SOCS2 | SOCS6 | SOD1 | SOD2 | SOGA3 | SORT1 | SOX2 | SP1 | SP100 | SP2 | SPEN | SPI1 | SPIN1 | SPINK7 | SPINT2 | SPOP | SPR | SPRTN | SPRY2 | SPTLC2 | SQSTM1 | SRC | SRI | SRPK1 | SRPK2 | SRSF12 | SRSF5 | SSB | STAG2 | STAT1 | STAT3 | STAT4 | STAT5A | STAT6 | STK11 | STK24 | STK25 | STK26 | STK3 | STK38 | STK40 | STOML2 | STRAP | STUB1 | STX5 | STYX | SUB1 | SUMO1 | SUMO2 | SUPT4H1 | SUPT5H | SUZ12 | SWAP70 | SYNJ2BP | TAB1 | TAB2 | TACO1 | TADA2B | TAF12 | TAF15 | TAF1A | TAGLN3 | TANK | TAOK3 | TARDBP | TAZ | TBL1X | TBRG4 | TCEA1 | TCEA2 | TCERG1 | TCF12 | TCF21 | TCF25 | TCF4 | TCOF1 | TCP1 | TCP10L | TDRD3 | TERF1 | TERF2 | TERF2IP | TFCP2 | TFDP1 | TFIP11 | TGFB1 | TGFBR3 | TGIF2LX | THAP11 | THAP7 | THBS1 | THRA | TIA1 | TIAL1 | TIMP1 | TIMP2 | TINF2 | TLE1 | TLE3 | TLR3 | TMBIM6 | TMEM173 | TMPO | TMTC3 | TNF | TNFAIP3 | TNFRSF1A | TNFRSF8 | TNFSF12 | TNFSF14 | TNIP2 | TNK2 | TNKS | TNKS2 | TNP1 | TNPO1 | TOE1 | TOLLIP | TOP1 | TOP2A | TP53 | TP63 | TP73 | TPCN2 | TPR | TRA2A | TRADD | TRAF1 | TRAF2 | TRAF3 | TRAF3IP1 | TRAF6 | TRAP1 | TREX1 | TRIAP1 | TRIB2 | TRIB3 | TRIM14 | TRIM21 | TRIM25 | TRIM28 | TRIM29 | TRIM32 | TRIM33 | TRIM34 | TRIP12 | TRIP13 | TRIP4 | TRUB2 | TSC22D1 | TSC22D3 | TSC22D4 | TSFM | TSG101 | TSKU | TSPYL2 | TTC5 | TULP3 | TWF1 | TWIST1 | TXN | TXNIP | TXNRD1 | TYMS | U2AF2 | UBA3 | UBB | UBC | UBE2B | UBE2C | UBE2D1 | UBE2D3 | UBE2I | UBE2J1 | UBE2K | UBE2L3 | UBE2N | UBE2O | UBE2S | UBE2V1 | UBE3A | UBQLN1 | UBQLN2 | UBQLN4 | UBR5 | UBXN1 | UCHL1 | UCHL5 | ULK1 | UNC119 | UNK | UNKL | UPF2 | UPF3B | UQCC1 | USP14 | USP15 | USP16 | USP17L2 | USP19 | USP2 | USP21 | USP4 | USP46 | USP47 | USP5 | USP50 | USP7 | USP8 | USP9X | USP9Y | VCP | VDAC1 | VDR | VGLL4 | VHL | VIM | VPS26A | VPS29 | VPS72 | VSTM2A | VTN | WARS | WAS | WBP2 | WDR35 | WDR41 | WDR48 | WDR77 | WDTC1 | WNK1 | WNT16 | WNT2 | WT1 | WTAP | WWOX | WWP2 | XBP1 | XIAP | XPO1 | XPO5 | XRCC1 | XRCC5 | XRCC6 | YAF2 | YAP1 | YEATS2 | YES1 | YTHDF1 | YWHAB | YWHAE | YWHAG | YWHAH | YWHAQ | YWHAZ | YY1 | ZBTB16 | ZBTB17 | ZBTB21 | ZBTB25 | ZBTB33 | ZBTB42 | ZBTB7A | ZBTB7B | ZC3H4 | ZFP41 | ZHX1 | ZIC1 | ZKSCAN1 | ZMPSTE24 | ZMYM5 | ZMYND8 | ZNF207 | ZNF217 | ZNF227 | ZNF24 | ZNF277 | ZNF354C | ZNF408 | ZNF420 | ZNF443 | ZNF517 | ZNF518A | ZNF592 | ZNF598 | ZNF618 | ZNF622 | ZNF646 | ZNF669 | ZNF696 | ZNF746 | ZNF747 | ZRANB2 | ZSCAN2 | ZSCAN29 | ZSCAN32 | ZZZ3 | GO:1904062 | 0.021472764770108 | 177/8243 | 90/3250 | 0.00120816067176465 | 1 | T | T | F | F | regulation of cation transmembrane transport | ABL1 | ACTN2 | ACTN4 | ADRB2 | AHNAK | AKAP9 | ANK3 | APP | ARF1 | ATG5 | ATP1B3 | BAX | BIN1 | CAB39 | CACNG2 | CACNG4 | CACNG5 | CALM1 | CALM2 | CALM3 | CAPN1 | CAV1 | CHCHD10 | CHP1 | COMMD1 | COX17 | CXCL11 | DAPK1 | DLG2 | DLG3 | DLG4 | DMD | DNAJC30 | FHL1 | FKBP1A | FKBP1B | FYN | G6PD | GLRX | GPD1L | GRIA1 | GSTO1 | HAP1 | HECW1 | HECW2 | HSPA2 | HTT | KCNAB1 | KCNAB2 | KCNIP2 | KIF5B | LRRC7 | MYO5A | NEDD4 | NOS1AP | OXSR1 | PARK7 | PCSK9 | PDE4D | PIK3CG | PLA2G6 | PLCG1 | PPARGC1A | PPIF | PRKACA | PRKCE | PSEN2 | PTEN | PTPN6 | RGN | RGS2 | S100A1 | SLC9A1 | SLC9A3R1 | SNCA | SNTA1 | SRI | STAC | STIM1 | STOM | SUMO1 | TGFB1 | THADA | TWIST1 | UBQLN1 | WNK1 | WNK4 | WWP2 | YWHAE | YWHAH | GO:1900180 | 0.0114036151886449 | 94/8243 | 52/3250 | 0.00123523843461454 | 1 | T | T | F | F | regulation of protein localization to nucleus | AKT1 | APOD | BAG3 | CCT2 | CCT3 | CCT4 | CCT5 | CCT6A | CCT7 | CCT8 | CD36 | CDH1 | CHP1 | CLDN18 | DCLK1 | DMAP1 | EIF2AK3 | FYN | GSK3B | HDAC3 | IPO5 | JUP | KAT7 | LMNA | MAPK1 | MAPK14 | MAVS | NF2 | NMD3 | PARK7 | PARP1 | PIK3R1 | PIN1 | PINX1 | PKIG | PLK1 | PRKCD | PSEN1 | RAN | SIN3A | SMAD3 | SMO | SRC | STK11 | TARDBP | TCP1 | TGFB1 | TPR | TRIM28 | TRIM29 | UBR5 | ZIC1 | GO:0034622 | 0.067572485745481 | 557/8243 | 254/3250 | 0.00124742289797052 | 1 | T | T | F | F | cellular protein-containing complex assembly | AAR2 | ACAD9 | ACD | ADRB2 | ADRM1 | AGO1 | AGO2 | ANKS4B | APCS | ARPC2 | ARPC4 | ATPAF2 | ATRX | ATXN2 | AXIN1 | BCS1L | BIN1 | BRD2 | C1QBP | CALR | CCT2 | CCT6B | CD2BP2 | CDC73 | CENPF | CENPJ | CENPV | CHAF1A | CHD1L | CHMP2A | CIRBP | CKAP5 | CLNS1A | CLTC | CLU | COA3 | COX11 | COX15 | COX17 | COX19 | COX20 | COX7A2 | CPSF7 | CRYAB | CSNK2B | CTTN | CUL4B | DAXX | DDB1 | DDB2 | DDX1 | DDX39B | DDX3X | DDX6 | DHX9 | DIAPH3 | DMD | DNAAF2 | DNAAF5 | DNM1L | DYNC1H1 | ECSIT | EIF2S1 | EIF3A | EIF3D | EIF3E | EIF3H | EIF3I | EIF4H | EIF5 | EIF6 | ERAL1 | ERCC5 | FGB | FKBP1A | G3BP1 | G3BP2 | GEMIN4 | GEMIN5 | GEMIN7 | GPX4 | GRB7 | H1F0 | H1FX | H2AFX | H3F3A | HCCS | HIST1H1A | HIST1H2BA | HIST1H3A | HIST1H4A | HIST2H3A | HIST3H3 | HLA-DRA | HMGB2 | HSF1 | HSP90AA1 | HSP90AB1 | HSPA4 | HSPD1 | IPO4 | ITGB3BP | LSM3 | LSM4 | LUC7L2 | LYRM7 | MAPT | MCM2 | MCTS1 | MDN1 | MED6 | MYH11 | NAP1L1 | NAP1L4 | NCBP1 | NCK1 | NDUFA10 | NDUFA13 | NDUFA5 | NDUFA8 | NDUFA9 | NDUFAF1 | NDUFAF2 | NDUFAF3 | NDUFAF4 | NDUFAF5 | NDUFAF7 | NDUFB1 | NDUFS1 | NDUFS2 | NDUFS3 | NDUFS6 | NDUFS7 | NDUFS8 | NDUFV1 | NDUFV3 | NPM1 | NUFIP1 | NUP107 | NUP153 | NUP205 | NUP93 | NUP98 | OBSL1 | OGFOD1 | OPA1 | OXA1L | PADI4 | PARP1 | PDE4DIP | PIAS1 | PIH1D1 | PIK3C2A | PIN1 | POLE3 | PRPF19 | PRPF8 | PSMD10 | PSMD11 | PSMD4 | PSMD9 | PSMG1 | PSMG4 | PSTPIP1 | PTGES3 | PTK2 | PXN | RAC1 | RAD23B | RAD51 | RAP1GDS1 | RBBP4 | RBX1 | RC3H1 | RIC3 | RPA2 | RPA3 | RPL10 | RPL12 | RPL23A | RPL24 | RPL38 | RPL6 | RPLP0 | RPLP0P6 | RPS10P5 | RPS14 | RPS19 | RRP7A | RRS1 | RTN4 | RUVBL1 | RUVBL2 | SART1 | SART3 | SBDS | SCO2 | SET | SF1 | SH3GL2 | SMAD2 | SMAD3 | SMAD4 | SMARCA4 | SMARCB1 | SMN1 | SNAP29 | SNCA | SNRPB | SNRPD1 | SNRPD3 | SNRPE | SRC | SRP19 | SRPK1 | SRPK2 | SRSF12 | SRSF5 | STRAP | STXBP1 | TAF12 | TAZ | TBCA | TCF4 | TCP1 | TEKT2 | TGFB1 | TGFBR3 | TIMM21 | TIMMDC1 | TM9SF4 | TMEM70 | TOMM20 | TP53 | TPR | TRAF2 | TSPYL2 | TTC19 | TXNL4A | UBB | UBC | UBE2C | UBE2K | UBE2S | UQCC1 | UQCRB | USP39 | USP4 | VBP1 | VTN | WAS | WDR77 | ZMYND10 | ZNF207 | GO:0010594 | 0.0129807109062235 | 107/8243 | 58/3250 | 0.00129498366748342 | 1 | T | T | F | F | regulation of endothelial cell migration | ABL1 | AKT1 | AKT3 | ALOX12 | ANXA1 | APOE | BCAR1 | CALR | CD40 | CSNK2B | DCN | ETS1 | FGF2 | FGFR1 | GLUL | GPI | HDAC5 | HDAC7 | HDAC9 | HIF1A | HMGB1 | HMOX1 | HSPB1 | ITGB1BP1 | ITGB3 | JUP | KDR | LGMN | MAP2K3 | MAP3K3 | MAPK14 | MECP2 | MEOX2 | NFE2L2 | NOS3 | NRP2 | P2RX4 | PDCD10 | PDCD6 | PIK3C2A | PLCG1 | PPARG | PRCP | PRKD1 | PRKD2 | PROX1 | PTK2 | RHOA | RHOJ | SIRT1 | SP1 | SP100 | STARD13 | STAT5A | SYNJ2BP | TBXA2R | TGFB1 | THBS1 | GO:0032392 | 0.00861336891908286 | 71/8243 | 41/3250 | 0.00130331411007465 | 1 | T | T | F | F | DNA geometric change | ANXA1 | CHD1L | CHD3 | CHD4 | CHD8 | CUL4B | DDB1 | DDB2 | DDX1 | DDX3X | DHX36 | DHX9 | DNA2 | FANCM | G3BP1 | GINS2 | GINS4 | HMGB1 | HMGB2 | HMGB3 | HNRNPA2B1 | MCM2 | MCM4 | MCM6 | MCM7 | MCM9 | MTERF1 | PARP1 | POT1 | RAD23B | RAD51 | RBX1 | RPA2 | RUVBL1 | RUVBL2 | TP53 | TREX1 | UBB | UBC | XRCC5 | XRCC6 | GO:0045597 | 0.0673298556350843 | 555/8243 | 253/3250 | 0.00130549683021116 | 1 | T | T | F | F | positive regulation of cell differentiation | ABL1 | ABL2 | ACTR2 | ADA | ADCYAP1 | AGER | AHI1 | AKT1 | ANXA1 | ANXA2 | AP3D1 | APBB1 | APOA1 | APOE | APP | AR | ARF1 | ARHGAP35 | ARHGDIA | ARHGEF7 | ARNT | ARPC2 | ARRB2 | ASCL1 | ATOH1 | AVIL | BCL2 | BCL6 | BIN1 | BMPR1A | BRAF | BTK | C1QA | C1QBP | CALR | CAMK1D | CAMK2B | CAPN2 | CARM1 | CASP8 | CBFB | CCR1 | CD27 | CD36 | CD74 | CDC42 | CDH5 | CDKL3 | CEBPA | CLIC1 | CREB1 | CRK | CRKL | CSNK1E | CTH | CTNNB1 | CTNNBIP1 | CUL7 | CYLD | DAB1 | DAB2 | DBN1 | DDX39B | DHX36 | DISC1 | DLG4 | DNM1L | DOCK5 | DVL1 | DVL2 | DVL3 | E2F1 | EP300 | EPHA4 | ETS1 | EZH2 | FADD | FAM129B | FGB | FGF2 | FGFR1 | FKBP1B | FLOT1 | FN1 | FOS | FOXA1 | FOXJ1 | FYN | GATA1 | GDI1 | GFAP | GNAS | GOLGA4 | GRIP1 | HAP1 | HAX1 | HDAC1 | HDAC2 | HIF1A | HIF1AN | HLX | HMGB1 | HMGB2 | HNRNPU | HOXA5 | HSF4 | HSP90AB1 | HSPA1B | HSPA5 | ID2 | ILK | IQGAP1 | ISG15 | JUN | JUNB | JUND | KDM1A | KDR | KLF5 | LEF1 | LGALS1 | LGALS3 | LIG4 | LIMS1 | LOXL2 | LPAR1 | LRRC7 | MAP1B | MAP2K1 | MAP3K13 | MAPK14 | MAPK6 | MAPK9 | MAPT | MED1 | METRN | METRNL | MME | MORF4L2 | MYOC | NAP1L1 | NCK1 | NCOA1 | NCOA3 | NELL1 | NEUROD1 | NF2 | NFATC2 | NFE2L2 | NIN | NIPBL | NME1 | NME2 | NTRK1 | NUMA1 | OBSL1 | OPA1 | P2RX4 | PA2G4 | PAFAH1B1 | PARP1 | PARP6 | PAX8 | PIAS1 | PIN1 | PLA2G10 | PLA2G2A | PLXNA1 | PLXNA2 | PLXNB2 | PNP | PPARD | PPARG | PPP1CC | PRKCZ | PRKD1 | PROX1 | PRPF19 | PSEN1 | PTEN | PTN | PTPRD | RAB11A | RAB21 | RAC1 | RAP1A | RARA | RASSF10 | RB1 | RBM4 | RBPJ | RELA | REST | RGS2 | RHOA | RIPK1 | RIPK2 | RPS6KA1 | RUNX1 | RUNX2 | S100A9 | SART1 | SDCBP | SEMA7A | SERPINB3 | SERPINE2 | SFN | SH3GL3 | SIRT1 | SMAD2 | SMAD3 | SMAD4 | SMAD5 | SMO | SMURF1 | SNAI2 | SNW1 | SNX3 | SOCS1 | SOCS2 | SOD2 | SOX2 | SPEN | STAT1 | STAT3 | STK11 | STK25 | STK3 | TCF12 | TCF4 | TGFB1 | TIMP2 | TMEM30A | TNF | TNFRSF12A | TNFSF14 | TP63 | TP73 | TRAF6 | TRIM32 | TUBB2B | TWF1 | TWF2 | TWIST1 | VDR | VHL | VSTM2A | XBP1 | XRCC5 | ZAP70 | ZBTB16 | ZBTB7B | ZMYND8 | GO:0009409 | 0.00376076671114885 | 31/8243 | 21/3250 | 0.00130770450760534 | 1 | T | T | F | F | response to cold | ACADVL | ADRB2 | CASP8 | CIRBP | EIF2AK3 | EIF2AK4 | FOS | FOXO1 | GMPR | HSP90AA1 | HSPA2 | HSPD1 | METRNL | NFKBIA | PPARG | PPARGC1A | PRKAA1 | RBM3 | SLC9A1 | THRA | VGF | GO:1901030 | 0.00376076671114885 | 31/8243 | 21/3250 | 0.00130770450760534 | 1 | T | T | F | F | positive regulation of mitochondrial outer membrane permeabilization involved in apoptotic signaling pathway | BAX | BBC3 | BCL2 | CASP8 | CHCHD10 | E2F1 | GSK3A | GSK3B | MAPK8 | PPP3CC | SFN | TFDP1 | TP53 | TP63 | TP73 | YWHAB | YWHAE | YWHAG | YWHAH | YWHAQ | YWHAZ | GO:0050851 | 0.0198956690525294 | 164/8243 | 84/3250 | 0.00131161455144112 | 1 | T | T | F | F | antigen receptor-mediated signaling pathway | ABL1 | BAX | BCAR1 | BCL10 | BCL2 | BRAF | BTK | BTN2A1 | BTRC | CD79B | CRKL | CUL1 | EIF2B1 | EIF2B3 | EIF2B5 | FBXW11 | FYN | HLA-DQA1 | HLA-DRA | HLA-DRB1 | HLA-DRB3 | HLA-DRB4 | HLA-DRB5 | HRAS | IGHV3-13 | IGHV3-23 | IGHV3-30 | IGLC7 | IKBKB | IKBKG | ITK | LAT | LCP2 | MAPK1 | NCK1 | NFATC2 | PAG1 | PAK2 | PDE4D | PIK3CA | PIK3R1 | PLCG1 | PRKD2 | PSEN1 | PSMA1 | PSMA2 | PSMA3 | PSMA5 | PSMA6 | PSMB5 | PSMB6 | PSMB7 | PSMB8 | PSMC1 | PSMC2 | PSMC3 | PSMC4 | PSMC5 | PSMC6 | PSMD1 | PSMD10 | PSMD11 | PSMD12 | PSMD14 | PSMD2 | PSMD4 | PSMD9 | PSME2 | PSME3 | PTPN22 | PTPN6 | RC3H1 | RELA | RIPK2 | RNF31 | SPG21 | STK11 | STOML2 | TAB2 | TRAF6 | UBE2N | WAS | WNK1 | ZAP70 | GO:0031577 | 0.002426301103967 | 20/8243 | 15/3250 | 0.00133751479168958 | 1 | T | T | F | F | spindle checkpoint | APC | ATM | AURKB | CDC20 | CENPF | IK | KLHL22 | MAD1L1 | MAD2L1 | PLK1 | SPDL1 | TPR | TRIP13 | ZNF207 | ZW10 | GO:0071174 | 0.002426301103967 | 20/8243 | 15/3250 | 0.00133751479168958 | 1 | T | T | F | F | mitotic spindle checkpoint | APC | ATM | AURKB | CDC20 | CENPF | IK | KLHL22 | MAD1L1 | MAD2L1 | PLK1 | SPDL1 | TPR | TRIP13 | ZNF207 | ZW10 | GO:0071108 | 0.002426301103967 | 20/8243 | 15/3250 | 0.00133751479168958 | 1 | T | T | F | F | protein K48-linked deubiquitination | CYLD | OTUB1 | OTUB2 | OTUD4 | OTUD5 | TNFAIP3 | USP17L2 | USP19 | USP20 | USP5 | USP50 | USP7 | USP8 | USP9X | VCPIP1 | GO:0032069 | 0.002426301103967 | 20/8243 | 15/3250 | 0.00133751479168958 | 1 | T | T | F | F | regulation of nuclease activity | ABCE1 | AKT1 | GZMA | HMGB1 | HMGB2 | NPM1 | OAS2 | PCNA | PRKCD | RPS3 | SIRT1 | TCEA1 | TERF1 | TERF2 | TMBIM6 | GO:0071173 | 0.002426301103967 | 20/8243 | 15/3250 | 0.00133751479168958 | 1 | T | T | F | F | spindle assembly checkpoint | APC | ATM | AURKB | CDC20 | CENPF | IK | KLHL22 | MAD1L1 | MAD2L1 | PLK1 | SPDL1 | TPR | TRIP13 | ZNF207 | ZW10 | GO:0007094 | 0.002426301103967 | 20/8243 | 15/3250 | 0.00133751479168958 | 1 | T | T | F | F | mitotic spindle assembly checkpoint | APC | ATM | AURKB | CDC20 | CENPF | IK | KLHL22 | MAD1L1 | MAD2L1 | PLK1 | SPDL1 | TPR | TRIP13 | ZNF207 | ZW10 | GO:1904064 | 0.00837073880868616 | 69/8243 | 40/3250 | 0.00134154397808587 | 1 | T | T | F | F | positive regulation of cation transmembrane transport | ABL1 | ACTN2 | ACTN4 | ADRB2 | AKAP9 | ANK3 | ARF1 | ATP1B3 | BAX | CACNG2 | CACNG4 | CALM1 | CALM2 | CALM3 | CHCHD10 | CHP1 | COX17 | CXCL11 | DMD | G6PD | GLRX | GSTO1 | HAP1 | HSPA2 | HTT | KCNIP2 | KIF5B | NOS1AP | PARK7 | PLCG1 | RGN | S100A1 | SLC9A1 | SNCA | SRI | STAC | STIM1 | SUMO1 | WNK1 | WNK4 | GO:1901570 | 0.00400339682154555 | 33/8243 | 22/3250 | 0.00138999671022725 | 1 | T | T | F | F | fatty acid derivative biosynthetic process | ACACA | ACAT1 | ACLY | ACSL1 | ACSL4 | ACSL5 | ALOX12 | ALOX5 | ALOX5AP | BDH1 | CBR4 | FAR2 | FASN | GCDH | GGT1 | GGTLC1 | HMGCL | HSD17B8 | LTC4S | PPT1 | SLC25A1 | TECR | GO:0006458 | 0.00400339682154555 | 33/8243 | 22/3250 | 0.00138999671022725 | 1 | T | T | F | F | 'de novo' protein folding | BAG1 | CCT2 | CD74 | DNAJB1 | DNAJC7 | FKBP1A | FKBP1B | GAK | HSPA14 | HSPA1B | HSPA1L | HSPA2 | HSPA5 | HSPA8 | HSPA9 | HSPD1 | HSPE1 | HSPH1 | PTGES3 | ST13 | TOR1A | UGGT1 | GO:0050804 | 0.0283877229164139 | 234/8243 | 115/3250 | 0.00139251371553203 | 1 | T | T | F | F | modulation of chemical synaptic transmission | ABAT | ABL1 | ADCYAP1 | ADORA2A | ADRB2 | AGER | AKAP9 | APOE | APP | ARF1 | ARRB2 | ATAD1 | ATP2A2 | BACE1 | BRAF | CACNA1A | CACNG2 | CACNG4 | CACNG5 | CALM1 | CALM3 | CAMK2A | CAMK2B | CDC20 | CDH1 | CDK5 | CEL | CHRM2 | CLSTN1 | CPLX1 | CPLX3 | CREB1 | DBN1 | DGKE | DLG4 | DNM1L | DVL1 | EGFR | EGLN1 | EIF2AK4 | EIF4A3 | EIF4E | EPHA4 | FLOT1 | FYN | GFAP | GIT1 | GLUL | GNAI2 | GRIA1 | GRID2 | GRIK2 | GSK3B | HAP1 | HRAS | HTR1B | ITPR3 | KIF5B | KRAS | LGMN | LRRK2 | MAPK1 | MAPT | MECP2 | MME | MPP2 | NCDN | NPTX1 | NRXN1 | NTF3 | NTRK1 | P2RX1 | PICK1 | PLA2G6 | PLCG1 | PREPL | PRKACA | PRKAR2B | PRKCE | PRKCZ | PSEN1 | PSMC5 | PTEN | PTN | PTPRA | RAB11A | RAB3GAP1 | RAB5A | RAP1A | RAP1B | RARA | RHOT1 | RNF19A | S100B | SEPT5 | SERPINE2 | SH3GL1 | SIPA1L1 | SNCA | SQSTM1 | SRC | STAT3 | STAU1 | STXBP1 | SYN1 | SYP | TNF | TOR1A | TUBB2B | USP46 | USP8 | VPS18 | YWHAG | YWHAH | ZMYND8 | GO:0046822 | 0.0106757248574548 | 88/8243 | 49/3250 | 0.00139657256458861 | 1 | T | T | F | F | regulation of nucleocytoplasmic transport | APOD | BAG3 | CD36 | CDH1 | CDK5 | CHP1 | DHX9 | DMAP1 | GSK3B | HDAC3 | IPO5 | JUP | KHDRBS1 | MAPK1 | MAPK14 | MAVS | MDM2 | NEDD4 | NUP153 | NUP214 | PARK7 | PIK3R1 | PKIG | PPM1A | PPP1CC | PPP1R12A | PRKACA | PRKCD | PSEN1 | PTPN14 | RAN | RANGAP1 | RBM4 | SFN | SMAD3 | SMO | SP100 | TARDBP | TGFB1 | TP53 | TPR | TRIM28 | TXN | UBR5 | XPO1 | XPO4 | XPO5 | YWHAE | ZIC1 | GO:1902533 | 0.0737595535605969 | 608/8243 | 275/3250 | 0.00143382931977613 | 1 | T | T | F | F | positive regulation of intracellular signal transduction | ABL1 | ACTN4 | ADA | ADCYAP1 | ADRB2 | AGER | AGO1 | AKR1C2 | AKT1 | AKT3 | APOA1 | APOE | APP | AR | ARRB1 | ARRB2 | ATM | AXIN1 | BAG4 | BAX | BCL10 | BCL2 | BCL2L1 | BIRC2 | BIRC3 | BIRC7 | BRAF | BRD4 | C1QBP | C1QTNF1 | CALM1 | CALM2 | CALM3 | CALR | CARD9 | CASP10 | CASP8 | CAT | CAV1 | CBL | CCDC22 | CCL21 | CCL22 | CCR1 | CD27 | CD36 | CD40 | CD40LG | CD74 | CD81 | CRK | CRKL | CTH | CTNNB1 | DAXX | DBNL | DCN | DDRGK1 | DDX1 | DHX36 | DNM1L | DUSP19 | DUSP22 | DUSP7 | DVL2 | DVL3 | EDA | EEF1D | EGFR | EP300 | EPHA4 | ERBB2 | ERBB3 | ESR1 | EZH2 | FADD | FASLG | FGB | FGF2 | FGFR1 | FGFR3 | FIS1 | FKBP1A | FYN | GADD45G | GJA1 | GNAI2 | GOLPH3 | GOLT1B | GPX1 | GRB2 | GSK3A | GSTO1 | HAP1 | HAVCR2 | HAX1 | HBEGF | HDAC3 | HEXIM1 | HINT1 | HMGB1 | HMOX1 | HRAS | HSP90AA1 | HSP90AB1 | HSPA1B | HTT | ICAM1 | IGF1R | IKBKB | IKBKE | IKBKG | ILK | INSR | IQGAP1 | ITGB1BP1 | JUN | KDR | KLHDC10 | KLHL22 | KRAS | KSR1 | LARS | LGALS1 | LIMS1 | LPAR1 | LPAR4 | LRRK1 | LRRK2 | LTBR | LURAP1 | MAP2K1 | MAP2K3 | MAP2K4 | MAP2K6 | MAP2K7 | MAP3K1 | MAP3K13 | MAP3K14 | MAP3K2 | MAP3K3 | MAP3K4 | MAP4K1 | MAPK1 | MAPK14 | MAPK3 | MAPK8IP3 | MAS1 | MAVS | MC1R | MIF | MLST8 | MYD88 | MYOC | NCK1 | NEDD4 | NELFE | NMNAT1 | NOD2 | NOS1AP | NPR1 | NQO2 | NTF3 | NTRK1 | P2RX2 | P2RX4 | PARK7 | PDCD10 | PDE6G | PEBP1 | PELI1 | PHB | PHB2 | PIH1D1 | PIK3CA | PIK3CG | PIK3R1 | PLA2G1B | PLA2G2A | PLA2G6 | PLEKHF1 | PPARD | PPM1A | PPP5C | PRDX2 | PRKAA1 | PRKCE | PRKCZ | PRKD1 | PRKD2 | PSAP | PSEN1 | PTEN | PTK2 | PTPN1 | PTPN22 | PTPN6 | PUM1 | RAC1 | RAF1 | RAP1A | RAP1B | RB1CC1 | RC3H1 | REL | RELA | RHOA | RIPK1 | RIPK2 | RIPK3 | RNF183 | RNF31 | RPL23 | RPS3 | RPTOR | RTN4 | S100A12 | S100A13 | S100A4 | S100A7 | S100A9 | S100B | SDCBP | SEC13 | SEMA7A | SEPT4 | SFPQ | SHC1 | SIAH1 | SIRT1 | SLAMF1 | SLC9A1 | SLC9A3R1 | SOD1 | SOX2 | SPRY2 | SRC | STK25 | STK3 | TAB1 | TAB2 | TAOK3 | TERF2IP | TFG | TGFB1 | TGM2 | THBS1 | TIMP2 | TLR3 | TMEM9B | TNF | TNFRSF1A | TNFSF14 | TNIP2 | TP53 | TP73 | TRADD | TRAF2 | TRAF6 | TRIM25 | TRIM32 | TRIP6 | TXN | UBB | UBC | UBE2I | UBE2N | UBE2V1 | USP17L2 | VAPA | VAV1 | WNT16 | ZAP70 | ZDHHC17 | ZNF622 | GO:0002220 | 0.0104330947470581 | 86/8243 | 48/3250 | 0.00145343420830408 | 1 | T | T | F | F | innate immune response activating cell surface receptor signaling pathway | BCL10 | BTRC | CARD9 | CD209 | CREBBP | CUL1 | EP300 | FBXW11 | FCN1 | FYN | HRAS | IKBKB | IKBKG | KRAS | PAK2 | PRKACA | PRKCD | PSMA1 | PSMA2 | PSMA3 | PSMA5 | PSMA6 | PSMB5 | PSMB6 | PSMB7 | PSMB8 | PSMC1 | PSMC2 | PSMC3 | PSMC4 | PSMC5 | PSMC6 | PSMD1 | PSMD10 | PSMD11 | PSMD12 | PSMD14 | PSMD2 | PSMD4 | PSMD9 | PSME2 | PSME3 | RAF1 | RELA | SRC | TAB1 | TAB2 | TRAF6 | GO:0032071 | 0.000849205386388451 | 7/8243 | 7/3250 | 0.00147531599635471 | 1 | T | T | F | F | regulation of endodeoxyribonuclease activity | AKT1 | GZMA | HMGB1 | NPM1 | PRKCD | RPS3 | SIRT1 | GO:0010870 | 0.000849205386388451 | 7/8243 | 7/3250 | 0.00147531599635471 | 1 | T | T | F | F | positive regulation of receptor biosynthetic process | HDAC1 | HDAC2 | HDAC6 | HIF1A | HNRNPK | HOXA5 | NR1H3 | GO:0006086 | 0.000849205386388451 | 7/8243 | 7/3250 | 0.00147531599635471 | 1 | T | T | F | F | acetyl-CoA biosynthetic process from pyruvate | DLAT | DLD | MPC2 | PDHA1 | PDHA2 | PDHB | PDHX | GO:0007205 | 0.0012131505519835 | 10/8243 | 9/3250 | 0.00147682499701499 | 1 | T | T | F | F | protein kinase C-activating G protein-coupled receptor signaling pathway | ADORA2A | AZU1 | DGKE | DGKZ | GAP43 | HTR1B | PARD3 | PICK1 | PRKD3 | GO:0006103 | 0.0012131505519835 | 10/8243 | 9/3250 | 0.00147682499701499 | 1 | T | T | F | F | 2-oxoglutarate metabolic process | DLD | DLST | GOT1 | GOT2 | GPT2 | IDH1 | IDH2 | L2HGDH | OGDH | GO:0070262 | 0.0012131505519835 | 10/8243 | 9/3250 | 0.00147682499701499 | 1 | T | T | F | F | peptidyl-serine dephosphorylation | DUSP1 | NCK1 | PPP1CA | PPP2CA | PPP2CB | PPP2R1A | PPP2R2A | PPP2R2D | PPP5C | GO:0071380 | 0.0012131505519835 | 10/8243 | 9/3250 | 0.00147682499701499 | 1 | T | T | F | F | cellular response to prostaglandin E stimulus | ACACA | ADCY6 | AKAP8 | AKT1 | GNB1 | PPARG | PRKAA1 | PRKAA2 | PRKCE | GO:1905564 | 0.0012131505519835 | 10/8243 | 9/3250 | 0.00147682499701499 | 1 | T | T | F | F | positive regulation of vascular endothelial cell proliferation | AKT3 | FGF2 | FGFR1 | HMGB1 | ITGA4 | PLCG1 | SIRT6 | SP1 | STAT3 | GO:0010952 | 0.0154070120101905 | 127/8243 | 67/3250 | 0.001478332017621 | 1 | T | T | F | F | positive regulation of peptidase activity | ADRM1 | AGER | AIFM1 | ALOX12 | APP | ARRB1 | BAX | BBC3 | BCL10 | BCL2L13 | CASP10 | CASP8 | CASP9 | CAV1 | CLPX | CYCS | DAPK1 | DDRGK1 | DDX3X | DIABLO | EGLN3 | EIF2AK3 | EPHA4 | FADD | FASLG | FIS1 | FN1 | FYN | HMGB1 | HSF1 | HSPD1 | HSPE1 | HTRA2 | IFI16 | LGMN | MAPK14 | MAPK3 | MAPT | MBP | MYC | NDUFA13 | P2RX1 | PDCD6 | PML | PPARG | PRELID1 | PSMD14 | PSME2 | PSME3 | REST | RHOA | RIPK1 | RIPK2 | RPS3 | S100A9 | SERPINB3 | SIRT1 | SMAD3 | SNCA | STAT3 | TANK | TNF | TRADD | TRAF2 | USP50 | VCP | WDR35 | GO:0090559 | 0.00740021836709936 | 61/8243 | 36/3250 | 0.00148340356000185 | 1 | T | T | F | F | regulation of membrane permeability | ACAA2 | ATF2 | BAX | BBC3 | BCL2 | BCL2L1 | CAMK2A | CASP8 | CHCHD10 | E2F1 | EYA2 | GSK3A | GSK3B | HK2 | MAPK8 | PDCD6IP | PPIF | PPP3CC | RHOT1 | RHOT2 | SFN | SLC25A5 | SLC35F6 | SLC9A1 | STAT3 | TFDP1 | THEM4 | TP53 | TP63 | TP73 | YWHAB | YWHAE | YWHAG | YWHAH | YWHAQ | YWHAZ | GO:0030308 | 0.0122528205750334 | 101/8243 | 55/3250 | 0.00148366875047918 | 1 | T | T | F | F | negative regulation of cell growth | APBB1 | APBB2 | BCL11A | BCL2 | BCL6 | CCAR2 | CDA | CDK5 | CDK5R1 | CDKL3 | CDKN1A | CDKN2C | CRYAB | CTDP1 | DDX3X | DNAJB2 | EAF2 | ENO1 | ESR2 | FBP1 | FHL1 | G6PD | GDF9 | GJA1 | GSK3A | HNF4A | HSPA1B | ING1 | MYL2 | NDUFA13 | NDUFS3 | NPR1 | PHB | PML | PPARD | PPARG | PPP2CA | PPP2R1A | PPT1 | RGS2 | RTN4 | SEMA7A | SERPINE2 | SIPA1 | SIRT1 | SMAD3 | SMAD4 | SMARCA2 | SMARCA4 | STK11 | TGFB1 | TP53 | TSPYL2 | WT1 | YY1 | GO:1901028 | 0.00448865704233895 | 37/8243 | 24/3250 | 0.00151232209809998 | 1 | T | T | F | F | regulation of mitochondrial outer membrane permeabilization involved in apoptotic signaling pathway | ACAA2 | BAX | BBC3 | BCL2 | CASP8 | CHCHD10 | E2F1 | GSK3A | GSK3B | MAPK8 | PPP3CC | SFN | SLC25A5 | SLC35F6 | TFDP1 | TP53 | TP63 | TP73 | YWHAB | YWHAE | YWHAG | YWHAH | YWHAQ | YWHAZ | GO:2000113 | 0.106999878684945 | 882/8243 | 389/3250 | 0.00155129791395867 | 1 | T | T | F | F | negative regulation of cellular macromolecule biosynthetic process | ACD | AEBP2 | AES | AGO1 | AGO2 | APBB1 | AR | ARHGAP35 | ASCL1 | ATF2 | ATRX | ATXN1 | AURKB | BACH2 | BAG3 | BASP1 | BCL11A | BCL6 | BCL6B | BCOR | BIRC5 | BMI1 | BRCA1 | BTRC | C1QBP | CALR | CAV1 | CBFA2T3 | CBFB | CBX4 | CBX7 | CBX8 | CBY1 | CCAR2 | CD36 | CDC73 | CDK2 | CDK5 | CDK5R1 | CEBPA | CENPF | CHCHD3 | CHD8 | CHEK2 | CIRBP | COPS2 | CREB1 | CREBBP | CRY1 | CRY2 | CTBP1 | CTCF | CTNNB1 | CTNNBIP1 | CUL3 | DAB2 | DAPK1 | DAXX | DCP2 | DDX3X | DHX36 | DMAP1 | DNAJA3 | DNAJB1 | DPF2 | DUSP1 | DUSP22 | E2F1 | E2F2 | E2F3 | E2F4 | E2F6 | EED | EHMT1 | EHMT2 | EID1 | EIF2AK3 | EIF2AK4 | EIF2S1 | EIF3E | EIF4A3 | EIF4E | EIF4E2 | EIF4EBP1 | EIF4ENIF1 | EIF6 | ELF3 | ENO1 | EP300 | EPRS | ERF | ESR1 | ESR2 | ESRRA | EZH2 | EZR | FAM129B | FASLG | FBP1 | FBXW11 | FGFR1 | FHL2 | FOXA1 | FOXJ1 | FOXK1 | FOXM1 | FOXO1 | FTO | FXR2 | GAPDH | GATA1 | GPS2 | GRB7 | GSK3A | GSK3B | H1F0 | H3F3A | HBEGF | HBZ | HDAC1 | HDAC2 | HDAC3 | HDAC4 | HDAC5 | HDAC6 | HDAC7 | HDAC9 | HDGF | HEXIM1 | HIF1AN | HIST1H3A | HIST1H4A | HIST2H3A | HIVEP1 | HMGB1 | HMGB2 | HNRNPA1 | HNRNPA2B1 | HNRNPD | HNRNPU | HSF1 | HSF4 | HSPA8 | ID2 | IFI16 | IFNA2 | IGBP1 | IGF2BP1 | IKZF5 | ILF3 | IREB2 | IRF1 | IRF7 | JAK3 | JUN | JUNB | JUND | KANK2 | KAT5 | KAT7 | KAT8 | KDM1A | KDM5B | KDM5C | KHDRBS1 | KLF5 | L3MBTL1 | LEF1 | LGR4 | LIMS1 | LOXL2 | MAF1 | MBD3 | MDFI | MDM2 | MDM4 | MECP2 | MED1 | MEPCE | MITF | MORF4L1 | MORF4L2 | MOV10 | MSH6 | MTA1 | MTA3 | MXD1 | MYBBP1A | MYC | NACC1 | NCK1 | NCOA2 | NCOR1 | NCOR2 | NDUFA13 | NEDD4 | NELFB | NELFCD | NELFE | NFATC2 | NIPBL | NKRF | NKX2-1 | NOSTRIN | NR0B2 | NR1H2 | NR1H3 | NR1I2 | NR3C1 | PA2G4 | PARP1 | PATZ1 | PAWR | PAX9 | PCGF1 | PCGF6 | PER1 | PER2 | PHB | PHB2 | PHF10 | PHF21A | PIAS1 | PIAS3 | PINX1 | PKIG | PLK1 | PML | POT1 | POU5F1 | PPARD | PPARG | PPID | PPM1A | PPM1D | PRDX5 | PRMT2 | PRMT6 | PROX1 | PSEN1 | PSMC5 | PSMD10 | PTPRK | PUM1 | PUS7 | QARS | RARA | RARG | RASD1 | RB1 | RBM3 | RBM4 | RBPJ | RC3H1 | REL | RELA | REST | RFC1 | RGN | RGS2 | RIF1 | RING1 | RNF139 | RNF2 | RNF8 | RPL10 | RPL23 | RPS14 | RPS3 | RTF1 | RUNX1 | RUNX1T1 | RUNX2 | RXRA | S100A1 | S100A11 | SARS | SET | SFMBT1 | SFPQ | SHC1 | SHMT1 | SIN3A | SIRT1 | SIRT5 | SIRT6 | SIRT7 | SKI | SMAD2 | SMAD3 | SMAD4 | SMAD5 | SMARCA2 | SMARCA4 | SMARCC2 | SMARCE1 | SMC1A | SMC3 | SMO | SMURF2 | SNAI2 | SNCA | SNW1 | SNX6 | SOX2 | SP100 | SP2 | SPEN | SPI1 | SQSTM1 | SRC | STAG2 | STAT1 | STAT3 | STAT6 | STRAP | SUMO1 | SUPT4H1 | SUPT5H | SUZ12 | TAGLN3 | TBL1X | TCERG1 | TCF21 | TCF25 | TCP10L | TERF1 | TERF2 | TGFB1 | THAP11 | THAP7 | THRA | TIA1 | TINF2 | TLE1 | TMBIM6 | TNF | TNP1 | TP53 | TP63 | TPR | TRAF6 | TRIB3 | TRIM28 | TRIM29 | TRIM33 | TSC22D4 | TSG101 | TSPYL2 | TWIST1 | TXN | TXNIP | TYMS | UBA3 | UBB | UBC | UBE2D1 | UBE2D3 | UBE2I | UNK | USP2 | USP47 | USP9X | VDR | VHL | VPS72 | WDTC1 | WT1 | WWOX | WWP2 | XPO1 | XRCC5 | XRCC6 | YAF2 | YEATS2 | YWHAB | YWHAQ | YY1 | ZBTB16 | ZBTB21 | ZBTB33 | ZBTB42 | ZBTB7A | ZBTB7B | ZC3H4 | ZHX1 | ZNF217 | ZNF24 | ZNF746 | GO:0006974 | 0.0640543491447289 | 528/8243 | 241/3250 | 0.00155460077560908 | 1 | T | T | F | F | cellular response to DNA damage stimulus | ABL1 | ACD | ACTL6A | AKT1 | APBB1 | APC | APEX1 | APEX2 | ATAD5 | ATF2 | ATM | ATRX | BABAM1 | BAG6 | BAX | BCL2 | BCL2L1 | BCL2L2 | BCL6 | BRCA1 | BRD4 | CARM1 | CASP2 | CASP3 | CASP9 | CBL | CCAR2 | CCDC155 | CCNB1 | CDC14B | CDC25C | CDC5L | CDK2 | CDK9 | CDKN1A | CHAF1A | CHD1L | CHEK2 | COPS2 | COPS5 | COPS6 | CRIP1 | CRY1 | CRY2 | CSNK1E | CUL4B | DDB1 | DDB2 | DDIT4 | DDX1 | DMAP1 | DNA2 | DNAJA1 | DYRK2 | E2F1 | E2F4 | EGLN3 | EIF2AK4 | EP300 | ERCC5 | EYA2 | EYA4 | FANCA | FANCC | FANCG | FANCI | FANCM | FBXO6 | FEN1 | FOXM1 | FOXO1 | FZR1 | GINS2 | GINS4 | GNL1 | GRB2 | H2AFX | HERC2 | HIST1H4A | HIST3H3 | HMOX1 | HSF1 | HTRA2 | HUWE1 | IFI16 | IKBKE | IKBKG | INO80B | INO80C | IRF7 | ISG15 | KAT5 | KDM2A | KDM4D | LIG4 | MAP2K6 | MAPK1 | MAPK14 | MAPK15 | MAPK3 | MC1R | MCM7 | MCM9 | MCTS1 | MDC1 | MDM2 | MDM4 | MGMT | MICA | MIF | MLH1 | MORF4L1 | MORF4L2 | MPG | MSH6 | MTA1 | MYC | MYO6 | NDRG1 | NEDD4 | NEK1 | NFATC2 | NIPBL | NPLOC4 | NPM1 | NSMCE4A | NUPR1 | OTUB1 | PALB2 | PARK7 | PARP1 | PAXIP1 | PCNA | PIK3R1 | PLK1 | PML | PNKP | POLD1 | POLE2 | POLE3 | POLM | POLR2A | POLR2E | POLR2L | PPIE | PPM1D | PPP5C | PRKDC | PRMT6 | PRPF19 | PSEN1 | PSMD14 | PTTG1 | RAD21 | RAD23A | RAD23B | RAD51 | RAD51AP1 | RBBP6 | RBM17 | RBX1 | RFC1 | RFC2 | RFC3 | RFC4 | RFC5 | RIF1 | RNASEH2A | RNF8 | RPA2 | RPA3 | RPAIN | RPS3 | RRM2B | RUVBL1 | RUVBL2 | SFN | SFPQ | SIRT1 | SIRT6 | SMC1A | SMC3 | SNW1 | SP100 | SPRTN | STK11 | STUB1 | SUMO1 | TANK | TAOK3 | TCEA1 | TDP1 | TERF2 | TERF2IP | TFDP1 | TFIP11 | TMEM189-UBE2V1 | TNF | TNFRSF1A | TNP1 | TOP2A | TP53 | TP63 | TP73 | TRAF6 | TREX1 | TRIAP1 | TRIM25 | TRIM28 | TRIP12 | TRIP13 | TTC5 | UBA1 | UBB | UBC | UBE2A | UBE2B | UBE2D3 | UBE2L6 | UBE2N | UBE2V1 | UBE2W | UBR5 | UCHL5 | USP16 | USP28 | USP47 | USP7 | VCP | WDR48 | XIAP | XRCC1 | XRCC3 | XRCC5 | XRCC6 | YAP1 | YY1 | ZBTB42 | ZBTB7A | ZFYVE26 | ZMPSTE24 | GO:0019395 | 0.00667232803590926 | 55/8243 | 33/3250 | 0.00156696644023611 | 1 | T | T | F | F | fatty acid oxidation | ABCD3 | ACAA1 | ACAA2 | ACADS | ACADVL | ACAT1 | ACAT2 | ACOX1 | ALOX12 | CPT1A | CPT2 | CROT | DECR1 | ECH1 | ECHDC2 | ECHS1 | ECI1 | ECI2 | EHHADH | ETFA | ETFB | ETFDH | GCDH | HADH | HIBCH | HSD17B4 | IVD | MAPK14 | MCAT | PPARD | PPARG | PPARGC1A | PRKAA1 | GO:0032355 | 0.00994783452626471 | 82/8243 | 46/3250 | 0.00157145533228411 | 1 | T | T | F | F | response to estradiol | AIFM1 | ANXA1 | ARNT2 | ARPC1B | ASS1 | CALR | CASP3 | CASP8 | CASP9 | CAT | CRYAB | CSN1S1 | CTNNB1 | CYP1A2 | DUSP1 | EEF2 | EGFR | ENDOG | ESR1 | ESRRA | ETS1 | EZH2 | FOXA1 | GGT1 | GPI | GPX4 | GSTP1 | H2AFZ | HNRNPD | HSF1 | KAT5 | MBD3 | NCOA1 | NCOA3 | NQO1 | PCNA | PPARGC1A | PTEN | PTN | RARA | RUVBL2 | SOCS2 | STAT3 | STXBP1 | TGFB1 | TXNIP | GO:0001817 | 0.0452505155889846 | 373/8243 | 175/3250 | 0.00157387906104628 | 1 | T | T | F | F | regulation of cytokine production | ABL1 | ACE2 | ADCYAP1 | AGER | AKAP8 | ANXA1 | APOA1 | APOD | APP | ARNT | ARRB1 | ARRB2 | ATF2 | ATG5 | AZU1 | BCL10 | BCL6 | BIRC2 | BIRC3 | BRCA1 | BTK | C1QBP | CAPN2 | CARD9 | CD2 | CD36 | CD40 | CD40LG | CD6 | CD74 | CLU | CREB1 | CREBBP | CRP | CTNNB1 | CXCL6 | CYLD | DDX1 | DDX3X | DHX36 | DHX9 | EIF2AK3 | EP300 | ERRFI1 | EZR | FADD | FAM49B | FCN1 | FGFR1 | FLOT1 | FN1 | FOXJ1 | GAPDH | GBP5 | GLMN | GSTP1 | HAVCR2 | HDAC2 | HDAC7 | HDAC9 | HIF1A | HK1 | HMGB1 | HMGB2 | HMOX1 | HRAS | HSF1 | HSP90AB1 | HSPA1B | HSPB1 | HSPD1 | IFI16 | IFNA2 | IGF2BP1 | IKBKE | IQGAP1 | IRF1 | IRF4 | IRF7 | ISG15 | ITCH | ITGAV | JAK3 | KAT5 | LEF1 | LGR4 | LRRK2 | LTBP1 | LUM | LURAP1 | MAP2K3 | MAPK14 | MAPK3 | MAVS | MBP | MC1R | MIF | MYD88 | NFKB2 | NOD2 | NOS2 | NPLOC4 | ORM1 | OTUD5 | PANX3 | PARK7 | PCBP2 | PDE4D | PELI1 | PER1 | PIK3R1 | PIN1 | PML | POLR2E | POLR2L | PPM1B | PPP1R11 | PRKCZ | PRKD2 | PRKDC | PTPN22 | PTPN6 | PUM1 | RAC1 | RARA | REL | RELA | RIPK1 | RIPK2 | RIPK3 | RPS3 | RUNX1 | SARS | SEMA7A | SLAMF1 | SMAD3 | SMAD4 | SNAI2 | SOCS1 | SOD1 | SPTBN1 | SRC | STAT1 | STAT3 | STAT6 | SULF2 | TBC1D23 | TGFB1 | THBS1 | TIA1 | TLR3 | TMEM173 | TNF | TNFAIP3 | TNFRSF8 | TRAF2 | TRAF3 | TRAF3IP1 | TRAF6 | TREX1 | TRIB2 | TRIM21 | TRIM25 | TRIM32 | TWIST1 | UBASH3A | UBE2J1 | UBE2L6 | USP17L2 | USP50 | VSIG4 | XBP1 | XRCC5 | XRCC6 | YY1 | GO:0044839 | 0.0135872861822152 | 112/8243 | 60/3250 | 0.00157863324454815 | 1 | T | T | F | F | cell cycle G2/M phase transition | ACTR1A | AKAP8 | AKAP9 | BTRC | CALM2 | CALM3 | CCNB1 | CCNY | CCP110 | CDC25A | CDC25B | CDC25C | CDK2 | CDKN1A | CENPJ | CEP135 | CEP152 | CEP290 | CEP57 | CEP70 | CHEK2 | CKAP5 | CNTRL | CSNK1E | CUL1 | DCTN1 | DYNC1H1 | DYNC1I2 | DYNLL1 | FBXL12 | FBXW11 | FGFR1OP | FOXM1 | HSP90AA1 | KDM8 | KHDRBS1 | LATS1 | MELK | OFD1 | OPTN | PAFAH1B1 | PCM1 | PLK1 | PPM1D | PPME1 | PPP1CB | PPP1R12A | PPP1R12B | PPP2R1A | PPP2R2A | PRKACA | PRKAR2B | TUBA1A | TUBA4A | TUBB | TUBB4A | TUBB4B | TUBG1 | YWHAE | YWHAG | GO:0045841 | 0.0026689312143637 | 22/8243 | 16/3250 | 0.00158482538646846 | 1 | T | T | F | F | negative regulation of mitotic metaphase/anaphase transition | APC | ATM | AURKB | CDC20 | CENPF | IK | KLHL22 | MAD1L1 | MAD2L1 | PLK1 | RAD21 | SPDL1 | TPR | TRIP13 | ZNF207 | ZW10 | GO:0043537 | 0.0026689312143637 | 22/8243 | 16/3250 | 0.00158482538646846 | 1 | T | T | F | F | negative regulation of blood vessel endothelial cell migration | APOE | CSNK2B | FGF2 | HDAC5 | HMGB1 | ITGB1BP1 | JUP | MECP2 | MEOX2 | PDCD10 | PPARG | RHOA | STARD13 | TBXA2R | TGFB1 | THBS1 | GO:1902100 | 0.0026689312143637 | 22/8243 | 16/3250 | 0.00158482538646846 | 1 | T | T | F | F | negative regulation of metaphase/anaphase transition of cell cycle | APC | ATM | AURKB | CDC20 | CENPF | IK | KLHL22 | MAD1L1 | MAD2L1 | PLK1 | RAD21 | SPDL1 | TPR | TRIP13 | ZNF207 | ZW10 | GO:0070542 | 0.00618706781511586 | 51/8243 | 31/3250 | 0.00160375538868701 | 1 | T | T | F | F | response to fatty acid | ACACA | ACSL1 | ADCY6 | AKAP8 | AKR1C2 | AKT1 | ASS1 | CAT | CCL21 | CCNB1 | CD36 | CDK4 | CPT1A | CREB1 | E2F1 | FABP3 | GNB1 | HMGCL | INSIG2 | NME1 | NME2 | PPARG | PPARGC1A | PPP5C | PRKAA1 | PRKAA2 | PRKCE | SRC | TGFBR3 | XRCC5 | YY1 | GO:0051851 | 0.00618706781511586 | 51/8243 | 31/3250 | 0.00160375538868701 | 1 | T | T | F | F | modification by host of symbiont morphology or physiology | ANXA2 | APCS | APOE | AZU1 | CALM1 | CALM2 | CALM3 | CCNT1 | CFL1 | CTDP1 | EIF2AK4 | EP300 | GAPDH | HDAC1 | JUN | LEF1 | PC | POU2F3 | PPIB | PSMC3 | RAB9A | REST | SMARCA4 | SMARCB1 | SMC3 | SNW1 | SP1 | STOM | SUGT1 | TARDBP | VAPA | GO:0032981 | 0.00521654737352906 | 43/8243 | 27/3250 | 0.00160492635630224 | 1 | T | T | F | F | mitochondrial respiratory chain complex I assembly | ACAD9 | BCS1L | ECSIT | NDUFA10 | NDUFA13 | NDUFA5 | NDUFA8 | NDUFA9 | NDUFAF1 | NDUFAF2 | NDUFAF3 | NDUFAF4 | NDUFAF5 | NDUFAF7 | NDUFB1 | NDUFS1 | NDUFS2 | NDUFS3 | NDUFS6 | NDUFS7 | NDUFS8 | NDUFV1 | NDUFV3 | OXA1L | TAZ | TIMM21 | TIMMDC1 | GO:0008637 | 0.00521654737352906 | 43/8243 | 27/3250 | 0.00160492635630224 | 1 | T | T | F | F | apoptotic mitochondrial changes | ACAA2 | AKT1 | ATF2 | BAX | BBC3 | BCL2 | BCL2L1 | CAMK2A | CCAR2 | CLU | DNM1L | EYA2 | FIS1 | GGCT | HK2 | HSPD1 | JUN | NDUFS1 | PPIF | PPP2CB | RHOT1 | RHOT2 | SFN | SLC25A4 | SOD2 | THEM4 | TIMM50 | GO:0010257 | 0.00521654737352906 | 43/8243 | 27/3250 | 0.00160492635630224 | 1 | T | T | F | F | NADH dehydrogenase complex assembly | ACAD9 | BCS1L | ECSIT | NDUFA10 | NDUFA13 | NDUFA5 | NDUFA8 | NDUFA9 | NDUFAF1 | NDUFAF2 | NDUFAF3 | NDUFAF4 | NDUFAF5 | NDUFAF7 | NDUFB1 | NDUFS1 | NDUFS2 | NDUFS3 | NDUFS6 | NDUFS7 | NDUFS8 | NDUFV1 | NDUFV3 | OXA1L | TAZ | TIMM21 | TIMMDC1 | GO:0006635 | 0.00545917748392576 | 45/8243 | 28/3250 | 0.0016159179643859 | 1 | T | T | F | F | fatty acid beta-oxidation | ABCD3 | ACAA1 | ACAA2 | ACADS | ACADVL | ACAT1 | ACAT2 | ACOX1 | CPT1A | CPT2 | CROT | DECR1 | ECH1 | ECHDC2 | ECHS1 | ECI1 | ECI2 | EHHADH | ETFA | ETFB | ETFDH | GCDH | HADH | HIBCH | HSD17B4 | IVD | MCAT | PPARD | GO:1990542 | 0.00570180759432246 | 47/8243 | 29/3250 | 0.00161874362781729 | 1 | T | T | F | F | mitochondrial transmembrane transport | ACACA | AFG3L2 | CPT1A | CPT2 | CYC1 | DNAJC19 | GRPEL1 | HSPD1 | LETM1 | MICU1 | MICU2 | MPC2 | OPA1 | PAM16 | PRKAA2 | PRKAB2 | ROMO1 | SFXN1 | SLC25A1 | SLC25A13 | SLC25A22 | SLC25A32 | STOML2 | TIMM21 | TIMM23 | TIMM44 | TIMM50 | TOMM20 | TOMM40 | GO:0044264 | 0.00570180759432246 | 47/8243 | 29/3250 | 0.00161874362781729 | 1 | T | T | F | F | cellular polysaccharide metabolic process | AGL | AKT1 | AKT2 | B3GNT3 | CALM1 | GAA | GBE1 | GSK3A | GSK3B | GYG1 | GYG2 | GYS1 | HS2ST1 | PER2 | PFKM | PGM1 | PGM2 | PHKA1 | PPP1CA | PPP1CB | PPP1CC | PPP1R2 | PPP1R3B | PPP1R3C | PYGB | PYGL | STBD1 | STK40 | UGP2 | GO:1903749 | 0.00570180759432246 | 47/8243 | 29/3250 | 0.00161874362781729 | 1 | T | T | F | F | positive regulation of establishment of protein localization to mitochondrion | BBC3 | BCL2 | CASP8 | CSNK2A2 | E2F1 | GSK3A | HSPA1L | HTRA2 | HUWE1 | MAPK8 | NPEPPS | PPP3CC | PRKAA1 | RHOU | RNF31 | SAE1 | SFN | TFDP1 | TP53 | TP63 | TP73 | UBE2D3 | UBE2L3 | YWHAB | YWHAE | YWHAG | YWHAH | YWHAQ | YWHAZ | GO:0051881 | 0.00570180759432246 | 47/8243 | 29/3250 | 0.00161874362781729 | 1 | T | T | F | F | regulation of mitochondrial membrane potential | ABL1 | ADORA2A | AKT1 | AKT2 | ALOX12 | BAX | BCL2 | BCL2L1 | CLIC1 | DCN | GOT1 | KDR | LRRK2 | MAPT | MLLT11 | MYOC | NDUFS1 | NNT | PARK7 | PARP1 | PPA2 | PRDX3 | PRELID1 | PYCR1 | SOD1 | SRC | STOML2 | UBB | VCP | GO:0031328 | 0.150066723280359 | 1237/8243 | 535/3250 | 0.00163232583617906 | 1 | T | T | F | F | positive regulation of cellular biosynthetic process | ABHD14B | ABL1 | ACD | ACTA2 | ACTL6A | ACTN1 | ACTN2 | ACTN4 | ACTR2 | ACTR3 | ADCYAP1 | ADRB2 | AGAP2 | AGER | AGO1 | AGO2 | AHI1 | AICDA | AIRE | AKT1 | AKT2 | ANKRD49 | ANXA1 | APBB1 | APEX1 | APOE | APP | AR | ARF4 | ARID3B | ARID4B | ARNT | ARNT2 | ARRB1 | ARRB2 | ASCL1 | ASS1 | ATAD5 | ATF2 | ATF6 | ATF6B | ATM | ATOH1 | ATRX | AURKB | AXIN1 | AZU1 | BANP | BCL10 | BIRC2 | BMPR1A | BRCA1 | BRD4 | BTRC | BUD31 | C1QBP | CAND1 | CAPN2 | CARM1 | CBFB | CCAR2 | CCL21 | CCNT1 | CCNT2 | CCT2 | CCT3 | CCT4 | CCT5 | CCT6A | CCT7 | CCT8 | CD36 | CD40 | CD74 | CD81 | CDC5L | CDC73 | CDH1 | CDK2 | CDK4 | CDK8 | CDK9 | CEBPA | CEBPE | CEP290 | CHCHD10 | CHCHD2 | CHD8 | CHEK2 | CHP1 | CIRBP | CLU | COA3 | COPS5 | CREB1 | CREB3 | CREBBP | CTCF | CTNNB1 | DAB2 | DAXX | DBF4B | DCN | DDRGK1 | DDX39B | DDX3X | DHX36 | DHX9 | DNA2 | DPF2 | DTX1 | DVL1 | DVL2 | DVL3 | DYRK2 | E2F1 | E2F2 | E2F3 | E2F4 | E2F6 | EAF2 | EBF1 | ECD | EEF2 | EGFR | EGLN1 | EIF2AK3 | EIF2AK4 | EIF2B5 | EIF3D | EIF3E | EIF4A3 | EIF5A | EIF6 | ELAVL1 | ELF3 | ELF4 | ENO1 | EP300 | ERBB2 | ERG | ERLIN2 | ESR1 | ESR2 | ESRRA | ESRRB | ESRRG | ETS1 | EXOSC9 | FABP3 | FADD | FAF1 | FAM129B | FBXW11 | FGF2 | FGFR1 | FHL2 | FOS | FOXA1 | FOXJ1 | FOXK1 | FOXK2 | FOXM1 | FOXO1 | FUBP3 | FUS | FXR2 | GABPB1 | GATA1 | GLMN | GOLGA2 | GPS2 | GRIP1 | GTF2A1 | GTF2F1 | GTF2F2 | H1F0 | H1FX | H2AFZ | HAX1 | HDAC1 | HDAC2 | HDAC3 | HDAC4 | HDAC5 | HELZ2 | HEXB | HIF1A | HIST1H1A | HMGB1 | HMGB2 | HMGB3 | HMOX1 | HNF1A | HNF4A | HNF4G | HNRNPA1 | HNRNPA2B1 | HNRNPAB | HNRNPD | HNRNPK | HNRNPL | HNRNPU | HOXA5 | HOXB5 | HOXB9 | HOXD10 | HRAS | HSF1 | HSF4 | HSP90AA1 | HSP90AB1 | HSPA5 | HSPB1 | HTATIP2 | ICAM1 | ID2 | IFI16 | IFNA2 | IKBKB | IKBKG | IKZF5 | ILF2 | ILF3 | ILK | ING1 | ING5 | INSIG1 | INSIG2 | INSR | IRF1 | IRF4 | IRF7 | ITGB1BP1 | JMJD6 | JUN | JUNB | JUND | JUP | KAT5 | KAT7 | KAT8 | KDM1A | KDM8 | KHDRBS1 | KLF5 | KLF6 | KMT2A | KMT2B | KMT2C | LARP4 | LEF1 | LGR4 | LMO2 | LMO7 | LPIN3 | LRP6 | LUM | MAP2K1 | MAP2K3 | MAP2K4 | MAP2K7 | MAP3K2 | MAP3K4 | MAPK1 | MAPK14 | MAPK15 | MAPK3 | MAPK7 | MAS1 | MAVS | MC1R | MECP2 | MED1 | MED12 | MED14 | MED17 | MED20 | MED21 | MED23 | MED24 | MED31 | MED4 | MED6 | MEOX2 | METTL23 | MITF | MLLT11 | MLLT3 | MORF4L2 | MRPL12 | MTA1 | MTA3 | MYC | MYO6 | NAMPT | NANOG | NCK1 | NCOA1 | NCOA2 | NCOA3 | NCOA4 | NEK7 | NELFE | NEUROD1 | NFATC1 | NFATC2 | NFE2L2 | NFKB2 | NFKBIA | NIF3L1 | NIPBL | NKRF | NKX2-1 | NMD3 | NME2 | NOD2 | NOS1AP | NOS2 | NOS3 | NPM1 | NR1H2 | NR1H3 | NR1I2 | NR3C1 | NR4A1 | NR5A2 | NUFIP1 | NUP85 | NUP98 | NUPR1 | OGT | ONECUT1 | P2RX4 | PABPC1 | PAIP1 | PARK7 | PARP1 | PATZ1 | PAWR | PAX8 | PAX9 | PAXIP1 | PCBD1 | PCBP1 | PCGF5 | PCNA | PER1 | PER2 | PFKM | PHB | PHF10 | PIAS1 | PIH1D1 | PIK3R1 | PIN1 | PLA2G1B | PLA2G6 | PLSCR1 | PML | PNKP | POLDIP3 | POT1 | POU2F3 | POU5F1 | PPARD | PPARG | PPARGC1A | PPM1A | PPP1R12A | PRKAA1 | PRKCD | PRKD1 | PRKD2 | PRKDC | PRMT2 | PROX1 | PSEN1 | PSMC2 | PSMC3 | PSMC4 | PSMC5 | PSMC6 | PSMD9 | PTGES2 | PTGES3 | RAB3GAP1 | RAC1 | RAF1 | RAN | RARA | RARG | RB1 | RBM3 | RBMX | RBPJ | REL | RELA | REST | RFC1 | RFC2 | RFC3 | RFC4 | RFC5 | RFXANK | RGN | RHOA | RIPK1 | RIPK2 | RIPK3 | RMND1 | RNF4 | RPS4X | RPS6KA1 | RPS6KB2 | RPTOR | RTF1 | RUNX1 | RUNX2 | RUVBL1 | RUVBL2 | RXRA | RXRB | RXRG | SFPQ | SHC1 | SIN3A | SIRT1 | SIRT7 | SKI | SLC9A1 | SMAD2 | SMAD3 | SMAD4 | SMAD5 | SMAD9 | SMARCA2 | SMARCA4 | SMARCB1 | SMARCC1 | SMARCC2 | SMARCE1 | SMO | SNCA | SNW1 | SOAT1 | SOX2 | SP1 | SP100 | SPI1 | SPIN1 | SQSTM1 | SRC | STAT1 | STAT3 | STAT4 | STAT6 | STOML2 | SUB1 | SUMO2 | SUPT4H1 | SUPT5H | TADA2B | TAF12 | TAF15 | TARDBP | TAZ | TBL1X | TCEA2 | TCERG1 | TCF12 | TCF21 | TCF4 | TCP1 | TDRD3 | TFCP2 | TFDP1 | TGFB1 | THBS1 | THRA | TLR3 | TMEM173 | TNF | TNFRSF1A | TNFRSF8 | TNIP2 | TNKS | TNKS2 | TOP2A | TP53 | TP63 | TP73 | TRAF6 | TRIAP1 | TRIM28 | TRIM32 | TRIP4 | TRUB2 | TSG101 | TTC5 | TWIST1 | UBB | UBC | UBE2L3 | UBE2V1 | UPF3B | UQCC1 | USP16 | USP21 | VCP | VDR | VHL | VIM | WAS | WBP2 | WDR77 | WNT2 | WT1 | WWOX | WWP2 | XBP1 | XRCC5 | XRCC6 | YAF2 | YAP1 | YES1 | YTHDF1 | YWHAH | YY1 | ZBTB16 | ZBTB17 | ZBTB7B | ZIC1 | ZNF24 | ZNF746 | GO:0071236 | 0.00970520441586801 | 80/8243 | 45/3250 | 0.00163244340899687 | 1 | T | T | F | F | cellular response to antibiotic | ABL1 | ACTR2 | ADCY6 | AIFM1 | ANXA1 | APEX1 | CBX8 | CFTR | CRIP1 | DAXX | ETS1 | EZH2 | FABP1 | FOXO1 | GNAI1 | HDAC2 | HDAC6 | HNRNPA1 | HSF1 | HSPA5 | ITPR2 | MAPK7 | MDM2 | NFE2L2 | NQO1 | PARK7 | PCNA | PPIF | PRKAA1 | PRKCD | PRKCE | PTEN | RELA | RIPK1 | RIPK3 | RPL23 | RPS3 | SIRT1 | SLC9A1 | SPI1 | SRC | STAT6 | TNFAIP3 | TP53 | ZNF277 | GO:0007162 | 0.0175906830037608 | 145/8243 | 75/3250 | 0.00164403045593689 | 1 | T | T | F | F | negative regulation of cell adhesion | ABAT | ABL1 | ACTN4 | ADORA2A | ADTRP | AKT1 | ALOX12 | ANXA1 | APOA1 | APOD | ARHGDIA | ASS1 | BCL6 | C1QTNF1 | CASP3 | CBFB | CCL21 | CD74 | CDH1 | DAB1 | DTX1 | DUSP1 | DUSP22 | ERBB2 | ERBB3 | FOXJ1 | GLMN | HAVCR2 | HLX | HMGB1 | IFNA2 | IRF1 | ITCH | ITGB1BP1 | JAK3 | LGALS1 | LGALS3 | MAD1L1 | MAPK7 | MBP | MYOC | NF2 | PAG1 | PAWR | PELI1 | PIK3R1 | PLXNA1 | PLXNA2 | PLXNB2 | PRDX2 | PRKAR1A | PRKCD | PTEN | PTK2 | PTPN22 | PTPN6 | PTPRO | RC3H1 | RDX | RHOA | RUNX1 | SDC4 | SERPINE2 | SIPA1 | SNAI2 | SOCS1 | SOCS6 | SPINT2 | SRC | SWAP70 | TGFB1 | THBS1 | VSIG4 | WNK1 | ZBTB7B | GO:1902176 | 0.00181972582797525 | 15/8243 | 12/3250 | 0.00164771772190459 | 1 | T | T | F | F | negative regulation of oxidative stress-induced intrinsic apoptotic signaling pathway | AKT1 | BAG5 | GPX1 | HIF1A | HSPB1 | HTRA2 | MAPK7 | NFE2L2 | PARK7 | SIRT1 | SOD2 | TRAP1 | GO:0008283 | 0.0467062962513648 | 385/8243 | 180/3250 | 0.00165334811337835 | 1 | T | T | F | F | cell population proliferation | ABL1 | AKT1 | ANXA7 | APOA1 | AR | ASCL1 | ATAD5 | AURKB | BABAM1 | BAX | BCAR1 | BCL2 | BCL2L1 | BCL2L2 | BLZF1 | BTRC | BYSL | CD40 | CD40LG | CD74 | CDC16 | CDC25A | CDC25C | CDC27 | CDK5 | CDK5R1 | CDK9 | CDV3 | CENPF | CHRM3 | CKS2 | CLK1 | CLU | COL4A3BP | COPS2 | CRIP1 | CSE1L | CTNNB1 | CTPS1 | CUL1 | CUL5 | CYP1A1 | DAB2 | DAZAP1 | DBN1 | DDIT4 | DUSP22 | ECD | EEF2 | EGFR | EIF2S2 | ELF4 | ERBB2 | ERG | ESR1 | FABP7 | FAM83D | FGF2 | FGFR3 | FIGNL1 | FKBP1B | FSCN1 | FYN | GINS4 | GLUL | GNAI2 | GNB1 | GOLPH3 | GPX1 | GSTP1 | HDGF | HLCS | HMOX1 | HPRT1 | HRAS | HSPD1 | ID2 | IFI16 | IFNA2 | IGF2BP1 | IGSF8 | ILK | IMPDH2 | ITGB1BP1 | JUNB | KHDRBS1 | KIF2C | LEF1 | LGR4 | LIG4 | LOXL2 | MAP2K1 | MAP4K1 | MAPK1 | MAS1 | MATK | MCM7 | MDM4 | MED1 | MELK | MIF | MORF4L1 | MSN | MTSS1 | MXD1 | NELFB | NF2 | NUDC | NUPR1 | OGFOD1 | PA2G4 | PAFAH1B1 | PALB2 | PCNA | PDXK | PIK3CG | PIM1 | PLK1 | PLXNB2 | POC1B | PPARD | PPP1R8 | PRDX1 | PRDX2 | PRKAR1A | PRKCD | PRKD1 | PROX1 | PRPF19 | PSEN1 | PSMG1 | PTEN | PTPN6 | RAC1 | RAF1 | RAP1B | RC3H1 | RIPK2 | RPGRIP1 | RPL23A | RPS6KA1 | RRM1 | RUNX1 | RXRA | S100B | SART3 | SBDS | SDCBP2 | SIPA1 | SMAD4 | SRC | SRGAP2 | STAT1 | STAT3 | STAT6 | STIL | TFDP1 | TGFB1 | TGFBR3 | TNF | TNFSF14 | TP53 | TP63 | TWIST1 | TXLNA | TXN | TXNRD1 | UBE2L3 | UCHL1 | USP28 | USP8 | USPL1 | VTN | WDR12 | WNT16 | WNT2 | XBP1 | XRCC5 | YAP1 | YME1L1 | GO:0000086 | 0.0133446560718185 | 110/8243 | 59/3250 | 0.00165564582984323 | 1 | T | T | F | F | G2/M transition of mitotic cell cycle | ACTR1A | AKAP9 | BTRC | CALM2 | CALM3 | CCNB1 | CCNY | CCP110 | CDC25A | CDC25B | CDC25C | CDK2 | CDKN1A | CENPJ | CEP135 | CEP152 | CEP290 | CEP57 | CEP70 | CHEK2 | CKAP5 | CNTRL | CSNK1E | CUL1 | DCTN1 | DYNC1H1 | DYNC1I2 | DYNLL1 | FBXL12 | FBXW11 | FGFR1OP | FOXM1 | HSP90AA1 | KDM8 | KHDRBS1 | LATS1 | MELK | OFD1 | OPTN | PAFAH1B1 | PCM1 | PLK1 | PPM1D | PPME1 | PPP1CB | PPP1R12A | PPP1R12B | PPP2R1A | PPP2R2A | PRKACA | PRKAR2B | TUBA1A | TUBA4A | TUBB | TUBB4A | TUBB4B | TUBG1 | YWHAE | YWHAG | GO:0022414 | 0.0834647579764649 | 688/8243 | 308/3250 | 0.00165584242381651 | 1 | T | T | F | F | reproductive process | ABAT | ACOX1 | ACSL4 | ACTR1A | ACTR2 | ACTR3 | ADA | ADCYAP1 | AFF4 | AKR1B1 | AKT1 | ALDOA | ANKRD49 | ANXA1 | APOL2 | APP | AR | ARID4B | ARNT | ARRB1 | ARRB2 | ATM | ATRX | BAG6 | BASP1 | BAX | BCL2 | BCL2L1 | BCL2L2 | BCL6 | BIRC2 | BIRC3 | BMPR1A | BRD2 | BSG | BYSL | CALR | CAPN2 | CASP2 | CASP3 | CASP8 | CBL | CCDC155 | CCDC36 | CCNB1 | CCT2 | CCT3 | CCT4 | CCT5 | CCT6B | CCT7 | CCT8 | CDC25B | CDC25C | CDK16 | CDK2 | CEBPA | CEP57 | CFTR | CKS2 | CLGN | CLIC4 | COMT | CRIP1 | CRKL | CSNK2A2 | CTNNB1 | CTSV | CUL7 | CYP1A1 | DAZAP1 | DBH | DCN | DDX6 | DEDD | DHX36 | DIAPH3 | DLD | DMRT3 | DNAJA1 | DNAJC19 | DUSP13 | DYNLL1 | E2F1 | EGFR | EGLN1 | EIF2B5 | EIF2S2 | EIF4H | ESR1 | ESRRB | ETS1 | FAM9B | FANCA | FANCC | FANCG | FANCM | FIGNL1 | FOS | FOXA1 | FOXJ1 | GAPDHS | GATA1 | GDF9 | GGT1 | GJA1 | GLRB | GNPDA1 | GOLGA2 | GORASP2 | GOT2 | GPX4 | GRB2 | GTSF1 | H2AFX | HAVCR2 | HERC2 | HEXB | HIF1A | HIST1H1A | HIST1H2BA | HMGB2 | HNF4A | HOXD10 | HS6ST1 | HSD17B4 | HSF1 | HSP90AB1 | HSPA1L | HSPA2 | HSPA5 | HTRA1 | ICAM1 | IDH1 | IGFBP7 | IGSF8 | INSR | JUNB | KDM5B | KHDRBS1 | KMT2B | KRAS | LDHC | LEF1 | LGR4 | LRP6 | LRRK2 | MAP2K1 | MAP2K6 | MAP3K4 | MAPK1 | MAPK14 | MAPK3 | MAS1 | MCM9 | MDFI | MED1 | MGST1 | MLH1 | MME | MSH6 | MTNR1A | NAMPT | NCAPH2 | NCOA1 | NCOA4 | NIPBL | NKX2-1 | NOS3 | NSUN2 | NTRK1 | NUMA1 | NUP107 | NUPR1 | OAZ3 | OVGP1 | P2RX1 | PAFAH1B1 | PARK7 | PATZ1 | PCNA | PEBP1 | PGAM2 | PGK2 | PGM3 | PHB2 | PHC2 | PIAS1 | PICK1 | PLA2G6 | PLK1 | PPARD | PPARG | PPAT | PPP2CA | PPP2R1A | PRDX3 | PRDX4 | PRKACA | PRKAG1 | PRKAR1A | PRKDC | PSAP | PTEN | PTK2 | PTN | PTTG1 | PUM1 | RAD21 | RAD23B | RAD51 | RAI14 | RARA | RARG | RBPJ | RECK | RGN | RGS2 | RHBDD1 | RNF2 | RNF8 | RPA2 | RRM1 | RTCB | RUVBL1 | RXRA | SDC1 | SEPT12 | SEPT2 | SEPT4 | SEPT6 | SEPT7 | SERPINB5 | SERPINE2 | SF1 | SFMBT1 | SGPL1 | SIAH1 | SIRT1 | SMAD4 | SMAD5 | SMARCA2 | SMARCC1 | SMC1A | SMC3 | SNRPA1 | SOD1 | SORD | SPACA1 | SPACA3 | SPATA2 | SPATA20 | SPATA24 | SPIN1 | SPINT2 | SRC | SRPK1 | STAG2 | STAT3 | STK11 | STK3 | STRBP | STXBP1 | SUN2 | TCF21 | TCP1 | TEKT2 | TEKT4 | TERF1 | TESK2 | TGFB1 | THRA | TIAL1 | TLR3 | TNP1 | TOP2A | TOP3A | TP63 | TRIM28 | TRIP13 | TSGA10 | TTLL1 | TUBB8 | TUBG1 | TUBGCP3 | UBAP2L | UBB | UBE2A | UBE2B | UBE2J1 | UMPS | USP42 | USP9X | USP9Y | VASH2 | VDAC2 | VDR | VGF | WDR19 | WDR48 | WDR77 | WNT2 | WT1 | YY1 | ZBTB16 | ZFP41 | ZSCAN2 | ZW10 | GO:0010959 | 0.0243843260948684 | 201/8243 | 100/3250 | 0.00168938104024594 | 1 | T | T | F | F | regulation of metal ion transport | ABL1 | ACTN2 | ACTN4 | ADCYAP1 | ADORA2A | ADRB2 | AHCYL1 | AHNAK | AKAP9 | AKT1 | ANK3 | ANXA2 | ARF1 | ARRB2 | ATG5 | ATP1A1 | ATP1B3 | BAX | BCL2 | BIN1 | CAB39 | CALM1 | CALM2 | CALM3 | CAMK2A | CAMK2B | CAV1 | CCR1 | CHP1 | COMMD1 | CTNNB1 | CXCL11 | DMD | EPPIN | FHL1 | FKBP1A | FKBP1B | FYN | G6PD | GLRX | GNAI2 | GNAO1 | GPD1L | GSTO1 | HAP1 | HECW1 | HECW2 | HSPA2 | HTT | ICAM1 | KCNAB1 | KCNAB2 | KCNIP2 | KIF5B | LGALS3 | MYO5A | NEDD4 | NOS1AP | NOS3 | OXSR1 | P2RX1 | P2RX2 | P2RX4 | PACSIN3 | PCSK9 | PDE4D | PER1 | PIK3CG | PLA2G1B | PLA2G6 | PLCG1 | PML | PRKACA | PRKCE | PSEN2 | PTEN | PTPN6 | RGN | RHOA | S100A1 | SARAF | SERPINE2 | SLC9A1 | SLC9A3R1 | SNCA | SNTA1 | SRI | STAC | STIM1 | STOM | SUMO1 | TGFB1 | THADA | TMBIM6 | UBQLN1 | WNK1 | WNK4 | WWP2 | YWHAE | YWHAH | GO:1901216 | 0.00921994419507461 | 76/8243 | 43/3250 | 0.00175777614321509 | 1 | T | T | F | F | positive regulation of neuron death | ABL1 | AIFM1 | AIMP2 | ASCL1 | ATF2 | ATM | BACE1 | BAX | C1QA | CAPN2 | CASP2 | CASP3 | CASP8 | CASP9 | CDC34 | CDK5 | CDK5R1 | CLU | CTNNB1 | DAXX | DDIT4 | EIF2S1 | FASLG | FIS1 | FOS | FYN | GRIK2 | GSK3B | HDAC4 | JUN | MAP2K4 | MAPT | NQO1 | NQO2 | PARP1 | PCSK9 | PIN1 | RHOA | SNCA | SRPK2 | TNF | UBE2M | ZNF746 | GO:0051179 | 0.325488293097173 | 2683/8243 | 1119/3250 | 0.00178453945861002 | 1 | T | T | F | F | localization | A2M | ABCB7 | ABCB8 | ABCD3 | ABCE1 | ABCG1 | ABHD17A | ABL1 | ACAA1 | ACACA | ACD | ACE2 | ACKR2 | ACLY | ACO1 | ACOX1 | ACSL1 | ACSL4 | ACTB | ACTG1 | ACTN1 | ACTN2 | ACTN4 | ACTR1A | ACTR2 | ACTR3 | ADA | ADCYAP1 | ADD3 | ADORA2A | ADPRH | ADRB2 | AFG3L2 | AFM | AGAP1 | AGAP2 | AGER | AGK | AGL | AGR2 | AHCYL1 | AHI1 | AHSG | AKAP8 | AKAP9 | AKT1 | AKT2 | AKTIP | ALB | ALDOA | ALDOC | ALOX5 | ANK1 | ANK3 | ANKS4B | ANP32A | ANXA1 | ANXA11 | ANXA2 | ANXA5 | ANXA6 | AP1B1 | AP1G1 | AP1M1 | AP3D1 | AP3M1 | AP4M1 | AP4S1 | APEH | APLP1 | APOA1 | APOD | APOE | APOL2 | APP | APPBP2 | APRT | ARF1 | ARF4 | ARF5 | ARFGAP1 | ARFIP1 | ARHGAP1 | ARL4D | ARL5B | ARMC8 | ARPC5 | ARR3 | ARRB1 | ARRB2 | ASGR1 | ASGR2 | ASPSCR1 | ATCAY | ATG4C | ATL3 | ATM | ATOX1 | ATP12A | ATP13A2 | ATP1A1 | ATP1B3 | ATP2A2 | ATP2B2 | ATP4A | ATP6V1A | ATP6V1B1 | ATP6V1C1 | ATP6V1D | ATP6V1E1 | ATP9A | ATRX | ATXN1 | ATXN2 | AUP1 | AURKB | AZU1 | BACH2 | BAG3 | BAG4 | BAG6 | BANF1 | BANP | BAX | BCL2 | BCL2L1 | BCL6 | BDKRB2 | BIN1 | BIRC5 | BLZF1 | BRAF | BSG | BSPRY | BTK | C16orf70 | CA14 | CAB39 | CACNA1A | CACNG2 | CACNG4 | CACNG5 | CALM1 | CALM3 | CALR | CAMK2A | CAND1 | CANX | CAPN1 | CAPZA1 | CAPZA2 | CAPZB | CARM1 | CAT | CAV1 | CBL | CBLB | CBY1 | CCDC120 | CCDC155 | CCDC22 | CCDC68 | CCDC88A | CCHCR1 | CCL21 | CCNB1 | CCP110 | CCR1 | CCR5 | CCS | CCT2 | CCT3 | CCT4 | CCT5 | CCT6B | CCT7 | CCT8 | CD109 | CD2 | CD209 | CD36 | CD40LG | CD6 | CD74 | CD81 | CDA | CDC37 | CDC42 | CDC42SE2 | CDH1 | CDK16 | CDK2 | CDK5 | CEBPE | CEL | CENPF | CENPJ | CEP128 | CEP135 | CEP152 | CEP290 | CEP55 | CEP57 | CEP70 | CEP89 | CFTR | CHCHD10 | CHMP2A | CHP1 | CHRM3 | CHRNA9 | CHRND | CHRNE | CHRNG | CKAP4 | CKAP5 | CLDN17 | CLIC1 | CLIC3 | CLIC4 | CLNS1A | CLSTN1 | CLTC | CLU | CLUH | CNNM1 | CNTRL | COG3 | COL4A3BP | COMMD1 | COPA | COPB1 | COPE | COPG1 | COPS5 | COTL1 | COX11 | COX15 | COX17 | COX5A | COX6A1 | COX6C | COX7A2 | CPLX1 | CPLX3 | CPNE3 | CPT1A | CPT2 | CREB1 | CROCC | CROT | CRY1 | CSE1L | CSN1S1 | CSNK1E | CSNK2B | CTCF | CTNNB1 | CTSA | CTSC | CTSD | CTTN | CUL3 | CUL5 | CUTA | CUTC | CXCL8 | CYB5R1 | CYB5R3 | CYC1 | DAB1 | DAB2 | DBN1 | DBNL | DCLK1 | DCTN1 | DDOST | DDX1 | DDX19A | DDX19B | DDX39A | DDX39B | DDX3X | DEGS1 | DENND1A | DENND4C | DERA | DERL1 | DHRS4 | DHX9 | DLG2 | DLG3 | DLG4 | DMD | DNAJA1 | DNAJA3 | DNAJC19 | DNM1L | DOCK7 | DUSP16 | DVL1 | DVL2 | DYNC1H1 | DYNC1I1 | DYNC1I2 | DYNC1LI1 | DYNLL1 | DYNLT1 | ECH1 | ECI2 | EEF1A1 | EEF2 | EGFR | EHD4 | EHHADH | EIF2S1 | EIF2S2 | EIF4A3 | EIF4E | EIF4ENIF1 | EIF5A | EIF6 | EPRS | ERLIN2 | ESR1 | EXOC6 | EZH2 | EZR | F5 | F9 | FABP1 | FABP3 | FABP5 | FAF1 | FAF2 | FAM129B | FAM3C | FAM49B | FAM83D | FAM83H | FAM98A | FASLG | FBF1 | FBXO7 | FCGR1A | FCN1 | FERMT2 | FGB | FGF2 | FGFR1OP | FGL2 | FIS1 | FKBP1A | FKBP1B | FLNB | FLOT1 | FN1 | FOLR1 | FRAS1 | FTH1 | FTL | FYN | FYTTD1 | G3BP2 | GAA | GABRA6 | GABRR1 | GAD2 | GAK | GALNS | GAPVD1 | GBP2 | GBP5 | GDI1 | GDI2 | GEMIN4 | GEMIN5 | GEMIN7 | GGA1 | GHDC | GJA1 | GLA | GLRB | GLS | GLS2 | GLUL | GNS | GOLGA2 | GOLGA4 | GOLPH3 | GOLT1B | GOPC | GORASP2 | GOT2 | GPI | GPR107 | GRAMD1A | GRB2 | GRIA1 | GRID2 | GRIK2 | GRIP1 | GRPEL1 | GSTK1 | GSTP1 | GUSB | GYG1 | HAP1 | HBZ | HCN1 | HCN2 | HCN3 | HDAC6 | HEATR3 | HERC2 | HEXB | HGS | HID1 | HIF1A | HK1 | HK2 | HMGB1 | HMGCL | HNF1A | HNRNPA1 | HNRNPA1L2 | HNRNPA2B1 | HNRNPU | HPX | HRAS | HRNR | HSD17B4 | HSF1 | HSP90AA1 | HSP90AB1 | HSP90B1 | HSPA1B | HSPA2 | HSPA4 | HSPA5 | HSPA8 | HSPB1 | HSPD1 | HSPG2 | HSPH1 | HTATIP2 | HTR1B | HTR3A | HTR3E | HTT | HUWE1 | IBTK | ICK | IDE | IDH1 | IFIT1 | IFT122 | IGF2BP1 | IGHV3-13 | IGHV3-23 | IGHV3-30 | IGKV3-20 | IGLC7 | IK | IKBKB | IKBKG | ILF2 | IMPDH2 | INSIG1 | INSR | IPO11 | IPO4 | IPO5 | IPO7 | IPO8 | IPO9 | IQCB1 | IQGAP1 | IREB2 | ISLR | ITGA4 | ITGAV | ITGB1BP1 | ITGB3 | ITPR1 | ITPR2 | ITPR3 | JUP | KCMF1 | KCNA4 | KCNAB1 | KCNAB2 | KCNIP2 | KCNK1 | KCNK16 | KCNS3 | KEAP1 | KIF11 | KIF13B | KIF2C | KIF5B | KIF5C | KLC1 | KLC2 | KLHL20 | KPNA1 | KPNA2 | KPNB1 | LARS | LASP1 | LAT | LATS1 | LCP2 | LEMD2 | LETM1 | LGALS3 | LGALS3BP | LIMA1 | LIMS1 | LMAN2L | LMNA | LOXL2 | LRP6 | LRPPRC | LRRC7 | LRRK2 | LRSAM1 | LTBP1 | MAL2 | MAN2B1 | MAP1B | MAPK1 | MAPK14 | MAPK15 | MAPK3 | MAPK8IP3 | MAPK9 | MAPT | MARCH5 | MASP1 | MCC | MCFD2 | MCM3AP | MCM9 | MCOLN3 | MDFI | MDM2 | MECP2 | MED1 | MGST1 | MIB1 | MICU1 | MICU2 | MIF | MKS1 | MLH1 | MME | MMGT1 | MMRN1 | MPC2 | MPPE1 | MTCH2 | MTX1 | MYC | MYO1B | MYO1C | MYO1D | MYO1E | MYO5A | MYO6 | NAPRT | NBAS | NCBP1 | NCOA1 | NCOA2 | NCOA4 | NDUFA13 | NDUFA9 | NDUFS7 | NEDD4 | NEDD8 | NEUROD1 | NFKBIA | NGB | NHLRC3 | NIN | NIPBL | NIT2 | NMB | NMD3 | NME1 | NME2 | NME4 | NNT | NOD2 | NOS2 | NOSTRIN | NPLOC4 | NPM1 | NPR1 | NR1H3 | NR1I2 | NRXN1 | NSFL1C | NUBP1 | NUDT19 | NUMA1 | NUP107 | NUP153 | NUP160 | NUP205 | NUP214 | NUP85 | NUP88 | NUP93 | NUP98 | NUSAP1 | NUTF2 | NXF1 | NXF2 | NXT2 | OAZ3 | OBSL1 | OFD1 | OLA1 | OPA1 | OPTN | ORM1 | OSBPL1A | OXA1L | P2RX1 | P2RX2 | P2RX4 | PA2G4 | PACS1 | PACS2 | PACSIN3 | PAFAH1B1 | PAM16 | PANX3 | PARD3 | PARK7 | PCM1 | PCSK9 | PCTP | PDCD10 | PDCD6 | PDCD6IP | PDIA4 | PDXK | PEF1 | PEX19 | PEX5 | PFKL | PGAM1 | PGM1 | PGM2 | PGRMC1 | PHB | PHB2 | PI4K2A | PICK1 | PIGU | PIH1D1 | PIK3C2A | PIK3CG | PIK3R1 | PIKFYVE | PINX1 | PITPNA | PITPNB | PKM | PLA2G10 | PLA2G12A | PLA2G1B | PLA2G2A | PLAUR | PLCG1 | PLEKHF1 | PLEKHF2 | PLIN3 | PLK1 | PLSCR1 | PLXNA2 | PML | PMPCA | PMPCB | PNP | POLDIP3 | POLR2M | POM121 | POT1 | PPARD | PPARG | PPAT | PPIA | PPID | PPIE | PPP2R1A | PPT1 | PQLC1 | PRCP | PRDX4 | PRDX6 | PRELID1 | PRG2 | PRKAA2 | PRKAB2 | PRKACA | PRKAG1 | PRKAR2B | PRKCD | PRKCE | PRKCZ | PRKD1 | PROZ | PRPF19 | PSAP | PSEN1 | PSEN2 | PSMA1 | PSMA2 | PSMA3 | PSMA5 | PSMA6 | PSMB5 | PSMB6 | PSMB7 | PSMB8 | PSMC1 | PSMC2 | PSMC3 | PSMC4 | PSMC5 | PSMC6 | PSMD1 | PSMD10 | PSMD11 | PSMD12 | PSMD14 | PSMD2 | PSMD4 | PSMD9 | PSME2 | PSME3 | PSTPIP1 | PTGES2 | PTK2 | PTPN23 | PTPN6 | PTPRK | PYGB | PYGL | QSOX1 | RAB10 | RAB11A | RAB11B | RAB11FIP2 | RAB18 | RAB1A | RAB1B | RAB21 | RAB24 | RAB29 | RAB2A | RAB31 | RAB32 | RAB35 | RAB3GAP1 | RAB3IP | RAB5A | RAB5B | RAB7A | RAB9A | RABGEF1 | RABL2A | RABL3 | RAC1 | RAD21 | RAE1 | RAF1 | RAN | RANBP3 | RANGAP1 | RAP1A | RAP1B | RAPGEF4 | RARA | RASSF9 | RB1 | RBM8A | RBPJ | RDX | RHOA | RHOJ | RHOT1 | RHOT2 | RHOU | RIC3 | RNASE3 | RNF126 | RNF185 | ROMO1 | RP2 | RPA2 | RPAIN | RPGRIP1L | RPL10 | RPL10A | RPL12 | RPL18A | RPL22 | RPL23 | RPL23A | RPL24 | RPL30 | RPL37A | RPL38 | RPL6 | RPL7 | RPL7A | RPL9 | RPLP0 | RPLP1 | RPLP2 | RPS12 | RPS13 | RPS14 | RPS15A | RPS19 | RPS2 | RPS20 | RPS24 | RPS29 | RPS3 | RPS4X | RPS8 | RRS1 | RTKN | RTN4 | RUVBL2 | RXRA | S100A11 | S100A12 | S100A13 | S100A6 | S100A7 | S100A9 | S100P | SARAF | SCARA3 | SCCPDH | SCFD1 | SCG2 | SCG3 | SCLT1 | SCO2 | SCYL2 | SDCBP | SDCBP2 | SEC13 | SEC16A | SEC22C | SEC24B | SEC24C | SEC31A | SEC62 | SEC63 | SEL1L | SELENBP1 | SELL | SERPINB3 | SERPINB6 | SERPINE2 | SFPQ | SFXN1 | SFXN4 | SGTA | SGTB | SH3GL3 | SH3KBP1 | SIN3A | SLAMF1 | SLC12A2 | SLC15A1 | SLC16A1 | SLC25A1 | SLC25A10 | SLC25A11 | SLC25A13 | SLC25A22 | SLC25A24 | SLC25A3 | SLC25A32 | SLC25A4 | SLC25A41 | SLC25A5 | SLC25A6 | SLC2A5 | SLC2A9 | SLC35F6 | SLC37A3 | SLC39A4 | SLC39A9 | SLC3A2 | SLC7A1 | SLC9A1 | SLC9A3R1 | SMAD2 | SMG5 | SMG6 | SMN1 | SMO | SMURF1 | SNAP29 | SNCA | SNF8 | SNRPB | SNRPD1 | SNRPD3 | SNRPE | SNX12 | SNX17 | SNX2 | SNX21 | SNX24 | SNX3 | SNX6 | SOAT1 | SOCS2 | SOD1 | SORT1 | SP100 | SPDL1 | SPICE1 | SPRY2 | SPTAN1 | SPTBN1 | SPTBN2 | SQSTM1 | SRC | SRI | SRP14 | SRP19 | SRSF11 | SRSF5 | SSB | SSX2IP | STARD7 | STAT3 | STAT5A | STBD1 | STIL | STIM1 | STK11 | STK25 | STOM | STOML2 | STX5 | STXBP1 | STXBP2 | STYX | SUMO1 | SUN2 | SYN1 | SYNE2 | SYNJ2BP | SYP | SYVN1 | TAGLN2 | TANGO2 | TBC1D15 | TBC1D22B | TBC1D23 | TBC1D4 | TCIRG1 | TCP1 | TCTN2 | TCTN3 | TERF1 | TERF2 | TERF2IP | TEX264 | TFG | TGFB1 | TGM2 | THBS1 | THOC2 | THOC6 | THOC7 | TIA1 | TIMM13 | TIMM21 | TIMM23 | TIMM44 | TIMM50 | TIMP1 | TIMP2 | TINF2 | TM9SF4 | TMED9 | TMEM173 | TMEM216 | TMEM30A | TNF | TNFAIP3 | TNFRSF1A | TNK2 | TNKS | TNKS2 | TNPO1 | TOLLIP | TOMM20 | TOMM22 | TOMM40 | TOR1A | TP53 | TPCN2 | TPD52 | TPR | TRAF3IP1 | TRAPPC8 | TREX1 | TRIAP1 | TRIM23 | TRIM9 | TRIP4 | TSC22D3 | TSG101 | TSPAN17 | TTYH3 | TUBA1A | TUBA1C | TUBA4A | TUBB | TUBB4A | TUBB4B | TUBG1 | TULP3 | TWF1 | TWF2 | TXLNA | TXN | TXNDC5 | TXNIP | U2AF2 | UBB | UBC | UBE2B | UBE2D1 | UBE2D2 | UBE2D3 | UBE2O | UBL4A | UBR4 | UCHL1 | UGT8 | ULK1 | UMPS | UNC119 | UPF2 | UPF3B | USP20 | USP4 | USP9X | VAPA | VAPB | VAT1 | VCL | VCP | VDAC1 | VDAC2 | VDAC3 | VDR | VGF | VPS13B | VPS18 | VPS26A | VPS29 | VPS37D | VPS4B | VPS8 | VTA1 | VTN | WAS | WBP2 | WDR1 | WDR19 | WDR35 | WDR45B | WIPI1 | WIPI2 | WNK1 | WNK4 | WWP2 | XBP1 | XPO1 | XPO4 | XPO5 | XPO7 | XPOT | XRCC5 | XRCC6 | YKT6 | YME1L1 | YWHAB | YWHAE | YWHAG | YWHAH | YWHAZ | ZBTB16 | ZBTB7A | ZBTB7B | ZDHHC17 | ZDHHC23 | ZFAND6 | ZMPSTE24 | ZW10 | ZWINT | GO:0070423 | 0.0029115613247604 | 24/8243 | 17/3250 | 0.00180531825081198 | 1 | T | T | F | F | nucleotide-binding oligomerization domain containing signaling pathway | CASP8 | CYLD | IKBKG | ITCH | MAP2K6 | NFKBIA | NOD2 | RELA | RIPK2 | TAB1 | TAB2 | TNFAIP3 | TRAF6 | UBB | UBC | UBE2N | UBE2V1 | GO:1900739 | 0.0029115613247604 | 24/8243 | 17/3250 | 0.00180531825081198 | 1 | T | T | F | F | regulation of protein insertion into mitochondrial membrane involved in apoptotic signaling pathway | BBC3 | BCL2 | CASP8 | E2F1 | MAPK8 | PPP3CC | SFN | TFDP1 | TP53 | TP63 | TP73 | YWHAB | YWHAE | YWHAG | YWHAH | YWHAQ | YWHAZ | GO:1900740 | 0.0029115613247604 | 24/8243 | 17/3250 | 0.00180531825081198 | 1 | T | T | F | F | positive regulation of protein insertion into mitochondrial membrane involved in apoptotic signaling pathway | BBC3 | BCL2 | CASP8 | E2F1 | MAPK8 | PPP3CC | SFN | TFDP1 | TP53 | TP63 | TP73 | YWHAB | YWHAE | YWHAG | YWHAH | YWHAQ | YWHAZ | GO:0046685 | 0.0029115613247604 | 24/8243 | 17/3250 | 0.00180531825081198 | 1 | T | T | F | F | response to arsenic-containing substance | CDKN1A | CYP1A1 | DAXX | DDX3X | DHX36 | FECH | GSTO1 | GSTO2 | HMOX1 | HNRNPA1 | HSF1 | MKNK2 | PPIF | PTEN | RBM4 | RNF4 | VCP | GO:0071480 | 0.0029115613247604 | 24/8243 | 17/3250 | 0.00180531825081198 | 1 | T | T | F | F | cellular response to gamma radiation | ATM | BCL2L1 | CHEK2 | CRYAB | H2AFX | HRAS | HSF1 | HSPA5 | KDM1A | MDM2 | POLE3 | TP53 | TREX1 | XRCC5 | XRCC6 | YAP1 | ZMPSTE24 | GO:0031145 | 0.00897731408467791 | 74/8243 | 42/3250 | 0.00182179956137925 | 1 | T | T | F | F | anaphase-promoting complex-dependent catabolic process | ANAPC4 | AURKB | CCNB1 | CDC16 | CDC20 | CDC27 | CDK2 | CUL3 | FZR1 | MAD2L1 | PLK1 | PSMA1 | PSMA2 | PSMA3 | PSMA5 | PSMA6 | PSMB5 | PSMB6 | PSMB7 | PSMB8 | PSMC1 | PSMC2 | PSMC3 | PSMC4 | PSMC5 | PSMC6 | PSMD1 | PSMD10 | PSMD11 | PSMD12 | PSMD14 | PSMD2 | PSMD4 | PSMD9 | PSME2 | PSME3 | PTTG1 | UBB | UBC | UBE2C | UBE2D1 | UBE2S | GO:0006887 | 0.059929637267985 | 494/8243 | 226/3250 | 0.00186963928899599 | 1 | T | T | F | F | exocytosis | A2M | ACAA1 | ACLY | ACTN1 | ACTN2 | ACTN4 | ACTR2 | AGL | AHSG | ALB | ALDOA | ALDOC | ALOX5 | ANK1 | ANXA2 | ANXA5 | AP1M1 | APEH | APOA1 | APP | APRT | ARMC8 | ARPC5 | ATP6V1D | AZU1 | CAB39 | CALM1 | CAND1 | CAPN1 | CAT | CBL | CCR1 | CCT2 | CCT8 | CD109 | CD36 | CDA | CDK16 | CDK5 | CEP290 | CHP1 | CKAP4 | CLU | COPB1 | COPS5 | COTL1 | CPLX1 | CPLX3 | CPNE3 | CSNK2B | CTSA | CTSC | CTSD | CYB5R1 | CYB5R3 | DBNL | DDOST | DDX3X | DEGS1 | DERA | DYNC1H1 | DYNC1LI1 | DYNLL1 | DYNLT1 | EEF1A1 | EEF2 | EXOC6 | F5 | FABP5 | FAF2 | FAM3C | FAM49B | FCN1 | FGB | FGL2 | FN1 | FTH1 | FTL | GAA | GALNS | GDI2 | GHDC | GLA | GNS | GPI | GSTP1 | GUSB | GYG1 | HAP1 | HEXB | HMGB1 | HRNR | HSP90AA1 | HSP90AB1 | HSPA1B | HSPA8 | HUWE1 | IDH1 | ILF2 | IMPDH2 | IQGAP1 | ISLR | ITGAV | ITGB3 | JUP | KCMF1 | KCNAB2 | KPNB1 | LAT | LGALS3 | LGALS3BP | LRRC7 | MAN2B1 | MAPK1 | MAPK14 | MGST1 | MIF | MME | MMRN1 | MYO5A | NAPRT | NHLRC3 | NIT2 | NME2 | OLA1 | ORM1 | P2RX1 | PA2G4 | PDXK | PFKL | PGAM1 | PGM1 | PGM2 | PGRMC1 | PI4K2A | PIK3C2A | PIK3CG | PKM | PLAUR | PNP | PPIA | PPIE | PRCP | PRDX4 | PRDX6 | PRG2 | PRKCD | PSAP | PSEN1 | PSMA2 | PSMA5 | PSMB7 | PSMC2 | PSMC3 | PSMD1 | PSMD11 | PSMD12 | PSMD14 | PSMD2 | PTGES2 | PTPN6 | PYGB | PYGL | QSOX1 | RAB10 | RAB11A | RAB11B | RAB11FIP2 | RAB18 | RAB24 | RAB31 | RAB5B | RAB7A | RAC1 | RAP1A | RAP1B | RAPGEF4 | RHOA | RNASE3 | S100A11 | S100A12 | S100A13 | S100A7 | S100A9 | S100P | SCCPDH | SCG3 | SDCBP | SELL | SERPINB3 | SERPINB6 | SLC2A5 | SNAP29 | SNCA | SOD1 | SPTAN1 | SRP14 | STBD1 | STOM | STXBP1 | STXBP2 | TAGLN2 | TCIRG1 | TEX264 | TGFB1 | THBS1 | TIMP1 | TIMP2 | TMEM173 | TMEM30A | TOLLIP | TRIM9 | TSG101 | TUBA4A | TUBB | TUBB4B | TXLNA | TXNDC5 | UBR4 | VAPA | VAT1 | VCL | VCP | WDR1 | XRCC5 | XRCC6 | GO:0045859 | 0.0622346233167536 | 513/8243 | 234/3250 | 0.00188857621190072 | 1 | T | T | F | F | regulation of protein kinase activity | ABL1 | ACSL1 | ACTB | ADCY6 | ADCYAP1 | ADORA2A | ADRB2 | AGER | AKT1 | APC | APOE | APP | ARRB1 | AXIN1 | AZU1 | BIRC7 | BRAF | CAB39 | CALM1 | CALM2 | CALM3 | CASP3 | CAV1 | CBL | CBLB | CBLC | CCDC88A | CCL21 | CCNB1 | CCNT1 | CCNT2 | CCNY | CCNYL1 | CD40 | CD40LG | CD74 | CD81 | CDC25A | CDC25B | CDC25C | CDC37 | CDK4 | CDK5 | CDK5R1 | CDKN1A | CDKN2C | CEBPA | CHORDC1 | CHP1 | CKS1B | CKS2 | CLU | CRK | CRKL | CSNK2B | DAB1 | DAXX | DBF4B | DBNDD2 | DBNL | DDX3X | DLG3 | DLG4 | DNAJA1 | DUSP1 | DUSP14 | DUSP16 | DUSP19 | DUSP22 | DUSP7 | DVL1 | DVL2 | DVL3 | EGFR | EPHA4 | ERBB2 | ERBB3 | ERRFI1 | EZH2 | FAF1 | FBXO7 | FGF2 | FGFR1 | FGFR1OP | FYN | GADD45G | GNAQ | GPS2 | GSTP1 | HEXIM1 | HGS | HRAS | HSP90AA1 | HSP90AB1 | HSPB1 | HTT | IBTK | IGF1R | IKBKG | ILK | INSR | IPO5 | IPO7 | IQGAP1 | ITGB1BP1 | ITGB3 | KRAS | KSR1 | LAT | LATS1 | LCP2 | LPAR1 | LRP6 | LRRK2 | MAP2K1 | MAP2K3 | MAP2K4 | MAP2K6 | MAP2K7 | MAP3K1 | MAP3K13 | MAP3K14 | MAP3K2 | MAP3K3 | MAP3K4 | MAP4K1 | MAPK1 | MAPK14 | MAPK3 | MAPK8IP3 | MAPKAP1 | MAS1 | MIF | MLLT1 | MLST8 | NCK1 | NEK1 | NF2 | NOD2 | NPM1 | NTF3 | NTRK1 | OXSR1 | PAK2 | PAK6 | PARK7 | PDCD10 | PDE6G | PIH1D1 | PIK3CA | PIK3CG | PKIG | PLA2G1B | PLK1 | PPP2CA | PPP2R1A | PRDX2 | PRKAA1 | PRKACA | PRKAG1 | PRKAR1A | PRKAR2B | PRKCD | PRKCZ | PROX1 | PRRC1 | PSEN1 | PSMD10 | PTEN | PTK2 | PTPN1 | PTPN22 | PTPN6 | QARS | RAF1 | RAP1A | RB1 | RGN | RGS2 | RHOA | RIPK1 | RIPK2 | RIPK3 | RPLP1 | RPS3 | RPTOR | S100A12 | SDC4 | SERPINB3 | SFN | SHC1 | SIRT1 | SLC9A3R1 | SMG8 | SNCA | SNF8 | SNX6 | SOCS1 | SOD1 | SPRY2 | SRC | STK11 | STK24 | STK25 | STK26 | STK3 | STK38 | TAB1 | TAB2 | TAOK3 | TGFB1 | THBS1 | TLR3 | TNF | TNFAIP3 | TP73 | TRAF2 | TRAF6 | TRIB2 | TRIB3 | TSG101 | TSPYL2 | TXN | UBB | UBC | UBE2N | UBE2V1 | UCHL1 | UNC119 | WARS | WNK1 | XRCC5 | XRCC6 | YWHAG | GO:0010608 | 0.0434307897610093 | 358/8243 | 168/3250 | 0.00191298842754358 | 1 | T | T | F | F | posttranscriptional regulation of gene expression | AARS | ACO1 | AGO1 | AGO2 | AIRE | AKT1 | AKT2 | ANP32A | APEX1 | APP | ATXN2 | C1QBP | CALR | CAV1 | CDC37 | CDK4 | CIRBP | COA3 | DAPK1 | DCP2 | DDX1 | DDX39B | DDX3X | DHX36 | DHX9 | DIS3 | E2F1 | EEF2 | EIF2AK3 | EIF2AK4 | EIF2B5 | EIF2S1 | EIF3D | EIF3E | EIF3H | EIF4A3 | EIF4E | EIF4E2 | EIF4EBP1 | EIF4ENIF1 | EIF4G3 | EIF4H | EIF5 | EIF5A | EIF5B | EIF6 | ELAVL1 | EPRS | ERBB2 | EXOSC1 | EXOSC5 | EXOSC6 | EXOSC9 | FTO | FXR2 | GAPDH | GEMIN5 | GRB7 | HELZ | HNRNPD | HNRNPL | HNRNPM | HNRNPU | HSPA8 | HSPB1 | IGF2BP1 | ILF3 | IREB2 | KHDRBS1 | LARP4 | LRPPRC | MAPK1 | MAPK14 | MAPK3 | MATR3 | MKNK2 | MOV10 | MTPN | MYD88 | NCBP1 | NCK1 | NPM1 | NUP214 | OGFOD1 | PA2G4 | PABPC1 | PABPC4 | PAIP1 | PDE12 | PER1 | PER2 | POLDIP3 | PPP1CA | PRKCD | PSMA1 | PSMA2 | PSMA3 | PSMA5 | PSMA6 | PSMB5 | PSMB6 | PSMB7 | PSMB8 | PSMC1 | PSMC2 | PSMC3 | PSMC4 | PSMC5 | PSMC6 | PSMD1 | PSMD10 | PSMD11 | PSMD12 | PSMD14 | PSMD2 | PSMD4 | PSMD9 | PSME2 | PSME3 | PUM1 | PUS7 | RARA | RBM3 | RBM4 | RBM8A | RC3H1 | RGS2 | RHOA | RMND1 | RNF139 | RPL10 | RPL38 | RPS14 | RPS3 | RPS4X | RPS6KA1 | RPS6KB2 | RXRA | SET | SHMT1 | SHMT2 | SMG6 | STAT3 | TACO1 | TARDBP | TBRG4 | TCOF1 | THBS1 | TIA1 | TNF | TNPO1 | TPR | TRAP1 | TRUB2 | TSFM | TYMS | UBB | UBC | UNK | UPF3B | UQCC1 | USP16 | VIM | XPO1 | YTHDF1 | YWHAB | YWHAZ | ZNF598 | GO:0051171 | 0.398398641271382 | 3284/8243 | 1358/3250 | 0.00195956500272556 | 1 | T | T | F | F | regulation of nitrogen compound metabolic process | A2M | AARS | ABAT | ABCE1 | ABCG1 | ABHD14B | ABL1 | ACD | ACO1 | ACSL1 | ACTA2 | ACTB | ACTL6A | ACTN1 | ACTN2 | ACTN4 | ACTR2 | ACTR3 | ADCY6 | ADCYAP1 | ADORA2A | ADRB2 | ADRM1 | ADTRP | AEBP2 | AES | AFF1 | AFF4 | AGER | AGO1 | AGO2 | AHCYL1 | AHI1 | AHNAK | AHSG | AICDA | AIFM1 | AIMP2 | AIRE | AKAP8 | AKAP9 | AKT1 | AKT2 | AKTIP | ALOX12 | ANKRD49 | ANP32A | ANXA2 | APBB1 | APBB2 | APBB3 | APC | APCS | APEX1 | APOA1 | APOD | APOE | APP | AR | ARAF | ARF4 | ARHGAP35 | ARID3B | ARID4B | ARNT | ARNT2 | ARR3 | ARRB1 | ARRB2 | ASCC1 | ASCC2 | ASCL1 | ASPSCR1 | ASS1 | ATAD5 | ATCAY | ATF2 | ATF6 | ATF6B | ATF7 | ATG5 | ATM | ATOH1 | ATP13A2 | ATRX | ATXN1 | ATXN2 | AURKB | AXIN1 | AZU1 | B3GAT3 | BABAM1 | BACH2 | BAG2 | BAG3 | BAG4 | BAG5 | BAG6 | BANP | BASP1 | BAX | BBC3 | BCAR3 | BCL10 | BCL11A | BCL2 | BCL2L13 | BCL6 | BCL6B | BCOR | BDKRB2 | BIN1 | BIRC2 | BIRC3 | BIRC5 | BIRC7 | BIRC8 | BLZF1 | BMI1 | BMPR1A | BOLA1 | BOLA3 | BRAF | BRCA1 | BRD2 | BRD3 | BRD4 | BTK | BTRC | BUD31 | C1QBP | C1QL4 | C1QTNF1 | C4A | CAB39 | CALM1 | CALM2 | CALM3 | CALR | CAMK1D | CAMK2A | CAND1 | CAPN2 | CARD9 | CARM1 | CASP10 | CASP3 | CASP8 | CASP9 | CAT | CAV1 | CBFA2T3 | CBFB | CBL | CBLB | CBLC | CBX4 | CBX7 | CBX8 | CBY1 | CCAR2 | CCDC22 | CCDC88A | CCL21 | CCL22 | CCNB1 | CCNT1 | CCNT2 | CCNY | CCNYL1 | CCR1 | CCT2 | CCT3 | CCT4 | CCT5 | CCT6A | CCT7 | CCT8 | CD109 | CD27 | CD36 | CD40 | CD40LG | CD74 | CD81 | CDA | CDC14B | CDC20 | CDC25A | CDC25B | CDC25C | CDC37 | CDC5L | CDC73 | CDH1 | CDK2 | CDK4 | CDK5 | CDK5R1 | CDK8 | CDK9 | CDKN1A | CDKN2C | CEBPA | CEBPE | CENPF | CENPJ | CEP290 | CHCHD10 | CHCHD2 | CHCHD3 | CHD3 | CHD4 | CHD8 | CHEK2 | CHORDC1 | CHP1 | CHRD | CHRNA9 | CIRBP | CKS1B | CKS2 | CLK1 | CLPX | CLTC | CLU | CNDP1 | COA3 | COMMD1 | COMT | COPS2 | COPS5 | CPSF7 | CREB1 | CREB3 | CREBBP | CRK | CRKL | CRY1 | CRY2 | CRYAB | CSNK1A1 | CSNK1E | CSNK2A2 | CSNK2B | CTBP1 | CTCF | CTDP1 | CTH | CTNNB1 | CTNNBIP1 | CTSC | CUL2 | CUL3 | CYCS | CYLD | DAB1 | DAB2 | DAPK1 | DAXX | DAZAP1 | DBF4B | DBNDD2 | DBNL | DCN | DCP2 | DCUN1D1 | DDA1 | DDIT4 | DDRGK1 | DDX1 | DDX39B | DDX3X | DERL1 | DHCR24 | DHX36 | DHX9 | DIABLO | DIS3 | DLG3 | DLG4 | DMAP1 | DMD | DMRT3 | DNAJA1 | DNAJA3 | DNAJB1 | DNAJB2 | DNAJC10 | DNAJC30 | DNM1L | DOCK7 | DPEP1 | DPF2 | DROSHA | DTX1 | DUSP1 | DUSP14 | DUSP16 | DUSP19 | DUSP22 | DUSP7 | DVL1 | DVL2 | DVL3 | E2F1 | E2F2 | E2F3 | E2F4 | E2F6 | EAF2 | EBF1 | ECD | EDA | EDEM1 | EED | EEF2 | EFEMP1 | EGFR | EGLN1 | EGLN3 | EHD4 | EHMT1 | EHMT2 | EID1 | EIF2AK3 | EIF2AK4 | EIF2B5 | EIF2S1 | EIF3A | EIF3D | EIF3E | EIF3H | EIF4A3 | EIF4E | EIF4E2 | EIF4EBP1 | EIF4ENIF1 | EIF4G3 | EIF4H | EIF5 | EIF5A | EIF5B | EIF6 | ELAVL1 | ELF3 | ELF4 | ENDOG | ENO1 | EP300 | EPHA4 | EPPIN | EPRS | ERBB2 | ERBB3 | ERF | ERG | ERLIN2 | ERRFI1 | ESR1 | ESR2 | ESRRA | ESRRB | ESRRG | ETS1 | EWSR1 | EXOSC1 | EXOSC5 | EXOSC6 | EXOSC9 | EYA2 | EYA4 | EZH2 | EZR | FABP1 | FABP3 | FADD | FAF1 | FAM129B | FAM192A | FAM83D | FANCA | FANCI | FANCM | FASLG | FBP1 | FBXO7 | FBXW11 | FGB | FGF2 | FGFR1 | FGFR1OP | FGFR3 | FHL2 | FIGNL1 | FIS1 | FKBP1A | FKBP1B | FKBP8 | FLOT1 | FN1 | FOS | FOXA1 | FOXD4 | FOXD4L6 | FOXI2 | FOXJ1 | FOXK1 | FOXK2 | FOXM1 | FOXO1 | FTO | FUBP1 | FUBP3 | FUS | FXR2 | FYN | FZR1 | G3BP2 | G6PD | GABPB1 | GADD45G | GAPDH | GATA1 | GDF9 | GEMIN5 | GLA | GLMN | GLO1 | GNA12 | GNAI2 | GNAQ | GOLGA2 | GPD1L | GPI | GPS2 | GPX1 | GRB2 | GRB7 | GRIK2 | GRIP1 | GSK3A | GSK3B | GSTP1 | GSTZ1 | GTF2A1 | GTF2F1 | GTF2F2 | GTF2I | GZMA | H1F0 | H1FX | H2AFX | H2AFZ | H3F3A | HAP1 | HAVCR2 | HAX1 | HBEGF | HBZ | HDAC1 | HDAC2 | HDAC3 | HDAC4 | HDAC5 | HDAC6 | HDAC7 | HDAC9 | HDGF | HELZ2 | HEXB | HEXIM1 | HGS | HIF1A | HIF1AN | HINT1 | HIST1H1A | HIST1H3A | HIST1H4A | HIST2H3A | HIVEP1 | HLX | HMGB1 | HMGB2 | HMGB3 | HMOX1 | HNF1A | HNF4A | HNF4G | HNRNPA1 | HNRNPA2B1 | HNRNPAB | HNRNPD | HNRNPF | HNRNPH1 | HNRNPK | HNRNPL | HNRNPM | HNRNPU | HOXA5 | HOXB5 | HOXB6 | HOXB9 | HOXD10 | HPRT1 | HPX | HRAS | HSF1 | HSF4 | HSP90AA1 | HSP90AB1 | HSP90AB3P | HSP90AB4P | HSP90B1 | HSPA1B | HSPA2 | HSPA5 | HSPA8 | HSPB1 | HSPBP1 | HSPD1 | HSPE1 | HTATIP2 | HTRA1 | HTRA2 | HTT | IBTK | ICAM1 | ID2 | IDE | IFI16 | IFNA2 | IGBP1 | IGF1R | IGF2BP1 | IKBKB | IKBKG | IKZF5 | ILF2 | ILF3 | ILK | ING1 | ING5 | INSIG1 | INSIG2 | INSR | IPO5 | IPO7 | IQGAP1 | IREB2 | IRF1 | IRF4 | IRF7 | ISG15 | ITCH | ITGAV | ITGB1BP1 | ITGB3 | ITGB3BP | JAK3 | JMJD6 | JUN | JUNB | JUND | JUP | KANK2 | KAT5 | KAT7 | KAT8 | KDM1A | KDM2A | KDM4D | KDM5B | KDM5C | KDM8 | KDR | KEAP1 | KHDRBS1 | KLF5 | KLF6 | KLHDC10 | KMT2A | KMT2B | KMT2C | KPNA1 | KPNA2 | KRAS | KSR1 | L3MBTL1 | L3MBTL2 | L3MBTL3 | L3MBTL4 | LAMP3 | LARP4 | LAT | LATS1 | LCP2 | LDLRAD4 | LEF1 | LEMD2 | LEMD3 | LGALS3 | LGMN | LGR4 | LIMS1 | LMNA | LMO2 | LMO7 | LOXL2 | LPAR1 | LPIN3 | LRIF1 | LRP6 | LRPPRC | LRRK1 | LRRK2 | LTBR | LUM | LZTR1 | MAD2L1 | MAF1 | MAP2K1 | MAP2K3 | MAP2K4 | MAP2K6 | MAP2K7 | MAP3K1 | MAP3K13 | MAP3K14 | MAP3K2 | MAP3K3 | MAP3K4 | MAP4K1 | MAPK1 | MAPK14 | MAPK15 | MAPK3 | MAPK7 | MAPK8 | MAPK8IP3 | MAPK9 | MAPKAP1 | MAPT | MAS1 | MAVS | MBD3 | MBP | MC1R | MDFI | MDM2 | MDM4 | ME1 | MECP2 | MED1 | MED12 | MED14 | MED17 | MED20 | MED21 | MED23 | MED24 | MED31 | MED4 | MED6 | MEOX2 | MEPCE | METTL23 | MGA | MGMT | MIF | MITF | MKNK2 | MLH1 | MLLT1 | MLLT11 | MLLT3 | MLST8 | MOB1B | MORF4L1 | MORF4L2 | MOV10 | MRGBP | MRPL12 | MSH6 | MSN | MTA1 | MTA3 | MTERF1 | MTPN | MXD1 | MYBBP1A | MYC | MYD88 | MYH6 | MYO6 | MYOC | NACC1 | NAMPT | NANOG | NBAS | NCBP1 | NCK1 | NCOA1 | NCOA2 | NCOA3 | NCOA4 | NCOR1 | NCOR2 | NDUFA13 | NEDD4 | NEDD8 | NEK1 | NEK7 | NELFB | NELFCD | NELFE | NELL1 | NEUROD1 | NF2 | NFATC1 | NFATC2 | NFE2L2 | NFKB2 | NFKBIA | NIF3L1 | NIPBL | NKRF | NKX2-1 | NLK | NMD3 | NME2 | NMNAT1 | NNT | NOD2 | NOS1AP | NOS2 | NOS3 | NOSTRIN | NPM1 | NQO1 | NQO2 | NR0B2 | NR1H2 | NR1H3 | NR1I2 | NR3C1 | NR4A1 | NR5A2 | NTF3 | NTRK1 | NUFIP1 | NUMA1 | NUP214 | NUP85 | NUP98 | NUPR1 | OAS2 | OAZ3 | OGFOD1 | OGT | ONECUT1 | OTUB1 | OTUD4 | OXSR1 | P2RX1 | P2RX4 | PA2G4 | PABPC1 | PABPC4 | PACSIN3 | PAFAH1B1 | PAIP1 | PAK2 | PAK6 | PAM16 | PAPLN | PARD3 | PARD6A | PARK7 | PARP1 | PARP14 | PATZ1 | PAWR | PAX8 | PAX9 | PAXIP1 | PCBD1 | PCBP1 | PCGF1 | PCGF5 | PCGF6 | PCNA | PCSK9 | PDCD10 | PDCD6 | PDE12 | PDE4D | PDE6G | PDK2 | PEBP1 | PEF1 | PELI1 | PER1 | PER2 | PFKM | PFN1 | PGAM1 | PHB | PHB2 | PHF10 | PHF20L1 | PHF21A | PIAS1 | PIAS3 | PIH1D1 | PIK3CA | PIK3CG | PIK3R1 | PIK3R3 | PIM1 | PIN1 | PINX1 | PIR | PKIG | PLA2G10 | PLA2G1B | PLA2G2A | PLA2G6 | PLAUR | PLGRKT | PLK1 | PLSCR1 | PLXNB2 | PML | PNKP | POLDIP3 | POLR2A | POLR2L | POT1 | POU2F3 | POU5F1 | PPARD | PPARG | PPARGC1A | PPIB | PPID | PPIE | PPM1A | PPM1D | PPME1 | PPP1CA | PPP1R11 | PPP1R12A | PPP1R14A | PPP1R16A | PPP1R2 | PPP1R3B | PPP1R3C | PPP1R8 | PPP2CA | PPP2R1A | PPP2R1B | PPP2R2A | PPP2R2D | PPP2R5D | PPP4R2 | PPP6R1 | PRDX1 | PRDX2 | PRDX3 | PRDX5 | PRDX6 | PRELID1 | PRKAA1 | PRKAA2 | PRKACA | PRKAG1 | PRKAR1A | PRKAR2B | PRKCD | PRKCE | PRKCZ | PRKD1 | PRKD2 | PRKDC | PRMT2 | PRMT3 | PRMT6 | PROX1 | PRPF19 | PRRC1 | PSAP | PSEN1 | PSMA1 | PSMA2 | PSMA3 | PSMA5 | PSMA6 | PSMB5 | PSMB6 | PSMB7 | PSMB8 | PSMC1 | PSMC2 | PSMC3 | PSMC4 | PSMC5 | PSMC6 | PSMD1 | PSMD10 | PSMD11 | PSMD12 | PSMD14 | PSMD2 | PSMD4 | PSMD9 | PSME2 | PSME3 | PTEN | PTGES2 | PTGES3 | PTK2 | PTN | PTPN1 | PTPN14 | PTPN22 | PTPN6 | PTPRK | PTPRR | PTTG1 | PUM1 | PUS7 | QARS | QRICH1 | RAB1A | RAB1B | RAB3GAP1 | RAC1 | RAD21 | RAD23A | RAD23B | RAD51 | RAD51AP1 | RAF1 | RAN | RANBP9 | RAP1A | RAP1B | RARA | RARG | RASD1 | RB1 | RB1CC1 | RBM17 | RBM25 | RBM3 | RBM4 | RBM8A | RBMX | RBPJ | RBX1 | RC3H1 | RDX | RECK | REL | RELA | REST | RFC1 | RFC2 | RFC3 | RFC4 | RFC5 | RFXANK | RGN | RGS2 | RHBDD1 | RHOA | RIF1 | RING1 | RIOK1 | RIPK1 | RIPK2 | RIPK3 | RIPK4 | RMND1 | RNF139 | RNF185 | RNF19A | RNF2 | RNF25 | RNF31 | RNF4 | RNF8 | RPA2 | RPL10 | RPL23 | RPL38 | RPL6 | RPLP1 | RPS13 | RPS14 | RPS2 | RPS3 | RPS4X | RPS6KA1 | RPS6KB2 | RPTOR | RRM2 | RTF1 | RUNX1 | RUNX1T1 | RUNX2 | RUVBL1 | RUVBL2 | RXRA | RXRB | RXRG | S100A1 | S100A12 | S100A7 | S100A9 | SAG | SAP18 | SARS | SART3 | SCML2 | SDC4 | SDCBP | SEMA7A | SEPT4 | SERPINB2 | SERPINB3 | SERPINB5 | SERPINB6 | SERPINB8 | SERPINE2 | SERPINH1 | SET | SETBP1 | SETDB1 | SF1 | SFMBT1 | SFN | SFPQ | SGTA | SH2B1 | SHC1 | SHMT1 | SHMT2 | SIN3A | SIRT1 | SIRT3 | SIRT5 | SIRT6 | SIRT7 | SKI | SLAMF1 | SLC9A1 | SLC9A3R1 | SLTM | SMAD2 | SMAD3 | SMAD4 | SMAD5 | SMAD9 | SMARCA2 | SMARCA4 | SMARCB1 | SMARCC1 | SMARCC2 | SMARCE1 | SMG5 | SMG6 | SMG8 | SMO | SMURF1 | SMURF2 | SNAI2 | SNCA | SND1 | SNF8 | SNRPA | SNTA1 | SNW1 | SNX12 | SNX6 | SOAT1 | SOCS1 | SOD1 | SOD2 | SOX2 | SP1 | SP100 | SP2 | SPEN | SPI1 | SPIN1 | SPINK7 | SPINT2 | SPOP | SPRTN | SPRY2 | SQSTM1 | SRC | SRI | SRPK1 | SRPK2 | SRSF12 | SRSF5 | STAT1 | STAT3 | STAT4 | STAT5A | STAT6 | STK11 | STK24 | STK25 | STK26 | STK3 | STK38 | STK40 | STRAP | STUB1 | STYX | SUB1 | SUMO1 | SUMO2 | SUPT4H1 | SUPT5H | SUZ12 | SWAP70 | SYNJ2BP | TAB1 | TAB2 | TACO1 | TADA2B | TAF12 | TAF15 | TAGLN3 | TANK | TAOK3 | TARDBP | TAZ | TBL1X | TBRG4 | TCEA1 | TCEA2 | TCERG1 | TCF12 | TCF21 | TCF25 | TCF4 | TCOF1 | TCP1 | TCP10L | TDRD3 | TERF1 | TERF2 | TERF2IP | TFCP2 | TFDP1 | TFIP11 | TGFB1 | TGFBR3 | TGIF2LX | THAP11 | THAP7 | THBS1 | THRA | TIA1 | TIAL1 | TIMP1 | TIMP2 | TINF2 | TLE1 | TLE3 | TLR3 | TMBIM6 | TMEM173 | TMPO | TMTC3 | TNF | TNFAIP3 | TNFRSF1A | TNFRSF8 | TNFSF14 | TNIP2 | TNK2 | TNKS | TNKS2 | TNP1 | TNPO1 | TOE1 | TOLLIP | TOP2A | TP53 | TP63 | TP73 | TPR | TRA2A | TRADD | TRAF1 | TRAF2 | TRAF3 | TRAF3IP1 | TRAF6 | TRAP1 | TRIAP1 | TRIB2 | TRIB3 | TRIM14 | TRIM21 | TRIM25 | TRIM28 | TRIM29 | TRIM32 | TRIM33 | TRIM34 | TRIP12 | TRIP13 | TRIP4 | TRUB2 | TSC22D1 | TSC22D3 | TSC22D4 | TSFM | TSG101 | TSPYL2 | TTC5 | TULP3 | TWF1 | TWIST1 | TXN | TXNIP | TYMS | U2AF2 | UBA3 | UBB | UBC | UBE2B | UBE2C | UBE2D1 | UBE2D3 | UBE2I | UBE2J1 | UBE2K | UBE2L3 | UBE2N | UBE2O | UBE2S | UBE2V1 | UBE3A | UBQLN1 | UBQLN2 | UBQLN4 | UBR5 | UBXN1 | UCHL1 | UCHL5 | UNC119 | UNK | UNKL | UPF3B | UQCC1 | USP14 | USP15 | USP16 | USP17L2 | USP19 | USP2 | USP21 | USP4 | USP47 | USP5 | USP50 | USP7 | USP9X | USP9Y | VCP | VDR | VGLL4 | VHL | VIM | VPS72 | VTN | WARS | WAS | WBP2 | WDR35 | WDR48 | WDR77 | WDTC1 | WNK1 | WNT16 | WNT2 | WT1 | WTAP | WWOX | WWP2 | XBP1 | XIAP | XPO1 | XRCC1 | XRCC5 | XRCC6 | YAF2 | YAP1 | YEATS2 | YES1 | YTHDF1 | YWHAB | YWHAE | YWHAG | YWHAH | YWHAQ | YWHAZ | YY1 | ZBTB16 | ZBTB17 | ZBTB21 | ZBTB25 | ZBTB33 | ZBTB42 | ZBTB7A | ZBTB7B | ZC3H4 | ZFP41 | ZHX1 | ZIC1 | ZKSCAN1 | ZMPSTE24 | ZMYM5 | ZMYND8 | ZNF207 | ZNF217 | ZNF227 | ZNF24 | ZNF277 | ZNF354C | ZNF408 | ZNF420 | ZNF443 | ZNF517 | ZNF518A | ZNF592 | ZNF598 | ZNF618 | ZNF622 | ZNF646 | ZNF669 | ZNF696 | ZNF746 | ZNF747 | ZRANB2 | ZSCAN2 | ZSCAN29 | ZSCAN32 | ZZZ3 | GO:0071345 | 0.0344534756763314 | 284/8243 | 136/3250 | 0.00196675673917348 | 1 | T | T | F | F | cellular response to cytokine stimulus | ACTG1 | ACTR2 | ACTR3 | AKT1 | ASS1 | ATIC | BAG4 | BCAT2 | BRCA1 | BSPRY | BTK | CAPN2 | CCL21 | CCL22 | CD40 | CDC34 | CDC42EP4 | CDC5L | CDK4 | CDK9 | CEBPA | CREB1 | CRKL | CTH | CXCL8 | DAPK1 | DHX9 | DTX1 | DUSP1 | EED | EPRS | FASN | FGB | FLNB | GAPDH | GBP2 | GBP3 | GBP5 | GBP6 | GFPT2 | GNPNAT1 | GPD1 | GSK3A | GSK3B | HAX1 | HDAC4 | HDGF | HIF1A | HK2 | HNRNPU | HSP90AB1 | HSPA5 | HTRA2 | ICAM1 | IFI16 | IFIT1 | IFNAR1 | IKBKB | IL3RA | IMPDH2 | IRF1 | ITGA4 | KDM5B | KEAP1 | KIF5B | KLF5 | LEF1 | MAPK1 | MAPK14 | MAPK3 | MAPK8 | MAT2A | MCM2 | MME | MYO1C | NDUFA13 | NFE2L2 | NOS2 | NR5A2 | NRP2 | OTUB1 | OXSR1 | P4HB | PDCD10 | PDE12 | PDIA3 | PFKP | PHB | PLA2G10 | PML | PPARGC1A | PRPF8 | PTPN14 | PTPN23 | PTPN4 | PTPN6 | RAD23B | RARG | RBMX | RC3H1 | RELA | RHOA | RIF1 | RIPK1 | RPS3 | SHMT1 | SIRT1 | SLC12A2 | SLC25A5 | SMAD3 | SPRY2 | ST3GAL6 | STAT1 | STAT3 | STIP1 | STXBP1 | STXBP2 | TANK | THBS1 | TLR3 | TMEM173 | TPR | TRADD | TRAF6 | TREX1 | TRIM25 | UBE2K | VCAM1 | VIM | WAS | WDR35 | WNK1 | XBP1 | XRCC5 | YY1 | ZFAND6 | GO:0046903 | 0.0787334708237292 | 649/8243 | 291/3250 | 0.00198139403026462 | 1 | T | T | F | F | secretion | A2M | ACAA1 | ACLY | ACTN1 | ACTN2 | ACTN4 | ACTR2 | ADA | ADCYAP1 | AGL | AGR2 | AHSG | ALB | ALDOA | ALDOC | ALOX5 | ANK1 | ANXA1 | ANXA2 | ANXA5 | AP1M1 | APEH | APOA1 | APP | APRT | ARL4D | ARMC8 | ARPC5 | ATP6V1D | AZU1 | BDKRB2 | BTK | CAB39 | CALM1 | CAND1 | CANX | CAPN1 | CAT | CAV1 | CBL | CCR1 | CCT2 | CCT8 | CD109 | CD36 | CD40LG | CDA | CDK16 | CDK5 | CEL | CEP290 | CHP1 | CHRM3 | CKAP4 | CLU | COPA | COPB1 | COPG1 | COPS5 | COTL1 | CPLX1 | CPLX3 | CPNE3 | CREB1 | CSNK2B | CTSA | CTSC | CTSD | CYB5R1 | CYB5R3 | DBNL | DDOST | DDX3X | DEGS1 | DERA | DVL1 | DYNC1H1 | DYNC1LI1 | DYNLL1 | DYNLT1 | EEF1A1 | EEF2 | EXOC6 | F5 | FABP5 | FAF2 | FAM129B | FAM3C | FAM49B | FCN1 | FGB | FGL2 | FKBP1B | FN1 | FTH1 | FTL | GAA | GAD2 | GALNS | GBP5 | GDI2 | GHDC | GLA | GLS | GLS2 | GNS | GOLPH3 | GOT2 | GPI | GSTP1 | GUSB | GYG1 | HAP1 | HEXB | HIF1A | HK2 | HMGB1 | HNF1A | HRNR | HSP90AA1 | HSP90AB1 | HSPA1B | HSPA8 | HUWE1 | IDH1 | ILF2 | IMPDH2 | IQGAP1 | ISLR | ITGAV | ITGB3 | JUP | KCMF1 | KCNAB2 | KPNB1 | LAT | LCP2 | LGALS3 | LGALS3BP | LRRC7 | MAN2B1 | MAPK1 | MAPK14 | MECP2 | MED1 | MGST1 | MIF | MME | MMRN1 | MTX1 | MYO5A | NAPRT | NCOA1 | NEUROD1 | NHLRC3 | NIT2 | NMB | NME1 | NME2 | NOD2 | NOS2 | NPR1 | NRXN1 | OLA1 | ORM1 | P2RX1 | PA2G4 | PAFAH1B1 | PARK7 | PDIA4 | PDXK | PFKL | PGAM1 | PGM1 | PGM2 | PGRMC1 | PI4K2A | PIK3C2A | PIK3CG | PKM | PLA2G10 | PLA2G12A | PLA2G1B | PLA2G2A | PLAUR | PNP | PPAT | PPIA | PPIE | PPT1 | PRCP | PRDX4 | PRDX6 | PRG2 | PRKCD | PSAP | PSEN1 | PSMA2 | PSMA5 | PSMB7 | PSMC2 | PSMC3 | PSMD1 | PSMD11 | PSMD12 | PSMD14 | PSMD2 | PTGES2 | PTPN6 | PYGB | PYGL | QSOX1 | RAB10 | RAB11A | RAB11B | RAB11FIP2 | RAB18 | RAB1A | RAB24 | RAB31 | RAB5B | RAB7A | RAC1 | RAF1 | RAP1A | RAP1B | RAPGEF4 | RBPJ | RHOA | RNASE3 | S100A11 | S100A12 | S100A13 | S100A7 | S100A9 | S100P | SCCPDH | SCG2 | SCG3 | SDCBP | SEL1L | SELL | SERPINB3 | SERPINB6 | SERPINE2 | SLC2A5 | SLC9A3R1 | SMAD2 | SNAP29 | SNCA | SOCS2 | SOD1 | SPTAN1 | SRP14 | STAT5A | STBD1 | STOM | STXBP1 | STXBP2 | SYN1 | TAGLN2 | TANGO2 | TCIRG1 | TEX264 | TGFB1 | THBS1 | TIMP1 | TIMP2 | TMEM173 | TMEM30A | TOLLIP | TPD52 | TRIM9 | TSG101 | TUBA4A | TUBB | TUBB4B | TXLNA | TXNDC5 | UBR4 | UMPS | VAPA | VAT1 | VCL | VCP | VDR | VGF | WDR1 | XRCC5 | XRCC6 | ZBTB7B | GO:0060627 | 0.0406405434914473 | 335/8243 | 158/3250 | 0.00199258275163959 | 1 | T | T | F | F | regulation of vesicle-mediated transport | ABL1 | ABL2 | ACTN2 | ACTN4 | ADORA2A | AHI1 | AHSG | AKT2 | ANXA1 | ANXA2 | AP1G1 | APOA1 | APOE | APP | ARF1 | ARFGAP1 | ARHGAP1 | ARRB1 | ARRB2 | ATAD1 | ATG5 | ATP13A2 | ATP2A2 | ATXN2 | AZU1 | BACE1 | BIN1 | BRAF | BTBD9 | C4A | CALM1 | CALM3 | CALR | CAMK1D | CAMK2A | CAV1 | CBL | CCL21 | CD22 | CD36 | CDK5 | CFTR | CHMP2A | CHRM2 | CLU | CPLX1 | CPLX3 | DAB2 | DLG4 | DNM1L | DVL1 | EHD4 | EZR | FGB | FLOT1 | GIT1 | GNAI2 | GOPC | GSK3B | HAP1 | HGS | HIST1H1A | HMGB1 | HMOX1 | HNRNPK | HTR1B | IL13RA2 | INSIG1 | ITGAV | ITGB3 | KIF5B | LGALS3 | LRRK2 | LRSAM1 | LYPLA1 | MAP2K1 | MAPK1 | MAPK15 | MAPK3 | MIB1 | MSN | NR1H2 | NR1H3 | NTF3 | P2RX1 | PACSIN3 | PCSK9 | PDCD6IP | PICK1 | PLA2G6 | PPARG | PPP3CC | PPT1 | PREPL | PRKAA1 | PRKD1 | PSEN1 | PTEN | PTPN1 | PTPN23 | RAB10 | RAB11A | RAB11B | RAB21 | RAB29 | RAB31 | RAB3GAP1 | RAB5A | RAB5B | RAB7A | RAB9A | RAC1 | RAP1A | RAP1B | RAPGEF4 | RDX | REST | RNF139 | SCFD1 | SCYL2 | SDC1 | SDC4 | SDCBP | SEC24B | SEPT1 | SEPT4 | SEPT5 | SEPT8 | SH3GL1 | SH3GL2 | SH3GL3 | SNCA | SNF8 | SNX12 | SNX17 | SNX3 | SNX6 | SOD1 | SPACA3 | SRC | STXBP1 | STXBP2 | SYN1 | SYNJ2BP | SYP | TBC1D4 | TGFB1 | TNF | TNK2 | TOR1A | TRIM9 | TSG101 | UBQLN2 | UNC119 | USP46 | VPS18 | VPS4B | VTN | GO:0015949 | 0.0031541914351571 | 26/8243 | 18/3250 | 0.00199662855307278 | 1 | T | T | F | F | nucleobase-containing small molecule interconversion | AK1 | AK2 | AK4 | CMPK1 | CTPS1 | DUT | GLRX | GMPR | GSR | NME1 | NME2 | NME4 | NUDT13 | RRM1 | RRM2 | RRM2B | TXNRD1 | TYMS | GO:0009156 | 0.0031541914351571 | 26/8243 | 18/3250 | 0.00199662855307278 | 1 | T | T | F | F | ribonucleoside monophosphate biosynthetic process | ADA | ADK | ADSL | ADSS | APRT | ATIC | CMPK1 | GART | GMPS | HPRT1 | IMPDH2 | PAICS | PFAS | PPAT | RFK | UCK2 | UMPS | UPP2 | GO:0046148 | 0.0031541914351571 | 26/8243 | 18/3250 | 0.00199662855307278 | 1 | T | T | F | F | pigment biosynthetic process | ADA | ALAS1 | AP3D1 | APRT | COX15 | FECH | GART | GMPS | HMBS | HPRT1 | MC1R | MTHFD1 | MYO5A | PAICS | PPAT | PPOX | PRTFDC1 | SHMT1 | GO:1903793 | 0.0031541914351571 | 26/8243 | 18/3250 | 0.00199662855307278 | 1 | T | T | F | F | positive regulation of anion transport | ABAT | ACE2 | ADORA2A | APOA1 | APOE | CFTR | FABP3 | MAP2K6 | MIF | P2RX4 | PLA2G10 | PLA2G6 | PRELID1 | PRKCD | PSEN1 | RAB3GAP1 | STXBP1 | TRIAP1 | GO:0045765 | 0.0201382991629261 | 166/8243 | 84/3250 | 0.00207088291821392 | 1 | T | T | F | F | regulation of angiogenesis | ABL1 | AGO1 | AGO2 | AKT3 | ANXA1 | ATF2 | BRCA1 | CCR3 | CD40 | CDH5 | COL4A2 | CTNNB1 | CXCL8 | DCN | E2F2 | EGLN1 | ERBB2 | ETS1 | FAM129B | FASLG | FGF2 | FKBPL | GLUL | GTF2I | HDAC5 | HDAC7 | HDAC9 | HGS | HIF1A | HK2 | HMOX1 | HOXA5 | HSPB1 | HSPG2 | HTATIP2 | ITGB1BP1 | JAK1 | JUP | KDR | MAP3K3 | MAPK7 | MECP2 | MEOX2 | NFE2L2 | NOS3 | NPR1 | PDCD10 | PDCD6 | PGK1 | PIK3C2A | PKM | PLCG1 | PML | PPARG | PRKD1 | PRKD2 | PTN | RECK | RHOA | RHOJ | RUNX1 | S100A1 | SARS | SIRT1 | SIRT6 | SP1 | SP100 | SPRY2 | STARD13 | STAT1 | STAT3 | STIM1 | SYNJ2BP | TBXA2R | THBS1 | THBS2 | TLR3 | TNFAIP3 | TNFRSF12A | TNFSF12 | TWIST1 | VASH2 | WARS | XBP1 | GO:0031323 | 0.430304500788548 | 3547/8243 | 1462/3250 | 0.0020723138207984 | 1 | T | T | F | F | regulation of cellular metabolic process | A2M | AARS | ABAT | ABCE1 | ABCG1 | ABHD14B | ABL1 | ABL2 | ACACA | ACADVL | ACD | ACE2 | ACLY | ACO1 | ACSL1 | ACTA2 | ACTB | ACTL6A | ACTN1 | ACTN2 | ACTN4 | ACTR2 | ACTR3 | ADCY6 | ADCYAP1 | ADORA2A | ADRB2 | ADRM1 | ADTRP | AEBP2 | AES | AFF1 | AFF4 | AGAP2 | AGER | AGO1 | AGO2 | AHCYL1 | AHI1 | AHNAK | AHSG | AICDA | AIFM1 | AIMP2 | AIRE | AKAP8 | AKAP9 | AKT1 | AKT2 | AKTIP | ALOX12 | ANKRD49 | ANP32A | ANXA1 | ANXA2 | APBB1 | APBB2 | APBB3 | APC | APCS | APEX1 | APOA1 | APOD | APOE | APP | AR | ARAF | ARF1 | ARF4 | ARHGAP35 | ARID3B | ARID4B | ARNT | ARNT2 | ARR3 | ARRB1 | ARRB2 | ASCC1 | ASCC2 | ASCL1 | ASPSCR1 | ASS1 | ATAD1 | ATAD5 | ATCAY | ATF2 | ATF6 | ATF6B | ATF7 | ATG5 | ATM | ATOH1 | ATP13A2 | ATP1A1 | ATP6V1A | ATP6V1B1 | ATP6V1C1 | ATP6V1D | ATP6V1E1 | ATRX | ATXN1 | ATXN2 | AURKB | AXIN1 | AZU1 | B3GAT3 | BABAM1 | BACH2 | BAG2 | BAG3 | BAG4 | BAG5 | BAG6 | BANP | BASP1 | BAX | BBC3 | BCAR3 | BCL10 | BCL11A | BCL2 | BCL2L13 | BCL6 | BCL6B | BCOR | BDKRB2 | BIN1 | BIRC2 | BIRC3 | BIRC5 | BIRC7 | BIRC8 | BLZF1 | BMI1 | BMPR1A | BOLA1 | BOLA3 | BRAF | BRCA1 | BRD2 | BRD3 | BRD4 | BTK | BTRC | BUD31 | C1QBP | C1QL4 | C1QTNF1 | C4A | CAB39 | CALCOCO2 | CALM1 | CALM2 | CALM3 | CALR | CAMK1D | CAMK2A | CAND1 | CAPN1 | CAPN2 | CAPNS1 | CARD9 | CARM1 | CASP10 | CASP3 | CASP8 | CASP9 | CAT | CAV1 | CBFA2T3 | CBFB | CBL | CBLB | CBLC | CBX4 | CBX7 | CBX8 | CBY1 | CCAR2 | CCDC22 | CCDC8 | CCDC88A | CCL21 | CCL22 | CCNB1 | CCNT1 | CCNT2 | CCNY | CCNYL1 | CCR1 | CCT2 | CCT3 | CCT4 | CCT5 | CCT6A | CCT7 | CCT8 | CD109 | CD27 | CD2BP2 | CD36 | CD40 | CD40LG | CD74 | CD81 | CDA | CDC14B | CDC20 | CDC25A | CDC25B | CDC25C | CDC37 | CDC5L | CDC73 | CDH1 | CDK2 | CDK4 | CDK5 | CDK5R1 | CDK8 | CDK9 | CDKN1A | CDKN2C | CEBPA | CEBPE | CENPF | CENPJ | CEP290 | CHCHD10 | CHCHD2 | CHCHD3 | CHD3 | CHD4 | CHD8 | CHEK2 | CHORDC1 | CHP1 | CHRD | CHRNA9 | CIRBP | CISD1 | CISD2 | CKS1B | CKS2 | CLK1 | CLPX | CLU | CNDP1 | COA3 | COMMD1 | COMT | COPS2 | COPS5 | COQ3 | COQ7 | COX11 | COX17 | COX7A2 | CPSF7 | CPT1A | CREB1 | CREB3 | CREBBP | CRK | CRKL | CRP | CRY1 | CRY2 | CRYAB | CSNK1A1 | CSNK1E | CSNK2A2 | CSNK2B | CTBP1 | CTCF | CTDP1 | CTH | CTNNB1 | CTNNBIP1 | CTSA | CTSC | CTTN | CUL2 | CUL3 | CYCS | CYLD | DAB1 | DAB2 | DAPK1 | DAXX | DAZAP1 | DBF4B | DBNDD2 | DBNL | DCN | DCP2 | DCUN1D1 | DDA1 | DDIT4 | DDRGK1 | DDX1 | DDX39B | DDX3X | DERL1 | DGKZ | DHCR24 | DHX36 | DHX9 | DIABLO | DIS3 | DLG2 | DLG3 | DLG4 | DMAP1 | DMD | DMRT3 | DNA2 | DNAJA1 | DNAJA3 | DNAJB1 | DNAJB2 | DNAJC10 | DNAJC30 | DNM1L | DOCK7 | DPEP1 | DPF2 | DROSHA | DTX1 | DUSP1 | DUSP12 | DUSP14 | DUSP16 | DUSP19 | DUSP22 | DUSP7 | DVL1 | DVL2 | DVL3 | DYNLL1 | DYRK2 | E2F1 | E2F2 | E2F3 | E2F4 | E2F6 | EAF2 | EBF1 | ECD | EDA | EDEM1 | EED | EEF1A1 | EEF1A2 | EEF2 | EFEMP1 | EGFR | EGLN1 | EGLN3 | EHD4 | EHMT1 | EHMT2 | EID1 | EIF2AK3 | EIF2AK4 | EIF2B5 | EIF2S1 | EIF3A | EIF3D | EIF3E | EIF3H | EIF4A3 | EIF4E | EIF4E2 | EIF4EBP1 | EIF4ENIF1 | EIF4G3 | EIF4H | EIF5 | EIF5A | EIF5B | EIF6 | ELAVL1 | ELF3 | ELF4 | ENDOG | ENO1 | EP300 | EPHA4 | EPM2A | EPPIN | EPRS | ERBB2 | ERBB3 | ERF | ERG | ERLIN2 | ERRFI1 | ESR1 | ESR2 | ESRRA | ESRRB | ESRRG | ETS1 | EWSR1 | EXOSC1 | EXOSC5 | EXOSC6 | EXOSC9 | EYA2 | EYA4 | EZH2 | EZR | FABP1 | FABP3 | FABP5 | FADD | FAF1 | FAM129B | FAM192A | FAM3C | FAM83D | FANCA | FANCI | FANCM | FASLG | FASN | FBP1 | FBXO7 | FBXW11 | FGB | FGF2 | FGFR1 | FGFR1OP | FGFR3 | FHL2 | FIGNL1 | FIS1 | FKBP1A | FKBP1B | FKBP8 | FLOT1 | FN1 | FOS | FOXA1 | FOXD4 | FOXD4L6 | FOXI2 | FOXJ1 | FOXK1 | FOXK2 | FOXM1 | FOXO1 | FTO | FUBP1 | FUBP3 | FUS | FXR2 | FYN | FZR1 | G3BP2 | G6PD | GABPB1 | GADD45G | GAPDH | GAPDHS | GATA1 | GDF9 | GEMIN5 | GFAP | GLA | GLMN | GLO1 | GNA12 | GNAI2 | GNAI3 | GNAQ | GOLGA2 | GPD1 | GPD1L | GPI | GPS2 | GPSM1 | GPX1 | GRB2 | GRB7 | GRIK2 | GRIP1 | GSK3A | GSK3B | GSTP1 | GSTZ1 | GTF2A1 | GTF2F1 | GTF2F2 | GTF2I | GZMA | H1F0 | H1FX | H2AFX | H2AFZ | H3F3A | HAP1 | HAVCR2 | HAX1 | HBEGF | HBZ | HDAC1 | HDAC2 | HDAC3 | HDAC4 | HDAC5 | HDAC6 | HDAC7 | HDAC9 | HDGF | HELZ2 | HERC1 | HEXB | HEXIM1 | HGS | HIF1A | HIF1AN | HINT1 | HIST1H1A | HIST1H3A | HIST1H4A | HIST2H3A | HIVEP1 | HK2 | HLX | HMGB1 | HMGB2 | HMGB3 | HMOX1 | HNF1A | HNF4A | HNF4G | HNRNPA1 | HNRNPA2B1 | HNRNPAB | HNRNPD | HNRNPF | HNRNPH1 | HNRNPK | HNRNPL | HNRNPM | HNRNPU | HOXA5 | HOXB5 | HOXB6 | HOXB9 | HOXD10 | HPRT1 | HPX | HRAS | HSF1 | HSF4 | HSP90AA1 | HSP90AB1 | HSP90AB3P | HSP90AB4P | HSP90B1 | HSPA1B | HSPA2 | HSPA5 | HSPA8 | HSPB1 | HSPBP1 | HSPD1 | HSPE1 | HTATIP2 | HTRA1 | HTRA2 | HTT | IBTK | ICAM1 | ID2 | IDE | IDH1 | IFI16 | IFNA2 | IGBP1 | IGF1R | IGF2BP1 | IKBKB | IKBKG | IKZF5 | ILF2 | ILF3 | ILK | ING1 | ING5 | INSIG1 | INSIG2 | INSR | IPO5 | IPO7 | IQGAP1 | IREB2 | IRF1 | IRF4 | IRF7 | ISG15 | ITCH | ITGAV | ITGB1BP1 | ITGB3 | ITGB3BP | ITPR1 | JAK3 | JMJD6 | JUN | JUNB | JUND | JUP | KANK2 | KAT5 | KAT7 | KAT8 | KDM1A | KDM2A | KDM4D | KDM5B | KDM5C | KDM8 | KDR | KEAP1 | KHDRBS1 | KLF5 | KLF6 | KLHDC10 | KLHL22 | KMT2A | KMT2B | KMT2C | KPNA1 | KPNA2 | KRAS | KSR1 | L3MBTL1 | L3MBTL2 | L3MBTL3 | L3MBTL4 | LAMP3 | LARP4 | LARS | LAT | LATS1 | LCP2 | LDLRAD4 | LEF1 | LEMD2 | LEMD3 | LGALS3 | LGMN | LGR4 | LIMS1 | LMNA | LMO2 | LMO7 | LOXL2 | LPAR1 | LPIN3 | LRIF1 | LRP6 | LRPPRC | LRRK1 | LRRK2 | LRSAM1 | LTBR | LUM | LZTR1 | MAD2L1 | MAF1 | MAP2K1 | MAP2K3 | MAP2K4 | MAP2K6 | MAP2K7 | MAP3K1 | MAP3K13 | MAP3K14 | MAP3K2 | MAP3K3 | MAP3K4 | MAP4K1 | MAPK1 | MAPK14 | MAPK15 | MAPK3 | MAPK7 | MAPK8 | MAPK8IP3 | MAPK9 | MAPKAP1 | MAPT | MAS1 | MAVS | MBD3 | MBP | MC1R | MCM7 | MDFI | MDM2 | MDM4 | ME1 | MECP2 | MED1 | MED12 | MED14 | MED17 | MED20 | MED21 | MED23 | MED24 | MED31 | MED4 | MED6 | MEOX2 | MEPCE | METTL23 | MGA | MGMT | MIF | MITF | MKNK2 | MLH1 | MLLT1 | MLLT11 | MLLT3 | MLST8 | MOB1B | MORF4L1 | MORF4L2 | MOV10 | MRGBP | MRPL12 | MSH6 | MSN | MTA1 | MTA3 | MTERF1 | MTPN | MXD1 | MYBBP1A | MYC | MYD88 | MYH6 | MYO1D | MYO6 | MYOC | NACC1 | NAMPT | NANOG | NBAS | NCBP1 | NCK1 | NCOA1 | NCOA2 | NCOA3 | NCOA4 | NCOR1 | NCOR2 | NDUFA13 | NEDD4 | NEDD8 | NEK1 | NEK7 | NELFB | NELFCD | NELFE | NELL1 | NEUROD1 | NF2 | NFATC1 | NFATC2 | NFE2L2 | NFKB2 | NFKBIA | NIF3L1 | NIPBL | NKRF | NKX2-1 | NLK | NMD3 | NME2 | NMNAT1 | NNT | NOD2 | NOS1AP | NOS2 | NOS3 | NOSTRIN | NPM1 | NQO1 | NQO2 | NR0B2 | NR1H2 | NR1H3 | NR1I2 | NR3C1 | NR4A1 | NR5A2 | NRBF2 | NTF3 | NTRK1 | NUFIP1 | NUMA1 | NUP107 | NUP153 | NUP160 | NUP205 | NUP214 | NUP85 | NUP88 | NUP93 | NUP98 | NUPR1 | OAS2 | OAZ3 | OGFOD1 | OGT | ONECUT1 | OPTN | OTUB1 | OTUD4 | OXSR1 | P2RX1 | P2RX4 | PA2G4 | PABPC1 | PABPC4 | PACSIN3 | PAFAH1B1 | PAIP1 | PAK2 | PAK6 | PAM16 | PAPLN | PARD3 | PARD6A | PARK7 | PARP1 | PARP14 | PATZ1 | PAWR | PAX8 | PAX9 | PAXIP1 | PCBD1 | PCBP1 | PCGF1 | PCGF5 | PCGF6 | PCNA | PCSK9 | PDCD10 | PDCD6 | PDE12 | PDE4D | PDE6G | PDK2 | PEBP1 | PEF1 | PELI1 | PER1 | PER2 | PFKM | PFN1 | PGAM1 | PGK1 | PGK2 | PGP | PHB | PHB2 | PHF10 | PHF20L1 | PHF21A | PIAS1 | PIAS3 | PICK1 | PIH1D1 | PIK3CA | PIK3CG | PIK3R1 | PIK3R3 | PIKFYVE | PIM1 | PIN1 | PINX1 | PIP4K2A | PIR | PKIG | PLA2G10 | PLA2G1B | PLA2G2A | PLA2G6 | PLAUR | PLEKHF1 | PLGRKT | PLK1 | PLSCR1 | PLXNB2 | PML | PNKP | POLDIP2 | POLDIP3 | POLR2A | POLR2L | POM121 | POT1 | POU2F3 | POU5F1 | PPARD | PPARG | PPARGC1A | PPIB | PPID | PPIE | PPIF | PPM1A | PPM1D | PPME1 | PPP1CA | PPP1CB | PPP1R11 | PPP1R12A | PPP1R14A | PPP1R16A | PPP1R2 | PPP1R3B | PPP1R3C | PPP1R8 | PPP2CA | PPP2R1A | PPP2R1B | PPP2R2A | PPP2R2D | PPP2R5D | PPP4R2 | PPP6R1 | PRCP | PRDX1 | PRDX2 | PRDX3 | PRDX5 | PRDX6 | PRELID1 | PRKAA1 | PRKAA2 | PRKAB2 | PRKACA | PRKAG1 | PRKAR1A | PRKAR2B | PRKCD | PRKCE | PRKCZ | PRKD1 | PRKD2 | PRKDC | PRMT2 | PRMT3 | PRMT6 | PROX1 | PRPF19 | PRRC1 | PSAP | PSEN1 | PSMA1 | PSMA2 | PSMA3 | PSMA5 | PSMA6 | PSMB5 | PSMB6 | PSMB7 | PSMB8 | PSMC1 | PSMC2 | PSMC3 | PSMC4 | PSMC5 | PSMC6 | PSMD1 | PSMD10 | PSMD11 | PSMD12 | PSMD14 | PSMD2 | PSMD4 | PSMD9 | PSME2 | PSME3 | PTEN | PTGES2 | PTGES3 | PTK2 | PTN | PTPN1 | PTPN14 | PTPN22 | PTPN6 | PTPRK | PTPRR | PTTG1 | PUM1 | PUS7 | QARS | QRICH1 | QSOX1 | RAB1A | RAB1B | RAB29 | RAB3GAP1 | RAB5A | RAC1 | RAD21 | RAD23A | RAD23B | RAD51 | RAD51AP1 | RAE1 | RAF1 | RAN | RANBP9 | RAP1A | RAP1B | RARA | RARG | RASD1 | RB1 | RB1CC1 | RBBP6 | RBM17 | RBM25 | RBM3 | RBM4 | RBM8A | RBMX | RBPJ | RBX1 | RC3H1 | RDX | RECK | REL | RELA | REST | RFC1 | RFC2 | RFC3 | RFC4 | RFC5 | RFXANK | RGN | RGS2 | RHBDD1 | RHOA | RIF1 | RING1 | RIOK1 | RIPK1 | RIPK2 | RIPK3 | RIPK4 | RMND1 | RNF139 | RNF185 | RNF19A | RNF2 | RNF25 | RNF31 | RNF4 | RNF8 | ROMO1 | RPA2 | RPL10 | RPL23 | RPL38 | RPL6 | RPLP1 | RPS13 | RPS14 | RPS2 | RPS3 | RPS4X | RPS6KA1 | RPS6KB2 | RPTOR | RRM2 | RTF1 | RUNX1 | RUNX1T1 | RUNX2 | RUVBL1 | RUVBL2 | RXRA | RXRB | RXRG | S100A1 | S100A11 | S100A12 | S100A7 | S100A9 | SAG | SAP18 | SARS | SART3 | SCFD1 | SCML2 | SCYL2 | SDC4 | SDCBP | SEC13 | SEMA7A | SEPT4 | SERPINB2 | SERPINB3 | SERPINB5 | SERPINB6 | SERPINB8 | SERPINE2 | SERPINH1 | SET | SETBP1 | SETDB1 | SF1 | SFMBT1 | SFN | SFPQ | SGTA | SH2B1 | SH3GL2 | SHC1 | SHMT1 | SHMT2 | SIN3A | SIRT1 | SIRT3 | SIRT5 | SIRT6 | SIRT7 | SKI | SLAMF1 | SLAMF8 | SLC9A1 | SLC9A3R1 | SLTM | SMAD2 | SMAD3 | SMAD4 | SMAD5 | SMAD9 | SMARCA2 | SMARCA4 | SMARCB1 | SMARCC1 | SMARCC2 | SMARCE1 | SMC1A | SMC3 | SMG5 | SMG6 | SMG8 | SMO | SMURF1 | SMURF2 | SNAI2 | SNCA | SND1 | SNF8 | SNRPA | SNTA1 | SNW1 | SNX12 | SNX6 | SOAT1 | SOCS1 | SOCS2 | SOCS6 | SOD1 | SOD2 | SOGA3 | SOX2 | SP1 | SP100 | SP2 | SPEN | SPI1 | SPIN1 | SPINK7 | SPINT2 | SPRTN | SPRY2 | SPTLC2 | SQSTM1 | SRC | SRI | SRPK1 | SRPK2 | SRSF12 | SRSF5 | STAG2 | STAT1 | STAT3 | STAT4 | STAT5A | STAT6 | STK11 | STK24 | STK25 | STK26 | STK3 | STK38 | STK40 | STOML2 | STRAP | STUB1 | STYX | SUB1 | SUMO1 | SUMO2 | SUPT4H1 | SUPT5H | SUZ12 | SWAP70 | SYNJ2BP | TAB1 | TAB2 | TACO1 | TADA2B | TAF12 | TAF15 | TAGLN3 | TANK | TAOK3 | TARDBP | TAZ | TBL1X | TBRG4 | TCEA1 | TCEA2 | TCERG1 | TCF12 | TCF21 | TCF25 | TCF4 | TCOF1 | TCP1 | TCP10L | TDRD3 | TERF1 | TERF2 | TERF2IP | TFCP2 | TFDP1 | TFIP11 | TGFB1 | TGFBR3 | TGIF2LX | THAP11 | THAP7 | THBS1 | THRA | TIA1 | TIAL1 | TIMP1 | TIMP2 | TINF2 | TLE1 | TLE3 | TLR3 | TMBIM6 | TMEM173 | TMPO | TMTC3 | TNF | TNFAIP3 | TNFRSF1A | TNFRSF8 | TNFSF14 | TNIP2 | TNK2 | TNKS | TNKS2 | TNP1 | TNPO1 | TOE1 | TOLLIP | TOP2A | TP53 | TP63 | TP73 | TPCN2 | TPR | TRA2A | TRADD | TRAF1 | TRAF2 | TRAF3 | TRAF3IP1 | TRAF6 | TRAP1 | TREX1 | TRIAP1 | TRIB2 | TRIB3 | TRIM14 | TRIM21 | TRIM25 | TRIM28 | TRIM29 | TRIM32 | TRIM33 | TRIM34 | TRIP12 | TRIP13 | TRIP4 | TRUB2 | TSC22D1 | TSC22D3 | TSC22D4 | TSFM | TSG101 | TSPYL2 | TTC5 | TULP3 | TWF1 | TWIST1 | TXN | TXNIP | TYMS | U2AF2 | UBA3 | UBB | UBC | UBE2B | UBE2C | UBE2D1 | UBE2D3 | UBE2I | UBE2J1 | UBE2K | UBE2L3 | UBE2N | UBE2O | UBE2S | UBE2V1 | UBE3A | UBQLN1 | UBQLN2 | UBQLN4 | UBR5 | UBXN1 | UCHL1 | UCHL5 | ULK1 | UNC119 | UNK | UNKL | UPF3B | UQCC1 | USP14 | USP15 | USP16 | USP17L2 | USP19 | USP2 | USP21 | USP4 | USP46 | USP47 | USP5 | USP50 | USP7 | USP9X | USP9Y | VCP | VDAC1 | VDR | VGLL4 | VHL | VIM | VPS26A | VPS29 | VPS72 | VTN | WARS | WAS | WBP2 | WDR35 | WDR41 | WDR48 | WDR77 | WDTC1 | WNK1 | WNT16 | WNT2 | WT1 | WTAP | WWOX | WWP2 | XBP1 | XIAP | XPO1 | XRCC1 | XRCC5 | XRCC6 | YAF2 | YAP1 | YEATS2 | YES1 | YTHDF1 | YWHAB | YWHAE | YWHAG | YWHAH | YWHAQ | YWHAZ | YY1 | ZBTB16 | ZBTB17 | ZBTB21 | ZBTB25 | ZBTB33 | ZBTB42 | ZBTB7A | ZBTB7B | ZC3H4 | ZFP41 | ZHX1 | ZIC1 | ZKSCAN1 | ZMPSTE24 | ZMYM5 | ZMYND8 | ZNF207 | ZNF217 | ZNF227 | ZNF24 | ZNF277 | ZNF354C | ZNF408 | ZNF420 | ZNF443 | ZNF517 | ZNF518A | ZNF592 | ZNF598 | ZNF618 | ZNF622 | ZNF646 | ZNF669 | ZNF696 | ZNF746 | ZNF747 | ZRANB2 | ZSCAN2 | ZSCAN29 | ZSCAN32 | ZZZ3 | GO:0051641 | 0.173601843988839 | 1431/8243 | 613/3250 | 0.00208689107891352 | 1 | T | T | F | F | cellular localization | ABCD3 | ABCE1 | ABCG1 | ABHD17A | ACAA1 | ACACA | ACD | ACOX1 | ACTN2 | ACTN4 | ACTR1A | ACTR2 | ACTR3 | ADPRH | ADRB2 | AGER | AGK | AHCYL1 | AHI1 | AKAP9 | AKT1 | AKT2 | AKTIP | ALB | ANK1 | ANK3 | ANP32A | ANXA2 | AP1B1 | AP1G1 | AP1M1 | AP3D1 | AP3M1 | AP4M1 | AP4S1 | APOE | APP | APPBP2 | ARF1 | ARF4 | ARF5 | ARFGAP1 | ARFIP1 | ARHGAP1 | ARL4D | ARL5B | ASPSCR1 | ATCAY | ATG4C | ATL3 | ATM | ATP1B3 | ATP2A2 | ATP6V1D | ATRX | ATXN1 | AUP1 | AURKB | BACH2 | BAG3 | BAG4 | BAG6 | BANF1 | BANP | BAX | BIN1 | BLZF1 | BRAF | BSG | BTK | C16orf70 | CACNG2 | CACNG4 | CACNG5 | CALM1 | CALM3 | CALR | CAPZA1 | CAPZA2 | CAPZB | CARM1 | CAT | CAV1 | CBL | CBLB | CCDC155 | CCDC22 | CCDC88A | CCHCR1 | CCNB1 | CCP110 | CD2 | CD74 | CD81 | CDC37 | CDC42 | CDH1 | CDK5 | CENPF | CENPJ | CEP135 | CEP152 | CEP290 | CEP55 | CEP57 | CEP70 | CEP89 | CFTR | CHCHD10 | CHMP2A | CHP1 | CKAP5 | CLSTN1 | CLTC | CLU | CLUH | CNTRL | COG3 | COL4A3BP | COPA | COPB1 | COPE | COPG1 | CPLX1 | CPLX3 | CPT1A | CPT2 | CROCC | CROT | CSE1L | CSNK1E | CTCF | CTNNB1 | CTSA | CTSC | CTTN | CUL3 | CYC1 | DAB1 | DCLK1 | DCTN1 | DDX1 | DDX19B | DDX39A | DDX39B | DDX3X | DENND1A | DENND4C | DERL1 | DHRS4 | DHX9 | DLG2 | DLG3 | DLG4 | DMD | DNAJA1 | DNAJA3 | DNAJC19 | DNM1L | DOCK7 | DUSP16 | DVL1 | DVL2 | DYNC1H1 | DYNC1I1 | DYNC1I2 | DYNC1LI1 | DYNLL1 | DYNLT1 | ECH1 | ECI2 | EGFR | EHD4 | EHHADH | EIF4A3 | EIF4E | EIF5A | EIF6 | ESR1 | EXOC6 | EZH2 | EZR | F5 | F9 | FABP3 | FAF2 | FAM83D | FAM83H | FAM98A | FBF1 | FBXO7 | FCN1 | FERMT2 | FGFR1OP | FIS1 | FLOT1 | FOLR1 | FYTTD1 | GAK | GBP2 | GBP5 | GEMIN4 | GEMIN5 | GEMIN7 | GGA1 | GLRB | GOLGA2 | GOLGA4 | GOLPH3 | GOPC | GORASP2 | GRIA1 | GRID2 | GRIK2 | GRIP1 | GRPEL1 | GSTK1 | HAP1 | HDAC6 | HEATR3 | HERC2 | HGS | HID1 | HIF1A | HK1 | HK2 | HMGCL | HNRNPA1 | HNRNPA2B1 | HNRNPU | HSD17B4 | HSP90AA1 | HSP90B1 | HSPA4 | HSPA5 | HSPA8 | HSPB1 | HSPD1 | HTATIP2 | HTT | ICK | IDE | IDH1 | IFIT1 | IFT122 | IK | IKBKB | IKBKG | IPO11 | IPO4 | IPO5 | IPO7 | IPO8 | IPO9 | IQCB1 | ITGA4 | ITGB1BP1 | JUP | KIF13B | KIF2C | KIF5B | KIF5C | KLHL20 | KPNA1 | KPNA2 | KPNB1 | LARS | LAT | LATS1 | LEMD2 | LMAN2L | LMNA | LRP6 | LRPPRC | LRRC7 | LRRK2 | LRSAM1 | MAP1B | MAPK15 | MAPK8IP3 | MAPT | MARCH5 | MCFD2 | MCM3AP | MCM9 | MDM2 | MKS1 | MLH1 | MPPE1 | MTCH2 | MYO1C | MYO1D | MYO5A | MYO6 | NCBP1 | NCOA4 | NDUFA13 | NEDD4 | NEUROD1 | NIN | NIPBL | NMD3 | NOS2 | NPLOC4 | NPM1 | NRXN1 | NSFL1C | NUBP1 | NUDT19 | NUMA1 | NUP107 | NUP153 | NUP160 | NUP205 | NUP214 | NUP85 | NUP88 | NUP93 | NUP98 | NUSAP1 | NUTF2 | NXF1 | NXF2 | NXT2 | OBSL1 | OFD1 | OPA1 | OPTN | OXA1L | PACS1 | PACS2 | PAFAH1B1 | PAM16 | PARD3 | PCM1 | PDCD10 | PDCD6 | PDCD6IP | PEF1 | PEX19 | PEX5 | PHB2 | PICK1 | PIGU | PIH1D1 | PIK3CG | PIK3R1 | PIKFYVE | PINX1 | PLEKHF1 | PLK1 | PLXNA2 | PML | POLDIP3 | POLR2M | POM121 | POT1 | PPP2R1A | PQLC1 | PRKAA2 | PRKAB2 | PRKACA | PRKAG1 | PRKAR2B | PRKCZ | PROZ | PRPF19 | PSEN2 | PTK2 | PTPN23 | PTPRK | RAB10 | RAB11A | RAB11B | RAB18 | RAB1A | RAB1B | RAB21 | RAB24 | RAB29 | RAB2A | RAB31 | RAB32 | RAB35 | RAB3GAP1 | RAB3IP | RAB5A | RAB5B | RAB7A | RAB9A | RABGEF1 | RABL2A | RABL3 | RAC1 | RAD21 | RAE1 | RAN | RANBP3 | RASSF9 | RB1 | RBM8A | RDX | RHOT1 | RHOT2 | RIC3 | RNF126 | ROMO1 | RPA2 | RPAIN | RPGRIP1L | RPL10 | RPL10A | RPL12 | RPL18A | RPL22 | RPL23 | RPL23A | RPL24 | RPL30 | RPL37A | RPL38 | RPL6 | RPL7 | RPL7A | RPL9 | RPLP0 | RPLP1 | RPLP2 | RPS12 | RPS13 | RPS14 | RPS15A | RPS19 | RPS2 | RPS20 | RPS24 | RPS29 | RPS3 | RPS4X | RPS8 | RRS1 | RTKN | RTN4 | RUVBL2 | S100A13 | SCFD1 | SCG3 | SCLT1 | SCYL2 | SDCBP | SDCBP2 | SEC13 | SEC16A | SEC22C | SEC24B | SEC24C | SEC31A | SEC62 | SEC63 | SEL1L | SFPQ | SGTA | SGTB | SIN3A | SLC25A6 | SLC9A3R1 | SMG5 | SMG6 | SMN1 | SMO | SMURF1 | SNAP29 | SNCA | SNF8 | SNRPB | SNRPD1 | SNRPD3 | SNRPE | SNX17 | SNX2 | SNX6 | SOD1 | SORT1 | SPDL1 | SPICE1 | SPRY2 | SPTAN1 | SPTBN1 | SPTBN2 | SQSTM1 | SRP14 | SRP19 | SRSF11 | SRSF5 | SSB | SSX2IP | STAT3 | STBD1 | STIL | STK11 | STK25 | STOML2 | STX5 | STXBP1 | STXBP2 | STYX | SUMO1 | SUN2 | SYN1 | SYNE2 | SYNJ2BP | SYVN1 | TBC1D15 | TBC1D22B | TBC1D23 | TBC1D4 | TCTN2 | TCTN3 | TERF1 | TERF2 | TERF2IP | TFG | TGFB1 | THOC2 | THOC6 | THOC7 | TIA1 | TIMM13 | TIMM21 | TIMM23 | TIMM44 | TIMM50 | TINF2 | TM9SF4 | TMED9 | TMEM216 | TMEM30A | TNF | TNFAIP3 | TNFRSF1A | TNKS | TNKS2 | TNPO1 | TOLLIP | TOMM20 | TOMM22 | TOMM40 | TOR1A | TPR | TRAF3IP1 | TRAPPC8 | TRIM23 | TRIM9 | TSG101 | TSPAN17 | TUBA1A | TUBA1C | TUBA4A | TUBB | TUBB4A | TUBB4B | TUBG1 | TULP3 | TXNIP | U2AF2 | UBB | UBC | UBE2B | UBE2D1 | UBE2D2 | UBE2D3 | UBE2O | UBL4A | UCHL1 | UGT8 | UPF2 | UPF3B | USP4 | USP9X | VAPA | VAPB | VCL | VCP | VPS18 | VPS26A | VPS29 | VPS37D | VPS4B | VTA1 | WAS | WDR19 | WDR35 | WDR45B | WIPI1 | WIPI2 | XPO1 | XPO4 | XPO5 | XPO7 | XPOT | XRCC5 | YKT6 | YWHAB | YWHAE | YWHAG | YWHAH | YWHAZ | ZBTB16 | ZBTB7A | ZDHHC23 | ZFAND6 | ZMPSTE24 | ZW10 | GO:0009612 | 0.0137086012374136 | 113/8243 | 60/3250 | 0.00208878778596568 | 1 | T | T | F | F | response to mechanical stimulus | ACTA1 | ACTG1 | AKT1 | ASNS | ATP1A1 | BACE1 | BAG3 | BCL10 | CASP2 | CASP8 | CCNB1 | CD40 | CHRNA9 | DCN | DMD | EGFR | ENDOG | ETS1 | FADD | FOS | FYN | GOT1 | GPI | HDAC4 | IRF1 | JUN | JUNB | JUND | JUP | LTBR | MAP2K4 | MAP3K1 | MAP3K14 | MAP3K2 | MAPK14 | MAPK3 | MAPK8 | MAPK9 | MTPN | MYD88 | NFKBIA | NTRK1 | PPARG | RAC1 | RAF1 | RELA | RHOA | SCEL | SERPINE2 | SLC9A1 | SRC | STAT1 | TGFB1 | THBS1 | TLR3 | TNFRSF1A | TNFRSF8 | TNFSF14 | TXNIP | USP53 | GO:0002223 | 0.0103117796918598 | 85/8243 | 47/3250 | 0.00210411878703345 | 1 | T | T | F | F | stimulatory C-type lectin receptor signaling pathway | BCL10 | BTRC | CARD9 | CD209 | CREBBP | CUL1 | EP300 | FBXW11 | FYN | HRAS | IKBKB | IKBKG | KRAS | PAK2 | PRKACA | PRKCD | PSMA1 | PSMA2 | PSMA3 | PSMA5 | PSMA6 | PSMB5 | PSMB6 | PSMB7 | PSMB8 | PSMC1 | PSMC2 | PSMC3 | PSMC4 | PSMC5 | PSMC6 | PSMD1 | PSMD10 | PSMD11 | PSMD12 | PSMD14 | PSMD2 | PSMD4 | PSMD9 | PSME2 | PSME3 | RAF1 | RELA | SRC | TAB1 | TAB2 | TRAF6 | GO:0070987 | 0.00206235593837195 | 17/8243 | 13/3250 | 0.00214069613974608 | 1 | T | T | F | F | error-free translesion synthesis | NPLOC4 | PCNA | RFC1 | RFC2 | RFC3 | RFC4 | RFC5 | RPA2 | RPA3 | SPRTN | UBB | UBC | VCP | GO:1902036 | 0.00776416353269441 | 64/8243 | 37/3250 | 0.0021457733445894 | 1 | T | T | F | F | regulation of hematopoietic stem cell differentiation | ABL1 | CBFB | GATA1 | ITCH | KMT2A | LMO2 | NFE2L2 | PSMA1 | PSMA2 | PSMA3 | PSMA5 | PSMA6 | PSMB5 | PSMB6 | PSMB7 | PSMB8 | PSMC1 | PSMC2 | PSMC3 | PSMC4 | PSMC5 | PSMC6 | PSMD1 | PSMD10 | PSMD11 | PSMD12 | PSMD14 | PSMD2 | PSMD4 | PSMD9 | PSME2 | PSME3 | PUS7 | RUNX1 | TCF12 | TP73 | YAP1 | GO:0032388 | 0.0188038335557443 | 155/8243 | 79/3250 | 0.00214750946032449 | 1 | T | T | F | F | positive regulation of intracellular transport | ACTN2 | AKT2 | ANK3 | ANXA2 | ARF1 | B3GAT3 | BAG3 | CDH1 | CDK5 | CDK5R1 | CEP290 | CHP1 | CSNK2A2 | DAB2 | DHX9 | DMAP1 | DNM1L | DYNC1H1 | EDEM1 | ERBB2 | EZR | FIS1 | FYN | GSK3A | GSK3B | HDAC3 | HRAS | HSPA1L | HTRA2 | HUWE1 | IPO5 | ITGB1BP1 | JUP | KHDRBS1 | KIF5B | MAPK1 | MAPK14 | MAVS | MDM2 | MSN | MYO1C | NEDD4 | NPEPPS | NUMA1 | PCM1 | PDCD10 | PIK3R1 | PPM1A | PRKAA1 | PRKACA | PRKCD | PSEN1 | PTPN23 | RAB21 | RAB29 | RAN | RDX | RHOU | RNF31 | SAE1 | SEC16A | SFN | SMAD3 | SMO | STOM | STXBP1 | TARDBP | TGFB1 | TM9SF4 | TMEM30A | TP53 | TPR | TRIM28 | UBE2D3 | UBE2L3 | UBR5 | XPO4 | YWHAE | ZIC1 | GO:0002753 | 0.0033968215455538 | 28/8243 | 19/3250 | 0.00215847677574273 | 1 | T | T | F | F | cytoplasmic pattern recognition receptor signaling pathway | CASP8 | CYLD | IKBKG | IRF7 | ITCH | MAP2K6 | NFKBIA | NOD2 | RELA | RIPK2 | TAB1 | TAB2 | TMEM173 | TNFAIP3 | TRAF6 | UBB | UBC | UBE2N | UBE2V1 | GO:0009163 | 0.0033968215455538 | 28/8243 | 19/3250 | 0.00215847677574273 | 1 | T | T | F | F | nucleoside biosynthetic process | ADA | ADK | AMD1 | APRT | CDA | DCK | GMPS | HPRT1 | IMPDH2 | MTAP | NME1 | NME2 | NME4 | PRTFDC1 | TK1 | TK2 | UCK2 | UMPS | UPP2 | GO:0010950 | 0.0134659711270169 | 111/8243 | 59/3250 | 0.0021937772000648 | 1 | T | T | F | F | positive regulation of endopeptidase activity | ADRM1 | AGER | AIFM1 | ALOX12 | ARRB1 | BAX | BBC3 | BCL10 | BCL2L13 | CASP10 | CASP8 | CASP9 | CYCS | DAPK1 | DDX3X | DIABLO | EGLN3 | EIF2AK3 | EPHA4 | FADD | FASLG | FIS1 | FYN | HMGB1 | HSF1 | HSPD1 | HSPE1 | HTRA2 | IFI16 | LGMN | MAPT | MBP | MYC | NDUFA13 | P2RX1 | PDCD6 | PML | PPARG | PRELID1 | PSMD14 | PSME2 | PSME3 | REST | RHOA | RIPK1 | RIPK2 | RPS3 | S100A9 | SERPINB3 | SIRT1 | SMAD3 | SNCA | STAT3 | TNF | TRADD | TRAF2 | USP50 | VCP | WDR35 | GO:0044262 | 0.0118888754094383 | 98/8243 | 53/3250 | 0.00219556084627829 | 1 | T | T | F | F | cellular carbohydrate metabolic process | AGL | AKR1B1 | AKT1 | AKT2 | B3GNT3 | BRAF | CALM1 | COQ2 | COQ3 | FBP1 | FBP2 | GAA | GBE1 | GOT1 | GSK3A | GSK3B | GUSB | GYG1 | GYG2 | GYS1 | HK1 | HK2 | HS2ST1 | IDH1 | IDH2 | IMPA1 | IMPA2 | ISYNA1 | MECP2 | MTMR7 | NAGK | PER2 | PFKM | PGD | PGM1 | PGM2 | PGP | PHKA1 | PPP1CA | PPP1CB | PPP1CC | PPP1R2 | PPP1R3B | PPP1R3C | PTEN | PYGB | PYGL | RPE | SORD | STBD1 | STK40 | TPI1 | UGP2 | GO:0051336 | 0.0905010311779692 | 746/8243 | 331/3250 | 0.00223327810601281 | 1 | T | T | F | F | regulation of hydrolase activity | A2M | ABCE1 | ABL1 | ABL2 | ADCYAP1 | ADORA2A | ADPRH | ADRM1 | AGAP1 | AGAP2 | AGER | AHSA1 | AHSG | AIFM1 | AKT1 | AKT2 | ALOX12 | ANXA1 | APCS | APOA1 | APP | ARF4 | ARFGAP1 | ARHGAP1 | ARHGAP19 | ARHGAP26 | ARHGAP35 | ARHGDIA | ARHGEF7 | ARRB1 | ARRB2 | ATP13A2 | ATP1B3 | B3GAT3 | BAG4 | BAX | BBC3 | BCL10 | BCL2L13 | BCL6 | BIN1 | BIRC2 | BIRC3 | BIRC5 | BIRC7 | BIRC8 | C4A | CALM1 | CALM2 | CALM3 | CASP10 | CASP8 | CASP9 | CAV1 | CCDC8 | CCL21 | CCL22 | CD109 | CD27 | CD2BP2 | CD40 | CHP1 | CLPX | CRK | CRKL | CRY2 | CRYAB | CYCS | DAPK1 | DDRGK1 | DDX3X | DENND1A | DHCR24 | DHX9 | DIABLO | DLG2 | DLG3 | DNAJA1 | DNAJA2 | DNAJB1 | DNAJB11 | DNAJB2 | DNAJC10 | DNAJC19 | DNAJC9 | DNM1L | DOCK7 | DPEP1 | DVL2 | DVL3 | DYNLL1 | EGFR | EGLN1 | EGLN3 | EIF2AK3 | EIF5 | ELMOD3 | EPHA4 | EPPIN | ERBB2 | ERRFI1 | ESR1 | EZH2 | FABP1 | FADD | FASLG | FGF2 | FGFR1 | FGFR3 | FIS1 | FKBP1A | FKBP1B | FN1 | FOXJ1 | FYN | GAPDH | GAPVD1 | GDI1 | GDI2 | GIT1 | GNA13 | GNA15 | GNAI2 | GNAO1 | GNAQ | GPI | GPS2 | GPSM1 | GPX1 | GSK3B | GZMA | HDAC1 | HDAC9 | HMGB1 | HMGB2 | HNRNPU | HRAS | HSF1 | HSP90AB1 | HSP90B1 | HSPA2 | HSPD1 | HSPE1 | HTRA2 | HTT | ICAM1 | IFI16 | IFIT1 | IGBP1 | IKBKB | IPO5 | IQGAP1 | ITGB1BP1 | ITK | JUN | LAMP3 | LARS | LEF1 | LGALS3 | LGMN | LIMS1 | LPAR1 | LPAR2 | LRRK2 | MAPK14 | MAPK3 | MAPK7 | MAPK8 | MAPT | MBP | MCM2 | MDM2 | MGMT | MSH6 | MYC | MYH6 | MYO1D | NDUFA13 | NOS3 | NPM1 | NR1H2 | NR1H3 | NTF3 | NTRK1 | OAS2 | OXA1L | P2RX1 | PAFAH1B1 | PAK2 | PAM16 | PAPLN | PARK7 | PCNA | PDCD6 | PEBP1 | PFN1 | PGAM5 | PIH1D1 | PIN1 | PLA2G1B | PLAUR | PLCG1 | PLSCR1 | PLXNA1 | PLXNA2 | PLXNB2 | PML | POT1 | PPARG | PPIF | PPME1 | PPP1R11 | PPP1R12A | PPP1R14A | PPP1R16A | PPP1R2 | PPP1R3B | PPP1R3C | PPP1R8 | PPP2R1A | PPP2R1B | PPP2R2A | PPP2R2D | PPP2R5D | PPP4R2 | PPP6R1 | PPT1 | PRDX3 | PRDX5 | PRELID1 | PRKCD | PRKCZ | PRKD1 | PRKD2 | PSAP | PSMA3 | PSMB8 | PSMD14 | PSME2 | PSME3 | PTK2 | PTN | PTTG1 | RAB3GAP1 | RAF1 | RANBP3 | RANGAP1 | RAP1A | RAP1GDS1 | RDX | RECK | REST | RGN | RGS1 | RGS2 | RGS20 | RHOA | RIC8B | RIPK1 | RIPK2 | RIPK3 | RP2 | RPA2 | RPS3 | RPS6KA1 | RSU1 | RTKN | S100A9 | SAG | SERPINB2 | SERPINB3 | SERPINB5 | SERPINB6 | SERPINB8 | SERPINE2 | SERPINH1 | SET | SFN | SIPA1 | SIPA1L1 | SIRT1 | SMAD3 | SNCA | SOD1 | SORT1 | SOX2 | SPINK7 | SPINT2 | SPRY2 | SRC | SRGAP2 | SRGAP3 | STARD13 | STAT3 | SUMO1 | SYDE1 | TANK | TBC1D15 | TBC1D22B | TBC1D4 | TCEA1 | TERF1 | TERF2 | THADA | THBS1 | TIMP1 | TIMP2 | TMBIM6 | TNF | TNFSF14 | TNK2 | TP53 | TP63 | TPM1 | TPM2 | TRADD | TRAF2 | TRIAP1 | UBXN1 | UCHL5 | USP14 | USP17L2 | USP47 | USP50 | VAV1 | VCP | VTN | WDR35 | WNK1 | XIAP | YWHAE | GO:1901983 | 0.00727890331190101 | 60/8243 | 35/3250 | 0.00227036862146975 | 1 | T | T | F | F | regulation of protein acetylation | ARRB1 | ATG5 | BRCA1 | CTBP1 | CTCF | EID1 | GSK3B | HDAC2 | HDAC5 | KAT5 | KAT7 | KMT2A | MAPK3 | MECP2 | PARK7 | PAXIP1 | PIH1D1 | PPARGC1A | PRKAA1 | PRKAA2 | RUVBL2 | SET | SIN3A | SIRT1 | SMAD4 | SMARCB1 | SNAI2 | SNCA | SPI1 | TADA2B | TERF2IP | TGFB1 | TWIST1 | WBP2 | ZMPSTE24 | GO:0043549 | 0.0681790610214728 | 562/8243 | 254/3250 | 0.00227299303347027 | 1 | T | T | F | F | regulation of kinase activity | ABL1 | ACSL1 | ACTB | ADCY6 | ADCYAP1 | ADORA2A | ADRB2 | AGAP2 | AGER | AHSG | AKT1 | APC | APOE | APP | ARRB1 | AXIN1 | AZU1 | BCL10 | BIRC7 | BRAF | CAB39 | CALM1 | CALM2 | CALM3 | CASP3 | CAV1 | CBL | CBLB | CBLC | CCDC88A | CCL21 | CCNB1 | CCNT1 | CCNT2 | CCNY | CCNYL1 | CD40 | CD40LG | CD74 | CD81 | CDC25A | CDC25B | CDC25C | CDC37 | CDK4 | CDK5 | CDK5R1 | CDKN1A | CDKN2C | CEBPA | CHORDC1 | CHP1 | CKS1B | CKS2 | CLU | COX11 | CRK | CRKL | CSNK2B | DAB1 | DAXX | DBF4B | DBNDD2 | DBNL | DDX3X | DGKZ | DLG3 | DLG4 | DNAJA1 | DUSP1 | DUSP12 | DUSP14 | DUSP16 | DUSP19 | DUSP22 | DUSP7 | DVL1 | DVL2 | DVL3 | EEF1A2 | EGFR | EPHA4 | ERBB2 | ERBB3 | ERRFI1 | EZH2 | FAF1 | FBXO7 | FGF2 | FGFR1 | FGFR1OP | FGFR3 | FYN | GADD45G | GNAQ | GPS2 | GSTP1 | HEXIM1 | HGS | HNRNPU | HRAS | HSP90AA1 | HSP90AB1 | HSPB1 | HTT | IBTK | IGF1R | IKBKG | ILK | INSR | IPO5 | IPO7 | IQGAP1 | ITGB1BP1 | ITGB3 | KRAS | KSR1 | LAT | LATS1 | LCP2 | LPAR1 | LRP6 | LRRK2 | MAP2K1 | MAP2K3 | MAP2K4 | MAP2K6 | MAP2K7 | MAP3K1 | MAP3K13 | MAP3K14 | MAP3K2 | MAP3K3 | MAP3K4 | MAP4K1 | MAPK1 | MAPK14 | MAPK3 | MAPK8IP3 | MAPKAP1 | MAPT | MAS1 | MIF | MLLT1 | MLST8 | MOB1B | NCK1 | NEK1 | NF2 | NOD2 | NPM1 | NRBF2 | NTF3 | NTRK1 | OXSR1 | PAK2 | PAK6 | PARK7 | PDCD10 | PDE6G | PIH1D1 | PIK3CA | PIK3CG | PIK3R1 | PIK3R3 | PKIG | PLA2G1B | PLK1 | PPP2CA | PPP2R1A | PRDX2 | PRDX3 | PRKAA1 | PRKACA | PRKAG1 | PRKAR1A | PRKAR2B | PRKCD | PRKCZ | PRKD1 | PROX1 | PRRC1 | PSEN1 | PSMD10 | PTEN | PTK2 | PTPN1 | PTPN22 | PTPN6 | QARS | RAF1 | RAP1A | RB1 | RGN | RGS2 | RHOA | RIPK1 | RIPK2 | RIPK3 | RPLP1 | RPS3 | RPTOR | S100A12 | SDC4 | SERPINB3 | SFN | SHC1 | SIRT1 | SLAMF8 | SLC9A3R1 | SMG8 | SNCA | SNF8 | SNX6 | SOCS1 | SOCS2 | SOCS6 | SOD1 | SPRY2 | SRC | STK11 | STK24 | STK25 | STK26 | STK3 | STK38 | TAB1 | TAB2 | TAOK3 | TGFB1 | THBS1 | TLR3 | TNF | TNFAIP3 | TP73 | TRAF2 | TRAF6 | TRIB2 | TRIB3 | TSG101 | TSPYL2 | TXN | UBB | UBC | UBE2N | UBE2V1 | UCHL1 | UNC119 | WARS | WNK1 | XRCC5 | XRCC6 | YWHAG | ZNF622 | GO:1904646 | 0.0036394516559505 | 30/8243 | 20/3250 | 0.00229188398137303 | 1 | T | T | F | F | cellular response to amyloid-beta | ADRB2 | AGER | APP | BACE1 | CACNA1A | CD36 | CDK5 | EPHA4 | FYN | GSK3B | ICAM1 | IGF1R | ITGA4 | LGMN | NAMPT | NTRK1 | PARP1 | PSEN1 | SNX6 | VCAM1 | GO:1901659 | 0.0036394516559505 | 30/8243 | 20/3250 | 0.00229188398137303 | 1 | T | T | F | F | glycosyl compound biosynthetic process | ADA | ADK | AMD1 | APRT | CDA | DCK | GMPS | HPRT1 | IMPDH2 | LCMT2 | MTAP | NME1 | NME2 | NME4 | PRTFDC1 | TK1 | TK2 | UCK2 | UMPS | UPP2 | GO:0006936 | 0.0132233410166202 | 109/8243 | 58/3250 | 0.00230381274306014 | 1 | T | T | F | F | muscle contraction | ACTA1 | ACTA2 | ACTG2 | ALDOA | ATP2A2 | BDKRB2 | CALD1 | CALM1 | CHRM3 | CHRND | CHRNE | CHRNG | COL4A3BP | CRYAB | DES | DMD | EEF2 | FKBP1B | GAA | GJA1 | GNAO1 | ITGB5 | KCNIP2 | MAP2K3 | MAP2K6 | MYH11 | MYH6 | MYL12A | MYL12B | MYL2 | MYL6 | MYL9 | NDUFS6 | OBSL1 | P2RX2 | PDE4D | PGAM2 | PIK3C2A | PIK3CA | PLA2G6 | PTGER3 | PXN | RAP1GDS1 | SMAD5 | SNTA1 | STAC | SULF2 | TAZ | TMOD3 | TMOD4 | TNNT1 | TPCN2 | TPM1 | TPM2 | TPM3 | TPM4 | TRIM63 | VCL | GO:2001237 | 0.00958388936066966 | 79/8243 | 44/3250 | 0.00238230999075115 | 1 | T | T | F | F | negative regulation of extrinsic apoptotic signaling pathway | AKT1 | AR | BCL2 | BCL2L1 | BRCA1 | CASP8 | COL2A1 | CTTN | EYA2 | EYA4 | FADD | FASLG | FGB | FYN | GATA1 | GPX1 | GSTP1 | HMGB2 | HMOX1 | HSPA1B | HTT | ICAM1 | ITGAV | LGALS3 | LMNA | MAPK7 | NOS3 | PARK7 | PRDX2 | PSME3 | RAF1 | RB1CC1 | RELA | RIPK1 | SCG2 | SNAI2 | SRC | THBS1 | TNF | TNFAIP3 | TRADD | TRAF2 | YAP1 | ZC3HC1 | GO:0090175 | 0.00958388936066966 | 79/8243 | 44/3250 | 0.00238230999075115 | 1 | T | T | F | F | regulation of establishment of planar polarity | ABL1 | ARRB2 | CDC42 | CLTC | DAB2 | DVL1 | DVL2 | DVL3 | MED12 | MKS1 | MLLT3 | NPHP3 | PARD6A | PFN1 | PSMA1 | PSMA2 | PSMA3 | PSMA5 | PSMA6 | PSMB5 | PSMB6 | PSMB7 | PSMB8 | PSMC1 | PSMC2 | PSMC3 | PSMC4 | PSMC5 | PSMC6 | PSMD1 | PSMD10 | PSMD11 | PSMD12 | PSMD14 | PSMD2 | PSMD4 | PSMD9 | PSME2 | PSME3 | RAC1 | RHOA | SEC24B | SMURF1 | SMURF2 | GO:0034440 | 0.00679364309110761 | 56/8243 | 33/3250 | 0.00238579461493838 | 1 | T | T | F | F | lipid oxidation | ABCD3 | ACAA1 | ACAA2 | ACADS | ACADVL | ACAT1 | ACAT2 | ACOX1 | ALOX12 | CPT1A | CPT2 | CROT | DECR1 | ECH1 | ECHDC2 | ECHS1 | ECI1 | ECI2 | EHHADH | ETFA | ETFB | ETFDH | GCDH | HADH | HIBCH | HSD17B4 | IVD | MAPK14 | MCAT | PPARD | PPARG | PPARGC1A | PRKAA1 | GO:0042307 | 0.0038820817663472 | 32/8243 | 21/3250 | 0.00239863452918503 | 1 | T | T | F | F | positive regulation of protein import into nucleus | BAG3 | CDH1 | DMAP1 | HDAC3 | IPO5 | JUP | MAPK1 | MAPK14 | MAVS | PIK3R1 | PRKCD | PSEN1 | RAN | SMAD3 | SMO | TARDBP | TGFB1 | TPR | TRIM28 | UBR5 | ZIC1 | GO:0006873 | 0.0379716122770836 | 313/8243 | 148/3250 | 0.00240629416123412 | 1 | T | T | F | F | cellular ion homeostasis | ABCB7 | ABL1 | ABL2 | ACKR2 | ACO1 | ADCYAP1 | AFG3L2 | ANXA2 | ANXA6 | ANXA7 | APOE | APP | ARF1 | ATOX1 | ATP12A | ATP13A2 | ATP1A1 | ATP1B3 | ATP2A2 | ATP2B2 | ATP4A | ATP6V1A | ATP6V1B1 | ATP6V1C1 | ATP6V1D | ATP6V1E1 | BAX | BCL2 | BDKRB2 | C1QTNF1 | CACNA1A | CALM1 | CALM2 | CALM3 | CALR | CAND1 | CAV1 | CCDC22 | CCL21 | CCR1 | CCR3 | CCR4 | CCR5 | CD36 | CD40 | CDK5 | CFTR | CHP1 | CHRNA9 | CKB | CLIC4 | COX19 | CUL1 | CXCL11 | DLG4 | EGLN1 | ESR1 | FASLG | FGF2 | FIS1 | FKBP1B | FTH1 | FTL | GLP1R | GLRX3 | GNA13 | GNA15 | GNB1 | GOT1 | GRIA1 | GRIK2 | GSTO1 | GTF2I | HAP1 | HEXB | HIF1A | HMGB1 | HMOX1 | HPX | HTR1B | HTT | IBTK | IMMT | IREB2 | ITGAV | ITPR1 | ITPR2 | ITPR3 | KCTD17 | LETM1 | LPAR1 | LPAR2 | LPAR4 | LRP6 | LRRK2 | MAPK1 | MAPK3 | MCOLN3 | MICU1 | MICU2 | MYC | MYO5A | NCOA4 | NEDD8 | NMB | NUBP1 | P2RX1 | P2RX2 | P2RX4 | PIK3CG | PLA2G1B | PLA2G6 | PLCG1 | PML | PPT1 | PRKACA | PRKCE | PSEN1 | PTGER3 | PTGIR | RAB7A | RAP1GDS1 | RGN | RIC3 | SCO2 | SLAMF8 | SLC39A4 | SLC9A1 | SLC9A3R1 | SMAD3 | SMAD4 | SNCA | SOD1 | SRI | STIM1 | STOML2 | SWAP70 | TBXA2R | TCIRG1 | TGFB1 | TGM2 | THADA | TM9SF4 | TMBIM6 | TPCN2 | VAPB | VDR | YWHAE | GO:0031334 | 0.019410408831736 | 160/8243 | 81/3250 | 0.00242490732623238 | 1 | T | T | F | F | positive regulation of protein complex assembly | ACTR2 | ACTR3 | AKAP9 | ARPC1A | ARPC1B | ARPC2 | ARPC3 | ARPC4 | ARPC5 | ATM | BAG4 | BAIAP2 | BAIAP2L1 | BAX | BIN1 | CAND1 | CAPG | CCL21 | CD36 | CDC42EP3 | CDC42EP4 | CDK5R1 | CHD1L | CLU | CREB1 | CTTN | CUL4B | DCTN1 | DDB1 | DDB2 | ERCC5 | ESR1 | FAF1 | FCHSD1 | FSCN1 | GBP5 | GRB2 | GSK3B | HDAC6 | HSP90AA1 | HSPA1B | ICAM1 | IDE | LCP1 | MAPK9 | MAPT | MECP2 | MSN | MYO1C | NCK1 | NIN | NR1H2 | NUMA1 | PAN2 | PARP1 | PFN1 | PIH1D1 | PRKCE | PSMC2 | PSMC3 | PSMC4 | PSMC5 | PSMC6 | RAC1 | RBX1 | RHOA | RPA2 | RPA3 | RPS3 | SRC | STUB1 | SUMO1 | TERF1 | TGFB1 | TNF | TP53 | USP50 | VASP | VCP | WARS | WAS | GO:0006281 | 0.0405192284362489 | 334/8243 | 157/3250 | 0.0024371763716165 | 1 | T | T | F | F | DNA repair | ABL1 | ACTL6A | APBB1 | APEX1 | APEX2 | ATM | ATRX | BABAM1 | BRCA1 | CCDC155 | CDC14B | CDC5L | CDK2 | CDK9 | CHAF1A | CHD1L | CHEK2 | COPS2 | COPS5 | COPS6 | CRY2 | CSNK1E | CUL4B | DDB1 | DDB2 | DDX1 | DMAP1 | DNA2 | EP300 | ERCC5 | EYA2 | EYA4 | FANCA | FANCC | FANCG | FANCI | FANCM | FBXO6 | FEN1 | FOXM1 | FZR1 | GINS2 | GINS4 | H2AFX | HERC2 | HIST1H4A | HIST3H3 | HSF1 | HUWE1 | INO80B | INO80C | ISG15 | KAT5 | KDM2A | KDM4D | LIG4 | MC1R | MCM9 | MDC1 | MGMT | MLH1 | MORF4L1 | MORF4L2 | MPG | MSH6 | MTA1 | NIPBL | NPLOC4 | NPM1 | NSMCE4A | OTUB1 | PALB2 | PARK7 | PARP1 | PAXIP1 | PCNA | PNKP | POLD1 | POLE2 | POLM | POLR2A | POLR2E | POLR2L | PPIE | PPP5C | PRKDC | PRMT6 | PRPF19 | PSMD14 | PTTG1 | RAD21 | RAD23A | RAD23B | RAD51 | RAD51AP1 | RBM17 | RBX1 | RFC1 | RFC2 | RFC3 | RFC4 | RFC5 | RIF1 | RNASEH2A | RNF8 | RPA2 | RPA3 | RPAIN | RPS3 | RRM2B | RUVBL1 | RUVBL2 | SFPQ | SIRT1 | SIRT6 | SMC1A | SMC3 | SPRTN | STUB1 | SUMO1 | TAOK3 | TCEA1 | TDP1 | TMEM189-UBE2V1 | TNP1 | TP53 | TP73 | TREX1 | TRIM25 | TRIM28 | TRIP12 | TRIP13 | TTC5 | UBB | UBC | UBE2A | UBE2B | UBE2D3 | UBE2L6 | UBE2N | UBE2V1 | UBE2W | UBR5 | UCHL5 | USP28 | USP47 | USP7 | VCP | WDR48 | XRCC1 | XRCC3 | XRCC5 | XRCC6 | YY1 | ZBTB7A | ZFYVE26 | ZMPSTE24 | GO:0070585 | 0.00655101298071091 | 54/8243 | 32/3250 | 0.00243823033748896 | 1 | T | T | F | F | protein localization to mitochondrion | AGK | AKT1 | BAX | CALM1 | CALM3 | DNAJA1 | DNAJC19 | DNM1L | FBXO7 | FIS1 | GRPEL1 | HK1 | HK2 | HSP90AA1 | HSPA4 | HSPD1 | LRRK2 | MARCH5 | MTCH2 | NDUFA13 | OXA1L | PAM16 | ROMO1 | SLC25A6 | TIMM13 | TIMM21 | TIMM23 | TIMM44 | TIMM50 | TOMM20 | TOMM22 | TOMM40 | GO:0090239 | 0.0014557806623802 | 12/8243 | 10/3250 | 0.00245677507517693 | 1 | T | T | F | F | regulation of histone H4 acetylation | ARRB1 | ATG5 | BRCA1 | CTBP1 | KAT7 | PIH1D1 | SIRT1 | SMARCB1 | SPI1 | ZMPSTE24 | GO:1905562 | 0.0014557806623802 | 12/8243 | 10/3250 | 0.00245677507517693 | 1 | T | T | F | F | regulation of vascular endothelial cell proliferation | AKT3 | FGF2 | FGFR1 | HMGB1 | ITGA4 | PLCG1 | PPARG | SIRT6 | SP1 | STAT3 | GO:0006760 | 0.0014557806623802 | 12/8243 | 10/3250 | 0.00245677507517693 | 1 | T | T | F | F | folic acid-containing compound metabolic process | ATIC | FOLH1 | FOLR1 | FPGS | MTHFD1 | MTRR | SHMT1 | SHMT2 | SLC25A32 | TYMS | GO:0009070 | 0.0014557806623802 | 12/8243 | 10/3250 | 0.00245677507517693 | 1 | T | T | F | F | serine family amino acid biosynthetic process | CBS | CTH | GGT1 | MTHFD1 | PHGDH | PSAT1 | SEPHS1 | SEPHS2 | SHMT1 | SHMT2 | GO:0008535 | 0.0014557806623802 | 12/8243 | 10/3250 | 0.00245677507517693 | 1 | T | T | F | F | respiratory chain complex IV assembly | BCS1L | COA3 | COX11 | COX15 | COX17 | COX19 | COX20 | OXA1L | SCO2 | TIMM21 | GO:0048384 | 0.0014557806623802 | 12/8243 | 10/3250 | 0.00245677507517693 | 1 | T | T | F | F | retinoic acid receptor signaling pathway | ACTN4 | CRKL | ESRRG | NR1H2 | PML | RARA | RARG | RXRA | RXRG | SNW1 | GO:0010523 | 0.0014557806623802 | 12/8243 | 10/3250 | 0.00245677507517693 | 1 | T | T | F | F | negative regulation of calcium ion transport into cytosol | BCL2 | CALM1 | CALM2 | CALM3 | FKBP1A | FKBP1B | GSTO1 | SRI | TGFB1 | TMBIM6 | GO:0043101 | 0.0014557806623802 | 12/8243 | 10/3250 | 0.00245677507517693 | 1 | T | T | F | F | purine-containing compound salvage | ADA | ADK | APRT | DCK | DGUOK | GMPR | HPRT1 | MTAP | PNP | PRTFDC1 | GO:0010766 | 0.0014557806623802 | 12/8243 | 10/3250 | 0.00245677507517693 | 1 | T | T | F | F | negative regulation of sodium ion transport | COMMD1 | HECW1 | HECW2 | NEDD4 | PCSK9 | PRKCE | SERPINE2 | SLC9A3R1 | WNK1 | WNK4 | GO:0031056 | 0.0143151765134053 | 118/8243 | 62/3250 | 0.00247527814771357 | 1 | T | T | F | F | regulation of histone modification | AKAP8 | ARRB1 | ATG5 | ATM | ATRX | BCL6 | BCOR | BRCA1 | BRD4 | CCNB1 | CDK9 | CTBP1 | CTCF | CTNNB1 | EED | EID1 | HDAC5 | KAT7 | KDM1A | KDM4D | KMT2A | KMT2B | LMNA | LRRK2 | MAPK3 | MAPK8 | MECP2 | NELFE | NIPBL | OGT | OTUB1 | PAXIP1 | PIH1D1 | PPARGC1A | PRKD1 | PRKD2 | PRMT6 | RIF1 | RTF1 | RUVBL2 | SART3 | SET | SIN3A | SIRT1 | SKI | SMAD4 | SMARCB1 | SNAI2 | SNCA | SNW1 | SPI1 | TADA2B | TGFB1 | TRIP12 | TWIST1 | UBE2B | UBE2N | UBR5 | USP17L2 | WBP2 | ZBTB7B | ZMPSTE24 | GO:0033047 | 0.00630838287031421 | 52/8243 | 31/3250 | 0.00248595515292886 | 1 | T | T | F | F | regulation of mitotic sister chromatid segregation | ANAPC4 | APC | ATM | ATRX | AURKB | CCNB1 | CDC20 | CDC27 | CENPF | CUL3 | DUSP1 | DYNC1LI1 | HECW2 | HNRNPU | IK | KLHL22 | MAD1L1 | MAD2L1 | NIPBL | NUMA1 | PLK1 | PTTG1 | RAD21 | RB1 | SPDL1 | TNKS | TPR | TRIP13 | XRCC3 | ZNF207 | ZW10 | GO:0010565 | 0.0111609850782482 | 92/8243 | 50/3250 | 0.00252126082123784 | 1 | T | T | F | F | regulation of cellular ketone metabolic process | ACADVL | AKT1 | AKT2 | ATCAY | CAV1 | COMT | COQ3 | CPT1A | FABP1 | FABP3 | GSTZ1 | NCOR2 | NQO1 | OAZ3 | PDK2 | PPARG | PPARGC1A | PRKCE | PROX1 | PSMA1 | PSMA2 | PSMA3 | PSMA5 | PSMA6 | PSMB5 | PSMB6 | PSMB7 | PSMB8 | PSMC1 | PSMC2 | PSMC3 | PSMC4 | PSMC5 | PSMC6 | PSMD1 | PSMD10 | PSMD11 | PSMD12 | PSMD14 | PSMD2 | PSMD4 | PSMD9 | PSME2 | PSME3 | REST | SIRT1 | SIRT5 | SNCA | TREX1 | TWIST1 | GO:0006875 | 0.0332403251243479 | 274/8243 | 131/3250 | 0.00253751280118704 | 1 | T | T | F | F | cellular metal ion homeostasis | ABCB7 | ABL1 | ABL2 | ACKR2 | ACO1 | ADCYAP1 | AFG3L2 | ANXA2 | ANXA6 | ANXA7 | APOE | APP | ARF1 | ATOX1 | ATP12A | ATP13A2 | ATP1A1 | ATP1B3 | ATP2A2 | ATP2B2 | ATP4A | ATP6V1A | BAX | BCL2 | BDKRB2 | C1QTNF1 | CACNA1A | CALM1 | CALM2 | CALM3 | CALR | CAND1 | CAV1 | CCDC22 | CCL21 | CCR1 | CCR3 | CCR4 | CCR5 | CD36 | CD40 | CDK5 | CHRNA9 | COX19 | CUL1 | CXCL11 | DLG4 | EGLN1 | ESR1 | FASLG | FGF2 | FIS1 | FKBP1B | FTH1 | FTL | GLP1R | GLRX3 | GNA13 | GNA15 | GNB1 | GOT1 | GRIA1 | GRIK2 | GSTO1 | GTF2I | HAP1 | HEXB | HIF1A | HMGB1 | HMOX1 | HPX | HTR1B | HTT | IBTK | IMMT | IREB2 | ITGAV | ITPR1 | ITPR2 | ITPR3 | KCTD17 | LETM1 | LPAR1 | LPAR2 | LPAR4 | LRP6 | MCOLN3 | MICU1 | MICU2 | MYC | MYO5A | NCOA4 | NEDD8 | NMB | NUBP1 | P2RX1 | P2RX2 | P2RX4 | PIK3CG | PLA2G1B | PLA2G6 | PLCG1 | PML | PRKACA | PRKCE | PSEN1 | PTGER3 | PTGIR | RAP1GDS1 | RGN | RIC3 | SCO2 | SLC39A4 | SLC9A1 | SMAD3 | SMAD4 | SNCA | SOD1 | SRI | STIM1 | STOML2 | SWAP70 | TBXA2R | TGFB1 | TGM2 | THADA | TMBIM6 | TPCN2 | VAPB | VDR | YWHAE | GO:0009141 | 0.0043673419871406 | 36/8243 | 23/3250 | 0.00254108618828383 | 1 | T | T | F | F | nucleoside triphosphate metabolic process | ADCYAP1 | AK1 | AK2 | AK4 | ATP6V1A | ATP6V1B1 | CLPX | DGUOK | FIGNL1 | GNAI3 | HSPA1B | HSPA8 | LRRK2 | MYH6 | NDUFS1 | NMNAT1 | OLA1 | OPA1 | PARP1 | RAN | RHOA | RRM2B | VCP | GO:0035601 | 0.00582312264952081 | 48/8243 | 29/3250 | 0.00256294572757259 | 1 | T | T | F | F | protein deacylation | ABHD17A | HDAC1 | HDAC11 | HDAC2 | HDAC3 | HDAC4 | HDAC5 | HDAC6 | HDAC7 | HDAC9 | LYPLA1 | LYPLA2 | MORF4L1 | MORF4L2 | MTA1 | MTA3 | PER1 | PER2 | PHB | PPT1 | REST | SFPQ | SIN3A | SIRT1 | SIRT3 | SIRT5 | SIRT6 | SIRT7 | TBL1X | GO:1903902 | 0.00582312264952081 | 48/8243 | 29/3250 | 0.00256294572757259 | 1 | T | T | F | F | positive regulation of viral life cycle | ANXA2 | CD74 | CHMP2A | DDB1 | DDX3X | IFIT1 | LGALS1 | NR5A2 | P4HB | PABPC1 | PC | PDE12 | PKN2 | PPIA | PPIB | PPID | PPIE | RAD23A | SMC3 | SRPK1 | SRPK2 | STAU1 | STOM | TOP2A | TRIM21 | TSG101 | VAPA | VAPB | VPS4B | GO:0006521 | 0.00582312264952081 | 48/8243 | 29/3250 | 0.00256294572757259 | 1 | T | T | F | F | regulation of cellular amino acid metabolic process | ATCAY | COMT | NQO1 | OAZ3 | PSMA1 | PSMA2 | PSMA3 | PSMA5 | PSMA6 | PSMB5 | PSMB6 | PSMB7 | PSMB8 | PSMC1 | PSMC2 | PSMC3 | PSMC4 | PSMC5 | PSMC6 | PSMD1 | PSMD10 | PSMD11 | PSMD12 | PSMD14 | PSMD2 | PSMD4 | PSMD9 | PSME2 | PSME3 | GO:0005976 | 0.00582312264952081 | 48/8243 | 29/3250 | 0.00256294572757259 | 1 | T | T | F | F | polysaccharide metabolic process | AGL | AKT1 | AKT2 | B3GNT3 | CALM1 | GAA | GBE1 | GSK3A | GSK3B | GYG1 | GYG2 | GYS1 | HS2ST1 | PER2 | PFKM | PGM1 | PGM2 | PHKA1 | PPP1CA | PPP1CB | PPP1CC | PPP1R2 | PPP1R3B | PPP1R3C | PYGB | PYGL | STBD1 | STK40 | UGP2 | GO:1905818 | 0.00582312264952081 | 48/8243 | 29/3250 | 0.00256294572757259 | 1 | T | T | F | F | regulation of chromosome separation | ANAPC4 | APC | ATM | AURKB | CCNB1 | CDC20 | CDC27 | CENPF | CSNK2A2 | CUL3 | DUSP1 | DYNC1LI1 | HECW2 | IK | KLHL22 | MAD1L1 | MAD2L1 | MAPK15 | NUMA1 | PLK1 | PTTG1 | RAD21 | RB1 | SPDL1 | TPR | TRIP13 | XRCC3 | ZNF207 | ZW10 | GO:2001235 | 0.0167414776173723 | 138/8243 | 71/3250 | 0.00257828990535357 | 1 | T | T | F | F | positive regulation of apoptotic signaling pathway | ADORA2A | BAX | BBC3 | BCL10 | BCL2 | BCL2L1 | CASP2 | CASP8 | CAV1 | CHCHD10 | CTSC | CYLD | DNM1L | E2F1 | FADD | FAF1 | FIS1 | GSK3A | GSK3B | HTRA2 | ING5 | LTBR | MAPK8 | MAPK9 | MLLT11 | NCK1 | PAK2 | PARK7 | PDIA3 | PLA2G6 | PLAUR | PLEKHF1 | PML | PPIF | PPP1CA | PPP2R1A | PPP2R1B | PPP3CC | PRKCD | PTEN | RIPK1 | RIPK3 | RNF183 | RPS3 | S100A9 | SEPT4 | SFN | SFPQ | SIAH1 | SIRT1 | SLC9A3R1 | SMAD3 | SOD1 | STK3 | TFDP1 | THBS1 | TNFRSF12A | TNFSF12 | TP53 | TP63 | TP73 | TRAF2 | UBB | WDR35 | WWOX | YWHAB | YWHAE | YWHAG | YWHAH | YWHAQ | YWHAZ | GO:0042181 | 0.00230498604876865 | 19/8243 | 14/3250 | 0.0025874039454813 | 1 | T | T | F | F | ketone biosynthetic process | COQ2 | COQ3 | COQ4 | COQ5 | COQ6 | COQ7 | COQ9 | FDXR | GPI | NDUFA9 | PDSS1 | PDSS2 | TPI1 | UBIAD1 | GO:0060078 | 0.004852602207934 | 40/8243 | 25/3250 | 0.0026045071905396 | 1 | T | T | F | F | regulation of postsynaptic membrane potential | ADCYAP1 | ADORA2A | AKT1 | ARRB2 | CDK5 | CHRNA9 | CHRND | CHRNE | CHRNG | GABRA6 | GABRR1 | GLRB | GRIA1 | GRID2 | GRIK2 | GSK3A | GSK3B | LRRK2 | MECP2 | MPP2 | P2RX1 | P2RX2 | P2RX4 | RAB3GAP1 | SNCA | GO:0051092 | 0.0154070120101905 | 127/8243 | 66/3250 | 0.00261180696631026 | 1 | T | T | F | F | positive regulation of NF-kappaB transcription factor activity | AGER | APP | AR | BCL10 | BTK | CAMK2A | CARM1 | CAT | CAV1 | CD36 | CD40 | CD40LG | CLU | CTH | DDRGK1 | DHX9 | EDA | FLOT1 | HSPA1B | ICAM1 | IKBKB | IKBKG | KRAS | MAP3K13 | MTPN | MYD88 | NFKB2 | NOD2 | NPM1 | NTRK1 | PLA2G1B | PRDX3 | PRKCZ | PRKD1 | PRKD2 | PSMA6 | RELA | RIPK1 | RIPK2 | RIPK3 | RIPK4 | RNF25 | RNF31 | RPS3 | S100A12 | S100A9 | STAT3 | TAB1 | TAB2 | TERF2IP | TGFB1 | TLR3 | TNF | TNFRSF8 | TRADD | TRAF1 | TRAF2 | TRAF6 | TRIM14 | TRIM25 | TRIM32 | UBB | UBC | UBE2N | UBE2V1 | ZBTB7A | GO:0030003 | 0.0374863520562902 | 309/8243 | 146/3250 | 0.00265881819965948 | 1 | T | T | F | F | cellular cation homeostasis | ABCB7 | ABL1 | ABL2 | ACKR2 | ACO1 | ADCYAP1 | AFG3L2 | ANXA2 | ANXA6 | ANXA7 | APOE | APP | ARF1 | ATOX1 | ATP12A | ATP13A2 | ATP1A1 | ATP1B3 | ATP2A2 | ATP2B2 | ATP4A | ATP6V1A | ATP6V1B1 | ATP6V1C1 | ATP6V1D | ATP6V1E1 | BAX | BCL2 | BDKRB2 | C1QTNF1 | CACNA1A | CALM1 | CALM2 | CALM3 | CALR | CAND1 | CAV1 | CCDC22 | CCL21 | CCR1 | CCR3 | CCR4 | CCR5 | CD36 | CD40 | CDK5 | CFTR | CHP1 | CHRNA9 | CLIC4 | COX19 | CUL1 | CXCL11 | DLG4 | EGLN1 | ESR1 | FASLG | FGF2 | FIS1 | FKBP1B | FTH1 | FTL | GLP1R | GLRX3 | GNA13 | GNA15 | GNB1 | GOT1 | GRIA1 | GRIK2 | GSTO1 | GTF2I | HAP1 | HEXB | HIF1A | HMGB1 | HMOX1 | HPX | HTR1B | HTT | IBTK | IMMT | IREB2 | ITGAV | ITPR1 | ITPR2 | ITPR3 | KCTD17 | LETM1 | LPAR1 | LPAR2 | LPAR4 | LRP6 | LRRK2 | MAPK1 | MAPK3 | MCOLN3 | MICU1 | MICU2 | MYC | MYO5A | NCOA4 | NEDD8 | NMB | NUBP1 | P2RX1 | P2RX2 | P2RX4 | PIK3CG | PLA2G1B | PLA2G6 | PLCG1 | PML | PPT1 | PRKACA | PRKCE | PSEN1 | PTGER3 | PTGIR | RAB7A | RAP1GDS1 | RGN | RIC3 | SCO2 | SLAMF8 | SLC39A4 | SLC9A1 | SMAD3 | SMAD4 | SNCA | SOD1 | SRI | STIM1 | STOML2 | SWAP70 | TBXA2R | TCIRG1 | TGFB1 | TGM2 | THADA | TM9SF4 | TMBIM6 | TPCN2 | VAPB | VDR | YWHAE | GO:0051250 | 0.00885599902947956 | 73/8243 | 41/3250 | 0.0026832429181739 | 1 | T | T | F | F | negative regulation of lymphocyte activation | ADORA2A | ANXA1 | ATM | BCL6 | BTK | CASP3 | CBFB | CD74 | DTX1 | DUSP22 | ERBB2 | FBXO7 | FOXJ1 | GLMN | HAVCR2 | HLX | HMGB1 | ID2 | IFNA2 | IRF1 | ITCH | JAK3 | LGALS3 | MAD1L1 | MICA | PAG1 | PAWR | PELI1 | PRDX2 | PRKAR1A | PTPN22 | PTPN6 | RC3H1 | RUNX1 | SDC4 | SOCS1 | SOCS6 | TGFB1 | TNFAIP3 | VSIG4 | ZBTB7B | GO:0051099 | 0.0164988475069756 | 136/8243 | 70/3250 | 0.00271604281806714 | 1 | T | T | F | F | positive regulation of binding | ABL1 | ACD | AKTIP | AMFR | ANXA2 | APOE | APP | ARHGEF7 | ARRB1 | CALM2 | CALM3 | CAV1 | CDK5 | CDK9 | CRBN | CTNNB1 | DDRGK1 | DERL1 | DHX9 | EIF2S1 | EIF3D | EIF3E | EP300 | EPHA4 | FAM129B | FKBP1A | FLOT1 | GPSM1 | GSK3B | H1F0 | HIST1H2BA | HMGB1 | HMGB2 | HNRNPL | HSF1 | IRF4 | KDM1A | KDM4D | LGALS3 | LRRK2 | NCBP1 | NEUROD1 | NIPBL | NMD3 | NME1 | PARK7 | PARP1 | PIN1 | PINX1 | PLAUR | PPARG | PPP2CA | PPP2CB | PSEN1 | RAN | RARA | RB1 | RIPK2 | SKI | STK3 | STMN1 | TGFB1 | TMEM173 | TRAF2 | TRAF6 | TRIB3 | TRIM28 | TWIST1 | TXN | VTN | GO:0071375 | 0.0138299162926119 | 114/8243 | 60/3250 | 0.00273745633893718 | 1 | T | T | F | F | cellular response to peptide hormone stimulus | ADCY6 | AKT1 | AKT2 | ANXA5 | APEX1 | APRT | ASS1 | CAV1 | CDC5L | CDK4 | CREB1 | CRK | DENND4C | EPRS | ERRFI1 | FOXO1 | FYN | GLP1R | GOT1 | GSK3A | GSTP1 | HDAC5 | HDAC9 | HSF1 | INSR | LPIN3 | MDM2 | MYO5A | NFE2L2 | NR4A1 | PARP1 | PCSK9 | PIK3R1 | PKM | PLA2G1B | PPARG | PPAT | PRKACA | PRKAR1A | PRKAR2B | PRKCD | PRKCZ | PRKDC | PTEN | RAB10 | RAB31 | RANGAP1 | RAP1B | RELA | SLC9A1 | SP1 | SRC | SRSF5 | STAT1 | TBC1D4 | TGFB1 | TRIB3 | WDTC1 | XBP1 | YWHAG | GO:0010506 | 0.029115613247604 | 240/8243 | 116/3250 | 0.00276267255132201 | 1 | T | T | F | F | regulation of autophagy | ABL1 | ABL2 | ADRB2 | AKT1 | ATM | ATP13A2 | ATP6V1A | ATP6V1B1 | ATP6V1C1 | ATP6V1D | ATP6V1E1 | BAG3 | BCL2 | CALCOCO2 | CAPN1 | CAPNS1 | CASP3 | CDC37 | CDK5 | CDK5R1 | CISD2 | CSNK2A2 | CTSA | CTTN | DAPK1 | DCN | DNM1L | EEF1A1 | EEF1A2 | EIF2AK4 | EIF4E | EIF4G3 | EP300 | EPM2A | FBXO7 | FOXO1 | GAPDH | GFAP | GNAI3 | GOLGA2 | GPSM1 | GSK3A | HAX1 | HDAC6 | HERC1 | HIF1A | HK2 | HMGB1 | HMOX1 | HSPB1 | HTRA2 | HTT | IFI16 | IKBKG | ITPR1 | KAT8 | KDR | KLHL22 | LAMP3 | LARS | LRRK2 | LRSAM1 | MAPK15 | MAPK3 | MAPK8 | MAPT | MLST8 | NAMPT | NEDD4 | OPTN | PARK7 | PIK3CA | PIKFYVE | PIP4K2A | PLEKHF1 | POLDIP2 | PRKAA1 | PRKAA2 | PRKAB2 | PRKACA | PRKAG1 | PRKD1 | PSAP | PTK2 | PTPN22 | QSOX1 | RAB1B | RAB3GAP1 | RAB5A | RB1CC1 | RIPK2 | RPTOR | SCFD1 | SIRT1 | SMURF1 | SNCA | SNX6 | SOGA3 | SPTLC2 | STAT3 | STK11 | SUPT5H | TAB2 | TPCN2 | TRIM21 | UBQLN1 | UBQLN2 | UBQLN4 | UCHL1 | ULK1 | VDAC1 | VPS26A | VPS29 | WDR41 | XBP1 | ZMPSTE24 | GO:0016055 | 0.0271745723644304 | 224/8243 | 109/3250 | 0.00277288542022818 | 1 | T | T | F | F | Wnt signaling pathway | AES | AGO1 | AGO2 | APC | ARRB2 | AXIN1 | BTRC | CALM1 | CAMK2A | CCAR2 | CCNY | CDC42 | CDC73 | CHD8 | CLTC | CSNK1A1 | CSNK1E | CSNK2A2 | CSNK2B | CTNNB1 | CTNNBIP1 | CTNND1 | CUL1 | CUL3 | CYLD | DAB2 | DDB1 | DDX3X | DISC1 | DVL1 | DVL2 | DVL3 | FBXW11 | FERMT2 | GNAO1 | GNB1 | GRK5 | GSK3A | GSK3B | LEF1 | LGR4 | LRP6 | LRRK2 | MCC | MED12 | MITF | MOV10 | MYH6 | MYOC | NFATC1 | NLK | NPHP3 | PARD6A | PDE6G | PFN1 | PRKAA1 | PRKAA2 | PSMA1 | PSMA2 | PSMA3 | PSMA5 | PSMA6 | PSMB5 | PSMB6 | PSMB7 | PSMB8 | PSMC1 | PSMC2 | PSMC3 | PSMC4 | PSMC5 | PSMC6 | PSMD1 | PSMD10 | PSMD11 | PSMD12 | PSMD14 | PSMD2 | PSMD4 | PSMD9 | PSME2 | PSME3 | PTEN | RAC1 | RARG | RBX1 | RECK | RHOA | RTF1 | SDC1 | SLC9A3R1 | SMURF1 | SMURF2 | SNX3 | SPIN1 | STK11 | TLE1 | TLE3 | TNKS | TNKS2 | UBB | UBC | VPS26A | VPS29 | WNT16 | WNT2 | WWOX | XIAP | ZBTB33 | GO:0097327 | 0.00861336891908286 | 71/8243 | 40/3250 | 0.00278788113666692 | 1 | T | T | F | F | response to antineoplastic agent | ASNS | ASS1 | ATRX | BRCA1 | CASP9 | CDH1 | CDK4 | CTNNB1 | DAXX | DDIT4 | EGFR | EIF4E | EIF4EBP1 | ERRFI1 | FBXO32 | FECH | FOXO1 | HCN3 | HMOX1 | HNRNPA1 | HNRNPD | HSF1 | ICAM1 | JUP | KAT7 | KDM3B | MAP4K1 | NR3C1 | NUCB1 | OPA1 | PCNA | PRKAA1 | PTGR1 | RAD51 | RAP1A | RPL18A | TGFB1 | TREX1 | USP8 | WDR35 | GO:0031058 | 0.00861336891908286 | 71/8243 | 40/3250 | 0.00278788113666692 | 1 | T | T | F | F | positive regulation of histone modification | AKAP8 | ARRB1 | ATM | BCL6 | BRCA1 | BRD4 | CCNB1 | CDK9 | CTBP1 | CTNNB1 | EED | KAT7 | KDM1A | KMT2A | LMNA | LRRK2 | MAPK3 | MECP2 | NELFE | NIPBL | OGT | PAXIP1 | PIH1D1 | PPARGC1A | PRKD1 | PRKD2 | RIF1 | RTF1 | RUVBL2 | SART3 | SIRT1 | SMAD4 | SMARCB1 | SNAI2 | SNW1 | TADA2B | TGFB1 | UBE2N | WBP2 | ZBTB7B | GO:0034613 | 0.0846779085284484 | 698/8243 | 310/3250 | 0.00286605936265382 | 1 | T | T | F | F | cellular protein localization | ABHD17A | ACAA1 | ACD | ACOX1 | ACTN2 | AGER | AGK | AHI1 | AKT1 | AKT2 | ANK1 | ANK3 | ANXA2 | AP3D1 | AP3M1 | AP4M1 | APOE | ARF4 | ARL5B | ATG4C | ATM | ATP1B3 | ATP6V1D | ATRX | AURKB | BAG4 | BAG6 | BANP | BAX | BLZF1 | BRAF | BSG | C16orf70 | CACNG2 | CACNG4 | CACNG5 | CALM1 | CALM3 | CALR | CARM1 | CAT | CAV1 | CBLB | CD81 | CDH1 | CDK5 | CHCHD10 | CLSTN1 | CLU | COG3 | CROCC | CROT | CSE1L | CTCF | CTNNB1 | DDX1 | DDX3X | DENND4C | DHRS4 | DHX9 | DLG2 | DLG3 | DLG4 | DMD | DNAJA1 | DNAJA3 | DNAJC19 | DNM1L | DVL1 | DVL2 | ECH1 | ECI2 | EGFR | EHHADH | ESR1 | EZH2 | EZR | FAM83D | FAM83H | FBXO7 | FCN1 | FERMT2 | FIS1 | FLOT1 | GAK | GBP2 | GBP5 | GGA1 | GLRB | GOLGA4 | GOLPH3 | GORASP2 | GRID2 | GRIK2 | GRIP1 | GRPEL1 | GSTK1 | HEATR3 | HGS | HK1 | HK2 | HMGCL | HNRNPU | HSD17B4 | HSP90AA1 | HSPA4 | HSPA8 | HSPD1 | IDE | IDH1 | IFT122 | IK | IKBKB | IPO11 | IPO4 | IPO5 | IPO7 | IPO8 | IPO9 | ITGA4 | ITGB1BP1 | JUP | KPNA1 | KPNA2 | KPNB1 | LARS | LATS1 | LEMD2 | LMNA | LRP6 | LRRC7 | LRRK2 | LRSAM1 | MAPK15 | MARCH5 | MCM9 | MDM2 | MTCH2 | MYO1C | MYO5A | NCOA4 | NDUFA13 | NEDD4 | NIPBL | NOS2 | NRXN1 | NUBP1 | NUDT19 | NUP107 | NUP153 | NUP214 | NUP85 | NUP88 | NUP93 | NUP98 | NUTF2 | NXT2 | OBSL1 | OPTN | OXA1L | PACS1 | PACS2 | PAM16 | PARD3 | PCM1 | PEX19 | PEX5 | PHB2 | PICK1 | PIGU | PIH1D1 | PIK3R1 | PIKFYVE | PINX1 | PLEKHF1 | PLK1 | PML | POM121 | POT1 | PRKCZ | PRPF19 | PTPRK | RAB10 | RAB11A | RAB11B | RAB29 | RAB31 | RAB32 | RAB35 | RAB3GAP1 | RAB3IP | RAB7A | RABGEF1 | RAD21 | RAN | RB1 | RDX | RIC3 | ROMO1 | RPA2 | RPAIN | RPL10 | RPL10A | RPL12 | RPL18A | RPL22 | RPL23 | RPL23A | RPL24 | RPL30 | RPL37A | RPL38 | RPL6 | RPL7 | RPL7A | RPL9 | RPLP0 | RPLP1 | RPLP2 | RPS12 | RPS13 | RPS14 | RPS15A | RPS19 | RPS2 | RPS20 | RPS24 | RPS29 | RPS3 | RPS4X | RPS8 | RRS1 | RTKN | RTN4 | RUVBL2 | SCG3 | SDCBP | SEC16A | SEC62 | SEC63 | SGTA | SGTB | SIN3A | SLC25A6 | SLC9A3R1 | SMO | SMURF1 | SNF8 | SPDL1 | SPTBN1 | SRP14 | SRP19 | SSB | STAT3 | STIL | STXBP1 | SUMO1 | SYNJ2BP | TERF2 | TERF2IP | TIA1 | TIMM13 | TIMM21 | TIMM23 | TIMM44 | TIMM50 | TINF2 | TM9SF4 | TMEM30A | TNF | TNFAIP3 | TNFRSF1A | TNKS | TNKS2 | TNPO1 | TOLLIP | TOMM20 | TOMM22 | TOMM40 | TOR1A | TPR | TRAPPC8 | TSPAN17 | TULP3 | TXNIP | UBB | UBC | UBE2D1 | UBE2D2 | UBE2D3 | UBL4A | UGT8 | USP4 | USP9X | VAPA | VCL | VPS37D | WDR19 | WDR35 | WDR45B | WIPI1 | WIPI2 | XPO1 | XRCC5 | YWHAE | ZBTB16 | ZBTB7A | ZDHHC23 | ZFAND6 | ZW10 | GO:0043254 | 0.0361518864491083 | 298/8243 | 141/3250 | 0.00292017011317059 | 1 | T | T | F | F | regulation of protein complex assembly | ABL1 | ACTR2 | ACTR3 | ADD3 | AKAP9 | ALB | APOE | ARF1 | ARPC1A | ARPC1B | ARPC2 | ARPC3 | ARPC4 | ARPC5 | ATM | ATXN2 | AVIL | BAG4 | BAIAP2 | BAIAP2L1 | BAX | BIN1 | BIRC2 | CAND1 | CAPG | CAPZA1 | CAPZA2 | CAPZB | CCL21 | CD36 | CDC42 | CDC42EP3 | CDC42EP4 | CDK5R1 | CHD1L | CLU | CRBN | CREB1 | CRYAB | CTNNBIP1 | CTTN | CUL4B | DCTN1 | DDB1 | DDB2 | DDX3X | DNM1L | EIF4EBP1 | EP300 | ERCC5 | ESR1 | FAF1 | FCHSD1 | FSCN1 | GBP5 | GFAP | GRB2 | GSK3B | HAX1 | HDAC6 | HIST3H3 | HMGB1 | HSP90AA1 | HSPA1B | HSPA8 | ICAM1 | IDE | LATS1 | LCP1 | LDLRAD4 | MAPK15 | MAPK9 | MAPT | MECP2 | MSN | MTPN | MYO1C | NCK1 | NIN | NR1H2 | NUMA1 | PAN2 | PARK7 | PARP1 | PEX5 | PFN1 | PICK1 | PIH1D1 | PPM1A | PRKAA1 | PRKAA2 | PRKCD | PRKCE | PRKCZ | PSMC2 | PSMC3 | PSMC4 | PSMC5 | PSMC6 | RAC1 | RAF1 | RB1 | RBX1 | RDX | RHOA | RNF4 | RPA2 | RPA3 | RPS3 | SENP6 | SEPT8 | SNCA | SPTAN1 | SPTBN1 | SPTBN2 | SRC | STMN1 | STUB1 | STXBP1 | SUMO1 | TERF1 | TFIP11 | TGFB1 | THRA | TMOD3 | TMOD4 | TNF | TP53 | TRAF3IP1 | TRIM9 | TUBB4A | TWF1 | TWF2 | ULK1 | USP50 | VASP | VCP | VDAC2 | VPS18 | WARS | WAS | GO:0034250 | 0.0120101904646367 | 99/8243 | 53/3250 | 0.00293742066716488 | 1 | T | T | F | F | positive regulation of cellular amide metabolic process | APP | C1QBP | CASP3 | CDK4 | CIRBP | CLU | COA3 | DDX39B | DDX3X | DHX36 | DHX9 | EEF2 | EIF2AK4 | EIF2B5 | EIF3D | EIF3E | EIF4A3 | EIF5A | EIF6 | ELAVL1 | EPHA4 | ERBB2 | FXR2 | GSK3A | HNRNPD | HNRNPL | KHDRBS1 | LARP4 | MAPK1 | MAPK3 | NCK1 | NFE2L2 | NPM1 | PABPC1 | PAIP1 | PLA2G6 | POLDIP3 | PRKCD | RBM3 | RHOA | RMND1 | RPS4X | RPS6KB2 | RXRA | THBS1 | TNF | TNFRSF1A | TRUB2 | UPF3B | UQCC1 | USP16 | VIM | YTHDF1 | GO:0009605 | 0.101662016256217 | 838/8243 | 368/3250 | 0.00293883006576018 | 1 | T | T | F | F | response to external stimulus | ABCE1 | ABL1 | ACAT1 | ACSL1 | ACSL4 | ACTA1 | ACTA2 | ACTG1 | ADA | ADCYAP1 | ADH5 | ADSL | AFG3L2 | AGL | AHCY | AICDA | AIFM1 | AKAP8 | AKT1 | ALB | APOA1 | APOE | ARF1 | ARHGAP35 | ARRB1 | ARRB2 | ASCL1 | ASGR1 | ASNS | ASS1 | ATG5 | ATP1A1 | ATP2A2 | AZU1 | BACE1 | BAG3 | BAIAP2 | BAIAP2L1 | BANF1 | BCKDHB | BCL10 | BCL11A | BCL2 | BCL2L1 | BTK | CABP4 | CACNG2 | CAPN2 | CARD9 | CASP2 | CASP3 | CASP8 | CASP9 | CAT | CAV1 | CBL | CBS | CCDC130 | CCL22 | CCNB1 | CCR4 | CCR5 | CCT5 | CD36 | CD40 | CD6 | CD79B | CDC73 | CDK19 | CDK4 | CDKN1A | CEBPE | CFL1 | CHRM2 | CHRNA9 | CLU | COMT | COTL1 | CREB1 | CRK | CRP | CRY1 | CRY2 | CSMD1 | CTSV | CXCL11 | CXCL5 | CXCL6 | CXCL8 | CYP1A1 | CYP1A2 | DAD1 | DCLK1 | DCN | DCTPP1 | DDIT4 | DDX1 | DDX3X | DHX36 | DMD | DNM1L | DROSHA | EEF1G | EEF2 | EGFR | EHMT2 | EIF2AK3 | EIF2AK4 | EIF2S1 | EIF4EBP1 | ENDOG | ENO1 | EPPIN | ETS1 | EXOSC5 | FADD | FASLG | FGB | FIS1 | FKBP1B | FKBP5 | FOLR1 | FOS | FOXO1 | FYN | G6PD | GAPDH | GBP3 | GBP5 | GBP6 | GGT1 | GLRB | GLUL | GNAI2 | GNAQ | GNB1 | GNG12 | GOT1 | GPI | GPX1 | GRK1 | GSS | GSTP1 | GTF2F1 | GZMA | HAVCR2 | HDAC2 | HDAC4 | HDAC5 | HLCS | HMGB1 | HMGB2 | HMGCL | HMOX1 | HNRNPA1 | HNRNPL | HRAS | HSF1 | HSP90B1 | HSPA5 | HSPA8 | HSPB1 | ICAM1 | ID2 | IFI16 | IFIT1 | IFITM3 | IFNA2 | IFNAR1 | IGHV3-13 | IGHV3-23 | IGHV3-30 | IGKV3-20 | IGLC7 | IKBKB | IKBKE | IKBKG | ILF3 | IREB2 | IRF1 | IRF4 | IRF7 | ISG15 | ITCH | JUN | JUNB | JUND | JUP | KYNU | LARS | LDHA | LGALS8 | LRRK2 | LSM5 | LTBR | LYG2 | LYZL1 | LYZL2 | MAP2K4 | MAP3K1 | MAP3K14 | MAP3K2 | MAPK1 | MAPK14 | MAPK3 | MAPK8 | MAPK9 | MAVS | MBD3 | MDM2 | MECP2 | MGMT | MGST1 | MICA | MLH1 | MTA1 | MTPN | MYBBP1A | MYD88 | NAMPT | NCK1 | NCOA1 | NFE2L2 | NFKB2 | NFKBIA | NNT | NOD2 | NOS2 | NOS3 | NQO1 | NR1H2 | NR1H3 | NTRK1 | OAS2 | OGT | OPA1 | OPTN | OTC | OTUD5 | OXCT1 | P2RX1 | P2RX2 | P2RX4 | PCBP2 | PCNA | PCSK9 | PDE12 | PDE4D | PDE6G | PDK2 | PELI1 | PER1 | PER2 | PICK1 | PKM | PLA2G1B | PLA2G2A | PLA2G6 | PLSCR1 | PML | PPARD | PPARG | PPARGC1A | PPM1D | PPP1CA | PPP1CB | PPP1CC | PPP1R11 | PRDX2 | PRDX3 | PRG2 | PRKAA1 | PRKAA2 | PRKCD | PRKD1 | PROX1 | PRPF8 | PSMA2 | PTEN | PTGES | PTGIR | PTN | PTPN22 | PYGL | RAB1A | RAB29 | RAC1 | RAF1 | RARA | RBM4 | RBPJ | RELA | RGS1 | RHO | RHOA | RIPK2 | RNASE3 | ROMO1 | RPL30 | RPS15A | RPS19 | RPTOR | S100A12 | S100A7 | S100A9 | SAG | SCEL | SELL | SERPINE2 | SETDB1 | SHC1 | SIPA1 | SIRT1 | SIRT5 | SIRT6 | SLAMF8 | SLC16A1 | SLC25A6 | SLC39A4 | SLC9A1 | SMAD3 | SNCA | SNX3 | SOD1 | SORD | SPACA3 | SRC | ST13 | STAT1 | STK24 | STK26 | STMN1 | TBXA2R | TGFB1 | THBS1 | TLR3 | TMBIM6 | TMEM173 | TNF | TNFAIP3 | TNFRSF1A | TNFRSF8 | TNFSF14 | TNIP2 | TNKS | TP53 | TPCN2 | TRAF6 | TREX1 | TRIM25 | TRIM34 | TRIM41 | TXN2 | TXNIP | TYMS | ULK1 | UNC119 | USP2 | USP46 | USP53 | VCAM1 | VGF | VIM | WDR35 | WDR45B | WIPI1 | WIPI2 | XBP1 | GO:0055082 | 0.0446439403129928 | 368/8243 | 171/3250 | 0.00294731321560065 | 1 | T | T | F | F | cellular chemical homeostasis | ABCB7 | ABL1 | ABL2 | ACKR2 | ACO1 | ADCYAP1 | AFG3L2 | ANXA2 | ANXA6 | ANXA7 | APOE | APP | ARF1 | ATOX1 | ATP12A | ATP13A2 | ATP1A1 | ATP1B3 | ATP2A2 | ATP2B2 | ATP4A | ATP6V1A | ATP6V1B1 | ATP6V1C1 | ATP6V1D | ATP6V1E1 | BAX | BCL2 | BDKRB2 | C1QTNF1 | CACNA1A | CALM1 | CALM2 | CALM3 | CALR | CAND1 | CAV1 | CCDC22 | CCL21 | CCR1 | CCR3 | CCR4 | CCR5 | CD36 | CD40 | CDK5 | CFTR | CHP1 | CHRNA9 | CKB | CLIC4 | COX19 | CUL1 | CXCL11 | DLG4 | EGLN1 | ENDOG | ESR1 | FASLG | FGF2 | FIS1 | FKBP1B | FOXO1 | FTH1 | FTL | GLP1R | GLRX3 | GNA13 | GNA15 | GNB1 | GOT1 | GRIA1 | GRIK2 | GSTO1 | GTF2I | HAP1 | HEXB | HIF1A | HK1 | HK2 | HMGB1 | HMOX1 | HPX | HTR1B | HTT | IBTK | ICAM1 | IGF1R | IMMT | IREB2 | ITGAV | ITPR1 | ITPR2 | ITPR3 | KCTD17 | LETM1 | LGALS1 | LPAR1 | LPAR2 | LPAR4 | LRP6 | LRRK2 | MAPK1 | MAPK3 | MCOLN3 | MICU1 | MICU2 | MYC | MYO5A | NCOA4 | NEDD8 | NEUROD1 | NMB | NME1 | NME2 | NUBP1 | OPA1 | P2RX1 | P2RX2 | P2RX4 | PIK3CA | PIK3CG | PIK3R1 | PLA2G1B | PLA2G6 | PLCG1 | PML | PPARGC1A | PPT1 | PRKAA1 | PRKAA2 | PRKACA | PRKCE | PSEN1 | PTGER3 | PTGIR | RAB7A | RAP1A | RAP1GDS1 | RGN | RIC3 | RTN4 | SCO2 | SIN3A | SIRT1 | SLAMF8 | SLC39A4 | SLC9A1 | SLC9A3R1 | SMAD3 | SMAD4 | SNCA | SOD1 | SRI | STIM1 | STOML2 | SWAP70 | TBXA2R | TCIRG1 | TGFB1 | TGM2 | THADA | TM9SF4 | TMBIM6 | TPCN2 | VAPB | VDR | WDR35 | WDTC1 | XBP1 | YWHAE | GO:1901687 | 0.00254761615916535 | 21/8243 | 15/3250 | 0.00297707914439723 | 1 | T | T | F | F | glutathione derivative biosynthetic process | AKR1A1 | ESD | GSTA1 | GSTA2 | GSTA3 | GSTA4 | GSTK1 | GSTM4 | GSTO1 | GSTO2 | GSTP1 | GSTT1 | GSTT2B | GSTZ1 | MGST1 | GO:1903321 | 0.00812810869828946 | 67/8243 | 38/3250 | 0.00300209996112043 | 1 | T | T | F | F | negative regulation of protein modification by small protein conjugation or removal | ABL1 | AKT1 | ARRB1 | ARRB2 | ATG5 | BAG2 | BAG5 | CAV1 | CDK5 | CHP1 | CRY1 | CTNNB1 | DNAJA1 | FYN | GLMN | GPS2 | HSPA1B | ISG15 | MAD2L1 | OGT | OTUB1 | PARK7 | PER2 | PIAS3 | PINX1 | PRKCE | PRMT3 | PSEN1 | RELA | RPL23 | RPS3 | TNFAIP3 | TRIM21 | TRIP12 | U2AF2 | UBR5 | UBXN1 | USP4 | GO:0033138 | 0.00788547858789276 | 65/8243 | 37/3250 | 0.00311095309220395 | 1 | T | T | F | F | positive regulation of peptidyl-serine phosphorylation | AKAP9 | AKT1 | APP | ARAF | ARRB1 | ARRB2 | AXIN1 | BAG4 | BCAR3 | BCL2 | BRAF | CAV1 | DOCK7 | EGFR | HAX1 | HDAC6 | HSP90AA1 | HSP90AB1 | IFNA2 | LATS1 | MAPKAP1 | MIF | NTF3 | PARK7 | PDCD10 | PIK3CA | PRKD1 | PRKD2 | RAF1 | RIPK2 | RPTOR | SNCA | SPRY2 | TERF2IP | TGFB1 | TNF | TXN | GO:1905269 | 0.00994783452626471 | 82/8243 | 45/3250 | 0.00315704997238169 | 1 | T | T | F | F | positive regulation of chromatin organization | AKAP8 | ARRB1 | ATM | BCL6 | BRCA1 | BRD4 | CCNB1 | CDK9 | CTBP1 | CTNNB1 | EED | KAT7 | KDM1A | KMT2A | LMNA | LRRK2 | MAPK3 | MECP2 | NELFE | NIPBL | OGT | PAXIP1 | PIH1D1 | PPARGC1A | PRKD1 | PRKD2 | RIF1 | RTF1 | RUVBL2 | SART3 | SETDB1 | SIN3A | SIRT1 | SIRT6 | SMAD4 | SMARCB1 | SNAI2 | SNW1 | TADA2B | TGFB1 | TPR | TRIM28 | UBE2N | WBP2 | ZBTB7B | GO:0045786 | 0.0515588984592988 | 425/8243 | 195/3250 | 0.00319020772432086 | 1 | T | T | F | F | negative regulation of cell cycle | ABL1 | ADCYAP1 | APBB1 | APBB2 | APC | ATF2 | ATM | ATRX | AURKB | BABAM1 | BAX | BCL2 | BCL2L1 | BCL6 | BMI1 | BRCA1 | CAB39 | CALR | CARM1 | CASP2 | CASP3 | CCNB1 | CDC14B | CDC20 | CDC25C | CDC73 | CDK2 | CDK4 | CDK5 | CDK9 | CDKN1A | CDKN2C | CEBPA | CENPF | CGRRF1 | CHEK2 | CHMP2A | CTNNB1 | CUL1 | CUL2 | CUL3 | CUL5 | CXCL8 | DHCR24 | DNA2 | DUSP1 | E2F1 | E2F4 | E2F6 | EED | EGFR | EHMT1 | EHMT2 | EIF2AK4 | EP300 | EZH2 | FBXO6 | FBXO7 | FHL1 | FZR1 | H2AFX | HEXIM1 | HRAS | HSP90AB1 | HTRA2 | IK | ILK | IPO5 | IPO7 | IRF1 | KANK2 | KHDRBS1 | KLHL22 | L3MBTL2 | LATS1 | MAD1L1 | MAD2L1 | MAP2K1 | MAP2K6 | MAPK14 | MDC1 | MDM1 | MDM2 | MDM4 | MGA | MIF | MLST8 | MSH6 | MYC | NAE1 | NPM1 | NR4A1 | NSUN2 | NUBP1 | NUDT6 | NUPR1 | PA2G4 | PCGF6 | PCNA | PHC3 | PINX1 | PLK1 | PML | PPM1A | PRCC | PRKAA1 | PRKAA2 | PRKAB2 | PRKAG1 | PRKAR1A | PRKDC | PRMT2 | PSMA1 | PSMA2 | PSMA3 | PSMA5 | PSMA6 | PSMB5 | PSMB6 | PSMB7 | PSMB8 | PSMC1 | PSMC2 | PSMC3 | PSMC4 | PSMC5 | PSMC6 | PSMD1 | PSMD10 | PSMD11 | PSMD12 | PSMD14 | PSMD2 | PSMD4 | PSMD9 | PSME2 | PSME3 | PTEN | PTPRK | PTTG1 | RAD21 | RAD51 | RB1 | RBBP4 | RBX1 | RING1 | RIPK1 | RNF2 | RPA2 | RPL23 | RPL24 | RPS6KA2 | RPTOR | RRM2 | SEPT7 | SFN | SIPA1 | SLC9A3R1 | SMAD3 | SMC1A | SMC3 | SOX2 | SPDL1 | STAG2 | STK11 | SUZ12 | TAOK3 | TBRG4 | TERF1 | TERF2 | TFDP1 | TGFB1 | THBS1 | TIMP2 | TNF | TNFAIP3 | TNKS | TOP2A | TP53 | TP73 | TPR | TRIAP1 | TRIM35 | TRIP13 | TSG101 | TSPYL2 | USP17L2 | USP28 | USP47 | YAF2 | ZBTB17 | ZFYVE19 | ZNF207 | ZW10 | ZWINT | GO:0071900 | 0.0424602693194225 | 350/8243 | 163/3250 | 0.00326554003191375 | 1 | T | T | F | F | regulation of protein serine/threonine kinase activity | ABL1 | ACSL1 | ACTB | ADRB2 | AGER | AKT1 | APC | APOE | ARRB1 | BIRC7 | BRAF | CAB39 | CALM1 | CALM2 | CALM3 | CASP3 | CAV1 | CBLC | CCNB1 | CCNT1 | CCNT2 | CCNY | CCNYL1 | CD40 | CD40LG | CD74 | CD81 | CDC25A | CDC25C | CDC37 | CDK4 | CDK5R1 | CDKN1A | CDKN2C | CEBPA | CHORDC1 | CKS1B | CKS2 | CRK | CRKL | DAXX | DBF4B | DBNL | DDX3X | DNAJA1 | DUSP1 | DUSP14 | DUSP16 | DUSP19 | DUSP22 | DUSP7 | DVL2 | DVL3 | EGFR | EPHA4 | ERBB2 | EZH2 | FBXO7 | FGF2 | FGFR1 | GADD45G | GPS2 | GSTP1 | HEXIM1 | HGS | HRAS | HSPB1 | HTT | IGF1R | IKBKG | ILK | INSR | IPO5 | IPO7 | IQGAP1 | KRAS | KSR1 | LATS1 | LPAR1 | LRP6 | LRRK2 | MAP2K1 | MAP2K3 | MAP2K4 | MAP2K6 | MAP2K7 | MAP3K1 | MAP3K13 | MAP3K14 | MAP3K2 | MAP3K3 | MAP3K4 | MAP4K1 | MAPK1 | MAPK14 | MAPK3 | MAPK8IP3 | MIF | MLST8 | NOD2 | NTF3 | NTRK1 | PDCD10 | PDE6G | PIH1D1 | PIK3CG | PKIG | PLA2G1B | PLK1 | PPP2CA | PPP2R1A | PRDX2 | PRKAA1 | PRKAG1 | PRKAR1A | PRKAR2B | PRKCD | PROX1 | PSEN1 | PSMD10 | PTEN | PTPN1 | PTPN22 | PTPN6 | RAF1 | RGS2 | RHOA | RIPK1 | RIPK2 | RPS3 | RPTOR | S100A12 | SERPINB3 | SFN | SHC1 | SIRT1 | SNCA | SNF8 | SOD1 | SPRY2 | SRC | STK3 | STK38 | TAB1 | TAB2 | TAOK3 | TGFB1 | THBS1 | TNF | TNFAIP3 | TP73 | TRAF2 | TRAF6 | TRIB2 | TRIB3 | TSG101 | UBB | UBC | UBE2N | UBE2V1 | UCHL1 | WNK1 | YWHAG | GO:0006753 | 0.0168627926725707 | 139/8243 | 71/3250 | 0.00326723713106727 | 1 | T | T | F | F | nucleoside phosphate metabolic process | ABHD14B | ACACA | ACAT1 | ACLY | ACOT13 | ACOT7 | ACOT9 | ADCYAP1 | ADSS | AK1 | AK2 | AK4 | ATP6V1A | ATP6V1B1 | CLPX | CMPK1 | CROT | DBI | DCK | DCXR | DGUOK | DLG2 | DLST | FAR2 | FASN | FIGNL1 | G6PD | GMPR | GNAI3 | GPD1L | HMGCL | HPRT1 | HSD17B4 | HSPA1B | HSPA8 | IDH1 | IDH2 | KCNAB2 | LDHA | LRRK2 | MDH1 | MDH2 | MVD | MYH6 | NDUFS1 | NME1 | NME2 | NME4 | NMNAT1 | NMNAT3 | NMRK2 | NNT | NT5C2 | OGDH | OLA1 | OPA1 | PARK7 | PARP1 | PNKP | RAN | REXO2 | RHOA | RNASEH2B | RRM2B | SUCLA2 | SUCLG2 | SULT1A3 | THEM4 | TPST2 | TYMS | VCP | GO:0045860 | 0.0416110639330341 | 343/8243 | 160/3250 | 0.00327230929066027 | 1 | T | T | F | F | positive regulation of protein kinase activity | ABL1 | ACSL1 | ADCY6 | ADCYAP1 | ADRB2 | AGER | AKT1 | ARRB1 | AXIN1 | AZU1 | BIRC7 | BRAF | CAB39 | CALM1 | CALM2 | CALM3 | CCDC88A | CCL21 | CCNB1 | CCNT1 | CCNT2 | CCNY | CCNYL1 | CD40 | CD40LG | CD74 | CD81 | CDC25B | CDK5 | CDK5R1 | CDKN1A | CKS1B | CKS2 | CLU | CRK | CRKL | DAB1 | DAXX | DBF4B | DBNL | DDX3X | DLG3 | DLG4 | DUSP19 | DUSP7 | DVL2 | DVL3 | EGFR | EPHA4 | ERBB2 | ERBB3 | EZH2 | FGF2 | FGFR1 | FYN | GADD45G | HRAS | HSP90AA1 | HSP90AB1 | IKBKG | ILK | INSR | IQGAP1 | ITGB1BP1 | ITGB3 | KRAS | LAT | LCP2 | LPAR1 | LRRK2 | MAP2K1 | MAP2K3 | MAP2K4 | MAP2K6 | MAP2K7 | MAP3K1 | MAP3K13 | MAP3K14 | MAP3K2 | MAP3K3 | MAP3K4 | MAP4K1 | MAPK1 | MAPK14 | MAPK3 | MAPK8IP3 | MAPKAP1 | MAS1 | MIF | MLST8 | NEK1 | NOD2 | NTF3 | NTRK1 | OXSR1 | PAK2 | PAK6 | PARK7 | PDCD10 | PDE6G | PIH1D1 | PIK3CA | PIK3CG | PLA2G1B | PPP2CA | PRDX2 | PRKAA1 | PRKACA | PRKAG1 | PRKAR1A | PRKAR2B | PRKCD | PRKCZ | PROX1 | PRRC1 | PSEN1 | PSMD10 | PTK2 | PTPN1 | RAF1 | RAP1A | RHOA | RIPK1 | RIPK2 | RIPK3 | RPLP1 | RPS3 | RPTOR | S100A12 | SDC4 | SHC1 | SIRT1 | SNCA | SOD1 | SPRY2 | SRC | STK11 | STK24 | STK25 | STK26 | STK3 | TAB1 | TAB2 | TAOK3 | TGFB1 | THBS1 | TLR3 | TNF | TP73 | TRAF2 | TRAF6 | TXN | UBB | UBC | UBE2N | UBE2V1 | UNC119 | WNK1 | XRCC5 | XRCC6 | GO:0002479 | 0.00740021836709936 | 61/8243 | 35/3250 | 0.00332974466576519 | 1 | T | T | F | F | antigen processing and presentation of exogenous peptide antigen via MHC class I, TAP-dependent | CALR | CD36 | FCGR1A | HLA-A | HLA-B | IKBKB | IKBKG | ITGAV | ITGB5 | PDIA3 | PSMA1 | PSMA2 | PSMA3 | PSMA5 | PSMA6 | PSMB5 | PSMB6 | PSMB7 | PSMB8 | PSMC1 | PSMC2 | PSMC3 | PSMC4 | PSMC5 | PSMC6 | PSMD1 | PSMD10 | PSMD11 | PSMD12 | PSMD14 | PSMD2 | PSMD4 | PSMD9 | PSME2 | PSME3 | GO:1903047 | 0.0536212543976708 | 442/8243 | 202/3250 | 0.00338915871480574 | 1 | T | T | F | F | mitotic cell cycle process | ACD | ACTR1A | AKAP8 | AKAP9 | ANK3 | APC | ARF1 | ATF2 | ATM | AURKB | BANF1 | BAX | BCL2L1 | BIRC5 | BRCA1 | BRD4 | BTRC | CALM2 | CALM3 | CAMK2A | CARM1 | CASP2 | CCNB1 | CCNY | CCP110 | CDC20 | CDC25A | CDC25B | CDC25C | CDC27 | CDC34 | CDK2 | CDK4 | CDKN1A | CDKN2C | CENPF | CENPJ | CEP135 | CEP152 | CEP290 | CEP55 | CEP57 | CEP70 | CFL1 | CHEK2 | CHMP2A | CKAP5 | CKS2 | CNTRL | CNTROB | CSNK1E | CTDP1 | CUL1 | CUL2 | CUL3 | CUL4B | CUL5 | CUL7 | DCTN1 | DNA2 | DYNC1H1 | DYNC1I2 | DYNLL1 | DYNLT1 | E2F1 | E2F4 | E2F6 | EIF4E | EIF4EBP1 | EP300 | ESRRB | FBXL12 | FBXW11 | FGFR1OP | FOXM1 | GOLGA2 | GSPT1 | HRAS | HSP90AA1 | HTT | ID2 | IK | KDM8 | KHDRBS1 | KIF11 | KIF2C | KLHL22 | KPNB1 | LATS1 | LMNA | MAD1L1 | MAD2L1 | MCM2 | MCM3 | MCM4 | MCM5 | MCM6 | MCM7 | MDC1 | MDM2 | MDM4 | MECP2 | MELK | MSH6 | MYC | NAE1 | NCAPH2 | NIPBL | NSFL1C | NSL1 | NUMA1 | NUSAP1 | OFD1 | OPTN | PAFAH1B1 | PCM1 | PCNA | PDCD6IP | PIAS1 | PINX1 | PLK1 | PML | POLA2 | POLE2 | POLE3 | PPAT | PPM1D | PPME1 | PPP1CB | PPP1R12A | PPP1R12B | PPP2CA | PPP2R1A | PPP2R2A | PPP2R2D | PPP6C | PRCC | PRKACA | PRKAR2B | PRKDC | RAB11A | RAB35 | RAD51 | RAN | RB1 | RCC1 | RHOA | RHOU | RPA2 | RPA3 | RPL24 | RRM2 | RRS1 | RTFDC1 | RTKN | SBDS | SEPT6 | SFN | SIRT7 | SMC1A | SPC25 | SPDL1 | SPICE1 | SPRY2 | SPTBN1 | STIL | STMN1 | SUN2 | TAOK3 | TBCE | TFDP1 | TGFB1 | TNKS | TOP2A | TP53 | TPR | TRIAP1 | TRIP13 | TTC19 | TUBA1A | TUBA4A | TUBB | TUBB4A | TUBB4B | TUBG1 | TYMS | UBE2C | UBE2S | UNC119 | USP16 | USP17L2 | USP26 | USP8 | VCP | VPS4B | YWHAE | YWHAG | ZFYVE19 | ZFYVE26 | ZNF207 | ZW10 | ZWINT | GO:1904874 | 0.0016984107727769 | 14/8243 | 11/3250 | 0.00339209697666855 | 1 | T | T | F | F | positive regulation of telomerase RNA localization to Cajal body | CCT2 | CCT3 | CCT4 | CCT5 | CCT6A | CCT7 | CCT8 | NOP10 | RUVBL1 | RUVBL2 | TCP1 | GO:1901522 | 0.0016984107727769 | 14/8243 | 11/3250 | 0.00339209697666855 | 1 | T | T | F | F | positive regulation of transcription from RNA polymerase II promoter involved in cellular response to chemical stimulus | CREB3 | EP300 | HIF1A | NFE2L2 | RBPJ | RELA | RUNX2 | SMAD4 | SMAD5 | SMAD9 | XBP1 | GO:0071287 | 0.00109183549678515 | 9/8243 | 8/3250 | 0.00339867659018678 | 1 | T | T | F | F | cellular response to manganese ion | APP | ATP13A2 | BACE1 | EIF2AK3 | EIF2S1 | HSPA5 | IREB2 | LRRK2 | GO:0045717 | 0.00109183549678515 | 9/8243 | 8/3250 | 0.00339867659018678 | 1 | T | T | F | F | negative regulation of fatty acid biosynthetic process | ACADVL | BRCA1 | ERLIN2 | INSIG1 | INSIG2 | SIRT1 | TRIB3 | WDTC1 | GO:0098761 | 0.00109183549678515 | 9/8243 | 8/3250 | 0.00339867659018678 | 1 | T | T | F | F | cellular response to interleukin-7 | ATIC | BTK | CRKL | HDGF | P4HB | PDIA3 | RAD23B | STIP1 | GO:0072350 | 0.00109183549678515 | 9/8243 | 8/3250 | 0.00339867659018678 | 1 | T | T | F | F | tricarboxylic acid metabolic process | ACLY | ACO1 | ACO2 | IDH1 | IDH2 | IDH3B | IDH3G | IREB2 | GO:1904871 | 0.00109183549678515 | 9/8243 | 8/3250 | 0.00339867659018678 | 1 | T | T | F | F | positive regulation of protein localization to Cajal body | CCT2 | CCT3 | CCT4 | CCT5 | CCT6A | CCT7 | CCT8 | TCP1 | GO:0007271 | 0.00109183549678515 | 9/8243 | 8/3250 | 0.00339867659018678 | 1 | T | T | F | F | synaptic transmission, cholinergic | ADORA2A | APOE | CHRM3 | CHRND | CHRNE | CHRNG | NQO1 | RIC3 | GO:0032070 | 0.00109183549678515 | 9/8243 | 8/3250 | 0.00339867659018678 | 1 | T | T | F | F | regulation of deoxyribonuclease activity | AKT1 | GZMA | HMGB1 | NPM1 | PCNA | PRKCD | RPS3 | SIRT1 | GO:1904869 | 0.00109183549678515 | 9/8243 | 8/3250 | 0.00339867659018678 | 1 | T | T | F | F | regulation of protein localization to Cajal body | CCT2 | CCT3 | CCT4 | CCT5 | CCT6A | CCT7 | CCT8 | TCP1 | GO:0098760 | 0.00109183549678515 | 9/8243 | 8/3250 | 0.00339867659018678 | 1 | T | T | F | F | response to interleukin-7 | ATIC | BTK | CRKL | HDGF | P4HB | PDIA3 | RAD23B | STIP1 | GO:0044089 | 0.0379716122770836 | 313/8243 | 147/3250 | 0.00343358710384357 | 1 | T | T | F | F | positive regulation of cellular component biogenesis | ABL1 | ACE2 | ACTR2 | ACTR3 | AKAP9 | APC | APOA1 | ARHGAP35 | ARPC1A | ARPC1B | ARPC2 | ARPC3 | ARPC4 | ARPC5 | ATM | ATP13A2 | BAG4 | BAIAP2 | BAIAP2L1 | BAX | BIN1 | BRAF | CAND1 | CAPG | CAV1 | CCDC88A | CCL21 | CCP110 | CD36 | CDC34 | CDC42 | CDC42EP3 | CDC42EP4 | CDK5R1 | CENPJ | CEP135 | CHD1L | CHMP2A | CLSTN1 | CLU | CREB1 | CROCC | CTTN | CUL4B | DCTN1 | DDB1 | DDB2 | DYNC1H1 | DYNLL1 | ERCC5 | ESR1 | FAF1 | FAM98A | FCHSD1 | FLOT1 | FSCN1 | GBP5 | GRB2 | GRID2 | GSK3B | HAP1 | HDAC4 | HDAC6 | HGS | HRAS | HSF1 | HSP90AA1 | HSPA1B | HTT | ICAM1 | IDE | IQGAP1 | ITGB1BP1 | KCTD17 | KDR | LCP1 | LIMS1 | LPAR1 | LRSAM1 | MAPK15 | MAPK9 | MAPT | MECP2 | MSN | MYO1C | MYOC | NCK1 | NF2 | NIN | NR1H2 | NRXN1 | NTRK1 | NUMA1 | PAN2 | PARP1 | PDCD6IP | PFN1 | PIH1D1 | PIP4K2A | PRKCE | PROX1 | PSMC2 | PSMC3 | PSMC4 | PSMC5 | PSMC6 | PTPRD | PXN | RAB3GAP1 | RAB7A | RAC1 | RBX1 | RHOA | RIOK1 | RPA2 | RPA3 | RPS3 | SDC1 | SDC4 | SDCBP | SEPT7 | SEPT9 | SIRT6 | SMAD3 | SNF8 | SRC | STUB1 | SUMO1 | SWAP70 | TERF1 | TGFB1 | THBS2 | TNF | TP53 | TPM1 | TPR | TSG101 | TWF2 | ULK1 | USP50 | VASP | VCP | VPS4B | WARS | WAS | ZMYND10 | ZMYND8 | GO:0031397 | 0.00715758825670266 | 59/8243 | 34/3250 | 0.00343843831690437 | 1 | T | T | F | F | negative regulation of protein ubiquitination | ABL1 | AKT1 | ARRB1 | ARRB2 | ATG5 | BAG2 | BAG5 | CAV1 | CDK5 | CHP1 | CRY1 | DNAJA1 | FYN | GLMN | GPS2 | HSPA1B | ISG15 | MAD2L1 | OGT | OTUB1 | PARK7 | PER2 | PINX1 | PRKCE | PRMT3 | PSEN1 | RPL23 | RPS3 | TNFAIP3 | TRIP12 | U2AF2 | UBR5 | UBXN1 | USP4 | GO:0071396 | 0.0289942981924057 | 239/8243 | 115/3250 | 0.00346441269004298 | 1 | T | T | F | F | cellular response to lipid | ABL1 | ABL2 | ACACA | ADCY6 | ADCYAP1 | ADTRP | AICDA | AIFM1 | AKAP8 | AKR1C2 | AKT1 | ALOX12 | ANXA1 | AR | ASS1 | ATM | ATP1A1 | CAPN2 | CASP9 | CCNB1 | CCR5 | CD36 | CD40 | CDC73 | CDK19 | CDK4 | CEBPE | CFTR | CPT1A | CREB1 | CXCL11 | CXCL5 | CXCL6 | CXCL8 | DDIT4 | DNM1L | E2F1 | EGFR | EIF4E | EIF4EBP1 | ERRFI1 | ESR1 | FBXO32 | FECH | FIS1 | FOXO1 | GNAI1 | GNB1 | GRAMD1A | GSTP1 | H2AFZ | HAVCR2 | HDAC2 | HDAC5 | HMGB1 | HMGB2 | HNRNPD | HNRNPU | HSF1 | HTRA2 | ICAM1 | INSIG1 | IREB2 | KAT5 | LPAR1 | LRP6 | MAP4K1 | MAPK14 | MAPK8 | MED1 | MSN | NCOA3 | NDUFA13 | NME1 | NME2 | NOD2 | NOS2 | NR1H2 | NR1H3 | NR3C1 | PDE4D | PHB2 | PPARD | PPARG | PPARGC1A | PRKAA1 | PRKAA2 | PRKCE | PRPF8 | PTN | RARA | RARG | RELA | REST | RHOA | RUVBL2 | SMO | SNW1 | SRC | TBXA2R | TGFB1 | TNFAIP3 | TNIP2 | TRAF6 | TRIM41 | TWF2 | UBE2L3 | USP8 | VIM | WDR35 | WNT2 | XBP1 | XRCC5 | YAP1 | YES1 | GO:0050679 | 0.0126167657406284 | 104/8243 | 55/3250 | 0.0035174550331856 | 1 | T | T | F | F | positive regulation of epithelial cell proliferation | AKT1 | AKT3 | AR | ARNT | BMPR1A | CCR3 | CTNNB1 | EGFR | ERBB2 | FGF2 | FGFR1 | GLUL | HIF1A | HMGB1 | HMGB2 | HMOX1 | HRAS | HTRA1 | ITGA4 | ITGB3 | JUN | KDM5B | KDR | LAMB1 | MED1 | MYC | NME1 | NME2 | NOD2 | NR4A1 | NRP2 | PDCD6 | PLCG1 | PRKD1 | PRKD2 | PROX1 | PTN | RPTOR | RTN4 | SCG2 | SIRT1 | SIRT6 | SMO | SP1 | STAT3 | STAT5A | TGFB1 | TNFAIP3 | TNFSF12 | TP63 | TWIST1 | VASH2 | WDR48 | WNT2 | XBP1 | GO:0033238 | 0.00691495814630596 | 57/8243 | 33/3250 | 0.00354564283765874 | 1 | T | T | F | F | regulation of cellular amine metabolic process | ABAT | ATCAY | COMT | HPRT1 | NQO1 | OAZ3 | PEBP1 | PSMA1 | PSMA2 | PSMA3 | PSMA5 | PSMA6 | PSMB5 | PSMB6 | PSMB7 | PSMB8 | PSMC1 | PSMC2 | PSMC3 | PSMC4 | PSMC5 | PSMC6 | PSMD1 | PSMD10 | PSMD11 | PSMD12 | PSMD14 | PSMD2 | PSMD4 | PSMD9 | PSME2 | PSME3 | SNCA | GO:0070848 | 0.0240203809292733 | 198/8243 | 97/3250 | 0.003576990192869 | 1 | T | T | F | F | response to growth factor | AKT1 | ANXA1 | APP | ASCL1 | BAG4 | BCAR1 | BMPR1A | BRAF | CAT | CAV1 | CBL | CDC34 | CDC5L | COL2A1 | COL4A2 | CPNE3 | CREB1 | CRK | CRKL | CTNNB1 | CXCL8 | DUSP22 | E2F1 | EEF1A1 | EEF2 | EGFR | EHD4 | EIF4A3 | ERBB2 | ERRFI1 | FASLG | FYN | GSTP1 | HDAC2 | HDAC6 | HSPA5 | HSPB1 | HTR3A | HTRA2 | INSR | IQGAP1 | IREB2 | ITGB1BP1 | KDM5B | KDR | LGMN | LIMS1 | LUM | MAP2K3 | MAPK1 | MAPK14 | MAPK3 | MAPK7 | MAPT | MCM7 | MDM2 | MED1 | MYC | NR3C1 | NR4A1 | NTRK1 | PAX9 | PLCG1 | PPARGC1A | PPM1A | PRKD1 | PRKD2 | PTEN | PTN | RAB35 | RAP1A | RDX | RELA | RIPK1 | RUNX2 | SH3GL2 | SHC1 | SMAD3 | SMAD4 | SMAD9 | SNAI2 | SNCA | SOX2 | SPINT2 | SPRY2 | SRC | TGFB1 | THBS1 | TPR | TWF2 | TWIST1 | USP8 | VCAM1 | VSTM2A | WNT2 | WWOX | YES1 | GO:2000144 | 0.00303287637995875 | 25/8243 | 17/3250 | 0.00357756127772502 | 1 | T | T | F | F | positive regulation of DNA-templated transcription, initiation | CAND1 | CREB1 | CTNNB1 | DHX36 | ESR1 | HNF1A | JUN | MITF | PAXIP1 | PSMC2 | PSMC3 | PSMC4 | PSMC5 | PSMC6 | SUB1 | TP53 | TWIST1 | GO:0051085 | 0.00303287637995875 | 25/8243 | 17/3250 | 0.00357756127772502 | 1 | T | T | F | F | chaperone cofactor-dependent protein refolding | BAG1 | CD74 | DNAJB1 | DNAJC7 | GAK | HSPA14 | HSPA1B | HSPA1L | HSPA2 | HSPA5 | HSPA8 | HSPA9 | HSPE1 | HSPH1 | PTGES3 | ST13 | TOR1A | GO:0035872 | 0.00303287637995875 | 25/8243 | 17/3250 | 0.00357756127772502 | 1 | T | T | F | F | nucleotide-binding domain, leucine rich repeat containing receptor signaling pathway | CASP8 | CYLD | IKBKG | ITCH | MAP2K6 | NFKBIA | NOD2 | RELA | RIPK2 | TAB1 | TAB2 | TNFAIP3 | TRAF6 | UBB | UBC | UBE2N | UBE2V1 | GO:0046824 | 0.00667232803590926 | 55/8243 | 32/3250 | 0.00365042358582813 | 1 | T | T | F | F | positive regulation of nucleocytoplasmic transport | BAG3 | CDH1 | DHX9 | DMAP1 | GSK3B | HDAC3 | IPO5 | JUP | KHDRBS1 | MAPK1 | MAPK14 | MAVS | MDM2 | NEDD4 | PIK3R1 | PPM1A | PRKACA | PRKCD | PSEN1 | RAN | SFN | SMAD3 | SMO | TARDBP | TGFB1 | TP53 | TPR | TRIM28 | UBR5 | XPO4 | YWHAE | ZIC1 | GO:0009890 | 0.118160863763193 | 974/8243 | 423/3250 | 0.0037240739747116 | 1 | T | T | F | F | negative regulation of biosynthetic process | ACADVL | ACD | AEBP2 | AES | AGO1 | AGO2 | APBB1 | APEX1 | APOE | AR | ARHGAP35 | ASCL1 | ATF2 | ATP1A1 | ATRX | ATXN1 | AURKB | BACH2 | BAG3 | BASP1 | BCL11A | BCL6 | BCL6B | BCOR | BIRC5 | BMI1 | BRCA1 | BTRC | C1QBP | CALR | CAV1 | CBFA2T3 | CBFB | CBX4 | CBX7 | CBX8 | CBY1 | CCAR2 | CD36 | CDC73 | CDK2 | CDK5 | CDK5R1 | CEBPA | CENPF | CENPJ | CHCHD3 | CHD8 | CHEK2 | CIRBP | CLTC | COPS2 | CREB1 | CREBBP | CRY1 | CRY2 | CTBP1 | CTCF | CTNNB1 | CTNNBIP1 | CUL3 | DAB2 | DAPK1 | DAXX | DCP2 | DDX3X | DHX36 | DMAP1 | DNAJA3 | DNAJB1 | DPF2 | DUSP1 | DUSP22 | E2F1 | E2F2 | E2F3 | E2F4 | E2F6 | EED | EHMT1 | EHMT2 | EID1 | EIF2AK3 | EIF2AK4 | EIF2S1 | EIF3E | EIF4A3 | EIF4E | EIF4E2 | EIF4EBP1 | EIF4ENIF1 | EIF6 | ELF3 | ENO1 | EP300 | EPRS | ERF | ERLIN2 | ERRFI1 | ESR1 | ESR2 | ESRRA | EZH2 | EZR | FAM129B | FAM3C | FASLG | FBP1 | FBXW11 | FGFR1 | FHL2 | FOXA1 | FOXJ1 | FOXK1 | FOXM1 | FOXO1 | FTO | FXR2 | FYN | GAPDH | GATA1 | GLA | GOT1 | GPS2 | GRB7 | GSK3A | GSK3B | GSTP1 | H1F0 | H3F3A | HBEGF | HBZ | HDAC1 | HDAC2 | HDAC3 | HDAC4 | HDAC5 | HDAC6 | HDAC7 | HDAC9 | HDGF | HEXIM1 | HIF1AN | HIST1H3A | HIST1H4A | HIST2H3A | HIVEP1 | HMGB1 | HMGB2 | HNRNPA1 | HNRNPA2B1 | HNRNPD | HNRNPU | HSF1 | HSF4 | HSPA8 | ID2 | IFI16 | IFNA2 | IGBP1 | IGF2BP1 | IKZF5 | ILF3 | INSIG1 | INSIG2 | IREB2 | IRF1 | IRF7 | ITGAV | ITGB3 | JAK3 | JUN | JUNB | JUND | KANK2 | KAT5 | KAT7 | KAT8 | KDM1A | KDM5B | KDM5C | KHDRBS1 | KLF5 | L3MBTL1 | LEF1 | LGR4 | LIMS1 | LOXL2 | MAF1 | MBD3 | MDFI | MDM2 | MDM4 | MECP2 | MED1 | MEPCE | MITF | MORF4L1 | MORF4L2 | MOV10 | MSH6 | MTA1 | MTA3 | MXD1 | MYBBP1A | MYC | NACC1 | NANOG | NCK1 | NCOA2 | NCOR1 | NCOR2 | NDUFA13 | NEDD4 | NELFB | NELFCD | NELFE | NFATC2 | NIF3L1 | NIPBL | NKRF | NKX2-1 | NOSTRIN | NR0B2 | NR1H2 | NR1H3 | NR1I2 | NR3C1 | PA2G4 | PARK7 | PARP1 | PATZ1 | PAWR | PAX9 | PCGF1 | PCGF6 | PER1 | PER2 | PGP | PHB | PHB2 | PHF10 | PHF21A | PIAS1 | PIAS3 | PINX1 | PKIG | PLK1 | PML | POT1 | POU5F1 | PPARD | PPARG | PPID | PPM1A | PPM1D | PRDX5 | PRKAA1 | PRMT2 | PRMT6 | PROX1 | PSEN1 | PSMC5 | PSMD10 | PTPRK | PUM1 | PUS7 | QARS | RAP1A | RARA | RARG | RASD1 | RB1 | RBM3 | RBM4 | RBPJ | RC3H1 | REL | RELA | REST | RFC1 | RGN | RGS2 | RHOA | RIF1 | RING1 | RNF139 | RNF2 | RNF8 | RPL10 | RPL23 | RPS14 | RPS3 | RTF1 | RUNX1 | RUNX1T1 | RUNX2 | RUVBL2 | RXRA | S100A1 | S100A11 | SAP18 | SARS | SET | SF1 | SFMBT1 | SFPQ | SHC1 | SHMT1 | SIN3A | SIRT1 | SIRT5 | SIRT6 | SIRT7 | SKI | SMAD2 | SMAD3 | SMAD4 | SMAD5 | SMARCA2 | SMARCA4 | SMARCC2 | SMARCE1 | SMC1A | SMC3 | SMO | SMURF2 | SNAI2 | SNCA | SNW1 | SNX6 | SOD1 | SOX2 | SP100 | SP2 | SPEN | SPI1 | SQSTM1 | SRC | STAG2 | STAT1 | STAT3 | STAT6 | STRAP | SUMO1 | SUPT4H1 | SUPT5H | SUZ12 | TAGLN3 | TBL1X | TCERG1 | TCF21 | TCF25 | TCP10L | TERF1 | TERF2 | TGFB1 | THAP11 | THAP7 | THRA | TIA1 | TINF2 | TLE1 | TLE3 | TMBIM6 | TNF | TNP1 | TP53 | TP63 | TPR | TRAF6 | TRAP1 | TRIB2 | TRIB3 | TRIM28 | TRIM29 | TRIM33 | TSC22D4 | TSG101 | TSPYL2 | TWIST1 | TXN | TXNIP | TYMS | UBA3 | UBB | UBC | UBE2D1 | UBE2D3 | UBE2I | UNK | USP2 | USP47 | USP9X | VDR | VHL | VPS72 | WDR35 | WDTC1 | WT1 | WWOX | WWP2 | XPO1 | XRCC5 | XRCC6 | YAF2 | YAP1 | YEATS2 | YWHAB | YWHAQ | YY1 | ZBTB16 | ZBTB21 | ZBTB33 | ZBTB42 | ZBTB7A | ZBTB7B | ZC3H4 | ZHX1 | ZMYND8 | ZNF217 | ZNF24 | ZNF746 | GO:0003008 | 0.0750940191677787 | 619/8243 | 276/3250 | 0.00373261676343738 | 1 | T | T | F | F | system process | AARS | ABL1 | ABLIM1 | ACE2 | ACO1 | ACTA1 | ACTA2 | ACTG2 | ACTR2 | ADCY6 | ADCYAP1 | ADH5 | ADORA2A | ADRB2 | AGER | AKR1B1 | AKT1 | ALDH7A1 | ALDOA | ALOX12 | AMFR | AOC2 | APOE | APP | ARF4 | ARHGAP35 | ARR3 | ATAD1 | ATF6 | ATG5 | ATP1A1 | ATP2A2 | ATP2B2 | ATP6V1B1 | ATXN1 | AZU1 | BCL2 | BDKRB2 | BIRC5 | BRAF | BTBD9 | C1QL1 | CABP4 | CACNG2 | CACNG4 | CACNG5 | CALD1 | CALM1 | CASP3 | CAV1 | CD36 | CDK5 | CEL | CHRM3 | CHRNA9 | CHRND | CHRNE | CHRNG | COL18A1 | COL2A1 | COL4A3BP | COMT | COPA | CREB1 | CRP | CRYAB | CRYZ | CSMD1 | CTNNBIP1 | CYB5R3 | DBH | DCTN1 | DES | DLG2 | DLG4 | DMD | DMRT3 | DNAJC19 | DNM1L | DOCK5 | E2F4 | EEF2 | EFEMP1 | EGFR | EIF2AK4 | EIF4A3 | EP300 | EYA4 | EZH2 | EZR | F5 | FABP1 | FBXO11 | FEN1 | FGB | FGFR1 | FKBP1B | FOS | FYN | GAA | GABRA6 | GABRR1 | GJA1 | GLP1R | GLRB | GLS | GNAI2 | GNAO1 | GNB1 | GPI | GPX1 | GRHPR | GRIA1 | GRK1 | HDAC2 | HDAC4 | HERC1 | HEXB | HIF1A | HLA-DRA | HMOX1 | HNF1A | HOXA5 | HOXD10 | HSF4 | HTR1B | HTR3A | HTR3E | HTT | ICAM1 | IGKV3-20 | INSR | IREB2 | ITGB1BP1 | ITGB5 | ITPR3 | JUN | KCNAB1 | KCNAB2 | KCNIP2 | KIFC3 | KMT2B | KRAS | LEF1 | LGMN | LIMA1 | LUM | MAP2K3 | MAP2K6 | MAPK1 | MAPK3 | MAPT | MBP | MC1R | MECP2 | MEOX2 | METTL23 | MME | MYH11 | MYH6 | MYL12A | MYL12B | MYL2 | MYL6 | MYL9 | MYO1E | MYO5A | MYO6 | MYOC | NDUFS6 | NIPBL | NIPSNAP1 | NOB1 | NOD2 | NOS3 | NPR1 | NQO2 | NRXN1 | NTF3 | NTRK1 | OAT | OBSL1 | OPA1 | OR5F1 | P2RX1 | P2RX2 | P2RX4 | PAFAH1B1 | PDCL | PDE4D | PDE6G | PER2 | PGAM2 | PIAS1 | PIK3C2A | PIK3CA | PITPNA | PLA2G6 | PPARD | PPARGC1A | PPT1 | PRKACA | PRKAR1A | PRKAR2B | PRKCZ | PSAP | PSEN1 | PTEN | PTGER3 | PTN | PTPRO | PXN | RAB11A | RAB11FIP2 | RAP1GDS1 | RDH11 | RDH12 | RDH5 | RGS2 | RHO | RHOA | RP2 | RPGRIP1 | RPL38 | RRM2B | S100B | SERPINB6 | SLC2A5 | SLC9A3R1 | SMAD5 | SNAI2 | SNTA1 | SOD1 | SOD2 | SPRY2 | SRC | STAC | STAT1 | STK40 | STRAP | SULF2 | TAS2R60 | TAZ | TBL1X | TBXA2R | TGFB1 | THRA | TIMM13 | TMOD3 | TMOD4 | TNF | TNNT1 | TPCN2 | TPM1 | TPM2 | TPM3 | TPM4 | TREX1 | TRIM63 | UBA5 | UBA6 | UCHL1 | UNC119 | USP53 | VCL | VDAC1 | VDAC3 | VDR | WDR1 | WDR19 | WDR35 | WNK4 | YAP1 | YES1 | ZFAND5 | ZMPSTE24 | GO:0006189 | 0.000727890331190101 | 6/8243 | 6/3250 | 0.00374604742972087 | 1 | T | T | F | F | 'de novo' IMP biosynthetic process | ADSL | ATIC | GART | PAICS | PFAS | PPAT | GO:1901163 | 0.000727890331190101 | 6/8243 | 6/3250 | 0.00374604742972087 | 1 | T | T | F | F | regulation of trophoblast cell migration | AGO2 | C1QBP | CALR | SMURF2 | SYDE1 | TIMP1 | GO:1902525 | 0.000727890331190101 | 6/8243 | 6/3250 | 0.00374604742972087 | 1 | T | T | F | F | regulation of protein monoubiquitination | BIRC2 | FANCM | PDCD6 | PEF1 | UBB | WDR48 | GO:2000618 | 0.000727890331190101 | 6/8243 | 6/3250 | 0.00374604742972087 | 1 | T | T | F | F | regulation of histone H4-K16 acetylation | ATG5 | BRCA1 | PIH1D1 | SIRT1 | SMARCB1 | ZMPSTE24 | GO:0006554 | 0.000727890331190101 | 6/8243 | 6/3250 | 0.00374604742972087 | 1 | T | T | F | F | lysine catabolic process | AASS | ALDH7A1 | DLD | DLST | GCDH | OGDH | GO:2000756 | 0.00642969792551256 | 53/8243 | 31/3250 | 0.0037516872527381 | 1 | T | T | F | F | regulation of peptidyl-lysine acetylation | ARRB1 | ATG5 | BRCA1 | CTBP1 | CTCF | EID1 | HDAC2 | HDAC5 | KAT7 | KMT2A | MAPK3 | MECP2 | PAXIP1 | PIH1D1 | PPARGC1A | PRKAA1 | PRKAA2 | RUVBL2 | SET | SIN3A | SIRT1 | SMAD4 | SMARCB1 | SNAI2 | SNCA | SPI1 | TADA2B | TGFB1 | TWIST1 | WBP2 | ZMPSTE24 | GO:0010595 | 0.00897731408467791 | 74/8243 | 41/3250 | 0.0037535210281491 | 1 | T | T | F | F | positive regulation of endothelial cell migration | ABL1 | AKT1 | AKT3 | ALOX12 | ANXA1 | BCAR1 | CALR | CD40 | ETS1 | FGF2 | FGFR1 | GPI | HDAC7 | HDAC9 | HIF1A | HMGB1 | HMOX1 | HSPB1 | ITGB1BP1 | ITGB3 | KDR | LGMN | MAP2K3 | MAP3K3 | MAPK14 | NFE2L2 | NOS3 | NRP2 | P2RX4 | PDCD6 | PIK3C2A | PLCG1 | PRKD1 | PRKD2 | PROX1 | RHOJ | SIRT1 | SP1 | STAT5A | TGFB1 | THBS1 | GO:0001934 | 0.0698774717942497 | 576/8243 | 258/3250 | 0.0037581689012796 | 1 | T | T | F | F | positive regulation of protein phosphorylation | ABL1 | ACSL1 | ADCY6 | ADCYAP1 | ADRB2 | ADTRP | AGER | AKAP9 | AKT1 | AKT2 | AKTIP | ANXA2 | APOE | APP | AR | ARAF | ARRB1 | ARRB2 | ATM | AXIN1 | AZU1 | BAG4 | BCAR3 | BCL2 | BIRC7 | BMPR1A | BRAF | C1QTNF1 | CAB39 | CALM1 | CALM2 | CALM3 | CARD9 | CAV1 | CCDC88A | CCL21 | CCL22 | CCNB1 | CCNT1 | CCNT2 | CCNY | CCNYL1 | CCR1 | CD27 | CD36 | CD40 | CD40LG | CD74 | CD81 | CDC25B | CDK5 | CDK5R1 | CDK9 | CDKN1A | CHEK2 | CKS1B | CKS2 | CLU | CRK | CRKL | CSNK2B | CTNNB1 | DAB1 | DAB2 | DAXX | DBF4B | DBNL | DDRGK1 | DDX3X | DLG3 | DLG4 | DOCK7 | DUSP19 | DUSP22 | DUSP7 | DVL1 | DVL2 | DVL3 | EGFR | EHD4 | EPHA4 | ERBB2 | ERBB3 | EZH2 | FGB | FGF2 | FGFR1 | FGFR3 | FLOT1 | FYN | GADD45G | GATA1 | GDF9 | GNAI2 | HAVCR2 | HAX1 | HBEGF | HDAC2 | HDAC3 | HDAC6 | HMGB1 | HPX | HRAS | HSF1 | HSP90AA1 | HSP90AB1 | HSPA2 | ICAM1 | IFNA2 | IGF1R | IKBKG | ILK | INSR | IQGAP1 | ITGB1BP1 | ITGB3 | JUN | KDR | KLHDC10 | KRAS | KSR1 | LAT | LATS1 | LCP2 | LPAR1 | LRRK1 | LRRK2 | LTBR | MAP2K1 | MAP2K3 | MAP2K4 | MAP2K6 | MAP2K7 | MAP3K1 | MAP3K13 | MAP3K14 | MAP3K2 | MAP3K3 | MAP3K4 | MAP4K1 | MAPK1 | MAPK14 | MAPK3 | MAPK8IP3 | MAPKAP1 | MAS1 | MAVS | MIF | MLST8 | NEK1 | NELFE | NMNAT1 | NOD2 | NQO2 | NTF3 | NTRK1 | OXSR1 | PAK2 | PAK6 | PARK7 | PARP14 | PDCD10 | PDE6G | PHB | PHB2 | PIH1D1 | PIK3CA | PIK3CG | PIK3R3 | PIN1 | PLA2G1B | PLA2G2A | PLA2G6 | PLAUR | PLK1 | PPP2CA | PRDX2 | PRKAA1 | PRKACA | PRKAG1 | PRKAR1A | PRKAR2B | PRKCD | PRKCE | PRKCZ | PRKD1 | PRKD2 | PROX1 | PRRC1 | PSAP | PSEN1 | PSMD10 | PTEN | PTK2 | PTPN1 | PTPN22 | RAC1 | RAF1 | RAP1A | RAP1B | RB1CC1 | RHOA | RIPK1 | RIPK2 | RIPK3 | RPLP1 | RPS3 | RPTOR | S100A12 | S100A7 | SDC4 | SDCBP | SEMA7A | SHC1 | SIRT1 | SLAMF1 | SMAD4 | SNCA | SOD1 | SOX2 | SPRY2 | SQSTM1 | SRC | STAT3 | STK11 | STK24 | STK25 | STK26 | STK3 | TAB1 | TAB2 | TAOK3 | TERF2IP | TGFB1 | THBS1 | TIMP2 | TLR3 | TNF | TNFRSF1A | TNK2 | TP53 | TP73 | TRAF2 | TRAF6 | TXN | UBB | UBC | UBE2K | UBE2N | UBE2V1 | UNC119 | VTN | WNK1 | WNT16 | XRCC5 | XRCC6 | YES1 | ZNF622 | GO:0019219 | 0.0482833919689433 | 398/8243 | 183/3250 | 0.00376953683173036 | 1 | T | T | F | F | regulation of nucleobase-containing compound metabolic process | ABCE1 | ACD | ACTR2 | AHCYL1 | AHNAK | AIFM1 | AKT1 | APBB1 | ATAD5 | ATM | ATRX | AURKB | BABAM1 | BAX | BCL6 | BRCA1 | C1QBP | CBX8 | CCNB1 | CCNT1 | CCT2 | CCT3 | CCT4 | CCT5 | CCT6A | CCT7 | CCT8 | CD40 | CDA | CDC73 | CDK2 | CDK9 | CIRBP | CLK1 | CPSF7 | CTNNB1 | DAZAP1 | DCP2 | DHX36 | DHX9 | DNM1L | DROSHA | EGFR | ENDOG | ESR1 | EXOSC6 | EYA2 | EYA4 | FAM129B | FIGNL1 | FOXM1 | FUS | GSK3A | GZMA | H1F0 | H1FX | H2AFX | HIST1H1A | HMGB1 | HMGB2 | HNRNPA1 | HNRNPA2B1 | HNRNPD | HNRNPF | HNRNPH1 | HNRNPK | HNRNPL | HNRNPU | HRAS | HSF1 | JMJD6 | KDM1A | KDM4D | KHDRBS1 | KMT2A | KPNA1 | KPNA2 | MAP2K1 | MAP2K7 | MAP3K4 | MAPK1 | MAPK15 | MAPK3 | MBD3 | ME1 | MECP2 | MGMT | MLH1 | MSH6 | MYC | NCBP1 | NCOR1 | NCOR2 | NEK7 | NELFE | NMNAT1 | NPM1 | NUP98 | OAS2 | OTUB1 | OTUD4 | PAM16 | PARP1 | PAXIP1 | PCNA | PIK3CA | PIK3R1 | PINX1 | PML | PNKP | POLR2A | POT1 | PPARGC1A | PPP4R2 | PRDX6 | PRKCD | PRPF19 | RAD51 | RAD51AP1 | RBM17 | RBM25 | RBM3 | RBM4 | RBM8A | RBMX | RC3H1 | RELA | REST | RIF1 | RIOK1 | RIPK1 | RNF8 | RPA2 | RPS13 | RPS3 | SAP18 | SART3 | SIRT1 | SIRT6 | SLTM | SMG5 | SMG6 | SNRPA | SNW1 | SPI1 | SRC | SRPK1 | SRPK2 | SRSF12 | SRSF5 | STAT6 | SUPT5H | TCEA1 | TCP1 | TERF1 | TERF2 | TERF2IP | TFIP11 | TGFB1 | TIA1 | TINF2 | TMBIM6 | TNKS | TNKS2 | TP53 | TRA2A | TRIM28 | TRIP12 | TWIST1 | U2AF2 | UBE2B | UBE2N | UBE2V1 | UBR5 | USP7 | USP9X | WAS | WT1 | WTAP | XRCC1 | XRCC5 | ZBTB7A | ZMPSTE24 | GO:0038128 | 0.00327550649035545 | 27/8243 | 18/3250 | 0.00379358125231733 | 1 | T | T | F | F | ERBB2 signaling pathway | CDC37 | CPNE3 | CUL5 | EGFR | ERBB2 | ERBB3 | GRB2 | GRB7 | HBEGF | HSP90AA1 | MATK | MYOC | PIK3CA | PIK3R1 | PTPRR | SHC1 | SRC | STUB1 | GO:0016310 | 0.089409195681184 | 737/8243 | 325/3250 | 0.0038271487930869 | 1 | T | T | F | F | phosphorylation | ABL1 | ABL2 | ACTN2 | ADCK2 | ADK | AGK | AK1 | AK2 | AK4 | AKT1 | AKT2 | AKT3 | APOA1 | APP | ARAF | ATM | ATP13A2 | AURKB | BCL2 | BIRC5 | BMPR1A | BRAF | BRD4 | BTK | BTRC | CAB39 | CALM1 | CAMK1D | CAMK2A | CAMK2B | CAMKV | CCNB1 | CCNT1 | CCR5 | CDC25B | CDK15 | CDK16 | CDK19 | CDK2 | CDK4 | CDK5 | CDK5R1 | CDK8 | CDK9 | CDKL3 | CFL1 | CHCHD10 | CHEK2 | CKB | CKM | CKMT1A | CLK1 | CMPK1 | COL4A3BP | COPS2 | CPNE3 | CREB1 | CRKL | CRY1 | CRYAB | CSNK1A1 | CSNK1E | CSNK2A2 | CSNK2B | CTBP1 | CUL1 | CUL3 | CYC1 | DAPK1 | DCAKD | DCK | DCLK1 | DGKE | DGKZ | DGUOK | DLG2 | DLG3 | DLG4 | DUSP7 | DYRK2 | EFEMP1 | EGFR | EIF2AK3 | EIF2AK4 | EIF2S1 | EPHA4 | EPHA6 | EPHB6 | ERBB2 | ERBB3 | ERG | FAM20B | FBXW11 | FGF2 | FGFR1 | FGFR3 | FYN | GAK | GPS2 | GRB2 | GRK1 | GRK5 | GSK3A | GSK3B | HBEGF | HIPK4 | HK1 | HK2 | HRAS | HSF1 | ICK | IGF1R | IKBKB | IKBKE | IKBKG | IL3RA | ILF3 | ILK | IMPA1 | IMPA2 | INSR | IP6K1 | ITGAV | ITK | JAK1 | JAK3 | KBTBD7 | KDR | KRAS | KSR1 | LAT | LATS1 | LRRC7 | LRRK1 | LRRK2 | MAP2K1 | MAP2K3 | MAP2K4 | MAP2K6 | MAP2K7 | MAP3K1 | MAP3K13 | MAP3K14 | MAP3K2 | MAP3K3 | MAP3K4 | MAP4K1 | MAPK1 | MAPK14 | MAPK15 | MAPK3 | MAPK4 | MAPK6 | MAPK7 | MAPK8 | MAPK9 | MAST1 | MAST3 | MATK | MBP | MED1 | MELK | MKNK2 | MYC | NAGK | NEK1 | NEK7 | NLK | NME1 | NME2 | NME4 | NMRK2 | NOD2 | NPR1 | NTRK1 | OXSR1 | PAK2 | PAK6 | PAPSS1 | PAPSS2 | PDK2 | PDXK | PEBP1 | PFKM | PGK1 | PGK2 | PHKA1 | PI4K2A | PICK1 | PIK3C2A | PIK3CA | PIK3CG | PIK3R1 | PIK3R3 | PIKFYVE | PIM1 | PIP4K2A | PIPSL | PKN2 | PLK1 | PML | PNKP | PPM1D | PPP5C | PRDX4 | PRKAA1 | PRKAA2 | PRKAB2 | PRKACA | PRKAG1 | PRKCD | PRKCE | PRKCZ | PRKD1 | PRKD2 | PRKD3 | PRKDC | PSMA1 | PSMA2 | PSMA3 | PSMA5 | PSMA6 | PSMB5 | PSMB6 | PSMB7 | PSMB8 | PSMC1 | PSMC2 | PSMC3 | PSMC4 | PSMC5 | PSMC6 | PSMD1 | PSMD10 | PSMD11 | PSMD12 | PSMD14 | PSMD2 | PSMD4 | PSMD9 | PSME2 | PSME3 | PTK2 | PTPN6 | PTPRA | RAF1 | RANBP9 | RARA | RBX1 | RFK | RHO | RIOK1 | RIPK1 | RIPK2 | RIPK3 | RIPK4 | RPS6KA1 | RPS6KA2 | RPS6KB2 | SCG2 | SCYL2 | SEPHS1 | SEPHS2 | SHC1 | SMAD2 | SMAD5 | SOCS1 | SOCS2 | SOCS6 | SPTAN1 | SPTBN1 | SPTBN2 | SQSTM1 | SRC | SRPK1 | SRPK2 | ST3GAL1 | STAT3 | STK11 | STK24 | STK25 | STK26 | STK3 | STK38 | STK40 | STOML2 | SULT1A3 | TAB1 | TAB2 | TAOK3 | TBRG4 | TESK2 | TGFB1 | TGFBR3 | TK1 | TK2 | TLR3 | TNF | TNIP2 | TNK2 | TNKS | TOLLIP | TOP1 | TP53RK | TRAF6 | TRIB2 | TRIB3 | TRIM28 | TWF1 | UBB | UBC | UBE2N | UBE2V1 | UCK2 | ULK1 | UQCRB | UQCRC2 | USP15 | WNK1 | WNK4 | YES1 | YWHAB | YWHAE | ZAP70 | ZMPSTE24 | GO:0070301 | 0.00618706781511586 | 51/8243 | 30/3250 | 0.00384815926095613 | 1 | T | T | F | F | cellular response to hydrogen peroxide | ABL1 | AIFM1 | ANXA1 | APEX1 | CBX8 | ETS1 | EZH2 | FABP1 | FOXO1 | HDAC2 | HDAC6 | HSF1 | MAPK7 | MDM2 | NFE2L2 | NQO1 | PARK7 | PCNA | PPIF | PRKAA1 | PRKCD | RELA | RIPK1 | RIPK3 | RPS3 | SIRT1 | SRC | STAT6 | TNFAIP3 | ZNF277 | GO:0042327 | 0.0733956083950018 | 605/8243 | 270/3250 | 0.00388089525528459 | 1 | T | T | F | F | positive regulation of phosphorylation | ABL1 | ACSL1 | ADCY6 | ADCYAP1 | ADRB2 | ADTRP | AGAP2 | AGER | AKAP9 | AKT1 | AKT2 | AKTIP | ANXA2 | APOE | APP | AR | ARAF | ARRB1 | ARRB2 | ATM | AXIN1 | AZU1 | BAG4 | BCAR3 | BCL10 | BCL2 | BIRC7 | BMPR1A | BRAF | C1QTNF1 | CAB39 | CALM1 | CALM2 | CALM3 | CARD9 | CAV1 | CCDC88A | CCL21 | CCL22 | CCNB1 | CCNT1 | CCNT2 | CCNY | CCNYL1 | CCR1 | CD27 | CD36 | CD40 | CD40LG | CD74 | CD81 | CDC25B | CDK5 | CDK5R1 | CDK9 | CDKN1A | CHEK2 | CKS1B | CKS2 | CLU | CRK | CRKL | CSNK2B | CTNNB1 | DAB1 | DAB2 | DAXX | DBF4B | DBNL | DDRGK1 | DDX3X | DGKZ | DLG3 | DLG4 | DOCK7 | DUSP12 | DUSP19 | DUSP22 | DUSP7 | DVL1 | DVL2 | DVL3 | EEF1A2 | EGFR | EHD4 | EPHA4 | ERBB2 | ERBB3 | EZH2 | FGB | FGF2 | FGFR1 | FGFR3 | FLOT1 | FYN | GADD45G | GATA1 | GDF9 | GLMN | GNAI2 | HAVCR2 | HAX1 | HBEGF | HDAC2 | HDAC3 | HDAC6 | HMGB1 | HPX | HRAS | HSF1 | HSP90AA1 | HSP90AB1 | HSPA2 | ICAM1 | IFNA2 | IGF1R | IKBKG | ILK | INSR | IQGAP1 | ITGB1BP1 | ITGB3 | JUN | KDR | KLHDC10 | KRAS | KSR1 | LAT | LATS1 | LCP2 | LPAR1 | LRRK1 | LRRK2 | LTBR | MAP2K1 | MAP2K3 | MAP2K4 | MAP2K6 | MAP2K7 | MAP3K1 | MAP3K13 | MAP3K14 | MAP3K2 | MAP3K3 | MAP3K4 | MAP4K1 | MAPK1 | MAPK14 | MAPK3 | MAPK8IP3 | MAPKAP1 | MAPT | MAS1 | MAVS | MIF | MLST8 | MOB1B | NEK1 | NELFE | NMNAT1 | NOD2 | NQO2 | NTF3 | NTRK1 | OXSR1 | PAK2 | PAK6 | PARK7 | PARP14 | PDCD10 | PDE6G | PGK1 | PGK2 | PHB | PHB2 | PIH1D1 | PIK3CA | PIK3CG | PIK3R3 | PIN1 | PLA2G1B | PLA2G2A | PLA2G6 | PLAUR | PLK1 | PPP2CA | PRDX2 | PRKAA1 | PRKACA | PRKAG1 | PRKAR1A | PRKAR2B | PRKCD | PRKCE | PRKCZ | PRKD1 | PRKD2 | PROX1 | PRRC1 | PSAP | PSEN1 | PSMD10 | PTEN | PTGES3 | PTK2 | PTPN1 | PTPN22 | RAC1 | RAF1 | RAP1A | RAP1B | RB1CC1 | RHOA | RIPK1 | RIPK2 | RIPK3 | RPLP1 | RPS3 | RPTOR | S100A12 | S100A7 | SDC4 | SDCBP | SEMA7A | SHC1 | SIRT1 | SLAMF1 | SMAD4 | SNCA | SOD1 | SOX2 | SPRY2 | SQSTM1 | SRC | STAT3 | STK11 | STK24 | STK25 | STK26 | STK3 | TAB1 | TAB2 | TAOK3 | TERF2IP | TGFB1 | THBS1 | TIMP2 | TLR3 | TNF | TNFRSF1A | TNK2 | TP53 | TP73 | TRAF2 | TRAF6 | TXN | UBB | UBC | UBE2K | UBE2N | UBE2V1 | UNC119 | VCP | VTN | WNK1 | WNT16 | XRCC5 | XRCC6 | YES1 | ZNF622 | GO:0098732 | 0.00594443770471916 | 49/8243 | 29/3250 | 0.00393835841651306 | 1 | T | T | F | F | macromolecule deacylation | ABHD17A | HDAC1 | HDAC11 | HDAC2 | HDAC3 | HDAC4 | HDAC5 | HDAC6 | HDAC7 | HDAC9 | LYPLA1 | LYPLA2 | MORF4L1 | MORF4L2 | MTA1 | MTA3 | PER1 | PER2 | PHB | PPT1 | REST | SFPQ | SIN3A | SIRT1 | SIRT3 | SIRT5 | SIRT6 | SIRT7 | TBL1X | GO:0045620 | 0.00351813660075215 | 29/8243 | 19/3250 | 0.00395969910942201 | 1 | T | T | F | F | negative regulation of lymphocyte differentiation | ANXA1 | BCL6 | CBFB | CD74 | DTX1 | ERBB2 | FBXO7 | FOXJ1 | HLX | HMGB1 | ID2 | IFNA2 | IRF1 | JAK3 | PRDX2 | RC3H1 | RUNX1 | SOCS1 | ZBTB7B | GO:0045839 | 0.00351813660075215 | 29/8243 | 19/3250 | 0.00395969910942201 | 1 | T | T | F | F | negative regulation of mitotic nuclear division | APC | ATM | ATRX | AURKB | CDC20 | CENPF | IK | KLHL22 | MAD1L1 | MAD2L1 | PLK1 | PTTG1 | RAD21 | SPDL1 | TNKS | TPR | TRIP13 | ZNF207 | ZW10 | GO:0051084 | 0.00351813660075215 | 29/8243 | 19/3250 | 0.00395969910942201 | 1 | T | T | F | F | 'de novo' posttranslational protein folding | BAG1 | CCT2 | CD74 | DNAJB1 | DNAJC7 | GAK | HSPA14 | HSPA1B | HSPA1L | HSPA2 | HSPA5 | HSPA8 | HSPA9 | HSPE1 | HSPH1 | PTGES3 | ST13 | TOR1A | UGGT1 | GO:0010558 | 0.112944316389664 | 931/8243 | 405/3250 | 0.00396858720881948 | 1 | T | T | F | F | negative regulation of macromolecule biosynthetic process | ACD | AEBP2 | AES | AGO1 | AGO2 | APBB1 | APEX1 | AR | ARHGAP35 | ASCL1 | ATF2 | ATRX | ATXN1 | AURKB | BACH2 | BAG3 | BASP1 | BCL11A | BCL6 | BCL6B | BCOR | BIRC5 | BMI1 | BRCA1 | BTRC | C1QBP | CALR | CAV1 | CBFA2T3 | CBFB | CBX4 | CBX7 | CBX8 | CBY1 | CCAR2 | CD36 | CDC73 | CDK2 | CDK5 | CDK5R1 | CEBPA | CENPF | CENPJ | CHCHD3 | CHD8 | CHEK2 | CIRBP | CLTC | COPS2 | CREB1 | CREBBP | CRY1 | CRY2 | CTBP1 | CTCF | CTNNB1 | CTNNBIP1 | CUL3 | DAB2 | DAPK1 | DAXX | DCP2 | DDX3X | DHX36 | DMAP1 | DNAJA3 | DNAJB1 | DPF2 | DUSP1 | DUSP22 | E2F1 | E2F2 | E2F3 | E2F4 | E2F6 | EED | EHMT1 | EHMT2 | EID1 | EIF2AK3 | EIF2AK4 | EIF2S1 | EIF3E | EIF4A3 | EIF4E | EIF4E2 | EIF4EBP1 | EIF4ENIF1 | EIF6 | ELF3 | ENO1 | EP300 | EPRS | ERF | ERRFI1 | ESR1 | ESR2 | ESRRA | EZH2 | EZR | FAM129B | FASLG | FBP1 | FBXW11 | FGFR1 | FHL2 | FOXA1 | FOXJ1 | FOXK1 | FOXM1 | FOXO1 | FTO | FXR2 | GAPDH | GATA1 | GPS2 | GRB7 | GSK3A | GSK3B | GSTP1 | H1F0 | H3F3A | HBEGF | HBZ | HDAC1 | HDAC2 | HDAC3 | HDAC4 | HDAC5 | HDAC6 | HDAC7 | HDAC9 | HDGF | HEXIM1 | HIF1AN | HIST1H3A | HIST1H4A | HIST2H3A | HIVEP1 | HMGB1 | HMGB2 | HNRNPA1 | HNRNPA2B1 | HNRNPD | HNRNPU | HSF1 | HSF4 | HSPA8 | ID2 | IFI16 | IFNA2 | IGBP1 | IGF2BP1 | IKZF5 | ILF3 | IREB2 | IRF1 | IRF7 | ITGAV | ITGB3 | JAK3 | JUN | JUNB | JUND | KANK2 | KAT5 | KAT7 | KAT8 | KDM1A | KDM5B | KDM5C | KHDRBS1 | KLF5 | L3MBTL1 | LEF1 | LGR4 | LIMS1 | LOXL2 | MAF1 | MBD3 | MDFI | MDM2 | MDM4 | MECP2 | MED1 | MEPCE | MITF | MORF4L1 | MORF4L2 | MOV10 | MSH6 | MTA1 | MTA3 | MXD1 | MYBBP1A | MYC | NACC1 | NANOG | NCK1 | NCOA2 | NCOR1 | NCOR2 | NDUFA13 | NEDD4 | NELFB | NELFCD | NELFE | NFATC2 | NIF3L1 | NIPBL | NKRF | NKX2-1 | NOSTRIN | NR0B2 | NR1H2 | NR1H3 | NR1I2 | NR3C1 | PA2G4 | PARP1 | PATZ1 | PAWR | PAX9 | PCGF1 | PCGF6 | PER1 | PER2 | PHB | PHB2 | PHF10 | PHF21A | PIAS1 | PIAS3 | PINX1 | PKIG | PLK1 | PML | POT1 | POU5F1 | PPARD | PPARG | PPID | PPM1A | PPM1D | PRDX5 | PRMT2 | PRMT6 | PROX1 | PSEN1 | PSMC5 | PSMD10 | PTPRK | PUM1 | PUS7 | QARS | RARA | RARG | RASD1 | RB1 | RBM3 | RBM4 | RBPJ | RC3H1 | REL | RELA | REST | RFC1 | RGN | RGS2 | RIF1 | RING1 | RNF139 | RNF2 | RNF8 | RPL10 | RPL23 | RPS14 | RPS3 | RTF1 | RUNX1 | RUNX1T1 | RUNX2 | RUVBL2 | RXRA | S100A1 | S100A11 | SAP18 | SARS | SET | SF1 | SFMBT1 | SFPQ | SHC1 | SHMT1 | SIN3A | SIRT1 | SIRT5 | SIRT6 | SIRT7 | SKI | SMAD2 | SMAD3 | SMAD4 | SMAD5 | SMARCA2 | SMARCA4 | SMARCC2 | SMARCE1 | SMC1A | SMC3 | SMO | SMURF2 | SNAI2 | SNCA | SNW1 | SNX6 | SOX2 | SP100 | SP2 | SPEN | SPI1 | SQSTM1 | SRC | STAG2 | STAT1 | STAT3 | STAT6 | STRAP | SUMO1 | SUPT4H1 | SUPT5H | SUZ12 | TAGLN3 | TBL1X | TCERG1 | TCF21 | TCF25 | TCP10L | TERF1 | TERF2 | TGFB1 | THAP11 | THAP7 | THRA | TIA1 | TINF2 | TLE1 | TLE3 | TMBIM6 | TNF | TNP1 | TP53 | TP63 | TPR | TRAF6 | TRIB2 | TRIB3 | TRIM28 | TRIM29 | TRIM33 | TSC22D4 | TSG101 | TSPYL2 | TWIST1 | TXN | TXNIP | TYMS | UBA3 | UBB | UBC | UBE2D1 | UBE2D3 | UBE2I | UNK | USP2 | USP47 | USP9X | VDR | VHL | VPS72 | WDTC1 | WT1 | WWOX | WWP2 | XPO1 | XRCC5 | XRCC6 | YAF2 | YAP1 | YEATS2 | YWHAB | YWHAQ | YY1 | ZBTB16 | ZBTB21 | ZBTB33 | ZBTB42 | ZBTB7A | ZBTB7B | ZC3H4 | ZHX1 | ZMYND8 | ZNF217 | ZNF24 | ZNF746 | GO:0045892 | 0.0912289215091593 | 752/8243 | 331/3250 | 0.00402704027648477 | 1 | T | T | F | F | negative regulation of transcription, DNA-templated | AEBP2 | AES | APBB1 | AR | ARHGAP35 | ASCL1 | ATF2 | ATRX | ATXN1 | AURKB | BACH2 | BAG3 | BASP1 | BCL11A | BCL6 | BCL6B | BCOR | BIRC5 | BMI1 | BRCA1 | BTRC | C1QBP | CALR | CAV1 | CBFA2T3 | CBFB | CBX4 | CBX7 | CBX8 | CBY1 | CCAR2 | CD36 | CDC73 | CDK2 | CDK5 | CDK5R1 | CEBPA | CENPF | CHCHD3 | CHD8 | COPS2 | CREB1 | CREBBP | CRY1 | CRY2 | CTBP1 | CTCF | CTNNB1 | CTNNBIP1 | CUL3 | DAB2 | DAXX | DMAP1 | DNAJA3 | DNAJB1 | DPF2 | DUSP22 | E2F1 | E2F2 | E2F3 | E2F4 | E2F6 | EED | EHMT1 | EHMT2 | EID1 | ELF3 | ENO1 | EP300 | ERF | ESR1 | ESR2 | ESRRA | EZH2 | EZR | FAM129B | FASLG | FBP1 | FBXW11 | FGFR1 | FHL2 | FOXA1 | FOXJ1 | FOXK1 | FOXM1 | FOXO1 | GATA1 | GPS2 | H1F0 | H3F3A | HBZ | HDAC1 | HDAC2 | HDAC3 | HDAC4 | HDAC5 | HDAC6 | HDAC7 | HDAC9 | HDGF | HEXIM1 | HIF1AN | HIST1H3A | HIST1H4A | HIST2H3A | HIVEP1 | HMGB1 | HMGB2 | HNRNPA2B1 | HNRNPU | HSF1 | HSF4 | HSPA8 | ID2 | IFI16 | IFNA2 | IGBP1 | IKZF5 | ILF3 | IRF1 | IRF7 | JUN | JUNB | JUND | KANK2 | KAT5 | KAT7 | KAT8 | KDM1A | KDM5B | KDM5C | KHDRBS1 | KLF5 | L3MBTL1 | LEF1 | LGR4 | LIMS1 | LOXL2 | MAF1 | MBD3 | MDFI | MDM2 | MDM4 | MECP2 | MED1 | MEPCE | MITF | MORF4L1 | MORF4L2 | MTA1 | MTA3 | MXD1 | MYBBP1A | MYC | NACC1 | NCK1 | NCOA2 | NCOR1 | NCOR2 | NDUFA13 | NEDD4 | NELFB | NELFCD | NELFE | NFATC2 | NIPBL | NKRF | NKX2-1 | NOSTRIN | NR0B2 | NR1H2 | NR1H3 | NR1I2 | NR3C1 | PA2G4 | PARP1 | PATZ1 | PAWR | PAX9 | PCGF1 | PCGF6 | PER1 | PER2 | PHB | PHB2 | PHF10 | PHF21A | PIAS1 | PIAS3 | PKIG | PLK1 | PML | POU5F1 | PPARD | PPARG | PPID | PPM1A | PPM1D | PRDX5 | PRMT2 | PRMT6 | PROX1 | PSEN1 | PSMC5 | PSMD10 | PTPRK | QARS | RARA | RARG | RASD1 | RB1 | RBPJ | REL | RELA | REST | RFC1 | RIF1 | RING1 | RNF2 | RNF8 | RPL10 | RPL23 | RPS14 | RTF1 | RUNX1 | RUNX1T1 | RUNX2 | RXRA | S100A1 | SARS | SET | SFMBT1 | SFPQ | SHC1 | SIN3A | SIRT1 | SIRT5 | SIRT6 | SIRT7 | SKI | SMAD2 | SMAD3 | SMAD4 | SMAD5 | SMARCA2 | SMARCA4 | SMARCC2 | SMARCE1 | SMO | SMURF2 | SNAI2 | SNCA | SNW1 | SNX6 | SOX2 | SP100 | SP2 | SPEN | SPI1 | SQSTM1 | SRC | STAT1 | STAT3 | STAT6 | STRAP | SUMO1 | SUPT4H1 | SUPT5H | SUZ12 | TAGLN3 | TBL1X | TCERG1 | TCF21 | TCF25 | TCP10L | TGFB1 | THAP11 | THAP7 | THRA | TLE1 | TMBIM6 | TNF | TNP1 | TP53 | TP63 | TPR | TRAF6 | TRIB3 | TRIM28 | TRIM29 | TRIM33 | TSC22D4 | TSG101 | TWIST1 | TXN | TXNIP | UBA3 | UBB | UBC | UBE2D1 | UBE2D3 | UBE2I | USP2 | USP47 | USP9X | VDR | VHL | VPS72 | WDTC1 | WT1 | WWOX | WWP2 | XPO1 | XRCC5 | XRCC6 | YAF2 | YEATS2 | YWHAB | YWHAQ | YY1 | ZBTB16 | ZBTB21 | ZBTB33 | ZBTB42 | ZBTB7A | ZBTB7B | ZC3H4 | ZHX1 | ZNF217 | ZNF24 | ZNF746 | GO:1904645 | 0.00376076671114885 | 31/8243 | 20/3250 | 0.00408130120641127 | 1 | T | T | F | F | response to amyloid-beta | ADRB2 | AGER | APP | BACE1 | CACNA1A | CD36 | CDK5 | EPHA4 | FYN | GSK3B | ICAM1 | IGF1R | ITGA4 | LGMN | NAMPT | NTRK1 | PARP1 | PSEN1 | SNX6 | VCAM1 | GO:0033146 | 0.00376076671114885 | 31/8243 | 20/3250 | 0.00408130120641127 | 1 | T | T | F | F | regulation of intracellular estrogen receptor signaling pathway | AR | AXIN1 | BRCA1 | CARM1 | CBFB | DDRGK1 | ESR1 | FOXA1 | KANK2 | LATS1 | MED1 | PARP1 | PHB2 | RUNX1 | SRC | TP63 | UBA5 | UFM1 | WBP2 | YAP1 | GO:0009066 | 0.00376076671114885 | 31/8243 | 20/3250 | 0.00408130120641127 | 1 | T | T | F | F | aspartate family amino acid metabolic process | AASDHPPT | AASS | ADSS | ALDH7A1 | ASNS | ASS1 | DLD | DLST | ENOPH1 | GCDH | GOT1 | GOT1L1 | GOT2 | MTAP | MTHFD1 | MTRR | NIT2 | OGDH | PHGDH | PLOD2 | GO:0051784 | 0.00376076671114885 | 31/8243 | 20/3250 | 0.00408130120641127 | 1 | T | T | F | F | negative regulation of nuclear division | APC | ATM | ATRX | AURKB | CDC20 | CENPF | IK | KLHL22 | MAD1L1 | MAD2L1 | PLK1 | PRKAR1A | PTTG1 | RAD21 | SPDL1 | TNKS | TPR | TRIP13 | ZNF207 | ZW10 | GO:0010332 | 0.00545917748392576 | 45/8243 | 27/3250 | 0.0040928082890509 | 1 | T | T | F | F | response to gamma radiation | ATM | BAX | BCL2 | BCL2L1 | CBL | CHEK2 | CRYAB | GPX1 | H2AFX | HRAS | HSF1 | HSPA5 | KDM1A | LIG4 | MDM2 | MYC | PARP1 | PML | POLE3 | PRKAA1 | PRKDC | TP53 | TREX1 | XRCC5 | XRCC6 | YAP1 | ZMPSTE24 | GO:0006006 | 0.00545917748392576 | 45/8243 | 27/3250 | 0.0040928082890509 | 1 | T | T | F | F | glucose metabolic process | AKR1A1 | AKT1 | AKT2 | ALDH5A1 | APOD | CPT1A | DCXR | DLAT | FABP5 | G6PD | GAA | GDPGP1 | MAPK14 | ONECUT1 | PDHA1 | PDHA2 | PDHB | PDK2 | PGM1 | PGM2 | PGM5 | PIK3CA | PPARD | PRKAA1 | SORD | TNF | WDTC1 | GO:0045595 | 0.125682397185491 | 1036/8243 | 448/3250 | 0.00409535977979014 | 1 | T | T | F | F | regulation of cell differentiation | ABCG1 | ABL1 | ABL2 | ACTN4 | ACTR2 | ADA | ADCY6 | ADCYAP1 | AGER | AGO1 | AHI1 | AKT1 | ANXA1 | ANXA2 | AP3D1 | APBB1 | APC | APCS | APOA1 | APOE | APP | AR | ARF1 | ARHGAP35 | ARHGDIA | ARHGEF7 | ARNT | ARPC2 | ARRB2 | ASCL1 | ATOH1 | AVIL | BAG5 | BCL11A | BCL2 | BCL6 | BIN1 | BIRC2 | BMPR1A | BRAF | BTK | C1QA | C1QBP | C1QL4 | CALR | CAMK1D | CAMK2A | CAMK2B | CAPN2 | CARM1 | CASP8 | CAV1 | CBFB | CCDC88A | CCR1 | CD109 | CD2 | CD27 | CD36 | CD74 | CDC20 | CDC42 | CDC73 | CDH5 | CDK5 | CDK5R1 | CDK9 | CDKL3 | CEBPA | CFL1 | CHRD | CLDN18 | CLIC1 | CMTM5 | COL5A1 | CREB1 | CREBBP | CRK | CRKL | CRMP1 | CRP | CSNK1E | CTDP1 | CTH | CTNNB1 | CTNNBIP1 | CTSL | CTSV | CTTN | CUL7 | CYLD | DAB1 | DAB2 | DBN1 | DDX39B | DDX6 | DGUOK | DHX36 | DISC1 | DLG4 | DNM1L | DOCK5 | DOCK7 | DPF2 | DROSHA | DTX1 | DVL1 | DVL2 | DVL3 | DYNLT1 | E2F1 | EFEMP1 | EGFR | EID1 | EIF2AK4 | EIF4E | EIF4ENIF1 | EIF6 | EP300 | EPHA4 | ERBB2 | ERRFI1 | ESRRA | ESRRB | ETS1 | EZH2 | FADD | FAM129B | FANCA | FBXO7 | FGB | FGF2 | FGFR1 | FKBP1B | FLOT1 | FN1 | FOS | FOXA1 | FOXJ1 | FOXO1 | FTO | FYN | G6PD | GAK | GATA1 | GDI1 | GFAP | GNAS | GOLGA4 | GPS2 | GRID2 | GRIP1 | GSK3A | GSK3B | H3F3A | HAP1 | HAX1 | HDAC1 | HDAC2 | HDAC3 | HDAC4 | HDAC5 | HDAC6 | HDAC7 | HDAC9 | HECW1 | HECW2 | HIF1A | HIF1AN | HIST1H3A | HIST1H4A | HIST2H3A | HLX | HMGB1 | HMGB2 | HNRNPU | HOXA5 | HSF4 | HSP90AB1 | HSPA1B | HSPA5 | HSPA9 | ID2 | IDH2 | IFNA2 | IKBKB | ILK | INSIG1 | IQCB1 | IQGAP1 | IRF1 | IRF4 | IRF7 | ISG15 | ITCH | ITGAV | ITGB1BP1 | ITGB3 | JAK3 | JUN | JUNB | JUND | KDM1A | KDR | KEAP1 | KIF13B | KLF5 | KMT2A | KMT2B | KMT2C | KRAS | L3MBTL1 | LDLRAD4 | LEF1 | LGALS1 | LGALS3 | LIG4 | LIMS1 | LMO2 | LOXL2 | LPAR1 | LRRC7 | LRRK2 | LSM1 | MAP1B | MAP2K1 | MAP3K13 | MAPK1 | MAPK14 | MAPK6 | MAPK9 | MAPT | MDM2 | MECP2 | MED1 | METRN | METRNL | MEX3C | MIB1 | MITF | MME | MORF4L2 | MOV10 | MYC | MYL9 | MYOC | NANOG | NAP1L1 | NCK1 | NCOA1 | NCOA3 | NEDD4 | NELFB | NELL1 | NEUROD1 | NF2 | NFATC1 | NFATC2 | NFE2L2 | NFKBIA | NIN | NIPBL | NKX2-1 | NME1 | NME2 | NMRK2 | NPHP3 | NR1H2 | NR1H3 | NTF3 | NTRK1 | NUMA1 | OBSL1 | OPA1 | P2RX4 | PA2G4 | PAFAH1B1 | PARP1 | PARP6 | PAX8 | PCM1 | PDLIM5 | PER2 | PIAS1 | PIAS3 | PIK3R1 | PIN1 | PLA2G10 | PLA2G2A | PLXNA1 | PLXNA2 | PLXNB2 | PNP | PPARD | PPARG | PPP1CC | PPP1R12A | PPP2CA | PPP2R1A | PRDX2 | PRELID1 | PRKACA | PRKCZ | PRKD1 | PRMT6 | PROX1 | PRPF19 | PSEN1 | PSMA1 | PSMA2 | PSMA3 | PSMA5 | PSMA6 | PSMB5 | PSMB6 | PSMB7 | PSMB8 | PSMC1 | PSMC2 | PSMC3 | PSMC4 | PSMC5 | PSMC6 | PSMD1 | PSMD10 | PSMD11 | PSMD12 | PSMD14 | PSMD2 | PSMD4 | PSMD9 | PSME2 | PSME3 | PTEN | PTK2 | PTN | PTPN6 | PTPRD | PTPRF | PTPRO | PUS7 | RAB11A | RAB21 | RAB29 | RAC1 | RAF1 | RAP1A | RARA | RARG | RASSF10 | RB1 | RBM4 | RBPJ | RC3H1 | RECK | RELA | REST | RGS2 | RHOA | RIPK1 | RIPK2 | RPS19 | RPS6KA1 | RTN4 | RTN4IP1 | RUNX1 | RUNX1T1 | RUNX2 | S100A9 | S100B | SARM1 | SART1 | SDCBP | SEMA7A | SERPINB3 | SERPINE2 | SFN | SH3GL3 | SIN3A | SIPA1L1 | SIRT1 | SKI | SLAMF8 | SMAD2 | SMAD3 | SMAD4 | SMAD5 | SMO | SMURF1 | SNAI2 | SNW1 | SNX3 | SOCS1 | SOCS2 | SOD1 | SOD2 | SORT1 | SOX2 | SPEN | SPI1 | SPRY2 | SRGAP2 | STAT1 | STAT3 | STK11 | STK24 | STK25 | STK3 | STRAP | SUZ12 | TCF12 | TCF4 | TGFB1 | THBS1 | TIMP2 | TLR3 | TMEM30A | TNF | TNFRSF12A | TNFRSF1A | TNFSF14 | TNN | TP63 | TP73 | TRAF6 | TRIB2 | TRIB3 | TRIM32 | TRIP4 | TUBB2B | TWF1 | TWF2 | TWIST1 | VDR | VHL | VIM | VSTM2A | WDR1 | XBP1 | XRCC5 | YAP1 | YWHAG | YWHAH | YY1 | ZAP70 | ZBTB16 | ZBTB7B | ZMYND8 | GO:0033157 | 0.0213514497149096 | 176/8243 | 87/3250 | 0.0041053902393532 | 1 | T | T | F | F | regulation of intracellular protein transport | AKT2 | ANK3 | APOD | ATAD1 | ATP13A2 | B3GAT3 | BAG3 | BAG4 | CD36 | CDH1 | CDK5 | CDK5R1 | CEP290 | CHP1 | CSNK2A2 | DMAP1 | EDEM1 | ERBB2 | FIS1 | FYN | GDI1 | GSK3A | GSK3B | HAX1 | HDAC3 | HRAS | HSPA1L | HTRA2 | HUWE1 | INSIG1 | IPO5 | ITGB1BP1 | ITGB3 | JUP | KIF5B | LCP1 | LRRK2 | MAPK1 | MAPK14 | MAVS | MDM2 | MYO1C | NPEPPS | NUCB1 | PARK7 | PCM1 | PDCD10 | PIK3R1 | PKIG | PPM1A | PRKAA1 | PRKACA | PRKCD | PSEN1 | PTPN1 | PTPN14 | RAB29 | RAN | RANGAP1 | RHOU | RNF31 | SAE1 | SEC16A | SFN | SMAD3 | SMO | SP100 | STOM | TARDBP | TGFB1 | TM9SF4 | TMEM30A | TP53 | TPR | TRIM28 | TXN | UBE2D3 | UBE2J1 | UBE2L3 | UBR5 | USP17L2 | USP46 | XPO1 | XPO4 | XPO5 | YWHAE | ZIC1 | GO:0071363 | 0.0213514497149096 | 176/8243 | 87/3250 | 0.0041053902393532 | 1 | T | T | F | F | cellular response to growth factor stimulus | AKT1 | ANXA1 | APP | BAG4 | BCAR1 | BMPR1A | BRAF | CAT | CAV1 | CBL | CDC5L | COL2A1 | COL4A2 | CPNE3 | CREB1 | CRK | CRKL | CTNNB1 | CXCL8 | DUSP22 | E2F1 | EEF1A1 | EEF2 | EGFR | EHD4 | EIF4A3 | ERBB2 | ERRFI1 | FYN | GSTP1 | HDAC2 | HSPA5 | HSPB1 | HTR3A | HTRA2 | INSR | IQGAP1 | IREB2 | ITGB1BP1 | KDM5B | KDR | LGMN | LIMS1 | MAP2K3 | MAPK14 | MAPK7 | MAPT | MCM7 | MDM2 | MED1 | NR3C1 | NR4A1 | NTRK1 | PAX9 | PLCG1 | PPARGC1A | PPM1A | PRKD1 | PRKD2 | PTEN | PTN | RAB35 | RAP1A | RDX | RELA | RIPK1 | RUNX2 | SH3GL2 | SHC1 | SMAD3 | SMAD4 | SMAD9 | SNAI2 | SNCA | SPINT2 | SPRY2 | SRC | TGFB1 | THBS1 | TWF2 | TWIST1 | USP8 | VCAM1 | VSTM2A | WNT2 | WWOX | YES1 | GO:0006810 | 0.278418051680213 | 2295/8243 | 958/3250 | 0.00411287355446587 | 1 | T | T | F | F | transport | A2M | ABCB7 | ABCB8 | ABCD3 | ABCE1 | ABCG1 | ABL1 | ACAA1 | ACACA | ACD | ACE2 | ACKR2 | ACLY | ACO1 | ACOX1 | ACSL1 | ACSL4 | ACTB | ACTG1 | ACTN1 | ACTN2 | ACTN4 | ACTR1A | ACTR2 | ADA | ADCYAP1 | ADD3 | ADORA2A | ADPRH | ADRB2 | AFG3L2 | AFM | AGAP1 | AGAP2 | AGER | AGK | AGL | AGR2 | AHCYL1 | AHI1 | AHSG | AKAP8 | AKT1 | AKT2 | AKTIP | ALB | ALDOA | ALDOC | ALOX5 | ANK1 | ANK3 | ANP32A | ANXA1 | ANXA11 | ANXA2 | ANXA5 | ANXA6 | AP1B1 | AP1G1 | AP1M1 | AP3D1 | AP3M1 | AP4M1 | AP4S1 | APEH | APLP1 | APOA1 | APOD | APOE | APOL2 | APP | APPBP2 | APRT | ARF1 | ARF4 | ARF5 | ARFGAP1 | ARFIP1 | ARHGAP1 | ARL4D | ARL5B | ARMC8 | ARPC5 | ARR3 | ARRB1 | ARRB2 | ASGR1 | ASGR2 | ASPSCR1 | ATG4C | ATL3 | ATOX1 | ATP12A | ATP13A2 | ATP1A1 | ATP1B3 | ATP2A2 | ATP2B2 | ATP4A | ATP6V1A | ATP6V1B1 | ATP6V1C1 | ATP6V1D | ATP6V1E1 | ATP9A | ATXN1 | ATXN2 | AUP1 | AZU1 | BACH2 | BAG3 | BANF1 | BAX | BCL2 | BCL2L1 | BDKRB2 | BIN1 | BLZF1 | BSPRY | BTK | C16orf70 | CA14 | CAB39 | CACNA1A | CACNG2 | CACNG4 | CACNG5 | CALM1 | CALR | CAMK2A | CAND1 | CANX | CAPN1 | CAPZA1 | CAPZA2 | CAPZB | CAT | CAV1 | CBL | CBLB | CCDC22 | CCDC88A | CCHCR1 | CCL21 | CCR1 | CCR5 | CCS | CCT2 | CCT3 | CCT4 | CCT5 | CCT6B | CCT7 | CCT8 | CD109 | CD209 | CD36 | CD40LG | CD6 | CD74 | CD81 | CDA | CDC37 | CDC42 | CDC42SE2 | CDK16 | CDK2 | CDK5 | CEBPE | CEL | CENPF | CEP290 | CFTR | CHMP2A | CHP1 | CHRM3 | CHRNA9 | CHRND | CHRNE | CHRNG | CKAP4 | CKAP5 | CLDN17 | CLIC1 | CLIC3 | CLIC4 | CLNS1A | CLSTN1 | CLTC | CLU | CNNM1 | COG3 | COL4A3BP | COMMD1 | COPA | COPB1 | COPE | COPG1 | COPS5 | COTL1 | COX11 | COX15 | COX17 | COX5A | COX6A1 | COX6C | COX7A2 | CPLX1 | CPLX3 | CPNE3 | CPT1A | CPT2 | CREB1 | CROT | CSE1L | CSN1S1 | CSNK1E | CSNK2B | CTNNB1 | CTSA | CTSC | CTSD | CTTN | CUL3 | CUL5 | CUTC | CXCL8 | CYB5R1 | CYB5R3 | CYC1 | DAB2 | DBNL | DCLK1 | DCTN1 | DDOST | DDX19A | DDX19B | DDX39A | DDX39B | DDX3X | DEGS1 | DENND1A | DENND4C | DERA | DERL1 | DHRS4 | DHX9 | DLG2 | DNAJA1 | DNAJC19 | DNM1L | DOCK7 | DUSP16 | DVL1 | DYNC1H1 | DYNC1I1 | DYNC1I2 | DYNC1LI1 | DYNLL1 | DYNLT1 | ECH1 | ECI2 | EEF1A1 | EEF2 | EHD4 | EHHADH | EIF2S1 | EIF2S2 | EIF4A3 | EIF4E | EIF4ENIF1 | EIF5A | EIF6 | EPRS | ERLIN2 | EXOC6 | EZR | F5 | F9 | FABP1 | FABP3 | FABP5 | FAF2 | FAM129B | FAM3C | FAM49B | FASLG | FBXO7 | FCGR1A | FCN1 | FGB | FGF2 | FGL2 | FIS1 | FKBP1A | FKBP1B | FLOT1 | FN1 | FOLR1 | FRAS1 | FTH1 | FTL | FYN | FYTTD1 | G3BP2 | GAA | GABRA6 | GABRR1 | GAD2 | GAK | GALNS | GAPVD1 | GBP5 | GDI1 | GDI2 | GEMIN4 | GEMIN5 | GEMIN7 | GGA1 | GHDC | GJA1 | GLA | GLRB | GLS | GLS2 | GLUL | GNS | GOLGA2 | GOLGA4 | GOLPH3 | GOLT1B | GOPC | GOT2 | GPI | GPR107 | GRAMD1A | GRB2 | GRIA1 | GRID2 | GRIK2 | GRIP1 | GRPEL1 | GSTK1 | GSTP1 | GUSB | GYG1 | HAP1 | HBZ | HCN1 | HCN2 | HCN3 | HDAC6 | HEATR3 | HERC2 | HEXB | HGS | HID1 | HIF1A | HK2 | HMGB1 | HMGCL | HNF1A | HNRNPA1 | HNRNPA1L2 | HNRNPA2B1 | HNRNPU | HPX | HRAS | HRNR | HSD17B4 | HSF1 | HSP90AA1 | HSP90AB1 | HSP90B1 | HSPA1B | HSPA2 | HSPA4 | HSPA5 | HSPA8 | HSPB1 | HSPD1 | HSPG2 | HSPH1 | HTATIP2 | HTR1B | HTR3A | HTR3E | HTT | HUWE1 | IBTK | ICK | IDE | IDH1 | IFIT1 | IFT122 | IGF2BP1 | IGHV3-13 | IGHV3-23 | IGHV3-30 | IGKV3-20 | IGLC7 | ILF2 | IMPDH2 | INSR | IPO11 | IPO4 | IPO5 | IPO7 | IPO8 | IPO9 | IQGAP1 | IREB2 | ISLR | ITGA4 | ITGAV | ITGB1BP1 | ITGB3 | ITPR1 | ITPR2 | ITPR3 | JUP | KCMF1 | KCNA4 | KCNAB1 | KCNAB2 | KCNIP2 | KCNK1 | KCNK16 | KCNS3 | KIF11 | KIF13B | KIF2C | KIF5B | KIF5C | KLC1 | KLC2 | KLHL20 | KPNA1 | KPNA2 | KPNB1 | LARS | LASP1 | LAT | LCP2 | LETM1 | LGALS3 | LGALS3BP | LIMA1 | LMAN2L | LMNA | LOXL2 | LRP6 | LRPPRC | LRRC7 | LRRK2 | LRSAM1 | MAL2 | MAN2B1 | MAP1B | MAPK1 | MAPK14 | MAPK15 | MAPK3 | MAPK8IP3 | MAPT | MASP1 | MCFD2 | MCM3AP | MCOLN3 | MECP2 | MED1 | MGST1 | MIB1 | MICU1 | MICU2 | MIF | MME | MMGT1 | MMRN1 | MPC2 | MPPE1 | MTX1 | MYC | MYO1B | MYO1C | MYO1D | MYO1E | MYO5A | MYO6 | NAPRT | NBAS | NCBP1 | NCOA1 | NCOA2 | NCOA4 | NDUFA13 | NDUFA9 | NDUFS7 | NEDD4 | NEUROD1 | NGB | NHLRC3 | NIT2 | NMB | NMD3 | NME1 | NME2 | NME4 | NNT | NOD2 | NOS2 | NOSTRIN | NPLOC4 | NPM1 | NPR1 | NR1H3 | NR1I2 | NRXN1 | NUDT19 | NUP107 | NUP153 | NUP160 | NUP205 | NUP214 | NUP85 | NUP88 | NUP93 | NUP98 | NUTF2 | NXF1 | NXF2 | NXT2 | OAZ3 | OLA1 | OPA1 | OPTN | ORM1 | OSBPL1A | OXA1L | P2RX1 | P2RX2 | P2RX4 | PA2G4 | PACSIN3 | PAFAH1B1 | PAM16 | PANX3 | PARD3 | PARK7 | PCM1 | PCSK9 | PCTP | PDCD6 | PDCD6IP | PDIA4 | PDXK | PEF1 | PEX19 | PEX5 | PFKL | PGAM1 | PGM1 | PGM2 | PGRMC1 | PHB | PHB2 | PI4K2A | PICK1 | PIK3C2A | PIK3CG | PIK3R1 | PIKFYVE | PITPNA | PITPNB | PKM | PLA2G10 | PLA2G12A | PLA2G1B | PLA2G2A | PLAUR | PLCG1 | PLEKHF2 | PLIN3 | PLSCR1 | PML | PMPCA | PMPCB | PNP | POLDIP3 | POM121 | PPARD | PPARG | PPAT | PPIA | PPID | PPIE | PPT1 | PQLC1 | PRCP | PRDX4 | PRDX6 | PRELID1 | PRG2 | PRKAA2 | PRKAB2 | PRKAG1 | PRKCD | PRKCE | PRKCZ | PRKD1 | PROZ | PSAP | PSEN1 | PSEN2 | PSMA1 | PSMA2 | PSMA3 | PSMA5 | PSMA6 | PSMB5 | PSMB6 | PSMB7 | PSMB8 | PSMC1 | PSMC2 | PSMC3 | PSMC4 | PSMC5 | PSMC6 | PSMD1 | PSMD10 | PSMD11 | PSMD12 | PSMD14 | PSMD2 | PSMD4 | PSMD9 | PSME2 | PSME3 | PSTPIP1 | PTGES2 | PTK2 | PTPN23 | PTPN6 | PYGB | PYGL | QSOX1 | RAB10 | RAB11A | RAB11B | RAB11FIP2 | RAB18 | RAB1A | RAB1B | RAB21 | RAB24 | RAB29 | RAB2A | RAB31 | RAB32 | RAB35 | RAB3IP | RAB5A | RAB5B | RAB7A | RAB9A | RABGEF1 | RABL2A | RABL3 | RAC1 | RAE1 | RAF1 | RAN | RANBP3 | RAP1A | RAP1B | RAPGEF4 | RARA | RASSF9 | RBM8A | RBPJ | RHOA | RHOJ | RHOT1 | RHOT2 | RHOU | RNASE3 | RNF126 | RNF185 | ROMO1 | RP2 | RPAIN | RPL10 | RPL10A | RPL12 | RPL18A | RPL22 | RPL23 | RPL23A | RPL24 | RPL30 | RPL37A | RPL38 | RPL6 | RPL7 | RPL7A | RPL9 | RPLP0 | RPLP1 | RPLP2 | RPS12 | RPS13 | RPS14 | RPS15A | RPS19 | RPS2 | RPS20 | RPS24 | RPS29 | RPS3 | RPS4X | RPS8 | RRS1 | RXRA | S100A11 | S100A12 | S100A13 | S100A6 | S100A7 | S100A9 | S100P | SARAF | SCARA3 | SCCPDH | SCFD1 | SCG2 | SCG3 | SCO2 | SCYL2 | SDCBP | SDCBP2 | SEC13 | SEC16A | SEC22C | SEC24B | SEC24C | SEC31A | SEC62 | SEC63 | SEL1L | SELENBP1 | SELL | SERPINB3 | SERPINB6 | SERPINE2 | SFPQ | SFXN1 | SFXN4 | SGTA | SGTB | SH3GL3 | SH3KBP1 | SLAMF1 | SLC12A2 | SLC15A1 | SLC16A1 | SLC25A1 | SLC25A10 | SLC25A11 | SLC25A13 | SLC25A22 | SLC25A24 | SLC25A3 | SLC25A32 | SLC25A4 | SLC25A41 | SLC25A5 | SLC25A6 | SLC2A5 | SLC2A9 | SLC35F6 | SLC37A3 | SLC39A4 | SLC39A9 | SLC3A2 | SLC7A1 | SLC9A1 | SLC9A3R1 | SMAD2 | SMG5 | SMG6 | SMN1 | SMURF1 | SNAP29 | SNCA | SNF8 | SNRPB | SNRPD1 | SNRPD3 | SNRPE | SNX12 | SNX17 | SNX2 | SNX21 | SNX24 | SNX3 | SNX6 | SOAT1 | SOCS2 | SOD1 | SORT1 | SPTAN1 | SPTBN1 | SPTBN2 | SQSTM1 | SRC | SRI | SRP14 | SRP19 | SRSF11 | SRSF5 | SSB | SSX2IP | STARD7 | STAT3 | STAT5A | STBD1 | STIM1 | STOM | STOML2 | STX5 | STXBP1 | STXBP2 | STYX | SUN2 | SYN1 | SYNE2 | SYNJ2BP | SYP | SYVN1 | TAGLN2 | TANGO2 | TBC1D15 | TBC1D22B | TBC1D23 | TBC1D4 | TCIRG1 | TCP1 | TERF2 | TEX264 | TFG | TGFB1 | TGM2 | THBS1 | THOC2 | THOC6 | THOC7 | TIMM13 | TIMM21 | TIMM23 | TIMM44 | TIMM50 | TIMP1 | TIMP2 | TM9SF4 | TMED9 | TMEM173 | TMEM30A | TNK2 | TNKS | TNPO1 | TOLLIP | TOMM20 | TOMM22 | TOMM40 | TOR1A | TPCN2 | TPD52 | TPR | TRAF3IP1 | TRAPPC8 | TREX1 | TRIAP1 | TRIM23 | TRIM9 | TRIP4 | TSC22D3 | TSG101 | TTYH3 | TUBA1A | TUBA1C | TUBA4A | TUBB | TUBB4B | TXLNA | TXNDC5 | TXNIP | U2AF2 | UBB | UBC | UBE2D1 | UBE2D2 | UBE2D3 | UBE2O | UBR4 | UCHL1 | UMPS | UNC119 | UPF2 | UPF3B | USP20 | USP9X | VAPA | VAPB | VAT1 | VCL | VCP | VDAC1 | VDAC2 | VDAC3 | VDR | VGF | VPS13B | VPS18 | VPS26A | VPS29 | VPS37D | VPS4B | VPS8 | VTA1 | VTN | WAS | WDR1 | WDR19 | WDR35 | WNK1 | WNK4 | WWP2 | XBP1 | XPO1 | XPO4 | XPO5 | XPO7 | XPOT | XRCC5 | XRCC6 | YKT6 | YME1L1 | YWHAB | YWHAE | YWHAG | YWHAH | ZBTB7B | ZDHHC17 | ZDHHC23 | ZFAND6 | ZMPSTE24 | ZW10 | GO:0060255 | 0.424481378139027 | 3499/8243 | 1438/3250 | 0.00413806940054753 | 1 | T | T | F | F | regulation of macromolecule metabolic process | A2M | AARS | ABCE1 | ABCG1 | ABHD14B | ABL1 | ACD | ACO1 | ACSL1 | ACTA1 | ACTA2 | ACTB | ACTG2 | ACTL6A | ACTN1 | ACTN2 | ACTN4 | ACTR2 | ACTR3 | ADCY6 | ADCYAP1 | ADORA2A | ADRB2 | ADRM1 | ADTRP | AEBP2 | AES | AFF1 | AFF4 | AGER | AGO1 | AGO2 | AGR2 | AHCYL1 | AHI1 | AHNAK | AHSG | AICDA | AIFM1 | AIMP2 | AIRE | AKAP8 | AKAP9 | AKT1 | AKT2 | AKTIP | ALOX12 | ANK3 | ANKRD49 | ANP32A | ANXA2 | ANXA7 | APBB1 | APBB2 | APBB3 | APC | APCS | APEX1 | APOA1 | APOD | APOE | APP | AR | ARAF | ARF1 | ARF4 | ARHGAP35 | ARID3B | ARID4B | ARNT | ARNT2 | ARR3 | ARRB1 | ARRB2 | ASCC1 | ASCC2 | ASCL1 | ASPSCR1 | ATAD1 | ATAD5 | ATF2 | ATF6 | ATF6B | ATF7 | ATG5 | ATM | ATOH1 | ATP13A2 | ATP6V1B1 | ATRX | ATXN1 | ATXN2 | AURKB | AXIN1 | AZU1 | B3GAT3 | BABAM1 | BACH2 | BAG2 | BAG3 | BAG4 | BAG5 | BAG6 | BANP | BASP1 | BAX | BBC3 | BCAR3 | BCL10 | BCL11A | BCL2 | BCL2L13 | BCL6 | BCL6B | BCOR | BDKRB2 | BIN1 | BIRC2 | BIRC3 | BIRC5 | BIRC7 | BIRC8 | BLZF1 | BMI1 | BMPR1A | BOLA1 | BOLA3 | BRAF | BRCA1 | BRD2 | BRD3 | BRD4 | BTK | BTRC | BUD31 | C1QBP | C1QL4 | C1QTNF1 | C4A | CAB39 | CALM1 | CALM2 | CALM3 | CALR | CAMK1D | CAMK2A | CAND1 | CARD9 | CARM1 | CASP10 | CASP3 | CASP8 | CASP9 | CAT | CAV1 | CBFA2T3 | CBFB | CBL | CBLB | CBLC | CBX4 | CBX7 | CBX8 | CBY1 | CCAR2 | CCDC22 | CCDC88A | CCL21 | CCL22 | CCNB1 | CCNT1 | CCNT2 | CCNY | CCNYL1 | CCR1 | CCT2 | CCT3 | CCT4 | CCT5 | CCT6A | CCT7 | CCT8 | CD109 | CD27 | CD36 | CD40 | CD40LG | CD74 | CD81 | CDC14B | CDC20 | CDC25A | CDC25B | CDC25C | CDC37 | CDC5L | CDC73 | CDH1 | CDK2 | CDK4 | CDK5 | CDK5R1 | CDK8 | CDK9 | CDKN1A | CDKN2C | CEBPA | CEBPE | CENPF | CENPJ | CEP290 | CHCHD10 | CHCHD2 | CHCHD3 | CHD3 | CHD4 | CHD8 | CHEK2 | CHORDC1 | CHP1 | CHRD | CHRNA9 | CIRBP | CKS1B | CKS2 | CLK1 | CLPX | CLTC | CLU | CNDP1 | COA3 | COL2A1 | COMMD1 | COPS2 | COPS5 | COQ7 | CPSF7 | CREB1 | CREB3 | CREBBP | CRIP1 | CRK | CRKL | CRP | CRY1 | CRY2 | CRYAB | CSNK1A1 | CSNK1E | CSNK2A2 | CSNK2B | CTBP1 | CTCF | CTDP1 | CTH | CTNNB1 | CTNNBIP1 | CTSC | CUL2 | CUL3 | CYCS | CYLD | CYP1A2 | DAB1 | DAB2 | DAPK1 | DAXX | DAZAP1 | DBF4B | DBNDD2 | DBNL | DCN | DCP2 | DCUN1D1 | DDA1 | DDIT4 | DDRGK1 | DDX1 | DDX39B | DDX3X | DDX6 | DERL1 | DHCR24 | DHX36 | DHX9 | DIABLO | DIS3 | DLG3 | DLG4 | DMAP1 | DMD | DMRT3 | DNA2 | DNAJA1 | DNAJA3 | DNAJB1 | DNAJB2 | DNAJC10 | DOCK7 | DPEP1 | DPF2 | DROSHA | DTX1 | DUSP1 | DUSP14 | DUSP16 | DUSP19 | DUSP22 | DUSP7 | DVL1 | DVL2 | DVL3 | DYRK2 | E2F1 | E2F2 | E2F3 | E2F4 | E2F6 | EAF2 | EBF1 | ECD | EDA | EDC4 | EDEM1 | EED | EEF2 | EFEMP1 | EGFR | EGLN1 | EGLN3 | EHD4 | EHMT1 | EHMT2 | EID1 | EIF2AK3 | EIF2AK4 | EIF2B5 | EIF2S1 | EIF3A | EIF3D | EIF3E | EIF3H | EIF4A3 | EIF4E | EIF4E2 | EIF4EBP1 | EIF4ENIF1 | EIF4G3 | EIF4H | EIF5 | EIF5A | EIF5B | EIF6 | ELAVL1 | ELF3 | ELF4 | ENDOG | ENO1 | EP300 | EPHA4 | EPPIN | EPRS | ERBB2 | ERBB3 | ERF | ERG | ERLIN2 | ERRFI1 | ESR1 | ESR2 | ESRRA | ESRRB | ESRRG | ETS1 | EWSR1 | EXOSC1 | EXOSC5 | EXOSC6 | EXOSC9 | EYA2 | EYA4 | EZH2 | EZR | FABP1 | FADD | FAF1 | FAM129B | FAM192A | FAM83D | FAM98A | FANCA | FANCI | FANCM | FASLG | FBP1 | FBXO7 | FBXW11 | FGB | FGF2 | FGFR1 | FGFR1OP | FGFR3 | FHL2 | FIGNL1 | FIS1 | FKBP1A | FKBP1B | FKBP8 | FLOT1 | FN1 | FOS | FOXA1 | FOXD4 | FOXD4L6 | FOXI2 | FOXJ1 | FOXK1 | FOXK2 | FOXM1 | FOXO1 | FTO | FUBP1 | FUBP3 | FUS | FXR2 | FYN | FZR1 | G3BP2 | G6PD | GABPB1 | GADD45G | GAPDH | GATA1 | GDF9 | GEMIN5 | GFPT1 | GLMN | GLO1 | GNA12 | GNAI2 | GNAQ | GNAS | GOLGA2 | GPD1L | GPI | GPS2 | GPX1 | GRB2 | GRB7 | GRIK2 | GRIP1 | GSK3A | GSK3B | GSPT1 | GSPT2 | GSTP1 | GTF2A1 | GTF2F1 | GTF2F2 | GTF2I | GZMA | H1F0 | H1FX | H2AFX | H2AFZ | H3F3A | HAP1 | HAVCR2 | HAX1 | HBEGF | HBZ | HDAC1 | HDAC2 | HDAC3 | HDAC4 | HDAC5 | HDAC6 | HDAC7 | HDAC9 | HDGF | HELZ | HELZ2 | HEXB | HEXIM1 | HGS | HIF1A | HIF1AN | HINT1 | HIST1H1A | HIST1H3A | HIST1H4A | HIST2H3A | HIVEP1 | HLX | HMGB1 | HMGB2 | HMGB3 | HMOX1 | HNF1A | HNF4A | HNF4G | HNRNPA1 | HNRNPA2B1 | HNRNPAB | HNRNPD | HNRNPF | HNRNPH1 | HNRNPK | HNRNPL | HNRNPM | HNRNPU | HOXA5 | HOXB5 | HOXB6 | HOXB9 | HOXD10 | HPX | HRAS | HSF1 | HSF4 | HSP90AA1 | HSP90AB1 | HSP90AB3P | HSP90AB4P | HSP90B1 | HSPA1B | HSPA2 | HSPA5 | HSPA8 | HSPB1 | HSPBP1 | HSPD1 | HSPE1 | HSPH1 | HTATIP2 | HTRA1 | HTRA2 | HTT | IBTK | ICAM1 | ICK | ID2 | IDE | IFI16 | IFNA2 | IGBP1 | IGF1R | IGF2BP1 | IKBKB | IKBKG | IKZF5 | ILF2 | ILF3 | ILK | ING1 | ING5 | INSIG1 | INSIG2 | INSR | IPO5 | IPO7 | IPO8 | IQGAP1 | IREB2 | IRF1 | IRF4 | IRF7 | ISG15 | ITCH | ITGAV | ITGB1BP1 | ITGB3 | ITGB3BP | JAK3 | JMJD6 | JUN | JUNB | JUND | JUP | KANK2 | KAT5 | KAT7 | KAT8 | KDM1A | KDM2A | KDM4D | KDM5B | KDM5C | KDM8 | KDR | KEAP1 | KHDRBS1 | KLF5 | KLF6 | KLHDC10 | KMT2A | KMT2B | KMT2C | KPNA1 | KPNA2 | KRAS | KSR1 | L3MBTL1 | L3MBTL2 | L3MBTL3 | L3MBTL4 | LAMP3 | LARP4 | LAT | LATS1 | LCP2 | LDLRAD4 | LEF1 | LEMD2 | LEMD3 | LGALS3 | LGMN | LGR4 | LIMS1 | LMNA | LMO2 | LMO7 | LOXL2 | LPAR1 | LPIN3 | LRIF1 | LRP6 | LRPPRC | LRRK1 | LRRK2 | LSM1 | LSM3 | LSM4 | LSM5 | LTBR | LUM | LZTR1 | MAD2L1 | MAF1 | MAP2K1 | MAP2K3 | MAP2K4 | MAP2K6 | MAP2K7 | MAP3K1 | MAP3K13 | MAP3K14 | MAP3K2 | MAP3K3 | MAP3K4 | MAP4K1 | MAPK1 | MAPK14 | MAPK15 | MAPK3 | MAPK4 | MAPK6 | MAPK7 | MAPK8 | MAPK8IP3 | MAPK9 | MAPKAP1 | MAPT | MAS1 | MATR3 | MAVS | MBD3 | MBP | MC1R | MDFI | MDM2 | MDM4 | MECP2 | MED1 | MED12 | MED14 | MED17 | MED20 | MED21 | MED23 | MED24 | MED31 | MED4 | MED6 | MEOX2 | MEPCE | METTL23 | MGA | MGMT | MIF | MITF | MKNK2 | MLH1 | MLLT1 | MLLT11 | MLLT3 | MLST8 | MOB1B | MORF4L1 | MORF4L2 | MOV10 | MRGBP | MRPL12 | MSH6 | MSN | MTA1 | MTA3 | MTERF1 | MTPN | MXD1 | MYBBP1A | MYC | MYD88 | MYH6 | MYO1C | MYO6 | MYOC | NACC1 | NAMPT | NANOG | NBAS | NCBP1 | NCK1 | NCOA1 | NCOA2 | NCOA3 | NCOA4 | NCOR1 | NCOR2 | NDUFA13 | NEDD4 | NEDD8 | NEK1 | NEK7 | NELFB | NELFCD | NELFE | NELL1 | NEUROD1 | NF2 | NFATC1 | NFATC2 | NFE2L2 | NFKB2 | NFKBIA | NIF3L1 | NIPBL | NKRF | NKX2-1 | NLK | NMD3 | NME1 | NME2 | NMNAT1 | NNT | NOD2 | NOS1AP | NOS2 | NOS3 | NOSTRIN | NPM1 | NQO2 | NR0B2 | NR1H2 | NR1H3 | NR1I2 | NR3C1 | NR4A1 | NR5A2 | NTF3 | NTRK1 | NUFIP1 | NUMA1 | NUP107 | NUP153 | NUP160 | NUP205 | NUP214 | NUP85 | NUP88 | NUP93 | NUP98 | NUPR1 | OAS2 | OGFOD1 | OGT | ONECUT1 | OPTN | OTUB1 | OTUD4 | OXSR1 | P2RX1 | PA2G4 | PABPC1 | PABPC4 | PACSIN3 | PAFAH1B1 | PAIP1 | PAK2 | PAK6 | PAM16 | PAN2 | PAPLN | PARD3 | PARD6A | PARK7 | PARP1 | PARP14 | PATZ1 | PAWR | PAX8 | PAX9 | PAXIP1 | PC | PCBD1 | PCBP1 | PCGF1 | PCGF5 | PCGF6 | PCNA | PCSK9 | PDCD10 | PDCD6 | PDE12 | PDE4D | PDE6G | PEBP1 | PEF1 | PELI1 | PER1 | PER2 | PFKM | PFN1 | PGAM1 | PHB | PHB2 | PHF10 | PHF20L1 | PHF21A | PHGDH | PIAS1 | PIAS3 | PICK1 | PIH1D1 | PIK3CA | PIK3CG | PIK3R1 | PIK3R3 | PIM1 | PIN1 | PINX1 | PIR | PKIG | PLA2G10 | PLA2G1B | PLA2G2A | PLA2G6 | PLAUR | PLGRKT | PLK1 | PLSCR1 | PLXNB2 | PML | PNKP | POLDIP3 | POLR2A | POLR2E | POLR2L | POM121 | POT1 | POU2F3 | POU5F1 | PPARD | PPARG | PPARGC1A | PPIB | PPID | PPIE | PPM1A | PPM1D | PPME1 | PPP1CA | PPP1CB | PPP1CC | PPP1R11 | PPP1R12A | PPP1R14A | PPP1R16A | PPP1R2 | PPP1R3B | PPP1R3C | PPP1R8 | PPP2CA | PPP2CB | PPP2R1A | PPP2R1B | PPP2R2A | PPP2R2D | PPP2R5D | PPP4R2 | PPP6R1 | PRDX1 | PRDX2 | PRDX3 | PRDX5 | PRDX6 | PRELID1 | PRKAA1 | PRKAA2 | PRKACA | PRKAG1 | PRKAR1A | PRKAR2B | PRKCD | PRKCE | PRKCZ | PRKD1 | PRKD2 | PRKDC | PRMT2 | PRMT3 | PRMT6 | PROX1 | PRPF19 | PRRC1 | PSAP | PSEN1 | PSMA1 | PSMA2 | PSMA3 | PSMA5 | PSMA6 | PSMB5 | PSMB6 | PSMB7 | PSMB8 | PSMC1 | PSMC2 | PSMC3 | PSMC4 | PSMC5 | PSMC6 | PSMD1 | PSMD10 | PSMD11 | PSMD12 | PSMD14 | PSMD2 | PSMD4 | PSMD9 | PSME2 | PSME3 | PTEN | PTGES2 | PTGES3 | PTK2 | PTN | PTPN1 | PTPN14 | PTPN22 | PTPN6 | PTPRK | PTPRR | PTRH2 | PTTG1 | PUM1 | PUS7 | QARS | QRICH1 | RAB1A | RAB1B | RAB29 | RAB3GAP1 | RAC1 | RAD21 | RAD23A | RAD23B | RAD51 | RAD51AP1 | RAE1 | RAF1 | RAN | RANBP9 | RAP1A | RAP1B | RARA | RARG | RASD1 | RB1 | RB1CC1 | RBBP4 | RBBP6 | RBM17 | RBM25 | RBM3 | RBM4 | RBM8A | RBMX | RBPJ | RBX1 | RC3H1 | RDX | RECK | REL | RELA | REST | RFC1 | RFC2 | RFC3 | RFC4 | RFC5 | RFXANK | RGN | RGS2 | RHBDD1 | RHOA | RIF1 | RING1 | RIOK1 | RIPK1 | RIPK2 | RIPK3 | RIPK4 | RMND1 | RNASEH2B | RNF139 | RNF185 | RNF19A | RNF2 | RNF25 | RNF31 | RNF4 | RNF8 | RPA2 | RPL10 | RPL10A | RPL12 | RPL18A | RPL22 | RPL23 | RPL23A | RPL24 | RPL30 | RPL37A | RPL38 | RPL6 | RPL7 | RPL7A | RPL9 | RPLP0 | RPLP1 | RPLP2 | RPS12 | RPS13 | RPS14 | RPS15A | RPS19 | RPS2 | RPS20 | RPS24 | RPS29 | RPS3 | RPS4X | RPS6KA1 | RPS6KB2 | RPS8 | RPTOR | RRM2 | RTF1 | RUNX1 | RUNX1T1 | RUNX2 | RUVBL1 | RUVBL2 | RXRA | RXRB | RXRG | S100A1 | S100A11 | S100A12 | S100A7 | S100A9 | SAG | SAMD7 | SAP18 | SARS | SART3 | SCML2 | SCYL2 | SDC4 | SDCBP | SEC13 | SEMA7A | SEPT4 | SERPINB2 | SERPINB3 | SERPINB5 | SERPINB6 | SERPINB8 | SERPINE2 | SERPINH1 | SET | SETBP1 | SETDB1 | SF1 | SFMBT1 | SFN | SFPQ | SGTA | SH2B1 | SH3GL2 | SHC1 | SHMT1 | SHMT2 | SIN3A | SIRT1 | SIRT3 | SIRT5 | SIRT6 | SIRT7 | SKI | SKIV2L | SLAMF1 | SLC9A1 | SLC9A3R1 | SLTM | SMAD2 | SMAD3 | SMAD4 | SMAD5 | SMAD9 | SMARCA2 | SMARCA4 | SMARCB1 | SMARCC1 | SMARCC2 | SMARCE1 | SMC1A | SMC3 | SMG5 | SMG6 | SMG8 | SMO | SMURF1 | SMURF2 | SNAI2 | SNCA | SND1 | SNF8 | SNRPA | SNTA1 | SNW1 | SNX12 | SNX6 | SOAT1 | SOCS1 | SOD1 | SOD2 | SORT1 | SOX2 | SP1 | SP100 | SP2 | SPEN | SPI1 | SPIN1 | SPINK7 | SPINT2 | SPOP | SPRTN | SPRY2 | SQSTM1 | SRC | SRI | SRPK1 | SRPK2 | SRSF12 | SRSF5 | SSB | STAG2 | STAT1 | STAT3 | STAT4 | STAT5A | STAT6 | STK11 | STK24 | STK25 | STK26 | STK3 | STK38 | STK40 | STOML2 | STRAP | STUB1 | STYX | SUB1 | SUMO1 | SUMO2 | SUPT4H1 | SUPT5H | SUZ12 | SWAP70 | SYNJ2BP | TAB1 | TAB2 | TACO1 | TADA2B | TAF12 | TAF15 | TAF1A | TAGLN3 | TANK | TAOK3 | TARDBP | TAZ | TBL1X | TBRG4 | TCEA1 | TCEA2 | TCERG1 | TCF12 | TCF21 | TCF25 | TCF4 | TCOF1 | TCP1 | TCP10L | TDRD3 | TERF1 | TERF2 | TERF2IP | TFCP2 | TFDP1 | TFIP11 | TGFB1 | TGFBR3 | TGIF2LX | THAP11 | THAP7 | THBS1 | THRA | TIA1 | TIAL1 | TIMP1 | TIMP2 | TINF2 | TLE1 | TLE3 | TLR3 | TMBIM6 | TMEM173 | TMPO | TMTC3 | TNF | TNFAIP3 | TNFRSF1A | TNFRSF8 | TNFSF14 | TNIP2 | TNK2 | TNKS | TNKS2 | TNP1 | TNPO1 | TOE1 | TOLLIP | TOP1 | TOP2A | TP53 | TP63 | TP73 | TPR | TRA2A | TRADD | TRAF1 | TRAF2 | TRAF3 | TRAF3IP1 | TRAF6 | TRAP1 | TREX1 | TRIAP1 | TRIB2 | TRIB3 | TRIM14 | TRIM21 | TRIM25 | TRIM28 | TRIM29 | TRIM32 | TRIM33 | TRIM34 | TRIP12 | TRIP13 | TRIP4 | TRUB2 | TSC22D1 | TSC22D3 | TSC22D4 | TSFM | TSG101 | TSKU | TSPYL2 | TTC5 | TULP3 | TWF1 | TWIST1 | TXN | TXNIP | TYMS | U2AF2 | UBA3 | UBB | UBC | UBE2B | UBE2C | UBE2D1 | UBE2D3 | UBE2I | UBE2J1 | UBE2K | UBE2L3 | UBE2N | UBE2O | UBE2S | UBE2V1 | UBE3A | UBQLN1 | UBQLN2 | UBQLN4 | UBR5 | UBXN1 | UCHL1 | UCHL5 | UNC119 | UNK | UNKL | UPF2 | UPF3B | UQCC1 | USP14 | USP15 | USP16 | USP17L2 | USP19 | USP2 | USP21 | USP4 | USP46 | USP47 | USP5 | USP50 | USP7 | USP9X | USP9Y | VCP | VDR | VGLL4 | VHL | VIM | VPS72 | VSTM2A | VTN | WARS | WAS | WBP2 | WDR35 | WDR48 | WDR77 | WDTC1 | WNK1 | WNT16 | WNT2 | WT1 | WTAP | WWOX | WWP2 | XBP1 | XIAP | XPO1 | XPO5 | XRCC1 | XRCC5 | XRCC6 | YAF2 | YAP1 | YEATS2 | YES1 | YTHDF1 | YWHAB | YWHAE | YWHAG | YWHAH | YWHAQ | YWHAZ | YY1 | ZBTB16 | ZBTB17 | ZBTB21 | ZBTB25 | ZBTB33 | ZBTB42 | ZBTB7A | ZBTB7B | ZC3H4 | ZFP41 | ZHX1 | ZIC1 | ZKSCAN1 | ZMPSTE24 | ZMYM5 | ZMYND8 | ZNF207 | ZNF217 | ZNF227 | ZNF24 | ZNF277 | ZNF354C | ZNF408 | ZNF420 | ZNF443 | ZNF517 | ZNF518A | ZNF592 | ZNF598 | ZNF618 | ZNF622 | ZNF646 | ZNF669 | ZNF696 | ZNF746 | ZNF747 | ZRANB2 | ZSCAN2 | ZSCAN29 | ZSCAN32 | ZZZ3 | GO:0051402 | 0.00400339682154555 | 33/8243 | 21/3250 | 0.00416375071233206 | 1 | T | T | F | F | neuron apoptotic process | AIFM1 | AKT2 | APP | BAX | BCL2 | BCL2L1 | CASP3 | CDK5 | DIABLO | ERBB3 | FADD | GAPDH | GRIK2 | HSPA5 | LIG4 | NAE1 | PSEN1 | RB1 | SIAH1 | TP63 | USP53 | GO:0060079 | 0.00400339682154555 | 33/8243 | 21/3250 | 0.00416375071233206 | 1 | T | T | F | F | excitatory postsynaptic potential | ADORA2A | AKT1 | ARRB2 | CDK5 | CHRNA9 | CHRND | CHRNE | CHRNG | GLRB | GRID2 | GRIK2 | GSK3A | GSK3B | LRRK2 | MECP2 | MPP2 | P2RX1 | P2RX2 | P2RX4 | RAB3GAP1 | SNCA | GO:1902099 | 0.00497391726313235 | 41/8243 | 25/3250 | 0.00419791551649218 | 1 | T | T | F | F | regulation of metaphase/anaphase transition of cell cycle | ANAPC4 | APC | ATM | AURKB | CCNB1 | CDC20 | CENPF | CUL3 | DUSP1 | DYNC1LI1 | HECW2 | IK | KLHL22 | MAD1L1 | MAD2L1 | MAPK15 | PLK1 | RAD21 | RB1 | SPDL1 | TPR | TRIP13 | XRCC3 | ZNF207 | ZW10 | GO:0042769 | 0.00424602693194225 | 35/8243 | 22/3250 | 0.00421214617062903 | 1 | T | T | F | F | DNA damage response, detection of DNA damage | CUL4B | DDB1 | DNAJA1 | NEK1 | PARP1 | PCNA | PNKP | POLD1 | RBX1 | RFC1 | RFC2 | RFC3 | RFC4 | RFC5 | RPA2 | RPA3 | RPS3 | TREX1 | UBB | UBC | UBE2B | WDR48 | GO:0009144 | 0.00424602693194225 | 35/8243 | 22/3250 | 0.00421214617062903 | 1 | T | T | F | F | purine nucleoside triphosphate metabolic process | ADCYAP1 | AK1 | AK2 | AK4 | ATP6V1A | ATP6V1B1 | CLPX | DGUOK | FIGNL1 | GNAI3 | HSPA1B | HSPA8 | LRRK2 | MYH6 | NDUFS1 | NMNAT1 | OLA1 | OPA1 | PARP1 | RAN | RHOA | VCP | GO:0009250 | 0.0019410408831736 | 16/8243 | 12/3250 | 0.00422278657501416 | 1 | T | T | F | F | glucan biosynthetic process | AGL | AKT1 | AKT2 | GBE1 | GYG1 | GYG2 | GYS1 | PER2 | PGM1 | PGM2 | PPP1R3C | UGP2 | GO:0090201 | 0.0019410408831736 | 16/8243 | 12/3250 | 0.00422278657501416 | 1 | T | T | F | F | negative regulation of release of cytochrome c from mitochondria | AKT1 | ARRB2 | BCL2L1 | CLU | GPX1 | LMNA | OPA1 | PAM16 | PPIF | PRELID1 | PSMD10 | TRIAP1 | GO:0032892 | 0.0019410408831736 | 16/8243 | 12/3250 | 0.00422278657501416 | 1 | T | T | F | F | positive regulation of organic acid transport | ABAT | ACE2 | ADORA2A | FABP3 | MAP2K6 | MIF | P2RX4 | PLA2G10 | PLA2G6 | PSEN1 | RAB3GAP1 | STXBP1 | GO:0042451 | 0.0019410408831736 | 16/8243 | 12/3250 | 0.00422278657501416 | 1 | T | T | F | F | purine nucleoside biosynthetic process | ADA | ADK | AMD1 | APRT | GMPS | HPRT1 | IMPDH2 | MTAP | NME1 | NME2 | NME4 | PRTFDC1 | GO:0005978 | 0.0019410408831736 | 16/8243 | 12/3250 | 0.00422278657501416 | 1 | T | T | F | F | glycogen biosynthetic process | AGL | AKT1 | AKT2 | GBE1 | GYG1 | GYG2 | GYS1 | PER2 | PGM1 | PGM2 | PPP1R3C | UGP2 | GO:2000641 | 0.0019410408831736 | 16/8243 | 12/3250 | 0.00422278657501416 | 1 | T | T | F | F | regulation of early endosome to late endosome transport | DAB2 | EZR | MAP2K1 | MAPK1 | MAPK3 | MSN | PTPN23 | RAB21 | RDX | SNX12 | SNX3 | SRC | GO:0042455 | 0.0019410408831736 | 16/8243 | 12/3250 | 0.00422278657501416 | 1 | T | T | F | F | ribonucleoside biosynthetic process | ADA | ADK | AMD1 | APRT | GMPS | HPRT1 | IMPDH2 | MTAP | NME1 | NME2 | NME4 | PRTFDC1 | GO:0006541 | 0.0019410408831736 | 16/8243 | 12/3250 | 0.00422278657501416 | 1 | T | T | F | F | glutamine metabolic process | ALDH5A1 | ASL | ASNS | CTPS1 | GFPT1 | GFPT2 | GLUL | GMPS | MECP2 | NIT2 | PFAS | PHGDH | GO:0046129 | 0.0019410408831736 | 16/8243 | 12/3250 | 0.00422278657501416 | 1 | T | T | F | F | purine ribonucleoside biosynthetic process | ADA | ADK | AMD1 | APRT | GMPS | HPRT1 | IMPDH2 | MTAP | NME1 | NME2 | NME4 | PRTFDC1 | GO:0045333 | 0.00473128715273565 | 39/8243 | 24/3250 | 0.00422518028389986 | 1 | T | T | F | F | cellular respiration | ADSL | CAT | COX15 | COX20 | COX5A | COX6A1 | COX6C | CYCS | CYP1A2 | LYRM7 | MDH2 | NDUFAF2 | NDUFS1 | NDUFS7 | NDUFS8 | NDUFV1 | OXA1L | PPARGC1A | SDHB | SIRT3 | SLC25A13 | TBRG4 | UQCRB | UQCRC2 | GO:0042440 | 0.00448865704233895 | 37/8243 | 23/3250 | 0.00423119862216356 | 1 | T | T | F | F | pigment metabolic process | ADA | ALAS1 | AP3D1 | APRT | BCL2 | BLVRB | COX15 | FECH | GART | GMPS | HMBS | HMOX1 | HPRT1 | HPX | MC1R | MTHFD1 | MYO5A | PAICS | PPARGC1A | PPAT | PPOX | PRTFDC1 | SHMT1 | GO:0072332 | 0.00448865704233895 | 37/8243 | 23/3250 | 0.00423119862216356 | 1 | T | T | F | F | intrinsic apoptotic signaling pathway by p53 class mediator | ATAD5 | BAG6 | BAX | CHEK2 | DDIT4 | DYRK2 | E2F1 | E2F2 | EP300 | HINT1 | IFI16 | MYBBP1A | NUPR1 | PDK2 | PML | SIRT1 | SNW1 | STK11 | TP53 | TP63 | TP73 | USP28 | WWOX | GO:0051817 | 0.0100691495814631 | 83/8243 | 45/3250 | 0.00429732340584894 | 1 | T | T | F | F | modification of morphology or physiology of other organism involved in symbiotic interaction | ANXA2 | APCS | APOE | AZU1 | BCL2L1 | CALM1 | CALM2 | CALM3 | CASP8 | CCNT1 | CD209 | CFL1 | CTDP1 | EIF2AK4 | EP300 | GAPDH | HDAC1 | INSR | ITGAV | JUN | KPNA1 | KPNA2 | KPNB1 | LEF1 | PC | POU2F3 | PPIB | PSMC3 | RAB9A | REST | RXRA | SLC25A6 | SMAD3 | SMARCA4 | SMARCB1 | SMC3 | SNW1 | SP1 | STOM | SUGT1 | TARDBP | TGFB1 | TYMS | VAPA | VAPB | GO:0097711 | 0.0100691495814631 | 83/8243 | 45/3250 | 0.00429732340584894 | 1 | T | T | F | F | ciliary basal body-plasma membrane docking | ACTR1A | AHI1 | AKAP9 | CCP110 | CENPJ | CEP135 | CEP152 | CEP290 | CEP57 | CEP70 | CEP89 | CKAP5 | CNTRL | CSNK1E | DCTN1 | DYNC1H1 | DYNC1I2 | DYNLL1 | FBF1 | FGFR1OP | HSP90AA1 | IQCB1 | MKS1 | OFD1 | PAFAH1B1 | PCM1 | PLK1 | PPP2R1A | PRKACA | PRKAR2B | RAB11A | RAB3IP | RPGRIP1L | SCLT1 | TCTN2 | TCTN3 | TMEM216 | TUBA1A | TUBA4A | TUBB | TUBB4A | TUBB4B | TUBG1 | YWHAE | YWHAG | GO:0043154 | 0.00800679364309111 | 66/8243 | 37/3250 | 0.00442434914239967 | 1 | T | T | F | F | negative regulation of cysteine-type endopeptidase activity involved in apoptotic process | ADORA2A | AKT1 | ARRB1 | ARRB2 | BIRC2 | BIRC3 | BIRC7 | BIRC8 | CD27 | CRYAB | DDX3X | DHCR24 | DPEP1 | FABP1 | GPI | GPX1 | IGBP1 | LAMP3 | LEF1 | MDM2 | PAK2 | PARK7 | PIH1D1 | PLAUR | PRDX3 | PRDX5 | RAF1 | RPS6KA1 | SFN | SNCA | SRC | THBS1 | TNFSF14 | TRIAP1 | USP47 | XIAP | YWHAE | GO:1905114 | 0.0277811476404222 | 229/8243 | 110/3250 | 0.00448205157982742 | 1 | T | T | F | F | cell surface receptor signaling pathway involved in cell-cell signaling | AES | AGO1 | AGO2 | APC | ARRB2 | AXIN1 | BTRC | CALM1 | CAMK2A | CCAR2 | CCNY | CDC42 | CDC73 | CHD8 | CLTC | CSNK1A1 | CSNK1E | CSNK2A2 | CSNK2B | CTNNB1 | CTNNBIP1 | CTNND1 | CUL1 | CUL3 | CYLD | DAB2 | DDB1 | DDX3X | DISC1 | DVL1 | DVL2 | DVL3 | FBXW11 | FERMT2 | GNAO1 | GNB1 | GRK5 | GSK3A | GSK3B | LEF1 | LGR4 | LRP6 | LRRK2 | MCC | MED12 | MITF | MOV10 | MYH6 | MYOC | NFATC1 | NLK | NPHP3 | PARD6A | PDE6G | PFN1 | PRKAA1 | PRKAA2 | PSMA1 | PSMA2 | PSMA3 | PSMA5 | PSMA6 | PSMB5 | PSMB6 | PSMB7 | PSMB8 | PSMC1 | PSMC2 | PSMC3 | PSMC4 | PSMC5 | PSMC6 | PSMD1 | PSMD10 | PSMD11 | PSMD12 | PSMD14 | PSMD2 | PSMD4 | PSMD9 | PSME2 | PSME3 | PTEN | RAC1 | RARG | RBX1 | RECK | RHOA | RTF1 | SDC1 | SLC9A3R1 | SMO | SMURF1 | SMURF2 | SNX3 | SPIN1 | STK11 | TLE1 | TLE3 | TNKS | TNKS2 | UBB | UBC | VPS26A | VPS29 | WNT16 | WNT2 | WWOX | XIAP | ZBTB33 | GO:0009117 | 0.0164988475069756 | 136/8243 | 69/3250 | 0.00457012017394246 | 1 | T | T | F | F | nucleotide metabolic process | ABHD14B | ACACA | ACAT1 | ACLY | ACOT13 | ACOT7 | ACOT9 | ADCYAP1 | ADSS | AK1 | AK2 | AK4 | ATP6V1A | ATP6V1B1 | CLPX | CMPK1 | CROT | DBI | DCK | DCXR | DGUOK | DLG2 | DLST | FAR2 | FASN | FIGNL1 | G6PD | GMPR | GNAI3 | GPD1L | HMGCL | HPRT1 | HSD17B4 | HSPA1B | HSPA8 | IDH1 | IDH2 | KCNAB2 | LDHA | LRRK2 | MDH1 | MDH2 | MVD | MYH6 | NDUFS1 | NME1 | NME2 | NME4 | NMNAT1 | NMNAT3 | NMRK2 | NNT | NT5C2 | OGDH | OLA1 | OPA1 | PARK7 | PARP1 | RAN | REXO2 | RHOA | RNASEH2B | SUCLA2 | SUCLG2 | SULT1A3 | THEM4 | TPST2 | TYMS | VCP | GO:0001101 | 0.0247482712604634 | 204/8243 | 99/3250 | 0.00467167206712595 | 1 | T | T | F | F | response to acid chemical | ABL2 | ACACA | ACSL1 | ADCY6 | AIFM1 | AKAP8 | AKR1B1 | AKR1C2 | AKT1 | ASCL1 | ASNS | ASS1 | ATF2 | ATM | BCL11A | BCL2L1 | CAPN2 | CASP3 | CAT | CCL21 | CCNB1 | CD36 | CDK4 | CFL1 | COL18A1 | COL4A1 | COL6A1 | CPT1A | CREB1 | DUSP1 | E2F1 | EEF2 | EGFR | FABP3 | FIS1 | FOLR1 | FOXO1 | FYN | GLRB | GNB1 | GSS | GSTP1 | HDAC2 | HLCS | HMGCL | HNRNPD | HSF1 | HTRA2 | ICAM1 | IGFBP7 | INSIG2 | IPO5 | IREB2 | KDR | KLHL22 | LARS | MGMT | NCOA1 | NDUFA13 | NME1 | NME2 | NQO1 | NTRK1 | OPA1 | OTC | PCNA | PHB2 | PPARG | PPARGC1A | PPP5C | PRKAA1 | PRKAA2 | PRKCE | PTGES | PTN | RARA | RARG | RELA | RHOA | RPTOR | RXRA | SHMT1 | SHMT2 | SIPA1 | SNW1 | SOCS1 | SRC | TGFBR3 | TMBIM6 | TNF | TOMM20 | TWF2 | TYMS | WNT2 | XBP1 | XRCC5 | YAP1 | YES1 | YY1 | GO:0046649 | 0.0247482712604634 | 204/8243 | 99/3250 | 0.00467167206712595 | 1 | T | T | F | F | lymphocyte activation | ABL1 | ADA | AICDA | ANXA1 | ATAD5 | BAG6 | BAX | BCL2 | BCL6 | BRAF | BTK | CASP8 | CBFB | CD2 | CD22 | CD40 | CD40LG | CD79B | CTNNB1 | CTPS1 | DDOST | DNAJA3 | EIF2AK4 | ELF4 | EP300 | EXOSC6 | FADD | FKBP1A | FKBP1B | FYN | GPS2 | HDAC4 | HDAC5 | HDAC9 | HMGB1 | HNF1A | HPRT1 | HSPD1 | ICAM1 | ID2 | IFNA2 | IGBP1 | IMPDH2 | IRF1 | IRF4 | ITGA4 | ITK | JAK3 | JMJD6 | KIF13B | KIR3DS1 | KLF6 | LAT | LCP1 | LEF1 | LGALS1 | LIG4 | MICA | MLH1 | MSH6 | MSN | NCAPH2 | NCK1 | NEDD4 | NTRK1 | ONECUT1 | PATZ1 | PIK3CG | PIK3R1 | POLM | PRDX1 | PRDX2 | PRKCD | PRKDC | PSEN1 | PTPN22 | RAB29 | RBPJ | RC3H1 | RIPK2 | RIPK3 | RNF8 | RUNX2 | SLAMF1 | SMAD3 | SPI1 | STOML2 | SWAP70 | TGFB1 | TNFSF14 | TPD52 | TREML2 | TXLNA | VAV1 | VCAM1 | WAS | ZAP70 | ZBTB7A | ZBTB7B | GO:0070727 | 0.0852844838044401 | 703/8243 | 310/3250 | 0.00470921456848191 | 1 | T | T | F | F | cellular macromolecule localization | ABHD17A | ACAA1 | ACD | ACOX1 | ACTN2 | AGER | AGK | AHI1 | AKT1 | AKT2 | ANK1 | ANK3 | ANXA2 | AP3D1 | AP3M1 | AP4M1 | APOE | ARF4 | ARL5B | ATG4C | ATM | ATP1B3 | ATP6V1D | ATRX | AURKB | BAG4 | BAG6 | BANP | BAX | BLZF1 | BRAF | BSG | C16orf70 | CACNG2 | CACNG4 | CACNG5 | CALM1 | CALM3 | CALR | CARM1 | CAT | CAV1 | CBLB | CD81 | CDH1 | CDK5 | CHCHD10 | CLSTN1 | CLU | COG3 | CROCC | CROT | CSE1L | CTCF | CTNNB1 | DDX1 | DDX3X | DENND4C | DHRS4 | DHX9 | DLG2 | DLG3 | DLG4 | DMD | DNAJA1 | DNAJA3 | DNAJC19 | DNM1L | DVL1 | DVL2 | ECH1 | ECI2 | EGFR | EHHADH | ESR1 | EZH2 | EZR | FAM83D | FAM83H | FBXO7 | FCN1 | FERMT2 | FIS1 | FLOT1 | GAK | GBP2 | GBP5 | GGA1 | GLRB | GOLGA4 | GOLPH3 | GORASP2 | GRID2 | GRIK2 | GRIP1 | GRPEL1 | GSTK1 | HEATR3 | HGS | HK1 | HK2 | HMGCL | HNRNPU | HSD17B4 | HSP90AA1 | HSPA4 | HSPA8 | HSPD1 | IDE | IDH1 | IFT122 | IK | IKBKB | IPO11 | IPO4 | IPO5 | IPO7 | IPO8 | IPO9 | ITGA4 | ITGB1BP1 | JUP | KPNA1 | KPNA2 | KPNB1 | LARS | LATS1 | LEMD2 | LMNA | LRP6 | LRRC7 | LRRK2 | LRSAM1 | MAPK15 | MARCH5 | MCM9 | MDM2 | MTCH2 | MYO1C | MYO5A | NCOA4 | NDUFA13 | NEDD4 | NIPBL | NOS2 | NRXN1 | NUBP1 | NUDT19 | NUP107 | NUP153 | NUP214 | NUP85 | NUP88 | NUP93 | NUP98 | NUTF2 | NXT2 | OBSL1 | OPTN | OXA1L | PACS1 | PACS2 | PAM16 | PARD3 | PCM1 | PEX19 | PEX5 | PHB2 | PICK1 | PIGU | PIH1D1 | PIK3R1 | PIKFYVE | PINX1 | PLEKHF1 | PLK1 | PML | POM121 | POT1 | PRKCZ | PRPF19 | PTPRK | RAB10 | RAB11A | RAB11B | RAB29 | RAB31 | RAB32 | RAB35 | RAB3GAP1 | RAB3IP | RAB7A | RABGEF1 | RAD21 | RAN | RB1 | RDX | RIC3 | ROMO1 | RPA2 | RPAIN | RPL10 | RPL10A | RPL12 | RPL18A | RPL22 | RPL23 | RPL23A | RPL24 | RPL30 | RPL37A | RPL38 | RPL6 | RPL7 | RPL7A | RPL9 | RPLP0 | RPLP1 | RPLP2 | RPS12 | RPS13 | RPS14 | RPS15A | RPS19 | RPS2 | RPS20 | RPS24 | RPS29 | RPS3 | RPS4X | RPS8 | RRS1 | RTKN | RTN4 | RUVBL2 | SCG3 | SDCBP | SEC16A | SEC62 | SEC63 | SGTA | SGTB | SIN3A | SLC25A6 | SLC9A3R1 | SMO | SMURF1 | SNF8 | SPDL1 | SPTBN1 | SRP14 | SRP19 | SSB | STAT3 | STIL | STXBP1 | SUMO1 | SYNJ2BP | TERF2 | TERF2IP | TIA1 | TIMM13 | TIMM21 | TIMM23 | TIMM44 | TIMM50 | TINF2 | TM9SF4 | TMEM30A | TNF | TNFAIP3 | TNFRSF1A | TNKS | TNKS2 | TNPO1 | TOLLIP | TOMM20 | TOMM22 | TOMM40 | TOR1A | TPR | TRAPPC8 | TSPAN17 | TULP3 | TXNIP | UBB | UBC | UBE2D1 | UBE2D2 | UBE2D3 | UBL4A | UGT8 | USP4 | USP9X | VAPA | VCL | VPS37D | WDR19 | WDR35 | WDR45B | WIPI1 | WIPI2 | XPO1 | XRCC5 | YWHAE | ZBTB16 | ZBTB7A | ZDHHC23 | ZFAND6 | ZW10 | GO:0050801 | 0.0479194468033483 | 395/8243 | 181/3250 | 0.00470928251612342 | 1 | T | T | F | F | ion homeostasis | ABCB7 | ABCG1 | ABL1 | ABL2 | ACKR2 | ACO1 | ADCYAP1 | AFG3L2 | ALOX12 | ANK3 | ANXA2 | ANXA6 | ANXA7 | AP3D1 | APOA1 | APOE | APP | ARF1 | ATOX1 | ATP12A | ATP13A2 | ATP1A1 | ATP1B3 | ATP2A2 | ATP2B2 | ATP4A | ATP6V1A | ATP6V1B1 | ATP6V1C1 | ATP6V1D | ATP6V1E1 | BAX | BCL2 | BDKRB2 | BTBD9 | C1QTNF1 | CACNA1A | CALM1 | CALM2 | CALM3 | CALR | CAND1 | CAV1 | CCDC22 | CCL21 | CCR1 | CCR3 | CCR4 | CCR5 | CD36 | CD40 | CDK5 | CFTR | CHP1 | CHRNA9 | CKB | CLIC4 | COMMD1 | COX11 | COX19 | CUL1 | CUTC | CXCL11 | DCN | DLG4 | EGFR | EGLN1 | ESR1 | FABP3 | FASLG | FGF2 | FHL1 | FIS1 | FKBP1B | FTH1 | FTL | GLP1R | GLRX3 | GNA13 | GNA15 | GNB1 | GOT1 | GPD1L | GRIA1 | GRIK2 | GSTO1 | GTF2I | HAP1 | HEXB | HIF1A | HMGB1 | HMOX1 | HNF4A | HPX | HTR1B | HTT | IBTK | IMMT | IREB2 | ITGAV | ITPR1 | ITPR2 | ITPR3 | KCTD17 | KDR | LETM1 | LPAR1 | LPAR2 | LPAR4 | LRP6 | LRRK2 | MAPK1 | MAPK3 | MCOLN3 | MICU1 | MICU2 | MLLT11 | MYC | MYO5A | MYOC | NCOA4 | NEDD8 | NMB | NUBP1 | OTC | P2RX1 | P2RX2 | P2RX4 | PARP1 | PDK2 | PIK3CG | PLA2G1B | PLA2G6 | PLCG1 | PML | POLD1 | PPT1 | PRKAA1 | PRKAA2 | PRKACA | PRKCE | PSEN1 | PTGER3 | PTGIR | RAB7A | RAP1GDS1 | RGN | RIC3 | SCO2 | SFXN1 | SFXN4 | SIRT1 | SLAMF8 | SLC12A2 | SLC39A4 | SLC9A1 | SLC9A3R1 | SMAD3 | SMAD4 | SNCA | SOD1 | SRC | SRI | STIM1 | STOML2 | SWAP70 | TBXA2R | TCIRG1 | TGFB1 | TGM2 | THADA | TM9SF4 | TMBIM6 | TPCN2 | VAPB | VDR | WNK1 | WNK4 | XBP1 | XIAP | YWHAE | GO:1901991 | 0.0206235593837195 | 170/8243 | 84/3250 | 0.00481236603124682 | 1 | T | T | F | F | negative regulation of mitotic cell cycle phase transition | APC | ATM | AURKB | BAX | BCL2 | BRCA1 | CARM1 | CASP2 | CCNB1 | CDC20 | CDC25C | CDC73 | CDK2 | CDK4 | CDKN1A | CDKN2C | CENPF | CHEK2 | CUL1 | E2F1 | E2F4 | EP300 | EZH2 | FBXO7 | FHL1 | IK | KANK2 | KLHL22 | MAD1L1 | MAD2L1 | MDM2 | MDM4 | MSH6 | NAE1 | PCNA | PINX1 | PLK1 | PML | PRKDC | PRMT2 | PSMA1 | PSMA2 | PSMA3 | PSMA5 | PSMA6 | PSMB5 | PSMB6 | PSMB7 | PSMB8 | PSMC1 | PSMC2 | PSMC3 | PSMC4 | PSMC5 | PSMC6 | PSMD1 | PSMD10 | PSMD11 | PSMD12 | PSMD14 | PSMD2 | PSMD4 | PSMD9 | PSME2 | PSME3 | PTEN | RAD21 | RB1 | RBX1 | RIPK1 | RPA2 | SEPT7 | SFN | SPDL1 | TAOK3 | TFDP1 | TP53 | TPR | TRIAP1 | TRIP13 | USP47 | ZFYVE19 | ZNF207 | ZW10 | GO:0003006 | 0.038820817663472 | 320/8243 | 149/3250 | 0.00483037736670216 | 1 | T | T | F | F | developmental process involved in reproduction | ADA | ADCYAP1 | AFF4 | AKT1 | ANXA1 | AR | ARID4B | ARNT | ATRX | BASP1 | BAX | BCL2 | BCL2L1 | BIRC2 | BMPR1A | BSG | CASP2 | CASP8 | CBL | CCDC155 | CCDC36 | CCNB1 | CDC25B | CEBPA | CEP57 | CFTR | CRIP1 | CRKL | CTNNB1 | CTSV | CUL7 | DAZAP1 | DCN | DDX6 | DLD | DMRT3 | DNAJC19 | DYNLL1 | EGFR | EGLN1 | EIF2B5 | EIF2S2 | ESR1 | ESRRB | FAM9B | FANCA | FANCC | FANCG | FOXA1 | GATA1 | GDF9 | GRB2 | HEXB | HIF1A | HMGB2 | HNF4A | HS6ST1 | HSD17B4 | HSF1 | HSP90AB1 | HSPA2 | HTRA1 | ICAM1 | IDH1 | INSR | JUNB | KDM5B | KMT2B | LGR4 | LRP6 | MAP2K1 | MAP3K4 | MAPK1 | MAPK14 | MAPK3 | MAS1 | MDFI | MED1 | MGST1 | MLH1 | MME | NCOA1 | NCOA4 | NIPBL | NKX2-1 | NSUN2 | NTRK1 | NUP107 | NUPR1 | PAFAH1B1 | PATZ1 | PEBP1 | PHB2 | PPARD | PPARG | PRDX3 | PRDX4 | PRKACA | PRKDC | PSAP | PTEN | PTK2 | RARA | RARG | RBPJ | RHBDD1 | RNF2 | RNF8 | RRM1 | RTCB | RXRA | SDC1 | SEPT4 | SERPINB5 | SERPINE2 | SF1 | SGPL1 | SMAD4 | SMAD5 | SMARCA2 | SMARCC1 | SOD1 | SPACA1 | SPINT2 | SRC | STK3 | STRBP | STXBP1 | TCF21 | TGFB1 | TIAL1 | TLR3 | TNP1 | TP63 | TRIM28 | TRIP13 | TTLL1 | TUBB8 | UBB | UBE2B | UBE2J1 | VASH2 | VDR | VGF | WDR19 | WDR48 | WDR77 | WNT2 | WT1 | GO:0032469 | 0.0021836709935703 | 18/8243 | 13/3250 | 0.00492724489003032 | 1 | T | T | F | F | endoplasmic reticulum calcium ion homeostasis | APP | ATP2A2 | BAX | BCL2 | FIS1 | ITPR1 | KCTD17 | PML | PSEN1 | RAP1GDS1 | TGM2 | THADA | TMBIM6 | GO:0046112 | 0.0021836709935703 | 18/8243 | 13/3250 | 0.00492724489003032 | 1 | T | T | F | F | nucleobase biosynthetic process | ADA | APRT | CMPK1 | CTPS1 | GART | GMPS | HPRT1 | MTHFD1 | PAICS | PPAT | PRTFDC1 | SHMT1 | UMPS | GO:0060261 | 0.0021836709935703 | 18/8243 | 13/3250 | 0.00492724489003032 | 1 | T | T | F | F | positive regulation of transcription initiation from RNA polymerase II promoter | CAND1 | CREB1 | DHX36 | ESR1 | HNF1A | PAXIP1 | PSMC2 | PSMC3 | PSMC4 | PSMC5 | PSMC6 | SUB1 | TP53 | GO:0001890 | 0.0021836709935703 | 18/8243 | 13/3250 | 0.00492724489003032 | 1 | T | T | F | F | placenta development | ADA | BIRC2 | CUL7 | DCN | HSP90AB1 | HTRA1 | MAP3K4 | MAPK14 | MME | PPARG | PTK2 | RTCB | SOD1 | GO:0007339 | 0.0021836709935703 | 18/8243 | 13/3250 | 0.00492724489003032 | 1 | T | T | F | F | binding of sperm to zona pellucida | ALDOA | CCT2 | CCT3 | CCT4 | CCT5 | CCT7 | CCT8 | CLGN | HSPA1L | OVGP1 | TCP1 | UBAP2L | VDAC2 | GO:0045724 | 0.0021836709935703 | 18/8243 | 13/3250 | 0.00492724489003032 | 1 | T | T | F | F | positive regulation of cilium assembly | ARHGAP35 | CCDC88A | CCP110 | CENPJ | CEP135 | CROCC | DYNLL1 | HAP1 | HTT | KCTD17 | SEPT7 | SEPT9 | ZMYND10 | GO:1901655 | 0.00727890331190101 | 60/8243 | 34/3250 | 0.00496591407246666 | 1 | T | T | F | F | cellular response to ketone | ACACA | ADCY6 | AIFM1 | AKAP8 | AKR1B1 | AKR1C2 | AKT1 | AR | ASS1 | CASP9 | CDC5L | CDK4 | CFTR | DDIT4 | EGFR | EIF4E | EIF4EBP1 | ERRFI1 | FBXO32 | FECH | FOXO1 | GNAI1 | GNB1 | ICAM1 | MAP4K1 | MSN | NR3C1 | PPARG | PRKAA1 | PRKAA2 | PRKCE | SRC | TGFB1 | USP8 | GO:0031098 | 0.0109183549678515 | 90/8243 | 48/3250 | 0.00498021478654856 | 1 | T | T | F | F | stress-activated protein kinase signaling cascade | AKR1B1 | BTRC | CRKL | CRYAB | CUL1 | FBXW11 | GPS2 | IKBKB | IKBKG | KAT7 | MAP2K1 | MAP2K3 | MAP2K4 | MAP2K6 | MAP2K7 | MAP3K1 | MAP3K13 | MAP3K14 | MAP3K2 | MAP3K3 | MAP3K4 | MAP4K1 | MAPK1 | MAPK14 | MAPK3 | MAPK8 | MAPK9 | NEK1 | NOD2 | OXSR1 | PAK2 | PAK6 | RHOA | RIPK2 | STK24 | STK25 | STK26 | STK3 | TAB1 | TAB2 | TAOK3 | TNF | TNIP2 | TRAF6 | UBB | UBC | UBE2N | UBE2V1 | GO:2000651 | 0.00133446560718185 | 11/8243 | 9/3250 | 0.00526076940675165 | 1 | T | F | F | F | positive regulation of sodium ion transmembrane transporter activity | ACTN4 | ANK3 | CHP1 | DMD | GLRX | KIF5B | SLC9A1 | WNK1 | WNK4 | GO:0070203 | 0.00133446560718185 | 11/8243 | 9/3250 | 0.00526076940675165 | 1 | T | F | F | F | regulation of establishment of protein localization to telomere | CCT2 | CCT3 | CCT4 | CCT5 | CCT6A | CCT7 | CCT8 | TCP1 | TERF1 | GO:0090085 | 0.00133446560718185 | 11/8243 | 9/3250 | 0.00526076940675165 | 1 | T | F | F | F | regulation of protein deubiquitination | ITCH | OTUD4 | PARK7 | SART3 | TANK | TRIM21 | UBXN1 | VCP | WDR48 | GO:0070202 | 0.00133446560718185 | 11/8243 | 9/3250 | 0.00526076940675165 | 1 | T | F | F | F | regulation of establishment of protein localization to chromosome | CCT2 | CCT3 | CCT4 | CCT5 | CCT6A | CCT7 | CCT8 | TCP1 | TERF1 | GO:0045787 | 0.0323911197379595 | 267/8243 | 126/3250 | 0.00528874928712973 | 1 | T | F | F | F | positive regulation of cell cycle | ABL1 | AKT1 | ANAPC4 | ANXA1 | APEX1 | APP | ASCL1 | ASNS | ATAD5 | ATM | ATRX | AURKB | BAX | BRCA1 | BRD4 | CALR | CARM1 | CASP2 | CCNB1 | CCNT1 | CCNT2 | CCNY | CCNYL1 | CDC14B | CDC25A | CDC25B | CDC25C | CDC42 | CDC73 | CDK2 | CDK4 | CDK5R1 | CDK9 | CDKN1A | CENPJ | CENPV | CHEK2 | CKS1B | CKS2 | CUL3 | CUL4B | CYP1A1 | DBF4B | DDRGK1 | DDX39B | DDX3X | DYNC1H1 | DYNC1LI1 | E2F1 | E2F4 | EGFR | EIF4E | EIF4EBP1 | EP300 | EZH2 | FAM83D | FEN1 | FGFR1 | FOXA1 | HNRNPU | HSF1 | HSP90AB1 | HSPA2 | ID2 | INSR | LGMN | LRP6 | MAD2L1 | MAPK15 | MDM2 | MDM4 | MECP2 | MED1 | MEPCE | MIF | MTA3 | MYBBP1A | NIPBL | NPM1 | NSFL1C | NUMA1 | NUSAP1 | PAFAH1B1 | PCNA | PEBP1 | PHB2 | PKN2 | PML | POLDIP2 | PRKACA | PRKCE | PROX1 | PSMD10 | RAB11A | RAD21 | RAD23A | RARA | RB1 | RDX | RHOA | RPL23 | RPS15A | RPTOR | SFN | SFPQ | SH2B1 | SIN3A | SRC | SRPK2 | SVIL | TFDP1 | TGFB1 | TMOD3 | TP53 | TP73 | TPR | TRIAP1 | TRIM21 | TRIM32 | UBE2B | UBE2C | USP19 | USP2 | VPS4B | XRCC3 | ZBTB17 | GO:0030855 | 0.0231711755428849 | 191/8243 | 93/3250 | 0.00532460328271985 | 1 | T | F | F | F | epithelial cell differentiation | ABL1 | ACADVL | AGR2 | AIMP2 | AKR1C2 | AKT1 | AKT2 | ALDOC | ANXA1 | ANXA2 | ANXA7 | AR | ASCL1 | BASP1 | CASP3 | CBR1 | CDC42 | CES1 | CLIC4 | COL4A1 | CPT1A | CREB1 | CTNNB1 | CTSV | CYP1A1 | DAB2 | E2F4 | ELF3 | EZR | FOXA1 | FZR1 | GSTA1 | GSTA2 | GSTK1 | HDAC1 | HDAC2 | HIF1A | HNF4A | HNRNPH3 | HOXA5 | HOXB5 | ID2 | JUN | KDM5B | KDR | LATS1 | LEF1 | LGALS3 | MAP2K1 | MCOLN3 | MED1 | MGMT | MSN | MTSS1 | NEUROD1 | NF2 | NKX2-1 | NR5A2 | PAX8 | PCNA | PGK1 | PIH1D1 | PPARG | PROX1 | PSAP | PTN | PTPRO | RAB10 | RBPJ | RHOA | RXRA | S100A7 | SCEL | SLC9A3R1 | SMAD3 | SMAD4 | SMO | SPRR2A | TAGLN | TAGLN2 | TCF21 | TGFB1 | THRA | TMEM231 | TOLLIP | TP63 | TSG101 | TXNIP | VDAC1 | WDR77 | WNT16 | WT1 | YAP1 | GO:0030518 | 0.00679364309110761 | 56/8243 | 32/3250 | 0.00533659554698976 | 1 | T | F | F | F | intracellular steroid hormone receptor signaling pathway | AR | BRCA1 | CALR | CARM1 | CTNNB1 | DAXX | DNAJA1 | ESR1 | ESR2 | FHL2 | GRIP1 | KAT5 | MED1 | NCOA1 | NCOA3 | NCOA4 | NEDD4 | NR3C1 | PHB | PIAS1 | PPARGC1A | RAN | RB1 | RHOA | RNF4 | SRC | TRIP4 | UBE3A | UBR5 | WBP2 | YAP1 | YWHAH | GO:0002433 | 0.00885599902947956 | 73/8243 | 40/3250 | 0.00540728408901568 | 1 | T | F | F | F | immune response-regulating cell surface receptor signaling pathway involved in phagocytosis | ABL1 | ACTB | ACTG1 | ACTR2 | ACTR3 | ARPC1A | ARPC1B | ARPC2 | ARPC3 | ARPC4 | ARPC5 | BAIAP2 | CDC42 | CRK | FCGR1A | FYN | GRB2 | HSP90AA1 | HSP90AB1 | IGHV3-13 | IGHV3-23 | IGHV3-30 | IGKV3-20 | IGLC7 | MAPK1 | MAPK3 | MYO1C | NCK1 | PIK3CA | PIK3R1 | PLA2G6 | PLCG1 | PRKCD | PRKCE | PTK2 | RAC1 | SRC | VAV1 | WAS | YES1 | GO:0038096 | 0.00885599902947956 | 73/8243 | 40/3250 | 0.00540728408901568 | 1 | T | F | F | F | Fc-gamma receptor signaling pathway involved in phagocytosis | ABL1 | ACTB | ACTG1 | ACTR2 | ACTR3 | ARPC1A | ARPC1B | ARPC2 | ARPC3 | ARPC4 | ARPC5 | BAIAP2 | CDC42 | CRK | FCGR1A | FYN | GRB2 | HSP90AA1 | HSP90AB1 | IGHV3-13 | IGHV3-23 | IGHV3-30 | IGKV3-20 | IGLC7 | MAPK1 | MAPK3 | MYO1C | NCK1 | PIK3CA | PIK3R1 | PLA2G6 | PLCG1 | PRKCD | PRKCE | PTK2 | RAC1 | SRC | VAV1 | WAS | YES1 | GO:0003012 | 0.0168627926725707 | 139/8243 | 70/3250 | 0.00541670412157959 | 1 | T | F | F | F | muscle system process | ACTA1 | ACTA2 | ACTG2 | ALDOA | ATP1A1 | ATP2A2 | BDKRB2 | CALD1 | CALM1 | CHRM3 | CHRND | CHRNE | CHRNG | COL4A3BP | CRYAB | DES | DMD | EEF2 | EZH2 | FKBP1B | GAA | GJA1 | GNAO1 | HDAC2 | HDAC4 | HMOX1 | IREB2 | ITGB5 | KCNIP2 | MAP2K3 | MAP2K6 | MYH11 | MYH6 | MYL12A | MYL12B | MYL2 | MYL6 | MYL9 | MYOC | NDUFS6 | NOS3 | OBSL1 | P2RX2 | P2RX4 | PDE4D | PGAM2 | PIK3C2A | PIK3CA | PLA2G6 | PPARGC1A | PTGER3 | PXN | RAP1GDS1 | RGS2 | SMAD5 | SNTA1 | SOD1 | STAC | SULF2 | TAZ | TMOD3 | TMOD4 | TNNT1 | TPCN2 | TPM1 | TPM2 | TPM3 | TPM4 | TRIM63 | VCL | GO:0050678 | 0.0240203809292733 | 198/8243 | 96/3250 | 0.00546429522458417 | 1 | T | F | F | F | regulation of epithelial cell proliferation | AKT1 | AKT3 | APOE | AR | ARNT | ATF2 | BAX | BMPR1A | CAV1 | CCR3 | CD109 | CDC73 | CTNNB1 | EAF2 | EGFR | EPPK1 | ERBB2 | ERRFI1 | FGF2 | FGFR1 | GLUL | GPX1 | HIF1A | HMGB1 | HMGB2 | HMOX1 | HOXA5 | HRAS | HTRA1 | IFT122 | ITGA4 | ITGB3 | JUN | KDM5B | KDR | LAMB1 | LIMS1 | MCC | MED1 | MTSS1 | MYC | NME1 | NME2 | NOD2 | NR4A1 | NRP2 | PDCD10 | PDCD6 | PHB2 | PLCG1 | PPARD | PPARG | PRKD1 | PRKD2 | PRKDC | PROX1 | PTEN | PTN | PTPRK | RB1 | RGN | RPTOR | RTN4 | SCG2 | SERPINB5 | SFN | SIRT1 | SIRT6 | SMAD3 | SMO | SNAI2 | SOX2 | SP1 | STAT1 | STAT3 | STAT5A | STK11 | STRAP | SULF2 | SYNJ2BP | TGFB1 | TGFBR3 | THBS1 | TINF2 | TNF | TNFAIP3 | TNFSF12 | TP63 | TWIST1 | VASH2 | VDR | WDR48 | WDR77 | WNT2 | XBP1 | YAP1 | GO:0051348 | 0.0240203809292733 | 198/8243 | 96/3250 | 0.00546429522458417 | 1 | T | F | F | F | negative regulation of transferase activity | ABL1 | ADORA2A | AHSG | AKT1 | APC | APOE | BAG2 | BAG5 | CASP3 | CAV1 | CBL | CBLB | CBLC | CDKN1A | CDKN2C | CEBPA | CHORDC1 | CHP1 | COX11 | DBNDD2 | DNAJA1 | DUSP1 | DUSP14 | DUSP16 | DUSP19 | DUSP22 | DUSP7 | DVL1 | ERRFI1 | FBXO7 | FGFR1OP | GNAQ | GPS2 | GSK3A | GSK3B | GSTP1 | HEXIM1 | HNRNPU | HSPB1 | IBTK | IGF1R | ILK | IPO5 | IPO7 | ITGB1BP1 | LATS1 | LRP6 | MAD2L1 | MAPT | MLLT1 | NCK1 | NF2 | NPM1 | PAK2 | PARK7 | PINX1 | PKIG | PLK1 | POT1 | PPARG | PPP2CA | PPP2R1A | PRDX3 | PRKAR1A | PRKAR2B | PRKCD | PSEN1 | PTEN | PTPN1 | PTPN22 | PTPN6 | QARS | RB1 | RGN | RGS2 | RPL23 | RPTOR | SERPINB3 | SFN | SIRT1 | SNX6 | SOCS1 | SPRY2 | SRC | STK38 | TAOK3 | TERF1 | TNFAIP3 | TP53 | TRIB2 | TRIB3 | TSG101 | UCHL1 | WARS | WNK1 | YWHAG | GO:0035036 | 0.002426301103967 | 20/8243 | 14/3250 | 0.00550381197105402 | 1 | T | F | F | F | sperm-egg recognition | ALDOA | CCT2 | CCT3 | CCT4 | CCT5 | CCT7 | CCT8 | CLGN | HSPA1L | OVGP1 | SPACA3 | TCP1 | UBAP2L | VDAC2 | GO:0010614 | 0.002426301103967 | 20/8243 | 14/3250 | 0.00550381197105402 | 1 | T | F | F | F | negative regulation of cardiac muscle hypertrophy | CTDP1 | ERRFI1 | FOXO1 | G6PD | GLRX3 | GSK3A | LMNA | P2RX4 | RGS2 | SMAD3 | SMAD4 | TNFRSF1A | TRIM63 | YY1 | GO:1901984 | 0.002426301103967 | 20/8243 | 14/3250 | 0.00550381197105402 | 1 | T | F | F | F | negative regulation of protein acetylation | ATG5 | BRCA1 | CTBP1 | GSK3B | HDAC2 | MECP2 | PARK7 | PIH1D1 | SET | SIN3A | SIRT1 | SNCA | SPI1 | TWIST1 | GO:0002028 | 0.00655101298071091 | 54/8243 | 31/3250 | 0.00552199874715389 | 1 | T | F | F | F | regulation of sodium ion transport | ACTN4 | ADRB2 | AHCYL1 | AKT1 | ANK3 | ARF1 | ATP1A1 | ATP1B3 | CHP1 | COMMD1 | DMD | GLRX | GPD1L | HECW1 | HECW2 | KIF5B | NEDD4 | NOS3 | P2RX4 | PCSK9 | PER1 | PRKCE | SERPINE2 | SLC9A1 | SLC9A3R1 | SNTA1 | STOM | TGFB1 | WNK1 | WNK4 | YWHAH | GO:0045639 | 0.00630838287031421 | 52/8243 | 30/3250 | 0.00570571031733953 | 1 | T | F | F | F | positive regulation of myeloid cell differentiation | ARNT | CASP8 | CCR1 | CD74 | CREB1 | CTNNBIP1 | ETS1 | FADD | FOS | GATA1 | GNAS | HAX1 | HIF1A | HMGB2 | HOXA5 | HSPA1B | ID2 | ISG15 | JUN | LEF1 | MAPK14 | MED1 | RB1 | RIPK1 | RUNX1 | STAT1 | STAT3 | TGFB1 | TNF | TRAF6 | GO:0050794 | 0.658255489506248 | 5426/8243 | 2193/3250 | 0.00570817402533735 | 1 | T | F | F | F | regulation of cellular process | A2M | AARS | ABAT | ABCE1 | ABCG1 | ABHD14B | ABHD17A | ABL1 | ABL2 | ACAA2 | ACACA | ACADVL | ACD | ACE2 | ACKR2 | ACLY | ACO1 | ACSL1 | ACTA2 | ACTB | ACTG1 | ACTL6A | ACTN1 | ACTN2 | ACTN4 | ACTR1A | ACTR2 | ACTR3 | ADA | ADCY6 | ADCYAP1 | ADD3 | ADORA2A | ADRB2 | ADRM1 | ADTRP | AEBP2 | AES | AFF1 | AFF4 | AGAP2 | AGER | AGO1 | AGO2 | AGR2 | AGTRAP | AHCYL1 | AHI1 | AHNAK | AHSG | AICDA | AIFM1 | AIMP2 | AIRE | AKAP8 | AKAP9 | AKR1B1 | AKR1C2 | AKT1 | AKT2 | AKT3 | AKTIP | ALB | ALDOA | ALOX12 | ALOX5 | AMFR | ANAPC4 | ANK1 | ANK3 | ANKRD49 | ANP32A | ANXA1 | ANXA2 | ANXA5 | ANXA6 | ANXA7 | AP1G1 | AP3D1 | APBB1 | APBB2 | APBB3 | APC | APCS | APEX1 | API5 | APLP1 | APOA1 | APOD | APOE | APP | AR | ARAF | ARF1 | ARF4 | ARFGAP1 | ARFIP1 | ARHGAP1 | ARHGAP19 | ARHGAP26 | ARHGAP35 | ARHGDIA | ARHGEF7 | ARHGEF9 | ARID3B | ARID4B | ARNT | ARNT2 | ARPC1A | ARPC1B | ARPC2 | ARPC3 | ARPC4 | ARPC5 | ARR3 | ARRB1 | ARRB2 | ASB15 | ASB16 | ASB6 | ASB9 | ASCC1 | ASCC2 | ASCL1 | ASGR2 | ASNS | ASPSCR1 | ASS1 | ATAD1 | ATAD3A | ATAD5 | ATCAY | ATF2 | ATF6 | ATF6B | ATF7 | ATG5 | ATL3 | ATM | ATOH1 | ATOX1 | ATP13A2 | ATP1A1 | ATP2A2 | ATP6V1A | ATP6V1B1 | ATP6V1C1 | ATP6V1D | ATP6V1E1 | ATRX | ATXN1 | ATXN2 | AURKB | AVIL | AXIN1 | AZU1 | B3GAT3 | BABAM1 | BACE1 | BACH2 | BAG1 | BAG2 | BAG3 | BAG4 | BAG5 | BAG6 | BAIAP2 | BAIAP2L1 | BANP | BASP1 | BAX | BBC3 | BCAR1 | BCAR3 | BCL10 | BCL11A | BCL2 | BCL2L1 | BCL2L13 | BCL2L2 | BCL6 | BCL6B | BCOR | BDKRB2 | BIN1 | BIRC2 | BIRC3 | BIRC5 | BIRC7 | BIRC8 | BLZF1 | BMI1 | BMPR1A | BNIPL | BOLA1 | BOLA3 | BRAF | BRCA1 | BRD2 | BRD3 | BRD4 | BSG | BTBD9 | BTK | BTN2A1 | BTRC | BUD31 | C1QA | C1QBP | C1QL4 | C1QTNF1 | C4A | CA8 | CAB39 | CABP4 | CACNA1A | CACNG2 | CACNG4 | CACNG5 | CALCOCO2 | CALM1 | CALM2 | CALM3 | CALR | CAMK1D | CAMK2A | CAMK2B | CAMKV | CAND1 | CANX | CAP2 | CAPG | CAPN1 | CAPN2 | CAPNS1 | CAPZA1 | CAPZA2 | CAPZB | CARD9 | CARM1 | CASP10 | CASP2 | CASP3 | CASP8 | CASP9 | CAT | CAV1 | CBFA2T3 | CBFB | CBL | CBLB | CBLC | CBX4 | CBX7 | CBX8 | CBY1 | CCAR2 | CCDC22 | CCDC36 | CCDC68 | CCDC8 | CCDC88A | CCL21 | CCL22 | CCNB1 | CCNDBP1 | CCNT1 | CCNT2 | CCNY | CCNYL1 | CCP110 | CCR1 | CCR3 | CCR4 | CCR5 | CCT2 | CCT3 | CCT4 | CCT5 | CCT6A | CCT7 | CCT8 | CD109 | CD2 | CD209 | CD22 | CD27 | CD2BP2 | CD36 | CD40 | CD40LG | CD6 | CD74 | CD79B | CD81 | CDA | CDC14B | CDC16 | CDC20 | CDC25A | CDC25B | CDC25C | CDC27 | CDC34 | CDC37 | CDC42 | CDC42EP3 | CDC42EP4 | CDC42SE2 | CDC5L | CDC73 | CDH1 | CDH5 | CDK15 | CDK16 | CDK19 | CDK2 | CDK4 | CDK5 | CDK5R1 | CDK8 | CDK9 | CDKL3 | CDKN1A | CDKN2C | CEBPA | CEBPE | CEL | CENPF | CENPJ | CENPV | CEP135 | CEP152 | CEP290 | CEP55 | CEP57 | CEP70 | CFL1 | CFL2 | CFTR | CGN | CGRRF1 | CHCHD10 | CHCHD2 | CHCHD3 | CHD1L | CHD3 | CHD4 | CHD8 | CHEK2 | CHGB | CHMP2A | CHORDC1 | CHP1 | CHRD | CHRM2 | CHRM3 | CHRNA9 | CHRND | CHRNE | CHRNG | CIRBP | CISD1 | CISD2 | CKAP5 | CKS1B | CKS2 | CLDN18 | CLEC11A | CLIC1 | CLIC3 | CLIC4 | CLK1 | CLNK | CLPX | CLSTN1 | CLTC | CLU | CMTM5 | CNDP1 | CNTRL | CNTROB | COA3 | COL18A1 | COL2A1 | COL4A1 | COL4A2 | COL4A3BP | COL5A1 | COLGALT1 | COMMD1 | COMT | COPA | COPS2 | COPS5 | COQ3 | COQ7 | CORT | COX11 | COX17 | COX7A2 | CPLX1 | CPLX3 | CPNE3 | CPSF7 | CPT1A | CRBN | CREB1 | CREB3 | CREBBP | CRIP1 | CRIP2 | CRK | CRKL | CRMP1 | CROCC | CRP | CRY1 | CRY2 | CRYAB | CSNK1A1 | CSNK1E | CSNK2A2 | CSNK2B | CTBP1 | CTCF | CTDP1 | CTH | CTNNB1 | CTNNBIP1 | CTNND1 | CTSA | CTSC | CTSL | CTSV | CTTN | CUL1 | CUL2 | CUL3 | CUL4B | CUL5 | CUL7 | CXCL11 | CXCL5 | CXCL6 | CXCL8 | CYCS | CYLD | CYP1A1 | DAB1 | DAB2 | DAD1 | DAP3 | DAPK1 | DAPP1 | DAXX | DAZAP1 | DBF4B | DBH | DBN1 | DBNDD2 | DBNL | DCLK1 | DCN | DCP2 | DCTN1 | DCUN1D1 | DDA1 | DDB1 | DDB2 | DDIT4 | DDRGK1 | DDX1 | DDX39B | DDX3X | DDX6 | DEDD | DENND1A | DEPDC7 | DERL1 | DGKE | DGKZ | DGUOK | DHCR24 | DHX36 | DHX9 | DIABLO | DIDO1 | DIS3 | DISC1 | DLD | DLG2 | DLG3 | DLG4 | DMAP1 | DMD | DMRT3 | DNA2 | DNAJA1 | DNAJA2 | DNAJA3 | DNAJA4 | DNAJB1 | DNAJB11 | DNAJB2 | DNAJC10 | DNAJC30 | DNAJC7 | DNM1L | DOCK5 | DOCK7 | DOK1 | DPEP1 | DPF2 | DPYSL5 | DRG2 | DROSHA | DSTN | DTX1 | DTX2 | DUSP1 | DUSP12 | DUSP14 | DUSP16 | DUSP19 | DUSP22 | DUSP7 | DVL1 | DVL2 | DVL3 | DYNC1H1 | DYNC1I2 | DYNC1LI1 | DYNLL1 | DYNLT1 | DYRK2 | E2F1 | E2F2 | E2F3 | E2F4 | E2F6 | EAF2 | EBF1 | ECD | EDA | EDEM1 | EED | EEF1A1 | EEF1A2 | EEF1D | EEF2 | EFEMP1 | EGFR | EGLN1 | EGLN3 | EHD4 | EHMT1 | EHMT2 | EID1 | EIF2AK3 | EIF2AK4 | EIF2B1 | EIF2B3 | EIF2B5 | EIF2S1 | EIF3A | EIF3D | EIF3E | EIF3H | EIF4A3 | EIF4E | EIF4E2 | EIF4EBP1 | EIF4ENIF1 | EIF4G3 | EIF4H | EIF5 | EIF5A | EIF5B | EIF6 | ELAVL1 | ELF3 | ELF4 | ENDOG | ENO1 | EP300 | EPHA4 | EPHA6 | EPHB6 | EPM2A | EPPIN | EPPK1 | EPRS | ERBB2 | ERBB3 | ERCC5 | ERF | ERG | ERLIN2 | ERRFI1 | ESR1 | ESR2 | ESRRA | ESRRB | ESRRG | ETS1 | EVC2 | EWSR1 | EXOSC1 | EXOSC5 | EXOSC6 | EXOSC9 | EXTL2 | EYA2 | EYA4 | EZH2 | EZR | FABP1 | FABP3 | FABP5 | FABP7 | FADD | FAF1 | FAM129B | FAM192A | FAM19A3 | FAM3C | FAM49B | FAM83D | FAM83H | FAM98A | FANCA | FANCI | FANCM | FASLG | FASN | FBP1 | FBRS | FBXL12 | FBXO6 | FBXO7 | FBXW11 | FBXW5 | FCGR1A | FCHSD1 | FCN1 | FEN1 | FERMT2 | FGB | FGF2 | FGFR1 | FGFR1OP | FGFR3 | FHL1 | FHL2 | FIBP | FIGNL1 | FIS1 | FKBP14 | FKBP1A | FKBP1B | FKBP8 | FLNB | FLOT1 | FN1 | FOLR1 | FOS | FOXA1 | FOXD4 | FOXD4L6 | FOXI2 | FOXJ1 | FOXK1 | FOXK2 | FOXM1 | FOXO1 | FSCN1 | FSD1 | FTH1 | FTO | FUBP1 | FUBP3 | FUS | FXR2 | FYN | FZR1 | G3BP1 | G3BP2 | G6PD | GABPB1 | GABRA6 | GABRR1 | GADD45G | GAK | GAP43 | GAPDH | GAPDHS | GAPVD1 | GATA1 | GBP2 | GBP5 | GDF9 | GDI1 | GDI2 | GEMIN5 | GFAP | GFPT1 | GIGYF1 | GIT1 | GJA1 | GKAP1 | GLA | GLMN | GLO1 | GLP1R | GLRB | GLRX | GLRX3 | GLRX5 | GLS2 | GLUL | GMDS | GNA12 | GNA13 | GNA15 | GNAI1 | GNAI2 | GNAI3 | GNAO1 | GNAQ | GNAS | GNAZ | GNB1 | GNB2 | GNG12 | GNG5 | GNL1 | GOLGA2 | GOLGA4 | GOLPH3 | GOLT1B | GOPC | GOT1 | GPC5 | GPD1 | GPD1L | GPI | GPS2 | GPSM1 | GPX1 | GPX4 | GRB2 | GRB7 | GRIA1 | GRID2 | GRIK2 | GRIP1 | GRK1 | GRK5 | GSK3A | GSK3B | GSR | GSTA2 | GSTO1 | GSTP1 | GSTZ1 | GTF2A1 | GTF2F1 | GTF2F2 | GTF2I | GZMA | H1F0 | H1FX | H2AFX | H2AFZ | H3F3A | HADH | HAP1 | HAVCR2 | HAX1 | HBEGF | HBZ | HDAC1 | HDAC2 | HDAC3 | HDAC4 | HDAC5 | HDAC6 | HDAC7 | HDAC9 | HDGF | HEBP1 | HECW1 | HECW2 | HELZ2 | HERC1 | HEXB | HEXIM1 | HGS | HIF1A | HIF1AN | HINT1 | HIPK4 | HIST1H1A | HIST1H2AB | HIST1H3A | HIST1H4A | HIST2H3A | HIST3H3 | HIVEP1 | HK1 | HK2 | HLA-A | HLA-B | HLA-DQA1 | HLA-DRA | HLA-DRB1 | HLA-DRB3 | HLA-DRB4 | HLA-DRB5 | HLX | HMGB1 | HMGB2 | HMGB3 | HMMR | HMOX1 | HNF1A | HNF4A | HNF4G | HNRNPA1 | HNRNPA2B1 | HNRNPAB | HNRNPD | HNRNPDL | HNRNPF | HNRNPH1 | HNRNPK | HNRNPL | HNRNPM | HNRNPU | HOXA5 | HOXB5 | HOXB6 | HOXB9 | HOXD10 | HPRT1 | HPX | HRAS | HSF1 | HSF4 | HSP90AA1 | HSP90AB1 | HSP90AB3P | HSP90AB4P | HSP90B1 | HSPA1B | HSPA1L | HSPA2 | HSPA5 | HSPA8 | HSPA9 | HSPB1 | HSPBP1 | HSPD1 | HSPE1 | HSPH1 | HTATIP2 | HTR1B | HTR3A | HTR3E | HTRA1 | HTRA2 | HTT | HUWE1 | HYPK | IBTK | ICAM1 | ICK | ID2 | IDE | IDH1 | IDH2 | IFI16 | IFIT1 | IFITM3 | IFNA2 | IFNAR1 | IFT122 | IGBP1 | IGF1R | IGF2BP1 | IGFBP7 | IGHV3-13 | IGHV3-23 | IGHV3-30 | IGKV3-20 | IGLC7 | IK | IKBKB | IKBKE | IKBKG | IKZF5 | IL13RA2 | IL32 | IL3RA | ILF2 | ILF3 | ILK | IMPA1 | IMPA2 | ING1 | ING5 | INSIG1 | INSIG2 | INSR | IPO5 | IPO7 | IPO8 | IQCB1 | IQGAP1 | IREB2 | IRF1 | IRF4 | IRF7 | ISG15 | ITCH | ITGA4 | ITGA7 | ITGAV | ITGB1BP1 | ITGB3 | ITGB3BP | ITGB5 | ITK | ITPR1 | ITPR2 | ITPR3 | JAK1 | JAK3 | JMJD6 | JUN | JUNB | JUND | JUP | KANK2 | KAT5 | KAT7 | KAT8 | KBTBD7 | KCNIP2 | KCNS3 | KCTD17 | KDM1A | KDM2A | KDM4D | KDM5B | KDM5C | KDM8 | KDR | KEAP1 | KHDRBS1 | KIF11 | KIF13B | KIF2C | KIF5B | KIR3DL2 | KLF5 | KLF6 | KLHDC10 | KLHL20 | KLHL22 | KLRC2 | KMT2A | KMT2B | KMT2C | KPNA1 | KPNA2 | KPNB1 | KRAS | KRT17 | KSR1 | L3MBTL1 | L3MBTL2 | L3MBTL3 | L3MBTL4 | LAMB1 | LAMP3 | LANCL1 | LARP4 | LARS | LASP1 | LAT | LATS1 | LCP1 | LCP2 | LDHA | LDLRAD4 | LEF1 | LEMD2 | LEMD3 | LETM1 | LGALS1 | LGALS3 | LGALS3BP | LGMN | LGR4 | LIG4 | LIMA1 | LIMS1 | LMNA | LMNB1 | LMO2 | LMO7 | LOXL2 | LPAR1 | LPAR2 | LPAR4 | LPIN3 | LRFN1 | LRIF1 | LRP6 | LRPPRC | LRRC7 | LRRK1 | LRRK2 | LRRN2 | LRSAM1 | LSM1 | LTBP1 | LTBR | LUM | LURAP1 | LYPLA1 | LZTR1 | MAD1L1 | MAD2L1 | MAF1 | MAGI1 | MAOA | MAP1B | MAP2K1 | MAP2K3 | MAP2K4 | MAP2K6 | MAP2K7 | MAP3K1 | MAP3K13 | MAP3K14 | MAP3K2 | MAP3K3 | MAP3K4 | MAP4K1 | MAPK1 | MAPK14 | MAPK15 | MAPK3 | MAPK4 | MAPK6 | MAPK7 | MAPK8 | MAPK8IP3 | MAPK9 | MAPKAP1 | MAPT | MARCH5 | MARCKSL1 | MAS1 | MAST1 | MAST3 | MATK | MAVS | MBD3 | MBP | MC1R | MCC | MCM2 | MCM7 | MCOLN3 | MCTS1 | MDC1 | MDFI | MDM1 | MDM2 | MDM4 | ME1 | MECP2 | MED1 | MED12 | MED14 | MED17 | MED20 | MED21 | MED23 | MED24 | MED31 | MED4 | MED6 | MELK | MEOX2 | MEPCE | METAP2 | METRN | METRNL | METTL23 | MEX3C | MGA | MGMT | MIB1 | MICA | MIF | MITF | MKNK2 | MKS1 | MLH1 | MLLT1 | MLLT11 | MLLT3 | MLST8 | MME | MOB1A | MOB1B | MORF4L1 | MORF4L2 | MOV10 | MPC2 | MPP2 | MRGBP | MRPL12 | MSH6 | MSN | MTA1 | MTA3 | MTAP | MTCH2 | MTERF1 | MTNR1A | MTPN | MTSS1 | MVD | MXD1 | MYBBP1A | MYC | MYD88 | MYH6 | MYL2 | MYL9 | MYO1C | MYO1D | MYO1E | MYO5A | MYO6 | MYOC | NACC1 | NAE1 | NAMPT | NANOG | NAP1L1 | NBAS | NCBP1 | NCDN | NCK1 | NCOA1 | NCOA2 | NCOA3 | NCOA4 | NCOR1 | NCOR2 | NDRG1 | NDUFA13 | NDUFAF2 | NDUFS3 | NEDD4 | NEDD8 | NEK1 | NEK7 | NELFB | NELFCD | NELFE | NELL1 | NEUROD1 | NF2 | NFATC1 | NFATC2 | NFE2L2 | NFKB2 | NFKBIA | NIF3L1 | NIN | NIPBL | NKRF | NKX2-1 | NLK | NMB | NMD3 | NME1 | NME2 | NMNAT1 | NMRK2 | NNT | NOD2 | NOS1AP | NOS2 | NOS3 | NOSTRIN | NPEPPS | NPHP3 | NPLOC4 | NPM1 | NPR1 | NPTX1 | NQO1 | NQO2 | NR0B2 | NR1H2 | NR1H3 | NR1I2 | NR3C1 | NR4A1 | NR5A2 | NRBF2 | NRP2 | NRXN1 | NSFL1C | NSMCE4A | NSUN2 | NTF3 | NTRK1 | NUBP1 | NUDT6 | NUFIP1 | NUMA1 | NUP107 | NUP153 | NUP160 | NUP205 | NUP214 | NUP85 | NUP88 | NUP93 | NUP98 | NUPR1 | NUSAP1 | OAS2 | OAZ3 | OBSL1 | OFD1 | OGFOD1 | OGT | ONECUT1 | OPA1 | OPTN | OR5F1 | ORM1 | OTUB1 | OTUD4 | OVGP1 | OXCT1 | OXSR1 | P2RX1 | P2RX2 | P2RX4 | P4HB | PA2G4 | PABPC1 | PABPC4 | PACSIN3 | PAFAH1B1 | PAG1 | PAIP1 | PAK2 | PAK6 | PALB2 | PAM16 | PAN2 | PANX3 | PAPLN | PARD3 | PARD6A | PARK7 | PARP1 | PARP14 | PARP6 | PATZ1 | PAWR | PAX8 | PAX9 | PAXIP1 | PCBD1 | PCBP1 | PCGF1 | PCGF5 | PCGF6 | PCM1 | PCNA | PCSK9 | PDCD10 | PDCD6 | PDCD6IP | PDCL | PDE12 | PDE4D | PDE4DIP | PDE6G | PDF | PDIA3 | PDIA4 | PDIA6 | PDK2 | PDLIM5 | PEBP1 | PEF1 | PELI1 | PER1 | PER2 | PEX11B | PEX5 | PFDN2 | PFKL | PFKM | PFN1 | PGAM1 | PGAM2 | PGK1 | PGK2 | PGP | PHB | PHB2 | PHC3 | PHF10 | PHF20L1 | PHF21A | PIAS1 | PIAS3 | PICK1 | PIGU | PIH1D1 | PIK3C2A | PIK3CA | PIK3CG | PIK3R1 | PIK3R3 | PIKFYVE | PIM1 | PIN1 | PINX1 | PIP4K2A | PIR | PITPNA | PKIG | PKN2 | PLA2G10 | PLA2G1B | PLA2G2A | PLA2G6 | PLAUR | PLCG1 | PLEKHF1 | PLGRKT | PLK1 | PLSCR1 | PLXNA1 | PLXNA2 | PLXNB2 | PML | PNKP | PNMA1 | PNP | POLDIP2 | POLDIP3 | POLR2A | POLR2E | POLR2L | POLR2M | POM121 | POT1 | POU2F3 | POU5F1 | PPARD | PPARG | PPARGC1A | PPEF1 | PPIA | PPIB | PPID | PPIE | PPIF | PPM1A | PPM1B | PPM1D | PPME1 | PPP1CA | PPP1CB | PPP1CC | PPP1R11 | PPP1R12A | PPP1R12B | PPP1R14A | PPP1R16A | PPP1R2 | PPP1R3B | PPP1R3C | PPP1R8 | PPP2CA | PPP2CB | PPP2R1A | PPP2R1B | PPP2R2A | PPP2R2D | PPP2R5D | PPP3CC | PPP4R2 | PPP5C | PPP6R1 | PPT1 | PRCC | PRCP | PRDX1 | PRDX2 | PRDX3 | PRDX4 | PRDX5 | PRDX6 | PRELID1 | PREPL | PRKAA1 | PRKAA2 | PRKAB2 | PRKACA | PRKAG1 | PRKAR1A | PRKAR2B | PRKCD | PRKCE | PRKCSH | PRKCZ | PRKD1 | PRKD2 | PRKD3 | PRKDC | PRMT2 | PRMT3 | PRMT6 | PROX1 | PRPF19 | PRPF40A | PRRC1 | PSAP | PSEN1 | PSEN2 | PSMA1 | PSMA2 | PSMA3 | PSMA5 | PSMA6 | PSMB5 | PSMB6 | PSMB7 | PSMB8 | PSMC1 | PSMC2 | PSMC3 | PSMC4 | PSMC5 | PSMC6 | PSMD1 | PSMD10 | PSMD11 | PSMD12 | PSMD14 | PSMD2 | PSMD4 | PSMD9 | PSME2 | PSME3 | PSTPIP1 | PTCRA | PTEN | PTGER3 | PTGES | PTGES2 | PTGES3 | PTGIR | PTK2 | PTN | PTPMT1 | PTPN1 | PTPN14 | PTPN22 | PTPN23 | PTPN6 | PTPRA | PTPRD | PTPRF | PTPRK | PTPRO | PTPRR | PTRH2 | PTTG1 | PUM1 | PUS7 | PXDN | PXN | PYCR1 | QARS | QRICH1 | QSOX1 | RAB10 | RAB11A | RAB11B | RAB18 | RAB1A | RAB1B | RAB21 | RAB24 | RAB29 | RAB2A | RAB31 | RAB32 | RAB35 | RAB3GAP1 | RAB5A | RAB5B | RAB7A | RAB9A | RABL2A | RABL3 | RAC1 | RAD21 | RAD23A | RAD23B | RAD51 | RAD51AP1 | RADIL | RAE1 | RAF1 | RAN | RANBP9 | RANGAP1 | RAP1A | RAP1B | RAPGEF4 | RARA | RARG | RASD1 | RASL11B | RASSF10 | RASSF7 | RASSF9 | RB1 | RB1CC1 | RBBP4 | RBBP6 | RBM17 | RBM25 | RBM3 | RBM4 | RBM8A | RBMX | RBPJ | RBX1 | RC3H1 | RCC1 | RDH11 | RDX | RECK | REL | RELA | REST | RFC1 | RFC2 | RFC3 | RFC4 | RFC5 | RFXANK | RGN | RGS1 | RGS2 | RGS20 | RHBDD1 | RHO | RHOA | RHOJ | RHOT1 | RHOT2 | RHOU | RHPN1 | RIC8B | RIF1 | RING1 | RIOK1 | RIPK1 | RIPK2 | RIPK3 | RIPK4 | RIT1 | RMND1 | RNASEH2B | RNF126 | RNF139 | RNF183 | RNF185 | RNF19A | RNF2 | RNF25 | RNF31 | RNF4 | RNF8 | ROMO1 | RPA2 | RPA3 | RPGRIP1L | RPL10 | RPL23 | RPL24 | RPL38 | RPL6 | RPLP0 | RPLP1 | RPS13 | RPS14 | RPS15A | RPS19 | RPS2 | RPS3 | RPS4X | RPS6KA1 | RPS6KA2 | RPS6KB2 | RPTOR | RRM2 | RRM2B | RRS1 | RSU1 | RTF1 | RTKN | RTN4 | RTN4IP1 | RUNX1 | RUNX1T1 | RUNX2 | RUVBL1 | RUVBL2 | RXRA | RXRB | RXRG | S100A1 | S100A11 | S100A12 | S100A13 | S100A4 | S100A6 | S100A7 | S100A9 | S100B | SAE1 | SAG | SAP18 | SARM1 | SARS | SART1 | SART3 | SAT1 | SCARA3 | SCEL | SCFD1 | SCG2 | SCML2 | SCO2 | SCYL2 | SDC1 | SDC4 | SDCBP | SDCBP2 | SEC13 | SEC24B | SEC31A | SEL1L | SEMA7A | SENP6 | SEPT1 | SEPT11 | SEPT2 | SEPT4 | SEPT5 | SEPT7 | SEPT8 | SEPT9 | SERPINB2 | SERPINB3 | SERPINB5 | SERPINB6 | SERPINB8 | SERPINE2 | SERPINH1 | SET | SETBP1 | SETDB1 | SF1 | SFMBT1 | SFN | SFPQ | SGPL1 | SGTA | SGTB | SH2B1 | SH3GL1 | SH3GL2 | SH3GL3 | SH3KBP1 | SHC1 | SHKBP1 | SHMT1 | SHMT2 | SIAH1 | SIN3A | SIPA1 | SIPA1L1 | SIRT1 | SIRT3 | SIRT5 | SIRT6 | SIRT7 | SKA1 | SKA3 | SKI | SLAMF1 | SLAMF8 | SLC12A2 | SLC16A1 | SLC25A24 | SLC25A4 | SLC25A5 | SLC25A6 | SLC35F6 | SLC39A4 | SLC9A1 | SLC9A3R1 | SLTM | SMAD2 | SMAD3 | SMAD4 | SMAD5 | SMAD9 | SMARCA2 | SMARCA4 | SMARCB1 | SMARCC1 | SMARCC2 | SMARCE1 | SMC1A | SMC3 | SMG5 | SMG6 | SMG8 | SMO | SMURF1 | SMURF2 | SNAI2 | SNCA | SND1 | SNF8 | SNRPA | SNRPA1 | SNTA1 | SNW1 | SNX12 | SNX17 | SNX3 | SNX6 | SOAT1 | SOCS1 | SOCS2 | SOCS6 | SOD1 | SOD2 | SOGA3 | SORT1 | SOX2 | SP1 | SP100 | SP2 | SPACA3 | SPATA2 | SPDL1 | SPEN | SPERT | SPG21 | SPI1 | SPICE1 | SPIN1 | SPINK7 | SPINT2 | SPRTN | SPRY2 | SPTAN1 | SPTBN1 | SPTBN2 | SPTLC2 | SQSTM1 | SRC | SRGAP2 | SRGAP3 | SRI | SRPK1 | SRPK2 | SRPRB | SRSF12 | SRSF5 | SSX2IP | ST13 | STAC | STAG2 | STARD13 | STAT1 | STAT3 | STAT4 | STAT5A | STAT6 | STAU1 | STIL | STK11 | STK24 | STK25 | STK26 | STK3 | STK38 | STK40 | STMN1 | STOML2 | STRAP | STUB1 | STX5 | STXBP1 | STXBP2 | STYX | SUB1 | SULF2 | SULT1A3 | SUMO1 | SUMO2 | SUN2 | SUPT4H1 | SUPT5H | SUSD5 | SUZ12 | SVIL | SWAP70 | SYDE1 | SYN1 | SYNE2 | SYNJ2BP | SYP | SYVN1 | TAB1 | TAB2 | TACO1 | TADA2B | TAF12 | TAF15 | TAGLN3 | TALDO1 | TANK | TAOK3 | TARDBP | TAS2R41 | TAS2R60 | TAS2R7 | TAZ | TBC1D15 | TBC1D22B | TBC1D4 | TBL1X | TBRG4 | TBXA2R | TCEA1 | TCEA2 | TCERG1 | TCF12 | TCF21 | TCF25 | TCF4 | TCIRG1 | TCOF1 | TCP1 | TCP10L | TCTN2 | TCTN3 | TDRD3 | TERF1 | TERF2 | TERF2IP | TES | TFCP2 | TFDP1 | TFG | TFIP11 | TGFB1 | TGFBR3 | TGIF2LX | TGM2 | THAP11 | THAP7 | THBS1 | THBS2 | THEM4 | THOC6 | THRA | TIA1 | TIAL1 | TIMP1 | TIMP2 | TINF2 | TLE1 | TLE3 | TLR3 | TMBIM6 | TMED9 | TMEM17 | TMEM173 | TMEM219 | TMEM231 | TMEM30A | TMEM9B | TMOD3 | TMOD4 | TMPO | TMTC3 | TMX2 | TMX4 | TNF | TNFAIP3 | TNFRSF12A | TNFRSF1A | TNFRSF8 | TNFSF12 | TNFSF14 | TNIP2 | TNK2 | TNKS | TNKS2 | TNN | TNP1 | TNPO1 | TOE1 | TOLLIP | TOMM22 | TOP2A | TOP3A | TOR1A | TP53 | TP53RK | TP63 | TP73 | TPCN2 | TPM1 | TPR | TPTE | TRA2A | TRADD | TRAF1 | TRAF2 | TRAF3 | TRAF3IP1 | TRAF6 | TRAP1 | TREX1 | TRIAP1 | TRIB2 | TRIB3 | TRIM14 | TRIM21 | TRIM25 | TRIM28 | TRIM29 | TRIM32 | TRIM33 | TRIM34 | TRIM35 | TRIM63 | TRIM9 | TRIP12 | TRIP13 | TRIP4 | TRIP6 | TRUB2 | TSC22D1 | TSC22D3 | TSC22D4 | TSFM | TSG101 | TSKU | TSPAN17 | TSPYL2 | TTC5 | TUBA1A | TUBA4A | TUBB | TUBB2B | TUBB4A | TUBB4B | TUBG1 | TUFT1 | TULP3 | TWF1 | TWF2 | TWIST1 | TXLNA | TXN | TXN2 | TXNDC11 | TXNDC15 | TXNDC17 | TXNDC5 | TXNIP | TXNRD1 | TYMS | U2AF2 | UBA3 | UBA5 | UBASH3A | UBB | UBC | UBE2A | UBE2B | UBE2C | UBE2D1 | UBE2D2 | UBE2D3 | UBE2I | UBE2J1 | UBE2K | UBE2L3 | UBE2M | UBE2N | UBE2O | UBE2S | UBE2V1 | UBE3A | UBQLN1 | UBQLN2 | UBQLN4 | UBR5 | UBXN1 | UCHL1 | UCHL5 | UFM1 | ULK1 | UNC119 | UNK | UNKL | UPF3B | UQCC1 | USP14 | USP15 | USP16 | USP17L2 | USP19 | USP2 | USP20 | USP21 | USP28 | USP4 | USP42 | USP46 | USP47 | USP5 | USP50 | USP7 | USP8 | USP9X | USP9Y | VAC14 | VAPA | VAPB | VASH2 | VASP | VAT1 | VAV1 | VCAM1 | VCL | VCP | VDAC1 | VDAC2 | VDAC3 | VDR | VGF | VGLL4 | VHL | VIM | VN1R1 | VPS18 | VPS26A | VPS29 | VPS4B | VPS72 | VSIG4 | VSTM2A | VTN | WARS | WAS | WBP2 | WDFY1 | WDR1 | WDR12 | WDR19 | WDR35 | WDR41 | WDR48 | WDR77 | WDTC1 | WIPI1 | WNK1 | WNK4 | WNT16 | WNT2 | WSB1 | WT1 | WTAP | WWOX | WWP2 | XAF1 | XBP1 | XIAP | XPO1 | XPO5 | XRCC1 | XRCC3 | XRCC5 | XRCC6 | YAF2 | YAP1 | YEATS2 | YES1 | YME1L1 | YTHDF1 | YWHAB | YWHAE | YWHAG | YWHAH | YWHAQ | YWHAZ | YY1 | ZAP70 | ZBTB16 | ZBTB17 | ZBTB21 | ZBTB25 | ZBTB33 | ZBTB42 | ZBTB7A | ZBTB7B | ZC3H4 | ZC3HC1 | ZDHHC17 | ZFAND5 | ZFAND6 | ZFP41 | ZFYVE19 | ZFYVE26 | ZHX1 | ZIC1 | ZKSCAN1 | ZMPSTE24 | ZMYM5 | ZMYND10 | ZMYND8 | ZNF207 | ZNF217 | ZNF227 | ZNF24 | ZNF277 | ZNF354C | ZNF408 | ZNF420 | ZNF443 | ZNF517 | ZNF518A | ZNF592 | ZNF598 | ZNF618 | ZNF622 | ZNF646 | ZNF669 | ZNF696 | ZNF746 | ZNF747 | ZRANB2 | ZSCAN2 | ZSCAN29 | ZSCAN32 | ZW10 | ZWINT | ZZZ3 | GO:0030100 | 0.016620162562174 | 137/8243 | 69/3250 | 0.00571326765138447 | 1 | T | F | F | F | regulation of endocytosis | ABL1 | ABL2 | ACTN4 | AHI1 | ANXA2 | APOE | APP | ARF1 | ARFGAP1 | ARRB1 | ARRB2 | ATAD1 | ATXN2 | BIN1 | BTBD9 | CALM1 | CALM3 | CAV1 | CBL | CCL21 | CD22 | CD36 | CLU | DAB2 | DLG4 | DNM1L | EHD4 | FLOT1 | HIST1H1A | HNRNPK | ITGB3 | LGALS3 | LRRK2 | LRSAM1 | MIB1 | NR1H2 | NR1H3 | NTF3 | PACSIN3 | PCSK9 | PICK1 | PPARG | PPP3CC | PPT1 | PRKD1 | PSEN1 | PTPN1 | RAB21 | RAB31 | RAB5A | RAB5B | RAC1 | SCYL2 | SDCBP | SH3GL1 | SH3GL2 | SH3GL3 | SNCA | SNX12 | SNX17 | SRC | SYNJ2BP | TNK2 | TOR1A | TSG101 | UBQLN2 | UNC119 | USP46 | VTN | GO:0048232 | 0.021836709935703 | 180/8243 | 88/3250 | 0.00574461144491603 | 1 | T | F | F | F | male gamete generation | ACOX1 | ACTR1A | ANKRD49 | AR | ARID4B | ATRX | BAG6 | BCL2L1 | BCL2L2 | BCL6 | BIRC3 | BRD2 | CALR | CCDC155 | CCDC36 | CCNB1 | CCT6B | CDC25C | CDK16 | CRKL | CSNK2A2 | CTSV | DAZAP1 | DEDD | DHX36 | DIAPH3 | DNAJA1 | DUSP13 | E2F1 | FAM9B | FOXJ1 | GGT1 | GJA1 | GORASP2 | GPX4 | GTSF1 | H2AFX | HERC2 | HIST1H1A | HSF1 | HSPA2 | KHDRBS1 | LGR4 | LRRK2 | MAS1 | MLH1 | OAZ3 | PATZ1 | PGAM2 | PGM3 | PHC2 | PIAS1 | PRDX4 | PRKAG1 | PTTG1 | PUM1 | RAD23B | RAI14 | RARA | RGN | RGS2 | RUVBL1 | SEPT12 | SEPT2 | SEPT6 | SEPT7 | SFMBT1 | SGPL1 | SIAH1 | SIRT1 | SMAD4 | SNRPA1 | SOD1 | SPATA2 | SPATA20 | SPATA24 | STK11 | TESK2 | TP63 | TRIP13 | TSGA10 | UBE2B | USP42 | USP9Y | WDR48 | YY1 | ZFP41 | ZSCAN2 | GO:0007283 | 0.021836709935703 | 180/8243 | 88/3250 | 0.00574461144491603 | 1 | T | F | F | F | spermatogenesis | ACOX1 | ACTR1A | ANKRD49 | AR | ARID4B | ATRX | BAG6 | BCL2L1 | BCL2L2 | BCL6 | BIRC3 | BRD2 | CALR | CCDC155 | CCDC36 | CCNB1 | CCT6B | CDC25C | CDK16 | CRKL | CSNK2A2 | CTSV | DAZAP1 | DEDD | DHX36 | DIAPH3 | DNAJA1 | DUSP13 | E2F1 | FAM9B | FOXJ1 | GGT1 | GJA1 | GORASP2 | GPX4 | GTSF1 | H2AFX | HERC2 | HIST1H1A | HSF1 | HSPA2 | KHDRBS1 | LGR4 | LRRK2 | MAS1 | MLH1 | OAZ3 | PATZ1 | PGAM2 | PGM3 | PHC2 | PIAS1 | PRDX4 | PRKAG1 | PTTG1 | PUM1 | RAD23B | RAI14 | RARA | RGN | RGS2 | RUVBL1 | SEPT12 | SEPT2 | SEPT6 | SEPT7 | SFMBT1 | SGPL1 | SIAH1 | SIRT1 | SMAD4 | SNRPA1 | SOD1 | SPATA2 | SPATA20 | SPATA24 | STK11 | TESK2 | TP63 | TRIP13 | TSGA10 | UBE2B | USP42 | USP9Y | WDR48 | YY1 | ZFP41 | ZSCAN2 | GO:0062012 | 0.0265679970884387 | 219/8243 | 105/3250 | 0.00579843383752674 | 1 | T | F | F | F | regulation of small molecule metabolic process | ABCG1 | ACACA | ACADVL | AKT1 | AKT2 | ANXA1 | APOE | ATCAY | BRCA1 | C1QTNF1 | CAV1 | CDA | COMT | COQ3 | COX11 | CPT1A | CRY1 | DNM1L | DUSP12 | DYRK2 | EIF6 | EP300 | ERLIN2 | ESRRB | FABP1 | FABP3 | FABP5 | FAM3C | FASN | FBP1 | FDPS | FOXO1 | GGPS1 | GSK3A | GSK3B | GSTZ1 | INSIG1 | INSIG2 | INSR | KPNB1 | MAS1 | ME1 | MVD | NCOA2 | NCOR2 | NQO1 | NR1H2 | NR1H3 | OAZ3 | OGT | PARK7 | PDK2 | PGP | PPARG | PPARGC1A | PPP1CA | PPP1CB | PPP1R3B | PRKAA1 | PRKAA2 | PRKAB2 | PRKCE | PROX1 | PSMA1 | PSMA2 | PSMA3 | PSMA5 | PSMA6 | PSMB5 | PSMB6 | PSMB7 | PSMB8 | PSMC1 | PSMC2 | PSMC3 | PSMC4 | PSMC5 | PSMC6 | PSMD1 | PSMD10 | PSMD11 | PSMD12 | PSMD14 | PSMD2 | PSMD4 | PSMD9 | PSME2 | PSME3 | RAN | REST | RGN | SIRT1 | SIRT5 | SNAI2 | SNCA | SOD1 | SP1 | SRC | TNF | TREX1 | TRIB3 | TWIST1 | VDR | WDTC1 | ZMPSTE24 | GO:0034248 | 0.0327550649035545 | 270/8243 | 127/3250 | 0.0058676338072361 | 1 | T | F | F | F | regulation of cellular amide metabolic process | AARS | ACO1 | AGO1 | AGO2 | AIRE | AKT1 | AKT2 | APOE | APP | ATXN2 | BIN1 | C1QBP | CALR | CASP3 | CDK4 | CIRBP | CLU | COA3 | DAPK1 | DDX1 | DDX39B | DDX3X | DHX36 | DHX9 | EEF2 | EIF2AK3 | EIF2AK4 | EIF2B5 | EIF2S1 | EIF3D | EIF3E | EIF3H | EIF4A3 | EIF4E | EIF4E2 | EIF4EBP1 | EIF4ENIF1 | EIF4G3 | EIF4H | EIF5 | EIF5A | EIF5B | EIF6 | ELAVL1 | EPHA4 | EPRS | ERBB2 | FTO | FXR2 | GAPDH | GEMIN5 | GRB7 | GSK3A | GSTZ1 | HAP1 | HNRNPD | HNRNPL | HSPB1 | IGF2BP1 | ILF3 | IREB2 | KHDRBS1 | LARP4 | LRPPRC | MAPK1 | MAPK3 | MKNK2 | MOV10 | MTPN | NCBP1 | NCK1 | NFE2L2 | NPM1 | OGFOD1 | PA2G4 | PABPC1 | PAIP1 | PDK2 | PER1 | PER2 | PGAM1 | PLA2G6 | POLDIP3 | PPP1CA | PRKAA1 | PRKCD | PUM1 | PUS7 | RARA | RBM3 | RBM4 | RBM8A | RC3H1 | RGS2 | RHOA | RMND1 | RNF139 | RPL10 | RPL38 | RPS14 | RPS3 | RPS4X | RPS6KA1 | RPS6KB2 | RXRA | SHMT1 | SHMT2 | SNCA | STAT3 | TACO1 | TCOF1 | THBS1 | TIA1 | TNF | TNFRSF1A | TPR | TRAP1 | TRUB2 | TSFM | TYMS | UNK | UPF3B | UQCC1 | USP16 | VIM | YTHDF1 | ZNF598 | GO:0006913 | 0.0246269562052651 | 203/8243 | 98/3250 | 0.00588788052434687 | 1 | T | F | F | F | nucleocytoplasmic transport | ABCE1 | AHCYL1 | AKT1 | ANP32A | ATXN1 | BACH2 | CALR | CBLB | CCHCR1 | CDK5 | CHP1 | CSE1L | DDX19B | DDX39A | DDX39B | DUSP16 | EIF4A3 | EIF4E | EIF5A | EIF6 | FYTTD1 | GEMIN4 | GEMIN5 | GEMIN7 | HEATR3 | HNRNPA1 | HNRNPA2B1 | HTATIP2 | IPO11 | IPO4 | IPO5 | IPO7 | IPO8 | IPO9 | KPNA1 | KPNA2 | KPNB1 | LMNA | MCM3AP | NCBP1 | NEUROD1 | NMD3 | NPM1 | NUP107 | NUP153 | NUP160 | NUP205 | NUP214 | NUP85 | NUP88 | NUP93 | NUP98 | NUTF2 | NXF1 | NXF2 | NXT2 | PHB2 | PIK3R1 | PML | POLDIP3 | POM121 | PRKAG1 | RAE1 | RAN | RANBP3 | RBM8A | RPAIN | RPL23 | RRS1 | SEC13 | SMG5 | SMG6 | SMN1 | SMURF1 | SNRPB | SNRPD1 | SNRPD3 | SNRPE | SRSF11 | SRSF5 | SSB | STAT3 | STYX | TGFB1 | THOC2 | THOC6 | THOC7 | TNPO1 | TPR | TXNIP | U2AF2 | UPF2 | UPF3B | XPO1 | XPO4 | XPO5 | XPO7 | XPOT | GO:2000117 | 0.00837073880868616 | 69/8243 | 38/3250 | 0.00591261758033799 | 1 | T | F | F | F | negative regulation of cysteine-type endopeptidase activity | ADORA2A | AKT1 | ARRB1 | ARRB2 | BIRC2 | BIRC3 | BIRC7 | BIRC8 | CD27 | CRYAB | DDX3X | DHCR24 | DPEP1 | FABP1 | GPI | GPX1 | IFI16 | IGBP1 | LAMP3 | LEF1 | MDM2 | PAK2 | PARK7 | PIH1D1 | PLAUR | PRDX3 | PRDX5 | RAF1 | RPS6KA1 | SFN | SNCA | SRC | THBS1 | TNFSF14 | TRIAP1 | USP47 | XIAP | YWHAE | GO:0032869 | 0.00837073880868616 | 69/8243 | 38/3250 | 0.00591261758033799 | 1 | T | F | F | F | cellular response to insulin stimulus | AKT1 | AKT2 | APRT | CDK4 | DENND4C | EPRS | ERRFI1 | FOXO1 | GOT1 | GSK3A | GSTP1 | HDAC5 | HDAC9 | INSR | LPIN3 | MYO5A | PARP1 | PCSK9 | PIK3R1 | PKM | PLA2G1B | PPARG | PPAT | PRKCZ | PRKDC | PTEN | RAB10 | RAB31 | SLC9A1 | SP1 | SRC | SRSF5 | STAT1 | TBC1D4 | TRIB3 | WDTC1 | XBP1 | YWHAG | GO:0010972 | 0.00837073880868616 | 69/8243 | 38/3250 | 0.00591261758033799 | 1 | T | F | F | F | negative regulation of G2/M transition of mitotic cell cycle | ATM | AURKB | BRCA1 | CUL1 | FHL1 | MSH6 | NAE1 | PINX1 | PSMA1 | PSMA2 | PSMA3 | PSMA5 | PSMA6 | PSMB5 | PSMB6 | PSMB7 | PSMB8 | PSMC1 | PSMC2 | PSMC3 | PSMC4 | PSMC5 | PSMC6 | PSMD1 | PSMD10 | PSMD11 | PSMD12 | PSMD14 | PSMD2 | PSMD4 | PSMD9 | PSME2 | PSME3 | RAD21 | RBX1 | TAOK3 | USP47 | ZFYVE19 | GO:1901342 | 0.0226859153220915 | 187/8243 | 91/3250 | 0.00591420770524706 | 1 | T | F | F | F | regulation of vasculature development | ABL1 | AGO1 | AGO2 | AKT3 | ANXA1 | ATF2 | BRCA1 | CCR3 | CD40 | CDH5 | COL4A2 | CTH | CTNNB1 | CXCL8 | DCN | E2F2 | EGLN1 | ERBB2 | ETS1 | FAM129B | FASLG | FGF2 | FKBPL | GLUL | GTF2I | HDAC5 | HDAC7 | HDAC9 | HGS | HIF1A | HIF1AN | HK2 | HMOX1 | HOXA5 | HSPB1 | HSPG2 | HTATIP2 | ITGB1BP1 | JAK1 | JUP | KDR | MAP3K3 | MAPK7 | MECP2 | MEOX2 | NFATC1 | NFATC2 | NFE2L2 | NOS3 | NPR1 | PDCD10 | PDCD6 | PGK1 | PIK3C2A | PKM | PLCG1 | PML | PPARG | PRKD1 | PRKD2 | PTN | RAP1A | RECK | RHOA | RHOJ | RUNX1 | S100A1 | SARS | SIRT1 | SIRT6 | SOD2 | SP1 | SP100 | SPRY2 | STARD13 | STAT1 | STAT3 | STIM1 | SYNJ2BP | TBXA2R | THBS1 | THBS2 | TLR3 | TNFAIP3 | TNFRSF12A | TNFSF12 | TWIST1 | VASH2 | WARS | WT1 | XBP1 | GO:0034620 | 0.0026689312143637 | 22/8243 | 15/3250 | 0.00596048005430457 | 1 | T | F | F | F | cellular response to unfolded protein | BAG3 | BAX | DAXX | HSF1 | HSPA14 | HSPA1B | HSPA1L | HSPA2 | HSPA5 | HSPA8 | HSPA9 | OPTN | PTPN1 | RHBDD1 | TMBIM6 | GO:0035094 | 0.0026689312143637 | 22/8243 | 15/3250 | 0.00596048005430457 | 1 | T | F | F | F | response to nicotine | ABAT | BCL2 | CASP3 | CHRND | CHRNE | CHRNG | CREB1 | HDAC2 | HMOX1 | KCNK1 | MAPK1 | NTRK1 | RELA | TNF | VCAM1 | GO:0071276 | 0.0026689312143637 | 22/8243 | 15/3250 | 0.00596048005430457 | 1 | T | F | F | F | cellular response to cadmium ion | AKT1 | CYP1A2 | DAXX | EGFR | FOS | HMOX1 | HSF1 | JUN | MAPK1 | MAPK3 | MAPK8 | MAPK9 | SOD1 | SUMO1 | UBE2D3 | GO:0035065 | 0.00582312264952081 | 48/8243 | 28/3250 | 0.00606124088151127 | 1 | T | F | F | F | regulation of histone acetylation | ARRB1 | ATG5 | BRCA1 | CTBP1 | CTCF | EID1 | HDAC5 | KAT7 | KMT2A | MAPK3 | MECP2 | PAXIP1 | PIH1D1 | PPARGC1A | RUVBL2 | SET | SIN3A | SIRT1 | SMAD4 | SMARCB1 | SNAI2 | SNCA | SPI1 | TADA2B | TGFB1 | TWIST1 | WBP2 | ZMPSTE24 | GO:2000300 | 0.00582312264952081 | 48/8243 | 28/3250 | 0.00606124088151127 | 1 | T | F | F | F | regulation of synaptic vesicle exocytosis | ADORA2A | ATP2A2 | BACE1 | BRAF | CALM1 | CALM3 | CAMK2A | CDK5 | CHRM2 | CPLX1 | CPLX3 | DNM1L | DVL1 | GIT1 | GSK3B | HTR1B | LRRK2 | P2RX1 | PREPL | RAB3GAP1 | RAB5A | RAP1A | RAP1B | SEPT5 | STXBP1 | SYN1 | SYP | VPS18 | GO:0045934 | 0.0139512313478103 | 115/8243 | 59/3250 | 0.00615033057461177 | 1 | T | F | F | F | negative regulation of nucleobase-containing compound metabolic process | ABCE1 | ACD | ATM | BCL6 | C1QBP | CCNT1 | CDA | CDK9 | DCP2 | ESR1 | GSK3A | GZMA | H1F0 | H1FX | HIST1H1A | HNRNPA1 | HNRNPA2B1 | HNRNPK | HNRNPL | HNRNPU | HSF1 | KMT2A | MLH1 | MSH6 | NCOR1 | NCOR2 | NELFE | NMNAT1 | OTUB1 | PAM16 | PARP1 | PINX1 | POT1 | RBMX | RIF1 | RPS13 | RPS3 | SAP18 | SMG6 | SRC | SRSF12 | SUPT5H | TERF1 | TERF2 | TERF2IP | TFIP11 | TGFB1 | TINF2 | TMBIM6 | TNKS | TNKS2 | TRIM28 | TRIP12 | TWIST1 | U2AF2 | UBR5 | XRCC1 | XRCC5 | ZMPSTE24 | GO:0016052 | 0.00812810869828946 | 67/8243 | 37/3250 | 0.00617845646600349 | 1 | T | F | F | F | carbohydrate catabolic process | AGL | ALDOA | ALDOC | CALM1 | DERA | ENO1 | ENO2 | ENO3 | GAA | GALE | GAPDH | GAPDHS | GPI | GUSB | HEXB | HK1 | HK2 | LDHA | MAN2B1 | OGDH | PFKL | PFKM | PFKP | PGAM1 | PGAM2 | PGK1 | PGK2 | PGM1 | PGM2 | PHKA1 | PKM | PYGB | PYGL | RPE | SORD | STBD1 | TPI1 | GO:0045937 | 0.0757005944437705 | 624/8243 | 276/3250 | 0.00621941408065992 | 1 | T | F | F | F | positive regulation of phosphate metabolic process | ABL1 | ACSL1 | ADCY6 | ADCYAP1 | ADRB2 | ADTRP | AGAP2 | AGER | AKAP9 | AKT1 | AKT2 | AKTIP | ANXA2 | APOE | APP | AR | ARAF | ARRB1 | ARRB2 | ATM | AXIN1 | AZU1 | BAG4 | BCAR3 | BCL10 | BCL2 | BIRC7 | BMPR1A | BRAF | C1QTNF1 | CAB39 | CALM1 | CALM2 | CALM3 | CARD9 | CAV1 | CCDC88A | CCL21 | CCL22 | CCNB1 | CCNT1 | CCNT2 | CCNY | CCNYL1 | CCR1 | CD27 | CD36 | CD40 | CD40LG | CD74 | CD81 | CDC25B | CDK5 | CDK5R1 | CDK9 | CDKN1A | CHEK2 | CKS1B | CKS2 | CLU | CRK | CRKL | CSNK2B | CTNNB1 | DAB1 | DAB2 | DAXX | DBF4B | DBNL | DDRGK1 | DDX3X | DGKZ | DLG3 | DLG4 | DOCK7 | DUSP12 | DUSP19 | DUSP22 | DUSP7 | DVL1 | DVL2 | DVL3 | EEF1A2 | EGFR | EHD4 | EPHA4 | ERBB2 | ERBB3 | EZH2 | FGB | FGF2 | FGFR1 | FGFR3 | FLOT1 | FYN | GADD45G | GATA1 | GDF9 | GLMN | GNAI2 | HAVCR2 | HAX1 | HBEGF | HDAC2 | HDAC3 | HDAC6 | HMGB1 | HPX | HRAS | HSF1 | HSP90AA1 | HSP90AB1 | HSPA2 | ICAM1 | IFNA2 | IGF1R | IKBKG | ILK | INSR | IQGAP1 | ITGB1BP1 | ITGB3 | JUN | KDR | KLHDC10 | KRAS | KSR1 | LAT | LATS1 | LCP2 | LPAR1 | LRRK1 | LRRK2 | LTBR | MAP2K1 | MAP2K3 | MAP2K4 | MAP2K6 | MAP2K7 | MAP3K1 | MAP3K13 | MAP3K14 | MAP3K2 | MAP3K3 | MAP3K4 | MAP4K1 | MAPK1 | MAPK14 | MAPK3 | MAPK8IP3 | MAPKAP1 | MAPT | MAS1 | MAVS | MIF | MLST8 | MOB1B | NEK1 | NELFE | NMNAT1 | NOD2 | NQO2 | NTF3 | NTRK1 | OXSR1 | PAK2 | PAK6 | PARK7 | PARP14 | PDCD10 | PDE6G | PGK1 | PGK2 | PHB | PHB2 | PIH1D1 | PIK3CA | PIK3CG | PIK3R3 | PIN1 | PLA2G1B | PLA2G2A | PLA2G6 | PLAUR | PLK1 | PPP1R12A | PPP2CA | PPP2R5D | PRDX2 | PRKAA1 | PRKACA | PRKAG1 | PRKAR1A | PRKAR2B | PRKCD | PRKCE | PRKCZ | PRKD1 | PRKD2 | PROX1 | PRRC1 | PSAP | PSEN1 | PSMD10 | PTEN | PTGES3 | PTK2 | PTPN1 | PTPN22 | RAC1 | RAF1 | RAP1A | RAP1B | RB1CC1 | RGN | RHOA | RIPK1 | RIPK2 | RIPK3 | RPLP1 | RPS3 | RPTOR | S100A12 | S100A7 | SDC4 | SDCBP | SEMA7A | SHC1 | SIRT1 | SLAMF1 | SMAD3 | SMAD4 | SNCA | SOD1 | SOX2 | SPRY2 | SQSTM1 | SRC | STAT3 | STK11 | STK24 | STK25 | STK26 | STK3 | STOML2 | TAB1 | TAB2 | TAOK3 | TAZ | TERF2IP | TGFB1 | THBS1 | TIMP2 | TLR3 | TNF | TNFRSF1A | TNK2 | TP53 | TP73 | TRAF2 | TRAF6 | TXN | UBB | UBC | UBE2K | UBE2N | UBE2V1 | UNC119 | VCP | VTN | WNK1 | WNT16 | XRCC5 | XRCC6 | YES1 | ZNF622 | GO:0010562 | 0.0757005944437705 | 624/8243 | 276/3250 | 0.00621941408065992 | 1 | T | F | F | F | positive regulation of phosphorus metabolic process | ABL1 | ACSL1 | ADCY6 | ADCYAP1 | ADRB2 | ADTRP | AGAP2 | AGER | AKAP9 | AKT1 | AKT2 | AKTIP | ANXA2 | APOE | APP | AR | ARAF | ARRB1 | ARRB2 | ATM | AXIN1 | AZU1 | BAG4 | BCAR3 | BCL10 | BCL2 | BIRC7 | BMPR1A | BRAF | C1QTNF1 | CAB39 | CALM1 | CALM2 | CALM3 | CARD9 | CAV1 | CCDC88A | CCL21 | CCL22 | CCNB1 | CCNT1 | CCNT2 | CCNY | CCNYL1 | CCR1 | CD27 | CD36 | CD40 | CD40LG | CD74 | CD81 | CDC25B | CDK5 | CDK5R1 | CDK9 | CDKN1A | CHEK2 | CKS1B | CKS2 | CLU | CRK | CRKL | CSNK2B | CTNNB1 | DAB1 | DAB2 | DAXX | DBF4B | DBNL | DDRGK1 | DDX3X | DGKZ | DLG3 | DLG4 | DOCK7 | DUSP12 | DUSP19 | DUSP22 | DUSP7 | DVL1 | DVL2 | DVL3 | EEF1A2 | EGFR | EHD4 | EPHA4 | ERBB2 | ERBB3 | EZH2 | FGB | FGF2 | FGFR1 | FGFR3 | FLOT1 | FYN | GADD45G | GATA1 | GDF9 | GLMN | GNAI2 | HAVCR2 | HAX1 | HBEGF | HDAC2 | HDAC3 | HDAC6 | HMGB1 | HPX | HRAS | HSF1 | HSP90AA1 | HSP90AB1 | HSPA2 | ICAM1 | IFNA2 | IGF1R | IKBKG | ILK | INSR | IQGAP1 | ITGB1BP1 | ITGB3 | JUN | KDR | KLHDC10 | KRAS | KSR1 | LAT | LATS1 | LCP2 | LPAR1 | LRRK1 | LRRK2 | LTBR | MAP2K1 | MAP2K3 | MAP2K4 | MAP2K6 | MAP2K7 | MAP3K1 | MAP3K13 | MAP3K14 | MAP3K2 | MAP3K3 | MAP3K4 | MAP4K1 | MAPK1 | MAPK14 | MAPK3 | MAPK8IP3 | MAPKAP1 | MAPT | MAS1 | MAVS | MIF | MLST8 | MOB1B | NEK1 | NELFE | NMNAT1 | NOD2 | NQO2 | NTF3 | NTRK1 | OXSR1 | PAK2 | PAK6 | PARK7 | PARP14 | PDCD10 | PDE6G | PGK1 | PGK2 | PHB | PHB2 | PIH1D1 | PIK3CA | PIK3CG | PIK3R3 | PIN1 | PLA2G1B | PLA2G2A | PLA2G6 | PLAUR | PLK1 | PPP1R12A | PPP2CA | PPP2R5D | PRDX2 | PRKAA1 | PRKACA | PRKAG1 | PRKAR1A | PRKAR2B | PRKCD | PRKCE | PRKCZ | PRKD1 | PRKD2 | PROX1 | PRRC1 | PSAP | PSEN1 | PSMD10 | PTEN | PTGES3 | PTK2 | PTPN1 | PTPN22 | RAC1 | RAF1 | RAP1A | RAP1B | RB1CC1 | RGN | RHOA | RIPK1 | RIPK2 | RIPK3 | RPLP1 | RPS3 | RPTOR | S100A12 | S100A7 | SDC4 | SDCBP | SEMA7A | SHC1 | SIRT1 | SLAMF1 | SMAD3 | SMAD4 | SNCA | SOD1 | SOX2 | SPRY2 | SQSTM1 | SRC | STAT3 | STK11 | STK24 | STK25 | STK26 | STK3 | STOML2 | TAB1 | TAB2 | TAOK3 | TAZ | TERF2IP | TGFB1 | THBS1 | TIMP2 | TLR3 | TNF | TNFRSF1A | TNK2 | TP53 | TP73 | TRAF2 | TRAF6 | TXN | UBB | UBC | UBE2K | UBE2N | UBE2V1 | UNC119 | VCP | VTN | WNK1 | WNT16 | XRCC5 | XRCC6 | YES1 | ZNF622 | GO:0043525 | 0.0055804925391241 | 46/8243 | 27/3250 | 0.00622881253180178 | 1 | T | F | F | F | positive regulation of neuron apoptotic process | AIFM1 | ASCL1 | ATF2 | ATM | BACE1 | BAX | CASP2 | CASP3 | CASP9 | CDC34 | CDK5 | CDK5R1 | CTNNB1 | FASLG | FIS1 | GRIK2 | HDAC4 | JUN | MAP2K4 | NQO1 | NQO2 | PCSK9 | PIN1 | RHOA | SRPK2 | TNF | UBE2M | GO:0009060 | 0.0029115613247604 | 24/8243 | 16/3250 | 0.00630935827498641 | 1 | T | F | F | F | aerobic respiration | ADSL | CAT | COX15 | COX20 | COX5A | COX6A1 | COX6C | CYCS | MDH2 | NDUFS7 | NDUFS8 | OXA1L | SDHB | SIRT3 | UQCRB | UQCRC2 | GO:0010965 | 0.0053378624287274 | 44/8243 | 26/3250 | 0.0063860829848 | 1 | T | F | F | F | regulation of mitotic sister chromatid separation | ANAPC4 | APC | ATM | AURKB | CCNB1 | CDC20 | CDC27 | CENPF | CUL3 | DUSP1 | DYNC1LI1 | HECW2 | IK | KLHL22 | MAD1L1 | MAD2L1 | PLK1 | PTTG1 | RAD21 | RB1 | SPDL1 | TPR | TRIP13 | XRCC3 | ZNF207 | ZW10 | GO:0048609 | 0.0322698046827611 | 266/8243 | 125/3250 | 0.00647988185306269 | 1 | T | F | F | F | multicellular organismal reproductive process | ACOX1 | ACSL4 | ACTR1A | AKR1B1 | AKT1 | ANKRD49 | ANXA1 | APOL2 | APP | AR | ARID4B | ATRX | BAG6 | BCL2L1 | BCL2L2 | BCL6 | BIRC3 | BRD2 | CALR | CCDC155 | CCDC36 | CCNB1 | CCT6B | CDC25C | CDK16 | COMT | CRKL | CSNK2A2 | CTSV | CYP1A1 | DAZAP1 | DBH | DEDD | DHX36 | DIAPH3 | DNAJA1 | DUSP13 | E2F1 | EGFR | ESR1 | ETS1 | FAM9B | FOXJ1 | GDF9 | GGT1 | GJA1 | GORASP2 | GPX4 | GTSF1 | H2AFX | HAVCR2 | HERC2 | HEXB | HIST1H1A | HSF1 | HSPA2 | JUNB | KHDRBS1 | KMT2B | LGR4 | LRRK2 | MAS1 | MCM9 | MED1 | MLH1 | MTNR1A | NCOA1 | NOS3 | OAZ3 | P2RX1 | PATZ1 | PCNA | PGAM2 | PGM3 | PHC2 | PIAS1 | PLA2G6 | PPAT | PRDX3 | PRDX4 | PRKAG1 | PTEN | PTN | PTTG1 | PUM1 | RAD23B | RAI14 | RARA | RGN | RGS2 | RUVBL1 | RXRA | SEPT12 | SEPT2 | SEPT6 | SEPT7 | SERPINE2 | SFMBT1 | SGPL1 | SIAH1 | SIRT1 | SMAD4 | SNRPA1 | SOD1 | SPATA2 | SPATA20 | SPATA24 | SPIN1 | SRC | STK11 | TESK2 | THRA | TP63 | TRIP13 | TSGA10 | UBE2A | UBE2B | USP42 | USP9X | USP9Y | VDR | WDR48 | YY1 | ZFP41 | ZSCAN2 | GO:0071869 | 0.0031541914351571 | 26/8243 | 17/3250 | 0.00656376753535038 | 1 | T | F | F | F | response to catecholamine | ABL1 | ADCY6 | APLP1 | APP | GNB1 | HCN3 | HDAC2 | LRRK2 | MAPK1 | MAPK3 | PDE4D | PPARGC1A | PRKACA | SIN3A | SLC9A1 | SNCA | SULT1A3 | GO:0071867 | 0.0031541914351571 | 26/8243 | 17/3250 | 0.00656376753535038 | 1 | T | F | F | F | response to monoamine | ABL1 | ADCY6 | APLP1 | APP | GNB1 | HCN3 | HDAC2 | LRRK2 | MAPK1 | MAPK3 | PDE4D | PPARGC1A | PRKACA | SIN3A | SLC9A1 | SNCA | SULT1A3 | GO:0044273 | 0.0031541914351571 | 26/8243 | 17/3250 | 0.00656376753535038 | 1 | T | F | F | F | sulfur compound catabolic process | ACAT1 | ACOT7 | AHCY | CBS | CTH | DCN | GALNS | GGT1 | GGTLC1 | GNS | HEXA | HEXB | LUM | MPST | MTRR | TXN2 | VCAN | GO:0043124 | 0.0031541914351571 | 26/8243 | 17/3250 | 0.00656376753535038 | 1 | T | F | F | F | negative regulation of I-kappaB kinase/NF-kappaB signaling | ABL1 | CASP8 | CCDC22 | ESR1 | GSTP1 | HDAC1 | OPTN | PER1 | PPM1A | PPM1B | RHOA | RIPK1 | SIRT1 | STAT1 | TANK | TLE1 | TNFAIP3 | GO:0006984 | 0.0031541914351571 | 26/8243 | 17/3250 | 0.00656376753535038 | 1 | T | F | F | F | ER-nucleus signaling pathway | ASNS | ATF6 | ATF6B | ATP2A2 | CALR | CXCL8 | EIF2AK3 | EIF2S1 | ERLIN2 | GSK3B | HSP90B1 | HSPA5 | INSIG1 | INSIG2 | NFE2L2 | TP53 | XBP1 | GO:0045806 | 0.0031541914351571 | 26/8243 | 17/3250 | 0.00656376753535038 | 1 | T | F | F | F | negative regulation of endocytosis | APP | ATXN2 | CAV1 | DLG4 | LGALS3 | LRSAM1 | NR1H2 | NR1H3 | PACSIN3 | PCSK9 | PRKD1 | PSEN1 | RAC1 | SDCBP | SH3GL3 | UBQLN2 | UNC119 | GO:1902042 | 0.0031541914351571 | 26/8243 | 17/3250 | 0.00656376753535038 | 1 | T | F | F | F | negative regulation of extrinsic apoptotic signaling pathway via death domain receptors | BCL2L1 | BRCA1 | CASP8 | FADD | FASLG | FGB | GPX1 | HMGB2 | HMOX1 | ICAM1 | NOS3 | PARK7 | RAF1 | RIPK1 | TNFAIP3 | TRADD | TRAF2 | GO:0035666 | 0.0031541914351571 | 26/8243 | 17/3250 | 0.00656376753535038 | 1 | T | F | F | F | TRIF-dependent toll-like receptor signaling pathway | BIRC2 | BIRC3 | CASP8 | FADD | IKBKB | IKBKE | IKBKG | IRF7 | RIPK1 | TANK | TLR3 | TRAF3 | UBB | UBC | UBE2D1 | UBE2D2 | UBE2D3 | GO:0032479 | 0.0110396700230499 | 91/8243 | 48/3250 | 0.00656734384124296 | 1 | T | F | F | F | regulation of type I interferon production | CREBBP | CTNNB1 | CYLD | DDX3X | DHX36 | DHX9 | EP300 | FLOT1 | HAVCR2 | HMGB1 | HMGB2 | HSPD1 | IFI16 | IKBKE | IRF1 | IRF7 | ISG15 | ITCH | MAVS | MYD88 | NFKB2 | NPLOC4 | OTUD5 | PCBP2 | PIN1 | POLR2E | POLR2L | PPM1B | PRKDC | PTPN22 | REL | RELA | RIPK2 | STAT1 | STAT6 | TLR3 | TMEM173 | TNFAIP3 | TRAF3 | TRAF3IP1 | TREX1 | TRIM21 | TRIM25 | TRIM32 | UBE2L6 | XRCC5 | XRCC6 | YY1 | GO:0006139 | 0.160621133082616 | 1324/8243 | 563/3250 | 0.00660831226639882 | 1 | T | F | F | F | nucleobase-containing compound metabolic process | AARS | AARSD1 | ABHD14B | ABL1 | ACACA | ACAT1 | ACD | ACLY | ACOT13 | ACOT7 | ACOT9 | ACTL6A | ADA | ADCYAP1 | ADK | ADSS | AGO1 | AGO2 | AHCY | AHCYL1 | AICDA | AIMP2 | AK1 | AK2 | AK4 | ALDH6A1 | AMD1 | APBB1 | APEX1 | APEX2 | APP | APRT | ARID4B | ARNT | ASCC1 | ASCC2 | ASPDH | ATAD5 | ATIC | ATM | ATP6V1A | ATP6V1B1 | ATRX | ATXN1 | ATXN2 | BABAM1 | BANF1 | BRCA1 | BTRC | BUD13 | BUD31 | BYSL | C1QBP | C9orf64 | CARS | CASP3 | CBS | CCAR2 | CCDC155 | CCDC36 | CD2BP2 | CD40LG | CDA | CDC14B | CDC34 | CDC5L | CDC73 | CDK2 | CDK9 | CHAF1A | CHD1L | CHEK2 | CLPX | CMPK1 | COPS2 | COPS5 | COPS6 | CPSF7 | CRMP1 | CROT | CRY2 | CSNK1E | CSTF2T | CT45A5 | CTCF | CTPS1 | CUL4B | CWC15 | DARS | DBI | DCK | DCP2 | DCTPP1 | DCXR | DDB1 | DDB2 | DDX1 | DDX39A | DDX39B | DDX3X | DERA | DGUOK | DHX15 | DHX36 | DHX9 | DIS3 | DLG2 | DLST | DMAP1 | DNA2 | DNASE2 | DNASE2B | DROSHA | DUS3L | DUT | ECD | EDC4 | EED | EFTUD2 | EHMT1 | EHMT2 | EIF4A3 | ELAC2 | ELAVL1 | ELAVL4 | ENDOG | ENTPD7 | EP300 | EPRS | ERCC5 | EXOSC1 | EXOSC5 | EXOSC6 | EXOSC9 | EXTL2 | EYA2 | EYA4 | EZH2 | FANCA | FANCC | FANCG | FANCI | FANCM | FAR2 | FARSB | FASN | FBXO6 | FEN1 | FIGNL1 | FOS | FOXM1 | FPGS | FTO | FTSJ3 | FUS | FZR1 | G3BP1 | G6PD | GAR1 | GARS | GDA | GEMIN4 | GEMIN5 | GEMIN7 | GFPT1 | GFPT2 | GINS2 | GINS3 | GINS4 | GLRX | GMDS | GMPR | GMPS | GNAI3 | GNAS | GPD1L | GPX1 | GSR | GTF2F1 | GTF2F2 | H1F0 | H2AFX | HARS | HELZ2 | HERC2 | HIF1A | HINT1 | HIST1H4A | HIST3H3 | HMGB1 | HMGB2 | HMGB3 | HMGCL | HNRNPA1 | HNRNPA1L2 | HNRNPA2B1 | HNRNPD | HNRNPF | HNRNPH1 | HNRNPH2 | HNRNPH3 | HNRNPK | HNRNPL | HNRNPM | HNRNPU | HPRT1 | HSD17B10 | HSD17B4 | HSF1 | HSP90AA1 | HSP90AB1 | HSPA1B | HSPA8 | HSPD1 | HUWE1 | IARS | IDH1 | IDH2 | IK | IMP4 | IMPDH2 | INO80B | INO80C | ISG15 | ITPA | JMJD6 | KAT5 | KCNAB2 | KDM1A | KDM2A | KDM4D | KHDRBS1 | KIAA0391 | KPNA1 | KPNA2 | KPNB1 | LACTB2 | LARP7 | LARS | LCMT2 | LDHA | LEF1 | LGALS3 | LIG4 | LMO2 | LRRK2 | LSM1 | LSM12 | LSM3 | LSM4 | LSM5 | LSM8 | MAPK1 | MAPT | MC1R | MCM2 | MCM3 | MCM4 | MCM5 | MCM6 | MCM7 | MCM9 | MDC1 | MDH1 | MDH2 | MDN1 | MECP2 | MED1 | MEPCE | MGMT | MLH1 | MORF4L1 | MORF4L2 | MOV10 | MPG | MPHOSPH6 | MRM1 | MSH6 | MTA1 | MTAP | MTRR | MVD | MYH6 | NCBP1 | NDUFS1 | NIPBL | NME1 | NME2 | NME4 | NMNAT1 | NMNAT3 | NMRK2 | NNT | NOB1 | NOP10 | NOP56 | NPLOC4 | NPM1 | NSMCE4A | NSUN2 | NSUN6 | NT5C2 | NUDT13 | NUDT18 | NUDT3 | NUDT9 | NUFIP1 | OAS2 | OGDH | OLA1 | OPA1 | OTUB1 | PA2G4 | PABPC1 | PABPC3 | PABPC4 | PALB2 | PAN2 | PARK7 | PARP1 | PAXIP1 | PCBP1 | PCBP2 | PCF11 | PCGF5 | PCGF6 | PCNA | PDE12 | PDE4D | PICK1 | PIH1D1 | PINX1 | PNKP | PNP | POLA2 | POLD1 | POLDIP3 | POLE2 | POLE3 | POLM | POLR2A | POLR2E | POLR2L | POT1 | POU5F1 | PPA1 | PPA2 | PPARD | PPARGC1A | PPAT | PPIE | PPIL3 | PPM1D | PPP1R8 | PPP2CA | PPP2R1A | PPP4R2 | PPP5C | PRCC | PRKACA | PRKDC | PRMT6 | PRPF19 | PRPF40A | PRPF8 | PRTFDC1 | PSMD14 | PTGES3 | PTTG1 | PUM1 | PUS1 | PUS7 | QARS | RAD21 | RAD23A | RAD23B | RAD51 | RAD51AP1 | RAN | RARS2 | RBBP6 | RBM17 | RBM25 | RBM3 | RBM4 | RBM8A | RBMX | RBX1 | REXO2 | RFC1 | RFC2 | RFC3 | RFC4 | RFC5 | RHOA | RIF1 | RIOK1 | RNASE3 | RNASEH2A | RNASEH2B | RNF8 | RPA2 | RPA3 | RPAIN | RPL10A | RPL7 | RPL7A | RPP14 | RPS14 | RPS19 | RPS2 | RPS24 | RPS3 | RPS8 | RRM1 | RRM2 | RRM2B | RRP7A | RRS1 | RTCB | RTFDC1 | RUVBL1 | RUVBL2 | SAP18 | SARS | SART1 | SART3 | SBDS | SCAF1 | SCO2 | SENP3 | SEPHS1 | SEPHS2 | SF1 | SF3B6 | SFPQ | SIRT1 | SIRT6 | SMAD2 | SMAD3 | SMARCB1 | SMC1A | SMC3 | SMG5 | SMG6 | SND1 | SNRNP27 | SNRPA | SNRPA1 | SNRPB | SNRPB2 | SNRPD1 | SNRPD3 | SNRPE | SNW1 | SP100 | SPRTN | SRPK1 | SRPK2 | SRSF11 | SRSF12 | SRSF5 | SSB | STAT3 | STUB1 | SUCLA2 | SUCLG2 | SULT1A3 | SUMO1 | SUPT5H | SWAP70 | TAOK3 | TARDBP | TARS | TARS2 | TATDN1 | TBRG4 | TCEA1 | TDP1 | TERF1 | TERF2 | TERF2IP | TFCP2 | TFIP11 | THADA | THEM4 | THOC2 | THOC6 | THOC7 | TINF2 | TK1 | TK2 | TMEM189-UBE2V1 | TNP1 | TOE1 | TOP1 | TOP2A | TOP3A | TP53 | TP53RK | TP73 | TPST2 | TRA2A | TREX1 | TRIM25 | TRIM28 | TRIP12 | TRIP13 | TRMT44 | TRMT6 | TRMT61B | TRNT1 | TRUB2 | TSN | TTC5 | TXNL4A | TXNRD1 | TYMS | U2AF2 | UBB | UBC | UBE2A | UBE2B | UBE2D3 | UBE2L6 | UBE2N | UBE2V1 | UBE2W | UBR5 | UCHL5 | UCK2 | UGGT1 | UGP2 | UMPS | UPF3B | UPP2 | USP14 | USP28 | USP39 | USP47 | USP7 | UTP14A | UTP6 | VARS | VCP | WARS | WDR12 | WDR4 | WDR48 | WT1 | WTAP | XPO5 | XRCC1 | XRCC3 | XRCC5 | XRCC6 | YBEY | YY1 | ZBTB7A | ZFYVE26 | ZMPSTE24 | ZNF638 | ZRANB2 | GO:0051240 | 0.114278781996846 | 942/8243 | 407/3250 | 0.00662167156079199 | 1 | T | F | F | F | positive regulation of multicellular organismal process | ABAT | ABL1 | ABL2 | ACE2 | ACSL1 | ACTR2 | ADA | ADCYAP1 | ADORA2A | ADRB2 | AGER | AGO2 | AKT1 | AKT3 | ALOX12 | ANXA1 | ANXA2 | AP3D1 | APBB1 | APC | APOE | APP | AR | ARF1 | ARHGAP35 | ARHGDIA | ARNT | ARRB2 | ASCL1 | ATAD5 | ATF2 | ATG5 | ATOH1 | ATP1A1 | ATP2A2 | AVIL | AZU1 | BASP1 | BAX | BCAR1 | BCL10 | BCL2 | BCL6 | BIN1 | BMPR1A | BRAF | BRCA1 | BTK | C1QA | C1QBP | C1QTNF1 | CALR | CAMK1D | CAMK2B | CARD9 | CASP8 | CAV1 | CBFB | CCNB1 | CCR1 | CCR3 | CD2 | CD27 | CD36 | CD40 | CD40LG | CD6 | CD74 | CDC20 | CDH5 | CDK9 | CDKL3 | CEBPA | CHRM3 | CLIC1 | CLSTN1 | CLU | CPT2 | CREB1 | CREBBP | CRKL | CSNK1E | CTH | CTNNB1 | CTNNBIP1 | CTTN | CUL7 | CXCL8 | CYLD | DAB1 | DAB2 | DBH | DBN1 | DDX1 | DDX39B | DDX3X | DECR1 | DHX36 | DHX9 | DISC1 | DLG4 | DNM1L | DOCK5 | DVL1 | DVL2 | DVL3 | DYNC1H1 | E2F1 | EGFR | EHMT1 | EIF2AK3 | ENO1 | EP300 | EPHA4 | ERBB3 | ESRRB | ESRRG | ETS1 | EXOSC6 | EZH2 | EZR | FABP5 | FADD | FAM129B | FAM49B | FCN1 | FGB | FGF2 | FGFR1 | FH | FKBP1B | FLOT1 | FN1 | FOS | FOXA1 | FOXJ1 | FYN | GADD45G | GAPDH | GATA1 | GBP5 | GDI1 | GFAP | GJA1 | GLMN | GNAI2 | GNAS | GOLGA4 | GPI | GRID2 | GRIP1 | GSK3A | GSTO1 | HADH | HAP1 | HAVCR2 | HAX1 | HBEGF | HDAC1 | HDAC2 | HDAC3 | HDAC4 | HDAC6 | HDAC7 | HDAC9 | HIF1A | HIF1AN | HK1 | HK2 | HLX | HMGB1 | HMGB2 | HMOX1 | HOXA5 | HRAS | HSF1 | HSPA1B | HSPA5 | HSPB1 | HSPD1 | ICAM1 | ID2 | IFI16 | IGF1R | ILK | IQGAP1 | IRF1 | IRF4 | IRF7 | ISG15 | ITCH | ITGB1BP1 | ITGB3 | JAK1 | JUN | JUP | KDM1A | KDM5B | KDR | LEF1 | LGALS1 | LGALS3 | LGMN | LGR4 | LIG4 | LOXL2 | LPAR1 | LRRC7 | LRRK2 | LUM | LURAP1 | MAP1B | MAP2K1 | MAP2K3 | MAP3K13 | MAP3K3 | MAPK1 | MAPK14 | MAPK3 | MAPK6 | MAPT | MAVS | MBP | MECP2 | MED1 | METRN | MIF | MLH1 | MME | MSH6 | MTPN | MYC | MYD88 | NAP1L1 | NCK1 | NCOA1 | NCOA3 | NELL1 | NEUROD1 | NFE2L2 | NFKB2 | NIN | NIPBL | NME1 | NME2 | NOD2 | NOS1AP | NOS3 | NPR1 | NR1H2 | NRP2 | NRXN1 | NTRK1 | NUMA1 | OBSL1 | OGT | OPA1 | ORM1 | P2RX4 | PAFAH1B1 | PANX3 | PARD3 | PARK7 | PARP1 | PARP6 | PAX8 | PAXIP1 | PDCD6 | PDE4D | PELI1 | PER2 | PFN1 | PIK3C2A | PIK3R1 | PIM1 | PIN1 | PKM | PLCG1 | PLXNA1 | PLXNA2 | PLXNB2 | PNP | POLR2E | POLR2L | PPARG | PPARGC1A | PPIB | PPP1CC | PRDX2 | PRKAA1 | PRKAB2 | PRKCE | PRKCZ | PRKD1 | PRKD2 | PRKDC | PROX1 | PRPF19 | PSEN1 | PTEN | PTGER3 | PTK2 | PTN | PTPN22 | PTPRD | PUM1 | RAB11A | RAB21 | RAP1A | RARA | RASSF10 | RB1 | RBPJ | RELA | REST | RGS2 | RHOA | RHOJ | RIF1 | RIPK1 | RIPK2 | RPS19 | RPS3 | RTN4 | RUNX1 | RUNX2 | S100A1 | S100A9 | S100B | SART1 | SDCBP | SEMA7A | SERPINB3 | SERPINE2 | SH3GL3 | SIRT1 | SIRT6 | SLC9A1 | SMAD2 | SMAD3 | SMAD4 | SMAD5 | SMO | SMURF1 | SMURF2 | SNW1 | SNX3 | SOCS1 | SOCS2 | SOD1 | SOD2 | SP1 | SPEN | SPTBN1 | SRC | STAT1 | STAT3 | STAT5A | STAT6 | STIM1 | STK11 | STK25 | SULF2 | SYDE1 | TBC1D23 | TBXA2R | TCF12 | TCF4 | TGFB1 | TGFBR3 | THBS1 | THBS2 | THRA | TIMP2 | TLR3 | TMEM173 | TMEM30A | TNF | TNFAIP3 | TNFRSF12A | TNFRSF8 | TNFSF12 | TP63 | TP73 | TPM1 | TRADD | TRAF2 | TRAF6 | TRIM32 | TUBB2B | TWF1 | TWF2 | TWIST1 | USP17L2 | USP50 | VASH2 | VDR | WNT2 | WT1 | XBP1 | XRCC5 | XRCC6 | YAP1 | ZAP70 | ZBTB16 | ZBTB7B | ZMYND8 | GO:0030071 | 0.004852602207934 | 40/8243 | 24/3250 | 0.00665618992387699 | 1 | T | F | F | F | regulation of mitotic metaphase/anaphase transition | ANAPC4 | APC | ATM | AURKB | CCNB1 | CDC20 | CENPF | CUL3 | DUSP1 | DYNC1LI1 | HECW2 | IK | KLHL22 | MAD1L1 | MAD2L1 | PLK1 | RAD21 | RB1 | SPDL1 | TPR | TRIP13 | XRCC3 | ZNF207 | ZW10 | GO:0051169 | 0.0249909013708601 | 206/8243 | 99/3250 | 0.00665841330836578 | 1 | T | F | F | F | nuclear transport | ABCE1 | AHCYL1 | AKT1 | ANP32A | ATXN1 | BACH2 | BANF1 | CALR | CBLB | CCHCR1 | CDK5 | CHP1 | CSE1L | DDX19B | DDX39A | DDX39B | DUSP16 | EIF4A3 | EIF4E | EIF5A | EIF6 | FYTTD1 | GEMIN4 | GEMIN5 | GEMIN7 | HEATR3 | HNRNPA1 | HNRNPA2B1 | HTATIP2 | IPO11 | IPO4 | IPO5 | IPO7 | IPO8 | IPO9 | KPNA1 | KPNA2 | KPNB1 | LMNA | MCM3AP | NCBP1 | NEUROD1 | NMD3 | NPM1 | NUP107 | NUP153 | NUP160 | NUP205 | NUP214 | NUP85 | NUP88 | NUP93 | NUP98 | NUTF2 | NXF1 | NXF2 | NXT2 | PHB2 | PIK3R1 | PML | POLDIP3 | POM121 | PRKAG1 | RAE1 | RAN | RANBP3 | RBM8A | RPAIN | RPL23 | RRS1 | SEC13 | SMG5 | SMG6 | SMN1 | SMURF1 | SNRPB | SNRPD1 | SNRPD3 | SNRPE | SRSF11 | SRSF5 | SSB | STAT3 | STYX | TGFB1 | THOC2 | THOC6 | THOC7 | TNPO1 | TPR | TXNIP | U2AF2 | UPF2 | UPF3B | XPO1 | XPO4 | XPO5 | XPO7 | XPOT | GO:1902750 | 0.00946257430547131 | 78/8243 | 42/3250 | 0.00667773035289591 | 1 | T | F | F | F | negative regulation of cell cycle G2/M phase transition | ATM | AURKB | BABAM1 | BRCA1 | CDC14B | CUL1 | FHL1 | FZR1 | MSH6 | NAE1 | PINX1 | PLK1 | PSMA1 | PSMA2 | PSMA3 | PSMA5 | PSMA6 | PSMB5 | PSMB6 | PSMB7 | PSMB8 | PSMC1 | PSMC2 | PSMC3 | PSMC4 | PSMC5 | PSMC6 | PSMD1 | PSMD10 | PSMD11 | PSMD12 | PSMD14 | PSMD2 | PSMD4 | PSMD9 | PSME2 | PSME3 | RAD21 | RBX1 | TAOK3 | USP47 | ZFYVE19 | GO:0032508 | 0.00764284847749606 | 63/8243 | 35/3250 | 0.00673561738942376 | 1 | T | F | F | F | DNA duplex unwinding | ANXA1 | CHD1L | CHD3 | CHD4 | CHD8 | CUL4B | DDB1 | DDB2 | DDX1 | DDX3X | DHX36 | DHX9 | DNA2 | FANCM | G3BP1 | GINS2 | GINS4 | MCM2 | MCM4 | MCM6 | MCM7 | MCM9 | PARP1 | POT1 | RAD23B | RAD51 | RBX1 | RPA2 | RUVBL1 | RUVBL2 | TREX1 | UBB | UBC | XRCC5 | XRCC6 | GO:0002756 | 0.0033968215455538 | 28/8243 | 18/3250 | 0.00673676931016162 | 1 | T | F | F | F | MyD88-independent toll-like receptor signaling pathway | BIRC2 | BIRC3 | CASP8 | FADD | IKBKB | IKBKE | IKBKG | IRF7 | PRKCE | RIPK1 | TANK | TLR3 | TRAF3 | UBB | UBC | UBE2D1 | UBE2D2 | UBE2D3 | GO:0033144 | 0.0033968215455538 | 28/8243 | 18/3250 | 0.00673676931016162 | 1 | T | F | F | F | negative regulation of intracellular steroid hormone receptor signaling pathway | BRCA1 | CALR | CRY1 | CRY2 | DAB2 | HDAC1 | KANK2 | NCOR1 | NCOR2 | PER1 | PHB | PHB2 | RHOA | SIRT1 | SMARCA4 | TCF21 | TP63 | ZBTB7A | GO:0035967 | 0.0033968215455538 | 28/8243 | 18/3250 | 0.00673676931016162 | 1 | T | F | F | F | cellular response to topologically incorrect protein | BAG3 | BAX | DAXX | HDAC6 | HSF1 | HSPA14 | HSPA1B | HSPA1L | HSPA2 | HSPA5 | HSPA8 | HSPA9 | OPTN | PTPN1 | RHBDD1 | STUB1 | TMBIM6 | UBE2W | GO:0061448 | 0.0033968215455538 | 28/8243 | 18/3250 | 0.00673676931016162 | 1 | T | F | F | F | connective tissue development | ACAT1 | ANXA2 | ATF2 | CDK4 | COL5A1 | FTO | ID2 | NAMPT | OXCT1 | PAXIP1 | PIK3CA | PPARD | PPARGC1A | RARA | SPTLC2 | UBB | XBP1 | ZMPSTE24 | GO:0051443 | 0.0033968215455538 | 28/8243 | 18/3250 | 0.00673676931016162 | 1 | T | F | F | F | positive regulation of ubiquitin-protein transferase activity | AXIN1 | BMI1 | CDC14B | CDC20 | DCUN1D1 | FZR1 | GOLGA2 | PIN1 | PLK1 | PTEN | RAB1A | RPS2 | STUB1 | TRIB3 | UBE2C | UBE2L3 | UBE2N | UBE2S | GO:0048857 | 0.0046099720975373 | 38/8243 | 23/3250 | 0.00676071022268839 | 1 | T | F | F | F | neural nucleus development | ACTB | ASCL1 | BASP1 | BCL2 | CALM1 | CALM2 | CALM3 | CDC42 | CKB | DYNLL1 | G6PD | HSPA5 | MAPKAP1 | MBP | NDUFS3 | NKX2-1 | RHOA | S100A1 | SEC16A | SEC24B | YWHAE | YWHAH | YWHAQ | GO:2000379 | 0.0046099720975373 | 38/8243 | 23/3250 | 0.00676071022268839 | 1 | T | F | F | F | positive regulation of reactive oxygen species metabolic process | ACE2 | CDKN1A | CRP | EGFR | GNAI2 | GNAI3 | GRB2 | GSTP1 | MAPK14 | MAPT | NFE2L2 | NQO2 | PARK7 | PRKCD | RGN | RIPK1 | RIPK3 | ROMO1 | SOD1 | TGFB1 | THBS1 | TNF | TP53 | GO:0030834 | 0.0043673419871406 | 36/8243 | 22/3250 | 0.00683809757143621 | 1 | T | F | F | F | regulation of actin filament depolymerization | ACTN2 | ADD3 | AVIL | CAPG | CAPZA1 | CAPZA2 | CAPZB | CFL1 | CFL2 | DSTN | LIMA1 | MTPN | RDX | SPTAN1 | SPTBN1 | SPTBN2 | SWAP70 | TMOD3 | TMOD4 | TWF1 | TWF2 | WDR1 | GO:0045070 | 0.0036394516559505 | 30/8243 | 19/3250 | 0.00684047069422146 | 1 | T | F | F | F | positive regulation of viral genome replication | DDB1 | DDX3X | IFIT1 | NR5A2 | PABPC1 | PDE12 | PKN2 | PPIA | PPIB | PPID | PPIE | RAD23A | SRPK1 | SRPK2 | STAU1 | STOM | TOP2A | VAPA | VAPB | GO:0010596 | 0.0036394516559505 | 30/8243 | 19/3250 | 0.00684047069422146 | 1 | T | F | F | F | negative regulation of endothelial cell migration | APOE | CSNK2B | DCN | FGF2 | HDAC5 | HMGB1 | ITGB1BP1 | JUP | MECP2 | MEOX2 | PDCD10 | PPARG | RHOA | SP100 | STARD13 | SYNJ2BP | TBXA2R | TGFB1 | THBS1 | GO:0032890 | 0.0036394516559505 | 30/8243 | 19/3250 | 0.00684047069422146 | 1 | T | F | F | F | regulation of organic acid transport | ABAT | ACE2 | ADORA2A | AKT1 | AKT2 | FABP3 | MAP2K6 | MIF | NCOR1 | P2RX4 | PER2 | PLA2G10 | PLA2G6 | PSEN1 | RAB3GAP1 | RGS2 | SNCA | STXBP1 | THBS1 | GO:0006027 | 0.0036394516559505 | 30/8243 | 19/3250 | 0.00684047069422146 | 1 | T | F | F | F | glycosaminoglycan catabolic process | CHP1 | DCN | FGF2 | GALNS | GNS | GPC5 | GUSB | HEXA | HEXB | HMMR | HSPG2 | LUM | LYG2 | SDC1 | SDC4 | SGSH | SLC9A1 | TGFB1 | VCAN | GO:2000191 | 0.00157709571757855 | 13/8243 | 10/3250 | 0.00687312576333624 | 1 | T | F | F | F | regulation of fatty acid transport | AKT1 | AKT2 | FABP3 | MAP2K6 | MIF | NCOR1 | P2RX4 | PLA2G10 | PLA2G6 | THBS1 | GO:0071379 | 0.00157709571757855 | 13/8243 | 10/3250 | 0.00687312576333624 | 1 | T | F | F | F | cellular response to prostaglandin stimulus | ACACA | ADCY6 | AKAP8 | AKR1C2 | AKT1 | GNB1 | PPARG | PRKAA1 | PRKAA2 | PRKCE | GO:0097194 | 0.00157709571757855 | 13/8243 | 10/3250 | 0.00687312576333624 | 1 | T | F | F | F | execution phase of apoptosis | AKT1 | BBC3 | CASP10 | CASP2 | CASP3 | CASP7 | CASP8 | DNM1L | HTRA2 | STK24 | GO:0070266 | 0.00157709571757855 | 13/8243 | 10/3250 | 0.00687312576333624 | 1 | T | F | F | F | necroptotic process | BIRC2 | CYLD | DNM1L | FASLG | PGAM5 | PPIF | PYGL | RIPK1 | RIPK3 | TLR3 | GO:0090151 | 0.00157709571757855 | 13/8243 | 10/3250 | 0.00687312576333624 | 1 | T | F | F | F | establishment of protein localization to mitochondrial membrane | AGK | CALM1 | CALM3 | HSP90AA1 | HSPA4 | NDUFA13 | OXA1L | ROMO1 | TIMM13 | TOMM22 | GO:0030150 | 0.00157709571757855 | 13/8243 | 10/3250 | 0.00687312576333624 | 1 | T | F | F | F | protein import into mitochondrial matrix | DNAJC19 | GRPEL1 | PAM16 | ROMO1 | TIMM21 | TIMM23 | TIMM44 | TIMM50 | TOMM20 | TOMM40 | GO:1904591 | 0.0041247118767439 | 34/8243 | 21/3250 | 0.00688217517008753 | 1 | T | F | F | F | positive regulation of protein import | BAG3 | CDH1 | DMAP1 | HDAC3 | IPO5 | JUP | MAPK1 | MAPK14 | MAVS | PIK3R1 | PRKCD | PSEN1 | RAN | SMAD3 | SMO | TARDBP | TGFB1 | TPR | TRIM28 | UBR5 | ZIC1 | GO:0009205 | 0.0041247118767439 | 34/8243 | 21/3250 | 0.00688217517008753 | 1 | T | F | F | F | purine ribonucleoside triphosphate metabolic process | ADCYAP1 | AK1 | AK2 | AK4 | ATP6V1A | ATP6V1B1 | CLPX | FIGNL1 | GNAI3 | HSPA1B | HSPA8 | LRRK2 | MYH6 | NDUFS1 | NMNAT1 | OLA1 | OPA1 | PARP1 | RAN | RHOA | VCP | GO:0009199 | 0.0041247118767439 | 34/8243 | 21/3250 | 0.00688217517008753 | 1 | T | F | F | F | ribonucleoside triphosphate metabolic process | ADCYAP1 | AK1 | AK2 | AK4 | ATP6V1A | ATP6V1B1 | CLPX | FIGNL1 | GNAI3 | HSPA1B | HSPA8 | LRRK2 | MYH6 | NDUFS1 | NMNAT1 | OLA1 | OPA1 | PARP1 | RAN | RHOA | VCP | GO:0071398 | 0.0041247118767439 | 34/8243 | 21/3250 | 0.00688217517008753 | 1 | T | F | F | F | cellular response to fatty acid | ACACA | ADCY6 | AKAP8 | AKR1C2 | AKT1 | ASS1 | CCNB1 | CDK4 | CPT1A | CREB1 | E2F1 | GNB1 | NME1 | NME2 | PPARG | PPARGC1A | PRKAA1 | PRKAA2 | PRKCE | SRC | XRCC5 | GO:0060688 | 0.0038820817663472 | 32/8243 | 20/3250 | 0.00688574566849544 | 1 | T | F | F | F | regulation of morphogenesis of a branching structure | ABL1 | AR | BCL11A | CTNNB1 | ESR1 | FGFR1 | FKBPL | LGR4 | LRRK2 | MAP3K13 | PAX8 | PHB2 | RTN4 | RXRA | SIRT6 | SMO | SNAI2 | TGFB1 | TNF | WNT2 | GO:0090224 | 0.0038820817663472 | 32/8243 | 20/3250 | 0.00688574566849544 | 1 | T | F | F | F | regulation of spindle organization | CHMP2A | CLTC | DCTN1 | DYNC1H1 | FSD1 | GNAI1 | HNRNPU | HSPA1B | MAPK15 | NUMA1 | PDCD6IP | PLK1 | RAE1 | RNF4 | SENP6 | SMC1A | SMC3 | STAG2 | TPR | VPS4B | GO:0006026 | 0.0038820817663472 | 32/8243 | 20/3250 | 0.00688574566849544 | 1 | T | F | F | F | aminoglycan catabolic process | CHP1 | DCN | FGF2 | GALNS | GNS | GPC5 | GUSB | HEXA | HEXB | HMMR | HSPG2 | LUM | LYG2 | OVGP1 | SDC1 | SDC4 | SGSH | SLC9A1 | TGFB1 | VCAN | GO:0050866 | 0.012131505519835 | 100/8243 | 52/3250 | 0.0069450572973727 | 1 | T | F | F | F | negative regulation of cell activation | ABAT | ADORA2A | ALOX12 | ANXA1 | APOE | ATM | BCL6 | BTK | C1QTNF1 | CASP3 | CBFB | CD74 | DTX1 | DUSP22 | ERBB2 | FAM19A3 | FBXO7 | FOXJ1 | GLMN | HAVCR2 | HLX | HMGB1 | HMOX1 | ID2 | IFNA2 | IL13RA2 | IRF1 | ITCH | JAK3 | LGALS3 | MAD1L1 | MICA | NOS3 | NR1H3 | PAG1 | PAWR | PELI1 | PRDX2 | PRKAR1A | PRKCD | PTPN22 | PTPN6 | RC3H1 | RUNX1 | SDC4 | SERPINE2 | SOCS1 | SOCS6 | TGFB1 | TNFAIP3 | VSIG4 | ZBTB7B | GO:0022407 | 0.0283877229164139 | 234/8243 | 111/3250 | 0.00700709464724482 | 1 | T | F | F | F | regulation of cell-cell adhesion | ABAT | ABL1 | ADA | ADORA2A | ADTRP | AGER | AKT1 | ALOX12 | ANK3 | ANXA1 | AP3D1 | APOA1 | ASS1 | BCL10 | BCL6 | C1QTNF1 | CASP3 | CAV1 | CBFB | CCL21 | CD209 | CD27 | CD40LG | CD6 | CD74 | CDC42 | CDH1 | CYLD | DNAJA3 | DTX1 | DUSP22 | ERBB2 | ETS1 | FADD | FAM49B | FGB | FLOT1 | FOXA1 | FOXJ1 | FYN | GLMN | GRB2 | HAVCR2 | HLX | HMGB1 | HSPD1 | HSPH1 | ICAM1 | IFNA2 | IRF1 | ITCH | ITGA4 | JAK3 | LEF1 | LGALS1 | LGALS3 | MAD1L1 | MAPK14 | MAPK7 | MBP | NCK1 | NF2 | NOD2 | PAG1 | PAK2 | PAWR | PELI1 | PIK3CA | PIK3R1 | PNP | PRDX2 | PRKAR1A | PRKCD | PRKCZ | PTK2 | PTPN22 | PTPN23 | PTPN6 | PTPRR | RAC1 | RARA | RC3H1 | RDX | RELA | RHOA | RIPK2 | RPS3 | RUNX1 | SART1 | SDC4 | SERPINE2 | SNAI2 | SOCS1 | SOCS6 | SOX2 | SPINT2 | SRC | SWAP70 | TGFB1 | TNF | TNFSF14 | TRAF6 | VAV1 | VCAM1 | VSIG4 | WNK1 | XBP1 | YES1 | ZAP70 | ZBTB16 | ZBTB7B | GO:0045471 | 0.00921994419507461 | 76/8243 | 41/3250 | 0.00700795618602771 | 1 | T | F | F | F | response to ethanol | ABAT | ACTR2 | ADCYAP1 | BIRC2 | CASP8 | CAT | CBL | CD27 | CLDN18 | DMAP1 | EEF2 | EIF4EBP1 | FECH | FYN | G6PD | GOT2 | GSTP1 | HTR1B | HTR3A | ICAM1 | ITPR2 | MGMT | NQO1 | OXCT1 | PEBP1 | PRKAA1 | PRKCE | PSMD14 | PTEN | RARA | RGS2 | RHOA | RPL10A | SETDB1 | SOD1 | SPI1 | STAT3 | TBXA2R | TUFM | TYMS | VCAM1 | GO:0006606 | 0.00740021836709936 | 61/8243 | 34/3250 | 0.00702627554760077 | 1 | T | F | F | F | protein import into nucleus | AKT1 | CBLB | CSE1L | HEATR3 | IPO11 | IPO4 | IPO5 | IPO7 | IPO8 | IPO9 | KPNA1 | KPNA2 | KPNB1 | LMNA | NUP107 | NUP153 | NUP214 | NUP85 | NUP88 | NUP93 | NUP98 | NUTF2 | NXT2 | PHB2 | PIK3R1 | PML | POM121 | RAN | RPAIN | RPL23 | STAT3 | TNPO1 | TPR | TXNIP | GO:0040017 | 0.0385781875530753 | 318/8243 | 147/3250 | 0.0070303565082127 | 1 | T | F | F | F | positive regulation of locomotion | ABL1 | ACTN4 | AGER | AGO2 | AKT1 | AKT2 | AKT3 | ALOX12 | ANXA1 | APC | APP | ARHGEF7 | ATM | AZU1 | BAG4 | BCAR1 | BCL2 | C1QBP | CALR | CAMK1D | CAV1 | CCL21 | CCR1 | CCR4 | CD40 | CD74 | CHMP2A | COL18A1 | CPNE3 | CREB3 | CRK | CRKL | CXCL8 | DAB2 | DDB1 | DDRGK1 | DOCK5 | DOCK7 | EGFR | ETS1 | FADD | FAM83H | FGF2 | FGFR1 | FGFR1OP | FN1 | GNAI2 | GPI | GRB7 | HBEGF | HDAC4 | HDAC6 | HDAC7 | HDAC9 | HIF1A | HMGB1 | HMOX1 | HRAS | HSPA5 | HSPB1 | ICAM1 | IGF1R | ILK | INSR | IQGAP1 | ITGA4 | ITGAV | ITGB1BP1 | ITGB3 | JUN | KDR | LAMB1 | LEF1 | LGALS3 | LGMN | LPAR1 | MAP2K3 | MAP3K3 | MAPK1 | MAPK14 | MAPK3 | MDM2 | MYO1C | MYOC | NFE2L2 | NIPBL | NOS3 | NRP2 | NTF3 | ONECUT1 | OXSR1 | P2RX4 | PC | PDCD10 | PDCD6 | PFN1 | PIK3C2A | PIK3R1 | PIK3R3 | PLCG1 | PRKCE | PRKD1 | PRKD2 | PROX1 | PTK2 | RAB11A | RAC1 | RDX | RHOA | RHOJ | RTN4 | S100A7 | SCG2 | SDCBP | SEMA7A | SERPINB3 | SIRT1 | SMAD3 | SMC3 | SMO | SMURF2 | SNAI2 | SOD2 | SP1 | SPRY2 | SRC | STAT3 | STAT5A | SUN2 | SWAP70 | SYDE1 | SYNE2 | TGFB1 | THBS1 | TNF | TNFSF14 | TRADD | TRIM32 | TRIP6 | TSG101 | TUBB2B | TWIST1 | VAPA | VPS4B | VTN | WNK1 | XBP1 | GO:0010632 | 0.0178333131141575 | 147/8243 | 73/3250 | 0.00705791432734052 | 1 | T | F | F | F | regulation of epithelial cell migration | ABL1 | AKT1 | AKT3 | ALOX12 | ANXA1 | APOE | BCAR1 | CALR | CD40 | CSNK2B | DCN | DOCK5 | EPPK1 | ETS1 | FGF2 | FGFR1 | GLUL | GPI | HBEGF | HDAC5 | HDAC6 | HDAC7 | HDAC9 | HIF1A | HMGB1 | HMOX1 | HSPB1 | ITGB1BP1 | ITGB3 | JUN | JUP | KDR | LGMN | MAP2K3 | MAP3K3 | MAPK14 | MCC | MECP2 | MEOX2 | NFE2L2 | NOS3 | NRP2 | P2RX4 | PDCD10 | PDCD6 | PFN1 | PIK3C2A | PLCG1 | PPARG | PRCP | PRKCE | PRKD1 | PRKD2 | PROX1 | PTEN | PTK2 | PTPN23 | PTPRR | RAB11A | RHOA | RHOJ | RTN4 | SIRT1 | SP1 | SP100 | SRC | STARD13 | STAT5A | STRAP | SYNJ2BP | TBXA2R | TGFB1 | THBS1 | GO:0071241 | 0.0156496421205872 | 129/8243 | 65/3250 | 0.00707456357099432 | 1 | T | F | F | F | cellular response to inorganic substance | AIFM1 | AKT1 | ALOX5AP | ANK3 | ANXA5 | APP | ATP13A2 | ATRX | BACE1 | BRAF | CALR | CCNB1 | CDH1 | CDK2 | CEBPA | CLIC4 | CPNE3 | CREB1 | CRK | CYP1A1 | CYP1A2 | DAXX | DLG2 | DLG4 | DPEP1 | EGFR | EIF2AK3 | EIF2S1 | EIF4A3 | ENDOG | FBP1 | FOS | FOXO1 | FUS | HMOX1 | HNRNPD | HSF1 | HSPA5 | ID2 | IQGAP1 | IREB2 | JUN | JUNB | LGMN | LIG4 | LRRK2 | MAPK1 | MAPK3 | MAPK8 | MAPK9 | NQO1 | P2RX4 | PARP1 | PPARGC1A | PPIF | PRKAA1 | PRKAA2 | SLC25A24 | SNCA | SOD1 | SUMO1 | TRAF2 | TREX1 | UBE2D3 | WNK1 | GO:0006874 | 0.0255974766468519 | 211/8243 | 101/3250 | 0.00712491662434192 | 1 | T | F | F | F | cellular calcium ion homeostasis | ABL1 | ABL2 | ACKR2 | ADCYAP1 | AFG3L2 | ANXA2 | ANXA6 | ANXA7 | APOE | APP | ATP13A2 | ATP2A2 | ATP2B2 | BAX | BCL2 | BDKRB2 | C1QTNF1 | CACNA1A | CALM1 | CALM2 | CALM3 | CALR | CAV1 | CCL21 | CCR1 | CCR3 | CCR4 | CCR5 | CD36 | CD40 | CDK5 | CHRNA9 | CXCL11 | DLG4 | ESR1 | FASLG | FGF2 | FIS1 | FKBP1B | GLP1R | GNA13 | GNA15 | GNB1 | GOT1 | GRIA1 | GRIK2 | GSTO1 | GTF2I | HAP1 | HEXB | HMGB1 | HTR1B | HTT | IBTK | IMMT | ITGAV | ITPR1 | ITPR2 | ITPR3 | KCTD17 | LETM1 | LPAR1 | LPAR2 | LPAR4 | LRP6 | MCOLN3 | MICU1 | MICU2 | MYO5A | NMB | P2RX1 | P2RX2 | P2RX4 | PIK3CG | PLA2G1B | PLA2G6 | PLCG1 | PML | PRKACA | PRKCE | PSEN1 | PTGER3 | PTGIR | RAP1GDS1 | RGN | RIC3 | SMAD3 | SNCA | SRI | STIM1 | STOML2 | SWAP70 | TBXA2R | TGFB1 | TGM2 | THADA | TMBIM6 | TPCN2 | VAPB | VDR | YWHAE | GO:0034504 | 0.0118888754094383 | 98/8243 | 51/3250 | 0.00731772344191364 | 1 | T | F | F | F | protein localization to nucleus | AKT1 | BANP | CALR | CBLB | CHCHD10 | CSE1L | DVL1 | GBP2 | HEATR3 | IPO11 | IPO4 | IPO5 | IPO7 | IPO8 | IPO9 | KPNA1 | KPNA2 | KPNB1 | LMNA | MDM2 | NUP107 | NUP153 | NUP214 | NUP85 | NUP88 | NUP93 | NUP98 | NUTF2 | NXT2 | PHB2 | PIK3R1 | PIKFYVE | PINX1 | PLK1 | PML | POM121 | RAN | RPAIN | RPL23 | RRS1 | SMO | STAT3 | SUMO1 | TNPO1 | TOR1A | TPR | TXNIP | XPO1 | YWHAE | ZBTB16 | ZBTB7A | GO:0009410 | 0.0118888754094383 | 98/8243 | 51/3250 | 0.00731772344191364 | 1 | T | F | F | F | response to xenobiotic stimulus | ADA | ADORA2A | ASS1 | CACNG4 | CALM1 | CALM3 | CASP9 | CHEK2 | DBH | DDIT4 | E2F1 | EFTUD2 | EGFR | EIF4E | EIF4EBP1 | ERRFI1 | FADD | FBXO32 | FECH | FOXO1 | GNAO1 | GOT2 | GPI | GPX1 | GSS | HDAC2 | HDAC5 | HDAC9 | HPRT1 | HSF1 | HSPA5 | HTR1B | HTR3A | ICAM1 | MCM7 | MDM2 | NR3C1 | PCNA | PPARG | PPARGC1A | PPP2R2A | PRKAA1 | PRKAR2B | PRKCE | RB1 | RELA | RGS2 | SOD1 | TDO2 | TGFB1 | USP8 | GO:1905897 | 0.00715758825670266 | 59/8243 | 33/3250 | 0.00732431384800417 | 1 | T | F | F | F | regulation of response to endoplasmic reticulum stress | AGR2 | ATF6 | ATF6B | BAG6 | BAX | BCL2L1 | CAV1 | CLU | COPS5 | CREB3 | EDEM1 | EIF2AK3 | HSPA5 | LRRK2 | NCK1 | NFE2L2 | OPA1 | PARK7 | PIK3R1 | PTPN1 | RNF183 | RNF185 | SGTA | SIRT1 | SYVN1 | TMBIM6 | UBE2J1 | UBQLN1 | UBQLN2 | UBXN1 | USP14 | USP19 | XBP1 | GO:0038094 | 0.00897731408467791 | 74/8243 | 40/3250 | 0.00735288769007436 | 1 | T | F | F | F | Fc-gamma receptor signaling pathway | ABL1 | ACTB | ACTG1 | ACTR2 | ACTR3 | ARPC1A | ARPC1B | ARPC2 | ARPC3 | ARPC4 | ARPC5 | BAIAP2 | CDC42 | CRK | FCGR1A | FYN | GRB2 | HSP90AA1 | HSP90AB1 | IGHV3-13 | IGHV3-23 | IGHV3-30 | IGKV3-20 | IGLC7 | MAPK1 | MAPK3 | MYO1C | NCK1 | PIK3CA | PIK3R1 | PLA2G6 | PLCG1 | PRKCD | PRKCE | PTK2 | RAC1 | SRC | VAV1 | WAS | YES1 | GO:0040012 | 0.0666019653038942 | 549/8243 | 244/3250 | 0.0074938536817588 | 1 | T | F | F | F | regulation of locomotion | ABL1 | ABL2 | ACTN4 | ADA | ADORA2A | ADTRP | AGER | AGO2 | AIRE | AKT1 | AKT2 | AKT3 | ALOX12 | ANXA1 | ANXA5 | APC | APEX1 | APOD | APOE | APP | ARHGEF7 | ATM | AZU1 | BAG4 | BCAR1 | BCL2 | BMPR1A | BRAF | C1QBP | CALR | CAMK1D | CAMK2A | CAMK2B | CAV1 | CCL21 | CCR1 | CCR4 | CD40 | CD74 | CD81 | CDH1 | CDK5 | CHMP2A | CHRD | CLIC4 | COL18A1 | CPNE3 | CREB3 | CRK | CRKL | CSNK2B | CXCL8 | DAB2 | DCN | DDB1 | DDRGK1 | DOCK5 | DOCK7 | DPEP1 | DUSP1 | DUSP22 | EGFR | EPPIN | EPPK1 | ERBB2 | ERBB3 | ETS1 | FADD | FAM83H | FBXO7 | FGF2 | FGFR1 | FGFR1OP | FN1 | GLUL | GNA12 | GNA13 | GNAI2 | GPI | GRB7 | GSTP1 | HBEGF | HDAC4 | HDAC5 | HDAC6 | HDAC7 | HDAC9 | HIF1A | HMGB1 | HMOX1 | HRAS | HSPA5 | HSPB1 | ICAM1 | IDH2 | IGF1R | ILK | INSR | IQGAP1 | ITGA4 | ITGAV | ITGB1BP1 | ITGB3 | JUN | JUP | KDR | LAMB1 | LDLRAD4 | LEF1 | LGALS3 | LGMN | LMNA | LPAR1 | LRRK2 | MAP2K3 | MAP3K3 | MAPK1 | MAPK14 | MAPK15 | MAPK3 | MCC | MDM2 | MECP2 | MEOX2 | MIF | MITF | MSN | MYO1C | MYOC | NCK1 | NF2 | NFE2L2 | NIPBL | NKX2-1 | NOS3 | NRP2 | NTF3 | ONECUT1 | OXSR1 | P2RX4 | PC | PDCD10 | PDCD6 | PFN1 | PIK3C2A | PIK3R1 | PIK3R3 | PIN1 | PKN2 | PLCG1 | PLXNA1 | PLXNA2 | PLXNB2 | PML | PPARD | PPARG | PPARGC1A | PRCP | PRKCE | PRKD1 | PRKD2 | PROX1 | PTEN | PTK2 | PTN | PTPN23 | PTPRK | PTPRO | PTPRR | RAB11A | RAC1 | RAF1 | RDX | RECK | RGN | RHOA | RHOJ | RIPK3 | RTN4 | S100A7 | SCG2 | SDC4 | SDCBP | SEMA7A | SEPT7 | SERPINB3 | SERPINE2 | SIRT1 | SLAMF8 | SLC9A3R1 | SMAD3 | SMC3 | SMO | SMURF2 | SNAI2 | SNCA | SOD2 | SP1 | SP100 | SPINT2 | SPRY2 | SRC | SRGAP2 | SRGAP3 | SSX2IP | STARD13 | STAT3 | STAT5A | STK24 | STK26 | STRAP | SUN2 | SWAP70 | SYDE1 | SYNE2 | SYNJ2BP | TBXA2R | TGFB1 | THBS1 | TIMP1 | TNF | TNFSF14 | TNN | TPM1 | TRADD | TRIM21 | TRIM25 | TRIM28 | TRIM32 | TRIM35 | TRIP6 | TSG101 | TUBB2B | TWIST1 | USP14 | USP17L2 | VAPA | VCL | VPS4B | VTN | WAS | WNK1 | XBP1 | ZMYND8 | GO:0002695 | 0.0103117796918598 | 85/8243 | 45/3250 | 0.00764985736260259 | 1 | T | F | F | F | negative regulation of leukocyte activation | ADORA2A | ANXA1 | ATM | BCL6 | BTK | CASP3 | CBFB | CD74 | DTX1 | DUSP22 | ERBB2 | FAM19A3 | FBXO7 | FOXJ1 | GLMN | HAVCR2 | HLX | HMGB1 | HMOX1 | ID2 | IFNA2 | IL13RA2 | IRF1 | ITCH | JAK3 | LGALS3 | MAD1L1 | MICA | NR1H3 | PAG1 | PAWR | PELI1 | PRDX2 | PRKAR1A | PTPN22 | PTPN6 | RC3H1 | RUNX1 | SDC4 | SOCS1 | SOCS6 | TGFB1 | TNFAIP3 | VSIG4 | ZBTB7B | GO:1901532 | 0.00873468397428121 | 72/8243 | 39/3250 | 0.00771292765280553 | 1 | T | F | F | F | regulation of hematopoietic progenitor cell differentiation | ABL1 | CBFB | DHX36 | DPF2 | GATA1 | ITCH | KMT2A | LMO2 | NFE2L2 | PSMA1 | PSMA2 | PSMA3 | PSMA5 | PSMA6 | PSMB5 | PSMB6 | PSMB7 | PSMB8 | PSMC1 | PSMC2 | PSMC3 | PSMC4 | PSMC5 | PSMC6 | PSMD1 | PSMD10 | PSMD11 | PSMD12 | PSMD14 | PSMD2 | PSMD4 | PSMD9 | PSME2 | PSME3 | PUS7 | RUNX1 | TCF12 | TP73 | YAP1 | GO:0097191 | 0.00873468397428121 | 72/8243 | 39/3250 | 0.00771292765280553 | 1 | T | F | F | F | extrinsic apoptotic signaling pathway | BAG3 | BAX | BCL2 | BCL2L1 | BCL2L2 | CASP2 | CASP3 | CASP8 | CASP9 | CD27 | DAPK1 | DAXX | DDX3X | DEDD | DIABLO | ERBB3 | EYA2 | FADD | FASLG | FGFR3 | GSK3A | GSK3B | ITGAV | MKNK2 | MLLT11 | NDUFA13 | PIK3R1 | PML | RIPK1 | SMAD3 | SORT1 | TGFB1 | TLR3 | TNF | TNFRSF12A | TNFRSF1A | TNFSF12 | TRADD | WWOX | GO:1901989 | 0.00873468397428121 | 72/8243 | 39/3250 | 0.00771292765280553 | 1 | T | F | F | F | positive regulation of cell cycle phase transition | AKT1 | ANAPC4 | ANXA1 | APEX1 | APP | ATAD5 | BRD4 | CCNB1 | CDC25A | CDC25B | CDC25C | CDC73 | CDK4 | CUL3 | CUL4B | CYP1A1 | DBF4B | DDRGK1 | DDX3X | EGFR | EZH2 | FAM83D | HSPA2 | MAPK15 | MDM2 | MECP2 | MEPCE | MTA3 | NPM1 | PHB2 | RAB11A | RB1 | RDX | RPTOR | SIN3A | TGFB1 | TMOD3 | UBE2C | VPS4B | GO:1901033 | 0.000970520441586801 | 8/8243 | 7/3250 | 0.00773609421255088 | 1 | T | F | F | F | positive regulation of response to reactive oxygen species | ENDOG | HDAC6 | PARK7 | PAWR | RGN | RIPK1 | TNF | GO:1990000 | 0.000970520441586801 | 8/8243 | 7/3250 | 0.00773609421255088 | 1 | T | F | F | F | amyloid fibril formation | APP | CD36 | FKBP1A | MAPT | MDM2 | RIPK1 | RIPK3 | GO:0150011 | 0.000970520441586801 | 8/8243 | 7/3250 | 0.00773609421255088 | 1 | T | F | F | F | regulation of neuron projection arborization | DLG4 | DVL1 | DVL2 | DVL3 | GRIP1 | MAP3K13 | MOV10 | GO:0009157 | 0.000970520441586801 | 8/8243 | 7/3250 | 0.00773609421255088 | 1 | T | F | F | F | deoxyribonucleoside monophosphate biosynthetic process | DCK | DGUOK | DUT | SHMT1 | TK1 | TK2 | TYMS | GO:0046655 | 0.000970520441586801 | 8/8243 | 7/3250 | 0.00773609421255088 | 1 | T | F | F | F | folic acid metabolic process | FOLR1 | FPGS | MTHFD1 | MTRR | SHMT1 | SHMT2 | SLC25A32 | GO:0038166 | 0.000970520441586801 | 8/8243 | 7/3250 | 0.00773609421255088 | 1 | T | F | F | F | angiotensin-activated signaling pathway | ACTN2 | AGTRAP | AHCYL1 | CAMK2A | CAV1 | MAS1 | SRC | GO:0050847 | 0.000970520441586801 | 8/8243 | 7/3250 | 0.00773609421255088 | 1 | T | F | F | F | progesterone receptor signaling pathway | NEDD4 | PHB | SRC | UBE3A | UBR5 | WBP2 | YAP1 | GO:0030388 | 0.000970520441586801 | 8/8243 | 7/3250 | 0.00773609421255088 | 1 | T | F | F | F | fructose 1,6-bisphosphate metabolic process | ALDOA | ALDOC | FBP1 | FBP2 | PFKL | PFKM | PFKP | GO:0010649 | 0.000970520441586801 | 8/8243 | 7/3250 | 0.00773609421255088 | 1 | T | F | F | F | regulation of cell communication by electrical coupling | ANK3 | CALM1 | CALM2 | CALM3 | CAV1 | PDE4D | SRI | GO:0006188 | 0.000970520441586801 | 8/8243 | 7/3250 | 0.00773609421255088 | 1 | T | F | F | F | IMP biosynthetic process | ADSL | ATIC | GART | HPRT1 | PAICS | PFAS | PPAT | GO:0007204 | 0.0151643818997938 | 125/8243 | 63/3250 | 0.00787498701451475 | 1 | T | F | F | F | positive regulation of cytosolic calcium ion concentration | ABL1 | ABL2 | ACKR2 | ADCYAP1 | BAX | BDKRB2 | C1QTNF1 | CACNA1A | CALM1 | CALM2 | CALM3 | CAV1 | CCL21 | CCR1 | CCR3 | CCR4 | CCR5 | CD36 | CDK5 | CHRNA9 | CXCL11 | DLG4 | ESR1 | FASLG | FGF2 | FIS1 | FKBP1B | GLP1R | GNA13 | GNA15 | GNB1 | GSTO1 | HAP1 | HMGB1 | HTT | IBTK | ITGAV | ITPR1 | ITPR2 | ITPR3 | LETM1 | LPAR1 | LPAR2 | LPAR4 | LRP6 | MCOLN3 | NMB | P2RX2 | P2RX4 | PIK3CG | PLA2G1B | PLA2G6 | PLCG1 | PRKCE | PTGER3 | PTGIR | RIC3 | SNCA | SRI | SWAP70 | TBXA2R | TGM2 | TPCN2 | GO:0043280 | 0.0100691495814631 | 83/8243 | 44/3250 | 0.00804793966710051 | 1 | T | F | F | F | positive regulation of cysteine-type endopeptidase activity involved in apoptotic process | AIFM1 | ALOX12 | ARRB1 | BAX | BBC3 | BCL10 | BCL2L13 | CASP10 | CASP8 | CASP9 | CYCS | DAPK1 | DDX3X | DIABLO | EGLN3 | EIF2AK3 | FADD | FASLG | FIS1 | HMGB1 | HSF1 | HSPD1 | HSPE1 | HTRA2 | MAPT | MYC | NDUFA13 | P2RX1 | PDCD6 | PML | PPARG | REST | RHOA | RIPK1 | RPS3 | S100A9 | SIRT1 | SMAD3 | SNCA | TNF | TRADD | TRAF2 | VCP | WDR35 | GO:0002221 | 0.0114036151886449 | 94/8243 | 49/3250 | 0.00812434139893718 | 1 | T | F | F | F | pattern recognition receptor signaling pathway | BCL10 | BIRC2 | BIRC3 | BTK | CASP8 | CD36 | CTSL | CYLD | FADD | FCN1 | FGB | HAVCR2 | HMGB1 | HSP90B1 | HSPD1 | IKBKB | IKBKE | IKBKG | IRF7 | ITCH | LGMN | MAP2K6 | MAP3K1 | MYD88 | NFKBIA | NOD2 | PRKCE | RELA | RIPK1 | RIPK2 | S100A1 | S100A9 | SCARA3 | TAB1 | TAB2 | TANK | TLR3 | TMEM173 | TNFAIP3 | TNIP2 | TRAF3 | TRAF6 | UBB | UBC | UBE2D1 | UBE2D2 | UBE2D3 | UBE2N | UBE2V1 | GO:0045930 | 0.0276598325852238 | 228/8243 | 108/3250 | 0.00815657639280866 | 1 | T | F | F | F | negative regulation of mitotic cell cycle | ABL1 | APC | ATF2 | ATM | ATRX | AURKB | BAX | BCL2 | BCL2L1 | BCL6 | BRCA1 | CARM1 | CASP2 | CCNB1 | CDC14B | CDC20 | CDC25C | CDC73 | CDK2 | CDK4 | CDKN1A | CDKN2C | CENPF | CHEK2 | CTNNB1 | CUL1 | DUSP1 | E2F1 | E2F4 | EGFR | EP300 | EZH2 | FBXO7 | FHL1 | HRAS | IK | KANK2 | KLHL22 | MAD1L1 | MAD2L1 | MDC1 | MDM2 | MDM4 | MSH6 | NAE1 | PCNA | PINX1 | PLK1 | PML | PRCC | PRKDC | PRMT2 | PSMA1 | PSMA2 | PSMA3 | PSMA5 | PSMA6 | PSMB5 | PSMB6 | PSMB7 | PSMB8 | PSMC1 | PSMC2 | PSMC3 | PSMC4 | PSMC5 | PSMC6 | PSMD1 | PSMD10 | PSMD11 | PSMD12 | PSMD14 | PSMD2 | PSMD4 | PSMD9 | PSME2 | PSME3 | PTEN | PTTG1 | RAD21 | RB1 | RBX1 | RIPK1 | RPA2 | RPL24 | SEPT7 | SFN | SLC9A3R1 | SMAD3 | SPDL1 | TAOK3 | TFDP1 | TGFB1 | TIMP2 | TNF | TNKS | TOP2A | TP53 | TPR | TRIAP1 | TRIM35 | TRIP13 | USP17L2 | USP47 | ZFYVE19 | ZNF207 | ZW10 | ZWINT | GO:0071902 | 0.0276598325852238 | 228/8243 | 108/3250 | 0.00815657639280866 | 1 | T | F | F | F | positive regulation of protein serine/threonine kinase activity | ACSL1 | ADRB2 | AGER | AKT1 | ARRB1 | BIRC7 | BRAF | CAB39 | CALM1 | CALM2 | CALM3 | CCNB1 | CCNT1 | CCNT2 | CCNY | CCNYL1 | CD40 | CD40LG | CD74 | CD81 | CDK5R1 | CKS1B | CKS2 | CRK | CRKL | DAXX | DBF4B | DBNL | DDX3X | DUSP19 | DUSP7 | DVL2 | DVL3 | EGFR | EPHA4 | ERBB2 | EZH2 | FGF2 | FGFR1 | GADD45G | HRAS | IKBKG | ILK | INSR | IQGAP1 | KRAS | LPAR1 | LRRK2 | MAP2K1 | MAP2K3 | MAP2K4 | MAP2K6 | MAP2K7 | MAP3K1 | MAP3K13 | MAP3K14 | MAP3K2 | MAP3K3 | MAP3K4 | MAP4K1 | MAPK1 | MAPK14 | MAPK3 | MAPK8IP3 | MIF | MLST8 | NOD2 | NTF3 | NTRK1 | PDCD10 | PDE6G | PIH1D1 | PIK3CG | PLA2G1B | PPP2CA | PRDX2 | PRKAA1 | PROX1 | PSEN1 | PSMD10 | PTPN1 | RAF1 | RHOA | RIPK1 | RIPK2 | RPS3 | RPTOR | S100A12 | SHC1 | SIRT1 | SNCA | SOD1 | SPRY2 | SRC | STK3 | TAB1 | TAB2 | TAOK3 | TGFB1 | THBS1 | TNF | TP73 | TRAF2 | TRAF6 | UBB | UBC | UBE2N | UBE2V1 | GO:0070198 | 0.00181972582797525 | 15/8243 | 11/3250 | 0.00818913992734303 | 1 | T | F | F | F | protein localization to chromosome, telomeric region | ACD | ATM | ATRX | PINX1 | POT1 | TERF2 | TERF2IP | TINF2 | TNKS | TNKS2 | XRCC5 | GO:0043174 | 0.00181972582797525 | 15/8243 | 11/3250 | 0.00818913992734303 | 1 | T | F | F | F | nucleoside salvage | ADK | APRT | CDA | DCK | HPRT1 | MTAP | PRTFDC1 | TK1 | TK2 | UCK2 | UPP2 | GO:0035067 | 0.00181972582797525 | 15/8243 | 11/3250 | 0.00818913992734303 | 1 | T | F | F | F | negative regulation of histone acetylation | ATG5 | BRCA1 | CTBP1 | MECP2 | PIH1D1 | SET | SIN3A | SIRT1 | SNCA | SPI1 | TWIST1 | GO:0051480 | 0.0181972582797525 | 150/8243 | 74/3250 | 0.00819263766869646 | 1 | T | F | F | F | regulation of cytosolic calcium ion concentration | ABL1 | ABL2 | ACKR2 | ADCYAP1 | ANXA2 | ATP2B2 | BAX | BDKRB2 | C1QTNF1 | CACNA1A | CALM1 | CALM2 | CALM3 | CAV1 | CCL21 | CCR1 | CCR3 | CCR4 | CCR5 | CD36 | CDK5 | CHRNA9 | CXCL11 | DLG4 | ESR1 | FASLG | FGF2 | FIS1 | FKBP1B | GLP1R | GNA13 | GNA15 | GNB1 | GOT1 | GRIA1 | GSTO1 | GTF2I | HAP1 | HMGB1 | HTR1B | HTT | IBTK | ITGAV | ITPR1 | ITPR2 | ITPR3 | LETM1 | LPAR1 | LPAR2 | LPAR4 | LRP6 | MCOLN3 | MYO5A | NMB | P2RX1 | P2RX2 | P2RX4 | PIK3CG | PLA2G1B | PLA2G6 | PLCG1 | PRKACA | PRKCE | PTGER3 | PTGIR | RIC3 | SMAD3 | SNCA | SRI | SWAP70 | TBXA2R | TGM2 | TPCN2 | YWHAE | GO:0051094 | 0.0936552226131263 | 772/8243 | 336/3250 | 0.00824301308513021 | 1 | T | F | F | F | positive regulation of developmental process | ABL1 | ABL2 | ACTR2 | ADA | ADCYAP1 | ADRB2 | AGER | AGO2 | AGR2 | AHI1 | AKT1 | AKT3 | ALOX12 | ANXA1 | ANXA2 | AP3D1 | APBB1 | APOA1 | APOE | APP | AR | ARF1 | ARHGAP35 | ARHGDIA | ARHGEF7 | ARNT | ARPC2 | ARRB2 | ASCL1 | ATAD5 | ATOH1 | AVIL | BASP1 | BAX | BCL2 | BCL6 | BIN1 | BMPR1A | BRAF | BRCA1 | BTK | C1QA | C1QBP | CALR | CAMK1D | CAMK2B | CAPN2 | CARM1 | CASP8 | CBFB | CCNB1 | CCR1 | CCR3 | CD27 | CD36 | CD40 | CD74 | CDC20 | CDC42 | CDH5 | CDKL3 | CEBPA | CLIC1 | CLSTN1 | CREB1 | CRK | CRKL | CSNK1E | CTH | CTNNB1 | CTNNBIP1 | CUL7 | CXCL8 | CYLD | DAB1 | DAB2 | DBN1 | DCN | DDX39B | DHX36 | DISC1 | DLG4 | DNM1L | DOCK5 | DVL1 | DVL2 | DVL3 | E2F1 | EP300 | EPHA4 | ERBB3 | ESRRB | ETS1 | EXOSC6 | EZH2 | EZR | FADD | FAM129B | FGB | FGF2 | FGFR1 | FIS1 | FKBP1B | FLOT1 | FN1 | FOS | FOXA1 | FOXJ1 | FYN | GATA1 | GDI1 | GFAP | GNAS | GOLGA4 | GRID2 | GRIP1 | HAP1 | HAX1 | HDAC1 | HDAC2 | HDAC7 | HDAC9 | HIF1A | HIF1AN | HK2 | HLX | HMGB1 | HMGB2 | HMOX1 | HNRNPU | HOXA5 | HSF1 | HSF4 | HSP90AB1 | HSPA1B | HSPA5 | HSPB1 | ID2 | ILK | INSR | IQGAP1 | ISG15 | ITCH | JAK1 | JUN | JUNB | JUND | JUP | KDM1A | KDM5B | KDR | KLF5 | KRAS | LEF1 | LGALS1 | LGALS3 | LGR4 | LIG4 | LIMS1 | LMNA | LOXL2 | LPAR1 | LRRC7 | MAP1B | MAP2K1 | MAP3K13 | MAP3K3 | MAPK1 | MAPK14 | MAPK6 | MAPK9 | MAPT | MARCH5 | MECP2 | MED1 | METRN | METRNL | MLH1 | MME | MORF4L2 | MSH6 | MYC | MYOC | NAP1L1 | NCK1 | NCOA1 | NCOA3 | NELL1 | NEUROD1 | NF2 | NFATC2 | NFE2L2 | NIN | NIPBL | NME1 | NME2 | NOS3 | NRXN1 | NTRK1 | NUMA1 | OBSL1 | OPA1 | P2RX4 | PA2G4 | PAFAH1B1 | PARD3 | PARP1 | PARP6 | PAWR | PAX8 | PAXIP1 | PDCD6 | PIAS1 | PIK3C2A | PIM1 | PIN1 | PKM | PLA2G10 | PLA2G2A | PLCG1 | PLXNA1 | PLXNA2 | PLXNB2 | PNP | PPARD | PPARG | PPARGC1A | PPIB | PPP1CC | PRKAA1 | PRKCZ | PRKD1 | PRKD2 | PRKDC | PROX1 | PRPF19 | PSEN1 | PTEN | PTN | PTPRD | RAB11A | RAB21 | RAC1 | RAP1A | RARA | RASSF10 | RB1 | RBM4 | RBPJ | RELA | REST | RGS2 | RHOA | RHOJ | RIF1 | RIPK1 | RIPK2 | RPS6KA1 | RTN4 | RUNX1 | RUNX2 | S100A1 | S100A9 | S100B | SART1 | SDCBP | SEMA7A | SERPINB3 | SERPINE2 | SFN | SH3GL3 | SIRT1 | SIRT6 | SMAD2 | SMAD3 | SMAD4 | SMAD5 | SMO | SMURF1 | SMURF2 | SNAI2 | SNW1 | SNX3 | SOCS1 | SOCS2 | SOD2 | SOX2 | SP1 | SPEN | SRC | STAT1 | STAT3 | STAT6 | STIM1 | STK11 | STK25 | STK3 | SYDE1 | TBXA2R | TCF12 | TCF4 | TGFB1 | TGFBR3 | THBS1 | THBS2 | TIMP2 | TLR3 | TMEM30A | TNF | TNFAIP3 | TNFRSF12A | TNFRSF1A | TNFSF12 | TNFSF14 | TP63 | TP73 | TRADD | TRAF6 | TRIM32 | TUBB2B | TWF1 | TWF2 | TWIST1 | VASH2 | VDR | VHL | VSTM2A | WNT2 | WT1 | XBP1 | XRCC5 | YAP1 | ZAP70 | ZBTB16 | ZBTB7B | ZMYND8 | GO:0038127 | 0.00642969792551256 | 53/8243 | 30/3250 | 0.0082544360538328 | 1 | T | F | F | F | ERBB signaling pathway | ABL1 | AKT1 | ARF4 | BCAR1 | CBL | CDC37 | CPNE3 | CUL5 | EFEMP1 | EGFR | ERBB2 | ERBB3 | GRB2 | GRB7 | HBEGF | HSP90AA1 | IQGAP1 | MAPK1 | MATK | MYOC | PIK3C2A | PIK3CA | PIK3R1 | PLCG1 | PTK2 | PTPRR | SHC1 | SRC | STUB1 | TGFB1 | GO:0097237 | 0.00982651947106636 | 81/8243 | 43/3250 | 0.00846608648938071 | 1 | T | F | F | F | cellular response to toxic substance | ABL1 | ACTB | AIFM1 | ANXA1 | APEX1 | CBX8 | DAXX | ETS1 | EZH2 | FABP1 | FIS1 | FOXO1 | HDAC2 | HDAC6 | HMOX1 | HNRNPA1 | HSF1 | ITPR2 | KDR | MAPK7 | MDM2 | NFE2L2 | NQO1 | NTRK1 | PARK7 | PCNA | PPARGC1A | PPIF | PRKAA1 | PRKCD | PRKCE | PTEN | RELA | RIPK1 | RIPK3 | RPS3 | SIRT1 | SPI1 | SRC | STAT6 | TNF | TNFAIP3 | ZNF277 | GO:0017144 | 0.0198956690525294 | 164/8243 | 80/3250 | 0.00876892626793114 | 1 | T | F | F | F | drug metabolic process | ACAT1 | ACSS2 | ADA | ADCYAP1 | ADH1A | ADH1B | ADH5 | AHCY | AHCYL1 | AK1 | AK2 | AK4 | AKR1A1 | AKR1B1 | AKR1B10 | AKR1C2 | ALDH1B1 | AMD1 | ATP6V1A | ATP6V1B1 | BDH1 | CAT | CBR1 | CBR4 | CKB | CKM | CKMT1A | CLPX | COMT | CYP1A1 | CYP1A2 | CYP2C18 | DBH | DPEP1 | EGFR | ESD | FIGNL1 | FOLR1 | FPGS | GPX1 | HBZ | HPRT1 | HSPA1B | HSPA8 | IDE | MAOA | MMAB | MTHFD1 | MTRR | MYH6 | NDUFS1 | NMNAT1 | NPR1 | NR1I2 | NT5C2 | OLA1 | OVGP1 | OXCT1 | PAFAH1B1 | PARK7 | PARP1 | PDXK | PRDX1 | PRDX2 | PRDX3 | PRDX4 | PRDX5 | PRDX6 | PXDN | RFK | SAT2 | SHMT1 | SHMT2 | SLC25A32 | SOD1 | SULT1A3 | TYMS | VCP | VKORC1 | XPNPEP1 | GO:0006367 | 0.0157709571757855 | 130/8243 | 65/3250 | 0.00879022715797158 | 1 | T | F | F | F | transcription initiation from RNA polymerase II promoter | AR | BAX | CBFB | CCNB1 | CDK4 | CDK8 | CDK9 | CDKN1A | CREBBP | E2F2 | E2F3 | ESR1 | ESR2 | ESRRA | ESRRB | ESRRG | GTF2A1 | GTF2E2 | GTF2F1 | GTF2F2 | GTF2I | HNF4A | HNF4G | MECP2 | MED1 | MED12 | MED14 | MED17 | MED20 | MED23 | MED24 | MED31 | MED4 | MED6 | NR0B2 | NR1H2 | NR1H3 | NR1I2 | NR3C1 | NR4A1 | NR5A2 | NRBF2 | POLR2A | POLR2E | POLR2L | PPARD | PPARG | PPARGC1A | PPM1D | PTEN | RARA | RARG | RBPJ | RUNX2 | RXRA | RXRB | RXRG | SNW1 | TAF12 | TAF15 | TCF4 | THRA | TRIM28 | VDR | YAP1 | GO:0033674 | 0.0453718306441829 | 374/8243 | 170/3250 | 0.00881621014662025 | 1 | T | F | F | F | positive regulation of kinase activity | ABL1 | ACSL1 | ADCY6 | ADCYAP1 | ADRB2 | AGAP2 | AGER | AKT1 | ARRB1 | AXIN1 | AZU1 | BCL10 | BIRC7 | BRAF | CAB39 | CALM1 | CALM2 | CALM3 | CCDC88A | CCL21 | CCNB1 | CCNT1 | CCNT2 | CCNY | CCNYL1 | CD40 | CD40LG | CD74 | CD81 | CDC25B | CDK5 | CDK5R1 | CDKN1A | CKS1B | CKS2 | CLU | CRK | CRKL | DAB1 | DAXX | DBF4B | DBNL | DDX3X | DGKZ | DLG3 | DLG4 | DUSP12 | DUSP19 | DUSP7 | DVL2 | DVL3 | EEF1A2 | EGFR | EPHA4 | ERBB2 | ERBB3 | EZH2 | FGF2 | FGFR1 | FGFR3 | FYN | GADD45G | HRAS | HSP90AA1 | HSP90AB1 | IKBKG | ILK | INSR | IQGAP1 | ITGB1BP1 | ITGB3 | KRAS | LAT | LCP2 | LPAR1 | LRRK2 | MAP2K1 | MAP2K3 | MAP2K4 | MAP2K6 | MAP2K7 | MAP3K1 | MAP3K13 | MAP3K14 | MAP3K2 | MAP3K3 | MAP3K4 | MAP4K1 | MAPK1 | MAPK14 | MAPK3 | MAPK8IP3 | MAPKAP1 | MAPT | MAS1 | MIF | MLST8 | MOB1B | NEK1 | NOD2 | NTF3 | NTRK1 | OXSR1 | PAK2 | PAK6 | PARK7 | PDCD10 | PDE6G | PIH1D1 | PIK3CA | PIK3CG | PLA2G1B | PPP2CA | PRDX2 | PRKAA1 | PRKACA | PRKAG1 | PRKAR1A | PRKAR2B | PRKCD | PRKCZ | PRKD1 | PROX1 | PRRC1 | PSEN1 | PSMD10 | PTK2 | PTPN1 | RAF1 | RAP1A | RHOA | RIPK1 | RIPK2 | RIPK3 | RPLP1 | RPS3 | RPTOR | S100A12 | SDC4 | SHC1 | SIRT1 | SNCA | SOD1 | SPRY2 | SRC | STK11 | STK24 | STK25 | STK26 | STK3 | TAB1 | TAB2 | TAOK3 | TGFB1 | THBS1 | TLR3 | TNF | TP73 | TRAF2 | TRAF6 | TXN | UBB | UBC | UBE2N | UBE2V1 | UNC119 | WNK1 | XRCC5 | XRCC6 | ZNF622 | GO:0036294 | 0.00958388936066966 | 79/8243 | 42/3250 | 0.00890519951999699 | 1 | T | F | F | F | cellular response to decreased oxygen levels | ACAA2 | AIFM1 | AKT1 | BCL2 | CBL | CCNB1 | DNM1L | E2F1 | EIF4EBP1 | ENDOG | FABP1 | GNB1 | HIF1A | HMOX1 | ICAM1 | IREB2 | LMNA | MDM2 | MDM4 | MYC | NAMPT | NDRG1 | NPEPPS | OPA1 | P4HB | PGK1 | PHB2 | PICK1 | PPARD | PPARGC1A | PRKAA1 | PRKCE | PTEN | PTN | S100B | SIRT1 | SLC9A1 | SRC | STUB1 | TP53 | TWIST1 | UBQLN1 | GO:0051983 | 0.00958388936066966 | 79/8243 | 42/3250 | 0.00890519951999699 | 1 | T | F | F | F | regulation of chromosome segregation | ANAPC4 | APC | ATM | ATRX | AURKB | CCDC36 | CCNB1 | CDC20 | CDC27 | CDC42 | CENPF | CSNK2A2 | CTCF | CTNNB1 | CUL3 | DUSP1 | DYNC1H1 | DYNC1LI1 | FEN1 | HECW2 | HNRNPU | IK | KIF2C | KLHL22 | MAD1L1 | MAD2L1 | MAPK15 | NIPBL | NUMA1 | PLK1 | PTTG1 | PUM1 | RAD21 | RB1 | SFPQ | SPDL1 | TNKS | TPR | TRIP13 | XRCC3 | ZNF207 | ZW10 | GO:0031327 | 0.116219822880019 | 958/8243 | 412/3250 | 0.00894267544693855 | 1 | T | F | F | F | negative regulation of cellular biosynthetic process | ACADVL | ACD | AEBP2 | AES | AGO1 | AGO2 | APBB1 | APEX1 | AR | ARHGAP35 | ASCL1 | ATF2 | ATP1A1 | ATRX | ATXN1 | AURKB | BACH2 | BAG3 | BASP1 | BCL11A | BCL6 | BCL6B | BCOR | BIRC5 | BMI1 | BRCA1 | BTRC | C1QBP | CALR | CAV1 | CBFA2T3 | CBFB | CBX4 | CBX7 | CBX8 | CBY1 | CCAR2 | CD36 | CDC73 | CDK2 | CDK5 | CDK5R1 | CEBPA | CENPF | CENPJ | CHCHD3 | CHD8 | CHEK2 | CIRBP | COPS2 | CREB1 | CREBBP | CRY1 | CRY2 | CTBP1 | CTCF | CTNNB1 | CTNNBIP1 | CUL3 | DAB2 | DAPK1 | DAXX | DCP2 | DDX3X | DHX36 | DMAP1 | DNAJA3 | DNAJB1 | DPF2 | DUSP1 | DUSP22 | E2F1 | E2F2 | E2F3 | E2F4 | E2F6 | EED | EHMT1 | EHMT2 | EID1 | EIF2AK3 | EIF2AK4 | EIF2S1 | EIF3E | EIF4A3 | EIF4E | EIF4E2 | EIF4EBP1 | EIF4ENIF1 | EIF6 | ELF3 | ENO1 | EP300 | EPRS | ERF | ERLIN2 | ERRFI1 | ESR1 | ESR2 | ESRRA | EZH2 | EZR | FAM129B | FAM3C | FASLG | FBP1 | FBXW11 | FGFR1 | FHL2 | FOXA1 | FOXJ1 | FOXK1 | FOXM1 | FOXO1 | FTO | FXR2 | FYN | GAPDH | GATA1 | GLA | GPS2 | GRB7 | GSK3A | GSK3B | H1F0 | H3F3A | HBEGF | HBZ | HDAC1 | HDAC2 | HDAC3 | HDAC4 | HDAC5 | HDAC6 | HDAC7 | HDAC9 | HDGF | HEXIM1 | HIF1AN | HIST1H3A | HIST1H4A | HIST2H3A | HIVEP1 | HMGB1 | HMGB2 | HNRNPA1 | HNRNPA2B1 | HNRNPD | HNRNPU | HSF1 | HSF4 | HSPA8 | ID2 | IFI16 | IFNA2 | IGBP1 | IGF2BP1 | IKZF5 | ILF3 | INSIG1 | INSIG2 | IREB2 | IRF1 | IRF7 | JAK3 | JUN | JUNB | JUND | KANK2 | KAT5 | KAT7 | KAT8 | KDM1A | KDM5B | KDM5C | KHDRBS1 | KLF5 | L3MBTL1 | LEF1 | LGR4 | LIMS1 | LOXL2 | MAF1 | MBD3 | MDFI | MDM2 | MDM4 | MECP2 | MED1 | MEPCE | MITF | MORF4L1 | MORF4L2 | MOV10 | MSH6 | MTA1 | MTA3 | MXD1 | MYBBP1A | MYC | NACC1 | NANOG | NCK1 | NCOA2 | NCOR1 | NCOR2 | NDUFA13 | NEDD4 | NELFB | NELFCD | NELFE | NFATC2 | NIF3L1 | NIPBL | NKRF | NKX2-1 | NOSTRIN | NR0B2 | NR1H2 | NR1H3 | NR1I2 | NR3C1 | PA2G4 | PARP1 | PATZ1 | PAWR | PAX9 | PCGF1 | PCGF6 | PER1 | PER2 | PGP | PHB | PHB2 | PHF10 | PHF21A | PIAS1 | PIAS3 | PINX1 | PKIG | PLK1 | PML | POT1 | POU5F1 | PPARD | PPARG | PPID | PPM1A | PPM1D | PRDX5 | PRKAA1 | PRMT2 | PRMT6 | PROX1 | PSEN1 | PSMC5 | PSMD10 | PTPRK | PUM1 | PUS7 | QARS | RARA | RARG | RASD1 | RB1 | RBM3 | RBM4 | RBPJ | RC3H1 | REL | RELA | REST | RFC1 | RGN | RGS2 | RIF1 | RING1 | RNF139 | RNF2 | RNF8 | RPL10 | RPL23 | RPS14 | RPS3 | RTF1 | RUNX1 | RUNX1T1 | RUNX2 | RUVBL2 | RXRA | S100A1 | S100A11 | SAP18 | SARS | SET | SF1 | SFMBT1 | SFPQ | SHC1 | SHMT1 | SIN3A | SIRT1 | SIRT5 | SIRT6 | SIRT7 | SKI | SMAD2 | SMAD3 | SMAD4 | SMAD5 | SMARCA2 | SMARCA4 | SMARCC2 | SMARCE1 | SMC1A | SMC3 | SMO | SMURF2 | SNAI2 | SNCA | SNW1 | SNX6 | SOX2 | SP100 | SP2 | SPEN | SPI1 | SQSTM1 | SRC | STAG2 | STAT1 | STAT3 | STAT6 | STRAP | SUMO1 | SUPT4H1 | SUPT5H | SUZ12 | TAGLN3 | TBL1X | TCERG1 | TCF21 | TCF25 | TCP10L | TERF1 | TERF2 | TGFB1 | THAP11 | THAP7 | THRA | TIA1 | TINF2 | TLE1 | TLE3 | TMBIM6 | TNF | TNP1 | TP53 | TP63 | TPR | TRAF6 | TRIB2 | TRIB3 | TRIM28 | TRIM29 | TRIM33 | TSC22D4 | TSG101 | TSPYL2 | TWIST1 | TXN | TXNIP | TYMS | UBA3 | UBB | UBC | UBE2D1 | UBE2D3 | UBE2I | UNK | USP2 | USP47 | USP9X | VDR | VHL | VPS72 | WDR35 | WDTC1 | WT1 | WWOX | WWP2 | XPO1 | XRCC5 | XRCC6 | YAF2 | YAP1 | YEATS2 | YWHAB | YWHAQ | YY1 | ZBTB16 | ZBTB21 | ZBTB33 | ZBTB42 | ZBTB7A | ZBTB7B | ZC3H4 | ZHX1 | ZMYND8 | ZNF217 | ZNF24 | ZNF746 | GO:1902115 | 0.0188038335557443 | 155/8243 | 76/3250 | 0.00896159430821565 | 1 | T | F | F | F | regulation of organelle assembly | ARHGAP35 | ARHGEF9 | ATG5 | ATXN2 | BRCA1 | CAPG | CCDC88A | CCP110 | CENPJ | CEP135 | CHMP2A | CNTROB | CROCC | CYLD | DYNC1H1 | DYNLL1 | EZR | FSCN1 | GAP43 | GRID2 | HAP1 | HNRNPU | HSPA1B | HTT | KCTD17 | LCP1 | LRFN1 | LRRK2 | LRSAM1 | MAPK15 | MAPK9 | MDM1 | MSN | NPM1 | NRXN1 | NUMA1 | PAN2 | PDCD6IP | PIKFYVE | PIP4K2A | PLK1 | PRKAA1 | PRKAA2 | PROX1 | PTPRD | RAB1B | RAB3GAP1 | RAB5A | RDX | RHOA | RNF4 | SCFD1 | SDC1 | SDC4 | SDCBP | SENP6 | SEPT7 | SEPT9 | SMAD4 | SMC1A | SMC3 | SPICE1 | SRC | STAG2 | STIL | SYNE2 | TBC1D15 | TBC1D22B | TNF | TPR | TSG101 | UBQLN2 | ULK1 | VDAC3 | VPS4B | ZMYND10 | GO:2000146 | 0.0188038335557443 | 155/8243 | 76/3250 | 0.00896159430821565 | 1 | T | F | F | F | negative regulation of cell motility | ADA | ADTRP | AKT1 | APEX1 | APOD | APOE | BCL2 | BMPR1A | BRAF | CALR | CCL21 | CDH1 | CHRD | CLIC4 | CRK | CSNK2B | DCN | DPEP1 | DUSP1 | DUSP22 | EPPIN | EPPK1 | FGF2 | GSTP1 | HDAC5 | HMGB1 | HMOX1 | IDH2 | ILK | ITGB1BP1 | JUP | LDLRAD4 | MAPK15 | MCC | MECP2 | MEOX2 | MIF | MITF | NF2 | NFE2L2 | NKX2-1 | PDCD10 | PIN1 | PPARD | PPARG | PPARGC1A | PTEN | PTN | PTPN23 | PTPRK | PTPRR | RECK | RGN | RHOA | SEPT7 | SLAMF8 | SLC9A3R1 | SP100 | SPINT2 | SRGAP2 | SRGAP3 | STARD13 | STAT3 | STK24 | STK26 | STRAP | SYNJ2BP | TBXA2R | TGFB1 | THBS1 | TIMP1 | TNN | TPM1 | VCL | WAS | ZMYND8 | GO:0045931 | 0.0133446560718185 | 110/8243 | 56/3250 | 0.00913935447960568 | 1 | T | F | F | F | positive regulation of mitotic cell cycle | ABL1 | AKT1 | ANAPC4 | ANXA1 | APEX1 | APP | ASNS | BRD4 | CCNB1 | CDC25A | CDC25B | CDC25C | CDK4 | CUL3 | CUL4B | CYP1A1 | DBF4B | DDX3X | DYNC1LI1 | EGFR | EIF4E | EIF4EBP1 | FGFR1 | FOXA1 | HNRNPU | HSF1 | HSPA2 | INSR | LGMN | MAD2L1 | MDM2 | MECP2 | MEPCE | MTA3 | NIPBL | NSFL1C | NUMA1 | NUSAP1 | PAFAH1B1 | PEBP1 | PHB2 | PKN2 | POLDIP2 | RAB11A | RB1 | RDX | RPTOR | SH2B1 | SIN3A | TGFB1 | TMOD3 | TPR | UBE2C | USP2 | VPS4B | XRCC3 | GO:0097306 | 0.00570180759432246 | 47/8243 | 27/3250 | 0.00920994169346241 | 1 | T | F | F | F | cellular response to alcohol | ACACA | ADCY6 | AIFM1 | AKAP8 | AKR1C2 | AKT1 | BRCA1 | CDH1 | CDK4 | CFTR | CTNNB1 | GNAI1 | GNB1 | GRAMD1A | IREB2 | ITPR2 | JUP | LRP6 | MAP4K1 | PPARG | PRKAA1 | PRKAA2 | PRKCE | PTEN | RAD51 | SMO | SPI1 | GO:2000757 | 0.00206235593837195 | 17/8243 | 12/3250 | 0.00921980361961473 | 1 | T | F | F | F | negative regulation of peptidyl-lysine acetylation | ATG5 | BRCA1 | CTBP1 | HDAC2 | MECP2 | PIH1D1 | SET | SIN3A | SIRT1 | SNCA | SPI1 | TWIST1 | GO:1904872 | 0.00206235593837195 | 17/8243 | 12/3250 | 0.00921980361961473 | 1 | T | F | F | F | regulation of telomerase RNA localization to Cajal body | CCT2 | CCT3 | CCT4 | CCT5 | CCT6A | CCT7 | CCT8 | DCP2 | NOP10 | RUVBL1 | RUVBL2 | TCP1 | GO:0006469 | 0.0196530389421327 | 162/8243 | 79/3250 | 0.00924642492652064 | 1 | T | F | F | F | negative regulation of protein kinase activity | ABL1 | ADORA2A | AKT1 | APC | APOE | CASP3 | CAV1 | CBL | CBLB | CBLC | CDKN1A | CDKN2C | CEBPA | CHORDC1 | CHP1 | DBNDD2 | DNAJA1 | DUSP1 | DUSP14 | DUSP16 | DUSP19 | DUSP22 | DUSP7 | DVL1 | ERRFI1 | FBXO7 | FGFR1OP | GNAQ | GPS2 | GSTP1 | HEXIM1 | HSPB1 | IBTK | IGF1R | ILK | IPO5 | IPO7 | ITGB1BP1 | LATS1 | LRP6 | MLLT1 | NCK1 | NF2 | NPM1 | PAK2 | PARK7 | PKIG | PLK1 | PPP2CA | PPP2R1A | PRKAR1A | PRKAR2B | PRKCD | PSEN1 | PTEN | PTPN1 | PTPN22 | PTPN6 | QARS | RB1 | RGN | RGS2 | RPTOR | SERPINB3 | SFN | SIRT1 | SNX6 | SOCS1 | SPRY2 | STK38 | TAOK3 | TNFAIP3 | TRIB2 | TRIB3 | TSG101 | UCHL1 | WARS | WNK1 | YWHAG | GO:0051282 | 0.00776416353269441 | 64/8243 | 35/3250 | 0.00931071315736759 | 1 | T | F | F | F | regulation of sequestering of calcium ion | ABL1 | ANXA5 | ANXA6 | ATG5 | BAX | CALM1 | CALM2 | CALM3 | CCL21 | CCR5 | CXCL11 | DMD | FASLG | FGF2 | FKBP1A | FKBP1B | GSTO1 | HAP1 | HTT | IBTK | ITPR1 | ITPR2 | ITPR3 | LETM1 | MCOLN3 | MYO5A | PDE4D | PLCG1 | PRKACA | PRKCE | PTPN6 | SNCA | SRI | TGFB1 | TPCN2 | GO:0042590 | 0.00776416353269441 | 64/8243 | 35/3250 | 0.00931071315736759 | 1 | T | F | F | F | antigen processing and presentation of exogenous peptide antigen via MHC class I | CALR | CD36 | FCGR1A | HLA-A | HLA-B | IKBKB | IKBKG | ITGAV | ITGB5 | PDIA3 | PSMA1 | PSMA2 | PSMA3 | PSMA5 | PSMA6 | PSMB5 | PSMB6 | PSMB7 | PSMB8 | PSMC1 | PSMC2 | PSMC3 | PSMC4 | PSMC5 | PSMC6 | PSMD1 | PSMD10 | PSMD11 | PSMD12 | PSMD14 | PSMD2 | PSMD4 | PSMD9 | PSME2 | PSME3 | GO:1902679 | 0.0955962634962999 | 788/8243 | 342/3250 | 0.00931507660559897 | 1 | T | F | F | F | negative regulation of RNA biosynthetic process | AEBP2 | AES | APBB1 | APEX1 | AR | ARHGAP35 | ASCL1 | ATF2 | ATRX | ATXN1 | AURKB | BACH2 | BAG3 | BASP1 | BCL11A | BCL6 | BCL6B | BCOR | BIRC5 | BMI1 | BRCA1 | BTRC | C1QBP | CALR | CAV1 | CBFA2T3 | CBFB | CBX4 | CBX7 | CBX8 | CBY1 | CCAR2 | CD36 | CDC73 | CDK2 | CDK5 | CDK5R1 | CEBPA | CENPF | CENPJ | CHCHD3 | CHD8 | COPS2 | CREB1 | CREBBP | CRY1 | CRY2 | CTBP1 | CTCF | CTNNB1 | CTNNBIP1 | CUL3 | DAB2 | DAXX | DMAP1 | DNAJA3 | DNAJB1 | DPF2 | DUSP22 | E2F1 | E2F2 | E2F3 | E2F4 | E2F6 | EED | EHMT1 | EHMT2 | EID1 | ELF3 | ENO1 | EP300 | ERF | ESR1 | ESR2 | ESRRA | EZH2 | EZR | FAM129B | FASLG | FBP1 | FBXW11 | FGFR1 | FHL2 | FOXA1 | FOXJ1 | FOXK1 | FOXM1 | FOXO1 | GATA1 | GPS2 | H1F0 | H3F3A | HBZ | HDAC1 | HDAC2 | HDAC3 | HDAC4 | HDAC5 | HDAC6 | HDAC7 | HDAC9 | HDGF | HEXIM1 | HIF1AN | HIST1H3A | HIST1H4A | HIST2H3A | HIVEP1 | HMGB1 | HMGB2 | HNRNPA2B1 | HNRNPU | HSF1 | HSF4 | HSPA8 | ID2 | IFI16 | IFNA2 | IGBP1 | IKZF5 | ILF3 | IRF1 | IRF7 | JUN | JUNB | JUND | KANK2 | KAT5 | KAT7 | KAT8 | KDM1A | KDM5B | KDM5C | KHDRBS1 | KLF5 | L3MBTL1 | LEF1 | LGR4 | LIMS1 | LOXL2 | MAF1 | MBD3 | MDFI | MDM2 | MDM4 | MECP2 | MED1 | MEPCE | MITF | MORF4L1 | MORF4L2 | MTA1 | MTA3 | MXD1 | MYBBP1A | MYC | NACC1 | NANOG | NCK1 | NCOA2 | NCOR1 | NCOR2 | NDUFA13 | NEDD4 | NELFB | NELFCD | NELFE | NFATC2 | NIF3L1 | NIPBL | NKRF | NKX2-1 | NOSTRIN | NR0B2 | NR1H2 | NR1H3 | NR1I2 | NR3C1 | PA2G4 | PARP1 | PATZ1 | PAWR | PAX9 | PCGF1 | PCGF6 | PER1 | PER2 | PHB | PHB2 | PHF10 | PHF21A | PIAS1 | PIAS3 | PKIG | PLK1 | PML | POU5F1 | PPARD | PPARG | PPID | PPM1A | PPM1D | PRDX5 | PRMT2 | PRMT6 | PROX1 | PSEN1 | PSMC5 | PSMD10 | PTPRK | QARS | RARA | RARG | RASD1 | RB1 | RBPJ | REL | RELA | REST | RFC1 | RGN | RIF1 | RING1 | RNF2 | RNF8 | RPL10 | RPL23 | RPS14 | RTF1 | RUNX1 | RUNX1T1 | RUNX2 | RUVBL2 | RXRA | S100A1 | SAP18 | SARS | SET | SF1 | SFMBT1 | SFPQ | SHC1 | SIN3A | SIRT1 | SIRT5 | SIRT6 | SIRT7 | SKI | SMAD2 | SMAD3 | SMAD4 | SMAD5 | SMARCA2 | SMARCA4 | SMARCC2 | SMARCE1 | SMO | SMURF2 | SNAI2 | SNCA | SNW1 | SNX6 | SOX2 | SP100 | SP2 | SPEN | SPI1 | SQSTM1 | SRC | STAT1 | STAT3 | STAT6 | STRAP | SUMO1 | SUPT4H1 | SUPT5H | SUZ12 | TAGLN3 | TBL1X | TCERG1 | TCF21 | TCF25 | TCP10L | TGFB1 | THAP11 | THAP7 | THRA | TLE1 | TLE3 | TMBIM6 | TNF | TNP1 | TP53 | TP63 | TPR | TRAF6 | TRIB3 | TRIM28 | TRIM29 | TRIM33 | TSC22D4 | TSG101 | TWIST1 | TXN | TXNIP | UBA3 | UBB | UBC | UBE2D1 | UBE2D3 | UBE2I | USP2 | USP47 | USP9X | VDR | VHL | VPS72 | WDTC1 | WT1 | WWOX | WWP2 | XPO1 | XRCC5 | XRCC6 | YAF2 | YAP1 | YEATS2 | YWHAB | YWHAQ | YY1 | ZBTB16 | ZBTB21 | ZBTB33 | ZBTB42 | ZBTB7A | ZBTB7B | ZC3H4 | ZHX1 | ZMYND8 | ZNF217 | ZNF24 | ZNF746 | GO:0035567 | 0.0120101904646367 | 99/8243 | 51/3250 | 0.00940346069291605 | 1 | T | F | F | F | non-canonical Wnt signaling pathway | AGO1 | AGO2 | ARRB2 | CALM1 | CAMK2A | CDC42 | CLTC | CTNNB1 | DVL1 | DVL2 | DVL3 | GNAO1 | GNB1 | LEF1 | MED12 | MOV10 | MYOC | NFATC1 | NLK | PARD6A | PDE6G | PFN1 | PSMA1 | PSMA2 | PSMA3 | PSMA5 | PSMA6 | PSMB5 | PSMB6 | PSMB7 | PSMB8 | PSMC1 | PSMC2 | PSMC3 | PSMC4 | PSMC5 | PSMC6 | PSMD1 | PSMD10 | PSMD11 | PSMD12 | PSMD14 | PSMD2 | PSMD4 | PSMD9 | PSME2 | PSME3 | RAC1 | RHOA | SMURF1 | SMURF2 | GO:0032940 | 0.072425087953415 | 597/8243 | 263/3250 | 0.00944652783742514 | 1 | T | F | F | F | secretion by cell | A2M | ACAA1 | ACLY | ACTN1 | ACTN2 | ACTN4 | ACTR2 | ADCYAP1 | AGL | AHSG | ALB | ALDOA | ALDOC | ALOX5 | ANK1 | ANXA1 | ANXA2 | ANXA5 | AP1M1 | APEH | APOA1 | APP | APRT | ARL4D | ARMC8 | ARPC5 | ATP6V1D | AZU1 | BTK | CAB39 | CALM1 | CAND1 | CANX | CAPN1 | CAT | CBL | CCR1 | CCT2 | CCT8 | CD109 | CD36 | CD40LG | CDA | CDK16 | CDK5 | CEP290 | CHP1 | CKAP4 | CLU | COPB1 | COPG1 | COPS5 | COTL1 | CPLX1 | CPLX3 | CPNE3 | CSNK2B | CTSA | CTSC | CTSD | CYB5R1 | CYB5R3 | DBNL | DDOST | DDX3X | DEGS1 | DERA | DVL1 | DYNC1H1 | DYNC1LI1 | DYNLL1 | DYNLT1 | EEF1A1 | EEF2 | EXOC6 | F5 | FABP5 | FAF2 | FAM129B | FAM3C | FAM49B | FCN1 | FGB | FGL2 | FKBP1B | FN1 | FTH1 | FTL | GAA | GAD2 | GALNS | GBP5 | GDI2 | GHDC | GLA | GLS | GLS2 | GNS | GOLPH3 | GPI | GSTP1 | GUSB | GYG1 | HAP1 | HEXB | HMGB1 | HNF1A | HRNR | HSP90AA1 | HSP90AB1 | HSPA1B | HSPA8 | HUWE1 | IDH1 | ILF2 | IMPDH2 | IQGAP1 | ISLR | ITGAV | ITGB3 | JUP | KCMF1 | KCNAB2 | KPNB1 | LAT | LCP2 | LGALS3 | LGALS3BP | LRRC7 | MAN2B1 | MAPK1 | MAPK14 | MECP2 | MGST1 | MIF | MME | MMRN1 | MYO5A | NAPRT | NEUROD1 | NHLRC3 | NIT2 | NME2 | NOD2 | NOS2 | NRXN1 | OLA1 | ORM1 | P2RX1 | PA2G4 | PAFAH1B1 | PARK7 | PDIA4 | PDXK | PFKL | PGAM1 | PGM1 | PGM2 | PGRMC1 | PI4K2A | PIK3C2A | PIK3CG | PKM | PLAUR | PNP | PPIA | PPIE | PPT1 | PRCP | PRDX4 | PRDX6 | PRG2 | PRKCD | PSAP | PSEN1 | PSMA2 | PSMA5 | PSMB7 | PSMC2 | PSMC3 | PSMD1 | PSMD11 | PSMD12 | PSMD14 | PSMD2 | PTGES2 | PTPN6 | PYGB | PYGL | QSOX1 | RAB10 | RAB11A | RAB11B | RAB11FIP2 | RAB18 | RAB1A | RAB24 | RAB31 | RAB5B | RAB7A | RAC1 | RAF1 | RAP1A | RAP1B | RAPGEF4 | RBPJ | RHOA | RNASE3 | S100A11 | S100A12 | S100A13 | S100A7 | S100A9 | S100P | SCCPDH | SCG2 | SCG3 | SDCBP | SEL1L | SELL | SERPINB3 | SERPINB6 | SERPINE2 | SLC2A5 | SMAD2 | SNAP29 | SNCA | SOD1 | SPTAN1 | SRP14 | STBD1 | STOM | STXBP1 | STXBP2 | SYN1 | TAGLN2 | TANGO2 | TCIRG1 | TEX264 | TGFB1 | THBS1 | TIMP1 | TIMP2 | TMEM173 | TMEM30A | TOLLIP | TRIM9 | TSG101 | TUBA4A | TUBB | TUBB4B | TXLNA | TXNDC5 | UBR4 | VAPA | VAT1 | VCL | VCP | VGF | WDR1 | XRCC5 | XRCC6 | ZBTB7B | GO:0006915 | 0.052650733956084 | 434/8243 | 195/3250 | 0.00944993245443172 | 1 | T | F | F | F | apoptotic process | ADORA2A | AIFM1 | AIMP2 | AKT1 | AKT2 | AKTIP | APBB1 | API5 | APLP1 | APP | ARRB1 | ATCAY | ATG5 | AXIN1 | BAG1 | BAG6 | BAX | BBC3 | BCL10 | BCL2 | BCL2L1 | BCL2L13 | BIRC2 | BIRC3 | BIRC5 | BIRC7 | BIRC8 | BNIPL | BRCA1 | C1QBP | CASP10 | CASP2 | CASP3 | CASP7 | CASP8 | CASP9 | CD2 | CDK5 | CRKL | CRYAB | CSE1L | CSNK2A2 | CTSC | CTSL | CUL3 | CYCS | DAB2 | DAD1 | DAPK1 | DAXX | DHCR24 | DIABLO | DNAJA3 | DPEP1 | DPF2 | DUSP22 | DYNLL1 | E2F1 | E2F2 | EAF2 | EGLN3 | EIF5A | EP300 | ERBB3 | EVA1A | FADD | FAF1 | FASLG | FIS1 | FKBP8 | FOXO1 | GADD45G | GAPDH | GHITM | GNB1 | GRIK2 | GRK5 | GZMA | HSPA5 | HTATIP2 | HTT | IFNA2 | IKBKG | IRF1 | ITCH | ITGB3BP | KANK2 | KMT2A | LEF1 | LGALS1 | LGALS7 | LIG4 | LTBR | MAP2K4 | MAP2K6 | MAP2K7 | MAPK1 | MAPK14 | MAPK3 | MCM2 | MELK | MYC | MYD88 | NAE1 | NFKBIA | NGB | NR3C1 | OGT | OPA1 | P2RX1 | PACS2 | PAK2 | PAK6 | PARP1 | PAWR | PCSK9 | PDCD6IP | PIK3CA | PIK3CG | PIM1 | PKN2 | PLEKHF1 | PLSCR1 | PPARD | PPID | PPP2CA | PPP2R1A | PPP2R1B | PRDX3 | PRDX5 | PRELID1 | PRKCD | PRKCE | PRKD1 | PSEN1 | PSMD10 | PSME3 | PTEN | PTPN6 | PTRH2 | RAD21 | RAF1 | RASSF7 | RB1 | RFK | RHBDD1 | RIPK1 | RIPK2 | RNF183 | RPS3 | RPS6KA1 | RTKN | RTN4 | S100A9 | SEPT4 | SH3KBP1 | SIAH1 | SLC25A6 | SLTM | SNCA | SPI1 | SQSTM1 | STK11 | STK26 | STK3 | TCTN3 | TFDP1 | THOC6 | TIA1 | TIAL1 | TMEM173 | TMEM219 | TNF | TNFAIP3 | TNFSF12 | TNFSF14 | TNIP2 | TP53 | TP63 | TRADD | TRAF1 | TRAF3 | TRIAP1 | TRIM35 | TRIM69 | UBE2D3 | USP17L2 | USP53 | VDAC1 | XAF1 | XIAP | ZBTB16 | ZFAND6 | ZMPSTE24 | ZNF443 | GO:0002544 | 0.000606575275991751 | 5/8243 | 5/3250 | 0.00950999652577865 | 1 | T | F | F | F | chronic inflammatory response | AHCY | PTGES | THBS1 | TNF | VCAM1 | GO:0050930 | 0.000606575275991751 | 5/8243 | 5/3250 | 0.00950999652577865 | 1 | T | F | F | F | induction of positive chemotaxis | AGER | AZU1 | CXCL8 | NTF3 | SCG2 | GO:0061732 | 0.000606575275991751 | 5/8243 | 5/3250 | 0.00950999652577865 | 1 | T | F | F | F | mitochondrial acetyl-CoA biosynthetic process from pyruvate | DLD | MPC2 | PDHA1 | PDHB | PDHX | GO:2001138 | 0.000606575275991751 | 5/8243 | 5/3250 | 0.00950999652577865 | 1 | T | F | F | F | regulation of phospholipid transport | APOA1 | APOE | PRELID1 | PRKCD | TRIAP1 | GO:0090077 | 0.000606575275991751 | 5/8243 | 5/3250 | 0.00950999652577865 | 1 | T | F | F | F | foam cell differentiation | EP300 | PPARG | SOAT1 | STAT1 | TGFB1 | GO:0006544 | 0.000606575275991751 | 5/8243 | 5/3250 | 0.00950999652577865 | 1 | T | F | F | F | glycine metabolic process | GART | PHGDH | SHMT1 | SHMT2 | SLC25A32 | GO:0110095 | 0.000606575275991751 | 5/8243 | 5/3250 | 0.00950999652577865 | 1 | T | F | F | F | cellular detoxification of aldehyde | AKR1A1 | AKR1B10 | PARK7 | RDH11 | RDH12 | GO:1902527 | 0.000606575275991751 | 5/8243 | 5/3250 | 0.00950999652577865 | 1 | T | F | F | F | positive regulation of protein monoubiquitination | BIRC2 | FANCM | PDCD6 | PEF1 | UBB | GO:0010742 | 0.000606575275991751 | 5/8243 | 5/3250 | 0.00950999652577865 | 1 | T | F | F | F | macrophage derived foam cell differentiation | EP300 | PPARG | SOAT1 | STAT1 | TGFB1 | GO:0060539 | 0.000606575275991751 | 5/8243 | 5/3250 | 0.00950999652577865 | 1 | T | F | F | F | diaphragm development | ASS1 | BASP1 | KCNAB1 | TCF21 | WT1 | GO:0006166 | 0.000606575275991751 | 5/8243 | 5/3250 | 0.00950999652577865 | 1 | T | F | F | F | purine ribonucleoside salvage | ADK | APRT | HPRT1 | MTAP | PRTFDC1 | GO:2001140 | 0.000606575275991751 | 5/8243 | 5/3250 | 0.00950999652577865 | 1 | T | F | F | F | positive regulation of phospholipid transport | APOA1 | APOE | PRELID1 | PRKCD | TRIAP1 | GO:0090241 | 0.000606575275991751 | 5/8243 | 5/3250 | 0.00950999652577865 | 1 | T | F | F | F | negative regulation of histone H4 acetylation | ATG5 | CTBP1 | PIH1D1 | SIRT1 | SPI1 | GO:0071447 | 0.000606575275991751 | 5/8243 | 5/3250 | 0.00950999652577865 | 1 | T | F | F | F | cellular response to hydroperoxide | CD36 | DAPK1 | MGST1 | PRKCD | PRKD1 | GO:0006102 | 0.000606575275991751 | 5/8243 | 5/3250 | 0.00950999652577865 | 1 | T | F | F | F | isocitrate metabolic process | ACO2 | IDH1 | IDH2 | IDH3B | IDH3G | GO:0016056 | 0.000606575275991751 | 5/8243 | 5/3250 | 0.00950999652577865 | 1 | T | F | F | F | rhodopsin mediated signaling pathway | GNB1 | GRK1 | PDE6G | RHO | SAG | GO:0043388 | 0.00545917748392576 | 45/8243 | 26/3250 | 0.00952424835795324 | 1 | T | F | F | F | positive regulation of DNA binding | ACD | CALM2 | CALM3 | CTNNB1 | DDRGK1 | EP300 | FAM129B | H1F0 | HMGB1 | HMGB2 | IRF4 | NEUROD1 | NIPBL | NME1 | PARK7 | PARP1 | PINX1 | PLAUR | PPARG | RB1 | SKI | TGFB1 | TRAF6 | TRIM28 | TWIST1 | TXN | GO:1903522 | 0.0174693679485624 | 144/8243 | 71/3250 | 0.00961898284281514 | 1 | T | F | F | F | regulation of blood circulation | ACE2 | ADA | AGER | AHCYL1 | AKAP9 | AKT1 | ATP1A1 | ATP1B3 | ATP2A2 | ATP2B2 | BDKRB2 | BIN1 | CACNG2 | CACNG4 | CACNG5 | CALM1 | CALM2 | CALM3 | CAV1 | CHRM2 | CHRM3 | DBH | DES | DMD | DOCK5 | EGFR | FGB | FKBP1B | GAA | GLP1R | GLRX3 | GNAO1 | GPD1L | GSK3A | GSTO1 | HBEGF | HDAC4 | ICAM1 | ITPR1 | ITPR2 | ITPR3 | JUP | KCNIP2 | KCNK1 | MDM2 | MYH6 | MYL2 | NOS1AP | NOS3 | NPR1 | P2RX1 | P2RX4 | PDE4D | PEBP1 | PER2 | PIK3CG | PRKACA | RGS2 | RHOA | S100A1 | SLC9A1 | SNTA1 | SRI | STIM1 | SUMO1 | TBXA2R | THRA | TMEM65 | TPM1 | YWHAE | ZMPSTE24 | GO:1903507 | 0.0953536333859032 | 786/8243 | 341/3250 | 0.00965700743109281 | 1 | T | F | F | F | negative regulation of nucleic acid-templated transcription | AEBP2 | AES | APBB1 | APEX1 | AR | ARHGAP35 | ASCL1 | ATF2 | ATRX | ATXN1 | AURKB | BACH2 | BAG3 | BASP1 | BCL11A | BCL6 | BCL6B | BCOR | BIRC5 | BMI1 | BRCA1 | BTRC | C1QBP | CALR | CAV1 | CBFA2T3 | CBFB | CBX4 | CBX7 | CBX8 | CBY1 | CCAR2 | CD36 | CDC73 | CDK2 | CDK5 | CDK5R1 | CEBPA | CENPF | CENPJ | CHCHD3 | CHD8 | COPS2 | CREB1 | CREBBP | CRY1 | CRY2 | CTBP1 | CTCF | CTNNB1 | CTNNBIP1 | CUL3 | DAB2 | DAXX | DMAP1 | DNAJA3 | DNAJB1 | DPF2 | DUSP22 | E2F1 | E2F2 | E2F3 | E2F4 | E2F6 | EED | EHMT1 | EHMT2 | EID1 | ELF3 | ENO1 | EP300 | ERF | ESR1 | ESR2 | ESRRA | EZH2 | EZR | FAM129B | FASLG | FBP1 | FBXW11 | FGFR1 | FHL2 | FOXA1 | FOXJ1 | FOXK1 | FOXM1 | FOXO1 | GATA1 | GPS2 | H1F0 | H3F3A | HBZ | HDAC1 | HDAC2 | HDAC3 | HDAC4 | HDAC5 | HDAC6 | HDAC7 | HDAC9 | HDGF | HEXIM1 | HIF1AN | HIST1H3A | HIST1H4A | HIST2H3A | HIVEP1 | HMGB1 | HMGB2 | HNRNPA2B1 | HNRNPU | HSF1 | HSF4 | HSPA8 | ID2 | IFI16 | IFNA2 | IGBP1 | IKZF5 | ILF3 | IRF1 | IRF7 | JUN | JUNB | JUND | KANK2 | KAT5 | KAT7 | KAT8 | KDM1A | KDM5B | KDM5C | KHDRBS1 | KLF5 | L3MBTL1 | LEF1 | LGR4 | LIMS1 | LOXL2 | MAF1 | MBD3 | MDFI | MDM2 | MDM4 | MECP2 | MED1 | MEPCE | MITF | MORF4L1 | MORF4L2 | MTA1 | MTA3 | MXD1 | MYBBP1A | MYC | NACC1 | NANOG | NCK1 | NCOA2 | NCOR1 | NCOR2 | NDUFA13 | NEDD4 | NELFB | NELFCD | NELFE | NFATC2 | NIF3L1 | NIPBL | NKRF | NKX2-1 | NOSTRIN | NR0B2 | NR1H2 | NR1H3 | NR1I2 | NR3C1 | PA2G4 | PARP1 | PATZ1 | PAWR | PAX9 | PCGF1 | PCGF6 | PER1 | PER2 | PHB | PHB2 | PHF10 | PHF21A | PIAS1 | PIAS3 | PKIG | PLK1 | PML | POU5F1 | PPARD | PPARG | PPID | PPM1A | PPM1D | PRDX5 | PRMT2 | PRMT6 | PROX1 | PSEN1 | PSMC5 | PSMD10 | PTPRK | QARS | RARA | RARG | RASD1 | RB1 | RBPJ | REL | RELA | REST | RFC1 | RIF1 | RING1 | RNF2 | RNF8 | RPL10 | RPL23 | RPS14 | RTF1 | RUNX1 | RUNX1T1 | RUNX2 | RUVBL2 | RXRA | S100A1 | SAP18 | SARS | SET | SF1 | SFMBT1 | SFPQ | SHC1 | SIN3A | SIRT1 | SIRT5 | SIRT6 | SIRT7 | SKI | SMAD2 | SMAD3 | SMAD4 | SMAD5 | SMARCA2 | SMARCA4 | SMARCC2 | SMARCE1 | SMO | SMURF2 | SNAI2 | SNCA | SNW1 | SNX6 | SOX2 | SP100 | SP2 | SPEN | SPI1 | SQSTM1 | SRC | STAT1 | STAT3 | STAT6 | STRAP | SUMO1 | SUPT4H1 | SUPT5H | SUZ12 | TAGLN3 | TBL1X | TCERG1 | TCF21 | TCF25 | TCP10L | TGFB1 | THAP11 | THAP7 | THRA | TLE1 | TLE3 | TMBIM6 | TNF | TNP1 | TP53 | TP63 | TPR | TRAF6 | TRIB3 | TRIM28 | TRIM29 | TRIM33 | TSC22D4 | TSG101 | TWIST1 | TXN | TXNIP | UBA3 | UBB | UBC | UBE2D1 | UBE2D3 | UBE2I | USP2 | USP47 | USP9X | VDR | VHL | VPS72 | WDTC1 | WT1 | WWOX | WWP2 | XPO1 | XRCC5 | XRCC6 | YAF2 | YAP1 | YEATS2 | YWHAB | YWHAQ | YY1 | ZBTB16 | ZBTB21 | ZBTB33 | ZBTB42 | ZBTB7A | ZBTB7B | ZC3H4 | ZHX1 | ZMYND8 | ZNF217 | ZNF24 | ZNF746 | GO:0044255 | 0.0471915564721582 | 389/8243 | 176/3250 | 0.00967177079958357 | 1 | T | F | F | F | cellular lipid metabolic process | AASDH | ABCD3 | ACAA1 | ACAA2 | ACAD9 | ACADS | ACADSB | ACADVL | ACAT1 | ACAT2 | ACOX1 | ACSL1 | ACSL4 | ACSL5 | ADH1A | ADH1B | ADH5 | ADTRP | AGK | AKR1B1 | AKR1B10 | AKR1C2 | ALDH5A1 | ALDH8A1 | ALOX12 | ALOX5 | ALOX5AP | APOA1 | APOE | ATP6V1B1 | B4GALT3 | BAX | CAT | CAV1 | CBR1 | CD36 | CEL | CES1 | COL4A3BP | CPT1A | CPT2 | CROT | CTSA | CYP1A1 | CYP1A2 | CYP2C18 | DBI | DECR1 | DEGS1 | DGKE | DGKZ | DHRS7B | DLD | DPM1 | ECH1 | ECHDC2 | ECHS1 | ECI1 | ECI2 | EGFR | EHHADH | ETFA | ETFB | ETFDH | FABP1 | FABP3 | FABP5 | FABP7 | FASN | GCDH | GGPS1 | GGT1 | GLA | GPC5 | GPX1 | GPX4 | GSTA1 | GSTP1 | HADH | HEXA | HEXB | HIBCH | HSD17B4 | HSPG2 | HTRA2 | IDI2 | IMPA1 | IMPA2 | INSIG1 | INSIG2 | IP6K1 | IVD | KDSR | LPIN3 | LTC4S | LYPLA1 | LYPLA2 | MAPK14 | MAPK3 | MCAT | MECP2 | MPPE1 | MTMR7 | NDUFS6 | NKX2-1 | NR1H2 | NUDT19 | P2RX1 | PAFAH1B1 | PCSK9 | PER2 | PGP | PI4K2A | PIGU | PIK3C2A | PIK3CA | PIK3CG | PIK3R1 | PIK3R3 | PIKFYVE | PIP4K2A | PIPSL | PLA2G10 | PLA2G12A | PLA2G1B | PLA2G2A | PLA2G6 | PLCG1 | PPARD | PPARG | PPARGC1A | PPP2CA | PPP2R1A | PPT1 | PRDX6 | PRKAA1 | PRKAR2B | PRKD1 | PRKD2 | PRKD3 | PSAP | PTEN | PTGDS | PTGES | PTGES2 | PTGES3 | PTGR1 | RBP1 | RDH11 | RDH12 | RDH13 | RDH14 | RDH5 | RHO | SCCPDH | SDC1 | SDC4 | SEL1L | SGPL1 | SIRT1 | SNCA | SOAT1 | SOCS1 | SOCS2 | SOCS6 | SPTLC2 | ST3GAL6 | ST6GALNAC5 | ST8SIA4 | TAZ | THEM4 | TNFRSF1A | UGT8 | VAPA | VAPB | ZMPSTE24 | GO:0006476 | 0.00521654737352906 | 43/8243 | 25/3250 | 0.00983123125816994 | 1 | T | F | F | F | protein deacetylation | HDAC1 | HDAC11 | HDAC2 | HDAC3 | HDAC4 | HDAC5 | HDAC6 | HDAC7 | HDAC9 | MORF4L1 | MORF4L2 | MTA1 | MTA3 | PER1 | PER2 | PHB | REST | SFPQ | SIN3A | SIRT1 | SIRT3 | SIRT5 | SIRT6 | SIRT7 | TBL1X | GO:0014823 | 0.00521654737352906 | 43/8243 | 25/3250 | 0.00983123125816994 | 1 | T | F | F | F | response to activity | ADSL | CAT | CBL | COL4A2 | CREB1 | FIS1 | GOT2 | HADH | HDAC5 | HIF1A | HSF1 | IREB2 | MAS1 | METRNL | OPA1 | OXCT1 | PEBP1 | PPARD | PPARGC1A | PRKAA1 | PRKAA2 | PRKDC | PTEN | PTN | TOMM20 | GO:0002504 | 0.00909862913987626 | 75/8243 | 40/3250 | 0.00985002974641066 | 1 | T | F | F | F | antigen processing and presentation of peptide or polysaccharide antigen via MHC class II | ACTR1A | AP1B1 | AP1G1 | AP1M1 | ARF1 | CANX | CAPZA1 | CAPZA2 | CAPZB | CD74 | CLTC | CTSD | CTSL | CTSV | DCTN1 | DYNC1H1 | DYNC1I1 | DYNC1I2 | DYNC1LI1 | DYNLL1 | HLA-DQA1 | HLA-DRA | HLA-DRB1 | HLA-DRB3 | HLA-DRB4 | HLA-DRB5 | KIF11 | KIF2C | KLC1 | KLC2 | LGMN | OSBPL1A | RAB7A | SEC13 | SEC24B | SEC24C | SEC31A | SH3GL2 | SPTBN2 | TRAF6 | GO:0019886 | 0.00909862913987626 | 75/8243 | 40/3250 | 0.00985002974641066 | 1 | T | F | F | F | antigen processing and presentation of exogenous peptide antigen via MHC class II | ACTR1A | AP1B1 | AP1G1 | AP1M1 | ARF1 | CANX | CAPZA1 | CAPZA2 | CAPZB | CD74 | CLTC | CTSD | CTSL | CTSV | DCTN1 | DYNC1H1 | DYNC1I1 | DYNC1I2 | DYNC1LI1 | DYNLL1 | HLA-DQA1 | HLA-DRA | HLA-DRB1 | HLA-DRB3 | HLA-DRB4 | HLA-DRB5 | KIF11 | KIF2C | KLC1 | KLC2 | LGMN | OSBPL1A | RAB7A | SEC13 | SEC24B | SEC24C | SEC31A | SH3GL2 | SPTBN2 | TRAF6 | GO:0002431 | 0.00909862913987626 | 75/8243 | 40/3250 | 0.00985002974641066 | 1 | T | F | F | F | Fc receptor mediated stimulatory signaling pathway | ABL1 | ACTB | ACTG1 | ACTR2 | ACTR3 | ARPC1A | ARPC1B | ARPC2 | ARPC3 | ARPC4 | ARPC5 | BAIAP2 | CDC42 | CRK | FCGR1A | FYN | GRB2 | HSP90AA1 | HSP90AB1 | IGHV3-13 | IGHV3-23 | IGHV3-30 | IGKV3-20 | IGLC7 | MAPK1 | MAPK3 | MYO1C | NCK1 | PIK3CA | PIK3R1 | PLA2G6 | PLCG1 | PRKCD | PRKCE | PTK2 | RAC1 | SRC | VAV1 | WAS | YES1 | GO:0002474 | 0.00909862913987626 | 75/8243 | 40/3250 | 0.00985002974641066 | 1 | T | F | F | F | antigen processing and presentation of peptide antigen via MHC class I | CALR | CANX | CD36 | FCGR1A | HLA-A | HLA-B | IKBKB | IKBKG | ITGAV | ITGB5 | PDIA3 | PSMA1 | PSMA2 | PSMA3 | PSMA5 | PSMA6 | PSMB5 | PSMB6 | PSMB7 | PSMB8 | PSMC1 | PSMC2 | PSMC3 | PSMC4 | PSMC5 | PSMC6 | PSMD1 | PSMD10 | PSMD11 | PSMD12 | PSMD14 | PSMD2 | PSMD4 | PSMD9 | PSME2 | PSME3 | SEC13 | SEC24B | SEC24C | SEC31A | GO:0002495 | 0.00909862913987626 | 75/8243 | 40/3250 | 0.00985002974641066 | 1 | T | F | F | F | antigen processing and presentation of peptide antigen via MHC class II | ACTR1A | AP1B1 | AP1G1 | AP1M1 | ARF1 | CANX | CAPZA1 | CAPZA2 | CAPZB | CD74 | CLTC | CTSD | CTSL | CTSV | DCTN1 | DYNC1H1 | DYNC1I1 | DYNC1I2 | DYNC1LI1 | DYNLL1 | HLA-DQA1 | HLA-DRA | HLA-DRB1 | HLA-DRB3 | HLA-DRB4 | HLA-DRB5 | KIF11 | KIF2C | KLC1 | KLC2 | LGMN | OSBPL1A | RAB7A | SEC13 | SEC24B | SEC24C | SEC31A | SH3GL2 | SPTBN2 | TRAF6 | GO:0030968 | 0.00909862913987626 | 75/8243 | 40/3250 | 0.00985002974641066 | 1 | T | F | F | F | endoplasmic reticulum unfolded protein response | ACADVL | AMFR | ARFGAP1 | ASNS | ATF6 | ATF6B | CALR | CREB3 | CTH | CUL7 | CXCL8 | DCTN1 | DERL1 | DNAJB11 | EDEM1 | EIF2AK3 | EIF2S1 | EXTL2 | FKBP14 | GFPT1 | GSK3A | HDGF | HSP90B1 | HSPA5 | LMNA | NFE2L2 | PDIA6 | PTPN1 | SEC31A | SHC1 | SRPRB | STUB1 | SULT1A3 | SYVN1 | TSPYL2 | VAPB | VCP | WIPI1 | XBP1 | ZBTB17 | GO:0043604 | 0.0258401067572486 | 213/8243 | 101/3250 | 0.00991223289426327 | 1 | T | F | F | F | amide biosynthetic process | ACACA | ACAT1 | ACLY | ACSL1 | ACSL4 | ACSL5 | ACSS2 | AGK | AGO2 | AKT1 | ASL | ASS1 | B4GALT3 | CBR4 | CEBPA | CNDP2 | COPS5 | DARS | DEGS1 | DLAT | DLD | DMD | DRG2 | EEF1A1 | EEF1A1P5 | EEF1A2 | EGFR | EIF4H | FARSB | FASN | GCDH | GEMIN5 | GGCT | GGT1 | GGTLC1 | GSPT1 | GSPT2 | GSS | HAGH | HARS | HSD17B8 | HSPA14 | LARP4 | MPC2 | MRPL28 | MRPL43 | MRPL9 | MRRF | NMRAL1 | OTC | P2RX1 | PABPC4 | PDF | PDHA1 | PDHA2 | PDHB | PDHX | PPT1 | RARS2 | RMND1 | RPL10 | RPL10A | RPL12 | RPL18A | RPL22 | RPL23 | RPL23A | RPL24 | RPL30 | RPL36AL | RPL37A | RPL38 | RPL6 | RPL7 | RPL7A | RPL9 | RPLP0 | RPLP0P6 | RPLP1 | RPLP2 | RPS12 | RPS13 | RPS14 | RPS15A | RPS19 | RPS2 | RPS20 | RPS24 | RPS29 | RPS3 | RPS4X | RPS6KB2 | RPS8 | SARS | SLC25A1 | SPTLC2 | ST8SIA4 | TARS | TECR | UGT8 | WARS | GO:0060334 | 0.00230498604876865 | 19/8243 | 13/3250 | 0.00999690026985134 | 1 | T | F | F | F | regulation of interferon-gamma-mediated signaling pathway | CDC37 | HPX | HSP90AB1 | JAK1 | MED1 | NR1H2 | NR1H3 | PARP14 | PIAS1 | PPARG | SOCS1 | STAT1 | SUMO1 | GO:0060330 | 0.00230498604876865 | 19/8243 | 13/3250 | 0.00999690026985134 | 1 | T | F | F | F | regulation of response to interferon-gamma | CDC37 | HPX | HSP90AB1 | JAK1 | MED1 | NR1H2 | NR1H3 | PARP14 | PIAS1 | PPARG | SOCS1 | STAT1 | SUMO1 | GO:0035924 | 0.00230498604876865 | 19/8243 | 13/3250 | 0.00999690026985134 | 1 | T | F | F | F | cellular response to vascular endothelial growth factor stimulus | AKT1 | ANXA1 | HSPB1 | ITGB1BP1 | KDR | MAP2K3 | MAPK14 | NR4A1 | PRKD1 | PRKD2 | RELA | SPRY2 | VCAM1 | GO:0043153 | 0.00230498604876865 | 19/8243 | 13/3250 | 0.00999690026985134 | 1 | T | F | F | F | entrainment of circadian clock by photoperiod | CRY1 | CRY2 | ID2 | MTA1 | PER1 | PER2 | PML | PPP1CA | PPP1CB | PPP1CC | RBM4 | TP53 | USP2 | GO:0050806 | 0.0104330947470581 | 86/8243 | 45/3250 | 0.0100148731891595 | 1 | T | F | F | F | positive regulation of synaptic transmission | ABAT | ADCYAP1 | ADORA2A | ADRB2 | AGER | APP | ARRB2 | BRAF | CACNG2 | CACNG4 | CACNG5 | CDK5 | CLSTN1 | CREB1 | DLG4 | DNM1L | DVL1 | EGFR | EIF2AK4 | FLOT1 | GFAP | GLUL | GRIK2 | HAP1 | ITPR3 | KIF5B | LGMN | MAPK1 | MECP2 | MME | MPP2 | NRXN1 | NTRK1 | PRKCE | PRKCZ | PTEN | PTN | RAB3GAP1 | S100B | SERPINE2 | SNCA | SQSTM1 | STAU1 | STXBP1 | TNF | GO:0019637 | 0.0326337498483562 | 269/8243 | 125/3250 | 0.0100667951685675 | 1 | T | F | F | F | organophosphate metabolic process | ABHD14B | ACACA | ACAT1 | ACLY | ACOT13 | ACOT7 | ACOT9 | ADCYAP1 | ADSS | AK1 | AK2 | AK4 | ALDH5A1 | ALDOA | ALDOC | ATP6V1A | ATP6V1B1 | CALM1 | CKM | CLPX | CMPK1 | CROT | DBI | DCK | DCXR | DGUOK | DLG2 | DLST | FAR2 | FASN | FBP1 | FBP2 | FIGNL1 | G6PD | GFPT1 | GFPT2 | GMPR | GNAI3 | GPD1 | GPD1L | GPI | GPX4 | HK1 | HK2 | HMGCL | HPRT1 | HSD17B4 | HSPA1B | HSPA8 | IDH1 | IDH2 | IDI2 | IMPA1 | IMPA2 | IP6K1 | ISYNA1 | KCNAB2 | LDHA | LRRK2 | MDH1 | MDH2 | MECP2 | MOCOS | MTMR7 | MVD | MYH6 | NDUFS1 | NKX2-1 | NME1 | NME2 | NME4 | NMNAT1 | NMNAT3 | NMRK2 | NNT | NT5C2 | NUDT3 | OGDH | OLA1 | OPA1 | PAFAH1B1 | PARK7 | PARP1 | PCSK9 | PFKL | PFKM | PFKP | PGM3 | PGP | PI4K2A | PIK3C2A | PIK3CA | PIK3CG | PIK3R1 | PIK3R3 | PIKFYVE | PIP4K2A | PIPSL | PLA2G10 | PLA2G12A | PLA2G1B | PLA2G2A | PLA2G6 | PLCG1 | PNKP | PTEN | RAN | REXO2 | RHOA | RNASEH2B | RRM2B | SNCA | SOCS1 | SOCS2 | SOCS6 | SUCLA2 | SUCLG2 | SULT1A3 | TALDO1 | TAZ | THEM4 | TPST2 | TYMS | UGP2 | VCP | GO:0050863 | 0.0230498604876865 | 190/8243 | 91/3250 | 0.0100782991862985 | 1 | T | F | F | F | regulation of T cell activation | ABL1 | ADA | ADORA2A | AGER | AKT1 | ANXA1 | AP3D1 | BCL10 | BCL6 | BRAF | CASP3 | CAV1 | CBFB | CCL21 | CD2 | CD209 | CD27 | CD40LG | CD6 | CD74 | CDC42 | CTNNB1 | CYLD | DNAJA3 | DROSHA | DTX1 | DUSP22 | ERBB2 | FADD | FAM49B | FANCA | FOXJ1 | FYN | GLMN | GRB2 | HAVCR2 | HLX | HMGB1 | HSPD1 | HSPH1 | IFNA2 | IRF1 | IRF4 | ITCH | JAK3 | LAT | LGALS1 | LGALS3 | MAD1L1 | NCK1 | NFATC2 | NOD2 | PAG1 | PAK2 | PAWR | PELI1 | PIK3CA | PIK3R1 | PNP | PRDX2 | PRELID1 | PRKAR1A | PRKCZ | PTPN22 | PTPN6 | RAC1 | RARA | RC3H1 | RHOA | RIPK2 | RIPK3 | RPS3 | RUNX1 | SART1 | SDC4 | SOCS1 | SOCS6 | SOD1 | SRC | TGFB1 | TNFSF14 | TRAF6 | TREX1 | VAV1 | VCAM1 | VSIG4 | XBP1 | YES1 | ZAP70 | ZBTB16 | ZBTB7B | GO:0048146 | 0.00497391726313235 | 41/8243 | 24/3250 | 0.0101266949696199 | 1 | T | F | F | F | positive regulation of fibroblast proliferation | ANXA2 | BMI1 | CCNB1 | CD74 | CDK4 | CDKN1A | DHX9 | E2F1 | EGFR | ESR1 | FBRS | FN1 | JUN | LIG4 | MIF | MYC | PLA2G1B | PML | PRKDC | RNASEH2B | S100A6 | SIRT6 | TGFB1 | WNT2 | GO:0072593 | 0.00497391726313235 | 41/8243 | 24/3250 | 0.0101266949696199 | 1 | T | F | F | F | reactive oxygen species metabolic process | ALOX12 | BCL2 | CCS | DDIT4 | EGFR | FANCC | GLS2 | LRRK2 | NDUFA13 | NDUFAF2 | NDUFS1 | NDUFS3 | NNT | NOS2 | NOS3 | NQO1 | PARK7 | PRDX1 | PRDX2 | RFK | SH3PXD2A | SOD1 | SOD2 | VAV1 | GO:0033673 | 0.0211088196045129 | 174/8243 | 84/3250 | 0.0102440446453811 | 1 | T | F | F | F | negative regulation of kinase activity | ABL1 | ADORA2A | AHSG | AKT1 | APC | APOE | CASP3 | CAV1 | CBL | CBLB | CBLC | CDKN1A | CDKN2C | CEBPA | CHORDC1 | CHP1 | COX11 | DBNDD2 | DNAJA1 | DUSP1 | DUSP14 | DUSP16 | DUSP19 | DUSP22 | DUSP7 | DVL1 | ERRFI1 | FBXO7 | FGFR1OP | GNAQ | GPS2 | GSTP1 | HEXIM1 | HNRNPU | HSPB1 | IBTK | IGF1R | ILK | IPO5 | IPO7 | ITGB1BP1 | LATS1 | LRP6 | MAPT | MLLT1 | NCK1 | NF2 | NPM1 | PAK2 | PARK7 | PKIG | PLK1 | PPP2CA | PPP2R1A | PRDX3 | PRKAR1A | PRKAR2B | PRKCD | PSEN1 | PTEN | PTPN1 | PTPN22 | PTPN6 | QARS | RB1 | RGN | RGS2 | RPTOR | SERPINB3 | SFN | SIRT1 | SNX6 | SOCS1 | SPRY2 | STK38 | TAOK3 | TNFAIP3 | TRIB2 | TRIB3 | TSG101 | UCHL1 | WARS | WNK1 | YWHAG | GO:0008016 | 0.0139512313478103 | 115/8243 | 58/3250 | 0.010306357990918 | 1 | T | F | F | F | regulation of heart contraction | ACE2 | ADA | AHCYL1 | AKAP9 | ATP1A1 | ATP1B3 | ATP2A2 | ATP2B2 | BIN1 | CACNG2 | CACNG4 | CACNG5 | CALM1 | CALM2 | CALM3 | CAV1 | CHRM2 | DES | DMD | FKBP1B | GAA | GLP1R | GLRX3 | GNAO1 | GPD1L | GSK3A | GSTO1 | HBEGF | HDAC4 | ITPR1 | ITPR2 | ITPR3 | JUP | KCNIP2 | KCNK1 | MDM2 | MYH6 | MYL2 | NOS1AP | NOS3 | NPR1 | P2RX4 | PDE4D | PEBP1 | PIK3CG | PRKACA | RGS2 | S100A1 | SLC9A1 | SNTA1 | SRI | STIM1 | SUMO1 | THRA | TMEM65 | TPM1 | YWHAE | ZMPSTE24 | GO:0042752 | 0.00885599902947956 | 73/8243 | 39/3250 | 0.0103576364466772 | 1 | T | F | F | F | regulation of circadian rhythm | ADA | ADORA2A | BTRC | CCAR2 | CREB1 | CRY1 | CRY2 | CSNK1E | EZH2 | FBXW11 | GNAQ | HNRNPD | ID2 | MAPK8 | MAPK9 | MTA1 | NKX2-1 | NR1H3 | PER1 | PER2 | PML | PPARG | PPARGC1A | PPP1CA | PPP1CB | PPP1CC | PRKAA1 | PRKAA2 | PRKDC | PROX1 | PTGDS | RBM4 | SFPQ | SIN3A | TOP2A | TP53 | UBE3A | USP2 | USP9X | GO:1901988 | 0.0219580249909014 | 181/8243 | 87/3250 | 0.0104437615066068 | 1 | T | F | F | F | negative regulation of cell cycle phase transition | APC | ATM | AURKB | BABAM1 | BAX | BCL2 | BRCA1 | CARM1 | CASP2 | CCNB1 | CDC14B | CDC20 | CDC25C | CDC73 | CDK2 | CDK4 | CDKN1A | CDKN2C | CENPF | CHEK2 | CUL1 | E2F1 | E2F4 | EP300 | EZH2 | FBXO7 | FHL1 | FZR1 | IK | KANK2 | KLHL22 | MAD1L1 | MAD2L1 | MDM2 | MDM4 | MSH6 | NAE1 | PCNA | PINX1 | PLK1 | PML | PRKDC | PRMT2 | PSMA1 | PSMA2 | PSMA3 | PSMA5 | PSMA6 | PSMB5 | PSMB6 | PSMB7 | PSMB8 | PSMC1 | PSMC2 | PSMC3 | PSMC4 | PSMC5 | PSMC6 | PSMD1 | PSMD10 | PSMD11 | PSMD12 | PSMD14 | PSMD2 | PSMD4 | PSMD9 | PSME2 | PSME3 | PTEN | RAD21 | RB1 | RBX1 | RIPK1 | RPA2 | SEPT7 | SFN | SPDL1 | TAOK3 | TFDP1 | TP53 | TPR | TRIAP1 | TRIP13 | USP47 | ZFYVE19 | ZNF207 | ZW10 | GO:0090344 | 0.00254761615916535 | 21/8243 | 14/3250 | 0.0105572776242927 | 1 | T | F | F | F | negative regulation of cell aging | ABL1 | AKT3 | BCL6 | FOXM1 | FZR1 | MAP3K3 | MARCH5 | MIF | NAMPT | PRKDC | PTEN | SIRT1 | TERF2 | TWIST1 | GO:1903649 | 0.00254761615916535 | 21/8243 | 14/3250 | 0.0105572776242927 | 1 | T | F | F | F | regulation of cytoplasmic transport | DAB2 | EZR | LRRK2 | MAP2K1 | MAPK1 | MAPK3 | MSN | PTPN23 | RAB21 | RAB29 | RDX | SNX12 | SNX3 | SRC | GO:0060765 | 0.00254761615916535 | 21/8243 | 14/3250 | 0.0105572776242927 | 1 | T | F | F | F | regulation of androgen receptor signaling pathway | ARRB2 | DAB2 | EP300 | HDAC1 | HDAC6 | NCOR1 | NCOR2 | PARK7 | PHB | PRMT2 | SIRT1 | SMARCA4 | TCF21 | ZBTB7A | GO:0009069 | 0.00254761615916535 | 21/8243 | 14/3250 | 0.0105572776242927 | 1 | T | F | F | F | serine family amino acid metabolic process | CBS | CTH | GART | GGT1 | MPST | MTHFD1 | PHGDH | PSAT1 | SARS | SEPHS1 | SEPHS2 | SHMT1 | SHMT2 | SLC25A32 | GO:0014741 | 0.00254761615916535 | 21/8243 | 14/3250 | 0.0105572776242927 | 1 | T | F | F | F | negative regulation of muscle hypertrophy | CTDP1 | ERRFI1 | FOXO1 | G6PD | GLRX3 | GSK3A | LMNA | P2RX4 | RGS2 | SMAD3 | SMAD4 | TNFRSF1A | TRIM63 | YY1 | GO:0007589 | 0.00448865704233895 | 37/8243 | 22/3250 | 0.0106615493637153 | 1 | T | F | F | F | body fluid secretion | AGR2 | ANXA2 | APRT | CAV1 | CEL | CHRM3 | COPA | CREB1 | GOT2 | HIF1A | HK2 | MED1 | MTX1 | NCOA1 | NME1 | NPR1 | PPAT | SOCS2 | STAT5A | UMPS | VDR | ZBTB7B | GO:1902305 | 0.00448865704233895 | 37/8243 | 22/3250 | 0.0106615493637153 | 1 | T | F | F | F | regulation of sodium ion transmembrane transport | ACTN4 | ANK3 | ARF1 | ATP1B3 | CHP1 | COMMD1 | DMD | GLRX | GPD1L | HECW1 | HECW2 | KIF5B | NEDD4 | PCSK9 | PRKCE | SLC9A1 | SLC9A3R1 | SNTA1 | STOM | WNK1 | WNK4 | YWHAH | GO:0051272 | 0.0372437219458935 | 307/8243 | 141/3250 | 0.0106713876054707 | 1 | T | F | F | F | positive regulation of cellular component movement | ABL1 | ACTN4 | AGER | AGO2 | AKT1 | AKT2 | AKT3 | ALOX12 | ANXA1 | APC | APP | ARHGEF7 | ATM | BAG4 | BCAR1 | BCL2 | BCL6 | C1QBP | CALR | CAMK1D | CAV1 | CCL21 | CCR1 | CD40 | CD74 | COL18A1 | CPNE3 | CREB3 | CRK | CRKL | CXCL8 | DAB2 | DDRGK1 | DOCK5 | DOCK7 | EGFR | ETS1 | FADD | FAM83H | FGF2 | FGFR1 | FGFR1OP | FN1 | GNAI2 | GPI | GRB7 | HBEGF | HDAC4 | HDAC6 | HDAC7 | HDAC9 | HIF1A | HMGB1 | HMOX1 | HRAS | HSPA5 | HSPB1 | ICAM1 | IGF1R | ILK | INSR | IQGAP1 | ITGA4 | ITGAV | ITGB1BP1 | ITGB3 | JUN | KDR | LAMB1 | LEF1 | LGALS3 | LGMN | LPAR1 | MAP2K3 | MAP3K3 | MAPK1 | MAPK14 | MAPK3 | MDM2 | MYO1C | MYOC | NFE2L2 | NIPBL | NOS1AP | NOS3 | NRP2 | NTF3 | ONECUT1 | OXSR1 | P2RX4 | PDCD10 | PDCD6 | PFN1 | PIK3C2A | PIK3R1 | PIK3R3 | PLCG1 | PRKCE | PRKD1 | PRKD2 | PROX1 | PTK2 | RAB11A | RAC1 | RDX | RHOA | RHOJ | RPS19 | RTN4 | S100A7 | SDCBP | SEMA7A | SERPINB3 | SIRT1 | SMAD3 | SMO | SMURF2 | SNAI2 | SOD2 | SP1 | SPRY2 | SRC | STAT3 | STAT5A | STMN1 | SUN2 | SWAP70 | SYDE1 | SYNE2 | TGFB1 | THBS1 | TNF | TNFSF14 | TRADD | TRIM32 | TRIP6 | TUBB2B | TWIST1 | VTN | WNK1 | XBP1 | GO:0050848 | 0.00703627320150431 | 58/8243 | 32/3250 | 0.0106790543657774 | 1 | T | F | F | F | regulation of calcium-mediated signaling | ADA | CALM1 | CALM2 | CALM3 | CD22 | CHP1 | DMD | DYRK2 | ERBB3 | FHL2 | FKBP1A | FKBP1B | GSK3B | GSTO1 | HAP1 | HINT1 | HTT | ITPR1 | LRRK2 | MAPK7 | MAPT | MYO5A | P2RX2 | P2RX4 | PDE4D | PDK2 | PRKACA | RGN | SLC9A1 | SRI | TNF | ZAP70 | GO:1902579 | 0.00703627320150431 | 58/8243 | 32/3250 | 0.0106790543657774 | 1 | T | F | F | F | multi-organism localization | CAV1 | CD209 | DDX39B | DYNLT1 | IFIT1 | KPNA1 | KPNB1 | NUP107 | NUP153 | NUP160 | NUP205 | NUP214 | NUP85 | NUP88 | NUP93 | NUP98 | POM121 | RAB29 | RAB7A | RAE1 | RAN | SEC13 | TCP1 | THOC2 | THOC6 | THOC7 | TPR | TSG101 | UBB | UBC | VPS37D | XPO1 | GO:0044766 | 0.00703627320150431 | 58/8243 | 32/3250 | 0.0106790543657774 | 1 | T | F | F | F | multi-organism transport | CAV1 | CD209 | DDX39B | DYNLT1 | IFIT1 | KPNA1 | KPNB1 | NUP107 | NUP153 | NUP160 | NUP205 | NUP214 | NUP85 | NUP88 | NUP93 | NUP98 | POM121 | RAB29 | RAB7A | RAE1 | RAN | SEC13 | TCP1 | THOC2 | THOC6 | THOC7 | TPR | TSG101 | UBB | UBC | VPS37D | XPO1 | GO:0017158 | 0.00703627320150431 | 58/8243 | 32/3250 | 0.0106790543657774 | 1 | T | F | F | F | regulation of calcium ion-dependent exocytosis | ADORA2A | ARF1 | ATP2A2 | BACE1 | BRAF | CALM1 | CALM3 | CAMK2A | CDK5 | CHRM2 | CPLX1 | CPLX3 | DNM1L | DVL1 | GIT1 | GNAI2 | GSK3B | HTR1B | LRRK2 | P2RX1 | PREPL | RAB3GAP1 | RAB5A | RAP1A | RAP1B | REST | SEPT5 | STXBP1 | SYN1 | SYP | TRIM9 | VPS18 | GO:1901796 | 0.016984107727769 | 140/8243 | 69/3250 | 0.0107082451306361 | 1 | T | F | F | F | regulation of signal transduction by p53 class mediator | AKT1 | ATAD5 | ATM | AURKB | BANP | BCL2 | BDKRB2 | BRCA1 | CD74 | CDK2 | CDK5 | CDK5R1 | CHD3 | CHD4 | CHEK2 | CSNK2A2 | CSNK2B | DNA2 | DYRK2 | EHMT1 | EHMT2 | EP300 | HDAC1 | HDAC2 | HEXIM1 | HIPK4 | HNRNPK | ING5 | KAT5 | KDM1A | L3MBTL1 | MAPK14 | MBD3 | MDM2 | MDM4 | MIF | PIN1 | PML | PRKAA1 | PRKAA2 | PRKAB2 | PRKAG1 | PRMT6 | PSMD10 | RBBP4 | RFC2 | RFC3 | RFC4 | RFC5 | RPA2 | RPA3 | RPL23 | RRM2B | RRS1 | SIRT1 | SNAI2 | STK11 | TAF12 | TAF15 | TOP3A | TP53 | TP53RK | TP63 | TP73 | TRIAP1 | TTC5 | TWIST1 | UBB | ZMPSTE24 | GO:0051235 | 0.0126167657406284 | 104/8243 | 53/3250 | 0.0107389420880919 | 1 | T | F | F | F | maintenance of location | AKAP9 | AKT1 | ALB | ANK3 | APOA1 | APOE | CALR | CAV1 | CCDC22 | CD36 | CDK5 | CRY1 | DBN1 | EZR | FAF1 | FLNB | FTH1 | FTL | G3BP2 | GAA | GOLPH3 | GOPC | HEXB | HK1 | HK2 | HNRNPU | HSP90B1 | HSPA5 | INSIG1 | JUP | KEAP1 | LATS1 | LTBP1 | MDFI | NFKBIA | PAFAH1B1 | PARK7 | PML | POLR2M | PSMD10 | RANGAP1 | S100A7 | S100A9 | SOAT1 | SP100 | SRI | SUN2 | SYNE2 | TNF | TWF1 | TWF2 | TXN | YWHAB | GO:2001239 | 0.00424602693194225 | 35/8243 | 21/3250 | 0.0108872186692143 | 1 | T | F | F | F | regulation of extrinsic apoptotic signaling pathway in absence of ligand | AKT1 | BCL2 | BCL2L1 | COL2A1 | EYA2 | EYA4 | FGFR1 | FYN | GATA1 | HSPA1B | HTRA2 | MAPK7 | PPP1CA | PPP2R1A | PPP2R1B | PRDX2 | RIPK1 | SNAI2 | TNF | WWOX | ZC3HC1 | GO:0009166 | 0.00424602693194225 | 35/8243 | 21/3250 | 0.0108872186692143 | 1 | T | F | F | F | nucleotide catabolic process | ACAT1 | ACOT7 | ADA | ASPDH | DCTPP1 | DERA | DUT | GDA | GPX1 | HINT1 | HPRT1 | ITPA | NT5C2 | NUDT18 | NUDT3 | NUDT9 | PDE4D | PNP | PRTFDC1 | UPP2 | VCP | GO:0060071 | 0.00861336891908286 | 71/8243 | 38/3250 | 0.0108899769508318 | 1 | T | F | F | F | Wnt signaling pathway, planar cell polarity pathway | ARRB2 | CDC42 | CLTC | DVL1 | DVL2 | DVL3 | MED12 | PARD6A | PFN1 | PSMA1 | PSMA2 | PSMA3 | PSMA5 | PSMA6 | PSMB5 | PSMB6 | PSMB7 | PSMB8 | PSMC1 | PSMC2 | PSMC3 | PSMC4 | PSMC5 | PSMC6 | PSMD1 | PSMD10 | PSMD11 | PSMD12 | PSMD14 | PSMD2 | PSMD4 | PSMD9 | PSME2 | PSME3 | RAC1 | RHOA | SMURF1 | SMURF2 | GO:0017015 | 0.00861336891908286 | 71/8243 | 38/3250 | 0.0108899769508318 | 1 | T | F | F | F | regulation of transforming growth factor beta receptor signaling pathway | CAV1 | CD109 | CREBBP | DAB2 | EP300 | FOLR1 | GOT1 | HSP90AB1 | HSPA5 | HTRA1 | LDLRAD4 | LEMD3 | LTBP1 | NKX2-1 | ONECUT1 | PIN1 | PPM1A | RASL11B | SDCBP | SIRT1 | SKI | SMAD2 | SMAD3 | SMAD4 | SMURF1 | SMURF2 | SNW1 | SNX6 | STK11 | STRAP | STUB1 | TGFB1 | TGFBR3 | THBS1 | TRIM33 | UBB | UBC | ZBTB7A | GO:0090169 | 0.00279024626956205 | 23/8243 | 15/3250 | 0.0109363834208058 | 1 | T | F | F | F | regulation of spindle assembly | CHMP2A | DYNC1H1 | HNRNPU | HSPA1B | MAPK15 | NUMA1 | PDCD6IP | PLK1 | RNF4 | SENP6 | SMC1A | SMC3 | STAG2 | TPR | VPS4B | GO:1901031 | 0.00279024626956205 | 23/8243 | 15/3250 | 0.0109363834208058 | 1 | T | F | F | F | regulation of response to reactive oxygen species | CD36 | ENDOG | HDAC6 | LRRK2 | NFE2L2 | PARK7 | PAWR | PSAP | PYCR1 | RGN | RIPK1 | STK26 | TNF | TRAP1 | TXN | GO:0042398 | 0.00279024626956205 | 23/8243 | 15/3250 | 0.0109363834208058 | 1 | T | F | F | F | cellular modified amino acid biosynthetic process | ALDH7A1 | ALDH9A1 | ATIC | CHDH | CKM | CNDP2 | FPGS | GART | GGCT | GGT1 | GSS | HAGH | MTHFD1 | PLSCR1 | SHMT1 | GO:0006278 | 0.00279024626956205 | 23/8243 | 15/3250 | 0.0109363834208058 | 1 | T | F | F | F | RNA-dependent DNA biosynthetic process | GAR1 | HSP90AA1 | HSP90AB1 | NOP10 | PINX1 | POT1 | PPIA | PTGES3 | RFC1 | RPA2 | SMG5 | SMG6 | TERF1 | TERF2 | TERF2IP | GO:0043457 | 0.00279024626956205 | 23/8243 | 15/3250 | 0.0109363834208058 | 1 | T | F | F | F | regulation of cellular respiration | CBFA2T3 | CCNB1 | CISD1 | HIF1A | IDE | NOS2 | PARK7 | PIK3CA | PPARGC1A | PRELID1 | SHMT2 | SNCA | TRAP1 | TREX1 | VCP | GO:0006195 | 0.00279024626956205 | 23/8243 | 15/3250 | 0.0109363834208058 | 1 | T | F | F | F | purine nucleotide catabolic process | ACAT1 | ACOT7 | ADA | GDA | GPX1 | HINT1 | HPRT1 | ITPA | NT5C2 | NUDT18 | NUDT3 | NUDT9 | PDE4D | PNP | PRTFDC1 | GO:1903037 | 0.0217153948805047 | 179/8243 | 86/3250 | 0.0110033619506231 | 1 | T | F | F | F | regulation of leukocyte cell-cell adhesion | ADA | ADORA2A | ADTRP | AGER | AKT1 | ANXA1 | AP3D1 | ASS1 | BCL10 | BCL6 | CASP3 | CAV1 | CBFB | CCL21 | CD209 | CD27 | CD40LG | CD6 | CD74 | CDC42 | CYLD | DNAJA3 | DTX1 | DUSP22 | ERBB2 | ETS1 | FADD | FAM49B | FOXJ1 | FYN | GLMN | GRB2 | HAVCR2 | HLX | HMGB1 | HSPD1 | HSPH1 | ICAM1 | IFNA2 | IRF1 | ITCH | ITGA4 | JAK3 | LGALS1 | LGALS3 | MAD1L1 | NCK1 | NOD2 | PAG1 | PAK2 | PAWR | PELI1 | PIK3CA | PIK3R1 | PNP | PRDX2 | PRKAR1A | PRKCZ | PTPN22 | PTPN6 | RAC1 | RARA | RC3H1 | RELA | RHOA | RIPK2 | RPS3 | RUNX1 | SART1 | SDC4 | SOCS1 | SOCS6 | SRC | TGFB1 | TNF | TNFSF14 | TRAF6 | VAV1 | VCAM1 | VSIG4 | WNK1 | XBP1 | YES1 | ZAP70 | ZBTB16 | ZBTB7B | GO:2000142 | 0.00400339682154555 | 33/8243 | 20/3250 | 0.0110734302163591 | 1 | T | F | F | F | regulation of DNA-templated transcription, initiation | CAND1 | CREB1 | CTNNB1 | CTNNBIP1 | DHX36 | ESR1 | HMGB1 | HNF1A | JUN | MITF | PAXIP1 | PSMC2 | PSMC3 | PSMC4 | PSMC5 | PSMC6 | SUB1 | THRA | TP53 | TWIST1 | GO:0034314 | 0.0012131505519835 | 10/8243 | 8/3250 | 0.0110860829627927 | 1 | T | F | F | F | Arp2/3 complex-mediated actin nucleation | ACTR2 | ACTR3 | ARPC1A | ARPC1B | ARPC2 | ARPC3 | ARPC4 | ARPC5 | GO:1904994 | 0.0012131505519835 | 10/8243 | 8/3250 | 0.0110860829627927 | 1 | T | F | F | F | regulation of leukocyte adhesion to vascular endothelial cell | CCL21 | ETS1 | ICAM1 | ITGA4 | RELA | RHOA | TNF | TRAF6 | GO:0051280 | 0.0012131505519835 | 10/8243 | 8/3250 | 0.0110860829627927 | 1 | T | F | F | F | negative regulation of release of sequestered calcium ion into cytosol | CALM1 | CALM2 | CALM3 | FKBP1A | FKBP1B | GSTO1 | SRI | TGFB1 | GO:0030214 | 0.0012131505519835 | 10/8243 | 8/3250 | 0.0110860829627927 | 1 | T | F | F | F | hyaluronan catabolic process | CHP1 | FGF2 | GUSB | HEXA | HEXB | HMMR | SLC9A1 | TGFB1 | GO:0051645 | 0.0012131505519835 | 10/8243 | 8/3250 | 0.0110860829627927 | 1 | T | F | F | F | Golgi localization | CDC42 | COPG1 | DAB1 | PDCD10 | SLC9A3R1 | STK11 | STK25 | YWHAZ | GO:0060134 | 0.0012131505519835 | 10/8243 | 8/3250 | 0.0110860829627927 | 1 | T | F | F | F | prepulse inhibition | ADORA2A | BACE1 | CHD8 | DVL1 | FABP7 | GRID2 | NRXN1 | PTEN | GO:0043650 | 0.0012131505519835 | 10/8243 | 8/3250 | 0.0110860829627927 | 1 | T | F | F | F | dicarboxylic acid biosynthetic process | GLS | GLS2 | GLUD2 | GOT1 | GOT1L1 | GOT2 | HAAO | KYNU | GO:0033617 | 0.0012131505519835 | 10/8243 | 8/3250 | 0.0110860829627927 | 1 | T | F | F | F | mitochondrial respiratory chain complex IV assembly | BCS1L | COA3 | COX17 | COX19 | COX20 | OXA1L | SCO2 | TIMM21 | GO:0099640 | 0.0012131505519835 | 10/8243 | 8/3250 | 0.0110860829627927 | 1 | T | F | F | F | axo-dendritic protein transport | DLG2 | HNRNPU | HSPB1 | KIF5B | KIF5C | MAPK8IP3 | SFPQ | TERF2 | GO:2001028 | 0.0012131505519835 | 10/8243 | 8/3250 | 0.0110860829627927 | 1 | T | F | F | F | positive regulation of endothelial cell chemotaxis | FGF2 | FGFR1 | HSPB1 | KDR | LGMN | P2RX4 | PRKD1 | PRKD2 | GO:0019430 | 0.0012131505519835 | 10/8243 | 8/3250 | 0.0110860829627927 | 1 | T | F | F | F | removal of superoxide radicals | CCS | FANCC | NOS3 | NQO1 | PRDX1 | PRDX2 | SOD1 | SOD2 | GO:0071803 | 0.0012131505519835 | 10/8243 | 8/3250 | 0.0110860829627927 | 1 | T | F | F | F | positive regulation of podosome assembly | CAPG | FSCN1 | LCP1 | MAPK9 | MSN | RHOA | SRC | TNF | GO:1904294 | 0.0012131505519835 | 10/8243 | 8/3250 | 0.0110860829627927 | 1 | T | F | F | F | positive regulation of ERAD pathway | BAG6 | CAV1 | EDEM1 | NFE2L2 | RNF185 | SGTA | UBQLN1 | UBQLN2 | GO:0051284 | 0.0012131505519835 | 10/8243 | 8/3250 | 0.0110860829627927 | 1 | T | F | F | F | positive regulation of sequestering of calcium ion | CALM1 | CALM2 | CALM3 | FKBP1A | FKBP1B | GSTO1 | SRI | TGFB1 | GO:1903624 | 0.0012131505519835 | 10/8243 | 8/3250 | 0.0110860829627927 | 1 | T | F | F | F | regulation of DNA catabolic process | AIFM1 | ATM | BAX | ENDOG | HSF1 | NMNAT1 | PAM16 | RGN | GO:1904851 | 0.0012131505519835 | 10/8243 | 8/3250 | 0.0110860829627927 | 1 | T | F | F | F | positive regulation of establishment of protein localization to telomere | CCT2 | CCT3 | CCT4 | CCT5 | CCT6A | CCT7 | CCT8 | TCP1 | GO:0032502 | 0.312750212301347 | 2578/8243 | 1064/3250 | 0.0111461808138276 | 1 | T | F | F | F | developmental process | A2M | AARS | ABAT | ABL1 | ABL2 | ABLIM1 | ACADVL | ACAT1 | ACD | ACKR2 | ACO2 | ACSL4 | ACTA1 | ACTA2 | ACTB | ACTL6A | ACTN1 | ACTN2 | ADA | ADAMTS4 | ADCYAP1 | ADORA2A | ADRB2 | AES | AFF4 | AFG3L2 | AGER | AGR2 | AHI1 | AHSG | AICDA | AIMP2 | AIRE | AK4 | AKR1B1 | AKR1C2 | AKT1 | AKT2 | AKT3 | ALDH5A1 | ALDH6A1 | ALDOC | ALOX12 | AMFR | ANK3 | ANKRD49 | ANKS4B | ANXA1 | ANXA2 | ANXA6 | ANXA7 | AP1B1 | APBB1 | APC | APEX1 | APLP1 | APOA1 | APOD | APOE | APOL2 | APP | AR | ARF4 | ARHGAP26 | ARHGAP35 | ARHGEF7 | ARID4B | ARNT | ARNT2 | ARPC5 | ARRB2 | ASCL1 | ASGR2 | ASNS | ASS1 | ATF2 | ATF6 | ATG5 | ATIC | ATM | ATOH1 | ATP2A2 | ATP2B2 | ATP6V1B1 | ATRX | ATXN1 | ATXN10 | AURKB | AVIL | AXIN1 | BAG3 | BAG6 | BAIAP2 | BANP | BASP1 | BAX | BCAR3 | BCL10 | BCL2 | BCL2L1 | BCL6 | BCOR | BIRC2 | BMI1 | BMPR1A | BRAF | BRCA1 | BSG | BTK | BTRC | BYSL | C1QA | C1QL1 | CABP4 | CALM1 | CALM2 | CALM3 | CALR | CAMK2A | CAND1 | CANX | CAP2 | CAPN2 | CAPZB | CARM1 | CASP2 | CASP3 | CASP8 | CASP9 | CAT | CAV1 | CBFA2T3 | CBFB | CBL | CBR1 | CBY1 | CCDC120 | CCDC155 | CCDC36 | CCDC85C | CCHCR1 | CCL21 | CCNB1 | CCNT2 | CD109 | CD40LG | CD79B | CDC25B | CDC42 | CDC73 | CDH1 | CDH5 | CDK4 | CDK5 | CDK5R1 | CDKL3 | CDKN1A | CDKN2C | CEBPA | CEBPE | CENPF | CEP290 | CEP55 | CEP57 | CES1 | CFL1 | CFL2 | CFTR | CHD8 | CHEK2 | CHRD | CHRM2 | CHRM3 | CHRNA9 | CHRND | CISD2 | CKB | CLDN18 | CLIC4 | CLTC | CLU | COL12A1 | COL14A1 | COL18A1 | COL2A1 | COL4A1 | COL4A2 | COL4A3BP | COL5A1 | COL6A1 | COL6A2 | COLEC10 | COMT | COPS2 | COX17 | CPT1A | CREB1 | CREBBP | CRIP1 | CRIP2 | CRK | CRKL | CRMP1 | CRYAB | CSNK1A1 | CSNK2A2 | CSNK2B | CTBP1 | CTNNB1 | CTNNBIP1 | CTNND1 | CTSC | CTSV | CTTN | CUL1 | CUL3 | CUL7 | CXCL8 | CYLD | CYP1A1 | CYP1A2 | DAB1 | DAB2 | DAD1 | DAZAP1 | DBNL | DCAF7 | DCLK1 | DCN | DCTN1 | DDIT4 | DDRGK1 | DDX1 | DDX39B | DDX6 | DGCR6 | DHCR24 | DHRS7B | DHX9 | DLD | DLG3 | DLG4 | DMD | DMRT3 | DNAJA3 | DNAJB1 | DNAJC19 | DNAJC30 | DNASE2 | DNM1L | DOCK7 | DPF2 | DPPA4 | DPYSL5 | DTX1 | DUSP22 | DVL1 | DVL2 | DYM | DYNLL1 | E2F1 | E2F4 | EBF1 | EDA | EED | EEF2 | EFEMP1 | EGFR | EGLN1 | EID1 | EIF2AK3 | EIF2AK4 | EIF2B1 | EIF2B3 | EIF2B5 | EIF2S1 | EIF2S2 | EIF4A3 | EIF4E | EIF4E2 | EIF4EBP1 | EIF4ENIF1 | EIF4H | ELAVL1 | ELAVL3 | ELF3 | ELF4 | ENDOG | ENO3 | EP300 | EPHA4 | ERBB2 | ERBB3 | ERF | ERG | ERRFI1 | ESR1 | ESRRA | ESRRB | ETS1 | EYA2 | EYA4 | EZH2 | EZR | FABP5 | FABP7 | FADD | FAM129B | FAM3C | FAM83D | FAM83H | FAM9B | FANCA | FANCC | FANCG | FASLG | FASN | FERMT2 | FGF2 | FGFR1 | FGFR3 | FHL1 | FHL2 | FIGNL1 | FIS1 | FKBP1A | FKBP8 | FLNB | FLOT1 | FN1 | FOLR1 | FOS | FOXA1 | FOXD4 | FOXD4L6 | FOXI2 | FOXJ1 | FOXK1 | FOXO1 | FPGS | FRAS1 | FREM2 | FTO | FYN | FZR1 | G6PD | GAA | GADD45G | GAP43 | GART | GATA1 | GDA | GDF9 | GFAP | GJA1 | GLMN | GLO1 | GLRB | GLRX5 | GLUL | GNA12 | GNA13 | GNAI3 | GNAO1 | GNAS | GNB1 | GNG12 | GNPNAT1 | GORASP2 | GOT1 | GPI | GPS2 | GPSM1 | GPX1 | GPX4 | GRB2 | GRID2 | GRIP1 | GRK5 | GSK3A | GSK3B | GSS | GSTA1 | GSTA2 | GSTK1 | GSTP1 | GTSF1 | GYS1 | H2AFX | HAP1 | HBEGF | HBZ | HCCS | HCN1 | HDAC1 | HDAC11 | HDAC2 | HDAC4 | HDAC5 | HDAC6 | HDAC7 | HDAC9 | HERC1 | HEXB | HEXIM1 | HIF1A | HLX | HMGB1 | HMGB2 | HMGB3 | HMGCL | HMOX1 | HNF1A | HNF4A | HNRNPAB | HNRNPD | HNRNPH3 | HNRNPU | HOXA5 | HOXB5 | HOXB6 | HOXB9 | HOXD10 | HPRT1 | HRAS | HS6ST1 | HSD17B4 | HSF1 | HSF4 | HSP90AA1 | HSP90AB1 | HSPA2 | HSPA5 | HSPA9 | HSPE1 | HSPG2 | HTATIP2 | HTRA1 | HTRA2 | HUWE1 | IARS | ICAM1 | ICK | ID2 | IDH1 | IFI16 | IFNA2 | IFT122 | IGF1R | IGF2BP1 | IGSF21 | IGSF8 | ILK | IMPDH2 | INSIG1 | INSIG2 | INSR | IQGAP1 | IREB2 | IRF1 | IRF4 | ITGA4 | ITGA7 | ITGAV | ITGB1BP1 | ITGB3 | ITGB5 | ITK | JAK3 | JMJD6 | JUN | JUNB | KANK2 | KAT8 | KCNAB1 | KCNAB2 | KDM1A | KDM5B | KDR | KEAP1 | KIF5B | KLF5 | KLF6 | KMT2A | KMT2B | KRAS | KRT17 | KRT31 | KRT71 | KYNU | L3MBTL1 | L3MBTL3 | LAMB1 | LATS1 | LCP1 | LDHA | LEF1 | LEMD2 | LEMD3 | LGALS1 | LGALS3 | LGR4 | LIG4 | LIMS1 | LMBR1 | LMNA | LMO2 | LOXL2 | LPAR1 | LRP6 | LRRK1 | LRRK2 | LSM1 | LTBP1 | LTBR | LUM | LZTR1 | MAD1L1 | MAP1B | MAP2K1 | MAP2K4 | MAP3K3 | MAP3K4 | MAPK1 | MAPK14 | MAPK3 | MAPK8 | MAPK8IP3 | MAPK9 | MAPKAP1 | MAPT | MAS1 | MATK | MBD3 | MBP | MC1R | MCM2 | MCOLN3 | MDFI | MDM1 | MDM2 | MECP2 | MED1 | MED12 | MED14 | MED20 | MED21 | MED31 | MELK | MEOX2 | METRN | METRNL | MEX3C | MGMT | MGST1 | MIB1 | MIF | MITF | MKNK2 | MKS1 | MLH1 | MME | MPST | MSN | MTHFD1 | MTPN | MTSS1 | MXD1 | MYBBP1A | MYC | MYH11 | MYH6 | MYL2 | MYL6 | MYO1D | MYO1E | MYO5A | MYOC | NAMPT | NANOG | NCAPH2 | NCOA1 | NCOA3 | NCOA4 | NDUFS3 | NDUFS6 | NEBL | NEDD4 | NEDD8 | NELFB | NELL1 | NEUROD1 | NF2 | NFATC2 | NFE2L2 | NFKB2 | NID1 | NIF3L1 | NIN | NIPBL | NKX2-1 | NME1 | NOS3 | NOSIP | NPHP3 | NPM1 | NPTX1 | NQO1 | NR0B2 | NR1H2 | NR1H3 | NR1I2 | NR4A1 | NR5A2 | NRP2 | NSUN2 | NTF3 | NTRK1 | NUDC | NUP107 | NUP160 | NUP85 | NUP93 | NUPR1 | NXF2 | OBSL1 | OGDH | ONECUT1 | OPA1 | OTC | OXCT1 | P2RX2 | PABPC4 | PADI4 | PAFAH1B1 | PAK2 | PALB2 | PAPSS1 | PAPSS2 | PARD3 | PARP1 | PATZ1 | PAX8 | PAX9 | PAXIP1 | PCDHA3 | PCDHA4 | PCM1 | PCNA | PCSK9 | PDCD10 | PDCD6 | PDE4D | PDLIM5 | PEBP1 | PEF1 | PER2 | PFAS | PFN1 | PGK1 | PGM3 | PGM5 | PHB | PHB2 | PHC2 | PHC3 | PHF10 | PHGDH | PHOSPHO1 | PIAS1 | PICK1 | PIH1D1 | PIK3CA | PIK3CG | PIK3R1 | PIKFYVE | PIM1 | PIN1 | PIP4K2A | PIR | PITPNA | PKM | PLCG1 | PLS3 | PLXNA1 | PLXNA2 | PLXNB2 | PML | POLDIP2 | POLM | POLR2A | POLR2E | POLR2L | POU2F3 | POU5F1 | PPARD | PPARG | PPARGC1A | PPAT | PPIB | PPP1CA | PPP1CC | PPP2CA | PPP2R5D | PPP3CC | PPT1 | PRCP | PRDX1 | PRDX2 | PRDX3 | PRDX4 | PRELID1 | PRKACA | PRKAR1A | PRKCD | PRKCZ | PRKD1 | PRKD2 | PRKDC | PRMT2 | PRMT6 | PROX1 | PRRC2C | PSAP | PSEN1 | PSMB8 | PSMC2 | PSMC3 | PSMC4 | PSMD11 | PTEN | PTK2 | PTN | PTPN14 | PTPN22 | PTPN6 | PTPRD | PTPRF | PTPRO | PTPRR | PUM1 | QARS | QDPR | RAB10 | RAB18 | RAB1A | RAB1B | RAB3GAP1 | RAC1 | RAD23B | RADIL | RAF1 | RAI14 | RAP1A | RAP1B | RARA | RARG | RB1 | RB1CC1 | RBBP6 | RBM4 | RBMX | RBPJ | RC3H1 | RDH13 | RDH14 | RDX | RECK | RELA | REST | RGN | RGS2 | RHBDD1 | RHO | RHOA | RHOJ | RIF1 | RING1 | RIPK3 | RIPK4 | RNASEH2B | RNF2 | RNF8 | ROMO1 | RP2 | RPGRIP1 | RPGRIP1L | RPL10 | RPL24 | RPL38 | RPN2 | RPS14 | RPS19 | RPS4X | RRM1 | RRM2B | RRP7A | RRS1 | RTCB | RTF1 | RTL1 | RTN4 | RUNX1 | RUNX2 | RXRA | S100A1 | S100A4 | S100A6 | S100A7 | S100B | SART3 | SAT1 | SBDS | SCARA3 | SCEL | SCFD1 | SCG2 | SCML2 | SCO2 | SDC1 | SDC4 | SDCBP2 | SDHA | SEC16A | SEC24B | SEC24C | SEC63 | SEMA7A | SEPT12 | SEPT4 | SEPT6 | SEPT7 | SERF1A | SERPINB5 | SERPINE2 | SERPINH1 | SF1 | SF3B6 | SFMBT1 | SFN | SFXN1 | SGPL1 | SH3GL1 | SH3GL2 | SH3GL3 | SH3PXD2A | SHC1 | SIAH1 | SIN3A | SIRT1 | SIRT3 | SIRT6 | SKI | SLAMF8 | SLC12A2 | SLC9A1 | SLC9A3R1 | SMAD2 | SMAD3 | SMAD4 | SMAD5 | SMARCA2 | SMARCA4 | SMARCB1 | SMARCC1 | SMARCC2 | SMARCE1 | SMC1A | SMC3 | SMN1 | SMO | SMURF1 | SNAI2 | SNCA | SND1 | SNX17 | SNX2 | SOAT1 | SOCS1 | SOCS2 | SOD1 | SORT1 | SOX2 | SP2 | SPACA1 | SPATA2 | SPATA20 | SPATA24 | SPI1 | SPIN1 | SPINT2 | SPRR2A | SPRY2 | SPTBN2 | SPTLC2 | SQSTM1 | SRC | SRGAP2 | SRI | SRPK2 | SRSF5 | ST8SIA4 | STARD13 | STAT1 | STAT3 | STAT6 | STIL | STIM1 | STK11 | STK24 | STK26 | STK3 | STK40 | STMN1 | STRBP | STXBP1 | SULF2 | SUMO1 | SVIL | SYN1 | SYP | TAB1 | TAB2 | TAGLN | TAGLN2 | TAGLN3 | TAZ | TBC1D23 | TCF12 | TCF21 | TCF25 | TCOF1 | TERF2 | TFDP1 | TFIP11 | TGFB1 | TGFBR3 | TGM2 | THBS1 | THOC2 | THOC6 | THRA | TIAL1 | TIMP1 | TIMP2 | TLE1 | TLE3 | TLR3 | TMEM223 | TMEM231 | TMEM65 | TMOD3 | TMOD4 | TNF | TNFRSF12A | TNFRSF1A | TNFSF12 | TNK2 | TNKS2 | TNN | TNP1 | TOLLIP | TOP2A | TP53 | TP63 | TP73 | TPD52 | TPI1 | TPM1 | TPM4 | TRAF3IP1 | TRAF6 | TREX1 | TRIM28 | TRIM32 | TRIP13 | TSG101 | TSKU | TTLL1 | TUBB3 | TUBB8 | TUFT1 | TULP3 | TWIST1 | TXNIP | TXNRD1 | TYMS | UBA6 | UBB | UBE2B | UBE2J1 | UBE2V1 | UBE3A | UCHL1 | UCHL5 | UFC1 | UFM1 | UGDH | UGT8 | ULK1 | UNC119 | UNK | UPF2 | UQCRQ | USP2 | USP42 | USP7 | USP9X | VASH2 | VASP | VAV1 | VCAM1 | VCAN | VCL | VDAC1 | VDR | VGF | VHL | VIM | VKORC1 | VPS72 | VSTM2A | VTN | WARS | WAS | WDR1 | WDR19 | WDR35 | WDR48 | WDR77 | WDTC1 | WNK1 | WNK4 | WNT16 | WNT2 | WT1 | WTAP | WWOX | XBP1 | XIRP2 | XRCC1 | XRCC5 | XRCC6 | XYLT1 | YAP1 | YES1 | YWHAE | YWHAH | YWHAQ | YY1 | ZAP70 | ZBTB16 | ZBTB17 | ZBTB42 | ZBTB7A | ZBTB7B | ZFAND5 | ZFP41 | ZFYVE19 | ZHX1 | ZIC1 | ZMPSTE24 | ZNF488 | ZSCAN2 | GO:0045944 | 0.0881960451292005 | 727/8243 | 316/3250 | 0.0111510910844816 | 1 | T | F | F | F | positive regulation of transcription by RNA polymerase II | ABHD14B | ABL1 | ACTR2 | ACTR3 | ADCYAP1 | ADRB2 | AGO1 | AGO2 | AHI1 | AIRE | AKT1 | APBB1 | APP | AR | ARF4 | ARID3B | ARID4B | ARNT | ARNT2 | ARRB1 | ASCL1 | ATF2 | ATF6 | ATF6B | ATM | ATOH1 | ATRX | BMPR1A | BRCA1 | BRD4 | CAND1 | CARM1 | CBFB | CCNT1 | CCNT2 | CD40 | CD81 | CDC5L | CDC73 | CDK8 | CDK9 | CEBPA | CEBPE | CHCHD2 | CHD8 | COPS5 | CREB1 | CREB3 | CREBBP | CTCF | CTNNB1 | DCN | DDRGK1 | DDX3X | DHX36 | DHX9 | DPF2 | E2F1 | E2F2 | E2F3 | E2F4 | E2F6 | EAF2 | EBF1 | ECD | EGFR | EGLN1 | ELF3 | ELF4 | EP300 | ERG | ERLIN2 | ESR1 | ESRRA | ESRRB | ESRRG | ETS1 | EXOSC9 | FADD | FGF2 | FOS | FOXA1 | FOXJ1 | FOXK2 | FOXM1 | FOXO1 | FUBP3 | GABPB1 | GATA1 | GPS2 | GTF2F1 | GTF2F2 | H2AFZ | HAX1 | HDAC1 | HDAC2 | HDAC3 | HDAC4 | HDAC5 | HELZ2 | HEXB | HIF1A | HMGB1 | HMGB2 | HMGB3 | HNF1A | HNF4A | HNF4G | HNRNPK | HNRNPU | HOXA5 | HOXB5 | HOXB9 | HOXD10 | HRAS | HSF1 | HSF4 | HSPA5 | IFI16 | IKBKB | IKBKG | IKZF5 | INSIG1 | INSIG2 | IRF1 | IRF4 | IRF7 | ITGB1BP1 | JMJD6 | JUN | JUNB | JUND | JUP | KAT5 | KAT7 | KAT8 | KDM1A | KLF5 | KLF6 | KMT2A | KMT2C | LEF1 | LMO2 | LMO7 | LPIN3 | LRP6 | LUM | MAPK14 | MAPK3 | MAPK7 | MAVS | MC1R | MED1 | MED12 | MED14 | MED17 | MED21 | MED6 | MEOX2 | METTL23 | MITF | MORF4L2 | MYC | MYO6 | NAMPT | NANOG | NCK1 | NCOA1 | NCOA2 | NCOA3 | NELFE | NEUROD1 | NFATC1 | NFATC2 | NFE2L2 | NFKB2 | NFKBIA | NIPBL | NKRF | NKX2-1 | NME2 | NOD2 | NPM1 | NR1H2 | NR1H3 | NR1I2 | NR3C1 | NR4A1 | NR5A2 | NUFIP1 | OGT | ONECUT1 | PARK7 | PARP1 | PATZ1 | PAX8 | PAX9 | PAXIP1 | PCBP1 | PCGF5 | PER1 | PFKM | PHF10 | PIK3R1 | PIN1 | PLA2G1B | PLSCR1 | POU2F3 | POU5F1 | PPARD | PPARG | PPARGC1A | PPP1R12A | PRKD1 | PRKD2 | PRKDC | PROX1 | PSMC2 | PSMC3 | PSMC4 | PSMC5 | PSMC6 | RAF1 | RARA | RARG | RB1 | RBMX | RBPJ | REL | RELA | REST | RFXANK | RIPK1 | RIPK2 | RNF4 | RPS6KA1 | RTF1 | RUNX1 | RUNX2 | RUVBL2 | RXRA | RXRB | RXRG | SFPQ | SIN3A | SIRT1 | SKI | SLC9A1 | SMAD2 | SMAD3 | SMAD4 | SMAD5 | SMAD9 | SMARCA2 | SMARCA4 | SMARCB1 | SMARCC1 | SMO | SNW1 | SOX2 | SP1 | SPI1 | SQSTM1 | STAT1 | STAT3 | STAT4 | STAT6 | SUB1 | SUMO2 | SUPT4H1 | SUPT5H | TARDBP | TBL1X | TCEA2 | TCF12 | TCF21 | TCF4 | TFCP2 | TFDP1 | TGFB1 | THRA | TLR3 | TMEM173 | TNF | TNFRSF1A | TNIP2 | TNKS | TOP2A | TP53 | TP63 | TP73 | TRAF6 | TRIAP1 | TTC5 | TWIST1 | UBB | UBC | USP16 | VDR | WAS | WBP2 | WNT2 | WT1 | WWOX | WWP2 | XBP1 | XRCC6 | YAP1 | YES1 | YY1 | ZBTB16 | ZBTB17 | ZBTB7B | ZIC1 | ZNF24 | ZNF746 | GO:1902751 | 0.00303287637995875 | 25/8243 | 16/3250 | 0.0111658862701291 | 1 | T | F | F | F | positive regulation of cell cycle G2/M phase transition | APP | ATAD5 | BRD4 | CCNB1 | CDC25A | CDC25B | CDC25C | CDK4 | DBF4B | HSPA2 | MECP2 | MTA3 | NPM1 | RAB11A | SIN3A | VPS4B | GO:0072524 | 0.00303287637995875 | 25/8243 | 16/3250 | 0.0111658862701291 | 1 | T | F | F | F | pyridine-containing compound metabolic process | DCXR | G6PD | GPD1L | IDH1 | IDH2 | KCNAB2 | LDHA | MDH1 | MDH2 | NMNAT1 | NMNAT3 | NMRK2 | NNT | OGDH | PDXK | VCP | GO:0071868 | 0.00303287637995875 | 25/8243 | 16/3250 | 0.0111658862701291 | 1 | T | F | F | F | cellular response to monoamine stimulus | ABL1 | ADCY6 | APLP1 | APP | GNB1 | HCN3 | HDAC2 | LRRK2 | MAPK1 | MAPK3 | PDE4D | PRKACA | SIN3A | SLC9A1 | SNCA | SULT1A3 | GO:0071870 | 0.00303287637995875 | 25/8243 | 16/3250 | 0.0111658862701291 | 1 | T | F | F | F | cellular response to catecholamine stimulus | ABL1 | ADCY6 | APLP1 | APP | GNB1 | HCN3 | HDAC2 | LRRK2 | MAPK1 | MAPK3 | PDE4D | PRKACA | SIN3A | SLC9A1 | SNCA | SULT1A3 | GO:1904353 | 0.00303287637995875 | 25/8243 | 16/3250 | 0.0111658862701291 | 1 | T | F | F | F | regulation of telomere capping | ATM | AURKB | HNRNPD | MAP2K7 | MAP3K4 | MAPK1 | MAPK15 | MAPK3 | NEK7 | PNKP | SMG6 | TERF2 | TNKS | TNKS2 | USP7 | XRCC1 | GO:0120162 | 0.00679364309110761 | 56/8243 | 31/3250 | 0.011166989515347 | 1 | T | F | F | F | positive regulation of cold-induced thermogenesis | ACSL1 | ADCYAP1 | ADRB2 | APC | CAV1 | CD36 | CPT2 | DBH | DECR1 | DYNC1H1 | EHMT1 | ESRRG | FABP5 | FH | GADD45G | GJA1 | GNAS | HADH | HDAC3 | HSF1 | IGF1R | IRF4 | KDM1A | OGT | PER2 | PPARGC1A | PRKAB2 | SIRT6 | STAT6 | THRA | ZBTB7B | GO:0040008 | 0.0479194468033483 | 395/8243 | 178/3250 | 0.0111854155990512 | 1 | T | F | F | F | regulation of growth | ABL1 | ACTL6A | ADRB2 | AFG3L2 | AGR2 | AKT1 | APBB1 | APBB2 | APOE | APP | AR | ARMC10 | ATAD3A | BASP1 | BCAR1 | BCL11A | BCL2 | BCL2L1 | BCL6 | BLZF1 | BMPR1A | BNIPL | CARM1 | CCAR2 | CCNB1 | CD36 | CDA | CDC42 | CDK4 | CDK5 | CDK5R1 | CDKL3 | CDKN1A | CDKN2C | CLSTN1 | CREB1 | CRKL | CRYAB | CTDP1 | CTTN | DDX39B | DDX3X | DMAP1 | DNAJB2 | EAF2 | EGFR | ENO1 | EPPK1 | ERBB2 | ESR2 | EXOSC9 | EZR | FBP1 | FGF2 | FGFR1 | FGFR1OP | FGFR3 | FHL1 | FN1 | FTO | G6PD | GAP43 | GDF9 | GDI1 | GJA1 | GOLGA4 | GSK3A | GSK3B | H3F3A | HBEGF | HIF1A | HLX | HNF4A | HSF1 | HSPA1B | HTRA2 | IGFBP7 | ILK | ING1 | ING5 | INSR | ITCH | KAT5 | KLHL22 | LATS1 | LEF1 | LGMN | MAP1B | MAP3K13 | MAPK1 | MAPK14 | MAPT | MCTS1 | MORF4L1 | MORF4L2 | MRGBP | MTPN | MYH6 | MYL2 | NCBP1 | NDUFA13 | NDUFS3 | NIPBL | NPR1 | NUBP1 | PAFAH1B1 | PAK2 | PHB | PIK3CA | PIM1 | PIN1 | PML | PPARD | PPARG | PPIB | PPP2CA | PPP2R1A | PPT1 | PRKDC | PROX1 | PSMD10 | PTEN | PTK2 | RAB11A | RAB21 | RB1 | RBPJ | RGS2 | RHOA | RIPK1 | RPS6KA1 | RPTOR | RTN4 | RUVBL1 | RUVBL2 | S100A9 | SDCBP | SEMA7A | SERPINE2 | SFN | SGPL1 | SHC1 | SIPA1 | SIRT1 | SLC9A1 | SMAD3 | SMAD4 | SMARCA2 | SMARCA4 | SMO | SMURF1 | SOCS1 | SOCS2 | SOCS6 | SOD1 | SQSTM1 | STAT3 | STAT5A | STK11 | STK3 | TGFB1 | TGFBR3 | TKT | TNF | TNFRSF12A | TNKS2 | TP53 | TP73 | TRIM32 | TSG101 | TSPYL2 | TWF2 | USP47 | WNT2 | WT1 | YAP1 | YY1 | ZMPSTE24 | GO:0021762 | 0.00376076671114885 | 31/8243 | 19/3250 | 0.0112091557165996 | 1 | T | F | F | F | substantia nigra development | ACTB | BASP1 | CALM1 | CALM2 | CALM3 | CDC42 | CKB | DYNLL1 | G6PD | HSPA5 | MAPKAP1 | MBP | NDUFS3 | RHOA | S100A1 | SEC16A | YWHAE | YWHAH | YWHAQ | GO:0090049 | 0.00376076671114885 | 31/8243 | 19/3250 | 0.0112091557165996 | 1 | T | F | F | F | regulation of cell migration involved in sprouting angiogenesis | ABL1 | AKT3 | ANXA1 | FGF2 | HDAC5 | HDAC7 | HDAC9 | HMOX1 | ITGB1BP1 | KDR | MAP3K3 | MEOX2 | PDCD10 | PIK3C2A | RHOA | RHOJ | STARD13 | TBXA2R | THBS1 | GO:1903202 | 0.00376076671114885 | 31/8243 | 19/3250 | 0.0112091557165996 | 1 | T | F | F | F | negative regulation of oxidative stress-induced cell death | AKT1 | BAG5 | CTNNB1 | FBXO7 | FYN | GPX1 | HIF1A | HSPB1 | HTRA2 | LRRK2 | MAPK7 | NFE2L2 | PARK7 | PSAP | PYCR1 | SIRT1 | SOD2 | TRAP1 | TXN | GO:1900408 | 0.00376076671114885 | 31/8243 | 19/3250 | 0.0112091557165996 | 1 | T | F | F | F | negative regulation of cellular response to oxidative stress | AKT1 | BAG5 | CTNNB1 | FBXO7 | FYN | GPX1 | HIF1A | HSPB1 | HTRA2 | LRRK2 | MAPK7 | NFE2L2 | PARK7 | PSAP | PYCR1 | SIRT1 | SOD2 | TRAP1 | TXN | GO:0032370 | 0.00376076671114885 | 31/8243 | 19/3250 | 0.0112091557165996 | 1 | T | F | F | F | positive regulation of lipid transport | ABCG1 | ANXA2 | APOA1 | APOE | C1QTNF1 | FABP3 | GPS2 | MAP2K6 | MIF | NFKBIA | NR1H2 | NR1H3 | P2RX4 | PLA2G10 | PLA2G6 | PRELID1 | PRKCD | SIRT1 | TRIAP1 | GO:1902883 | 0.00376076671114885 | 31/8243 | 19/3250 | 0.0112091557165996 | 1 | T | F | F | F | negative regulation of response to oxidative stress | AKT1 | BAG5 | CTNNB1 | FBXO7 | FYN | GPX1 | HIF1A | HSPB1 | HTRA2 | LRRK2 | MAPK7 | NFE2L2 | PARK7 | PSAP | PYCR1 | SIRT1 | SOD2 | TRAP1 | TXN | GO:0048662 | 0.00327550649035545 | 27/8243 | 17/3250 | 0.0112730842165007 | 1 | T | F | F | F | negative regulation of smooth muscle cell proliferation | APOD | CDKN1A | COMT | CTNNBIP1 | GSTP1 | HMOX1 | ILK | NPR1 | PPARD | PPARG | PPARGC1A | PTEN | PTGIR | SF1 | SOD2 | TNFAIP3 | TPM1 | GO:0032570 | 0.00327550649035545 | 27/8243 | 17/3250 | 0.0112730842165007 | 1 | T | F | F | F | response to progesterone | CAV1 | CSN1S1 | FOS | GPI | NCOA1 | NCOA2 | NR1H3 | PTN | RELA | SRC | TGFB1 | THBS1 | TXNIP | TYMS | UBE3A | WBP2 | YAP1 | GO:0002931 | 0.00327550649035545 | 27/8243 | 17/3250 | 0.0112730842165007 | 1 | T | F | F | F | response to ischemia | AIFM1 | BCL2 | CAMK2A | CASP9 | CAV1 | EEF2 | EIF4EBP1 | HK2 | MAP2K6 | P2RX2 | P2RX4 | PER2 | PPARGC1A | PPIF | SQSTM1 | STUB1 | UCHL1 | GO:0045581 | 0.00327550649035545 | 27/8243 | 17/3250 | 0.0112730842165007 | 1 | T | F | F | F | negative regulation of T cell differentiation | ANXA1 | BCL6 | CBFB | CD74 | DTX1 | ERBB2 | FOXJ1 | HLX | HMGB1 | IFNA2 | IRF1 | JAK3 | PRDX2 | RC3H1 | RUNX1 | SOCS1 | ZBTB7B | GO:0043094 | 0.00351813660075215 | 29/8243 | 18/3250 | 0.0112810660022449 | 1 | T | F | F | F | cellular metabolic compound salvage | ADA | ADK | APRT | CDA | DCK | DGUOK | ENOPH1 | GMPR | HPRT1 | MTAP | NAPRT | PDXK | PNP | PRTFDC1 | TK1 | TK2 | UCK2 | UPP2 | GO:0043331 | 0.00351813660075215 | 29/8243 | 18/3250 | 0.0112810660022449 | 1 | T | F | F | F | response to dsRNA | CARD9 | CAV1 | DDX1 | DHX36 | DHX9 | FLOT1 | IFIT1 | IFNA2 | MAPK1 | MAPK3 | MAVS | NFKBIA | PDE12 | PELI1 | RIPK2 | SLC3A2 | TLR3 | TMEM173 | GO:0006296 | 0.00351813660075215 | 29/8243 | 18/3250 | 0.0112810660022449 | 1 | T | F | F | F | nucleotide-excision repair, DNA incision, 5'-to lesion | CHD1L | CUL4B | DDB1 | DDB2 | ERCC5 | PARP1 | PCNA | POLD1 | RBX1 | RFC1 | RFC2 | RFC3 | RFC4 | RFC5 | RPA2 | RPA3 | UBB | UBC | GO:0032205 | 0.00351813660075215 | 29/8243 | 18/3250 | 0.0112810660022449 | 1 | T | F | F | F | negative regulation of telomere maintenance | ACD | ATM | DCP2 | HNRNPA1 | HNRNPU | PARP1 | PINX1 | POT1 | SMG6 | SRC | TERF1 | TERF2 | TERF2IP | TINF2 | TNKS | TNKS2 | XRCC1 | XRCC5 | GO:0051606 | 0.0123741356302317 | 102/8243 | 52/3250 | 0.0113336648742962 | 1 | T | F | F | F | detection of stimulus | ABCG1 | ARRB1 | ARRB2 | BACE1 | CABP4 | CALM1 | CALM2 | CALM3 | CHRNA9 | CUL4B | DDB1 | DNAJA1 | FYN | GNAQ | GNB1 | GRK1 | JUP | KCNIP2 | NEK1 | NOD2 | NTRK1 | OR5F1 | P2RX2 | PARP1 | PCNA | PDE6G | PNKP | POLD1 | PPEF1 | RBX1 | RFC1 | RFC2 | RFC3 | RFC4 | RFC5 | RHO | RPA2 | RPA3 | RPS3 | SAG | SERPINE2 | SMO | STIM1 | TAS2R41 | TAS2R60 | TAS2R7 | TREX1 | UBB | UBC | UBE2B | UNC119 | WDR48 | GO:0051271 | 0.0206235593837195 | 170/8243 | 82/3250 | 0.0113788228825447 | 1 | T | F | F | F | negative regulation of cellular component movement | ACTN1 | ACTN4 | ADA | ADTRP | AKT1 | APEX1 | APOD | APOE | BCL2 | BIN1 | BMPR1A | BRAF | CALR | CCL21 | CDH1 | CHRD | CLIC4 | CRK | CSNK2B | DCN | DPEP1 | DUSP1 | DUSP22 | EPPIN | EPPK1 | FGF2 | GSTP1 | HDAC5 | HMGB1 | HMOX1 | IDH2 | ILK | ITGB1BP1 | JUP | LDLRAD4 | MAPK15 | MCC | MECP2 | MEOX2 | MIF | MITF | NF2 | NFE2L2 | NKX2-1 | PDCD10 | PIN1 | PPARD | PPARG | PPARGC1A | PTEN | PTN | PTPN23 | PTPRK | PTPRO | PTPRR | RECK | RGN | RHOA | SEMA7A | SEPT7 | SLAMF8 | SLC9A3R1 | SP100 | SPINT2 | SRGAP2 | SRGAP3 | STARD13 | STAT3 | STK24 | STK26 | STRAP | SYNJ2BP | TBXA2R | TGFB1 | TGFBR3 | THBS1 | TIMP1 | TNN | TPM1 | VCL | WAS | ZMYND8 | GO:0072329 | 0.00837073880868616 | 69/8243 | 37/3250 | 0.0114480042395871 | 1 | T | F | F | F | monocarboxylic acid catabolic process | ABCD3 | ACAA1 | ACAA2 | ACADS | ACADVL | ACAT1 | ACAT2 | ACOX1 | ADTRP | AKR1A1 | CEL | CPT1A | CPT2 | CROT | DCXR | DECR1 | ECH1 | ECHDC2 | ECHS1 | ECI1 | ECI2 | EHHADH | ETFA | ETFB | ETFDH | FAH | GCDH | HADH | HIBCH | HSD17B4 | IVD | LDHD | LPIN3 | MCAT | NUDT19 | PPARD | SORD | GO:0001558 | 0.0276598325852238 | 228/8243 | 107/3250 | 0.0117086107333494 | 1 | T | F | F | F | regulation of cell growth | ABL1 | AKT1 | APBB1 | APBB2 | APOE | ATAD3A | BCAR1 | BCL11A | BCL2 | BCL6 | BLZF1 | CCAR2 | CDA | CDC42 | CDK5 | CDK5R1 | CDKL3 | CDKN1A | CDKN2C | CLSTN1 | CRKL | CRYAB | CTDP1 | CTTN | DDX39B | DDX3X | DNAJB2 | EAF2 | EGFR | ENO1 | ERBB2 | ESR2 | EXOSC9 | FBP1 | FGFR1OP | FHL1 | FN1 | G6PD | GDF9 | GDI1 | GJA1 | GOLGA4 | GSK3A | GSK3B | H3F3A | HBEGF | HNF4A | HSPA1B | IGFBP7 | ILK | ING1 | ITCH | KLHL22 | LEF1 | MAP1B | MAP3K13 | MAPT | MTPN | MYL2 | NCBP1 | NDUFA13 | NDUFS3 | NPR1 | NUBP1 | PAFAH1B1 | PHB | PIN1 | PML | PPARD | PPARG | PPP2CA | PPP2R1A | PPT1 | PSMD10 | RAB11A | RAB21 | RB1 | RGS2 | RHOA | RPS6KA1 | RPTOR | RTN4 | S100A9 | SDCBP | SEMA7A | SERPINE2 | SFN | SIPA1 | SIRT1 | SLC9A1 | SMAD3 | SMAD4 | SMARCA2 | SMARCA4 | SMURF1 | SOCS2 | STK11 | TGFB1 | TNFRSF12A | TP53 | TRIM32 | TSG101 | TSPYL2 | TWF2 | USP47 | WT1 | YY1 | GO:0006629 | 0.0624772534271503 | 515/8243 | 228/3250 | 0.0117153909724148 | 1 | T | F | F | F | lipid metabolic process | AASDH | ABCD3 | ABCG1 | ACAA1 | ACAA2 | ACAD8 | ACAD9 | ACADS | ACADSB | ACADVL | ACAT1 | ACAT2 | ACBD3 | ACLY | ACOX1 | ACSL1 | ACSL4 | ACSL5 | ADH1A | ADH1B | ADH5 | ADTRP | AGK | AKR1B1 | AKR1B10 | AKR1B15 | AKR1C2 | ALDH5A1 | ALDH8A1 | ALOX12 | ALOX5 | ALOX5AP | APOA1 | APOD | APOE | APOL2 | APP | ATP6V1B1 | B4GALT3 | BAX | BTN2A1 | CAT | CAV1 | CBR1 | CD36 | CEBPA | CEL | CES1 | CFTR | CLU | COL4A3BP | COMT | CPNE3 | CPT1A | CPT2 | CRK | CRKL | CROT | CTSA | CYB5R1 | CYB5R3 | CYP1A1 | CYP1A2 | CYP2C18 | DBI | DECR1 | DEGS1 | DGKE | DGKZ | DHCR24 | DHRS4 | DHRS7B | DLD | DPM1 | ECH1 | ECHDC2 | ECHS1 | ECI1 | ECI2 | EGFR | EHHADH | ERLIN2 | ESR1 | ETFA | ETFB | ETFDH | FABP1 | FABP3 | FABP5 | FABP7 | FAR2 | FASN | FDPS | FDXR | FGFR1 | G6PD | GCDH | GGPS1 | GGT1 | GLA | GPC5 | GPX1 | GPX4 | GSTA1 | GSTP1 | HADH | HEXA | HEXB | HIBCH | HMGCL | HNF4A | HSD17B10 | HSD17B14 | HSD17B4 | HSD17B6 | HSD17B8 | HSD3B7 | HSPG2 | HTRA2 | IDI2 | IMPA1 | IMPA2 | INSIG1 | INSIG2 | IP6K1 | IVD | KDSR | LGMN | LIMA1 | LPIN3 | LTC4S | LYPLA1 | LYPLA2 | MAPK14 | MAPK3 | MCAT | MECP2 | MED1 | MPPE1 | MTMR7 | MVD | NDUFS6 | NKX2-1 | NR0B2 | NR1H2 | NR1H3 | NR1I2 | NR5A2 | NUDT19 | OSBPL1A | P2RX1 | PAFAH1B1 | PC | PCSK9 | PER2 | PGP | PI4K2A | PIGU | PIK3C2A | PIK3CA | PIK3CG | PIK3R1 | PIK3R3 | PIKFYVE | PIP4K2A | PIPSL | PITPNA | PITPNB | PLA2G10 | PLA2G12A | PLA2G1B | PLA2G2A | PLA2G6 | PLCG1 | PPARD | PPARG | PPARGC1A | PPP2CA | PPP2R1A | PPT1 | PRDX6 | PRKAA1 | PRKAA2 | PRKAR2B | PRKD1 | PRKD2 | PRKD3 | PSAP | PTEN | PTGDS | PTGES | PTGES2 | PTGES3 | PTGR1 | RBP1 | RDH11 | RDH12 | RDH13 | RDH14 | RDH5 | RHO | RXRA | SCCPDH | SDC1 | SDC4 | SEL1L | SGPL1 | SIRT1 | SLC16A1 | SNCA | SOAT1 | SOCS1 | SOCS2 | SOCS6 | SPTLC2 | ST3GAL6 | ST6GALNAC5 | ST8SIA4 | SULT1A3 | TAZ | THEM4 | TNFRSF1A | UGT8 | VAPA | VAPB | VDR | WWOX | ZMPSTE24 | GO:0007276 | 0.0240203809292733 | 198/8243 | 94/3250 | 0.012050776628688 | 1 | T | F | F | F | gamete generation | ACOX1 | ACTR1A | ANKRD49 | AR | ARID4B | ATRX | BAG6 | BCL2L1 | BCL2L2 | BCL6 | BIRC3 | BRD2 | CALR | CCDC155 | CCDC36 | CCNB1 | CCT6B | CDC25C | CDK16 | CRKL | CSNK2A2 | CTSV | DAZAP1 | DEDD | DHX36 | DIAPH3 | DNAJA1 | DUSP13 | E2F1 | FAM9B | FOXJ1 | GDF9 | GGT1 | GJA1 | GORASP2 | GPX4 | GTSF1 | H2AFX | HERC2 | HEXB | HIST1H1A | HSF1 | HSPA2 | KHDRBS1 | LGR4 | LRRK2 | MAS1 | MCM9 | MLH1 | OAZ3 | PATZ1 | PGAM2 | PGM3 | PHC2 | PIAS1 | PRDX4 | PRKAG1 | PTTG1 | PUM1 | RAD23B | RAI14 | RARA | RGN | RGS2 | RUVBL1 | SEPT12 | SEPT2 | SEPT6 | SEPT7 | SFMBT1 | SGPL1 | SIAH1 | SIRT1 | SMAD4 | SNRPA1 | SOD1 | SPATA2 | SPATA20 | SPATA24 | SPIN1 | SRC | STK11 | TESK2 | TP63 | TRIP13 | TSGA10 | UBE2B | USP42 | USP9X | USP9Y | WDR48 | YY1 | ZFP41 | ZSCAN2 | GO:0006109 | 0.0154070120101905 | 127/8243 | 63/3250 | 0.0120791865479066 | 1 | T | F | F | F | regulation of carbohydrate metabolic process | AKT1 | AKT2 | ARNT | C1QTNF1 | CBFA2T3 | CLTC | COX11 | CRY1 | DDIT4 | DUSP12 | DYRK2 | ECD | EIF6 | EP300 | ESRRB | FAM3C | FBP1 | FOXO1 | GAPDHS | GPD1 | GSK3A | GSK3B | HDAC4 | HIF1A | INSR | MAS1 | NCOA2 | NCOR1 | NUP107 | NUP153 | NUP160 | NUP205 | NUP214 | NUP85 | NUP88 | NUP93 | NUP98 | OGT | PDK2 | PGAM1 | PGP | POM121 | PPARGC1A | PPP1CA | PPP1CB | PPP1R3B | PRKAA1 | PRKAA2 | PRKAG1 | PRKCE | RAE1 | RGN | SEC13 | SIRT1 | SIRT6 | SNCA | SRC | STAT3 | TGFB1 | TPR | TREX1 | ZBTB7A | ZMPSTE24 | GO:0009991 | 0.0373650370010918 | 308/8243 | 141/3250 | 0.012157763263043 | 1 | T | F | F | F | response to extracellular stimulus | ACAT1 | ACSL1 | ACSL4 | ACTA1 | ADA | ADCYAP1 | ADSL | AGL | AHCY | AIFM1 | AKT1 | ALB | APOA1 | APOE | ARHGAP35 | ASCL1 | ASGR1 | ASNS | ASS1 | ATG5 | BCKDHB | BCL10 | BCL11A | BCL2 | CAT | CBL | CBS | CD40 | CDKN1A | COMT | CREB1 | CTSV | CYP1A1 | DAD1 | DCTPP1 | DNM1L | EEF2 | EGFR | EHMT2 | EIF2AK3 | EIF2AK4 | EIF2S1 | EIF4EBP1 | FIS1 | FKBP1B | FOLR1 | FOS | FOXO1 | FYN | G6PD | GLUL | GNAI2 | GSS | GSTP1 | HLCS | HMGCL | HMOX1 | HNRNPA1 | HNRNPL | HSF1 | HSP90B1 | HSPA5 | HSPA8 | ICAM1 | IFI16 | IREB2 | JUN | KYNU | LARS | LDHA | LRRK2 | MAPK1 | MAPK3 | MAPK8 | MBD3 | MDM2 | MGMT | MYBBP1A | NAMPT | NCOA1 | NFE2L2 | NNT | NOD2 | NQO1 | NTRK1 | OGT | OPA1 | OTC | OXCT1 | P2RX1 | P2RX2 | P2RX4 | PCNA | PCSK9 | PDK2 | PICK1 | PKM | PPARD | PPARG | PPARGC1A | PPM1D | PRKAA1 | PRKAA2 | PRKD1 | PROX1 | PTEN | PTN | RARA | RELA | RPS19 | RPTOR | SELL | SETDB1 | SIPA1 | SIRT1 | SIRT5 | SIRT6 | SLC16A1 | SLC39A4 | SOD1 | SORD | SRC | STAT1 | STK24 | STK26 | TBXA2R | TGFB1 | TMBIM6 | TNKS | TP53 | TPCN2 | TRIM25 | TXN2 | TYMS | ULK1 | VCAM1 | VGF | WDR45B | WIPI1 | WIPI2 | XBP1 | GO:1901214 | 0.0265679970884387 | 219/8243 | 103/3250 | 0.0122752037185833 | 1 | T | F | F | F | regulation of neuron death | AARS | ABL1 | ADORA2A | AGAP2 | AIFM1 | AIMP2 | AKT1 | APOE | ARRB1 | ARRB2 | ASCL1 | ATF2 | ATM | ATP13A2 | BACE1 | BAX | BCL2 | BCL2L1 | BRAF | C1QA | CAPN2 | CASP2 | CASP3 | CASP8 | CASP9 | CBL | CDC34 | CDK5 | CDK5R1 | CHP1 | CLU | CREB1 | CTNNB1 | DAXX | DDIT4 | DHCR24 | EGLN1 | EGLN3 | EIF2S1 | ERBB3 | FASLG | FBXO7 | FIS1 | FOS | FYN | G6PD | GPI | GPX1 | GRID2 | GRIK2 | GSK3B | HDAC4 | HIF1A | HMOX1 | HRAS | HSF1 | HTRA2 | IKBKG | ILK | JUN | KIR3DL2 | KRAS | LANCL1 | LGMN | LIG4 | LRRK2 | MAP2K4 | MAPT | MECP2 | NAE1 | NMNAT1 | NQO1 | NQO2 | NTF3 | NTRK1 | PARK7 | PARP1 | PCSK9 | PIK3CA | PIN1 | PPARGC1A | PPT1 | PSEN1 | PSMC1 | RAB29 | REL | RHOA | SARM1 | SET | SIRT1 | SNCA | SNX6 | SOD1 | SOD2 | SRPK2 | STAT3 | STXBP1 | SULT1A3 | TNF | TRAF2 | UBB | UBE2M | ZNF746 | GO:0006886 | 0.0721824578430183 | 595/8243 | 261/3250 | 0.0123267530216251 | 1 | T | F | F | F | intracellular protein transport | ABCE1 | ACAA1 | ACD | ACOX1 | ADPRH | AGK | AHCYL1 | AKT1 | AKT2 | AP1B1 | AP1G1 | AP1M1 | AP3D1 | AP3M1 | AP4M1 | AP4S1 | APPBP2 | ARF1 | ARF4 | ARF5 | ARFIP1 | ARL4D | ARL5B | ASPSCR1 | ATG4C | AUP1 | BAG3 | CACNG2 | CACNG4 | CACNG5 | CALR | CAT | CBLB | CCHCR1 | CD74 | CDC37 | CDK5 | CHP1 | CLSTN1 | CLTC | CLU | COG3 | COPA | COPB1 | COPG1 | CROT | CSE1L | CTSA | CTTN | DERL1 | DHRS4 | DLG2 | DNAJC19 | DUSP16 | DYNLL1 | DYNLT1 | ECH1 | ECI2 | EHHADH | EIF5A | EIF6 | FAF2 | FBXO7 | FIS1 | GGA1 | GRIP1 | GRPEL1 | GSTK1 | HAP1 | HDAC6 | HEATR3 | HERC2 | HGS | HID1 | HMGCL | HNRNPU | HSD17B4 | HSP90AA1 | HSP90B1 | HSPA4 | HSPA5 | HSPA8 | HSPB1 | HSPD1 | ICK | IDE | IDH1 | IFIT1 | IFT122 | IPO11 | IPO4 | IPO5 | IPO7 | IPO8 | IPO9 | KIF13B | KIF5B | KIF5C | KPNA1 | KPNA2 | KPNB1 | LARS | LMNA | LRRC7 | MAPK8IP3 | MYO1C | MYO6 | NCOA4 | NDUFA13 | NEDD4 | NMD3 | NOS2 | NPLOC4 | NPM1 | NUDT19 | NUP107 | NUP153 | NUP214 | NUP85 | NUP88 | NUP93 | NUP98 | NUTF2 | NXT2 | OXA1L | PAM16 | PARD3 | PCM1 | PDCD6 | PEX19 | PEX5 | PHB2 | PICK1 | PIK3R1 | PML | POM121 | RAB10 | RAB11A | RAB11B | RAB18 | RAB1A | RAB1B | RAB21 | RAB24 | RAB29 | RAB2A | RAB31 | RAB32 | RAB35 | RAB3IP | RAB5A | RAB5B | RAB7A | RAB9A | RABGEF1 | RABL2A | RABL3 | RAN | RANBP3 | RASSF9 | ROMO1 | RPAIN | RPL10 | RPL10A | RPL12 | RPL18A | RPL22 | RPL23 | RPL23A | RPL24 | RPL30 | RPL37A | RPL38 | RPL6 | RPL7 | RPL7A | RPL9 | RPLP0 | RPLP1 | RPLP2 | RPS12 | RPS13 | RPS14 | RPS15A | RPS19 | RPS2 | RPS20 | RPS24 | RPS29 | RPS3 | RPS4X | RPS8 | RRS1 | SDCBP | SEC13 | SEC16A | SEC24B | SEC24C | SEC31A | SEC62 | SEC63 | SEL1L | SFPQ | SGTA | SGTB | SLC25A6 | SMURF1 | SNF8 | SNX17 | SNX2 | SNX6 | SRP14 | SRP19 | SSX2IP | STAT3 | STX5 | STYX | SYNJ2BP | SYVN1 | TBC1D15 | TBC1D22B | TBC1D4 | TERF2 | TGFB1 | TIMM13 | TIMM21 | TIMM23 | TIMM44 | TIMM50 | TMED9 | TNPO1 | TOMM20 | TOMM22 | TOMM40 | TPR | TRAF3IP1 | TRIM23 | TXNIP | UBB | UBC | UBE2D1 | UBE2D2 | UBE2D3 | USP9X | VCP | VPS18 | VPS26A | VPS29 | VPS37D | WDR19 | WDR35 | XPO1 | XPO4 | XPO5 | XPO7 | YWHAB | YWHAE | YWHAG | YWHAH | ZDHHC23 | ZFAND6 | GO:0030335 | 0.0356666262283149 | 294/8243 | 135/3250 | 0.012389493764685 | 1 | T | F | F | F | positive regulation of cell migration | ABL1 | ACTN4 | AGER | AGO2 | AKT1 | AKT2 | AKT3 | ALOX12 | ANXA1 | APC | APP | ARHGEF7 | ATM | BAG4 | BCAR1 | BCL2 | C1QBP | CALR | CAMK1D | CAV1 | CCL21 | CCR1 | CD40 | CD74 | COL18A1 | CPNE3 | CREB3 | CRK | CRKL | CXCL8 | DAB2 | DDRGK1 | DOCK5 | DOCK7 | EGFR | ETS1 | FADD | FAM83H | FGF2 | FGFR1 | FGFR1OP | FN1 | GNAI2 | GPI | GRB7 | HBEGF | HDAC4 | HDAC6 | HDAC7 | HDAC9 | HIF1A | HMGB1 | HMOX1 | HRAS | HSPA5 | HSPB1 | ICAM1 | IGF1R | ILK | INSR | IQGAP1 | ITGA4 | ITGAV | ITGB1BP1 | ITGB3 | JUN | KDR | LAMB1 | LEF1 | LGALS3 | LGMN | LPAR1 | MAP2K3 | MAP3K3 | MAPK1 | MAPK14 | MAPK3 | MDM2 | MYO1C | MYOC | NFE2L2 | NIPBL | NOS3 | NRP2 | NTF3 | ONECUT1 | OXSR1 | P2RX4 | PDCD10 | PDCD6 | PFN1 | PIK3C2A | PIK3R1 | PIK3R3 | PLCG1 | PRKCE | PRKD1 | PRKD2 | PROX1 | PTK2 | RAB11A | RAC1 | RDX | RHOA | RHOJ | RTN4 | S100A7 | SDCBP | SEMA7A | SERPINB3 | SIRT1 | SMAD3 | SMO | SMURF2 | SNAI2 | SOD2 | SP1 | SPRY2 | SRC | STAT3 | STAT5A | SUN2 | SWAP70 | SYDE1 | SYNE2 | TGFB1 | THBS1 | TNF | TNFSF14 | TRADD | TRIM32 | TRIP6 | VTN | WNK1 | XBP1 | GO:0045184 | 0.114400097052044 | 943/8243 | 404/3250 | 0.0126244926682106 | 1 | T | F | F | F | establishment of protein localization | ABCE1 | ABCG1 | ABL1 | ACAA1 | ACD | ACOX1 | ACTN4 | ADCYAP1 | ADPRH | AFM | AGAP1 | AGAP2 | AGK | AHCYL1 | AKAP8 | AKT1 | AKT2 | AKTIP | ANK3 | ANXA1 | AP1B1 | AP1G1 | AP1M1 | AP3D1 | AP3M1 | AP4M1 | AP4S1 | APOE | APPBP2 | ARF1 | ARF4 | ARF5 | ARFGAP1 | ARFIP1 | ARHGAP1 | ARL4D | ARL5B | ARRB1 | ARRB2 | ASPSCR1 | ATG4C | ATM | ATP6V1A | ATP6V1B1 | ATP6V1C1 | ATP6V1D | ATP6V1E1 | AUP1 | BAG3 | BAG6 | BAX | BLZF1 | BRAF | C16orf70 | CACNG2 | CACNG4 | CACNG5 | CALM1 | CALM3 | CALR | CANX | CAT | CBLB | CCDC22 | CCHCR1 | CCT6B | CD36 | CD40LG | CD74 | CDC37 | CDK5 | CENPF | CEP290 | CEP55 | CHMP2A | CHP1 | CLSTN1 | CLTC | CLU | COG3 | COMMD1 | COPA | COPB1 | COPE | COPG1 | CPLX1 | CPLX3 | CROT | CSE1L | CTSA | CTTN | DAB2 | DDX19A | DENND1A | DENND4C | DERL1 | DHRS4 | DLG2 | DLG4 | DNAJC19 | DUSP16 | DYNLL1 | DYNLT1 | ECH1 | ECI2 | EGFR | EHHADH | EIF4ENIF1 | EIF5A | EIF6 | EXOC6 | FAF2 | FBXO7 | FIS1 | FKBP1B | FRAS1 | GBP5 | GDI1 | GDI2 | GGA1 | GOLGA2 | GOLGA4 | GOLPH3 | GOLT1B | GOPC | GORASP2 | GRIP1 | GRPEL1 | GSTK1 | HAP1 | HDAC6 | HEATR3 | HERC2 | HGS | HID1 | HK1 | HK2 | HMGB1 | HMGCL | HNF1A | HNRNPU | HSD17B4 | HSP90AA1 | HSP90B1 | HSPA4 | HSPA5 | HSPA8 | HSPB1 | HSPD1 | ICK | IDE | IDH1 | IFIT1 | IFT122 | IPO11 | IPO4 | IPO5 | IPO7 | IPO8 | IPO9 | ITGAV | ITGB1BP1 | KIF13B | KIF5B | KIF5C | KLHL20 | KPNA1 | KPNA2 | KPNB1 | LARS | LCP2 | LIMS1 | LMAN2L | LMNA | LRRC7 | LRSAM1 | MAPK8IP3 | MCC | MCFD2 | MDM2 | MTX1 | MYO1C | MYO5A | MYO6 | NBAS | NCOA4 | NDUFA13 | NEDD4 | NEUROD1 | NIPBL | NMD3 | NOD2 | NOS2 | NPLOC4 | NPM1 | NUDT19 | NUP107 | NUP153 | NUP214 | NUP85 | NUP88 | NUP93 | NUP98 | NUTF2 | NXT2 | OPTN | OXA1L | PAFAH1B1 | PAM16 | PARD3 | PARK7 | PCM1 | PDCD6 | PDCD6IP | PDIA4 | PEX19 | PEX5 | PHB2 | PICK1 | PIH1D1 | PIK3R1 | PLEKHF2 | PLK1 | PML | PNP | POM121 | POT1 | PPARG | PPID | PPT1 | PSEN1 | PTPN23 | RAB10 | RAB11A | RAB11B | RAB11FIP2 | RAB18 | RAB1A | RAB1B | RAB21 | RAB24 | RAB29 | RAB2A | RAB31 | RAB32 | RAB35 | RAB3GAP1 | RAB3IP | RAB5A | RAB5B | RAB7A | RAB9A | RABGEF1 | RABL2A | RABL3 | RAF1 | RAN | RANBP3 | RAP1A | RAPGEF4 | RASSF9 | RBPJ | RDX | ROMO1 | RP2 | RPAIN | RPL10 | RPL10A | RPL12 | RPL18A | RPL22 | RPL23 | RPL23A | RPL24 | RPL30 | RPL37A | RPL38 | RPL6 | RPL7 | RPL7A | RPL9 | RPLP0 | RPLP1 | RPLP2 | RPS12 | RPS13 | RPS14 | RPS15A | RPS19 | RPS2 | RPS20 | RPS24 | RPS29 | RPS3 | RPS4X | RPS8 | RRS1 | RUVBL2 | S100A12 | S100A13 | SCFD1 | SCG2 | SDCBP | SEC13 | SEC16A | SEC22C | SEC24B | SEC24C | SEC31A | SEC62 | SEC63 | SEL1L | SELENBP1 | SFPQ | SGTA | SGTB | SLC15A1 | SLC25A6 | SMAD2 | SMURF1 | SNAP29 | SNF8 | SNX12 | SNX17 | SNX2 | SNX21 | SNX24 | SNX3 | SNX6 | SPTBN1 | SRP14 | SRP19 | SSX2IP | STAT3 | STX5 | STXBP1 | STXBP2 | STYX | SYNJ2BP | SYVN1 | TANGO2 | TBC1D15 | TBC1D22B | TBC1D4 | TCIRG1 | TCP1 | TERF2 | TGFB1 | TIMM13 | TIMM21 | TIMM23 | TIMM44 | TIMM50 | TMED9 | TNFAIP3 | TNKS | TNPO1 | TOMM20 | TOMM22 | TOMM40 | TOR1A | TPR | TRAF3IP1 | TREX1 | TRIM23 | TSG101 | TSPAN17 | TXNIP | UBB | UBC | UBE2D1 | UBE2D2 | UBE2D3 | UBL4A | UNC119 | USP9X | VCP | VGF | VPS13B | VPS18 | VPS26A | VPS29 | VPS37D | VPS4B | VPS8 | VTA1 | WBP2 | WDR19 | WDR35 | XBP1 | XPO1 | XPO4 | XPO5 | XPO7 | YKT6 | YWHAB | YWHAE | YWHAG | YWHAH | ZBTB7B | ZDHHC17 | ZDHHC23 | ZFAND6 | ZW10 | GO:0010634 | 0.0118888754094383 | 98/8243 | 50/3250 | 0.0126265765386332 | 1 | T | F | F | F | positive regulation of epithelial cell migration | ABL1 | AKT1 | AKT3 | ALOX12 | ANXA1 | BCAR1 | CALR | CD40 | DOCK5 | ETS1 | FGF2 | FGFR1 | GPI | HBEGF | HDAC6 | HDAC7 | HDAC9 | HIF1A | HMGB1 | HMOX1 | HSPB1 | ITGB1BP1 | ITGB3 | JUN | KDR | LGMN | MAP2K3 | MAP3K3 | MAPK14 | NFE2L2 | NOS3 | NRP2 | P2RX4 | PDCD6 | PFN1 | PIK3C2A | PLCG1 | PRKCE | PRKD1 | PRKD2 | PROX1 | RAB11A | RHOJ | RTN4 | SIRT1 | SP1 | SRC | STAT5A | TGFB1 | THBS1 | GO:0016999 | 0.00606575275991751 | 50/8243 | 28/3250 | 0.0127171190583256 | 1 | T | F | F | F | antibiotic metabolic process | ACSS2 | ADH1A | ADH1B | ADH5 | AKR1A1 | AKR1B1 | AKR1B10 | AKR1C2 | ALDH1B1 | CAT | CBR4 | CYP1A1 | CYP1A2 | DPEP1 | EGFR | ESD | GPX1 | HBZ | PARK7 | PRDX1 | PRDX2 | PRDX3 | PRDX4 | PRDX5 | PRDX6 | PXDN | SOD1 | SULT1A3 | GO:1902905 | 0.0151643818997938 | 125/8243 | 62/3250 | 0.0127506122446388 | 1 | T | F | F | F | positive regulation of supramolecular fiber organization | ABL1 | ACTN2 | ACTR2 | ACTR3 | AKAP9 | APOA1 | APOE | APP | ARPC1A | ARPC1B | ARPC2 | ARPC3 | ARPC4 | ARPC5 | BAG4 | BAIAP2 | BAIAP2L1 | BIN1 | BRAF | CCL21 | CDC42 | CDC42EP3 | CDC42EP4 | CDK5R1 | CFL1 | CFL2 | CLU | COLGALT1 | CTTN | DCTN1 | DSTN | FCHSD1 | GPX1 | GRB2 | HSPA1B | ICAM1 | ITGB1BP1 | LPAR1 | MAPT | MECP2 | MYO1C | MYOC | NCK1 | NF2 | NIN | NUMA1 | PFN1 | PRKCE | PROX1 | PSEN1 | PXN | RAC1 | RB1 | RHOA | RPS3 | SDC4 | SMAD3 | SWAP70 | TPM1 | VASP | WAS | WDR1 | GO:0051270 | 0.0655101298071091 | 540/8243 | 238/3250 | 0.0128637102016783 | 1 | T | F | F | F | regulation of cellular component movement | ABL1 | ABL2 | ACTN1 | ACTN4 | ADA | ADTRP | AGER | AGO2 | AIRE | AKT1 | AKT2 | AKT3 | ALOX12 | ANXA1 | ANXA5 | APC | APEX1 | APOD | APOE | APP | ARHGEF7 | ATM | ATP1A1 | BAG4 | BCAR1 | BCL2 | BCL6 | BIN1 | BMPR1A | BRAF | C1QBP | CALR | CAMK1D | CAMK2A | CAMK2B | CAV1 | CCL21 | CCR1 | CD40 | CD74 | CD81 | CDH1 | CDK5 | CHRD | CLIC4 | COL18A1 | CPNE3 | CREB3 | CRK | CRKL | CSNK2B | CXCL8 | DAB2 | DCN | DDRGK1 | DOCK5 | DOCK7 | DPEP1 | DUSP1 | DUSP22 | EGFR | EPPIN | EPPK1 | ERBB2 | ERBB3 | ETS1 | FADD | FAM83H | FGF2 | FGFR1 | FGFR1OP | FN1 | GLUL | GNA12 | GNA13 | GNAI2 | GPI | GRB7 | GSTP1 | HAP1 | HBEGF | HDAC4 | HDAC5 | HDAC6 | HDAC7 | HDAC9 | HIF1A | HMGB1 | HMOX1 | HRAS | HSPA5 | HSPB1 | ICAM1 | IDH2 | IGBP1 | IGF1R | ILK | INSR | IQGAP1 | ITGA4 | ITGAV | ITGB1BP1 | ITGB3 | JUN | JUP | KDR | LAMB1 | LDLRAD4 | LEF1 | LGALS3 | LGMN | LMNA | LPAR1 | MAP2K3 | MAP3K3 | MAPK1 | MAPK14 | MAPK15 | MAPK3 | MCC | MDM2 | MECP2 | MEOX2 | MIF | MITF | MSN | MYO1C | MYOC | NCK1 | NF2 | NFE2L2 | NIPBL | NKX2-1 | NOS1AP | NOS3 | NRP2 | NTF3 | ONECUT1 | OXSR1 | P2RX4 | PDCD10 | PDCD6 | PDE4D | PFN1 | PIK3C2A | PIK3R1 | PIK3R3 | PIN1 | PKN2 | PLCG1 | PLXNA1 | PLXNA2 | PLXNB2 | PPARD | PPARG | PPARGC1A | PRCP | PRKCE | PRKD1 | PRKD2 | PROX1 | PTEN | PTK2 | PTN | PTPN23 | PTPRK | PTPRO | PTPRR | RAB11A | RAC1 | RAF1 | RDX | RECK | RGN | RHOA | RHOJ | RHOT1 | RIPK3 | RPS19 | RTN4 | S100A7 | SDC4 | SDCBP | SEMA7A | SEPT7 | SERPINB3 | SERPINE2 | SIRT1 | SLAMF8 | SLC9A3R1 | SMAD3 | SMO | SMURF2 | SNAI2 | SOD2 | SP1 | SP100 | SPINT2 | SPRY2 | SRC | SRGAP2 | SRGAP3 | SRI | SSX2IP | STARD13 | STAT3 | STAT5A | STK24 | STK26 | STMN1 | STRAP | SUMO1 | SUN2 | SWAP70 | SYDE1 | SYNE2 | SYNJ2BP | TBXA2R | TGFB1 | TGFBR3 | THBS1 | TIMP1 | TNF | TNFSF14 | TNN | TPM1 | TRADD | TRIM32 | TRIP6 | TUBB2B | TWIST1 | USP17L2 | VCL | VTN | WAS | WNK1 | XBP1 | ZMYND8 | GO:0055074 | 0.026325366978042 | 217/8243 | 102/3250 | 0.0129066815757812 | 1 | T | F | F | F | calcium ion homeostasis | ABL1 | ABL2 | ACKR2 | ADCYAP1 | AFG3L2 | ANXA2 | ANXA6 | ANXA7 | APOE | APP | ATP13A2 | ATP2A2 | ATP2B2 | ATP6V1B1 | BAX | BCL2 | BDKRB2 | C1QTNF1 | CACNA1A | CALM1 | CALM2 | CALM3 | CALR | CAV1 | CCL21 | CCR1 | CCR3 | CCR4 | CCR5 | CD36 | CD40 | CDK5 | CHRNA9 | CXCL11 | DLG4 | ESR1 | FASLG | FGF2 | FIS1 | FKBP1B | GLP1R | GNA13 | GNA15 | GNB1 | GOT1 | GRIA1 | GRIK2 | GSTO1 | GTF2I | HAP1 | HEXB | HMGB1 | HTR1B | HTT | IBTK | IMMT | ITGAV | ITPR1 | ITPR2 | ITPR3 | KCTD17 | LETM1 | LPAR1 | LPAR2 | LPAR4 | LRP6 | MCOLN3 | MICU1 | MICU2 | MYO5A | NMB | P2RX1 | P2RX2 | P2RX4 | PIK3CG | PLA2G1B | PLA2G6 | PLCG1 | PML | PRKACA | PRKCE | PSEN1 | PTGER3 | PTGIR | RAP1GDS1 | RGN | RIC3 | SMAD3 | SNCA | SRI | STIM1 | STOML2 | SWAP70 | TBXA2R | TGFB1 | TGM2 | THADA | TMBIM6 | TPCN2 | VAPB | VDR | YWHAE | GO:0033135 | 0.0105544098022565 | 87/8243 | 45/3250 | 0.0129547787917257 | 1 | T | F | F | F | regulation of peptidyl-serine phosphorylation | AKAP9 | AKT1 | APP | ARAF | ARRB1 | ARRB2 | AXIN1 | BAG4 | BAX | BCAR3 | BCL2 | BDKRB2 | BRAF | CAV1 | DDIT4 | DMD | DOCK7 | EGFR | GPD1L | HAX1 | HDAC6 | HSP90AA1 | HSP90AB1 | IFNA2 | LATS1 | MAPKAP1 | MIF | NCK1 | NTF3 | PARK7 | PDCD10 | PDE4D | PIK3CA | PRKAA1 | PRKD1 | PRKD2 | RAF1 | RIPK2 | RPTOR | SNCA | SPRY2 | TERF2IP | TGFB1 | TNF | TXN | GO:0000122 | 0.067572485745481 | 557/8243 | 245/3250 | 0.0130336434289322 | 1 | T | F | F | F | negative regulation of transcription by RNA polymerase II | AEBP2 | AES | APBB1 | AR | ARHGAP35 | ASCL1 | ATF2 | ATRX | ATXN1 | AURKB | BACH2 | BAG3 | BCL11A | BCL6 | BCL6B | BCOR | BMI1 | C1QBP | CALR | CAV1 | CBFB | CBX4 | CBX7 | CBX8 | CD36 | CDC73 | CDK2 | CEBPA | CHCHD3 | CHD8 | COPS2 | CREBBP | CRY1 | CRY2 | CTBP1 | CTCF | CTNNB1 | CTNNBIP1 | CUL3 | DMAP1 | DNAJA3 | DNAJB1 | DPF2 | DUSP22 | E2F1 | E2F2 | E2F3 | E2F4 | E2F6 | EED | EHMT1 | EHMT2 | EID1 | ENO1 | EP300 | ERF | ESR1 | ESR2 | ESRRA | EZH2 | EZR | FASLG | FBP1 | FGFR1 | FHL2 | FOXA1 | FOXJ1 | FOXM1 | FOXO1 | GATA1 | GPS2 | H1F0 | HBZ | HDAC1 | HDAC2 | HDAC3 | HDAC4 | HDAC5 | HDAC7 | HDAC9 | HDGF | HEXIM1 | HIF1AN | HIVEP1 | HMGB1 | HNRNPA2B1 | HNRNPU | HSF1 | HSF4 | ID2 | IFI16 | IGBP1 | IKZF5 | IRF7 | JUN | JUNB | JUND | KANK2 | KAT5 | KDM1A | KLF5 | LEF1 | LOXL2 | MBD3 | MDFI | MDM2 | MDM4 | MECP2 | MED1 | MEPCE | MITF | MTA1 | MTA3 | MXD1 | MYC | NCK1 | NCOA2 | NCOR1 | NCOR2 | NEDD4 | NELFB | NELFE | NFATC2 | NIPBL | NKX2-1 | NR0B2 | NR1H2 | NR1H3 | NR3C1 | PARP1 | PAWR | PCGF1 | PCGF6 | PER1 | PER2 | PHB | PHF21A | PIAS1 | PIAS3 | PKIG | PLK1 | POU5F1 | PPARD | PPARG | PPID | PPM1A | PRMT6 | PROX1 | PSEN1 | PSMD10 | RARA | RARG | RB1 | RBPJ | REL | RELA | REST | RFC1 | RIF1 | RNF2 | RNF8 | RPL10 | RPL23 | RPS14 | RTF1 | RUNX1 | RXRA | S100A1 | SARS | SFPQ | SIN3A | SIRT1 | SIRT7 | SKI | SMAD2 | SMAD3 | SMAD4 | SMAD5 | SMARCA2 | SMARCA4 | SMO | SMURF2 | SNAI2 | SNCA | SNW1 | SOX2 | SP100 | SP2 | SPEN | SPI1 | SQSTM1 | STAT1 | STAT3 | STAT6 | STRAP | SUMO1 | SUPT4H1 | SUPT5H | SUZ12 | TAGLN3 | TBL1X | TCERG1 | TCF21 | TCF25 | TCP10L | TGFB1 | THAP11 | THRA | TMBIM6 | TNF | TP53 | TP63 | TPR | TRAF6 | TRIB3 | TRIM28 | TRIM29 | TRIM33 | TWIST1 | TXN | TXNIP | UBB | UBC | UBE2D1 | UBE2D3 | UBE2I | USP2 | USP9X | VDR | VHL | VPS72 | WDTC1 | WT1 | WWOX | WWP2 | XPO1 | YEATS2 | YY1 | ZBTB16 | ZBTB42 | ZBTB7A | ZBTB7B | ZHX1 | ZNF217 | ZNF746 | GO:0051649 | 0.136479437098144 | 1125/8243 | 478/3250 | 0.0131330831229525 | 1 | T | F | F | F | establishment of localization in cell | ABCD3 | ABCE1 | ABCG1 | ACAA1 | ACACA | ACD | ACOX1 | ACTB | ACTG1 | ACTN4 | ACTR1A | ACTR2 | ACTR3 | ADPRH | ADRB2 | AGK | AHCYL1 | AKT1 | AKT2 | AKTIP | ANK1 | ANK3 | ANP32A | AP1B1 | AP1G1 | AP1M1 | AP3D1 | AP3M1 | AP4M1 | AP4S1 | APOE | APP | APPBP2 | ARF1 | ARF4 | ARF5 | ARFGAP1 | ARFIP1 | ARHGAP1 | ARL4D | ARL5B | ASPSCR1 | ATG4C | ATL3 | ATP2A2 | ATXN1 | AUP1 | BACH2 | BAG3 | BANF1 | BAX | BIN1 | BTK | CACNG2 | CACNG4 | CACNG5 | CALM1 | CALM3 | CALR | CANX | CAPZA1 | CAPZA2 | CAPZB | CAT | CBL | CBLB | CCDC155 | CCDC22 | CCDC88A | CCHCR1 | CCL21 | CCNB1 | CCR5 | CD74 | CDC37 | CDC42 | CDK5 | CEL | CENPF | CEP55 | CHMP2A | CHP1 | CLSTN1 | CLTC | CLU | COG3 | COL4A3BP | COPA | COPB1 | COPE | COPG1 | CPLX1 | CPLX3 | CPT1A | CPT2 | CROT | CSE1L | CTNNB1 | CTSA | CTSC | CTTN | CUL3 | CYC1 | DCLK1 | DCTN1 | DDX19B | DDX39A | DDX39B | DENND1A | DERL1 | DHRS4 | DLG2 | DNAJC19 | DNM1L | DOCK7 | DUSP16 | DVL1 | DYNC1H1 | DYNC1I1 | DYNC1I2 | DYNC1LI1 | DYNLL1 | DYNLT1 | ECH1 | ECI2 | EHD4 | EHHADH | EIF4A3 | EIF4E | EIF5A | EIF6 | EZR | F5 | F9 | FABP3 | FAF2 | FAM83D | FASLG | FBXO7 | FGF2 | FIS1 | FKBP1B | FOLR1 | FYTTD1 | GAD2 | GAK | GEMIN4 | GEMIN5 | GEMIN7 | GGA1 | GOLGA2 | GOPC | GRIA1 | GRIP1 | GRPEL1 | GSTK1 | HAP1 | HDAC6 | HEATR3 | HERC2 | HGS | HID1 | HIF1A | HMGCL | HNRNPA1 | HNRNPA2B1 | HNRNPU | HSD17B4 | HSP90AA1 | HSP90B1 | HSPA4 | HSPA5 | HSPA8 | HSPB1 | HSPD1 | HTATIP2 | HTT | IBTK | ICK | IDE | IDH1 | IFIT1 | IFT122 | IKBKG | IPO11 | IPO4 | IPO5 | IPO7 | IPO8 | IPO9 | ITGA4 | ITPR1 | ITPR2 | ITPR3 | KIF13B | KIF2C | KIF5B | KIF5C | KLHL20 | KPNA1 | KPNA2 | KPNB1 | LARS | LAT | LETM1 | LMAN2L | LMNA | LRP6 | LRPPRC | LRRC7 | MAP1B | MAPK8IP3 | MAPT | MCFD2 | MCM3AP | MCOLN3 | MLH1 | MPPE1 | MYO1C | MYO1D | MYO5A | MYO6 | NCBP1 | NCOA4 | NDUFA13 | NEDD4 | NEUROD1 | NMD3 | NOS2 | NPLOC4 | NPM1 | NRXN1 | NSFL1C | NUDT19 | NUMA1 | NUP107 | NUP153 | NUP160 | NUP205 | NUP214 | NUP85 | NUP88 | NUP93 | NUP98 | NUSAP1 | NUTF2 | NXF1 | NXF2 | NXT2 | OPA1 | OXA1L | PAFAH1B1 | PAM16 | PARD3 | PARK7 | PCM1 | PDCD10 | PDCD6 | PDCD6IP | PEF1 | PEX19 | PEX5 | PHB2 | PICK1 | PIK3CG | PIK3R1 | PIKFYVE | PINX1 | PLCG1 | PML | POLDIP3 | POM121 | PPT1 | PQLC1 | PRKAA2 | PRKAB2 | PRKAG1 | PRKCE | PRKCZ | PROZ | PTK2 | PTPN23 | RAB10 | RAB11A | RAB11B | RAB11FIP2 | RAB18 | RAB1A | RAB1B | RAB21 | RAB24 | RAB29 | RAB2A | RAB31 | RAB32 | RAB35 | RAB3IP | RAB5A | RAB5B | RAB7A | RAB9A | RABGEF1 | RABL2A | RABL3 | RAE1 | RAF1 | RAN | RANBP3 | RASSF9 | RBM8A | RHOT1 | RHOT2 | RNF126 | ROMO1 | RPAIN | RPL10 | RPL10A | RPL12 | RPL18A | RPL22 | RPL23 | RPL23A | RPL24 | RPL30 | RPL37A | RPL38 | RPL6 | RPL7 | RPL7A | RPL9 | RPLP0 | RPLP1 | RPLP2 | RPS12 | RPS13 | RPS14 | RPS15A | RPS19 | RPS2 | RPS20 | RPS24 | RPS29 | RPS3 | RPS4X | RPS8 | RRS1 | S100A13 | SCYL2 | SDCBP | SDCBP2 | SEC13 | SEC16A | SEC22C | SEC24B | SEC24C | SEC31A | SEC62 | SEC63 | SEL1L | SFPQ | SGTA | SGTB | SLC25A6 | SLC9A3R1 | SMG5 | SMG6 | SMN1 | SMURF1 | SNCA | SNF8 | SNRPB | SNRPD1 | SNRPD3 | SNRPE | SNX17 | SNX2 | SNX6 | SOD1 | SORT1 | SPDL1 | SPICE1 | SPRY2 | SPTAN1 | SPTBN1 | SPTBN2 | SQSTM1 | SRP14 | SRP19 | SRSF11 | SRSF5 | SSB | SSX2IP | STAT3 | STBD1 | STK25 | STOML2 | STX5 | STXBP1 | STYX | SUN2 | SYN1 | SYNE2 | SYNJ2BP | SYP | SYVN1 | TBC1D15 | TBC1D22B | TBC1D23 | TBC1D4 | TERF1 | TERF2 | TFG | TGFB1 | THOC2 | THOC6 | THOC7 | TIMM13 | TIMM21 | TIMM23 | TIMM44 | TIMM50 | TMED9 | TNPO1 | TOMM20 | TOMM22 | TOMM40 | TOR1A | TPCN2 | TPR | TRAF3IP1 | TRAPPC8 | TRIM23 | TRIM9 | TSG101 | TUBA1A | TUBA1C | TUBB | TXNIP | U2AF2 | UBB | UBC | UBE2B | UBE2D1 | UBE2D2 | UBE2D3 | UBE2O | UCHL1 | UPF2 | UPF3B | USP9X | VAPA | VAPB | VCP | VPS18 | VPS26A | VPS29 | VPS37D | VPS4B | VTA1 | WAS | WDR19 | WDR35 | XPO1 | XPO4 | XPO5 | XPO7 | XPOT | YKT6 | YWHAB | YWHAE | YWHAG | YWHAH | YWHAZ | ZDHHC23 | ZFAND6 | ZMPSTE24 | ZW10 | ZWINT | GO:0008219 | 0.0632051437583404 | 521/8243 | 230/3250 | 0.0131837417938507 | 1 | T | F | F | F | cell death | ADORA2A | AIFM1 | AIMP2 | AKT1 | AKT2 | AKTIP | APBB1 | API5 | APLP1 | APP | ARRB1 | ATCAY | ATG5 | AXIN1 | BAG1 | BAG5 | BAG6 | BAX | BBC3 | BCL10 | BCL2 | BCL2L1 | BCL2L13 | BIRC2 | BIRC3 | BIRC5 | BIRC7 | BIRC8 | BNIPL | BRCA1 | C1QBP | CACNA1A | CAPN1 | CAPNS1 | CASP10 | CASP2 | CASP3 | CASP7 | CASP8 | CASP9 | CBL | CD2 | CDK5 | CRKL | CRYAB | CSE1L | CSNK2A2 | CTSC | CTSL | CTSV | CUL3 | CYCS | CYLD | DAB2 | DAD1 | DAPK1 | DAXX | DHCR24 | DHX9 | DIABLO | DNAJA3 | DNM1L | DPEP1 | DPF2 | DUSP22 | DYNLL1 | E2F1 | E2F2 | EAF2 | EGLN3 | EIF5A | ENDOG | EP300 | ERBB3 | EVA1A | FADD | FAF1 | FASLG | FIS1 | FKBP8 | FOXO1 | GADD45G | GAPDH | GHITM | GNB1 | GRIK2 | GRK5 | GZMA | HMOX1 | HSPA5 | HTATIP2 | HTT | IFNA2 | IKBKG | IRF1 | ITCH | ITGB3BP | JUP | KANK2 | KMT2A | KRT17 | KRT31 | KRT40 | KRT71 | LEF1 | LGALS1 | LGALS7 | LIG4 | LRRK2 | LTBR | MAP2K4 | MAP2K6 | MAP2K7 | MAPK1 | MAPK14 | MAPK3 | MCM2 | MELK | MYC | MYD88 | NAE1 | NAMPT | NFKBIA | NGB | NR3C1 | NTRK1 | OGT | OPA1 | OPTN | P2RX1 | PACS2 | PAK2 | PAK6 | PARP1 | PAWR | PCSK9 | PDCD6IP | PGAM5 | PIK3CA | PIK3CG | PIM1 | PKM | PKN2 | PLEKHF1 | PLSCR1 | PPARD | PPID | PPIF | PPP2CA | PPP2R1A | PPP2R1B | PRDX3 | PRDX5 | PRELID1 | PRKCD | PRKCE | PRKD1 | PRKD2 | PRKDC | PSEN1 | PSMD10 | PSME3 | PTEN | PTGER3 | PTPN6 | PTRH2 | PYGL | RAD21 | RAF1 | RASSF7 | RB1 | RFK | RHBDD1 | RIPK1 | RIPK2 | RIPK3 | RNF183 | RPS3 | RPS6KA1 | RTKN | RTN4 | S100A9 | SEPT4 | SH3KBP1 | SIAH1 | SLC25A6 | SLC9A1 | SLTM | SNCA | SPATA2 | SPI1 | SPRR2A | SQSTM1 | STK11 | STK26 | STK3 | TCTN3 | TFDP1 | THOC6 | TIA1 | TIAL1 | TLR3 | TMEM173 | TMEM219 | TNF | TNFAIP3 | TNFSF12 | TNFSF14 | TNIP2 | TOP1 | TP53 | TP63 | TRADD | TRAF1 | TRAF2 | TRAF3 | TRIAP1 | TRIM35 | TRIM69 | UBE2D3 | USP17L2 | USP53 | VAPA | VDAC1 | XAF1 | XIAP | ZBTB16 | ZFAND6 | ZMPSTE24 | ZNF443 | GO:0120036 | 0.0400339682154555 | 330/8243 | 150/3250 | 0.0133215185923756 | 1 | T | F | F | F | plasma membrane bounded cell projection organization | ABL1 | ABLIM1 | ACTN2 | ACTR2 | ACTR3 | ADCYAP1 | AGER | AHI1 | APOA1 | APOD | APOE | APP | ARF1 | ARHGAP35 | ARHGEF7 | ARPC5 | ATCAY | ATP6V1D | ATXN10 | AVIL | BAG4 | BCL2 | CASP3 | CBY1 | CCDC88A | CCL21 | CCP110 | CDC42 | CDH1 | CDK16 | CDK5 | CDK5R1 | CENPJ | CEP290 | CEP89 | CRK | CRKL | CROCC | CTTN | CUL4B | DAB1 | DBNL | DCTN1 | DISC1 | DLG4 | DOCK7 | DVL1 | DYNLL1 | E2F4 | EPHA4 | EZR | FAM161B | FBF1 | FOLR1 | FOPNL | FOXJ1 | FSCN1 | FYN | GAP43 | GFAP | GNAO1 | GOLPH3 | GRIP1 | GSK3B | HDAC2 | HDAC6 | HERC1 | HMGB1 | ICK | IFT122 | IGF1R | INSR | IQCB1 | JUN | KLF5 | LAMB1 | LGMN | LIMA1 | LPAR1 | MAP1B | MAPK8IP3 | MAPT | MECP2 | MKS1 | MTSS1 | MYOC | NCDN | NCK1 | NEDD4 | NEK1 | NPHP3 | NUP85 | OFD1 | ONECUT1 | PCM1 | PHGDH | PICK1 | POC1B | PSEN1 | PTEN | PTPN23 | PTPRF | PTPRK | PTPRO | RAB11A | RAB1A | RAB35 | RAC1 | RAP1A | RAP1B | RB1 | RDX | RPGRIP1L | RTN4 | SCLT1 | SDC4 | SDCBP | SEC24B | SEPT2 | SEPT6 | SEPT7 | SH2B1 | SH3GL2 | SLC9A3R1 | SNAP29 | SNX2 | SOD1 | SPERT | SRGAP2 | SSX2IP | STK26 | STMN1 | TBC1D23 | TCTN2 | TCTN3 | TEKT2 | TEKT4 | TMEM17 | TMEM216 | TMEM231 | TOR1A | TPM1 | TRIM32 | UBXN10 | ULK1 | USP21 | VAPA | VCL | WDR35 | ZMYND10 | GO:2001056 | 0.0116462452990416 | 96/8243 | 49/3250 | 0.0133289085267094 | 1 | T | F | F | F | positive regulation of cysteine-type endopeptidase activity | AIFM1 | ALOX12 | ARRB1 | BAX | BBC3 | BCL10 | BCL2L13 | CASP10 | CASP8 | CASP9 | CYCS | DAPK1 | DDX3X | DIABLO | EGLN3 | EIF2AK3 | FADD | FASLG | FIS1 | FYN | HMGB1 | HSF1 | HSPD1 | HSPE1 | HTRA2 | IFI16 | LGMN | MAPT | MYC | NDUFA13 | P2RX1 | PDCD6 | PML | PPARG | REST | RHOA | RIPK1 | RIPK2 | RPS3 | S100A9 | SIRT1 | SMAD3 | SNCA | TNF | TRADD | TRAF2 | USP50 | VCP | WDR35 | GO:0030030 | 0.0536212543976708 | 442/8243 | 197/3250 | 0.0134327274472005 | 1 | T | F | F | F | cell projection organization | ABL1 | ABLIM1 | ACTN2 | ACTR2 | ACTR3 | ADCYAP1 | ADORA2A | AFG3L2 | AGER | AHI1 | AKT1 | ANK3 | APBB1 | APOA1 | APOD | APOE | APP | ARF1 | ARHGAP35 | ARHGEF7 | ARPC5 | ATCAY | ATP6V1D | ATXN10 | AVIL | BAG4 | BAIAP2 | BCL2 | CAMK2A | CASP3 | CBY1 | CCDC88A | CCL21 | CCP110 | CDC42 | CDH1 | CDK16 | CDK5 | CDK5R1 | CENPJ | CEP290 | CEP89 | CREB1 | CRK | CRKL | CROCC | CTTN | CUL3 | CUL4B | DAB1 | DBNL | DCLK1 | DCTN1 | DISC1 | DLG4 | DNAH9 | DOCK7 | DVL1 | DYNLL1 | E2F4 | EGFR | EPHA4 | EZR | FAM161B | FBF1 | FLOT1 | FOLR1 | FOPNL | FOXJ1 | FSCN1 | FYN | GAP43 | GFAP | GNAO1 | GOLPH3 | GRIP1 | GSK3B | HAP1 | HDAC2 | HDAC6 | HERC1 | HMGB1 | HPRT1 | HSP90AA1 | HSP90AB1 | ICK | IFT122 | IGF1R | ILK | INSR | IQCB1 | ITGA4 | JUN | KCTD17 | KLF5 | LAMB1 | LGMN | LIMA1 | LPAR1 | LRRK2 | MAP1B | MAPK8IP3 | MAPT | MECP2 | MKS1 | MTSS1 | MYOC | NCDN | NCK1 | NEDD4 | NEK1 | NIN | NPHP3 | NPTX1 | NTF3 | NTRK1 | NUBP1 | NUBP2 | NUP85 | OFD1 | ONECUT1 | PAFAH1B1 | PARD3 | PCM1 | PDCL | PHGDH | PICK1 | PITPNA | PKN2 | POC1B | PSEN1 | PTEN | PTK2 | PTPN23 | PTPRF | PTPRK | PTPRO | RAB10 | RAB11A | RAB1A | RAB35 | RAC1 | RAP1A | RAP1B | RB1 | RDX | RHOA | RHOJ | RHOU | RPGRIP1L | RTN4 | S100A6 | S100B | SCLT1 | SDC4 | SDCBP | SEC24B | SEPT2 | SEPT6 | SEPT7 | SH2B1 | SH3GL2 | SLC9A3R1 | SNAP29 | SNX2 | SOD1 | SPERT | SRGAP2 | SSX2IP | STK11 | STK24 | STK26 | STMN1 | TBC1D23 | TCTN2 | TCTN3 | TEKT2 | TEKT4 | TMEM17 | TMEM216 | TMEM231 | TNN | TOR1A | TPM1 | TRIM32 | TSGA10 | TSKU | TWF2 | UBB | UBXN10 | UGT8 | ULK1 | USP21 | VAPA | VCL | WDR35 | ZMYND10 | GO:0031667 | 0.0351813660075215 | 290/8243 | 133/3250 | 0.0136057633296518 | 1 | T | F | F | F | response to nutrient levels | ACAT1 | ACSL1 | ACSL4 | ADA | ADCYAP1 | ADSL | AGL | AHCY | AIFM1 | AKT1 | ALB | APOA1 | APOE | ASCL1 | ASNS | ASS1 | ATG5 | BCKDHB | BCL10 | BCL11A | BCL2 | CAT | CBL | CD40 | CDKN1A | COMT | CREB1 | CTSV | CYP1A1 | DAD1 | DNM1L | EEF2 | EGFR | EHMT2 | EIF2AK3 | EIF2AK4 | EIF2S1 | EIF4EBP1 | FIS1 | FKBP1B | FOLR1 | FOXO1 | FYN | G6PD | GLUL | GNAI2 | GSS | GSTP1 | HLCS | HMGCL | HMOX1 | HNRNPA1 | HNRNPL | HSF1 | HSP90B1 | HSPA5 | HSPA8 | ICAM1 | IFI16 | IREB2 | JUN | KYNU | LARS | LDHA | LRRK2 | MAPK1 | MAPK3 | MAPK8 | MBD3 | MDM2 | MGMT | MYBBP1A | NAMPT | NCOA1 | NFE2L2 | NNT | NOD2 | NQO1 | NTRK1 | OGT | OPA1 | OTC | OXCT1 | P2RX1 | P2RX2 | P2RX4 | PCNA | PCSK9 | PDK2 | PICK1 | PKM | PPARD | PPARG | PPARGC1A | PPM1D | PRKAA1 | PRKAA2 | PRKD1 | PROX1 | PTEN | PTN | RARA | RELA | RPTOR | SELL | SETDB1 | SIRT1 | SIRT5 | SIRT6 | SLC16A1 | SLC39A4 | SOD1 | SORD | SRC | STAT1 | STK24 | STK26 | TBXA2R | TGFB1 | TMBIM6 | TNKS | TP53 | TPCN2 | TRIM25 | TXN2 | TYMS | ULK1 | VCAM1 | VGF | WDR45B | WIPI1 | WIPI2 | XBP1 | GO:0120161 | 0.0103117796918598 | 85/8243 | 44/3250 | 0.0136678857818748 | 1 | T | F | F | F | regulation of cold-induced thermogenesis | ACOT13 | ACSL1 | ADCYAP1 | ADRB2 | APC | CAV1 | CD36 | CPT2 | DBH | DECR1 | DOCK7 | DYNC1H1 | EHMT1 | ESRRG | FABP5 | FH | GADD45G | GJA1 | GNAS | HADH | HDAC3 | HSF1 | IGF1R | IP6K1 | IRF4 | KDM1A | LGR4 | MAP2K6 | NR1H2 | NR1H3 | OGT | PCTP | PER2 | PGAM5 | PPARGC1A | PRKAB2 | RB1 | RBPJ | SIRT6 | STAT6 | STK11 | THRA | TLE3 | ZBTB7B | GO:0016233 | 0.0014557806623802 | 12/8243 | 9/3250 | 0.0136727524015442 | 1 | T | F | F | F | telomere capping | ACD | HIST1H4A | HIST3H3 | POT1 | PRKDC | TERF1 | TERF2 | TERF2IP | TINF2 | GO:0038111 | 0.0014557806623802 | 12/8243 | 9/3250 | 0.0136727524015442 | 1 | T | F | F | F | interleukin-7-mediated signaling pathway | HIST1H3A | HIST2H3A | JAK1 | JAK3 | SMARCA4 | SOCS1 | SOCS2 | STAT3 | STAT5A | GO:0007602 | 0.0014557806623802 | 12/8243 | 9/3250 | 0.0136727524015442 | 1 | T | F | F | F | phototransduction | ARRB1 | CABP4 | GNAQ | GNB1 | GRK1 | PDE6G | RHO | SAG | UNC119 | GO:1901380 | 0.0014557806623802 | 12/8243 | 9/3250 | 0.0136727524015442 | 1 | T | F | F | F | negative regulation of potassium ion transmembrane transport | ACTN2 | ANK3 | BIN1 | CAB39 | CAV1 | KCNAB1 | OXSR1 | PTEN | SUMO1 | GO:1904668 | 0.0014557806623802 | 12/8243 | 9/3250 | 0.0136727524015442 | 1 | T | F | F | F | positive regulation of ubiquitin protein ligase activity | CDC14B | CDC20 | FZR1 | GOLGA2 | PLK1 | PTEN | RAB1A | UBE2C | UBE2S | GO:0010155 | 0.0014557806623802 | 12/8243 | 9/3250 | 0.0136727524015442 | 1 | T | F | F | F | regulation of proton transport | ACTN4 | CHCHD10 | CHP1 | COX17 | DNAJC30 | PARK7 | PPIF | SLC9A3R1 | TWIST1 | GO:2001026 | 0.0014557806623802 | 12/8243 | 9/3250 | 0.0136727524015442 | 1 | T | F | F | F | regulation of endothelial cell chemotaxis | FGF2 | FGFR1 | HSPB1 | KDR | LGMN | P2RX4 | PRKD1 | PRKD2 | THBS1 | GO:0060766 | 0.0014557806623802 | 12/8243 | 9/3250 | 0.0136727524015442 | 1 | T | F | F | F | negative regulation of androgen receptor signaling pathway | DAB2 | HDAC1 | NCOR1 | NCOR2 | PHB | SIRT1 | SMARCA4 | TCF21 | ZBTB7A | GO:0048878 | 0.0693922115734563 | 572/8243 | 251/3250 | 0.0136982467982423 | 1 | F | F | F | F | chemical homeostasis | ABCB7 | ABCG1 | ABL1 | ABL2 | ACACA | ACKR2 | ACO1 | ACOX1 | ADCY6 | ADCYAP1 | AFG3L2 | AKR1B1 | AKT1 | ALOX12 | ANK3 | ANXA2 | ANXA6 | ANXA7 | AP3D1 | APOA1 | APOE | APP | ARF1 | ASGR2 | ASPSCR1 | ATOX1 | ATP12A | ATP13A2 | ATP1A1 | ATP1B3 | ATP2A2 | ATP2B2 | ATP4A | ATP6V1A | ATP6V1B1 | ATP6V1C1 | ATP6V1D | ATP6V1E1 | BAX | BCL2 | BDKRB2 | BTBD9 | C1QTNF1 | CACNA1A | CALM1 | CALM2 | CALM3 | CALR | CAND1 | CAV1 | CCDC22 | CCL21 | CCR1 | CCR3 | CCR4 | CCR5 | CD36 | CD40 | CDK5 | CEBPA | CFTR | CHP1 | CHRNA9 | CKB | CLIC4 | COL4A3BP | COMMD1 | COX11 | COX19 | CRY1 | CRY2 | CSMD1 | CUL1 | CUTC | CXCL11 | DBH | DCN | DDB1 | DLG4 | EGFR | EGLN1 | ENDOG | ESR1 | FABP3 | FABP5 | FASLG | FGF2 | FHL1 | FIS1 | FKBP1B | FOXA1 | FOXO1 | FTH1 | FTL | GLP1R | GLRX3 | GNA13 | GNA15 | GNB1 | GOT1 | GPD1L | GPI | GRIA1 | GRIK2 | GSTO1 | GTF2I | HAP1 | HDAC9 | HEXB | HIF1A | HK1 | HK2 | HMGB1 | HMOX1 | HNF1A | HNF4A | HPX | HRNR | HTR1B | HTT | IBTK | ICAM1 | IGF1R | IMMT | INSIG1 | INSR | IREB2 | ITGAV | ITPR1 | ITPR2 | ITPR3 | KCTD17 | KDR | LETM1 | LGALS1 | LIMA1 | LPAR1 | LPAR2 | LPAR4 | LRP6 | LRRK2 | MAPK1 | MAPK3 | MCOLN3 | MICU1 | MICU2 | MLLT11 | MYC | MYO5A | MYOC | NCOA4 | NEDD8 | NEUROD1 | NMB | NME1 | NME2 | NNT | NR1H2 | NR1H3 | NR5A2 | NUBP1 | OPA1 | OTC | P2RX1 | P2RX2 | P2RX4 | PARK7 | PARP1 | PCSK9 | PDK2 | PFKM | PIK3CA | PIK3CG | PIK3R1 | PLA2G10 | PLA2G1B | PLA2G6 | PLCG1 | PML | POLD1 | PPARG | PPARGC1A | PPT1 | PRCP | PRKAA1 | PRKAA2 | PRKACA | PRKAR1A | PRKAR2B | PRKCE | PSEN1 | PTGER3 | PTGIR | PYGL | RAB11A | RAB11FIP2 | RAB7A | RAP1A | RAP1GDS1 | RBP1 | RGN | RIC3 | RTN4 | SCO2 | SFN | SFXN1 | SFXN4 | SIN3A | SIRT1 | SIRT6 | SLAMF8 | SLC12A2 | SLC16A1 | SLC39A4 | SLC9A1 | SLC9A3R1 | SMAD3 | SMAD4 | SNCA | SOAT1 | SOD1 | SOD2 | SRC | SRI | STAT1 | STAT3 | STAU1 | STIM1 | STK11 | STMN1 | STOML2 | SWAP70 | TBXA2R | TCIRG1 | TGFB1 | TGM2 | THADA | TM9SF4 | TMBIM6 | TP63 | TPCN2 | VAPB | VDR | VGF | WDR35 | WDTC1 | WNK1 | WNK4 | XBP1 | XIAP | YWHAE | GO:0030512 | 0.0055804925391241 | 46/8243 | 26/3250 | 0.0138083149319218 | 1 | F | F | F | F | negative regulation of transforming growth factor beta receptor signaling pathway | CAV1 | CD109 | HSPA5 | HTRA1 | LDLRAD4 | LEMD3 | LTBP1 | NKX2-1 | ONECUT1 | PIN1 | PPM1A | RASL11B | SIRT1 | SKI | SMAD2 | SMAD3 | SMURF1 | SMURF2 | SNX6 | STRAP | STUB1 | TGFB1 | TGFBR3 | UBB | UBC | ZBTB7A | GO:0030433 | 0.0055804925391241 | 46/8243 | 26/3250 | 0.0138083149319218 | 1 | F | F | F | F | ubiquitin-dependent ERAD pathway | AMFR | AUP1 | BAG6 | DERL1 | DNAJB2 | DNAJC10 | EDEM1 | ERLIN2 | FAF2 | FBXO6 | HSP90B1 | HSPA5 | NPLOC4 | PSMC6 | RNF185 | SEL1L | SGTA | SGTB | STUB1 | SYVN1 | TRIM25 | UBE2J1 | UBQLN1 | UBQLN2 | USP19 | VCP | GO:0051924 | 0.0157709571757855 | 130/8243 | 64/3250 | 0.0140172750396474 | 1 | F | F | F | F | regulation of calcium ion transport | ABL1 | ADORA2A | AHNAK | ANXA2 | ARRB2 | ATG5 | BAX | BCL2 | BIN1 | CALM1 | CALM2 | CALM3 | CAMK2A | CAMK2B | CAV1 | CCR1 | CTNNB1 | CXCL11 | DMD | EPPIN | FKBP1A | FKBP1B | FYN | G6PD | GNAI2 | GNAO1 | GSTO1 | HAP1 | HSPA2 | HTT | ICAM1 | LGALS3 | MYO5A | NOS1AP | NOS3 | P2RX1 | P2RX2 | P2RX4 | PACSIN3 | PDE4D | PIK3CG | PLA2G1B | PLA2G6 | PLCG1 | PML | PRKACA | PRKCE | PSEN2 | PTPN6 | RGN | RHOA | S100A1 | SARAF | SLC9A1 | SNCA | SRI | STAC | STIM1 | SUMO1 | TGFB1 | THADA | TMBIM6 | UBQLN1 | YWHAE | GO:0090068 | 0.0241416959844717 | 199/8243 | 94/3250 | 0.0141505338294089 | 1 | F | F | F | F | positive regulation of cell cycle process | AKT1 | ANAPC4 | ANXA1 | APEX1 | APP | ATAD5 | ATM | ATRX | AURKB | BAX | BRCA1 | BRD4 | CARM1 | CASP2 | CCNB1 | CDC14B | CDC25A | CDC25B | CDC25C | CDC42 | CDC73 | CDK2 | CDK4 | CDK5R1 | CDKN1A | CENPJ | CENPV | CHEK2 | CUL3 | CUL4B | CYP1A1 | DBF4B | DDRGK1 | DDX3X | DYNC1H1 | DYNC1LI1 | E2F1 | E2F4 | EGFR | EP300 | EZH2 | FAM83D | FEN1 | FGFR1 | HNRNPU | HSPA2 | ID2 | INSR | MAD2L1 | MAPK15 | MDM2 | MDM4 | MECP2 | MED1 | MEPCE | MTA3 | MYBBP1A | NIPBL | NPM1 | NSFL1C | NUMA1 | NUSAP1 | PCNA | PEBP1 | PHB2 | PKN2 | PML | PRKACA | PRKCE | RAB11A | RAD21 | RB1 | RDX | RHOA | RPL23 | RPTOR | SFN | SFPQ | SH2B1 | SIN3A | SVIL | TFDP1 | TGFB1 | TMOD3 | TP53 | TP73 | TPR | TRIAP1 | UBE2B | UBE2C | USP19 | VPS4B | XRCC3 | ZBTB17 | GO:0034764 | 0.0124954506854301 | 103/8243 | 52/3250 | 0.0142694783337421 | 1 | F | F | F | F | positive regulation of transmembrane transport | ABL1 | ACTN2 | ACTN4 | ADRB2 | AKAP9 | AKT1 | AKT2 | ANK3 | ARF1 | ATP1B3 | BAX | BRAF | CACNG2 | CACNG4 | CALM1 | CALM2 | CALM3 | CFTR | CHCHD10 | CHP1 | COX17 | CXCL11 | DMD | G6PD | GLRX | GSTO1 | HAP1 | HSPA2 | HTR3A | HTT | INSR | KCNIP2 | KIF5B | MAPK14 | NFE2L2 | NOS1AP | PARK7 | PIK3R1 | PLCG1 | PSEN1 | RAP1A | RGN | S100A1 | SLC9A1 | SLC9A3R1 | SNCA | SRI | STAC | STIM1 | SUMO1 | WNK1 | WNK4 | GO:0010212 | 0.0135872861822152 | 112/8243 | 56/3250 | 0.0143043183839324 | 1 | F | F | F | F | response to ionizing radiation | ANXA1 | ATM | BABAM1 | BAX | BCL2 | BCL2L1 | BRCA1 | CASP3 | CBL | CDKN1A | CHEK2 | CRYAB | EEF1D | FIGNL1 | GPX1 | GRB2 | H2AFX | HRAS | HSF1 | HSPA5 | ICAM1 | IFI16 | KAT5 | KDM1A | KDM4D | LIG4 | MAPK14 | MDM2 | MGMT | MTA1 | MYC | NAMPT | NIPBL | PARP1 | PAXIP1 | PML | POLE3 | PRKAA1 | PRKDC | RAD51 | RAD51AP1 | RNF8 | RRM1 | SIRT1 | SNAI2 | STK11 | TANK | TGFB1 | TP53 | TREX1 | USP28 | VCAM1 | XRCC5 | XRCC6 | YAP1 | ZMPSTE24 | GO:0006303 | 0.0053378624287274 | 44/8243 | 25/3250 | 0.0143643210915785 | 1 | F | F | F | F | double-strand break repair via nonhomologous end joining | ATM | BABAM1 | BRCA1 | H2AFX | HERC2 | HIST1H4A | HIST3H3 | KAT5 | KDM2A | LIG4 | MDC1 | MLH1 | PAXIP1 | POLM | PRKDC | PRPF19 | PSMD14 | RIF1 | RNF8 | SUMO1 | UBE2N | XRCC1 | XRCC5 | XRCC6 | ZBTB7A | GO:0042306 | 0.0053378624287274 | 44/8243 | 25/3250 | 0.0143643210915785 | 1 | F | F | F | F | regulation of protein import into nucleus | APOD | BAG3 | CD36 | CDH1 | CHP1 | DMAP1 | HDAC3 | IPO5 | JUP | MAPK1 | MAPK14 | MAVS | PIK3R1 | PKIG | PRKCD | PSEN1 | RAN | SMAD3 | SMO | TARDBP | TGFB1 | TPR | TRIM28 | UBR5 | ZIC1 | GO:0021766 | 0.0053378624287274 | 44/8243 | 25/3250 | 0.0143643210915785 | 1 | F | F | F | F | hippocampus development | CASP3 | CDK5 | CDK5R1 | CRK | CRKL | DAB1 | EIF2B3 | EIF2B5 | EZH2 | GSK3B | HDAC1 | KIF5B | MAS1 | NCOA1 | NF2 | NKX2-1 | NME1 | OGDH | PAFAH1B1 | PEBP1 | RARA | UBA6 | UQCRQ | XRCC1 | YWHAE | GO:0051438 | 0.0053378624287274 | 44/8243 | 25/3250 | 0.0143643210915785 | 1 | F | F | F | F | regulation of ubiquitin-protein transferase activity | ABL1 | AXIN1 | BAG2 | BAG5 | BMI1 | CDC14B | CDC20 | DCUN1D1 | FZR1 | GOLGA2 | MAD2L1 | PARK7 | PIN1 | PLK1 | PSEN1 | PTEN | RAB1A | RPL23 | RPS2 | STUB1 | TRIB3 | UBE2C | UBE2L3 | UBE2N | UBE2S | GO:0036498 | 0.0053378624287274 | 44/8243 | 25/3250 | 0.0143643210915785 | 1 | F | F | F | F | IRE1-mediated unfolded protein response | ACADVL | ARFGAP1 | CUL7 | DCTN1 | DNAJB11 | EDEM1 | EXTL2 | FKBP14 | GFPT1 | GSK3A | HDGF | HSPA5 | LMNA | PDIA6 | PTPN1 | SEC31A | SHC1 | SRPRB | SULT1A3 | SYVN1 | TSPYL2 | VAPB | WIPI1 | XBP1 | ZBTB17 | GO:0010633 | 0.0053378624287274 | 44/8243 | 25/3250 | 0.0143643210915785 | 1 | F | F | F | F | negative regulation of epithelial cell migration | APOE | CSNK2B | DCN | EPPK1 | FGF2 | HDAC5 | HMGB1 | ITGB1BP1 | JUP | MCC | MECP2 | MEOX2 | PDCD10 | PPARG | PTEN | PTPN23 | PTPRR | RHOA | SP100 | STARD13 | STRAP | SYNJ2BP | TBXA2R | TGFB1 | THBS1 | GO:0015908 | 0.0053378624287274 | 44/8243 | 25/3250 | 0.0143643210915785 | 1 | F | F | F | F | fatty acid transport | ABCD3 | ACACA | ACSL1 | ACSL4 | ANXA1 | APOE | BDKRB2 | CD36 | CPT1A | CPT2 | CROT | EPRS | FABP1 | FABP3 | GOT2 | NMB | NOS2 | PLA2G10 | PLA2G12A | PLA2G1B | PLA2G2A | PPARD | PPARG | PRKAA2 | PRKAB2 | GO:0098771 | 0.0427028994298192 | 352/8243 | 159/3250 | 0.0144062254750896 | 1 | F | F | F | F | inorganic ion homeostasis | ABCB7 | ABL1 | ABL2 | ACKR2 | ACO1 | ADCYAP1 | AFG3L2 | ANK3 | ANXA2 | ANXA6 | ANXA7 | APOE | APP | ARF1 | ATOX1 | ATP12A | ATP13A2 | ATP1A1 | ATP1B3 | ATP2A2 | ATP2B2 | ATP4A | ATP6V1A | ATP6V1B1 | ATP6V1C1 | ATP6V1D | ATP6V1E1 | BAX | BCL2 | BDKRB2 | BTBD9 | C1QTNF1 | CACNA1A | CALM1 | CALM2 | CALM3 | CALR | CAND1 | CAV1 | CCDC22 | CCL21 | CCR1 | CCR3 | CCR4 | CCR5 | CD36 | CD40 | CDK5 | CFTR | CHP1 | CHRNA9 | CKB | CLIC4 | COMMD1 | COX11 | COX19 | CUL1 | CUTC | CXCL11 | DLG4 | EGFR | EGLN1 | ESR1 | FASLG | FGF2 | FIS1 | FKBP1B | FTH1 | FTL | GLP1R | GLRX3 | GNA13 | GNA15 | GNB1 | GOT1 | GRIA1 | GRIK2 | GSTO1 | GTF2I | HAP1 | HEXB | HIF1A | HMGB1 | HMOX1 | HPX | HTR1B | HTT | IBTK | IMMT | IREB2 | ITGAV | ITPR1 | ITPR2 | ITPR3 | KCTD17 | LETM1 | LPAR1 | LPAR2 | LPAR4 | LRP6 | LRRK2 | MAPK1 | MAPK3 | MCOLN3 | MICU1 | MICU2 | MYC | MYO5A | NCOA4 | NEDD8 | NMB | NUBP1 | P2RX1 | P2RX2 | P2RX4 | PDK2 | PIK3CG | PLA2G1B | PLA2G6 | PLCG1 | PML | PPT1 | PRKACA | PRKCE | PSEN1 | PTGER3 | PTGIR | RAB7A | RAP1GDS1 | RGN | RIC3 | SCO2 | SFXN1 | SFXN4 | SLAMF8 | SLC12A2 | SLC39A4 | SLC9A1 | SLC9A3R1 | SMAD3 | SMAD4 | SNCA | SOD1 | SRI | STIM1 | STOML2 | SWAP70 | TBXA2R | TCIRG1 | TGFB1 | TGM2 | THADA | TM9SF4 | TMBIM6 | TPCN2 | VAPB | VDR | XIAP | YWHAE | GO:1903844 | 0.00873468397428121 | 72/8243 | 38/3250 | 0.0144407967543439 | 1 | F | F | F | F | regulation of cellular response to transforming growth factor beta stimulus | CAV1 | CD109 | CREBBP | DAB2 | EP300 | FOLR1 | GOT1 | HSP90AB1 | HSPA5 | HTRA1 | LDLRAD4 | LEMD3 | LTBP1 | NKX2-1 | ONECUT1 | PIN1 | PPM1A | RASL11B | SDCBP | SIRT1 | SKI | SMAD2 | SMAD3 | SMAD4 | SMURF1 | SMURF2 | SNW1 | SNX6 | STK11 | STRAP | STUB1 | TGFB1 | TGFBR3 | THBS1 | TRIM33 | UBB | UBC | ZBTB7A | GO:0009062 | 0.00715758825670266 | 59/8243 | 32/3250 | 0.0146550431275042 | 1 | F | F | F | F | fatty acid catabolic process | ABCD3 | ACAA1 | ACAA2 | ACADS | ACADVL | ACAT1 | ACAT2 | ACOX1 | ADTRP | CEL | CPT1A | CPT2 | CROT | DECR1 | ECH1 | ECHDC2 | ECHS1 | ECI1 | ECI2 | EHHADH | ETFA | ETFB | ETFDH | GCDH | HADH | HIBCH | HSD17B4 | IVD | LPIN3 | MCAT | NUDT19 | PPARD | GO:0006301 | 0.0050952323183307 | 42/8243 | 24/3250 | 0.01492253444914 | 1 | F | F | F | F | postreplication repair | BRCA1 | ISG15 | NPLOC4 | PCNA | POLD1 | POLE2 | RFC1 | RFC2 | RFC3 | RFC4 | RFC5 | RPA2 | RPA3 | SPRTN | TMEM189-UBE2V1 | TRIM25 | UBB | UBC | UBE2A | UBE2B | UBE2L6 | UBE2N | UBE2V1 | VCP | GO:0016575 | 0.0050952323183307 | 42/8243 | 24/3250 | 0.01492253444914 | 1 | F | F | F | F | histone deacetylation | HDAC1 | HDAC11 | HDAC2 | HDAC3 | HDAC4 | HDAC5 | HDAC6 | HDAC7 | HDAC9 | MORF4L1 | MORF4L2 | MTA1 | MTA3 | PER1 | PER2 | PHB | REST | SFPQ | SIN3A | SIRT1 | SIRT3 | SIRT6 | SIRT7 | TBL1X | GO:0097549 | 0.0050952323183307 | 42/8243 | 24/3250 | 0.01492253444914 | 1 | F | F | F | F | chromatin organization involved in negative regulation of transcription | BMI1 | CHRAC1 | CTBP1 | EED | EZH2 | H1F0 | H3F3A | HDAC1 | HDAC5 | HIST1H3A | HIST1H4A | HIST2H3A | HMGB1 | MBD3 | MECP2 | MORF4L1 | MORF4L2 | PCGF1 | POLE3 | PPM1D | RIF1 | SIRT1 | SIRT5 | TNP1 | GO:0030336 | 0.0174693679485624 | 144/8243 | 70/3250 | 0.0149298829559087 | 1 | F | F | F | F | negative regulation of cell migration | ADA | ADTRP | AKT1 | APEX1 | APOD | APOE | BCL2 | BMPR1A | BRAF | CALR | CCL21 | CDH1 | CHRD | CLIC4 | CSNK2B | DCN | DPEP1 | DUSP1 | DUSP22 | EPPK1 | FGF2 | GSTP1 | HDAC5 | HMGB1 | HMOX1 | IDH2 | ILK | ITGB1BP1 | JUP | LDLRAD4 | MAPK15 | MCC | MECP2 | MEOX2 | MIF | MITF | NF2 | NFE2L2 | NKX2-1 | PDCD10 | PPARD | PPARG | PPARGC1A | PTEN | PTN | PTPN23 | PTPRK | PTPRR | RECK | RHOA | SEPT7 | SLAMF8 | SLC9A3R1 | SP100 | SRGAP2 | SRGAP3 | STARD13 | STAT3 | STK24 | STK26 | STRAP | SYNJ2BP | TBXA2R | TGFB1 | THBS1 | TIMP1 | TNN | TPM1 | VCL | ZMYND8 | GO:1903708 | 0.0133446560718185 | 110/8243 | 55/3250 | 0.0151057342424181 | 1 | F | F | F | F | positive regulation of hemopoiesis | ADA | AGER | ANXA1 | AP3D1 | ARNT | BCL6 | BTK | CASP8 | CBFB | CCR1 | CD27 | CD74 | CREB1 | CTNNBIP1 | CYLD | DHX36 | ETS1 | FADD | FOS | GATA1 | GNAS | HAX1 | HIF1A | HLX | HMGB1 | HMGB2 | HOXA5 | HSPA1B | ID2 | ISG15 | JUN | LEF1 | LGALS1 | LGALS3 | MAPK14 | MED1 | PNP | PRKCZ | RARA | RB1 | RHOA | RIPK1 | RIPK2 | RUNX1 | SART1 | SOCS1 | STAT1 | STAT3 | TGFB1 | TNF | TRAF6 | XBP1 | ZAP70 | ZBTB16 | ZBTB7B | GO:0098693 | 0.00849205386388451 | 70/8243 | 37/3250 | 0.0152147849745507 | 1 | F | F | F | F | regulation of synaptic vesicle cycle | ADORA2A | ATP2A2 | BACE1 | BRAF | BTBD9 | CALM1 | CALM3 | CAMK2A | CDK5 | CDK5R1 | CHRM2 | CPLX1 | CPLX3 | DNM1L | DVL1 | GIT1 | GSK3B | HTR1B | LRRK2 | NRXN1 | P2RX1 | PPP3CC | PREPL | PTEN | RAB3GAP1 | RAB5A | RAP1A | RAP1B | RAPGEF4 | SEPT5 | SH3GL1 | SNCA | STXBP1 | SYN1 | SYP | TOR1A | VPS18 | GO:0006839 | 0.00982651947106636 | 81/8243 | 42/3250 | 0.015216625763277 | 1 | F | F | F | F | mitochondrial transport | ACACA | AFG3L2 | AGK | CPT1A | CPT2 | CYC1 | DNAJC19 | FBXO7 | FIS1 | GRPEL1 | HSP90AA1 | HSPA4 | HSPD1 | LETM1 | MICU1 | MICU2 | MPC2 | NDUFA13 | OPA1 | OXA1L | PAM16 | PRKAA2 | PRKAB2 | PSEN1 | ROMO1 | SFXN1 | SLC25A1 | SLC25A10 | SLC25A13 | SLC25A22 | SLC25A24 | SLC25A32 | SLC25A6 | STOML2 | TIMM13 | TIMM21 | TIMM23 | TIMM44 | TIMM50 | TOMM20 | TOMM22 | TOMM40 | GO:0061640 | 0.00691495814630596 | 57/8243 | 31/3250 | 0.0153853437274084 | 1 | F | F | F | F | cytoskeleton-dependent cytokinesis | ACTR2 | ACTR3 | ANK3 | APC | CEP55 | CFL1 | CUL7 | NUSAP1 | PDCD6IP | PLK1 | RAB35 | RTKN | SEPT1 | SEPT10 | SEPT11 | SEPT12 | SEPT14 | SEPT2 | SEPT3 | SEPT4 | SEPT5 | SEPT6 | SEPT7 | SEPT8 | SEPT9 | SPTBN1 | STMN1 | TTC19 | UNC119 | USP8 | ZFYVE26 | GO:0001938 | 0.00691495814630596 | 57/8243 | 31/3250 | 0.0153853437274084 | 1 | F | F | F | F | positive regulation of endothelial cell proliferation | AKT1 | AKT3 | ARNT | CCR3 | FGF2 | FGFR1 | HIF1A | HMGB1 | HMGB2 | HMOX1 | ITGA4 | ITGB3 | JUN | KDR | NR4A1 | NRP2 | PDCD6 | PLCG1 | PRKD1 | PRKD2 | PROX1 | RPTOR | SCG2 | SIRT1 | SIRT6 | SP1 | STAT3 | STAT5A | TNFSF12 | VASH2 | WNT2 | GO:0000910 | 0.00691495814630596 | 57/8243 | 31/3250 | 0.0153853437274084 | 1 | F | F | F | F | cytokinesis | ACTR2 | ACTR3 | ANK3 | APC | CEP55 | CFL1 | CUL7 | NUSAP1 | PDCD6IP | PLK1 | RAB35 | RTKN | SEPT1 | SEPT10 | SEPT11 | SEPT12 | SEPT14 | SEPT2 | SEPT3 | SEPT4 | SEPT5 | SEPT6 | SEPT7 | SEPT8 | SEPT9 | SPTBN1 | STMN1 | TTC19 | UNC119 | USP8 | ZFYVE26 | GO:0010823 | 0.004852602207934 | 40/8243 | 23/3250 | 0.0154781771845208 | 1 | F | F | F | F | negative regulation of mitochondrion organization | ACAA2 | AKT1 | ARRB2 | BAG3 | BAG4 | BCL2L1 | CLU | DNM1L | GPX1 | HTRA2 | LMNA | LRRK2 | MAPT | OPA1 | PAM16 | PPARGC1A | PPIF | PRELID1 | PSMD10 | SLC25A5 | SLC35F6 | TRIAP1 | VAT1 | GO:0034063 | 0.0016984107727769 | 14/8243 | 10/3250 | 0.015575697729966 | 1 | F | F | F | F | stress granule assembly | ATXN2 | CIRBP | DDX3X | DYNC1H1 | EIF2S1 | G3BP1 | G3BP2 | GRB7 | MAPT | OGFOD1 | GO:0006730 | 0.0016984107727769 | 14/8243 | 10/3250 | 0.015575697729966 | 1 | F | F | F | F | one-carbon metabolic process | AHCY | AHCYL1 | FPGS | MAT2A | MAT2B | MTHFD1 | SFXN1 | SHMT1 | SHMT2 | TYMS | GO:0090083 | 0.0016984107727769 | 14/8243 | 10/3250 | 0.015575697729966 | 1 | F | F | F | F | regulation of inclusion body assembly | BAG5 | CDC34 | CLU | DNAJA4 | DNAJB1 | DNAJB2 | HSF1 | HSPA1B | HSPA2 | PSMC5 | GO:0002026 | 0.0016984107727769 | 14/8243 | 10/3250 | 0.015575697729966 | 1 | F | F | F | F | regulation of the force of heart contraction | ATP1A1 | ATP2A2 | CAV1 | GAA | GLRX3 | MYH6 | MYL2 | NOS3 | PEBP1 | SLC9A1 | GO:0009084 | 0.0016984107727769 | 14/8243 | 10/3250 | 0.015575697729966 | 1 | F | F | F | F | glutamine family amino acid biosynthetic process | ASL | ASS1 | GLS | GLS2 | GLUD2 | GLUL | OAT | OTC | PYCR1 | PYCR2 | GO:0060444 | 0.0016984107727769 | 14/8243 | 10/3250 | 0.015575697729966 | 1 | F | F | F | F | branching involved in mammary gland duct morphogenesis | BTRC | ESR1 | KDM5B | LRP6 | MED1 | PHB2 | PML | SRC | TGFB1 | VDR | GO:0031333 | 0.0109183549678515 | 90/8243 | 46/3250 | 0.0156871371182379 | 1 | F | F | F | F | negative regulation of protein complex assembly | ADD3 | ALB | AVIL | BIRC2 | CAPG | CAPZA1 | CAPZA2 | CAPZB | CDC42 | CLU | CRBN | CRYAB | CTNNBIP1 | DDX3X | EIF4EBP1 | EP300 | GSK3B | HIST3H3 | HMGB1 | LDLRAD4 | PARK7 | PEX5 | PFN1 | PPM1A | PRKCD | PRKCZ | RAF1 | RDX | SNCA | SPTAN1 | SPTBN1 | SPTBN2 | SRC | STMN1 | STXBP1 | TFIP11 | THRA | TMOD3 | TMOD4 | TRAF3IP1 | TRIM9 | TUBB4A | TWF1 | TWF2 | ULK1 | VDAC2 | GO:0055080 | 0.0422176392090258 | 348/8243 | 157/3250 | 0.015735851325628 | 1 | F | F | F | F | cation homeostasis | ABCB7 | ABL1 | ABL2 | ACKR2 | ACO1 | ADCYAP1 | AFG3L2 | ANK3 | ANXA2 | ANXA6 | ANXA7 | APOE | APP | ARF1 | ATOX1 | ATP12A | ATP13A2 | ATP1A1 | ATP1B3 | ATP2A2 | ATP2B2 | ATP4A | ATP6V1A | ATP6V1B1 | ATP6V1C1 | ATP6V1D | ATP6V1E1 | BAX | BCL2 | BDKRB2 | BTBD9 | C1QTNF1 | CACNA1A | CALM1 | CALM2 | CALM3 | CALR | CAND1 | CAV1 | CCDC22 | CCL21 | CCR1 | CCR3 | CCR4 | CCR5 | CD36 | CD40 | CDK5 | CFTR | CHP1 | CHRNA9 | CLIC4 | COMMD1 | COX11 | COX19 | CUL1 | CUTC | CXCL11 | DLG4 | EGFR | EGLN1 | ESR1 | FASLG | FGF2 | FIS1 | FKBP1B | FTH1 | FTL | GLP1R | GLRX3 | GNA13 | GNA15 | GNB1 | GOT1 | GRIA1 | GRIK2 | GSTO1 | GTF2I | HAP1 | HEXB | HIF1A | HMGB1 | HMOX1 | HPX | HTR1B | HTT | IBTK | IMMT | IREB2 | ITGAV | ITPR1 | ITPR2 | ITPR3 | KCTD17 | LETM1 | LPAR1 | LPAR2 | LPAR4 | LRP6 | LRRK2 | MAPK1 | MAPK3 | MCOLN3 | MICU1 | MICU2 | MYC | MYO5A | NCOA4 | NEDD8 | NMB | NUBP1 | P2RX1 | P2RX2 | P2RX4 | PDK2 | PIK3CG | PLA2G1B | PLA2G6 | PLCG1 | PML | PPT1 | PRKACA | PRKCE | PSEN1 | PTGER3 | PTGIR | RAB7A | RAP1GDS1 | RGN | RIC3 | SCO2 | SFXN1 | SFXN4 | SLAMF8 | SLC12A2 | SLC39A4 | SLC9A1 | SMAD3 | SMAD4 | SNCA | SOD1 | SRI | STIM1 | STOML2 | SWAP70 | TBXA2R | TCIRG1 | TGFB1 | TGM2 | THADA | TM9SF4 | TMBIM6 | TPCN2 | VAPB | VDR | XIAP | YWHAE | GO:0043405 | 0.0279024626956205 | 230/8243 | 107/3250 | 0.0157451935884963 | 1 | F | F | F | F | regulation of MAP kinase activity | AGER | APOE | ARRB1 | BIRC7 | BRAF | CAV1 | CBLC | CD40 | CD40LG | CD74 | CD81 | CRK | CRKL | DAXX | DBNL | DNAJA1 | DUSP1 | DUSP14 | DUSP16 | DUSP19 | DUSP22 | DUSP7 | DVL2 | DVL3 | EGFR | EPHA4 | ERBB2 | EZH2 | FGF2 | FGFR1 | GADD45G | GPS2 | GSTP1 | HGS | HRAS | IGF1R | IKBKG | ILK | INSR | IQGAP1 | KRAS | KSR1 | LPAR1 | LRRK2 | MAP2K1 | MAP2K3 | MAP2K4 | MAP2K6 | MAP2K7 | MAP3K1 | MAP3K13 | MAP3K14 | MAP3K2 | MAP3K3 | MAP3K4 | MAP4K1 | MAPK1 | MAPK14 | MAPK3 | MAPK8IP3 | MIF | NOD2 | NTF3 | NTRK1 | PDCD10 | PDE6G | PIK3CG | PLA2G1B | PPP2CA | PPP2R1A | PRDX2 | PRKAA1 | PRKCD | PSEN1 | PTPN1 | PTPN22 | PTPN6 | RAF1 | RGS2 | RIPK1 | RIPK2 | RPS3 | S100A12 | SERPINB3 | SHC1 | SNF8 | SOD1 | SPRY2 | SRC | STK38 | TAB1 | TAB2 | TAOK3 | TGFB1 | THBS1 | TNF | TP73 | TRAF2 | TRAF6 | TRIB2 | TRIB3 | TSG101 | UBB | UBC | UBE2N | UBE2V1 | UCHL1 | GO:0045926 | 0.0172267378381657 | 142/8243 | 69/3250 | 0.015750088899904 | 1 | F | F | F | F | negative regulation of growth | ADRB2 | APBB1 | APBB2 | BCL11A | BCL2 | BCL6 | CCAR2 | CD36 | CDA | CDK5 | CDK5R1 | CDKL3 | CDKN1A | CDKN2C | CRYAB | CTDP1 | DDX3X | DNAJB2 | EAF2 | ENO1 | ESR2 | FBP1 | FGFR3 | FHL1 | G6PD | GDF9 | GJA1 | GSK3A | HIF1A | HNF4A | HSPA1B | ING1 | ING5 | LGMN | MYL2 | NDUFA13 | NDUFS3 | NPR1 | PHB | PML | PPARD | PPARG | PPP2CA | PPP2R1A | PPT1 | PTEN | PTK2 | RGS2 | RIPK1 | RTN4 | SEMA7A | SERPINE2 | SIPA1 | SIRT1 | SMAD3 | SMAD4 | SMARCA2 | SMARCA4 | SOCS2 | SQSTM1 | STK11 | STK3 | TGFB1 | TNF | TP53 | TP73 | TSPYL2 | WT1 | YY1 | GO:0044087 | 0.0719398277326216 | 593/8243 | 259/3250 | 0.0159522334171629 | 1 | F | F | F | F | regulation of cellular component biogenesis | ABL1 | ACE2 | ACTR2 | ACTR3 | ADD3 | AKAP9 | ALB | APC | APOA1 | APOD | APOE | APP | ARF1 | ARHGAP35 | ARHGEF9 | ARPC1A | ARPC1B | ARPC2 | ARPC3 | ARPC4 | ARPC5 | ATG5 | ATM | ATP13A2 | ATXN2 | AVIL | BAG4 | BAG5 | BAIAP2 | BAIAP2L1 | BAX | BIN1 | BIRC2 | BRAF | BRCA1 | CAND1 | CAPG | CAPZA1 | CAPZA2 | CAPZB | CAV1 | CBFB | CCDC88A | CCL21 | CCP110 | CD36 | CDC34 | CDC42 | CDC42EP3 | CDC42EP4 | CDK5R1 | CENPJ | CEP135 | CHD1L | CHMP2A | CLSTN1 | CLU | CNTROB | CRBN | CREB1 | CROCC | CRYAB | CTNNBIP1 | CTTN | CUL4B | CYLD | DCTN1 | DDB1 | DDB2 | DDX3X | DNAJA4 | DNAJB1 | DNAJB2 | DNM1L | DUSP22 | DYNC1H1 | DYNLL1 | EIF4EBP1 | EP300 | ERCC5 | ESR1 | EZR | FAF1 | FAM98A | FCHSD1 | FLOT1 | FSCN1 | GAP43 | GBP5 | GFAP | GRB2 | GRID2 | GSK3B | HAP1 | HAX1 | HDAC4 | HDAC6 | HGS | HIST3H3 | HMGB1 | HNRNPU | HRAS | HSF1 | HSP90AA1 | HSPA1B | HSPA2 | HSPA8 | HTT | ICAM1 | IDE | IKBKB | IQGAP1 | ITGB1BP1 | KCTD17 | KDR | KLF5 | LATS1 | LCP1 | LDLRAD4 | LIMS1 | LPAR1 | LRFN1 | LRRK2 | LRSAM1 | MAPK15 | MAPK9 | MAPT | MDM1 | MECP2 | MSN | MTPN | MYO1C | MYOC | NCK1 | NF2 | NIN | NPM1 | NR1H2 | NRXN1 | NTRK1 | NUMA1 | P2RX4 | PAN2 | PARK7 | PARP1 | PDCD6IP | PDLIM5 | PEX5 | PFN1 | PICK1 | PIH1D1 | PIK3R1 | PIKFYVE | PIP4K2A | PLA2G6 | PLK1 | PPM1A | PRKAA1 | PRKAA2 | PRKACA | PRKCD | PRKCE | PRKCZ | PROX1 | PSMC2 | PSMC3 | PSMC4 | PSMC5 | PSMC6 | PTEN | PTK2 | PTPRD | PXN | RAB1B | RAB3GAP1 | RAB5A | RAB7A | RAC1 | RAF1 | RAP1A | RAP1B | RB1 | RBX1 | RDX | REST | RHOA | RIOK1 | RNF4 | RPA2 | RPA3 | RPS3 | RUNX1 | SCFD1 | SDC1 | SDC4 | SDCBP | SENP6 | SEPT7 | SEPT8 | SEPT9 | SIRT6 | SLC9A1 | SMAD3 | SMAD4 | SMC1A | SMC3 | SNAI2 | SNCA | SNF8 | SPICE1 | SPTAN1 | SPTBN1 | SPTBN2 | SRC | STAG2 | STIL | STMN1 | STUB1 | STXBP1 | SUMO1 | SWAP70 | SYNE2 | TBC1D15 | TBC1D22B | TERF1 | TERF2 | TFIP11 | TGFB1 | THBS1 | THBS2 | THRA | TMOD3 | TMOD4 | TNF | TNFRSF1A | TP53 | TPM1 | TPR | TRAF3IP1 | TRIM9 | TSG101 | TUBB4A | TWF1 | TWF2 | UBQLN2 | ULK1 | USP17L2 | USP50 | VASP | VCP | VDAC2 | VDAC3 | VPS18 | VPS4B | WARS | WAS | XRCC5 | ZMYND10 | ZMYND8 | GO:0071103 | 0.0131020259614218 | 108/8243 | 54/3250 | 0.015953564952334 | 1 | F | F | F | F | DNA conformation change | ABL1 | AIFM1 | AKAP8 | ANXA1 | CHD1L | CHD3 | CHD4 | CHD8 | CUL4B | DDB1 | DDB2 | DDX1 | DDX3X | DHX36 | DHX9 | DNA2 | FANCM | G3BP1 | GINS2 | GINS4 | H1F0 | H1FX | HIST1H1A | HMGB1 | HMGB2 | HMGB3 | HNRNPA2B1 | MCM2 | MCM4 | MCM6 | MCM7 | MCM9 | MTERF1 | NCAPH2 | NIPBL | NUSAP1 | PARP1 | POT1 | RAD23B | RAD51 | RBX1 | RPA2 | RUVBL1 | RUVBL2 | SRPK1 | TOP1 | TOP2A | TOP3A | TP53 | TREX1 | UBB | UBC | XRCC5 | XRCC6 | GO:0031057 | 0.0046099720975373 | 38/8243 | 22/3250 | 0.0160252031827147 | 1 | F | F | F | F | negative regulation of histone modification | ATG5 | BCOR | BRCA1 | CTBP1 | KDM1A | KDM4D | MECP2 | OTUB1 | PIH1D1 | PRMT6 | SET | SIN3A | SIRT1 | SKI | SMARCB1 | SNCA | SPI1 | TRIP12 | TWIST1 | UBE2B | UBR5 | USP17L2 | GO:0043392 | 0.0046099720975373 | 38/8243 | 22/3250 | 0.0160252031827147 | 1 | F | F | F | F | negative regulation of DNA binding | CTNNBIP1 | E2F1 | GATA1 | GZMA | HDAC2 | HMOX1 | ID2 | IFI16 | JUN | KDM1A | LEF1 | MDFI | NFKBIA | PER2 | PSEN1 | SIN3A | SMO | SP100 | SRI | SUMO1 | SUMO3 | TNKS | GO:0034637 | 0.0046099720975373 | 38/8243 | 22/3250 | 0.0160252031827147 | 1 | F | F | F | F | cellular carbohydrate biosynthetic process | AGL | AKR1B1 | AKT1 | AKT2 | B3GNT3 | FBP1 | FBP2 | GBE1 | GOT1 | GYG1 | GYG2 | GYS1 | HS2ST1 | IMPA1 | IMPA2 | ISYNA1 | PER2 | PGM1 | PGM2 | PGP | PPP1R3C | UGP2 | GO:0031146 | 0.00824942375348781 | 68/8243 | 36/3250 | 0.0160296757717868 | 1 | F | F | F | F | SCF-dependent proteasomal ubiquitin-dependent protein catabolic process | BTRC | CUL1 | CUL2 | CUL3 | CUL4B | CUL5 | FBXL12 | FBXO6 | FBXW11 | FBXW5 | PSMA1 | PSMA2 | PSMA3 | PSMA5 | PSMA6 | PSMB5 | PSMB6 | PSMB7 | PSMB8 | PSMC1 | PSMC2 | PSMC3 | PSMC4 | PSMC5 | PSMC6 | PSMD1 | PSMD10 | PSMD11 | PSMD12 | PSMD14 | PSMD2 | PSMD4 | PSMD9 | PSME2 | PSME3 | RBX1 | GO:0036503 | 0.00667232803590926 | 55/8243 | 30/3250 | 0.0161466219149005 | 1 | F | F | F | F | ERAD pathway | AMFR | AUP1 | BAG6 | DERL1 | DNAJB2 | DNAJC10 | EDEM1 | ERLIN2 | FAF2 | FBXO6 | HSP90B1 | HSPA5 | NPLOC4 | PSMC6 | RHBDD1 | RNF139 | RNF185 | SEL1L | SGTA | SGTB | STUB1 | SYVN1 | TOR1A | TRIM25 | UBE2J1 | UBQLN1 | UBQLN2 | UGGT1 | USP19 | VCP | GO:0010522 | 0.00667232803590926 | 55/8243 | 30/3250 | 0.0161466219149005 | 1 | F | F | F | F | regulation of calcium ion transport into cytosol | ABL1 | ATG5 | BAX | BCL2 | CALM1 | CALM2 | CALM3 | CAV1 | CXCL11 | DMD | FKBP1A | FKBP1B | FYN | GSTO1 | HAP1 | HTT | MYO5A | P2RX2 | P2RX4 | PDE4D | PLA2G1B | PLCG1 | PML | PRKACA | PRKCE | PTPN6 | SNCA | SRI | TGFB1 | TMBIM6 | GO:0048513 | 0.0843139633628533 | 695/8243 | 301/3250 | 0.0161700904642194 | 1 | F | F | F | F | animal organ development | ABL1 | ACAT1 | ACO2 | ACTL6A | ADA | ADCYAP1 | AHI1 | AK4 | AKT1 | ANXA1 | ANXA2 | AP1B1 | APOA1 | APOD | AR | ARF4 | ARHGAP35 | ARNT | ARNT2 | ARRB2 | ASCL1 | ASNS | ASS1 | ATF6 | ATIC | ATXN1 | BAG3 | BAG6 | BASP1 | BAX | BCL2 | BCL2L1 | BCOR | BIRC2 | BMI1 | BMPR1A | BRAF | CASP2 | CASP3 | CASP9 | CAV1 | CBFB | CBL | CDH1 | CDK4 | CDK5R1 | CDKN1A | CEBPA | CENPF | CEP290 | CHD8 | COL2A1 | COL4A1 | COL5A1 | COX17 | CREB1 | CRIP1 | CRIP2 | CRKL | CRYAB | CSNK2A2 | CSNK2B | CTNNB1 | CTNND1 | CUL7 | CXCL8 | CYP1A1 | CYP1A2 | DCN | DDIT4 | DDRGK1 | DDX39B | DHCR24 | DMD | DNAJC19 | DNAJC30 | DVL2 | DYM | EDA | EED | EFEMP1 | EGFR | EIF2S2 | EIF4E | EIF4EBP1 | EP300 | ERBB2 | ERBB3 | ESR1 | ESRRA | ESRRB | ETS1 | EZH2 | FADD | FANCA | FASN | FHL1 | FKBP8 | FOXJ1 | FOXK1 | FPGS | FRAS1 | FREM2 | GATA1 | GJA1 | GLRX5 | GNAI3 | GNAS | GNPNAT1 | GSTP1 | GYS1 | HAP1 | HBEGF | HDAC9 | HEXIM1 | HIF1A | HLX | HMGB2 | HMGCL | HMOX1 | HNF1A | HNRNPD | HOXA5 | HOXB9 | HS6ST1 | HSF1 | HSF4 | HSP90AB1 | HSPG2 | HTRA1 | ID2 | IDH1 | IFI16 | INSR | IREB2 | ITGA7 | JMJD6 | JUN | JUNB | KCNAB1 | KDR | KMT2A | KRAS | L3MBTL1 | LCP1 | LDHA | LEF1 | LGR4 | LUM | MAD1L1 | MAP2K1 | MAP3K4 | MAPK1 | MAPK14 | MAPK3 | MAS1 | MBD3 | MED1 | MED12 | MELK | MITF | MKNK2 | MKS1 | MME | MPST | MTHFD1 | MYH6 | MYL2 | MYO1E | MYOC | NCOA1 | NCOA4 | NFKB2 | NIPBL | NKX2-1 | NOS3 | NPHP3 | NR0B2 | NRP2 | NUP107 | NUPR1 | ONECUT1 | OTC | OXCT1 | PALB2 | PAPSS2 | PATZ1 | PAX8 | PCNA | PCSK9 | PGM3 | PHGDH | PIK3CA | PKM | PLS3 | PLXNB2 | PPARD | PPARG | PPAT | PPIB | PPP1CA | PPP3CC | PPT1 | PRDX2 | PRDX4 | PRKDC | PROX1 | PSEN1 | PTEN | PTK2 | PTN | QARS | QDPR | RAB18 | RAB3GAP1 | RAD23B | RAF1 | RAP1A | RARA | RARG | RB1CC1 | RBBP6 | RBPJ | RC3H1 | RELA | RGN | RGS2 | RIPK3 | RPGRIP1L | RPL10 | RRM1 | RRM2B | RTCB | RUNX1 | RUNX2 | RXRA | SBDS | SCO2 | SEC63 | SEPT4 | SGPL1 | SHC1 | SIRT1 | SKI | SMAD2 | SMAD3 | SMAD4 | SMAD5 | SMARCC1 | SOD1 | SOX2 | SRC | SRI | SRSF5 | STIL | STK3 | STMN1 | SULF2 | TAB1 | TAB2 | TAGLN | TAZ | TBC1D23 | TCF12 | TCF21 | TCF25 | TGFB1 | TGFBR3 | THRA | TIMP1 | TLR3 | TMEM231 | TNF | TP73 | TRAF3IP1 | TREX1 | TULP3 | TWIST1 | TYMS | UBB | UBE3A | UFC1 | UFM1 | UPF2 | USP2 | VASH2 | VKORC1 | VTN | WDR19 | WDR35 | WDR48 | WNT2 | WT1 | XBP1 | XRCC5 | XRCC6 | YAP1 | ZBTB16 | ZBTB42 | ZIC1 | ZMPSTE24 | GO:0008285 | 0.0480407618585466 | 396/8243 | 177/3250 | 0.0163345743822094 | 1 | F | F | F | F | negative regulation of cell population proliferation | ADCYAP1 | ADORA2A | AIMP2 | APC | APOD | APOE | APP | AR | ATF2 | ATM | BAX | BCL2 | BCL6 | BNIPL | BTK | C1QL4 | CASP3 | CAV1 | CBFA2T3 | CD109 | CDC73 | CDKN1A | CDKN2C | CEBPA | CGRRF1 | COL18A1 | COMT | CSNK2B | CTBP1 | CTCF | CTNNB1 | CTNNBIP1 | CUL1 | CUL2 | CUL5 | CXCL8 | DLG3 | DNAJA3 | DNAJB2 | DUSP1 | E2F1 | E2F3 | EAF2 | EPPK1 | ERBB2 | ETS1 | FABP3 | FABP7 | FAM129B | FOXJ1 | FTH1 | GATA1 | GLMN | GSTP1 | HAVCR2 | HDAC4 | HGS | HIST1H2AB | HMOX1 | HNF4A | HRAS | HSF1 | HSPA1B | ID2 | IDH2 | IFT122 | IGFBP7 | ILK | ING1 | ING5 | IRF1 | ITCH | ITGB1BP1 | JUN | KANK2 | LMNA | MAD1L1 | MAP2K1 | MCC | MDM4 | MED1 | MED31 | MTSS1 | MYC | NDRG1 | NELL1 | NF2 | NME1 | NOS3 | NPM1 | NPR1 | NTRK1 | NUDT6 | NUPR1 | PAWR | PDCD10 | PELI1 | PER2 | PHB | PHB2 | PINX1 | PML | PPARD | PPARG | PPARGC1A | PPM1D | PRDX4 | PRKAR1A | PROX1 | PTEN | PTGES | PTGIR | PTN | PTPN14 | PTPN6 | PTPRK | RAF1 | RARA | RARG | RB1 | RBBP4 | RBPJ | RC3H1 | REST | RGN | RIPK1 | RNF139 | RPS6KA2 | S100A11 | SCG2 | SDC4 | SERPINE2 | SF1 | SFN | SIRT6 | SKI | SLC9A3R1 | SMAD2 | SMAD3 | SMAD4 | SMARCA2 | SNAI2 | SOD2 | SOX2 | SPINT2 | SPRY2 | STAT1 | STAT3 | STK11 | STK3 | STRAP | SYNJ2BP | TES | TFDP1 | TGFB1 | TGFBR3 | THBS1 | TIMP2 | TINF2 | TNF | TNFAIP3 | TNFRSF8 | TNN | TP53 | TP73 | TPM1 | TRIM32 | TSG101 | VDR | VHL | VSIG4 | WARS | WDR77 | WT1 | ZBTB16 | ZBTB17 | ZBTB7B | GO:2000145 | 0.0611427878199685 | 504/8243 | 222/3250 | 0.016410106115039 | 1 | F | F | F | F | regulation of cell motility | ABL1 | ABL2 | ACTN4 | ADA | ADTRP | AGER | AGO2 | AIRE | AKT1 | AKT2 | AKT3 | ALOX12 | ANXA1 | ANXA5 | APC | APEX1 | APOD | APOE | APP | ARHGEF7 | ATM | BAG4 | BCAR1 | BCL2 | BMPR1A | BRAF | C1QBP | CALR | CAMK1D | CAMK2A | CAMK2B | CAV1 | CCL21 | CCR1 | CD40 | CD74 | CD81 | CDH1 | CDK5 | CHRD | CLIC4 | COL18A1 | CPNE3 | CREB3 | CRK | CRKL | CSNK2B | CXCL8 | DAB2 | DCN | DDRGK1 | DOCK5 | DOCK7 | DPEP1 | DUSP1 | DUSP22 | EGFR | EPPIN | EPPK1 | ERBB2 | ERBB3 | ETS1 | FADD | FAM83H | FGF2 | FGFR1 | FGFR1OP | FN1 | GLUL | GNA12 | GNA13 | GNAI2 | GPI | GRB7 | GSTP1 | HBEGF | HDAC4 | HDAC5 | HDAC6 | HDAC7 | HDAC9 | HIF1A | HMGB1 | HMOX1 | HRAS | HSPA5 | HSPB1 | ICAM1 | IDH2 | IGF1R | ILK | INSR | IQGAP1 | ITGA4 | ITGAV | ITGB1BP1 | ITGB3 | JUN | JUP | KDR | LAMB1 | LDLRAD4 | LEF1 | LGALS3 | LGMN | LMNA | LPAR1 | MAP2K3 | MAP3K3 | MAPK1 | MAPK14 | MAPK15 | MAPK3 | MCC | MDM2 | MECP2 | MEOX2 | MIF | MITF | MSN | MYO1C | MYOC | NCK1 | NF2 | NFE2L2 | NIPBL | NKX2-1 | NOS3 | NRP2 | NTF3 | ONECUT1 | OXSR1 | P2RX4 | PDCD10 | PDCD6 | PFN1 | PIK3C2A | PIK3R1 | PIK3R3 | PIN1 | PKN2 | PLCG1 | PLXNA1 | PLXNA2 | PLXNB2 | PPARD | PPARG | PPARGC1A | PRCP | PRKCE | PRKD1 | PRKD2 | PROX1 | PTEN | PTK2 | PTN | PTPN23 | PTPRK | PTPRR | RAB11A | RAC1 | RAF1 | RDX | RECK | RGN | RHOA | RHOJ | RIPK3 | RTN4 | S100A7 | SDC4 | SDCBP | SEMA7A | SEPT7 | SERPINB3 | SERPINE2 | SIRT1 | SLAMF8 | SLC9A3R1 | SMAD3 | SMO | SMURF2 | SNAI2 | SOD2 | SP1 | SP100 | SPINT2 | SPRY2 | SRC | SRGAP2 | SRGAP3 | SSX2IP | STARD13 | STAT3 | STAT5A | STK24 | STK26 | STRAP | SUN2 | SWAP70 | SYDE1 | SYNE2 | SYNJ2BP | TBXA2R | TGFB1 | THBS1 | TIMP1 | TNF | TNFSF14 | TNN | TPM1 | TRADD | TRIM32 | TRIP6 | TWIST1 | USP17L2 | VCL | VTN | WAS | WNK1 | XBP1 | ZMYND8 | GO:0070303 | 0.0043673419871406 | 36/8243 | 21/3250 | 0.0165559882060554 | 1 | F | F | F | F | negative regulation of stress-activated protein kinase signaling cascade | AKT1 | CYLD | DNAJA1 | DUSP1 | DUSP19 | EZR | FOXM1 | FOXO1 | GPS2 | GSTP1 | HDAC3 | IGBP1 | ITCH | MYC | NCOR1 | PAFAH1B1 | PER1 | PTPN22 | QARS | SERPINB3 | TAOK3 | GO:0032873 | 0.0043673419871406 | 36/8243 | 21/3250 | 0.0165559882060554 | 1 | F | F | F | F | negative regulation of stress-activated MAPK cascade | AKT1 | CYLD | DNAJA1 | DUSP1 | DUSP19 | EZR | FOXM1 | FOXO1 | GPS2 | GSTP1 | HDAC3 | IGBP1 | ITCH | MYC | NCOR1 | PAFAH1B1 | PER1 | PTPN22 | QARS | SERPINB3 | TAOK3 | GO:1901292 | 0.0043673419871406 | 36/8243 | 21/3250 | 0.0165559882060554 | 1 | F | F | F | F | nucleoside phosphate catabolic process | ACAT1 | ACOT7 | ADA | ASPDH | DCTPP1 | DERA | DUT | GDA | GPX1 | HINT1 | HPRT1 | ITPA | NT5C2 | NUDT18 | NUDT3 | NUDT9 | PDE4D | PNP | PRTFDC1 | UPP2 | VCP | GO:0005996 | 0.00800679364309111 | 66/8243 | 35/3250 | 0.0168874559433872 | 1 | F | F | F | F | monosaccharide metabolic process | AKR1A1 | AKT1 | AKT2 | ALDH5A1 | ALDOA | ALDOC | APOD | CPT1A | DCXR | DLAT | FABP5 | FBP1 | FBP2 | G6PD | GAA | GDPGP1 | MAN2B1 | MAPK14 | MPI | ONECUT1 | PDHA1 | PDHA2 | PDHB | PDK2 | PGM1 | PGM2 | PGM5 | PIK3CA | PPARD | PPARGC1A | PRKAA1 | RPIA | SORD | TNF | WDTC1 | GO:0043408 | 0.0544704597840592 | 449/8243 | 199/3250 | 0.0168897300729519 | 1 | F | F | F | F | regulation of MAPK cascade | ABL1 | ADCYAP1 | ADRB2 | AGER | AKT1 | APOE | APP | AR | ARRB1 | ARRB2 | AXIN1 | BIRC7 | BRAF | C1QL4 | C1QTNF1 | CARD9 | CAV1 | CBLC | CCL21 | CCL22 | CCR1 | CD27 | CD36 | CD40 | CD40LG | CD74 | CD81 | CHRNA9 | COPS5 | CRK | CRKL | CTNNB1 | CYLD | DAXX | DBNL | DNAJA1 | DUSP1 | DUSP14 | DUSP16 | DUSP19 | DUSP22 | DUSP7 | DVL2 | DVL3 | EGFR | EIF3A | EPHA4 | ERBB2 | ERRFI1 | EZH2 | EZR | FAM83D | FGB | FGF2 | FGFR1 | FGFR3 | FN1 | FOXM1 | FOXO1 | GADD45G | GDF9 | GNAI2 | GPS2 | GRB2 | GRIK2 | GSTP1 | HAVCR2 | HDAC3 | HGS | HMGB1 | HRAS | ICAM1 | IGBP1 | IGF1R | IKBKG | ILK | INSR | IQGAP1 | ITCH | ITGB1BP1 | JUN | KDR | KLHDC10 | KRAS | KSR1 | LEMD2 | LPAR1 | LRRK2 | LTBR | MAP2K1 | MAP2K3 | MAP2K4 | MAP2K6 | MAP2K7 | MAP3K1 | MAP3K13 | MAP3K14 | MAP3K2 | MAP3K3 | MAP3K4 | MAP4K1 | MAPK1 | MAPK14 | MAPK3 | MAPK8IP3 | MIF | MYC | MYOC | NCOR1 | NELFE | NF2 | NMNAT1 | NOD2 | NQO2 | NTF3 | NTRK1 | PAFAH1B1 | PAK2 | PAK6 | PDCD10 | PDE6G | PEBP1 | PER1 | PHB | PHB2 | PIK3CG | PIN1 | PLA2G1B | PLA2G2A | PPP2CA | PPP2R1A | PRDX1 | PRDX2 | PRKAA1 | PRKCD | PRKCE | PRKCZ | PRKD2 | PSAP | PSEN1 | PSMD10 | PTEN | PTPN1 | PTPN22 | PTPN6 | PTPRR | QARS | RAF1 | RANBP9 | RAP1A | RAP1B | RB1CC1 | RGS2 | RIPK1 | RIPK2 | RPS3 | S100A12 | S100A7 | SDCBP | SEMA7A | SERPINB3 | SHC1 | SIRT3 | SLAMF1 | SLC9A3R1 | SMAD4 | SNF8 | SOD1 | SOX2 | SPRY2 | SRC | STK25 | STK3 | STK38 | STK40 | STYX | SYNJ2BP | TAB1 | TAB2 | TAOK3 | TGFB1 | TGFBR3 | THBS1 | TIMP2 | TLR3 | TNF | TP73 | TRAF2 | TRAF6 | TRIB2 | TRIB3 | TSG101 | UBB | UBC | UBE2N | UBE2V1 | UCHL1 | WNT16 | ZNF622 | GO:0070265 | 0.0019410408831736 | 16/8243 | 11/3250 | 0.0169151173024488 | 1 | F | F | F | F | necrotic cell death | BIRC2 | CYLD | DNM1L | FASLG | PGAM5 | PPIF | PYGL | RIPK1 | RIPK3 | TLR3 | TRAF2 | GO:1904355 | 0.0019410408831736 | 16/8243 | 11/3250 | 0.0169151173024488 | 1 | F | F | F | F | positive regulation of telomere capping | AURKB | HNRNPD | MAP2K7 | MAP3K4 | MAPK1 | MAPK15 | MAPK3 | NEK7 | PNKP | TNKS | TNKS2 | GO:0046426 | 0.0019410408831736 | 16/8243 | 11/3250 | 0.0169151173024488 | 1 | F | F | F | F | negative regulation of receptor signaling pathway via JAK-STAT | CAV1 | DAB1 | HGS | NEUROD1 | NF2 | PARP14 | PPP2CA | PPP2R1A | SOCS1 | SOCS2 | VHL | GO:0010467 | 0.0019410408831736 | 16/8243 | 11/3250 | 0.0169151173024488 | 1 | F | F | F | F | gene expression | EDA | ELAC2 | GOLPH3 | HSD17B10 | KIAA0391 | LAT | PUS1 | TBRG4 | TRMT61B | TRNT1 | ZBTB25 | GO:0043267 | 0.0019410408831736 | 16/8243 | 11/3250 | 0.0169151173024488 | 1 | F | F | F | F | negative regulation of potassium ion transport | ACTN2 | ADCYAP1 | ANK3 | BIN1 | CAB39 | CAV1 | KCNAB1 | NOS3 | OXSR1 | PTEN | SUMO1 | GO:0060544 | 0.0019410408831736 | 16/8243 | 11/3250 | 0.0169151173024488 | 1 | F | F | F | F | regulation of necroptotic process | BIRC2 | BIRC3 | CASP8 | CAV1 | CYLD | FADD | PELI1 | RIPK1 | RIPK3 | SLC25A4 | SPATA2 | GO:0014003 | 0.0019410408831736 | 16/8243 | 11/3250 | 0.0169151173024488 | 1 | F | F | F | F | oligodendrocyte development | ASCL1 | EIF2B1 | EIF2B3 | EIF2B5 | GSTP1 | HDAC11 | ID2 | LPAR1 | MED12 | TGFB1 | ZNF488 | GO:1904893 | 0.0019410408831736 | 16/8243 | 11/3250 | 0.0169151173024488 | 1 | F | F | F | F | negative regulation of receptor signaling pathway via STAT | CAV1 | DAB1 | HGS | NEUROD1 | NF2 | PARP14 | PPP2CA | PPP2R1A | SOCS1 | SOCS2 | VHL | GO:0097300 | 0.0019410408831736 | 16/8243 | 11/3250 | 0.0169151173024488 | 1 | F | F | F | F | programmed necrotic cell death | BIRC2 | CYLD | DNM1L | FASLG | PGAM5 | PPIF | PYGL | RIPK1 | RIPK3 | TLR3 | TRAF2 | GO:0055065 | 0.0379716122770836 | 313/8243 | 142/3250 | 0.0169209991772067 | 1 | F | F | F | F | metal ion homeostasis | ABCB7 | ABL1 | ABL2 | ACKR2 | ACO1 | ADCYAP1 | AFG3L2 | ANK3 | ANXA2 | ANXA6 | ANXA7 | APOE | APP | ARF1 | ATOX1 | ATP12A | ATP13A2 | ATP1A1 | ATP1B3 | ATP2A2 | ATP2B2 | ATP4A | ATP6V1A | ATP6V1B1 | BAX | BCL2 | BDKRB2 | BTBD9 | C1QTNF1 | CACNA1A | CALM1 | CALM2 | CALM3 | CALR | CAND1 | CAV1 | CCDC22 | CCL21 | CCR1 | CCR3 | CCR4 | CCR5 | CD36 | CD40 | CDK5 | CHRNA9 | COMMD1 | COX11 | COX19 | CUL1 | CUTC | CXCL11 | DLG4 | EGFR | EGLN1 | ESR1 | FASLG | FGF2 | FIS1 | FKBP1B | FTH1 | FTL | GLP1R | GLRX3 | GNA13 | GNA15 | GNB1 | GOT1 | GRIA1 | GRIK2 | GSTO1 | GTF2I | HAP1 | HEXB | HIF1A | HMGB1 | HMOX1 | HPX | HTR1B | HTT | IBTK | IMMT | IREB2 | ITGAV | ITPR1 | ITPR2 | ITPR3 | KCTD17 | LETM1 | LPAR1 | LPAR2 | LPAR4 | LRP6 | MCOLN3 | MICU1 | MICU2 | MYC | MYO5A | NCOA4 | NEDD8 | NMB | NUBP1 | P2RX1 | P2RX2 | P2RX4 | PIK3CG | PLA2G1B | PLA2G6 | PLCG1 | PML | PRKACA | PRKCE | PSEN1 | PTGER3 | PTGIR | RAP1GDS1 | RGN | RIC3 | SCO2 | SFXN1 | SFXN4 | SLC12A2 | SLC39A4 | SLC9A1 | SMAD3 | SMAD4 | SNCA | SOD1 | SRI | STIM1 | STOML2 | SWAP70 | TBXA2R | TGFB1 | TGM2 | THADA | TMBIM6 | TPCN2 | VAPB | VDR | XIAP | YWHAE | GO:0032482 | 0.0041247118767439 | 34/8243 | 20/3250 | 0.0170609374253959 | 1 | F | F | F | F | Rab protein signal transduction | GDI1 | RAB10 | RAB11A | RAB11B | RAB18 | RAB1A | RAB1B | RAB21 | RAB24 | RAB29 | RAB2A | RAB31 | RAB32 | RAB35 | RAB5A | RAB5B | RAB7A | RAB9A | RABL2A | RABL3 | GO:0031648 | 0.0041247118767439 | 34/8243 | 20/3250 | 0.0170609374253959 | 1 | F | F | F | F | protein destabilization | BTRC | CDC73 | CREBBP | CUL3 | DERL1 | EP300 | FBXW11 | HTT | MDM2 | PLK1 | PRKDC | RAD23A | RNF139 | SIAH1 | SIRT1 | SIRT6 | SNCA | SRC | TRIM21 | XBP1 | GO:0015909 | 0.0041247118767439 | 34/8243 | 20/3250 | 0.0170609374253959 | 1 | F | F | F | F | long-chain fatty acid transport | ABCD3 | ACACA | ACSL1 | ANXA1 | APOE | BDKRB2 | CD36 | CPT1A | CPT2 | EPRS | FABP1 | FABP3 | NMB | PLA2G10 | PLA2G12A | PLA2G1B | PLA2G2A | PPARG | PRKAA2 | PRKAB2 | GO:0090342 | 0.0041247118767439 | 34/8243 | 20/3250 | 0.0170609374253959 | 1 | F | F | F | F | regulation of cell aging | ABL1 | AKT3 | BCL6 | BMPR1A | FOXM1 | FZR1 | KRAS | LMNA | MAP3K3 | MARCH5 | MIF | NAMPT | PAWR | PRKDC | PTEN | SIRT1 | TERF2 | TWIST1 | ZMPSTE24 | ZNF277 | GO:0048468 | 0.0417323789882324 | 344/8243 | 155/3250 | 0.0171825836658793 | 1 | F | F | F | F | cell development | ACTA1 | ACTA2 | ACTN2 | AFF4 | AFG3L2 | AGER | AKT1 | ANXA2 | APP | ARID4B | ASCL1 | ATG5 | ATRX | BAX | BCL2 | BCL2L1 | BCL6 | BMPR1A | CABP4 | CCDC155 | CCDC36 | CCNB1 | CDK5 | CEP290 | CEP57 | CRK | CRKL | CTNNB1 | DMD | DYNLL1 | E2F4 | EIF2AK3 | EIF2B1 | EIF2B3 | EIF2B5 | EP300 | ESR1 | EZR | FAM9B | FANCC | FANCG | FASN | FGFR1 | FHL2 | FLNB | FOXJ1 | GATA1 | GDF9 | GFAP | GPX1 | GSTP1 | HCN1 | HDAC11 | HDAC4 | HEXB | HNF4A | HNRNPU | HOXD10 | HPRT1 | HS6ST1 | HSD17B4 | HSF4 | HSPA2 | HTRA2 | ICAM1 | ID2 | IQGAP1 | JMJD6 | KDM1A | KLF5 | KRAS | LMNA | LPAR1 | LRRK1 | MAPK1 | MAPK3 | MAPK8 | MAPK9 | MAPT | MED1 | MED12 | MLH1 | MSN | MYH11 | MYH6 | MYL2 | MYO1E | NEBL | NEUROD1 | NFATC2 | NSUN2 | NTRK1 | OBSL1 | OGDH | ONECUT1 | P2RX2 | PABPC4 | PAFAH1B1 | PDCD6 | PDE4D | PEF1 | PHGDH | PICK1 | PIP4K2A | PPT1 | PSAP | PSEN1 | PTPN6 | RAB1A | RAB1B | RAP1A | RAP1B | RARA | RARG | RDH13 | RDX | RNF2 | RNF8 | RPGRIP1 | RUNX1 | RUNX2 | SDC1 | SERPINH1 | SFN | SKI | SMAD3 | SMAD5 | SMARCA2 | SMO | SNAI2 | SRC | STRBP | SULF2 | SYN1 | TCOF1 | TGFB1 | TGFBR3 | THOC2 | TIAL1 | TMOD3 | TNN | TNP1 | TP63 | TRAF6 | TRIP13 | UBB | UBE2J1 | UCHL1 | UQCRQ | VIM | WNK1 | WT1 | XBP1 | ZMPSTE24 | ZNF488 | GO:0006417 | 0.029115613247604 | 240/8243 | 111/3250 | 0.017213668185781 | 1 | F | F | F | F | regulation of translation | AARS | ACO1 | AGO1 | AGO2 | AIRE | AKT1 | AKT2 | APP | ATXN2 | C1QBP | CALR | CDK4 | CIRBP | COA3 | DAPK1 | DDX1 | DDX39B | DDX3X | DHX36 | DHX9 | EEF2 | EIF2AK3 | EIF2AK4 | EIF2B5 | EIF2S1 | EIF3D | EIF3E | EIF3H | EIF4A3 | EIF4E | EIF4E2 | EIF4EBP1 | EIF4ENIF1 | EIF4G3 | EIF4H | EIF5 | EIF5A | EIF5B | EIF6 | ELAVL1 | EPRS | ERBB2 | FTO | FXR2 | GAPDH | GEMIN5 | GRB7 | HNRNPD | HNRNPL | HSPB1 | IGF2BP1 | ILF3 | IREB2 | KHDRBS1 | LARP4 | LRPPRC | MAPK1 | MAPK3 | MKNK2 | MOV10 | MTPN | NCBP1 | NCK1 | NPM1 | OGFOD1 | PA2G4 | PABPC1 | PAIP1 | PER1 | PER2 | POLDIP3 | PPP1CA | PUM1 | PUS7 | RARA | RBM3 | RBM4 | RBM8A | RC3H1 | RGS2 | RHOA | RMND1 | RNF139 | RPL10 | RPL38 | RPS14 | RPS3 | RPS4X | RPS6KA1 | RPS6KB2 | RXRA | SHMT1 | SHMT2 | STAT3 | TACO1 | TCOF1 | THBS1 | TIA1 | TNF | TPR | TRAP1 | TRUB2 | TSFM | TYMS | UNK | UPF3B | UQCC1 | USP16 | VIM | YTHDF1 | ZNF598 | GO:2000147 | 0.0362732015043067 | 299/8243 | 136/3250 | 0.017368684404413 | 1 | F | F | F | F | positive regulation of cell motility | ABL1 | ACTN4 | AGER | AGO2 | AKT1 | AKT2 | AKT3 | ALOX12 | ANXA1 | APC | APP | ARHGEF7 | ATM | BAG4 | BCAR1 | BCL2 | C1QBP | CALR | CAMK1D | CAV1 | CCL21 | CCR1 | CD40 | CD74 | COL18A1 | CPNE3 | CREB3 | CRK | CRKL | CXCL8 | DAB2 | DDRGK1 | DOCK5 | DOCK7 | EGFR | ETS1 | FADD | FAM83H | FGF2 | FGFR1 | FGFR1OP | FN1 | GNAI2 | GPI | GRB7 | HBEGF | HDAC4 | HDAC6 | HDAC7 | HDAC9 | HIF1A | HMGB1 | HMOX1 | HRAS | HSPA5 | HSPB1 | ICAM1 | IGF1R | ILK | INSR | IQGAP1 | ITGA4 | ITGAV | ITGB1BP1 | ITGB3 | JUN | KDR | LAMB1 | LEF1 | LGALS3 | LGMN | LPAR1 | MAP2K3 | MAP3K3 | MAPK1 | MAPK14 | MAPK3 | MDM2 | MYO1C | MYOC | NFE2L2 | NIPBL | NOS3 | NRP2 | NTF3 | ONECUT1 | OXSR1 | P2RX4 | PDCD10 | PDCD6 | PFN1 | PIK3C2A | PIK3R1 | PIK3R3 | PLCG1 | PRKCE | PRKD1 | PRKD2 | PROX1 | PTK2 | RAB11A | RAC1 | RDX | RHOA | RHOJ | RTN4 | S100A7 | SDCBP | SEMA7A | SERPINB3 | SIRT1 | SMAD3 | SMO | SMURF2 | SNAI2 | SOD2 | SP1 | SPRY2 | SRC | STAT3 | STAT5A | SUN2 | SWAP70 | SYDE1 | SYNE2 | TGFB1 | THBS1 | TNF | TNFSF14 | TRADD | TRIM32 | TRIP6 | TWIST1 | VTN | WNK1 | XBP1 | GO:0032075 | 0.000849205386388451 | 7/8243 | 6/3250 | 0.0173704360298398 | 1 | F | F | F | F | positive regulation of nuclease activity | AKT1 | HMGB2 | PCNA | PRKCD | RPS3 | TCEA1 | GO:0006107 | 0.000849205386388451 | 7/8243 | 6/3250 | 0.0173704360298398 | 1 | F | F | F | F | oxaloacetate metabolic process | ACLY | GOT1 | GOT2 | MDH1 | MDH2 | NIT2 | GO:0036500 | 0.000849205386388451 | 7/8243 | 6/3250 | 0.0173704360298398 | 1 | F | F | F | F | ATF6-mediated unfolded protein response | ATF6 | ATF6B | CALR | HSP90B1 | HSPA5 | XBP1 | GO:0034101 | 0.000849205386388451 | 7/8243 | 6/3250 | 0.0173704360298398 | 1 | F | F | F | F | erythrocyte homeostasis | GPI | HMOX1 | HOXB6 | IREB2 | PRDX1 | RPS24 | GO:2000193 | 0.000849205386388451 | 7/8243 | 6/3250 | 0.0173704360298398 | 1 | F | F | F | F | positive regulation of fatty acid transport | FABP3 | MAP2K6 | MIF | P2RX4 | PLA2G10 | PLA2G6 | GO:0046130 | 0.000849205386388451 | 7/8243 | 6/3250 | 0.0173704360298398 | 1 | F | F | F | F | purine ribonucleoside catabolic process | ADA | AHCY | HPRT1 | NUDT18 | PNP | PRTFDC1 | GO:0150012 | 0.000849205386388451 | 7/8243 | 6/3250 | 0.0173704360298398 | 1 | F | F | F | F | positive regulation of neuron projection arborization | DLG4 | DVL1 | DVL2 | DVL3 | GRIP1 | MAP3K13 | GO:0051683 | 0.000849205386388451 | 7/8243 | 6/3250 | 0.0173704360298398 | 1 | F | F | F | F | establishment of Golgi localization | CDC42 | COPG1 | PDCD10 | SLC9A3R1 | STK25 | YWHAZ | GO:0050667 | 0.000849205386388451 | 7/8243 | 6/3250 | 0.0173704360298398 | 1 | F | F | F | F | homocysteine metabolic process | CBS | CTH | DPEP1 | MPST | MTHFD1 | MTRR | GO:0070344 | 0.000849205386388451 | 7/8243 | 6/3250 | 0.0173704360298398 | 1 | F | F | F | F | regulation of fat cell proliferation | E2F1 | E2F3 | FTO | PER2 | TFDP1 | VSTM2A | GO:0006206 | 0.000849205386388451 | 7/8243 | 6/3250 | 0.0173704360298398 | 1 | F | F | F | F | pyrimidine nucleobase metabolic process | ALDH6A1 | CDA | MAPK1 | RRM1 | SCO2 | TYMS | GO:0090646 | 0.000849205386388451 | 7/8243 | 6/3250 | 0.0173704360298398 | 1 | F | F | F | F | mitochondrial tRNA processing | ELAC2 | HSD17B10 | KIAA0391 | PUS1 | TRMT61B | TRNT1 | GO:0060020 | 0.000849205386388451 | 7/8243 | 6/3250 | 0.0173704360298398 | 1 | F | F | F | F | Bergmann glial cell differentiation | ABL1 | GFAP | MAP2K1 | MAPK1 | MAPK3 | VIM | GO:0034982 | 0.000849205386388451 | 7/8243 | 6/3250 | 0.0173704360298398 | 1 | F | F | F | F | mitochondrial protein processing | AFG3L2 | PMPCA | PMPCB | STOML2 | UQCRC2 | YME1L1 | GO:0043247 | 0.000849205386388451 | 7/8243 | 6/3250 | 0.0173704360298398 | 1 | F | F | F | F | telomere maintenance in response to DNA damage | ACD | APEX1 | RIF1 | TERF2 | TERF2IP | TFIP11 | GO:1901978 | 0.000849205386388451 | 7/8243 | 6/3250 | 0.0173704360298398 | 1 | F | F | F | F | positive regulation of cell cycle checkpoint | CCAR2 | DYNC1LI1 | MAD2L1 | PROX1 | TPR | XRCC3 | GO:0006152 | 0.000849205386388451 | 7/8243 | 6/3250 | 0.0173704360298398 | 1 | F | F | F | F | purine nucleoside catabolic process | ADA | AHCY | HPRT1 | NUDT18 | PNP | PRTFDC1 | GO:1903069 | 0.000849205386388451 | 7/8243 | 6/3250 | 0.0173704360298398 | 1 | F | F | F | F | regulation of ER-associated ubiquitin-dependent protein catabolic process | CAV1 | NFE2L2 | SGTA | UBQLN1 | UBQLN2 | USP14 | GO:1902857 | 0.000849205386388451 | 7/8243 | 6/3250 | 0.0173704360298398 | 1 | F | F | F | F | positive regulation of non-motile cilium assembly | CENPJ | CEP135 | DYNLL1 | HAP1 | SEPT7 | SEPT9 | GO:1901844 | 0.000849205386388451 | 7/8243 | 6/3250 | 0.0173704360298398 | 1 | F | F | F | F | regulation of cell communication by electrical coupling involved in cardiac conduction | CALM1 | CALM2 | CALM3 | CAV1 | PDE4D | SRI | GO:0051901 | 0.000849205386388451 | 7/8243 | 6/3250 | 0.0173704360298398 | 1 | F | F | F | F | positive regulation of mitochondrial depolarization | ALOX12 | DCN | KDR | MLLT11 | MYOC | PARP1 | GO:0051342 | 0.000849205386388451 | 7/8243 | 6/3250 | 0.0173704360298398 | 1 | F | F | F | F | regulation of cyclic-nucleotide phosphodiesterase activity | CALM1 | CALM2 | CALM3 | EGLN1 | MAPK7 | RGN | GO:0006739 | 0.000849205386388451 | 7/8243 | 6/3250 | 0.0173704360298398 | 1 | F | F | F | F | NADP metabolic process | DCXR | G6PD | IDH1 | IDH2 | KCNAB2 | NNT | GO:0046683 | 0.0104330947470581 | 86/8243 | 44/3250 | 0.0174948513554941 | 1 | F | F | F | F | response to organophosphorus | AKAP9 | APEX1 | APP | ASS1 | BIRC2 | BRAF | BSG | CARM1 | CFTR | DUSP1 | EZR | FOS | GPD1 | HCN1 | HCN2 | HCN3 | HSP90B1 | HSPA5 | ITPR2 | JUN | JUNB | JUND | KDM1A | LDHA | LPAR1 | NME1 | P2RX1 | P2RX2 | P2RX4 | PEBP1 | PER1 | PIK3CG | PTEN | RAP1A | RAP1B | RELA | RFC3 | SDC1 | SELL | SOD1 | STAT1 | TYMS | VGF | WT1 | GO:0071248 | 0.0137086012374136 | 113/8243 | 56/3250 | 0.0176700693853538 | 1 | F | F | F | F | cellular response to metal ion | AKT1 | ALOX5AP | ANK3 | ANXA5 | APP | ATP13A2 | BACE1 | BRAF | CALR | CCNB1 | CDH1 | CEBPA | CLIC4 | CPNE3 | CREB1 | CYP1A1 | CYP1A2 | DAXX | DLG2 | DLG4 | DPEP1 | EGFR | EIF2AK3 | EIF2S1 | ENDOG | FBP1 | FOS | FUS | HMOX1 | HSF1 | HSPA5 | ID2 | IQGAP1 | IREB2 | JUN | JUNB | LGMN | LIG4 | LRRK2 | MAPK1 | MAPK3 | MAPK8 | MAPK9 | NQO1 | P2RX4 | PARP1 | PPARGC1A | PPIF | PRKAA1 | PRKAA2 | SLC25A24 | SNCA | SOD1 | SUMO1 | UBE2D3 | WNK1 | GO:1902803 | 0.00618706781511586 | 51/8243 | 28/3250 | 0.0177627586123754 | 1 | F | F | F | F | regulation of synaptic vesicle transport | ADORA2A | ATP2A2 | BACE1 | BRAF | CALM1 | CALM3 | CAMK2A | CDK5 | CHRM2 | CPLX1 | CPLX3 | DNM1L | DVL1 | GIT1 | GSK3B | HTR1B | LRRK2 | P2RX1 | PREPL | RAB3GAP1 | RAB5A | RAP1A | RAP1B | SEPT5 | STXBP1 | SYN1 | SYP | VPS18 | GO:0060359 | 0.00618706781511586 | 51/8243 | 28/3250 | 0.0177627586123754 | 1 | F | F | F | F | response to ammonium ion | ABL1 | ADA | ADSS | ASS1 | CACNG4 | CASP3 | CASP7 | EFTUD2 | FADD | GNAO1 | GOT2 | GPI | HCN3 | HDAC2 | HDAC5 | HNRNPD | HSPA5 | HTR1B | HTR3A | LRRK2 | MAPK1 | MAPK3 | MDM2 | PPP2R2A | PRKCE | RELA | SIN3A | SULT1A3 | GO:0007249 | 0.00618706781511586 | 51/8243 | 28/3250 | 0.0177627586123754 | 1 | F | F | F | F | I-kappaB kinase/NF-kappaB signaling | AKT1 | BCL10 | BIRC2 | BIRC3 | BTK | CARD9 | IKBKB | IKBKG | MAP3K14 | NFKBIA | REL | RELA | RIPK1 | RIPK2 | RIPK3 | RNF31 | TAB1 | TAB2 | TANK | TLR3 | TNF | TNFRSF1A | TNIP2 | TRADD | TRAF2 | TRAF6 | UBB | UBC | GO:0034502 | 0.00618706781511586 | 51/8243 | 28/3250 | 0.0177627586123754 | 1 | F | F | F | F | protein localization to chromosome | ACD | ATM | ATRX | AURKB | CARM1 | CTCF | ESR1 | EZH2 | IK | LEMD2 | MCM9 | NIPBL | PIH1D1 | PINX1 | PLK1 | POT1 | RAD21 | RB1 | RPA2 | RUVBL2 | SPDL1 | TERF2 | TERF2IP | TINF2 | TNKS | TNKS2 | XRCC5 | ZW10 | GO:0016925 | 0.00618706781511586 | 51/8243 | 28/3250 | 0.0177627586123754 | 1 | F | F | F | F | protein sumoylation | BCL11A | CBX4 | HDAC4 | MDM2 | NUP107 | NUP153 | NUP160 | NUP205 | NUP214 | NUP85 | NUP88 | NUP93 | NUP98 | PIAS1 | PIAS3 | POM121 | RAE1 | RANGAP1 | SAE1 | SENP6 | SUMO1 | SUMO1P1 | SUMO2 | SUMO3 | TPR | TRIM28 | UBA2 | UBE2I | GO:0019318 | 0.00776416353269441 | 64/8243 | 34/3250 | 0.0177901661536988 | 1 | F | F | F | F | hexose metabolic process | AKR1A1 | AKT1 | AKT2 | ALDH5A1 | ALDOA | ALDOC | APOD | CPT1A | DCXR | DLAT | FABP5 | FBP1 | FBP2 | G6PD | GAA | GDPGP1 | MAN2B1 | MAPK14 | MPI | ONECUT1 | PDHA1 | PDHA2 | PDHB | PDK2 | PGM1 | PGM2 | PGM5 | PIK3CA | PPARD | PPARGC1A | PRKAA1 | SORD | TNF | WDTC1 | GO:0006851 | 0.0021836709935703 | 18/8243 | 12/3250 | 0.0178049210788018 | 1 | F | F | F | F | mitochondrial calcium ion transmembrane transport | AFG3L2 | LETM1 | MICU1 | MICU2 | OPA1 | PHB | PHB2 | PMPCA | PMPCB | STOML2 | VDAC1 | YME1L1 | GO:0009145 | 0.0021836709935703 | 18/8243 | 12/3250 | 0.0178049210788018 | 1 | F | F | F | F | purine nucleoside triphosphate biosynthetic process | ADK | ALDOA | CYC1 | DNAJC30 | IMPDH2 | LDHC | NME1 | NME2 | NME4 | SLC25A13 | STOML2 | TGFB1 | GO:0046949 | 0.0021836709935703 | 18/8243 | 12/3250 | 0.0178049210788018 | 1 | F | F | F | F | fatty-acyl-CoA biosynthetic process | ACACA | ACLY | ACSL1 | ACSL4 | ACSL5 | CBR4 | FASN | GCDH | HSD17B8 | PPT1 | SLC25A1 | TECR | GO:0051899 | 0.0021836709935703 | 18/8243 | 12/3250 | 0.0178049210788018 | 1 | F | F | F | F | membrane depolarization | ABL1 | ADORA2A | CACNA1A | CACNG2 | CACNG4 | CAV1 | HCN2 | JUN | P2RX4 | PARK7 | PRKCZ | YWHAH | GO:0001836 | 0.0021836709935703 | 18/8243 | 12/3250 | 0.0178049210788018 | 1 | F | F | F | F | release of cytochrome c from mitochondria | BAX | BBC3 | BCL2 | BCL2L1 | CLU | DNM1L | FIS1 | GGCT | JUN | SFN | SOD2 | TIMM50 | GO:2000679 | 0.0021836709935703 | 18/8243 | 12/3250 | 0.0178049210788018 | 1 | F | F | F | F | positive regulation of transcription regulatory region DNA binding | CTNNB1 | DDRGK1 | EP300 | FAM129B | H1F0 | NEUROD1 | PARK7 | PARP1 | RB1 | TGFB1 | TRAF6 | TWIST1 | GO:0001936 | 0.00909862913987626 | 75/8243 | 39/3250 | 0.0178828533187667 | 1 | F | F | F | F | regulation of endothelial cell proliferation | AKT1 | AKT3 | APOE | ARNT | CAV1 | CCR3 | FGF2 | FGFR1 | HIF1A | HMGB1 | HMGB2 | HMOX1 | ITGA4 | ITGB3 | JUN | KDR | NR4A1 | NRP2 | PDCD10 | PDCD6 | PLCG1 | PPARG | PRKD1 | PRKD2 | PROX1 | RPTOR | SCG2 | SIRT1 | SIRT6 | SP1 | STAT1 | STAT3 | STAT5A | SYNJ2BP | THBS1 | TNF | TNFSF12 | VASH2 | WNT2 | GO:0009988 | 0.0036394516559505 | 30/8243 | 18/3250 | 0.0179419589642559 | 1 | F | F | F | F | cell-cell recognition | ALDOA | CCL21 | CCT2 | CCT3 | CCT4 | CCT5 | CCT7 | CCT8 | CD209 | CD6 | CLGN | HSPA1L | MSN | OVGP1 | SPACA3 | TCP1 | UBAP2L | VDAC2 | GO:0019985 | 0.0036394516559505 | 30/8243 | 18/3250 | 0.0179419589642559 | 1 | F | F | F | F | translesion synthesis | ISG15 | NPLOC4 | PCNA | POLD1 | POLE2 | RFC1 | RFC2 | RFC3 | RFC4 | RFC5 | RPA2 | RPA3 | SPRTN | TRIM25 | UBB | UBC | UBE2L6 | VCP | GO:0097366 | 0.0036394516559505 | 30/8243 | 18/3250 | 0.0179419589642559 | 1 | F | F | F | F | response to bronchodilator | ADORA2A | AIFM1 | CALM1 | CALM3 | CDK2 | CRK | DBH | DPEP1 | EGLN1 | FOXO1 | HDAC2 | HDAC9 | HNRNPD | HPRT1 | ICAM1 | RGS2 | SOD1 | TRAF2 | GO:2000649 | 0.0036394516559505 | 30/8243 | 18/3250 | 0.0179419589642559 | 1 | F | F | F | F | regulation of sodium ion transmembrane transporter activity | ACTN4 | ANK3 | CHP1 | DMD | GLRX | GPD1L | HECW1 | HECW2 | KIF5B | NEDD4 | PCSK9 | PRKCE | SLC9A1 | SLC9A3R1 | STOM | WNK1 | WNK4 | YWHAH | GO:0033683 | 0.0036394516559505 | 30/8243 | 18/3250 | 0.0179419589642559 | 1 | F | F | F | F | nucleotide-excision repair, DNA incision | CHD1L | CUL4B | DDB1 | DDB2 | ERCC5 | PARP1 | PCNA | POLD1 | RBX1 | RFC1 | RFC2 | RFC3 | RFC4 | RFC5 | RPA2 | RPA3 | UBB | UBC | GO:0002763 | 0.0036394516559505 | 30/8243 | 18/3250 | 0.0179419589642559 | 1 | F | F | F | F | positive regulation of myeloid leukocyte differentiation | CASP8 | CCR1 | CD74 | CREB1 | CTNNBIP1 | FADD | FOS | GNAS | HAX1 | ID2 | JUN | LEF1 | RB1 | RIPK1 | RUNX1 | TGFB1 | TNF | TRAF6 | GO:0051640 | 0.0414897488778357 | 342/8243 | 154/3250 | 0.0179528481648977 | 1 | F | F | F | F | organelle localization | ABCE1 | ACTN4 | ACTR1A | ACTR2 | ACTR3 | AHI1 | AKAP9 | ALB | AP3D1 | AP3M1 | ATCAY | BIN1 | CBL | CCDC155 | CCNB1 | CCP110 | CDC42 | CDK5 | CENPF | CENPJ | CEP135 | CEP152 | CEP290 | CEP55 | CEP57 | CEP70 | CEP89 | CFTR | CHMP2A | CKAP5 | CLUH | CNTRL | COPG1 | CPLX1 | CPLX3 | CROCC | CSNK1E | CTNNB1 | CUL3 | DAB1 | DCTN1 | DNM1L | DOCK7 | DYNC1H1 | DYNC1I1 | DYNC1I2 | DYNLL1 | DYNLT1 | EIF6 | EXOC6 | EZR | FAM83D | FAM98A | FBF1 | FGFR1OP | HAP1 | HDAC6 | HIF1A | HSP90AA1 | HTT | IKBKG | IQCB1 | ITGA4 | KIF2C | KIF5B | KIF5C | KPNB1 | LAT | LRPPRC | LRRK2 | MAP1B | MAPT | MKS1 | MLH1 | MYO1C | MYO5A | NIN | NMD3 | NPM1 | NSFL1C | NUBP1 | NUMA1 | NUP88 | NUP98 | NUSAP1 | OFD1 | OPA1 | PAFAH1B1 | PARD3 | PCM1 | PDCD10 | PDCD6IP | PIK3CG | PINX1 | PLK1 | PLXNA2 | POLR2M | PPP2R1A | PRKACA | PRKAR2B | PRKCZ | PSEN2 | PTK2 | RAB10 | RAB11A | RAB11B | RAB1A | RAB3IP | RAN | RHOT1 | RHOT2 | RPGRIP1L | RRS1 | S100A13 | SCFD1 | SCLT1 | SLC9A3R1 | SNAP29 | SNCA | SPDL1 | SPICE1 | SPRY2 | STK11 | STK25 | STX5 | STXBP1 | STXBP2 | SUN2 | SYN1 | SYNE2 | SYNJ2BP | TCTN2 | TCTN3 | TERF1 | TMEM216 | TOR1A | TRIM9 | TUBA1A | TUBA4A | TUBB | TUBB4A | TUBB4B | TUBG1 | UBB | UBE2B | UCHL1 | VPS18 | VPS4B | XPO1 | YKT6 | YWHAE | YWHAG | YWHAZ | ZW10 | GO:0060236 | 0.0033968215455538 | 28/8243 | 17/3250 | 0.0182837527081375 | 1 | F | F | F | F | regulation of mitotic spindle organization | CHMP2A | CLTC | DCTN1 | DYNC1H1 | FSD1 | GNAI1 | HNRNPU | HSPA1B | NUMA1 | PDCD6IP | PLK1 | RAE1 | SMC1A | SMC3 | STAG2 | TPR | VPS4B | GO:1905898 | 0.0033968215455538 | 28/8243 | 17/3250 | 0.0182837527081375 | 1 | F | F | F | F | positive regulation of response to endoplasmic reticulum stress | AGR2 | ATF6 | BAG6 | BAX | CAV1 | EDEM1 | NCK1 | NFE2L2 | PIK3R1 | PTPN1 | RNF183 | RNF185 | SGTA | SIRT1 | UBQLN1 | UBQLN2 | XBP1 | GO:0051031 | 0.0033968215455538 | 28/8243 | 17/3250 | 0.0182837527081375 | 1 | F | F | F | F | tRNA transport | NUP107 | NUP153 | NUP160 | NUP205 | NUP214 | NUP85 | NUP88 | NUP93 | NUP98 | POM121 | RAE1 | RAN | SEC13 | SSB | TOMM20 | TPR | XPOT | GO:1904357 | 0.002426301103967 | 20/8243 | 13/3250 | 0.0183412071106847 | 1 | F | F | F | F | negative regulation of telomere maintenance via telomere lengthening | ACD | DCP2 | HNRNPA1 | HNRNPU | PARP1 | PINX1 | POT1 | SRC | TERF1 | TERF2 | TINF2 | TNKS | TNKS2 | GO:0006939 | 0.002426301103967 | 20/8243 | 13/3250 | 0.0183412071106847 | 1 | F | F | F | F | smooth muscle contraction | ACTA2 | BDKRB2 | CHRM3 | FKBP1B | MYH11 | P2RX2 | PDE4D | PIK3C2A | PLA2G6 | PTGER3 | RAP1GDS1 | SULF2 | TPCN2 | GO:0006297 | 0.002426301103967 | 20/8243 | 13/3250 | 0.0183412071106847 | 1 | F | F | F | F | nucleotide-excision repair, DNA gap filling | LIG4 | PCNA | POLD1 | RFC1 | RFC2 | RFC3 | RFC4 | RFC5 | RPA2 | RPA3 | UBB | UBC | XRCC1 | GO:0006282 | 0.0101904646366614 | 84/8243 | 43/3250 | 0.018478158076722 | 1 | F | F | F | F | regulation of DNA repair | ACTR2 | APBB1 | BABAM1 | BRCA1 | CBX8 | CDK9 | DHX9 | EGFR | EYA2 | EYA4 | FIGNL1 | FOXM1 | FUS | H2AFX | HMGB1 | HSF1 | KDM1A | KDM4D | MGMT | OTUB1 | PARP1 | PCNA | PML | PPP4R2 | RAD51 | RAD51AP1 | RIF1 | RNF8 | RPA2 | RPS3 | SIRT1 | SIRT6 | TERF2 | TERF2IP | TFIP11 | TRIM28 | TRIP12 | TWIST1 | UBE2N | UBE2V1 | UBR5 | WAS | XRCC1 | GO:0060260 | 0.0031541914351571 | 26/8243 | 16/3250 | 0.0185292762456302 | 1 | F | F | F | F | regulation of transcription initiation from RNA polymerase II promoter | CAND1 | CREB1 | CTNNBIP1 | DHX36 | ESR1 | HMGB1 | HNF1A | PAXIP1 | PSMC2 | PSMC3 | PSMC4 | PSMC5 | PSMC6 | SUB1 | THRA | TP53 | GO:0042304 | 0.0031541914351571 | 26/8243 | 16/3250 | 0.0185292762456302 | 1 | F | F | F | F | regulation of fatty acid biosynthetic process | ACADVL | ANXA1 | BRCA1 | EIF6 | ERLIN2 | FABP5 | INSIG1 | INSIG2 | NR1H2 | NR1H3 | PRKAA2 | PRKAB2 | RGN | SIRT1 | TRIB3 | WDTC1 | GO:0051283 | 0.0031541914351571 | 26/8243 | 16/3250 | 0.0185292762456302 | 1 | F | F | F | F | negative regulation of sequestering of calcium ion | ANXA5 | ANXA6 | CCL21 | CCR5 | FASLG | FGF2 | FKBP1B | IBTK | ITPR1 | ITPR2 | ITPR3 | LETM1 | MCOLN3 | PLCG1 | PRKCE | TPCN2 | GO:0072523 | 0.0031541914351571 | 26/8243 | 16/3250 | 0.0185292762456302 | 1 | F | F | F | F | purine-containing compound catabolic process | ACAT1 | ACOT7 | ADA | AHCY | GDA | GPX1 | HINT1 | HPRT1 | ITPA | NT5C2 | NUDT18 | NUDT3 | NUDT9 | PDE4D | PNP | PRTFDC1 | GO:0046034 | 0.0031541914351571 | 26/8243 | 16/3250 | 0.0185292762456302 | 1 | F | F | F | F | ATP metabolic process | ADCYAP1 | AK1 | AK2 | AK4 | ATP6V1A | ATP6V1B1 | CLPX | FIGNL1 | HSPA1B | HSPA8 | MYH6 | NDUFS1 | NMNAT1 | OLA1 | PARP1 | VCP | GO:0106027 | 0.0031541914351571 | 26/8243 | 16/3250 | 0.0185292762456302 | 1 | F | F | F | F | neuron projection organization | APP | ARF1 | CDC42 | CTTN | DCTN1 | DLG4 | DVL1 | FYN | GSK3B | HDAC6 | IGF1R | INSR | LGMN | PICK1 | PSEN1 | PTEN | GO:0043330 | 0.0031541914351571 | 26/8243 | 16/3250 | 0.0185292762456302 | 1 | F | F | F | F | response to exogenous dsRNA | CARD9 | CAV1 | DDX1 | DHX36 | DHX9 | FLOT1 | IFIT1 | IFNA2 | MAPK1 | MAPK3 | MAVS | NFKBIA | RIPK2 | SLC3A2 | TLR3 | TMEM173 | GO:0007595 | 0.0031541914351571 | 26/8243 | 16/3250 | 0.0185292762456302 | 1 | F | F | F | F | lactation | APRT | CAV1 | CREB1 | GOT2 | HIF1A | HK2 | MED1 | MTX1 | NCOA1 | NME1 | PPAT | SOCS2 | STAT5A | UMPS | VDR | ZBTB7B | GO:1902680 | 0.123983986412714 | 1022/8243 | 434/3250 | 0.0185887563159026 | 1 | F | F | F | F | positive regulation of RNA biosynthetic process | ABHD14B | ABL1 | ACTA2 | ACTL6A | ACTN1 | ACTN2 | ACTN4 | ACTR2 | ACTR3 | ADCYAP1 | ADRB2 | AGO1 | AGO2 | AHI1 | AICDA | AIRE | AKT1 | ANKRD49 | APBB1 | APEX1 | APOE | APP | AR | ARF4 | ARID3B | ARID4B | ARNT | ARNT2 | ARRB1 | ASCL1 | ATF2 | ATF6 | ATF6B | ATM | ATOH1 | ATRX | AXIN1 | BANP | BCL10 | BIRC2 | BMPR1A | BRCA1 | BRD4 | BTRC | BUD31 | CAND1 | CARM1 | CBFB | CCAR2 | CCNT1 | CCNT2 | CD40 | CD74 | CD81 | CDC5L | CDC73 | CDH1 | CDK2 | CDK8 | CDK9 | CEBPA | CEBPE | CEP290 | CHCHD10 | CHCHD2 | CHD8 | CHEK2 | COPS5 | CREB1 | CREB3 | CREBBP | CTCF | CTNNB1 | DAB2 | DAXX | DCN | DDRGK1 | DDX39B | DDX3X | DHX36 | DHX9 | DPF2 | DTX1 | DVL1 | DVL2 | DVL3 | E2F1 | E2F2 | E2F3 | E2F4 | E2F6 | EAF2 | EBF1 | ECD | EGFR | EGLN1 | EIF2AK3 | ELF3 | ELF4 | EP300 | ERBB2 | ERG | ERLIN2 | ESR1 | ESR2 | ESRRA | ESRRB | ESRRG | ETS1 | EXOSC9 | FADD | FAM129B | FBXW11 | FGF2 | FHL2 | FOS | FOXA1 | FOXJ1 | FOXK1 | FOXK2 | FOXM1 | FOXO1 | FUBP3 | FUS | GABPB1 | GATA1 | GPS2 | GRIP1 | GTF2A1 | GTF2F1 | GTF2F2 | H1F0 | H1FX | H2AFZ | HAX1 | HDAC1 | HDAC2 | HDAC3 | HDAC4 | HDAC5 | HELZ2 | HEXB | HIF1A | HIST1H1A | HMGB1 | HMGB2 | HMGB3 | HNF1A | HNF4A | HNF4G | HNRNPAB | HNRNPD | HNRNPK | HNRNPU | HOXA5 | HOXB5 | HOXB9 | HOXD10 | HRAS | HSF1 | HSF4 | HSPA5 | HTATIP2 | ID2 | IFI16 | IFNA2 | IKBKB | IKBKG | IKZF5 | ILF2 | ILF3 | ILK | ING1 | ING5 | INSIG1 | INSIG2 | INSR | IRF1 | IRF4 | IRF7 | ITGB1BP1 | JMJD6 | JUN | JUNB | JUND | JUP | KAT5 | KAT7 | KAT8 | KDM1A | KDM8 | KLF5 | KLF6 | KMT2A | KMT2B | KMT2C | LEF1 | LGR4 | LMO2 | LMO7 | LPIN3 | LRP6 | LUM | MAP2K1 | MAP2K3 | MAP2K7 | MAP3K2 | MAPK1 | MAPK14 | MAPK3 | MAPK7 | MAVS | MC1R | MECP2 | MED1 | MED12 | MED14 | MED17 | MED20 | MED21 | MED23 | MED24 | MED31 | MED4 | MED6 | MEOX2 | METTL23 | MITF | MLLT11 | MLLT3 | MORF4L2 | MRPL12 | MTA1 | MTA3 | MYC | MYO6 | NAMPT | NANOG | NCK1 | NCOA1 | NCOA2 | NCOA3 | NCOA4 | NELFE | NEUROD1 | NFATC1 | NFATC2 | NFE2L2 | NFKB2 | NFKBIA | NIF3L1 | NIPBL | NKRF | NKX2-1 | NMD3 | NME2 | NOD2 | NPM1 | NR1H2 | NR1H3 | NR1I2 | NR3C1 | NR4A1 | NR5A2 | NUFIP1 | NUP85 | NUP98 | NUPR1 | OGT | ONECUT1 | PARK7 | PARP1 | PATZ1 | PAX8 | PAX9 | PAXIP1 | PCBD1 | PCBP1 | PCGF5 | PER1 | PER2 | PFKM | PHB | PHF10 | PIAS1 | PIH1D1 | PIK3R1 | PIN1 | PLA2G1B | PLSCR1 | PML | POU2F3 | POU5F1 | PPARD | PPARG | PPARGC1A | PPM1A | PPP1R12A | PRKAA1 | PRKD1 | PRKD2 | PRKDC | PRMT2 | PROX1 | PSEN1 | PSMC2 | PSMC3 | PSMC4 | PSMC5 | PSMC6 | PSMD9 | PTGES2 | RAF1 | RAN | RARA | RARG | RB1 | RBMX | RBPJ | REL | RELA | REST | RFC1 | RFXANK | RIPK1 | RIPK2 | RIPK3 | RNF4 | RPS6KA1 | RPTOR | RTF1 | RUNX1 | RUNX2 | RUVBL1 | RUVBL2 | RXRA | RXRB | RXRG | SFPQ | SHC1 | SIN3A | SIRT1 | SIRT7 | SKI | SLC9A1 | SMAD2 | SMAD3 | SMAD4 | SMAD5 | SMAD9 | SMARCA2 | SMARCA4 | SMARCB1 | SMARCC1 | SMARCC2 | SMARCE1 | SMO | SNW1 | SOX2 | SP1 | SP100 | SPI1 | SPIN1 | SQSTM1 | SRC | STAT1 | STAT3 | STAT4 | STAT6 | SUB1 | SUMO2 | SUPT4H1 | SUPT5H | TADA2B | TAF12 | TAF15 | TARDBP | TBL1X | TCEA2 | TCERG1 | TCF12 | TCF21 | TCF4 | TDRD3 | TFCP2 | TFDP1 | TGFB1 | THRA | TLR3 | TMEM173 | TNF | TNFRSF1A | TNIP2 | TNKS | TOP2A | TP53 | TP63 | TP73 | TRAF6 | TRIAP1 | TRIM28 | TRIM32 | TRIP4 | TSG101 | TTC5 | TWIST1 | UBB | UBC | UBE2L3 | UBE2V1 | USP16 | USP21 | VDR | VHL | WAS | WBP2 | WDR77 | WNT2 | WT1 | WWOX | WWP2 | XBP1 | XRCC6 | YAF2 | YAP1 | YES1 | YWHAH | YY1 | ZBTB16 | ZBTB17 | ZBTB7B | ZIC1 | ZNF24 | ZNF746 | GO:2001038 | 0.0026689312143637 | 22/8243 | 14/3250 | 0.0186016733719697 | 1 | F | F | F | F | regulation of cellular response to drug | ENDOG | HDAC6 | KMT2A | LRRK2 | NCOA1 | NFE2L2 | PARK7 | PAWR | PSAP | PYCR1 | RIPK1 | STK26 | TRAP1 | TXN | GO:0009649 | 0.0026689312143637 | 22/8243 | 14/3250 | 0.0186016733719697 | 1 | F | F | F | F | entrainment of circadian clock | CRY1 | CRY2 | GNAQ | ID2 | MTA1 | PER1 | PER2 | PML | PPP1CA | PPP1CB | PPP1CC | RBM4 | TP53 | USP2 | GO:0070536 | 0.0026689312143637 | 22/8243 | 14/3250 | 0.0186016733719697 | 1 | F | F | F | F | protein K63-linked deubiquitination | BABAM1 | CYLD | OTUB2 | OTUD4 | OTUD5 | PSMD14 | SHMT2 | SPATA2 | TNFAIP3 | USP16 | USP17L2 | USP20 | USP50 | USP8 | GO:2000404 | 0.0026689312143637 | 22/8243 | 14/3250 | 0.0186016733719697 | 1 | F | F | F | F | regulation of T cell migration | AIRE | APOD | APP | CCL21 | CRK | CRKL | FADD | ITGA4 | OXSR1 | RHOA | RIPK3 | S100A7 | TNFSF14 | WNK1 | GO:0050849 | 0.0026689312143637 | 22/8243 | 14/3250 | 0.0186016733719697 | 1 | F | F | F | F | negative regulation of calcium-mediated signaling | CALM1 | CALM2 | CALM3 | CD22 | CHP1 | DYRK2 | FHL2 | FKBP1A | FKBP1B | GSK3B | GSTO1 | ITPR1 | MAPK7 | SRI | GO:0072655 | 0.00594443770471916 | 49/8243 | 27/3250 | 0.0186171827996793 | 1 | F | F | F | F | establishment of protein localization to mitochondrion | AGK | AKT1 | BAX | CALM1 | CALM3 | DNAJC19 | FBXO7 | FIS1 | GRPEL1 | HK1 | HK2 | HSP90AA1 | HSPA4 | HSPD1 | NDUFA13 | OXA1L | PAM16 | ROMO1 | SLC25A6 | TIMM13 | TIMM21 | TIMM23 | TIMM44 | TIMM50 | TOMM20 | TOMM22 | TOMM40 | GO:0046496 | 0.0029115613247604 | 24/8243 | 15/3250 | 0.0186480919974079 | 1 | F | F | F | F | nicotinamide nucleotide metabolic process | DCXR | G6PD | GPD1L | IDH1 | IDH2 | KCNAB2 | LDHA | MDH1 | MDH2 | NMNAT1 | NMNAT3 | NMRK2 | NNT | OGDH | VCP | GO:0032435 | 0.0029115613247604 | 24/8243 | 15/3250 | 0.0186480919974079 | 1 | F | F | F | F | negative regulation of proteasomal ubiquitin-dependent protein catabolic process | BAG5 | BAG6 | CCAR2 | DDRGK1 | HSP90AB1 | OGT | PARK7 | SDCBP | SGTA | SMARCC1 | STYX | UBXN1 | UCHL5 | USP14 | USP7 | GO:0019362 | 0.0029115613247604 | 24/8243 | 15/3250 | 0.0186480919974079 | 1 | F | F | F | F | pyridine nucleotide metabolic process | DCXR | G6PD | GPD1L | IDH1 | IDH2 | KCNAB2 | LDHA | MDH1 | MDH2 | NMNAT1 | NMNAT3 | NMRK2 | NNT | OGDH | VCP | GO:0035456 | 0.0029115613247604 | 24/8243 | 15/3250 | 0.0186480919974079 | 1 | F | F | F | F | response to interferon-beta | CAPN2 | CDC34 | HTRA2 | IFI16 | IFITM3 | IKBKE | IRF1 | NDUFA13 | PLSCR1 | STAT1 | TLR3 | TMEM173 | TREX1 | UBE2K | XAF1 | GO:0042771 | 0.0029115613247604 | 24/8243 | 15/3250 | 0.0186480919974079 | 1 | F | F | F | F | intrinsic apoptotic signaling pathway in response to DNA damage by p53 class mediator | ATAD5 | BAG6 | CHEK2 | DDIT4 | DYRK2 | EP300 | IFI16 | NUPR1 | PML | SIRT1 | SNW1 | TP53 | TP63 | TP73 | USP28 | GO:0014074 | 0.0112823001334466 | 93/8243 | 47/3250 | 0.0187618331501844 | 1 | F | F | F | F | response to purine-containing compound | ADORA2A | ADSS | AKAP9 | APEX1 | APP | ASS1 | BIRC2 | BRAF | BSG | CARM1 | CFTR | DUSP1 | EZR | FOS | GPD1 | HCN1 | HCN2 | HCN3 | HDAC2 | HSP90B1 | HSPA5 | ITPR2 | JUN | JUNB | JUND | KDM1A | LDHA | NME1 | P2RX1 | P2RX2 | P2RX4 | PEBP1 | PER1 | PIK3CG | PPARG | PPARGC1A | PRKAA1 | PTEN | RAP1A | RAP1B | RELA | SDC1 | SELL | SOD1 | STAT1 | VGF | WT1 | GO:0043488 | 0.0173480528933641 | 143/8243 | 69/3250 | 0.01892212472989 | 1 | F | F | F | F | regulation of mRNA stability | AKT1 | ANP32A | APEX1 | CIRBP | DCP2 | DHX36 | DHX9 | DIS3 | E2F1 | ELAVL1 | EXOSC1 | EXOSC5 | EXOSC6 | EXOSC9 | FTO | FXR2 | HNRNPD | HNRNPM | HNRNPU | HSPA8 | HSPB1 | IGF2BP1 | MAPK14 | MOV10 | MYD88 | NPM1 | NUP214 | PABPC1 | PABPC4 | PAIP1 | PDE12 | PRKCD | PSMA1 | PSMA2 | PSMA3 | PSMA5 | PSMA6 | PSMB5 | PSMB6 | PSMB7 | PSMB8 | PSMC1 | PSMC2 | PSMC3 | PSMC4 | PSMC5 | PSMC6 | PSMD1 | PSMD10 | PSMD11 | PSMD12 | PSMD14 | PSMD2 | PSMD4 | PSMD9 | PSME2 | PSME3 | PUM1 | RC3H1 | SET | TARDBP | TBRG4 | TNPO1 | UBB | UBC | VIM | XPO1 | YWHAB | YWHAZ | GO:0006468 | 0.0763071697197622 | 629/8243 | 273/3250 | 0.0191096785446453 | 1 | F | F | F | F | protein phosphorylation | ABL1 | ABL2 | ACTN2 | ADCK2 | AKT1 | AKT2 | AKT3 | APOA1 | APP | ARAF | ATM | ATP13A2 | AURKB | BCL2 | BIRC5 | BMPR1A | BRAF | BRD4 | BTK | BTRC | CAB39 | CALM1 | CAMK1D | CAMK2A | CAMK2B | CAMKV | CCNB1 | CCNT1 | CCR5 | CDC25B | CDK15 | CDK16 | CDK19 | CDK2 | CDK4 | CDK5 | CDK5R1 | CDK8 | CDK9 | CDKL3 | CFL1 | CHEK2 | CLK1 | COPS2 | CPNE3 | CREB1 | CRKL | CRY1 | CRYAB | CSNK1A1 | CSNK1E | CSNK2A2 | CSNK2B | CTBP1 | CUL1 | CUL3 | DAPK1 | DCLK1 | DGUOK | DLG2 | DLG3 | DLG4 | DUSP7 | DYRK2 | EFEMP1 | EGFR | EIF2AK3 | EIF2AK4 | EIF2S1 | EPHA4 | EPHA6 | EPHB6 | ERBB2 | ERBB3 | ERG | FAM20B | FBXW11 | FGF2 | FGFR1 | FGFR3 | FYN | GAK | GPS2 | GRB2 | GRK1 | GRK5 | GSK3A | GSK3B | HBEGF | HIPK4 | HRAS | HSF1 | ICK | IGF1R | IKBKB | IKBKE | IKBKG | IL3RA | ILF3 | ILK | INSR | ITGAV | ITK | JAK1 | JAK3 | KBTBD7 | KDR | KRAS | KSR1 | LAT | LATS1 | LRRC7 | LRRK1 | LRRK2 | MAP2K1 | MAP2K3 | MAP2K4 | MAP2K6 | MAP2K7 | MAP3K1 | MAP3K13 | MAP3K14 | MAP3K2 | MAP3K3 | MAP3K4 | MAP4K1 | MAPK1 | MAPK14 | MAPK15 | MAPK3 | MAPK4 | MAPK6 | MAPK7 | MAPK8 | MAPK9 | MAST1 | MAST3 | MATK | MBP | MED1 | MELK | MKNK2 | MYC | NEK1 | NEK7 | NLK | NME2 | NOD2 | NPR1 | NTRK1 | OXSR1 | PAK2 | PAK6 | PDK2 | PEBP1 | PHKA1 | PICK1 | PIK3CA | PIK3CG | PIK3R1 | PIM1 | PKN2 | PLK1 | PML | PPM1D | PPP5C | PRDX4 | PRKAA1 | PRKAA2 | PRKAB2 | PRKACA | PRKAG1 | PRKCD | PRKCE | PRKCZ | PRKD1 | PRKD2 | PRKD3 | PRKDC | PSMA1 | PSMA2 | PSMA3 | PSMA5 | PSMA6 | PSMB5 | PSMB6 | PSMB7 | PSMB8 | PSMC1 | PSMC2 | PSMC3 | PSMC4 | PSMC5 | PSMC6 | PSMD1 | PSMD10 | PSMD11 | PSMD12 | PSMD14 | PSMD2 | PSMD4 | PSMD9 | PSME2 | PSME3 | PTK2 | PTPN6 | PTPRA | RAF1 | RANBP9 | RARA | RBX1 | RHO | RIOK1 | RIPK1 | RIPK2 | RIPK3 | RIPK4 | RPS6KA1 | RPS6KA2 | RPS6KB2 | SCG2 | SCYL2 | SHC1 | SMAD2 | SMAD5 | SPTAN1 | SPTBN1 | SPTBN2 | SQSTM1 | SRC | SRPK1 | SRPK2 | ST3GAL1 | STK11 | STK24 | STK25 | STK26 | STK3 | STK38 | STK40 | SULT1A3 | TAB1 | TAB2 | TAOK3 | TBRG4 | TESK2 | TGFB1 | TGFBR3 | TLR3 | TNF | TNIP2 | TNK2 | TNKS | TOP1 | TP53RK | TRAF6 | TRIB2 | TRIB3 | TRIM28 | TWF1 | UBB | UBC | UBE2N | UBE2V1 | ULK1 | USP15 | WNK1 | WNK4 | YES1 | YWHAB | YWHAE | ZAP70 | ZMPSTE24 | GO:0008284 | 0.065146184641514 | 537/8243 | 235/3250 | 0.0191445466318879 | 1 | F | F | F | F | positive regulation of cell population proliferation | ABL1 | ADA | ADCYAP1 | AGER | AKR1B1 | AKR1C2 | AKT1 | AKT2 | AKT3 | ALOX12 | ANXA1 | ANXA2 | AR | ARNT | ARNT2 | ARRB1 | ASCL1 | ATAD5 | ATF2 | BCL2 | BCL2L1 | BCL6 | BIRC5 | BMI1 | BMPR1A | CALR | CAPN1 | CAPNS1 | CARM1 | CBX8 | CCNB1 | CCR3 | CD209 | CD40 | CD40LG | CD6 | CD74 | CD81 | CDC20 | CDC25B | CDK2 | CDK4 | CDKN1A | CHRD | CLEC11A | COL18A1 | COX17 | CRIP2 | CRKL | CTNNB1 | CUL3 | CXCL5 | DBF4B | DDRGK1 | DDX39B | DHX9 | DISC1 | DNAJA2 | DNAJA3 | E2F1 | E2F3 | EGFR | EIF5A | ERBB2 | ERBB3 | ESR1 | ETS1 | EZH2 | FADD | FAM98A | FASLG | FBRS | FGF2 | FGFR1 | FGFR1OP | FGFR3 | FN1 | FOXM1 | FZR1 | GATA1 | GDF9 | GFAP | GLUL | GNAI2 | GNAI3 | GNG5 | GRK5 | HAVCR2 | HBEGF | HDAC1 | HDAC2 | HDAC4 | HIF1A | HLX | HMGB1 | HMGB2 | HMOX1 | HNRNPU | HRAS | HSF1 | HSF4 | HTR1B | HTRA1 | ID2 | IGF1R | ILK | INSR | ITGA4 | ITGAV | ITGB1BP1 | ITGB3 | JAK3 | JUN | KDM1A | KDM5B | KDR | KLF5 | KRAS | LAMB1 | LEF1 | LGMN | LIG4 | MAP3K3 | MAPK1 | MAPK14 | MAPK15 | MARCKSL1 | MAS1 | MATK | MCTS1 | MDM2 | MECP2 | MED1 | MIF | MTA3 | MVD | MYC | NACC1 | NAMPT | NANOG | NAP1L1 | NCK1 | NFATC2 | NMB | NME1 | NME2 | NOD2 | NPM1 | NQO2 | NR4A1 | NRP2 | NTF3 | PDCD10 | PDCD6 | PDF | PELI1 | PIM1 | PLA2G1B | PLCG1 | PML | PNP | PPARD | PPARGC1A | PPP1CC | PRDX3 | PRKAA1 | PRKCZ | PRKD1 | PRKD2 | PRKDC | PROX1 | PTEN | PTK2 | PTN | PTPN6 | RARA | RARG | RASSF10 | RBPJ | RELA | RIPK2 | RNASEH2B | ROMO1 | RPL23 | RPS15A | RPS3 | RPS4X | RPTOR | RTN4 | RUNX2 | S100A13 | S100A6 | S100B | SCG2 | SDCBP | SERPINB3 | SHC1 | SHMT2 | SIRT1 | SIRT6 | SLAMF1 | SLC25A5 | SLC35F6 | SMAD4 | SMO | SP1 | SRPK2 | STAT1 | STAT3 | STAT5A | SUZ12 | TBRG4 | TCIRG1 | TGFB1 | TGFBR3 | TGM2 | THBS1 | TIAL1 | TIMP1 | TNF | TNFAIP3 | TNFSF12 | TP63 | TRAF6 | TWIST1 | UBE2A | VASH2 | VCAM1 | VSTM2A | WDR48 | WDR77 | WNT2 | XBP1 | YAP1 | ZAP70 | GO:1901019 | 0.00570180759432246 | 47/8243 | 26/3250 | 0.0195015389415039 | 1 | F | F | F | F | regulation of calcium ion transmembrane transporter activity | AHNAK | CALM1 | CALM2 | CALM3 | DMD | FKBP1A | FKBP1B | GSTO1 | HAP1 | HSPA2 | HTT | MYO5A | NOS1AP | PDE4D | PLA2G6 | PRKACA | RGN | S100A1 | SLC9A1 | SRI | STAC | STIM1 | SUMO1 | THADA | UBQLN1 | YWHAE | GO:1903845 | 0.00570180759432246 | 47/8243 | 26/3250 | 0.0195015389415039 | 1 | F | F | F | F | negative regulation of cellular response to transforming growth factor beta stimulus | CAV1 | CD109 | HSPA5 | HTRA1 | LDLRAD4 | LEMD3 | LTBP1 | NKX2-1 | ONECUT1 | PIN1 | PPM1A | RASL11B | SIRT1 | SKI | SMAD2 | SMAD3 | SMURF1 | SMURF2 | SNX6 | STRAP | STUB1 | TGFB1 | TGFBR3 | UBB | UBC | ZBTB7A | GO:0009100 | 0.00570180759432246 | 47/8243 | 26/3250 | 0.0195015389415039 | 1 | F | F | F | F | glycoprotein metabolic process | AMFR | B3GAT3 | COL2A1 | DSE | EDEM1 | EXTL2 | GANAB | GLCE | HIF1A | HS2ST1 | HS6ST1 | MAN2B1 | MPI | PARK7 | PPARD | PRKCSH | RNF139 | RNF185 | SEL1L | SGSH | ST8SIA4 | SULF2 | SYVN1 | UGDH | UGGT1 | XYLT1 | GO:1904589 | 0.00570180759432246 | 47/8243 | 26/3250 | 0.0195015389415039 | 1 | F | F | F | F | regulation of protein import | APOD | BAG3 | CD36 | CDH1 | CHP1 | DMAP1 | HDAC3 | HSPA8 | IPO5 | JUP | MAPK1 | MAPK14 | MAVS | PIK3R1 | PKIG | PRKCD | PSEN1 | RAN | SMAD3 | SMO | TARDBP | TGFB1 | TPR | TRIM28 | UBR5 | ZIC1 | GO:0050769 | 0.0340895305107364 | 281/8243 | 128/3250 | 0.0195085765648822 | 1 | F | F | F | F | positive regulation of neurogenesis | ABL2 | ACTR2 | ADCYAP1 | AGER | APBB1 | APOE | APP | ARF1 | ARHGAP35 | ARHGDIA | ASCL1 | ATOH1 | AVIL | BCL2 | BCL6 | BIN1 | BRAF | C1QA | CAMK1D | CAMK2B | CDKL3 | CRKL | CSNK1E | CTNNB1 | CUL7 | DAB1 | DBN1 | DHX36 | DISC1 | DLG4 | DNM1L | DVL1 | DVL2 | DVL3 | E2F1 | EP300 | EPHA4 | EZH2 | FGFR1 | FKBP1B | FN1 | FOXA1 | FYN | GDI1 | GFAP | GOLGA4 | GRIP1 | HAP1 | HDAC1 | HDAC2 | HIF1A | HSPA5 | ID2 | ILK | IQGAP1 | KDM1A | LIG4 | LPAR1 | LRRC7 | MAP1B | MAP2K1 | MAP3K13 | MAPK6 | MAPT | METRN | MME | NAP1L1 | NCK1 | NCOA1 | NEUROD1 | NFE2L2 | NIN | NIPBL | NME1 | NME2 | NTRK1 | OBSL1 | OPA1 | P2RX4 | PAFAH1B1 | PARP6 | PLXNA1 | PLXNA2 | PLXNB2 | PPARG | PPP1CC | PRKD1 | PROX1 | PRPF19 | PSEN1 | PTEN | PTN | PTPRD | RAB11A | RAB21 | RAP1A | RARA | RASSF10 | RELA | REST | RGS2 | RHOA | S100A9 | SEMA7A | SERPINE2 | SH3GL3 | SMO | SMURF1 | SNW1 | SNX3 | SOCS2 | SPEN | STK11 | STK25 | TCF12 | TCF4 | TGFB1 | TIMP2 | TMEM30A | TNF | TNFRSF12A | TP73 | TRIM32 | TUBB2B | TWF1 | TWF2 | XRCC5 | ZMYND8 | GO:0048545 | 0.0162562173965789 | 134/8243 | 65/3250 | 0.0195182740559138 | 1 | F | F | F | F | response to steroid hormone | ACTA1 | ADCYAP1 | ADTRP | AGL | AIFM1 | ANXA1 | AR | ASS1 | ATP1A1 | BCL2 | CALM1 | CALM3 | CASP3 | CASP9 | CAV1 | CDKN1A | CSN1S1 | CTSV | DDIT4 | DUSP1 | EGFR | EIF4E | EIF4EBP1 | ERRFI1 | FBXO32 | FECH | FOS | FOXO1 | GOT1 | GPI | GSTP1 | HMGB2 | HNRNPU | HTR1B | ICAM1 | IDH1 | IGFBP7 | KRAS | MDM2 | MED1 | NCOA1 | NCOA2 | NR1H3 | NR3C1 | PARP1 | PCNA | PEBP1 | PTN | RELA | REST | RHOA | S100B | SDC1 | SRC | TGFB1 | THBS1 | TNF | TRIM63 | TXNIP | TYMS | UBE2L3 | UBE3A | USP8 | WBP2 | YAP1 | GO:0006914 | 0.019046463666141 | 157/8243 | 75/3250 | 0.0195662073090928 | 1 | F | F | F | F | autophagy | ABL1 | ANXA7 | ATG2B | ATG4C | ATG5 | BAG3 | CALCOCO2 | CHMP2A | CISD2 | CLTC | CSNK2A2 | CSNK2B | DYNLL1 | EVA1A | FBXO7 | FIS1 | FOXO1 | FUNDC2 | HAP1 | HDAC6 | HGS | HMGB1 | HSP90AA1 | HSPA8 | IFI16 | LGALS8 | LRRK2 | LRSAM1 | MTMR14 | NRBF2 | OPTN | PARK7 | PGAM5 | PPARGC1A | PRKAA1 | PRKAA2 | PRKAB2 | PRKAG1 | PTPN22 | RAB1A | RAB1B | RAB24 | RAB7A | RB1CC1 | RNF185 | S100A9 | SNF8 | SQSTM1 | SRC | STBD1 | STK11 | STUB1 | TCIRG1 | TMBIM6 | TOLLIP | TOMM20 | TOMM22 | TOMM40 | TP53 | TRAPPC8 | TSG101 | UBQLN1 | UBQLN4 | ULK1 | VCP | VDAC1 | VPS18 | VPS37D | VPS4B | VTA1 | WDR41 | WDR45B | WIPI1 | WIPI2 | XBP1 | GO:0061919 | 0.019046463666141 | 157/8243 | 75/3250 | 0.0195662073090928 | 1 | F | F | F | F | process utilizing autophagic mechanism | ABL1 | ANXA7 | ATG2B | ATG4C | ATG5 | BAG3 | CALCOCO2 | CHMP2A | CISD2 | CLTC | CSNK2A2 | CSNK2B | DYNLL1 | EVA1A | FBXO7 | FIS1 | FOXO1 | FUNDC2 | HAP1 | HDAC6 | HGS | HMGB1 | HSP90AA1 | HSPA8 | IFI16 | LGALS8 | LRRK2 | LRSAM1 | MTMR14 | NRBF2 | OPTN | PARK7 | PGAM5 | PPARGC1A | PRKAA1 | PRKAA2 | PRKAB2 | PRKAG1 | PTPN22 | RAB1A | RAB1B | RAB24 | RAB7A | RB1CC1 | RNF185 | S100A9 | SNF8 | SQSTM1 | SRC | STBD1 | STK11 | STUB1 | TCIRG1 | TMBIM6 | TOLLIP | TOMM20 | TOMM22 | TOMM40 | TP53 | TRAPPC8 | TSG101 | UBQLN1 | UBQLN4 | ULK1 | VCP | VDAC1 | VPS18 | VPS37D | VPS4B | VTA1 | WDR41 | WDR45B | WIPI1 | WIPI2 | XBP1 | GO:1903900 | 0.0132233410166202 | 109/8243 | 54/3250 | 0.0197149306236079 | 1 | F | F | F | F | regulation of viral life cycle | AICDA | ANXA2 | APCS | BANF1 | BCL2 | CD74 | CHMP2A | CXCL8 | DDB1 | DDX3X | EIF2AK4 | FCN1 | IFI16 | IFIT1 | IFITM3 | IFNA2 | ILF3 | ISG15 | LAMP3 | LGALS1 | MAVS | NR5A2 | P4HB | PABPC1 | PC | PDE12 | PKN2 | PLSCR1 | PML | PPIA | PPIB | PPID | PPIE | PROX1 | RAD23A | SETDB1 | SMC3 | SNF8 | SNX3 | SRPK1 | SRPK2 | STAU1 | STOM | TNF | TOP2A | TRIM21 | TRIM25 | TRIM28 | TRIM32 | TRIM35 | TSG101 | VAPA | VAPB | VPS4B | GO:0009059 | 0.104452262525779 | 861/8243 | 368/3250 | 0.0197244157826969 | 1 | F | F | F | F | macromolecule biosynthetic process | ACE2 | ADRM1 | AFF4 | AGL | AGO2 | AKT1 | AKT2 | APOA1 | APOE | AR | ARHGAP35 | ARNT | ARRB1 | ARRB2 | ATAD5 | ATF6B | ATM | ATOH1 | B3GAT3 | B3GNT3 | B4GALT3 | BAX | BCL10 | BRCA1 | CBFB | CCDC88A | CCNB1 | CCNT1 | CCNT2 | CDC73 | CDK2 | CDK4 | CDK8 | CDK9 | CDKN1A | CEBPA | CEBPE | CENPF | CHAF1A | CHRAC1 | COL4A2 | COL5A1 | COPS2 | COPS5 | CPSF7 | CREB1 | CREBBP | CSTF2T | CTDP1 | DAP3 | DARS | DCN | DHX9 | DIDO1 | DNA2 | DNAJA3 | DRG2 | DSE | DTX1 | DUT | DVL1 | DVL2 | E2F1 | E2F2 | E2F3 | EAF2 | ECD | EEF1A1 | EEF1A1P5 | EEF1A2 | EEF1B2 | EEF1D | EEF1G | EEF2 | EGFR | EIF4H | EIF5A | ELF3 | ERAL1 | ESR1 | ESR2 | ESRRA | ESRRB | ESRRG | ETS1 | EXTL2 | FAM20B | FARSB | FEN1 | FOS | FUBP1 | FUBP3 | GAR1 | GATA1 | GBE1 | GEMIN5 | GGT1 | GLCE | GOLPH3 | GPC5 | GSPT1 | GSPT2 | GTF2A1 | GTF2E2 | GTF2F1 | GTF2F2 | GTF2I | GTF3C2 | GYG1 | GYG2 | GYS1 | HARS | HEXA | HIF1A | HIST1H4A | HNF4A | HNF4G | HOXB9 | HS2ST1 | HS6ST1 | HSF1 | HSP90AA1 | HSP90AB1 | HSPA14 | HSPG2 | ILF2 | ING5 | IRF1 | IRF7 | ISG15 | JUN | KAT5 | KAT7 | KLF5 | KMT2A | LARP4 | LEF1 | LIG4 | LMO2 | LUM | MAPK3 | MCM2 | MCM3 | MCM4 | MCM5 | MCM6 | MCM7 | MCM9 | MCMBP | MECP2 | MED1 | MED12 | MED14 | MED17 | MED20 | MED21 | MED23 | MED24 | MED31 | MED4 | MED6 | MITF | MLLT1 | MLLT3 | MPI | MRPL12 | MRPL14 | MRPL19 | MRPL28 | MRPL38 | MRPL4 | MRPL43 | MRPL45 | MRPL46 | MRPL9 | MRPS5 | MRRF | MTERF1 | NAP1L1 | NCBP1 | NCOA1 | NCOR1 | NELFB | NELFCD | NELFE | NFATC1 | NFE2L2 | NOP10 | NPLOC4 | NR0B2 | NR1H2 | NR1H3 | NR1I2 | NR3C1 | NR4A1 | NR5A2 | NRBF2 | NUP98 | OXA1L | PABPC4 | PARP1 | PAWR | PAX8 | PCF11 | PCNA | PDF | PER2 | PGM1 | PGM2 | PHB | PINX1 | PIR | PNKP | POLA2 | POLD1 | POLE2 | POLE3 | POLM | POLR2A | POLR2E | POLR2L | POLR2M | POT1 | POU5F1 | PPARD | PPARG | PPARGC1A | PPIA | PPM1D | PPP1R3C | PPP5C | PTEN | PTGES3 | PTMA | PTMS | PTTG1 | RARA | RARG | RARS2 | RBBP4 | RBBP6 | RBMX | RBPJ | RELA | REPIN1 | RFC1 | RFC2 | RFC3 | RFC4 | RFC5 | RMND1 | RNASEH2A | RPA2 | RPA3 | RPAIN | RPL10 | RPL10A | RPL12 | RPL18A | RPL22 | RPL23 | RPL23A | RPL24 | RPL30 | RPL36AL | RPL37A | RPL38 | RPL6 | RPL7 | RPL7A | RPL9 | RPLP0 | RPLP0P6 | RPLP1 | RPLP2 | RPS12 | RPS13 | RPS14 | RPS15A | RPS19 | RPS2 | RPS20 | RPS24 | RPS29 | RPS3 | RPS4X | RPS6KB2 | RPS8 | RRM1 | RRM2 | RRM2B | RTF1 | RUNX2 | RXRA | RXRB | RXRG | SARS | SCAF1 | SDC1 | SDC4 | SET | SIN3A | SIRT1 | SIRT7 | SKI | SMAD4 | SMG5 | SMG6 | SMN1 | SNRPB | SNRPD3 | SNRPE | SNW1 | SP1 | SPI1 | SPIN1 | SPRTN | ST3GAL1 | ST3GAL6 | STAT3 | SUPT4H1 | SUPT5H | TAF12 | TAF15 | TAF1A | TARDBP | TARS | TCEA1 | TCEA2 | TCERG1 | TCF4 | TERF1 | TERF2 | TERF2IP | TFCP2 | THRA | TK1 | TK2 | TNF | TOP1 | TOP3A | TP53 | TREX1 | TRIM25 | TRIM28 | TRIM29 | TRIP13 | TSC22D1 | TSFM | TUFM | TYMS | UBB | UBC | UBE2L6 | UGDH | UGP2 | USPL1 | VCAN | VCP | VDR | WARS | XBP1 | XYLT1 | YAP1 | ZBTB7A | ZBTB7B | ZNF217 | ZNF598 | GO:1905477 | 0.0110396700230499 | 91/8243 | 46/3250 | 0.0198240459310292 | 1 | F | F | F | F | positive regulation of protein localization to membrane | AGR2 | AKT1 | AKT2 | ANK3 | BBC3 | BCL2 | CACNG2 | CASP8 | CDK5 | CDK5R1 | CHP1 | E2F1 | EGFR | ERBB2 | EZR | FIS1 | FYN | HRAS | ITGB1BP1 | KIF5B | LGALS3 | MAPK8 | MYO1C | PIK3R1 | PPP3CC | PRKCE | PTN | RAB11A | RAB11FIP2 | SFN | SPTBN1 | SQSTM1 | STAC | STOM | TFDP1 | TGFB1 | TNF | TP53 | TP63 | TP73 | YWHAB | YWHAE | YWHAG | YWHAH | YWHAQ | YWHAZ | GO:0071456 | 0.00861336891908286 | 71/8243 | 37/3250 | 0.0199211937062407 | 1 | F | F | F | F | cellular response to hypoxia | ACAA2 | AKT1 | BCL2 | CCNB1 | E2F1 | EIF4EBP1 | ENDOG | FABP1 | GNB1 | HIF1A | HMOX1 | ICAM1 | IREB2 | LMNA | MDM2 | MDM4 | MYC | NDRG1 | NPEPPS | OPA1 | P4HB | PGK1 | PHB2 | PPARD | PPARGC1A | PRKAA1 | PRKCE | PTEN | PTN | S100B | SIRT1 | SLC9A1 | SRC | STUB1 | TP53 | TWIST1 | UBQLN1 | GO:0051241 | 0.0787334708237292 | 649/8243 | 281/3250 | 0.0200467034447077 | 1 | F | F | F | F | negative regulation of multicellular organismal process | ABAT | ACOT13 | ADA | ADCY6 | ADCYAP1 | ADRB2 | ADTRP | AGER | AGO1 | AHSG | AKAP8 | ALOX12 | ANXA1 | ANXA2 | ANXA5 | APCS | APOA1 | APOD | APOE | APP | ARHGDIA | ARRB1 | ARRB2 | ASCL1 | ATF2 | ATP1A1 | ATP2A2 | BAG5 | BCL11A | BCL2 | BCL6 | BCOR | BIN1 | BMPR1A | BTK | C1QBP | C1QTNF1 | CALR | CARM1 | CBFB | CCR1 | CD74 | CDC73 | CDK5 | CDK5R1 | CDKL3 | CHRD | CLDN18 | COL4A2 | COL5A1 | COMT | CRMP1 | CRY1 | CSNK2B | CTDP1 | CTNNB1 | CYLD | DAB1 | DCN | DDX6 | DGUOK | DOCK5 | DOCK7 | DPF2 | DTX1 | DYNLT1 | E2F2 | EAF2 | EFEMP1 | EGFR | EIF2AK3 | EIF2AK4 | EIF4A3 | EIF4E | EIF4ENIF1 | EPHA4 | EPPK1 | ERBB2 | ERRFI1 | EZR | FAM129B | FASLG | FBXO7 | FGB | FGF2 | FGFR1 | FKBP1B | FN1 | FOXA1 | FOXJ1 | FOXO1 | G6PD | GAK | GATA1 | GDI1 | GFAP | GLRX3 | GNAI2 | GSK3A | GSK3B | GSTP1 | GTF2I | HAVCR2 | HDAC2 | HDAC4 | HDAC5 | HDAC7 | HDAC9 | HGS | HIF1A | HIST1H4A | HLX | HMGB1 | HMOX1 | HOXA5 | HSF1 | HSP90AB1 | HSPA9 | HSPG2 | ID2 | IDH2 | IFNA2 | IFT122 | IKBKE | IP6K1 | IRF1 | ISG15 | ITCH | ITGB1BP1 | JAK3 | JUP | KAT5 | LDLRAD4 | LEF1 | LGALS1 | LGMN | LGR4 | LMNA | LOXL2 | LPAR1 | LRRK2 | LSM1 | MAP2K6 | MAVS | MC1R | MCC | MDM2 | MECP2 | MED1 | MEOX2 | MIB1 | MYC | NFATC1 | NFATC2 | NFE2L2 | NFKBIA | NKX2-1 | NME1 | NME2 | NOS3 | NPLOC4 | NPR1 | NR1H2 | NR1H3 | ORM1 | OTUD5 | P2RX4 | PAFAH1B1 | PAX8 | PCBP2 | PCM1 | PCSK9 | PCTP | PDCD10 | PDE4D | PGAM5 | PGK1 | PHB2 | PIAS3 | PIK3CG | PIK3R1 | PIN1 | PLAUR | PML | PPARG | PPM1B | PPP1R11 | PPP2CA | PRDX2 | PRDX4 | PRKACA | PRKCD | PSEN1 | PTEN | PTGER3 | PTK2 | PTN | PTPN22 | PTPN23 | PTPN6 | PTPRO | PTPRR | RAB29 | RAC1 | RARA | RARG | RB1 | RBPJ | RC3H1 | REL | REST | RGN | RGS2 | RHOA | RIPK1 | RPS19 | RTN4 | RUNX1 | S100B | SARS | SEMA7A | SERPINB2 | SERPINE2 | SFMBT1 | SKI | SLAMF1 | SLAMF8 | SMAD3 | SMAD4 | SMO | SMURF1 | SNAI2 | SOCS1 | SOCS2 | SOD1 | SOX2 | SP100 | SPRY2 | SRGAP2 | SRI | STARD13 | STAT1 | STAT3 | STK11 | STK3 | STRAP | SYNJ2BP | TBXA2R | TGFB1 | THBS1 | THBS2 | TIA1 | TIMP1 | TLE3 | TLR3 | TNF | TNFAIP3 | TNFRSF1A | TNN | TNNT1 | TP63 | TP73 | TRAF3IP1 | TRIB2 | TRIM25 | TRIM63 | TULP3 | TWIST1 | UBE2L6 | USP19 | VIM | VSIG4 | VTN | WDR77 | WNK1 | WNK4 | WT1 | YWHAH | YY1 | ZBTB16 | ZBTB7B | GO:0072503 | 0.026689312143637 | 220/8243 | 102/3250 | 0.0201104899027148 | 1 | F | F | F | F | cellular divalent inorganic cation homeostasis | ABL1 | ABL2 | ACKR2 | ADCYAP1 | AFG3L2 | ANXA2 | ANXA6 | ANXA7 | APOE | APP | ATP13A2 | ATP2A2 | ATP2B2 | BAX | BCL2 | BDKRB2 | C1QTNF1 | CACNA1A | CALM1 | CALM2 | CALM3 | CALR | CAV1 | CCL21 | CCR1 | CCR3 | CCR4 | CCR5 | CD36 | CD40 | CDK5 | CHRNA9 | CXCL11 | DLG4 | ESR1 | FASLG | FGF2 | FIS1 | FKBP1B | GLP1R | GNA13 | GNA15 | GNB1 | GOT1 | GRIA1 | GRIK2 | GSTO1 | GTF2I | HAP1 | HEXB | HMGB1 | HTR1B | HTT | IBTK | IMMT | ITGAV | ITPR1 | ITPR2 | ITPR3 | KCTD17 | LETM1 | LPAR1 | LPAR2 | LPAR4 | LRP6 | MCOLN3 | MICU1 | MICU2 | MYO5A | NMB | P2RX1 | P2RX2 | P2RX4 | PIK3CG | PLA2G1B | PLA2G6 | PLCG1 | PML | PRKACA | PRKCE | PSEN1 | PTGER3 | PTGIR | RAP1GDS1 | RGN | RIC3 | SLC39A4 | SMAD3 | SNCA | SRI | STIM1 | STOML2 | SWAP70 | TBXA2R | TGFB1 | TGM2 | THADA | TMBIM6 | TPCN2 | VAPB | VDR | YWHAE | GO:1903508 | 0.123862671357515 | 1021/8243 | 433/3250 | 0.0205008861419905 | 1 | F | F | F | F | positive regulation of nucleic acid-templated transcription | ABHD14B | ABL1 | ACTA2 | ACTL6A | ACTN1 | ACTN2 | ACTN4 | ACTR2 | ACTR3 | ADCYAP1 | ADRB2 | AGO1 | AGO2 | AHI1 | AICDA | AIRE | AKT1 | ANKRD49 | APBB1 | APEX1 | APOE | APP | AR | ARF4 | ARID3B | ARID4B | ARNT | ARNT2 | ARRB1 | ASCL1 | ATF2 | ATF6 | ATF6B | ATM | ATOH1 | ATRX | AXIN1 | BANP | BCL10 | BIRC2 | BMPR1A | BRCA1 | BRD4 | BTRC | BUD31 | CAND1 | CARM1 | CBFB | CCAR2 | CCNT1 | CCNT2 | CD40 | CD74 | CD81 | CDC5L | CDC73 | CDH1 | CDK2 | CDK8 | CDK9 | CEBPA | CEBPE | CEP290 | CHCHD10 | CHCHD2 | CHD8 | CHEK2 | COPS5 | CREB1 | CREB3 | CREBBP | CTCF | CTNNB1 | DAB2 | DAXX | DCN | DDRGK1 | DDX39B | DDX3X | DHX36 | DHX9 | DPF2 | DTX1 | DVL1 | DVL2 | DVL3 | E2F1 | E2F2 | E2F3 | E2F4 | E2F6 | EAF2 | EBF1 | ECD | EGFR | EGLN1 | EIF2AK3 | ELF3 | ELF4 | EP300 | ERBB2 | ERG | ERLIN2 | ESR1 | ESR2 | ESRRA | ESRRB | ESRRG | ETS1 | EXOSC9 | FADD | FAM129B | FBXW11 | FGF2 | FHL2 | FOS | FOXA1 | FOXJ1 | FOXK1 | FOXK2 | FOXM1 | FOXO1 | FUBP3 | FUS | GABPB1 | GATA1 | GPS2 | GRIP1 | GTF2A1 | GTF2F1 | GTF2F2 | H1F0 | H1FX | H2AFZ | HAX1 | HDAC1 | HDAC2 | HDAC3 | HDAC4 | HDAC5 | HELZ2 | HEXB | HIF1A | HIST1H1A | HMGB1 | HMGB2 | HMGB3 | HNF1A | HNF4A | HNF4G | HNRNPAB | HNRNPD | HNRNPK | HNRNPU | HOXA5 | HOXB5 | HOXB9 | HOXD10 | HRAS | HSF1 | HSF4 | HSPA5 | HTATIP2 | ID2 | IFI16 | IFNA2 | IKBKB | IKBKG | IKZF5 | ILF2 | ILF3 | ILK | ING1 | ING5 | INSIG1 | INSIG2 | INSR | IRF1 | IRF4 | IRF7 | ITGB1BP1 | JMJD6 | JUN | JUNB | JUND | JUP | KAT5 | KAT7 | KAT8 | KDM1A | KDM8 | KLF5 | KLF6 | KMT2A | KMT2B | KMT2C | LEF1 | LGR4 | LMO2 | LMO7 | LPIN3 | LRP6 | LUM | MAP2K1 | MAP2K3 | MAP2K7 | MAP3K2 | MAPK1 | MAPK14 | MAPK3 | MAPK7 | MAVS | MC1R | MECP2 | MED1 | MED12 | MED14 | MED17 | MED20 | MED21 | MED23 | MED24 | MED31 | MED4 | MED6 | MEOX2 | METTL23 | MITF | MLLT11 | MLLT3 | MORF4L2 | MRPL12 | MTA1 | MTA3 | MYC | MYO6 | NAMPT | NANOG | NCK1 | NCOA1 | NCOA2 | NCOA3 | NCOA4 | NELFE | NEUROD1 | NFATC1 | NFATC2 | NFE2L2 | NFKB2 | NFKBIA | NIF3L1 | NIPBL | NKRF | NKX2-1 | NME2 | NOD2 | NPM1 | NR1H2 | NR1H3 | NR1I2 | NR3C1 | NR4A1 | NR5A2 | NUFIP1 | NUP85 | NUP98 | NUPR1 | OGT | ONECUT1 | PARK7 | PARP1 | PATZ1 | PAX8 | PAX9 | PAXIP1 | PCBD1 | PCBP1 | PCGF5 | PER1 | PER2 | PFKM | PHB | PHF10 | PIAS1 | PIH1D1 | PIK3R1 | PIN1 | PLA2G1B | PLSCR1 | PML | POU2F3 | POU5F1 | PPARD | PPARG | PPARGC1A | PPM1A | PPP1R12A | PRKAA1 | PRKD1 | PRKD2 | PRKDC | PRMT2 | PROX1 | PSEN1 | PSMC2 | PSMC3 | PSMC4 | PSMC5 | PSMC6 | PSMD9 | PTGES2 | RAF1 | RAN | RARA | RARG | RB1 | RBMX | RBPJ | REL | RELA | REST | RFC1 | RFXANK | RIPK1 | RIPK2 | RIPK3 | RNF4 | RPS6KA1 | RPTOR | RTF1 | RUNX1 | RUNX2 | RUVBL1 | RUVBL2 | RXRA | RXRB | RXRG | SFPQ | SHC1 | SIN3A | SIRT1 | SIRT7 | SKI | SLC9A1 | SMAD2 | SMAD3 | SMAD4 | SMAD5 | SMAD9 | SMARCA2 | SMARCA4 | SMARCB1 | SMARCC1 | SMARCC2 | SMARCE1 | SMO | SNW1 | SOX2 | SP1 | SP100 | SPI1 | SPIN1 | SQSTM1 | SRC | STAT1 | STAT3 | STAT4 | STAT6 | SUB1 | SUMO2 | SUPT4H1 | SUPT5H | TADA2B | TAF12 | TAF15 | TARDBP | TBL1X | TCEA2 | TCERG1 | TCF12 | TCF21 | TCF4 | TDRD3 | TFCP2 | TFDP1 | TGFB1 | THRA | TLR3 | TMEM173 | TNF | TNFRSF1A | TNIP2 | TNKS | TOP2A | TP53 | TP63 | TP73 | TRAF6 | TRIAP1 | TRIM28 | TRIM32 | TRIP4 | TSG101 | TTC5 | TWIST1 | UBB | UBC | UBE2L3 | UBE2V1 | USP16 | USP21 | VDR | VHL | WAS | WBP2 | WDR77 | WNT2 | WT1 | WWOX | WWP2 | XBP1 | XRCC6 | YAF2 | YAP1 | YES1 | YWHAH | YY1 | ZBTB16 | ZBTB17 | ZBTB7B | ZIC1 | ZNF24 | ZNF746 | GO:0010628 | 0.149581463059566 | 1233/8243 | 519/3250 | 0.020658901397794 | 1 | F | F | F | F | positive regulation of gene expression | ABHD14B | ABL1 | ACTA1 | ACTA2 | ACTB | ACTG2 | ACTR2 | ACTR3 | ADCYAP1 | ADRB2 | ADTRP | AGO1 | AGO2 | AGR2 | AHI1 | AICDA | AIRE | AKT1 | ALOX12 | ANK3 | ANKRD49 | APBB1 | APOE | APP | AR | ARF4 | ARID3B | ARID4B | ARNT | ARNT2 | ARRB1 | ARRB2 | ASCL1 | ATF2 | ATF6 | ATF6B | ATM | ATOH1 | ATRX | AXIN1 | AZU1 | BAG2 | BANP | BCL10 | BCL11A | BMPR1A | BRAF | BRCA1 | BRD4 | BTRC | C1QBP | C1QTNF1 | CALR | CAND1 | CARM1 | CAV1 | CBFB | CCNB1 | CCNT1 | CCNT2 | CD36 | CD40 | CD74 | CD81 | CDC5L | CDC73 | CDH1 | CDK2 | CDK4 | CDK8 | CDK9 | CEBPA | CEBPE | CEP290 | CHCHD10 | CHCHD2 | CHD8 | CHEK2 | CIRBP | CLU | COA3 | COPS5 | CREB1 | CREB3 | CREBBP | CRP | CTCF | CTNNB1 | DAB2 | DAZAP1 | DCN | DDRGK1 | DDX39B | DDX3X | DHX36 | DHX9 | DPF2 | DROSHA | DVL1 | DVL2 | DVL3 | E2F1 | E2F2 | E2F3 | E2F4 | E2F6 | EAF2 | EBF1 | ECD | EDA | EEF2 | EGFR | EGLN1 | EIF2AK3 | EIF2AK4 | EIF2B5 | EIF3D | EIF3E | EIF4A3 | EIF5A | EIF6 | ELAVL1 | ELF3 | ELF4 | ENO1 | EP300 | ERBB2 | ERBB3 | ERG | ERLIN2 | ESR1 | ESR2 | ESRRA | ESRRB | ESRRG | ETS1 | EXOSC9 | EZR | FADD | FAM129B | FAM98A | FBXW11 | FGF2 | FHL2 | FN1 | FOS | FOXA1 | FOXJ1 | FOXK1 | FOXK2 | FOXM1 | FOXO1 | FUBP1 | FUBP3 | FXR2 | GABPB1 | GATA1 | GPS2 | GRIP1 | GSK3A | GSK3B | GTF2F1 | GTF2F2 | H1F0 | H1FX | H2AFZ | HAX1 | HDAC1 | HDAC2 | HDAC3 | HDAC4 | HDAC5 | HELZ2 | HEXB | HGS | HIF1A | HIST1H1A | HMGB1 | HMGB2 | HMGB3 | HNF1A | HNF4A | HNF4G | HNRNPAB | HNRNPD | HNRNPK | HNRNPL | HNRNPU | HOXA5 | HOXB5 | HOXB9 | HOXD10 | HRAS | HSF1 | HSF4 | HSPA1B | HSPA5 | ID2 | IFI16 | IFNA2 | IGF2BP1 | IKBKB | IKBKG | IKZF5 | ILF2 | ILF3 | ILK | ING1 | ING5 | INSIG1 | INSIG2 | INSR | IRF1 | IRF4 | IRF7 | ITGB1BP1 | JMJD6 | JUN | JUNB | JUND | JUP | KAT5 | KAT7 | KAT8 | KDM1A | KDM5B | KDM8 | KHDRBS1 | KLF5 | KLF6 | KMT2A | KMT2B | KMT2C | KRAS | LAMP3 | LARP4 | LEF1 | LGR4 | LIMS1 | LMNA | LMO2 | LMO7 | LPIN3 | LRP6 | LUM | MAP2K1 | MAP2K3 | MAP2K7 | MAP3K2 | MAPK1 | MAPK14 | MAPK3 | MAPK7 | MAPK8 | MAPK9 | MAVS | MC1R | MDM2 | MECP2 | MED1 | MED12 | MED14 | MED17 | MED21 | MED24 | MED4 | MED6 | MEOX2 | METTL23 | MITF | MLLT11 | MLLT3 | MORF4L2 | MOV10 | MRPL12 | MSN | MYBBP1A | MYC | MYD88 | MYO1C | MYO6 | NAMPT | NANOG | NBAS | NCBP1 | NCK1 | NCOA1 | NCOA2 | NCOA3 | NCOA4 | NCOR1 | NCOR2 | NELFE | NEUROD1 | NFATC1 | NFATC2 | NFE2L2 | NFKB2 | NFKBIA | NIF3L1 | NIPBL | NKRF | NKX2-1 | NME2 | NOD2 | NOS1AP | NOS3 | NPM1 | NR0B2 | NR1H2 | NR1H3 | NR1I2 | NR3C1 | NR4A1 | NR5A2 | NUFIP1 | NUP85 | NUP98 | OGT | ONECUT1 | PABPC1 | PAIP1 | PARK7 | PARP1 | PATZ1 | PAWR | PAX8 | PAX9 | PAXIP1 | PCBD1 | PCBP1 | PCGF5 | PDCD10 | PER1 | PFKM | PHB | PHF10 | PIAS1 | PIAS3 | PIH1D1 | PIK3R1 | PIK3R3 | PIN1 | PLA2G1B | PLGRKT | PLSCR1 | POLDIP3 | POLR2A | POLR2E | POLR2L | POU2F3 | POU5F1 | PPARD | PPARG | PPARGC1A | PPM1A | PPP1R12A | PRDX6 | PRKAA1 | PRKAG1 | PRKD1 | PRKD2 | PRKDC | PRMT2 | PROX1 | PRPF19 | PSEN1 | PSMC2 | PSMC3 | PSMC4 | PSMC5 | PSMC6 | PSMD9 | PTEN | PTGES2 | PTPN22 | RAB3GAP1 | RAF1 | RAN | RARA | RARG | RB1 | RBM3 | RBMX | RBPJ | RDX | REL | RELA | REST | RFC1 | RFXANK | RHBDD1 | RHOA | RIOK1 | RIPK1 | RIPK2 | RMND1 | RNF4 | RPL23 | RPS3 | RPS4X | RPS6KA1 | RPS6KB2 | RPTOR | RTF1 | RUNX1 | RUNX2 | RUVBL2 | RXRA | RXRB | RXRG | SFPQ | SHC1 | SIN3A | SIRT1 | SIRT7 | SKI | SLC9A1 | SMAD2 | SMAD3 | SMAD4 | SMAD5 | SMAD9 | SMARCA2 | SMARCA4 | SMARCB1 | SMARCC1 | SMARCC2 | SMO | SNF8 | SNW1 | SOX2 | SP1 | SP100 | SPI1 | SPIN1 | SPRY2 | SQSTM1 | SRC | SRPK2 | SRSF5 | STAT1 | STAT3 | STAT4 | STAT6 | SUB1 | SUMO2 | SUPT4H1 | SUPT5H | TAF15 | TAF1A | TARDBP | TBL1X | TCEA2 | TCF12 | TCF21 | TCF4 | TERF2 | TFCP2 | TFDP1 | TGFB1 | THBS1 | THRA | TLE1 | TLR3 | TMEM173 | TNF | TNFRSF1A | TNIP2 | TNKS | TNP1 | TOP2A | TP53 | TP63 | TP73 | TRA2A | TRAF6 | TRIAP1 | TRIM28 | TRIP4 | TRUB2 | TTC5 | TWIST1 | U2AF2 | UBB | UBC | UBE2V1 | UBR5 | UPF3B | UQCC1 | USP16 | USP21 | VDR | VHL | VIM | VSTM2A | WARS | WAS | WBP2 | WNT16 | WNT2 | WT1 | WWOX | WWP2 | XBP1 | XRCC6 | YAF2 | YAP1 | YES1 | YTHDF1 | YWHAH | YY1 | ZBTB16 | ZBTB17 | ZBTB7B | ZIC1 | ZMPSTE24 | ZNF24 | ZNF746 | GO:0043434 | 0.0230498604876865 | 190/8243 | 89/3250 | 0.021287360328879 | 1 | F | F | F | F | response to peptide hormone | ADCY6 | AKT1 | AKT2 | ANXA1 | ANXA5 | APEX1 | APRT | ASS1 | BRAF | BSG | CAT | CAV1 | CDC5L | CDK4 | CREB1 | CRK | CRY1 | CYC1 | DENND4C | EIF2B1 | EIF2B3 | EIF2B5 | EIF6 | EPRS | ERRFI1 | FABP3 | FOXO1 | FYN | GLP1R | GNAI1 | GOT1 | GOT2 | GSK3A | GSTP1 | HADH | HDAC5 | HDAC9 | HSF1 | ICAM1 | INSIG2 | INSR | IQGAP1 | LPIN3 | LRP6 | MDM2 | MYO5A | NFE2L2 | NME2 | NR4A1 | OGT | OTC | PARP1 | PCSK9 | PIK3R1 | PKM | PLA2G1B | PPARG | PPAT | PRKACA | PRKAR1A | PRKAR2B | PRKCD | PRKCZ | PRKDC | PTEN | QDPR | RAB10 | RAB31 | RANGAP1 | RAP1B | RBBP4 | RELA | SIRT1 | SLC9A1 | SORT1 | SP1 | SRC | SRSF5 | STAT1 | TBC1D4 | TGFB1 | TGFBR3 | TIMP1 | TRIB3 | VGF | WDTC1 | XBP1 | YWHAG | ZBTB7B | GO:1903169 | 0.00946257430547131 | 78/8243 | 40/3250 | 0.0217864627325365 | 1 | F | F | F | F | regulation of calcium ion transmembrane transport | ABL1 | AHNAK | ATG5 | BAX | BIN1 | CALM1 | CALM2 | CALM3 | CXCL11 | DMD | FKBP1A | FKBP1B | FYN | G6PD | GSTO1 | HAP1 | HSPA2 | HTT | MYO5A | NOS1AP | PDE4D | PIK3CG | PLA2G6 | PLCG1 | PRKACA | PRKCE | PSEN2 | PTPN6 | RGN | S100A1 | SLC9A1 | SNCA | SRI | STAC | STIM1 | SUMO1 | TGFB1 | THADA | UBQLN1 | YWHAE | GO:0008104 | 0.150915928666748 | 1244/8243 | 523/3250 | 0.0221183582283959 | 1 | F | F | F | F | protein localization | ABCE1 | ABCG1 | ABHD17A | ABL1 | ACAA1 | ACD | ACOX1 | ACTN2 | ACTN4 | ADCYAP1 | ADPRH | AFM | AGAP1 | AGAP2 | AGER | AGK | AHCYL1 | AHI1 | AKAP8 | AKT1 | AKT2 | AKTIP | ANK1 | ANK3 | ANKS4B | ANXA1 | ANXA2 | AP1B1 | AP1G1 | AP1M1 | AP3D1 | AP3M1 | AP4M1 | AP4S1 | APOE | APPBP2 | ARF1 | ARF4 | ARF5 | ARFGAP1 | ARFIP1 | ARHGAP1 | ARL4D | ARL5B | ARRB1 | ARRB2 | ASPSCR1 | ATG4C | ATM | ATP1B3 | ATP6V1A | ATP6V1B1 | ATP6V1C1 | ATP6V1D | ATP6V1E1 | ATRX | AUP1 | AURKB | BAG3 | BAG4 | BAG6 | BANP | BAX | BCL6 | BIRC5 | BLZF1 | BRAF | BSG | C16orf70 | CACNG2 | CACNG4 | CACNG5 | CALM1 | CALM3 | CALR | CANX | CARM1 | CAT | CAV1 | CBLB | CBY1 | CCDC120 | CCDC22 | CCDC68 | CCHCR1 | CCT6B | CD36 | CD40LG | CD74 | CD81 | CDC37 | CDH1 | CDK5 | CENPF | CEP128 | CEP290 | CEP55 | CHCHD10 | CHMP2A | CHP1 | CLSTN1 | CLTC | CLU | COG3 | COMMD1 | COPA | COPB1 | COPE | COPG1 | CPLX1 | CPLX3 | CROCC | CROT | CSE1L | CTCF | CTNNB1 | CTSA | CTTN | CUTA | DAB2 | DDX1 | DDX19A | DDX3X | DENND1A | DENND4C | DERL1 | DHRS4 | DHX9 | DLG2 | DLG3 | DLG4 | DMD | DNAJA1 | DNAJA3 | DNAJC19 | DNM1L | DUSP16 | DVL1 | DVL2 | DYNLL1 | DYNLT1 | ECH1 | ECI2 | EGFR | EHHADH | EIF4ENIF1 | EIF5A | EIF6 | ESR1 | EXOC6 | EZH2 | EZR | FAF2 | FAM83D | FAM83H | FBXO7 | FCN1 | FERMT2 | FIS1 | FKBP1B | FLOT1 | FRAS1 | GAK | GBP2 | GBP5 | GDI1 | GDI2 | GGA1 | GLRB | GOLGA2 | GOLGA4 | GOLPH3 | GOLT1B | GOPC | GORASP2 | GRID2 | GRIK2 | GRIP1 | GRPEL1 | GSTK1 | HAP1 | HCN1 | HDAC6 | HEATR3 | HERC2 | HGS | HID1 | HK1 | HK2 | HMGB1 | HMGCL | HNF1A | HNRNPU | HSD17B4 | HSP90AA1 | HSP90B1 | HSPA4 | HSPA5 | HSPA8 | HSPB1 | HSPD1 | ICK | IDE | IDH1 | IFIT1 | IFT122 | IK | IKBKB | IPO11 | IPO4 | IPO5 | IPO7 | IPO8 | IPO9 | ITGA4 | ITGAV | ITGB1BP1 | JUP | KIF13B | KIF5B | KIF5C | KLHL20 | KPNA1 | KPNA2 | KPNB1 | LARS | LATS1 | LCP2 | LEMD2 | LIMS1 | LMAN2L | LMNA | LRP6 | LRRC7 | LRRK2 | LRSAM1 | MAPK15 | MAPK8IP3 | MAPK9 | MARCH5 | MCC | MCFD2 | MCM9 | MDM2 | MECP2 | MTCH2 | MTX1 | MYO1C | MYO5A | MYO6 | NBAS | NCOA4 | NDUFA13 | NEDD4 | NEDD8 | NEUROD1 | NIN | NIPBL | NMD3 | NOD2 | NOS2 | NPLOC4 | NPM1 | NRXN1 | NUBP1 | NUDT19 | NUP107 | NUP153 | NUP214 | NUP85 | NUP88 | NUP93 | NUP98 | NUTF2 | NXT2 | OBSL1 | OPTN | OXA1L | PACS1 | PACS2 | PAFAH1B1 | PAM16 | PARD3 | PARK7 | PCM1 | PDCD6 | PDCD6IP | PDIA4 | PEX19 | PEX5 | PHB2 | PICK1 | PIGU | PIH1D1 | PIK3R1 | PIKFYVE | PINX1 | PLEKHF1 | PLEKHF2 | PLK1 | PML | PNP | POM121 | POT1 | PPARG | PPID | PPT1 | PRKCZ | PRPF19 | PSEN1 | PTPN23 | PTPRK | RAB10 | RAB11A | RAB11B | RAB11FIP2 | RAB18 | RAB1A | RAB1B | RAB21 | RAB24 | RAB29 | RAB2A | RAB31 | RAB32 | RAB35 | RAB3GAP1 | RAB3IP | RAB5A | RAB5B | RAB7A | RAB9A | RABGEF1 | RABL2A | RABL3 | RAD21 | RAF1 | RAN | RANBP3 | RAP1A | RAPGEF4 | RASSF9 | RB1 | RBPJ | RDX | RIC3 | ROMO1 | RP2 | RPA2 | RPAIN | RPL10 | RPL10A | RPL12 | RPL18A | RPL22 | RPL23 | RPL23A | RPL24 | RPL30 | RPL37A | RPL38 | RPL6 | RPL7 | RPL7A | RPL9 | RPLP0 | RPLP1 | RPLP2 | RPS12 | RPS13 | RPS14 | RPS15A | RPS19 | RPS2 | RPS20 | RPS24 | RPS29 | RPS3 | RPS4X | RPS8 | RRS1 | RTKN | RTN4 | RUVBL2 | S100A12 | S100A13 | SCFD1 | SCG2 | SCG3 | SDCBP | SEC13 | SEC16A | SEC22C | SEC24B | SEC24C | SEC31A | SEC62 | SEC63 | SEL1L | SELENBP1 | SFPQ | SGTA | SGTB | SIN3A | SLC15A1 | SLC25A6 | SLC9A3R1 | SMAD2 | SMO | SMURF1 | SNAP29 | SNF8 | SNX12 | SNX17 | SNX2 | SNX21 | SNX24 | SNX3 | SNX6 | SPDL1 | SPTBN1 | SQSTM1 | SRP14 | SRP19 | SSB | SSX2IP | STAT3 | STIL | STX5 | STXBP1 | STXBP2 | STYX | SUMO1 | SYNJ2BP | SYVN1 | TANGO2 | TBC1D15 | TBC1D22B | TBC1D4 | TCIRG1 | TCP1 | TERF2 | TERF2IP | TGFB1 | TIA1 | TIMM13 | TIMM21 | TIMM23 | TIMM44 | TIMM50 | TINF2 | TM9SF4 | TMED9 | TMEM30A | TNF | TNFAIP3 | TNFRSF1A | TNKS | TNKS2 | TNPO1 | TOLLIP | TOMM20 | TOMM22 | TOMM40 | TOR1A | TP53 | TPR | TRAF3IP1 | TRAPPC8 | TREX1 | TRIM23 | TSG101 | TSPAN17 | TULP3 | TXNIP | UBB | UBC | UBE2D1 | UBE2D2 | UBE2D3 | UBL4A | UGT8 | ULK1 | UNC119 | USP4 | USP9X | VAPA | VCL | VCP | VGF | VPS13B | VPS18 | VPS26A | VPS29 | VPS37D | VPS4B | VPS8 | VTA1 | WBP2 | WDR19 | WDR35 | WDR45B | WIPI1 | WIPI2 | WNK4 | XBP1 | XPO1 | XPO4 | XPO5 | XPO7 | XRCC5 | YKT6 | YWHAB | YWHAE | YWHAG | YWHAH | ZBTB16 | ZBTB7A | ZBTB7B | ZDHHC17 | ZDHHC23 | ZFAND6 | ZW10 | GO:0045727 | 0.0105544098022565 | 87/8243 | 44/3250 | 0.0221405070017641 | 1 | F | F | F | F | positive regulation of translation | C1QBP | CDK4 | CIRBP | COA3 | DDX39B | DDX3X | DHX36 | DHX9 | EEF2 | EIF2AK4 | EIF2B5 | EIF3D | EIF3E | EIF4A3 | EIF5A | EIF6 | ELAVL1 | ERBB2 | FXR2 | HNRNPD | HNRNPL | KHDRBS1 | LARP4 | MAPK1 | MAPK3 | NCK1 | NPM1 | PABPC1 | PAIP1 | POLDIP3 | RBM3 | RHOA | RMND1 | RPS4X | RPS6KB2 | RXRA | THBS1 | TNF | TRUB2 | UPF3B | UQCC1 | USP16 | VIM | YTHDF1 | GO:0051962 | 0.0365158316147034 | 301/8243 | 136/3250 | 0.022151935313527 | 1 | F | F | F | F | positive regulation of nervous system development | ABL2 | ACTR2 | ADCYAP1 | AGER | APBB1 | APOE | APP | ARF1 | ARHGAP35 | ARHGDIA | ASCL1 | ATOH1 | AVIL | BCL2 | BCL6 | BIN1 | BRAF | C1QA | CAMK1D | CAMK2B | CDC20 | CDKL3 | CLSTN1 | CRKL | CSNK1E | CTNNB1 | CUL7 | DAB1 | DBN1 | DHX36 | DISC1 | DLG4 | DNM1L | DVL1 | DVL2 | DVL3 | E2F1 | EP300 | EPHA4 | EZH2 | FGFR1 | FKBP1B | FN1 | FOXA1 | FYN | GDI1 | GFAP | GOLGA4 | GRID2 | GRIP1 | HAP1 | HDAC1 | HDAC2 | HIF1A | HSPA5 | ID2 | ILK | IQGAP1 | KDM1A | LIG4 | LPAR1 | LRRC7 | MAP1B | MAP2K1 | MAP3K13 | MAPK6 | MAPT | MECP2 | METRN | MME | NAP1L1 | NCK1 | NCOA1 | NEUROD1 | NFE2L2 | NIN | NIPBL | NME1 | NME2 | NRXN1 | NTRK1 | OBSL1 | OPA1 | P2RX4 | PAFAH1B1 | PARD3 | PARP6 | PLXNA1 | PLXNA2 | PLXNB2 | PPARG | PPP1CC | PRKD1 | PROX1 | PRPF19 | PSEN1 | PTEN | PTN | PTPRD | RAB11A | RAB21 | RAP1A | RARA | RASSF10 | RELA | REST | RGS2 | RHOA | S100A9 | S100B | SEMA7A | SERPINE2 | SH3GL3 | SMO | SMURF1 | SNW1 | SNX3 | SOCS2 | SPEN | STK11 | STK25 | TCF12 | TCF4 | TGFB1 | THBS2 | TIMP2 | TMEM30A | TNF | TNFRSF12A | TP73 | TRIM32 | TUBB2B | TWF1 | TWF2 | XRCC5 | ZMYND8 | GO:0007584 | 0.016620162562174 | 137/8243 | 66/3250 | 0.0222082703531645 | 1 | F | F | F | F | response to nutrient | ACSL1 | ACSL4 | ADA | ADSL | AGL | AHCY | AIFM1 | APOA1 | ASCL1 | ASS1 | BCKDHB | BCL11A | CAT | CD40 | CREB1 | CYP1A1 | DAD1 | EEF2 | EGFR | FKBP1B | FOLR1 | FYN | GNAI2 | GSTP1 | HLCS | HMGCL | HMOX1 | HSF1 | HSP90B1 | KYNU | LDHA | MDM2 | MGMT | NCOA1 | NNT | NOD2 | NQO1 | OGT | OPA1 | OTC | OXCT1 | P2RX1 | P2RX2 | P2RX4 | PCNA | PDK2 | PKM | PPARD | PPARG | PTEN | PTN | RARA | RELA | SELL | SETDB1 | SOD1 | STAT1 | TBXA2R | TGFB1 | TMBIM6 | TNKS | TPCN2 | TRIM25 | TYMS | VCAM1 | XBP1 | GO:0090066 | 0.0302074487443892 | 249/8243 | 114/3250 | 0.0223791135692186 | 1 | F | F | F | F | regulation of anatomical structure size | ACE2 | ACTA2 | ACTN2 | ACTR2 | ACTR3 | ADCY6 | ADCYAP1 | ADD3 | ADORA2A | ADRB2 | AKT1 | AKT3 | ALOX12 | ANXA7 | APOE | ARF1 | ARHGAP35 | ARPC1A | ARPC1B | ARPC2 | ARPC3 | ARPC4 | ARPC5 | ATG5 | ATP13A2 | AVIL | BAG4 | BAIAP2 | BAIAP2L1 | BDKRB2 | BIN1 | CAPG | CAPZA1 | CAPZA2 | CAPZB | CAV1 | CCL21 | CDC42EP3 | CDC42EP4 | CDK4 | CFL1 | CFL2 | CHRM3 | CLNS1A | CREB1 | CRP | CTTN | DBH | DOCK5 | DSTN | E2F4 | EGFR | EZR | FCHSD1 | FGB | GPX1 | GRB2 | HAX1 | HMOX1 | HNF1A | HTR1B | ICAM1 | ITGB1BP1 | KDM1A | LARS | LATS1 | LIMA1 | LRRK2 | MSN | MTPN | MYO1C | NCK1 | NOS3 | NPR1 | P2RX1 | PER2 | PEX11B | PFN1 | PICK1 | PIK3C2A | PLA2G6 | PPARD | PPP1CA | PRKCD | PRKCE | PTEN | RAB11A | RAB5A | RAC1 | RAP1GDS1 | RARG | RB1CC1 | RDX | RHOA | RPTOR | SLC12A2 | SLC9A3R1 | SNF8 | SOD2 | SPTAN1 | SPTBN1 | SPTBN2 | SWAP70 | TBXA2R | TMOD3 | TMOD4 | TWF1 | TWF2 | VASP | VAV1 | WAS | WDR1 | WDR35 | WDTC1 | GO:0051292 | 0.00109183549678515 | 9/8243 | 7/3250 | 0.0229170558909863 | 1 | F | F | F | F | nuclear pore complex assembly | NUP107 | NUP153 | NUP205 | NUP93 | NUP98 | RTN4 | TPR | GO:1902306 | 0.00109183549678515 | 9/8243 | 7/3250 | 0.0229170558909863 | 1 | F | F | F | F | negative regulation of sodium ion transmembrane transport | COMMD1 | HECW1 | HECW2 | NEDD4 | PCSK9 | PRKCE | SLC9A3R1 | GO:0060315 | 0.00109183549678515 | 9/8243 | 7/3250 | 0.0229170558909863 | 1 | F | F | F | F | negative regulation of ryanodine-sensitive calcium-release channel activity | CALM1 | CALM2 | CALM3 | FKBP1A | FKBP1B | GSTO1 | SRI | GO:1900112 | 0.00109183549678515 | 9/8243 | 7/3250 | 0.0229170558909863 | 1 | F | F | F | F | regulation of histone H3-K9 trimethylation | ATRX | KDM4D | LMNA | MECP2 | PIH1D1 | SIRT1 | SMARCB1 | GO:0007143 | 0.00109183549678515 | 9/8243 | 7/3250 | 0.0229170558909863 | 1 | F | F | F | F | female meiotic nuclear division | CDC25B | HSF1 | PPP2R1A | PRKAR1A | TOP2A | TRIP13 | UBB | GO:0001702 | 0.00109183549678515 | 9/8243 | 7/3250 | 0.0229170558909863 | 1 | F | F | F | F | gastrulation with mouth forming second | CHRD | CTNNB1 | LRP6 | RNF2 | SMAD4 | UGDH | ZBTB17 | GO:0060742 | 0.00109183549678515 | 9/8243 | 7/3250 | 0.0229170558909863 | 1 | F | F | F | F | epithelial cell differentiation involved in prostate gland development | AR | CTNNB1 | FOXA1 | PSAP | RXRA | TP63 | WDR77 | GO:0034214 | 0.00109183549678515 | 9/8243 | 7/3250 | 0.0229170558909863 | 1 | F | F | F | F | protein hexamerization | LETM1 | MAT2A | OAT | PANX3 | UGDH | VCP | YME1L1 | GO:0033539 | 0.00109183549678515 | 9/8243 | 7/3250 | 0.0229170558909863 | 1 | F | F | F | F | fatty acid beta-oxidation using acyl-CoA dehydrogenase | ACADS | ACADVL | ETFA | ETFB | ETFDH | GCDH | IVD | GO:1904996 | 0.00109183549678515 | 9/8243 | 7/3250 | 0.0229170558909863 | 1 | F | F | F | F | positive regulation of leukocyte adhesion to vascular endothelial cell | ETS1 | ICAM1 | ITGA4 | RELA | RHOA | TNF | TRAF6 | GO:1902065 | 0.00109183549678515 | 9/8243 | 7/3250 | 0.0229170558909863 | 1 | F | F | F | F | response to L-glutamate | AIFM1 | BCL11A | CREB1 | FYN | OPA1 | PCNA | TMBIM6 | GO:1904181 | 0.00109183549678515 | 9/8243 | 7/3250 | 0.0229170558909863 | 1 | F | F | F | F | positive regulation of membrane depolarization | ALOX12 | ANK3 | DCN | KDR | MLLT11 | MYOC | PARP1 | GO:0010839 | 0.00109183549678515 | 9/8243 | 7/3250 | 0.0229170558909863 | 1 | F | F | F | F | negative regulation of keratinocyte proliferation | CD109 | EPPK1 | MED1 | PTPRK | SFN | SNAI2 | VDR | GO:0060966 | 0.00921994419507461 | 76/8243 | 39/3250 | 0.0230213289505694 | 1 | F | F | F | F | regulation of gene silencing by RNA | AGO1 | AGO2 | DHX9 | EGFR | ELAVL1 | ESR1 | H3F3A | HIST1H3A | HIST1H4A | HIST2H3A | IPO8 | MAP2K1 | NCOR1 | NCOR2 | NUP107 | NUP153 | NUP160 | NUP205 | NUP214 | NUP85 | NUP88 | NUP93 | NUP98 | POLR2A | POLR2E | POLR2L | POM121 | POU5F1 | PPARG | PUM1 | RAE1 | RIPK1 | SEC13 | STAT3 | TGFB1 | TP53 | TPR | XPO5 | ZMPSTE24 | GO:0060147 | 0.00921994419507461 | 76/8243 | 39/3250 | 0.0230213289505694 | 1 | F | F | F | F | regulation of posttranscriptional gene silencing | AGO1 | AGO2 | DHX9 | EGFR | ELAVL1 | ESR1 | H3F3A | HIST1H3A | HIST1H4A | HIST2H3A | IPO8 | MAP2K1 | NCOR1 | NCOR2 | NUP107 | NUP153 | NUP160 | NUP205 | NUP214 | NUP85 | NUP88 | NUP93 | NUP98 | POLR2A | POLR2E | POLR2L | POM121 | POU5F1 | PPARG | PUM1 | RAE1 | RIPK1 | SEC13 | STAT3 | TGFB1 | TP53 | TPR | XPO5 | ZMPSTE24 | GO:1902106 | 0.00655101298071091 | 54/8243 | 29/3250 | 0.0230512924160239 | 1 | F | F | F | F | negative regulation of leukocyte differentiation | ANXA1 | APCS | BCL6 | CBFB | CD74 | CLDN18 | CTNNB1 | DTX1 | ERBB2 | FBXO7 | FOXJ1 | HLX | HMGB1 | ID2 | IFNA2 | IRF1 | JAK3 | MYC | NME1 | NME2 | PIAS3 | PIK3R1 | PRDX2 | RARA | RC3H1 | RUNX1 | SOCS1 | TLR3 | ZBTB7B | GO:0012501 | 0.0604148974887784 | 498/8243 | 218/3250 | 0.0231251376181322 | 1 | F | F | F | F | programmed cell death | ADORA2A | AIFM1 | AIMP2 | AKT1 | AKT2 | AKTIP | APBB1 | API5 | APLP1 | APP | ARRB1 | ATCAY | ATG5 | AXIN1 | BAG1 | BAG6 | BAX | BBC3 | BCL10 | BCL2 | BCL2L1 | BCL2L13 | BIRC2 | BIRC3 | BIRC5 | BIRC7 | BIRC8 | BNIPL | BRCA1 | C1QBP | CAPN1 | CAPNS1 | CASP10 | CASP2 | CASP3 | CASP7 | CASP8 | CASP9 | CD2 | CDK5 | CRKL | CRYAB | CSE1L | CSNK2A2 | CTSC | CTSL | CTSV | CUL3 | CYCS | CYLD | DAB2 | DAD1 | DAPK1 | DAXX | DHCR24 | DHX9 | DIABLO | DNAJA3 | DNM1L | DPEP1 | DPF2 | DUSP22 | DYNLL1 | E2F1 | E2F2 | EAF2 | EGLN3 | EIF5A | EP300 | ERBB3 | EVA1A | FADD | FAF1 | FASLG | FIS1 | FKBP8 | FOXO1 | GADD45G | GAPDH | GHITM | GNB1 | GRIK2 | GRK5 | GZMA | HSPA5 | HTATIP2 | HTT | IFNA2 | IKBKG | IRF1 | ITCH | ITGB3BP | JUP | KANK2 | KMT2A | KRT17 | KRT31 | KRT40 | KRT71 | LEF1 | LGALS1 | LGALS7 | LIG4 | LTBR | MAP2K4 | MAP2K6 | MAP2K7 | MAPK1 | MAPK14 | MAPK3 | MCM2 | MELK | MYC | MYD88 | NAE1 | NFKBIA | NGB | NR3C1 | NTRK1 | OGT | OPA1 | P2RX1 | PACS2 | PAK2 | PAK6 | PARP1 | PAWR | PCSK9 | PDCD6IP | PGAM5 | PIK3CA | PIK3CG | PIM1 | PKM | PKN2 | PLEKHF1 | PLSCR1 | PPARD | PPID | PPIF | PPP2CA | PPP2R1A | PPP2R1B | PRDX3 | PRDX5 | PRELID1 | PRKCD | PRKCE | PRKD1 | PRKDC | PSEN1 | PSMD10 | PSME3 | PTEN | PTPN6 | PTRH2 | PYGL | RAD21 | RAF1 | RASSF7 | RB1 | RFK | RHBDD1 | RIPK1 | RIPK2 | RIPK3 | RNF183 | RPS3 | RPS6KA1 | RTKN | RTN4 | S100A9 | SEPT4 | SH3KBP1 | SIAH1 | SLC25A6 | SLTM | SNCA | SPATA2 | SPI1 | SPRR2A | SQSTM1 | STK11 | STK26 | STK3 | TCTN3 | TFDP1 | THOC6 | TIA1 | TIAL1 | TLR3 | TMEM173 | TMEM219 | TNF | TNFAIP3 | TNFSF12 | TNFSF14 | TNIP2 | TOP1 | TP53 | TP63 | TRADD | TRAF1 | TRAF2 | TRAF3 | TRIAP1 | TRIM35 | TRIM69 | UBE2D3 | USP17L2 | USP53 | VDAC1 | XAF1 | XIAP | ZBTB16 | ZFAND6 | ZMPSTE24 | ZNF443 | GO:0010770 | 0.0124954506854301 | 103/8243 | 51/3250 | 0.0232530872864949 | 1 | F | F | F | F | positive regulation of cell morphogenesis involved in differentiation | ABL1 | ACTR2 | APOA1 | ARHGDIA | ARHGEF7 | ARPC2 | BRAF | C1QBP | CALR | CAMK2B | CDC42 | CDKL3 | CRK | CRKL | CUL7 | DBN1 | DHX36 | DNM1L | DOCK5 | EPHA4 | FGB | FN1 | GDI1 | GOLGA4 | ILK | LIMS1 | MAP1B | MAP2K1 | MAP3K13 | MAPT | METRN | MYOC | NIN | OBSL1 | OPA1 | PAFAH1B1 | PARP6 | PLXNA1 | PLXNA2 | PLXNB2 | PTPRD | RAB11A | RAB21 | RAC1 | RHOA | SEMA7A | STK11 | STK25 | TNFRSF12A | TUBB2B | TWF2 | GO:0006342 | 0.00473128715273565 | 39/8243 | 22/3250 | 0.0232977155831039 | 1 | F | F | F | F | chromatin silencing | BMI1 | CTBP1 | EED | EZH2 | H1F0 | H3F3A | HDAC1 | HDAC5 | HIST1H3A | HIST1H4A | HIST2H3A | HMGB1 | MBD3 | MECP2 | MORF4L1 | MORF4L2 | PCGF1 | PPM1D | RIF1 | SIRT1 | SIRT5 | TNP1 | GO:0032768 | 0.00473128715273565 | 39/8243 | 22/3250 | 0.0232977155831039 | 1 | F | F | F | F | regulation of monooxygenase activity | AKT1 | APOE | CALM1 | CALM3 | CAV1 | EGFR | GLA | HIF1A | HSP90AA1 | KRAS | LYPLA1 | NOS1AP | NOS3 | NOSIP | NOSTRIN | PARK7 | S100A1 | SNCA | SPR | TERF2 | TNF | VDR | GO:0042770 | 0.0103117796918598 | 85/8243 | 43/3250 | 0.0234029606643412 | 1 | F | F | F | F | signal transduction in response to DNA damage | ABL1 | ATAD5 | ATM | ATRX | BABAM1 | BAX | BRCA1 | CARM1 | CASP2 | CASP9 | CCNB1 | CDC14B | CDC25C | CDC5L | CDK2 | CDKN1A | CHEK2 | E2F1 | E2F4 | EP300 | FOXM1 | FZR1 | GRB2 | KAT5 | MAPK14 | MDM2 | MDM4 | MIF | MYO6 | NDRG1 | PAXIP1 | PCNA | PLK1 | PML | PPM1D | PRKDC | PRPF19 | SFN | SP100 | TFDP1 | TP53 | TREX1 | TRIAP1 | GO:0030334 | 0.057503336164018 | 474/8243 | 208/3250 | 0.0234182557645156 | 1 | F | F | F | F | regulation of cell migration | ABL1 | ACTN4 | ADA | ADTRP | AGER | AGO2 | AIRE | AKT1 | AKT2 | AKT3 | ALOX12 | ANXA1 | APC | APEX1 | APOD | APOE | APP | ARHGEF7 | ATM | BAG4 | BCAR1 | BCL2 | BMPR1A | BRAF | C1QBP | CALR | CAMK1D | CAMK2A | CAMK2B | CAV1 | CCL21 | CCR1 | CD40 | CD74 | CDH1 | CDK5 | CHRD | CLIC4 | COL18A1 | CPNE3 | CREB3 | CRK | CRKL | CSNK2B | CXCL8 | DAB2 | DCN | DDRGK1 | DOCK5 | DOCK7 | DPEP1 | DUSP1 | DUSP22 | EGFR | EPPK1 | ETS1 | FADD | FAM83H | FGF2 | FGFR1 | FGFR1OP | FN1 | GLUL | GNA12 | GNA13 | GNAI2 | GPI | GRB7 | GSTP1 | HBEGF | HDAC4 | HDAC5 | HDAC6 | HDAC7 | HDAC9 | HIF1A | HMGB1 | HMOX1 | HRAS | HSPA5 | HSPB1 | ICAM1 | IDH2 | IGF1R | ILK | INSR | IQGAP1 | ITGA4 | ITGAV | ITGB1BP1 | ITGB3 | JUN | JUP | KDR | LAMB1 | LDLRAD4 | LEF1 | LGALS3 | LGMN | LMNA | LPAR1 | MAP2K3 | MAP3K3 | MAPK1 | MAPK14 | MAPK15 | MAPK3 | MCC | MDM2 | MECP2 | MEOX2 | MIF | MITF | MSN | MYO1C | MYOC | NCK1 | NF2 | NFE2L2 | NIPBL | NKX2-1 | NOS3 | NRP2 | NTF3 | ONECUT1 | OXSR1 | P2RX4 | PDCD10 | PDCD6 | PFN1 | PIK3C2A | PIK3R1 | PIK3R3 | PLCG1 | PLXNA1 | PLXNA2 | PLXNB2 | PPARD | PPARG | PPARGC1A | PRCP | PRKCE | PRKD1 | PRKD2 | PROX1 | PTEN | PTK2 | PTN | PTPN23 | PTPRK | PTPRR | RAB11A | RAC1 | RDX | RECK | RHOA | RHOJ | RIPK3 | RTN4 | S100A7 | SDC4 | SDCBP | SEMA7A | SEPT7 | SERPINB3 | SERPINE2 | SIRT1 | SLAMF8 | SLC9A3R1 | SMAD3 | SMO | SMURF2 | SNAI2 | SOD2 | SP1 | SP100 | SPRY2 | SRC | SRGAP2 | SRGAP3 | STARD13 | STAT3 | STAT5A | STK24 | STK26 | STRAP | SUN2 | SWAP70 | SYDE1 | SYNE2 | SYNJ2BP | TBXA2R | TGFB1 | THBS1 | TIMP1 | TNF | TNFSF14 | TNN | TPM1 | TRADD | TRIM32 | TRIP6 | USP17L2 | VCL | VTN | WNK1 | XBP1 | ZMYND8 | GO:0051591 | 0.00788547858789276 | 65/8243 | 34/3250 | 0.0234334140364982 | 1 | F | F | F | F | response to cAMP | AKAP9 | APEX1 | APP | ASS1 | BIRC2 | BRAF | BSG | CARM1 | CFTR | DUSP1 | EZR | FOS | GPD1 | HCN1 | HCN2 | HCN3 | HSPA5 | ITPR2 | JUN | JUNB | JUND | KDM1A | LDHA | NME1 | PEBP1 | PER1 | PIK3CG | RAP1A | RAP1B | RELA | SDC1 | STAT1 | VGF | WT1 | GO:0007610 | 0.0331190100691496 | 273/8243 | 124/3250 | 0.0234342857876265 | 1 | F | F | F | F | behavior | ABAT | ABL2 | ACE2 | ACTR2 | ADCYAP1 | ADORA2A | AGER | AHCY | AMFR | ANXA7 | APOE | APP | APRT | ARF4 | ARRB2 | ASL | ATAD1 | ATP6V1B1 | ATXN1 | BCL2 | BRAF | BTBD9 | C12orf10 | C1QL1 | CAPN2 | CASP3 | CDK5 | CHD8 | COMT | CPT1A | CREB1 | DAB1 | DBH | DCTN1 | DLG4 | DMRT3 | DNAJC9 | DVL1 | EGFR | EIF2AK4 | EIF4A3 | EIF4E | EP300 | EPHA4 | FADD | FEN1 | FOS | FYN | GAA | GLP1R | GLRB | GLS | GNAO1 | GPI | GRIA1 | GRIK2 | HDAC2 | HEXB | HIF1A | HOXD10 | HPRT1 | HTR1B | HTRA2 | HTT | ID2 | INSR | ITPR3 | JUN | KCNAB1 | KMT2B | KRAS | LGMN | LRRK2 | MAPK1 | MAPT | MCOLN3 | MECP2 | MME | MTA1 | MTNR1A | NCOA1 | NCOA2 | NKX2-1 | NQO2 | NRXN1 | NTF3 | NTRK1 | P2RX2 | P2RX4 | PAFAH1B1 | PARK7 | PCM1 | PIAS1 | PLA2G6 | PPT1 | PRKAA1 | PRKAR2B | PRKCE | PRKCZ | PSAP | PSEN1 | PTEN | PTN | PUM1 | S100B | SEPT5 | SERPINE2 | SLC16A1 | SNCA | SOD1 | SPTBN2 | STAT3 | STRBP | THBS1 | THRA | TP53 | UBA6 | UCHL1 | USP2 | USP46 | VDAC1 | VDAC3 | ZIC1 | ZMPSTE24 | GO:0072331 | 0.0114036151886449 | 94/8243 | 47/3250 | 0.0234521915674815 | 1 | F | F | F | F | signal transduction by p53 class mediator | ATAD5 | ATM | ATRX | BAG6 | BAX | BRCA1 | CARM1 | CASP2 | CCNB1 | CDC25C | CDK2 | CDKN1A | CHEK2 | DDIT4 | DYRK2 | E2F1 | E2F2 | E2F4 | EP300 | FOXM1 | HINT1 | IFI16 | KAT5 | MDM2 | MDM4 | MIF | MYBBP1A | MYO6 | NDRG1 | NUPR1 | PAXIP1 | PCNA | PDK2 | PML | PPM1D | SFN | SIRT1 | SNW1 | SP100 | STK11 | TFDP1 | TP53 | TP63 | TP73 | TRIAP1 | USP28 | WWOX | GO:0071453 | 0.0114036151886449 | 94/8243 | 47/3250 | 0.0234521915674815 | 1 | F | F | F | F | cellular response to oxygen levels | ACAA2 | AIFM1 | AKT1 | ATP6V1A | BCL2 | CAV1 | CBL | CCNB1 | DNM1L | E2F1 | EIF4EBP1 | ENDOG | FABP1 | FOXO1 | GNB1 | HIF1A | HMOX1 | ICAM1 | IREB2 | LMNA | LPAR1 | MDM2 | MDM4 | MYC | NAMPT | NDRG1 | NPEPPS | OPA1 | P4HB | PGK1 | PHB2 | PICK1 | PPARD | PPARG | PPARGC1A | PRKAA1 | PRKCE | PTEN | PTN | S100B | SIRT1 | SLC9A1 | SRC | STUB1 | TP53 | TWIST1 | UBQLN1 | GO:0046907 | 0.120829794977557 | 996/8243 | 422/3250 | 0.0234767242343438 | 1 | F | F | F | F | intracellular transport | ABCD3 | ABCE1 | ABCG1 | ACAA1 | ACACA | ACD | ACOX1 | ACTN4 | ACTR1A | ACTR2 | ADPRH | ADRB2 | AGK | AHCYL1 | AKT1 | AKT2 | AKTIP | ANK1 | ANK3 | ANP32A | AP1B1 | AP1G1 | AP1M1 | AP3D1 | AP3M1 | AP4M1 | AP4S1 | APOE | APP | APPBP2 | ARF1 | ARF4 | ARF5 | ARFGAP1 | ARFIP1 | ARHGAP1 | ARL4D | ARL5B | ASPSCR1 | ATG4C | ATL3 | ATP2A2 | ATXN1 | AUP1 | BACH2 | BAG3 | BANF1 | BIN1 | BTK | CACNG2 | CACNG4 | CACNG5 | CALR | CAPZA1 | CAPZA2 | CAPZB | CAT | CBL | CBLB | CCDC22 | CCDC88A | CCHCR1 | CD74 | CDC37 | CDC42 | CDK5 | CHMP2A | CHP1 | CLSTN1 | CLTC | CLU | COG3 | COL4A3BP | COPA | COPB1 | COPE | COPG1 | CPLX1 | CPLX3 | CPT1A | CPT2 | CROT | CSE1L | CTNNB1 | CTSA | CTSC | CTTN | CUL3 | CYC1 | DCLK1 | DCTN1 | DDX19B | DDX39A | DDX39B | DENND1A | DERL1 | DHRS4 | DLG2 | DNAJC19 | DNM1L | DOCK7 | DUSP16 | DYNC1H1 | DYNC1I1 | DYNC1I2 | DYNC1LI1 | DYNLL1 | DYNLT1 | ECH1 | ECI2 | EHD4 | EHHADH | EIF4A3 | EIF4E | EIF5A | EIF6 | F5 | F9 | FABP3 | FAF2 | FBXO7 | FIS1 | FOLR1 | FYTTD1 | GAK | GEMIN4 | GEMIN5 | GEMIN7 | GGA1 | GOLGA2 | GOPC | GRIA1 | GRIP1 | GRPEL1 | GSTK1 | HAP1 | HDAC6 | HEATR3 | HERC2 | HGS | HID1 | HIF1A | HMGCL | HNRNPA1 | HNRNPA2B1 | HNRNPU | HSD17B4 | HSP90AA1 | HSP90B1 | HSPA4 | HSPA5 | HSPA8 | HSPB1 | HSPD1 | HTATIP2 | HTT | ICK | IDE | IDH1 | IFIT1 | IFT122 | IPO11 | IPO4 | IPO5 | IPO7 | IPO8 | IPO9 | KIF13B | KIF5B | KIF5C | KLHL20 | KPNA1 | KPNA2 | KPNB1 | LARS | LAT | LMAN2L | LMNA | LRP6 | LRPPRC | LRRC7 | MAP1B | MAPK8IP3 | MAPT | MCFD2 | MCM3AP | MPPE1 | MYO1C | MYO1D | MYO5A | MYO6 | NCBP1 | NCOA4 | NDUFA13 | NEDD4 | NEUROD1 | NMD3 | NOS2 | NPLOC4 | NPM1 | NUDT19 | NUP107 | NUP153 | NUP160 | NUP205 | NUP214 | NUP85 | NUP88 | NUP93 | NUP98 | NUTF2 | NXF1 | NXF2 | NXT2 | OPA1 | OXA1L | PAFAH1B1 | PAM16 | PARD3 | PCM1 | PDCD6 | PEF1 | PEX19 | PEX5 | PHB2 | PICK1 | PIK3CG | PIK3R1 | PIKFYVE | PML | POLDIP3 | POM121 | PQLC1 | PRKAA2 | PRKAB2 | PRKAG1 | PRKCZ | PROZ | PTK2 | PTPN23 | RAB10 | RAB11A | RAB11B | RAB18 | RAB1A | RAB1B | RAB21 | RAB24 | RAB29 | RAB2A | RAB31 | RAB32 | RAB35 | RAB3IP | RAB5A | RAB5B | RAB7A | RAB9A | RABGEF1 | RABL2A | RABL3 | RAE1 | RAN | RANBP3 | RASSF9 | RBM8A | RHOT1 | RHOT2 | RNF126 | ROMO1 | RPAIN | RPL10 | RPL10A | RPL12 | RPL18A | RPL22 | RPL23 | RPL23A | RPL24 | RPL30 | RPL37A | RPL38 | RPL6 | RPL7 | RPL7A | RPL9 | RPLP0 | RPLP1 | RPLP2 | RPS12 | RPS13 | RPS14 | RPS15A | RPS19 | RPS2 | RPS20 | RPS24 | RPS29 | RPS3 | RPS4X | RPS8 | RRS1 | S100A13 | SCYL2 | SDCBP | SDCBP2 | SEC13 | SEC16A | SEC22C | SEC24B | SEC24C | SEC31A | SEC62 | SEC63 | SEL1L | SFPQ | SGTA | SGTB | SLC25A6 | SLC9A3R1 | SMG5 | SMG6 | SMN1 | SMURF1 | SNCA | SNF8 | SNRPB | SNRPD1 | SNRPD3 | SNRPE | SNX17 | SNX2 | SNX6 | SOD1 | SORT1 | SPTAN1 | SPTBN1 | SPTBN2 | SQSTM1 | SRP14 | SRP19 | SRSF11 | SRSF5 | SSB | SSX2IP | STAT3 | STBD1 | STOML2 | STX5 | STYX | SUN2 | SYNE2 | SYNJ2BP | SYVN1 | TBC1D15 | TBC1D22B | TBC1D23 | TBC1D4 | TERF2 | TFG | TGFB1 | THOC2 | THOC6 | THOC7 | TIMM13 | TIMM21 | TIMM23 | TIMM44 | TIMM50 | TMED9 | TNPO1 | TOMM20 | TOMM22 | TOMM40 | TOR1A | TPR | TRAF3IP1 | TRAPPC8 | TRIM23 | TRIM9 | TSG101 | TUBA1A | TUBA1C | TUBB | TXNIP | U2AF2 | UBB | UBC | UBE2D1 | UBE2D2 | UBE2D3 | UBE2O | UCHL1 | UPF2 | UPF3B | USP9X | VAPA | VAPB | VCP | VPS18 | VPS26A | VPS29 | VPS37D | VPS4B | VTA1 | WAS | WDR19 | WDR35 | XPO1 | XPO4 | XPO5 | XPO7 | XPOT | YKT6 | YWHAB | YWHAE | YWHAG | YWHAH | ZDHHC23 | ZFAND6 | ZMPSTE24 | ZW10 | GO:0043409 | 0.0144364915686037 | 119/8243 | 58/3250 | 0.0236013391083844 | 1 | F | F | F | F | negative regulation of MAPK cascade | ABL1 | AKT1 | APOE | ARRB1 | C1QL4 | CAV1 | CBLC | CHRNA9 | CYLD | DNAJA1 | DUSP1 | DUSP14 | DUSP16 | DUSP19 | DUSP22 | DUSP7 | EIF3A | ERRFI1 | EZR | FOXM1 | FOXO1 | GPS2 | GSTP1 | HDAC3 | IGBP1 | IGF1R | ITCH | ITGB1BP1 | LEMD2 | MYC | NCOR1 | NF2 | PAFAH1B1 | PEBP1 | PER1 | PHB | PIN1 | PPP2CA | PPP2R1A | PRKCD | PSMD10 | PTEN | PTPN1 | PTPN22 | PTPN6 | PTPRR | QARS | RANBP9 | RGS2 | SERPINB3 | SIRT3 | SLC9A3R1 | SMAD4 | SPRY2 | STK38 | SYNJ2BP | TAOK3 | UCHL1 | GO:0061024 | 0.0531359941768774 | 438/8243 | 193/3250 | 0.0237341857782759 | 1 | F | F | F | F | membrane organization | ACAA2 | ACTB | ACTG1 | ACTR2 | ACTR3 | ADRB2 | AFG3L2 | AGK | AKT1 | AKT2 | ANK3 | ANXA2 | ANXA6 | ANXA7 | AP3D1 | ARF1 | ARPC1A | ARPC2 | ARPC3 | ARPC4 | ARPC5 | ARRB1 | ARRB2 | ATF2 | ATP2A2 | ATP9A | AURKB | BAIAP2 | BAIAP2L1 | BANF1 | BAX | BBC3 | BCL2 | BCL2L1 | BIN1 | CACNG2 | CACNG4 | CACNG5 | CALM1 | CALM3 | CALR | CAMK2A | CASP8 | CAV1 | CBL | CCDC88A | CCNB1 | CCR5 | CD2 | CD36 | CDC42 | CEL | CEP55 | CFTR | CHCHD10 | CHCHD3 | CHMP2A | CHP1 | CHRM2 | CLSTN1 | CLTC | CLU | COL4A3BP | CTTN | DAB2 | DCTN1 | DNM1L | DVL2 | E2F1 | EGFR | EYA2 | FCGR1A | FCHSD1 | FLOT1 | GAK | GAPVD1 | GNAI3 | GOLPH3 | GRAMD1A | GRB2 | GRIP1 | GSK3A | GSK3B | HBEGF | HGS | HK2 | HSP90AA1 | HSPA4 | HSPA8 | IGHV3-13 | IGHV3-23 | IGHV3-30 | IGLC7 | IMMT | KCNIP2 | KIF5C | LEMD2 | LEMD3 | LETM1 | LMNA | LRRC7 | MAPK8 | MBP | MTSS1 | MYOC | NDRG1 | NDUFA13 | NRXN1 | NSFL1C | NUP93 | OPA1 | OXA1L | PACSIN3 | PAFAH1B1 | PDCD6IP | PICK1 | PIK3C2A | PLK1 | PLSCR1 | PMP2 | PPIA | PPIF | PPP2CA | PPP2R1A | PPP2R2A | PPP3CC | PPT1 | PQLC1 | PRELID1 | PRKCD | PSAP | PTEN | PTPRD | RAB11A | RAB5A | RAB7A | RHOA | RHOT1 | RHOT2 | ROMO1 | RTN4 | SAMD9 | SCLT1 | SEC13 | SEC31A | SFN | SH3GL2 | SH3KBP1 | SLC25A5 | SLC35F6 | SLC9A1 | SMURF1 | SNAP29 | SNCA | SNX3 | SOD1 | SPACA3 | SPTBN1 | STAT3 | STX5 | SUN2 | SYP | TARDBP | TAZ | TFDP1 | TGOLN2 | THBS1 | THEM4 | TIMM13 | TIMM50 | TMEM11 | TMEM30A | TOMM22 | TOR1A | TP53 | TP63 | TP73 | TRIAP1 | UBB | UBC | VAPA | VAPB | VCPIP1 | VPS4B | VPS8 | YKT6 | YWHAB | YWHAE | YWHAG | YWHAH | YWHAQ | YWHAZ | ZMPSTE24 | GO:0009052 | 0.0004852602207934 | 4/8243 | 4/3250 | 0.0241382813850766 | 1 | F | F | F | F | pentose-phosphate shunt, non-oxidative branch | RPE | RPIA | TALDO1 | TKT | GO:1904885 | 0.0004852602207934 | 4/8243 | 4/3250 | 0.0241382813850766 | 1 | F | F | F | F | beta-catenin destruction complex assembly | APC | AXIN1 | CSNK1A1 | GSK3B | GO:0009092 | 0.0004852602207934 | 4/8243 | 4/3250 | 0.0241382813850766 | 1 | F | F | F | F | homoserine metabolic process | CBS | CTH | MPST | MTHFD1 | GO:1902576 | 0.0004852602207934 | 4/8243 | 4/3250 | 0.0241382813850766 | 1 | F | F | F | F | negative regulation of nuclear cell cycle DNA replication | BCL6 | CHEK2 | TERF1 | TERF2 | GO:0043096 | 0.0004852602207934 | 4/8243 | 4/3250 | 0.0241382813850766 | 1 | F | F | F | F | purine nucleobase salvage | ADA | APRT | HPRT1 | PRTFDC1 | GO:0032364 | 0.0004852602207934 | 4/8243 | 4/3250 | 0.0241382813850766 | 1 | F | F | F | F | oxygen homeostasis | EGLN1 | HIF1A | NNT | SOD2 | GO:0090186 | 0.0004852602207934 | 4/8243 | 4/3250 | 0.0241382813850766 | 1 | F | F | F | F | regulation of pancreatic juice secretion | NR1H2 | NR1H3 | WNK1 | WNK4 | GO:0090261 | 0.0004852602207934 | 4/8243 | 4/3250 | 0.0241382813850766 | 1 | F | F | F | F | positive regulation of inclusion body assembly | CDC34 | CLU | HSF1 | PSMC5 | GO:0017014 | 0.0004852602207934 | 4/8243 | 4/3250 | 0.0241382813850766 | 1 | F | F | F | F | protein nitrosylation | ADH5 | GAPDH | NOS2 | S100A9 | GO:2000172 | 0.0004852602207934 | 4/8243 | 4/3250 | 0.0241382813850766 | 1 | F | F | F | F | regulation of branching morphogenesis of a nerve | BCL11A | LRRK2 | MAP3K13 | RTN4 | GO:0018119 | 0.0004852602207934 | 4/8243 | 4/3250 | 0.0241382813850766 | 1 | F | F | F | F | peptidyl-cysteine S-nitrosylation | ADH5 | GAPDH | NOS2 | S100A9 | GO:0002905 | 0.0004852602207934 | 4/8243 | 4/3250 | 0.0241382813850766 | 1 | F | F | F | F | regulation of mature B cell apoptotic process | ADA | BCL10 | CD74 | MIF | GO:1903553 | 0.0004852602207934 | 4/8243 | 4/3250 | 0.0241382813850766 | 1 | F | F | F | F | positive regulation of extracellular exosome assembly | PDCD6IP | SDC1 | SDC4 | SDCBP | GO:0006167 | 0.0004852602207934 | 4/8243 | 4/3250 | 0.0241382813850766 | 1 | F | F | F | F | AMP biosynthetic process | ADK | ADSL | ADSS | APRT | GO:0034349 | 0.0004852602207934 | 4/8243 | 4/3250 | 0.0241382813850766 | 1 | F | F | F | F | glial cell apoptotic process | CASP3 | CASP9 | RB1 | TP53 | GO:0045625 | 0.0004852602207934 | 4/8243 | 4/3250 | 0.0241382813850766 | 1 | F | F | F | F | regulation of T-helper 1 cell differentiation | ANXA1 | HLX | JAK3 | RIPK2 | GO:0035900 | 0.0004852602207934 | 4/8243 | 4/3250 | 0.0241382813850766 | 1 | F | F | F | F | response to isolation stress | ACO2 | HRAS | KRAS | LGALS1 | GO:0060789 | 0.0004852602207934 | 4/8243 | 4/3250 | 0.0241382813850766 | 1 | F | F | F | F | hair follicle placode formation | CTNNB1 | EDA | HDAC1 | HDAC2 | GO:0072716 | 0.0004852602207934 | 4/8243 | 4/3250 | 0.0241382813850766 | 1 | F | F | F | F | response to actinomycin D | KAT7 | MDM2 | RPL23 | TP53 | GO:0038033 | 0.0004852602207934 | 4/8243 | 4/3250 | 0.0241382813850766 | 1 | F | F | F | F | positive regulation of endothelial cell chemotaxis by VEGF-activated vascular endothelial growth factor receptor signaling pathway | HSPB1 | KDR | PRKD1 | PRKD2 | GO:0006574 | 0.0004852602207934 | 4/8243 | 4/3250 | 0.0241382813850766 | 1 | F | F | F | F | valine catabolic process | ACAD8 | ALDH6A1 | HIBADH | HIBCH | GO:0002069 | 0.0004852602207934 | 4/8243 | 4/3250 | 0.0241382813850766 | 1 | F | F | F | F | columnar/cuboidal epithelial cell maturation | CDKN1A | HIF1A | HOXA5 | TYMS | GO:1905908 | 0.0004852602207934 | 4/8243 | 4/3250 | 0.0241382813850766 | 1 | F | F | F | F | positive regulation of amyloid fibril formation | APOE | APP | CLU | PSEN1 | GO:0002906 | 0.0004852602207934 | 4/8243 | 4/3250 | 0.0241382813850766 | 1 | F | F | F | F | negative regulation of mature B cell apoptotic process | ADA | BCL10 | CD74 | MIF | GO:0090114 | 0.0004852602207934 | 4/8243 | 4/3250 | 0.0241382813850766 | 1 | F | F | F | F | COPII-coated vesicle budding | SEC13 | SEC31A | VAPA | VAPB | GO:1903626 | 0.0004852602207934 | 4/8243 | 4/3250 | 0.0241382813850766 | 1 | F | F | F | F | positive regulation of DNA catabolic process | ATM | BAX | ENDOG | HSF1 | GO:0006531 | 0.0004852602207934 | 4/8243 | 4/3250 | 0.0241382813850766 | 1 | F | F | F | F | aspartate metabolic process | ADSS | ASS1 | GOT1 | GOT2 | GO:1901995 | 0.0004852602207934 | 4/8243 | 4/3250 | 0.0241382813850766 | 1 | F | F | F | F | positive regulation of meiotic cell cycle phase transition | CDC25A | CDC25B | CDC25C | MAPK15 | GO:0019346 | 0.0004852602207934 | 4/8243 | 4/3250 | 0.0241382813850766 | 1 | F | F | F | F | transsulfuration | CBS | CTH | MPST | MTHFD1 | GO:0030043 | 0.0004852602207934 | 4/8243 | 4/3250 | 0.0241382813850766 | 1 | F | F | F | F | actin filament fragmentation | CFL1 | CFL2 | DSTN | WDR1 | GO:1901165 | 0.0004852602207934 | 4/8243 | 4/3250 | 0.0241382813850766 | 1 | F | F | F | F | positive regulation of trophoblast cell migration | AGO2 | C1QBP | SMURF2 | SYDE1 | GO:0032077 | 0.0004852602207934 | 4/8243 | 4/3250 | 0.0241382813850766 | 1 | F | F | F | F | positive regulation of deoxyribonuclease activity | AKT1 | PCNA | PRKCD | RPS3 | GO:0051097 | 0.0004852602207934 | 4/8243 | 4/3250 | 0.0241382813850766 | 1 | F | F | F | F | negative regulation of helicase activity | IFIT1 | MCM2 | SIRT1 | TP53 | GO:0099576 | 0.0004852602207934 | 4/8243 | 4/3250 | 0.0241382813850766 | 1 | F | F | F | F | regulation of protein catabolic process at postsynapse, modulating synaptic transmission | CDH1 | EGLN1 | RNF19A | USP8 | GO:0030035 | 0.0004852602207934 | 4/8243 | 4/3250 | 0.0241382813850766 | 1 | F | F | F | F | microspike assembly | ABL1 | ACTN2 | FSCN1 | MTSS1 | GO:0098943 | 0.0004852602207934 | 4/8243 | 4/3250 | 0.0241382813850766 | 1 | F | F | F | F | neurotransmitter receptor transport, postsynaptic endosome to lysosome | AP3D1 | CACNG2 | CACNG4 | CACNG5 | GO:0032909 | 0.0004852602207934 | 4/8243 | 4/3250 | 0.0241382813850766 | 1 | F | F | F | F | regulation of transforming growth factor beta2 production | ATF2 | HIF1A | SMAD3 | SMAD4 | GO:0090168 | 0.0004852602207934 | 4/8243 | 4/3250 | 0.0241382813850766 | 1 | F | F | F | F | Golgi reassembly | PDCD10 | STK25 | VCPIP1 | YWHAZ | GO:0060574 | 0.0004852602207934 | 4/8243 | 4/3250 | 0.0241382813850766 | 1 | F | F | F | F | intestinal epithelial cell maturation | CDKN1A | HIF1A | HOXA5 | TYMS | GO:0034627 | 0.0004852602207934 | 4/8243 | 4/3250 | 0.0241382813850766 | 1 | F | F | F | F | 'de novo' NAD biosynthetic process | HAAO | KYNU | NMNAT1 | NMNAT3 | GO:1903463 | 0.0004852602207934 | 4/8243 | 4/3250 | 0.0241382813850766 | 1 | F | F | F | F | regulation of mitotic cell cycle DNA replication | BCL6 | CHEK2 | FGFR1 | ZMPSTE24 | GO:0031848 | 0.0004852602207934 | 4/8243 | 4/3250 | 0.0241382813850766 | 1 | F | F | F | F | protection from non-homologous end joining at telomere | ACD | TERF2 | TERF2IP | TFIP11 | GO:0006101 | 0.0004852602207934 | 4/8243 | 4/3250 | 0.0241382813850766 | 1 | F | F | F | F | citrate metabolic process | ACLY | ACO1 | ACO2 | IREB2 | GO:2000152 | 0.0004852602207934 | 4/8243 | 4/3250 | 0.0241382813850766 | 1 | F | F | F | F | regulation of ubiquitin-specific protease activity | PARK7 | TANK | UBXN1 | VCP | GO:0006108 | 0.0004852602207934 | 4/8243 | 4/3250 | 0.0241382813850766 | 1 | F | F | F | F | malate metabolic process | FH | MDH1 | MDH2 | ME1 | GO:0097167 | 0.0004852602207934 | 4/8243 | 4/3250 | 0.0241382813850766 | 1 | F | F | F | F | circadian regulation of translation | HNRNPD | PER1 | PER2 | RBM4 | GO:0019322 | 0.0004852602207934 | 4/8243 | 4/3250 | 0.0241382813850766 | 1 | F | F | F | F | pentose biosynthetic process | G6PD | PGD | TALDO1 | TKT | GO:0009403 | 0.0004852602207934 | 4/8243 | 4/3250 | 0.0241382813850766 | 1 | F | F | F | F | toxin biosynthetic process | CBS | CTH | CYP1A2 | MPST | GO:0070316 | 0.00448865704233895 | 37/8243 | 21/3250 | 0.0242924391866334 | 1 | F | F | F | F | regulation of G0 to G1 transition | BMI1 | BRCA1 | E2F1 | E2F6 | EED | EHMT1 | EHMT2 | EZH2 | L3MBTL2 | MED1 | MGA | PCGF6 | PHC3 | RAD51 | RBBP4 | RING1 | RNF2 | RRM2 | SUZ12 | TFDP1 | YAF2 | GO:0050434 | 0.00448865704233895 | 37/8243 | 21/3250 | 0.0242924391866334 | 1 | F | F | F | F | positive regulation of viral transcription | CCNT1 | CDK9 | CTDP1 | DHX9 | EP300 | GTF2F1 | GTF2F2 | JUN | LEF1 | NELFB | NELFCD | NELFE | POLR2A | POLR2E | POLR2L | SMARCA4 | SMARCB1 | SNW1 | SP1 | SUPT4H1 | SUPT5H | GO:0090199 | 0.00448865704233895 | 37/8243 | 21/3250 | 0.0242924391866334 | 1 | F | F | F | F | regulation of release of cytochrome c from mitochondria | AKT1 | ARRB2 | BAX | BBC3 | BCL2L1 | CHCHD10 | CLU | DNM1L | GPX1 | LMNA | MLLT11 | OPA1 | PAM16 | PLA2G6 | PLAUR | PPIF | PRELID1 | PSMD10 | TP53 | TRIAP1 | WDR35 | GO:0018208 | 0.00448865704233895 | 37/8243 | 21/3250 | 0.0242924391866334 | 1 | F | F | F | F | peptidyl-proline modification | EGLN1 | EGLN3 | FKBP14 | FKBP1A | FKBP1B | FKBP2 | FKBP5 | FKBP7 | FKBP8 | NTMT1 | OGFOD1 | P4HA1 | P4HA2 | P4HB | PIN1 | PPIA | PPIB | PPID | PPIE | PPIF | PPIL3 | GO:0050732 | 0.00448865704233895 | 37/8243 | 21/3250 | 0.0242924391866334 | 1 | F | F | F | F | negative regulation of peptidyl-tyrosine phosphorylation | CAV1 | CBL | CBLB | CBLC | DUSP22 | ERRFI1 | FGFR1OP | IBTK | LRRK1 | NF2 | NTF3 | PARP14 | PPP2CA | PPP2R1A | PRKCD | PRKCZ | PSEN1 | PTPN6 | SNX6 | SOCS1 | TSG101 | GO:0060964 | 0.00897731408467791 | 74/8243 | 38/3250 | 0.0243292192445738 | 1 | F | F | F | F | regulation of gene silencing by miRNA | AGO1 | AGO2 | DHX9 | EGFR | ELAVL1 | ESR1 | H3F3A | HIST1H3A | HIST1H4A | HIST2H3A | IPO8 | MAP2K1 | NCOR1 | NCOR2 | NUP107 | NUP153 | NUP160 | NUP205 | NUP214 | NUP85 | NUP88 | NUP93 | NUP98 | POLR2A | POLR2E | POLR2L | POM121 | POU5F1 | PPARG | PUM1 | RAE1 | RIPK1 | SEC13 | STAT3 | TGFB1 | TP53 | TPR | ZMPSTE24 | GO:1902105 | 0.0189251486109426 | 156/8243 | 74/3250 | 0.0243833497497621 | 1 | F | F | F | F | regulation of leukocyte differentiation | ABL1 | ADA | AGER | ANXA1 | AP3D1 | APCS | BCL6 | BRAF | BTK | CASP8 | CBFB | CCR1 | CD2 | CD27 | CD74 | CLDN18 | CREB1 | CTNNB1 | CTNNBIP1 | CYLD | DROSHA | DTX1 | ERBB2 | ESRRA | FADD | FANCA | FBXO7 | FOS | FOXJ1 | GNAS | HAX1 | HLX | HMGB1 | ID2 | IFNA2 | IRF1 | IRF4 | IRF7 | JAK3 | JUN | LEF1 | LGALS1 | LGALS3 | MITF | MYC | NFATC2 | NME1 | NME2 | PIAS3 | PIK3R1 | PNP | PRDX2 | PRELID1 | PRKCZ | PTPN6 | RARA | RB1 | RC3H1 | RHOA | RIPK1 | RIPK2 | RUNX1 | SART1 | SLAMF8 | SOCS1 | SOD1 | TGFB1 | TLR3 | TNF | TRAF6 | XBP1 | ZAP70 | ZBTB16 | ZBTB7B | GO:0035821 | 0.0122528205750334 | 101/8243 | 50/3250 | 0.0245740811929232 | 1 | F | F | F | F | modification of morphology or physiology of other organism | ANXA2 | APCS | APOE | AZU1 | BCL2L1 | CALM1 | CALM2 | CALM3 | CASP8 | CCNT1 | CD209 | CFL1 | CTDP1 | EIF2AK4 | EP300 | GAPDH | HDAC1 | INSR | ITGAV | JUN | KPNA1 | KPNA2 | KPNB1 | LEF1 | PC | POU2F3 | PPIB | PSMC3 | RAB9A | REST | ROMO1 | RPL30 | RPS19 | RXRA | S100A12 | S100A9 | SLC25A6 | SMAD3 | SMARCA4 | SMARCB1 | SMC3 | SNW1 | SP1 | STOM | SUGT1 | TARDBP | TGFB1 | TYMS | VAPA | VAPB | GO:0001649 | 0.00764284847749606 | 63/8243 | 33/3250 | 0.024739769129541 | 1 | F | F | F | F | osteoblast differentiation | AKT1 | CAT | CBFB | CHRD | CLTC | COL6A1 | DHX9 | FASN | FHL2 | FIGNL1 | HNRNPU | HSD17B4 | HSPE1 | IARS | JUNB | LEF1 | LGR4 | MYBBP1A | MYOC | PHB | PSMC2 | RBMX | RDH14 | RUNX2 | SEMA7A | SMO | SNAI2 | SND1 | SOX2 | TPM4 | TWIST1 | VCAN | WWOX | GO:0051656 | 0.0223219701564964 | 184/8243 | 86/3250 | 0.0247574488665251 | 1 | F | F | F | F | establishment of organelle localization | ABCE1 | ACTN4 | ACTR2 | ACTR3 | AP3D1 | AP3M1 | CBL | CCDC155 | CCNB1 | CDC42 | CDK5 | CENPF | CEP55 | CHMP2A | COPG1 | CPLX1 | CPLX3 | CROCC | CTNNB1 | CUL3 | DCTN1 | DOCK7 | DYNC1H1 | DYNC1I1 | DYNLT1 | EIF6 | EZR | FAM83D | HAP1 | HIF1A | HTT | IKBKG | ITGA4 | KIF2C | KIF5B | KIF5C | KPNB1 | LAT | LRPPRC | MAP1B | MAPT | MLH1 | MYO1C | MYO5A | NMD3 | NPM1 | NSFL1C | NUMA1 | NUP88 | NUSAP1 | OPA1 | PAFAH1B1 | PARD3 | PCM1 | PDCD10 | PDCD6IP | PIK3CG | PINX1 | PRKCZ | PTK2 | RAB11A | RAB11B | RAB1A | RAN | RHOT1 | RHOT2 | RRS1 | S100A13 | SLC9A3R1 | SNCA | SPDL1 | SPICE1 | SPRY2 | STK25 | SUN2 | SYNE2 | TERF1 | TOR1A | TRIM9 | UBB | UBE2B | UCHL1 | VPS4B | XPO1 | YWHAZ | ZW10 | GO:0051592 | 0.0111609850782482 | 92/8243 | 46/3250 | 0.0247913989875534 | 1 | F | F | F | F | response to calcium ion | ACTG1 | AHCYL1 | ALOX5AP | ANXA11 | ANXA5 | ANXA7 | BRAF | CACNG2 | CALM1 | CALM2 | CALM3 | CAV1 | CLIC4 | CPNE3 | DPEP1 | DUSP1 | EGFR | ENDOG | FGB | FOS | FUS | GDI1 | HNRNPD | HSPA5 | IQGAP1 | ITPR3 | JUN | JUNB | KCNIP2 | LGMN | NEDD4 | PDCD6 | PEBP1 | PEF1 | PPIF | PRKAA1 | PRKAA2 | PTGES | SDC1 | SEC31A | SLC25A13 | SLC25A24 | STIM1 | THBS1 | TXNIP | WNK1 | GO:0018193 | 0.0690282664078612 | 569/8243 | 247/3250 | 0.0248189033945857 | 1 | F | F | F | F | peptidyl-amino acid modification | ABL1 | ABL2 | ACTL6A | ADH5 | AKT1 | AKT2 | AKT3 | APBB1 | APOA1 | ARID4B | ARRB1 | ASGR1 | ASGR2 | ATF2 | ATM | ATP13A2 | AURKB | BAG6 | BCL11A | BCL2 | BTK | CAB39 | CAMK2A | CARM1 | CBX4 | CCNB1 | CDK2 | CDK5 | CDK5R1 | CHEK2 | CLK1 | CREBBP | CSNK1A1 | CSNK1E | CSNK2A2 | CSNK2B | CTH | DAD1 | DCLK1 | DCN | DDOST | DMAP1 | DPF2 | DPM1 | DYRK2 | EED | EEF2KMT | EFEMP1 | EGFR | EGLN1 | EGLN3 | EHMT1 | EHMT2 | EIF2AK3 | EP300 | EPHA4 | EPHA6 | EPHB6 | ERBB2 | ERBB3 | EZH2 | FBXO11 | FGFR1 | FGFR3 | FKBP14 | FKBP1A | FKBP1B | FKBP2 | FKBP5 | FKBP7 | FKBP8 | FOLH1 | FYN | GAPDH | GLRX5 | GSK3A | GSK3B | HDAC4 | HDAC6 | HDAC9 | HIF1AN | HIPK4 | HMBS | IGF1R | IKBKB | IKBKE | ILK | ING5 | INSR | IRF4 | ITK | JAK1 | JAK3 | JMJD6 | KAT5 | KAT7 | KAT8 | KDR | KMT2A | KMT2B | KMT2C | LATS1 | LEF1 | LOXL2 | LRRK2 | MAP2K1 | MAP2K3 | MAP2K4 | MAP2K6 | MAP2K7 | MAP3K13 | MAP4K1 | MAPK1 | MAPK14 | MAPK3 | MAPK7 | MAPK8 | MAPK9 | MAST1 | MAST3 | MATK | MBD3 | MCFD2 | MDM2 | MECP2 | MELK | METAP2 | MKNK2 | MORF4L1 | MORF4L2 | MRGBP | NCOA1 | NCOA3 | NDUFAF5 | NDUFAF7 | NEK1 | NLK | NME2 | NOS2 | NTMT1 | NTRK1 | NUP107 | NUP153 | NUP160 | NUP205 | NUP214 | NUP85 | NUP88 | NUP93 | NUP98 | OGFOD1 | OGT | OXSR1 | P4HA1 | P4HA2 | P4HB | PADI4 | PAK2 | PARK7 | PARP1 | PAXIP1 | PDF | PER1 | PHF10 | PIAS1 | PIAS3 | PIN1 | PKN2 | PLK1 | PLOD2 | POLE3 | POM121 | PPIA | PPIB | PPID | PPIE | PPIF | PPIL3 | PRDX3 | PRKAA1 | PRKAA2 | PRKACA | PRKCD | PRKCE | PRKCZ | PRKD1 | PRKD2 | PRKDC | PRMT2 | PRMT3 | PRMT6 | PTK2 | PTPN6 | RAE1 | RANGAP1 | RIPK1 | RIPK2 | RPN1 | RPN2 | RTF1 | RUVBL1 | RUVBL2 | S100A9 | SAE1 | SENP3 | SENP6 | SENP7 | SETDB1 | SIRT1 | SIRT3 | SIRT5 | SPI1 | SRC | STK38 | SUMO1 | SUMO1P1 | SUMO2 | SUMO3 | SUZ12 | SYVN1 | TADA2B | TAF12 | TESK2 | TGM2 | TNK2 | TNKS | TOP1 | TPR | TPST2 | TRIM28 | TTLL1 | TWF1 | UBA2 | UBE2I | UBE2J1 | UGGT1 | ULK1 | USPL1 | VCP | VKORC1 | WDR82 | WNK1 | YEATS2 | YES1 | ZAP70 | ZDHHC23 | ZMPSTE24 | GO:0009755 | 0.014193861458207 | 117/8243 | 57/3250 | 0.0249230957635236 | 1 | F | F | F | F | hormone-mediated signaling pathway | ACSL1 | AR | BRCA1 | CALR | CARM1 | CSNK2B | CTNNB1 | DAXX | DNAJA1 | ESR1 | ESR2 | ESRRA | ESRRB | ESRRG | FHL2 | GRIP1 | HNF4A | HNF4G | KAT5 | LATS1 | LEF1 | LGR4 | MED1 | NCOA1 | NCOA3 | NCOA4 | NEDD4 | NR0B2 | NR1H2 | NR1H3 | NR1I2 | NR3C1 | NR4A1 | NR5A2 | PHB | PIAS1 | PPARD | PPARG | PPARGC1A | RAN | RARA | RARG | RB1 | RHOA | RNF4 | RXRA | RXRB | RXRG | SRC | THRA | TRIP4 | UBE3A | UBR5 | VDR | WBP2 | YAP1 | YWHAH | GO:0070613 | 0.00424602693194225 | 35/8243 | 20/3250 | 0.0252925624334984 | 1 | F | F | F | F | regulation of protein processing | ANXA2 | BAG2 | BIRC2 | BIRC3 | BIRC7 | BIRC8 | ENO1 | LRRK2 | MDM2 | PLGRKT | PRKACA | RHBDD1 | RNF139 | SERPINE2 | SNX12 | SRC | THBS1 | TNP1 | USP17L2 | XIAP | GO:0043487 | 0.0178333131141575 | 147/8243 | 70/3250 | 0.0254453322538037 | 1 | F | F | F | F | regulation of RNA stability | AKT1 | ANP32A | APEX1 | CIRBP | DCP2 | DHX36 | DHX9 | DIS3 | E2F1 | ELAVL1 | EXOSC1 | EXOSC5 | EXOSC6 | EXOSC9 | FTO | FXR2 | HNRNPD | HNRNPM | HNRNPU | HSPA8 | HSPB1 | IGF2BP1 | MAPK14 | MOV10 | MYD88 | NPM1 | NUP214 | PABPC1 | PABPC4 | PAIP1 | PDE12 | PRKCD | PSMA1 | PSMA2 | PSMA3 | PSMA5 | PSMA6 | PSMB5 | PSMB6 | PSMB7 | PSMB8 | PSMC1 | PSMC2 | PSMC3 | PSMC4 | PSMC5 | PSMC6 | PSMD1 | PSMD10 | PSMD11 | PSMD12 | PSMD14 | PSMD2 | PSMD4 | PSMD9 | PSME2 | PSME3 | PUM1 | RC3H1 | SET | SMG6 | TARDBP | TBRG4 | TNPO1 | UBB | UBC | VIM | XPO1 | YWHAB | YWHAZ | GO:0040013 | 0.0220793400460997 | 182/8243 | 85/3250 | 0.0260348040987362 | 1 | F | F | F | F | negative regulation of locomotion | ADA | ADORA2A | ADTRP | AKT1 | APEX1 | APOD | APOE | BCL2 | BMPR1A | BRAF | CALR | CCL21 | CDH1 | CHRD | CLIC4 | CRK | CSNK2B | DCN | DPEP1 | DUSP1 | DUSP22 | EPPIN | EPPK1 | FGF2 | GSTP1 | HDAC5 | HMGB1 | HMOX1 | IDH2 | ILK | ITGB1BP1 | JUP | LDLRAD4 | MAPK15 | MCC | MECP2 | MEOX2 | MIF | MITF | NF2 | NFE2L2 | NKX2-1 | PDCD10 | PIN1 | PML | PPARD | PPARG | PPARGC1A | PTEN | PTN | PTPN23 | PTPRK | PTPRO | PTPRR | RECK | RGN | RHOA | SEMA7A | SEPT7 | SLAMF8 | SLC9A3R1 | SP100 | SPINT2 | SRGAP2 | SRGAP3 | STARD13 | STAT3 | STK24 | STK26 | STRAP | SYNJ2BP | TBXA2R | TGFB1 | THBS1 | TIMP1 | TNN | TPM1 | TRIM21 | TRIM25 | TRIM28 | TRIM32 | TRIM35 | VCL | WAS | ZMYND8 | GO:0030168 | 0.00740021836709936 | 61/8243 | 32/3250 | 0.026120502476792 | 1 | F | F | F | F | platelet activation | ACTN1 | ARRB1 | ARRB2 | CD40 | CD40LG | DGKE | DGKZ | FYN | GNA12 | GNA13 | GNA15 | GNAQ | GNB1 | ITGB3 | ITPR1 | ITPR2 | ITPR3 | LCP2 | MAPK1 | MAPK3 | P2RX1 | PIK3CA | PIK3CG | PIK3R1 | PLSCR1 | PRKCD | PRKCE | PTPN6 | RAF1 | SRC | VAV1 | YWHAZ | GO:1901992 | 0.00740021836709936 | 61/8243 | 32/3250 | 0.026120502476792 | 1 | F | F | F | F | positive regulation of mitotic cell cycle phase transition | AKT1 | ANAPC4 | ANXA1 | APEX1 | APP | BRD4 | CCNB1 | CDC25A | CDC25B | CDC25C | CDK4 | CUL3 | CUL4B | CYP1A1 | DBF4B | DDX3X | EGFR | HSPA2 | MDM2 | MECP2 | MEPCE | MTA3 | PHB2 | RAB11A | RB1 | RDX | RPTOR | SIN3A | TGFB1 | TMOD3 | UBE2C | VPS4B | GO:0042177 | 0.0109183549678515 | 90/8243 | 45/3250 | 0.0262107619393001 | 1 | F | F | F | F | negative regulation of protein catabolic process | AGAP2 | ANXA2 | ATP13A2 | BAG5 | BAG6 | BANP | CCAR2 | CSNK2A2 | DDRGK1 | EGFR | EPHA4 | FAM192A | FYN | HSP90AB1 | HSP90AB3P | HSP90AB4P | LAMP3 | MAD2L1 | MDM4 | NELL1 | NOS2 | OGT | PARK7 | PHB | PIN1 | PML | PSEN1 | RELA | RPL23 | SDCBP | SERPINE2 | SGTA | SMAD3 | SMARCC1 | SNX12 | SNX3 | STYX | TIMP1 | TIMP2 | UBE2J1 | UBXN1 | UCHL5 | USP14 | USP19 | USP7 | GO:0050999 | 0.00400339682154555 | 33/8243 | 19/3250 | 0.0262877397755693 | 1 | F | F | F | F | regulation of nitric-oxide synthase activity | AKT1 | APOE | CALM1 | CALM3 | CAV1 | EGFR | GLA | HIF1A | HSP90AA1 | KRAS | LYPLA1 | NOS1AP | NOS3 | NOSIP | NOSTRIN | S100A1 | SPR | TERF2 | TNF | GO:0051926 | 0.00400339682154555 | 33/8243 | 19/3250 | 0.0262877397755693 | 1 | F | F | F | F | negative regulation of calcium ion transport | BCL2 | BIN1 | CALM1 | CALM2 | CALM3 | EPPIN | FKBP1A | FKBP1B | GNAO1 | GSTO1 | ICAM1 | NOS3 | PACSIN3 | SRI | TGFB1 | THADA | TMBIM6 | UBQLN1 | YWHAE | GO:0010466 | 0.0167414776173723 | 138/8243 | 66/3250 | 0.0264956481683345 | 1 | F | F | F | F | negative regulation of peptidase activity | A2M | ADORA2A | AHSG | AKT1 | APP | ARRB1 | ARRB2 | BIN1 | BIRC2 | BIRC3 | BIRC5 | BIRC7 | BIRC8 | C4A | CD109 | CD27 | CRYAB | DDX3X | DHCR24 | DPEP1 | EPPIN | FABP1 | GAPDH | GPI | GPX1 | IFI16 | IGBP1 | LAMP3 | LEF1 | MDM2 | PAK2 | PAPLN | PARK7 | PEBP1 | PIH1D1 | PLAUR | PRDX3 | PRDX5 | PTTG1 | RAF1 | RECK | RPS6KA1 | SERPINB2 | SERPINB3 | SERPINB5 | SERPINB6 | SERPINB8 | SERPINE2 | SERPINH1 | SFN | SNCA | SPINK7 | SPINT2 | SRC | THBS1 | TIMP1 | TIMP2 | TNFSF14 | TRIAP1 | UBXN1 | UCHL5 | USP14 | USP47 | VTN | XIAP | YWHAE | GO:0060396 | 0.00133446560718185 | 11/8243 | 8/3250 | 0.0266202524458589 | 1 | F | F | F | F | growth hormone receptor signaling pathway | JAK3 | PIK3R1 | PTK2 | PTPN1 | PXN | SOCS2 | STAT3 | STAT5A | GO:0010875 | 0.00133446560718185 | 11/8243 | 8/3250 | 0.0266202524458589 | 1 | F | F | F | F | positive regulation of cholesterol efflux | ABCG1 | APOA1 | APOE | GPS2 | NFKBIA | NR1H2 | NR1H3 | SIRT1 | GO:0001659 | 0.00133446560718185 | 11/8243 | 8/3250 | 0.0266202524458589 | 1 | F | F | F | F | temperature homeostasis | ACADVL | ADRB2 | DBH | FOXO1 | FTO | GPX1 | PPARGC1A | STAT3 | GO:0033688 | 0.00133446560718185 | 11/8243 | 8/3250 | 0.0266202524458589 | 1 | F | F | F | F | regulation of osteoblast proliferation | ABL1 | BCL2 | GATA1 | ITGAV | NELL1 | RHOA | SMAD3 | TNN | GO:0006098 | 0.00133446560718185 | 11/8243 | 8/3250 | 0.0266202524458589 | 1 | F | F | F | F | pentose-phosphate shunt | DERA | G6PD | PGD | PGM2 | RPE | RPIA | TALDO1 | TKT | GO:1901017 | 0.00133446560718185 | 11/8243 | 8/3250 | 0.0266202524458589 | 1 | F | F | F | F | negative regulation of potassium ion transmembrane transporter activity | ACTN2 | ANK3 | CAB39 | CAV1 | KCNAB1 | OXSR1 | PTEN | SUMO1 | GO:0009068 | 0.00133446560718185 | 11/8243 | 8/3250 | 0.0266202524458589 | 1 | F | F | F | F | aspartate family amino acid catabolic process | AASS | ALDH7A1 | DLD | DLST | GCDH | GOT1 | GOT2 | OGDH | GO:1903350 | 0.00133446560718185 | 11/8243 | 8/3250 | 0.0266202524458589 | 1 | F | F | F | F | response to dopamine | ABL1 | HCN3 | HDAC2 | LRRK2 | MAPK1 | MAPK3 | SIN3A | SULT1A3 | GO:0095500 | 0.00133446560718185 | 11/8243 | 8/3250 | 0.0266202524458589 | 1 | F | F | F | F | acetylcholine receptor signaling pathway | CDK5R1 | CHRM2 | CHRM3 | CRKL | GNA15 | GNAI2 | GNAQ | GNB1 | GO:1904816 | 0.00133446560718185 | 11/8243 | 8/3250 | 0.0266202524458589 | 1 | F | F | F | F | positive regulation of protein localization to chromosome, telomeric region | CCT2 | CCT3 | CCT4 | CCT5 | CCT6A | CCT7 | CCT8 | TCP1 | GO:0010745 | 0.00133446560718185 | 11/8243 | 8/3250 | 0.0266202524458589 | 1 | F | F | F | F | negative regulation of macrophage derived foam cell differentiation | ABCG1 | CRP | ITGAV | ITGB3 | NFKBIA | NR1H2 | NR1H3 | PPARG | GO:0044794 | 0.00133446560718185 | 11/8243 | 8/3250 | 0.0266202524458589 | 1 | F | F | F | F | positive regulation by host of viral process | ANXA2 | APOE | CFL1 | PC | PPIB | SMC3 | STOM | VAPA | GO:0043518 | 0.00133446560718185 | 11/8243 | 8/3250 | 0.0266202524458589 | 1 | F | F | F | F | negative regulation of DNA damage response, signal transduction by p53 class mediator | CD74 | KDM1A | MDM2 | MIF | PSMD10 | SIRT1 | SNAI2 | TWIST1 | GO:1903831 | 0.00133446560718185 | 11/8243 | 8/3250 | 0.0266202524458589 | 1 | F | F | F | F | signal transduction involved in cellular response to ammonium ion | CDK5R1 | CHRM2 | CHRM3 | CRKL | GNA15 | GNAI2 | GNAQ | GNB1 | GO:0045838 | 0.00133446560718185 | 11/8243 | 8/3250 | 0.0266202524458589 | 1 | F | F | F | F | positive regulation of membrane potential | AKT1 | AKT2 | ANK3 | GLRX | NNT | PIAS3 | STOML2 | VCP | GO:0045947 | 0.00133446560718185 | 11/8243 | 8/3250 | 0.0266202524458589 | 1 | F | F | F | F | negative regulation of translational initiation | AGO2 | EIF2AK3 | EIF2AK4 | EIF2S1 | EIF3E | EIF4EBP1 | RBM4 | TPR | GO:0048512 | 0.00133446560718185 | 11/8243 | 8/3250 | 0.0266202524458589 | 1 | F | F | F | F | circadian behavior | AHCY | BTBD9 | ID2 | MTA1 | NCOA2 | PTEN | TP53 | USP2 | GO:0060965 | 0.00133446560718185 | 11/8243 | 8/3250 | 0.0266202524458589 | 1 | F | F | F | F | negative regulation of gene silencing by miRNA | ELAVL1 | ESR1 | NCOR1 | NCOR2 | POU5F1 | PPARG | TGFB1 | ZMPSTE24 | GO:1900273 | 0.00133446560718185 | 11/8243 | 8/3250 | 0.0266202524458589 | 1 | F | F | F | F | positive regulation of long-term synaptic potentiation | ADORA2A | APP | CREB1 | EIF2AK4 | LGMN | MME | SQSTM1 | STAU1 | GO:1903351 | 0.00133446560718185 | 11/8243 | 8/3250 | 0.0266202524458589 | 1 | F | F | F | F | cellular response to dopamine | ABL1 | HCN3 | HDAC2 | LRRK2 | MAPK1 | MAPK3 | SIN3A | SULT1A3 | GO:0034401 | 0.00582312264952081 | 48/8243 | 26/3250 | 0.0268799461437695 | 1 | F | F | F | F | chromatin organization involved in regulation of transcription | BMI1 | CHRAC1 | CTBP1 | CTNNB1 | EED | EZH2 | H1F0 | H3F3A | HDAC1 | HDAC5 | HIST1H3A | HIST1H4A | HIST2H3A | HMGB1 | KMT2B | MBD3 | MECP2 | MORF4L1 | MORF4L2 | PCGF1 | POLE3 | PPM1D | RIF1 | SIRT1 | SIRT5 | TNP1 | GO:0032101 | 0.0501031177969186 | 413/8243 | 182/3250 | 0.0273982211454023 | 1 | F | F | F | F | regulation of response to external stimulus | ABAT | ACE2 | ADA | ADCYAP1 | ADORA2A | ADTRP | AGER | AHSG | ALOX12 | ANXA1 | ANXA2 | ANXA5 | APCS | APOA1 | APOD | APOE | APP | ATM | AZU1 | BACE1 | BCL6 | BIRC2 | BIRC3 | BRAF | BRD4 | BTK | C1QA | C1QBP | C1QTNF1 | CALM1 | CALR | CAMK1D | CAV1 | CCL21 | CCR1 | CCR4 | CD109 | CD36 | CD6 | CD74 | CDH5 | CDK19 | CDKN1A | CEBPA | CHD8 | CREB3 | CRK | CXCL6 | CXCL8 | CYLD | DHX9 | DROSHA | DUSP1 | DVL1 | EGFR | EIF2AK4 | EPHA4 | EPPK1 | ESR1 | ETS1 | FABP7 | FAM19A3 | FANCA | FGB | FGF2 | FGFR1 | FKBP1B | GBP5 | GGT1 | GPS2 | GPX1 | GPX4 | GRID2 | GRK1 | GSTP1 | HAVCR2 | HMGB1 | HSPB1 | HTRA1 | IFIT1 | ITCH | KANK2 | KDR | LGMN | LPAR1 | LRRK2 | LRSAM1 | MAP2K1 | MAPK14 | MAPK3 | MAPK7 | MAS1 | MAVS | MED1 | METAP2 | METRNL | MIF | MMP26 | MYD88 | NFE2L2 | NFKBIA | NOD2 | NOS2 | NOS3 | NPLOC4 | NR1H3 | NRXN1 | NTF3 | OPTN | OXSR1 | P2RX4 | PARK7 | PCBP2 | PDE6G | PER1 | PIK3CG | PLA2G2A | PLAUR | PPARD | PPARG | PPEF1 | PPM1B | PRDX2 | PRKCD | PRKD1 | PRKD2 | PSMA1 | PSMA6 | PTEN | PTGER3 | PTPRF | PTPRO | PUM1 | RAC1 | RB1 | RELA | RHO | RIPK2 | RPS19 | S100A12 | S100A7 | S100A9 | SAG | SCG2 | SEMA7A | SERPINB2 | SERPINE2 | SIN3A | SLAMF8 | SMAD3 | SNAI2 | SNCA | SNW1 | SNX6 | SOD1 | SPATA2 | STAT1 | STK24 | SWAP70 | TBC1D23 | TBXA2R | TGFB1 | TGM2 | THBS1 | TLR3 | TMEM173 | TNF | TNFAIP3 | TNFRSF1A | TNFSF14 | TRADD | TRAF3 | TRAF3IP1 | TRAF6 | TREX1 | TUBB2B | USP14 | USP17L2 | VTN | WNK1 | XIAP | ZMPSTE24 | GO:0019693 | 0.01176756035424 | 97/8243 | 48/3250 | 0.0274559655503348 | 1 | F | F | F | F | ribose phosphate metabolic process | ABHD14B | ACACA | ACAT1 | ACLY | ACOT13 | ACOT7 | ACOT9 | ADCYAP1 | ADSS | AK1 | AK2 | AK4 | ATP6V1A | ATP6V1B1 | CLPX | CROT | DBI | DLG2 | DLST | FAR2 | FASN | FIGNL1 | GMPR | GNAI3 | HMGCL | HPRT1 | HSD17B4 | HSPA1B | HSPA8 | LRRK2 | MVD | MYH6 | NDUFS1 | NMNAT1 | NT5C2 | OGDH | OLA1 | OPA1 | PARP1 | RAN | RHOA | RNASEH2B | SUCLA2 | SUCLG2 | SULT1A3 | THEM4 | TPST2 | VCP | GO:0006163 | 0.01176756035424 | 97/8243 | 48/3250 | 0.0274559655503348 | 1 | F | F | F | F | purine nucleotide metabolic process | ABHD14B | ACACA | ACAT1 | ACLY | ACOT13 | ACOT7 | ACOT9 | ADCYAP1 | ADSS | AK1 | AK2 | AK4 | ATP6V1A | ATP6V1B1 | CLPX | CROT | DBI | DGUOK | DLG2 | DLST | FAR2 | FASN | FIGNL1 | GMPR | GNAI3 | HMGCL | HPRT1 | HSD17B4 | HSPA1B | HSPA8 | LRRK2 | MVD | MYH6 | NDUFS1 | NMNAT1 | NT5C2 | OGDH | OLA1 | OPA1 | PARP1 | RAN | RHOA | SUCLA2 | SUCLG2 | SULT1A3 | THEM4 | TPST2 | VCP | GO:0009259 | 0.01176756035424 | 97/8243 | 48/3250 | 0.0274559655503348 | 1 | F | F | F | F | ribonucleotide metabolic process | ABHD14B | ACACA | ACAT1 | ACLY | ACOT13 | ACOT7 | ACOT9 | ADCYAP1 | ADSS | AK1 | AK2 | AK4 | ATP6V1A | ATP6V1B1 | CLPX | CROT | DBI | DLG2 | DLST | FAR2 | FASN | FIGNL1 | GMPR | GNAI3 | HMGCL | HPRT1 | HSD17B4 | HSPA1B | HSPA8 | LRRK2 | MVD | MYH6 | NDUFS1 | NMNAT1 | NT5C2 | OGDH | OLA1 | OPA1 | PARP1 | RAN | RHOA | RNASEH2B | SUCLA2 | SUCLG2 | SULT1A3 | THEM4 | TPST2 | VCP | GO:0051093 | 0.0641756641999272 | 529/8243 | 230/3250 | 0.0275557399631495 | 1 | F | F | F | F | negative regulation of developmental process | ABCG1 | ABL1 | ACTN4 | ADCY6 | ADCYAP1 | ADRB2 | AGO1 | AHSG | AKT3 | ANXA1 | ANXA2 | APCS | APOE | APP | ARHGDIA | ASCL1 | ATF2 | BAG5 | BCL11A | BCL2 | BCL6 | BCOR | BMPR1A | C1QL4 | CALR | CARM1 | CAV1 | CBFB | CCR1 | CD74 | CDC73 | CDK5 | CDK5R1 | CDKL3 | CDKN1A | CHRD | CLDN18 | CMTM5 | COL4A2 | COL5A1 | CRMP1 | CRP | CTDP1 | CTNNB1 | DAB1 | DCN | DDX6 | DGUOK | DNM1L | DPF2 | DTX1 | DYNLT1 | E2F1 | E2F2 | EAF2 | EFEMP1 | EGFR | EIF2AK3 | EIF2AK4 | EIF4E | EIF4ENIF1 | EPHA4 | ERBB2 | ESRRB | EZH2 | FAM129B | FASLG | FBXO7 | FGFR3 | FOXA1 | FOXJ1 | FOXM1 | FOXO1 | FZR1 | G6PD | GAK | GATA1 | GDI1 | GFAP | GPS2 | GSK3A | GSK3B | GTF2I | HDAC1 | HDAC2 | HDAC3 | HDAC4 | HDAC5 | HDAC7 | HGS | HIF1A | HIST1H4A | HLX | HMGB1 | HNRNPU | HOXA5 | HSPA9 | HSPG2 | ID2 | IDH2 | IFNA2 | IFT122 | INSIG1 | IQCB1 | IRF1 | ITGAV | ITGB1BP1 | ITGB3 | JAK3 | KRAS | LDLRAD4 | LEF1 | LGALS1 | LGMN | LOXL2 | LPAR1 | LRRK2 | LSM1 | MAP3K3 | MAPK1 | MAPT | MARCH5 | MDM2 | MECP2 | MED1 | MEOX2 | MIB1 | MIF | MYC | NAMPT | NELFB | NFATC1 | NFATC2 | NFE2L2 | NFKBIA | NKX2-1 | NME1 | NME2 | NMRK2 | NOS3 | NPHP3 | NPR1 | NR1H2 | NR1H3 | PAFAH1B1 | PAX8 | PCM1 | PDCD10 | PGK1 | PHB2 | PIAS3 | PIK3R1 | PML | PPARD | PPARG | PPARGC1A | PPP2CA | PRDX2 | PRKACA | PRKDC | PSEN1 | PTEN | PTK2 | PTN | PTPRO | RAB29 | RARA | RARG | RB1 | RBPJ | RC3H1 | REST | RGN | RGS2 | RHOA | RIPK1 | RTN4 | RUNX1 | RUNX1T1 | S100B | SARS | SEMA7A | SFMBT1 | SIRT1 | SKI | SMAD3 | SMAD4 | SMO | SNAI2 | SOCS1 | SOCS2 | SORT1 | SOX2 | SPRY2 | SRGAP2 | STARD13 | STAT1 | STAT3 | STK11 | STK3 | STRAP | SUZ12 | SYNJ2BP | TBXA2R | TERF2 | TGFB1 | THBS1 | THBS2 | TIMP1 | TLR3 | TNF | TNN | TP63 | TP73 | TRAF3IP1 | TRIB2 | TRIB3 | TULP3 | TWIST1 | USP19 | VAT1 | VIM | WDR77 | WT1 | XBP1 | YAP1 | YWHAH | YY1 | ZBTB16 | ZBTB7B | GO:1903533 | 0.00715758825670266 | 59/8243 | 31/3250 | 0.0275798739452442 | 1 | F | F | F | F | regulation of protein targeting | AKT2 | ANK3 | BAG3 | BAG4 | CDK5 | CDK5R1 | CHP1 | CSNK2A2 | ERBB2 | FIS1 | FYN | GDI1 | GSK3A | HAX1 | HRAS | HSPA1L | HTRA2 | HUWE1 | ITGB1BP1 | LRRK2 | MYO1C | NPEPPS | NUCB1 | PRKAA1 | RHOU | RNF31 | SAE1 | STOM | UBE2D3 | UBE2L3 | USP17L2 | GO:2000241 | 0.00958388936066966 | 79/8243 | 40/3250 | 0.0276628113499139 | 1 | F | F | F | F | regulation of reproductive process | ADA | AGO2 | ANXA5 | AR | C1QBP | CALR | CCDC36 | CDC20 | CDC25A | CDC25B | CDC25C | CTNNB1 | DUSP1 | EAF2 | EPPIN | ESR1 | FZR1 | GDF9 | HDAC2 | HDAC4 | INSR | MAPK15 | NUPR1 | OVGP1 | PPP2R1A | PRDX4 | PRKAR1A | RGN | RHBDD1 | RXRA | SMURF2 | SRC | STK11 | STK3 | SYDE1 | TIMP1 | TRIP13 | UBE2B | WDR77 | WT1 | GO:0010951 | 0.0164988475069756 | 136/8243 | 65/3250 | 0.0279428402212519 | 1 | F | F | F | F | negative regulation of endopeptidase activity | A2M | ADORA2A | AHSG | AKT1 | APP | ARRB1 | ARRB2 | BIN1 | BIRC2 | BIRC3 | BIRC5 | BIRC7 | BIRC8 | C4A | CD109 | CD27 | CRYAB | DDX3X | DHCR24 | DPEP1 | EPPIN | FABP1 | GAPDH | GPI | GPX1 | IFI16 | IGBP1 | LAMP3 | LEF1 | MDM2 | PAK2 | PAPLN | PARK7 | PEBP1 | PIH1D1 | PLAUR | PRDX3 | PRDX5 | PTTG1 | RAF1 | RECK | RPS6KA1 | SERPINB2 | SERPINB3 | SERPINB5 | SERPINB6 | SERPINB8 | SERPINE2 | SERPINH1 | SFN | SNCA | SPINK7 | SPINT2 | SRC | THBS1 | TIMP1 | TIMP2 | TNFSF14 | TRIAP1 | UCHL5 | USP14 | USP47 | VTN | XIAP | YWHAE | GO:0072666 | 0.00351813660075215 | 29/8243 | 17/3250 | 0.0282042255413888 | 1 | F | F | F | F | establishment of protein localization to vacuole | AP3D1 | AP3M1 | AP4M1 | CACNG2 | CACNG4 | CACNG5 | CLU | HGS | HSPA8 | LARS | NCOA4 | NEDD4 | RAB7A | SMURF1 | SNF8 | TNFAIP3 | VPS37D | GO:0030521 | 0.00351813660075215 | 29/8243 | 17/3250 | 0.0282042255413888 | 1 | F | F | F | F | androgen receptor signaling pathway | AR | BRCA1 | CTNNB1 | DAXX | DNAJA1 | FHL2 | GRIP1 | KAT5 | NCOA1 | NCOA3 | NCOA4 | PIAS1 | PPARGC1A | RAN | RB1 | RHOA | RNF4 | GO:0032922 | 0.0055804925391241 | 46/8243 | 25/3250 | 0.028278813290448 | 1 | F | F | F | F | circadian regulation of gene expression | CRY1 | CRY2 | CSNK1E | GFPT1 | HDAC1 | HDAC2 | HNRNPU | ID2 | KMT2A | LGR4 | MTA1 | MYBBP1A | NAMPT | NCOA2 | OGT | PER1 | PER2 | PML | PPARGC1A | PPP1CA | PPP1CB | PPP1CC | SIRT1 | TOP1 | USP2 | GO:0000726 | 0.0055804925391241 | 46/8243 | 25/3250 | 0.028278813290448 | 1 | F | F | F | F | non-recombinational repair | ATM | BABAM1 | BRCA1 | H2AFX | HERC2 | HIST1H4A | HIST3H3 | KAT5 | KDM2A | LIG4 | MDC1 | MLH1 | PAXIP1 | POLM | PRKDC | PRPF19 | PSMD14 | RIF1 | RNF8 | SUMO1 | UBE2N | XRCC1 | XRCC5 | XRCC6 | ZBTB7A | GO:0048708 | 0.00157709571757855 | 13/8243 | 9/3250 | 0.0289719123371164 | 1 | F | F | F | F | astrocyte differentiation | ABL1 | EIF2B5 | GFAP | MAP2K1 | MAPK1 | MAPK3 | S100B | STAT3 | VIM | GO:0046931 | 0.00157709571757855 | 13/8243 | 9/3250 | 0.0289719123371164 | 1 | F | F | F | F | pore complex assembly | CCT3 | CCT8 | NUP107 | NUP153 | NUP205 | NUP93 | NUP98 | RTN4 | TPR | GO:0043277 | 0.00157709571757855 | 13/8243 | 9/3250 | 0.0289719123371164 | 1 | F | F | F | F | apoptotic cell clearance | CD36 | HMGB1 | ITGAV | ITGB3 | NR1H3 | RARA | TGM2 | TREX1 | TXNDC5 | GO:0051023 | 0.00157709571757855 | 13/8243 | 9/3250 | 0.0289719123371164 | 1 | F | F | F | F | regulation of immunoglobulin secretion | CD22 | CD40 | CD40LG | GPI | TMBIM6 | TNF | TRAF2 | TRAF6 | XBP1 | GO:1904814 | 0.00157709571757855 | 13/8243 | 9/3250 | 0.0289719123371164 | 1 | F | F | F | F | regulation of protein localization to chromosome, telomeric region | CCT2 | CCT3 | CCT4 | CCT5 | CCT6A | CCT7 | CCT8 | TCP1 | TERF1 | GO:0035994 | 0.00157709571757855 | 13/8243 | 9/3250 | 0.0289719123371164 | 1 | F | F | F | F | response to muscle stretch | DMD | FOS | GPI | JUN | MAPK14 | NFKBIA | RAF1 | RELA | SLC9A1 | GO:0031065 | 0.00157709571757855 | 13/8243 | 9/3250 | 0.0289719123371164 | 1 | F | F | F | F | positive regulation of histone deacetylation | AKAP8 | BCL6 | CTBP1 | LRRK2 | NIPBL | PRKD1 | PRKD2 | TGFB1 | ZBTB7B | GO:0006409 | 0.00327550649035545 | 27/8243 | 16/3250 | 0.0290837373788928 | 1 | F | F | F | F | tRNA export from nucleus | NUP107 | NUP153 | NUP160 | NUP205 | NUP214 | NUP85 | NUP88 | NUP93 | NUP98 | POM121 | RAE1 | RAN | SEC13 | SSB | TPR | XPOT | GO:0051290 | 0.00327550649035545 | 27/8243 | 16/3250 | 0.0290837373788928 | 1 | F | F | F | F | protein heterotetramerization | ANXA2 | CBR4 | CD74 | CPSF7 | FARSB | HIST1H3A | HIST1H4A | HIST3H3 | HSD17B8 | INSR | PDSS1 | PDSS2 | RCC1 | RRM1 | RRM2 | XRCC6 | GO:1901016 | 0.00327550649035545 | 27/8243 | 16/3250 | 0.0290837373788928 | 1 | F | F | F | F | regulation of potassium ion transmembrane transporter activity | ACTN2 | AKAP9 | ANK3 | ATP1B3 | CAB39 | CAV1 | FHL1 | KCNAB1 | KCNIP2 | NEDD4 | NOS1AP | OXSR1 | PTEN | SUMO1 | WWP2 | YWHAE | GO:2000758 | 0.00327550649035545 | 27/8243 | 16/3250 | 0.0290837373788928 | 1 | F | F | F | F | positive regulation of peptidyl-lysine acetylation | ARRB1 | BRCA1 | KAT7 | MAPK3 | PAXIP1 | PIH1D1 | PPARGC1A | PRKAA1 | PRKAA2 | RUVBL2 | SMAD4 | SMARCB1 | SNAI2 | TADA2B | TGFB1 | WBP2 | GO:0046782 | 0.00691495814630596 | 57/8243 | 30/3250 | 0.0291223603613703 | 1 | F | F | F | F | regulation of viral transcription | CCNT1 | CDK9 | CTDP1 | DHX9 | EP300 | GTF2F1 | GTF2F2 | HDAC1 | IFITM3 | JUN | LEF1 | NELFB | NELFCD | NELFE | POLR2A | POLR2E | POLR2L | POU2F3 | PSMC3 | REST | SMARCA4 | SMARCB1 | SNW1 | SP1 | SUPT4H1 | SUPT5H | TARDBP | TRIM14 | TRIM21 | TRIM32 | GO:1904031 | 0.00303287637995875 | 25/8243 | 15/3250 | 0.0298714005883456 | 1 | F | F | F | F | positive regulation of cyclin-dependent protein kinase activity | AKT1 | CCNB1 | CCNT1 | CCNT2 | CCNY | CCNYL1 | CDK5R1 | CDKN1A | CKS1B | CKS2 | EGFR | HSP90AB1 | PROX1 | PSMD10 | SRC | GO:0006733 | 0.00303287637995875 | 25/8243 | 15/3250 | 0.0298714005883456 | 1 | F | F | F | F | oxidoreduction coenzyme metabolic process | DCXR | G6PD | GPD1L | IDH1 | IDH2 | KCNAB2 | LDHA | MDH1 | MDH2 | NMNAT1 | NMNAT3 | NMRK2 | NNT | OGDH | VCP | GO:0050856 | 0.00303287637995875 | 25/8243 | 15/3250 | 0.0298714005883456 | 1 | F | F | F | F | regulation of T cell receptor signaling pathway | ADA | BCL10 | CYLD | DUSP22 | EZR | LGALS3 | PAWR | PRKD2 | PTPN22 | PTPN6 | RAB29 | RC3H1 | RELA | RPS3 | UBASH3A | GO:0061462 | 0.00303287637995875 | 25/8243 | 15/3250 | 0.0298714005883456 | 1 | F | F | F | F | protein localization to lysosome | AP3D1 | AP3M1 | AP4M1 | CACNG2 | CACNG4 | CACNG5 | CD81 | CLU | HGS | HSPA8 | LARS | NCOA4 | NEDD4 | RAB7A | RTN4 | GO:2000279 | 0.00303287637995875 | 25/8243 | 15/3250 | 0.0298714005883456 | 1 | F | F | F | F | negative regulation of DNA biosynthetic process | ACD | DCP2 | DUSP1 | FAM129B | HNRNPA1 | HNRNPU | PINX1 | POT1 | PPARG | RGN | SRC | TERF1 | TERF2 | TINF2 | TP53 | GO:0046716 | 0.00181972582797525 | 15/8243 | 10/3250 | 0.0303488133354955 | 1 | F | F | F | F | muscle cell cellular homeostasis | ALDOA | BAG3 | CFL2 | DMD | GAA | HIF1A | PFKM | SOD1 | TGFB1 | TRIM32 | GO:1902254 | 0.00181972582797525 | 15/8243 | 10/3250 | 0.0303488133354955 | 1 | F | F | F | F | negative regulation of intrinsic apoptotic signaling pathway by p53 class mediator | ATAD5 | BCL2 | BDKRB2 | CD74 | KDM1A | MDM2 | MIF | RRM2B | SIRT1 | TRIAP1 | GO:0008631 | 0.00181972582797525 | 15/8243 | 10/3250 | 0.0303488133354955 | 1 | F | F | F | F | intrinsic apoptotic signaling pathway in response to oxidative stress | BCL2 | DIABLO | GPX1 | MELK | PDCD10 | PML | PRKCD | STK24 | STK25 | ZNF622 | GO:0060314 | 0.00181972582797525 | 15/8243 | 10/3250 | 0.0303488133354955 | 1 | F | F | F | F | regulation of ryanodine-sensitive calcium-release channel activity | CALM1 | CALM2 | CALM3 | DMD | FKBP1A | FKBP1B | GSTO1 | PDE4D | PRKACA | SRI | GO:0009112 | 0.00181972582797525 | 15/8243 | 10/3250 | 0.0303488133354955 | 1 | F | F | F | F | nucleobase metabolic process | ALDH6A1 | CDA | GDA | GMPR | HPRT1 | KDM1A | MAPK1 | RRM1 | SCO2 | TYMS | GO:0032211 | 0.00181972582797525 | 15/8243 | 10/3250 | 0.0303488133354955 | 1 | F | F | F | F | negative regulation of telomere maintenance via telomerase | ACD | DCP2 | HNRNPA1 | HNRNPU | PINX1 | POT1 | SRC | TERF1 | TERF2 | TINF2 | GO:0060612 | 0.00279024626956205 | 23/8243 | 14/3250 | 0.0305254577404699 | 1 | F | F | F | F | adipose tissue development | ACAT1 | ATF2 | CDK4 | FTO | ID2 | NAMPT | OXCT1 | PAXIP1 | PIK3CA | PPARD | PPARGC1A | SPTLC2 | UBB | XBP1 | GO:0010765 | 0.00279024626956205 | 23/8243 | 14/3250 | 0.0305254577404699 | 1 | F | F | F | F | positive regulation of sodium ion transport | ACTN4 | AHCYL1 | AKT1 | ANK3 | ARF1 | ATP1B3 | CHP1 | DMD | GLRX | GPD1L | KIF5B | SLC9A1 | WNK1 | WNK4 | GO:0010971 | 0.00279024626956205 | 23/8243 | 14/3250 | 0.0305254577404699 | 1 | F | F | F | F | positive regulation of G2/M transition of mitotic cell cycle | APP | BRD4 | CCNB1 | CDC25A | CDC25B | CDC25C | CDK4 | DBF4B | HSPA2 | MECP2 | MTA3 | RAB11A | SIN3A | VPS4B | GO:0052472 | 0.00279024626956205 | 23/8243 | 14/3250 | 0.0305254577404699 | 1 | F | F | F | F | modulation by host of symbiont transcription | CCNT1 | CTDP1 | EP300 | HDAC1 | JUN | LEF1 | POU2F3 | PSMC3 | REST | SMARCA4 | SMARCB1 | SNW1 | SP1 | TARDBP | GO:0043921 | 0.00279024626956205 | 23/8243 | 14/3250 | 0.0305254577404699 | 1 | F | F | F | F | modulation by host of viral transcription | CCNT1 | CTDP1 | EP300 | HDAC1 | JUN | LEF1 | POU2F3 | PSMC3 | REST | SMARCA4 | SMARCB1 | SNW1 | SP1 | TARDBP | GO:1902895 | 0.00279024626956205 | 23/8243 | 14/3250 | 0.0305254577404699 | 1 | F | F | F | F | positive regulation of pri-miRNA transcription by RNA polymerase II | BMPR1A | FOS | HIF1A | JUN | KLF5 | NR3C1 | RELA | SMAD3 | SMARCA4 | SPI1 | STAT3 | TGFB1 | TP53 | WT1 | GO:0050789 | 0.696105786728133 | 5738/8243 | 2301/3250 | 0.0306343745319478 | 1 | F | F | F | F | regulation of biological process | A2M | AARS | ABAT | ABCE1 | ABCG1 | ABHD14B | ABHD17A | ABI3BP | ABL1 | ABL2 | ACAA2 | ACACA | ACADVL | ACD | ACE2 | ACKR2 | ACLY | ACO1 | ACOT13 | ACOX1 | ACSL1 | ACTA1 | ACTA2 | ACTB | ACTG1 | ACTG2 | ACTL6A | ACTN1 | ACTN2 | ACTN4 | ACTR1A | ACTR2 | ACTR3 | ADA | ADCY6 | ADCYAP1 | ADD3 | ADORA2A | ADRB2 | ADRM1 | ADTRP | AEBP2 | AES | AFF1 | AFF4 | AFG3L2 | AGAP2 | AGER | AGO1 | AGO2 | AGR2 | AGTRAP | AHCYL1 | AHI1 | AHNAK | AHSG | AICDA | AIFM1 | AIMP2 | AIRE | AKAP8 | AKAP9 | AKR1B1 | AKR1C2 | AKT1 | AKT2 | AKT3 | AKTIP | ALAS1 | ALB | ALDOA | ALOX12 | ALOX5 | AMFR | ANAPC4 | ANK1 | ANK3 | ANKRD49 | ANP32A | ANXA1 | ANXA2 | ANXA5 | ANXA6 | ANXA7 | AP1G1 | AP3D1 | APBB1 | APBB2 | APBB3 | APC | APCS | APEX1 | API5 | APLP1 | APOA1 | APOD | APOE | APP | AR | ARAF | ARF1 | ARF4 | ARFGAP1 | ARFIP1 | ARHGAP1 | ARHGAP19 | ARHGAP26 | ARHGAP35 | ARHGDIA | ARHGEF7 | ARHGEF9 | ARID3B | ARID4B | ARMC10 | ARNT | ARNT2 | ARPC1A | ARPC1B | ARPC2 | ARPC3 | ARPC4 | ARPC5 | ARR3 | ARRB1 | ARRB2 | ASB15 | ASB16 | ASB6 | ASB9 | ASCC1 | ASCC2 | ASCL1 | ASGR2 | ASNS | ASPSCR1 | ASS1 | ATAD1 | ATAD3A | ATAD5 | ATCAY | ATF2 | ATF6 | ATF6B | ATF7 | ATG5 | ATL3 | ATM | ATOH1 | ATOX1 | ATP13A2 | ATP1A1 | ATP1B3 | ATP2A2 | ATP2B2 | ATP6V1A | ATP6V1B1 | ATP6V1C1 | ATP6V1D | ATP6V1E1 | ATRX | ATXN1 | ATXN2 | AURKB | AVIL | AXIN1 | AZU1 | B3GAT3 | BABAM1 | BACE1 | BACH2 | BAG1 | BAG2 | BAG3 | BAG4 | BAG5 | BAG6 | BAIAP2 | BAIAP2L1 | BANF1 | BANP | BASP1 | BAX | BBC3 | BCAR1 | BCAR3 | BCL10 | BCL11A | BCL2 | BCL2L1 | BCL2L13 | BCL2L2 | BCL6 | BCL6B | BCOR | BDKRB2 | BIN1 | BIRC2 | BIRC3 | BIRC5 | BIRC7 | BIRC8 | BLZF1 | BMI1 | BMPR1A | BNIPL | BOLA1 | BOLA3 | BRAF | BRCA1 | BRD2 | BRD3 | BRD4 | BSG | BTBD9 | BTK | BTN2A1 | BTRC | BUD31 | C1QA | C1QBP | C1QL4 | C1QTNF1 | C1R | C4A | CA8 | CAB39 | CABP4 | CACNA1A | CACNG2 | CACNG4 | CACNG5 | CALCOCO2 | CALM1 | CALM2 | CALM3 | CALR | CAMK1D | CAMK2A | CAMK2B | CAMKV | CAND1 | CANX | CAP2 | CAPG | CAPN1 | CAPN2 | CAPNS1 | CAPZA1 | CAPZA2 | CAPZB | CARD9 | CARM1 | CASP10 | CASP2 | CASP3 | CASP8 | CASP9 | CAT | CAV1 | CBFA2T3 | CBFB | CBL | CBLB | CBLC | CBX4 | CBX7 | CBX8 | CBY1 | CCAR2 | CCDC22 | CCDC36 | CCDC68 | CCDC8 | CCDC88A | CCL21 | CCL22 | CCNB1 | CCNDBP1 | CCNT1 | CCNT2 | CCNY | CCNYL1 | CCP110 | CCR1 | CCR3 | CCR4 | CCR5 | CCS | CCT2 | CCT3 | CCT4 | CCT5 | CCT6A | CCT7 | CCT8 | CD109 | CD2 | CD209 | CD22 | CD27 | CD2BP2 | CD36 | CD40 | CD40LG | CD6 | CD74 | CD79B | CD81 | CDA | CDC14B | CDC16 | CDC20 | CDC25A | CDC25B | CDC25C | CDC27 | CDC34 | CDC37 | CDC42 | CDC42EP3 | CDC42EP4 | CDC42SE2 | CDC5L | CDC73 | CDH1 | CDH5 | CDK15 | CDK16 | CDK19 | CDK2 | CDK4 | CDK5 | CDK5R1 | CDK8 | CDK9 | CDKL3 | CDKN1A | CDKN2C | CEBPA | CEBPE | CEL | CENPF | CENPJ | CENPV | CEP135 | CEP152 | CEP290 | CEP55 | CEP57 | CEP70 | CFL1 | CFL2 | CFTR | CGN | CGRRF1 | CHCHD10 | CHCHD2 | CHCHD3 | CHD1L | CHD3 | CHD4 | CHD8 | CHEK2 | CHGB | CHMP2A | CHORDC1 | CHP1 | CHRD | CHRM2 | CHRM3 | CHRNA9 | CHRND | CHRNE | CHRNG | CIRBP | CISD1 | CISD2 | CKAP5 | CKS1B | CKS2 | CLDN18 | CLEC11A | CLIC1 | CLIC3 | CLIC4 | CLK1 | CLNK | CLPX | CLSTN1 | CLTC | CLU | CMTM5 | CNDP1 | CNTRL | CNTROB | COA3 | COL18A1 | COL2A1 | COL4A1 | COL4A2 | COL4A3BP | COL5A1 | COLEC10 | COLGALT1 | COMMD1 | COMT | COPA | COPS2 | COPS5 | COQ3 | COQ7 | CORT | COX11 | COX17 | COX7A2 | CPLX1 | CPLX3 | CPNE3 | CPSF7 | CPT1A | CPT2 | CRBN | CREB1 | CREB3 | CREBBP | CRIP1 | CRIP2 | CRK | CRKL | CRMP1 | CROCC | CRP | CRY1 | CRY2 | CRYAB | CSNK1A1 | CSNK1E | CSNK2A2 | CSNK2B | CTBP1 | CTCF | CTDP1 | CTH | CTNNB1 | CTNNBIP1 | CTNND1 | CTSA | CTSC | CTSL | CTSV | CTTN | CUL1 | CUL2 | CUL3 | CUL4B | CUL5 | CUL7 | CXCL11 | CXCL5 | CXCL6 | CXCL8 | CYCS | CYLD | CYP1A1 | CYP1A2 | DAB1 | DAB2 | DAD1 | DAP3 | DAPK1 | DAPP1 | DAXX | DAZAP1 | DBF4B | DBH | DBN1 | DBNDD2 | DBNL | DCLK1 | DCN | DCP2 | DCTN1 | DCUN1D1 | DDA1 | DDB1 | DDB2 | DDIT4 | DDRGK1 | DDX1 | DDX39B | DDX3X | DDX6 | DECR1 | DEDD | DENND1A | DEPDC7 | DERL1 | DES | DGKE | DGKZ | DGUOK | DHCR24 | DHX36 | DHX9 | DIABLO | DIDO1 | DIS3 | DISC1 | DLD | DLG2 | DLG3 | DLG4 | DMAP1 | DMD | DMRT3 | DNA2 | DNAJA1 | DNAJA2 | DNAJA3 | DNAJA4 | DNAJB1 | DNAJB11 | DNAJB2 | DNAJC10 | DNAJC30 | DNAJC7 | DNASE2 | DNM1L | DOCK5 | DOCK7 | DOK1 | DPEP1 | DPF2 | DPYSL5 | DRG2 | DROSHA | DSTN | DTX1 | DTX2 | DUSP1 | DUSP12 | DUSP14 | DUSP16 | DUSP19 | DUSP22 | DUSP7 | DVL1 | DVL2 | DVL3 | DYNC1H1 | DYNC1I2 | DYNC1LI1 | DYNLL1 | DYNLT1 | DYRK2 | E2F1 | E2F2 | E2F3 | E2F4 | E2F6 | EAF2 | EBF1 | ECD | ECSIT | EDA | EDC4 | EDEM1 | EED | EEF1A1 | EEF1A2 | EEF1D | EEF2 | EFEMP1 | EGFR | EGLN1 | EGLN3 | EHD4 | EHMT1 | EHMT2 | EID1 | EIF2AK3 | EIF2AK4 | EIF2B1 | EIF2B3 | EIF2B5 | EIF2S1 | EIF3A | EIF3D | EIF3E | EIF3H | EIF4A3 | EIF4E | EIF4E2 | EIF4EBP1 | EIF4ENIF1 | EIF4G3 | EIF4H | EIF5 | EIF5A | EIF5B | EIF6 | ELAVL1 | ELF3 | ELF4 | EMILIN2 | ENDOG | ENO1 | EP300 | EPHA4 | EPHA6 | EPHB6 | EPM2A | EPPIN | EPPK1 | EPRS | ERBB2 | ERBB3 | ERCC5 | ERF | ERG | ERLIN2 | ERRFI1 | ESR1 | ESR2 | ESRRA | ESRRB | ESRRG | ETS1 | EVC2 | EWSR1 | EXOSC1 | EXOSC5 | EXOSC6 | EXOSC9 | EXTL2 | EYA2 | EYA4 | EZH2 | EZR | FABP1 | FABP3 | FABP5 | FABP7 | FADD | FAF1 | FAM129B | FAM192A | FAM19A3 | FAM3C | FAM49B | FAM83D | FAM83H | FAM98A | FANCA | FANCI | FANCM | FASLG | FASN | FBP1 | FBRS | FBXL12 | FBXO6 | FBXO7 | FBXW11 | FBXW5 | FCGR1A | FCHSD1 | FCN1 | FDPS | FEN1 | FERMT2 | FGB | FGF2 | FGFR1 | FGFR1OP | FGFR3 | FH | FHL1 | FHL2 | FIBP | FIGNL1 | FIS1 | FKBP14 | FKBP1A | FKBP1B | FKBP8 | FKBPL | FLNB | FLOT1 | FN1 | FOLR1 | FOS | FOXA1 | FOXD4 | FOXD4L6 | FOXI2 | FOXJ1 | FOXK1 | FOXK2 | FOXM1 | FOXO1 | FSCN1 | FSD1 | FTH1 | FTO | FUBP1 | FUBP3 | FUS | FXR2 | FYN | FZR1 | G3BP1 | G3BP2 | G6PD | GAA | GABPB1 | GABRA6 | GABRR1 | GADD45G | GAK | GAP43 | GAPDH | GAPDHS | GAPVD1 | GATA1 | GBP2 | GBP5 | GDF9 | GDI1 | GDI2 | GEMIN5 | GFAP | GFPT1 | GGA1 | GGPS1 | GGT1 | GIGYF1 | GIT1 | GJA1 | GKAP1 | GLA | GLMN | GLO1 | GLP1R | GLRB | GLRX | GLRX3 | GLRX5 | GLS | GLS2 | GLUL | GMDS | GNA12 | GNA13 | GNA15 | GNAI1 | GNAI2 | GNAI3 | GNAO1 | GNAQ | GNAS | GNAZ | GNB1 | GNB2 | GNG12 | GNG5 | GNL1 | GOLGA2 | GOLGA4 | GOLPH3 | GOLT1B | GOPC | GOT1 | GPC5 | GPD1 | GPD1L | GPI | GPS2 | GPSM1 | GPX1 | GPX4 | GRB2 | GRB7 | GRIA1 | GRID2 | GRIK2 | GRIP1 | GRK1 | GRK5 | GSK3A | GSK3B | GSPT1 | GSPT2 | GSR | GSTA2 | GSTO1 | GSTP1 | GSTZ1 | GTF2A1 | GTF2F1 | GTF2F2 | GTF2I | GZMA | H1F0 | H1FX | H2AFX | H2AFZ | H3F3A | HADH | HAP1 | HAVCR2 | HAX1 | HBEGF | HBZ | HCN1 | HCN2 | HCN3 | HDAC1 | HDAC2 | HDAC3 | HDAC4 | HDAC5 | HDAC6 | HDAC7 | HDAC9 | HDGF | HEBP1 | HECW1 | HECW2 | HELZ | HELZ2 | HERC1 | HEXB | HEXIM1 | HGS | HIF1A | HIF1AN | HINT1 | HIPK4 | HIST1H1A | HIST1H2AB | HIST1H3A | HIST1H4A | HIST2H3A | HIST3H3 | HIVEP1 | HK1 | HK2 | HLA-A | HLA-B | HLA-DQA1 | HLA-DRA | HLA-DRB1 | HLA-DRB3 | HLA-DRB4 | HLA-DRB5 | HLX | HMGB1 | HMGB2 | HMGB3 | HMMR | HMOX1 | HNF1A | HNF4A | HNF4G | HNRNPA1 | HNRNPA2B1 | HNRNPAB | HNRNPD | HNRNPDL | HNRNPF | HNRNPH1 | HNRNPK | HNRNPL | HNRNPM | HNRNPU | HOXA5 | HOXB5 | HOXB6 | HOXB9 | HOXD10 | HPRT1 | HPX | HRAS | HSF1 | HSF4 | HSP90AA1 | HSP90AB1 | HSP90AB3P | HSP90AB4P | HSP90B1 | HSPA1B | HSPA1L | HSPA2 | HSPA5 | HSPA8 | HSPA9 | HSPB1 | HSPBP1 | HSPD1 | HSPE1 | HSPG2 | HSPH1 | HTATIP2 | HTR1B | HTR3A | HTR3E | HTRA1 | HTRA2 | HTT | HUWE1 | HYPK | IBTK | ICAM1 | ICK | ID2 | IDE | IDH1 | IDH2 | IFI16 | IFIT1 | IFITM3 | IFNA2 | IFNAR1 | IFT122 | IGBP1 | IGF1R | IGF2BP1 | IGFBP7 | IGHV3-13 | IGHV3-23 | IGHV3-30 | IGKV3-20 | IGLC7 | IK | IKBKB | IKBKE | IKBKG | IKZF5 | IL13RA2 | IL32 | IL3RA | ILF2 | ILF3 | ILK | IMPA1 | IMPA2 | ING1 | ING5 | INSIG1 | INSIG2 | INSR | IP6K1 | IPO5 | IPO7 | IPO8 | IQCB1 | IQGAP1 | IREB2 | IRF1 | IRF4 | IRF7 | ISG15 | ITCH | ITGA4 | ITGA7 | ITGAV | ITGB1BP1 | ITGB3 | ITGB3BP | ITGB5 | ITK | ITPR1 | ITPR2 | ITPR3 | JAK1 | JAK3 | JMJD6 | JUN | JUNB | JUND | JUP | KANK2 | KAT5 | KAT7 | KAT8 | KBTBD7 | KCNA4 | KCNAB1 | KCNAB2 | KCNIP2 | KCNK1 | KCNK16 | KCNS3 | KCTD17 | KDM1A | KDM2A | KDM4D | KDM5B | KDM5C | KDM8 | KDR | KEAP1 | KHDRBS1 | KIF11 | KIF13B | KIF2C | KIF5B | KIR3DL2 | KLF5 | KLF6 | KLHDC10 | KLHL20 | KLHL22 | KLRC2 | KMT2A | KMT2B | KMT2C | KPNA1 | KPNA2 | KPNB1 | KRAS | KRT17 | KSR1 | L3MBTL1 | L3MBTL2 | L3MBTL3 | L3MBTL4 | LAMB1 | LAMP3 | LANCL1 | LARP4 | LARS | LASP1 | LAT | LATS1 | LCP1 | LCP2 | LDHA | LDLRAD4 | LEF1 | LEMD2 | LEMD3 | LETM1 | LGALS1 | LGALS3 | LGALS3BP | LGMN | LGR4 | LIG4 | LIMA1 | LIMS1 | LMNA | LMNB1 | LMO2 | LMO7 | LOXL2 | LPAR1 | LPAR2 | LPAR4 | LPIN3 | LRFN1 | LRIF1 | LRP6 | LRPPRC | LRRC7 | LRRK1 | LRRK2 | LRRN2 | LRSAM1 | LSM1 | LSM3 | LSM4 | LSM5 | LTBP1 | LTBR | LUM | LURAP1 | LYPLA1 | LZTR1 | MAD1L1 | MAD2L1 | MAF1 | MAGI1 | MAOA | MAP1B | MAP2K1 | MAP2K3 | MAP2K4 | MAP2K6 | MAP2K7 | MAP3K1 | MAP3K13 | MAP3K14 | MAP3K2 | MAP3K3 | MAP3K4 | MAP4K1 | MAPK1 | MAPK14 | MAPK15 | MAPK3 | MAPK4 | MAPK6 | MAPK7 | MAPK8 | MAPK8IP3 | MAPK9 | MAPKAP1 | MAPT | MARCH5 | MARCKSL1 | MAS1 | MASP1 | MASP2 | MAST1 | MAST3 | MATK | MATR3 | MAVS | MBD3 | MBP | MC1R | MCC | MCM2 | MCM7 | MCOLN3 | MCTS1 | MDC1 | MDFI | MDM1 | MDM2 | MDM4 | ME1 | MECP2 | MED1 | MED12 | MED14 | MED17 | MED20 | MED21 | MED23 | MED24 | MED31 | MED4 | MED6 | MELK | MEOX2 | MEPCE | METAP2 | METRN | METRNL | METTL23 | MEX3C | MGA | MGMT | MIB1 | MICA | MIF | MITF | MKNK2 | MKS1 | MLH1 | MLLT1 | MLLT11 | MLLT3 | MLST8 | MME | MMP26 | MMRN1 | MOB1A | MOB1B | MORF4L1 | MORF4L2 | MOV10 | MPC2 | MPP2 | MRGBP | MRPL12 | MSH6 | MSN | MTA1 | MTA3 | MTAP | MTCH2 | MTERF1 | MTNR1A | MTPN | MTSS1 | MVD | MXD1 | MYBBP1A | MYC | MYD88 | MYH6 | MYL2 | MYL9 | MYO1C | MYO1D | MYO1E | MYO5A | MYO6 | MYOC | NACC1 | NAE1 | NAMPT | NANOG | NAP1L1 | NBAS | NCBP1 | NCDN | NCK1 | NCOA1 | NCOA2 | NCOA3 | NCOA4 | NCOR1 | NCOR2 | NDRG1 | NDUFA13 | NDUFAF2 | NDUFS3 | NEDD4 | NEDD8 | NEK1 | NEK7 | NELFB | NELFCD | NELFE | NELL1 | NEUROD1 | NF2 | NFATC1 | NFATC2 | NFE2L2 | NFKB2 | NFKBIA | NID1 | NIF3L1 | NIN | NIPBL | NKRF | NKX2-1 | NLK | NMB | NMD3 | NME1 | NME2 | NMNAT1 | NMRK2 | NNT | NOD2 | NOP10 | NOS1AP | NOS2 | NOS3 | NOSIP | NOSTRIN | NPEPPS | NPHP3 | NPLOC4 | NPM1 | NPR1 | NPTX1 | NQO1 | NQO2 | NR0B2 | NR1H2 | NR1H3 | NR1I2 | NR3C1 | NR4A1 | NR5A2 | NRBF2 | NRP2 | NRXN1 | NSFL1C | NSMCE4A | NSUN2 | NTF3 | NTRK1 | NUBP1 | NUCB1 | NUDT6 | NUFIP1 | NUMA1 | NUP107 | NUP153 | NUP160 | NUP205 | NUP214 | NUP85 | NUP88 | NUP93 | NUP98 | NUPR1 | NUSAP1 | OAS2 | OAZ3 | OBSL1 | OFD1 | OGFOD1 | OGT | ONECUT1 | OPA1 | OPTN | OR5F1 | ORM1 | OTUB1 | OTUD4 | OTUD5 | OVGP1 | OXA1L | OXCT1 | OXSR1 | P2RX1 | P2RX2 | P2RX4 | P4HB | PA2G4 | PABPC1 | PABPC4 | PACSIN3 | PAFAH1B1 | PAG1 | PAIP1 | PAK2 | PAK6 | PALB2 | PAM16 | PAN2 | PANX3 | PAPLN | PARD3 | PARD6A | PARK7 | PARP1 | PARP14 | PARP6 | PATZ1 | PAWR | PAX8 | PAX9 | PAXIP1 | PC | PCBD1 | PCBP1 | PCBP2 | PCGF1 | PCGF5 | PCGF6 | PCM1 | PCNA | PCSK9 | PCTP | PDCD10 | PDCD6 | PDCD6IP | PDCL | PDE12 | PDE4D | PDE4DIP | PDE6G | PDF | PDIA3 | PDIA4 | PDIA6 | PDK2 | PDLIM5 | PEBP1 | PEF1 | PELI1 | PER1 | PER2 | PEX11B | PEX5 | PFDN2 | PFKL | PFKM | PFN1 | PGAM1 | PGAM2 | PGAM5 | PGK1 | PGK2 | PGP | PHB | PHB2 | PHC3 | PHF10 | PHF20L1 | PHF21A | PHGDH | PHOSPHO1 | PIANP | PIAS1 | PIAS3 | PICK1 | PIGU | PIH1D1 | PIK3C2A | PIK3CA | PIK3CG | PIK3R1 | PIK3R3 | PIKFYVE | PIM1 | PIN1 | PINX1 | PIP4K2A | PIR | PITPNA | PKIG | PKM | PKN2 | PLA2G10 | PLA2G1B | PLA2G2A | PLA2G6 | PLAUR | PLCG1 | PLEKHF1 | PLGRKT | PLK1 | PLSCR1 | PLXNA1 | PLXNA2 | PLXNB2 | PML | PNKP | PNMA1 | PNP | POLDIP2 | POLDIP3 | POLR2A | POLR2E | POLR2L | POLR2M | POM121 | POT1 | POU2F3 | POU5F1 | PPARD | PPARG | PPARGC1A | PPEF1 | PPIA | PPIB | PPID | PPIE | PPIF | PPM1A | PPM1B | PPM1D | PPM1K | PPME1 | PPP1CA | PPP1CB | PPP1CC | PPP1R11 | PPP1R12A | PPP1R12B | PPP1R14A | PPP1R16A | PPP1R2 | PPP1R3B | PPP1R3C | PPP1R8 | PPP2CA | PPP2CB | PPP2R1A | PPP2R1B | PPP2R2A | PPP2R2D | PPP2R5D | PPP3CC | PPP4R2 | PPP5C | PPP6R1 | PPT1 | PRCC | PRCP | PRDX1 | PRDX2 | PRDX3 | PRDX4 | PRDX5 | PRDX6 | PRELID1 | PREPL | PRKAA1 | PRKAA2 | PRKAB2 | PRKACA | PRKAG1 | PRKAR1A | PRKAR2B | PRKCD | PRKCE | PRKCSH | PRKCZ | PRKD1 | PRKD2 | PRKD3 | PRKDC | PRMT2 | PRMT3 | PRMT6 | PROX1 | PRPF19 | PRPF40A | PRRC1 | PSAP | PSEN1 | PSEN2 | PSMA1 | PSMA2 | PSMA3 | PSMA5 | PSMA6 | PSMB5 | PSMB6 | PSMB7 | PSMB8 | PSMC1 | PSMC2 | PSMC3 | PSMC4 | PSMC5 | PSMC6 | PSMD1 | PSMD10 | PSMD11 | PSMD12 | PSMD14 | PSMD2 | PSMD4 | PSMD9 | PSME2 | PSME3 | PSTPIP1 | PTCRA | PTEN | PTGDS | PTGER3 | PTGES | PTGES2 | PTGES3 | PTGIR | PTK2 | PTN | PTPMT1 | PTPN1 | PTPN14 | PTPN22 | PTPN23 | PTPN6 | PTPRA | PTPRD | PTPRF | PTPRK | PTPRO | PTPRR | PTRH2 | PTTG1 | PUM1 | PUS7 | PXDN | PXN | PYCR1 | QARS | QRICH1 | QSOX1 | RAB10 | RAB11A | RAB11B | RAB11FIP2 | RAB18 | RAB1A | RAB1B | RAB21 | RAB24 | RAB29 | RAB2A | RAB31 | RAB32 | RAB35 | RAB3GAP1 | RAB5A | RAB5B | RAB7A | RAB9A | RABL2A | RABL3 | RAC1 | RAD21 | RAD23A | RAD23B | RAD51 | RAD51AP1 | RADIL | RAE1 | RAF1 | RAN | RANBP9 | RANGAP1 | RAP1A | RAP1B | RAPGEF4 | RARA | RARG | RASD1 | RASL11B | RASSF10 | RASSF7 | RASSF9 | RB1 | RB1CC1 | RBBP4 | RBBP6 | RBM17 | RBM25 | RBM3 | RBM4 | RBM8A | RBMX | RBPJ | RBX1 | RC3H1 | RCC1 | RDH11 | RDX | RECK | REL | RELA | REST | RFC1 | RFC2 | RFC3 | RFC4 | RFC5 | RFK | RFXANK | RGN | RGS1 | RGS2 | RGS20 | RHBDD1 | RHO | RHOA | RHOJ | RHOT1 | RHOT2 | RHOU | RHPN1 | RIC8B | RIF1 | RING1 | RIOK1 | RIPK1 | RIPK2 | RIPK3 | RIPK4 | RIT1 | RMND1 | RNASEH2B | RNF126 | RNF139 | RNF183 | RNF185 | RNF19A | RNF2 | RNF25 | RNF31 | RNF4 | RNF8 | ROMO1 | RPA2 | RPA3 | RPGRIP1L | RPL10 | RPL10A | RPL12 | RPL18A | RPL22 | RPL23 | RPL23A | RPL24 | RPL30 | RPL37A | RPL38 | RPL6 | RPL7 | RPL7A | RPL9 | RPLP0 | RPLP1 | RPLP2 | RPS12 | RPS13 | RPS14 | RPS15A | RPS19 | RPS2 | RPS20 | RPS24 | RPS29 | RPS3 | RPS4X | RPS6KA1 | RPS6KA2 | RPS6KB2 | RPS8 | RPTOR | RRM2 | RRM2B | RRS1 | RSU1 | RTF1 | RTKN | RTN4 | RTN4IP1 | RUNX1 | RUNX1T1 | RUNX2 | RUVBL1 | RUVBL2 | RXRA | RXRB | RXRG | S100A1 | S100A11 | S100A12 | S100A13 | S100A4 | S100A6 | S100A7 | S100A9 | S100B | SAE1 | SAG | SAMD7 | SAP18 | SARAF | SARM1 | SARS | SART1 | SART3 | SAT1 | SCARA3 | SCEL | SCFD1 | SCG2 | SCML2 | SCO2 | SCYL2 | SDC1 | SDC4 | SDCBP | SDCBP2 | SEC13 | SEC16A | SEC24B | SEC31A | SEL1L | SELL | SEMA7A | SENP6 | SEPT1 | SEPT11 | SEPT2 | SEPT4 | SEPT5 | SEPT7 | SEPT8 | SEPT9 | SERPINB2 | SERPINB3 | SERPINB5 | SERPINB6 | SERPINB8 | SERPINE2 | SERPINH1 | SET | SETBP1 | SETDB1 | SF1 | SFMBT1 | SFN | SFPQ | SGPL1 | SGTA | SGTB | SH2B1 | SH3GL1 | SH3GL2 | SH3GL3 | SH3KBP1 | SHC1 | SHKBP1 | SHMT1 | SHMT2 | SIAH1 | SIN3A | SIPA1 | SIPA1L1 | SIRT1 | SIRT3 | SIRT5 | SIRT6 | SIRT7 | SKA1 | SKA3 | SKI | SKIV2L | SLAMF1 | SLAMF8 | SLC12A2 | SLC16A1 | SLC25A24 | SLC25A4 | SLC25A5 | SLC25A6 | SLC35F6 | SLC39A4 | SLC9A1 | SLC9A3R1 | SLTM | SMAD2 | SMAD3 | SMAD4 | SMAD5 | SMAD9 | SMARCA2 | SMARCA4 | SMARCB1 | SMARCC1 | SMARCC2 | SMARCE1 | SMC1A | SMC3 | SMG5 | SMG6 | SMG8 | SMO | SMURF1 | SMURF2 | SNAI2 | SNCA | SND1 | SNF8 | SNRPA | SNRPA1 | SNTA1 | SNW1 | SNX12 | SNX17 | SNX3 | SNX6 | SOAT1 | SOCS1 | SOCS2 | SOCS6 | SOD1 | SOD2 | SOGA3 | SORT1 | SOX2 | SP1 | SP100 | SP2 | SPACA3 | SPATA2 | SPDL1 | SPEN | SPERT | SPG21 | SPI1 | SPICE1 | SPIN1 | SPINK7 | SPINT2 | SPOP | SPR | SPRTN | SPRY2 | SPTAN1 | SPTBN1 | SPTBN2 | SPTLC2 | SQSTM1 | SRC | SRGAP2 | SRGAP3 | SRI | SRPK1 | SRPK2 | SRPRB | SRSF12 | SRSF5 | SSB | SSX2IP | ST13 | STAC | STAG2 | STARD13 | STAT1 | STAT3 | STAT4 | STAT5A | STAT6 | STAU1 | STIL | STIM1 | STK11 | STK24 | STK25 | STK26 | STK3 | STK38 | STK40 | STMN1 | STOM | STOML2 | STRAP | STUB1 | STX5 | STXBP1 | STXBP2 | STYX | SUB1 | SULF2 | SULT1A3 | SUMO1 | SUMO2 | SUN2 | SUPT4H1 | SUPT5H | SUSD5 | SUZ12 | SVIL | SWAP70 | SYDE1 | SYN1 | SYNE2 | SYNJ2BP | SYP | SYVN1 | TAB1 | TAB2 | TACO1 | TADA2B | TAF12 | TAF15 | TAF1A | TAGLN3 | TALDO1 | TANK | TAOK3 | TARDBP | TAS2R41 | TAS2R60 | TAS2R7 | TAZ | TBC1D15 | TBC1D22B | TBC1D23 | TBC1D4 | TBL1X | TBRG4 | TBXA2R | TCEA1 | TCEA2 | TCERG1 | TCF12 | TCF21 | TCF25 | TCF4 | TCIRG1 | TCOF1 | TCP1 | TCP10L | TCTN2 | TCTN3 | TDRD3 | TERF1 | TERF2 | TERF2IP | TES | TFCP2 | TFDP1 | TFG | TFIP11 | TGFB1 | TGFBR3 | TGIF2LX | TGM2 | THADA | THAP11 | THAP7 | THBS1 | THBS2 | THEM4 | THOC6 | THRA | TIA1 | TIAL1 | TIMP1 | TIMP2 | TINF2 | TKT | TLE1 | TLE3 | TLR3 | TM9SF4 | TMBIM6 | TMED9 | TMEM17 | TMEM173 | TMEM219 | TMEM231 | TMEM30A | TMEM65 | TMEM9B | TMOD3 | TMOD4 | TMPO | TMTC3 | TMX2 | TMX4 | TNF | TNFAIP3 | TNFRSF12A | TNFRSF1A | TNFRSF8 | TNFSF12 | TNFSF14 | TNIP2 | TNK2 | TNKS | TNKS2 | TNN | TNNT1 | TNP1 | TNPO1 | TOE1 | TOLLIP | TOMM22 | TOP1 | TOP2A | TOP3A | TOR1A | TP53 | TP53RK | TP63 | TP73 | TPCN2 | TPM1 | TPR | TPTE | TRA2A | TRADD | TRAF1 | TRAF2 | TRAF3 | TRAF3IP1 | TRAF6 | TRAP1 | TREML2 | TREX1 | TRIAP1 | TRIB2 | TRIB3 | TRIM14 | TRIM21 | TRIM25 | TRIM28 | TRIM29 | TRIM32 | TRIM33 | TRIM34 | TRIM35 | TRIM63 | TRIM9 | TRIP12 | TRIP13 | TRIP4 | TRIP6 | TRUB2 | TSC22D1 | TSC22D3 | TSC22D4 | TSFM | TSG101 | TSKU | TSPAN17 | TSPYL2 | TTC5 | TUBA1A | TUBA4A | TUBB | TUBB2B | TUBB4A | TUBB4B | TUBG1 | TUFT1 | TULP3 | TWF1 | TWF2 | TWIST1 | TXLNA | TXN | TXN2 | TXNDC11 | TXNDC15 | TXNDC17 | TXNDC5 | TXNIP | TXNRD1 | TYMS | U2AF2 | UBA3 | UBA5 | UBASH3A | UBB | UBC | UBE2A | UBE2B | UBE2C | UBE2D1 | UBE2D2 | UBE2D3 | UBE2I | UBE2J1 | UBE2K | UBE2L3 | UBE2L6 | UBE2M | UBE2N | UBE2O | UBE2S | UBE2V1 | UBE3A | UBQLN1 | UBQLN2 | UBQLN4 | UBR5 | UBXN1 | UCHL1 | UCHL5 | UFM1 | ULK1 | UNC119 | UNK | UNKL | UPF2 | UPF3B | UQCC1 | USP14 | USP15 | USP16 | USP17L2 | USP19 | USP2 | USP20 | USP21 | USP28 | USP4 | USP42 | USP46 | USP47 | USP5 | USP50 | USP7 | USP8 | USP9X | USP9Y | VAC14 | VAPA | VAPB | VASH2 | VASP | VAT1 | VAV1 | VCAM1 | VCL | VCP | VDAC1 | VDAC2 | VDAC3 | VDR | VGF | VGLL4 | VHL | VIM | VN1R1 | VPS18 | VPS26A | VPS29 | VPS4B | VPS72 | VSIG4 | VSTM2A | VTN | WARS | WAS | WBP2 | WDFY1 | WDR1 | WDR12 | WDR19 | WDR35 | WDR41 | WDR48 | WDR77 | WDTC1 | WIPI1 | WNK1 | WNK4 | WNT16 | WNT2 | WSB1 | WT1 | WTAP | WWOX | WWP2 | XAF1 | XBP1 | XIAP | XPO1 | XPO4 | XPO5 | XRCC1 | XRCC3 | XRCC5 | XRCC6 | YAF2 | YAP1 | YEATS2 | YES1 | YME1L1 | YRDC | YTHDF1 | YWHAB | YWHAE | YWHAG | YWHAH | YWHAQ | YWHAZ | YY1 | ZAP70 | ZBTB16 | ZBTB17 | ZBTB21 | ZBTB25 | ZBTB33 | ZBTB42 | ZBTB7A | ZBTB7B | ZC3H4 | ZC3HC1 | ZDHHC17 | ZFAND5 | ZFAND6 | ZFP41 | ZFYVE19 | ZFYVE26 | ZHX1 | ZIC1 | ZKSCAN1 | ZMPSTE24 | ZMYM5 | ZMYND10 | ZMYND8 | ZNF207 | ZNF217 | ZNF227 | ZNF24 | ZNF277 | ZNF354C | ZNF408 | ZNF420 | ZNF443 | ZNF517 | ZNF518A | ZNF592 | ZNF598 | ZNF618 | ZNF622 | ZNF646 | ZNF669 | ZNF696 | ZNF746 | ZNF747 | ZRANB2 | ZSCAN2 | ZSCAN29 | ZSCAN32 | ZW10 | ZWINT | ZZZ3 | GO:0009895 | 0.0253548465364552 | 209/8243 | 96/3250 | 0.0309066074707771 | 1 | F | F | F | F | negative regulation of catabolic process | AGAP2 | AKT1 | ANXA2 | ATP13A2 | BAG5 | BAG6 | BANP | BCL2 | CBFA2T3 | CCAR2 | CIRBP | CSNK2A2 | CTSA | DDIT4 | DDRGK1 | DHX36 | DHX9 | E2F1 | EGFR | EIF4E | EIF4G3 | ELAVL1 | EPHA4 | FAM192A | FBP1 | FYN | GOLGA2 | GSK3A | HDAC4 | HERC1 | HMOX1 | HNRNPD | HNRNPU | HSP90AB1 | HSP90AB3P | HSP90AB4P | HTRA2 | IGF2BP1 | KLHL22 | LAMP3 | LARS | LRPPRC | LRRK2 | MAD2L1 | MAPK14 | MDM4 | MYD88 | NAMPT | NBAS | NELL1 | NMNAT1 | NOS2 | OGT | PABPC1 | PAIP1 | PAM16 | PARK7 | PHB | PIK3CA | PIK3CG | PIN1 | PML | POLDIP2 | PPARGC1A | PRKAA1 | PSEN1 | PTK2 | PTPN22 | QSOX1 | RELA | RGN | RPL23 | SCFD1 | SDCBP | SERPINE2 | SGTA | SIRT6 | SMAD3 | SMARCC1 | SNCA | SNX12 | SNX3 | STAT3 | STYX | TAB2 | TARDBP | TIMP1 | TIMP2 | TNF | UBE2J1 | UBQLN4 | UBXN1 | UCHL5 | USP14 | USP19 | USP7 | GO:0009648 | 0.00254761615916535 | 21/8243 | 13/3250 | 0.0309900925258436 | 1 | F | F | F | F | photoperiodism | CRY1 | CRY2 | ID2 | MTA1 | PER1 | PER2 | PML | PPP1CA | PPP1CB | PPP1CC | RBM4 | TP53 | USP2 | GO:1901797 | 0.00254761615916535 | 21/8243 | 13/3250 | 0.0309900925258436 | 1 | F | F | F | F | negative regulation of signal transduction by p53 class mediator | ATAD5 | BCL2 | BDKRB2 | CD74 | KDM1A | MDM2 | MIF | PSMD10 | RRM2B | SIRT1 | SNAI2 | TRIAP1 | TWIST1 | GO:0070059 | 0.00254761615916535 | 21/8243 | 13/3250 | 0.0309900925258436 | 1 | F | F | F | F | intrinsic apoptotic signaling pathway in response to endoplasmic reticulum stress | AIFM1 | BAG6 | BAX | BBC3 | BCL2 | DNAJC10 | ITPR1 | PML | TMBIM6 | TRAF2 | TRIB3 | UBE2K | XBP1 | GO:0007004 | 0.00254761615916535 | 21/8243 | 13/3250 | 0.0309900925258436 | 1 | F | F | F | F | telomere maintenance via telomerase | GAR1 | HSP90AA1 | HSP90AB1 | NOP10 | PINX1 | POT1 | PTGES3 | RFC1 | RPA2 | SMG5 | SMG6 | TERF1 | TERF2IP | GO:0010800 | 0.00254761615916535 | 21/8243 | 13/3250 | 0.0309900925258436 | 1 | F | F | F | F | positive regulation of peptidyl-threonine phosphorylation | APP | AXIN1 | AZU1 | CAB39 | CALM1 | CALM2 | CALM3 | MAPK1 | PLK1 | RIPK2 | RPTOR | TGFB1 | UBE2K | GO:0044275 | 0.00254761615916535 | 21/8243 | 13/3250 | 0.0309900925258436 | 1 | F | F | F | F | cellular carbohydrate catabolic process | AGL | CALM1 | GAA | GUSB | PFKM | PGM1 | PGM2 | PHKA1 | PYGB | PYGL | SORD | STBD1 | TPI1 | GO:0051205 | 0.00254761615916535 | 21/8243 | 13/3250 | 0.0309900925258436 | 1 | F | F | F | F | protein insertion into membrane | AGK | BAG6 | BAX | EGFR | HSP90AA1 | HSPA4 | NDUFA13 | OXA1L | ROMO1 | SGTA | TIMM13 | TOMM22 | UBL4A | GO:1902107 | 0.0101904646366614 | 84/8243 | 42/3250 | 0.0310035521890049 | 1 | F | F | F | F | positive regulation of leukocyte differentiation | ADA | AGER | ANXA1 | AP3D1 | BCL6 | BTK | CASP8 | CBFB | CCR1 | CD27 | CD74 | CREB1 | CTNNBIP1 | CYLD | FADD | FOS | GNAS | HAX1 | HLX | HMGB1 | ID2 | JUN | LEF1 | LGALS1 | LGALS3 | PNP | PRKCZ | RARA | RB1 | RHOA | RIPK1 | RIPK2 | RUNX1 | SART1 | SOCS1 | TGFB1 | TNF | TRAF6 | XBP1 | ZAP70 | ZBTB16 | ZBTB7B | GO:0006816 | 0.0132233410166202 | 109/8243 | 53/3250 | 0.0310231044791089 | 1 | F | F | F | F | calcium ion transport | AFG3L2 | ANXA2 | ANXA5 | ANXA6 | ATP2A2 | ATP2B2 | BSPRY | CACNA1A | CACNG2 | CACNG4 | CACNG5 | CAMK2A | CAV1 | CCL21 | CCR1 | CCR5 | CDK5 | CHRNA9 | CUL5 | FASLG | FGF2 | FKBP1A | FKBP1B | FYN | IBTK | ITGAV | ITPR1 | ITPR2 | ITPR3 | LETM1 | MCOLN3 | MICU1 | MICU2 | OPA1 | P2RX4 | PHB | PHB2 | PLCG1 | PMPCA | PMPCB | PRKCE | PSEN1 | PSEN2 | SARAF | SLC3A2 | SRI | STIM1 | STOML2 | TPCN2 | VDAC1 | VDR | YME1L1 | ZMPSTE24 | GO:0030099 | 0.0132233410166202 | 109/8243 | 53/3250 | 0.0310231044791089 | 1 | F | F | F | F | myeloid cell differentiation | ACTN1 | CASP3 | CASP8 | CASP9 | CBFA2T3 | CBFB | CDC42 | CEBPA | CEBPE | CTNNB1 | DHRS7B | DNASE2 | EP300 | FASN | GATA1 | GLO1 | HSPA9 | ID2 | IFI16 | IREB2 | IRF4 | JAK3 | JUN | JUNB | KAT8 | L3MBTL3 | LEF1 | LTBR | MAPK14 | MED1 | MITF | PARP1 | PIR | PML | PPARG | PRDX3 | PSEN1 | PTPN6 | RB1 | RBPJ | RPS14 | RPS19 | SFXN1 | SIRT1 | SMAD5 | SPI1 | TGFB1 | TGFBR3 | THRA | TNF | TRAF6 | WDR1 | ZBTB16 | GO:0062098 | 0.00206235593837195 | 17/8243 | 11/3250 | 0.0310231923875006 | 1 | F | F | F | F | regulation of programmed necrotic cell death | BIRC2 | BIRC3 | CASP8 | CAV1 | CYLD | FADD | PELI1 | RIPK1 | RIPK3 | SLC25A4 | SPATA2 | GO:0090050 | 0.00206235593837195 | 17/8243 | 11/3250 | 0.0310231923875006 | 1 | F | F | F | F | positive regulation of cell migration involved in sprouting angiogenesis | ABL1 | AKT3 | ANXA1 | FGF2 | HDAC7 | HDAC9 | HMOX1 | KDR | MAP3K3 | PIK3C2A | RHOJ | GO:0060479 | 0.00206235593837195 | 17/8243 | 11/3250 | 0.0310231923875006 | 1 | F | F | F | F | lung cell differentiation | AGR2 | AIMP2 | ASCL1 | CREB1 | CTNNB1 | FOXA1 | HOXA5 | NKX2-1 | RBPJ | THRA | YAP1 | GO:0009206 | 0.00206235593837195 | 17/8243 | 11/3250 | 0.0310231923875006 | 1 | F | F | F | F | purine ribonucleoside triphosphate biosynthetic process | ALDOA | CYC1 | DNAJC30 | IMPDH2 | LDHC | NME1 | NME2 | NME4 | SLC25A13 | STOML2 | TGFB1 | GO:0046823 | 0.00206235593837195 | 17/8243 | 11/3250 | 0.0310231923875006 | 1 | F | F | F | F | negative regulation of nucleocytoplasmic transport | APOD | CD36 | CDK5 | CHP1 | NUP153 | PARK7 | PKIG | RANGAP1 | SP100 | TPR | TXN | GO:0007212 | 0.00206235593837195 | 17/8243 | 11/3250 | 0.0310231923875006 | 1 | F | F | F | F | dopamine receptor signaling pathway | ADCY6 | ARRB2 | GNA15 | GNAI3 | GNAO1 | GNAS | GNB1 | GSK3A | GSK3B | SLC9A3R1 | SULT1A3 | GO:0042276 | 0.00206235593837195 | 17/8243 | 11/3250 | 0.0310231923875006 | 1 | F | F | F | F | error-prone translesion synthesis | PCNA | POLE2 | RFC1 | RFC2 | RFC3 | RFC4 | RFC5 | RPA2 | RPA3 | UBB | UBC | GO:0051193 | 0.00230498604876865 | 19/8243 | 12/3250 | 0.0311900996082965 | 1 | F | F | F | F | regulation of cofactor metabolic process | COQ3 | FYN | GSTZ1 | HDAC6 | ME1 | NFE2L2 | NNT | PDK2 | PRDX2 | RAC1 | SNCA | STAT3 | GO:1903205 | 0.00230498604876865 | 19/8243 | 12/3250 | 0.0311900996082965 | 1 | F | F | F | F | regulation of hydrogen peroxide-induced cell death | ENDOG | HDAC6 | LRRK2 | NFE2L2 | PARK7 | PAWR | PSAP | PYCR1 | RIPK1 | STK26 | TRAP1 | TXN | GO:0045920 | 0.00230498604876865 | 19/8243 | 12/3250 | 0.0311900996082965 | 1 | F | F | F | F | negative regulation of exocytosis | ANXA1 | BRAF | GNAI2 | HMOX1 | IL13RA2 | RAB7A | RAP1A | RAP1B | REST | SNCA | TRIM9 | VPS4B | GO:0010288 | 0.00230498604876865 | 19/8243 | 12/3250 | 0.0311900996082965 | 1 | F | F | F | F | response to lead ion | ANXA5 | APP | BACE1 | CAT | CDK4 | FECH | MAPT | PPP1CA | PPP2CA | PPP2CB | PPP5C | QDPR | GO:0010743 | 0.00230498604876865 | 19/8243 | 12/3250 | 0.0311900996082965 | 1 | F | F | F | F | regulation of macrophage derived foam cell differentiation | ABCG1 | CD36 | CRP | ITGAV | ITGB3 | MAPK9 | NFKBIA | NR1H2 | NR1H3 | PLA2G10 | PLA2G2A | PPARG | GO:0051046 | 0.0514375834041004 | 424/8243 | 186/3250 | 0.031233484837385 | 1 | F | F | F | F | regulation of secretion | ABAT | ABL1 | ADA | ADCYAP1 | ADORA2A | ADTRP | AGER | ANXA1 | ANXA5 | AP1G1 | APBB1 | APOA1 | ARF1 | ARFIP1 | ARHGEF7 | ARRB1 | ATG5 | ATP13A2 | ATP2A2 | BACE1 | BRAF | C1QTNF1 | CACNA1A | CALM1 | CALM3 | CAMK2A | CD2 | CD22 | CD40 | CD40LG | CD74 | CDK16 | CDK5 | CFTR | CHMP2A | CHRM2 | COMT | CPLX1 | CPLX3 | CPT1A | CREB1 | CRP | CRY1 | DHX36 | DHX9 | DNM1L | DVL1 | DYNLL1 | EGFR | ERBB3 | EZR | FCN1 | FGB | FGFR1 | FKBP1B | FN1 | GAPDH | GDF9 | GIT1 | GLMN | GLP1R | GLUL | GNAI2 | GNAS | GOLPH3 | GPI | GSK3B | HADH | HAP1 | HAVCR2 | HDAC9 | HGS | HIF1A | HK1 | HMGB1 | HMOX1 | HNF4A | HTR1B | IDH2 | IFNA2 | IL13RA2 | ITPR1 | ITPR2 | ITPR3 | KCNS3 | KDM5B | KIF5B | LGR4 | LRRK2 | MAP2K6 | MAPK14 | MAPK3 | MAVS | MBP | MIF | MPC2 | MYO6 | NDUFAF2 | NEUROD1 | NMB | NOD2 | NOS2 | NPR1 | NR0B2 | NR1H2 | NR1H3 | OAS2 | ORM1 | OXCT1 | P2RX1 | P2RX4 | PANX3 | PAX8 | PDCD6IP | PER2 | PFKL | PFKM | PLA2G10 | PLA2G1B | PLA2G6 | PML | PPARD | PPIA | PPID | PPP1R11 | PREPL | PRKCE | PRKCZ | PSMD9 | PTGER3 | PTPN22 | PTPN23 | RAB10 | RAB21 | RAB3GAP1 | RAB5A | RAB7A | RAB9A | RAP1A | RAP1B | RAPGEF4 | REST | RHBDD1 | RHOT1 | RIPK2 | SDC1 | SDC4 | SDCBP | SEPT1 | SEPT4 | SEPT5 | SIRT3 | SLC16A1 | SLC25A4 | SLC25A5 | SLC25A6 | SLC9A3R1 | SMAD4 | SNCA | SNF8 | SNX6 | SOCS1 | SPTBN1 | SRC | SRI | STXBP1 | STXBP2 | SYN1 | SYP | TARDBP | TGFB1 | TMBIM6 | TNF | TNFAIP3 | TNFRSF1A | TRAF2 | TRAF6 | TRIM9 | TSG101 | TWIST1 | USP50 | VPS18 | VPS4B | WNK1 | WNK4 | XBP1 | GO:0007167 | 0.0514375834041004 | 424/8243 | 186/3250 | 0.031233484837385 | 1 | F | F | F | F | enzyme linked receptor protein signaling pathway | ABL1 | ACTB | ACTG1 | ACTR2 | ACTR3 | AHI1 | AKT1 | AKT2 | APC | APOA1 | ARF4 | ARHGEF7 | ARPC1A | ARPC1B | ARPC2 | ARPC3 | ARPC4 | ARPC5 | ARRB2 | ATP6V1A | ATP6V1B1 | ATP6V1C1 | ATP6V1D | ATP6V1E1 | BAIAP2 | BCAR1 | BDKRB2 | BMPR1A | CASP3 | CBL | CDC37 | CDC42 | CDH5 | CDK5R1 | CEP57 | CGN | CHRD | CLNK | COL4A1 | COL4A2 | CPNE3 | CREB1 | CRK | CRKL | CUL5 | DDIT4 | DOK1 | DUSP22 | EFEMP1 | EGFR | EIF2AK3 | EIF4EBP1 | EPHA4 | EPHA6 | EPHB6 | ERBB2 | ERBB3 | FERMT2 | FGF2 | FGFR1 | FGFR3 | FIBP | FOS | FOXO1 | FYN | GDF9 | GIGYF1 | GIT1 | GRB2 | GRB7 | GSK3A | GTF2F1 | GTF2F2 | HAP1 | HBEGF | HNRNPA1 | HNRNPF | HNRNPH1 | HNRNPM | HRAS | HSP90AA1 | HSPB1 | IDE | IGF1R | INSR | IQGAP1 | ITGAV | ITGB3 | ITGB5 | JAK3 | JUN | KDR | LCP2 | LEF1 | LTBP1 | MAPK1 | MAPK14 | MAPK3 | MATK | MTSS1 | MYH6 | MYO1C | MYO1E | MYOC | NAMPT | NCBP1 | NCK1 | NLK | NPR1 | NRP2 | NTF3 | NTRK1 | PAK2 | PARD3 | PARD6A | PARP1 | PDCD6 | PDK2 | PIK3C2A | PIK3CA | PIK3R1 | PIK3R3 | PLCG1 | PML | POLR2A | POLR2E | POLR2L | PRKCZ | PRKD1 | PRKD2 | PSEN1 | PSEN2 | PTEN | PTK2 | PTN | PTPN1 | PTPRA | PTPRD | PTPRF | PTPRK | PTPRR | PXN | RAC1 | RAF1 | RHOA | RUNX2 | SDCBP | SGPL1 | SH2B1 | SHC1 | SIPA1L1 | SIRT1 | SKI | SMAD2 | SMAD3 | SMAD4 | SMAD5 | SMAD9 | SMARCC1 | SMURF1 | SMURF2 | SOCS2 | SORT1 | SRC | STAT3 | STAT5A | STMN1 | STUB1 | SULF2 | TAB1 | TCIRG1 | TGFB1 | TGFBR3 | TIA1 | TIAL1 | TXNIP | UBB | UBC | UBE2D1 | UBE2D3 | USP15 | USP9X | USP9Y | VAV1 | YES1 | ZFAND5 | GO:1902041 | 0.0050952323183307 | 42/8243 | 23/3250 | 0.0312663874535026 | 1 | F | F | F | F | regulation of extrinsic apoptotic signaling pathway via death domain receptors | BCL2L1 | BRCA1 | CASP8 | FADD | FAF1 | FASLG | FGB | GPX1 | HMGB2 | HMOX1 | ICAM1 | LGALS3 | NOS3 | PARK7 | PTEN | RAF1 | RIPK1 | SP100 | STK3 | THBS1 | TNFAIP3 | TRADD | TRAF2 | GO:0045637 | 0.0177119980589591 | 146/8243 | 69/3250 | 0.0316526203158362 | 1 | F | F | F | F | regulation of myeloid cell differentiation | AGO1 | APCS | ARNT | CASP8 | CBFB | CCR1 | CD74 | CDC73 | CLDN18 | CREB1 | CREBBP | CTNNB1 | CTNNBIP1 | EIF6 | EP300 | ESRRA | ETS1 | FADD | FOS | GATA1 | GNAS | H3F3A | HAX1 | HDAC1 | HIF1A | HIST1H3A | HIST1H4A | HIST2H3A | HMGB2 | HOXA5 | HSPA1B | HSPA9 | ID2 | IRF7 | ISG15 | JUN | KMT2A | KMT2B | KMT2C | L3MBTL1 | LEF1 | LGALS3 | MAPK14 | MED1 | MITF | MOV10 | MYC | MYL9 | NFKBIA | NME1 | NME2 | PIAS3 | PIK3R1 | PRMT6 | RARA | RARG | RB1 | RIPK1 | RUNX1 | SIN3A | SPI1 | STAT1 | STAT3 | TGFB1 | THBS1 | TLR3 | TNF | TRAF6 | ZBTB16 | GO:0042110 | 0.0140725464030086 | 116/8243 | 56/3250 | 0.0317548584240754 | 1 | F | F | F | F | T cell activation | ABL1 | ADA | ANXA1 | BAX | BCL2 | BRAF | CASP8 | CD2 | CTNNB1 | CTPS1 | DDOST | DNAJA3 | EIF2AK4 | ELF4 | FADD | FKBP1A | FKBP1B | FYN | HMGB1 | HSPD1 | ICAM1 | IFNA2 | IRF1 | IRF4 | ITK | JMJD6 | KIF13B | LAT | LCP1 | LEF1 | LIG4 | MICA | MSN | NCAPH2 | NCK1 | NEDD4 | PATZ1 | PIK3CG | PRDX2 | PRKDC | PSEN1 | PTPN22 | RAB29 | RC3H1 | RIPK2 | RIPK3 | RUNX2 | SMAD3 | STOML2 | TGFB1 | TNFSF14 | TREML2 | VAV1 | WAS | ZAP70 | ZBTB7B | GO:0006513 | 0.00642969792551256 | 53/8243 | 28/3250 | 0.032475666828628 | 1 | F | F | F | F | protein monoubiquitination | BCOR | BMI1 | CBL | CDC73 | CUL1 | CUL3 | CUL4B | DDB1 | DDB2 | HUWE1 | KLHL22 | NEDD4 | PCGF1 | PCGF5 | PCGF6 | RBX1 | RING1 | RNF126 | RNF2 | TRIM21 | TRIM25 | TSG101 | UBE2B | UBE2D3 | UBE2O | UBE2R2 | UBE2W | ZNF598 | GO:0010639 | 0.0328763799587529 | 271/8243 | 122/3250 | 0.0326422189900138 | 1 | F | F | F | F | negative regulation of organelle organization | ACAA2 | ACD | ADD3 | AICDA | AKT1 | APC | ARRB2 | ATG5 | ATM | ATRX | AURKB | AVIL | BAG3 | BAG4 | BCL2L1 | BCOR | BRCA1 | CAPG | CAPZA1 | CAPZA2 | CAPZB | CCP110 | CDC20 | CENPF | CHMP2A | CLU | CTBP1 | DCP2 | DNM1L | GPX1 | H1F0 | H1FX | HDAC6 | HIST1H1A | HNRNPA1 | HNRNPU | HTRA2 | IK | INSIG1 | KDM1A | KDM4D | KLHL22 | LIMA1 | LMNA | LRRK2 | MAD1L1 | MAD2L1 | MAPT | MCM2 | MDM1 | MECP2 | MYOC | NPM1 | NUBP1 | OPA1 | OTUB1 | PAM16 | PARP1 | PFN1 | PICK1 | PIH1D1 | PINX1 | PLK1 | POT1 | PPARGC1A | PPIF | PRELID1 | PRKAR1A | PRKCD | PRMT6 | PSMD10 | PTTG1 | RAB7A | RAD21 | RDX | SCFD1 | SET | SIN3A | SIRT1 | SKI | SLC25A5 | SLC35F6 | SMAD4 | SMARCB1 | SMG6 | SNCA | SPDL1 | SPI1 | SPTAN1 | SPTBN1 | SPTBN2 | SRC | STMN1 | SWAP70 | TBC1D4 | TERF1 | TERF2 | TERF2IP | TINF2 | TMOD3 | TMOD4 | TNKS | TNKS2 | TOP2A | TPR | TRIAP1 | TRIM9 | TRIP12 | TRIP13 | TUBB4A | TWF1 | TWF2 | TWIST1 | UBE2B | UBR5 | USP17L2 | VAT1 | WAS | XRCC1 | XRCC5 | ZNF207 | ZW10 | GO:1901222 | 0.00885599902947956 | 73/8243 | 37/3250 | 0.0327455927750168 | 1 | F | F | F | F | regulation of NIK/NF-kappaB signaling | ACTN4 | AGER | AGO1 | APP | BIRC2 | CALR | CD27 | CYLD | EDA | EGFR | EP300 | EZR | FBXW11 | HAVCR2 | HDAC7 | HMGB1 | ILK | LIMS1 | MAS1 | NFKBIA | NOD2 | PPM1A | PPM1B | PRDX1 | PTPN22 | RC3H1 | RELA | RHOA | RPS3 | TERF2IP | TLR3 | TNF | TNFSF14 | TRADD | TRAF2 | TRAF6 | TRIP6 | GO:0044782 | 0.0129807109062235 | 107/8243 | 52/3250 | 0.0327770925471054 | 1 | F | F | F | F | cilium organization | ABLIM1 | ACTR2 | ACTR3 | AHI1 | ATP6V1D | ATXN10 | AVIL | CBY1 | CCP110 | CENPJ | CEP290 | CEP89 | CROCC | DCTN1 | DISC1 | DYNLL1 | E2F4 | FAM161B | FBF1 | FOPNL | FOXJ1 | HDAC6 | ICK | IFT122 | IQCB1 | MKS1 | NEK1 | NPHP3 | OFD1 | ONECUT1 | PCM1 | POC1B | PTPN23 | RAB1A | RPGRIP1L | SCLT1 | SEPT2 | SEPT6 | SEPT7 | SNAP29 | SPERT | SSX2IP | TCTN2 | TCTN3 | TEKT2 | TEKT4 | TMEM17 | TMEM216 | TMEM231 | UBXN10 | WDR35 | ZMYND10 | GO:0031346 | 0.0274172024748271 | 226/8243 | 103/3250 | 0.0329338842452841 | 1 | F | F | F | F | positive regulation of cell projection organization | ABL2 | ACTR2 | ADCYAP1 | APBB1 | APC | APOE | ARF1 | ARHGAP35 | ARHGDIA | ARHGEF7 | ARPC2 | AVIL | BRAF | CAMK1D | CAMK2B | CCDC88A | CCL21 | CCP110 | CDC42 | CDC42EP3 | CDC42EP4 | CDKL3 | CENPJ | CEP135 | CROCC | CUL7 | DBN1 | DHX36 | DLG4 | DNM1L | DVL1 | DVL2 | DVL3 | DYNLL1 | EP300 | EPHA4 | EZH2 | FAM98A | FGFR1 | FKBP1B | FN1 | FSCN1 | FYN | GDI1 | GOLGA4 | GRIP1 | HAP1 | HDAC4 | HRAS | HSPA5 | HTT | ILK | IQGAP1 | KCTD17 | KDM1A | LPAR1 | LRRC7 | MAP1B | MAP2K1 | MAP3K13 | MAPK6 | MAPT | METRN | NCK1 | NFE2L2 | NIN | NME1 | NME2 | NTRK1 | OBSL1 | OPA1 | PAFAH1B1 | PARP6 | PFN1 | PLXNA1 | PLXNA2 | PLXNB2 | PRKD1 | PSEN1 | PTN | PTPRD | RAB11A | RAB21 | RAC1 | RAP1A | RGS2 | RHOA | S100A9 | SEMA7A | SEPT7 | SEPT9 | SMURF1 | SNX3 | SRC | STK11 | STK25 | TMEM30A | TNFRSF12A | TUBB2B | TWF1 | TWF2 | ZMYND10 | ZMYND8 | GO:0010720 | 0.0395487079946621 | 326/8243 | 145/3250 | 0.032988242644302 | 1 | F | F | F | F | positive regulation of cell development | ABL1 | ABL2 | ACTR2 | ADCYAP1 | AGER | APBB1 | APOA1 | APOE | APP | ARF1 | ARHGAP35 | ARHGDIA | ARHGEF7 | ARPC2 | ASCL1 | ATOH1 | AVIL | BCL2 | BCL6 | BIN1 | BRAF | C1QA | C1QBP | CALR | CAMK1D | CAMK2B | CDC42 | CDH5 | CDKL3 | CRK | CRKL | CSNK1E | CTNNB1 | CUL7 | DAB1 | DBN1 | DDX39B | DHX36 | DISC1 | DLG4 | DNM1L | DOCK5 | DVL1 | DVL2 | DVL3 | E2F1 | EP300 | EPHA4 | EZH2 | FAM129B | FGB | FGFR1 | FKBP1B | FN1 | FOXA1 | FYN | GDI1 | GFAP | GOLGA4 | GRIP1 | HAP1 | HDAC1 | HDAC2 | HIF1A | HSPA5 | ID2 | ILK | IQGAP1 | KDM1A | LIG4 | LIMS1 | LPAR1 | LRRC7 | MAP1B | MAP2K1 | MAP3K13 | MAPK6 | MAPT | METRN | MME | MYOC | NAP1L1 | NCK1 | NCOA1 | NEUROD1 | NFE2L2 | NIN | NIPBL | NME1 | NME2 | NTRK1 | OBSL1 | OPA1 | P2RX4 | PAFAH1B1 | PARP6 | PIN1 | PLXNA1 | PLXNA2 | PLXNB2 | PPARG | PPP1CC | PRKD1 | PROX1 | PRPF19 | PSEN1 | PTEN | PTN | PTPRD | RAB11A | RAB21 | RAC1 | RAP1A | RARA | RASSF10 | RELA | REST | RGS2 | RHOA | S100A9 | SEMA7A | SERPINE2 | SH3GL3 | SMO | SMURF1 | SNW1 | SNX3 | SOCS2 | SPEN | STK11 | STK25 | TCF12 | TCF4 | TGFB1 | TIMP2 | TMEM30A | TNF | TNFRSF12A | TP73 | TRIM32 | TUBB2B | TWF1 | TWF2 | XRCC5 | ZMYND8 | GO:0072507 | 0.0280237777508189 | 231/8243 | 105/3250 | 0.0340770921456765 | 1 | F | F | F | F | divalent inorganic cation homeostasis | ABL1 | ABL2 | ACKR2 | ADCYAP1 | AFG3L2 | ANK3 | ANXA2 | ANXA6 | ANXA7 | APOE | APP | ATP13A2 | ATP2A2 | ATP2B2 | ATP6V1B1 | BAX | BCL2 | BDKRB2 | C1QTNF1 | CACNA1A | CALM1 | CALM2 | CALM3 | CALR | CAV1 | CCL21 | CCR1 | CCR3 | CCR4 | CCR5 | CD36 | CD40 | CDK5 | CHRNA9 | CXCL11 | DLG4 | EGFR | ESR1 | FASLG | FGF2 | FIS1 | FKBP1B | GLP1R | GNA13 | GNA15 | GNB1 | GOT1 | GRIA1 | GRIK2 | GSTO1 | GTF2I | HAP1 | HEXB | HMGB1 | HTR1B | HTT | IBTK | IMMT | ITGAV | ITPR1 | ITPR2 | ITPR3 | KCTD17 | LETM1 | LPAR1 | LPAR2 | LPAR4 | LRP6 | MCOLN3 | MICU1 | MICU2 | MYO5A | NMB | P2RX1 | P2RX2 | P2RX4 | PIK3CG | PLA2G1B | PLA2G6 | PLCG1 | PML | PRKACA | PRKCE | PSEN1 | PTGER3 | PTGIR | RAP1GDS1 | RGN | RIC3 | SLC39A4 | SMAD3 | SNCA | SRI | STIM1 | STOML2 | SWAP70 | TBXA2R | TGFB1 | TGM2 | THADA | TMBIM6 | TPCN2 | VAPB | VDR | YWHAE | GO:0097435 | 0.0303287637995875 | 250/8243 | 113/3250 | 0.0342074240256137 | 1 | F | F | F | F | supramolecular fiber organization | ABL1 | ACTA1 | ACTN1 | ACTN4 | ACTR2 | ACTR3 | ADD3 | ALDOA | ANXA2 | APP | ARF1 | ARPC1A | ARPC1B | ARPC2 | ARPC3 | ARPC4 | ARPC5 | ARRB1 | BAIAP2 | BAIAP2L1 | BCAR1 | BCL2 | CAP2 | CCDC155 | CCDC88A | CD36 | CDC42 | CENPJ | CFL1 | CFL2 | CHP1 | CKAP5 | COL12A1 | COL14A1 | COL2A1 | COL5A1 | CTTN | CUL3 | DBN1 | DES | DIAPH3 | DSTN | DVL1 | DYNC1H1 | EFEMP2 | EPPK1 | EZR | FIGNL1 | FKBP1A | FSCN1 | FSD1 | GFAP | HSP90AB1 | HSP90B1 | ILK | ITGB5 | KIF2C | KPNB1 | KRT71 | LCP1 | LIMA1 | LOXL2 | LUM | MAPT | MDM2 | MYH11 | MYH6 | MYL2 | MYO1B | NCK1 | NEBL | NUMA1 | OBSL1 | P4HA1 | PAFAH1B1 | PAWR | PCM1 | PDCD10 | PGM5 | PIN1 | PLA2G1B | PLOD2 | PLS3 | PROX1 | PSTPIP1 | RAB11A | RAC1 | RHOA | RHOJ | RHOU | RIPK1 | RIPK3 | SERPINH1 | SNCA | SRC | STMN1 | SWAP70 | TMOD3 | TMOD4 | TNXB | TPM1 | TPM2 | TPM3 | TPM4 | TUBG1 | TUBGCP3 | TWF1 | TWF2 | VASP | VIM | WAS | WDR1 | ZNF207 | GO:2000736 | 0.0107970399126532 | 89/8243 | 44/3250 | 0.0343316127836605 | 1 | F | F | F | F | regulation of stem cell differentiation | ABL1 | BMPR1A | CBFB | DHX36 | ESRRB | GATA1 | HNRNPU | ITCH | KMT2A | LMO2 | NELFB | NFE2L2 | PSMA1 | PSMA2 | PSMA3 | PSMA5 | PSMA6 | PSMB5 | PSMB6 | PSMB7 | PSMB8 | PSMC1 | PSMC2 | PSMC3 | PSMC4 | PSMC5 | PSMC6 | PSMD1 | PSMD10 | PSMD11 | PSMD12 | PSMD14 | PSMD2 | PSMD4 | PSMD9 | PSME2 | PSME3 | PUS7 | REST | RUNX1 | STAT3 | TCF12 | TP73 | YAP1 | GO:2001238 | 0.0046099720975373 | 38/8243 | 21/3250 | 0.0345049071916939 | 1 | F | F | F | F | positive regulation of extrinsic apoptotic signaling pathway | BCL10 | CAV1 | CYLD | FADD | FAF1 | HTRA2 | LTBR | PAK2 | PDIA3 | PML | PPP1CA | PPP2R1A | PPP2R1B | PTEN | RIPK1 | STK3 | THBS1 | TNFRSF12A | TNFSF12 | TRAF2 | WWOX | GO:0009743 | 0.0127380807958268 | 105/8243 | 51/3250 | 0.0346342043352244 | 1 | F | F | F | F | response to carbohydrate | CASP3 | CAT | COL6A2 | CTSV | EIF2B1 | EIF2B3 | EIF2B5 | ENDOG | FIS1 | FKBP1B | GLUL | GOT1 | GSTP1 | HNF4A | ICAM1 | IGF1R | KAT7 | LDHA | LGALS1 | MAP2K4 | MAP2K6 | ME1 | NAMPT | NDUFA9 | NEUROD1 | NME1 | NME2 | NR0B2 | OPA1 | P2RX2 | PFKL | PIK3CA | PPARD | PPARGC1A | PRKAA1 | PRKAA2 | PRKACA | PTEN | RAP1A | RAP1B | RHOA | SARM1 | SIN3A | SLC2A5 | SMAD2 | TGFB1 | THBS1 | TXN2 | TXNIP | WDR35 | XBP1 | GO:0010556 | 0.284362489384933 | 2344/8243 | 961/3250 | 0.0349113640164464 | 1 | F | F | F | F | regulation of macromolecule biosynthetic process | AARS | ABCG1 | ABHD14B | ABL1 | ACD | ACO1 | ACTA2 | ACTL6A | ACTN1 | ACTN2 | ACTN4 | ACTR2 | ACTR3 | ADCYAP1 | ADORA2A | ADRB2 | AEBP2 | AES | AFF1 | AFF4 | AGER | AGO1 | AGO2 | AHI1 | AICDA | AIRE | AKT1 | AKT2 | ANKRD49 | APBB1 | APBB2 | APBB3 | APEX1 | APOE | APP | AR | ARF4 | ARHGAP35 | ARID3B | ARID4B | ARNT | ARNT2 | ARRB1 | ARRB2 | ASCC1 | ASCC2 | ASCL1 | ATAD5 | ATF2 | ATF6 | ATF6B | ATF7 | ATM | ATOH1 | ATRX | ATXN1 | ATXN2 | AURKB | AXIN1 | AZU1 | BACH2 | BAG3 | BANP | BASP1 | BCL10 | BCL11A | BCL2 | BCL6 | BCL6B | BCOR | BIRC2 | BIRC5 | BLZF1 | BMI1 | BMPR1A | BOLA1 | BOLA3 | BRCA1 | BRD2 | BRD3 | BRD4 | BTK | BTRC | BUD31 | C1QBP | CALR | CAMK1D | CAMK2A | CAND1 | CARD9 | CARM1 | CAT | CAV1 | CBFA2T3 | CBFB | CBX4 | CBX7 | CBX8 | CBY1 | CCAR2 | CCDC22 | CCDC88A | CCL21 | CCNT1 | CCNT2 | CCT2 | CCT3 | CCT4 | CCT5 | CCT6A | CCT7 | CCT8 | CD36 | CD40 | CD40LG | CD74 | CD81 | CDC5L | CDC73 | CDH1 | CDK2 | CDK4 | CDK5 | CDK5R1 | CDK8 | CDK9 | CDKN1A | CEBPA | CEBPE | CENPF | CENPJ | CEP290 | CHCHD10 | CHCHD2 | CHCHD3 | CHD3 | CHD4 | CHD8 | CHEK2 | CHP1 | CHRD | CIRBP | CKS2 | CLTC | CLU | COA3 | COMMD1 | COPS2 | COPS5 | CREB1 | CREB3 | CREBBP | CRK | CRY1 | CRY2 | CTBP1 | CTCF | CTDP1 | CTH | CTNNB1 | CTNNBIP1 | CUL2 | CUL3 | CYLD | DAB2 | DAPK1 | DAXX | DBF4B | DCN | DCP2 | DDRGK1 | DDX1 | DDX39B | DDX3X | DHX36 | DHX9 | DMAP1 | DMRT3 | DNA2 | DNAJA3 | DNAJB1 | DPF2 | DTX1 | DUSP1 | DUSP22 | DVL1 | DVL2 | DVL3 | DYRK2 | E2F1 | E2F2 | E2F3 | E2F4 | E2F6 | EAF2 | EBF1 | ECD | EDA | EED | EEF2 | EFEMP1 | EGFR | EGLN1 | EGLN3 | EHMT1 | EHMT2 | EID1 | EIF2AK3 | EIF2AK4 | EIF2B5 | EIF2S1 | EIF3D | EIF3E | EIF3H | EIF4A3 | EIF4E | EIF4E2 | EIF4EBP1 | EIF4ENIF1 | EIF4G3 | EIF4H | EIF5 | EIF5A | EIF5B | EIF6 | ELAVL1 | ELF3 | ELF4 | ENO1 | EP300 | EPRS | ERBB2 | ERF | ERG | ERLIN2 | ERRFI1 | ESR1 | ESR2 | ESRRA | ESRRB | ESRRG | ETS1 | EWSR1 | EXOSC9 | EZH2 | EZR | FADD | FAF1 | FAM129B | FANCA | FASLG | FBP1 | FBXW11 | FGF2 | FGFR1 | FHL2 | FKBP8 | FLOT1 | FOS | FOXA1 | FOXD4 | FOXD4L6 | FOXI2 | FOXJ1 | FOXK1 | FOXK2 | FOXM1 | FOXO1 | FTO | FUBP1 | FUBP3 | FUS | FXR2 | G3BP2 | GABPB1 | GAPDH | GATA1 | GDF9 | GEMIN5 | GLMN | GLO1 | GOLGA2 | GPS2 | GRB7 | GRIP1 | GSK3A | GSK3B | GSTP1 | GTF2A1 | GTF2F1 | GTF2F2 | GTF2I | H1F0 | H1FX | H2AFZ | H3F3A | HAVCR2 | HAX1 | HBEGF | HBZ | HDAC1 | HDAC2 | HDAC3 | HDAC4 | HDAC5 | HDAC6 | HDAC7 | HDAC9 | HDGF | HELZ2 | HEXB | HEXIM1 | HIF1A | HIF1AN | HINT1 | HIST1H1A | HIST1H3A | HIST1H4A | HIST2H3A | HIVEP1 | HLX | HMGB1 | HMGB2 | HMGB3 | HMOX1 | HNF1A | HNF4A | HNF4G | HNRNPA1 | HNRNPA2B1 | HNRNPAB | HNRNPD | HNRNPK | HNRNPL | HNRNPU | HOXA5 | HOXB5 | HOXB6 | HOXB9 | HOXD10 | HRAS | HSF1 | HSF4 | HSP90AA1 | HSP90AB1 | HSPA1B | HSPA5 | HSPA8 | HSPB1 | HSPD1 | HSPH1 | HTATIP2 | HTRA1 | ICAM1 | ID2 | IFI16 | IFNA2 | IGBP1 | IGF2BP1 | IKBKB | IKBKG | IKZF5 | ILF2 | ILF3 | ILK | ING1 | ING5 | INSIG1 | INSIG2 | INSR | IREB2 | IRF1 | IRF4 | IRF7 | ITCH | ITGAV | ITGB1BP1 | ITGB3 | ITGB3BP | JAK3 | JMJD6 | JUN | JUNB | JUND | JUP | KANK2 | KAT5 | KAT7 | KAT8 | KDM1A | KDM2A | KDM5B | KDM5C | KDM8 | KDR | KEAP1 | KHDRBS1 | KLF5 | KLF6 | KMT2A | KMT2B | KMT2C | KRAS | L3MBTL1 | L3MBTL2 | L3MBTL3 | L3MBTL4 | LARP4 | LEF1 | LEMD2 | LEMD3 | LGR4 | LIMS1 | LMO2 | LMO7 | LOXL2 | LPIN3 | LRIF1 | LRP6 | LRPPRC | LRRK2 | LUM | LZTR1 | MAF1 | MAP2K1 | MAP2K3 | MAP2K4 | MAP2K6 | MAP2K7 | MAP3K13 | MAP3K2 | MAP3K4 | MAPK1 | MAPK14 | MAPK15 | MAPK3 | MAPK7 | MAPK8 | MAPK9 | MAS1 | MAVS | MBD3 | MC1R | MDFI | MDM2 | MDM4 | MECP2 | MED1 | MED12 | MED14 | MED17 | MED20 | MED21 | MED23 | MED24 | MED31 | MED4 | MED6 | MEOX2 | MEPCE | METTL23 | MGA | MITF | MKNK2 | MLLT1 | MLLT11 | MLLT3 | MORF4L1 | MORF4L2 | MOV10 | MRGBP | MRPL12 | MSH6 | MTA1 | MTA3 | MTERF1 | MTPN | MXD1 | MYBBP1A | MYC | MYD88 | MYH6 | MYO6 | NACC1 | NAMPT | NANOG | NCBP1 | NCK1 | NCOA1 | NCOA2 | NCOA3 | NCOA4 | NCOR1 | NCOR2 | NDUFA13 | NEDD4 | NEDD8 | NEK7 | NELFB | NELFCD | NELFE | NEUROD1 | NFATC1 | NFATC2 | NFE2L2 | NFKB2 | NFKBIA | NIF3L1 | NIPBL | NKRF | NKX2-1 | NLK | NMD3 | NME2 | NOD2 | NOSTRIN | NPM1 | NR0B2 | NR1H2 | NR1H3 | NR1I2 | NR3C1 | NR4A1 | NR5A2 | NTRK1 | NUFIP1 | NUMA1 | NUP85 | NUP98 | NUPR1 | OGFOD1 | OGT | ONECUT1 | PA2G4 | PABPC1 | PAIP1 | PAK6 | PARK7 | PARP1 | PATZ1 | PAWR | PAX8 | PAX9 | PAXIP1 | PCBD1 | PCBP1 | PCGF1 | PCGF5 | PCGF6 | PCNA | PELI1 | PER1 | PER2 | PFKM | PFN1 | PHB | PHB2 | PHF10 | PHF20L1 | PHF21A | PIAS1 | PIAS3 | PIH1D1 | PIK3R1 | PIM1 | PIN1 | PINX1 | PIR | PKIG | PLA2G10 | PLA2G1B | PLA2G6 | PLK1 | PLSCR1 | PML | PNKP | POLDIP3 | POLR2A | POLR2L | POT1 | POU2F3 | POU5F1 | PPARD | PPARG | PPARGC1A | PPID | PPIE | PPM1A | PPM1D | PPP1CA | PPP1CB | PPP1R12A | PPP1R3B | PPP2CA | PPP2R1A | PRDX2 | PRDX3 | PRDX5 | PRKAA1 | PRKAR1A | PRKCD | PRKCZ | PRKD1 | PRKD2 | PRKDC | PRMT2 | PRMT3 | PRMT6 | PROX1 | PSEN1 | PSMA1 | PSMA2 | PSMA3 | PSMA5 | PSMA6 | PSMB5 | PSMB6 | PSMB7 | PSMB8 | PSMC1 | PSMC2 | PSMC3 | PSMC4 | PSMC5 | PSMC6 | PSMD1 | PSMD10 | PSMD11 | PSMD12 | PSMD14 | PSMD2 | PSMD4 | PSMD9 | PSME2 | PSME3 | PTEN | PTGES2 | PTGES3 | PTPN14 | PTPRK | PTTG1 | PUM1 | PUS7 | QARS | QRICH1 | RAB3GAP1 | RAC1 | RAD21 | RAF1 | RAN | RARA | RARG | RASD1 | RB1 | RBBP6 | RBM3 | RBM4 | RBM8A | RBMX | RBPJ | RBX1 | RC3H1 | REL | RELA | REST | RFC1 | RFC2 | RFC3 | RFC4 | RFC5 | RFXANK | RGN | RGS2 | RHOA | RIF1 | RING1 | RIPK1 | RIPK2 | RIPK3 | RIPK4 | RMND1 | RNF139 | RNF2 | RNF25 | RNF31 | RNF4 | RNF8 | RPL10 | RPL23 | RPL38 | RPL6 | RPS14 | RPS3 | RPS4X | RPS6KA1 | RPS6KB2 | RPTOR | RRM2 | RTF1 | RUNX1 | RUNX1T1 | RUNX2 | RUVBL1 | RUVBL2 | RXRA | RXRB | RXRG | S100A1 | S100A11 | S100A12 | S100A9 | SAP18 | SARS | SCML2 | SET | SETBP1 | SETDB1 | SF1 | SFMBT1 | SFPQ | SH2B1 | SHC1 | SHMT1 | SHMT2 | SIN3A | SIRT1 | SIRT5 | SIRT6 | SIRT7 | SKI | SLC9A1 | SLTM | SMAD2 | SMAD3 | SMAD4 | SMAD5 | SMAD9 | SMARCA2 | SMARCA4 | SMARCB1 | SMARCC1 | SMARCC2 | SMARCE1 | SMC1A | SMC3 | SMG5 | SMG6 | SMO | SMURF1 | SMURF2 | SNAI2 | SNCA | SND1 | SNF8 | SNW1 | SNX6 | SOAT1 | SOD2 | SOX2 | SP1 | SP100 | SP2 | SPEN | SPI1 | SPIN1 | SQSTM1 | SRC | SRI | STAG2 | STAT1 | STAT3 | STAT4 | STAT5A | STAT6 | STK3 | STOML2 | STRAP | SUB1 | SUMO1 | SUMO2 | SUPT4H1 | SUPT5H | SUZ12 | TAB1 | TAB2 | TACO1 | TADA2B | TAF12 | TAF15 | TAGLN3 | TARDBP | TBL1X | TCEA1 | TCEA2 | TCERG1 | TCF12 | TCF21 | TCF25 | TCF4 | TCOF1 | TCP1 | TCP10L | TDRD3 | TERF1 | TERF2 | TERF2IP | TFCP2 | TFDP1 | TGFB1 | TGFBR3 | TGIF2LX | THAP11 | THAP7 | THBS1 | THRA | TIA1 | TIAL1 | TINF2 | TLE1 | TLE3 | TLR3 | TMBIM6 | TMEM173 | TMPO | TNF | TNFAIP3 | TNFRSF1A | TNFRSF8 | TNIP2 | TNKS | TNKS2 | TNP1 | TOE1 | TOP2A | TP53 | TP63 | TP73 | TPR | TRADD | TRAF1 | TRAF2 | TRAF3 | TRAF6 | TRAP1 | TRIAP1 | TRIB2 | TRIB3 | TRIM14 | TRIM21 | TRIM25 | TRIM28 | TRIM29 | TRIM32 | TRIM33 | TRIM34 | TRIP13 | TRIP4 | TRUB2 | TSC22D1 | TSC22D3 | TSC22D4 | TSFM | TSG101 | TSPYL2 | TTC5 | TULP3 | TWIST1 | TXN | TXNIP | TYMS | UBA3 | UBB | UBC | UBE2D1 | UBE2D3 | UBE2I | UBE2J1 | UBE2L3 | UBE2N | UBE2O | UBE2V1 | UNK | UNKL | UPF3B | UQCC1 | USP15 | USP16 | USP2 | USP21 | USP47 | USP7 | USP9X | USP9Y | VDR | VGLL4 | VHL | VIM | VPS72 | WAS | WBP2 | WDR77 | WDTC1 | WNT2 | WT1 | WWOX | WWP2 | XBP1 | XIAP | XPO1 | XRCC5 | XRCC6 | YAF2 | YAP1 | YEATS2 | YES1 | YTHDF1 | YWHAB | YWHAH | YWHAQ | YY1 | ZBTB16 | ZBTB17 | ZBTB21 | ZBTB25 | ZBTB33 | ZBTB42 | ZBTB7A | ZBTB7B | ZC3H4 | ZFP41 | ZHX1 | ZIC1 | ZKSCAN1 | ZMPSTE24 | ZMYM5 | ZMYND8 | ZNF207 | ZNF217 | ZNF227 | ZNF24 | ZNF277 | ZNF354C | ZNF408 | ZNF420 | ZNF443 | ZNF517 | ZNF518A | ZNF592 | ZNF598 | ZNF618 | ZNF622 | ZNF646 | ZNF669 | ZNF696 | ZNF746 | ZNF747 | ZRANB2 | ZSCAN2 | ZSCAN29 | ZSCAN32 | ZZZ3 | GO:0015031 | 0.107849084071333 | 889/8243 | 376/3250 | 0.0350280747443222 | 1 | F | F | F | F | protein transport | ABCE1 | ABCG1 | ACAA1 | ACD | ACOX1 | ACTN4 | ADCYAP1 | ADPRH | AFM | AGAP1 | AGAP2 | AGK | AHCYL1 | AKAP8 | AKT1 | AKT2 | AKTIP | ANK3 | ANXA1 | AP1B1 | AP1G1 | AP1M1 | AP3D1 | AP3M1 | AP4M1 | AP4S1 | APOE | APPBP2 | ARF1 | ARF4 | ARF5 | ARFGAP1 | ARFIP1 | ARHGAP1 | ARL4D | ARL5B | ARRB1 | ARRB2 | ASPSCR1 | ATG4C | ATP6V1A | ATP6V1B1 | ATP6V1C1 | ATP6V1D | ATP6V1E1 | AUP1 | BAG3 | BLZF1 | C16orf70 | CACNG2 | CACNG4 | CACNG5 | CALR | CANX | CAT | CBLB | CCDC22 | CCHCR1 | CCT6B | CD36 | CD40LG | CD74 | CDC37 | CDK5 | CENPF | CEP290 | CHMP2A | CHP1 | CLSTN1 | CLTC | CLU | COG3 | COMMD1 | COPA | COPB1 | COPE | COPG1 | CPLX1 | CPLX3 | CROT | CSE1L | CTSA | CTTN | DAB2 | DDX19A | DENND1A | DENND4C | DERL1 | DHRS4 | DLG2 | DNAJC19 | DUSP16 | DYNLL1 | DYNLT1 | ECH1 | ECI2 | EHHADH | EIF4ENIF1 | EIF5A | EIF6 | EXOC6 | FAF2 | FBXO7 | FIS1 | FKBP1B | FRAS1 | GBP5 | GDI1 | GDI2 | GGA1 | GOLGA2 | GOLGA4 | GOLPH3 | GOLT1B | GOPC | GRIP1 | GRPEL1 | GSTK1 | HAP1 | HDAC6 | HEATR3 | HERC2 | HGS | HID1 | HMGB1 | HMGCL | HNF1A | HNRNPU | HSD17B4 | HSP90AA1 | HSP90B1 | HSPA4 | HSPA5 | HSPA8 | HSPB1 | HSPD1 | ICK | IDE | IDH1 | IFIT1 | IFT122 | IPO11 | IPO4 | IPO5 | IPO7 | IPO8 | IPO9 | ITGAV | ITGB1BP1 | KIF13B | KIF5B | KIF5C | KLHL20 | KPNA1 | KPNA2 | KPNB1 | LARS | LCP2 | LMAN2L | LMNA | LRRC7 | LRSAM1 | MAPK8IP3 | MCFD2 | MTX1 | MYO1C | MYO5A | MYO6 | NBAS | NCOA4 | NDUFA13 | NEDD4 | NEUROD1 | NMD3 | NOD2 | NOS2 | NPLOC4 | NPM1 | NUDT19 | NUP107 | NUP153 | NUP214 | NUP85 | NUP88 | NUP93 | NUP98 | NUTF2 | NXT2 | OPTN | OXA1L | PAFAH1B1 | PAM16 | PARD3 | PARK7 | PCM1 | PDCD6 | PDCD6IP | PDIA4 | PEX19 | PEX5 | PHB2 | PICK1 | PIK3R1 | PLEKHF2 | PML | PNP | POM121 | PPARG | PPID | PPT1 | PSEN1 | PTPN23 | RAB10 | RAB11A | RAB11B | RAB11FIP2 | RAB18 | RAB1A | RAB1B | RAB21 | RAB24 | RAB29 | RAB2A | RAB31 | RAB32 | RAB35 | RAB3IP | RAB5A | RAB5B | RAB7A | RAB9A | RABGEF1 | RABL2A | RABL3 | RAF1 | RAN | RANBP3 | RAP1A | RAPGEF4 | RASSF9 | RBPJ | ROMO1 | RP2 | RPAIN | RPL10 | RPL10A | RPL12 | RPL18A | RPL22 | RPL23 | RPL23A | RPL24 | RPL30 | RPL37A | RPL38 | RPL6 | RPL7 | RPL7A | RPL9 | RPLP0 | RPLP1 | RPLP2 | RPS12 | RPS13 | RPS14 | RPS15A | RPS19 | RPS2 | RPS20 | RPS24 | RPS29 | RPS3 | RPS4X | RPS8 | RRS1 | S100A12 | S100A13 | SCFD1 | SCG2 | SDCBP | SEC13 | SEC16A | SEC22C | SEC24B | SEC24C | SEC31A | SEC62 | SEC63 | SEL1L | SELENBP1 | SFPQ | SGTA | SGTB | SLC15A1 | SLC25A6 | SMAD2 | SMURF1 | SNAP29 | SNF8 | SNX12 | SNX17 | SNX2 | SNX21 | SNX24 | SNX3 | SNX6 | SPTBN1 | SRP14 | SRP19 | SSX2IP | STAT3 | STX5 | STXBP1 | STXBP2 | STYX | SYNJ2BP | SYVN1 | TANGO2 | TBC1D15 | TBC1D22B | TBC1D4 | TCIRG1 | TCP1 | TERF2 | TGFB1 | TIMM13 | TIMM21 | TIMM23 | TIMM44 | TIMM50 | TMED9 | TNKS | TNPO1 | TOMM20 | TOMM22 | TOMM40 | TOR1A | TPR | TRAF3IP1 | TRIM23 | TSG101 | TXNIP | UBB | UBC | UBE2D1 | UBE2D2 | UBE2D3 | UNC119 | USP9X | VCP | VGF | VPS13B | VPS18 | VPS26A | VPS29 | VPS37D | VPS4B | VPS8 | VTA1 | WDR19 | WDR35 | XBP1 | XPO1 | XPO4 | XPO5 | XPO7 | YKT6 | YWHAB | YWHAE | YWHAG | YWHAH | ZBTB7B | ZDHHC17 | ZDHHC23 | ZFAND6 | ZW10 | GO:0009150 | 0.0116462452990416 | 96/8243 | 47/3250 | 0.0355868794660319 | 1 | F | F | F | F | purine ribonucleotide metabolic process | ABHD14B | ACACA | ACAT1 | ACLY | ACOT13 | ACOT7 | ACOT9 | ADCYAP1 | ADSS | AK1 | AK2 | AK4 | ATP6V1A | ATP6V1B1 | CLPX | CROT | DBI | DLG2 | DLST | FAR2 | FASN | FIGNL1 | GMPR | GNAI3 | HMGCL | HPRT1 | HSD17B4 | HSPA1B | HSPA8 | LRRK2 | MVD | MYH6 | NDUFS1 | NMNAT1 | NT5C2 | OGDH | OLA1 | OPA1 | PARP1 | RAN | RHOA | SUCLA2 | SUCLG2 | SULT1A3 | THEM4 | TPST2 | VCP | GO:0006955 | 0.0568967608880262 | 469/8243 | 204/3250 | 0.0357603293547196 | 1 | F | F | F | F | immune response | ACKR2 | AGER | AHCY | AIRE | AKAP8 | ANXA1 | APCS | APP | AZU1 | BCL10 | BCL2 | BCL6 | BMPR1A | BTK | C1QA | C1QBP | C1R | C4A | CAPZA1 | CAPZA2 | CARD9 | CCL21 | CCL22 | CCR1 | CCR3 | CCR4 | CCR5 | CD209 | CD27 | CD6 | CD74 | CD79B | CD81 | CHST4 | CLNK | CLU | COL4A3BP | COLEC10 | CRIP1 | CRP | CTSC | CTSL | CXCL11 | CXCL5 | CXCL6 | CXCL8 | CYLD | DBNL | DDX1 | DDX3X | DHX36 | DHX9 | ECSIT | EDA | EIF2AK4 | ELF4 | EPPIN | ETS1 | EXOSC9 | FADD | FASLG | FCGR1A | FCN1 | FGB | FOXJ1 | FTH1 | FYN | GAPDH | GBP2 | GBP6 | GNL1 | GPI | GZMA | HAVCR2 | HEXIM1 | HLA-A | HLA-B | HLA-DQA1 | HLA-DRA | HLA-DRB3 | HLA-DRB4 | HMGB1 | HMGB2 | HMGB3 | HPRT1 | HRAS | HSPD1 | ICAM1 | IFI16 | IFITM3 | IFNA2 | IGF1R | IGHV3-13 | IGHV3-23 | IGHV3-30 | IGKV3-20 | IGLC7 | IKBKB | IKBKE | IKBKG | IL13RA2 | IL32 | IPO7 | IRF7 | ITCH | ITK | JAK3 | KIR3DS1 | KLRC2 | LAMP3 | LAT | LCP2 | LGALS3 | LGR4 | LTBR | MAP3K14 | MASP1 | MASP2 | MATR3 | MAVS | MBP | MICA | MIF | NEDD4 | NFKB2 | NOD2 | NOS2 | OPTN | OTUB1 | OTUD4 | PADI4 | PAG1 | PARP14 | PCBP2 | PIK3CG | PLA2G1B | PLA2G2A | PLA2G6 | PML | PNMA1 | PNP | PPARG | PPP6C | PRDX1 | PRELID1 | PRKCD | PRKD1 | PRKD2 | PRKDC | PSTPIP1 | PTPN6 | PXDN | RBPJ | RGS1 | RIPK2 | RNASE3 | ROMO1 | RPL30 | RPS19 | S100A12 | S100A7 | S100A9 | S100B | SARM1 | SCARA3 | SEMA7A | SFPQ | SLAMF1 | SMAD3 | SP2 | SRPK1 | SRPK2 | TCF12 | TGFB1 | TGFBR3 | THBS1 | TLR3 | TMEM173 | TNF | TNFSF12 | TNFSF14 | TOLLIP | TRAF3 | TRAF6 | TREX1 | TRIM14 | TRIM21 | TRIM23 | TRIM25 | TRIM28 | TRIM29 | TRIM32 | TRIM35 | TUBB | TUBB4B | USP14 | VAV1 | VSIG4 | VTN | WAS | XBP1 | XRCC5 | XRCC6 | ZAP70 | GO:0016525 | 0.00727890331190101 | 60/8243 | 31/3250 | 0.0359617685389913 | 1 | F | F | F | F | negative regulation of angiogenesis | AGO1 | ATF2 | COL4A2 | CTNNB1 | DCN | E2F2 | FAM129B | FASLG | GTF2I | HDAC5 | HGS | HOXA5 | HSPG2 | ITGB1BP1 | MECP2 | MEOX2 | NPR1 | PDCD10 | PGK1 | PML | PPARG | PTN | RHOA | SARS | SPRY2 | STARD13 | STAT1 | SYNJ2BP | TBXA2R | THBS1 | THBS2 | GO:0070317 | 0.0043673419871406 | 36/8243 | 20/3250 | 0.0362132802177017 | 1 | F | F | F | F | negative regulation of G0 to G1 transition | BMI1 | BRCA1 | E2F1 | E2F6 | EED | EHMT1 | EHMT2 | EZH2 | L3MBTL2 | MGA | PCGF6 | PHC3 | RAD51 | RBBP4 | RING1 | RNF2 | RRM2 | SUZ12 | TFDP1 | YAF2 | GO:0007031 | 0.00594443770471916 | 49/8243 | 26/3250 | 0.0362204612282031 | 1 | F | F | F | F | peroxisome organization | ABCD3 | ACAA1 | ACOX1 | CAT | CROT | DHRS4 | ECH1 | ECI2 | EHHADH | GSTK1 | HMGCL | HSD17B4 | IDE | IDH1 | NOS2 | NUDT19 | PEX11B | PEX19 | PEX5 | UBB | UBC | UBE2D1 | UBE2D2 | UBE2D3 | USP9X | ZFAND6 | GO:0033036 | 0.152856969549921 | 1260/8243 | 526/3250 | 0.0363044691658993 | 1 | F | F | F | F | macromolecule localization | ABCE1 | ABCG1 | ABHD17A | ABL1 | ACAA1 | ACD | ACOX1 | ACTN2 | ACTN4 | ADCYAP1 | ADPRH | AFM | AGAP1 | AGAP2 | AGER | AGK | AHCYL1 | AHI1 | AKAP8 | AKT1 | AKT2 | AKTIP | ANK1 | ANK3 | ANKS4B | ANXA1 | ANXA2 | AP1B1 | AP1G1 | AP1M1 | AP3D1 | AP3M1 | AP4M1 | AP4S1 | APOE | APPBP2 | ARF1 | ARF4 | ARF5 | ARFGAP1 | ARFIP1 | ARHGAP1 | ARL4D | ARL5B | ARRB1 | ARRB2 | ASPSCR1 | ATG4C | ATM | ATP1B3 | ATP6V1A | ATP6V1B1 | ATP6V1C1 | ATP6V1D | ATP6V1E1 | ATRX | AUP1 | AURKB | BAG3 | BAG4 | BAG6 | BANP | BAX | BCL6 | BIRC5 | BLZF1 | BRAF | BSG | C16orf70 | CACNG2 | CACNG4 | CACNG5 | CALM1 | CALM3 | CALR | CANX | CARM1 | CAT | CAV1 | CBLB | CBY1 | CCDC120 | CCDC22 | CCDC68 | CCHCR1 | CCT2 | CCT4 | CCT6B | CD36 | CD40LG | CD74 | CD81 | CDC37 | CDH1 | CDK5 | CENPF | CEP128 | CEP290 | CEP55 | CHCHD10 | CHMP2A | CHP1 | CLSTN1 | CLTC | CLU | COG3 | COMMD1 | COPA | COPB1 | COPE | COPG1 | CPLX1 | CPLX3 | CROCC | CROT | CSE1L | CTCF | CTNNB1 | CTSA | CTTN | CUTA | DAB2 | DDX1 | DDX19A | DDX3X | DENND1A | DENND4C | DERL1 | DHRS4 | DHX9 | DLG2 | DLG3 | DLG4 | DMD | DNAJA1 | DNAJA3 | DNAJC19 | DNM1L | DUSP16 | DVL1 | DVL2 | DYNLL1 | DYNLT1 | ECH1 | ECI2 | EGFR | EHHADH | EIF4ENIF1 | EIF5A | EIF6 | ESR1 | EXOC6 | EZH2 | EZR | FAF2 | FAM83D | FAM83H | FBXO7 | FCN1 | FERMT2 | FIS1 | FKBP1B | FLOT1 | FRAS1 | GAK | GBP2 | GBP5 | GDI1 | GDI2 | GGA1 | GLRB | GOLGA2 | GOLGA4 | GOLPH3 | GOLT1B | GOPC | GORASP2 | GRID2 | GRIK2 | GRIP1 | GRPEL1 | GSTK1 | HAP1 | HCN1 | HDAC6 | HEATR3 | HERC2 | HGS | HID1 | HK1 | HK2 | HMGB1 | HMGCL | HNF1A | HNRNPU | HSD17B4 | HSP90AA1 | HSP90B1 | HSPA4 | HSPA5 | HSPA8 | HSPB1 | HSPD1 | ICK | IDE | IDH1 | IFIT1 | IFT122 | IK | IKBKB | IPO11 | IPO4 | IPO5 | IPO7 | IPO8 | IPO9 | ITGA4 | ITGAV | ITGB1BP1 | JUP | KIF13B | KIF5B | KIF5C | KLHL20 | KPNA1 | KPNA2 | KPNB1 | LARS | LATS1 | LCP2 | LEMD2 | LIMS1 | LMAN2L | LMNA | LRP6 | LRRC7 | LRRK2 | LRSAM1 | MAPK15 | MAPK8IP3 | MAPK9 | MARCH5 | MCC | MCFD2 | MCM9 | MDM2 | MECP2 | MTCH2 | MTX1 | MYO1C | MYO5A | MYO6 | NBAS | NCOA4 | NDUFA13 | NEDD4 | NEDD8 | NEUROD1 | NIN | NIPBL | NMD3 | NOD2 | NOS2 | NPLOC4 | NPM1 | NRXN1 | NUBP1 | NUDT19 | NUP107 | NUP153 | NUP214 | NUP85 | NUP88 | NUP93 | NUP98 | NUTF2 | NXT2 | OBSL1 | OPTN | OXA1L | PACS1 | PACS2 | PAFAH1B1 | PAM16 | PARD3 | PARK7 | PCM1 | PDCD6 | PDCD6IP | PDIA4 | PEX19 | PEX5 | PHB2 | PICK1 | PIGU | PIH1D1 | PIK3R1 | PIKFYVE | PINX1 | PLEKHF1 | PLEKHF2 | PLK1 | PML | PNP | POM121 | POT1 | PPARG | PPID | PPT1 | PRKCZ | PRPF19 | PSEN1 | PTPN23 | PTPRK | RAB10 | RAB11A | RAB11B | RAB11FIP2 | RAB18 | RAB1A | RAB1B | RAB21 | RAB24 | RAB29 | RAB2A | RAB31 | RAB32 | RAB35 | RAB3GAP1 | RAB3IP | RAB5A | RAB5B | RAB7A | RAB9A | RABGEF1 | RABL2A | RABL3 | RAD21 | RAF1 | RAN | RANBP3 | RAP1A | RAPGEF4 | RASSF9 | RB1 | RBPJ | RDX | RIC3 | ROMO1 | RP2 | RPA2 | RPAIN | RPL10 | RPL10A | RPL12 | RPL18A | RPL22 | RPL23 | RPL23A | RPL24 | RPL30 | RPL37A | RPL38 | RPL6 | RPL7 | RPL7A | RPL9 | RPLP0 | RPLP1 | RPLP2 | RPS12 | RPS13 | RPS14 | RPS15A | RPS19 | RPS2 | RPS20 | RPS24 | RPS29 | RPS3 | RPS4X | RPS8 | RRS1 | RTKN | RTN4 | RUVBL2 | S100A12 | S100A13 | SCFD1 | SCG2 | SCG3 | SDCBP | SEC13 | SEC16A | SEC22C | SEC24B | SEC24C | SEC31A | SEC62 | SEC63 | SEL1L | SELENBP1 | SFPQ | SGTA | SGTB | SIN3A | SLC15A1 | SLC25A6 | SLC9A3R1 | SMAD2 | SMO | SMURF1 | SNAP29 | SNF8 | SNX12 | SNX17 | SNX2 | SNX21 | SNX24 | SNX3 | SNX6 | SPDL1 | SPTBN1 | SQSTM1 | SRP14 | SRP19 | SSB | SSX2IP | STAT3 | STIL | STOML2 | STX5 | STXBP1 | STXBP2 | STYX | SUMO1 | SYNJ2BP | SYVN1 | TANGO2 | TBC1D15 | TBC1D22B | TBC1D4 | TCIRG1 | TCP1 | TERF2 | TERF2IP | TGFB1 | TIA1 | TIMM13 | TIMM21 | TIMM23 | TIMM44 | TIMM50 | TINF2 | TM9SF4 | TMED9 | TMEM30A | TNF | TNFAIP3 | TNFRSF1A | TNKS | TNKS2 | TNPO1 | TOLLIP | TOMM20 | TOMM22 | TOMM40 | TOR1A | TP53 | TPR | TRAF3IP1 | TRAPPC8 | TREX1 | TRIM23 | TSG101 | TSPAN17 | TULP3 | TXNIP | UBB | UBC | UBE2D1 | UBE2D2 | UBE2D3 | UBL4A | UGT8 | ULK1 | UNC119 | USP4 | USP9X | VAPA | VCL | VCP | VGF | VPS13B | VPS18 | VPS26A | VPS29 | VPS37D | VPS4B | VPS8 | VTA1 | WBP2 | WDR19 | WDR35 | WDR45B | WIPI1 | WIPI2 | WNK4 | XBP1 | XPO1 | XPO4 | XPO5 | XPO7 | XRCC5 | YKT6 | YWHAB | YWHAE | YWHAG | YWHAH | ZBTB16 | ZBTB7A | ZBTB7B | ZDHHC17 | ZDHHC23 | ZFAND6 | ZW10 | GO:0002685 | 0.0105544098022565 | 87/8243 | 43/3250 | 0.036318665604723 | 1 | F | F | F | F | regulation of leukocyte migration | ADA | ADTRP | AGER | AIRE | AKT1 | ANXA1 | APOD | APP | C1QBP | CALR | CAMK1D | CCL21 | CCR1 | CD74 | CREB3 | CRK | CRKL | CXCL8 | DUSP1 | FADD | HMGB1 | HMOX1 | ICAM1 | ITGA4 | LGALS3 | LGMN | MAPK14 | MAPK3 | MIF | MSN | OXSR1 | P2RX4 | RAC1 | RHOA | RIPK3 | S100A7 | SLAMF8 | SWAP70 | TGFB1 | THBS1 | TNF | TNFSF14 | WNK1 | GO:0032147 | 0.0260827368676453 | 215/8243 | 98/3250 | 0.0366131188772961 | 1 | F | F | F | F | activation of protein kinase activity | ABL1 | ADCY6 | ADRB2 | AKT1 | ARRB1 | AXIN1 | BIRC7 | BRAF | CAB39 | CCDC88A | CD40LG | CD74 | CD81 | CRK | CRKL | DAXX | DBF4B | DBNL | DUSP19 | DUSP7 | EGFR | FGF2 | GADD45G | IKBKG | INSR | ITGB1BP1 | ITGB3 | LPAR1 | LRRK2 | MAP2K1 | MAP2K3 | MAP2K4 | MAP2K6 | MAP2K7 | MAP3K1 | MAP3K13 | MAP3K14 | MAP3K2 | MAP3K3 | MAP3K4 | MAP4K1 | MAPK1 | MAPK14 | MAPK3 | MAPK8IP3 | MAPKAP1 | MAS1 | MLST8 | NEK1 | NOD2 | NTF3 | NTRK1 | OXSR1 | PAK2 | PAK6 | PARK7 | PDE6G | PIK3CA | PLA2G1B | PRDX2 | PRKAA1 | PRKACA | PRKAR1A | PRKAR2B | PRKCD | PRKCZ | PRRC1 | PSEN1 | PTPN1 | RAF1 | RIPK1 | RIPK2 | RIPK3 | RPLP1 | RPTOR | SHC1 | SOD1 | SRC | STK11 | STK24 | STK25 | STK26 | STK3 | TAB1 | TAB2 | TAOK3 | THBS1 | TLR3 | TNF | TP73 | TRAF2 | TRAF6 | TXN | UBB | UBC | UBE2N | UBE2V1 | WNK1 | GO:0051053 | 0.00837073880868616 | 69/8243 | 35/3250 | 0.0366767473733619 | 1 | F | F | F | F | negative regulation of DNA metabolic process | ACD | ATM | BCL6 | DCP2 | GZMA | H1F0 | H1FX | HIST1H1A | HNRNPA1 | HNRNPU | HSF1 | KMT2A | MLH1 | MSH6 | OTUB1 | PARP1 | PINX1 | POT1 | RIF1 | RPS3 | SMG6 | SRC | TERF1 | TERF2 | TERF2IP | TFIP11 | TINF2 | TNKS | TNKS2 | TRIM28 | TRIP12 | TWIST1 | UBR5 | XRCC1 | XRCC5 | GO:0050921 | 0.00837073880868616 | 69/8243 | 35/3250 | 0.0366767473733619 | 1 | F | F | F | F | positive regulation of chemotaxis | AGER | AZU1 | C1QBP | CALR | CAMK1D | CCL21 | CCR1 | CCR4 | CD74 | CREB3 | CXCL8 | FGF2 | FGFR1 | HMGB1 | HSPB1 | KDR | LGMN | LPAR1 | MAPK14 | MAPK3 | NTF3 | OXSR1 | P2RX4 | PRKD1 | PRKD2 | RAC1 | S100A7 | SCG2 | SMAD3 | SWAP70 | TGFB1 | THBS1 | TNFSF14 | TUBB2B | WNK1 | GO:0016241 | 0.0161349023413806 | 133/8243 | 63/3250 | 0.0368131320269374 | 1 | F | F | F | F | regulation of macroautophagy | ADRB2 | AKT1 | ATP13A2 | ATP6V1A | ATP6V1B1 | ATP6V1C1 | ATP6V1D | ATP6V1E1 | BAG3 | CALCOCO2 | CAPN1 | CAPNS1 | CASP3 | CDC37 | CDK5 | CDK5R1 | DCN | EPM2A | GAPDH | GNAI3 | GPSM1 | HDAC6 | HIF1A | HMOX1 | HTRA2 | HTT | IKBKG | KDR | LRRK2 | LRSAM1 | MAPK3 | MAPK8 | MLST8 | NEDD4 | OPTN | PIK3CA | PIKFYVE | PIP4K2A | POLDIP2 | PRKAA1 | PRKAA2 | PRKAB2 | PRKACA | PRKAG1 | QSOX1 | RAB1B | RAB3GAP1 | RAB5A | RB1CC1 | RIPK2 | RPTOR | SCFD1 | SIRT1 | SMURF1 | SNX6 | SPTLC2 | SUPT5H | UBQLN1 | UBQLN2 | UBQLN4 | UCHL1 | ULK1 | VPS26A | GO:0045861 | 0.0252335314812568 | 208/8243 | 95/3250 | 0.0370414622608992 | 1 | F | F | F | F | negative regulation of proteolysis | A2M | ADORA2A | AHSG | AKT1 | APP | ARRB1 | ARRB2 | BAG5 | BAG6 | BIN1 | BIRC2 | BIRC3 | BIRC5 | BIRC7 | BIRC8 | C4A | CCAR2 | CD109 | CD27 | CDK5 | CRYAB | CSNK2A2 | DDRGK1 | DDX3X | DHCR24 | DPEP1 | EPHA4 | EPPIN | FABP1 | FAM192A | GAPDH | GPI | GPX1 | HDAC6 | HSP90AB1 | HSP90AB3P | HSP90AB4P | IDE | IFI16 | IGBP1 | LAMP3 | LEF1 | LRRK2 | MDM2 | NR1H2 | OGT | PAK2 | PAPLN | PARK7 | PEBP1 | PIH1D1 | PLAUR | PML | PRDX3 | PRDX5 | PSEN1 | PTTG1 | RAF1 | RECK | RPL23 | RPS6KA1 | SDCBP | SERPINB2 | SERPINB3 | SERPINB5 | SERPINB6 | SERPINB8 | SERPINE2 | SERPINH1 | SFN | SGTA | SMARCC1 | SNCA | SNX12 | SPINK7 | SPINT2 | SRC | STYX | THBS1 | TIMP1 | TIMP2 | TNFSF14 | TRIAP1 | TRIM21 | UBE2J1 | UBXN1 | UCHL5 | USP14 | USP17L2 | USP19 | USP47 | USP7 | VTN | XIAP | YWHAE | GO:0070302 | 0.0195317238869344 | 161/8243 | 75/3250 | 0.037123399754668 | 1 | F | F | F | F | regulation of stress-activated protein kinase signaling cascade | AGER | AKT1 | APP | AXIN1 | BIRC7 | CARD9 | CCL21 | CD27 | CD40LG | COPS5 | CYLD | DAXX | DBNL | DNAJA1 | DUSP1 | DUSP19 | DUSP22 | DVL2 | DVL3 | EGFR | EPHA4 | EZR | FOXM1 | FOXO1 | GADD45G | GPS2 | GRIK2 | GSTP1 | HDAC3 | HMGB1 | HRAS | IGBP1 | IGF1R | ITCH | KLHDC10 | LTBR | MAP2K1 | MAP2K4 | MAP2K7 | MAP3K13 | MAP3K2 | MAP3K3 | MAP3K4 | MAP4K1 | MAPK1 | MAPK3 | MAPK8IP3 | MYC | NCOR1 | NOD2 | PAFAH1B1 | PDCD10 | PER1 | PRDX1 | PTPN1 | PTPN22 | QARS | RB1CC1 | RIPK1 | RIPK2 | RPS3 | SDCBP | SERPINB3 | SLAMF1 | STK25 | STK3 | TAOK3 | TGFBR3 | TLR3 | TNF | TRAF2 | TRAF6 | WNT16 | ZMPSTE24 | ZNF622 | GO:0006470 | 0.0186825185005459 | 154/8243 | 72/3250 | 0.0371717234217079 | 1 | F | F | F | F | protein dephosphorylation | ACP1 | BCL2 | BTRC | CDC14B | CDC25A | CDC25B | CDC25C | CTDP1 | CYCS | DAPP1 | DUSP1 | DUSP12 | DUSP13 | DUSP14 | DUSP16 | DUSP19 | DUSP22 | DUSP7 | EYA2 | EYA4 | FBXW11 | MTMR14 | MTMR7 | MYH6 | NCK1 | PGAM5 | PGP | PPA2 | PPEF1 | PPM1A | PPM1B | PPM1D | PPM1H | PPM1K | PPP1CA | PPP1CB | PPP1CC | PPP1R12A | PPP1R3B | PPP1R3C | PPP2CA | PPP2CB | PPP2R1A | PPP2R1B | PPP2R2A | PPP2R2D | PPP2R5D | PPP3CC | PPP4R2 | PPP5C | PPP6C | PPTC7 | PTEN | PTPMT1 | PTPN1 | PTPN14 | PTPN21 | PTPN22 | PTPN23 | PTPN4 | PTPN6 | PTPRA | PTPRD | PTPRF | PTPRK | PTPRO | PTPRR | STK11 | STYX | TAB1 | TIMM50 | TPTE | GO:0032868 | 0.0133446560718185 | 110/8243 | 53/3250 | 0.0374050405569141 | 1 | F | F | F | F | response to insulin | AKT1 | AKT2 | APRT | CAT | CDK4 | CRY1 | DENND4C | EIF6 | EPRS | ERRFI1 | FABP3 | FOXO1 | GOT1 | GOT2 | GSK3A | GSTP1 | HADH | HDAC5 | HDAC9 | ICAM1 | INSIG2 | INSR | LPIN3 | MYO5A | OGT | OTC | PARP1 | PCSK9 | PIK3R1 | PKM | PLA2G1B | PPARG | PPAT | PRKCZ | PRKDC | PTEN | RAB10 | RAB31 | RELA | SIRT1 | SLC9A1 | SORT1 | SP1 | SRC | SRSF5 | STAT1 | TBC1D4 | TRIB3 | VGF | WDTC1 | XBP1 | YWHAG | ZBTB7B | GO:0051187 | 0.0041247118767439 | 34/8243 | 19/3250 | 0.0379750227526046 | 1 | F | F | F | F | cofactor catabolic process | ACAT1 | ACOT7 | AHCY | ASPDH | BLVRB | CAT | GGT1 | GGTLC1 | GPX1 | HBZ | HMOX1 | PRDX1 | PRDX2 | PRDX3 | PRDX4 | PRDX5 | PRDX6 | PXDN | VCP | GO:0006766 | 0.00703627320150431 | 58/8243 | 30/3250 | 0.0380511095244022 | 1 | F | F | F | F | vitamin metabolic process | AASDHPPT | AKR1A1 | CBR1 | CYB5R3 | CYP1A1 | FGFR1 | FOLR1 | FPGS | GSTO1 | GSTO2 | HLCS | LGMN | MCCC1 | MMAB | MTHFD1 | MTRR | PC | PDXK | PPARD | PSAT1 | RBP1 | RFK | RGN | RXRA | SHMT1 | SHMT2 | SLC25A32 | UBIAD1 | VDR | VKORC1 | GO:0045739 | 0.00570180759432246 | 47/8243 | 25/3250 | 0.0382529886870887 | 1 | F | F | F | F | positive regulation of DNA repair | ACTR2 | APBB1 | BABAM1 | BRCA1 | CBX8 | DHX9 | EGFR | EYA2 | EYA4 | FOXM1 | FUS | H2AFX | HMGB1 | KDM4D | MGMT | PARP1 | PCNA | RIF1 | RNF8 | RPS3 | SIRT1 | TRIM28 | UBE2N | WAS | XRCC1 | GO:1903670 | 0.00570180759432246 | 47/8243 | 25/3250 | 0.0382529886870887 | 1 | F | F | F | F | regulation of sprouting angiogenesis | ABL1 | AKT3 | ANXA1 | E2F2 | FGF2 | GLUL | HDAC5 | HDAC7 | HDAC9 | HMOX1 | ITGB1BP1 | JAK1 | KDR | MAP3K3 | MEOX2 | PDCD10 | PIK3C2A | PKM | RHOA | RHOJ | S100A1 | STARD13 | SYNJ2BP | TBXA2R | THBS1 | GO:0043574 | 0.00570180759432246 | 47/8243 | 25/3250 | 0.0382529886870887 | 1 | F | F | F | F | peroxisomal transport | ABCD3 | ACAA1 | ACOX1 | CAT | CROT | DHRS4 | ECH1 | ECI2 | EHHADH | GSTK1 | HMGCL | HSD17B4 | IDE | IDH1 | NOS2 | NUDT19 | PEX19 | PEX5 | UBB | UBC | UBE2D1 | UBE2D2 | UBE2D3 | USP9X | ZFAND6 | GO:0097527 | 0.000727890331190101 | 6/8243 | 5/3250 | 0.0383297420058745 | 1 | F | F | F | F | necroptotic signaling pathway | FADD | FASLG | RIPK1 | TLR3 | TNF | GO:0038113 | 0.000727890331190101 | 6/8243 | 5/3250 | 0.0383297420058745 | 1 | F | F | F | F | interleukin-9-mediated signaling pathway | JAK1 | JAK3 | STAT1 | STAT3 | STAT5A | GO:1903748 | 0.000727890331190101 | 6/8243 | 5/3250 | 0.0383297420058745 | 1 | F | F | F | F | negative regulation of establishment of protein localization to mitochondrion | BAG3 | BAG4 | DNAJA1 | LRRK2 | MAPT | GO:0006105 | 0.000727890331190101 | 6/8243 | 5/3250 | 0.0383297420058745 | 1 | F | F | F | F | succinate metabolic process | ALDH5A1 | SDHA | SDHB | SUCLA2 | SUCLG2 | GO:0032835 | 0.000727890331190101 | 6/8243 | 5/3250 | 0.0383297420058745 | 1 | F | F | F | F | glomerulus development | BCL2 | LGR4 | PTPRO | TCF21 | WT1 | GO:2000121 | 0.000727890331190101 | 6/8243 | 5/3250 | 0.0383297420058745 | 1 | F | F | F | F | regulation of removal of superoxide radicals | CD36 | NFE2L2 | PARK7 | RGN | TNF | GO:0044849 | 0.000727890331190101 | 6/8243 | 5/3250 | 0.0383297420058745 | 1 | F | F | F | F | estrous cycle | ANXA1 | ETS1 | NCOA1 | PCNA | PTN | GO:0007183 | 0.000727890331190101 | 6/8243 | 5/3250 | 0.0383297420058745 | 1 | F | F | F | F | SMAD protein complex assembly | FKBP1A | SMAD2 | SMAD3 | SMAD4 | TGFB1 | GO:2001040 | 0.000727890331190101 | 6/8243 | 5/3250 | 0.0383297420058745 | 1 | F | F | F | F | positive regulation of cellular response to drug | ENDOG | HDAC6 | KMT2A | PAWR | RIPK1 | GO:0038114 | 0.000727890331190101 | 6/8243 | 5/3250 | 0.0383297420058745 | 1 | F | F | F | F | interleukin-21-mediated signaling pathway | JAK1 | JAK3 | STAT1 | STAT3 | STAT4 | GO:0033483 | 0.000727890331190101 | 6/8243 | 5/3250 | 0.0383297420058745 | 1 | F | F | F | F | gas homeostasis | CAV1 | EGLN1 | HIF1A | NNT | SOD2 | GO:0021794 | 0.000727890331190101 | 6/8243 | 5/3250 | 0.0383297420058745 | 1 | F | F | F | F | thalamus development | LRP6 | OGDH | PTN | SMO | UQCRQ | GO:0032305 | 0.000727890331190101 | 6/8243 | 5/3250 | 0.0383297420058745 | 1 | F | F | F | F | positive regulation of icosanoid secretion | MAP2K6 | MIF | P2RX4 | PLA2G10 | PLA2G6 | GO:0000012 | 0.000727890331190101 | 6/8243 | 5/3250 | 0.0383297420058745 | 1 | F | F | F | F | single strand break repair | LIG4 | SIRT1 | TDP1 | TNP1 | XRCC1 | GO:0072520 | 0.000727890331190101 | 6/8243 | 5/3250 | 0.0383297420058745 | 1 | F | F | F | F | seminiferous tubule development | AR | ATRX | SMAD4 | UBB | WDR48 | GO:0009074 | 0.000727890331190101 | 6/8243 | 5/3250 | 0.0383297420058745 | 1 | F | F | F | F | aromatic amino acid family catabolic process | FAH | GSTZ1 | HAAO | KYNU | TDO2 | GO:0006538 | 0.000727890331190101 | 6/8243 | 5/3250 | 0.0383297420058745 | 1 | F | F | F | F | glutamate catabolic process | GAD2 | GLUD2 | GLUL | GOT1 | GOT2 | GO:0034397 | 0.000727890331190101 | 6/8243 | 5/3250 | 0.0383297420058745 | 1 | F | F | F | F | telomere localization | CCDC155 | MLH1 | NUP98 | TERF1 | UBE2B | GO:0090336 | 0.000727890331190101 | 6/8243 | 5/3250 | 0.0383297420058745 | 1 | F | F | F | F | positive regulation of brown fat cell differentiation | HNRNPU | MAPK14 | METRNL | VSTM2A | ZBTB7B | GO:0032074 | 0.000727890331190101 | 6/8243 | 5/3250 | 0.0383297420058745 | 1 | F | F | F | F | negative regulation of nuclease activity | ABCE1 | GZMA | TERF1 | TERF2 | TMBIM6 | GO:0046785 | 0.000727890331190101 | 6/8243 | 5/3250 | 0.0383297420058745 | 1 | F | F | F | F | microtubule polymerization | CENPJ | CKAP5 | MAPT | PIN1 | ZNF207 | GO:0046487 | 0.000727890331190101 | 6/8243 | 5/3250 | 0.0383297420058745 | 1 | F | F | F | F | glyoxylate metabolic process | ALDH4A1 | GOT2 | GRHPR | IDH1 | IDH2 | GO:0021895 | 0.000727890331190101 | 6/8243 | 5/3250 | 0.0383297420058745 | 1 | F | F | F | F | cerebral cortex neuron differentiation | ASCL1 | HPRT1 | NKX2-1 | PAFAH1B1 | PSEN1 | GO:0090240 | 0.000727890331190101 | 6/8243 | 5/3250 | 0.0383297420058745 | 1 | F | F | F | F | positive regulation of histone H4 acetylation | ARRB1 | BRCA1 | KAT7 | PIH1D1 | SMARCB1 | GO:0002825 | 0.000727890331190101 | 6/8243 | 5/3250 | 0.0383297420058745 | 1 | F | F | F | F | regulation of T-helper 1 type immune response | ANXA1 | HAVCR2 | HLX | JAK3 | RIPK2 | GO:1905906 | 0.000727890331190101 | 6/8243 | 5/3250 | 0.0383297420058745 | 1 | F | F | F | F | regulation of amyloid fibril formation | APOE | APP | CLU | CRYAB | PSEN1 | GO:0035112 | 0.000727890331190101 | 6/8243 | 5/3250 | 0.0383297420058745 | 1 | F | F | F | F | genitalia morphogenesis | AR | CTNNB1 | LRP6 | NIPBL | TP63 | GO:0046826 | 0.000727890331190101 | 6/8243 | 5/3250 | 0.0383297420058745 | 1 | F | F | F | F | negative regulation of protein export from nucleus | CDK5 | PARK7 | RANGAP1 | SP100 | TXN | GO:0006559 | 0.000727890331190101 | 6/8243 | 5/3250 | 0.0383297420058745 | 1 | F | F | F | F | L-phenylalanine catabolic process | FAH | GSTZ1 | PAH | PCBD1 | QDPR | GO:0038089 | 0.000727890331190101 | 6/8243 | 5/3250 | 0.0383297420058745 | 1 | F | F | F | F | positive regulation of cell migration by vascular endothelial growth factor signaling pathway | HSPB1 | KDR | MYO1C | PRKD1 | PRKD2 | GO:1990001 | 0.000727890331190101 | 6/8243 | 5/3250 | 0.0383297420058745 | 1 | F | F | F | F | inhibition of cysteine-type endopeptidase activity involved in apoptotic process | BIRC2 | BIRC3 | BIRC7 | BIRC8 | XIAP | GO:0060538 | 0.000727890331190101 | 6/8243 | 5/3250 | 0.0383297420058745 | 1 | F | F | F | F | skeletal muscle organ development | ASS1 | BASP1 | KCNAB1 | TCF21 | WT1 | GO:0030836 | 0.000727890331190101 | 6/8243 | 5/3250 | 0.0383297420058745 | 1 | F | F | F | F | positive regulation of actin filament depolymerization | ACTN2 | CFL1 | CFL2 | DSTN | WDR1 | GO:1903551 | 0.000727890331190101 | 6/8243 | 5/3250 | 0.0383297420058745 | 1 | F | F | F | F | regulation of extracellular exosome assembly | PDCD6IP | SDC1 | SDC4 | SDCBP | TSG101 | GO:0042364 | 0.000727890331190101 | 6/8243 | 5/3250 | 0.0383297420058745 | 1 | F | F | F | F | water-soluble vitamin biosynthetic process | AKR1A1 | MMAB | PSAT1 | RFK | RGN | GO:0045628 | 0.000727890331190101 | 6/8243 | 5/3250 | 0.0383297420058745 | 1 | F | F | F | F | regulation of T-helper 2 cell differentiation | ANXA1 | BCL6 | HLX | PRKCZ | RARA | GO:0060081 | 0.000727890331190101 | 6/8243 | 5/3250 | 0.0383297420058745 | 1 | F | F | F | F | membrane hyperpolarization | ATP1A1 | CACNG2 | CFTR | PARK7 | PRKCZ | GO:1903071 | 0.000727890331190101 | 6/8243 | 5/3250 | 0.0383297420058745 | 1 | F | F | F | F | positive regulation of ER-associated ubiquitin-dependent protein catabolic process | CAV1 | NFE2L2 | SGTA | UBQLN1 | UBQLN2 | GO:0060271 | 0.0122528205750334 | 101/8243 | 49/3250 | 0.0386840750137739 | 1 | F | F | F | F | cilium assembly | ABLIM1 | ACTR2 | ACTR3 | AHI1 | ATP6V1D | ATXN10 | AVIL | CBY1 | CENPJ | CEP290 | CEP89 | DCTN1 | DISC1 | DYNLL1 | E2F4 | FBF1 | FOPNL | FOXJ1 | HDAC6 | ICK | IFT122 | IQCB1 | MKS1 | NEK1 | NPHP3 | OFD1 | ONECUT1 | PCM1 | POC1B | PTPN23 | RAB1A | RPGRIP1L | SCLT1 | SEPT2 | SEPT6 | SEPT7 | SNAP29 | SPERT | SSX2IP | TCTN2 | TCTN3 | TEKT2 | TEKT4 | TMEM17 | TMEM216 | TMEM231 | UBXN10 | WDR35 | ZMYND10 | GO:0071901 | 0.0122528205750334 | 101/8243 | 49/3250 | 0.0386840750137739 | 1 | F | F | F | F | negative regulation of protein serine/threonine kinase activity | ABL1 | AKT1 | APC | APOE | CASP3 | CAV1 | CBLC | CDKN1A | CDKN2C | CEBPA | CHORDC1 | DNAJA1 | DUSP1 | DUSP14 | DUSP16 | DUSP19 | DUSP22 | DUSP7 | FBXO7 | GPS2 | GSTP1 | HEXIM1 | HSPB1 | IPO5 | IPO7 | LATS1 | LRP6 | PKIG | PLK1 | PPP2CA | PPP2R1A | PRKAR1A | PRKAR2B | PRKCD | PTEN | PTPN1 | PTPN22 | PTPN6 | RGS2 | RPTOR | SERPINB3 | SFN | SIRT1 | SPRY2 | STK38 | TNFAIP3 | UCHL1 | WNK1 | YWHAG | GO:0015833 | 0.108940919568118 | 898/8243 | 379/3250 | 0.0388498668444432 | 1 | F | F | F | F | peptide transport | ABCE1 | ABCG1 | ACAA1 | ACD | ACOX1 | ACTN4 | ADCYAP1 | ADPRH | AFM | AGAP1 | AGAP2 | AGK | AHCYL1 | AKAP8 | AKT1 | AKT2 | AKTIP | ANK3 | ANXA1 | AP1B1 | AP1G1 | AP1M1 | AP3D1 | AP3M1 | AP4M1 | AP4S1 | APOE | APPBP2 | ARF1 | ARF4 | ARF5 | ARFGAP1 | ARFIP1 | ARHGAP1 | ARL4D | ARL5B | ARRB1 | ARRB2 | ASPSCR1 | ATG4C | ATP6V1A | ATP6V1B1 | ATP6V1C1 | ATP6V1D | ATP6V1E1 | AUP1 | BAG3 | BLZF1 | C16orf70 | CACNG2 | CACNG4 | CACNG5 | CALR | CANX | CAT | CBLB | CCDC22 | CCHCR1 | CCT6B | CD209 | CD36 | CD40LG | CD74 | CDC37 | CDK16 | CDK5 | CENPF | CEP290 | CHMP2A | CHP1 | CLSTN1 | CLTC | CLU | COG3 | COMMD1 | COPA | COPB1 | COPE | COPG1 | CPLX1 | CPLX3 | CROT | CSE1L | CTSA | CTTN | DAB2 | DDX19A | DENND1A | DENND4C | DERL1 | DHRS4 | DLG2 | DNAJC19 | DUSP16 | DYNLL1 | DYNLT1 | ECH1 | ECI2 | EHHADH | EIF4ENIF1 | EIF5A | EIF6 | EXOC6 | FAF2 | FBXO7 | FIS1 | FKBP1B | FRAS1 | GBP5 | GDI1 | GDI2 | GGA1 | GOLGA2 | GOLGA4 | GOLPH3 | GOLT1B | GOPC | GRIP1 | GRPEL1 | GSTK1 | HAP1 | HDAC6 | HEATR3 | HERC2 | HGS | HID1 | HMGB1 | HMGCL | HNF1A | HNRNPU | HSD17B4 | HSP90AA1 | HSP90B1 | HSPA4 | HSPA5 | HSPA8 | HSPB1 | HSPD1 | ICK | IDE | IDH1 | IFIT1 | IFT122 | IPO11 | IPO4 | IPO5 | IPO7 | IPO8 | IPO9 | ITGAV | ITGB1BP1 | KIF13B | KIF5B | KIF5C | KLHL20 | KPNA1 | KPNA2 | KPNB1 | LARS | LCP2 | LMAN2L | LMNA | LRRC7 | LRSAM1 | MAPK8IP3 | MCFD2 | MTX1 | MYO1C | MYO5A | MYO6 | NBAS | NCOA4 | NDUFA13 | NEDD4 | NEUROD1 | NMD3 | NOD2 | NOS2 | NPLOC4 | NPM1 | NUDT19 | NUP107 | NUP153 | NUP214 | NUP85 | NUP88 | NUP93 | NUP98 | NUTF2 | NXT2 | OPTN | OXA1L | PAFAH1B1 | PAM16 | PARD3 | PARK7 | PCM1 | PDCD6 | PDCD6IP | PDIA4 | PEX19 | PEX5 | PHB2 | PICK1 | PIK3R1 | PLEKHF2 | PML | PNP | POM121 | PPARG | PPID | PPT1 | PSEN1 | PTPN23 | RAB10 | RAB11A | RAB11B | RAB11FIP2 | RAB18 | RAB1A | RAB1B | RAB21 | RAB24 | RAB29 | RAB2A | RAB31 | RAB32 | RAB35 | RAB3IP | RAB5A | RAB5B | RAB7A | RAB9A | RABGEF1 | RABL2A | RABL3 | RAF1 | RAN | RANBP3 | RAP1A | RAPGEF4 | RASSF9 | RBPJ | ROMO1 | RP2 | RPAIN | RPL10 | RPL10A | RPL12 | RPL18A | RPL22 | RPL23 | RPL23A | RPL24 | RPL30 | RPL37A | RPL38 | RPL6 | RPL7 | RPL7A | RPL9 | RPLP0 | RPLP1 | RPLP2 | RPS12 | RPS13 | RPS14 | RPS15A | RPS19 | RPS2 | RPS20 | RPS24 | RPS29 | RPS3 | RPS4X | RPS8 | RRS1 | S100A12 | S100A13 | SCFD1 | SCG2 | SDCBP | SEC13 | SEC16A | SEC22C | SEC24B | SEC24C | SEC31A | SEC62 | SEC63 | SEL1L | SELENBP1 | SFPQ | SGTA | SGTB | SLC15A1 | SLC25A6 | SLC9A3R1 | SMAD2 | SMURF1 | SNAP29 | SNF8 | SNX12 | SNX17 | SNX2 | SNX21 | SNX24 | SNX3 | SNX6 | SPTBN1 | SRP14 | SRP19 | SSX2IP | STAT3 | STX5 | STXBP1 | STXBP2 | STYX | SYNJ2BP | SYVN1 | TANGO2 | TBC1D15 | TBC1D22B | TBC1D4 | TCIRG1 | TCP1 | TERF2 | TGFB1 | TIMM13 | TIMM21 | TIMM23 | TIMM44 | TIMM50 | TMED9 | TNKS | TNPO1 | TOMM20 | TOMM22 | TOMM40 | TOR1A | TPR | TRAF3IP1 | TRIM23 | TSG101 | TXNIP | UBB | UBC | UBE2D1 | UBE2D2 | UBE2D3 | UNC119 | USP9X | VCP | VGF | VPS13B | VPS18 | VPS26A | VPS29 | VPS37D | VPS4B | VPS8 | VTA1 | WDR19 | WDR35 | XBP1 | XPO1 | XPO4 | XPO5 | XPO7 | YKT6 | YWHAB | YWHAE | YWHAG | YWHAH | ZBTB7B | ZDHHC17 | ZDHHC23 | ZFAND6 | ZW10 | GO:0032872 | 0.0192890937765377 | 159/8243 | 74/3250 | 0.0390537029969227 | 1 | F | F | F | F | regulation of stress-activated MAPK cascade | AGER | AKT1 | APP | AXIN1 | BIRC7 | CARD9 | CCL21 | CD27 | CD40LG | COPS5 | CYLD | DAXX | DBNL | DNAJA1 | DUSP1 | DUSP19 | DUSP22 | DVL2 | DVL3 | EGFR | EPHA4 | EZR | FOXM1 | FOXO1 | GADD45G | GPS2 | GRIK2 | GSTP1 | HDAC3 | HMGB1 | HRAS | IGBP1 | IGF1R | ITCH | KLHDC10 | LTBR | MAP2K1 | MAP2K4 | MAP2K7 | MAP3K13 | MAP3K2 | MAP3K3 | MAP3K4 | MAP4K1 | MAPK1 | MAPK3 | MAPK8IP3 | MYC | NCOR1 | NOD2 | PAFAH1B1 | PDCD10 | PER1 | PRDX1 | PTPN1 | PTPN22 | QARS | RB1CC1 | RIPK1 | RIPK2 | RPS3 | SDCBP | SERPINB3 | SLAMF1 | STK25 | STK3 | TAOK3 | TGFBR3 | TLR3 | TNF | TRAF2 | TRAF6 | WNT16 | ZNF622 | GO:1902653 | 0.0038820817663472 | 32/8243 | 18/3250 | 0.0397824226685201 | 1 | F | F | F | F | secondary alcohol biosynthetic process | ACAA2 | ACAT2 | ACLY | APOA1 | APOE | CES1 | CFTR | CYB5R3 | DHCR24 | FDPS | G6PD | GGPS1 | IDI2 | INSIG1 | INSIG2 | MVD | PRKAA1 | PRKAA2 | GO:0006695 | 0.0038820817663472 | 32/8243 | 18/3250 | 0.0397824226685201 | 1 | F | F | F | F | cholesterol biosynthetic process | ACAA2 | ACAT2 | ACLY | APOA1 | APOE | CES1 | CFTR | CYB5R3 | DHCR24 | FDPS | G6PD | GGPS1 | IDI2 | INSIG1 | INSIG2 | MVD | PRKAA1 | PRKAA2 | GO:0009142 | 0.0038820817663472 | 32/8243 | 18/3250 | 0.0397824226685201 | 1 | F | F | F | F | nucleoside triphosphate biosynthetic process | ADK | AK1 | AK4 | ALDOA | CMPK1 | CTPS1 | CYC1 | DNAJC30 | IMPDH2 | LDHC | NME1 | NME2 | NME4 | SLC25A13 | STOML2 | TGFB1 | TYMS | UCK2 | GO:0072662 | 0.00545917748392576 | 45/8243 | 24/3250 | 0.040399645024276 | 1 | F | F | F | F | protein localization to peroxisome | ACAA1 | ACOX1 | CAT | CROT | DHRS4 | ECH1 | ECI2 | EHHADH | GSTK1 | HMGCL | HSD17B4 | IDE | IDH1 | NOS2 | NUDT19 | PEX19 | PEX5 | UBB | UBC | UBE2D1 | UBE2D2 | UBE2D3 | USP9X | ZFAND6 | GO:0072663 | 0.00545917748392576 | 45/8243 | 24/3250 | 0.040399645024276 | 1 | F | F | F | F | establishment of protein localization to peroxisome | ACAA1 | ACOX1 | CAT | CROT | DHRS4 | ECH1 | ECI2 | EHHADH | GSTK1 | HMGCL | HSD17B4 | IDE | IDH1 | NOS2 | NUDT19 | PEX19 | PEX5 | UBB | UBC | UBE2D1 | UBE2D2 | UBE2D3 | USP9X | ZFAND6 | GO:0006625 | 0.00545917748392576 | 45/8243 | 24/3250 | 0.040399645024276 | 1 | F | F | F | F | protein targeting to peroxisome | ACAA1 | ACOX1 | CAT | CROT | DHRS4 | ECH1 | ECI2 | EHHADH | GSTK1 | HMGCL | HSD17B4 | IDE | IDH1 | NOS2 | NUDT19 | PEX19 | PEX5 | UBB | UBC | UBE2D1 | UBE2D2 | UBE2D3 | USP9X | ZFAND6 | GO:0071496 | 0.0262040519228436 | 216/8243 | 98/3250 | 0.0415886025672619 | 1 | F | F | F | F | cellular response to external stimulus | AIFM1 | AKT1 | ALB | ARHGAP35 | ASGR1 | ASNS | ATG5 | ATP1A1 | BAG3 | BCL10 | BCL11A | BCL2 | CASP2 | CASP8 | CBL | CBS | CD40 | CDKN1A | COMT | CTSV | DCTPP1 | DNM1L | EGFR | EHMT2 | EIF2AK3 | EIF2AK4 | EIF2S1 | FADD | FOLR1 | FOS | FOXO1 | FYN | GLUL | GOT1 | GSTP1 | HDAC4 | HMOX1 | HNRNPA1 | HNRNPL | HSF1 | HSP90B1 | HSPA5 | HSPA8 | ICAM1 | IFI16 | IREB2 | IRF1 | JUN | LARS | LRRK2 | LTBR | MAP2K4 | MAP3K1 | MAP3K14 | MAP3K2 | MAPK1 | MAPK3 | MAPK8 | MDM2 | MTPN | MYBBP1A | MYD88 | NAMPT | NCOA1 | NFE2L2 | OPA1 | P2RX4 | PCSK9 | PDK2 | PICK1 | PPARG | PPM1D | PRKAA1 | PRKAA2 | PRKD1 | PTN | RAC1 | RPTOR | SIPA1 | SIRT1 | SLC39A4 | SLC9A1 | SOD1 | STK24 | STK26 | TGFB1 | TLR3 | TNFRSF1A | TNFRSF8 | TNFSF14 | TNKS | TP53 | TXN2 | ULK1 | WDR45B | WIPI1 | WIPI2 | XBP1 | GO:2000401 | 0.0036394516559505 | 30/8243 | 17/3250 | 0.0416241126037607 | 1 | F | F | F | F | regulation of lymphocyte migration | ADTRP | AIRE | AKT1 | APOD | APP | CCL21 | CRK | CRKL | FADD | ITGA4 | MSN | OXSR1 | RHOA | RIPK3 | S100A7 | TNFSF14 | WNK1 | GO:0090311 | 0.0036394516559505 | 30/8243 | 17/3250 | 0.0416241126037607 | 1 | F | F | F | F | regulation of protein deacetylation | AKAP8 | BCL6 | CCAR2 | CTBP1 | EP300 | LRRK2 | MAPK8 | MAPT | NIPBL | PRKAA1 | PRKAA2 | PRKD1 | PRKD2 | SKI | TGFB1 | USP17L2 | ZBTB7B | GO:0034284 | 0.0109183549678515 | 90/8243 | 44/3250 | 0.0420993831567086 | 1 | F | F | F | F | response to monosaccharide | CASP3 | CAT | COL6A2 | CTSV | EIF2B1 | EIF2B3 | EIF2B5 | ENDOG | FIS1 | FKBP1B | GLUL | GSTP1 | HNF4A | ICAM1 | IGF1R | LDHA | LGALS1 | NAMPT | NDUFA9 | NEUROD1 | NME1 | NME2 | NR0B2 | OPA1 | PFKL | PIK3CA | PPARD | PPARGC1A | PRKAA1 | PRKAA2 | PRKACA | PTEN | RAP1A | RHOA | SARM1 | SIN3A | SLC2A5 | SMAD2 | TGFB1 | THBS1 | TXN2 | TXNIP | WDR35 | XBP1 | GO:0075733 | 0.00521654737352906 | 43/8243 | 23/3250 | 0.0426660306153039 | 1 | F | F | F | F | intracellular transport of virus | CD209 | DYNLT1 | KPNA1 | KPNB1 | NUP107 | NUP153 | NUP160 | NUP205 | NUP214 | NUP85 | NUP88 | NUP93 | NUP98 | POM121 | RAE1 | RAN | SEC13 | TPR | TSG101 | UBB | UBC | VPS37D | XPO1 | GO:0009749 | 0.00982651947106636 | 81/8243 | 40/3250 | 0.0430431406363887 | 1 | F | F | F | F | response to glucose | CASP3 | COL6A2 | CTSV | EIF2B1 | EIF2B3 | EIF2B5 | ENDOG | FIS1 | FKBP1B | GLUL | HNF4A | ICAM1 | IGF1R | LDHA | LGALS1 | NDUFA9 | NEUROD1 | NME1 | NME2 | NR0B2 | OPA1 | PFKL | PIK3CA | PPARD | PPARGC1A | PRKAA1 | PRKAA2 | PRKACA | PTEN | RAP1A | RHOA | SARM1 | SIN3A | SMAD2 | TGFB1 | THBS1 | TXN2 | TXNIP | WDR35 | XBP1 | GO:0010468 | 0.31541914351571 | 2600/8243 | 1061/3250 | 0.0431513702289407 | 1 | F | F | F | F | regulation of gene expression | AARS | ABCG1 | ABHD14B | ABL1 | ACO1 | ACTA1 | ACTA2 | ACTB | ACTG2 | ACTL6A | ACTR2 | ACTR3 | ADCYAP1 | ADORA2A | ADRB2 | ADTRP | AEBP2 | AES | AFF1 | AFF4 | AGER | AGO1 | AGO2 | AGR2 | AHCYL1 | AHI1 | AHNAK | AICDA | AIRE | AKT1 | AKT2 | ALOX12 | ANK3 | ANKRD49 | ANP32A | ANXA2 | ANXA7 | APBB1 | APBB2 | APBB3 | APEX1 | APOE | APP | AR | ARF4 | ARHGAP35 | ARID3B | ARID4B | ARNT | ARNT2 | ARRB1 | ARRB2 | ASCC1 | ASCC2 | ASCL1 | ATF2 | ATF6 | ATF6B | ATF7 | ATM | ATOH1 | ATP6V1B1 | ATRX | ATXN1 | ATXN2 | AURKB | AXIN1 | AZU1 | BACH2 | BAG2 | BAG3 | BANP | BASP1 | BCL10 | BCL11A | BCL2 | BCL6 | BCL6B | BCOR | BIRC2 | BIRC3 | BIRC5 | BIRC7 | BIRC8 | BLZF1 | BMI1 | BMPR1A | BOLA1 | BOLA3 | BRAF | BRCA1 | BRD2 | BRD3 | BRD4 | BTK | BTRC | BUD31 | C1QBP | C1QTNF1 | CALR | CAMK1D | CAMK2A | CAND1 | CARM1 | CAT | CAV1 | CBFA2T3 | CBFB | CBX4 | CBX7 | CBX8 | CBY1 | CCAR2 | CCDC22 | CCNB1 | CCNT1 | CCNT2 | CCR1 | CD36 | CD40 | CD40LG | CD74 | CD81 | CDC37 | CDC5L | CDC73 | CDH1 | CDK2 | CDK4 | CDK5 | CDK5R1 | CDK8 | CDK9 | CDKN1A | CEBPA | CEBPE | CENPF | CEP290 | CHCHD10 | CHCHD2 | CHCHD3 | CHD3 | CHD4 | CHD8 | CHEK2 | CHP1 | CHRD | CIRBP | CKS2 | CLK1 | CLU | COA3 | COL2A1 | COMMD1 | COPS2 | COPS5 | COQ7 | CPSF7 | CREB1 | CREB3 | CREBBP | CRIP1 | CRK | CRKL | CRP | CRY1 | CRY2 | CRYAB | CSNK1E | CTBP1 | CTCF | CTDP1 | CTH | CTNNB1 | CTNNBIP1 | CUL2 | CUL3 | CYLD | CYP1A2 | DAB2 | DAPK1 | DAXX | DAZAP1 | DCN | DCP2 | DDRGK1 | DDX1 | DDX39B | DDX3X | DDX6 | DHX36 | DHX9 | DIS3 | DMAP1 | DMRT3 | DNAJA3 | DNAJB1 | DPF2 | DROSHA | DUSP22 | DVL1 | DVL2 | DVL3 | E2F1 | E2F2 | E2F3 | E2F4 | E2F6 | EAF2 | EBF1 | ECD | EDA | EDC4 | EED | EEF2 | EFEMP1 | EGFR | EGLN1 | EGLN3 | EHMT1 | EHMT2 | EID1 | EIF2AK3 | EIF2AK4 | EIF2B5 | EIF2S1 | EIF3D | EIF3E | EIF3H | EIF4A3 | EIF4E | EIF4E2 | EIF4EBP1 | EIF4ENIF1 | EIF4G3 | EIF4H | EIF5 | EIF5A | EIF5B | EIF6 | ELAVL1 | ELF3 | ELF4 | ENO1 | EP300 | EPRS | ERBB2 | ERBB3 | ERF | ERG | ERLIN2 | ESR1 | ESR2 | ESRRA | ESRRB | ESRRG | ETS1 | EWSR1 | EXOSC1 | EXOSC5 | EXOSC6 | EXOSC9 | EZH2 | EZR | FADD | FAF1 | FAM129B | FAM98A | FANCA | FASLG | FBP1 | FBXW11 | FGF2 | FGFR1 | FHL2 | FKBP8 | FLOT1 | FN1 | FOS | FOXA1 | FOXD4 | FOXD4L6 | FOXI2 | FOXJ1 | FOXK1 | FOXK2 | FOXM1 | FOXO1 | FTO | FUBP1 | FUBP3 | FUS | FXR2 | FYN | G3BP2 | GABPB1 | GAPDH | GATA1 | GDF9 | GEMIN5 | GFPT1 | GLMN | GLO1 | GPS2 | GPX1 | GRB7 | GRIP1 | GSK3A | GSK3B | GSPT1 | GSPT2 | GTF2F1 | GTF2F2 | GTF2I | H1F0 | H1FX | H2AFZ | H3F3A | HAVCR2 | HAX1 | HBZ | HDAC1 | HDAC2 | HDAC3 | HDAC4 | HDAC5 | HDAC6 | HDAC7 | HDAC9 | HDGF | HELZ | HELZ2 | HEXB | HEXIM1 | HGS | HIF1A | HIF1AN | HINT1 | HIST1H1A | HIST1H3A | HIST1H4A | HIST2H3A | HIVEP1 | HLX | HMGB1 | HMGB2 | HMGB3 | HMOX1 | HNF1A | HNF4A | HNF4G | HNRNPA1 | HNRNPA2B1 | HNRNPAB | HNRNPD | HNRNPF | HNRNPH1 | HNRNPK | HNRNPL | HNRNPM | HNRNPU | HOXA5 | HOXB5 | HOXB6 | HOXB9 | HOXD10 | HRAS | HSF1 | HSF4 | HSPA1B | HSPA5 | HSPA8 | HSPB1 | HSPD1 | HTATIP2 | HTRA1 | ICAM1 | ICK | ID2 | IFI16 | IFNA2 | IGBP1 | IGF2BP1 | IKBKB | IKBKG | IKZF5 | ILF2 | ILF3 | ILK | ING1 | ING5 | INSIG1 | INSIG2 | INSR | IPO8 | IREB2 | IRF1 | IRF4 | IRF7 | ITCH | ITGB1BP1 | ITGB3BP | JMJD6 | JUN | JUNB | JUND | JUP | KANK2 | KAT5 | KAT7 | KAT8 | KDM1A | KDM2A | KDM5B | KDM5C | KDM8 | KDR | KEAP1 | KHDRBS1 | KLF5 | KLF6 | KMT2A | KMT2B | KMT2C | KRAS | L3MBTL1 | L3MBTL2 | L3MBTL3 | L3MBTL4 | LAMP3 | LARP4 | LEF1 | LEMD2 | LEMD3 | LGMN | LGR4 | LIMS1 | LMNA | LMO2 | LMO7 | LOXL2 | LPIN3 | LRIF1 | LRP6 | LRPPRC | LRRK2 | LSM1 | LSM3 | LSM4 | LSM5 | LUM | LZTR1 | MAF1 | MAP2K1 | MAP2K3 | MAP2K7 | MAP3K13 | MAP3K2 | MAP3K4 | MAPK1 | MAPK14 | MAPK15 | MAPK3 | MAPK4 | MAPK6 | MAPK7 | MAPK8 | MAPK9 | MAPT | MATR3 | MAVS | MBD3 | MC1R | MDFI | MDM2 | MDM4 | MECP2 | MED1 | MED12 | MED14 | MED17 | MED20 | MED21 | MED23 | MED24 | MED31 | MED4 | MED6 | MEOX2 | MEPCE | METTL23 | MGA | MIF | MITF | MKNK2 | MLH1 | MLLT1 | MLLT11 | MLLT3 | MORF4L1 | MORF4L2 | MOV10 | MRGBP | MRPL12 | MSN | MTA1 | MTA3 | MTERF1 | MTPN | MXD1 | MYBBP1A | MYC | MYD88 | MYH6 | MYO1C | MYO6 | NACC1 | NAMPT | NANOG | NBAS | NCBP1 | NCK1 | NCOA1 | NCOA2 | NCOA3 | NCOA4 | NCOR1 | NCOR2 | NDUFA13 | NEDD4 | NEDD8 | NELFB | NELFCD | NELFE | NELL1 | NEUROD1 | NFATC1 | NFATC2 | NFE2L2 | NFKB2 | NFKBIA | NIF3L1 | NIPBL | NKRF | NKX2-1 | NLK | NME1 | NME2 | NOD2 | NOS1AP | NOS2 | NOS3 | NOSTRIN | NPM1 | NR0B2 | NR1H2 | NR1H3 | NR1I2 | NR3C1 | NR4A1 | NR5A2 | NTRK1 | NUFIP1 | NUMA1 | NUP107 | NUP153 | NUP160 | NUP205 | NUP214 | NUP85 | NUP88 | NUP93 | NUP98 | OGFOD1 | OGT | ONECUT1 | PA2G4 | PABPC1 | PABPC4 | PAIP1 | PAK6 | PAN2 | PARK7 | PARP1 | PARP14 | PATZ1 | PAWR | PAX8 | PAX9 | PAXIP1 | PC | PCBD1 | PCBP1 | PCGF1 | PCGF5 | PCGF6 | PCNA | PDCD10 | PDE12 | PELI1 | PER1 | PER2 | PFKM | PFN1 | PHB | PHB2 | PHF10 | PHF20L1 | PHF21A | PHGDH | PIAS1 | PIAS3 | PIH1D1 | PIK3CA | PIK3R1 | PIK3R3 | PIM1 | PIN1 | PKIG | PLA2G10 | PLA2G1B | PLGRKT | PLK1 | PLSCR1 | PML | POLDIP3 | POLR2A | POLR2E | POLR2L | POM121 | POU2F3 | POU5F1 | PPARD | PPARG | PPARGC1A | PPID | PPIE | PPM1A | PPM1D | PPP1CA | PPP1CB | PPP1CC | PPP1R12A | PPP2CA | PPP2CB | PPP2R1A | PPP2R2A | PRDX2 | PRDX3 | PRDX5 | PRDX6 | PRKAA1 | PRKAA2 | PRKACA | PRKAG1 | PRKAR1A | PRKCD | PRKCZ | PRKD1 | PRKD2 | PRKDC | PRMT2 | PRMT3 | PRMT6 | PROX1 | PRPF19 | PSEN1 | PSMA1 | PSMA2 | PSMA3 | PSMA5 | PSMA6 | PSMB5 | PSMB6 | PSMB7 | PSMB8 | PSMC1 | PSMC2 | PSMC3 | PSMC4 | PSMC5 | PSMC6 | PSMD1 | PSMD10 | PSMD11 | PSMD12 | PSMD14 | PSMD2 | PSMD4 | PSMD9 | PSME2 | PSME3 | PTEN | PTGES2 | PTPN22 | PTPRK | PTRH2 | PTTG1 | PUM1 | PUS7 | QARS | QRICH1 | RAB3GAP1 | RAD21 | RAE1 | RAF1 | RAN | RARA | RARG | RASD1 | RB1 | RBBP4 | RBM17 | RBM25 | RBM3 | RBM4 | RBM8A | RBMX | RBPJ | RBX1 | RC3H1 | RDX | REL | RELA | REST | RFC1 | RFXANK | RGS2 | RHBDD1 | RHOA | RIF1 | RING1 | RIOK1 | RIPK1 | RIPK2 | RIPK3 | RIPK4 | RMND1 | RNASEH2B | RNF139 | RNF2 | RNF25 | RNF31 | RNF4 | RNF8 | RPL10 | RPL10A | RPL12 | RPL18A | RPL22 | RPL23 | RPL23A | RPL24 | RPL30 | RPL37A | RPL38 | RPL6 | RPL7 | RPL7A | RPL9 | RPLP0 | RPLP1 | RPLP2 | RPS12 | RPS13 | RPS14 | RPS15A | RPS19 | RPS2 | RPS20 | RPS24 | RPS29 | RPS3 | RPS4X | RPS6KA1 | RPS6KB2 | RPS8 | RPTOR | RRM2 | RTF1 | RUNX1 | RUNX1T1 | RUNX2 | RUVBL1 | RUVBL2 | RXRA | RXRB | RXRG | S100A1 | S100A12 | S100A9 | SAMD7 | SAP18 | SARS | SART3 | SCML2 | SEC13 | SERPINE2 | SET | SETBP1 | SETDB1 | SFMBT1 | SFPQ | SHC1 | SHMT1 | SHMT2 | SIN3A | SIRT1 | SIRT5 | SIRT6 | SIRT7 | SKI | SKIV2L | SLC9A1 | SLTM | SMAD2 | SMAD3 | SMAD4 | SMAD5 | SMAD9 | SMARCA2 | SMARCA4 | SMARCB1 | SMARCC1 | SMARCC2 | SMARCE1 | SMG5 | SMG6 | SMG8 | SMO | SMURF1 | SMURF2 | SNAI2 | SNCA | SND1 | SNF8 | SNRPA | SNW1 | SNX12 | SNX6 | SOD2 | SORT1 | SOX2 | SP1 | SP100 | SP2 | SPEN | SPI1 | SPIN1 | SPRY2 | SQSTM1 | SRC | SRI | SRPK1 | SRPK2 | SRSF12 | SRSF5 | SSB | STAT1 | STAT3 | STAT4 | STAT5A | STAT6 | STK3 | STK40 | STRAP | SUB1 | SUMO1 | SUMO2 | SUPT4H1 | SUPT5H | SUZ12 | TAB1 | TAB2 | TACO1 | TADA2B | TAF12 | TAF15 | TAF1A | TAGLN3 | TARDBP | TAZ | TBL1X | TBRG4 | TCEA1 | TCEA2 | TCERG1 | TCF12 | TCF21 | TCF25 | TCF4 | TCOF1 | TCP10L | TERF1 | TERF2 | TERF2IP | TFCP2 | TFDP1 | TGFB1 | TGFBR3 | TGIF2LX | THAP11 | THAP7 | THBS1 | THRA | TIA1 | TIAL1 | TLE1 | TLE3 | TLR3 | TMBIM6 | TMEM173 | TMPO | TNF | TNFAIP3 | TNFRSF1A | TNFRSF8 | TNIP2 | TNKS | TNP1 | TNPO1 | TOE1 | TOP1 | TOP2A | TP53 | TP63 | TP73 | TPR | TRA2A | TRADD | TRAF1 | TRAF2 | TRAF3 | TRAF6 | TRAP1 | TREX1 | TRIAP1 | TRIB3 | TRIM14 | TRIM21 | TRIM25 | TRIM28 | TRIM29 | TRIM32 | TRIM33 | TRIM34 | TRIP4 | TRUB2 | TSC22D1 | TSC22D3 | TSC22D4 | TSFM | TSG101 | TSKU | TTC5 | TULP3 | TWIST1 | TXN | TXNIP | TYMS | U2AF2 | UBA3 | UBB | UBC | UBE2D1 | UBE2D3 | UBE2I | UBE2L3 | UBE2N | UBE2O | UBE2V1 | UBR5 | UNK | UNKL | UPF2 | UPF3B | UQCC1 | USP15 | USP16 | USP17L2 | USP2 | USP21 | USP47 | USP7 | USP9X | USP9Y | VDR | VGLL4 | VHL | VIM | VPS72 | VSTM2A | WARS | WAS | WBP2 | WDR35 | WDR77 | WDTC1 | WNT16 | WNT2 | WT1 | WTAP | WWOX | WWP2 | XBP1 | XIAP | XPO1 | XPO5 | XRCC5 | XRCC6 | YAF2 | YAP1 | YEATS2 | YES1 | YTHDF1 | YWHAB | YWHAH | YWHAQ | YWHAZ | YY1 | ZBTB16 | ZBTB17 | ZBTB21 | ZBTB25 | ZBTB33 | ZBTB42 | ZBTB7A | ZBTB7B | ZC3H4 | ZFP41 | ZHX1 | ZIC1 | ZKSCAN1 | ZMPSTE24 | ZMYM5 | ZMYND8 | ZNF207 | ZNF217 | ZNF227 | ZNF24 | ZNF277 | ZNF354C | ZNF408 | ZNF420 | ZNF443 | ZNF517 | ZNF518A | ZNF592 | ZNF598 | ZNF618 | ZNF622 | ZNF646 | ZNF669 | ZNF696 | ZNF746 | ZNF747 | ZRANB2 | ZSCAN2 | ZSCAN29 | ZSCAN32 | ZZZ3 | GO:0006310 | 0.0154070120101905 | 127/8243 | 60/3250 | 0.0431538514604505 | 1 | F | F | F | F | DNA recombination | ACTL6A | AICDA | APEX1 | APEX2 | ATAD5 | ATM | BRCA1 | CCDC155 | CCDC36 | CD40LG | ENDOG | EXOSC6 | FEN1 | GINS2 | GINS4 | H2AFX | HMGB1 | HMGB2 | HMGB3 | HSPD1 | INO80B | INO80C | KDM4D | LEF1 | LIG4 | MCM9 | MLH1 | MORF4L1 | MSH6 | NIPBL | NSMCE4A | PALB2 | PARP1 | PAXIP1 | POLM | PRKDC | PSMD14 | RAD21 | RAD51 | RAD51AP1 | RNF8 | RPA2 | RPA3 | RPAIN | RUVBL1 | RUVBL2 | SFPQ | SWAP70 | TREX1 | TRIP13 | TSN | UBE2N | UCHL5 | WDR48 | XRCC1 | XRCC3 | XRCC5 | XRCC6 | YY1 | ZFYVE26 | GO:1901998 | 0.0033968215455538 | 28/8243 | 16/3250 | 0.0434837169399407 | 1 | F | F | F | F | toxin transport | ABCG1 | CCT2 | CCT3 | CCT4 | CCT5 | CCT6B | CCT7 | CCT8 | DNAJA1 | GGA1 | HSPA5 | LRP6 | RAB29 | SCFD1 | TCP1 | TRIP4 | GO:0045740 | 0.0033968215455538 | 28/8243 | 16/3250 | 0.0434837169399407 | 1 | F | F | F | F | positive regulation of DNA replication | ATAD5 | ATRX | CDK2 | DBF4B | DHX9 | DNA2 | EGFR | FAF1 | FGFR1 | HRAS | JUN | MAP2K4 | MAS1 | PCNA | RAC1 | STOML2 | GO:0008625 | 0.0033968215455538 | 28/8243 | 16/3250 | 0.0434837169399407 | 1 | F | F | F | F | extrinsic apoptotic signaling pathway via death domain receptors | BAG3 | BAX | BCL2 | CASP8 | DAPK1 | DAXX | DDX3X | DEDD | DIABLO | FADD | FASLG | PIK3R1 | SORT1 | TNF | TNFRSF1A | TRADD | GO:0048168 | 0.0033968215455538 | 28/8243 | 16/3250 | 0.0434837169399407 | 1 | F | F | F | F | regulation of neuronal synaptic plasticity | APOE | APP | CAMK2A | CAMK2B | DBN1 | DLG4 | GRIK2 | HRAS | KRAS | NCDN | RAB11A | RAB3GAP1 | RAB5A | S100B | SNCA | SYP | GO:0006633 | 0.00764284847749606 | 63/8243 | 32/3250 | 0.043545036148885 | 1 | F | F | F | F | fatty acid biosynthetic process | ABCD3 | ACACA | ACLY | ACOT7 | ACSS2 | ALOX12 | ALOX5 | BRCA1 | CBR4 | CD74 | CYP1A1 | CYP1A2 | DEGS1 | FASN | GPX4 | GSTM4 | HSD17B8 | LTC4S | MCAT | MGST1 | MIF | MYO5A | PLA2G1B | PRKAA1 | PRKAA2 | PRKAB2 | PRKAG1 | PTGDS | PTGES | PTGES3 | TECR | XBP1 | GO:1901361 | 0.0378502972218852 | 312/8243 | 138/3250 | 0.0441490411841035 | 1 | F | F | F | F | organic cyclic compound catabolic process | ACAT1 | ACOT7 | ADA | AGO1 | AHCY | AICDA | ALDH6A1 | ALDH8A1 | APOE | ASPDH | ATM | BLVRB | CASP3 | CDA | CEL | COMT | CYP1A1 | CYP1A2 | DBH | DCP2 | DCTPP1 | DDX6 | DERA | DIS3 | DNA2 | DNASE2 | DNASE2B | DROSHA | DUT | EDC4 | EIF3E | EIF4A3 | ENDOG | ENTPD7 | EXOSC1 | EXOSC5 | EXOSC6 | EXOSC9 | FAH | FEN1 | GDA | GPX1 | GSPT1 | GSPT2 | GSTZ1 | H1F0 | HAAO | HELZ2 | HINT1 | HMGB1 | HMGB2 | HMOX1 | HNRNPD | HPRT1 | HSD17B14 | HSD17B6 | HSD3B7 | HSPA1B | ITPA | KPNA1 | KPNB1 | KYNU | LSM1 | LSM3 | LSM4 | LSM5 | MAOA | MLH1 | MTAP | NBAS | NCBP1 | NFE2L2 | NT5C2 | NUDT18 | NUDT3 | NUDT9 | OAS2 | PABPC1 | PABPC4 | PAN2 | PDE12 | PDE4D | PNP | PPP1R8 | PPP2CA | PPP2R1A | PPP2R2A | PRTFDC1 | RBM8A | RC3H1 | RNASE3 | RNASEH2A | RNASEH2B | RPL10 | RPL10A | RPL12 | RPL18A | RPL22 | RPL23 | RPL23A | RPL24 | RPL30 | RPL37A | RPL38 | RPL6 | RPL7 | RPL7A | RPL9 | RPLP0 | RPLP1 | RPLP2 | RPS12 | RPS13 | RPS14 | RPS15A | RPS19 | RPS2 | RPS20 | RPS24 | RPS29 | RPS3 | RPS4X | RPS8 | SKIV2L | SMG5 | SMG6 | SMG8 | SND1 | SNX17 | SSB | SULT1A3 | TDO2 | TREX1 | UPF2 | UPF3B | UPP2 | VCP | YWHAH | GO:0043406 | 0.0211088196045129 | 174/8243 | 80/3250 | 0.0445646237343198 | 1 | F | F | F | F | positive regulation of MAP kinase activity | AGER | ARRB1 | BIRC7 | BRAF | CD40 | CD40LG | CD74 | CD81 | CRK | CRKL | DAXX | DBNL | DUSP19 | DUSP7 | DVL2 | DVL3 | EGFR | EPHA4 | ERBB2 | EZH2 | FGF2 | FGFR1 | GADD45G | HRAS | IKBKG | ILK | INSR | IQGAP1 | KRAS | LPAR1 | LRRK2 | MAP2K1 | MAP2K3 | MAP2K4 | MAP2K6 | MAP2K7 | MAP3K1 | MAP3K13 | MAP3K14 | MAP3K2 | MAP3K3 | MAP3K4 | MAP4K1 | MAPK1 | MAPK14 | MAPK3 | MAPK8IP3 | MIF | NOD2 | NTF3 | NTRK1 | PDCD10 | PDE6G | PIK3CG | PLA2G1B | PRDX2 | PRKAA1 | PSEN1 | PTPN1 | RAF1 | RIPK1 | RIPK2 | RPS3 | S100A12 | SHC1 | SOD1 | SRC | TAB1 | TAB2 | TAOK3 | TGFB1 | THBS1 | TNF | TP73 | TRAF2 | TRAF6 | UBB | UBC | UBE2N | UBE2V1 | GO:1905954 | 0.00497391726313235 | 41/8243 | 22/3250 | 0.0450576888006422 | 1 | F | F | F | F | positive regulation of lipid localization | ABCG1 | ANXA2 | APOA1 | APOE | C1QTNF1 | CD36 | FABP3 | GPS2 | IKBKE | MAP2K6 | MIF | NFKBIA | NR1H2 | NR1H3 | P2RX4 | PLA2G10 | PLA2G6 | PRELID1 | PRKCD | SIRT1 | TRIAP1 | VSTM2A | GO:0016192 | 0.127502123013466 | 1051/8243 | 440/3250 | 0.0451117571630147 | 1 | F | F | F | F | vesicle-mediated transport | A2M | ABL1 | ACAA1 | ACKR2 | ACLY | ACTB | ACTG1 | ACTN1 | ACTN2 | ACTN4 | ACTR1A | ACTR2 | ADORA2A | ADRB2 | AGER | AGL | AHI1 | AHSG | AKTIP | ALB | ALDOA | ALDOC | ALOX5 | ANK1 | ANK3 | ANXA1 | ANXA11 | ANXA2 | ANXA5 | AP1B1 | AP1G1 | AP1M1 | AP3D1 | AP3M1 | AP4M1 | APEH | APLP1 | APOA1 | APOE | APP | APRT | ARF1 | ARF4 | ARF5 | ARFGAP1 | ARHGAP1 | ARL4D | ARL5B | ARMC8 | ARPC5 | ARR3 | ARRB1 | ARRB2 | ASGR1 | ASGR2 | ATL3 | ATP6V1D | ATP9A | AZU1 | BCL2L1 | BIN1 | BLZF1 | C16orf70 | CAB39 | CACNG2 | CACNG4 | CACNG5 | CALM1 | CALR | CAND1 | CANX | CAPN1 | CAPZA1 | CAPZA2 | CAPZB | CAT | CAV1 | CBL | CCDC22 | CCR1 | CCT2 | CCT8 | CD109 | CD209 | CD36 | CD6 | CD81 | CDA | CDC42 | CDC42SE2 | CDK16 | CDK5 | CEBPE | CEL | CEP290 | CHMP2A | CHP1 | CKAP4 | CLSTN1 | CLTC | CLU | COG3 | COMMD1 | COPA | COPB1 | COPE | COPG1 | COPS5 | COTL1 | CPLX1 | CPLX3 | CPNE3 | CSNK1E | CSNK2B | CTNNB1 | CTSA | CTSC | CTSD | CTTN | CUL3 | CXCL8 | CYB5R1 | CYB5R3 | DAB2 | DBNL | DCLK1 | DCTN1 | DDOST | DDX3X | DEGS1 | DENND1A | DERA | DNM1L | DYNC1H1 | DYNC1I1 | DYNC1I2 | DYNC1LI1 | DYNLL1 | DYNLT1 | EEF1A1 | EEF2 | EHD4 | EXOC6 | EZR | F5 | F9 | FABP5 | FAF2 | FAM3C | FAM49B | FCGR1A | FCN1 | FGB | FGL2 | FN1 | FOLR1 | FTH1 | FTL | GAA | GAK | GALNS | GAPVD1 | GDI2 | GGA1 | GHDC | GLA | GNS | GOLGA2 | GOLGA4 | GOLPH3 | GOLT1B | GOPC | GPI | GPR107 | GRB2 | GRIA1 | GRIP1 | GSTP1 | GUSB | GYG1 | HAP1 | HEXB | HGS | HMGB1 | HPX | HRAS | HRNR | HSP90AA1 | HSP90AB1 | HSP90B1 | HSPA1B | HSPA2 | HSPA8 | HSPG2 | HSPH1 | HTR1B | HTT | HUWE1 | IDH1 | IGHV3-13 | IGHV3-23 | IGHV3-30 | IGKV3-20 | IGLC7 | ILF2 | IMPDH2 | INSR | IQGAP1 | ISLR | ITGA4 | ITGAV | ITGB3 | JUP | KCMF1 | KCNAB2 | KIF11 | KIF2C | KIF5C | KLC1 | KLC2 | KLHL20 | KPNB1 | LAT | LGALS3 | LGALS3BP | LMAN2L | LOXL2 | LRP6 | LRRC7 | LRRK2 | LRSAM1 | MAL2 | MAN2B1 | MAPK1 | MAPK14 | MAPK3 | MAPK8IP3 | MASP1 | MCFD2 | MGST1 | MIB1 | MIF | MME | MMRN1 | MPPE1 | MYO1B | MYO1D | MYO1E | MYO5A | MYO6 | NAPRT | NBAS | NEDD4 | NHLRC3 | NIT2 | NME1 | NME2 | NOSTRIN | NR1H3 | OLA1 | OPTN | ORM1 | OSBPL1A | P2RX1 | PA2G4 | PACSIN3 | PDCD6 | PDXK | PEF1 | PFKL | PGAM1 | PGM1 | PGM2 | PGRMC1 | PI4K2A | PICK1 | PIK3C2A | PIK3CG | PIKFYVE | PITPNB | PKM | PLAUR | PLIN3 | PNP | PPIA | PPIE | PPT1 | PQLC1 | PRCP | PRDX4 | PRDX6 | PRG2 | PRKCD | PRKD1 | PROZ | PSAP | PSEN1 | PSMA2 | PSMA5 | PSMB7 | PSMC2 | PSMC3 | PSMD1 | PSMD11 | PSMD12 | PSMD14 | PSMD2 | PSTPIP1 | PTGES2 | PTPN23 | PTPN6 | PYGB | PYGL | QSOX1 | RAB10 | RAB11A | RAB11B | RAB11FIP2 | RAB18 | RAB1A | RAB1B | RAB24 | RAB29 | RAB2A | RAB31 | RAB32 | RAB35 | RAB5A | RAB5B | RAB7A | RAB9A | RABGEF1 | RAC1 | RAP1A | RAP1B | RAPGEF4 | RARA | RASSF9 | RHOA | RHOJ | RHOU | RNASE3 | RNF126 | RP2 | S100A11 | S100A12 | S100A13 | S100A7 | S100A9 | S100P | SCARA3 | SCCPDH | SCFD1 | SCG3 | SCYL2 | SDCBP | SEC16A | SEC22C | SEC24B | SEC24C | SEC31A | SELL | SERPINB3 | SERPINB6 | SH3GL3 | SH3KBP1 | SLAMF1 | SLC2A5 | SNAP29 | SNCA | SNF8 | SNX17 | SNX2 | SNX6 | SOD1 | SORT1 | SPTAN1 | SPTBN1 | SPTBN2 | SQSTM1 | SRC | SRP14 | STBD1 | STOM | STX5 | STXBP1 | STXBP2 | SYP | TAGLN2 | TBC1D23 | TBC1D4 | TCIRG1 | TEX264 | TFG | TGFB1 | TGM2 | THBS1 | TIMP1 | TIMP2 | TM9SF4 | TMED9 | TMEM173 | TMEM30A | TNK2 | TOLLIP | TOR1A | TRAPPC8 | TREX1 | TRIM23 | TRIM9 | TSG101 | TUBA4A | TUBB | TUBB4B | TXLNA | TXNDC5 | UBB | UBC | UBE2O | UBR4 | UNC119 | USP20 | VAPA | VAPB | VAT1 | VCL | VCP | VPS18 | VPS26A | VPS29 | VPS37D | VPS4B | VTA1 | VTN | WAS | WDR1 | XRCC5 | XRCC6 | YKT6 | ZW10 | GO:0021549 | 0.0031541914351571 | 26/8243 | 15/3250 | 0.0453379337826022 | 1 | F | F | F | F | cerebellum development | ABAT | ATIC | CDK5R1 | CKB | GART | HAP1 | HNRNPD | LPAR1 | MECP2 | NCOA1 | NEUROD1 | PPARGC1A | PSEN1 | PTN | RPGRIP1L | GO:1901099 | 0.0031541914351571 | 26/8243 | 15/3250 | 0.0453379337826022 | 1 | F | F | F | F | negative regulation of signal transduction in absence of ligand | AKT1 | BCL2 | BCL2L1 | COL2A1 | EYA2 | EYA4 | FYN | GATA1 | HSPA1B | MAPK7 | PRDX2 | RIPK1 | SNAI2 | TNF | ZC3HC1 | GO:0038083 | 0.0031541914351571 | 26/8243 | 15/3250 | 0.0453379337826022 | 1 | F | F | F | F | peptidyl-tyrosine autophosphorylation | ABL1 | ABL2 | BTK | EGFR | FYN | IGF1R | INSR | ITK | KDR | MAPK3 | NTRK1 | PTK2 | SRC | TNK2 | YES1 | GO:0030073 | 0.0031541914351571 | 26/8243 | 15/3250 | 0.0453379337826022 | 1 | F | F | F | F | insulin secretion | ADCYAP1 | ANXA1 | CPLX1 | CPLX3 | FKBP1B | HNF1A | MYO5A | NEUROD1 | PARK7 | RAB11B | RAB11FIP2 | RAF1 | RAPGEF4 | SMAD2 | VGF | GO:2001240 | 0.0031541914351571 | 26/8243 | 15/3250 | 0.0453379337826022 | 1 | F | F | F | F | negative regulation of extrinsic apoptotic signaling pathway in absence of ligand | AKT1 | BCL2 | BCL2L1 | COL2A1 | EYA2 | EYA4 | FYN | GATA1 | HSPA1B | MAPK7 | PRDX2 | RIPK1 | SNAI2 | TNF | ZC3HC1 | GO:1902275 | 0.0185612034453476 | 153/8243 | 71/3250 | 0.0454656718918122 | 1 | F | F | F | F | regulation of chromatin organization | AICDA | AKAP8 | ARRB1 | ATG5 | ATM | ATRX | BCL6 | BCOR | BRCA1 | BRD4 | CCNB1 | CDK9 | CTBP1 | CTCF | CTNNB1 | EED | EID1 | H1F0 | H1FX | HDAC5 | HIST1H1A | HNRNPU | KAT7 | KDM1A | KDM4D | KMT2A | KMT2B | LMNA | LRRK2 | MAPK3 | MAPK8 | MECP2 | NELFE | NIPBL | OGT | OTUB1 | PAXIP1 | PIH1D1 | PPARGC1A | PRKD1 | PRKD2 | PRMT6 | RIF1 | RTF1 | RUVBL2 | SART3 | SET | SETDB1 | SIN3A | SIRT1 | SIRT6 | SKI | SMAD4 | SMARCB1 | SNAI2 | SNCA | SNW1 | SPI1 | TADA2B | TGFB1 | TPR | TRIM28 | TRIP12 | TWIST1 | UBE2B | UBE2N | UBR5 | USP17L2 | WBP2 | ZBTB7B | ZMPSTE24 | GO:0019932 | 0.0185612034453476 | 153/8243 | 71/3250 | 0.0454656718918122 | 1 | F | F | F | F | second-messenger-mediated signaling | ACKR2 | ADCY6 | ADCYAP1 | ADORA2A | ADRB2 | APOE | ATP2A2 | AZU1 | BTK | CALM1 | CALM2 | CALM3 | CCR1 | CCR3 | CCR4 | CCR5 | CD36 | CXCL11 | CXCL8 | DMD | EGFR | EIF2AK3 | FIS1 | FKBP1B | GLP1R | GNA13 | GNAI2 | GNAQ | GNAS | GNB1 | GSTO1 | HDAC4 | ITPR1 | ITPR2 | ITPR3 | KDR | KSR1 | LAT | LGR4 | LRRK2 | MAPK7 | MC1R | NEUROD1 | NFATC1 | NFATC2 | NME2 | NOS2 | NOS3 | NPR1 | NR5A2 | P2RX4 | PDE4D | PLCG1 | PPP2CA | PPP2R1A | PPP3CC | PRKAA1 | PRKACA | PRKAR1A | PRKAR2B | PTGER3 | PTGIR | RAPGEF4 | RASD1 | SLC9A1 | SLC9A3R1 | SULT1A3 | TBXA2R | TPCN2 | VCAM1 | ZMPSTE24 | GO:0002224 | 0.00958388936066966 | 79/8243 | 39/3250 | 0.0455684719926052 | 1 | F | F | F | F | toll-like receptor signaling pathway | BCL10 | BIRC2 | BIRC3 | BTK | CASP8 | CD36 | CTSL | FADD | FGB | HAVCR2 | HMGB1 | HSP90B1 | HSPD1 | IKBKB | IKBKE | IKBKG | IRF7 | LGMN | MAP3K1 | MYD88 | NFKBIA | PRKCE | RIPK1 | RIPK2 | S100A1 | S100A9 | SCARA3 | TAB1 | TAB2 | TANK | TLR3 | TNIP2 | TRAF3 | TRAF6 | UBB | UBC | UBE2D1 | UBE2D2 | UBE2D3 | GO:0031175 | 0.0168627926725707 | 139/8243 | 65/3250 | 0.0456952386954458 | 1 | F | F | F | F | neuron projection development | ADCYAP1 | AGER | APOA1 | APOD | APOE | APP | ARHGAP35 | ATCAY | ATXN10 | BCL2 | CASP3 | CDC42 | CDH1 | CDK16 | CDK5 | CDK5R1 | CRK | CRKL | CUL4B | DAB1 | DLG4 | DOCK7 | DVL1 | EPHA4 | FOLR1 | GAP43 | GFAP | GNAO1 | GRIP1 | GSK3B | HDAC2 | HDAC6 | HERC1 | HMGB1 | JUN | LAMB1 | MAP1B | MAPK8IP3 | MAPT | MECP2 | MKS1 | MYOC | NCDN | NEDD4 | PHGDH | PTEN | PTPRF | PTPRK | RAB11A | RAB35 | RB1 | RTN4 | SDC4 | SEC24B | SEPT2 | SH3GL2 | SLC9A3R1 | SOD1 | STMN1 | TBC1D23 | TOR1A | TRIM32 | ULK1 | USP21 | VAPA | GO:2000181 | 0.00740021836709936 | 61/8243 | 31/3250 | 0.0461310768034275 | 1 | F | F | F | F | negative regulation of blood vessel morphogenesis | AGO1 | ATF2 | COL4A2 | CTNNB1 | DCN | E2F2 | FAM129B | FASLG | GTF2I | HDAC5 | HGS | HOXA5 | HSPG2 | ITGB1BP1 | MECP2 | MEOX2 | NPR1 | PDCD10 | PGK1 | PML | PPARG | PTN | RHOA | SARS | SPRY2 | STARD13 | STAT1 | SYNJ2BP | TBXA2R | THBS1 | THBS2 | GO:0007188 | 0.00849205386388451 | 70/8243 | 35/3250 | 0.0461706081653777 | 1 | F | F | F | F | adenylate cyclase-modulating G protein-coupled receptor signaling pathway | ADCY6 | ADCYAP1 | ADORA2A | ADRB2 | CCR3 | CHRM2 | CHRM3 | CORT | CXCL11 | GLP1R | GNA12 | GNA13 | GNA15 | GNAI1 | GNAI2 | GNAI3 | GNAO1 | GNAQ | GNAS | GNAZ | GNB1 | GRK5 | HTR1B | LGR4 | LPAR1 | MC1R | MTNR1A | NME2 | PDE4D | PSAP | PTGER3 | PTGIR | RGS1 | SLC9A3R1 | TBXA2R | GO:0006265 | 0.000970520441586801 | 8/8243 | 6/3250 | 0.046273461481657 | 1 | F | F | F | F | DNA topological change | HMGB1 | HMGB2 | RPA2 | TOP1 | TOP2A | TOP3A | GO:2000650 | 0.000970520441586801 | 8/8243 | 6/3250 | 0.046273461481657 | 1 | F | F | F | F | negative regulation of sodium ion transmembrane transporter activity | HECW1 | HECW2 | NEDD4 | PCSK9 | PRKCE | SLC9A3R1 | GO:0044458 | 0.000970520441586801 | 8/8243 | 6/3250 | 0.046273461481657 | 1 | F | F | F | F | motile cilium assembly | CENPJ | DYNLL1 | E2F4 | FOXJ1 | MKS1 | ZMYND10 | GO:0048312 | 0.000970520441586801 | 8/8243 | 6/3250 | 0.046273461481657 | 1 | F | F | F | F | intracellular distribution of mitochondria | CLUH | DNM1L | LRRK2 | MAPT | OPA1 | SYNJ2BP | GO:0030042 | 0.000970520441586801 | 8/8243 | 6/3250 | 0.046273461481657 | 1 | F | F | F | F | actin filament depolymerization | CFL1 | CFL2 | DSTN | TWF1 | TWF2 | WDR1 | GO:0006610 | 0.000970520441586801 | 8/8243 | 6/3250 | 0.046273461481657 | 1 | F | F | F | F | ribosomal protein import into nucleus | IPO11 | IPO4 | IPO5 | KPNB1 | RPL23 | TNPO1 | GO:0060009 | 0.000970520441586801 | 8/8243 | 6/3250 | 0.046273461481657 | 1 | F | F | F | F | Sertoli cell development | ARID4B | ATRX | HSD17B4 | ICAM1 | NTRK1 | SDC1 | GO:0045116 | 0.000970520441586801 | 8/8243 | 6/3250 | 0.046273461481657 | 1 | F | F | F | F | protein neddylation | DCUN1D1 | NAE1 | NEDD8 | RBX1 | UBA3 | UBE2M | GO:0035635 | 0.000970520441586801 | 8/8243 | 6/3250 | 0.046273461481657 | 1 | F | F | F | F | entry of bacterium into host cell | CBL | CDH1 | CTNNB1 | CTNND1 | GRB2 | SRC | GO:0010501 | 0.000970520441586801 | 8/8243 | 6/3250 | 0.046273461481657 | 1 | F | F | F | F | RNA secondary structure unwinding | AGO1 | AGO2 | DDX39B | DDX3X | DHX36 | DHX9 | GO:0061154 | 0.000970520441586801 | 8/8243 | 6/3250 | 0.046273461481657 | 1 | F | F | F | F | endothelial tube morphogenesis | CSNK2B | CTNNB1 | PRKD2 | RBPJ | RHOA | STARD13 | GO:0070424 | 0.000970520441586801 | 8/8243 | 6/3250 | 0.046273461481657 | 1 | F | F | F | F | regulation of nucleotide-binding oligomerization domain containing signaling pathway | BIRC2 | BIRC3 | HSPA1B | PTPN22 | TNFAIP3 | XIAP | GO:0050862 | 0.000970520441586801 | 8/8243 | 6/3250 | 0.046273461481657 | 1 | F | F | F | F | positive regulation of T cell receptor signaling pathway | ADA | CYLD | PRKD2 | RAB29 | RELA | RPS3 | GO:0019068 | 0.000970520441586801 | 8/8243 | 6/3250 | 0.046273461481657 | 1 | F | F | F | F | virion assembly | APOE | PPIA | RAB1A | RAB1B | UBB | UBC | GO:0003159 | 0.000970520441586801 | 8/8243 | 6/3250 | 0.046273461481657 | 1 | F | F | F | F | morphogenesis of an endothelium | CSNK2B | CTNNB1 | PRKD2 | RBPJ | RHOA | STARD13 | GO:0019226 | 0.000970520441586801 | 8/8243 | 6/3250 | 0.046273461481657 | 1 | F | F | F | F | transmission of nerve impulse | CACNG2 | CACNG4 | CACNG5 | DMRT3 | PAFAH1B1 | SOD1 | GO:1902510 | 0.000970520441586801 | 8/8243 | 6/3250 | 0.046273461481657 | 1 | F | F | F | F | regulation of apoptotic DNA fragmentation | AIFM1 | BAX | ENDOG | HSF1 | NMNAT1 | PAM16 | GO:0050872 | 0.000970520441586801 | 8/8243 | 6/3250 | 0.046273461481657 | 1 | F | F | F | F | white fat cell differentiation | CEBPA | CTBP1 | PER2 | PPARG | SIRT1 | SNAI2 | GO:1904714 | 0.000970520441586801 | 8/8243 | 6/3250 | 0.046273461481657 | 1 | F | F | F | F | regulation of chaperone-mediated autophagy | ATP13A2 | CTSA | EEF1A1 | EEF1A2 | GFAP | SNCA | GO:0035723 | 0.000970520441586801 | 8/8243 | 6/3250 | 0.046273461481657 | 1 | F | F | F | F | interleukin-15-mediated signaling pathway | GRB2 | JAK1 | JAK3 | SHC1 | STAT3 | STAT5A | GO:0006268 | 0.000970520441586801 | 8/8243 | 6/3250 | 0.046273461481657 | 1 | F | F | F | F | DNA unwinding involved in DNA replication | MCM2 | MCM4 | MCM6 | MCM7 | RAD51 | RPA2 | GO:0019471 | 0.000970520441586801 | 8/8243 | 6/3250 | 0.046273461481657 | 1 | F | F | F | F | 4-hydroxyproline metabolic process | EGLN1 | EGLN3 | P4HA1 | P4HA2 | P4HB | PRDX4 | GO:0010763 | 0.000970520441586801 | 8/8243 | 6/3250 | 0.046273461481657 | 1 | F | F | F | F | positive regulation of fibroblast migration | AKT1 | ARHGEF7 | BAG4 | PRKCE | TGFB1 | THBS1 | GO:1905383 | 0.000970520441586801 | 8/8243 | 6/3250 | 0.046273461481657 | 1 | F | F | F | F | protein localization to presynapse | DLG2 | HSPB1 | KIF5B | KIF5C | MAPK8IP3 | TERF2 | GO:2000643 | 0.000970520441586801 | 8/8243 | 6/3250 | 0.046273461481657 | 1 | F | F | F | F | positive regulation of early endosome to late endosome transport | DAB2 | EZR | MSN | PTPN23 | RAB21 | RDX | GO:0033235 | 0.000970520441586801 | 8/8243 | 6/3250 | 0.046273461481657 | 1 | F | F | F | F | positive regulation of protein sumoylation | ARNT | HDAC4 | PIAS1 | PIAS3 | TOLLIP | UBE2I | GO:0006975 | 0.000970520441586801 | 8/8243 | 6/3250 | 0.046273461481657 | 1 | F | F | F | F | DNA damage induced protein phosphorylation | ABL1 | ATM | CHEK2 | CRY1 | MAP2K6 | MAPK3 | GO:0051974 | 0.000970520441586801 | 8/8243 | 6/3250 | 0.046273461481657 | 1 | F | F | F | F | negative regulation of telomerase activity | PINX1 | POT1 | PPARG | SRC | TERF1 | TP53 | GO:0099641 | 0.000970520441586801 | 8/8243 | 6/3250 | 0.046273461481657 | 1 | F | F | F | F | anterograde axonal protein transport | DLG2 | HSPB1 | KIF5B | KIF5C | MAPK8IP3 | TERF2 | GO:0045955 | 0.000970520441586801 | 8/8243 | 6/3250 | 0.046273461481657 | 1 | F | F | F | F | negative regulation of calcium ion-dependent exocytosis | BRAF | GNAI2 | RAP1A | RAP1B | REST | TRIM9 | GO:0009889 | 0.296736625015164 | 2446/8243 | 999/3250 | 0.0463359440880267 | 1 | F | F | F | F | regulation of biosynthetic process | AARS | ABCG1 | ABHD14B | ABL1 | ACACA | ACADVL | ACD | ACO1 | ACTA2 | ACTL6A | ACTN1 | ACTN2 | ACTN4 | ACTR2 | ACTR3 | ADCYAP1 | ADORA2A | ADRB2 | AEBP2 | AES | AFF1 | AFF4 | AGAP2 | AGER | AGO1 | AGO2 | AHI1 | AICDA | AIRE | AKT1 | AKT2 | ANKRD49 | ANXA1 | APBB1 | APBB2 | APBB3 | APEX1 | APOA1 | APOE | APP | AR | ARF4 | ARHGAP35 | ARID3B | ARID4B | ARNT | ARNT2 | ARRB1 | ARRB2 | ASCC1 | ASCC2 | ASCL1 | ASS1 | ATAD5 | ATF2 | ATF6 | ATF6B | ATF7 | ATM | ATOH1 | ATP1A1 | ATRX | ATXN1 | ATXN2 | AURKB | AXIN1 | AZU1 | BACH2 | BAG3 | BANP | BASP1 | BCL10 | BCL11A | BCL2 | BCL6 | BCL6B | BCOR | BIRC2 | BIRC5 | BLZF1 | BMI1 | BMPR1A | BOLA1 | BOLA3 | BRCA1 | BRD2 | BRD3 | BRD4 | BTK | BTRC | BUD31 | C1QBP | CALR | CAMK1D | CAMK2A | CAND1 | CAPN2 | CARD9 | CARM1 | CAT | CAV1 | CBFA2T3 | CBFB | CBX4 | CBX7 | CBX8 | CBY1 | CCAR2 | CCDC22 | CCDC88A | CCL21 | CCNT1 | CCNT2 | CCT2 | CCT3 | CCT4 | CCT5 | CCT6A | CCT7 | CCT8 | CD36 | CD40 | CD40LG | CD74 | CD81 | CDC5L | CDC73 | CDH1 | CDK2 | CDK4 | CDK5 | CDK5R1 | CDK8 | CDK9 | CDKN1A | CEBPA | CEBPE | CENPF | CENPJ | CEP290 | CHCHD10 | CHCHD2 | CHCHD3 | CHD3 | CHD4 | CHD8 | CHEK2 | CHP1 | CHRD | CIRBP | CKS2 | CLTC | CLU | COA3 | COMMD1 | COPS2 | COPS5 | COQ3 | CREB1 | CREB3 | CREBBP | CRK | CRY1 | CRY2 | CTBP1 | CTCF | CTDP1 | CTH | CTNNB1 | CTNNBIP1 | CUL2 | CUL3 | CYLD | DAB2 | DAPK1 | DAXX | DBF4B | DCN | DCP2 | DDRGK1 | DDX1 | DDX39B | DDX3X | DHX36 | DHX9 | DMAP1 | DMRT3 | DNA2 | DNAJA3 | DNAJB1 | DNAJC30 | DPF2 | DTX1 | DUSP1 | DUSP22 | DVL1 | DVL2 | DVL3 | DYRK2 | E2F1 | E2F2 | E2F3 | E2F4 | E2F6 | EAF2 | EBF1 | ECD | EDA | EED | EEF2 | EFEMP1 | EGFR | EGLN1 | EGLN3 | EHMT1 | EHMT2 | EID1 | EIF2AK3 | EIF2AK4 | EIF2B5 | EIF2S1 | EIF3D | EIF3E | EIF3H | EIF4A3 | EIF4E | EIF4E2 | EIF4EBP1 | EIF4ENIF1 | EIF4G3 | EIF4H | EIF5 | EIF5A | EIF5B | EIF6 | ELAVL1 | ELF3 | ELF4 | ENO1 | EP300 | EPRS | ERBB2 | ERF | ERG | ERLIN2 | ERRFI1 | ESR1 | ESR2 | ESRRA | ESRRB | ESRRG | ETS1 | EWSR1 | EXOSC9 | EZH2 | EZR | FABP3 | FABP5 | FADD | FAF1 | FAM129B | FAM3C | FANCA | FASLG | FASN | FBP1 | FBXW11 | FDPS | FGF2 | FGFR1 | FHL2 | FKBP8 | FLOT1 | FOS | FOXA1 | FOXD4 | FOXD4L6 | FOXI2 | FOXJ1 | FOXK1 | FOXK2 | FOXM1 | FOXO1 | FTO | FUBP1 | FUBP3 | FUS | FXR2 | FYN | G3BP2 | GABPB1 | GAPDH | GATA1 | GDF9 | GEMIN5 | GGPS1 | GLA | GLMN | GLO1 | GOLGA2 | GOT1 | GPS2 | GRB7 | GRIP1 | GSK3A | GSK3B | GSTP1 | GSTZ1 | GTF2A1 | GTF2F1 | GTF2F2 | GTF2I | H1F0 | H1FX | H2AFZ | H3F3A | HAVCR2 | HAX1 | HBEGF | HBZ | HDAC1 | HDAC2 | HDAC3 | HDAC4 | HDAC5 | HDAC6 | HDAC7 | HDAC9 | HDGF | HELZ2 | HEXB | HEXIM1 | HIF1A | HIF1AN | HINT1 | HIST1H1A | HIST1H3A | HIST1H4A | HIST2H3A | HIVEP1 | HLX | HMGB1 | HMGB2 | HMGB3 | HMOX1 | HNF1A | HNF4A | HNF4G | HNRNPA1 | HNRNPA2B1 | HNRNPAB | HNRNPD | HNRNPK | HNRNPL | HNRNPU | HOXA5 | HOXB5 | HOXB6 | HOXB9 | HOXD10 | HRAS | HSF1 | HSF4 | HSP90AA1 | HSP90AB1 | HSPA1B | HSPA5 | HSPA8 | HSPB1 | HSPD1 | HSPH1 | HTATIP2 | HTRA1 | ICAM1 | ID2 | IDH1 | IFI16 | IFNA2 | IGBP1 | IGF2BP1 | IGFBP7 | IKBKB | IKBKG | IKZF5 | ILF2 | ILF3 | ILK | ING1 | ING5 | INSIG1 | INSIG2 | INSR | IREB2 | IRF1 | IRF4 | IRF7 | ITCH | ITGAV | ITGB1BP1 | ITGB3 | ITGB3BP | JAK3 | JMJD6 | JUN | JUNB | JUND | JUP | KANK2 | KAT5 | KAT7 | KAT8 | KDM1A | KDM2A | KDM5B | KDM5C | KDM8 | KDR | KEAP1 | KHDRBS1 | KLF5 | KLF6 | KMT2A | KMT2B | KMT2C | KPNB1 | KRAS | L3MBTL1 | L3MBTL2 | L3MBTL3 | L3MBTL4 | LARP4 | LEF1 | LEMD2 | LEMD3 | LGR4 | LIMS1 | LMO2 | LMO7 | LOXL2 | LPIN3 | LRIF1 | LRP6 | LRPPRC | LRRK2 | LUM | LZTR1 | MAF1 | MAP2K1 | MAP2K3 | MAP2K4 | MAP2K6 | MAP2K7 | MAP3K13 | MAP3K2 | MAP3K4 | MAPK1 | MAPK14 | MAPK15 | MAPK3 | MAPK7 | MAPK8 | MAPK9 | MAS1 | MAVS | MBD3 | MC1R | MDFI | MDM2 | MDM4 | MECP2 | MED1 | MED12 | MED14 | MED17 | MED20 | MED21 | MED23 | MED24 | MED31 | MED4 | MED6 | MEOX2 | MEPCE | METTL23 | MGA | MITF | MKNK2 | MLLT1 | MLLT11 | MLLT3 | MORF4L1 | MORF4L2 | MOV10 | MRGBP | MRPL12 | MSH6 | MTA1 | MTA3 | MTERF1 | MTPN | MVD | MXD1 | MYBBP1A | MYC | MYD88 | MYH6 | MYO6 | NACC1 | NAMPT | NANOG | NCBP1 | NCK1 | NCOA1 | NCOA2 | NCOA3 | NCOA4 | NCOR1 | NCOR2 | NDUFA13 | NEDD4 | NEDD8 | NEK7 | NELFB | NELFCD | NELFE | NEUROD1 | NFATC1 | NFATC2 | NFE2L2 | NFKB2 | NFKBIA | NIF3L1 | NIPBL | NKRF | NKX2-1 | NLK | NMD3 | NME2 | NOD2 | NOS1AP | NOS2 | NOS3 | NOSTRIN | NPM1 | NR0B2 | NR1H2 | NR1H3 | NR1I2 | NR3C1 | NR4A1 | NR5A2 | NTRK1 | NUFIP1 | NUMA1 | NUP85 | NUP98 | NUPR1 | OGFOD1 | OGT | ONECUT1 | P2RX4 | PA2G4 | PABPC1 | PAIP1 | PAK6 | PARK7 | PARP1 | PATZ1 | PAWR | PAX8 | PAX9 | PAXIP1 | PCBD1 | PCBP1 | PCGF1 | PCGF5 | PCGF6 | PCNA | PDK2 | PELI1 | PER1 | PER2 | PFKM | PFN1 | PGP | PHB | PHB2 | PHF10 | PHF20L1 | PHF21A | PIAS1 | PIAS3 | PIH1D1 | PIK3R1 | PIM1 | PIN1 | PINX1 | PIR | PKIG | PLA2G10 | PLA2G1B | PLA2G6 | PLK1 | PLSCR1 | PML | PNKP | POLDIP3 | POLR2A | POLR2L | POT1 | POU2F3 | POU5F1 | PPARD | PPARG | PPARGC1A | PPID | PPIE | PPM1A | PPM1D | PPP1CA | PPP1CB | PPP1R12A | PPP1R3B | PPP2CA | PPP2R1A | PRDX2 | PRDX3 | PRDX5 | PRKAA1 | PRKAA2 | PRKAB2 | PRKAR1A | PRKCD | PRKCZ | PRKD1 | PRKD2 | PRKDC | PRMT2 | PRMT3 | PRMT6 | PROX1 | PSEN1 | PSMA1 | PSMA2 | PSMA3 | PSMA5 | PSMA6 | PSMB5 | PSMB6 | PSMB7 | PSMB8 | PSMC1 | PSMC2 | PSMC3 | PSMC4 | PSMC5 | PSMC6 | PSMD1 | PSMD10 | PSMD11 | PSMD12 | PSMD14 | PSMD2 | PSMD4 | PSMD9 | PSME2 | PSME3 | PTEN | PTGES2 | PTGES3 | PTPN14 | PTPRK | PTTG1 | PUM1 | PUS7 | QARS | QRICH1 | RAB3GAP1 | RAC1 | RAD21 | RAF1 | RAN | RAP1A | RARA | RARG | RASD1 | RB1 | RBBP6 | RBM3 | RBM4 | RBM8A | RBMX | RBPJ | RBX1 | RC3H1 | REL | RELA | REST | RFC1 | RFC2 | RFC3 | RFC4 | RFC5 | RFXANK | RGN | RGS2 | RHOA | RIF1 | RING1 | RIPK1 | RIPK2 | RIPK3 | RIPK4 | RMND1 | RNF139 | RNF2 | RNF25 | RNF31 | RNF4 | RNF8 | RPL10 | RPL23 | RPL38 | RPL6 | RPS14 | RPS3 | RPS4X | RPS6KA1 | RPS6KB2 | RPTOR | RRM2 | RTF1 | RUNX1 | RUNX1T1 | RUNX2 | RUVBL1 | RUVBL2 | RXRA | RXRB | RXRG | S100A1 | S100A11 | S100A12 | S100A9 | SAP18 | SARS | SCML2 | SET | SETBP1 | SETDB1 | SF1 | SFMBT1 | SFPQ | SH2B1 | SHC1 | SHMT1 | SHMT2 | SIN3A | SIRT1 | SIRT5 | SIRT6 | SIRT7 | SKI | SLC9A1 | SLTM | SMAD2 | SMAD3 | SMAD4 | SMAD5 | SMAD9 | SMARCA2 | SMARCA4 | SMARCB1 | SMARCC1 | SMARCC2 | SMARCE1 | SMC1A | SMC3 | SMG5 | SMG6 | SMO | SMURF1 | SMURF2 | SNAI2 | SNCA | SND1 | SNF8 | SNW1 | SNX6 | SOAT1 | SOD1 | SOD2 | SOX2 | SP1 | SP100 | SP2 | SPEN | SPI1 | SPIN1 | SQSTM1 | SRC | SRI | STAG2 | STAT1 | STAT3 | STAT4 | STAT5A | STAT6 | STK3 | STOML2 | STRAP | SUB1 | SUMO1 | SUMO2 | SUPT4H1 | SUPT5H | SUZ12 | TAB1 | TAB2 | TACO1 | TADA2B | TAF12 | TAF15 | TAGLN3 | TARDBP | TAZ | TBL1X | TCEA1 | TCEA2 | TCERG1 | TCF12 | TCF21 | TCF25 | TCF4 | TCOF1 | TCP1 | TCP10L | TDRD3 | TERF1 | TERF2 | TERF2IP | TFCP2 | TFDP1 | TGFB1 | TGFBR3 | TGIF2LX | THAP11 | THAP7 | THBS1 | THRA | TIA1 | TIAL1 | TINF2 | TLE1 | TLE3 | TLR3 | TMBIM6 | TMEM173 | TMPO | TNF | TNFAIP3 | TNFRSF1A | TNFRSF8 | TNIP2 | TNKS | TNKS2 | TNP1 | TOE1 | TOP2A | TP53 | TP63 | TP73 | TPR | TRADD | TRAF1 | TRAF2 | TRAF3 | TRAF6 | TRAP1 | TREX1 | TRIAP1 | TRIB2 | TRIB3 | TRIM14 | TRIM21 | TRIM25 | TRIM28 | TRIM29 | TRIM32 | TRIM33 | TRIM34 | TRIP13 | TRIP4 | TRUB2 | TSC22D1 | TSC22D3 | TSC22D4 | TSFM | TSG101 | TSPYL2 | TTC5 | TULP3 | TWIST1 | TXN | TXNIP | TYMS | UBA3 | UBB | UBC | UBE2D1 | UBE2D3 | UBE2I | UBE2J1 | UBE2L3 | UBE2N | UBE2O | UBE2V1 | UNK | UNKL | UPF3B | UQCC1 | USP15 | USP16 | USP2 | USP21 | USP47 | USP7 | USP9X | USP9Y | VCP | VDR | VGLL4 | VHL | VIM | VPS72 | WAS | WBP2 | WDR35 | WDR77 | WDTC1 | WNT2 | WT1 | WWOX | WWP2 | XBP1 | XIAP | XPO1 | XRCC5 | XRCC6 | YAF2 | YAP1 | YEATS2 | YES1 | YTHDF1 | YWHAB | YWHAH | YWHAQ | YY1 | ZBTB16 | ZBTB17 | ZBTB21 | ZBTB25 | ZBTB33 | ZBTB42 | ZBTB7A | ZBTB7B | ZC3H4 | ZFP41 | ZHX1 | ZIC1 | ZKSCAN1 | ZMPSTE24 | ZMYM5 | ZMYND8 | ZNF207 | ZNF217 | ZNF227 | ZNF24 | ZNF277 | ZNF354C | ZNF408 | ZNF420 | ZNF443 | ZNF517 | ZNF518A | ZNF592 | ZNF598 | ZNF618 | ZNF622 | ZNF646 | ZNF669 | ZNF696 | ZNF746 | ZNF747 | ZRANB2 | ZSCAN2 | ZSCAN29 | ZSCAN32 | ZZZ3 | GO:0034645 | 0.0833434429212665 | 687/8243 | 292/3250 | 0.046624194297838 | 1 | F | F | F | F | cellular macromolecule biosynthetic process | AFF4 | AGL | AGO2 | AKT1 | AKT2 | APOA1 | APOE | AR | ARHGAP35 | ARNT | ARRB1 | ARRB2 | ATAD5 | ATF6B | ATM | ATOH1 | B3GAT3 | B3GNT3 | BRCA1 | CBFB | CCDC88A | CCNT1 | CCNT2 | CDC73 | CDK2 | CDK9 | CEBPA | CENPF | CHAF1A | CHRAC1 | COL4A2 | COPS2 | COPS5 | CREB1 | CTDP1 | DAP3 | DARS | DHX9 | DIDO1 | DNA2 | DNAJA3 | DRG2 | DSE | DTX1 | DUT | DVL1 | DVL2 | E2F1 | EAF2 | ECD | EEF1A1 | EEF1A1P5 | EEF1A2 | EEF1B2 | EEF1D | EEF1G | EEF2 | EGFR | EIF4H | EIF5A | ELF3 | ERAL1 | ESR1 | ETS1 | EXTL2 | FAM20B | FARSB | FEN1 | FOS | FUBP1 | FUBP3 | GAR1 | GATA1 | GBE1 | GEMIN5 | GGT1 | GLCE | GOLPH3 | GSPT1 | GSPT2 | GTF2A1 | GTF2E2 | GTF2F1 | GTF2F2 | GTF2I | GTF3C2 | GYG1 | GYG2 | GYS1 | HARS | HIF1A | HOXB9 | HS2ST1 | HSF1 | HSP90AA1 | HSP90AB1 | HSPA14 | ILF2 | ING5 | IRF1 | IRF7 | ISG15 | JUN | KAT5 | KAT7 | KLF5 | KMT2A | LARP4 | LEF1 | LIG4 | LMO2 | MCM2 | MCM3 | MCM4 | MCM5 | MCM6 | MCM7 | MCM9 | MCMBP | MED1 | MED20 | MED4 | MITF | MLLT1 | MLLT3 | MPI | MRPL12 | MRPL14 | MRPL19 | MRPL28 | MRPL38 | MRPL4 | MRPL43 | MRPL45 | MRPL46 | MRPL9 | MRPS5 | MRRF | NAP1L1 | NCBP1 | NCOA1 | NCOR1 | NELFB | NELFCD | NELFE | NFATC1 | NFE2L2 | NOP10 | NPLOC4 | NR3C1 | NUP98 | OXA1L | PABPC4 | PARP1 | PAX8 | PCNA | PDF | PER2 | PGM1 | PGM2 | PHB | PINX1 | PIR | PNKP | POLA2 | POLD1 | POLE2 | POLE3 | POLM | POLR2A | POLR2E | POLR2L | POT1 | POU5F1 | PPARD | PPARG | PPIA | PPP1R3C | PPP5C | PTGES3 | PTMA | PTMS | PTTG1 | RARS2 | RBBP4 | RBBP6 | RBMX | RELA | REPIN1 | RFC1 | RFC2 | RFC3 | RFC4 | RFC5 | RMND1 | RNASEH2A | RPA2 | RPA3 | RPAIN | RPL10 | RPL10A | RPL12 | RPL18A | RPL22 | RPL23 | RPL23A | RPL24 | RPL30 | RPL36AL | RPL37A | RPL38 | RPL6 | RPL7 | RPL7A | RPL9 | RPLP0 | RPLP0P6 | RPLP1 | RPLP2 | RPS12 | RPS13 | RPS14 | RPS15A | RPS19 | RPS2 | RPS20 | RPS24 | RPS29 | RPS3 | RPS4X | RPS6KB2 | RPS8 | RRM1 | RRM2 | RRM2B | RTF1 | SARS | SCAF1 | SET | SIN3A | SIRT1 | SIRT7 | SKI | SMAD4 | SMG5 | SMG6 | SP1 | SPI1 | SPIN1 | SPRTN | STAT3 | SUPT4H1 | SUPT5H | TAF12 | TAF15 | TAF1A | TARDBP | TARS | TCEA1 | TCERG1 | TERF1 | TERF2 | TERF2IP | TFCP2 | THRA | TK1 | TK2 | TOP1 | TOP3A | TP53 | TREX1 | TRIM25 | TRIM29 | TRIP13 | TSC22D1 | TSFM | TUFM | TYMS | UBB | UBC | UBE2L6 | UGDH | UGP2 | USPL1 | VCP | WARS | XBP1 | XYLT1 | ZBTB7A | ZBTB7B | ZNF217 | ZNF598 | GO:0009888 | 0.0396700230498605 | 327/8243 | 144/3250 | 0.0468012240688383 | 1 | F | F | F | F | tissue development | ACAT1 | ADA | AHI1 | ANXA2 | ANXA6 | APOD | ASCL1 | ASGR2 | ATF2 | ATP2A2 | BASP1 | BMPR1A | BSG | BTK | CAT | CAV1 | CCNB1 | CCNT2 | CDK4 | CDK5 | CFL2 | COL2A1 | COL5A1 | CTNNB1 | CTSV | DCN | DHCR24 | DVL1 | EIF2AK3 | ELF3 | ENO3 | EP300 | ERBB3 | ERRFI1 | EYA2 | FABP5 | FAM83H | FGFR1 | FGFR3 | FLNB | FOXA1 | FOXJ1 | FOXO1 | FTO | GAA | GAP43 | GPX1 | HDAC1 | HLX | HOXA5 | HOXD10 | ID2 | IFT122 | IGSF8 | INSR | ITGB1BP1 | JUNB | KANK2 | KCNAB1 | KDR | KRT17 | KRT31 | LGR4 | MAPK1 | MAPK14 | MAPK3 | MATK | MBD3 | MED1 | MED12 | MEOX2 | MTHFD1 | MTPN | MYL6 | NAMPT | NEDD4 | NF2 | NKX2-1 | NR5A2 | NRP2 | ONECUT1 | OXCT1 | PALB2 | PAX8 | PAX9 | PAXIP1 | PGM5 | PHGDH | PHOSPHO1 | PIK3CA | PKM | PLXNA2 | POU2F3 | PPARD | PPARGC1A | PPP2CA | PROX1 | PTEN | PTN | RARA | RARG | RBBP6 | RHOA | RPGRIP1L | RPL38 | S100A7 | SBDS | SCEL | SDC1 | SDC4 | SERPINE2 | SMAD2 | SMAD3 | SMAD4 | SMAD5 | SMO | SMURF1 | SNAI2 | SOX2 | SPRR2A | SPTLC2 | STAT1 | STIL | STIM1 | SVIL | TAZ | TCF21 | TFDP1 | TFIP11 | TGFB1 | TRAF3IP1 | TUFT1 | TULP3 | UBB | VDR | WAS | WNT2 | WT1 | XBP1 | YAP1 | ZBTB17 | ZBTB7B | ZFAND5 | ZMPSTE24 | GO:0009746 | 0.0104330947470581 | 86/8243 | 42/3250 | 0.0471293235788756 | 1 | F | F | F | F | response to hexose | CASP3 | COL6A2 | CTSV | EIF2B1 | EIF2B3 | EIF2B5 | ENDOG | FIS1 | FKBP1B | GLUL | HNF4A | ICAM1 | IGF1R | LDHA | LGALS1 | NAMPT | NDUFA9 | NEUROD1 | NME1 | NME2 | NR0B2 | OPA1 | PFKL | PIK3CA | PPARD | PPARGC1A | PRKAA1 | PRKAA2 | PRKACA | PTEN | RAP1A | RHOA | SARM1 | SIN3A | SLC2A5 | SMAD2 | TGFB1 | THBS1 | TXN2 | TXNIP | WDR35 | XBP1 | GO:1903313 | 0.0029115613247604 | 24/8243 | 14/3250 | 0.0471537697813246 | 1 | F | F | F | F | positive regulation of mRNA metabolic process | CCNB1 | CDC73 | CIRBP | DAZAP1 | DHX36 | HSF1 | NCBP1 | NUP98 | PRDX6 | PRPF19 | RBM3 | RBMX | SNW1 | TRA2A | GO:0033692 | 0.0029115613247604 | 24/8243 | 14/3250 | 0.0471537697813246 | 1 | F | F | F | F | cellular polysaccharide biosynthetic process | AGL | AKT1 | AKT2 | B3GNT3 | GBE1 | GYG1 | GYG2 | GYS1 | HS2ST1 | PER2 | PGM1 | PGM2 | PPP1R3C | UGP2 | GO:0010833 | 0.0029115613247604 | 24/8243 | 14/3250 | 0.0471537697813246 | 1 | F | F | F | F | telomere maintenance via telomere lengthening | GAR1 | HSP90AA1 | HSP90AB1 | NOP10 | PINX1 | POT1 | PTGES3 | RAD51 | RFC1 | RPA2 | SMG5 | SMG6 | TERF1 | TERF2IP | GO:0000271 | 0.0029115613247604 | 24/8243 | 14/3250 | 0.0471537697813246 | 1 | F | F | F | F | polysaccharide biosynthetic process | AGL | AKT1 | AKT2 | B3GNT3 | GBE1 | GYG1 | GYG2 | GYS1 | HS2ST1 | PER2 | PGM1 | PGM2 | PPP1R3C | UGP2 | GO:0043949 | 0.0029115613247604 | 24/8243 | 14/3250 | 0.0471537697813246 | 1 | F | F | F | F | regulation of cAMP-mediated signaling | ADCYAP1 | APLP1 | CALM1 | CALM2 | CALM3 | CDC34 | GNAI1 | GNAI2 | GSK3A | LPAR1 | PDE4D | PEBP1 | RGS2 | UBE2B | GO:0050685 | 0.0029115613247604 | 24/8243 | 14/3250 | 0.0471537697813246 | 1 | F | F | F | F | positive regulation of mRNA processing | CCNB1 | CDC73 | CIRBP | DAZAP1 | DHX36 | HSF1 | NCBP1 | NUP98 | PRDX6 | PRPF19 | RBM3 | RBMX | SNW1 | TRA2A | GO:0051209 | 0.0029115613247604 | 24/8243 | 14/3250 | 0.0471537697813246 | 1 | F | F | F | F | release of sequestered calcium ion into cytosol | CCL21 | CCR5 | FASLG | FGF2 | FKBP1B | IBTK | ITPR1 | ITPR2 | ITPR3 | LETM1 | MCOLN3 | PLCG1 | PRKCE | TPCN2 | GO:0051261 | 0.0029115613247604 | 24/8243 | 14/3250 | 0.0471537697813246 | 1 | F | F | F | F | protein depolymerization | CFL1 | CFL2 | CKAP5 | DSTN | GAK | HSPA8 | KIF2C | SH3GL1 | SH3GL2 | STMN1 | TWF1 | TWF2 | VPS4B | WDR1 | GO:0071675 | 0.0029115613247604 | 24/8243 | 14/3250 | 0.0471537697813246 | 1 | F | F | F | F | regulation of mononuclear cell migration | AGER | CCR1 | CREB3 | DUSP1 | HMGB1 | LGALS3 | LGMN | MAPK14 | MAPK3 | S100A7 | SLAMF8 | TGFB1 | THBS1 | TNF | GO:0052312 | 0.0029115613247604 | 24/8243 | 14/3250 | 0.0471537697813246 | 1 | F | F | F | F | modulation of transcription in other organism involved in symbiotic interaction | CCNT1 | CTDP1 | EP300 | HDAC1 | JUN | LEF1 | POU2F3 | PSMC3 | REST | SMARCA4 | SMARCB1 | SNW1 | SP1 | TARDBP | GO:0035066 | 0.0029115613247604 | 24/8243 | 14/3250 | 0.0471537697813246 | 1 | F | F | F | F | positive regulation of histone acetylation | ARRB1 | BRCA1 | KAT7 | MAPK3 | PAXIP1 | PIH1D1 | PPARGC1A | RUVBL2 | SMAD4 | SMARCB1 | SNAI2 | TADA2B | TGFB1 | WBP2 | GO:0090322 | 0.0029115613247604 | 24/8243 | 14/3250 | 0.0471537697813246 | 1 | F | F | F | F | regulation of superoxide metabolic process | CD36 | CRP | EGFR | GNAI2 | GNAI3 | GSTP1 | MAPT | NFE2L2 | PARK7 | PRKCD | RGN | SOD1 | TGFB1 | TNF | GO:0006261 | 0.0029115613247604 | 24/8243 | 14/3250 | 0.0471537697813246 | 1 | F | F | F | F | DNA-dependent DNA replication | ATAD5 | DNA2 | DNAJA3 | MCMBP | PNKP | POLD1 | POLE2 | RFC1 | RFC2 | RFC3 | RFC4 | RFC5 | RPAIN | RRM2B | GO:0042886 | 0.110518015285697 | 911/8243 | 383/3250 | 0.0471853672450196 | 1 | F | F | F | F | amide transport | ABCE1 | ABCG1 | ACAA1 | ACD | ACOX1 | ACTN4 | ADCYAP1 | ADPRH | AFM | AGAP1 | AGAP2 | AGK | AHCYL1 | AKAP8 | AKT1 | AKT2 | AKTIP | ANK3 | ANXA1 | AP1B1 | AP1G1 | AP1M1 | AP3D1 | AP3M1 | AP4M1 | AP4S1 | APOE | APPBP2 | ARF1 | ARF4 | ARF5 | ARFGAP1 | ARFIP1 | ARHGAP1 | ARL4D | ARL5B | ARRB1 | ARRB2 | ASPSCR1 | ATG4C | ATP6V1A | ATP6V1B1 | ATP6V1C1 | ATP6V1D | ATP6V1E1 | AUP1 | BAG3 | BLZF1 | C16orf70 | CACNG2 | CACNG4 | CACNG5 | CALR | CANX | CAT | CBLB | CCDC22 | CCHCR1 | CCT6B | CD209 | CD36 | CD40LG | CD74 | CDC37 | CDK16 | CDK5 | CENPF | CEP290 | CHMP2A | CHP1 | CLSTN1 | CLTC | CLU | COG3 | COL4A3BP | COMMD1 | COPA | COPB1 | COPE | COPG1 | CPLX1 | CPLX3 | CROT | CSE1L | CTSA | CTTN | DAB2 | DDX19A | DENND1A | DENND4C | DERL1 | DHRS4 | DLG2 | DNAJC19 | DUSP16 | DYNLL1 | DYNLT1 | ECH1 | ECI2 | EHHADH | EIF4ENIF1 | EIF5A | EIF6 | EXOC6 | FAF2 | FBXO7 | FIS1 | FKBP1B | FOLR1 | FRAS1 | GBP5 | GDI1 | GDI2 | GGA1 | GOLGA2 | GOLGA4 | GOLPH3 | GOLT1B | GOPC | GRIP1 | GRPEL1 | GSTK1 | HAP1 | HDAC6 | HEATR3 | HERC2 | HGS | HID1 | HMGB1 | HMGCL | HNF1A | HNRNPU | HSD17B4 | HSP90AA1 | HSP90B1 | HSPA4 | HSPA5 | HSPA8 | HSPB1 | HSPD1 | ICK | IDE | IDH1 | IFIT1 | IFT122 | IPO11 | IPO4 | IPO5 | IPO7 | IPO8 | IPO9 | ITGAV | ITGB1BP1 | KIF13B | KIF5B | KIF5C | KLHL20 | KPNA1 | KPNA2 | KPNB1 | LARS | LCP2 | LMAN2L | LMNA | LRRC7 | LRSAM1 | MAPK8IP3 | MCFD2 | MTX1 | MYO1C | MYO5A | MYO6 | NBAS | NCOA4 | NDUFA13 | NEDD4 | NEUROD1 | NMD3 | NOD2 | NOS2 | NPLOC4 | NPM1 | NUDT19 | NUP107 | NUP153 | NUP214 | NUP85 | NUP88 | NUP93 | NUP98 | NUTF2 | NXT2 | OPTN | OXA1L | PAFAH1B1 | PAM16 | PARD3 | PARK7 | PCM1 | PDCD6 | PDCD6IP | PDIA4 | PEX19 | PEX5 | PHB2 | PICK1 | PIK3R1 | PLEKHF2 | PML | PNP | POM121 | PPARG | PPID | PPT1 | PSAP | PSEN1 | PTPN23 | RAB10 | RAB11A | RAB11B | RAB11FIP2 | RAB18 | RAB1A | RAB1B | RAB21 | RAB24 | RAB29 | RAB2A | RAB31 | RAB32 | RAB35 | RAB3IP | RAB5A | RAB5B | RAB7A | RAB9A | RABGEF1 | RABL2A | RABL3 | RAF1 | RAN | RANBP3 | RAP1A | RAPGEF4 | RASSF9 | RBPJ | ROMO1 | RP2 | RPAIN | RPL10 | RPL10A | RPL12 | RPL18A | RPL22 | RPL23 | RPL23A | RPL24 | RPL30 | RPL37A | RPL38 | RPL6 | RPL7 | RPL7A | RPL9 | RPLP0 | RPLP1 | RPLP2 | RPS12 | RPS13 | RPS14 | RPS15A | RPS19 | RPS2 | RPS20 | RPS24 | RPS29 | RPS3 | RPS4X | RPS8 | RRS1 | S100A12 | S100A13 | SCFD1 | SCG2 | SDCBP | SEC13 | SEC16A | SEC22C | SEC24B | SEC24C | SEC31A | SEC62 | SEC63 | SEL1L | SELENBP1 | SFPQ | SGTA | SGTB | SLC15A1 | SLC25A32 | SLC25A6 | SLC9A3R1 | SMAD2 | SMURF1 | SNAP29 | SNF8 | SNX12 | SNX17 | SNX2 | SNX21 | SNX24 | SNX3 | SNX6 | SPTBN1 | SRP14 | SRP19 | SSX2IP | STAT3 | STX5 | STXBP1 | STXBP2 | STYX | SYNJ2BP | SYVN1 | TANGO2 | TBC1D15 | TBC1D22B | TBC1D4 | TCIRG1 | TCP1 | TERF2 | TGFB1 | TIMM13 | TIMM21 | TIMM23 | TIMM44 | TIMM50 | TMED9 | TNKS | TNPO1 | TOMM20 | TOMM22 | TOMM40 | TOR1A | TPR | TRAF3IP1 | TRIM23 | TSG101 | TXNIP | UBB | UBC | UBE2D1 | UBE2D2 | UBE2D3 | UNC119 | USP9X | VCP | VGF | VPS13B | VPS18 | VPS26A | VPS29 | VPS37D | VPS4B | VPS8 | VTA1 | WDR19 | WDR35 | XBP1 | XPO1 | XPO4 | XPO5 | XPO7 | YKT6 | YWHAB | YWHAE | YWHAG | YWHAH | ZBTB7B | ZDHHC17 | ZDHHC23 | ZFAND6 | ZW10 | GO:0051258 | 0.00473128715273565 | 39/8243 | 21/3250 | 0.0475799640684867 | 1 | F | F | F | F | protein polymerization | ARPC2 | ARPC4 | CENPJ | CHMP2A | CKAP5 | CTTN | DIAPH3 | DNM1L | FGB | GPX4 | MAPT | OPA1 | PIN1 | PSTPIP1 | RAC1 | UBE2C | UBE2K | UBE2S | VTN | WAS | ZNF207 | GO:0006638 | 0.00473128715273565 | 39/8243 | 21/3250 | 0.0475799640684867 | 1 | F | F | F | F | neutral lipid metabolic process | ACSL1 | ACSL4 | APOA1 | APOE | CAT | CAV1 | CPT1A | DGKE | DGKZ | FABP1 | FABP3 | FABP5 | FABP7 | GPX1 | INSIG1 | INSIG2 | LPIN3 | PCSK9 | SEL1L | SIRT1 | SNCA | GO:2001020 | 0.0183185733349509 | 151/8243 | 70/3250 | 0.047828078592513 | 1 | F | F | F | F | regulation of response to DNA damage stimulus | ABL1 | ACTR2 | APBB1 | ATAD5 | ATM | BABAM1 | BCL2 | BCL2L1 | BRCA1 | CASP9 | CBX8 | CCAR2 | CD74 | CDK9 | CHEK2 | CLU | CRY1 | DDX39B | DHX9 | EGFR | EYA2 | EYA4 | FIGNL1 | FOXM1 | FUS | H2AFX | HMGB1 | HNRNPK | HSF1 | KDM1A | KDM4D | MAPT | MDM2 | MGMT | MIF | MYC | NSMCE4A | OTUB1 | PARP1 | PAXIP1 | PCNA | PML | PPP4R2 | PRKCD | PSMD10 | RAD51 | RAD51AP1 | RIF1 | RNASEH2B | RNF8 | RPA2 | RPS3 | SIRT1 | SIRT6 | SNAI2 | TERF2 | TERF2IP | TFIP11 | TRIAP1 | TRIM28 | TRIM32 | TRIP12 | TWIST1 | UBE2N | UBE2V1 | UBR5 | USP47 | WAS | XRCC1 | ZMPSTE24 | GO:0090257 | 0.0157709571757855 | 130/8243 | 61/3250 | 0.0481207965820605 | 1 | F | F | F | F | regulation of muscle system process | ABAT | ACE2 | ADA | ADRB2 | ANXA6 | ATP1A1 | ATP2A2 | BIN1 | CALM1 | CALM2 | CALM3 | CAMK2B | CAV1 | CDK9 | CHRM2 | CHRM3 | CTDP1 | CTTN | DDX39B | DMD | DOCK5 | ENO1 | ERRFI1 | FKBP1B | FOXO1 | G6PD | GLRX3 | GSK3A | GSTO1 | HDAC4 | JUP | LMNA | MTPN | MYL2 | MYL9 | NOS1AP | NOS3 | P2RX1 | P2RX4 | PARP1 | PDE4D | PIK3CG | PIN1 | PPP1R12B | PRKACA | PTK2 | RGS2 | RHOA | SLC9A1 | SMAD3 | SMAD4 | SOD1 | SRI | SUMO1 | TBXA2R | TNFRSF1A | TNNT1 | TPM1 | TRIM63 | TWF1 | YY1 | GO:0051588 | 0.00934125925027296 | 77/8243 | 38/3250 | 0.0482521900339689 | 1 | F | F | F | F | regulation of neurotransmitter transport | ABAT | ADORA2A | ATP2A2 | BACE1 | BRAF | CALM1 | CALM3 | CAMK2A | CDK5 | CHRM2 | CPLX1 | CPLX3 | DNM1L | DVL1 | FLOT1 | GFAP | GIT1 | GSK3B | HTR1B | LRRK2 | P2RX1 | PER2 | PREPL | PSEN1 | RAB3GAP1 | RAB5A | RAP1A | RAP1B | RGS2 | RHOT1 | SEPT5 | SNCA | SNX6 | STXBP1 | SYN1 | SYP | TOR1A | VPS18 | GO:0048872 | 0.00934125925027296 | 77/8243 | 38/3250 | 0.0482521900339689 | 1 | F | F | F | F | homeostasis of number of cells | ABL1 | AKT3 | ANXA1 | BAX | BCL2 | CASP3 | CCR4 | CXCL6 | CYLD | EZH2 | FADD | FANCC | FH | GATA1 | GPI | HIF1A | HMOX1 | HOXB6 | IREB2 | JAK3 | KRAS | LAT | LGR4 | MTHFD1 | NOS3 | PRDX1 | PRDX2 | RC3H1 | RIPK3 | RPS24 | SART3 | SMO | SOD1 | TGFB1 | TNFAIP3 | TNFSF14 | UBAP2L | WDR48 | GO:2000112 | 0.275263860245056 | 2269/8243 | 928/3250 | 0.0486130528854984 | 1 | F | F | F | F | regulation of cellular macromolecule biosynthetic process | AARS | ABCG1 | ABHD14B | ABL1 | ACD | ACO1 | ACTA2 | ACTL6A | ACTR2 | ACTR3 | ADCYAP1 | ADORA2A | ADRB2 | AEBP2 | AES | AFF1 | AFF4 | AGER | AGO1 | AGO2 | AHI1 | AICDA | AIRE | AKT1 | AKT2 | ANKRD49 | APBB1 | APBB2 | APBB3 | APOE | APP | AR | ARF4 | ARHGAP35 | ARID3B | ARID4B | ARNT | ARNT2 | ARRB1 | ARRB2 | ASCC1 | ASCC2 | ASCL1 | ATAD5 | ATF2 | ATF6 | ATF6B | ATF7 | ATM | ATOH1 | ATRX | ATXN1 | ATXN2 | AURKB | AXIN1 | BACH2 | BAG3 | BANP | BASP1 | BCL10 | BCL11A | BCL2 | BCL6 | BCL6B | BCOR | BIRC5 | BLZF1 | BMI1 | BMPR1A | BOLA1 | BOLA3 | BRCA1 | BRD2 | BRD3 | BRD4 | BTK | BTRC | BUD31 | C1QBP | CALR | CAMK1D | CAMK2A | CAND1 | CARM1 | CAT | CAV1 | CBFA2T3 | CBFB | CBX4 | CBX7 | CBX8 | CBY1 | CCAR2 | CCDC22 | CCDC88A | CCL21 | CCNT1 | CCNT2 | CCT2 | CCT3 | CCT4 | CCT5 | CCT6A | CCT7 | CCT8 | CD36 | CD40 | CD40LG | CD74 | CD81 | CDC5L | CDC73 | CDH1 | CDK2 | CDK4 | CDK5 | CDK5R1 | CDK8 | CDK9 | CDKN1A | CEBPA | CEBPE | CENPF | CEP290 | CHCHD10 | CHCHD2 | CHCHD3 | CHD3 | CHD4 | CHD8 | CHEK2 | CHP1 | CHRD | CIRBP | CKS2 | CLU | COA3 | COMMD1 | COPS2 | COPS5 | CREB1 | CREB3 | CREBBP | CRK | CRY1 | CRY2 | CTBP1 | CTCF | CTDP1 | CTH | CTNNB1 | CTNNBIP1 | CUL2 | CUL3 | CYLD | DAB2 | DAPK1 | DAXX | DBF4B | DCN | DCP2 | DDRGK1 | DDX1 | DDX39B | DDX3X | DHX36 | DHX9 | DMAP1 | DMRT3 | DNA2 | DNAJA3 | DNAJB1 | DPF2 | DUSP1 | DUSP22 | DVL1 | DVL2 | DVL3 | DYRK2 | E2F1 | E2F2 | E2F3 | E2F4 | E2F6 | EAF2 | EBF1 | ECD | EDA | EED | EEF2 | EFEMP1 | EGFR | EGLN1 | EGLN3 | EHMT1 | EHMT2 | EID1 | EIF2AK3 | EIF2AK4 | EIF2B5 | EIF2S1 | EIF3D | EIF3E | EIF3H | EIF4A3 | EIF4E | EIF4E2 | EIF4EBP1 | EIF4ENIF1 | EIF4G3 | EIF4H | EIF5 | EIF5A | EIF5B | EIF6 | ELAVL1 | ELF3 | ELF4 | ENO1 | EP300 | EPRS | ERBB2 | ERF | ERG | ERLIN2 | ESR1 | ESR2 | ESRRA | ESRRB | ESRRG | ETS1 | EWSR1 | EXOSC9 | EZH2 | EZR | FADD | FAF1 | FAM129B | FANCA | FASLG | FBP1 | FBXW11 | FGF2 | FGFR1 | FHL2 | FKBP8 | FLOT1 | FOS | FOXA1 | FOXD4 | FOXD4L6 | FOXI2 | FOXJ1 | FOXK1 | FOXK2 | FOXM1 | FOXO1 | FTO | FUBP1 | FUBP3 | FUS | FXR2 | G3BP2 | GABPB1 | GAPDH | GATA1 | GDF9 | GEMIN5 | GLO1 | GOLGA2 | GPS2 | GRB7 | GRIP1 | GSK3A | GSK3B | GTF2F1 | GTF2F2 | GTF2I | H1F0 | H1FX | H2AFZ | H3F3A | HAVCR2 | HAX1 | HBEGF | HBZ | HDAC1 | HDAC2 | HDAC3 | HDAC4 | HDAC5 | HDAC6 | HDAC7 | HDAC9 | HDGF | HELZ2 | HEXB | HEXIM1 | HIF1A | HIF1AN | HINT1 | HIST1H1A | HIST1H3A | HIST1H4A | HIST2H3A | HIVEP1 | HLX | HMGB1 | HMGB2 | HMGB3 | HMOX1 | HNF1A | HNF4A | HNF4G | HNRNPA1 | HNRNPA2B1 | HNRNPAB | HNRNPD | HNRNPK | HNRNPL | HNRNPU | HOXA5 | HOXB5 | HOXB6 | HOXB9 | HOXD10 | HRAS | HSF1 | HSF4 | HSP90AA1 | HSP90AB1 | HSPA1B | HSPA5 | HSPA8 | HSPB1 | HSPD1 | HTATIP2 | HTRA1 | ICAM1 | ID2 | IFI16 | IFNA2 | IGBP1 | IGF2BP1 | IKBKB | IKBKG | IKZF5 | ILF2 | ILF3 | ILK | ING1 | ING5 | INSIG1 | INSIG2 | INSR | IREB2 | IRF1 | IRF4 | IRF7 | ITCH | ITGB1BP1 | ITGB3BP | JAK3 | JMJD6 | JUN | JUNB | JUND | JUP | KANK2 | KAT5 | KAT7 | KAT8 | KDM1A | KDM2A | KDM5B | KDM5C | KDM8 | KEAP1 | KHDRBS1 | KLF5 | KLF6 | KMT2A | KMT2B | KMT2C | KRAS | L3MBTL1 | L3MBTL2 | L3MBTL3 | L3MBTL4 | LARP4 | LEF1 | LEMD2 | LEMD3 | LGR4 | LIMS1 | LMO2 | LMO7 | LOXL2 | LPIN3 | LRIF1 | LRP6 | LRPPRC | LUM | LZTR1 | MAF1 | MAP2K1 | MAP2K3 | MAP2K4 | MAP2K7 | MAP3K13 | MAP3K2 | MAP3K4 | MAPK1 | MAPK14 | MAPK15 | MAPK3 | MAPK7 | MAPK8 | MAPK9 | MAS1 | MAVS | MBD3 | MC1R | MDFI | MDM2 | MDM4 | MECP2 | MED1 | MED12 | MED14 | MED17 | MED20 | MED21 | MED23 | MED24 | MED31 | MED4 | MED6 | MEOX2 | MEPCE | METTL23 | MGA | MITF | MKNK2 | MLLT1 | MLLT11 | MLLT3 | MORF4L1 | MORF4L2 | MOV10 | MRGBP | MRPL12 | MSH6 | MTA1 | MTA3 | MTERF1 | MTPN | MXD1 | MYBBP1A | MYC | MYD88 | MYH6 | MYO6 | NACC1 | NAMPT | NANOG | NCBP1 | NCK1 | NCOA1 | NCOA2 | NCOA3 | NCOA4 | NCOR1 | NCOR2 | NDUFA13 | NEDD4 | NEDD8 | NEK7 | NELFB | NELFCD | NELFE | NEUROD1 | NFATC1 | NFATC2 | NFE2L2 | NFKB2 | NFKBIA | NIF3L1 | NIPBL | NKRF | NKX2-1 | NLK | NME2 | NOD2 | NOSTRIN | NPM1 | NR0B2 | NR1H2 | NR1H3 | NR1I2 | NR3C1 | NR4A1 | NR5A2 | NTRK1 | NUFIP1 | NUMA1 | NUP85 | OGFOD1 | OGT | ONECUT1 | PA2G4 | PABPC1 | PAIP1 | PAK6 | PARK7 | PARP1 | PATZ1 | PAWR | PAX8 | PAX9 | PAXIP1 | PCBD1 | PCBP1 | PCGF1 | PCGF5 | PCGF6 | PCNA | PELI1 | PER1 | PER2 | PFKM | PFN1 | PHB | PHB2 | PHF10 | PHF20L1 | PHF21A | PIAS1 | PIAS3 | PIH1D1 | PIK3R1 | PIM1 | PIN1 | PINX1 | PKIG | PLA2G10 | PLA2G1B | PLK1 | PLSCR1 | PML | PNKP | POLDIP3 | POLR2A | POLR2L | POT1 | POU2F3 | POU5F1 | PPARD | PPARG | PPARGC1A | PPID | PPIE | PPM1A | PPM1D | PPP1CA | PPP1CB | PPP1R12A | PPP1R3B | PPP2CA | PPP2R1A | PRDX2 | PRDX3 | PRDX5 | PRKAA1 | PRKAR1A | PRKCZ | PRKD1 | PRKD2 | PRKDC | PRMT2 | PRMT3 | PRMT6 | PROX1 | PSEN1 | PSMA1 | PSMA2 | PSMA3 | PSMA5 | PSMA6 | PSMB5 | PSMB6 | PSMB7 | PSMB8 | PSMC1 | PSMC2 | PSMC3 | PSMC4 | PSMC5 | PSMC6 | PSMD1 | PSMD10 | PSMD11 | PSMD12 | PSMD14 | PSMD2 | PSMD4 | PSMD9 | PSME2 | PSME3 | PTEN | PTGES2 | PTGES3 | PTPRK | PTTG1 | PUM1 | PUS7 | QARS | QRICH1 | RAB3GAP1 | RAC1 | RAD21 | RAF1 | RAN | RARA | RARG | RASD1 | RB1 | RBBP6 | RBM3 | RBM4 | RBM8A | RBMX | RBPJ | RBX1 | RC3H1 | REL | RELA | REST | RFC1 | RFC2 | RFC3 | RFC4 | RFC5 | RFXANK | RGN | RGS2 | RHOA | RIF1 | RING1 | RIPK1 | RIPK2 | RIPK3 | RIPK4 | RMND1 | RNF139 | RNF2 | RNF25 | RNF31 | RNF4 | RNF8 | RPL10 | RPL23 | RPL38 | RPL6 | RPS14 | RPS3 | RPS4X | RPS6KA1 | RPS6KB2 | RPTOR | RRM2 | RTF1 | RUNX1 | RUNX1T1 | RUNX2 | RUVBL1 | RUVBL2 | RXRA | RXRB | RXRG | S100A1 | S100A11 | S100A12 | S100A9 | SAP18 | SARS | SCML2 | SET | SETBP1 | SETDB1 | SFMBT1 | SFPQ | SH2B1 | SHC1 | SHMT1 | SHMT2 | SIN3A | SIRT1 | SIRT5 | SIRT6 | SIRT7 | SKI | SLC9A1 | SLTM | SMAD2 | SMAD3 | SMAD4 | SMAD5 | SMAD9 | SMARCA2 | SMARCA4 | SMARCB1 | SMARCC1 | SMARCC2 | SMARCE1 | SMC1A | SMC3 | SMG5 | SMG6 | SMO | SMURF1 | SMURF2 | SNAI2 | SNCA | SNF8 | SNW1 | SNX6 | SOAT1 | SOD2 | SOX2 | SP1 | SP100 | SP2 | SPEN | SPI1 | SPIN1 | SQSTM1 | SRC | SRI | STAG2 | STAT1 | STAT3 | STAT4 | STAT5A | STAT6 | STK3 | STOML2 | STRAP | SUB1 | SUMO1 | SUMO2 | SUPT4H1 | SUPT5H | SUZ12 | TAB1 | TAB2 | TACO1 | TADA2B | TAF12 | TAF15 | TAGLN3 | TARDBP | TBL1X | TCEA1 | TCEA2 | TCERG1 | TCF12 | TCF21 | TCF25 | TCF4 | TCOF1 | TCP1 | TCP10L | TERF1 | TERF2 | TERF2IP | TFCP2 | TFDP1 | TGFB1 | TGFBR3 | TGIF2LX | THAP11 | THAP7 | THBS1 | THRA | TIA1 | TIAL1 | TINF2 | TLE1 | TLE3 | TLR3 | TMBIM6 | TMEM173 | TMPO | TNF | TNFAIP3 | TNFRSF1A | TNFRSF8 | TNIP2 | TNKS | TNKS2 | TNP1 | TOE1 | TOP2A | TP53 | TP63 | TP73 | TPR | TRADD | TRAF1 | TRAF2 | TRAF3 | TRAF6 | TRAP1 | TRIAP1 | TRIB3 | TRIM14 | TRIM21 | TRIM25 | TRIM28 | TRIM29 | TRIM32 | TRIM33 | TRIM34 | TRIP4 | TRUB2 | TSC22D1 | TSC22D3 | TSC22D4 | TSFM | TSG101 | TSPYL2 | TTC5 | TULP3 | TWIST1 | TXN | TXNIP | TYMS | UBA3 | UBB | UBC | UBE2D1 | UBE2D3 | UBE2I | UBE2L3 | UBE2N | UBE2O | UBE2V1 | UNK | UNKL | UPF3B | UQCC1 | USP15 | USP16 | USP2 | USP21 | USP47 | USP7 | USP9X | USP9Y | VDR | VGLL4 | VHL | VIM | VPS72 | WAS | WBP2 | WDR77 | WDTC1 | WNT2 | WT1 | WWOX | WWP2 | XBP1 | XIAP | XPO1 | XRCC5 | XRCC6 | YAF2 | YAP1 | YEATS2 | YES1 | YTHDF1 | YWHAB | YWHAH | YWHAQ | YY1 | ZBTB16 | ZBTB17 | ZBTB21 | ZBTB25 | ZBTB33 | ZBTB42 | ZBTB7A | ZBTB7B | ZC3H4 | ZFP41 | ZHX1 | ZIC1 | ZKSCAN1 | ZMPSTE24 | ZMYM5 | ZMYND8 | ZNF207 | ZNF217 | ZNF227 | ZNF24 | ZNF277 | ZNF354C | ZNF408 | ZNF420 | ZNF443 | ZNF517 | ZNF518A | ZNF592 | ZNF598 | ZNF618 | ZNF622 | ZNF646 | ZNF669 | ZNF696 | ZNF746 | ZNF747 | ZRANB2 | ZSCAN2 | ZSCAN29 | ZSCAN32 | ZZZ3 | GO:0046928 | 0.00715758825670266 | 59/8243 | 30/3250 | 0.0488834221923263 | 1 | F | F | F | F | regulation of neurotransmitter secretion | ADORA2A | ATP2A2 | BACE1 | BRAF | CALM1 | CALM3 | CAMK2A | CDK5 | CHRM2 | CPLX1 | CPLX3 | DNM1L | DVL1 | GIT1 | GSK3B | HTR1B | LRRK2 | P2RX1 | PREPL | RAB3GAP1 | RAB5A | RAP1A | RAP1B | RHOT1 | SEPT5 | SNCA | STXBP1 | SYN1 | SYP | VPS18 | GO:0090101 | 0.00715758825670266 | 59/8243 | 30/3250 | 0.0488834221923263 | 1 | F | F | F | F | negative regulation of transmembrane receptor protein serine/threonine kinase signaling pathway | ABL1 | CAV1 | CD109 | CHRD | HSPA5 | HTRA1 | LDLRAD4 | LEMD2 | LEMD3 | LTBP1 | NKX2-1 | ONECUT1 | PIN1 | PPM1A | RASL11B | SIRT1 | SKI | SMAD2 | SMAD3 | SMURF1 | SMURF2 | SNX6 | STRAP | STUB1 | TGFB1 | TGFBR3 | TRIM33 | UBB | UBC | ZBTB7A | GO:0046688 | 0.0026689312143637 | 22/8243 | 13/3250 | 0.0488844757486403 | 1 | F | F | F | F | response to copper ion | APP | BACE1 | CYP1A1 | CYP1A2 | DAXX | HSF1 | ICAM1 | IREB2 | LOXL2 | S100A13 | SNCA | SOD1 | SORD | GO:0033522 | 0.0026689312143637 | 22/8243 | 13/3250 | 0.0488844757486403 | 1 | F | F | F | F | histone H2A ubiquitination | BCOR | BMI1 | CUL4B | DDB1 | DDB2 | PCGF1 | PCGF5 | PCGF6 | RING1 | RNF2 | RNF8 | UBE2A | UBE2B | GO:1902235 | 0.0026689312143637 | 22/8243 | 13/3250 | 0.0488844757486403 | 1 | F | F | F | F | regulation of endoplasmic reticulum stress-induced intrinsic apoptotic signaling pathway | BCL2L1 | CREB3 | EIF2AK3 | LRRK2 | NCK1 | OPA1 | PARK7 | PTPN1 | RNF183 | SIRT1 | SYVN1 | TMBIM6 | XBP1 | GO:0035296 | 0.00824942375348781 | 68/8243 | 34/3250 | 0.0489167188654828 | 1 | F | F | F | F | regulation of tube diameter | ACE2 | ACTA2 | ADCY6 | ADCYAP1 | ADORA2A | ADRB2 | AKT1 | ALOX12 | APOE | ATG5 | BDKRB2 | CAV1 | CHRM3 | CRP | DBH | DOCK5 | EGFR | FGB | GPX1 | HMOX1 | HTR1B | ICAM1 | NOS3 | NPR1 | P2RX1 | PER2 | PIK3C2A | PLA2G6 | PPARD | RAP1GDS1 | RHOA | SOD2 | TBXA2R | WDR35 | GO:0097746 | 0.00824942375348781 | 68/8243 | 34/3250 | 0.0489167188654828 | 1 | F | F | F | F | regulation of blood vessel diameter | ACE2 | ACTA2 | ADCY6 | ADCYAP1 | ADORA2A | ADRB2 | AKT1 | ALOX12 | APOE | ATG5 | BDKRB2 | CAV1 | CHRM3 | CRP | DBH | DOCK5 | EGFR | FGB | GPX1 | HMOX1 | HTR1B | ICAM1 | NOS3 | NPR1 | P2RX1 | PER2 | PIK3C2A | PLA2G6 | PPARD | RAP1GDS1 | RHOA | SOD2 | TBXA2R | WDR35 | GO:0008277 | 0.00824942375348781 | 68/8243 | 34/3250 | 0.0489167188654828 | 1 | F | F | F | F | regulation of G protein-coupled receptor signaling pathway | ADA | ADCYAP1 | ADRB2 | APLP1 | ARRB2 | CALM1 | CRY1 | CXCL8 | DYNLT1 | GIT1 | GNAI2 | GRK1 | GRK5 | GSK3A | LRRK2 | METAP2 | PDCL | PDE6G | PHB | PPEF1 | RGS1 | RGS2 | RGS20 | RHO | RIC8B | RPGRIP1L | SAG | SNCA | STMN1 | SYP | TULP3 | UBQLN2 | USP20 | YWHAB | GO:0007017 | 0.0464636661409681 | 383/8243 | 167/3250 | 0.0491022426406451 | 1 | F | F | F | F | microtubule-based process | ACTR2 | ACTR3 | AKAP9 | AP3D1 | AP3M1 | APP | ATRX | AURKB | BAG3 | BIRC5 | BRCA1 | CCDC120 | CCDC155 | CCDC68 | CCDC69 | CCDC8 | CCDC88A | CCNB1 | CCP110 | CDC14B | CDC20 | CDC42 | CDK2 | CDK5 | CDK5R1 | CENPJ | CEP135 | CEP152 | CEP350 | CEP57 | CETN3 | CHD3 | CHEK2 | CHP1 | CKAP5 | CNTROB | COPG1 | CRMP1 | CROCC | CRYAB | CUL7 | DCTN1 | DISC1 | DLG2 | DNAAF5 | DNAH9 | DOCK7 | DVL1 | DYNC1H1 | DYNC1I1 | DYNC1I2 | DYNC1LI1 | DYNLL1 | DYNLT1 | E2F4 | EML4 | EZR | FGFR1OP | FIGNL1 | FOPNL | FSD1 | GAPDH | GOLGA2 | HAP1 | HDAC3 | HEPACAM2 | HIF1A | HNRNPU | HSPA8 | HSPB1 | HTT | ICK | IFT122 | KIF11 | KIF13B | KIF2C | KIF5B | KIF5C | KIF6 | KIFC3 | KLC1 | KLC2 | KPNB1 | LMNA | LRPPRC | MAP1B | MAPK8IP3 | MAPT | MECP2 | MLH1 | NCOR1 | NEK7 | NIN | NPHP3 | NPM1 | NSFL1C | NTMT1 | NUMA1 | NUSAP1 | OBSL1 | OFD1 | OPA1 | PAFAH1B1 | PARD3 | PARD6A | PCM1 | PIN1 | PLK1 | PPP1R12A | PPP2R1A | PRKCZ | PTK2 | RAB11A | RAB1A | RAB21 | RAN | RANBP9 | RCC1 | RHOA | RHOT1 | RHOT2 | RNF19A | RPS3 | SBDS | SFPQ | SLC16A1 | SOD1 | SPC25 | SPDL1 | SPICE1 | SPRY2 | SRGAP2 | SSX2IP | STIL | STMN1 | SUN2 | SYNE2 | TBCE | TEKT2 | TEKT4 | TERF2 | TNKS | TNPO1 | TRAF3IP1 | TTLL1 | TUBA1A | TUBA1C | TUBA4A | TUBB | TUBB2A | TUBB2B | TUBB3 | TUBB4A | TUBB4B | TUBB6 | TUBB8 | TUBG1 | TUBGCP3 | UBB | UBE2B | UCHL1 | VBP1 | VCP | WDR19 | WDR35 | ZNF207 | ZW10 | GO:1903317 | 0.00448865704233895 | 37/8243 | 20/3250 | 0.0502377990777555 | 1 | F | F | F | F | regulation of protein maturation | ANXA2 | BAG2 | BIRC2 | BIRC3 | BIRC7 | BIRC8 | ENO1 | LRRK2 | MDM2 | PLGRKT | PRKACA | RHBDD1 | RNF139 | SERPINE2 | SNX12 | SRC | THBS1 | TNP1 | USP17L2 | XIAP | GO:1901379 | 0.00448865704233895 | 37/8243 | 20/3250 | 0.0502377990777555 | 1 | F | F | F | F | regulation of potassium ion transmembrane transport | ACTN2 | AKAP9 | ANK3 | ATP1B3 | BIN1 | CAB39 | CAV1 | FHL1 | KCNAB1 | KCNAB2 | KCNIP2 | NEDD4 | NOS1AP | OXSR1 | PTEN | SUMO1 | WNK1 | WNK4 | WWP2 | YWHAE | GO:0000387 | 0.00448865704233895 | 37/8243 | 20/3250 | 0.0502377990777555 | 1 | F | F | F | F | spliceosomal snRNP assembly | AAR2 | CD2BP2 | CLNS1A | GEMIN4 | GEMIN5 | GEMIN7 | LSM4 | PRPF19 | PRPF8 | SART1 | SART3 | SMN1 | SNRPB | SNRPD1 | SNRPD3 | SNRPE | SRSF12 | STRAP | USP4 | WDR77 | GO:1904427 | 0.00448865704233895 | 37/8243 | 20/3250 | 0.0502377990777555 | 1 | F | F | F | F | positive regulation of calcium ion transmembrane transport | ABL1 | BAX | CALM1 | CALM2 | CALM3 | CXCL11 | G6PD | GSTO1 | HAP1 | HSPA2 | HTT | PLCG1 | RGN | S100A1 | SLC9A1 | SNCA | SRI | STAC | STIM1 | SUMO1 | GO:0070228 | 0.00448865704233895 | 37/8243 | 20/3250 | 0.0502377990777555 | 1 | F | F | F | F | regulation of lymphocyte apoptotic process | ADA | AURKB | BAX | BCL10 | BCL6 | BIRC7 | BTK | CD27 | CD74 | FADD | HIF1A | JAK3 | LGALS3 | MIF | PRELID1 | PTCRA | PTEN | RIPK3 | TP53 | TSC22D3 | GO:0090092 | 0.0138299162926119 | 114/8243 | 54/3250 | 0.0503479383936131 | 1 | F | F | F | F | regulation of transmembrane receptor protein serine/threonine kinase signaling pathway | ABL1 | BMPR1A | CAV1 | CD109 | CHRD | CREBBP | CSNK2B | DAB2 | EP300 | FKBP1A | FKBP8 | FOLR1 | GDF9 | GOT1 | HSP90AB1 | HSPA5 | HTRA1 | ILK | LDLRAD4 | LEMD2 | LEMD3 | LTBP1 | NKX2-1 | NUMA1 | NUP93 | ONECUT1 | PARP1 | PIN1 | PPM1A | RASL11B | RBPJ | SDCBP | SIRT1 | SKI | SMAD2 | SMAD3 | SMAD4 | SMURF1 | SMURF2 | SNW1 | SNX6 | SPTBN1 | STK11 | STRAP | STUB1 | TGFB1 | TGFBR3 | THBS1 | TRIM33 | UBB | UBC | UBE2O | XIAP | ZBTB7A | GO:0006029 | 0.002426301103967 | 20/8243 | 12/3250 | 0.0504634383552938 | 1 | F | F | F | F | proteoglycan metabolic process | B3GAT3 | COL2A1 | DSE | EXTL2 | GLCE | HS2ST1 | HS6ST1 | PPARD | SGSH | SULF2 | UGDH | XYLT1 | GO:0007007 | 0.002426301103967 | 20/8243 | 12/3250 | 0.0504634383552938 | 1 | F | F | F | F | inner mitochondrial membrane organization | AFG3L2 | AGK | CHCHD3 | IMMT | LETM1 | NDUFA13 | OPA1 | OXA1L | ROMO1 | TAZ | TIMM13 | TMEM11 | GO:2000378 | 0.002426301103967 | 20/8243 | 12/3250 | 0.0504634383552938 | 1 | F | F | F | F | negative regulation of reactive oxygen species metabolic process | ATG5 | BCL2 | BRCA1 | CRYAB | G6PD | HDAC6 | HIF1A | HK2 | PRDX2 | SIRT3 | SIRT5 | VDAC1 | GO:0031063 | 0.002426301103967 | 20/8243 | 12/3250 | 0.0504634383552938 | 1 | F | F | F | F | regulation of histone deacetylation | AKAP8 | BCL6 | CTBP1 | LRRK2 | MAPK8 | NIPBL | PRKD1 | PRKD2 | SKI | TGFB1 | USP17L2 | ZBTB7B | GO:0097061 | 0.002426301103967 | 20/8243 | 12/3250 | 0.0504634383552938 | 1 | F | F | F | F | dendritic spine organization | ARF1 | CDC42 | CTTN | DLG4 | DVL1 | FYN | HDAC6 | IGF1R | INSR | LGMN | PICK1 | PTEN | GO:1904666 | 0.002426301103967 | 20/8243 | 12/3250 | 0.0504634383552938 | 1 | F | F | F | F | regulation of ubiquitin protein ligase activity | BAG2 | CDC14B | CDC20 | FZR1 | GOLGA2 | MAD2L1 | PLK1 | PTEN | RAB1A | RPL23 | UBE2C | UBE2S | GO:0045744 | 0.002426301103967 | 20/8243 | 12/3250 | 0.0504634383552938 | 1 | F | F | F | F | negative regulation of G protein-coupled receptor signaling pathway | ADA | ADRB2 | APLP1 | ARRB2 | CRY1 | CXCL8 | GNAI2 | RGS2 | RPGRIP1L | SNCA | STMN1 | YWHAB | GO:1903170 | 0.002426301103967 | 20/8243 | 12/3250 | 0.0504634383552938 | 1 | F | F | F | F | negative regulation of calcium ion transmembrane transport | BIN1 | CALM1 | CALM2 | CALM3 | FKBP1A | FKBP1B | GSTO1 | SRI | TGFB1 | THADA | UBQLN1 | YWHAE | GO:0007213 | 0.0012131505519835 | 10/8243 | 7/3250 | 0.0505226593897189 | 1 | F | F | F | F | G protein-coupled acetylcholine receptor signaling pathway | CDK5R1 | CHRM2 | CHRM3 | GNA15 | GNAI2 | GNAQ | GNB1 | GO:0000963 | 0.0012131505519835 | 10/8243 | 7/3250 | 0.0505226593897189 | 1 | F | F | F | F | mitochondrial RNA processing | ELAC2 | HSD17B10 | KIAA0391 | PUS1 | TBRG4 | TRMT61B | TRNT1 | GO:0019915 | 0.0012131505519835 | 10/8243 | 7/3250 | 0.0505226593897189 | 1 | F | F | F | F | lipid storage | APOA1 | CAV1 | CD36 | CRY1 | HEXB | SOAT1 | TNF | GO:1903963 | 0.0012131505519835 | 10/8243 | 7/3250 | 0.0505226593897189 | 1 | F | F | F | F | arachidonate transport | ANXA1 | BDKRB2 | NMB | PLA2G10 | PLA2G12A | PLA2G1B | PLA2G2A | GO:0006754 | 0.0012131505519835 | 10/8243 | 7/3250 | 0.0505226593897189 | 1 | F | F | F | F | ATP biosynthetic process | ALDOA | CYC1 | DNAJC30 | LDHC | SLC25A13 | STOML2 | TGFB1 | GO:0042402 | 0.0012131505519835 | 10/8243 | 7/3250 | 0.0505226593897189 | 1 | F | F | F | F | cellular biogenic amine catabolic process | ALDH7A1 | CHDH | HAAO | KYNU | SAT1 | SAT2 | TDO2 | GO:0097201 | 0.0012131505519835 | 10/8243 | 7/3250 | 0.0505226593897189 | 1 | F | F | F | F | negative regulation of transcription from RNA polymerase II promoter in response to stress | BAG3 | DNAJB1 | HIF1AN | JUN | NCK1 | TMBIM6 | VHL | GO:0042559 | 0.0012131505519835 | 10/8243 | 7/3250 | 0.0505226593897189 | 1 | F | F | F | F | pteridine-containing compound biosynthetic process | ATIC | FPGS | GART | MTHFD1 | PCBD1 | QDPR | SPR | GO:1903332 | 0.0012131505519835 | 10/8243 | 7/3250 | 0.0505226593897189 | 1 | F | F | F | F | regulation of protein folding | BAG5 | DNAJB2 | HSPA5 | PDCL | SGTA | SGTB | ST13 | GO:0050482 | 0.0012131505519835 | 10/8243 | 7/3250 | 0.0505226593897189 | 1 | F | F | F | F | arachidonic acid secretion | ANXA1 | BDKRB2 | NMB | PLA2G10 | PLA2G12A | PLA2G1B | PLA2G2A | GO:0006040 | 0.0012131505519835 | 10/8243 | 7/3250 | 0.0505226593897189 | 1 | F | F | F | F | amino sugar metabolic process | CHST4 | EXTL2 | GFPT1 | GFPT2 | GNPNAT1 | NAGK | PGM3 | GO:0009263 | 0.0012131505519835 | 10/8243 | 7/3250 | 0.0505226593897189 | 1 | F | F | F | F | deoxyribonucleotide biosynthetic process | ADK | DUT | RRM1 | RRM2 | RRM2B | SHMT1 | TYMS | GO:0009310 | 0.0012131505519835 | 10/8243 | 7/3250 | 0.0505226593897189 | 1 | F | F | F | F | amine catabolic process | ALDH7A1 | CHDH | HAAO | KYNU | SAT1 | SAT2 | TDO2 | GO:0034139 | 0.0012131505519835 | 10/8243 | 7/3250 | 0.0505226593897189 | 1 | F | F | F | F | regulation of toll-like receptor 3 signaling pathway | CAV1 | FLOT1 | PELI1 | PTPN22 | TNFAIP3 | UBQLN1 | WDFY1 | GO:1900044 | 0.0012131505519835 | 10/8243 | 7/3250 | 0.0505226593897189 | 1 | F | F | F | F | regulation of protein K63-linked ubiquitination | BIRC2 | GPS2 | NOD2 | OTUB1 | PTPN22 | TRIP12 | UBR5 | GO:0140053 | 0.0012131505519835 | 10/8243 | 7/3250 | 0.0505226593897189 | 1 | F | F | F | F | mitochondrial gene expression | ELAC2 | HSD17B10 | KIAA0391 | PUS1 | TBRG4 | TRMT61B | TRNT1 | GO:0032785 | 0.0012131505519835 | 10/8243 | 7/3250 | 0.0505226593897189 | 1 | F | F | F | F | negative regulation of DNA-templated transcription, elongation | EZH2 | HNRNPU | NELFB | NELFE | RNF8 | SUPT4H1 | SUPT5H | GO:0051580 | 0.0012131505519835 | 10/8243 | 7/3250 | 0.0505226593897189 | 1 | F | F | F | F | regulation of neurotransmitter uptake | FLOT1 | GFAP | PER2 | PSEN1 | RGS2 | SNCA | TOR1A | GO:0045820 | 0.0012131505519835 | 10/8243 | 7/3250 | 0.0505226593897189 | 1 | F | F | F | F | negative regulation of glycolytic process | CBFA2T3 | DDIT4 | FBP1 | HDAC4 | PPARGC1A | SIRT6 | STAT3 | GO:0017157 | 0.0155283270653888 | 128/8243 | 60/3250 | 0.0507148289988798 | 1 | F | F | F | F | regulation of exocytosis | ADORA2A | ANXA1 | AP1G1 | ARF1 | ATP13A2 | ATP2A2 | BACE1 | BRAF | CALM1 | CALM3 | CAMK2A | CDK5 | CFTR | CHMP2A | CHRM2 | CPLX1 | CPLX3 | DNM1L | DVL1 | FGB | GIT1 | GNAI2 | GSK3B | HAP1 | HGS | HMOX1 | HTR1B | IL13RA2 | LRRK2 | P2RX1 | PDCD6IP | PLA2G6 | PREPL | RAB10 | RAB21 | RAB3GAP1 | RAB5A | RAB7A | RAB9A | RAP1A | RAP1B | RAPGEF4 | REST | SDC1 | SDC4 | SDCBP | SEPT1 | SEPT4 | SEPT5 | SNCA | SNF8 | SNX6 | STXBP1 | STXBP2 | SYN1 | SYP | TRIM9 | TSG101 | VPS18 | VPS4B | GO:0019216 | 0.0296008734683974 | 244/8243 | 109/3250 | 0.0516629183925551 | 1 | F | F | F | F | regulation of lipid metabolic process | ABCG1 | ACACA | ACADVL | ACOX1 | ACSL1 | AGAP2 | AKT1 | AKT2 | ALAS1 | ANXA1 | APOA1 | APOD | APOE | ARF1 | ATP1A1 | BRCA1 | CAPN2 | CARM1 | CAV1 | CCL21 | CD36 | CD81 | CDK4 | CPT1A | CPT2 | CREB1 | CREBBP | CYP1A1 | DGKZ | EEF1A2 | EIF6 | ERLIN2 | FABP1 | FABP3 | FABP5 | FASN | FDPS | FGF2 | FGFR3 | FHL2 | GGPS1 | GPS2 | GSTZ1 | HDAC3 | HELZ2 | HNF4A | ID2 | IDH1 | IGFBP7 | INSIG1 | INSIG2 | KPNB1 | ME1 | MED1 | MVD | NCOA1 | NCOA2 | NCOR1 | NCOR2 | NOD2 | NR1H2 | NR1H3 | NRBF2 | PDK2 | PIK3CG | PIK3R1 | PIK3R3 | PLA2G6 | PPARD | PPARG | PPARGC1A | PRKAA1 | PRKAA2 | PRKAB2 | PRKCD | PRKD1 | PROX1 | PSAP | PTK2 | RAN | RB1 | REST | RGN | RXRA | SF1 | SIN3A | SIRT1 | SNAI2 | SNCA | SOCS1 | SOCS2 | SOCS6 | SOD1 | SP1 | SRC | STOML2 | STUB1 | TAZ | TBL1X | TGFB1 | TNF | TNFRSF1A | TREX1 | TRIB3 | TWIST1 | TXNRD1 | VDR | WDTC1 | ZMPSTE24 | GO:0010499 | 0.0021836709935703 | 18/8243 | 11/3250 | 0.0517947615869254 | 1 | F | F | F | F | proteasomal ubiquitin-independent protein catabolic process | KEAP1 | NFE2L2 | PSMA1 | PSMA2 | PSMA3 | PSMA5 | PSMA6 | PSMB5 | PSMB6 | PSMB7 | PSMB8 | GO:0044030 | 0.0021836709935703 | 18/8243 | 11/3250 | 0.0517947615869254 | 1 | F | F | F | F | regulation of DNA methylation | BRCA1 | FAM129B | KMT2A | MBD3 | MECP2 | MYC | PARP1 | PIK3CA | SPI1 | WT1 | ZMPSTE24 | GO:0032527 | 0.0021836709935703 | 18/8243 | 11/3250 | 0.0517947615869254 | 1 | F | F | F | F | protein exit from endoplasmic reticulum | AUP1 | DERL1 | FAF2 | HSP90B1 | NPLOC4 | SEC13 | SEC16A | SEL1L | SYVN1 | TMED9 | VCP | GO:0009067 | 0.0021836709935703 | 18/8243 | 11/3250 | 0.0517947615869254 | 1 | F | F | F | F | aspartate family amino acid biosynthetic process | AASDHPPT | AASS | ASNS | ENOPH1 | GOT1 | GOT1L1 | GOT2 | MTAP | MTHFD1 | MTRR | PLOD2 | GO:0006471 | 0.0021836709935703 | 18/8243 | 11/3250 | 0.0517947615869254 | 1 | F | F | F | F | protein ADP-ribosylation | ARF4 | PARP1 | PARP12 | PARP14 | PARP6 | SIRT1 | SIRT3 | SIRT5 | SIRT6 | TNKS | TNKS2 | GO:0070670 | 0.0021836709935703 | 18/8243 | 11/3250 | 0.0517947615869254 | 1 | F | F | F | F | response to interleukin-4 | CDK4 | FASN | HSP90AB1 | HSPA5 | IMPDH2 | JAK3 | KEAP1 | LEF1 | MCM2 | PML | XBP1 | GO:0002687 | 0.00691495814630596 | 57/8243 | 29/3250 | 0.0518143009535653 | 1 | F | F | F | F | positive regulation of leukocyte migration | AGER | APP | C1QBP | CALR | CAMK1D | CCL21 | CCR1 | CD74 | CREB3 | CXCL8 | FADD | HMGB1 | ICAM1 | ITGA4 | LGALS3 | LGMN | MAPK14 | MAPK3 | OXSR1 | P2RX4 | RAC1 | RHOA | S100A7 | SWAP70 | TGFB1 | THBS1 | TNF | TNFSF14 | WNK1 | GO:1901615 | 0.0249909013708601 | 206/8243 | 93/3250 | 0.0524210058004926 | 1 | F | F | F | F | organic hydroxy compound metabolic process | ABCG1 | ACAA1 | ACAA2 | ACAT2 | ACLY | ACSS2 | ADH1A | ADH1B | ADH5 | AKR1A1 | AKR1B1 | AKR1B10 | AKR1C2 | ALDH1B1 | ALOX12 | AOC2 | APOA1 | APOE | APOL2 | APP | BCL2 | BTBD9 | CALM1 | CAT | CBR4 | CEBPA | CES1 | CFTR | COMT | COQ2 | COQ3 | CYB5R1 | CYB5R3 | CYP1A1 | DBH | DHCR24 | DHRS4 | DPM1 | ERLIN2 | FDPS | FDXR | FGF2 | FGFR1 | G6PD | GGPS1 | GOT1 | GPD1 | HPRT1 | HSD17B4 | HSD3B7 | IDI2 | IMPA1 | IMPA2 | INSIG1 | INSIG2 | IP6K1 | ISYNA1 | LGMN | LIMA1 | MC1R | MECP2 | MED1 | MTPN | MVD | MYO5A | NPR1 | NR0B2 | NR5A2 | NUDT3 | OSBPL1A | PAH | PCBD1 | PCSK9 | PGP | PLCG1 | PPARD | PRKAA1 | PRKAA2 | PSAT1 | PTEN | QDPR | RDH11 | RDH12 | RDH13 | RDH14 | RDH5 | RXRA | SNCA | SOAT1 | SORD | SPR | SPTLC2 | VDR | GO:0000096 | 0.0014557806623802 | 12/8243 | 8/3250 | 0.0525152525347518 | 1 | F | F | F | F | sulfur amino acid metabolic process | AHCY | CBS | CTH | DPEP1 | MPST | MTHFD1 | MTRR | NFS1 | GO:0007622 | 0.0014557806623802 | 12/8243 | 8/3250 | 0.0525152525347518 | 1 | F | F | F | F | rhythmic behavior | AHCY | BTBD9 | ID2 | MTA1 | NCOA2 | PTEN | TP53 | USP2 | GO:0003015 | 0.0014557806623802 | 12/8243 | 8/3250 | 0.0525152525347518 | 1 | F | F | F | F | heart process | ATG5 | DNM1L | FYN | GPX1 | MYL2 | SOD1 | TREX1 | YAP1 | GO:0030970 | 0.0014557806623802 | 12/8243 | 8/3250 | 0.0525152525347518 | 1 | F | F | F | F | retrograde protein transport, ER to cytosol | AUP1 | DERL1 | FAF2 | HSP90B1 | NPLOC4 | SEL1L | SYVN1 | VCP | GO:0060149 | 0.0014557806623802 | 12/8243 | 8/3250 | 0.0525152525347518 | 1 | F | F | F | F | negative regulation of posttranscriptional gene silencing | ELAVL1 | ESR1 | NCOR1 | NCOR2 | POU5F1 | PPARG | TGFB1 | ZMPSTE24 | GO:0071731 | 0.0014557806623802 | 12/8243 | 8/3250 | 0.0525152525347518 | 1 | F | F | F | F | response to nitric oxide | AIFM1 | CDK2 | CRK | DPEP1 | EGLN1 | FOXO1 | HNRNPD | TRAF2 | GO:0033750 | 0.0014557806623802 | 12/8243 | 8/3250 | 0.0525152525347518 | 1 | F | F | F | F | ribosome localization | ABCE1 | EIF6 | NMD3 | NPM1 | NUP88 | RAN | RRS1 | XPO1 | GO:0035970 | 0.0014557806623802 | 12/8243 | 8/3250 | 0.0525152525347518 | 1 | F | F | F | F | peptidyl-threonine dephosphorylation | DUSP1 | PPM1A | PPM1B | PPM1D | PPP1CA | PPP2CA | PPP2CB | PPP5C | GO:0045821 | 0.0014557806623802 | 12/8243 | 8/3250 | 0.0525152525347518 | 1 | F | F | F | F | positive regulation of glycolytic process | ARNT | ESRRB | GAPDHS | GPD1 | HIF1A | INSR | PRKAA1 | PRKAA2 | GO:0030225 | 0.0014557806623802 | 12/8243 | 8/3250 | 0.0525152525347518 | 1 | F | F | F | F | macrophage differentiation | CASP8 | CDC42 | CEBPA | CEBPE | L3MBTL3 | PARP1 | SIRT1 | SPI1 | GO:1903513 | 0.0014557806623802 | 12/8243 | 8/3250 | 0.0525152525347518 | 1 | F | F | F | F | endoplasmic reticulum to cytosol transport | AUP1 | DERL1 | FAF2 | HSP90B1 | NPLOC4 | SEL1L | SYVN1 | VCP | GO:0071428 | 0.0014557806623802 | 12/8243 | 8/3250 | 0.0525152525347518 | 1 | F | F | F | F | rRNA-containing ribonucleoprotein complex export from nucleus | ABCE1 | EIF6 | NMD3 | NPM1 | NUP88 | RAN | RRS1 | XPO1 | GO:1905048 | 0.0014557806623802 | 12/8243 | 8/3250 | 0.0525152525347518 | 1 | F | F | F | F | regulation of metallopeptidase activity | DDRGK1 | MAPK14 | MAPK3 | MBP | RECK | STAT3 | TIMP1 | TIMP2 | GO:0006536 | 0.0014557806623802 | 12/8243 | 8/3250 | 0.0525152525347518 | 1 | F | F | F | F | glutamate metabolic process | ALDH5A1 | ASL | FPGS | GGT1 | GLUD2 | GLUL | GOT1 | GOT2 | GO:0071426 | 0.0014557806623802 | 12/8243 | 8/3250 | 0.0525152525347518 | 1 | F | F | F | F | ribonucleoprotein complex export from nucleus | ABCE1 | EIF6 | NMD3 | NPM1 | NUP88 | RAN | RRS1 | XPO1 | GO:0060967 | 0.0014557806623802 | 12/8243 | 8/3250 | 0.0525152525347518 | 1 | F | F | F | F | negative regulation of gene silencing by RNA | ELAVL1 | ESR1 | NCOR1 | NCOR2 | POU5F1 | PPARG | TGFB1 | ZMPSTE24 | GO:0090051 | 0.0014557806623802 | 12/8243 | 8/3250 | 0.0525152525347518 | 1 | F | F | F | F | negative regulation of cell migration involved in sprouting angiogenesis | HDAC5 | ITGB1BP1 | MEOX2 | PDCD10 | RHOA | STARD13 | TBXA2R | THBS1 | GO:0032460 | 0.0014557806623802 | 12/8243 | 8/3250 | 0.0525152525347518 | 1 | F | F | F | F | negative regulation of protein oligomerization | ALB | CLU | CRBN | CRYAB | EP300 | HIST3H3 | PEX5 | SRC | GO:0000054 | 0.0014557806623802 | 12/8243 | 8/3250 | 0.0525152525347518 | 1 | F | F | F | F | ribosomal subunit export from nucleus | ABCE1 | EIF6 | NMD3 | NPM1 | NUP88 | RAN | RRS1 | XPO1 | GO:0072521 | 0.0127380807958268 | 105/8243 | 50/3250 | 0.0527078903034518 | 1 | F | F | F | F | purine-containing compound metabolic process | ABHD14B | ACACA | ACAT1 | ACLY | ACOT13 | ACOT7 | ACOT9 | ADCYAP1 | ADSS | AK1 | AK2 | AK4 | ATP6V1A | ATP6V1B1 | CLPX | CROT | DBI | DGUOK | DLG2 | DLST | FAR2 | FASN | FIGNL1 | GDA | GMPR | GNAI3 | HMGCL | HPRT1 | HSD17B4 | HSPA1B | HSPA8 | KDM1A | LRRK2 | MVD | MYH6 | NDUFS1 | NMNAT1 | NT5C2 | OGDH | OLA1 | OPA1 | PARP1 | RAN | RHOA | SUCLA2 | SUCLG2 | SULT1A3 | THEM4 | TPST2 | VCP | GO:0002208 | 0.0019410408831736 | 16/8243 | 10/3250 | 0.0527383067239004 | 1 | F | F | F | F | somatic diversification of immunoglobulins involved in immune response | AICDA | ATAD5 | CD40LG | EXOSC6 | HSPD1 | LIG4 | MLH1 | MSH6 | RNF8 | SWAP70 | GO:0005980 | 0.0019410408831736 | 16/8243 | 10/3250 | 0.0527383067239004 | 1 | F | F | F | F | glycogen catabolic process | AGL | CALM1 | GAA | PFKM | PGM1 | PGM2 | PHKA1 | PYGB | PYGL | STBD1 | GO:0090659 | 0.0019410408831736 | 16/8243 | 10/3250 | 0.0527383067239004 | 1 | F | F | F | F | walking behavior | ARRB2 | DAB1 | DMRT3 | EPHA4 | GLRB | HTRA2 | PSAP | UCHL1 | ZIC1 | ZMPSTE24 | GO:0009261 | 0.0019410408831736 | 16/8243 | 10/3250 | 0.0527383067239004 | 1 | F | F | F | F | ribonucleotide catabolic process | ACAT1 | ACOT7 | HINT1 | HPRT1 | ITPA | NUDT18 | NUDT3 | NUDT9 | PDE4D | PRTFDC1 | GO:0051000 | 0.0019410408831736 | 16/8243 | 10/3250 | 0.0527383067239004 | 1 | F | F | F | F | positive regulation of nitric-oxide synthase activity | AKT1 | APOE | CALM1 | CALM3 | HIF1A | KRAS | NOS1AP | S100A1 | TERF2 | TNF | GO:0051900 | 0.0019410408831736 | 16/8243 | 10/3250 | 0.0527383067239004 | 1 | F | F | F | F | regulation of mitochondrial depolarization | ALOX12 | BCL2 | DCN | GOT1 | KDR | LRRK2 | MLLT11 | MYOC | PARP1 | SRC | GO:0009154 | 0.0019410408831736 | 16/8243 | 10/3250 | 0.0527383067239004 | 1 | F | F | F | F | purine ribonucleotide catabolic process | ACAT1 | ACOT7 | HINT1 | HPRT1 | ITPA | NUDT18 | NUDT3 | NUDT9 | PDE4D | PRTFDC1 | GO:0009251 | 0.0019410408831736 | 16/8243 | 10/3250 | 0.0527383067239004 | 1 | F | F | F | F | glucan catabolic process | AGL | CALM1 | GAA | PFKM | PGM1 | PGM2 | PHKA1 | PYGB | PYGL | STBD1 | GO:0030878 | 0.0019410408831736 | 16/8243 | 10/3250 | 0.0527383067239004 | 1 | F | F | F | F | thyroid gland development | BRAF | HOXA5 | MAP2K1 | MAPK1 | MAPK3 | NKX2-1 | PAX8 | RAF1 | SMAD3 | THRA | GO:0045190 | 0.0019410408831736 | 16/8243 | 10/3250 | 0.0527383067239004 | 1 | F | F | F | F | isotype switching | AICDA | ATAD5 | CD40LG | EXOSC6 | HSPD1 | LIG4 | MLH1 | MSH6 | RNF8 | SWAP70 | GO:0030201 | 0.0019410408831736 | 16/8243 | 10/3250 | 0.0527383067239004 | 1 | F | F | F | F | heparan sulfate proteoglycan metabolic process | B3GAT3 | DSE | EXTL2 | GLCE | HS2ST1 | HS6ST1 | SGSH | SULF2 | UGDH | XYLT1 | GO:0060487 | 0.0019410408831736 | 16/8243 | 10/3250 | 0.0527383067239004 | 1 | F | F | F | F | lung epithelial cell differentiation | AGR2 | AIMP2 | ASCL1 | CREB1 | FOXA1 | HOXA5 | NKX2-1 | RBPJ | THRA | YAP1 | GO:0047497 | 0.0019410408831736 | 16/8243 | 10/3250 | 0.0527383067239004 | 1 | F | F | F | F | mitochondrion transport along microtubule | HAP1 | HIF1A | LRPPRC | MAP1B | MAPT | OPA1 | RHOT1 | RHOT2 | UBB | UCHL1 | GO:0032495 | 0.0019410408831736 | 16/8243 | 10/3250 | 0.0527383067239004 | 1 | F | F | F | F | response to muramyl dipeptide | CARD9 | MAPK14 | NFKBIA | NOD2 | PTPN22 | RELA | RIPK2 | TNFAIP3 | TRIM41 | VIM | GO:0034643 | 0.0019410408831736 | 16/8243 | 10/3250 | 0.0527383067239004 | 1 | F | F | F | F | establishment of mitochondrion localization, microtubule-mediated | HAP1 | HIF1A | LRPPRC | MAP1B | MAPT | OPA1 | RHOT1 | RHOT2 | UBB | UCHL1 | GO:0002204 | 0.0019410408831736 | 16/8243 | 10/3250 | 0.0527383067239004 | 1 | F | F | F | F | somatic recombination of immunoglobulin genes involved in immune response | AICDA | ATAD5 | CD40LG | EXOSC6 | HSPD1 | LIG4 | MLH1 | MSH6 | RNF8 | SWAP70 | GO:0048026 | 0.0019410408831736 | 16/8243 | 10/3250 | 0.0527383067239004 | 1 | F | F | F | F | positive regulation of mRNA splicing, via spliceosome | CIRBP | DAZAP1 | NCBP1 | NUP98 | PRDX6 | PRPF19 | RBM3 | RBMX | SNW1 | TRA2A | GO:0010882 | 0.0019410408831736 | 16/8243 | 10/3250 | 0.0527383067239004 | 1 | F | F | F | F | regulation of cardiac muscle contraction by calcium ion signaling | ATP2A2 | CALM1 | CALM2 | CALM3 | DMD | FKBP1B | GSTO1 | HDAC4 | PRKACA | SLC9A1 | GO:0042744 | 0.0019410408831736 | 16/8243 | 10/3250 | 0.0527383067239004 | 1 | F | F | F | F | hydrogen peroxide catabolic process | CAT | GPX1 | HBZ | PRDX1 | PRDX2 | PRDX3 | PRDX4 | PRDX5 | PRDX6 | PXDN | GO:0045580 | 0.00994783452626471 | 82/8243 | 40/3250 | 0.0527991474416028 | 1 | F | F | F | F | regulation of T cell differentiation | ABL1 | ADA | ANXA1 | AP3D1 | BCL6 | BRAF | CBFB | CD2 | CD27 | CD74 | CYLD | DROSHA | DTX1 | ERBB2 | FANCA | FOXJ1 | HLX | HMGB1 | IFNA2 | IRF1 | IRF4 | JAK3 | NFATC2 | PNP | PRDX2 | PRELID1 | PRKCZ | RARA | RC3H1 | RHOA | RIPK2 | RUNX1 | SART1 | SOCS1 | SOD1 | TGFB1 | XBP1 | ZAP70 | ZBTB16 | ZBTB7B | GO:0070588 | 0.00994783452626471 | 82/8243 | 40/3250 | 0.0527991474416028 | 1 | F | F | F | F | calcium ion transmembrane transport | AFG3L2 | ANXA2 | ANXA5 | ANXA6 | ATP2A2 | ATP2B2 | CACNA1A | CACNG2 | CACNG4 | CACNG5 | CCL21 | CCR5 | CHRNA9 | CUL5 | FASLG | FGF2 | FKBP1A | FKBP1B | IBTK | ITGAV | ITPR1 | ITPR2 | ITPR3 | LETM1 | MCOLN3 | MICU1 | MICU2 | OPA1 | P2RX4 | PHB | PHB2 | PLCG1 | PMPCA | PMPCB | PRKCE | PSEN1 | STOML2 | TPCN2 | VDAC1 | YME1L1 | GO:1990089 | 0.00424602693194225 | 35/8243 | 19/3250 | 0.0530354443812626 | 1 | F | F | F | F | response to nerve growth factor | AKT1 | APP | BRAF | CBL | CDC5L | CREB1 | CRK | E2F1 | EEF2 | EIF4A3 | HSPA5 | MAPT | NTRK1 | PTEN | PTN | RAB35 | RAP1A | SH3GL2 | USP8 | GO:0016126 | 0.00424602693194225 | 35/8243 | 19/3250 | 0.0530354443812626 | 1 | F | F | F | F | sterol biosynthetic process | ACAA2 | ACAT2 | ACLY | APOA1 | APOE | CES1 | CFTR | CYB5R1 | CYB5R3 | DHCR24 | FDPS | G6PD | GGPS1 | IDI2 | INSIG1 | INSIG2 | MVD | PRKAA1 | PRKAA2 | GO:0030509 | 0.00424602693194225 | 35/8243 | 19/3250 | 0.0530354443812626 | 1 | F | F | F | F | BMP signaling pathway | BMPR1A | CHRD | GDF9 | LEF1 | MAPK3 | MYH6 | RUNX2 | SKI | SMAD4 | SMAD5 | SMAD9 | SMURF1 | SMURF2 | TGFBR3 | UBE2D1 | UBE2D3 | USP15 | USP9X | USP9Y | GO:0033262 | 0.0016984107727769 | 14/8243 | 9/3250 | 0.0530850986298288 | 1 | F | F | F | F | regulation of nuclear cell cycle DNA replication | AICDA | ATRX | BCL6 | CHEK2 | DBF4B | FGFR1 | TERF1 | TERF2 | ZMPSTE24 | GO:1902884 | 0.0016984107727769 | 14/8243 | 9/3250 | 0.0530850986298288 | 1 | F | F | F | F | positive regulation of response to oxidative stress | ENDOG | HDAC6 | PARK7 | PAWR | RGN | RIPK1 | SFPQ | SOD1 | TNF | GO:0032373 | 0.0016984107727769 | 14/8243 | 9/3250 | 0.0530850986298288 | 1 | F | F | F | F | positive regulation of sterol transport | ABCG1 | ANXA2 | APOA1 | APOE | GPS2 | NFKBIA | NR1H2 | NR1H3 | SIRT1 | GO:0002437 | 0.0016984107727769 | 14/8243 | 9/3250 | 0.0530850986298288 | 1 | F | F | F | F | inflammatory response to antigenic stimulus | AHCY | CD6 | HMGB1 | HMGB2 | ICAM1 | PNMA1 | RBPJ | TNF | TREX1 | GO:0051131 | 0.0016984107727769 | 14/8243 | 9/3250 | 0.0530850986298288 | 1 | F | F | F | F | chaperone-mediated protein complex assembly | APCS | CCT2 | CCT6B | CLU | HSP90AA1 | HSP90AB1 | HSPA4 | HSPD1 | PTGES3 | GO:1902236 | 0.0016984107727769 | 14/8243 | 9/3250 | 0.0530850986298288 | 1 | F | F | F | F | negative regulation of endoplasmic reticulum stress-induced intrinsic apoptotic signaling pathway | BCL2L1 | CREB3 | LRRK2 | OPA1 | PARK7 | PTPN1 | SYVN1 | TMBIM6 | XBP1 | GO:0009583 | 0.0016984107727769 | 14/8243 | 9/3250 | 0.0530850986298288 | 1 | F | F | F | F | detection of light stimulus | ARRB1 | CABP4 | GNAQ | GNB1 | GRK1 | PDE6G | RHO | SAG | UNC119 | GO:0006309 | 0.0016984107727769 | 14/8243 | 9/3250 | 0.0530850986298288 | 1 | F | F | F | F | apoptotic DNA fragmentation | CASP3 | DNASE2 | DNASE2B | ENDOG | H1F0 | HMGB1 | HMGB2 | KPNA1 | KPNB1 | GO:0055070 | 0.0016984107727769 | 14/8243 | 9/3250 | 0.0530850986298288 | 1 | F | F | F | F | copper ion homeostasis | APP | ARF1 | ATOX1 | CCDC22 | COMMD1 | COX19 | CUTC | SCO2 | XIAP | GO:1900409 | 0.0016984107727769 | 14/8243 | 9/3250 | 0.0530850986298288 | 1 | F | F | F | F | positive regulation of cellular response to oxidative stress | ENDOG | HDAC6 | PARK7 | PAWR | RGN | RIPK1 | SFPQ | SOD1 | TNF | GO:0002902 | 0.0016984107727769 | 14/8243 | 9/3250 | 0.0530850986298288 | 1 | F | F | F | F | regulation of B cell apoptotic process | ADA | AURKB | BAX | BCL10 | BCL6 | BTK | CD74 | MIF | PTEN | GO:0032376 | 0.0016984107727769 | 14/8243 | 9/3250 | 0.0530850986298288 | 1 | F | F | F | F | positive regulation of cholesterol transport | ABCG1 | ANXA2 | APOA1 | APOE | GPS2 | NFKBIA | NR1H2 | NR1H3 | SIRT1 | GO:1902170 | 0.0016984107727769 | 14/8243 | 9/3250 | 0.0530850986298288 | 1 | F | F | F | F | cellular response to reactive nitrogen species | AIFM1 | CDK2 | CRK | DPEP1 | FOXO1 | HNRNPD | PPARGC1A | STAT6 | TRAF2 | GO:0032930 | 0.0016984107727769 | 14/8243 | 9/3250 | 0.0530850986298288 | 1 | F | F | F | F | positive regulation of superoxide anion generation | CRP | EGFR | GNAI2 | GNAI3 | GSTP1 | MAPT | PRKCD | SOD1 | TGFB1 | GO:0071677 | 0.0016984107727769 | 14/8243 | 9/3250 | 0.0530850986298288 | 1 | F | F | F | F | positive regulation of mononuclear cell migration | AGER | CCR1 | CREB3 | HMGB1 | LGALS3 | LGMN | S100A7 | TGFB1 | TNF | GO:0051249 | 0.0316632294067694 | 261/8243 | 116/3250 | 0.0531440962950124 | 1 | F | F | F | F | regulation of lymphocyte activation | ABL1 | ADA | ADORA2A | AGER | AKT1 | ANXA1 | AP1G1 | AP3D1 | ATAD5 | ATM | BCL10 | BCL2 | BCL6 | BRAF | BTK | CASP3 | CAV1 | CBFB | CCL21 | CD2 | CD209 | CD22 | CD27 | CD40 | CD40LG | CD6 | CD74 | CD81 | CDC42 | CTNNB1 | CYLD | DNAJA3 | DROSHA | DTX1 | DUSP22 | ERBB2 | EXOSC6 | FADD | FAM49B | FANCA | FBXO7 | FOXJ1 | FYN | GLMN | GRB2 | HAVCR2 | HLX | HMGB1 | HSPD1 | HSPH1 | ID2 | IFNA2 | IGHV3-13 | IGHV3-23 | IGHV3-30 | IGLC7 | IRF1 | IRF4 | ITCH | JAK3 | LAT | LGALS1 | LGALS3 | MAD1L1 | MICA | MIF | MLH1 | MSH6 | NCK1 | NFATC2 | NOD2 | PAG1 | PAK2 | PAWR | PAXIP1 | PELI1 | PIK3CA | PIK3R1 | PNP | PRDX2 | PRELID1 | PRKAR1A | PRKCZ | PTPN22 | PTPN6 | RAC1 | RARA | RC3H1 | RHOA | RIF1 | RIPK2 | RIPK3 | RPS3 | RUNX1 | SART1 | SDC4 | SLAMF8 | SOCS1 | SOCS6 | SOD1 | SRC | STAT6 | TGFB1 | TNFAIP3 | TNFSF14 | TNIP2 | TRAF6 | TREX1 | VAV1 | VCAM1 | VSIG4 | XBP1 | YES1 | ZAP70 | ZBTB16 | ZBTB7B | GO:0051168 | 0.0161349023413806 | 133/8243 | 62/3250 | 0.0533872934335141 | 1 | F | F | F | F | nuclear export | ABCE1 | AHCYL1 | ATXN1 | CALR | CCHCR1 | CHP1 | CSE1L | DDX19B | DDX39A | DDX39B | DUSP16 | EIF4A3 | EIF4E | EIF5A | EIF6 | FYTTD1 | HNRNPA1 | HNRNPA2B1 | MCM3AP | NCBP1 | NMD3 | NPM1 | NUP107 | NUP153 | NUP160 | NUP205 | NUP214 | NUP85 | NUP88 | NUP93 | NUP98 | NUTF2 | NXF1 | NXF2 | POLDIP3 | POM121 | RAE1 | RAN | RANBP3 | RBM8A | RRS1 | SEC13 | SMG5 | SMG6 | SMURF1 | SRSF11 | SRSF5 | SSB | STYX | TGFB1 | THOC2 | THOC6 | THOC7 | TPR | U2AF2 | UPF2 | UPF3B | XPO1 | XPO4 | XPO5 | XPO7 | XPOT | GO:0046794 | 0.0055804925391241 | 46/8243 | 24/3250 | 0.053622370551973 | 1 | F | F | F | F | transport of virus | CAV1 | CD209 | DYNLT1 | KPNA1 | KPNB1 | NUP107 | NUP153 | NUP160 | NUP205 | NUP214 | NUP85 | NUP88 | NUP93 | NUP98 | POM121 | RAE1 | RAN | SEC13 | TPR | TSG101 | UBB | UBC | VPS37D | XPO1 | GO:0043542 | 0.0055804925391241 | 46/8243 | 24/3250 | 0.053622370551973 | 1 | F | F | F | F | endothelial cell migration | ABL1 | ADTRP | AKT1 | APOA1 | FGF2 | GPX1 | KDR | LGALS8 | LOXL2 | NOS3 | NR4A1 | PAXIP1 | PIK3CA | PIK3R3 | PTEN | PTK2 | PXN | RHOA | S100A2 | S100P | SCG2 | STARD13 | STAT1 | TNFSF12 | GO:1902850 | 0.00885599902947956 | 73/8243 | 36/3250 | 0.0541400457135569 | 1 | F | F | F | F | microtubule cytoskeleton organization involved in mitosis | AURKB | BIRC5 | CCNB1 | CDC20 | CHEK2 | DCTN1 | DYNC1H1 | DYNLT1 | GOLGA2 | HTT | KIF11 | KPNB1 | MECP2 | NSFL1C | NUMA1 | NUSAP1 | OFD1 | PAFAH1B1 | RAB11A | RAN | RCC1 | RHOA | SBDS | SPC25 | SPDL1 | SPICE1 | SPRY2 | STIL | STMN1 | SUN2 | TBCE | TNKS | TUBG1 | VCP | ZNF207 | ZW10 | GO:0051493 | 0.0416110639330341 | 343/8243 | 150/3250 | 0.0542773769096128 | 1 | F | F | F | F | regulation of cytoskeleton organization | ABL1 | ABL2 | ACTN2 | ACTR2 | ACTR3 | ADD3 | AKAP9 | APC | APOA1 | ARF1 | ARHGAP35 | ARHGDIA | ARPC1A | ARPC1B | ARPC2 | ARPC3 | ARPC4 | ARPC5 | AVIL | BAG4 | BAIAP2 | BAIAP2L1 | BIN1 | BRAF | BRCA1 | CAPG | CAPN2 | CAPZA1 | CAPZA2 | CAPZB | CCDC88A | CCL21 | CDC42 | CDC42EP3 | CDC42EP4 | CDK5 | CDK5R1 | CENPJ | CEP70 | CFL1 | CFL2 | CHMP2A | CHORDC1 | CLIC4 | CLTC | CNTRL | CRK | CTNNB1 | CTTN | CYLD | DCTN1 | DSTN | DYNC1H1 | EZR | FBXW5 | FCHSD1 | FSCN1 | FSD1 | GNAI1 | GRB2 | GSK3B | HAX1 | HDAC6 | HNRNPU | HRAS | HSPA1B | ICAM1 | ILK | ITGB1BP1 | KIF11 | LATS1 | LIMA1 | LPAR1 | MAP1B | MAPK1 | MAPK15 | MAPK3 | MAPT | MDM1 | MECP2 | MLST8 | MTPN | MYO1C | MYOC | NCK1 | NF2 | NIN | NPM1 | NSFL1C | NTF3 | NUBP1 | NUMA1 | PAFAH1B1 | PDCD6IP | PFDN2 | PFN1 | PICK1 | PIK3R1 | PLK1 | PRKAA1 | PRKAA2 | PRKCD | PRKCE | PROX1 | PTK2 | PXN | RAC1 | RAE1 | RASSF7 | RDX | RHOA | RHOJ | RHOU | RNF4 | RPS3 | S100A9 | SDC4 | SENP6 | SKA1 | SKA3 | SLC9A1 | SMAD3 | SMAD4 | SMC1A | SMC3 | SNCA | SPICE1 | SPTAN1 | SPTBN1 | SPTBN2 | STAG2 | STIL | STMN1 | SWAP70 | SYDE1 | TGFB1 | TMOD3 | TMOD4 | TPM1 | TPR | TRAF3IP1 | TUBB4A | TWF1 | TWF2 | VASP | VPS4B | WAS | WDR1 | XPO1 | XRCC3 | GO:0050878 | 0.0270532573092321 | 223/8243 | 100/3250 | 0.0545812072265508 | 1 | F | F | F | F | regulation of body fluid levels | ABAT | ADA | ADCY6 | ADORA2A | ADTRP | AGR2 | AKR1B1 | ALOX12 | ANXA2 | ANXA5 | ANXA7 | APOE | APRT | ATG5 | ATP6V1B1 | C1QTNF1 | CAPZA1 | CAPZA2 | CAPZB | CAV1 | CD36 | CDC42 | CEL | CFTR | CHRM3 | COPA | CREB1 | F5 | F9 | FGB | FYN | GATA1 | GNA12 | GNAI2 | GNAQ | GOT2 | GPI | H3F3A | HDAC1 | HDAC2 | HIF1A | HIST1H3A | HIST2H3A | HK2 | HNF4A | HRNR | IFNA2 | IRF1 | ITGB3 | KDM1A | MED1 | MMRN1 | MTX1 | NCOA1 | NFE2L2 | NME1 | NOS3 | NPR1 | NR1H2 | NR1H3 | OAS2 | P2RX1 | P2RX2 | P2RX4 | PABPC4 | PAPSS2 | PDSS2 | PHF21A | PLAUR | PPAT | PRDX2 | PRKACA | PRKAR1A | PRKAR2B | PRKCD | PRKCE | PROZ | RAB11A | RAB11FIP2 | RAB5A | RAC1 | SERPINB2 | SERPINE2 | SFN | SH2B1 | SOCS2 | STAT5A | STMN1 | TBXA2R | THBS1 | TP63 | UMPS | VDR | VKORC1 | VTN | WAS | WNK1 | WNK4 | XBP1 | ZBTB7B | GO:0007569 | 0.00667232803590926 | 55/8243 | 28/3250 | 0.0549370467720581 | 1 | F | F | F | F | cell aging | ATM | BCL2 | CALR | CDKN1A | CHEK2 | DNAJA3 | H2AFX | HRAS | ICAM1 | ID2 | ILK | LIMS1 | MAP2K1 | MAPK14 | MIF | MME | NPM1 | OPA1 | PML | PRKCD | PRMT6 | ROMO1 | SIRT1 | SOD1 | TERF2 | TP53 | TP63 | WNT16 | GO:1903530 | 0.0477981317481499 | 394/8243 | 171/3250 | 0.0551923624533125 | 1 | F | F | F | F | regulation of secretion by cell | ABAT | ABL1 | ADCYAP1 | ADORA2A | ADTRP | AGER | ANXA1 | ANXA5 | AP1G1 | APBB1 | APOA1 | ARF1 | ARFIP1 | ARHGEF7 | ARRB1 | ATG5 | ATP13A2 | ATP2A2 | BACE1 | BRAF | C1QTNF1 | CACNA1A | CALM1 | CALM3 | CAMK2A | CD2 | CD22 | CD40 | CD40LG | CDK16 | CDK5 | CFTR | CHMP2A | CHRM2 | CPLX1 | CPLX3 | CPT1A | CREB1 | CRP | CRY1 | DHX36 | DHX9 | DNM1L | DVL1 | DYNLL1 | EGFR | EZR | FCN1 | FGB | FGFR1 | FKBP1B | FN1 | GAPDH | GDF9 | GIT1 | GLMN | GLP1R | GLUL | GNAI2 | GNAS | GOLPH3 | GPI | GSK3B | HADH | HAP1 | HAVCR2 | HDAC9 | HGS | HIF1A | HK1 | HMGB1 | HMOX1 | HNF4A | HTR1B | IDH2 | IFNA2 | IL13RA2 | ITPR1 | ITPR2 | ITPR3 | KCNS3 | KDM5B | KIF5B | LGR4 | LRRK2 | MAPK14 | MAPK3 | MAVS | MBP | MIF | MPC2 | NDUFAF2 | NEUROD1 | NMB | NOD2 | NOS2 | NR0B2 | NR1H2 | NR1H3 | ORM1 | OXCT1 | P2RX1 | PANX3 | PAX8 | PDCD6IP | PER2 | PFKL | PFKM | PLA2G1B | PLA2G6 | PML | PPARD | PPIA | PPID | PPP1R11 | PREPL | PRKCE | PRKCZ | PSMD9 | PTPN22 | PTPN23 | RAB10 | RAB21 | RAB3GAP1 | RAB5A | RAB7A | RAB9A | RAP1A | RAP1B | RAPGEF4 | REST | RHOT1 | RIPK2 | SDC1 | SDC4 | SDCBP | SEPT1 | SEPT4 | SEPT5 | SIRT3 | SLC16A1 | SLC25A4 | SLC25A5 | SLC25A6 | SMAD4 | SNCA | SNF8 | SNX6 | SOCS1 | SPTBN1 | SRC | SRI | STXBP1 | STXBP2 | SYN1 | SYP | TARDBP | TGFB1 | TMBIM6 | TNF | TNFAIP3 | TNFRSF1A | TRAF2 | TRAF6 | TRIM9 | TSG101 | TWIST1 | USP50 | VPS18 | VPS4B | XBP1 | GO:0002697 | 0.0299648186339925 | 247/8243 | 110/3250 | 0.0552947922894686 | 1 | F | F | F | F | regulation of immune effector process | A2M | AGER | ANXA1 | AP1G1 | APOA1 | ARRB2 | ATAD5 | ATG5 | BCL10 | BCL6 | BIRC2 | BIRC3 | BTK | C1QA | C1QBP | C1R | C4A | CD22 | CD36 | CD40 | CD40LG | CD74 | CD81 | CLU | CRK | CXCL6 | DDX1 | DHX36 | DHX9 | DUSP22 | EIF2AK4 | EXOSC6 | FADD | FAM49B | FOXJ1 | GPI | HAVCR2 | HK1 | HLA-A | HLA-B | HLX | HMGB1 | HMOX1 | HPX | HSPD1 | HTRA1 | IFIT1 | IFNA2 | IGHV3-13 | IGHV3-23 | IGHV3-30 | IGKV3-20 | IGLC7 | IL13RA2 | IRF4 | ITCH | JAK3 | LGALS3 | MAPK14 | MAPK3 | MAVS | MICA | MIF | MLH1 | MSH6 | NOD2 | NPLOC4 | PAXIP1 | PCBP2 | PHB | PHB2 | PPM1B | PRKCZ | PRKDC | PTPN6 | PUM1 | RAP1A | RARA | RC3H1 | RIF1 | RIPK2 | RIPK3 | RPS19 | SEMA7A | SIN3A | SLAMF8 | SNX6 | STAT1 | STAT6 | STXBP1 | STXBP2 | TGFB1 | TLR3 | TMBIM6 | TMEM173 | TNF | TNFAIP3 | TRAF2 | TRAF3 | TRAF3IP1 | TRAF6 | TREX1 | USP17L2 | VAV1 | VSIG4 | VTN | WAS | XBP1 | ZBTB7B | ZMPSTE24 | GO:0090288 | 0.00970520441586801 | 80/8243 | 39/3250 | 0.0559017099716317 | 1 | F | F | F | F | negative regulation of cellular response to growth factor stimulus | ABL1 | CAV1 | CD109 | CHRD | DCN | FAM129B | HGS | HSPA5 | HTRA1 | LDLRAD4 | LEMD2 | LEMD3 | LTBP1 | NEDD4 | NKX2-1 | ONECUT1 | PDCD6 | PIN1 | PPM1A | PTPN1 | RASL11B | SIRT1 | SKI | SMAD2 | SMAD3 | SMURF1 | SMURF2 | SNX6 | SPRY2 | STRAP | STUB1 | SULF2 | TGFB1 | TGFBR3 | THBS1 | TRIM33 | UBB | UBC | ZBTB7A | GO:1901985 | 0.00400339682154555 | 33/8243 | 18/3250 | 0.0559760421396563 | 1 | F | F | F | F | positive regulation of protein acetylation | ARRB1 | BRCA1 | KAT5 | KAT7 | MAPK3 | PAXIP1 | PIH1D1 | PPARGC1A | PRKAA1 | PRKAA2 | RUVBL2 | SMAD4 | SMARCB1 | SNAI2 | TADA2B | TERF2IP | TGFB1 | WBP2 | GO:0030705 | 0.0133446560718185 | 110/8243 | 52/3250 | 0.0560866304124817 | 1 | F | F | F | F | cytoskeleton-dependent intracellular transport | ACTN4 | AP3D1 | AP3M1 | APP | BAG3 | CCDC88A | CDC42 | COPG1 | DCTN1 | DLG2 | DYNC1H1 | DYNC1I1 | DYNLL1 | HAP1 | HIF1A | HNRNPU | HSPA8 | HSPB1 | HTT | ICK | IFT122 | KIF5B | KIF5C | LRPPRC | MAP1B | MAPK8IP3 | MAPT | MYO1C | MYO5A | OPA1 | PAFAH1B1 | PCM1 | PRKCZ | RAB1A | RAB21 | RHOT1 | RHOT2 | SFPQ | SOD1 | SSX2IP | SUN2 | SYNE2 | TERF2 | TNPO1 | TRAF3IP1 | TUBA1A | TUBA1C | TUBB | UBB | UCHL1 | WDR19 | WDR35 | GO:0003013 | 0.0133446560718185 | 110/8243 | 52/3250 | 0.0560866304124817 | 1 | F | F | F | F | circulatory system process | ACE2 | ACTA2 | ADCY6 | ADCYAP1 | ADORA2A | ADRB2 | AKT1 | ALOX12 | APOE | ARHGAP35 | ATG5 | AZU1 | BDKRB2 | CAV1 | CHRM3 | CRP | CTNNBIP1 | CYB5R3 | DBH | DNM1L | DOCK5 | E2F4 | EGFR | F5 | FGB | FYN | GPX1 | HMOX1 | HTR1B | ICAM1 | ITGB1BP1 | MEOX2 | MYL2 | NOS3 | NPR1 | P2RX1 | PER2 | PIK3C2A | PLA2G6 | PPARD | RAP1GDS1 | RHOA | SOD1 | SOD2 | SRC | STAT1 | TBXA2R | TGFB1 | TREX1 | WDR35 | YAP1 | YES1 | GO:0045893 | 0.115977192769623 | 956/8243 | 400/3250 | 0.0563410926717528 | 1 | F | F | F | F | positive regulation of transcription, DNA-templated | ABHD14B | ABL1 | ACTA2 | ACTR2 | ACTR3 | ADCYAP1 | ADRB2 | AGO1 | AGO2 | AHI1 | AICDA | AIRE | AKT1 | ANKRD49 | APBB1 | APOE | APP | AR | ARF4 | ARID3B | ARID4B | ARNT | ARNT2 | ARRB1 | ASCL1 | ATF2 | ATF6 | ATF6B | ATM | ATOH1 | ATRX | AXIN1 | BANP | BCL10 | BMPR1A | BRCA1 | BRD4 | BTRC | CAND1 | CARM1 | CBFB | CCNT1 | CCNT2 | CD40 | CD74 | CD81 | CDC5L | CDC73 | CDH1 | CDK2 | CDK8 | CDK9 | CEBPA | CEBPE | CEP290 | CHCHD10 | CHCHD2 | CHD8 | CHEK2 | COPS5 | CREB1 | CREB3 | CREBBP | CTCF | CTNNB1 | DAB2 | DCN | DDRGK1 | DDX39B | DDX3X | DHX36 | DHX9 | DPF2 | DVL1 | DVL2 | DVL3 | E2F1 | E2F2 | E2F3 | E2F4 | E2F6 | EAF2 | EBF1 | ECD | EGFR | EGLN1 | EIF2AK3 | ELF3 | ELF4 | EP300 | ERBB2 | ERG | ERLIN2 | ESR1 | ESR2 | ESRRA | ESRRB | ESRRG | ETS1 | EXOSC9 | FADD | FAM129B | FBXW11 | FGF2 | FHL2 | FOS | FOXA1 | FOXJ1 | FOXK1 | FOXK2 | FOXM1 | FOXO1 | FUBP3 | GABPB1 | GATA1 | GPS2 | GRIP1 | GTF2F1 | GTF2F2 | H1F0 | H1FX | H2AFZ | HAX1 | HDAC1 | HDAC2 | HDAC3 | HDAC4 | HDAC5 | HELZ2 | HEXB | HIF1A | HIST1H1A | HMGB1 | HMGB2 | HMGB3 | HNF1A | HNF4A | HNF4G | HNRNPAB | HNRNPD | HNRNPK | HNRNPU | HOXA5 | HOXB5 | HOXB9 | HOXD10 | HRAS | HSF1 | HSF4 | HSPA5 | ID2 | IFI16 | IFNA2 | IKBKB | IKBKG | IKZF5 | ILF2 | ILF3 | ILK | ING1 | ING5 | INSIG1 | INSIG2 | INSR | IRF1 | IRF4 | IRF7 | ITGB1BP1 | JMJD6 | JUN | JUNB | JUND | JUP | KAT5 | KAT7 | KAT8 | KDM1A | KDM8 | KLF5 | KLF6 | KMT2A | KMT2B | KMT2C | LEF1 | LGR4 | LMO2 | LMO7 | LPIN3 | LRP6 | LUM | MAP2K1 | MAP2K3 | MAP2K7 | MAP3K2 | MAPK1 | MAPK14 | MAPK3 | MAPK7 | MAVS | MC1R | MECP2 | MED1 | MED12 | MED14 | MED17 | MED21 | MED24 | MED4 | MED6 | MEOX2 | METTL23 | MITF | MLLT11 | MLLT3 | MORF4L2 | MRPL12 | MYC | MYO6 | NAMPT | NANOG | NCK1 | NCOA1 | NCOA2 | NCOA3 | NCOA4 | NELFE | NEUROD1 | NFATC1 | NFATC2 | NFE2L2 | NFKB2 | NFKBIA | NIF3L1 | NIPBL | NKRF | NKX2-1 | NME2 | NOD2 | NPM1 | NR1H2 | NR1H3 | NR1I2 | NR3C1 | NR4A1 | NR5A2 | NUFIP1 | NUP85 | OGT | ONECUT1 | PARK7 | PARP1 | PATZ1 | PAX8 | PAX9 | PAXIP1 | PCBD1 | PCBP1 | PCGF5 | PER1 | PFKM | PHB | PHF10 | PIAS1 | PIH1D1 | PIK3R1 | PIN1 | PLA2G1B | PLSCR1 | POU2F3 | POU5F1 | PPARD | PPARG | PPARGC1A | PPM1A | PPP1R12A | PRKAA1 | PRKD1 | PRKD2 | PRKDC | PRMT2 | PROX1 | PSEN1 | PSMC2 | PSMC3 | PSMC4 | PSMC5 | PSMC6 | PSMD9 | PTGES2 | RAF1 | RAN | RARA | RARG | RB1 | RBMX | RBPJ | REL | RELA | REST | RFC1 | RFXANK | RIPK1 | RIPK2 | RNF4 | RPS6KA1 | RPTOR | RTF1 | RUNX1 | RUNX2 | RUVBL2 | RXRA | RXRB | RXRG | SFPQ | SHC1 | SIN3A | SIRT1 | SIRT7 | SKI | SLC9A1 | SMAD2 | SMAD3 | SMAD4 | SMAD5 | SMAD9 | SMARCA2 | SMARCA4 | SMARCB1 | SMARCC1 | SMARCC2 | SMO | SNW1 | SOX2 | SP1 | SP100 | SPI1 | SPIN1 | SQSTM1 | SRC | STAT1 | STAT3 | STAT4 | STAT6 | SUB1 | SUMO2 | SUPT4H1 | SUPT5H | TAF15 | TARDBP | TBL1X | TCEA2 | TCF12 | TCF21 | TCF4 | TFCP2 | TFDP1 | TGFB1 | THRA | TLR3 | TMEM173 | TNF | TNFRSF1A | TNIP2 | TNKS | TOP2A | TP53 | TP63 | TP73 | TRAF6 | TRIAP1 | TRIM28 | TRIP4 | TTC5 | TWIST1 | UBB | UBC | UBE2V1 | USP16 | USP21 | VDR | VHL | WAS | WBP2 | WNT2 | WT1 | WWOX | WWP2 | XBP1 | XRCC6 | YAF2 | YAP1 | YES1 | YWHAH | YY1 | ZBTB16 | ZBTB17 | ZBTB7B | ZIC1 | ZNF24 | ZNF746 | GO:0009887 | 0.0320271745723644 | 264/8243 | 117/3250 | 0.056691727474471 | 1 | F | F | F | F | animal organ morphogenesis | ABLIM1 | AES | AKT3 | APLP1 | AR | ATF2 | BAX | BCL2 | BCOR | BMPR1A | BRAF | BSG | CARM1 | COL12A1 | COL14A1 | COL18A1 | COL2A1 | COL4A3BP | COL5A1 | COL6A1 | COL6A2 | CTNNB1 | CUL1 | CUL3 | DCN | DGCR6 | E2F4 | EDA | EFEMP1 | EGFR | EIF4A3 | EP300 | ERRFI1 | EZR | FANCC | FGF2 | FGFR1 | FGFR3 | FHL1 | FKBP1A | FRAS1 | GAA | GNAO1 | GNAS | HCCS | HDAC1 | HDAC2 | HLX | HOXA5 | HOXB5 | HOXB6 | HOXD10 | HRAS | HTRA1 | ID2 | IFT122 | INSR | KDM5B | LAMB1 | LEF1 | LRP6 | MAPK1 | MAPK3 | MDFI | MSN | MYO5A | NEUROD1 | NF2 | NIPBL | NPHP3 | NR5A2 | NRP2 | PAFAH1B1 | PALB2 | PAX9 | PSEN1 | PTEN | PTK2 | RARA | RARG | RELA | RHOA | RING1 | RUNX2 | SCARA3 | SDC1 | SERPINB5 | SGPL1 | SKI | SLC9A3R1 | SMAD2 | SMAD3 | SMAD4 | SMARCC1 | SMO | SNAI2 | STAT6 | STK40 | TAB1 | TCF21 | TGFB1 | TGFBR3 | THRA | TLE1 | TLE3 | TP63 | TRAF6 | TREX1 | TUFT1 | TULP3 | TWIST1 | VDR | WDR19 | WDR48 | WWOX | ZFAND5 | ZMPSTE24 | GO:0035304 | 0.0105544098022565 | 87/8243 | 42/3250 | 0.0572192710204823 | 1 | F | F | F | F | regulation of protein dephosphorylation | B3GAT3 | CALM1 | CALM2 | CALM3 | CRY2 | FKBP1A | FKBP1B | GNAI2 | GSK3B | HSP90AB1 | HSP90B1 | HTT | IGBP1 | IKBKB | LGALS3 | PIN1 | PPME1 | PPP1R11 | PPP1R12A | PPP1R14A | PPP1R16A | PPP1R2 | PPP1R3B | PPP1R3C | PPP1R8 | PPP2R1A | PPP2R1B | PPP2R2A | PPP2R2D | PPP2R5D | PPP4R2 | PPP6R1 | PRKCD | PTN | RGN | SAG | SET | SWAP70 | TGFB1 | TNF | YWHAB | YWHAE | GO:0001819 | 0.0282664078612156 | 233/8243 | 104/3250 | 0.0574799397626077 | 1 | F | F | F | F | positive regulation of cytokine production | ABL1 | ADCYAP1 | AGER | ANXA1 | APP | ARNT | ATF2 | AZU1 | BCL10 | BRCA1 | CARD9 | CD2 | CD36 | CD40 | CD40LG | CD6 | CD74 | CLU | CREB1 | CREBBP | CTNNB1 | DDX1 | DDX3X | DHX36 | DHX9 | EIF2AK3 | EP300 | FADD | FAM49B | FCN1 | FLOT1 | GAPDH | GBP5 | GLMN | HAVCR2 | HDAC2 | HIF1A | HK1 | HMGB1 | HMGB2 | HMOX1 | HRAS | HSPA1B | HSPB1 | HSPD1 | IFI16 | IRF1 | IRF4 | IRF7 | LRRK2 | LUM | LURAP1 | MAPK14 | MAPK3 | MAVS | MBP | MIF | MYD88 | NFKB2 | NOD2 | ORM1 | PANX3 | PARK7 | PDE4D | PELI1 | PIK3R1 | POLR2E | POLR2L | PRKCZ | PRKD2 | PRKDC | PTPN22 | PUM1 | RARA | RELA | RIPK1 | RIPK2 | RPS3 | RUNX1 | SEMA7A | SMAD3 | SOD1 | SPTBN1 | SRC | STAT1 | STAT3 | STAT6 | SULF2 | TBC1D23 | TGFB1 | THBS1 | TLR3 | TMEM173 | TNF | TNFRSF8 | TRAF2 | TRAF6 | TRIM32 | TWIST1 | USP17L2 | USP50 | XBP1 | XRCC5 | XRCC6 | GO:0050708 | 0.0297221885235958 | 245/8243 | 109/3250 | 0.0577393408322698 | 1 | F | F | F | F | regulation of protein secretion | ABAT | ABL1 | ADORA2A | ADTRP | AGER | ANXA1 | ANXA5 | APBB1 | APOA1 | ARF1 | ARFIP1 | ARRB1 | ATG5 | ATP13A2 | CACNA1A | CD2 | CD22 | CD40 | CD40LG | CDK16 | CFTR | CPT1A | CRP | DHX36 | DHX9 | DNM1L | DYNLL1 | EGFR | EZR | FCN1 | FGB | FKBP1B | FN1 | GAPDH | GLMN | GLP1R | GLUL | GOLPH3 | GPI | HADH | HAVCR2 | HDAC9 | HIF1A | HK1 | HMGB1 | HNF4A | IDH2 | IFNA2 | ITPR1 | ITPR2 | ITPR3 | KCNS3 | KIF5B | LGR4 | LRRK2 | MAPK14 | MAPK3 | MAVS | MBP | MIF | MPC2 | NDUFAF2 | NEUROD1 | NOD2 | NOS2 | NR0B2 | NR1H2 | NR1H3 | ORM1 | OXCT1 | PANX3 | PER2 | PFKL | PFKM | PLA2G1B | PLA2G6 | PML | PPARD | PPIA | PPID | PPP1R11 | PRKCE | PRKCZ | PSMD9 | PTPN22 | PTPN23 | RAP1A | RAPGEF4 | REST | RIPK2 | SIRT3 | SLC16A1 | SLC25A4 | SLC25A5 | SLC25A6 | SOCS1 | SPTBN1 | SRC | SRI | TARDBP | TGFB1 | TMBIM6 | TNF | TNFAIP3 | TRAF2 | TRAF6 | TWIST1 | USP50 | XBP1 | GO:1901224 | 0.00642969792551256 | 53/8243 | 27/3250 | 0.0582662291364386 | 1 | F | F | F | F | positive regulation of NIK/NF-kappaB signaling | ACTN4 | AGER | AGO1 | APP | CALR | CD27 | EDA | EGFR | EP300 | HAVCR2 | HMGB1 | ILK | LIMS1 | MAS1 | NOD2 | RC3H1 | RELA | RHOA | RPS3 | TERF2IP | TLR3 | TNF | TNFSF14 | TRADD | TRAF2 | TRAF6 | TRIP6 | GO:0032368 | 0.00752153342229771 | 62/8243 | 31/3250 | 0.0582689991185632 | 1 | F | F | F | F | regulation of lipid transport | ABCG1 | AKT1 | AKT2 | ANXA2 | APOA1 | APOE | C1QTNF1 | CRY1 | FABP3 | GDF9 | GPS2 | HNRNPK | ITGAV | ITGB3 | KDM5B | MAP2K6 | MIF | NCOR1 | NFKBIA | NR1H2 | NR1H3 | P2RX4 | PCSK9 | PLA2G10 | PLA2G6 | PPARG | PRELID1 | PRKCD | SIRT1 | THBS1 | TRIAP1 | GO:0048588 | 0.00752153342229771 | 62/8243 | 31/3250 | 0.0582689991185632 | 1 | F | F | F | F | developmental cell growth | ABL1 | APP | CDK5 | CDKL3 | CTNNB1 | DCLK1 | DVL1 | EIF2AK4 | GDF9 | HDAC6 | HSP90AA1 | HSP90AB1 | IQGAP1 | ITGA4 | MAP1B | MAP2K4 | MEX3C | NIN | NRP2 | PDLIM5 | PLXNA1 | PRKCZ | PRMT2 | RARG | SEMA7A | SH3GL2 | SIRT6 | STK11 | ULK1 | USP9X | VCL | GO:0032535 | 0.0213514497149096 | 176/8243 | 80/3250 | 0.0582775032722837 | 1 | F | F | F | F | regulation of cellular component size | ACTN2 | ACTR2 | ACTR3 | ADD3 | AKT1 | AKT3 | ANXA7 | ARF1 | ARHGAP35 | ARPC1A | ARPC1B | ARPC2 | ARPC3 | ARPC4 | ARPC5 | ATP13A2 | AVIL | BAG4 | BAIAP2 | BAIAP2L1 | BIN1 | CAPG | CAPZA1 | CAPZA2 | CAPZB | CCL21 | CDC42EP3 | CDC42EP4 | CDK4 | CFL1 | CFL2 | CLNS1A | CREB1 | CTTN | DSTN | E2F4 | EZR | FCHSD1 | GRB2 | HAX1 | ICAM1 | KDM1A | LARS | LATS1 | LIMA1 | MSN | MTPN | MYO1C | NCK1 | PEX11B | PFN1 | PICK1 | PPP1CA | PRKCD | PRKCE | PTEN | RAB11A | RAB5A | RAC1 | RARG | RB1CC1 | RDX | RHOA | RPTOR | SLC12A2 | SLC9A3R1 | SNF8 | SPTAN1 | SPTBN1 | SPTBN2 | SWAP70 | TMOD3 | TMOD4 | TWF1 | TWF2 | VASP | VAV1 | WAS | WDR1 | WDTC1 | GO:0051783 | 0.0164988475069756 | 136/8243 | 63/3250 | 0.0589491913297441 | 1 | F | F | F | F | regulation of nuclear division | ANAPC4 | APC | ATM | ATRX | AURKB | CALR | CCDC36 | CCDC8 | CCNB1 | CDC14B | CDC16 | CDC20 | CDC25C | CDC27 | CENPF | CHMP2A | CUL3 | CUL7 | DUSP1 | DYNC1LI1 | FBXW5 | FZR1 | HECW2 | HNRNPU | HSPA1B | IK | INSR | KIF11 | KLHL22 | L3MBTL1 | MAD1L1 | MAD2L1 | MAPK15 | NIPBL | NSFL1C | NUMA1 | NUSAP1 | OBSL1 | PDCD6IP | PEBP1 | PHB2 | PIN1 | PLK1 | PRKAR1A | PTTG1 | RAD21 | RB1 | RCC1 | SH2B1 | SMC1A | SMC3 | SPDL1 | STAG2 | TGFB1 | TNKS | TPR | TRIP13 | UBE2B | UBE2C | VPS4B | XRCC3 | ZNF207 | ZW10 | GO:0050714 | 0.0164988475069756 | 136/8243 | 63/3250 | 0.0589491913297441 | 1 | F | F | F | F | positive regulation of protein secretion | ABAT | ABL1 | ADORA2A | AGER | APBB1 | ARF1 | ARRB1 | ATP13A2 | CD2 | CFTR | DHX36 | DHX9 | DNM1L | DYNLL1 | EGFR | EZR | FCN1 | FGB | GAPDH | GLMN | GLUL | GOLPH3 | GPI | HAVCR2 | HIF1A | HK1 | HMGB1 | KIF5B | LRRK2 | MAPK14 | MAPK3 | MAVS | MBP | MIF | MPC2 | NOD2 | NR0B2 | NR1H2 | ORM1 | OXCT1 | PANX3 | PFKM | PLA2G1B | PLA2G6 | PPARD | PPIA | PPID | PRKCE | PRKCZ | PSMD9 | PTPN22 | PTPN23 | RIPK2 | SIRT3 | SPTBN1 | SRC | SRI | TARDBP | TGFB1 | TNF | TWIST1 | USP50 | XBP1 | GO:0002521 | 0.0205022443285212 | 169/8243 | 77/3250 | 0.059050340559771 | 1 | F | F | F | F | leukocyte differentiation | ABL1 | ADA | AICDA | ANXA1 | BCL2 | BCL6 | BRAF | CASP8 | CBFA2T3 | CBFB | CD40LG | CD79B | CDC42 | CEBPA | CEBPE | CTNNB1 | DHRS7B | DNAJA3 | EP300 | FADD | FASN | GATA1 | GLO1 | GPS2 | HDAC4 | HDAC5 | HDAC9 | HMGB1 | HNF1A | ID2 | IFI16 | IFNA2 | IREB2 | IRF1 | IRF4 | ITGA4 | ITK | JAK3 | JMJD6 | JUN | JUNB | KLF6 | L3MBTL3 | LEF1 | LGALS1 | LIG4 | LTBR | MAPK14 | MED1 | MITF | NCAPH2 | NTRK1 | ONECUT1 | PARP1 | PATZ1 | PIK3R1 | PIR | POLM | PPARG | PRKDC | PSEN1 | PTPN22 | RBPJ | RC3H1 | RIPK3 | RUNX2 | SIRT1 | SPI1 | TGFB1 | TNF | TPD52 | TRAF6 | VAV1 | VCAM1 | ZAP70 | ZBTB7A | ZBTB7B | GO:0032480 | 0.00376076671114885 | 31/8243 | 17/3250 | 0.059061025573797 | 1 | F | F | F | F | negative regulation of type I interferon production | CYLD | HAVCR2 | IKBKE | ISG15 | ITCH | MAVS | NPLOC4 | OTUD5 | PCBP2 | PIN1 | PPM1B | REL | TNFAIP3 | TRAF3IP1 | TRIM25 | UBE2L6 | YY1 | GO:0010799 | 0.00376076671114885 | 31/8243 | 17/3250 | 0.059061025573797 | 1 | F | F | F | F | regulation of peptidyl-threonine phosphorylation | APP | AXIN1 | AZU1 | CAB39 | CALM1 | CALM2 | CALM3 | DDIT4 | MAPK1 | PARD3 | PLK1 | PPP2R5D | RIPK2 | RPTOR | SPRY2 | TGFB1 | UBE2K | GO:0071229 | 0.0148004367341987 | 122/8243 | 57/3250 | 0.0593791456747116 | 1 | F | F | F | F | cellular response to acid chemical | ABL2 | ACACA | ADCY6 | AKAP8 | AKR1C2 | AKT1 | ASS1 | ATM | BCL11A | BCL2L1 | CAPN2 | CCNB1 | CDK4 | COL4A1 | COL6A1 | CPT1A | CREB1 | E2F1 | EGFR | FOLR1 | FYN | GNB1 | HDAC2 | HNRNPD | HSF1 | HTRA2 | IPO5 | IREB2 | KDR | KLHL22 | LARS | NDUFA13 | NME1 | NME2 | OPA1 | PHB2 | PPARG | PPARGC1A | PRKAA1 | PRKAA2 | PRKCE | RARA | RARG | RPTOR | SHMT1 | SHMT2 | SIPA1 | SNW1 | SOCS1 | SRC | TNF | TWF2 | WNT2 | XBP1 | XRCC5 | YAP1 | YES1 | GO:0051960 | 0.0609001577095718 | 502/8243 | 215/3250 | 0.0595880840214705 | 1 | F | F | F | F | regulation of nervous system development | ABL1 | ABL2 | ACTR2 | ADCY6 | ADCYAP1 | AGER | AKT1 | APBB1 | APOE | APP | ARF1 | ARHGAP35 | ARHGDIA | ARHGEF9 | ASCL1 | ATOH1 | AVIL | BAG5 | BCL11A | BCL2 | BCL6 | BIN1 | BMPR1A | BRAF | C1QA | CALR | CAMK1D | CAMK2A | CAMK2B | CARM1 | CCDC88A | CDC20 | CDK5 | CDK5R1 | CDKL3 | CFL1 | CLSTN1 | CREB1 | CRK | CRKL | CRMP1 | CSNK1E | CTNNB1 | CTTN | CUL7 | DAB1 | DBN1 | DDX6 | DGUOK | DHX36 | DISC1 | DLG4 | DNM1L | DOCK7 | DTX1 | DVL1 | DVL2 | DVL3 | DYNLT1 | E2F1 | EIF2AK3 | EIF2AK4 | EIF4E | EIF4ENIF1 | EP300 | EPHA4 | EZH2 | FBXO7 | FGFR1 | FKBP1B | FN1 | FOXA1 | FYN | GAK | GAP43 | GDI1 | GFAP | GOLGA4 | GRID2 | GRIP1 | GSK3B | HAP1 | HDAC1 | HDAC2 | HECW1 | HECW2 | HIF1A | HMGB2 | HSPA5 | ID2 | IDH2 | IFT122 | ILK | IQGAP1 | KDM1A | KIF13B | LGALS1 | LIG4 | LPAR1 | LRFN1 | LRRC7 | LRRK2 | LSM1 | MAP1B | MAP2K1 | MAP3K13 | MAPK6 | MAPT | MDM2 | MECP2 | MED1 | METRN | MIB1 | MKS1 | MME | MOV10 | NAP1L1 | NCK1 | NCOA1 | NEDD4 | NEUROD1 | NF2 | NFE2L2 | NIN | NIPBL | NME1 | NME2 | NPHP3 | NRXN1 | NTF3 | NTRK1 | OBSL1 | OPA1 | P2RX4 | PAFAH1B1 | PARD3 | PARP6 | PCM1 | PDLIM5 | PER2 | PLXNA1 | PLXNA2 | PLXNB2 | PPARG | PPP1CC | PRKACA | PRKD1 | PROX1 | PRPF19 | PSEN1 | PTEN | PTK2 | PTN | PTPRD | PTPRF | PTPRO | RAB11A | RAB21 | RAB29 | RAP1A | RARA | RARG | RASSF10 | RELA | REST | RGS2 | RHOA | RTN4 | RTN4IP1 | S100A9 | S100B | SARM1 | SEC24B | SEMA7A | SERPINE2 | SH3GL3 | SIPA1L1 | SKI | SMO | SMURF1 | SNCA | SNW1 | SNX3 | SOCS2 | SOX2 | SPEN | SRGAP2 | STAT3 | STK11 | STK24 | STK25 | TCF12 | TCF4 | TGFB1 | THBS2 | TIMP2 | TMEM30A | TNF | TNFRSF12A | TNN | TP73 | TRAF3IP1 | TRIM32 | TUBB2B | TULP3 | TWF1 | TWF2 | VIM | WDR1 | XRCC5 | YAP1 | YWHAG | YWHAH | YWHAZ | ZMYND8 | GO:0060284 | 0.0641756641999272 | 529/8243 | 226/3250 | 0.060178333782546 | 1 | F | F | F | F | regulation of cell development | ABL1 | ABL2 | ACTN4 | ACTR2 | ADCY6 | ADCYAP1 | AGER | AKT1 | APBB1 | APOA1 | APOE | APP | ARF1 | ARHGAP35 | ARHGDIA | ARHGEF7 | ARPC2 | ASCL1 | ATOH1 | AVIL | BAG5 | BCL11A | BCL2 | BCL6 | BIN1 | BMPR1A | BRAF | C1QA | C1QBP | CALR | CAMK1D | CAMK2A | CAMK2B | CARM1 | CCDC88A | CDC20 | CDC42 | CDH5 | CDK5 | CDK5R1 | CDKL3 | CFL1 | CLDN18 | CREB1 | CRK | CRKL | CRMP1 | CSNK1E | CTDP1 | CTNNB1 | CTTN | CUL7 | DAB1 | DBN1 | DDX39B | DDX6 | DGUOK | DHX36 | DISC1 | DLG4 | DNM1L | DOCK5 | DOCK7 | DTX1 | DVL1 | DVL2 | DVL3 | DYNLT1 | E2F1 | EIF2AK4 | EIF4E | EIF4ENIF1 | EP300 | EPHA4 | EZH2 | FAM129B | FBXO7 | FGB | FGFR1 | FKBP1B | FN1 | FOXA1 | FYN | G6PD | GAK | GDI1 | GFAP | GOLGA4 | GRID2 | GRIP1 | GSK3A | GSK3B | HAP1 | HDAC1 | HDAC2 | HDAC4 | HDAC9 | HECW1 | HECW2 | HIF1A | HMGB2 | HSPA5 | ID2 | IDH2 | IKBKB | ILK | IQGAP1 | ITGB1BP1 | KDM1A | KIF13B | LGALS1 | LIG4 | LIMS1 | LPAR1 | LRRC7 | LRRK2 | LSM1 | MAP1B | MAP2K1 | MAP3K13 | MAPK6 | MAPT | MDM2 | MED1 | METRN | MIB1 | MME | MOV10 | MYOC | NAP1L1 | NCK1 | NCOA1 | NEDD4 | NEUROD1 | NF2 | NFE2L2 | NIN | NIPBL | NME1 | NME2 | NTF3 | NTRK1 | OBSL1 | OPA1 | P2RX4 | PAFAH1B1 | PARP6 | PCM1 | PDLIM5 | PER2 | PIN1 | PLXNA1 | PLXNA2 | PLXNB2 | PPARG | PPP1CC | PPP1R12A | PPP2R1A | PRKD1 | PROX1 | PRPF19 | PSEN1 | PTEN | PTK2 | PTN | PTPRD | PTPRF | PTPRO | RAB11A | RAB21 | RAB29 | RAC1 | RAP1A | RARA | RASSF10 | RECK | RELA | REST | RGS2 | RHOA | RPS19 | RTN4 | RTN4IP1 | S100A9 | SARM1 | SEMA7A | SERPINE2 | SH3GL3 | SIPA1L1 | SKI | SLAMF8 | SMAD4 | SMO | SMURF1 | SNW1 | SNX3 | SOCS2 | SOX2 | SPEN | SRGAP2 | STAT3 | STK11 | STK24 | STK25 | TCF12 | TCF4 | TGFB1 | TIMP2 | TMEM30A | TNF | TNFRSF12A | TNFRSF1A | TNN | TP73 | TRIM32 | TUBB2B | TWF1 | TWF2 | VIM | WDR1 | XRCC5 | YAP1 | YWHAG | YWHAH | YY1 | ZMYND8 | GO:0055002 | 0.0050952323183307 | 42/8243 | 22/3250 | 0.0602281202819529 | 1 | F | F | F | F | striated muscle cell development | ACTA1 | ACTN2 | AFG3L2 | ATG5 | CCNB1 | DMD | FHL2 | GPX1 | HNRNPU | LMNA | MYH11 | MYH6 | MYL2 | NEBL | NFATC2 | OBSL1 | P2RX2 | SDC1 | SKI | SMO | UCHL1 | ZMPSTE24 | GO:0040014 | 0.0050952323183307 | 42/8243 | 22/3250 | 0.0602281202819529 | 1 | F | F | F | F | regulation of multicellular organism growth | ADRB2 | AFG3L2 | APP | BCL2 | CDK4 | CREB1 | EZR | FTO | HSF1 | HTRA2 | LGMN | NIPBL | PIK3CA | PPIB | SGPL1 | SMO | SOCS2 | SOD1 | STAT3 | STAT5A | TNKS2 | ZMPSTE24 | GO:0010611 | 0.0050952323183307 | 42/8243 | 22/3250 | 0.0602281202819529 | 1 | F | F | F | F | regulation of cardiac muscle hypertrophy | CDK9 | CTDP1 | DDX39B | ERRFI1 | FOXO1 | G6PD | GLRX3 | GSK3A | LMNA | MTPN | P2RX4 | PARP1 | PIN1 | PTK2 | RGS2 | SLC9A1 | SMAD3 | SMAD4 | TNFRSF1A | TRIM63 | TWF1 | YY1 | GO:0002637 | 0.0050952323183307 | 42/8243 | 22/3250 | 0.0602281202819529 | 1 | F | F | F | F | regulation of immunoglobulin production | ATAD5 | BCL6 | CD22 | CD40 | CD40LG | EXOSC6 | GPI | HPX | IL13RA2 | MLH1 | MSH6 | PAXIP1 | PRKDC | RIF1 | STAT6 | TGFB1 | TMBIM6 | TNF | TRAF2 | TRAF6 | TREX1 | XBP1 | GO:0051279 | 0.0050952323183307 | 42/8243 | 22/3250 | 0.0602281202819529 | 1 | F | F | F | F | regulation of release of sequestered calcium ion into cytosol | ABL1 | ATG5 | BAX | CALM1 | CALM2 | CALM3 | CXCL11 | DMD | FKBP1A | FKBP1B | GSTO1 | HAP1 | HTT | MYO5A | PDE4D | PLCG1 | PRKACA | PRKCE | PTPN6 | SNCA | SRI | TGFB1 | GO:0055117 | 0.0050952323183307 | 42/8243 | 22/3250 | 0.0602281202819529 | 1 | F | F | F | F | regulation of cardiac muscle contraction | ACE2 | ATP1A1 | ATP2A2 | BIN1 | CALM1 | CALM2 | CALM3 | CAV1 | DMD | FKBP1B | GSTO1 | HDAC4 | JUP | NOS1AP | P2RX4 | PDE4D | PIK3CG | PRKACA | RGS2 | SLC9A1 | SRI | SUMO1 | GO:0070936 | 0.0050952323183307 | 42/8243 | 22/3250 | 0.0602281202819529 | 1 | F | F | F | F | protein K48-linked ubiquitination | AMFR | CDC34 | ITCH | PELI1 | RNF126 | RNF4 | RNF8 | SYVN1 | TNFAIP3 | TRIM32 | UBE2A | UBE2B | UBE2C | UBE2D1 | UBE2D2 | UBE2D3 | UBE2H | UBE2K | UBE2Q2 | UBE2R2 | UBE3A | UBR5 | GO:0002248 | 0.00036394516559505 | 3/8243 | 3/3250 | 0.0612563716082966 | 1 | F | F | F | F | connective tissue replacement involved in inflammatory response wound healing | HIF1A | TGFB1 | TIMP1 | GO:0006569 | 0.00036394516559505 | 3/8243 | 3/3250 | 0.0612563716082966 | 1 | F | F | F | F | tryptophan catabolic process | HAAO | KYNU | TDO2 | GO:0060745 | 0.00036394516559505 | 3/8243 | 3/3250 | 0.0612563716082966 | 1 | F | F | F | F | mammary gland branching involved in pregnancy | ESR1 | MED1 | VDR | GO:1901069 | 0.00036394516559505 | 3/8243 | 3/3250 | 0.0612563716082966 | 1 | F | F | F | F | guanosine-containing compound catabolic process | HPRT1 | NUDT18 | PRTFDC1 | GO:0033590 | 0.00036394516559505 | 3/8243 | 3/3250 | 0.0612563716082966 | 1 | F | F | F | F | response to cobalamin | CD40 | EGFR | RELA | GO:0001554 | 0.00036394516559505 | 3/8243 | 3/3250 | 0.0612563716082966 | 1 | F | F | F | F | luteolysis | CASP2 | CASP3 | HSPA5 | GO:0038116 | 0.00036394516559505 | 3/8243 | 3/3250 | 0.0612563716082966 | 1 | F | F | F | F | chemokine (C-C motif) ligand 21 signaling pathway | CCL21 | OXSR1 | WNK1 | GO:0032488 | 0.00036394516559505 | 3/8243 | 3/3250 | 0.0612563716082966 | 1 | F | F | F | F | Cdc42 protein signal transduction | CDC42 | RHOU | WAS | GO:1904504 | 0.00036394516559505 | 3/8243 | 3/3250 | 0.0612563716082966 | 1 | F | F | F | F | positive regulation of lipophagy | ADRB2 | HTT | SPTLC2 | GO:2000110 | 0.00036394516559505 | 3/8243 | 3/3250 | 0.0612563716082966 | 1 | F | F | F | F | negative regulation of macrophage apoptotic process | CCR5 | IRF7 | NOD2 | GO:0035617 | 0.00036394516559505 | 3/8243 | 3/3250 | 0.0612563716082966 | 1 | F | F | F | F | stress granule disassembly | KIF5B | KLC1 | VCP | GO:0071475 | 0.00036394516559505 | 3/8243 | 3/3250 | 0.0612563716082966 | 1 | F | F | F | F | cellular hyperosmotic salinity response | AKR1B1 | XRCC5 | XRCC6 | GO:1903721 | 0.00036394516559505 | 3/8243 | 3/3250 | 0.0612563716082966 | 1 | F | F | F | F | positive regulation of I-kappaB phosphorylation | AKT1 | DDRGK1 | TRAF2 | GO:0044028 | 0.00036394516559505 | 3/8243 | 3/3250 | 0.0612563716082966 | 1 | F | F | F | F | DNA hypomethylation | FAM129B | PIK3CA | ZMPSTE24 | GO:0007144 | 0.00036394516559505 | 3/8243 | 3/3250 | 0.0612563716082966 | 1 | F | F | F | F | female meiosis I | CDC25B | TRIP13 | UBB | GO:0075206 | 0.00036394516559505 | 3/8243 | 3/3250 | 0.0612563716082966 | 1 | F | F | F | F | positive regulation by host of symbiont cAMP-mediated signal transduction | CALM1 | CALM2 | CALM3 | GO:2000275 | 0.00036394516559505 | 3/8243 | 3/3250 | 0.0612563716082966 | 1 | F | F | F | F | regulation of oxidative phosphorylation uncoupler activity | PARK7 | PPIF | TWIST1 | GO:0045541 | 0.00036394516559505 | 3/8243 | 3/3250 | 0.0612563716082966 | 1 | F | F | F | F | negative regulation of cholesterol biosynthetic process | APOE | ERLIN2 | SOD1 | GO:0090086 | 0.00036394516559505 | 3/8243 | 3/3250 | 0.0612563716082966 | 1 | F | F | F | F | negative regulation of protein deubiquitination | PARK7 | TRIM21 | UBXN1 | GO:0072717 | 0.00036394516559505 | 3/8243 | 3/3250 | 0.0612563716082966 | 1 | F | F | F | F | cellular response to actinomycin D | MDM2 | RPL23 | TP53 | GO:0070814 | 0.00036394516559505 | 3/8243 | 3/3250 | 0.0612563716082966 | 1 | F | F | F | F | hydrogen sulfide biosynthetic process | CBS | CTH | MPST | GO:1901388 | 0.00036394516559505 | 3/8243 | 3/3250 | 0.0612563716082966 | 1 | F | F | F | F | regulation of transforming growth factor beta activation | HSP90AB1 | ITGAV | LTBP1 | GO:0110032 | 0.00036394516559505 | 3/8243 | 3/3250 | 0.0612563716082966 | 1 | F | F | F | F | positive regulation of G2/MI transition of meiotic cell cycle | CDC25A | CDC25B | CDC25C | GO:1901537 | 0.00036394516559505 | 3/8243 | 3/3250 | 0.0612563716082966 | 1 | F | F | F | F | positive regulation of DNA demethylation | OTUD4 | USP7 | USP9X | GO:1905598 | 0.00036394516559505 | 3/8243 | 3/3250 | 0.0612563716082966 | 1 | F | F | F | F | negative regulation of low-density lipoprotein receptor activity | APP | PCSK9 | PSEN1 | GO:1902661 | 0.00036394516559505 | 3/8243 | 3/3250 | 0.0612563716082966 | 1 | F | F | F | F | positive regulation of glucose mediated signaling pathway | PIH1D1 | SMARCA4 | SMARCB1 | GO:0032415 | 0.00036394516559505 | 3/8243 | 3/3250 | 0.0612563716082966 | 1 | F | F | F | F | regulation of sodium:proton antiporter activity | ACTN4 | CHP1 | SLC9A3R1 | GO:0050748 | 0.00036394516559505 | 3/8243 | 3/3250 | 0.0612563716082966 | 1 | F | F | F | F | negative regulation of lipoprotein metabolic process | APOD | ITGAV | ITGB3 | GO:0032079 | 0.00036394516559505 | 3/8243 | 3/3250 | 0.0612563716082966 | 1 | F | F | F | F | positive regulation of endodeoxyribonuclease activity | AKT1 | PRKCD | RPS3 | GO:0006627 | 0.00036394516559505 | 3/8243 | 3/3250 | 0.0612563716082966 | 1 | F | F | F | F | protein processing involved in protein targeting to mitochondrion | PMPCA | PMPCB | UQCRC2 | GO:0033183 | 0.00036394516559505 | 3/8243 | 3/3250 | 0.0612563716082966 | 1 | F | F | F | F | negative regulation of histone ubiquitination | OTUB1 | TRIP12 | UBR5 | GO:1901315 | 0.00036394516559505 | 3/8243 | 3/3250 | 0.0612563716082966 | 1 | F | F | F | F | negative regulation of histone H2A K63-linked ubiquitination | OTUB1 | TRIP12 | UBR5 | GO:0090188 | 0.00036394516559505 | 3/8243 | 3/3250 | 0.0612563716082966 | 1 | F | F | F | F | negative regulation of pancreatic juice secretion | NR1H3 | WNK1 | WNK4 | GO:0051343 | 0.00036394516559505 | 3/8243 | 3/3250 | 0.0612563716082966 | 1 | F | F | F | F | positive regulation of cyclic-nucleotide phosphodiesterase activity | CALM1 | CALM2 | CALM3 | GO:1903093 | 0.00036394516559505 | 3/8243 | 3/3250 | 0.0612563716082966 | 1 | F | F | F | F | regulation of protein K48-linked deubiquitination | OTUD4 | PARK7 | UBXN1 | GO:0110030 | 0.00036394516559505 | 3/8243 | 3/3250 | 0.0612563716082966 | 1 | F | F | F | F | regulation of G2/MI transition of meiotic cell cycle | CDC25A | CDC25B | CDC25C | GO:0046040 | 0.00036394516559505 | 3/8243 | 3/3250 | 0.0612563716082966 | 1 | F | F | F | F | IMP metabolic process | ADSS | HPRT1 | NT5C2 | GO:0006740 | 0.00036394516559505 | 3/8243 | 3/3250 | 0.0612563716082966 | 1 | F | F | F | F | NADPH regeneration | G6PD | IDH1 | NNT | GO:0097709 | 0.00036394516559505 | 3/8243 | 3/3250 | 0.0612563716082966 | 1 | F | F | F | F | connective tissue replacement | HIF1A | TGFB1 | TIMP1 | GO:0046218 | 0.00036394516559505 | 3/8243 | 3/3250 | 0.0612563716082966 | 1 | F | F | F | F | indolalkylamine catabolic process | HAAO | KYNU | TDO2 | GO:1990182 | 0.00036394516559505 | 3/8243 | 3/3250 | 0.0612563716082966 | 1 | F | F | F | F | exosomal secretion | COPS5 | RAB11A | TSG101 | GO:0043461 | 0.00036394516559505 | 3/8243 | 3/3250 | 0.0612563716082966 | 1 | F | F | F | F | proton-transporting ATP synthase complex assembly | ATPAF2 | OXA1L | TMEM70 | GO:0045627 | 0.00036394516559505 | 3/8243 | 3/3250 | 0.0612563716082966 | 1 | F | F | F | F | positive regulation of T-helper 1 cell differentiation | ANXA1 | HLX | RIPK2 | GO:1902523 | 0.00036394516559505 | 3/8243 | 3/3250 | 0.0612563716082966 | 1 | F | F | F | F | positive regulation of protein K63-linked ubiquitination | BIRC2 | NOD2 | PTPN22 | GO:1904428 | 0.00036394516559505 | 3/8243 | 3/3250 | 0.0612563716082966 | 1 | F | F | F | F | negative regulation of tubulin deacetylation | MAPT | PRKAA1 | PRKAA2 | GO:0045629 | 0.00036394516559505 | 3/8243 | 3/3250 | 0.0612563716082966 | 1 | F | F | F | F | negative regulation of T-helper 2 cell differentiation | ANXA1 | BCL6 | HLX | GO:0003360 | 0.00036394516559505 | 3/8243 | 3/3250 | 0.0612563716082966 | 1 | F | F | F | F | brainstem development | ATIC | GART | SMAD4 | GO:0052526 | 0.00036394516559505 | 3/8243 | 3/3250 | 0.0612563716082966 | 1 | F | F | F | F | positive regulation by organism of signal transduction in other organism involved in symbiotic interaction | CALM1 | CALM2 | CALM3 | GO:1903719 | 0.00036394516559505 | 3/8243 | 3/3250 | 0.0612563716082966 | 1 | F | F | F | F | regulation of I-kappaB phosphorylation | AKT1 | DDRGK1 | TRAF2 | GO:0006532 | 0.00036394516559505 | 3/8243 | 3/3250 | 0.0612563716082966 | 1 | F | F | F | F | aspartate biosynthetic process | GOT1 | GOT1L1 | GOT2 | GO:0015853 | 0.00036394516559505 | 3/8243 | 3/3250 | 0.0612563716082966 | 1 | F | F | F | F | adenine transport | SLC25A4 | SLC25A5 | VDAC3 | GO:0044502 | 0.00036394516559505 | 3/8243 | 3/3250 | 0.0612563716082966 | 1 | F | F | F | F | positive regulation of signal transduction in other organism | CALM1 | CALM2 | CALM3 | GO:1901671 | 0.00036394516559505 | 3/8243 | 3/3250 | 0.0612563716082966 | 1 | F | F | F | F | positive regulation of superoxide dismutase activity | PARK7 | RGN | TNF | GO:0006046 | 0.00036394516559505 | 3/8243 | 3/3250 | 0.0612563716082966 | 1 | F | F | F | F | N-acetylglucosamine catabolic process | AMDHD2 | GNPDA1 | GNPDA2 | GO:1904502 | 0.00036394516559505 | 3/8243 | 3/3250 | 0.0612563716082966 | 1 | F | F | F | F | regulation of lipophagy | ADRB2 | HTT | SPTLC2 | GO:1903516 | 0.00036394516559505 | 3/8243 | 3/3250 | 0.0612563716082966 | 1 | F | F | F | F | regulation of single strand break repair | PARP1 | TERF2 | XRCC1 | GO:0075205 | 0.00036394516559505 | 3/8243 | 3/3250 | 0.0612563716082966 | 1 | F | F | F | F | modulation by host of symbiont cAMP-mediated signal transduction | CALM1 | CALM2 | CALM3 | GO:1904833 | 0.00036394516559505 | 3/8243 | 3/3250 | 0.0612563716082966 | 1 | F | F | F | F | positive regulation of removal of superoxide radicals | PARK7 | RGN | TNF | GO:0090494 | 0.00036394516559505 | 3/8243 | 3/3250 | 0.0612563716082966 | 1 | F | F | F | F | dopamine uptake | MAPK15 | PARK7 | SNCA | GO:1901314 | 0.00036394516559505 | 3/8243 | 3/3250 | 0.0612563716082966 | 1 | F | F | F | F | regulation of histone H2A K63-linked ubiquitination | OTUB1 | TRIP12 | UBR5 | GO:0019249 | 0.00036394516559505 | 3/8243 | 3/3250 | 0.0612563716082966 | 1 | F | F | F | F | lactate biosynthetic process | LDHC | PARK7 | PER2 | GO:1905553 | 0.00036394516559505 | 3/8243 | 3/3250 | 0.0612563716082966 | 1 | F | F | F | F | regulation of blood vessel branching | ABL1 | FKBPL | SIRT6 | GO:0050665 | 0.00036394516559505 | 3/8243 | 3/3250 | 0.0612563716082966 | 1 | F | F | F | F | hydrogen peroxide biosynthetic process | CYP1A1 | CYP1A2 | SOD1 | GO:0009120 | 0.00036394516559505 | 3/8243 | 3/3250 | 0.0612563716082966 | 1 | F | F | F | F | deoxyribonucleoside metabolic process | DGUOK | TK1 | TK2 | GO:0006106 | 0.00036394516559505 | 3/8243 | 3/3250 | 0.0612563716082966 | 1 | F | F | F | F | fumarate metabolic process | FH | GOT1 | GOT2 | GO:2000619 | 0.00036394516559505 | 3/8243 | 3/3250 | 0.0612563716082966 | 1 | F | F | F | F | negative regulation of histone H4-K16 acetylation | ATG5 | PIH1D1 | SIRT1 | GO:1905777 | 0.00036394516559505 | 3/8243 | 3/3250 | 0.0612563716082966 | 1 | F | F | F | F | regulation of exonuclease activity | TCEA1 | TERF1 | TERF2 | GO:0001922 | 0.00036394516559505 | 3/8243 | 3/3250 | 0.0612563716082966 | 1 | F | F | F | F | B-1 B cell homeostasis | ABL1 | HIF1A | TNFAIP3 | GO:1900186 | 0.00036394516559505 | 3/8243 | 3/3250 | 0.0612563716082966 | 1 | F | F | F | F | negative regulation of clathrin-dependent endocytosis | SH3GL3 | UBQLN2 | UNC119 | GO:0061198 | 0.00036394516559505 | 3/8243 | 3/3250 | 0.0612563716082966 | 1 | F | F | F | F | fungiform papilla formation | CTNNB1 | HDAC1 | HDAC2 | GO:0051194 | 0.00036394516559505 | 3/8243 | 3/3250 | 0.0612563716082966 | 1 | F | F | F | F | positive regulation of cofactor metabolic process | NFE2L2 | NNT | SNCA | GO:0010836 | 0.00036394516559505 | 3/8243 | 3/3250 | 0.0612563716082966 | 1 | F | F | F | F | negative regulation of protein ADP-ribosylation | PNKP | TINF2 | XRCC1 | GO:0003339 | 0.00036394516559505 | 3/8243 | 3/3250 | 0.0612563716082966 | 1 | F | F | F | F | regulation of mesenchymal to epithelial transition involved in metanephros morphogenesis | CTNNB1 | PAX8 | STAT1 | GO:1905232 | 0.00036394516559505 | 3/8243 | 3/3250 | 0.0612563716082966 | 1 | F | F | F | F | cellular response to L-glutamate | BCL11A | FYN | OPA1 | GO:1901896 | 0.00036394516559505 | 3/8243 | 3/3250 | 0.0612563716082966 | 1 | F | F | F | F | positive regulation of calcium-transporting ATPase activity | HSPA2 | RGN | SUMO1 | GO:0090238 | 0.00036394516559505 | 3/8243 | 3/3250 | 0.0612563716082966 | 1 | F | F | F | F | positive regulation of arachidonic acid secretion | MIF | PLA2G10 | PLA2G6 | GO:0055009 | 0.00036394516559505 | 3/8243 | 3/3250 | 0.0612563716082966 | 1 | F | F | F | F | atrial cardiac muscle tissue morphogenesis | MYH6 | PROX1 | WNT2 | GO:2000286 | 0.00036394516559505 | 3/8243 | 3/3250 | 0.0612563716082966 | 1 | F | F | F | F | receptor internalization involved in canonical Wnt signaling pathway | CAV1 | RAB5A | SCYL2 | GO:0090493 | 0.00036394516559505 | 3/8243 | 3/3250 | 0.0612563716082966 | 1 | F | F | F | F | catecholamine uptake | MAPK15 | PARK7 | SNCA | GO:0061890 | 0.00036394516559505 | 3/8243 | 3/3250 | 0.0612563716082966 | 1 | F | F | F | F | positive regulation of astrocyte activation | AGER | APP | C1QA | GO:0046084 | 0.00036394516559505 | 3/8243 | 3/3250 | 0.0612563716082966 | 1 | F | F | F | F | adenine biosynthetic process | APRT | GART | HPRT1 | GO:0106119 | 0.00036394516559505 | 3/8243 | 3/3250 | 0.0612563716082966 | 1 | F | F | F | F | negative reguation of sterol biosynthetic process | APOE | ERLIN2 | SOD1 | GO:0044029 | 0.00036394516559505 | 3/8243 | 3/3250 | 0.0612563716082966 | 1 | F | F | F | F | hypomethylation of CpG island | FAM129B | PIK3CA | ZMPSTE24 | GO:0052525 | 0.00036394516559505 | 3/8243 | 3/3250 | 0.0612563716082966 | 1 | F | F | F | F | positive regulation by host of symbiont signal transduction pathway | CALM1 | CALM2 | CALM3 | GO:0006591 | 0.00036394516559505 | 3/8243 | 3/3250 | 0.0612563716082966 | 1 | F | F | F | F | ornithine metabolic process | ASL | HNF4A | OTC | GO:0015851 | 0.00036394516559505 | 3/8243 | 3/3250 | 0.0612563716082966 | 1 | F | F | F | F | nucleobase transport | SLC25A4 | SLC25A5 | VDAC3 | GO:0006990 | 0.00036394516559505 | 3/8243 | 3/3250 | 0.0612563716082966 | 1 | F | F | F | F | positive regulation of transcription from RNA polymerase II promoter involved in unfolded protein response | CREB3 | EP300 | XBP1 | GO:0110096 | 0.00036394516559505 | 3/8243 | 3/3250 | 0.0612563716082966 | 1 | F | F | F | F | cellular response to aldehyde | AIFM1 | AKR1B1 | PARK7 | GO:0071873 | 0.00036394516559505 | 3/8243 | 3/3250 | 0.0612563716082966 | 1 | F | F | F | F | response to norepinephrine | APLP1 | APP | PPARGC1A | GO:0006863 | 0.00036394516559505 | 3/8243 | 3/3250 | 0.0612563716082966 | 1 | F | F | F | F | purine nucleobase transport | SLC25A4 | SLC25A5 | VDAC3 | GO:0002155 | 0.00036394516559505 | 3/8243 | 3/3250 | 0.0612563716082966 | 1 | F | F | F | F | regulation of thyroid hormone mediated signaling pathway | NCOA1 | PRCP | THRA | GO:0046719 | 0.00036394516559505 | 3/8243 | 3/3250 | 0.0612563716082966 | 1 | F | F | F | F | regulation by virus of viral protein levels in host cell | DDB1 | STAT1 | STAU1 | GO:0035093 | 0.00036394516559505 | 3/8243 | 3/3250 | 0.0612563716082966 | 1 | F | F | F | F | spermatogenesis, exchange of chromosomal proteins | HIST1H2BA | RNF8 | TNP1 | GO:1901668 | 0.00036394516559505 | 3/8243 | 3/3250 | 0.0612563716082966 | 1 | F | F | F | F | regulation of superoxide dismutase activity | PARK7 | RGN | TNF | GO:0043137 | 0.00036394516559505 | 3/8243 | 3/3250 | 0.0612563716082966 | 1 | F | F | F | F | DNA replication, removal of RNA primer | DNA2 | FEN1 | RNASEH2A | GO:0044340 | 0.00036394516559505 | 3/8243 | 3/3250 | 0.0612563716082966 | 1 | F | F | F | F | canonical Wnt signaling pathway involved in regulation of cell proliferation | CTNNB1 | DVL2 | LRP6 | GO:0071651 | 0.00036394516559505 | 3/8243 | 3/3250 | 0.0612563716082966 | 1 | F | F | F | F | positive regulation of chemokine (C-C motif) ligand 5 production | ADCYAP1 | DDX3X | MAVS | GO:1902512 | 0.00036394516559505 | 3/8243 | 3/3250 | 0.0612563716082966 | 1 | F | F | F | F | positive regulation of apoptotic DNA fragmentation | BAX | ENDOG | HSF1 | GO:0032803 | 0.00036394516559505 | 3/8243 | 3/3250 | 0.0612563716082966 | 1 | F | F | F | F | regulation of low-density lipoprotein particle receptor catabolic process | ANXA2 | APOE | PCSK9 | GO:0052470 | 0.00036394516559505 | 3/8243 | 3/3250 | 0.0612563716082966 | 1 | F | F | F | F | modulation by host of symbiont signal transduction pathway | CALM1 | CALM2 | CALM3 | GO:0089700 | 0.00036394516559505 | 3/8243 | 3/3250 | 0.0612563716082966 | 1 | F | F | F | F | protein kinase D signaling | PRKD1 | PRKD2 | PRKD3 | GO:0097680 | 0.00036394516559505 | 3/8243 | 3/3250 | 0.0612563716082966 | 1 | F | F | F | F | double-strand break repair via classical nonhomologous end joining | LIG4 | XRCC6 | ZBTB7A | GO:0019344 | 0.00036394516559505 | 3/8243 | 3/3250 | 0.0612563716082966 | 1 | F | F | F | F | cysteine biosynthetic process | CBS | CTH | GGT1 | GO:0042436 | 0.00036394516559505 | 3/8243 | 3/3250 | 0.0612563716082966 | 1 | F | F | F | F | indole-containing compound catabolic process | HAAO | KYNU | TDO2 | GO:0006543 | 0.00036394516559505 | 3/8243 | 3/3250 | 0.0612563716082966 | 1 | F | F | F | F | glutamine catabolic process | GLS | GLS2 | PPAT | GO:0042113 | 0.0111609850782482 | 92/8243 | 44/3250 | 0.0615061552915301 | 1 | F | F | F | F | B cell activation | ABL1 | ADA | AICDA | ATAD5 | BAX | BCL2 | BCL6 | BTK | CASP8 | CD22 | CD40 | CD40LG | CD79B | CTPS1 | EP300 | EXOSC6 | GPS2 | HDAC4 | HDAC5 | HDAC9 | HSPD1 | IFNA2 | IGBP1 | ITGA4 | JAK3 | KLF6 | LEF1 | LGALS1 | LIG4 | MLH1 | MSH6 | NTRK1 | ONECUT1 | PIK3R1 | POLM | PRKCD | RBPJ | RNF8 | SWAP70 | TPD52 | TXLNA | VCAM1 | ZAP70 | ZBTB7A | GO:0022411 | 0.0286303530268106 | 236/8243 | 105/3250 | 0.0615097216526026 | 1 | F | F | F | F | cellular component disassembly | A2M | ABCE1 | ADAMTS4 | APC | APEH | ATG2B | ATG5 | AXIN1 | BSG | C1QA | CAPG | CAPN1 | CAPN2 | CAPNS1 | CAV1 | CCNB1 | CDK5 | CFL1 | CFL2 | CHCHD10 | CHMP2A | CISD2 | CKAP5 | CSNK1A1 | CTNNB1 | CTSL | CTSV | DAP3 | DCTN1 | DDIT4 | DSTN | DVL1 | DVL2 | DVL3 | EPHA4 | ERAL1 | FBXO7 | FIS1 | FLOT1 | FUNDC2 | GAK | GOLGA2 | GSK3B | GSPT1 | GSPT2 | HDAC6 | HIST1H2BA | HSPA2 | HSPA8 | HTRA1 | KIF2C | KIF5B | KLC1 | LCP1 | LRP6 | MCTS1 | MRPL12 | MRPL14 | MRPL19 | MRPL28 | MRPL38 | MRPL4 | MRPL43 | MRPL45 | MRPL46 | MRPL9 | MRPS5 | MRRF | MYC | OXA1L | PAFAH1B1 | PLK1 | PPARGC1A | PPP1CA | RB1CC1 | RPL23 | SET | SH3GL1 | SH3GL2 | SMARCA4 | SMARCB1 | SMARCC1 | SMARCC2 | SMARCE1 | SNAI2 | SNAP29 | SQSTM1 | STMN1 | STX5 | TFIP11 | TIMP1 | TIMP2 | TNP1 | TPSAB1 | TSG101 | TWF1 | TWF2 | UBQLN1 | VCP | VPS4B | VTA1 | WDR1 | WDR45B | WIPI1 | WIPI2 | GO:0051495 | 0.0171054227829674 | 141/8243 | 65/3250 | 0.0616045215752457 | 1 | F | F | F | F | positive regulation of cytoskeleton organization | ABL1 | ACTN2 | ACTR2 | ACTR3 | AKAP9 | APOA1 | ARPC1A | ARPC1B | ARPC2 | ARPC3 | ARPC4 | ARPC5 | BAG4 | BAIAP2 | BAIAP2L1 | BIN1 | BRAF | CCL21 | CDC42 | CDC42EP3 | CDC42EP4 | CDK5 | CDK5R1 | CENPJ | CFL1 | CFL2 | CTTN | DCTN1 | DSTN | DYNC1H1 | FCHSD1 | GRB2 | HAX1 | HRAS | HSPA1B | ICAM1 | ITGB1BP1 | LPAR1 | MAPK15 | MAPT | MECP2 | MYO1C | MYOC | NCK1 | NF2 | NIN | NSFL1C | NTF3 | NUMA1 | PFDN2 | PFN1 | PRKCE | PROX1 | PXN | RAC1 | RHOA | RPS3 | SDC4 | SMAD3 | SWAP70 | TPM1 | VASP | VPS4B | WAS | WDR1 | GO:0010524 | 0.00351813660075215 | 29/8243 | 16/3250 | 0.0622892448148754 | 1 | F | F | F | F | positive regulation of calcium ion transport into cytosol | ABL1 | BAX | CALM1 | CALM2 | CALM3 | CAV1 | CXCL11 | GSTO1 | HAP1 | HTT | P2RX2 | P2RX4 | PLA2G1B | PLCG1 | SNCA | SRI | GO:0030835 | 0.00351813660075215 | 29/8243 | 16/3250 | 0.0622892448148754 | 1 | F | F | F | F | negative regulation of actin filament depolymerization | ADD3 | AVIL | CAPG | CAPZA1 | CAPZA2 | CAPZB | LIMA1 | RDX | SPTAN1 | SPTBN1 | SPTBN2 | SWAP70 | TMOD3 | TMOD4 | TWF1 | TWF2 | GO:0001961 | 0.00351813660075215 | 29/8243 | 16/3250 | 0.0622892448148754 | 1 | F | F | F | F | positive regulation of cytokine-mediated signaling pathway | CD74 | FADD | HIF1A | HPX | HSPA1B | IKBKE | IRF7 | MAVS | MED1 | PAFAH1B1 | PARP14 | RIPK1 | RIPK2 | TRAF2 | TRIM32 | UBE2K | GO:0045666 | 0.026325366978042 | 217/8243 | 97/3250 | 0.0624056015321956 | 1 | F | F | F | F | positive regulation of neuron differentiation | ABL2 | ACTR2 | ADCYAP1 | APBB1 | APOE | ARF1 | ARHGAP35 | ARHGDIA | ASCL1 | ATOH1 | AVIL | BCL2 | BCL6 | BRAF | CAMK1D | CAMK2B | CDKL3 | CSNK1E | CUL7 | DAB1 | DBN1 | DHX36 | DLG4 | DNM1L | DVL1 | DVL2 | DVL3 | EP300 | EPHA4 | EZH2 | FGFR1 | FKBP1B | FN1 | FOXA1 | FYN | GDI1 | GOLGA4 | GRIP1 | HSPA5 | ILK | IQGAP1 | KDM1A | LPAR1 | LRRC7 | MAP1B | MAP2K1 | MAP3K13 | MAPK6 | MAPT | METRN | NCK1 | NCOA1 | NEUROD1 | NFE2L2 | NIN | NME1 | NME2 | NTRK1 | OBSL1 | OPA1 | PAFAH1B1 | PARP6 | PLXNA1 | PLXNA2 | PLXNB2 | PRKD1 | PROX1 | PRPF19 | PSEN1 | PTEN | PTN | PTPRD | RAB11A | RAB21 | RAP1A | RARA | REST | RGS2 | RHOA | S100A9 | SEMA7A | SH3GL3 | SMURF1 | SNX3 | SOCS2 | STK11 | STK25 | TCF12 | TCF4 | TIMP2 | TMEM30A | TNFRSF12A | TRIM32 | TUBB2B | TWF1 | TWF2 | ZMYND8 | GO:0006275 | 0.00921994419507461 | 76/8243 | 37/3250 | 0.0627054371315644 | 1 | F | F | F | F | regulation of DNA replication | AICDA | ATAD5 | ATRX | BCL6 | CCDC88A | CDK2 | CHEK2 | DBF4B | DHX9 | DNA2 | EGFR | EHMT2 | FAF1 | FGFR1 | HRAS | JUN | MAP2K4 | MAS1 | MSH6 | PCNA | PPP2CA | PPP2R1A | RAC1 | RBBP6 | RFC2 | RFC3 | RFC4 | RFC5 | S100A11 | SMC1A | SMC3 | STAG2 | STOML2 | TERF1 | TERF2 | TSPYL2 | ZMPSTE24 | GO:0050877 | 0.0427028994298192 | 352/8243 | 153/3250 | 0.0636756805001033 | 1 | F | F | F | F | nervous system process | AARS | ABL1 | ABLIM1 | ACTR2 | ADCYAP1 | ADORA2A | AGER | ALDH7A1 | AMFR | AOC2 | APOE | APP | ARF4 | ARR3 | ATAD1 | ATF6 | ATP2B2 | ATP6V1B1 | ATXN1 | BIRC5 | BRAF | BTBD9 | C1QL1 | CABP4 | CACNG2 | CACNG4 | CACNG5 | CASP3 | CD36 | CDK5 | CHRNA9 | CHRND | CHRNE | CHRNG | COL18A1 | COL2A1 | COMT | CREB1 | CRYZ | CSMD1 | DBH | DCTN1 | DLG2 | DLG4 | DMRT3 | DNAJC19 | EFEMP1 | EGFR | EIF2AK4 | EIF4A3 | EP300 | EYA4 | FBXO11 | FEN1 | FGFR1 | FKBP1B | FOS | FYN | GAA | GABRA6 | GABRR1 | GLP1R | GLRB | GLS | GNB1 | GPI | GPX1 | GRIA1 | GRK1 | HERC1 | HEXB | HIF1A | HLA-DRA | HOXD10 | HSF4 | HTR3A | HTR3E | HTT | ICAM1 | INSR | ITPR3 | JUN | KCNAB1 | KCNAB2 | KIFC3 | KMT2B | KRAS | LEF1 | LGMN | LUM | MAPK1 | MAPK3 | MAPT | MBP | MC1R | MECP2 | METTL23 | MME | MYO5A | MYO6 | NIPBL | NIPSNAP1 | NOB1 | NQO2 | NRXN1 | NTF3 | NTRK1 | OAT | OPA1 | OR5F1 | P2RX2 | P2RX4 | PAFAH1B1 | PDCL | PDE6G | PIAS1 | PITPNA | PLA2G6 | PPT1 | PRKAR2B | PRKCZ | PSAP | PSEN1 | PTEN | PTN | RDH11 | RDH12 | RDH5 | RHO | RP2 | RPGRIP1 | RPL38 | S100B | SERPINB6 | SLC9A3R1 | SNAI2 | SOD1 | SPRY2 | TAS2R60 | TBL1X | THRA | TIMM13 | TNF | UBA5 | UBA6 | UCHL1 | UNC119 | USP53 | VDAC1 | VDAC3 | WDR1 | WDR19 | ZMPSTE24 | GO:0048771 | 0.004852602207934 | 40/8243 | 21/3250 | 0.0638550026275878 | 1 | F | F | F | F | tissue remodeling | ANXA1 | ATG5 | BAX | CAPN1 | CAV1 | DBH | ELF3 | GJA1 | HIF1A | HMGB1 | HTR1B | LGR4 | LRP6 | MDM2 | MITF | NOS3 | RBPJ | TGFB1 | TGM2 | TIMP1 | WNT16 | GO:0062014 | 0.004852602207934 | 40/8243 | 21/3250 | 0.0638550026275878 | 1 | F | F | F | F | negative regulation of small molecule metabolic process | ACADVL | AKT1 | APOE | ATCAY | BRCA1 | CDA | CRY1 | EP300 | ERLIN2 | FAM3C | GSK3A | INSIG1 | INSIG2 | PGP | PROX1 | REST | SIRT1 | SNAI2 | SOD1 | TRIB3 | WDTC1 | GO:0001505 | 0.0200169841077278 | 165/8243 | 75/3250 | 0.0650309654413663 | 1 | F | F | F | F | regulation of neurotransmitter levels | AASDH | ABAT | ADORA2A | AKT1 | ALDH5A1 | ALDH7A1 | ALDH9A1 | ASS1 | ATP2A2 | BACE1 | BRAF | BTBD9 | CALM1 | CALM3 | CAMK2A | CAV1 | CD36 | CDK5 | CHDH | CHRM2 | CLU | COMT | CPLX1 | CPLX3 | DBH | DNM1L | DVL1 | FLOT1 | GAD2 | GFAP | GIT1 | GLA | GLUL | GSK3B | HSP90AA1 | HSP90AB1 | HSPA8 | HTR1B | ICAM1 | INSR | LRRK2 | MAOA | NOS1AP | NOS2 | NOS3 | NQO1 | NRXN1 | P2RX1 | P2RX4 | PAH | PARK7 | PEBP1 | PER2 | PPT1 | PREPL | PSEN1 | RAB3GAP1 | RAB5A | RAC1 | RAP1A | RAP1B | RGN | RGS2 | RHOT1 | SEPT5 | SMAD3 | SNCA | SPR | STXBP1 | SYN1 | SYP | TNF | TOR1A | VPS18 | WDR35 | GO:0060191 | 0.00703627320150431 | 58/8243 | 29/3250 | 0.0655774923830708 | 1 | F | F | F | F | regulation of lipase activity | ABL1 | ABL2 | ANXA1 | APOA1 | ARF4 | EGFR | ESR1 | FGF2 | FGFR1 | FGFR3 | GNA13 | GNA15 | GNAQ | HDAC9 | HRAS | ITK | LPAR1 | LPAR2 | NR1H2 | NR1H3 | NTF3 | PLA2G1B | PLCG1 | PPT1 | PRKCZ | RGS2 | RHOA | SNCA | SORT1 | GO:0048010 | 0.00594443770471916 | 49/8243 | 25/3250 | 0.0656092937674172 | 1 | F | F | F | F | vascular endothelial growth factor receptor signaling pathway | BAIAP2 | BCAR1 | CDC42 | CRK | FYN | HSP90AA1 | HSPB1 | ITGAV | ITGB3 | KDR | MAPK14 | NCK1 | NRP2 | PAK2 | PDCD6 | PIK3CA | PIK3R1 | PRKD1 | PRKD2 | PTK2 | PXN | RAC1 | RHOA | SRC | VAV1 | GO:1903557 | 0.00594443770471916 | 49/8243 | 25/3250 | 0.0656092937674172 | 1 | F | F | F | F | positive regulation of tumor necrosis factor superfamily cytokine production | AGER | APP | AZU1 | CARD9 | CD2 | CD36 | CLU | DHX9 | FADD | HAVCR2 | HDAC2 | HMGB1 | HSPB1 | LRRK2 | MAVS | MIF | NOD2 | ORM1 | PIK3R1 | RIPK1 | RIPK2 | THBS1 | TLR3 | TNFRSF8 | TWIST1 | GO:0032760 | 0.00594443770471916 | 49/8243 | 25/3250 | 0.0656092937674172 | 1 | F | F | F | F | positive regulation of tumor necrosis factor production | AGER | APP | AZU1 | CARD9 | CD2 | CD36 | CLU | DHX9 | FADD | HAVCR2 | HDAC2 | HMGB1 | HSPB1 | LRRK2 | MAVS | MIF | NOD2 | ORM1 | PIK3R1 | RIPK1 | RIPK2 | THBS1 | TLR3 | TNFRSF8 | TWIST1 | GO:0032459 | 0.00327550649035545 | 27/8243 | 15/3250 | 0.0656556792871882 | 1 | F | F | F | F | regulation of protein oligomerization | ALB | APOE | BAX | CLU | CRBN | CRYAB | DNM1L | EP300 | HDAC6 | HIST3H3 | IDE | PEX5 | SRC | TP53 | USP50 | GO:0099173 | 0.00327550649035545 | 27/8243 | 15/3250 | 0.0656556792871882 | 1 | F | F | F | F | postsynapse organization | ACTN1 | ARF1 | CDC42 | CTTN | DLG4 | DVL1 | FYN | HDAC6 | IGF1R | INSR | LGMN | NDRG1 | PICK1 | PTEN | SPTBN2 | GO:0061001 | 0.00327550649035545 | 27/8243 | 15/3250 | 0.0656556792871882 | 1 | F | F | F | F | regulation of dendritic spine morphogenesis | ACTR2 | CAMK2B | CDK5 | CDK5R1 | CFL1 | DBN1 | DHX36 | DNM1L | EPHA4 | LRRK2 | OPA1 | PAFAH1B1 | PDLIM5 | PTEN | SIPA1L1 | GO:0050775 | 0.00327550649035545 | 27/8243 | 15/3250 | 0.0656556792871882 | 1 | F | F | F | F | positive regulation of dendrite morphogenesis | ACTR2 | CAMK2B | CDKL3 | CUL7 | DBN1 | DHX36 | DNM1L | EPHA4 | ILK | OBSL1 | OPA1 | PAFAH1B1 | PARP6 | PTPRD | RAB21 | GO:0030258 | 0.0143151765134053 | 118/8243 | 55/3250 | 0.0659753039182188 | 1 | F | F | F | F | lipid modification | ABCD3 | ACAA1 | ACAA2 | ACADS | ACADVL | ACAT1 | ACAT2 | ACOX1 | AGK | ALOX12 | CPT1A | CPT2 | CROT | CYP1A1 | DECR1 | DGKE | DGKZ | ECH1 | ECHDC2 | ECHS1 | ECI1 | ECI2 | EHHADH | ETFA | ETFB | ETFDH | GCDH | HADH | HIBCH | HSD17B4 | IMPA1 | IMPA2 | IP6K1 | IVD | MAPK14 | MCAT | MTMR7 | PI4K2A | PIK3C2A | PIK3CA | PIK3CG | PIK3R1 | PIK3R3 | PIKFYVE | PIP4K2A | PIPSL | PPARD | PPARG | PPARGC1A | PRKAA1 | PTEN | SOAT1 | SOCS1 | SOCS2 | SOCS6 | GO:0043666 | 0.00897731408467791 | 74/8243 | 36/3250 | 0.0664350683109009 | 1 | F | F | F | F | regulation of phosphoprotein phosphatase activity | B3GAT3 | CALM1 | CALM2 | CALM3 | CRY2 | FKBP1A | FKBP1B | GNAI2 | GSK3B | HSP90AB1 | HSP90B1 | HTT | IGBP1 | IKBKB | LGALS3 | PPME1 | PPP1R11 | PPP1R12A | PPP1R14A | PPP1R16A | PPP1R2 | PPP1R3B | PPP1R3C | PPP1R8 | PPP2R1A | PPP2R1B | PPP2R2A | PPP2R2D | PPP2R5D | PPP4R2 | PPP6R1 | PTN | RGN | SAG | SET | TNF | GO:0007265 | 0.0183185733349509 | 151/8243 | 69/3250 | 0.0667266326137107 | 1 | F | F | F | F | Ras protein signal transduction | ARHGAP1 | ARHGDIA | BCL6 | CDC42 | CDC42EP3 | CDC42EP4 | CDK2 | CDKN1A | CFL1 | CRKL | DBNL | DOK1 | FGF2 | G3BP1 | G3BP2 | GDI1 | GNA12 | GNA13 | GNB1 | GRB2 | HRAS | JUN | KPNB1 | KRAS | KSR1 | LAT | MAPK14 | PARK7 | PRKD1 | RAB10 | RAB11A | RAB11B | RAB18 | RAB1A | RAB1B | RAB21 | RAB24 | RAB29 | RAB2A | RAB31 | RAB32 | RAB35 | RAB5A | RAB5B | RAB7A | RAB9A | RABL2A | RABL3 | RAC1 | RAP1A | RAP1B | RB1 | RFXANK | RHOA | RHOJ | RHOT1 | RHOT2 | RHOU | RIT1 | RTKN | SETDB1 | SHC1 | SYNJ2BP | TP53 | TRIM28 | USP28 | USP50 | USP8 | WAS | GO:0031326 | 0.291762707752032 | 2405/8243 | 979/3250 | 0.0668060651307052 | 1 | F | F | F | F | regulation of cellular biosynthetic process | AARS | ABCG1 | ABHD14B | ABL1 | ACADVL | ACD | ACO1 | ACTA2 | ACTL6A | ACTN1 | ACTN2 | ACTN4 | ACTR2 | ACTR3 | ADCYAP1 | ADORA2A | ADRB2 | AEBP2 | AES | AFF1 | AFF4 | AGAP2 | AGER | AGO1 | AGO2 | AHI1 | AICDA | AIRE | AKT1 | AKT2 | ANKRD49 | ANXA1 | APBB1 | APBB2 | APBB3 | APEX1 | APOE | APP | AR | ARF4 | ARHGAP35 | ARID3B | ARID4B | ARNT | ARNT2 | ARRB1 | ARRB2 | ASCC1 | ASCC2 | ASCL1 | ASS1 | ATAD5 | ATF2 | ATF6 | ATF6B | ATF7 | ATM | ATOH1 | ATP1A1 | ATRX | ATXN1 | ATXN2 | AURKB | AXIN1 | AZU1 | BACH2 | BAG3 | BANP | BASP1 | BCL10 | BCL11A | BCL2 | BCL6 | BCL6B | BCOR | BIRC2 | BIRC5 | BLZF1 | BMI1 | BMPR1A | BOLA1 | BOLA3 | BRCA1 | BRD2 | BRD3 | BRD4 | BTK | BTRC | BUD31 | C1QBP | CALR | CAMK1D | CAMK2A | CAND1 | CAPN2 | CARD9 | CARM1 | CAT | CAV1 | CBFA2T3 | CBFB | CBX4 | CBX7 | CBX8 | CBY1 | CCAR2 | CCDC22 | CCDC88A | CCL21 | CCNT1 | CCNT2 | CCT2 | CCT3 | CCT4 | CCT5 | CCT6A | CCT7 | CCT8 | CD36 | CD40 | CD40LG | CD74 | CD81 | CDC5L | CDC73 | CDH1 | CDK2 | CDK4 | CDK5 | CDK5R1 | CDK8 | CDK9 | CDKN1A | CEBPA | CEBPE | CENPF | CENPJ | CEP290 | CHCHD10 | CHCHD2 | CHCHD3 | CHD3 | CHD4 | CHD8 | CHEK2 | CHP1 | CHRD | CIRBP | CKS2 | CLU | COA3 | COMMD1 | COPS2 | COPS5 | COQ3 | CREB1 | CREB3 | CREBBP | CRK | CRY1 | CRY2 | CTBP1 | CTCF | CTDP1 | CTH | CTNNB1 | CTNNBIP1 | CUL2 | CUL3 | CYLD | DAB2 | DAPK1 | DAXX | DBF4B | DCN | DCP2 | DDRGK1 | DDX1 | DDX39B | DDX3X | DHX36 | DHX9 | DMAP1 | DMRT3 | DNA2 | DNAJA3 | DNAJB1 | DNAJC30 | DPF2 | DTX1 | DUSP1 | DUSP22 | DVL1 | DVL2 | DVL3 | DYRK2 | E2F1 | E2F2 | E2F3 | E2F4 | E2F6 | EAF2 | EBF1 | ECD | EDA | EED | EEF2 | EFEMP1 | EGFR | EGLN1 | EGLN3 | EHMT1 | EHMT2 | EID1 | EIF2AK3 | EIF2AK4 | EIF2B5 | EIF2S1 | EIF3D | EIF3E | EIF3H | EIF4A3 | EIF4E | EIF4E2 | EIF4EBP1 | EIF4ENIF1 | EIF4G3 | EIF4H | EIF5 | EIF5A | EIF5B | EIF6 | ELAVL1 | ELF3 | ELF4 | ENO1 | EP300 | EPRS | ERBB2 | ERF | ERG | ERLIN2 | ERRFI1 | ESR1 | ESR2 | ESRRA | ESRRB | ESRRG | ETS1 | EWSR1 | EXOSC9 | EZH2 | EZR | FABP3 | FABP5 | FADD | FAF1 | FAM129B | FAM3C | FANCA | FASLG | FBP1 | FBXW11 | FGF2 | FGFR1 | FHL2 | FKBP8 | FLOT1 | FOS | FOXA1 | FOXD4 | FOXD4L6 | FOXI2 | FOXJ1 | FOXK1 | FOXK2 | FOXM1 | FOXO1 | FTO | FUBP1 | FUBP3 | FUS | FXR2 | FYN | G3BP2 | GABPB1 | GAPDH | GATA1 | GDF9 | GEMIN5 | GLA | GLMN | GLO1 | GOLGA2 | GPS2 | GRB7 | GRIP1 | GSK3A | GSK3B | GSTZ1 | GTF2A1 | GTF2F1 | GTF2F2 | GTF2I | H1F0 | H1FX | H2AFZ | H3F3A | HAVCR2 | HAX1 | HBEGF | HBZ | HDAC1 | HDAC2 | HDAC3 | HDAC4 | HDAC5 | HDAC6 | HDAC7 | HDAC9 | HDGF | HELZ2 | HEXB | HEXIM1 | HIF1A | HIF1AN | HINT1 | HIST1H1A | HIST1H3A | HIST1H4A | HIST2H3A | HIVEP1 | HLX | HMGB1 | HMGB2 | HMGB3 | HMOX1 | HNF1A | HNF4A | HNF4G | HNRNPA1 | HNRNPA2B1 | HNRNPAB | HNRNPD | HNRNPK | HNRNPL | HNRNPU | HOXA5 | HOXB5 | HOXB6 | HOXB9 | HOXD10 | HRAS | HSF1 | HSF4 | HSP90AA1 | HSP90AB1 | HSPA1B | HSPA5 | HSPA8 | HSPB1 | HSPD1 | HTATIP2 | HTRA1 | ICAM1 | ID2 | IDH1 | IFI16 | IFNA2 | IGBP1 | IGF2BP1 | IKBKB | IKBKG | IKZF5 | ILF2 | ILF3 | ILK | ING1 | ING5 | INSIG1 | INSIG2 | INSR | IREB2 | IRF1 | IRF4 | IRF7 | ITCH | ITGB1BP1 | ITGB3BP | JAK3 | JMJD6 | JUN | JUNB | JUND | JUP | KANK2 | KAT5 | KAT7 | KAT8 | KDM1A | KDM2A | KDM5B | KDM5C | KDM8 | KEAP1 | KHDRBS1 | KLF5 | KLF6 | KMT2A | KMT2B | KMT2C | KRAS | L3MBTL1 | L3MBTL2 | L3MBTL3 | L3MBTL4 | LARP4 | LEF1 | LEMD2 | LEMD3 | LGR4 | LIMS1 | LMO2 | LMO7 | LOXL2 | LPIN3 | LRIF1 | LRP6 | LRPPRC | LUM | LZTR1 | MAF1 | MAP2K1 | MAP2K3 | MAP2K4 | MAP2K7 | MAP3K13 | MAP3K2 | MAP3K4 | MAPK1 | MAPK14 | MAPK15 | MAPK3 | MAPK7 | MAPK8 | MAPK9 | MAS1 | MAVS | MBD3 | MC1R | MDFI | MDM2 | MDM4 | MECP2 | MED1 | MED12 | MED14 | MED17 | MED20 | MED21 | MED23 | MED24 | MED31 | MED4 | MED6 | MEOX2 | MEPCE | METTL23 | MGA | MITF | MKNK2 | MLLT1 | MLLT11 | MLLT3 | MORF4L1 | MORF4L2 | MOV10 | MRGBP | MRPL12 | MSH6 | MTA1 | MTA3 | MTERF1 | MTPN | MXD1 | MYBBP1A | MYC | MYD88 | MYH6 | MYO6 | NACC1 | NAMPT | NANOG | NCBP1 | NCK1 | NCOA1 | NCOA2 | NCOA3 | NCOA4 | NCOR1 | NCOR2 | NDUFA13 | NEDD4 | NEDD8 | NEK7 | NELFB | NELFCD | NELFE | NEUROD1 | NFATC1 | NFATC2 | NFE2L2 | NFKB2 | NFKBIA | NIF3L1 | NIPBL | NKRF | NKX2-1 | NLK | NMD3 | NME2 | NOD2 | NOS1AP | NOS2 | NOS3 | NOSTRIN | NPM1 | NR0B2 | NR1H2 | NR1H3 | NR1I2 | NR3C1 | NR4A1 | NR5A2 | NTRK1 | NUFIP1 | NUMA1 | NUP85 | NUP98 | NUPR1 | OGFOD1 | OGT | ONECUT1 | P2RX4 | PA2G4 | PABPC1 | PAIP1 | PAK6 | PARK7 | PARP1 | PATZ1 | PAWR | PAX8 | PAX9 | PAXIP1 | PCBD1 | PCBP1 | PCGF1 | PCGF5 | PCGF6 | PCNA | PDK2 | PELI1 | PER1 | PER2 | PFKM | PFN1 | PGP | PHB | PHB2 | PHF10 | PHF20L1 | PHF21A | PIAS1 | PIAS3 | PIH1D1 | PIK3R1 | PIM1 | PIN1 | PINX1 | PIR | PKIG | PLA2G10 | PLA2G1B | PLA2G6 | PLK1 | PLSCR1 | PML | PNKP | POLDIP3 | POLR2A | POLR2L | POT1 | POU2F3 | POU5F1 | PPARD | PPARG | PPARGC1A | PPID | PPIE | PPM1A | PPM1D | PPP1CA | PPP1CB | PPP1R12A | PPP1R3B | PPP2CA | PPP2R1A | PRDX2 | PRDX3 | PRDX5 | PRKAA1 | PRKAA2 | PRKAB2 | PRKAR1A | PRKCD | PRKCZ | PRKD1 | PRKD2 | PRKDC | PRMT2 | PRMT3 | PRMT6 | PROX1 | PSEN1 | PSMA1 | PSMA2 | PSMA3 | PSMA5 | PSMA6 | PSMB5 | PSMB6 | PSMB7 | PSMB8 | PSMC1 | PSMC2 | PSMC3 | PSMC4 | PSMC5 | PSMC6 | PSMD1 | PSMD10 | PSMD11 | PSMD12 | PSMD14 | PSMD2 | PSMD4 | PSMD9 | PSME2 | PSME3 | PTEN | PTGES2 | PTGES3 | PTPN14 | PTPRK | PTTG1 | PUM1 | PUS7 | QARS | QRICH1 | RAB3GAP1 | RAC1 | RAD21 | RAF1 | RAN | RARA | RARG | RASD1 | RB1 | RBBP6 | RBM3 | RBM4 | RBM8A | RBMX | RBPJ | RBX1 | RC3H1 | REL | RELA | REST | RFC1 | RFC2 | RFC3 | RFC4 | RFC5 | RFXANK | RGN | RGS2 | RHOA | RIF1 | RING1 | RIPK1 | RIPK2 | RIPK3 | RIPK4 | RMND1 | RNF139 | RNF2 | RNF25 | RNF31 | RNF4 | RNF8 | RPL10 | RPL23 | RPL38 | RPL6 | RPS14 | RPS3 | RPS4X | RPS6KA1 | RPS6KB2 | RPTOR | RRM2 | RTF1 | RUNX1 | RUNX1T1 | RUNX2 | RUVBL1 | RUVBL2 | RXRA | RXRB | RXRG | S100A1 | S100A11 | S100A12 | S100A9 | SAP18 | SARS | SCML2 | SET | SETBP1 | SETDB1 | SF1 | SFMBT1 | SFPQ | SH2B1 | SHC1 | SHMT1 | SHMT2 | SIN3A | SIRT1 | SIRT5 | SIRT6 | SIRT7 | SKI | SLC9A1 | SLTM | SMAD2 | SMAD3 | SMAD4 | SMAD5 | SMAD9 | SMARCA2 | SMARCA4 | SMARCB1 | SMARCC1 | SMARCC2 | SMARCE1 | SMC1A | SMC3 | SMG5 | SMG6 | SMO | SMURF1 | SMURF2 | SNAI2 | SNCA | SND1 | SNF8 | SNW1 | SNX6 | SOAT1 | SOD2 | SOX2 | SP1 | SP100 | SP2 | SPEN | SPI1 | SPIN1 | SQSTM1 | SRC | SRI | STAG2 | STAT1 | STAT3 | STAT4 | STAT5A | STAT6 | STK3 | STOML2 | STRAP | SUB1 | SUMO1 | SUMO2 | SUPT4H1 | SUPT5H | SUZ12 | TAB1 | TAB2 | TACO1 | TADA2B | TAF12 | TAF15 | TAGLN3 | TARDBP | TAZ | TBL1X | TCEA1 | TCEA2 | TCERG1 | TCF12 | TCF21 | TCF25 | TCF4 | TCOF1 | TCP1 | TCP10L | TDRD3 | TERF1 | TERF2 | TERF2IP | TFCP2 | TFDP1 | TGFB1 | TGFBR3 | TGIF2LX | THAP11 | THAP7 | THBS1 | THRA | TIA1 | TIAL1 | TINF2 | TLE1 | TLE3 | TLR3 | TMBIM6 | TMEM173 | TMPO | TNF | TNFAIP3 | TNFRSF1A | TNFRSF8 | TNIP2 | TNKS | TNKS2 | TNP1 | TOE1 | TOP2A | TP53 | TP63 | TP73 | TPR | TRADD | TRAF1 | TRAF2 | TRAF3 | TRAF6 | TRAP1 | TRIAP1 | TRIB2 | TRIB3 | TRIM14 | TRIM21 | TRIM25 | TRIM28 | TRIM29 | TRIM32 | TRIM33 | TRIM34 | TRIP13 | TRIP4 | TRUB2 | TSC22D1 | TSC22D3 | TSC22D4 | TSFM | TSG101 | TSPYL2 | TTC5 | TULP3 | TWIST1 | TXN | TXNIP | TYMS | UBA3 | UBB | UBC | UBE2D1 | UBE2D3 | UBE2I | UBE2J1 | UBE2L3 | UBE2N | UBE2O | UBE2V1 | UNK | UNKL | UPF3B | UQCC1 | USP15 | USP16 | USP2 | USP21 | USP47 | USP7 | USP9X | USP9Y | VCP | VDR | VGLL4 | VHL | VIM | VPS72 | WAS | WBP2 | WDR35 | WDR77 | WDTC1 | WNT2 | WT1 | WWOX | WWP2 | XBP1 | XIAP | XPO1 | XRCC5 | XRCC6 | YAF2 | YAP1 | YEATS2 | YES1 | YTHDF1 | YWHAB | YWHAH | YWHAQ | YY1 | ZBTB16 | ZBTB17 | ZBTB21 | ZBTB25 | ZBTB33 | ZBTB42 | ZBTB7A | ZBTB7B | ZC3H4 | ZFP41 | ZHX1 | ZIC1 | ZKSCAN1 | ZMPSTE24 | ZMYM5 | ZMYND8 | ZNF207 | ZNF217 | ZNF227 | ZNF24 | ZNF277 | ZNF354C | ZNF408 | ZNF420 | ZNF443 | ZNF517 | ZNF518A | ZNF592 | ZNF598 | ZNF618 | ZNF622 | ZNF646 | ZNF669 | ZNF696 | ZNF746 | ZNF747 | ZRANB2 | ZSCAN2 | ZSCAN29 | ZSCAN32 | ZZZ3 | GO:0010976 | 0.0206235593837195 | 170/8243 | 77/3250 | 0.0672417769750135 | 1 | F | F | F | F | positive regulation of neuron projection development | ABL2 | ACTR2 | ADCYAP1 | APBB1 | APOE | ARF1 | ARHGAP35 | ARHGDIA | AVIL | BRAF | CAMK1D | CAMK2B | CDKL3 | CUL7 | DBN1 | DHX36 | DLG4 | DNM1L | DVL1 | DVL2 | DVL3 | EP300 | EPHA4 | EZH2 | FGFR1 | FKBP1B | FN1 | FYN | GDI1 | GOLGA4 | GRIP1 | HSPA5 | ILK | IQGAP1 | KDM1A | LPAR1 | LRRC7 | MAP1B | MAP2K1 | MAP3K13 | MAPK6 | MAPT | METRN | NCK1 | NFE2L2 | NIN | NME1 | NME2 | NTRK1 | OBSL1 | OPA1 | PAFAH1B1 | PARP6 | PLXNA1 | PLXNA2 | PLXNB2 | PRKD1 | PSEN1 | PTN | PTPRD | RAB11A | RAB21 | RAP1A | RGS2 | RHOA | S100A9 | SEMA7A | SMURF1 | SNX3 | STK11 | STK25 | TMEM30A | TNFRSF12A | TUBB2B | TWF1 | TWF2 | ZMYND8 | GO:0006879 | 0.0046099720975373 | 38/8243 | 20/3250 | 0.0677187900627567 | 1 | F | F | F | F | cellular iron ion homeostasis | ABCB7 | ACO1 | ATP13A2 | ATP6V1A | CAND1 | CUL1 | EGLN1 | FTH1 | FTL | GLRX3 | HIF1A | HMOX1 | HPX | IREB2 | MYC | NCOA4 | NEDD8 | NUBP1 | SMAD4 | SOD1 | GO:0035308 | 0.0046099720975373 | 38/8243 | 20/3250 | 0.0677187900627567 | 1 | F | F | F | F | negative regulation of protein dephosphorylation | CRY2 | FKBP1A | FKBP1B | GNAI2 | GSK3B | IKBKB | LGALS3 | PPME1 | PPP1R11 | PPP1R14A | PPP1R2 | PPP1R8 | PTN | RGN | SAG | SET | SWAP70 | TNF | YWHAB | YWHAE | GO:0000731 | 0.0046099720975373 | 38/8243 | 20/3250 | 0.0677187900627567 | 1 | F | F | F | F | DNA synthesis involved in DNA repair | ISG15 | NPLOC4 | PCNA | POLD1 | POLE2 | RFC1 | RFC2 | RFC3 | RFC4 | RFC5 | RPA2 | RPA3 | SIRT1 | SPRTN | TREX1 | TRIM25 | UBB | UBC | UBE2L6 | VCP | GO:0032103 | 0.0197743539973311 | 163/8243 | 74/3250 | 0.068238820629241 | 1 | F | F | F | F | positive regulation of response to external stimulus | AGER | APP | AZU1 | BRAF | BTK | C1QA | C1QBP | CALR | CAMK1D | CCL21 | CCR1 | CCR4 | CD6 | CD74 | CDK19 | CEBPA | CREB3 | CXCL8 | DHX9 | EGFR | ETS1 | FAM19A3 | FGF2 | FGFR1 | FKBP1B | GBP5 | HAVCR2 | HMGB1 | HSPB1 | KDR | LGMN | LPAR1 | LRRK2 | LRSAM1 | MAPK14 | MAPK3 | MIF | MYD88 | NFKBIA | NOD2 | NTF3 | OPTN | OXSR1 | P2RX4 | PARK7 | PIK3CG | PLA2G2A | PRKD1 | PRKD2 | PTGER3 | PUM1 | RAC1 | RIPK2 | RPS19 | S100A12 | S100A7 | S100A9 | SCG2 | SMAD3 | SNCA | SNW1 | SWAP70 | TGFB1 | TGM2 | THBS1 | TLR3 | TNF | TNFRSF1A | TNFSF14 | TRADD | TRAF6 | TUBB2B | USP17L2 | WNK1 | GO:0016444 | 0.00303287637995875 | 25/8243 | 14/3250 | 0.0691495123906606 | 1 | F | F | F | F | somatic cell DNA recombination | AICDA | ATAD5 | CD40LG | EXOSC6 | HMGB1 | HMGB2 | HSPD1 | LEF1 | LIG4 | MLH1 | MSH6 | PRKDC | RNF8 | SWAP70 | GO:0002562 | 0.00303287637995875 | 25/8243 | 14/3250 | 0.0691495123906606 | 1 | F | F | F | F | somatic diversification of immune receptors via germline recombination within a single locus | AICDA | ATAD5 | CD40LG | EXOSC6 | HMGB1 | HMGB2 | HSPD1 | LEF1 | LIG4 | MLH1 | MSH6 | PRKDC | RNF8 | SWAP70 | GO:0070979 | 0.00303287637995875 | 25/8243 | 14/3250 | 0.0691495123906606 | 1 | F | F | F | F | protein K11-linked ubiquitination | ANAPC4 | CDC16 | CDC27 | FZR1 | RNF4 | UBE2A | UBE2B | UBE2C | UBE2D2 | UBE2D3 | UBE2H | UBE2L3 | UBE2S | UBE2W | GO:0060395 | 0.00303287637995875 | 25/8243 | 14/3250 | 0.0691495123906606 | 1 | F | F | F | F | SMAD protein signal transduction | FOS | GDF9 | HNF4A | JUN | NUP93 | SKI | SMAD2 | SMAD3 | SMAD4 | SMAD5 | SMAD9 | SUB1 | TGFB1 | VIM | GO:0051602 | 0.00303287637995875 | 25/8243 | 14/3250 | 0.0691495123906606 | 1 | F | F | F | F | response to electrical stimulus | ADSS | AKAP9 | EEF1A2 | HNRNPD | NQO1 | NTRK1 | OPA1 | PPARGC1A | PTEN | REST | S100A13 | SLC9A1 | SRC | TRIM63 | GO:0019748 | 0.00303287637995875 | 25/8243 | 14/3250 | 0.0691495123906606 | 1 | F | F | F | F | secondary metabolic process | AKR1A1 | AKR1B1 | AKR1B10 | AKR1C2 | BCL2 | CBR4 | CBS | CTH | CYP1A1 | CYP1A2 | MC1R | MPST | MYO5A | NFE2L2 | GO:0007088 | 0.0149217517893971 | 123/8243 | 57/3250 | 0.0692166531464471 | 1 | F | F | F | F | regulation of mitotic nuclear division | ANAPC4 | APC | ATM | ATRX | AURKB | CCDC8 | CCNB1 | CDC14B | CDC16 | CDC20 | CDC25C | CDC27 | CENPF | CHMP2A | CUL3 | CUL7 | DUSP1 | DYNC1LI1 | FBXW5 | HECW2 | HNRNPU | HSPA1B | IK | INSR | KIF11 | KLHL22 | L3MBTL1 | MAD1L1 | MAD2L1 | NIPBL | NSFL1C | NUMA1 | NUSAP1 | OBSL1 | PDCD6IP | PEBP1 | PHB2 | PIN1 | PLK1 | PTTG1 | RAD21 | RB1 | RCC1 | SH2B1 | SMC1A | SMC3 | SPDL1 | STAG2 | TGFB1 | TNKS | TPR | TRIP13 | UBE2C | VPS4B | XRCC3 | ZNF207 | ZW10 | GO:0007186 | 0.0337255853451413 | 278/8243 | 122/3250 | 0.0693892400079101 | 1 | F | F | F | F | G protein-coupled receptor signaling pathway | ACKR2 | ACTN2 | ADCY6 | ADCYAP1 | ADORA2A | ADRB2 | AGTRAP | AHCYL1 | AKR1C2 | AKT1 | ANXA1 | APOA1 | APOE | APP | ARHGEF7 | ARHGEF9 | ARRB1 | ARRB2 | AZU1 | BCAR1 | BDKRB2 | BTK | CALM1 | CALM2 | CALM3 | CAMK2A | CAV1 | CCL21 | CCL22 | CCR1 | CCR3 | CCR4 | CCR5 | CDK5R1 | CHRM2 | CHRM3 | CORT | CRKL | CXCL11 | CXCL5 | CXCL6 | CXCL8 | DGKE | DGKZ | ESR1 | EZR | FCN1 | GABRA6 | GABRR1 | GAP43 | GLP1R | GLRB | GNA12 | GNA13 | GNA15 | GNAI1 | GNAI2 | GNAI3 | GNAO1 | GNAQ | GNAS | GNAZ | GNB1 | GNB2 | GNG12 | GNG5 | GRK1 | GRK5 | GSK3A | GSK3B | HEBP1 | HTR1B | INSR | ITPR3 | LANCL1 | LGR4 | LPAR1 | LPAR2 | LPAR4 | MAS1 | MC1R | MTNR1A | NMB | NME2 | NPR1 | OR5F1 | PARD3 | PAX8 | PDE4D | PDE6G | PICK1 | PIK3CA | PIK3CG | PIK3R1 | PPARG | PRKD3 | PSAP | PTGER3 | PTGIR | PTPN6 | RAPGEF4 | RASD1 | RGS1 | RGS2 | RGS20 | RHO | RHOA | RIC8B | SAG | SLC12A2 | SLC9A3R1 | SMO | SORT1 | SRC | SULT1A3 | TAS2R41 | TAS2R60 | TAS2R7 | TBXA2R | TULP3 | VAV1 | VN1R1 | GO:0008064 | 0.0140725464030086 | 116/8243 | 54/3250 | 0.0695473989186609 | 1 | F | F | F | F | regulation of actin polymerization or depolymerization | ACTN2 | ACTR2 | ACTR3 | ADD3 | ARF1 | ARHGAP35 | ARPC1A | ARPC1B | ARPC2 | ARPC3 | ARPC4 | ARPC5 | AVIL | BAG4 | BAIAP2 | BAIAP2L1 | BIN1 | CAPG | CAPZA1 | CAPZA2 | CAPZB | CCL21 | CDC42EP3 | CDC42EP4 | CFL1 | CFL2 | CTTN | DSTN | FCHSD1 | GRB2 | HAX1 | ICAM1 | LATS1 | LIMA1 | MTPN | MYO1C | NCK1 | PFN1 | PICK1 | PRKCD | PRKCE | RDX | RHOA | SPTAN1 | SPTBN1 | SPTBN2 | SWAP70 | TMOD3 | TMOD4 | TWF1 | TWF2 | VASP | WAS | WDR1 | GO:0060759 | 0.0140725464030086 | 116/8243 | 54/3250 | 0.0695473989186609 | 1 | F | F | F | F | regulation of response to cytokine stimulus | ABCE1 | AES | APOA1 | BIRC2 | BIRC3 | CASP8 | CAV1 | CBFB | CD74 | CDC37 | CYLD | DHX9 | FADD | GPS2 | GSTP1 | HIF1A | HPX | HSP90AB1 | HSPA1B | IKBKB | IKBKE | IKBKG | IRF7 | JAK1 | MAPK7 | MAVS | MED1 | NR1H2 | NR1H3 | OTUD4 | PAFAH1B1 | PARP14 | PIAS1 | PPARG | PTPN1 | PTPN6 | PXDN | RIPK1 | RIPK2 | RNF31 | RUNX1 | SOCS1 | SPATA2 | STAT1 | SUMO1 | TNF | TNFAIP3 | TNFRSF1A | TRADD | TRAF1 | TRAF2 | TREX1 | TRIM32 | UBE2K | GO:0030832 | 0.0140725464030086 | 116/8243 | 54/3250 | 0.0695473989186609 | 1 | F | F | F | F | regulation of actin filament length | ACTN2 | ACTR2 | ACTR3 | ADD3 | ARF1 | ARHGAP35 | ARPC1A | ARPC1B | ARPC2 | ARPC3 | ARPC4 | ARPC5 | AVIL | BAG4 | BAIAP2 | BAIAP2L1 | BIN1 | CAPG | CAPZA1 | CAPZA2 | CAPZB | CCL21 | CDC42EP3 | CDC42EP4 | CFL1 | CFL2 | CTTN | DSTN | FCHSD1 | GRB2 | HAX1 | ICAM1 | LATS1 | LIMA1 | MTPN | MYO1C | NCK1 | PFN1 | PICK1 | PRKCD | PRKCE | RDX | RHOA | SPTAN1 | SPTBN1 | SPTBN2 | SWAP70 | TMOD3 | TMOD4 | TWF1 | TWF2 | VASP | WAS | WDR1 | GO:0071260 | 0.00679364309110761 | 56/8243 | 28/3250 | 0.069604814996919 | 1 | F | F | F | F | cellular response to mechanical stimulus | AKT1 | ATP1A1 | BAG3 | BCL10 | CASP2 | CASP8 | CD40 | EGFR | FADD | GOT1 | HDAC4 | IRF1 | LTBR | MAP2K4 | MAP3K1 | MAP3K14 | MAP3K2 | MAPK3 | MAPK8 | MTPN | MYD88 | RAC1 | SLC9A1 | TGFB1 | TLR3 | TNFRSF1A | TNFRSF8 | TNFSF14 | GO:0006919 | 0.00679364309110761 | 56/8243 | 28/3250 | 0.069604814996919 | 1 | F | F | F | F | activation of cysteine-type endopeptidase activity involved in apoptotic process | AIFM1 | BAX | BBC3 | BCL2L13 | CASP10 | CASP8 | CASP9 | CYCS | DIABLO | EGLN3 | EIF2AK3 | FADD | FASLG | HSPD1 | HSPE1 | MAPT | P2RX1 | PDCD6 | PML | PPARG | RIPK1 | S100A9 | SMAD3 | SNCA | TNF | TRADD | TRAF2 | VCP | GO:0032481 | 0.00679364309110761 | 56/8243 | 28/3250 | 0.069604814996919 | 1 | F | F | F | F | positive regulation of type I interferon production | CREBBP | CTNNB1 | DDX3X | DHX36 | DHX9 | EP300 | FLOT1 | HMGB1 | HMGB2 | HSPD1 | IFI16 | IRF1 | IRF7 | MAVS | MYD88 | NFKB2 | POLR2E | POLR2L | PRKDC | PTPN22 | RELA | RIPK2 | STAT1 | STAT6 | TLR3 | TMEM173 | XRCC5 | XRCC6 | GO:0001959 | 0.0132233410166202 | 109/8243 | 51/3250 | 0.0697104037185707 | 1 | F | F | F | F | regulation of cytokine-mediated signaling pathway | ABCE1 | APOA1 | BIRC2 | BIRC3 | CASP8 | CAV1 | CBFB | CD74 | CDC37 | CYLD | FADD | GPS2 | GSTP1 | HIF1A | HPX | HSP90AB1 | HSPA1B | IKBKB | IKBKE | IKBKG | IRF7 | JAK1 | MAVS | MED1 | NR1H2 | NR1H3 | OTUD4 | PAFAH1B1 | PARP14 | PIAS1 | PPARG | PTPN1 | PTPN6 | PXDN | RIPK1 | RIPK2 | RNF31 | RUNX1 | SOCS1 | SPATA2 | STAT1 | SUMO1 | TNF | TNFAIP3 | TNFRSF1A | TRADD | TRAF1 | TRAF2 | TREX1 | TRIM32 | UBE2K | GO:0035295 | 0.0132233410166202 | 109/8243 | 51/3250 | 0.0697104037185707 | 1 | F | F | F | F | tube development | ACAT1 | ADA | AHI1 | AKR1B1 | AR | ASS1 | ATRX | CAT | CCNB1 | CHD8 | CLDN18 | COL2A1 | CTNNB1 | CXCL8 | CYP1A1 | DAB1 | DVL1 | EGFR | FGFR1 | FOXJ1 | HOXA5 | IFT122 | ITGB3 | LGR4 | MED1 | MKS1 | NPHP3 | OTC | PAX8 | PHGDH | PLXNA2 | PROX1 | RARA | RB1 | SDC1 | SDC4 | SERPINE2 | SMAD2 | SMAD3 | SMAD4 | SMAD5 | SMO | STAT1 | STIL | TCF21 | TGFB1 | TNF | TRAF3IP1 | UBB | WDR48 | WT1 | GO:0002683 | 0.0293582433580007 | 242/8243 | 107/3250 | 0.0700394412702048 | 1 | F | F | F | F | negative regulation of immune system process | A2M | ADA | ADCYAP1 | ADORA2A | ADTRP | AKT1 | ANXA1 | APCS | APOA1 | APOD | ARRB2 | ATM | BCL10 | BCL6 | BTK | C1QBP | CASP3 | CBFB | CCL21 | CD22 | CD74 | CDC73 | CLDN18 | CRK | CTNNB1 | DPF2 | DTX1 | DUSP1 | DUSP22 | ERBB2 | EZR | FADD | FAM19A3 | FBXO7 | FOXJ1 | GLMN | GPS2 | GPX1 | HAVCR2 | HIST1H4A | HLX | HMGB1 | HMOX1 | HOXA5 | HSPA9 | HTRA1 | ID2 | IFI16 | IFNA2 | IL13RA2 | IRF1 | IRF4 | ITCH | JAK3 | LGALS3 | LGR4 | MAD1L1 | MICA | MIF | MYC | NFE2L2 | NFKBIA | NME1 | NME2 | NPLOC4 | NR1H2 | NR1H3 | OTUD4 | PAG1 | PARP14 | PAWR | PCBP2 | PELI1 | PIAS3 | PIK3R1 | PPARG | PPM1B | PRDX2 | PRKAR1A | PRKDC | PSMA1 | PTPN22 | PTPN6 | RARA | RC3H1 | RPS19 | RUNX1 | SARM1 | SDC4 | SLAMF8 | SOCS1 | SOCS6 | STAT6 | TGFB1 | THBS1 | TLR3 | TMBIM6 | TNF | TNFAIP3 | TRAF3IP1 | TREX1 | TSC22D3 | UBASH3A | UBQLN1 | VSIG4 | ZBTB16 | ZBTB7B | GO:1905952 | 0.00958388936066966 | 79/8243 | 38/3250 | 0.0718326547020091 | 1 | F | F | F | F | regulation of lipid localization | ABCG1 | AKT1 | AKT2 | ANXA2 | APOA1 | APOE | C1QTNF1 | CD36 | CRP | CRY1 | FABP3 | FTO | GDF9 | GPS2 | HNRNPK | IKBKE | ITGAV | ITGB3 | KDM5B | MAP2K6 | MIF | NCOR1 | NFKBIA | NR1H2 | NR1H3 | P2RX4 | PCSK9 | PLA2G10 | PLA2G6 | PPARD | PPARG | PRELID1 | PRKCD | SIRT1 | THBS1 | TNF | TRIAP1 | VSTM2A | GO:2000059 | 0.0043673419871406 | 36/8243 | 19/3250 | 0.0718366866815473 | 1 | F | F | F | F | negative regulation of ubiquitin-dependent protein catabolic process | BAG5 | BAG6 | CCAR2 | CSNK2A2 | DDRGK1 | HSP90AB1 | OGT | PARK7 | PML | PSEN1 | RPL23 | SDCBP | SGTA | SMARCC1 | STYX | UBXN1 | UCHL5 | USP14 | USP7 | GO:0031623 | 0.0043673419871406 | 36/8243 | 19/3250 | 0.0718366866815473 | 1 | F | F | F | F | receptor internalization | ARRB1 | ARRB2 | CACNG2 | CACNG4 | CACNG5 | CAV1 | CD36 | CD81 | CLTC | CXCL8 | EZR | GRB2 | GRIA1 | HTR1B | NEDD4 | RAB31 | RAB5A | SCYL2 | SNCA | GO:0021782 | 0.0043673419871406 | 36/8243 | 19/3250 | 0.0718366866815473 | 1 | F | F | F | F | glial cell development | AGER | APP | ASCL1 | CDK5 | EIF2B1 | EIF2B3 | EIF2B5 | GFAP | GSTP1 | HDAC11 | ID2 | KRAS | LPAR1 | MED12 | PHGDH | PICK1 | TGFB1 | VIM | ZNF488 | GO:0046622 | 0.0043673419871406 | 36/8243 | 19/3250 | 0.0718366866815473 | 1 | F | F | F | F | positive regulation of organ growth | AKT1 | BASP1 | BMPR1A | CCNB1 | DDX39B | FGF2 | FGFR1 | HLX | MAPK1 | MAPK14 | PIM1 | PIN1 | PROX1 | RBPJ | SMO | TGFBR3 | WNT2 | WT1 | YAP1 | GO:2000677 | 0.0043673419871406 | 36/8243 | 19/3250 | 0.0718366866815473 | 1 | F | F | F | F | regulation of transcription regulatory region DNA binding | CTNNB1 | DDRGK1 | EP300 | FAM129B | GATA1 | H1F0 | NEUROD1 | PARK7 | PARP1 | PER2 | PSEN1 | RB1 | SIN3A | SRI | TGFB1 | TRAF6 | TWIST1 | ZBTB7A | ZMPSTE24 | GO:0007173 | 0.0043673419871406 | 36/8243 | 19/3250 | 0.0718366866815473 | 1 | F | F | F | F | epidermal growth factor receptor signaling pathway | ABL1 | AKT1 | ARF4 | BCAR1 | CBL | EFEMP1 | EGFR | GRB2 | GRB7 | HBEGF | IQGAP1 | PIK3C2A | PIK3CA | PIK3R1 | PLCG1 | PTK2 | SHC1 | SRC | TGFB1 | GO:0043030 | 0.0043673419871406 | 36/8243 | 19/3250 | 0.0718366866815473 | 1 | F | F | F | F | regulation of macrophage activation | AGER | APP | ATM | C1QA | CD74 | CEBPA | FAM19A3 | HAVCR2 | HSPD1 | LRRK2 | MIF | NR1H3 | PLA2G10 | SNCA | SPACA3 | THBS1 | TNF | TNIP2 | VSIG4 | GO:0046328 | 0.0155283270653888 | 128/8243 | 59/3250 | 0.0723628528349284 | 1 | F | F | F | F | regulation of JNK cascade | AGER | AKT1 | APP | AXIN1 | BIRC7 | CARD9 | CCL21 | CD27 | CD40LG | COPS5 | CYLD | DAXX | DBNL | DNAJA1 | DUSP19 | DUSP22 | DVL2 | DVL3 | EGFR | EPHA4 | GADD45G | GPS2 | GRIK2 | GSTP1 | HDAC3 | HMGB1 | HRAS | IGF1R | ITCH | LTBR | MAP2K4 | MAP2K7 | MAP3K13 | MAP3K2 | MAP3K4 | MAP4K1 | MAPK8IP3 | NCOR1 | NOD2 | PAFAH1B1 | PER1 | PTPN1 | PTPN22 | RB1CC1 | RIPK1 | RIPK2 | RPS3 | SDCBP | SERPINB3 | SLAMF1 | STK3 | TAOK3 | TGFBR3 | TLR3 | TNF | TRAF2 | TRAF6 | WNT16 | ZNF622 | GO:0016049 | 0.00764284847749606 | 63/8243 | 31/3250 | 0.0725328382458093 | 1 | F | F | F | F | cell growth | ABL1 | APP | CDK5 | CDKL3 | CTNNB1 | DCLK1 | DVL1 | EIF2AK4 | GDF9 | HDAC6 | HSP90AA1 | HSP90AB1 | IQGAP1 | ITGA4 | MAP1B | MAP2K4 | MEX3C | NIN | NRP2 | PDLIM5 | PLXNA1 | PRKCZ | PRMT2 | RARG | SEMA7A | SH3GL2 | SIRT6 | STK11 | ULK1 | USP9X | VCL | GO:0048147 | 0.00279024626956205 | 23/8243 | 13/3250 | 0.07275119915935 | 1 | F | F | F | F | negative regulation of fibroblast proliferation | BAX | C1QL4 | CDC73 | FTH1 | GSTP1 | MED31 | MYC | NUPR1 | PAWR | PPARG | SKI | TP53 | TRIM32 | GO:0030049 | 0.00279024626956205 | 23/8243 | 13/3250 | 0.07275119915935 | 1 | F | F | F | F | muscle filament sliding | ACTA1 | ACTN2 | DES | DMD | MYH6 | MYL2 | MYL6 | TNNT1 | TPM1 | TPM2 | TPM3 | TPM4 | VIM | GO:0009201 | 0.00279024626956205 | 23/8243 | 13/3250 | 0.07275119915935 | 1 | F | F | F | F | ribonucleoside triphosphate biosynthetic process | ALDOA | CTPS1 | CYC1 | DNAJC30 | IMPDH2 | LDHC | NME1 | NME2 | NME4 | SLC25A13 | STOML2 | TGFB1 | UCK2 | GO:0010939 | 0.00279024626956205 | 23/8243 | 13/3250 | 0.07275119915935 | 1 | F | F | F | F | regulation of necrotic cell death | BIRC2 | BIRC3 | CASP8 | CAV1 | CYLD | FADD | FTH1 | PELI1 | PPIF | RIPK1 | RIPK3 | SLC25A4 | SPATA2 | GO:0045737 | 0.00279024626956205 | 23/8243 | 13/3250 | 0.07275119915935 | 1 | F | F | F | F | positive regulation of cyclin-dependent protein serine/threonine kinase activity | AKT1 | CCNB1 | CCNT1 | CCNT2 | CCNY | CCNYL1 | CDK5R1 | CKS1B | CKS2 | EGFR | PROX1 | PSMD10 | SRC | GO:0033275 | 0.00279024626956205 | 23/8243 | 13/3250 | 0.07275119915935 | 1 | F | F | F | F | actin-myosin filament sliding | ACTA1 | ACTN2 | DES | DMD | MYH6 | MYL2 | MYL6 | TNNT1 | TPM1 | TPM2 | TPM3 | TPM4 | VIM | GO:0018196 | 0.00279024626956205 | 23/8243 | 13/3250 | 0.07275119915935 | 1 | F | F | F | F | peptidyl-asparagine modification | ASGR1 | ASGR2 | DAD1 | DDOST | DPM1 | HIF1AN | MCFD2 | RPN1 | RPN2 | SYVN1 | UBE2J1 | UGGT1 | VCP | GO:0051569 | 0.00279024626956205 | 23/8243 | 13/3250 | 0.07275119915935 | 1 | F | F | F | F | regulation of histone H3-K4 methylation | BCOR | BRCA1 | CTNNB1 | KDM1A | KMT2A | KMT2B | NELFE | PAXIP1 | PIH1D1 | PRMT6 | RTF1 | SMAD4 | SNW1 | GO:0061900 | 0.00279024626956205 | 23/8243 | 13/3250 | 0.07275119915935 | 1 | F | F | F | F | glial cell activation | ADORA2A | APP | AZU1 | CLU | EGFR | JUN | MAPT | NAMPT | PSEN1 | SMO | SNCA | TLR3 | TNF | GO:0006446 | 0.00655101298071091 | 54/8243 | 27/3250 | 0.0739074651534406 | 1 | F | F | F | F | regulation of translational initiation | AGO2 | DDX1 | DDX3X | EIF2AK3 | EIF2AK4 | EIF2B5 | EIF2S1 | EIF3E | EIF3H | EIF4EBP1 | EIF4G3 | EIF4H | EIF5 | EIF5B | HSPB1 | KHDRBS1 | NCBP1 | NCK1 | NPM1 | PAIP1 | PPP1CA | RBM4 | RPS6KB2 | RXRA | TNF | TPR | YTHDF1 | GO:2000106 | 0.00655101298071091 | 54/8243 | 27/3250 | 0.0739074651534406 | 1 | F | F | F | F | regulation of leukocyte apoptotic process | ADA | ANXA1 | AURKB | BAX | BCL10 | BCL6 | BIRC7 | BTK | CCL21 | CCR5 | CD27 | CD74 | FADD | HIF1A | IRF7 | JAK3 | LGALS3 | MIF | NOD2 | PRELID1 | PTCRA | PTEN | RIPK3 | SIRT1 | TCP1 | TP53 | TSC22D3 | GO:0043266 | 0.00545917748392576 | 45/8243 | 23/3250 | 0.0739911863557403 | 1 | F | F | F | F | regulation of potassium ion transport | ACTN2 | ADCYAP1 | AKAP9 | ANK3 | ATP1B3 | BIN1 | CAB39 | CAV1 | FHL1 | KCNAB1 | KCNAB2 | KCNIP2 | KIF5B | NEDD4 | NOS1AP | NOS3 | OXSR1 | PTEN | SUMO1 | WNK1 | WNK4 | WWP2 | YWHAE | GO:0010517 | 0.00545917748392576 | 45/8243 | 23/3250 | 0.0739911863557403 | 1 | F | F | F | F | regulation of phospholipase activity | ABL1 | ABL2 | ANXA1 | ARF4 | EGFR | ESR1 | FGF2 | FGFR1 | FGFR3 | GNA13 | GNA15 | GNAQ | HRAS | ITK | LPAR1 | LPAR2 | NTF3 | PLA2G1B | PLCG1 | PPT1 | PRKCZ | RGS2 | SNCA | GO:0030031 | 0.0215940798253063 | 178/8243 | 80/3250 | 0.0749102492035725 | 1 | F | F | F | F | cell projection assembly | ABL1 | ABLIM1 | ACTN2 | ACTR2 | ACTR3 | AHI1 | ARHGEF7 | ATP6V1D | ATXN10 | AVIL | BAG4 | CBY1 | CCDC88A | CCL21 | CDC42 | CENPJ | CEP290 | CEP89 | CTTN | DBNL | DCTN1 | DISC1 | DYNLL1 | E2F4 | EZR | FBF1 | FOPNL | FOXJ1 | FSCN1 | GOLPH3 | HDAC6 | ICK | IFT122 | IQCB1 | KLF5 | LPAR1 | MKS1 | MTSS1 | NCK1 | NEK1 | NPHP3 | NUP85 | OFD1 | ONECUT1 | PCM1 | POC1B | PTPN23 | PTPRO | RAB1A | RAC1 | RAP1A | RAP1B | RDX | RHOJ | RHOU | RPGRIP1L | SCLT1 | SDCBP | SEPT2 | SEPT6 | SEPT7 | SH2B1 | SLC9A3R1 | SNAP29 | SPERT | SRGAP2 | SSX2IP | STK26 | TCTN2 | TCTN3 | TEKT2 | TEKT4 | TMEM17 | TMEM216 | TMEM231 | TSGA10 | UBXN10 | VCL | WDR35 | ZMYND10 | GO:0048856 | 0.208783209996361 | 1721/8243 | 705/3250 | 0.0752034876165819 | 1 | F | F | F | F | anatomical structure development | AARS | ABAT | ABL1 | ACAT1 | ACD | ACKR2 | ACO2 | ACSL4 | ACTA1 | ACTA2 | ACTB | ACTL6A | ACTN2 | ADA | ADAMTS4 | ADCYAP1 | ADORA2A | AES | AFF4 | AFG3L2 | AGER | AHI1 | AHSG | AK4 | AKR1B1 | AKT1 | ALDH5A1 | ANXA1 | ANXA2 | ANXA6 | AP1B1 | APLP1 | APOA1 | APOD | APOL2 | APP | AR | ARF4 | ARHGAP26 | ARHGAP35 | ARHGEF7 | ARID4B | ARNT | ARNT2 | ARPC5 | ARRB2 | ASCL1 | ASGR2 | ASNS | ASS1 | ATF2 | ATF6 | ATG5 | ATIC | ATOH1 | ATP2A2 | ATRX | ATXN1 | ATXN10 | AVIL | AXIN1 | BAG3 | BAG6 | BANP | BASP1 | BAX | BCL2 | BCL2L1 | BCL6 | BCOR | BIRC2 | BMI1 | BMPR1A | BRAF | BRCA1 | BSG | BTK | CABP4 | CALM1 | CALM2 | CALM3 | CAMK2A | CAPN2 | CASP2 | CASP3 | CASP9 | CAT | CAV1 | CBFB | CBL | CCDC120 | CCDC155 | CCDC36 | CCDC85C | CCHCR1 | CCNB1 | CCNT2 | CD109 | CDC42 | CDH1 | CDK4 | CDK5 | CDK5R1 | CDKN1A | CEBPA | CENPF | CEP290 | CEP55 | CEP57 | CFL2 | CHD8 | CHRD | CHRM2 | CHRM3 | CISD2 | CKB | CLDN18 | COL2A1 | COL4A1 | COL4A3BP | COL5A1 | COLEC10 | COX17 | CREB1 | CRIP1 | CRIP2 | CRK | CRKL | CRMP1 | CRYAB | CSNK2A2 | CSNK2B | CTNNB1 | CTNND1 | CTSV | CUL7 | CXCL8 | CYP1A1 | CYP1A2 | DAB1 | DAB2 | DAD1 | DAZAP1 | DCAF7 | DCLK1 | DCN | DCTN1 | DDIT4 | DDRGK1 | DDX1 | DDX39B | DHCR24 | DLG4 | DMD | DNAJB1 | DNAJC19 | DNAJC30 | DPF2 | DPPA4 | DPYSL5 | DUSP22 | DVL1 | DVL2 | DYM | DYNLL1 | E2F1 | E2F4 | EBF1 | EDA | EED | EFEMP1 | EGFR | EGLN1 | EIF2AK3 | EIF2B1 | EIF2B3 | EIF2B5 | EIF2S2 | EIF4E | EIF4E2 | EIF4EBP1 | ELAVL1 | ELAVL3 | ELF3 | ENDOG | ENO3 | EP300 | ERBB2 | ERBB3 | ERG | ERRFI1 | ESR1 | ESRRA | ESRRB | ETS1 | EYA2 | EYA4 | EZH2 | EZR | FABP5 | FABP7 | FADD | FAM3C | FAM83H | FAM9B | FANCA | FANCC | FANCG | FASN | FGF2 | FGFR1 | FGFR3 | FHL1 | FHL2 | FKBP8 | FLNB | FOLR1 | FOS | FOXA1 | FOXJ1 | FOXK1 | FOXO1 | FPGS | FRAS1 | FREM2 | FTO | FYN | G6PD | GAA | GADD45G | GAP43 | GART | GATA1 | GDA | GDF9 | GFAP | GJA1 | GLRB | GLRX5 | GNA12 | GNA13 | GNAI3 | GNAO1 | GNAS | GNB1 | GNG12 | GNPNAT1 | GPI | GPSM1 | GPX1 | GPX4 | GRIP1 | GSK3A | GSK3B | GSS | GSTP1 | GYS1 | H2AFX | HAP1 | HBEGF | HCN1 | HDAC1 | HDAC11 | HDAC2 | HDAC4 | HDAC9 | HEXB | HEXIM1 | HIF1A | HLX | HMGB2 | HMGB3 | HMGCL | HMOX1 | HNF1A | HNF4A | HNRNPD | HNRNPU | HOXA5 | HOXB9 | HOXD10 | HPRT1 | HS6ST1 | HSD17B4 | HSF1 | HSF4 | HSP90AB1 | HSPA2 | HSPA5 | HSPG2 | HTRA1 | HTRA2 | ICAM1 | ICK | ID2 | IDH1 | IFI16 | IFT122 | IGF1R | IGSF8 | ILK | IMPDH2 | INSIG1 | INSIG2 | INSR | IQGAP1 | IREB2 | ITGA7 | ITGB1BP1 | ITGB3 | JMJD6 | JUN | JUNB | KANK2 | KCNAB1 | KDM1A | KDR | KEAP1 | KIF5B | KLF5 | KMT2A | KMT2B | KRAS | KRT17 | KRT31 | L3MBTL1 | LCP1 | LDHA | LEF1 | LGR4 | LIG4 | LMNA | LMO2 | LPAR1 | LRP6 | LRRK1 | LRRK2 | LTBP1 | LUM | MAD1L1 | MAP1B | MAP2K1 | MAP3K3 | MAP3K4 | MAPK1 | MAPK14 | MAPK3 | MAPK8 | MAPK8IP3 | MAPK9 | MAPKAP1 | MAPT | MAS1 | MATK | MBD3 | MBP | MC1R | MCM2 | MDM1 | MDM2 | MECP2 | MED1 | MED12 | MED21 | MED31 | MELK | MEOX2 | MIB1 | MITF | MKNK2 | MKS1 | MLH1 | MME | MPST | MSN | MTHFD1 | MTPN | MXD1 | MYH11 | MYH6 | MYL2 | MYL6 | MYO1D | MYO1E | MYOC | NAMPT | NCOA1 | NCOA4 | NDUFS3 | NDUFS6 | NEBL | NEDD4 | NELL1 | NEUROD1 | NF2 | NFATC2 | NFKB2 | NID1 | NIPBL | NKX2-1 | NME1 | NOS3 | NOSIP | NPHP3 | NPTX1 | NR0B2 | NR1H2 | NR1H3 | NR1I2 | NR5A2 | NRP2 | NSUN2 | NTF3 | NTRK1 | NUDC | NUP107 | NUP160 | NUP85 | NUP93 | NUPR1 | NXF2 | OBSL1 | OGDH | ONECUT1 | OPA1 | OTC | OXCT1 | P2RX2 | PABPC4 | PAFAH1B1 | PAK2 | PALB2 | PAPSS1 | PAPSS2 | PATZ1 | PAX8 | PAX9 | PAXIP1 | PCDHA3 | PCDHA4 | PCNA | PCSK9 | PDCD6 | PDE4D | PEBP1 | PEF1 | PFAS | PGM3 | PGM5 | PHB2 | PHC2 | PHC3 | PHF10 | PHGDH | PHOSPHO1 | PICK1 | PIK3CA | PIM1 | PIP4K2A | PKM | PLCG1 | PLS3 | PLXNA1 | PLXNA2 | PLXNB2 | POU2F3 | POU5F1 | PPARD | PPARG | PPARGC1A | PPAT | PPIB | PPP1CA | PPP2CA | PPP2R5D | PPP3CC | PPT1 | PRDX1 | PRDX2 | PRDX3 | PRDX4 | PRELID1 | PRKDC | PROX1 | PSAP | PSEN1 | PSMC3 | PSMC4 | PTEN | PTK2 | PTN | PTPN6 | PTPRF | PTPRO | PTPRR | QARS | QDPR | RAB18 | RAB1A | RAB1B | RAB3GAP1 | RAD23B | RADIL | RAF1 | RAP1A | RAP1B | RARA | RARG | RB1 | RB1CC1 | RBBP6 | RBPJ | RC3H1 | RDH13 | RDX | RECK | RELA | RGN | RGS2 | RHO | RHOA | RIPK3 | RNASEH2B | RNF2 | RNF8 | RPGRIP1 | RPGRIP1L | RPL10 | RPL24 | RPL38 | RPS4X | RRM1 | RRM2B | RTCB | RTL1 | RUNX1 | RUNX2 | RXRA | S100A1 | S100A7 | S100B | SBDS | SCEL | SCO2 | SDC1 | SDC4 | SDCBP2 | SDHA | SEC16A | SEC24B | SEC24C | SEC63 | SEMA7A | SEPT4 | SERF1A | SERPINE2 | SERPINH1 | SFN | SGPL1 | SH3GL1 | SH3GL2 | SH3GL3 | SHC1 | SIAH1 | SIN3A | SIRT1 | SKI | SMAD2 | SMAD3 | SMAD4 | SMAD5 | SMARCA2 | SMARCA4 | SMARCB1 | SMARCC1 | SMARCC2 | SMN1 | SMO | SMURF1 | SNAI2 | SNX17 | SOCS2 | SOD1 | SORT1 | SOX2 | SPATA2 | SPATA20 | SPATA24 | SPIN1 | SPRR2A | SPTLC2 | SRC | SRGAP2 | SRI | SRSF5 | ST8SIA4 | STAT1 | STAT3 | STIL | STIM1 | STK11 | STK3 | STK40 | STMN1 | STRBP | SULF2 | SUMO1 | SVIL | SYN1 | TAB1 | TAB2 | TAGLN | TAGLN3 | TAZ | TBC1D23 | TCF12 | TCF21 | TCF25 | TCOF1 | TFDP1 | TFIP11 | TGFB1 | TGFBR3 | THOC2 | THOC6 | THRA | TIAL1 | TIMP1 | TIMP2 | TLE1 | TLR3 | TMEM223 | TMEM231 | TMEM65 | TMOD3 | TNF | TNFRSF1A | TNN | TNP1 | TP53 | TP63 | TP73 | TPI1 | TPM1 | TRAF3IP1 | TRAF6 | TREX1 | TRIP13 | TSKU | TUFT1 | TULP3 | TWIST1 | TYMS | UBA6 | UBB | UBE2B | UBE2J1 | UBE3A | UCHL1 | UCHL5 | UFC1 | UFM1 | UGT8 | ULK1 | UNC119 | UPF2 | UQCRQ | USP2 | USP7 | VASH2 | VCAN | VDR | VGF | VIM | VKORC1 | VTN | WAS | WDR19 | WDR35 | WDR48 | WDTC1 | WNK1 | WNT2 | WT1 | WTAP | XBP1 | XIRP2 | XRCC1 | XRCC5 | XRCC6 | XYLT1 | YAP1 | YWHAE | YWHAH | YWHAQ | ZBTB16 | ZBTB17 | ZBTB42 | ZBTB7B | ZFAND5 | ZFP41 | ZIC1 | ZMPSTE24 | ZNF488 | ZSCAN2 | GO:1990090 | 0.0041247118767439 | 34/8243 | 18/3250 | 0.0762271889503596 | 1 | F | F | F | F | cellular response to nerve growth factor stimulus | AKT1 | APP | BRAF | CBL | CDC5L | CREB1 | CRK | E2F1 | EEF2 | EIF4A3 | HSPA5 | MAPT | NTRK1 | PTEN | RAB35 | RAP1A | SH3GL2 | USP8 | GO:0035418 | 0.0041247118767439 | 34/8243 | 18/3250 | 0.0762271889503596 | 1 | F | F | F | F | protein localization to synapse | CACNG2 | CACNG4 | CACNG5 | CDK5 | CLSTN1 | DLG2 | DLG3 | DLG4 | GRIP1 | HSPB1 | KIF5B | KIF5C | LRRC7 | MAPK8IP3 | NRXN1 | RAB11A | SYNJ2BP | TERF2 | GO:1903524 | 0.0041247118767439 | 34/8243 | 18/3250 | 0.0762271889503596 | 1 | F | F | F | F | positive regulation of blood circulation | ACE2 | ADA | AKT1 | ATP1A1 | ATP2A2 | CAV1 | CHRM3 | DBH | EGFR | FGB | GSK3A | ICAM1 | NOS1AP | PDE4D | RGS2 | RHOA | TBXA2R | TPM1 | GO:0060070 | 0.0041247118767439 | 34/8243 | 18/3250 | 0.0762271889503596 | 1 | F | F | F | F | canonical Wnt signaling pathway | CSNK1E | CTNNB1 | DVL1 | DVL2 | DVL3 | LEF1 | LGR4 | LRP6 | LRRK2 | MED12 | MITF | MYH6 | PTEN | RARG | RECK | SDC1 | STK11 | WNT2 | GO:0002009 | 0.0184398883901492 | 152/8243 | 69/3250 | 0.0762614775554464 | 1 | F | F | F | F | morphogenesis of an epithelium | AHI1 | AIRE | AR | BCL2 | BTRC | CFL1 | CLIC4 | COL4A1 | CSNK2B | CTNNB1 | CTNNBIP1 | DVL1 | DVL2 | EGFR | EPHA4 | ESR1 | FGF2 | FGFR1 | FOLR1 | FOXA1 | FRAS1 | FREM2 | GNA13 | GRB2 | HIF1A | HOXA5 | ILK | KDM5B | KRAS | LEF1 | LGR4 | LRP6 | MED1 | MIB1 | MKS1 | MTSS1 | MYC | NKX2-1 | NPHP3 | PAX8 | PHB2 | PLXNA1 | PML | PPP1CA | PRKD2 | PROX1 | PSEN1 | RARG | RBPJ | RHOA | RIPK4 | SERPINB5 | SMAD3 | SMAD4 | SMO | SRC | STARD13 | STIL | TCF21 | TGFB1 | TGM2 | TP63 | TRAF3IP1 | TRIM28 | VCL | VDR | WNK4 | WT1 | YAP1 | GO:0048869 | 0.178211816086376 | 1469/8243 | 604/3250 | 0.0762898856867405 | 1 | F | F | F | F | cellular developmental process | A2M | ABL1 | ABL2 | ACADVL | ACTA1 | ACTA2 | ACTN1 | ACTN2 | ADA | ADORA2A | ADRB2 | AFF4 | AFG3L2 | AGER | AGR2 | AICDA | AIMP2 | AKR1C2 | AKT1 | AKT2 | ALDH6A1 | ALDOC | ANK3 | ANKRD49 | ANKS4B | ANXA1 | ANXA2 | ANXA6 | ANXA7 | APBB1 | APC | APP | AR | ARHGAP35 | ARHGEF7 | ARID4B | ARNT | ASCL1 | ATF2 | ATG5 | ATM | ATOH1 | ATP2B2 | ATRX | BAG6 | BAIAP2 | BASP1 | BAX | BCL2 | BCL2L1 | BCL6 | BMPR1A | BRAF | BTK | BYSL | C1QA | C1QL1 | CABP4 | CALR | CAMK2A | CAND1 | CAP2 | CAPZB | CASP2 | CASP3 | CASP8 | CASP9 | CAT | CAV1 | CBFA2T3 | CBFB | CBR1 | CBY1 | CCDC155 | CCDC36 | CCHCR1 | CCL21 | CCNB1 | CD40LG | CD79B | CDC25B | CDC42 | CDC73 | CDH1 | CDH5 | CDK5 | CDK5R1 | CDKN1A | CDKN2C | CEBPA | CEBPE | CENPF | CEP290 | CEP57 | CES1 | CFTR | CHEK2 | CHRD | CLIC4 | CLTC | CLU | COL12A1 | COL14A1 | COL18A1 | COL2A1 | COL4A1 | COL4A2 | COL4A3BP | COL6A1 | COL6A2 | COPS2 | CPT1A | CREB1 | CRK | CRKL | CSNK1A1 | CTBP1 | CTNNB1 | CTSV | CTTN | CUL3 | CUL7 | CYLD | CYP1A1 | DAB2 | DAZAP1 | DBNL | DCLK1 | DDIT4 | DDX6 | DHRS7B | DHX9 | DLD | DLG4 | DMD | DMRT3 | DNAJA3 | DNASE2 | DNM1L | DOCK7 | DTX1 | DVL1 | DYNLL1 | E2F4 | EDA | EEF2 | EGFR | EID1 | EIF2AK3 | EIF2B1 | EIF2B3 | EIF2B5 | ELAVL3 | ELF3 | ELF4 | EP300 | EPHA4 | ERBB2 | ERBB3 | ERF | ERG | ESR1 | ESRRB | ETS1 | EYA2 | EZR | FABP7 | FADD | FAM129B | FAM83D | FAM9B | FANCC | FANCG | FASLG | FASN | FERMT2 | FGF2 | FGFR1 | FGFR3 | FHL1 | FHL2 | FIGNL1 | FIS1 | FKBP8 | FLNB | FLOT1 | FN1 | FOS | FOXA1 | FOXD4 | FOXD4L6 | FOXI2 | FOXJ1 | FOXK1 | FOXO1 | FYN | FZR1 | G6PD | GADD45G | GAP43 | GATA1 | GDF9 | GFAP | GLMN | GLO1 | GNA12 | GNA13 | GORASP2 | GOT1 | GPS2 | GPSM1 | GPX1 | GRID2 | GRK5 | GSK3B | GSTA1 | GSTA2 | GSTK1 | GSTP1 | GTSF1 | H2AFX | HAP1 | HBZ | HCN1 | HDAC1 | HDAC11 | HDAC2 | HDAC4 | HDAC5 | HDAC6 | HDAC7 | HDAC9 | HERC1 | HEXB | HIF1A | HLX | HMGB1 | HNF1A | HNF4A | HNRNPAB | HNRNPD | HNRNPH3 | HNRNPU | HOXA5 | HOXB5 | HOXD10 | HPRT1 | HRAS | HS6ST1 | HSD17B4 | HSF4 | HSP90AA1 | HSP90AB1 | HSPA2 | HSPA5 | HSPA9 | HSPE1 | HSPG2 | HTATIP2 | HTRA1 | HTRA2 | HUWE1 | IARS | ICAM1 | ID2 | IFI16 | IFNA2 | ILK | IQGAP1 | IREB2 | IRF1 | IRF4 | ITGA4 | ITGA7 | ITGAV | ITGB1BP1 | ITGB3 | ITGB5 | ITK | JAK3 | JMJD6 | JUN | JUNB | KAT8 | KCNAB1 | KCNAB2 | KDM1A | KDM5B | KDR | KLF5 | KLF6 | KMT2B | KRAS | L3MBTL3 | LAMB1 | LATS1 | LEF1 | LEMD2 | LGALS1 | LGALS3 | LGR4 | LIG4 | LIMS1 | LMNA | LOXL2 | LPAR1 | LRP6 | LRRK1 | LRRK2 | LTBR | MAP1B | MAP2K1 | MAP3K4 | MAPK1 | MAPK14 | MAPK3 | MAPK8 | MAPK9 | MAPT | MCOLN3 | MDFI | MECP2 | MED1 | MED12 | MED20 | METRN | METRNL | MGMT | MGST1 | MIF | MITF | MLH1 | MME | MSN | MTPN | MTSS1 | MYBBP1A | MYH11 | MYH6 | MYL2 | MYO1E | MYO5A | MYOC | NANOG | NCAPH2 | NCOA3 | NDUFS6 | NEBL | NELFB | NELL1 | NEUROD1 | NF2 | NFATC2 | NFKB2 | NIF3L1 | NIN | NIPBL | NKX2-1 | NPM1 | NPTX1 | NR1H2 | NR1H3 | NR1I2 | NR4A1 | NR5A2 | NSUN2 | NTF3 | NTRK1 | NUPR1 | OBSL1 | OGDH | ONECUT1 | OPA1 | P2RX2 | PABPC4 | PAFAH1B1 | PARD3 | PARP1 | PATZ1 | PAX8 | PAXIP1 | PCNA | PCSK9 | PDCD6 | PDE4D | PEBP1 | PEF1 | PER2 | PGK1 | PHB | PHGDH | PIAS1 | PICK1 | PIH1D1 | PIK3R1 | PIN1 | PIP4K2A | PIR | PITPNA | PML | POLDIP2 | POLM | POU5F1 | PPARD | PPARG | PPARGC1A | PPP1CC | PPT1 | PRDX3 | PRKACA | PRKCD | PRKDC | PRMT6 | PROX1 | PRRC2C | PSAP | PSEN1 | PSMB8 | PSMC2 | PSMD11 | PTEN | PTK2 | PTN | PTPN22 | PTPN6 | PTPRD | PTPRO | PUM1 | RAB10 | RAB1A | RAB1B | RAC1 | RADIL | RAF1 | RAI14 | RAP1A | RAP1B | RARA | RARG | RB1 | RBM4 | RBMX | RBPJ | RC3H1 | RDH13 | RDH14 | RDX | REST | RGS2 | RHBDD1 | RHOA | RIPK3 | RNF2 | RNF8 | ROMO1 | RP2 | RPGRIP1 | RPS14 | RPS19 | RRS1 | RTF1 | RTN4 | RUNX1 | RUNX2 | RXRA | S100A4 | S100A6 | S100A7 | S100B | SART3 | SCARA3 | SCEL | SCFD1 | SDC1 | SEMA7A | SEPT12 | SEPT4 | SEPT6 | SEPT7 | SERPINH1 | SF1 | SFMBT1 | SFN | SFXN1 | SGPL1 | SIN3A | SIRT1 | SKI | SLAMF8 | SLC9A1 | SLC9A3R1 | SMAD2 | SMAD3 | SMAD4 | SMAD5 | SMARCA2 | SMARCE1 | SMO | SMURF1 | SNAI2 | SND1 | SNX2 | SOAT1 | SOCS1 | SOD1 | SORT1 | SOX2 | SPATA20 | SPATA24 | SPI1 | SPINT2 | SPRR2A | SPRY2 | SQSTM1 | SRC | SRGAP2 | SRPK2 | STAT1 | STAT3 | STAT6 | STK11 | STK24 | STK26 | STK3 | STMN1 | STRBP | SULF2 | SYN1 | TAGLN | TAGLN2 | TCF21 | TCOF1 | TERF2 | TGFB1 | TGFBR3 | THOC2 | THRA | TIAL1 | TMEM231 | TMOD3 | TNF | TNFSF12 | TNK2 | TNN | TNP1 | TOLLIP | TOP2A | TP53 | TP63 | TPD52 | TPM4 | TRAF6 | TREX1 | TRIM28 | TRIM32 | TRIP13 | TSG101 | TSKU | TUBB3 | TUBB8 | TULP3 | TWIST1 | TXNIP | TYMS | UBB | UBE2J1 | UBE2V1 | UCHL1 | UGT8 | UNK | UQCRQ | USP42 | VAV1 | VCAM1 | VCAN | VDAC1 | VDR | VHL | VIM | VSTM2A | VTN | WDR1 | WDR19 | WDR77 | WNK1 | WNT16 | WNT2 | WT1 | WWOX | XBP1 | XRCC5 | YAP1 | YES1 | YY1 | ZAP70 | ZBTB16 | ZBTB7A | ZBTB7B | ZFP41 | ZHX1 | ZIC1 | ZMPSTE24 | ZNF488 | ZSCAN2 | GO:0002082 | 0.00254761615916535 | 21/8243 | 12/3250 | 0.0764278994606574 | 1 | F | F | F | F | regulation of oxidative phosphorylation | CCNB1 | COX7A2 | DNAJC30 | PARK7 | PDE12 | PGK1 | PGK2 | PPIF | RHOA | SHMT2 | SNCA | VCP | GO:0097120 | 0.00254761615916535 | 21/8243 | 12/3250 | 0.0764278994606574 | 1 | F | F | F | F | receptor localization to synapse | CACNG2 | CACNG4 | CACNG5 | CLSTN1 | DLG2 | DLG3 | DLG4 | GRIP1 | KIF5C | LRRC7 | RAB11A | SYNJ2BP | GO:0045648 | 0.00254761615916535 | 21/8243 | 12/3250 | 0.0764278994606574 | 1 | F | F | F | F | positive regulation of erythrocyte differentiation | ARNT | ETS1 | GATA1 | HIF1A | HMGB2 | HSPA1B | ID2 | ISG15 | MAPK14 | MED1 | STAT1 | STAT3 | GO:0051220 | 0.00254761615916535 | 21/8243 | 12/3250 | 0.0764278994606574 | 1 | F | F | F | F | cytoplasmic sequestering of protein | CCDC22 | DBN1 | FAF1 | G3BP2 | GOPC | KEAP1 | LATS1 | MDFI | NFKBIA | PSMD10 | SRI | YWHAB | GO:0051281 | 0.00254761615916535 | 21/8243 | 12/3250 | 0.0764278994606574 | 1 | F | F | F | F | positive regulation of release of sequestered calcium ion into cytosol | ABL1 | BAX | CALM1 | CALM2 | CALM3 | CXCL11 | GSTO1 | HAP1 | HTT | PLCG1 | SNCA | SRI | GO:1904292 | 0.00254761615916535 | 21/8243 | 12/3250 | 0.0764278994606574 | 1 | F | F | F | F | regulation of ERAD pathway | BAG6 | CAV1 | EDEM1 | NFE2L2 | RNF185 | SGTA | UBE2J1 | UBQLN1 | UBQLN2 | UBXN1 | USP14 | USP19 | GO:2001258 | 0.00254761615916535 | 21/8243 | 12/3250 | 0.0764278994606574 | 1 | F | F | F | F | negative regulation of cation channel activity | ANK3 | CALM1 | CALM2 | CALM3 | CAV1 | FKBP1A | FKBP1B | GSTO1 | KCNAB1 | SRI | SUMO1 | UBQLN1 | GO:0048660 | 0.0110396700230499 | 91/8243 | 43/3250 | 0.0775136339026613 | 1 | F | F | F | F | regulation of smooth muscle cell proliferation | AKR1B1 | AKT1 | ALOX12 | APOD | BMPR1A | CDKN1A | COMT | CTNNB1 | CTNNBIP1 | DDX39B | EGFR | FGF2 | GNAI2 | GNAI3 | GSTP1 | HBEGF | HDAC4 | HMOX1 | HTR1B | ID2 | ILK | JUN | MDM2 | NAMPT | NPR1 | NQO2 | PPARD | PPARG | PPARGC1A | PRKDC | PTEN | PTGIR | SF1 | SOD2 | STAT1 | TGM2 | THBS1 | TNF | TNFAIP3 | TPM1 | TRAF6 | XRCC5 | XRCC6 | GO:0050773 | 0.0118888754094383 | 98/8243 | 46/3250 | 0.0776926475624341 | 1 | F | F | F | F | regulation of dendrite development | ACTR2 | APOE | ARF1 | BCL11A | CAMK1D | CAMK2B | CARM1 | CDC20 | CDK5 | CDK5R1 | CDKL3 | CFL1 | CRK | CRKL | CUL7 | DBN1 | DHX36 | DNM1L | EPHA4 | EZH2 | GSK3B | HDAC2 | HECW1 | HECW2 | ILK | IQGAP1 | LPAR1 | LRRK2 | MAPK6 | NEDD4 | OBSL1 | OPA1 | PAFAH1B1 | PARP6 | PDLIM5 | PSEN1 | PTEN | PTPRD | RAB21 | RHOA | RTN4IP1 | SARM1 | SIPA1L1 | STK11 | YWHAH | ZMYND8 | GO:0022406 | 0.0167414776173723 | 138/8243 | 63/3250 | 0.0783763213312872 | 1 | F | F | F | F | membrane docking | ACTR1A | AHI1 | AKAP9 | CCP110 | CENPJ | CEP135 | CEP152 | CEP290 | CEP57 | CEP70 | CEP89 | CFTR | CHP1 | CKAP5 | CNTRL | CSNK1E | DCTN1 | DYNC1H1 | DYNC1I2 | DYNLL1 | EXOC6 | EZR | FBF1 | FGFR1OP | HSP90AA1 | ICAM1 | IQCB1 | MKS1 | MSN | OFD1 | PAFAH1B1 | PCM1 | PLK1 | PPP2R1A | PRKACA | PRKAR2B | PSEN2 | RAB10 | RAB11A | RAB3IP | RAB7A | RPGRIP1L | SCFD1 | SCLT1 | SNAP29 | SNX3 | STX5 | STXBP1 | STXBP2 | TCTN2 | TCTN3 | TMEM216 | TUBA1A | TUBA4A | TUBB | TUBB4A | TUBB4B | TUBG1 | VCAM1 | VPS18 | YKT6 | YWHAE | YWHAG | GO:0043279 | 0.00630838287031421 | 52/8243 | 26/3250 | 0.078507968687006 | 1 | F | F | F | F | response to alkaloid | ADA | ADORA2A | BCL2L1 | CACNG4 | CASP3 | CASP7 | DHX15 | EFTUD2 | FADD | GNAO1 | GOT2 | GPI | HDAC2 | HDAC5 | HSPA5 | HTR1B | HTR3A | ICAM1 | MDM2 | PPARG | PPARGC1A | PPP2R2A | PRKAA1 | PRKCE | RAD51 | RELA | GO:0014743 | 0.00521654737352906 | 43/8243 | 22/3250 | 0.0786264999326574 | 1 | F | F | F | F | regulation of muscle hypertrophy | CDK9 | CTDP1 | DDX39B | ERRFI1 | FOXO1 | G6PD | GLRX3 | GSK3A | LMNA | MTPN | P2RX4 | PARP1 | PIN1 | PTK2 | RGS2 | SLC9A1 | SMAD3 | SMAD4 | TNFRSF1A | TRIM63 | TWF1 | YY1 | GO:0061013 | 0.019046463666141 | 157/8243 | 71/3250 | 0.0788143190019965 | 1 | F | F | F | F | regulation of mRNA catabolic process | AGO2 | AKT1 | ANP32A | APEX1 | CIRBP | DCP2 | DHX36 | DHX9 | DIS3 | E2F1 | ELAVL1 | EXOSC1 | EXOSC5 | EXOSC6 | EXOSC9 | FTO | FXR2 | HNRNPD | HNRNPM | HNRNPU | HSPA8 | HSPB1 | IGF2BP1 | MAPK14 | MOV10 | MYD88 | NBAS | NPM1 | NUP214 | PABPC1 | PABPC4 | PAIP1 | PDE12 | PRKCD | PSMA1 | PSMA2 | PSMA3 | PSMA5 | PSMA6 | PSMB5 | PSMB6 | PSMB7 | PSMB8 | PSMC1 | PSMC2 | PSMC3 | PSMC4 | PSMC5 | PSMC6 | PSMD1 | PSMD10 | PSMD11 | PSMD12 | PSMD14 | PSMD2 | PSMD4 | PSMD9 | PSME2 | PSME3 | PUM1 | RC3H1 | SET | TARDBP | TBRG4 | TNPO1 | UBB | UBC | VIM | XPO1 | YWHAB | YWHAZ | GO:0048259 | 0.00824942375348781 | 68/8243 | 33/3250 | 0.0791320932270457 | 1 | F | F | F | F | regulation of receptor-mediated endocytosis | AHI1 | ANXA2 | APP | ARF1 | ARRB1 | ARRB2 | ATAD1 | ATXN2 | CBL | CCL21 | CLU | DAB2 | DLG4 | FLOT1 | HIST1H1A | HNRNPK | ITGB3 | NTF3 | PCSK9 | PICK1 | PPT1 | PSEN1 | RAB21 | RAC1 | SCYL2 | SDCBP | SH3GL2 | SH3GL3 | TNK2 | UBQLN2 | UNC119 | USP46 | VTN | GO:0006352 | 0.0181972582797525 | 150/8243 | 68/3250 | 0.0800548012387344 | 1 | F | F | F | F | DNA-templated transcription, initiation | AR | BAX | CBFB | CCNB1 | CDK4 | CDK8 | CDK9 | CDKN1A | CREBBP | E2F2 | E2F3 | ESR1 | ESR2 | ESRRA | ESRRB | ESRRG | GTF2A1 | GTF2E2 | GTF2F1 | GTF2F2 | GTF2I | HIST1H4A | HNF4A | HNF4G | MAPK3 | MECP2 | MED1 | MED12 | MED14 | MED17 | MED20 | MED23 | MED24 | MED31 | MED4 | MED6 | NR0B2 | NR1H2 | NR1H3 | NR1I2 | NR3C1 | NR4A1 | NR5A2 | NRBF2 | POLR2A | POLR2E | POLR2L | PPARD | PPARG | PPARGC1A | PPM1D | PTEN | RARA | RARG | RBPJ | RUNX2 | RXRA | RXRB | RXRG | SNW1 | TAF12 | TAF15 | TAF1A | TCF4 | THRA | TRIM28 | VDR | YAP1 | GO:0043523 | 0.0181972582797525 | 150/8243 | 68/3250 | 0.0800548012387344 | 1 | F | F | F | F | regulation of neuron apoptotic process | AARS | ADORA2A | AGAP2 | AIFM1 | APOE | ARRB1 | ARRB2 | ASCL1 | ATF2 | ATM | BACE1 | BAX | BCL2 | BCL2L1 | BRAF | CASP2 | CASP3 | CASP9 | CDC34 | CDK5 | CDK5R1 | CTNNB1 | EGLN3 | ERBB3 | FASLG | FIS1 | FYN | G6PD | GPI | GPX1 | GRID2 | GRIK2 | HDAC4 | HIF1A | HMOX1 | HRAS | ILK | JUN | KRAS | LANCL1 | LGMN | LIG4 | MAP2K4 | MECP2 | NAE1 | NMNAT1 | NQO1 | NQO2 | NTF3 | NTRK1 | PARK7 | PARP1 | PCSK9 | PIK3CA | PIN1 | PPARGC1A | PPT1 | PSEN1 | RHOA | SET | SNCA | SNX6 | SOD1 | SOD2 | SRPK2 | STXBP1 | TNF | UBE2M | GO:0002262 | 0.00230498604876865 | 19/8243 | 11/3250 | 0.0801261718071755 | 1 | F | F | F | F | myeloid cell homeostasis | ANXA1 | BAX | FANCC | GPI | HMOX1 | HOXB6 | IREB2 | MTHFD1 | PRDX1 | RPS24 | SOD1 | GO:0032770 | 0.00230498604876865 | 19/8243 | 11/3250 | 0.0801261718071755 | 1 | F | F | F | F | positive regulation of monooxygenase activity | AKT1 | APOE | CALM1 | CALM3 | HIF1A | KRAS | NOS1AP | PARK7 | S100A1 | TERF2 | TNF | GO:0060348 | 0.00230498604876865 | 19/8243 | 11/3250 | 0.0801261718071755 | 1 | F | F | F | F | bone development | DYM | GJA1 | GNAS | MYOC | PAPSS2 | PLS3 | PPIB | SMAD5 | SULF2 | TULP3 | VKORC1 | GO:0051385 | 0.00230498604876865 | 19/8243 | 11/3250 | 0.0801261718071755 | 1 | F | F | F | F | response to mineralocorticoid | AIFM1 | CALM1 | CALM3 | CDKN1A | CSN1S1 | FOS | HTR1B | KRAS | PARP1 | PEBP1 | SRC | GO:0035458 | 0.00230498604876865 | 19/8243 | 11/3250 | 0.0801261718071755 | 1 | F | F | F | F | cellular response to interferon-beta | CAPN2 | CDC34 | HTRA2 | IFI16 | IRF1 | NDUFA13 | STAT1 | TLR3 | TMEM173 | TREX1 | UBE2K | GO:1905332 | 0.00230498604876865 | 19/8243 | 11/3250 | 0.0801261718071755 | 1 | F | F | F | F | positive regulation of morphogenesis of an epithelium | ABL1 | AHI1 | ALOX12 | AR | CTNNB1 | LGR4 | PAX8 | SIRT6 | SMO | TGFB1 | WNT2 | GO:0016447 | 0.00230498604876865 | 19/8243 | 11/3250 | 0.0801261718071755 | 1 | F | F | F | F | somatic recombination of immunoglobulin gene segments | AICDA | ATAD5 | CD40LG | EXOSC6 | HSPD1 | LIG4 | MLH1 | MSH6 | PRKDC | RNF8 | SWAP70 | GO:0031338 | 0.00230498604876865 | 19/8243 | 11/3250 | 0.0801261718071755 | 1 | F | F | F | F | regulation of vesicle fusion | AKT2 | ANXA1 | ANXA2 | CPLX1 | CPLX3 | KIF5B | SEPT8 | STXBP1 | TBC1D4 | TRIM9 | VPS18 | GO:0006801 | 0.00230498604876865 | 19/8243 | 11/3250 | 0.0801261718071755 | 1 | F | F | F | F | superoxide metabolic process | ALOX12 | CCS | FANCC | NOS2 | NOS3 | NQO1 | PRDX1 | PRDX2 | SH3PXD2A | SOD1 | SOD2 | GO:0043032 | 0.00230498604876865 | 19/8243 | 11/3250 | 0.0801261718071755 | 1 | F | F | F | F | positive regulation of macrophage activation | AGER | APP | C1QA | CEBPA | FAM19A3 | HAVCR2 | HSPD1 | LRRK2 | SPACA3 | THBS1 | TNIP2 | GO:0010762 | 0.00230498604876865 | 19/8243 | 11/3250 | 0.0801261718071755 | 1 | F | F | F | F | regulation of fibroblast migration | AKT1 | ARHGEF7 | BAG4 | BRAF | FGF2 | GNA12 | ITGB1BP1 | PRKCE | SDC4 | TGFB1 | THBS1 | GO:0045600 | 0.0038820817663472 | 32/8243 | 17/3250 | 0.0809101088669871 | 1 | F | F | F | F | positive regulation of fat cell differentiation | AKT1 | CARM1 | CEBPA | CREB1 | HNRNPU | ID2 | KLF5 | MAPK14 | METRNL | PPARD | PPARG | SNAI2 | STK3 | VSTM2A | XBP1 | ZBTB16 | ZBTB7B | GO:0032722 | 0.0038820817663472 | 32/8243 | 17/3250 | 0.0809101088669871 | 1 | F | F | F | F | positive regulation of chemokine production | ADCYAP1 | AGER | APP | AZU1 | CD74 | DDX3X | HAVCR2 | HIF1A | HMOX1 | MAVS | MBP | MIF | RIPK2 | TLR3 | TNF | TRIM32 | TWIST1 | GO:2001259 | 0.0038820817663472 | 32/8243 | 17/3250 | 0.0809101088669871 | 1 | F | F | F | F | positive regulation of cation channel activity | ACTN2 | ADRB2 | ANK3 | CACNG2 | CACNG4 | CALM1 | CALM2 | CALM3 | GSTO1 | HAP1 | HTT | KCNIP2 | KIF5B | NOS1AP | S100A1 | STAC | STIM1 | GO:0090317 | 0.0038820817663472 | 32/8243 | 17/3250 | 0.0809101088669871 | 1 | F | F | F | F | negative regulation of intracellular protein transport | APOD | BAG3 | BAG4 | CD36 | CDK5 | CHP1 | GDI1 | INSIG1 | ITGB1BP1 | LRRK2 | PARK7 | PKIG | RANGAP1 | SP100 | TXN | UBE2J1 | USP17L2 | GO:0097064 | 0.0038820817663472 | 32/8243 | 17/3250 | 0.0809101088669871 | 1 | F | F | F | F | ncRNA export from nucleus | NPM1 | NUP107 | NUP153 | NUP160 | NUP205 | NUP214 | NUP85 | NUP88 | NUP93 | NUP98 | POM121 | RAE1 | RAN | SEC13 | SSB | TPR | XPOT | GO:0043113 | 0.0038820817663472 | 32/8243 | 17/3250 | 0.0809101088669871 | 1 | F | F | F | F | receptor clustering | APOE | CDK5 | DLG2 | DLG3 | DLG4 | DNAJA3 | DVL1 | GLRB | GRIK2 | IFT122 | ITGA4 | ITGB1BP1 | LRRC7 | NRXN1 | PICK1 | SYNJ2BP | WDR19 | GO:0006120 | 0.0038820817663472 | 32/8243 | 17/3250 | 0.0809101088669871 | 1 | F | F | F | F | mitochondrial electron transport, NADH to ubiquinone | COQ9 | DLD | NDUFA10 | NDUFA13 | NDUFA5 | NDUFA8 | NDUFA9 | NDUFAF1 | NDUFB1 | NDUFS1 | NDUFS2 | NDUFS3 | NDUFS6 | NDUFS7 | NDUFS8 | NDUFV1 | NDUFV3 | GO:0065007 | 0.732015043066845 | 6034/8243 | 2407/3250 | 0.0810978415323155 | 1 | F | F | F | F | biological regulation | A2M | AARS | AARSD1 | AASDH | ABAT | ABCB7 | ABCE1 | ABCG1 | ABHD14B | ABHD17A | ABI3BP | ABL1 | ABL2 | ACAA2 | ACACA | ACADVL | ACD | ACE2 | ACKR2 | ACLY | ACO1 | ACOT13 | ACOX1 | ACSL1 | ACTA1 | ACTA2 | ACTB | ACTG1 | ACTG2 | ACTL6A | ACTN1 | ACTN2 | ACTN4 | ACTR1A | ACTR2 | ACTR3 | ADA | ADCY6 | ADCYAP1 | ADD3 | ADH1A | ADH1B | ADH5 | ADORA2A | ADPRH | ADRB2 | ADRM1 | ADTRP | AEBP2 | AES | AFF1 | AFF4 | AFG3L2 | AFM | AGAP1 | AGAP2 | AGER | AGO1 | AGO2 | AGR2 | AGTRAP | AHCYL1 | AHI1 | AHNAK | AHSA1 | AHSG | AICDA | AIFM1 | AIMP2 | AIRE | AKAP8 | AKAP9 | AKR1B1 | AKR1B10 | AKR1B15 | AKR1C2 | AKT1 | AKT2 | AKT3 | AKTIP | ALAS1 | ALB | ALDH5A1 | ALDH7A1 | ALDH8A1 | ALDH9A1 | ALDOA | ALOX12 | ALOX5 | ALOX5AP | AMFR | ANAPC4 | ANK1 | ANK3 | ANKRD49 | ANP32A | ANXA1 | ANXA2 | ANXA5 | ANXA6 | ANXA7 | AP1G1 | AP3D1 | APBB1 | APBB2 | APBB3 | APC | APCS | APEX1 | API5 | APLP1 | APOA1 | APOD | APOE | APP | APRT | AR | ARAF | ARF1 | ARF4 | ARFGAP1 | ARFIP1 | ARHGAP1 | ARHGAP19 | ARHGAP26 | ARHGAP35 | ARHGDIA | ARHGEF7 | ARHGEF9 | ARID3B | ARID4B | ARMC10 | ARNT | ARNT2 | ARPC1A | ARPC1B | ARPC2 | ARPC3 | ARPC4 | ARPC5 | ARR3 | ARRB1 | ARRB2 | ASB15 | ASB16 | ASB6 | ASB9 | ASCC1 | ASCC2 | ASCL1 | ASGR2 | ASNS | ASPSCR1 | ASS1 | ATAD1 | ATAD3A | ATAD5 | ATCAY | ATF2 | ATF6 | ATF6B | ATF7 | ATG5 | ATL3 | ATM | ATOH1 | ATOX1 | ATP12A | ATP13A2 | ATP1A1 | ATP1B3 | ATP2A2 | ATP2B2 | ATP4A | ATP6V1A | ATP6V1B1 | ATP6V1C1 | ATP6V1D | ATP6V1E1 | ATP9A | ATRX | ATXN1 | ATXN2 | AUP1 | AURKB | AVIL | AXIN1 | AZU1 | B3GAT3 | BABAM1 | BACE1 | BACH2 | BAG1 | BAG2 | BAG3 | BAG4 | BAG5 | BAG6 | BAIAP2 | BAIAP2L1 | BANF1 | BANP | BASP1 | BAX | BBC3 | BCAR1 | BCAR3 | BCAT2 | BCL10 | BCL11A | BCL2 | BCL2L1 | BCL2L13 | BCL2L2 | BCL6 | BCL6B | BCOR | BDKRB2 | BIN1 | BIRC2 | BIRC3 | BIRC5 | BIRC7 | BIRC8 | BLZF1 | BMI1 | BMPR1A | BNIPL | BOLA1 | BOLA3 | BRAF | BRCA1 | BRD2 | BRD3 | BRD4 | BSG | BTBD9 | BTK | BTN2A1 | BTRC | BUD31 | C1QA | C1QBP | C1QL4 | C1QTNF1 | C1R | C4A | CA8 | CAB39 | CABP4 | CACNA1A | CACNG2 | CACNG4 | CACNG5 | CALCOCO2 | CALM1 | CALM2 | CALM3 | CALR | CAMK1D | CAMK2A | CAMK2B | CAMKV | CAND1 | CANX | CAP2 | CAPG | CAPN1 | CAPN2 | CAPNS1 | CAPZA1 | CAPZA2 | CAPZB | CARD9 | CARM1 | CASP10 | CASP2 | CASP3 | CASP8 | CASP9 | CAT | CAV1 | CBFA2T3 | CBFB | CBL | CBLB | CBLC | CBX4 | CBX7 | CBX8 | CBY1 | CCAR2 | CCDC22 | CCDC36 | CCDC68 | CCDC8 | CCDC88A | CCL21 | CCL22 | CCNB1 | CCNDBP1 | CCNT1 | CCNT2 | CCNY | CCNYL1 | CCP110 | CCR1 | CCR3 | CCR4 | CCR5 | CCS | CCT2 | CCT3 | CCT4 | CCT5 | CCT6A | CCT7 | CCT8 | CD109 | CD2 | CD209 | CD22 | CD27 | CD2BP2 | CD36 | CD40 | CD40LG | CD6 | CD74 | CD79B | CD81 | CDA | CDC14B | CDC16 | CDC20 | CDC25A | CDC25B | CDC25C | CDC27 | CDC34 | CDC37 | CDC42 | CDC42EP3 | CDC42EP4 | CDC42SE2 | CDC5L | CDC73 | CDH1 | CDH5 | CDK15 | CDK16 | CDK19 | CDK2 | CDK4 | CDK5 | CDK5R1 | CDK8 | CDK9 | CDKL3 | CDKN1A | CDKN2C | CEBPA | CEBPE | CEL | CENPF | CENPJ | CENPV | CEP135 | CEP152 | CEP290 | CEP55 | CEP57 | CEP70 | CES1 | CFL1 | CFL2 | CFTR | CGN | CGRRF1 | CHCHD10 | CHCHD2 | CHCHD3 | CHD1L | CHD3 | CHD4 | CHD8 | CHDH | CHEK2 | CHGB | CHMP2A | CHORDC1 | CHP1 | CHRD | CHRM2 | CHRM3 | CHRNA9 | CHRND | CHRNE | CHRNG | CIRBP | CISD1 | CISD2 | CKAP5 | CKB | CKS1B | CKS2 | CLDN18 | CLEC11A | CLIC1 | CLIC3 | CLIC4 | CLK1 | CLNK | CLNS1A | CLPX | CLSTN1 | CLTC | CLU | CMTM5 | CNDP1 | CNTRL | CNTROB | COA3 | COG3 | COL18A1 | COL2A1 | COL4A1 | COL4A2 | COL4A3BP | COL5A1 | COLEC10 | COLGALT1 | COMMD1 | COMT | COPA | COPS2 | COPS5 | COQ3 | COQ7 | CORT | COX11 | COX17 | COX19 | COX6A1 | COX7A2 | CPLX1 | CPLX3 | CPNE3 | CPSF7 | CPT1A | CPT2 | CRBN | CREB1 | CREB3 | CREBBP | CRIP1 | CRIP2 | CRK | CRKL | CRMP1 | CROCC | CRP | CRY1 | CRY2 | CRYAB | CSMD1 | CSNK1A1 | CSNK1E | CSNK2A2 | CSNK2B | CTBP1 | CTCF | CTDP1 | CTH | CTNNB1 | CTNNBIP1 | CTNND1 | CTSA | CTSC | CTSL | CTSV | CTTN | CUL1 | CUL2 | CUL3 | CUL4B | CUL5 | CUL7 | CUTC | CXCL11 | CXCL5 | CXCL6 | CXCL8 | CYCS | CYLD | CYP1A1 | CYP1A2 | DAB1 | DAB2 | DAD1 | DAP3 | DAPK1 | DAPP1 | DAXX | DAZAP1 | DBF4B | DBH | DBN1 | DBNDD2 | DBNL | DCLK1 | DCN | DCP2 | DCTN1 | DCUN1D1 | DDA1 | DDB1 | DDB2 | DDIT4 | DDRGK1 | DDX1 | DDX39B | DDX3X | DDX6 | DECR1 | DEDD | DENND1A | DEPDC7 | DERL1 | DES | DGKE | DGKZ | DGUOK | DHCR24 | DHX36 | DHX9 | DIABLO | DIDO1 | DIS3 | DISC1 | DLD | DLG2 | DLG3 | DLG4 | DMAP1 | DMD | DMRT3 | DNA2 | DNAJA1 | DNAJA2 | DNAJA3 | DNAJA4 | DNAJB1 | DNAJB11 | DNAJB2 | DNAJC10 | DNAJC19 | DNAJC30 | DNAJC7 | DNAJC9 | DNASE2 | DNM1L | DOCK5 | DOCK7 | DOK1 | DPEP1 | DPF2 | DPYSL5 | DRG2 | DROSHA | DSTN | DTX1 | DTX2 | DUSP1 | DUSP12 | DUSP14 | DUSP16 | DUSP19 | DUSP22 | DUSP7 | DVL1 | DVL2 | DVL3 | DYNC1H1 | DYNC1I2 | DYNC1LI1 | DYNLL1 | DYNLT1 | DYRK2 | E2F1 | E2F2 | E2F3 | E2F4 | E2F6 | EAF2 | EBF1 | ECD | ECSIT | EDA | EDC4 | EDEM1 | EED | EEF1A1 | EEF1A2 | EEF1D | EEF2 | EFEMP1 | EGFR | EGLN1 | EGLN3 | EHD4 | EHMT1 | EHMT2 | EID1 | EIF2AK3 | EIF2AK4 | EIF2B1 | EIF2B3 | EIF2B5 | EIF2S1 | EIF3A | EIF3D | EIF3E | EIF3H | EIF4A3 | EIF4E | EIF4E2 | EIF4EBP1 | EIF4ENIF1 | EIF4G3 | EIF4H | EIF5 | EIF5A | EIF5B | EIF6 | ELAVL1 | ELF3 | ELF4 | ELMOD3 | EMILIN2 | ENDOG | ENO1 | EP300 | EPHA4 | EPHA6 | EPHB6 | EPM2A | EPPIN | EPPK1 | EPRS | ERBB2 | ERBB3 | ERCC5 | ERF | ERG | ERLIN2 | ERRFI1 | ESR1 | ESR2 | ESRRA | ESRRB | ESRRG | ETS1 | EVC2 | EWSR1 | EXOSC1 | EXOSC5 | EXOSC6 | EXOSC9 | EXTL2 | EYA2 | EYA4 | EZH2 | EZR | F5 | F9 | FABP1 | FABP3 | FABP5 | FABP7 | FADD | FAF1 | FAF2 | FAM129B | FAM192A | FAM19A3 | FAM3C | FAM49B | FAM83D | FAM83H | FAM98A | FANCA | FANCC | FANCI | FANCM | FASLG | FASN | FBP1 | FBRS | FBXL12 | FBXO6 | FBXO7 | FBXW11 | FBXW5 | FCGR1A | FCHSD1 | FCN1 | FDPS | FDXR | FEN1 | FERMT2 | FGB | FGF2 | FGFR1 | FGFR1OP | FGFR3 | FH | FHL1 | FHL2 | FIBP | FIGNL1 | FIS1 | FKBP14 | FKBP1A | FKBP1B | FKBP8 | FKBPL | FLNB | FLOT1 | FN1 | FOLR1 | FOS | FOXA1 | FOXD4 | FOXD4L6 | FOXI2 | FOXJ1 | FOXK1 | FOXK2 | FOXM1 | FOXO1 | FSCN1 | FSD1 | FTH1 | FTL | FTO | FUBP1 | FUBP3 | FUS | FXR2 | FYN | FZR1 | G3BP1 | G3BP2 | G6PD | GAA | GABPB1 | GABRA6 | GABRR1 | GAD2 | GADD45G | GAK | GAP43 | GAPDH | GAPDHS | GAPVD1 | GAR1 | GATA1 | GBP2 | GBP5 | GDF9 | GDI1 | GDI2 | GEMIN5 | GFAP | GFPT1 | GGA1 | GGPS1 | GGT1 | GIGYF1 | GIT1 | GJA1 | GKAP1 | GLA | GLMN | GLO1 | GLP1R | GLRB | GLRX | GLRX3 | GLRX5 | GLS | GLS2 | GLUL | GMDS | GNA12 | GNA13 | GNA15 | GNAI1 | GNAI2 | GNAI3 | GNAO1 | GNAQ | GNAS | GNAZ | GNB1 | GNB2 | GNG12 | GNG5 | GNL1 | GOLGA2 | GOLGA4 | GOLPH3 | GOLT1B | GOPC | GOT1 | GOT2 | GPC5 | GPD1 | GPD1L | GPI | GPS2 | GPSM1 | GPX1 | GPX4 | GRB2 | GRB7 | GRIA1 | GRID2 | GRIK2 | GRIP1 | GRK1 | GRK5 | GRPEL1 | GSK3A | GSK3B | GSPT1 | GSPT2 | GSR | GSTA2 | GSTO1 | GSTP1 | GSTZ1 | GTF2A1 | GTF2F1 | GTF2F2 | GTF2I | GZMA | H1F0 | H1FX | H2AFX | H2AFZ | H3F3A | HAAO | HADH | HAP1 | HAVCR2 | HAX1 | HBEGF | HBZ | HCN1 | HCN2 | HCN3 | HDAC1 | HDAC2 | HDAC3 | HDAC4 | HDAC5 | HDAC6 | HDAC7 | HDAC9 | HDGF | HEBP1 | HECW1 | HECW2 | HELZ | HELZ2 | HERC1 | HEXB | HEXIM1 | HGS | HIF1A | HIF1AN | HINT1 | HIPK4 | HIST1H1A | HIST1H2AB | HIST1H2BA | HIST1H3A | HIST1H4A | HIST2H3A | HIST3H3 | HIVEP1 | HK1 | HK2 | HLA-A | HLA-B | HLA-DQA1 | HLA-DRA | HLA-DRB1 | HLA-DRB3 | HLA-DRB4 | HLA-DRB5 | HLX | HMGB1 | HMGB2 | HMGB3 | HMMR | HMOX1 | HNF1A | HNF4A | HNF4G | HNRNPA1 | HNRNPA2B1 | HNRNPAB | HNRNPD | HNRNPDL | HNRNPF | HNRNPH1 | HNRNPK | HNRNPL | HNRNPM | HNRNPU | HOXA5 | HOXB5 | HOXB6 | HOXB9 | HOXD10 | HPRT1 | HPX | HRAS | HRNR | HSD17B14 | HSD17B4 | HSD17B6 | HSD17B8 | HSF1 | HSF4 | HSP90AA1 | HSP90AB1 | HSP90AB3P | HSP90AB4P | HSP90B1 | HSPA14 | HSPA1B | HSPA1L | HSPA2 | HSPA5 | HSPA8 | HSPA9 | HSPB1 | HSPB2 | HSPBP1 | HSPD1 | HSPE1 | HSPG2 | HSPH1 | HTATIP2 | HTR1B | HTR3A | HTR3E | HTRA1 | HTRA2 | HTT | HUWE1 | HYPK | IARS | IBTK | ICAM1 | ICK | ID2 | IDE | IDH1 | IDH2 | IFI16 | IFIT1 | IFITM3 | IFNA2 | IFNAR1 | IFT122 | IGBP1 | IGF1R | IGF2BP1 | IGFBP7 | IGHV3-13 | IGHV3-23 | IGHV3-30 | IGKV3-20 | IGLC7 | IK | IKBKB | IKBKE | IKBKG | IKZF5 | IL13RA2 | IL32 | IL3RA | ILF2 | ILF3 | ILK | IMMT | IMPA1 | IMPA2 | ING1 | ING5 | INSIG1 | INSIG2 | INSR | IP6K1 | IPO5 | IPO7 | IPO8 | IQCB1 | IQGAP1 | IREB2 | IRF1 | IRF4 | IRF7 | ISG15 | ITCH | ITGA4 | ITGA7 | ITGAV | ITGB1BP1 | ITGB3 | ITGB3BP | ITGB5 | ITK | ITPR1 | ITPR2 | ITPR3 | JAK1 | JAK3 | JMJD6 | JUN | JUNB | JUND | JUP | KANK2 | KAT5 | KAT7 | KAT8 | KBTBD7 | KCNA4 | KCNAB1 | KCNAB2 | KCNIP2 | KCNK1 | KCNK16 | KCNS3 | KCTD17 | KDM1A | KDM2A | KDM4D | KDM5B | KDM5C | KDM8 | KDR | KEAP1 | KHDRBS1 | KIF11 | KIF13B | KIF2C | KIF5B | KIR3DL2 | KLF5 | KLF6 | KLHDC10 | KLHL20 | KLHL22 | KLRC2 | KMT2A | KMT2B | KMT2C | KPNA1 | KPNA2 | KPNB1 | KRAS | KRT17 | KSR1 | L3MBTL1 | L3MBTL2 | L3MBTL3 | L3MBTL4 | LAMB1 | LAMP3 | LANCL1 | LARP4 | LARS | LASP1 | LAT | LATS1 | LCP1 | LCP2 | LDHA | LDLRAD4 | LEF1 | LEMD2 | LEMD3 | LETM1 | LGALS1 | LGALS3 | LGALS3BP | LGMN | LGR4 | LIG4 | LIMA1 | LIMS1 | LMNA | LMNB1 | LMO2 | LMO7 | LOXL2 | LPAR1 | LPAR2 | LPAR4 | LPIN3 | LRFN1 | LRIF1 | LRP6 | LRPPRC | LRRC7 | LRRK1 | LRRK2 | LRRN2 | LRSAM1 | LSM1 | LSM3 | LSM4 | LSM5 | LTBP1 | LTBR | LTC4S | LUM | LURAP1 | LYPLA1 | LZTR1 | MAD1L1 | MAD2L1 | MAF1 | MAGI1 | MAOA | MAP1B | MAP2K1 | MAP2K3 | MAP2K4 | MAP2K6 | MAP2K7 | MAP3K1 | MAP3K13 | MAP3K14 | MAP3K2 | MAP3K3 | MAP3K4 | MAP4K1 | MAPK1 | MAPK14 | MAPK15 | MAPK3 | MAPK4 | MAPK6 | MAPK7 | MAPK8 | MAPK8IP3 | MAPK9 | MAPKAP1 | MAPT | MARCH5 | MARCKSL1 | MAS1 | MASP1 | MASP2 | MAST1 | MAST3 | MAT2B | MATK | MATR3 | MAVS | MBD3 | MBP | MC1R | MCC | MCM2 | MCM7 | MCOLN3 | MCTS1 | MDC1 | MDFI | MDM1 | MDM2 | MDM4 | ME1 | MECP2 | MED1 | MED12 | MED14 | MED17 | MED20 | MED21 | MED23 | MED24 | MED31 | MED4 | MED6 | MELK | MEOX2 | MEPCE | METAP2 | METRN | METRNL | METTL23 | MEX3C | MGA | MGMT | MIB1 | MICA | MICU1 | MICU2 | MIF | MITF | MKNK2 | MKS1 | MLH1 | MLLT1 | MLLT11 | MLLT3 | MLST8 | MME | MMP26 | MMRN1 | MOB1A | MOB1B | MORF4L1 | MORF4L2 | MOV10 | MPC2 | MPP2 | MRGBP | MRPL12 | MSH6 | MSN | MTA1 | MTA3 | MTAP | MTCH2 | MTERF1 | MTHFD1 | MTNR1A | MTPN | MTRR | MTSS1 | MTX1 | MVD | MXD1 | MYBBP1A | MYC | MYD88 | MYH6 | MYL2 | MYL9 | MYO1C | MYO1D | MYO1E | MYO5A | MYO6 | MYOC | NACC1 | NAE1 | NAMPT | NANOG | NAP1L1 | NBAS | NCBP1 | NCDN | NCK1 | NCOA1 | NCOA2 | NCOA3 | NCOA4 | NCOR1 | NCOR2 | NDRG1 | NDUFA13 | NDUFAF2 | NDUFS1 | NDUFS3 | NEDD4 | NEDD8 | NEK1 | NEK7 | NELFB | NELFCD | NELFE | NELL1 | NEUROD1 | NF2 | NFATC1 | NFATC2 | NFE2L2 | NFKB2 | NFKBIA | NID1 | NIF3L1 | NIN | NIPBL | NKRF | NKX2-1 | NLK | NMB | NMD3 | NME1 | NME2 | NMNAT1 | NMRK2 | NNT | NOD2 | NOP10 | NOS1AP | NOS2 | NOS3 | NOSIP | NOSTRIN | NPEPPS | NPHP3 | NPLOC4 | NPM1 | NPR1 | NPTX1 | NQO1 | NQO2 | NR0B2 | NR1H2 | NR1H3 | NR1I2 | NR3C1 | NR4A1 | NR5A2 | NRBF2 | NRP2 | NRXN1 | NSFL1C | NSMCE4A | NSUN2 | NTF3 | NTRK1 | NUBP1 | NUCB1 | NUDT6 | NUFIP1 | NUMA1 | NUP107 | NUP153 | NUP160 | NUP205 | NUP214 | NUP85 | NUP88 | NUP93 | NUP98 | NUPR1 | NUSAP1 | OAS2 | OAZ3 | OBSL1 | OFD1 | OGFOD1 | OGT | ONECUT1 | OPA1 | OPTN | OR5F1 | ORM1 | OTC | OTUB1 | OTUD4 | OTUD5 | OVGP1 | OXA1L | OXCT1 | OXSR1 | P2RX1 | P2RX2 | P2RX4 | P4HB | PA2G4 | PABPC1 | PABPC4 | PACSIN3 | PAFAH1B1 | PAG1 | PAH | PAIP1 | PAK2 | PAK6 | PALB2 | PAM16 | PAN2 | PANX3 | PAPLN | PAPSS2 | PARD3 | PARD6A | PARK7 | PARP1 | PARP14 | PARP6 | PATZ1 | PAWR | PAX8 | PAX9 | PAXIP1 | PC | PCBD1 | PCBP1 | PCBP2 | PCGF1 | PCGF5 | PCGF6 | PCM1 | PCNA | PCSK9 | PCTP | PDCD10 | PDCD6 | PDCD6IP | PDCL | PDE12 | PDE4D | PDE4DIP | PDE6G | PDF | PDIA3 | PDIA4 | PDIA6 | PDK2 | PDLIM5 | PDSS2 | PEBP1 | PEF1 | PELI1 | PER1 | PER2 | PEX11B | PEX19 | PEX5 | PFDN2 | PFKL | PFKM | PFN1 | PGAM1 | PGAM2 | PGAM5 | PGK1 | PGK2 | PGP | PHB | PHB2 | PHC3 | PHF10 | PHF20L1 | PHF21A | PHGDH | PHOSPHO1 | PIANP | PIAS1 | PIAS3 | PICK1 | PIGU | PIH1D1 | PIK3C2A | PIK3CA | PIK3CG | PIK3R1 | PIK3R3 | PIKFYVE | PIM1 | PIN1 | PINX1 | PIP4K2A | PIR | PITPNA | PKIG | PKM | PKN2 | PLA2G10 | PLA2G1B | PLA2G2A | PLA2G6 | PLAUR | PLCG1 | PLEKHF1 | PLGRKT | PLK1 | PLSCR1 | PLXNA1 | PLXNA2 | PLXNB2 | PML | PNKP | PNMA1 | PNP | POC1B | POLA2 | POLD1 | POLDIP2 | POLDIP3 | POLE2 | POLE3 | POLR2A | POLR2E | POLR2L | POLR2M | POM121 | POT1 | POTEF | POTEI | POU2F3 | POU5F1 | PPA2 | PPARD | PPARG | PPARGC1A | PPAT | PPEF1 | PPIA | PPIB | PPID | PPIE | PPIF | PPM1A | PPM1B | PPM1D | PPM1K | PPME1 | PPP1CA | PPP1CB | PPP1CC | PPP1R11 | PPP1R12A | PPP1R12B | PPP1R14A | PPP1R16A | PPP1R2 | PPP1R3B | PPP1R3C | PPP1R8 | PPP2CA | PPP2CB | PPP2R1A | PPP2R1B | PPP2R2A | PPP2R2D | PPP2R5D | PPP3CC | PPP4R2 | PPP5C | PPP6R1 | PPT1 | PQLC1 | PRCC | PRCP | PRDX1 | PRDX2 | PRDX3 | PRDX4 | PRDX5 | PRDX6 | PRELID1 | PREPL | PRKAA1 | PRKAA2 | PRKAB2 | PRKACA | PRKAG1 | PRKAR1A | PRKAR2B | PRKCD | PRKCE | PRKCSH | PRKCZ | PRKD1 | PRKD2 | PRKD3 | PRKDC | PRMT2 | PRMT3 | PRMT6 | PROX1 | PROZ | PRPF19 | PRPF40A | PRRC1 | PSAP | PSEN1 | PSEN2 | PSMA1 | PSMA2 | PSMA3 | PSMA5 | PSMA6 | PSMB5 | PSMB6 | PSMB7 | PSMB8 | PSMC1 | PSMC2 | PSMC3 | PSMC4 | PSMC5 | PSMC6 | PSMD1 | PSMD10 | PSMD11 | PSMD12 | PSMD14 | PSMD2 | PSMD4 | PSMD9 | PSME2 | PSME3 | PSTPIP1 | PTCRA | PTEN | PTGDS | PTGER3 | PTGES | PTGES2 | PTGES3 | PTGIR | PTK2 | PTN | PTPMT1 | PTPN1 | PTPN14 | PTPN22 | PTPN23 | PTPN6 | PTPRA | PTPRD | PTPRF | PTPRK | PTPRO | PTPRR | PTRH2 | PTTG1 | PUM1 | PUS7 | PXDN | PXN | PYCR1 | PYGL | QARS | QRICH1 | QSOX1 | RAB10 | RAB11A | RAB11B | RAB11FIP2 | RAB18 | RAB1A | RAB1B | RAB21 | RAB24 | RAB29 | RAB2A | RAB31 | RAB32 | RAB35 | RAB3GAP1 | RAB5A | RAB5B | RAB7A | RAB9A | RABL2A | RABL3 | RAC1 | RAD21 | RAD23A | RAD23B | RAD51 | RAD51AP1 | RADIL | RAE1 | RAF1 | RAN | RANBP3 | RANBP9 | RANGAP1 | RAP1A | RAP1B | RAP1GDS1 | RAPGEF4 | RARA | RARG | RASD1 | RASL11B | RASSF10 | RASSF7 | RASSF9 | RB1 | RB1CC1 | RBBP4 | RBBP6 | RBM17 | RBM25 | RBM3 | RBM4 | RBM8A | RBMX | RBP1 | RBPJ | RBX1 | RC3H1 | RCC1 | RDH11 | RDH12 | RDH13 | RDH14 | RDH5 | RDX | RECK | REL | RELA | REST | RFC1 | RFC2 | RFC3 | RFC4 | RFC5 | RFK | RFXANK | RGN | RGS1 | RGS2 | RGS20 | RHBDD1 | RHO | RHOA | RHOJ | RHOT1 | RHOT2 | RHOU | RHPN1 | RIC3 | RIC8B | RIF1 | RING1 | RIOK1 | RIPK1 | RIPK2 | RIPK3 | RIPK4 | RIT1 | RMND1 | RNASEH2B | RNF126 | RNF139 | RNF183 | RNF185 | RNF19A | RNF2 | RNF25 | RNF31 | RNF4 | RNF8 | RNPEP | ROMO1 | RP2 | RPA2 | RPA3 | RPGRIP1L | RPL10 | RPL10A | RPL12 | RPL18A | RPL22 | RPL23 | RPL23A | RPL24 | RPL30 | RPL37A | RPL38 | RPL6 | RPL7 | RPL7A | RPL9 | RPLP0 | RPLP1 | RPLP2 | RPS12 | RPS13 | RPS14 | RPS15A | RPS19 | RPS2 | RPS20 | RPS24 | RPS29 | RPS3 | RPS4X | RPS6KA1 | RPS6KA2 | RPS6KB2 | RPS8 | RPTOR | RRM2 | RRM2B | RRS1 | RSU1 | RTF1 | RTKN | RTN4 | RTN4IP1 | RUNX1 | RUNX1T1 | RUNX2 | RUVBL1 | RUVBL2 | RXRA | RXRB | RXRG | S100A1 | S100A11 | S100A12 | S100A13 | S100A4 | S100A6 | S100A7 | S100A9 | S100B | SAE1 | SAG | SAMD7 | SAP18 | SARAF | SARM1 | SARS | SART1 | SART3 | SAT1 | SCARA3 | SCEL | SCFD1 | SCG2 | SCML2 | SCO2 | SCYL2 | SDC1 | SDC4 | SDCBP | SDCBP2 | SEC13 | SEC16A | SEC24B | SEC31A | SEL1L | SELL | SEMA7A | SENP6 | SEPT1 | SEPT11 | SEPT2 | SEPT4 | SEPT5 | SEPT7 | SEPT8 | SEPT9 | SERPINB2 | SERPINB3 | SERPINB5 | SERPINB6 | SERPINB8 | SERPINE2 | SERPINH1 | SET | SETBP1 | SETDB1 | SF1 | SFMBT1 | SFN | SFPQ | SFXN1 | SFXN4 | SGPL1 | SGTA | SGTB | SH2B1 | SH3GL1 | SH3GL2 | SH3GL3 | SH3KBP1 | SH3PXD2A | SHC1 | SHKBP1 | SHMT1 | SHMT2 | SIAH1 | SIN3A | SIPA1 | SIPA1L1 | SIRT1 | SIRT3 | SIRT5 | SIRT6 | SIRT7 | SKA1 | SKA3 | SKI | SKIV2L | SLAMF1 | SLAMF8 | SLC12A2 | SLC16A1 | SLC25A24 | SLC25A4 | SLC25A5 | SLC25A6 | SLC2A5 | SLC35F6 | SLC39A4 | SLC9A1 | SLC9A3R1 | SLTM | SMAD2 | SMAD3 | SMAD4 | SMAD5 | SMAD9 | SMARCA2 | SMARCA4 | SMARCB1 | SMARCC1 | SMARCC2 | SMARCE1 | SMC1A | SMC3 | SMG5 | SMG6 | SMG8 | SMO | SMURF1 | SMURF2 | SNAI2 | SNCA | SND1 | SNF8 | SNRPA | SNRPA1 | SNTA1 | SNW1 | SNX12 | SNX17 | SNX3 | SNX6 | SOAT1 | SOCS1 | SOCS2 | SOCS6 | SOD1 | SOD2 | SOGA3 | SORT1 | SOX2 | SP1 | SP100 | SP2 | SPACA3 | SPATA2 | SPDL1 | SPEN | SPERT | SPG21 | SPI1 | SPICE1 | SPIN1 | SPINK7 | SPINT2 | SPOP | SPR | SPRTN | SPRY2 | SPTAN1 | SPTBN1 | SPTBN2 | SPTLC2 | SQSTM1 | SRC | SRGAP2 | SRGAP3 | SRI | SRPK1 | SRPK2 | SRPRB | SRSF12 | SRSF5 | SSB | SSX2IP | ST13 | STAC | STAG2 | STARD13 | STAT1 | STAT3 | STAT4 | STAT5A | STAT6 | STAU1 | STIL | STIM1 | STK11 | STK24 | STK25 | STK26 | STK3 | STK38 | STK40 | STMN1 | STOM | STOML2 | STRAP | STUB1 | STX5 | STXBP1 | STXBP2 | STYX | SUB1 | SUGT1 | SULF2 | SULT1A3 | SUMO1 | SUMO2 | SUMO3 | SUN2 | SUPT4H1 | SUPT5H | SUSD5 | SUZ12 | SVIL | SWAP70 | SYDE1 | SYN1 | SYNE2 | SYNJ2BP | SYP | SYVN1 | TAB1 | TAB2 | TACO1 | TADA2B | TAF12 | TAF15 | TAF1A | TAGLN3 | TALDO1 | TANK | TAOK3 | TARDBP | TARS2 | TAS2R41 | TAS2R60 | TAS2R7 | TAZ | TBC1D15 | TBC1D22B | TBC1D23 | TBC1D4 | TBL1X | TBRG4 | TBXA2R | TCEA1 | TCEA2 | TCERG1 | TCF12 | TCF21 | TCF25 | TCF4 | TCIRG1 | TCOF1 | TCP1 | TCP10L | TCTN2 | TCTN3 | TDRD3 | TERF1 | TERF2 | TERF2IP | TES | TFCP2 | TFDP1 | TFG | TFIP11 | TGFB1 | TGFBR3 | TGIF2LX | TGM2 | THADA | THAP11 | THAP7 | THBS1 | THBS2 | THEM4 | THOC6 | THRA | TIA1 | TIAL1 | TIMP1 | TIMP2 | TINF2 | TKT | TLE1 | TLE3 | TLR3 | TM9SF4 | TMBIM6 | TMED9 | TMEM17 | TMEM173 | TMEM219 | TMEM231 | TMEM30A | TMEM65 | TMEM9B | TMOD3 | TMOD4 | TMPO | TMTC3 | TMX2 | TMX4 | TNF | TNFAIP3 | TNFRSF12A | TNFRSF1A | TNFRSF8 | TNFSF12 | TNFSF14 | TNIP2 | TNK2 | TNKS | TNKS2 | TNN | TNNT1 | TNP1 | TNPO1 | TOE1 | TOLLIP | TOMM22 | TOP1 | TOP2A | TOP3A | TOR1A | TP53 | TP53RK | TP63 | TP73 | TPCN2 | TPM1 | TPM2 | TPR | TPTE | TRA2A | TRADD | TRAF1 | TRAF2 | TRAF3 | TRAF3IP1 | TRAF6 | TRAP1 | TREML2 | TREX1 | TRIAP1 | TRIB2 | TRIB3 | TRIM14 | TRIM21 | TRIM23 | TRIM25 | TRIM28 | TRIM29 | TRIM32 | TRIM33 | TRIM34 | TRIM35 | TRIM63 | TRIM9 | TRIP12 | TRIP13 | TRIP4 | TRIP6 | TRUB2 | TSC22D1 | TSC22D3 | TSC22D4 | TSFM | TSG101 | TSKU | TSPAN17 | TSPYL2 | TTC5 | TUBA1A | TUBA4A | TUBB | TUBB2B | TUBB4A | TUBB4B | TUBG1 | TUFT1 | TULP3 | TWF1 | TWF2 | TWIST1 | TXLNA | TXN | TXN2 | TXNDC11 | TXNDC15 | TXNDC17 | TXNDC5 | TXNIP | TXNRD1 | TYMS | U2AF2 | UBA2 | UBA3 | UBA5 | UBAP2L | UBASH3A | UBB | UBC | UBE2A | UBE2B | UBE2C | UBE2D1 | UBE2D2 | UBE2D3 | UBE2I | UBE2J1 | UBE2K | UBE2L3 | UBE2L6 | UBE2M | UBE2N | UBE2O | UBE2S | UBE2V1 | UBE3A | UBQLN1 | UBQLN2 | UBQLN4 | UBR5 | UBXN1 | UCHL1 | UCHL5 | UFM1 | ULK1 | UMPS | UNC119 | UNK | UNKL | UPF2 | UPF3B | UQCC1 | USP14 | USP15 | USP16 | USP17L2 | USP19 | USP2 | USP20 | USP21 | USP28 | USP4 | USP42 | USP46 | USP47 | USP5 | USP50 | USP53 | USP7 | USP8 | USP9X | USP9Y | VAC14 | VAPA | VAPB | VARS | VASH2 | VASP | VAT1 | VAV1 | VCAM1 | VCL | VCP | VDAC1 | VDAC2 | VDAC3 | VDR | VGF | VGLL4 | VHL | VIM | VKORC1 | VN1R1 | VPS18 | VPS26A | VPS29 | VPS4B | VPS72 | VSIG4 | VSTM2A | VTN | WARS | WAS | WBP2 | WDFY1 | WDR1 | WDR12 | WDR19 | WDR35 | WDR41 | WDR48 | WDR77 | WDTC1 | WIPI1 | WNK1 | WNK4 | WNT16 | WNT2 | WSB1 | WT1 | WTAP | WWOX | WWP2 | XAF1 | XBP1 | XIAP | XPO1 | XPO4 | XPO5 | XRCC1 | XRCC3 | XRCC5 | XRCC6 | YAF2 | YAP1 | YEATS2 | YES1 | YME1L1 | YRDC | YTHDF1 | YWHAB | YWHAE | YWHAG | YWHAH | YWHAQ | YWHAZ | YY1 | ZAP70 | ZBTB16 | ZBTB17 | ZBTB21 | ZBTB25 | ZBTB33 | ZBTB42 | ZBTB7A | ZBTB7B | ZC3H4 | ZC3HC1 | ZDHHC17 | ZFAND5 | ZFAND6 | ZFP41 | ZFYVE19 | ZFYVE26 | ZHX1 | ZIC1 | ZKSCAN1 | ZMPSTE24 | ZMYM5 | ZMYND10 | ZMYND8 | ZNF207 | ZNF217 | ZNF227 | ZNF24 | ZNF277 | ZNF354C | ZNF408 | ZNF420 | ZNF443 | ZNF517 | ZNF518A | ZNF592 | ZNF598 | ZNF618 | ZNF622 | ZNF646 | ZNF669 | ZNF696 | ZNF746 | ZNF747 | ZRANB2 | ZSCAN2 | ZSCAN29 | ZSCAN32 | ZW10 | ZWINT | ZZZ3 | GO:0072330 | 0.00994783452626471 | 82/8243 | 39/3250 | 0.0814851552410554 | 1 | F | F | F | F | monocarboxylic acid biosynthetic process | ABCD3 | ACACA | ACLY | ACOT7 | ACSS2 | ALOX12 | ALOX5 | BRCA1 | CBR4 | CD74 | CYP1A1 | CYP1A2 | DEGS1 | FASN | GPX4 | GSTM4 | HSD17B4 | HSD17B8 | HSD3B7 | LDHC | LTC4S | MCAT | MGST1 | MIF | MYO5A | OSBPL1A | PARK7 | PER2 | PLA2G1B | PRKAA1 | PRKAA2 | PRKAB2 | PRKAG1 | PTGDS | PTGES | PTGES3 | RBP1 | TECR | XBP1 | GO:0071156 | 0.00994783452626471 | 82/8243 | 39/3250 | 0.0814851552410554 | 1 | F | F | F | F | regulation of cell cycle arrest | AKT2 | ATM | BAX | BIN1 | BRCA1 | CALR | CARM1 | CASP2 | CCNB1 | CDC25C | CDK2 | CDK4 | CDK5 | CDK5R1 | CDK9 | CDKN1A | CHEK2 | E2F1 | E2F4 | EP300 | FOXM1 | HSP90AB1 | ID2 | MDM2 | MDM4 | MIF | MYBBP1A | NEUROD1 | PCNA | PML | PRKACA | RPL23 | SFN | TFDP1 | TGFB1 | TP53 | TP73 | TRIAP1 | ZBTB17 | GO:0070374 | 0.0133446560718185 | 110/8243 | 51/3250 | 0.081486885729212 | 1 | F | F | F | F | positive regulation of ERK1 and ERK2 cascade | ABL1 | ADCYAP1 | AGER | APOE | APP | ARRB1 | ARRB2 | BRAF | CCL21 | CCL22 | CCR1 | CD36 | CD74 | CRKL | EGFR | FGB | FGF2 | FGFR3 | GNAI2 | HAVCR2 | HMGB1 | HRAS | ICAM1 | JUN | KDR | MAP2K1 | MAP2K7 | MAPK3 | MIF | NELFE | NOD2 | NQO2 | NTRK1 | PHB | PHB2 | PLA2G2A | PRKCZ | PRKD2 | PTEN | PTPN22 | RAP1A | RAP1B | RIPK2 | S100A7 | SEMA7A | SHC1 | SLAMF1 | SPRY2 | SRC | TGFB1 | TNF | GO:0032306 | 0.000606575275991751 | 5/8243 | 4/3250 | 0.0826514208221515 | 1 | F | F | F | F | regulation of prostaglandin secretion | MAP2K6 | MIF | P2RX4 | PLA2G10 | GO:1900264 | 0.000606575275991751 | 5/8243 | 4/3250 | 0.0826514208221515 | 1 | F | F | F | F | positive regulation of DNA-directed DNA polymerase activity | RFC2 | RFC3 | RFC4 | RFC5 | GO:0002084 | 0.000606575275991751 | 5/8243 | 4/3250 | 0.0826514208221515 | 1 | F | F | F | F | protein depalmitoylation | ABHD17A | LYPLA1 | LYPLA2 | PPT1 | GO:0001887 | 0.000606575275991751 | 5/8243 | 4/3250 | 0.0826514208221515 | 1 | F | F | F | F | selenium compound metabolic process | SCLY | SEPHS1 | SEPHS2 | TXNRD1 | GO:1990910 | 0.000606575275991751 | 5/8243 | 4/3250 | 0.0826514208221515 | 1 | F | F | F | F | response to hypobaric hypoxia | DNM1L | FIS1 | HSF1 | IREB2 | GO:0090267 | 0.000606575275991751 | 5/8243 | 4/3250 | 0.0826514208221515 | 1 | F | F | F | F | positive regulation of mitotic cell cycle spindle assembly checkpoint | DYNC1LI1 | MAD2L1 | TPR | XRCC3 | GO:1900864 | 0.000606575275991751 | 5/8243 | 4/3250 | 0.0826514208221515 | 1 | F | F | F | F | mitochondrial RNA modification | HSD17B10 | KIAA0391 | PUS1 | TRMT61B | GO:1901072 | 0.000606575275991751 | 5/8243 | 4/3250 | 0.0826514208221515 | 1 | F | F | F | F | glucosamine-containing compound catabolic process | AMDHD2 | GNPDA1 | GNPDA2 | OVGP1 | GO:0019953 | 0.000606575275991751 | 5/8243 | 4/3250 | 0.0826514208221515 | 1 | F | F | F | F | sexual reproduction | EIF4H | STAT3 | TNP1 | VGF | GO:0070141 | 0.000606575275991751 | 5/8243 | 4/3250 | 0.0826514208221515 | 1 | F | F | F | F | response to UV-A | AKT1 | EGFR | MME | PPID | GO:0098970 | 0.000606575275991751 | 5/8243 | 4/3250 | 0.0826514208221515 | 1 | F | F | F | F | postsynaptic neurotransmitter receptor diffusion trapping | CACNG2 | CACNG4 | CACNG5 | DLG4 | GO:2000304 | 0.000606575275991751 | 5/8243 | 4/3250 | 0.0826514208221515 | 1 | F | F | F | F | positive regulation of ceramide biosynthetic process | PLA2G6 | PRKCD | TNF | TNFRSF1A | GO:0140212 | 0.000606575275991751 | 5/8243 | 4/3250 | 0.0826514208221515 | 1 | F | F | F | F | regulation of long-chain fatty acid import into cell | AKT1 | AKT2 | FABP3 | THBS1 | GO:0046348 | 0.000606575275991751 | 5/8243 | 4/3250 | 0.0826514208221515 | 1 | F | F | F | F | amino sugar catabolic process | AMDHD2 | GNPDA1 | GNPDA2 | OVGP1 | GO:0090232 | 0.000606575275991751 | 5/8243 | 4/3250 | 0.0826514208221515 | 1 | F | F | F | F | positive regulation of spindle checkpoint | DYNC1LI1 | MAD2L1 | TPR | XRCC3 | GO:0032308 | 0.000606575275991751 | 5/8243 | 4/3250 | 0.0826514208221515 | 1 | F | F | F | F | positive regulation of prostaglandin secretion | MAP2K6 | MIF | P2RX4 | PLA2G10 | GO:0043376 | 0.000606575275991751 | 5/8243 | 4/3250 | 0.0826514208221515 | 1 | F | F | F | F | regulation of CD8-positive, alpha-beta T cell differentiation | CBFB | RUNX1 | SOCS1 | ZBTB7B | GO:0042420 | 0.000606575275991751 | 5/8243 | 4/3250 | 0.0826514208221515 | 1 | F | F | F | F | dopamine catabolic process | COMT | DBH | MAOA | SULT1A3 | GO:0022614 | 0.000606575275991751 | 5/8243 | 4/3250 | 0.0826514208221515 | 1 | F | F | F | F | membrane to membrane docking | EZR | ICAM1 | MSN | VCAM1 | GO:0090220 | 0.000606575275991751 | 5/8243 | 4/3250 | 0.0826514208221515 | 1 | F | F | F | F | chromosome localization to nuclear envelope involved in homologous chromosome segregation | CCDC155 | MLH1 | TERF1 | UBE2B | GO:0070900 | 0.000606575275991751 | 5/8243 | 4/3250 | 0.0826514208221515 | 1 | F | F | F | F | mitochondrial tRNA modification | HSD17B10 | KIAA0391 | PUS1 | TRMT61B | GO:2000270 | 0.000606575275991751 | 5/8243 | 4/3250 | 0.0826514208221515 | 1 | F | F | F | F | negative regulation of fibroblast apoptotic process | API5 | CHD8 | PIK3CA | PIK3CG | GO:1990564 | 0.000606575275991751 | 5/8243 | 4/3250 | 0.0826514208221515 | 1 | F | F | F | F | protein polyufmylation | DDRGK1 | UBA5 | UFC1 | UFM1 | GO:1900113 | 0.000606575275991751 | 5/8243 | 4/3250 | 0.0826514208221515 | 1 | F | F | F | F | negative regulation of histone H3-K9 trimethylation | KDM4D | PIH1D1 | SIRT1 | SMARCB1 | GO:0000056 | 0.000606575275991751 | 5/8243 | 4/3250 | 0.0826514208221515 | 1 | F | F | F | F | ribosomal small subunit export from nucleus | NPM1 | NUP88 | RAN | XPO1 | GO:0035891 | 0.000606575275991751 | 5/8243 | 4/3250 | 0.0826514208221515 | 1 | F | F | F | F | exit from host cell | PPIA | PPID | RAB7A | VPS4B | GO:2000480 | 0.000606575275991751 | 5/8243 | 4/3250 | 0.0826514208221515 | 1 | F | F | F | F | negative regulation of cAMP-dependent protein kinase activity | PKIG | PRKAR1A | PRKAR2B | SIRT1 | GO:0099628 | 0.000606575275991751 | 5/8243 | 4/3250 | 0.0826514208221515 | 1 | F | F | F | F | neurotransmitter receptor diffusion trapping | CACNG2 | CACNG4 | CACNG5 | DLG4 | GO:0052192 | 0.000606575275991751 | 5/8243 | 4/3250 | 0.0826514208221515 | 1 | F | F | F | F | movement in environment of other organism involved in symbiotic interaction | PPIA | PPID | RAB7A | VPS4B | GO:1905206 | 0.000606575275991751 | 5/8243 | 4/3250 | 0.0826514208221515 | 1 | F | F | F | F | positive regulation of hydrogen peroxide-induced cell death | ENDOG | HDAC6 | PAWR | RIPK1 | GO:0052126 | 0.000606575275991751 | 5/8243 | 4/3250 | 0.0826514208221515 | 1 | F | F | F | F | movement in host environment | PPIA | PPID | RAB7A | VPS4B | GO:0070841 | 0.000606575275991751 | 5/8243 | 4/3250 | 0.0826514208221515 | 1 | F | F | F | F | inclusion body assembly | BAG3 | HDAC6 | MAPT | VCP | GO:0019076 | 0.000606575275991751 | 5/8243 | 4/3250 | 0.0826514208221515 | 1 | F | F | F | F | viral release from host cell | PPIA | PPID | RAB7A | VPS4B | GO:0070562 | 0.000606575275991751 | 5/8243 | 4/3250 | 0.0826514208221515 | 1 | F | F | F | F | regulation of vitamin D receptor signaling pathway | KANK2 | MED1 | SNAI2 | SNW1 | GO:0009396 | 0.000606575275991751 | 5/8243 | 4/3250 | 0.0826514208221515 | 1 | F | F | F | F | folic acid-containing compound biosynthetic process | ATIC | FPGS | GART | MTHFD1 | GO:0051105 | 0.000606575275991751 | 5/8243 | 4/3250 | 0.0826514208221515 | 1 | F | F | F | F | regulation of DNA ligation | HMGB1 | RAD51 | TFIP11 | XRCC1 | GO:2000347 | 0.000606575275991751 | 5/8243 | 4/3250 | 0.0826514208221515 | 1 | F | F | F | F | positive regulation of hepatocyte proliferation | MED1 | PTN | TNFAIP3 | XBP1 | GO:1902741 | 0.000606575275991751 | 5/8243 | 4/3250 | 0.0826514208221515 | 1 | F | F | F | F | positive regulation of interferon-alpha secretion | DHX36 | DHX9 | HMGB1 | MAVS | GO:0032049 | 0.000606575275991751 | 5/8243 | 4/3250 | 0.0826514208221515 | 1 | F | F | F | F | cardiolipin biosynthetic process | PLA2G6 | PTPMT1 | TAMM41 | TAZ | GO:0060736 | 0.000606575275991751 | 5/8243 | 4/3250 | 0.0826514208221515 | 1 | F | F | F | F | prostate gland growth | AR | ESR1 | PSAP | PTEN | GO:1902739 | 0.000606575275991751 | 5/8243 | 4/3250 | 0.0826514208221515 | 1 | F | F | F | F | regulation of interferon-alpha secretion | DHX36 | DHX9 | HMGB1 | MAVS | GO:0042424 | 0.000606575275991751 | 5/8243 | 4/3250 | 0.0826514208221515 | 1 | F | F | F | F | catecholamine catabolic process | COMT | DBH | MAOA | SULT1A3 | GO:0046653 | 0.000606575275991751 | 5/8243 | 4/3250 | 0.0826514208221515 | 1 | F | F | F | F | tetrahydrofolate metabolic process | MTHFD1 | SHMT1 | SHMT2 | TYMS | GO:0098953 | 0.000606575275991751 | 5/8243 | 4/3250 | 0.0826514208221515 | 1 | F | F | F | F | receptor diffusion trapping | CACNG2 | CACNG4 | CACNG5 | DLG4 | GO:1990592 | 0.000606575275991751 | 5/8243 | 4/3250 | 0.0826514208221515 | 1 | F | F | F | F | protein K69-linked ufmylation | DDRGK1 | UBA5 | UFC1 | UFM1 | GO:1900262 | 0.000606575275991751 | 5/8243 | 4/3250 | 0.0826514208221515 | 1 | F | F | F | F | regulation of DNA-directed DNA polymerase activity | RFC2 | RFC3 | RFC4 | RFC5 | GO:1990314 | 0.000606575275991751 | 5/8243 | 4/3250 | 0.0826514208221515 | 1 | F | F | F | F | cellular response to insulin-like growth factor stimulus | CREB1 | CRK | PTEN | TGFB1 | GO:0061478 | 0.000606575275991751 | 5/8243 | 4/3250 | 0.0826514208221515 | 1 | F | F | F | F | response to platelet aggregation inhibitor | ACTB | ADCY6 | CFTR | GNAI1 | GO:0042256 | 0.000606575275991751 | 5/8243 | 4/3250 | 0.0826514208221515 | 1 | F | F | F | F | mature ribosome assembly | C1QBP | DDX3X | EIF6 | SBDS | GO:0072318 | 0.000606575275991751 | 5/8243 | 4/3250 | 0.0826514208221515 | 1 | F | F | F | F | clathrin coat disassembly | GAK | HSPA8 | SH3GL1 | SH3GL2 | GO:0032836 | 0.000606575275991751 | 5/8243 | 4/3250 | 0.0826514208221515 | 1 | F | F | F | F | glomerular basement membrane development | MYO1E | NID1 | SULF2 | WT1 | GO:0033689 | 0.000606575275991751 | 5/8243 | 4/3250 | 0.0826514208221515 | 1 | F | F | F | F | negative regulation of osteoblast proliferation | BCL2 | NELL1 | SMAD3 | TNN | GO:0035890 | 0.000606575275991751 | 5/8243 | 4/3250 | 0.0826514208221515 | 1 | F | F | F | F | exit from host | PPIA | PPID | RAB7A | VPS4B | GO:0006104 | 0.000606575275991751 | 5/8243 | 4/3250 | 0.0826514208221515 | 1 | F | F | F | F | succinyl-CoA metabolic process | DLST | OGDH | SUCLA2 | SUCLG2 | GO:0019262 | 0.000606575275991751 | 5/8243 | 4/3250 | 0.0826514208221515 | 1 | F | F | F | F | N-acetylneuraminate catabolic process | AMDHD2 | GNPDA1 | GNPDA2 | NAGK | GO:0070431 | 0.000606575275991751 | 5/8243 | 4/3250 | 0.0826514208221515 | 1 | F | F | F | F | nucleotide-binding oligomerization domain containing 2 signaling pathway | NFKBIA | NOD2 | RELA | RIPK2 | GO:0071680 | 0.000606575275991751 | 5/8243 | 4/3250 | 0.0826514208221515 | 1 | F | F | F | F | response to indole-3-methanol | BRCA1 | CDH1 | CTNNB1 | JUP | GO:2001301 | 0.000606575275991751 | 5/8243 | 4/3250 | 0.0826514208221515 | 1 | F | F | F | F | lipoxin biosynthetic process | ALOX12 | ALOX5 | ALOX5AP | LTC4S | GO:0019614 | 0.000606575275991751 | 5/8243 | 4/3250 | 0.0826514208221515 | 1 | F | F | F | F | catechol-containing compound catabolic process | COMT | DBH | MAOA | SULT1A3 | GO:0090154 | 0.000606575275991751 | 5/8243 | 4/3250 | 0.0826514208221515 | 1 | F | F | F | F | positive regulation of sphingolipid biosynthetic process | PLA2G6 | PRKCD | TNF | TNFRSF1A | GO:0052405 | 0.000606575275991751 | 5/8243 | 4/3250 | 0.0826514208221515 | 1 | F | F | F | F | negative regulation by host of symbiont molecular function | ANXA2 | APCS | RAB9A | SUGT1 | GO:2000646 | 0.000606575275991751 | 5/8243 | 4/3250 | 0.0826514208221515 | 1 | F | F | F | F | positive regulation of receptor catabolic process | APOE | ITCH | PCSK9 | PTPN1 | GO:0000003 | 0.000606575275991751 | 5/8243 | 4/3250 | 0.0826514208221515 | 1 | F | F | F | F | reproduction | EIF4H | STAT3 | TNP1 | VGF | GO:0032055 | 0.000606575275991751 | 5/8243 | 4/3250 | 0.0826514208221515 | 1 | F | F | F | F | negative regulation of translation in response to stress | EIF2AK3 | EIF2AK4 | EIF2S1 | RBM4 | GO:1902581 | 0.000606575275991751 | 5/8243 | 4/3250 | 0.0826514208221515 | 1 | F | F | F | F | multi-organism cellular localization | DYNLT1 | IFIT1 | NUP153 | RAB7A | GO:0035999 | 0.000606575275991751 | 5/8243 | 4/3250 | 0.0826514208221515 | 1 | F | F | F | F | tetrahydrofolate interconversion | MTHFD1 | SHMT1 | SHMT2 | TYMS | GO:1901535 | 0.000606575275991751 | 5/8243 | 4/3250 | 0.0826514208221515 | 1 | F | F | F | F | regulation of DNA demethylation | OTUD4 | TRIM28 | USP7 | USP9X | GO:2000617 | 0.000606575275991751 | 5/8243 | 4/3250 | 0.0826514208221515 | 1 | F | F | F | F | positive regulation of histone H3-K9 acetylation | BRCA1 | PIH1D1 | SMAD4 | SMARCB1 | GO:0036035 | 0.000606575275991751 | 5/8243 | 4/3250 | 0.0826514208221515 | 1 | F | F | F | F | osteoclast development | ANXA2 | LRRK1 | PAFAH1B1 | SRC | GO:0010737 | 0.000606575275991751 | 5/8243 | 4/3250 | 0.0826514208221515 | 1 | F | F | F | F | protein kinase A signaling | EZR | LCP1 | PRKACA | RDX | GO:0032261 | 0.000606575275991751 | 5/8243 | 4/3250 | 0.0826514208221515 | 1 | F | F | F | F | purine nucleotide salvage | ADA | ADK | APRT | HPRT1 | GO:0070345 | 0.000606575275991751 | 5/8243 | 4/3250 | 0.0826514208221515 | 1 | F | F | F | F | negative regulation of fat cell proliferation | E2F1 | E2F3 | PER2 | TFDP1 | GO:1902583 | 0.000606575275991751 | 5/8243 | 4/3250 | 0.0826514208221515 | 1 | F | F | F | F | multi-organism intracellular transport | DYNLT1 | IFIT1 | NUP153 | RAB7A | GO:0071681 | 0.000606575275991751 | 5/8243 | 4/3250 | 0.0826514208221515 | 1 | F | F | F | F | cellular response to indole-3-methanol | BRCA1 | CDH1 | CTNNB1 | JUP | GO:0072319 | 0.000606575275991751 | 5/8243 | 4/3250 | 0.0826514208221515 | 1 | F | F | F | F | vesicle uncoating | GAK | HSPA8 | SH3GL1 | SH3GL2 | GO:0019228 | 0.000606575275991751 | 5/8243 | 4/3250 | 0.0826514208221515 | 1 | F | F | F | F | neuronal action potential | ANK3 | GRIK2 | P2RX1 | P2RX4 | GO:0051572 | 0.000606575275991751 | 5/8243 | 4/3250 | 0.0826514208221515 | 1 | F | F | F | F | negative regulation of histone H3-K4 methylation | BCOR | BRCA1 | KDM1A | PRMT6 | GO:0060420 | 0.00606575275991751 | 50/8243 | 25/3250 | 0.0834312499417723 | 1 | F | F | F | F | regulation of heart growth | BASP1 | BMPR1A | CCNB1 | CTDP1 | DDX39B | FGF2 | FGFR1 | G6PD | GSK3A | MAPK1 | MAPK14 | MYH6 | PIM1 | PIN1 | PROX1 | PTEN | RBPJ | RGS2 | RIPK1 | TGFBR3 | TP73 | WNT2 | WT1 | YAP1 | YY1 | GO:1905475 | 0.0156496421205872 | 129/8243 | 59/3250 | 0.0834342326301825 | 1 | F | F | F | F | regulation of protein localization to membrane | ACTB | AGR2 | AKT1 | AKT2 | ANK3 | AR | BBC3 | BCL2 | BCL2L1 | CACNG2 | CASP8 | CDK5 | CDK5R1 | CHP1 | CLTC | CRKL | DAB2 | E2F1 | EGFR | ERBB2 | EZR | FIS1 | FYN | GDI1 | GOPC | HRAS | ITGB1BP1 | KIF5B | LGALS3 | LYPLA1 | MAPK8 | MYO1C | PIK3R1 | PPP3CC | PRKCE | PRKCZ | PTN | RAB11A | RAB11FIP2 | RAP1A | SFN | SPTBN1 | SQSTM1 | STAC | STOM | TFDP1 | TGFB1 | TNF | TP53 | TP63 | TP73 | USP17L2 | YWHAB | YWHAE | YWHAG | YWHAH | YWHAQ | YWHAZ | ZMYND8 | GO:1901799 | 0.00497391726313235 | 41/8243 | 21/3250 | 0.0835921821461177 | 1 | F | F | F | F | negative regulation of proteasomal protein catabolic process | BAG5 | BAG6 | CCAR2 | DDRGK1 | FAM192A | HSP90AB1 | HSP90AB3P | HSP90AB4P | LAMP3 | OGT | PARK7 | SDCBP | SGTA | SMARCC1 | STYX | UBE2J1 | UBXN1 | UCHL5 | USP14 | USP19 | USP7 | GO:0043038 | 0.00497391726313235 | 41/8243 | 21/3250 | 0.0835921821461177 | 1 | F | F | F | F | amino acid activation | AARS | AARSD1 | AASDH | AIMP2 | CARS | DARS | EPRS | FARSB | GARS | HARS | IARS | LARS | PPA1 | PPA2 | QARS | RARS2 | SARS | TARS | TARS2 | VARS | WARS | GO:0110110 | 0.00497391726313235 | 41/8243 | 21/3250 | 0.0835921821461177 | 1 | F | F | F | F | positive regulation of animal organ morphogenesis | AR | BASP1 | BAX | CTNNB1 | FGFR1 | LGR4 | MED1 | MYC | PAX8 | PIM1 | PTN | RBPJ | SMAD4 | SMO | TGFB1 | TNFAIP3 | TWIST1 | VDR | WNT2 | WT1 | XBP1 | GO:0048261 | 0.00206235593837195 | 17/8243 | 10/3250 | 0.083759898632637 | 1 | F | F | F | F | negative regulation of receptor-mediated endocytosis | APP | ATXN2 | DLG4 | PCSK9 | PSEN1 | RAC1 | SDCBP | SH3GL3 | UBQLN2 | UNC119 | GO:0010888 | 0.00206235593837195 | 17/8243 | 10/3250 | 0.083759898632637 | 1 | F | F | F | F | negative regulation of lipid storage | ABCG1 | CRP | ITGAV | ITGB3 | NFKBIA | NR1H2 | NR1H3 | PPARD | PPARG | TNF | GO:0031102 | 0.00206235593837195 | 17/8243 | 10/3250 | 0.083759898632637 | 1 | F | F | F | F | neuron projection regeneration | APOA1 | APOD | BCL2 | FOLR1 | GAP43 | GFAP | JUN | MAPK8IP3 | PTPRF | ULK1 | GO:0071353 | 0.00206235593837195 | 17/8243 | 10/3250 | 0.083759898632637 | 1 | F | F | F | F | cellular response to interleukin-4 | CDK4 | FASN | HSP90AB1 | HSPA5 | IMPDH2 | KEAP1 | LEF1 | MCM2 | PML | XBP1 | GO:0000272 | 0.00206235593837195 | 17/8243 | 10/3250 | 0.083759898632637 | 1 | F | F | F | F | polysaccharide catabolic process | AGL | CALM1 | GAA | PFKM | PGM1 | PGM2 | PHKA1 | PYGB | PYGL | STBD1 | GO:0044247 | 0.00206235593837195 | 17/8243 | 10/3250 | 0.083759898632637 | 1 | F | F | F | F | cellular polysaccharide catabolic process | AGL | CALM1 | GAA | PFKM | PGM1 | PGM2 | PHKA1 | PYGB | PYGL | STBD1 | GO:1901673 | 0.00206235593837195 | 17/8243 | 10/3250 | 0.083759898632637 | 1 | F | F | F | F | regulation of mitotic spindle assembly | CHMP2A | HNRNPU | HSPA1B | PDCD6IP | PLK1 | SMC1A | SMC3 | STAG2 | TPR | VPS4B | GO:0035987 | 0.00206235593837195 | 17/8243 | 10/3250 | 0.083759898632637 | 1 | F | F | F | F | endodermal cell differentiation | COL12A1 | COL4A2 | COL6A1 | FN1 | ITGA4 | ITGA7 | ITGAV | ITGB5 | LAMB1 | VTN | GO:0097066 | 0.00206235593837195 | 17/8243 | 10/3250 | 0.083759898632637 | 1 | F | F | F | F | response to thyroid hormone | AKR1B1 | ANXA2 | ANXA5 | CTSL | GATA1 | LMO2 | MED1 | PPARGC1A | RDX | TOMM20 | GO:0033032 | 0.00206235593837195 | 17/8243 | 10/3250 | 0.083759898632637 | 1 | F | F | F | F | regulation of myeloid cell apoptotic process | ANXA1 | BCL2 | CCR5 | IRF7 | MIF | NOD2 | PTEN | SIRT1 | TCP1 | THRA | GO:1901020 | 0.00206235593837195 | 17/8243 | 10/3250 | 0.083759898632637 | 1 | F | F | F | F | negative regulation of calcium ion transmembrane transporter activity | CALM1 | CALM2 | CALM3 | FKBP1A | FKBP1B | GSTO1 | SRI | THADA | UBQLN1 | YWHAE | GO:0035518 | 0.00206235593837195 | 17/8243 | 10/3250 | 0.083759898632637 | 1 | F | F | F | F | histone H2A monoubiquitination | BCOR | BMI1 | CUL4B | DDB1 | DDB2 | PCGF1 | PCGF5 | PCGF6 | RING1 | RNF2 | GO:0046639 | 0.00206235593837195 | 17/8243 | 10/3250 | 0.083759898632637 | 1 | F | F | F | F | negative regulation of alpha-beta T cell differentiation | ANXA1 | BCL6 | CBFB | HLX | HMGB1 | JAK3 | RC3H1 | RUNX1 | SOCS1 | ZBTB7B | GO:0051651 | 0.00800679364309111 | 66/8243 | 32/3250 | 0.0839304640189522 | 1 | F | F | F | F | maintenance of location in cell | AKAP9 | AKT1 | ALB | ANK3 | APOE | CAV1 | CDK5 | DBN1 | EZR | FLNB | FTH1 | FTL | GAA | GOLPH3 | HK1 | HK2 | HNRNPU | HSPA5 | INSIG1 | JUP | PAFAH1B1 | PARK7 | PML | POLR2M | RANGAP1 | SP100 | SRI | SUN2 | SYNE2 | TWF1 | TWF2 | TXN | GO:0120035 | 0.0469489263617615 | 387/8243 | 166/3250 | 0.0848080891316264 | 1 | F | F | F | F | regulation of plasma membrane bounded cell projection organization | ABL1 | ABL2 | ACTR2 | ADCY6 | ADCYAP1 | AKT1 | APBB1 | APC | APOE | ARF1 | ARHGAP35 | ARHGDIA | ARHGEF7 | ARPC2 | ATG5 | AVIL | BAG5 | BCL11A | BRAF | CAMK1D | CAMK2B | CAPZB | CARM1 | CAV1 | CCDC88A | CCL21 | CCP110 | CDC20 | CDC42 | CDC42EP3 | CDC42EP4 | CDK5 | CDK5R1 | CDKL3 | CENPJ | CEP135 | CFL1 | CNTROB | CRK | CRKL | CRMP1 | CROCC | CTTN | CUL7 | CYLD | DAB1 | DBN1 | DGUOK | DHX36 | DLG4 | DNM1L | DVL1 | DVL2 | DVL3 | DYNLL1 | EP300 | EPHA4 | EZH2 | EZR | FAM98A | FBXO7 | FGFR1 | FKBP1B | FN1 | FSCN1 | FYN | GAK | GAP43 | GDI1 | GFAP | GOLGA4 | GRID2 | GRIP1 | GSK3B | HAP1 | HDAC2 | HDAC4 | HECW1 | HECW2 | HRAS | HSPA5 | HTT | ICAM1 | ILK | IQGAP1 | KCTD17 | KDM1A | KIF13B | KLF5 | LGALS1 | LPAR1 | LRRC7 | LRRK2 | MAP1B | MAP2K1 | MAP3K13 | MAPK15 | MAPK6 | MAPT | MDM2 | METRN | MOV10 | NCK1 | NEDD4 | NFE2L2 | NIN | NME1 | NME2 | NTRK1 | OBSL1 | OPA1 | P2RX4 | PAFAH1B1 | PARP6 | PDLIM5 | PFN1 | PLXNA1 | PLXNA2 | PLXNB2 | PRKCD | PRKD1 | PSEN1 | PTEN | PTK2 | PTN | PTPRD | PTPRF | PTPRO | RAB11A | RAB21 | RAB29 | RAB5A | RAC1 | RAP1A | RDX | RGS2 | RHOA | RTN4 | RTN4IP1 | S100A9 | SARM1 | SEMA7A | SEPT7 | SEPT9 | SIPA1L1 | SMURF1 | SNX3 | SRC | ST
[truncated: 1,785,640 more chars]
